# Supplementary material for: Sunflower Hybrids and Inbred Lines Adopt Different Physiological Strategies and Proteome Responses to Cope with Water Deficit
Source: Biomolecules. 2023 Jul 12;13(7):1110. doi: 10.3390/biom13071110 (PMC10377273; doi:10.3390/biom13071110)
Supplement: Supplementary file 1 [file biomolecules-13-01110-s001.zip › biomolecules-2390314 - Supplementary Figures 2-7.pdf]

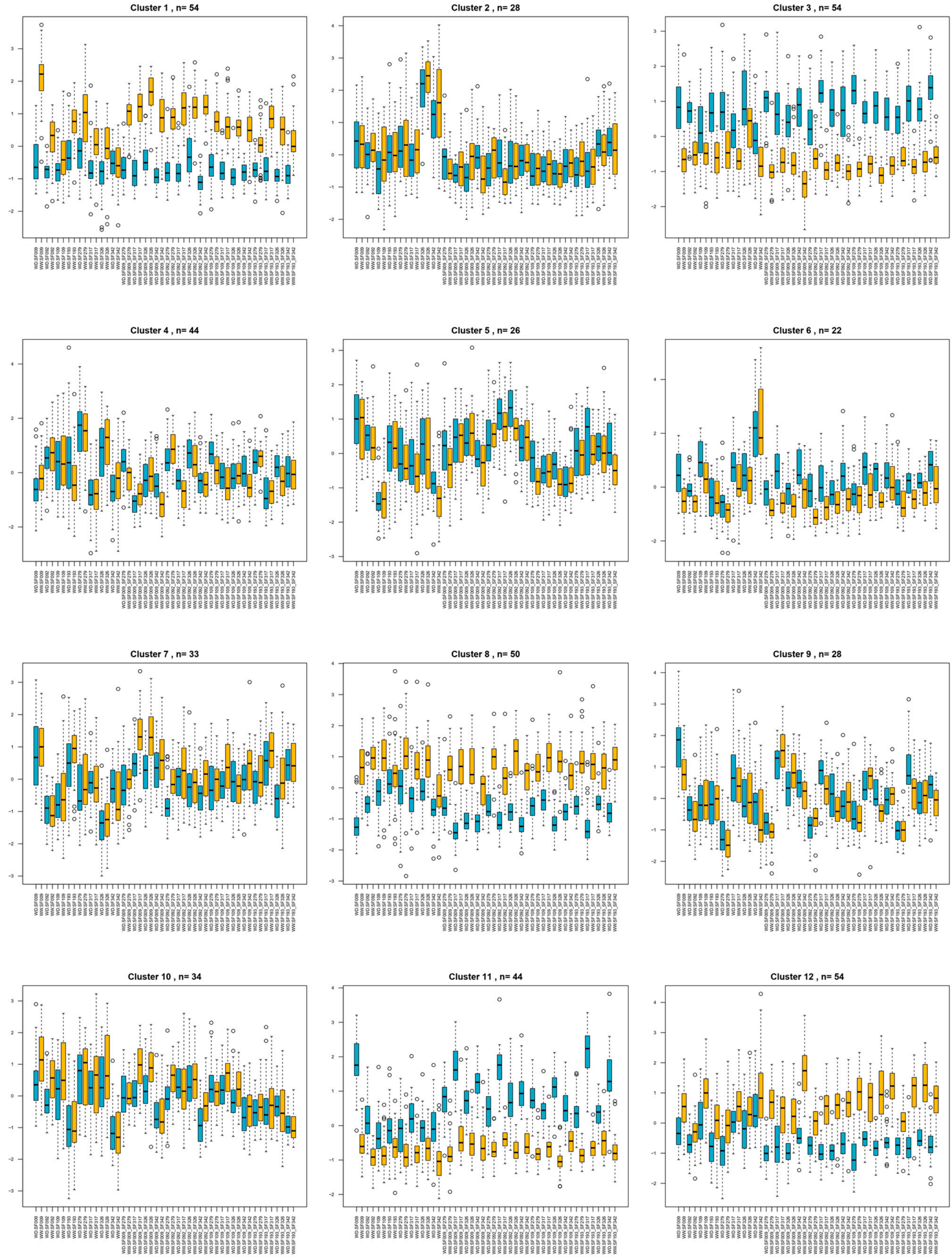

Figure S2. Variation of DAP clusters according to genotype and treatment (blue: well watered, yellow: water stressed).

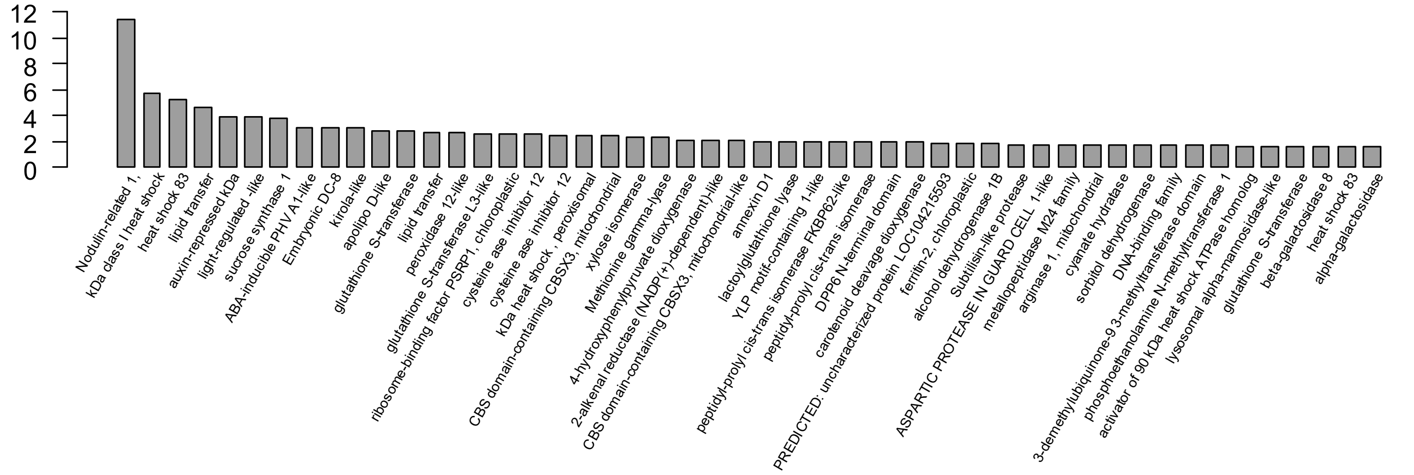

Figure S3. Fold change of the 50 most upregulated proteins in response to drought.

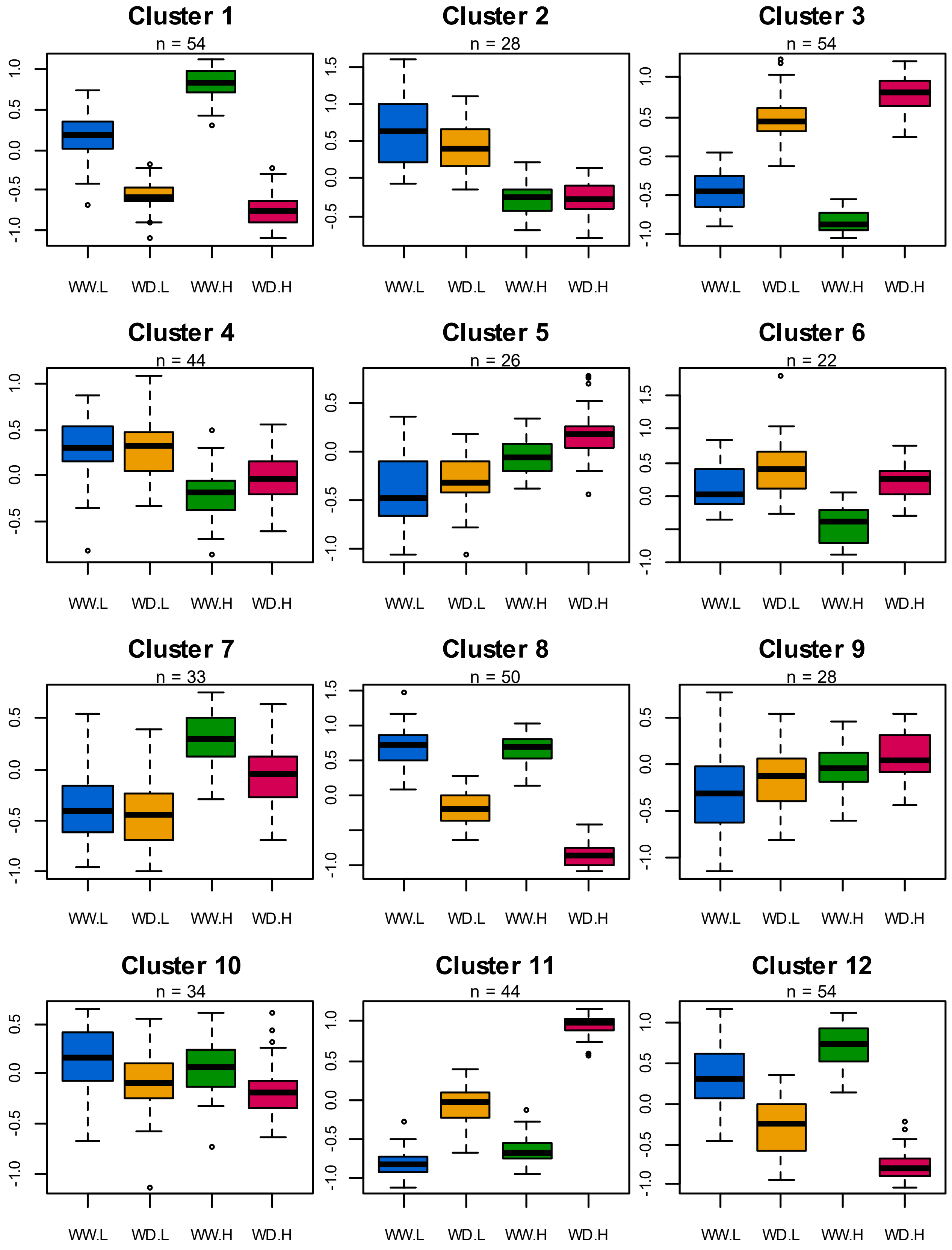

Figure S4. Response of all clusters to hybridity and water status. Hybridity status: hybrid vs line (H/L); water status: WD/WW. Lines-WW in blue, Lines-WD in orange, Hybrids-WW in green and Hybrids-WD in red (the SF009 line was excluded, see text).

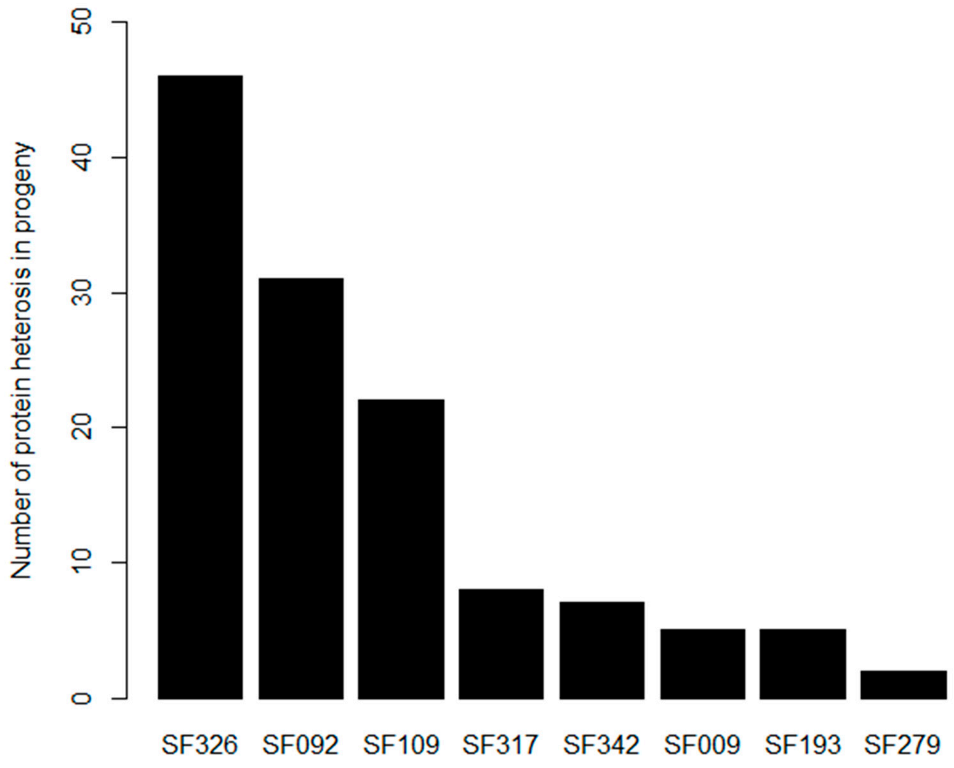

Figure S5. Number of proteins showing heterosis in the progeny

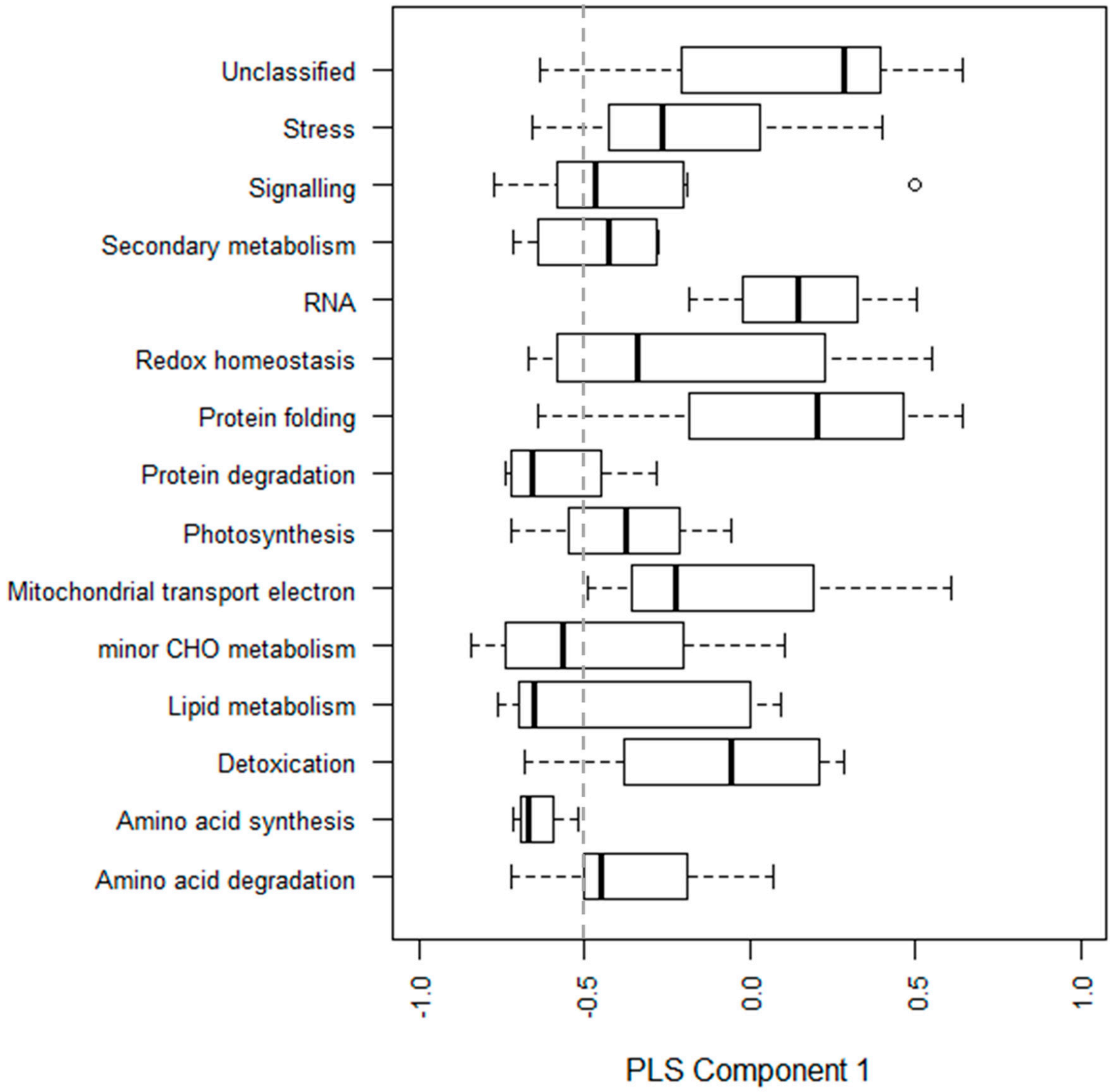

Figure S6. Correlation of DAPs with the first component of the PLS shown in Fig. 7. DAPs are grouped by functional category. The vertical dotted line separates proteins according to whether the correlation is greater or smaller than -0.5.

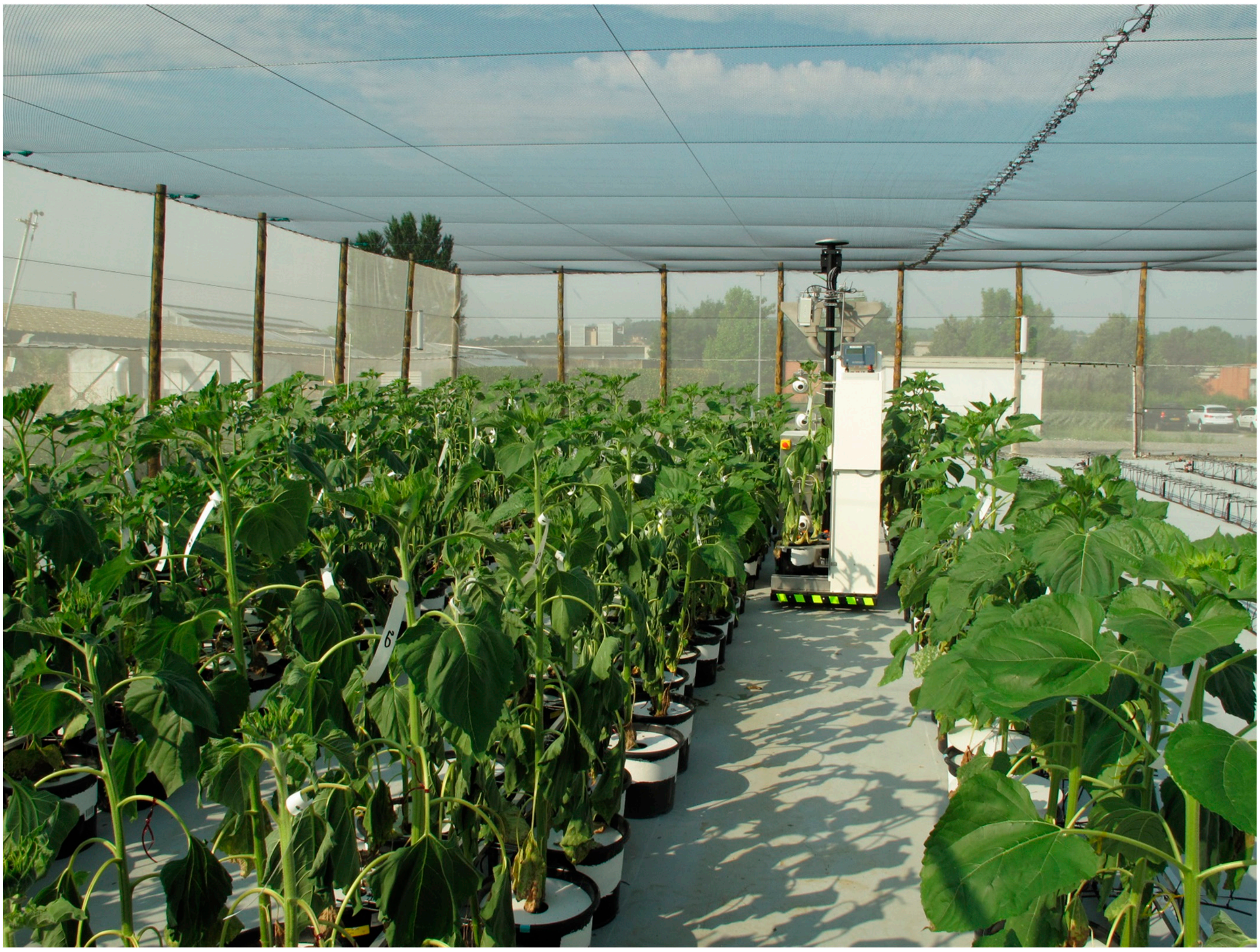

Figure S7. Overview of the 144 plants with surrounding protecting plants of the 13HP02 experiment on the Heliaphen platform at 44 DAG (end of the experiment)
