# Supplementary material for: Sunflower Hybrids and Inbred Lines Adopt Different Physiological Strategies and Proteome Responses to Cope with Water Deficit
Source: Biomolecules. 2023 Jul 12;13(7):1110. doi: 10.3390/biom13071110 (PMC10377273; doi:10.3390/biom13071110)
Supplement: Supplementary file 1 [file Fig-S1-boxprot-1211-newcolors-proteines-v3.pdf › Fig-S1-boxprot-1211-newcolors-proteines-v3.pdf]

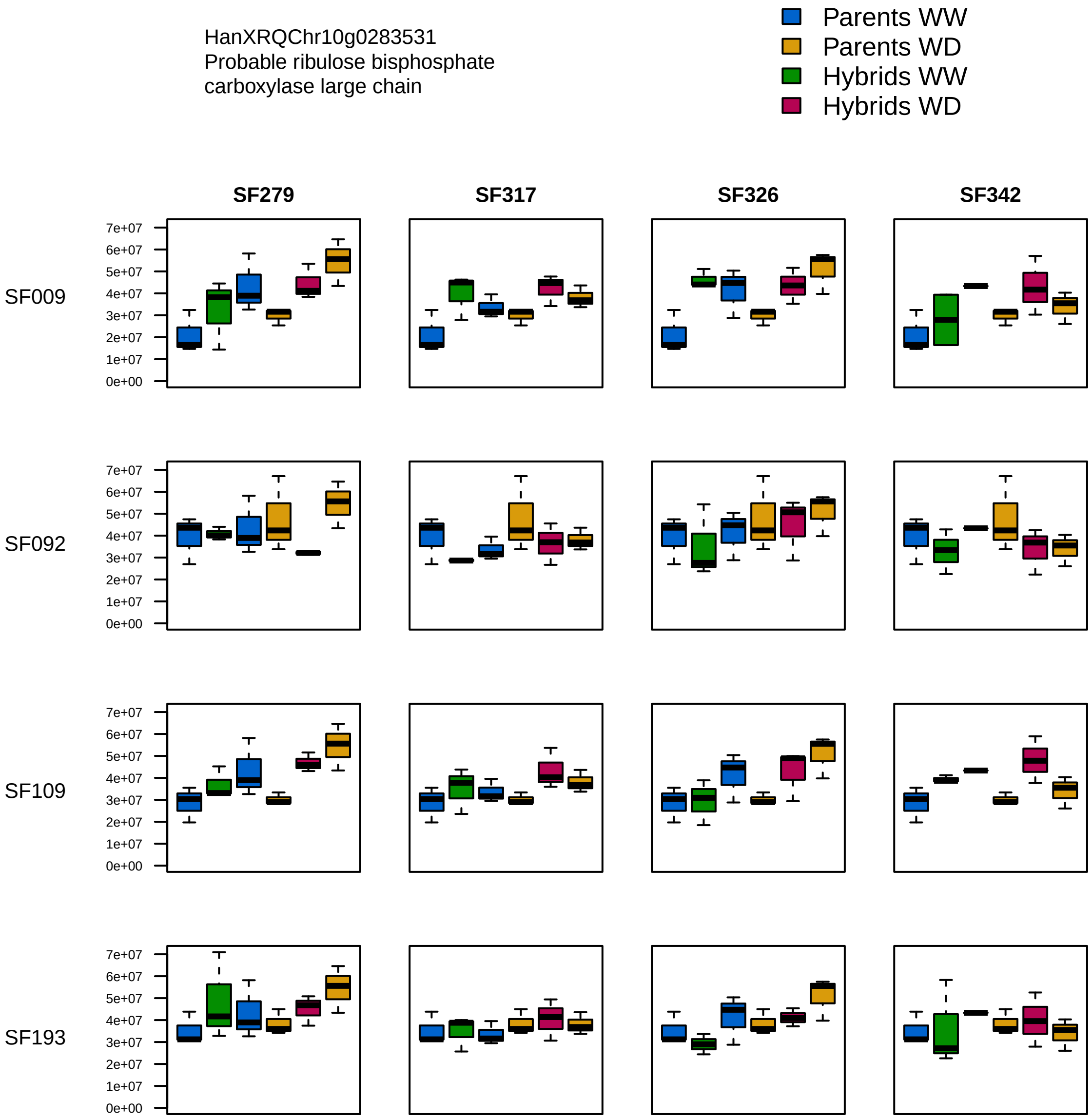
HanXRQChr10g0283531
Probable ribulose bisphosphate
carboxylase large chain
Parents WW
Parents WD
Hybrids WW
Hybrids WD
SF279
SF317
SF326
SF342
SF009
SF092
SF109
SF193
7e+07
6e+07
5e+07
4e+07
3e+07
2e+07
1e+07
0e+00

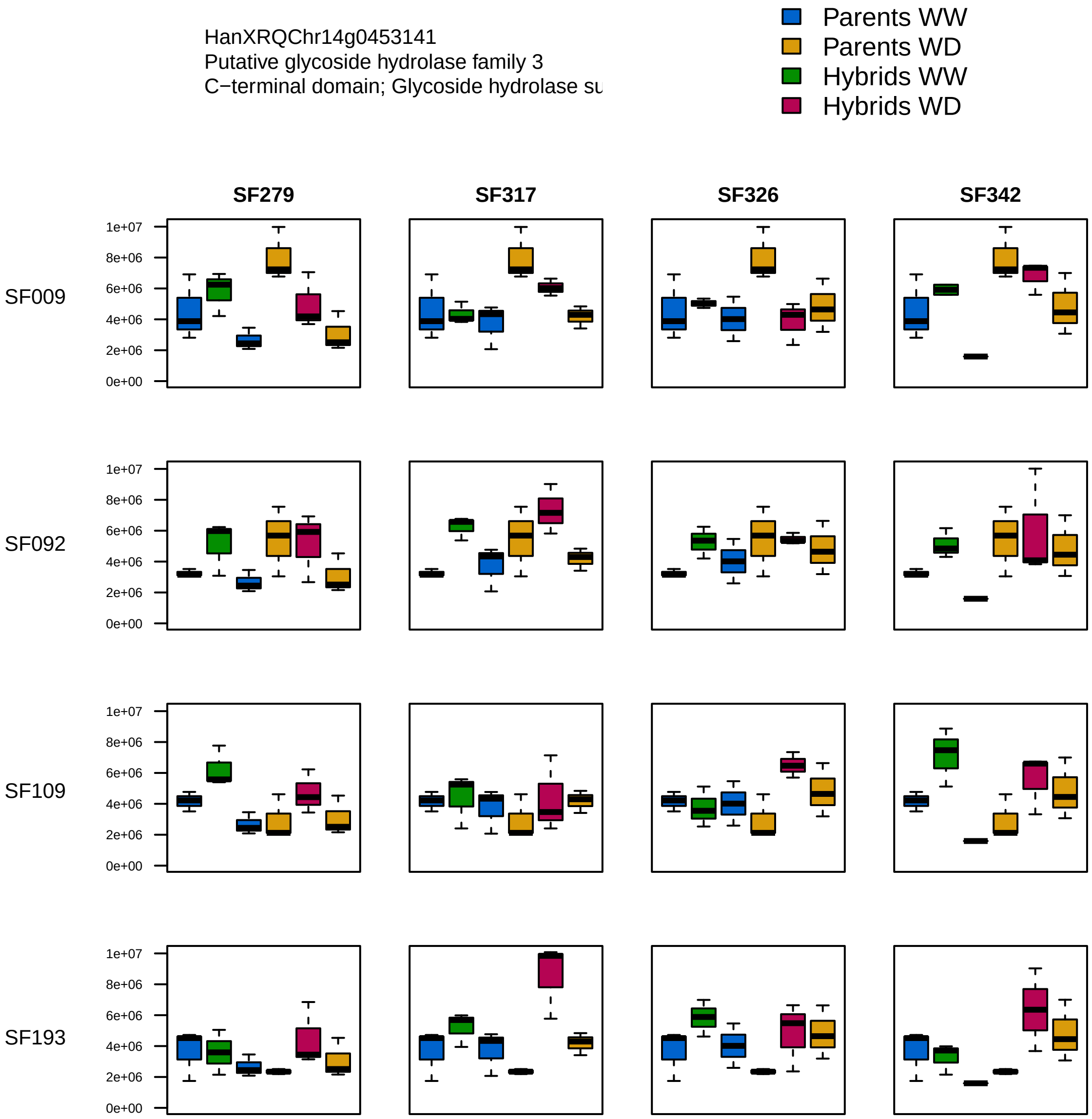
HanXRQChr14g0453141
Putative glycoside hydrolase family 3
C−terminal domain; Glycoside hydrolase su
Parents WW
Parents WD
Hybrids WW
Hybrids WD
SF279
SF317
SF326
SF342
SF009
SF092
SF109
SF193
1e+07
8e+06
6e+06
4e+06
2e+06
0e+00

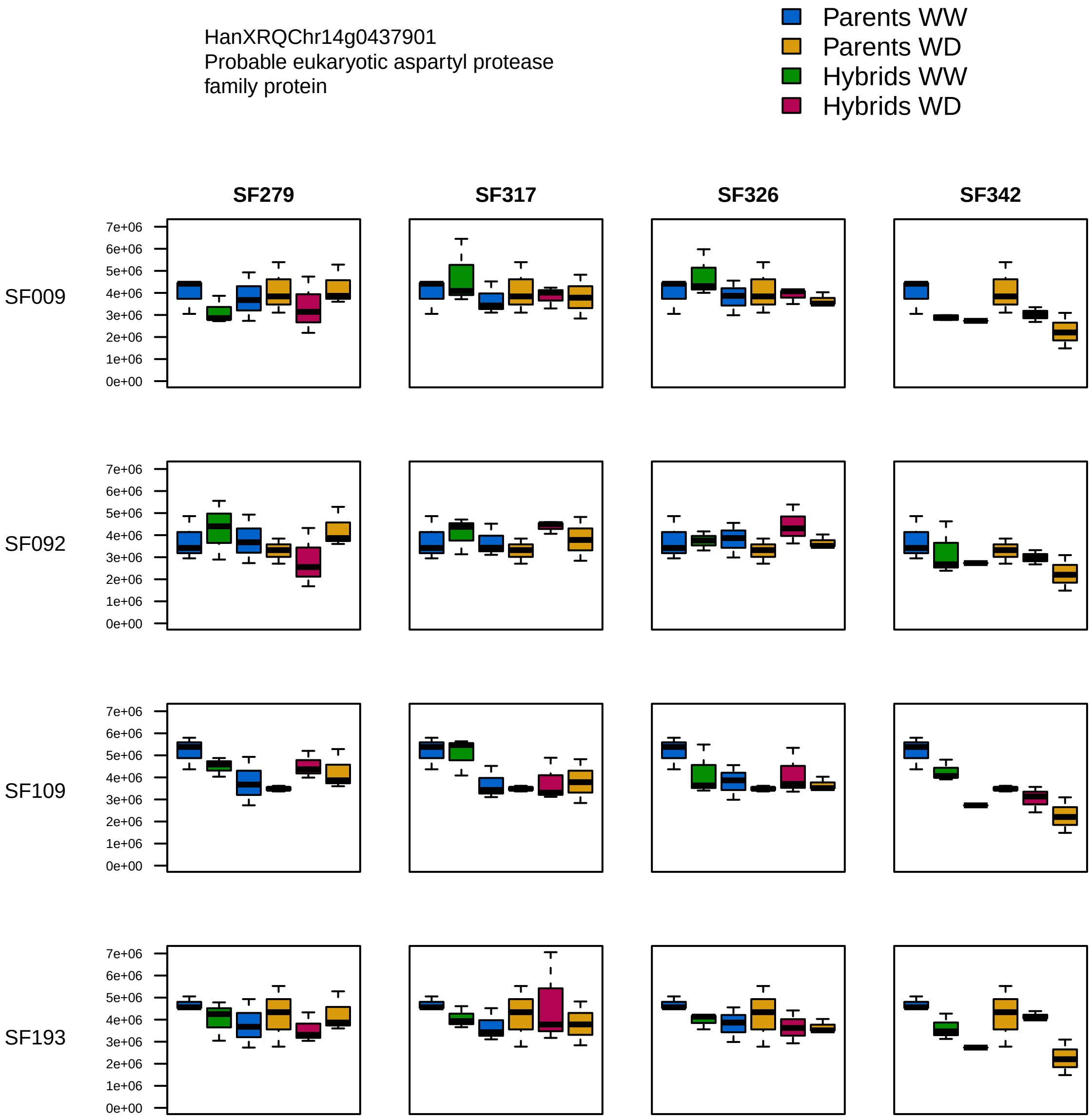

HanXRQChr14g0437901
Probable eukaryotic aspartyl protease
family protein
Parents WW
Parents WD
Hybrids WW
Hybrids WD
SF279
SF317
SF326
SF342
SF009
SF092
SF109
SF193
7e+06
6e+06
5e+06
4e+06
3e+06
2e+06
1e+06
0e+00

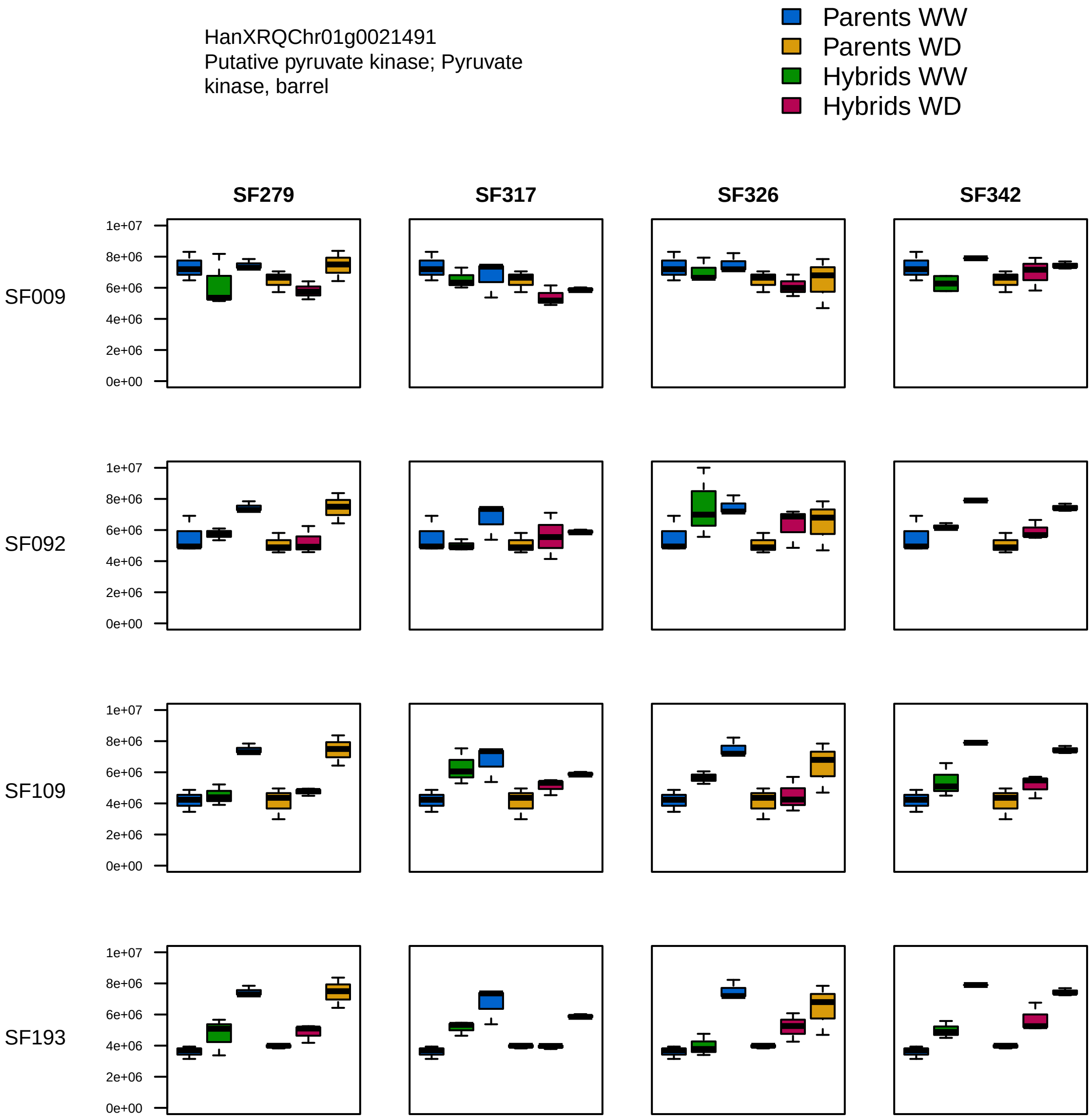

HanXRQChr01g0021491
Putative pyruvate kinase; Pyruvate kinase, barrel
Parents WW
Parents WD
Hybrids WW
Hybrids WD
SF279
SF317
SF326
SF342
SF009
SF092
SF109
SF193
1e+07
8e+06
6e+06
4e+06
2e+06
0e+00

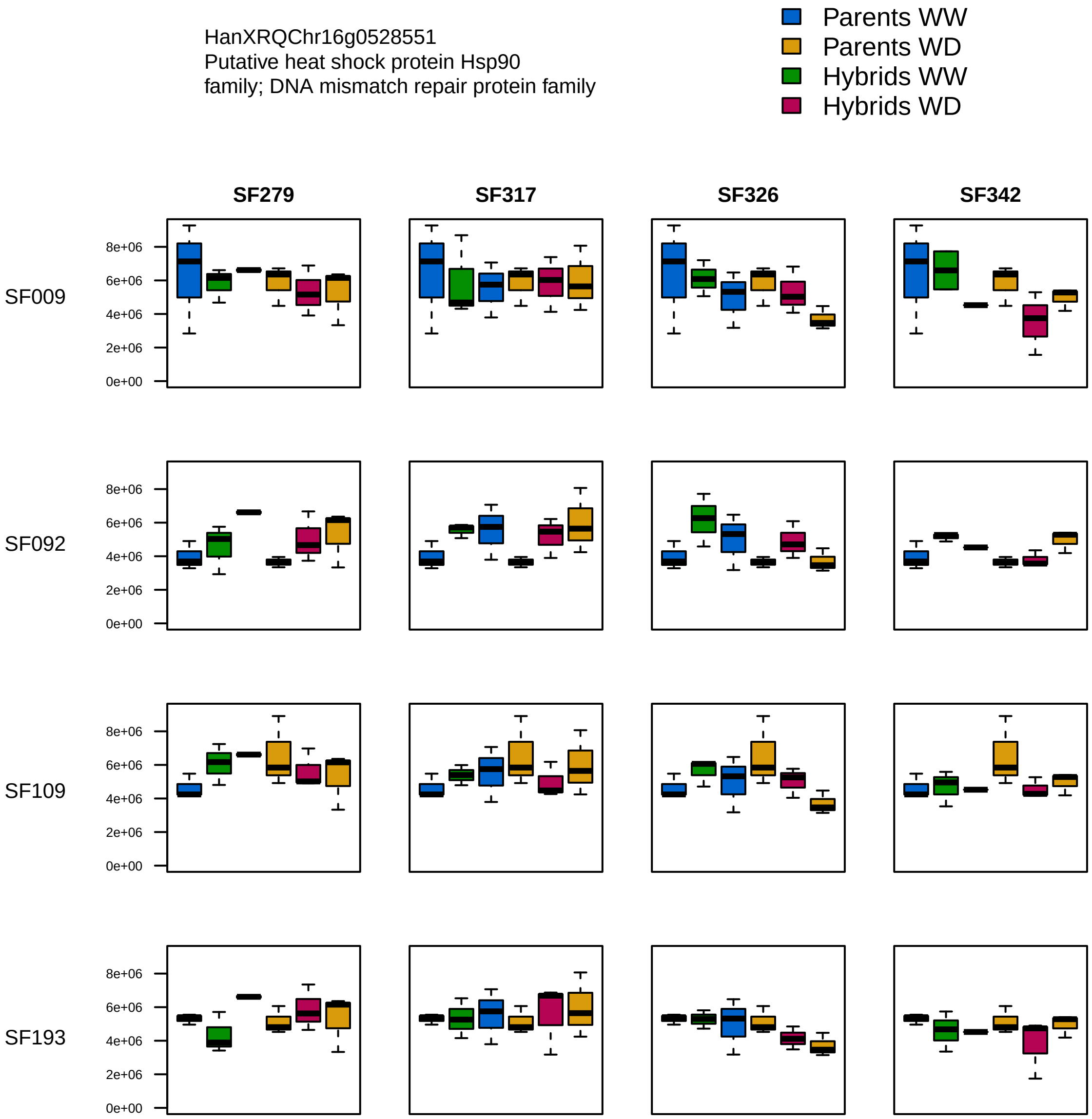
HanXRQChr16g0528551
Putative heat shock protein Hsp90
family; DNA mismatch repair protein family
Parents WW
Parents WD
Hybrids WW
Hybrids WD
SF279
SF317
SF326
SF342
SF009
SF092
SF109
SF193
8e+06
6e+06
4e+06
2e+06
0e+00

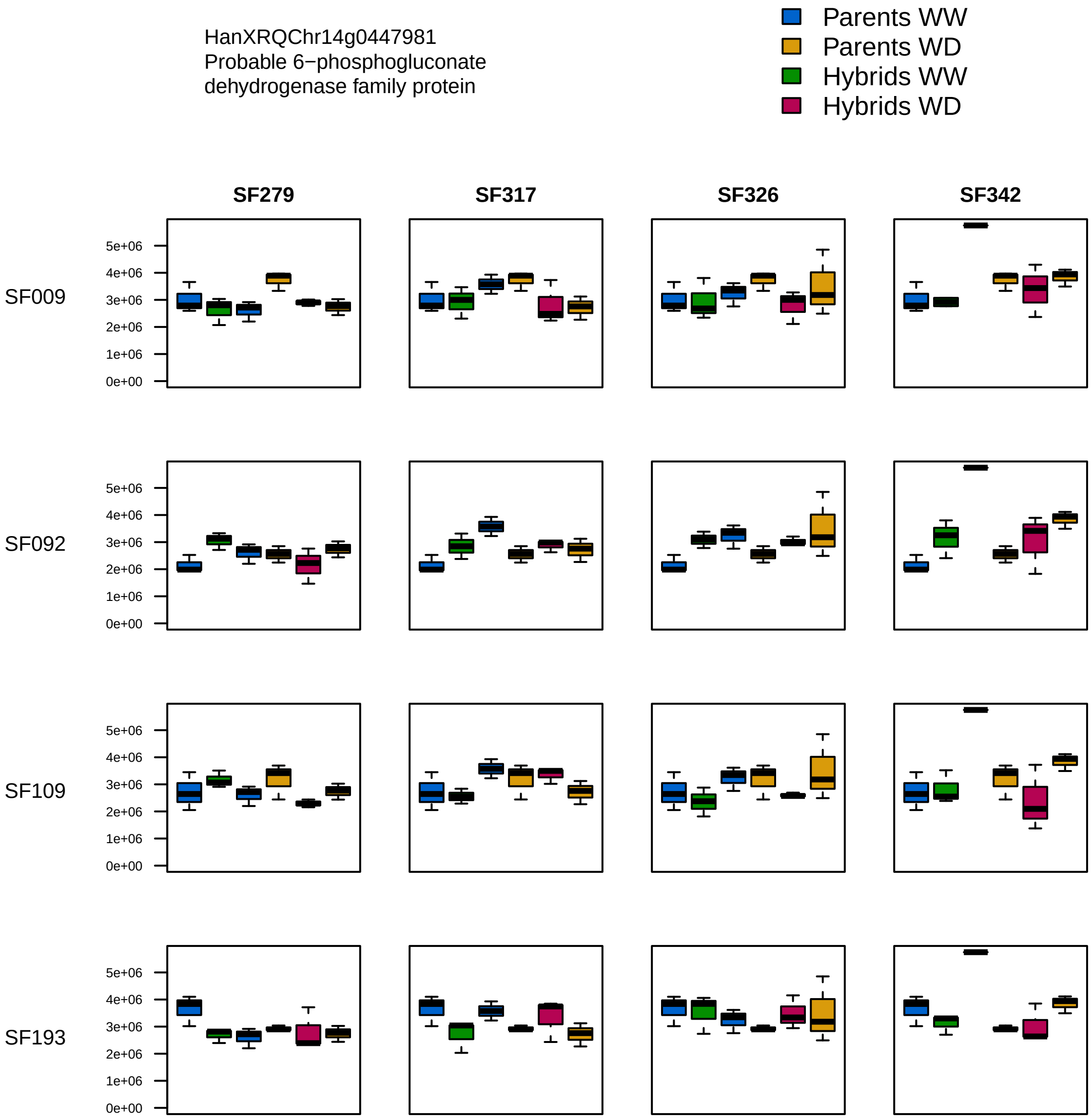

HanXRQChr14g0447981
Probable 6−phosphogluconate
dehydrogenase family protein
Parents WW
Parents WD
Hybrids WW
Hybrids WD
SF279
SF317
SF326
SF342
SF009
SF092
SF109
SF193
0e+00
1e+06
2e+06
3e+06
4e+06
5e+06

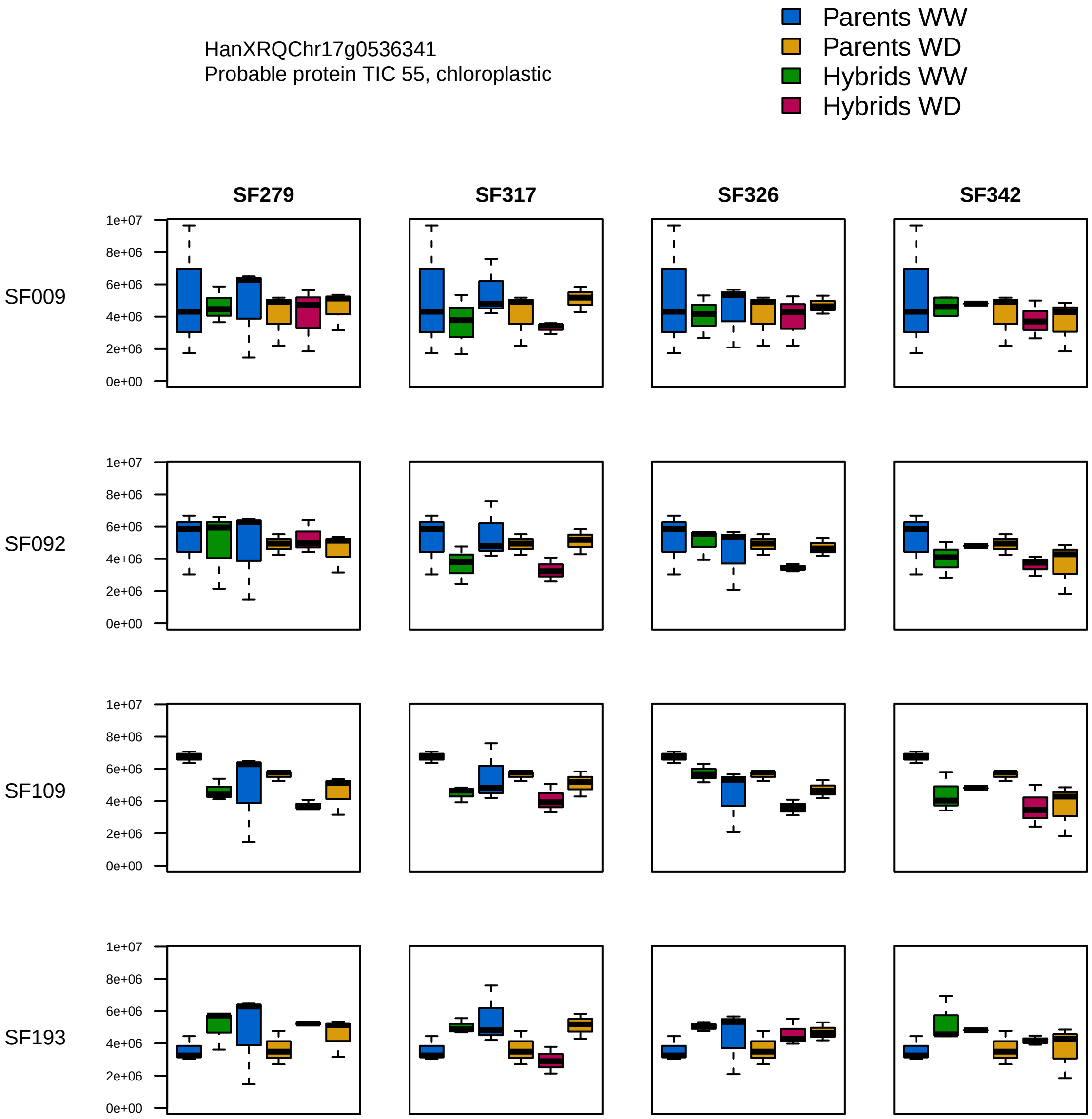

HanXRQChr17g0536341
Probable protein TIC 55, chloroplastic
Parents WW
Parents WD
Hybrids WW
Hybrids WD
SF279
SF317
SF326
SF342
SF009
SF092
SF109
SF193
1e+07
8e+06
6e+06
4e+06
2e+06
0e+00

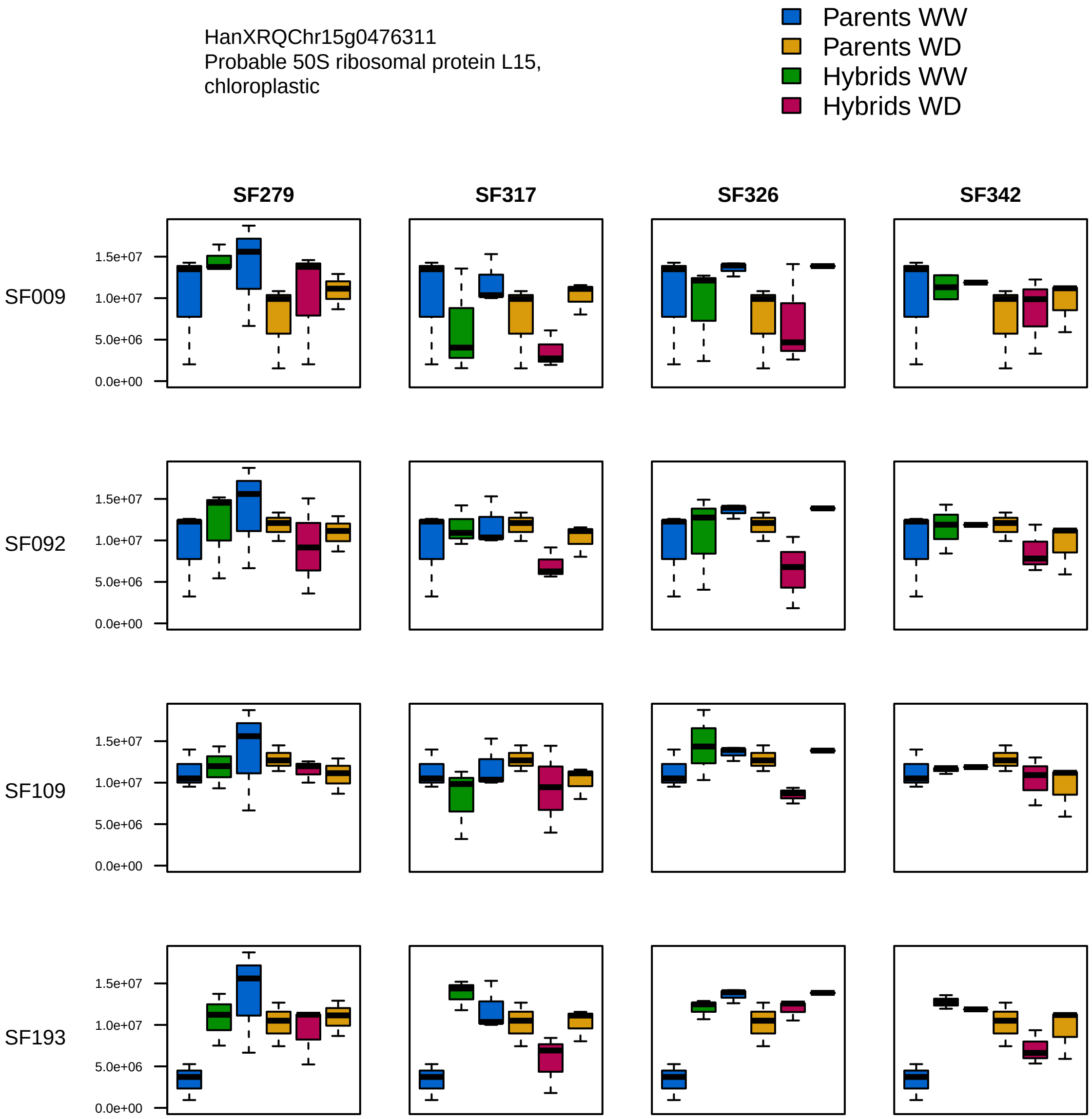
HanXRQChr15g0476311
Probable 50S ribosomal protein L15, chloroplastic
Parents WW
Parents WD
Hybrids WW
Hybrids WD
SF279
SF317
SF326
SF342
SF009
SF092
SF109
SF193
1.5e+07
1.0e+07
5.0e+06
0.0e+00

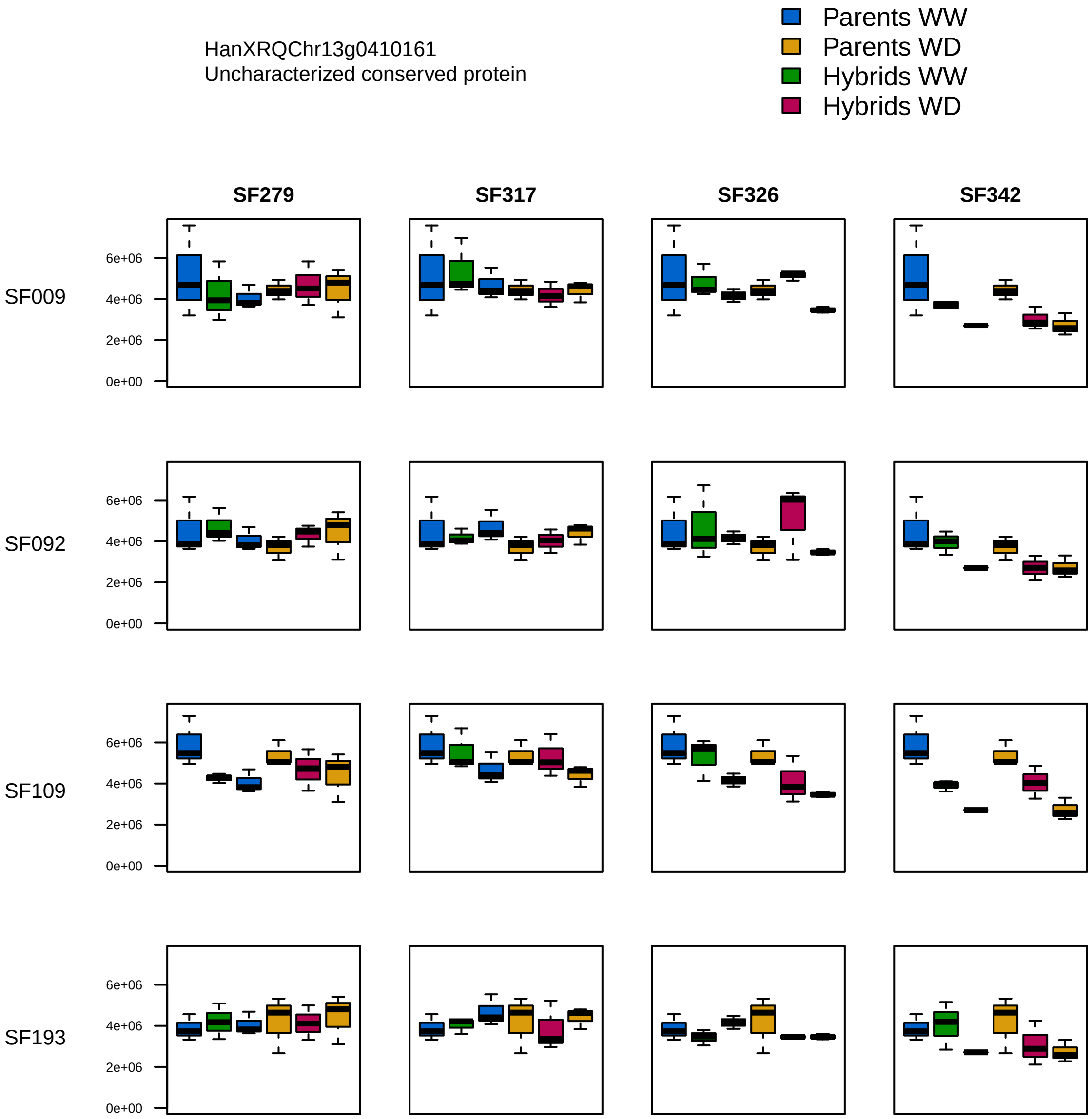

HanXRQChr13g0410161
Uncharacterized conserved protein
Parents WW
Parents WD
Hybrids WW
Hybrids WD
SF279
SF317
SF326
SF342
SF009
SF092
SF109
SF193
6e+06
4e+06
2e+06
0e+00

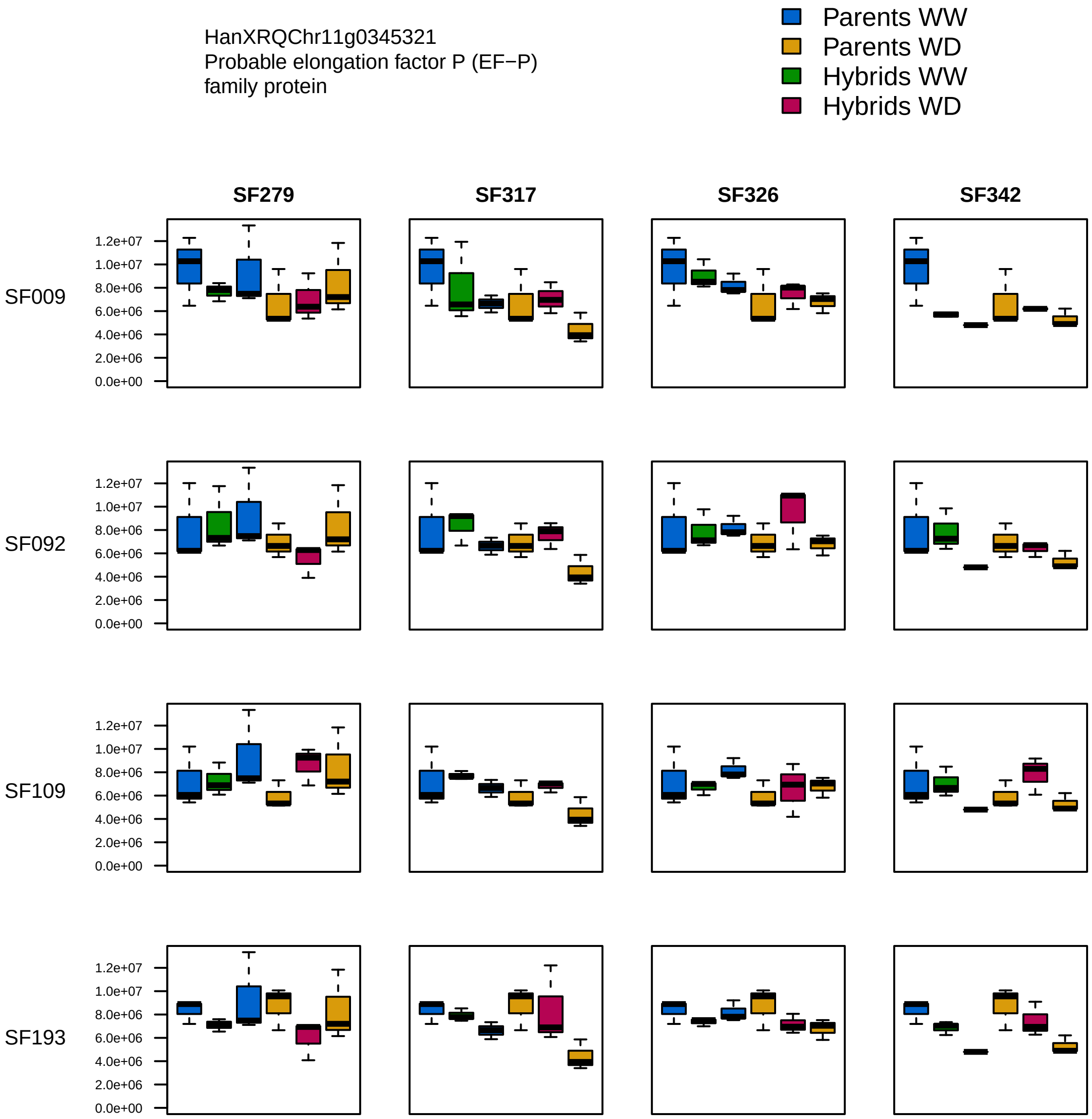
HanXRQChr11g0345321
Probable elongation factor P (EF−P)
family protein
Parents WW
Parents WD
Hybrids WW
Hybrids WD
SF279
SF317
SF326
SF342
SF009
SF092
SF109
SF193
1.2e+07
1.0e+07
8.0e+06
6.0e+06
4.0e+06
2.0e+06
0.0e+00

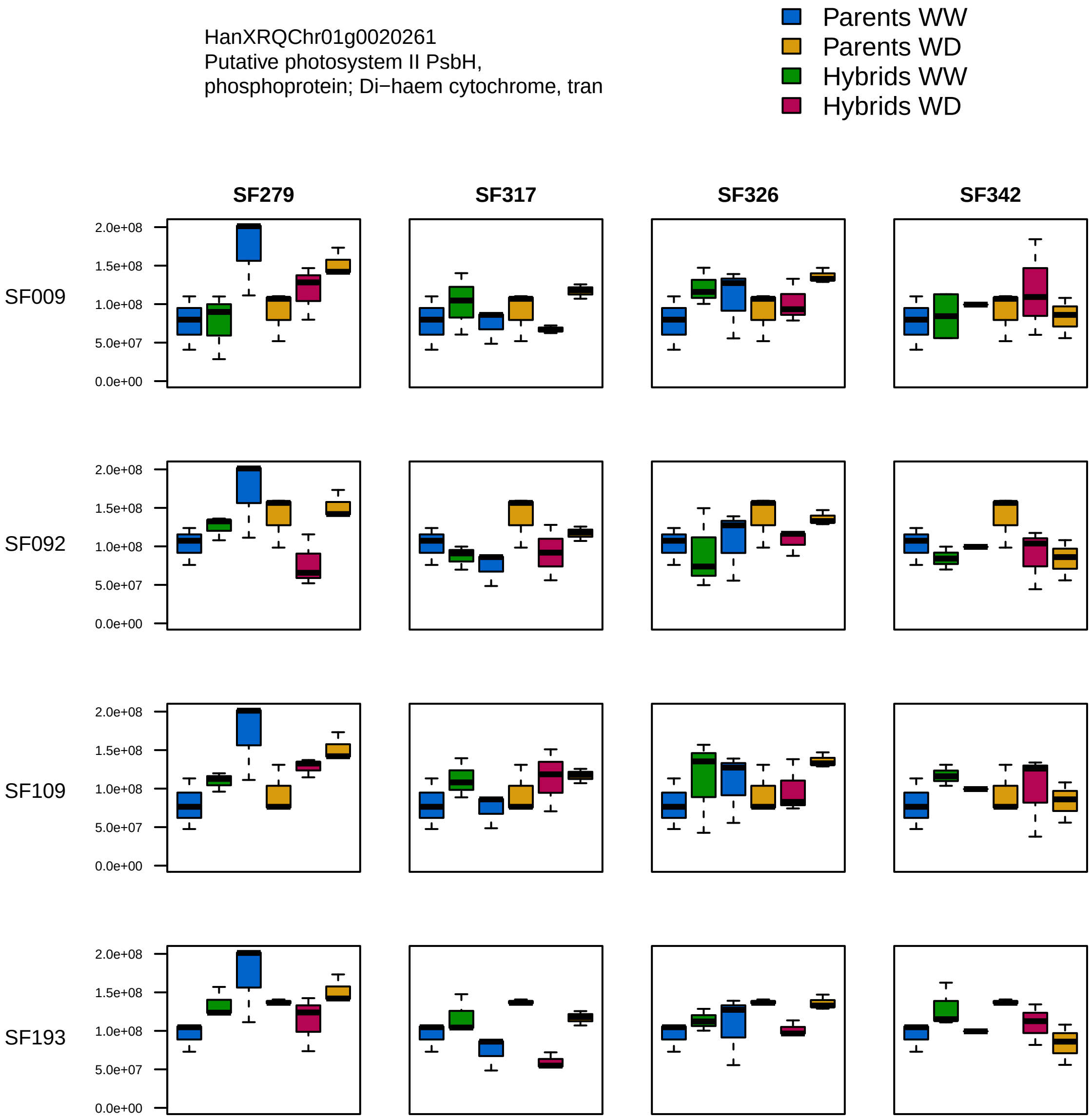

HanXRQChr01g0020261
Putative photosystem II PsbH,
phosphoprotein; Di−haem cytochrome, tran
Parents WW
Parents WD
Hybrids WW
Hybrids WD
SF279
SF317
SF326
SF342
SF009
SF092
SF109
SF193
2.0e+08
1.5e+08
1.0e+08
5.0e+07
0.0e+00

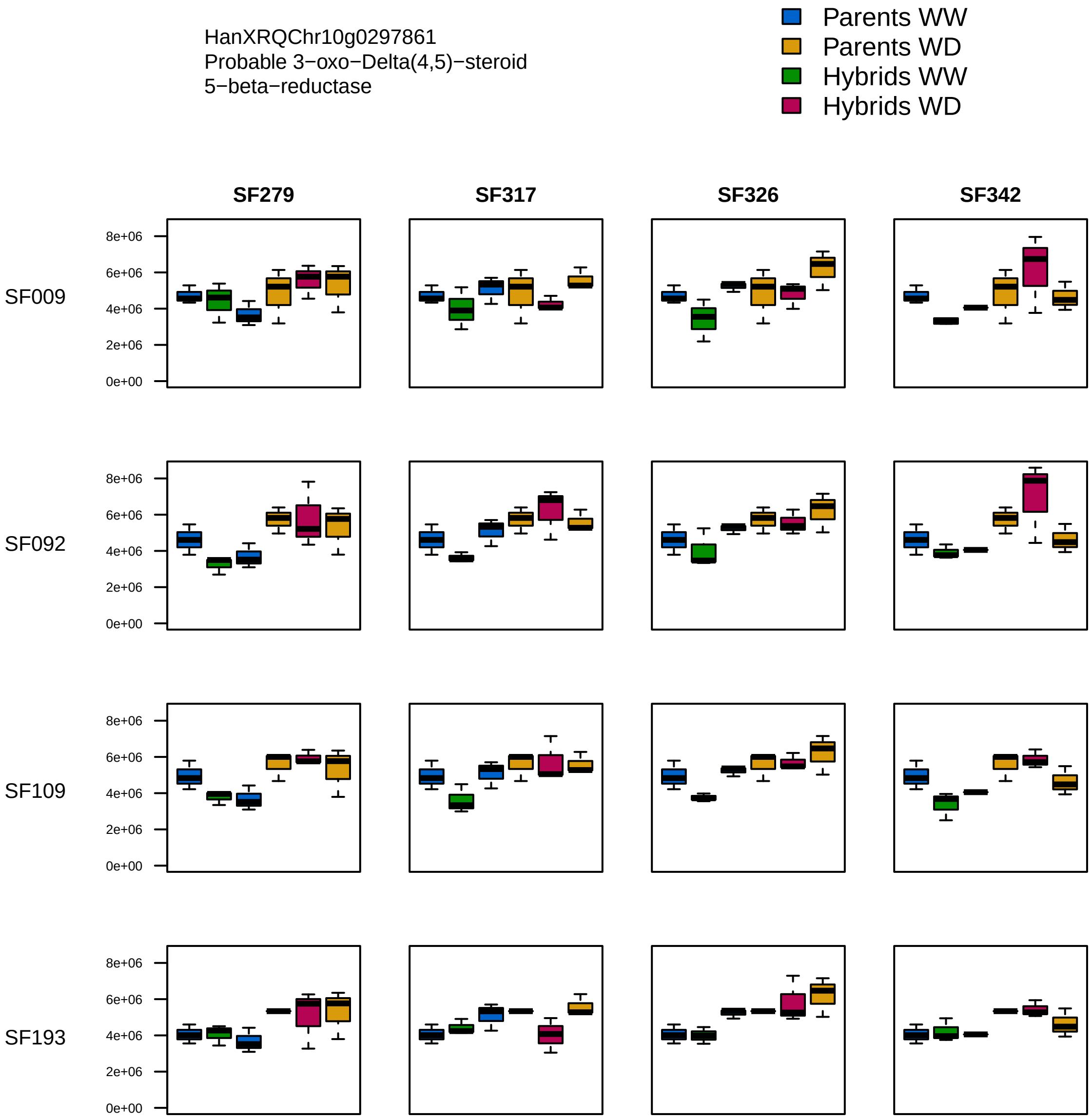
HanXRQChr10g0297861
Probable 3−oxo−Delta(4,5)−steroid
5−beta−reductase
Parents WW
Parents WD
Hybrids WW
Hybrids WD
SF279
SF317
SF326
SF342
SF009
SF092
SF109
SF193
8e+06
6e+06
4e+06
2e+06
0e+00

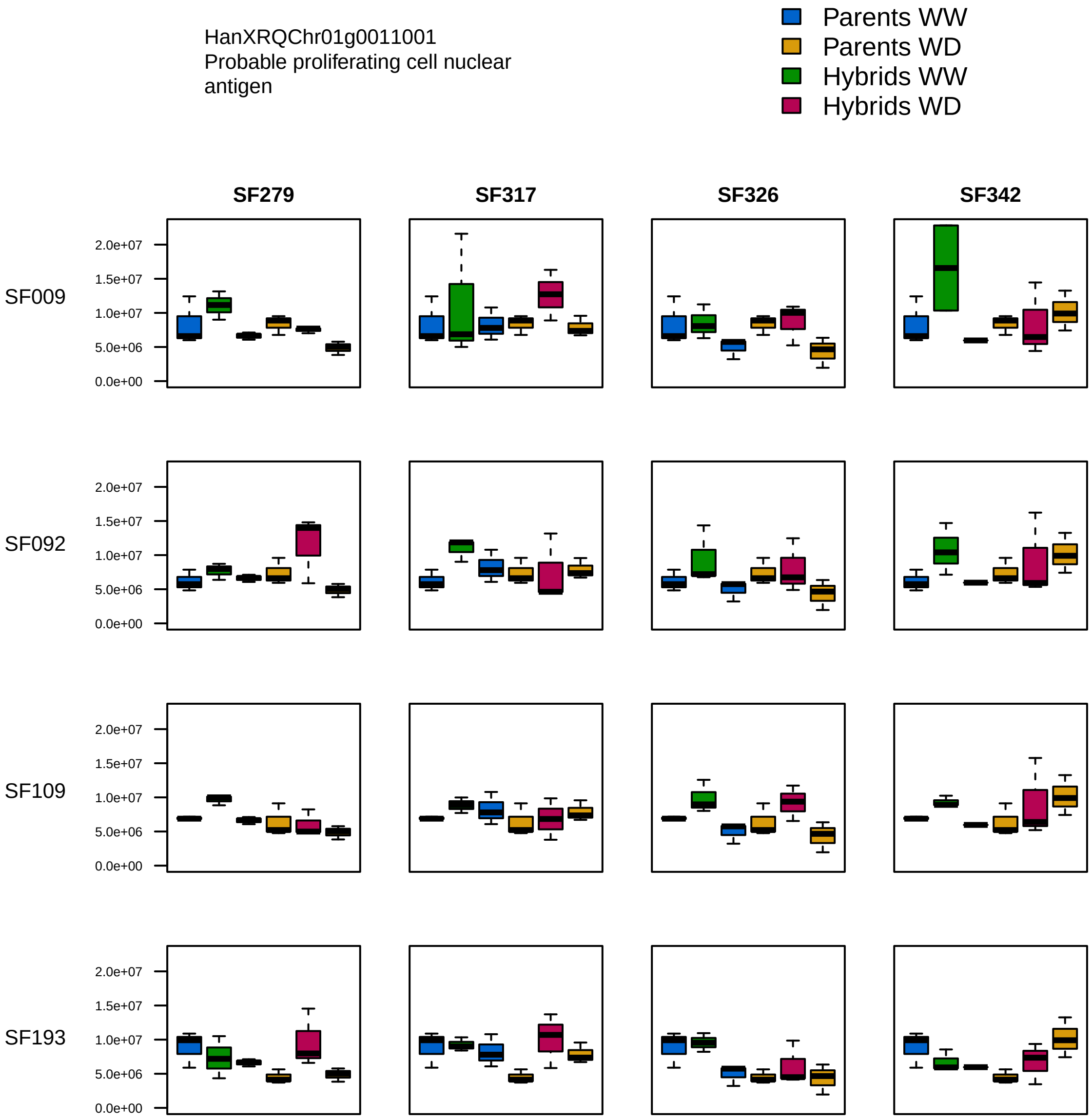
HanXRQChr01g0011001
Probable proliferating cell nuclear antigen
Parents WW
Parents WD
Hybrids WW
Hybrids WD
SF279
SF317
SF326
SF342
SF009
SF092
SF109
SF193
2.0e+07
1.5e+07
1.0e+07
5.0e+06
0.0e+00

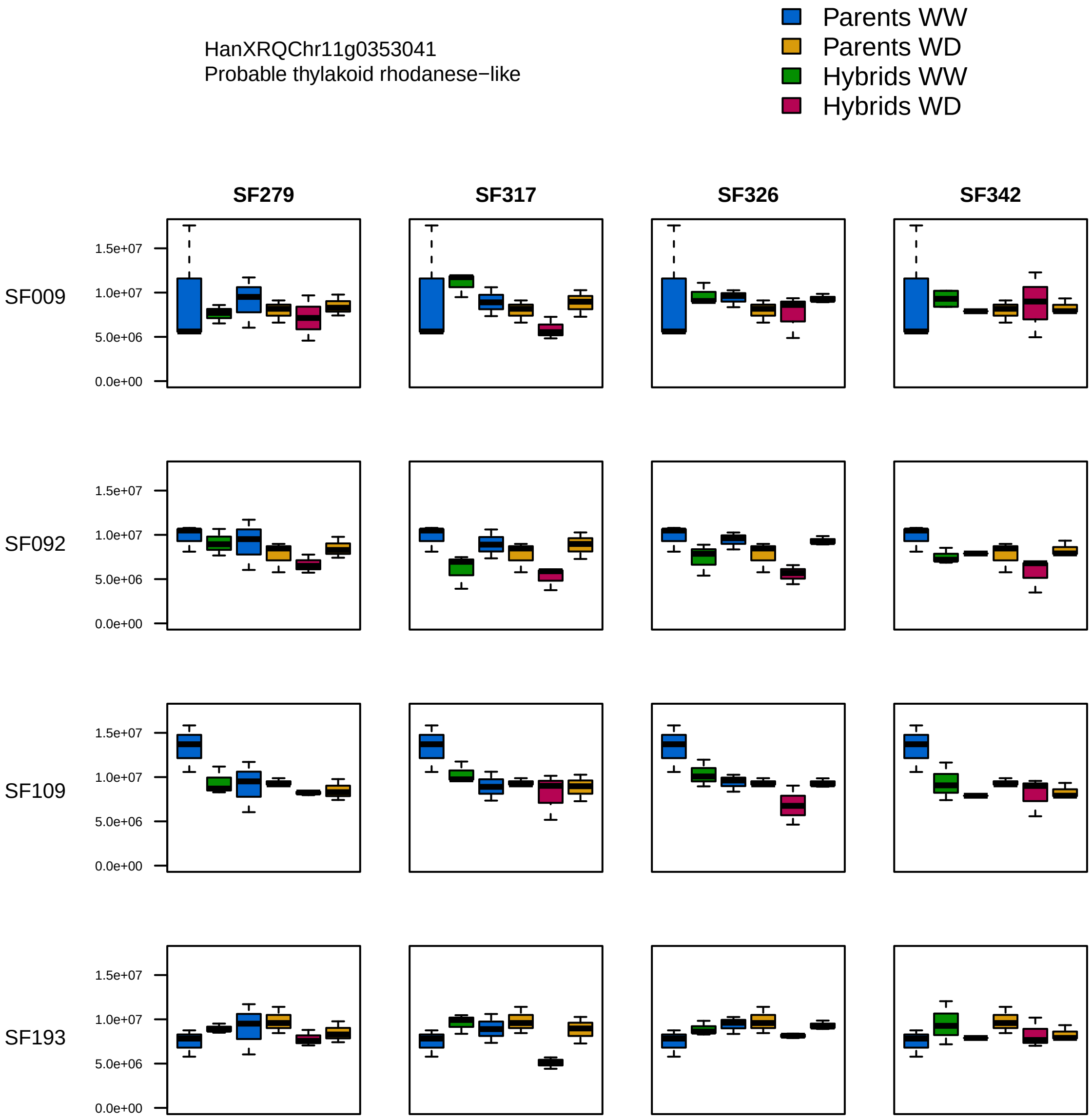
HanXRQChr11g0353041
Probable thylakoid rhodanese−like
Parents WW
Parents WD
Hybrids WW
Hybrids WD
SF279
SF317
SF326
SF342
SF009
SF092
SF109
SF193
1.5e+07
1.0e+07
5.0e+06
0.0e+00

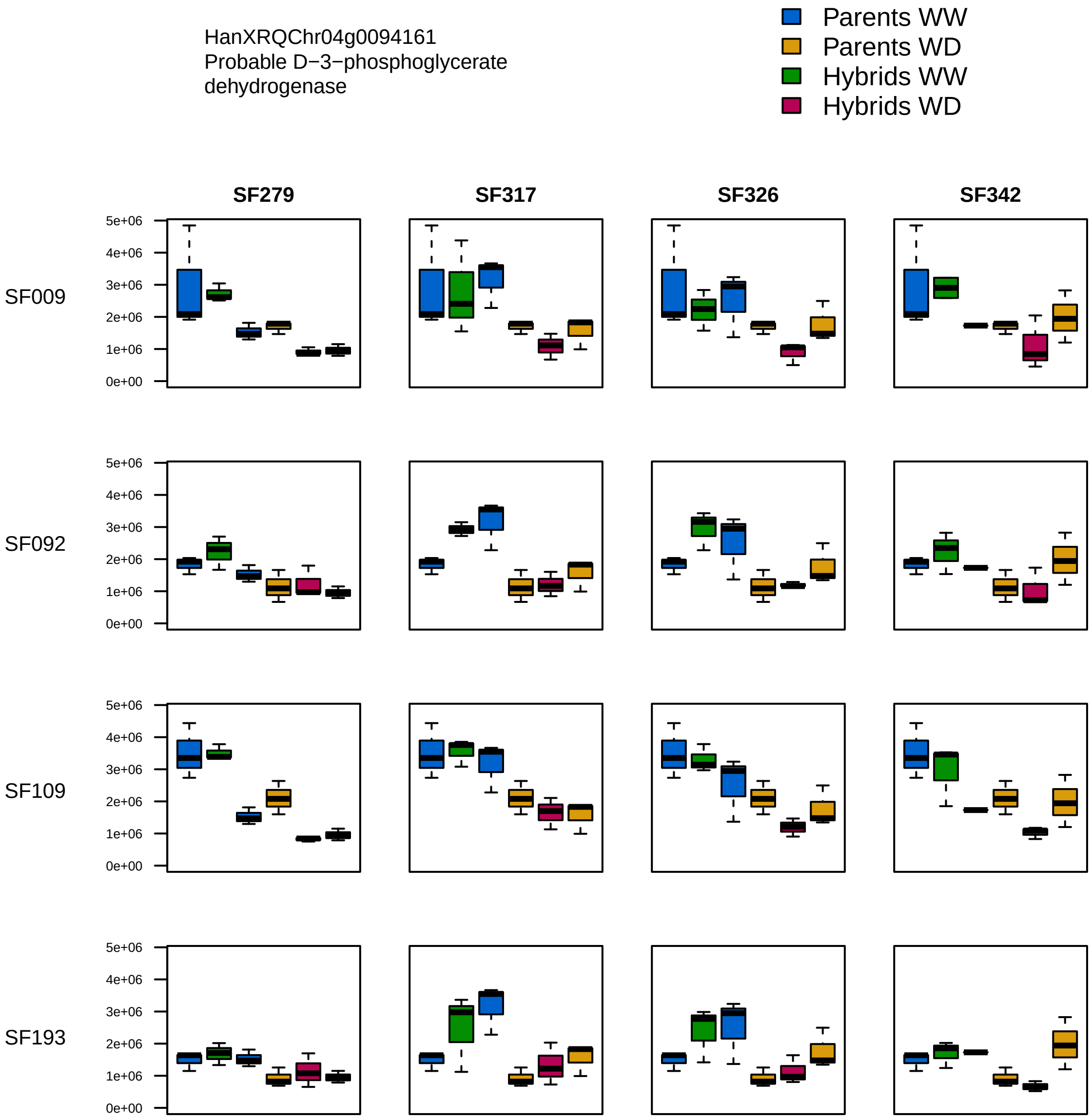
HanXRQChr04g0094161
Probable D−3−phosphoglycerate
dehydrogenase
Parents WW
Parents WD
Hybrids WW
Hybrids WD
SF279
SF317
SF326
SF342
SF009
SF092
SF109
SF193
5e+06
4e+06
3e+06
2e+06
1e+06
0e+00

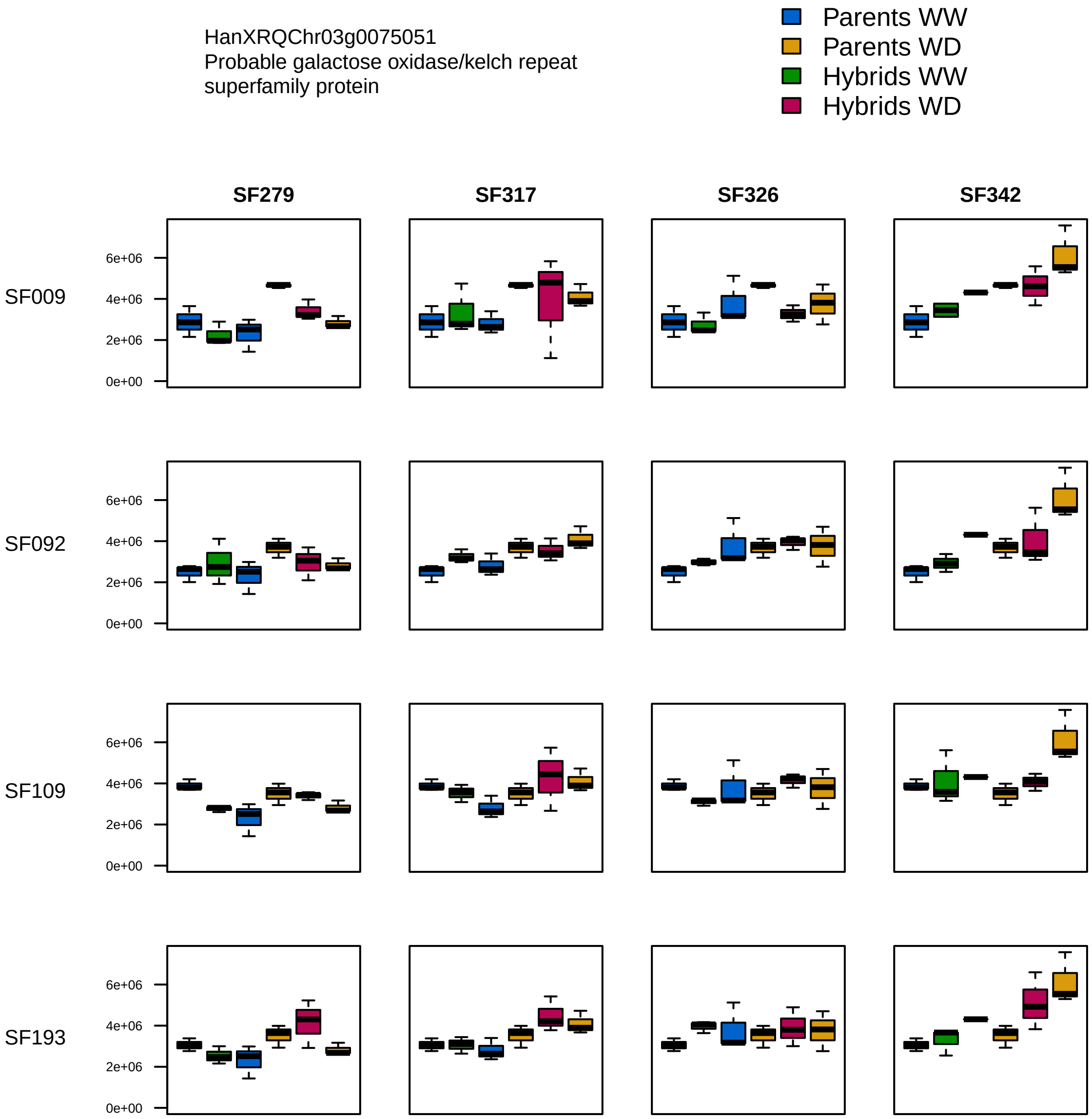

HanXRQChr03g0075051
Probable galactose oxidase/kelch repeat
superfamily protein
Parents WW
Parents WD
Hybrids WW
Hybrids WD
SF279
SF317
SF326
SF342
SF009
SF092
SF109
SF193
6e+06
4e+06
2e+06
0e+00

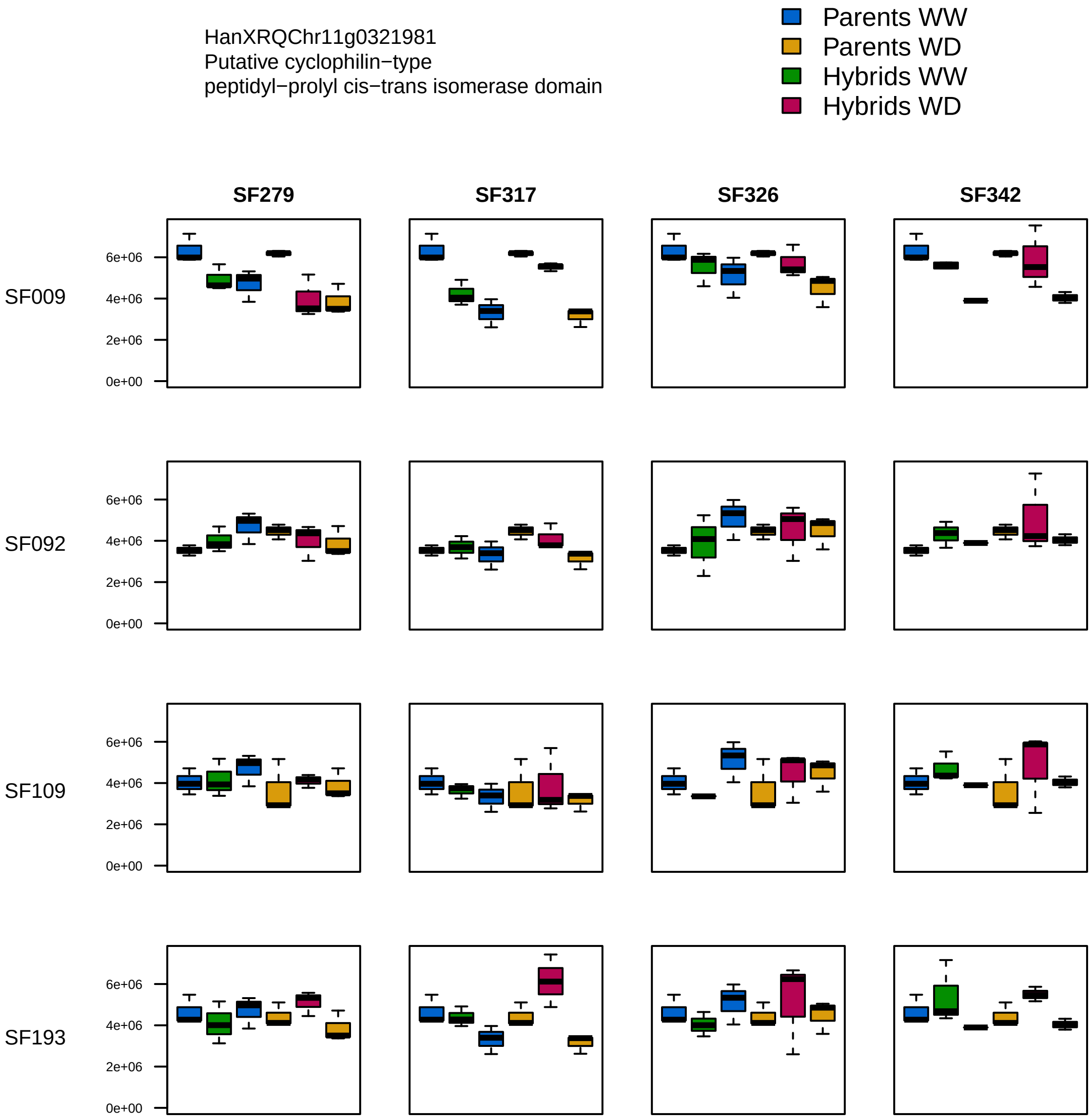
HanXRQChr11g0321981
Putative cyclophilin−type
peptidyl−prolyl cis−trans isomerase domain
Parents WW
Parents WD
Hybrids WW
Hybrids WD
SF279
SF317
SF326
SF342
SF009
SF092
SF109
SF193
0e+00
2e+06
4e+06
6e+06

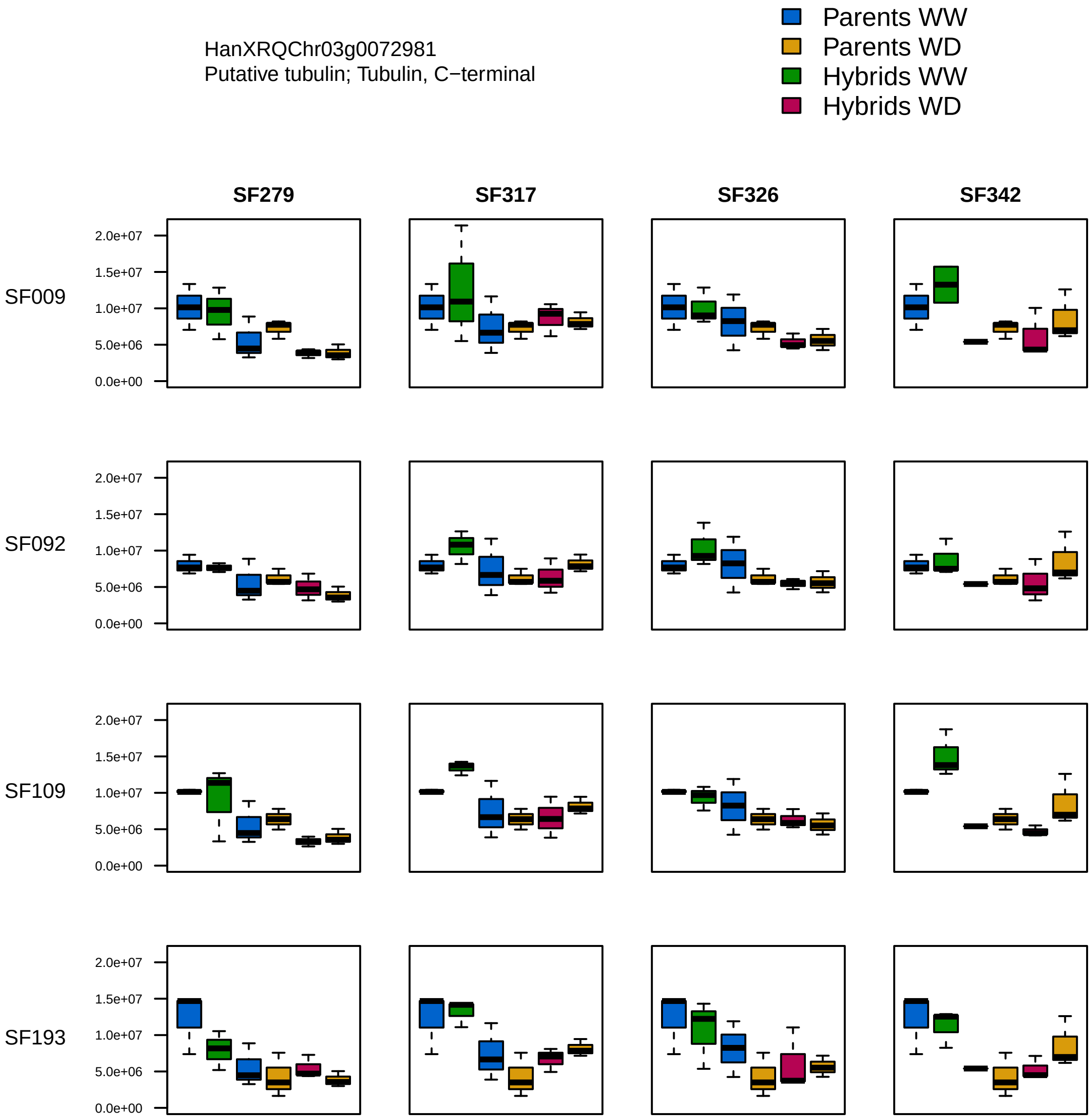

HanXRQChr03g0072981
Putative tubulin; Tubulin, C−terminal
Parents WW
Parents WD
Hybrids WW
Hybrids WD
SF279
SF317
SF326
SF342
SF009
SF092
SF109
SF193
2.0e+07
1.5e+07
1.0e+07
5.0e+06
0.0e+00

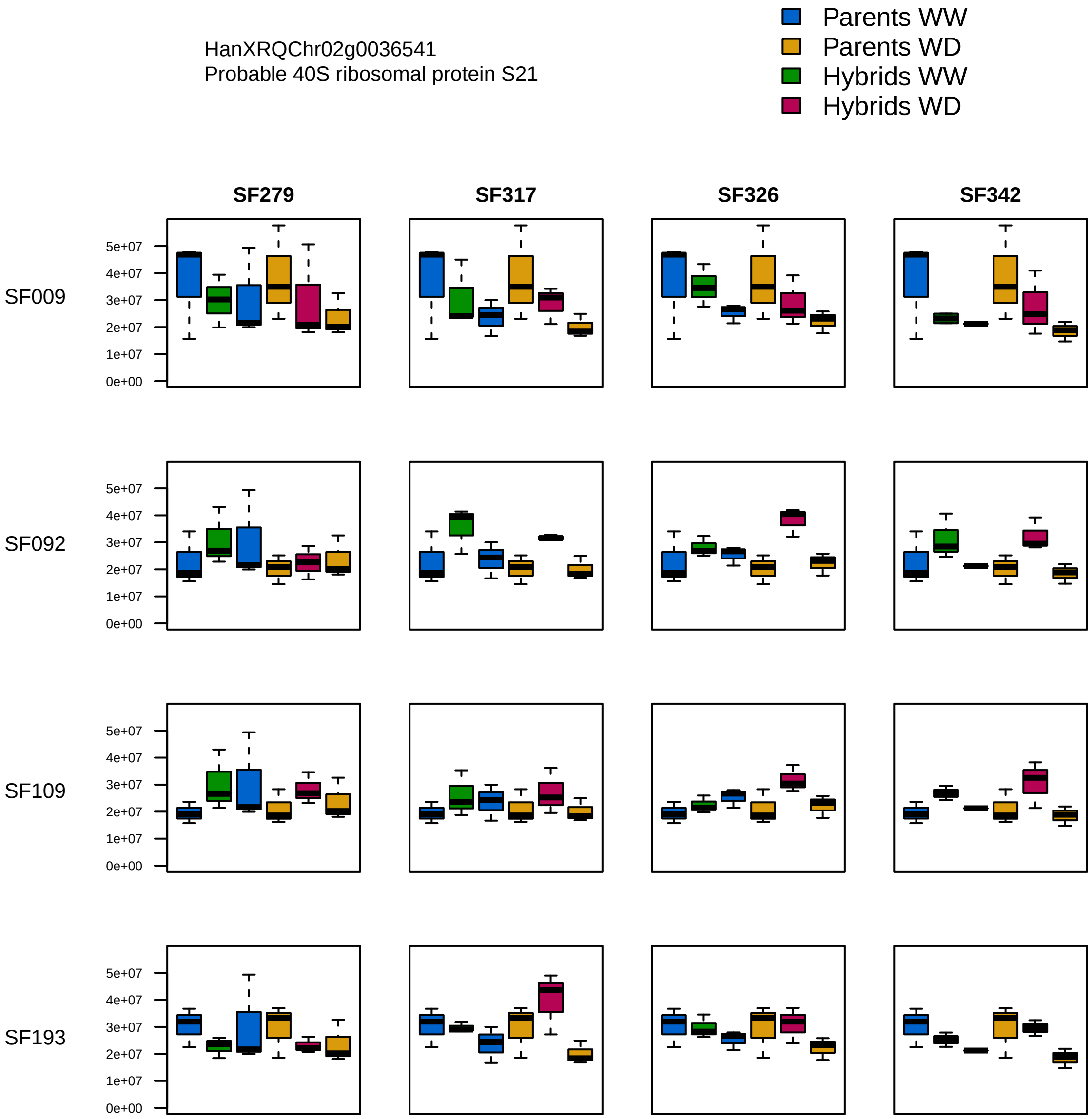

HanXRQChr02g0036541
Probable 40S ribosomal protein S21
Parents WW
Parents WD
Hybrids WW
Hybrids WD
SF279
SF317
SF326
SF342
SF009
SF092
SF109
SF193
5e+07
4e+07
3e+07
2e+07
1e+07
0e+00

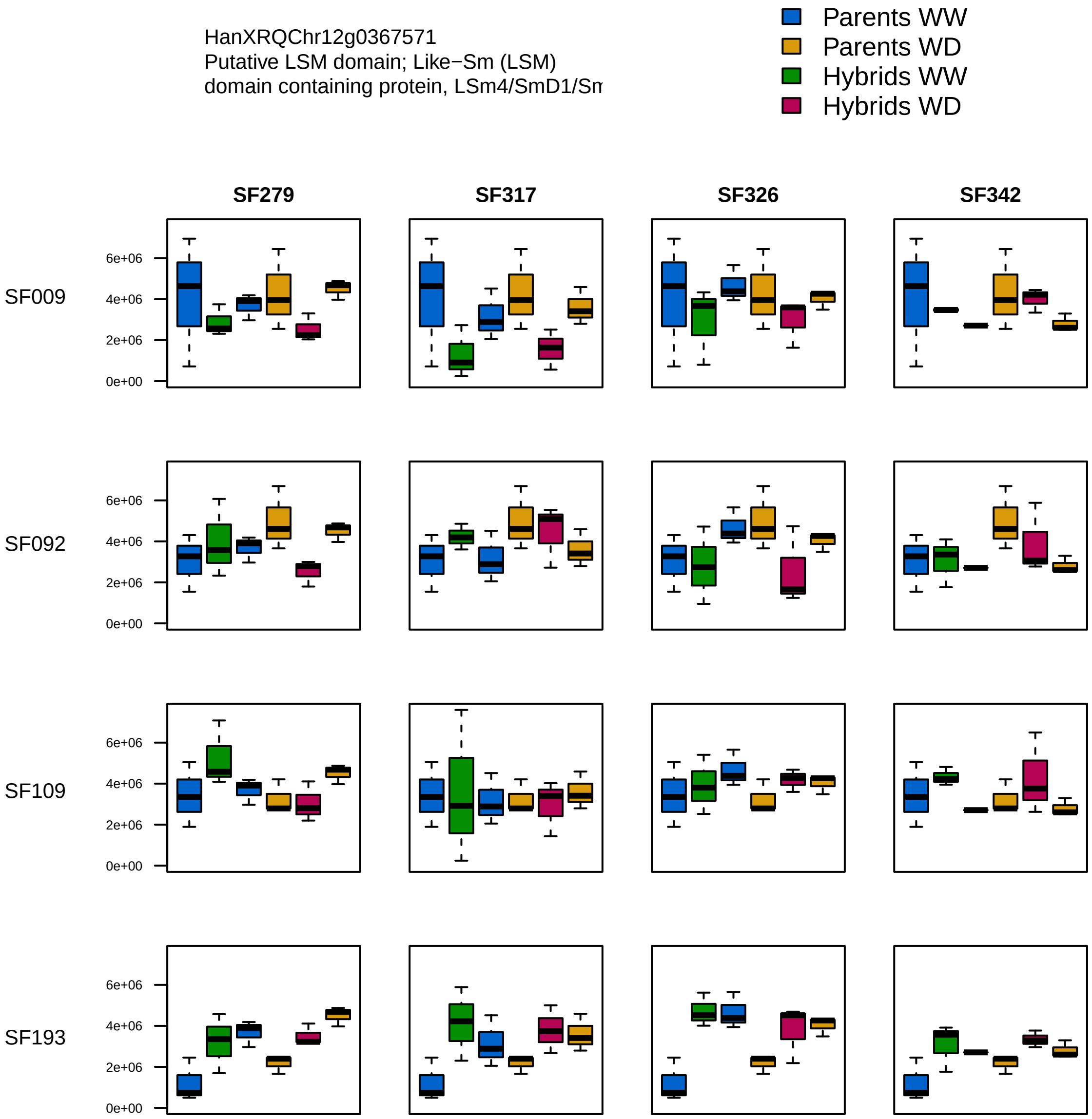
HanXRQChr12g0367571
Putative LSM domain; Like−Sm (LSM)
domain containing protein, LSm4/SmD1/Sm
Parents WW
Parents WD
Hybrids WW
Hybrids WD
SF279
SF317
SF326
SF342
SF009
SF092
SF109
SF193
0e+00
2e+06
4e+06
6e+06

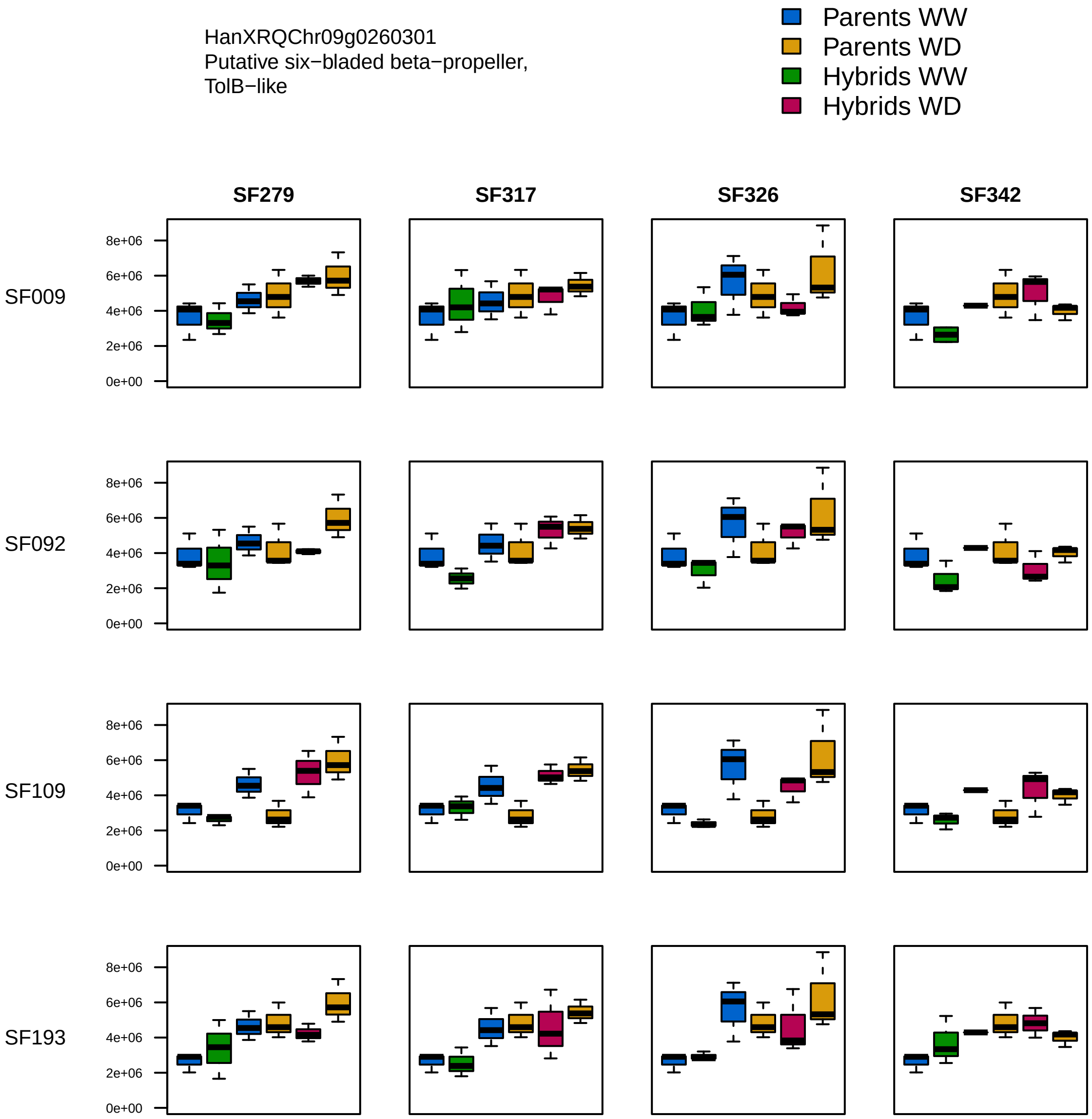
HanXRQChr09g0260301
Putative six−bladed beta−propeller,
TolB−like
Parents WW
Parents WD
Hybrids WW
Hybrids WD
SF279
SF317
SF326
SF342
SF009
SF092
SF109
SF193
8e+06
6e+06
4e+06
2e+06
0e+00

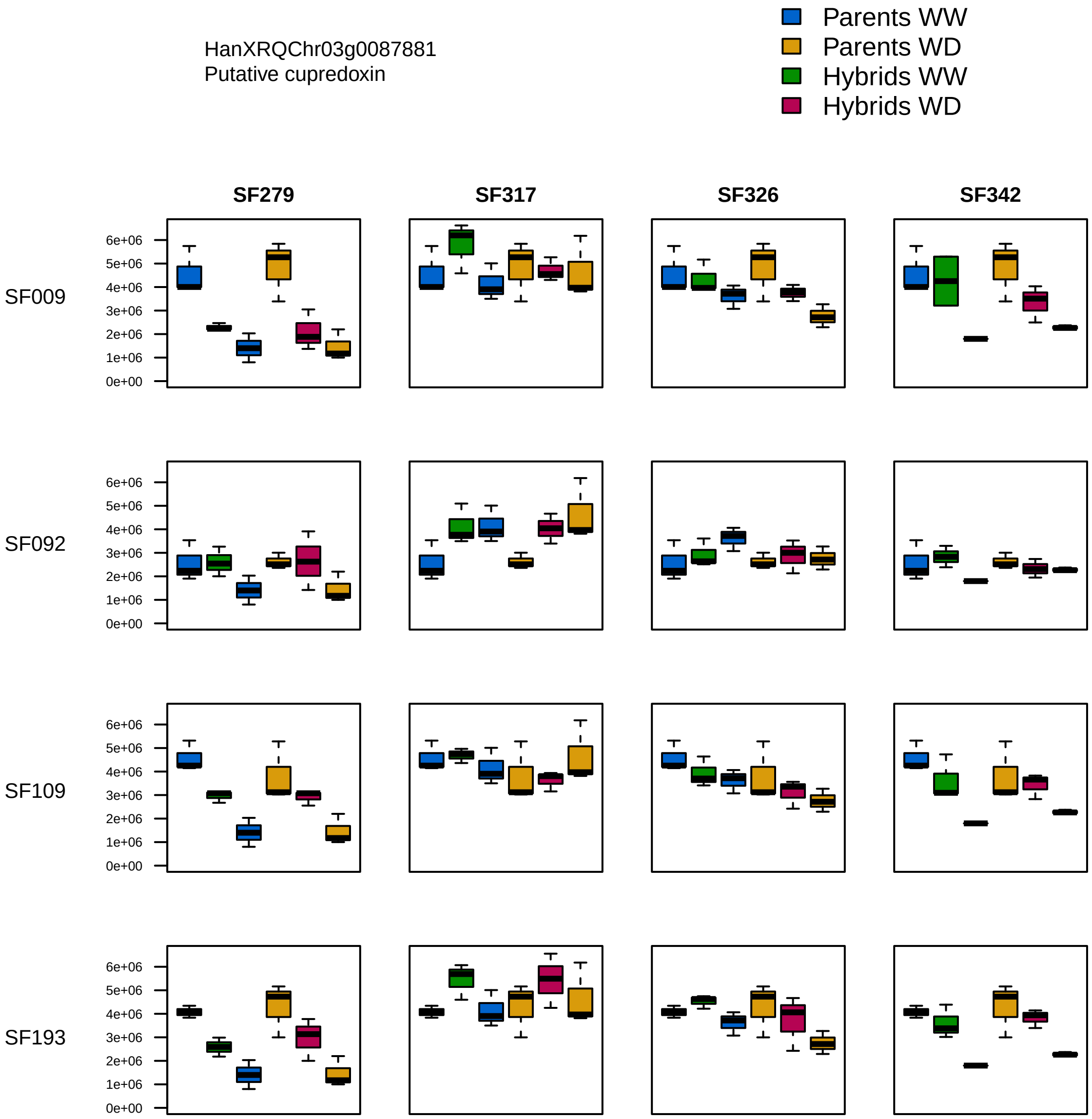
HanXRQChr03g0087881
Putative cupredoxin
Parents WW
Parents WD
Hybrids WW
Hybrids WD
SF279
SF317
SF326
SF342
SF009
SF092
SF109
SF193
6e+06
5e+06
4e+06
3e+06
2e+06
1e+06
0e+00

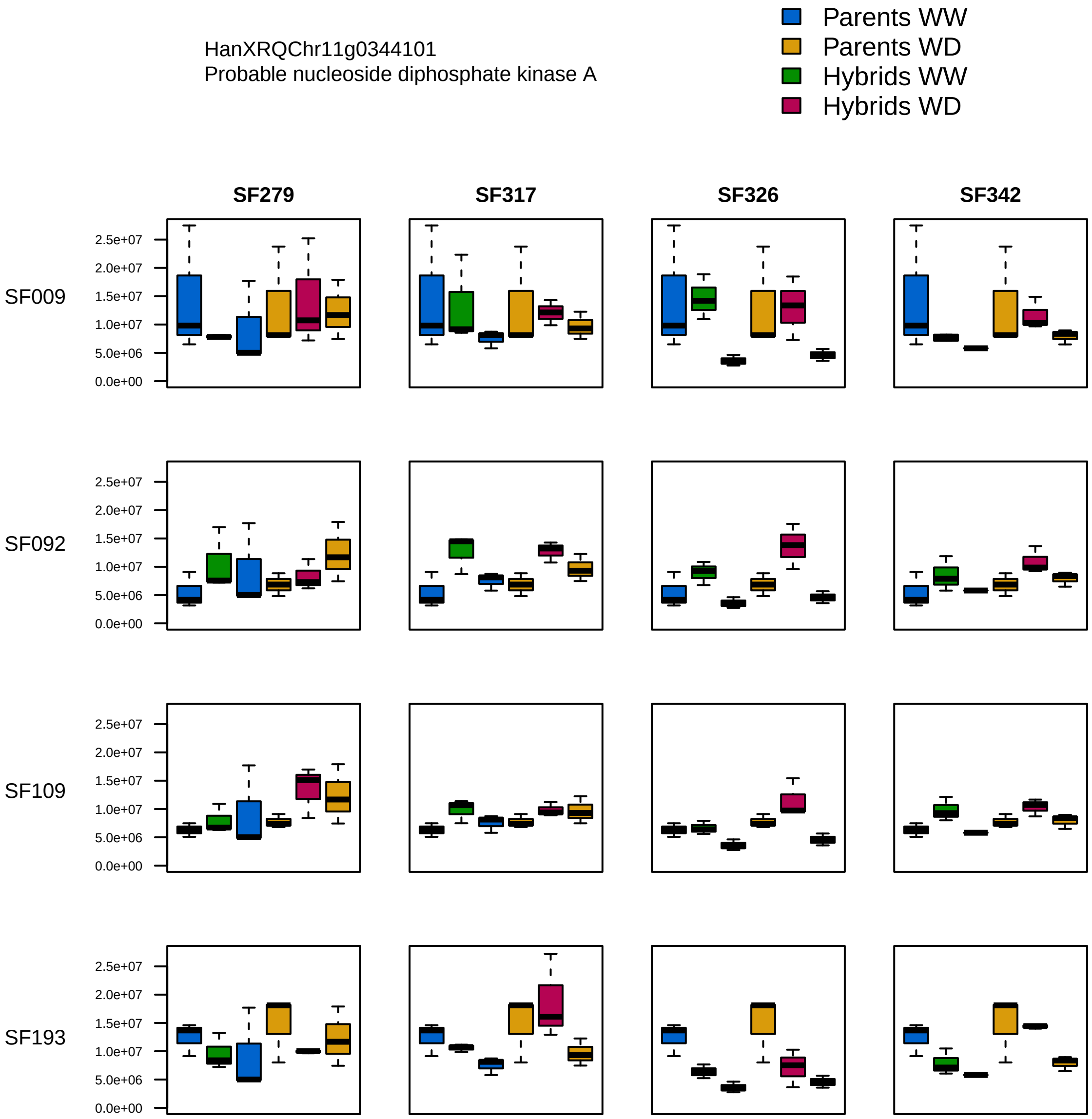
HanXRQChr11g0344101
Probable nucleoside diphosphate kinase A
Parents WW
Parents WD
Hybrids WW
Hybrids WD
SF279
SF317
SF326
SF342
SF009
SF092
SF109
SF193
2.5e+07
2.0e+07
1.5e+07
1.0e+07
5.0e+06
0.0e+00

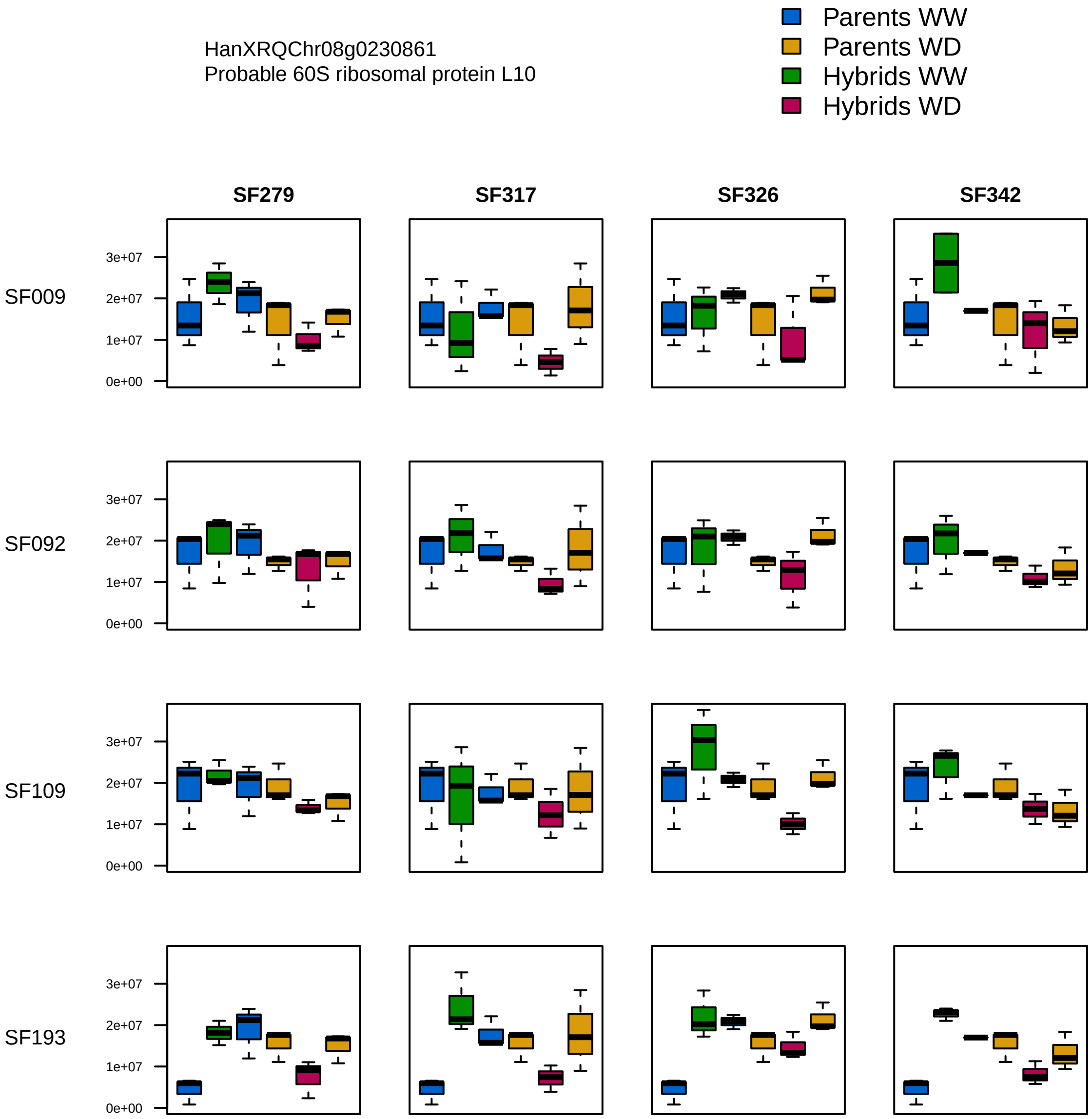
HanXRQChr08g0230861
Probable 60S ribosomal protein L10
Parents WW
Parents WD
Hybrids WW
Hybrids WD
SF279
SF317
SF326
SF342
SF009
SF092
SF109
SF193
3e+07
2e+07
1e+07
0e+00

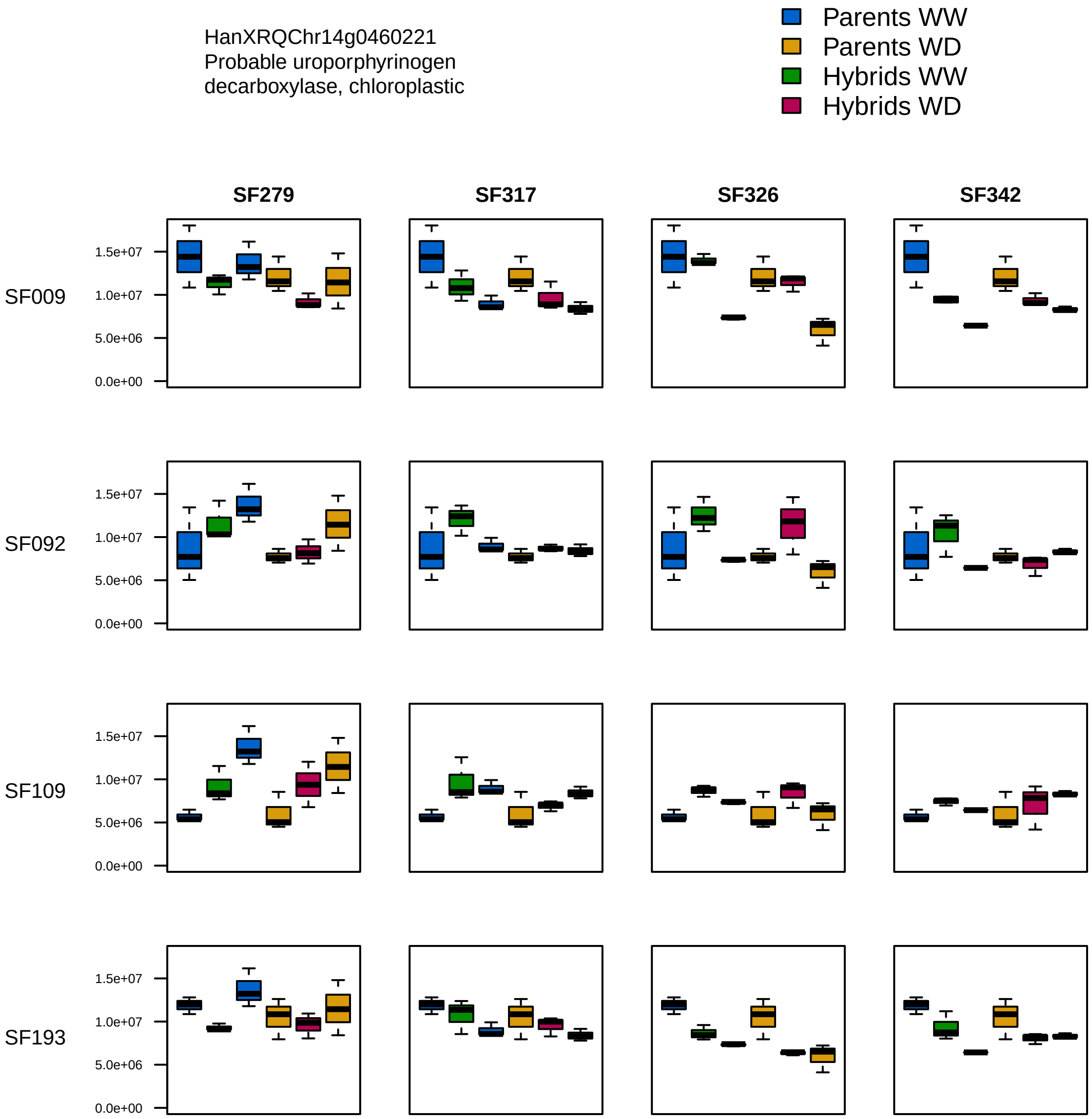
HanXRQChr14g0460221
Probable uroporphyrinogen
decarboxylase, chloroplastic
Parents WW
Parents WD
Hybrids WW
Hybrids WD
SF279
SF317
SF326
SF342
SF009
SF092
SF109
SF193
1.5e+07
1.0e+07
5.0e+06
0.0e+00

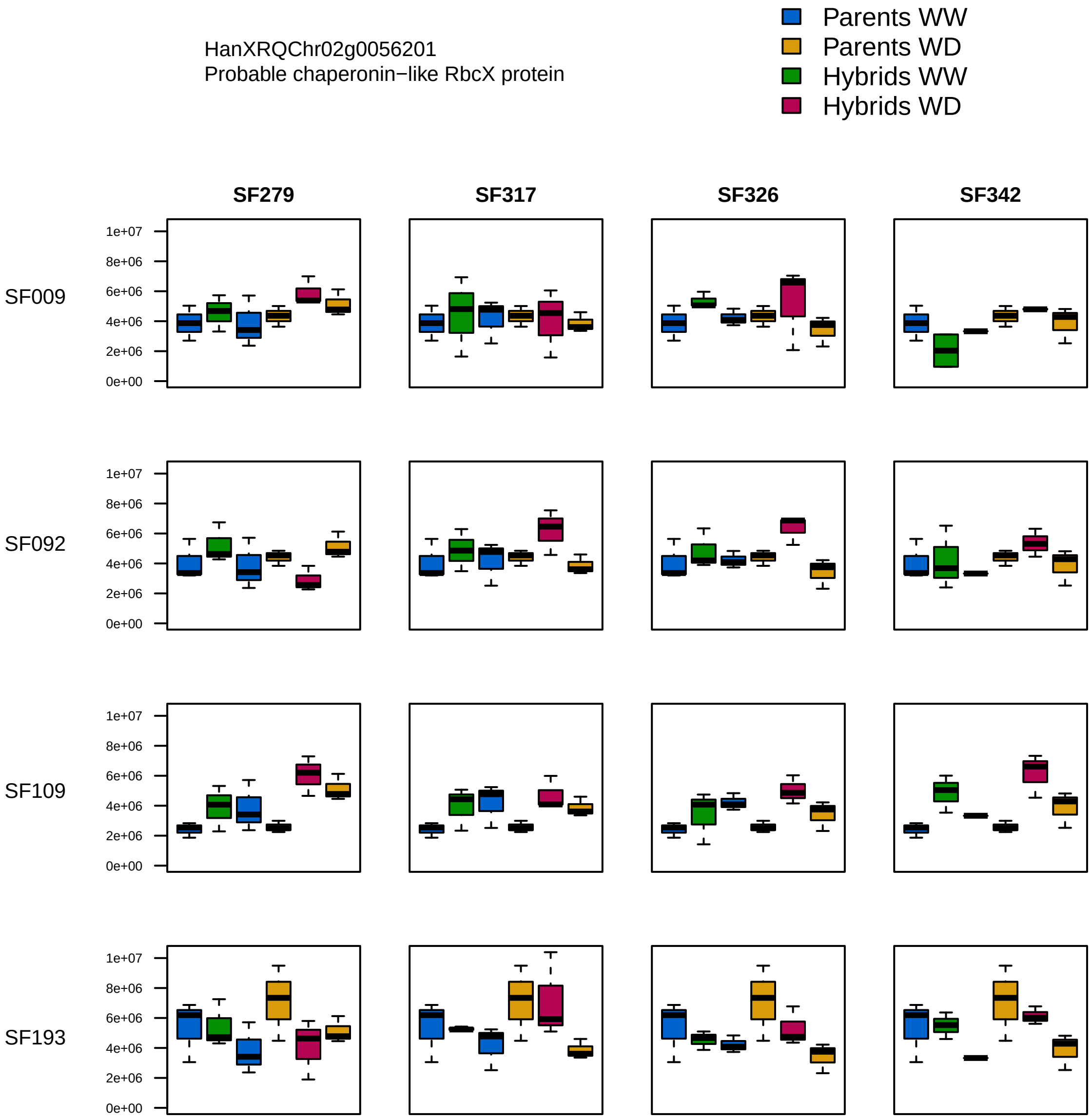
HanXRQChr02g0056201
Probable chaperonin−like RbcX protein
Parents WW
Parents WD
Hybrids WW
Hybrids WD
SF279
SF317
SF326
SF342
SF009
SF092
SF109
SF193
1e+07
8e+06
6e+06
4e+06
2e+06
0e+00

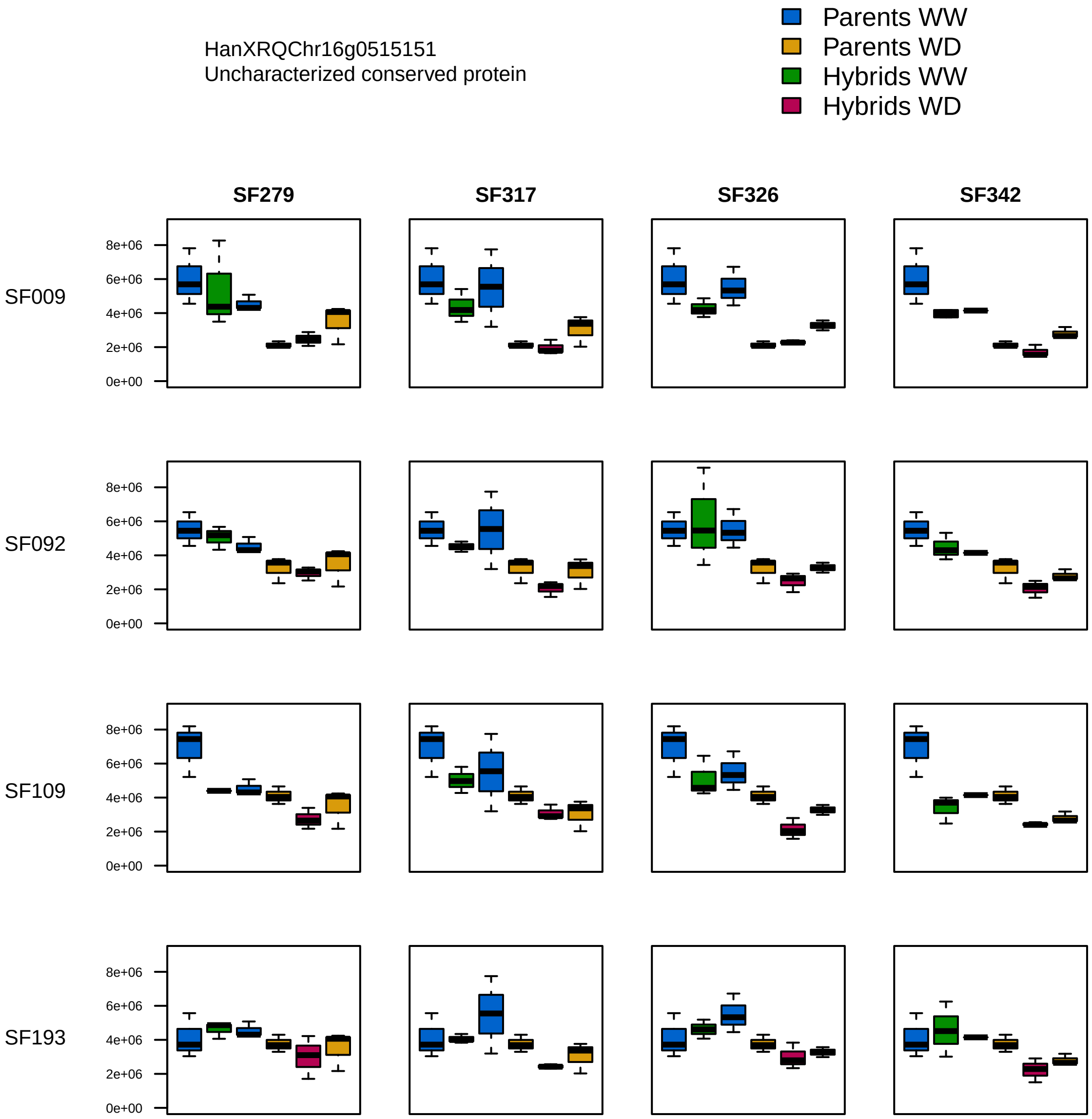
HanXRQChr16g0515151
Uncharacterized conserved protein
Parents WW
Parents WD
Hybrids WW
Hybrids WD
SF279
SF317
SF326
SF342
SF009
SF092
SF109
SF193
8e+06
6e+06
4e+06
2e+06
0e+00

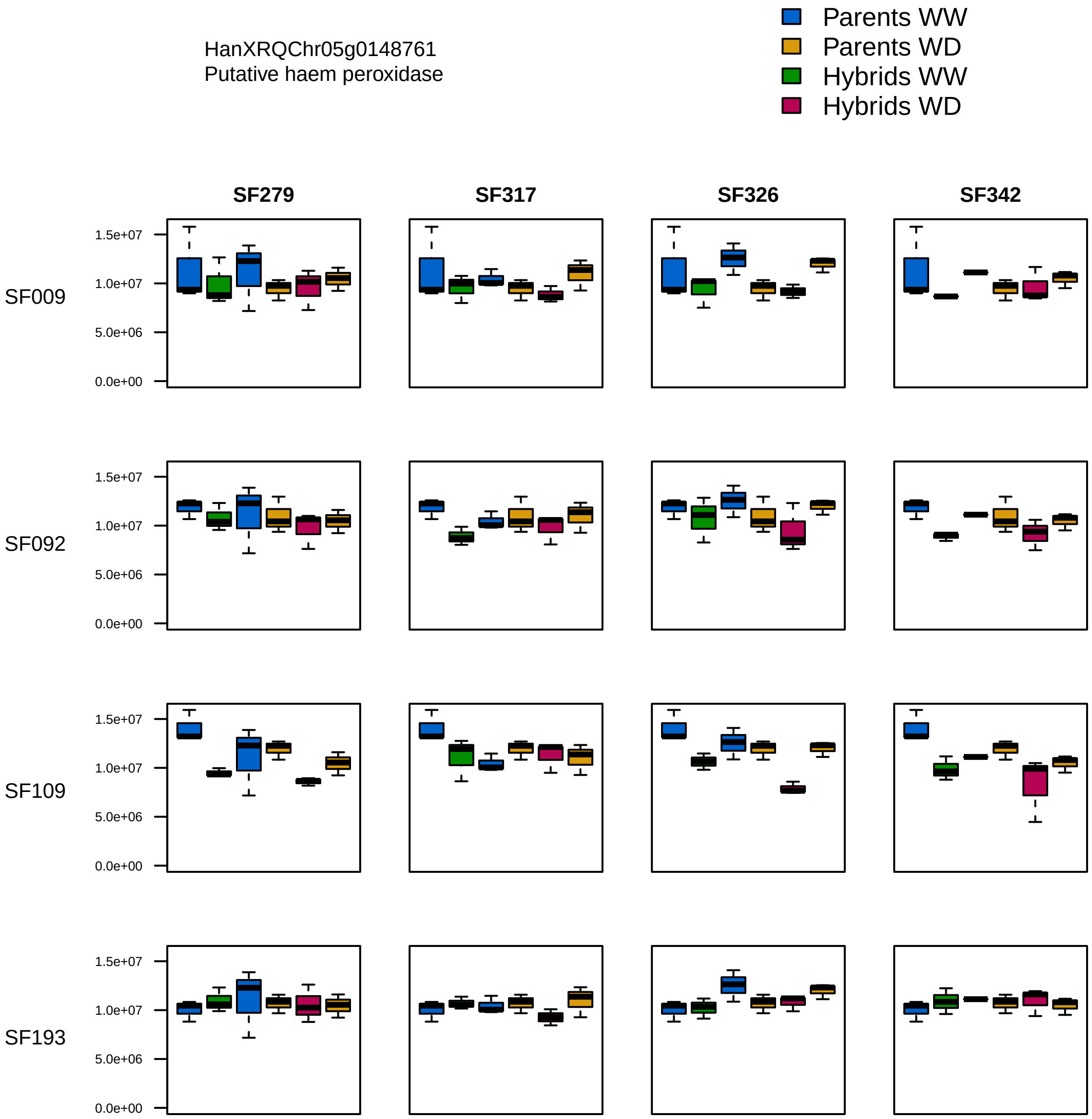
HanXRQChr05g0148761
Putative haem peroxidase
Parents WW
Parents WD
Hybrids WW
Hybrids WD
SF279
SF317
SF326
SF342
SF009
SF092
SF109
SF193
1.5e+07
1.0e+07
5.0e+06
0.0e+00

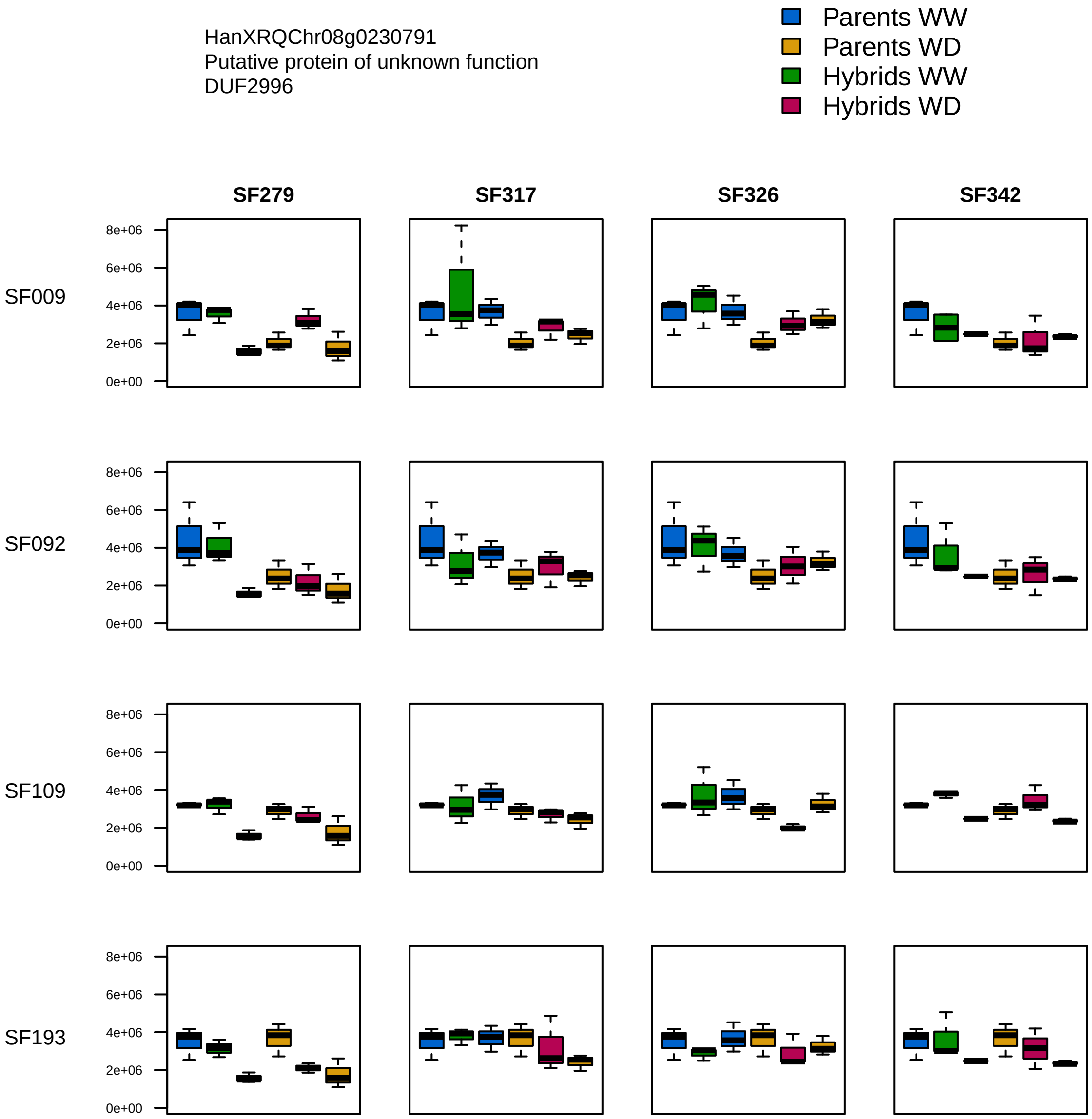

HanXRQChr08g0230791
Putative protein of unknown function
DUF2996
Parents WW
Parents WD
Hybrids WW
Hybrids WD
SF279
SF317
SF326
SF342
SF009
SF092
SF109
SF193
8e+06
6e+06
4e+06
2e+06
0e+00

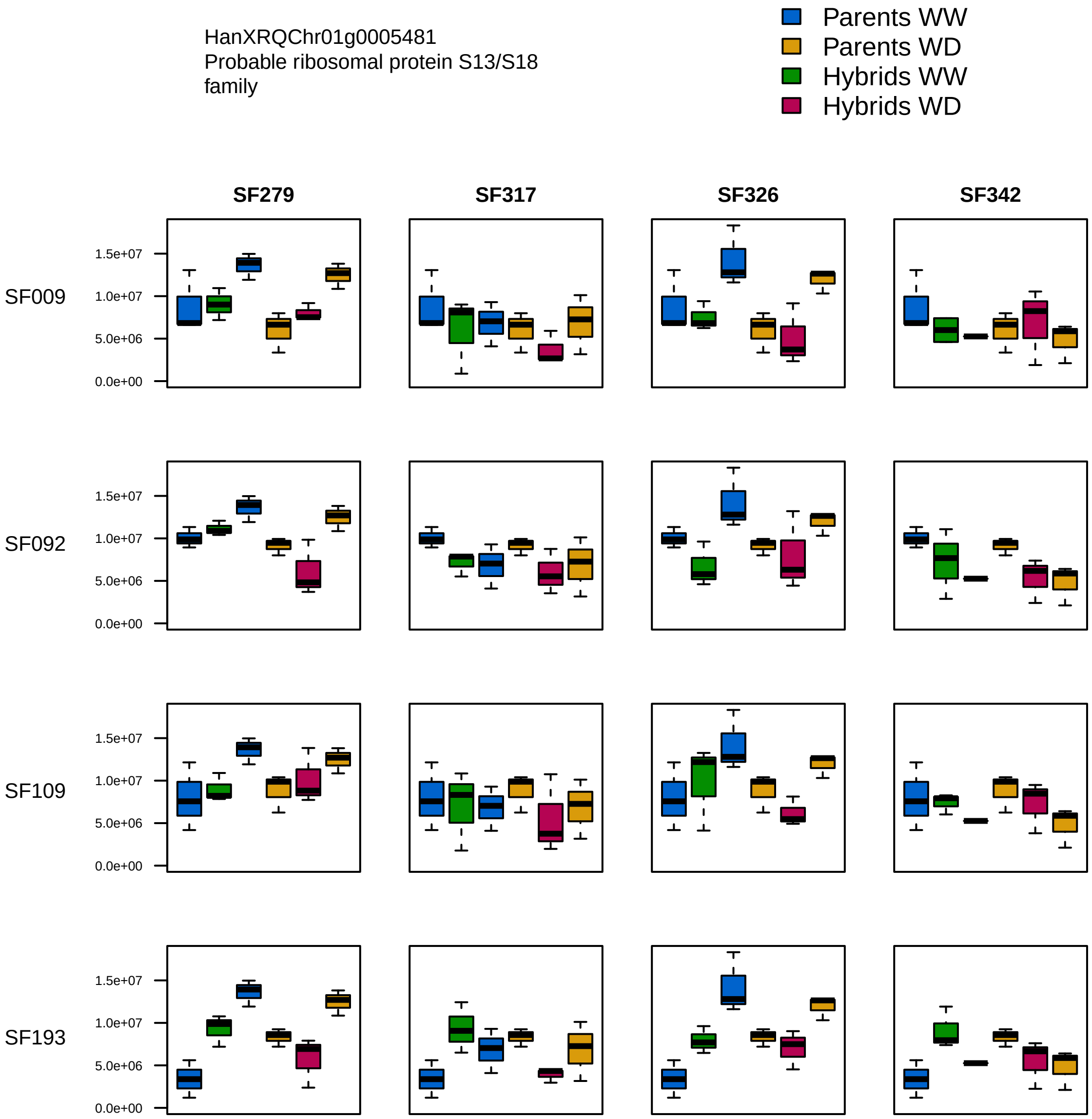
HanXRQChr01g0005481
Probable ribosomal protein S13/S18
family
Parents WW
Parents WD
Hybrids WW
Hybrids WD
SF279
SF317
SF326
SF342
SF009
SF092
SF109
SF193
1.5e+07
1.0e+07
5.0e+06
0.0e+00

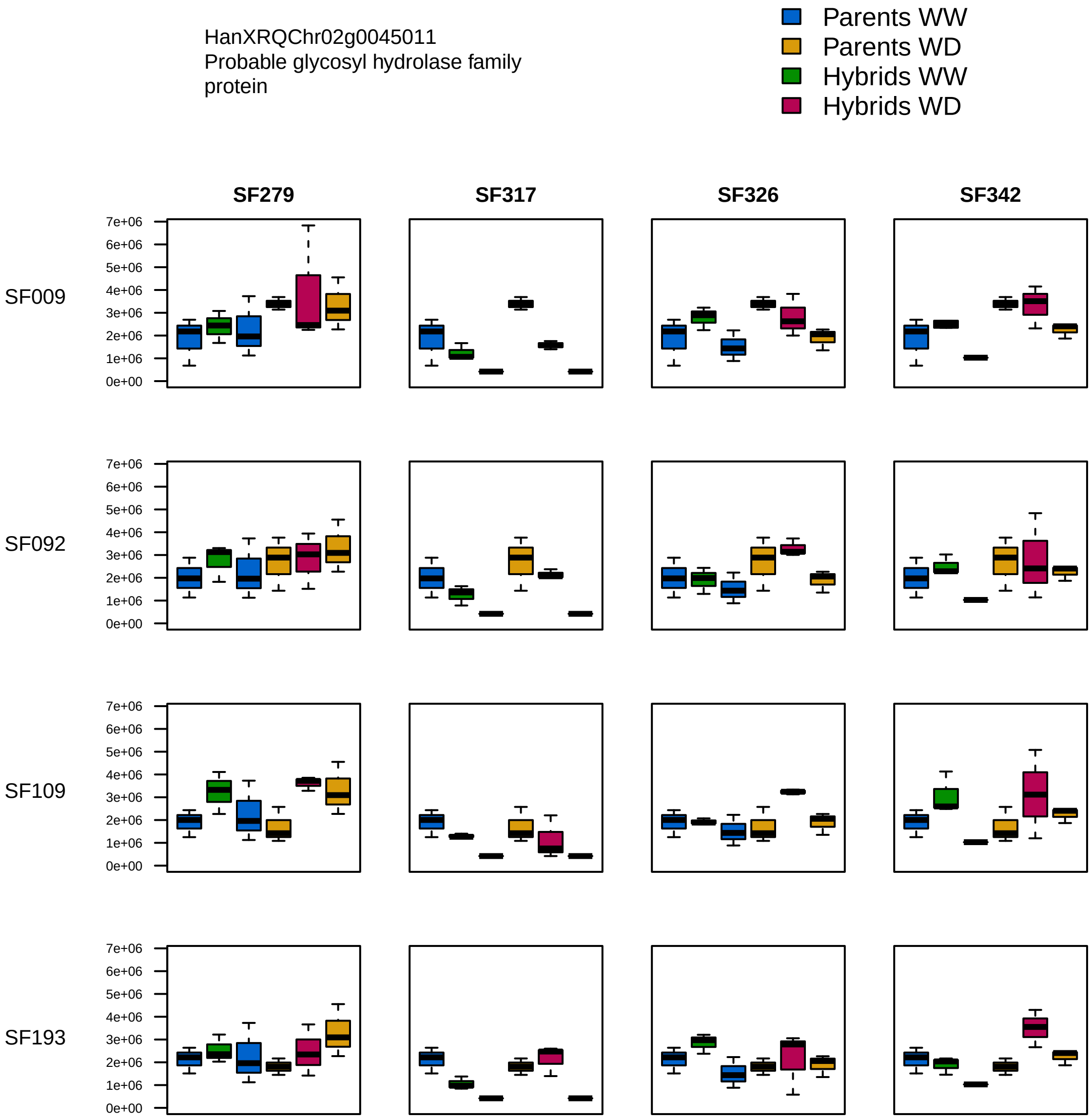

HanXRQChr02g0045011
Probable glycosyl hydrolase family protein
Parents WW
Parents WD
Hybrids WW
Hybrids WD
SF279
SF317
SF326
SF342
SF009
SF092
SF109
SF193
0e+00
1e+06
2e+06
3e+06
4e+06
5e+06
6e+06
7e+06

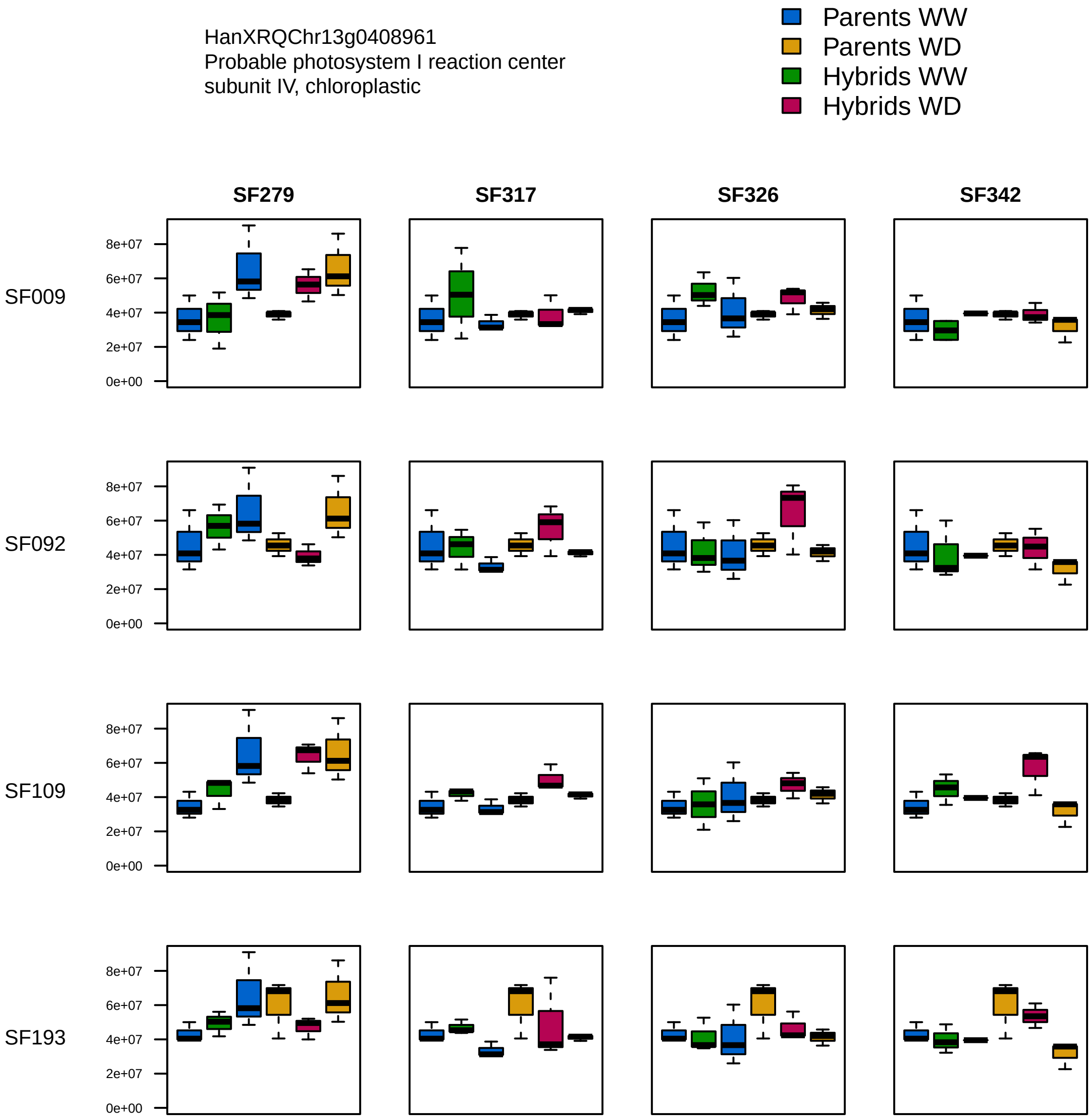
HanXRQChr13g0408961
Probable photosystem I reaction center
subunit IV, chloroplastic
Parents WW
Parents WD
Hybrids WW
Hybrids WD
SF279
SF317
SF326
SF342
SF009
SF092
SF109
SF193
8e+07
6e+07
4e+07
2e+07
0e+00

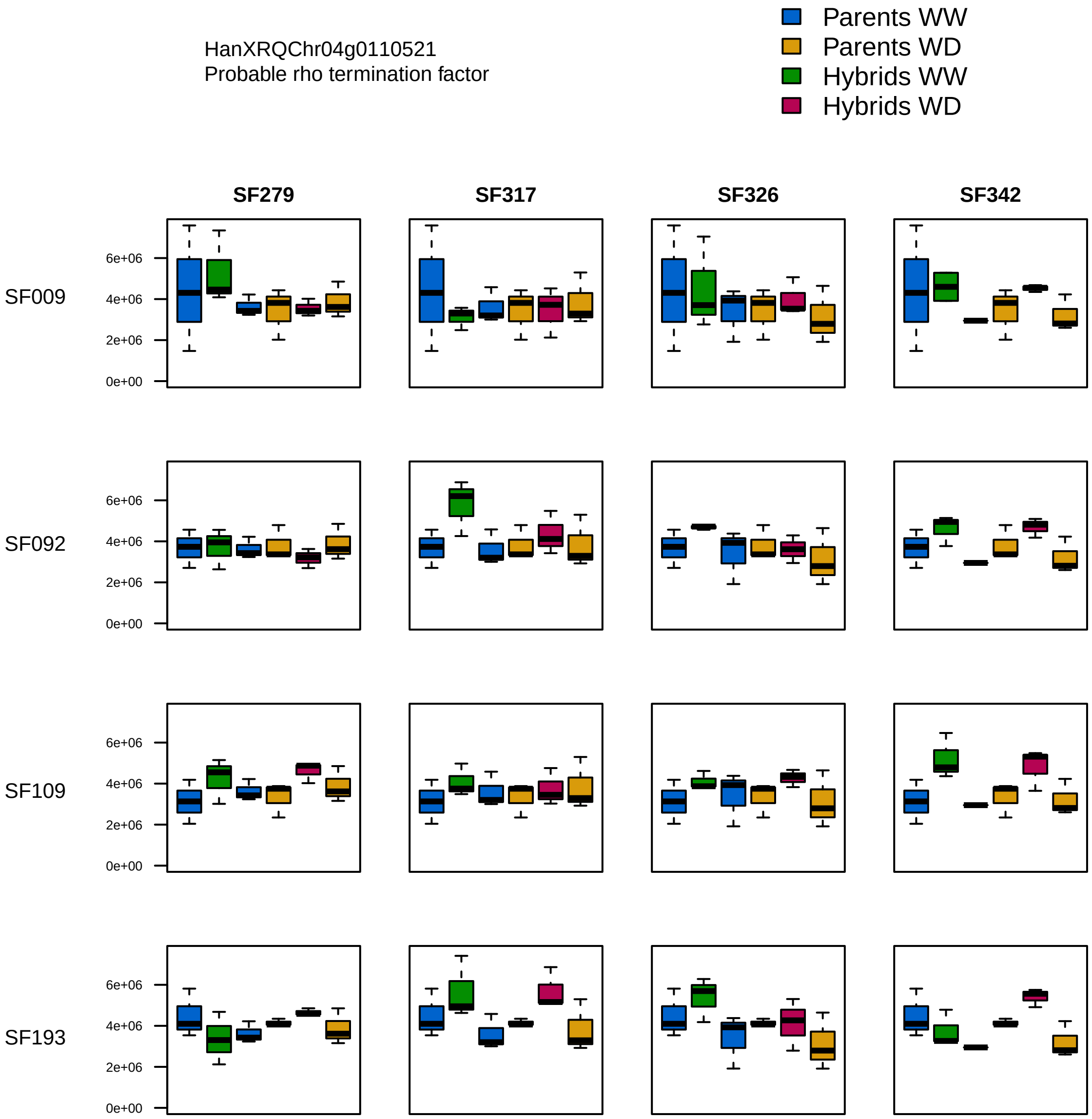

HanXRQChr04g0110521
Probable rho termination factor
Parents WW
Parents WD
Hybrids WW
Hybrids WD
SF279
SF317
SF326
SF342
SF009
SF092
SF109
SF193
0e+00
2e+06
4e+06
6e+06

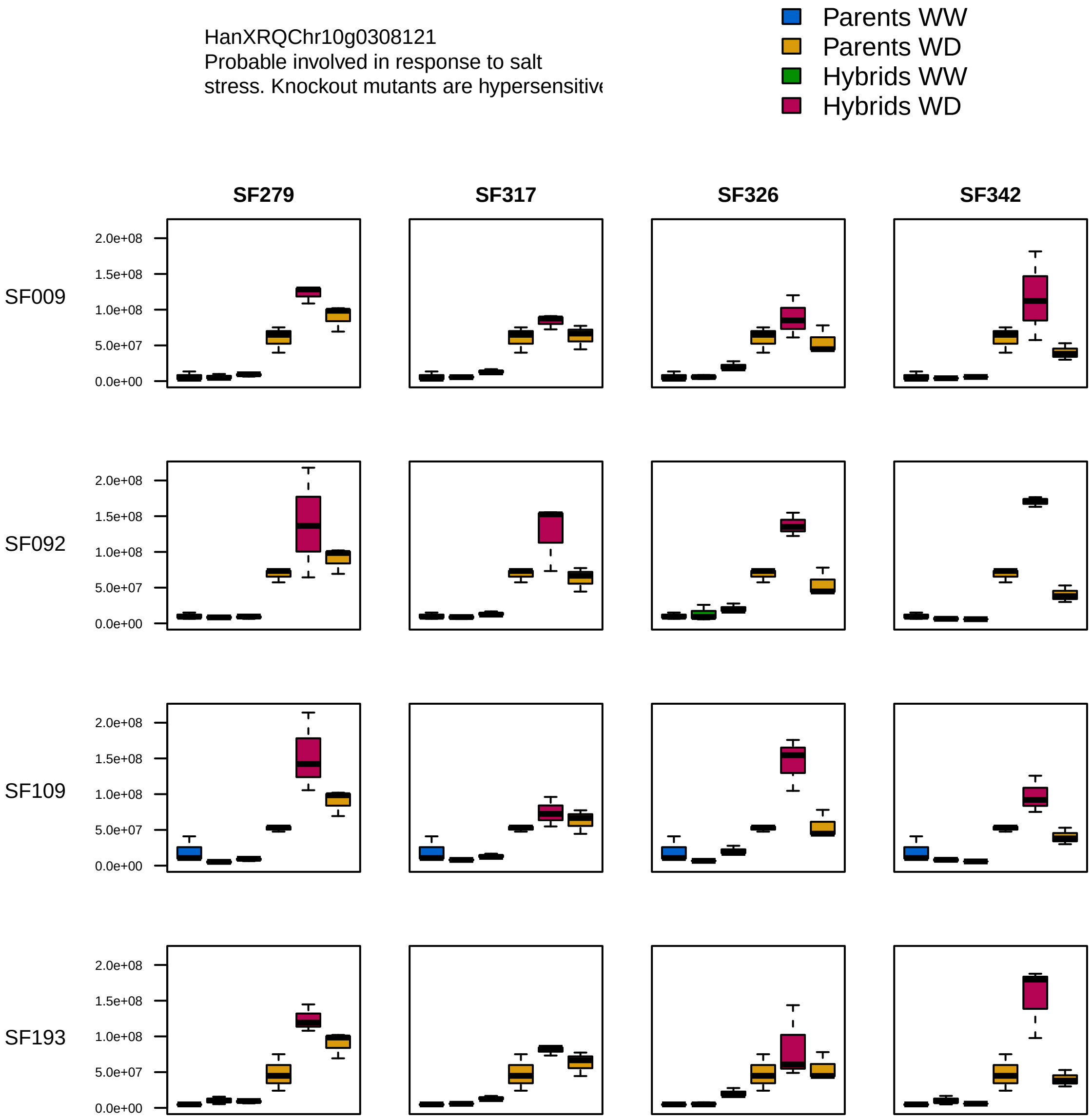
HanXRQChr10g0308121
Probable involved in response to salt
stress. Knockout mutants are hypersensitive
Parents WW
Parents WD
Hybrids WW
Hybrids WD
SF279
SF317
SF326
SF342
SF009
SF092
SF109
SF193
2.0e+08
1.5e+08
1.0e+08
5.0e+07
0.0e+00

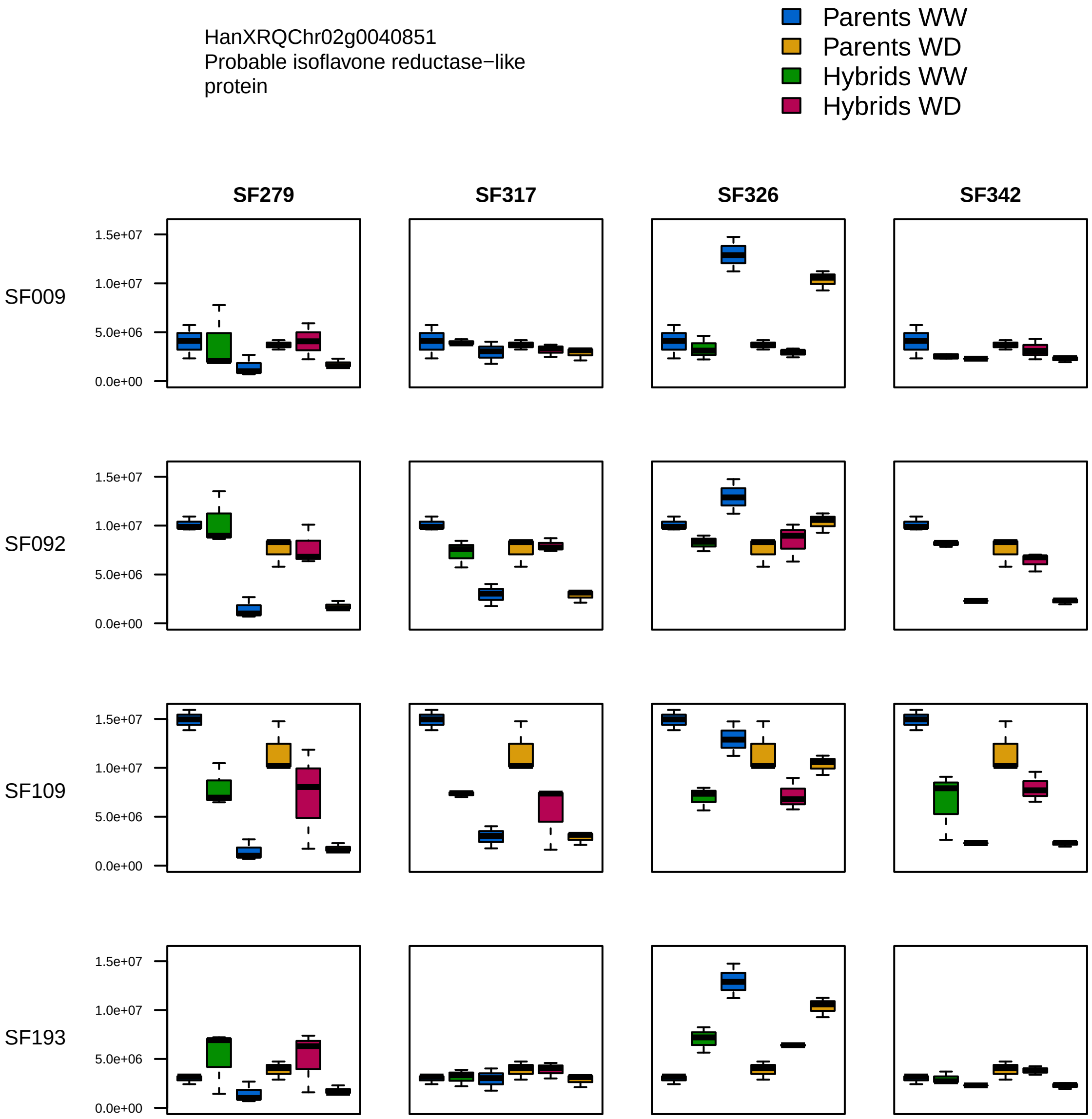
HanXRQChr02g0040851
Probable isoflavone reductase−like protein
Parents WW
Parents WD
Hybrids WW
Hybrids WD
SF279
SF317
SF326
SF342
SF009
SF092
SF109
SF193
1.5e+07
1.0e+07
5.0e+06
0.0e+00

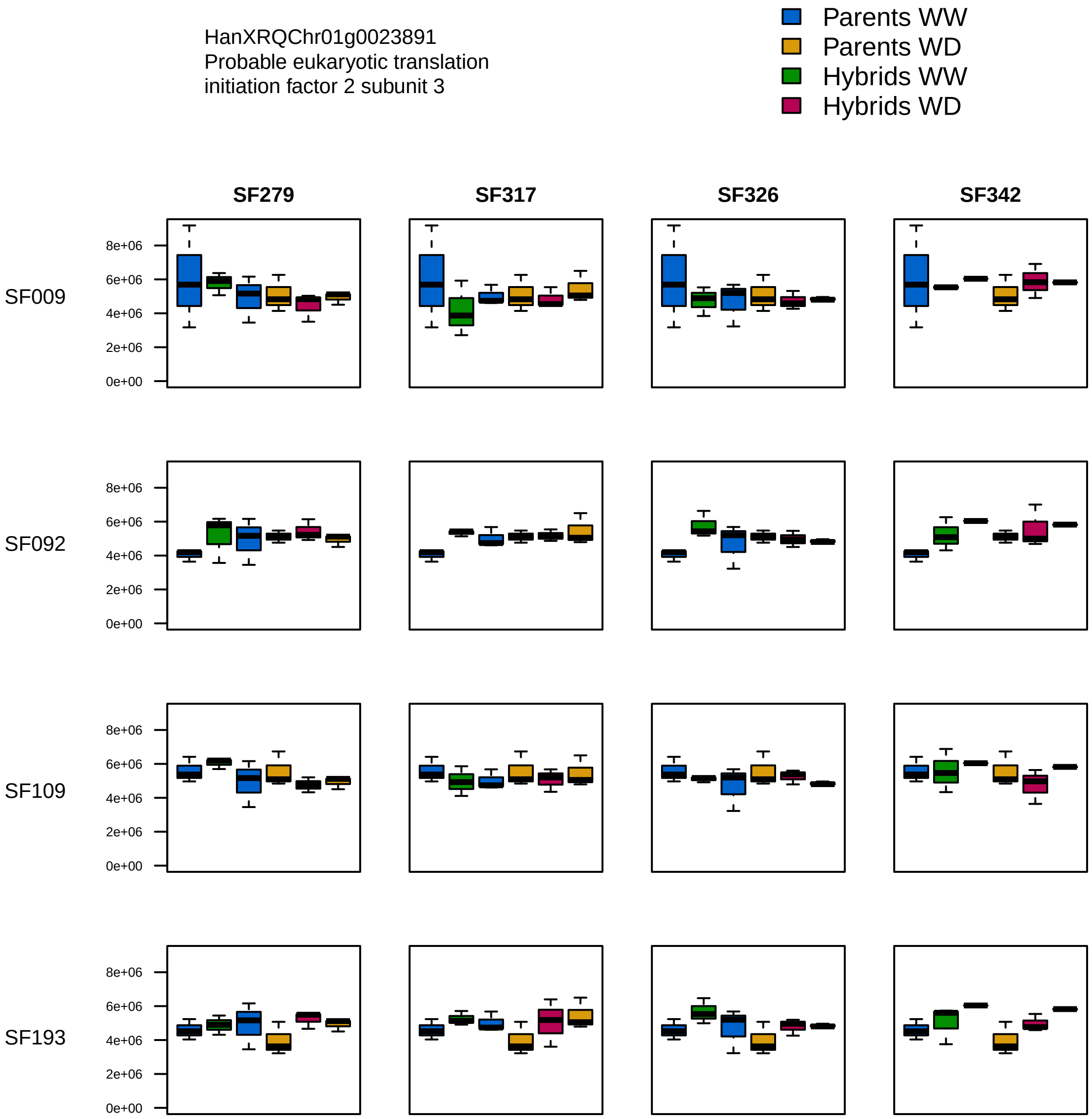
HanXRQChr01g0023891
Probable eukaryotic translation
initiation factor 2 subunit 3
Parents WW
Parents WD
Hybrids WW
Hybrids WD
SF279
SF317
SF326
SF342
SF009
SF092
SF109
SF193
8e+06
6e+06
4e+06
2e+06
0e+00

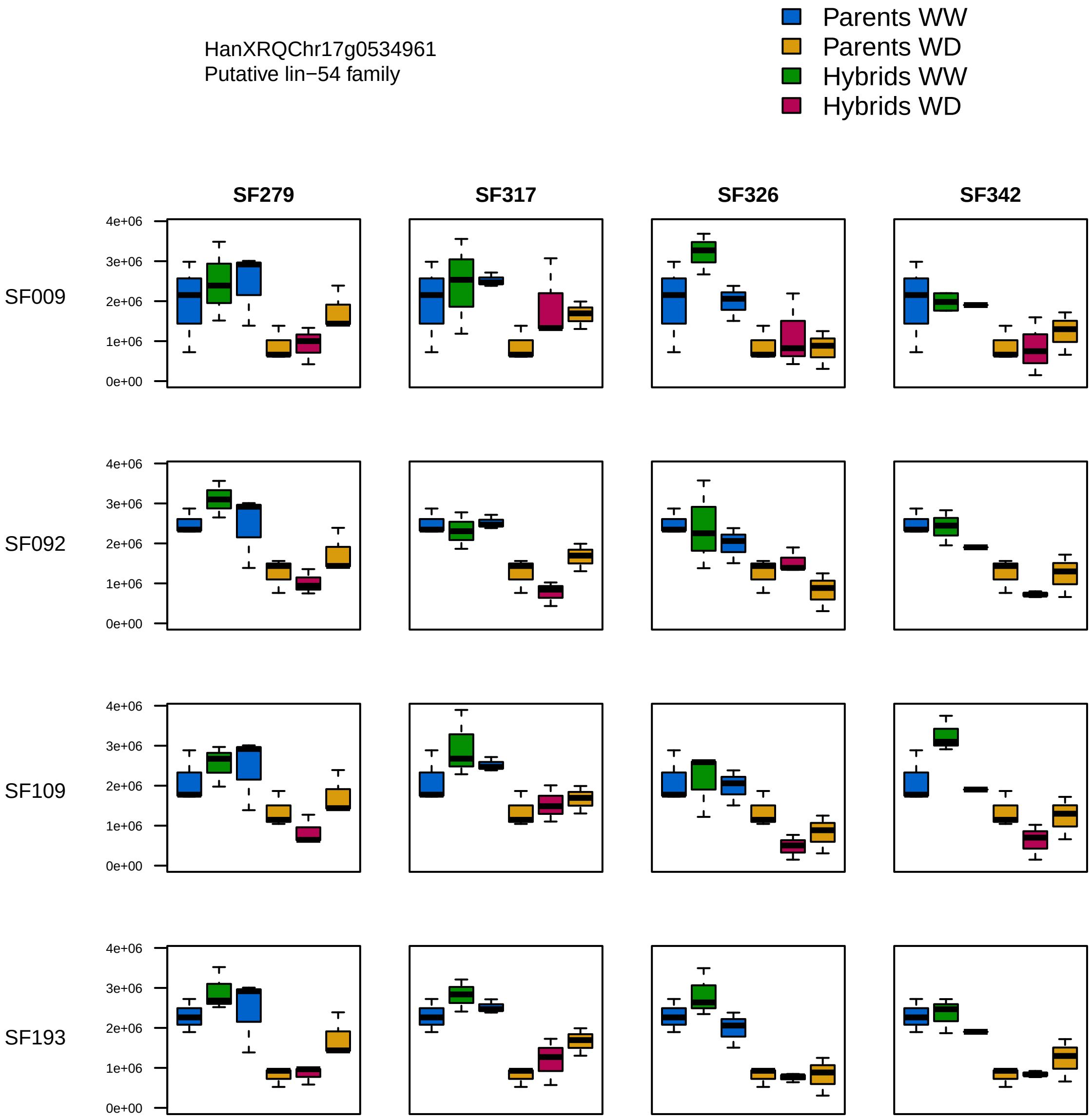

HanXRQChr17g0534961
Putative lin−54 family
Parents WW
Parents WD
Hybrids WW
Hybrids WD
SF279
SF317
SF326
SF342
SF009
SF092
SF109
SF193
0e+00
1e+06
2e+06
3e+06
4e+06

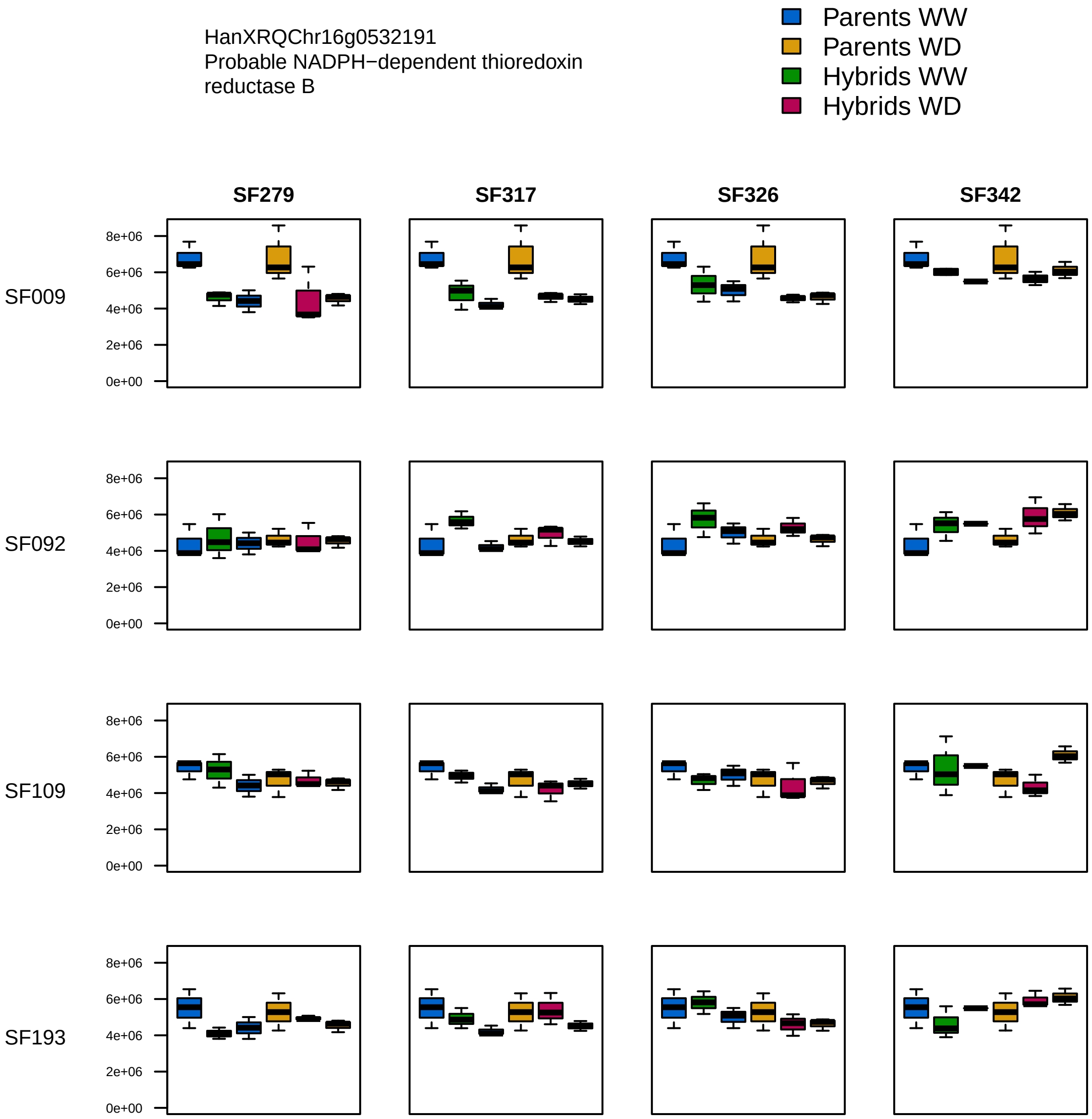
HanXRQChr16g0532191
Probable NADPH−dependent thioredoxin
reductase B
Parents WW
Parents WD
Hybrids WW
Hybrids WD
SF279
SF317
SF326
SF342
SF009
SF092
SF109
SF193
8e+06
6e+06
4e+06
2e+06
0e+00

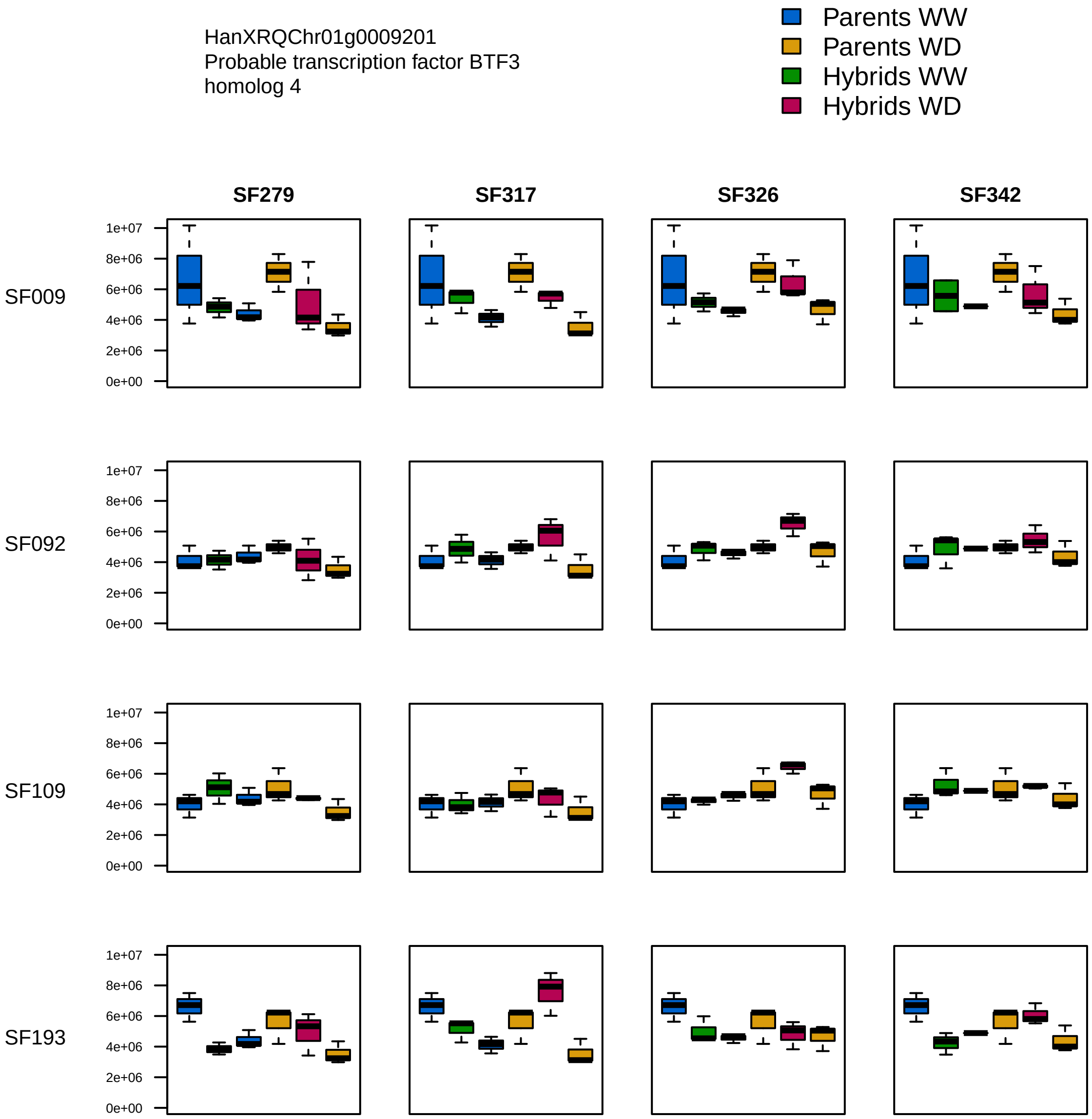
HanXRQChr01g0009201
Probable transcription factor BTF3
homolog 4
Parents WW
Parents WD
Hybrids WW
Hybrids WD
SF279
SF317
SF326
SF342
SF009
SF092
SF109
SF193
1e+07
8e+06
6e+06
4e+06
2e+06
0e+00

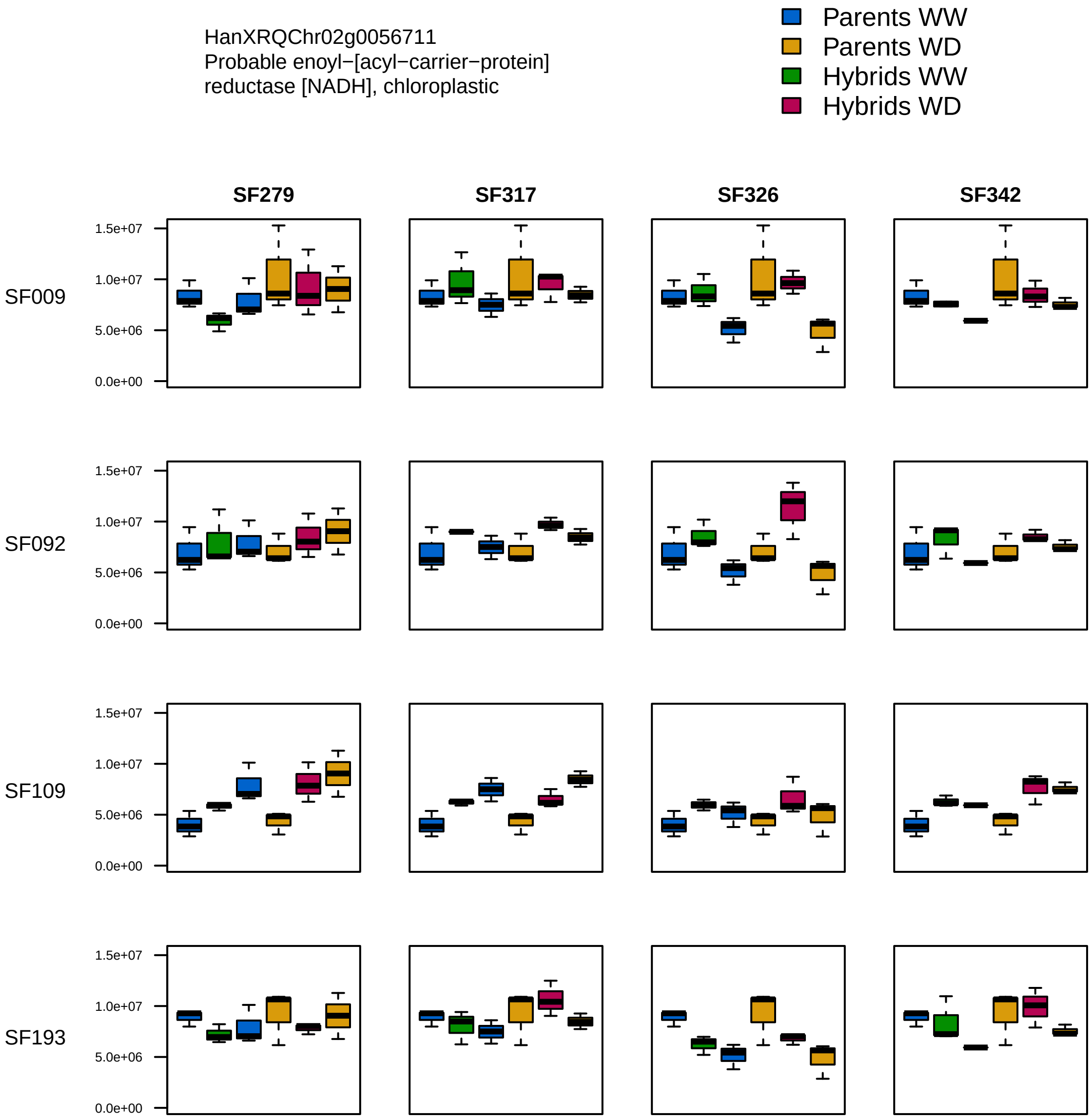
HanXRQChr02g0056711
Probable enoyl−[acyl−carrier−protein]
reductase [NADH], chloroplastic
Parents WW
Parents WD
Hybrids WW
Hybrids WD
SF279
SF317
SF326
SF342
SF009
SF092
SF109
SF193
1.5e+07
1.0e+07
5.0e+06
0.0e+00

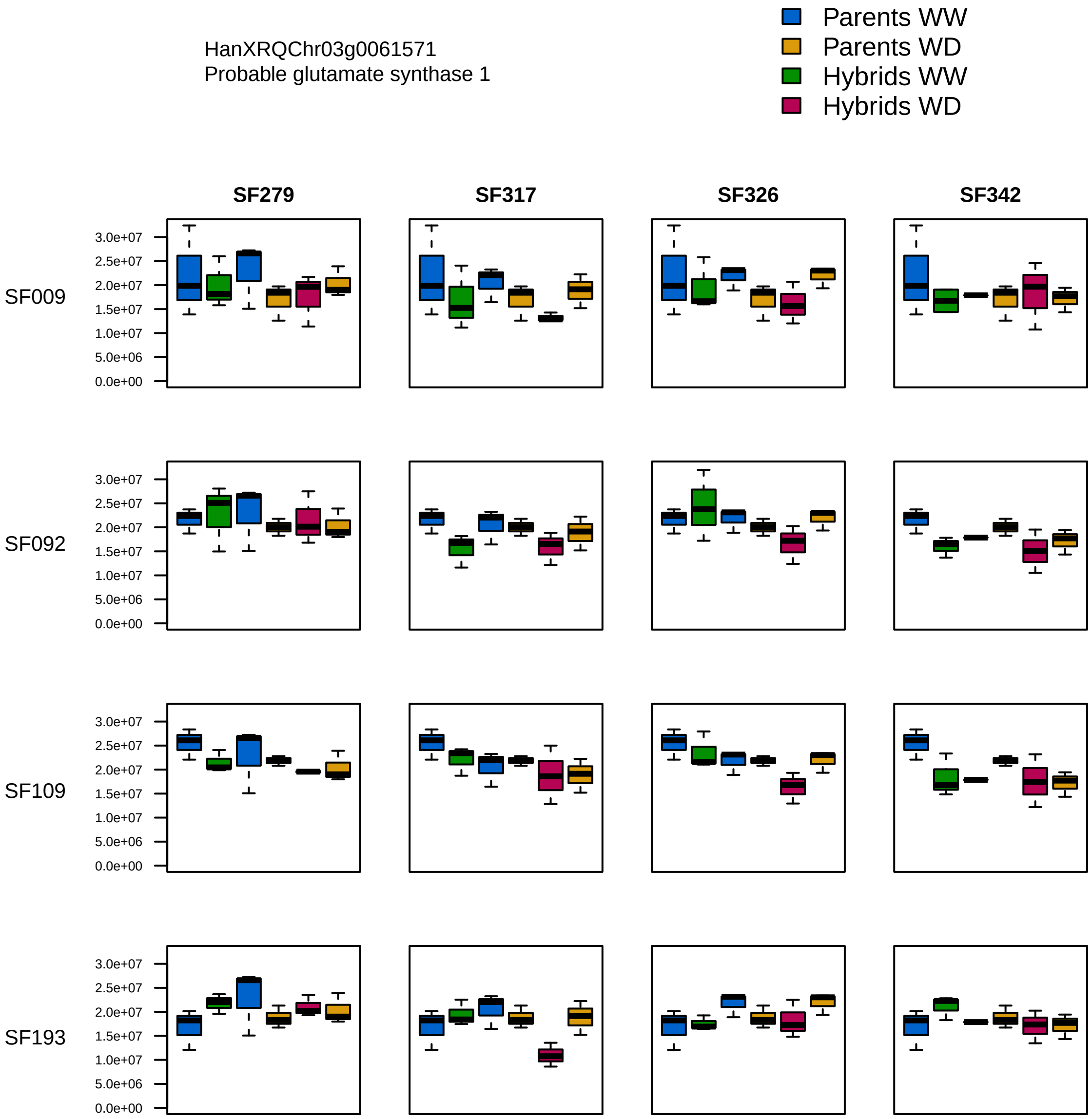
HanXRQChr03g0061571
Probable glutamate synthase 1
Parents WW
Parents WD
Hybrids WW
Hybrids WD
SF279
SF317
SF326
SF342
SF009
SF092
SF109
SF193
3.0e+07
2.5e+07
2.0e+07
1.5e+07
1.0e+07
5.0e+06
0.0e+00

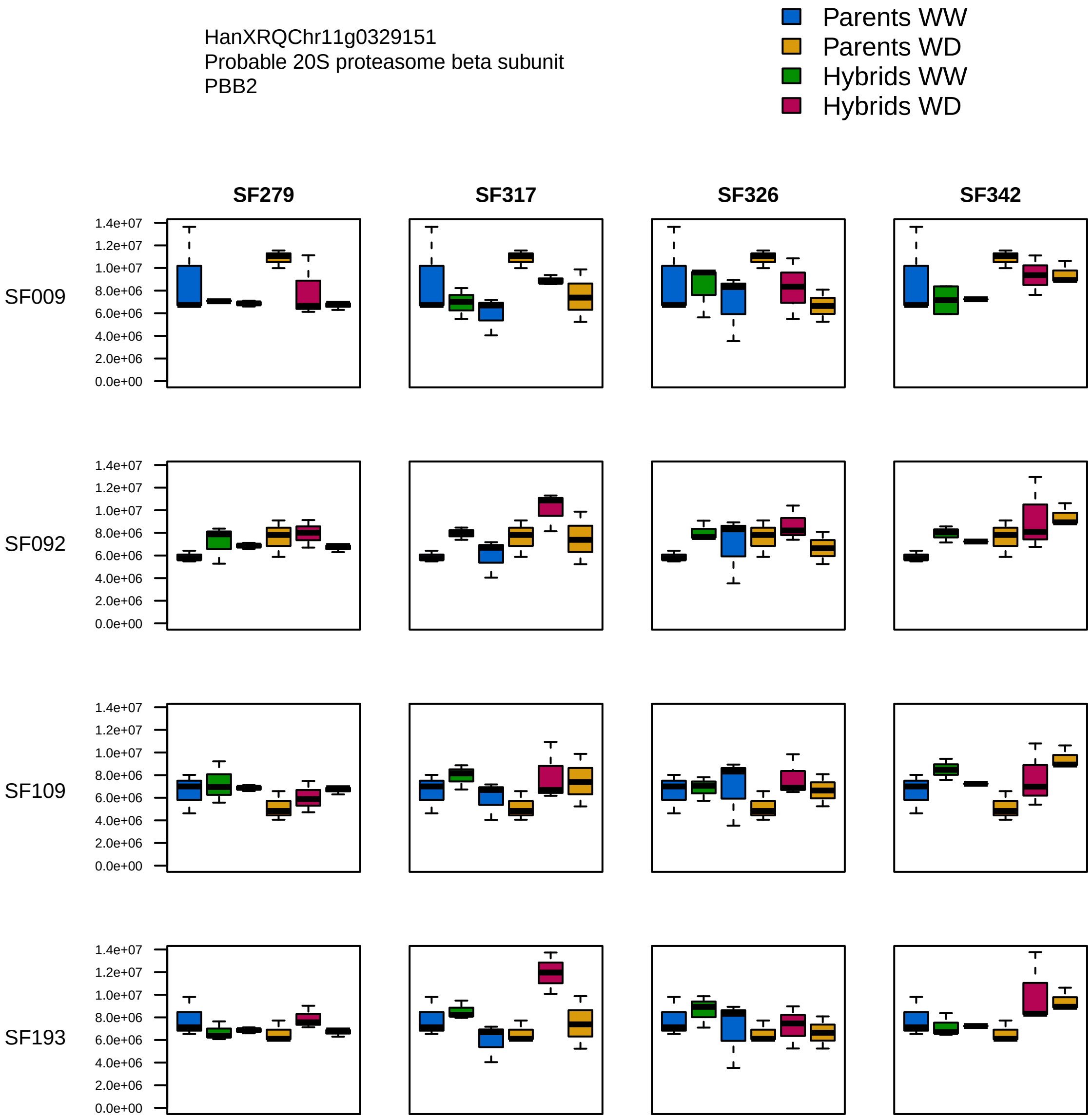
HanXRQChr11g0329151
Probable 20S proteasome beta subunit
PBB2
Parents WW
Parents WD
Hybrids WW
Hybrids WD
SF279
SF317
SF326
SF342
SF009
SF092
SF109
SF193
1.4e+07
1.2e+07
1.0e+07
8.0e+06
6.0e+06
4.0e+06
2.0e+06
0.0e+00

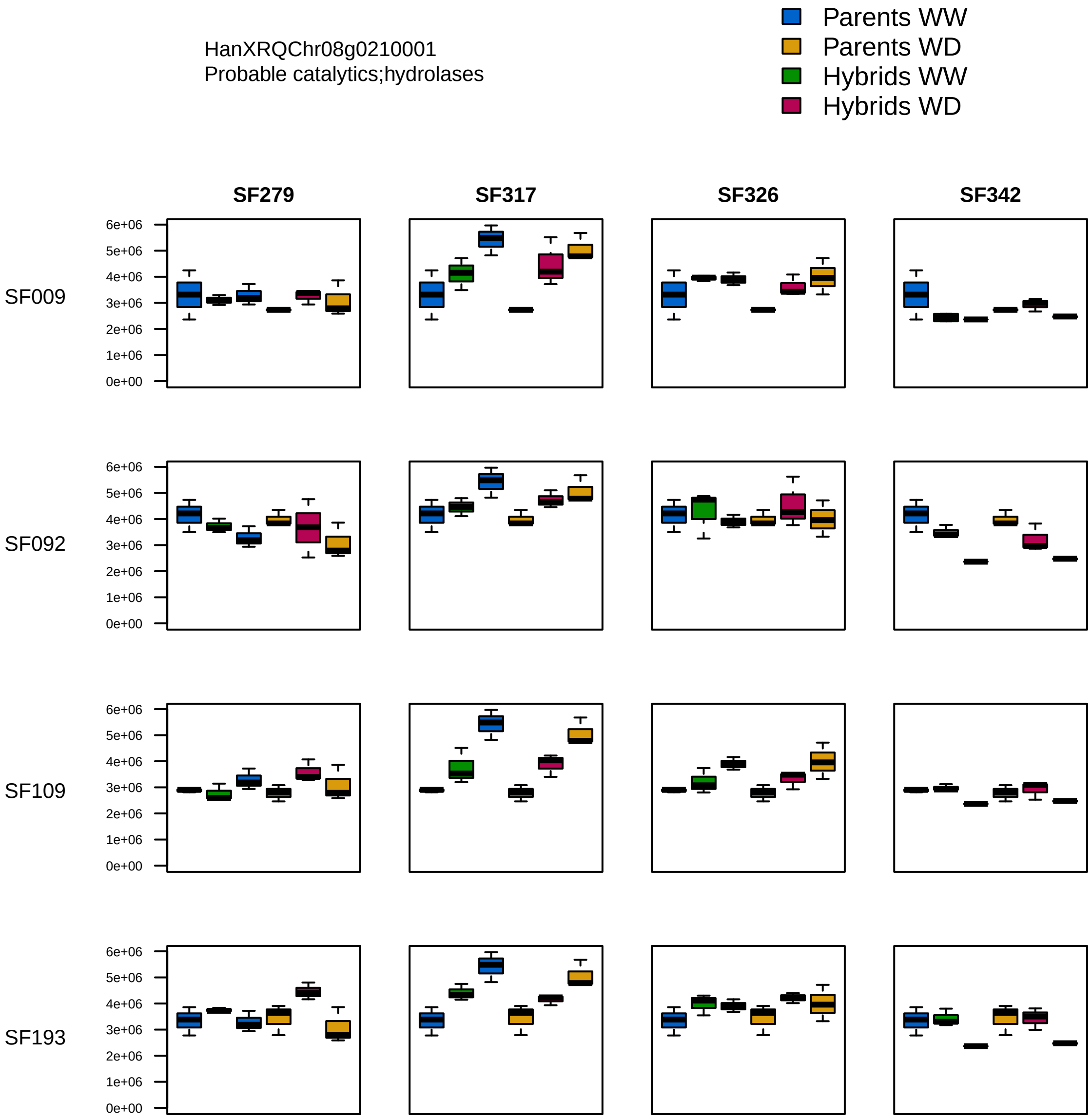
HanXRQChr08g0210001
Probable catalytics;hydrolases
Parents WW
Parents WD
Hybrids WW
Hybrids WD
SF279
SF317
SF326
SF342
SF009
SF092
SF109
SF193
0e+00
1e+06
2e+06
3e+06
4e+06
5e+06
6e+06

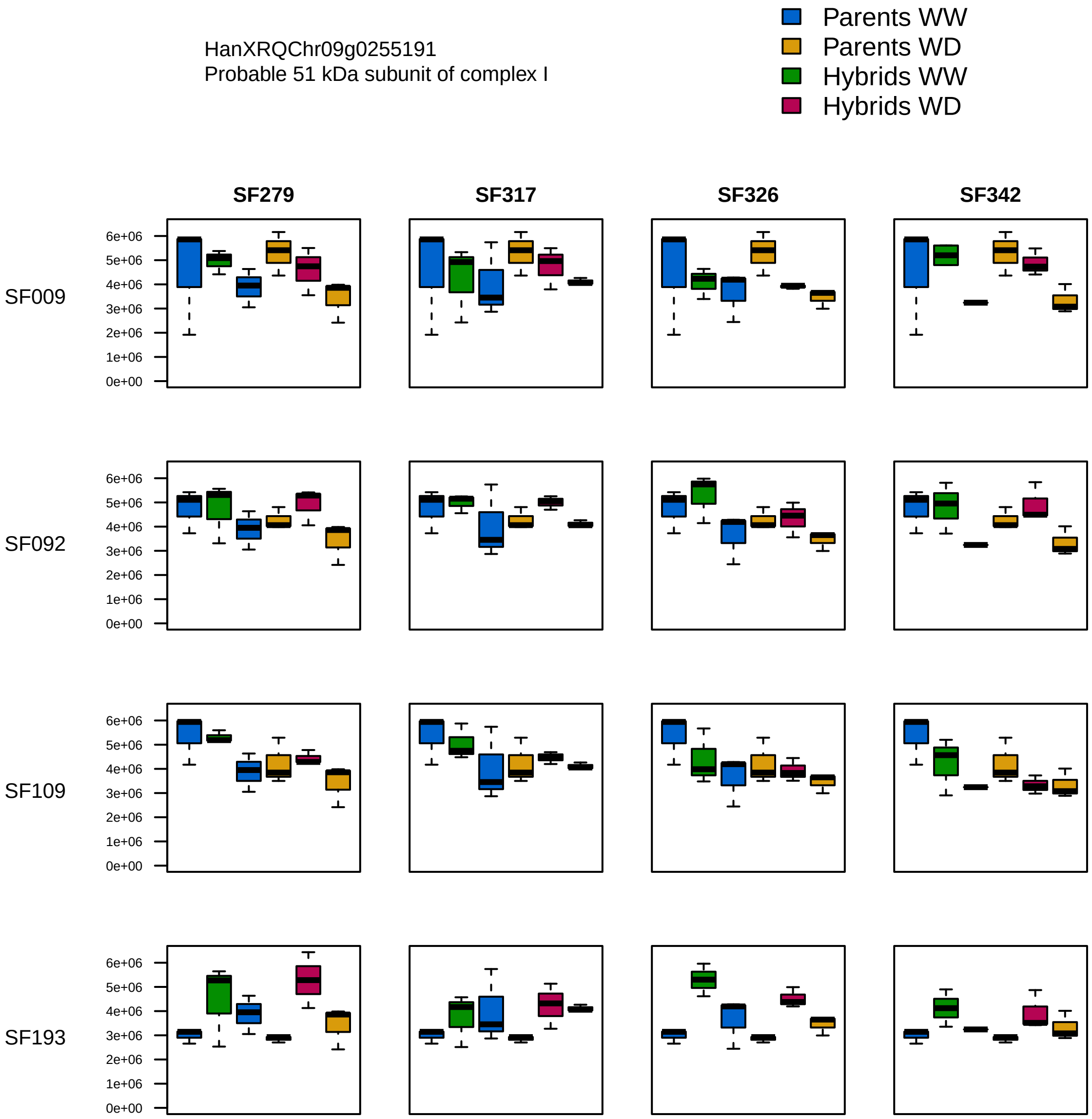
HanXRQChr09g0255191
Probable 51 kDa subunit of complex I
Parents WW
Parents WD
Hybrids WW
Hybrids WD
SF279
SF317
SF326
SF342
SF009
SF092
SF109
SF193
6e+06
5e+06
4e+06
3e+06
2e+06
1e+06
0e+00

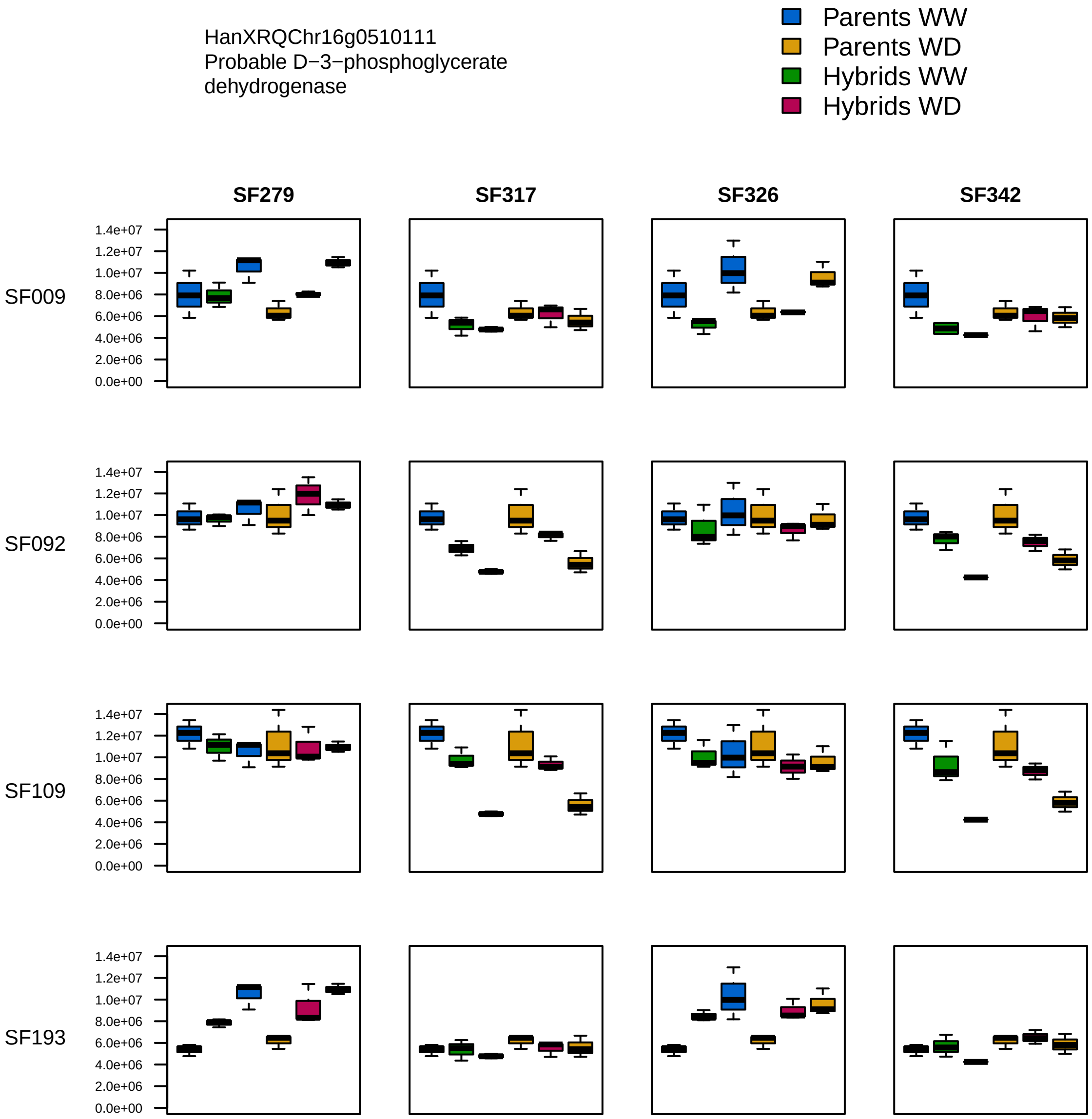
HanXRQChr16g0510111
Probable D−3−phosphoglycerate
dehydrogenase
Parents WW
Parents WD
Hybrids WW
Hybrids WD
SF279
SF317
SF326
SF342
SF009
SF092
SF109
SF193
1.4e+07
1.2e+07
1.0e+07
8.0e+06
6.0e+06
4.0e+06
2.0e+06
0.0e+00

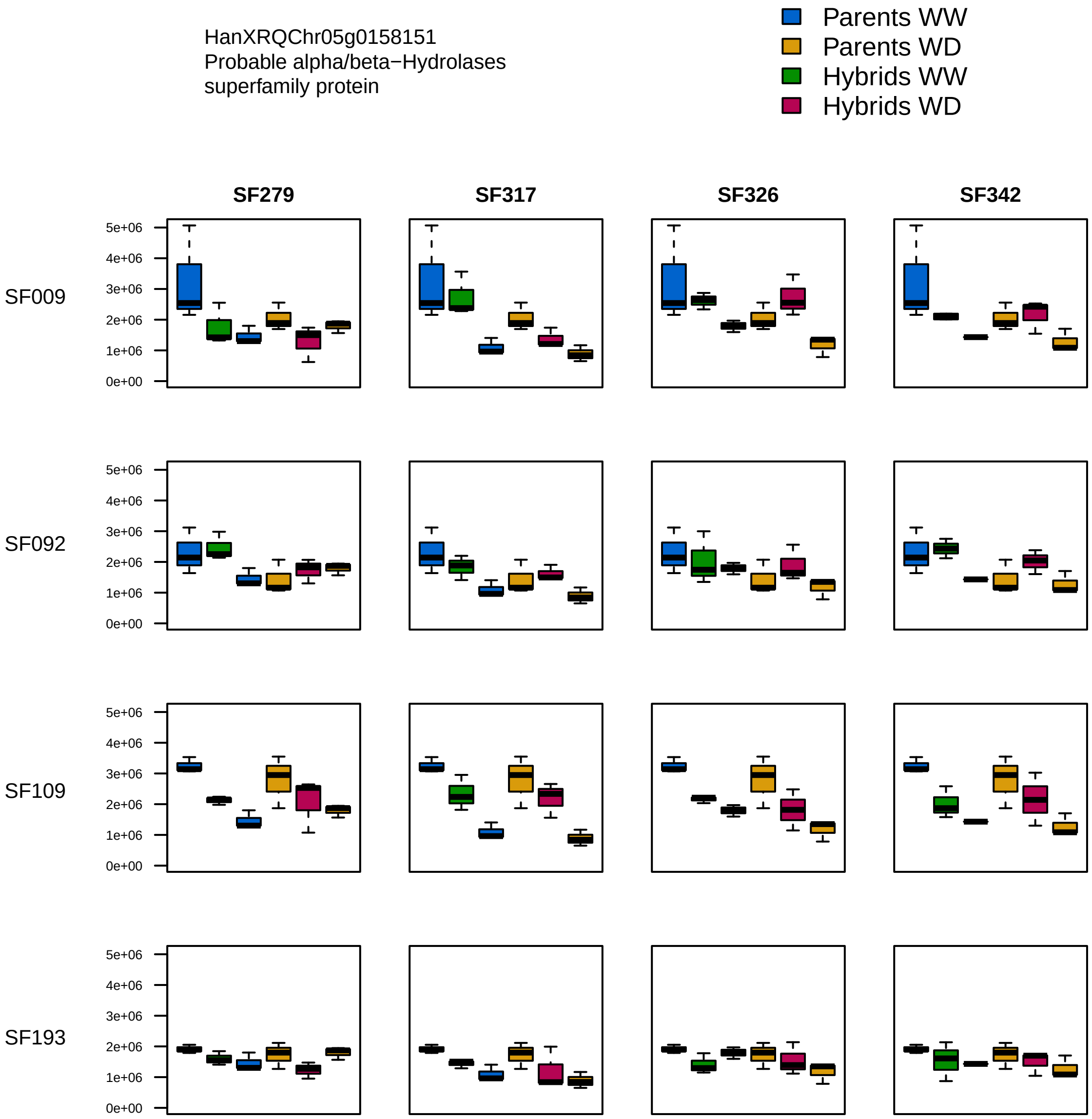
HanXRQChr05g0158151
Probable alpha/beta−Hydrolases
superfamily protein
Parents WW
Parents WD
Hybrids WW
Hybrids WD
SF279
SF317
SF326
SF342
SF009
SF092
SF109
SF193
0e+00
1e+06
2e+06
3e+06
4e+06
5e+06

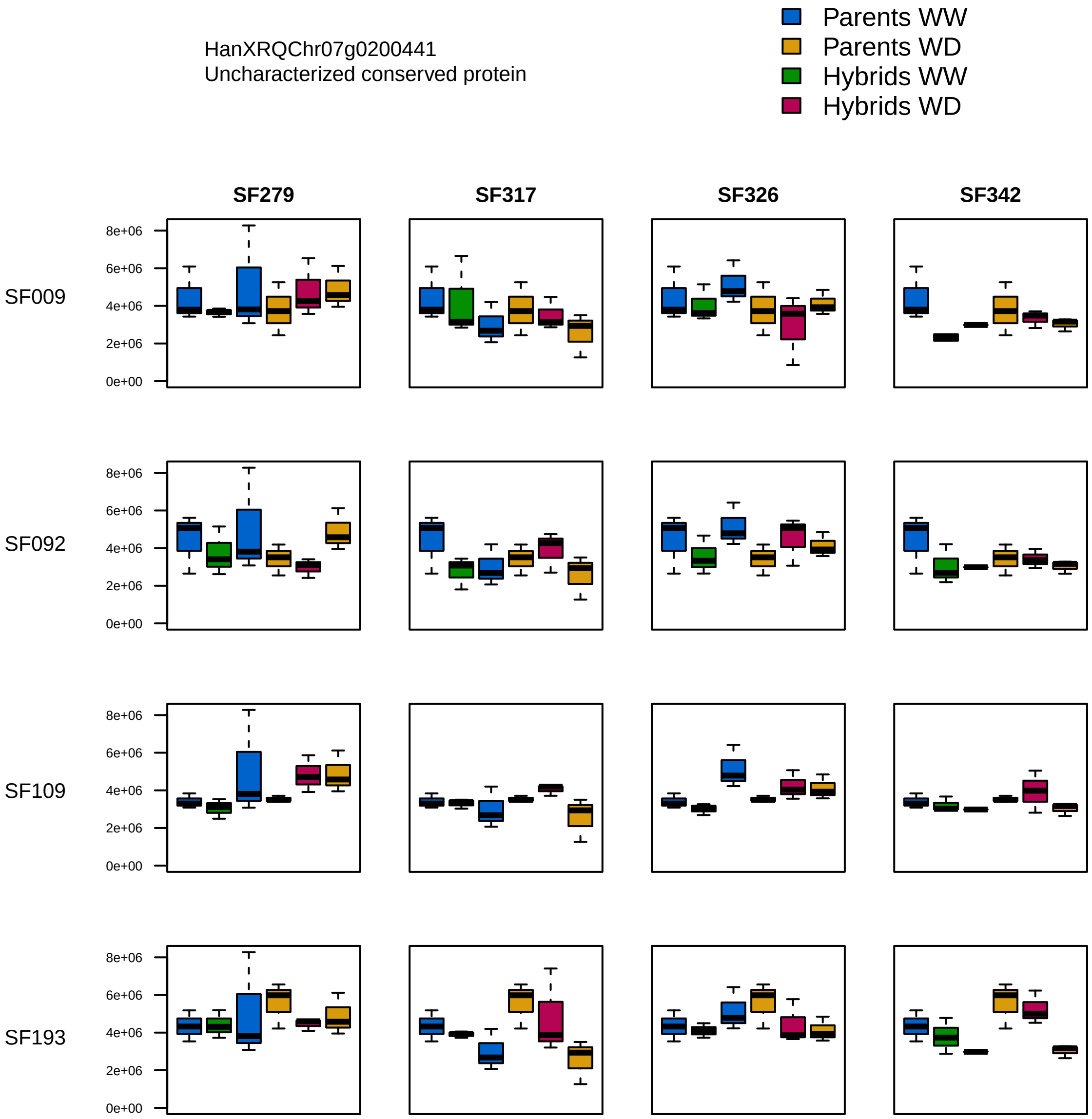

HanXRQChr07g0200441
Uncharacterized conserved protein
Parents WW
Parents WD
Hybrids WW
Hybrids WD
SF279
SF317
SF326
SF342
SF009
SF092
SF109
SF193
8e+06
6e+06
4e+06
2e+06
0e+00

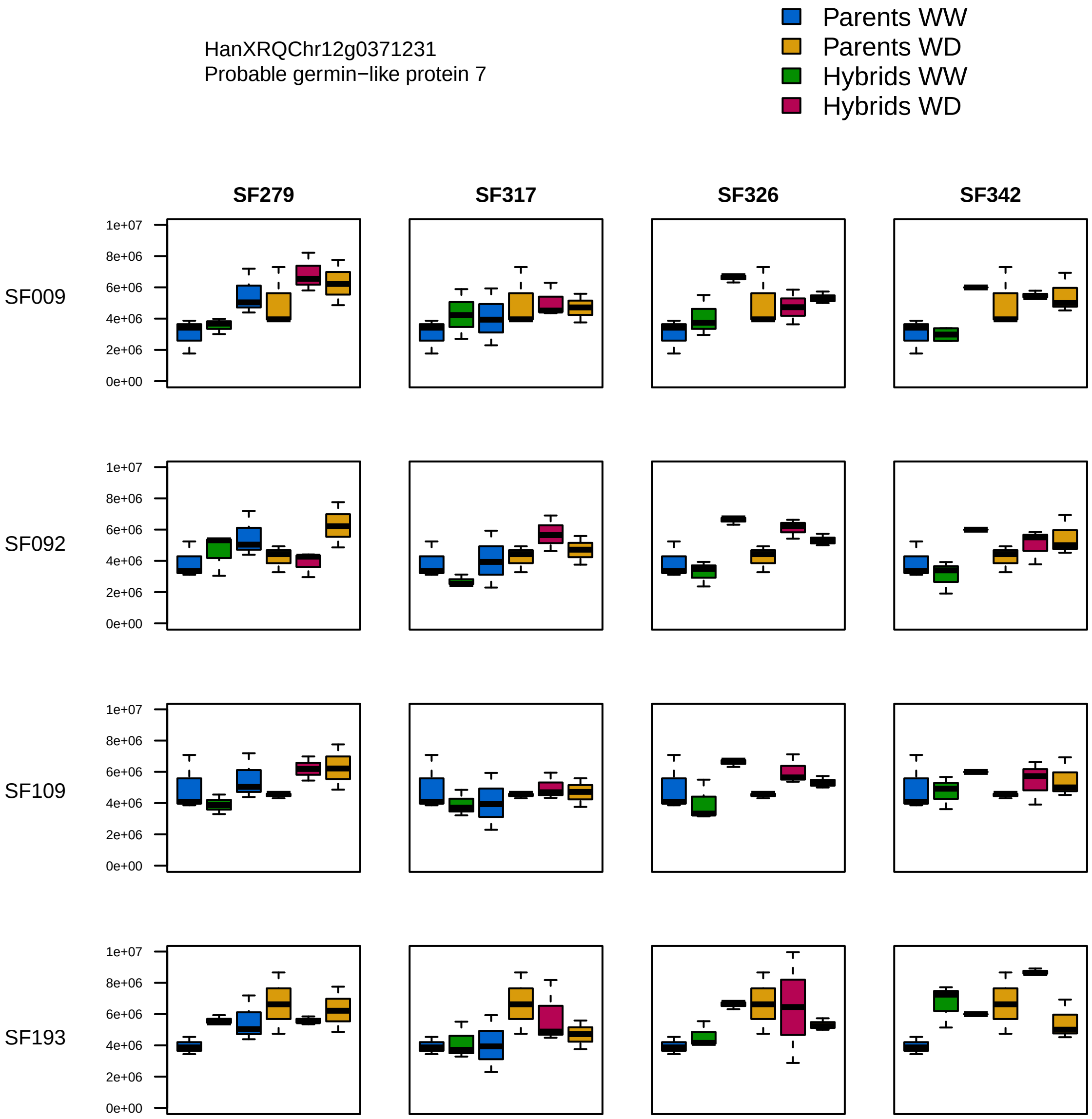

HanXRQChr12g0371231
Probable germin−like protein 7
Parents WW
Parents WD
Hybrids WW
Hybrids WD
SF279
SF317
SF326
SF342
SF009
SF092
SF109
SF193
0e+00
2e+06
4e+06
6e+06
8e+06
1e+07

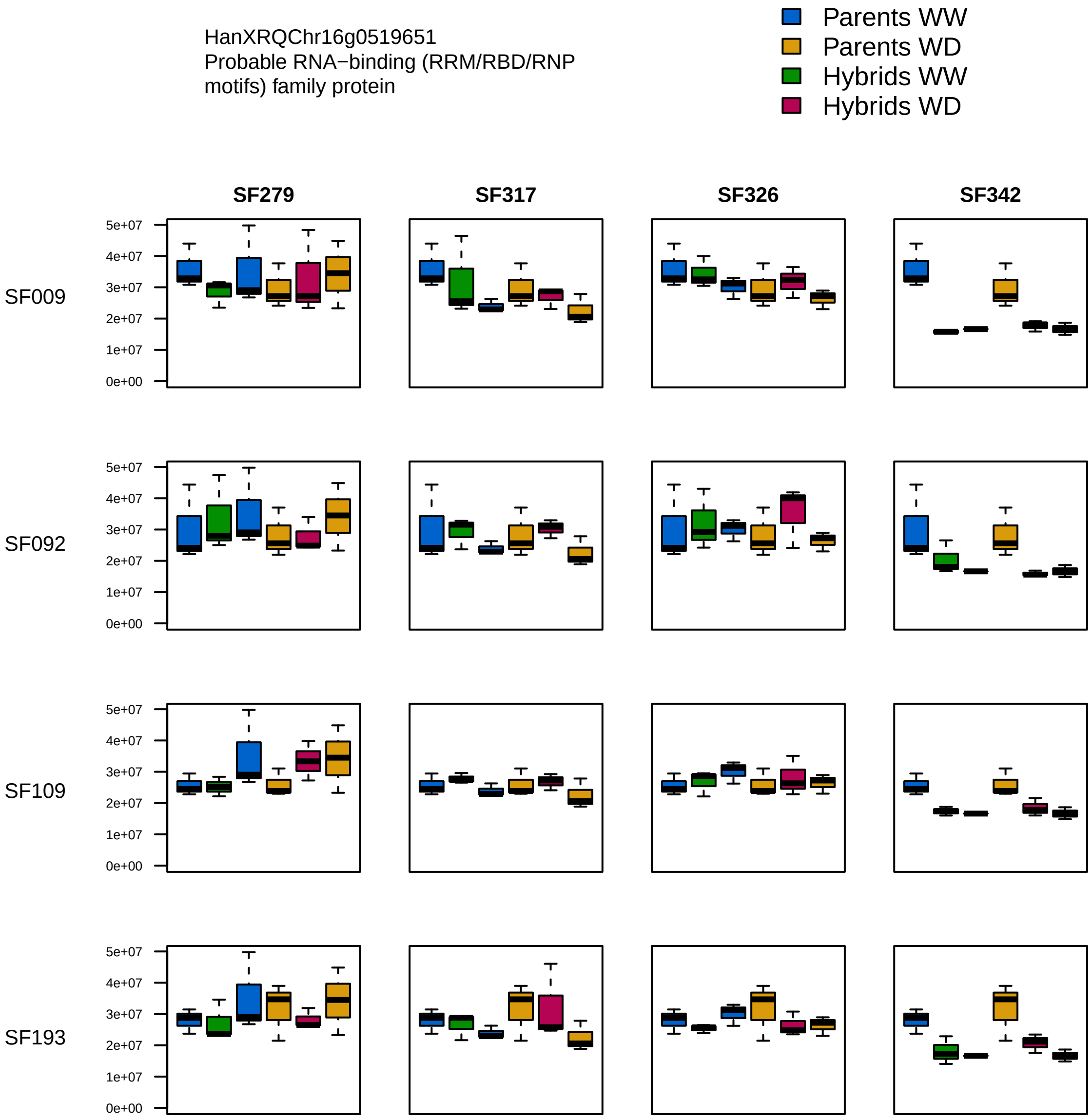

HanXRQChr16g0519651
Probable RNA−binding (RRM/RBD/RNP motifs) family protein
Parents WW
Parents WD
Hybrids WW
Hybrids WD
SF279
SF317
SF326
SF342
SF009
SF092
SF109
SF193
0e+00
1e+07
2e+07
3e+07
4e+07
5e+07

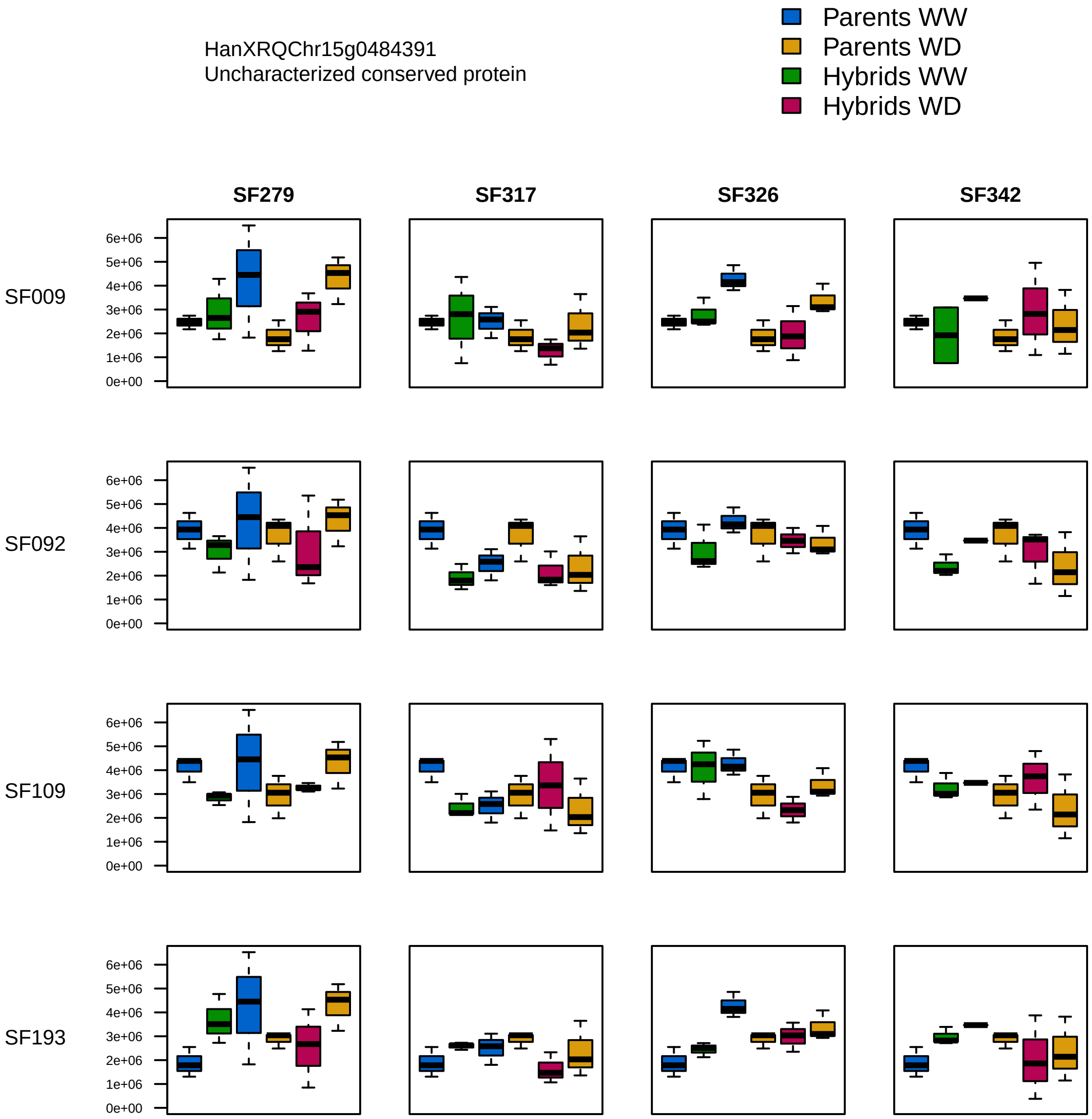
HanXRQChr15g0484391
Uncharacterized conserved protein
Parents WW
Parents WD
Hybrids WW
Hybrids WD
SF279
SF317
SF326
SF342
SF009
SF092
SF109
SF193
0e+00
1e+06
2e+06
3e+06
4e+06
5e+06
6e+06

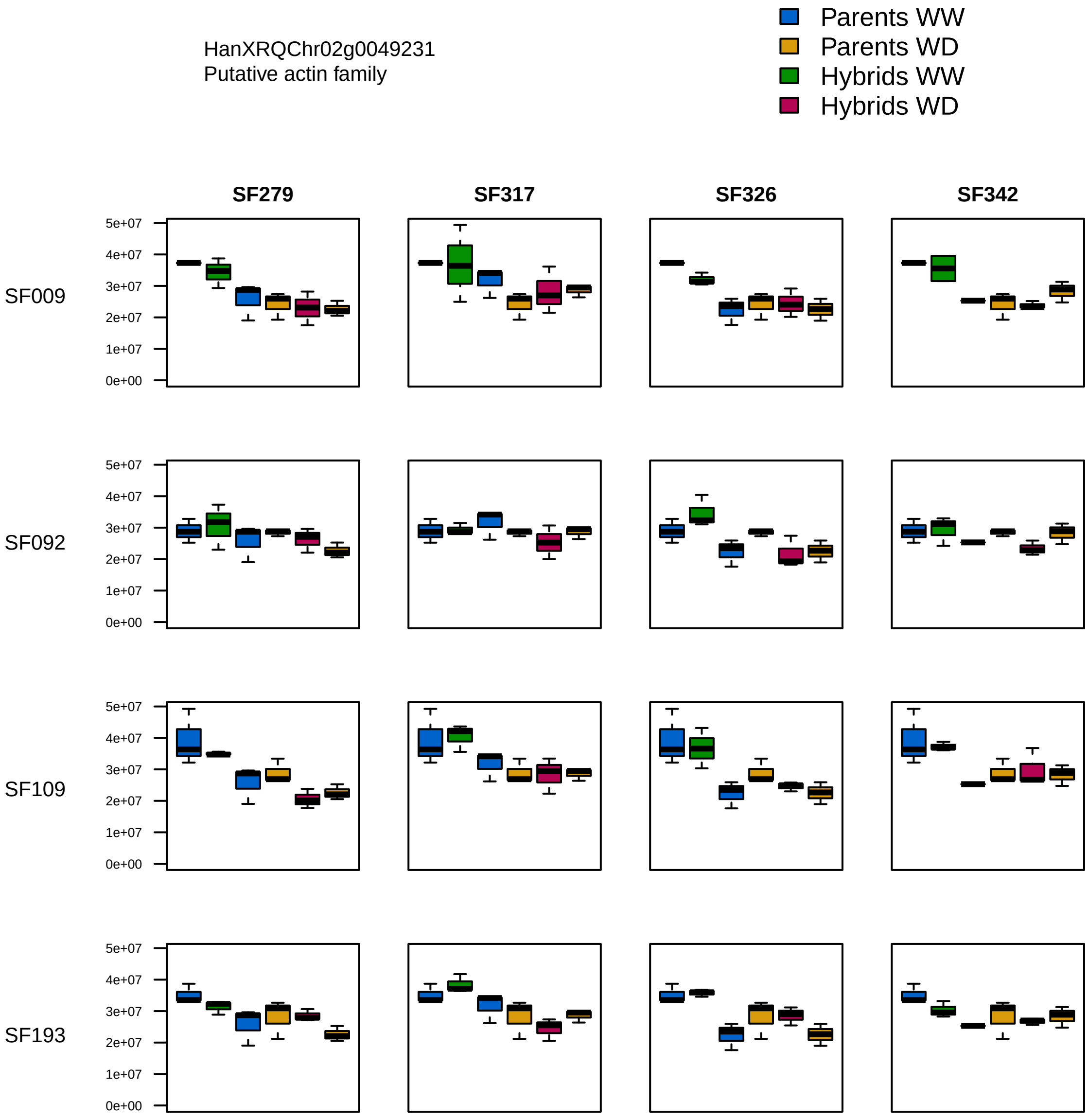
HanXRQChr02g0049231
Putative actin family
Parents WW
Parents WD
Hybrids WW
Hybrids WD
SF279
SF317
SF326
SF342
SF009
SF092
SF109
SF193
5e+07
4e+07
3e+07
2e+07
1e+07
0e+00

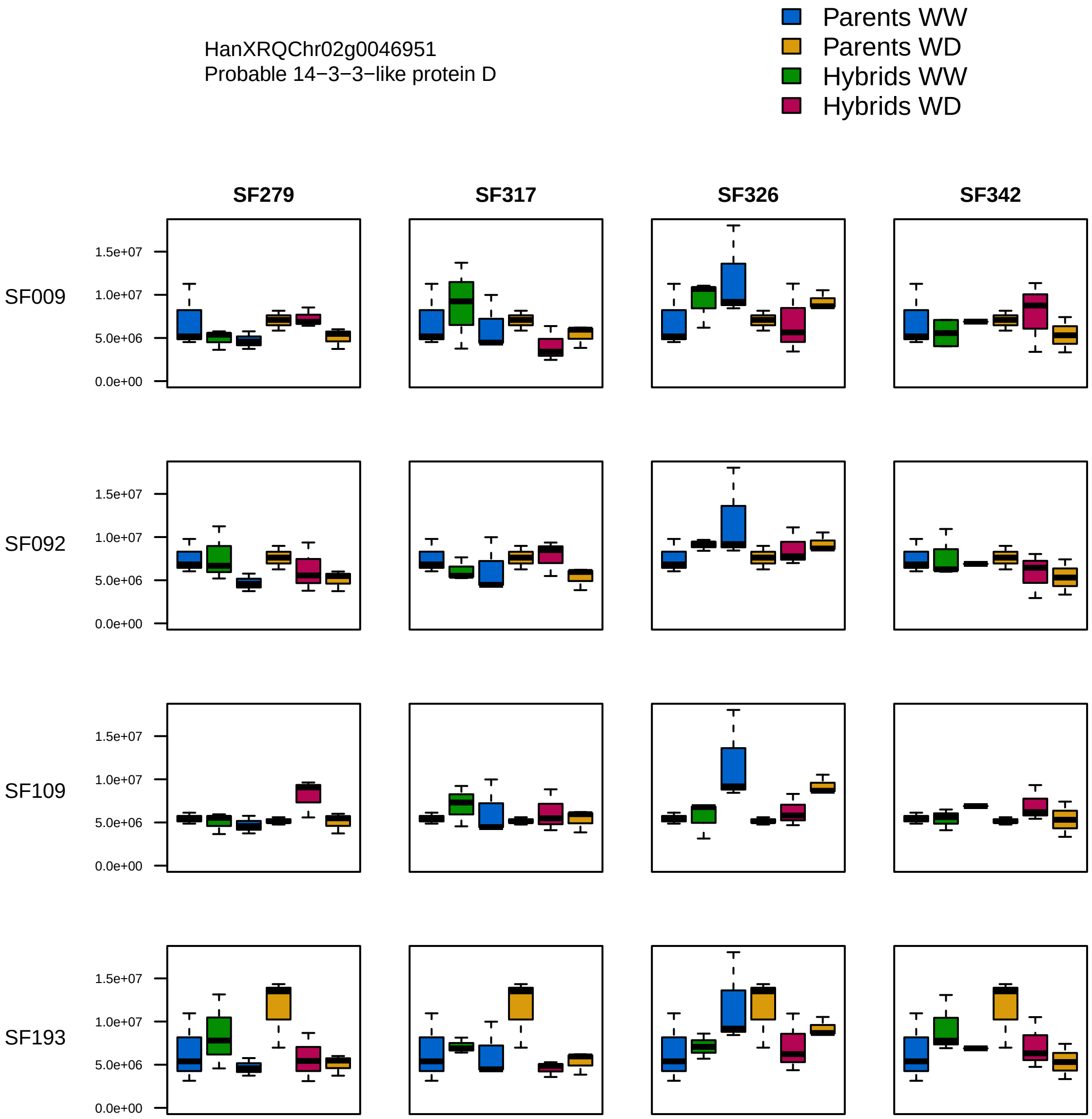

HanXRQChr02g0046951
Probable 14−3−3−like protein D
Parents WW
Parents WD
Hybrids WW
Hybrids WD
SF279
SF317
SF326
SF342
SF009
SF092
SF109
SF193
1.5e+07
1.0e+07
5.0e+06
0.0e+00

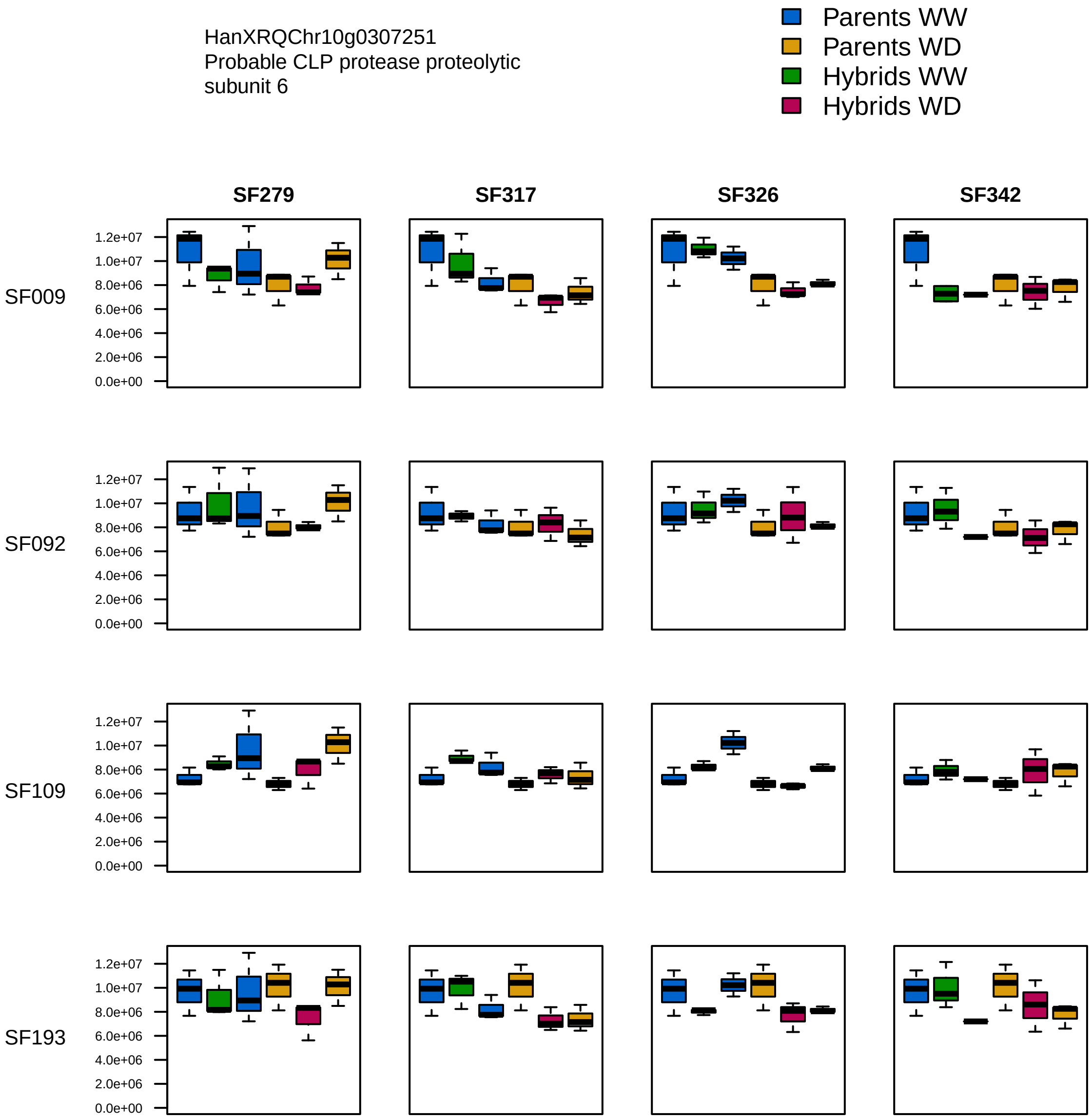

HanXRQChr10g0307251
Probable CLP protease proteolytic subunit 6
Parents WW
Parents WD
Hybrids WW
Hybrids WD
SF279
SF317
SF326
SF342
SF009
SF092
SF109
SF193
1.2e+07
1.0e+07
8.0e+06
6.0e+06
4.0e+06
2.0e+06
0.0e+00

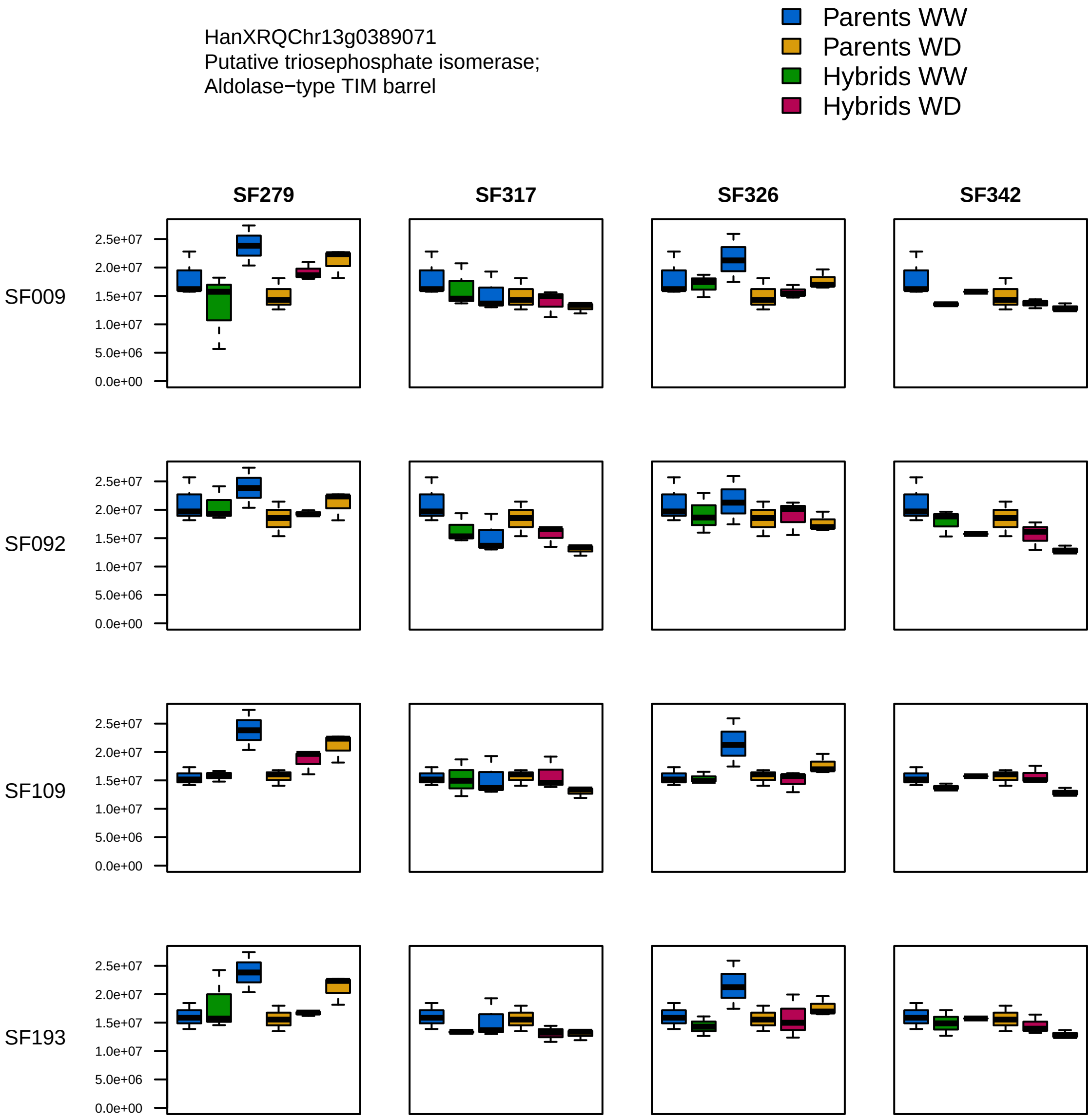
HanXRQChr13g0389071
Putative triosephosphate isomerase;
Aldolase−type TIM barrel
Parents WW
Parents WD
Hybrids WW
Hybrids WD
SF279
SF317
SF326
SF342
SF009
SF092
SF109
SF193
2.5e+07
2.0e+07
1.5e+07
1.0e+07
5.0e+06
0.0e+00

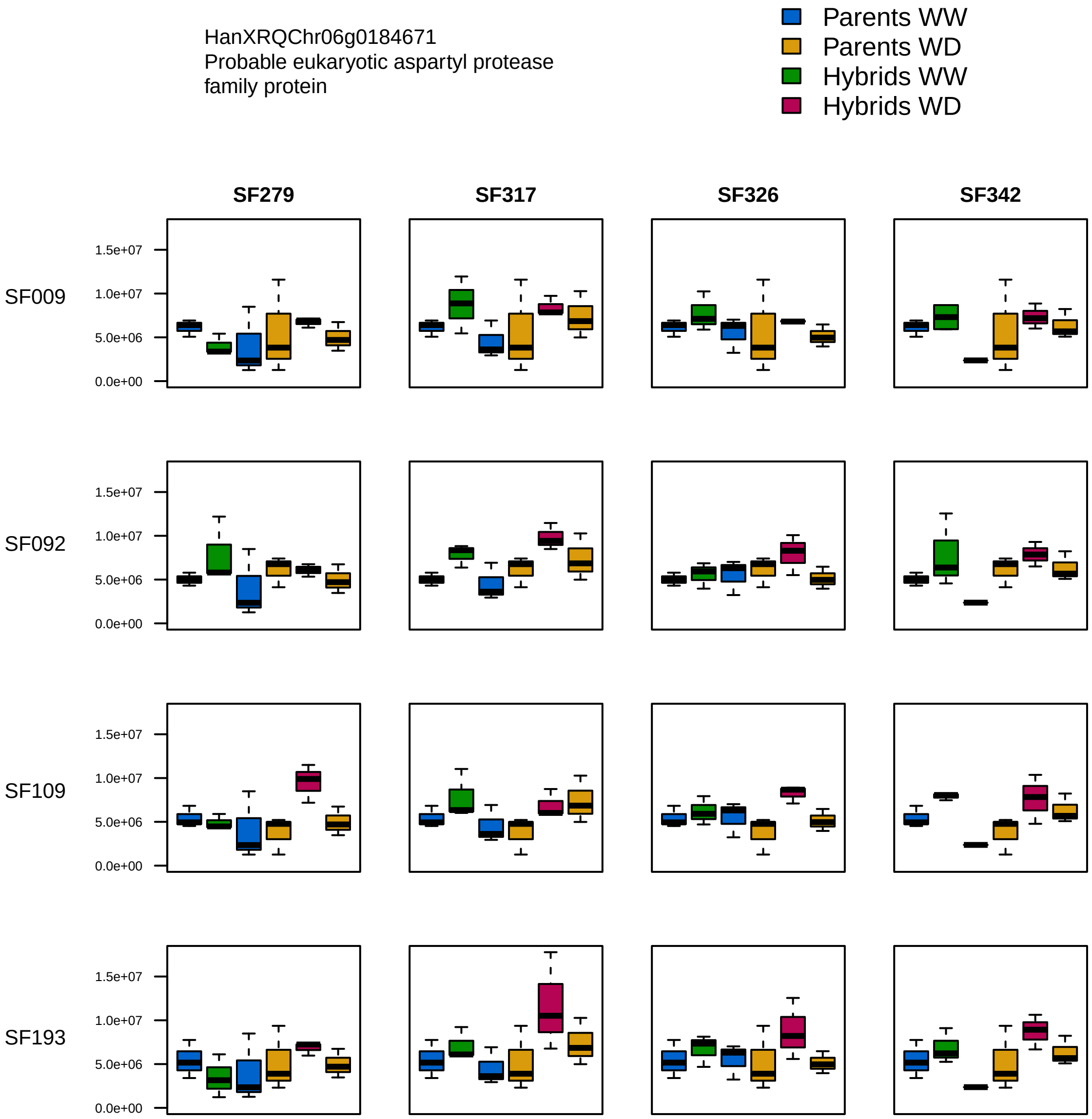
HanXRQChr06g0184671
Probable eukaryotic aspartyl protease family protein
Parents WW
Parents WD
Hybrids WW
Hybrids WD
SF279
SF317
SF326
SF342
SF009
SF092
SF109
SF193
1.5e+07
1.0e+07
5.0e+06
0.0e+00

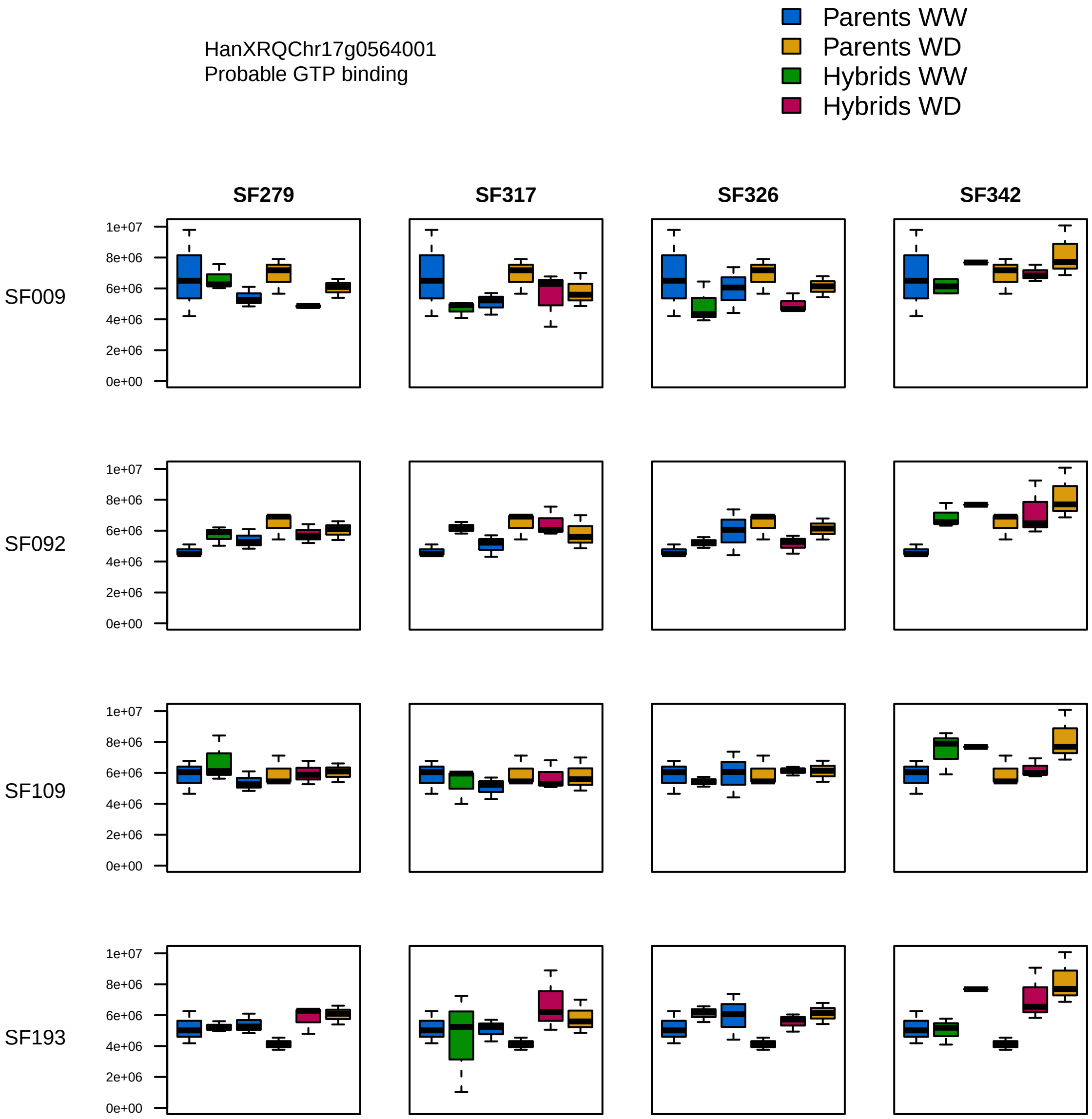
HanXRQChr17g0564001
Probable GTP binding
Parents WW
Parents WD
Hybrids WW
Hybrids WD
SF279
SF317
SF326
SF342
SF009
SF092
SF109
SF193
1e+07
8e+06
6e+06
4e+06
2e+06
0e+00

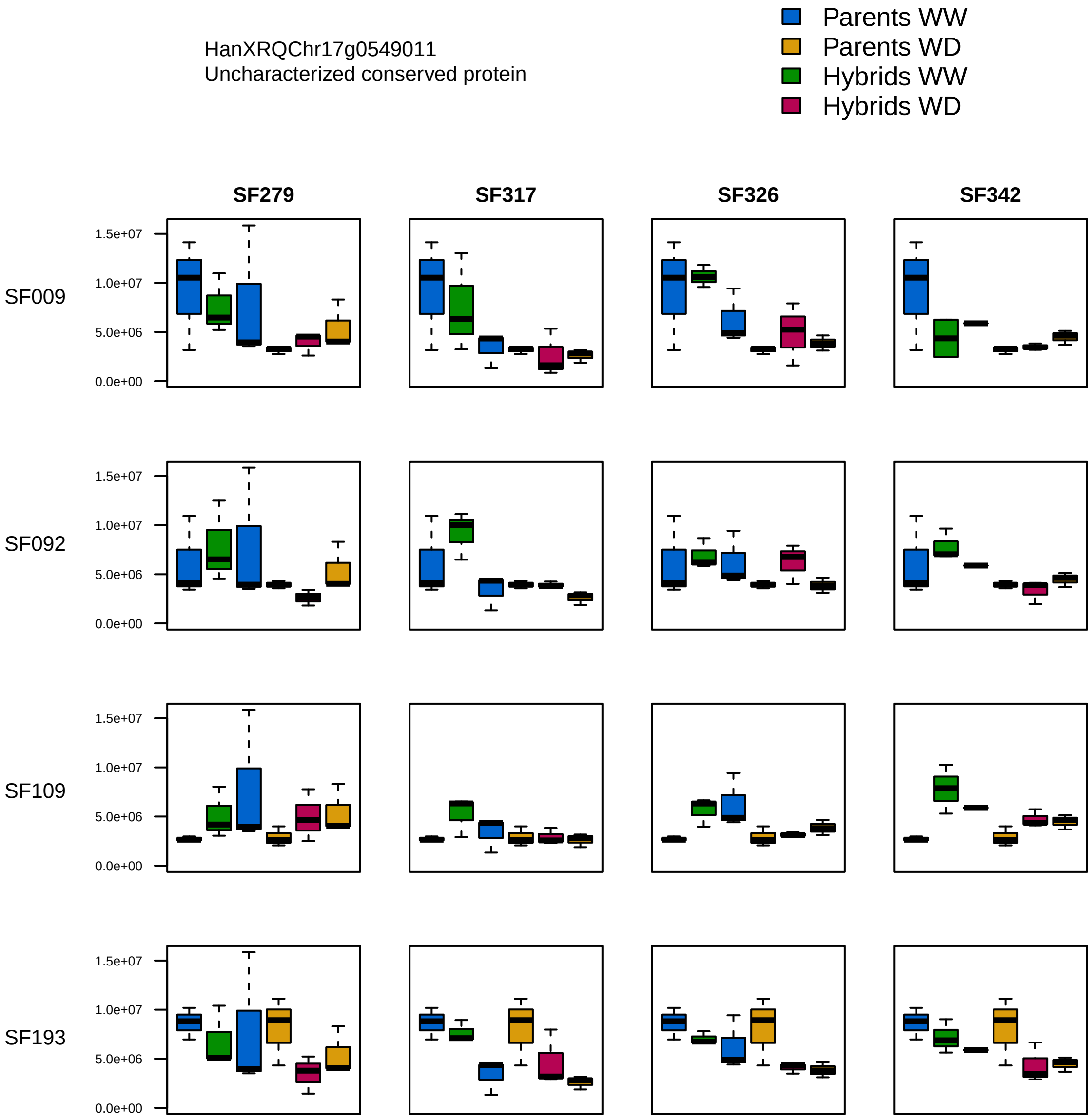
HanXRQChr17g0549011
Uncharacterized conserved protein
Parents WW
Parents WD
Hybrids WW
Hybrids WD
SF279
SF317
SF326
SF342
SF009
SF092
SF109
SF193
1.5e+07
1.0e+07
5.0e+06
0.0e+00

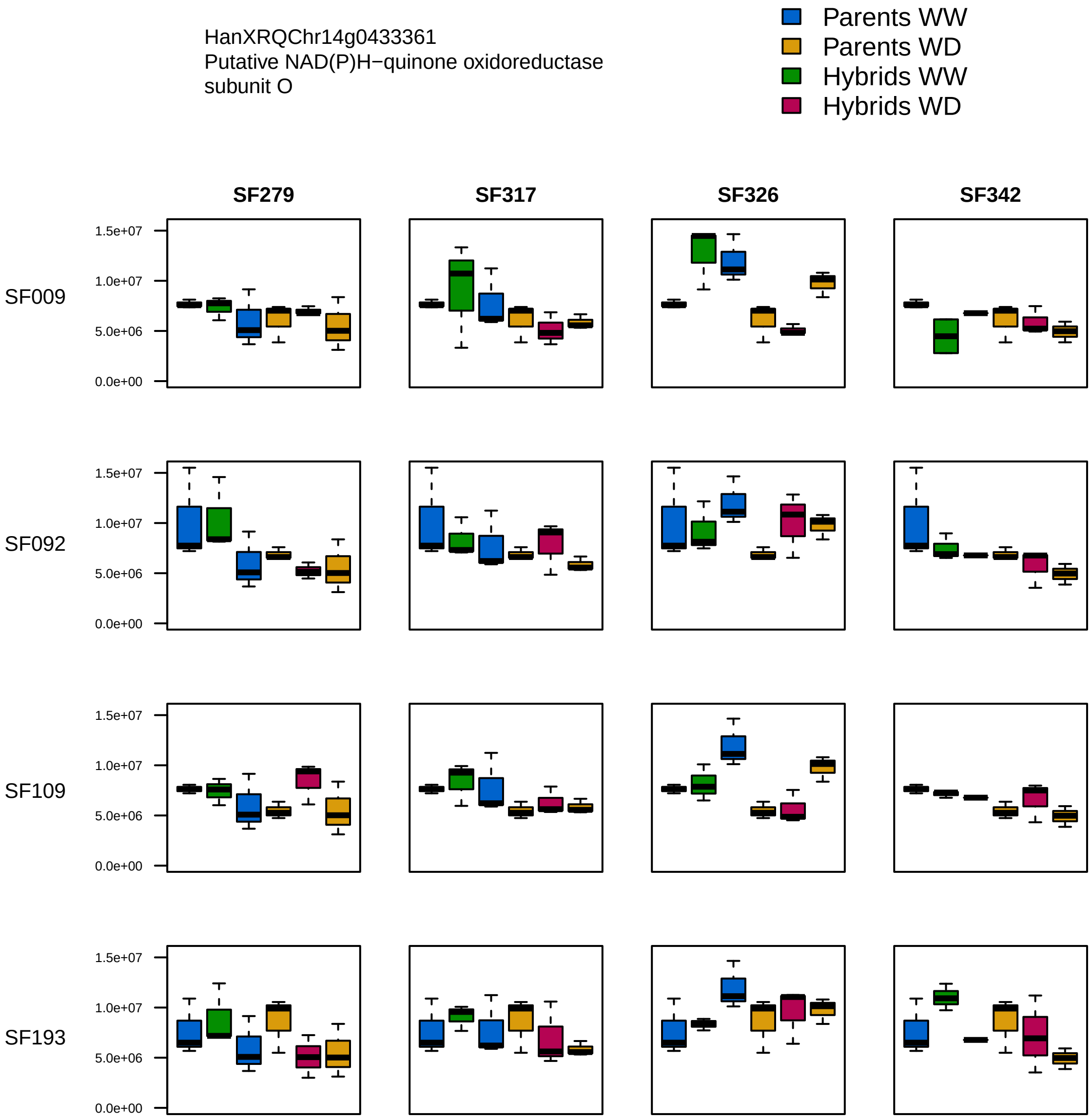
HanXRQChr14g0433361
Putative NAD(P)H−quinone oxidoreductase
subunit O
Parents WW
Parents WD
Hybrids WW
Hybrids WD
SF279
SF317
SF326
SF342
SF009
SF092
SF109
SF193
1.5e+07
1.0e+07
5.0e+06
0.0e+00

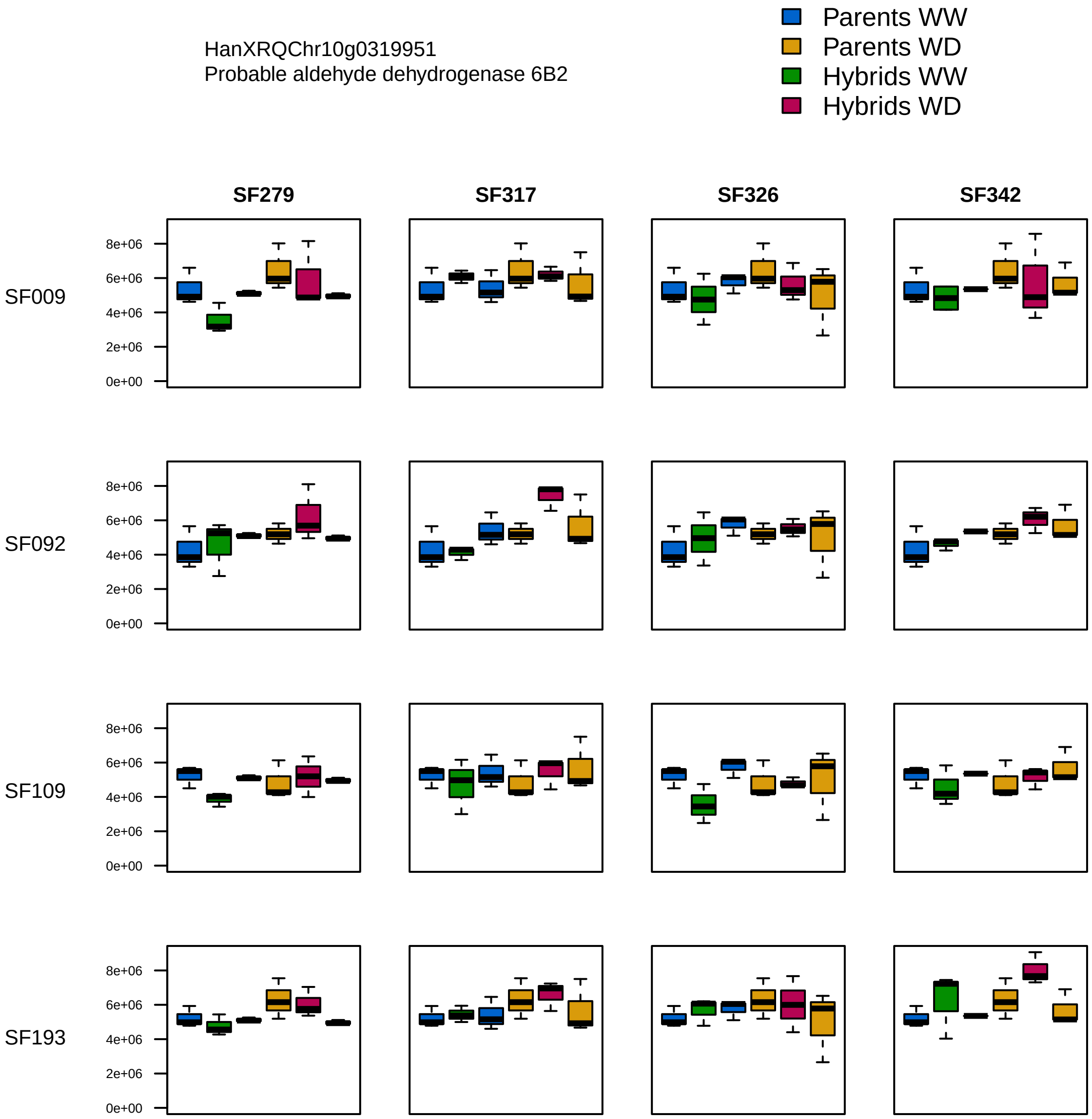
HanXRQChr10g0319951
Probable aldehyde dehydrogenase 6B2
Parents WW
Parents WD
Hybrids WW
Hybrids WD
SF279
SF317
SF326
SF342
SF009
SF092
SF109
SF193
8e+06
6e+06
4e+06
2e+06
0e+00

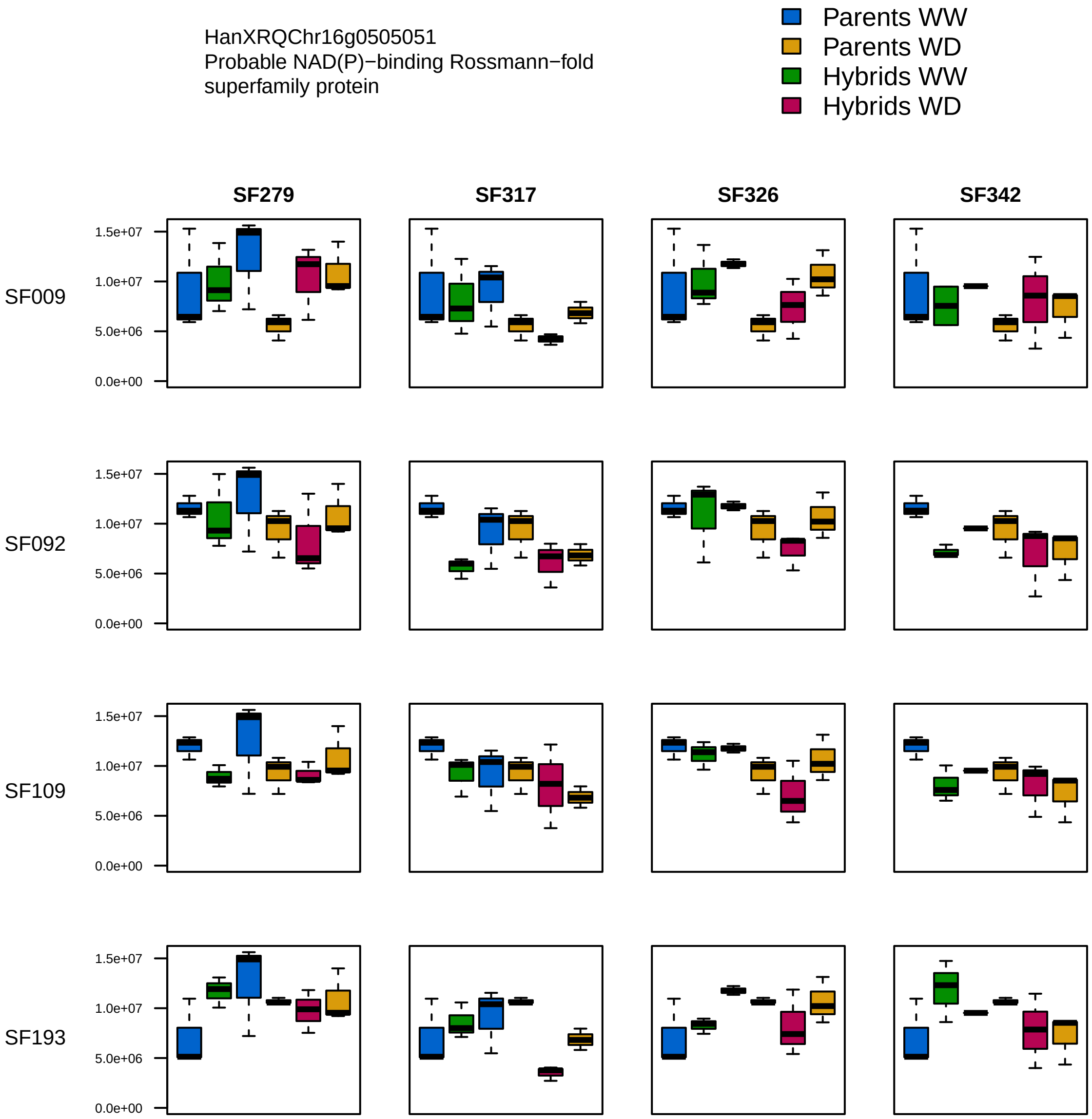
HanXRQChr16g0505051
Probable NAD(P)−binding Rossmann−fold
superfamily protein
Parents WW
Parents WD
Hybrids WW
Hybrids WD
SF279
SF317
SF326
SF342
SF009
SF092
SF109
SF193
1.5e+07
1.0e+07
5.0e+06
0.0e+00

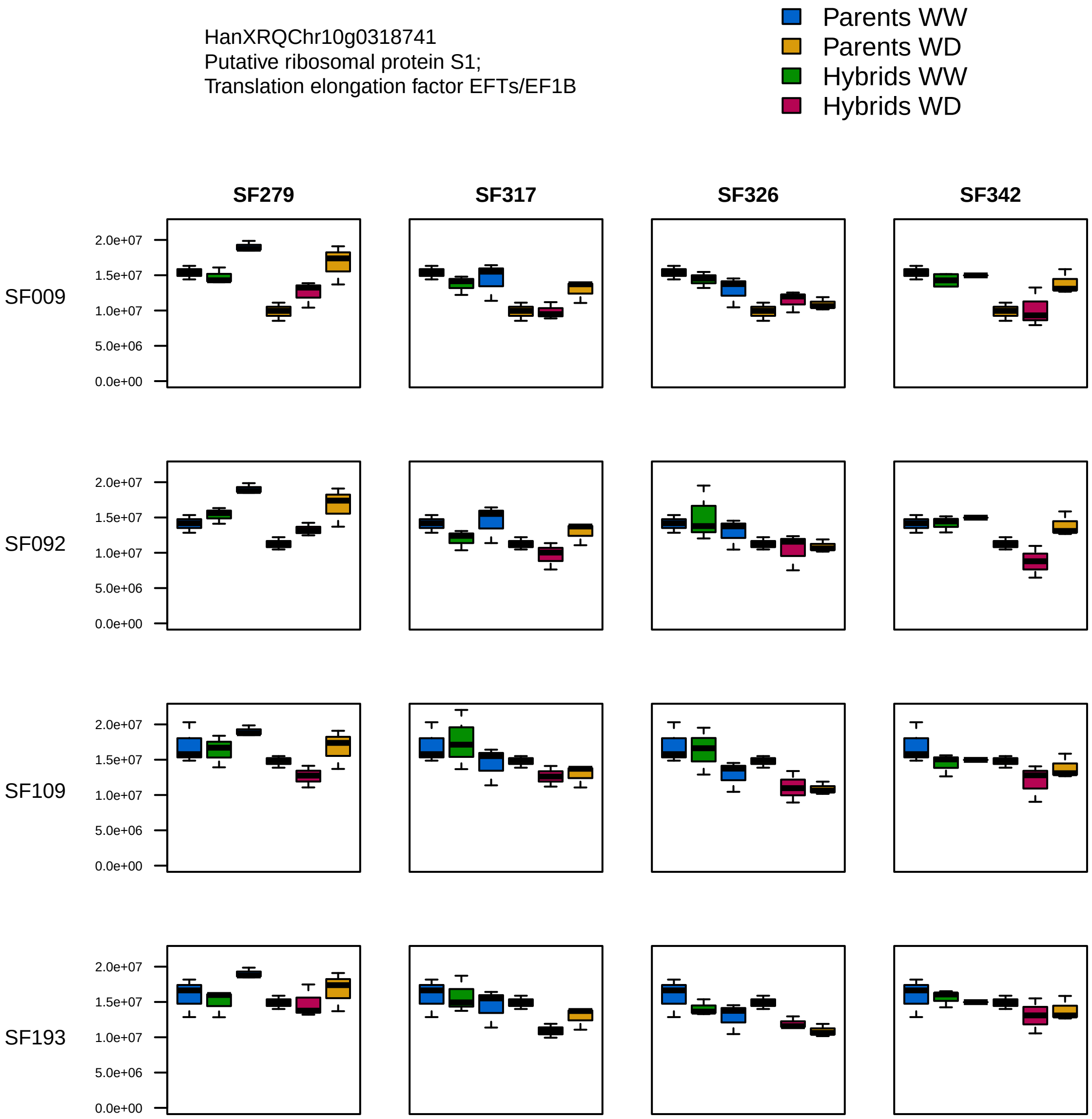
HanXRQChr10g0318741
Putative ribosomal protein S1;
Translation elongation factor EFTs/EF1B
Parents WW
Parents WD
Hybrids WW
Hybrids WD
SF279
SF317
SF326
SF342
SF009
SF092
SF109
SF193
2.0e+07
1.5e+07
1.0e+07
5.0e+06
0.0e+00

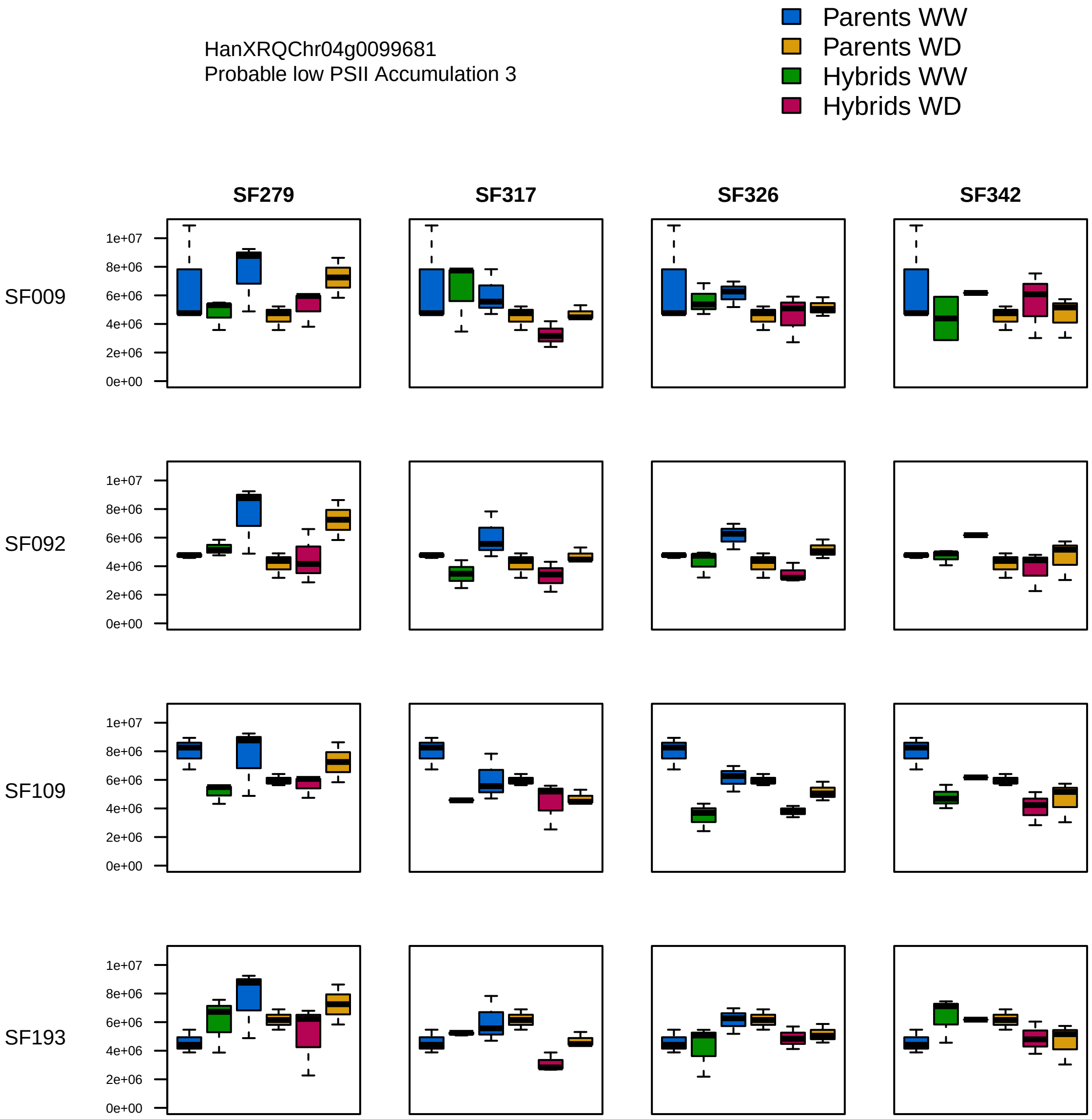

HanXRQChr04g0099681
Probable low PSII Accumulation 3
Parents WW
Parents WD
Hybrids WW
Hybrids WD
SF279
SF317
SF326
SF342
SF009
SF092
SF109
SF193
1e+07
8e+06
6e+06
4e+06
2e+06
0e+00

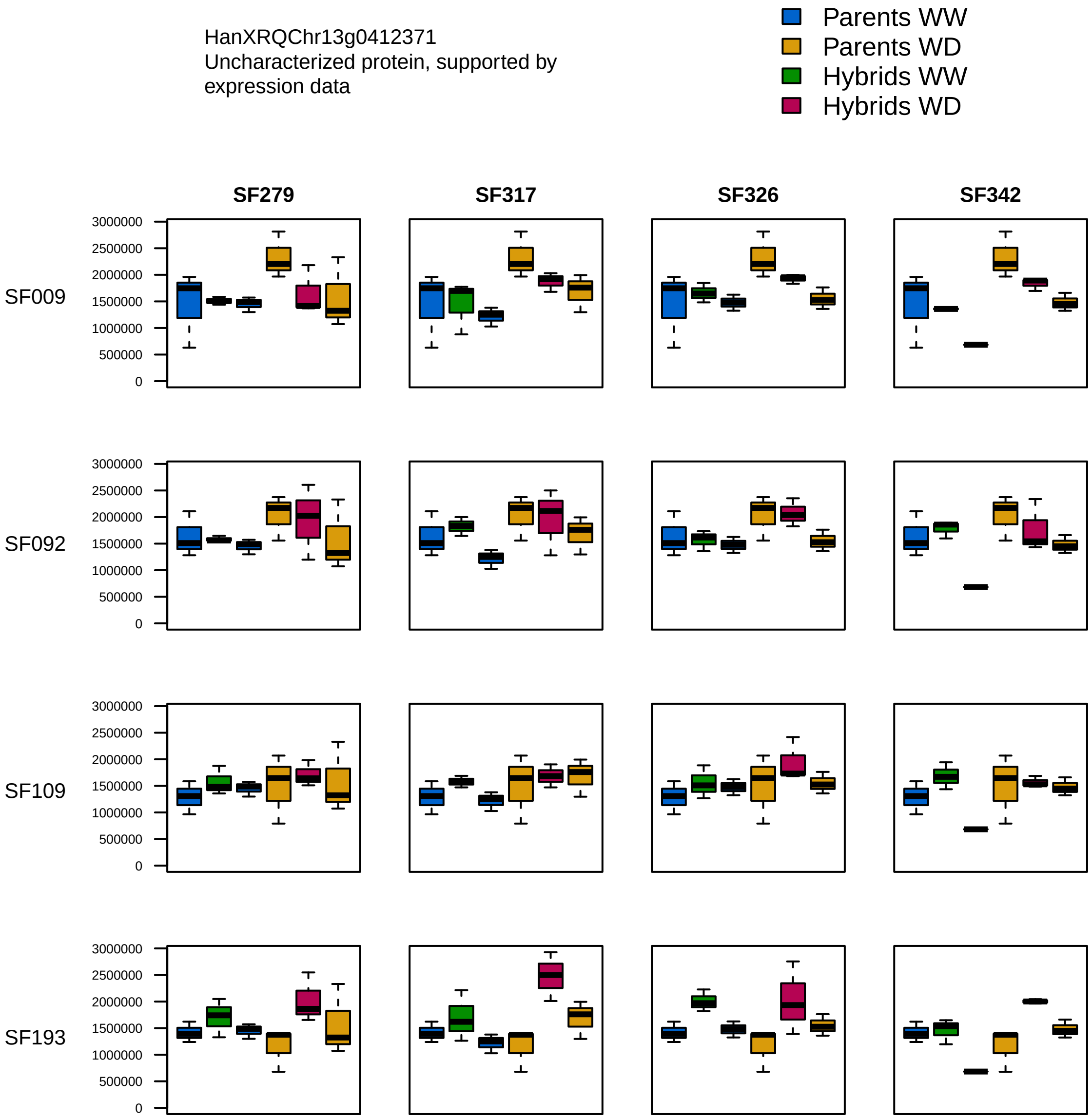
HanXRQChr13g0412371
Uncharacterized protein, supported by expression data
Parents WW
Parents WD
Hybrids WW
Hybrids WD
SF279
SF317
SF326
SF342
SF009
SF092
SF109
SF193
3000000
2500000
2000000
1500000
1000000
500000
0

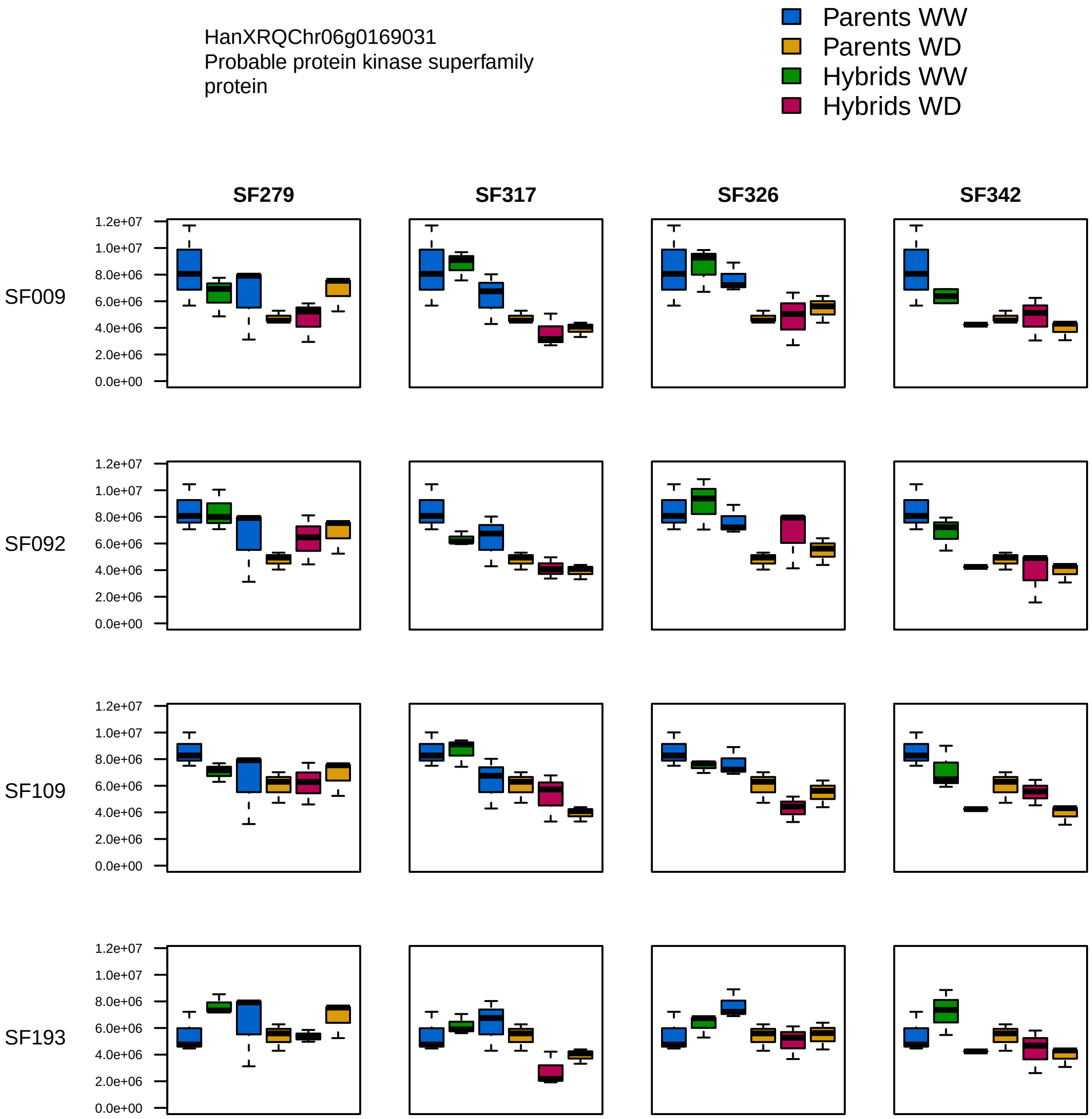

HanXRQChr06g0169031
Probable protein kinase superfamily protein
Parents WW
Parents WD
Hybrids WW
Hybrids WD
SF279
SF317
SF326
SF342
SF009
SF092
SF109
SF193
1.2e+07
1.0e+07
8.0e+06
6.0e+06
4.0e+06
2.0e+06
0.0e+00

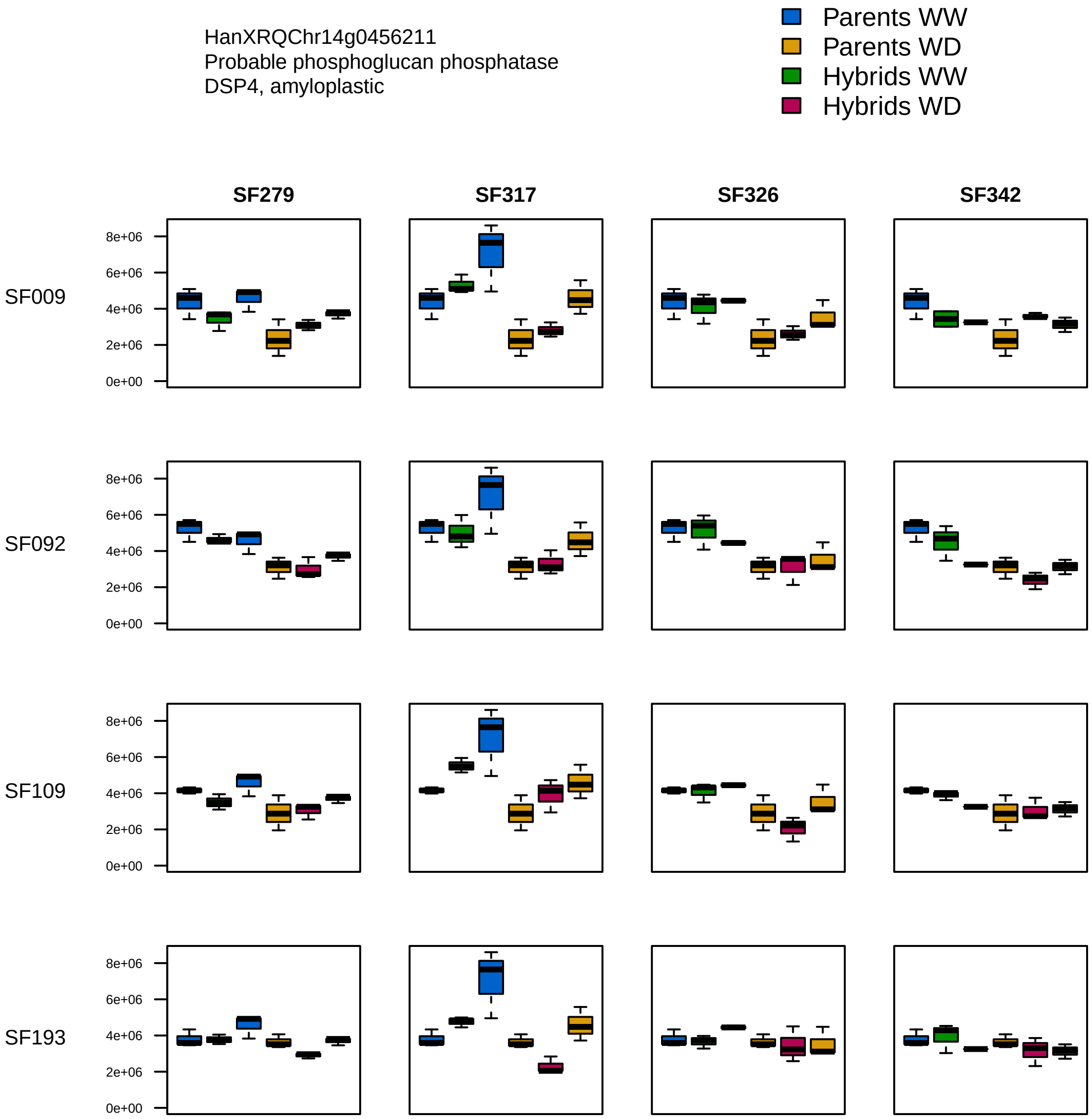
HanXRQChr14g0456211
Probable phosphoglucan phosphatase
DSP4, amyloplastic
Parents WW
Parents WD
Hybrids WW
Hybrids WD
SF279
SF317
SF326
SF342
SF009
SF092
SF109
SF193
8e+06
6e+06
4e+06
2e+06
0e+00

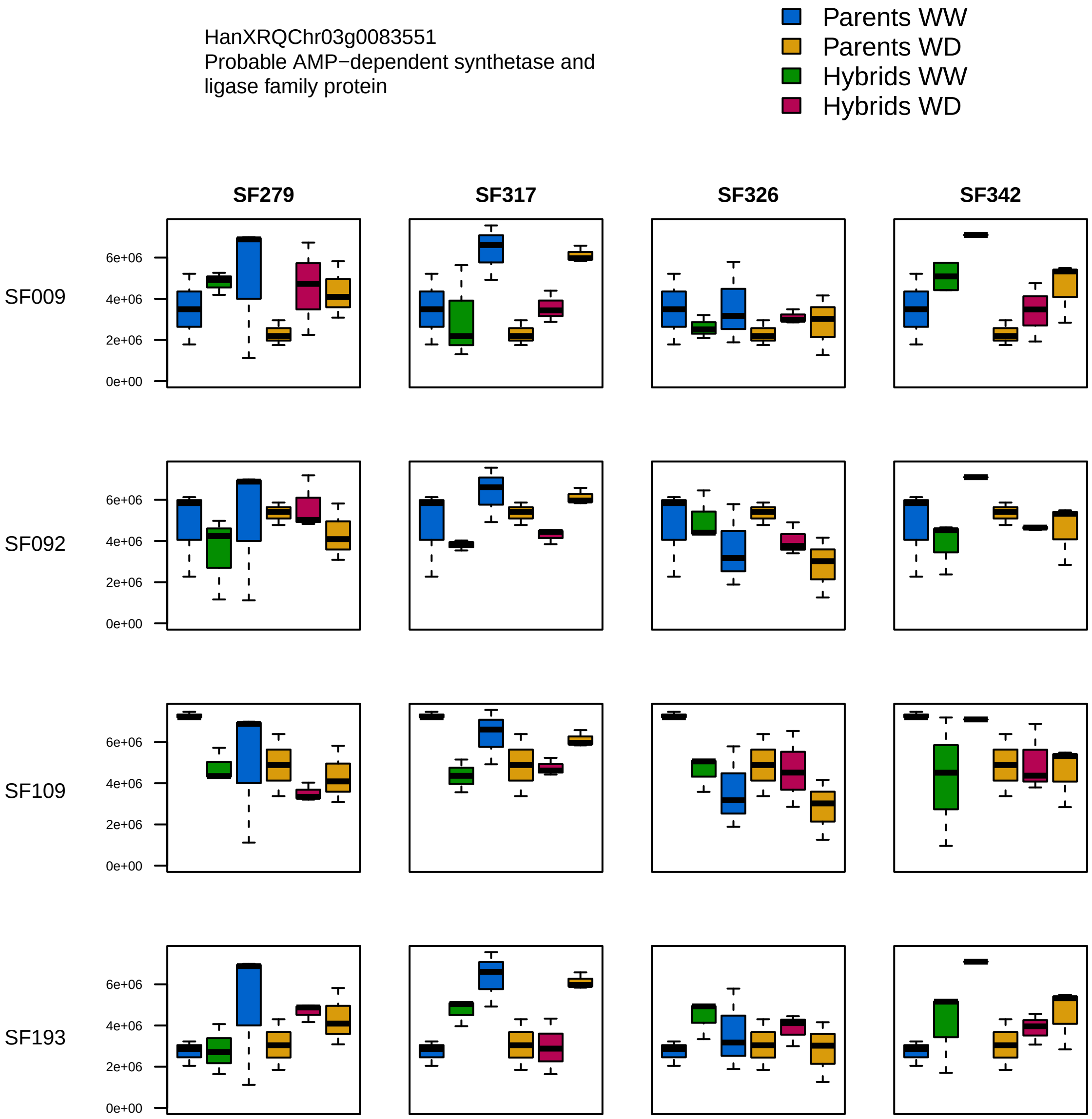

HanXRQChr03g0083551
Probable AMP−dependent synthetase and
ligase family protein
Parents WW
Parents WD
Hybrids WW
Hybrids WD
SF279
SF317
SF326
SF342
SF009
SF092
SF109
SF193
0e+00
2e+06
4e+06
6e+06

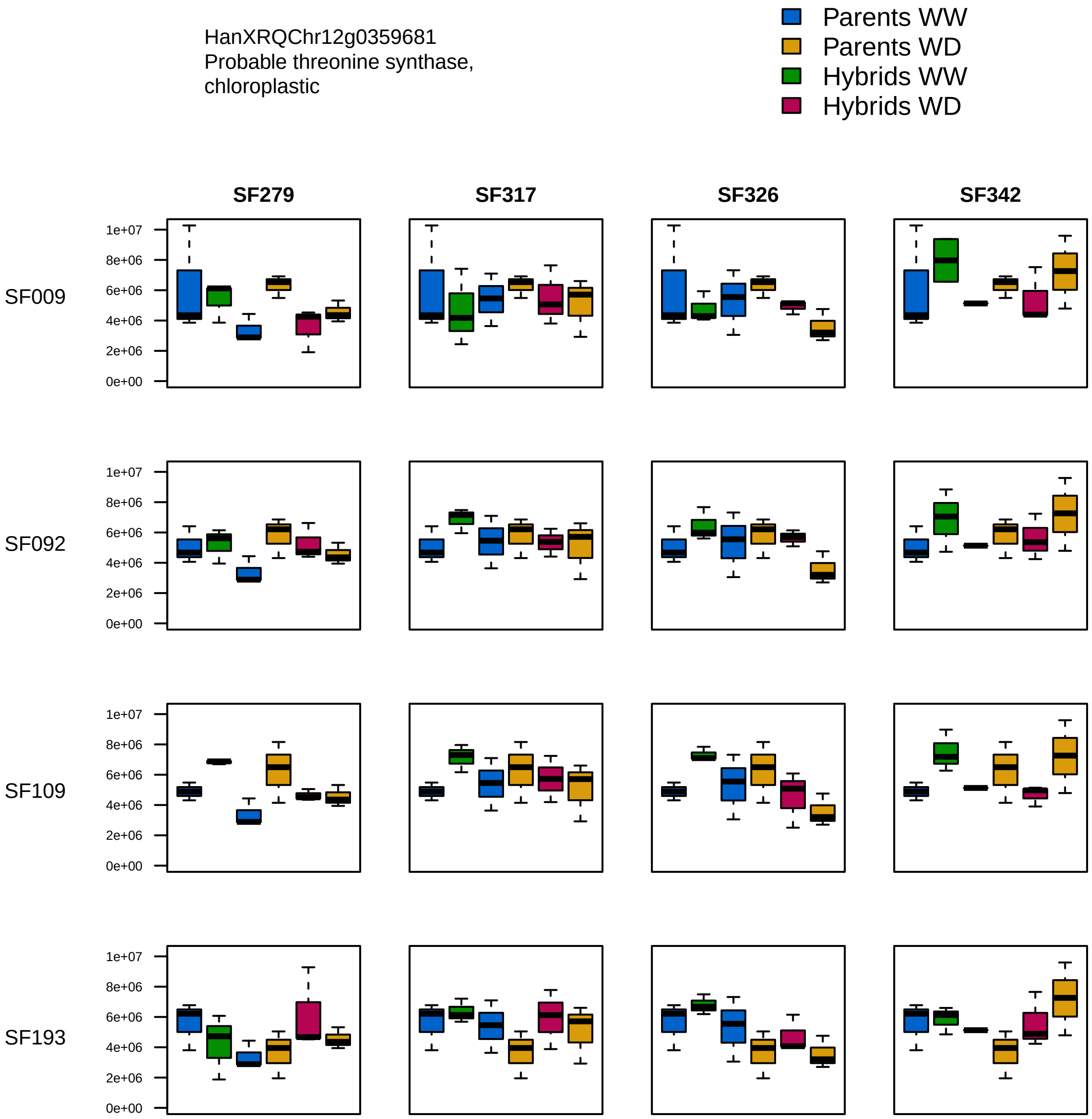
HanXRQChr12g0359681
Probable threonine synthase,
chloroplastic
Parents WW
Parents WD
Hybrids WW
Hybrids WD
SF279
SF317
SF326
SF342
SF009
SF092
SF109
SF193
1e+07
8e+06
6e+06
4e+06
2e+06
0e+00

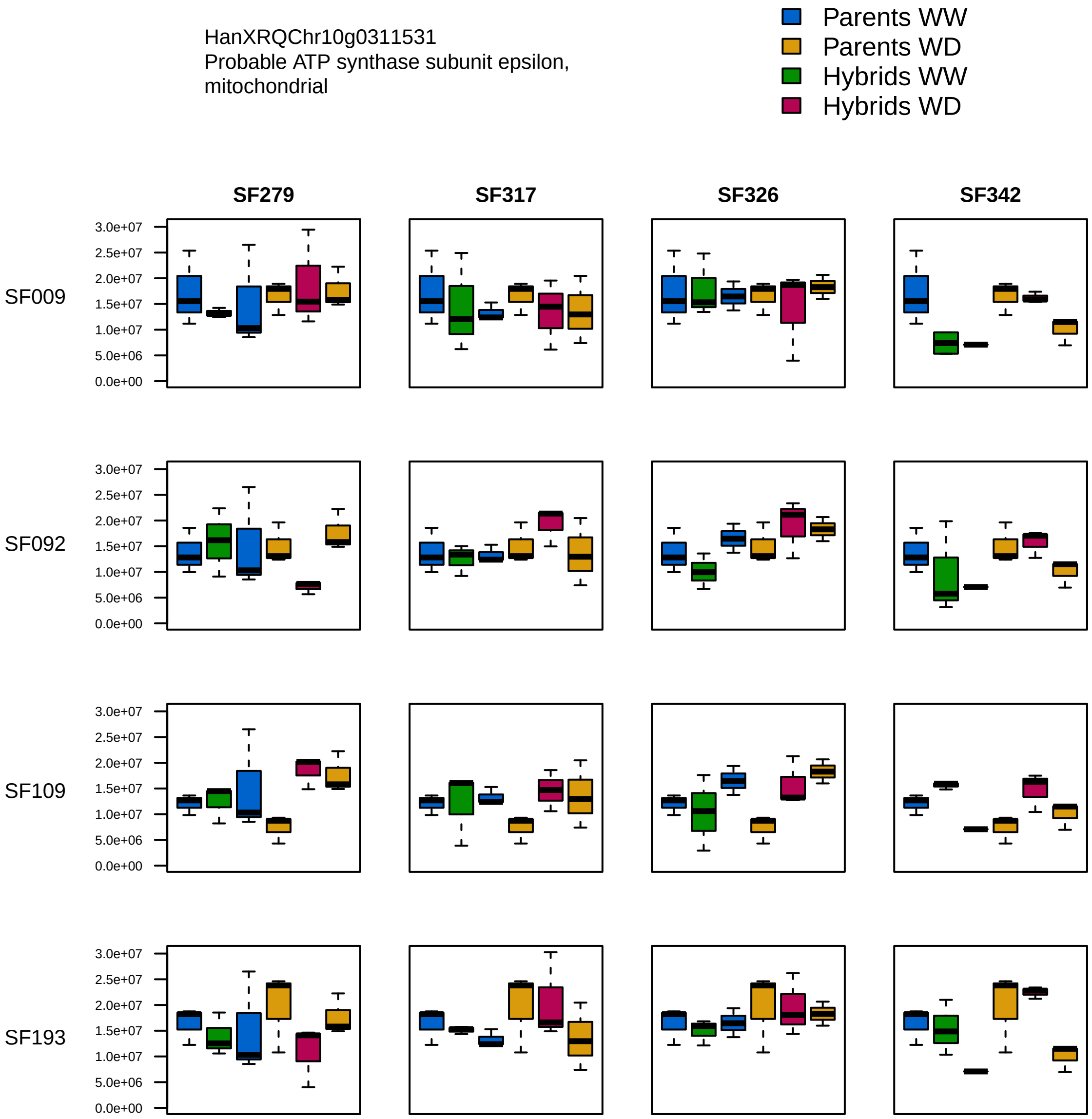
HanXRQChr10g0311531
Probable ATP synthase subunit epsilon, mitochondrial
Parents WW
Parents WD
Hybrids WW
Hybrids WD
SF279
SF317
SF326
SF342
SF009
SF092
SF109
SF193
3.0e+07
2.5e+07
2.0e+07
1.5e+07
1.0e+07
5.0e+06
0.0e+00

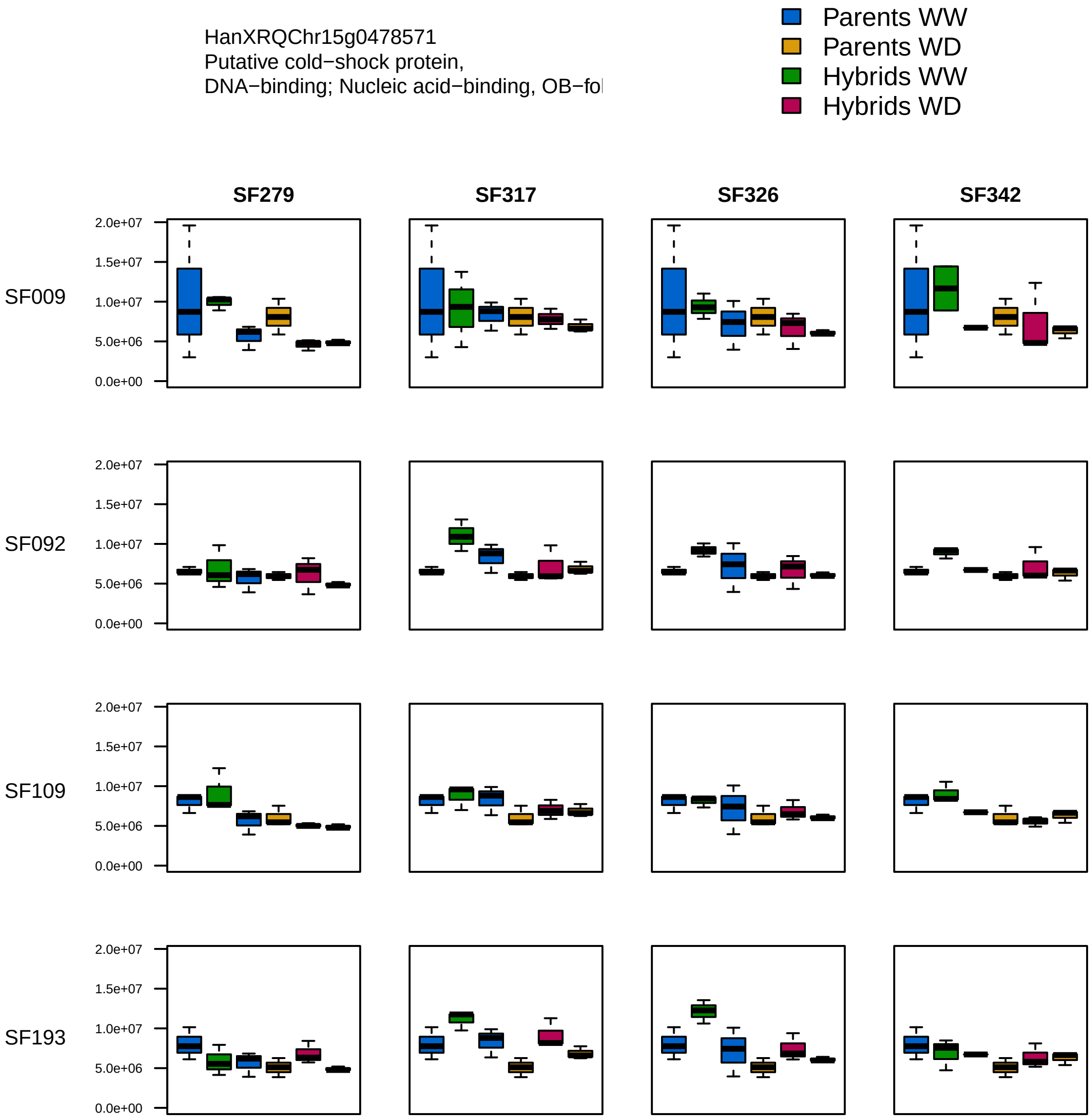
HanXRQChr15g0478571
Putative cold−shock protein,
DNA−binding; Nucleic acid−binding, OB−fol
Parents WW
Parents WD
Hybrids WW
Hybrids WD
SF279
SF317
SF326
SF342
SF009
SF092
SF109
SF193
2.0e+07
1.5e+07
1.0e+07
5.0e+06
0.0e+00

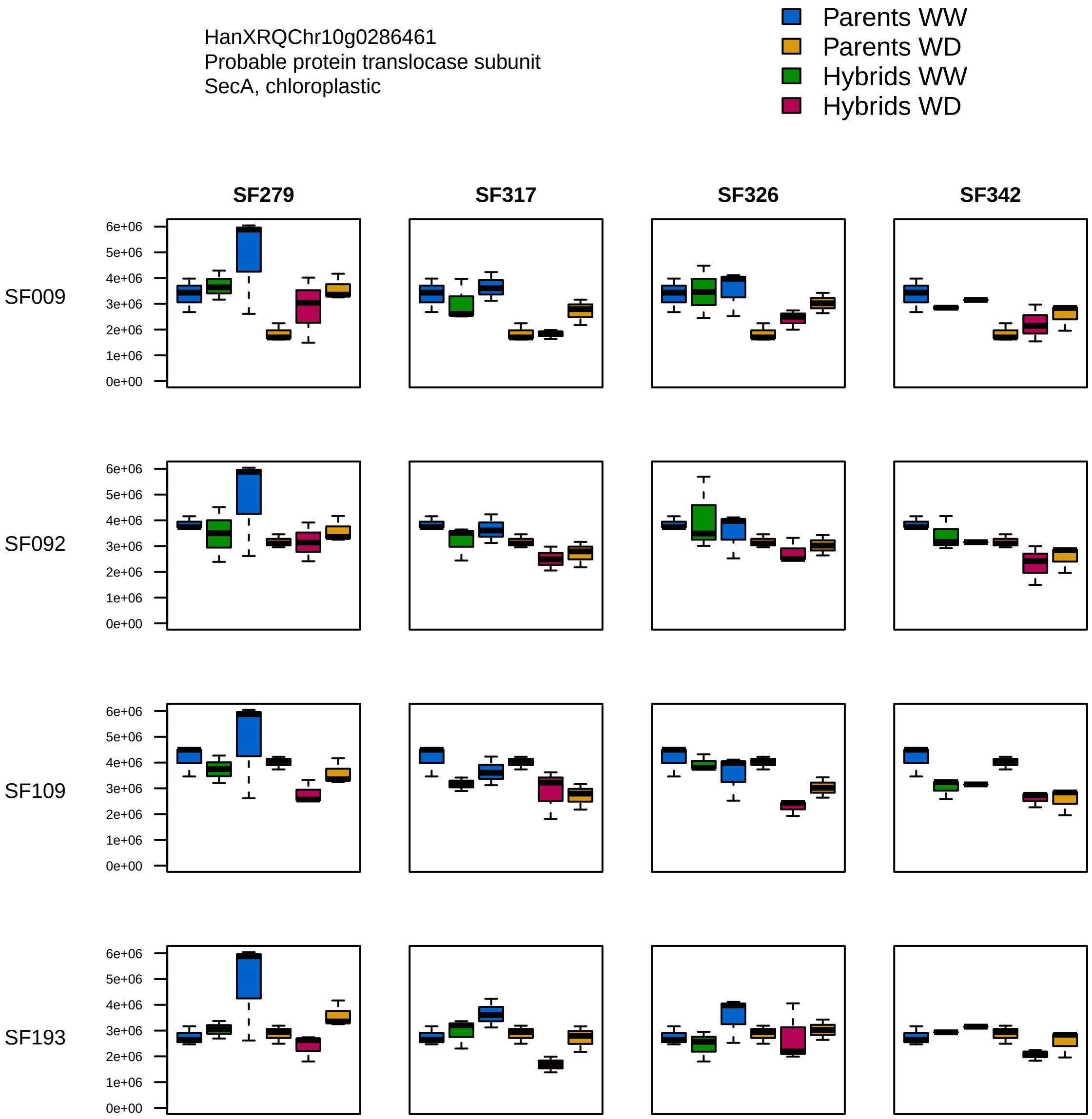
HanXRQChr10g0286461
Probable protein translocase subunit
SecA, chloroplastic
Parents WW
Parents WD
Hybrids WW
Hybrids WD
SF279
SF317
SF326
SF342
SF009
SF092
SF109
SF193
0e+00
1e+06
2e+06
3e+06
4e+06
5e+06
6e+06

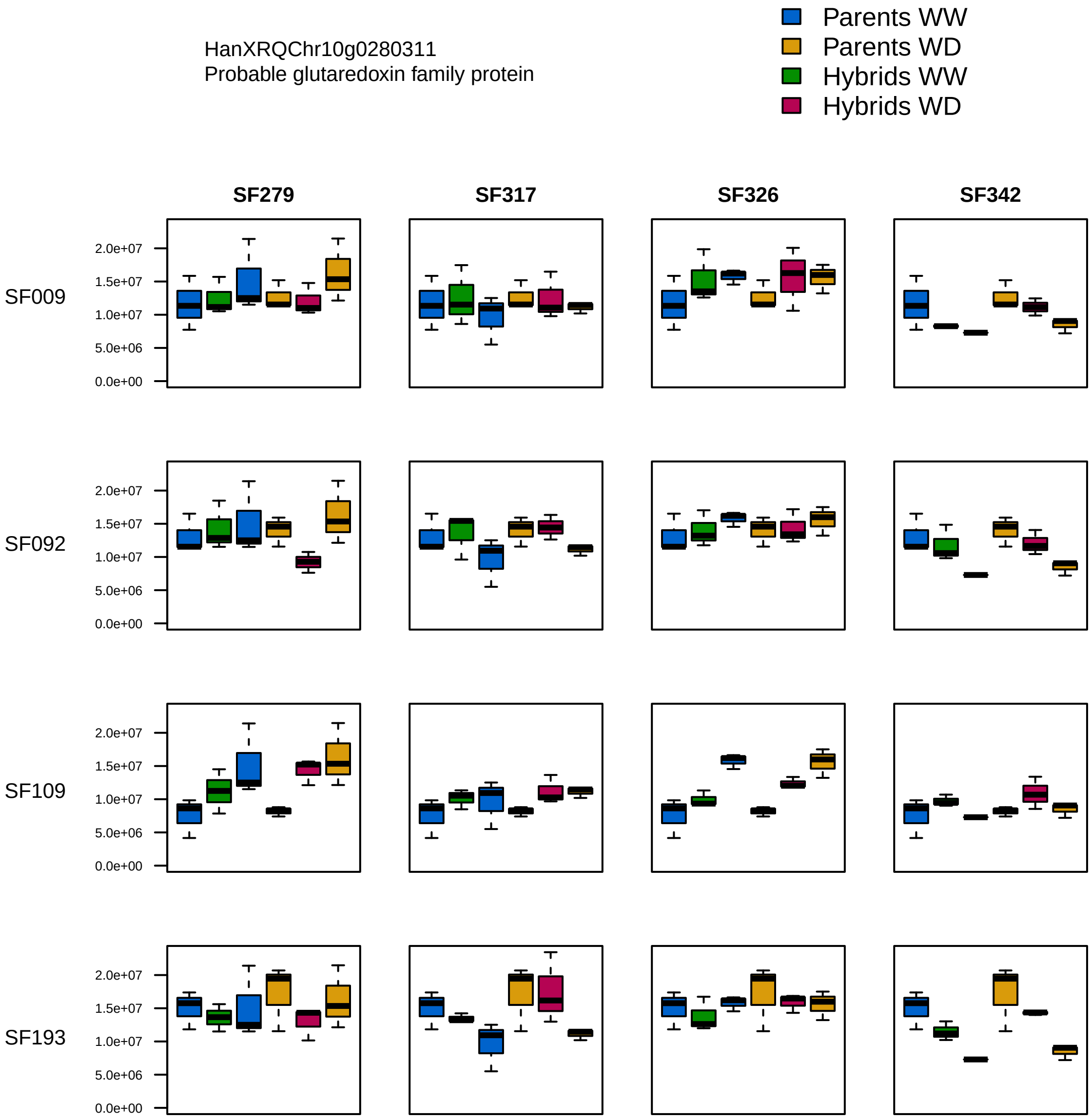
HanXRQChr10g0280311
Probable glutaredoxin family protein
Parents WW
Parents WD
Hybrids WW
Hybrids WD
SF279
SF317
SF326
SF342
SF009
SF092
SF109
SF193
2.0e+07
1.5e+07
1.0e+07
5.0e+06
0.0e+00

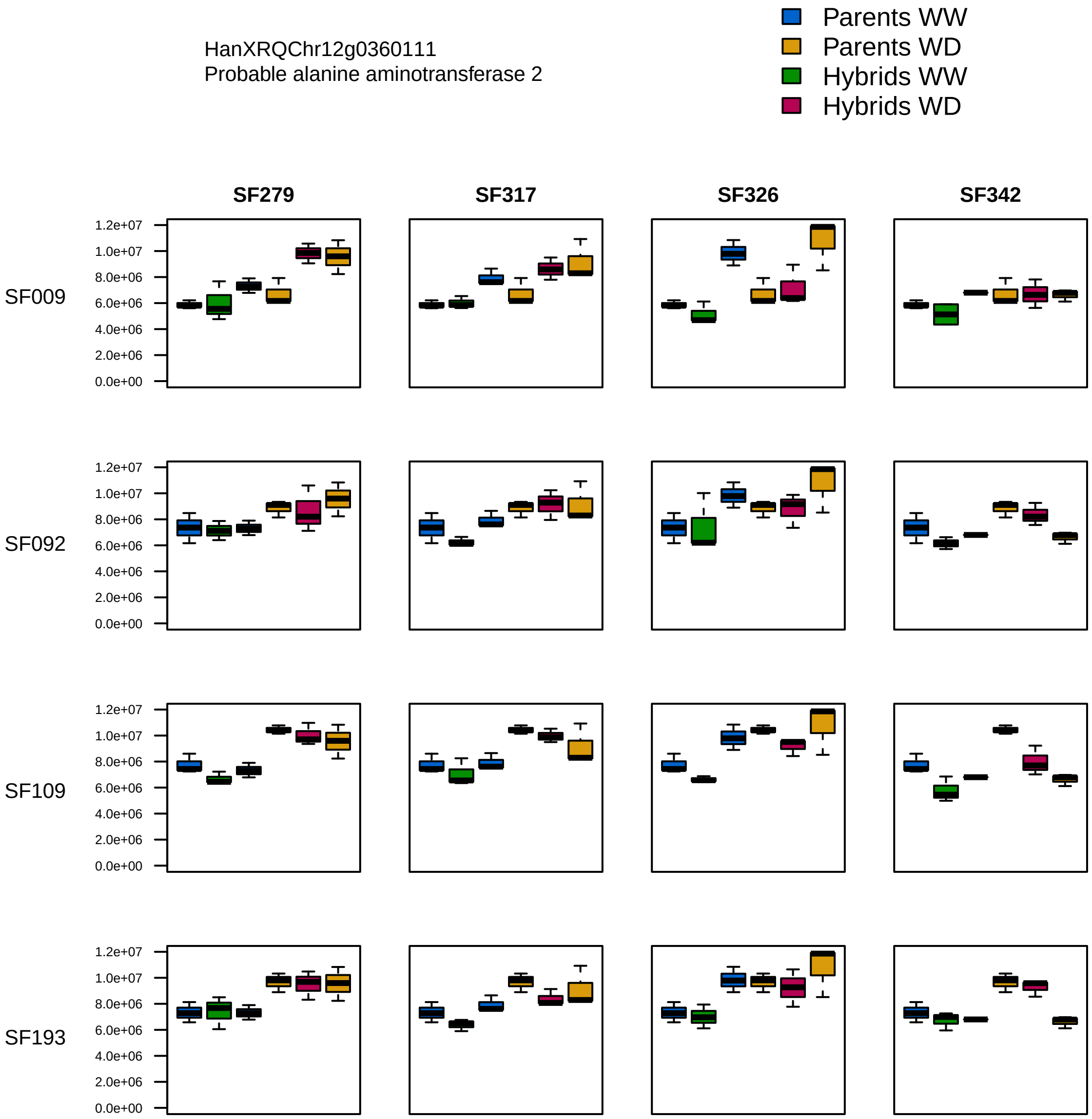
HanXRQChr12g0360111
Probable alanine aminotransferase 2
Parents WW
Parents WD
Hybrids WW
Hybrids WD
SF279
SF317
SF326
SF342
SF009
SF092
SF109
SF193
0.0e+00
2.0e+06
4.0e+06
6.0e+06
8.0e+06
1.0e+07
1.2e+07

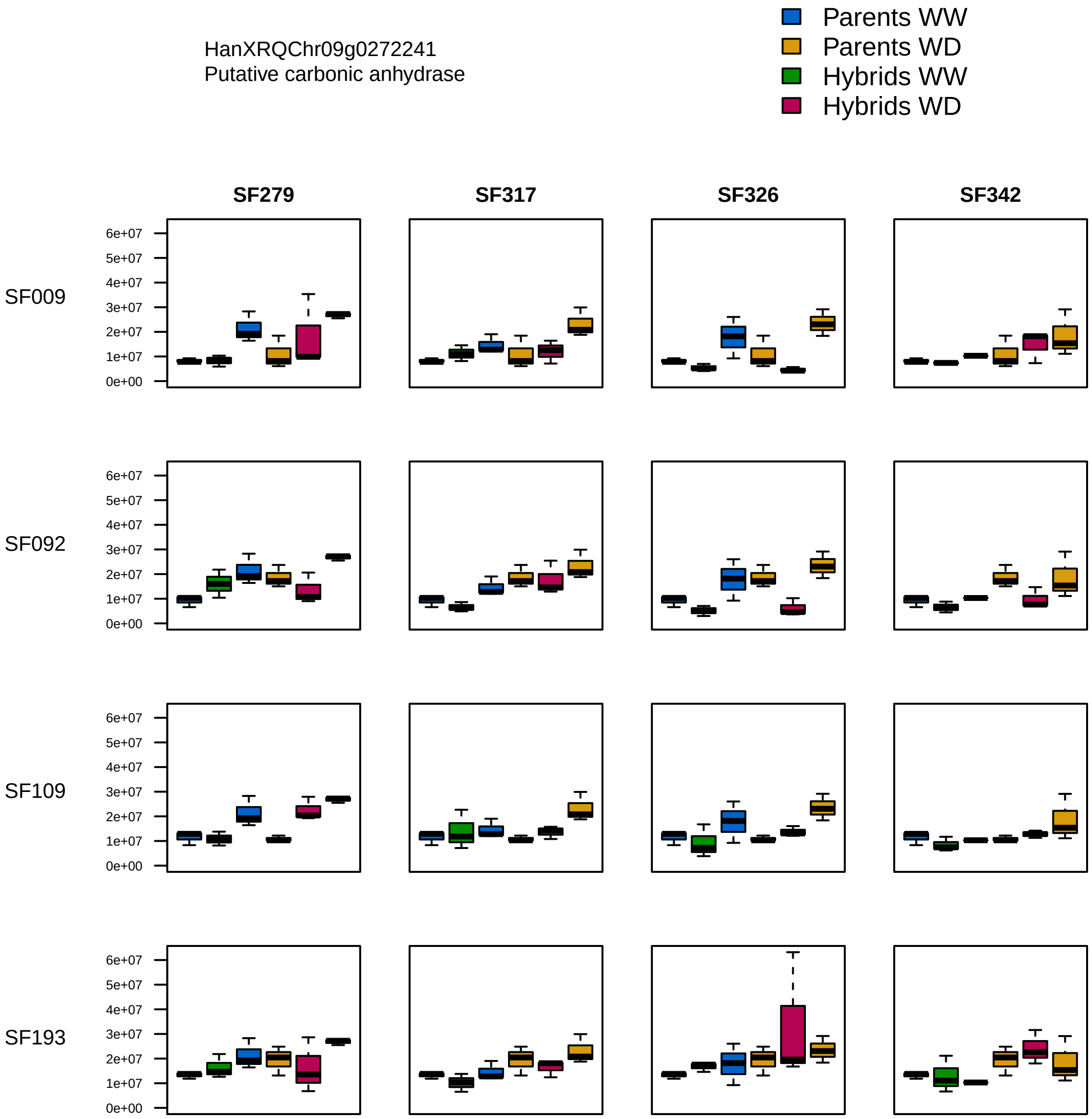
HanXRQChr09g0272241
Putative carbonic anhydrase
Parents WW
Parents WD
Hybrids WW
Hybrids WD
SF279
SF317
SF326
SF342
SF009
SF092
SF109
SF193
6e+07
5e+07
4e+07
3e+07
2e+07
1e+07
0e+00

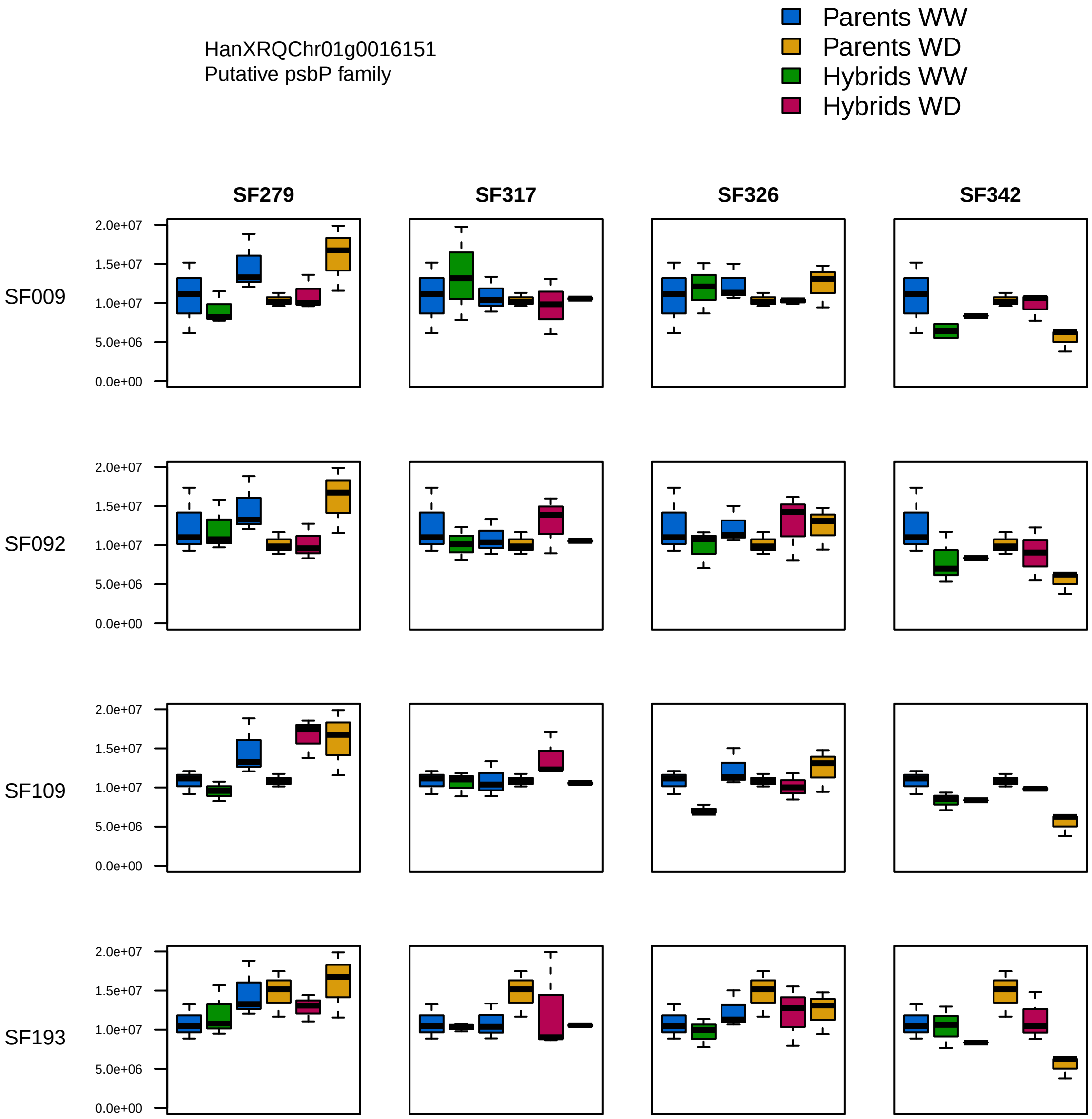
HanXRQChr01g0016151
Putative psbP family
Parents WW
Parents WD
Hybrids WW
Hybrids WD
SF279
SF317
SF326
SF342
SF009
SF092
SF109
SF193
2.0e+07
1.5e+07
1.0e+07
5.0e+06
0.0e+00

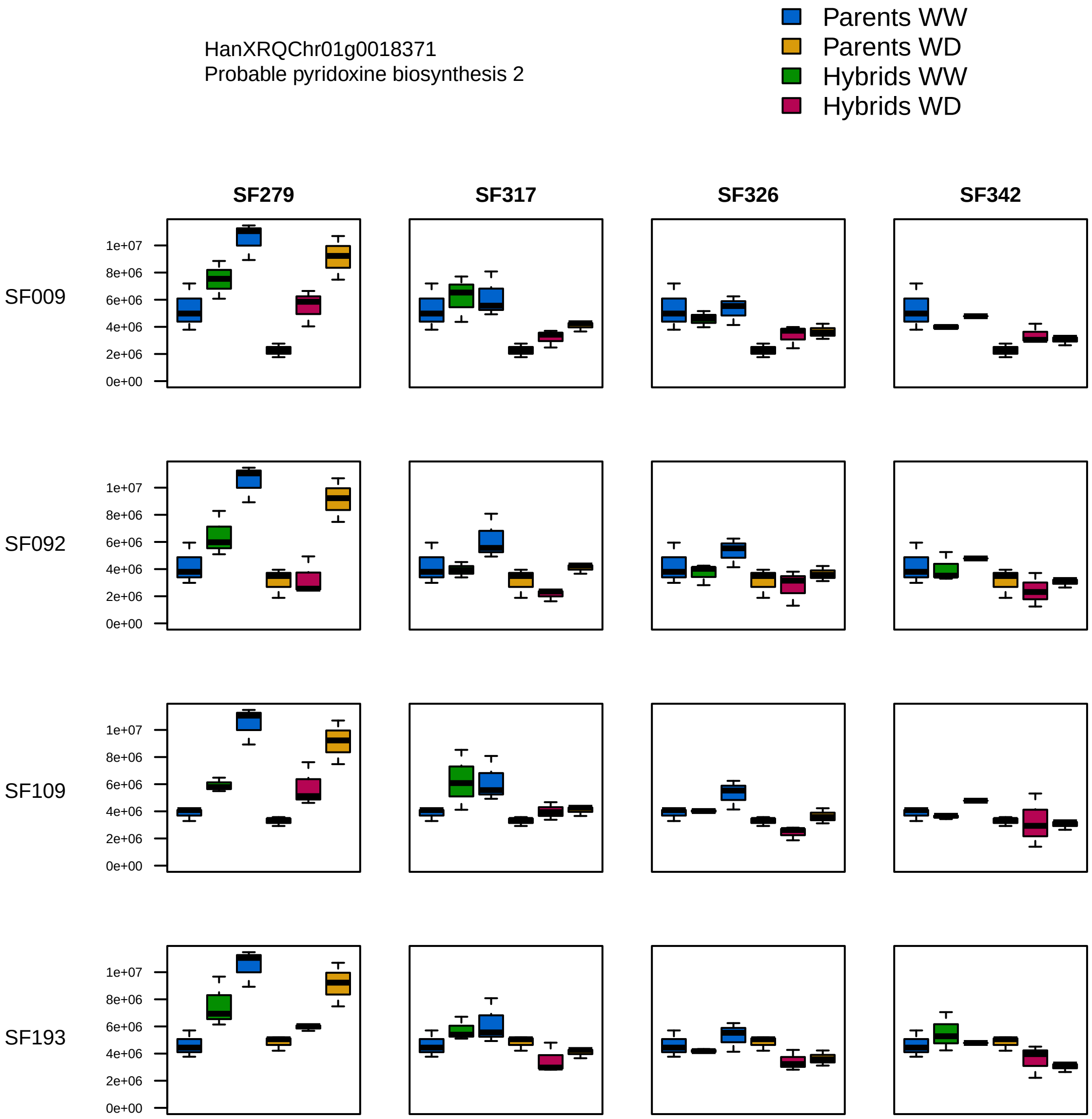
HanXRQChr01g0018371
Probable pyridoxine biosynthesis 2
Parents WW
Parents WD
Hybrids WW
Hybrids WD
SF279
SF317
SF326
SF342
SF009
SF092
SF109
SF193
1e+07
8e+06
6e+06
4e+06
2e+06
0e+00

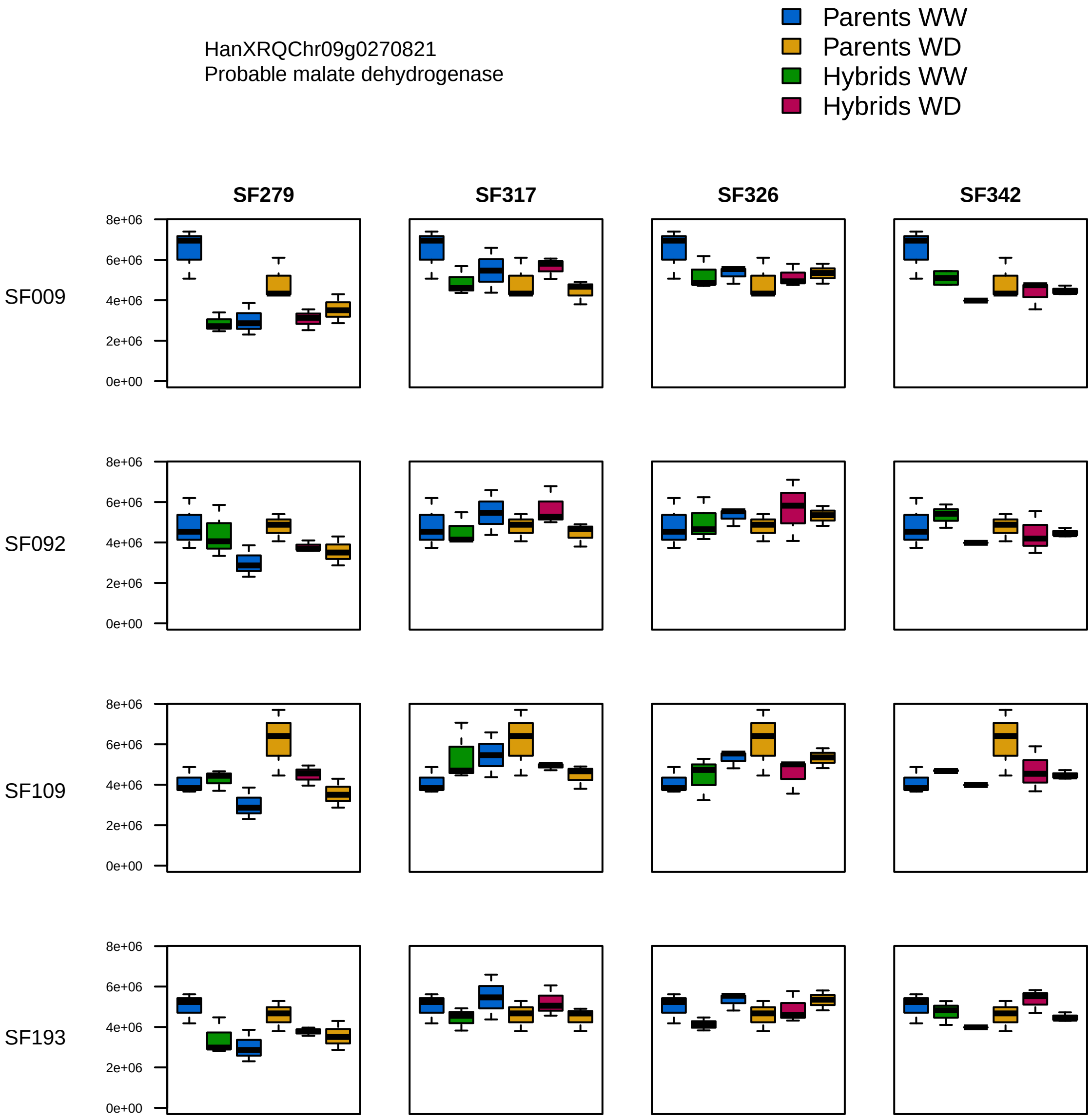
HanXRQChr09g0270821
Probable malate dehydrogenase
Parents WW
Parents WD
Hybrids WW
Hybrids WD
SF279
SF317
SF326
SF342
SF009
SF092
SF109
SF193
0e+00
2e+06
4e+06
6e+06
8e+06

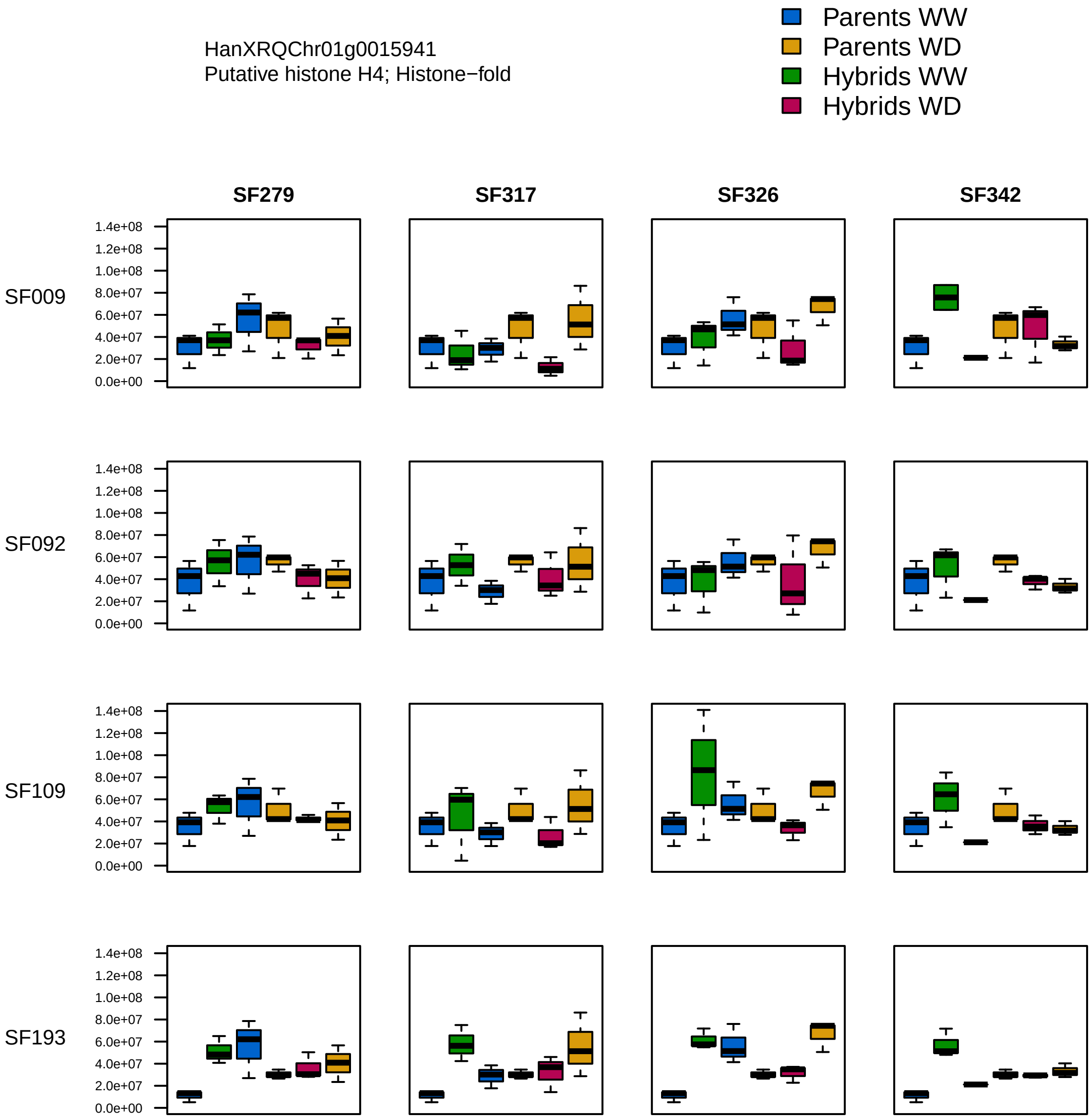
HanXRQChr01g0015941
Putative histone H4; Histone−fold
Parents WW
Parents WD
Hybrids WW
Hybrids WD
SF279
SF317
SF326
SF342
SF009
SF092
SF109
SF193
1.4e+08
1.2e+08
1.0e+08
8.0e+07
6.0e+07
4.0e+07
2.0e+07
0.0e+00

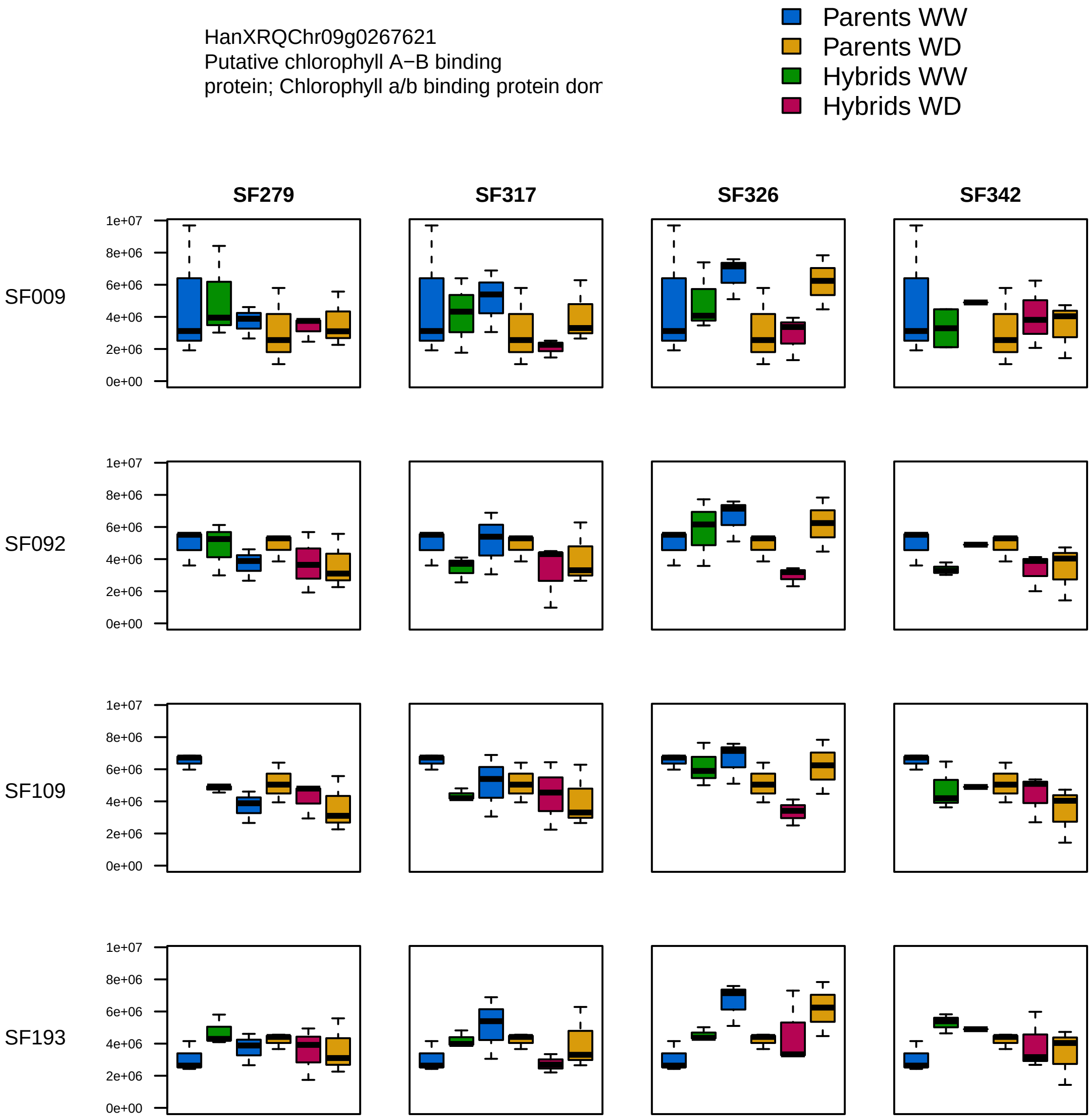
HanXRQChr09g0267621
Putative chlorophyll A−B binding
protein; Chlorophyll a/b binding protein dom
Parents WW
Parents WD
Hybrids WW
Hybrids WD
SF279
SF317
SF326
SF342
SF009
SF092
SF109
SF193
1e+07
8e+06
6e+06
4e+06
2e+06
0e+00

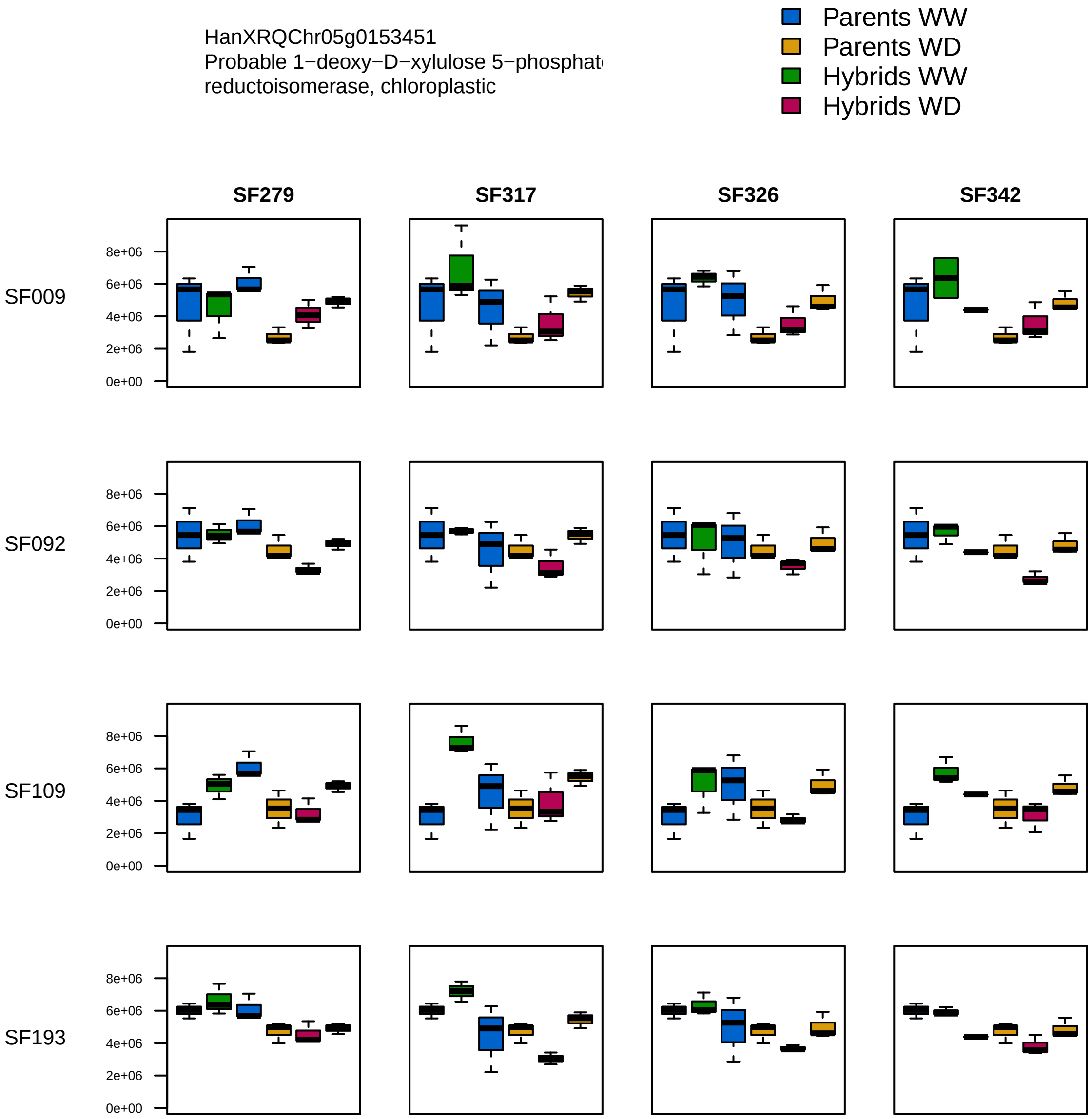
HanXRQChr05g0153451
Probable 1−deoxy−D−xylulose 5−phosphat
reductoisomerase, chloroplastic
Parents WW
Parents WD
Hybrids WW
Hybrids WD
SF279
SF317
SF326
SF342
SF009
SF092
SF109
SF193
8e+06
6e+06
4e+06
2e+06
0e+00

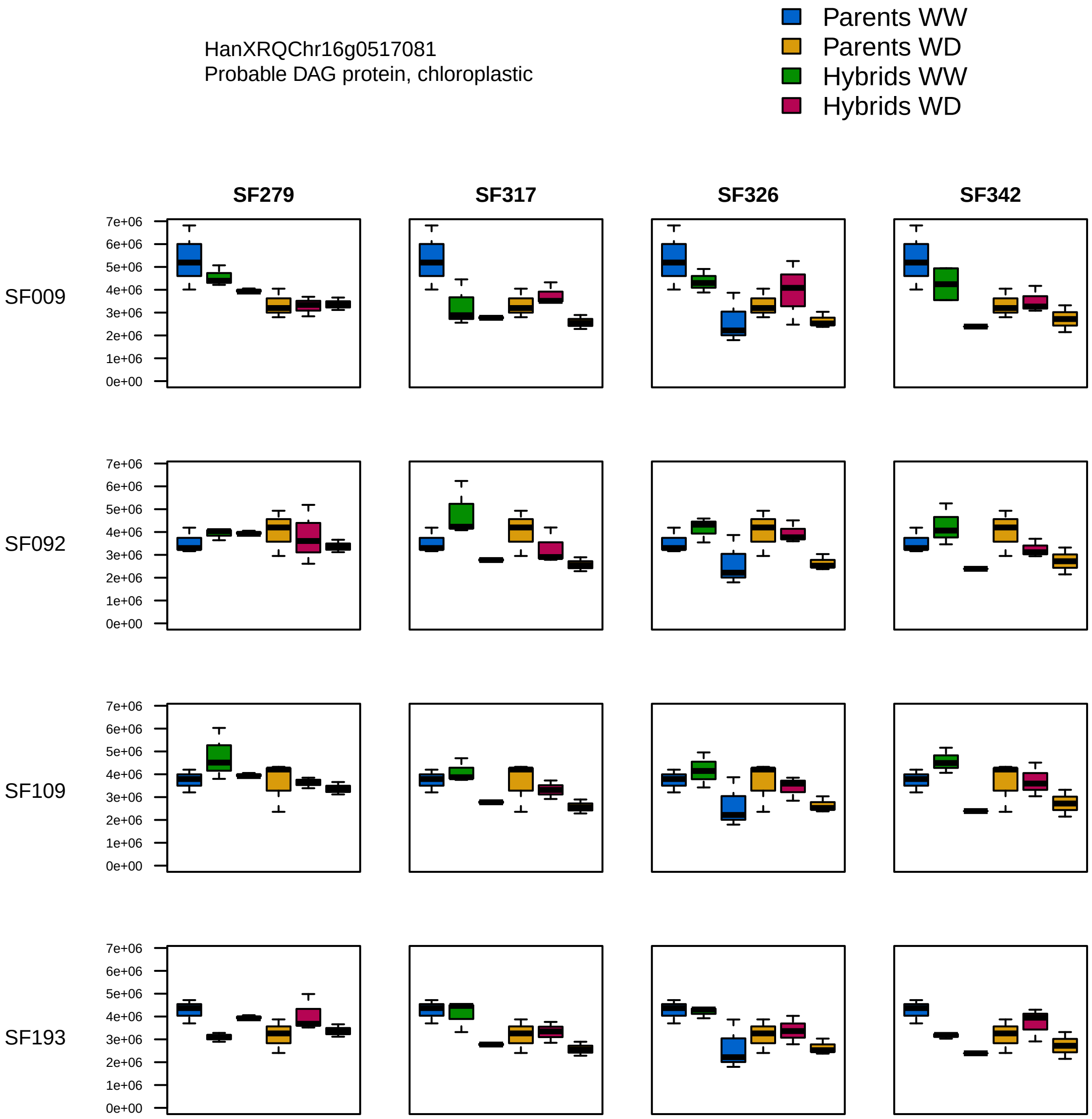
HanXRQChr16g0517081
Probable DAG protein, chloroplastic
Parents WW
Parents WD
Hybrids WW
Hybrids WD
SF279
SF317
SF326
SF342
SF009
SF092
SF109
SF193
7e+06
6e+06
5e+06
4e+06
3e+06
2e+06
1e+06
0e+00

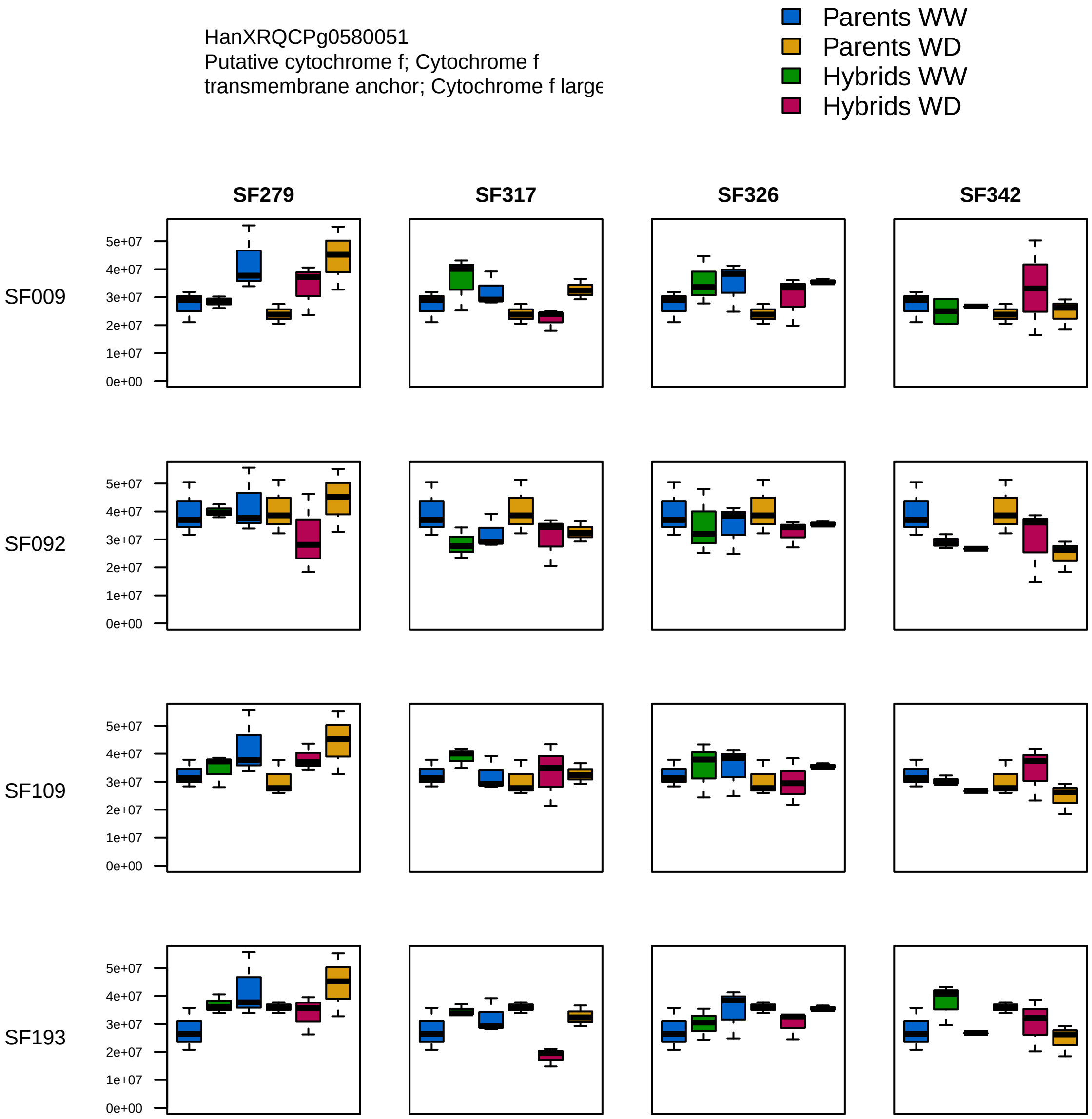
HanXRQCPg0580051
Putative cytochrome f; Cytochrome f transmembrane anchor; Cytochrome f large
Parents WW
Parents WD
Hybrids WW
Hybrids WD
SF279
SF317
SF326
SF342
SF009
SF092
SF109
SF193
0e+00
1e+07
2e+07
3e+07
4e+07
5e+07

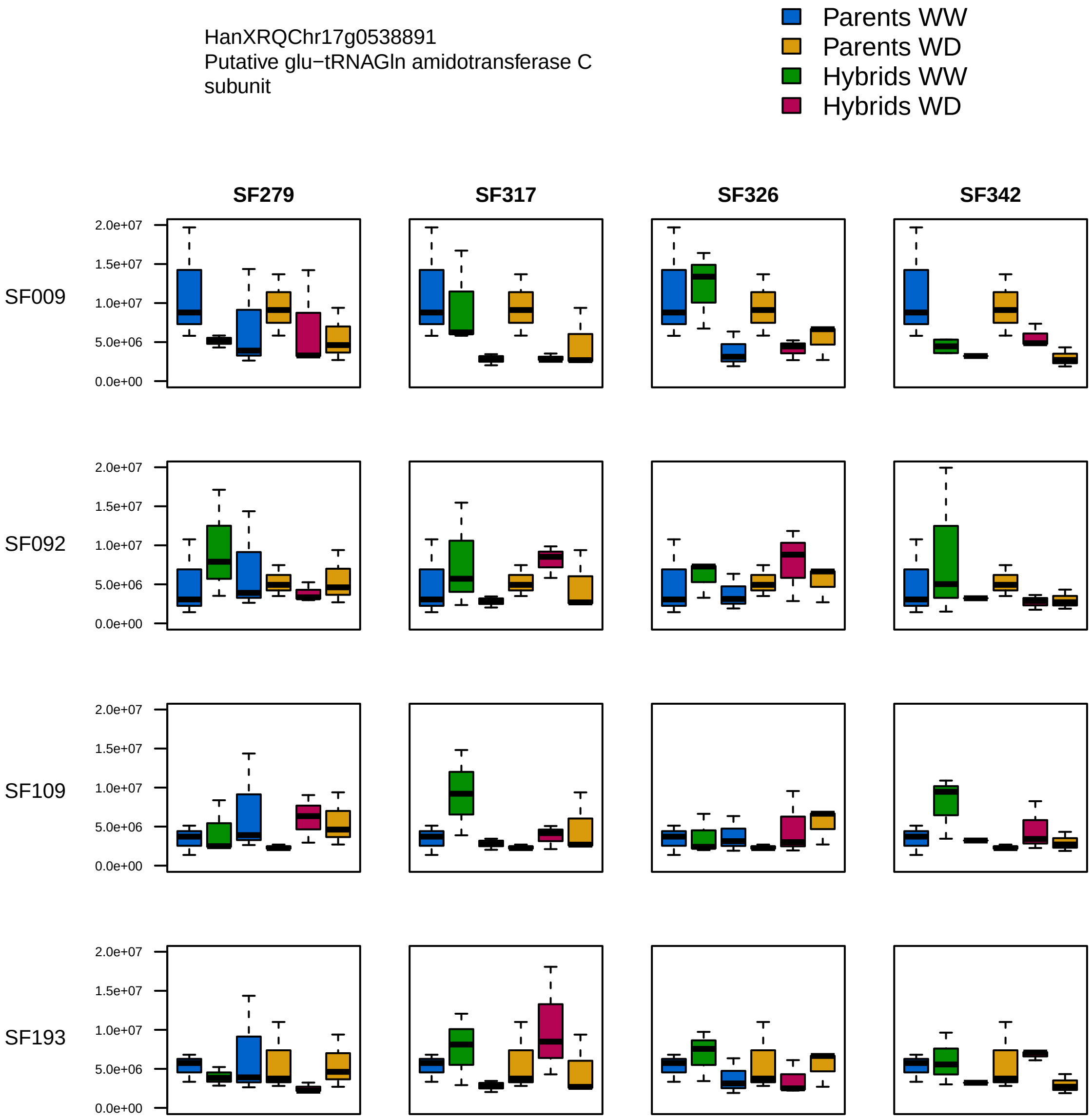
HanXRQChr17g0538891
Putative glu−tRNAGln amidotransferase C subunit
Parents WW
Parents WD
Hybrids WW
Hybrids WD
SF279
SF317
SF326
SF342
SF009
SF092
SF109
SF193
0.0e+00
5.0e+06
1.0e+07
1.5e+07
2.0e+07

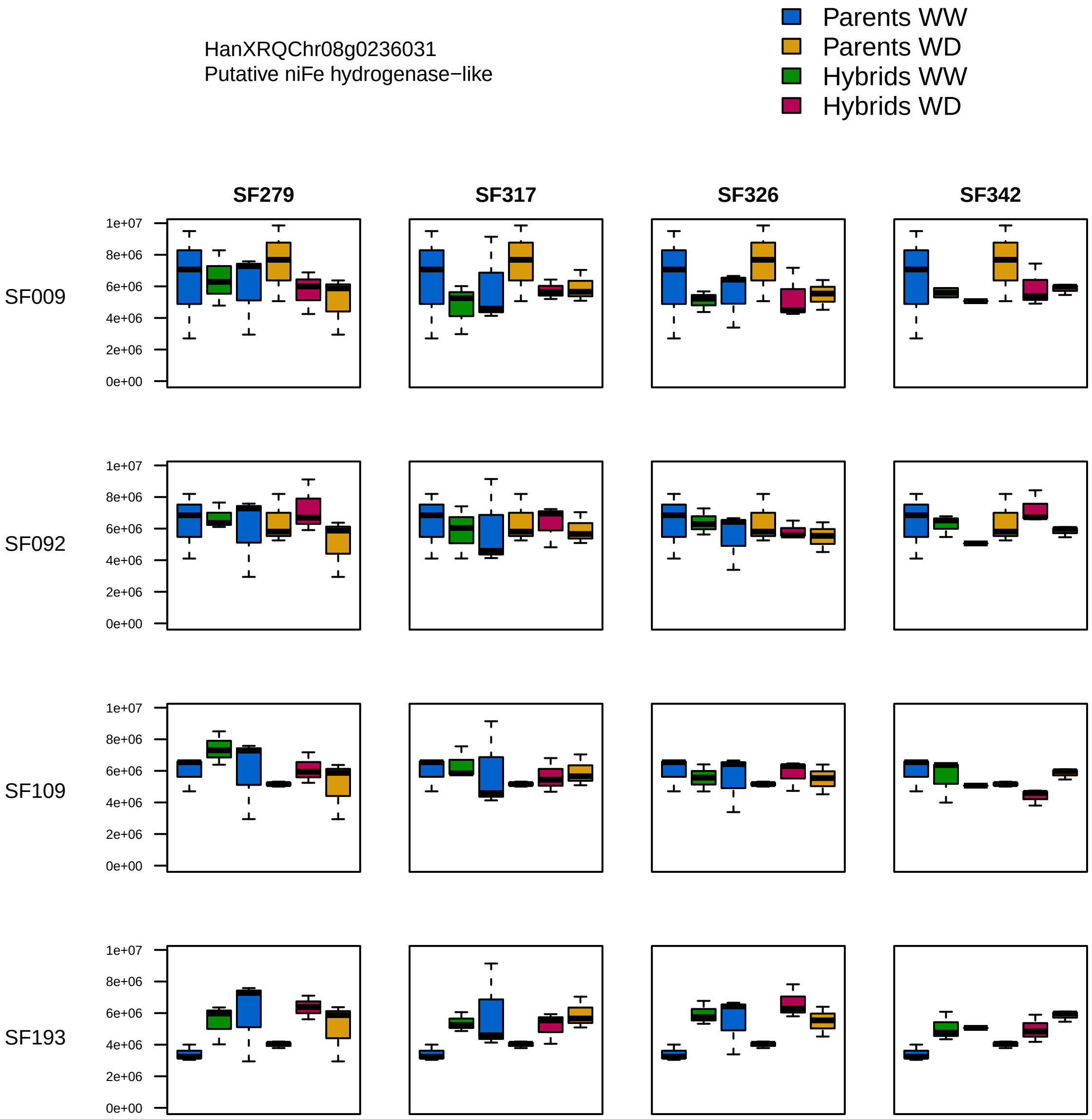

HanXRQChr08g0236031
Putative niFe hydrogenase−like
Parents WW
Parents WD
Hybrids WW
Hybrids WD
SF279
SF317
SF326
SF342
SF009
SF092
SF109
SF193
1e+07
8e+06
6e+06
4e+06
2e+06
0e+00

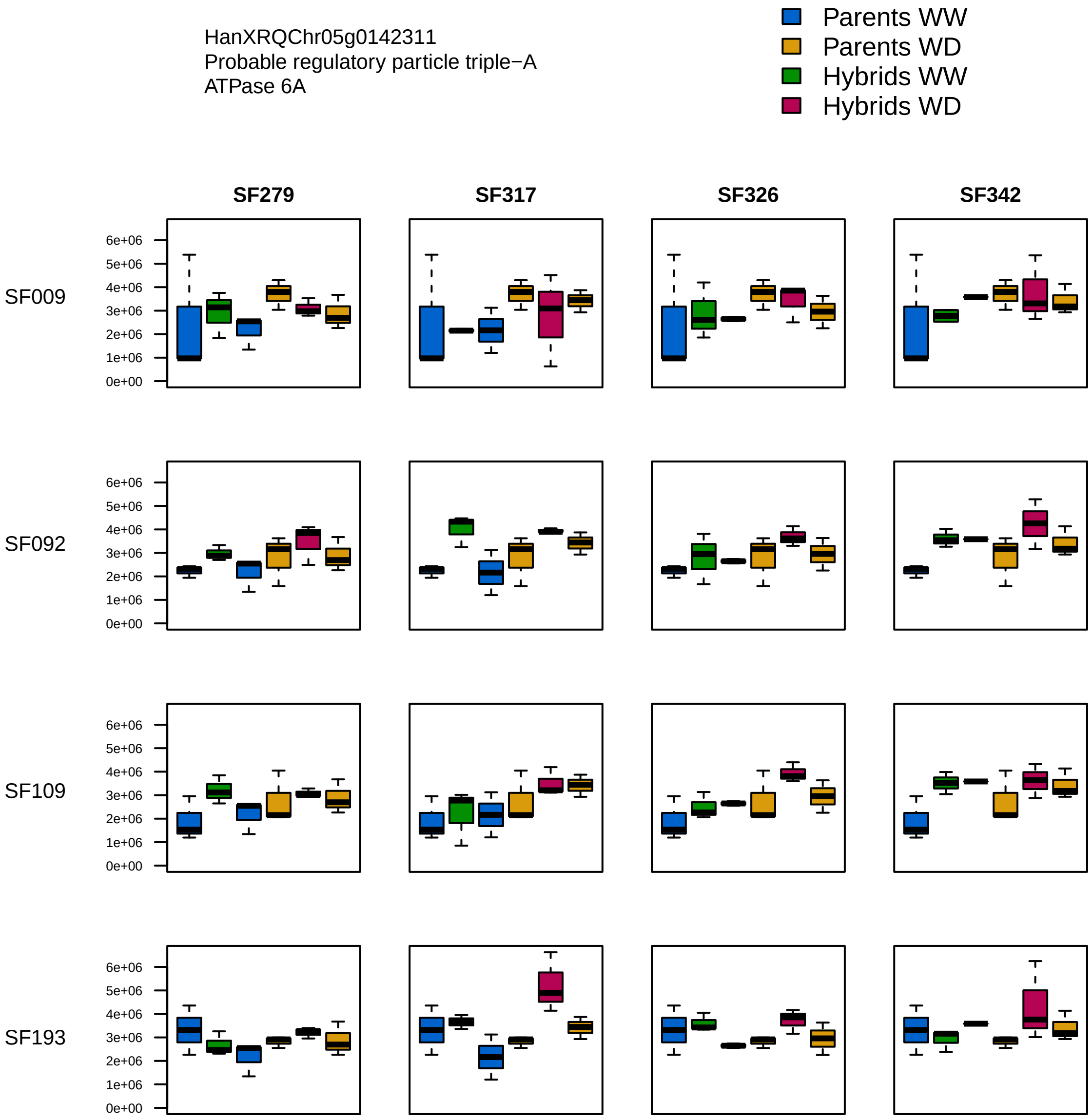
HanXRQChr05g0142311
Probable regulatory particle triple−A
ATPase 6A
Parents WW
Parents WD
Hybrids WW
Hybrids WD
SF279
SF317
SF326
SF342
SF009
SF092
SF109
SF193
6e+06
5e+06
4e+06
3e+06
2e+06
1e+06
0e+00

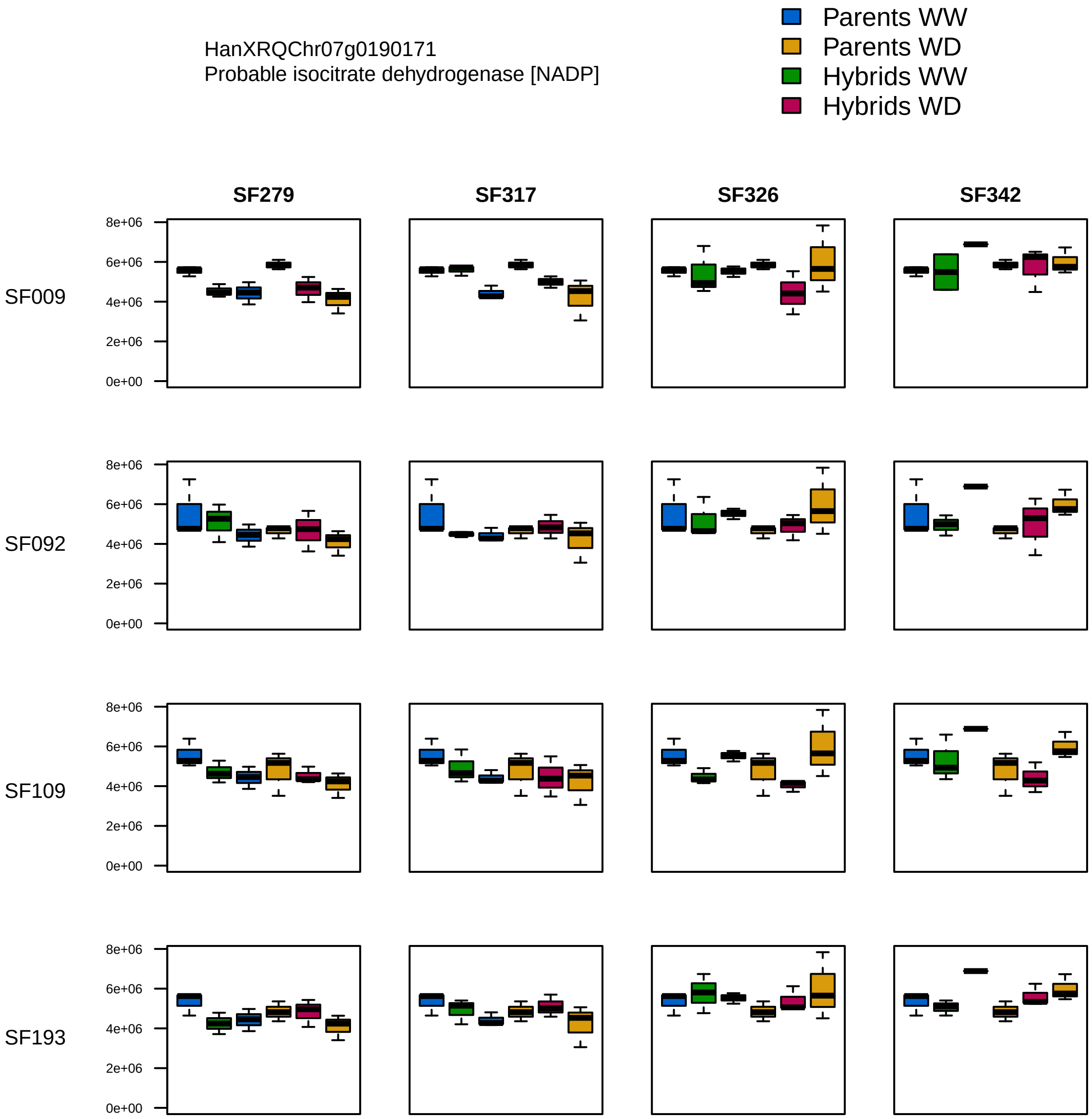

HanXRQChr07g0190171
Probable isocitrate dehydrogenase [NADP]
Parents WW
Parents WD
Hybrids WW
Hybrids WD
SF279
SF317
SF326
SF342
SF009
SF092
SF109
SF193
8e+06
6e+06
4e+06
2e+06
0e+00

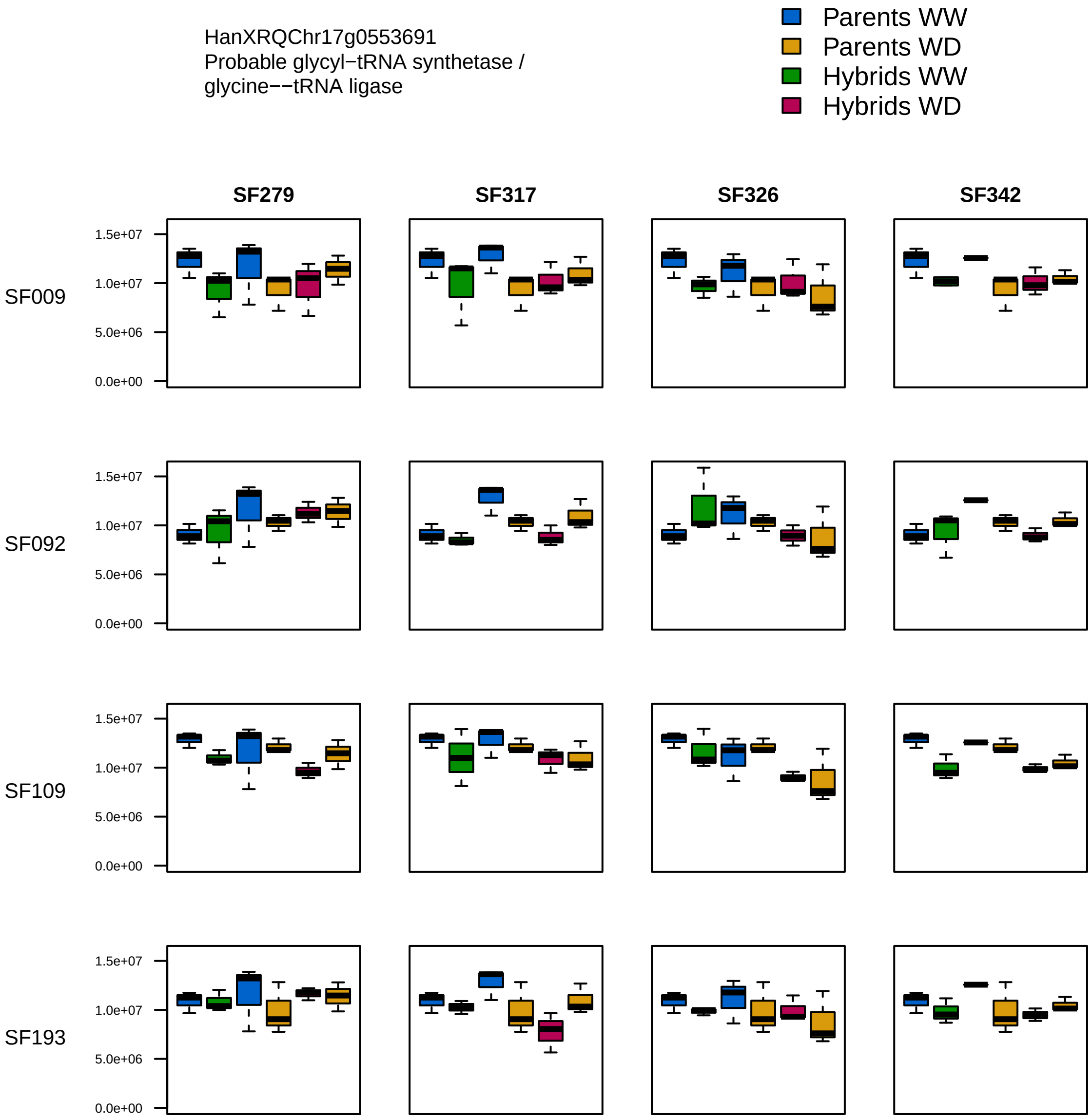
HanXRQChr17g0553691
Probable glycyl−tRNA synthetase /
glycine−−tRNA ligase
Parents WW
Parents WD
Hybrids WW
Hybrids WD
SF279
SF317
SF326
SF342
SF009
SF092
SF109
SF193
1.5e+07
1.0e+07
5.0e+06
0.0e+00

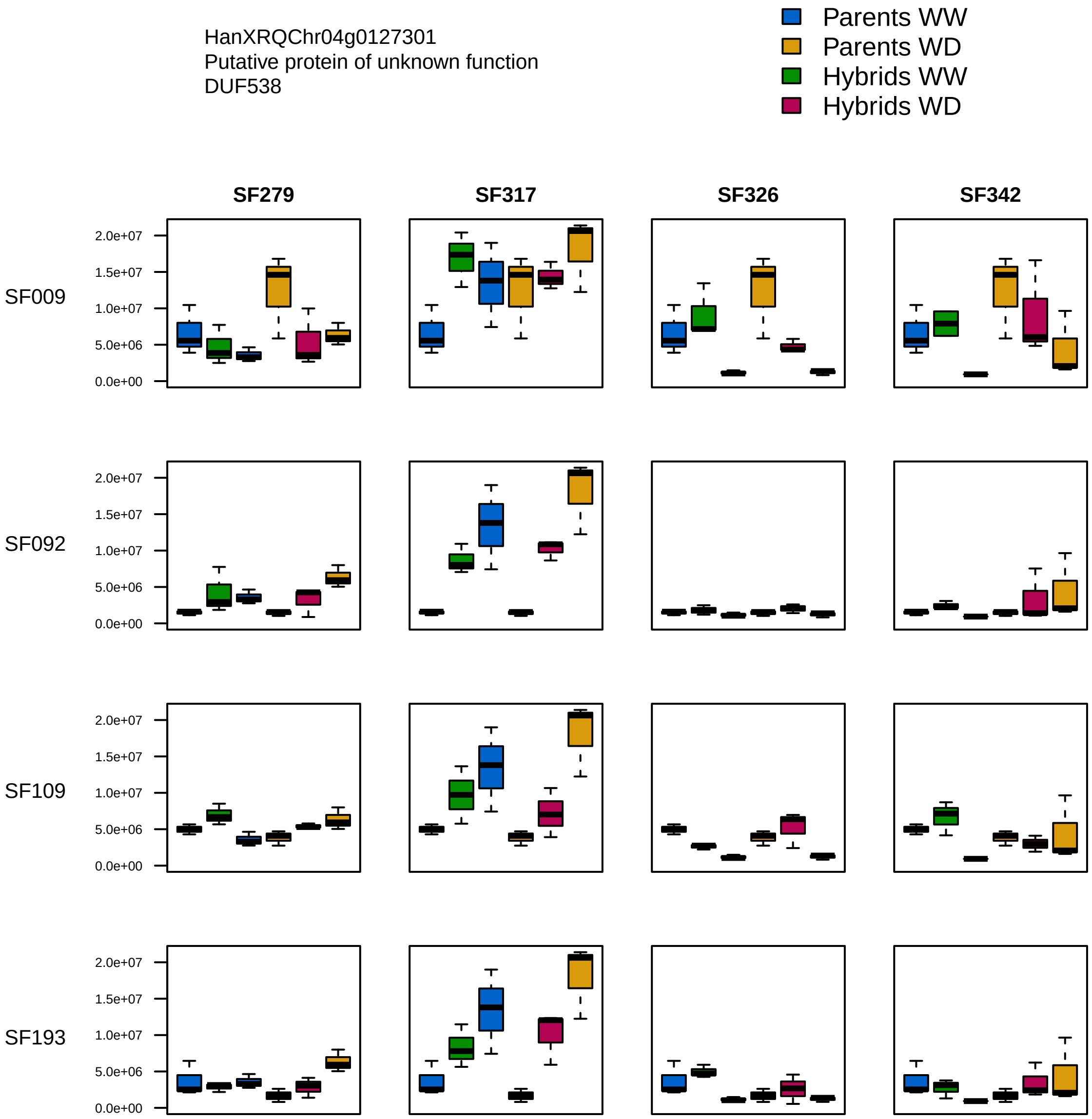
HanXRQChr04g0127301
Putative protein of unknown function
DUF538
Parents WW
Parents WD
Hybrids WW
Hybrids WD
SF279
SF317
SF326
SF342
SF009
SF092
SF109
SF193
2.0e+07
1.5e+07
1.0e+07
5.0e+06
0.0e+00

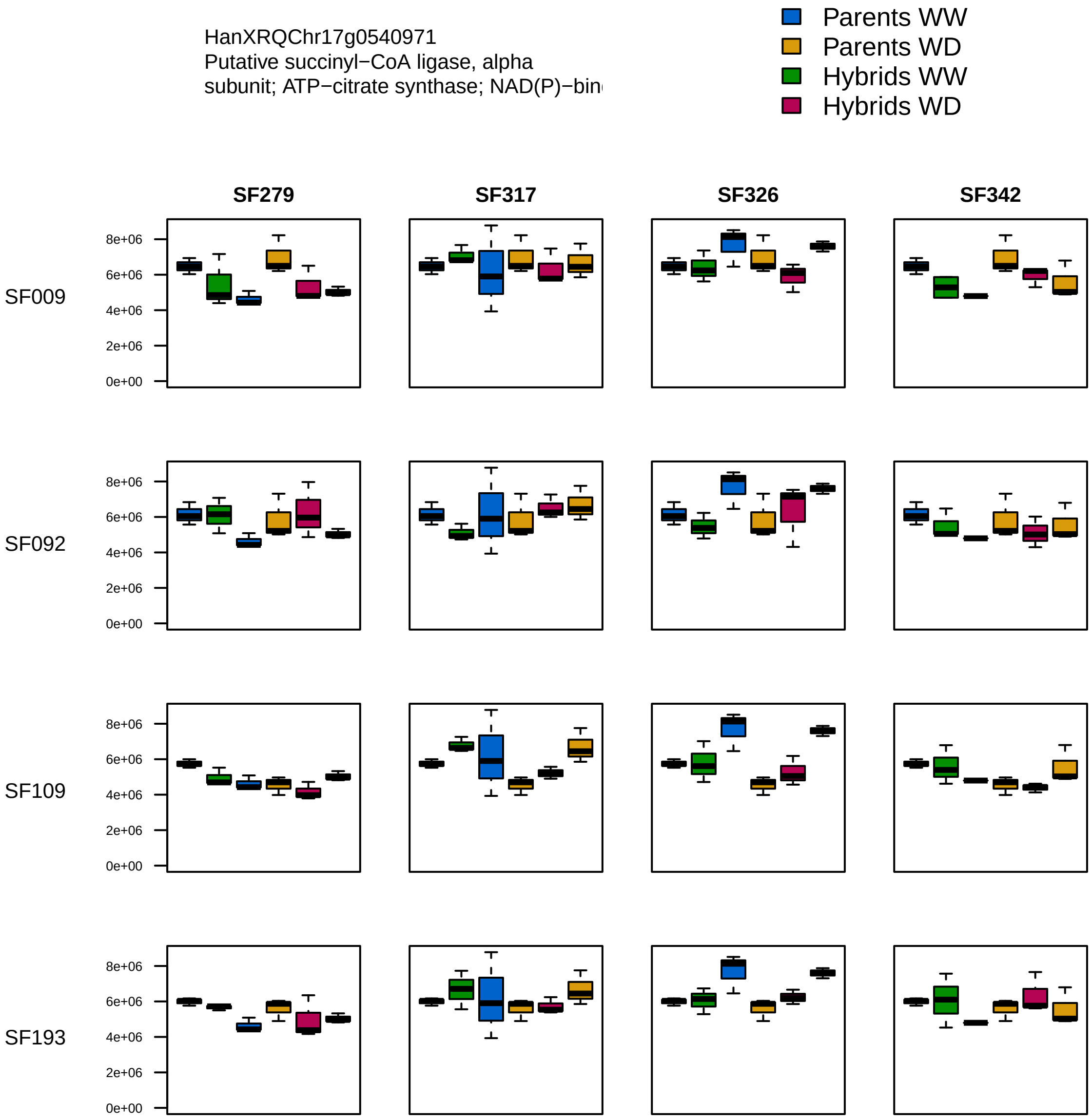

HanXRQChr17g0540971
Putative succinyl−CoA ligase, alpha
subunit; ATP−citrate synthase; NAD(P)−bin
Parents WW
Parents WD
Hybrids WW
Hybrids WD
SF279
SF317
SF326
SF342
SF009
SF092
SF109
SF193
8e+06
6e+06
4e+06
2e+06
0e+00

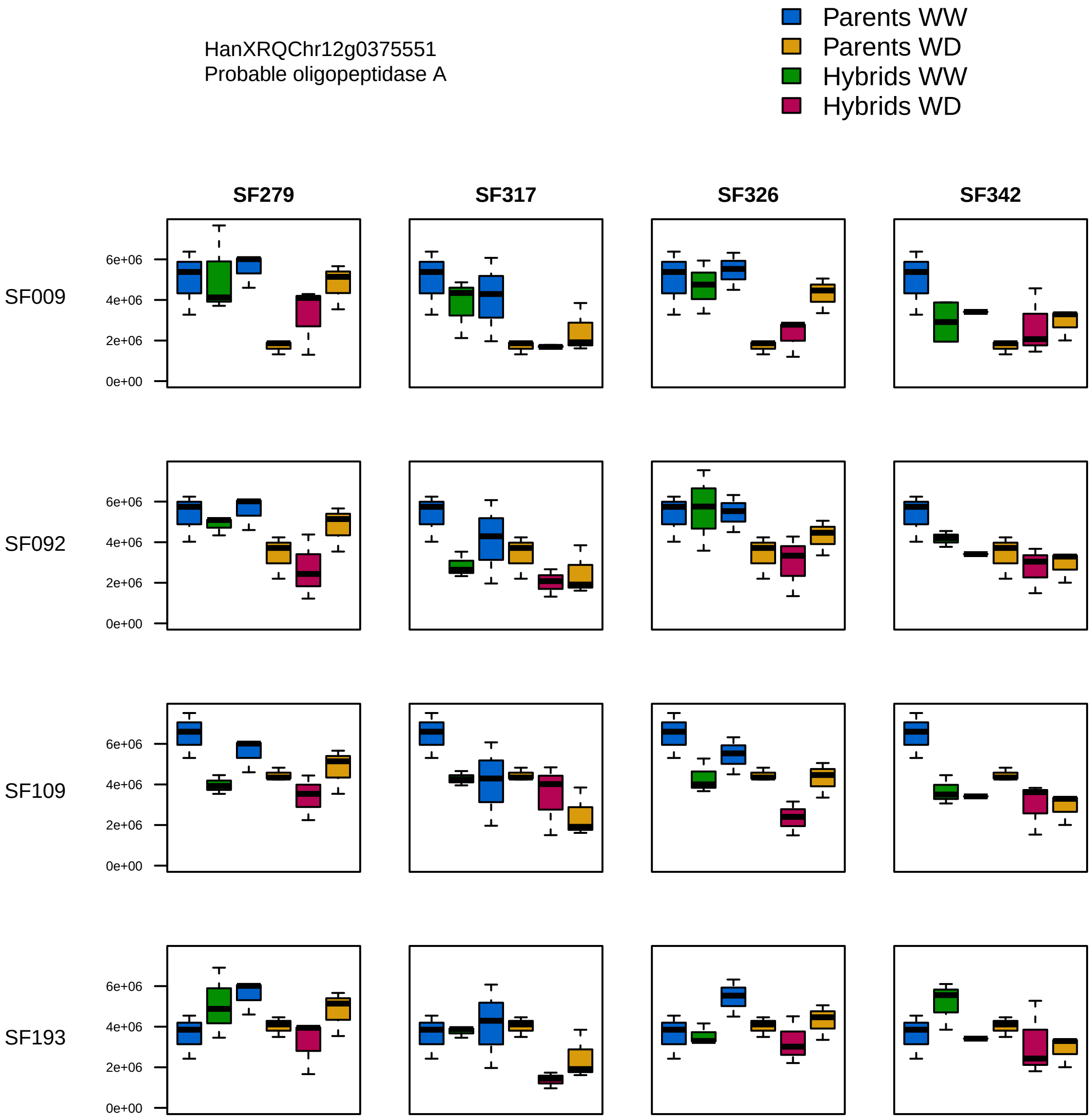
HanXRQChr12g0375551
Probable oligopeptidase A
Parents WW
Parents WD
Hybrids WW
Hybrids WD
SF279
SF317
SF326
SF342
SF009
SF092
SF109
SF193
6e+06
4e+06
2e+06
0e+00

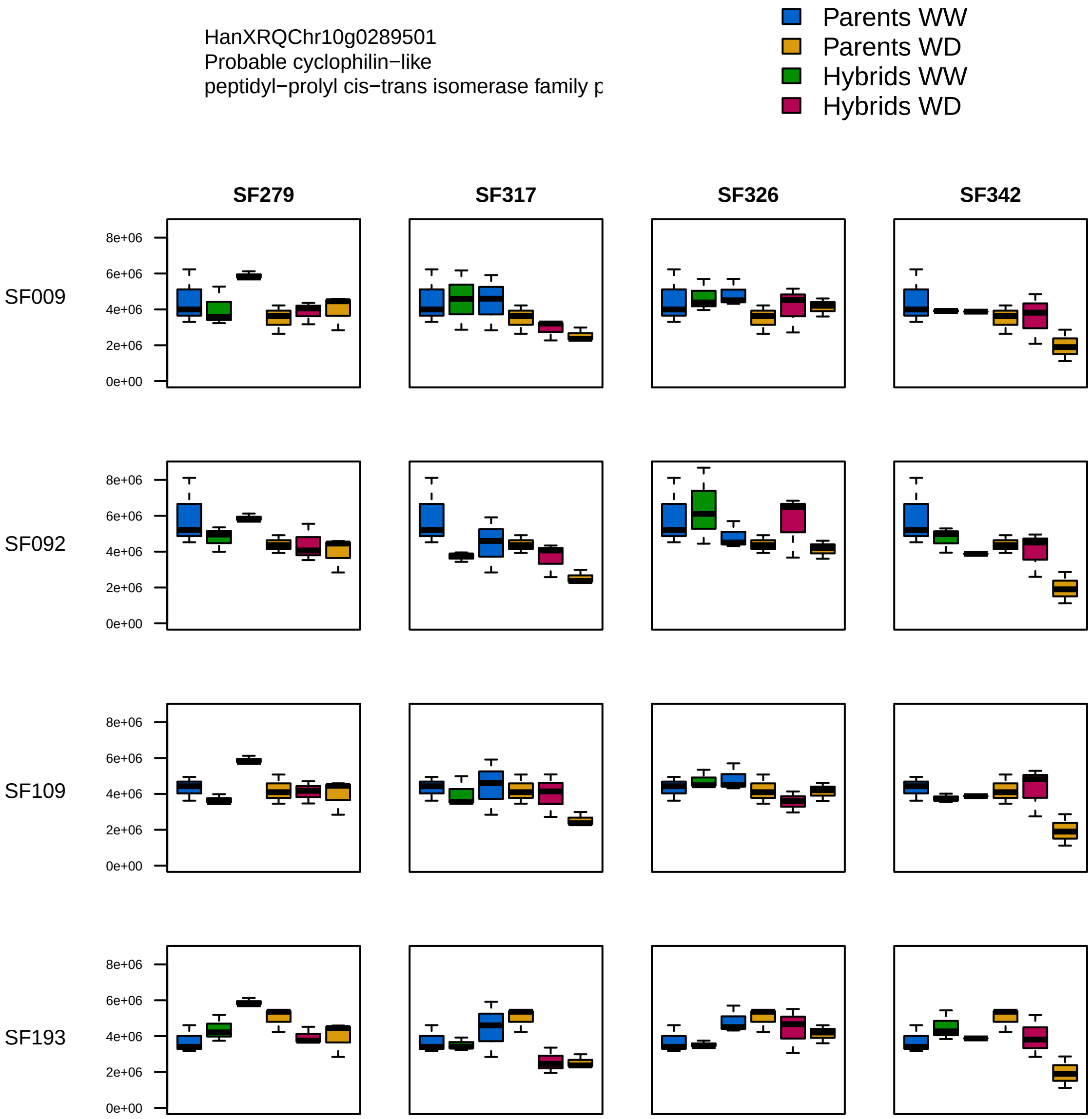
HanXRQChr10g0289501
Probable cyclophilin−like
peptidyl−prolyl cis−trans isomerase family p
Parents WW
Parents WD
Hybrids WW
Hybrids WD
SF279
SF317
SF326
SF342
SF009
SF092
SF109
SF193
8e+06
6e+06
4e+06
2e+06
0e+00

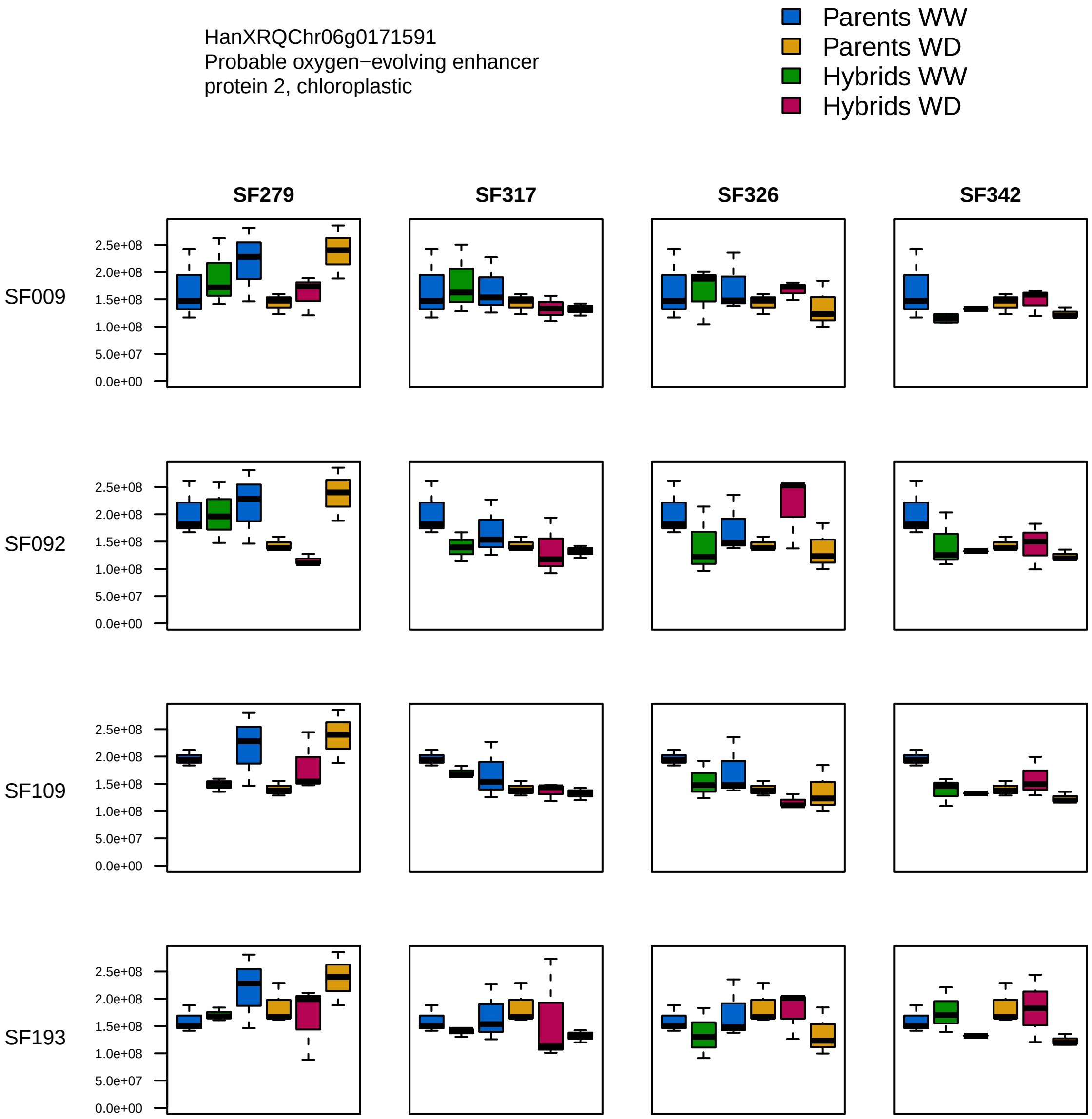

HanXRQChr06g0171591
Probable oxygen−evolving enhancer
protein 2, chloroplastic
Parents WW
Parents WD
Hybrids WW
Hybrids WD
SF279
SF317
SF326
SF342
SF009
SF092
SF109
SF193
2.5e+08
2.0e+08
1.5e+08
1.0e+08
5.0e+07
0.0e+00

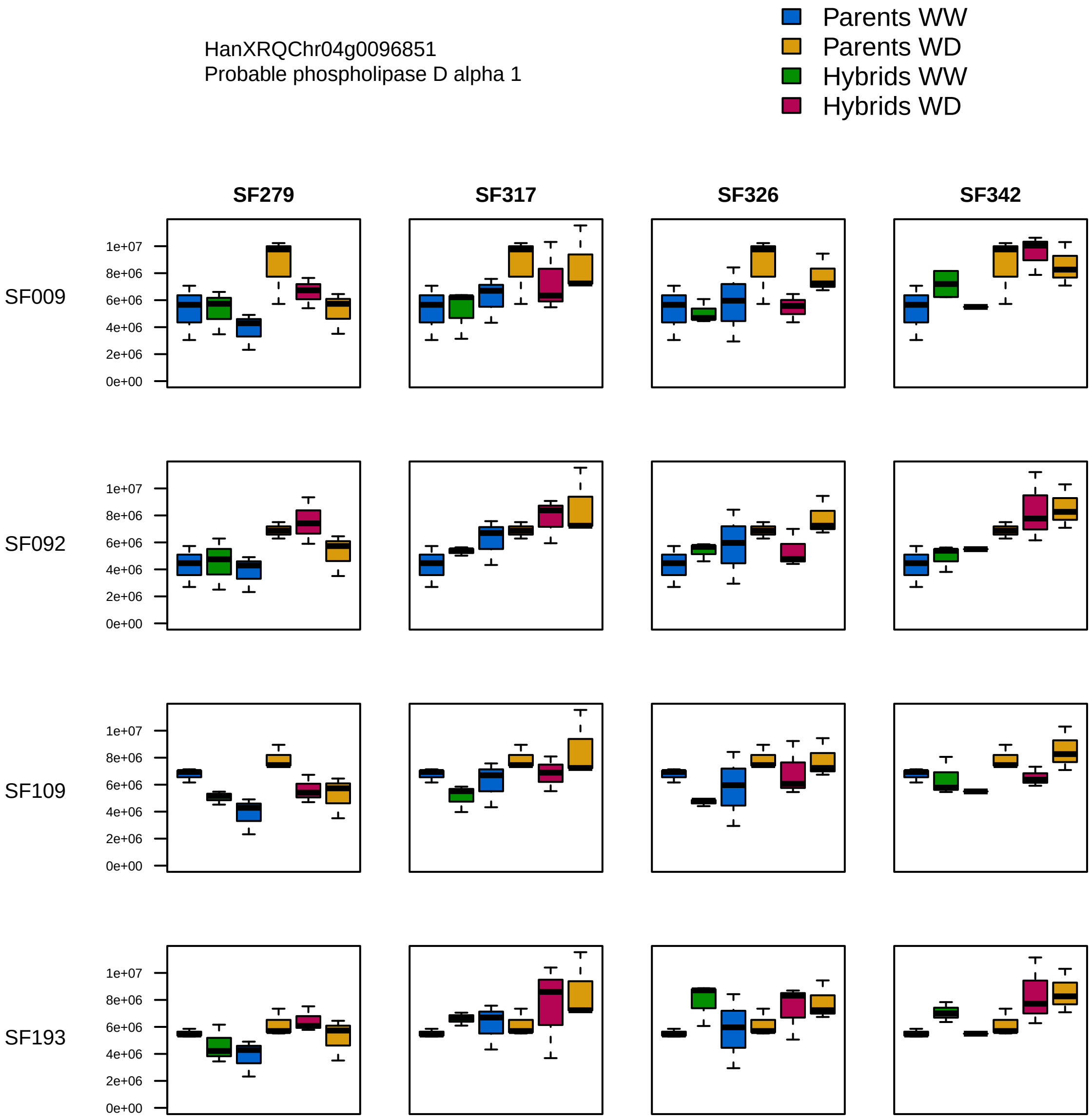

HanXRQChr04g0096851
Probable phospholipase D alpha 1
Parents WW
Parents WD
Hybrids WW
Hybrids WD
SF279
SF317
SF326
SF342
SF009
SF092
SF109
SF193
0e+00
2e+06
4e+06
6e+06
8e+06
1e+07

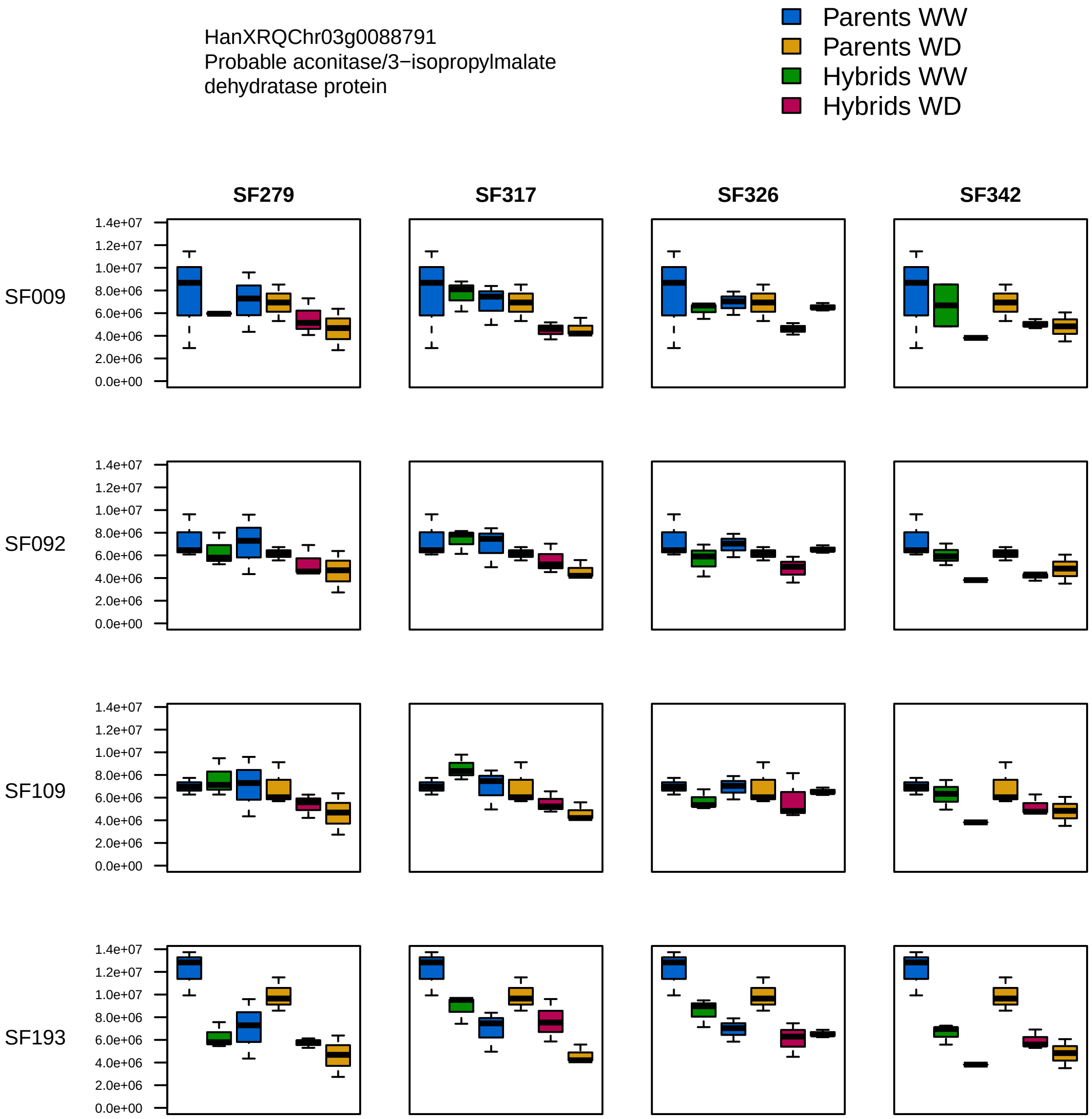

HanXRQChr03g0088791
Probable aconitase/3−isopropylmalate
dehydratase protein
Parents WW
Parents WD
Hybrids WW
Hybrids WD
SF279
SF317
SF326
SF342
SF009
SF092
SF109
SF193
1.4e+07
1.2e+07
1.0e+07
8.0e+06
6.0e+06
4.0e+06
2.0e+06
0.0e+00

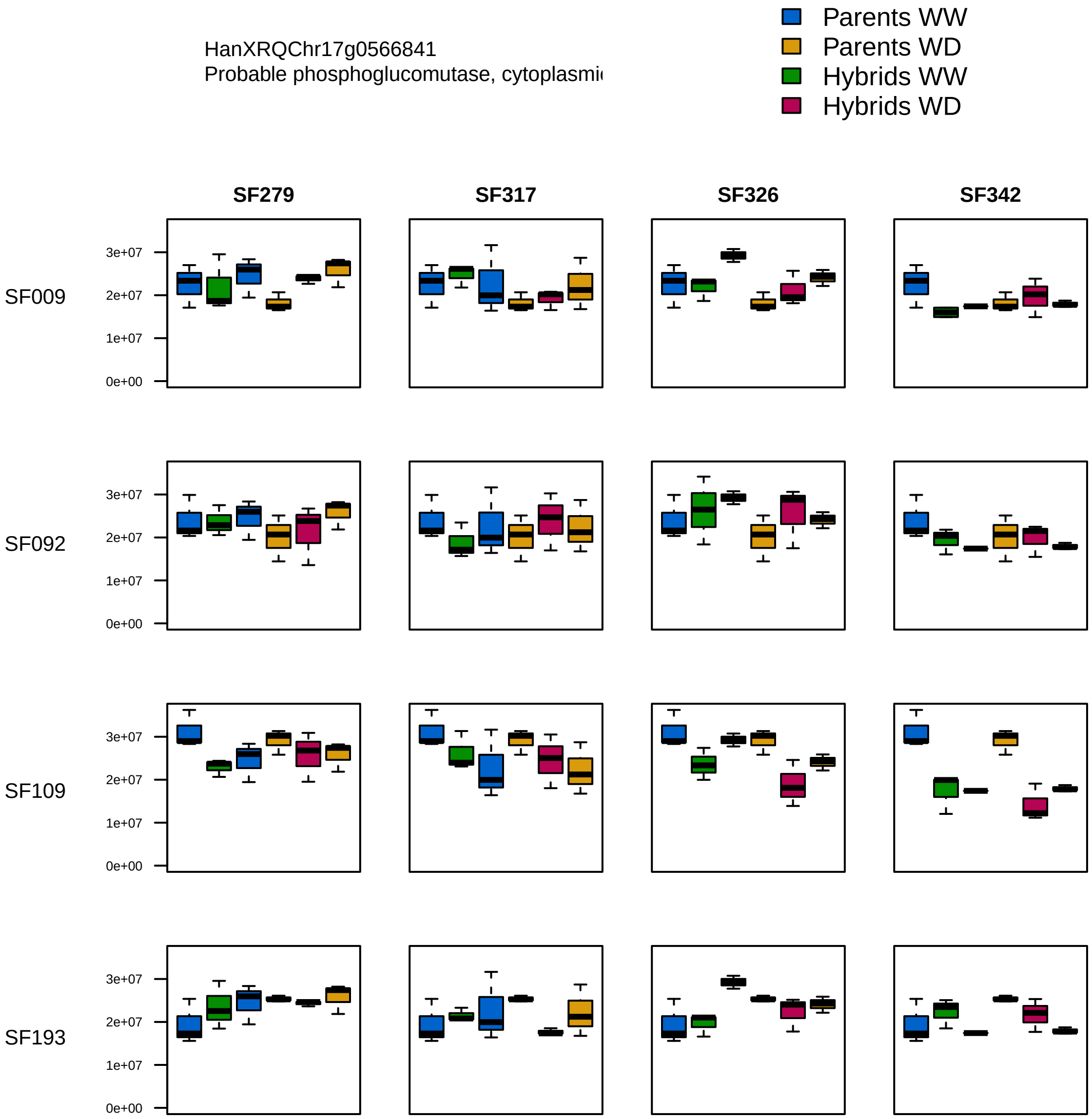

HanXRQChr17g0566841
Probable phosphoglucomutase, cytoplasmi
Parents WW
Parents WD
Hybrids WW
Hybrids WD
SF279
SF317
SF326
SF342
SF009
SF092
SF109
SF193
0e+00
1e+07
2e+07
3e+07

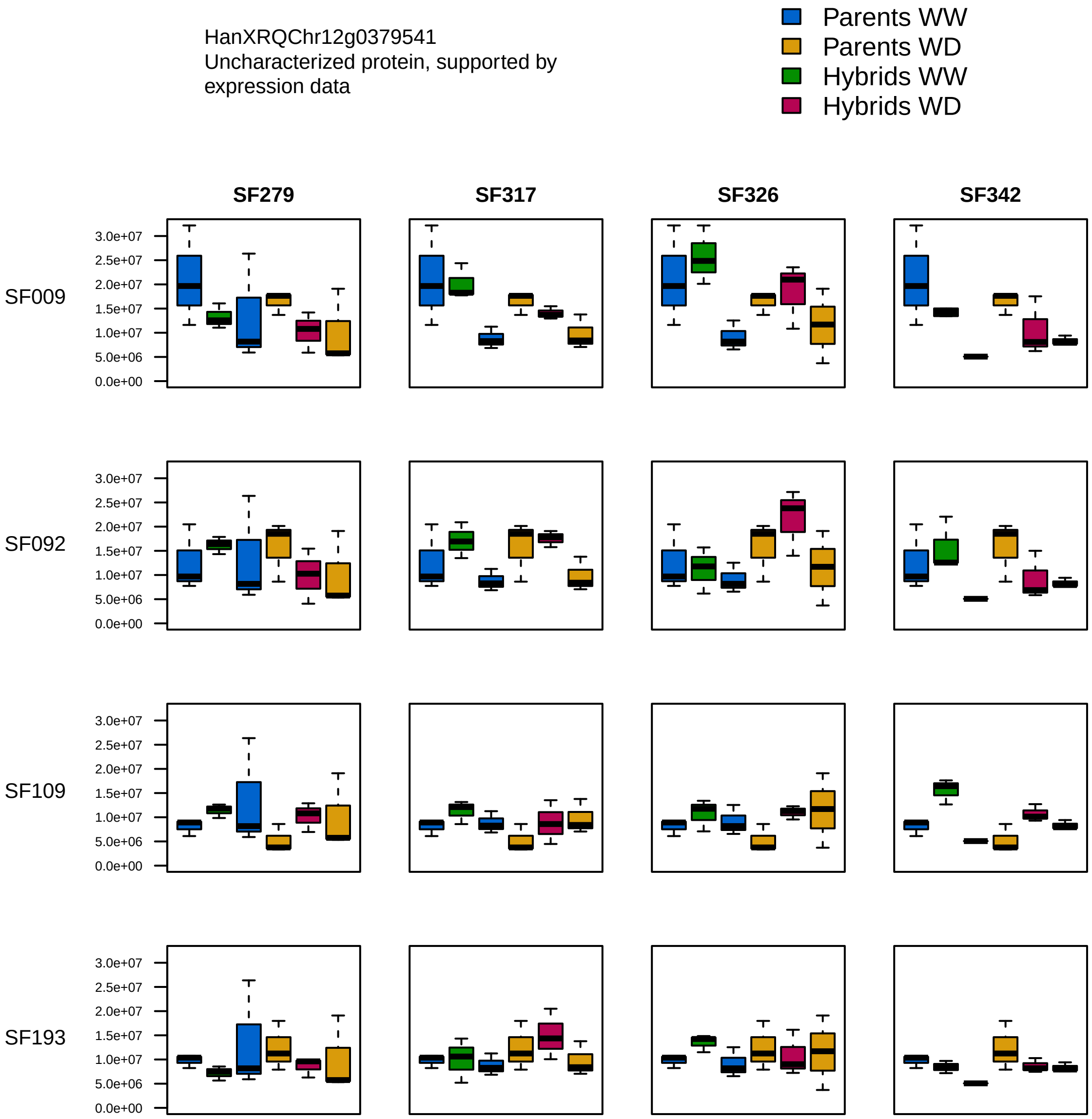

HanXRQChr12g0379541
Uncharacterized protein, supported by
expression data
Parents WW
Parents WD
Hybrids WW
Hybrids WD
SF279
SF317
SF326
SF342
SF009
SF092
SF109
SF193
3.0e+07
2.5e+07
2.0e+07
1.5e+07
1.0e+07
5.0e+06
0.0e+00

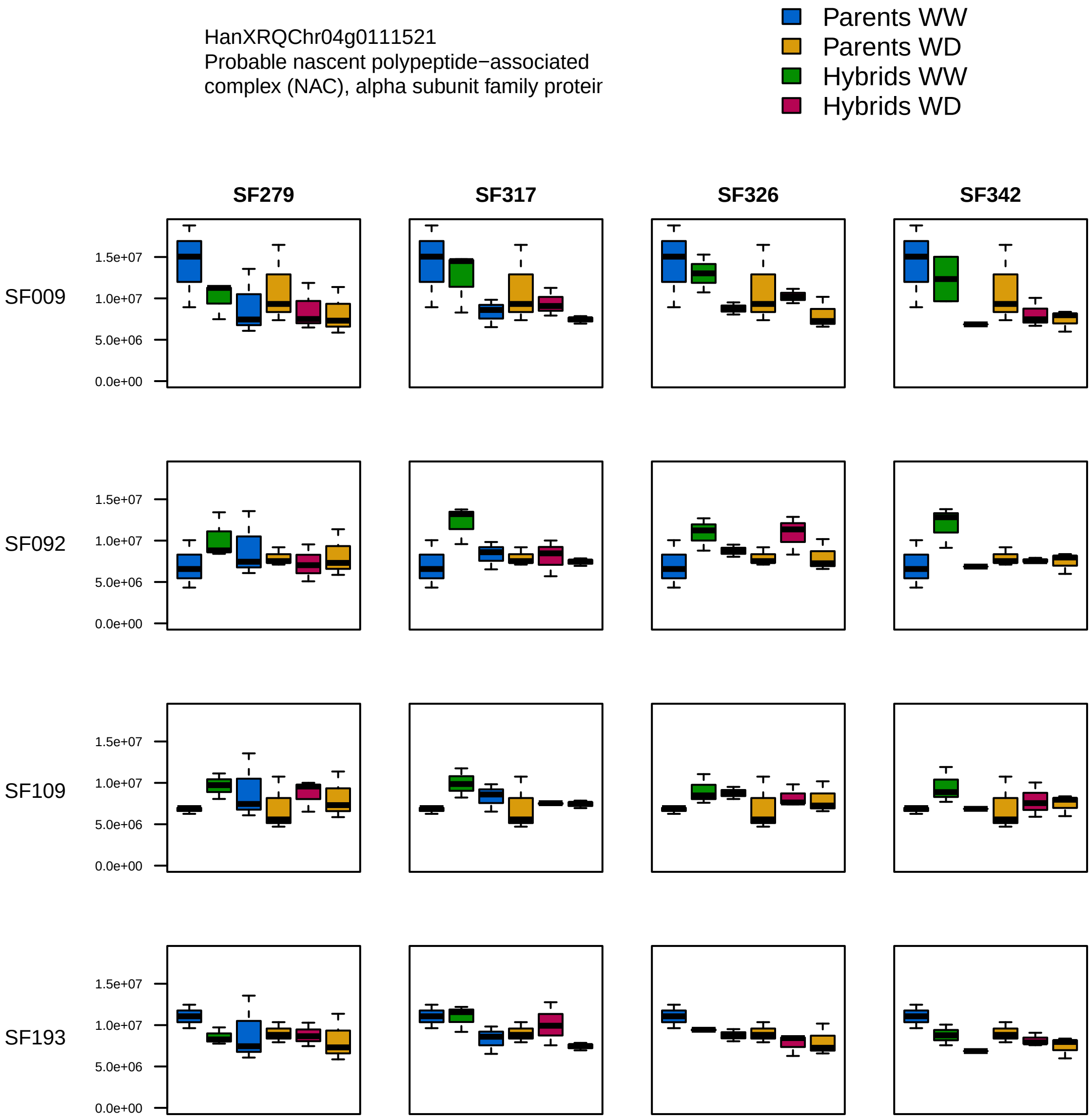
HanXRQChr04g0111521
Probable nascent polypeptide−associated
complex (NAC), alpha subunit family proteir
Parents WW
Parents WD
Hybrids WW
Hybrids WD
SF279
SF317
SF326
SF342
SF009
SF092
SF109
SF193
1.5e+07
1.0e+07
5.0e+06
0.0e+00

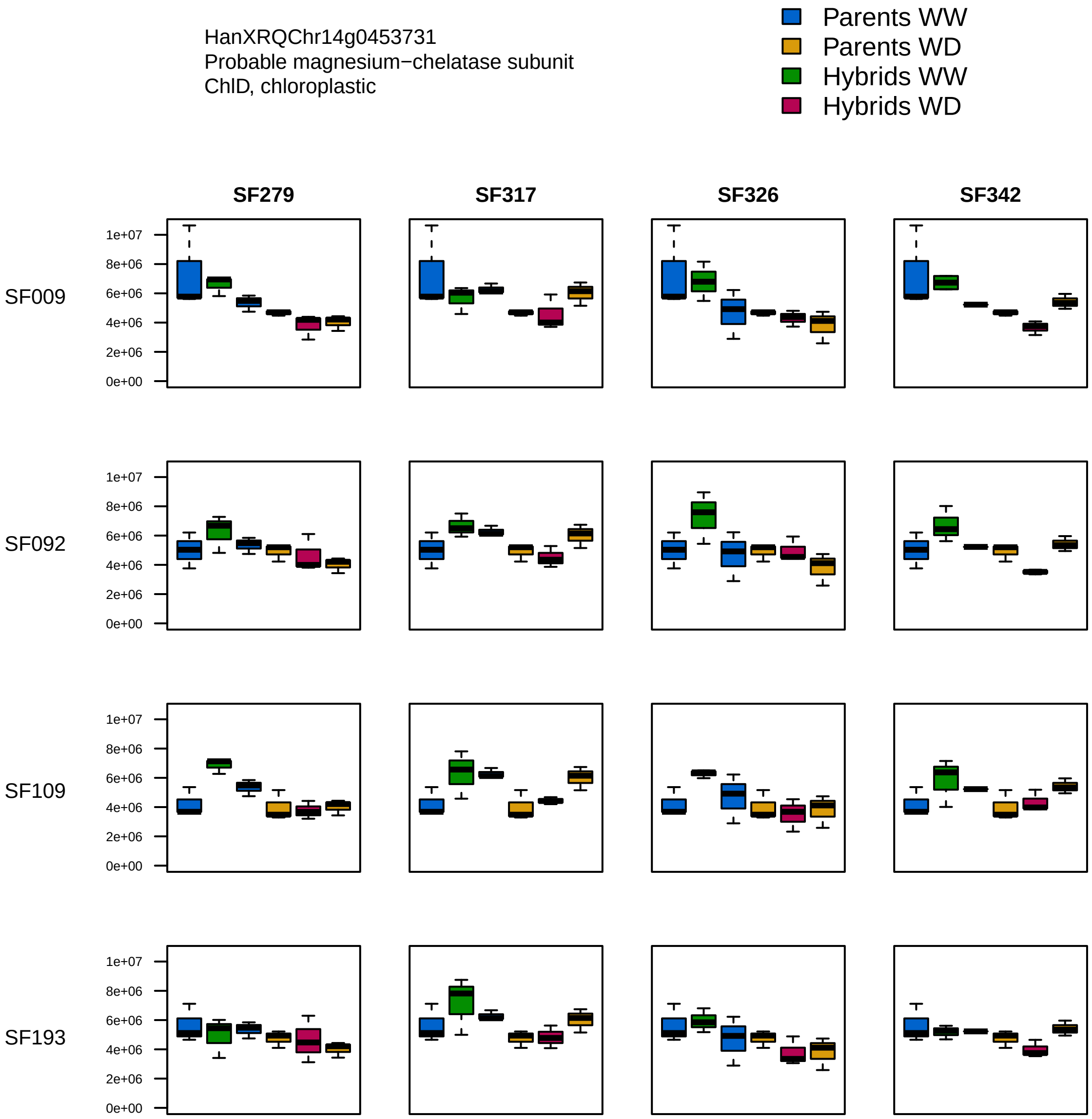
HanXRQChr14g0453731
Probable magnesium−chelatase subunit
ChlD, chloroplastic
Parents WW
Parents WD
Hybrids WW
Hybrids WD
SF279
SF317
SF326
SF342
SF009
SF092
SF109
SF193
1e+07
8e+06
6e+06
4e+06
2e+06
0e+00

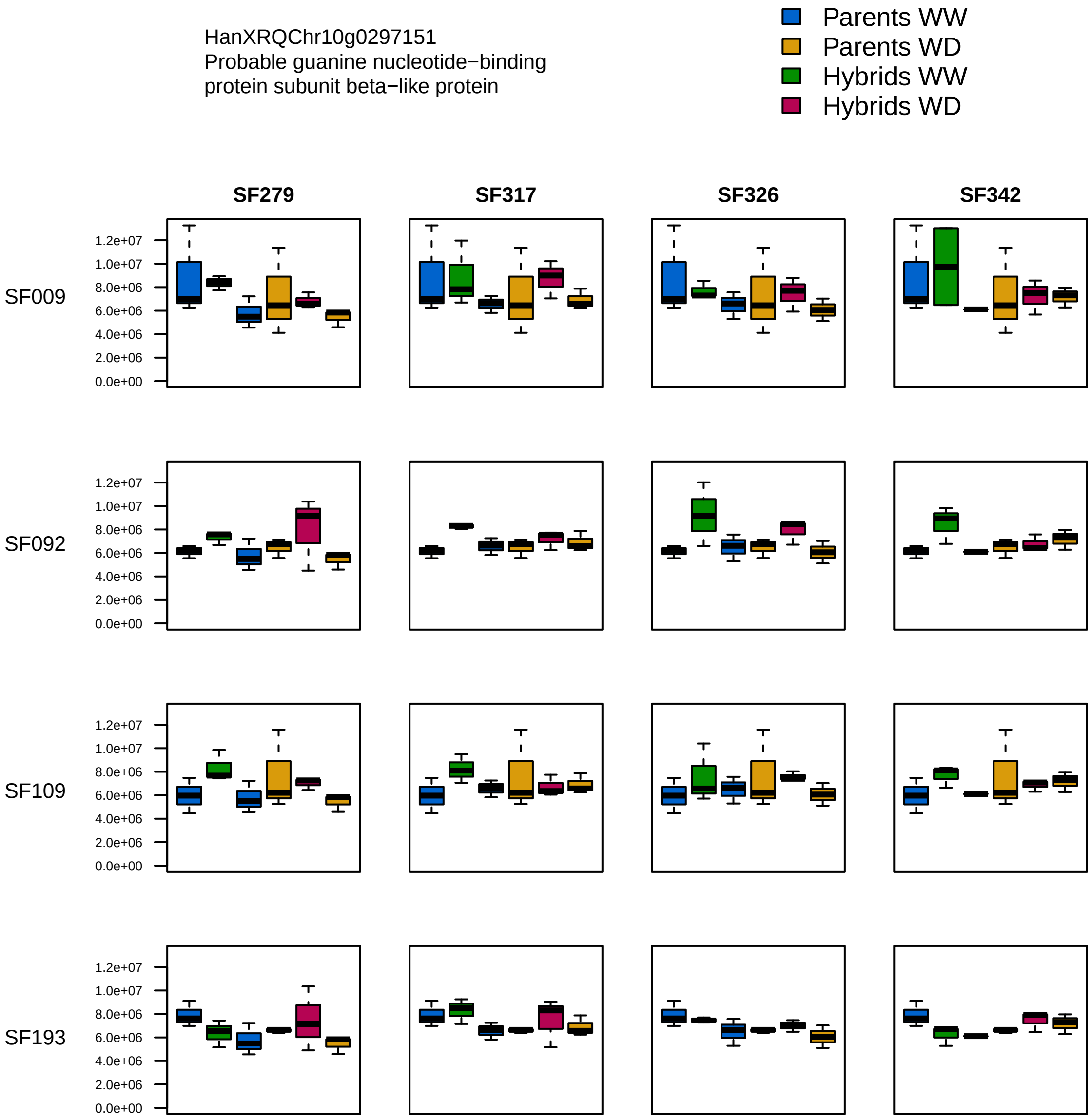
HanXRQChr10g0297151
Probable guanine nucleotide−binding
protein subunit beta−like protein
Parents WW
Parents WD
Hybrids WW
Hybrids WD
SF279
SF317
SF326
SF342
SF009
SF092
SF109
SF193
1.2e+07
1.0e+07
8.0e+06
6.0e+06
4.0e+06
2.0e+06
0.0e+00

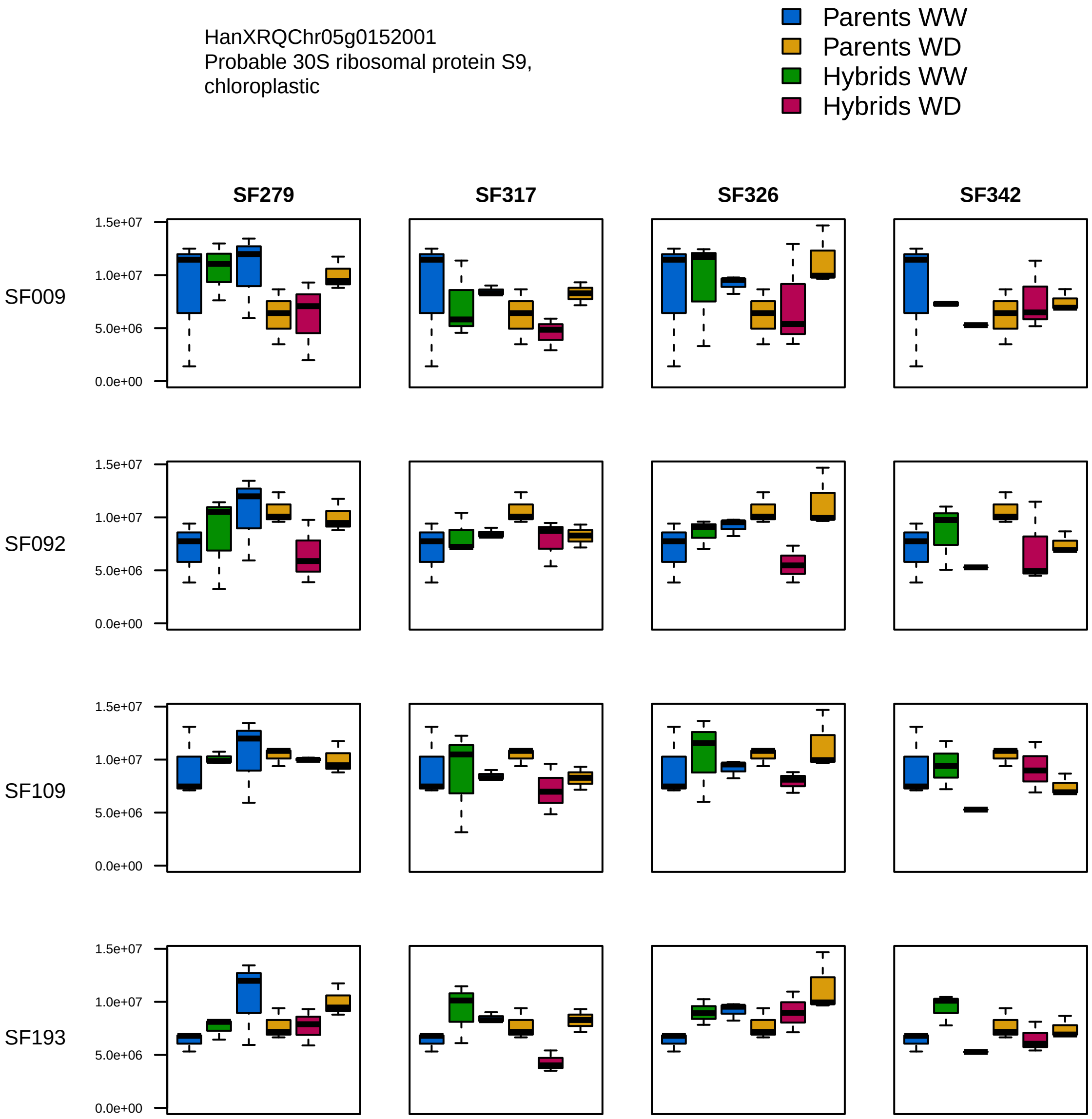
HanXRQChr05g0152001
Probable 30S ribosomal protein S9,
chloroplastic
Parents WW
Parents WD
Hybrids WW
Hybrids WD
SF279
SF317
SF326
SF342
SF009
SF092
SF109
SF193
1.5e+07
1.0e+07
5.0e+06
0.0e+00

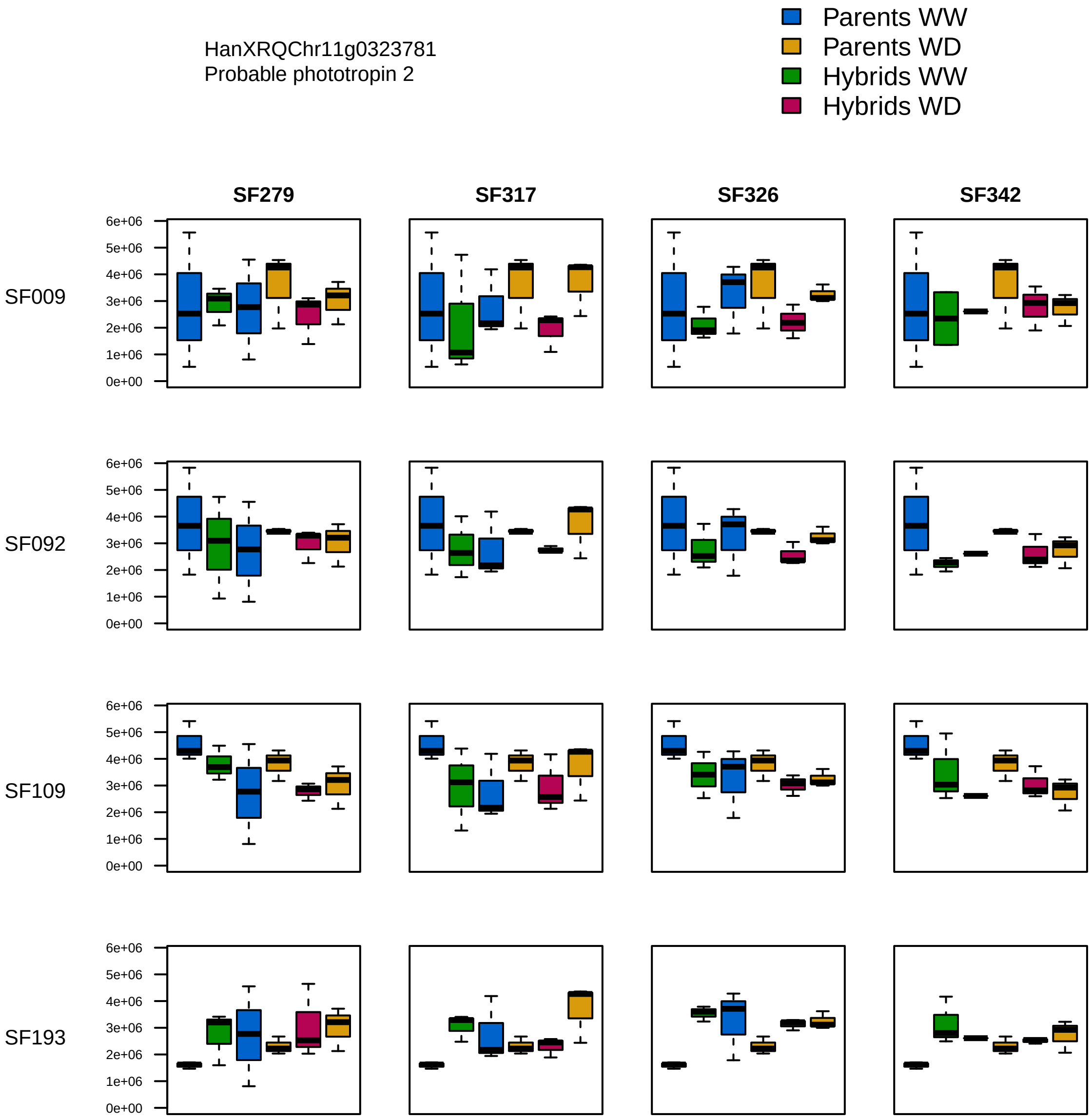
HanXRQChr11g0323781
Probable phototropin 2
Parents WW
Parents WD
Hybrids WW
Hybrids WD
SF279
SF317
SF326
SF342
SF009
SF092
SF109
SF193
0e+00
1e+06
2e+06
3e+06
4e+06
5e+06
6e+06

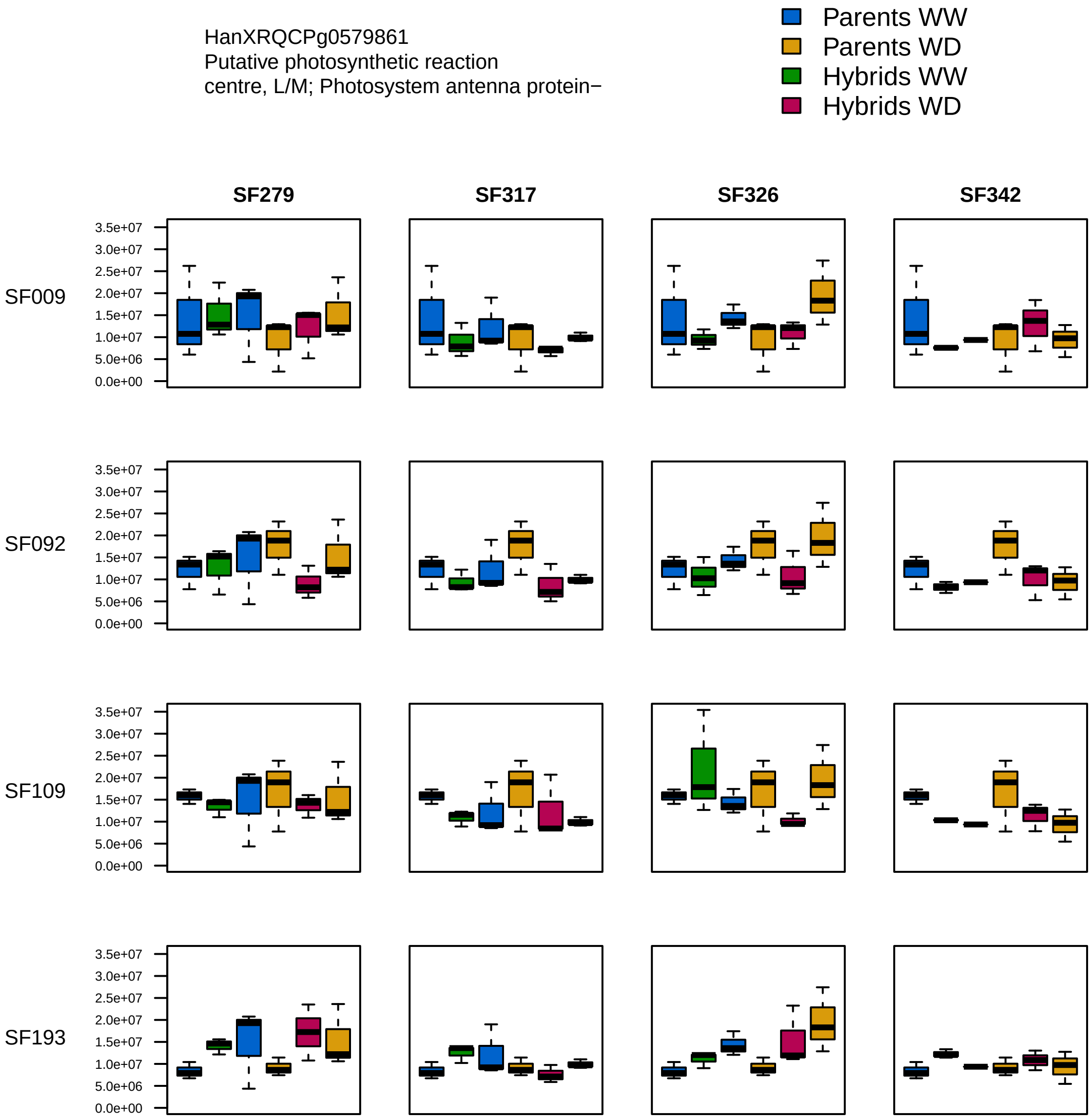
HanXRQCPg0579861
Putative photosynthetic reaction
centre, L/M; Photosystem antenna protein−
Parents WW
Parents WD
Hybrids WW
Hybrids WD
SF279
SF317
SF326
SF342
SF009
SF092
SF109
SF193
3.5e+07
3.0e+07
2.5e+07
2.0e+07
1.5e+07
1.0e+07
5.0e+06
0.0e+00

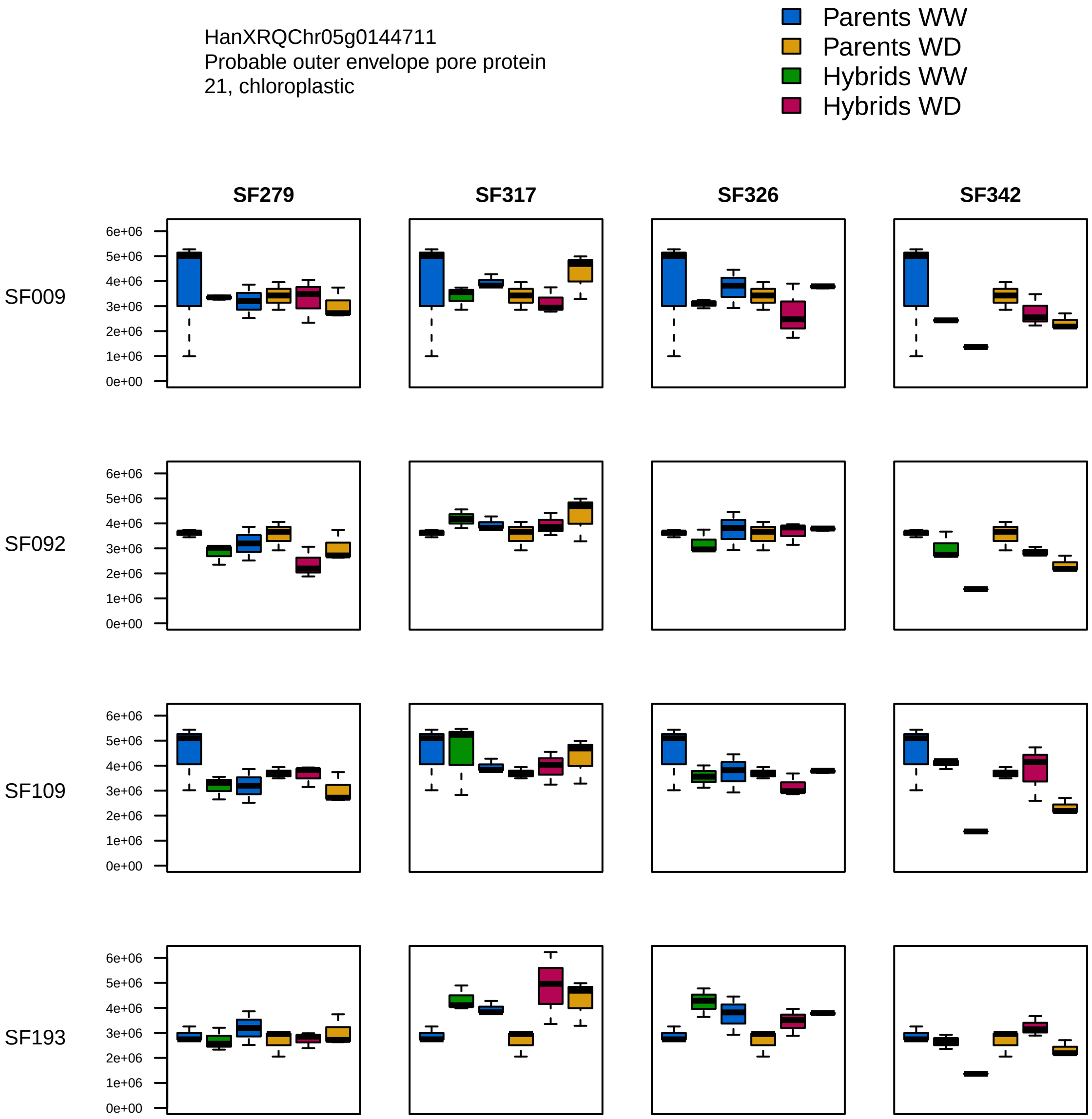
HanXRQChr05g0144711
Probable outer envelope pore protein
21, chloroplastic
Parents WW
Parents WD
Hybrids WW
Hybrids WD
SF279
SF317
SF326
SF342
SF009
SF092
SF109
SF193
0e+00
1e+06
2e+06
3e+06
4e+06
5e+06
6e+06

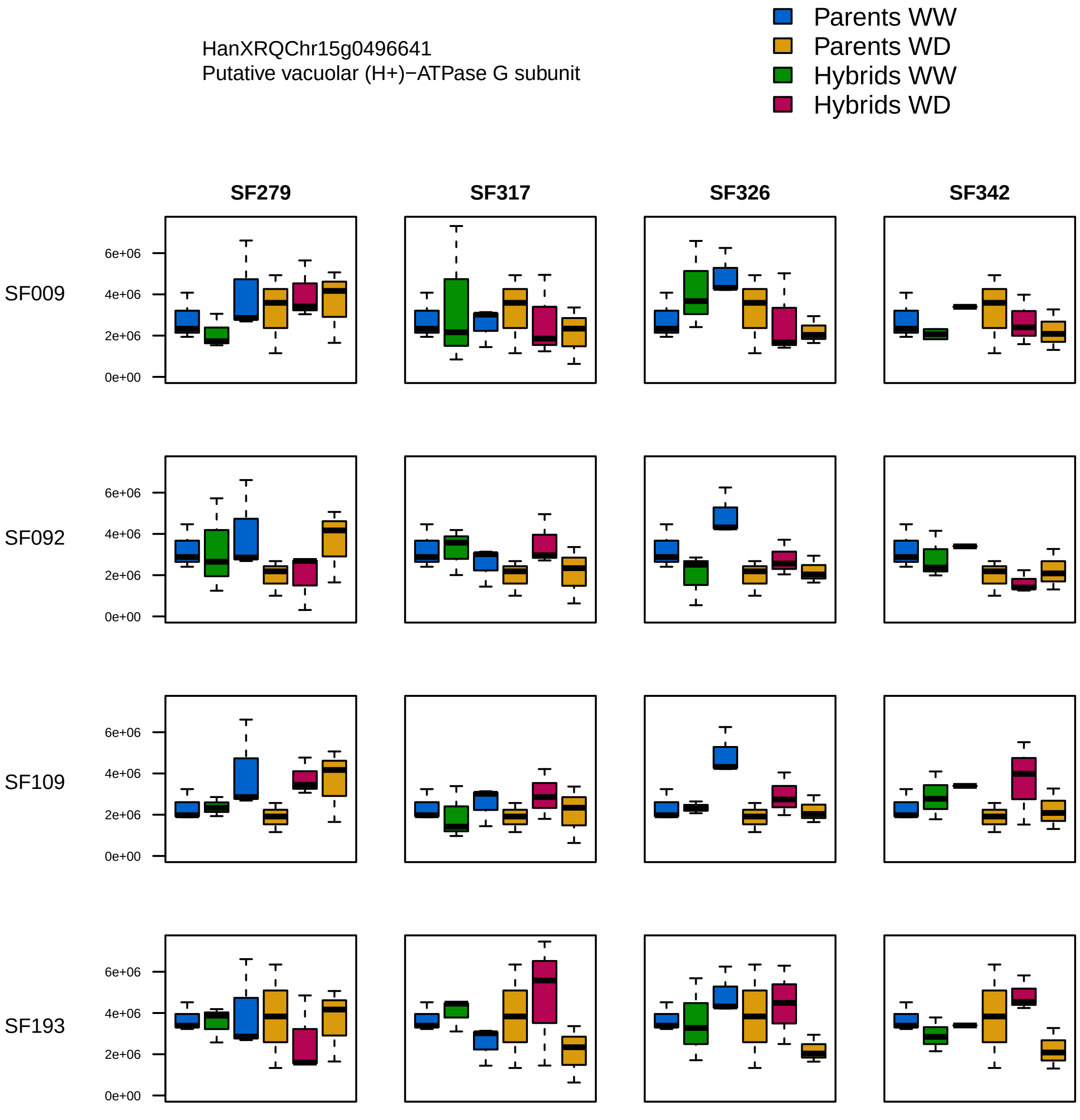
HanXRQChr15g0496641
Putative vacuolar (H+)−ATPase G subunit
Parents WW
Parents WD
Hybrids WW
Hybrids WD
SF279
SF317
SF326
SF342
SF009
SF092
SF109
SF193
6e+06
4e+06
2e+06
0e+00

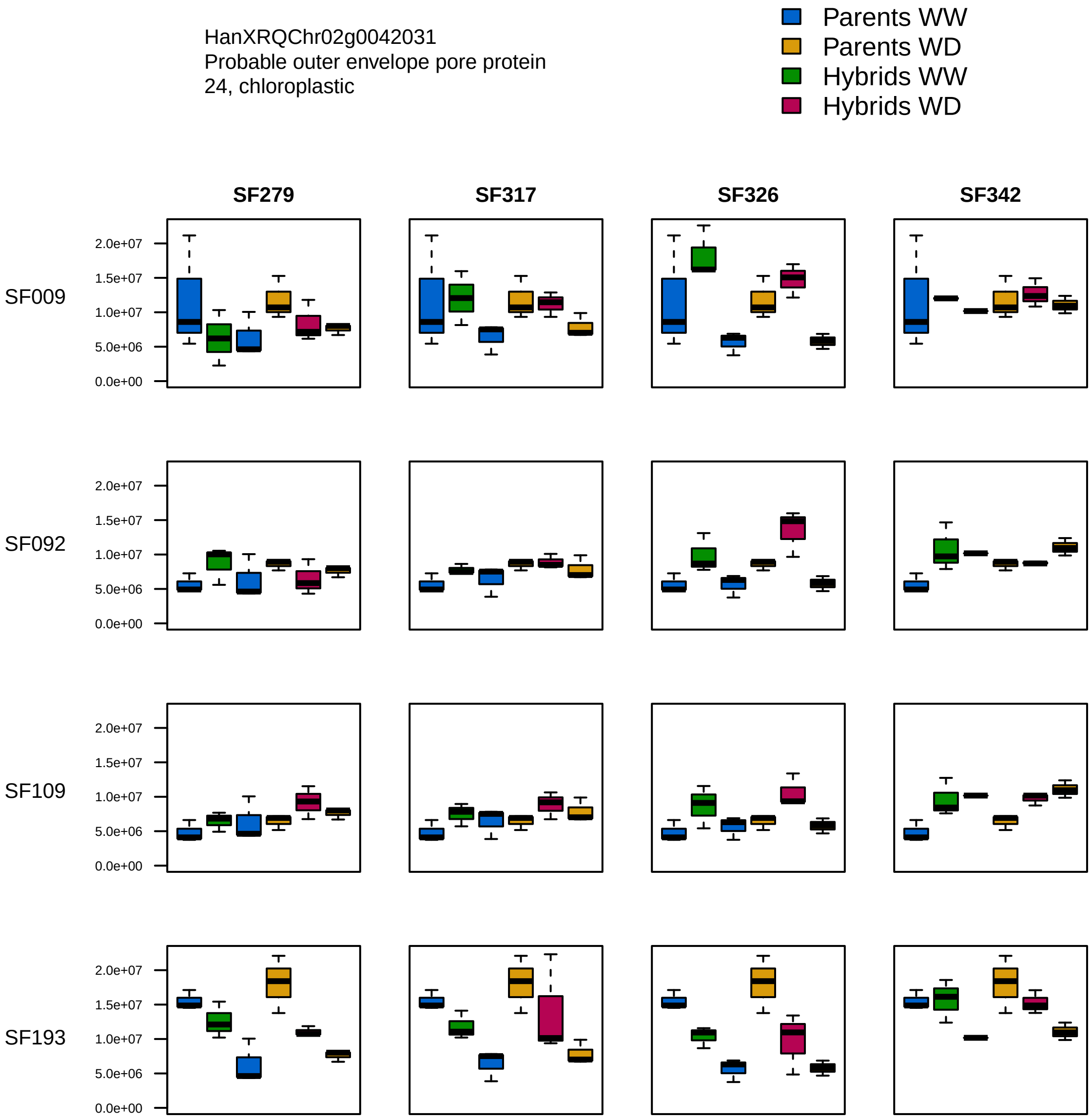

HanXRQChr02g0042031
Probable outer envelope pore protein
24, chloroplastic
Parents WW
Parents WD
Hybrids WW
Hybrids WD
SF279
SF317
SF326
SF342
SF009
SF092
SF109
SF193
2.0e+07
1.5e+07
1.0e+07
5.0e+06
0.0e+00

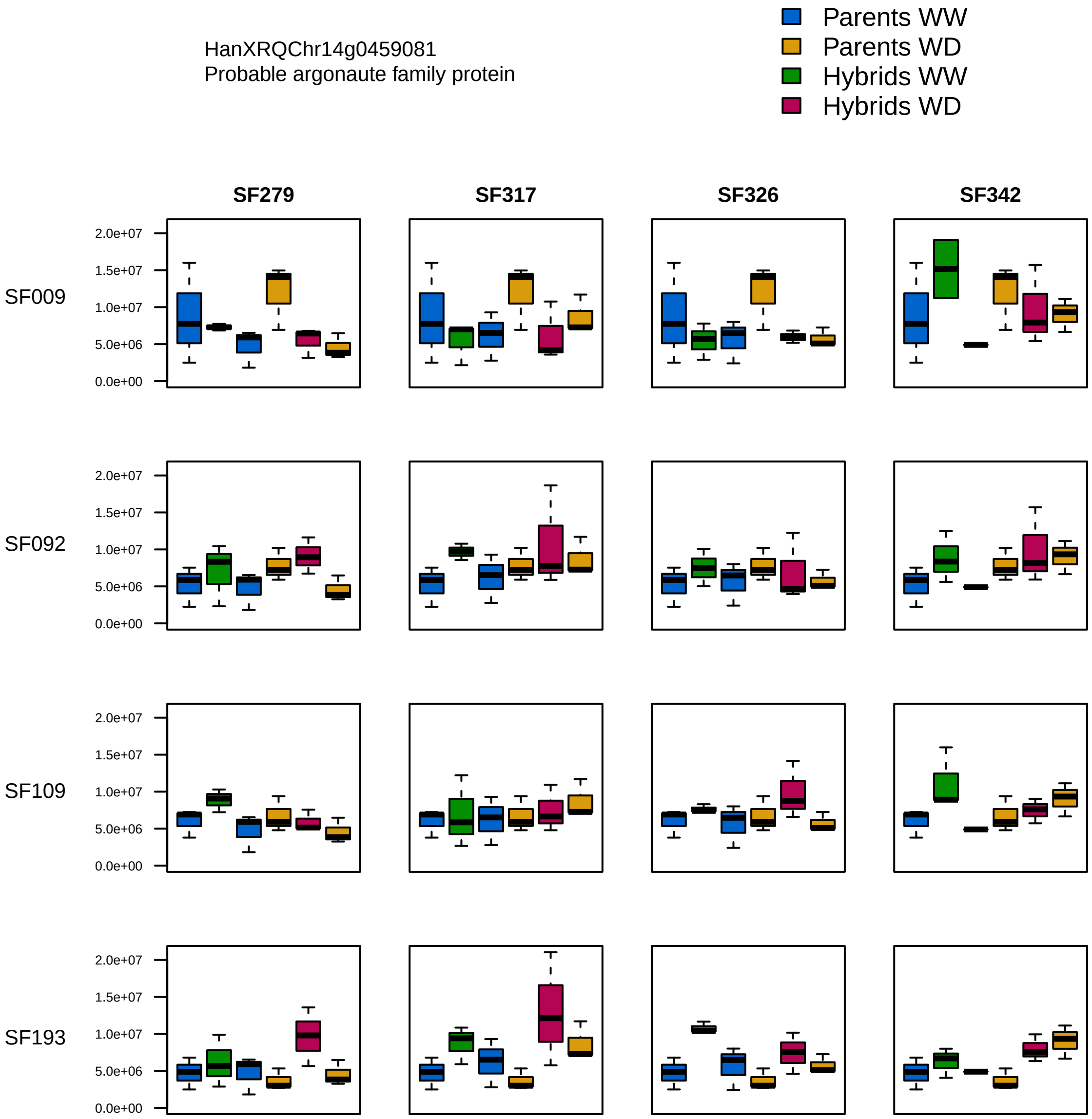
HanXRQChr14g0459081
Probable argonaute family protein
Parents WW
Parents WD
Hybrids WW
Hybrids WD
SF279
SF317
SF326
SF342
SF009
SF092
SF109
SF193
2.0e+07
1.5e+07
1.0e+07
5.0e+06
0.0e+00

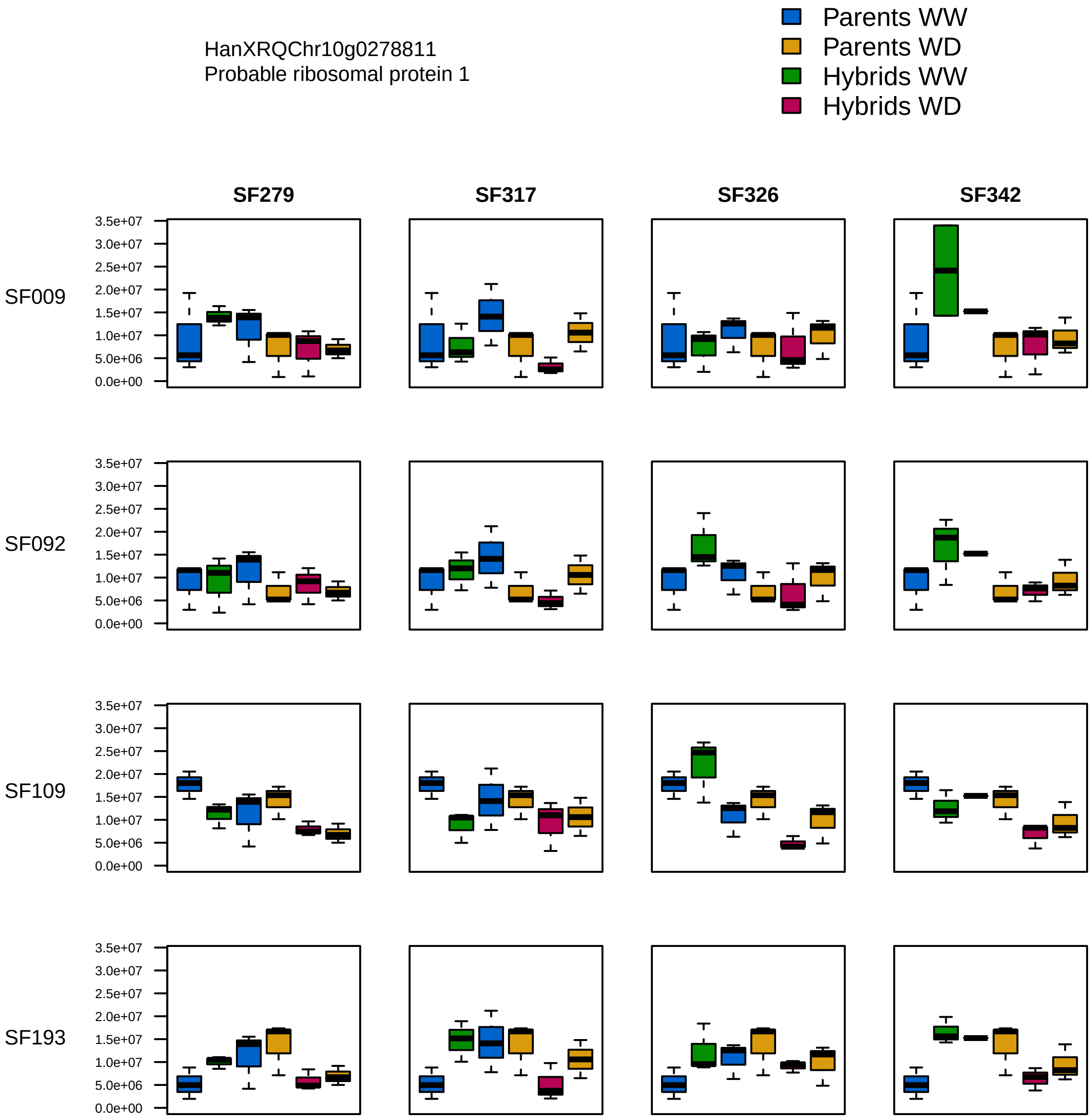
HanXRQChr10g0278811
Probable ribosomal protein 1
Parents WW
Parents WD
Hybrids WW
Hybrids WD
SF279
SF317
SF326
SF342
SF009
SF092
SF109
SF193
3.5e+07
3.0e+07
2.5e+07
2.0e+07
1.5e+07
1.0e+07
5.0e+06
0.0e+00

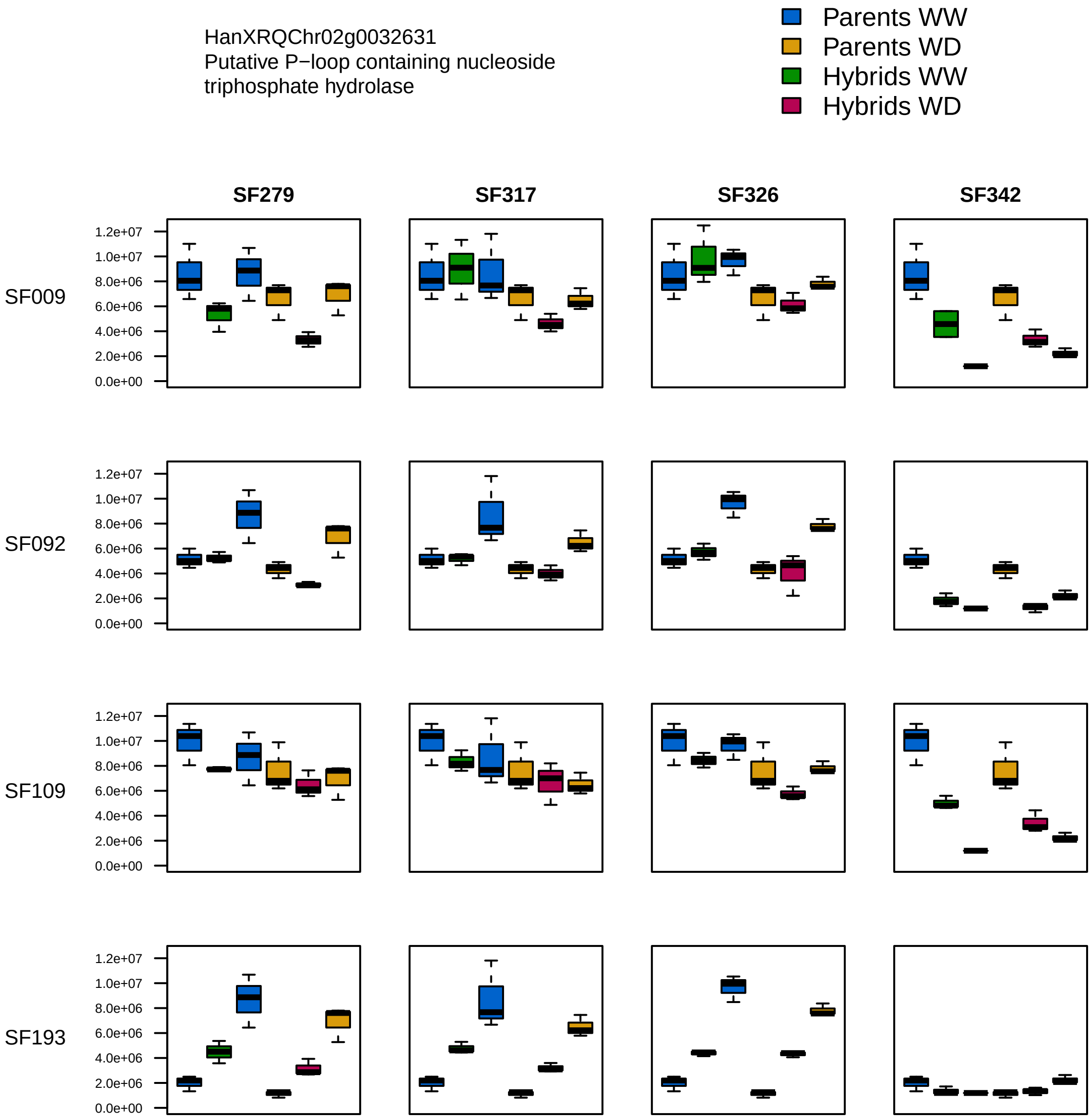
HanXRQChr02g0032631
Putative P−loop containing nucleoside
triphosphate hydrolase
Parents WW
Parents WD
Hybrids WW
Hybrids WD
SF279
SF317
SF326
SF342
SF009
SF092
SF109
SF193
1.2e+07
1.0e+07
8.0e+06
6.0e+06
4.0e+06
2.0e+06
0.0e+00

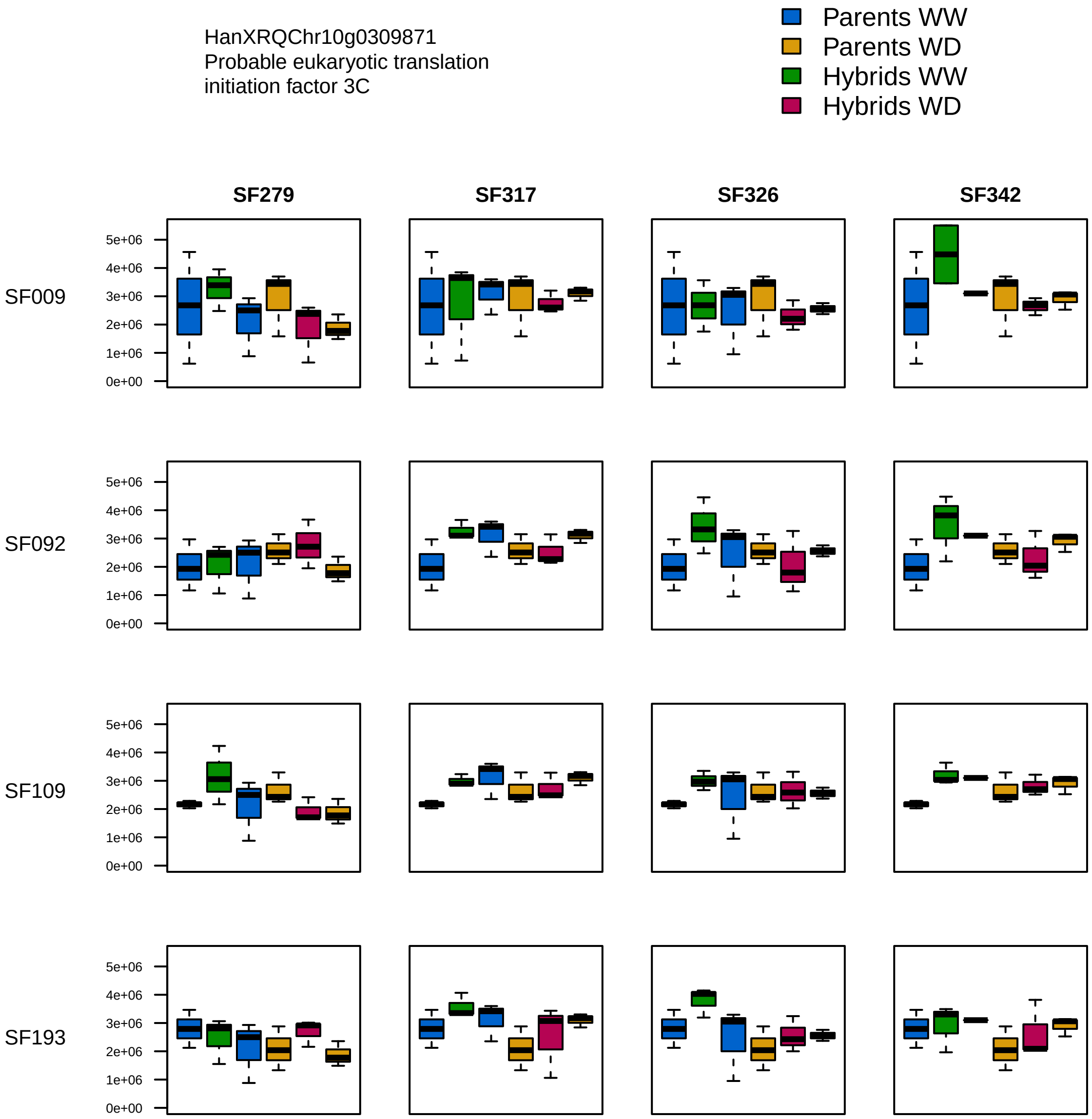

HanXRQChr10g0309871
Probable eukaryotic translation
initiation factor 3C
Parents WW
Parents WD
Hybrids WW
Hybrids WD
SF279
SF317
SF326
SF342
SF009
SF092
SF109
SF193
5e+06
4e+06
3e+06
2e+06
1e+06
0e+00

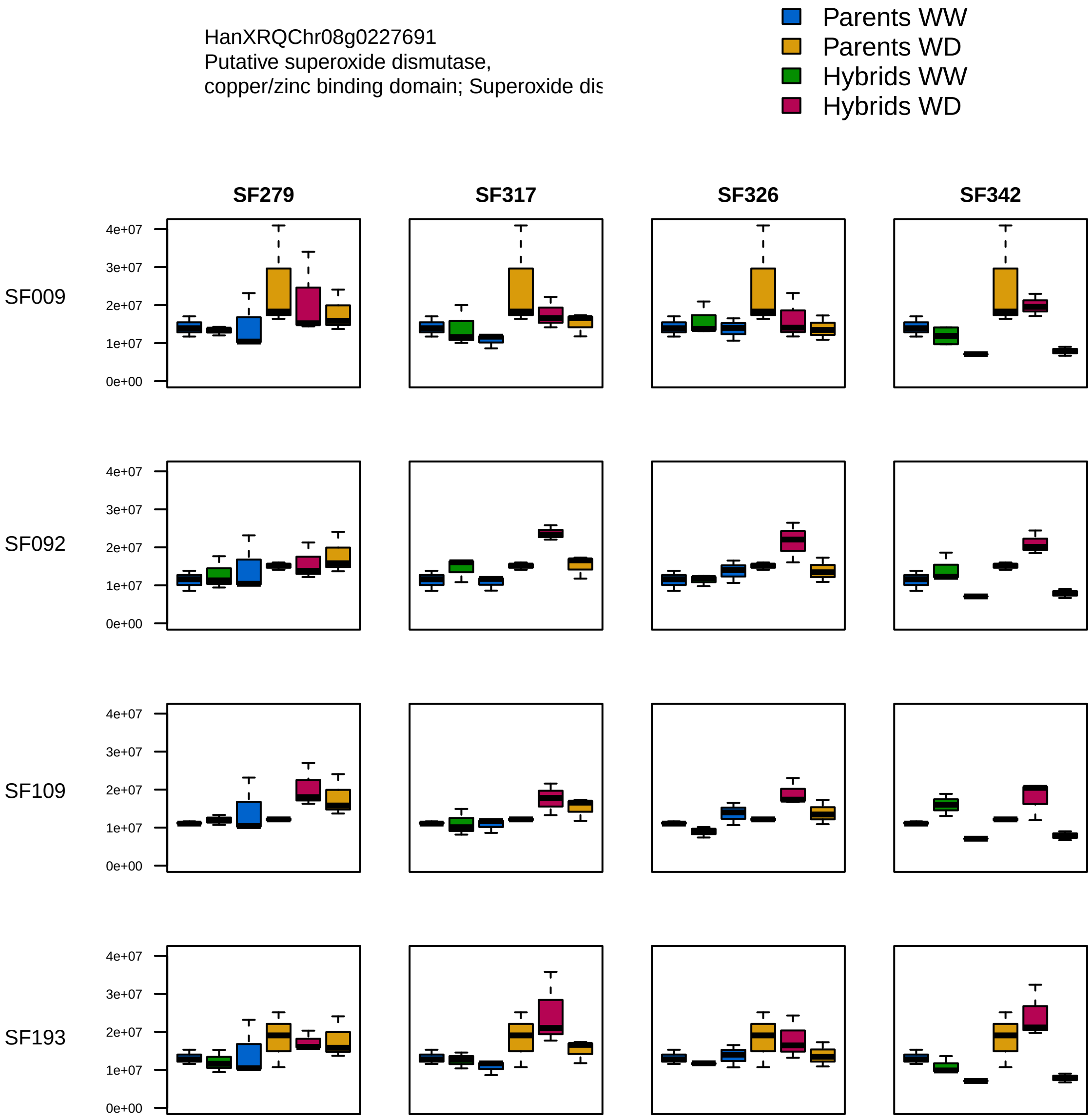
HanXRQChr08g0227691
Putative superoxide dismutase,
copper/zinc binding domain; Superoxide dis
Parents WW
Parents WD
Hybrids WW
Hybrids WD
SF279
SF317
SF326
SF342
SF009
SF092
SF109
SF193
4e+07
3e+07
2e+07
1e+07
0e+00

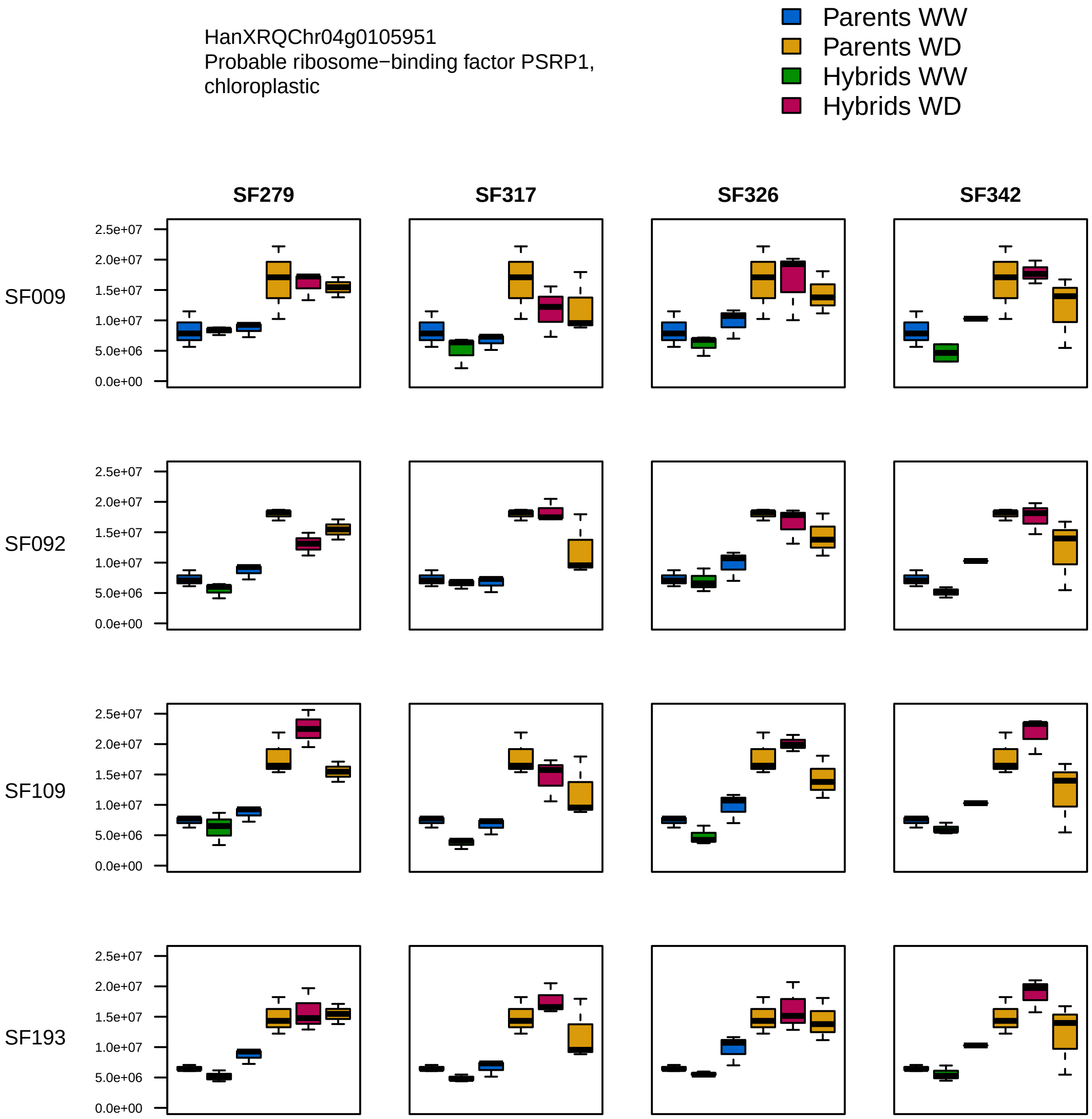

HanXRQChr04g0105951
Probable ribosome−binding factor PSRP1, chloroplastic
Parents WW
Parents WD
Hybrids WW
Hybrids WD
SF279
SF317
SF326
SF342
SF009
SF092
SF109
SF193
2.5e+07
2.0e+07
1.5e+07
1.0e+07
5.0e+06
0.0e+00

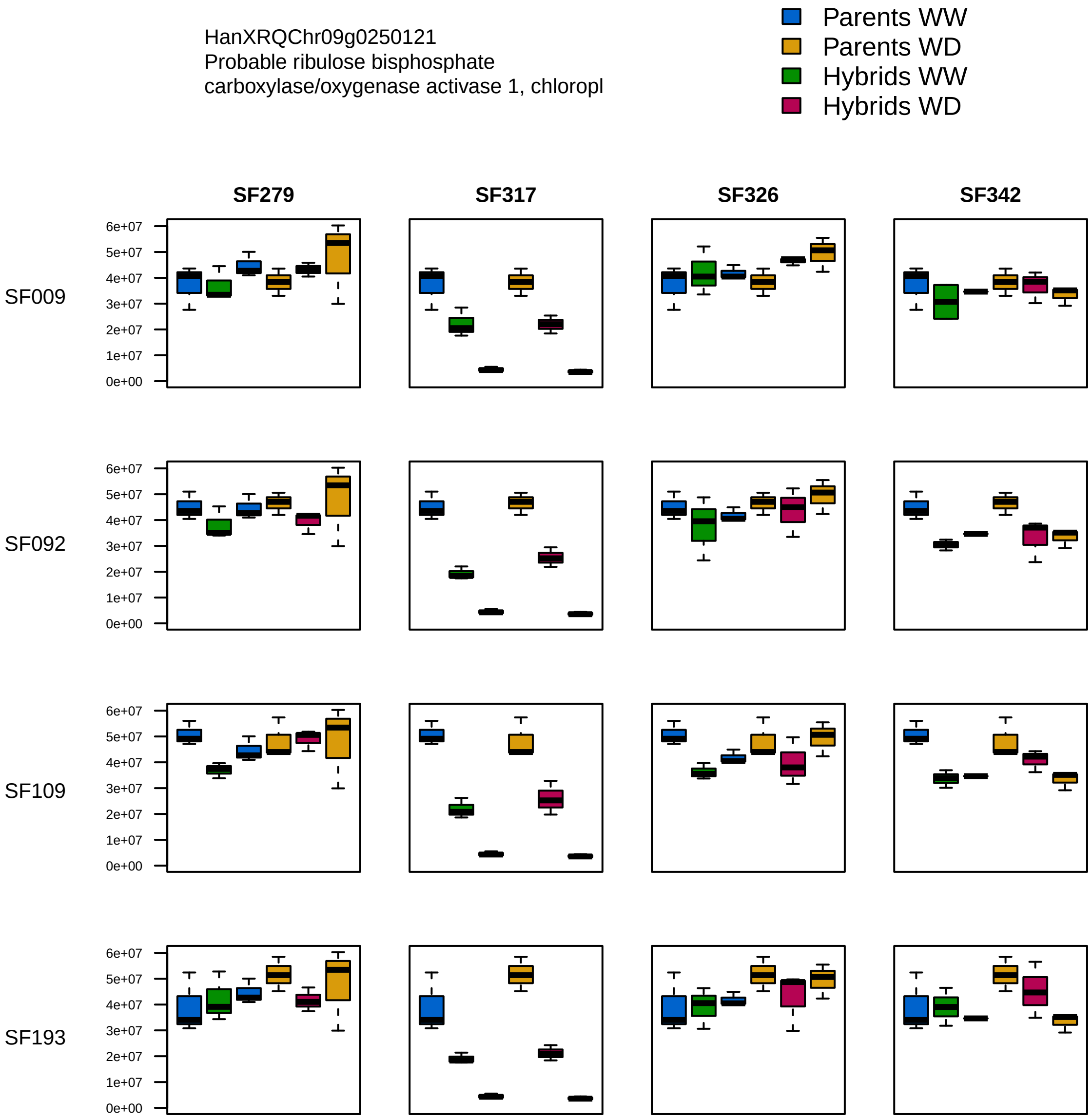

HanXRQChr09g0250121
Probable ribulose bisphosphate
carboxylase/oxygenase activase 1, chloropl
Parents WW
Parents WD
Hybrids WW
Hybrids WD
SF279
SF317
SF326
SF342
SF009
SF092
SF109
SF193
0e+00
1e+07
2e+07
3e+07
4e+07
5e+07
6e+07

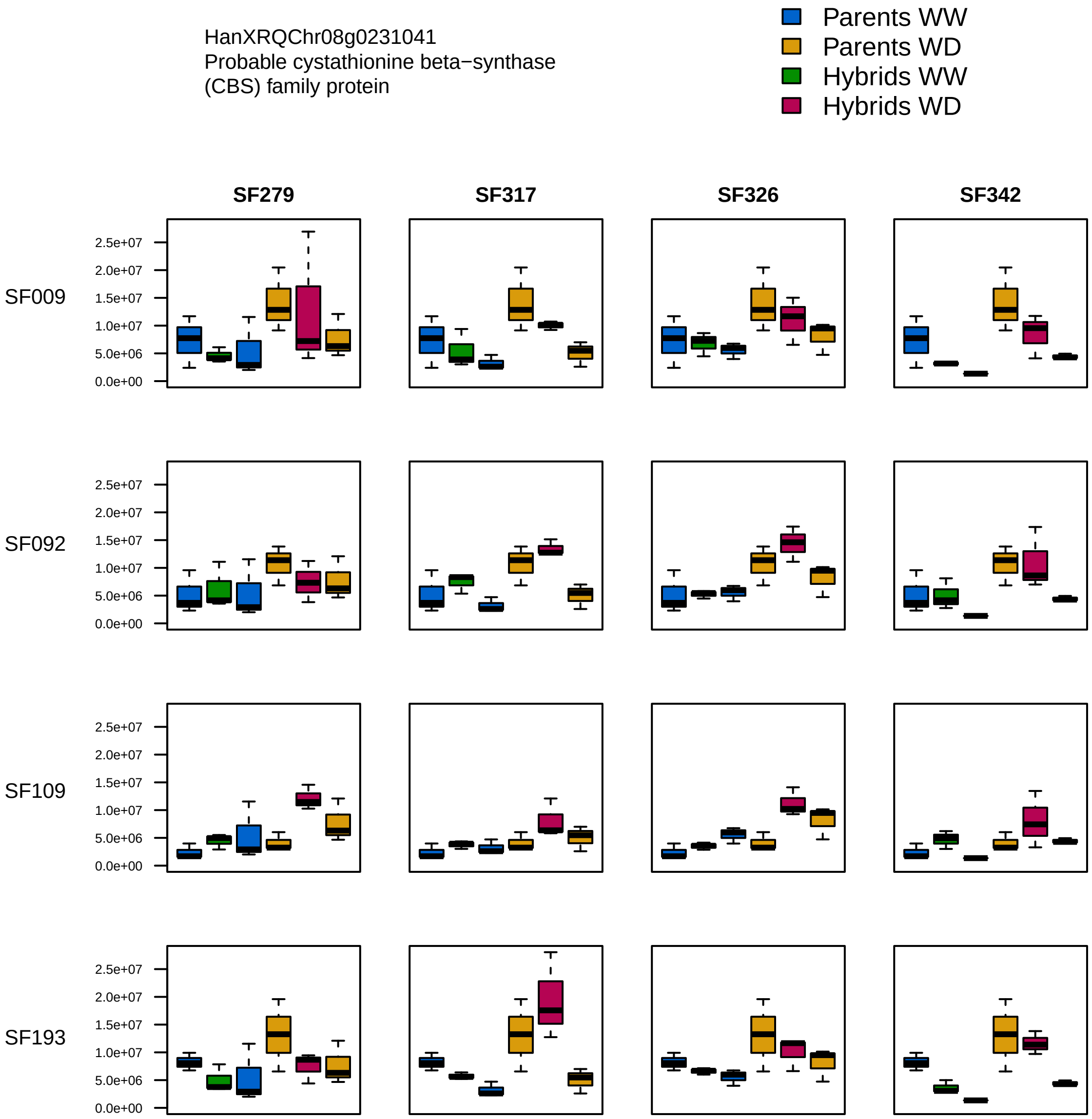

HanXRQChr08g0231041
Probable cystathionine beta−synthase
(CBS) family protein
Parents WW
Parents WD
Hybrids WW
Hybrids WD
SF279
SF317
SF326
SF342
SF009
SF092
SF109
SF193
2.5e+07
2.0e+07
1.5e+07
1.0e+07
5.0e+06
0.0e+00

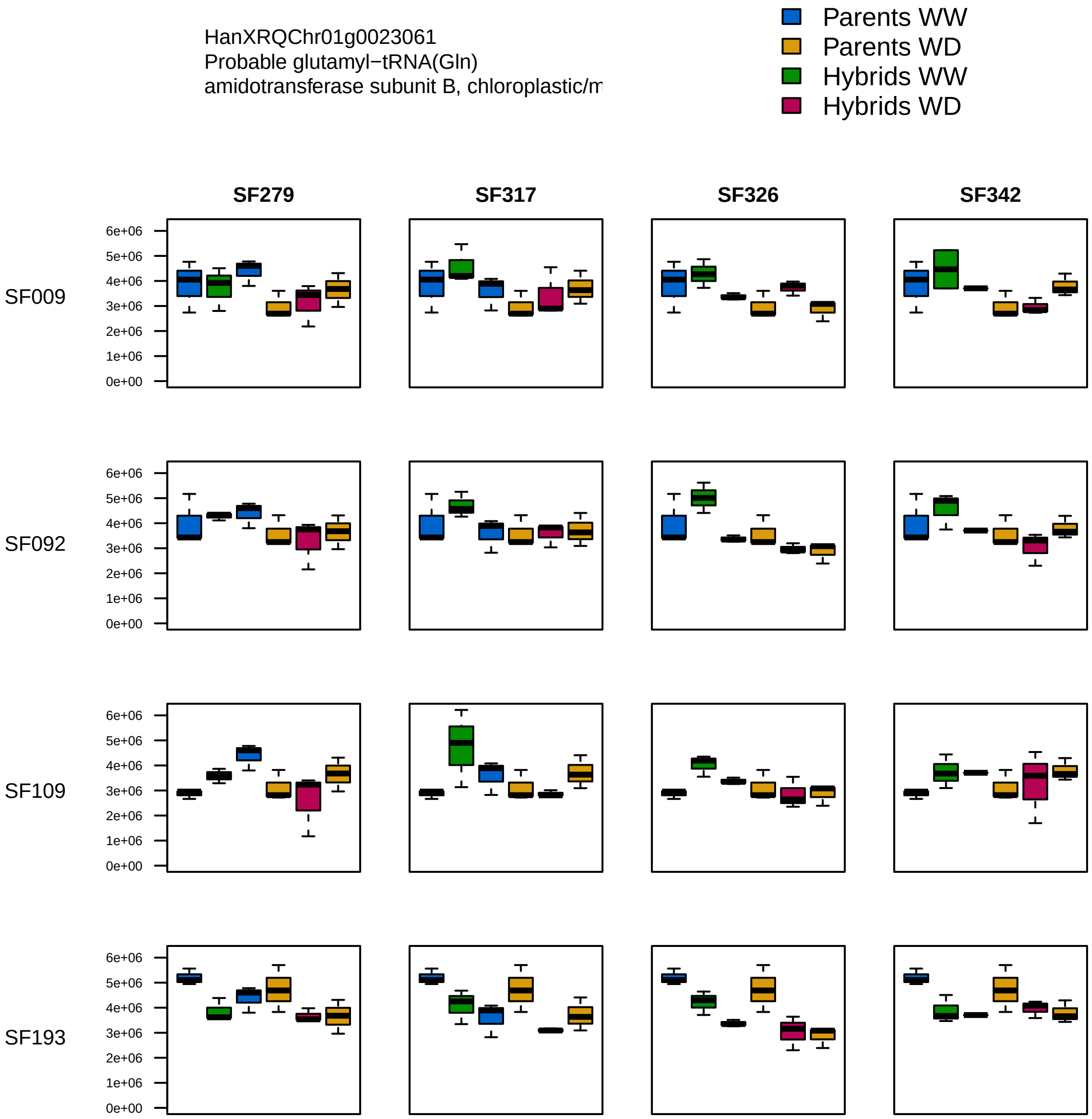

HanXRQChr01g0023061
Probable glutamyl−tRNA(Gln)
amidotransferase subunit B, chloroplastic/m
Parents WW
Parents WD
Hybrids WW
Hybrids WD
SF279
SF317
SF326
SF342
SF009
SF092
SF109
SF193
6e+06
5e+06
4e+06
3e+06
2e+06
1e+06
0e+00

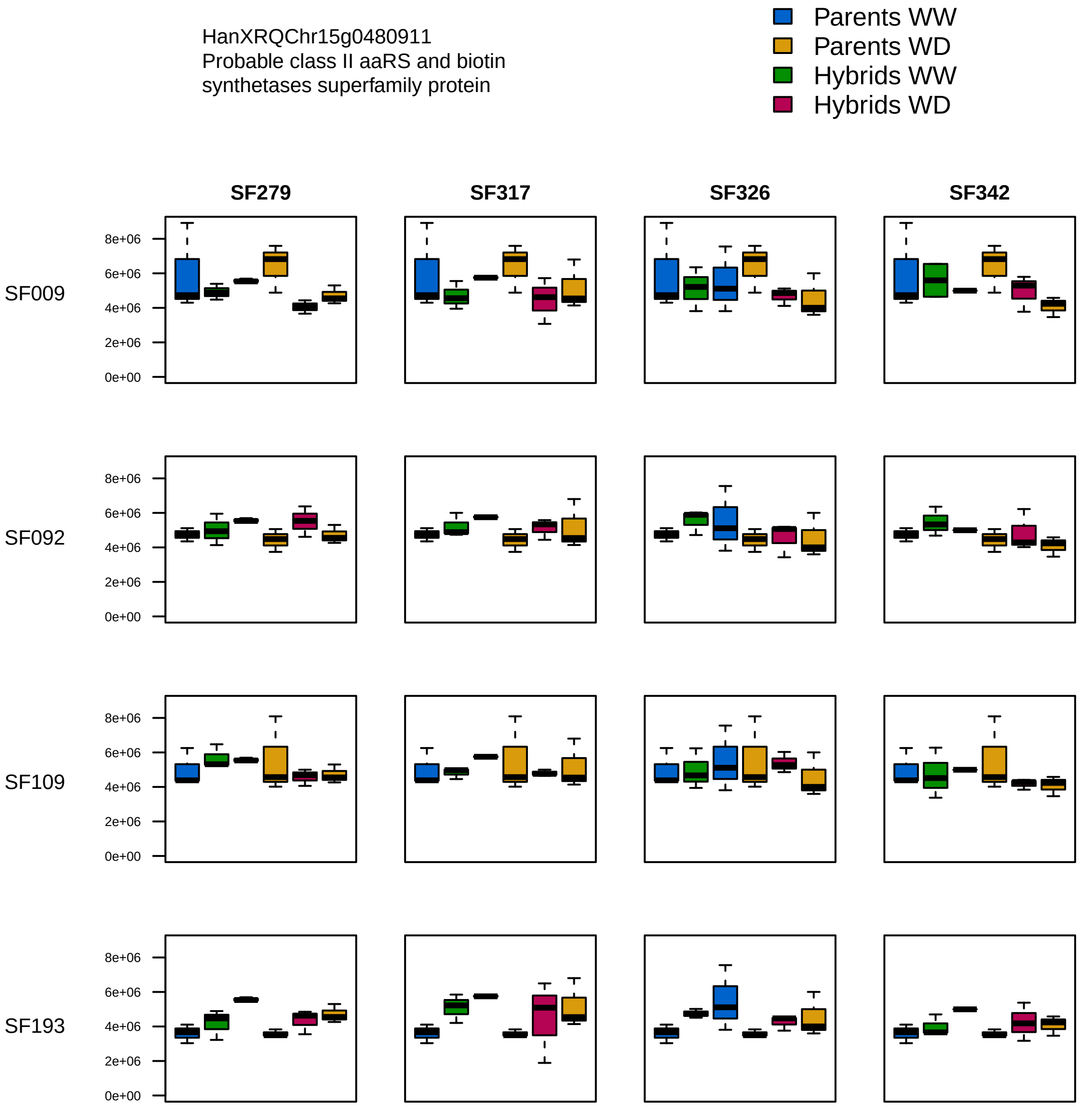

HanXRQChr15g0480911
Probable class II aaRS and biotin
synthetases superfamily protein
Parents WW
Parents WD
Hybrids WW
Hybrids WD
SF279
SF317
SF326
SF342
SF009
SF092
SF109
SF193
0e+00
2e+06
4e+06
6e+06
8e+06

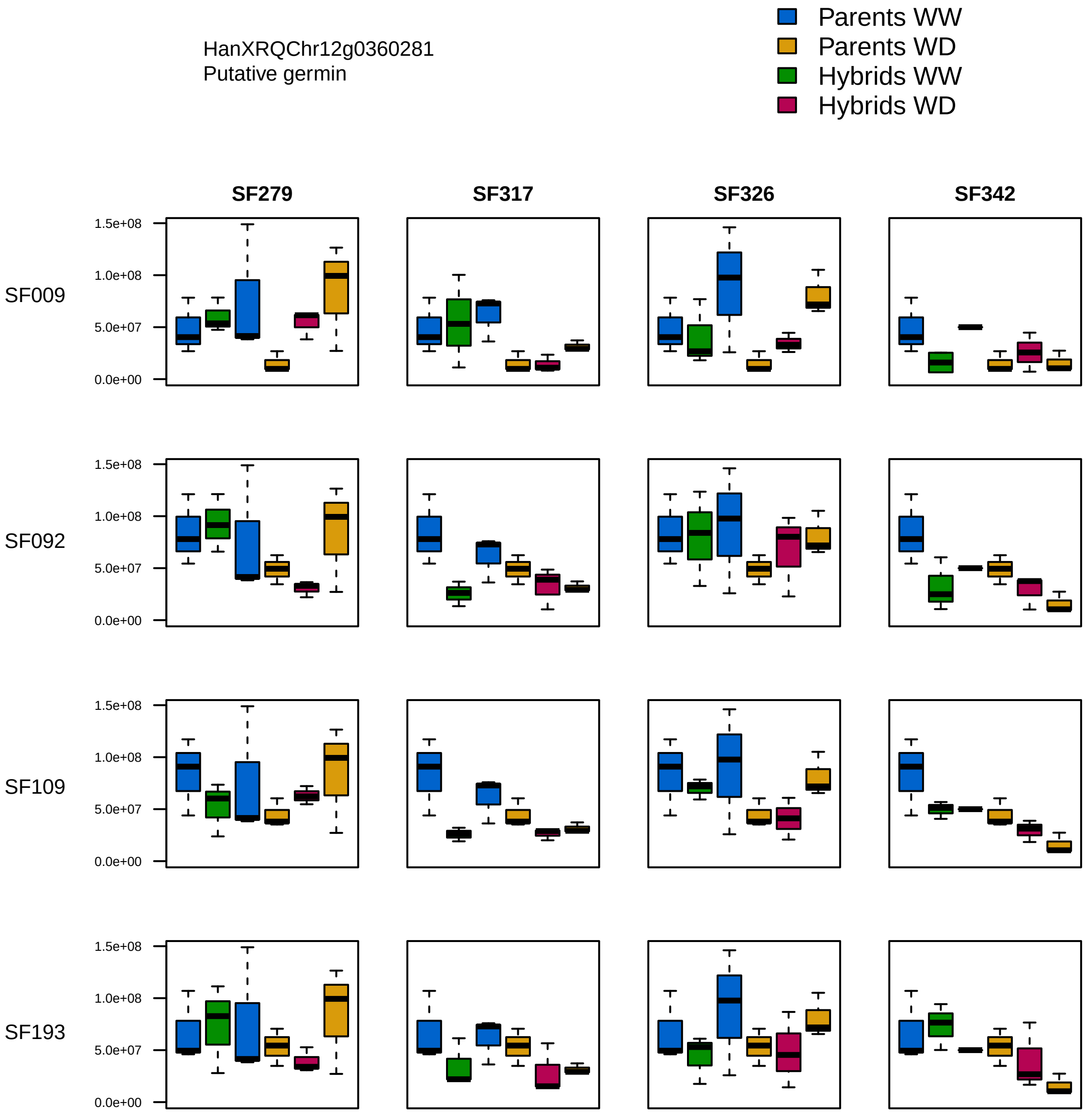
HanXRQChr12g0360281
Putative germin
Parents WW
Parents WD
Hybrids WW
Hybrids WD
SF279
SF317
SF326
SF342
SF009
SF092
SF109
SF193
1.5e+08
1.0e+08
5.0e+07
0.0e+00

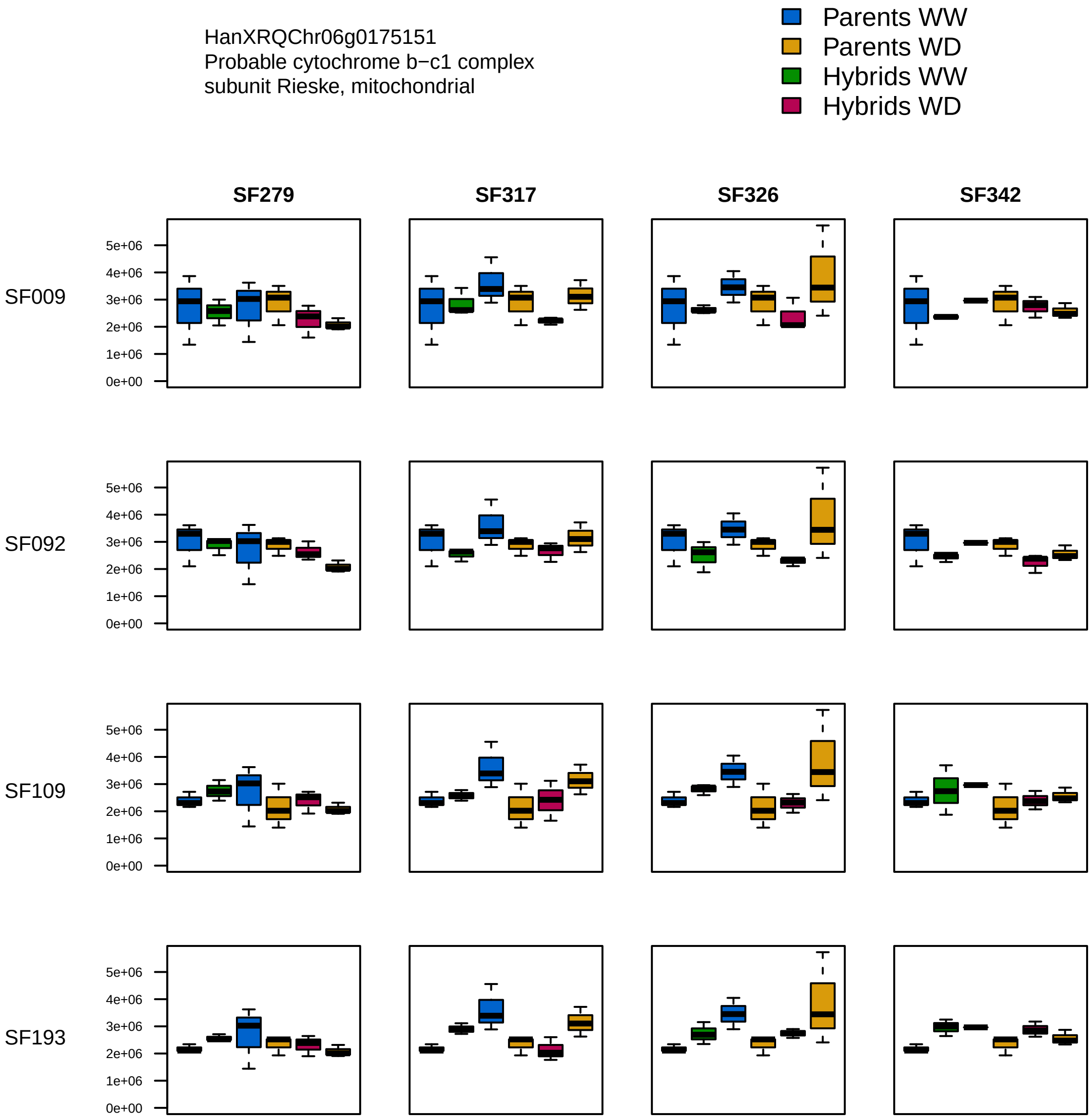
HanXRQChr06g0175151
Probable cytochrome b−c1 complex
subunit Rieske, mitochondrial
Parents WW
Parents WD
Hybrids WW
Hybrids WD
SF279
SF317
SF326
SF342
SF009
SF092
SF109
SF193
0e+00
1e+06
2e+06
3e+06
4e+06
5e+06

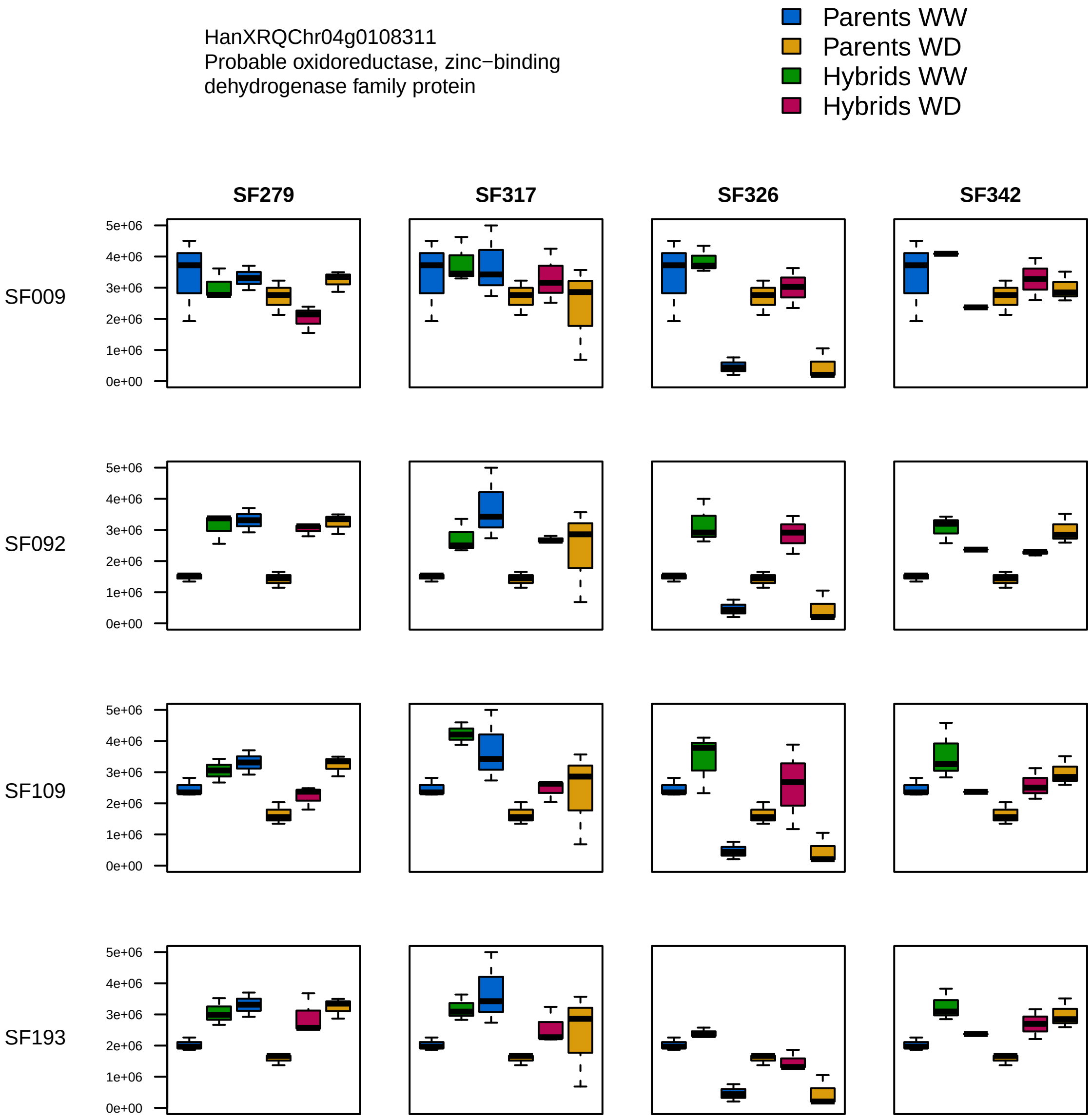
HanXRQChr04g0108311
Probable oxidoreductase, zinc−binding
dehydrogenase family protein
Parents WW
Parents WD
Hybrids WW
Hybrids WD
SF279
SF317
SF326
SF342
SF009
SF092
SF109
SF193
5e+06
4e+06
3e+06
2e+06
1e+06
0e+00

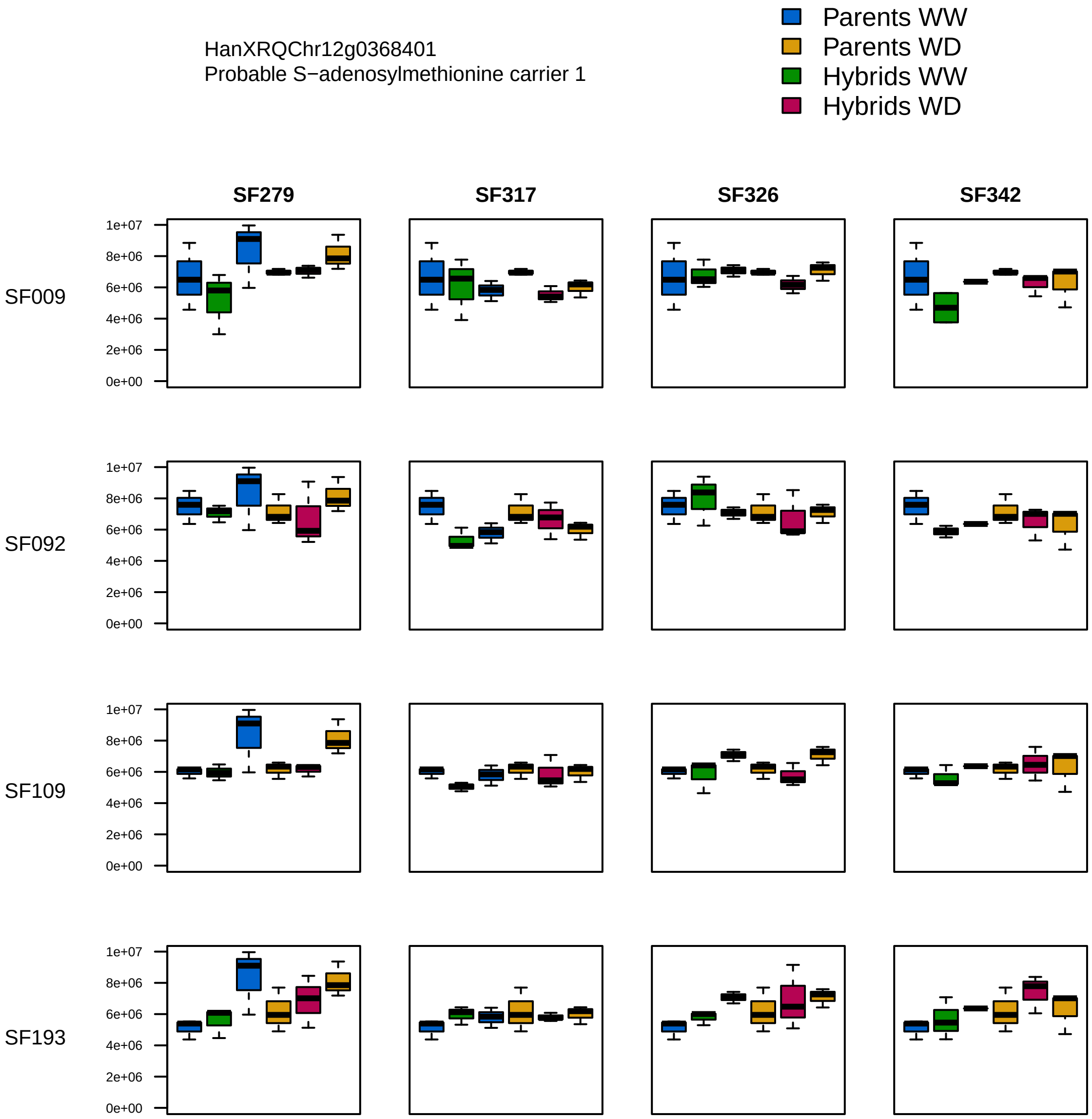
HanXRQChr12g0368401
Probable S−adenosylmethionine carrier 1
Parents WW
Parents WD
Hybrids WW
Hybrids WD
SF279
SF317
SF326
SF342
SF009
SF092
SF109
SF193
1e+07
8e+06
6e+06
4e+06
2e+06
0e+00

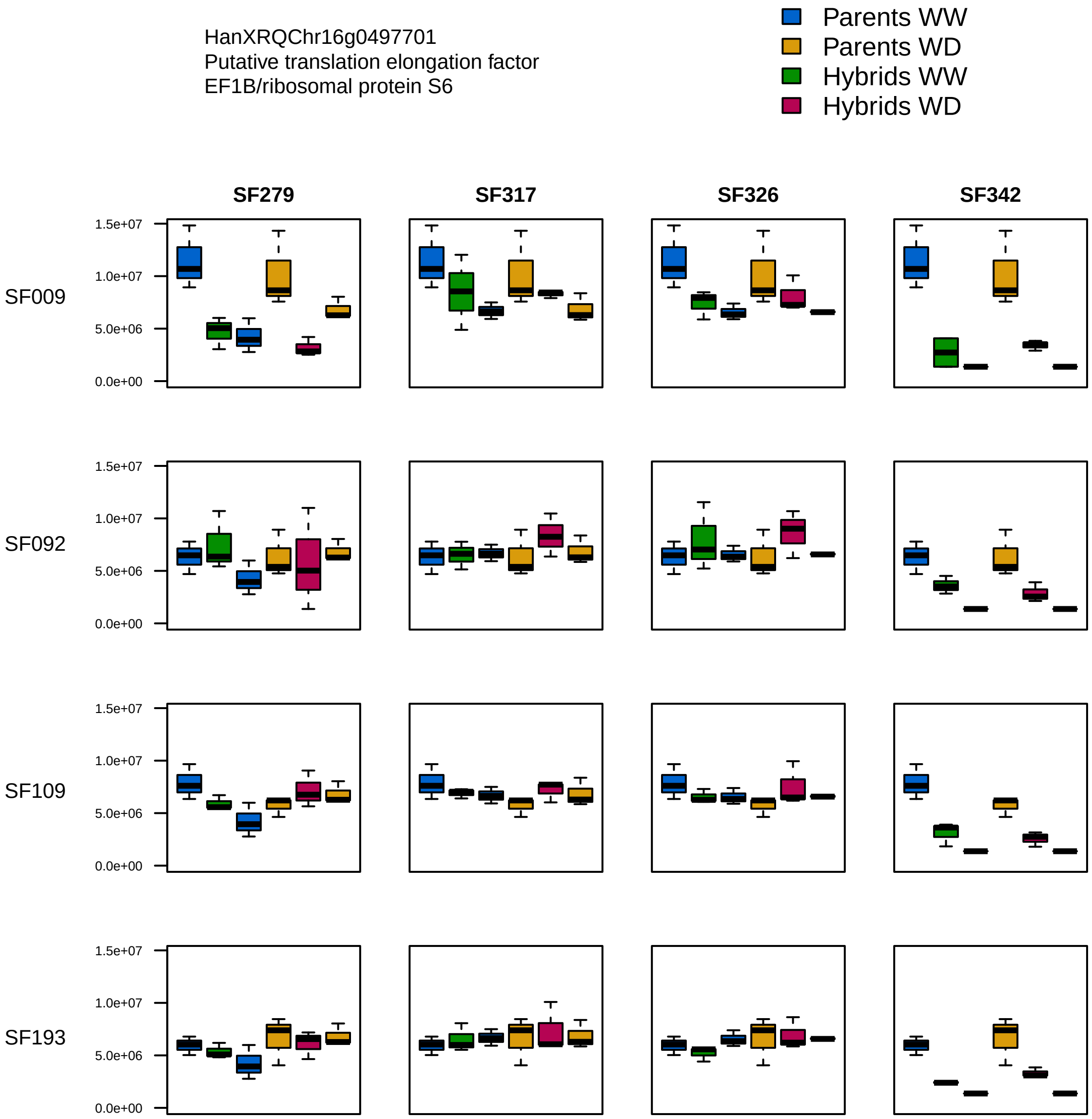
HanXRQChr16g0497701
Putative translation elongation factor
EF1B/ribosomal protein S6
Parents WW
Parents WD
Hybrids WW
Hybrids WD
SF279
SF317
SF326
SF342
SF009
SF092
SF109
SF193
1.5e+07
1.0e+07
5.0e+06
0.0e+00

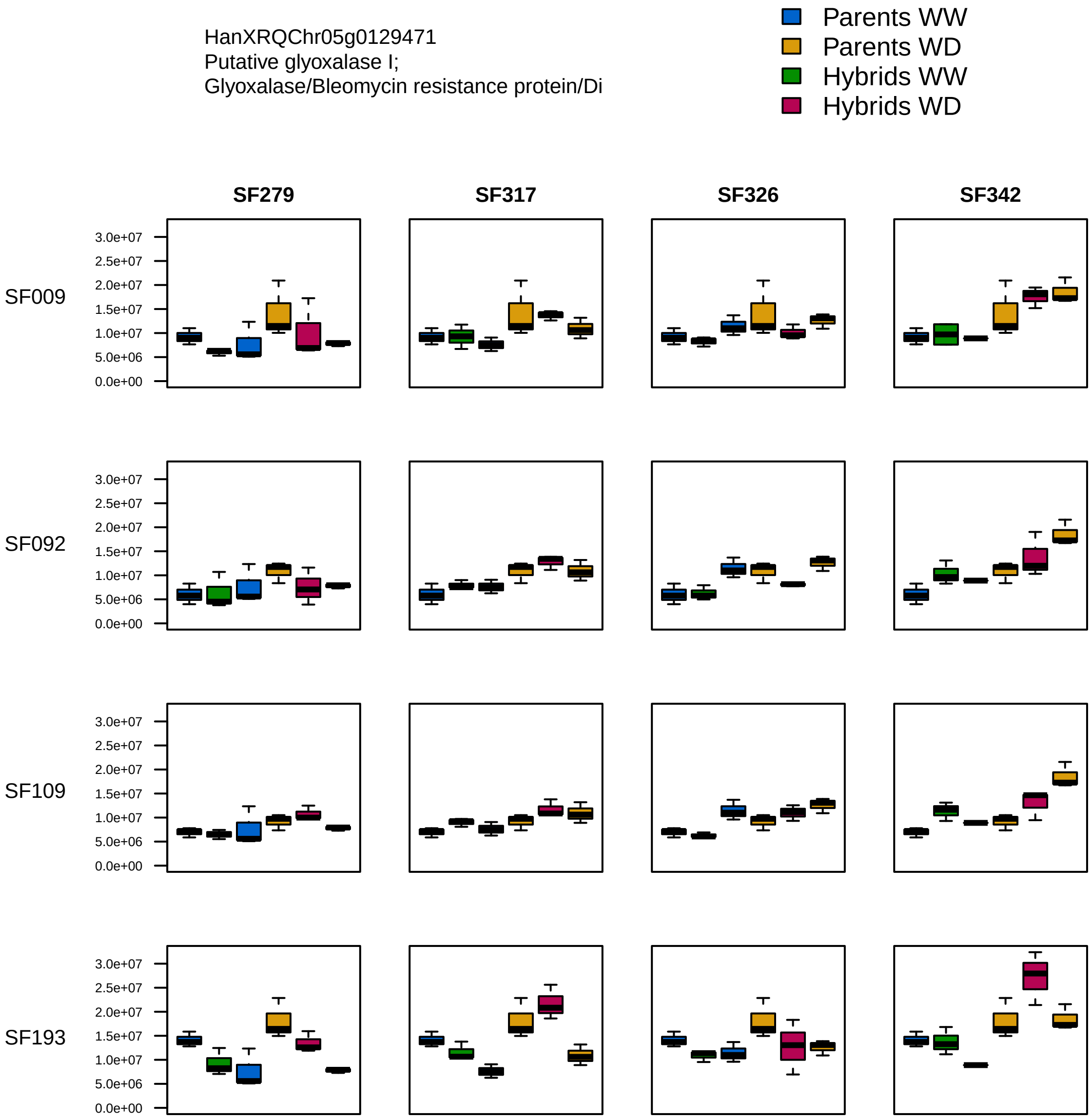
HanXRQChr05g0129471
Putative glyoxalase I;
Glyoxalase/Bleomycin resistance protein/Di
Parents WW
Parents WD
Hybrids WW
Hybrids WD
SF279
SF317
SF326
SF342
SF009
SF092
SF109
SF193
0.0e+00
5.0e+06
1.0e+07
1.5e+07
2.0e+07
2.5e+07
3.0e+07

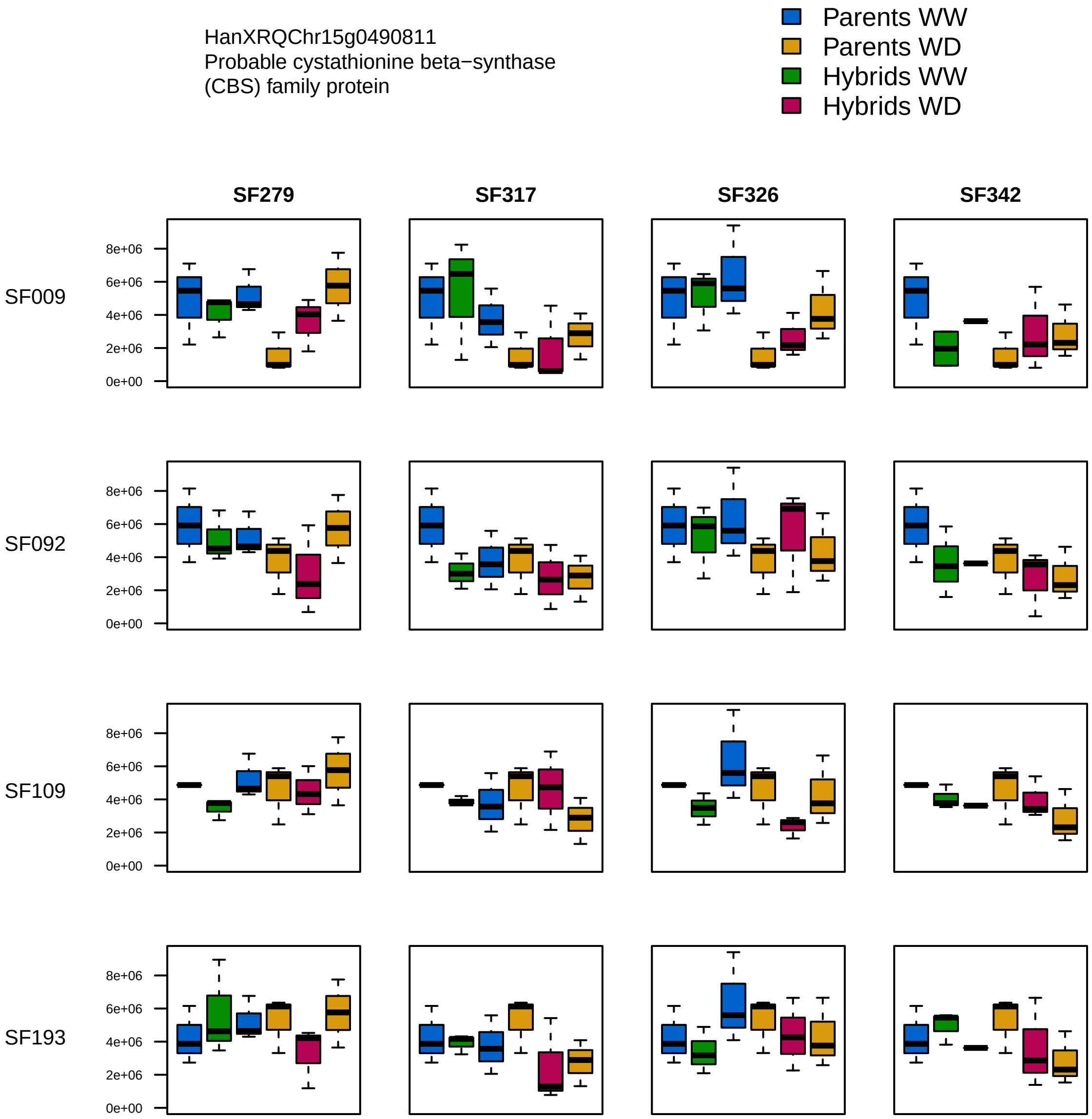
HanXRQChr15g0490811
Probable cystathionine beta−synthase
(CBS) family protein
Parents WW
Parents WD
Hybrids WW
Hybrids WD
SF279
SF317
SF326
SF342
SF009
SF092
SF109
SF193
0e+00
2e+06
4e+06
6e+06
8e+06

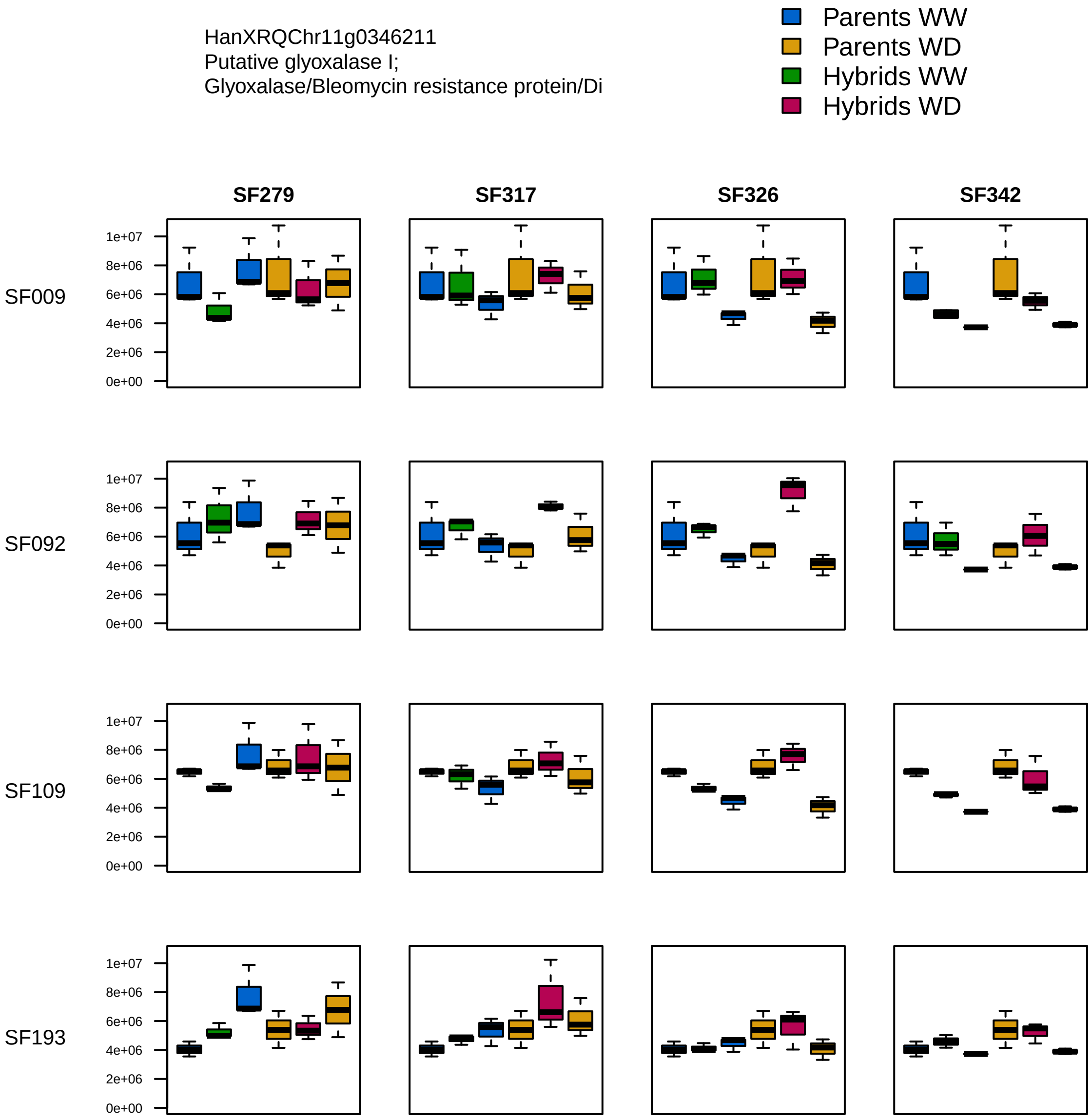
HanXRQChr11g0346211
Putative glyoxalase I;
Glyoxalase/Bleomycin resistance protein/Di
Parents WW
Parents WD
Hybrids WW
Hybrids WD
SF279
SF317
SF326
SF342
SF009
SF092
SF109
SF193
1e+07
8e+06
6e+06
4e+06
2e+06
0e+00

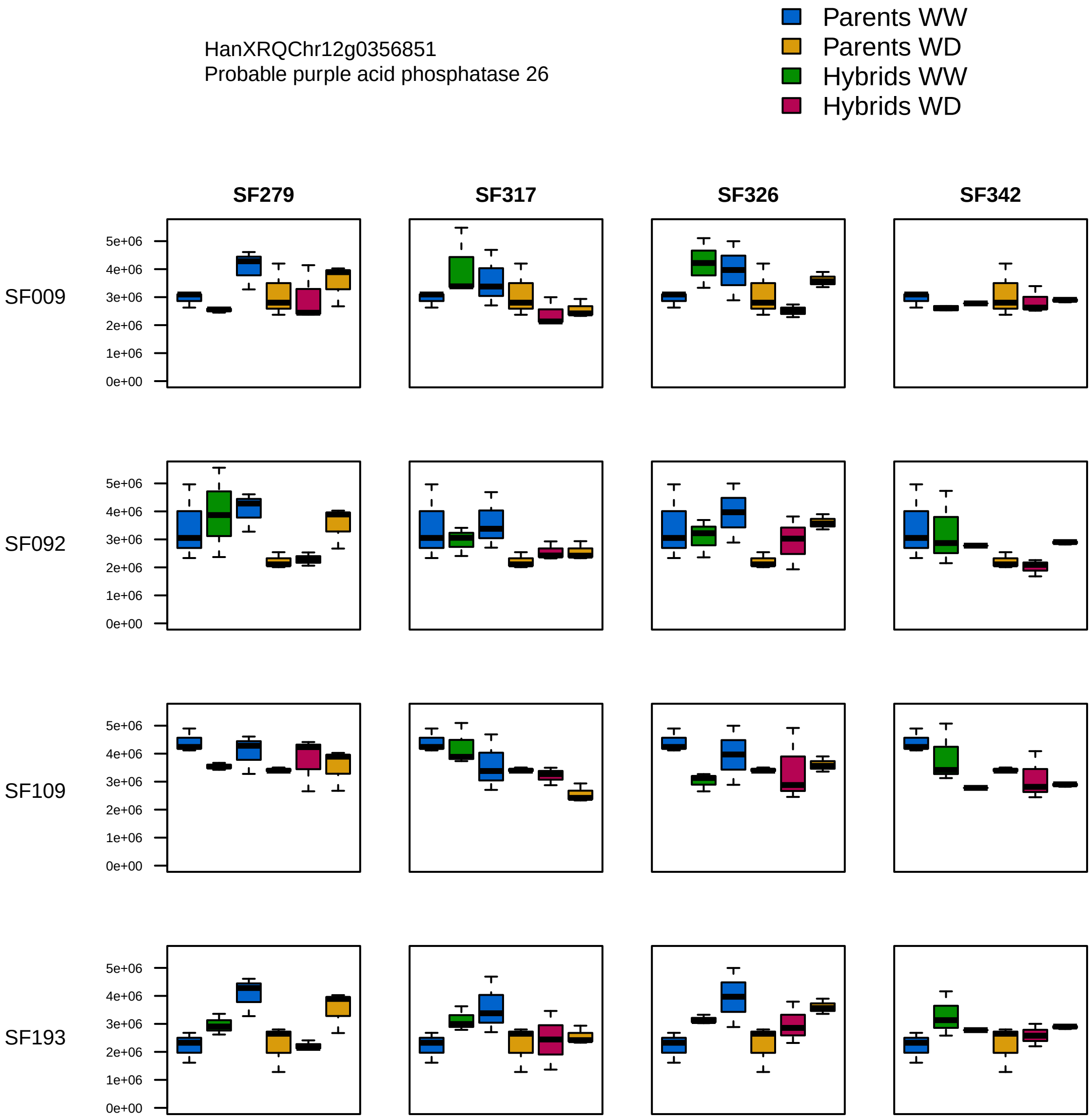
HanXRQChr12g0356851
Probable purple acid phosphatase 26
Parents WW
Parents WD
Hybrids WW
Hybrids WD
SF279
SF317
SF326
SF342
SF009
SF092
SF109
SF193
0e+00
1e+06
2e+06
3e+06
4e+06
5e+06

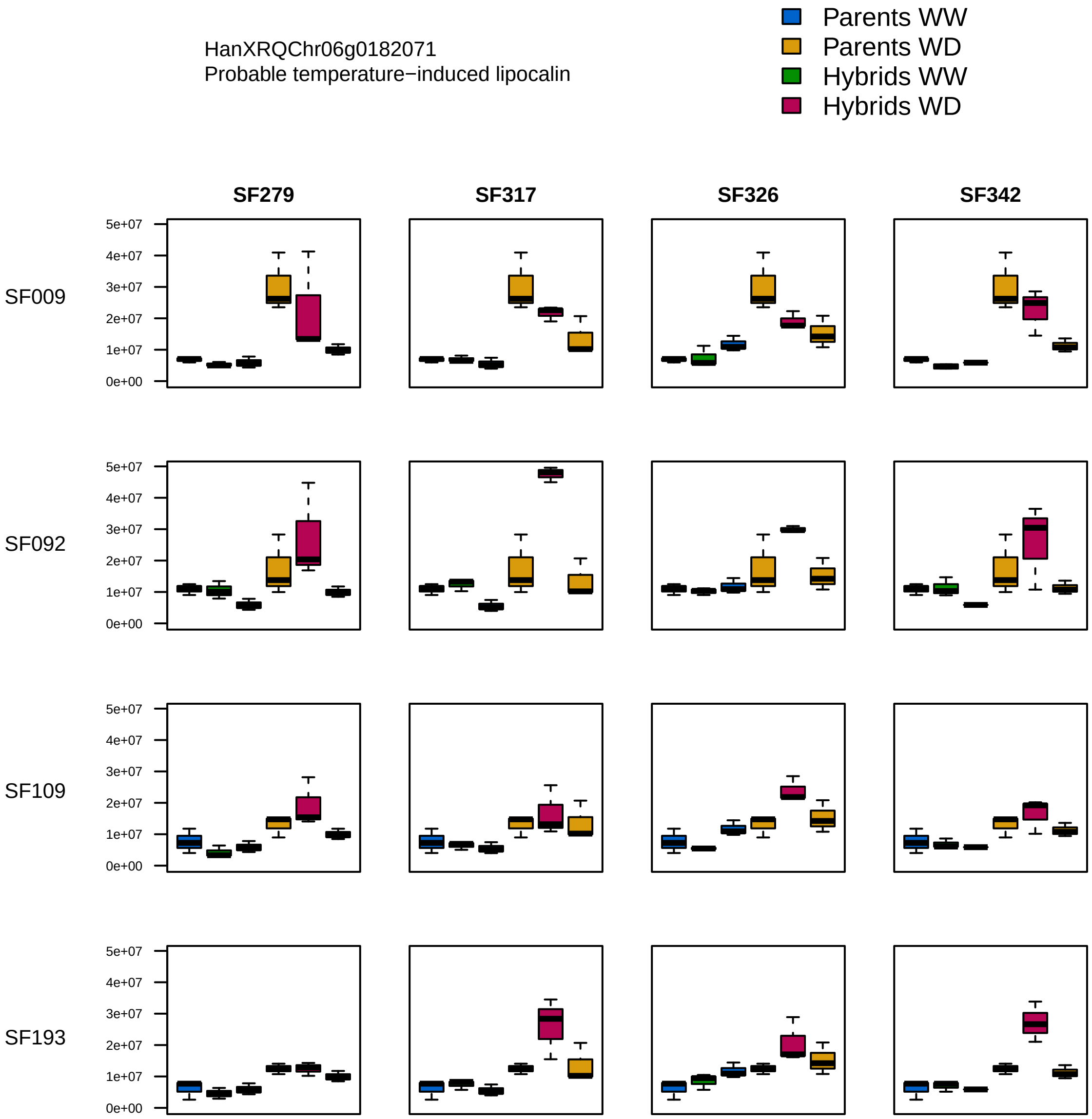

HanXRQChr06g0182071
Probable temperature−induced lipocalin
Parents WW
Parents WD
Hybrids WW
Hybrids WD
SF279
SF317
SF326
SF342
SF009
SF092
SF109
SF193
5e+07
4e+07
3e+07
2e+07
1e+07
0e+00

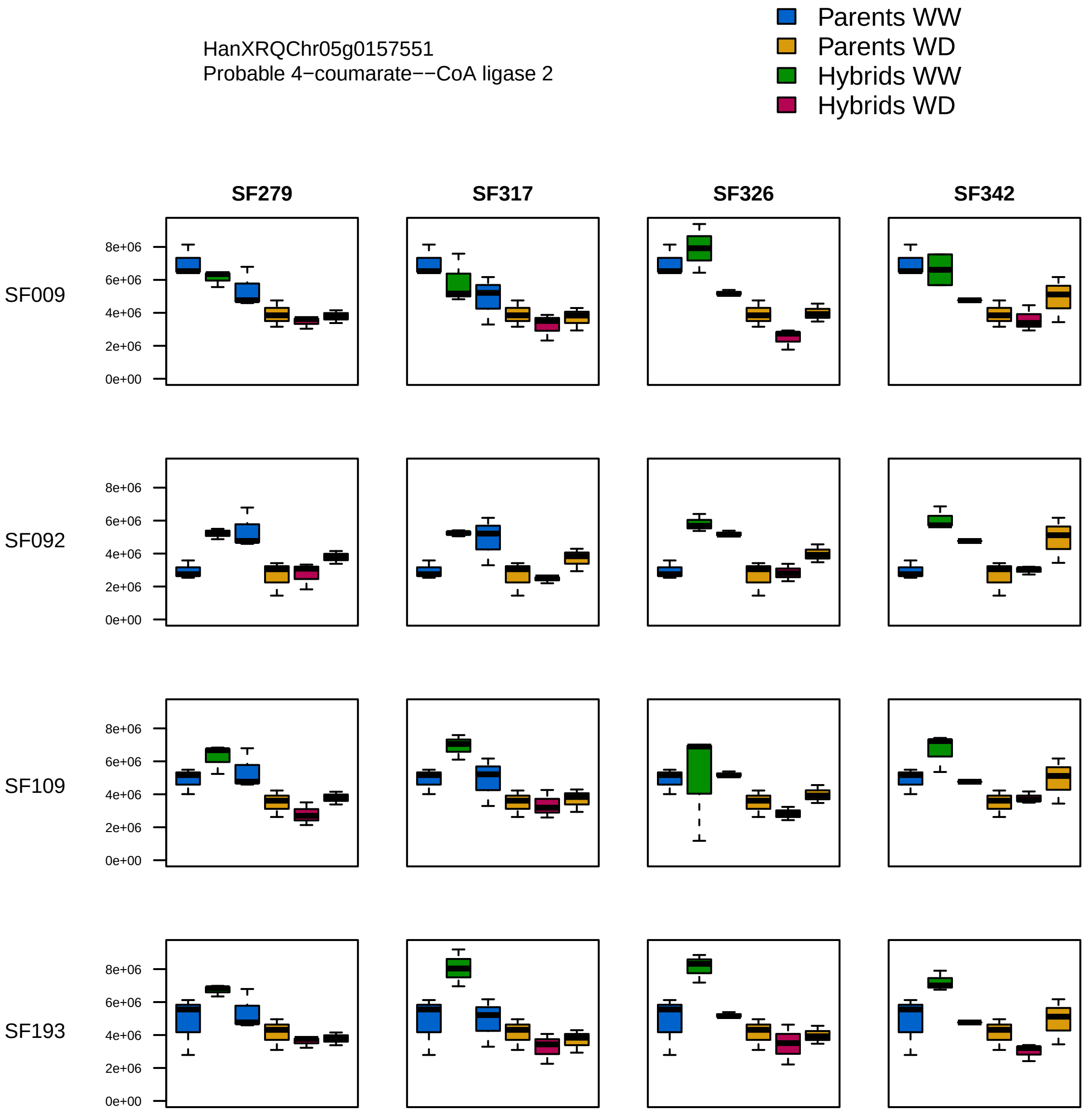

HanXRQChr05g0157551
Probable 4−coumarate−−CoA ligase 2
Parents WW
Parents WD
Hybrids WW
Hybrids WD
SF279
SF317
SF326
SF342
SF009
SF092
SF109
SF193
0e+00
2e+06
4e+06
6e+06
8e+06

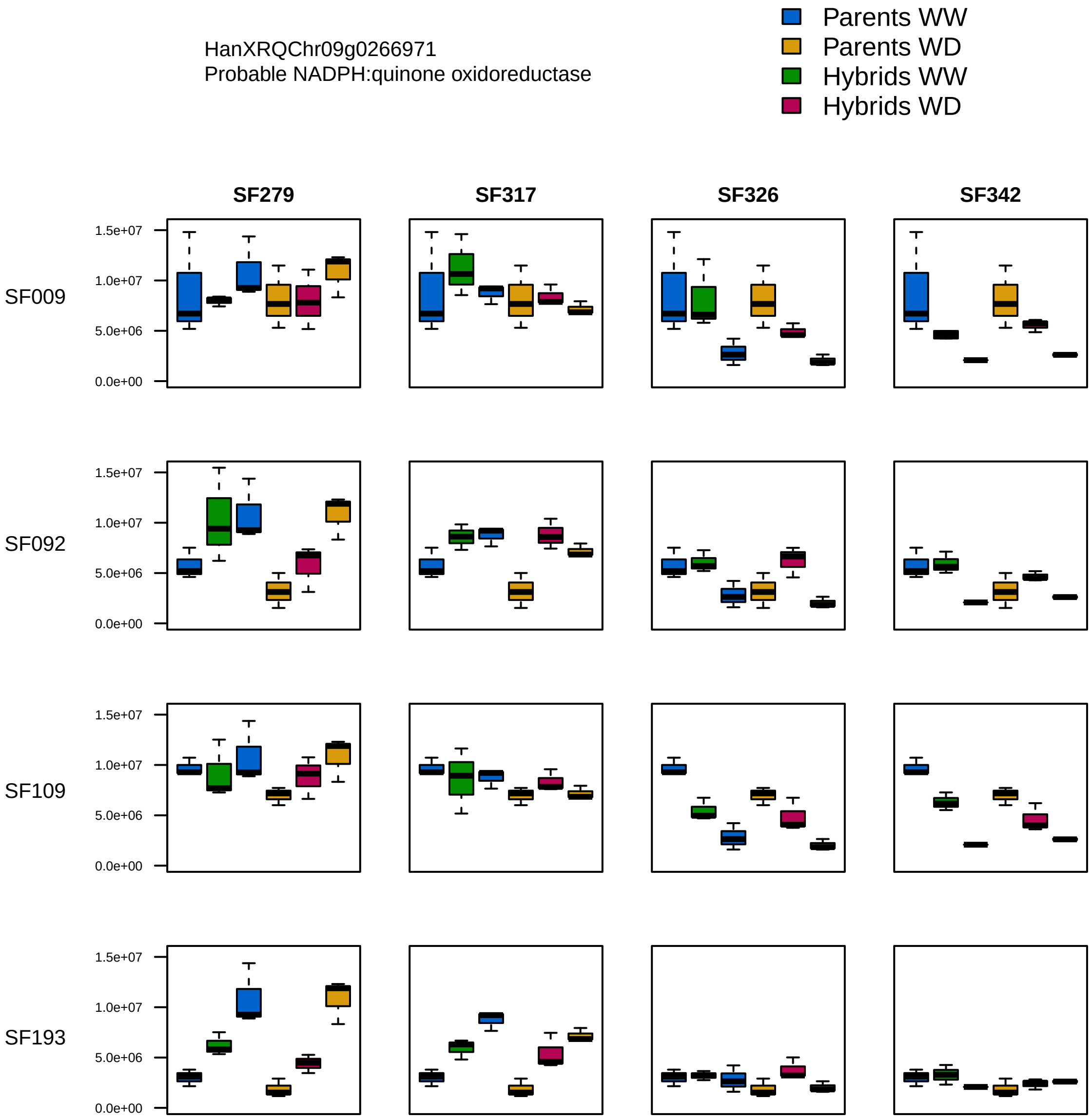
HanXRQChr09g0266971
Probable NADPH:quinone oxidoreductase
Parents WW
Parents WD
Hybrids WW
Hybrids WD
SF279
SF317
SF326
SF342
SF009
SF092
SF109
SF193
1.5e+07
1.0e+07
5.0e+06
0.0e+00

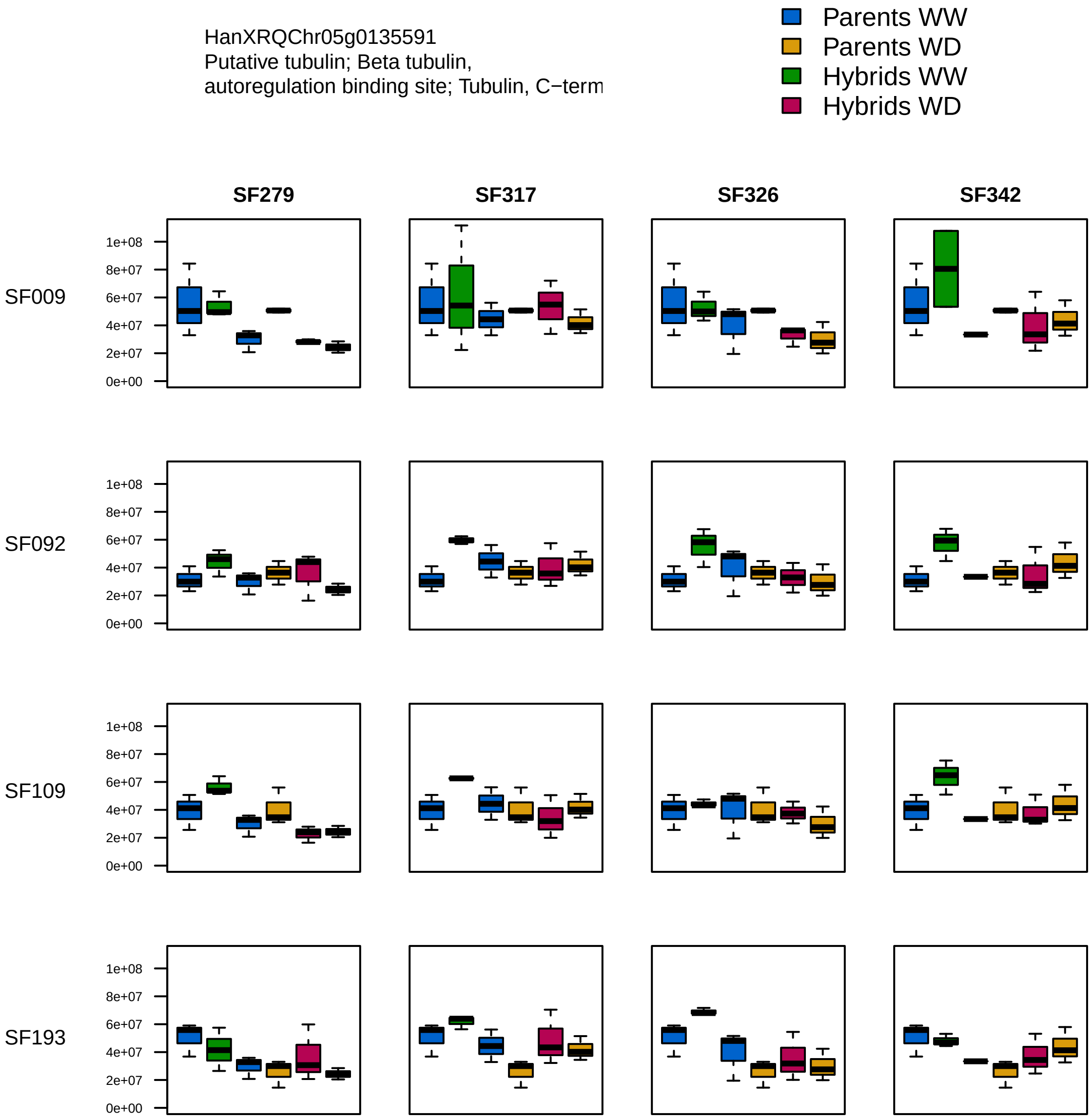
HanXRQChr05g0135591
Putative tubulin; Beta tubulin,
autoregulation binding site; Tubulin, C−term
Parents WW
Parents WD
Hybrids WW
Hybrids WD
SF279
SF317
SF326
SF342
SF009
SF092
SF109
SF193
1e+08
8e+07
6e+07
4e+07
2e+07
0e+00

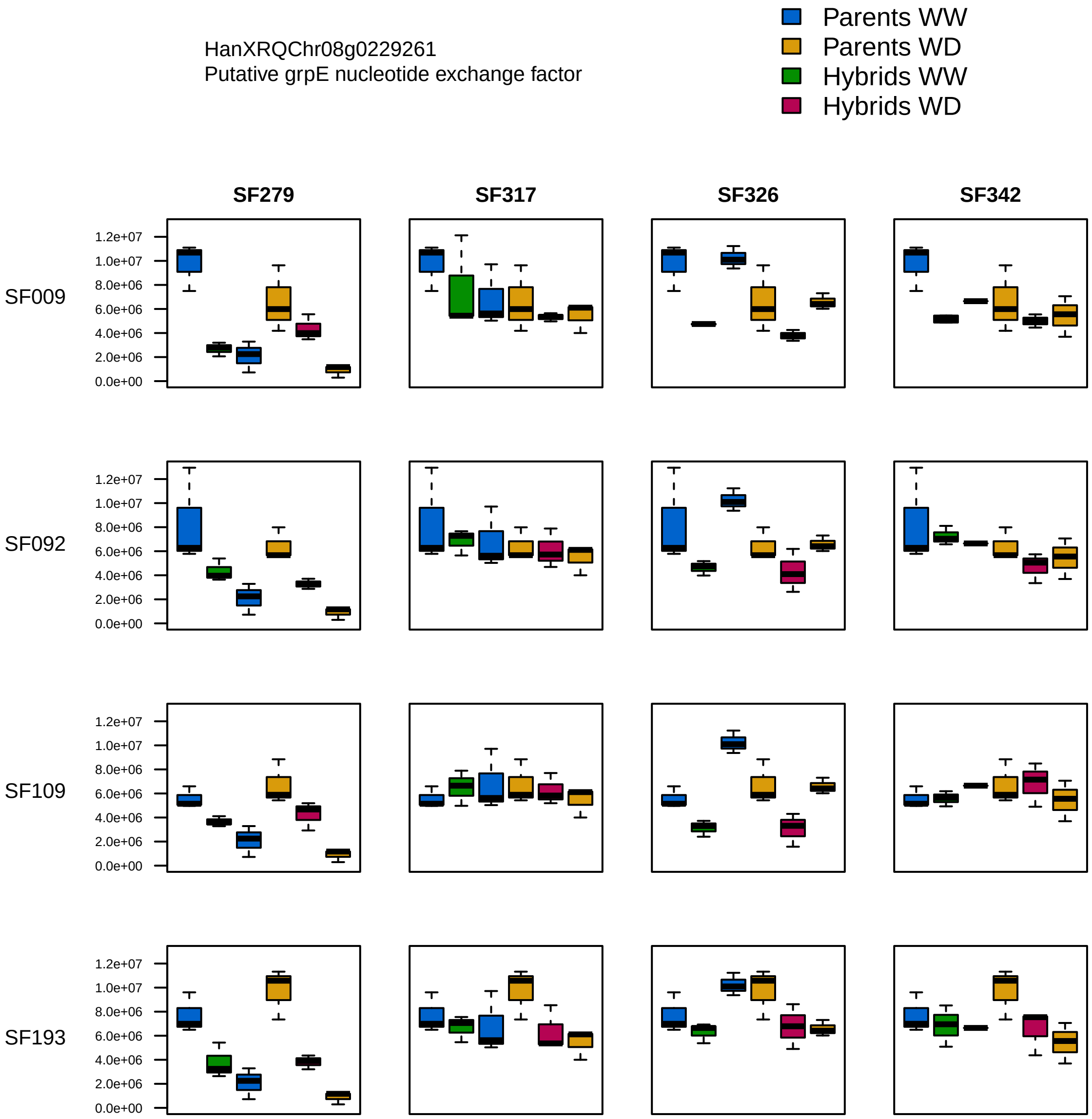
HanXRQChr08g0229261
Putative grpE nucleotide exchange factor
Parents WW
Parents WD
Hybrids WW
Hybrids WD
SF279
SF317
SF326
SF342
SF009
SF092
SF109
SF193
1.2e+07
1.0e+07
8.0e+06
6.0e+06
4.0e+06
2.0e+06
0.0e+00

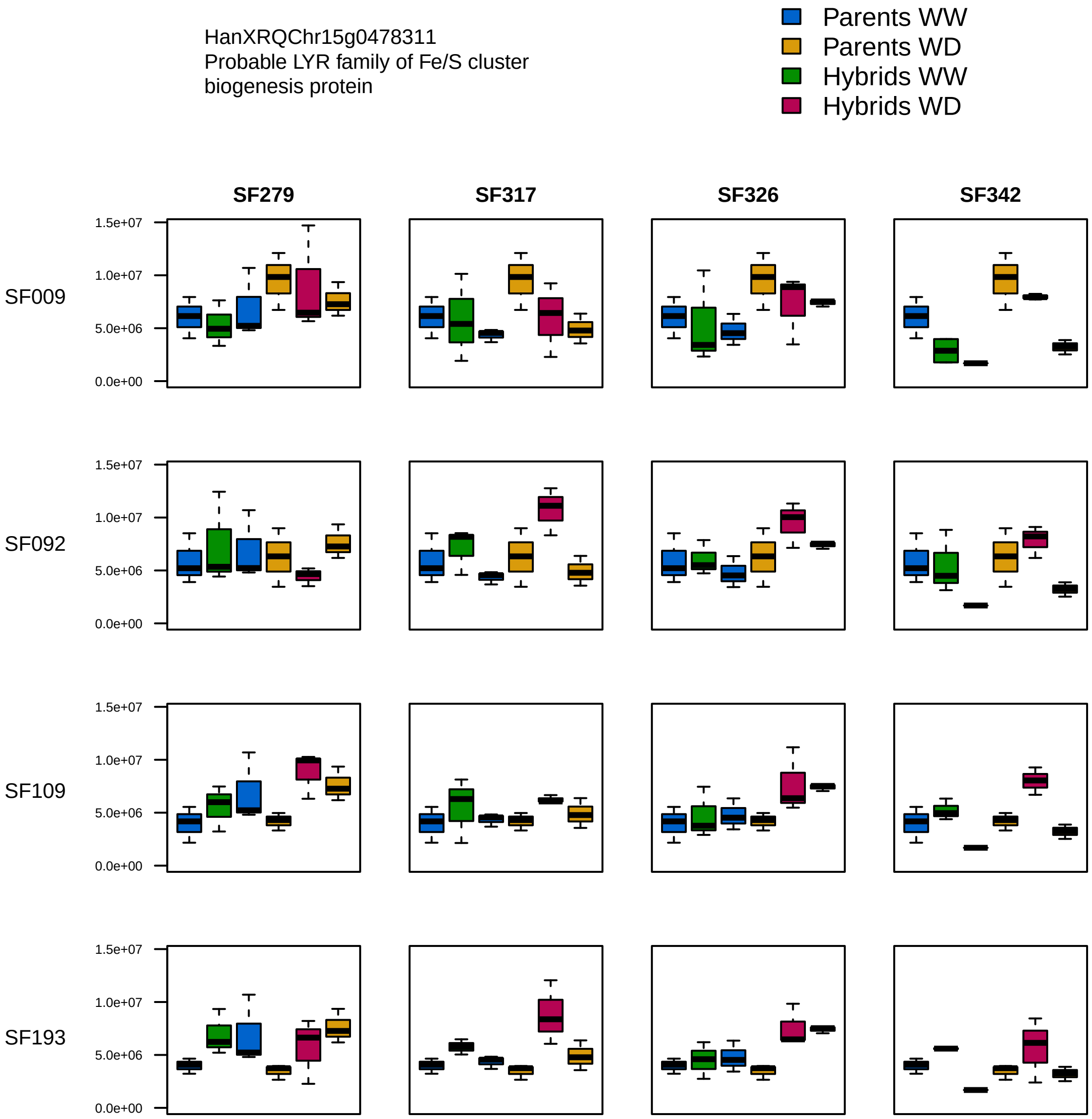
HanXRQChr15g0478311
Probable LYR family of Fe/S cluster
biogenesis protein
Parents WW
Parents WD
Hybrids WW
Hybrids WD
SF279
SF317
SF326
SF342
SF009
SF092
SF109
SF193
1.5e+07
1.0e+07
5.0e+06
0.0e+00

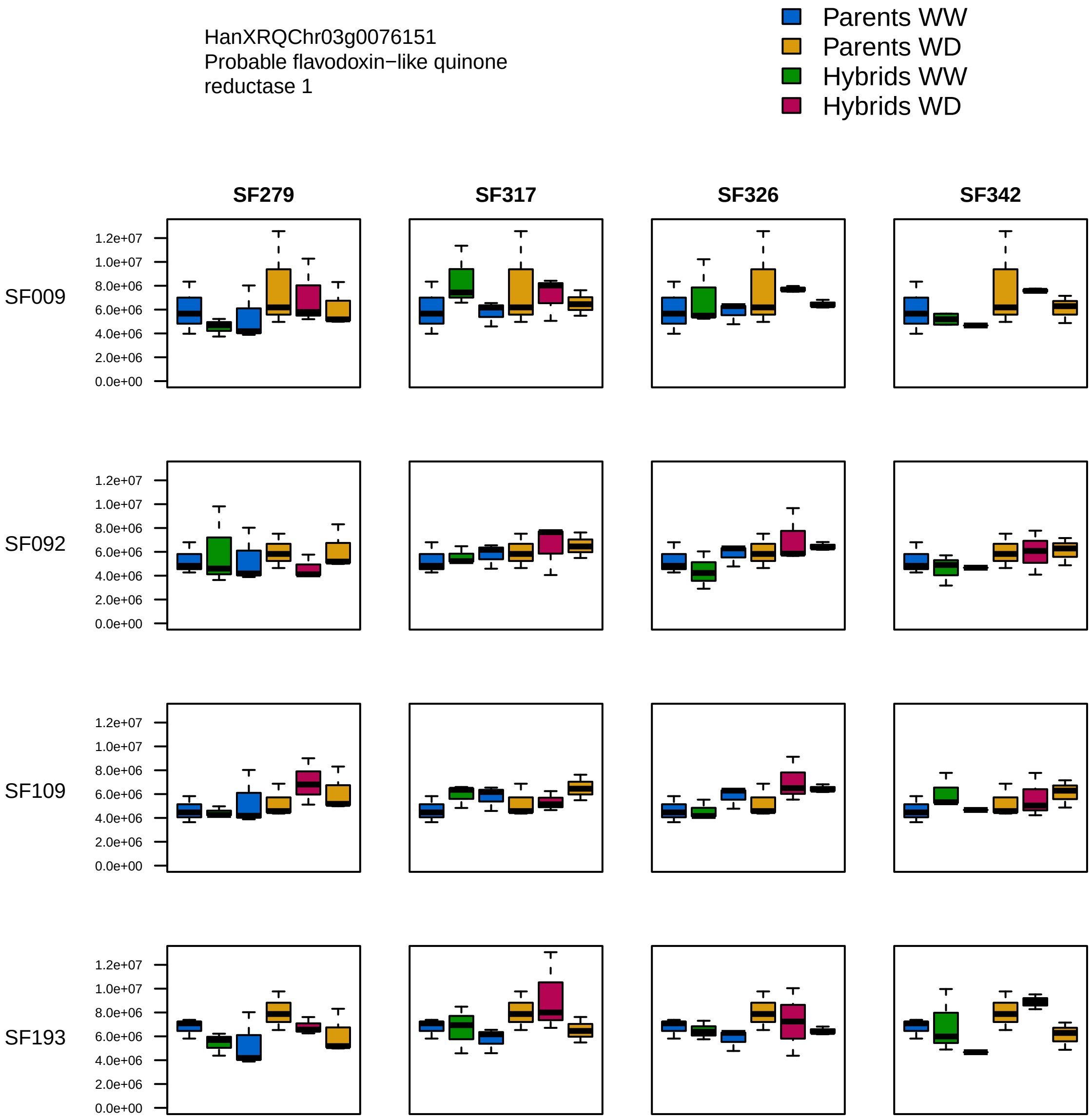
HanXRQChr03g0076151
Probable flavodoxin−like quinone reductase 1
Parents WW
Parents WD
Hybrids WW
Hybrids WD
SF279
SF317
SF326
SF342
SF009
SF092
SF109
SF193
0.0e+00
2.0e+06
4.0e+06
6.0e+06
8.0e+06
1.0e+07
1.2e+07

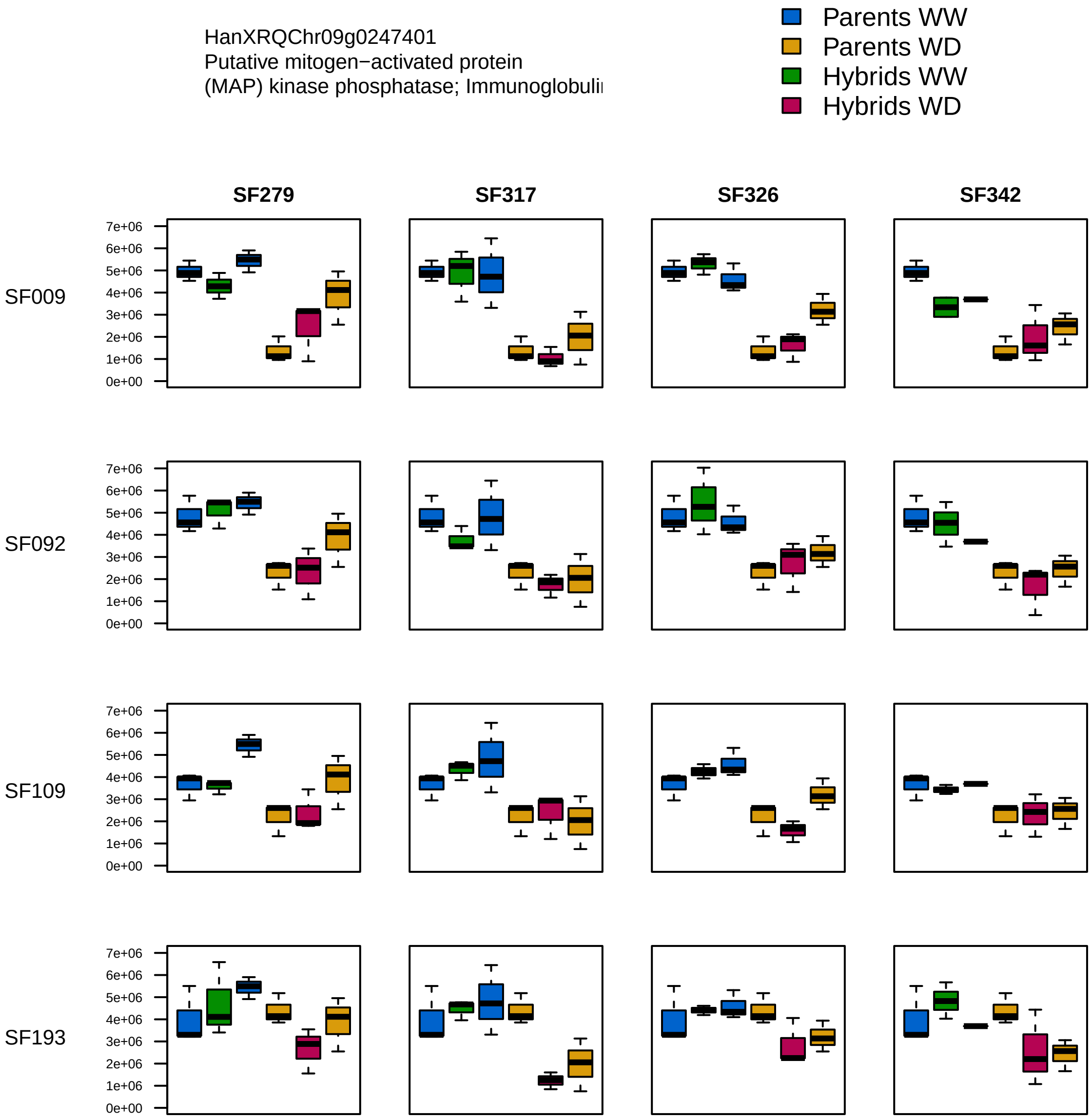
HanXRQChr09g0247401
Putative mitogen−activated protein
(MAP) kinase phosphatase; Immunoglobuli
Parents WW
Parents WD
Hybrids WW
Hybrids WD
SF279
SF317
SF326
SF342
SF009
SF092
SF109
SF193
7e+06
6e+06
5e+06
4e+06
3e+06
2e+06
1e+06
0e+00

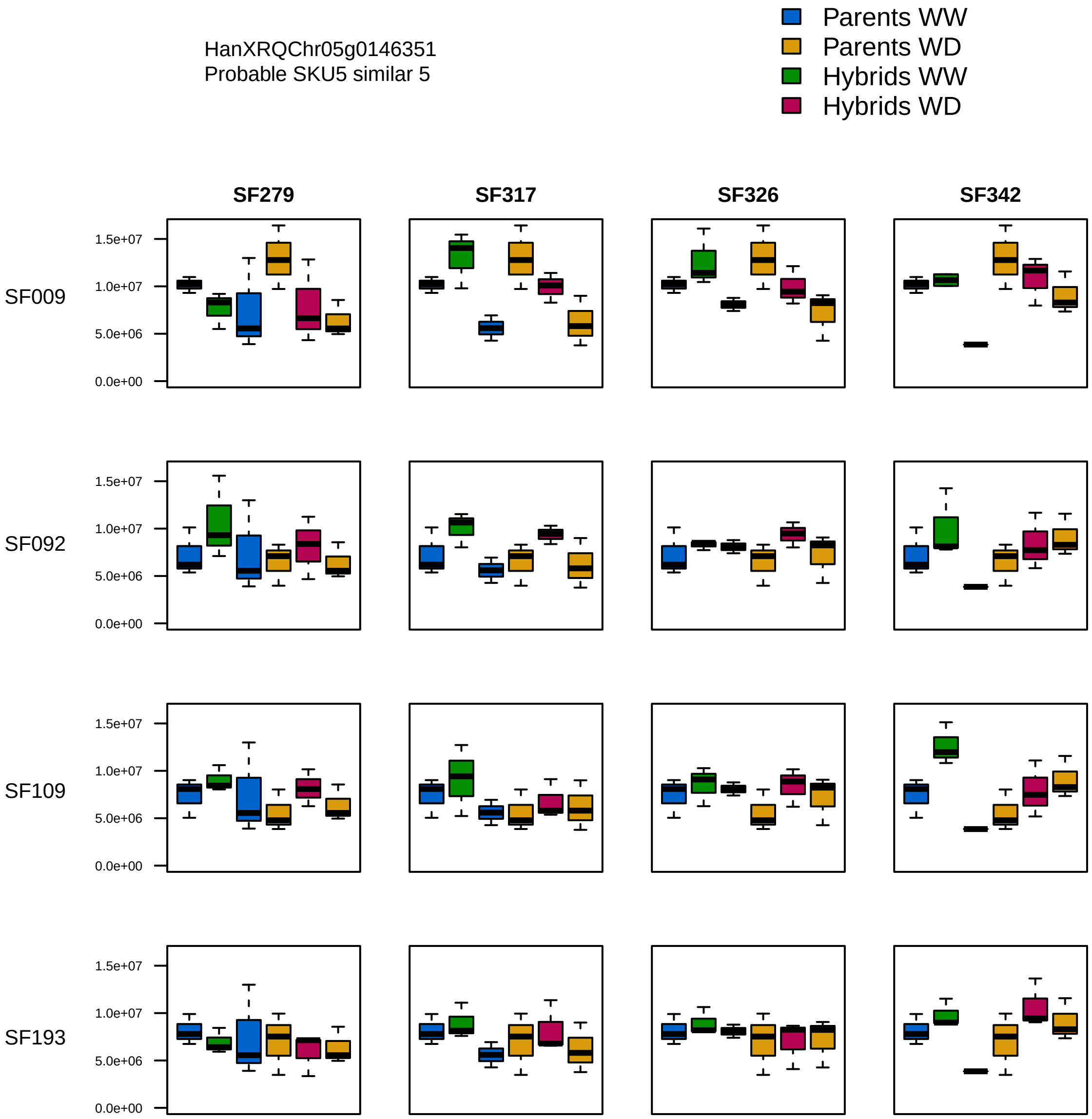
HanXRQChr05g0146351
Probable SKU5 similar 5
Parents WW
Parents WD
Hybrids WW
Hybrids WD
SF279
SF317
SF326
SF342
SF009
SF092
SF109
SF193
1.5e+07
1.0e+07
5.0e+06
0.0e+00

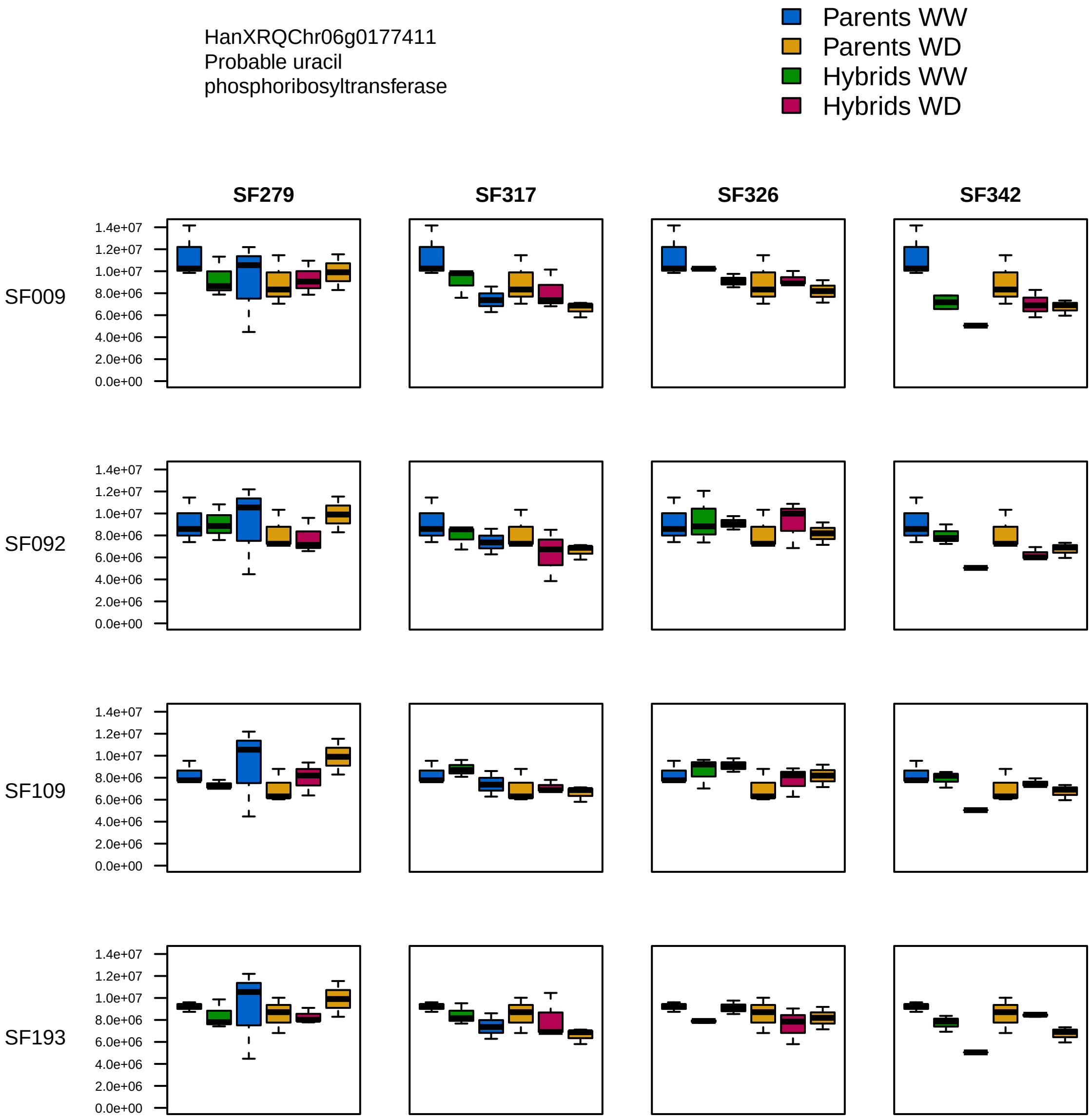
HanXRQChr06g0177411
Probable uracil
phosphoribosyltransferase
Parents WW
Parents WD
Hybrids WW
Hybrids WD
SF279
SF317
SF326
SF342
SF009
SF092
SF109
SF193
1.4e+07
1.2e+07
1.0e+07
8.0e+06
6.0e+06
4.0e+06
2.0e+06
0.0e+00

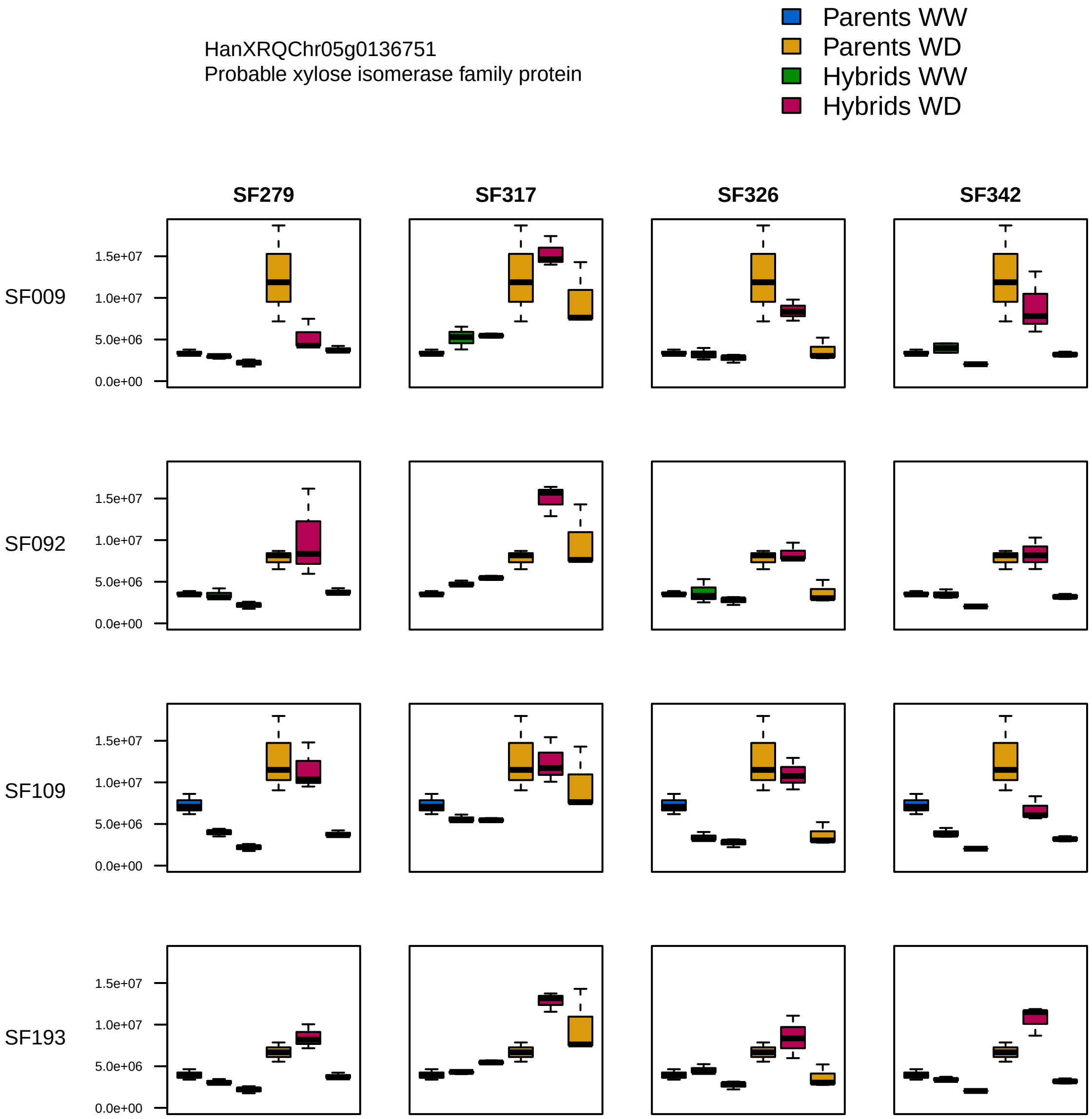
HanXRQChr05g0136751
Probable xylose isomerase family protein
Parents WW
Parents WD
Hybrids WW
Hybrids WD
SF279
SF317
SF326
SF342
SF009
SF092
SF109
SF193
0.0e+00
5.0e+06
1.0e+07
1.5e+07

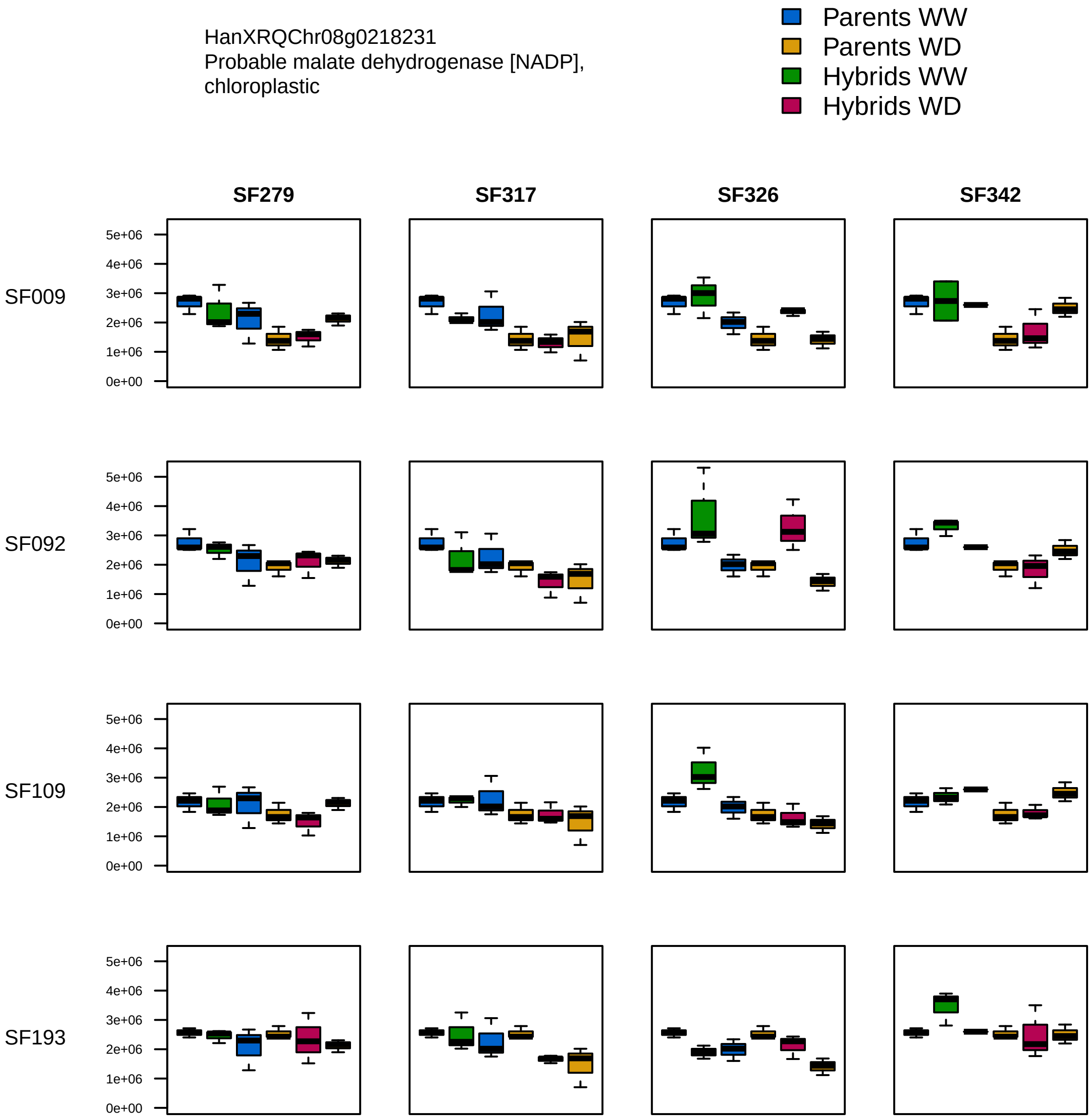
HanXRQChr08g0218231
Probable malate dehydrogenase [NADP], chloroplastic
Parents WW
Parents WD
Hybrids WW
Hybrids WD
SF279
SF317
SF326
SF342
SF009
SF092
SF109
SF193
0e+00
1e+06
2e+06
3e+06
4e+06
5e+06

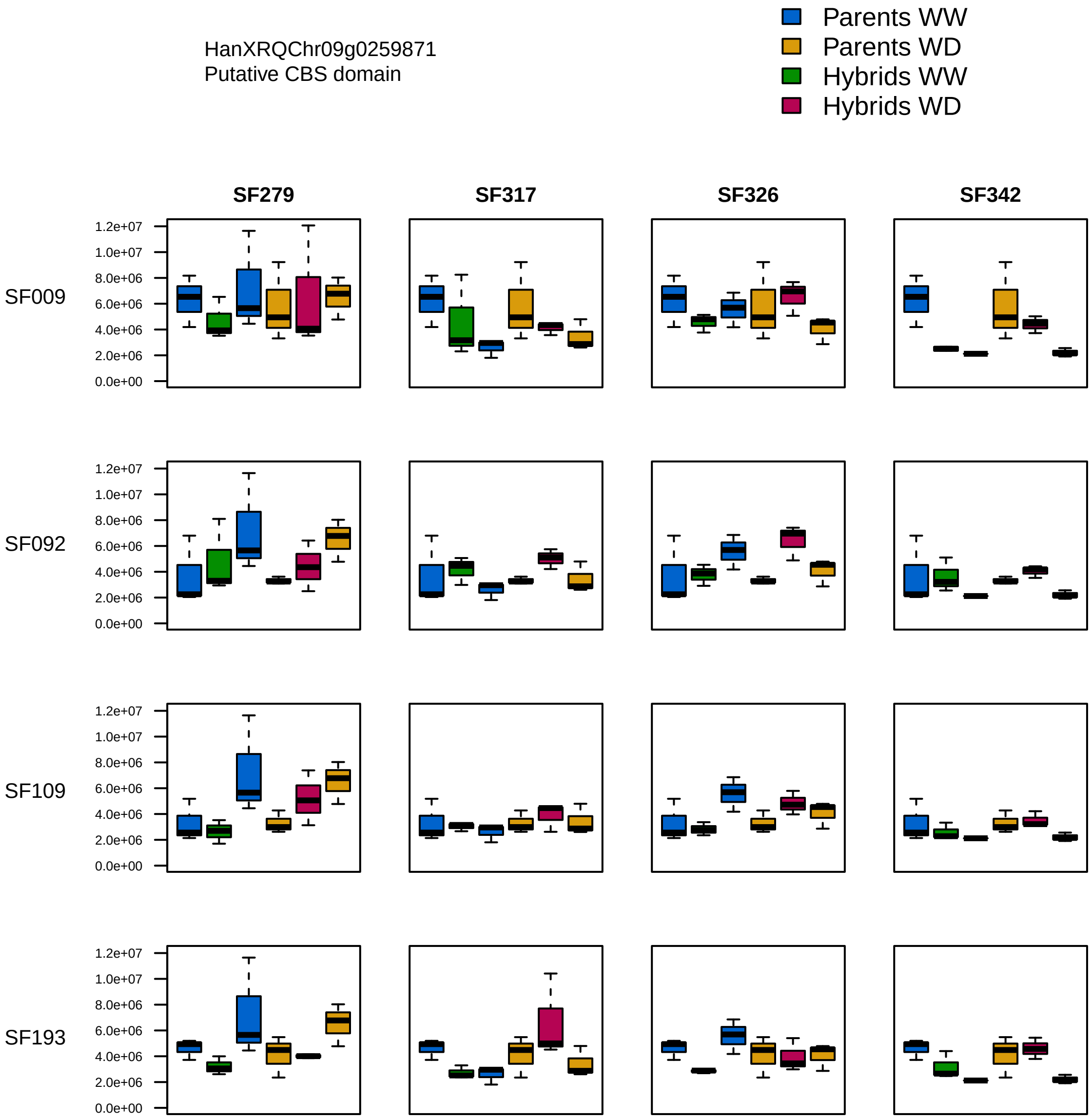
HanXRQChr09g0259871
Putative CBS domain
Parents WW
Parents WD
Hybrids WW
Hybrids WD
SF279
SF317
SF326
SF342
SF009
SF092
SF109
SF193
1.2e+07
1.0e+07
8.0e+06
6.0e+06
4.0e+06
2.0e+06
0.0e+00

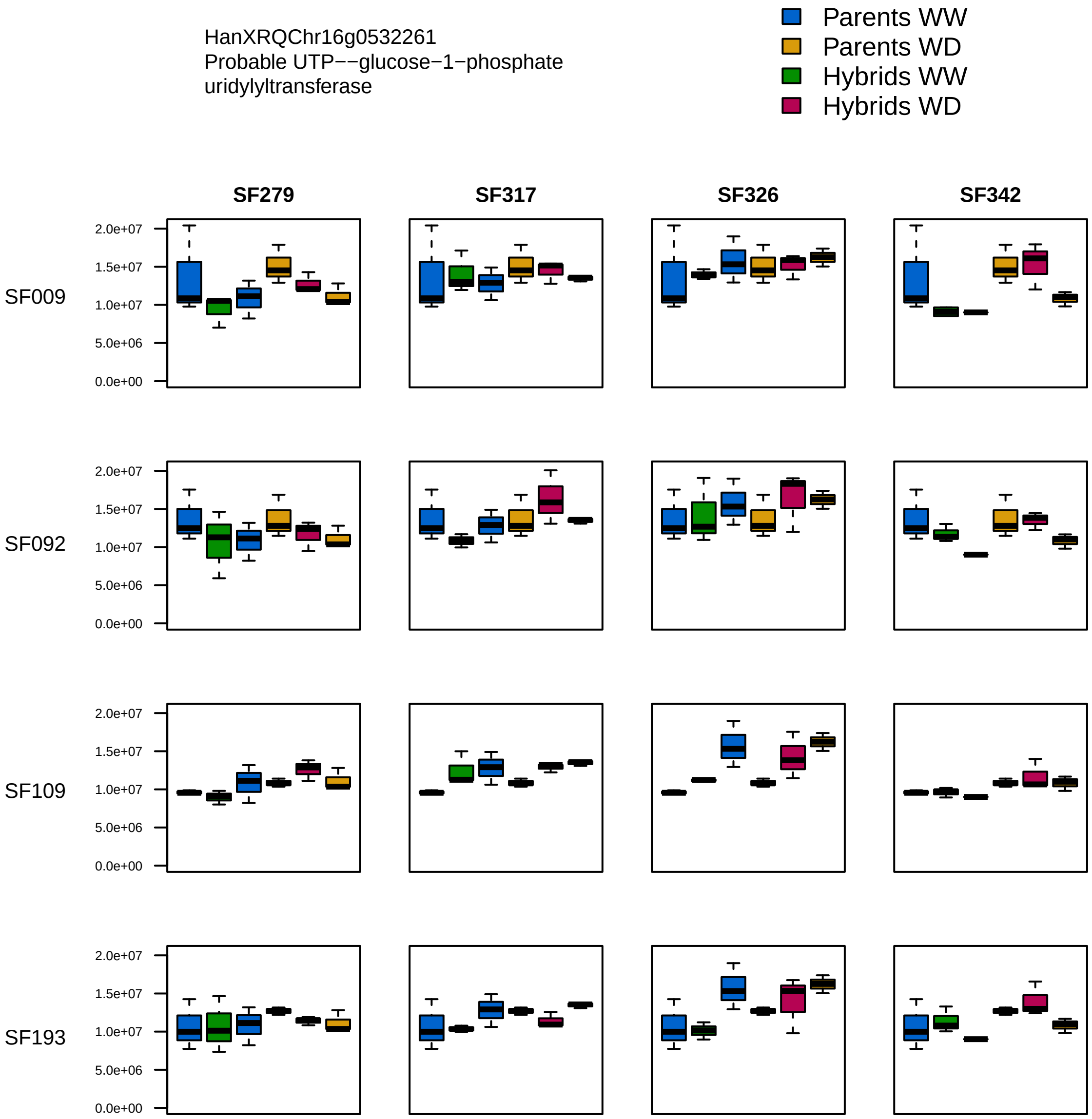
HanXRQChr16g0532261
Probable UTP−−glucose−1−phosphate
uridylyltransferase
Parents WW
Parents WD
Hybrids WW
Hybrids WD
SF279
SF317
SF326
SF342
SF009
SF092
SF109
SF193
0.0e+00
5.0e+06
1.0e+07
1.5e+07
2.0e+07

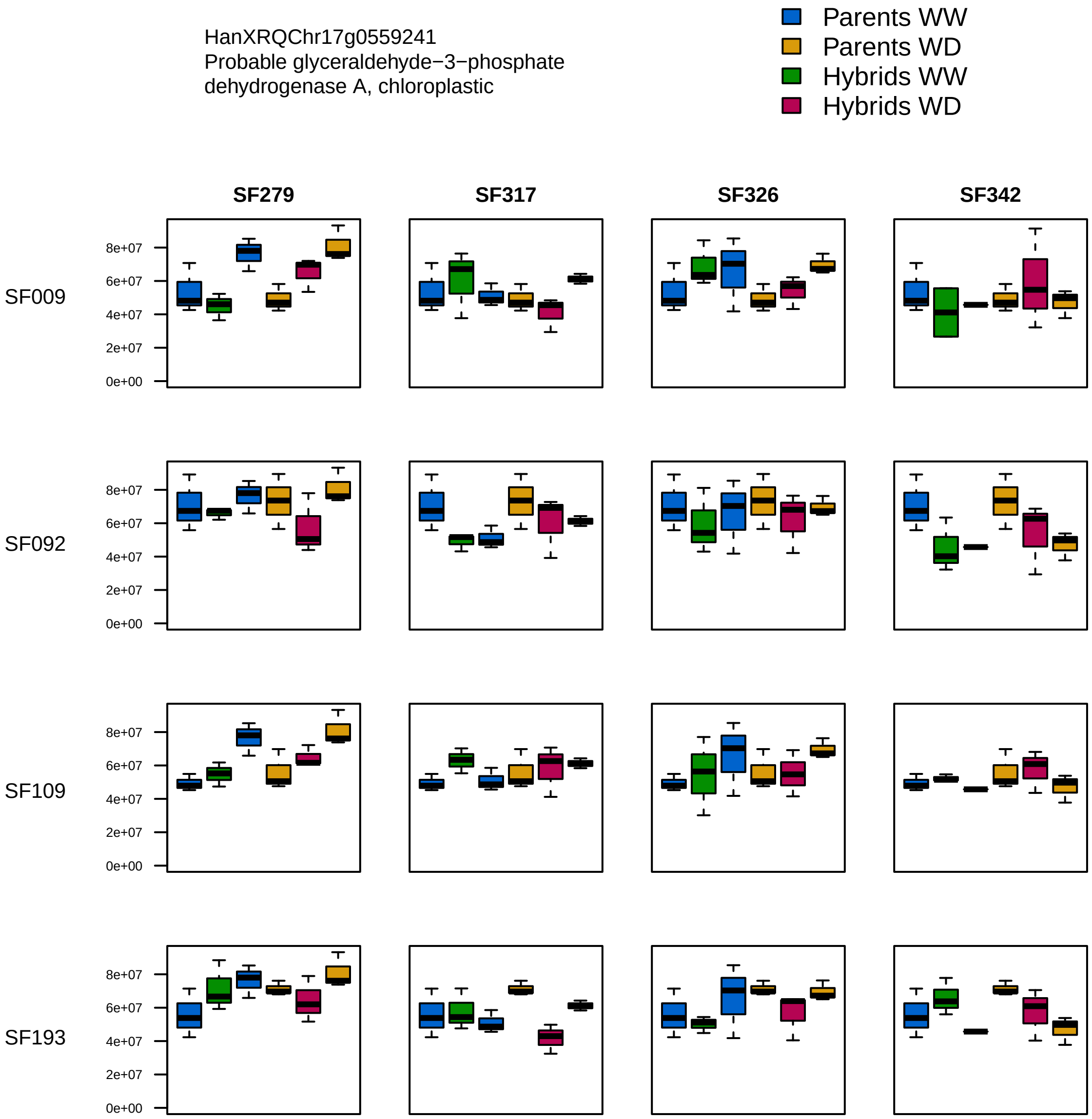

HanXRQChr17g0559241
Probable glyceraldehyde−3−phosphate
dehydrogenase A, chloroplastic
Parents WW
Parents WD
Hybrids WW
Hybrids WD
SF279
SF317
SF326
SF342
SF009
SF092
SF109
SF193
8e+07
6e+07
4e+07
2e+07
0e+00

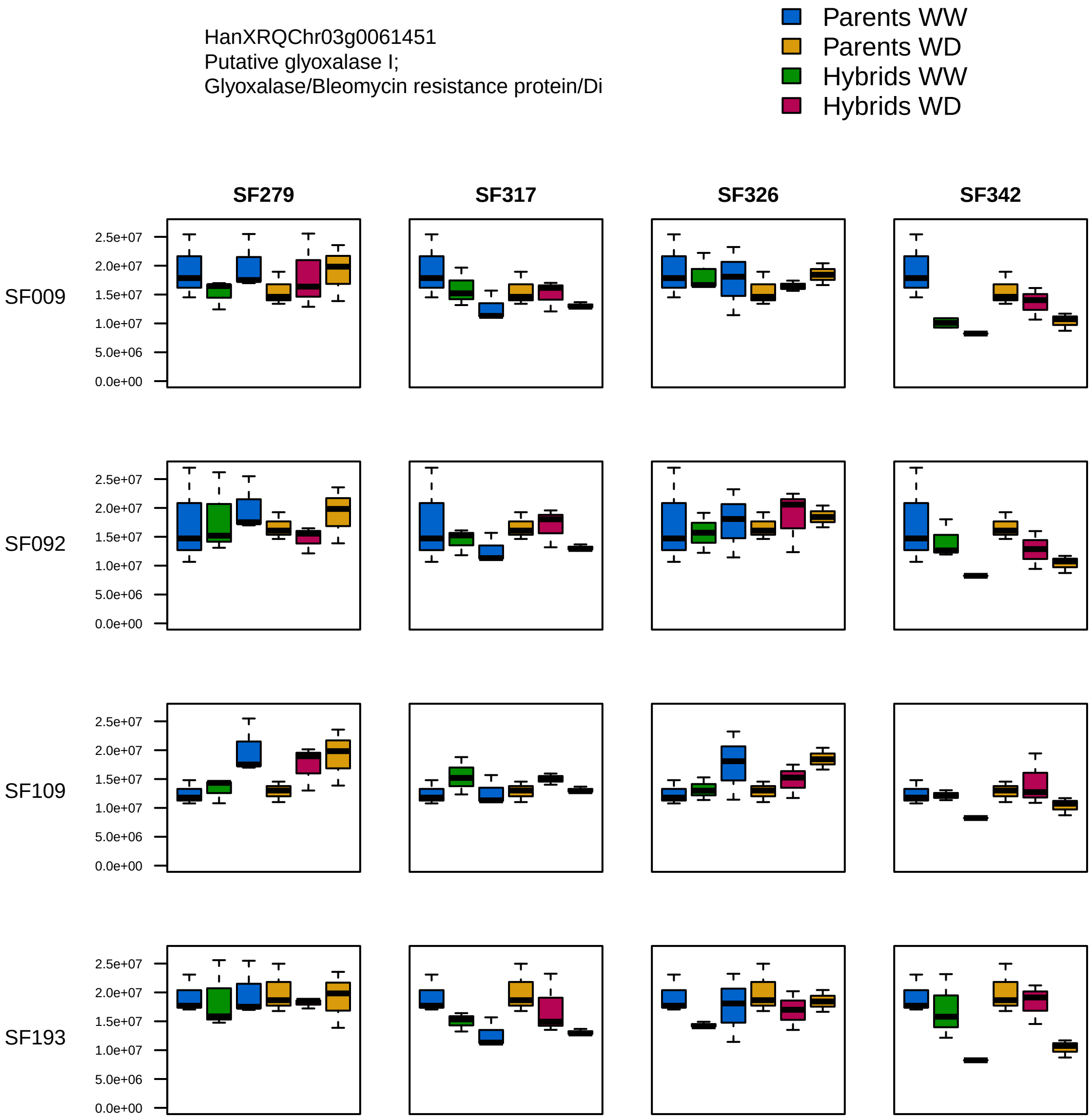

HanXRQChr03g0061451
Putative glyoxalase I;
Glyoxalase/Bleomycin resistance protein/Di
Parents WW
Parents WD
Hybrids WW
Hybrids WD
SF279
SF317
SF326
SF342
SF009
SF092
SF109
SF193
2.5e+07
2.0e+07
1.5e+07
1.0e+07
5.0e+06
0.0e+00

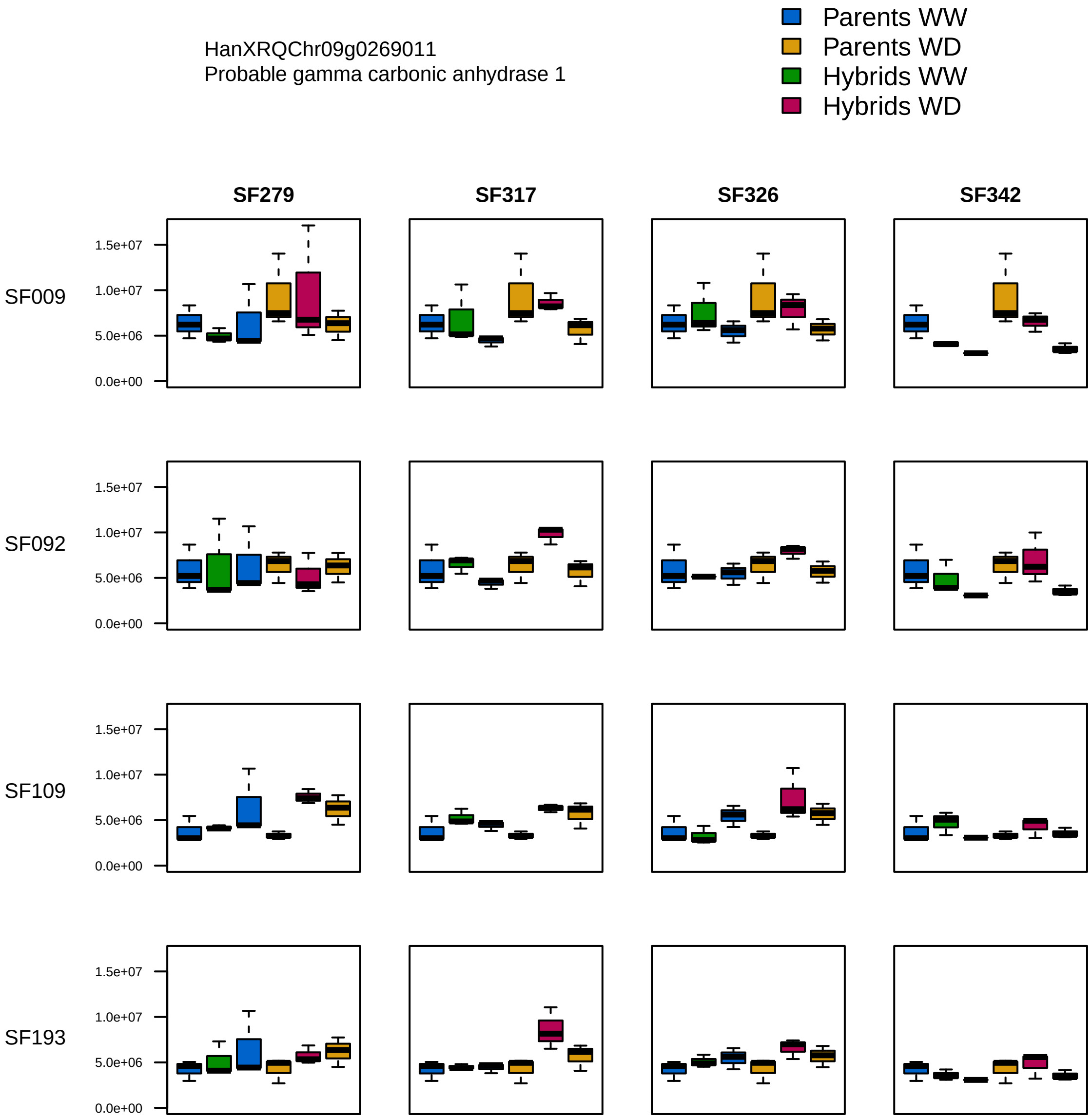
HanXRQChr09g0269011
Probable gamma carbonic anhydrase 1
Parents WW
Parents WD
Hybrids WW
Hybrids WD
SF279
SF317
SF326
SF342
SF009
SF092
SF109
SF193
1.5e+07
1.0e+07
5.0e+06
0.0e+00

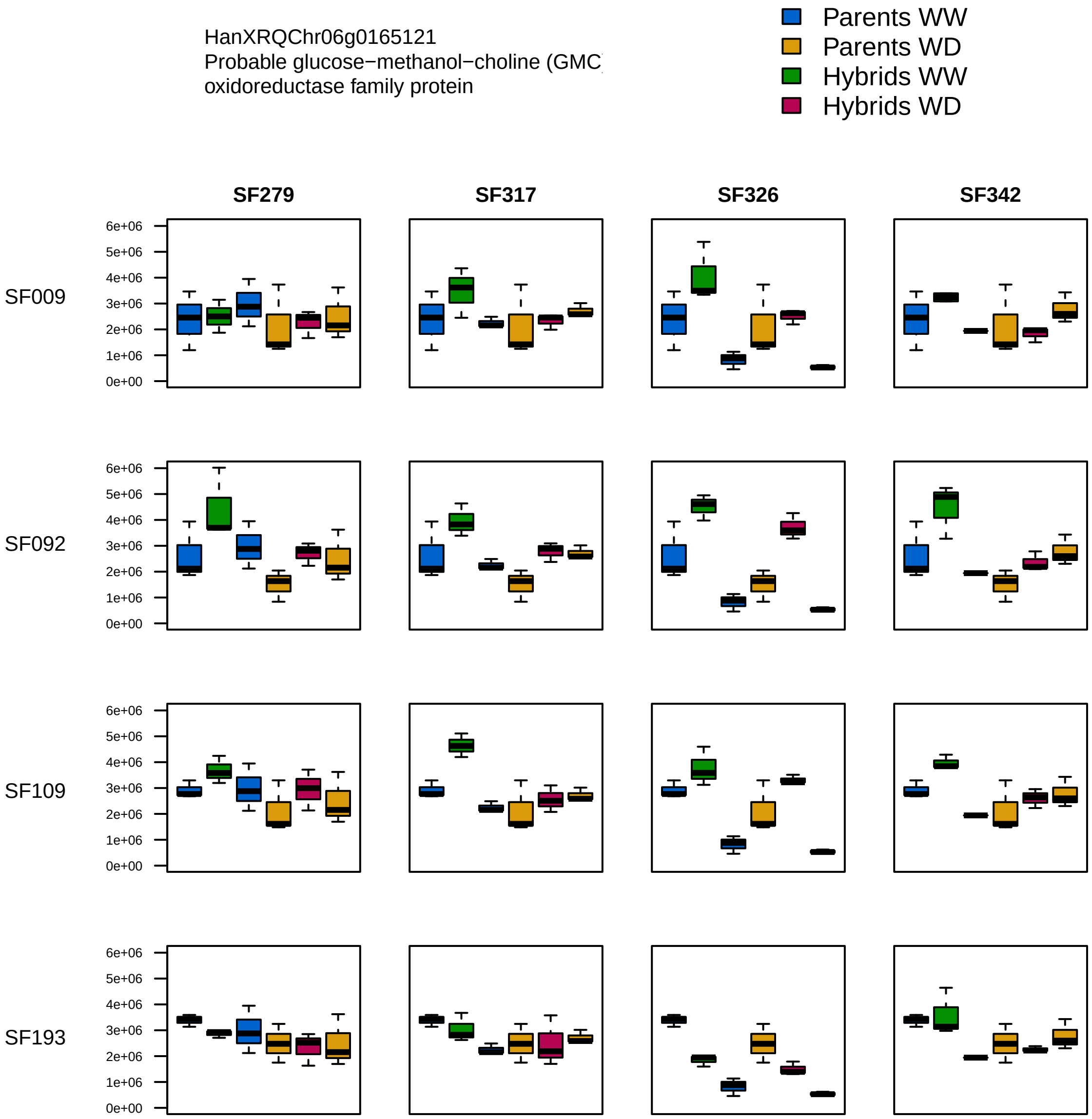
HanXRQChr06g0165121
Probable glucose−methanol−choline (GMC)
oxidoreductase family protein
Parents WW
Parents WD
Hybrids WW
Hybrids WD
SF279
SF317
SF326
SF342
SF009
SF092
SF109
SF193
6e+06
5e+06
4e+06
3e+06
2e+06
1e+06
0e+00

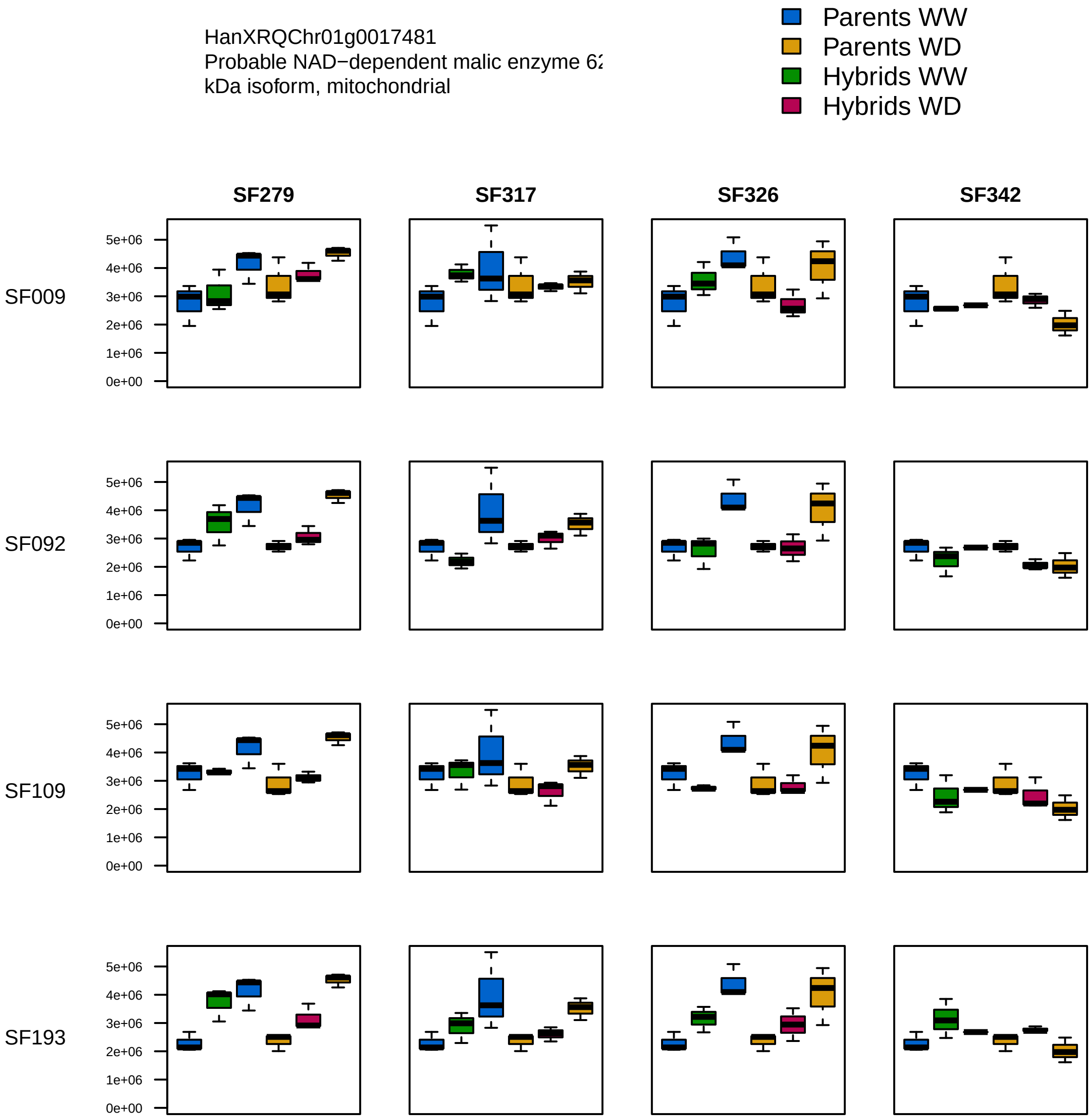

HanXRQChr01g0017481
Probable NAD−dependent malic enzyme 62
kDa isoform, mitochondrial
Parents WW
Parents WD
Hybrids WW
Hybrids WD
SF279
SF317
SF326
SF342
SF009
SF092
SF109
SF193
0e+00
1e+06
2e+06
3e+06
4e+06
5e+06

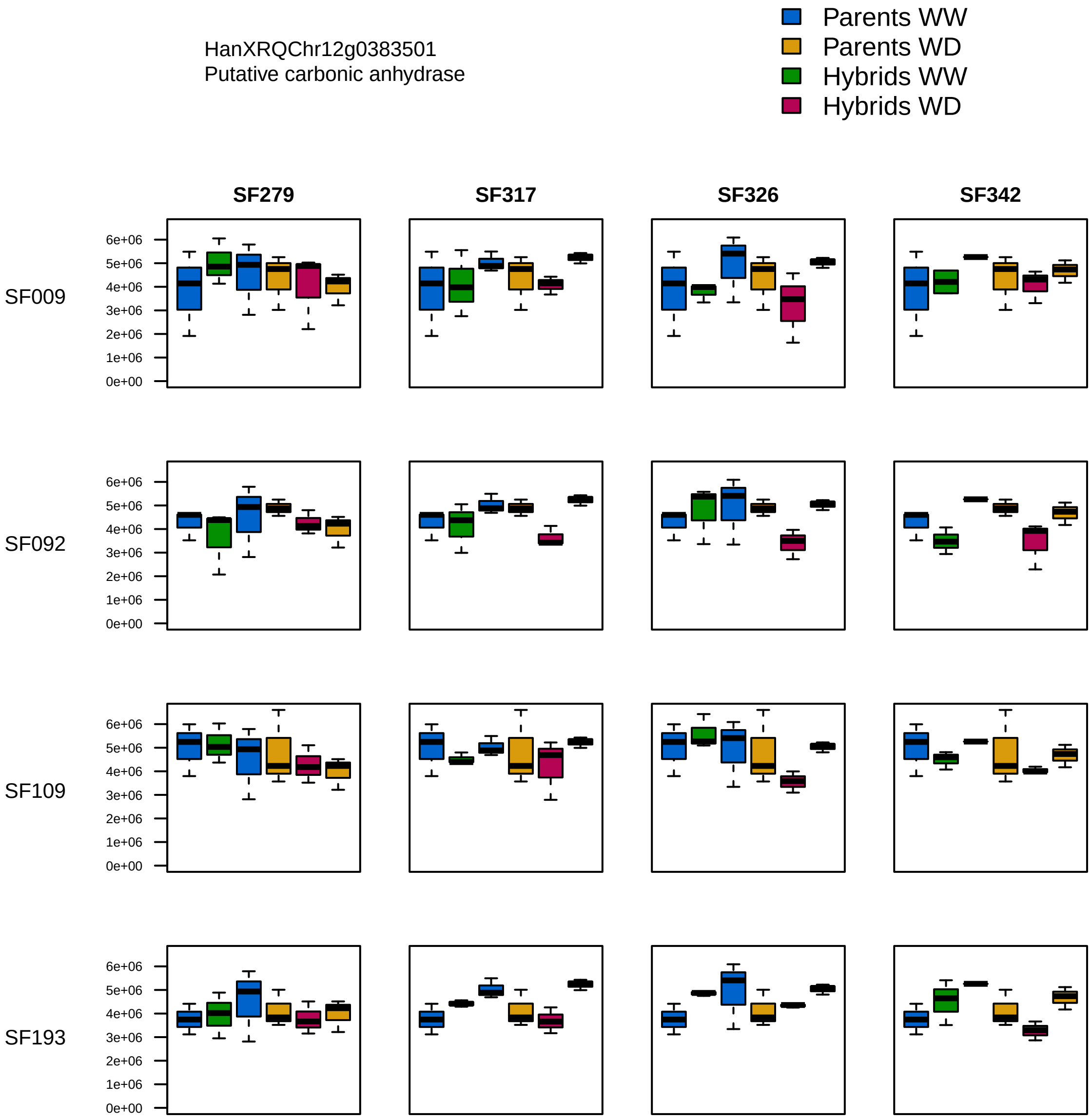

HanXRQChr12g0383501
Putative carbonic anhydrase
Parents WW
Parents WD
Hybrids WW
Hybrids WD
SF279
SF317
SF326
SF342
SF009
SF092
SF109
SF193
0e+00
1e+06
2e+06
3e+06
4e+06
5e+06
6e+06

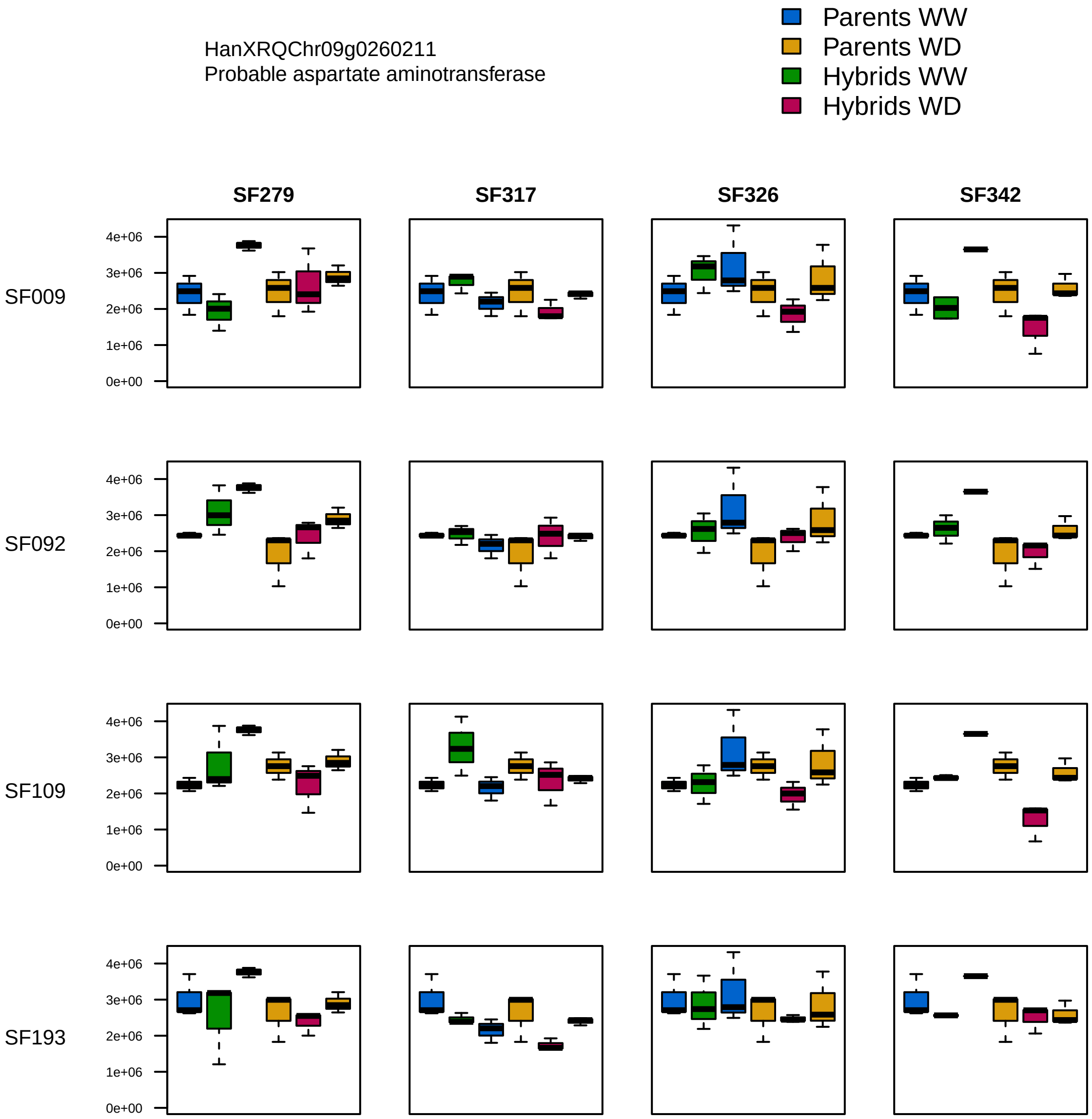
HanXRQChr09g0260211
Probable aspartate aminotransferase
Parents WW
Parents WD
Hybrids WW
Hybrids WD
SF279
SF317
SF326
SF342
SF009
SF092
SF109
SF193
4e+06
3e+06
2e+06
1e+06
0e+00

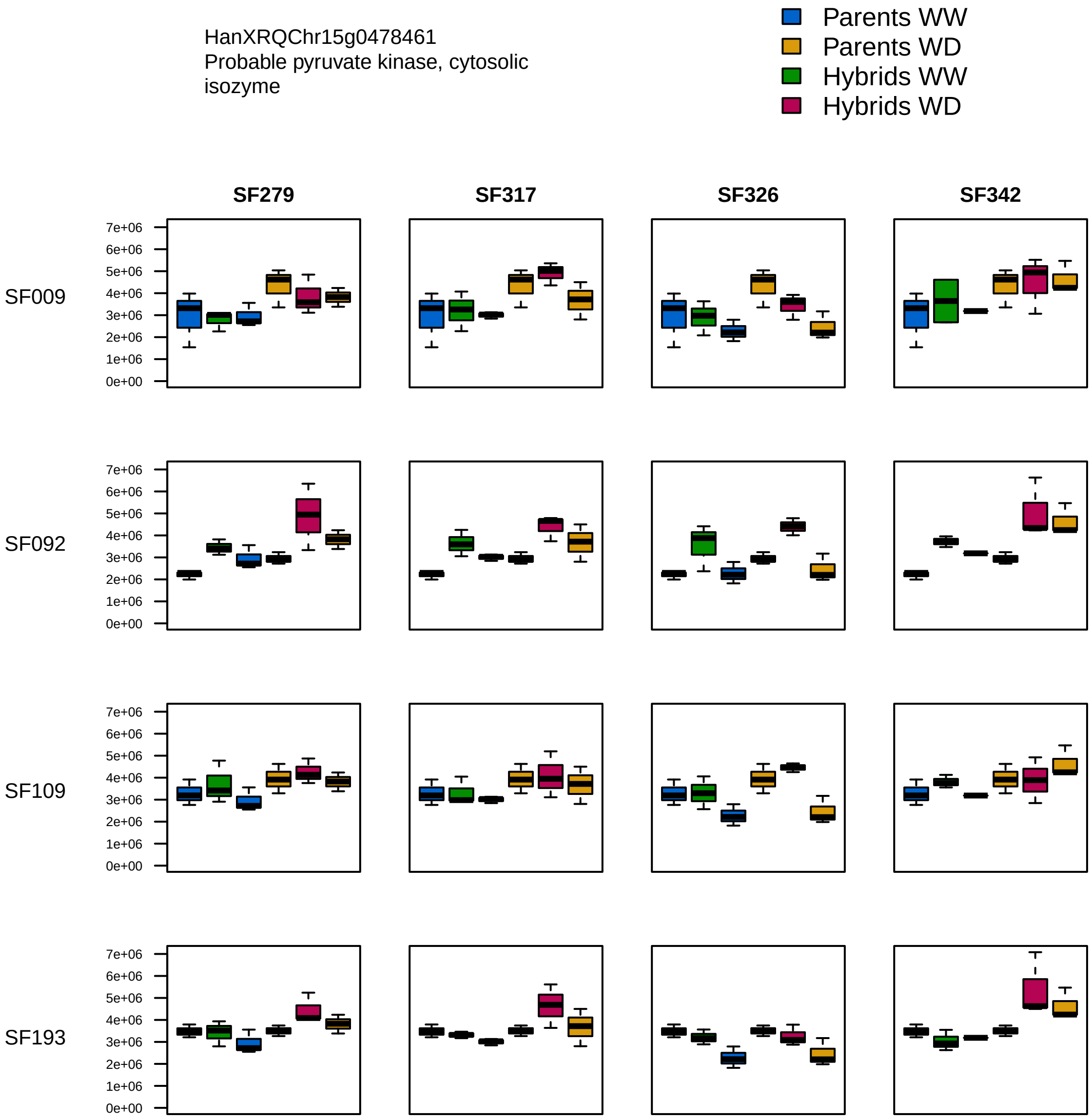
HanXRQChr15g0478461
Probable pyruvate kinase, cytosolic
isozyme
Parents WW
Parents WD
Hybrids WW
Hybrids WD
SF279
SF317
SF326
SF342
SF009
SF092
SF109
SF193
0e+00
1e+06
2e+06
3e+06
4e+06
5e+06
6e+06
7e+06

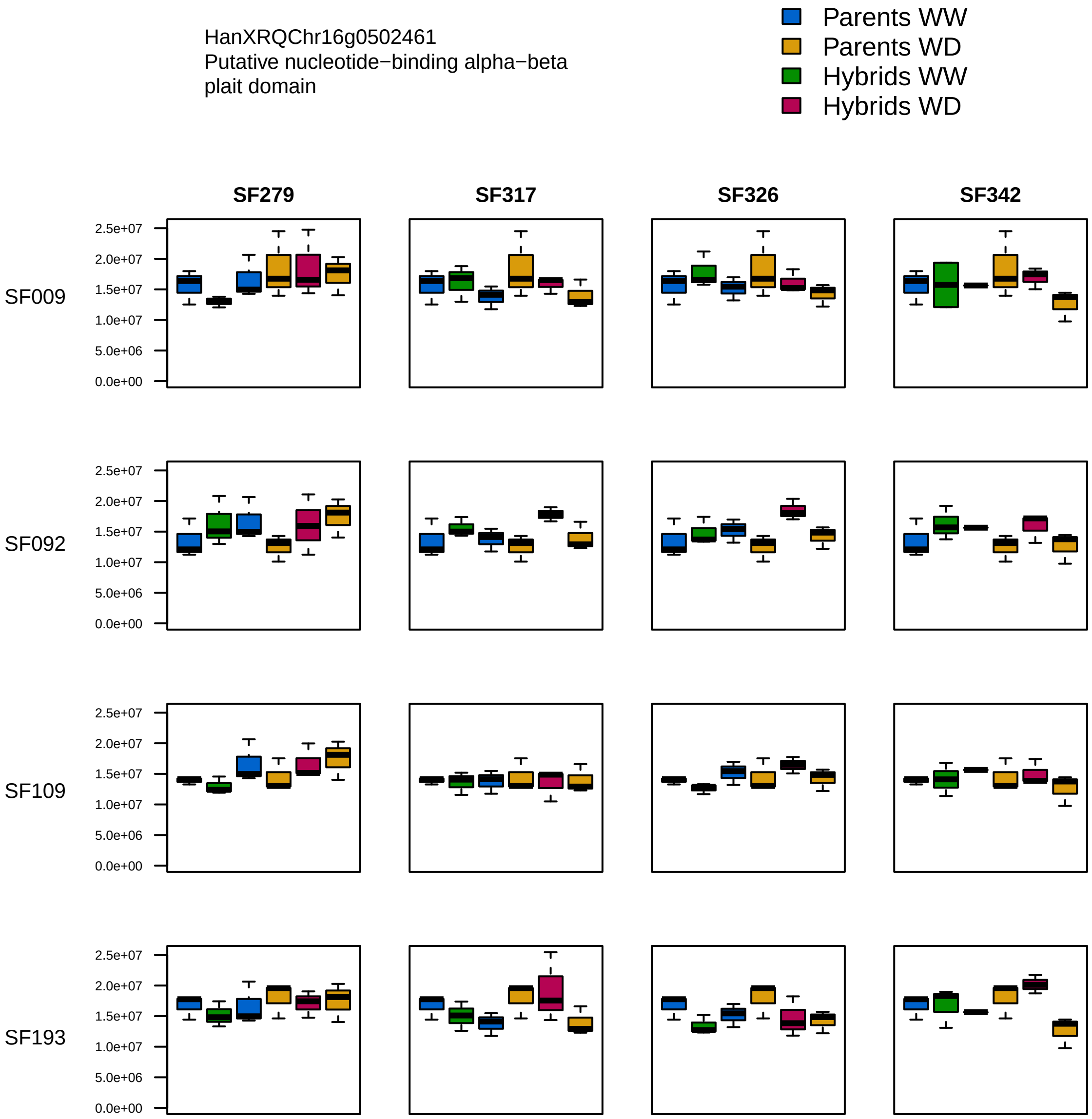
HanXRQChr16g0502461
Putative nucleotide−binding alpha−beta plait domain
Parents WW
Parents WD
Hybrids WW
Hybrids WD
SF279
SF317
SF326
SF342
SF009
SF092
SF109
SF193
2.5e+07
2.0e+07
1.5e+07
1.0e+07
5.0e+06
0.0e+00

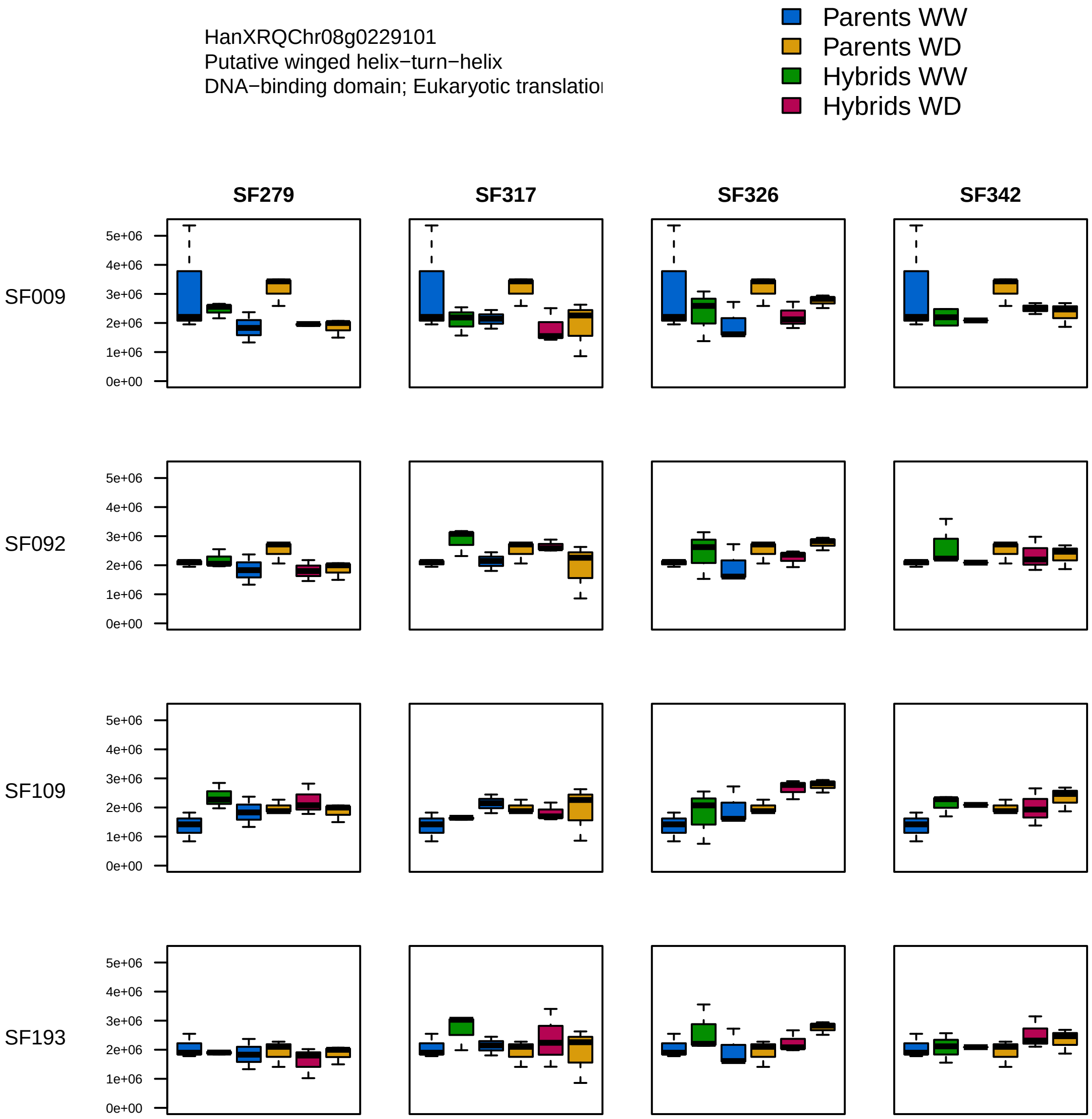

HanXRQChr08g0229101
Putative winged helix−turn−helix
DNA−binding domain; Eukaryotic translatio
Parents WW
Parents WD
Hybrids WW
Hybrids WD
SF279
SF317
SF326
SF342
SF009
SF092
SF109
SF193
0e+00
1e+06
2e+06
3e+06
4e+06
5e+06

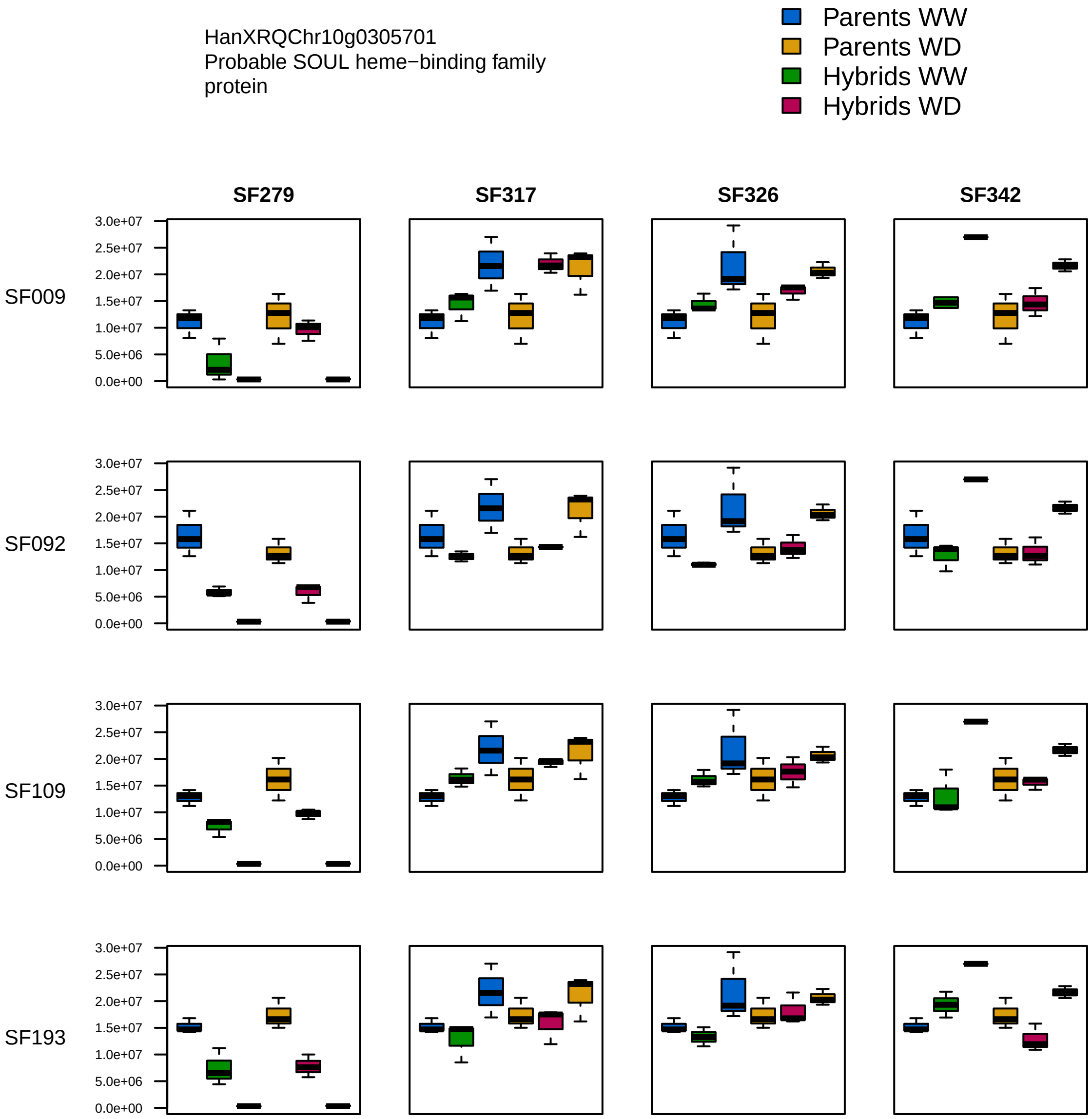
HanXRQChr10g0305701
Probable SOUL heme−binding family protein
Parents WW
Parents WD
Hybrids WW
Hybrids WD
SF279
SF317
SF326
SF342
SF009
SF092
SF109
SF193
3.0e+07
2.5e+07
2.0e+07
1.5e+07
1.0e+07
5.0e+06
0.0e+00

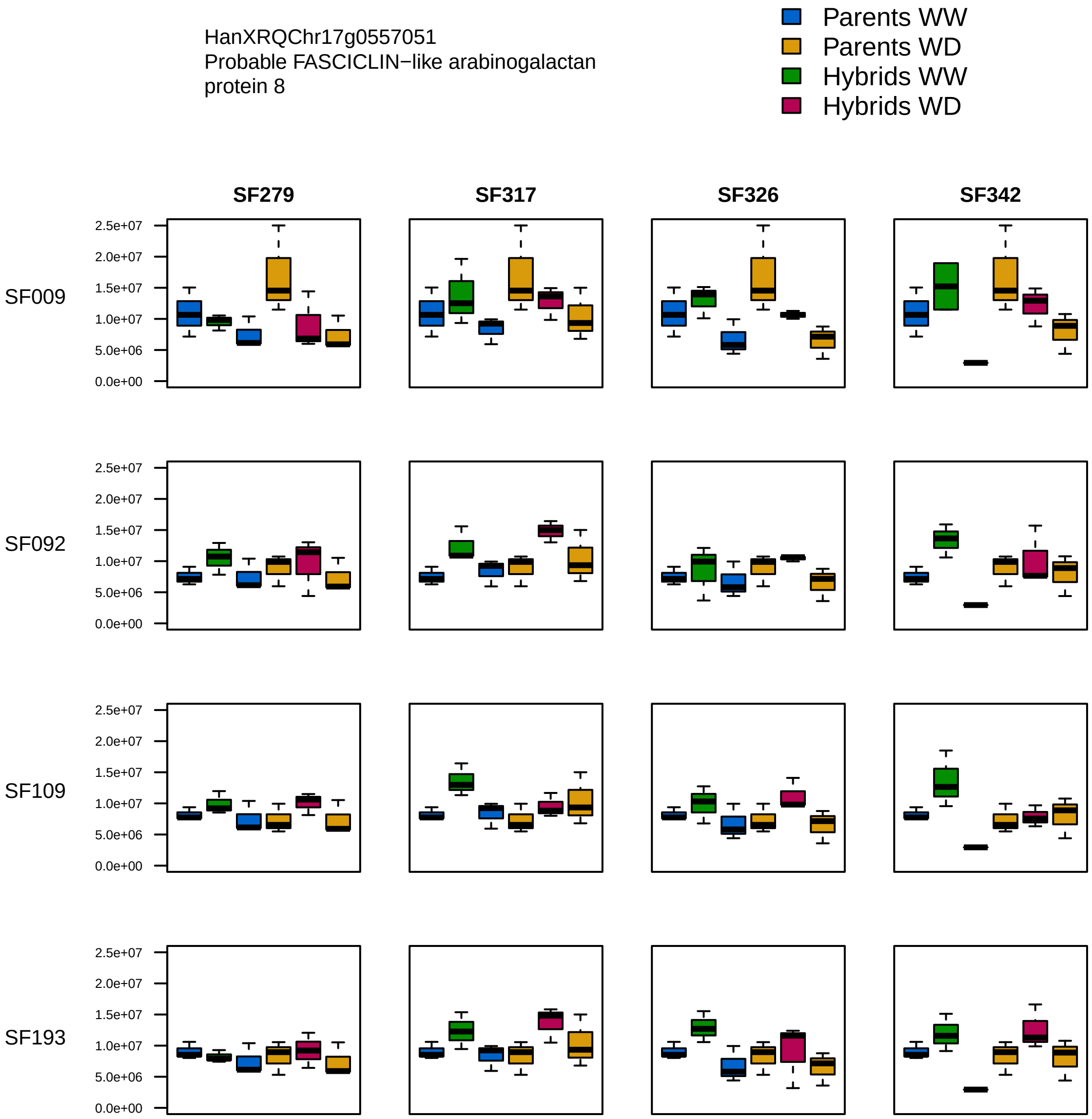
HanXRQChr17g0557051
Probable FASCICLIN−like arabinogalactan protein 8
Parents WW
Parents WD
Hybrids WW
Hybrids WD
SF279
SF317
SF326
SF342
SF009
SF092
SF109
SF193
2.5e+07
2.0e+07
1.5e+07
1.0e+07
5.0e+06
0.0e+00

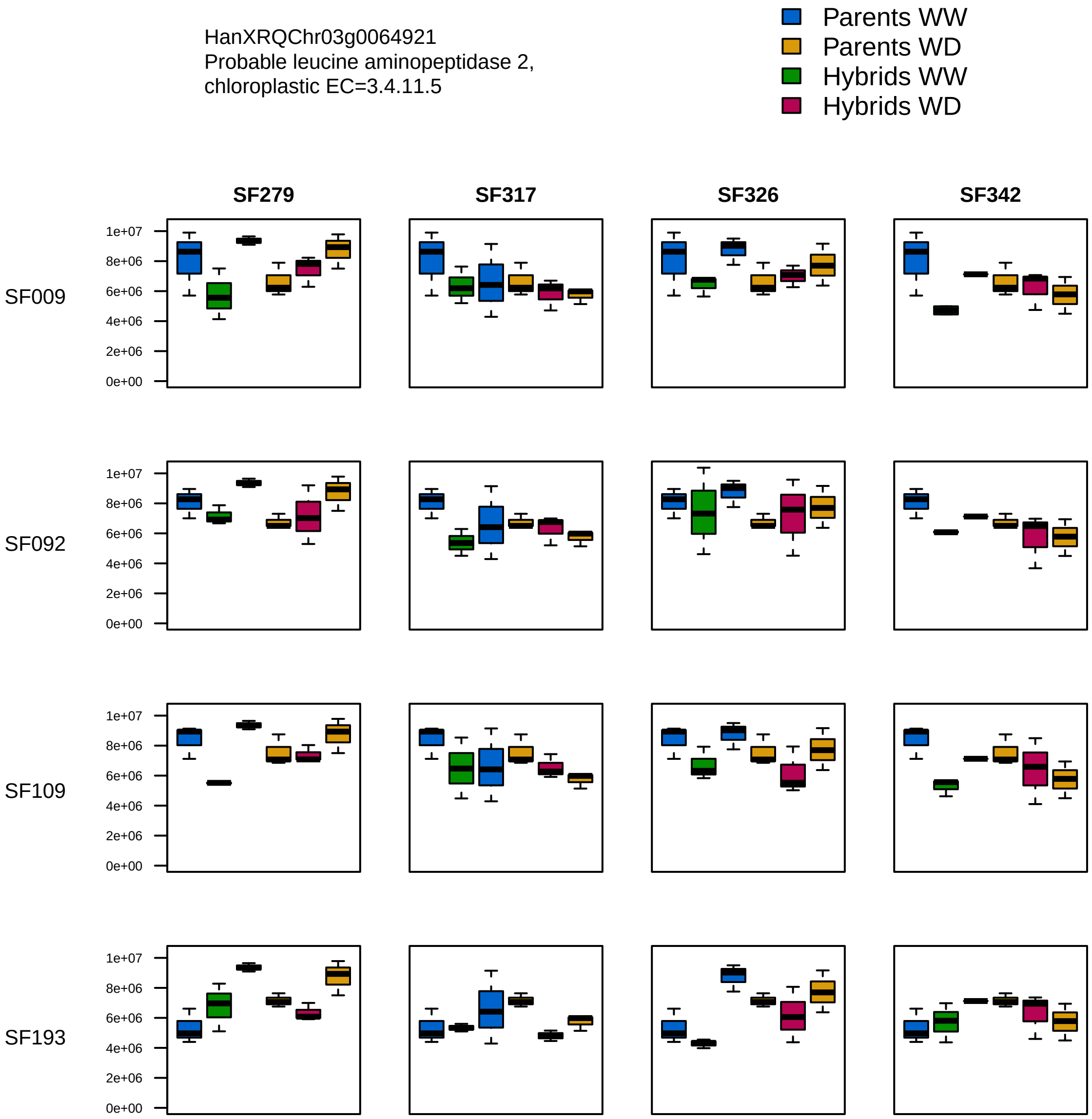

HanXRQChr03g0064921
Probable leucine aminopeptidase 2,
chloroplastic EC=3.4.11.5
Parents WW
Parents WD
Hybrids WW
Hybrids WD
SF279
SF317
SF326
SF342
SF009
SF092
SF109
SF193
1e+07
8e+06
6e+06
4e+06
2e+06
0e+00

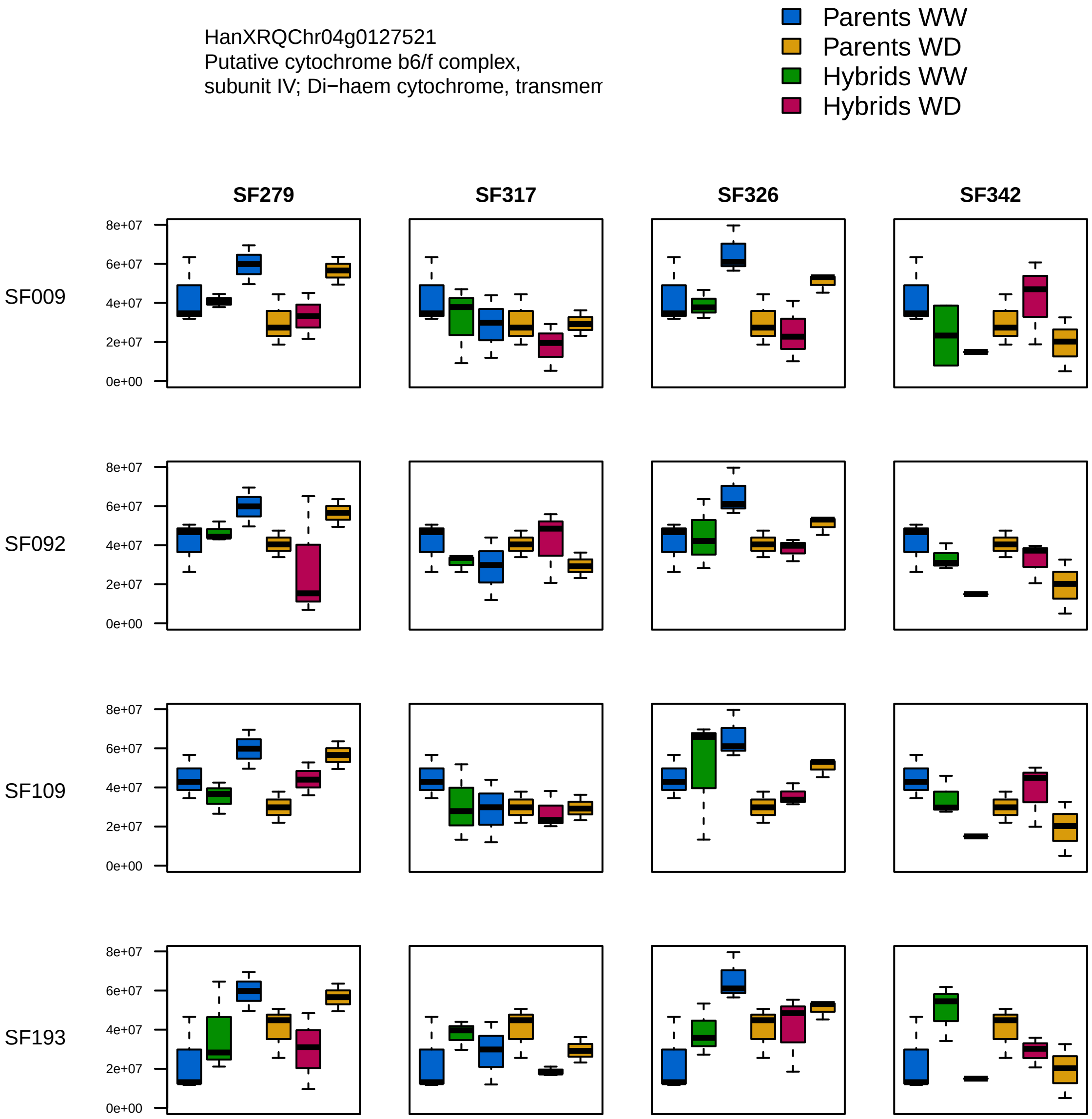
HanXRQChr04g0127521
Putative cytochrome b6/f complex,
subunit IV; Di−haem cytochrome, transmem
Parents WW
Parents WD
Hybrids WW
Hybrids WD
SF279
SF317
SF326
SF342
SF009
SF092
SF109
SF193
8e+07
6e+07
4e+07
2e+07
0e+00

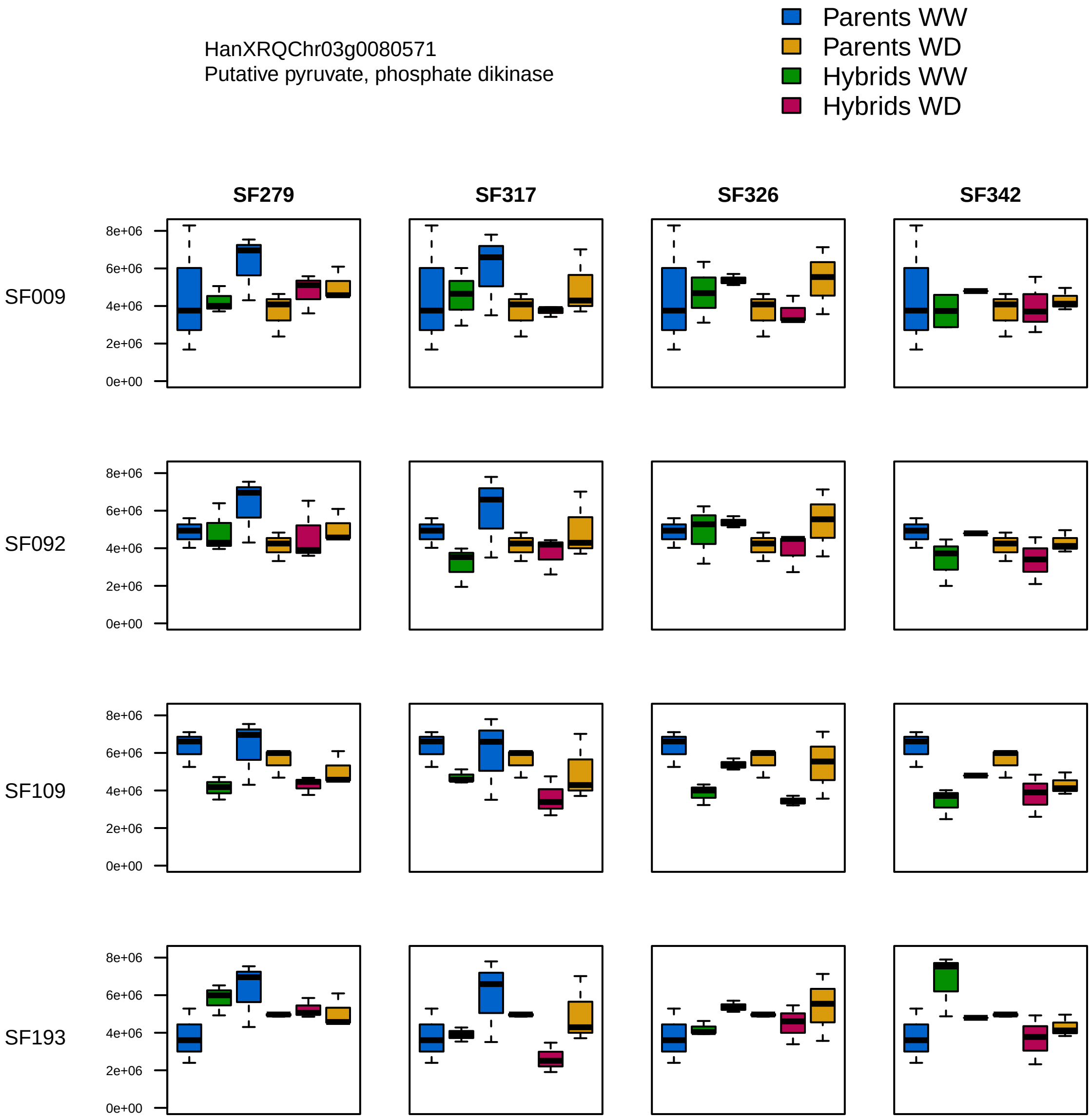
HanXRQChr03g0080571
Putative pyruvate, phosphate dikinase
Parents WW
Parents WD
Hybrids WW
Hybrids WD
SF279
SF317
SF326
SF342
SF009
SF092
SF109
SF193
8e+06
6e+06
4e+06
2e+06
0e+00

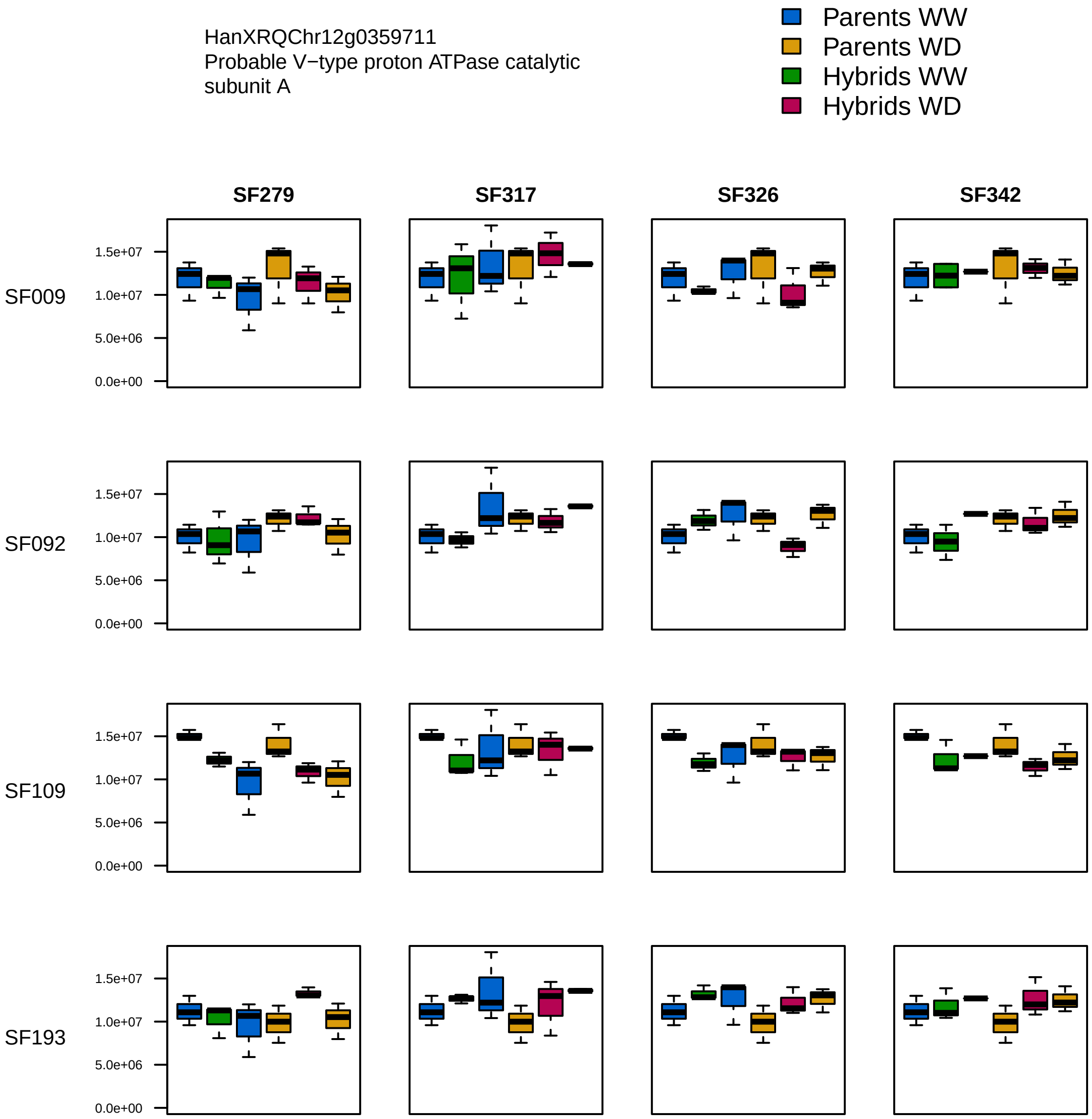
HanXRQChr12g0359711
Probable V−type proton ATPase catalytic subunit A
Parents WW
Parents WD
Hybrids WW
Hybrids WD
SF279
SF317
SF326
SF342
SF009
SF092
SF109
SF193
1.5e+07
1.0e+07
5.0e+06
0.0e+00

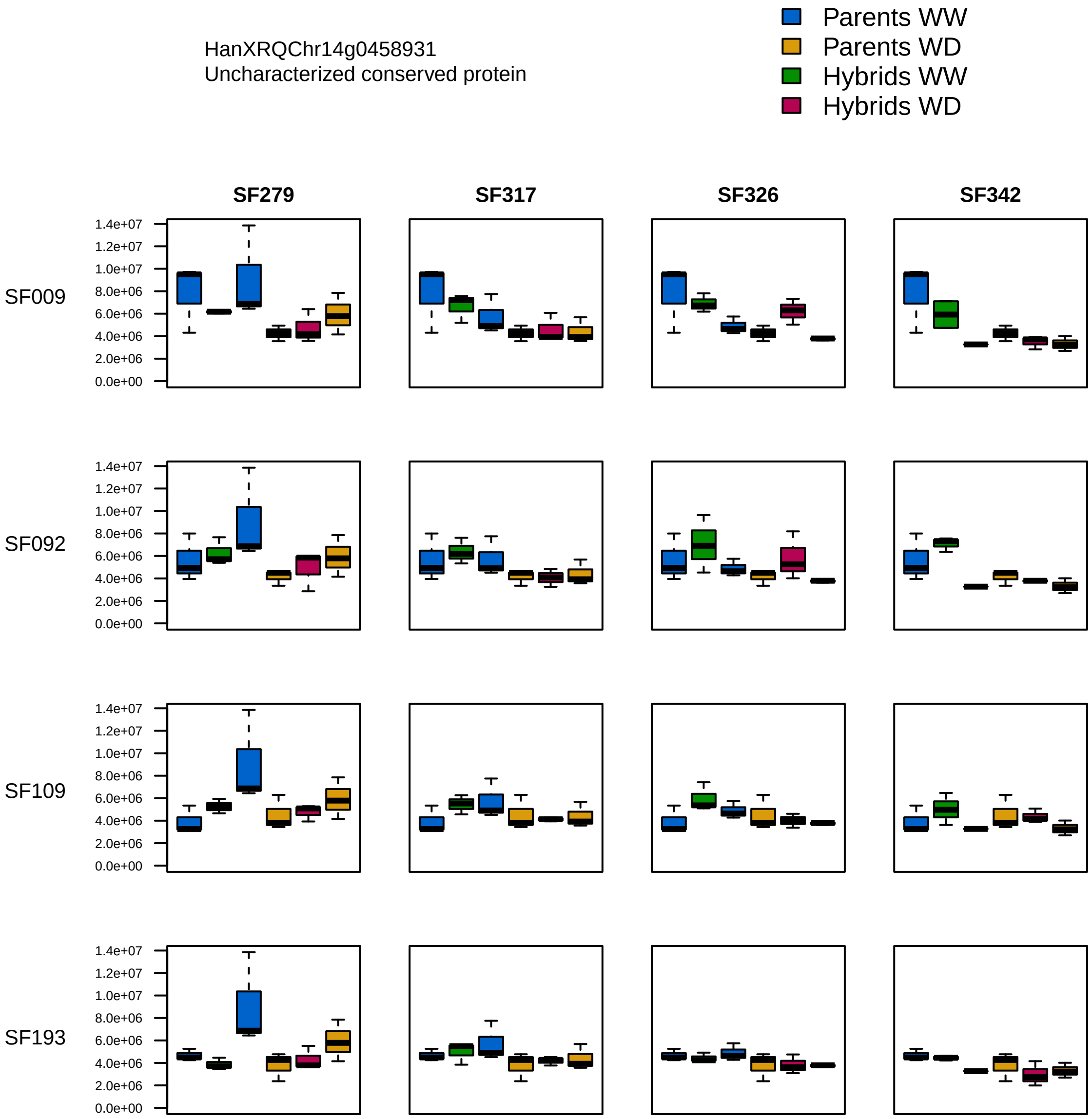
HanXRQChr14g0458931
Uncharacterized conserved protein
Parents WW
Parents WD
Hybrids WW
Hybrids WD
SF279
SF317
SF326
SF342
SF009
SF092
SF109
SF193
1.4e+07
1.2e+07
1.0e+07
8.0e+06
6.0e+06
4.0e+06
2.0e+06
0.0e+00

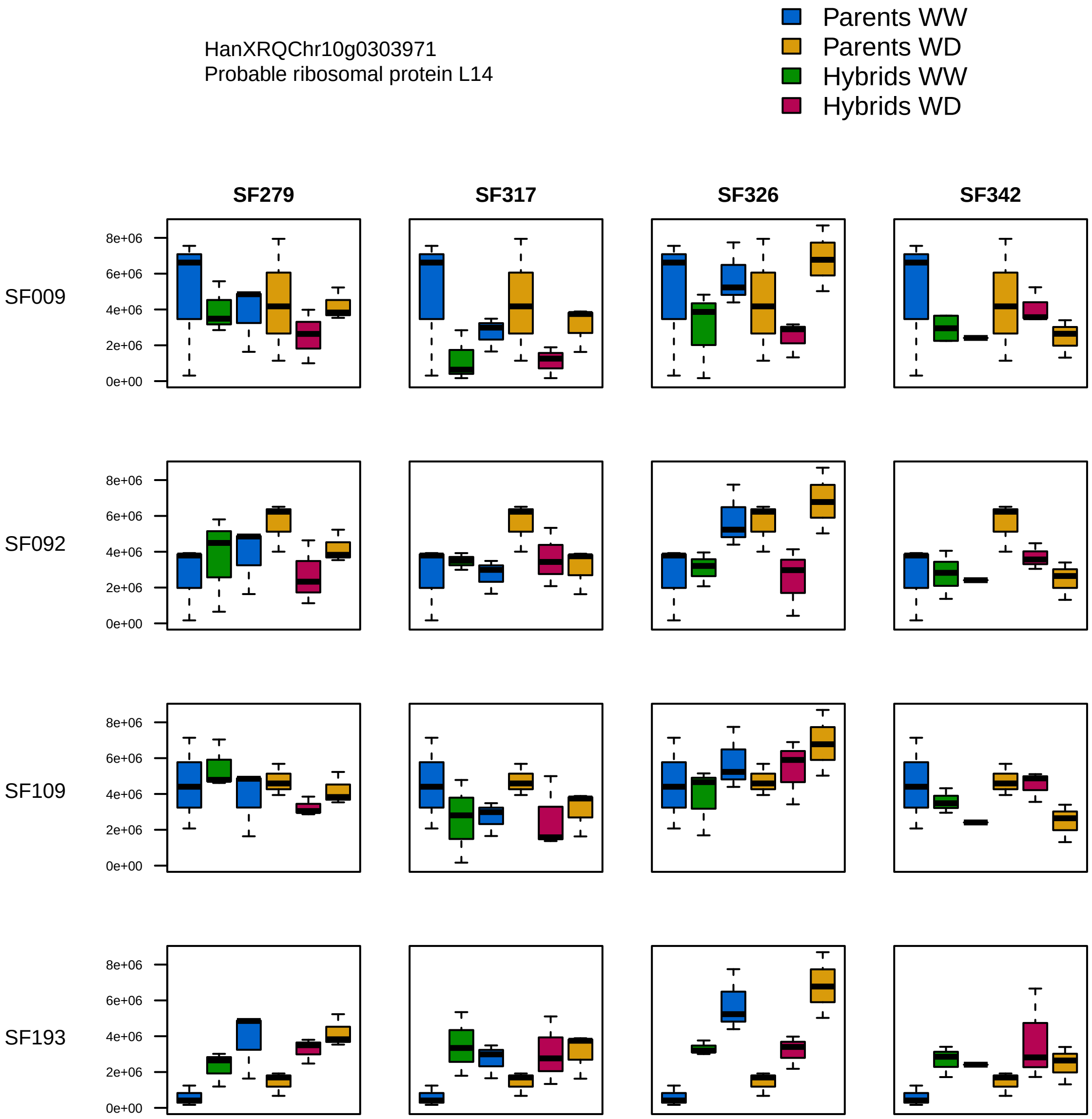
HanXRQChr10g0303971
Probable ribosomal protein L14
Parents WW
Parents WD
Hybrids WW
Hybrids WD
SF279
SF317
SF326
SF342
SF009
SF092
SF109
SF193
8e+06
6e+06
4e+06
2e+06
0e+00

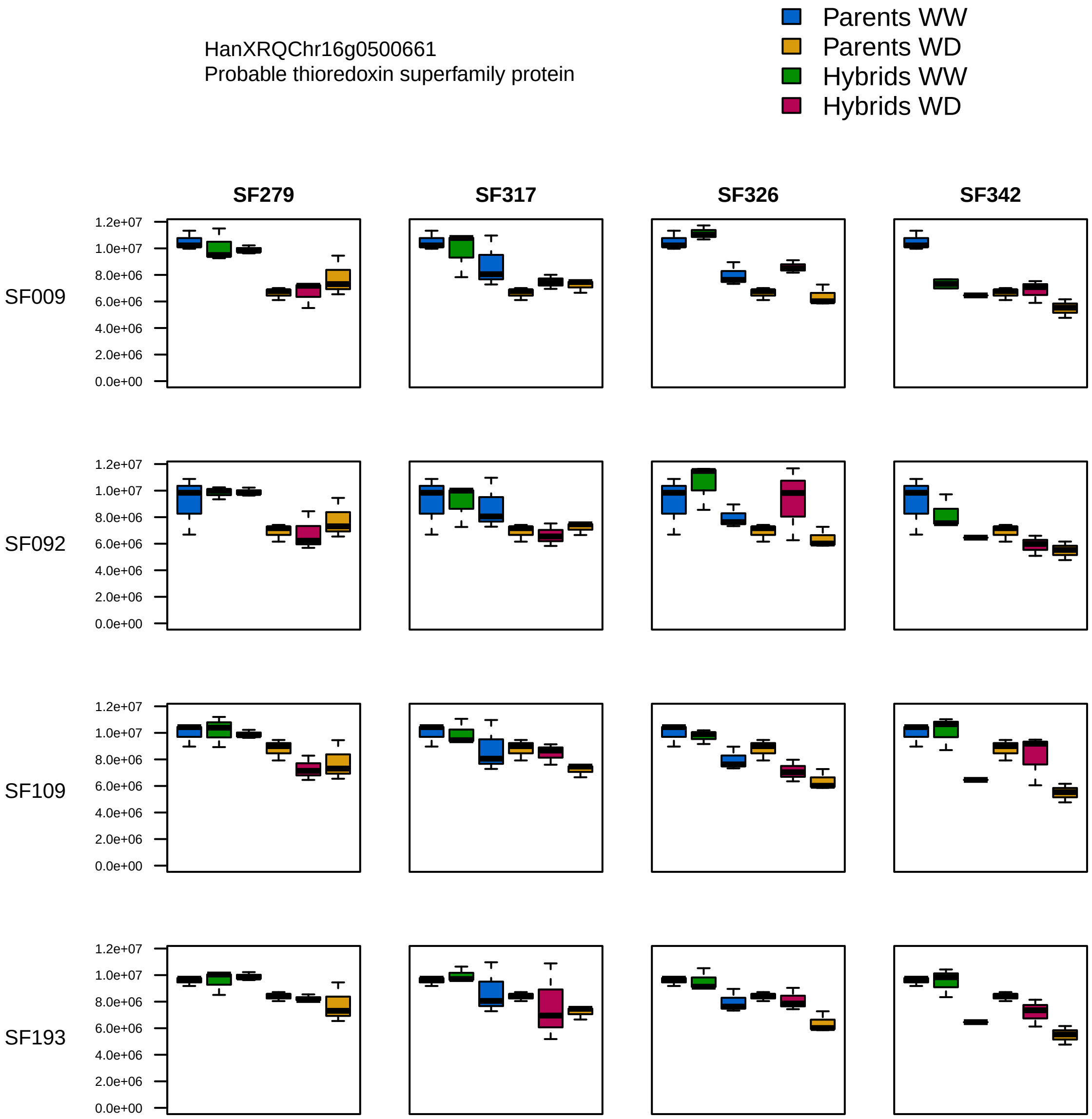
HanXRQChr16g0500661
Probable thioredoxin superfamily protein
Parents WW
Parents WD
Hybrids WW
Hybrids WD
SF279
SF317
SF326
SF342
SF009
SF092
SF109
SF193
1.2e+07
1.0e+07
8.0e+06
6.0e+06
4.0e+06
2.0e+06
0.0e+00

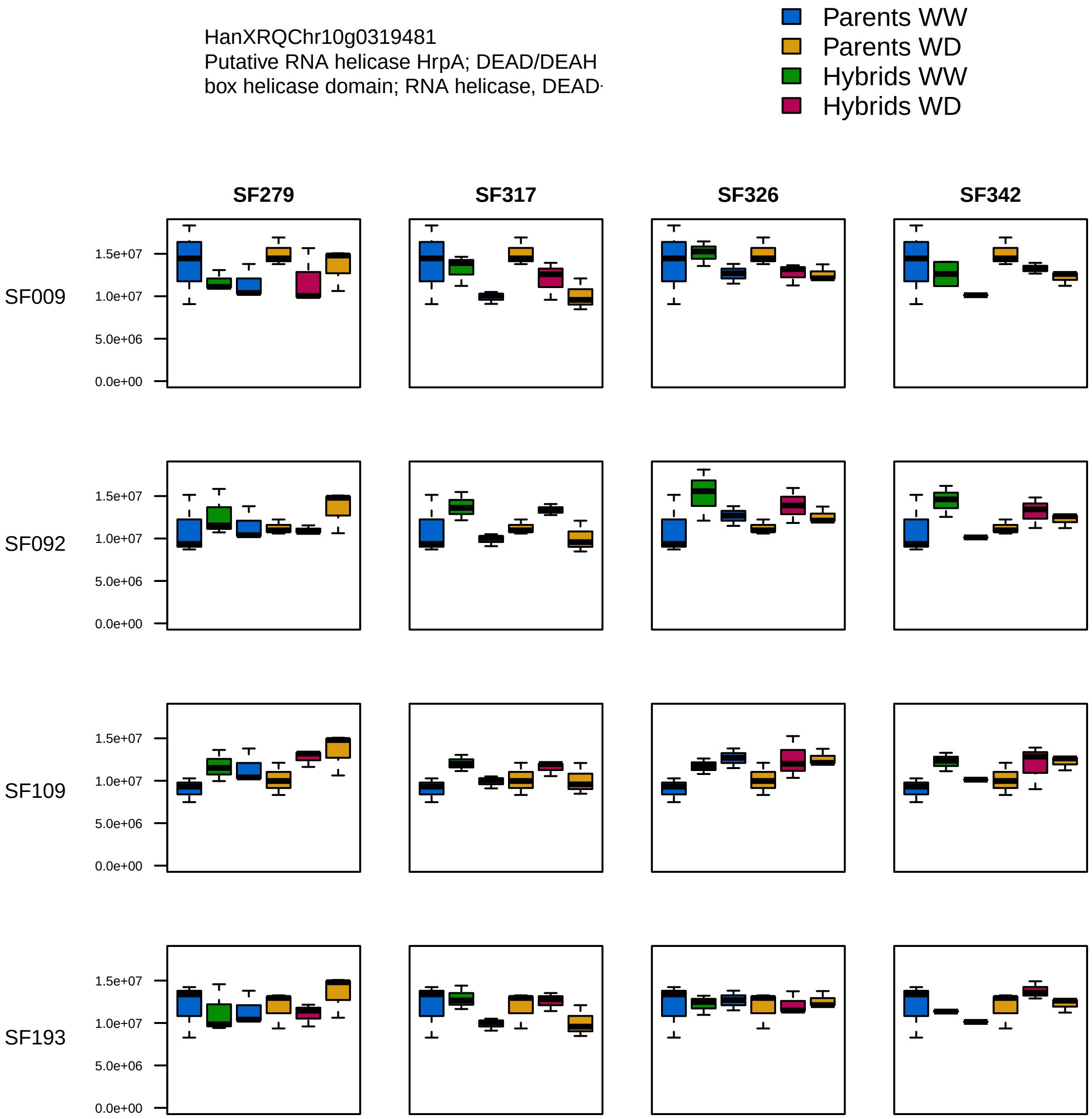
HanXRQChr10g0319481
Putative RNA helicase HrpA; DEAD/DEAH
box helicase domain; RNA helicase, DEAD-
Parents WW
Parents WD
Hybrids WW
Hybrids WD
SF279
SF317
SF326
SF342
SF009
SF092
SF109
SF193
1.5e+07
1.0e+07
5.0e+06
0.0e+00

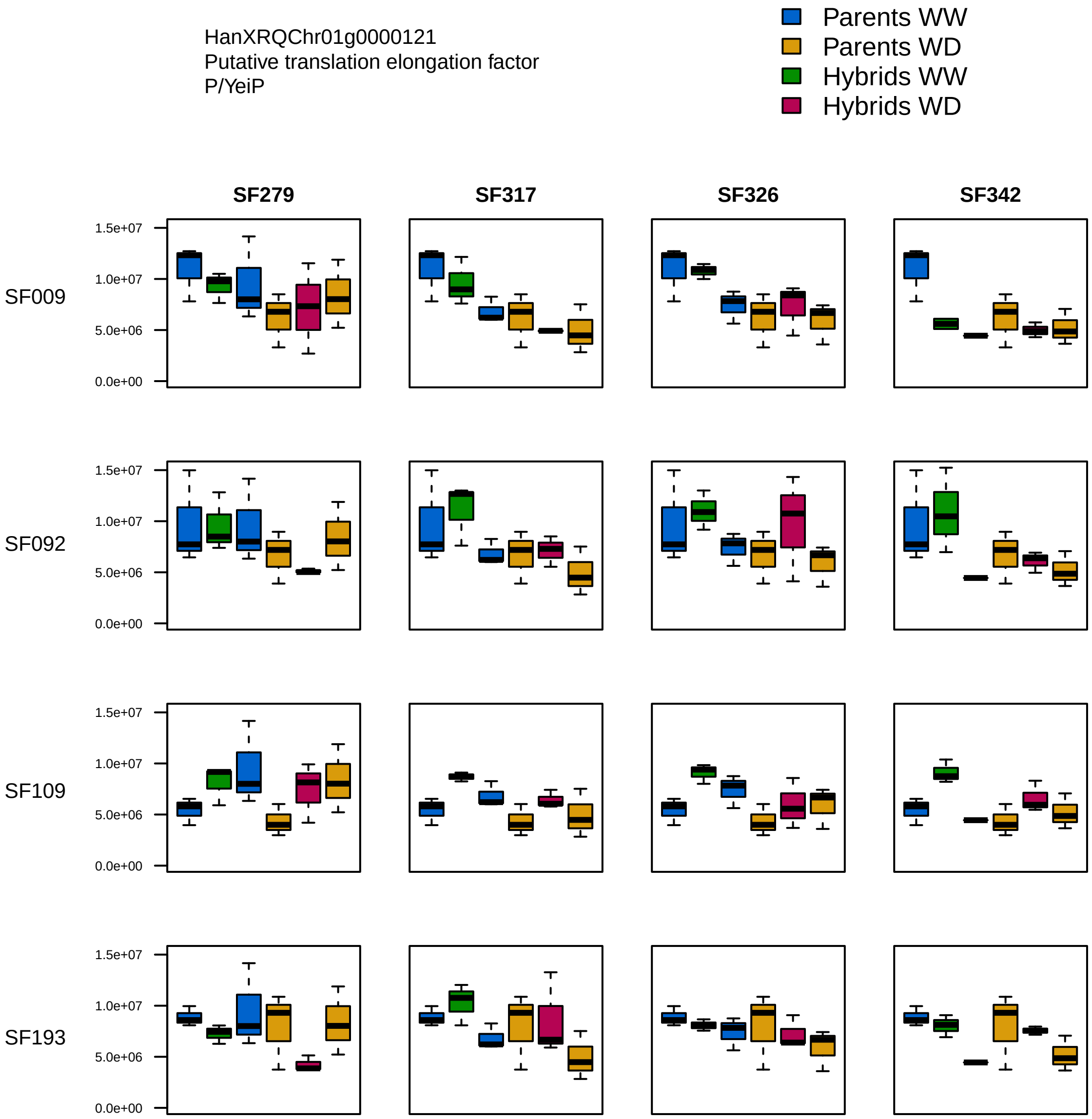
HanXRQChr01g0000121
Putative translation elongation factor
P/YeiP
Parents WW
Parents WD
Hybrids WW
Hybrids WD
SF279
SF317
SF326
SF342
SF009
SF092
SF109
SF193
1.5e+07
1.0e+07
5.0e+06
0.0e+00

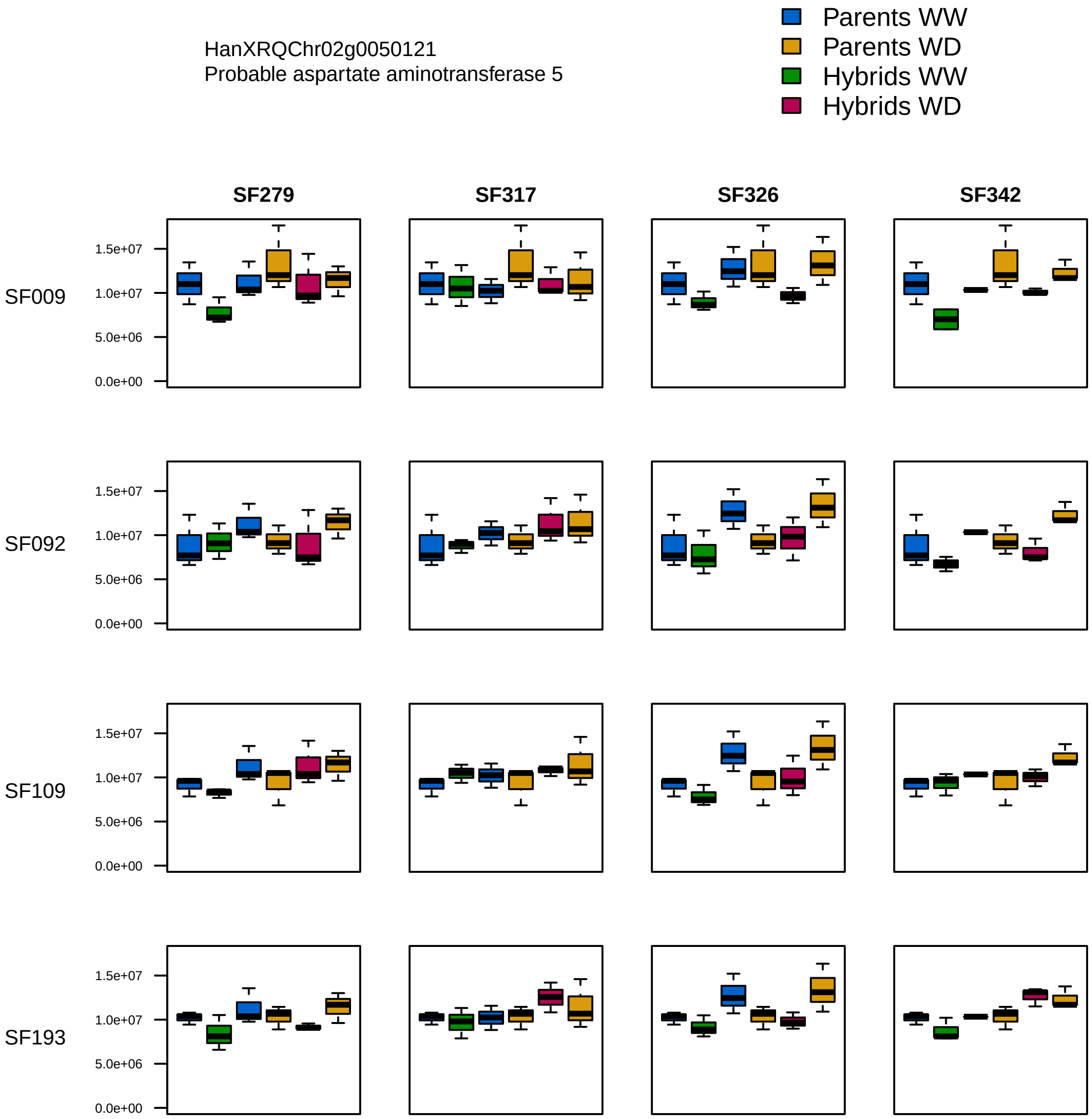

HanXRQChr02g0050121
Probable aspartate aminotransferase 5
Parents WW
Parents WD
Hybrids WW
Hybrids WD
SF279
SF317
SF326
SF342
SF009
SF092
SF109
SF193
1.5e+07
1.0e+07
5.0e+06
0.0e+00

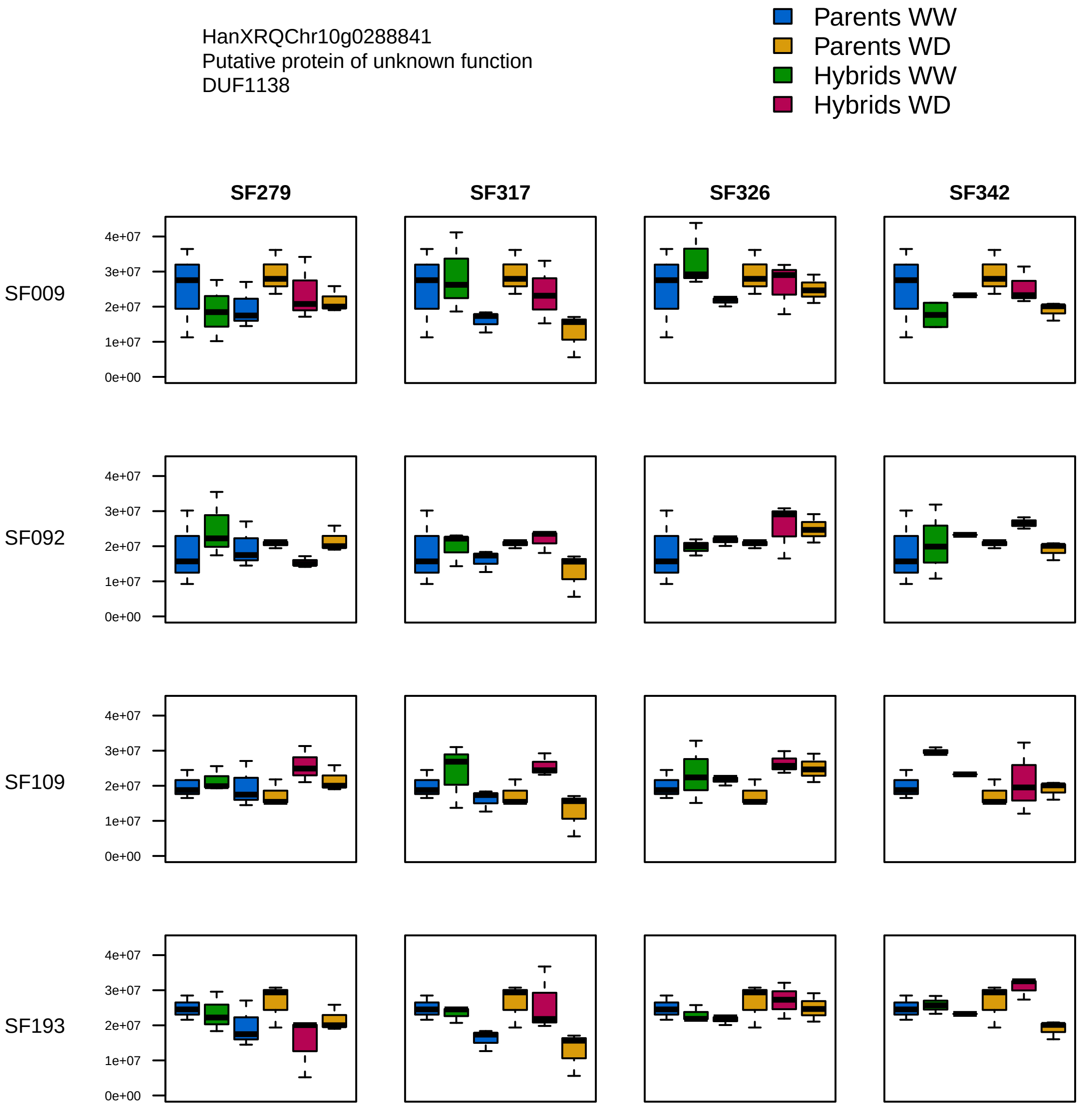

HanXRQChr10g0288841
Putative protein of unknown function
DUF1138
Parents WW
Parents WD
Hybrids WW
Hybrids WD
SF279
SF317
SF326
SF342
SF009
SF092
SF109
SF193
4e+07
3e+07
2e+07
1e+07
0e+00

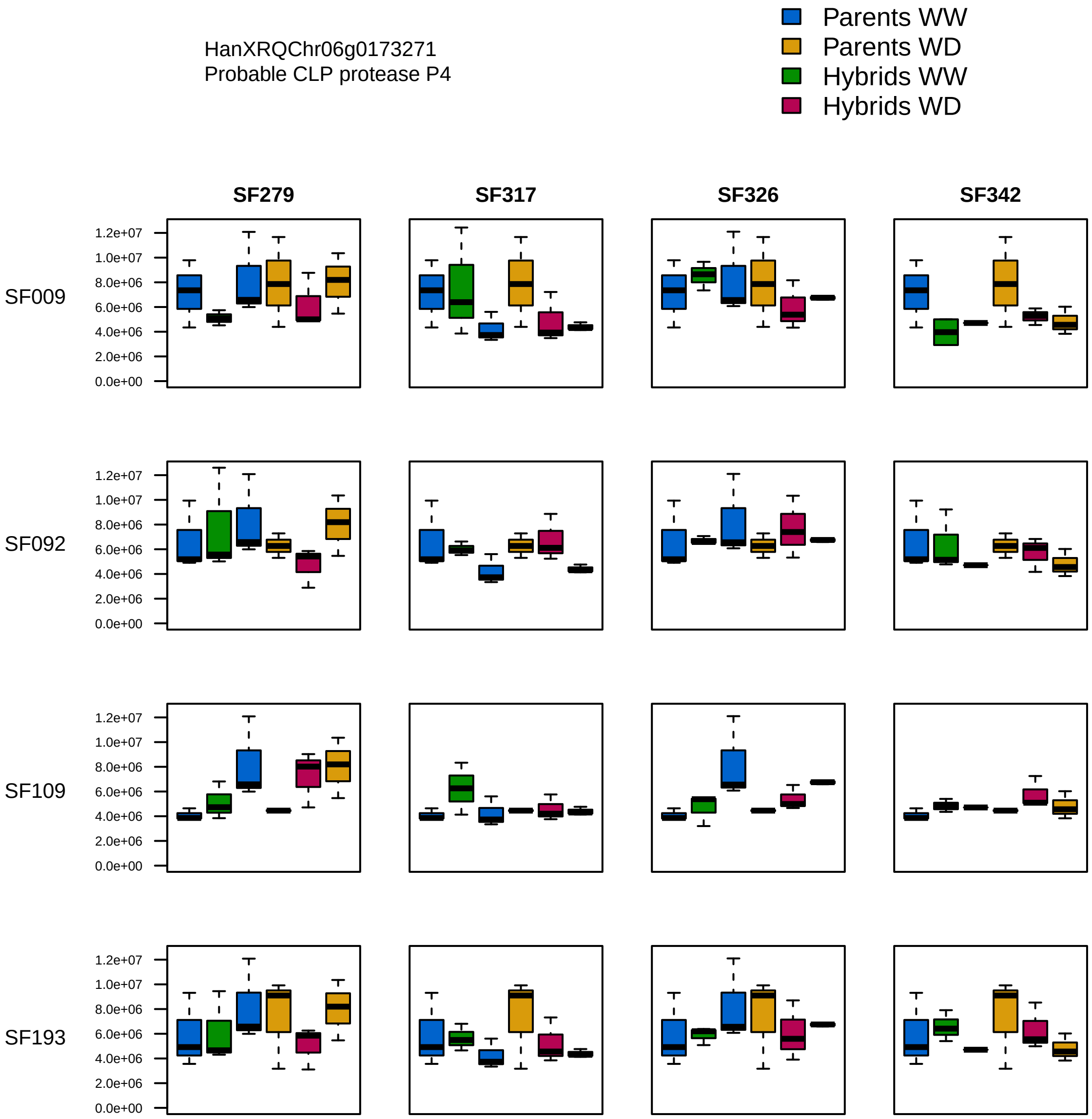
HanXRQChr06g0173271
Probable CLP protease P4
Parents WW
Parents WD
Hybrids WW
Hybrids WD
SF279
SF317
SF326
SF342
SF009
SF092
SF109
SF193
1.2e+07
1.0e+07
8.0e+06
6.0e+06
4.0e+06
2.0e+06
0.0e+00

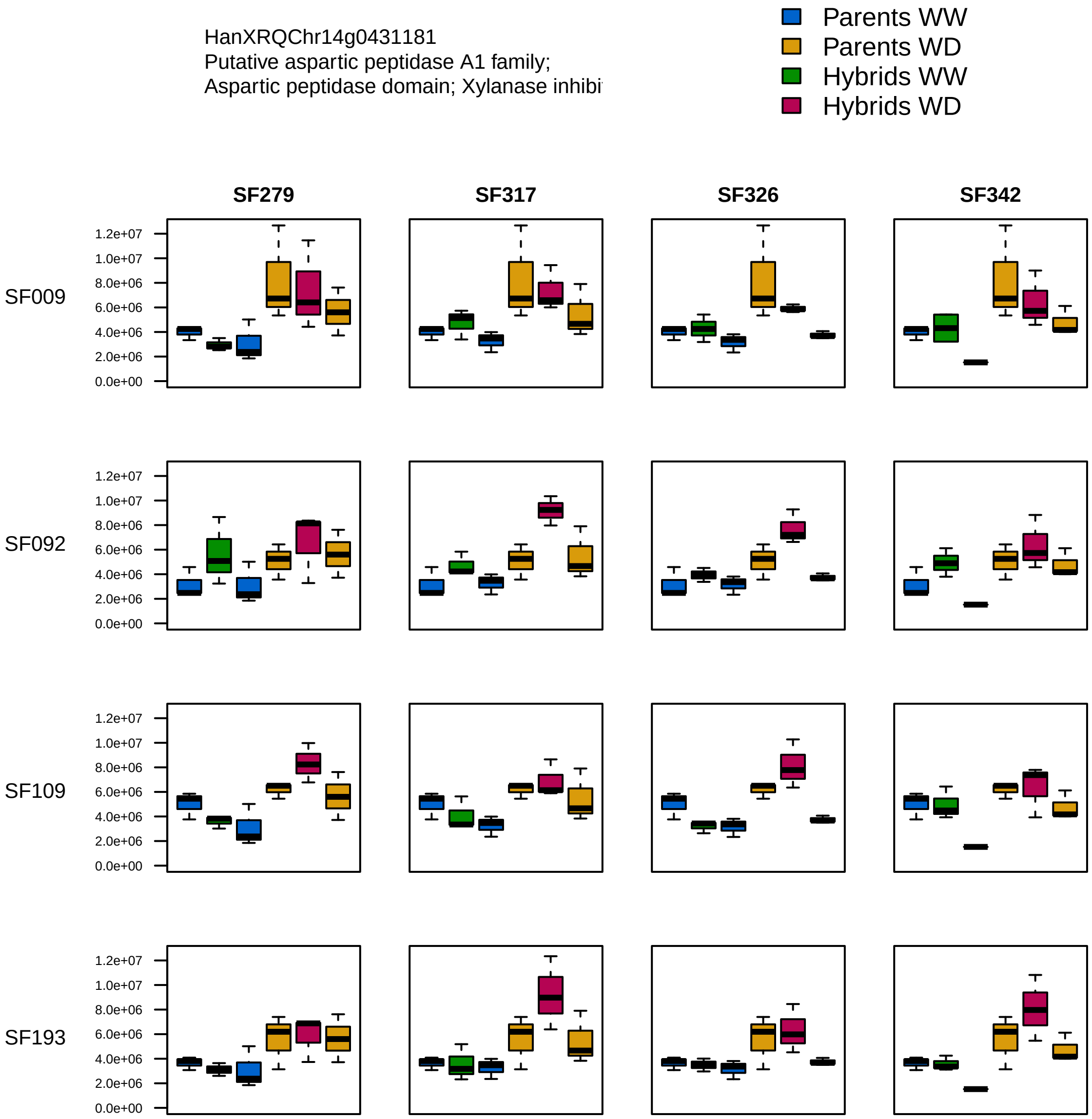

HanXRQChr14g0431181
Putative aspartic peptidase A1 family;
Aspartic peptidase domain; Xylanase inhibi
Parents WW
Parents WD
Hybrids WW
Hybrids WD
SF279
SF317
SF326
SF342
SF009
SF092
SF109
SF193
1.2e+07
1.0e+07
8.0e+06
6.0e+06
4.0e+06
2.0e+06
0.0e+00

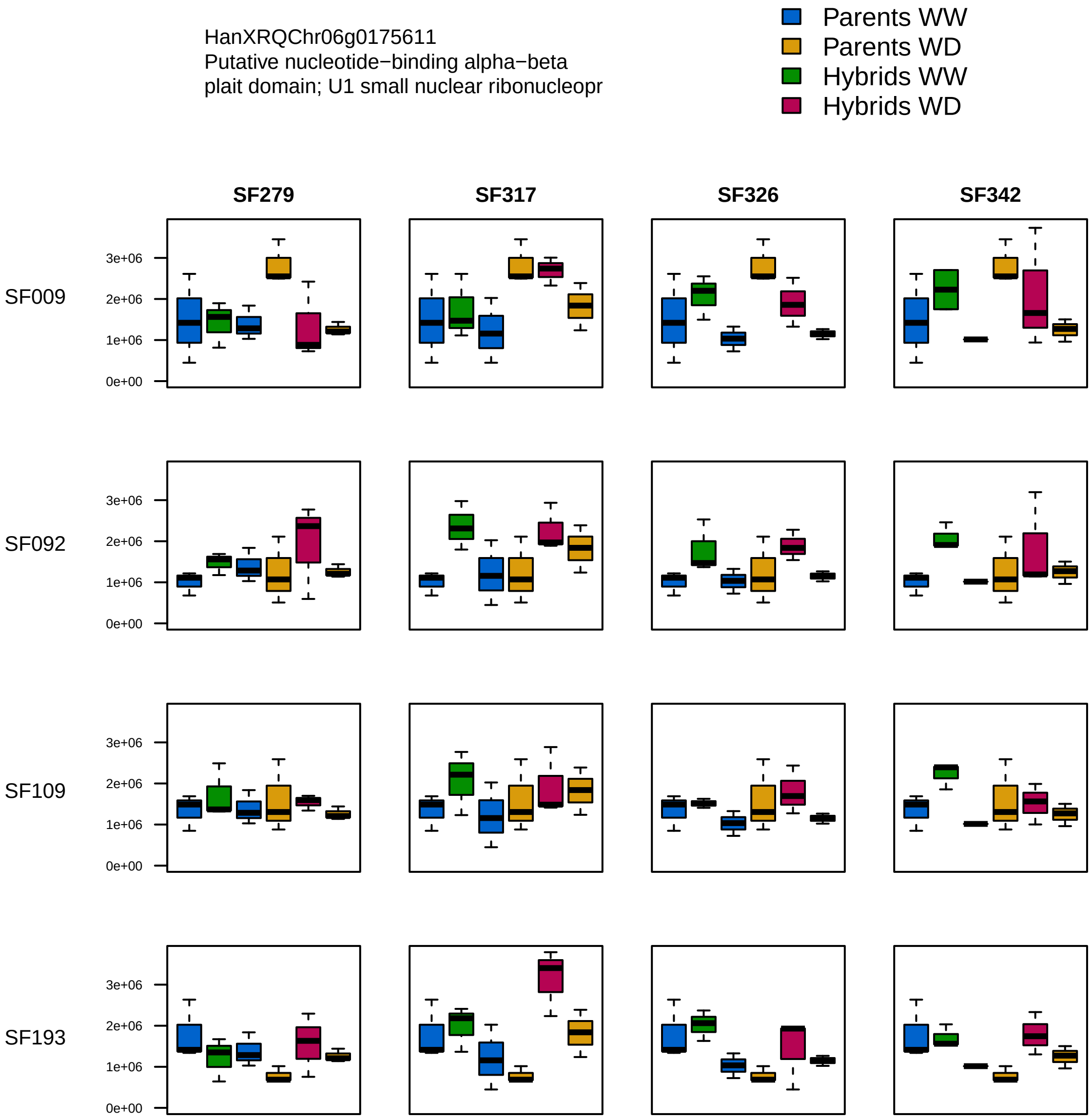
HanXRQChr06g0175611
Putative nucleotide−binding alpha−beta
plait domain; U1 small nuclear ribonucleopr
Parents WW
Parents WD
Hybrids WW
Hybrids WD
SF279
SF317
SF326
SF342
SF009
SF092
SF109
SF193
0e+00
1e+06
2e+06
3e+06

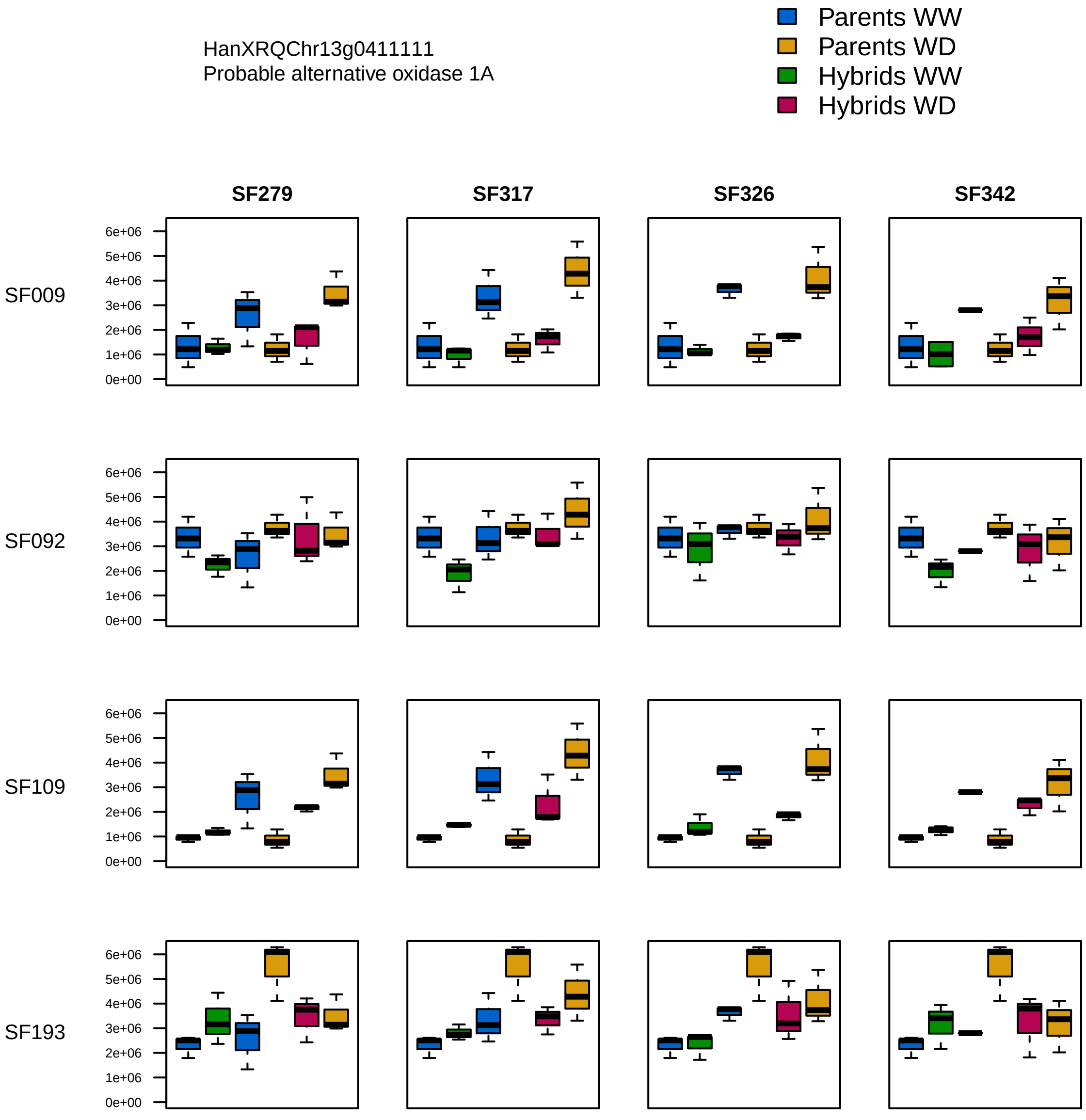
HanXRQChr13g0411111
Probable alternative oxidase 1A
Parents WW
Parents WD
Hybrids WW
Hybrids WD
SF279
SF317
SF326
SF342
SF009
SF092
SF109
SF193
6e+06
5e+06
4e+06
3e+06
2e+06
1e+06
0e+00

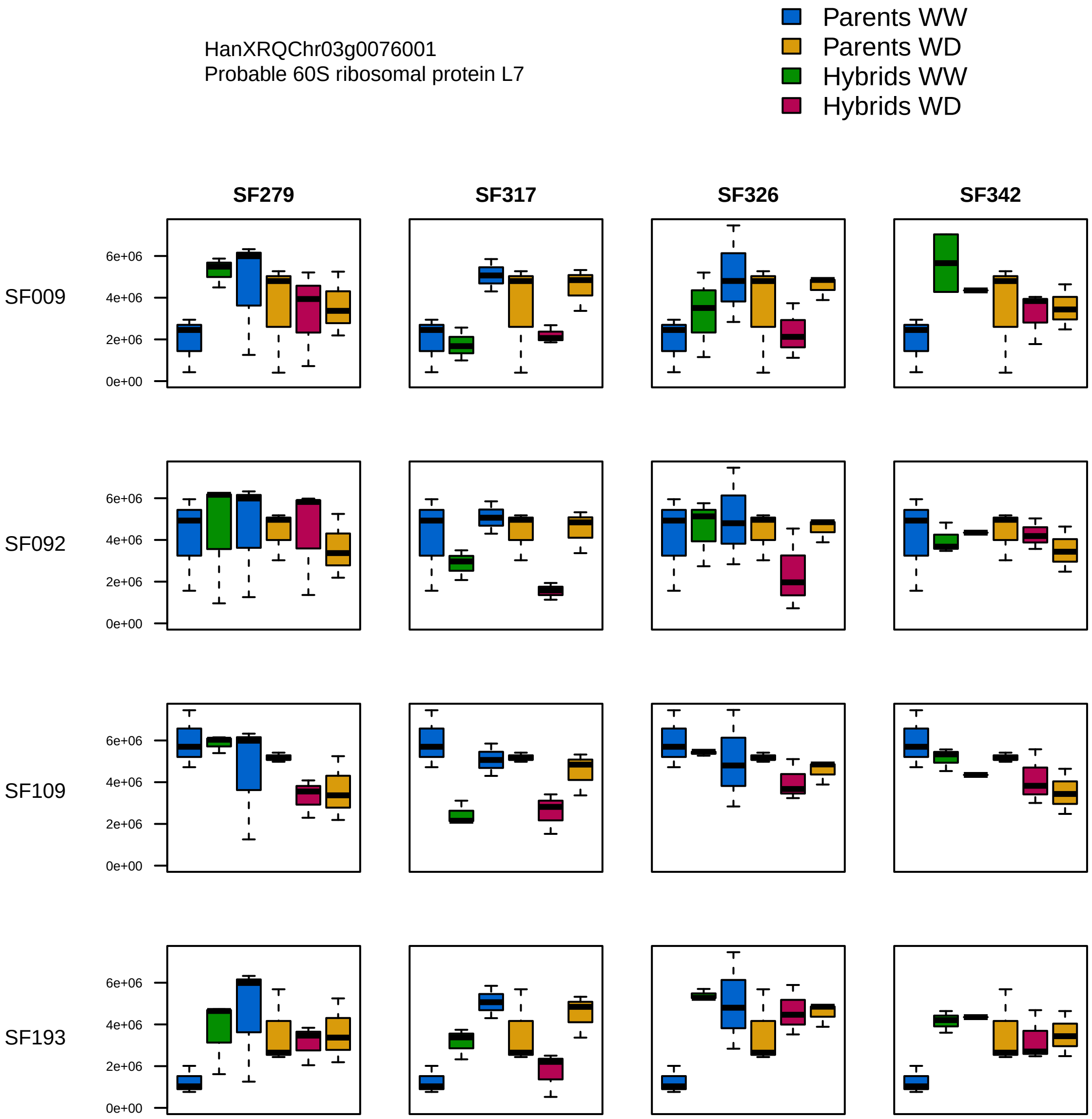
HanXRQChr03g0076001
Probable 60S ribosomal protein L7
Parents WW
Parents WD
Hybrids WW
Hybrids WD
SF279
SF317
SF326
SF342
SF009
SF092
SF109
SF193
6e+06
4e+06
2e+06
0e+00

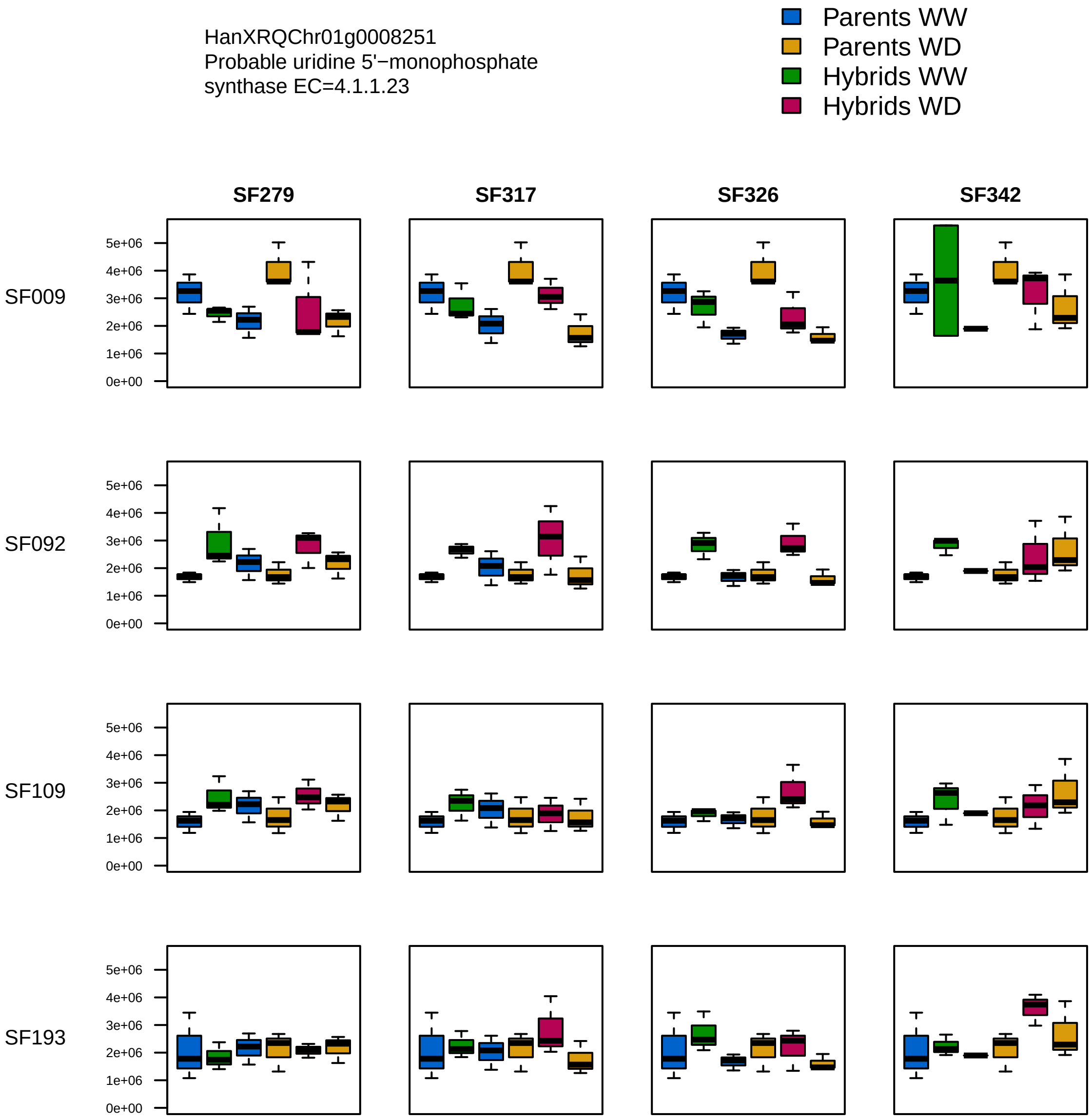
HanXRQChr01g0008251
Probable uridine 5'−monophosphate
synthase EC=4.1.1.23
Parents WW
Parents WD
Hybrids WW
Hybrids WD
SF279
SF317
SF326
SF342
SF009
SF092
SF109
SF193
0e+00
1e+06
2e+06
3e+06
4e+06
5e+06

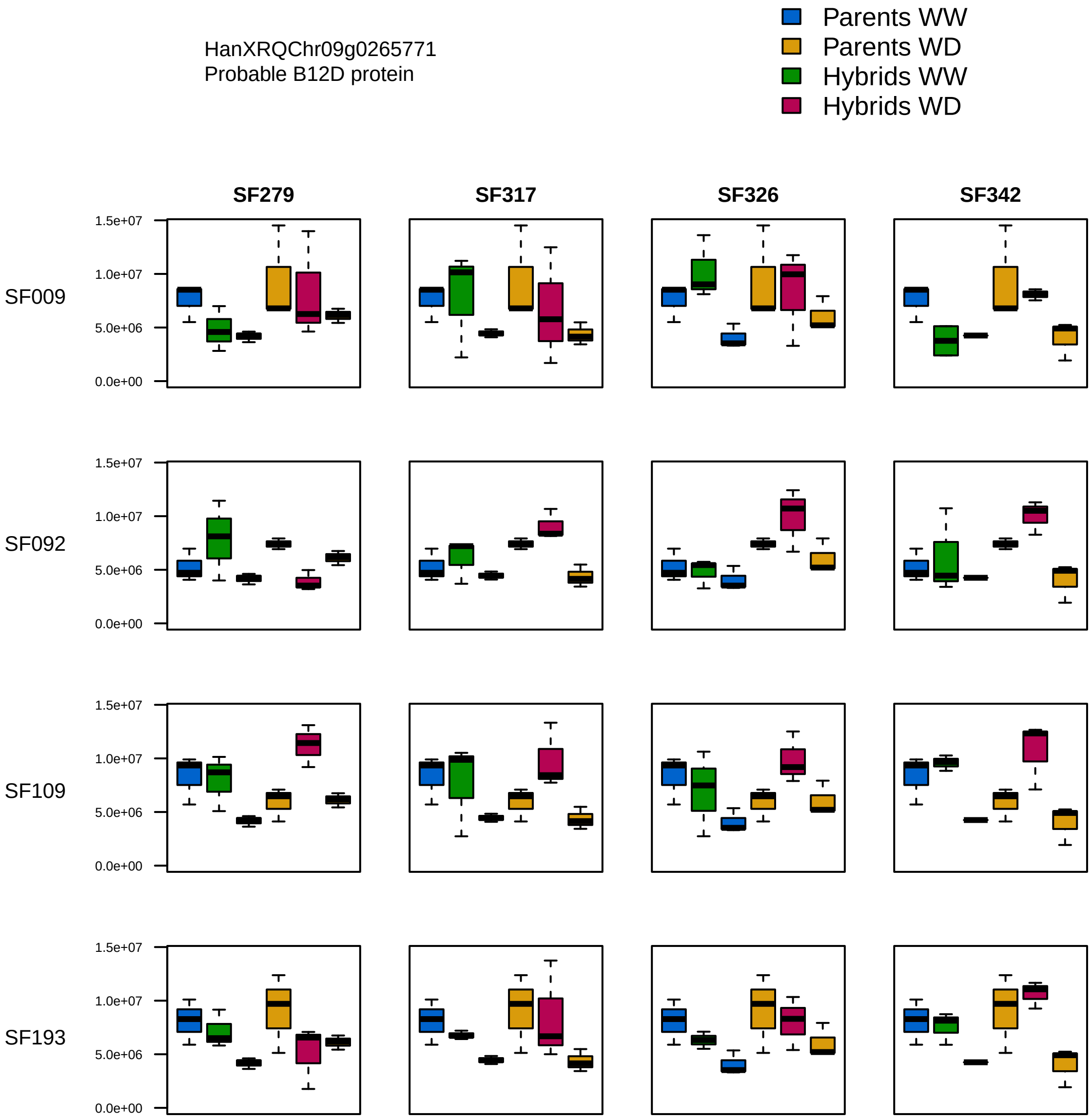

HanXRQChr09g0265771
Probable B12D protein
Parents WW
Parents WD
Hybrids WW
Hybrids WD
SF279
SF317
SF326
SF342
SF009
SF092
SF109
SF193
1.5e+07
1.0e+07
5.0e+06
0.0e+00

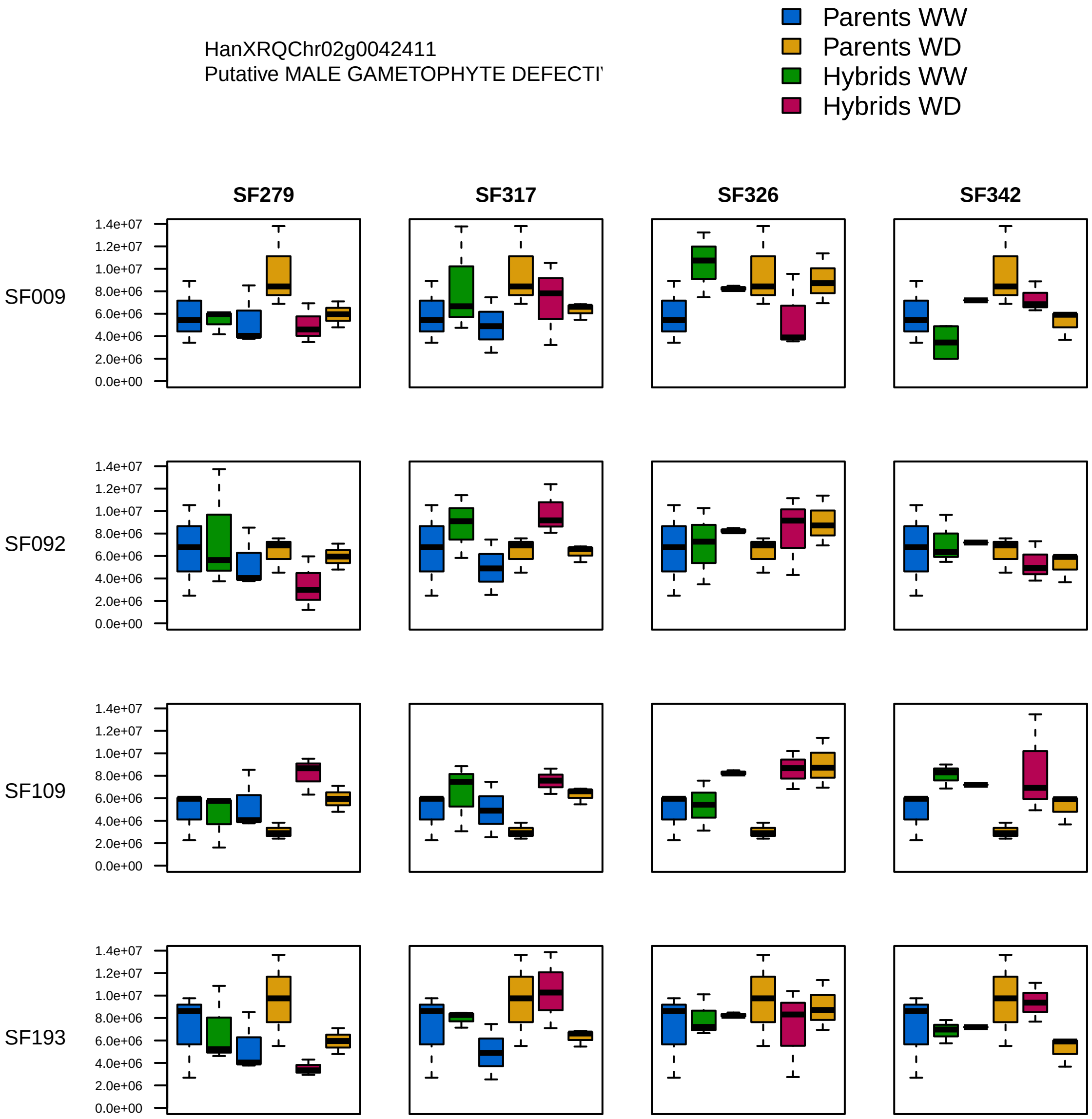

HanXRQChr02g0042411
Putative MALE GAMETOPHYTE DEFECTI
Parents WW
Parents WD
Hybrids WW
Hybrids WD
SF279
SF317
SF326
SF342
SF009
SF092
SF109
SF193
1.4e+07
1.2e+07
1.0e+07
8.0e+06
6.0e+06
4.0e+06
2.0e+06
0.0e+00

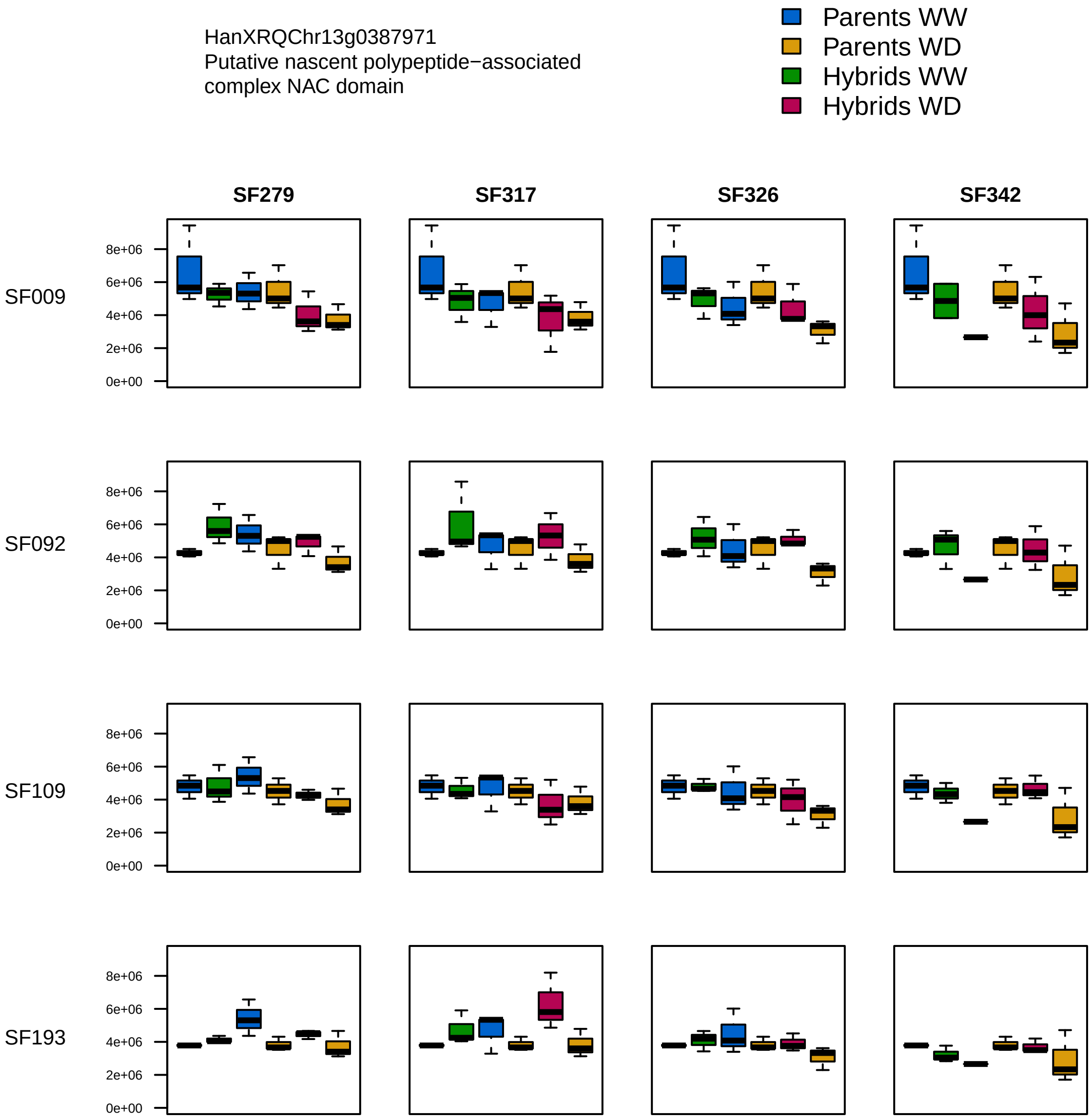

HanXRQChr13g0387971
Putative nascent polypeptide−associated
complex NAC domain
Parents WW
Parents WD
Hybrids WW
Hybrids WD
SF279
SF317
SF326
SF342
SF009
SF092
SF109
SF193
8e+06
6e+06
4e+06
2e+06
0e+00

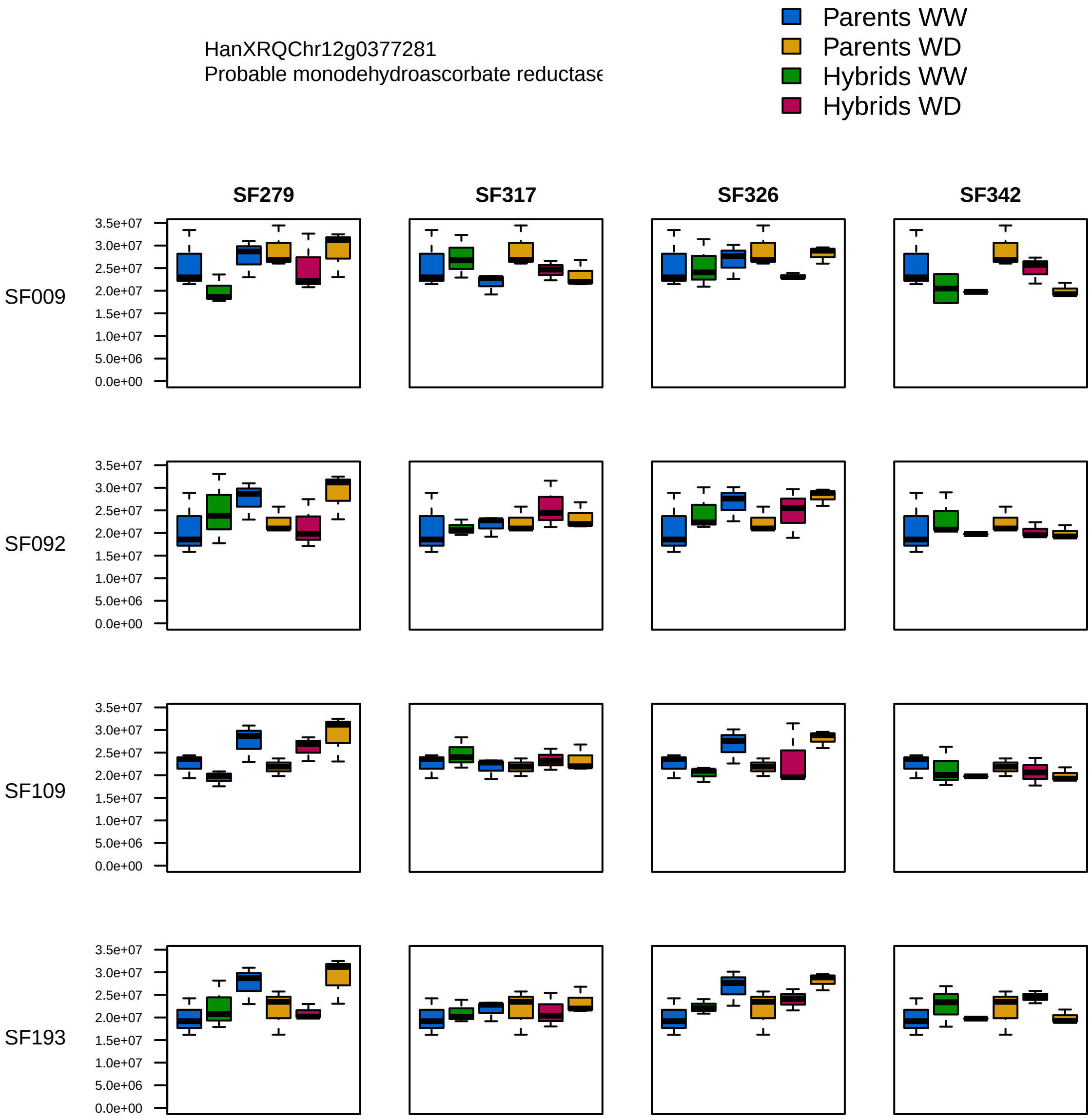
HanXRQChr12g0377281
Probable monodehydroascorbate reductase
Parents WW
Parents WD
Hybrids WW
Hybrids WD
SF279
SF317
SF326
SF342
SF009
SF092
SF109
SF193
3.5e+07
3.0e+07
2.5e+07
2.0e+07
1.5e+07
1.0e+07
5.0e+06
0.0e+00

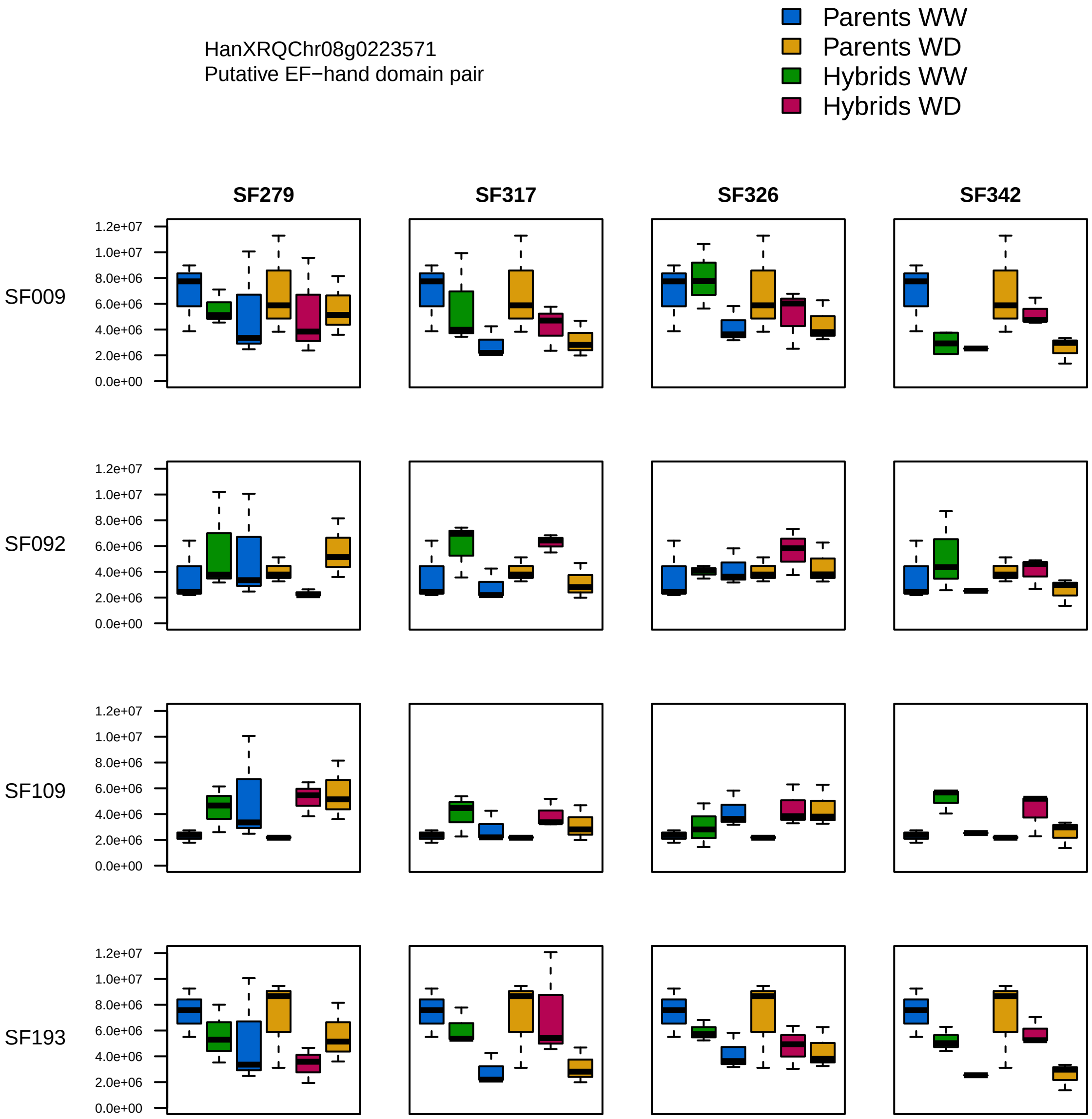
HanXRQChr08g0223571
Putative EF−hand domain pair
Parents WW
Parents WD
Hybrids WW
Hybrids WD
SF279
SF317
SF326
SF342
SF009
SF092
SF109
SF193
1.2e+07
1.0e+07
8.0e+06
6.0e+06
4.0e+06
2.0e+06
0.0e+00

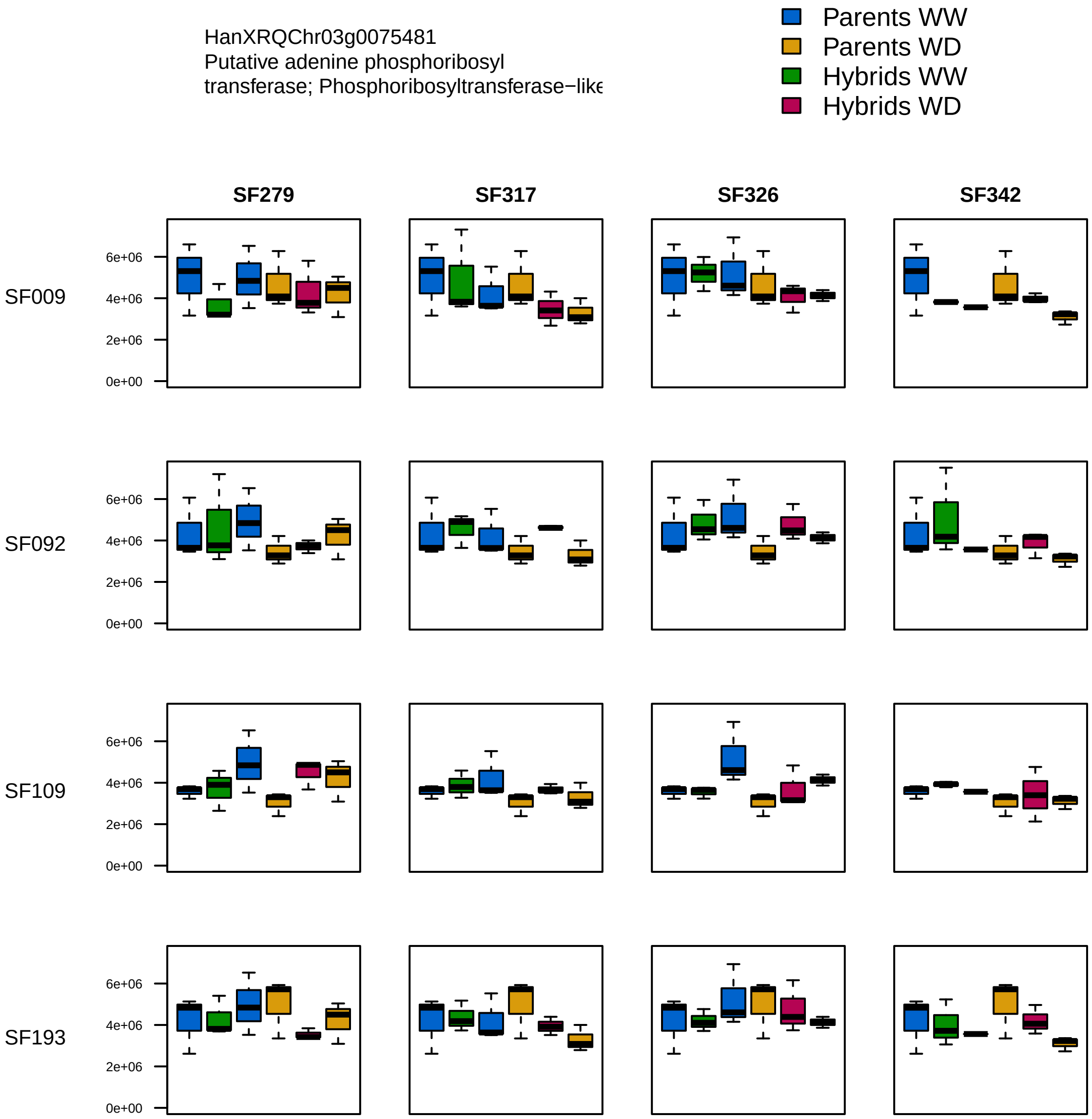
HanXRQChr03g0075481
Putative adenine phosphoribosyl
transferase; Phosphoribosyltransferase−like
Parents WW
Parents WD
Hybrids WW
Hybrids WD
SF279
SF317
SF326
SF342
SF009
SF092
SF109
SF193
0e+00
2e+06
4e+06
6e+06

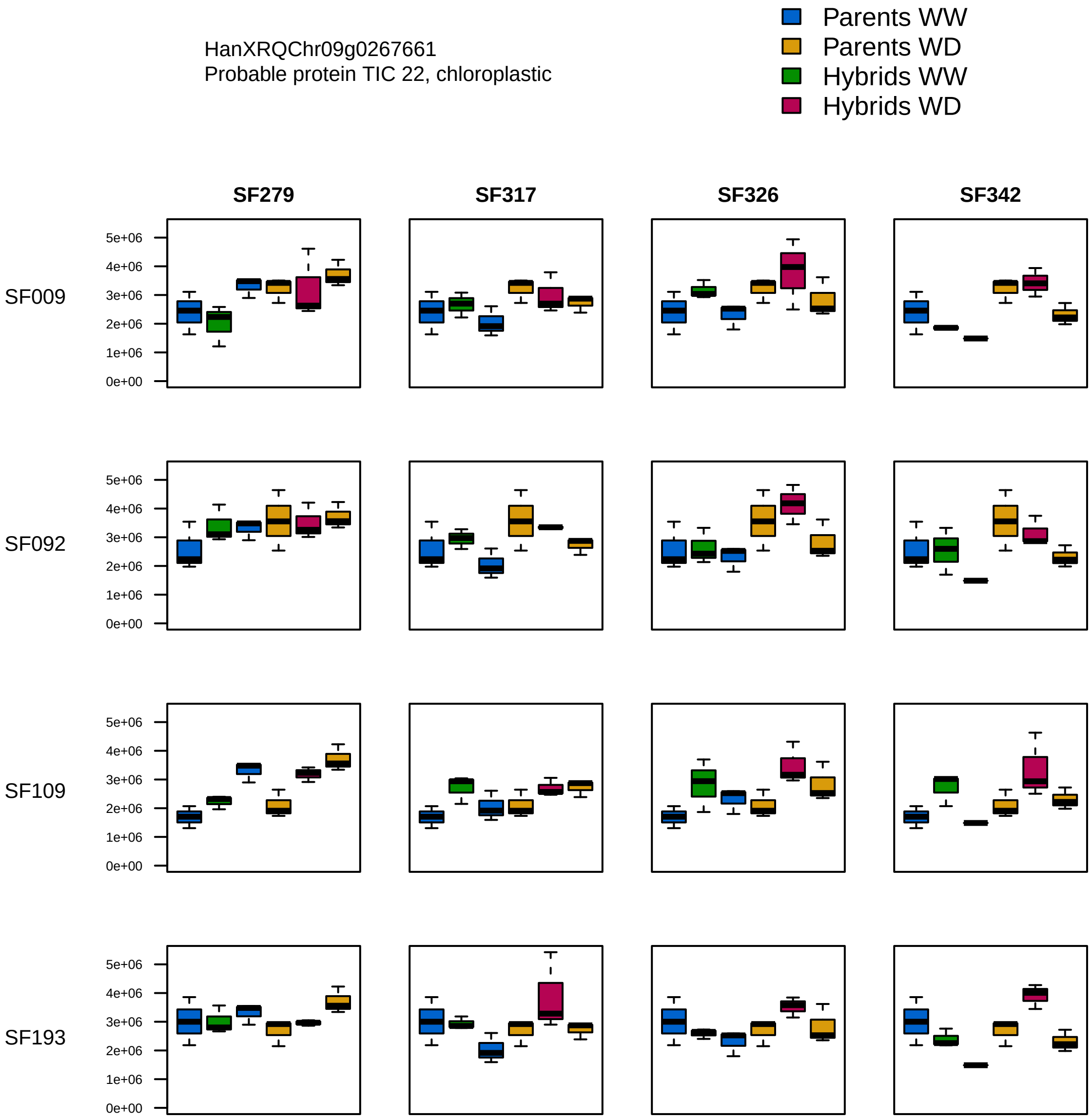
HanXRQChr09g0267661
Probable protein TIC 22, chloroplastic
Parents WW
Parents WD
Hybrids WW
Hybrids WD
SF279
SF317
SF326
SF342
SF009
SF092
SF109
SF193
5e+06
4e+06
3e+06
2e+06
1e+06
0e+00

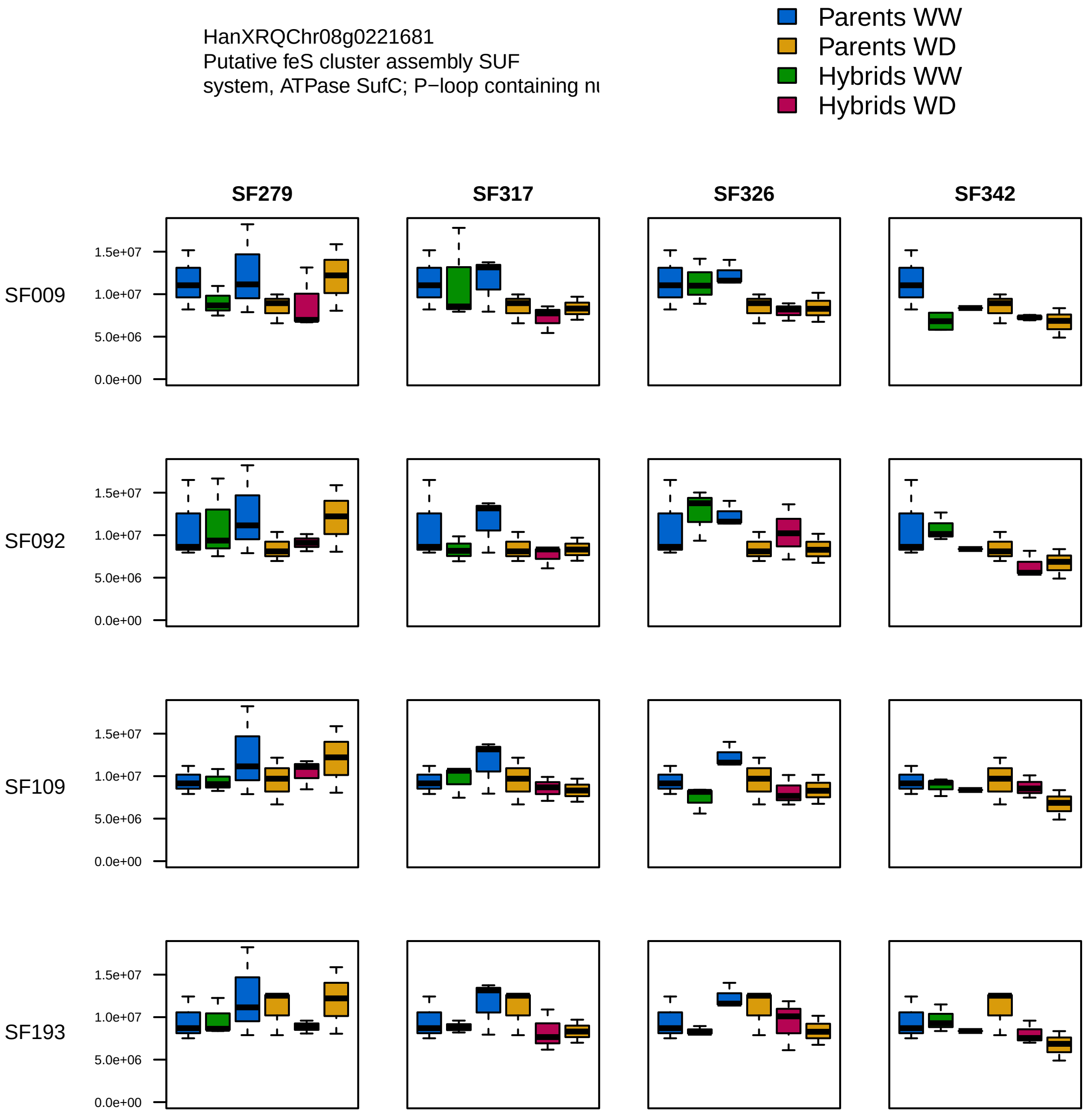

HanXRQChr08g0221681
Putative feS cluster assembly SUF
system, ATPase SufC; P−loop containing nu
Parents WW
Parents WD
Hybrids WW
Hybrids WD
SF279
SF317
SF326
SF342
SF009
SF092
SF109
SF193
0.0e+00
5.0e+06
1.0e+07
1.5e+07

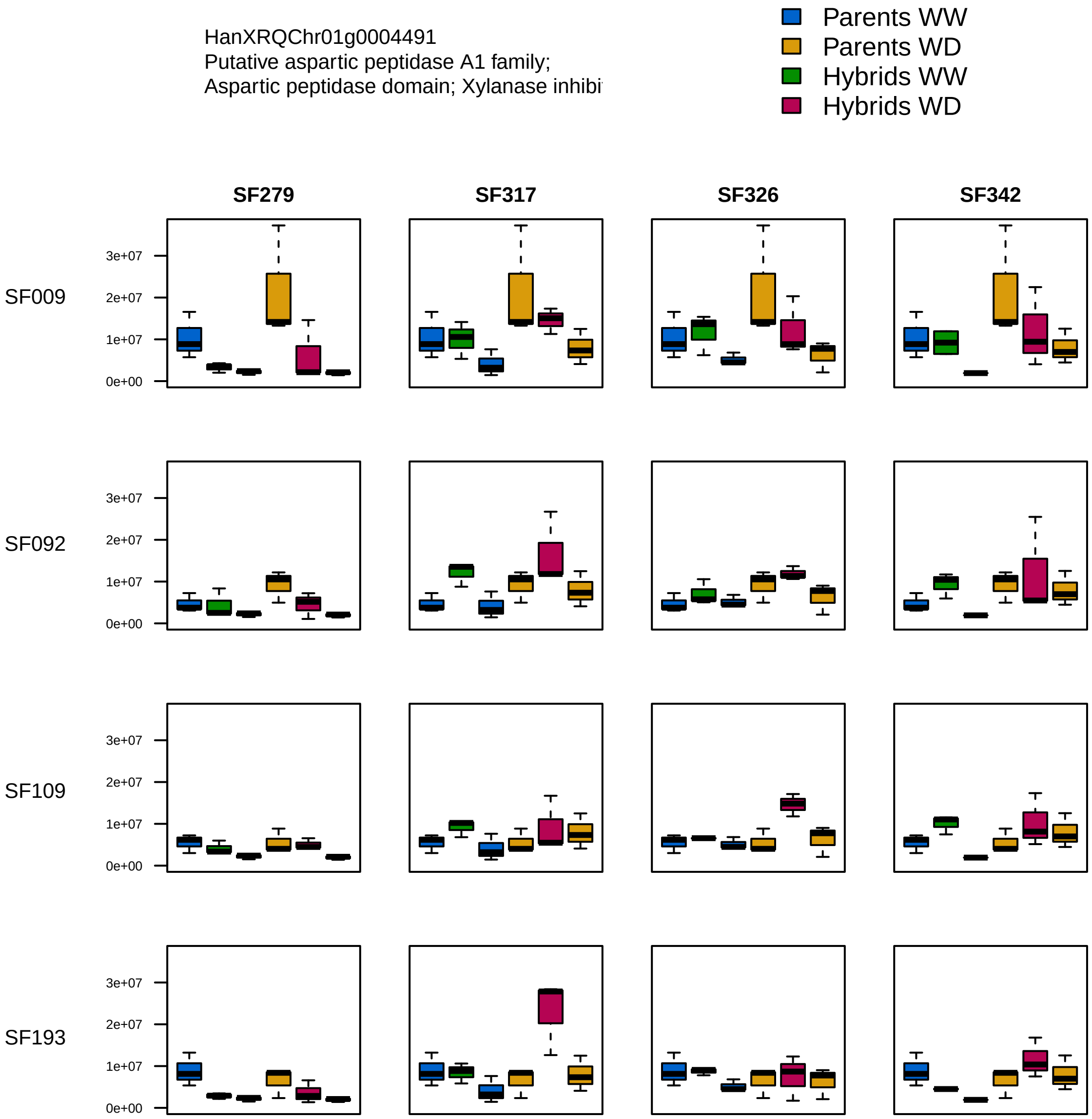
HanXRQChr01g0004491
Putative aspartic peptidase A1 family;
Aspartic peptidase domain; Xylanase inhibi
Parents WW
Parents WD
Hybrids WW
Hybrids WD
SF279
SF317
SF326
SF342
SF009
SF092
SF109
SF193
0e+00
1e+07
2e+07
3e+07

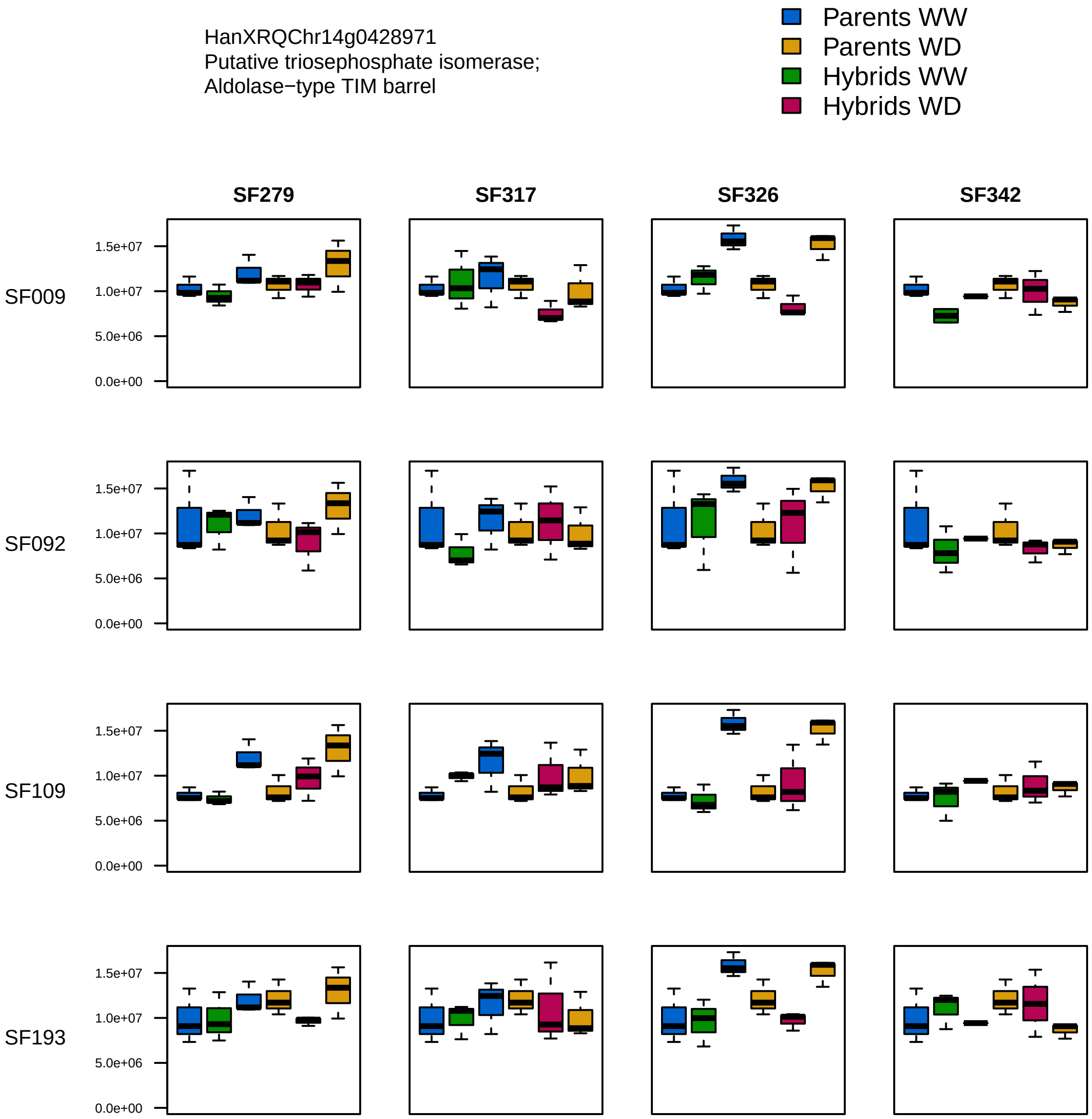

HanXRQChr14g0428971
Putative triosephosphate isomerase;
Aldolase−type TIM barrel
Parents WW
Parents WD
Hybrids WW
Hybrids WD
SF279
SF317
SF326
SF342
SF009
SF092
SF109
SF193
1.5e+07
1.0e+07
5.0e+06
0.0e+00

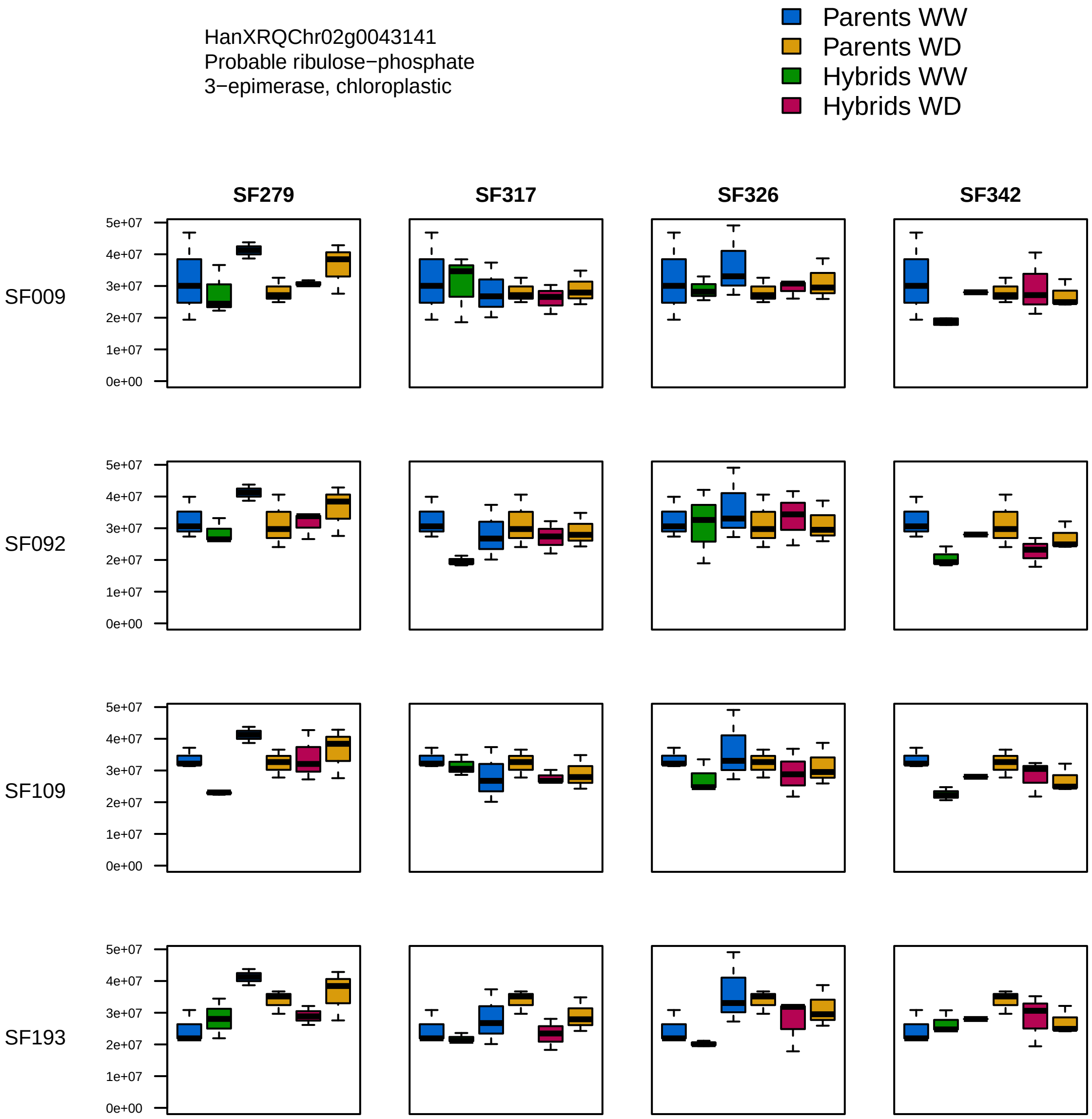
HanXRQChr02g0043141
Probable ribulose−phosphate
3−epimerase, chloroplastic
Parents WW
Parents WD
Hybrids WW
Hybrids WD
SF279
SF317
SF326
SF342
SF009
SF092
SF109
SF193
0e+00
1e+07
2e+07
3e+07
4e+07
5e+07

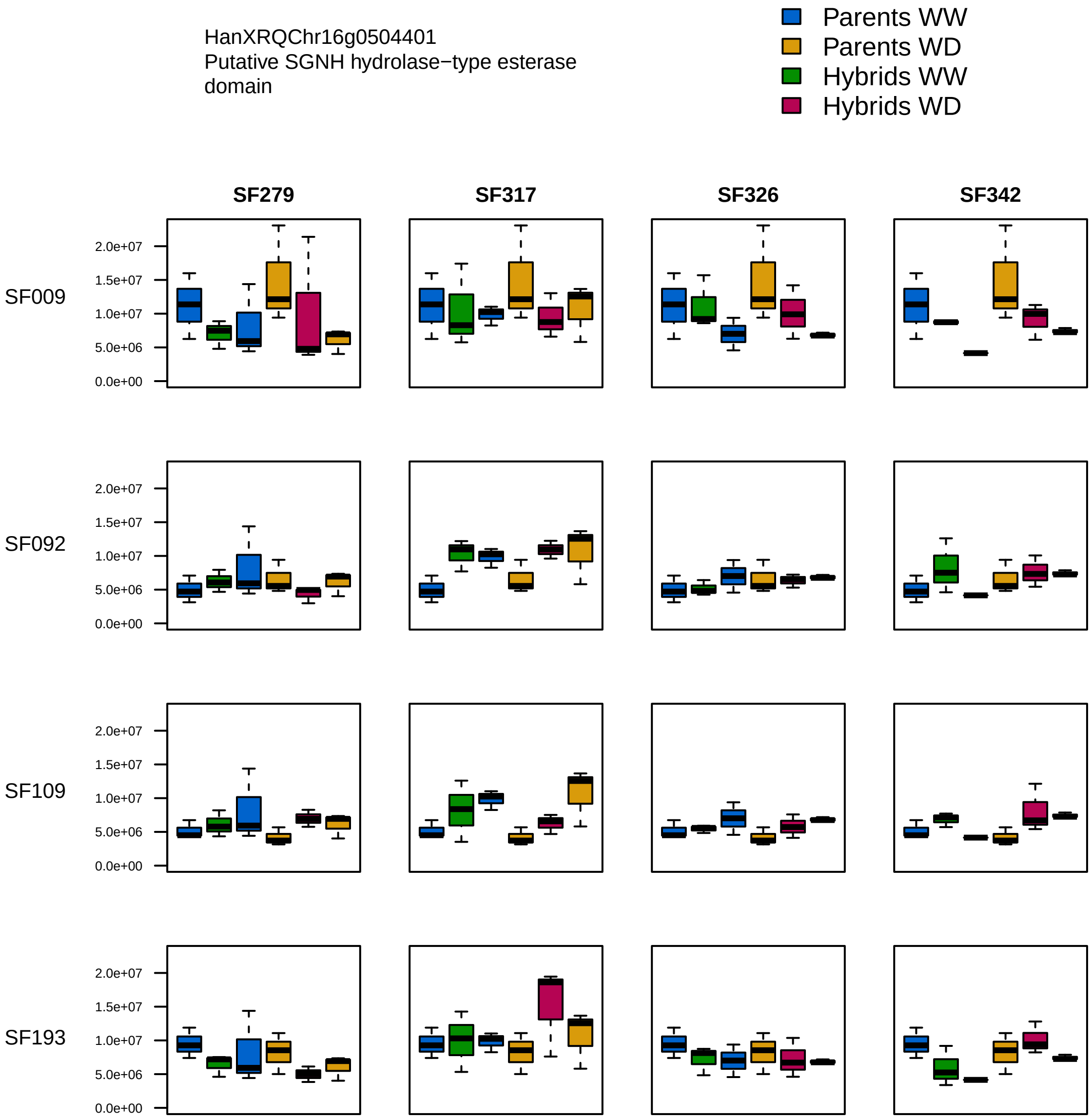

HanXRQChr16g0504401
Putative SGNH hydrolase−type esterase domain
Parents WW
Parents WD
Hybrids WW
Hybrids WD
SF279
SF317
SF326
SF342
SF009
SF092
SF109
SF193
0.0e+00
5.0e+06
1.0e+07
1.5e+07
2.0e+07

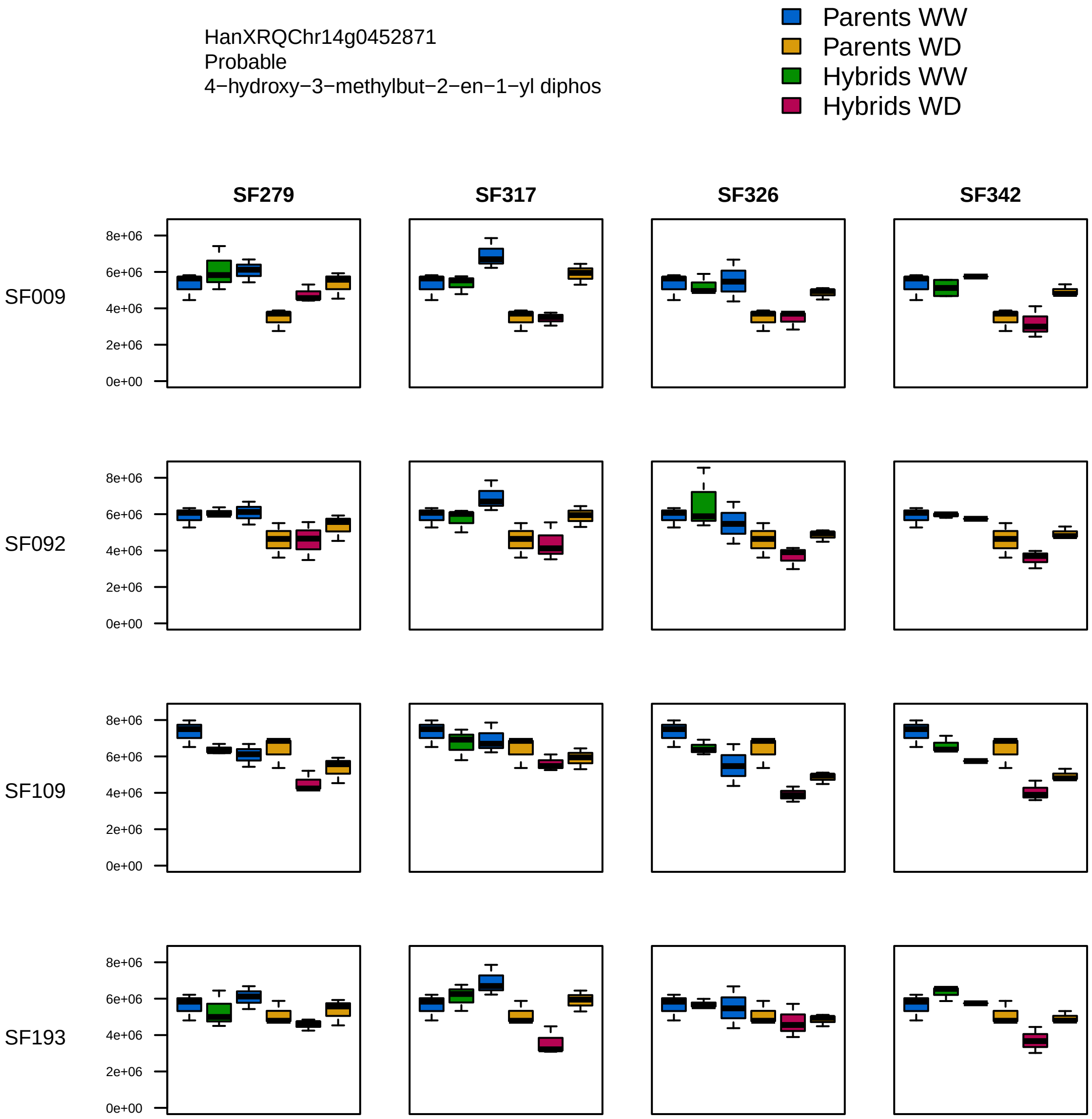
HanXRQChr14g0452871
Probable
4−hydroxy−3−methylbut−2−en−1−yl diphos
Parents WW
Parents WD
Hybrids WW
Hybrids WD
SF279
SF317
SF326
SF342
SF009
SF092
SF109
SF193
8e+06
6e+06
4e+06
2e+06
0e+00

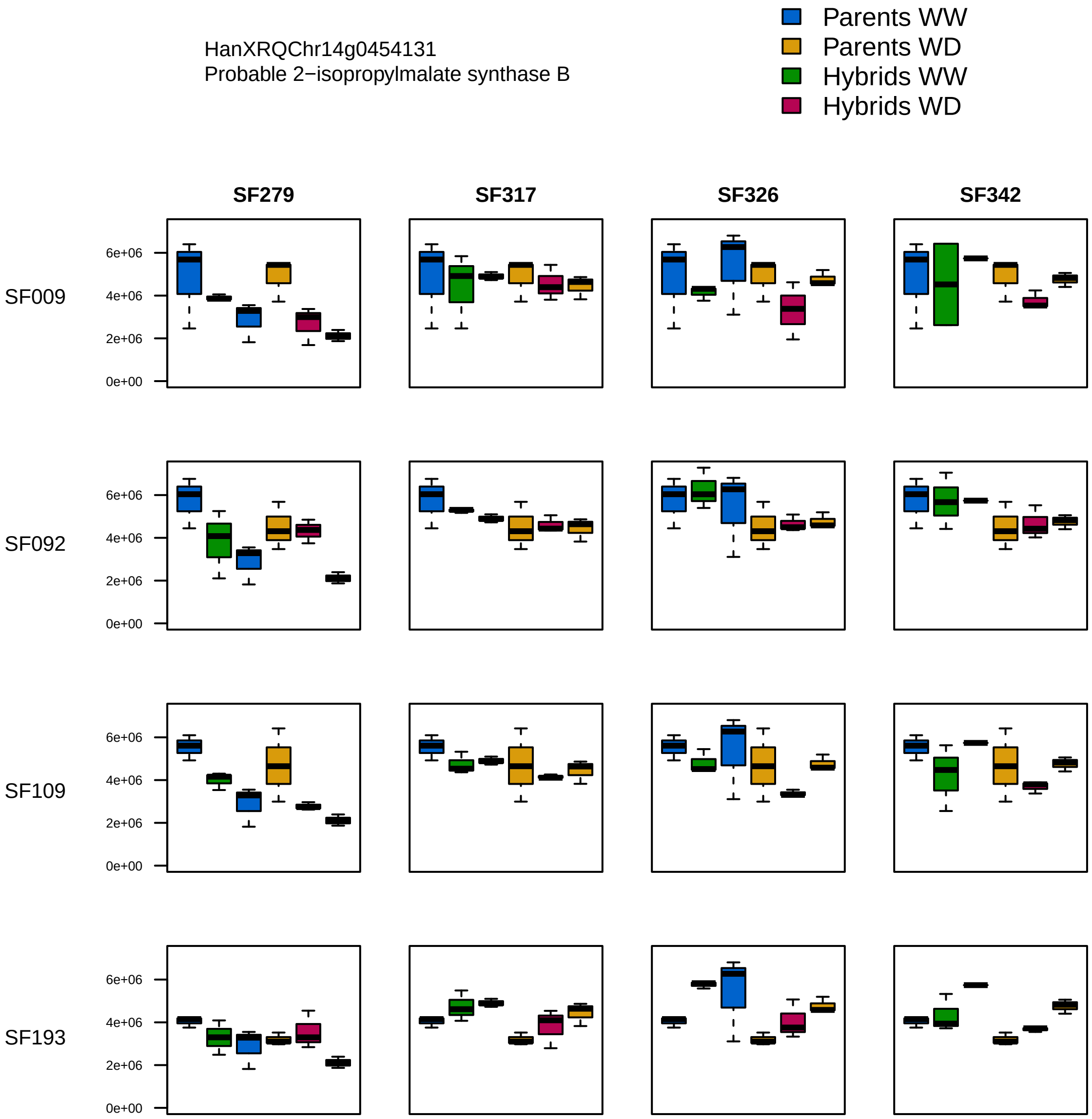
HanXRQChr14g0454131
Probable 2−isopropylmalate synthase B
Parents WW
Parents WD
Hybrids WW
Hybrids WD
SF279
SF317
SF326
SF342
SF009
SF092
SF109
SF193
0e+00
2e+06
4e+06
6e+06

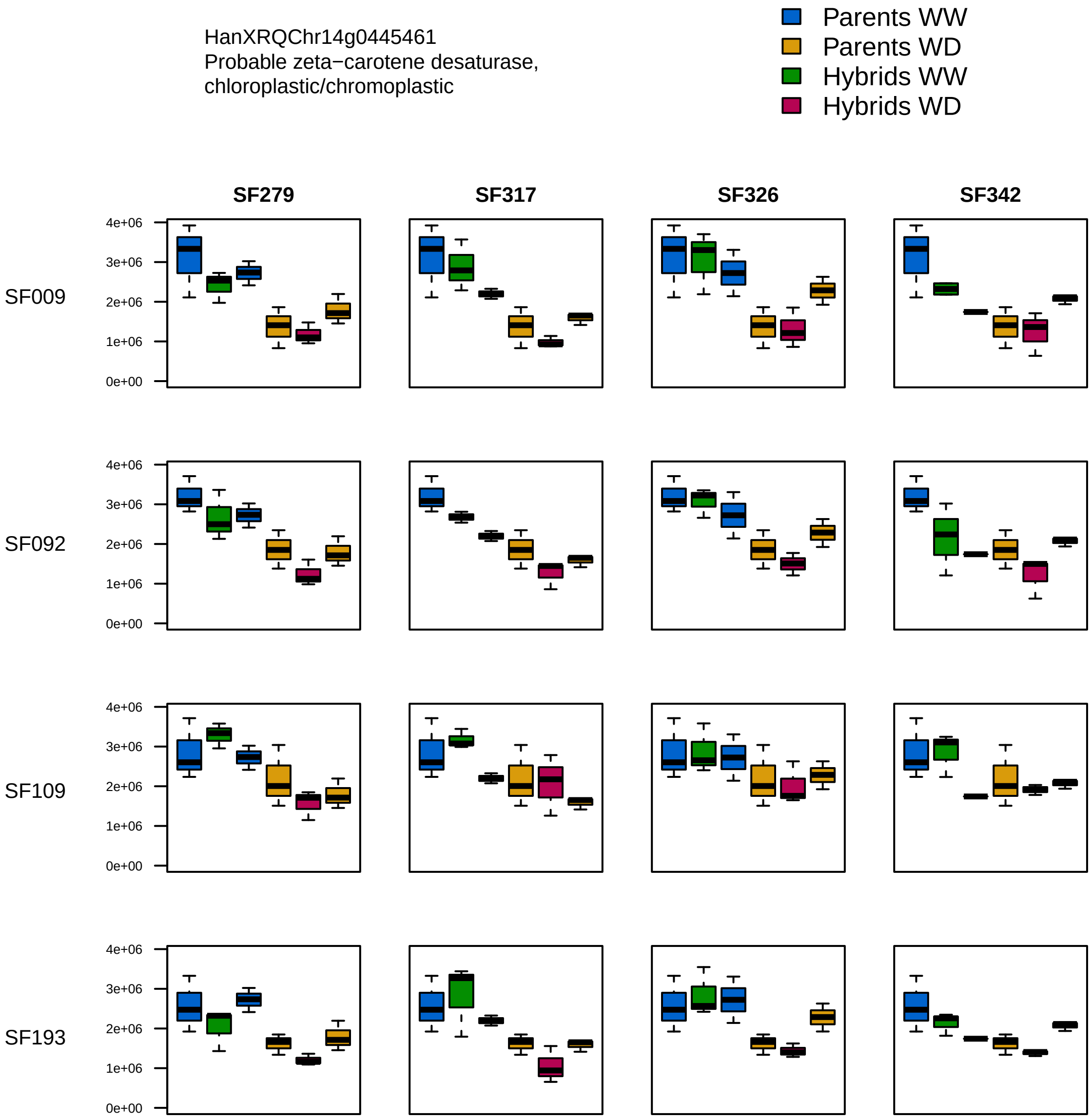
HanXRQChr14g0445461
Probable zeta−carotene desaturase,
chloroplastic/chromoplastic
Parents WW
Parents WD
Hybrids WW
Hybrids WD
SF279
SF317
SF326
SF342
SF009
SF092
SF109
SF193
4e+06
3e+06
2e+06
1e+06
0e+00

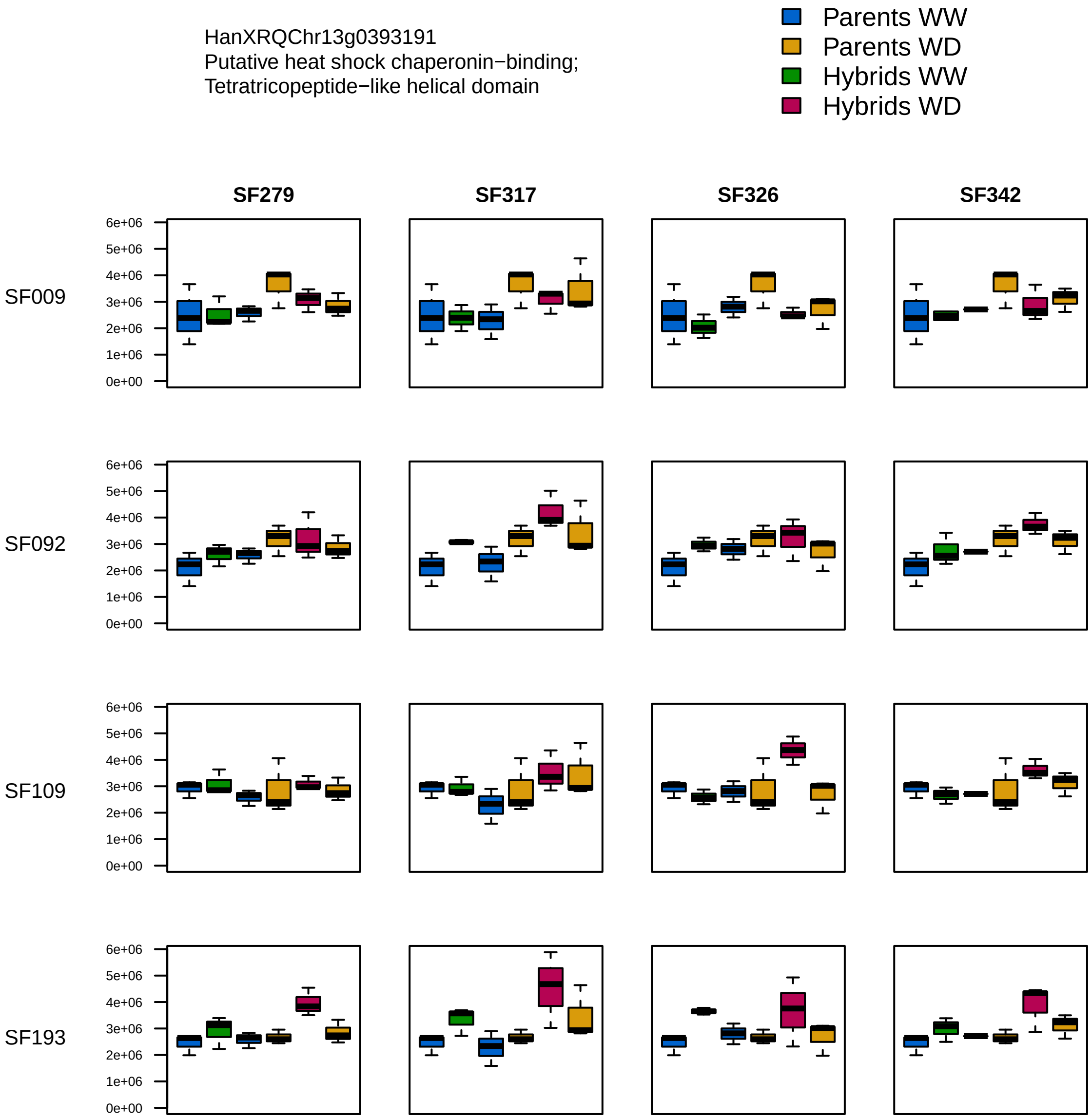

HanXRQChr13g0393191
Putative heat shock chaperonin−binding;
Tetratricopeptide−like helical domain
Parents WW
Parents WD
Hybrids WW
Hybrids WD
SF279
SF317
SF326
SF342
SF009
SF092
SF109
SF193
6e+06
5e+06
4e+06
3e+06
2e+06
1e+06
0e+00

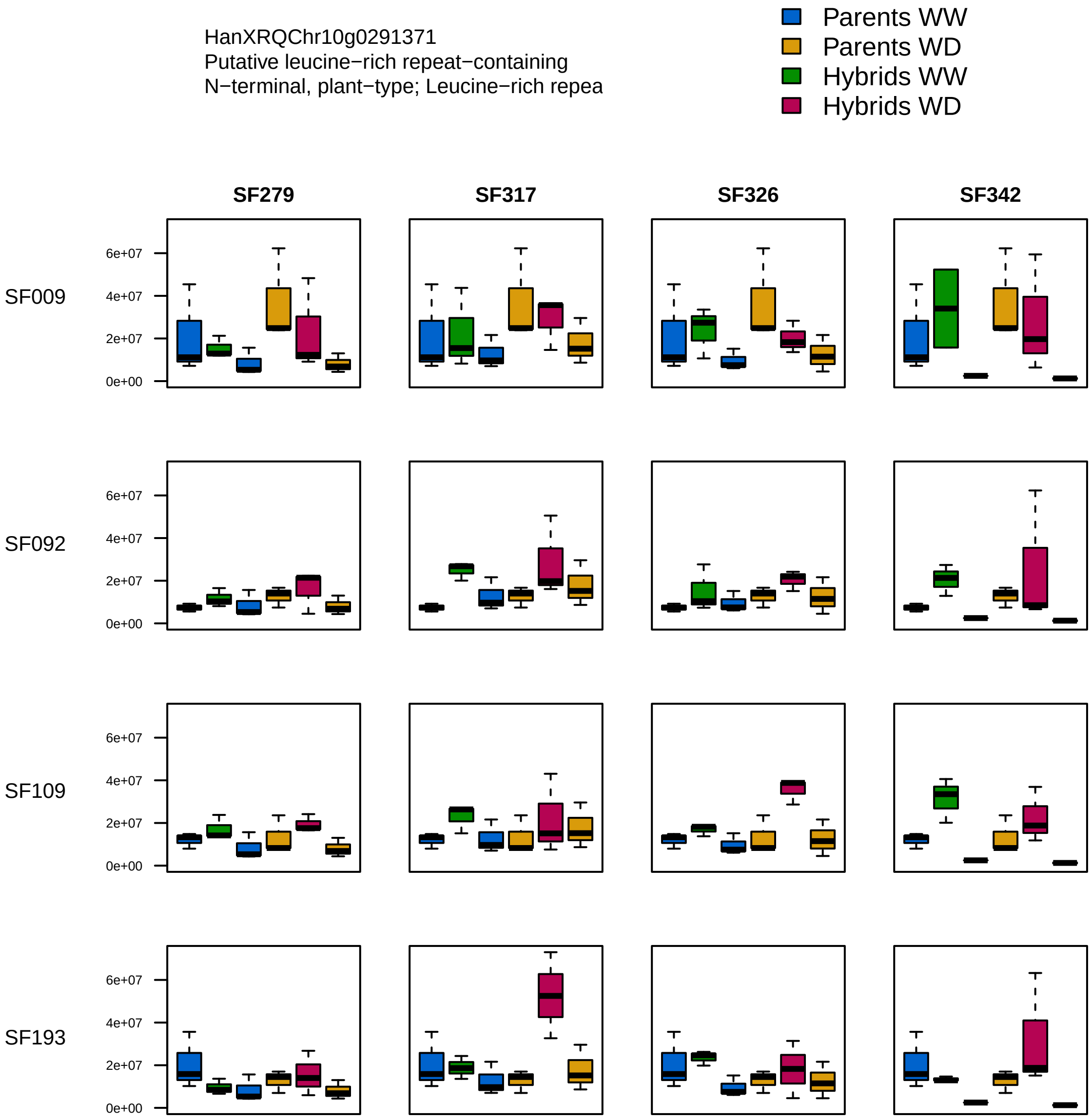

HanXRQChr10g0291371
Putative leucine−rich repeat−containing
N−terminal, plant−type; Leucine−rich repea
Parents WW
Parents WD
Hybrids WW
Hybrids WD
SF279
SF317
SF326
SF342
SF009
SF092
SF109
SF193
0e+00
2e+07
4e+07
6e+07

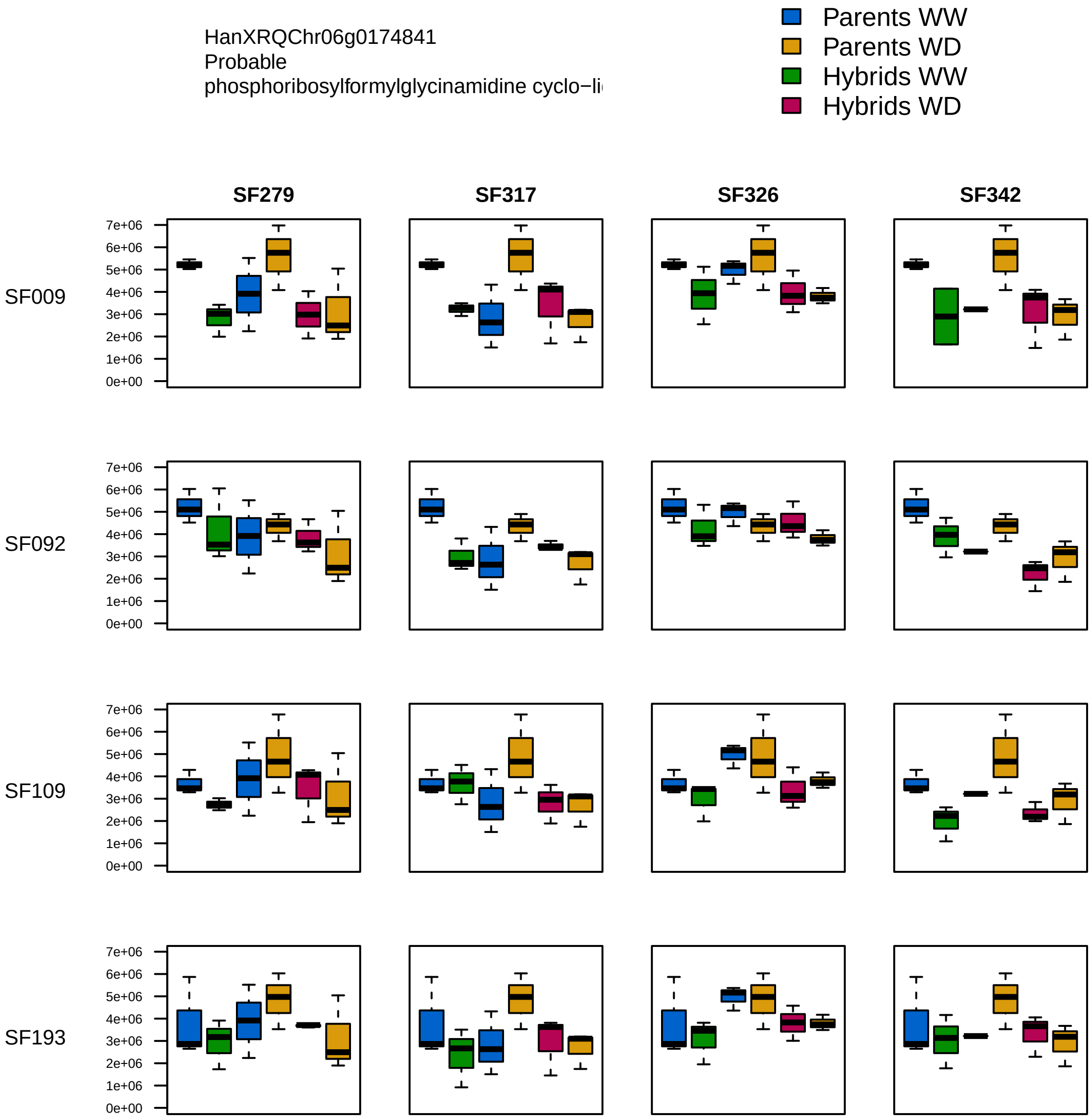

HanXRQChr06g0174841
Probable
phosphoribosylformylglycinamidine cyclo−li
Parents WW
Parents WD
Hybrids WW
Hybrids WD
SF279
SF317
SF326
SF342
SF009
SF092
SF109
SF193
7e+06
6e+06
5e+06
4e+06
3e+06
2e+06
1e+06
0e+00

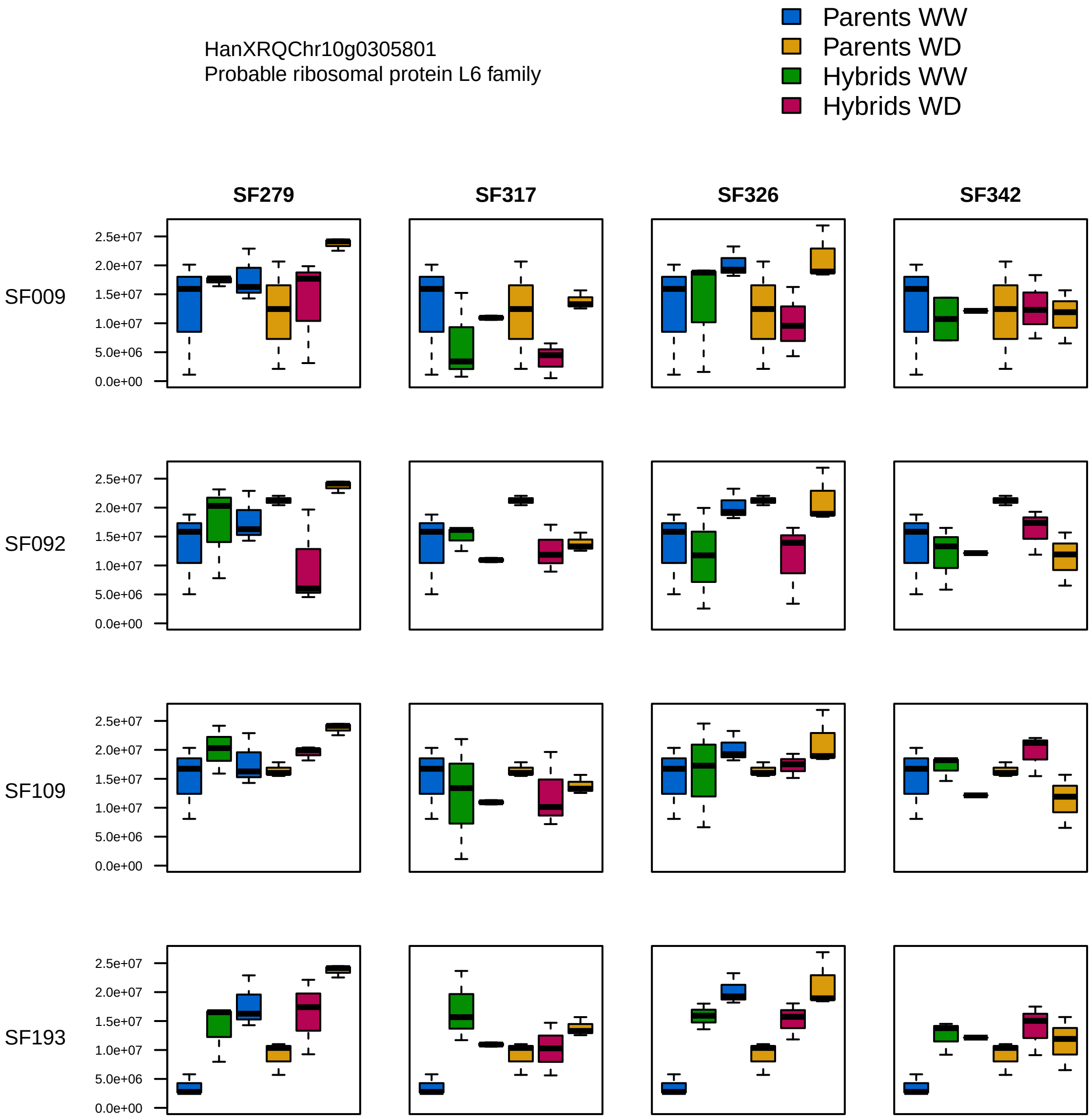
HanXRQChr10g0305801
Probable ribosomal protein L6 family
Parents WW
Parents WD
Hybrids WW
Hybrids WD
SF279
SF317
SF326
SF342
SF009
SF092
SF109
SF193
2.5e+07
2.0e+07
1.5e+07
1.0e+07
5.0e+06
0.0e+00

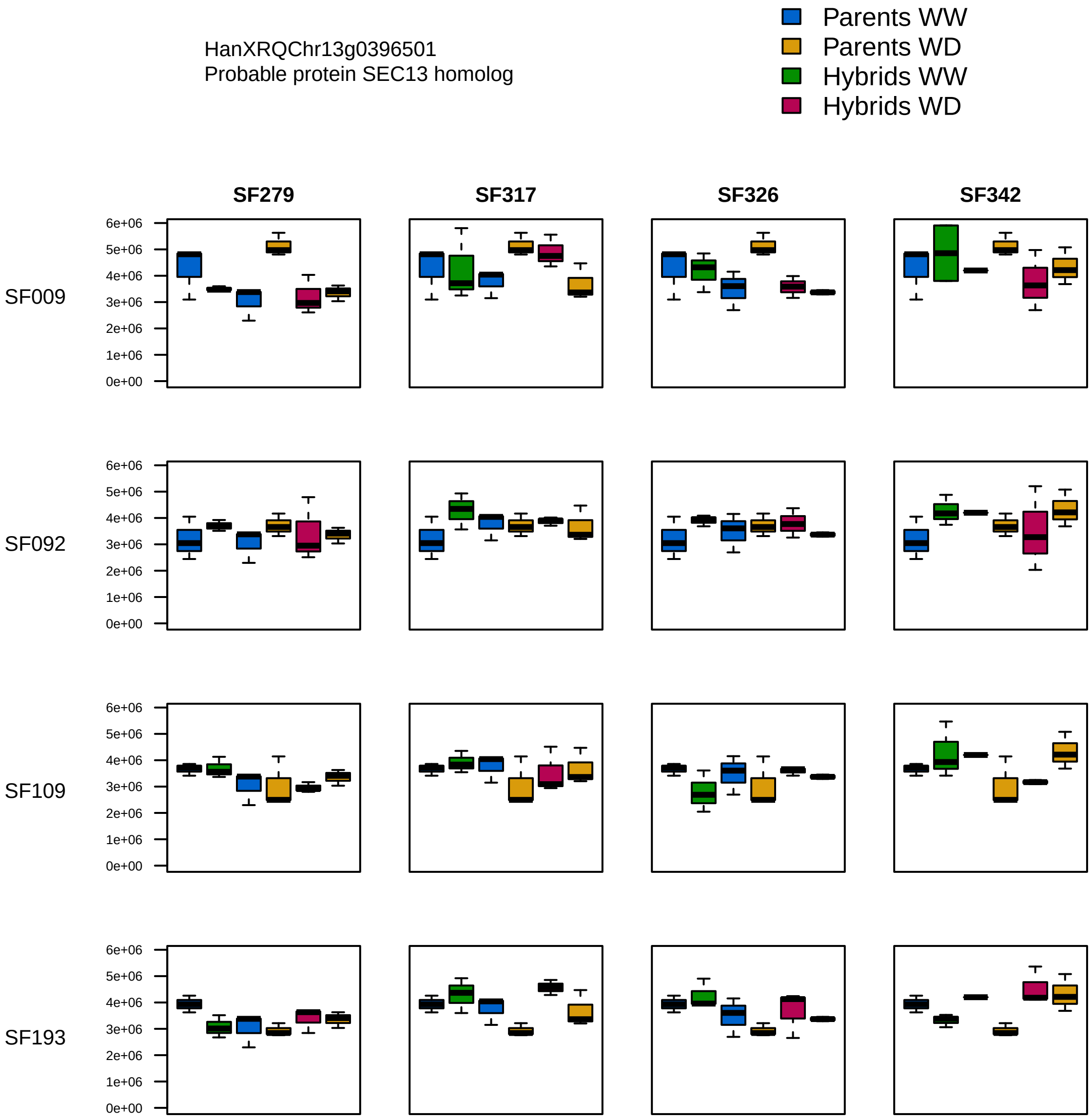

HanXRQChr13g0396501
Probable protein SEC13 homolog
Parents WW
Parents WD
Hybrids WW
Hybrids WD
SF279
SF317
SF326
SF342
SF009
SF092
SF109
SF193
0e+00
1e+06
2e+06
3e+06
4e+06
5e+06
6e+06

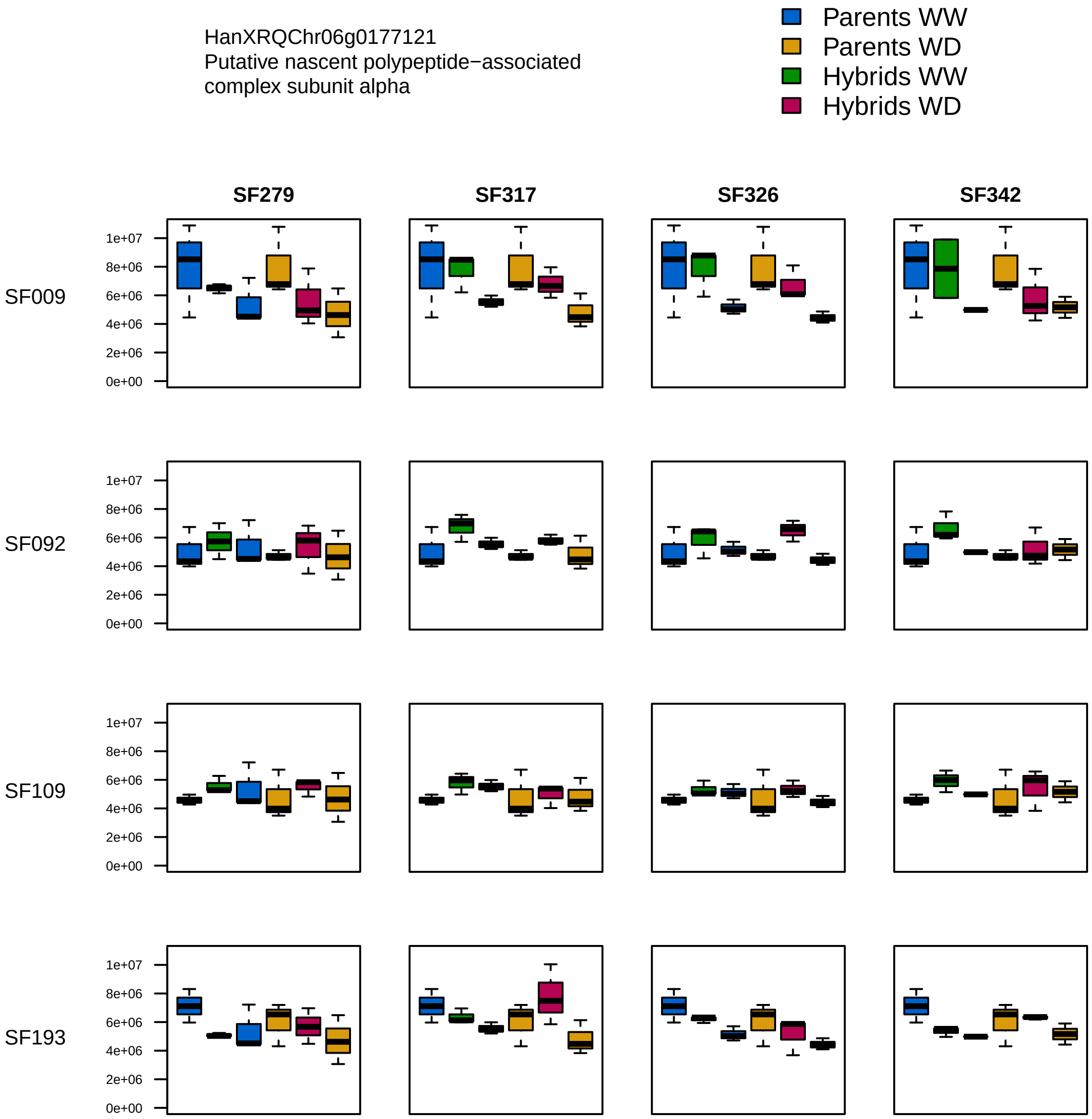
HanXRQChr06g0177121
Putative nascent polypeptide−associated complex subunit alpha
Parents WW
Parents WD
Hybrids WW
Hybrids WD
SF279
SF317
SF326
SF342
SF009
SF092
SF109
SF193
1e+07
8e+06
6e+06
4e+06
2e+06
0e+00

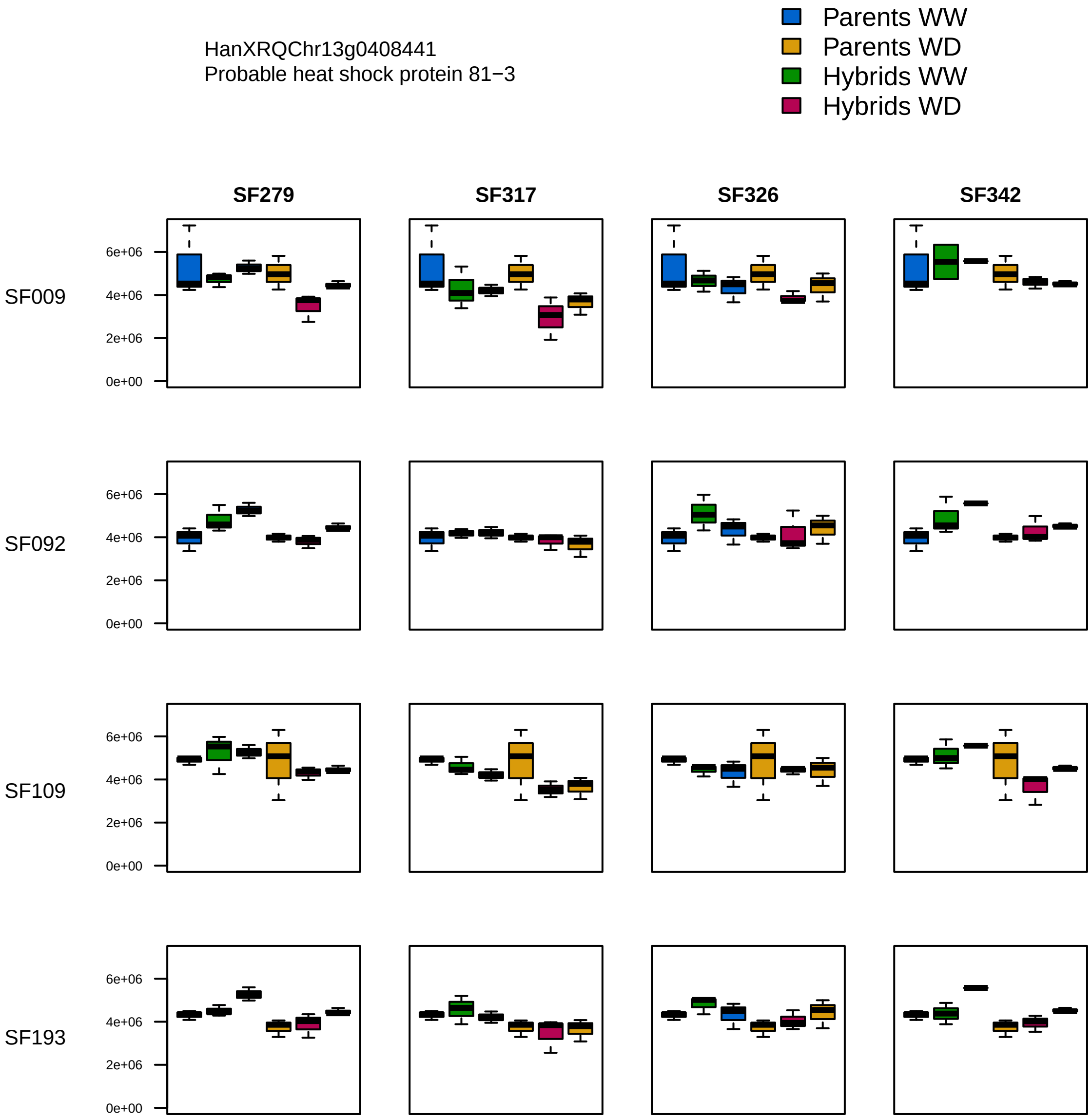
HanXRQChr13g0408441
Probable heat shock protein 81−3
Parents WW
Parents WD
Hybrids WW
Hybrids WD
SF279
SF317
SF326
SF342
SF009
SF092
SF109
SF193
0e+00
2e+06
4e+06
6e+06

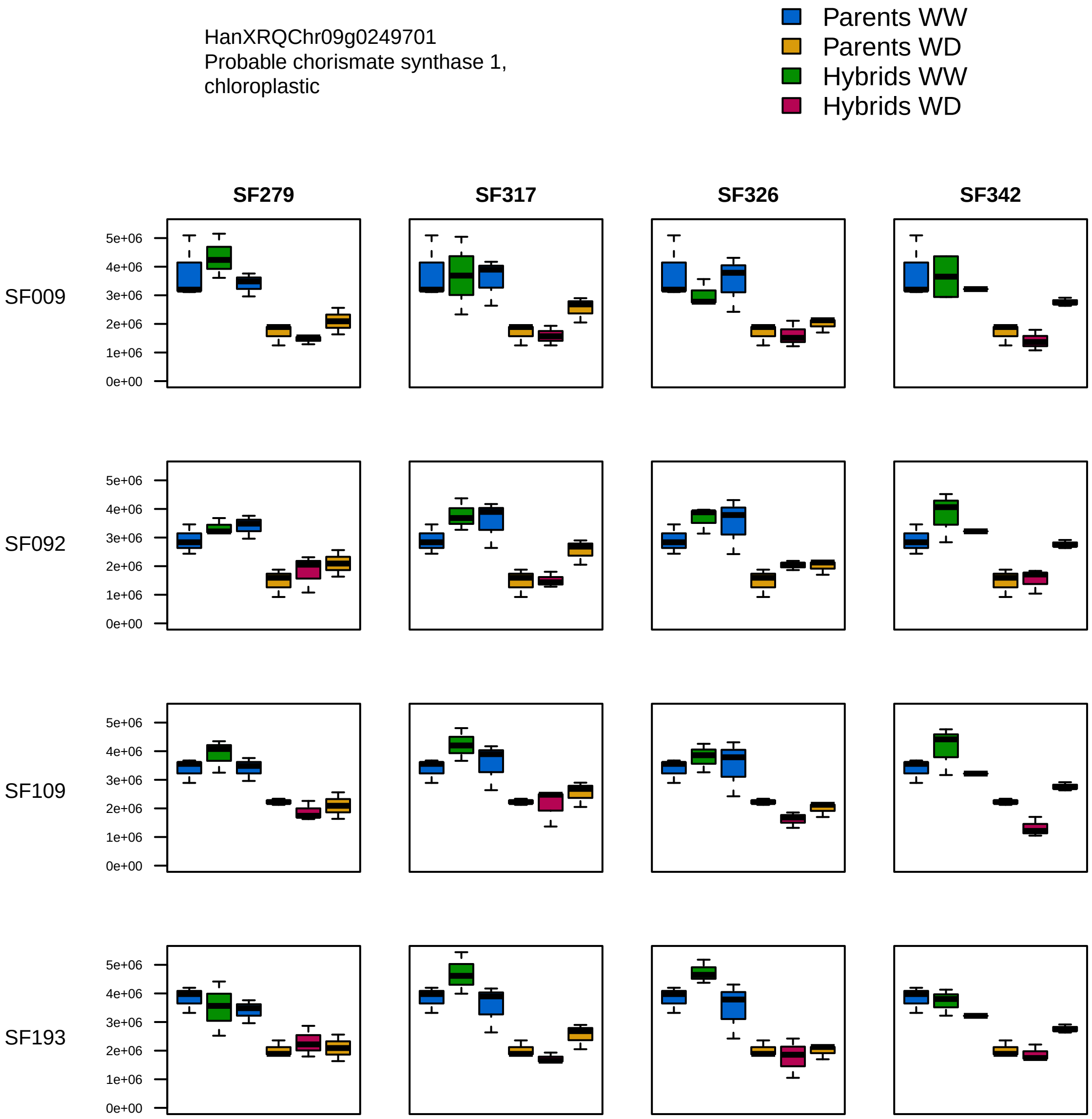
HanXRQChr09g0249701
Probable chorismate synthase 1,
chloroplastic
Parents WW
Parents WD
Hybrids WW
Hybrids WD
SF279
SF317
SF326
SF342
SF009
SF092
SF109
SF193
5e+06
4e+06
3e+06
2e+06
1e+06
0e+00

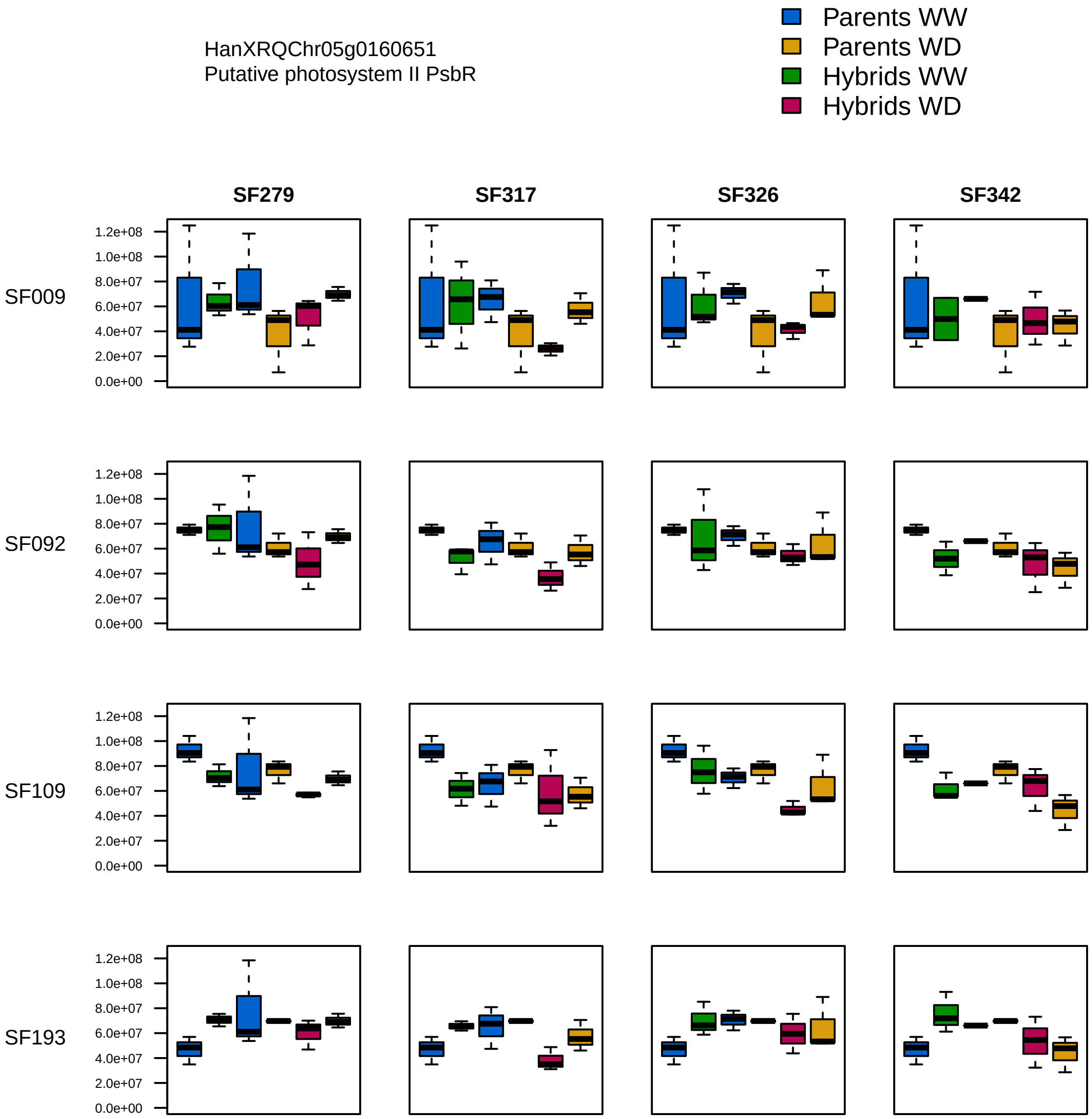
HanXRQChr05g0160651
Putative photosystem II PsbR
Parents WW
Parents WD
Hybrids WW
Hybrids WD
SF279
SF317
SF326
SF342
SF009
SF092
SF109
SF193
1.2e+08
1.0e+08
8.0e+07
6.0e+07
4.0e+07
2.0e+07
0.0e+00

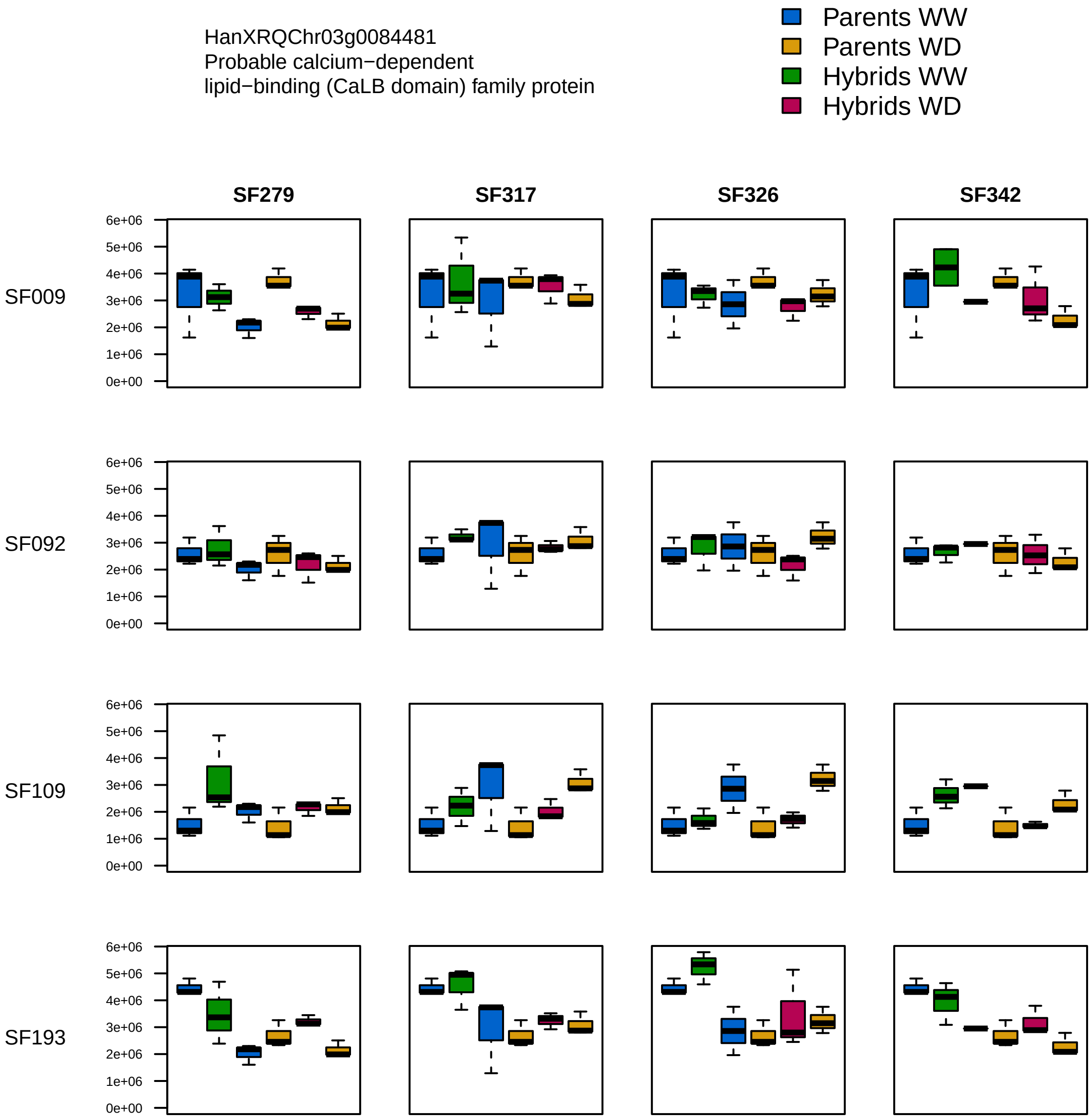
HanXRQChr03g0084481
Probable calcium−dependent
lipid−binding (CaLB domain) family protein
Parents WW
Parents WD
Hybrids WW
Hybrids WD
SF279
SF317
SF326
SF342
SF009
SF092
SF109
SF193
0e+00
1e+06
2e+06
3e+06
4e+06
5e+06
6e+06

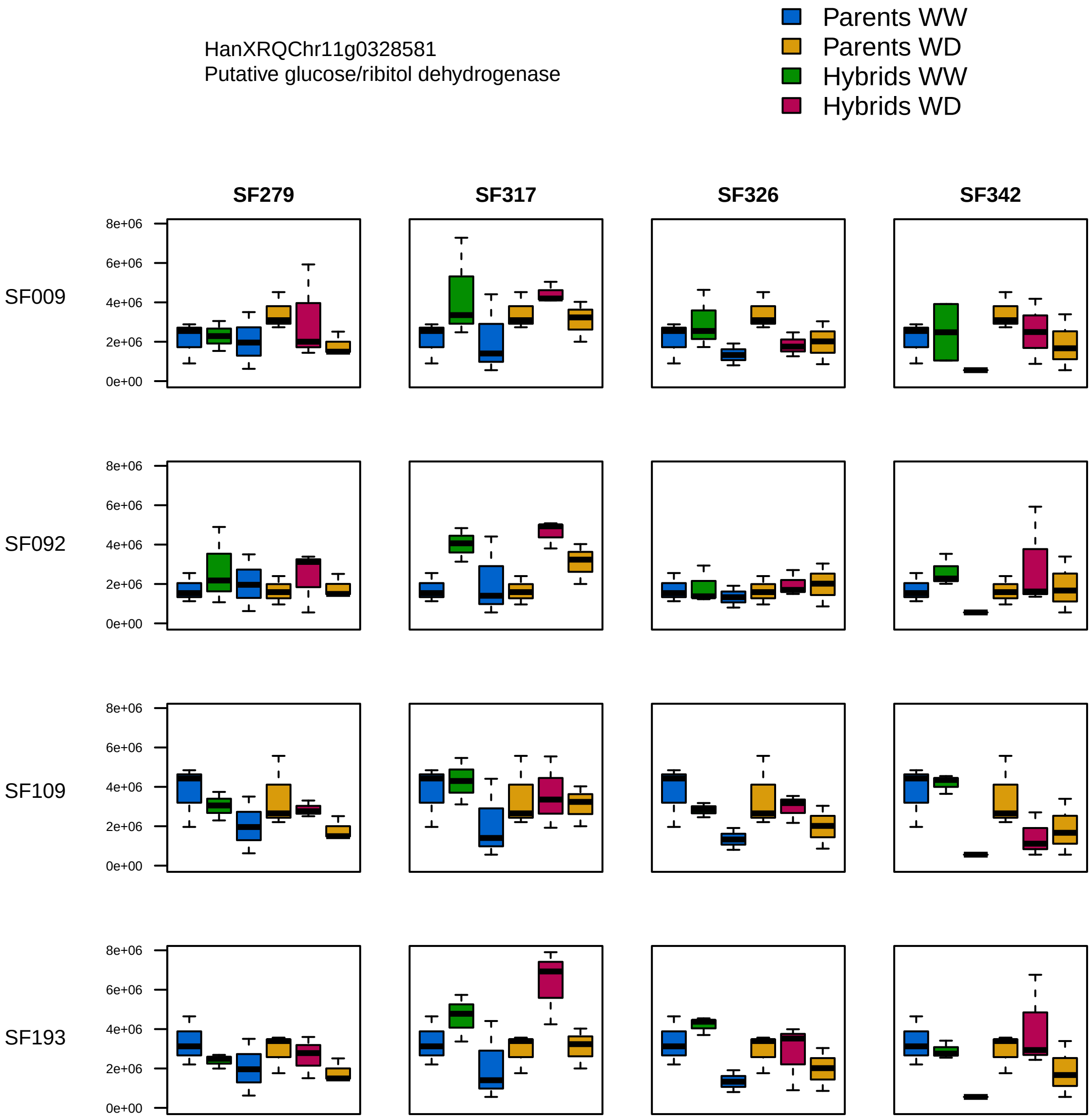
HanXRQChr11g0328581
Putative glucose/ribitol dehydrogenase
Parents WW
Parents WD
Hybrids WW
Hybrids WD
SF279
SF317
SF326
SF342
SF009
SF092
SF109
SF193
8e+06
6e+06
4e+06
2e+06
0e+00

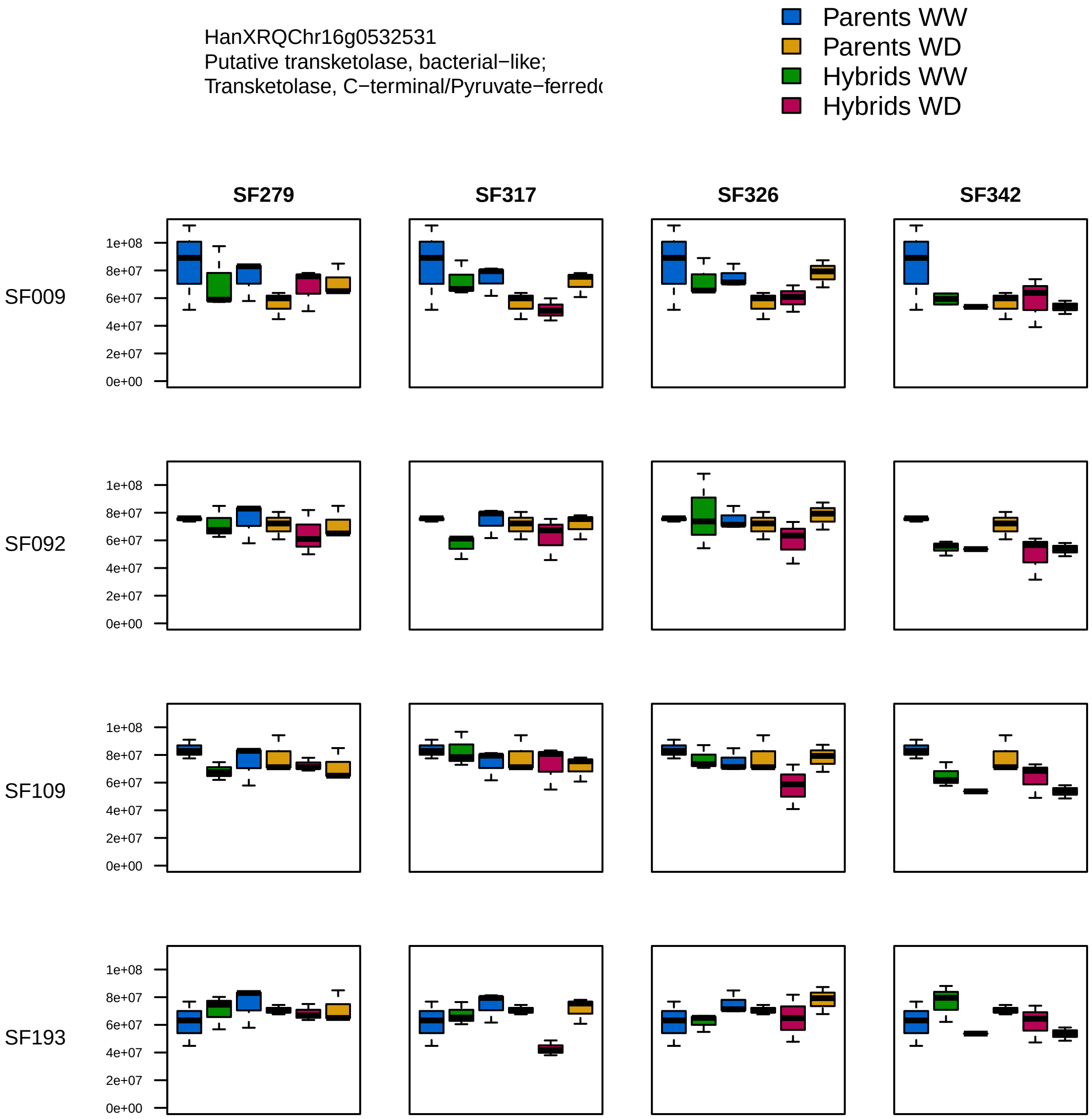

HanXRQChr16g0532531
Putative transketolase, bacterial−like;
Transketolase, C−terminal/Pyruvate−ferredo
Parents WW
Parents WD
Hybrids WW
Hybrids WD
SF279
SF317
SF326
SF342
SF009
SF092
SF109
SF193
1e+08
8e+07
6e+07
4e+07
2e+07
0e+00

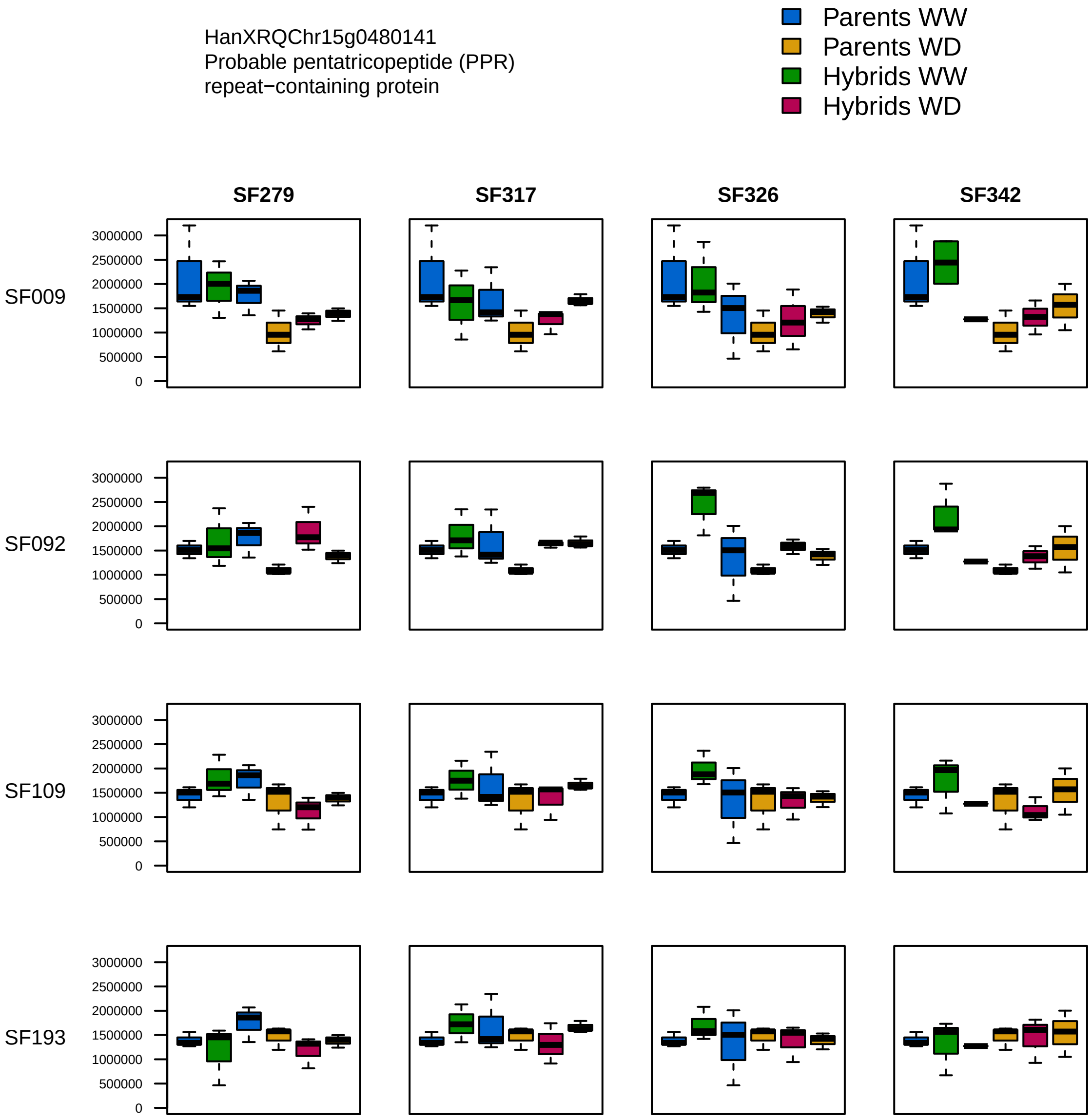
HanXRQChr15g0480141
Probable pentatricopeptide (PPR)
repeat−containing protein
Parents WW
Parents WD
Hybrids WW
Hybrids WD
SF279
SF317
SF326
SF342
SF009
SF092
SF109
SF193
3000000
2500000
2000000
1500000
1000000
500000
0

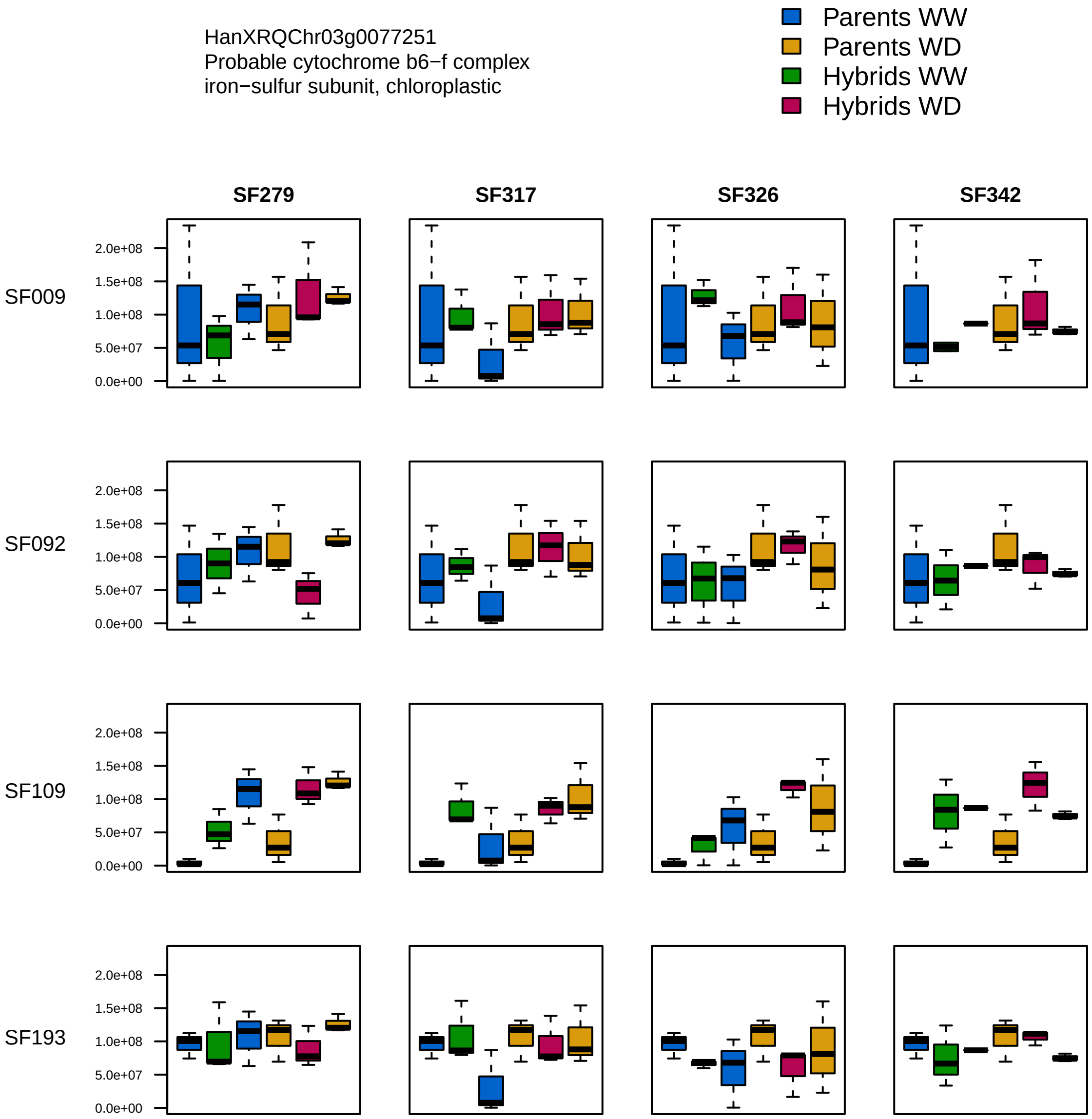
HanXRQChr03g0077251
Probable cytochrome b6−f complex
iron−sulfur subunit, chloroplastic
Parents WW
Parents WD
Hybrids WW
Hybrids WD
SF279
SF317
SF326
SF342
SF009
SF092
SF109
SF193
2.0e+08
1.5e+08
1.0e+08
5.0e+07
0.0e+00

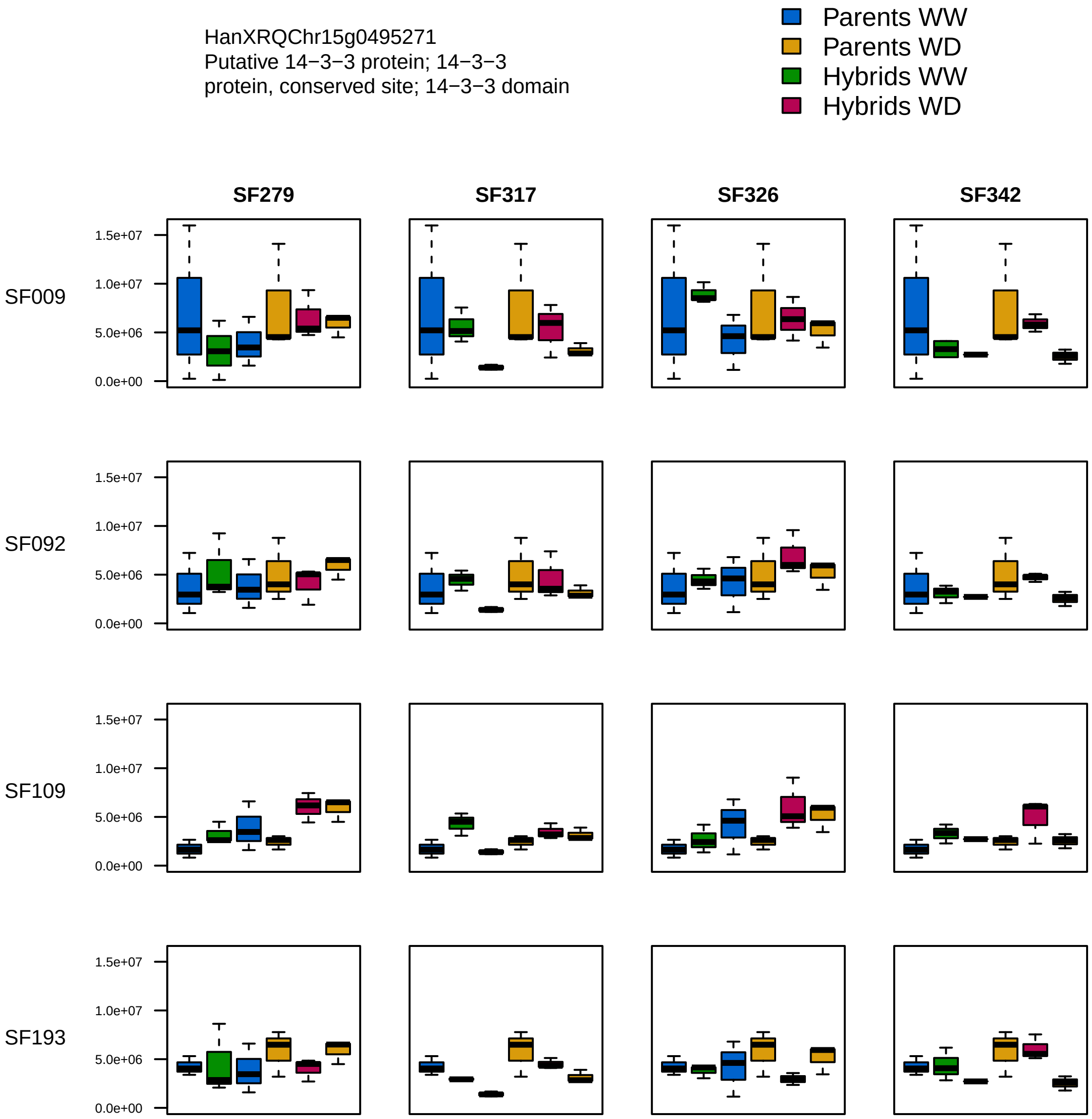
HanXRQChr15g0495271
Putative 14−3−3 protein; 14−3−3 protein, conserved site; 14−3−3 domain
Parents WW
Parents WD
Hybrids WW
Hybrids WD
SF279
SF317
SF326
SF342
SF009
SF092
SF109
SF193
0.0e+00
5.0e+06
1.0e+07
1.5e+07

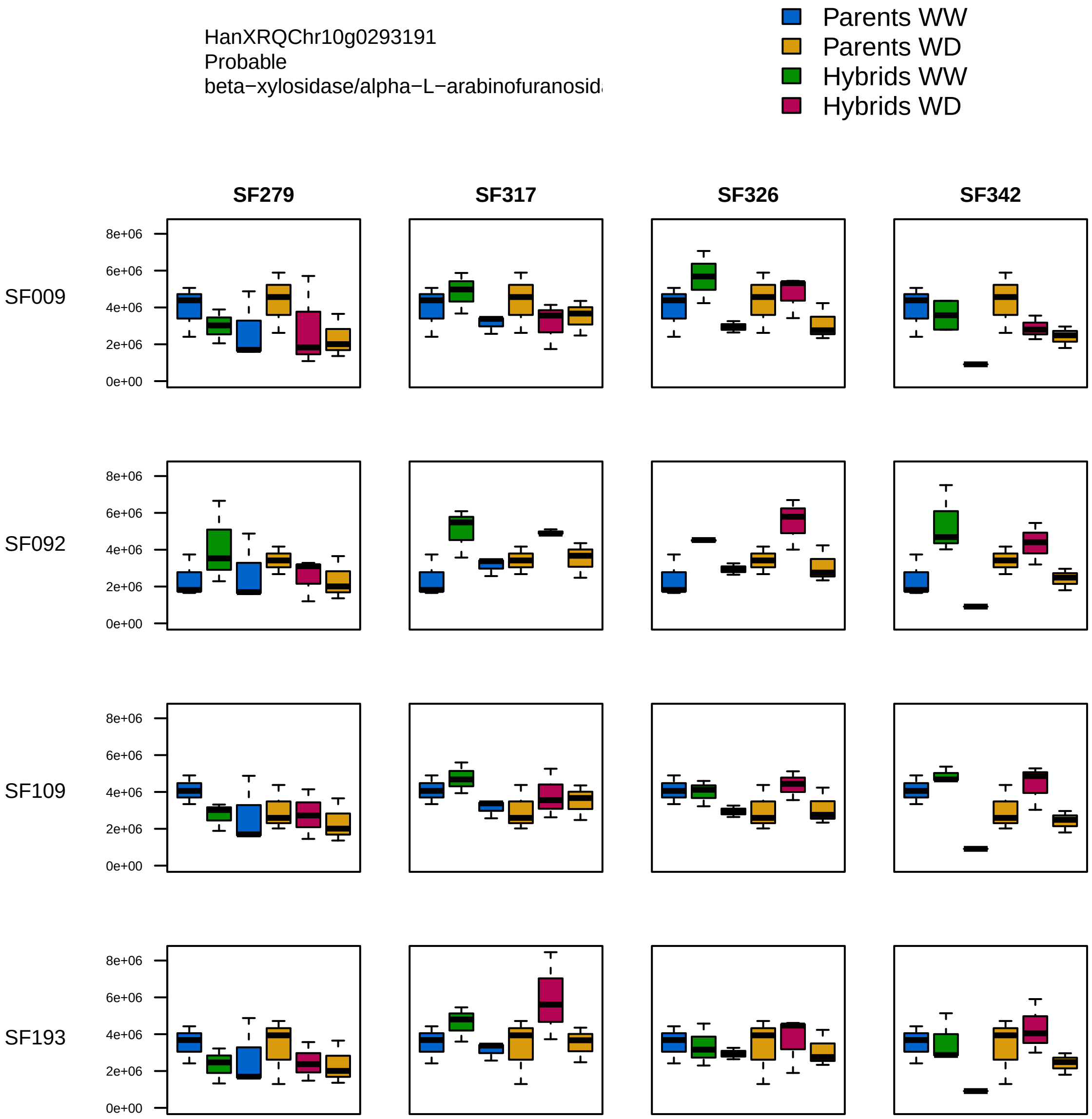

HanXRQChr10g0293191
Probable
beta−xylosidase/alpha−L−arabinofuranosida
Parents WW
Parents WD
Hybrids WW
Hybrids WD
SF279
SF317
SF326
SF342
SF009
SF092
SF109
SF193
8e+06
6e+06
4e+06
2e+06
0e+00

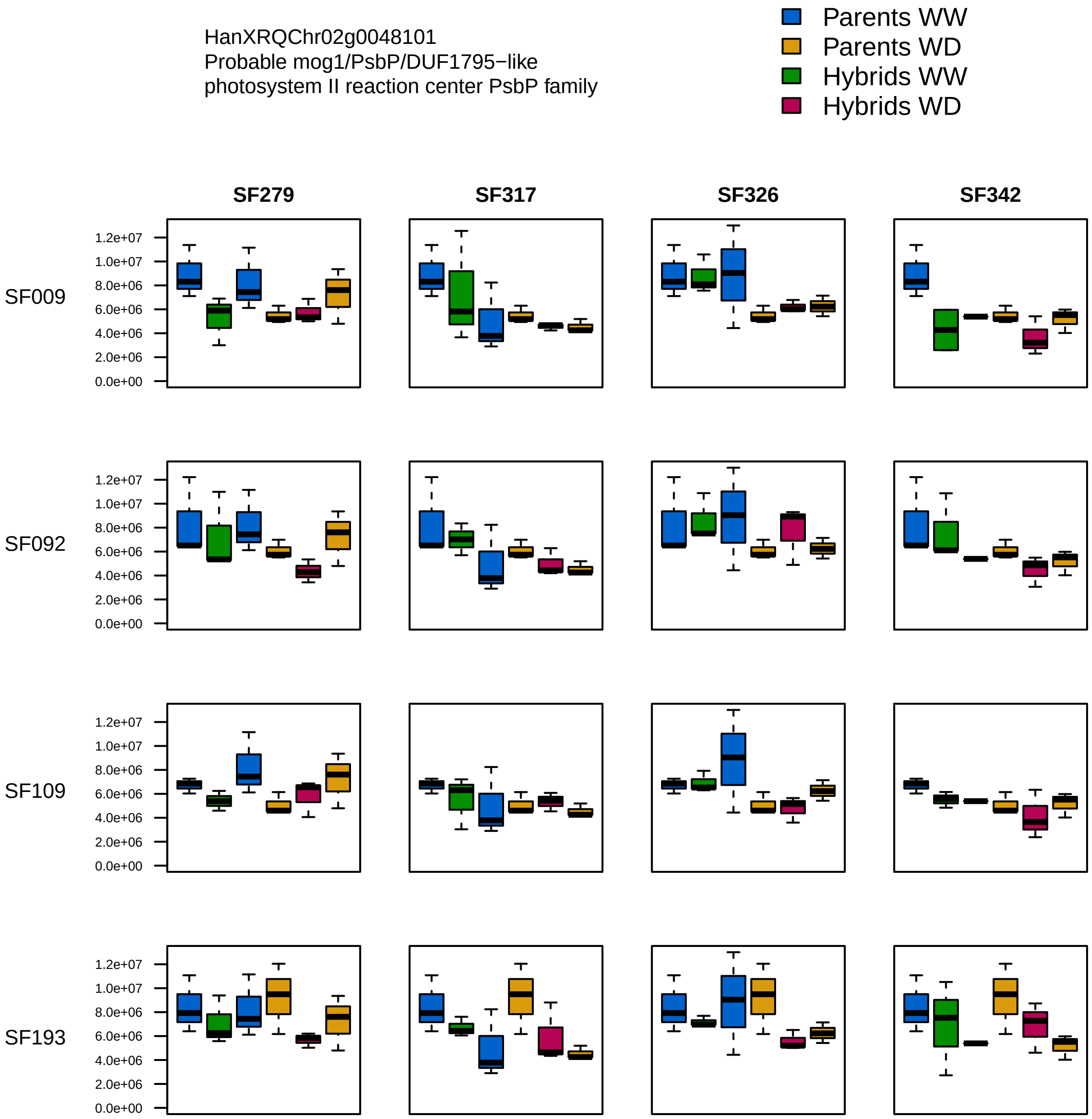

HanXRQChr02g0048101
Probable mog1/PsbP/DUF1795−like
photosystem II reaction center PsbP family
Parents WW
Parents WD
Hybrids WW
Hybrids WD
SF279
SF317
SF326
SF342
SF009
SF092
SF109
SF193
1.2e+07
1.0e+07
8.0e+06
6.0e+06
4.0e+06
2.0e+06
0.0e+00

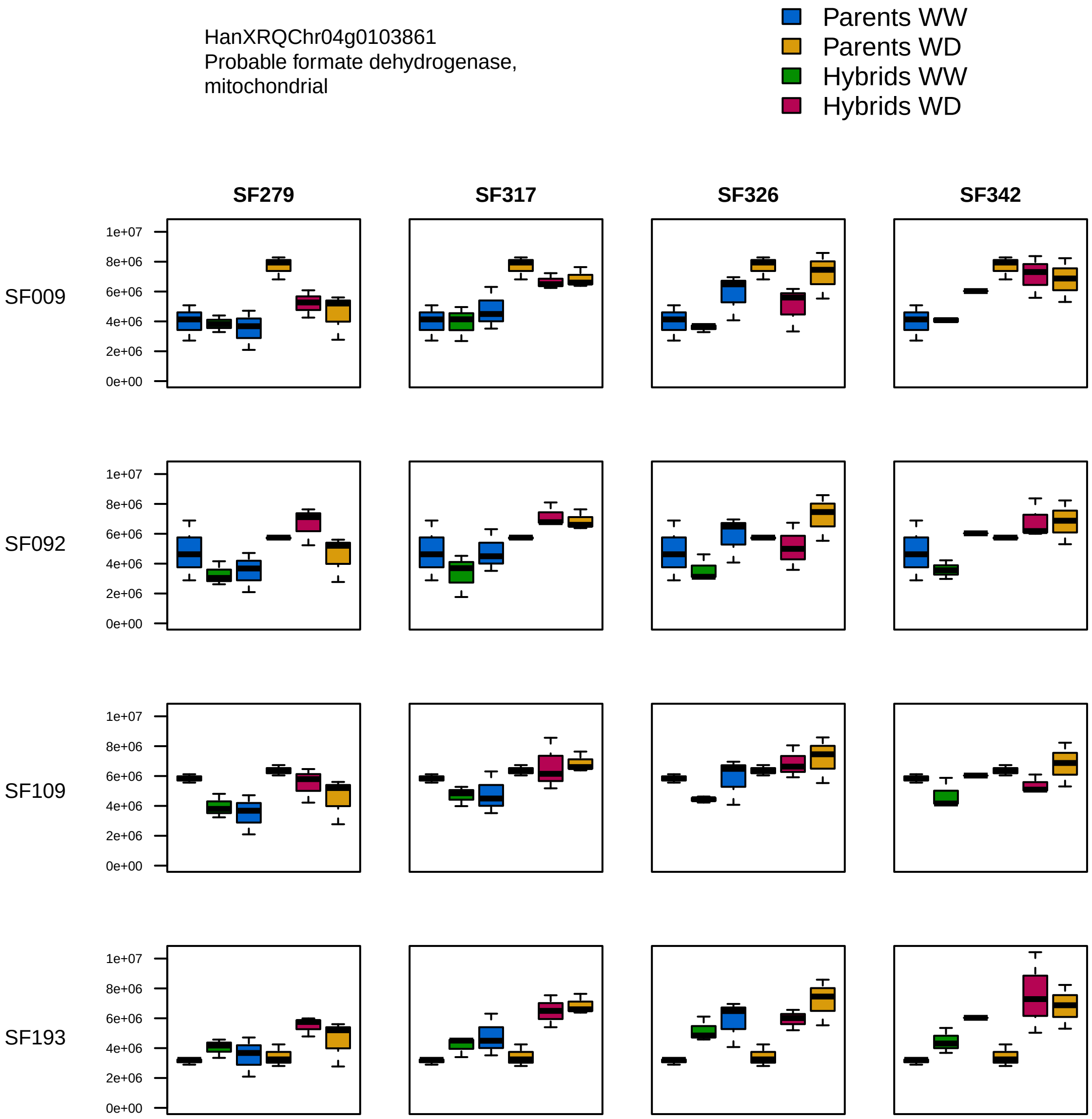
HanXRQChr04g0103861
Probable formate dehydrogenase, mitochondrial
Parents WW
Parents WD
Hybrids WW
Hybrids WD
SF279
SF317
SF326
SF342
SF009
SF092
SF109
SF193
1e+07
8e+06
6e+06
4e+06
2e+06
0e+00

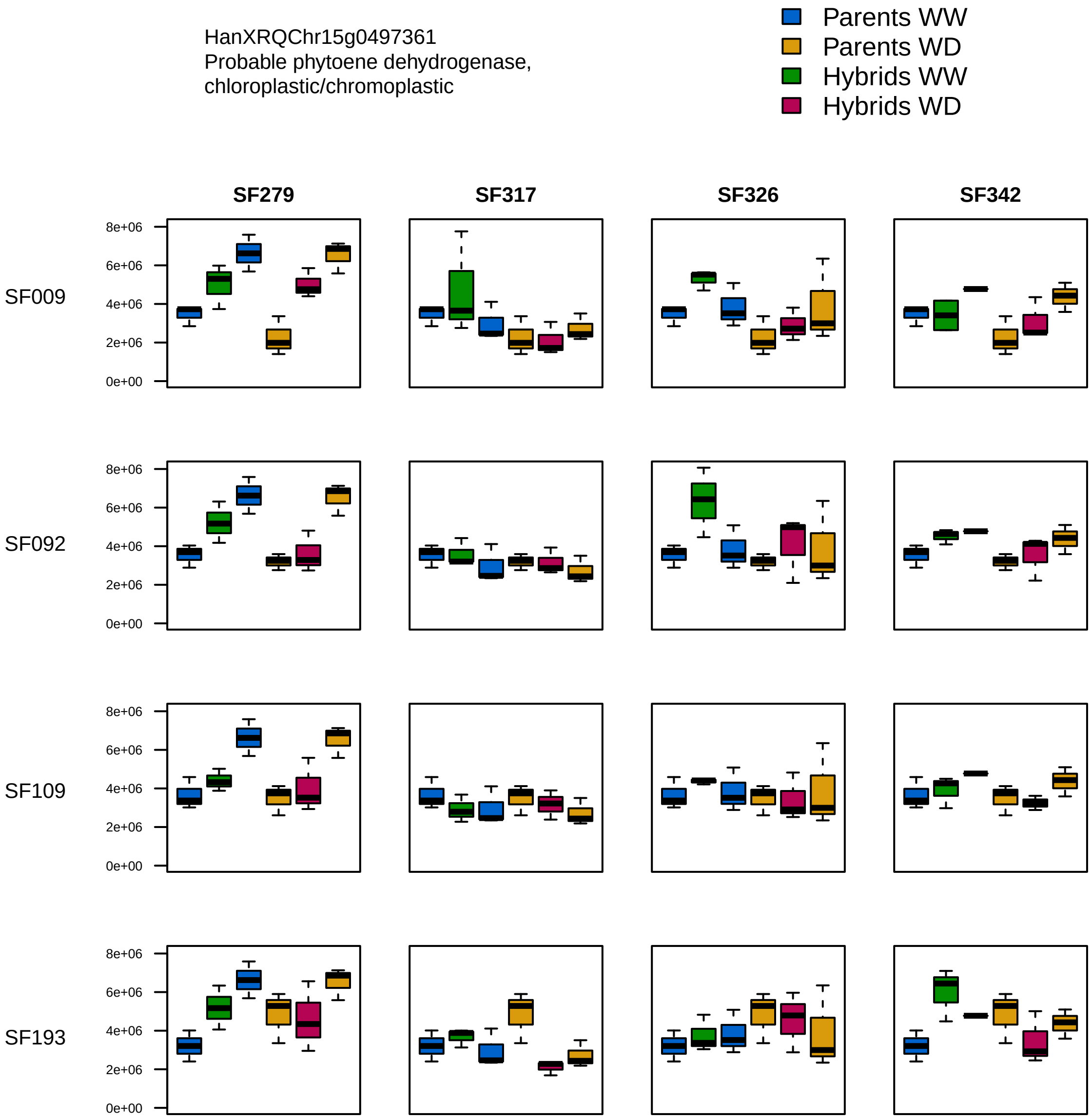

HanXRQChr15g0497361
Probable phytoene dehydrogenase, chloroplastic/chromoplastic
Parents WW
Parents WD
Hybrids WW
Hybrids WD
SF279
SF317
SF326
SF342
SF009
SF092
SF109
SF193
8e+06
6e+06
4e+06
2e+06
0e+00

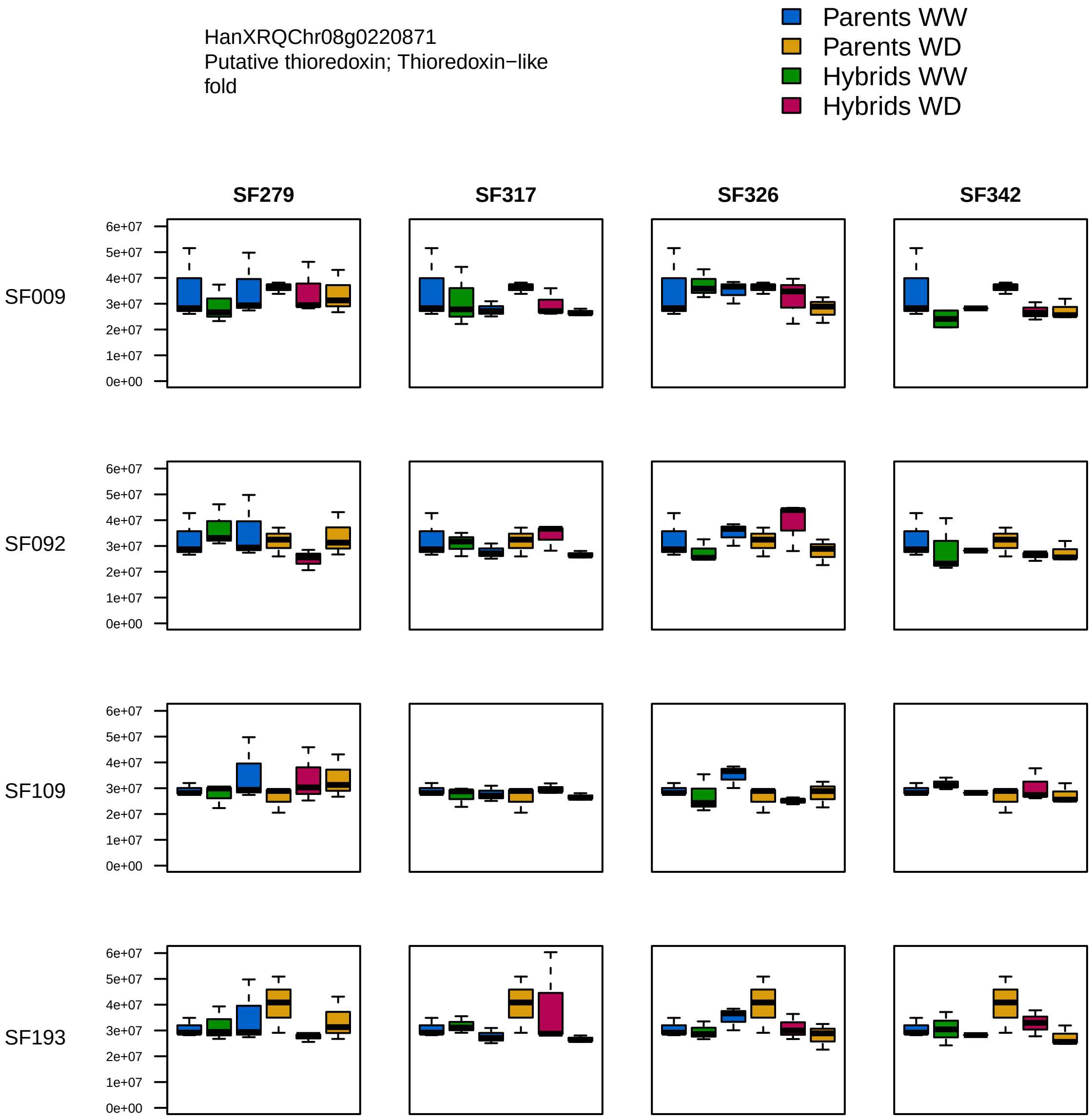

HanXRQChr08g0220871
Putative thioredoxin; Thioredoxin−like fold
Parents WW
Parents WD
Hybrids WW
Hybrids WD
SF279
SF317
SF326
SF342
SF009
SF092
SF109
SF193
6e+07
5e+07
4e+07
3e+07
2e+07
1e+07
0e+00

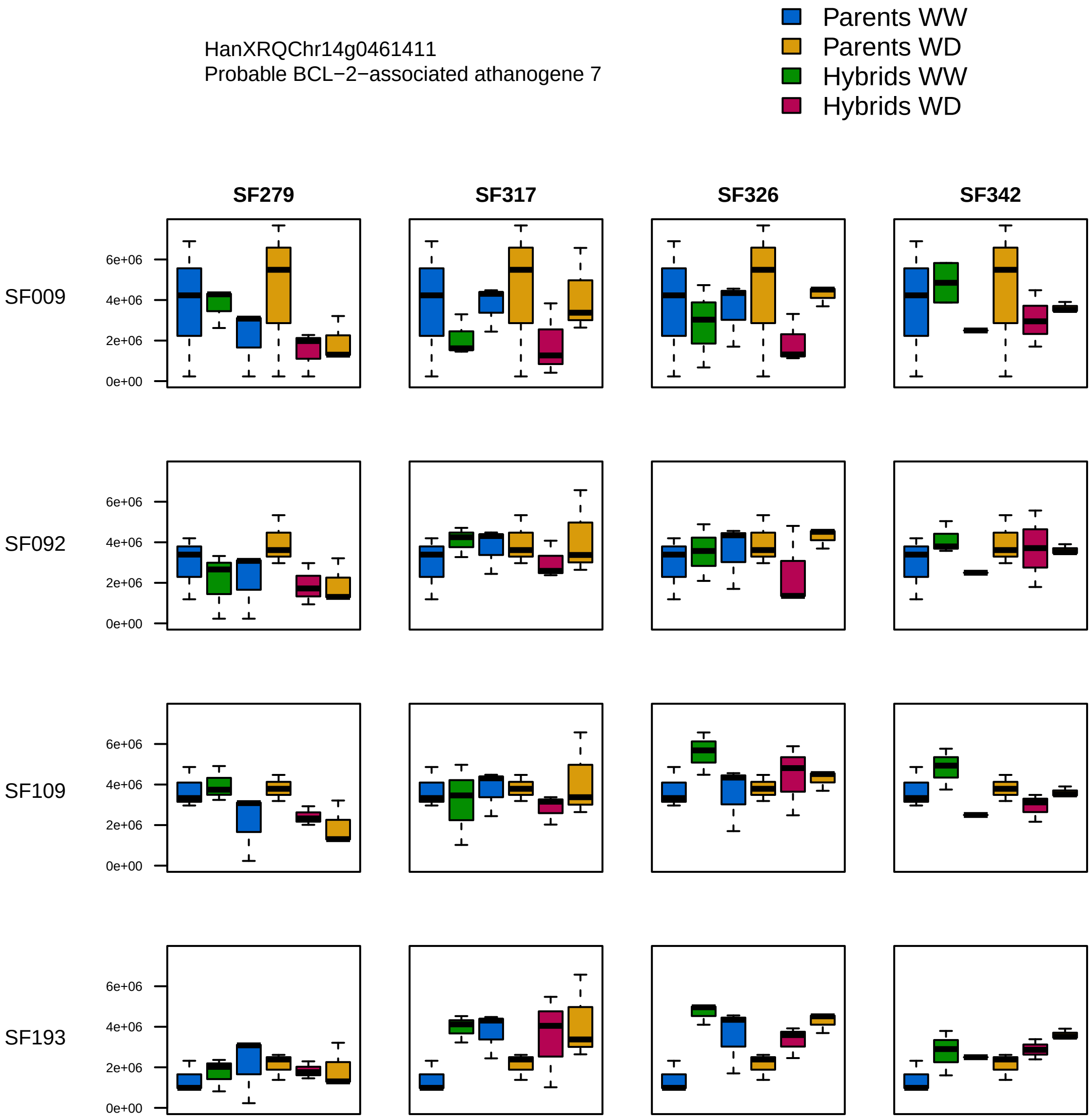

HanXRQChr14g0461411
Probable BCL−2−associated athanogene 7
Parents WW
Parents WD
Hybrids WW
Hybrids WD
SF279
SF317
SF326
SF342
SF009
SF092
SF109
SF193
0e+00
2e+06
4e+06
6e+06

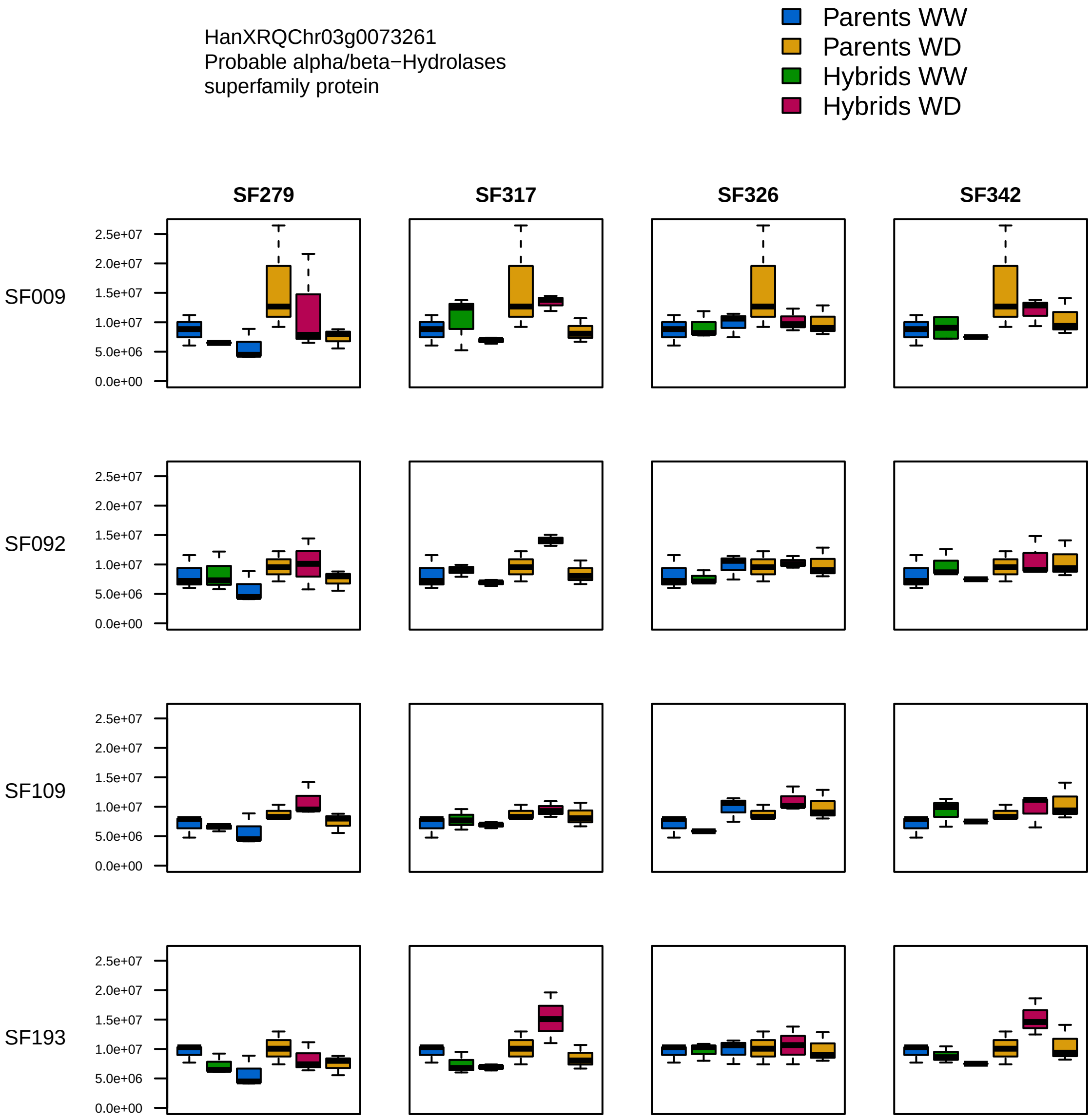

HanXRQChr03g0073261
Probable alpha/beta−Hydrolases
superfamily protein
Parents WW
Parents WD
Hybrids WW
Hybrids WD
SF279
SF317
SF326
SF342
SF009
SF092
SF109
SF193
2.5e+07
2.0e+07
1.5e+07
1.0e+07
5.0e+06
0.0e+00

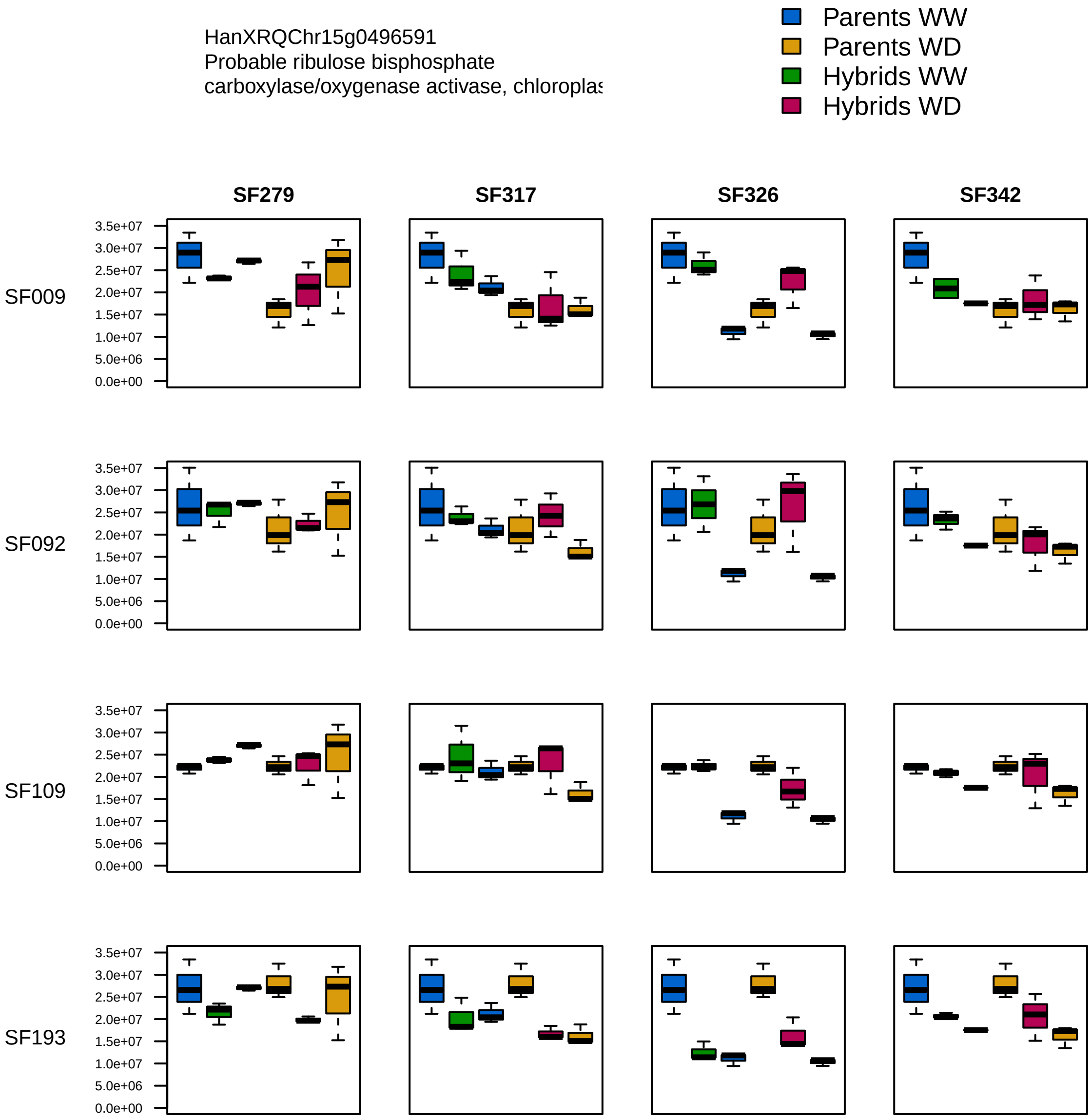

HanXRQChr15g0496591
Probable ribulose bisphosphate
carboxylase/oxygenase activase, chloroplas
Parents WW
Parents WD
Hybrids WW
Hybrids WD
SF279
SF317
SF326
SF342
SF009
SF092
SF109
SF193
3.5e+07
3.0e+07
2.5e+07
2.0e+07
1.5e+07
1.0e+07
5.0e+06
0.0e+00

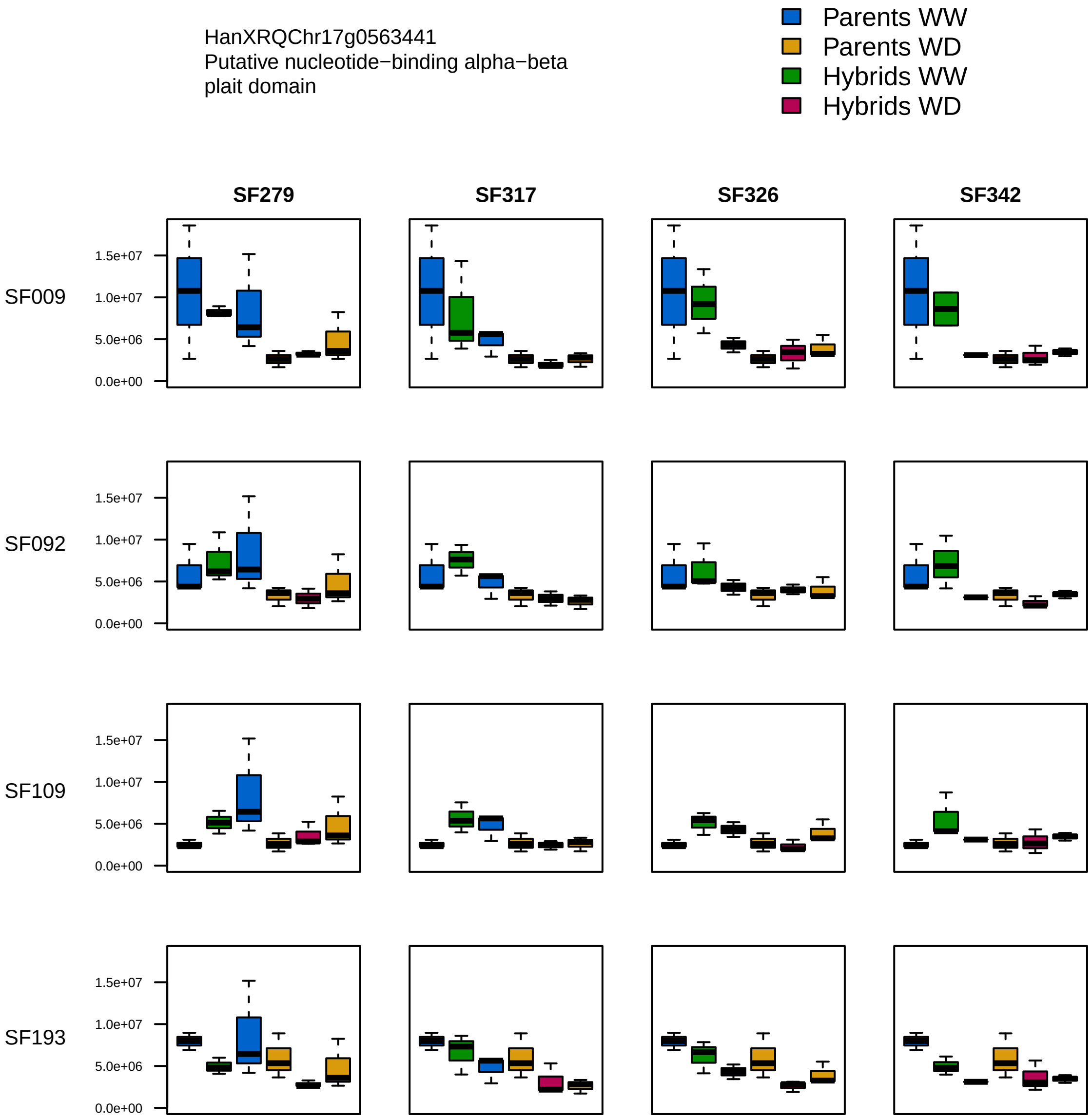

HanXRQChr17g0563441
Putative nucleotide−binding alpha−beta plait domain
Parents WW
Parents WD
Hybrids WW
Hybrids WD
SF279
SF317
SF326
SF342
SF009
SF092
SF109
SF193
1.5e+07
1.0e+07
5.0e+06
0.0e+00

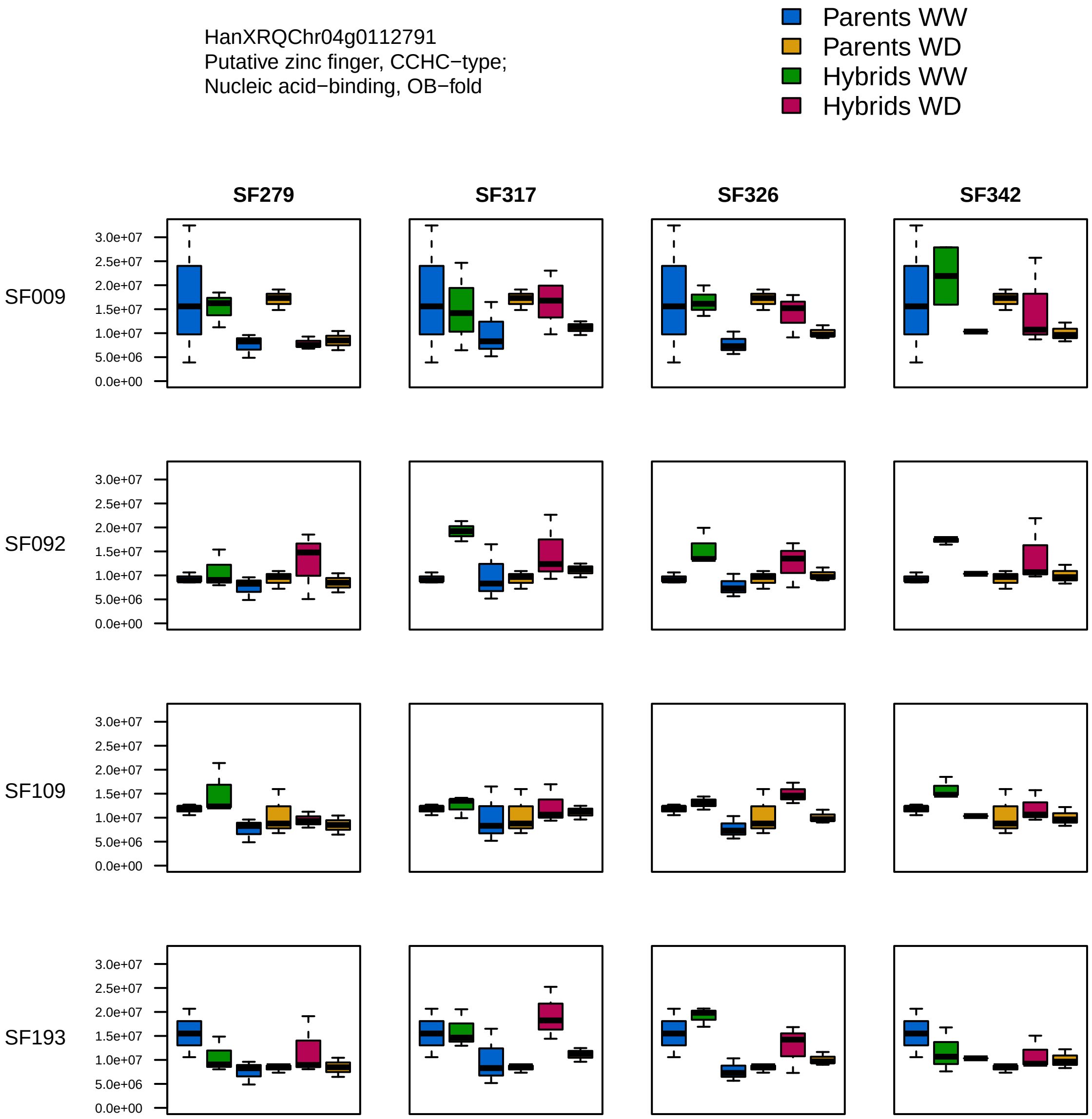
HanXRQChr04g0112791
Putative zinc finger, CCHC−type;
Nucleic acid−binding, OB−fold
Parents WW
Parents WD
Hybrids WW
Hybrids WD
SF279
SF317
SF326
SF342
SF009
SF092
SF109
SF193
3.0e+07
2.5e+07
2.0e+07
1.5e+07
1.0e+07
5.0e+06
0.0e+00

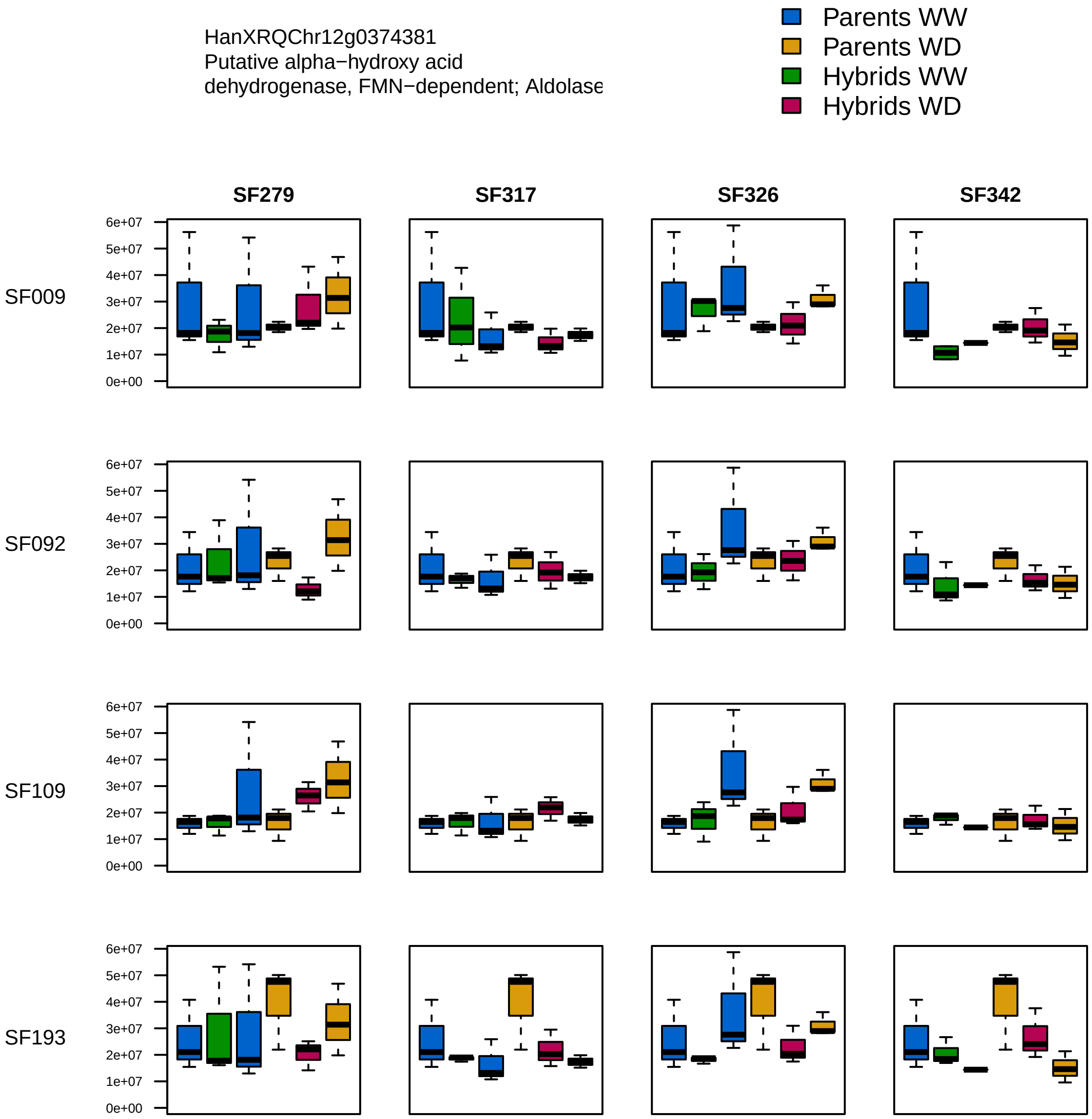
HanXRQChr12g0374381
Putative alpha−hydroxy acid
dehydrogenase, FMN−dependent; Aldolase
Parents WW
Parents WD
Hybrids WW
Hybrids WD
SF279
SF317
SF326
SF342
SF009
SF092
SF109
SF193
0e+00
1e+07
2e+07
3e+07
4e+07
5e+07
6e+07

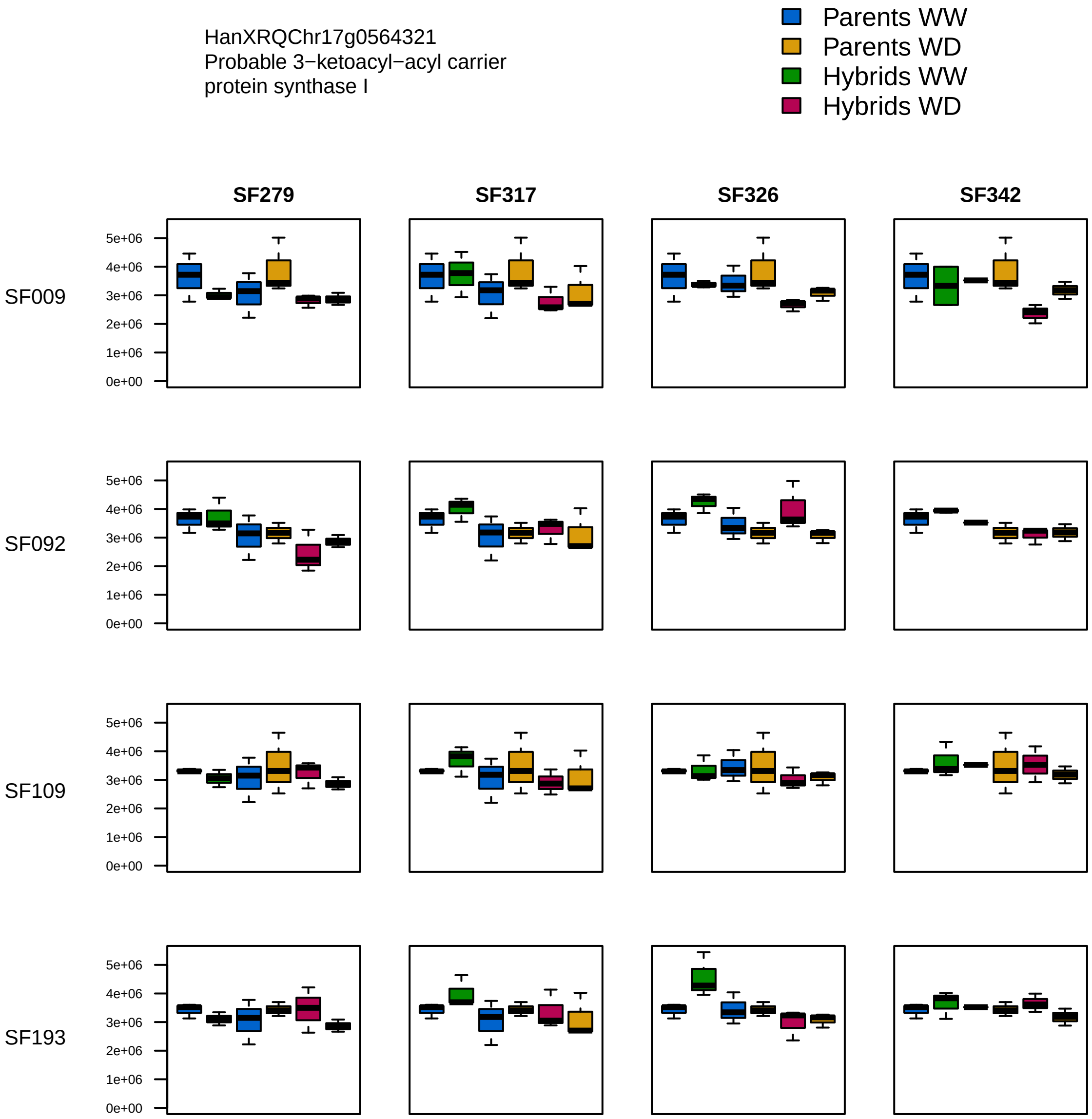
HanXRQChr17g0564321
Probable 3−ketoacyl−acyl carrier
protein synthase I
Parents WW
Parents WD
Hybrids WW
Hybrids WD
SF279
SF317
SF326
SF342
SF009
SF092
SF109
SF193
0e+00
1e+06
2e+06
3e+06
4e+06
5e+06

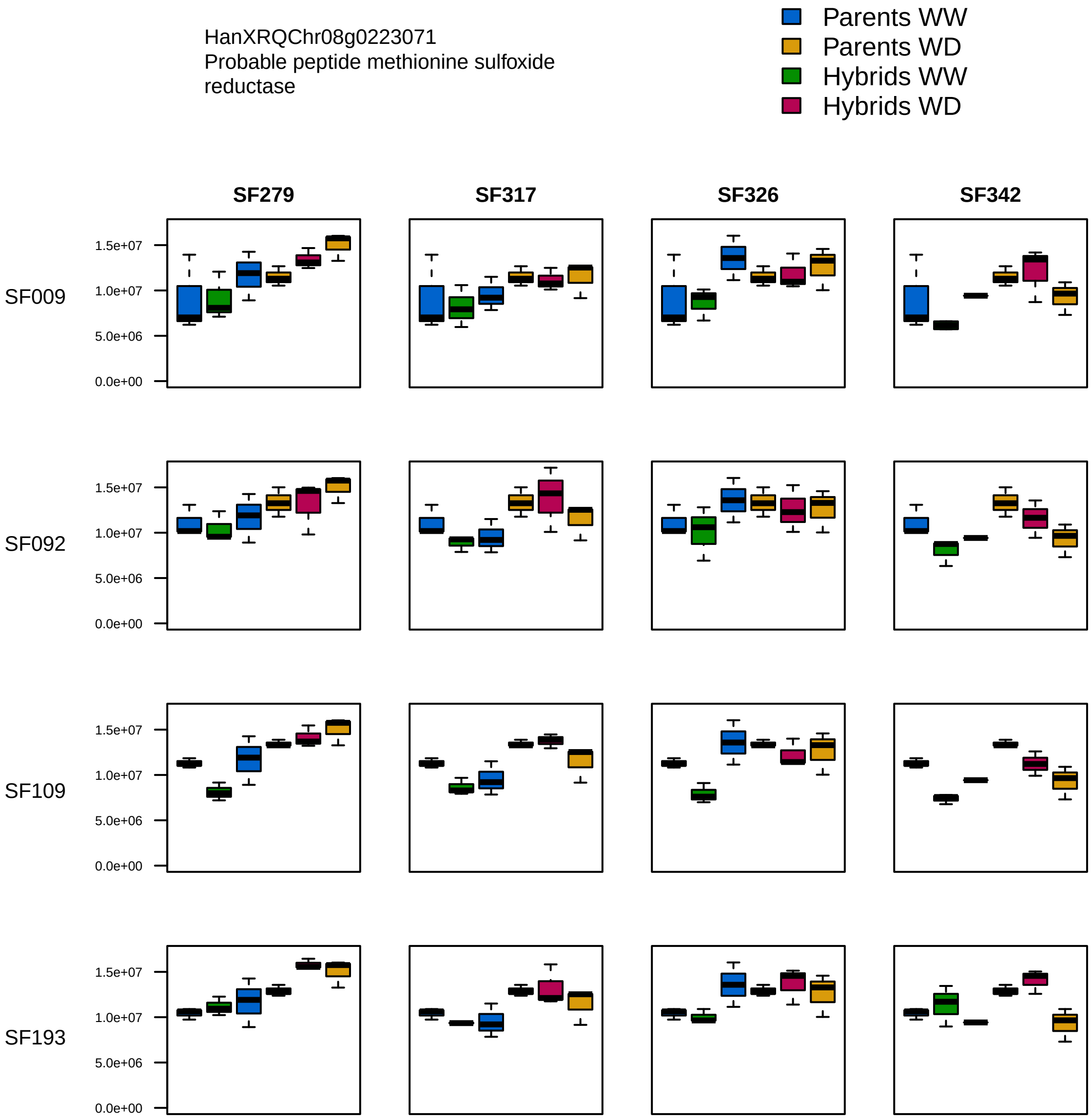

HanXRQChr08g0223071
Probable peptide methionine sulfoxide reductase
Parents WW
Parents WD
Hybrids WW
Hybrids WD
SF279
SF317
SF326
SF342
SF009
SF092
SF109
SF193
1.5e+07
1.0e+07
5.0e+06
0.0e+00

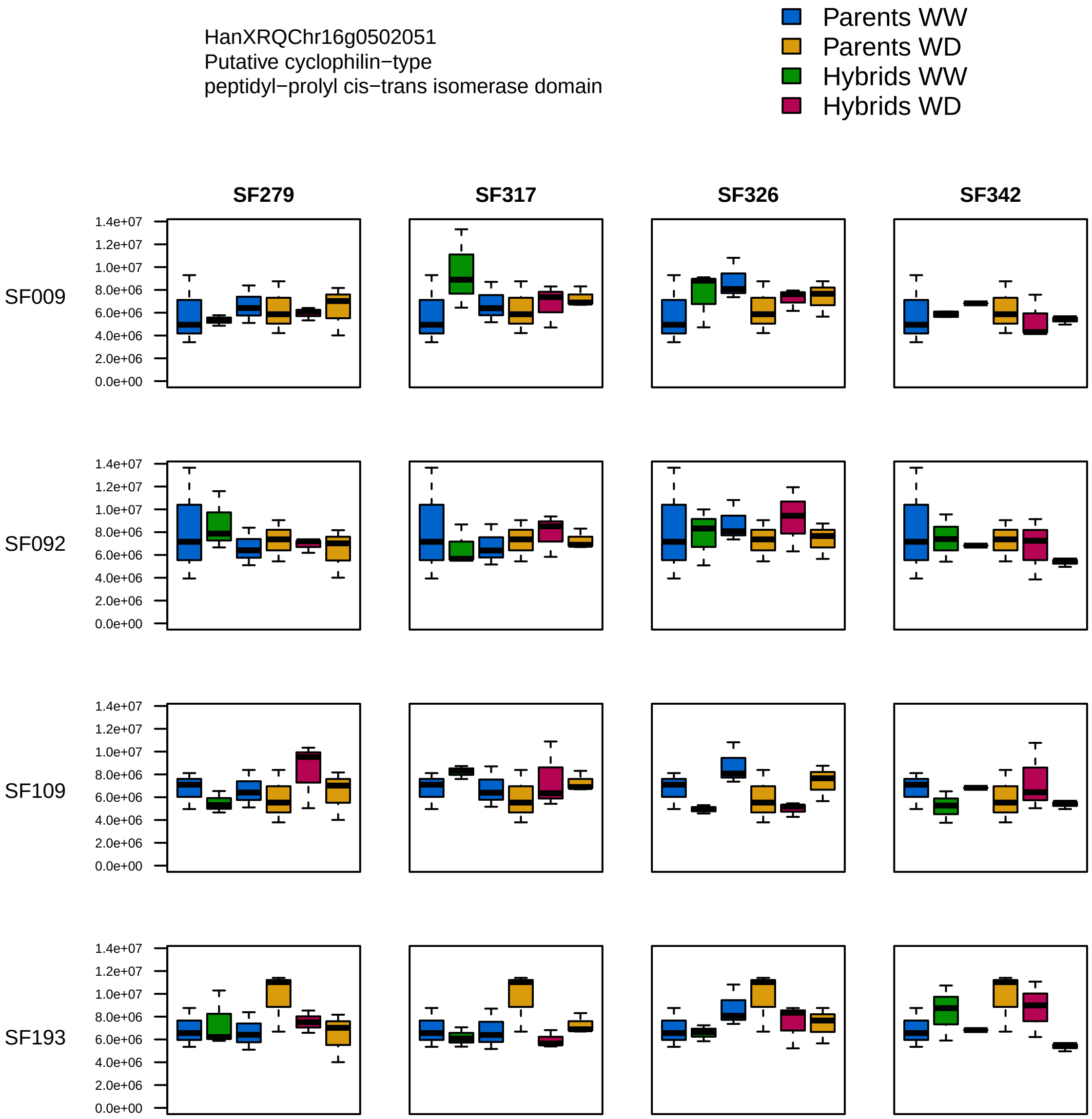
HanXRQChr16g0502051
Putative cyclophilin−type
peptidyl−prolyl cis−trans isomerase domain
Parents WW
Parents WD
Hybrids WW
Hybrids WD
SF279
SF317
SF326
SF342
SF009
SF092
SF109
SF193
1.4e+07
1.2e+07
1.0e+07
8.0e+06
6.0e+06
4.0e+06
2.0e+06
0.0e+00

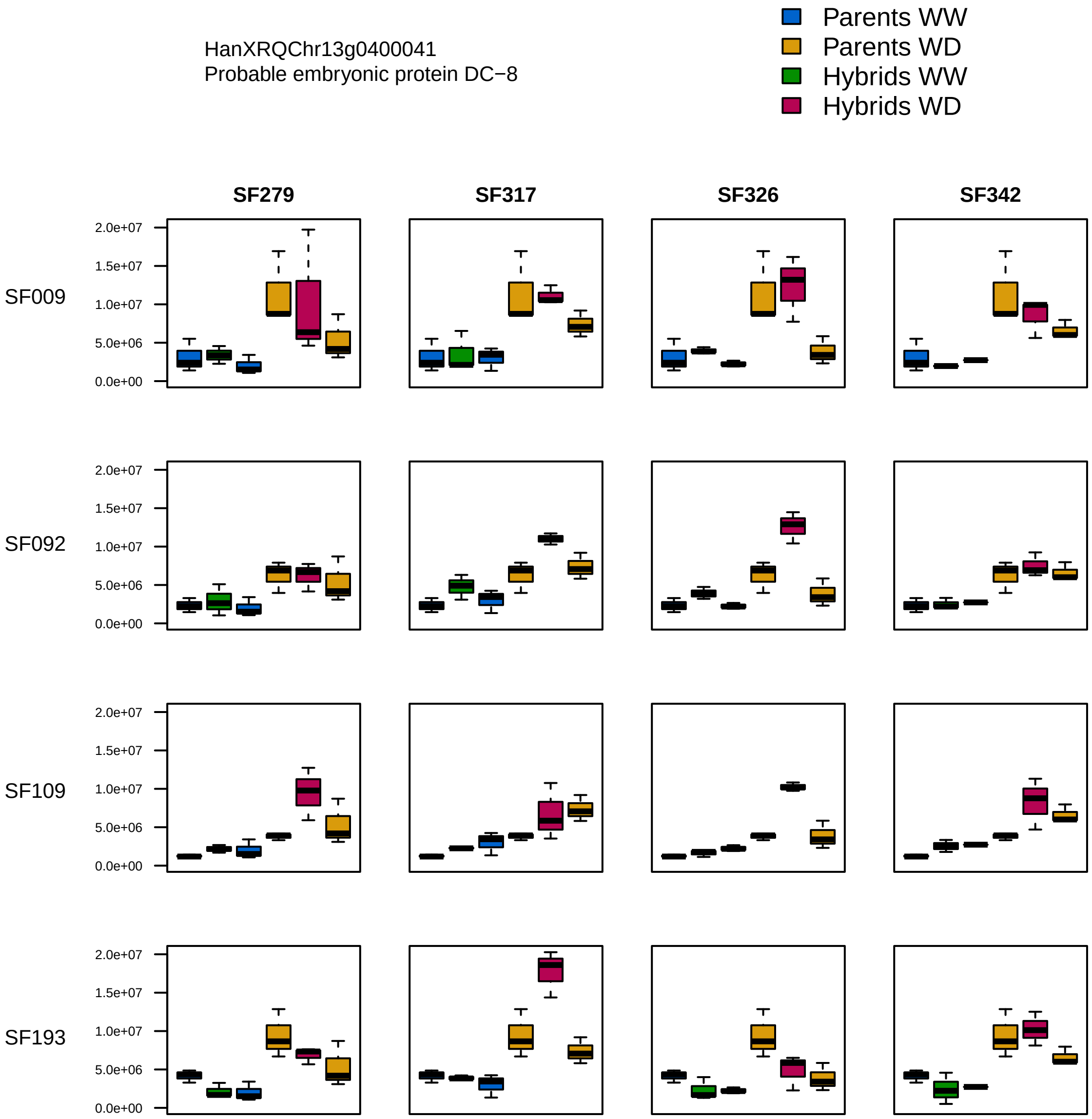
HanXRQChr13g0400041
Probable embryonic protein DC−8
Parents WW
Parents WD
Hybrids WW
Hybrids WD
SF279
SF317
SF326
SF342
SF009
SF092
SF109
SF193
0.0e+00
5.0e+06
1.0e+07
1.5e+07
2.0e+07

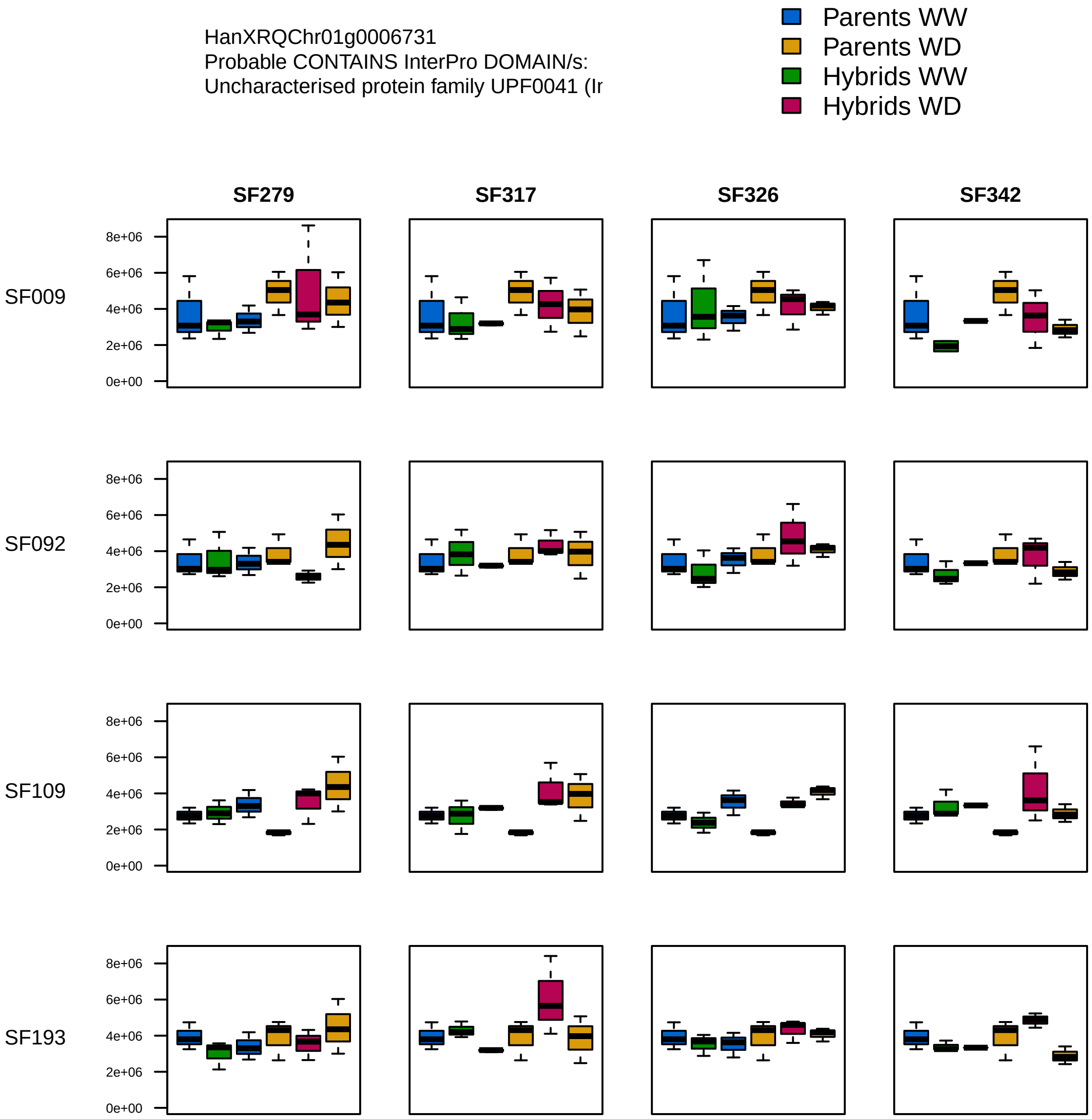
HanXRQChr01g0006731
Probable CONTAINS InterPro DOMAIN/s:
Uncharacterised protein family UPF0041 (Ir
Parents WW
Parents WD
Hybrids WW
Hybrids WD
SF279
SF317
SF326
SF342
SF009
SF092
SF109
SF193
8e+06
6e+06
4e+06
2e+06
0e+00

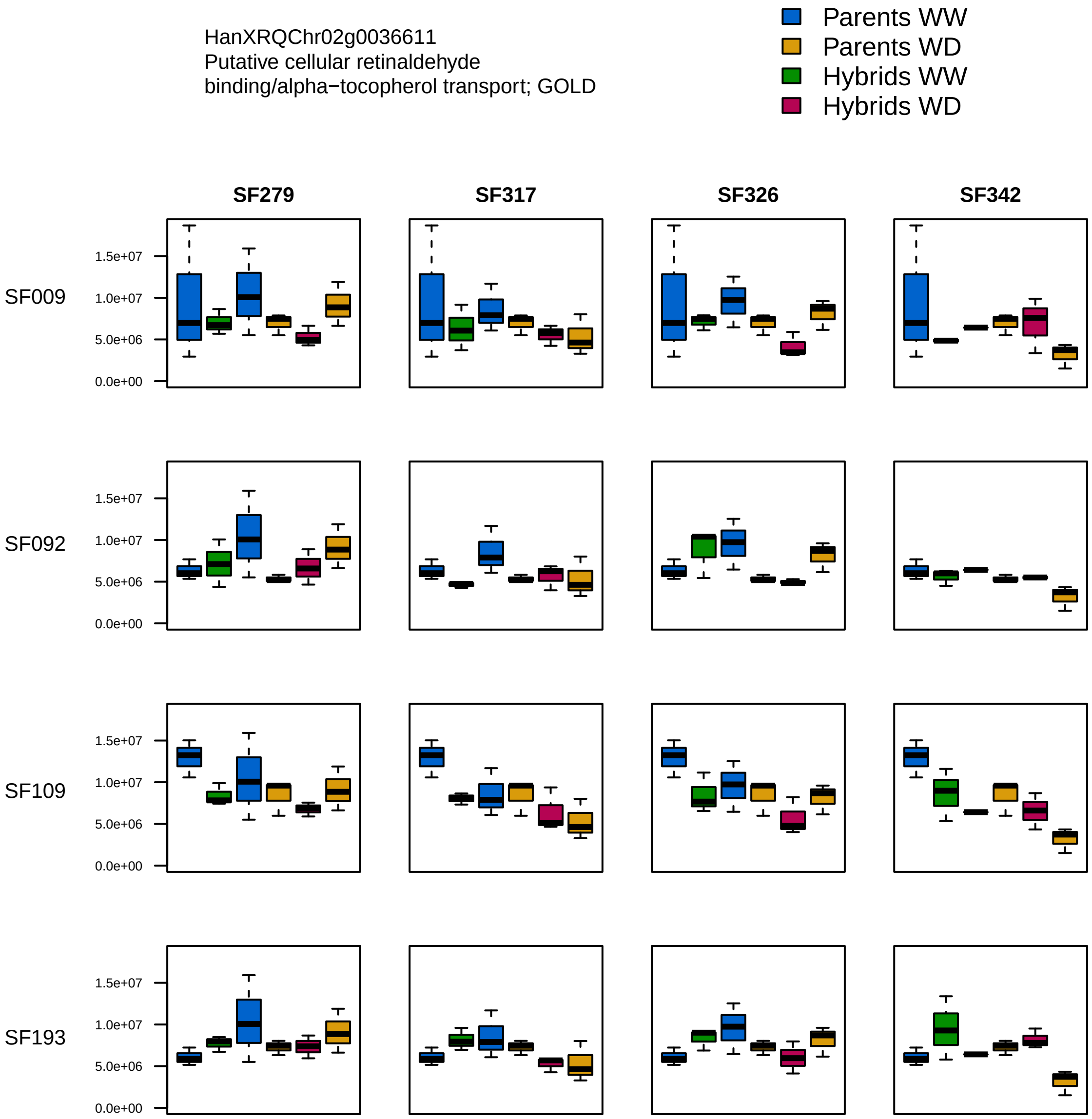
HanXRQChr02g0036611
Putative cellular retinaldehyde
binding/alpha−tocopherol transport; GOLD
Parents WW
Parents WD
Hybrids WW
Hybrids WD
SF279
SF317
SF326
SF342
SF009
SF092
SF109
SF193
1.5e+07
1.0e+07
5.0e+06
0.0e+00

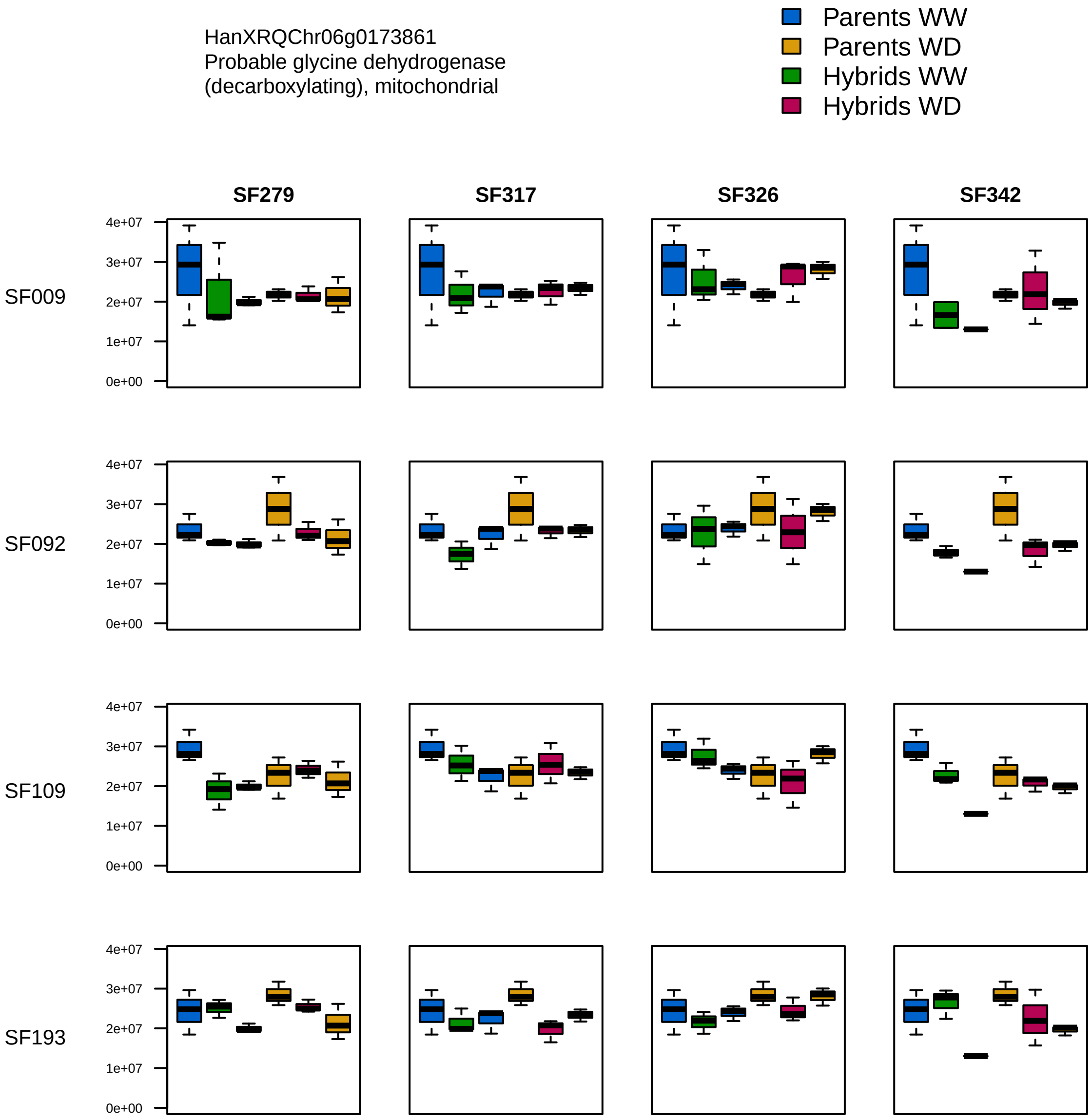
HanXRQChr06g0173861
Probable glycine dehydrogenase
(decarboxylating), mitochondrial
Parents WW
Parents WD
Hybrids WW
Hybrids WD
SF279
SF317
SF326
SF342
SF009
SF092
SF109
SF193
4e+07
3e+07
2e+07
1e+07
0e+00

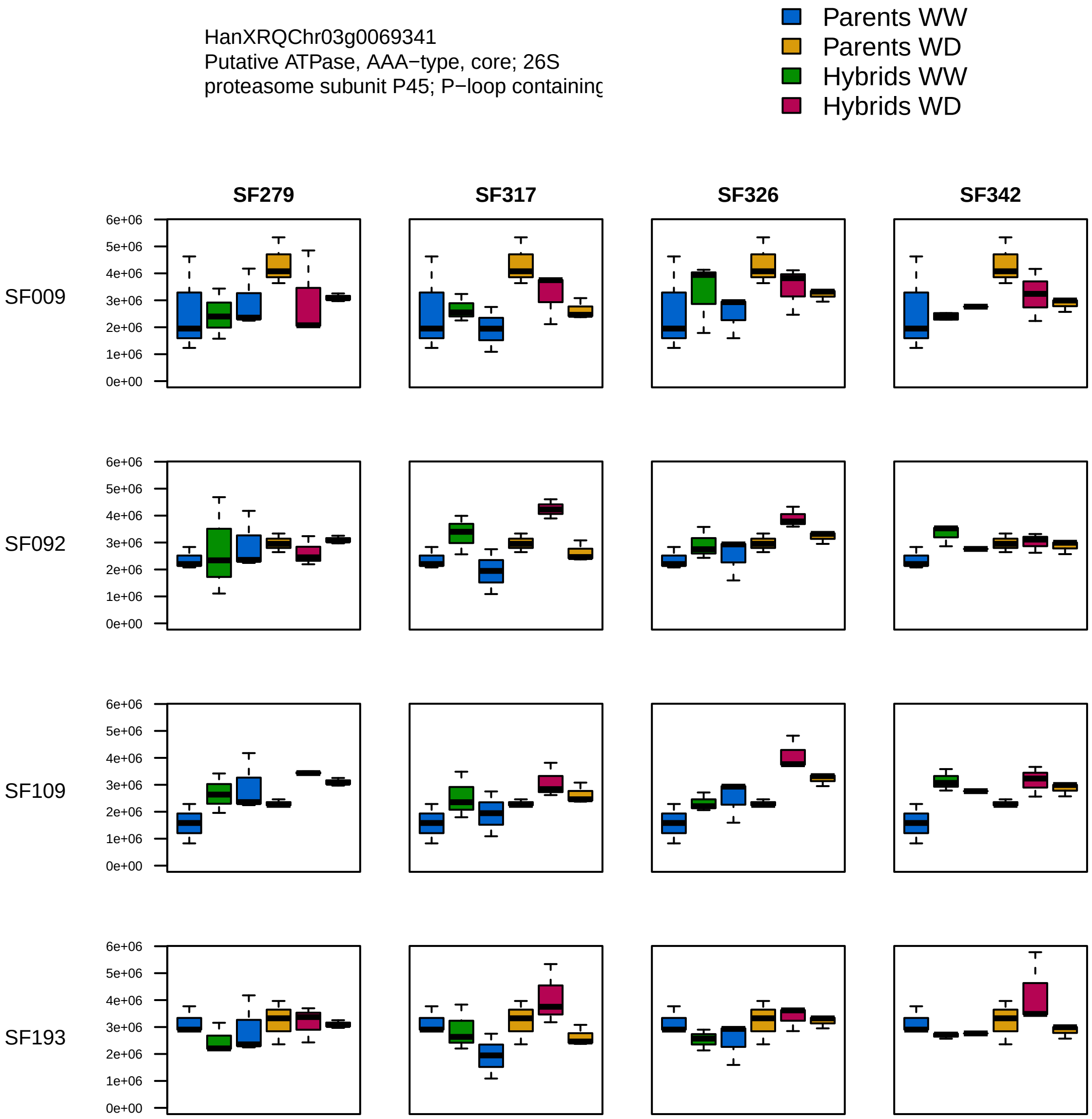

HanXRQChr03g0069341
Putative ATPase, AAA−type, core; 26S
proteasome subunit P45; P−loop containing
Parents WW
Parents WD
Hybrids WW
Hybrids WD
SF279
SF317
SF326
SF342
SF009
SF092
SF109
SF193
6e+06
5e+06
4e+06
3e+06
2e+06
1e+06
0e+00

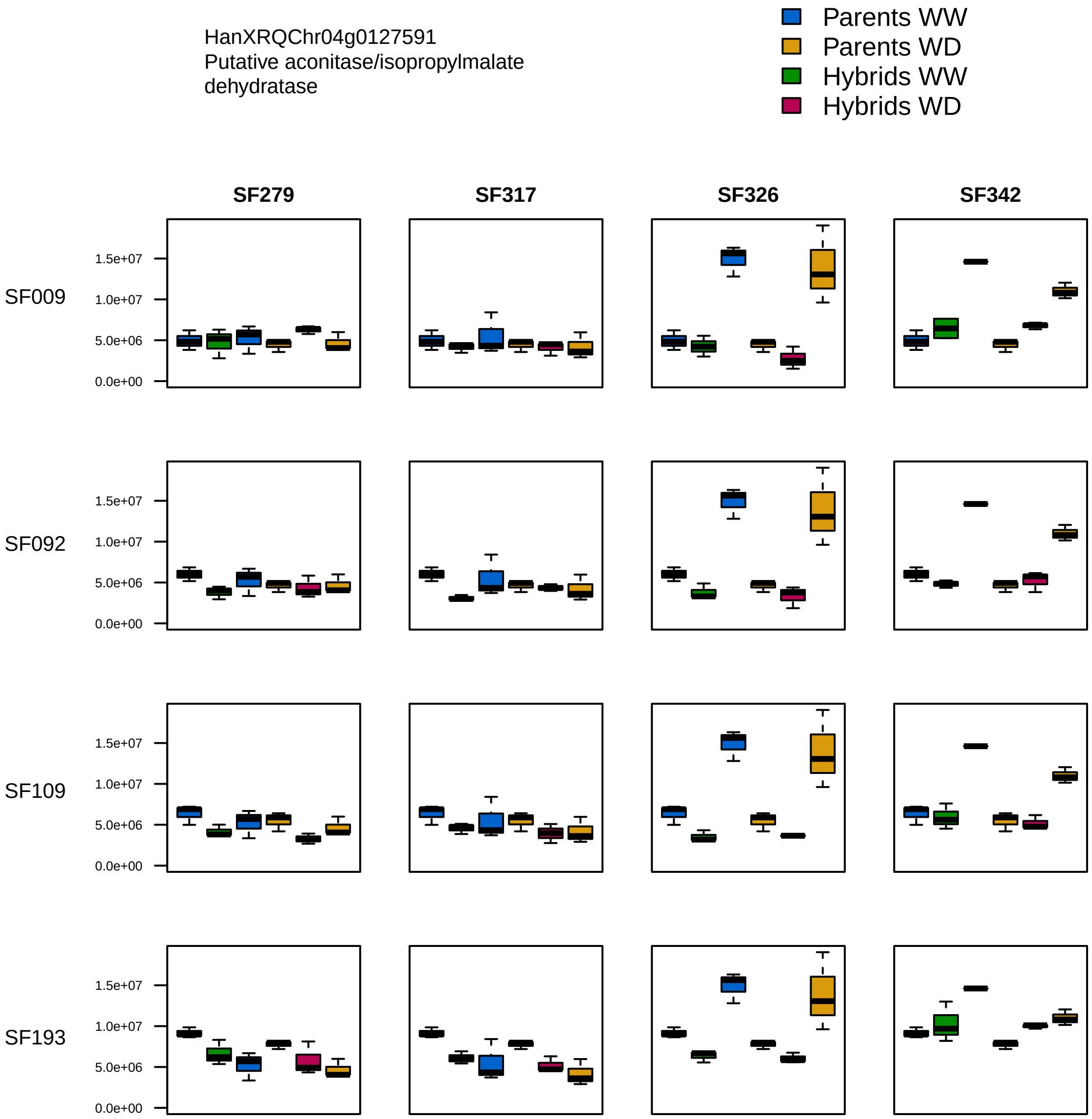
HanXRQChr04g0127591
Putative aconitase/isopropylmalate
dehydratase
Parents WW
Parents WD
Hybrids WW
Hybrids WD
SF279
SF317
SF326
SF342
SF009
SF092
SF109
SF193
0.0e+00
5.0e+06
1.0e+07
1.5e+07

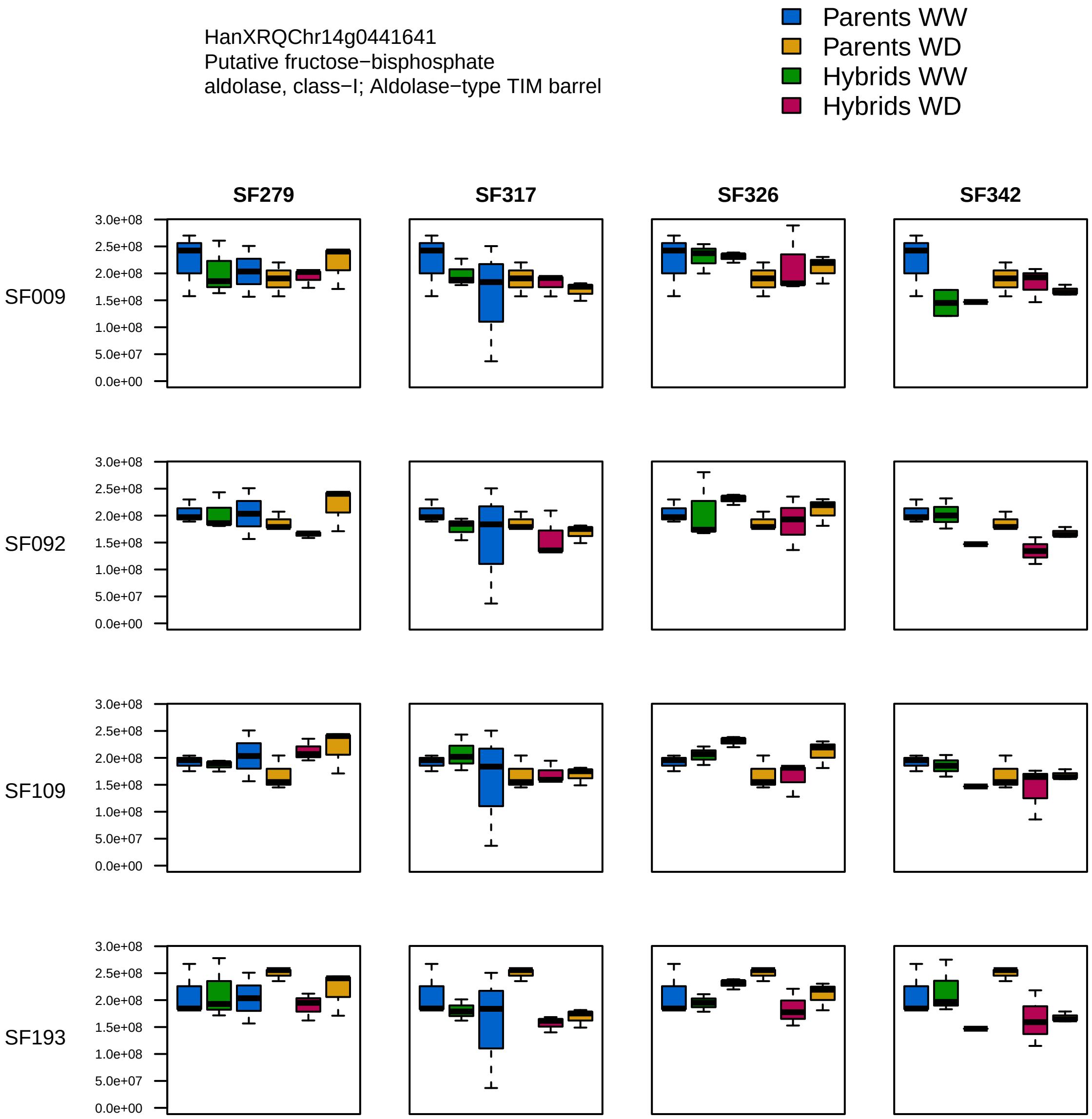

HanXRQChr14g0441641
Putative fructose−bisphosphate
aldolase, class−I; Aldolase−type TIM barrel
Parents WW
Parents WD
Hybrids WW
Hybrids WD
SF279
SF317
SF326
SF342
SF009
SF092
SF109
SF193
3.0e+08
2.5e+08
2.0e+08
1.5e+08
1.0e+08
5.0e+07
0.0e+00

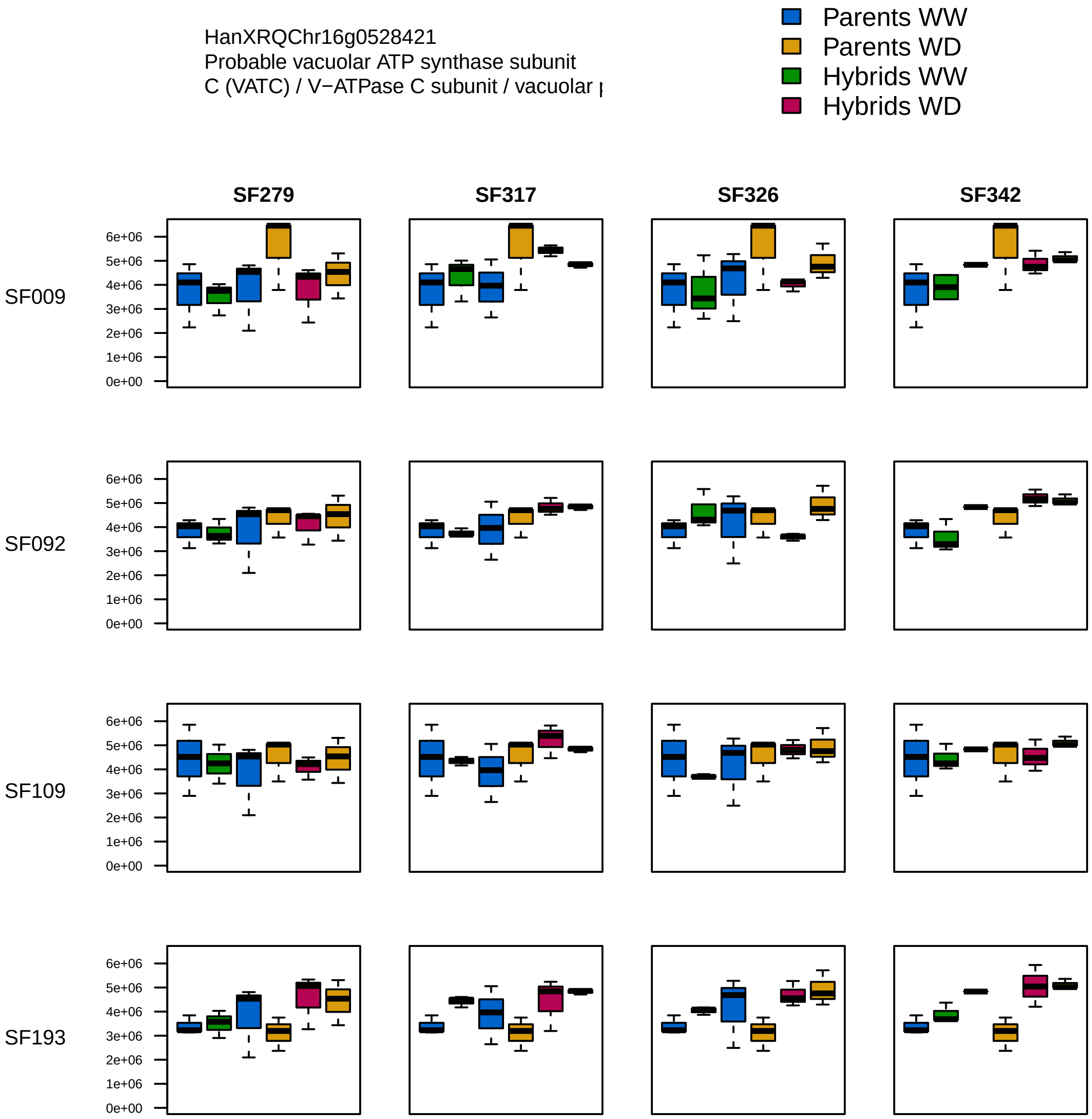
HanXRQChr16g0528421
Probable vacuolar ATP synthase subunit
C (VATC) / V−ATPase C subunit / vacuolar
Parents WW
Parents WD
Hybrids WW
Hybrids WD
SF279
SF317
SF326
SF342
SF009
SF092
SF109
SF193
6e+06
5e+06
4e+06
3e+06
2e+06
1e+06
0e+00

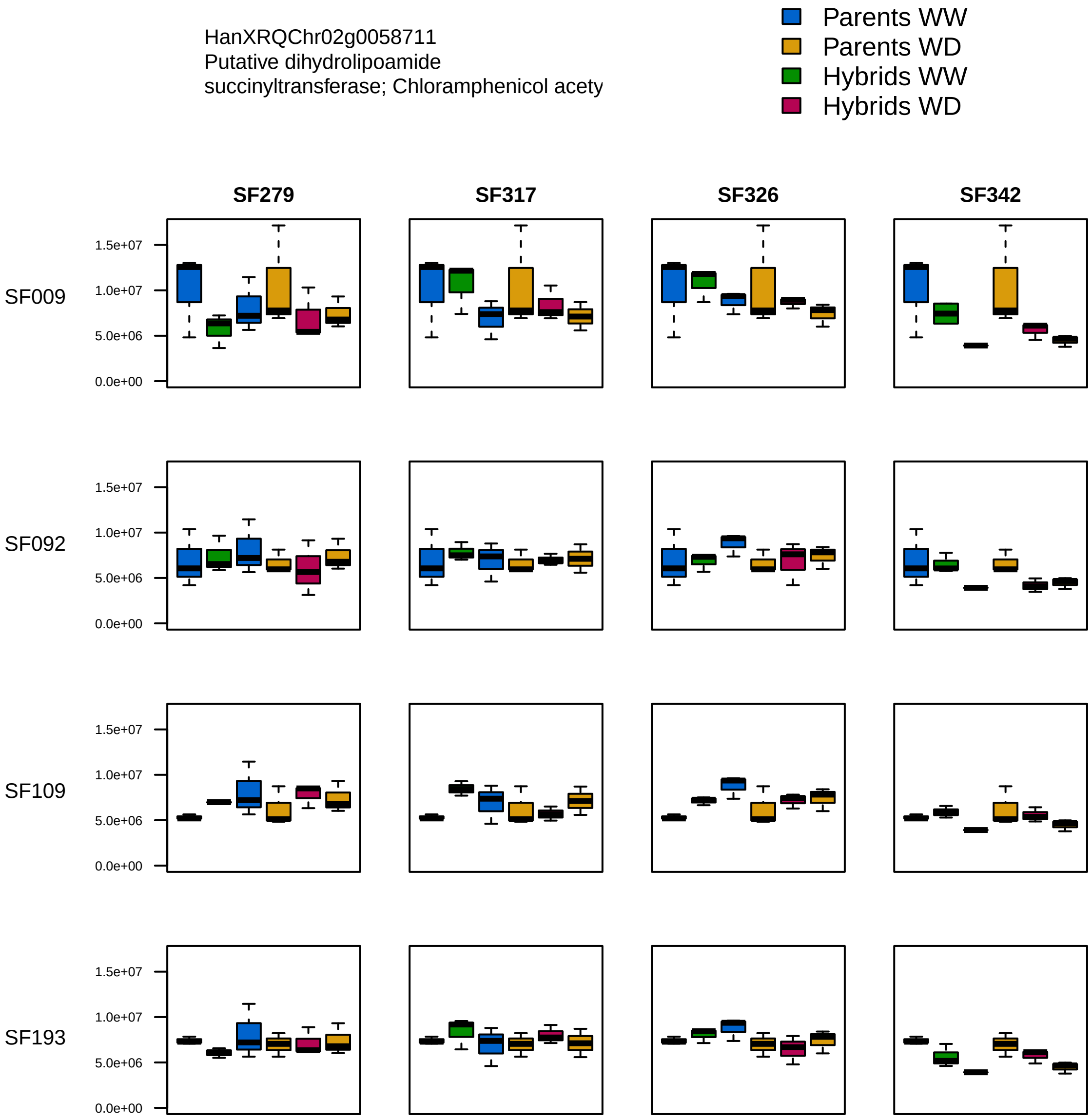
HanXRQChr02g0058711
Putative dihydrolipoamide
succinyltransferase; Chloramphenicol acety
Parents WW
Parents WD
Hybrids WW
Hybrids WD
SF279
SF317
SF326
SF342
SF009
SF092
SF109
SF193
1.5e+07
1.0e+07
5.0e+06
0.0e+00

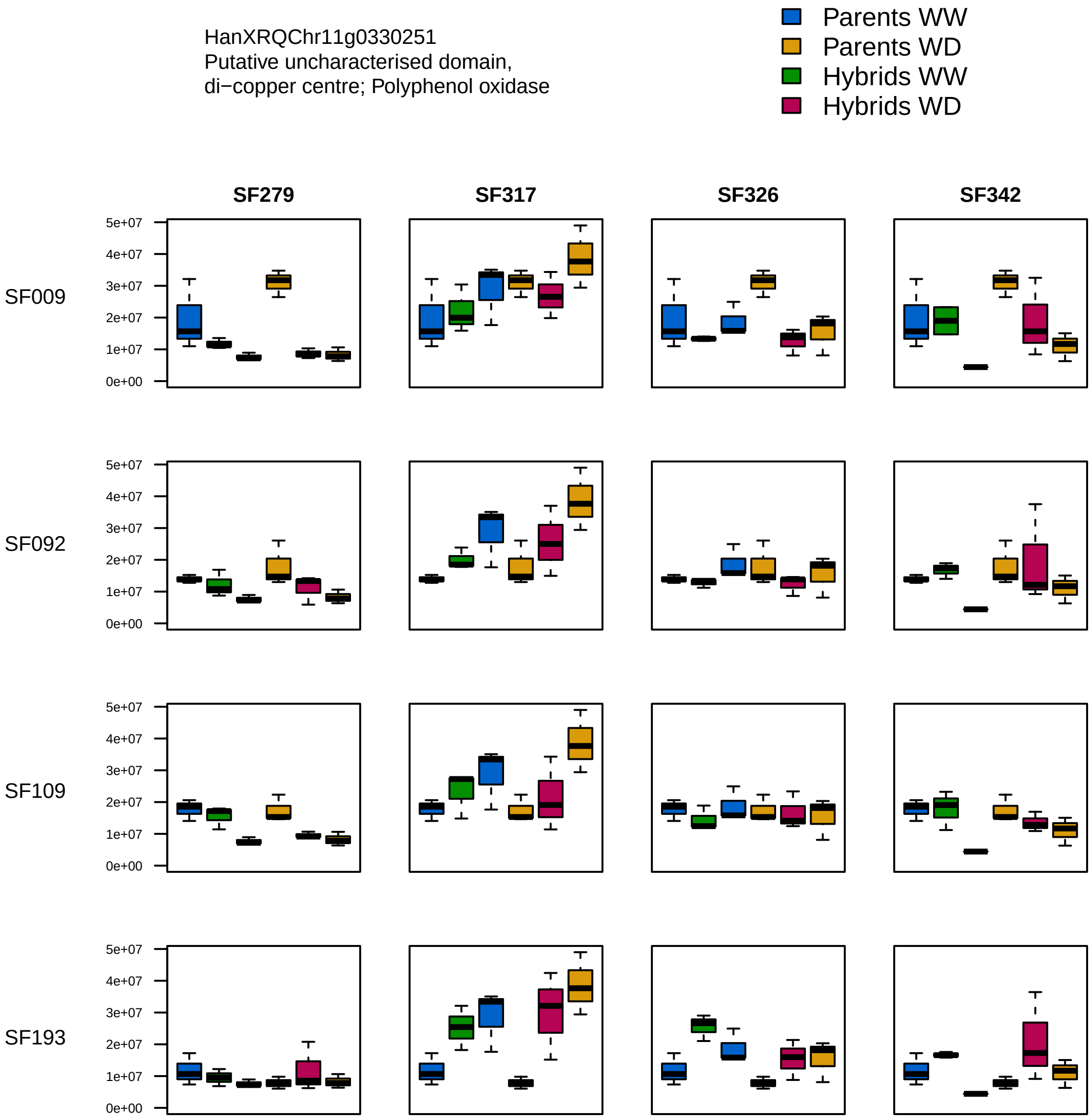
HanXRQChr11g0330251
Putative uncharacterised domain,
di−copper centre; Polyphenol oxidase
Parents WW
Parents WD
Hybrids WW
Hybrids WD
SF279
SF317
SF326
SF342
SF009
SF092
SF109
SF193
0e+00
1e+07
2e+07
3e+07
4e+07
5e+07

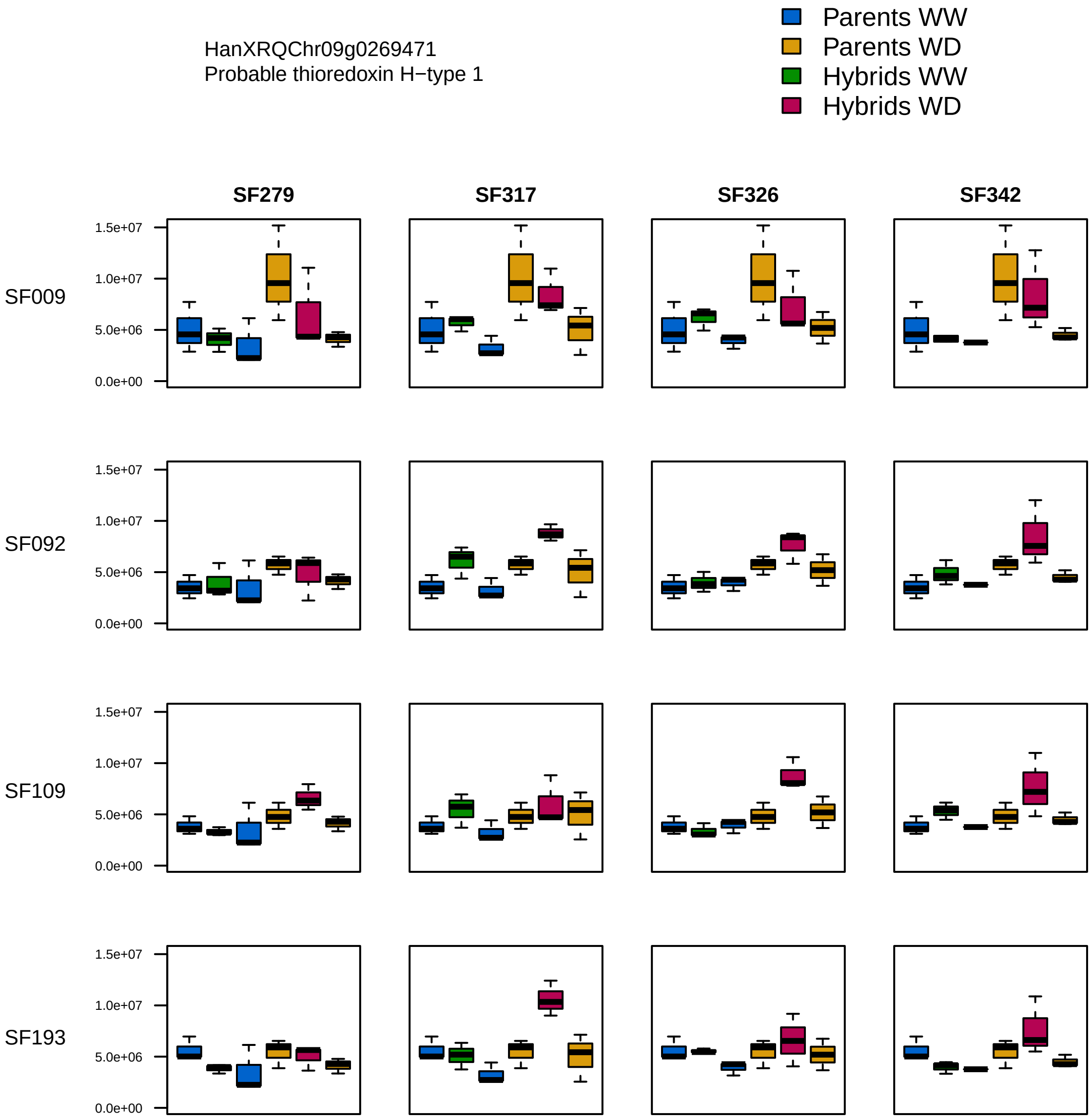
HanXRQChr09g0269471
Probable thioredoxin H−type 1
Parents WW
Parents WD
Hybrids WW
Hybrids WD
SF279
SF317
SF326
SF342
SF009
SF092
SF109
SF193
1.5e+07
1.0e+07
5.0e+06
0.0e+00

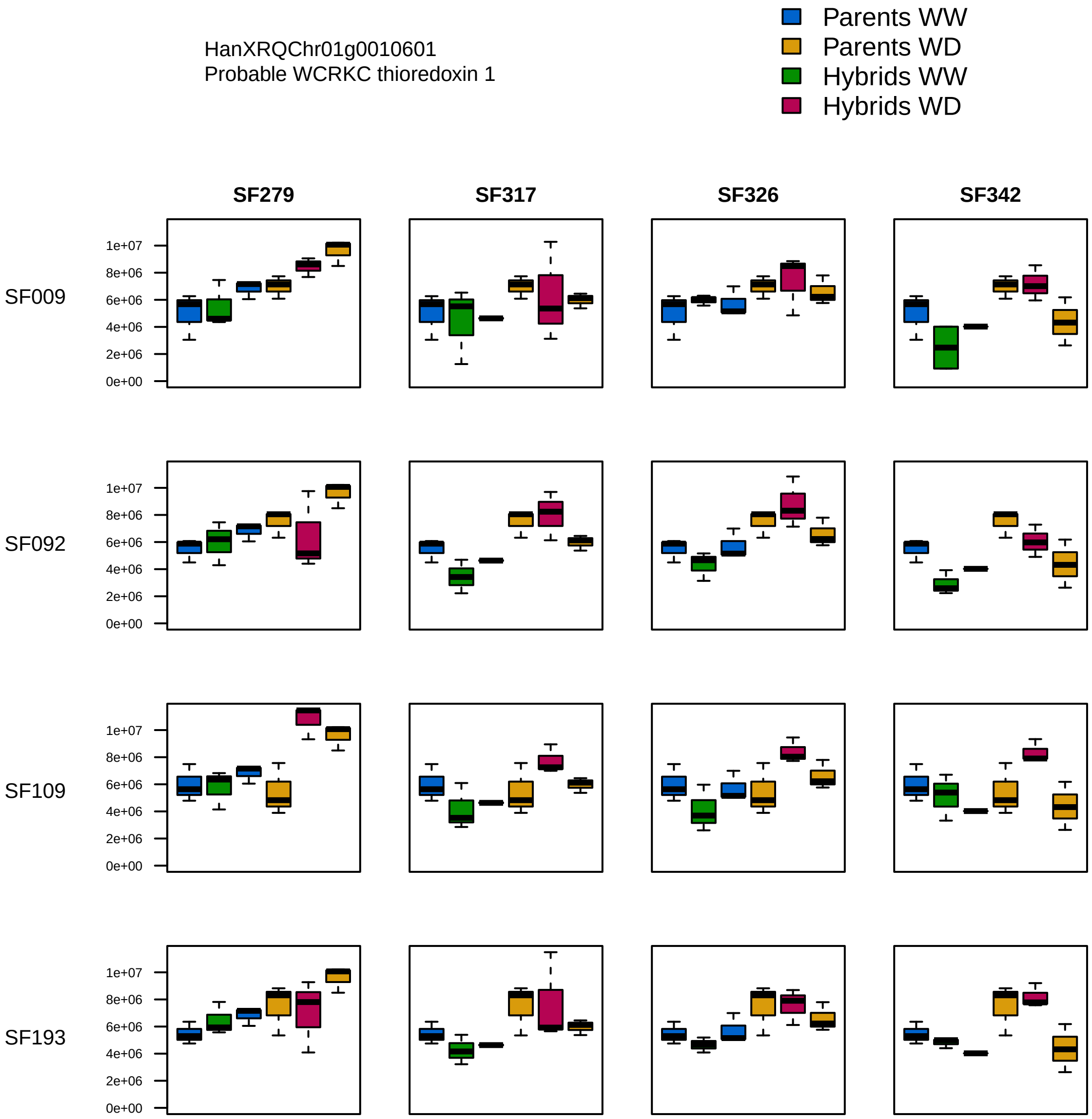
HanXRQChr01g0010601
Probable WCRKC thioredoxin 1
Parents WW
Parents WD
Hybrids WW
Hybrids WD
SF279
SF317
SF326
SF342
SF009
SF092
SF109
SF193
0e+00
2e+06
4e+06
6e+06
8e+06
1e+07

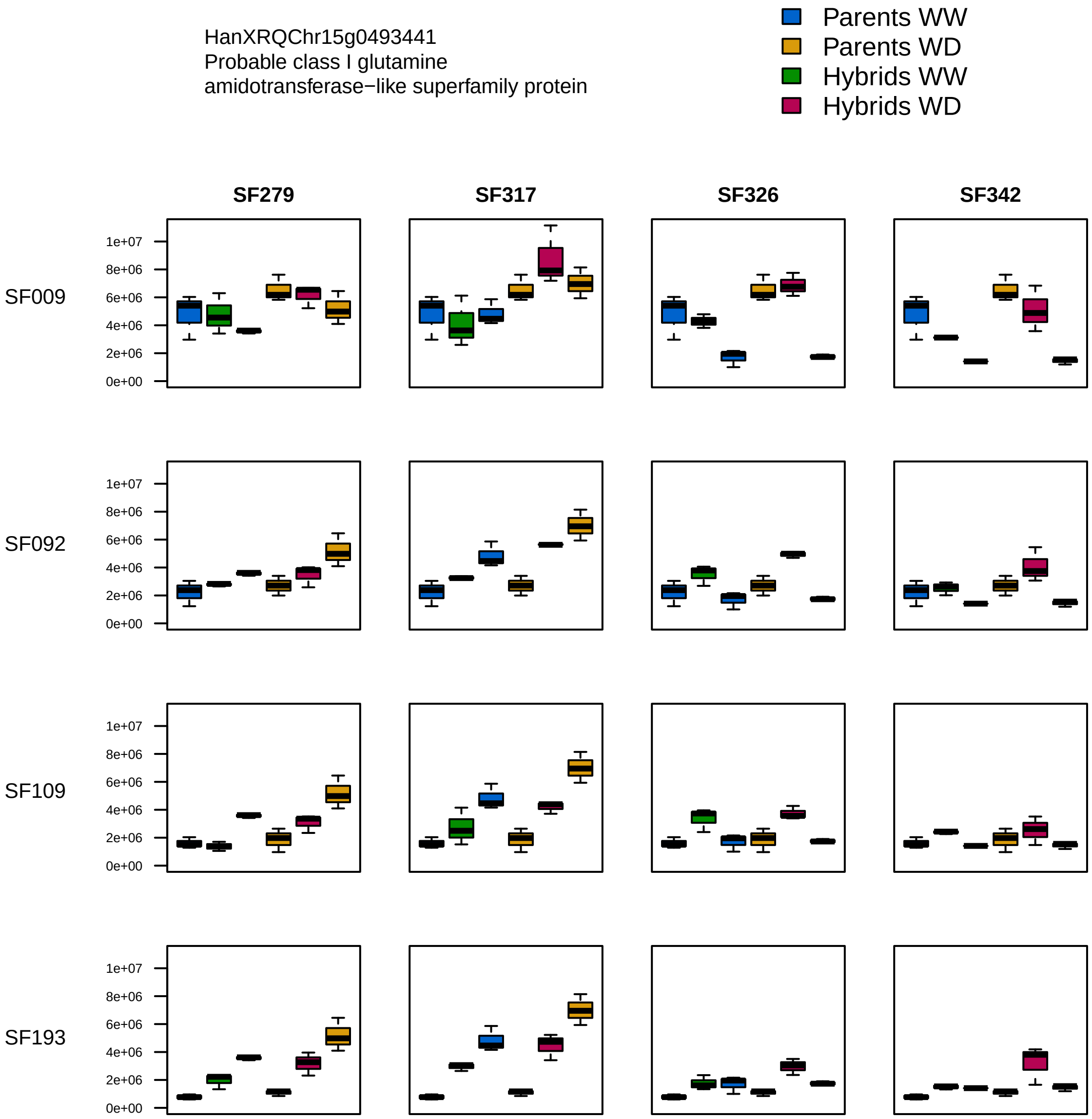
HanXRQChr15g0493441
Probable class I glutamine
amidotransferase−like superfamily protein
Parents WW
Parents WD
Hybrids WW
Hybrids WD
SF279
SF317
SF326
SF342
SF009
SF092
SF109
SF193
1e+07
8e+06
6e+06
4e+06
2e+06
0e+00

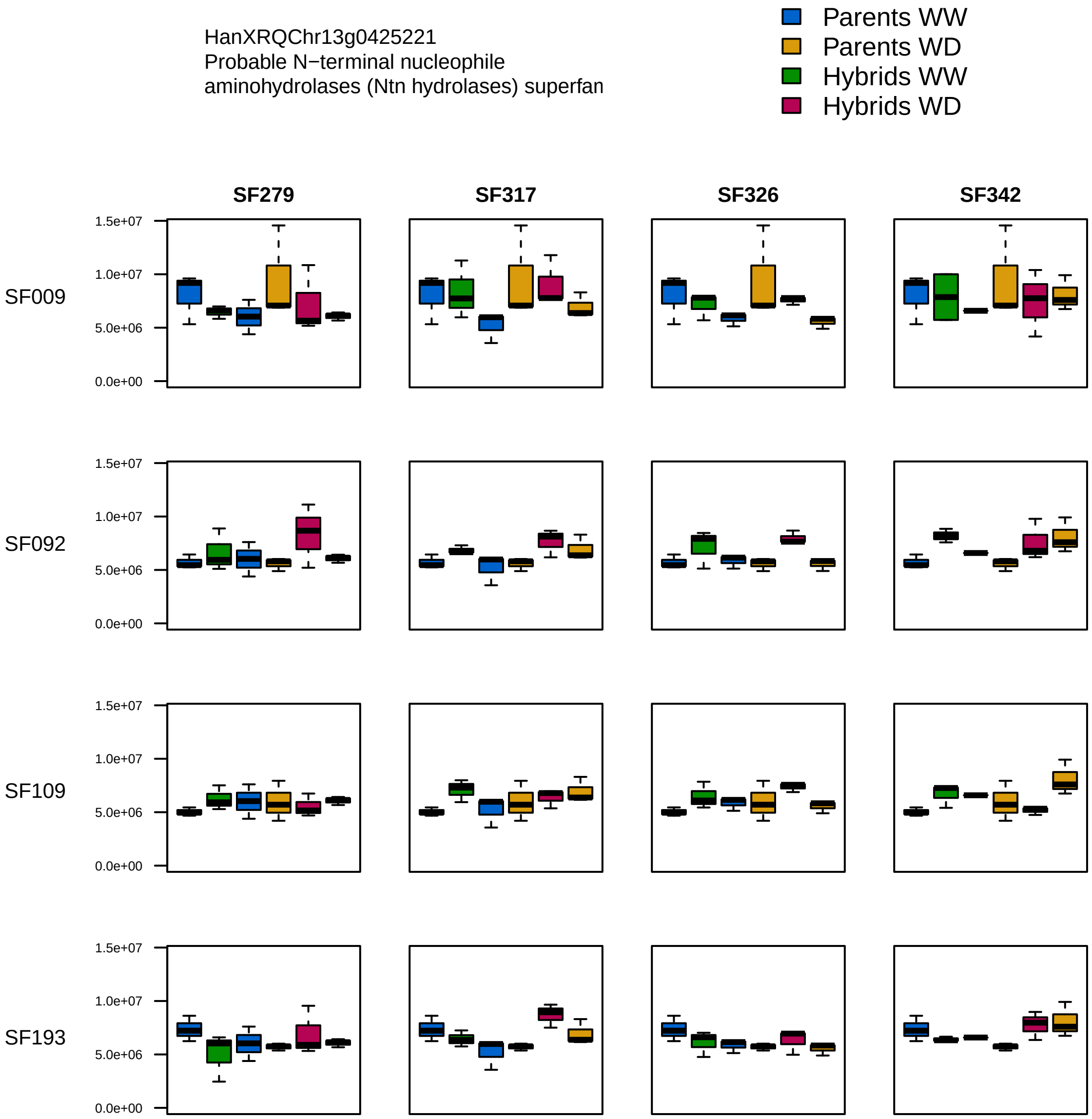
HanXRQChr13g0425221
Probable N−terminal nucleophile
aminohydrolases (Ntn hydrolases) superfan
Parents WW
Parents WD
Hybrids WW
Hybrids WD
SF279
SF317
SF326
SF342
SF009
SF092
SF109
SF193
1.5e+07
1.0e+07
5.0e+06
0.0e+00

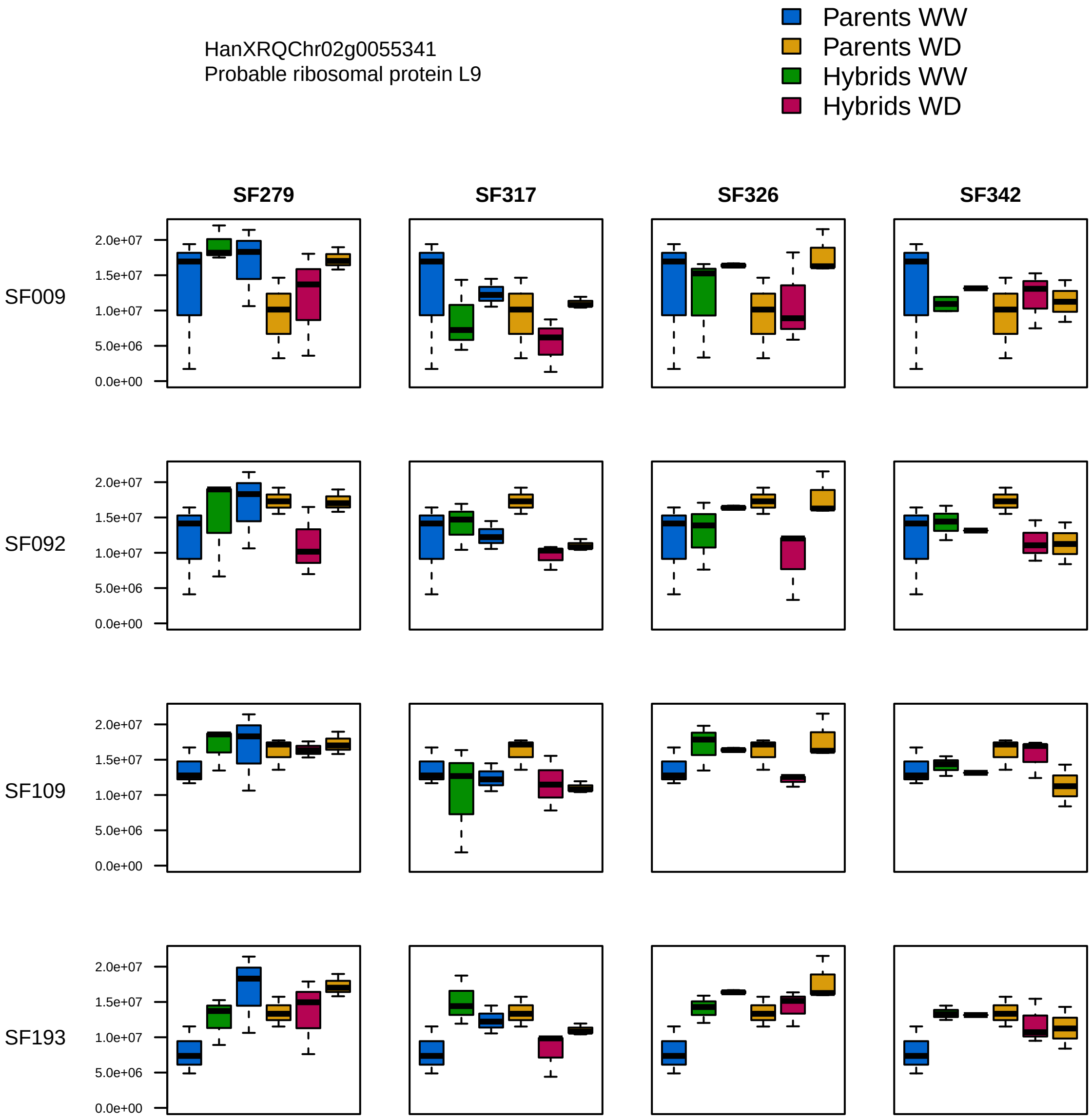
HanXRQChr02g0055341
Probable ribosomal protein L9
Parents WW
Parents WD
Hybrids WW
Hybrids WD
SF279
SF317
SF326
SF342
SF009
SF092
SF109
SF193
2.0e+07
1.5e+07
1.0e+07
5.0e+06
0.0e+00

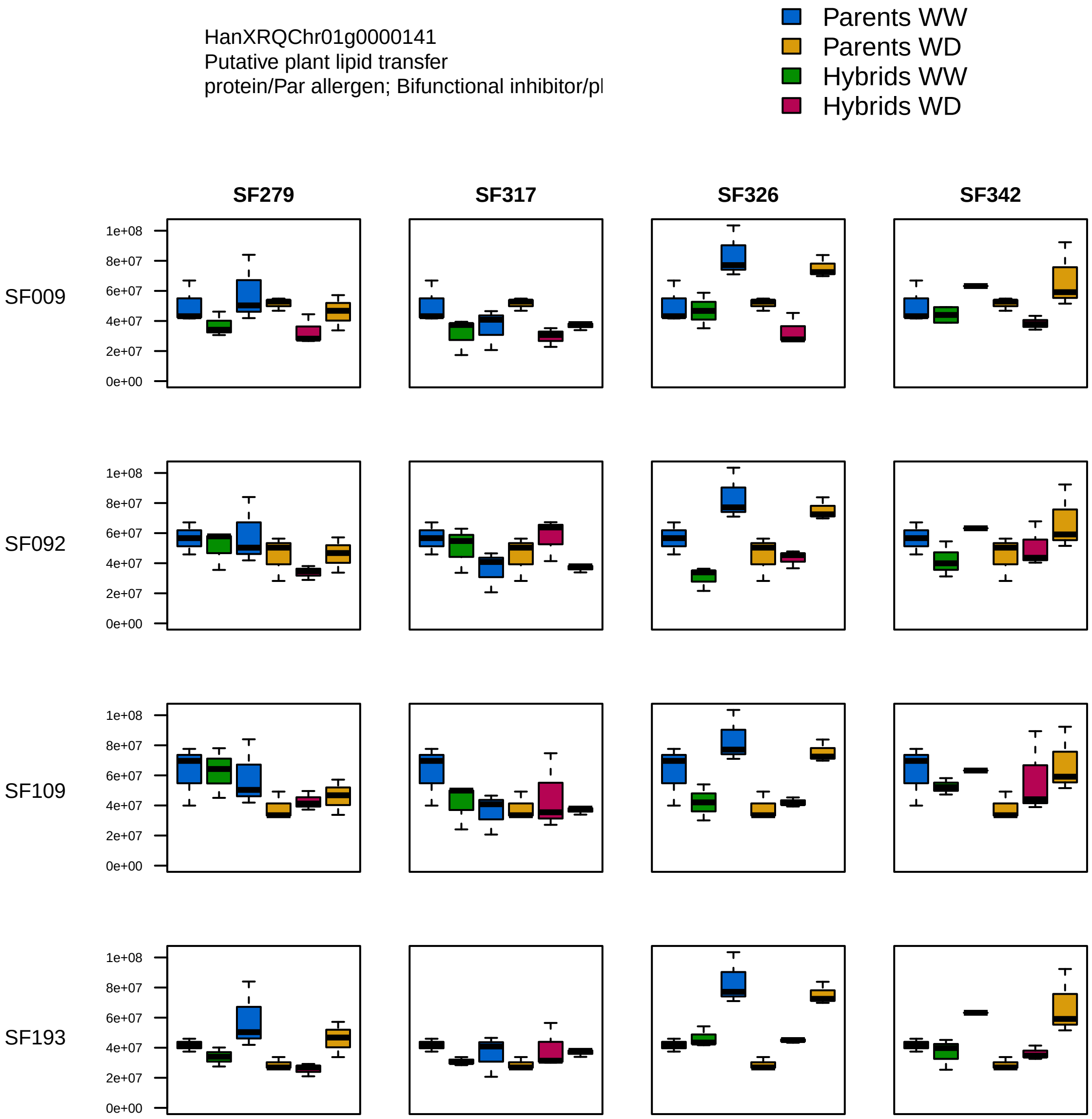
HanXRQChr01g0000141
Putative plant lipid transfer
protein/Par allergen; Bifunctional inhibitor/pl
Parents WW
Parents WD
Hybrids WW
Hybrids WD
SF279
SF317
SF326
SF342
SF009
SF092
SF109
SF193
1e+08
8e+07
6e+07
4e+07
2e+07
0e+00

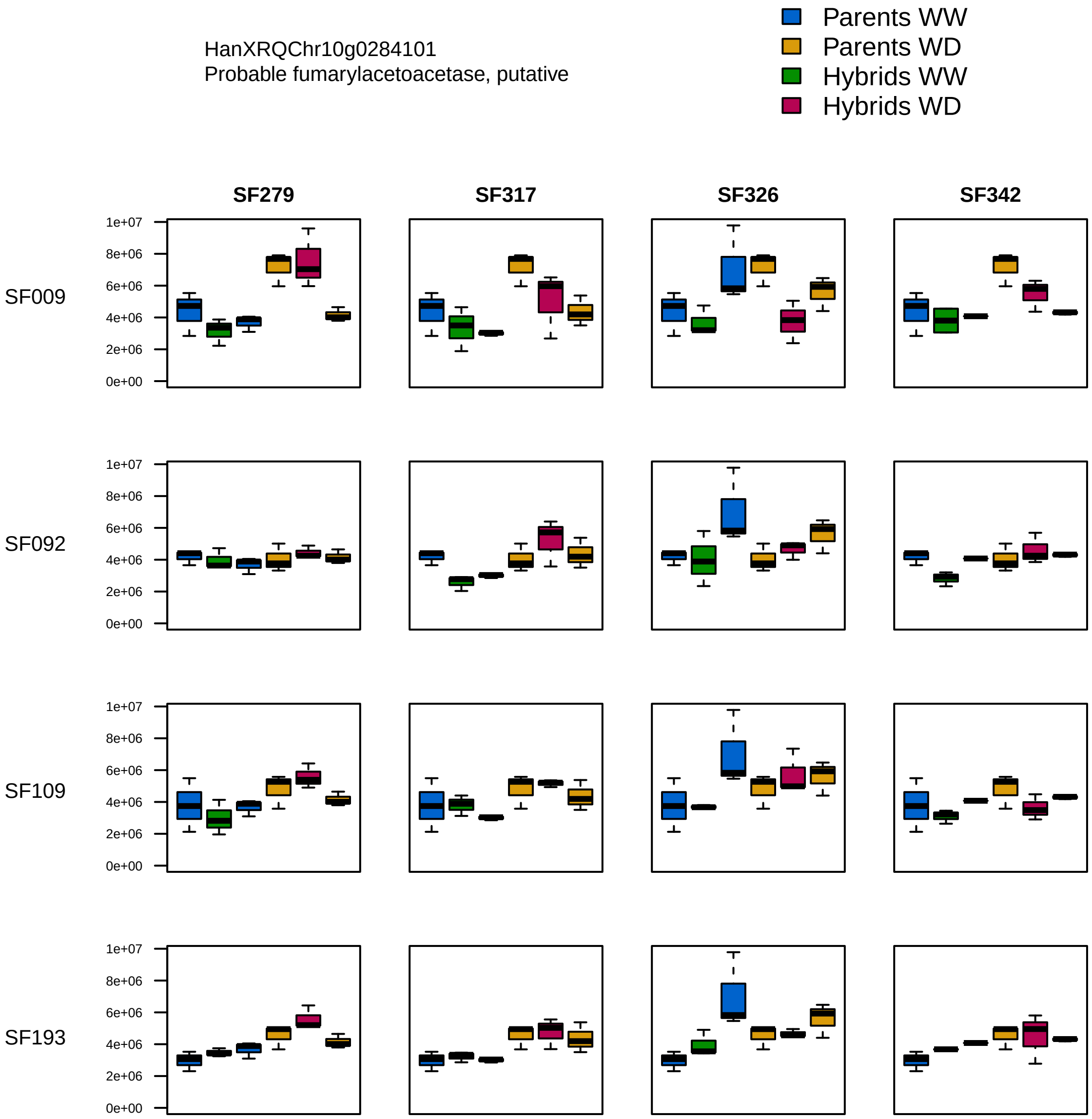
HanXRQChr10g0284101
Probable fumarylacetoacetase, putative
Parents WW
Parents WD
Hybrids WW
Hybrids WD
SF279
SF317
SF326
SF342
SF009
SF092
SF109
SF193
1e+07
8e+06
6e+06
4e+06
2e+06
0e+00

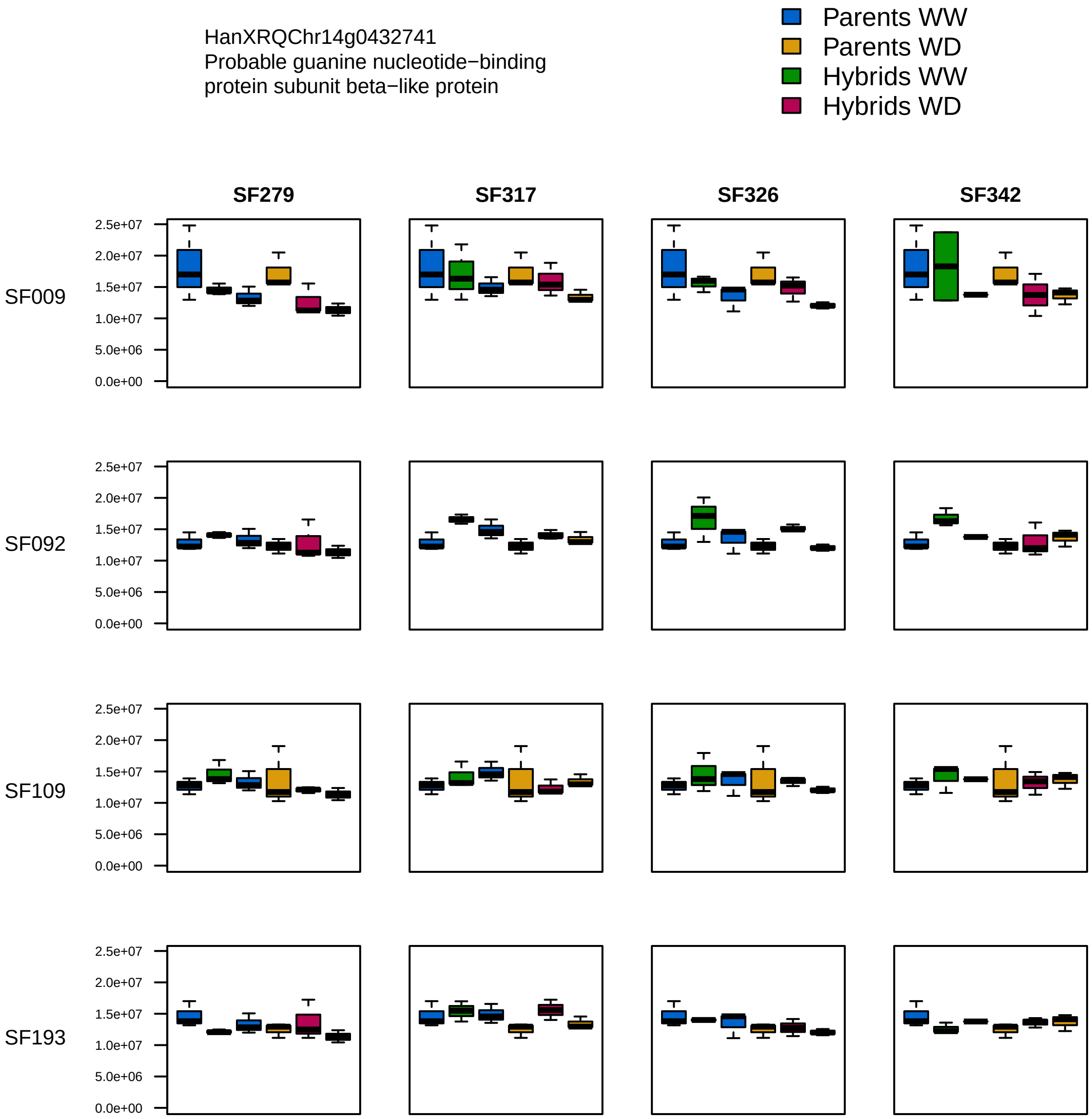
HanXRQChr14g0432741
Probable guanine nucleotide−binding
protein subunit beta−like protein
Parents WW
Parents WD
Hybrids WW
Hybrids WD
SF279
SF317
SF326
SF342
SF009
SF092
SF109
SF193
2.5e+07
2.0e+07
1.5e+07
1.0e+07
5.0e+06
0.0e+00

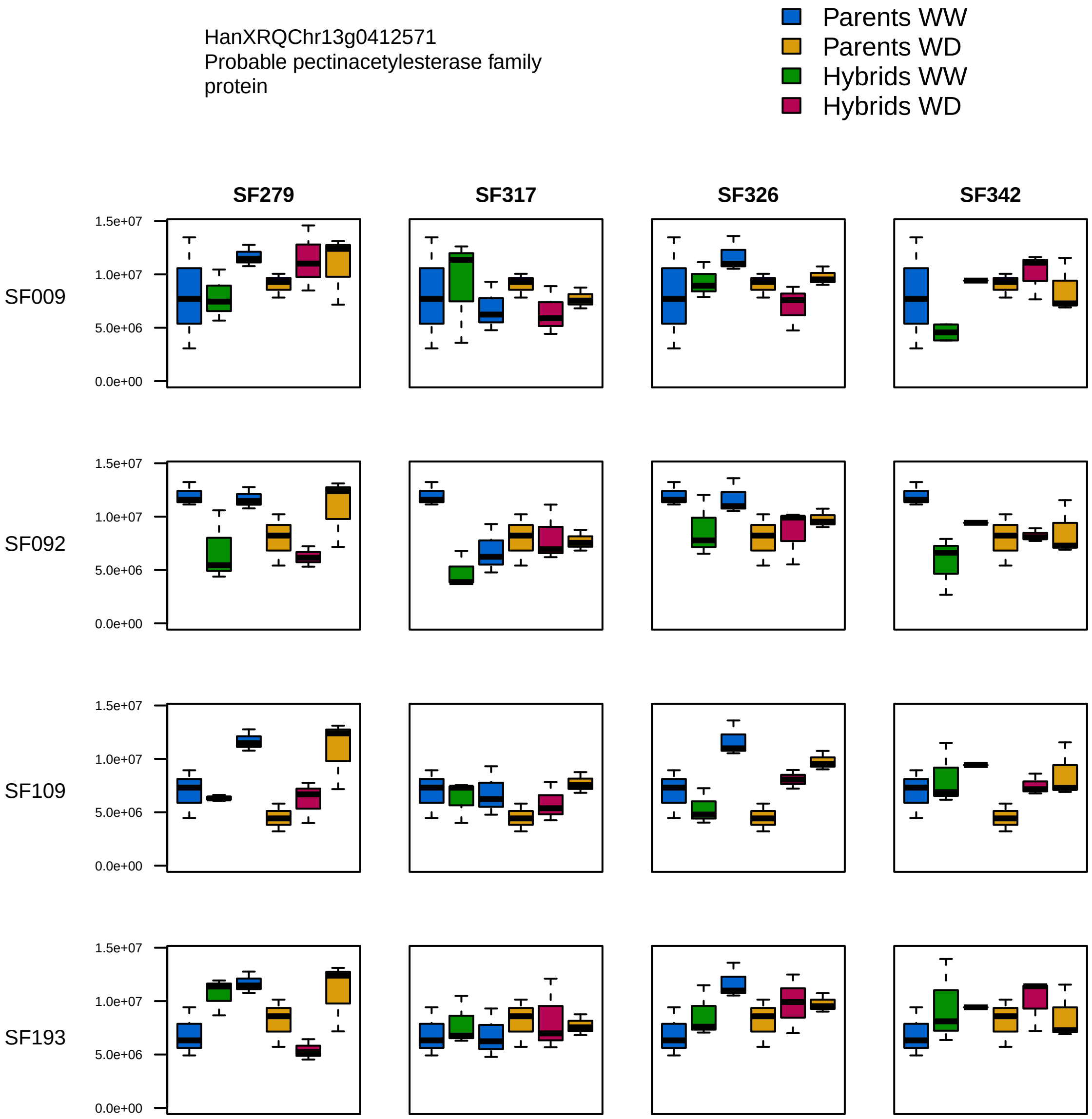

HanXRQChr13g0412571
Probable pectinacetylesterase family
protein
Parents WW
Parents WD
Hybrids WW
Hybrids WD
SF279
SF317
SF326
SF342
SF009
SF092
SF109
SF193
1.5e+07
1.0e+07
5.0e+06
0.0e+00

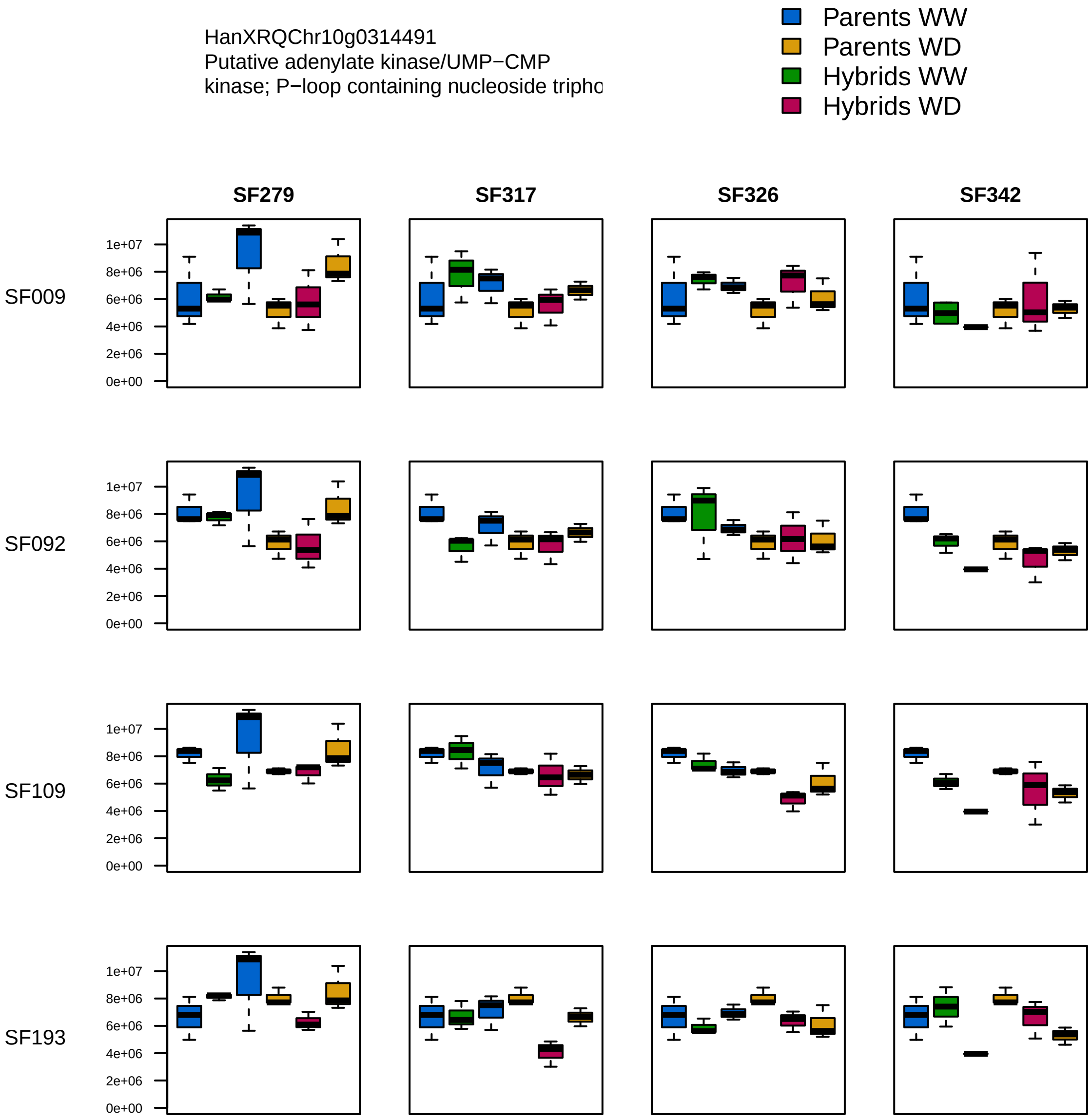
HanXRQChr10g0314491
Putative adenylate kinase/UMP−CMP
kinase; P−loop containing nucleoside tripho
Parents WW
Parents WD
Hybrids WW
Hybrids WD
SF279
SF317
SF326
SF342
SF009
SF092
SF109
SF193
1e+07
8e+06
6e+06
4e+06
2e+06
0e+00

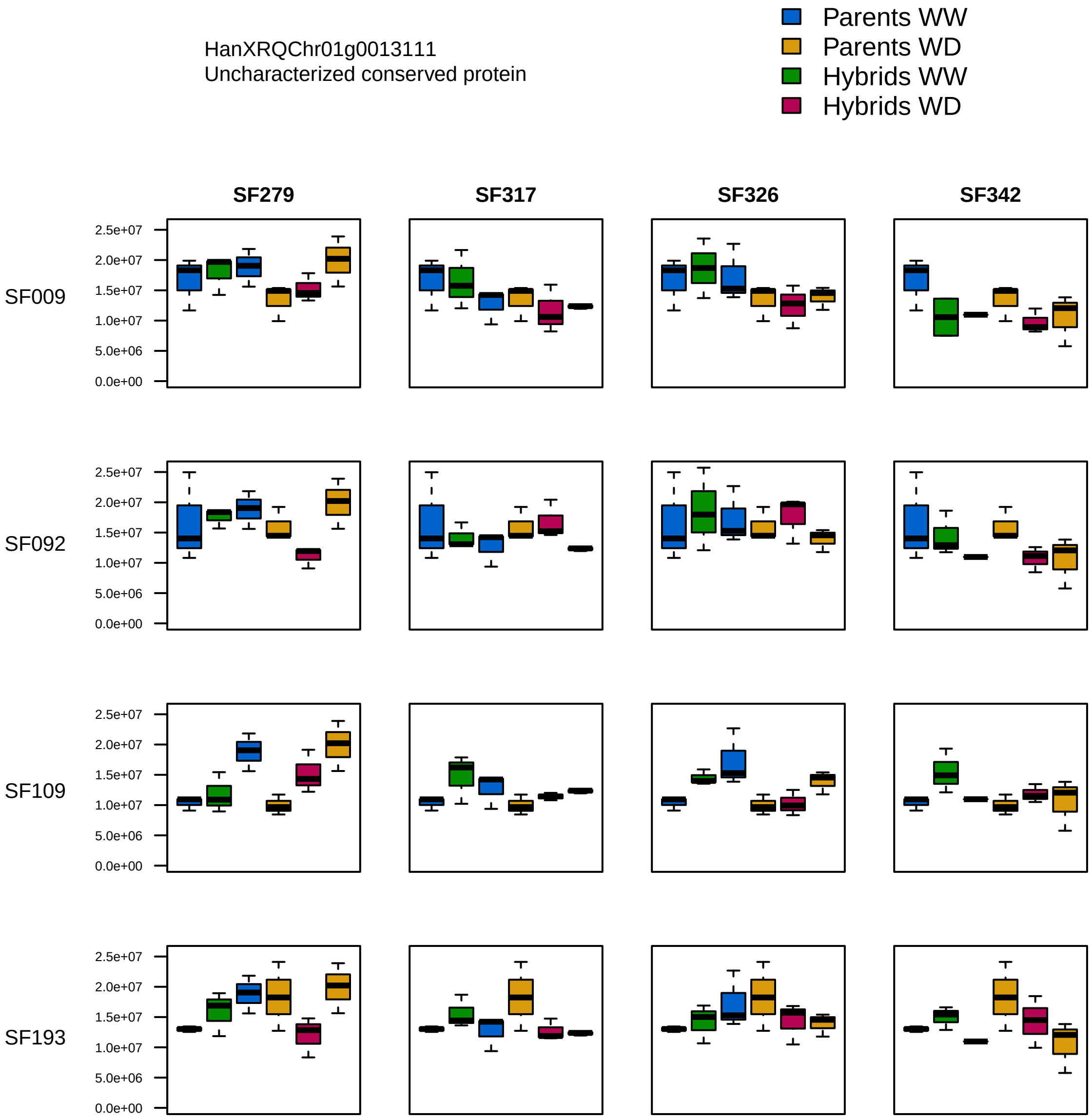
HanXRQChr01g0013111
Uncharacterized conserved protein
Parents WW
Parents WD
Hybrids WW
Hybrids WD
SF279
SF317
SF326
SF342
SF009
SF092
SF109
SF193
2.5e+07
2.0e+07
1.5e+07
1.0e+07
5.0e+06
0.0e+00

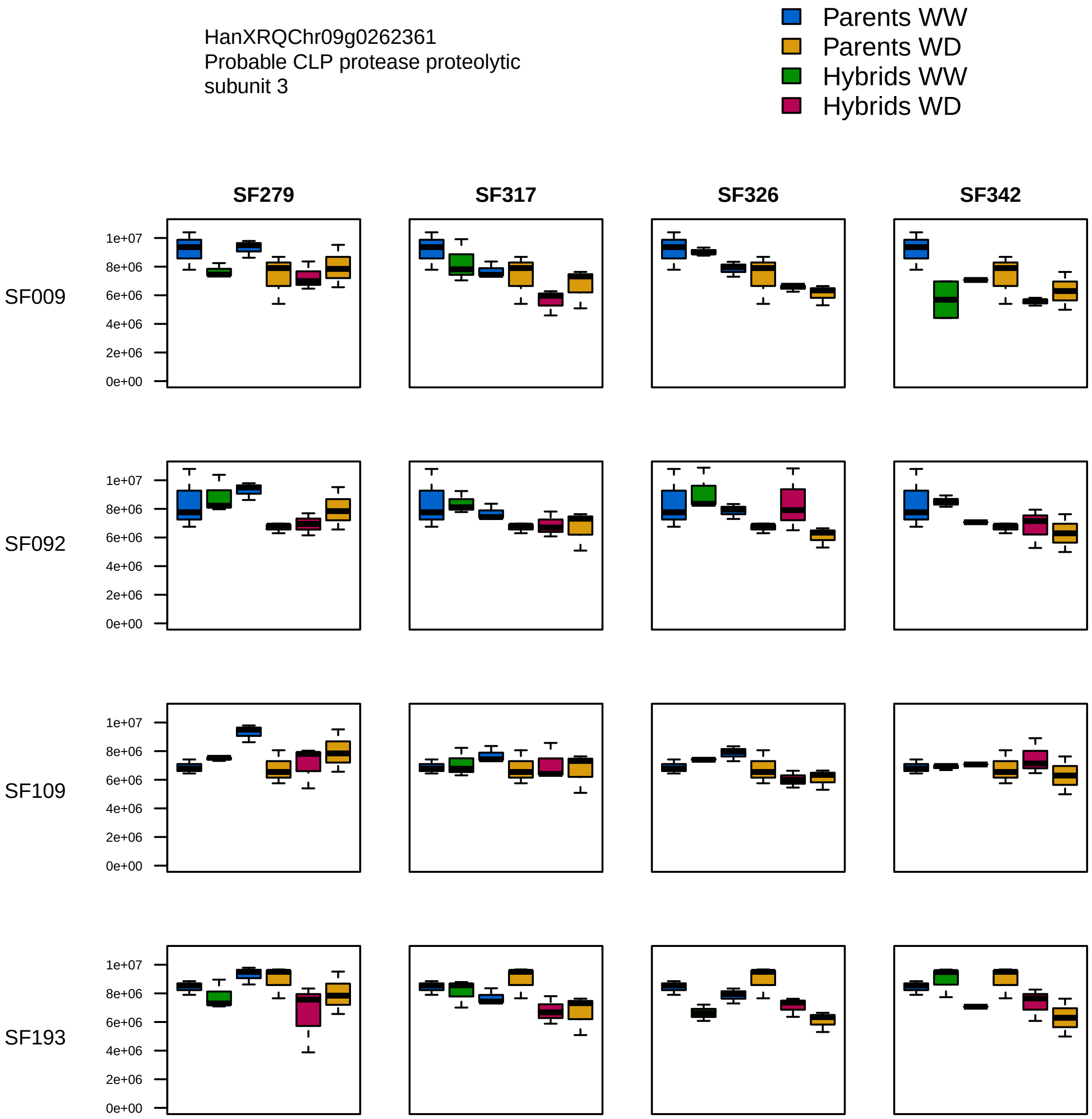

HanXRQChr09g0262361
Probable CLP protease proteolytic
subunit 3
Parents WW
Parents WD
Hybrids WW
Hybrids WD
SF279
SF317
SF326
SF342
SF009
SF092
SF109
SF193
1e+07
8e+06
6e+06
4e+06
2e+06
0e+00

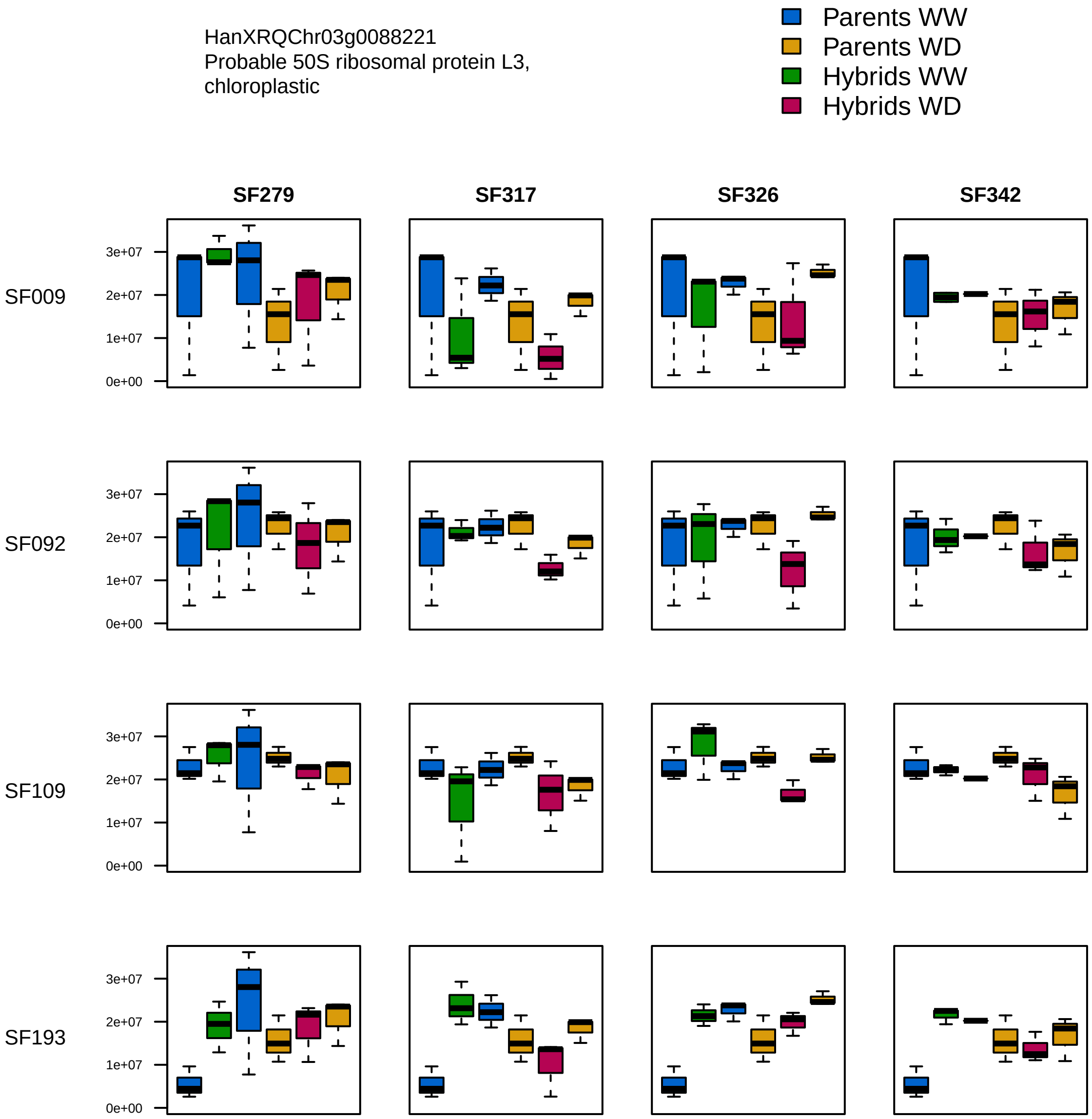
HanXRQChr03g0088221
Probable 50S ribosomal protein L3,
chloroplastic
Parents WW
Parents WD
Hybrids WW
Hybrids WD
SF279
SF317
SF326
SF342
SF009
SF092
SF109
SF193
0e+00
1e+07
2e+07
3e+07

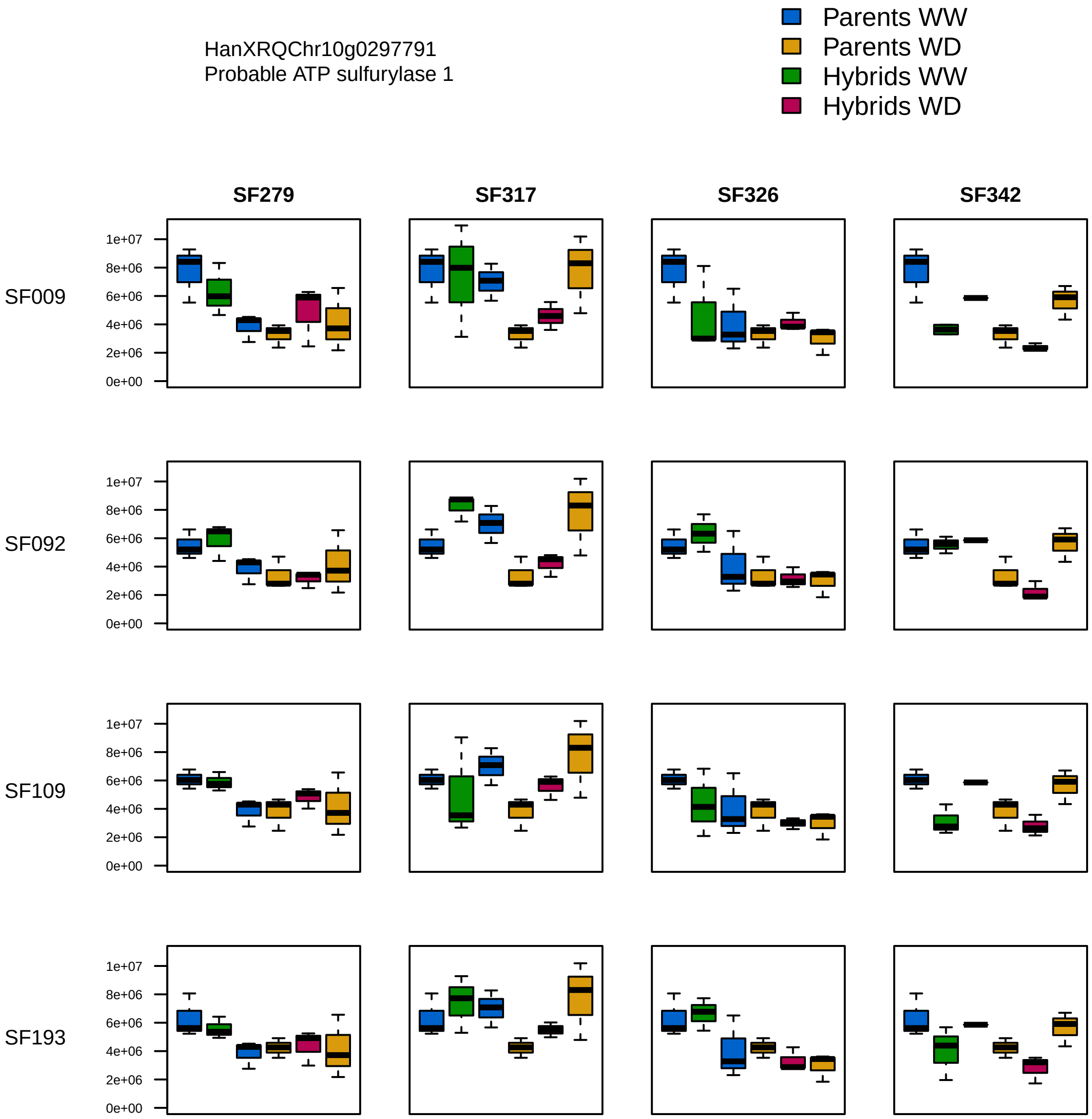
HanXRQChr10g0297791
Probable ATP sulfurylase 1
Parents WW
Parents WD
Hybrids WW
Hybrids WD
SF279
SF317
SF326
SF342
SF009
SF092
SF109
SF193
1e+07
8e+06
6e+06
4e+06
2e+06
0e+00

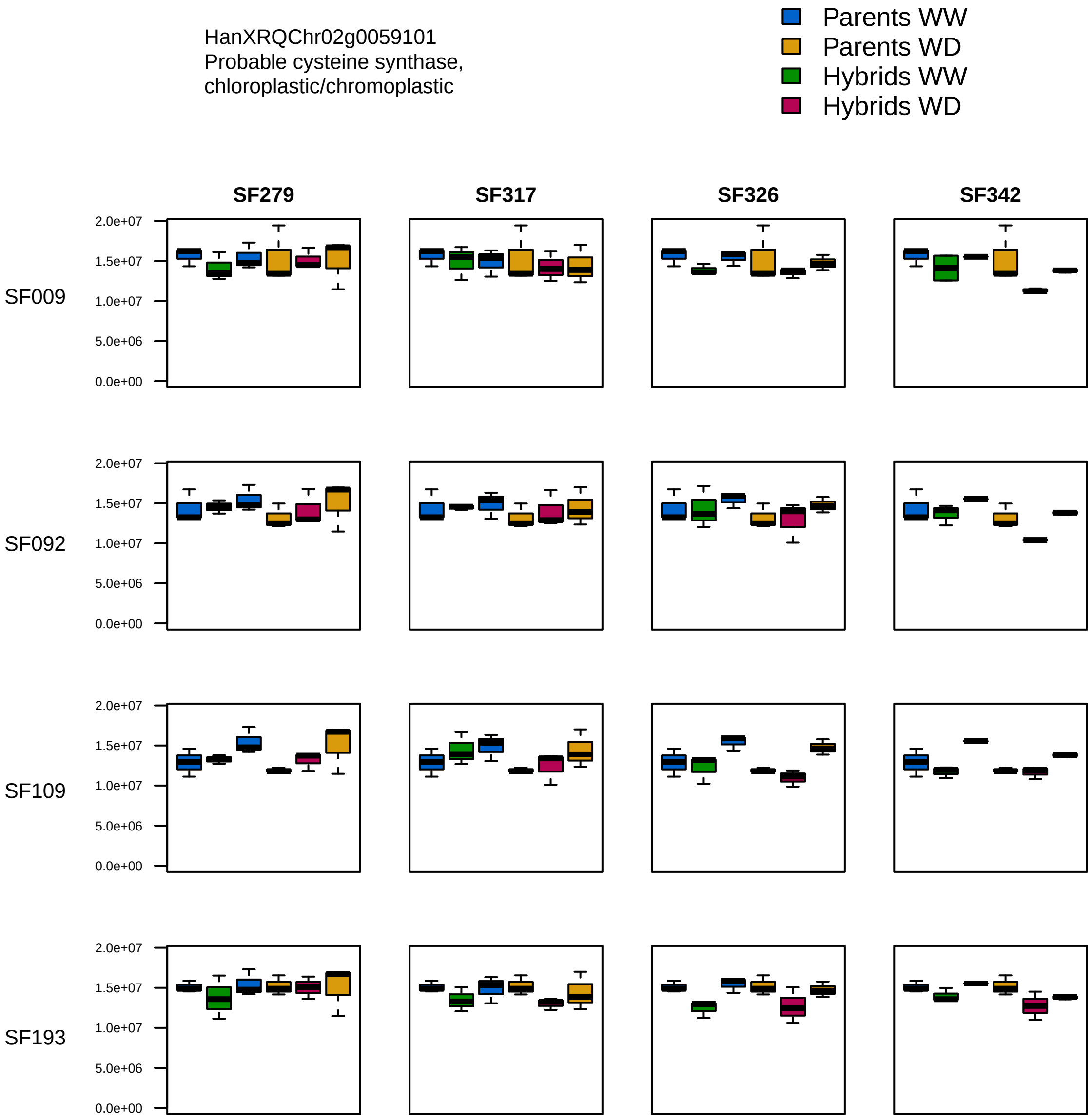

HanXRQChr02g0059101
Probable cysteine synthase, chloroplastic/chromoplastic
Parents WW
Parents WD
Hybrids WW
Hybrids WD
SF279
SF317
SF326
SF342
SF009
SF092
SF109
SF193
2.0e+07
1.5e+07
1.0e+07
5.0e+06
0.0e+00

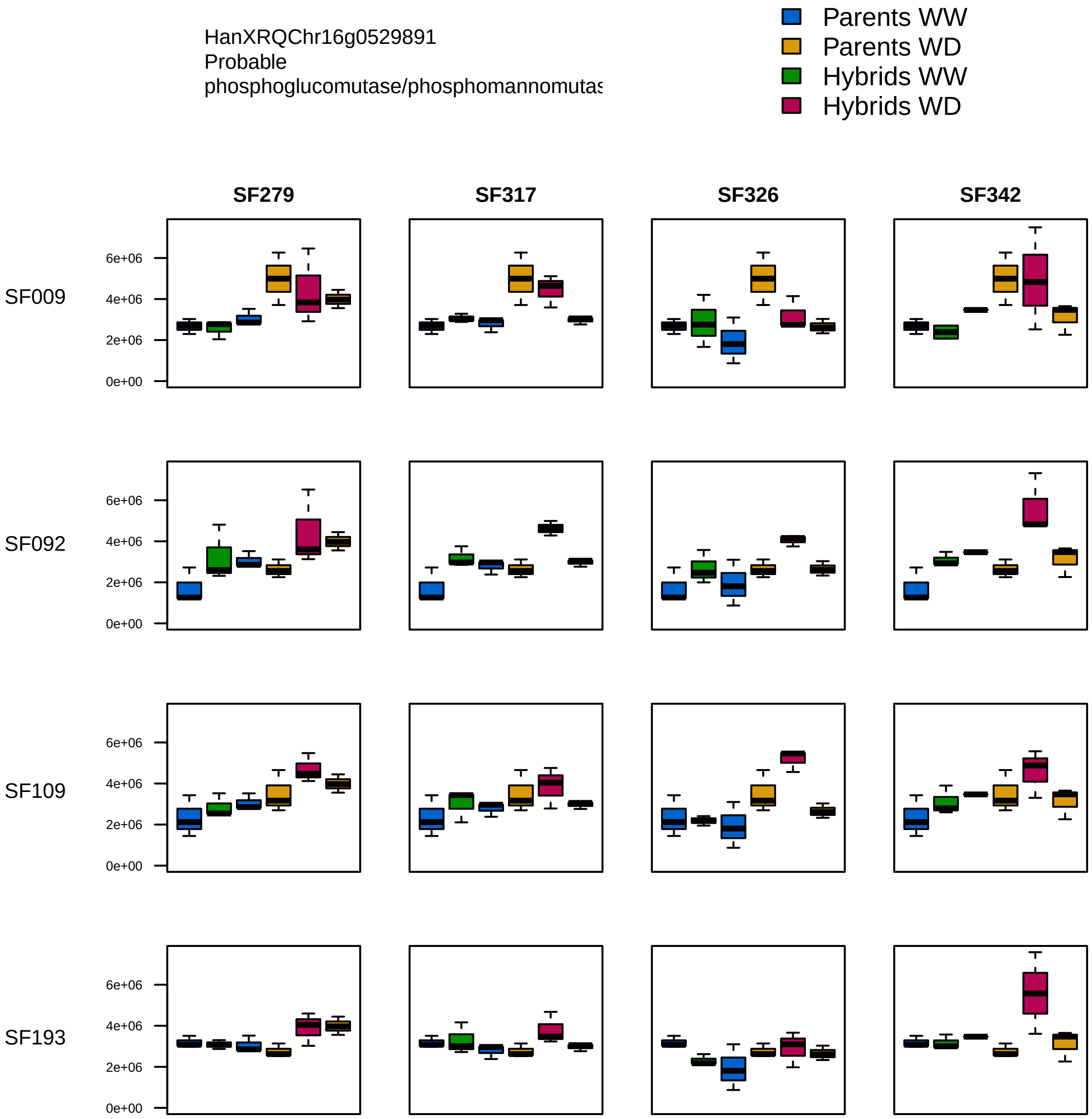

HanXRQChr16g0529891
Probable
phosphoglucomutase/phosphomannomutas
Parents WW
Parents WD
Hybrids WW
Hybrids WD
SF279
SF317
SF326
SF342
SF009
SF092
SF109
SF193
0e+00
2e+06
4e+06
6e+06

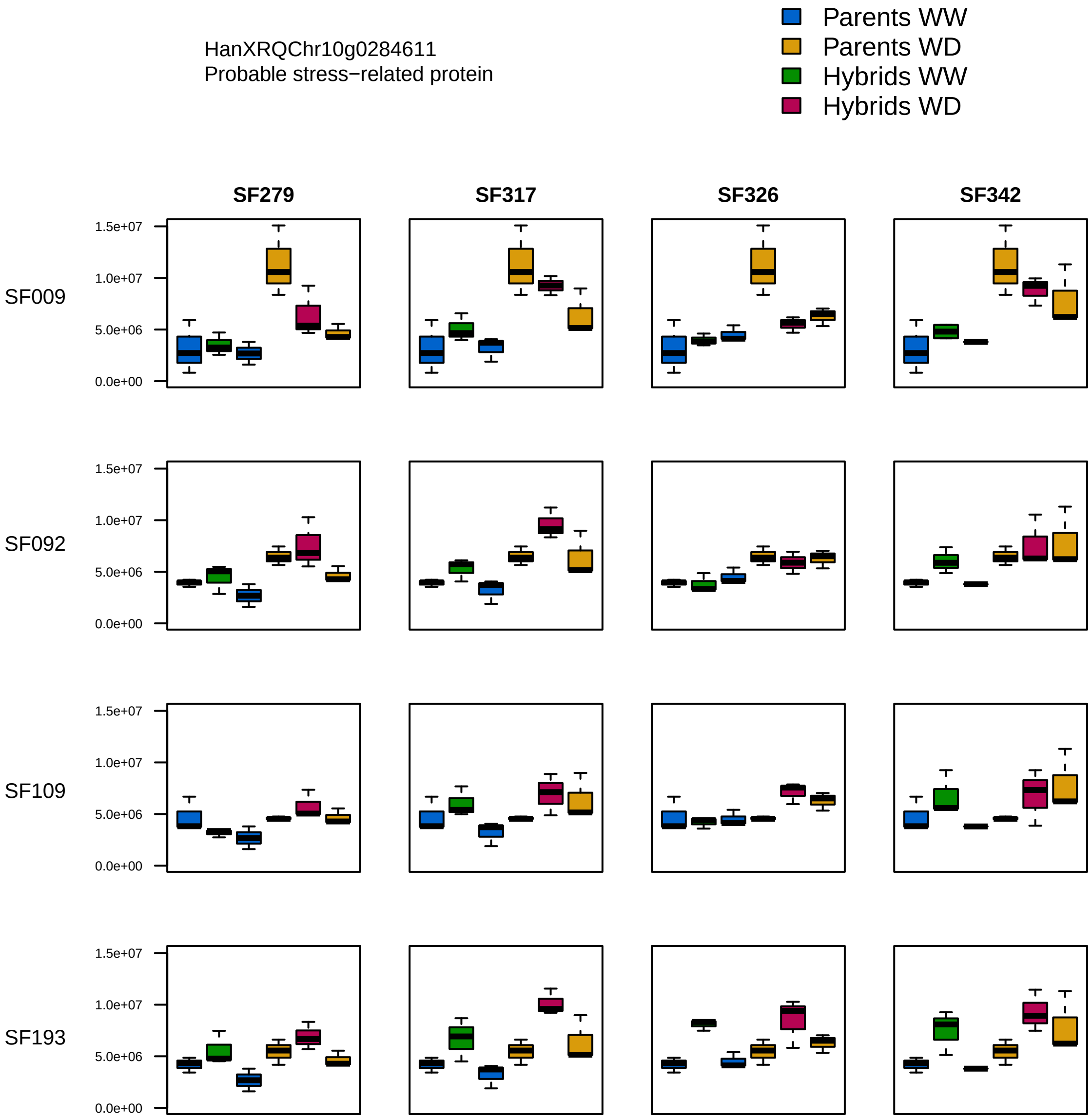

HanXRQChr10g0284611
Probable stress−related protein
Parents WW
Parents WD
Hybrids WW
Hybrids WD
SF279
SF317
SF326
SF342
SF009
SF092
SF109
SF193
1.5e+07
1.0e+07
5.0e+06
0.0e+00

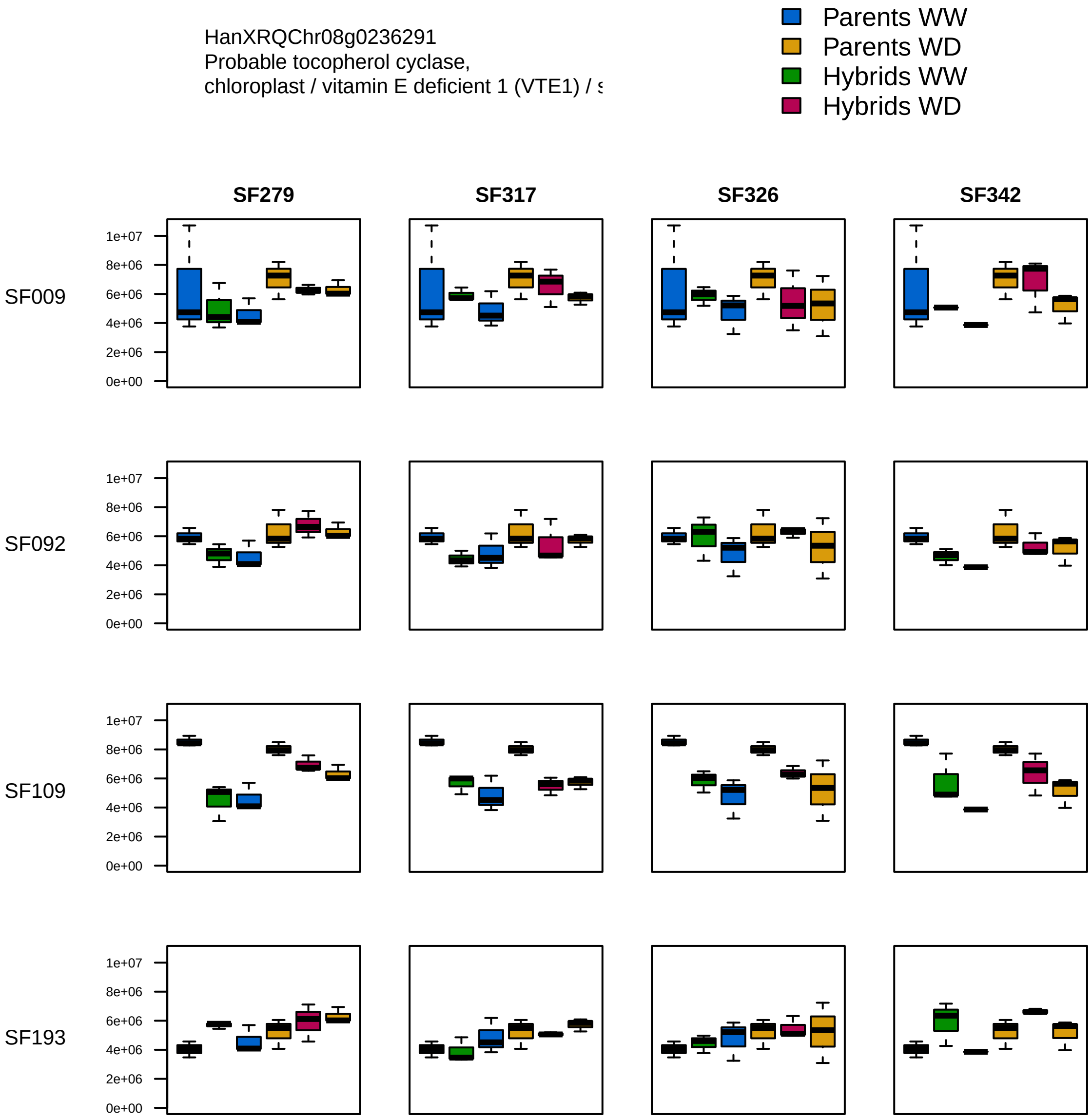
HanXRQChr08g0236291
Probable tocopherol cyclase,
chloroplast / vitamin E deficient 1 (VTE1) / s
Parents WW
Parents WD
Hybrids WW
Hybrids WD
SF279
SF317
SF326
SF342
SF009
SF092
SF109
SF193
1e+07
8e+06
6e+06
4e+06
2e+06
0e+00

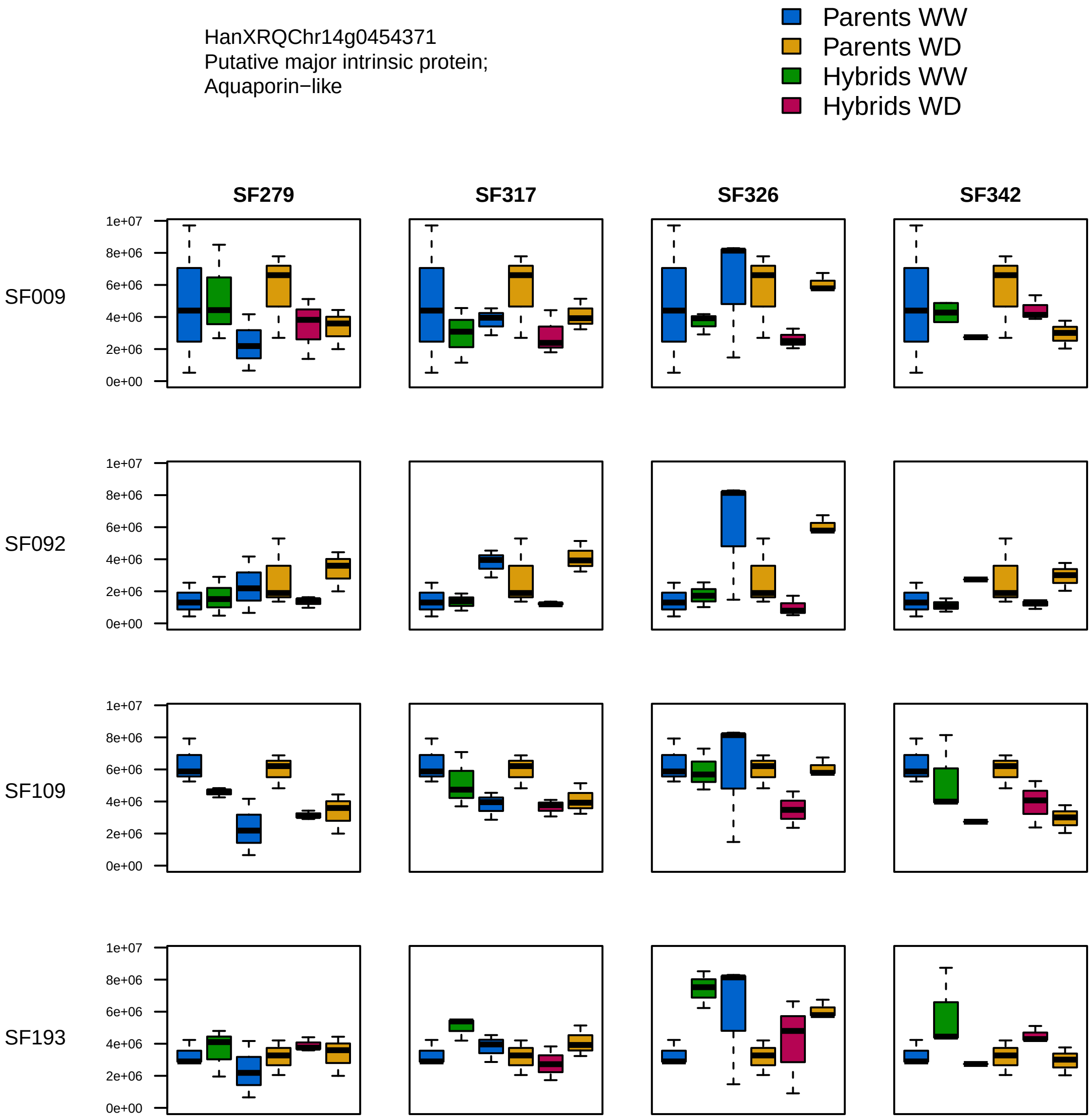
HanXRQChr14g0454371
Putative major intrinsic protein;
Aquaporin−like
Parents WW
Parents WD
Hybrids WW
Hybrids WD
SF279
SF317
SF326
SF342
SF009
SF092
SF109
SF193
1e+07
8e+06
6e+06
4e+06
2e+06
0e+00

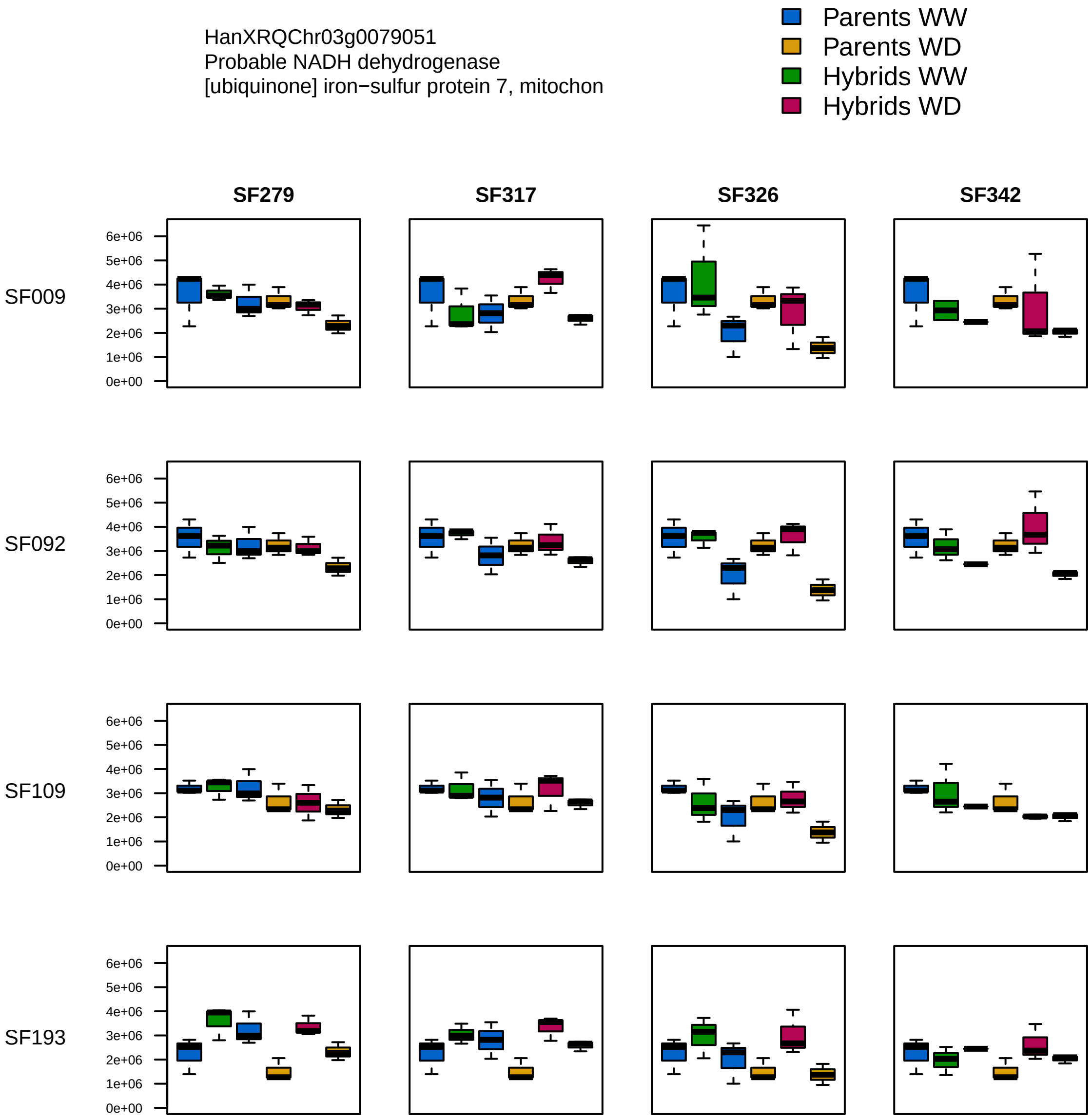
HanXRQChr03g0079051
Probable NADH dehydrogenase
[ubiquinone] iron−sulfur protein 7, mitochon
Parents WW
Parents WD
Hybrids WW
Hybrids WD
SF279
SF317
SF326
SF342
SF009
SF092
SF109
SF193
6e+06
5e+06
4e+06
3e+06
2e+06
1e+06
0e+00

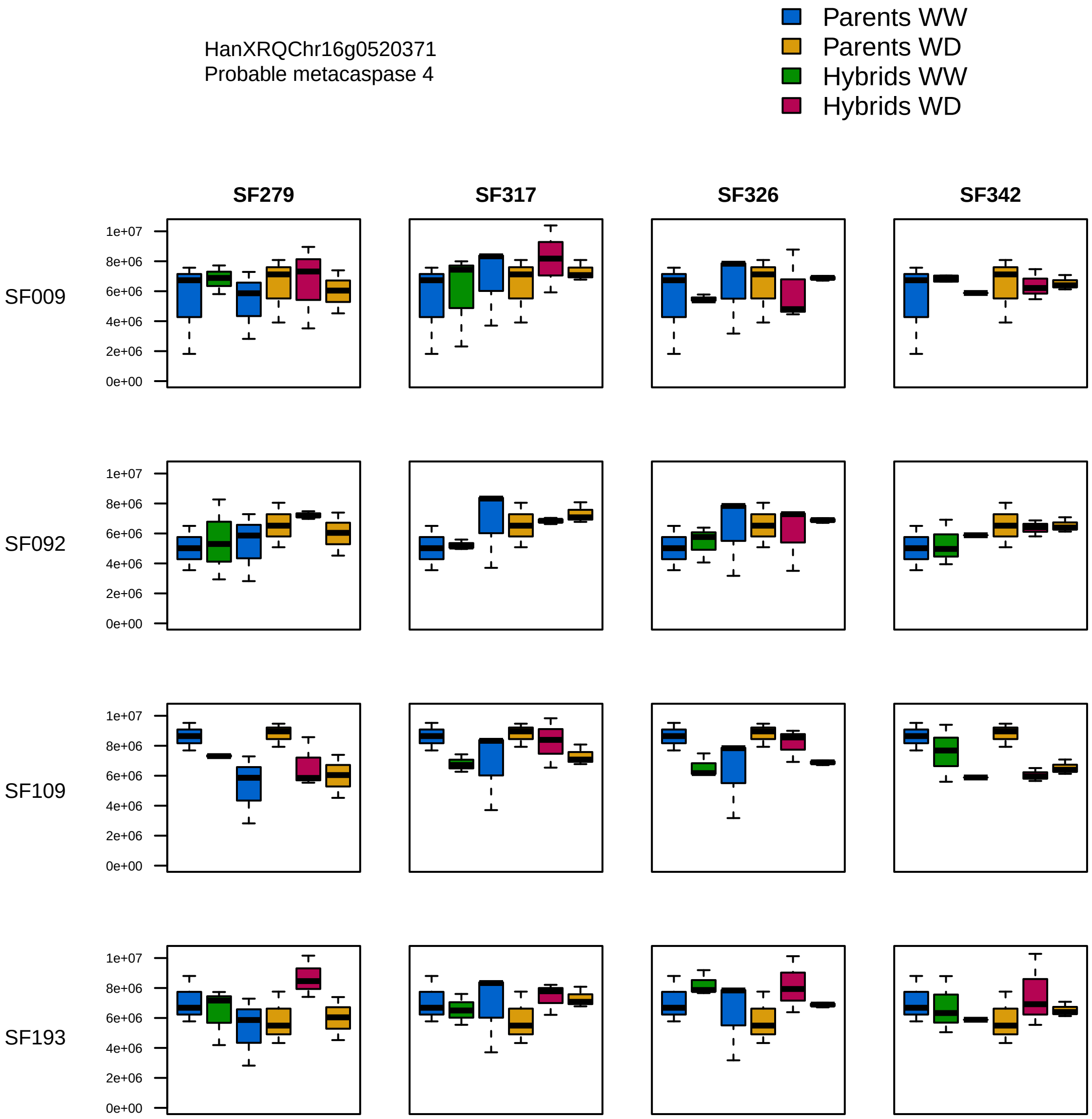
HanXRQChr16g0520371
Probable metacaspase 4
Parents WW
Parents WD
Hybrids WW
Hybrids WD
SF279
SF317
SF326
SF342
SF009
SF092
SF109
SF193
1e+07
8e+06
6e+06
4e+06
2e+06
0e+00

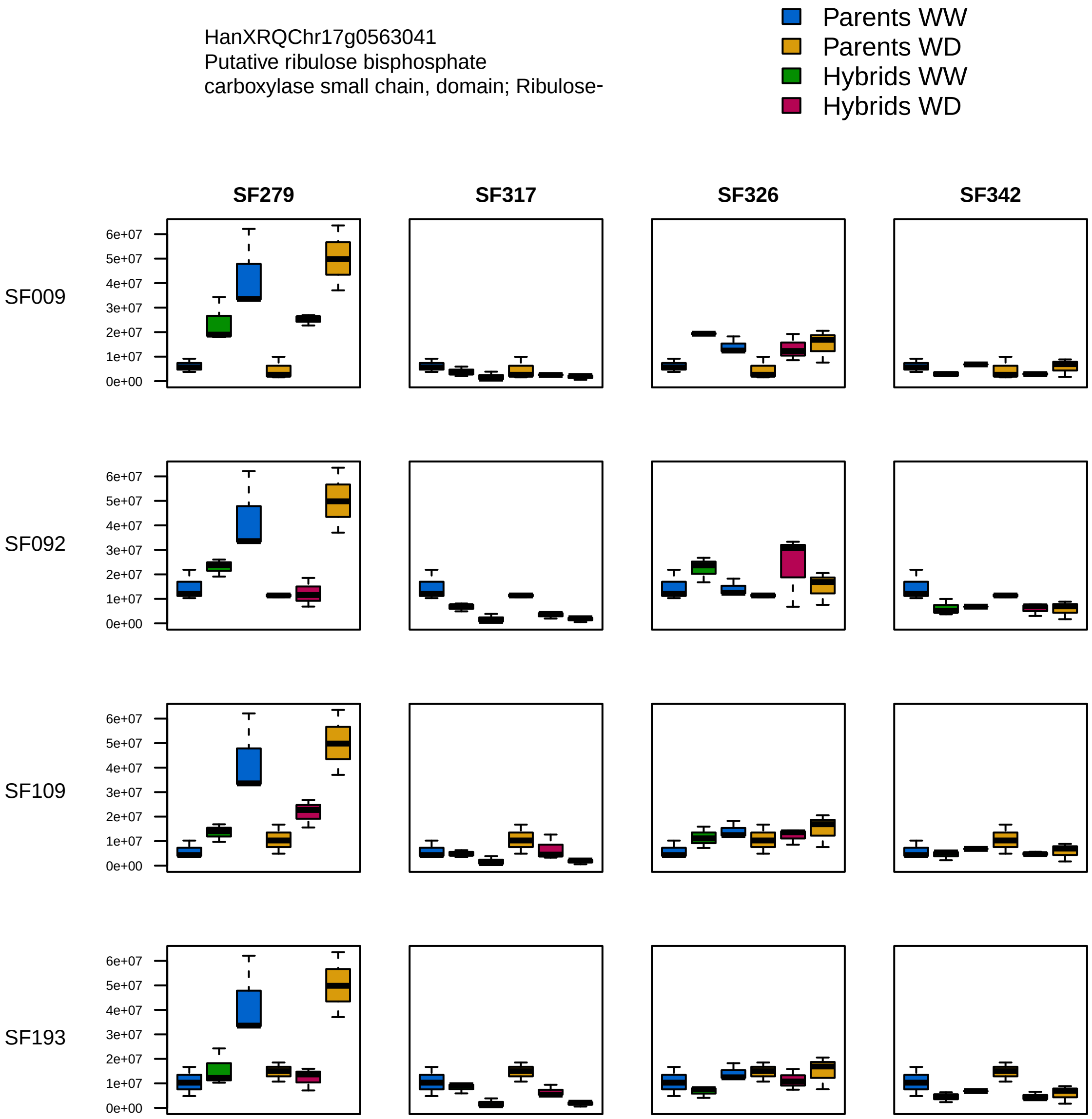
HanXRQChr17g0563041
Putative ribulose bisphosphate
carboxylase small chain, domain; Ribulose-
Parents WW
Parents WD
Hybrids WW
Hybrids WD
SF279
SF317
SF326
SF342
SF009
SF092
SF109
SF193
6e+07
5e+07
4e+07
3e+07
2e+07
1e+07
0e+00

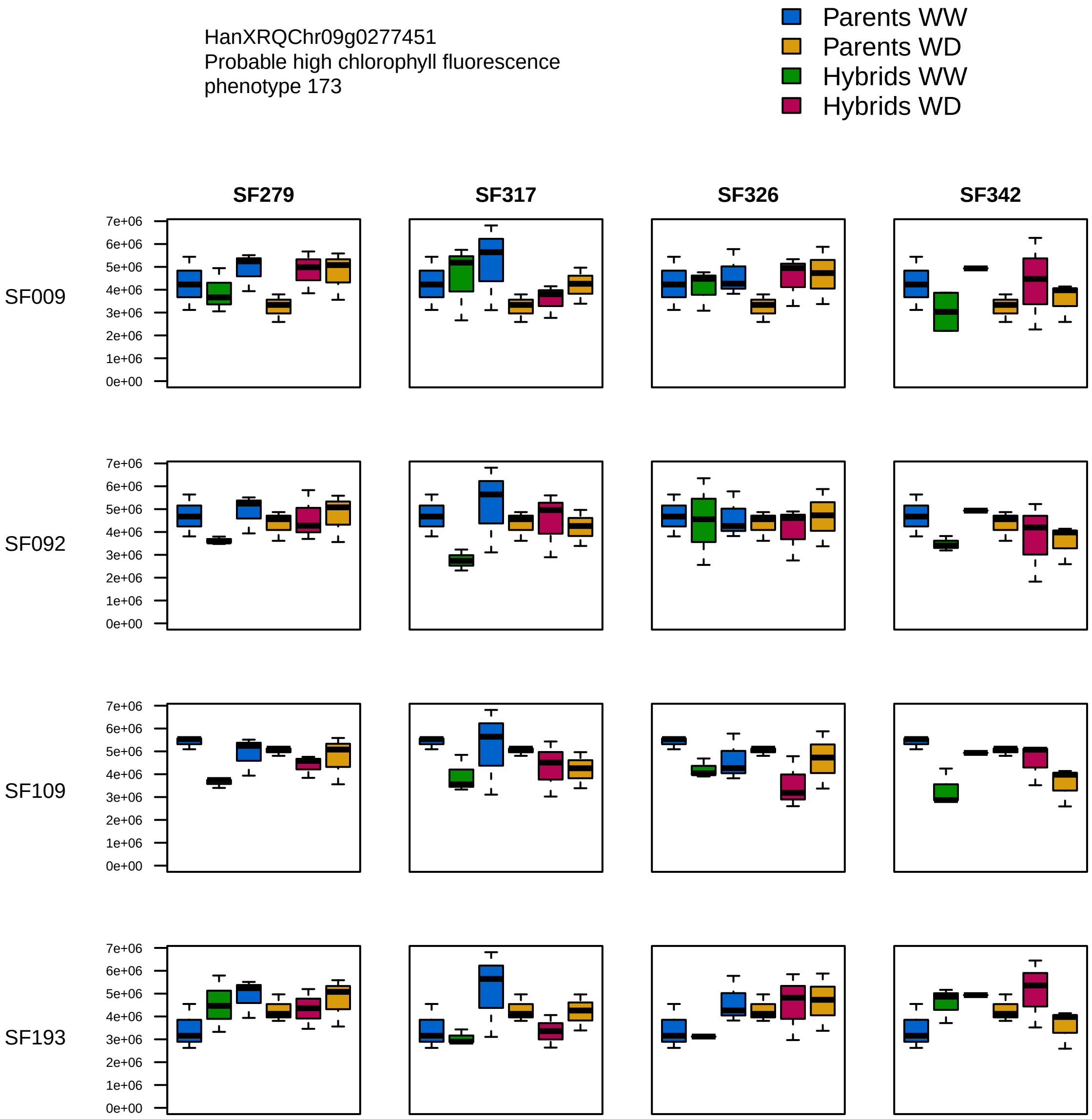

HanXRQChr09g0277451
Probable high chlorophyll fluorescence
phenotype 173
Parents WW
Parents WD
Hybrids WW
Hybrids WD
SF279
SF317
SF326
SF342
SF009
SF092
SF109
SF193
0e+00
1e+06
2e+06
3e+06
4e+06
5e+06
6e+06
7e+06

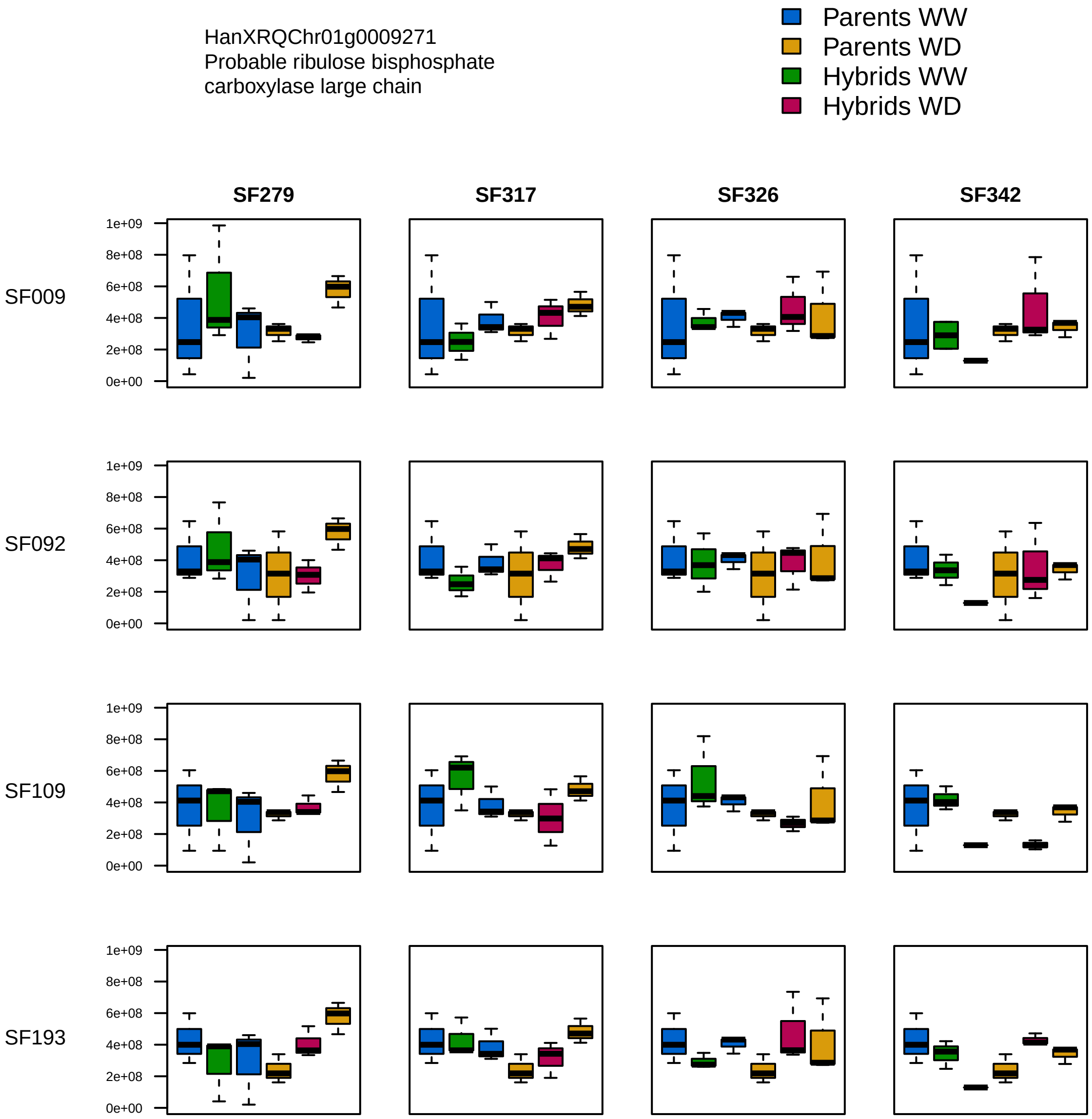
HanXRQChr01g0009271
Probable ribulose bisphosphate
carboxylase large chain
Parents WW
Parents WD
Hybrids WW
Hybrids WD
SF279
SF317
SF326
SF342
SF009
SF092
SF109
SF193
1e+09
8e+08
6e+08
4e+08
2e+08
0e+00

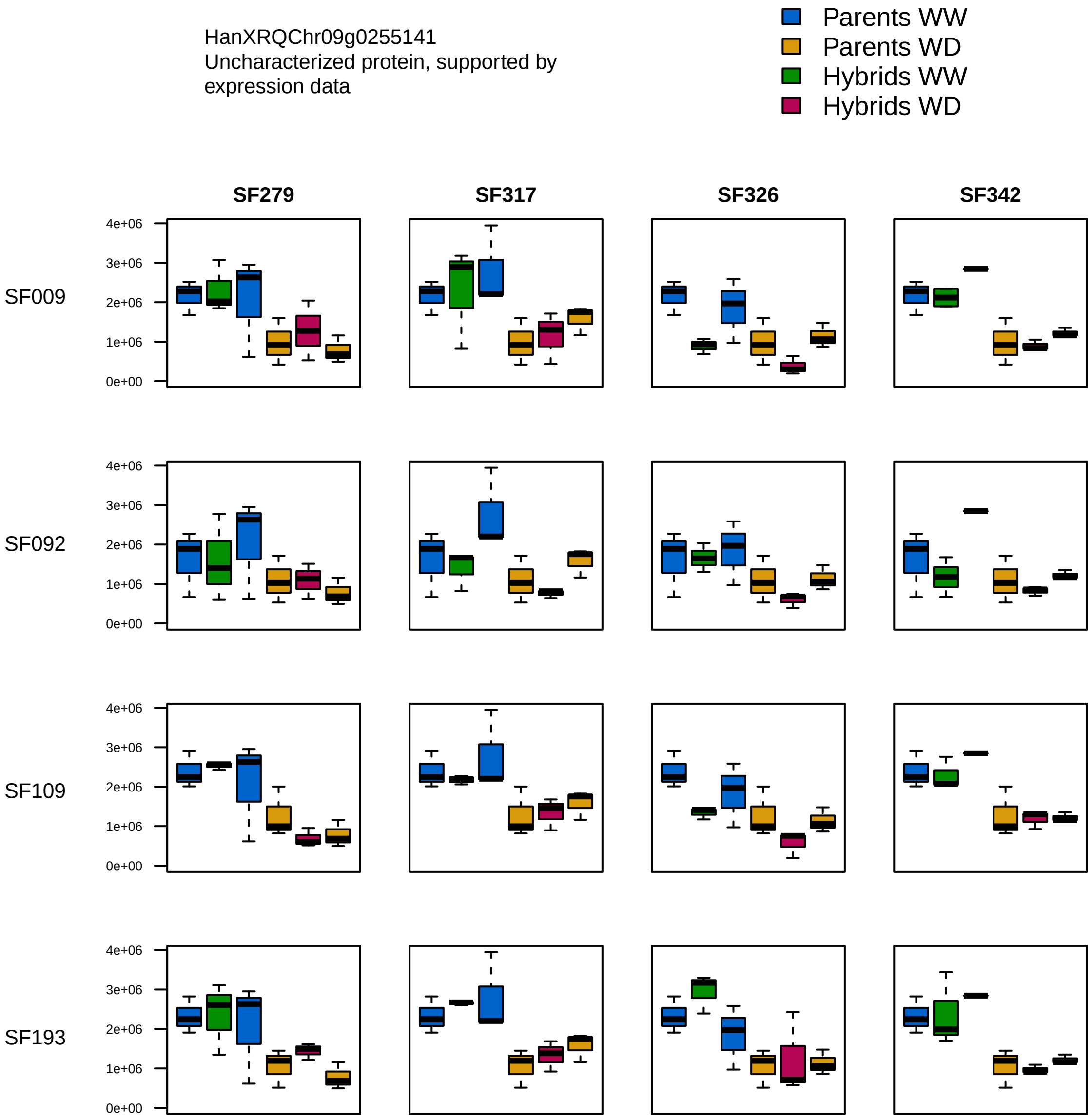
HanXRQChr09g0255141
Uncharacterized protein, supported by
expression data
Parents WW
Parents WD
Hybrids WW
Hybrids WD
SF279
SF317
SF326
SF342
SF009
SF092
SF109
SF193
4e+06
3e+06
2e+06
1e+06
0e+00

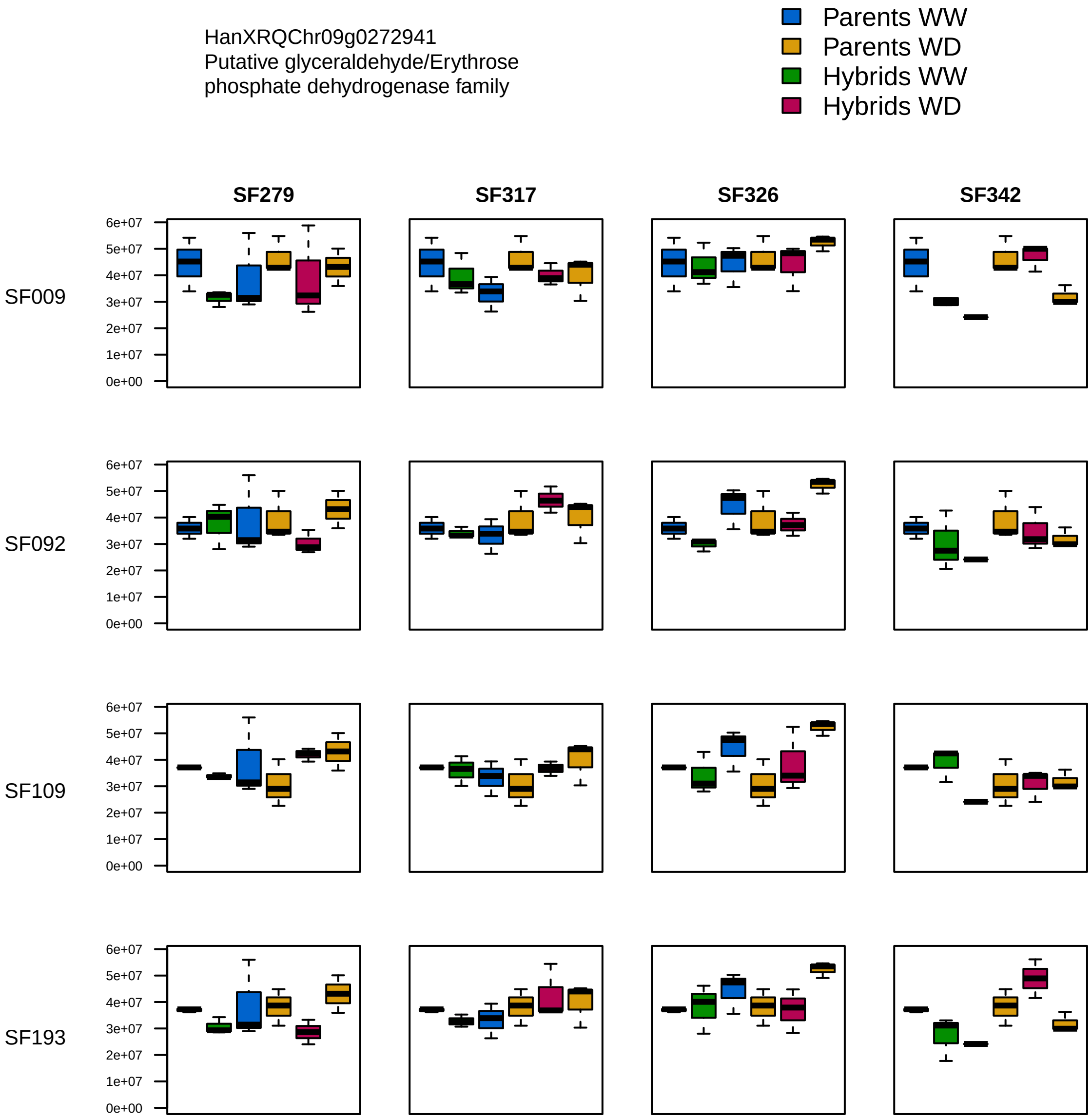
HanXRQChr09g0272941
Putative glyceraldehyde/Erythrose
phosphate dehydrogenase family
Parents WW
Parents WD
Hybrids WW
Hybrids WD
SF279
SF317
SF326
SF342
SF009
SF092
SF109
SF193
0e+00
1e+07
2e+07
3e+07
4e+07
5e+07
6e+07

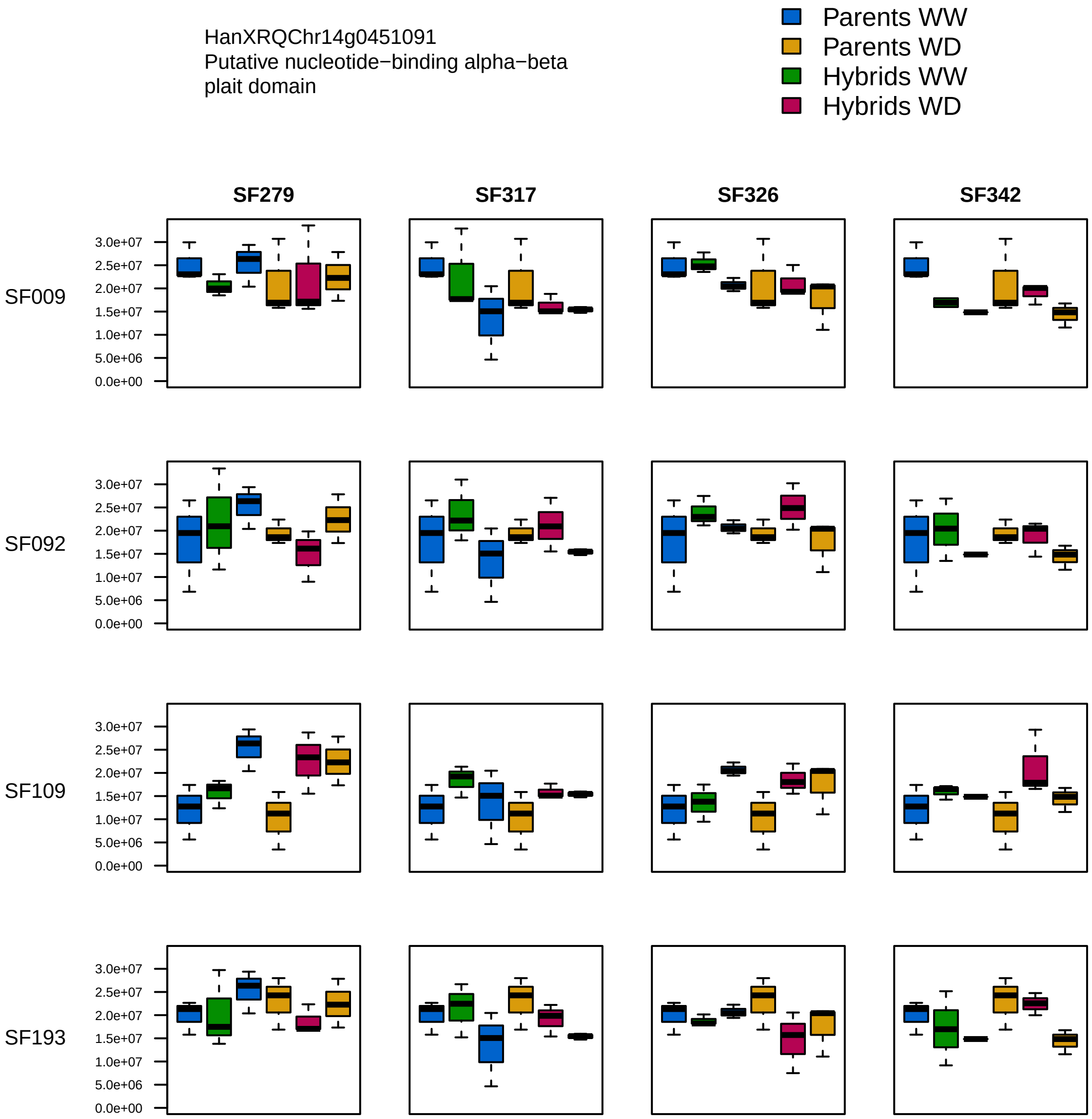
HanXRQChr14g0451091
Putative nucleotide−binding alpha−beta
plait domain
Parents WW
Parents WD
Hybrids WW
Hybrids WD
SF279
SF317
SF326
SF342
SF009
SF092
SF109
SF193
0.0e+00
5.0e+06
1.0e+07
1.5e+07
2.0e+07
2.5e+07
3.0e+07

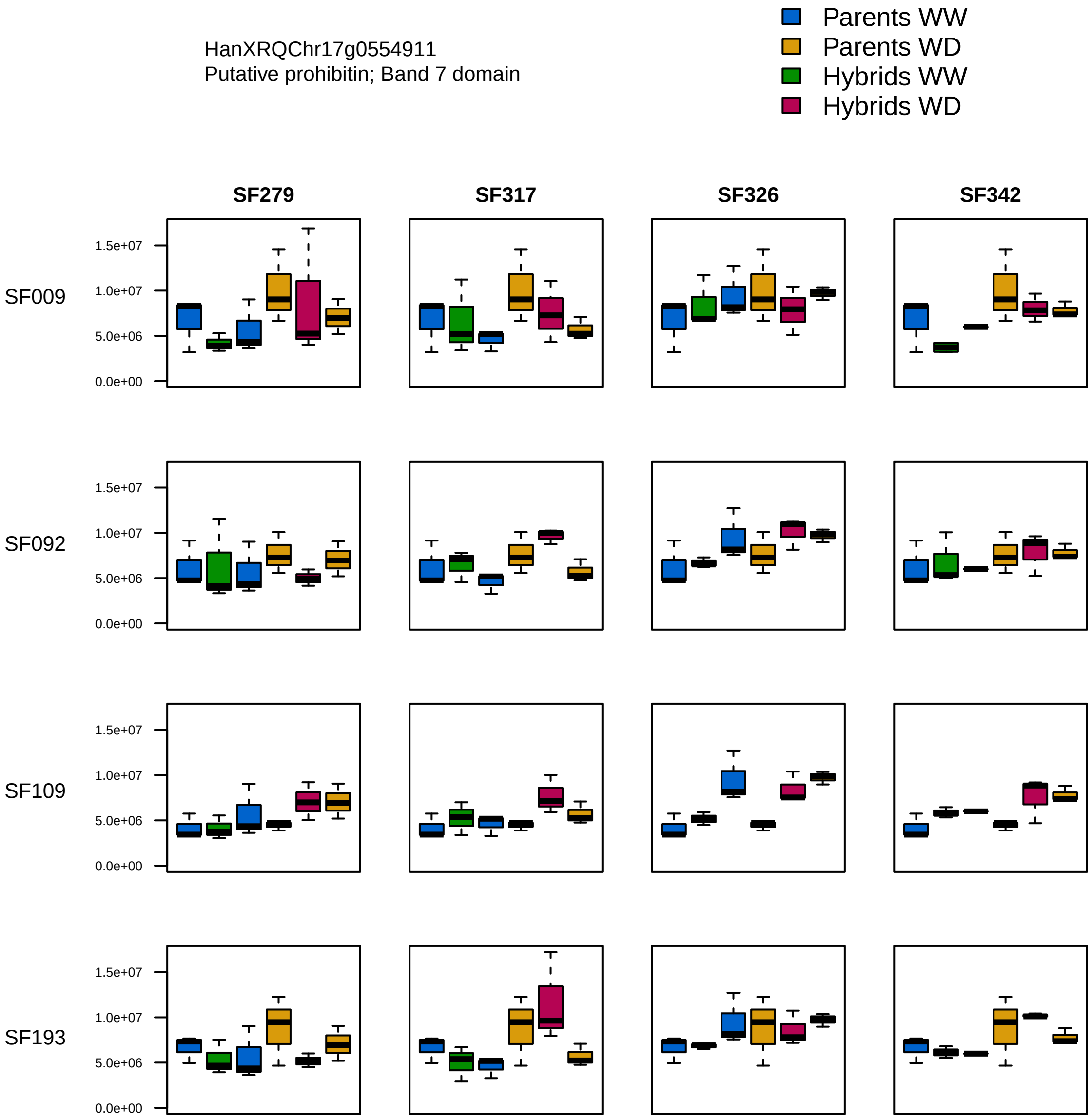
HanXRQChr17g0554911
Putative prohibitin; Band 7 domain
Parents WW
Parents WD
Hybrids WW
Hybrids WD
SF279
SF317
SF326
SF342
SF009
SF092
SF109
SF193
1.5e+07
1.0e+07
5.0e+06
0.0e+00

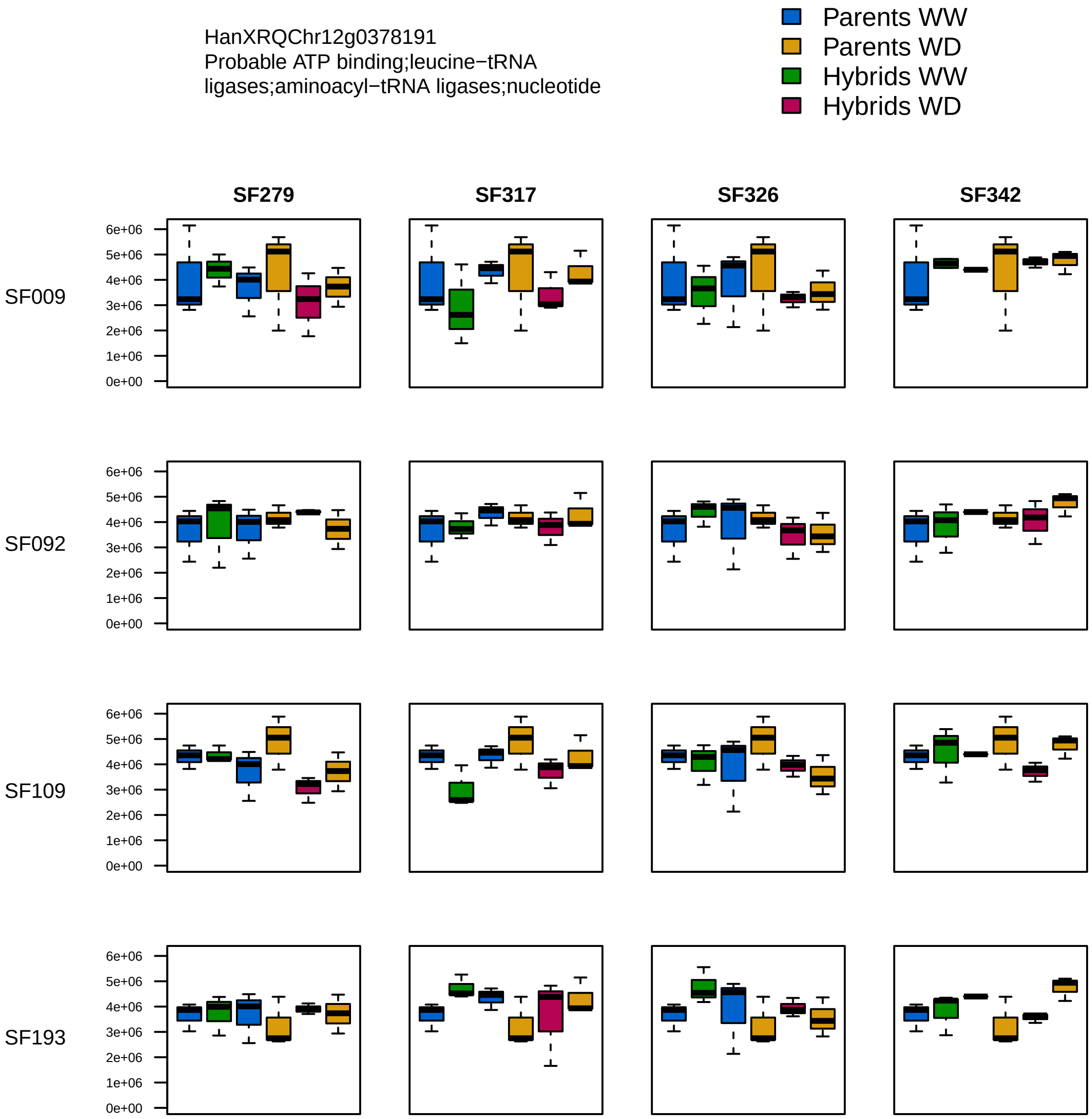

HanXRQChr12g0378191
Probable ATP binding;leucine−tRNA
ligases;aminoacyl−tRNA ligases;nucleotide
Parents WW
Parents WD
Hybrids WW
Hybrids WD
SF279
SF317
SF326
SF342
SF009
SF092
SF109
SF193
6e+06
5e+06
4e+06
3e+06
2e+06
1e+06
0e+00

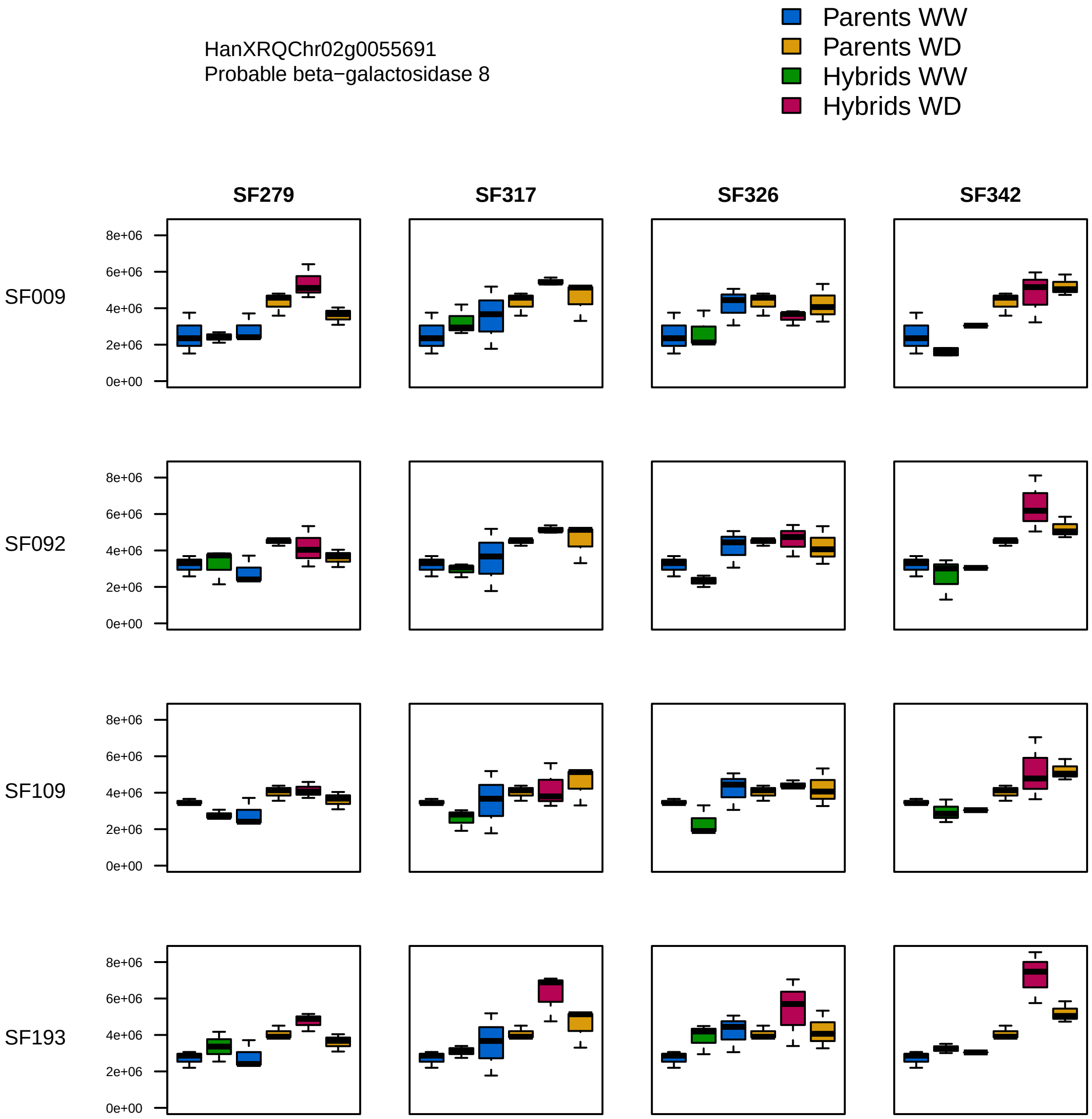
HanXRQChr02g0055691
Probable beta−galactosidase 8
Parents WW
Parents WD
Hybrids WW
Hybrids WD
SF279
SF317
SF326
SF342
SF009
SF092
SF109
SF193
8e+06
6e+06
4e+06
2e+06
0e+00

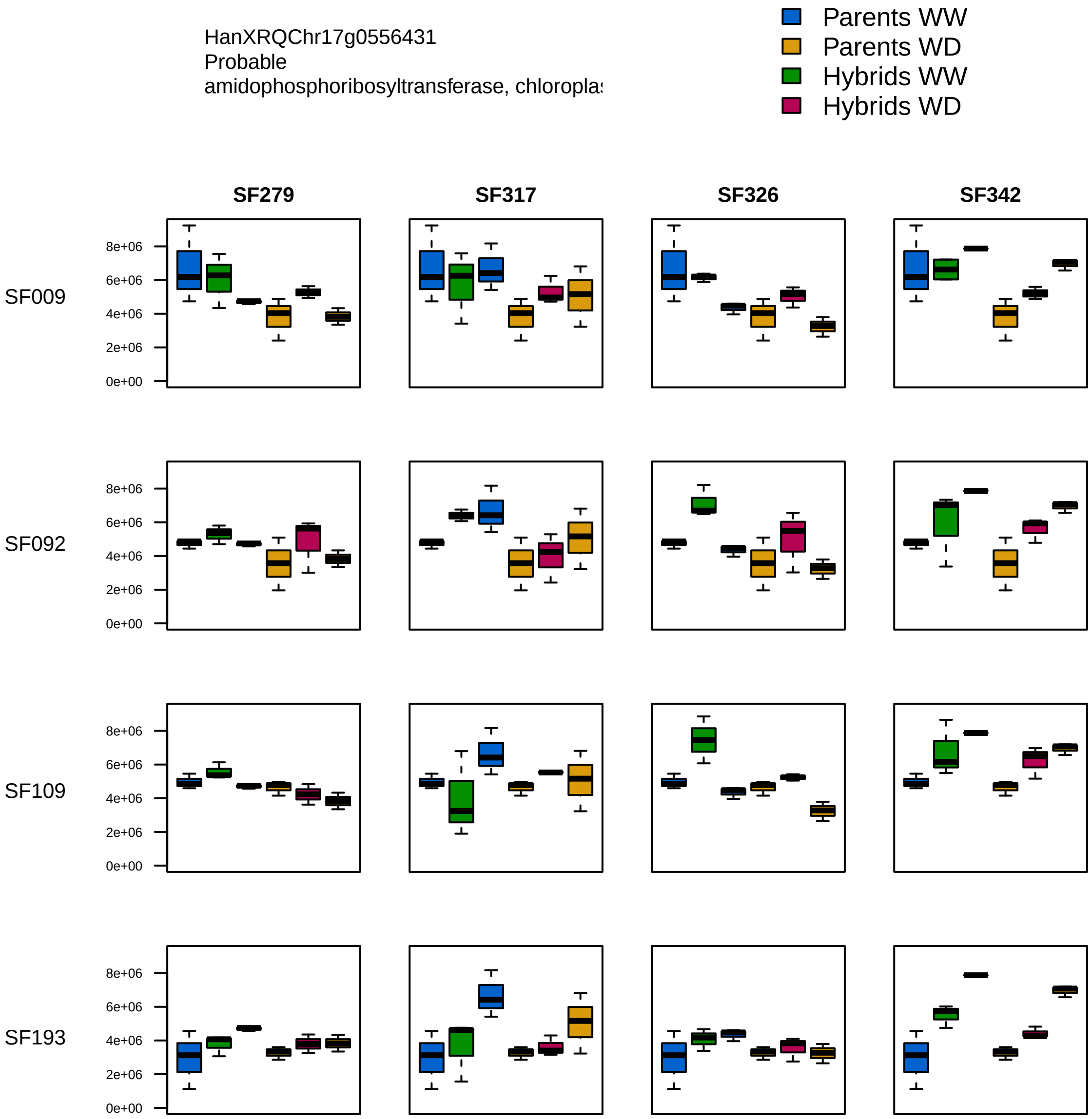
HanXRQChr17g0556431
Probable
amidophosphoribosyltransferase, chloropla
Parents WW
Parents WD
Hybrids WW
Hybrids WD
SF279
SF317
SF326
SF342
SF009
SF092
SF109
SF193
8e+06
6e+06
4e+06
2e+06
0e+00

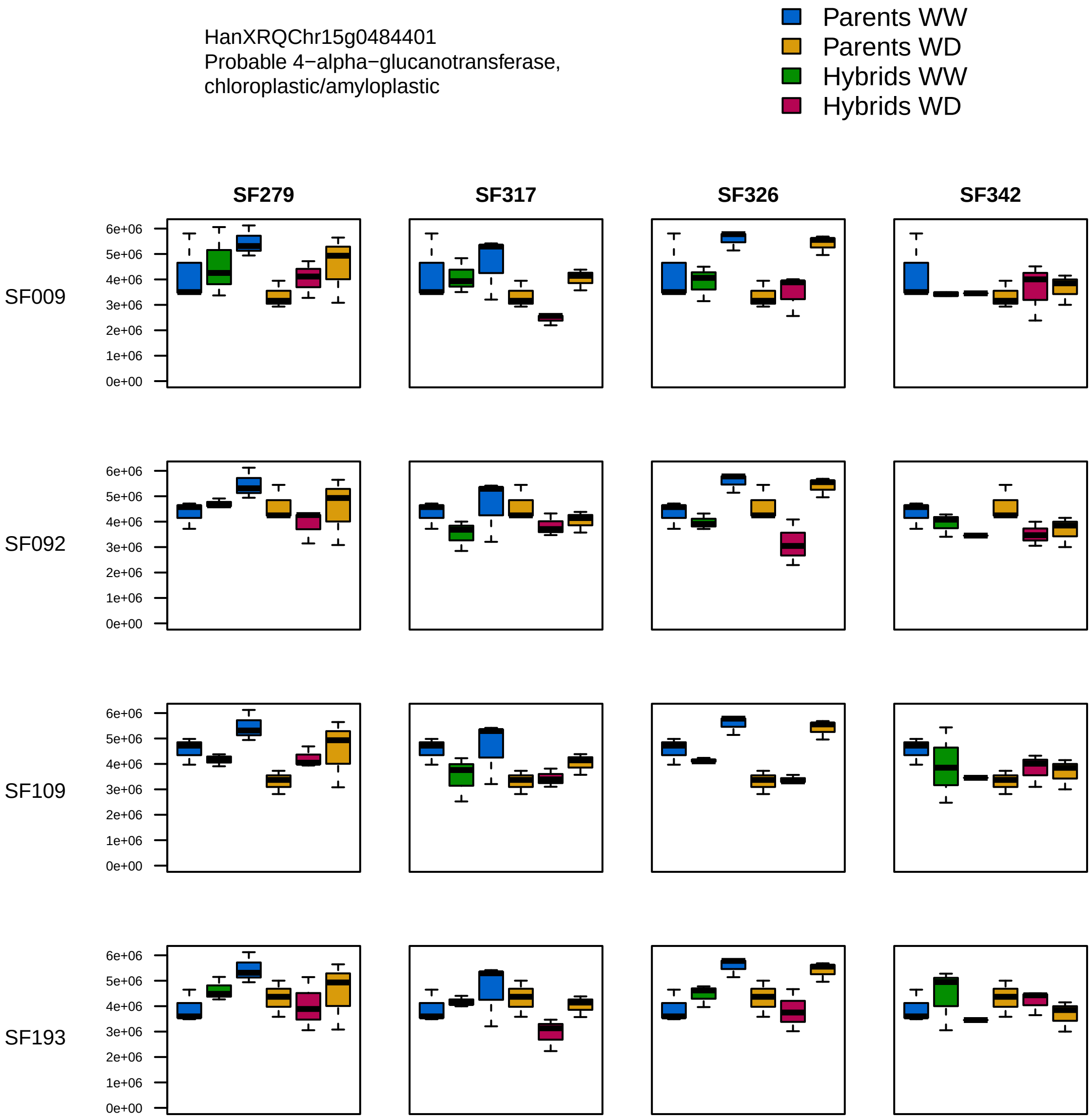
HanXRQChr15g0484401
Probable 4−alpha−glucanotransferase, chloroplastic/amyloplastic
Parents WW
Parents WD
Hybrids WW
Hybrids WD
SF279
SF317
SF326
SF342
SF009
SF092
SF109
SF193
6e+06
5e+06
4e+06
3e+06
2e+06
1e+06
0e+00

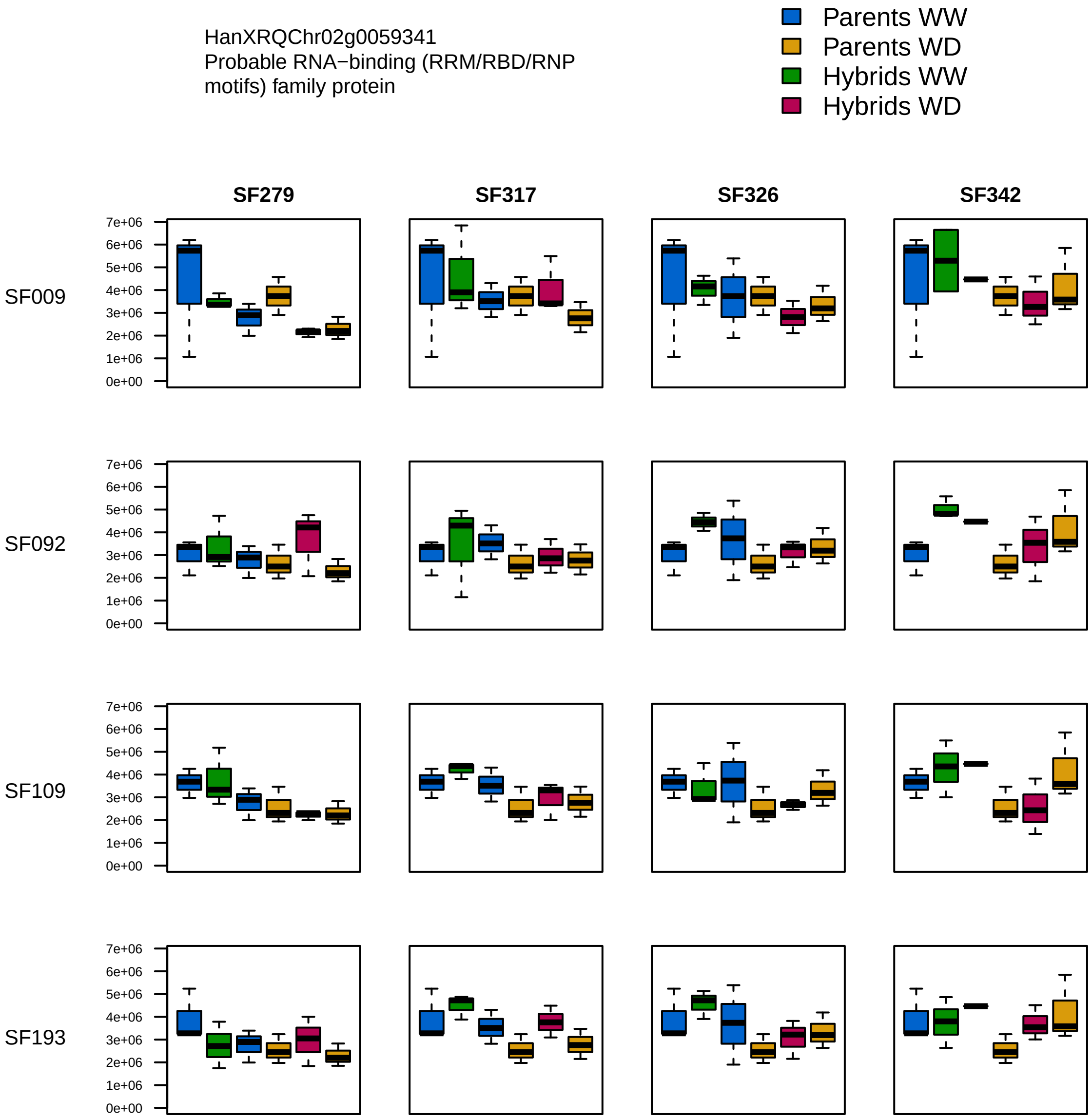
HanXRQChr02g0059341
Probable RNA−binding (RRM/RBD/RNP
motifs) family protein
Parents WW
Parents WD
Hybrids WW
Hybrids WD
SF279
SF317
SF326
SF342
SF009
SF092
SF109
SF193
7e+06
6e+06
5e+06
4e+06
3e+06
2e+06
1e+06
0e+00

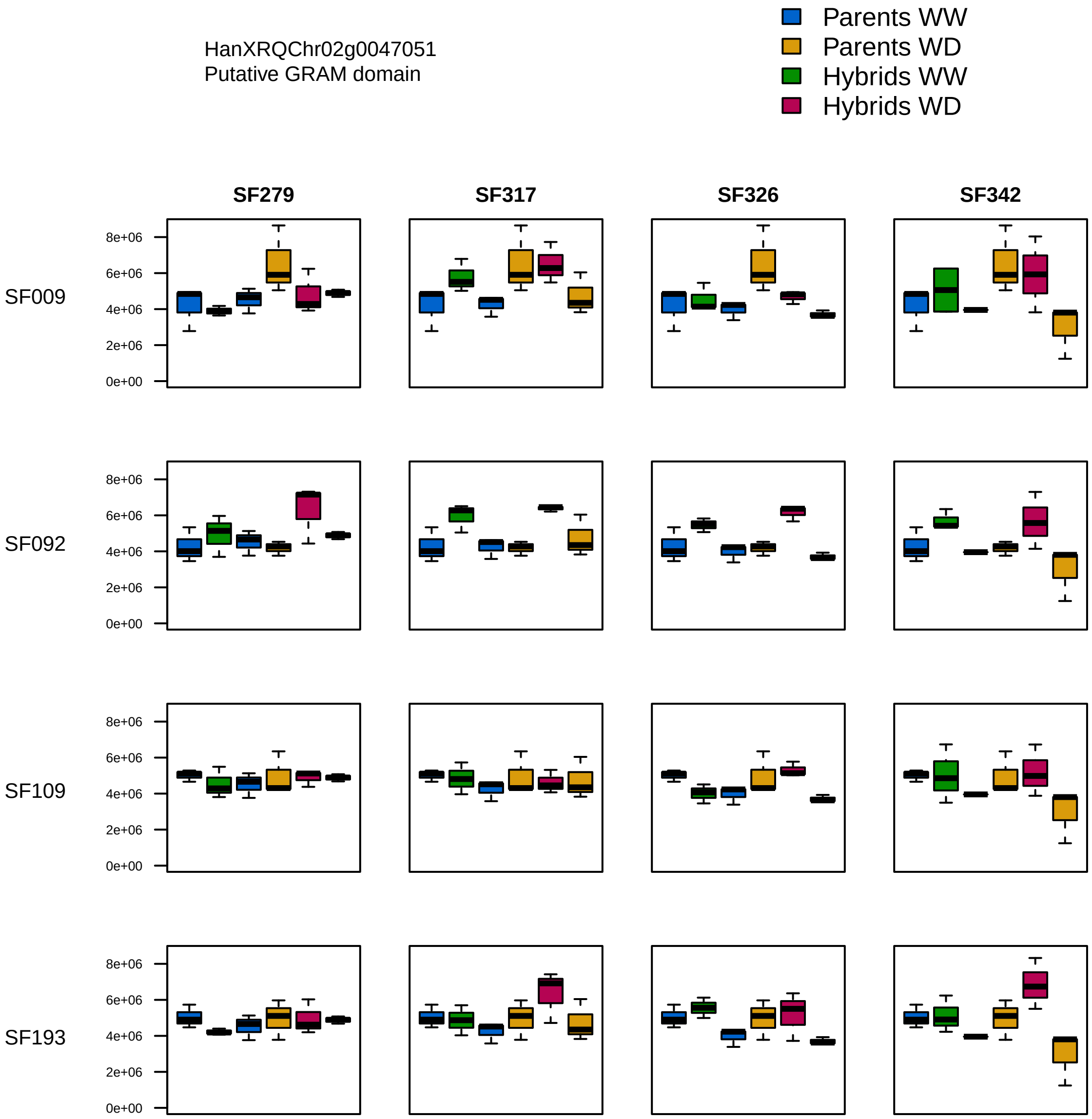
HanXRQChr02g0047051
Putative GRAM domain
Parents WW
Parents WD
Hybrids WW
Hybrids WD
SF279
SF317
SF326
SF342
SF009
SF092
SF109
SF193
8e+06
6e+06
4e+06
2e+06
0e+00

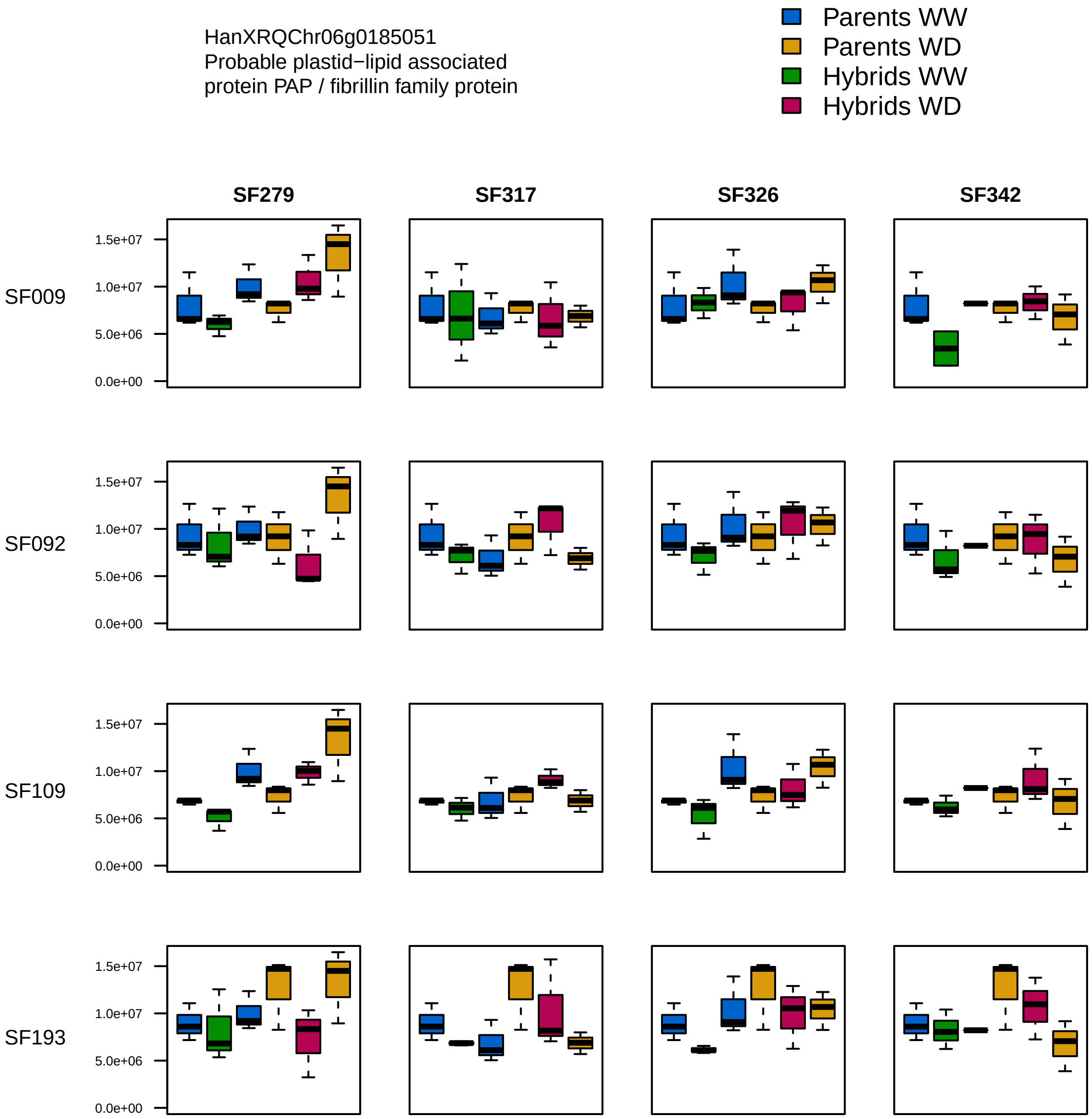
HanXRQChr06g0185051
Probable plastid−lipid associated
protein PAP / fibrillin family protein
Parents WW
Parents WD
Hybrids WW
Hybrids WD
SF279
SF317
SF326
SF342
SF009
SF092
SF109
SF193
1.5e+07
1.0e+07
5.0e+06
0.0e+00

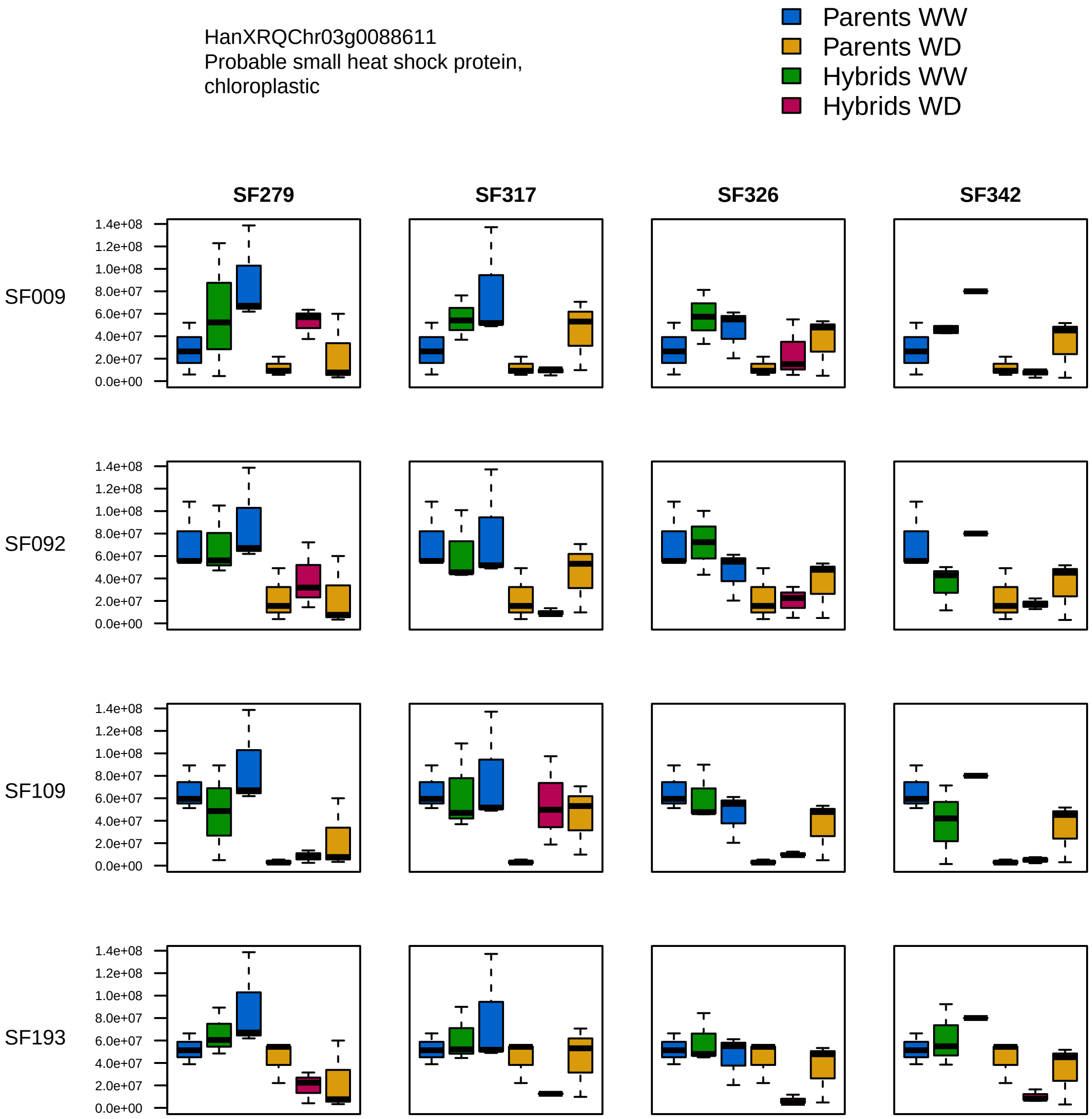
HanXRQChr03g0088611
Probable small heat shock protein,
chloroplastic
Parents WW
Parents WD
Hybrids WW
Hybrids WD
SF279
SF317
SF326
SF342
SF009
SF092
SF109
SF193
1.4e+08
1.2e+08
1.0e+08
8.0e+07
6.0e+07
4.0e+07
2.0e+07
0.0e+00

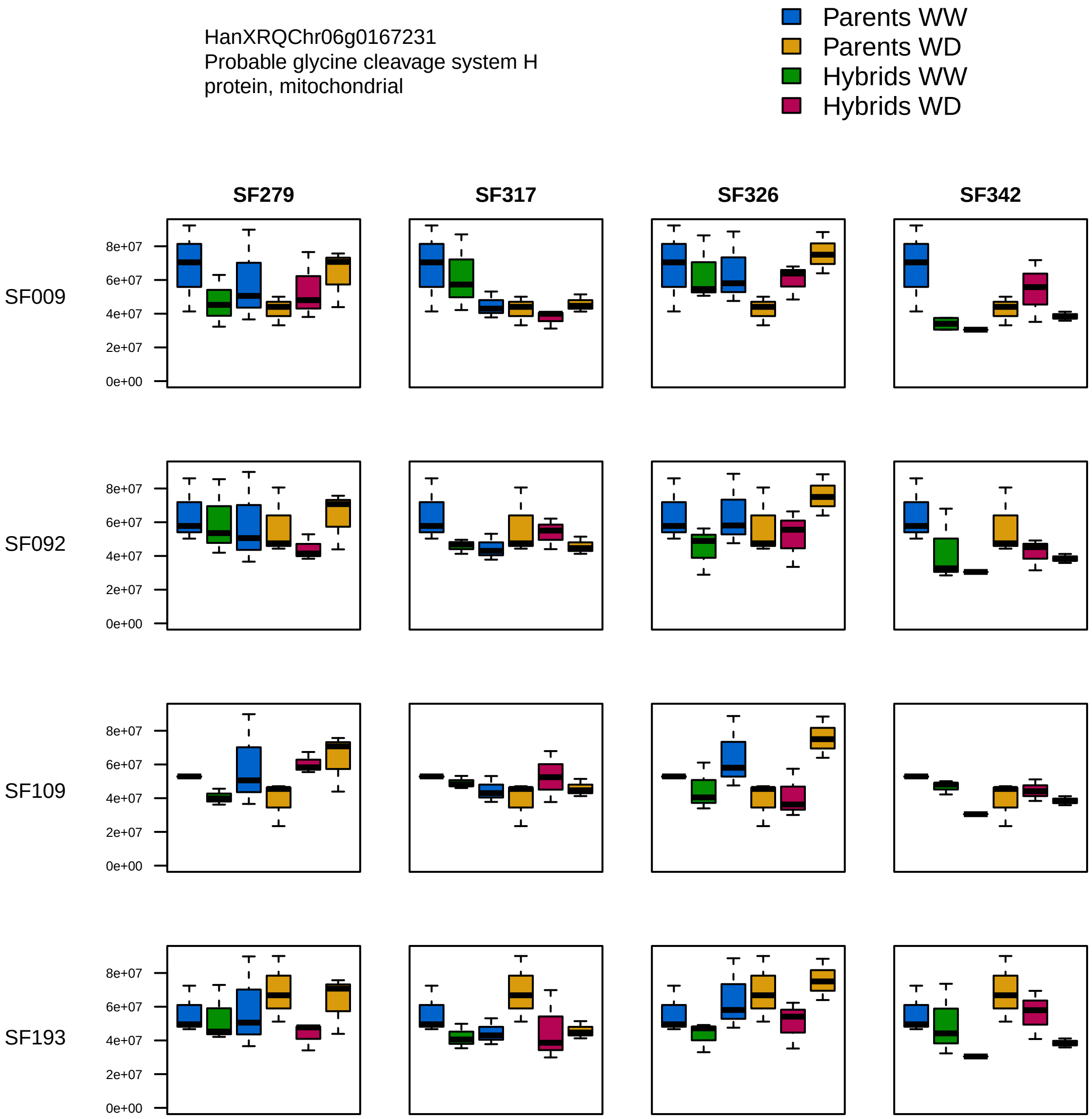
HanXRQChr06g0167231
Probable glycine cleavage system H
protein, mitochondrial
Parents WW
Parents WD
Hybrids WW
Hybrids WD
SF279
SF317
SF326
SF342
SF009
SF092
SF109
SF193
8e+07
6e+07
4e+07
2e+07
0e+00

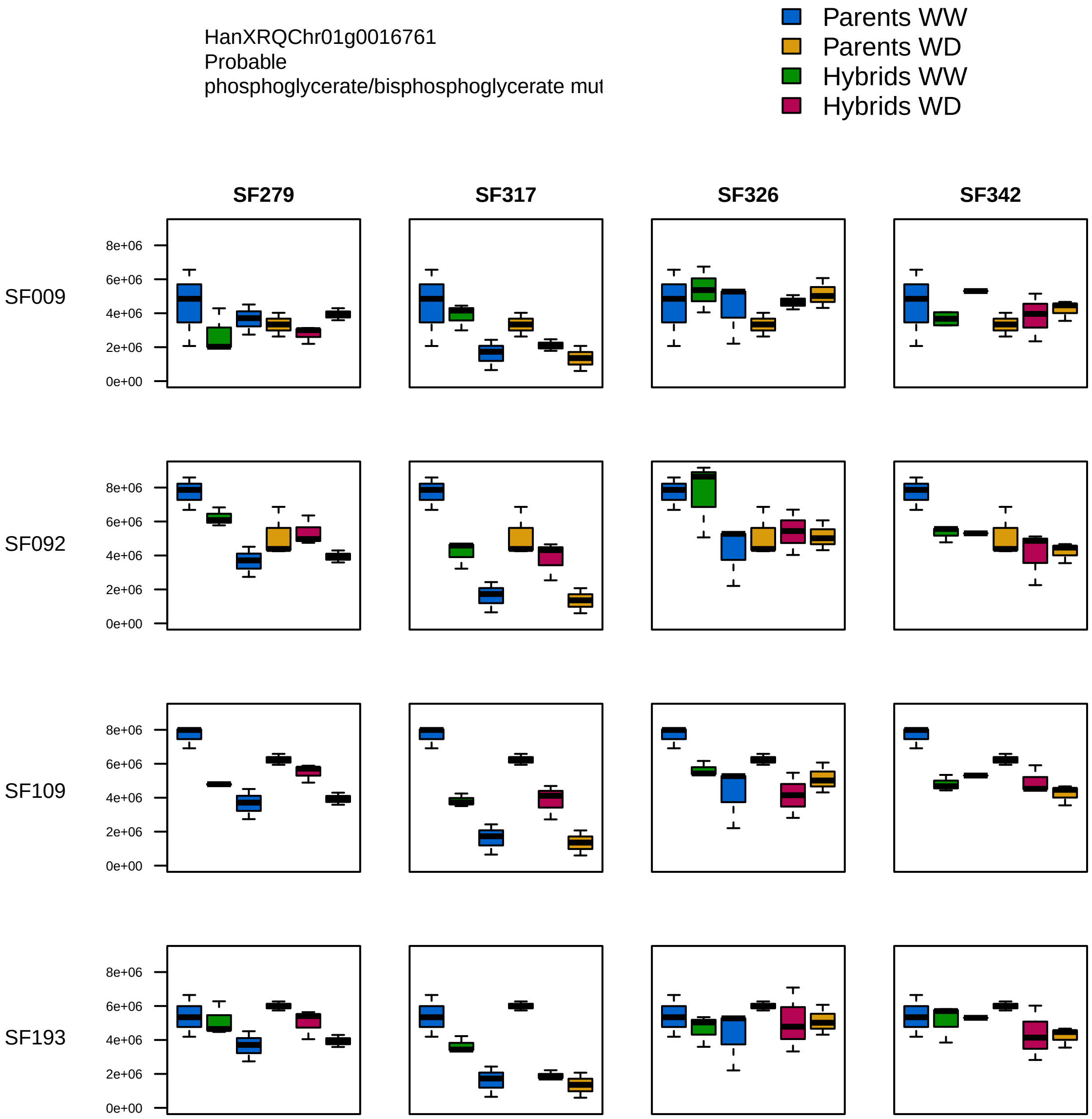

HanXRQChr01g0016761
Probable
phosphoglycerate/bisphosphoglycerate mut
Parents WW
Parents WD
Hybrids WW
Hybrids WD
SF279
SF317
SF326
SF342
SF009
SF092
SF109
SF193
8e+06
6e+06
4e+06
2e+06
0e+00

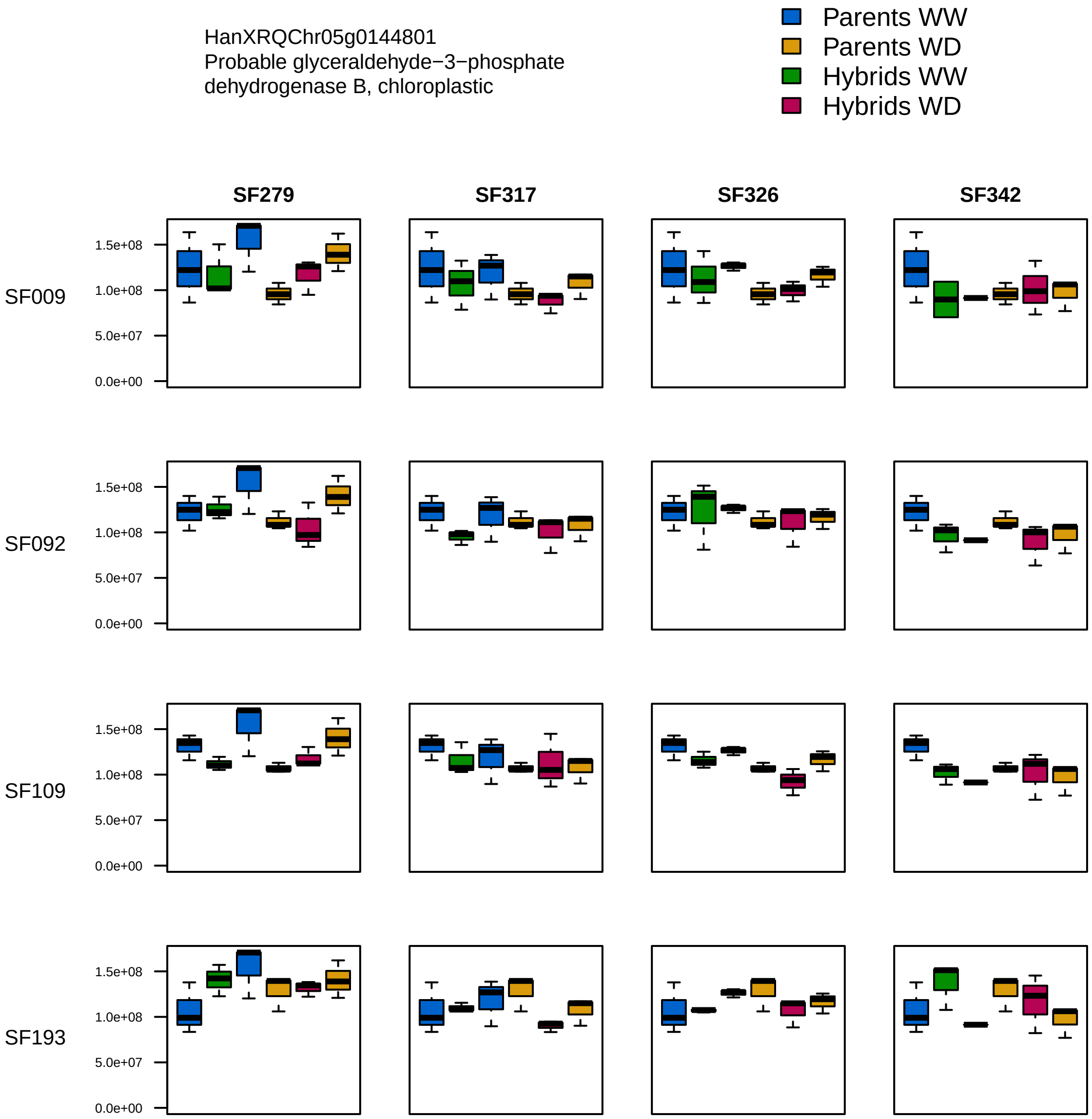

HanXRQChr05g0144801
Probable glyceraldehyde−3−phosphate
dehydrogenase B, chloroplastic
Parents WW
Parents WD
Hybrids WW
Hybrids WD
SF279
SF317
SF326
SF342
SF009
SF092
SF109
SF193
1.5e+08
1.0e+08
5.0e+07
0.0e+00

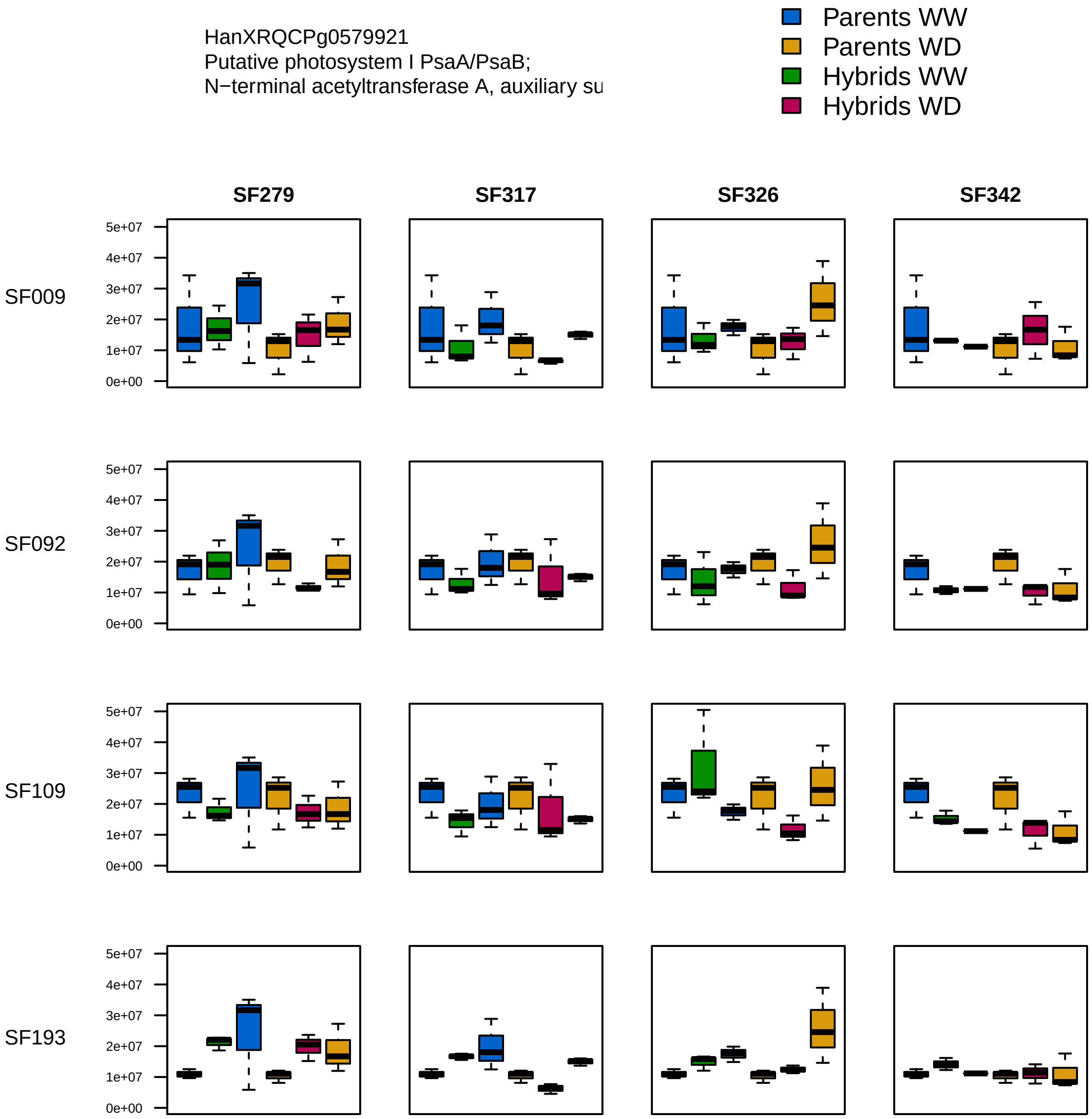
HanXRQCPg0579921
Putative photosystem I PsaA/PsaB;
N−terminal acetyltransferase A, auxiliary su
Parents WW
Parents WD
Hybrids WW
Hybrids WD
SF279
SF317
SF326
SF342
SF009
SF092
SF109
SF193
0e+00
1e+07
2e+07
3e+07
4e+07
5e+07

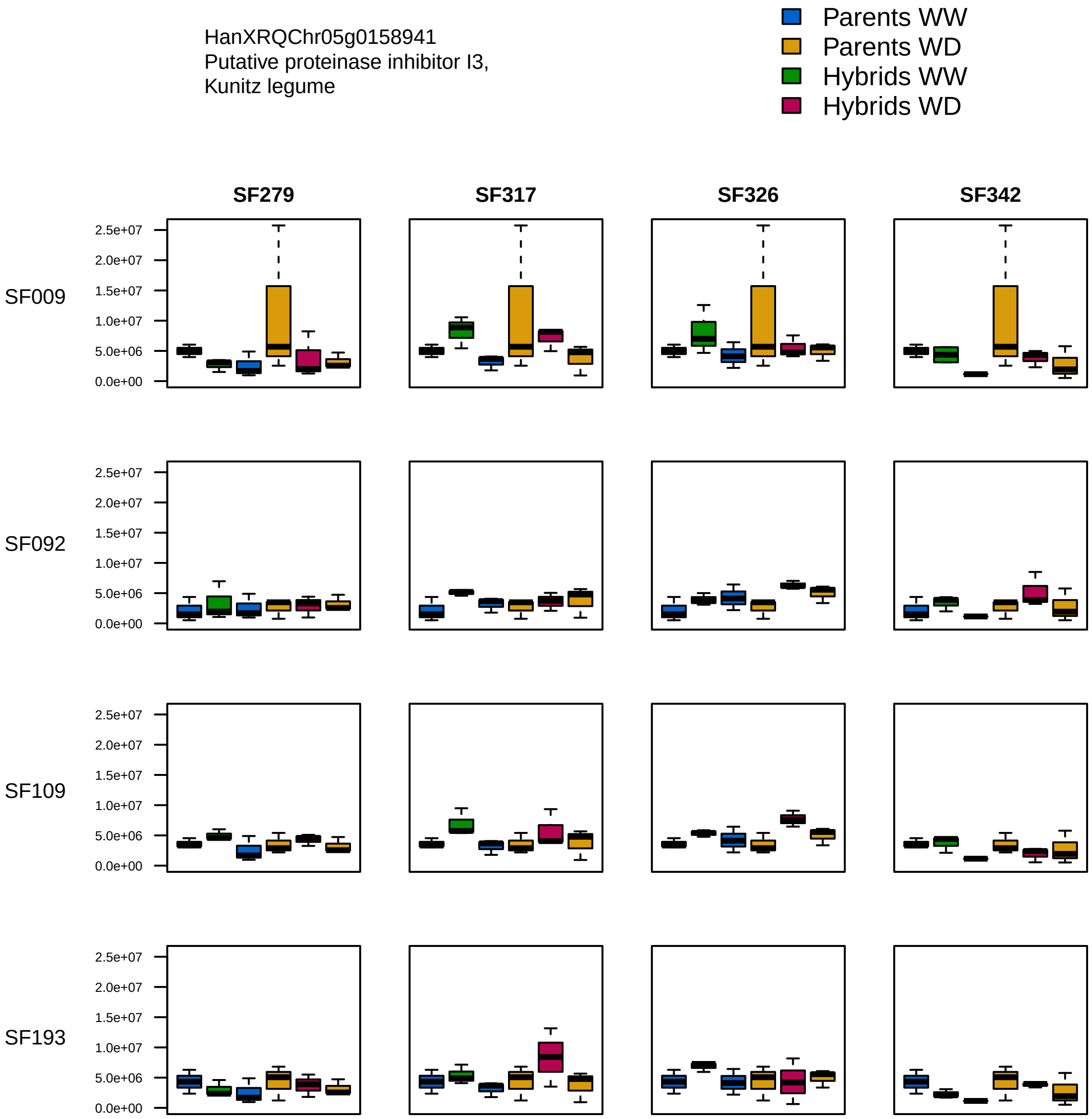
HanXRQChr05g0158941
Putative proteinase inhibitor I3,
Kunitz legume
Parents WW
Parents WD
Hybrids WW
Hybrids WD
SF279
SF317
SF326
SF342
SF009
SF092
SF109
SF193
2.5e+07
2.0e+07
1.5e+07
1.0e+07
5.0e+06
0.0e+00

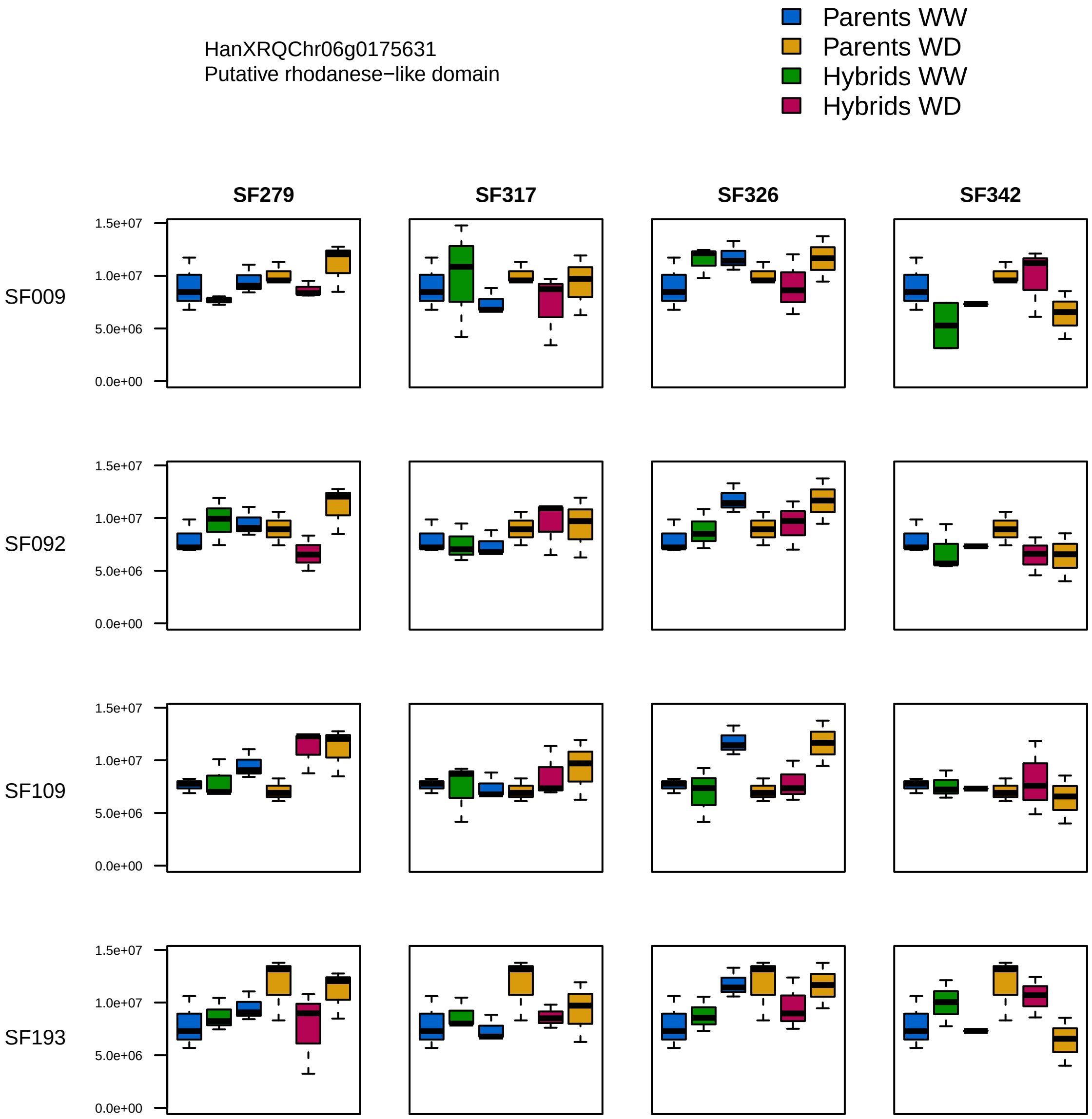

HanXRQChr06g0175631
Putative rhodanese−like domain
Parents WW
Parents WD
Hybrids WW
Hybrids WD
SF279
SF317
SF326
SF342
SF009
SF092
SF109
SF193
1.5e+07
1.0e+07
5.0e+06
0.0e+00

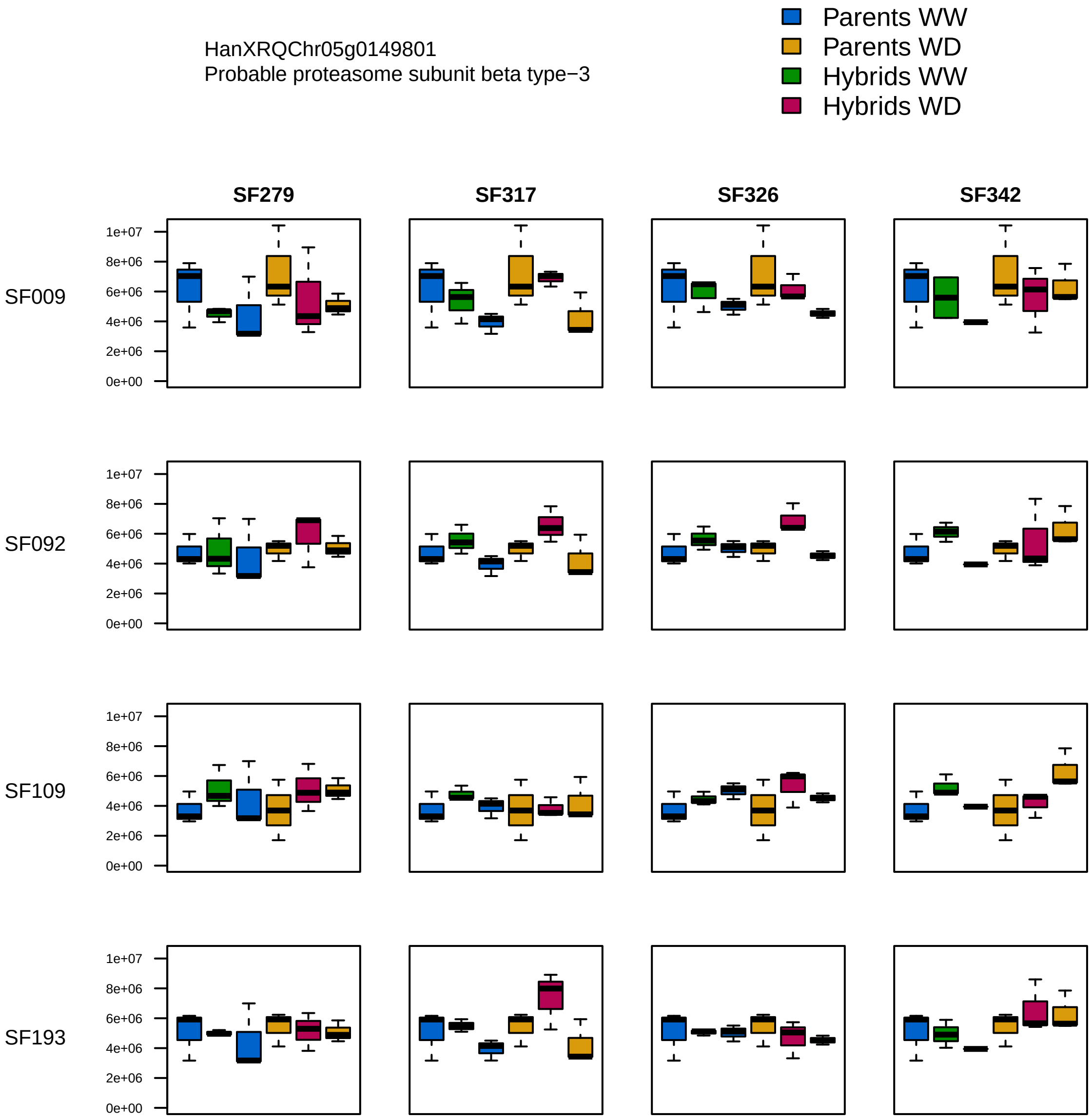

HanXRQChr05g0149801
Probable proteasome subunit beta type−3
Parents WW
Parents WD
Hybrids WW
Hybrids WD
SF279
SF317
SF326
SF342
SF009
SF092
SF109
SF193
1e+07
8e+06
6e+06
4e+06
2e+06
0e+00

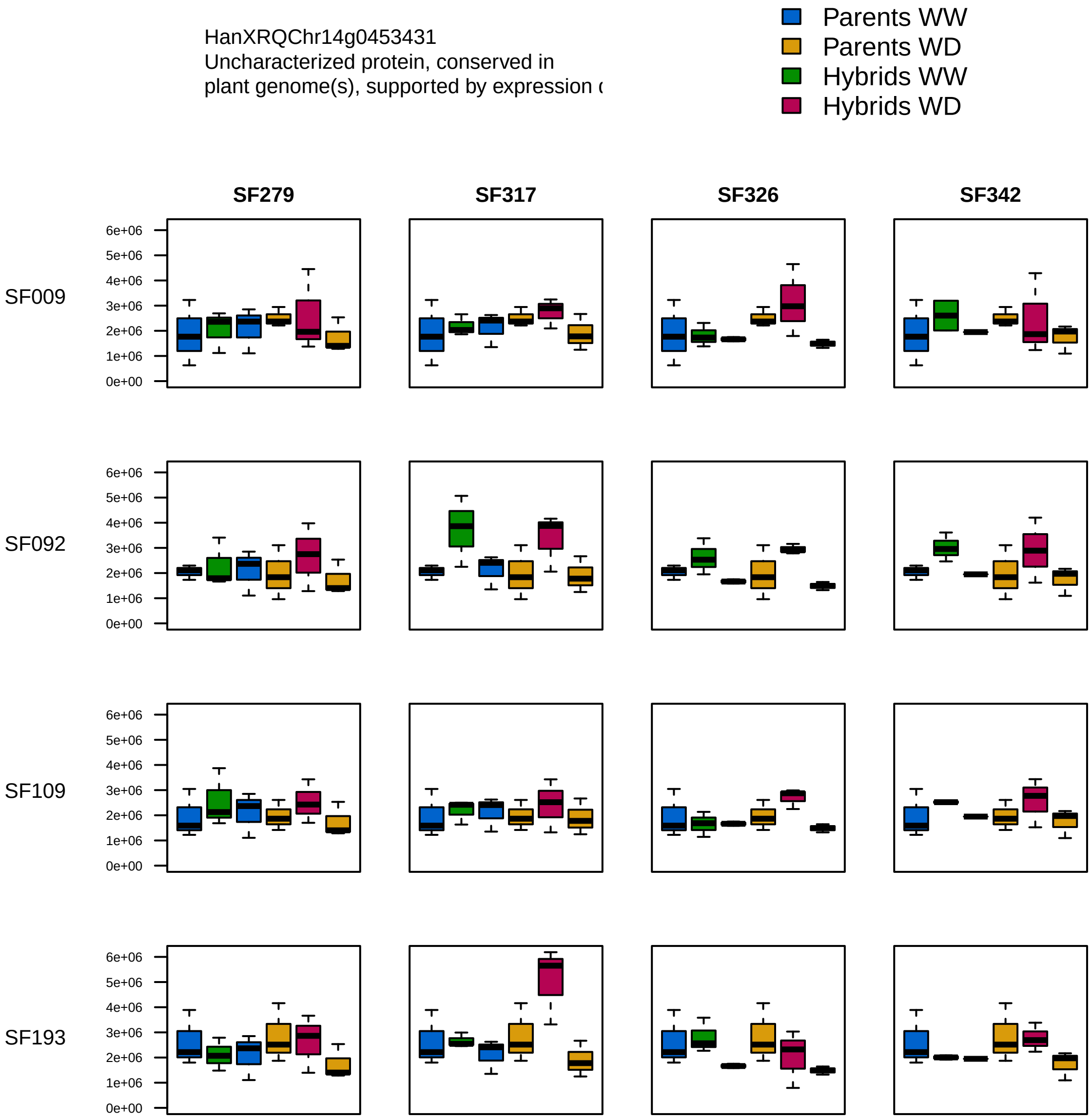

HanXRQChr14g0453431
Uncharacterized protein, conserved in
plant genome(s), supported by expression d
Parents WW
Parents WD
Hybrids WW
Hybrids WD
SF279
SF317
SF326
SF342
SF009
SF092
SF109
SF193
6e+06
5e+06
4e+06
3e+06
2e+06
1e+06
0e+00

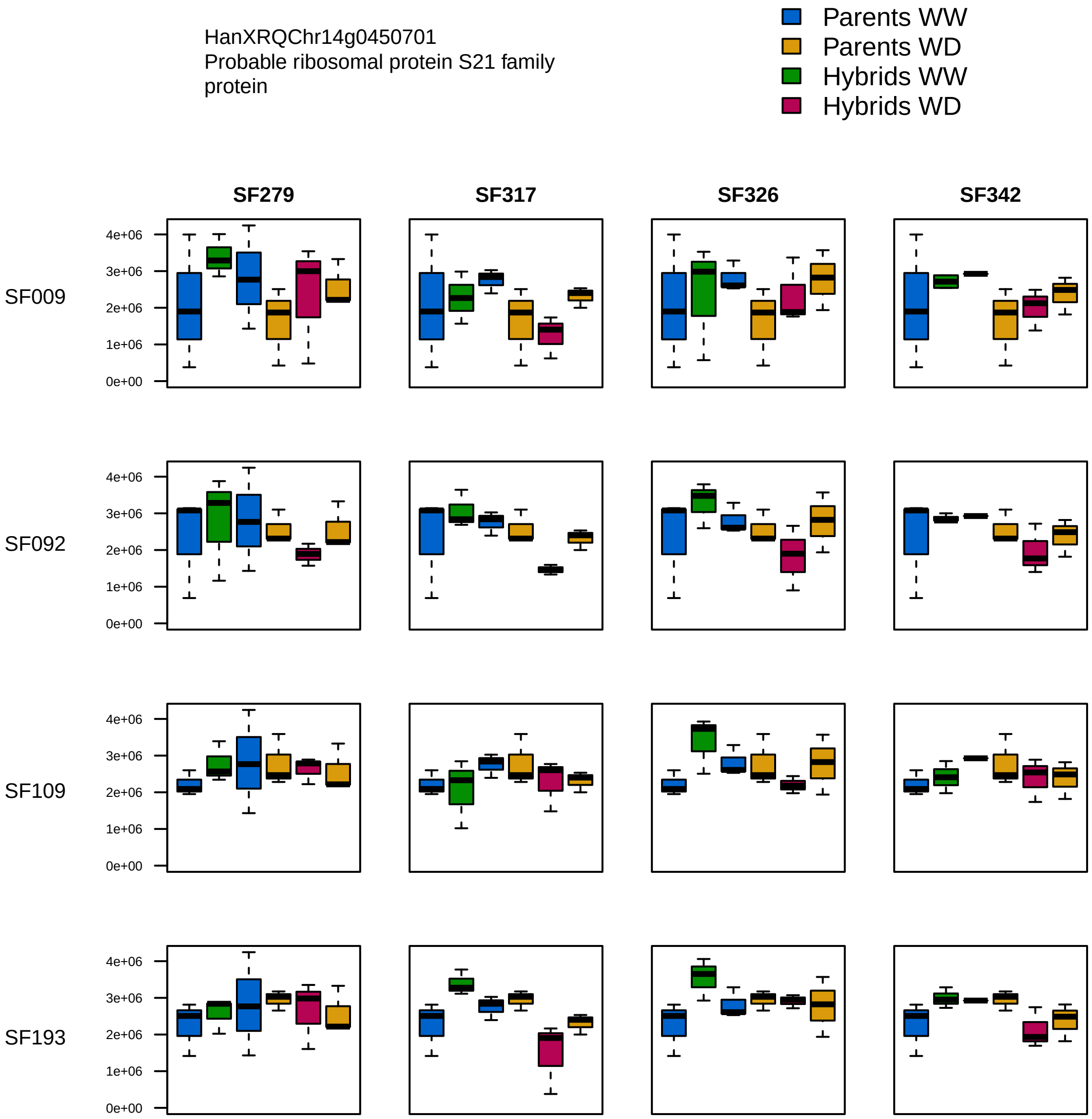

HanXRQChr14g0450701
Probable ribosomal protein S21 family protein
Parents WW
Parents WD
Hybrids WW
Hybrids WD
SF279
SF317
SF326
SF342
SF009
SF092
SF109
SF193
4e+06
3e+06
2e+06
1e+06
0e+00

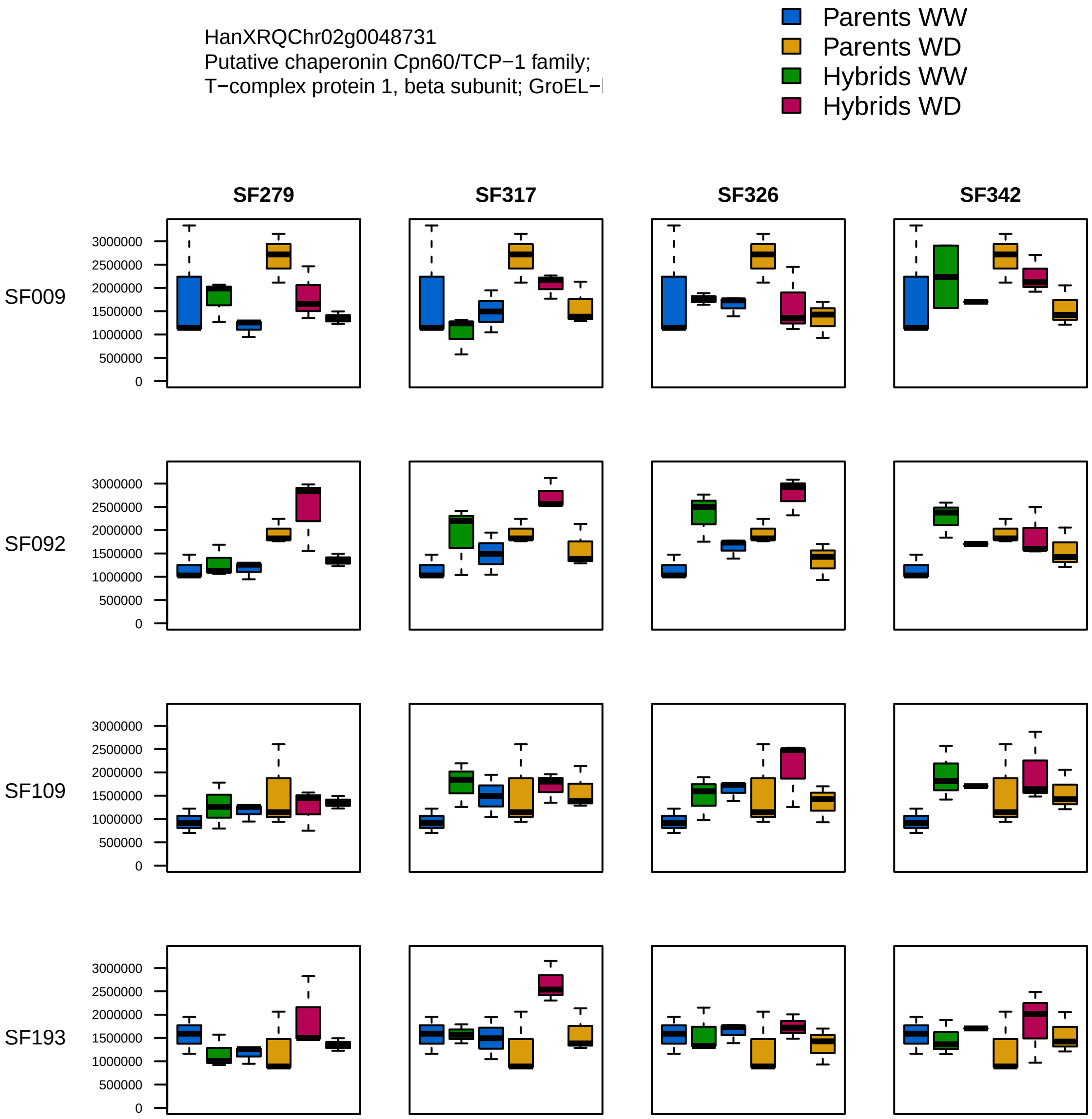
HanXRQChr02g0048731
Putative chaperonin Cpn60/TCP−1 family;
T−complex protein 1, beta subunit; GroEL−l
Parents WW
Parents WD
Hybrids WW
Hybrids WD
SF279
SF317
SF326
SF342
SF009
SF092
SF109
SF193
3000000
2500000
2000000
1500000
1000000
500000
0

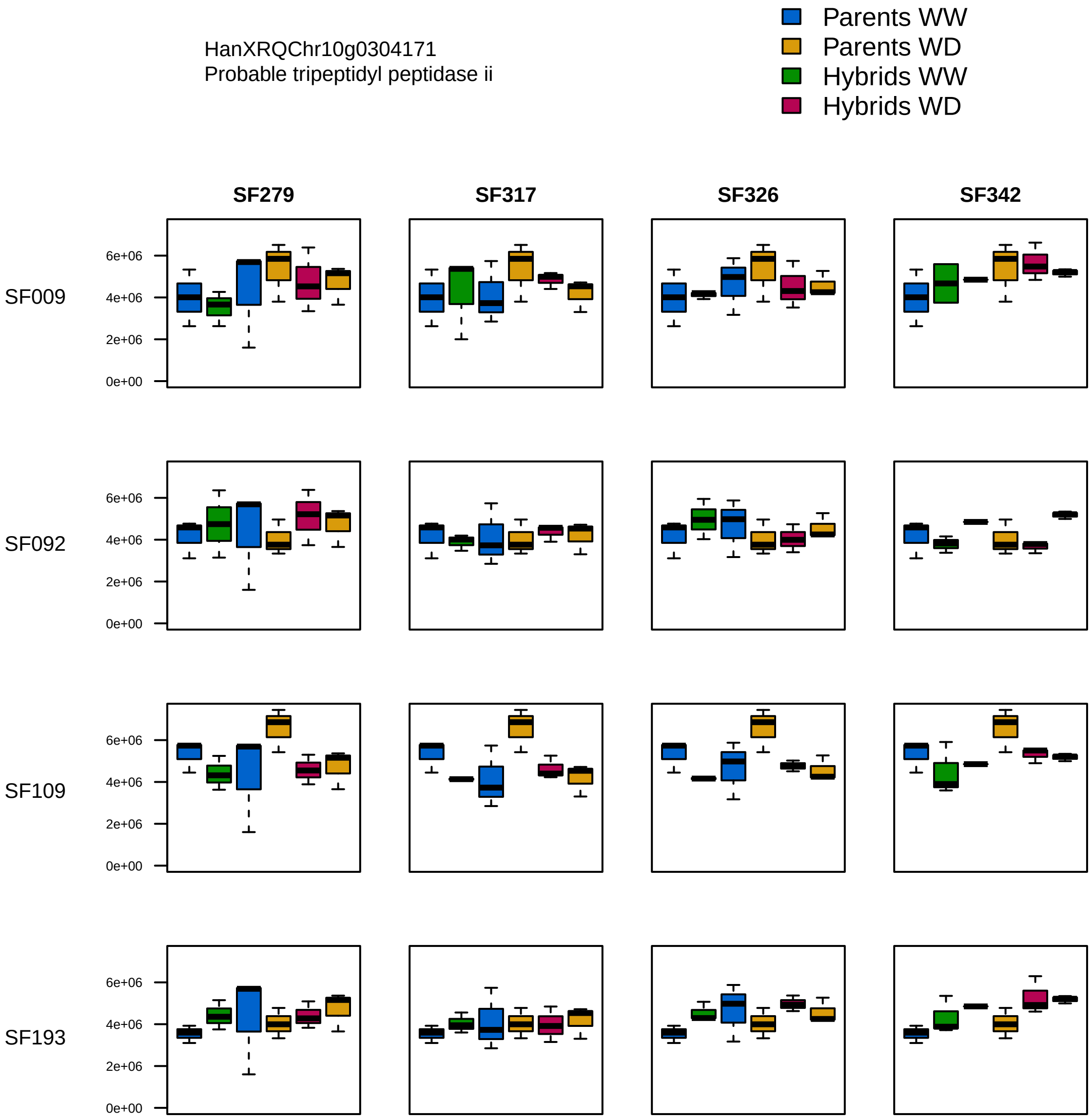

HanXRQChr10g0304171
Probable tripeptidyl peptidase ii
Parents WW
Parents WD
Hybrids WW
Hybrids WD
SF279
SF317
SF326
SF342
SF009
SF092
SF109
SF193
0e+00
2e+06
4e+06
6e+06

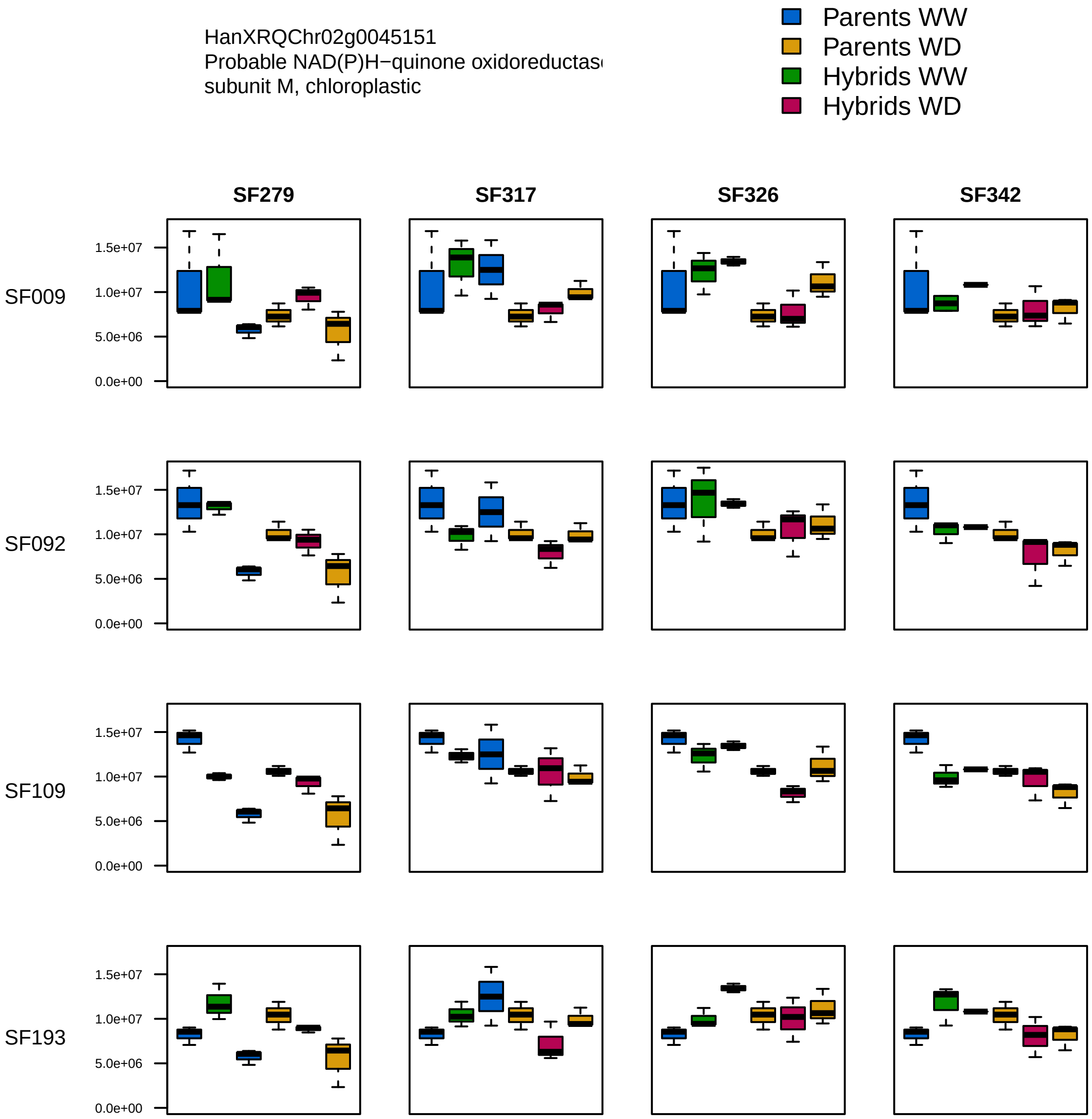

HanXRQChr02g0045151
Probable NAD(P)H−quinone oxidoreductas
subunit M, chloroplastic
Parents WW
Parents WD
Hybrids WW
Hybrids WD
SF279
SF317
SF326
SF342
SF009
SF092
SF109
SF193
1.5e+07
1.0e+07
5.0e+06
0.0e+00

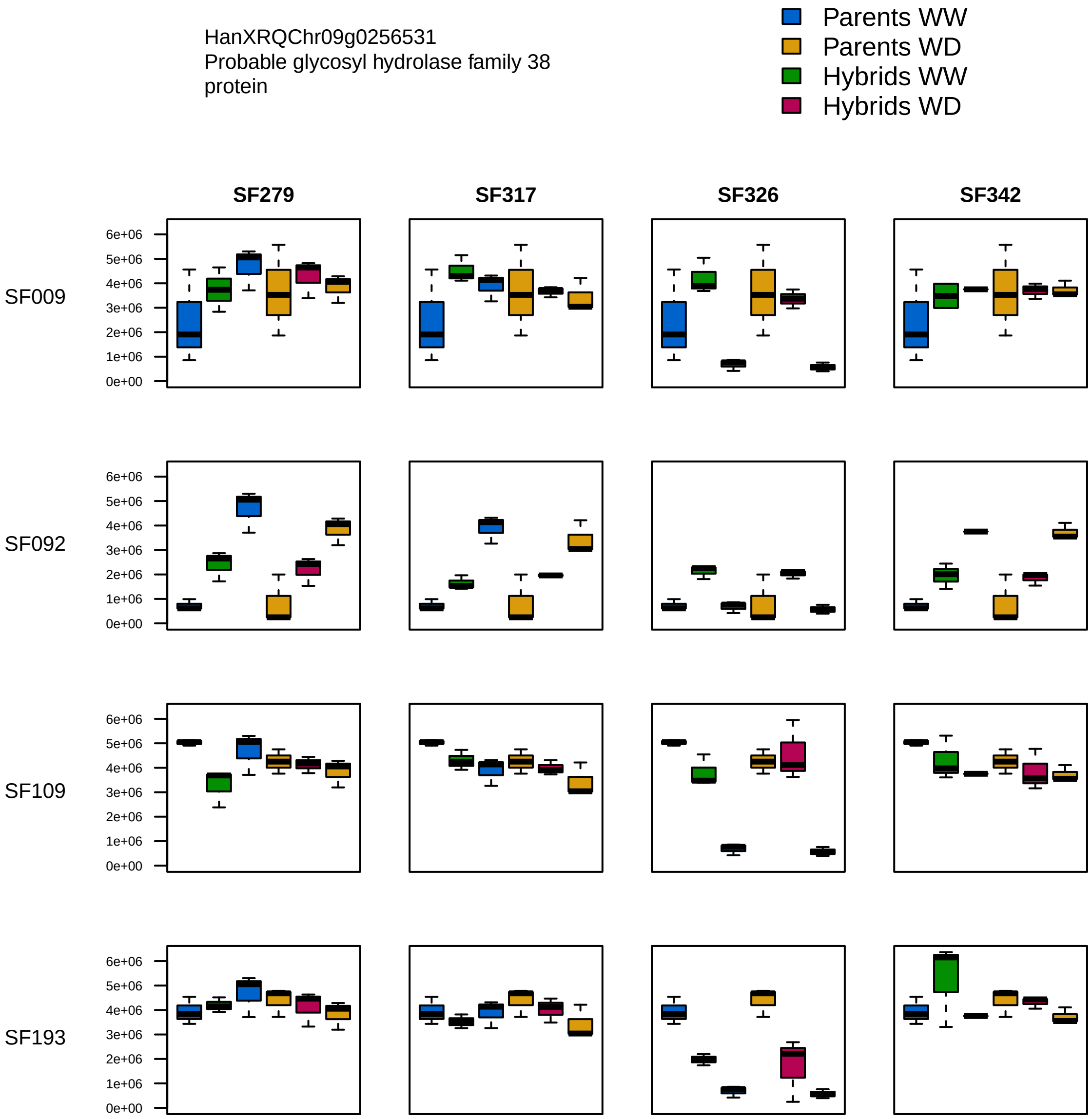
HanXRQChr09g0256531
Probable glycosyl hydrolase family 38
protein
Parents WW
Parents WD
Hybrids WW
Hybrids WD
SF279
SF317
SF326
SF342
SF009
SF092
SF109
SF193
6e+06
5e+06
4e+06
3e+06
2e+06
1e+06
0e+00

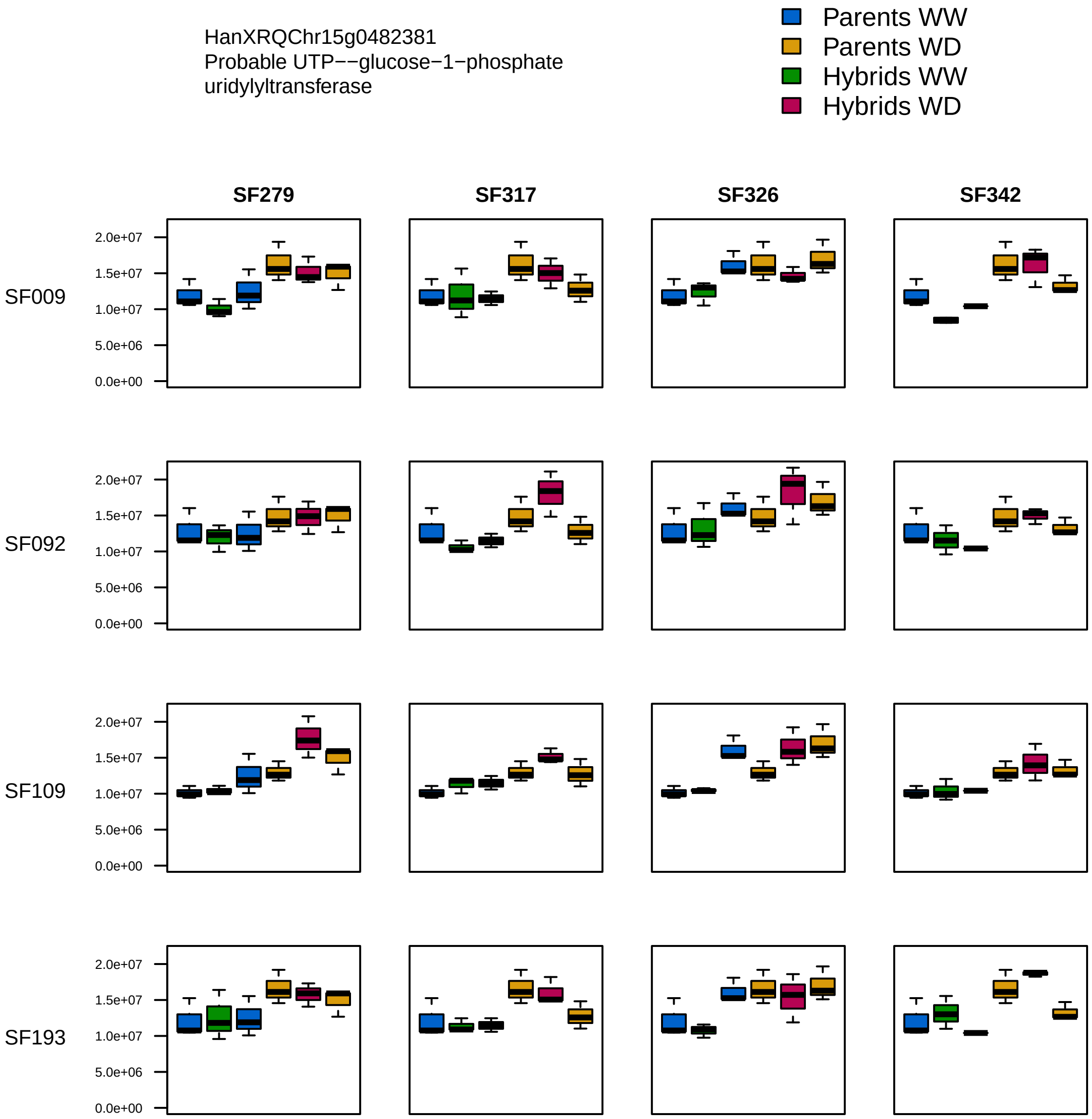
HanXRQChr15g0482381
Probable UTP−−glucose−1−phosphate
uridylyltransferase
Parents WW
Parents WD
Hybrids WW
Hybrids WD
SF279
SF317
SF326
SF342
SF009
SF092
SF109
SF193
2.0e+07
1.5e+07
1.0e+07
5.0e+06
0.0e+00

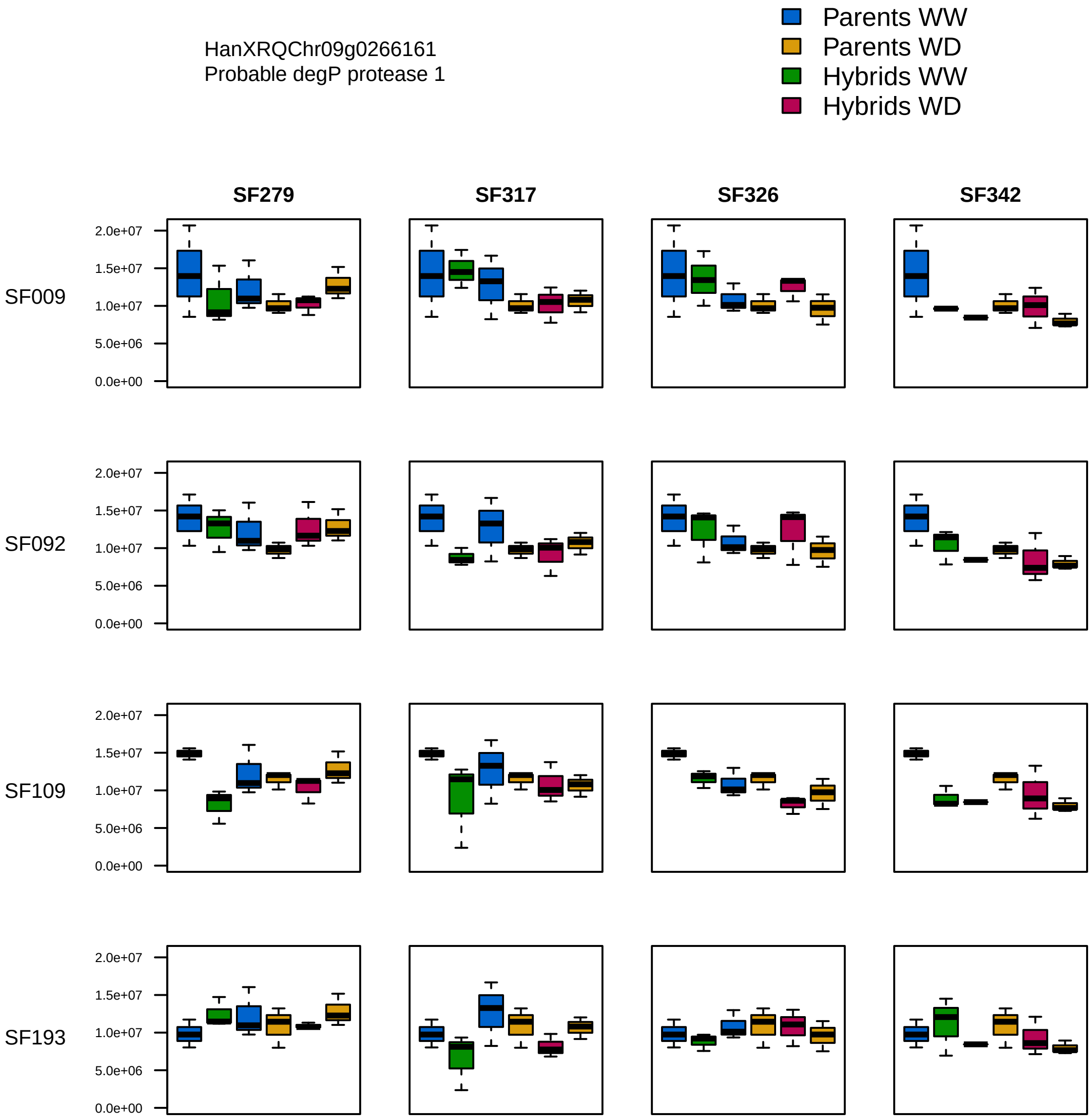

HanXRQChr09g0266161
Probable degP protease 1
Parents WW
Parents WD
Hybrids WW
Hybrids WD
SF279
SF317
SF326
SF342
SF009
SF092
SF109
SF193
2.0e+07
1.5e+07
1.0e+07
5.0e+06
0.0e+00

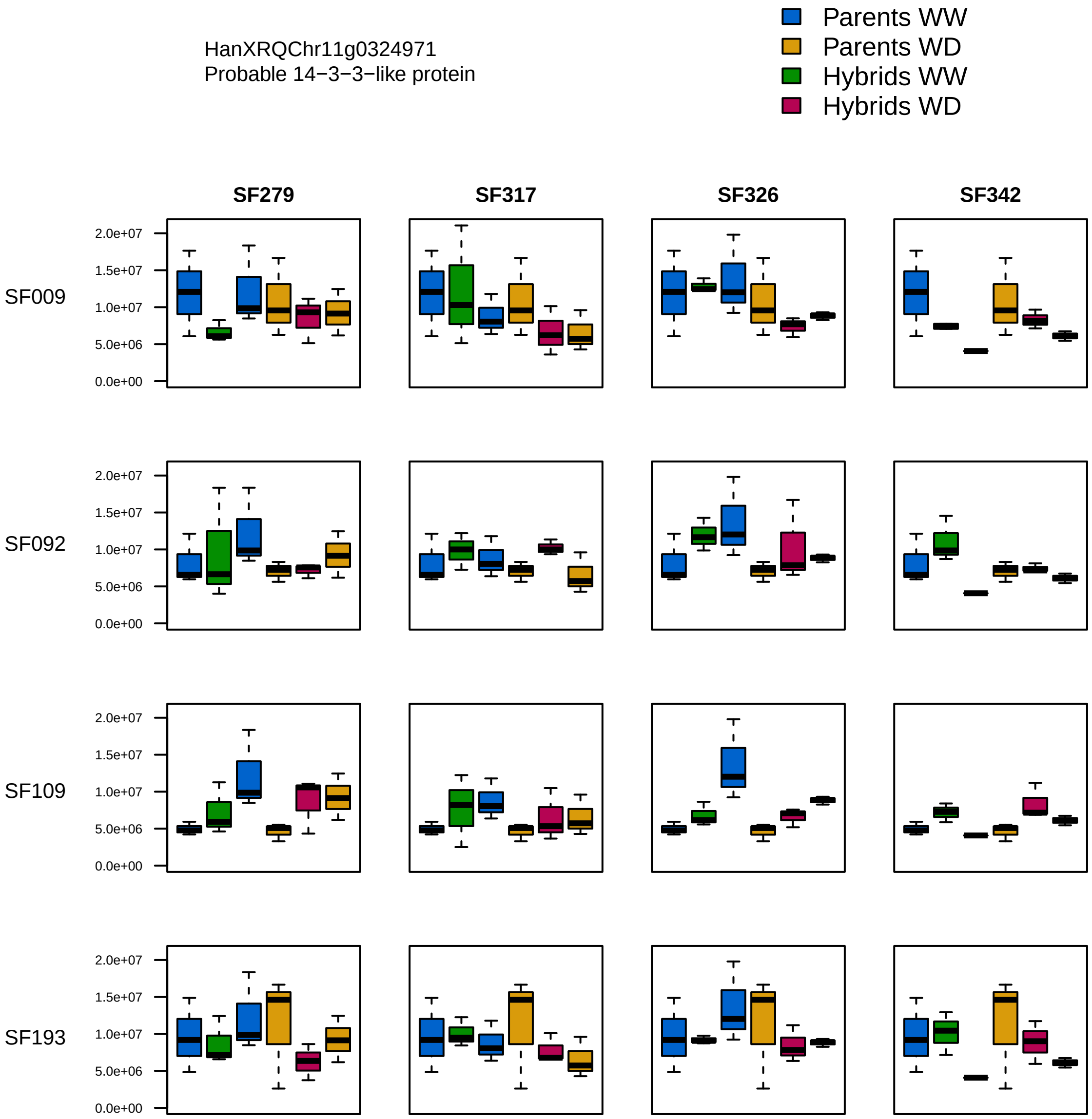
HanXRQChr11g0324971
Probable 14−3−3−like protein
Parents WW
Parents WD
Hybrids WW
Hybrids WD
SF279
SF317
SF326
SF342
SF009
SF092
SF109
SF193
2.0e+07
1.5e+07
1.0e+07
5.0e+06
0.0e+00

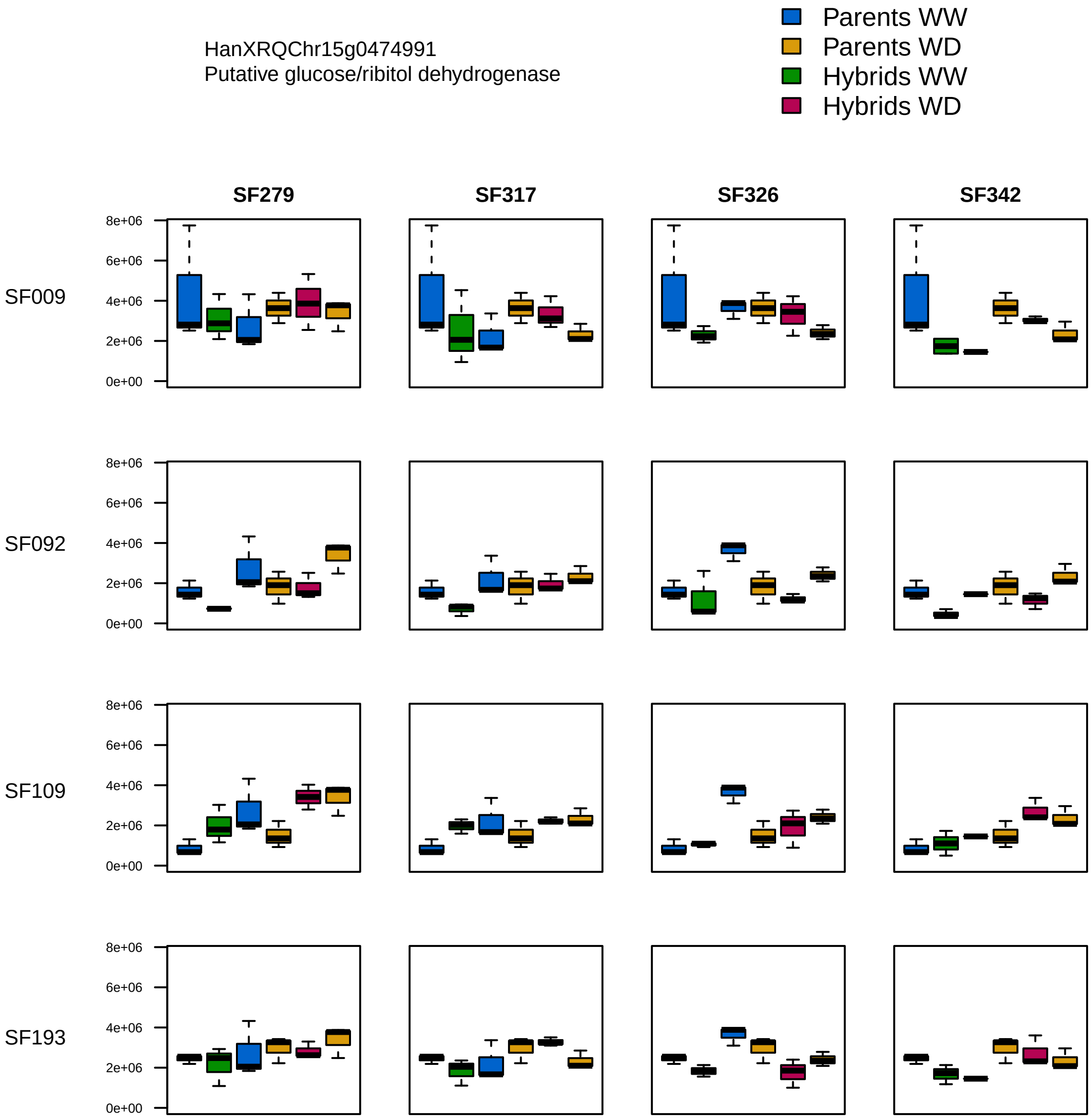
HanXRQChr15g0474991
Putative glucose/ribitol dehydrogenase
Parents WW
Parents WD
Hybrids WW
Hybrids WD
SF279
SF317
SF326
SF342
SF009
SF092
SF109
SF193
8e+06
6e+06
4e+06
2e+06
0e+00

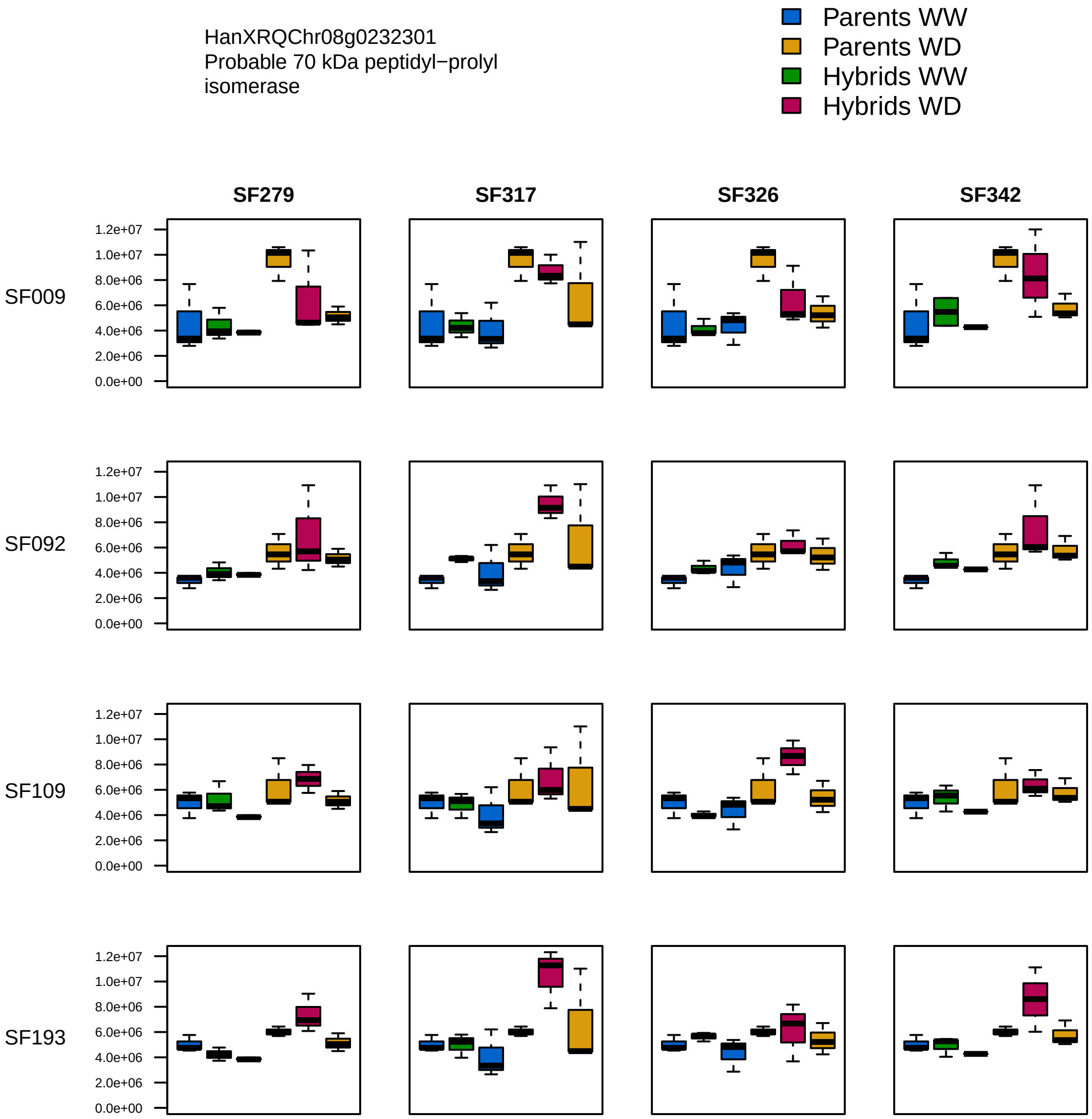

HanXRQChr08g0232301
Probable 70 kDa peptidyl−prolyl
isomerase
Parents WW
Parents WD
Hybrids WW
Hybrids WD
SF279
SF317
SF326
SF342
SF009
SF092
SF109
SF193
1.2e+07
1.0e+07
8.0e+06
6.0e+06
4.0e+06
2.0e+06
0.0e+00

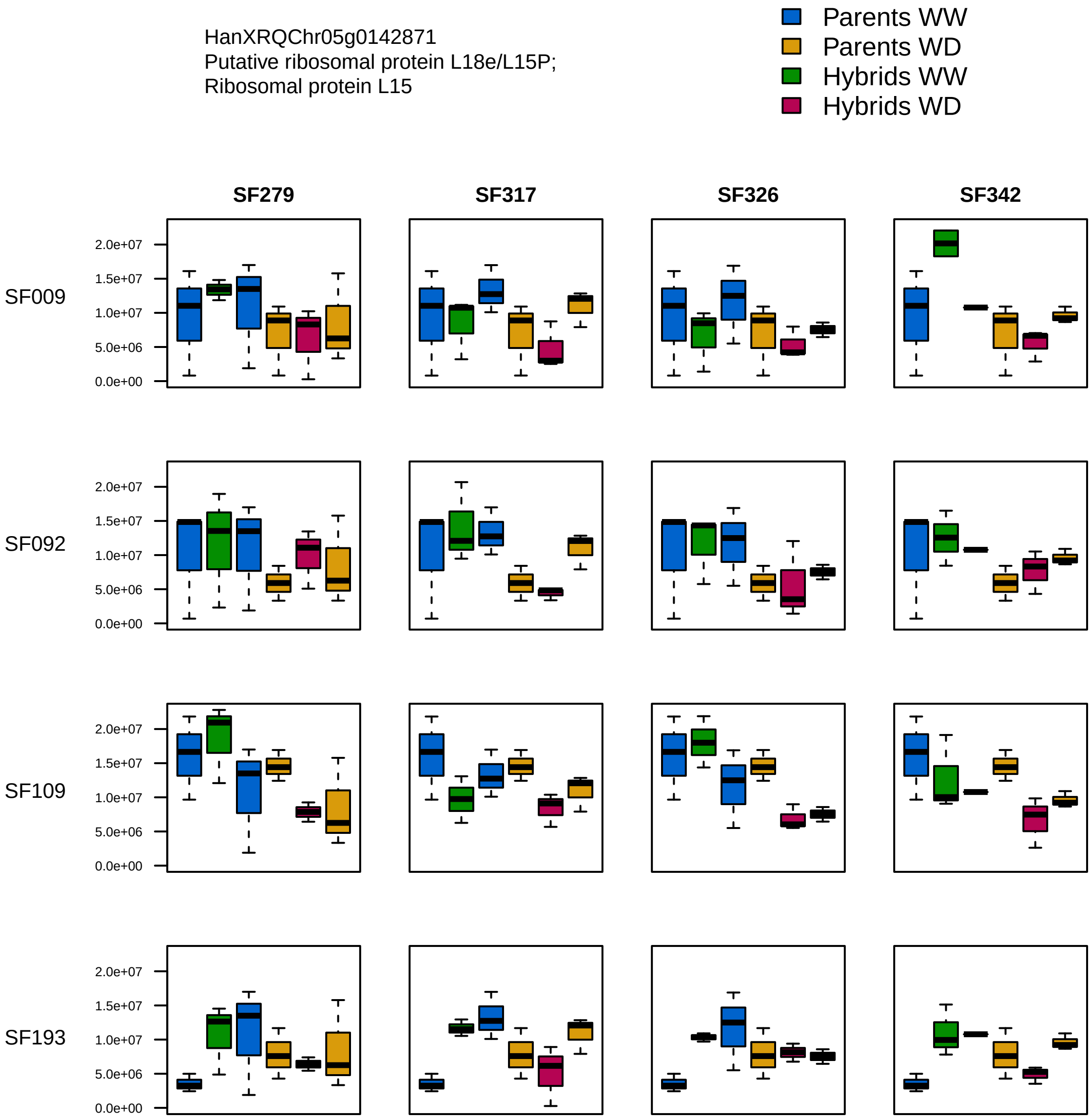
HanXRQChr05g0142871
Putative ribosomal protein L18e/L15P;
Ribosomal protein L15
Parents WW
Parents WD
Hybrids WW
Hybrids WD
SF279
SF317
SF326
SF342
SF009
SF092
SF109
SF193
2.0e+07
1.5e+07
1.0e+07
5.0e+06
0.0e+00

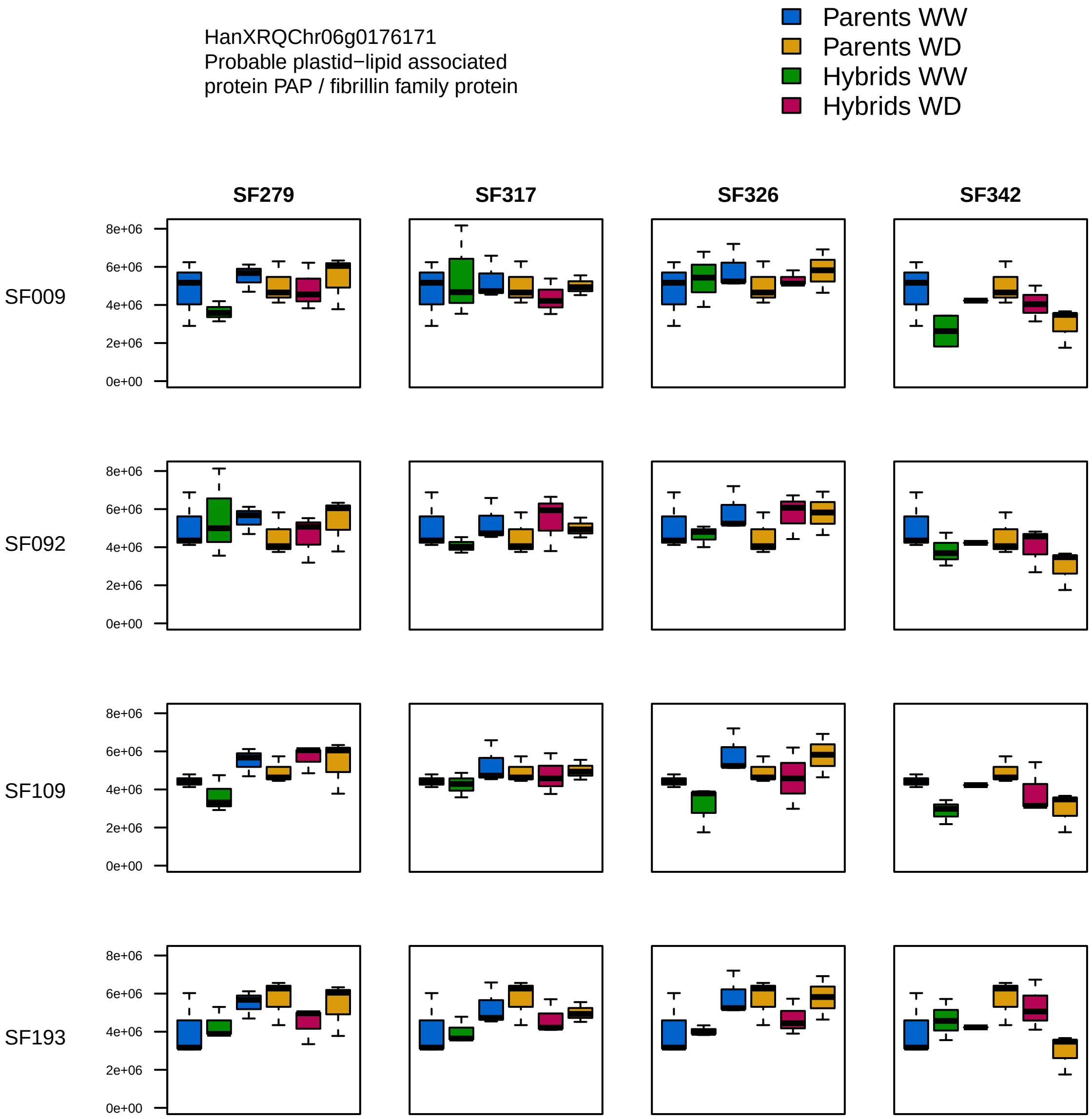
HanXRQChr06g0176171
Probable plastid−lipid associated
protein PAP / fibrillin family protein
Parents WW
Parents WD
Hybrids WW
Hybrids WD
SF279
SF317
SF326
SF342
SF009
SF092
SF109
SF193
0e+00
2e+06
4e+06
6e+06
8e+06

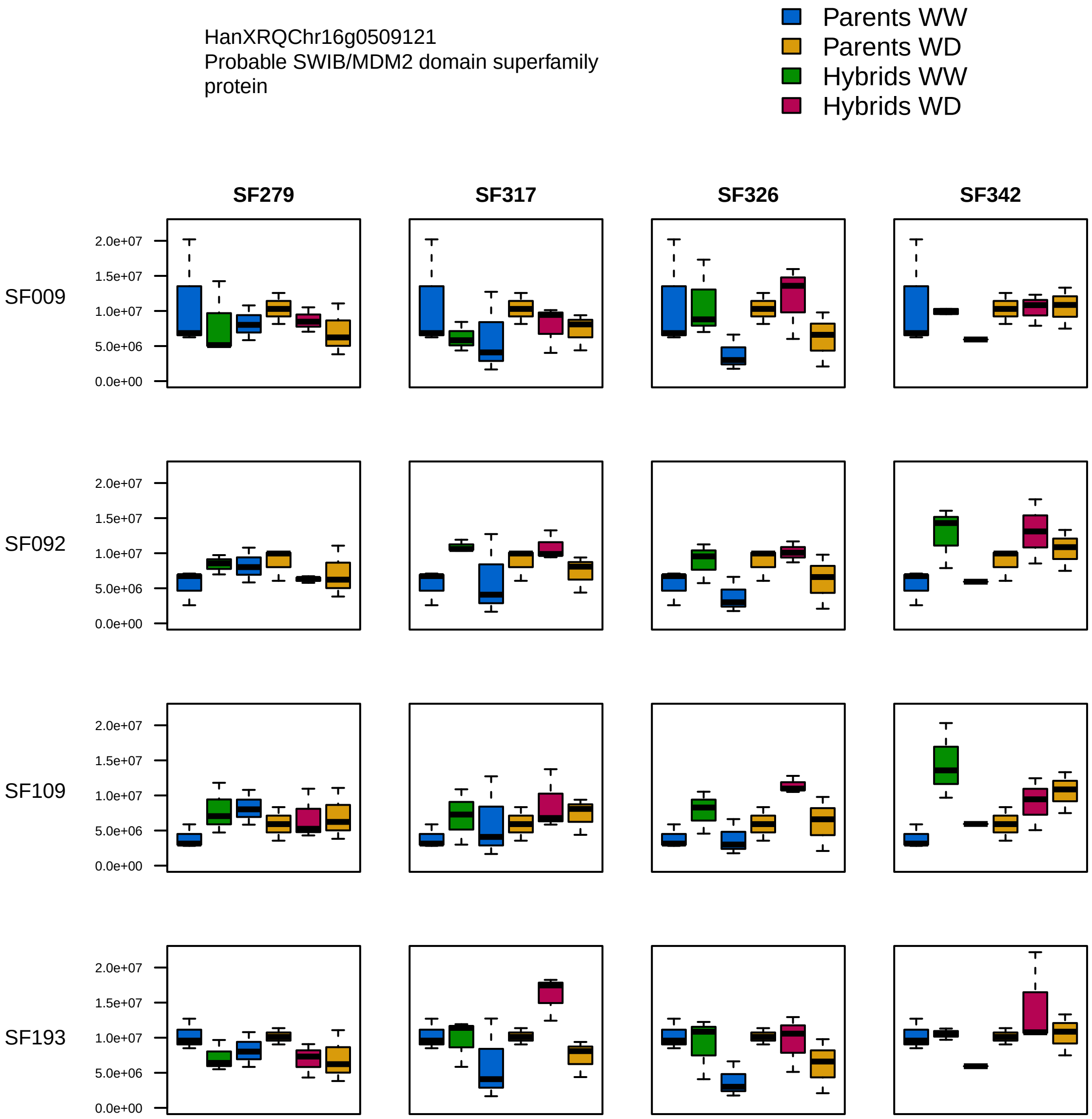

HanXRQChr16g0509121
Probable SWIB/MDM2 domain superfamily protein
Parents WW
Parents WD
Hybrids WW
Hybrids WD
SF279
SF317
SF326
SF342
SF009
SF092
SF109
SF193
2.0e+07
1.5e+07
1.0e+07
5.0e+06
0.0e+00

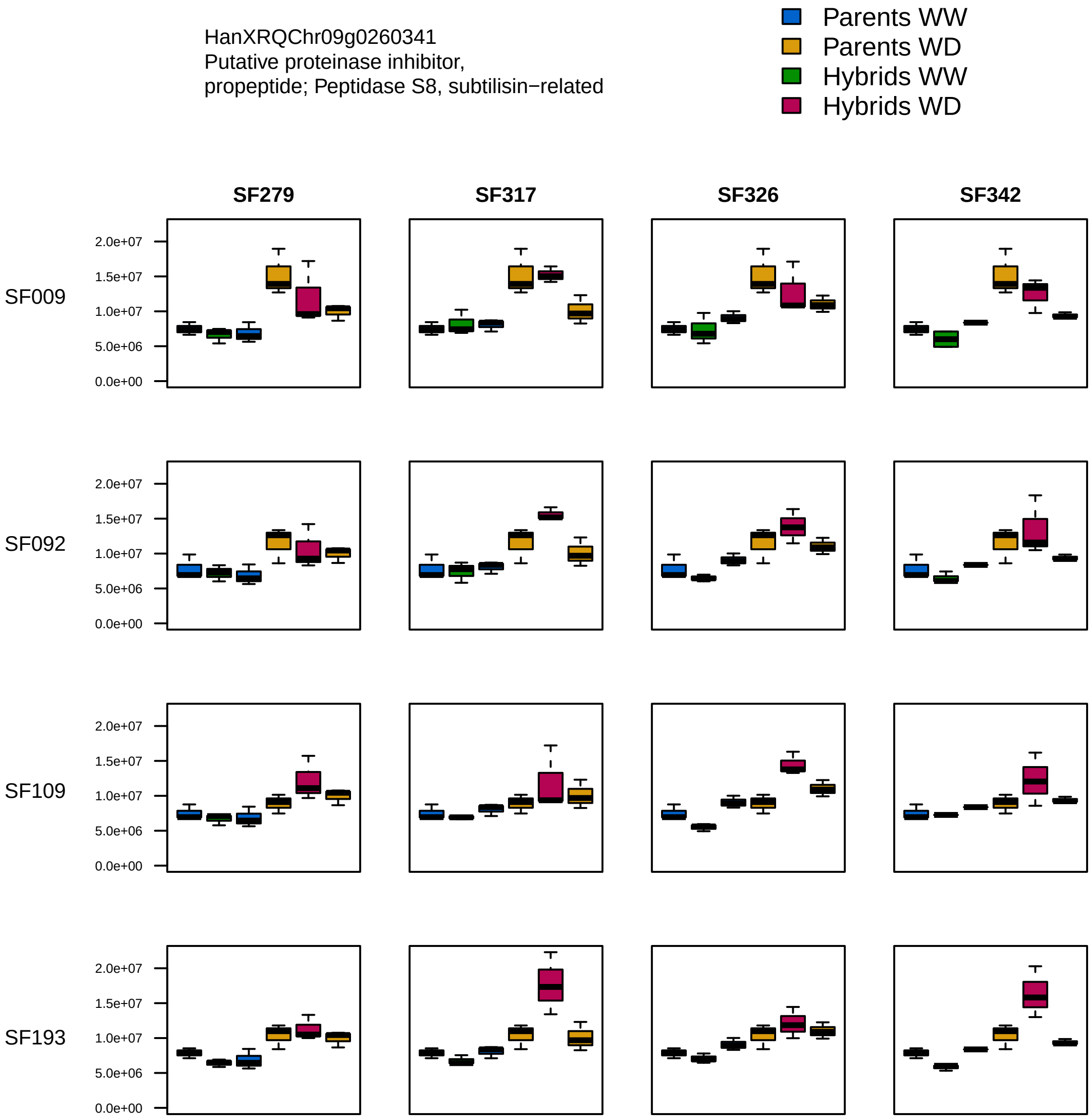

HanXRQChr09g0260341
Putative proteinase inhibitor,
propeptide; Peptidase S8, subtilisin−related
Parents WW
Parents WD
Hybrids WW
Hybrids WD
SF279
SF317
SF326
SF342
SF009
SF092
SF109
SF193
0.0e+00
5.0e+06
1.0e+07
1.5e+07
2.0e+07

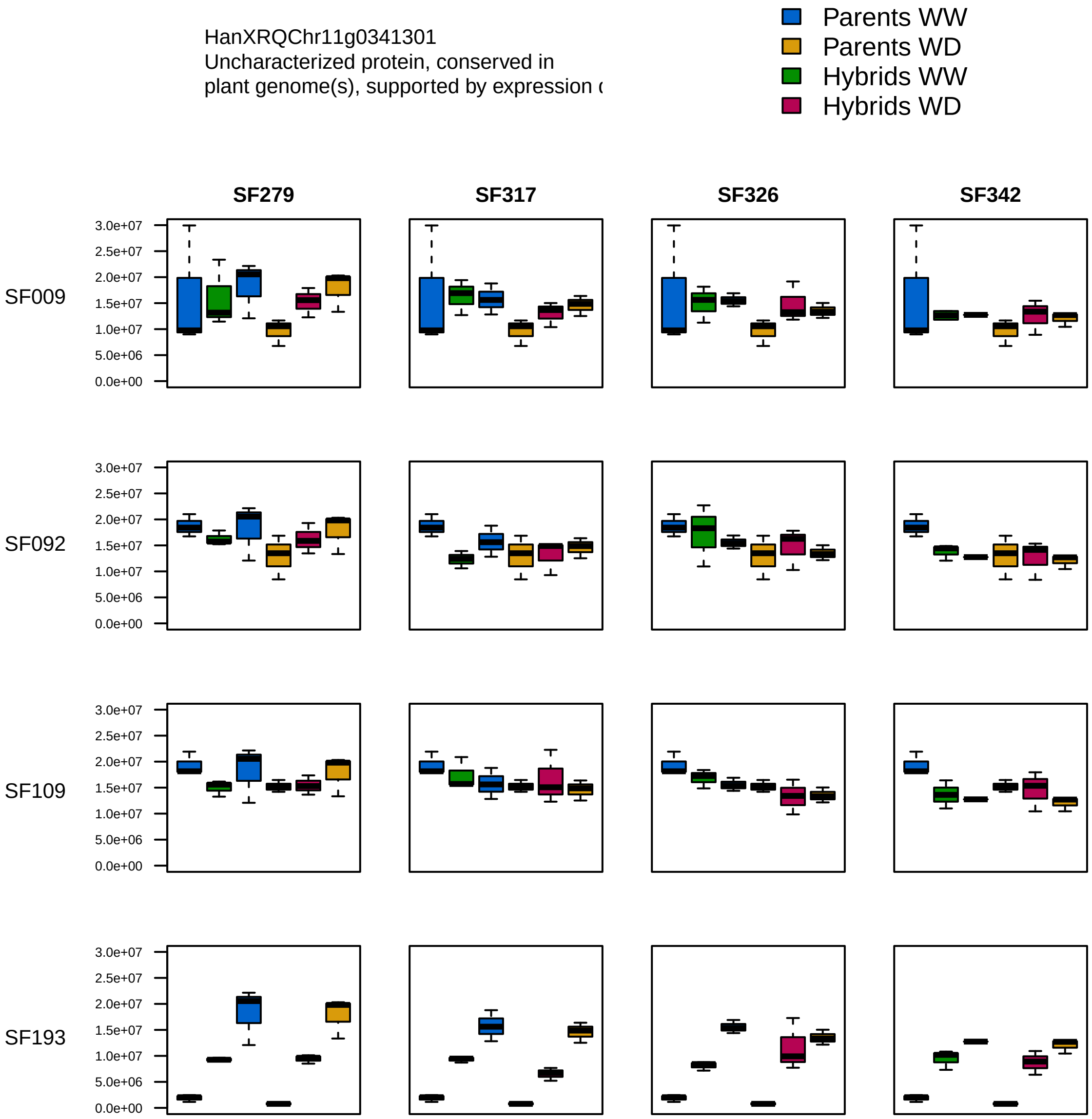

HanXRQChr11g0341301
Uncharacterized protein, conserved in
plant genome(s), supported by expression (
Parents WW
Parents WD
Hybrids WW
Hybrids WD
SF279
SF317
SF326
SF342
SF009
SF092
SF109
SF193
3.0e+07
2.5e+07
2.0e+07
1.5e+07
1.0e+07
5.0e+06
0.0e+00

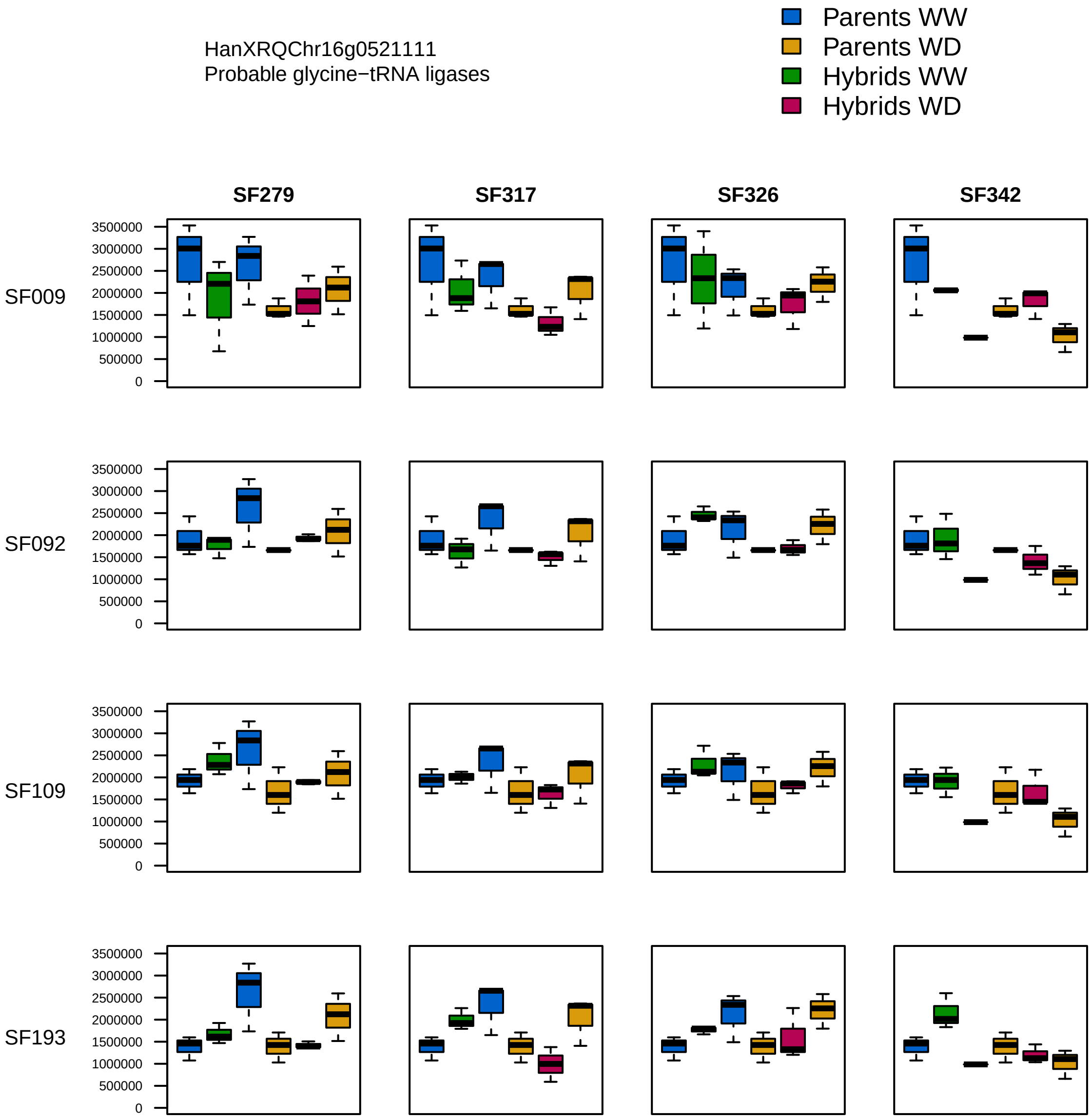
HanXRQChr16g0521111
Probable glycine−tRNA ligases
Parents WW
Parents WD
Hybrids WW
Hybrids WD
SF279
SF317
SF326
SF342
SF009
SF092
SF109
SF193
3500000
3000000
2500000
2000000
1500000
1000000
500000
0

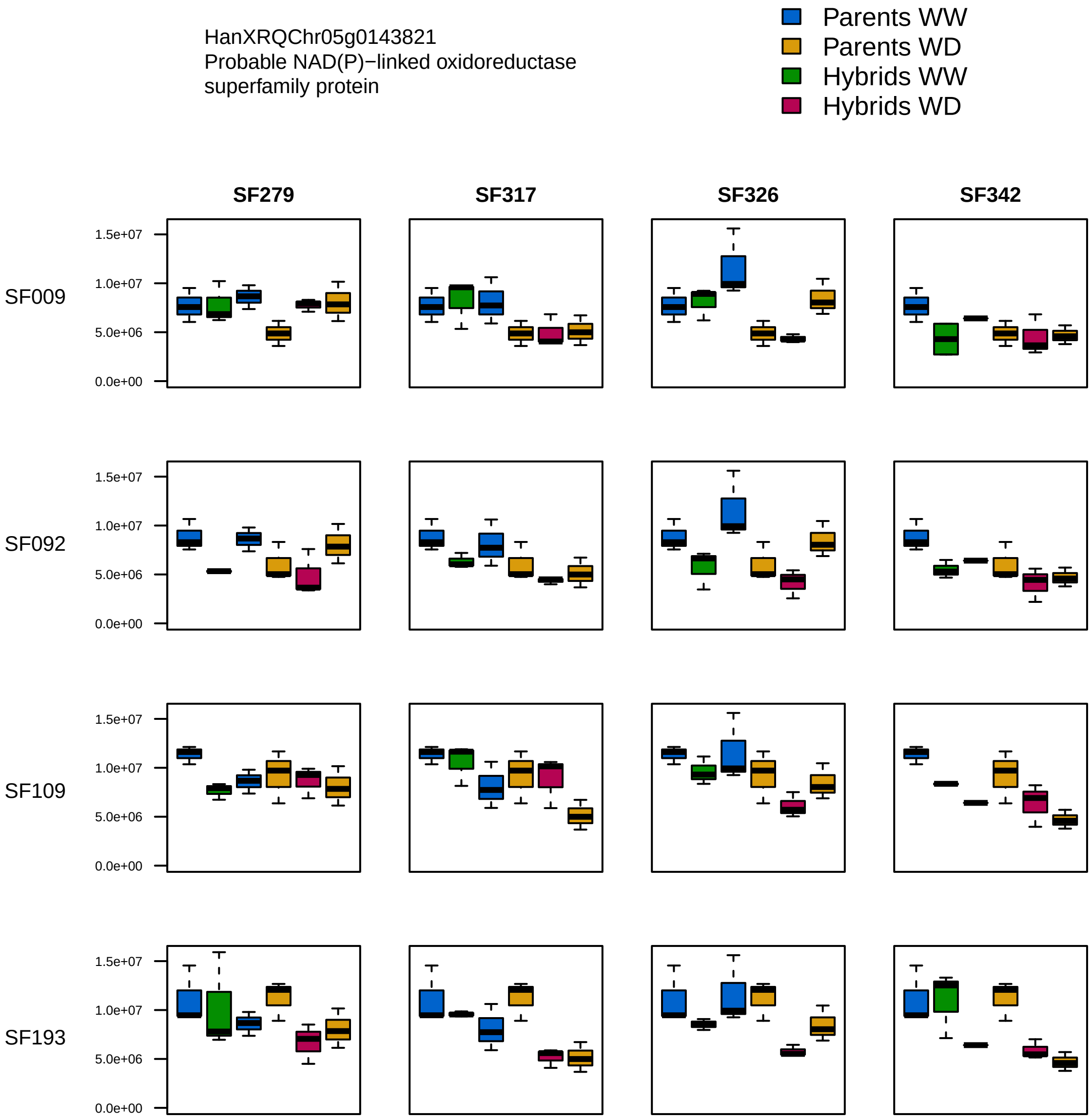
HanXRQChr05g0143821
Probable NAD(P)−linked oxidoreductase
superfamily protein
Parents WW
Parents WD
Hybrids WW
Hybrids WD
SF279
SF317
SF326
SF342
SF009
SF092
SF109
SF193
1.5e+07
1.0e+07
5.0e+06
0.0e+00

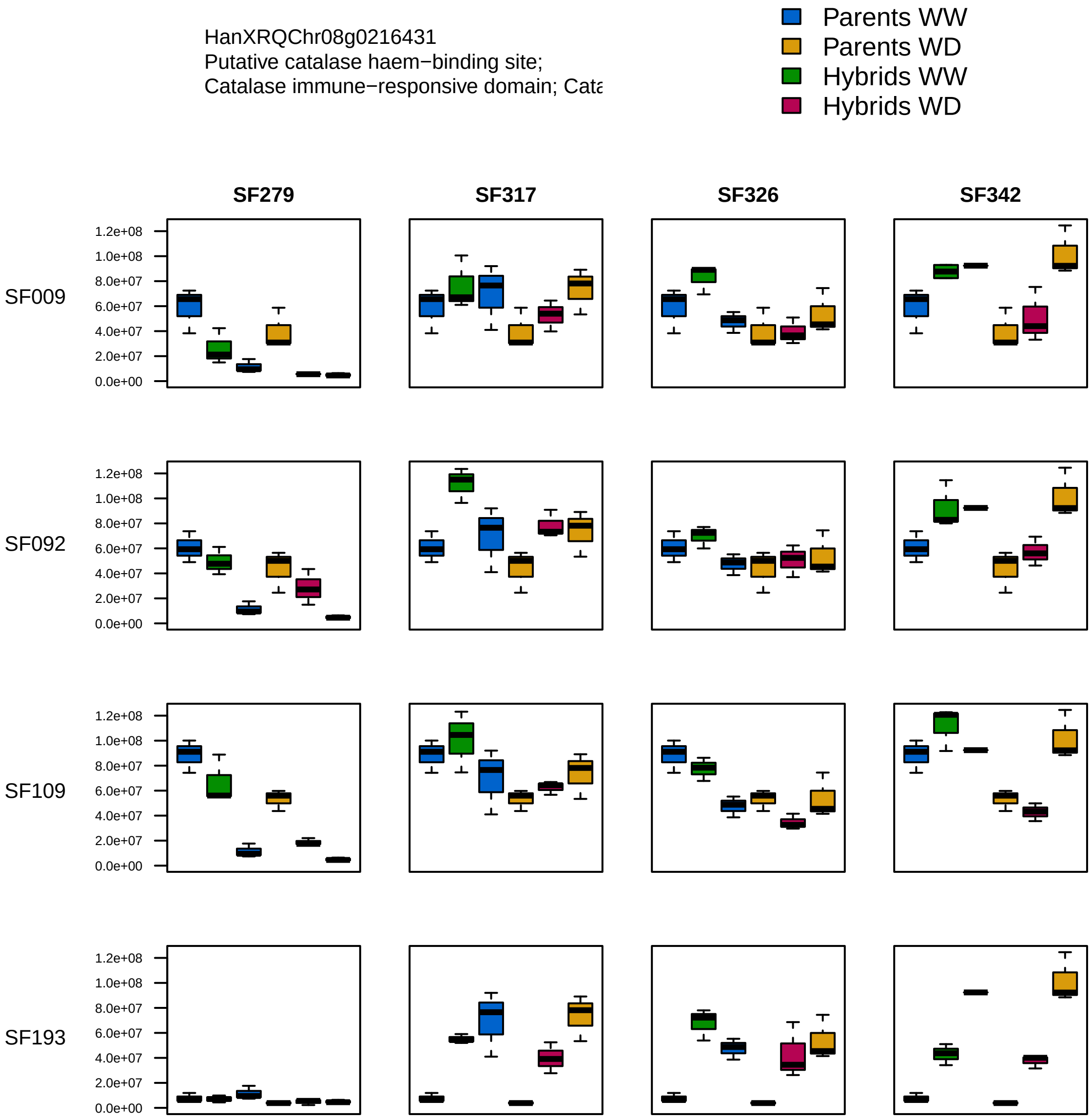
HanXRQChr08g0216431
Putative catalase haem−binding site;
Catalase immune−responsive domain; Cata
Parents WW
Parents WD
Hybrids WW
Hybrids WD
SF279
SF317
SF326
SF342
SF009
SF092
SF109
SF193
1.2e+08
1.0e+08
8.0e+07
6.0e+07
4.0e+07
2.0e+07
0.0e+00

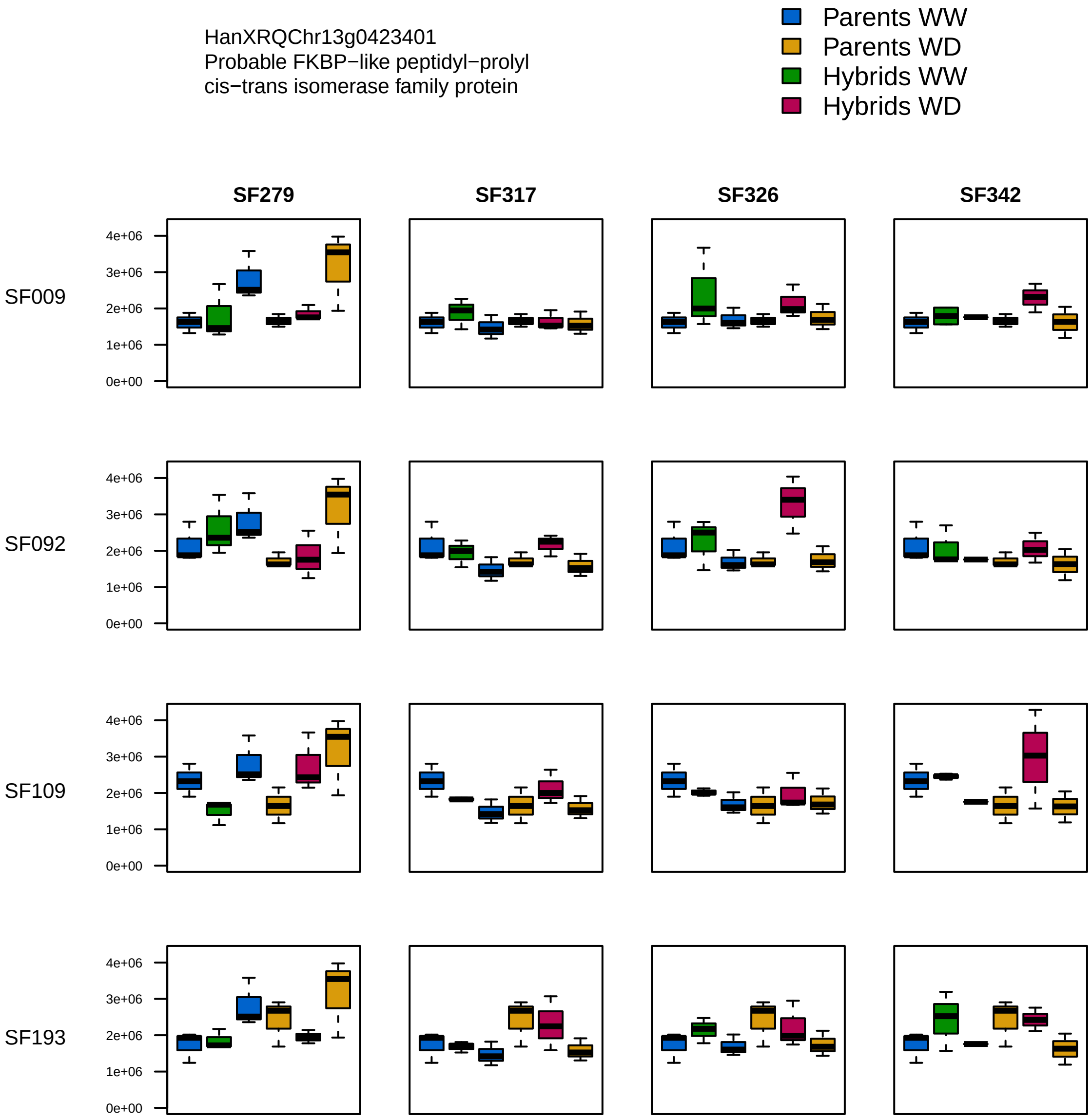

HanXRQChr13g0423401
Probable FKBP−like peptidyl−prolyl
cis−trans isomerase family protein
Parents WW
Parents WD
Hybrids WW
Hybrids WD
SF279
SF317
SF326
SF342
SF009
SF092
SF109
SF193
0e+00
1e+06
2e+06
3e+06
4e+06

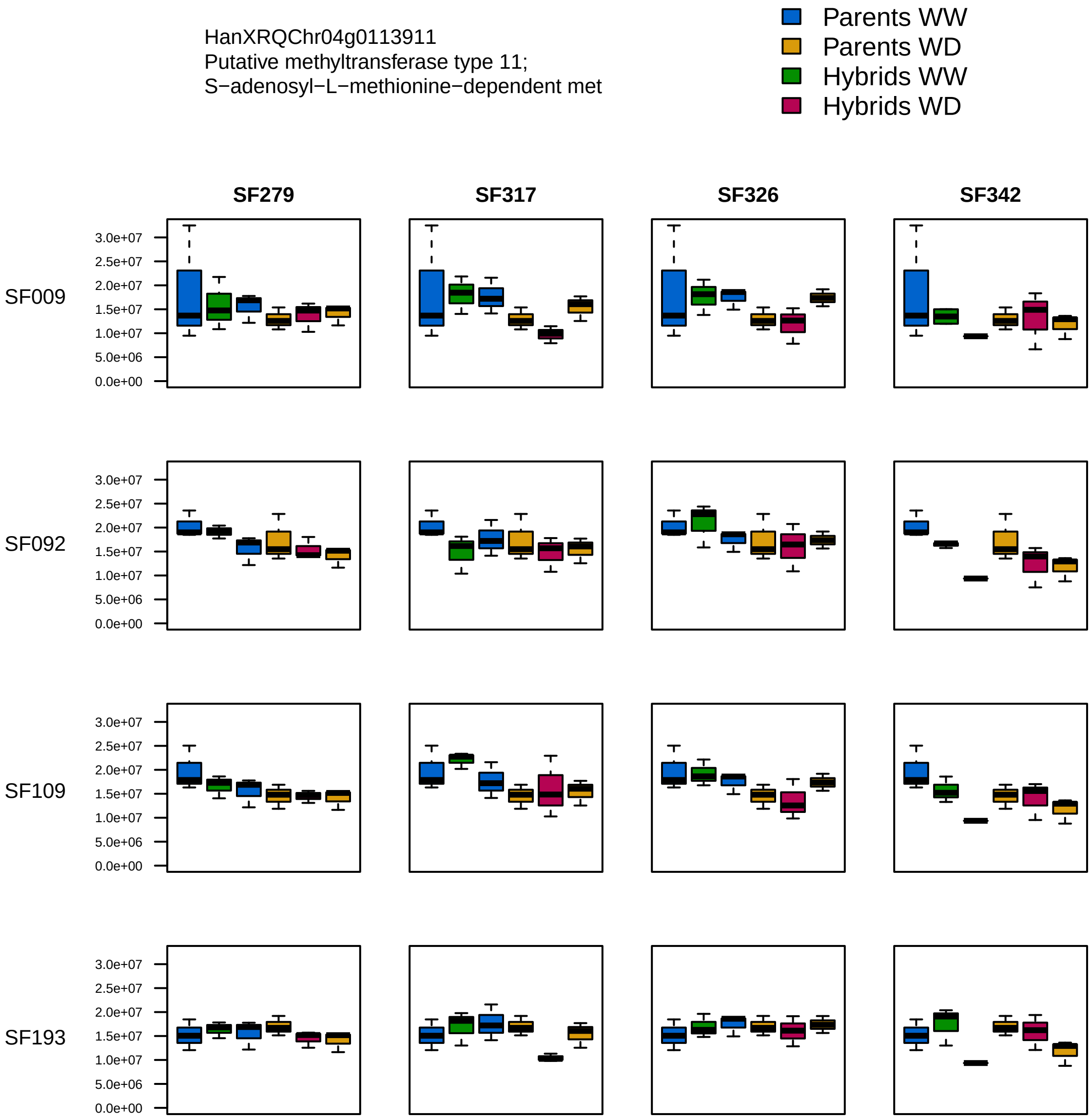
HanXRQChr04g0113911
Putative methyltransferase type 11;
S−adenosyl−L−methionine−dependent met
Parents WW
Parents WD
Hybrids WW
Hybrids WD
SF279
SF317
SF326
SF342
SF009
SF092
SF109
SF193
3.0e+07
2.5e+07
2.0e+07
1.5e+07
1.0e+07
5.0e+06
0.0e+00

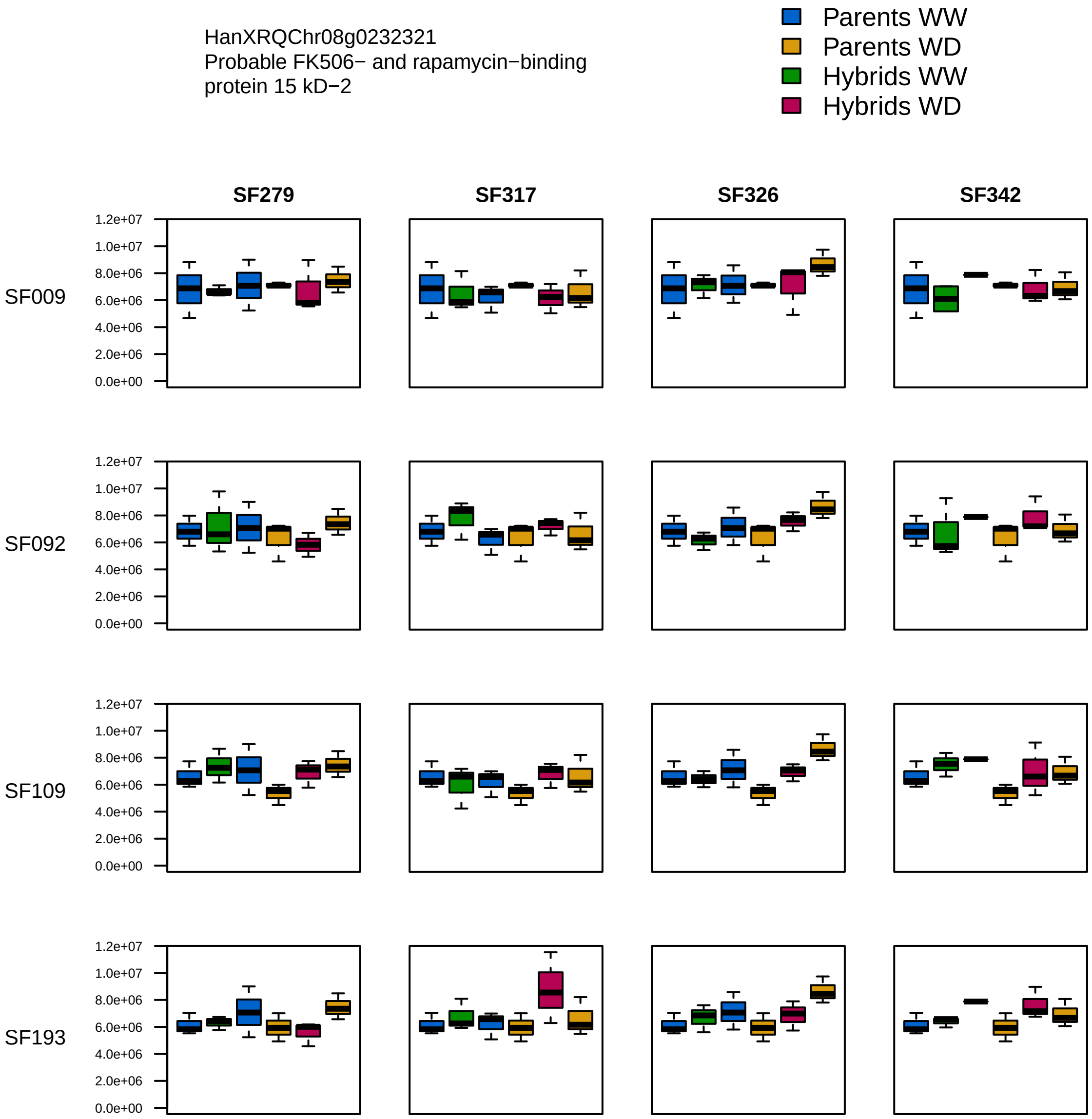
HanXRQChr08g0232321
Probable FK506− and rapamycin−binding
protein 15 kD−2
Parents WW
Parents WD
Hybrids WW
Hybrids WD
SF279
SF317
SF326
SF342
SF009
SF092
SF109
SF193
1.2e+07
1.0e+07
8.0e+06
6.0e+06
4.0e+06
2.0e+06
0.0e+00

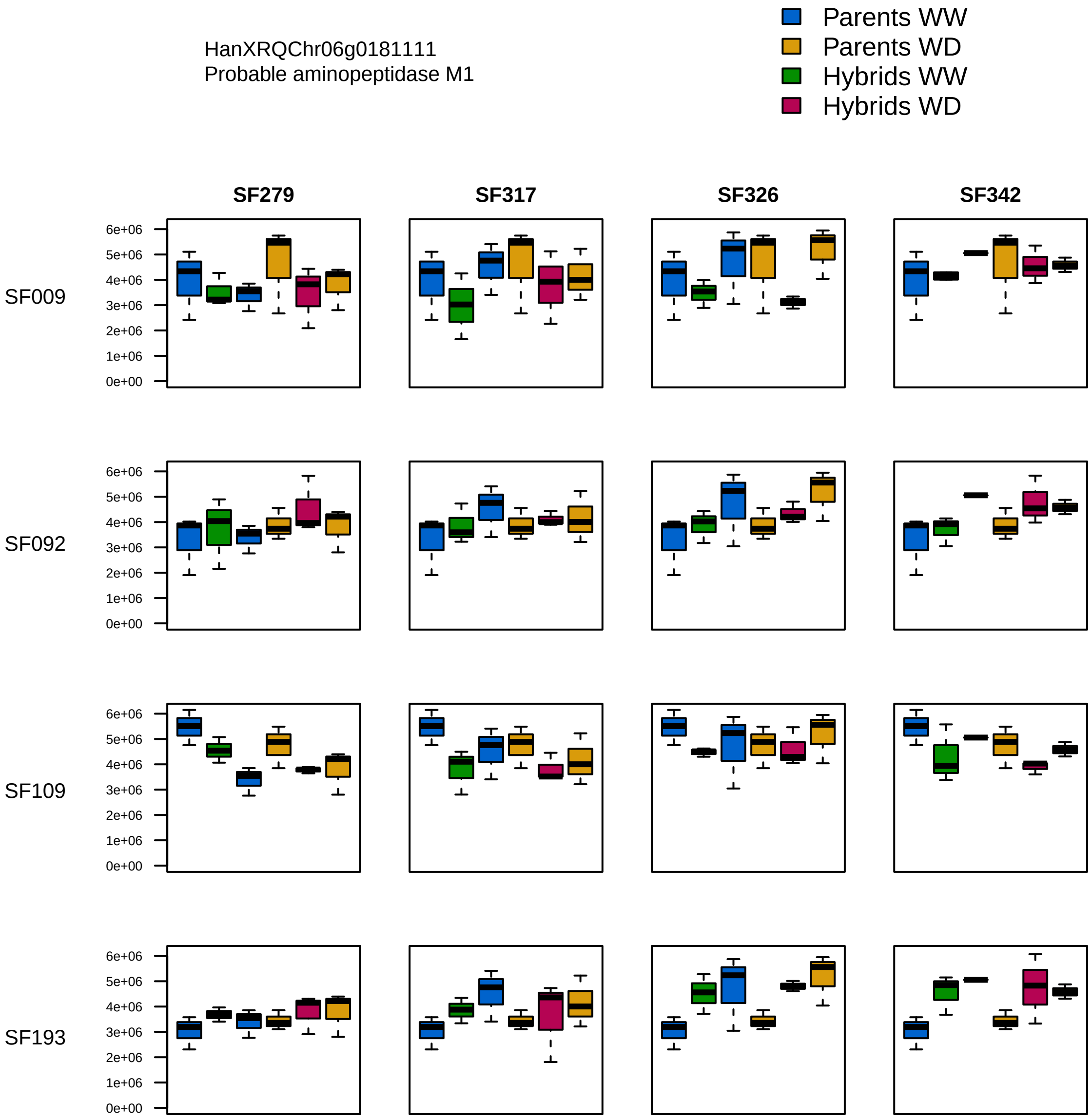
HanXRQChr06g0181111
Probable aminopeptidase M1
Parents WW
Parents WD
Hybrids WW
Hybrids WD
SF279
SF317
SF326
SF342
SF009
SF092
SF109
SF193
6e+06
5e+06
4e+06
3e+06
2e+06
1e+06
0e+00

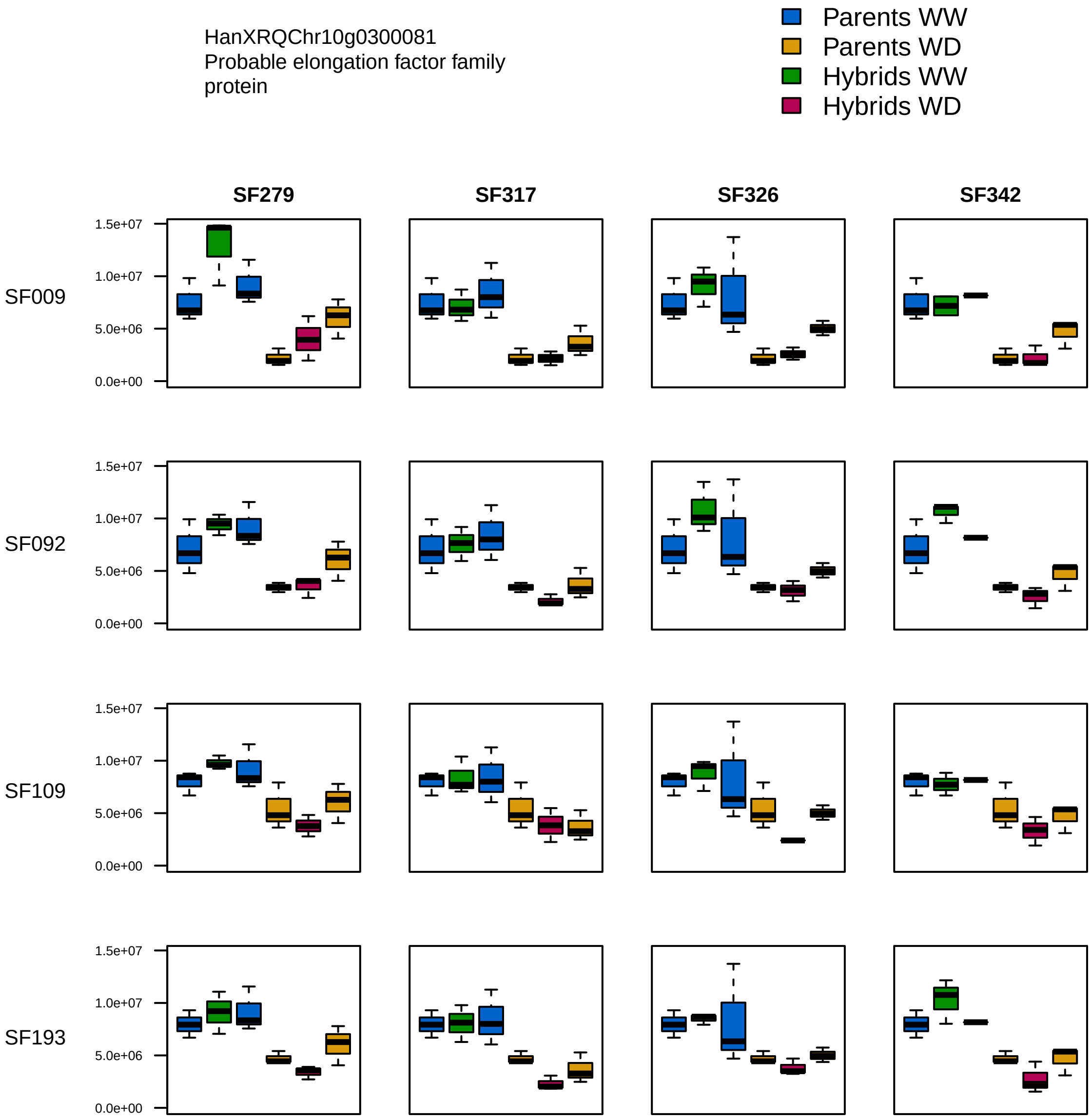

HanXRQChr10g0300081
Probable elongation factor family protein
Parents WW
Parents WD
Hybrids WW
Hybrids WD
SF279
SF317
SF326
SF342
SF009
SF092
SF109
SF193
1.5e+07
1.0e+07
5.0e+06
0.0e+00

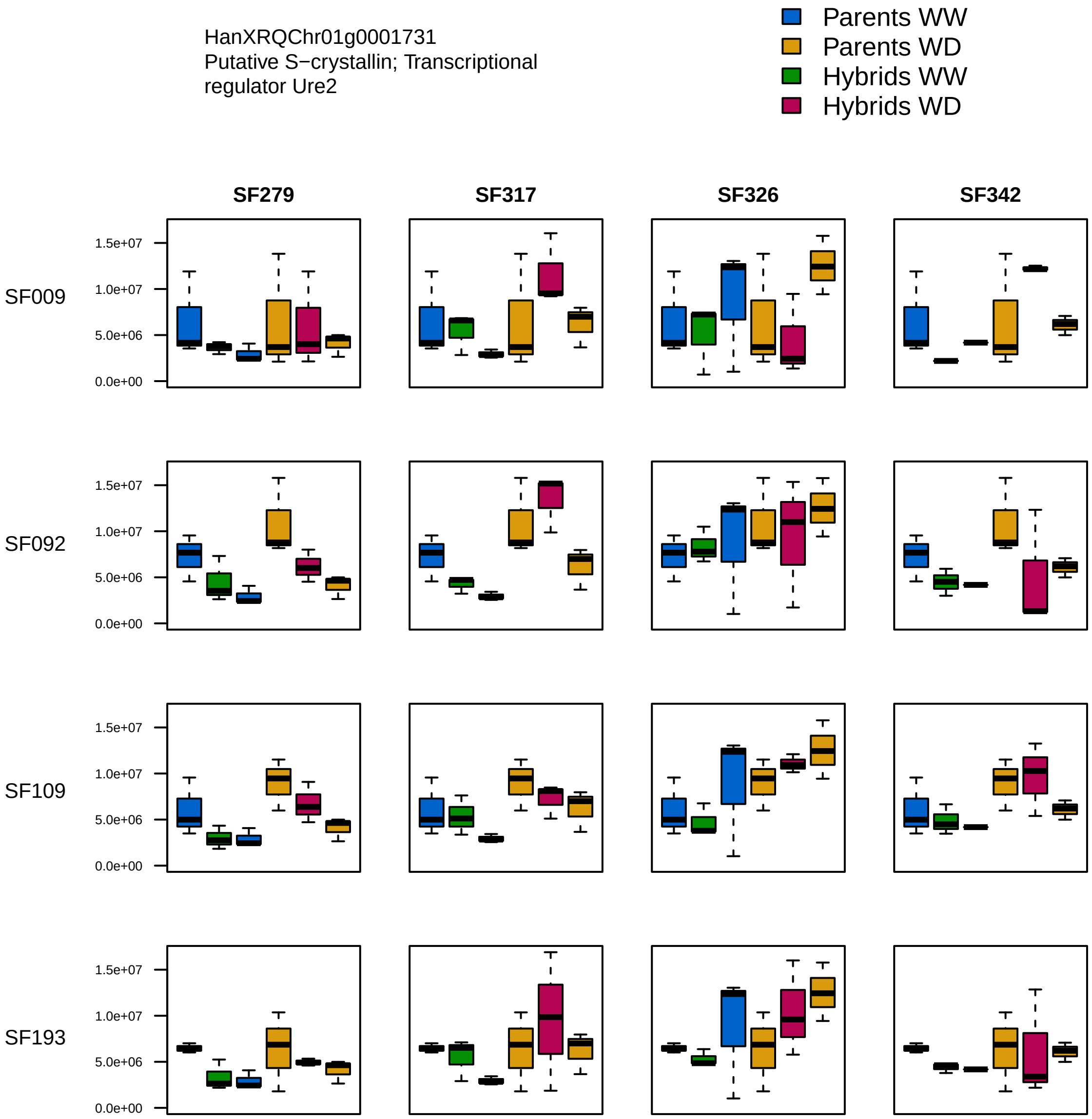
HanXRQChr01g0001731
Putative S−crystallin; Transcriptional regulator Ure2
Parents WW
Parents WD
Hybrids WW
Hybrids WD
SF279
SF317
SF326
SF342
SF009
SF092
SF109
SF193
0.0e+00
5.0e+06
1.0e+07
1.5e+07

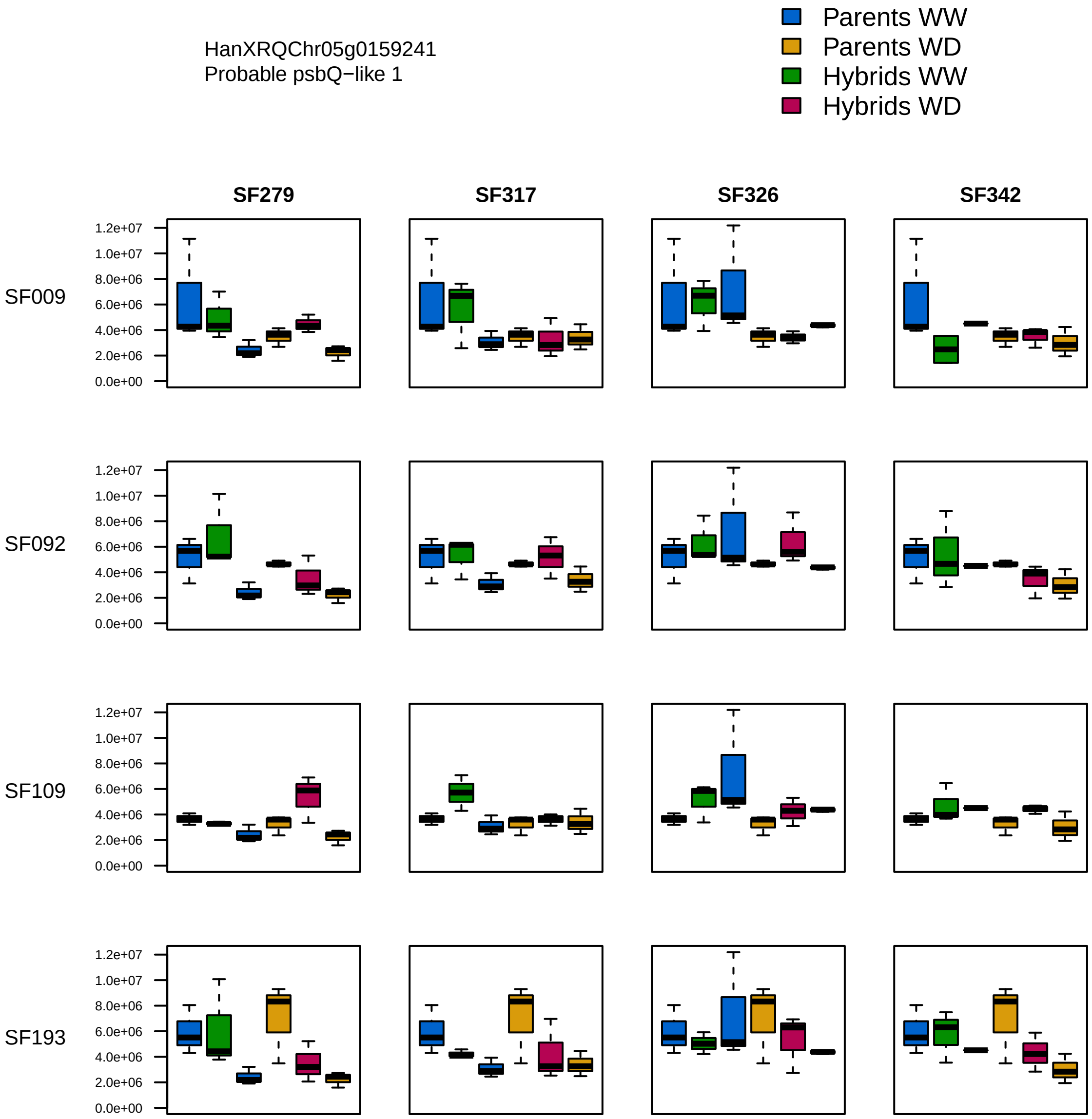

HanXRQChr05g0159241
Probable psbQ−like 1
Parents WW
Parents WD
Hybrids WW
Hybrids WD
SF279
SF317
SF326
SF342
SF009
SF092
SF109
SF193
1.2e+07
1.0e+07
8.0e+06
6.0e+06
4.0e+06
2.0e+06
0.0e+00

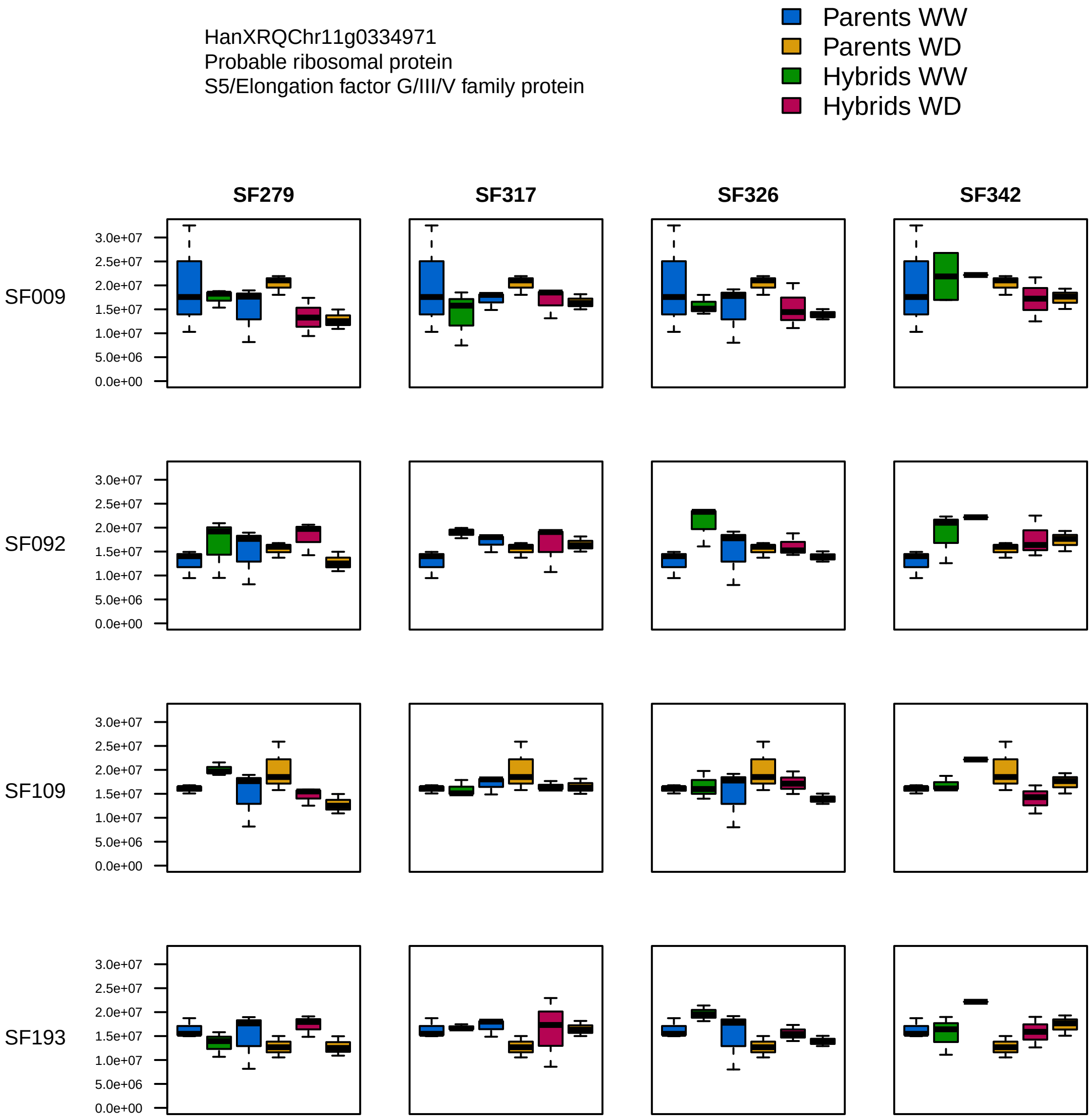
HanXRQChr11g0334971
Probable ribosomal protein
S5/Elongation factor G/III/V family protein
Parents WW
Parents WD
Hybrids WW
Hybrids WD
SF279
SF317
SF326
SF342
SF009
SF092
SF109
SF193
3.0e+07
2.5e+07
2.0e+07
1.5e+07
1.0e+07
5.0e+06
0.0e+00

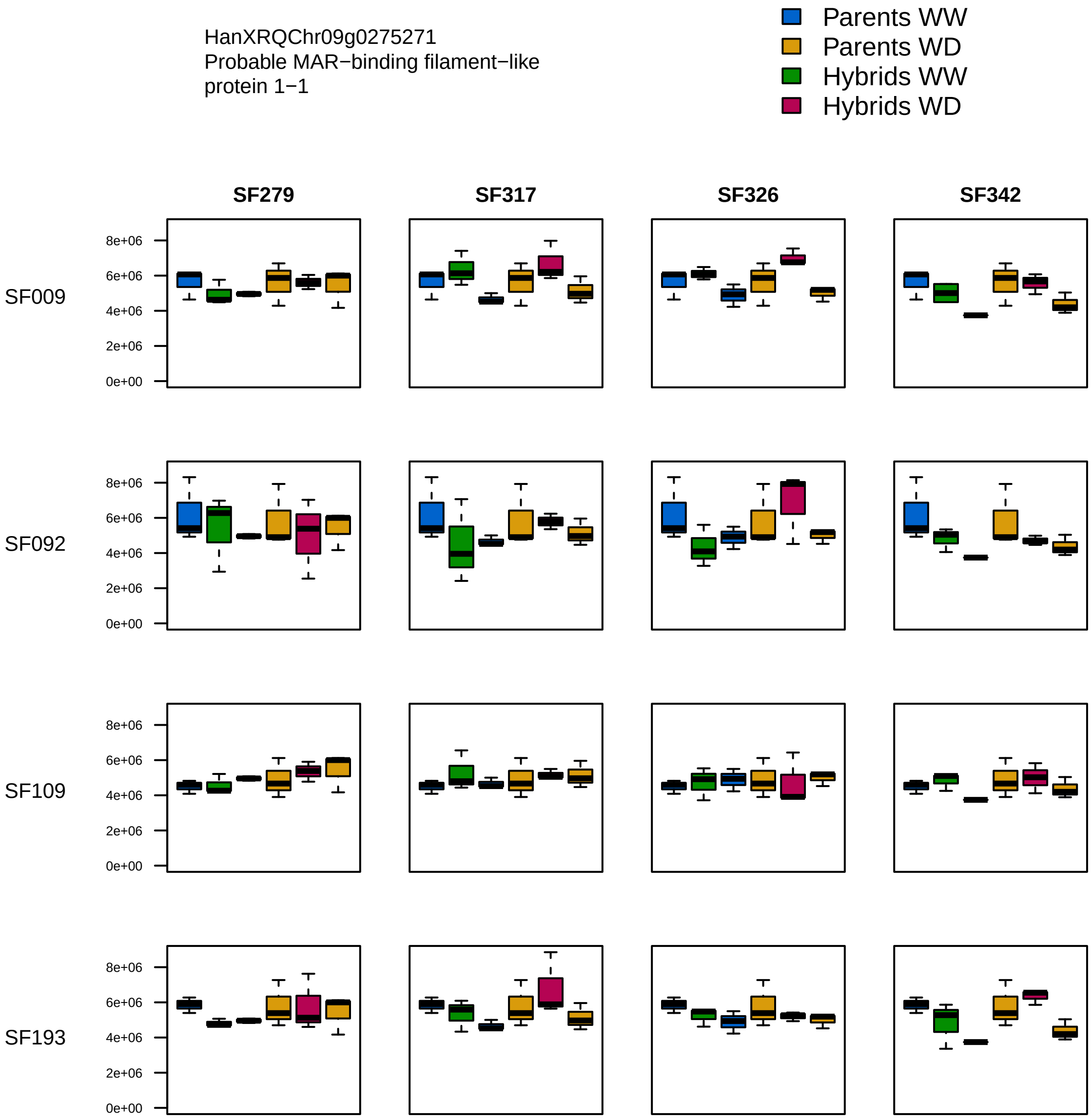
HanXRQChr09g0275271
Probable MAR−binding filament−like
protein 1−1
Parents WW
Parents WD
Hybrids WW
Hybrids WD
SF279
SF317
SF326
SF342
SF009
SF092
SF109
SF193
0e+00
2e+06
4e+06
6e+06
8e+06

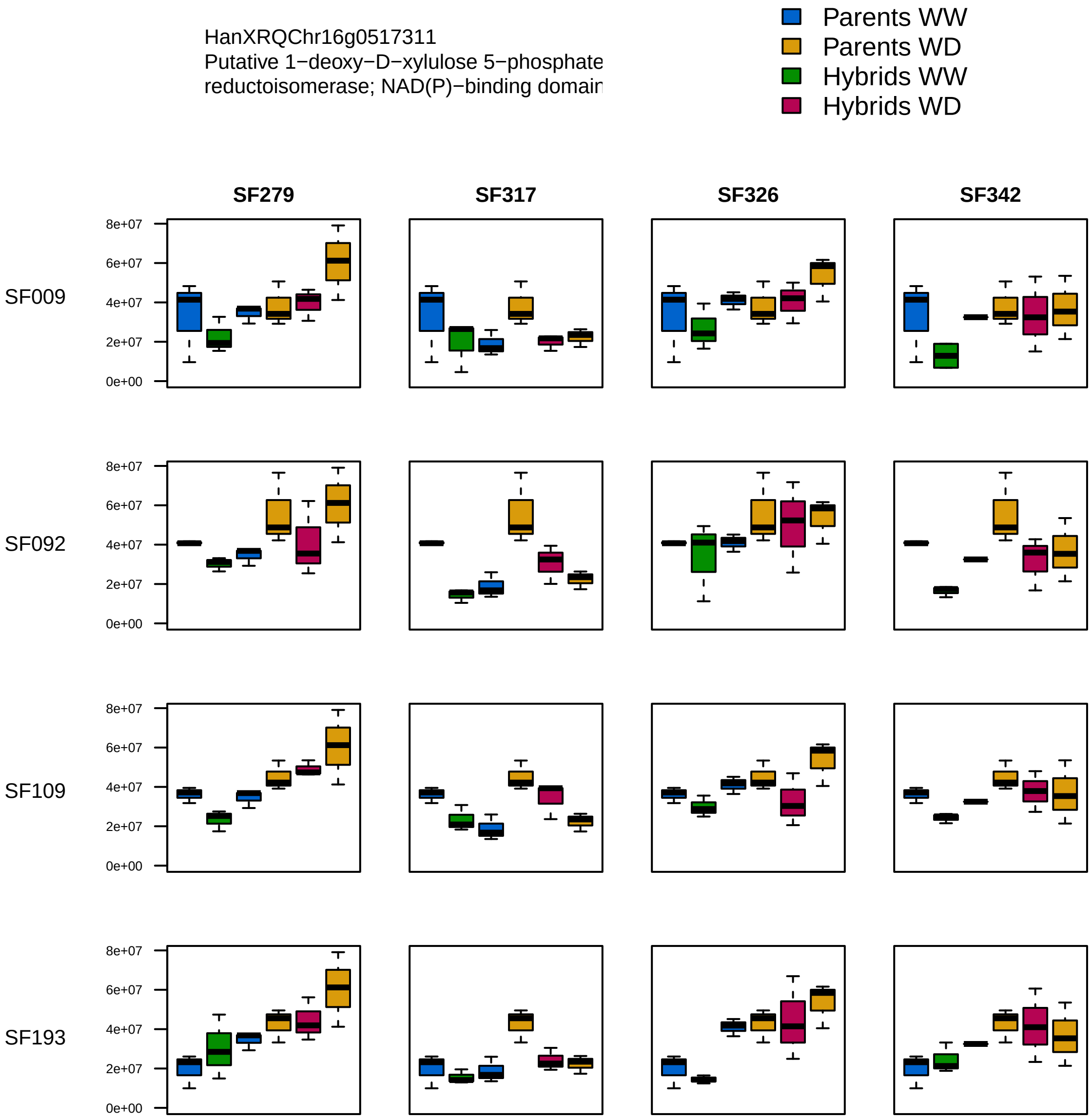

HanXRQChr16g0517311
Putative 1−deoxy−D−xylulose 5−phosphate
reductoisomerase; NAD(P)−binding domain
Parents WW
Parents WD
Hybrids WW
Hybrids WD
SF279
SF317
SF326
SF342
SF009
SF092
SF109
SF193
8e+07
6e+07
4e+07
2e+07
0e+00

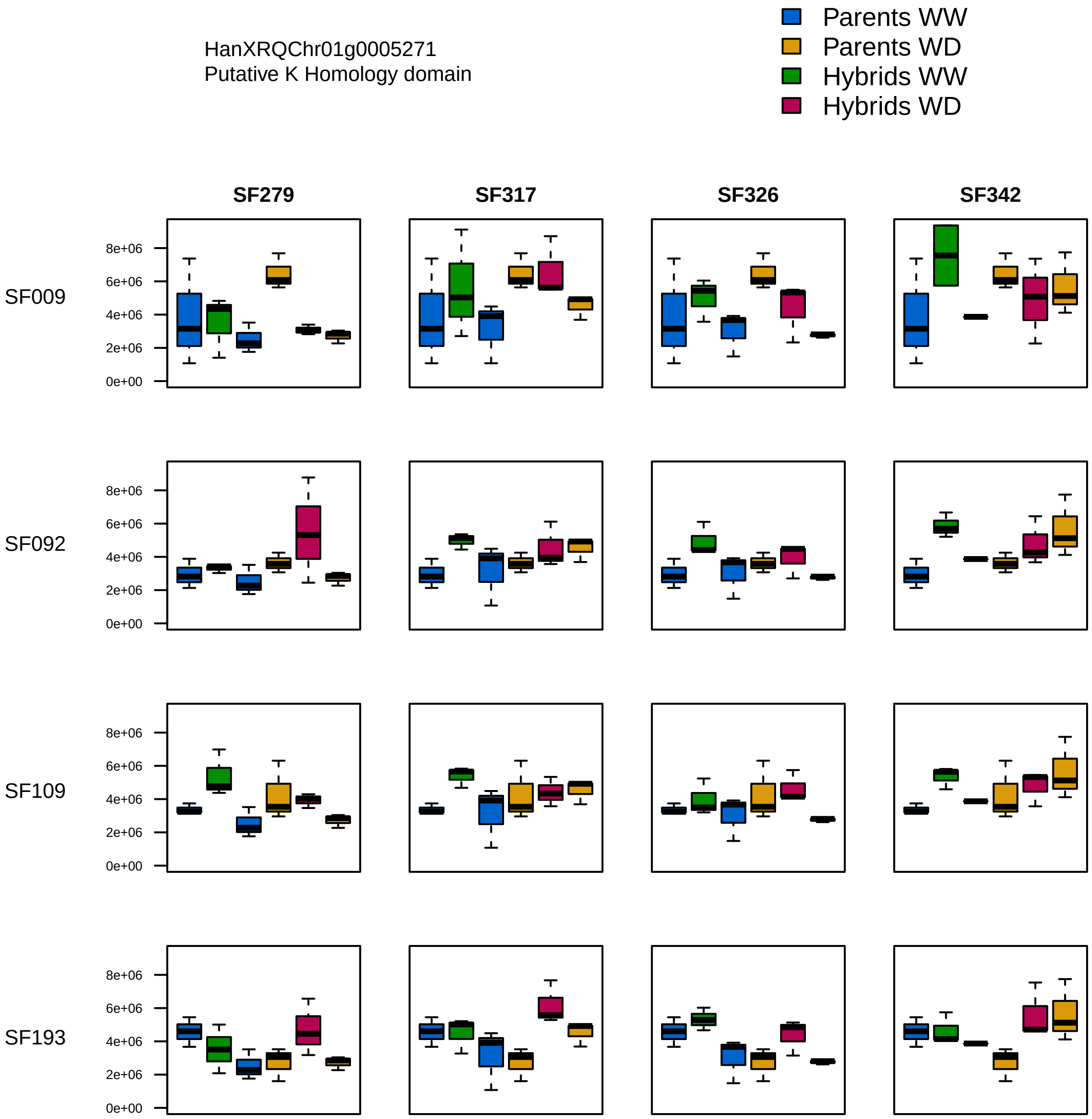

HanXRQChr01g0005271
Putative K Homology domain
Parents WW
Parents WD
Hybrids WW
Hybrids WD
SF279
SF317
SF326
SF342
SF009
SF092
SF109
SF193
8e+06
6e+06
4e+06
2e+06
0e+00

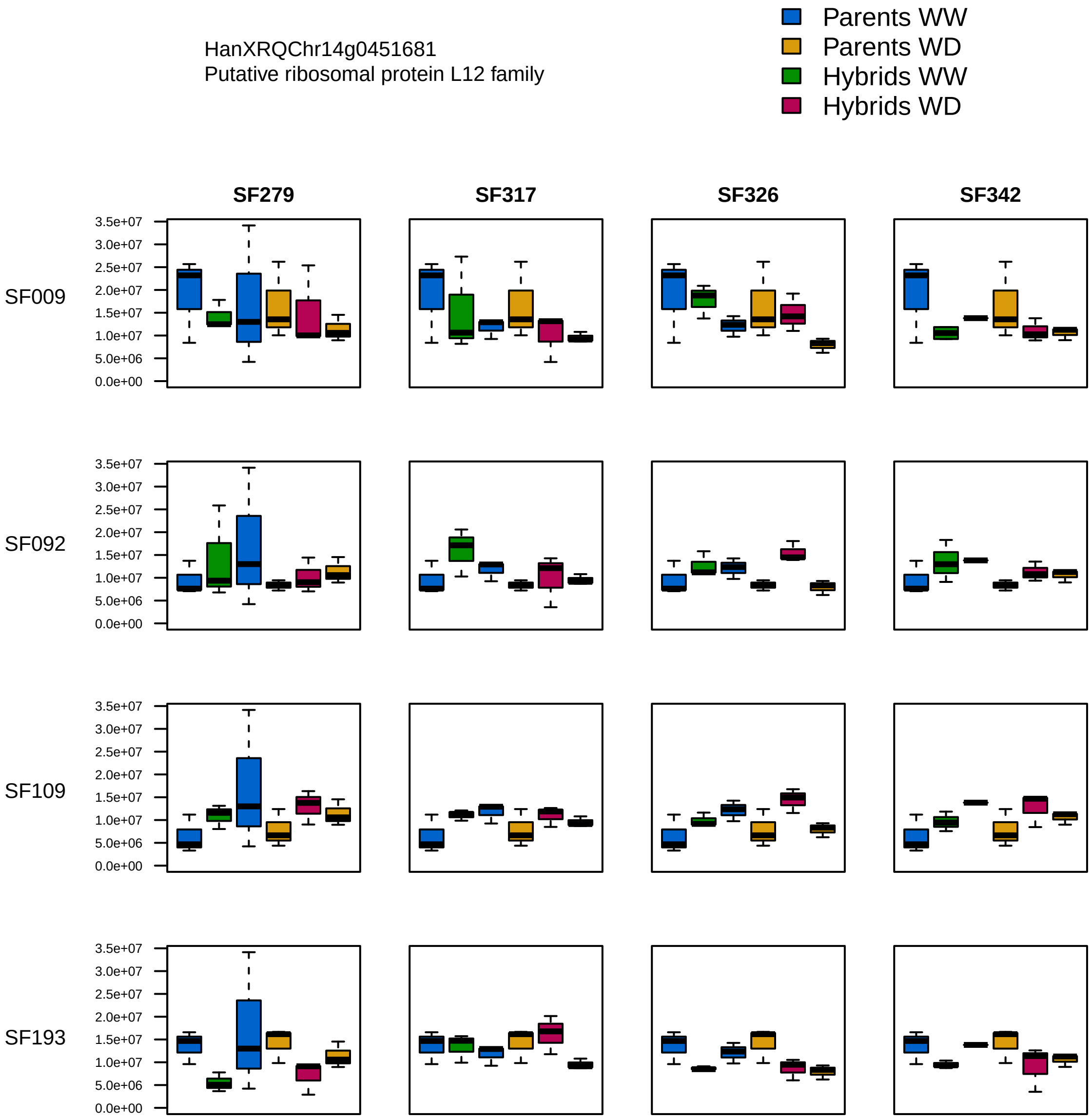
HanXRQChr14g0451681
Putative ribosomal protein L12 family
Parents WW
Parents WD
Hybrids WW
Hybrids WD
SF279
SF317
SF326
SF342
SF009
SF092
SF109
SF193
3.5e+07
3.0e+07
2.5e+07
2.0e+07
1.5e+07
1.0e+07
5.0e+06
0.0e+00

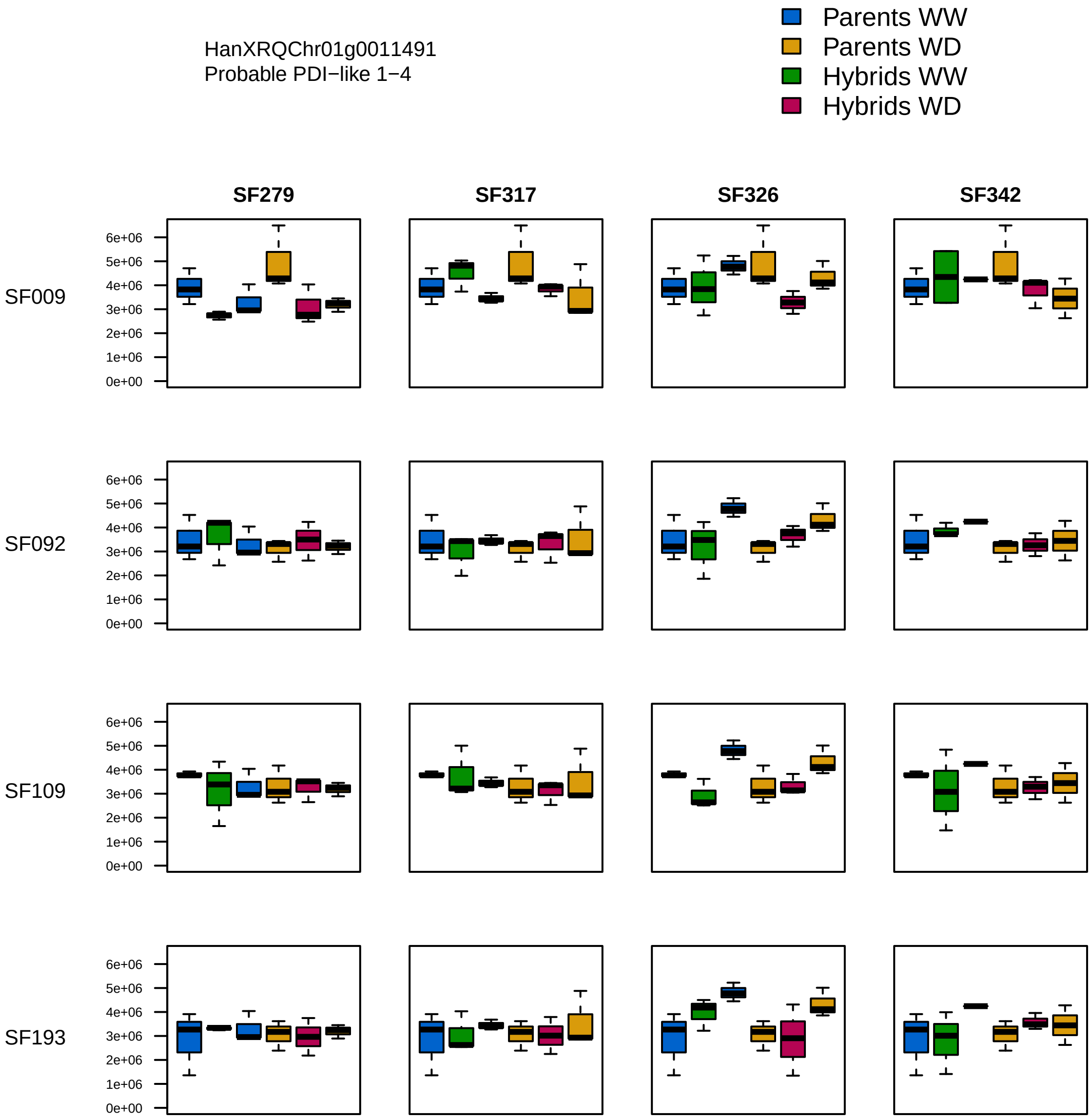
HanXRQChr01g0011491
Probable PDI−like 1−4
Parents WW
Parents WD
Hybrids WW
Hybrids WD
SF279
SF317
SF326
SF342
SF009
SF092
SF109
SF193
6e+06
5e+06
4e+06
3e+06
2e+06
1e+06
0e+00

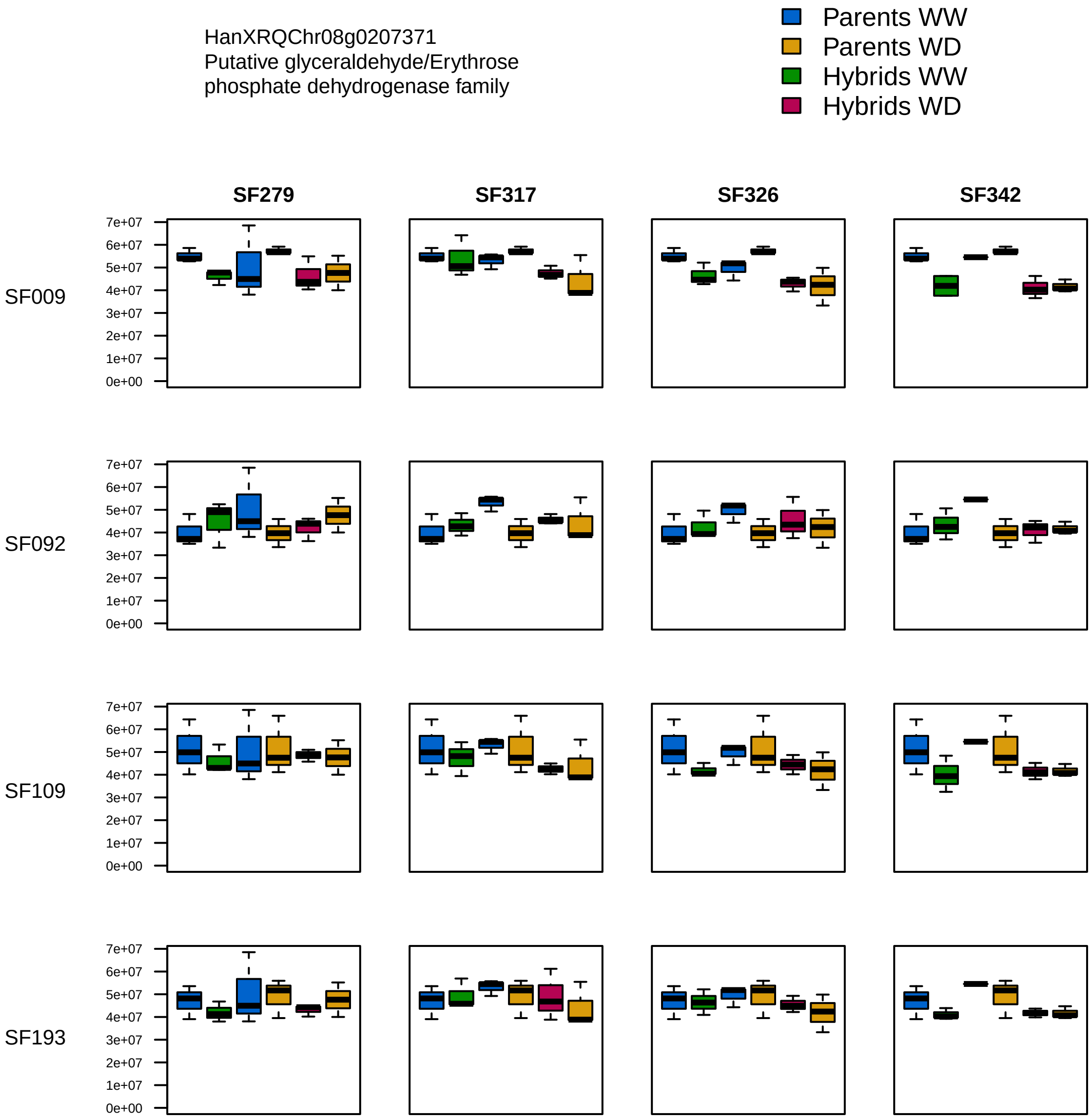
HanXRQChr08g0207371
Putative glyceraldehyde/Erythrose
phosphate dehydrogenase family
Parents WW
Parents WD
Hybrids WW
Hybrids WD
SF279
SF317
SF326
SF342
SF009
SF092
SF109
SF193
7e+07
6e+07
5e+07
4e+07
3e+07
2e+07
1e+07
0e+00

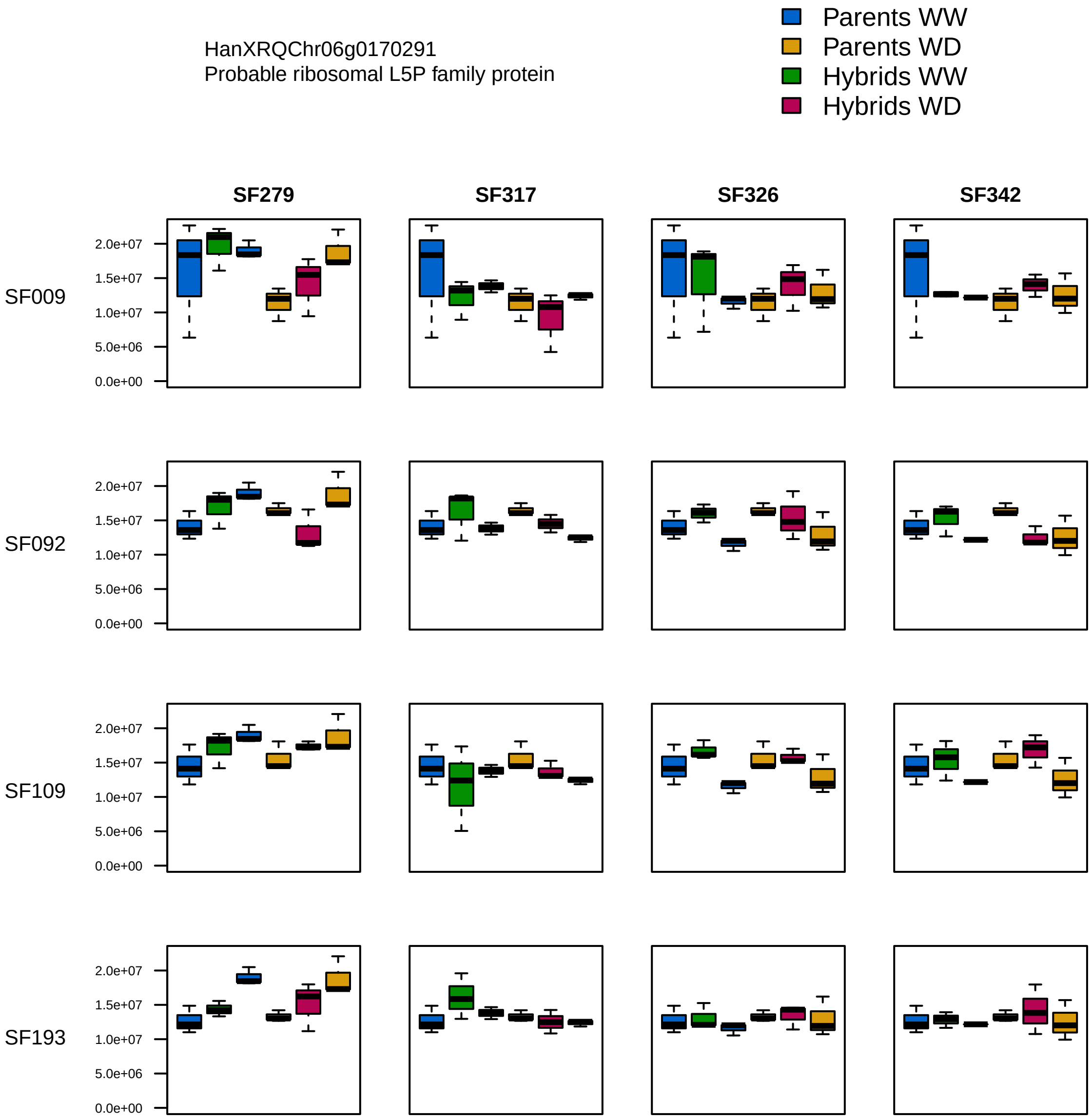
HanXRQChr06g0170291
Probable ribosomal L5P family protein
Parents WW
Parents WD
Hybrids WW
Hybrids WD
SF279
SF317
SF326
SF342
SF009
SF092
SF109
SF193
0.0e+00
5.0e+06
1.0e+07
1.5e+07
2.0e+07

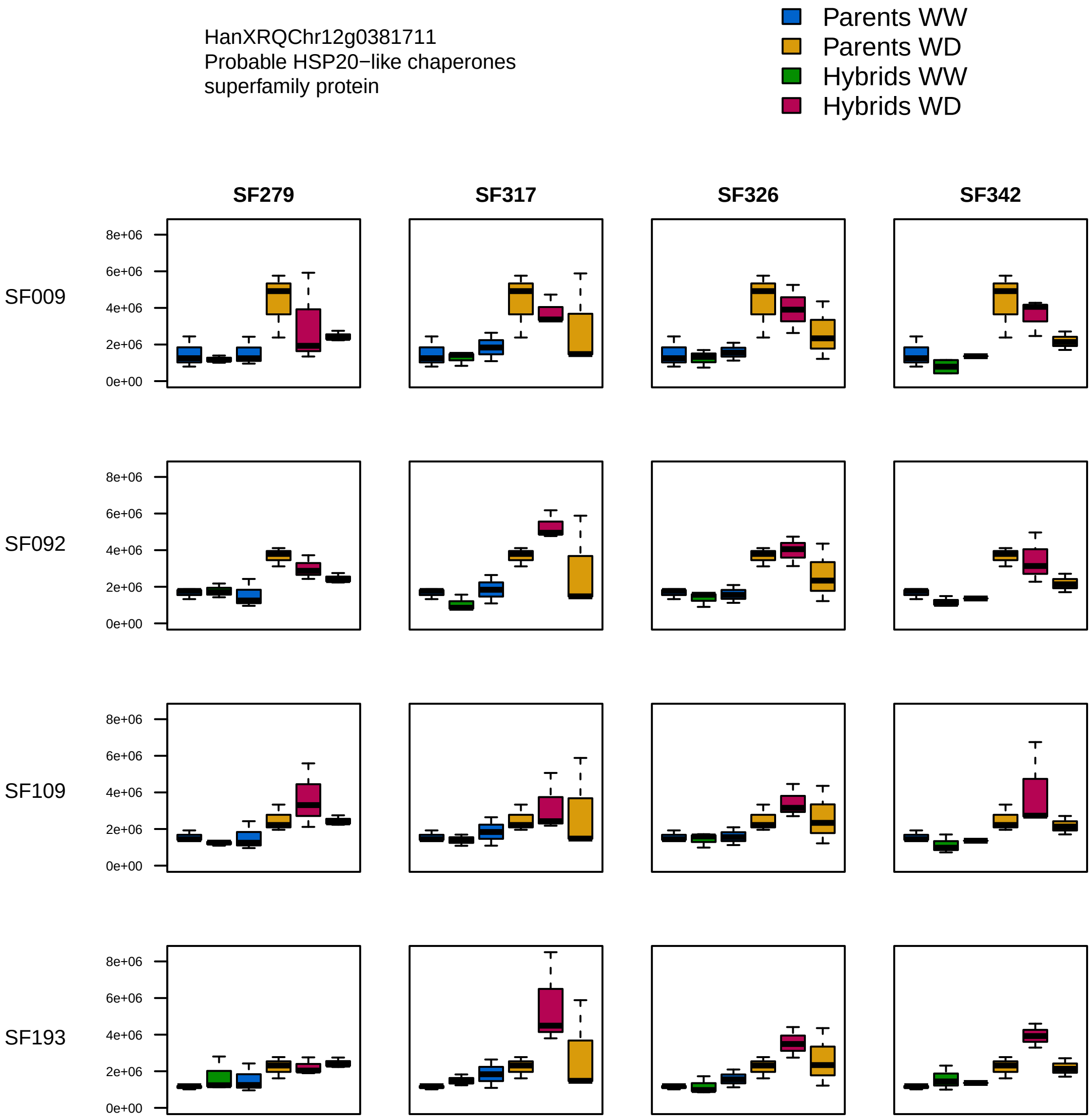

HanXRQChr12g0381711
Probable HSP20−like chaperones
superfamily protein
Parents WW
Parents WD
Hybrids WW
Hybrids WD
SF279
SF317
SF326
SF342
SF009
SF092
SF109
SF193
8e+06
6e+06
4e+06
2e+06
0e+00

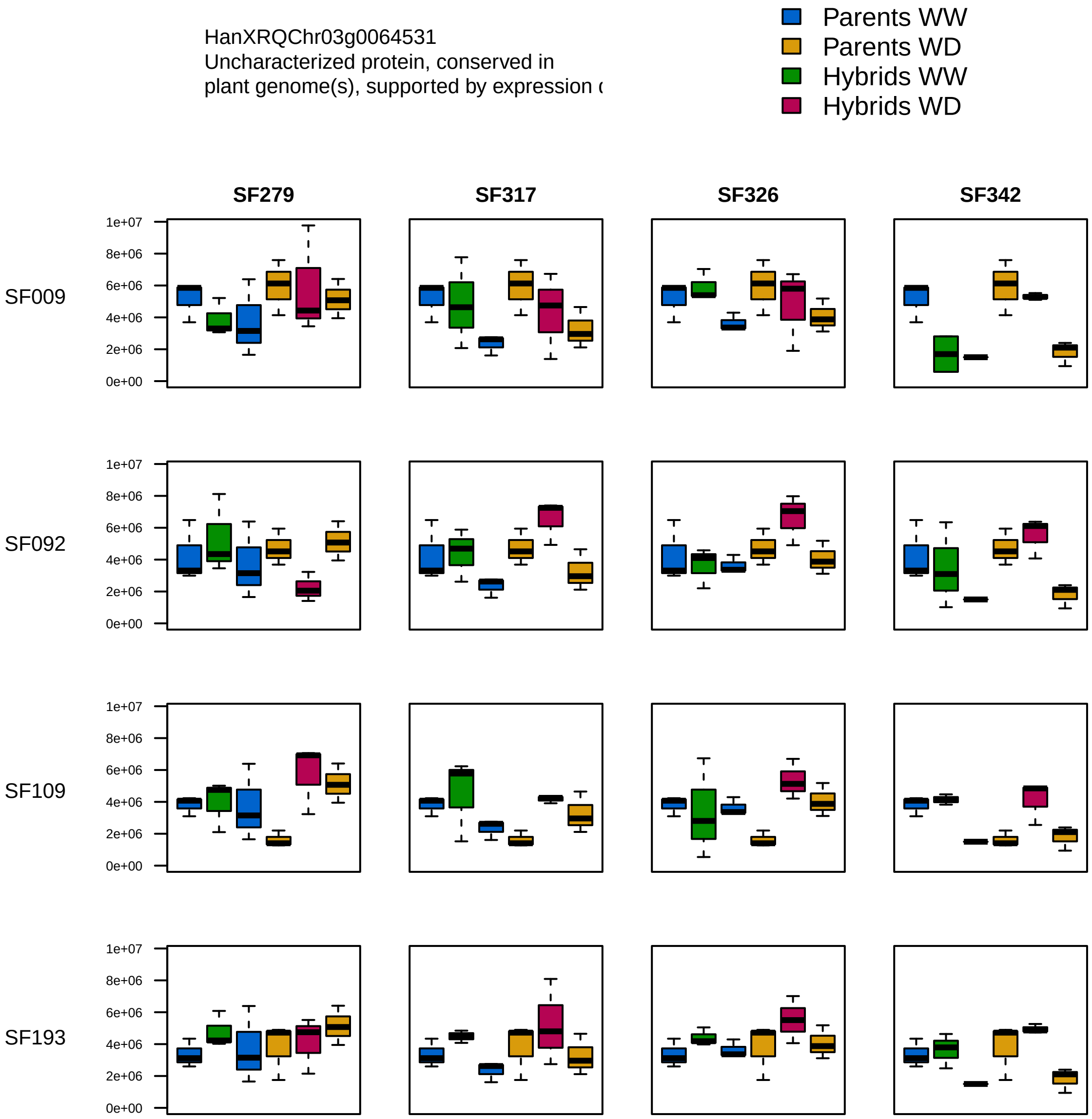
HanXRQChr03g0064531
Uncharacterized protein, conserved in
plant genome(s), supported by expression
Parents WW
Parents WD
Hybrids WW
Hybrids WD
SF279
SF317
SF326
SF342
SF009
SF092
SF109
SF193
1e+07
8e+06
6e+06
4e+06
2e+06
0e+00

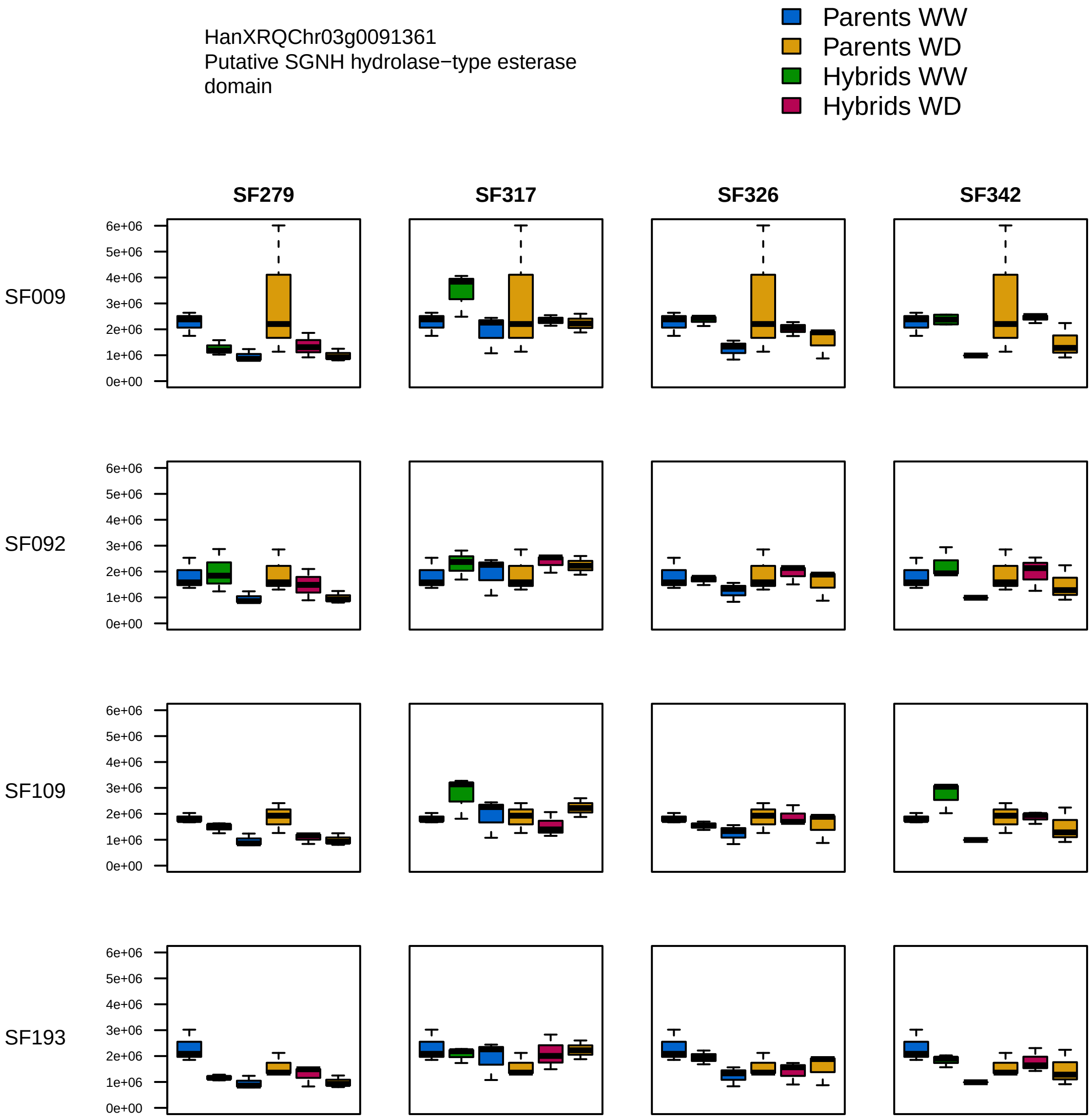

HanXRQChr03g0091361
Putative SGNH hydrolase−type esterase domain
Parents WW
Parents WD
Hybrids WW
Hybrids WD
SF279
SF317
SF326
SF342
SF009
SF092
SF109
SF193
6e+06
5e+06
4e+06
3e+06
2e+06
1e+06
0e+00

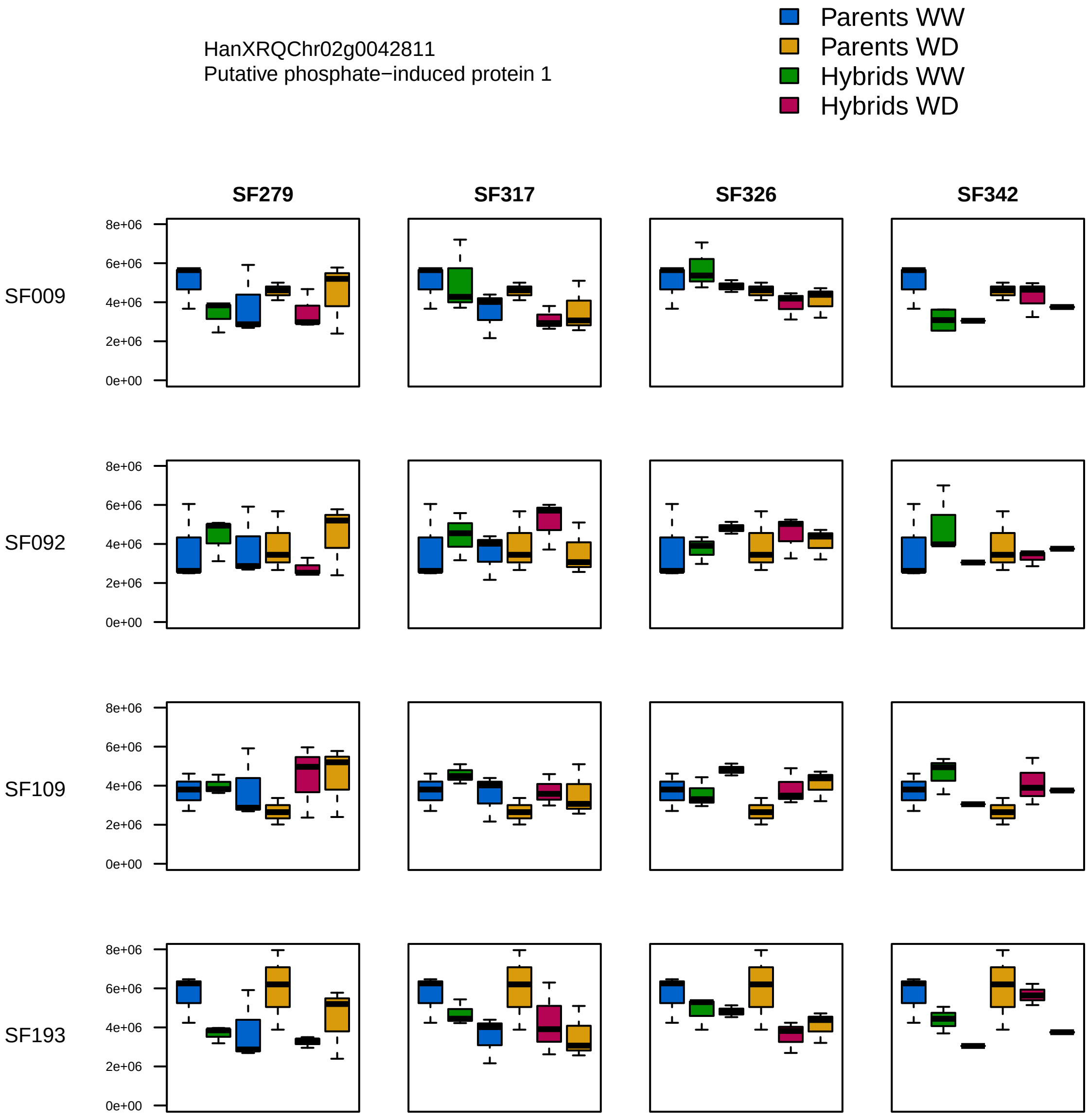
HanXRQChr02g0042811
Putative phosphate−induced protein 1
Parents WW
Parents WD
Hybrids WW
Hybrids WD
SF279
SF317
SF326
SF342
SF009
SF092
SF109
SF193
8e+06
6e+06
4e+06
2e+06
0e+00

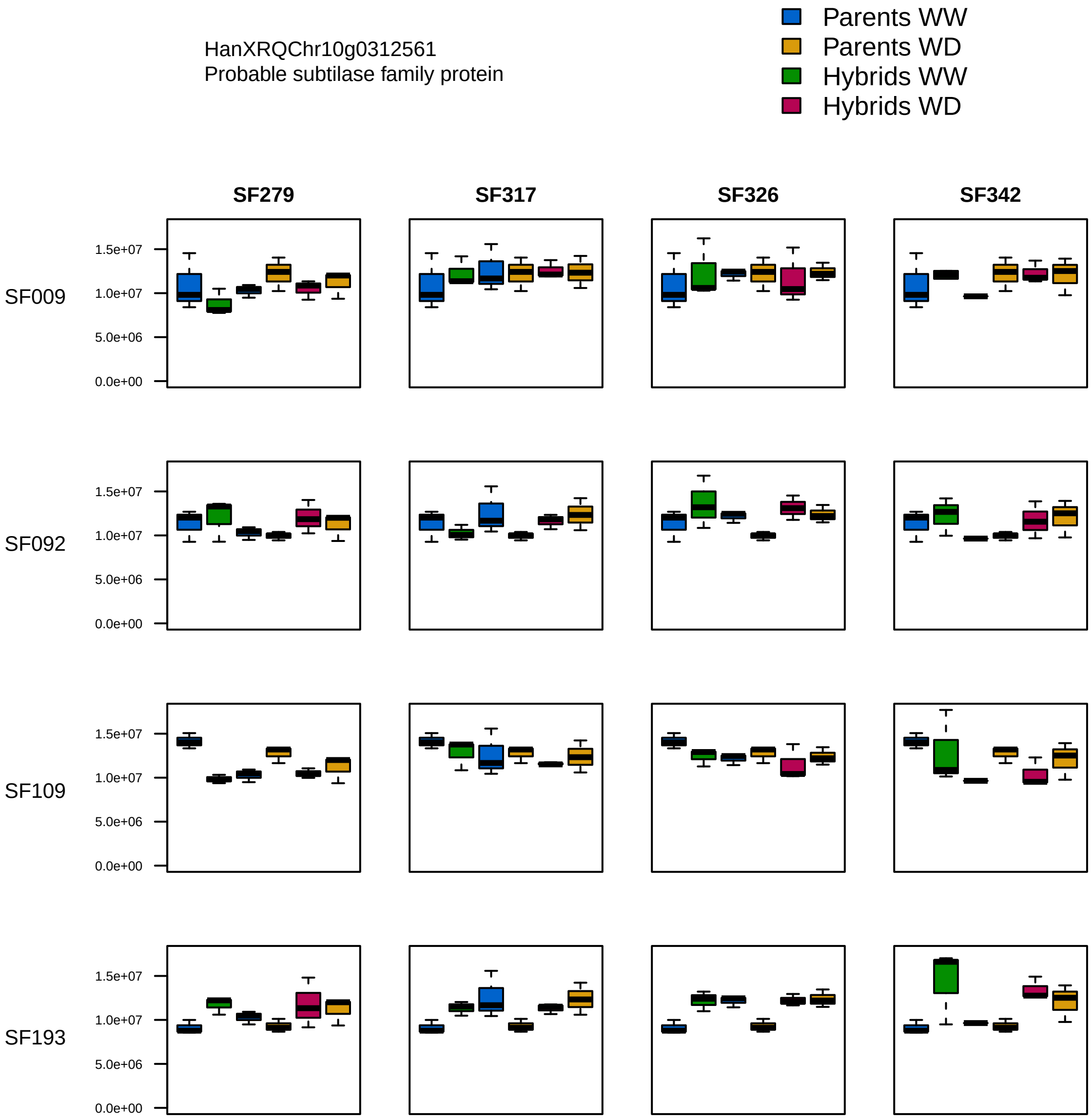
HanXRQChr10g0312561
Probable subtilase family protein
Parents WW
Parents WD
Hybrids WW
Hybrids WD
SF279
SF317
SF326
SF342
SF009
SF092
SF109
SF193
1.5e+07
1.0e+07
5.0e+06
0.0e+00

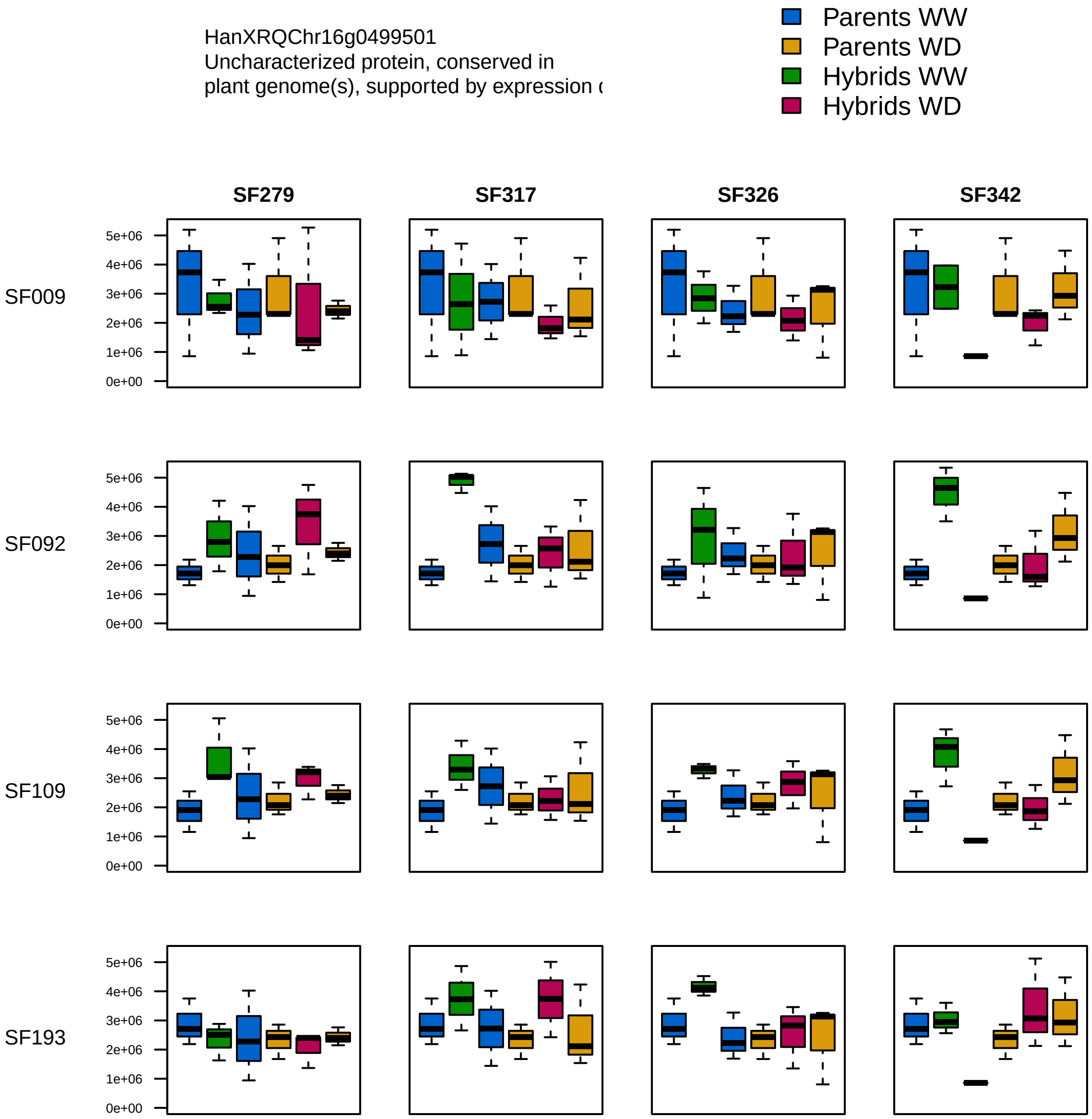
HanXRQChr16g0499501
Uncharacterized protein, conserved in
plant genome(s), supported by expression (
Parents WW
Parents WD
Hybrids WW
Hybrids WD
SF279
SF317
SF326
SF342
SF009
SF092
SF109
SF193
5e+06
4e+06
3e+06
2e+06
1e+06
0e+00

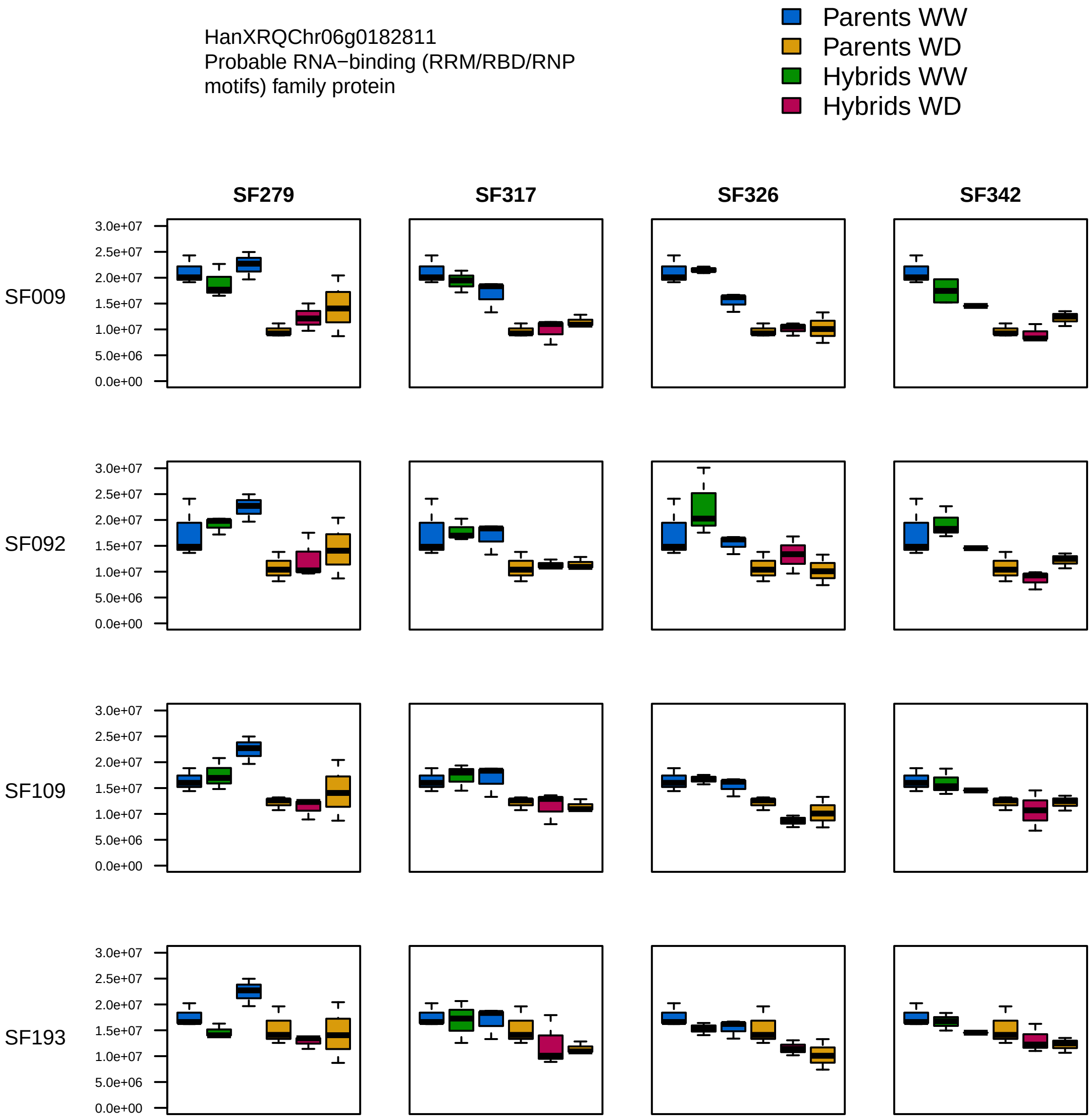

HanXRQChr06g0182811
Probable RNA−binding (RRM/RBD/RNP motifs) family protein
Parents WW
Parents WD
Hybrids WW
Hybrids WD
SF279
SF317
SF326
SF342
SF009
SF092
SF109
SF193
3.0e+07
2.5e+07
2.0e+07
1.5e+07
1.0e+07
5.0e+06
0.0e+00

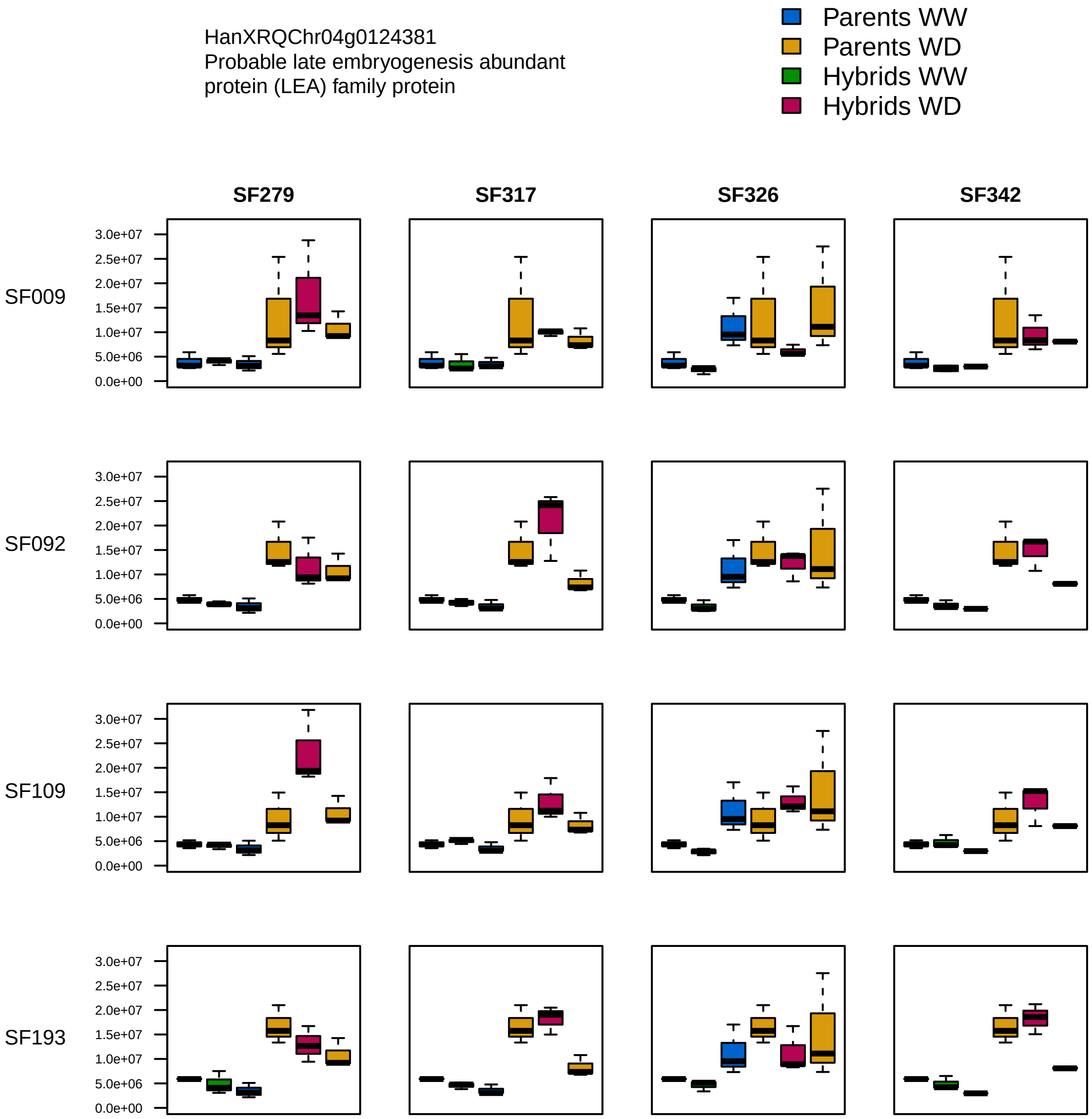

HanXRQChr04g0124381
Probable late embryogenesis abundant
protein (LEA) family protein
Parents WW
Parents WD
Hybrids WW
Hybrids WD
SF279
SF317
SF326
SF342
SF009
SF092
SF109
SF193
3.0e+07
2.5e+07
2.0e+07
1.5e+07
1.0e+07
5.0e+06
0.0e+00

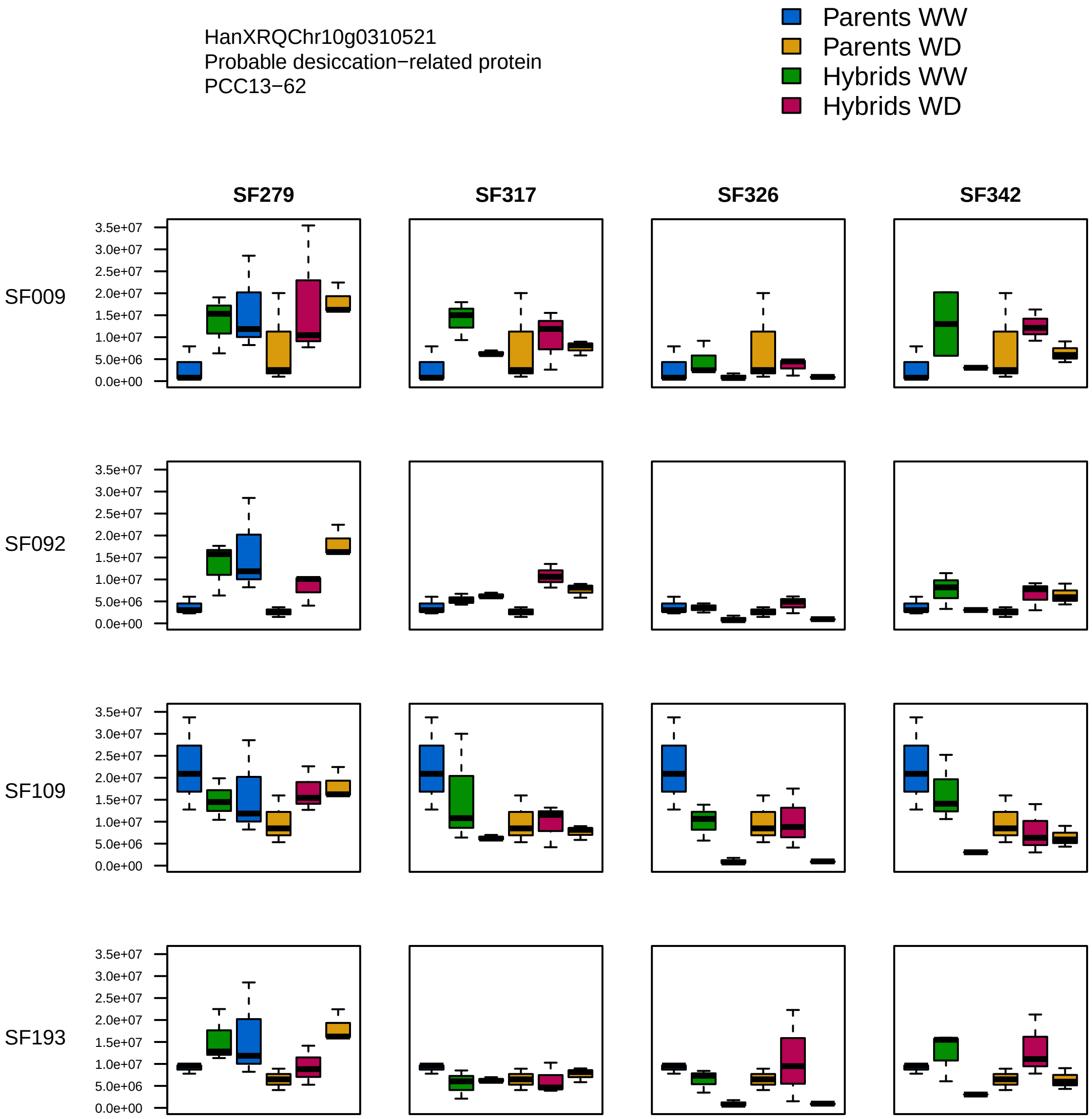

HanXRQChr10g0310521
Probable desiccation−related protein
PCC13−62
Parents WW
Parents WD
Hybrids WW
Hybrids WD
SF279
SF317
SF326
SF342
SF009
SF092
SF109
SF193
3.5e+07
3.0e+07
2.5e+07
2.0e+07
1.5e+07
1.0e+07
5.0e+06
0.0e+00

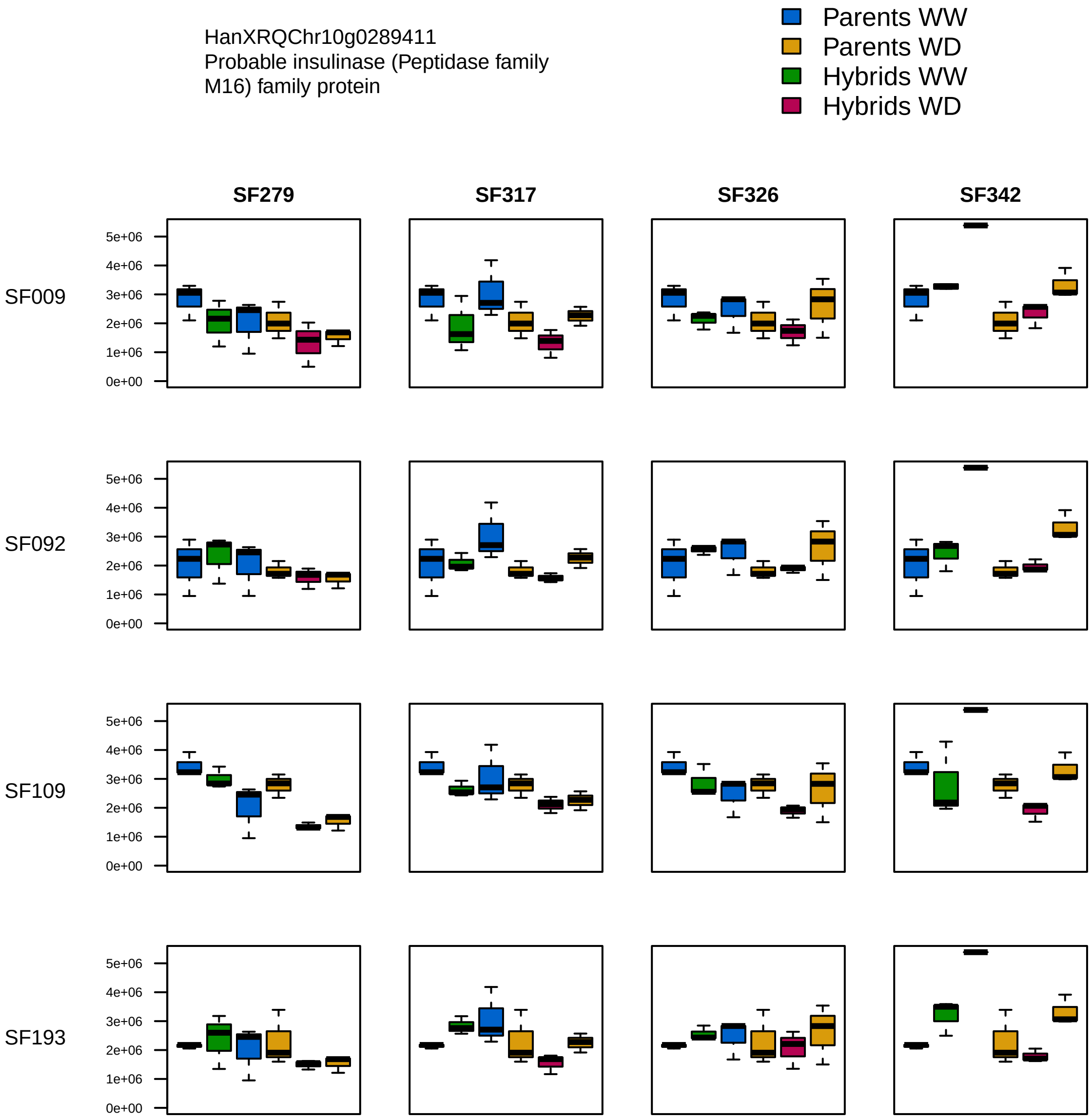
HanXRQChr10g0289411
Probable insulinase (Peptidase family M16) family protein
Parents WW
Parents WD
Hybrids WW
Hybrids WD
SF279
SF317
SF326
SF342
SF009
SF092
SF109
SF193
5e+06
4e+06
3e+06
2e+06
1e+06
0e+00

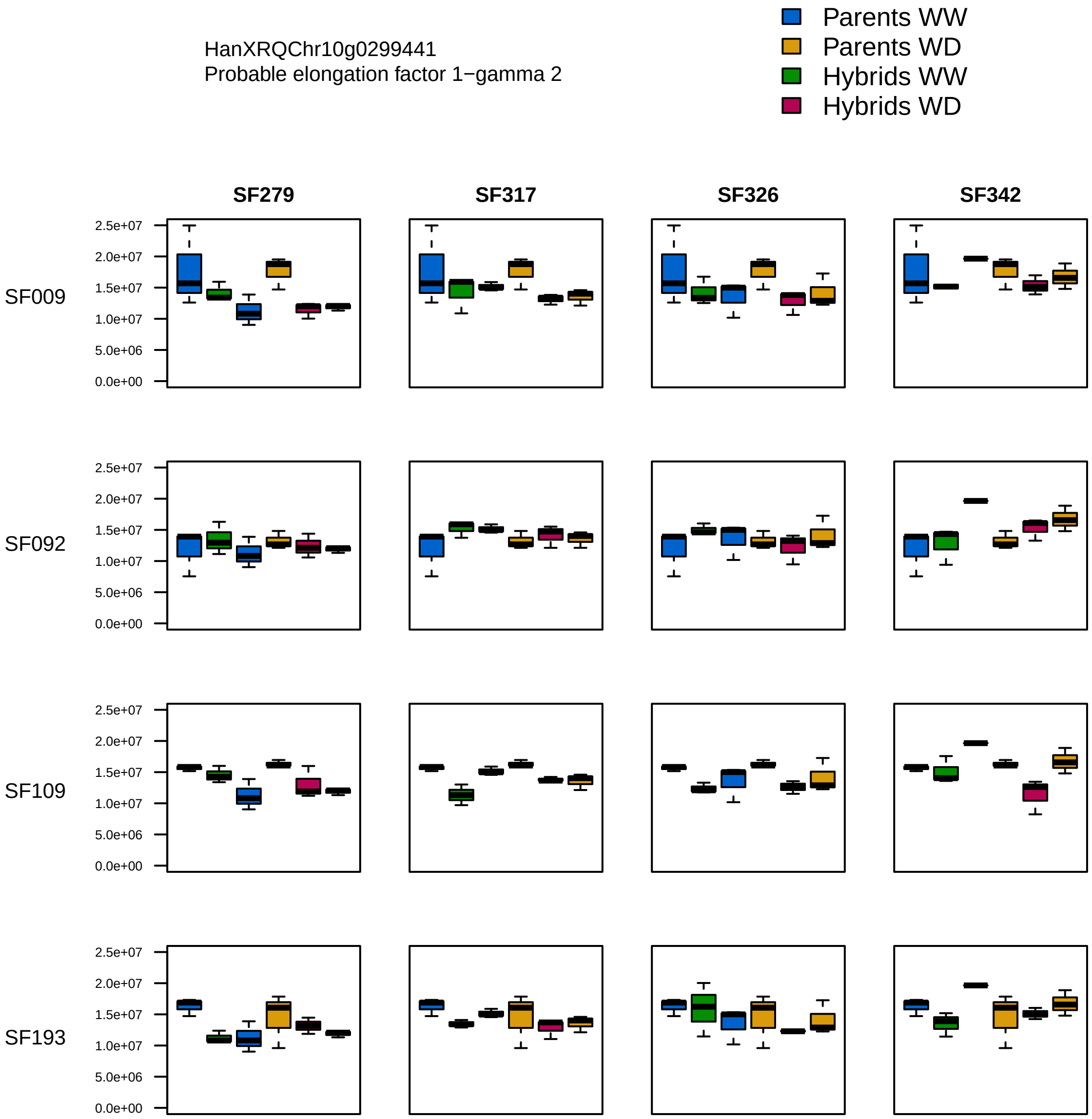
HanXRQChr10g0299441
Probable elongation factor 1−gamma 2
Parents WW
Parents WD
Hybrids WW
Hybrids WD
SF279
SF317
SF326
SF342
SF009
SF092
SF109
SF193
2.5e+07
2.0e+07
1.5e+07
1.0e+07
5.0e+06
0.0e+00

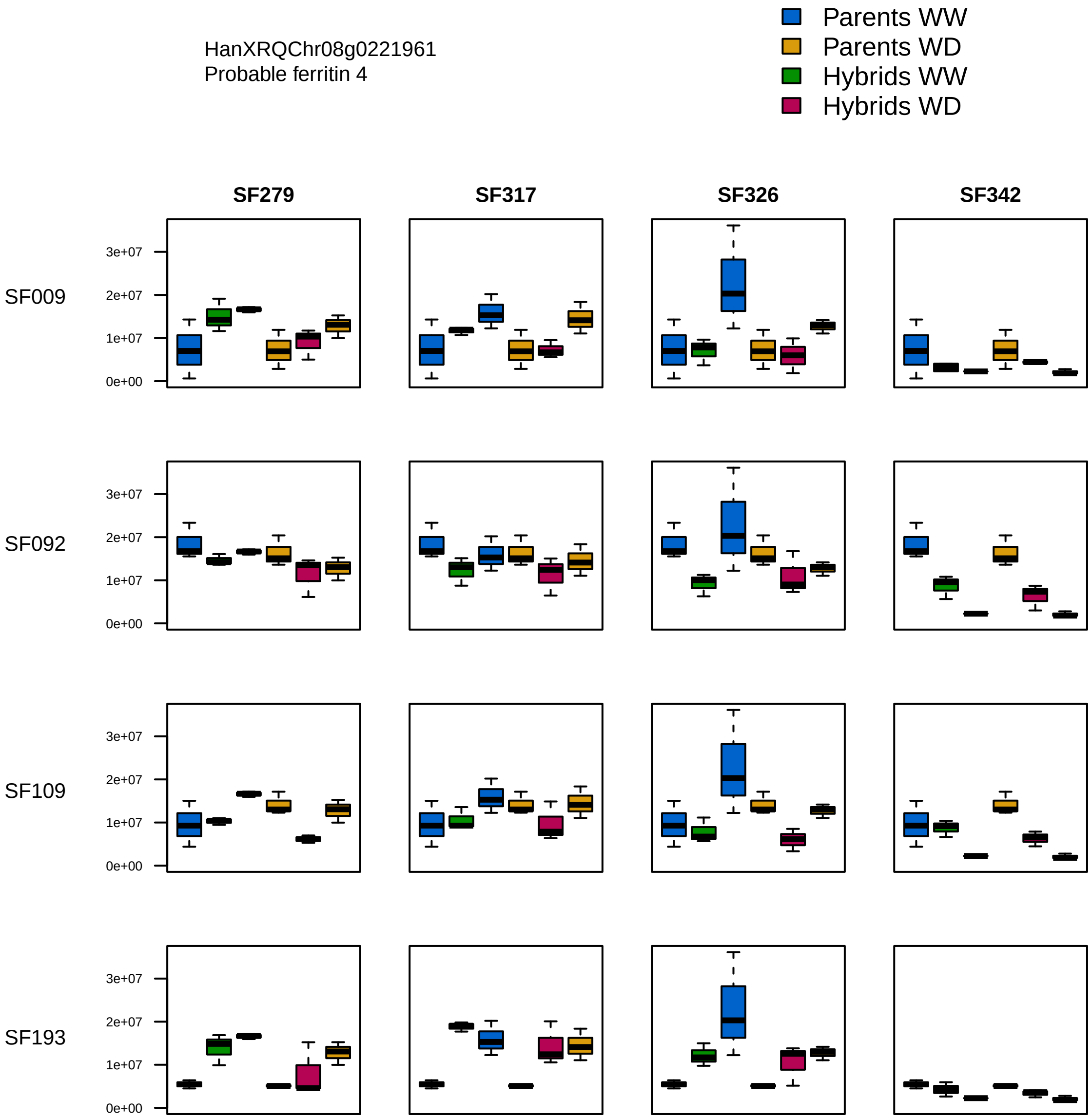
HanXRQChr08g0221961
Probable ferritin 4
Parents WW
Parents WD
Hybrids WW
Hybrids WD
SF279
SF317
SF326
SF342
SF009
SF092
SF109
SF193
3e+07
2e+07
1e+07
0e+00

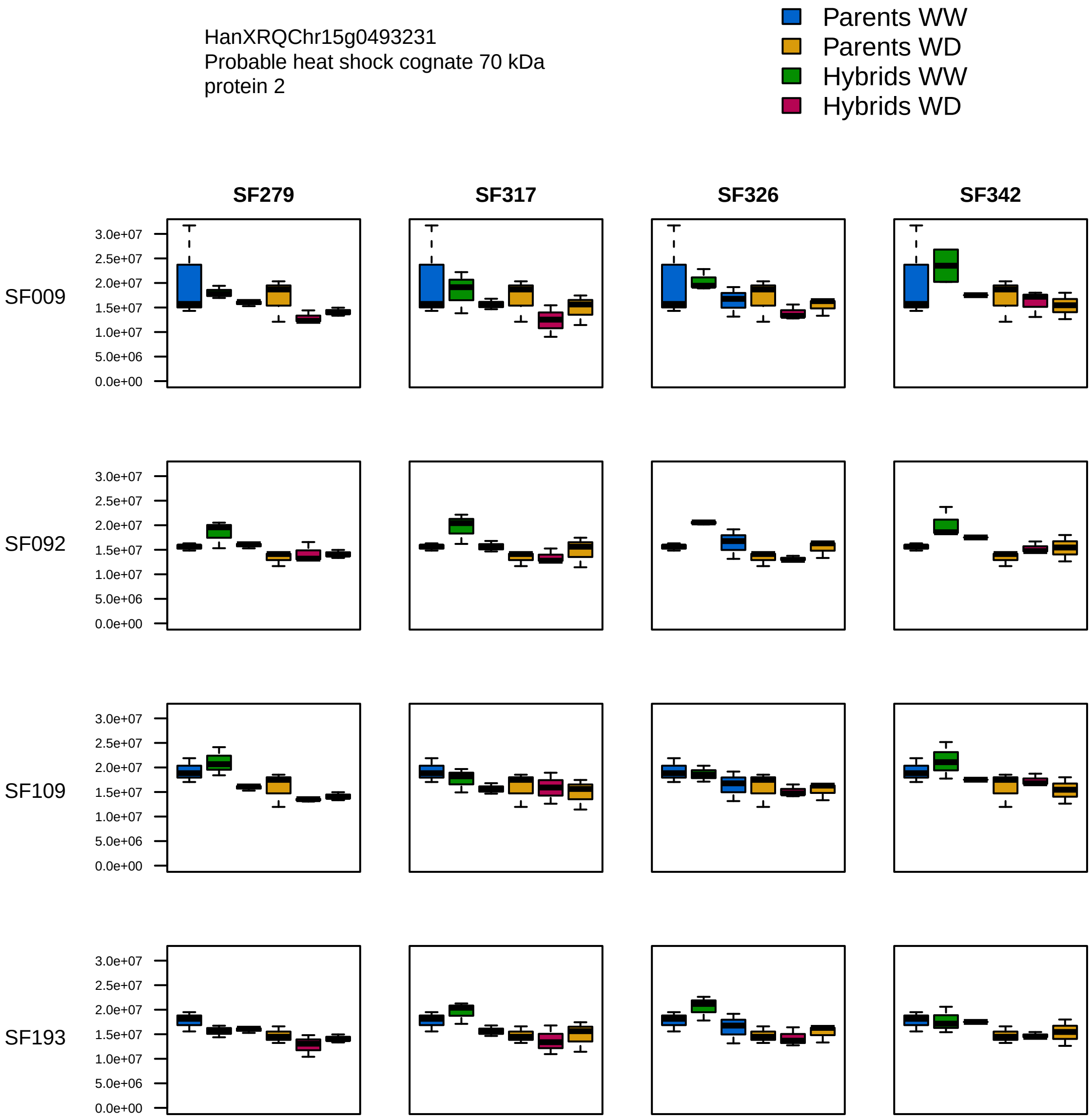
HanXRQChr15g0493231
Probable heat shock cognate 70 kDa
protein 2
Parents WW
Parents WD
Hybrids WW
Hybrids WD
SF279
SF317
SF326
SF342
SF009
SF092
SF109
SF193
0.0e+00
5.0e+06
1.0e+07
1.5e+07
2.0e+07
2.5e+07
3.0e+07

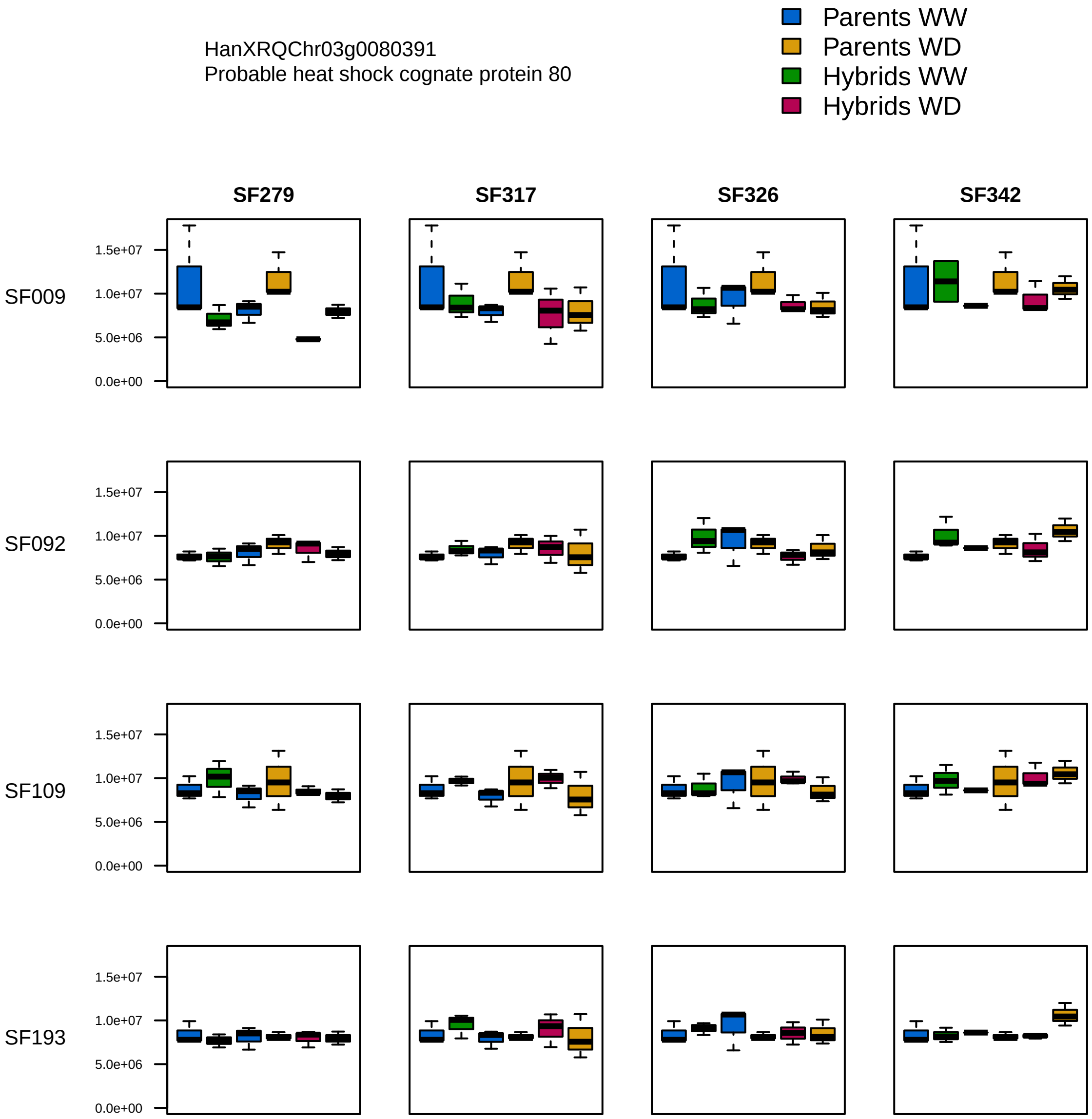
HanXRQChr03g0080391
Probable heat shock cognate protein 80
Parents WW
Parents WD
Hybrids WW
Hybrids WD
SF279
SF317
SF326
SF342
SF009
SF092
SF109
SF193
1.5e+07
1.0e+07
5.0e+06
0.0e+00

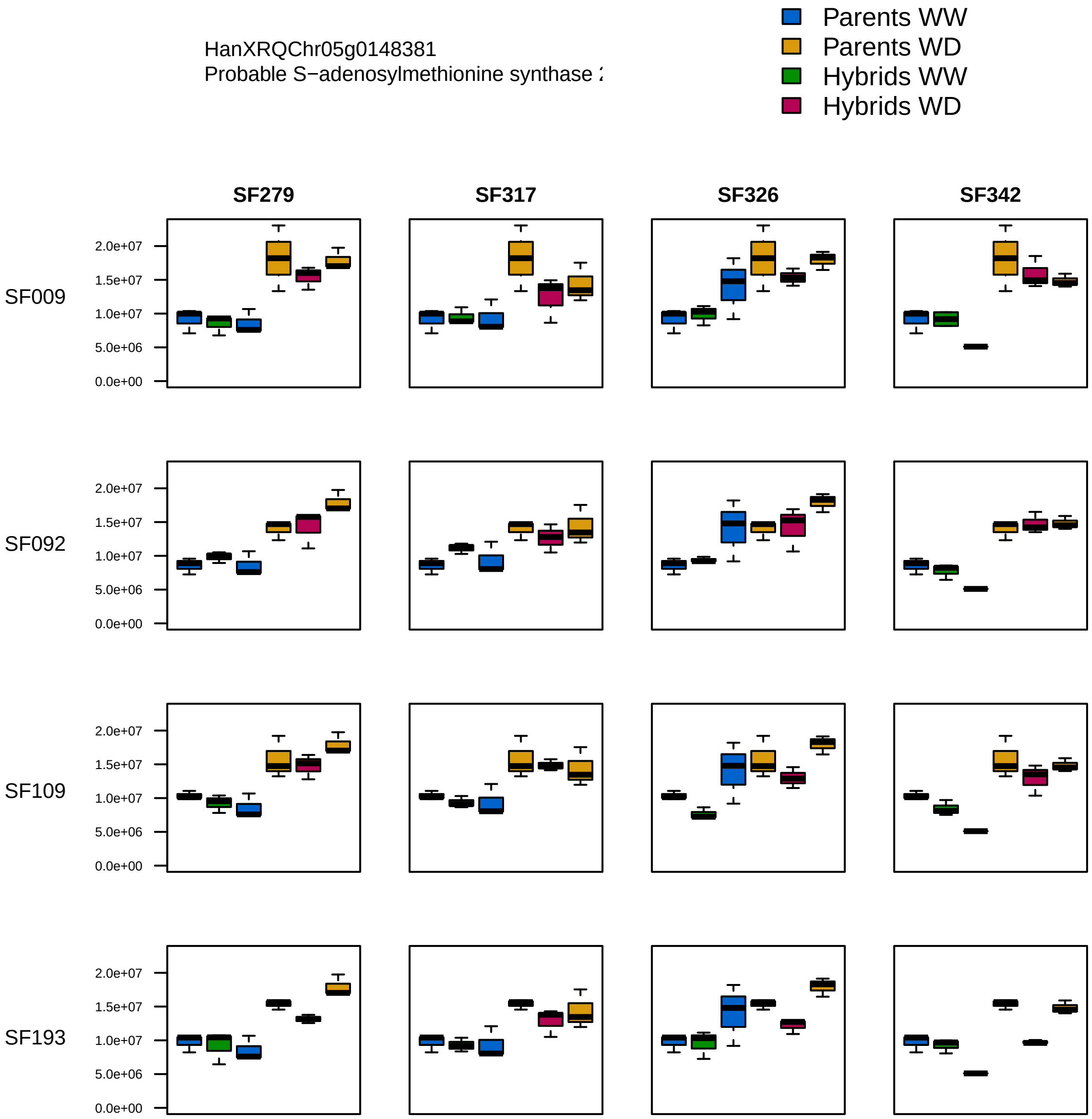
HanXRQChr05g0148381
Probable S−adenosylmethionine synthase 2
Parents WW
Parents WD
Hybrids WW
Hybrids WD
SF279
SF317
SF326
SF342
SF009
SF092
SF109
SF193
2.0e+07
1.5e+07
1.0e+07
5.0e+06
0.0e+00

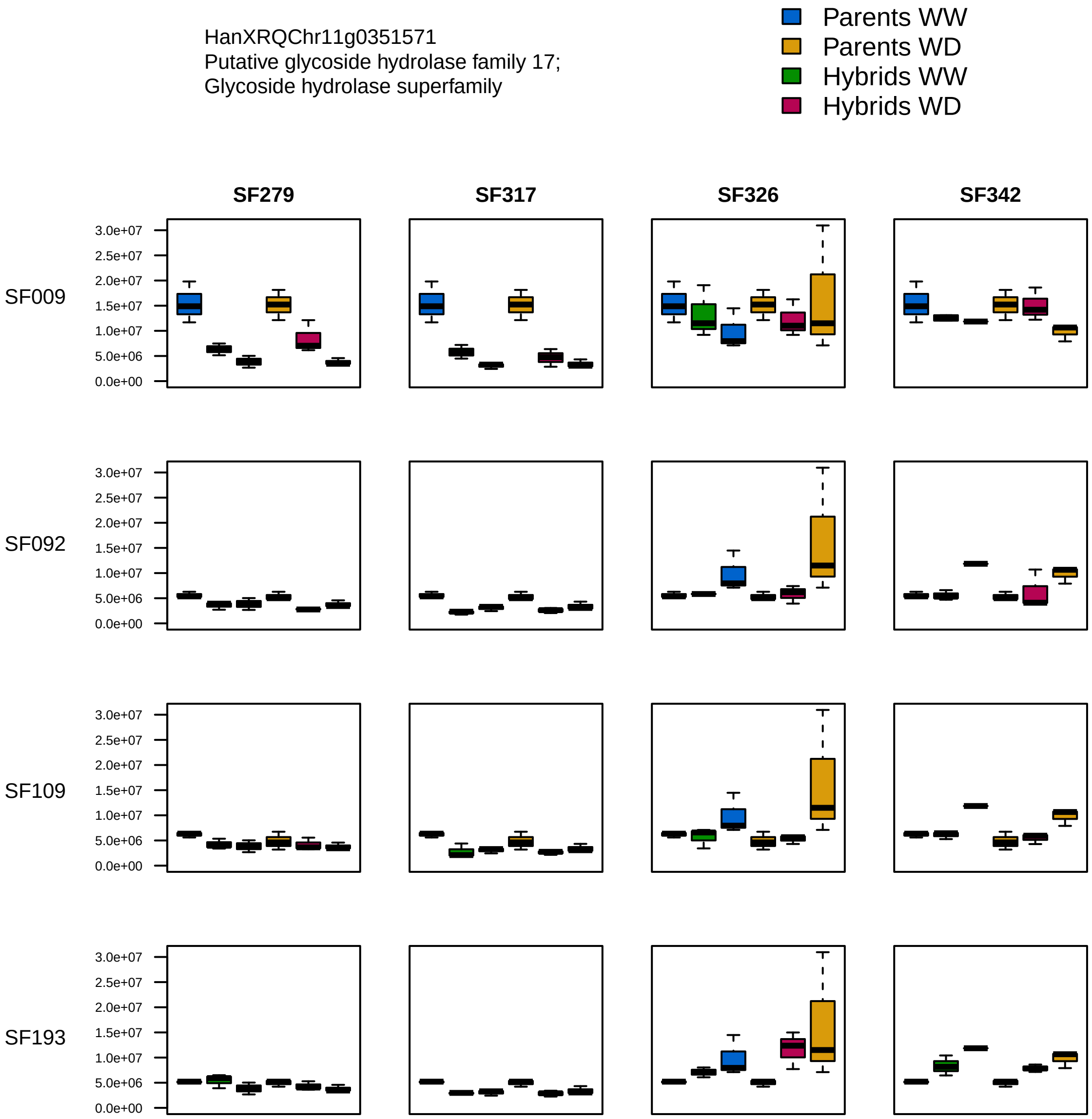

HanXRQChr11g0351571
Putative glycoside hydrolase family 17;
Glycoside hydrolase superfamily
Parents WW
Parents WD
Hybrids WW
Hybrids WD
SF279
SF317
SF326
SF342
SF009
SF092
SF109
SF193
3.0e+07
2.5e+07
2.0e+07
1.5e+07
1.0e+07
5.0e+06
0.0e+00

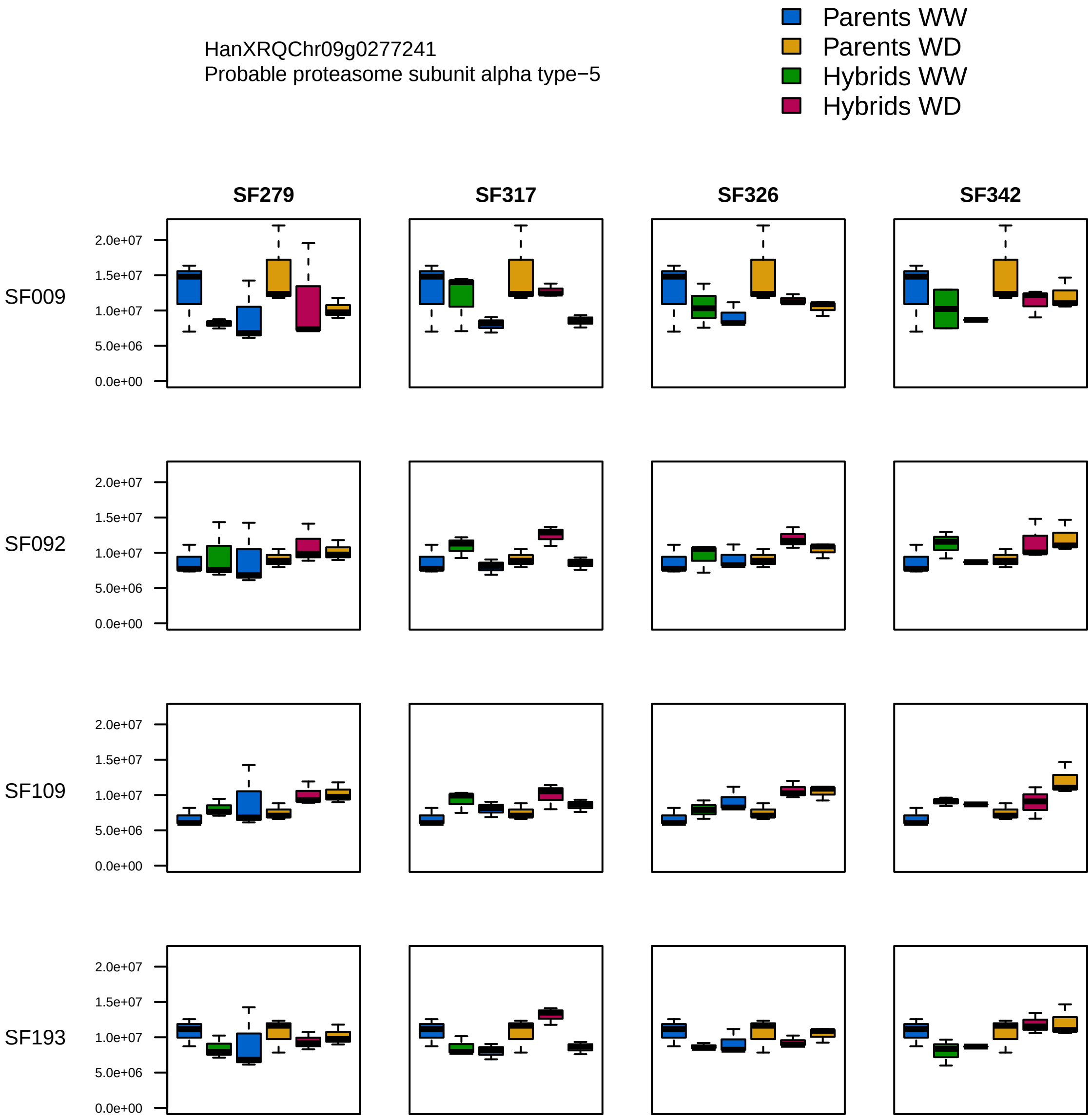
HanXRQChr09g0277241
Probable proteasome subunit alpha type−5
Parents WW
Parents WD
Hybrids WW
Hybrids WD
SF279
SF317
SF326
SF342
SF009
SF092
SF109
SF193
0.0e+00
5.0e+06
1.0e+07
1.5e+07
2.0e+07

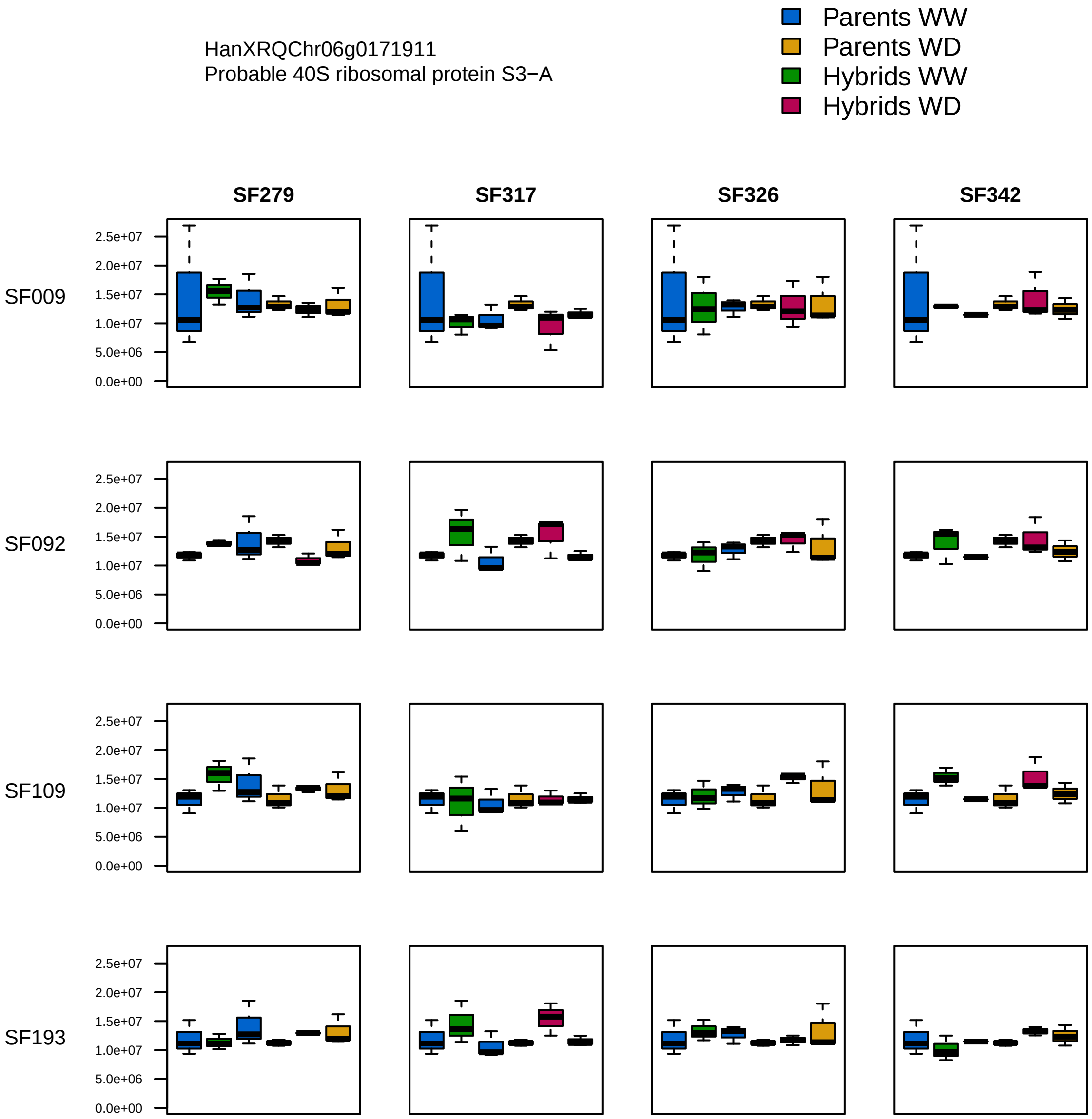

HanXRQChr06g0171911
Probable 40S ribosomal protein S3−A
Parents WW
Parents WD
Hybrids WW
Hybrids WD
SF279
SF317
SF326
SF342
SF009
SF092
SF109
SF193
2.5e+07
2.0e+07
1.5e+07
1.0e+07
5.0e+06
0.0e+00

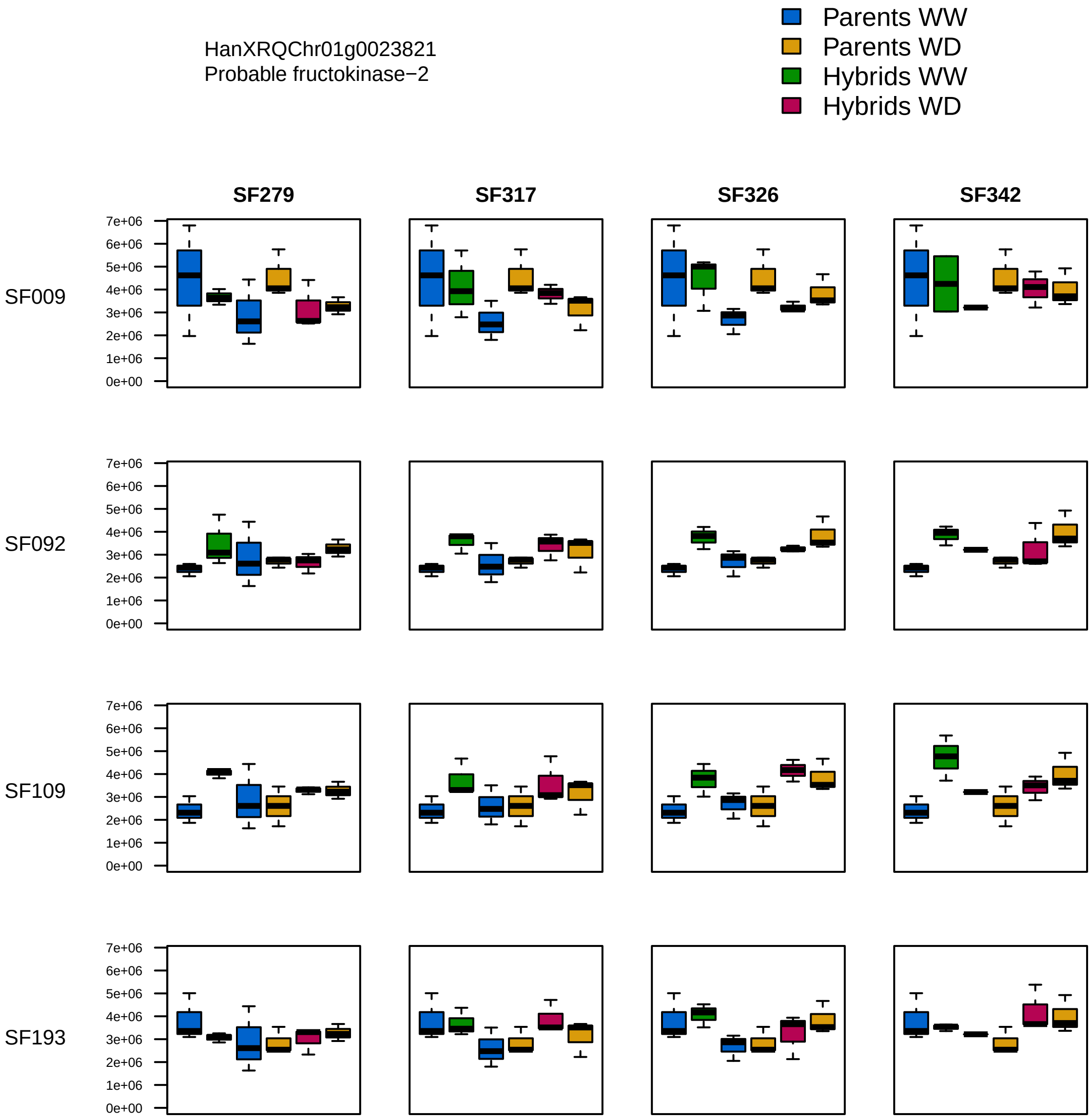
HanXRQChr01g0023821
Probable fructokinase−2
Parents WW
Parents WD
Hybrids WW
Hybrids WD
SF279
SF317
SF326
SF342
SF009
SF092
SF109
SF193
7e+06
6e+06
5e+06
4e+06
3e+06
2e+06
1e+06
0e+00

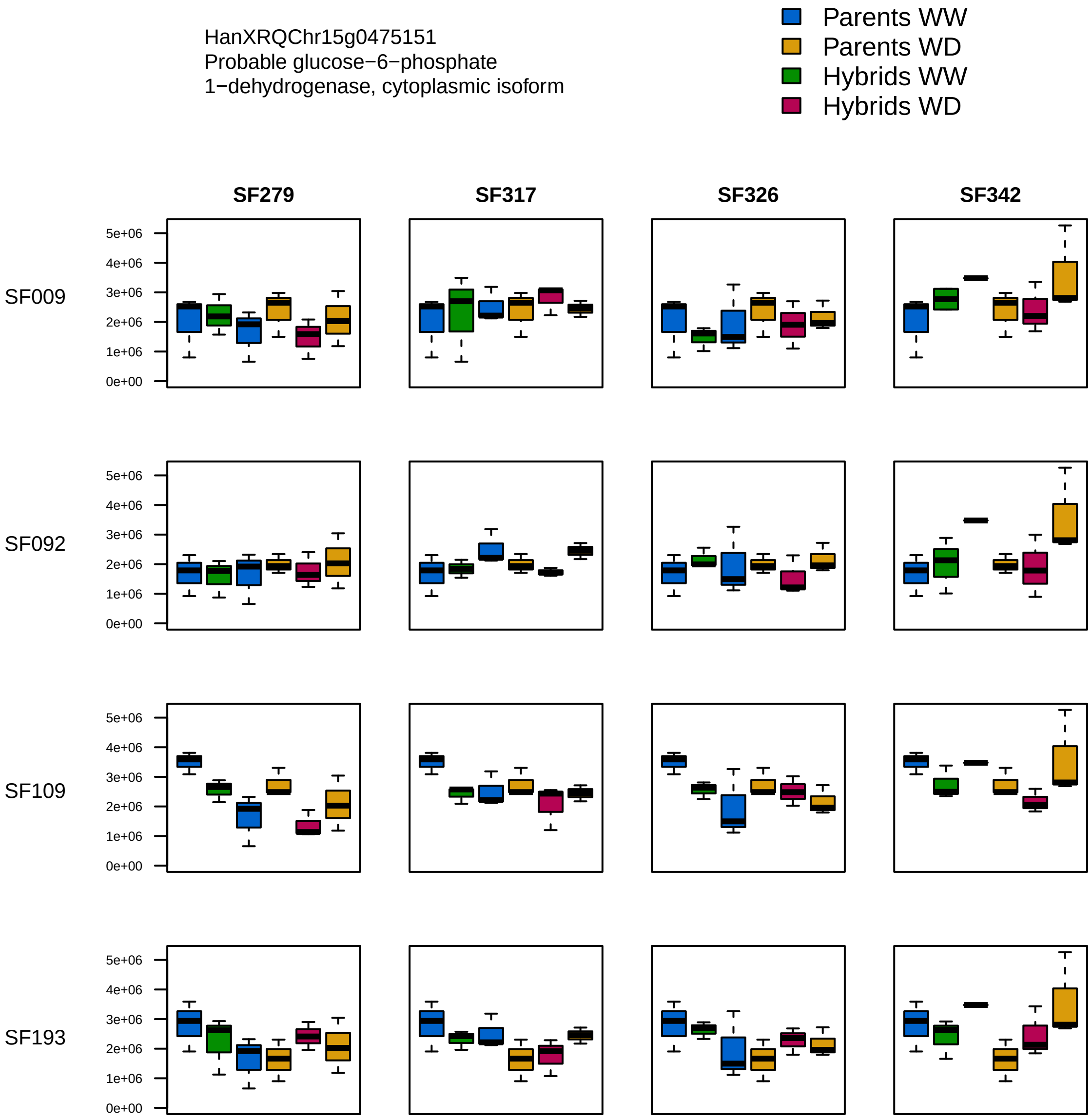

HanXRQChr15g0475151
Probable glucose−6−phosphate
1−dehydrogenase, cytoplasmic isoform
Parents WW
Parents WD
Hybrids WW
Hybrids WD
SF279
SF317
SF326
SF342
SF009
SF092
SF109
SF193
5e+06
4e+06
3e+06
2e+06
1e+06
0e+00

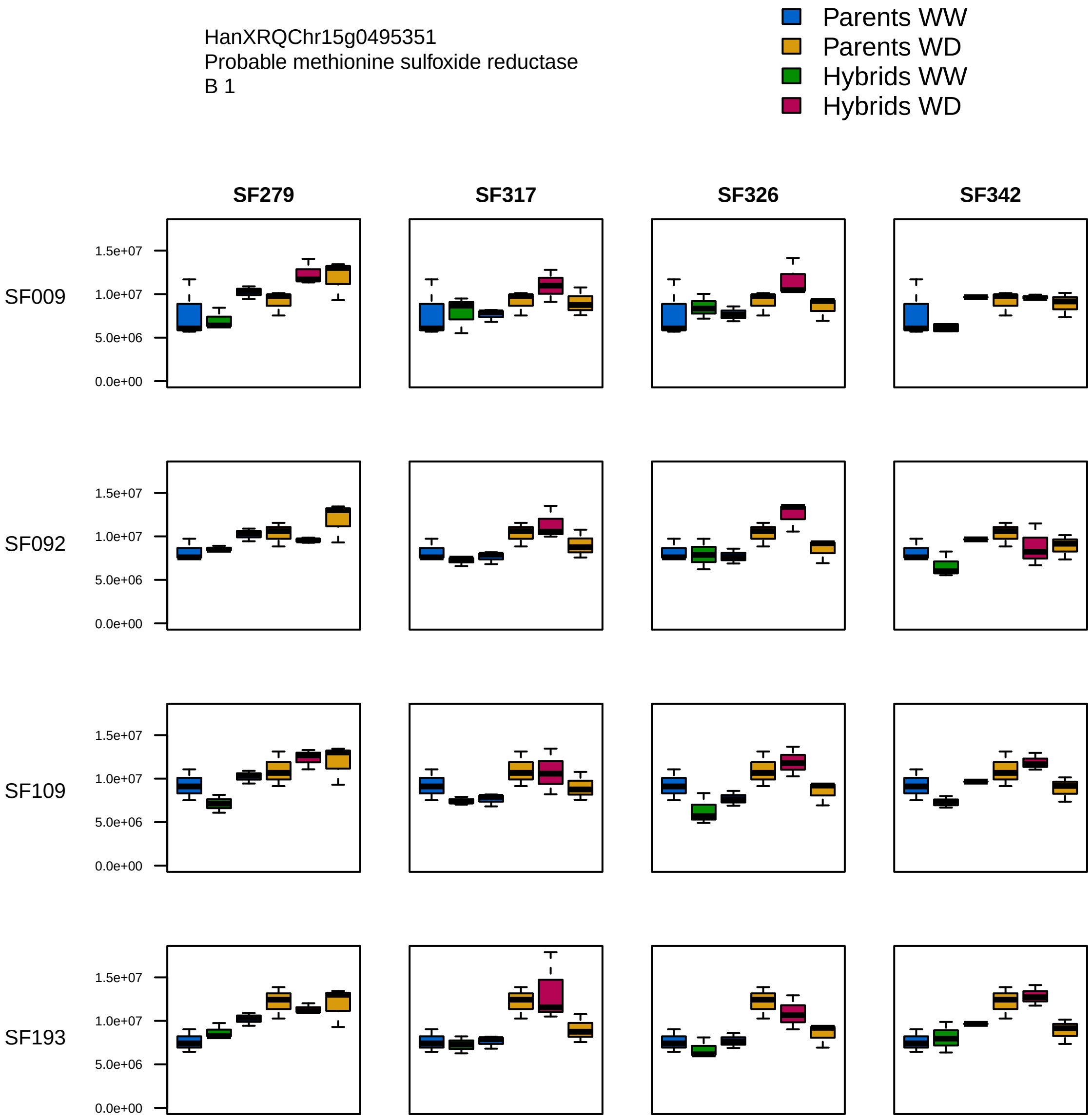
HanXRQChr15g0495351
Probable methionine sulfoxide reductase
B 1
Parents WW
Parents WD
Hybrids WW
Hybrids WD
SF279
SF317
SF326
SF342
SF009
SF092
SF109
SF193
0.0e+00
5.0e+06
1.0e+07
1.5e+07

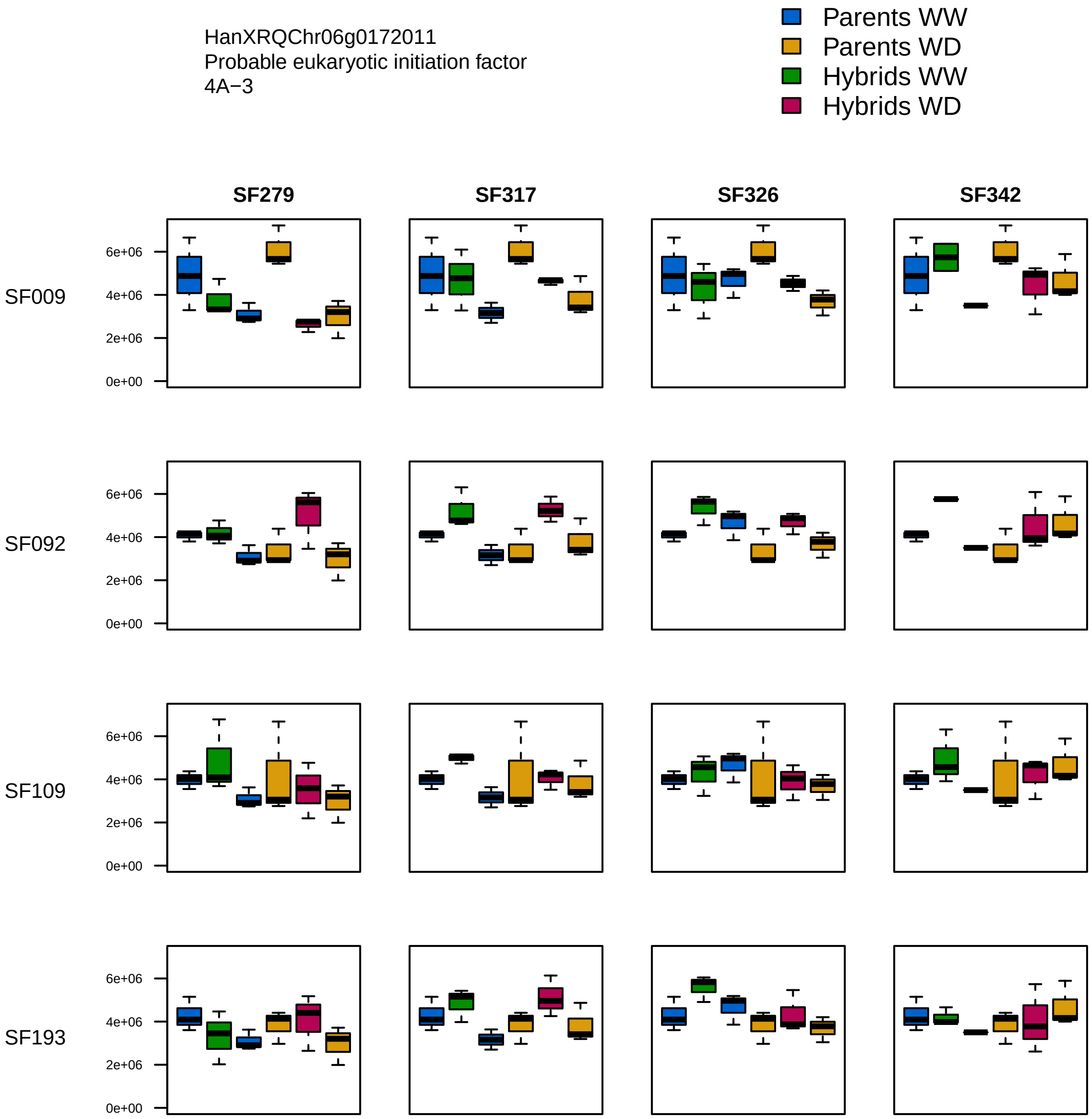
HanXRQChr06g0172011
Probable eukaryotic initiation factor
4A−3
Parents WW
Parents WD
Hybrids WW
Hybrids WD
SF279
SF317
SF326
SF342
SF009
SF092
SF109
SF193
0e+00
2e+06
4e+06
6e+06

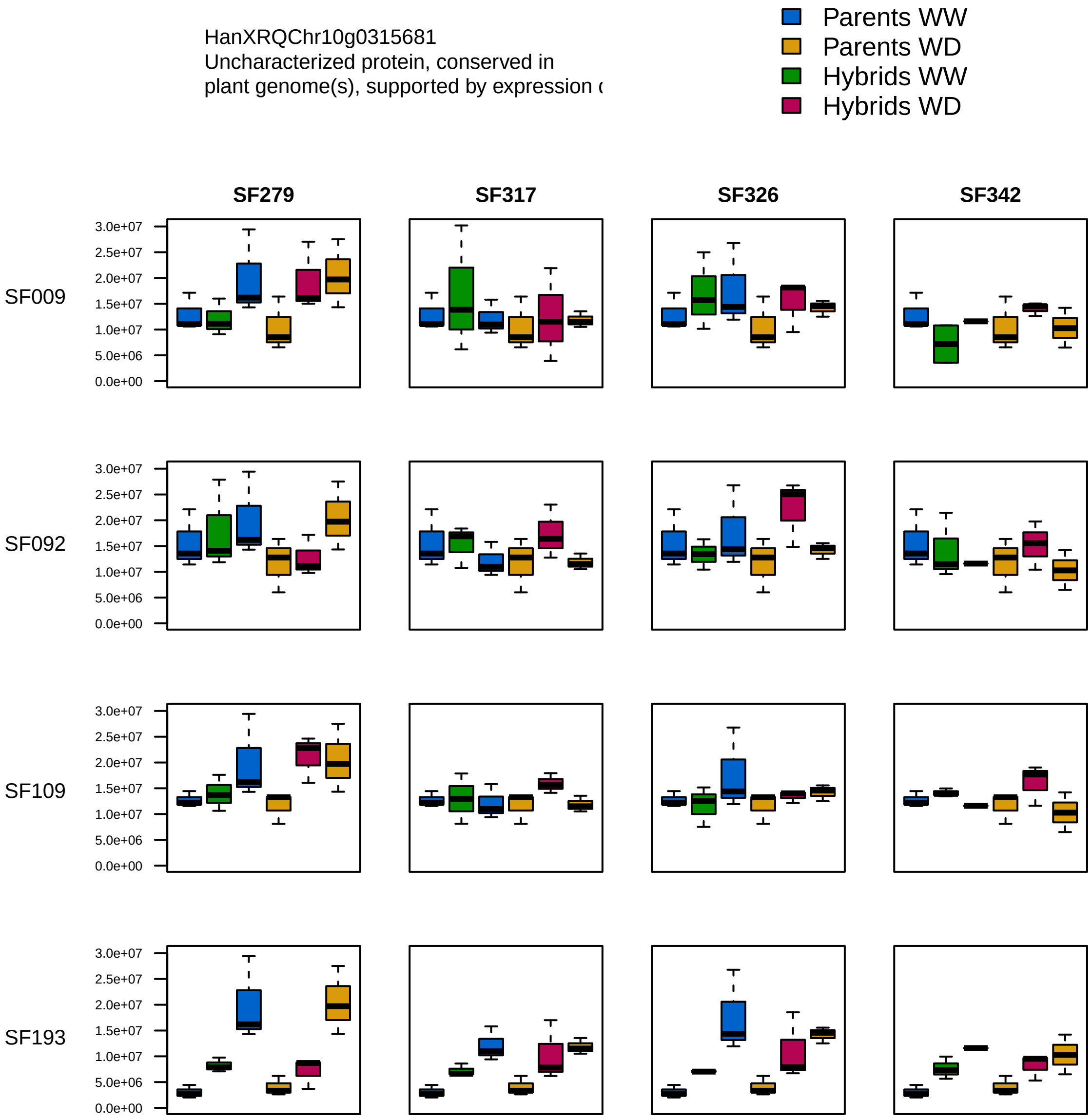
HanXRQChr10g0315681
Uncharacterized protein, conserved in
plant genome(s), supported by expression
Parents WW
Parents WD
Hybrids WW
Hybrids WD
SF279
SF317
SF326
SF342
SF009
SF092
SF109
SF193
3.0e+07
2.5e+07
2.0e+07
1.5e+07
1.0e+07
5.0e+06
0.0e+00

HanXRQChr01g0021711
Probable catalytics;carbohydrate kinases;phosphoglucan, water dikinases

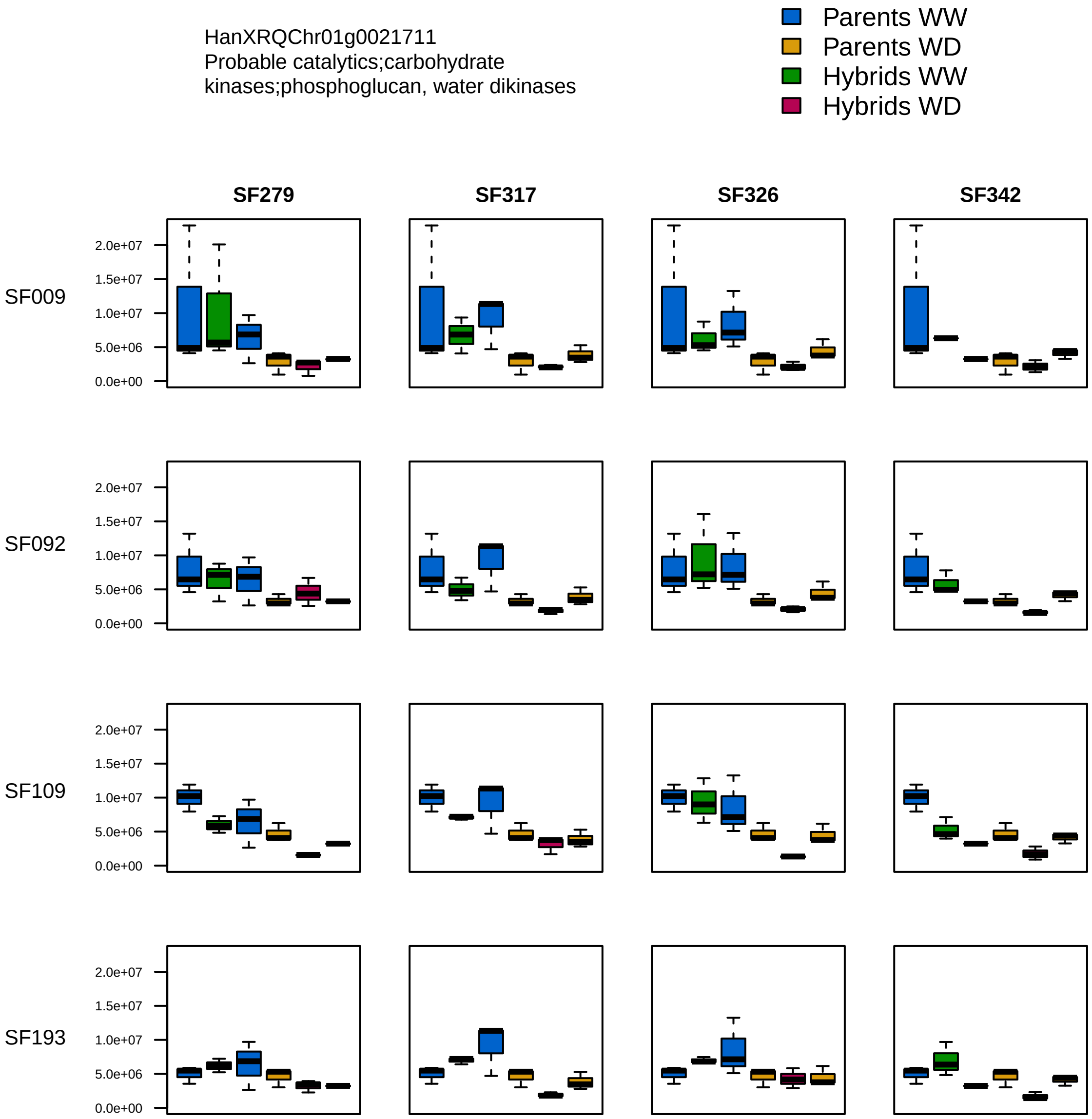

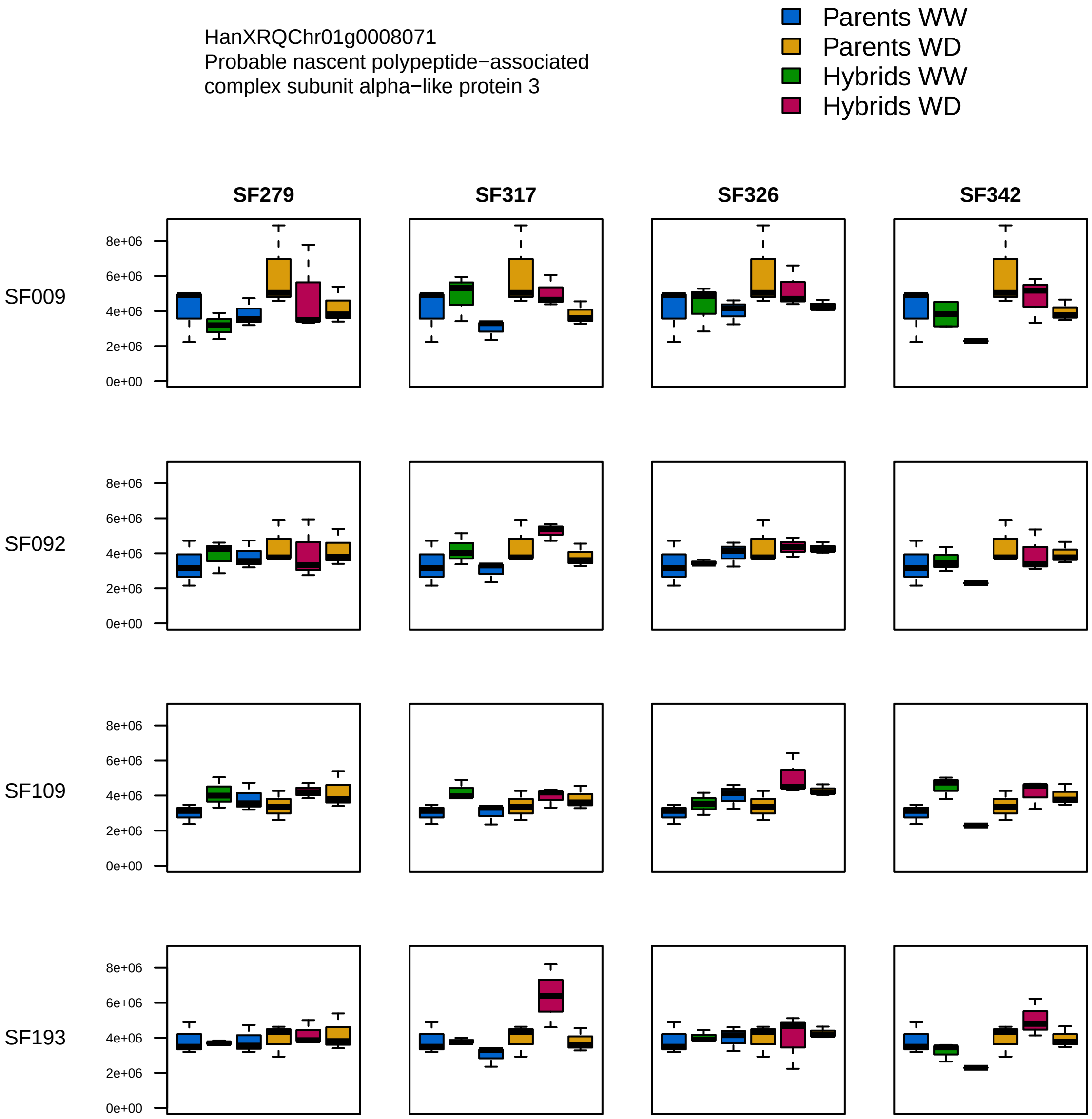

HanXRQChr01g0008071
Probable nascent polypeptide−associated
complex subunit alpha−like protein 3
Parents WW
Parents WD
Hybrids WW
Hybrids WD
SF279
SF317
SF326
SF342
SF009
SF092
SF109
SF193
8e+06
6e+06
4e+06
2e+06
0e+00

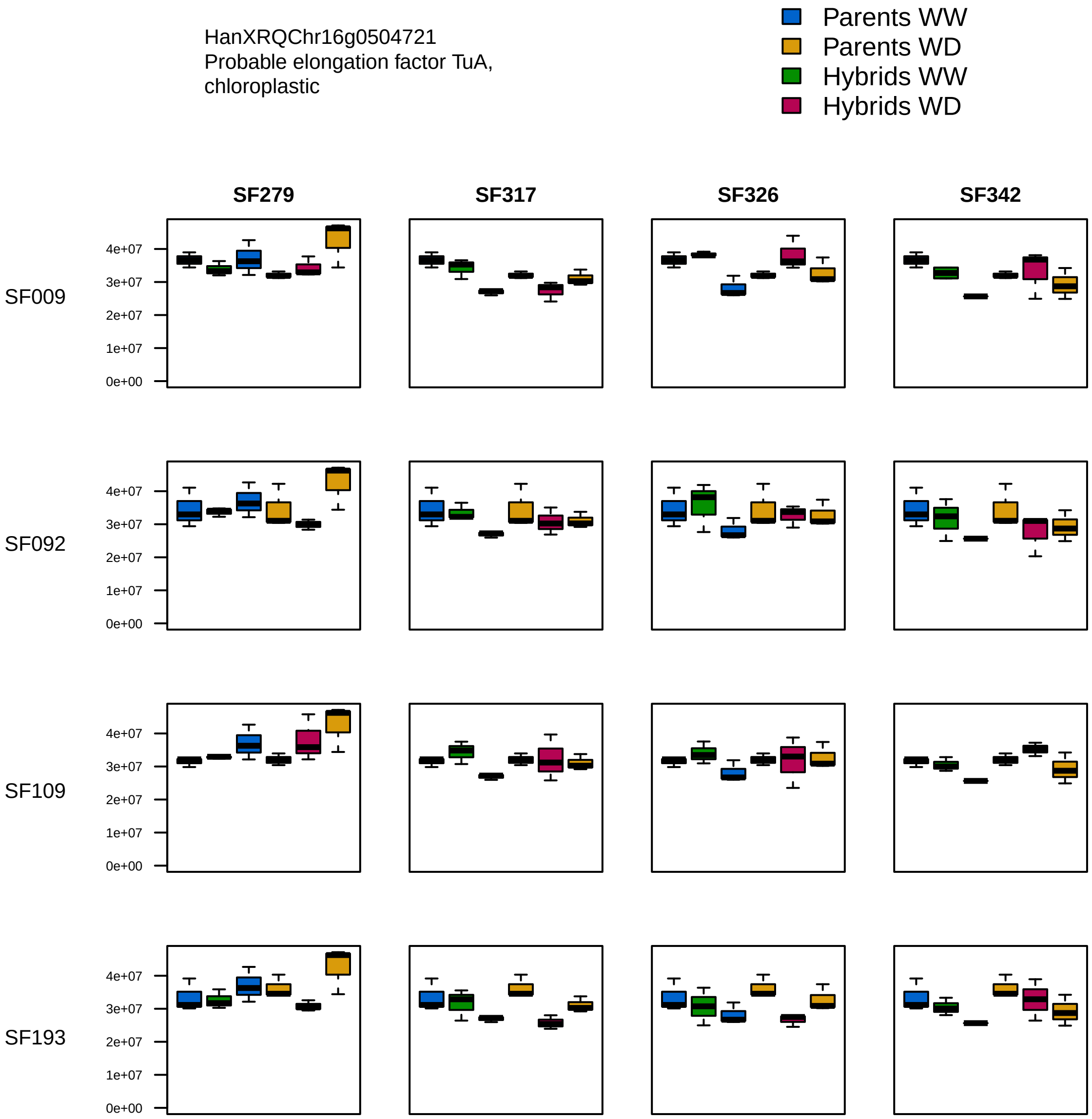

HanXRQChr16g0504721
Probable elongation factor TuA,
chloroplastic
Parents WW
Parents WD
Hybrids WW
Hybrids WD
SF279
SF317
SF326
SF342
SF009
SF092
SF109
SF193
4e+07
3e+07
2e+07
1e+07
0e+00

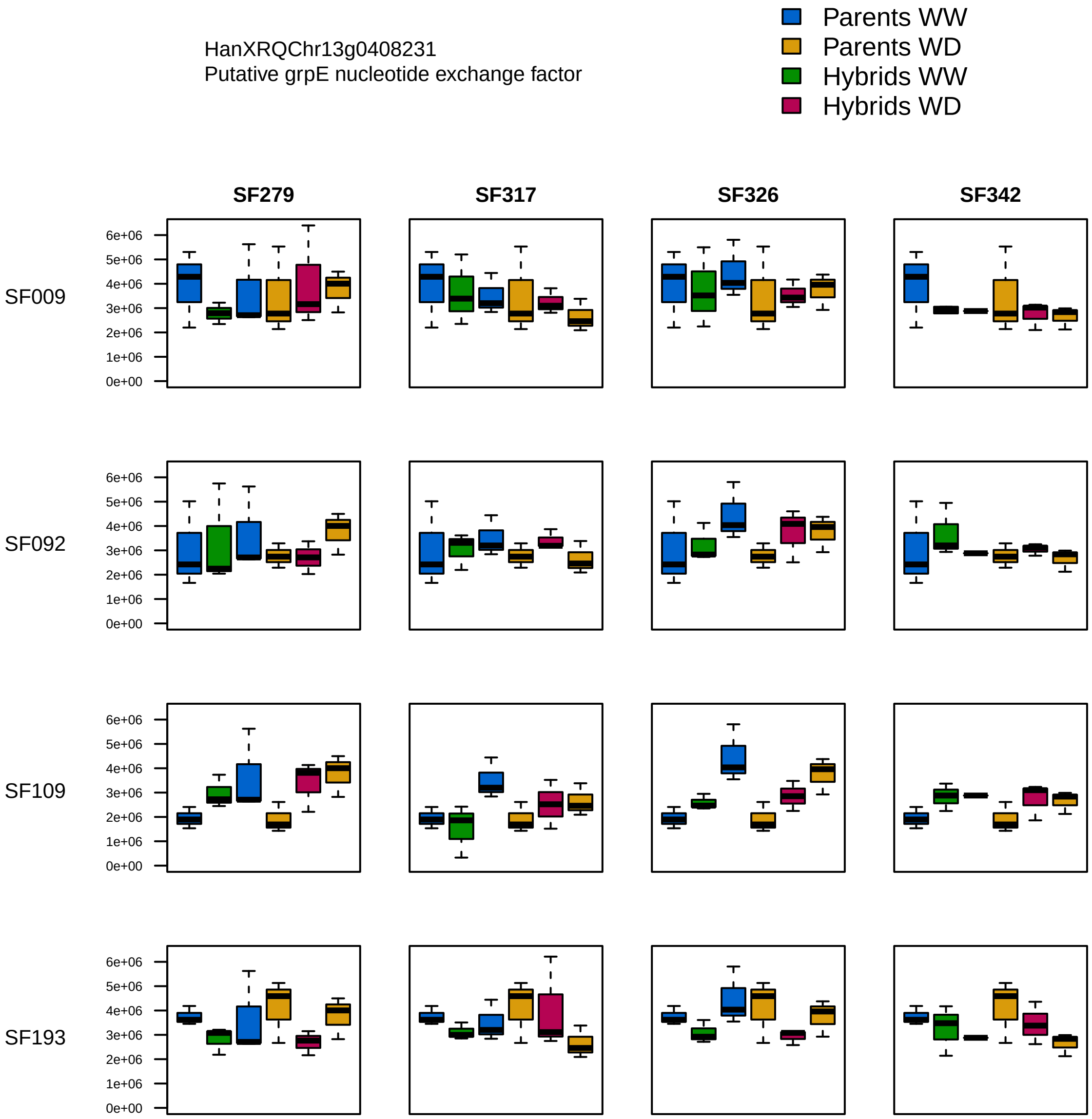
HanXRQChr13g0408231
Putative grpE nucleotide exchange factor
Parents WW
Parents WD
Hybrids WW
Hybrids WD
SF279
SF317
SF326
SF342
SF009
SF092
SF109
SF193
0e+00
1e+06
2e+06
3e+06
4e+06
5e+06
6e+06

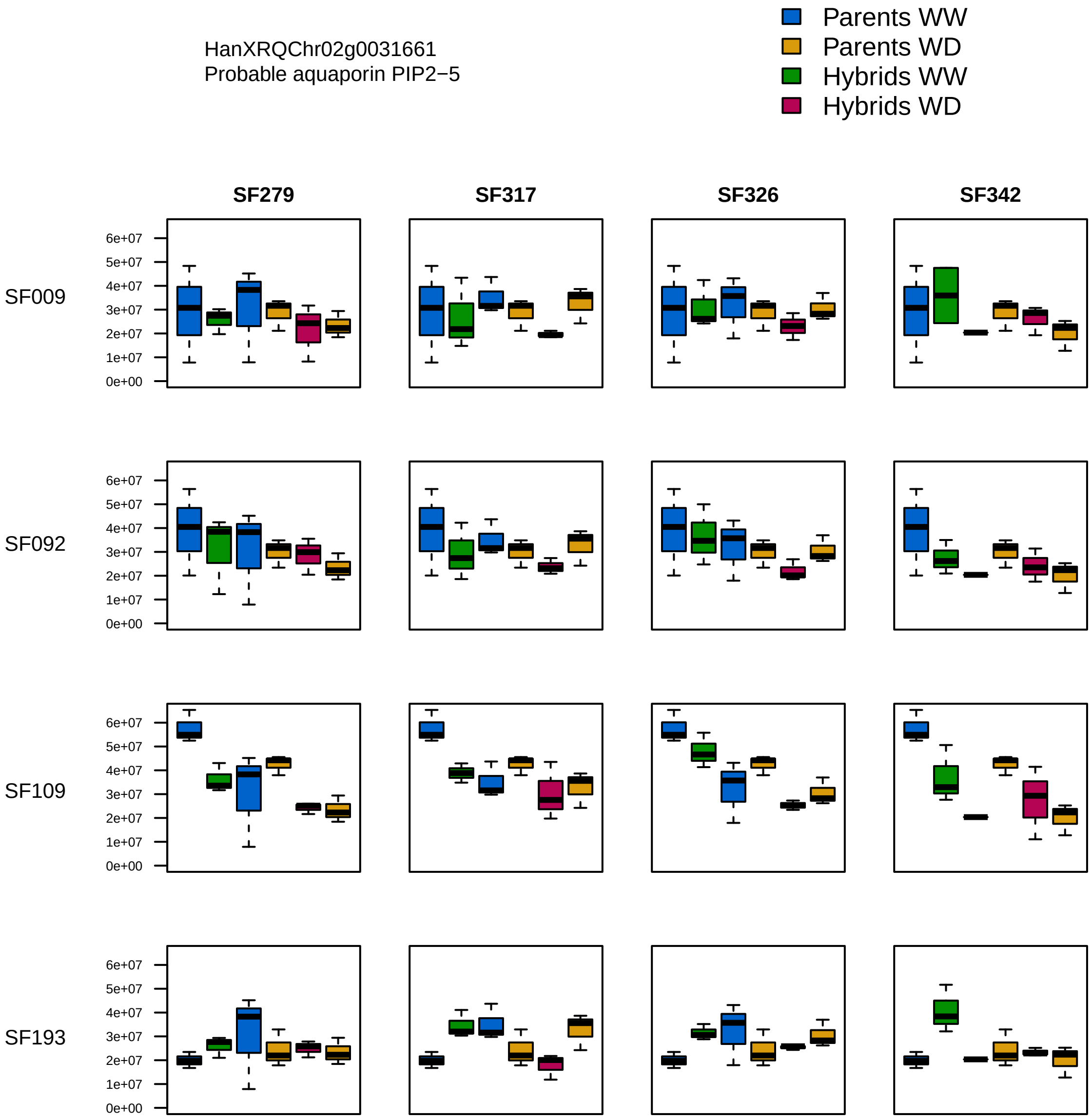

HanXRQChr02g0031661
Probable aquaporin PIP2−5
Parents WW
Parents WD
Hybrids WW
Hybrids WD
SF279
SF317
SF326
SF342
SF009
SF092
SF109
SF193
0e+00
1e+07
2e+07
3e+07
4e+07
5e+07
6e+07

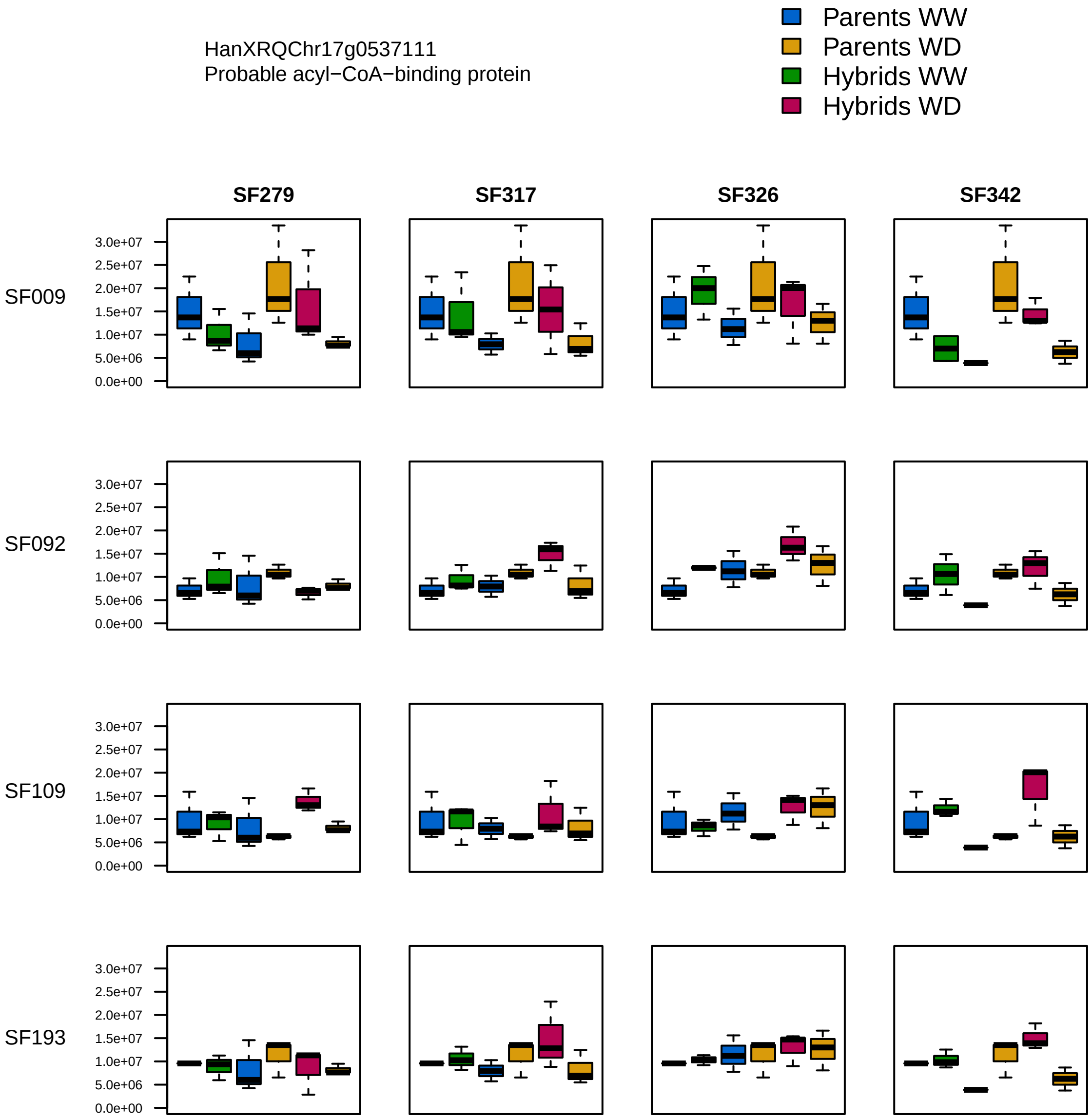
HanXRQChr17g0537111
Probable acyl−CoA−binding protein
Parents WW
Parents WD
Hybrids WW
Hybrids WD
SF279
SF317
SF326
SF342
SF009
SF092
SF109
SF193
3.0e+07
2.5e+07
2.0e+07
1.5e+07
1.0e+07
5.0e+06
0.0e+00

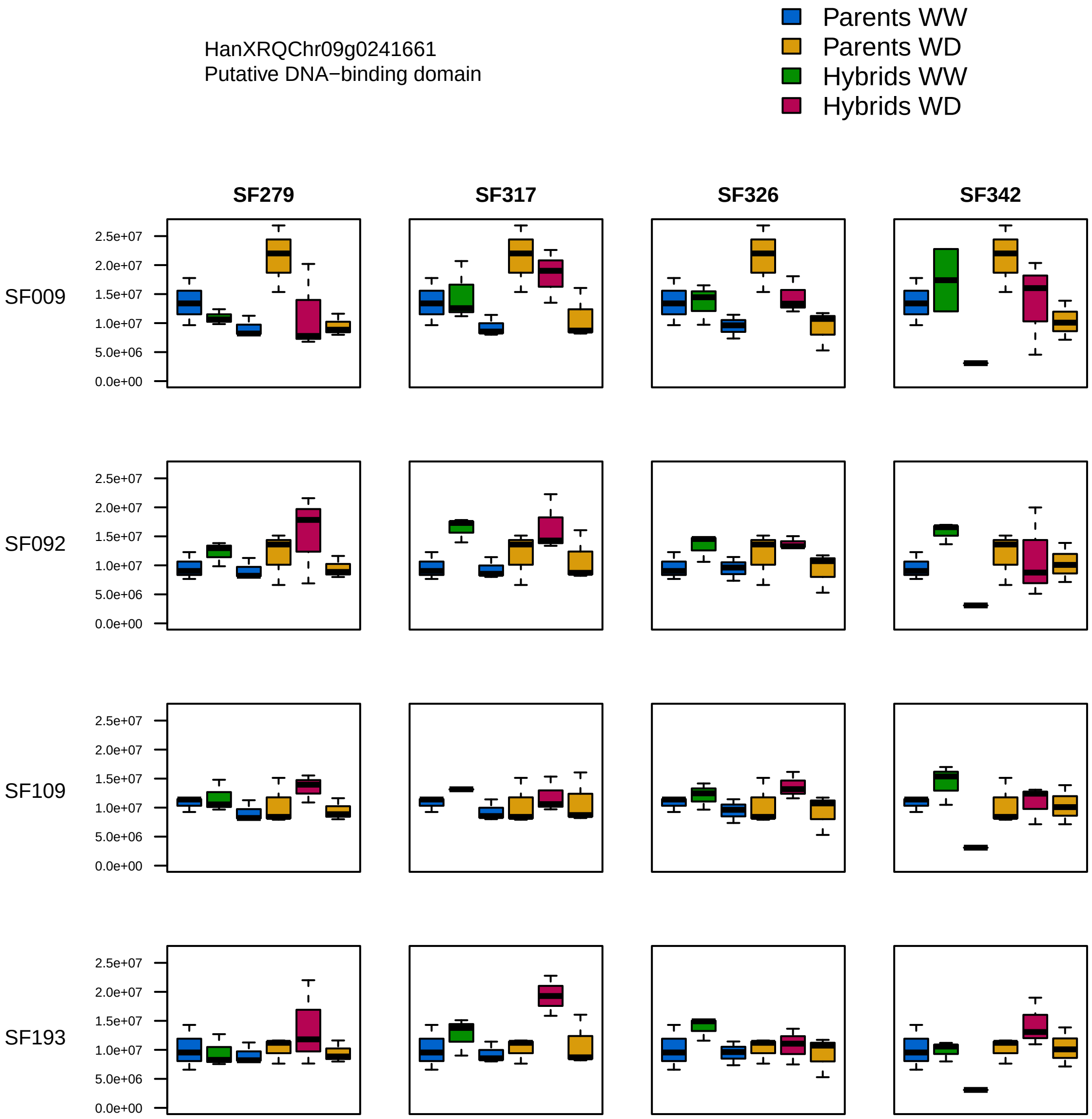

HanXRQChr09g0241661
Putative DNA−binding domain
Parents WW
Parents WD
Hybrids WW
Hybrids WD
SF279
SF317
SF326
SF342
SF009
SF092
SF109
SF193
2.5e+07
2.0e+07
1.5e+07
1.0e+07
5.0e+06
0.0e+00

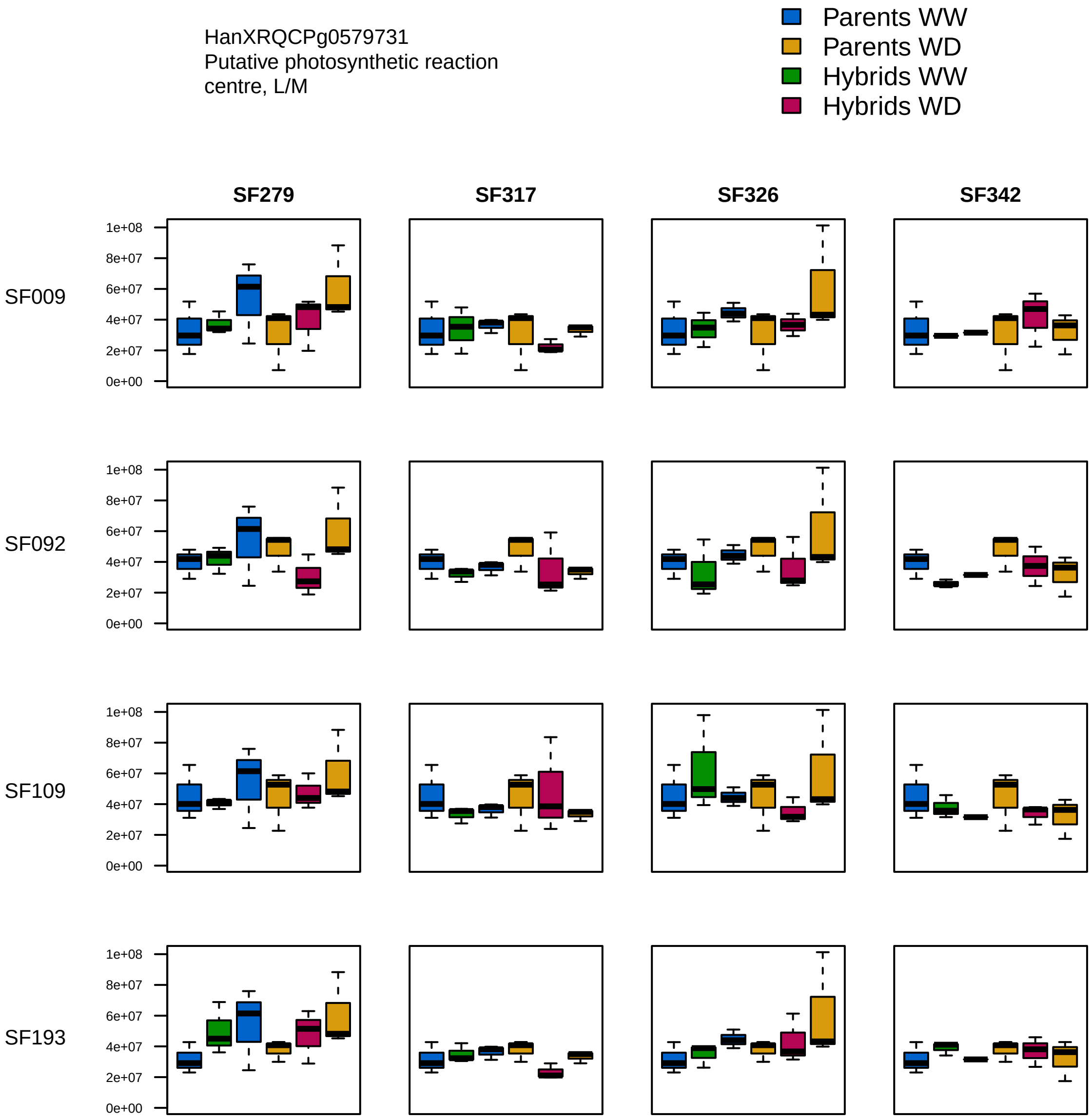

HanXRQCPg0579731
Putative photosynthetic reaction
centre, L/M
Parents WW
Parents WD
Hybrids WW
Hybrids WD
SF279
SF317
SF326
SF342
SF009
SF092
SF109
SF193
1e+08
8e+07
6e+07
4e+07
2e+07
0e+00

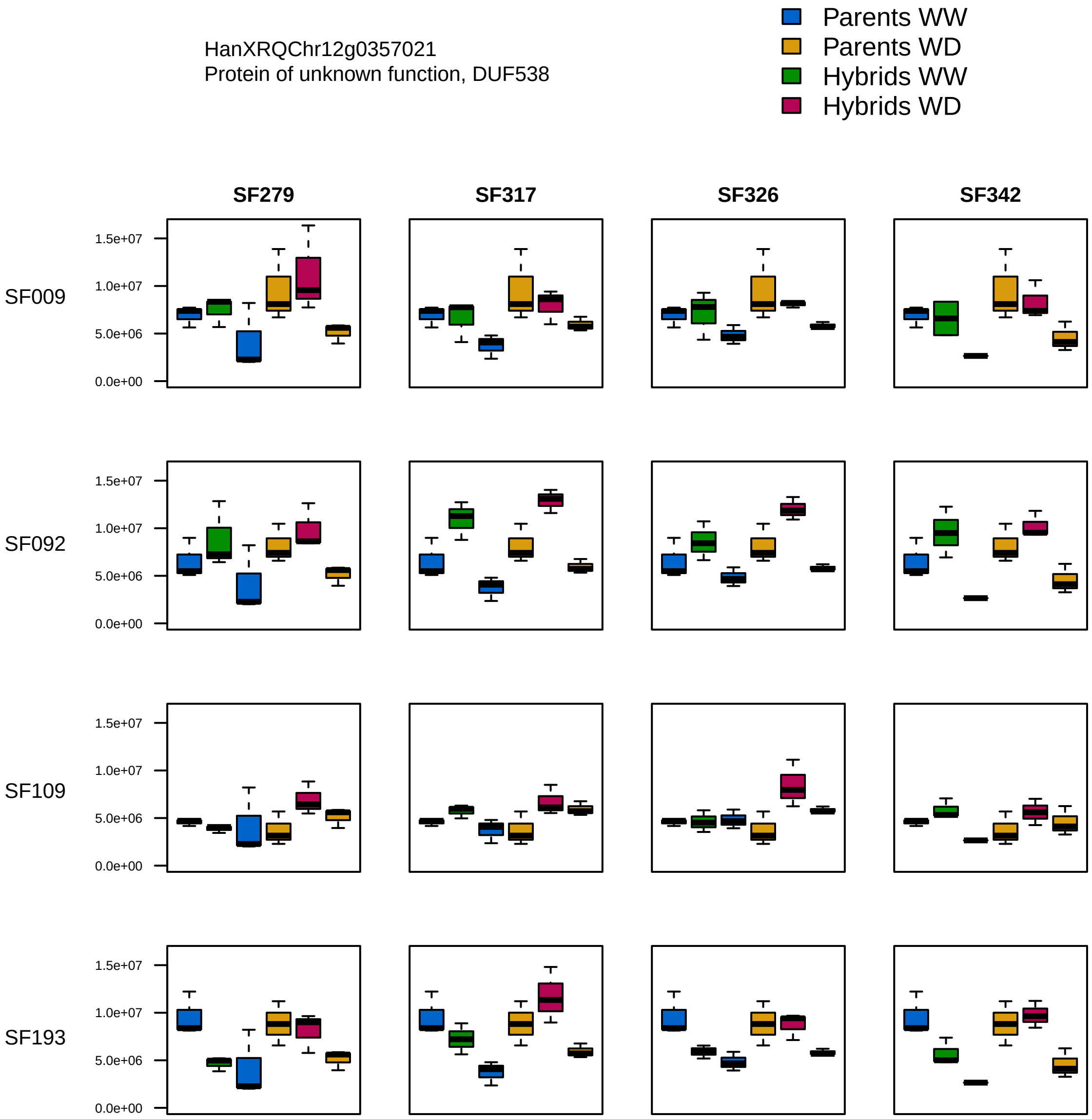
HanXRQChr12g0357021
Protein of unknown function, DUF538
Parents WW
Parents WD
Hybrids WW
Hybrids WD
SF279
SF317
SF326
SF342
SF009
SF092
SF109
SF193
1.5e+07
1.0e+07
5.0e+06
0.0e+00

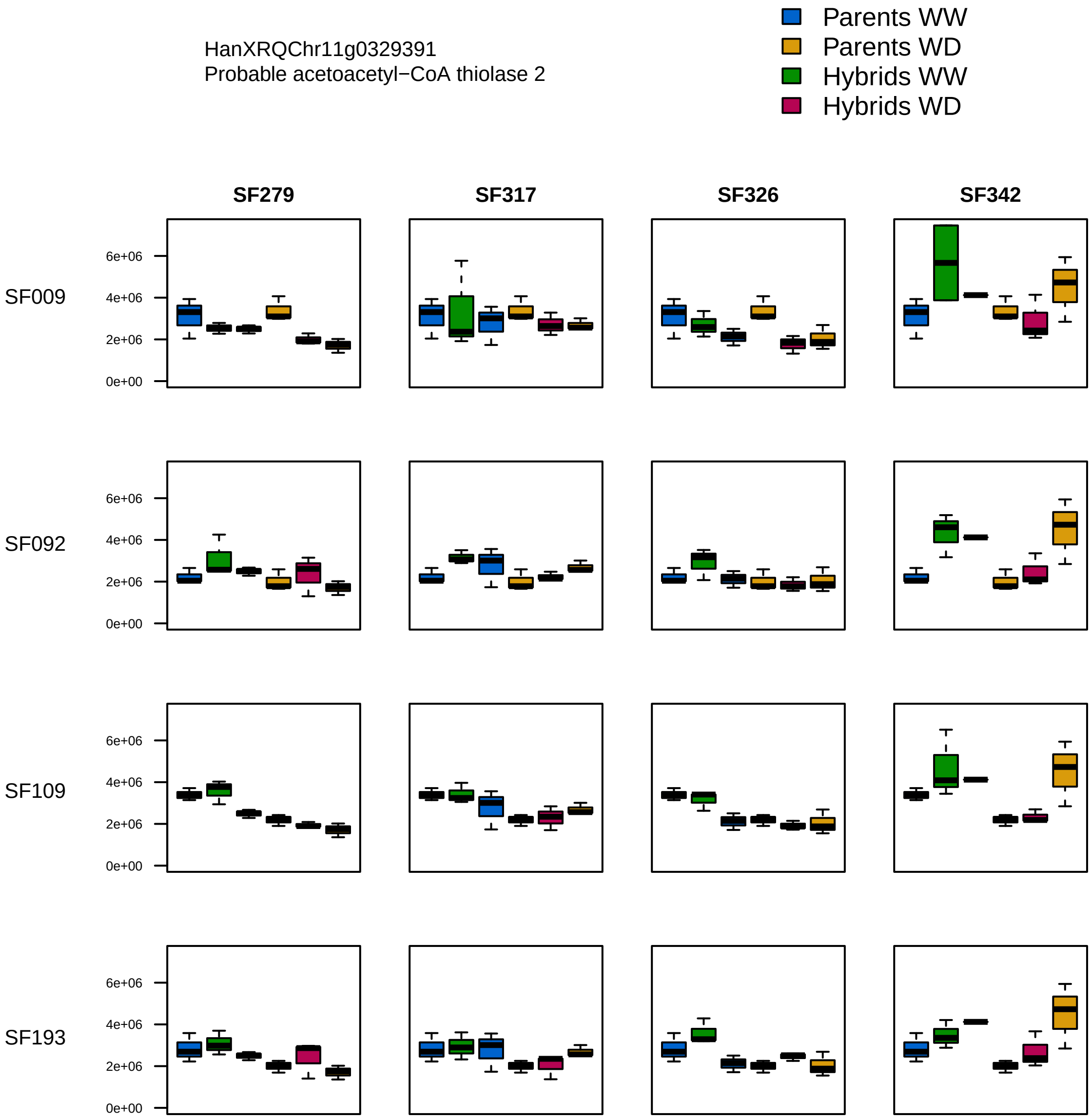

HanXRQChr11g0329391
Probable acetoacetyl−CoA thiolase 2
Parents WW
Parents WD
Hybrids WW
Hybrids WD
SF279
SF317
SF326
SF342
SF009
SF092
SF109
SF193
0e+00
2e+06
4e+06
6e+06

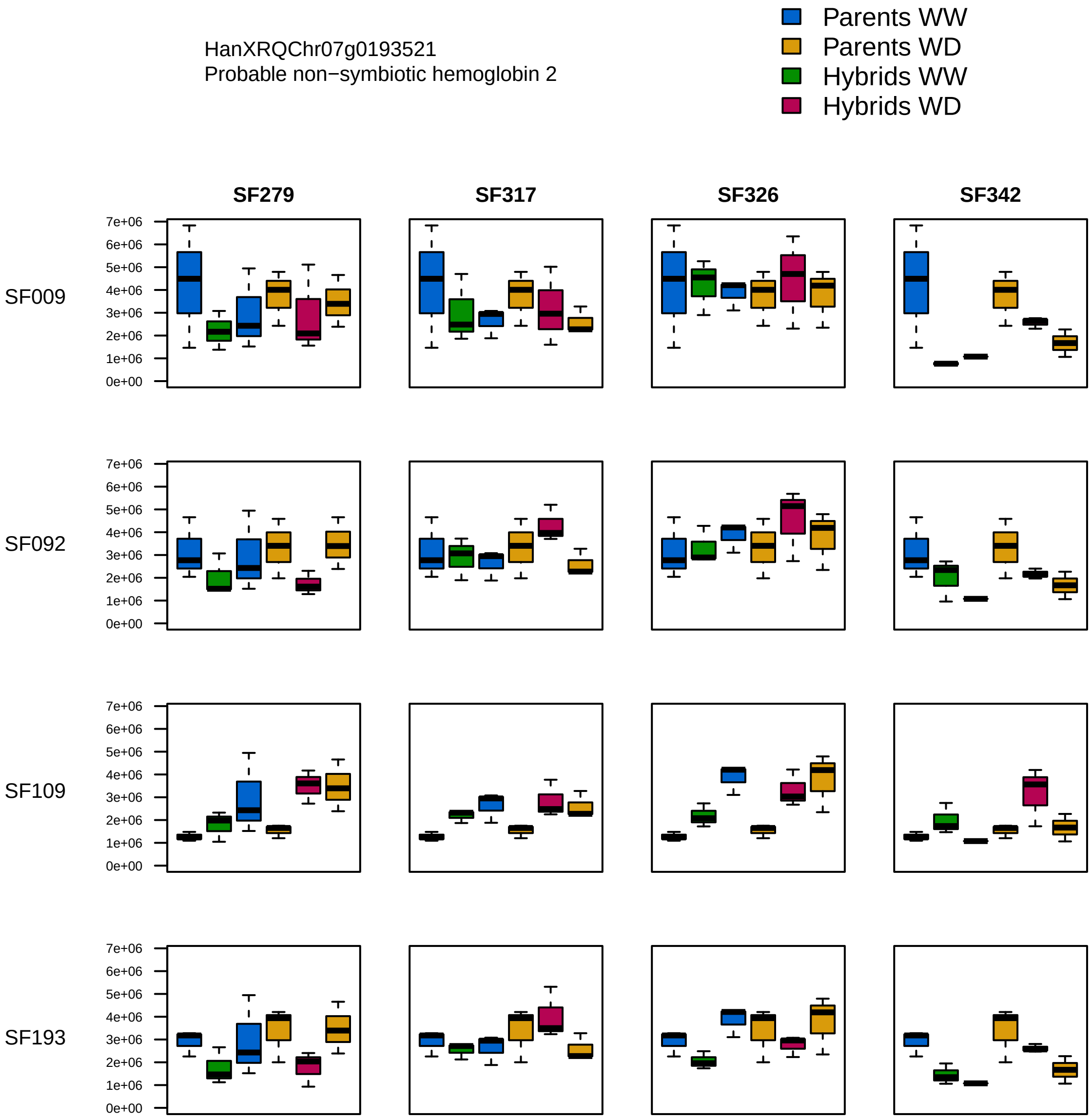
HanXRQChr07g0193521
Probable non−symbiotic hemoglobin 2
Parents WW
Parents WD
Hybrids WW
Hybrids WD
SF279
SF317
SF326
SF342
SF009
SF092
SF109
SF193
7e+06
6e+06
5e+06
4e+06
3e+06
2e+06
1e+06
0e+00

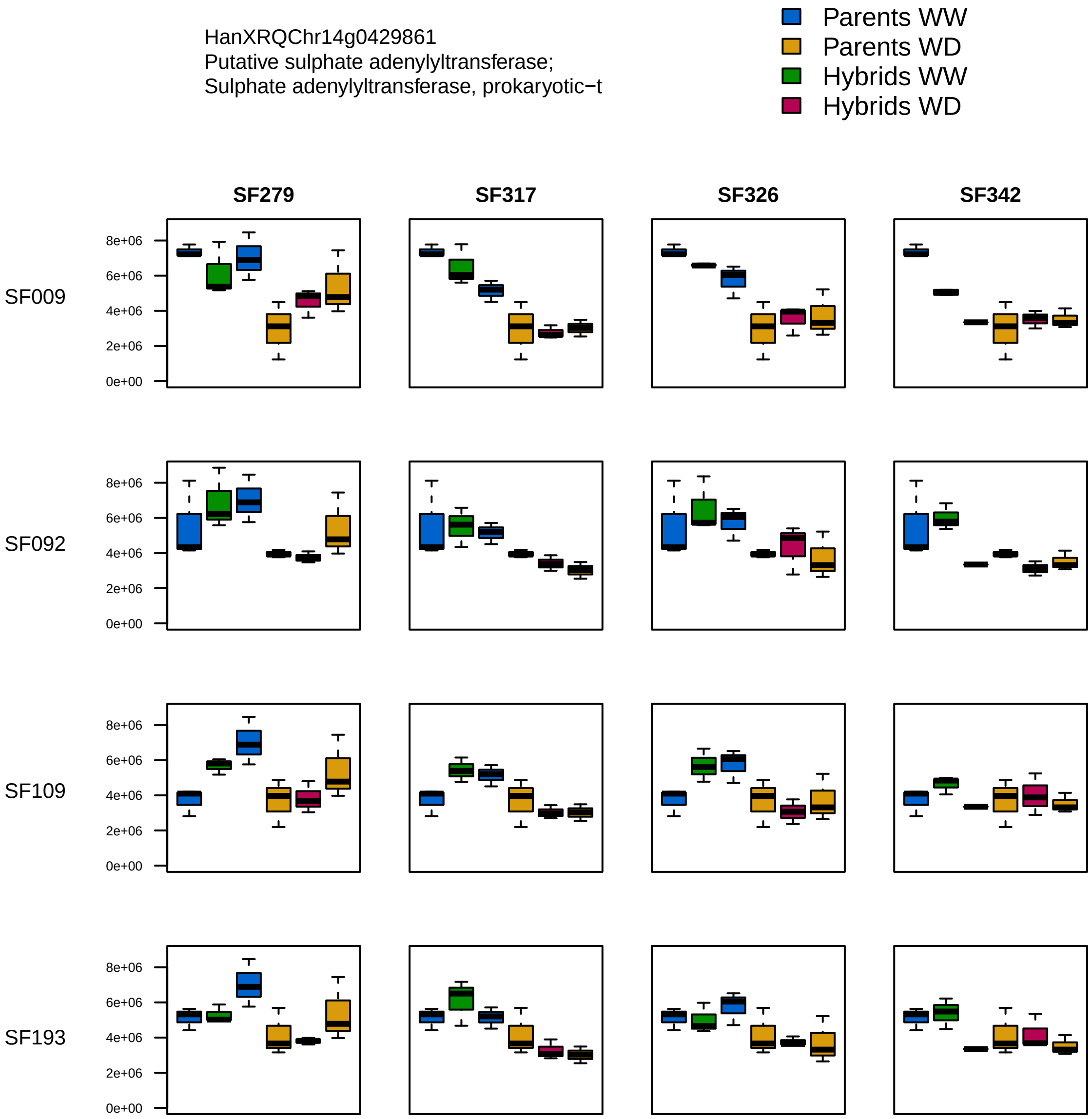
HanXRQChr14g0429861
Putative sulphate adenylyltransferase;
Sulphate adenylyltransferase, prokaryotic−t
Parents WW
Parents WD
Hybrids WW
Hybrids WD
SF279
SF317
SF326
SF342
SF009
SF092
SF109
SF193
0e+00
2e+06
4e+06
6e+06
8e+06

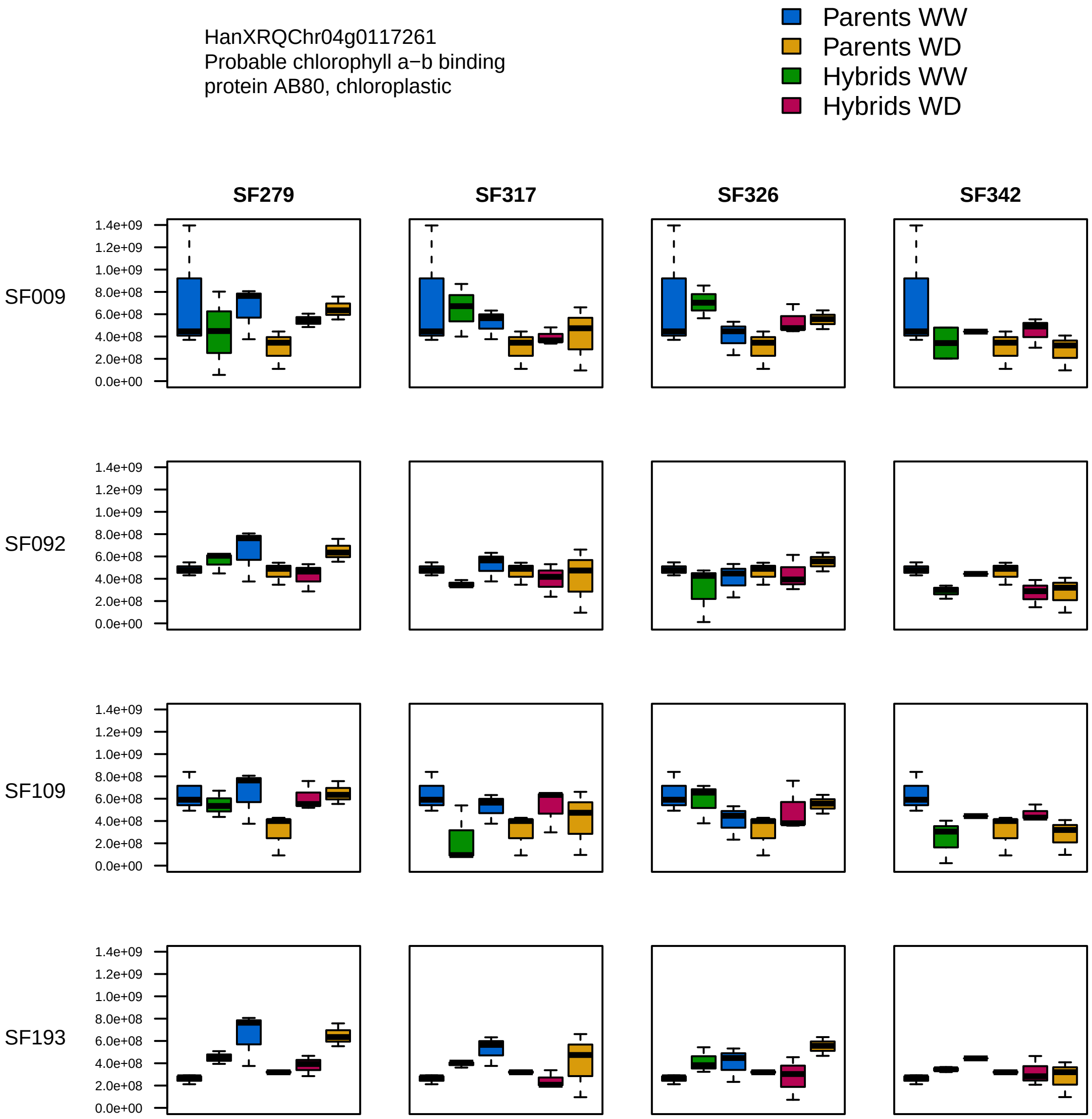
HanXRQChr04g0117261
Probable chlorophyll a−b binding
protein AB80, chloroplastic
Parents WW
Parents WD
Hybrids WW
Hybrids WD
SF279
SF317
SF326
SF342
SF009
SF092
SF109
SF193
1.4e+09
1.2e+09
1.0e+09
8.0e+08
6.0e+08
4.0e+08
2.0e+08
0.0e+00

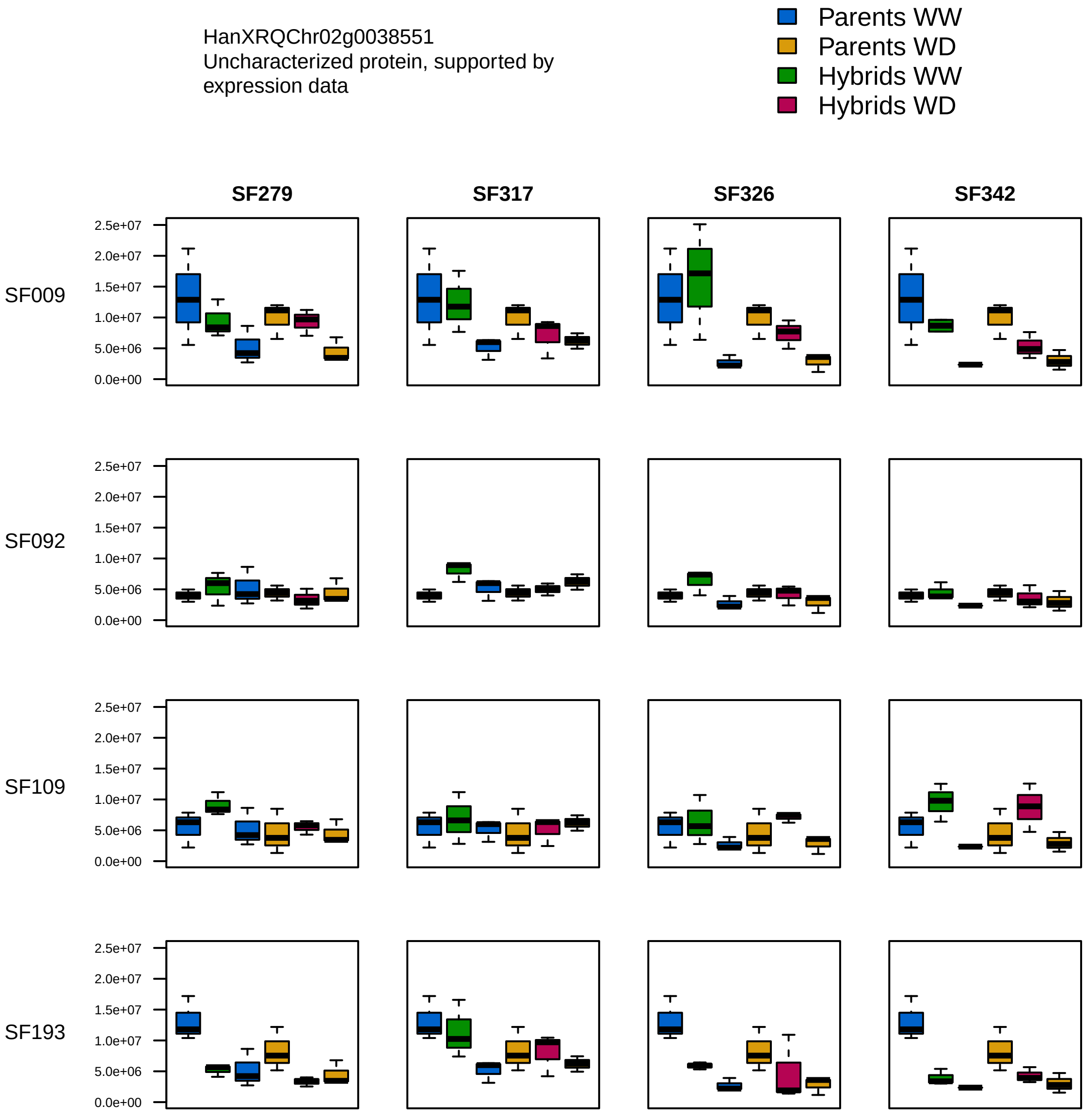
HanXRQChr02g0038551
Uncharacterized protein, supported by expression data
Parents WW
Parents WD
Hybrids WW
Hybrids WD
SF279
SF317
SF326
SF342
SF009
SF092
SF109
SF193
2.5e+07
2.0e+07
1.5e+07
1.0e+07
5.0e+06
0.0e+00

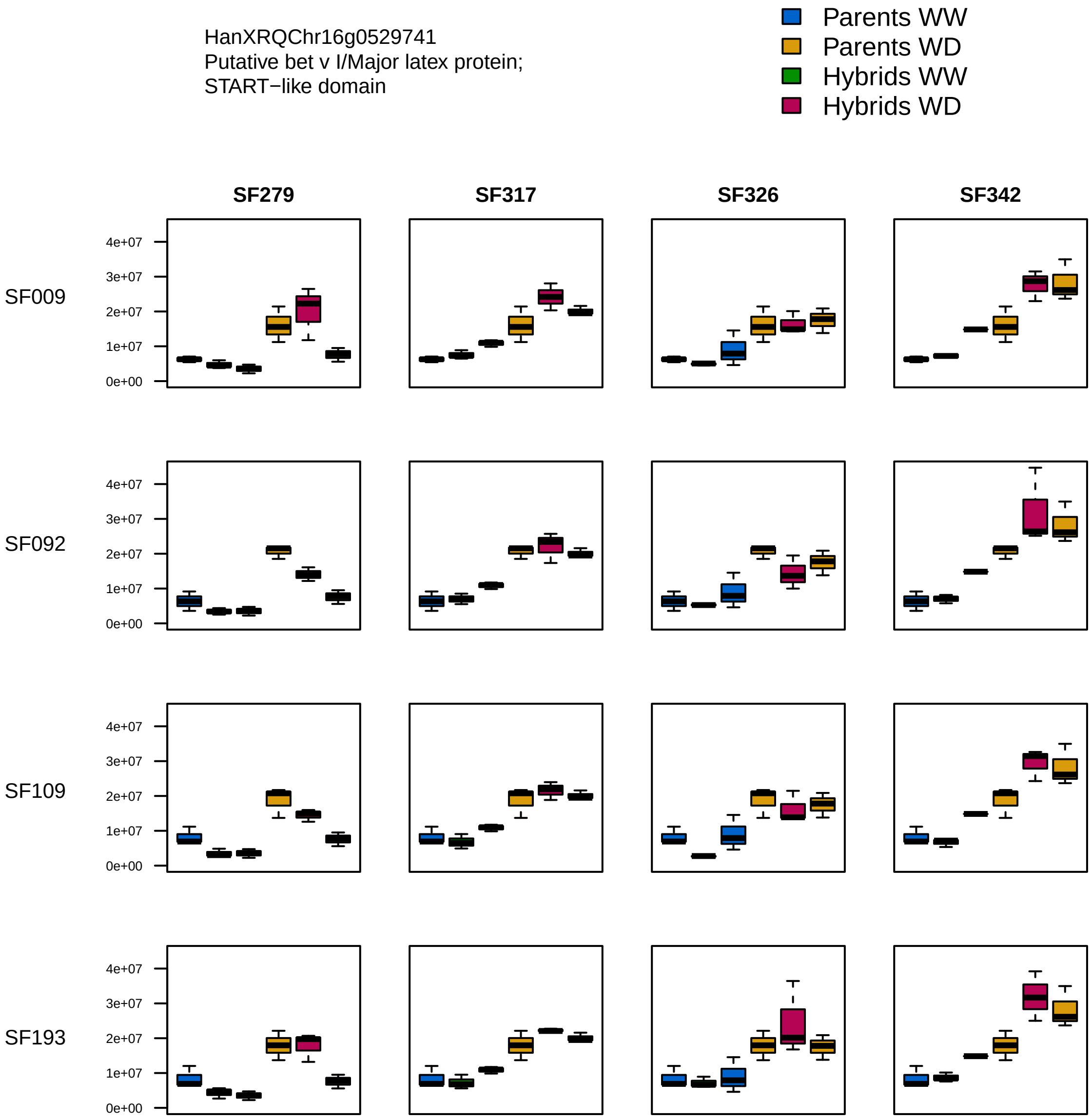

HanXRQChr16g0529741
Putative bet v I/Major latex protein;
START−like domain
Parents WW
Parents WD
Hybrids WW
Hybrids WD
SF279
SF317
SF326
SF342
SF009
SF092
SF109
SF193
0e+00
1e+07
2e+07
3e+07
4e+07

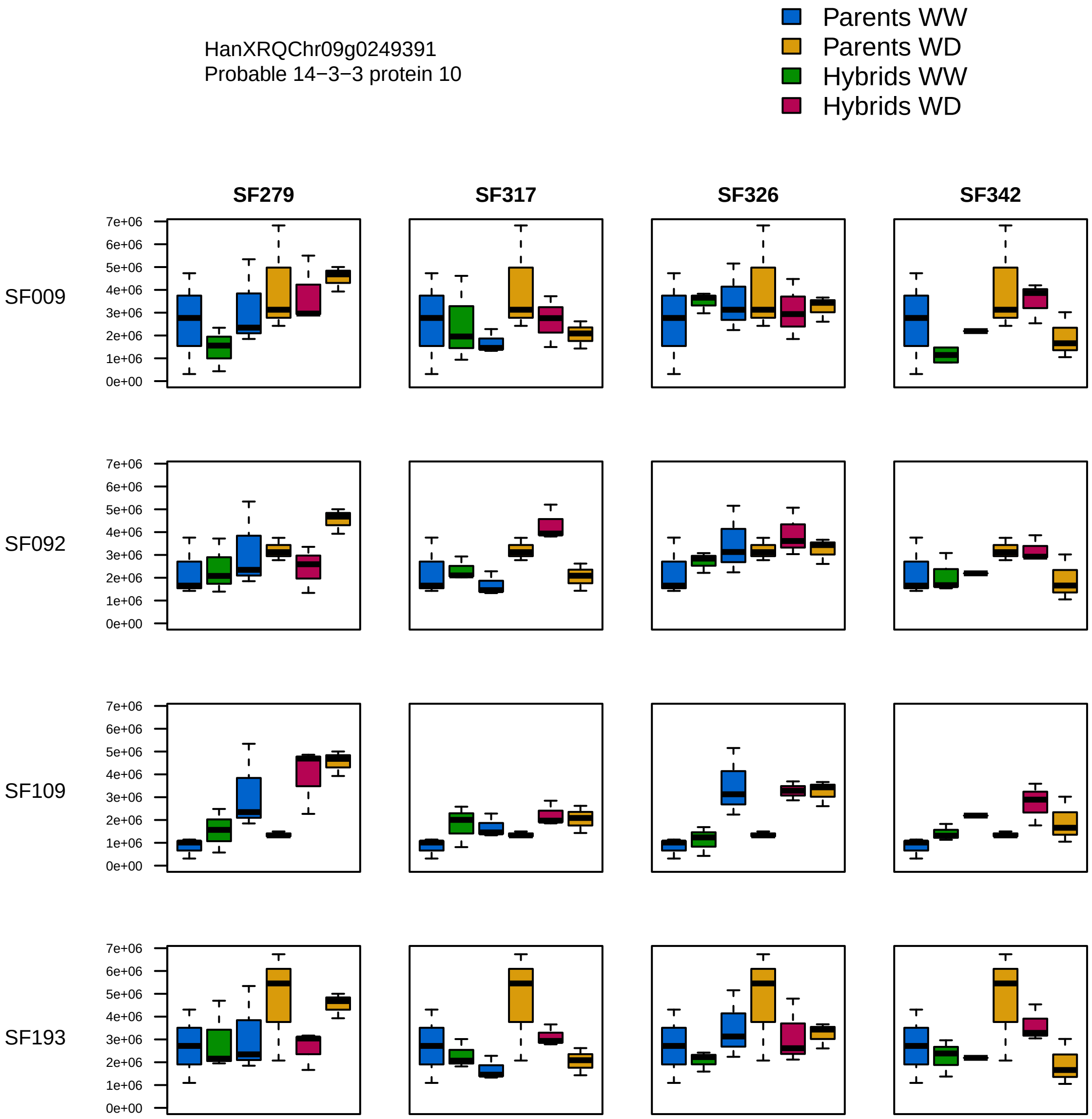

HanXRQChr09g0249391
Probable 14−3−3 protein 10
Parents WW
Parents WD
Hybrids WW
Hybrids WD
SF279
SF317
SF326
SF342
SF009
SF092
SF109
SF193
0e+00
1e+06
2e+06
3e+06
4e+06
5e+06
6e+06
7e+06

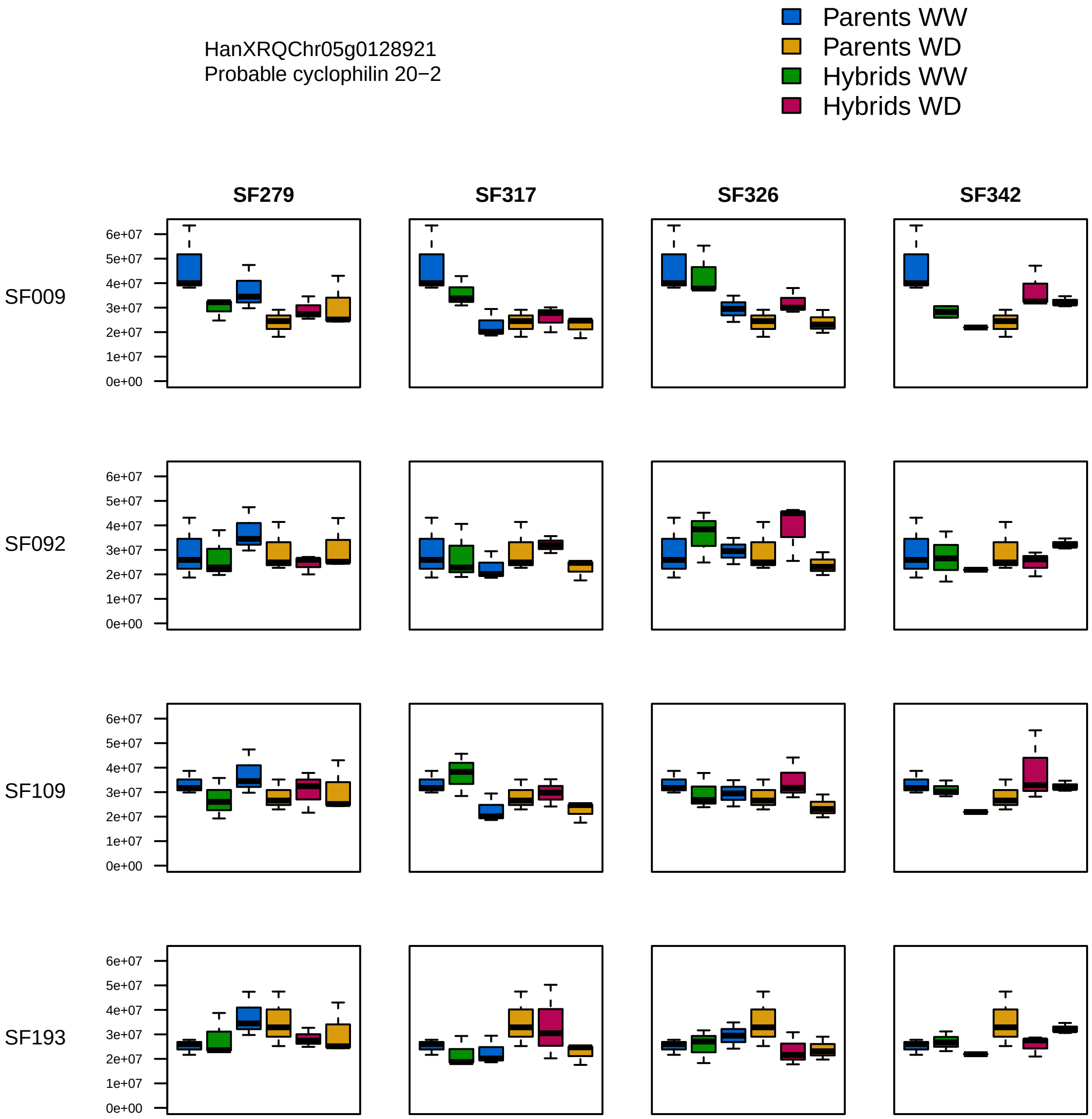
HanXRQChr05g0128921
Probable cyclophilin 20−2
Parents WW
Parents WD
Hybrids WW
Hybrids WD
SF279
SF317
SF326
SF342
SF009
SF092
SF109
SF193
6e+07
5e+07
4e+07
3e+07
2e+07
1e+07
0e+00

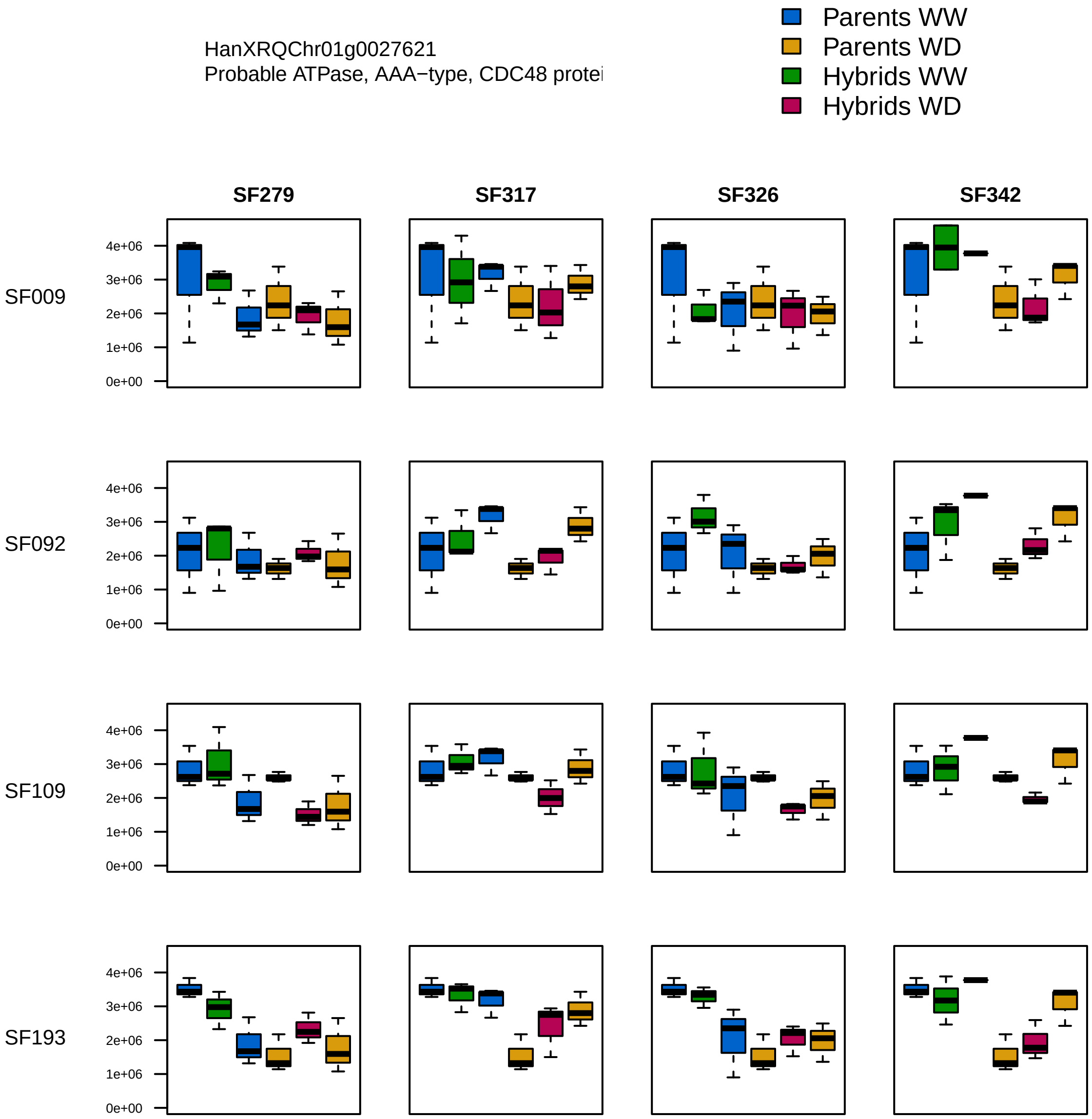
HanXRQChr01g0027621
Probable ATPase, AAA−type, CDC48 protei
Parents WW
Parents WD
Hybrids WW
Hybrids WD
SF279
SF317
SF326
SF342
SF009
SF092
SF109
SF193
4e+06
3e+06
2e+06
1e+06
0e+00

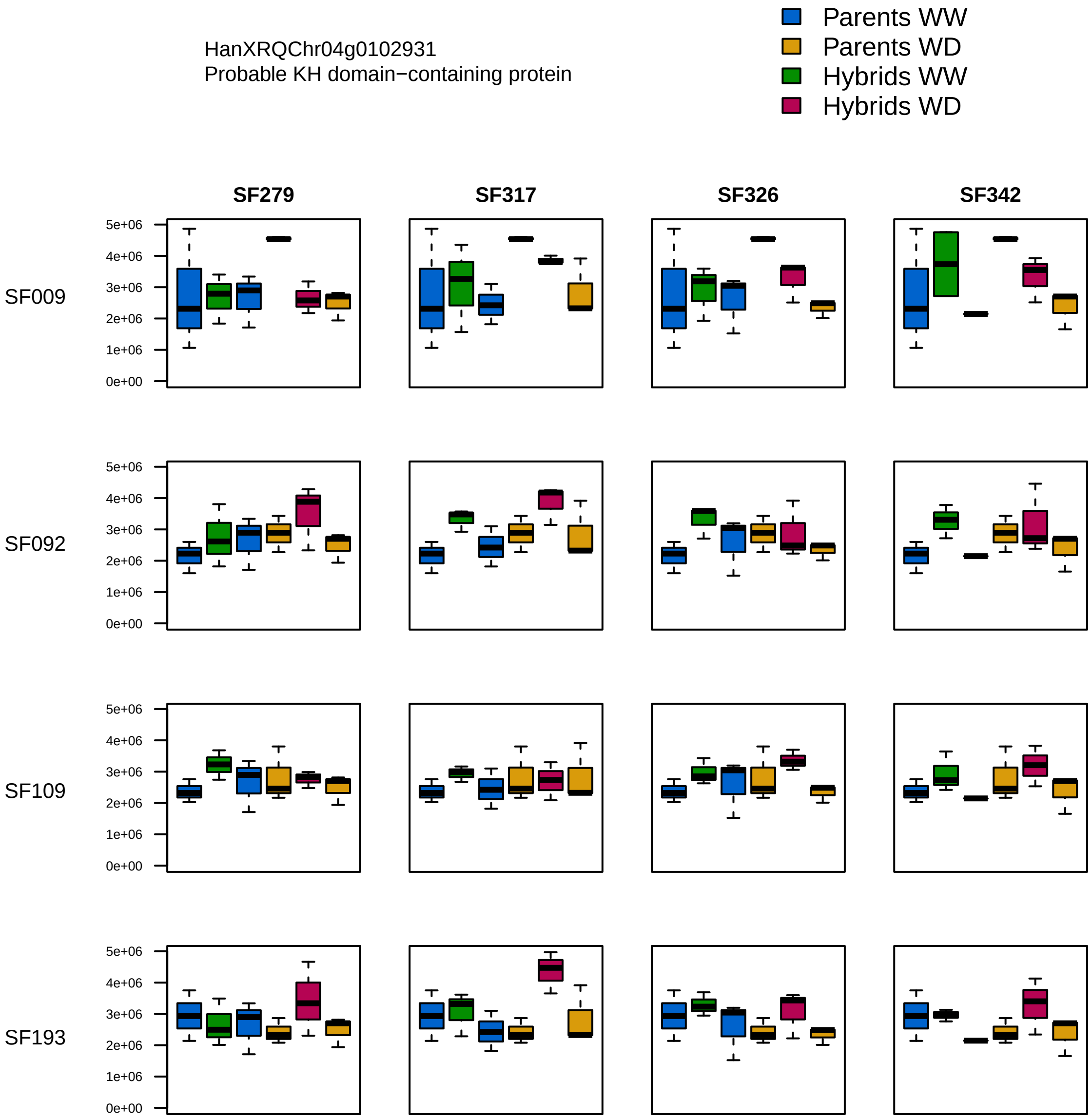
HanXRQChr04g0102931
Probable KH domain−containing protein
Parents WW
Parents WD
Hybrids WW
Hybrids WD
SF279
SF317
SF326
SF342
SF009
SF092
SF109
SF193
0e+00
1e+06
2e+06
3e+06
4e+06
5e+06

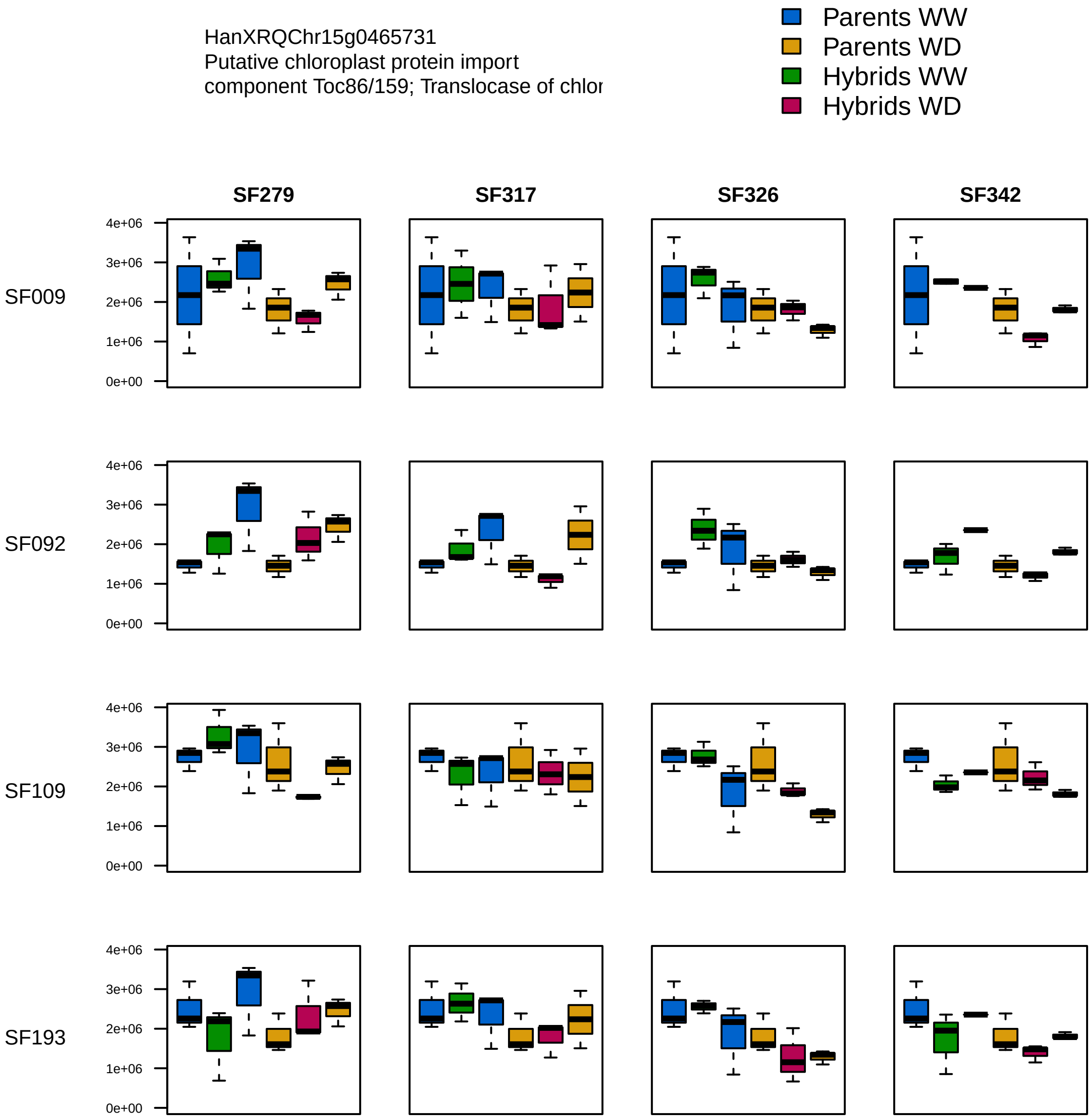
HanXRQChr15g0465731
Putative chloroplast protein import
component Toc86/159; Translocase of chlor
Parents WW
Parents WD
Hybrids WW
Hybrids WD
SF279
SF317
SF326
SF342
SF009
SF092
SF109
SF193
0e+00
1e+06
2e+06
3e+06
4e+06

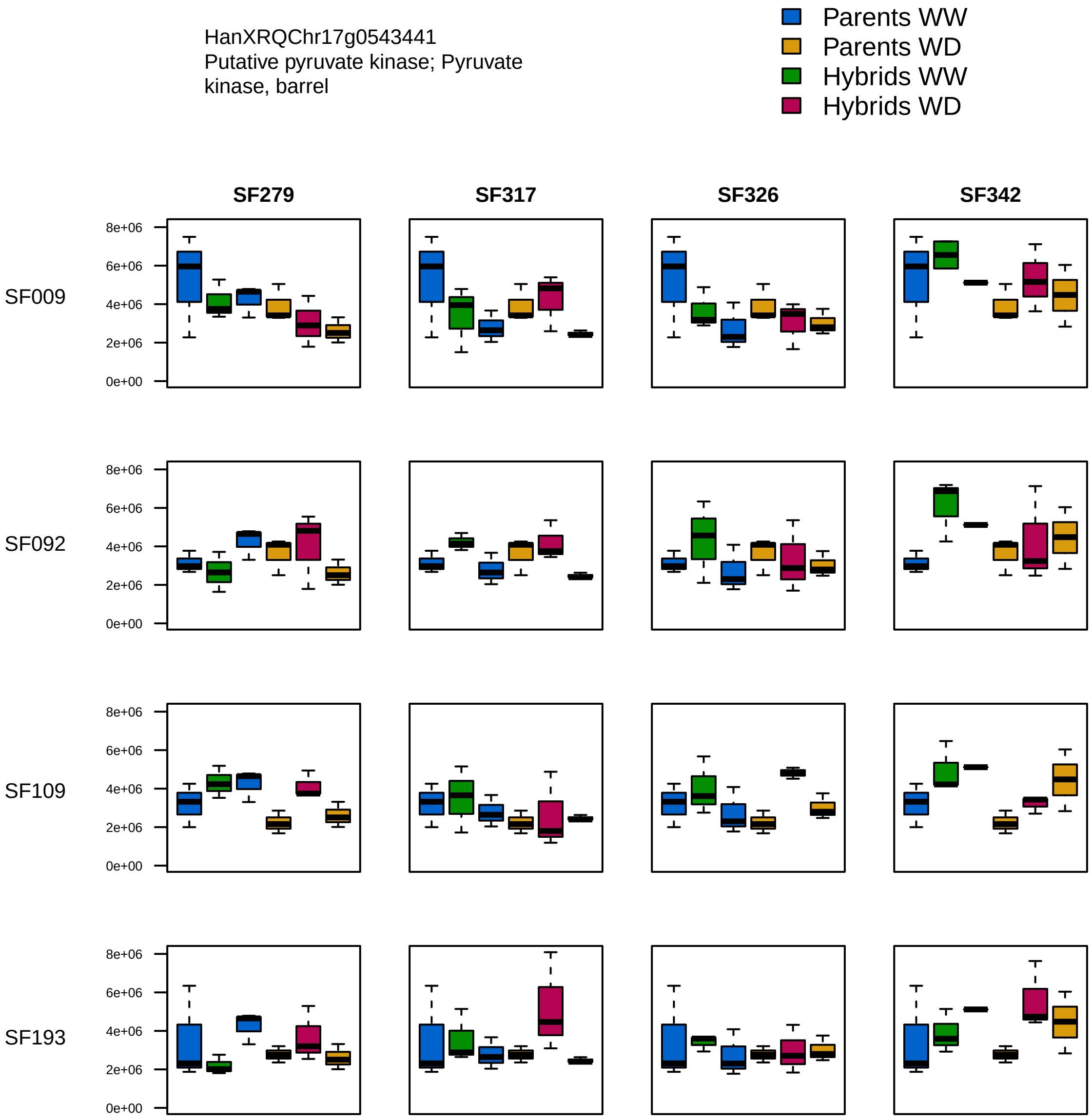
HanXRQChr17g0543441
Putative pyruvate kinase; Pyruvate kinase, barrel
Parents WW
Parents WD
Hybrids WW
Hybrids WD
SF279
SF317
SF326
SF342
SF009
SF092
SF109
SF193
8e+06
6e+06
4e+06
2e+06
0e+00

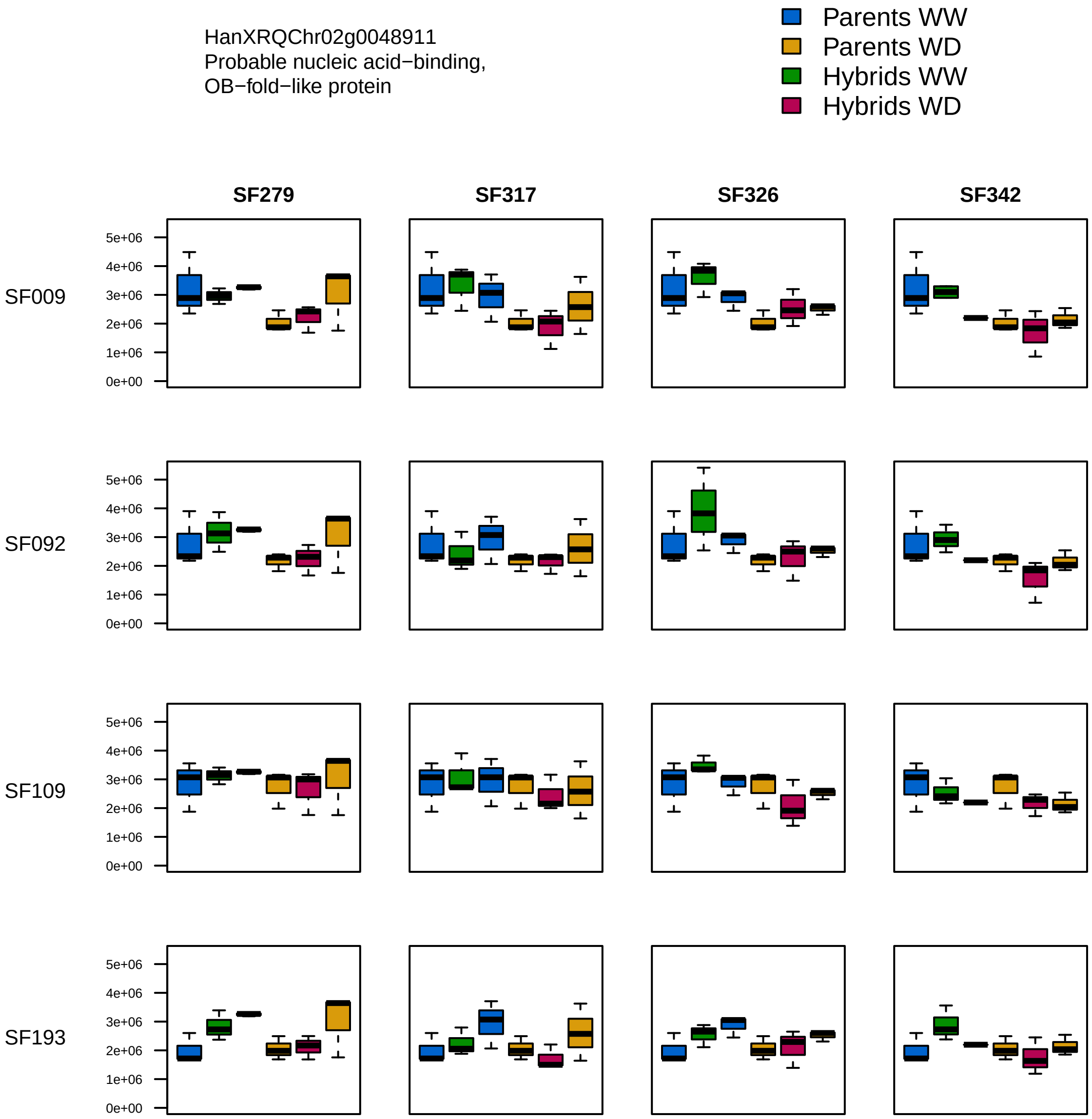
HanXRQChr02g0048911
Probable nucleic acid−binding,
OB−fold−like protein
Parents WW
Parents WD
Hybrids WW
Hybrids WD
SF279
SF317
SF326
SF342
SF009
SF092
SF109
SF193
0e+00
1e+06
2e+06
3e+06
4e+06
5e+06

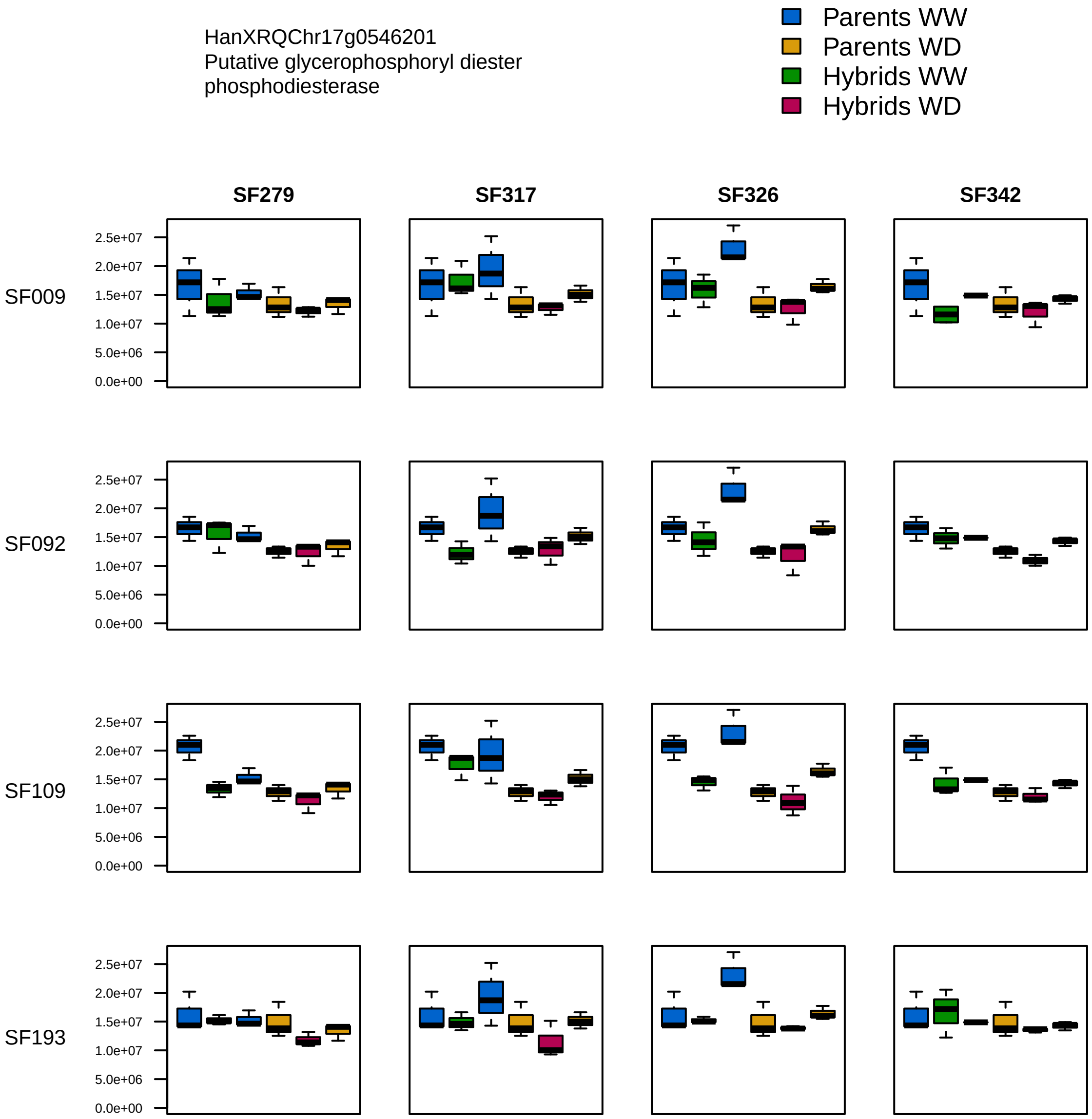

HanXRQChr17g0546201
Putative glycerophosphoryl diester phosphodiesterase
Parents WW
Parents WD
Hybrids WW
Hybrids WD
SF279
SF317
SF326
SF342
SF009
SF092
SF109
SF193
2.5e+07
2.0e+07
1.5e+07
1.0e+07
5.0e+06
0.0e+00

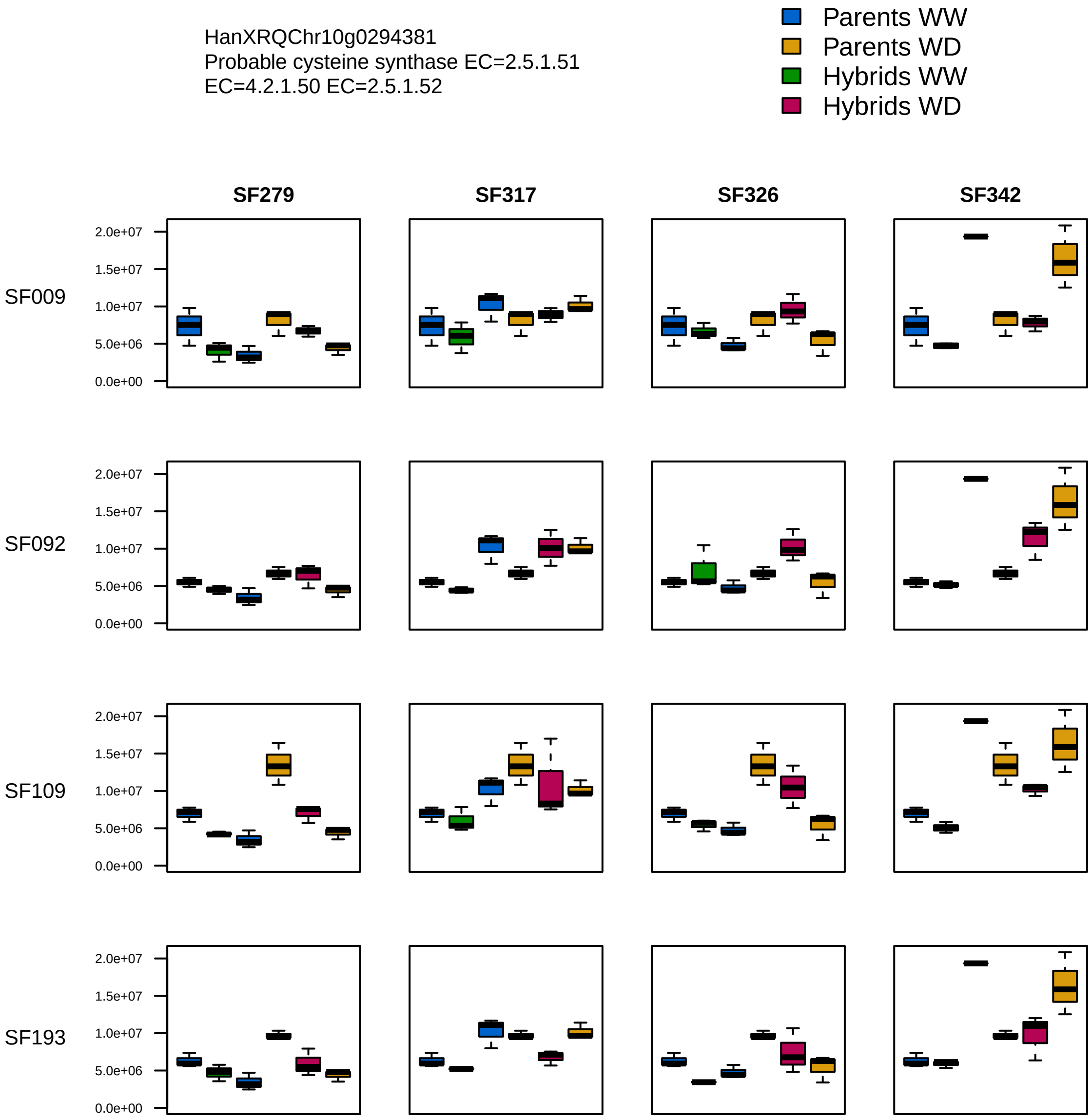
HanXRQChr10g0294381
Probable cysteine synthase EC=2.5.1.51
EC=4.2.1.50 EC=2.5.1.52
Parents WW
Parents WD
Hybrids WW
Hybrids WD
SF279
SF317
SF326
SF342
SF009
SF092
SF109
SF193
2.0e+07
1.5e+07
1.0e+07
5.0e+06
0.0e+00

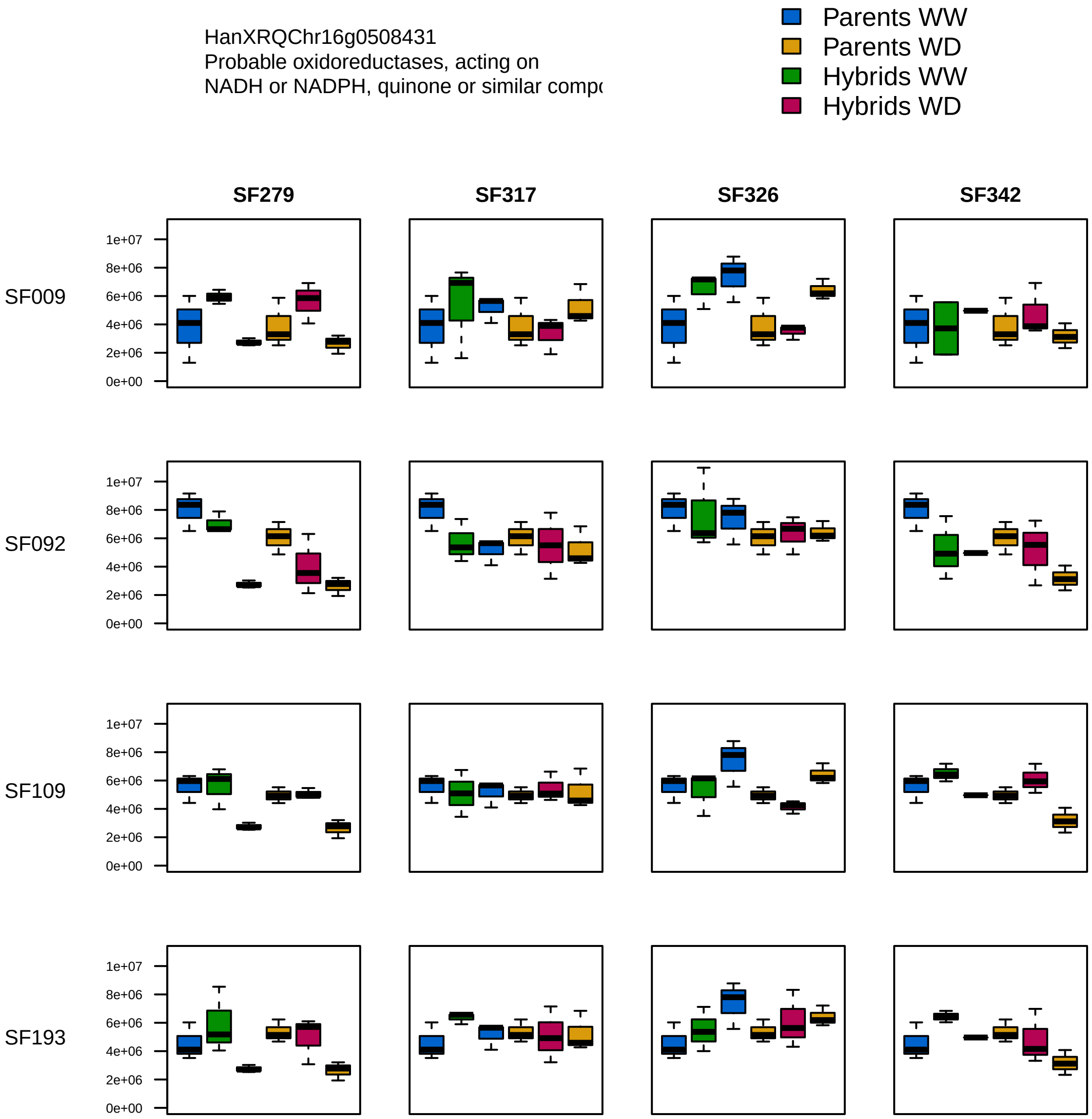

HanXRQChr16g0508431
Probable oxidoreductases, acting on
NADH or NADPH, quinone or similar compo
Parents WW
Parents WD
Hybrids WW
Hybrids WD
SF279
SF317
SF326
SF342
SF009
SF092
SF109
SF193
1e+07
8e+06
6e+06
4e+06
2e+06
0e+00

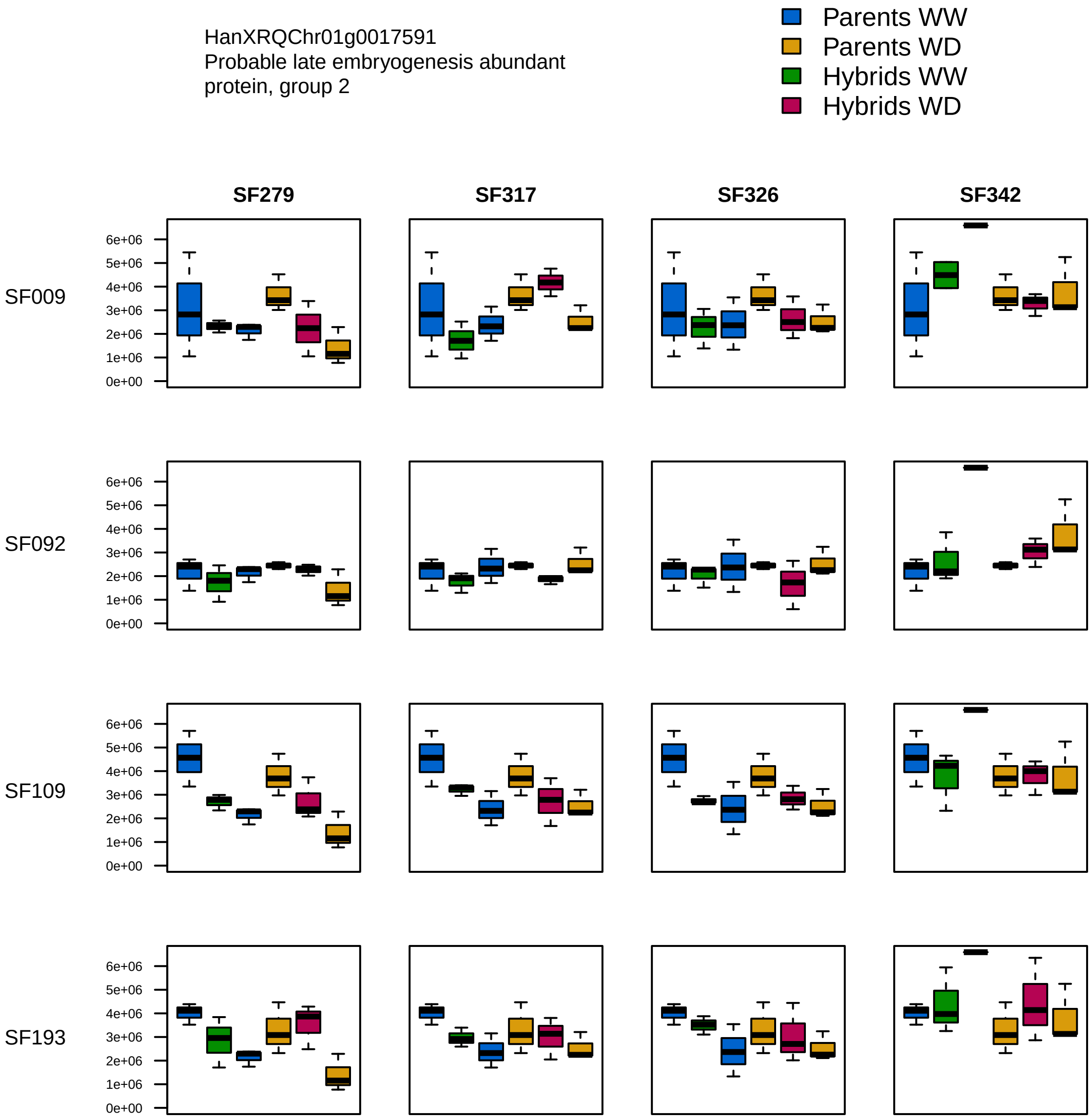
HanXRQChr01g0017591
Probable late embryogenesis abundant protein, group 2
Parents WW
Parents WD
Hybrids WW
Hybrids WD
SF279
SF317
SF326
SF342
SF009
SF092
SF109
SF193
6e+06
5e+06
4e+06
3e+06
2e+06
1e+06
0e+00

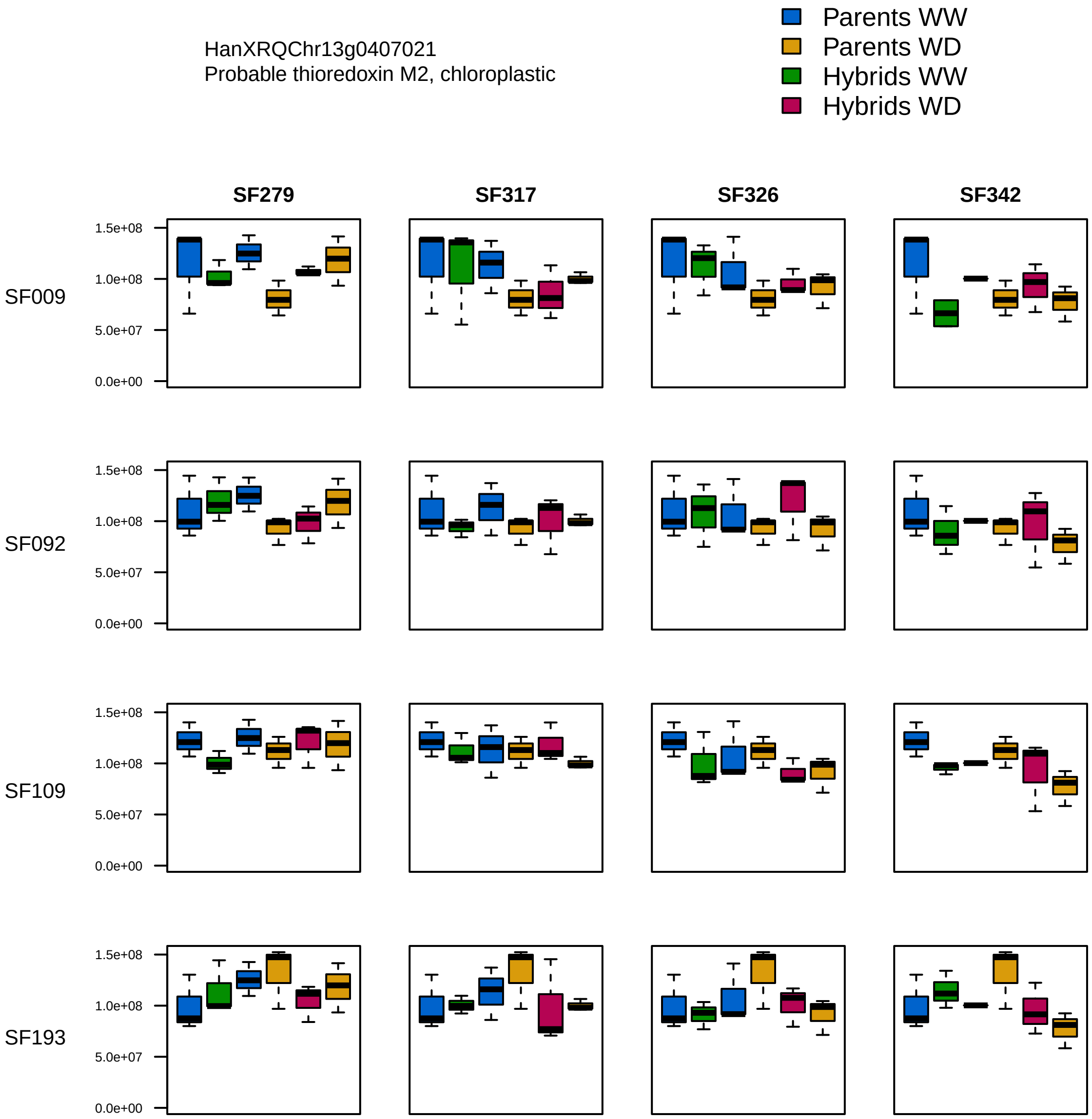
HanXRQChr13g0407021
Probable thioredoxin M2, chloroplastic
Parents WW
Parents WD
Hybrids WW
Hybrids WD
SF279
SF317
SF326
SF342
SF009
SF092
SF109
SF193
1.5e+08
1.0e+08
5.0e+07
0.0e+00

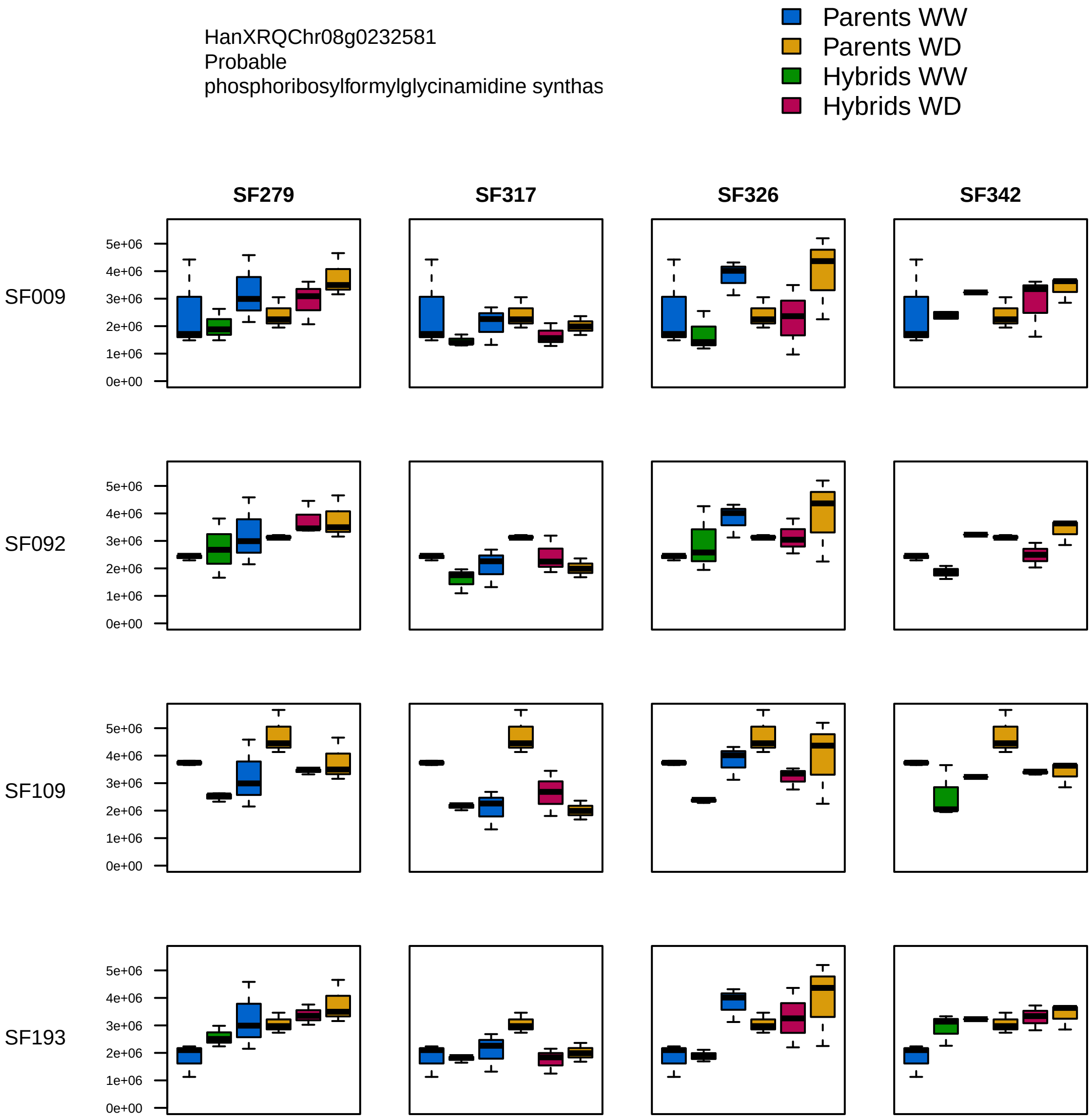

HanXRQChr08g0232581
Probable
phosphoribosylformylglycinamidine synthas
Parents WW
Parents WD
Hybrids WW
Hybrids WD
SF279
SF317
SF326
SF342
SF009
SF092
SF109
SF193
0e+00
1e+06
2e+06
3e+06
4e+06
5e+06

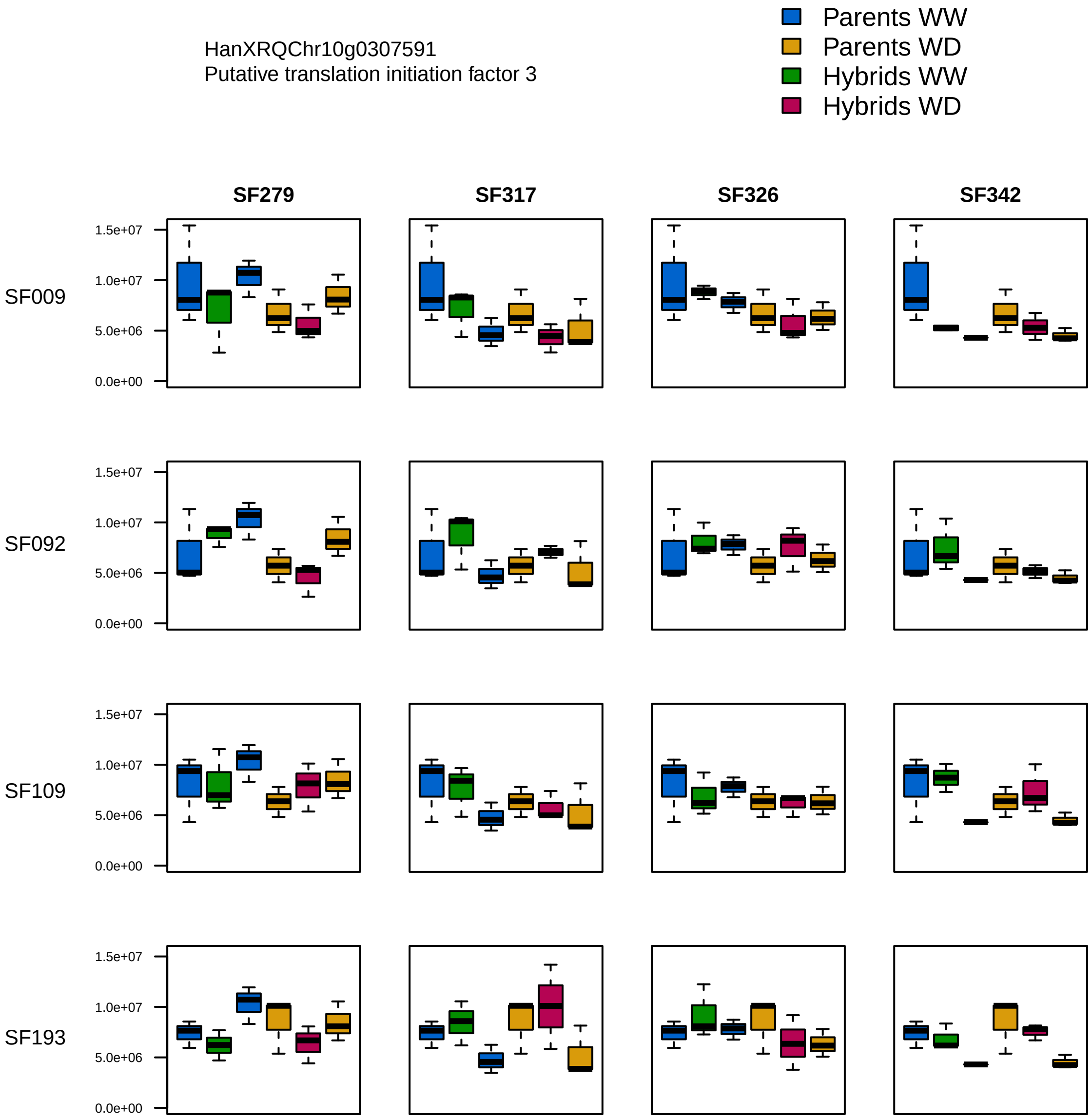
HanXRQChr10g0307591
Putative translation initiation factor 3
Parents WW
Parents WD
Hybrids WW
Hybrids WD
SF279
SF317
SF326
SF342
SF009
SF092
SF109
SF193
1.5e+07
1.0e+07
5.0e+06
0.0e+00

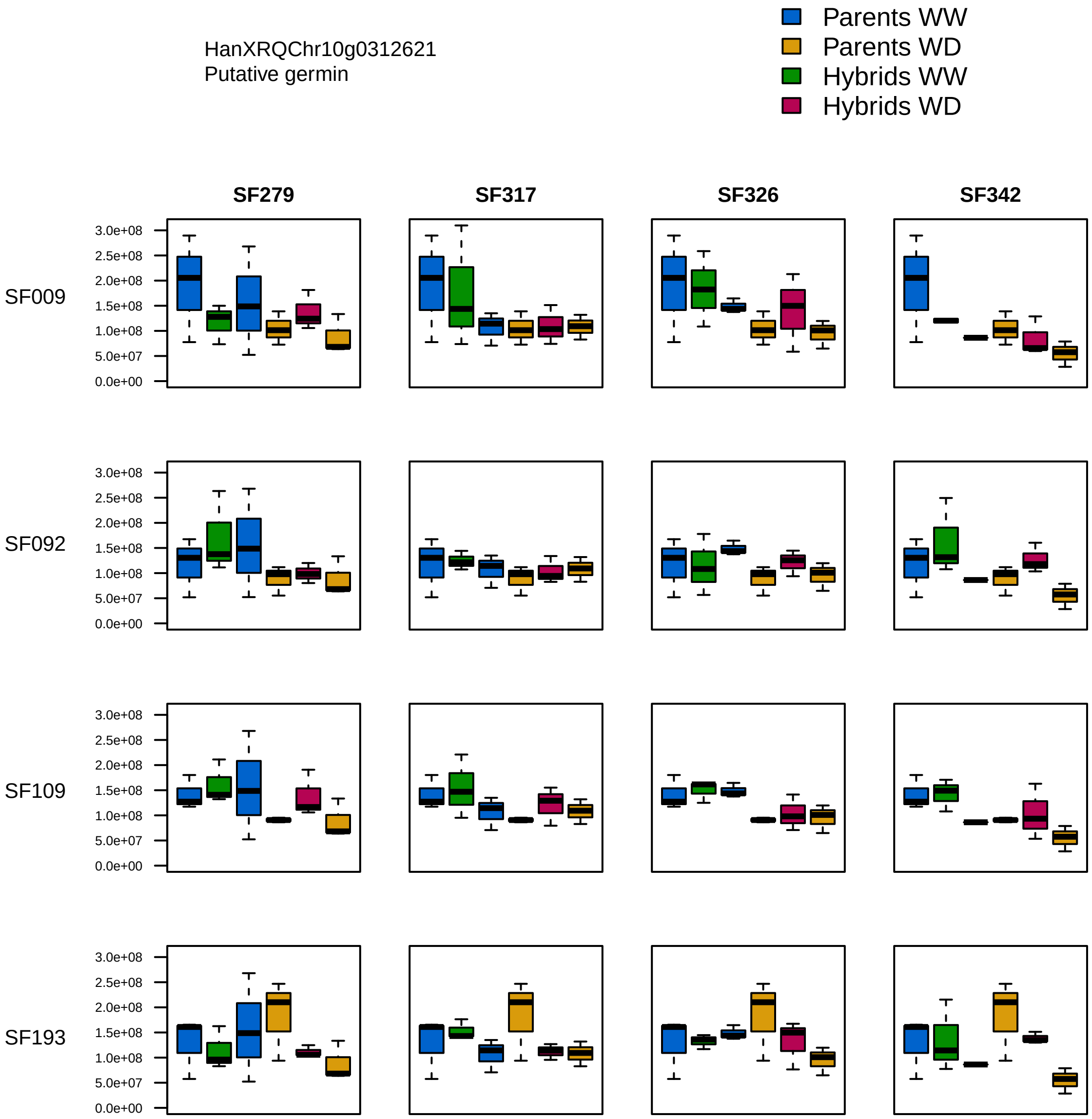
HanXRQChr10g0312621
Putative germin
Parents WW
Parents WD
Hybrids WW
Hybrids WD
SF279
SF317
SF326
SF342
SF009
SF092
SF109
SF193
3.0e+08
2.5e+08
2.0e+08
1.5e+08
1.0e+08
5.0e+07
0.0e+00

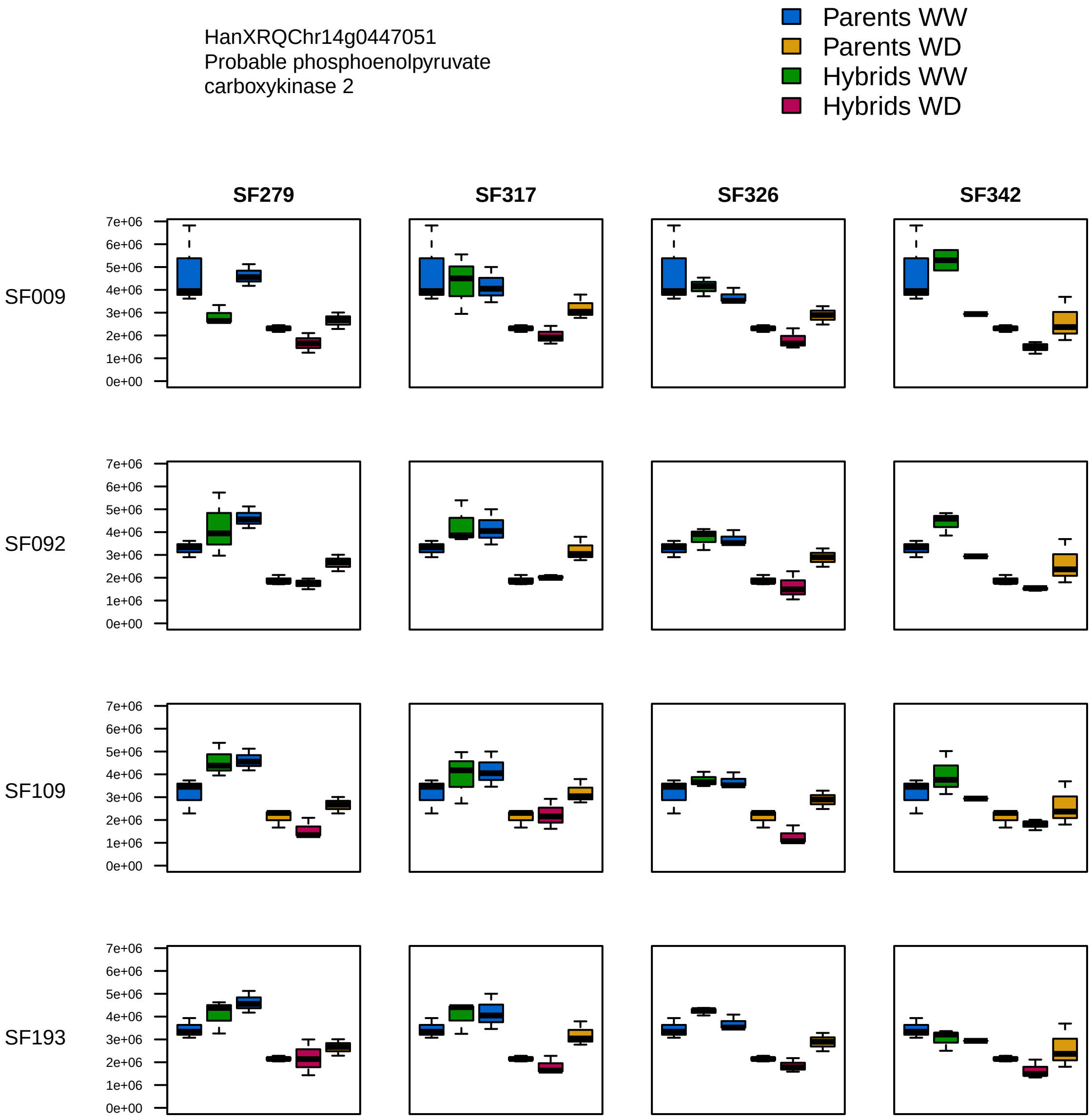
HanXRQChr14g0447051
Probable phosphoenolpyruvate
carboxykinase 2
Parents WW
Parents WD
Hybrids WW
Hybrids WD
SF279
SF317
SF326
SF342
SF009
SF092
SF109
SF193
7e+06
6e+06
5e+06
4e+06
3e+06
2e+06
1e+06
0e+00

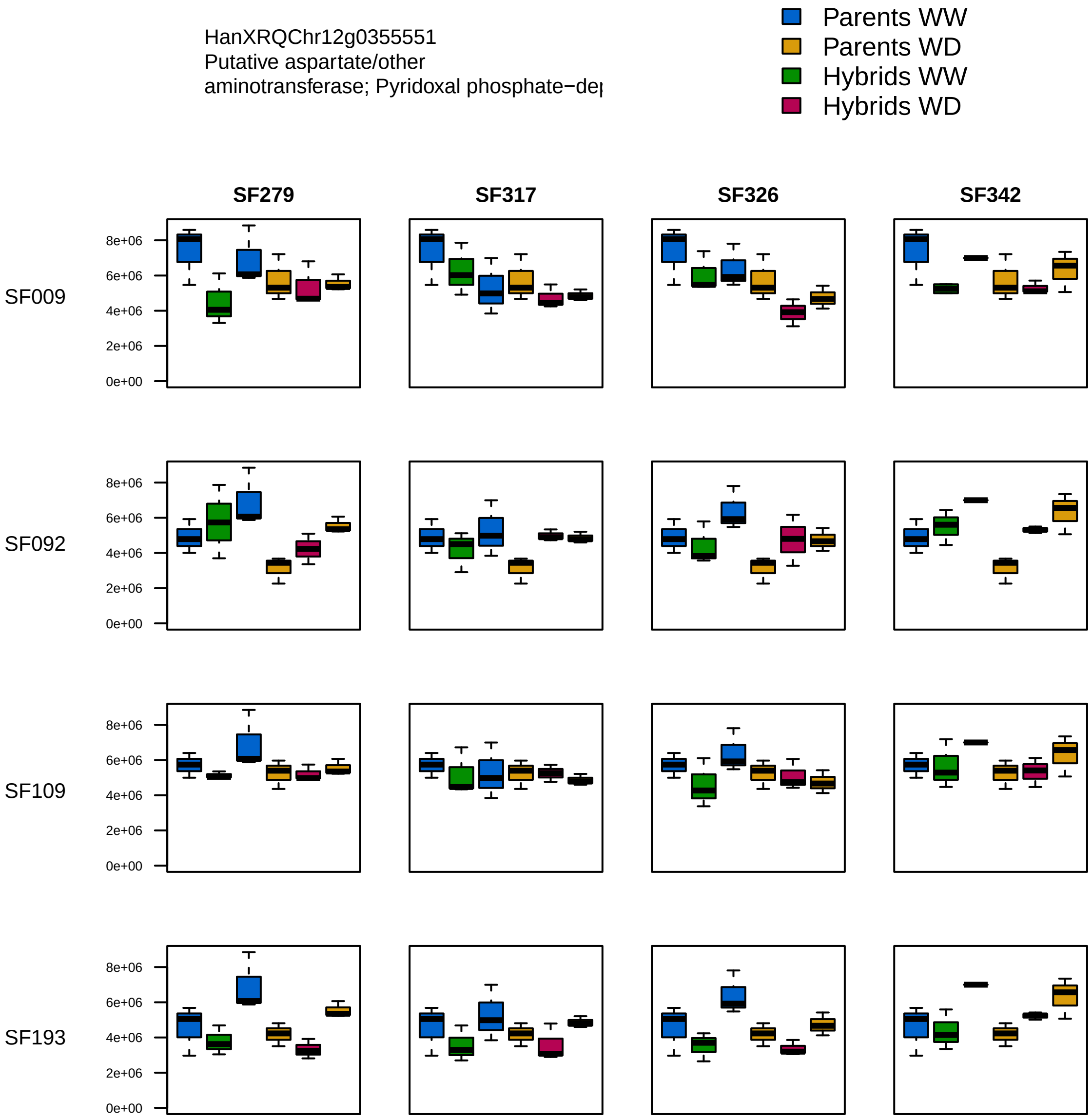
HanXRQChr12g0355551
Putative aspartate/other
aminotransferase; Pyridoxal phosphate−de[illegible]
Parents WW
Parents WD
Hybrids WW
Hybrids WD
SF279
SF317
SF326
SF342
SF009
SF092
SF109
SF193
0e+00
2e+06
4e+06
6e+06
8e+06

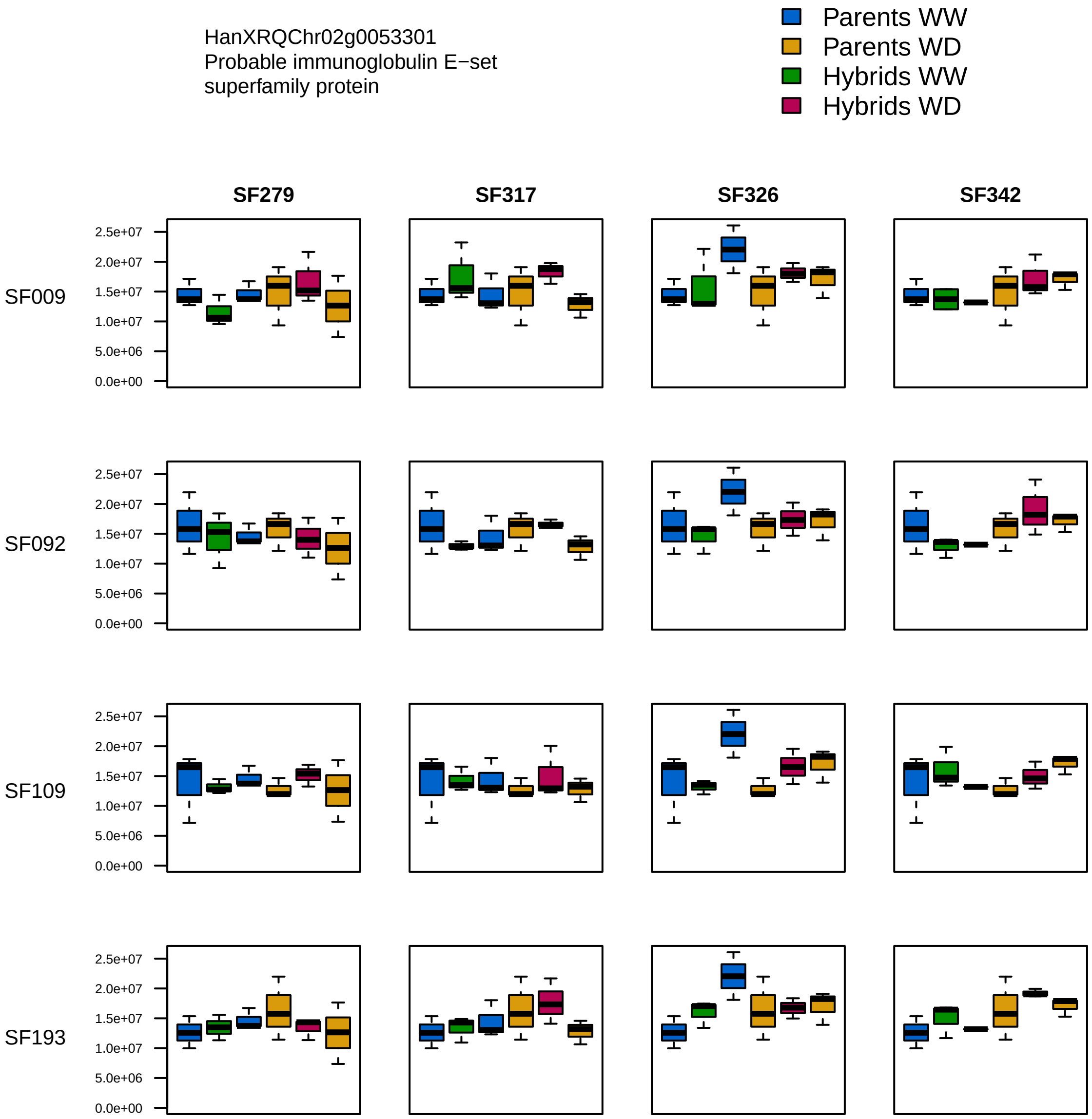
HanXRQChr02g0053301
Probable immunoglobulin E−set
superfamily protein
Parents WW
Parents WD
Hybrids WW
Hybrids WD
SF279
SF317
SF326
SF342
SF009
SF092
SF109
SF193
2.5e+07
2.0e+07
1.5e+07
1.0e+07
5.0e+06
0.0e+00

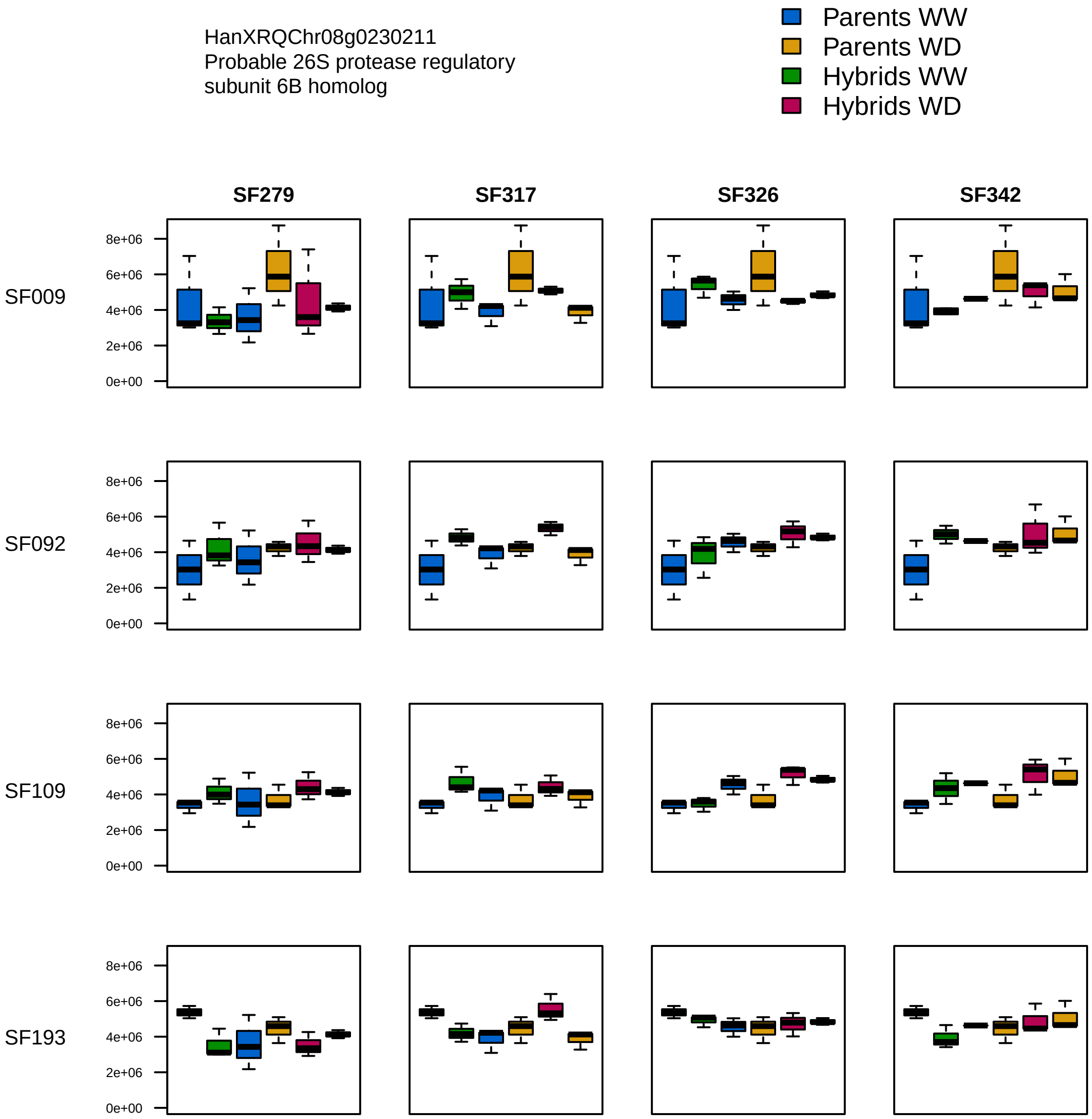
HanXRQChr08g0230211
Probable 26S protease regulatory
subunit 6B homolog
Parents WW
Parents WD
Hybrids WW
Hybrids WD
SF279
SF317
SF326
SF342
SF009
SF092
SF109
SF193
8e+06
6e+06
4e+06
2e+06
0e+00

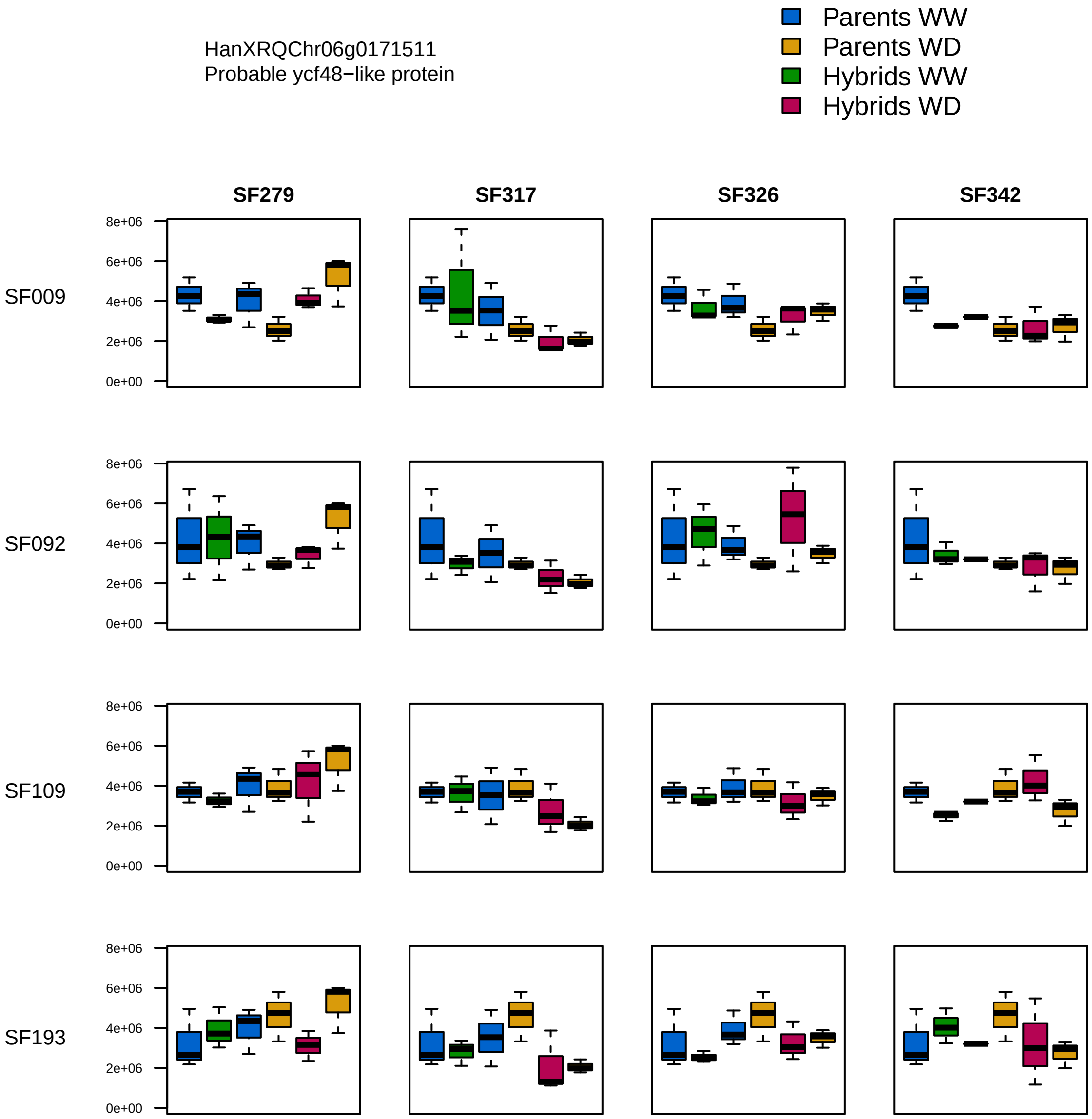
HanXRQChr06g0171511
Probable ycf48−like protein
Parents WW
Parents WD
Hybrids WW
Hybrids WD
SF279
SF317
SF326
SF342
SF009
SF092
SF109
SF193
8e+06
6e+06
4e+06
2e+06
0e+00

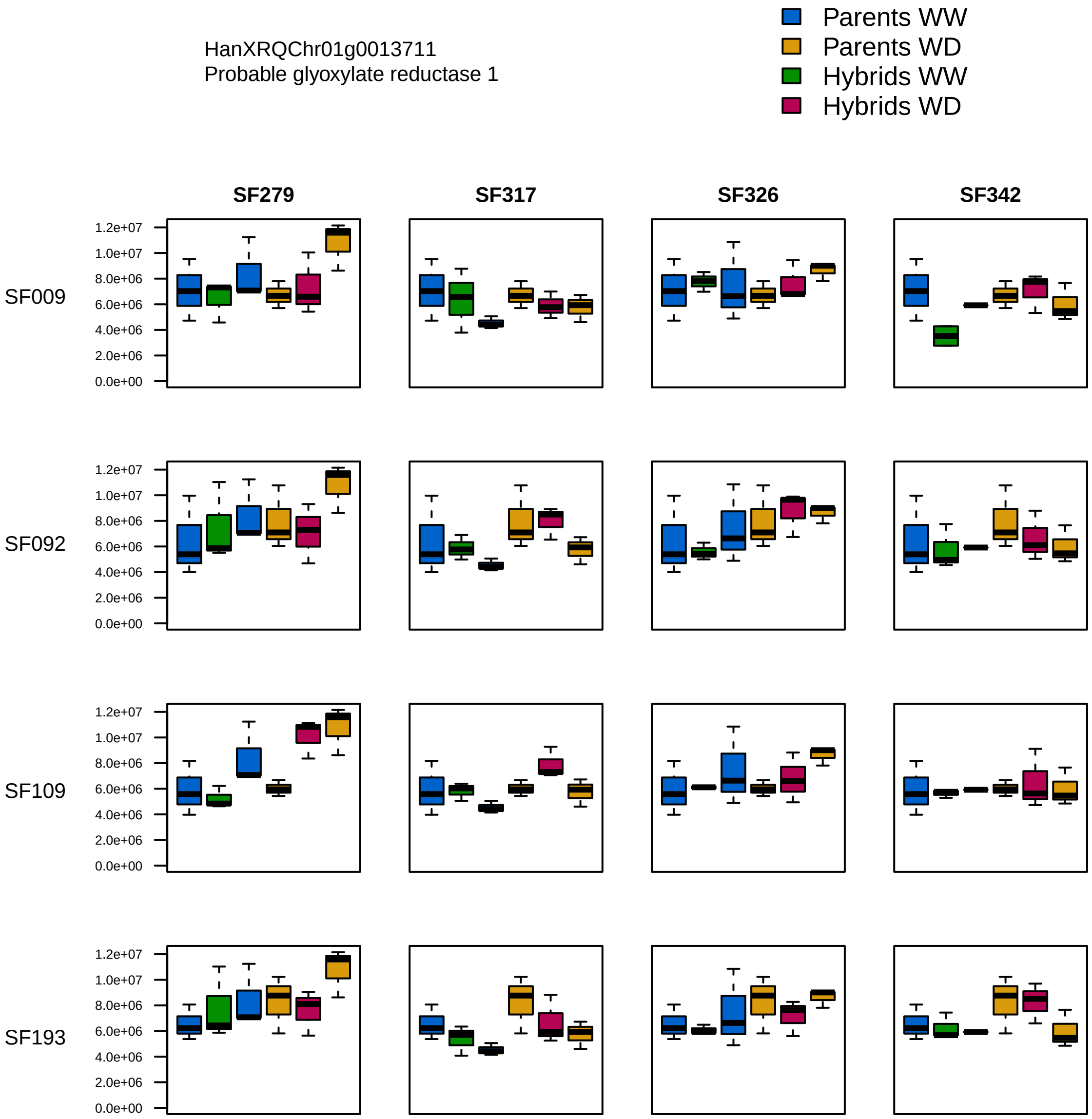
HanXRQChr01g0013711
Probable glyoxylate reductase 1
Parents WW
Parents WD
Hybrids WW
Hybrids WD
SF279
SF317
SF326
SF342
SF009
SF092
SF109
SF193
0.0e+00
2.0e+06
4.0e+06
6.0e+06
8.0e+06
1.0e+07
1.2e+07

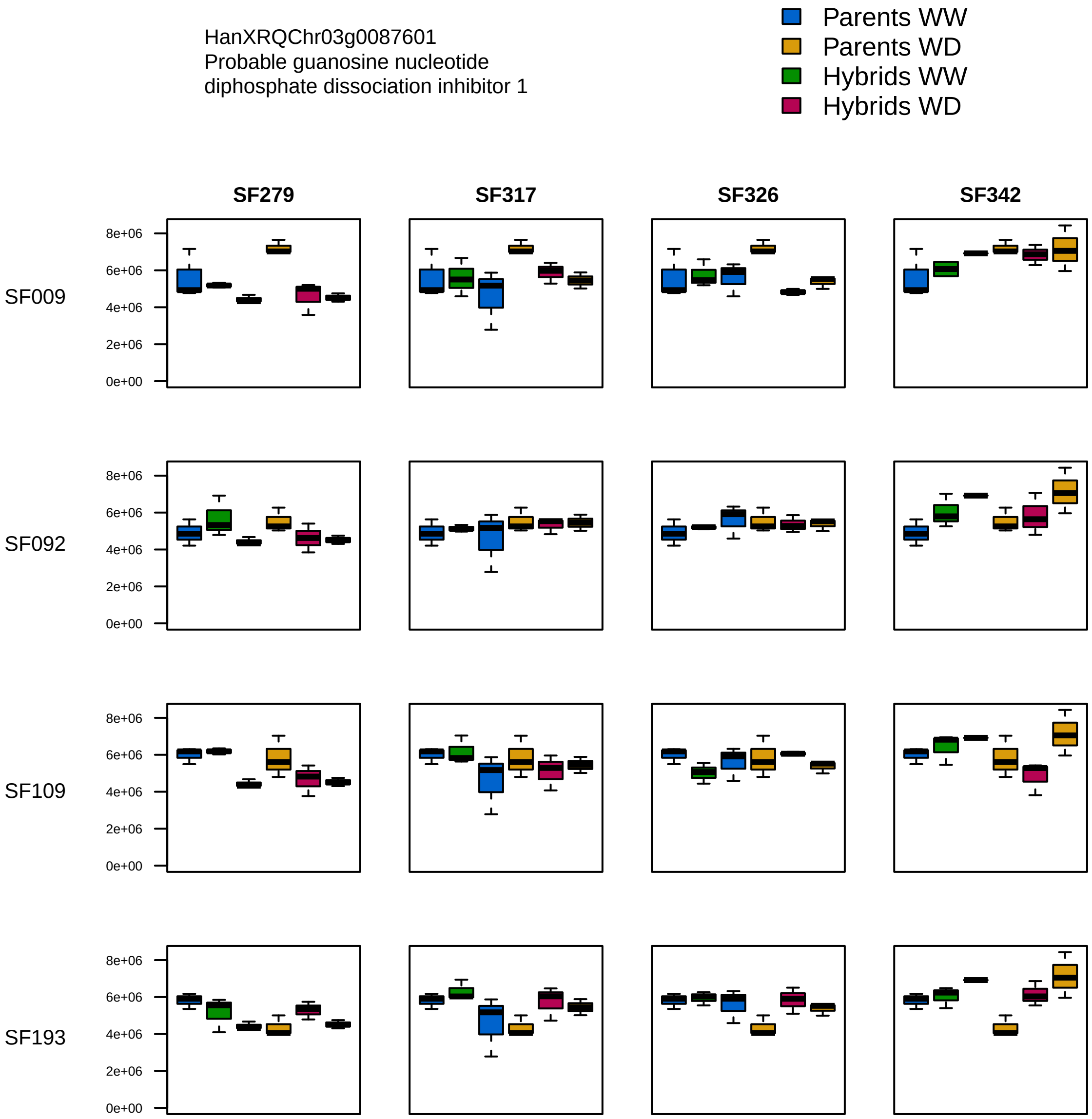
HanXRQChr03g0087601
Probable guanosine nucleotide diphosphate dissociation inhibitor 1
Parents WW
Parents WD
Hybrids WW
Hybrids WD
SF279
SF317
SF326
SF342
SF009
SF092
SF109
SF193
8e+06
6e+06
4e+06
2e+06
0e+00

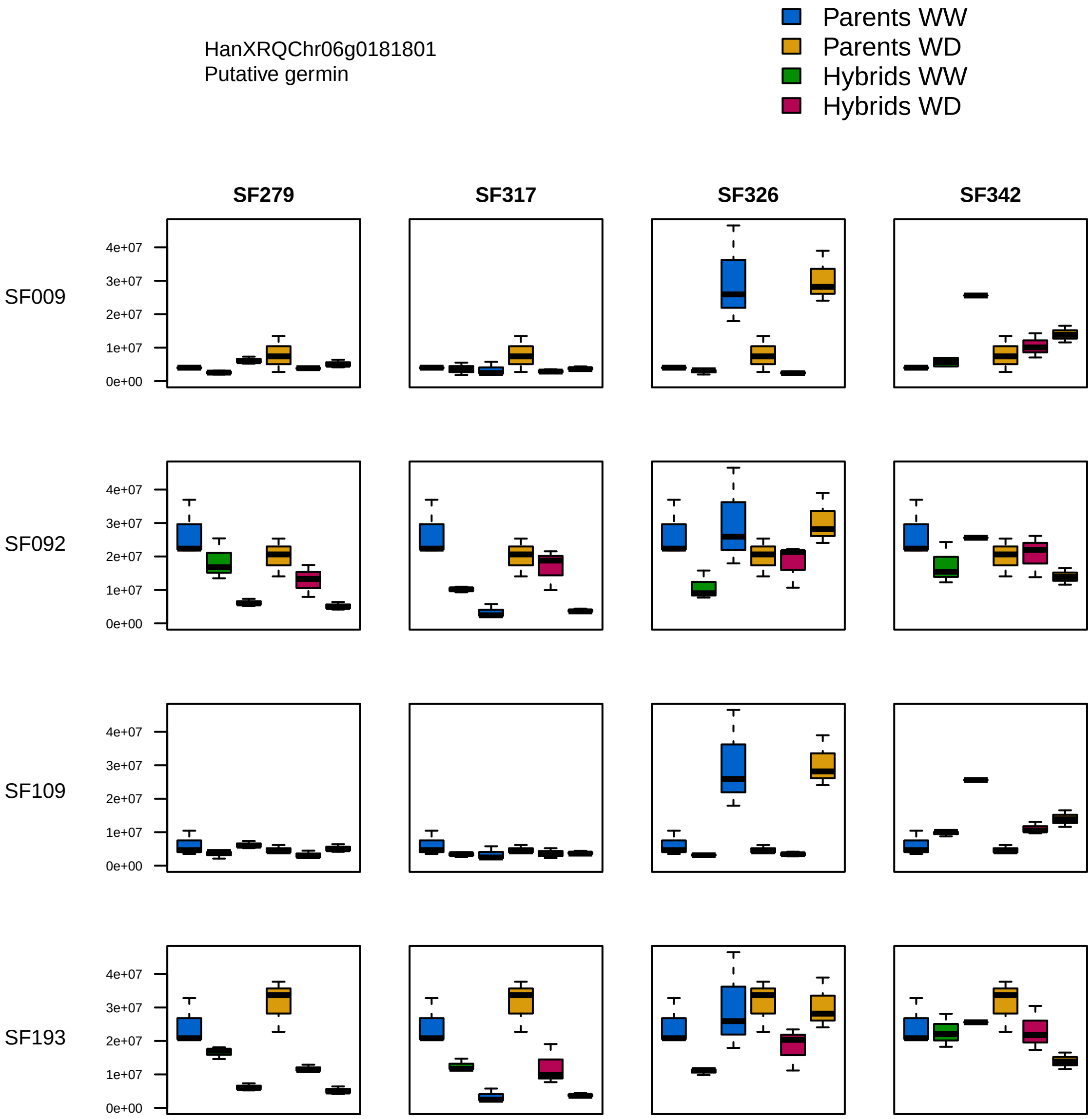
HanXRQChr06g0181801
Putative germin
Parents WW
Parents WD
Hybrids WW
Hybrids WD
SF279
SF317
SF326
SF342
SF009
SF092
SF109
SF193
4e+07
3e+07
2e+07
1e+07
0e+00

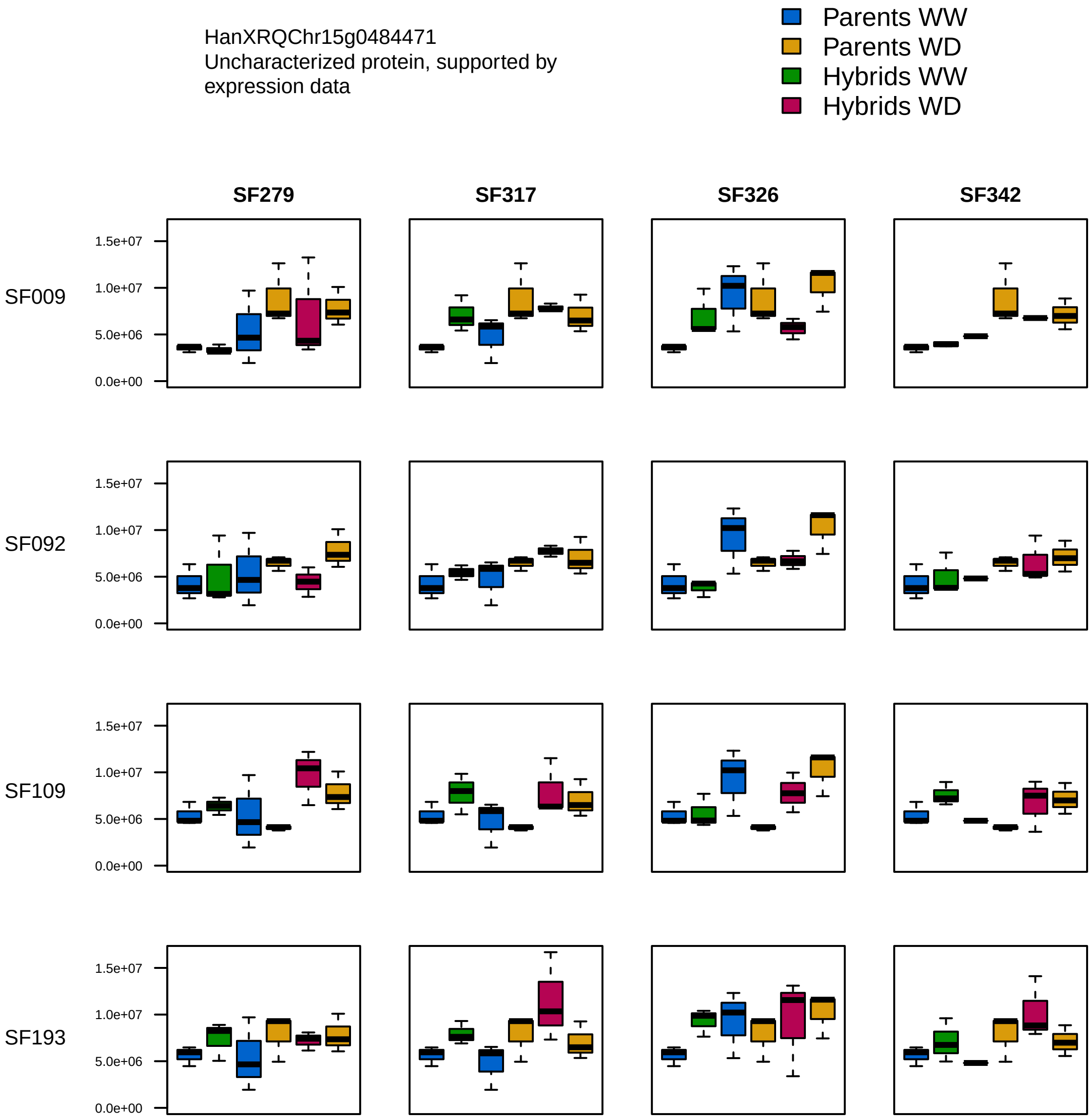
HanXRQChr15g0484471
Uncharacterized protein, supported by expression data
Parents WW
Parents WD
Hybrids WW
Hybrids WD
SF279
SF317
SF326
SF342
SF009
SF092
SF109
SF193
1.5e+07
1.0e+07
5.0e+06
0.0e+00

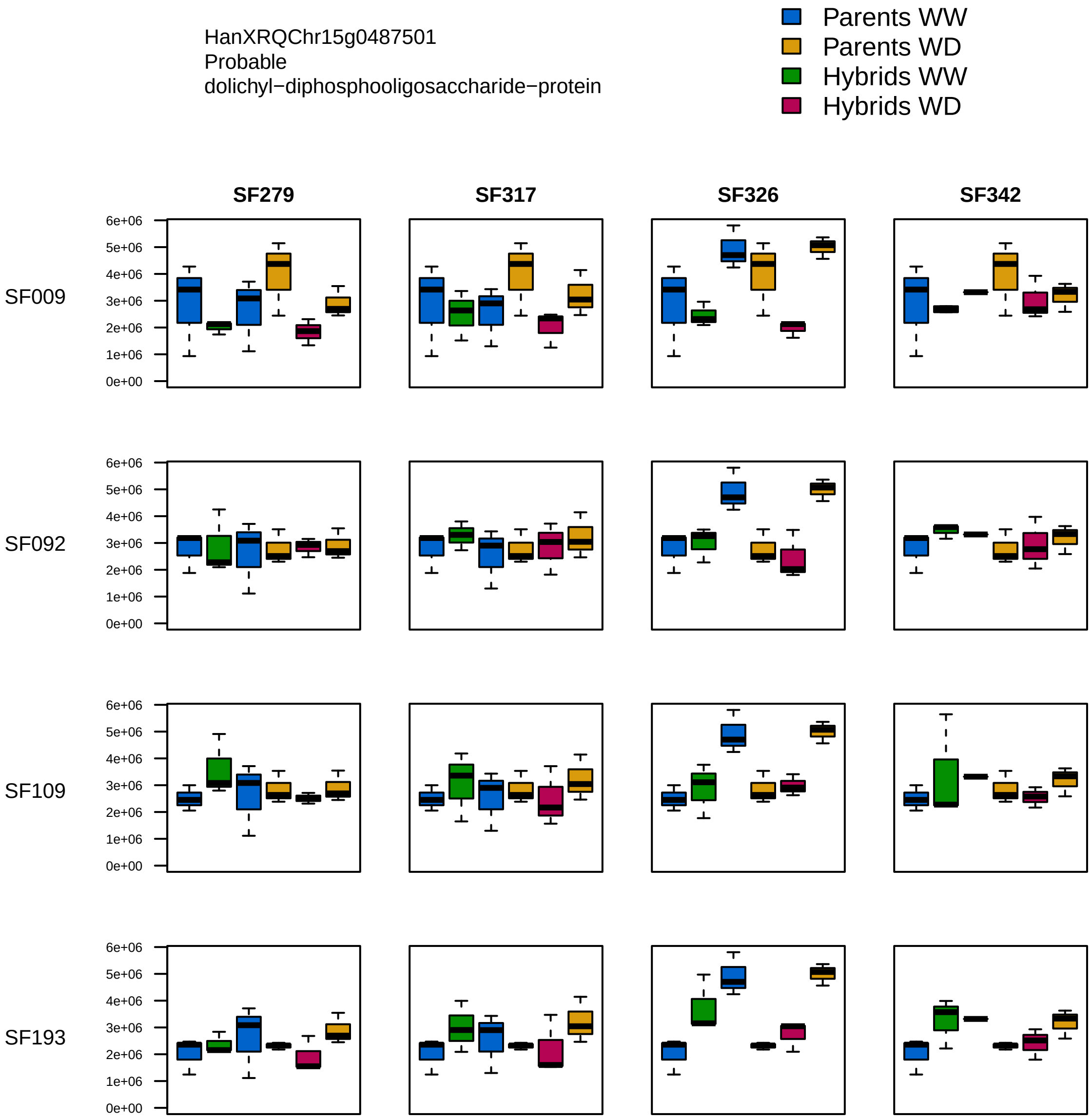
HanXRQChr15g0487501
Probable
dolichyl−diphosphooligosaccharide−protein
Parents WW
Parents WD
Hybrids WW
Hybrids WD
SF279
SF317
SF326
SF342
SF009
SF092
SF109
SF193
6e+06
5e+06
4e+06
3e+06
2e+06
1e+06
0e+00

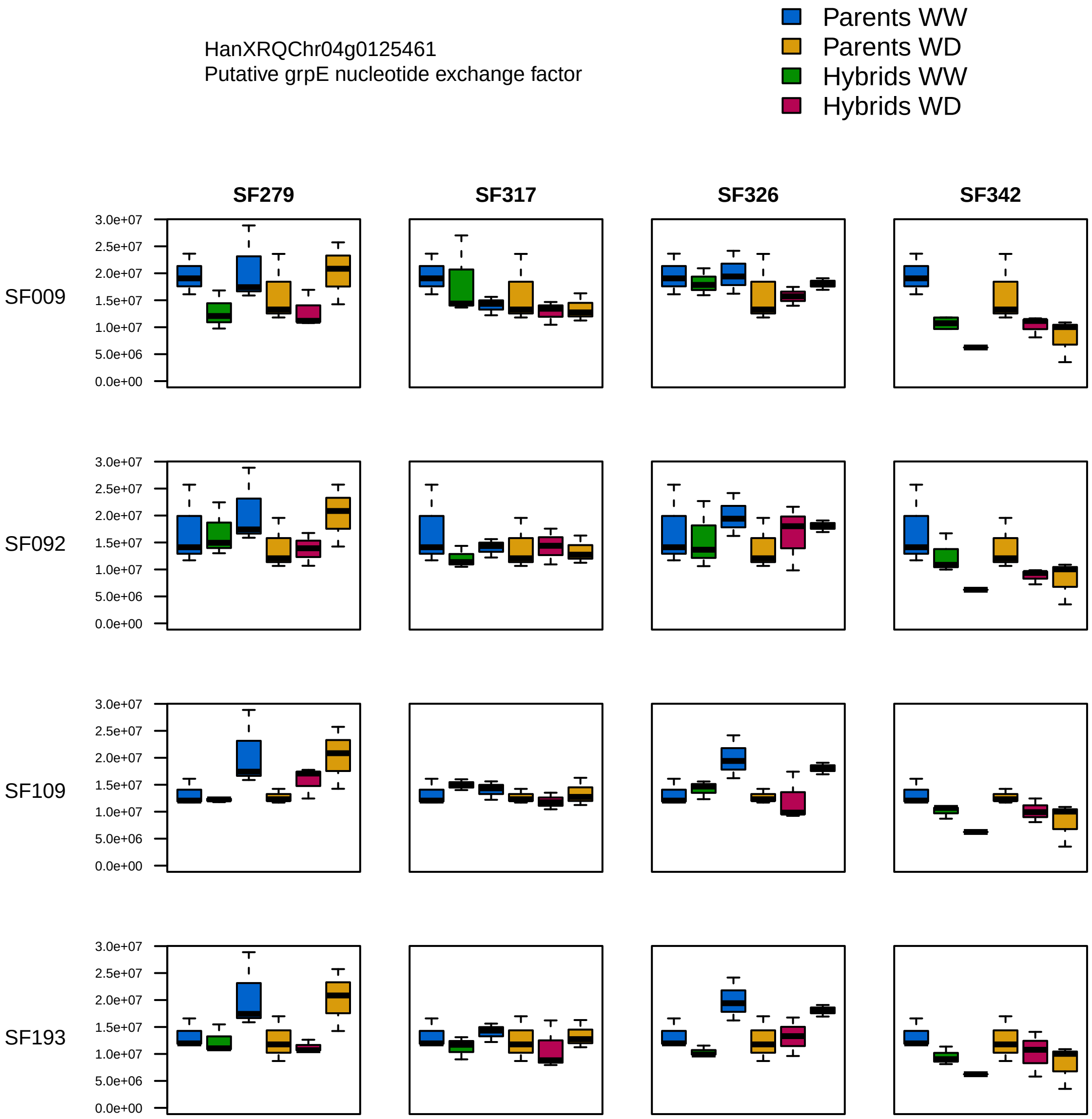
HanXRQChr04g0125461
Putative grpE nucleotide exchange factor
Parents WW
Parents WD
Hybrids WW
Hybrids WD
SF279
SF317
SF326
SF342
SF009
SF092
SF109
SF193
3.0e+07
2.5e+07
2.0e+07
1.5e+07
1.0e+07
5.0e+06
0.0e+00

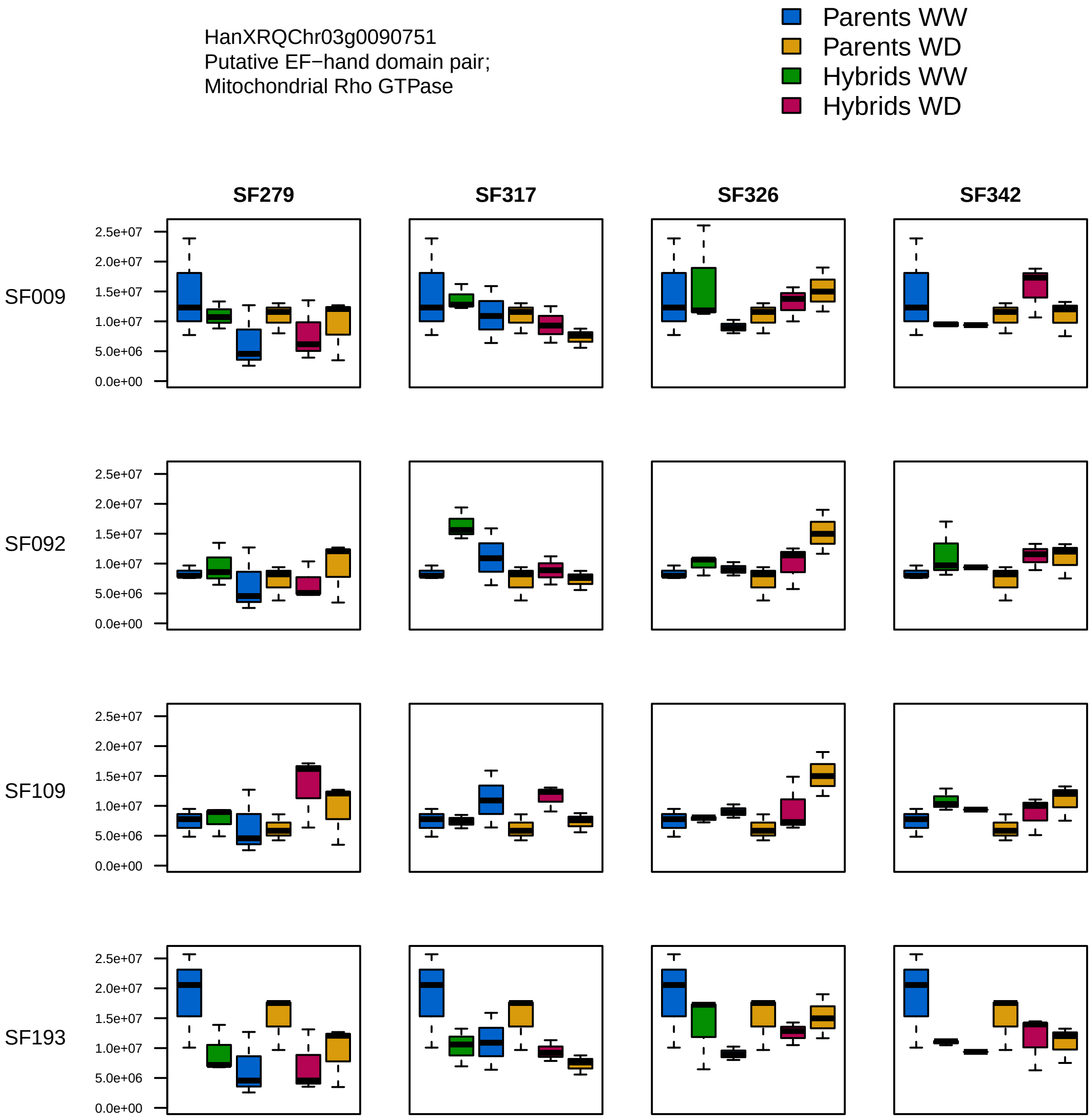
HanXRQChr03g0090751
Putative EF−hand domain pair;
Mitochondrial Rho GTPase
Parents WW
Parents WD
Hybrids WW
Hybrids WD
SF279
SF317
SF326
SF342
SF009
SF092
SF109
SF193
2.5e+07
2.0e+07
1.5e+07
1.0e+07
5.0e+06
0.0e+00

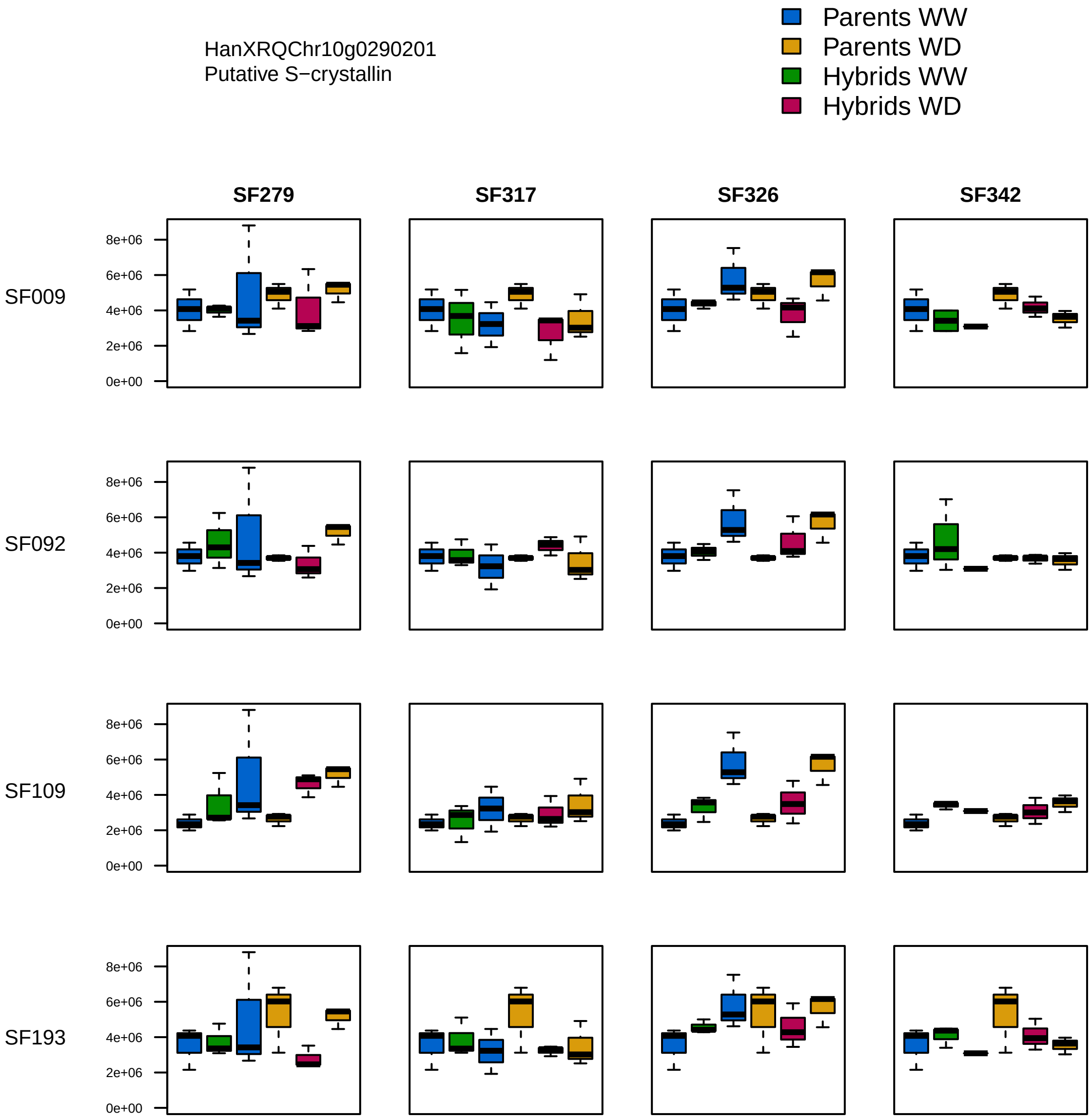

HanXRQChr10g0290201
Putative S−crystallin
Parents WW
Parents WD
Hybrids WW
Hybrids WD
SF279
SF317
SF326
SF342
SF009
SF092
SF109
SF193
8e+06
6e+06
4e+06
2e+06
0e+00

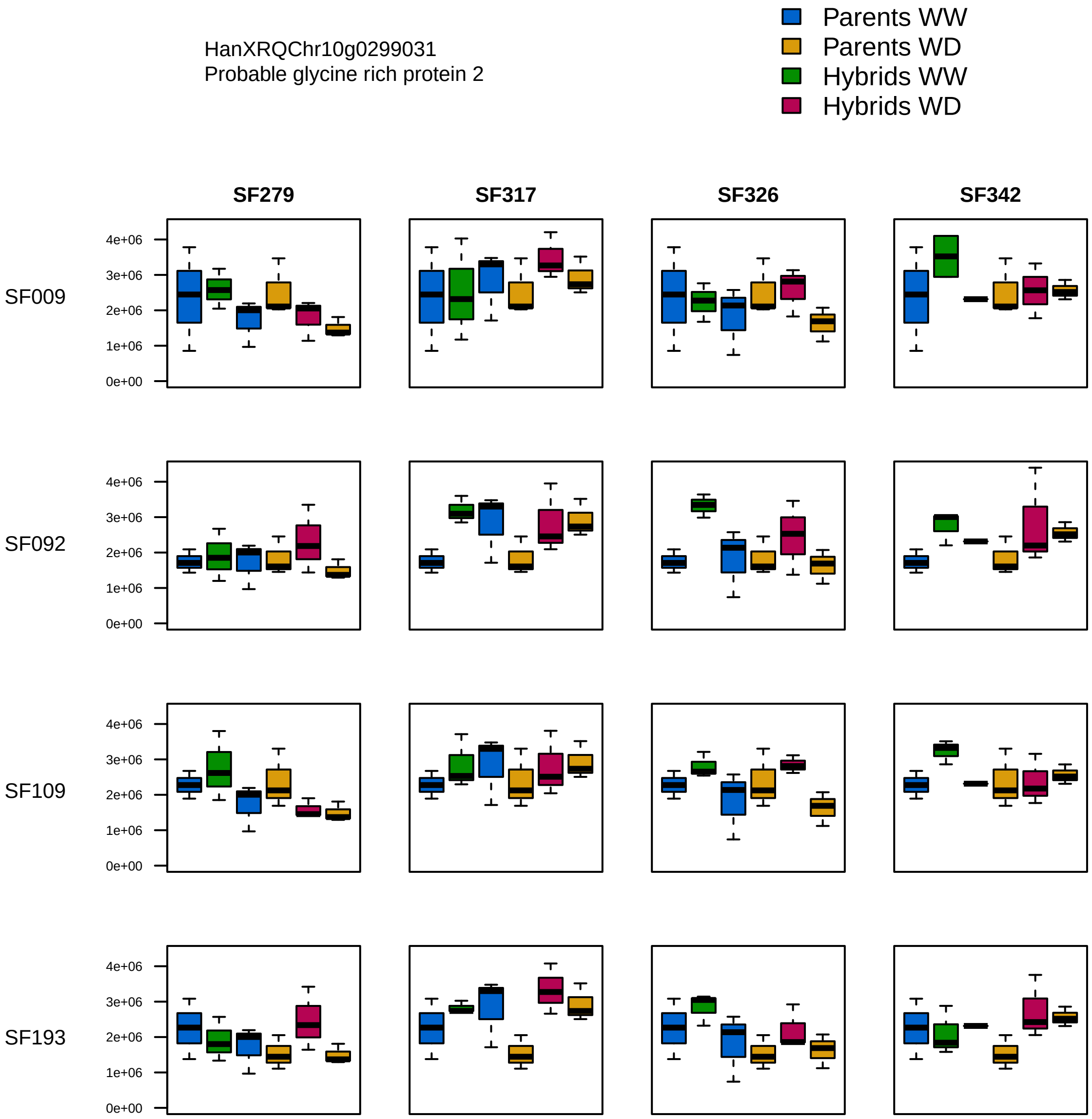
HanXRQChr10g0299031
Probable glycine rich protein 2
Parents WW
Parents WD
Hybrids WW
Hybrids WD
SF279
SF317
SF326
SF342
SF009
SF092
SF109
SF193
4e+06
3e+06
2e+06
1e+06
0e+00

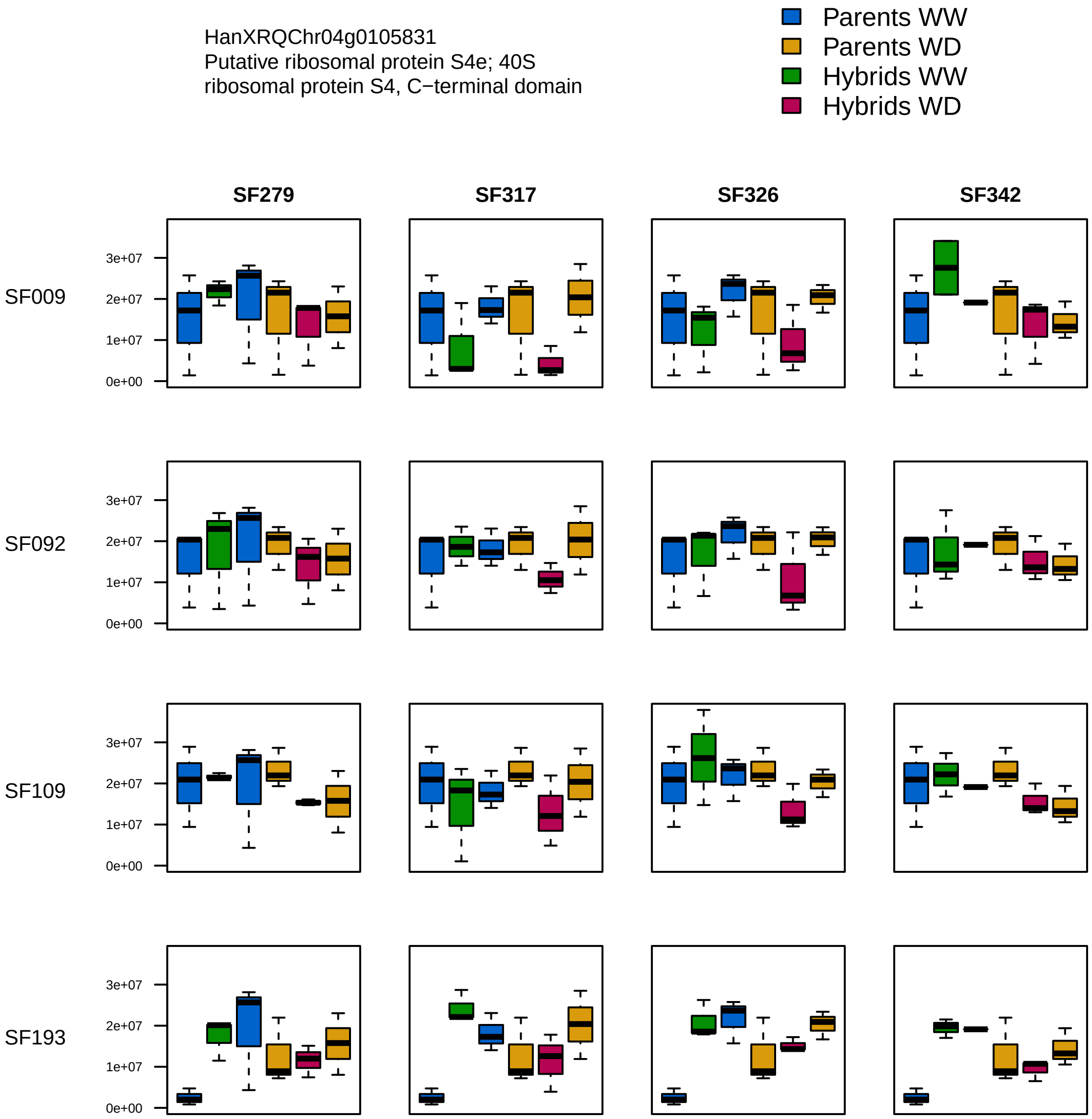

HanXRQChr04g0105831
Putative ribosomal protein S4e; 40S
ribosomal protein S4, C−terminal domain
Parents WW
Parents WD
Hybrids WW
Hybrids WD
SF279
SF317
SF326
SF342
SF009
SF092
SF109
SF193
3e+07
2e+07
1e+07
0e+00

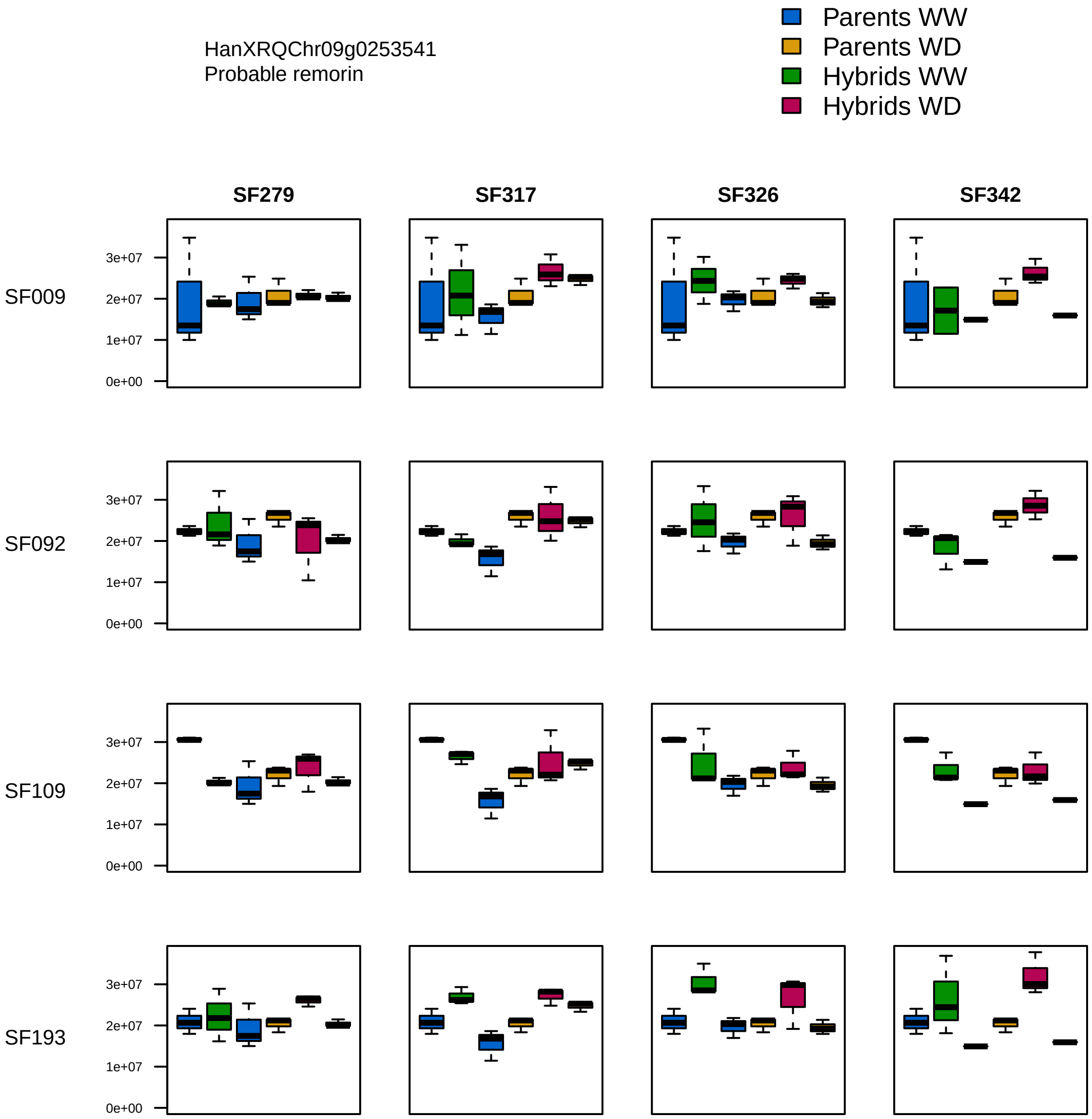

HanXRQChr09g0253541
Probable remorin
Parents WW
Parents WD
Hybrids WW
Hybrids WD
SF279
SF317
SF326
SF342
SF009
SF092
SF109
SF193
3e+07
2e+07
1e+07
0e+00

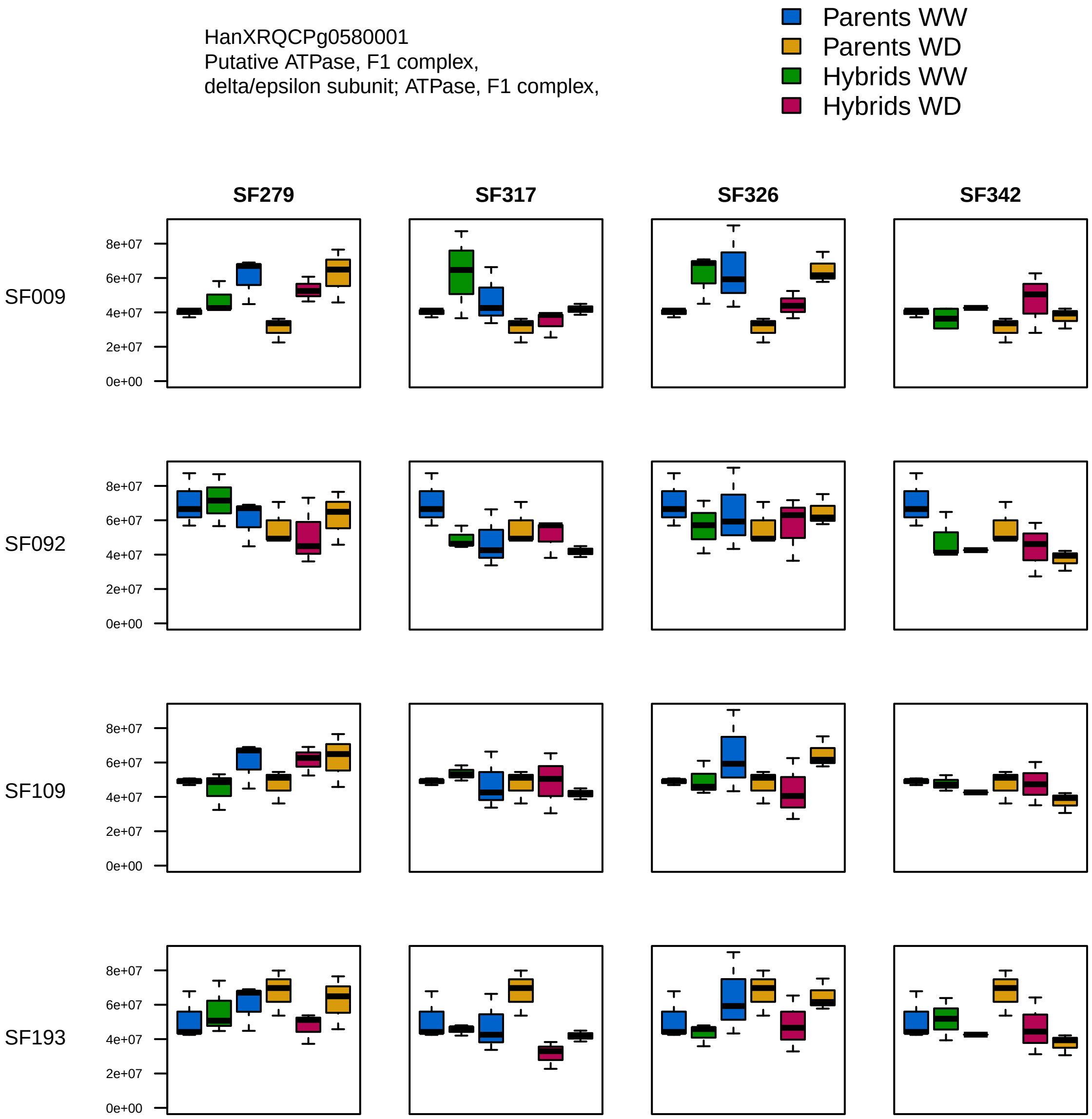
HanXRQCPg0580001
Putative ATPase, F1 complex,
delta/epsilon subunit; ATPase, F1 complex,
Parents WW
Parents WD
Hybrids WW
Hybrids WD
SF279
SF317
SF326
SF342
SF009
SF092
SF109
SF193
8e+07
6e+07
4e+07
2e+07
0e+00

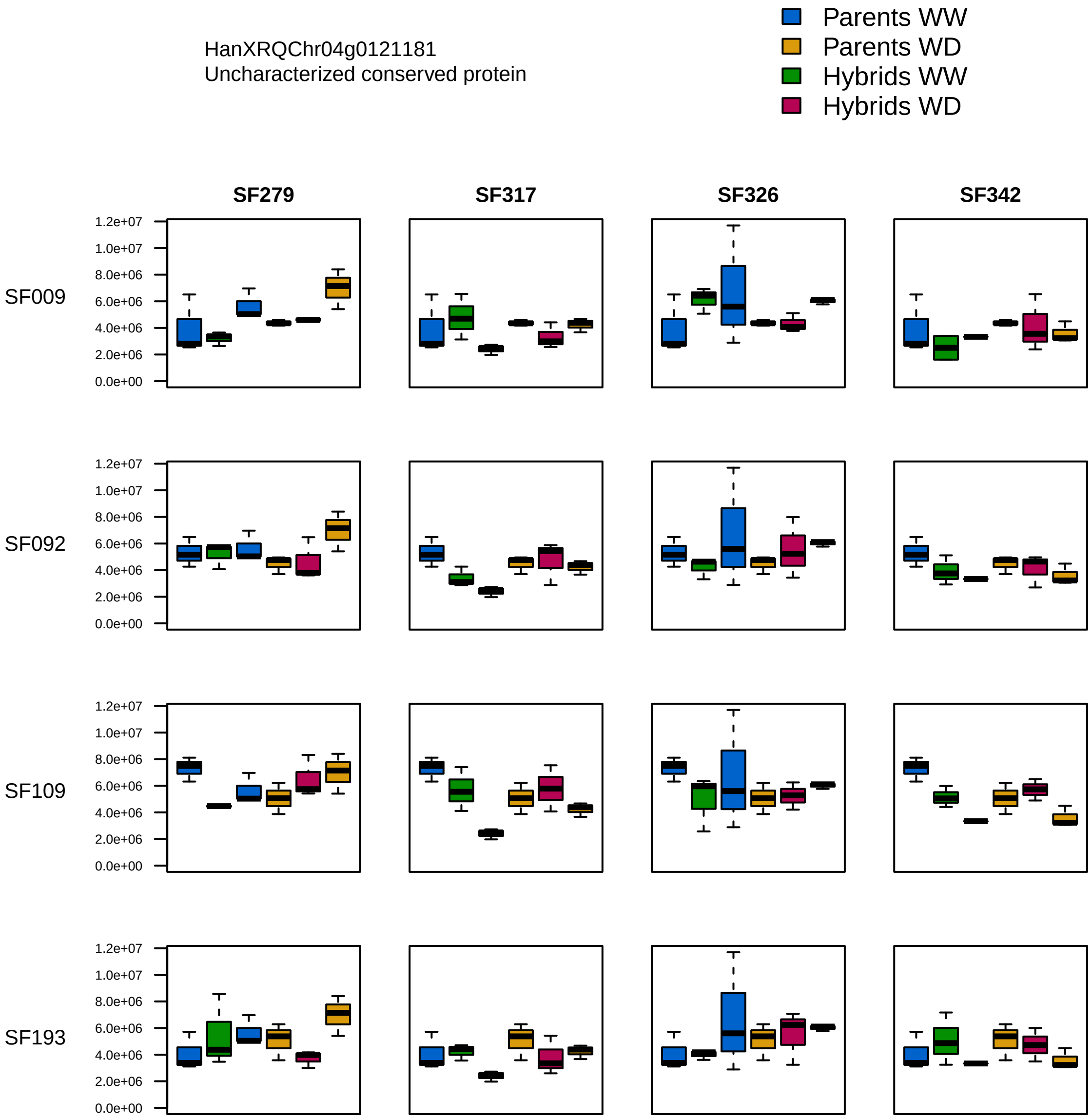
HanXRQChr04g0121181
Uncharacterized conserved protein
Parents WW
Parents WD
Hybrids WW
Hybrids WD
SF279
SF317
SF326
SF342
SF009
SF092
SF109
SF193
1.2e+07
1.0e+07
8.0e+06
6.0e+06
4.0e+06
2.0e+06
0.0e+00

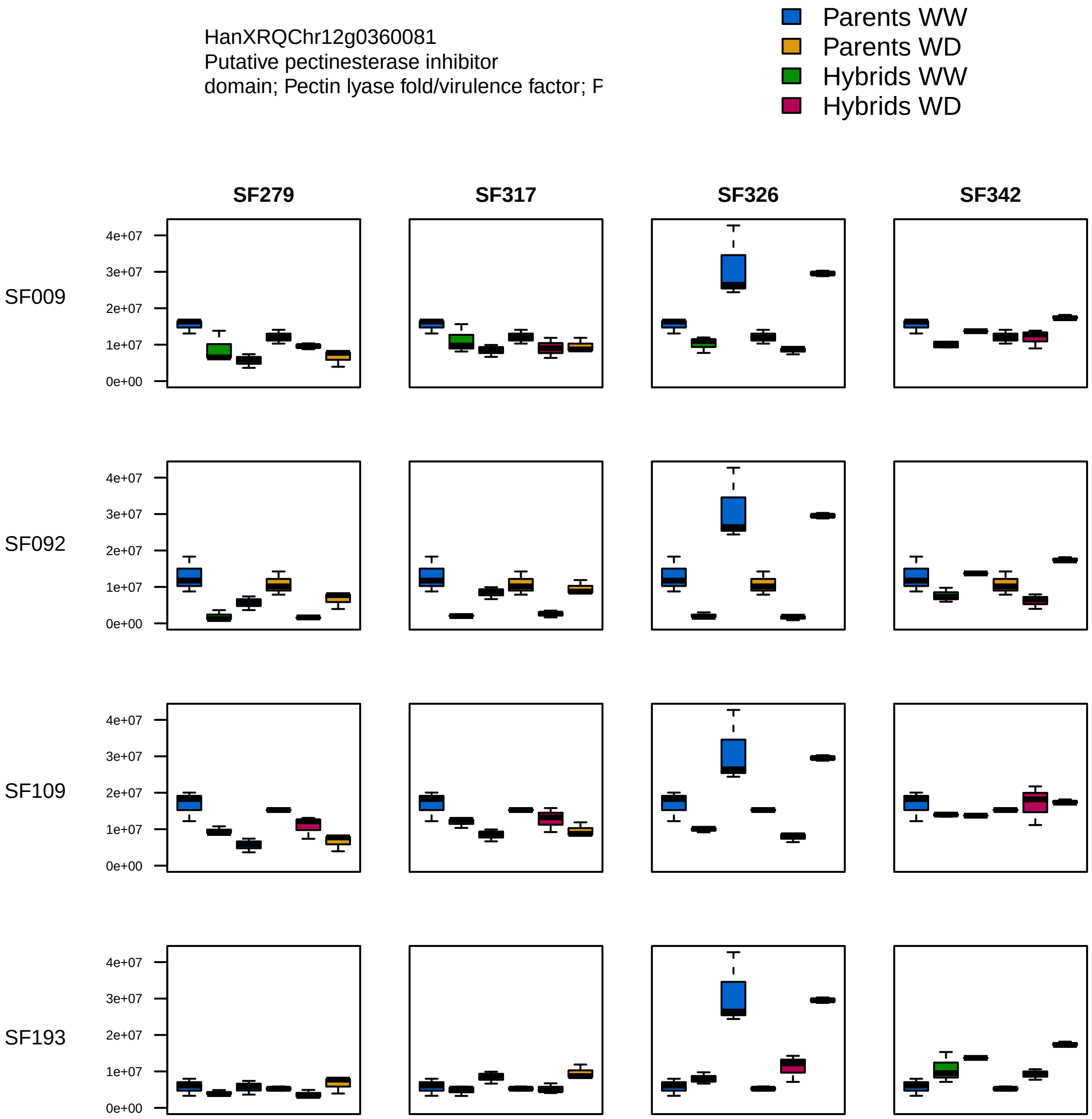
HanXRQChr12g0360081
Putative pectinesterase inhibitor
domain; Pectin lyase fold/virulence factor; P
Parents WW
Parents WD
Hybrids WW
Hybrids WD
SF279
SF317
SF326
SF342
SF009
SF092
SF109
SF193
0e+00
1e+07
2e+07
3e+07
4e+07

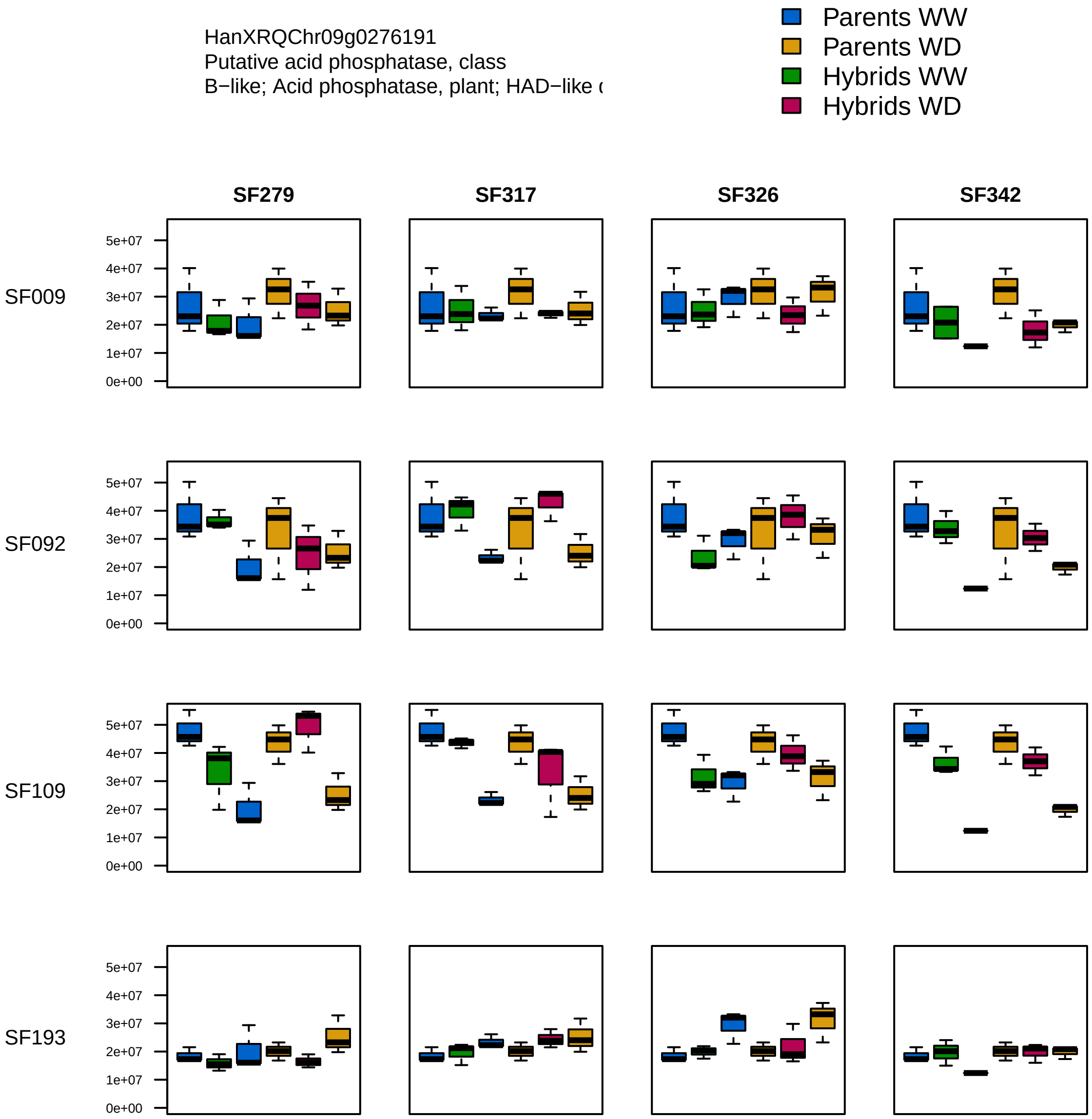
HanXRQChr09g0276191
Putative acid phosphatase, class
B−like; Acid phosphatase, plant; HAD−like (
Parents WW
Parents WD
Hybrids WW
Hybrids WD
SF279
SF317
SF326
SF342
SF009
SF092
SF109
SF193
5e+07
4e+07
3e+07
2e+07
1e+07
0e+00

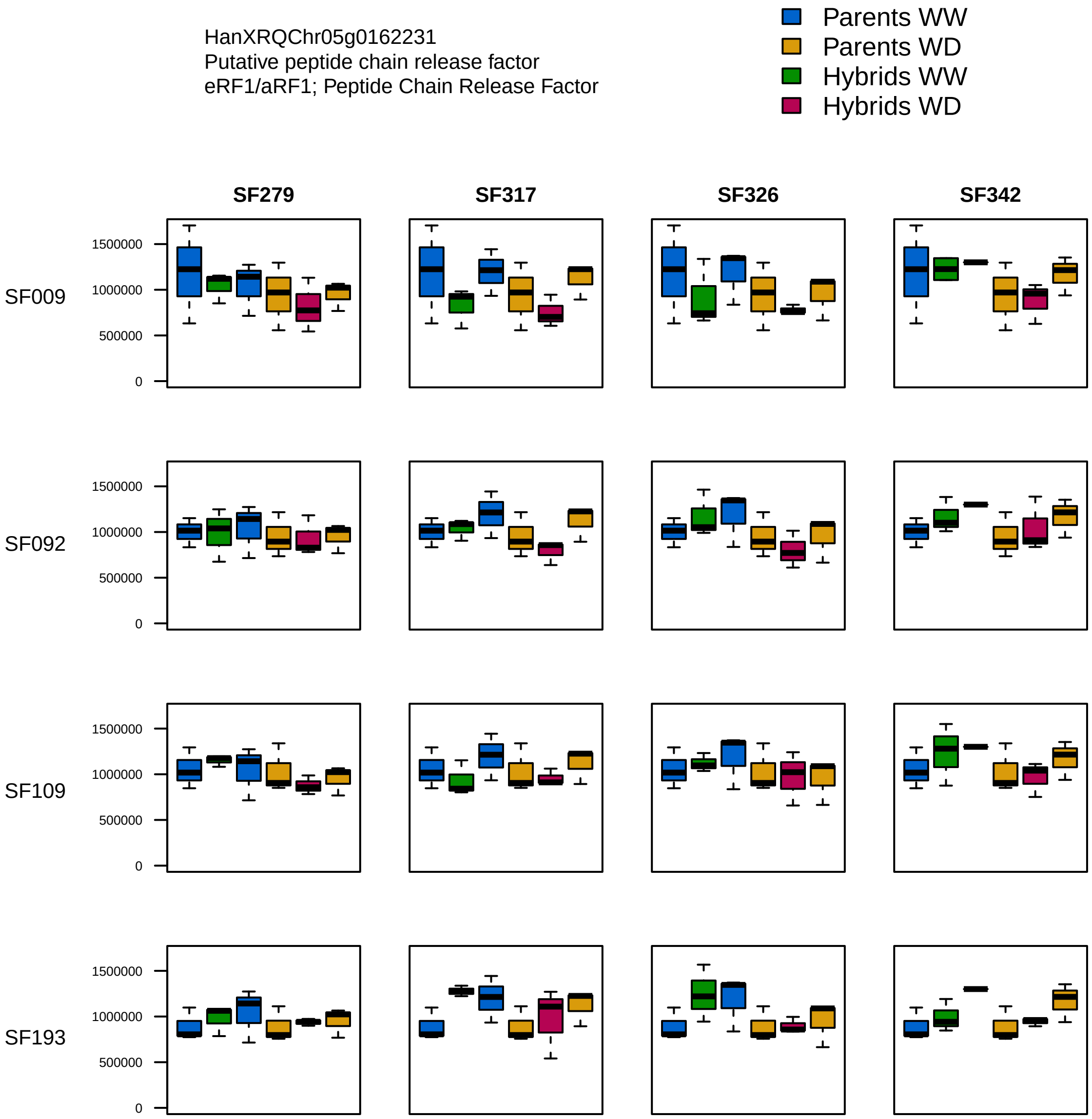

HanXRQChr05g0162231
Putative peptide chain release factor
eRF1/aRF1; Peptide Chain Release Factor
Parents WW
Parents WD
Hybrids WW
Hybrids WD
SF279
SF317
SF326
SF342
SF009
SF092
SF109
SF193
0
500000
1000000
1500000

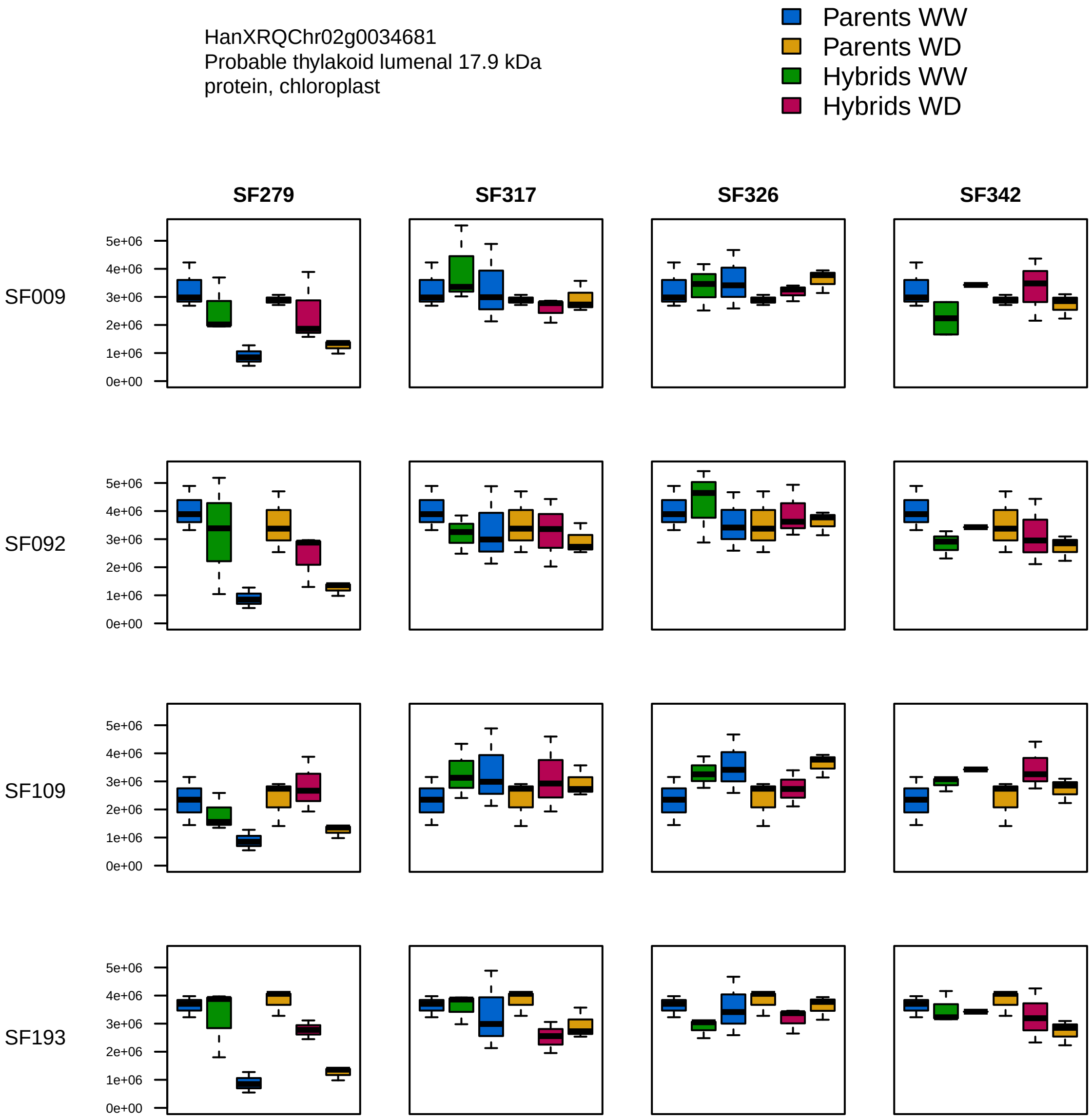

HanXRQChr02g0034681
Probable thylakoid lumenal 17.9 kDa
protein, chloroplast
Parents WW
Parents WD
Hybrids WW
Hybrids WD
SF279
SF317
SF326
SF342
SF009
SF092
SF109
SF193
0e+00
1e+06
2e+06
3e+06
4e+06
5e+06

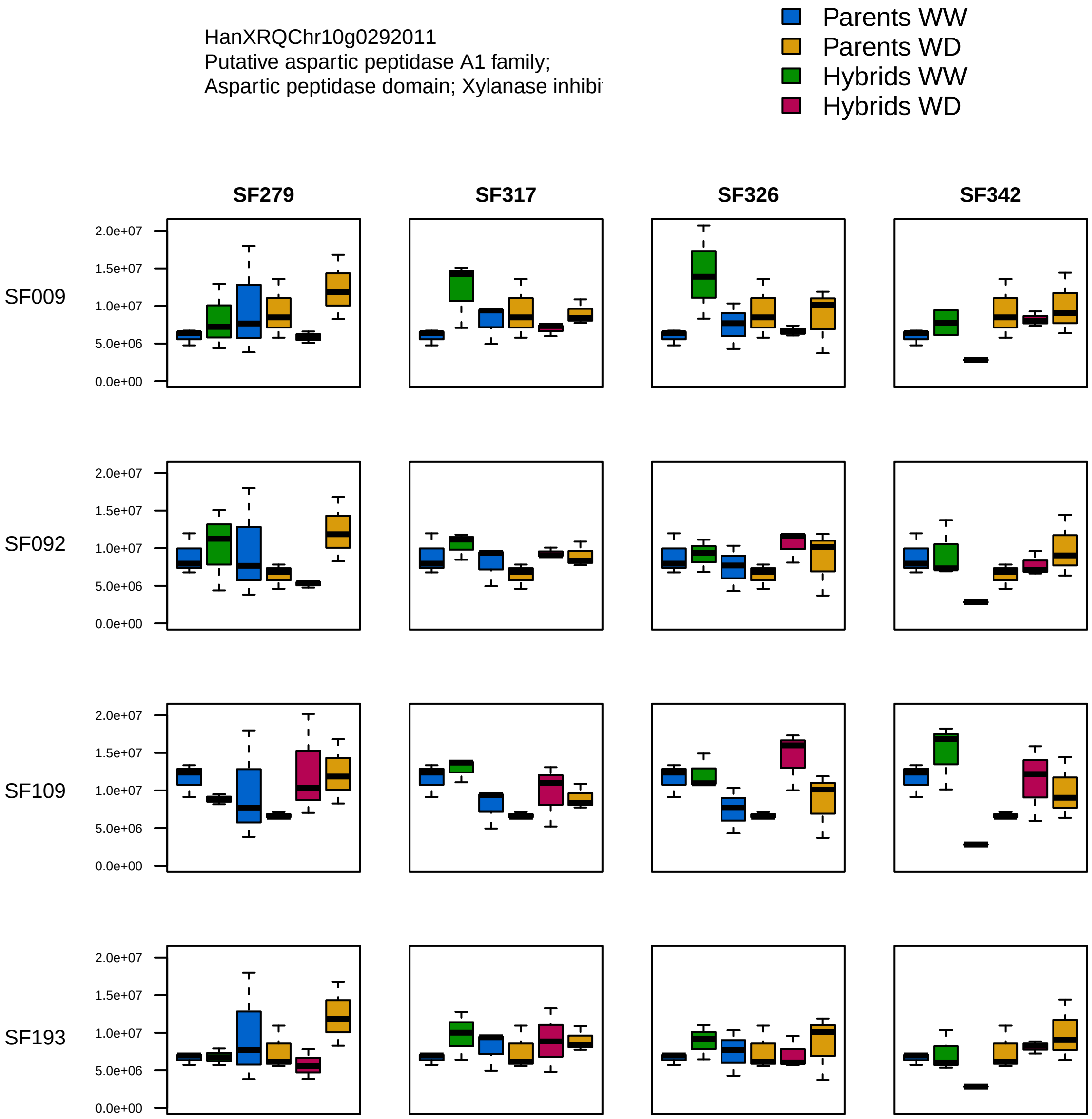
HanXRQChr10g0292011
Putative aspartic peptidase A1 family;
Aspartic peptidase domain; Xylanase inhibi
Parents WW
Parents WD
Hybrids WW
Hybrids WD
SF279
SF317
SF326
SF342
SF009
SF092
SF109
SF193
2.0e+07
1.5e+07
1.0e+07
5.0e+06
0.0e+00

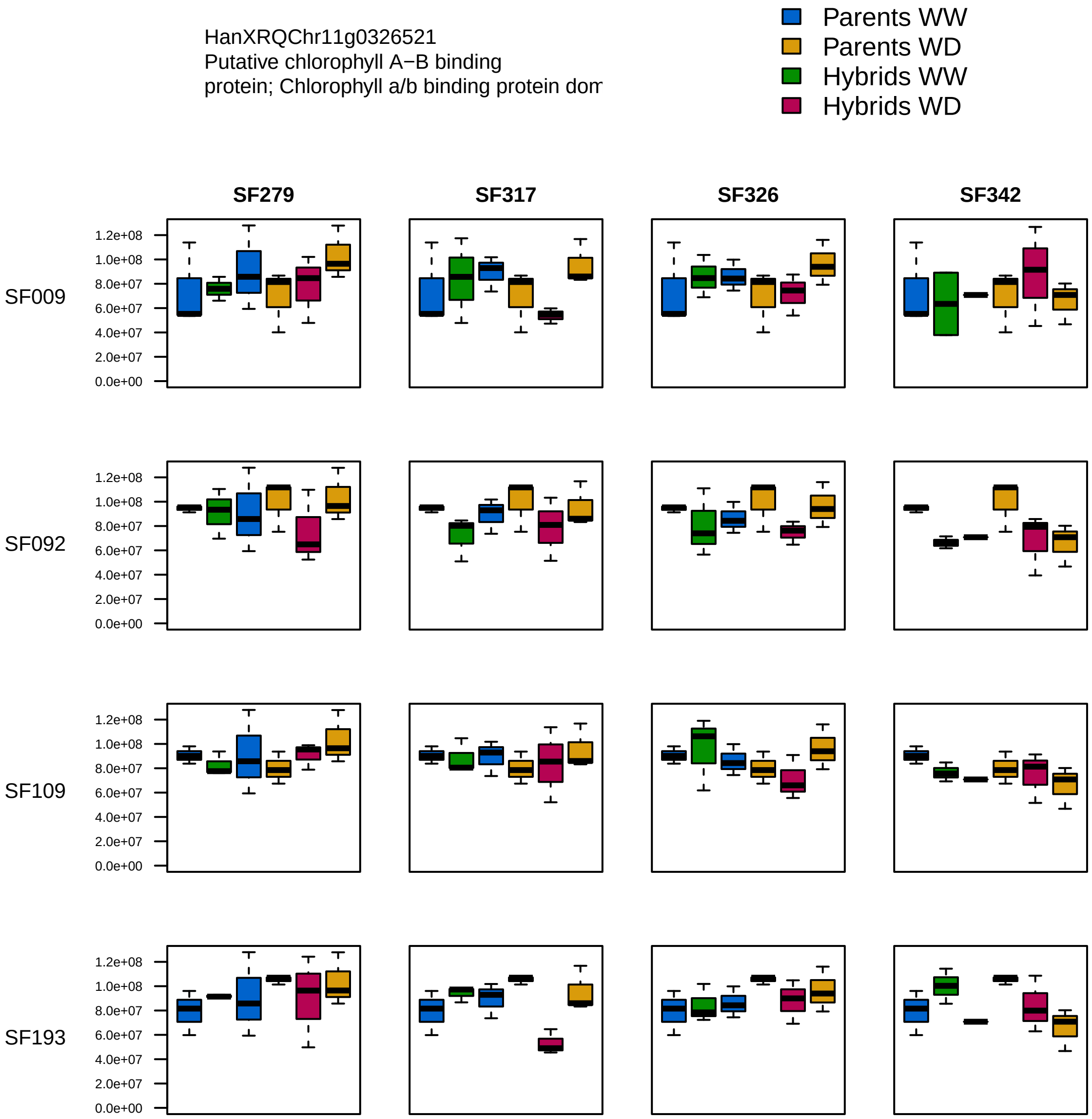
HanXRQChr11g0326521
Putative chlorophyll A−B binding
protein; Chlorophyll a/b binding protein dom
Parents WW
Parents WD
Hybrids WW
Hybrids WD
SF279
SF317
SF326
SF342
SF009
SF092
SF109
SF193
1.2e+08
1.0e+08
8.0e+07
6.0e+07
4.0e+07
2.0e+07
0.0e+00

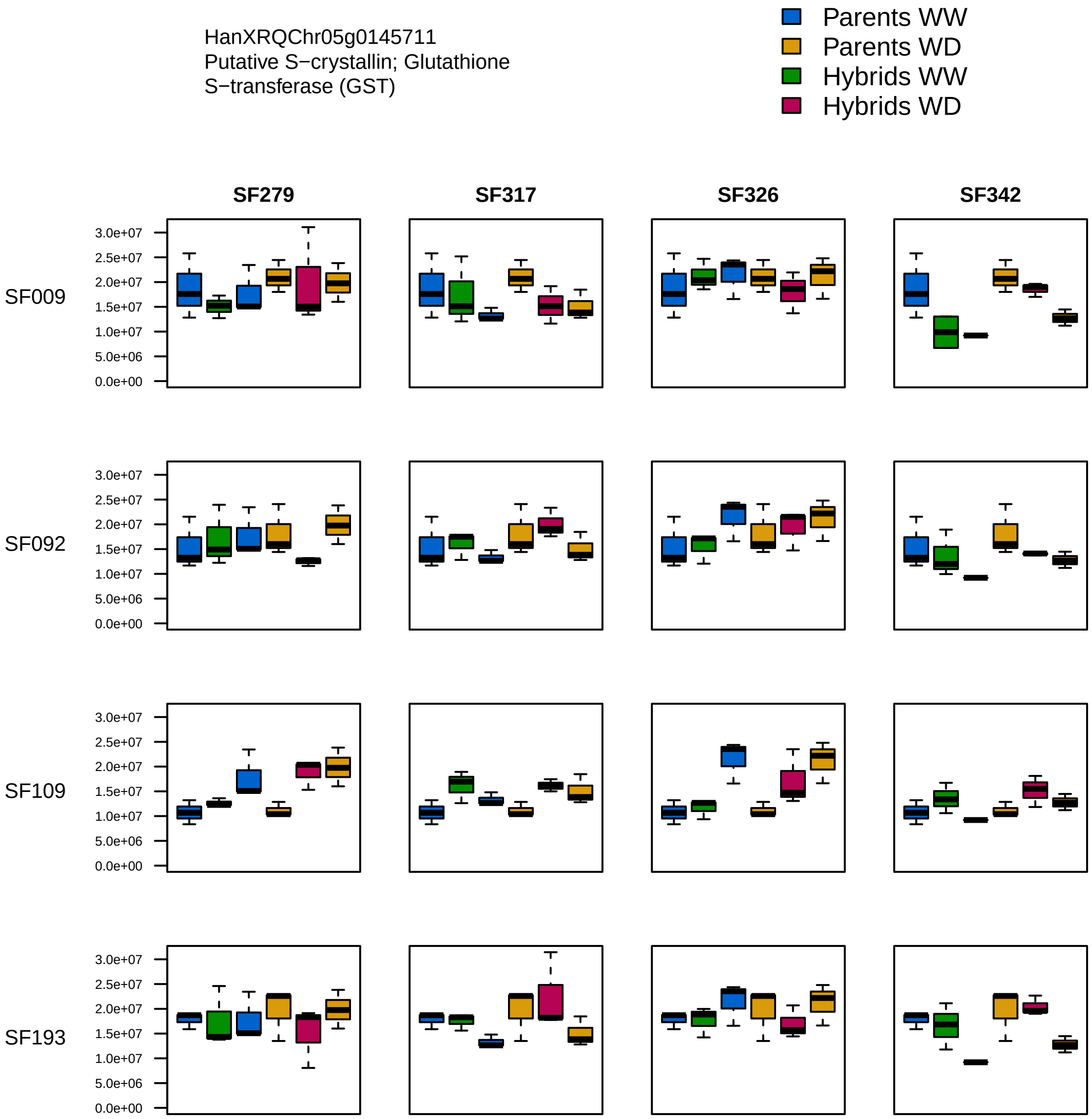
HanXRQChr05g0145711
Putative S−crystallin; Glutathione
S−transferase (GST)
Parents WW
Parents WD
Hybrids WW
Hybrids WD
SF279
SF317
SF326
SF342
SF009
SF092
SF109
SF193
3.0e+07
2.5e+07
2.0e+07
1.5e+07
1.0e+07
5.0e+06
0.0e+00

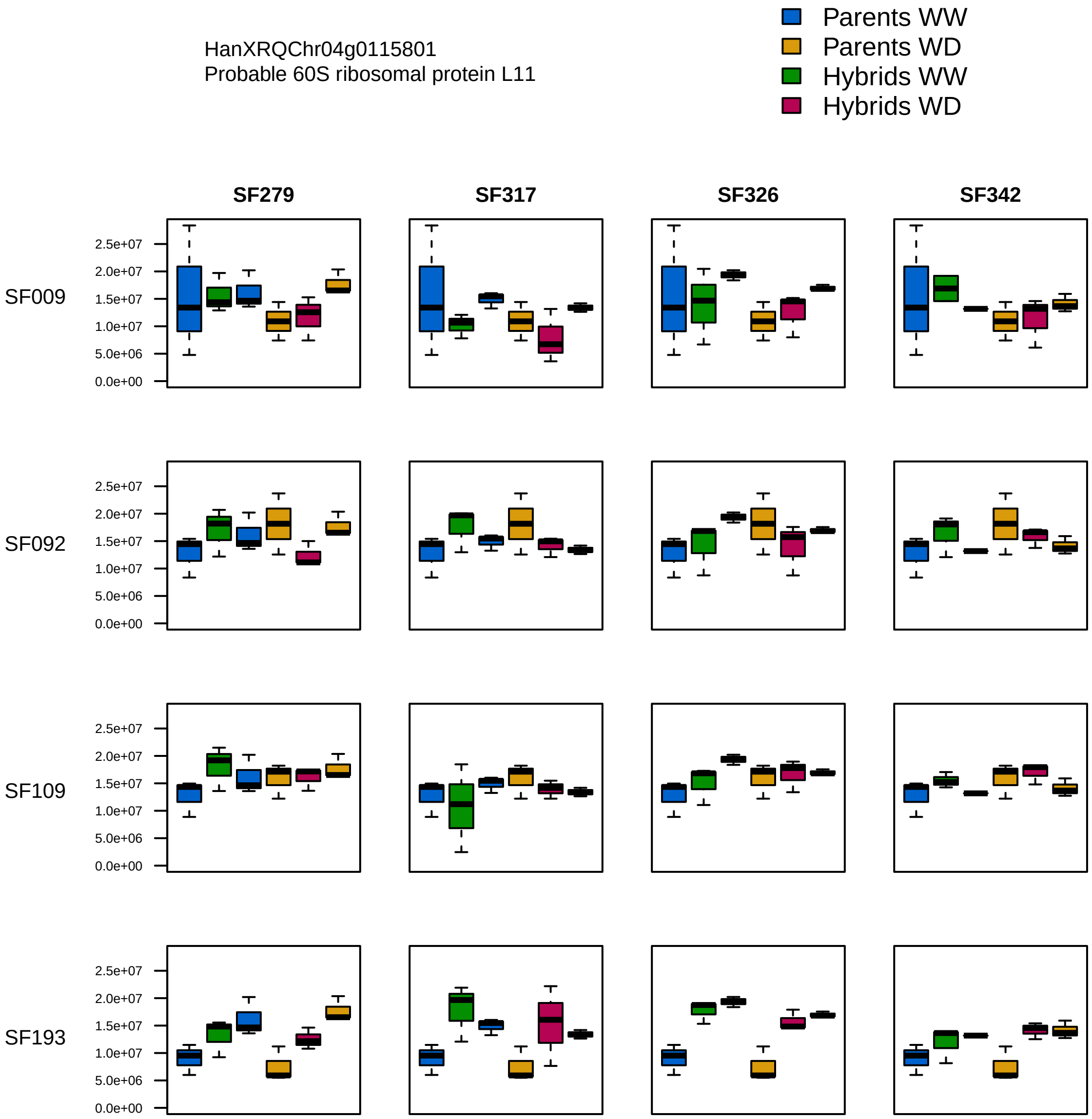
HanXRQChr04g0115801
Probable 60S ribosomal protein L11
Parents WW
Parents WD
Hybrids WW
Hybrids WD
SF279
SF317
SF326
SF342
SF009
SF092
SF109
SF193
2.5e+07
2.0e+07
1.5e+07
1.0e+07
5.0e+06
0.0e+00

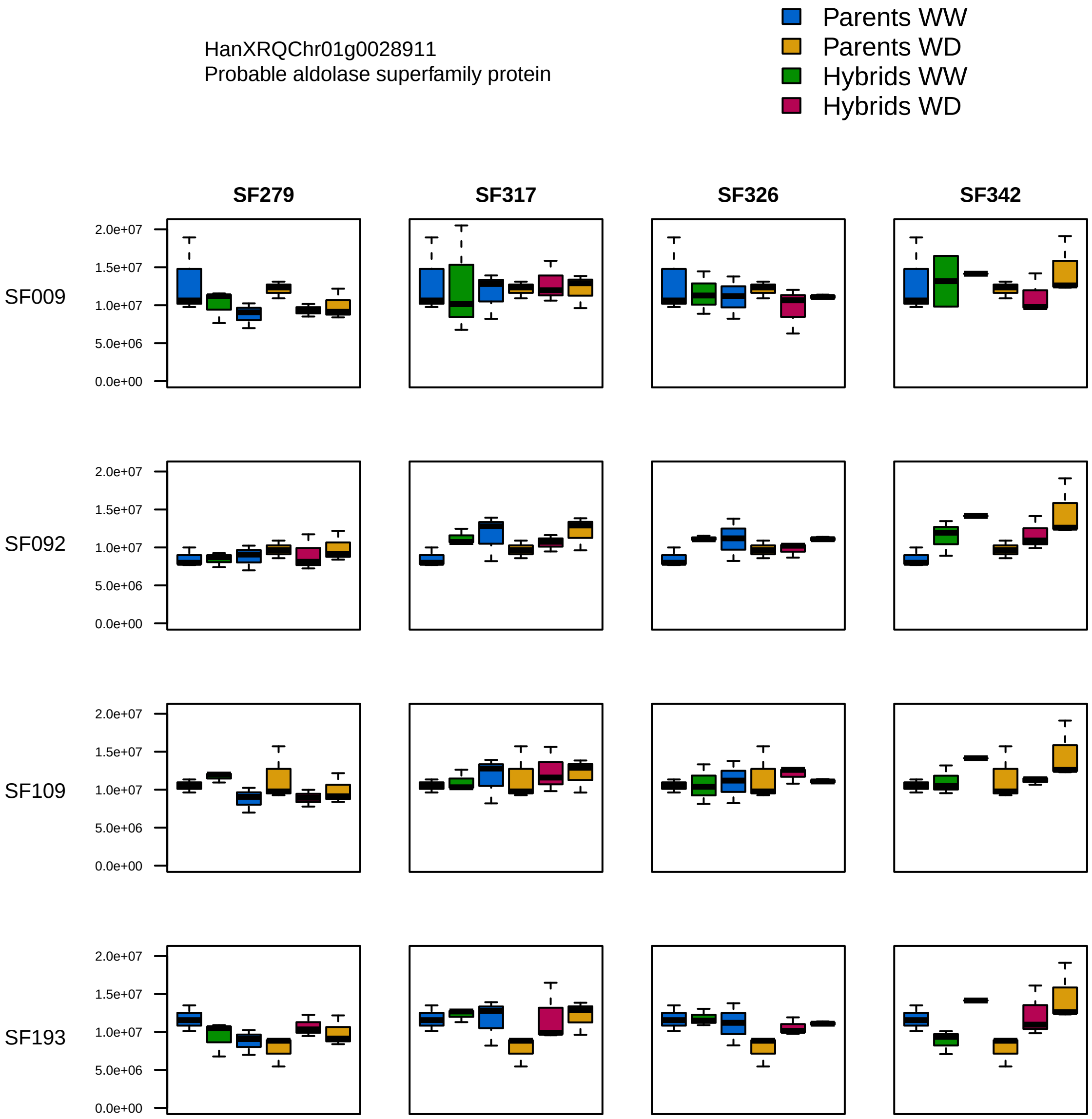
HanXRQChr01g0028911
Probable aldolase superfamily protein
Parents WW
Parents WD
Hybrids WW
Hybrids WD
SF279
SF317
SF326
SF342
SF009
SF092
SF109
SF193
2.0e+07
1.5e+07
1.0e+07
5.0e+06
0.0e+00

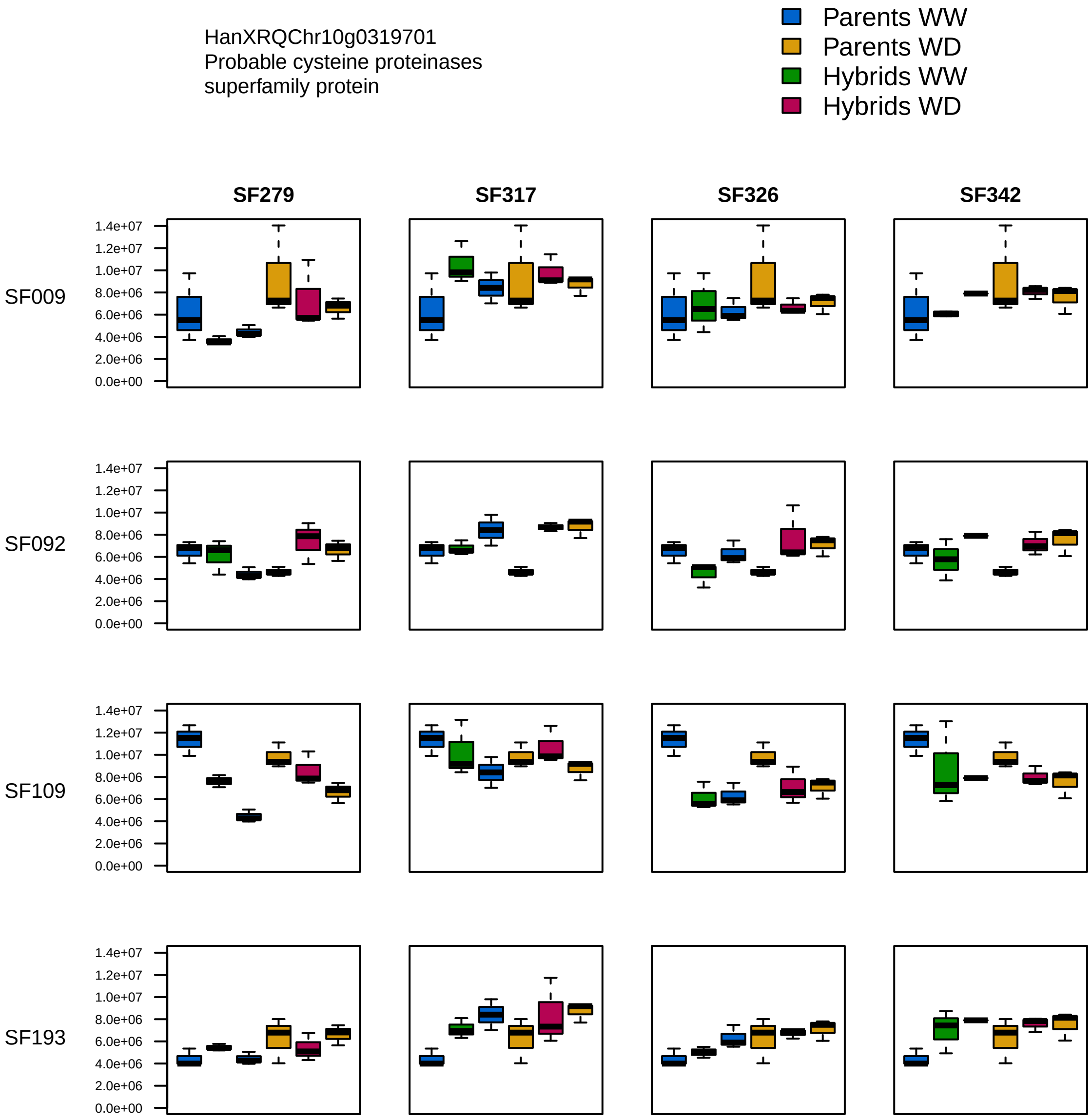
HanXRQChr10g0319701
Probable cysteine proteinases
superfamily protein
Parents WW
Parents WD
Hybrids WW
Hybrids WD
SF279
SF317
SF326
SF342
SF009
SF092
SF109
SF193
1.4e+07
1.2e+07
1.0e+07
8.0e+06
6.0e+06
4.0e+06
2.0e+06
0.0e+00

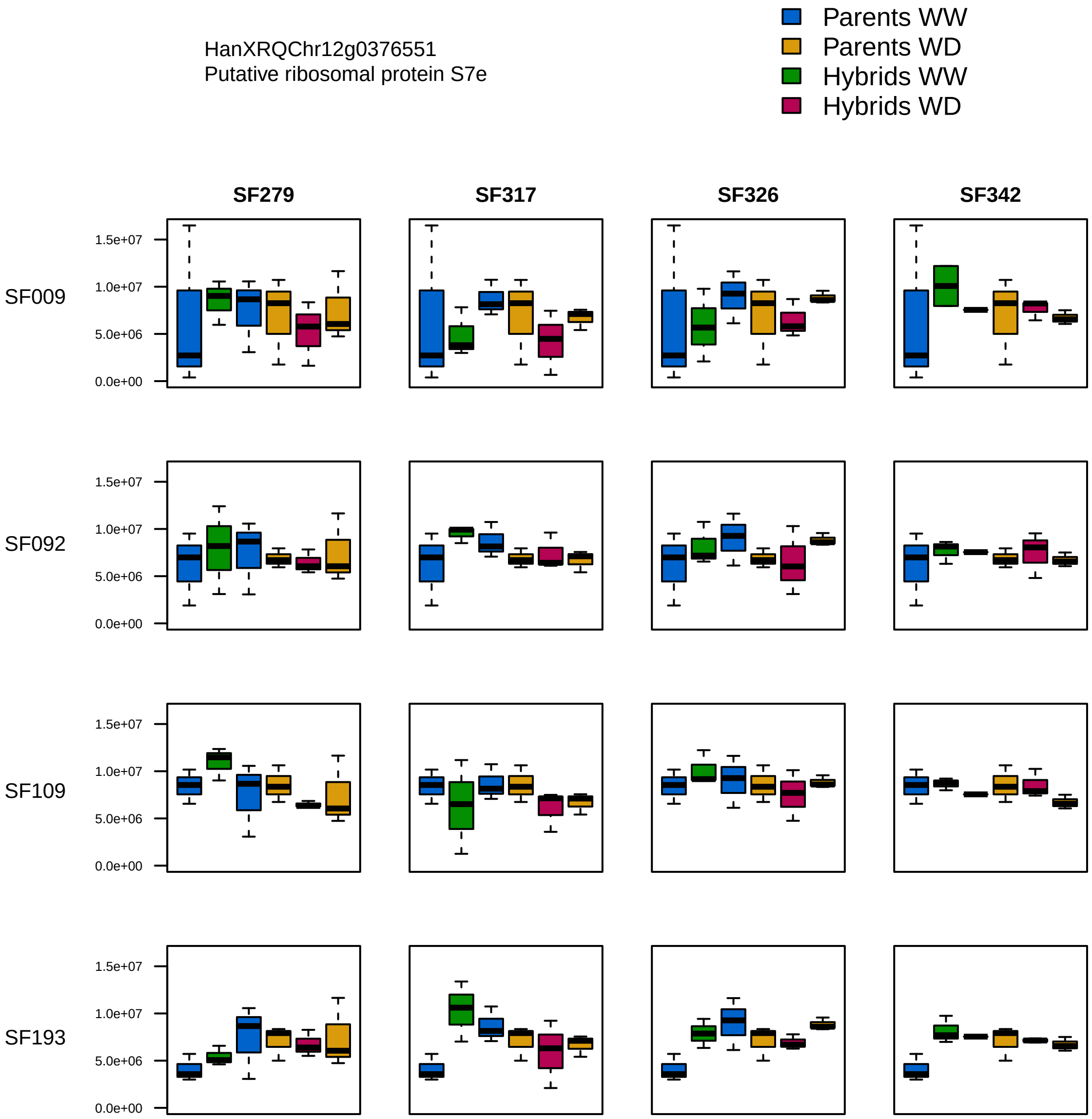

HanXRQChr12g0376551
Putative ribosomal protein S7e
Parents WW
Parents WD
Hybrids WW
Hybrids WD
SF279
SF317
SF326
SF342
SF009
SF092
SF109
SF193
1.5e+07
1.0e+07
5.0e+06
0.0e+00

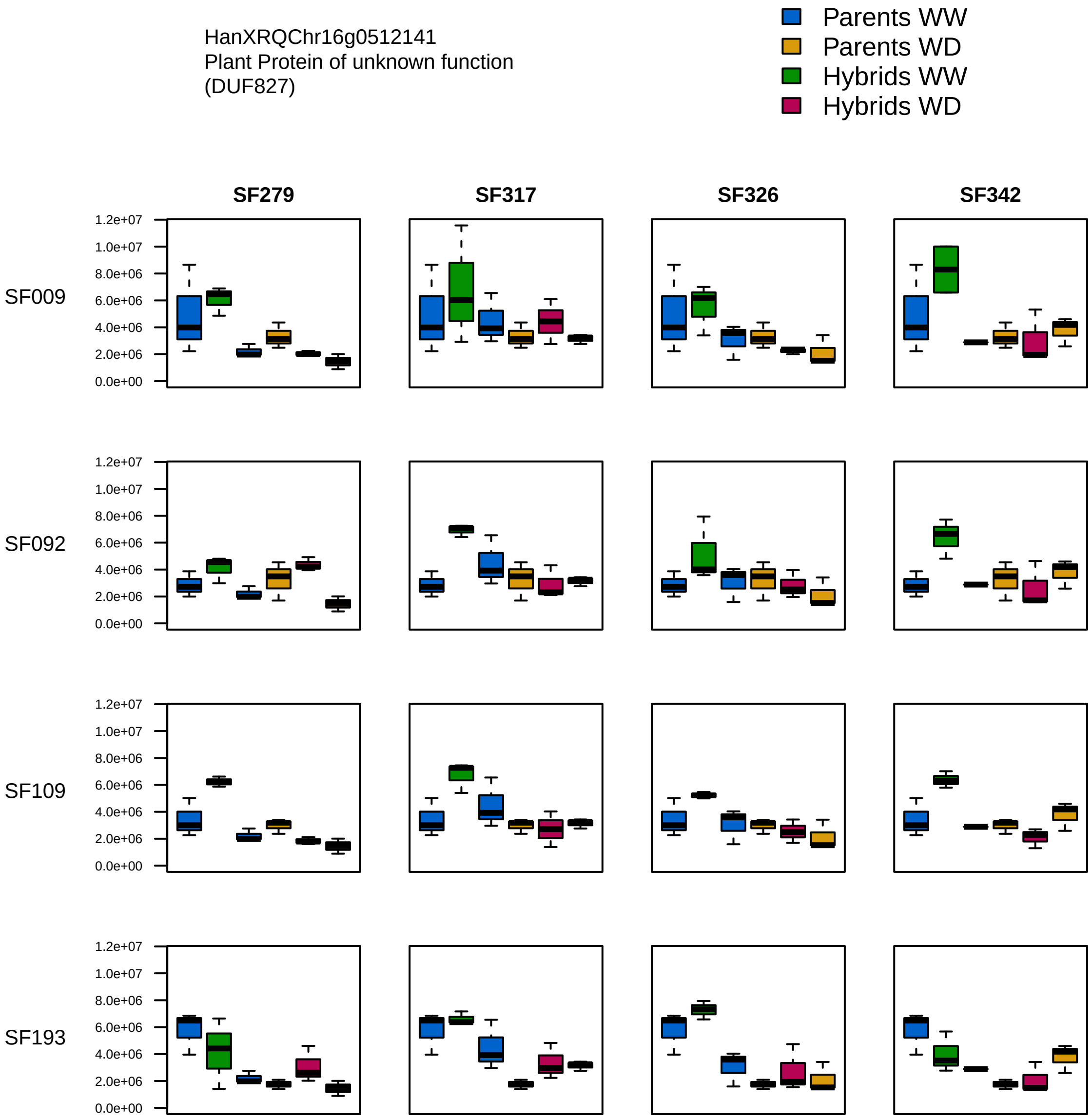
HanXRQChr16g0512141
Plant Protein of unknown function
(DUF827)
Parents WW
Parents WD
Hybrids WW
Hybrids WD
SF279
SF317
SF326
SF342
SF009
SF092
SF109
SF193
1.2e+07
1.0e+07
8.0e+06
6.0e+06
4.0e+06
2.0e+06
0.0e+00

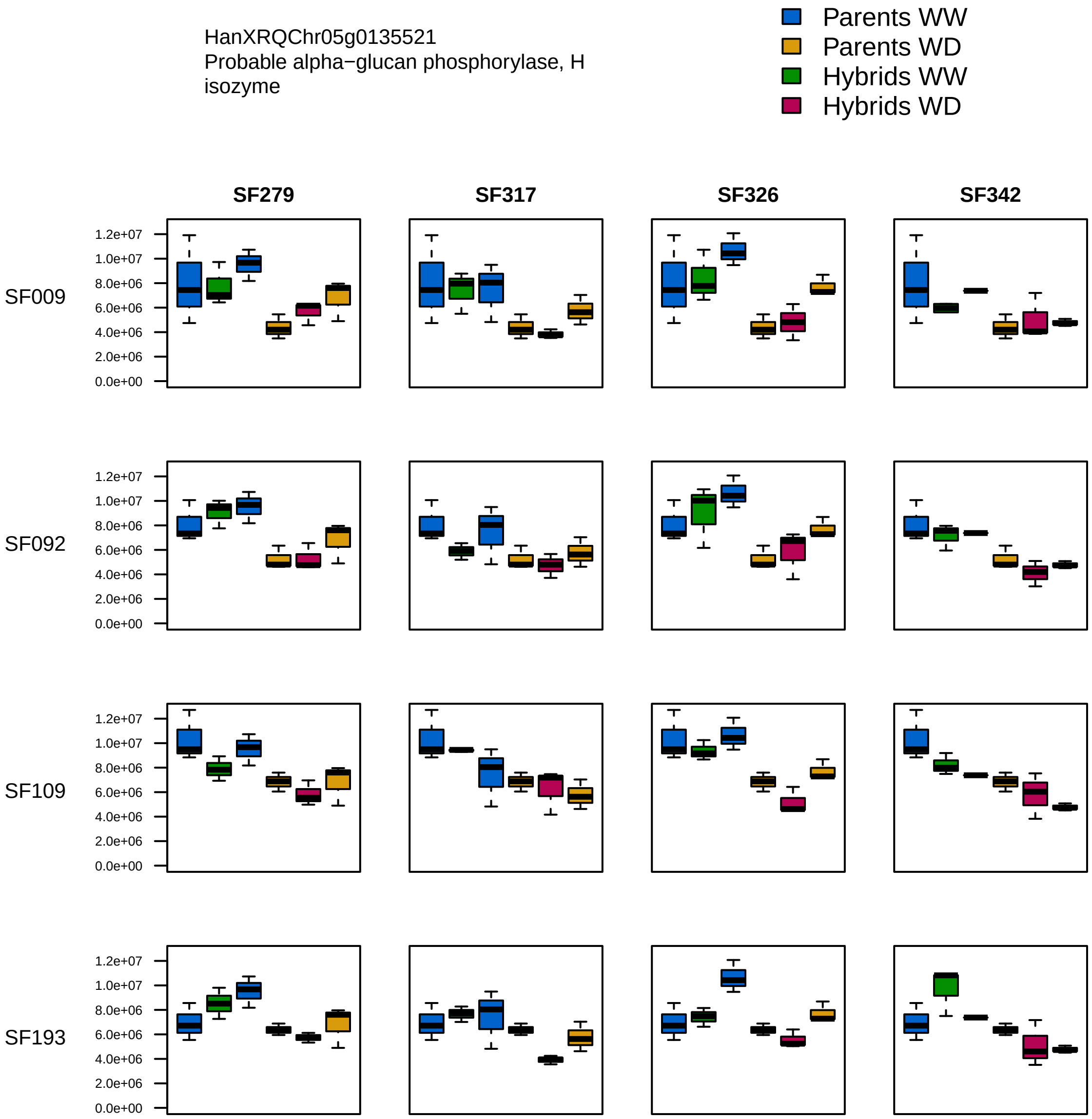
HanXRQChr05g0135521
Probable alpha−glucan phosphorylase, H isozyme
Parents WW
Parents WD
Hybrids WW
Hybrids WD
SF279
SF317
SF326
SF342
SF009
SF092
SF109
SF193
1.2e+07
1.0e+07
8.0e+06
6.0e+06
4.0e+06
2.0e+06
0.0e+00

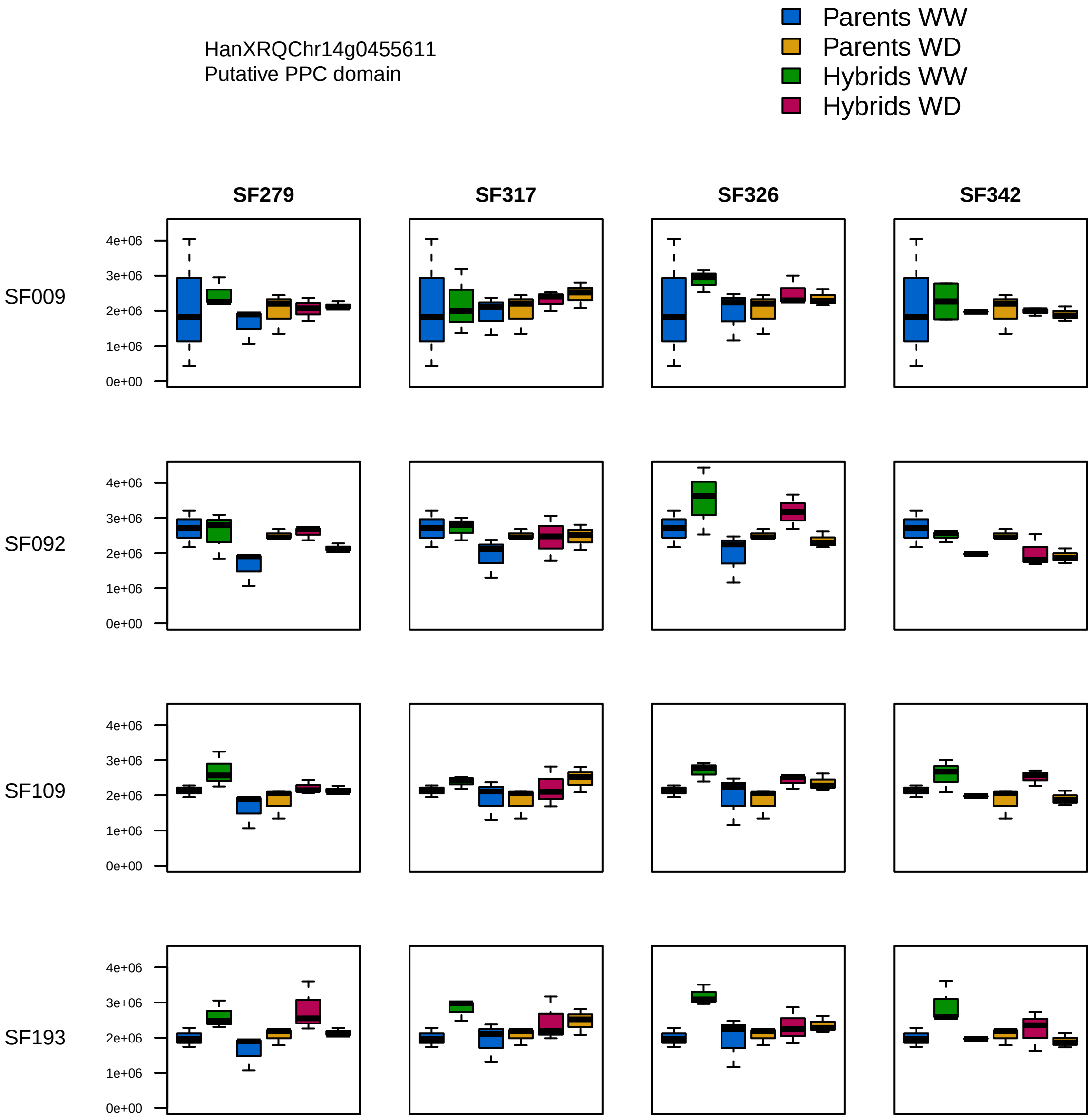
HanXRQChr14g0455611
Putative PPC domain
Parents WW
Parents WD
Hybrids WW
Hybrids WD
SF279
SF317
SF326
SF342
SF009
SF092
SF109
SF193
4e+06
3e+06
2e+06
1e+06
0e+00

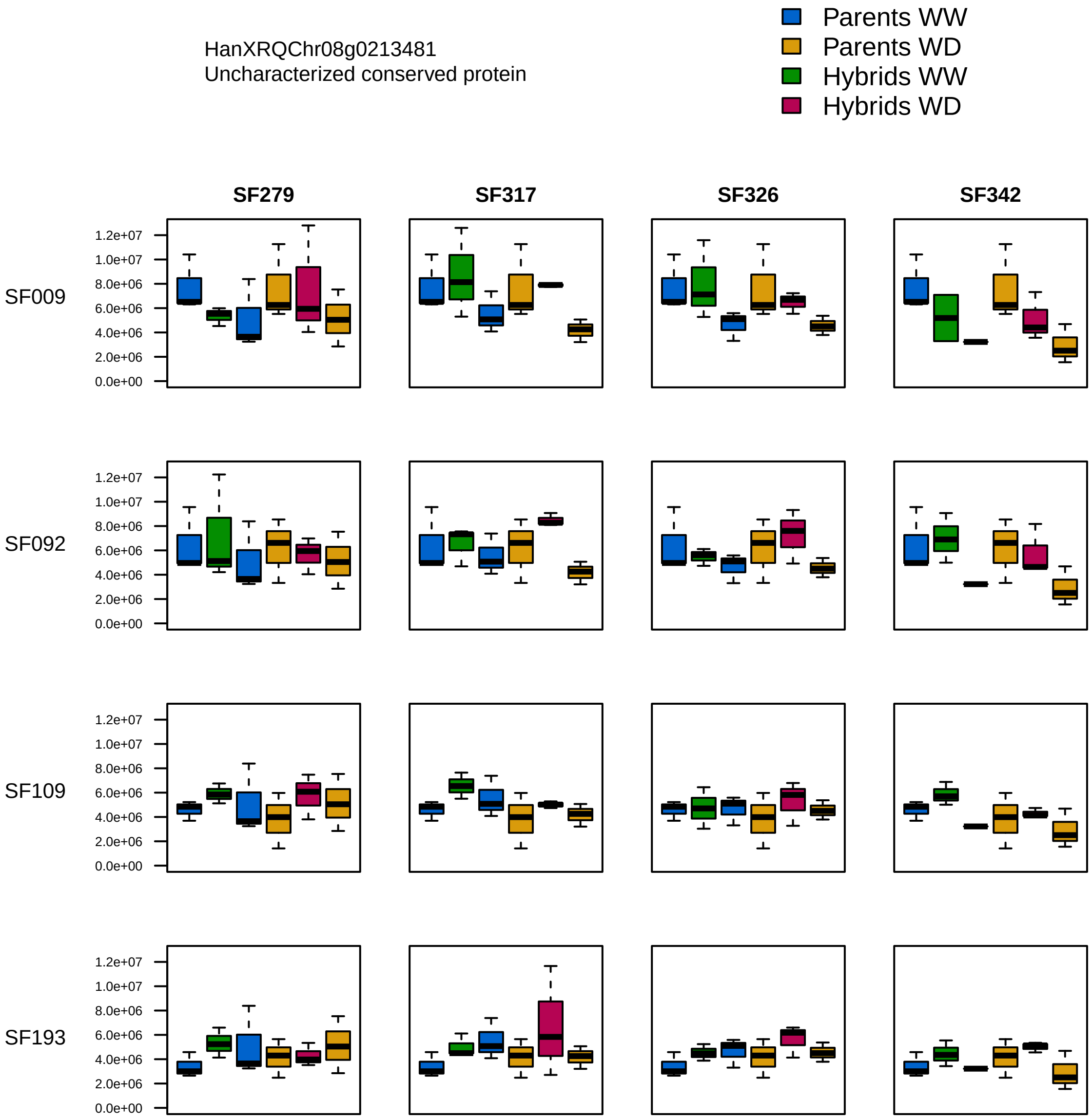
HanXRQChr08g0213481
Uncharacterized conserved protein
Parents WW
Parents WD
Hybrids WW
Hybrids WD
SF279
SF317
SF326
SF342
SF009
SF092
SF109
SF193
1.2e+07
1.0e+07
8.0e+06
6.0e+06
4.0e+06
2.0e+06
0.0e+00

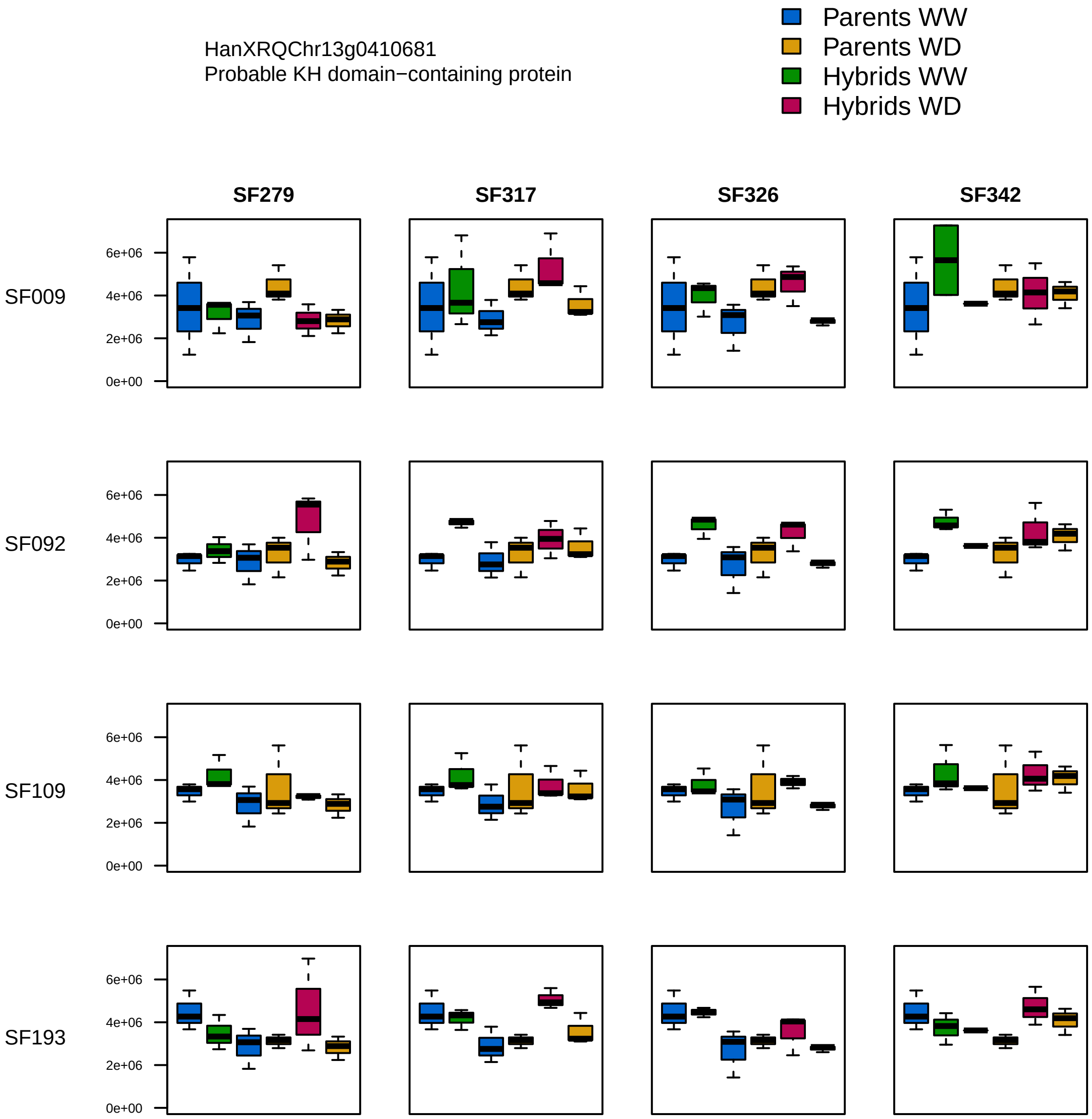

HanXRQChr13g0410681
Probable KH domain−containing protein
Parents WW
Parents WD
Hybrids WW
Hybrids WD
SF279
SF317
SF326
SF342
SF009
SF092
SF109
SF193
6e+06
4e+06
2e+06
0e+00

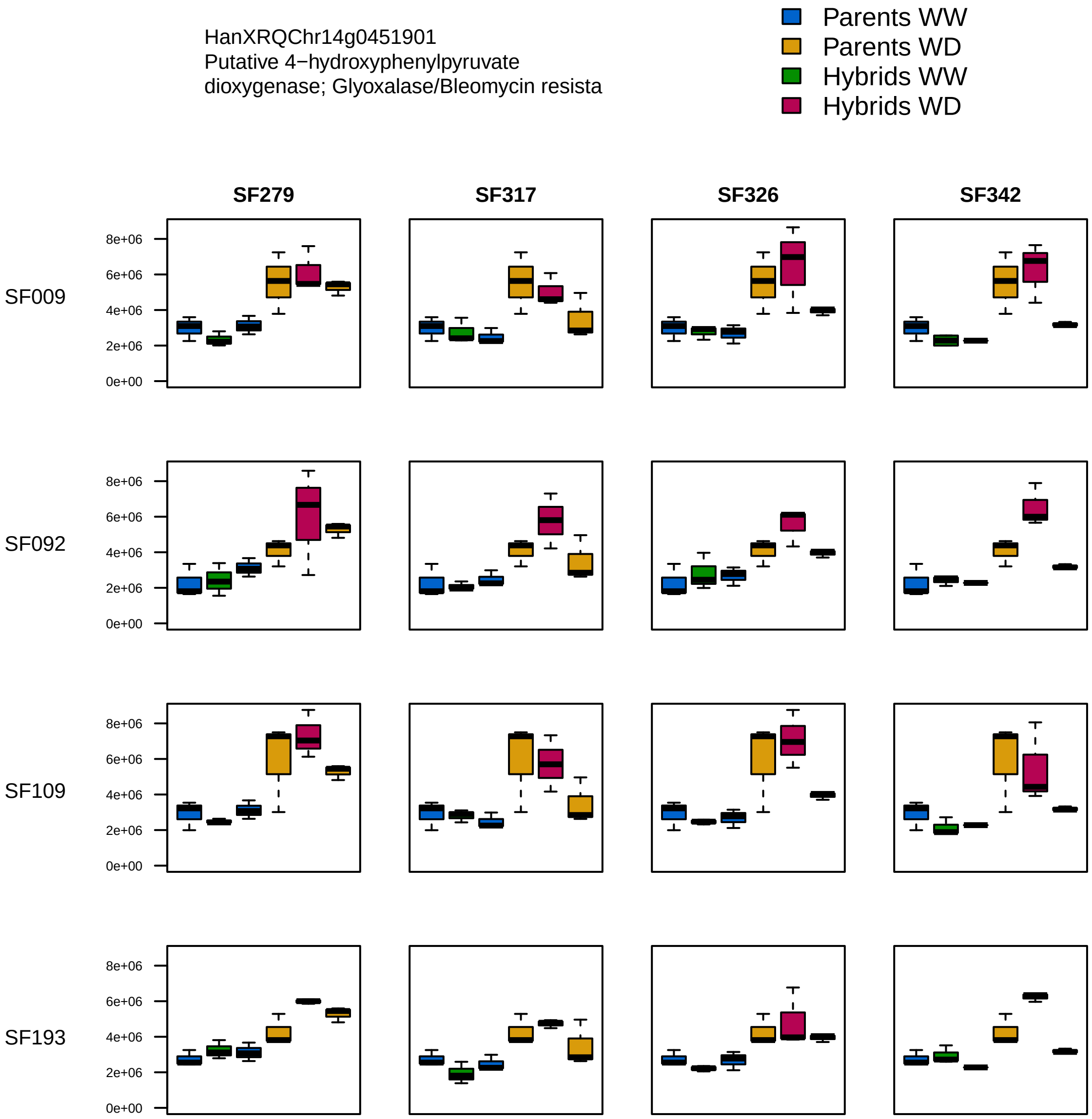

HanXRQChr14g0451901
Putative 4−hydroxyphenylpyruvate
dioxygenase; Glyoxalase/Bleomycin resista
Parents WW
Parents WD
Hybrids WW
Hybrids WD
SF279
SF317
SF326
SF342
SF009
SF092
SF109
SF193
0e+00
2e+06
4e+06
6e+06
8e+06

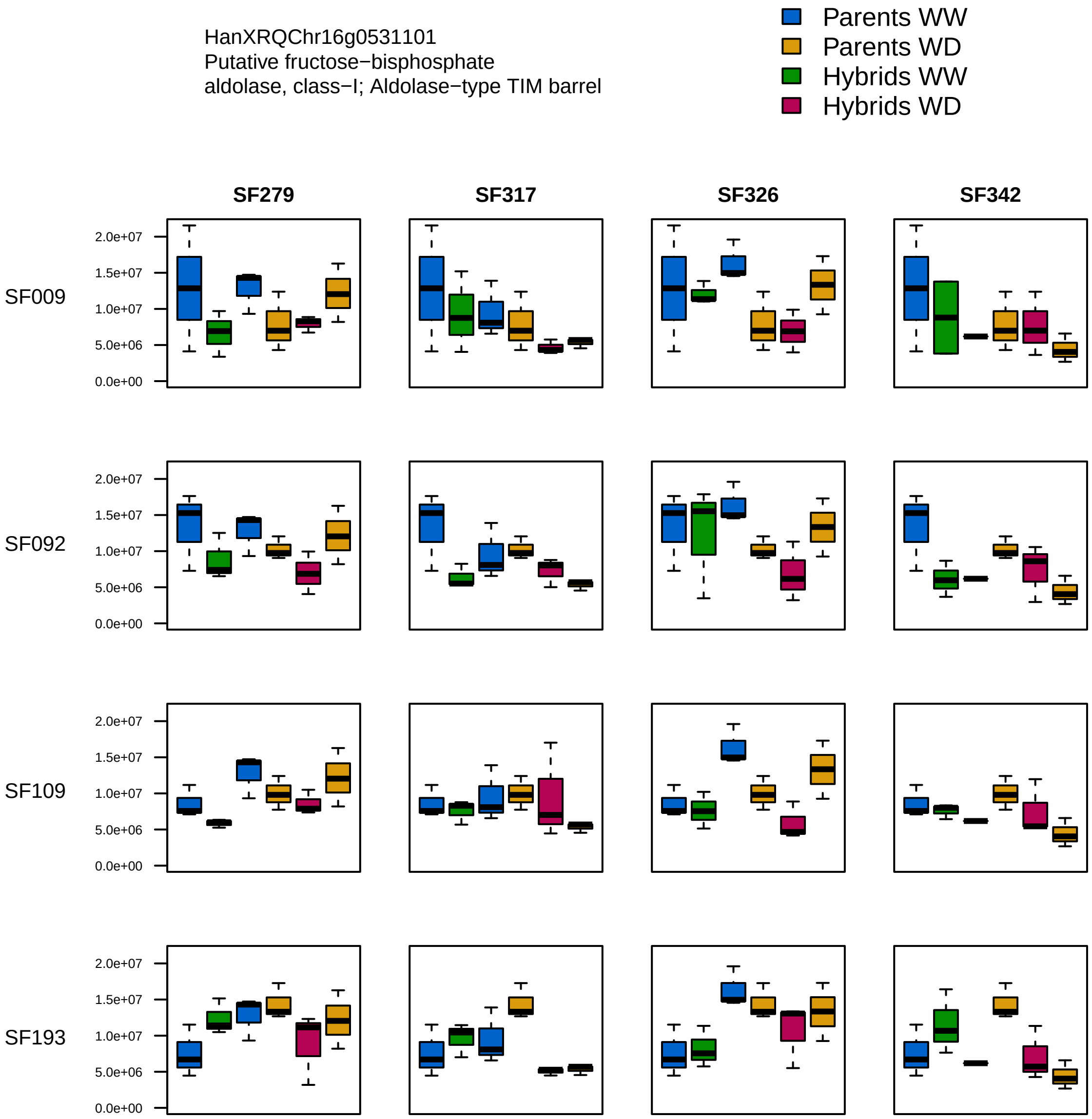
HanXRQChr16g0531101
Putative fructose−bisphosphate
aldolase, class−I; Aldolase−type TIM barrel
Parents WW
Parents WD
Hybrids WW
Hybrids WD
SF279
SF317
SF326
SF342
SF009
SF092
SF109
SF193
2.0e+07
1.5e+07
1.0e+07
5.0e+06
0.0e+00

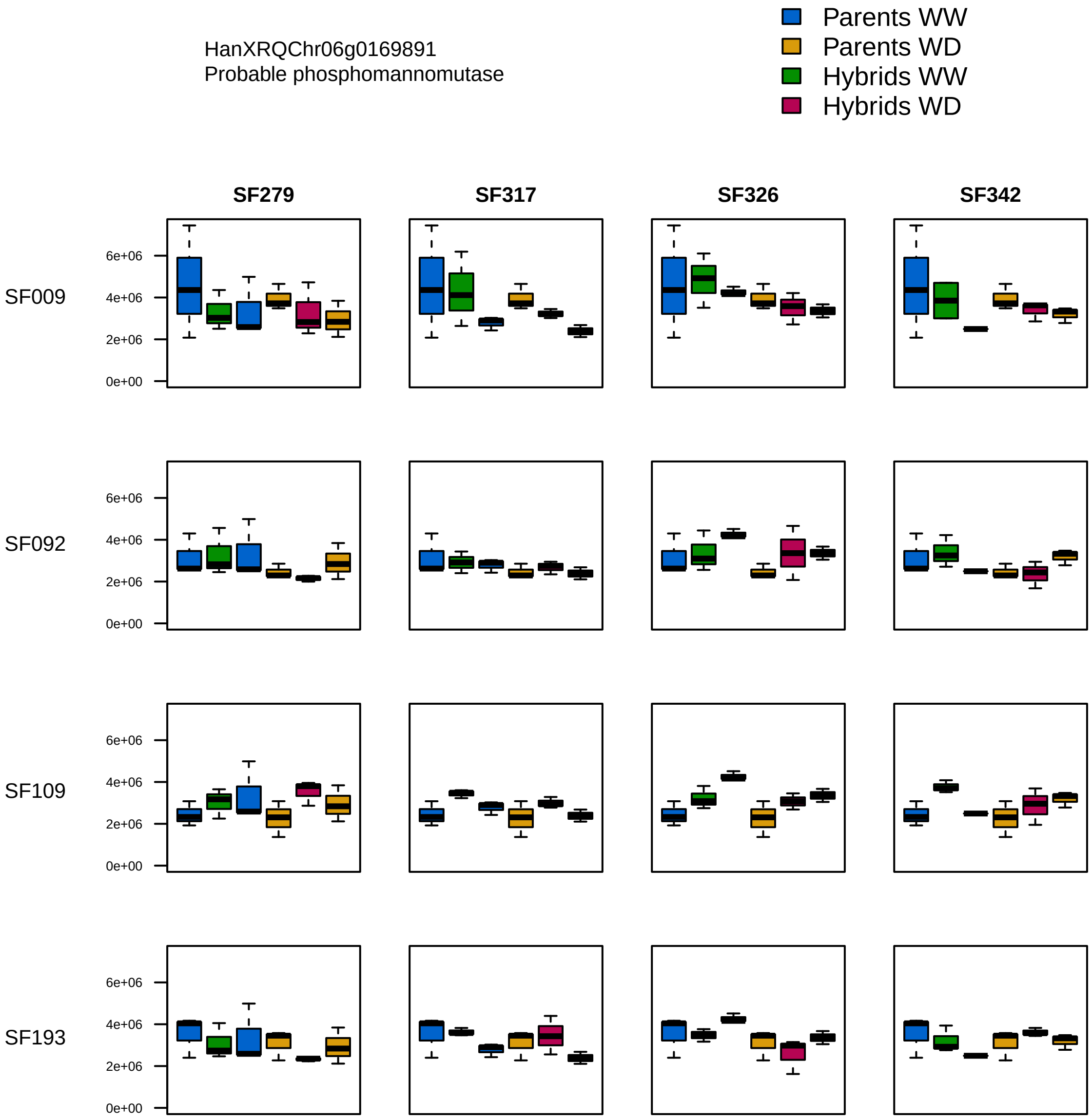
HanXRQChr06g0169891
Probable phosphomannomutase
Parents WW
Parents WD
Hybrids WW
Hybrids WD
SF279
SF317
SF326
SF342
SF009
SF092
SF109
SF193
6e+06
4e+06
2e+06
0e+00

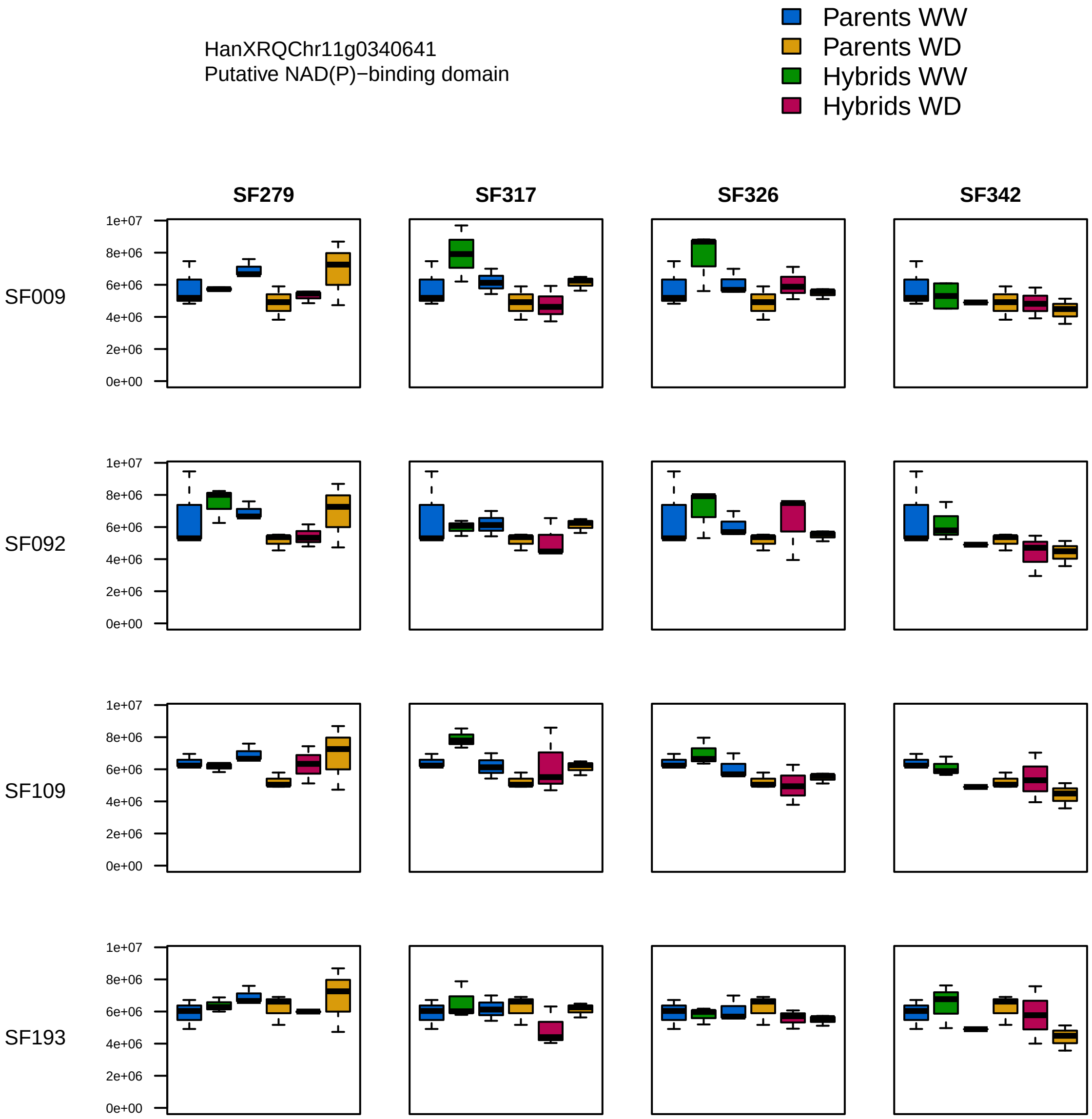
HanXRQChr11g0340641
Putative NAD(P)−binding domain
Parents WW
Parents WD
Hybrids WW
Hybrids WD
SF279
SF317
SF326
SF342
SF009
SF092
SF109
SF193
1e+07
8e+06
6e+06
4e+06
2e+06
0e+00

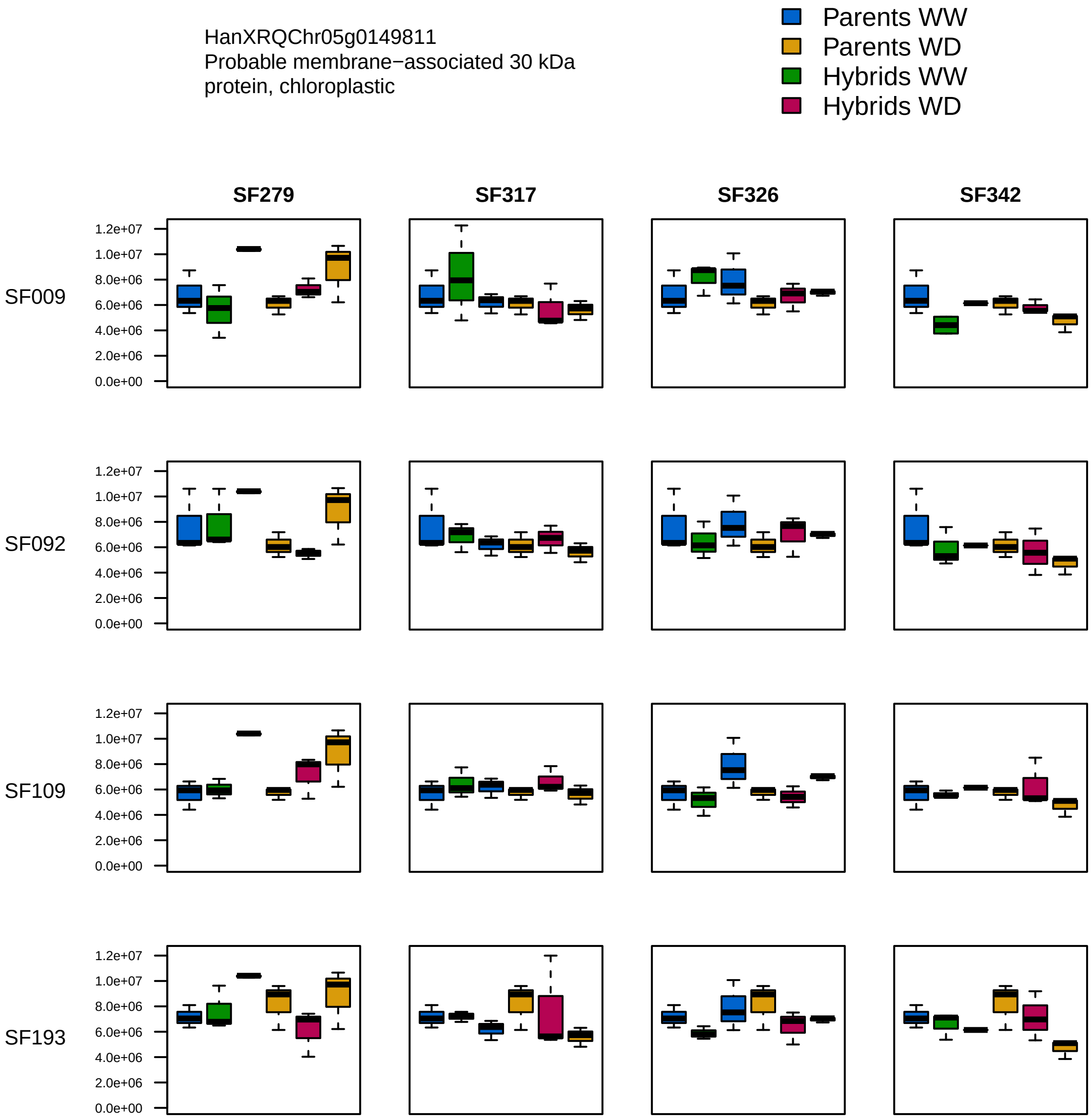
HanXRQChr05g0149811
Probable membrane−associated 30 kDa
protein, chloroplastic
Parents WW
Parents WD
Hybrids WW
Hybrids WD
SF279
SF317
SF326
SF342
SF009
SF092
SF109
SF193
1.2e+07
1.0e+07
8.0e+06
6.0e+06
4.0e+06
2.0e+06
0.0e+00

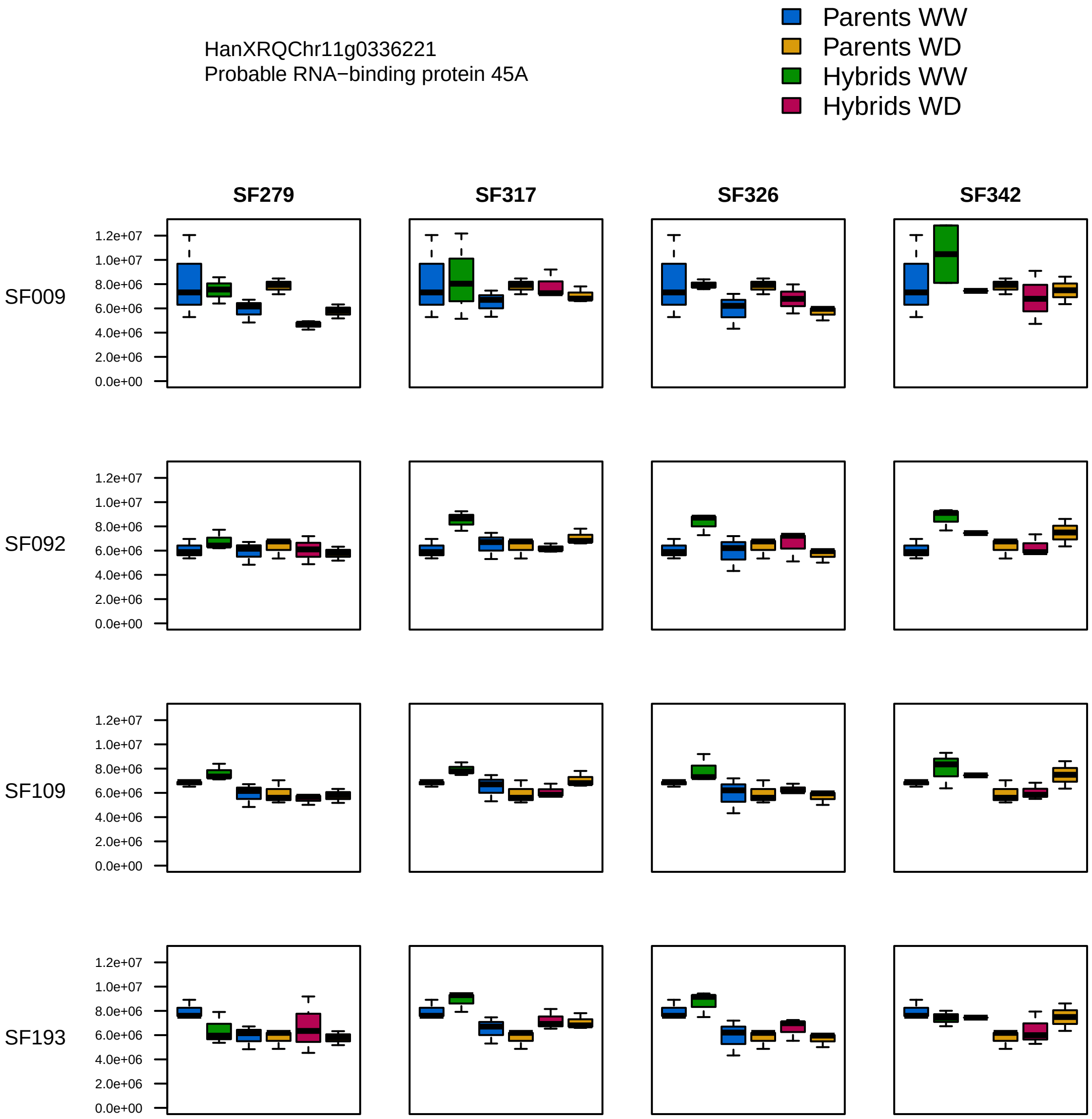
HanXRQChr11g0336221
Probable RNA−binding protein 45A
Parents WW
Parents WD
Hybrids WW
Hybrids WD
SF279
SF317
SF326
SF342
SF009
SF092
SF109
SF193
1.2e+07
1.0e+07
8.0e+06
6.0e+06
4.0e+06
2.0e+06
0.0e+00

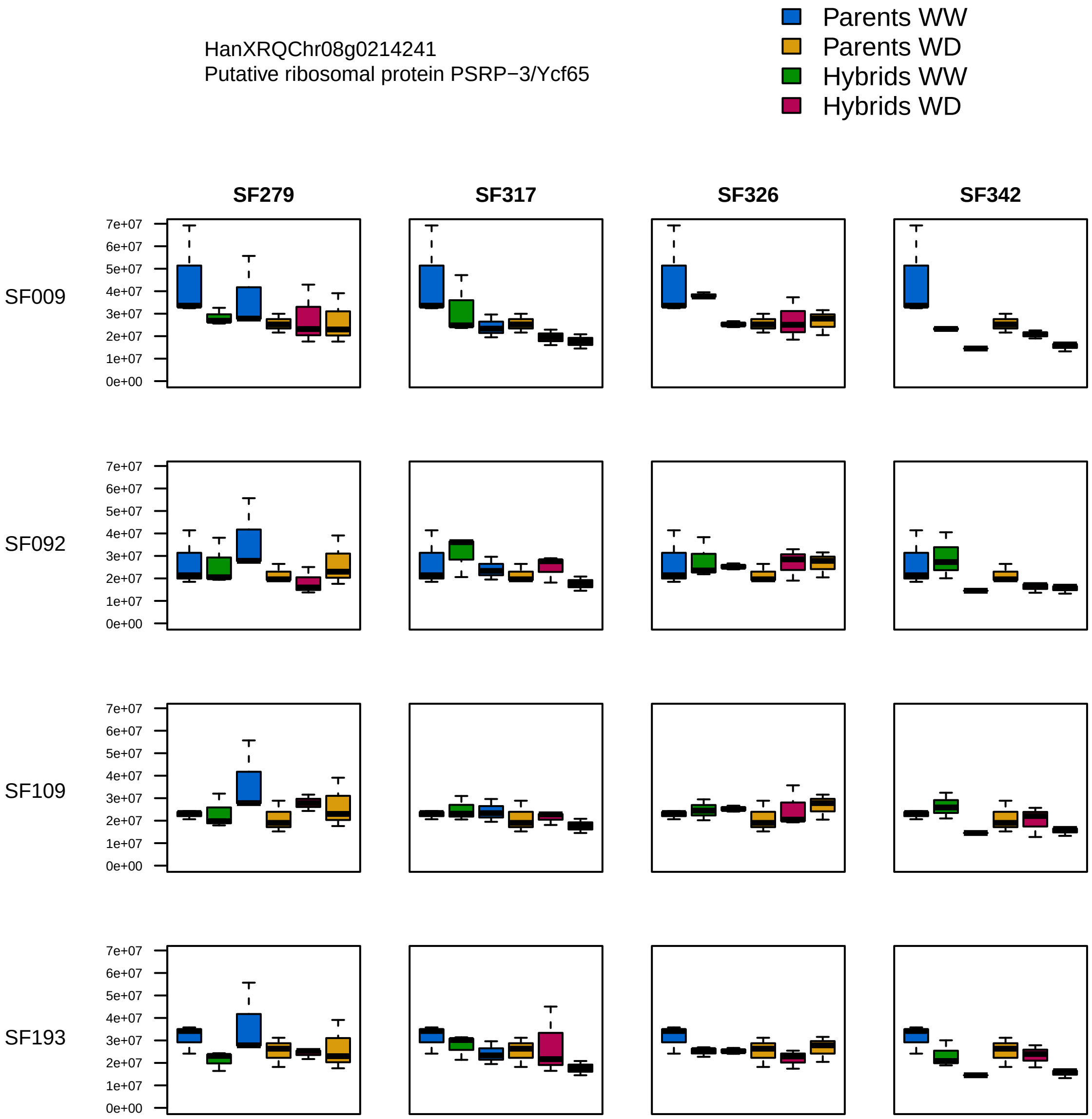
HanXRQChr08g0214241
Putative ribosomal protein PSRP−3/Ycf65
Parents WW
Parents WD
Hybrids WW
Hybrids WD
SF279
SF317
SF326
SF342
SF009
SF092
SF109
SF193
7e+07
6e+07
5e+07
4e+07
3e+07
2e+07
1e+07
0e+00

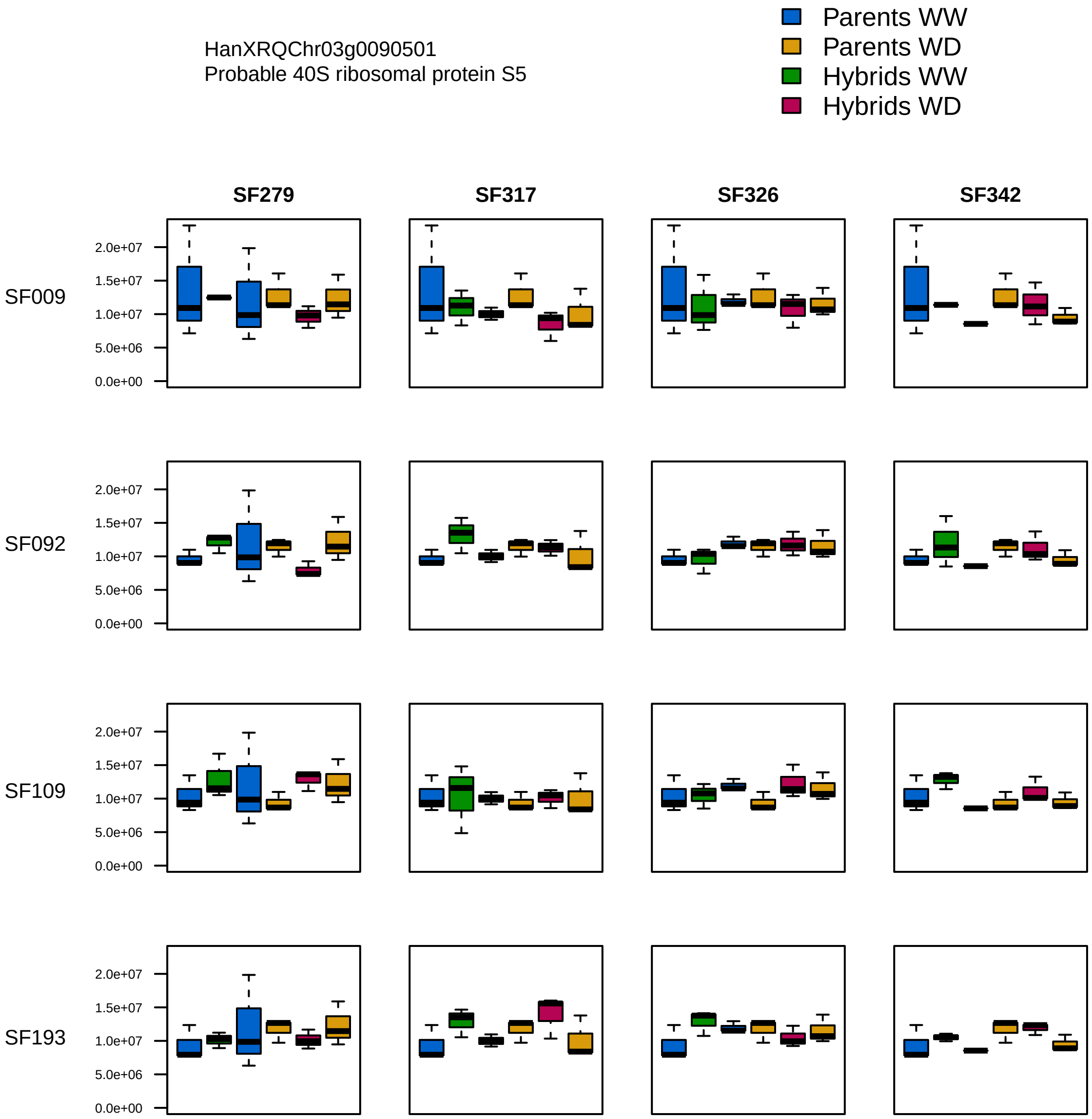
HanXRQChr03g0090501
Probable 40S ribosomal protein S5
Parents WW
Parents WD
Hybrids WW
Hybrids WD
SF279
SF317
SF326
SF342
SF009
SF092
SF109
SF193
2.0e+07
1.5e+07
1.0e+07
5.0e+06
0.0e+00

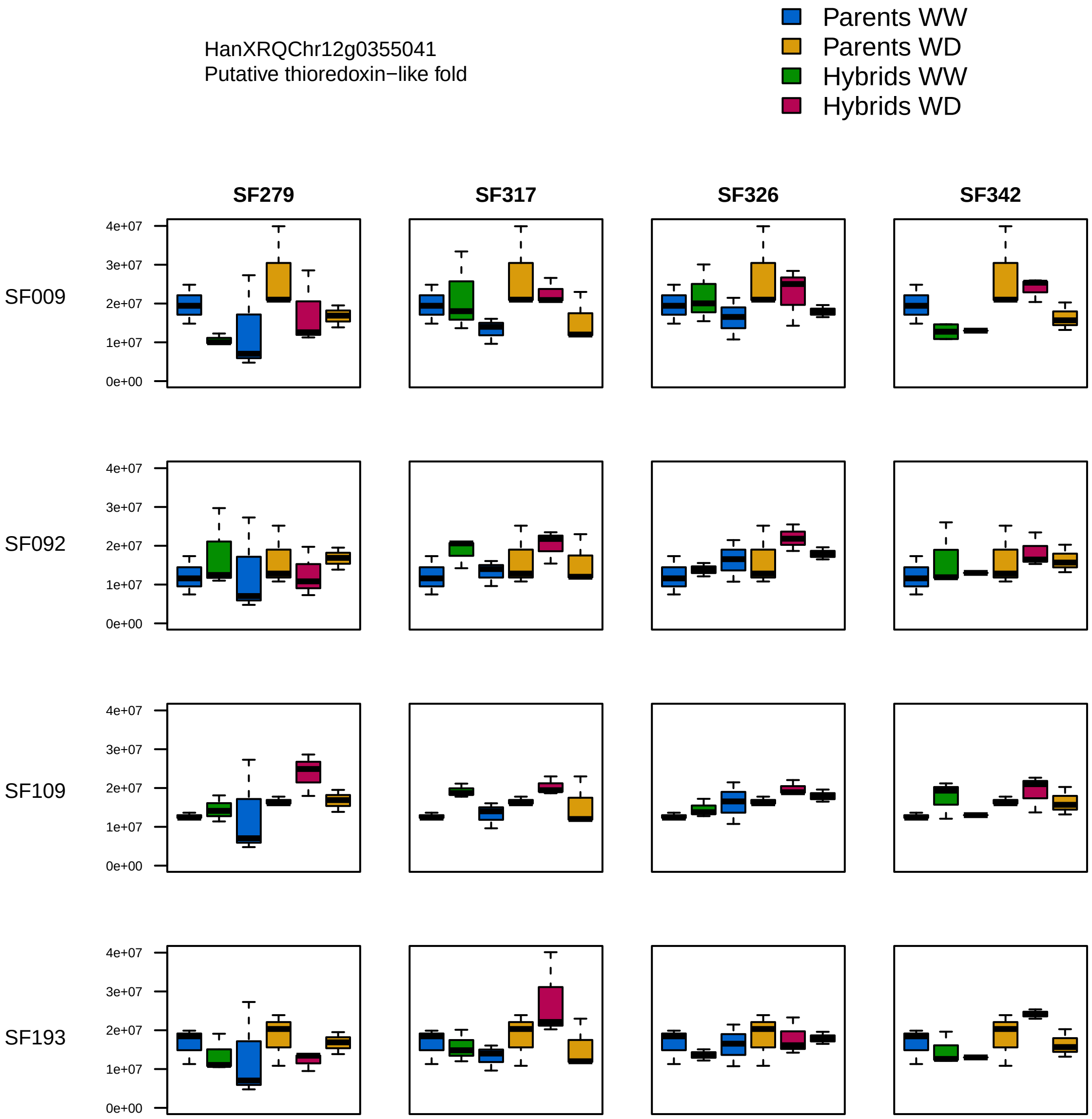
HanXRQChr12g0355041
Putative thioredoxin−like fold
Parents WW
Parents WD
Hybrids WW
Hybrids WD
SF279
SF317
SF326
SF342
SF009
SF092
SF109
SF193
4e+07
3e+07
2e+07
1e+07
0e+00

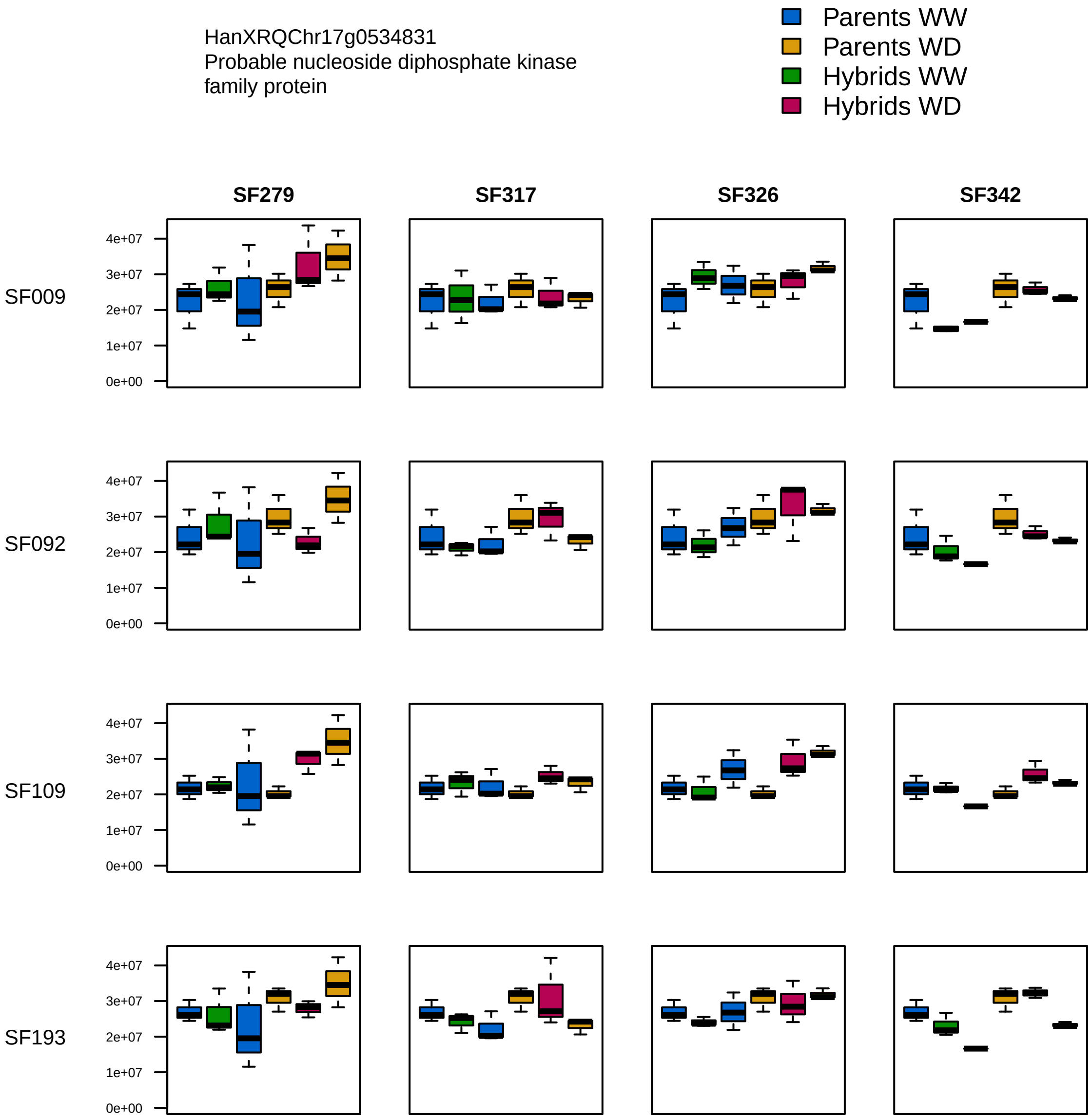
HanXRQChr17g0534831
Probable nucleoside diphosphate kinase
family protein
Parents WW
Parents WD
Hybrids WW
Hybrids WD
SF279
SF317
SF326
SF342
SF009
SF092
SF109
SF193
4e+07
3e+07
2e+07
1e+07
0e+00

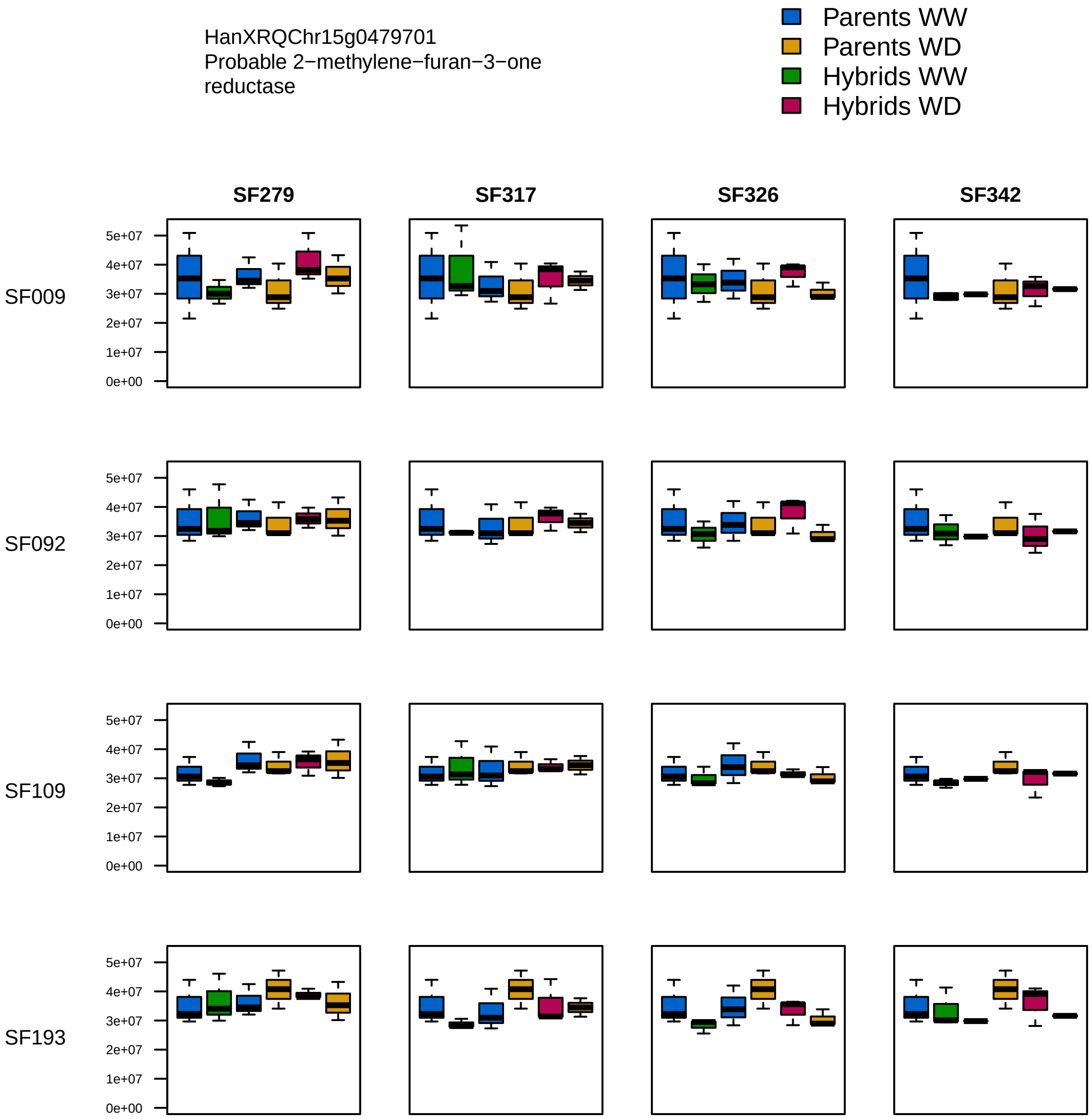
HanXRQChr15g0479701
Probable 2−methylene−furan−3−one
reductase
Parents WW
Parents WD
Hybrids WW
Hybrids WD
SF279
SF317
SF326
SF342
SF009
SF092
SF109
SF193
5e+07
4e+07
3e+07
2e+07
1e+07
0e+00

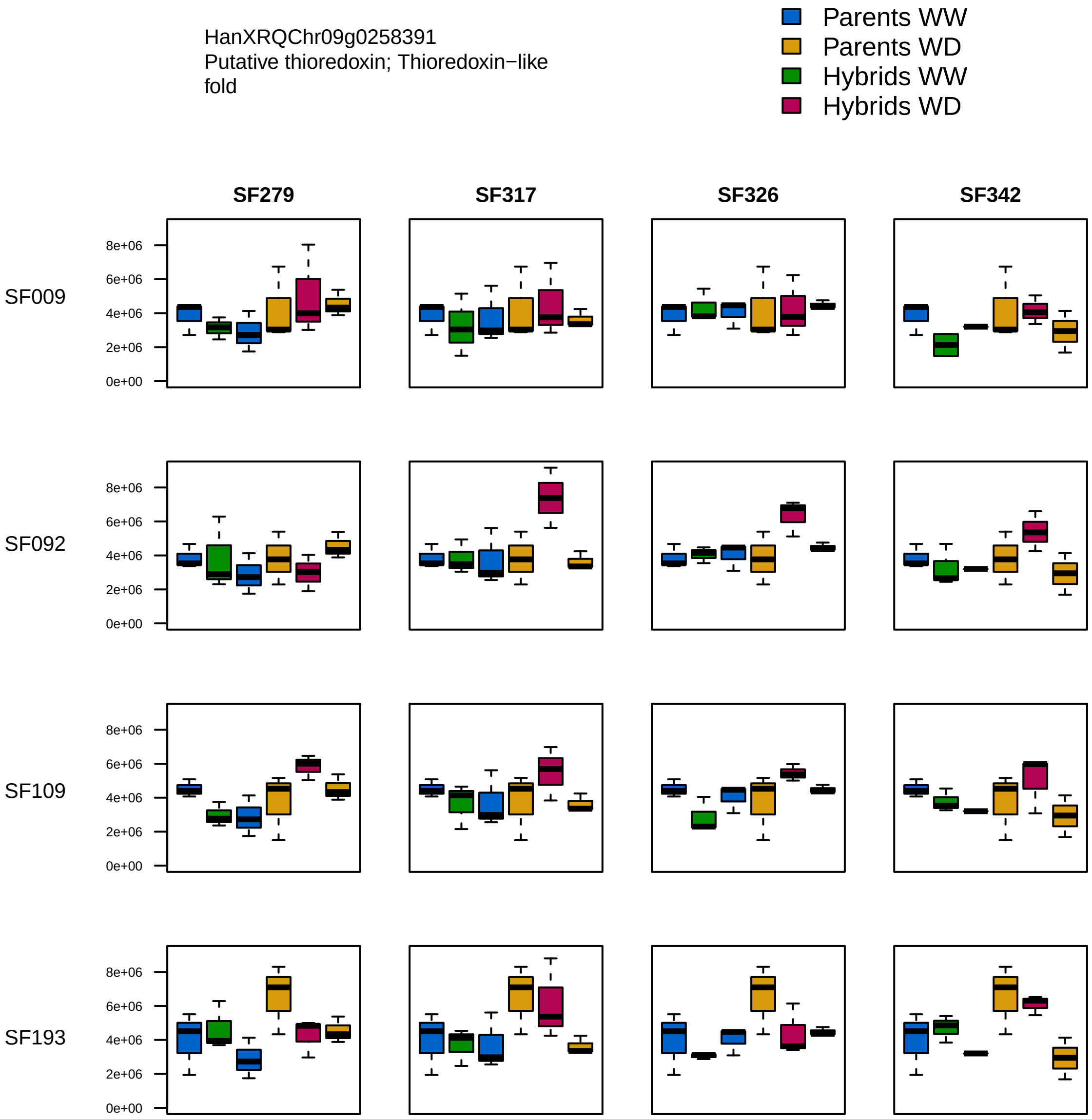

HanXRQChr09g0258391
Putative thioredoxin; Thioredoxin−like fold
Parents WW
Parents WD
Hybrids WW
Hybrids WD
SF279
SF317
SF326
SF342
SF009
SF092
SF109
SF193
8e+06
6e+06
4e+06
2e+06
0e+00

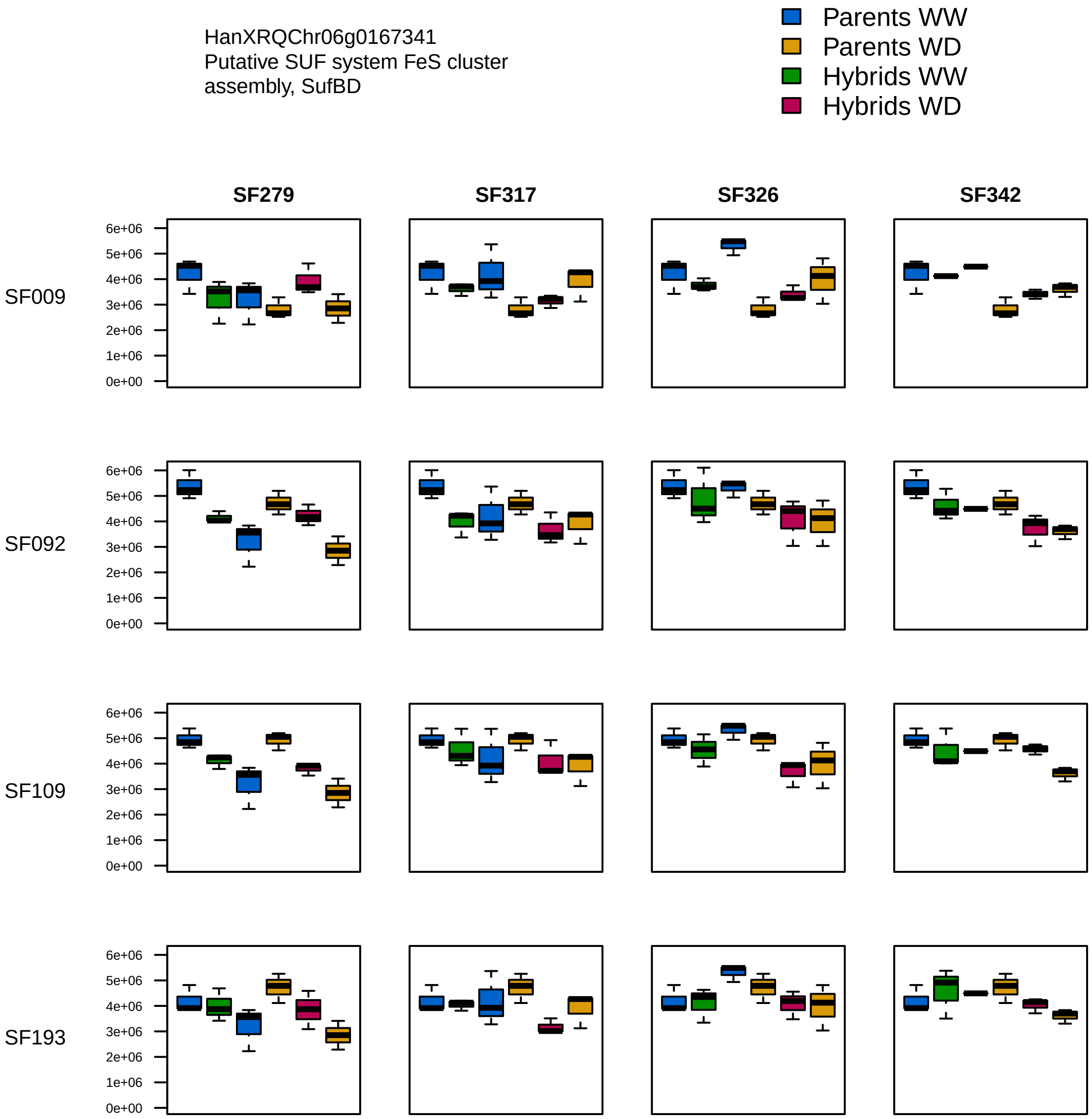
HanXRQChr06g0167341
Putative SUF system FeS cluster
assembly, SufBD
Parents WW
Parents WD
Hybrids WW
Hybrids WD
SF279
SF317
SF326
SF342
SF009
SF092
SF109
SF193
0e+00
1e+06
2e+06
3e+06
4e+06
5e+06
6e+06

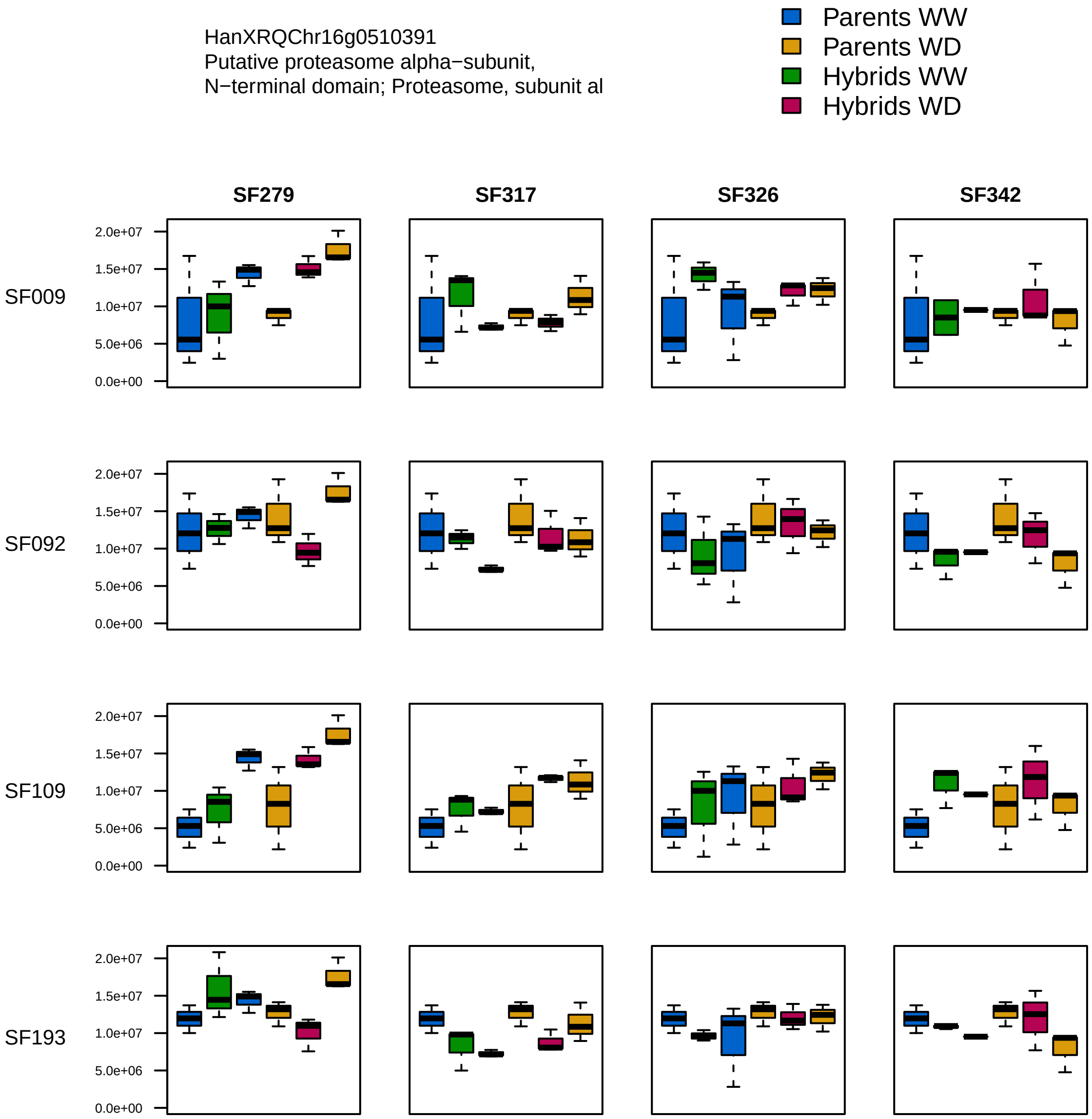

HanXRQChr16g0510391
Putative proteasome alpha−subunit,
N−terminal domain; Proteasome, subunit al
Parents WW
Parents WD
Hybrids WW
Hybrids WD
SF279
SF317
SF326
SF342
SF009
SF092
SF109
SF193
2.0e+07
1.5e+07
1.0e+07
5.0e+06
0.0e+00

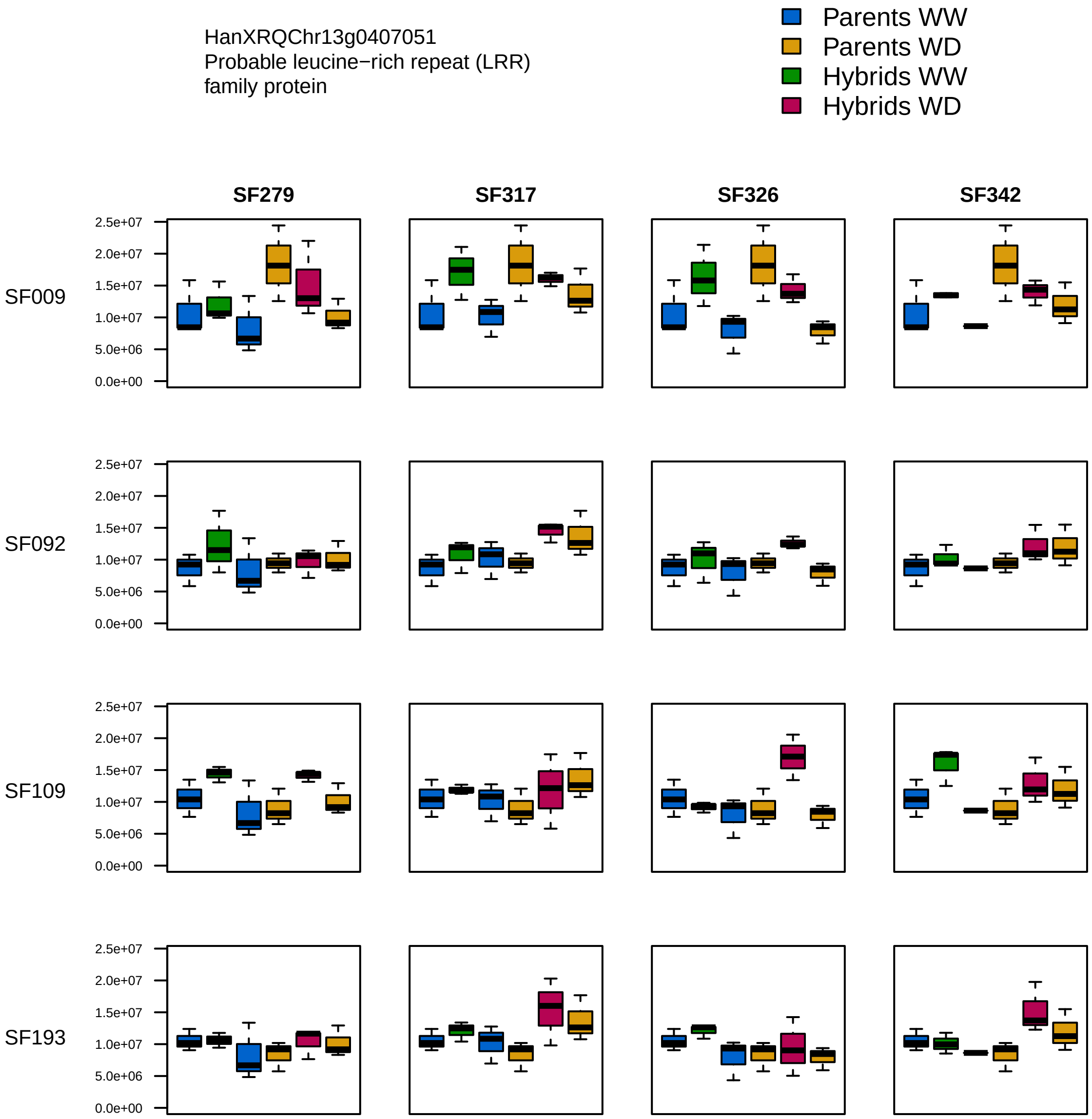
HanXRQChr13g0407051
Probable leucine−rich repeat (LRR)
family protein
Parents WW
Parents WD
Hybrids WW
Hybrids WD
SF279
SF317
SF326
SF342
SF009
SF092
SF109
SF193
2.5e+07
2.0e+07
1.5e+07
1.0e+07
5.0e+06
0.0e+00

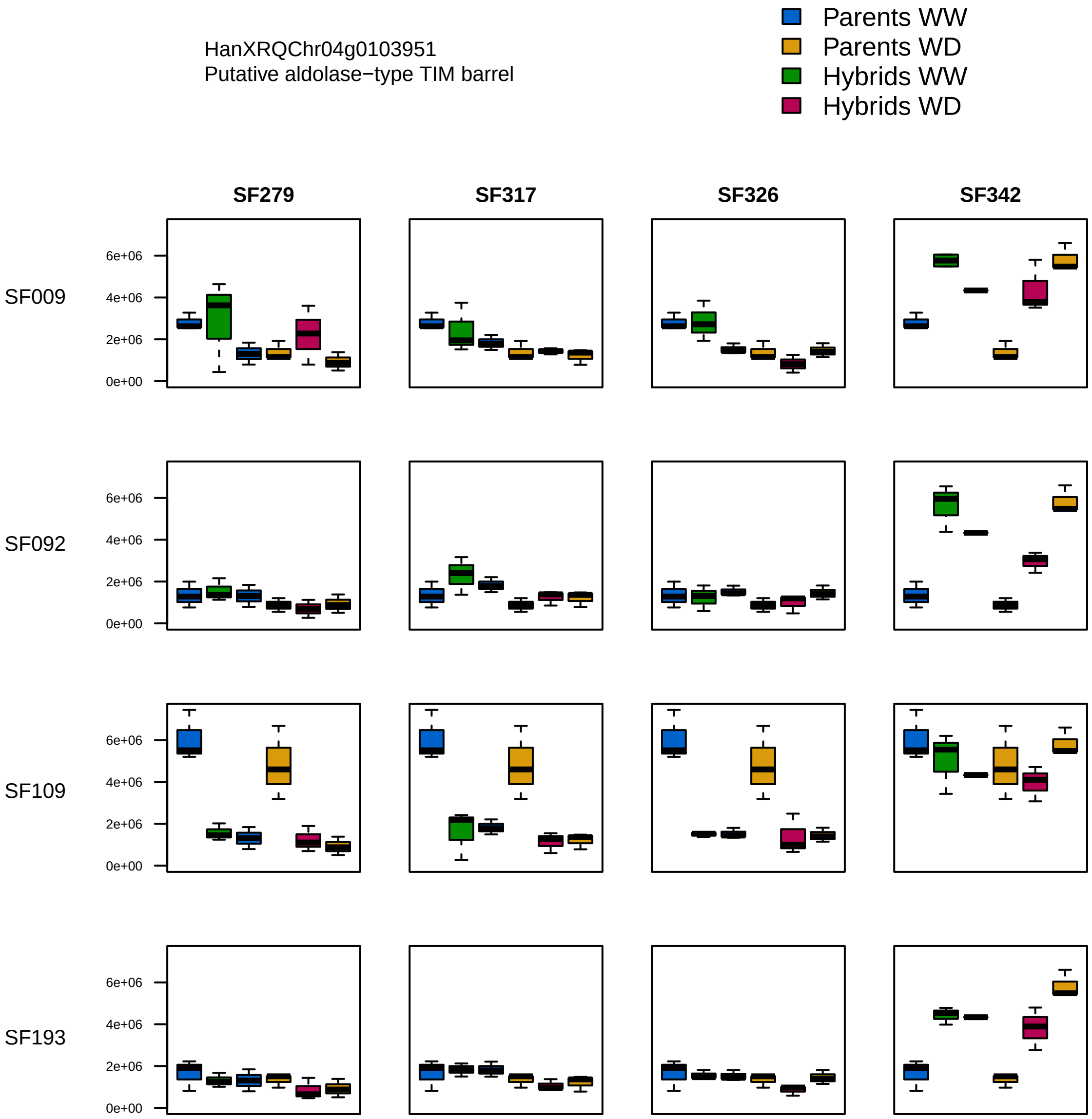

HanXRQChr04g0103951
Putative aldolase−type TIM barrel
Parents WW
Parents WD
Hybrids WW
Hybrids WD
SF279
SF317
SF326
SF342
SF009
SF092
SF109
SF193
6e+06
4e+06
2e+06
0e+00

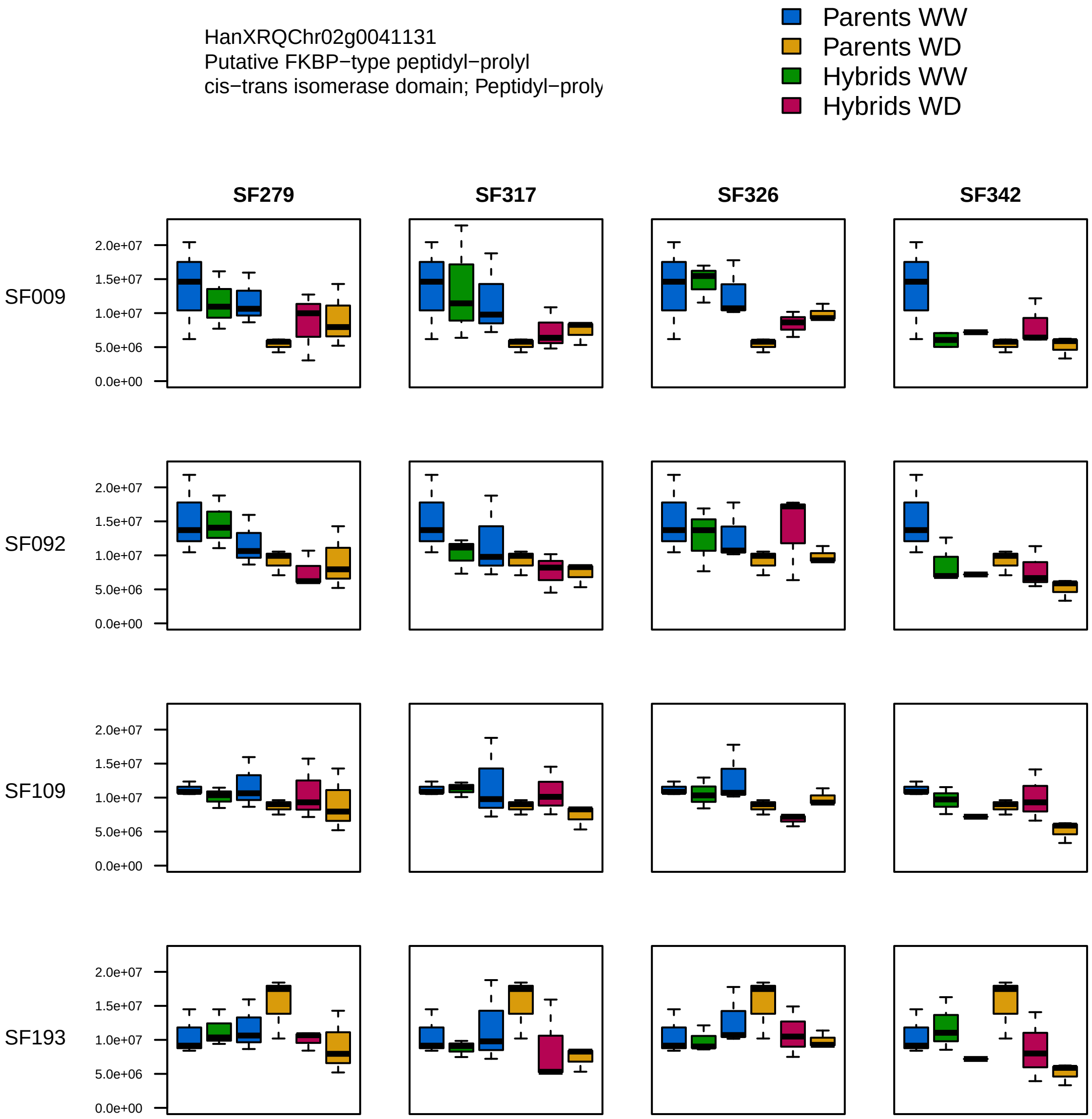
HanXRQChr02g0041131
Putative FKBP−type peptidyl−prolyl
cis−trans isomerase domain; Peptidyl−proly
Parents WW
Parents WD
Hybrids WW
Hybrids WD
SF279
SF317
SF326
SF342
SF009
SF092
SF109
SF193
2.0e+07
1.5e+07
1.0e+07
5.0e+06
0.0e+00

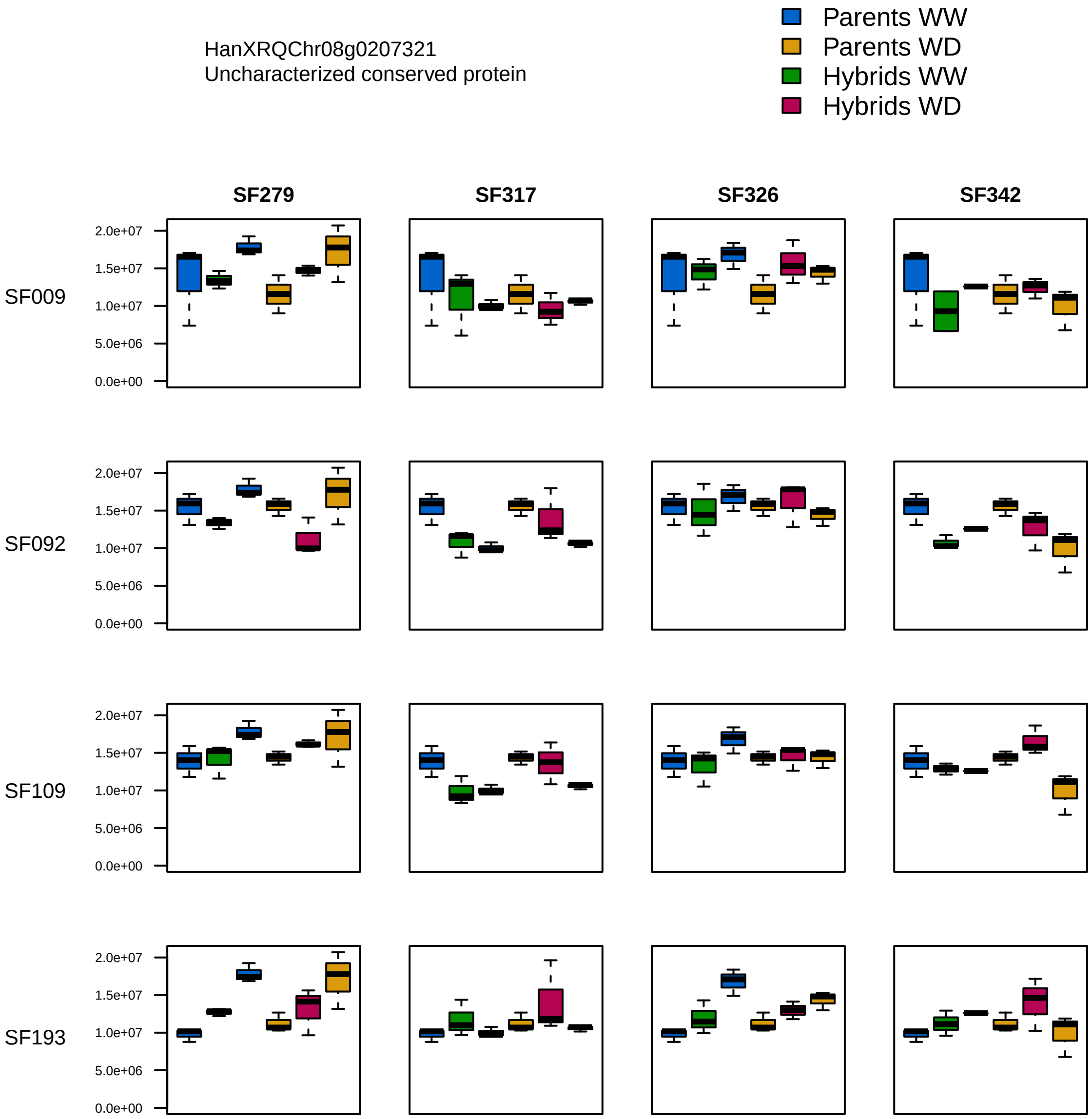
HanXRQChr08g0207321
Uncharacterized conserved protein
Parents WW
Parents WD
Hybrids WW
Hybrids WD
SF279
SF317
SF326
SF342
SF009
SF092
SF109
SF193
2.0e+07
1.5e+07
1.0e+07
5.0e+06
0.0e+00

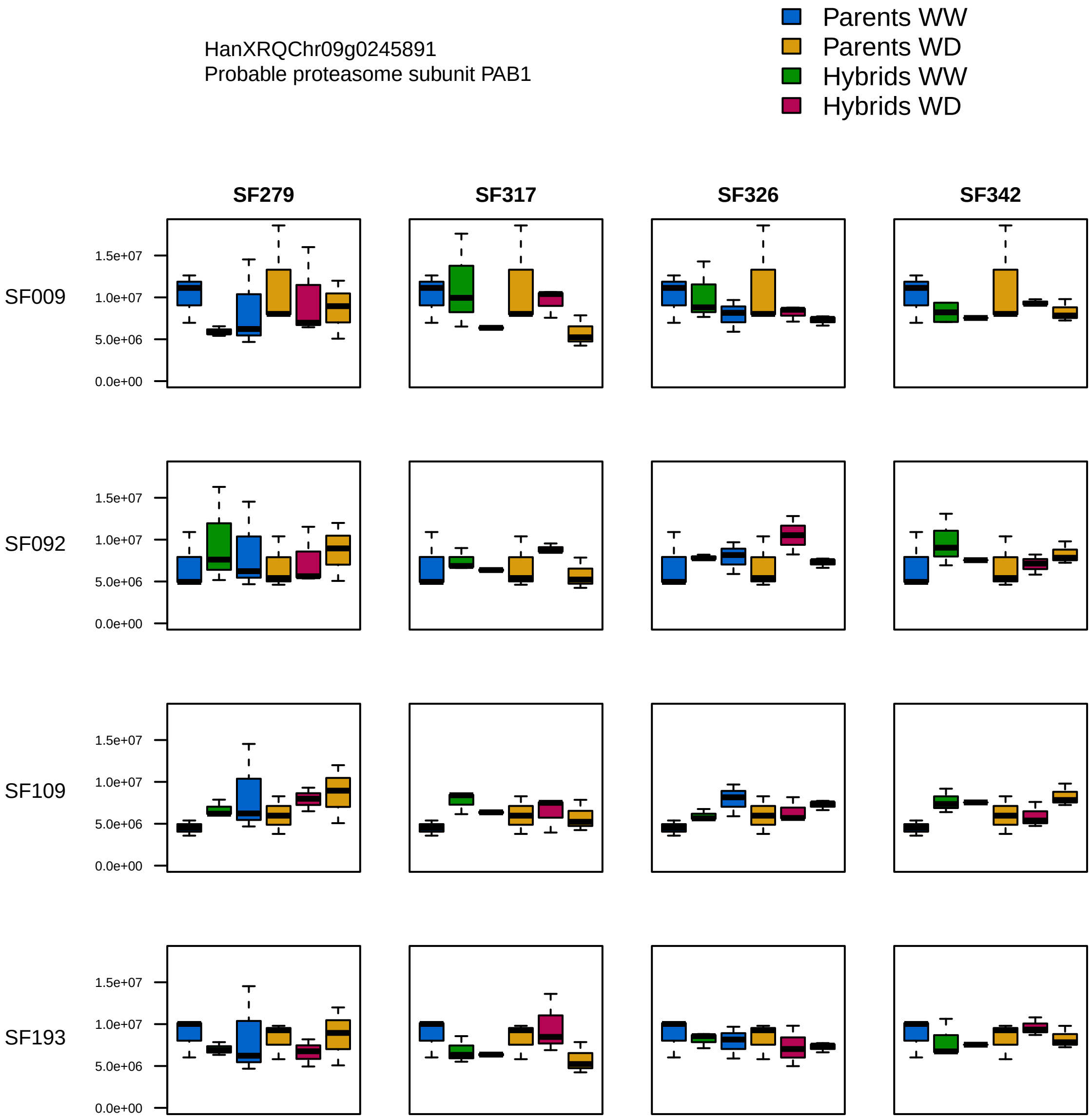
HanXRQChr09g0245891
Probable proteasome subunit PAB1
Parents WW
Parents WD
Hybrids WW
Hybrids WD
SF279
SF317
SF326
SF342
SF009
SF092
SF109
SF193
1.5e+07
1.0e+07
5.0e+06
0.0e+00

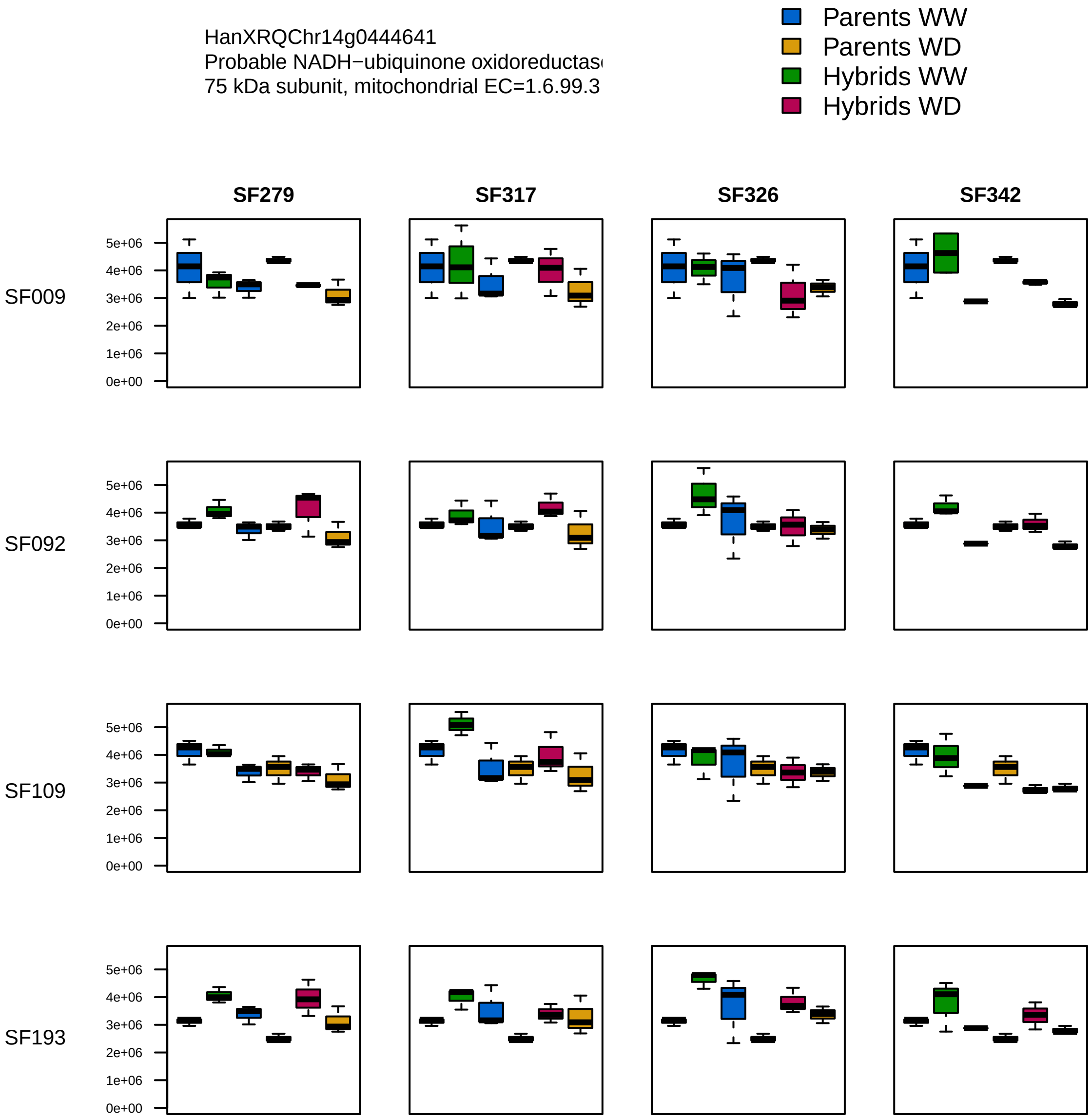

HanXRQChr14g0444641
Probable NADH−ubiquinone oxidoreductas
75 kDa subunit, mitochondrial EC=1.6.99.3
Parents WW
Parents WD
Hybrids WW
Hybrids WD
SF279
SF317
SF326
SF342
SF009
SF092
SF109
SF193
5e+06
4e+06
3e+06
2e+06
1e+06
0e+00

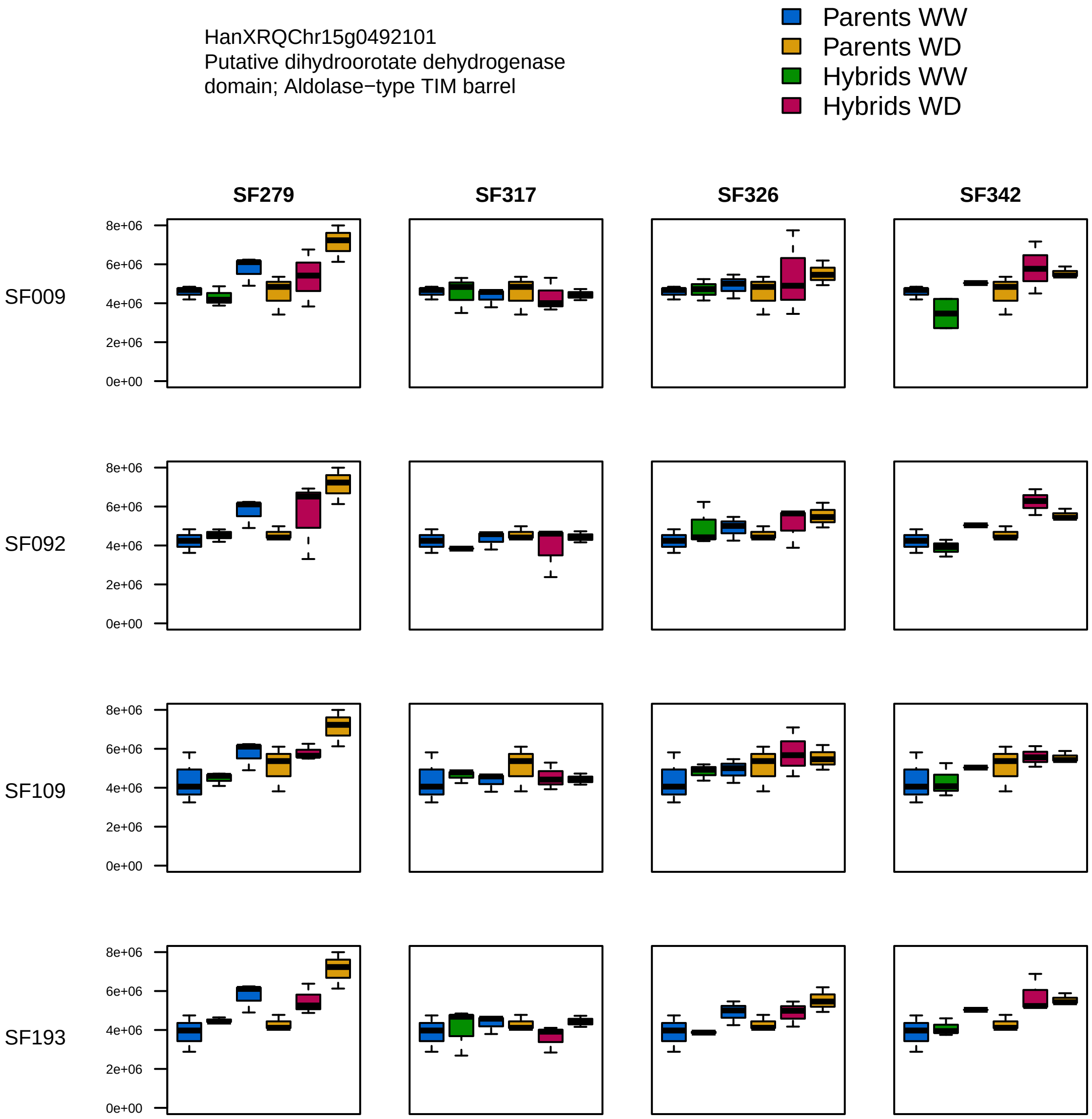
HanXRQChr15g0492101
Putative dihydroorotate dehydrogenase domain; Aldolase−type TIM barrel
Parents WW
Parents WD
Hybrids WW
Hybrids WD
SF279
SF317
SF326
SF342
SF009
SF092
SF109
SF193
8e+06
6e+06
4e+06
2e+06
0e+00

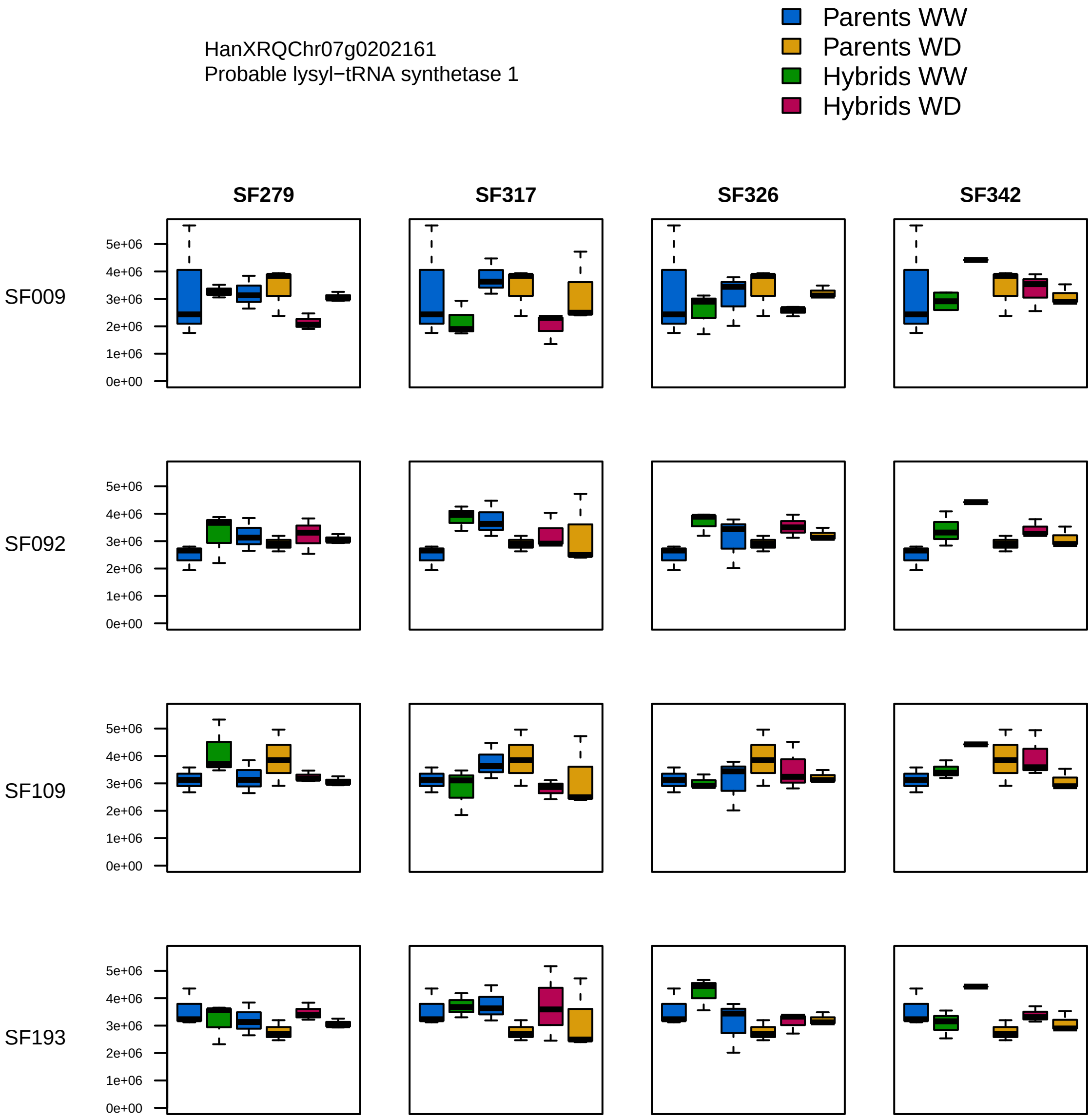

HanXRQChr07g0202161
Probable lysyl−tRNA synthetase 1
Parents WW
Parents WD
Hybrids WW
Hybrids WD
SF279
SF317
SF326
SF342
SF009
SF092
SF109
SF193
0e+00
1e+06
2e+06
3e+06
4e+06
5e+06

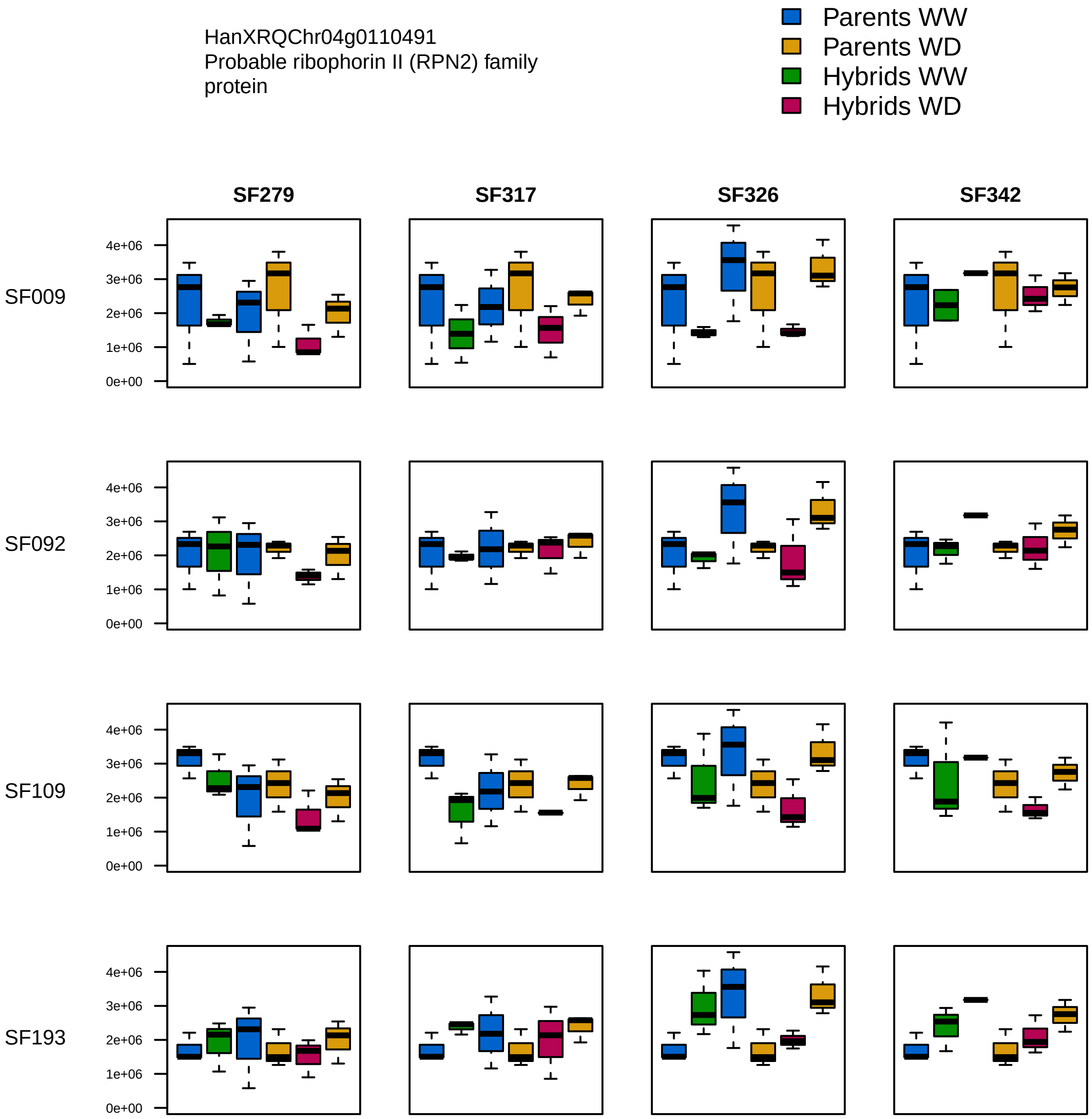
HanXRQChr04g0110491
Probable ribophorin II (RPN2) family protein
Parents WW
Parents WD
Hybrids WW
Hybrids WD
SF279
SF317
SF326
SF342
SF009
SF092
SF109
SF193
4e+06
3e+06
2e+06
1e+06
0e+00

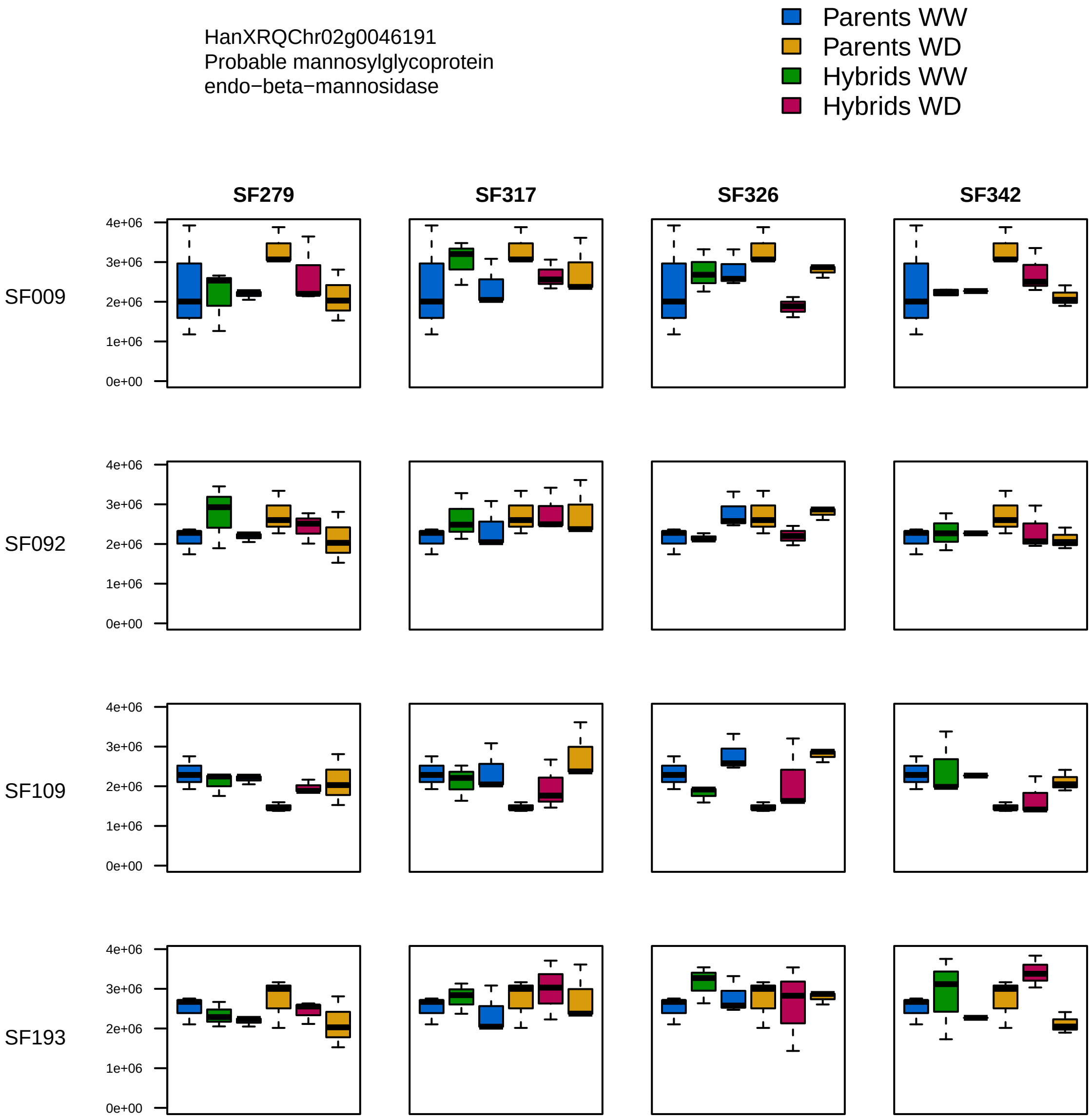

HanXRQChr02g0046191
Probable mannosylglycoprotein
endo−beta−mannosidase
Parents WW
Parents WD
Hybrids WW
Hybrids WD
SF279
SF317
SF326
SF342
SF009
SF092
SF109
SF193
4e+06
3e+06
2e+06
1e+06
0e+00

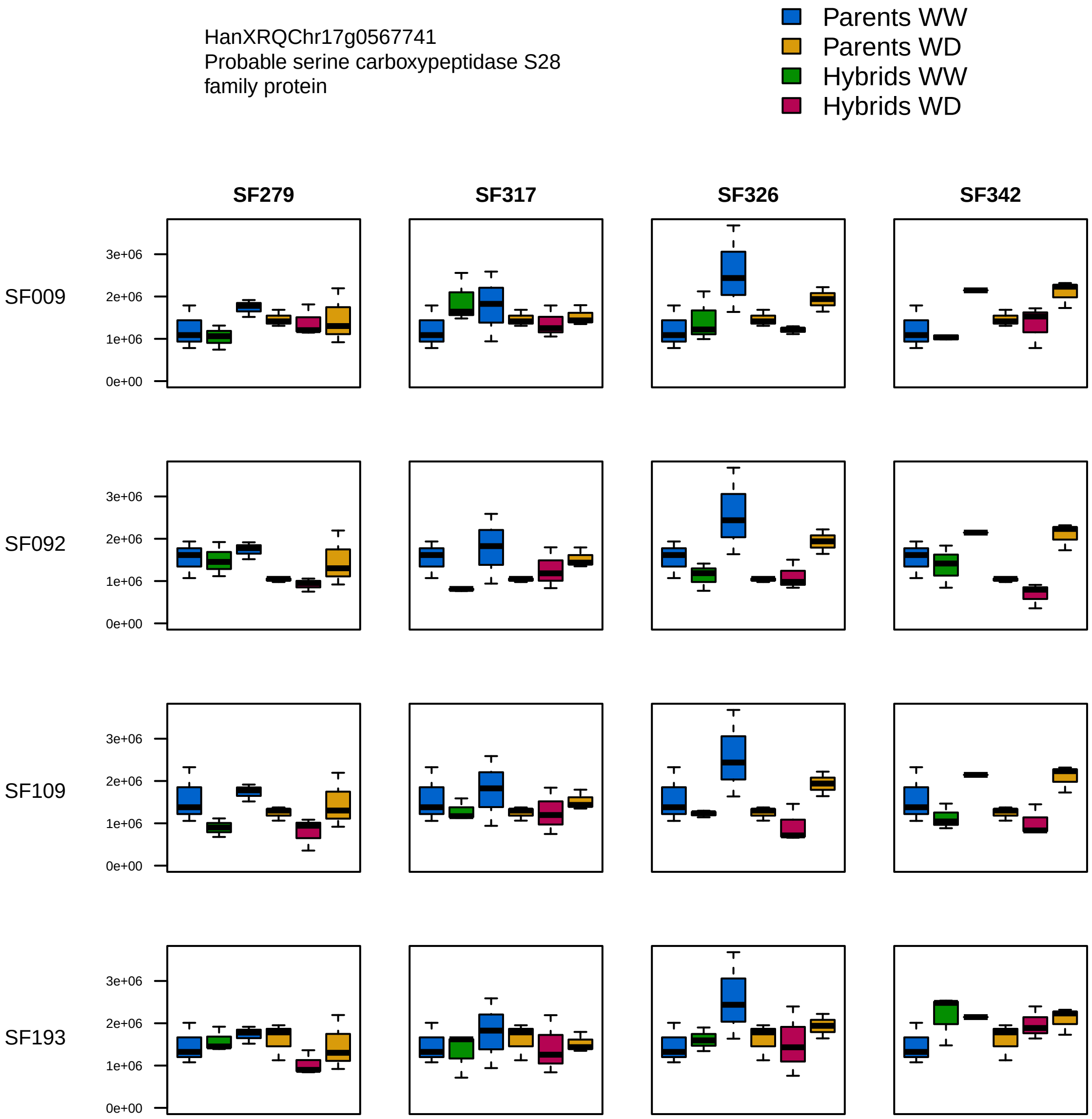
HanXRQChr17g0567741
Probable serine carboxypeptidase S28
family protein
Parents WW
Parents WD
Hybrids WW
Hybrids WD
SF279
SF317
SF326
SF342
SF009
SF092
SF109
SF193
3e+06
2e+06
1e+06
0e+00

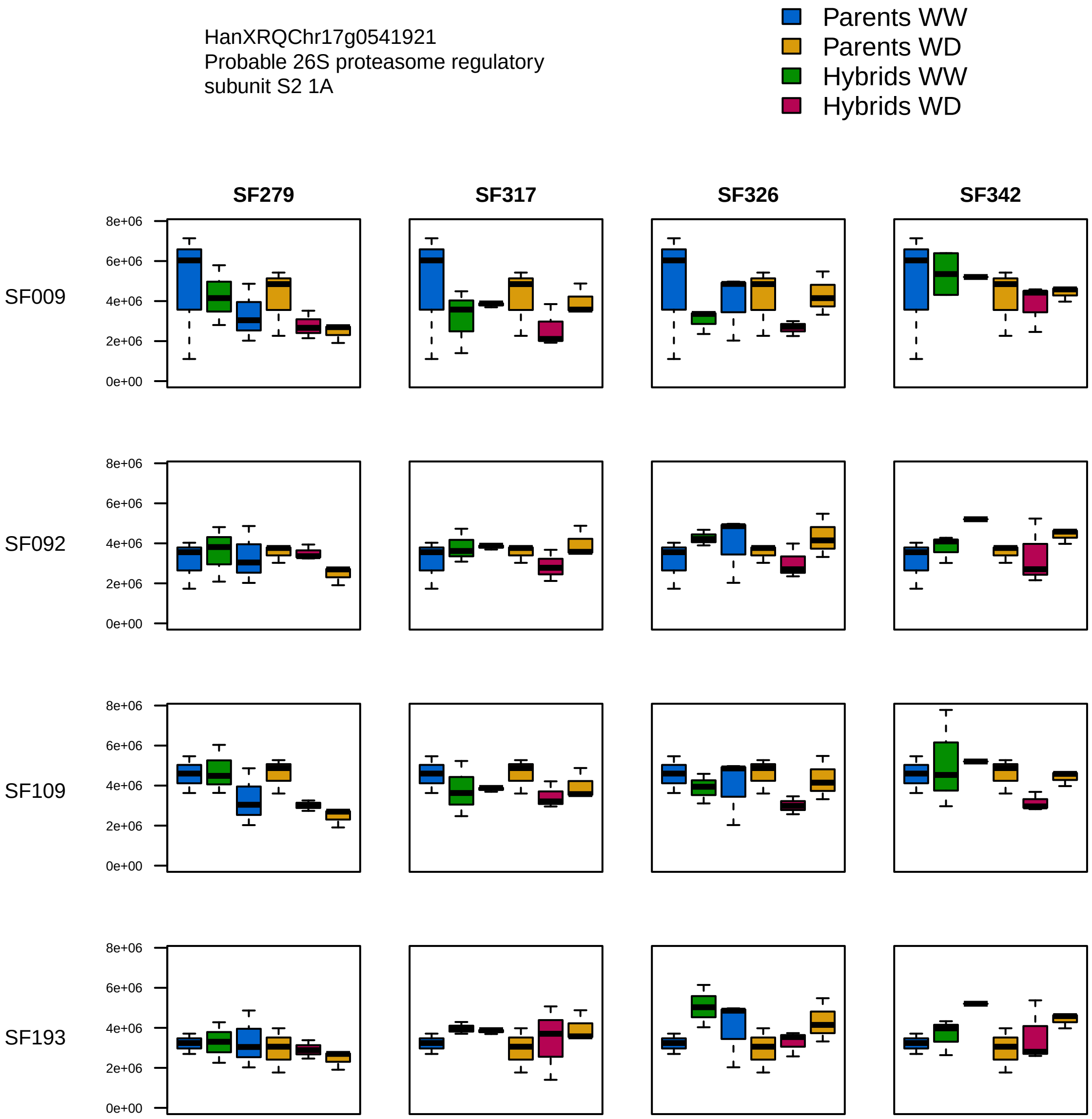

HanXRQChr17g0541921
Probable 26S proteasome regulatory
subunit S2 1A
Parents WW
Parents WD
Hybrids WW
Hybrids WD
SF279
SF317
SF326
SF342
SF009
SF092
SF109
SF193
8e+06
6e+06
4e+06
2e+06
0e+00

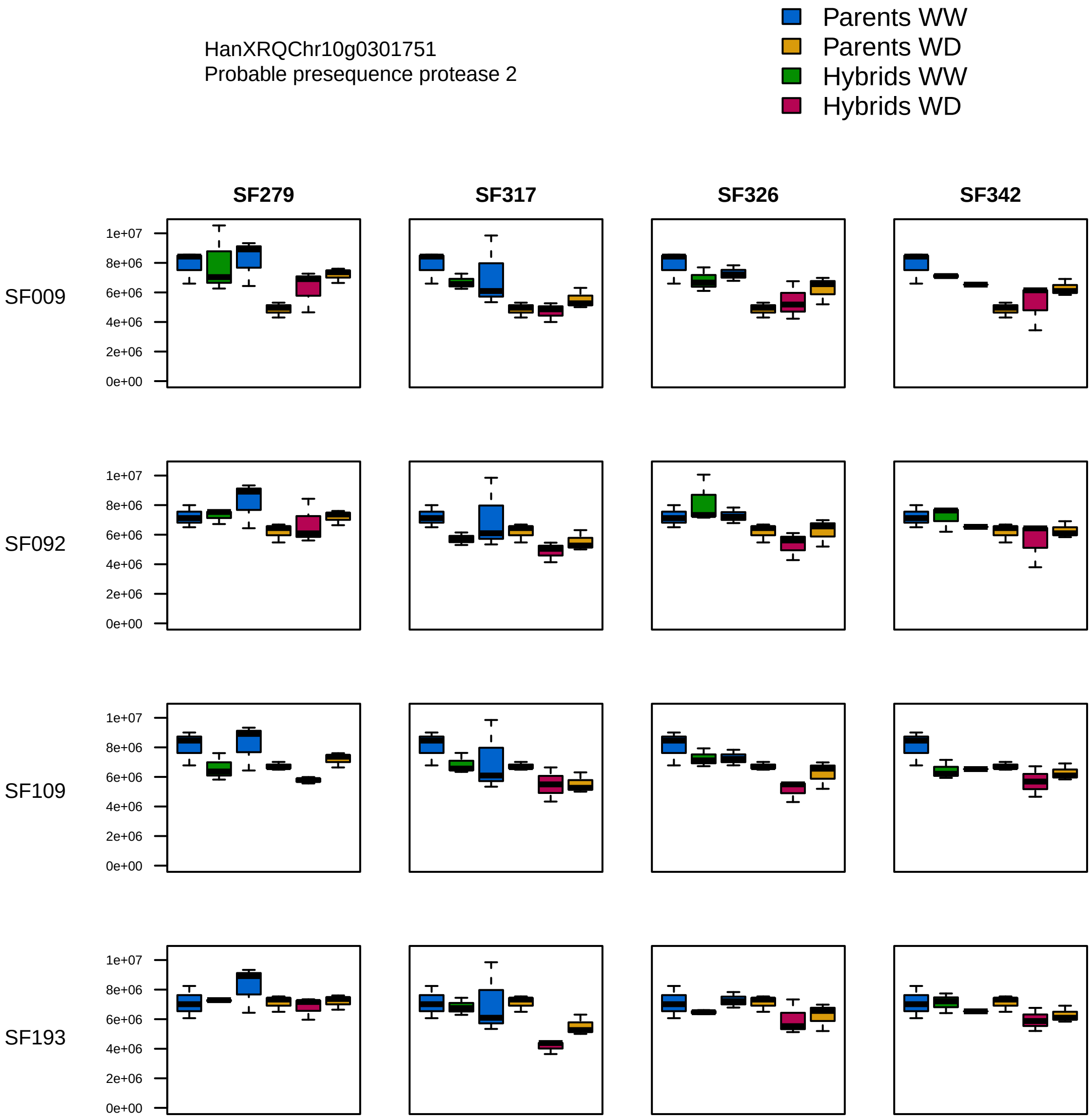
HanXRQChr10g0301751
Probable presequence protease 2
Parents WW
Parents WD
Hybrids WW
Hybrids WD
SF279
SF317
SF326
SF342
SF009
SF092
SF109
SF193
1e+07
8e+06
6e+06
4e+06
2e+06
0e+00

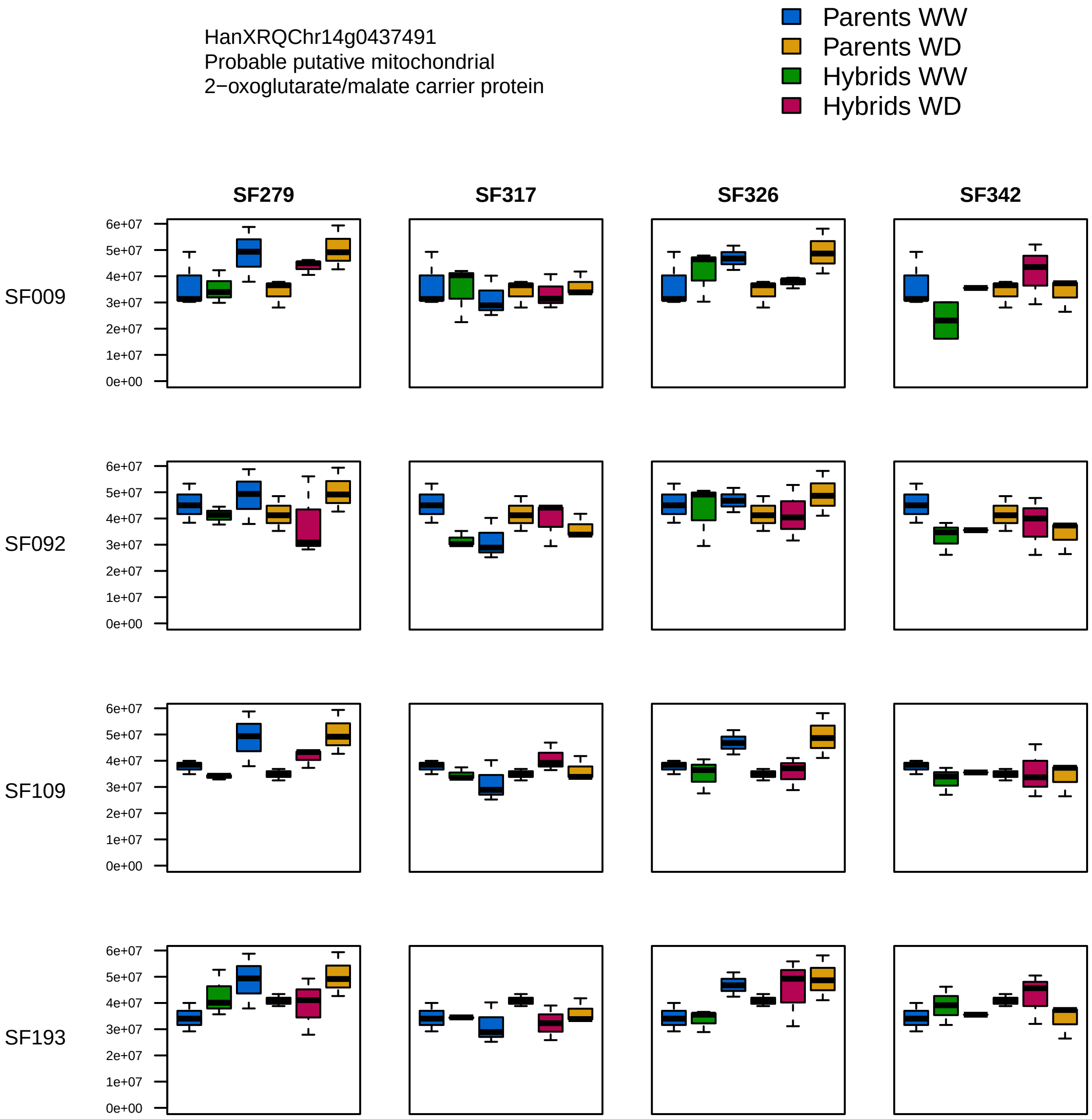
HanXRQChr14g0437491
Probable putative mitochondrial
2−oxoglutarate/malate carrier protein
Parents WW
Parents WD
Hybrids WW
Hybrids WD
SF279
SF317
SF326
SF342
SF009
SF092
SF109
SF193
6e+07
5e+07
4e+07
3e+07
2e+07
1e+07
0e+00

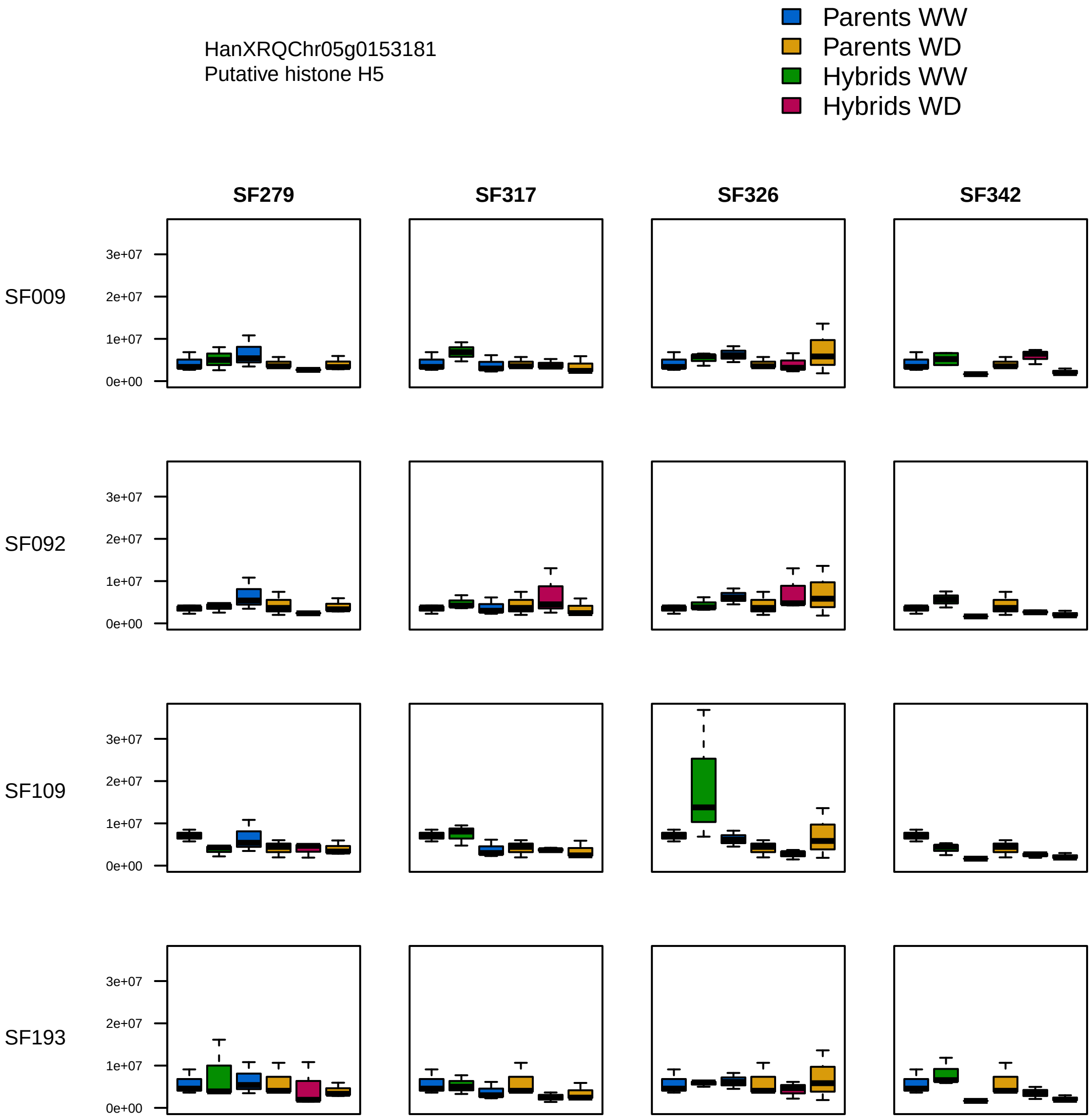
HanXRQChr05g0153181
Putative histone H5
Parents WW
Parents WD
Hybrids WW
Hybrids WD
SF279
SF317
SF326
SF342
SF009
SF092
SF109
SF193
0e+00
1e+07
2e+07
3e+07

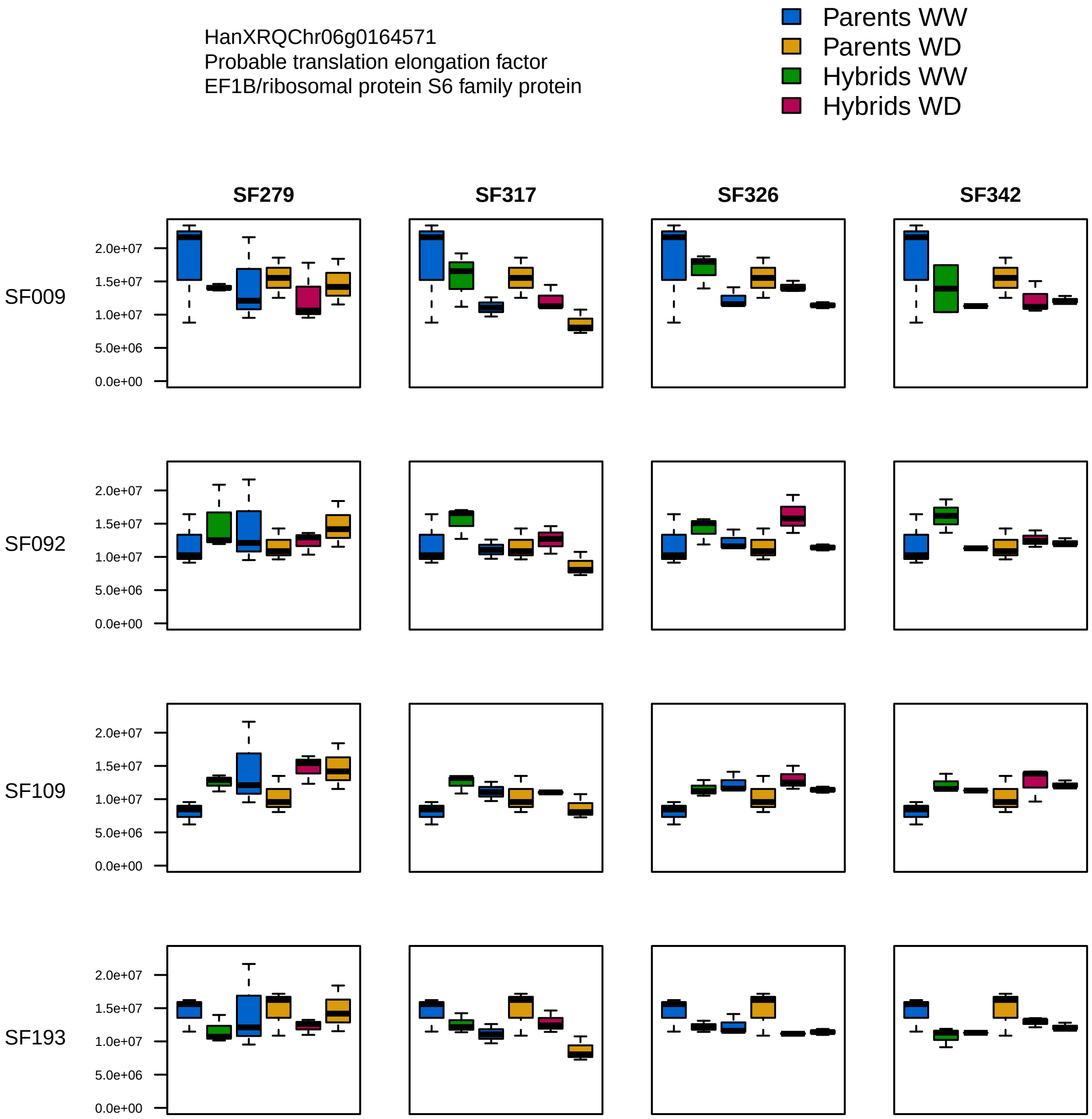
HanXRQChr06g0164571
Probable translation elongation factor
EF1B/ribosomal protein S6 family protein
Parents WW
Parents WD
Hybrids WW
Hybrids WD
SF279
SF317
SF326
SF342
SF009
SF092
SF109
SF193
2.0e+07
1.5e+07
1.0e+07
5.0e+06
0.0e+00

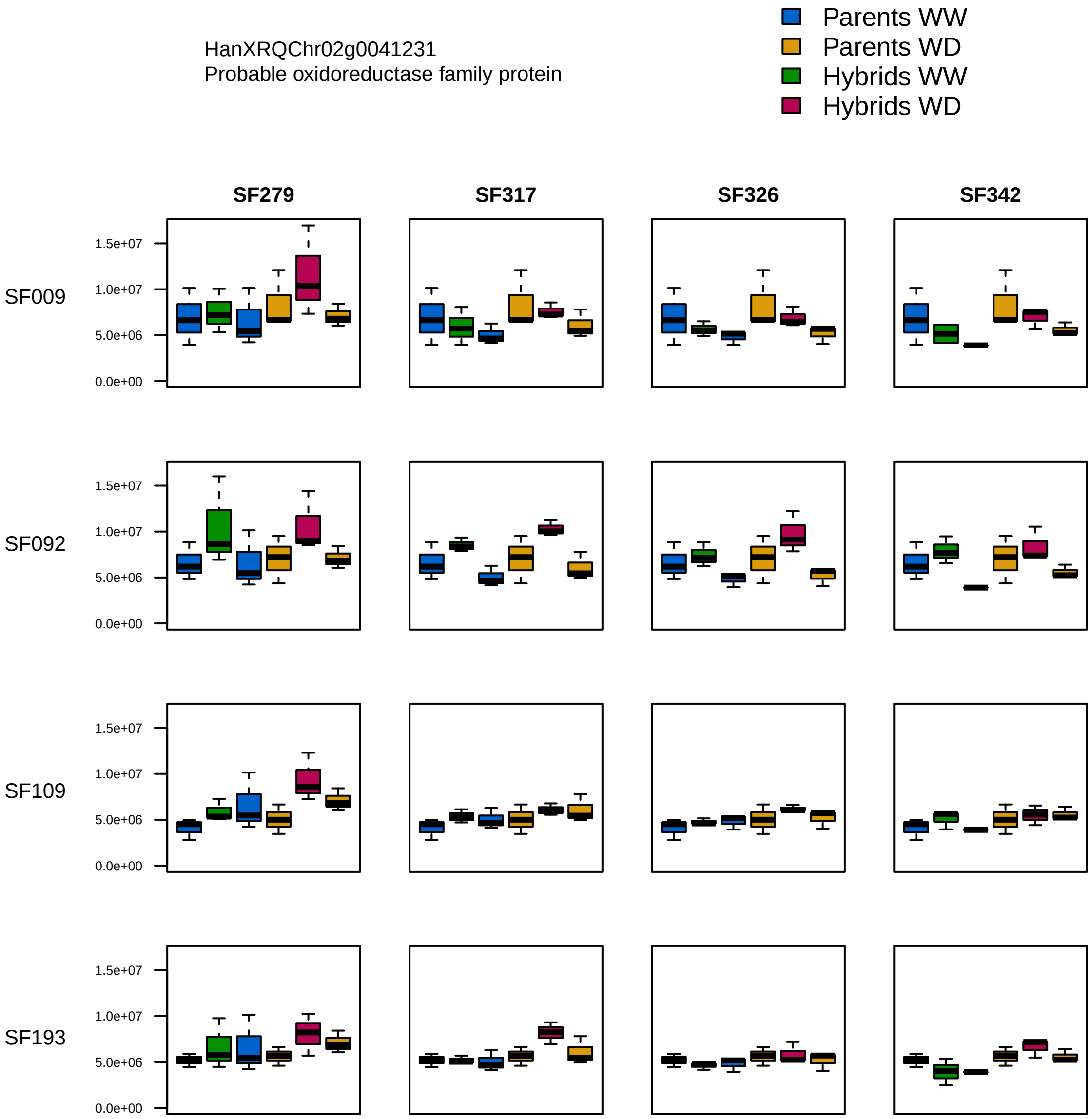
HanXRQChr02g0041231
Probable oxidoreductase family protein
Parents WW
Parents WD
Hybrids WW
Hybrids WD
SF279
SF317
SF326
SF342
SF009
SF092
SF109
SF193
1.5e+07
1.0e+07
5.0e+06
0.0e+00

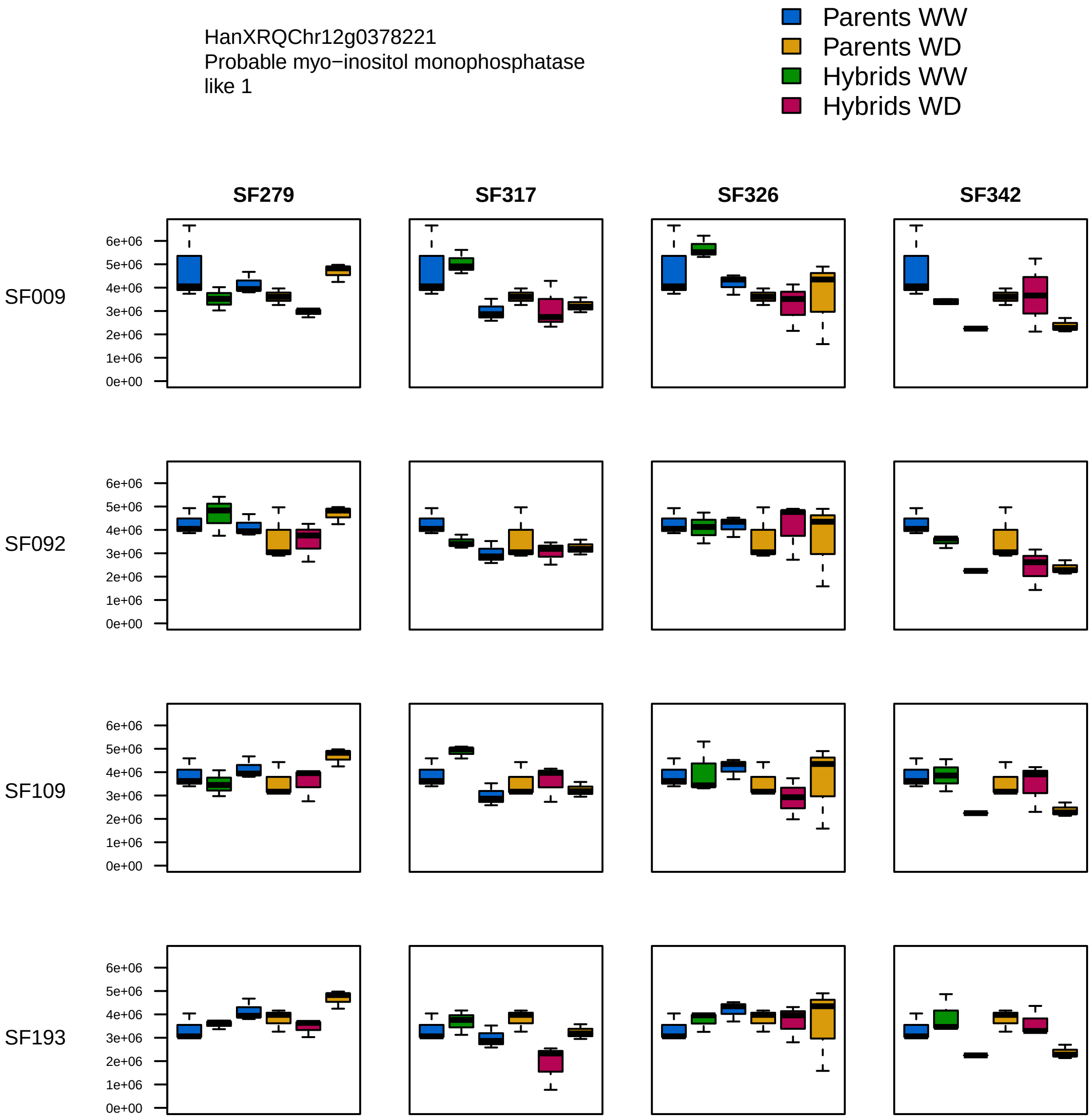

HanXRQChr12g0378221
Probable myo−inositol monophosphatase like 1
Parents WW
Parents WD
Hybrids WW
Hybrids WD
SF279
SF317
SF326
SF342
SF009
SF092
SF109
SF193
6e+06
5e+06
4e+06
3e+06
2e+06
1e+06
0e+00

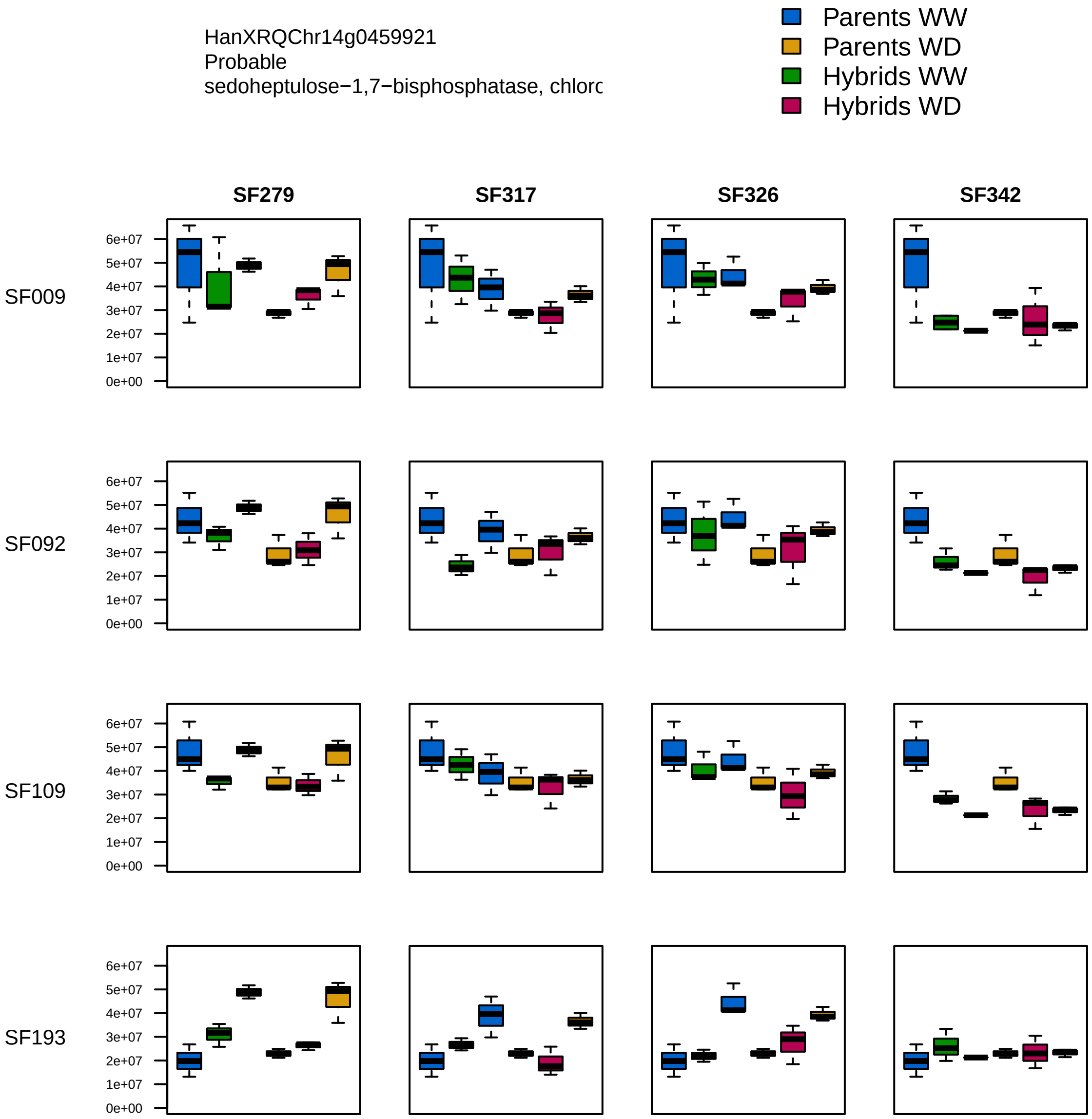

HanXRQChr14g0459921
Probable
sedoheptulose−1,7−bisphosphatase, chlorc
Parents WW
Parents WD
Hybrids WW
Hybrids WD
SF279
SF317
SF326
SF342
SF009
SF092
SF109
SF193
6e+07
5e+07
4e+07
3e+07
2e+07
1e+07
0e+00

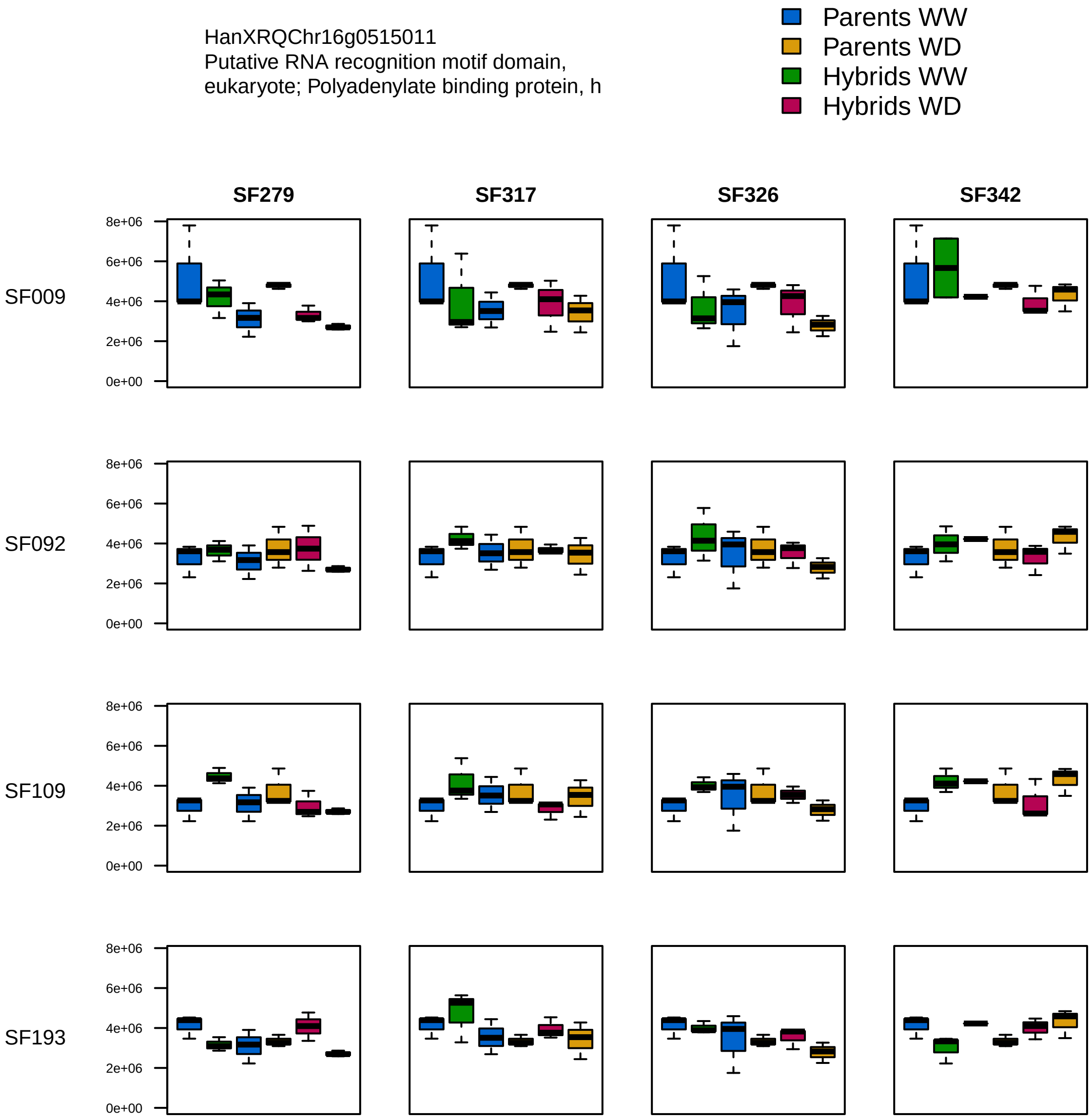

HanXRQChr16g0515011
Putative RNA recognition motif domain,
eukaryote; Polyadenylate binding protein, h
Parents WW
Parents WD
Hybrids WW
Hybrids WD
SF279
SF317
SF326
SF342
SF009
SF092
SF109
SF193
0e+00
2e+06
4e+06
6e+06
8e+06

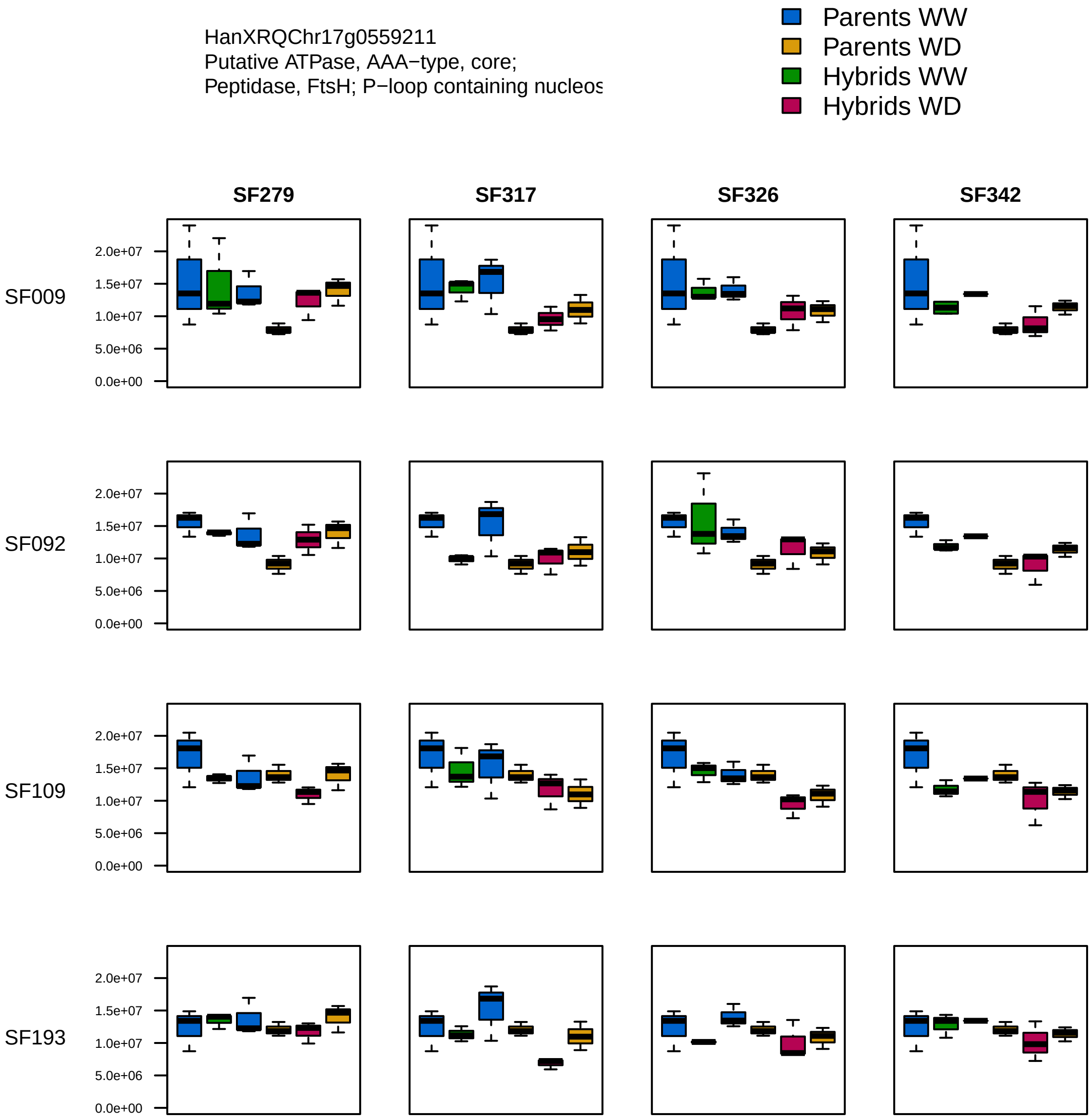
HanXRQChr17g0559211
Putative ATPase, AAA−type, core;
Peptidase, FtsH; P−loop containing nucleos
Parents WW
Parents WD
Hybrids WW
Hybrids WD
SF279
SF317
SF326
SF342
SF009
SF092
SF109
SF193
0.0e+00
5.0e+06
1.0e+07
1.5e+07
2.0e+07

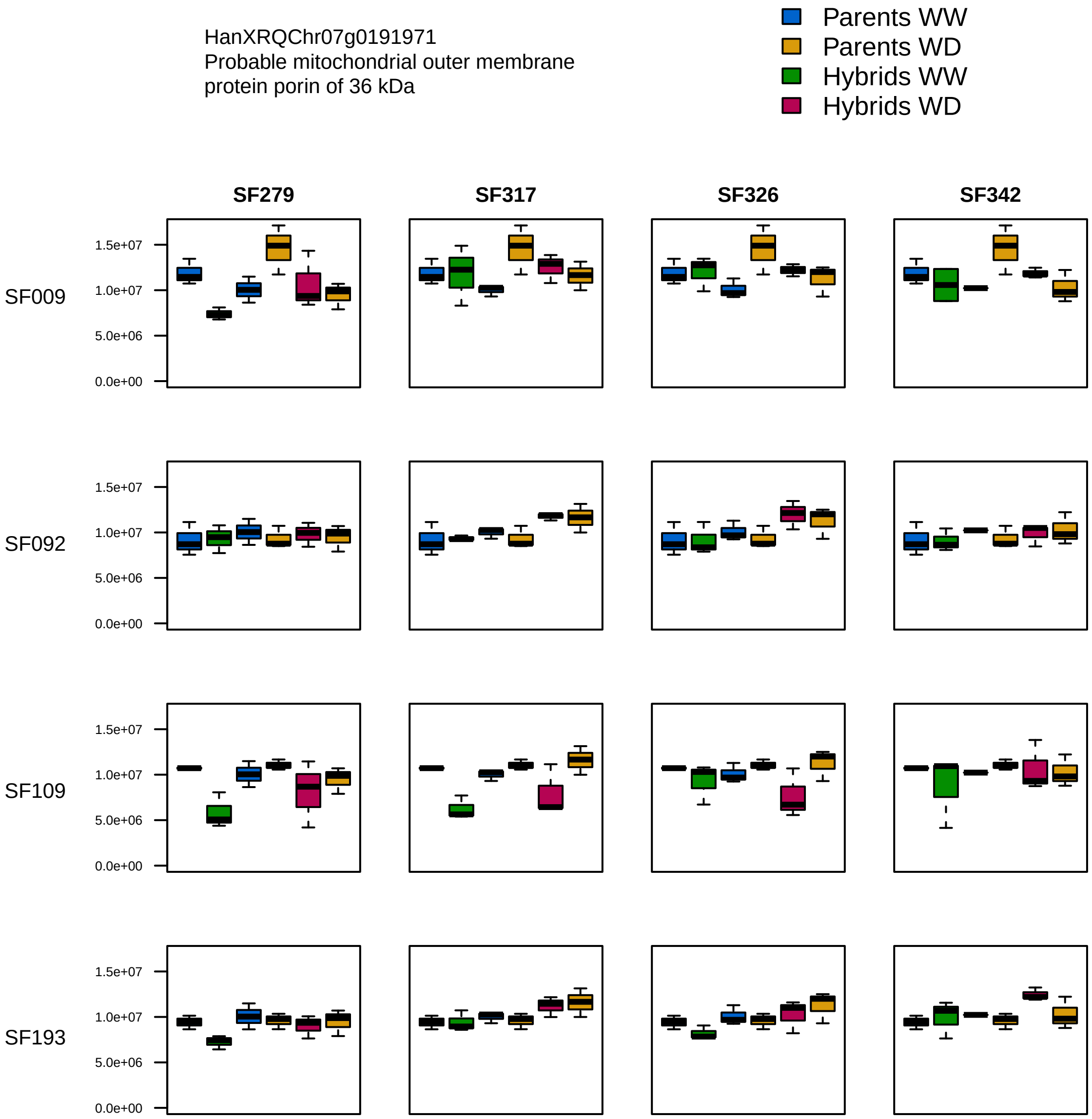

HanXRQChr07g0191971
Probable mitochondrial outer membrane
protein porin of 36 kDa
Parents WW
Parents WD
Hybrids WW
Hybrids WD
SF279
SF317
SF326
SF342
SF009
SF092
SF109
SF193
1.5e+07
1.0e+07
5.0e+06
0.0e+00

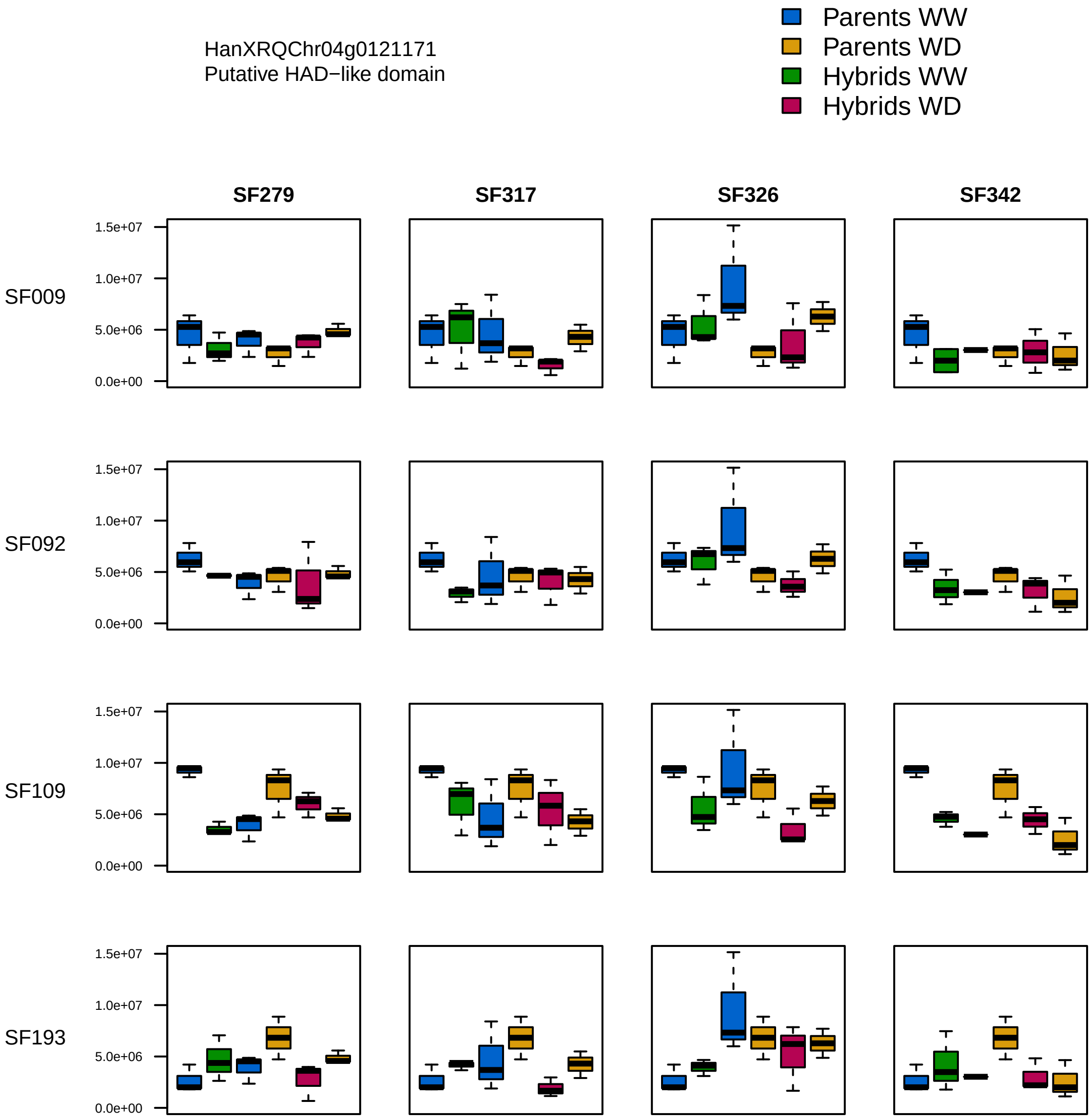

HanXRQChr04g0121171
Putative HAD−like domain
Parents WW
Parents WD
Hybrids WW
Hybrids WD
SF279
SF317
SF326
SF342
SF009
SF092
SF109
SF193
1.5e+07
1.0e+07
5.0e+06
0.0e+00

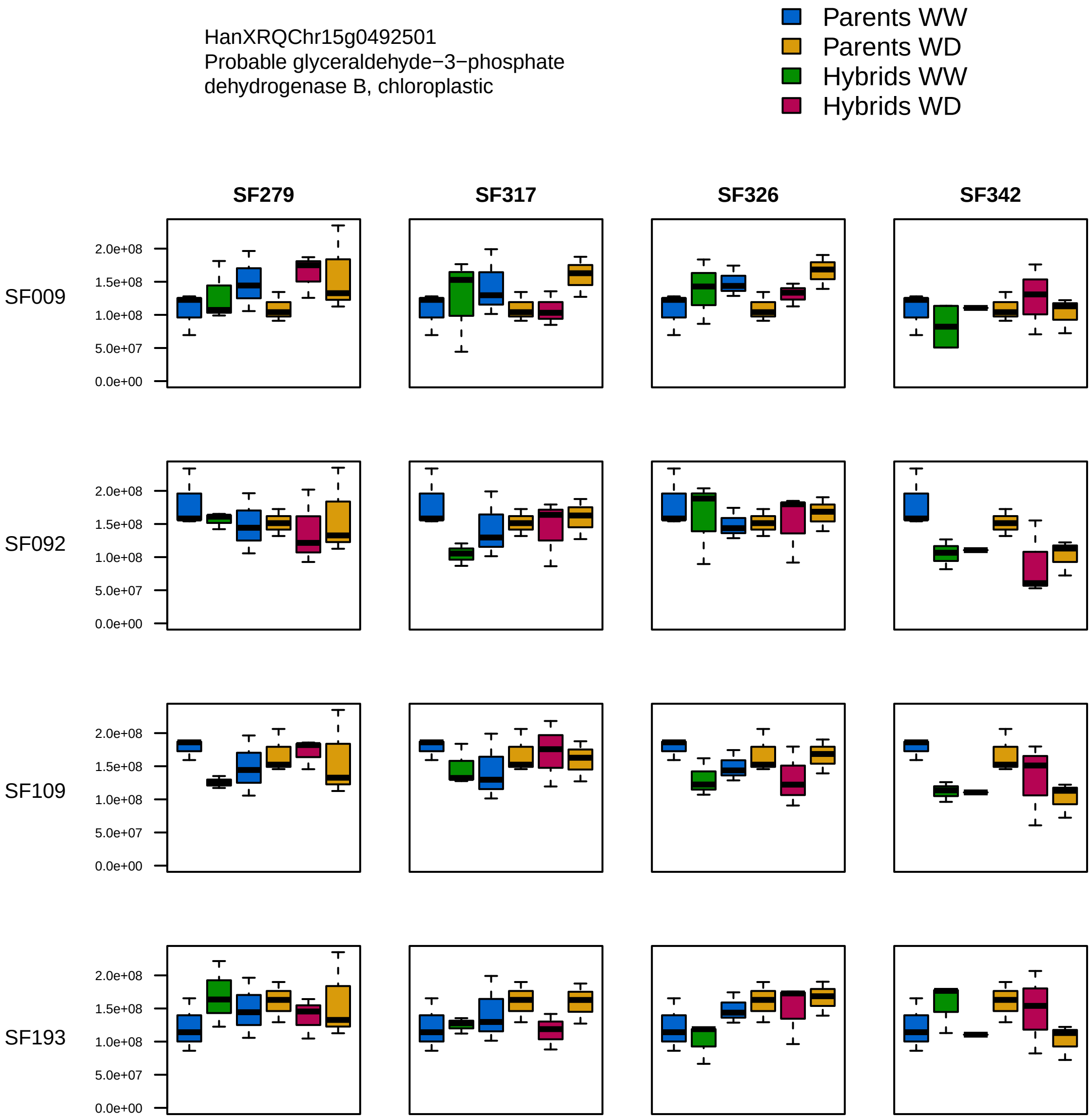
HanXRQChr15g0492501
Probable glyceraldehyde−3−phosphate
dehydrogenase B, chloroplastic
Parents WW
Parents WD
Hybrids WW
Hybrids WD
SF279
SF317
SF326
SF342
SF009
SF092
SF109
SF193
2.0e+08
1.5e+08
1.0e+08
5.0e+07
0.0e+00

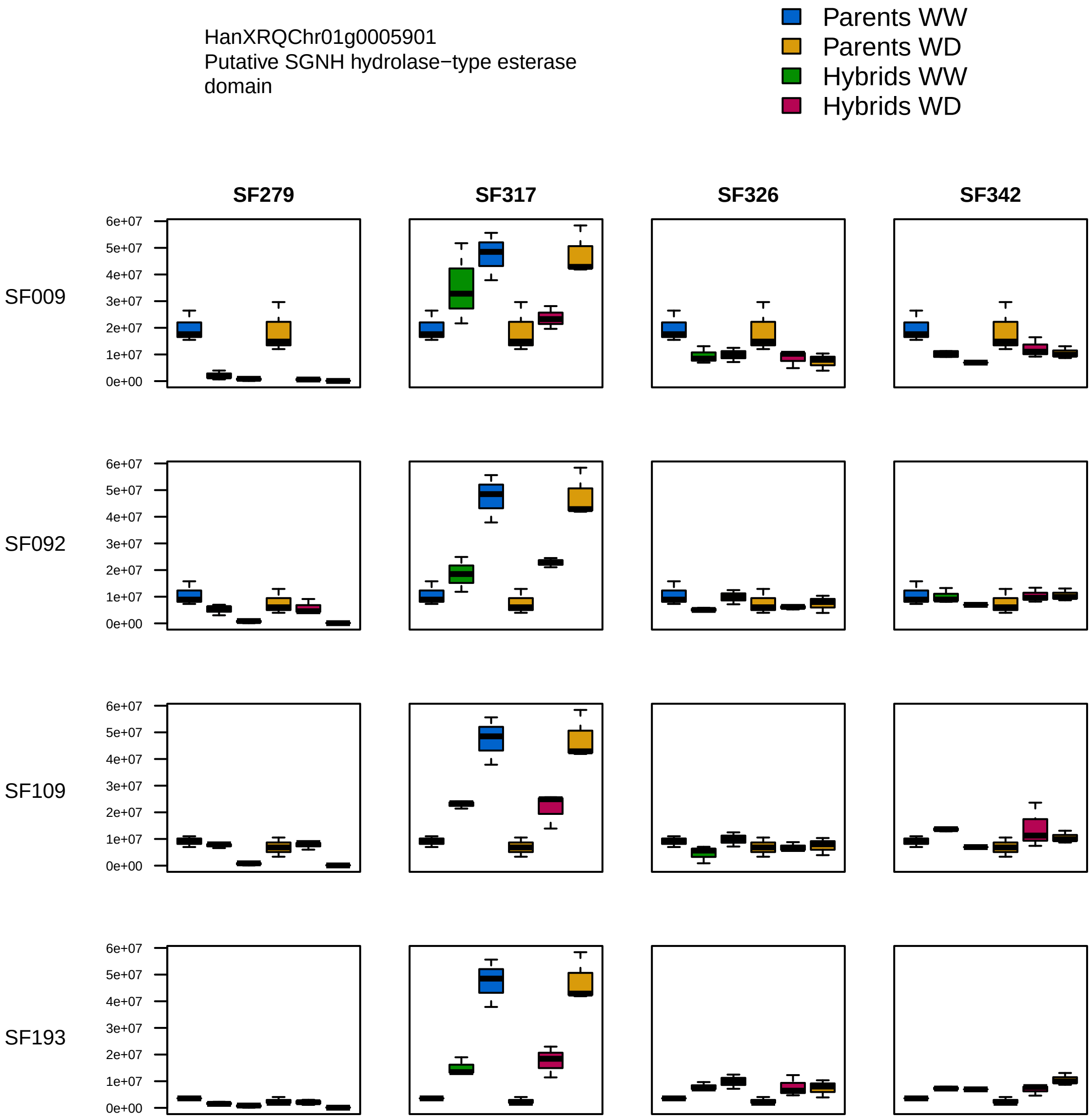
HanXRQChr01g0005901
Putative SGNH hydrolase−type esterase domain
Parents WW
Parents WD
Hybrids WW
Hybrids WD
SF279
SF317
SF326
SF342
SF009
SF092
SF109
SF193
6e+07
5e+07
4e+07
3e+07
2e+07
1e+07
0e+00

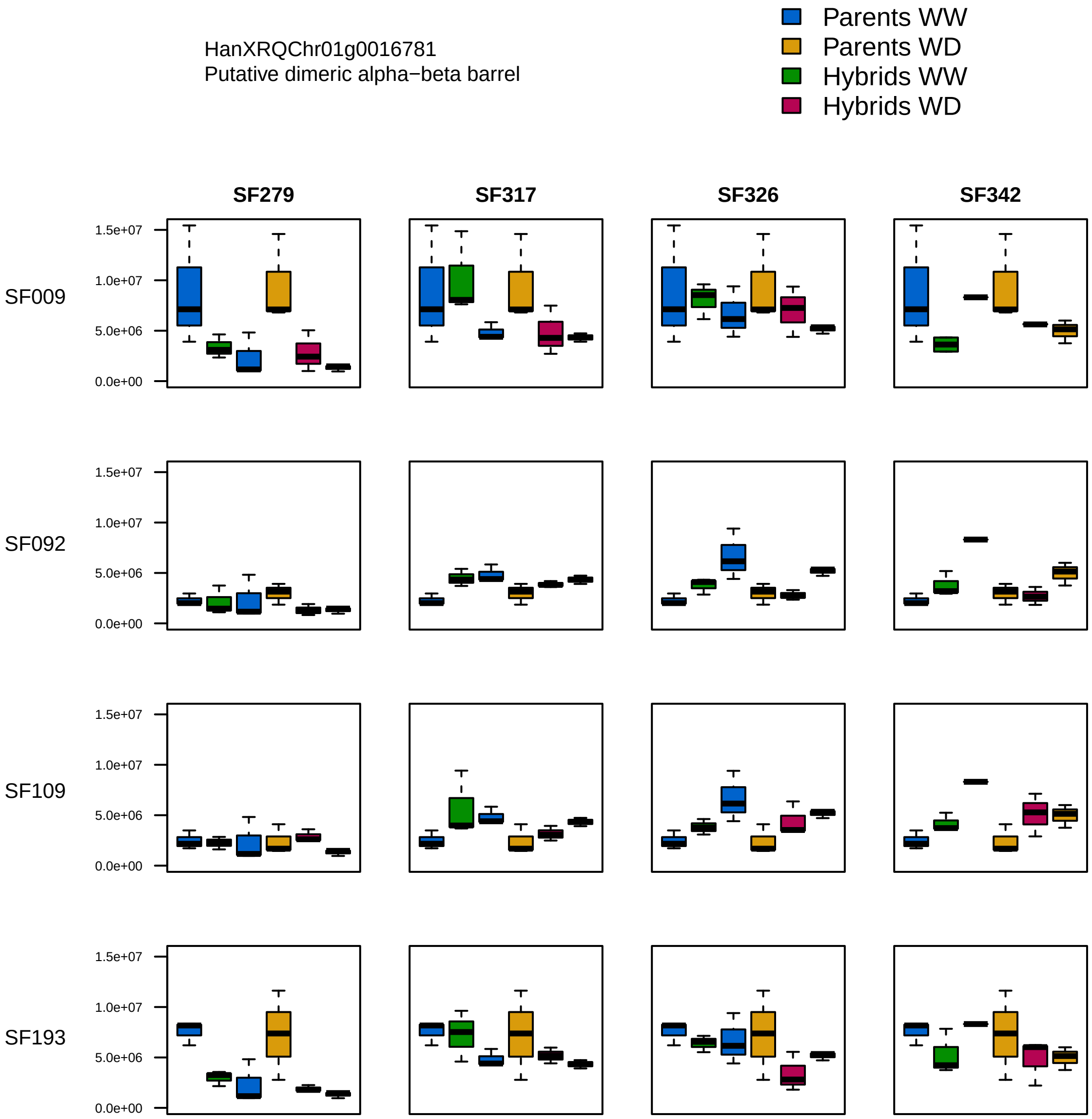

HanXRQChr01g0016781
Putative dimeric alpha−beta barrel
Parents WW
Parents WD
Hybrids WW
Hybrids WD
SF279
SF317
SF326
SF342
SF009
SF092
SF109
SF193
1.5e+07
1.0e+07
5.0e+06
0.0e+00

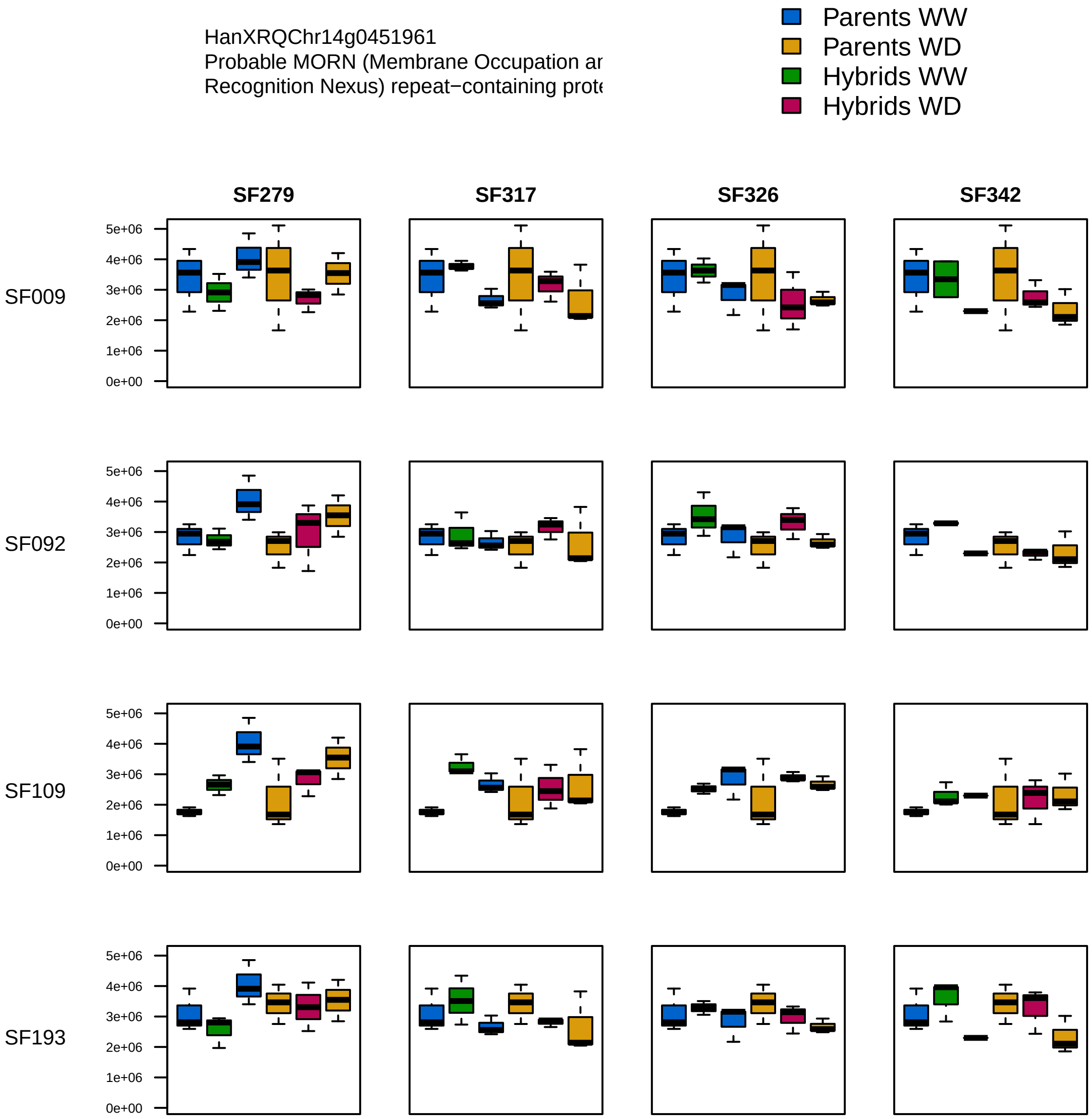
HanXRQChr14g0451961
Probable MORN (Membrane Occupation ar
Recognition Nexus) repeat−containing prote
Parents WW
Parents WD
Hybrids WW
Hybrids WD
SF279
SF317
SF326
SF342
SF009
SF092
SF109
SF193
5e+06
4e+06
3e+06
2e+06
1e+06
0e+00

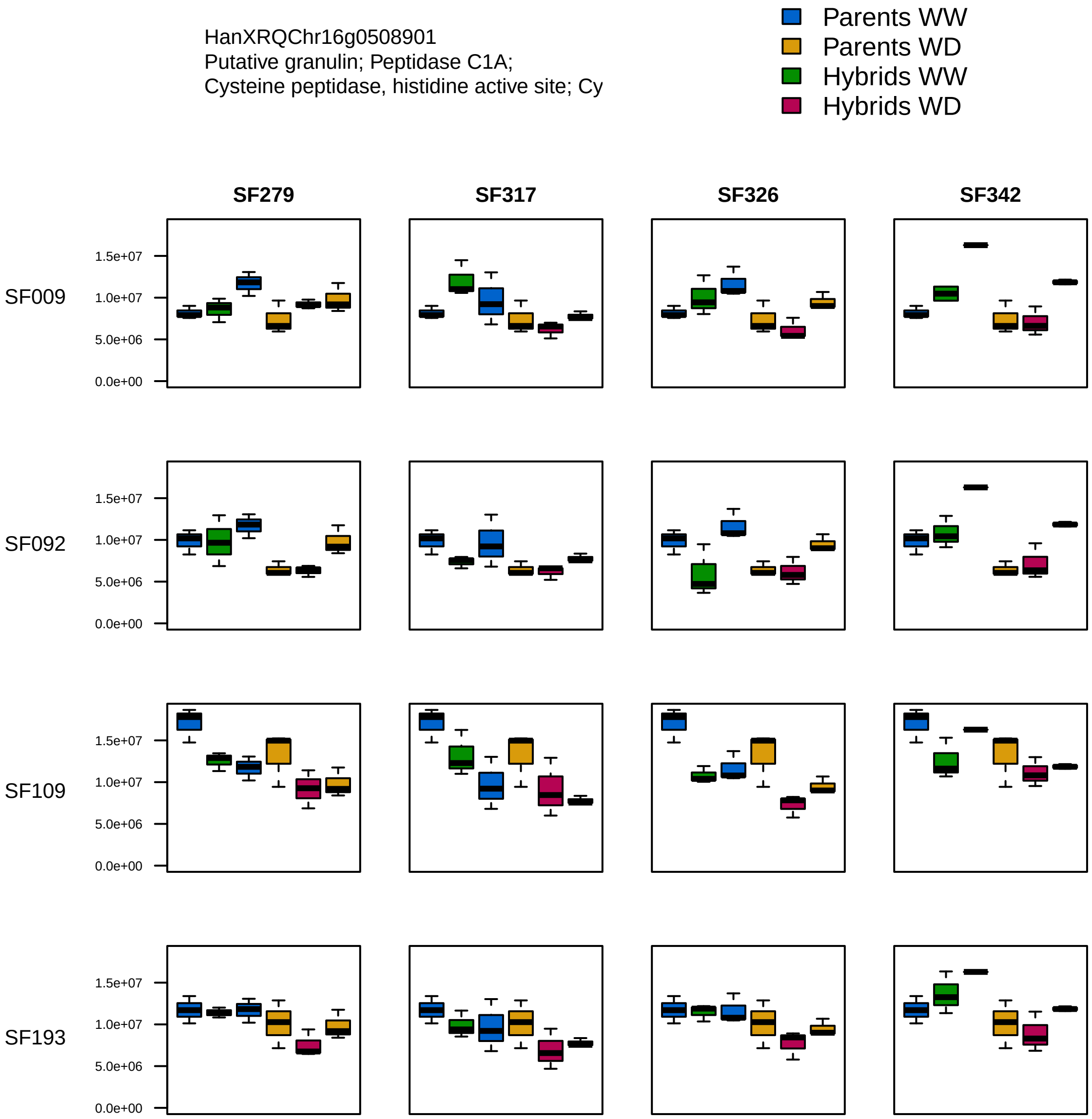

HanXRQChr16g0508901
Putative granulin; Peptidase C1A;
Cysteine peptidase, histidine active site; Cy
Parents WW
Parents WD
Hybrids WW
Hybrids WD
SF279
SF317
SF326
SF342
SF009
SF092
SF109
SF193
1.5e+07
1.0e+07
5.0e+06
0.0e+00

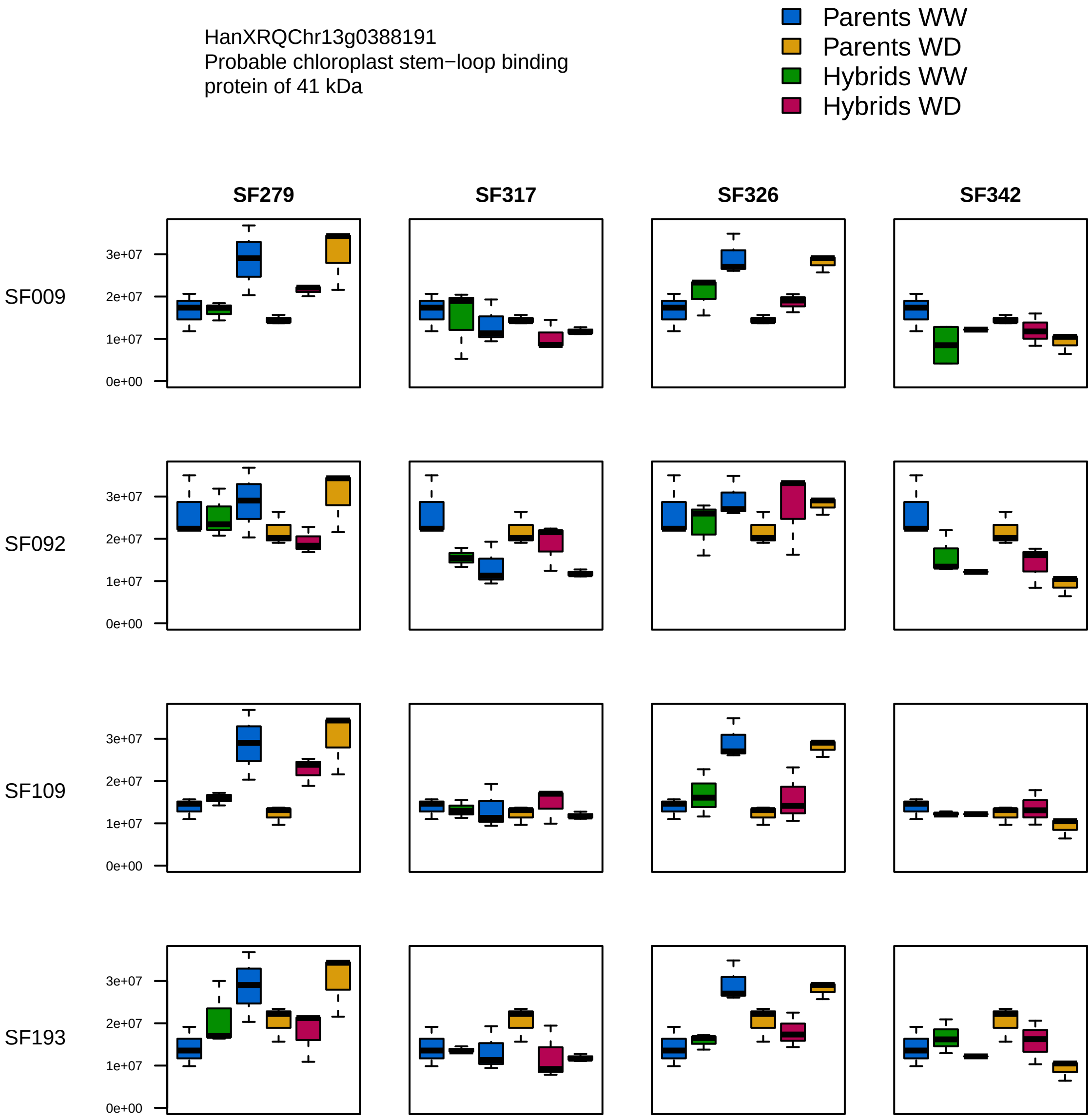
HanXRQChr13g0388191
Probable chloroplast stem−loop binding
protein of 41 kDa
Parents WW
Parents WD
Hybrids WW
Hybrids WD
SF279
SF317
SF326
SF342
SF009
SF092
SF109
SF193
0e+00
1e+07
2e+07
3e+07

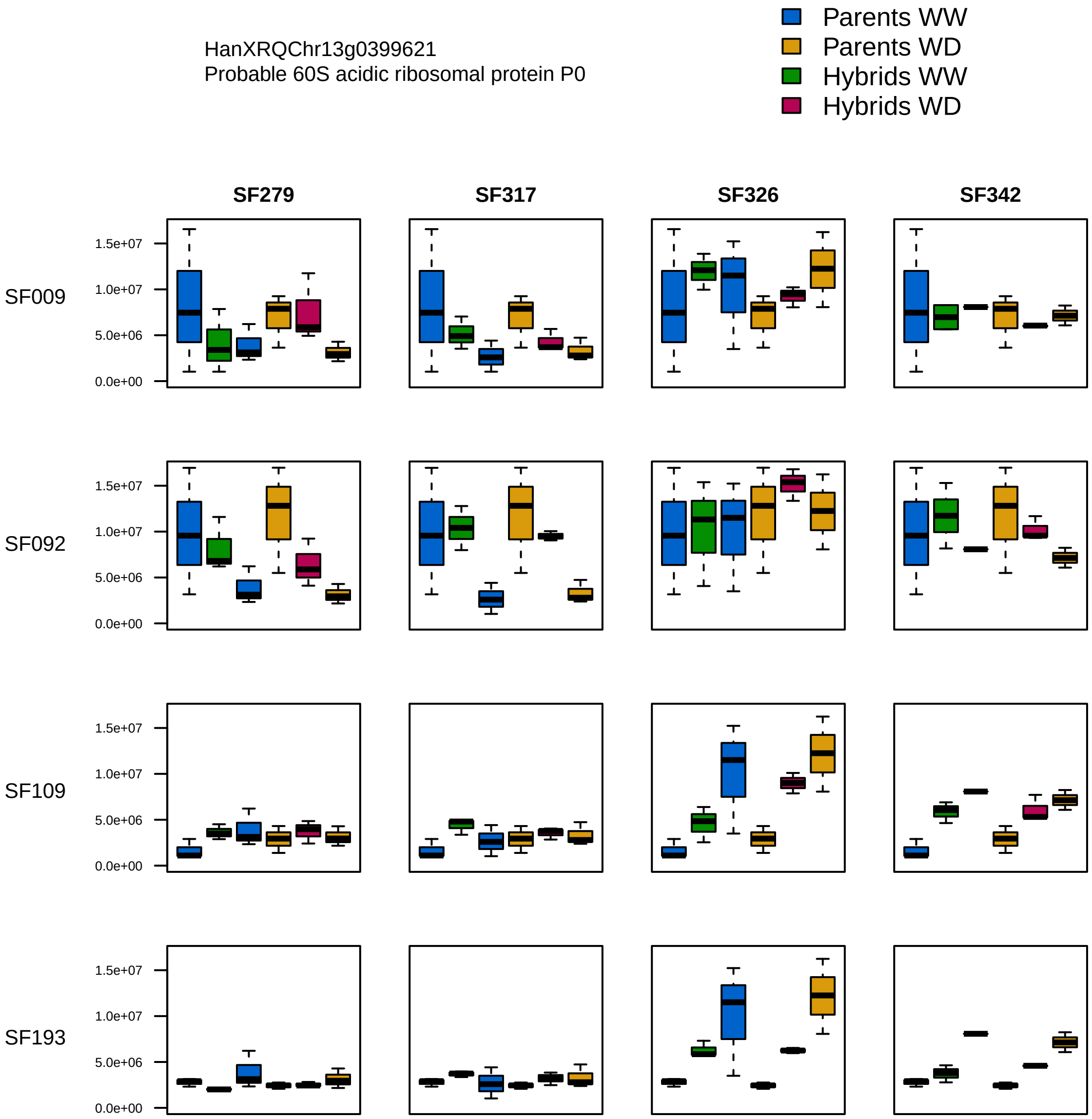
HanXRQChr13g0399621
Probable 60S acidic ribosomal protein P0
Parents WW
Parents WD
Hybrids WW
Hybrids WD
SF279
SF317
SF326
SF342
SF009
SF092
SF109
SF193
0.0e+00
5.0e+06
1.0e+07
1.5e+07

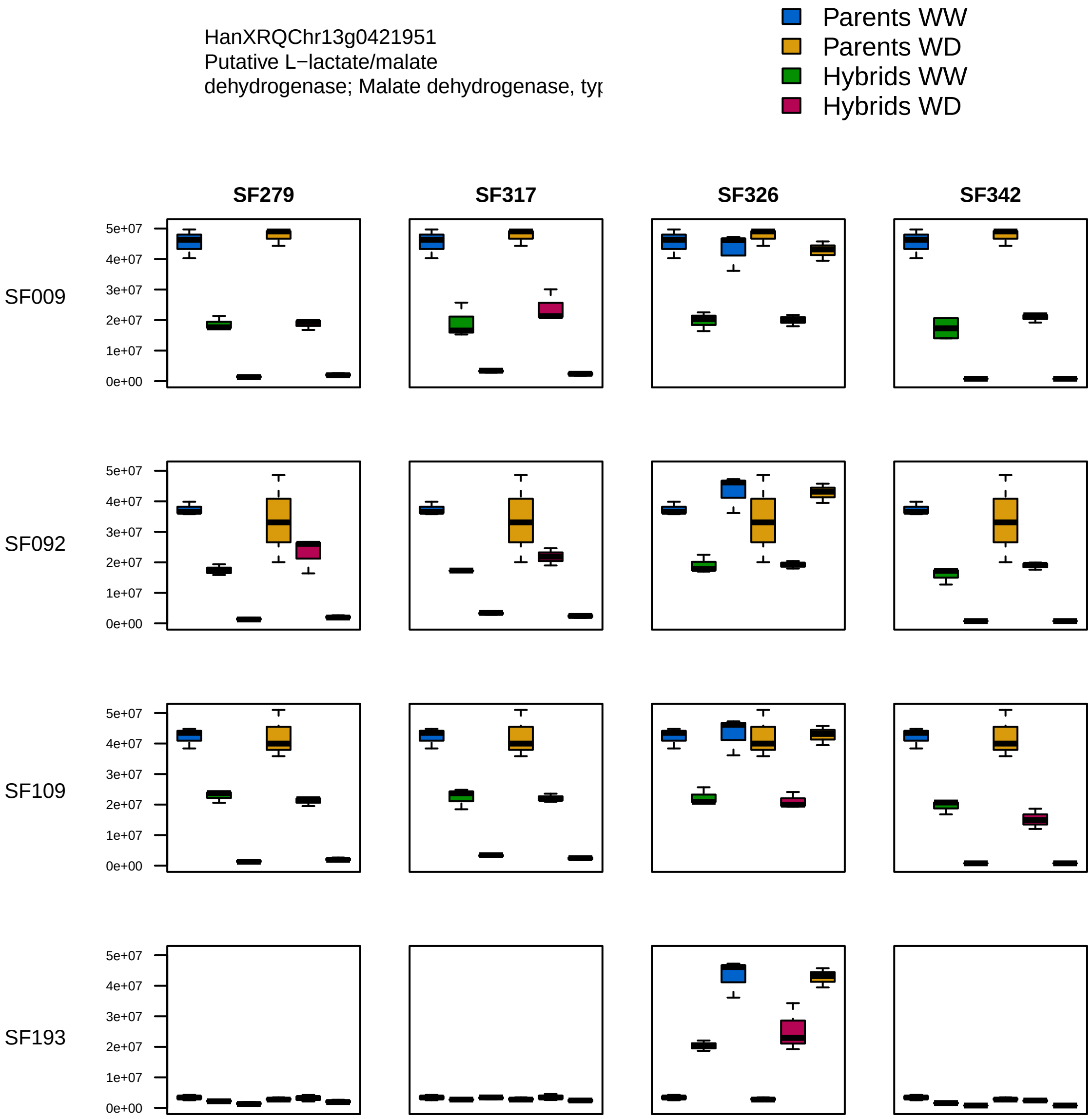
HanXRQChr13g0421951
Putative L−lactate/malate
dehydrogenase; Malate dehydrogenase, tyı
Parents WW
Parents WD
Hybrids WW
Hybrids WD
SF279
SF317
SF326
SF342
SF009
SF092
SF109
SF193
5e+07
4e+07
3e+07
2e+07
1e+07
0e+00

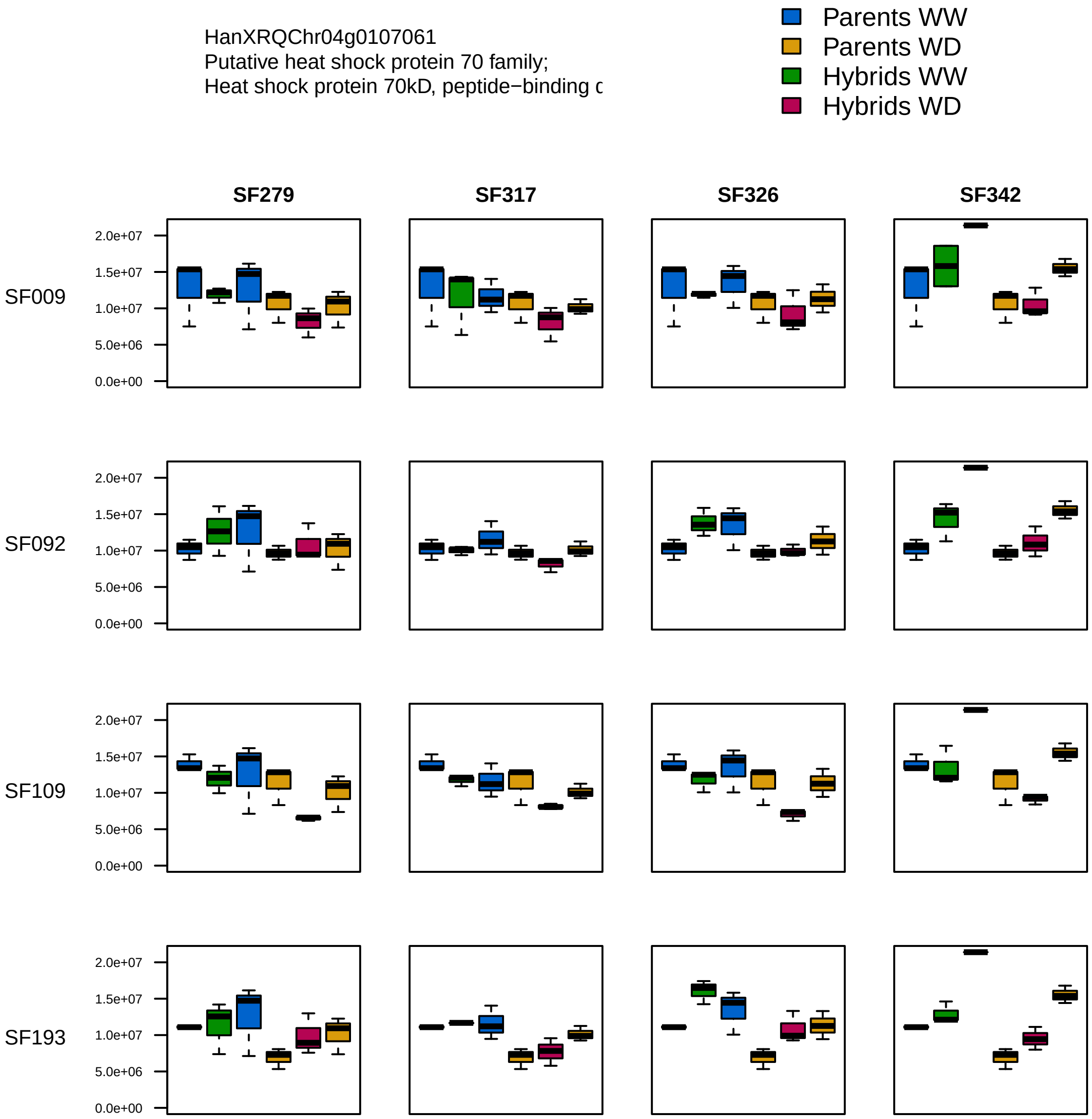

HanXRQChr04g0107061
Putative heat shock protein 70 family;
Heat shock protein 70kD, peptide−binding c
Parents WW
Parents WD
Hybrids WW
Hybrids WD
SF279
SF317
SF326
SF342
SF009
SF092
SF109
SF193
2.0e+07
1.5e+07
1.0e+07
5.0e+06
0.0e+00

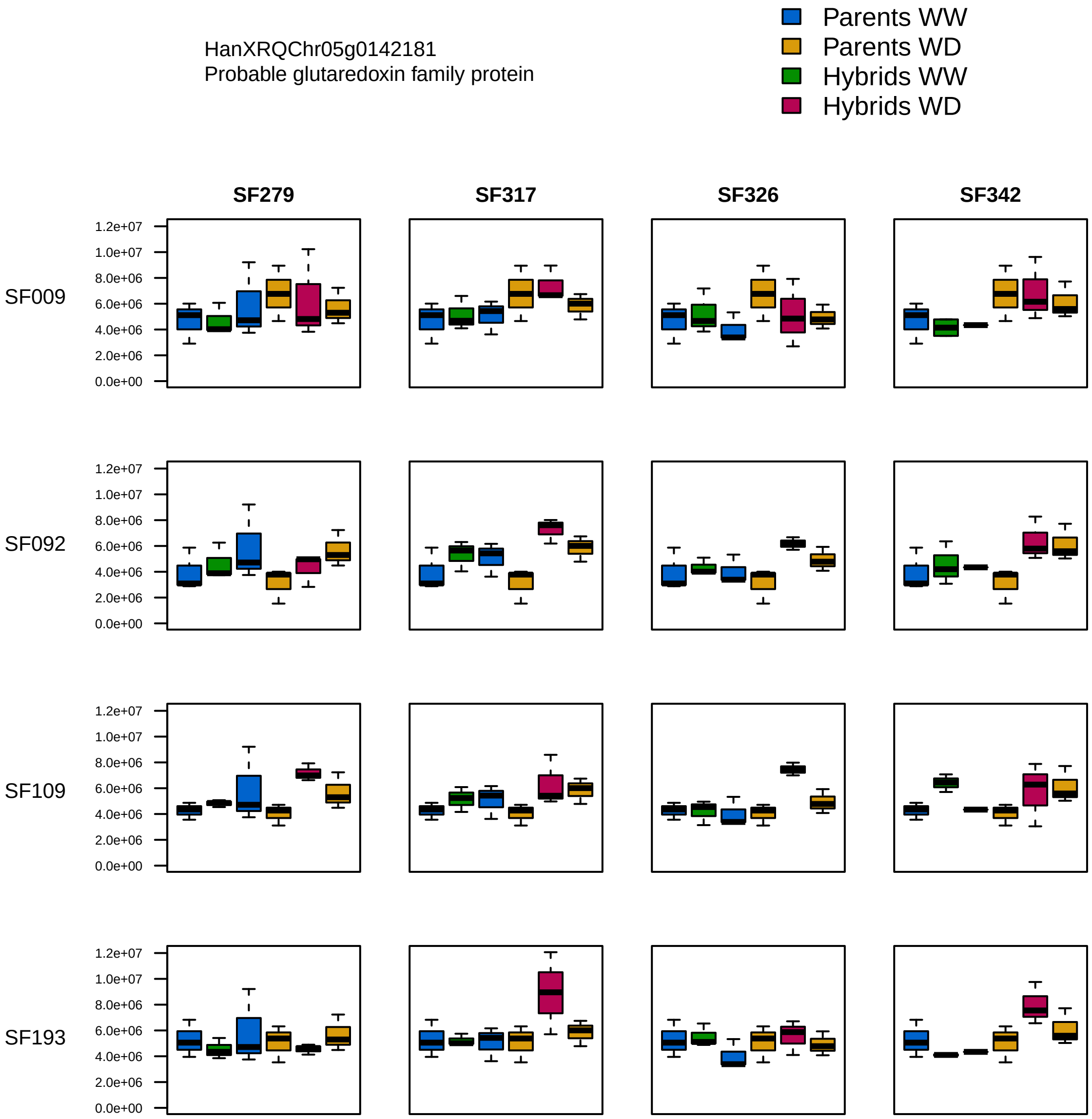

HanXRQChr05g0142181
Probable glutaredoxin family protein
Parents WW
Parents WD
Hybrids WW
Hybrids WD
SF279
SF317
SF326
SF342
SF009
SF092
SF109
SF193
1.2e+07
1.0e+07
8.0e+06
6.0e+06
4.0e+06
2.0e+06
0.0e+00

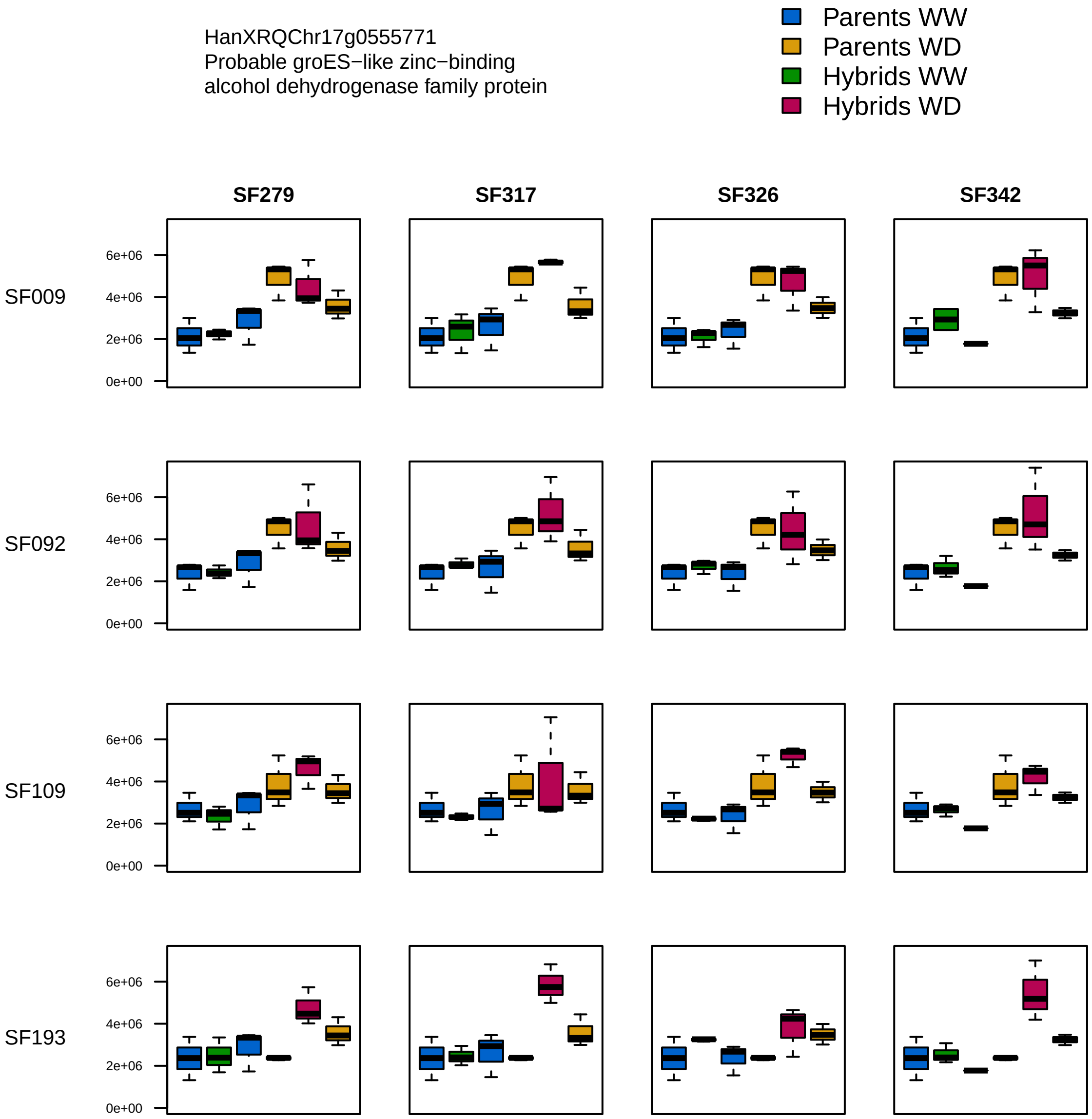

HanXRQChr17g0555771
Probable groES−like zinc−binding
alcohol dehydrogenase family protein
Parents WW
Parents WD
Hybrids WW
Hybrids WD
SF279
SF317
SF326
SF342
SF009
SF092
SF109
SF193
0e+00
2e+06
4e+06
6e+06

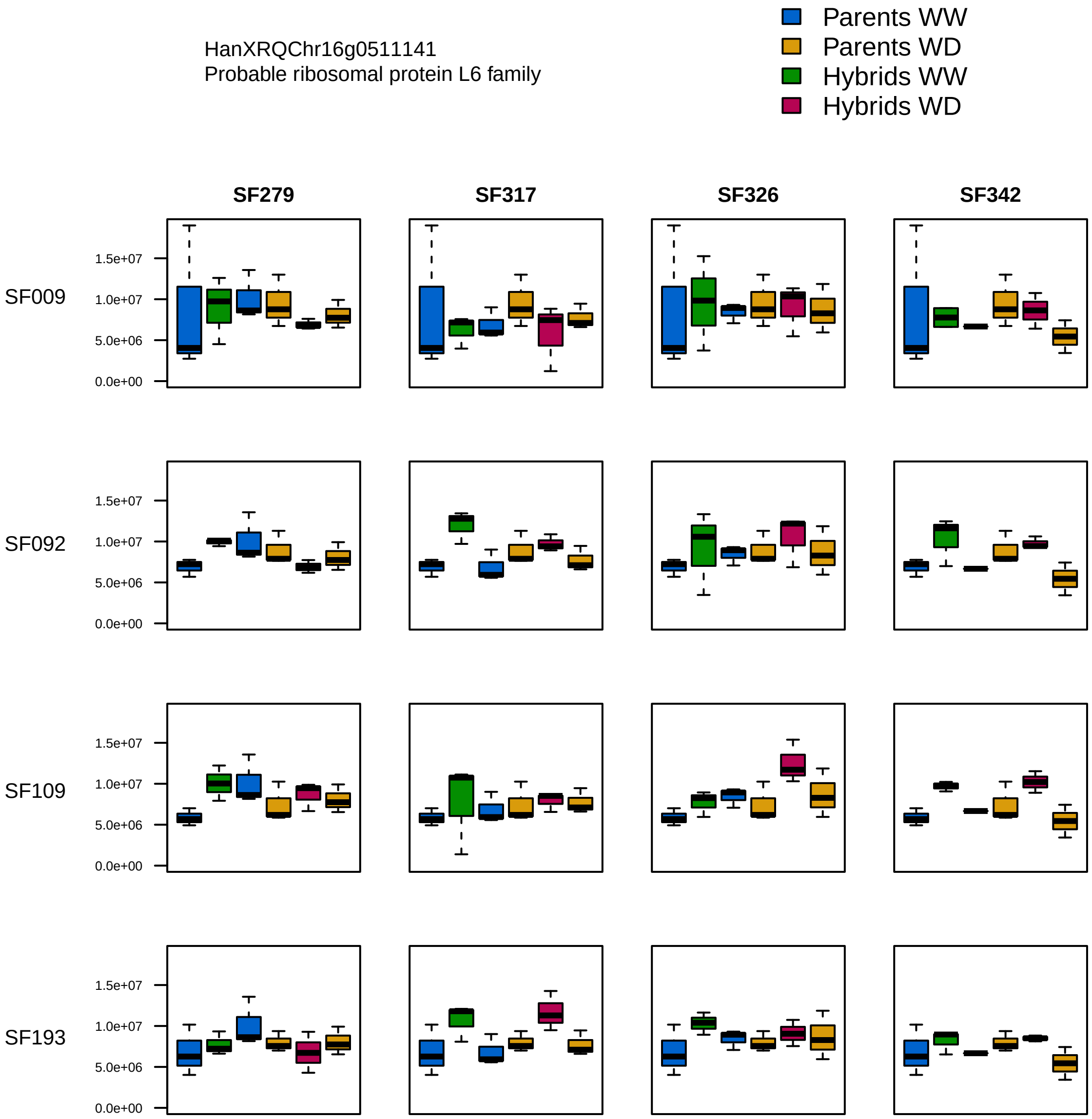
HanXRQChr16g0511141
Probable ribosomal protein L6 family
Parents WW
Parents WD
Hybrids WW
Hybrids WD
SF279
SF317
SF326
SF342
SF009
SF092
SF109
SF193
1.5e+07
1.0e+07
5.0e+06
0.0e+00

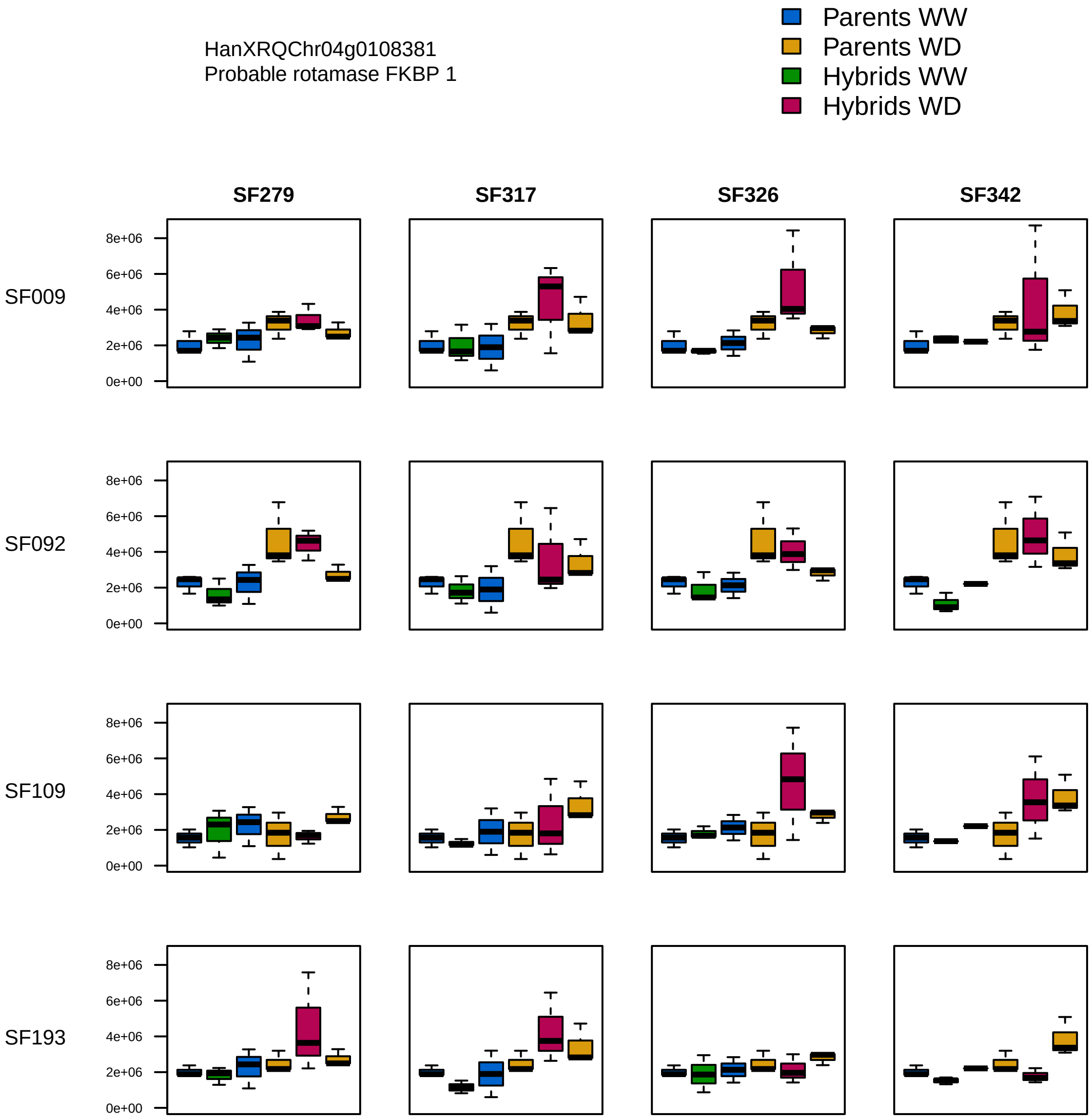
HanXRQChr04g0108381
Probable rotamase FKBP 1
Parents WW
Parents WD
Hybrids WW
Hybrids WD
SF279
SF317
SF326
SF342
SF009
SF092
SF109
SF193
0e+00
2e+06
4e+06
6e+06
8e+06

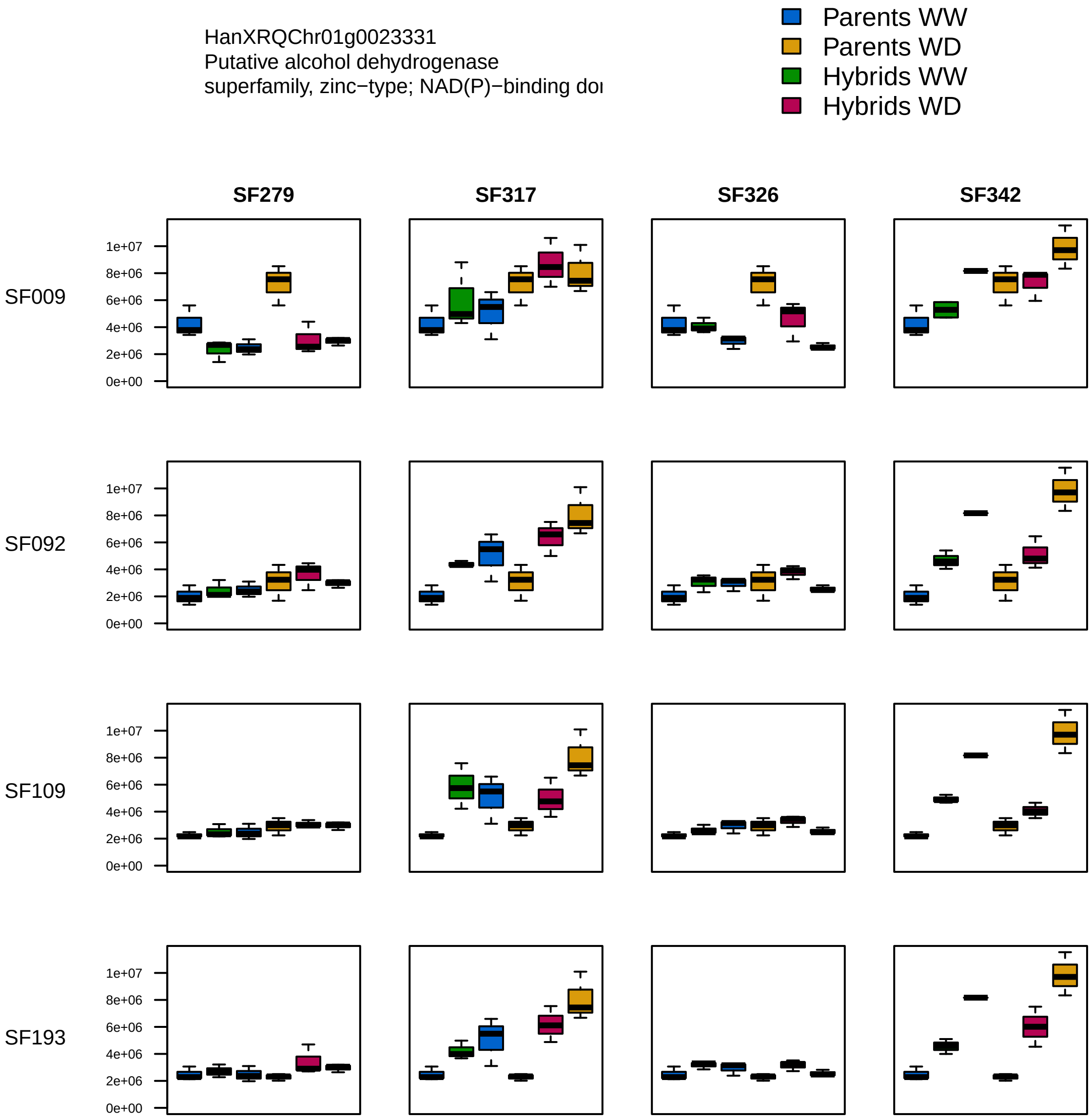
HanXRQChr01g0023331
Putative alcohol dehydrogenase
superfamily, zinc−type; NAD(P)−binding doı
Parents WW
Parents WD
Hybrids WW
Hybrids WD
SF279
SF317
SF326
SF342
SF009
SF092
SF109
SF193
1e+07
8e+06
6e+06
4e+06
2e+06
0e+00

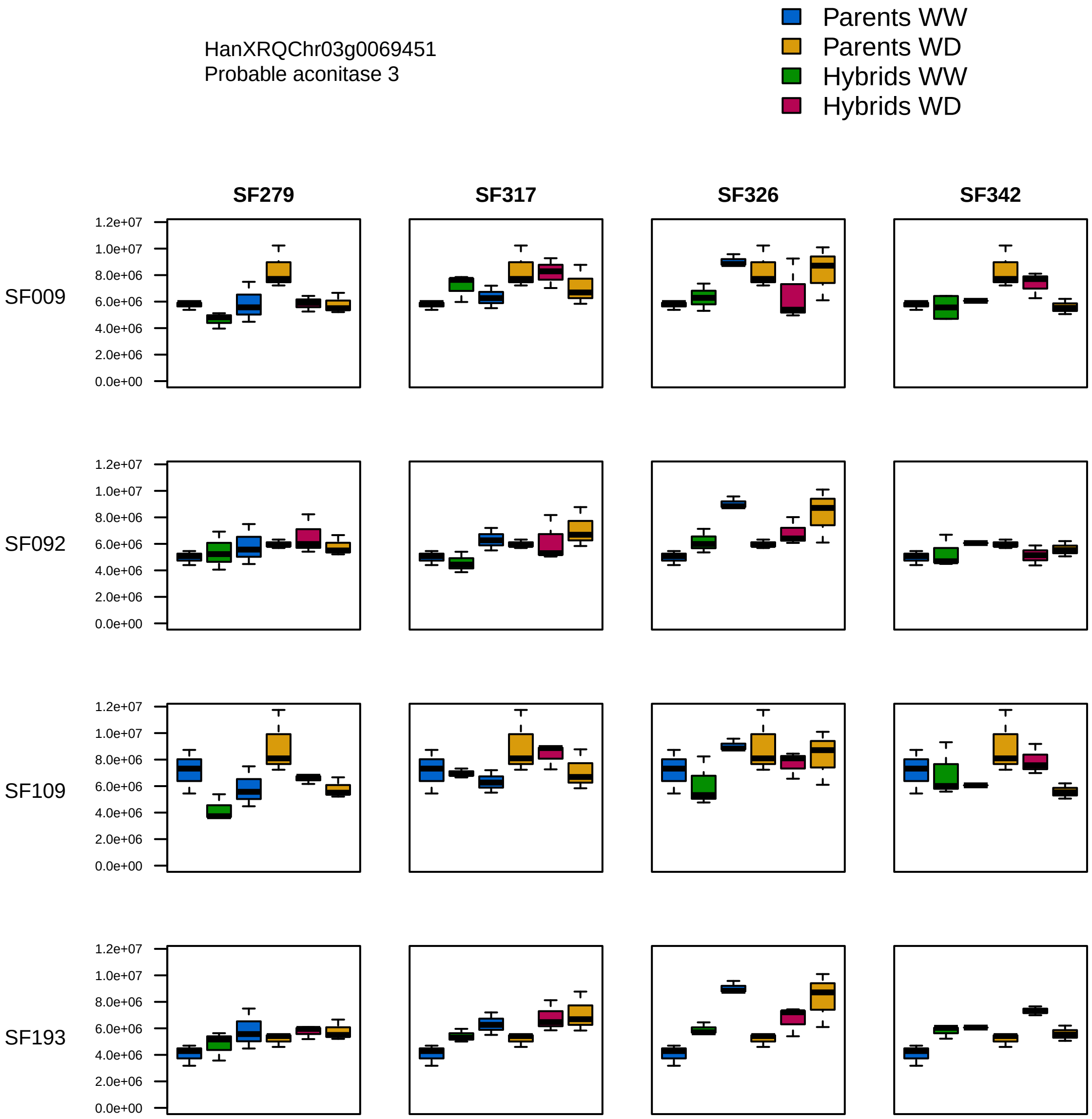
HanXRQChr03g0069451
Probable aconitase 3
Parents WW
Parents WD
Hybrids WW
Hybrids WD
SF279
SF317
SF326
SF342
SF009
SF092
SF109
SF193
1.2e+07
1.0e+07
8.0e+06
6.0e+06
4.0e+06
2.0e+06
0.0e+00

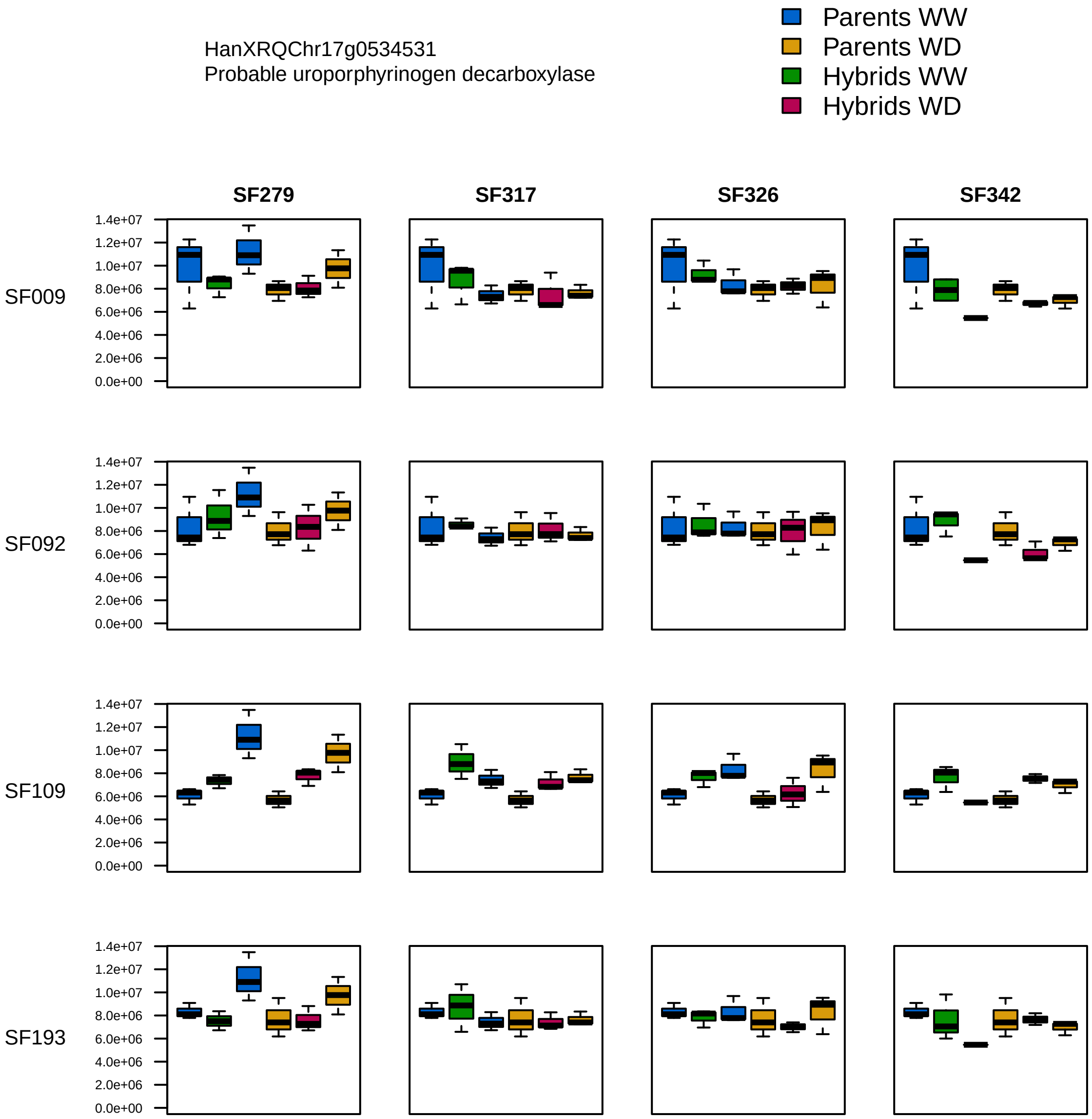
HanXRQChr17g0534531
Probable uroporphyrinogen decarboxylase
Parents WW
Parents WD
Hybrids WW
Hybrids WD
SF279
SF317
SF326
SF342
SF009
SF092
SF109
SF193
1.4e+07
1.2e+07
1.0e+07
8.0e+06
6.0e+06
4.0e+06
2.0e+06
0.0e+00

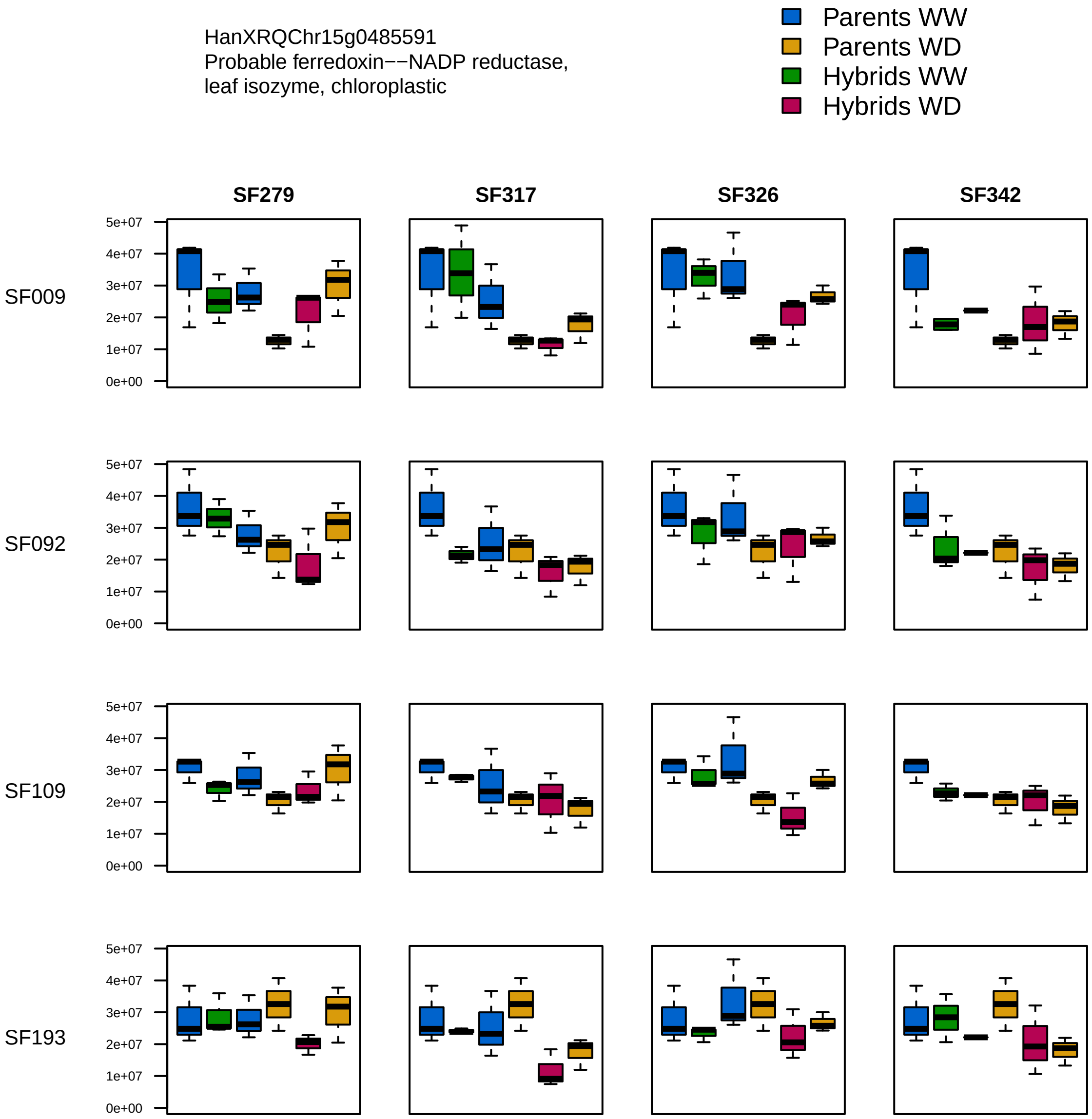
HanXRQChr15g0485591
Probable ferredoxin−−NADP reductase,
leaf isozyme, chloroplastic
Parents WW
Parents WD
Hybrids WW
Hybrids WD
SF279
SF317
SF326
SF342
SF009
SF092
SF109
SF193
5e+07
4e+07
3e+07
2e+07
1e+07
0e+00

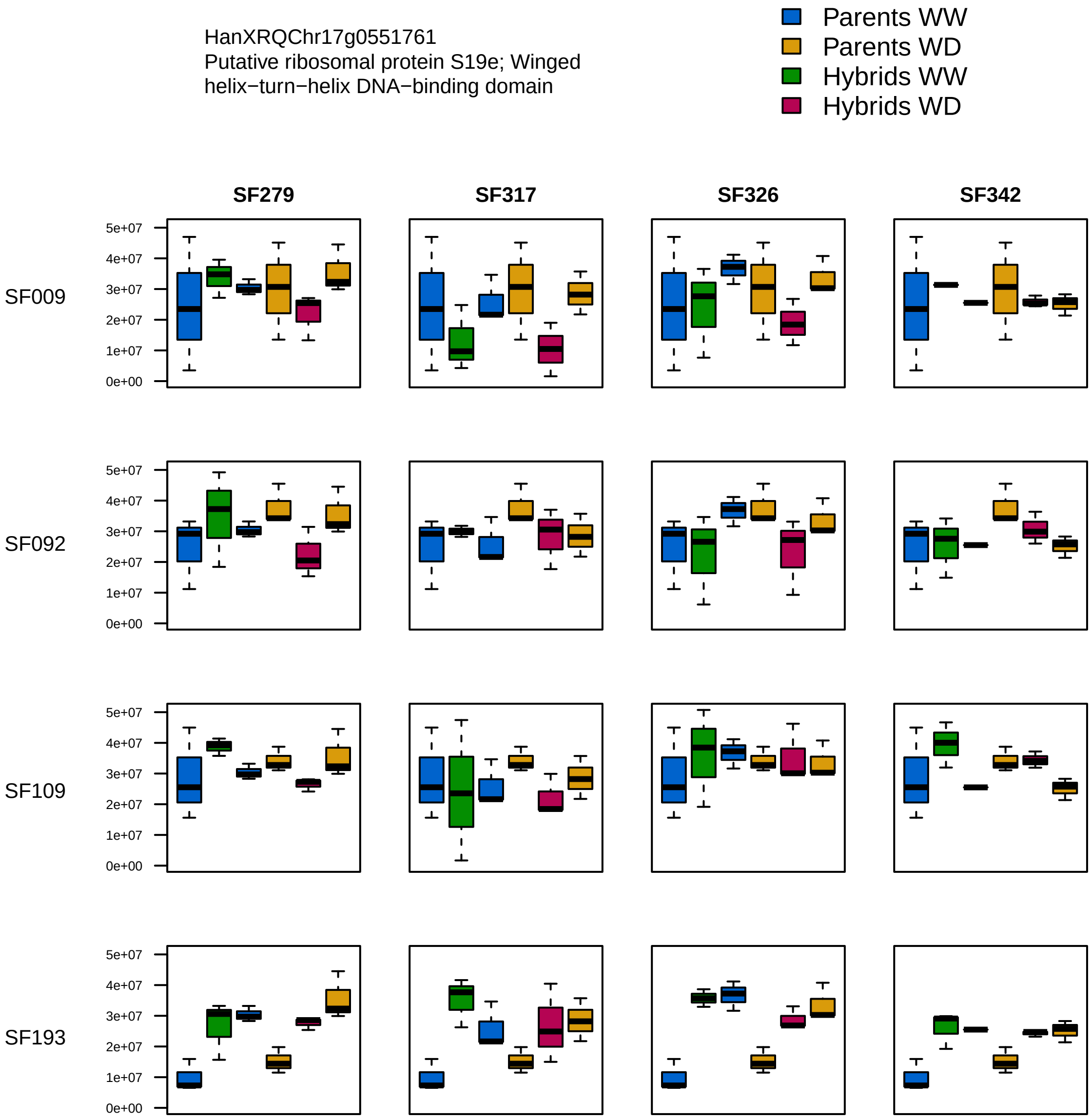

HanXRQChr17g0551761
Putative ribosomal protein S19e; Winged helix−turn−helix DNA−binding domain
Parents WW
Parents WD
Hybrids WW
Hybrids WD
SF279
SF317
SF326
SF342
SF009
SF092
SF109
SF193
5e+07
4e+07
3e+07
2e+07
1e+07
0e+00

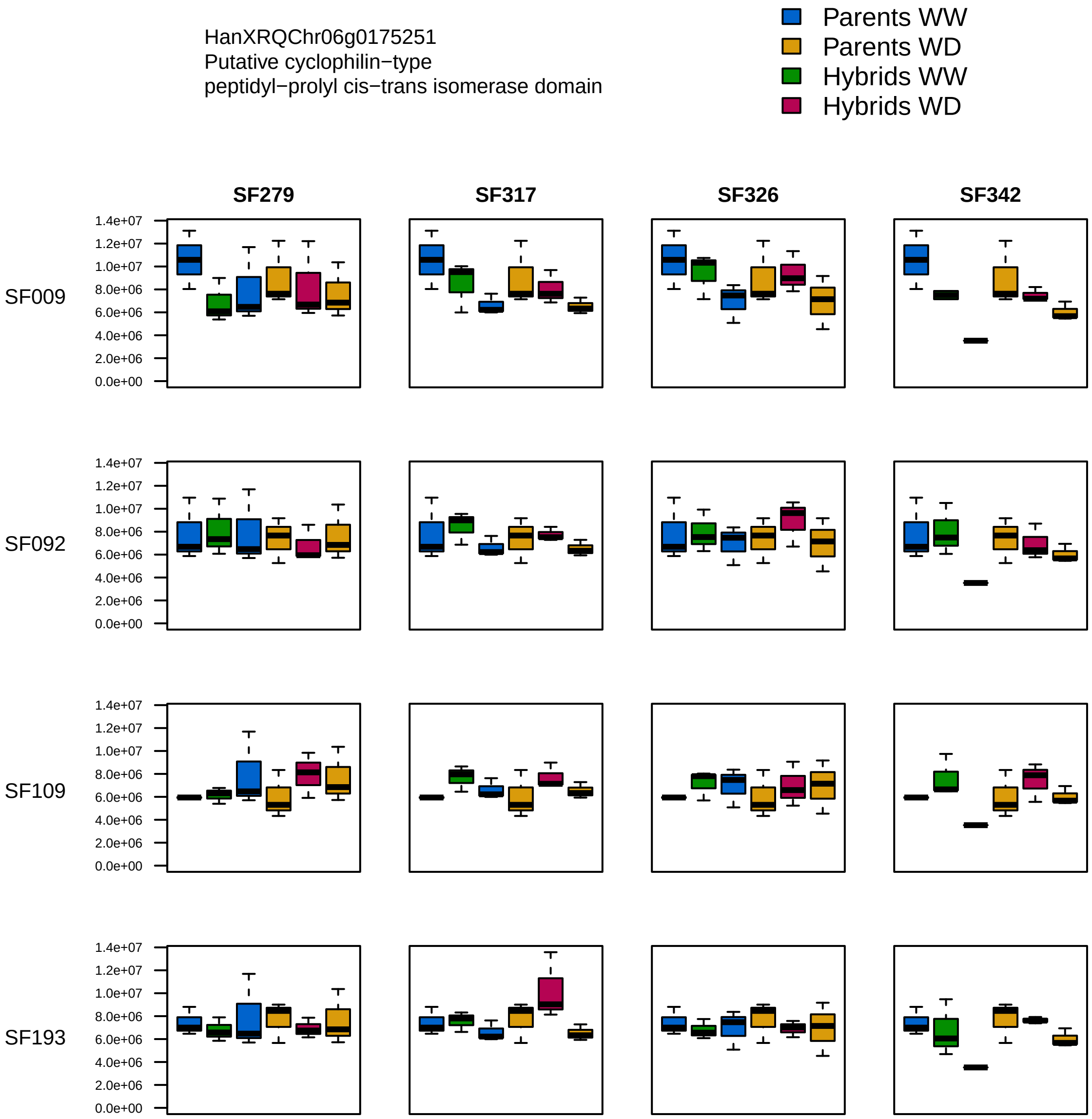

HanXRQChr06g0175251
Putative cyclophilin−type
peptidyl−prolyl cis−trans isomerase domain
Parents WW
Parents WD
Hybrids WW
Hybrids WD
SF279
SF317
SF326
SF342
SF009
SF092
SF109
SF193
1.4e+07
1.2e+07
1.0e+07
8.0e+06
6.0e+06
4.0e+06
2.0e+06
0.0e+00

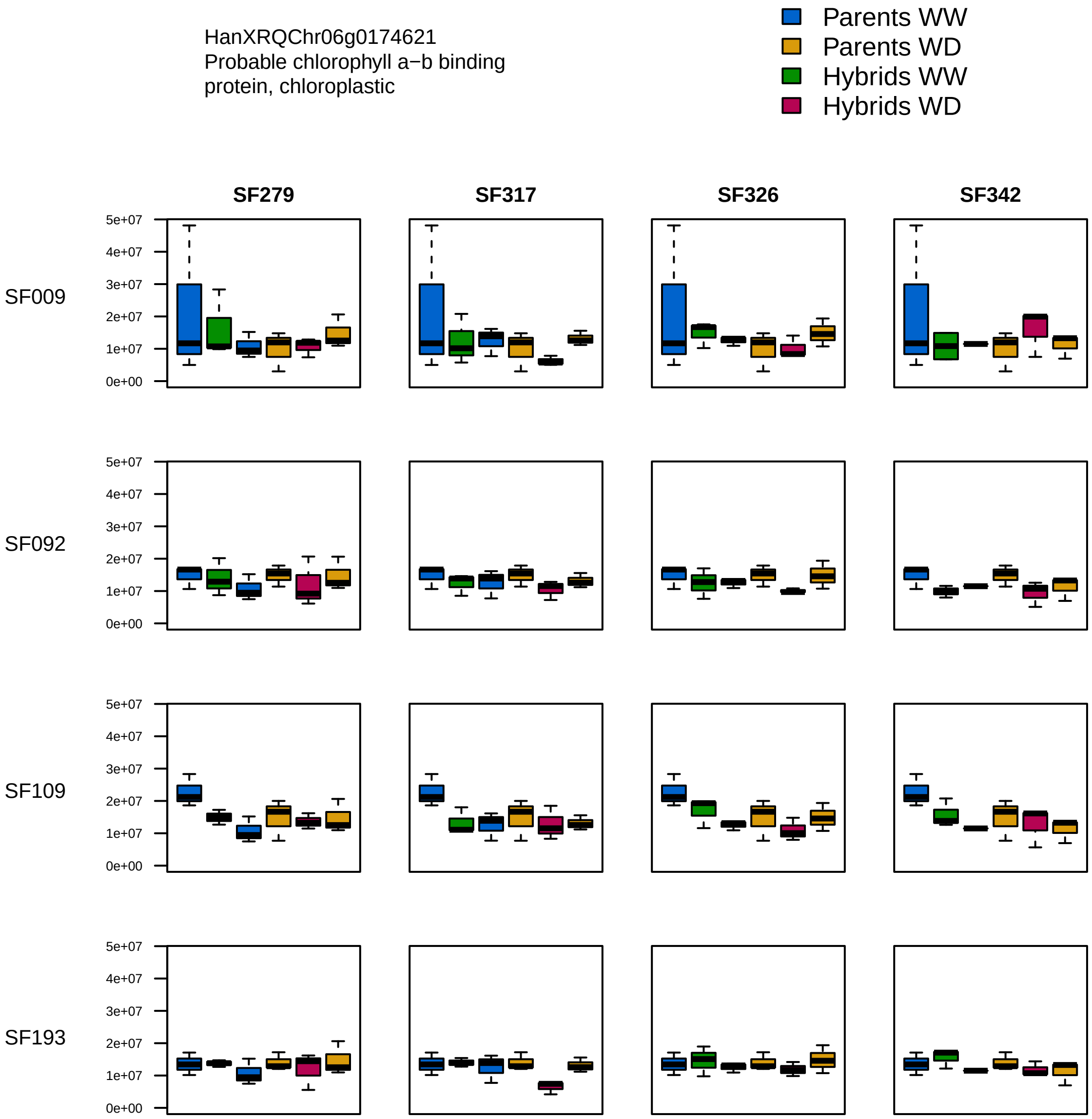

HanXRQChr06g0174621
Probable chlorophyll a−b binding
protein, chloroplastic
Parents WW
Parents WD
Hybrids WW
Hybrids WD
SF279
SF317
SF326
SF342
SF009
SF092
SF109
SF193
5e+07
4e+07
3e+07
2e+07
1e+07
0e+00

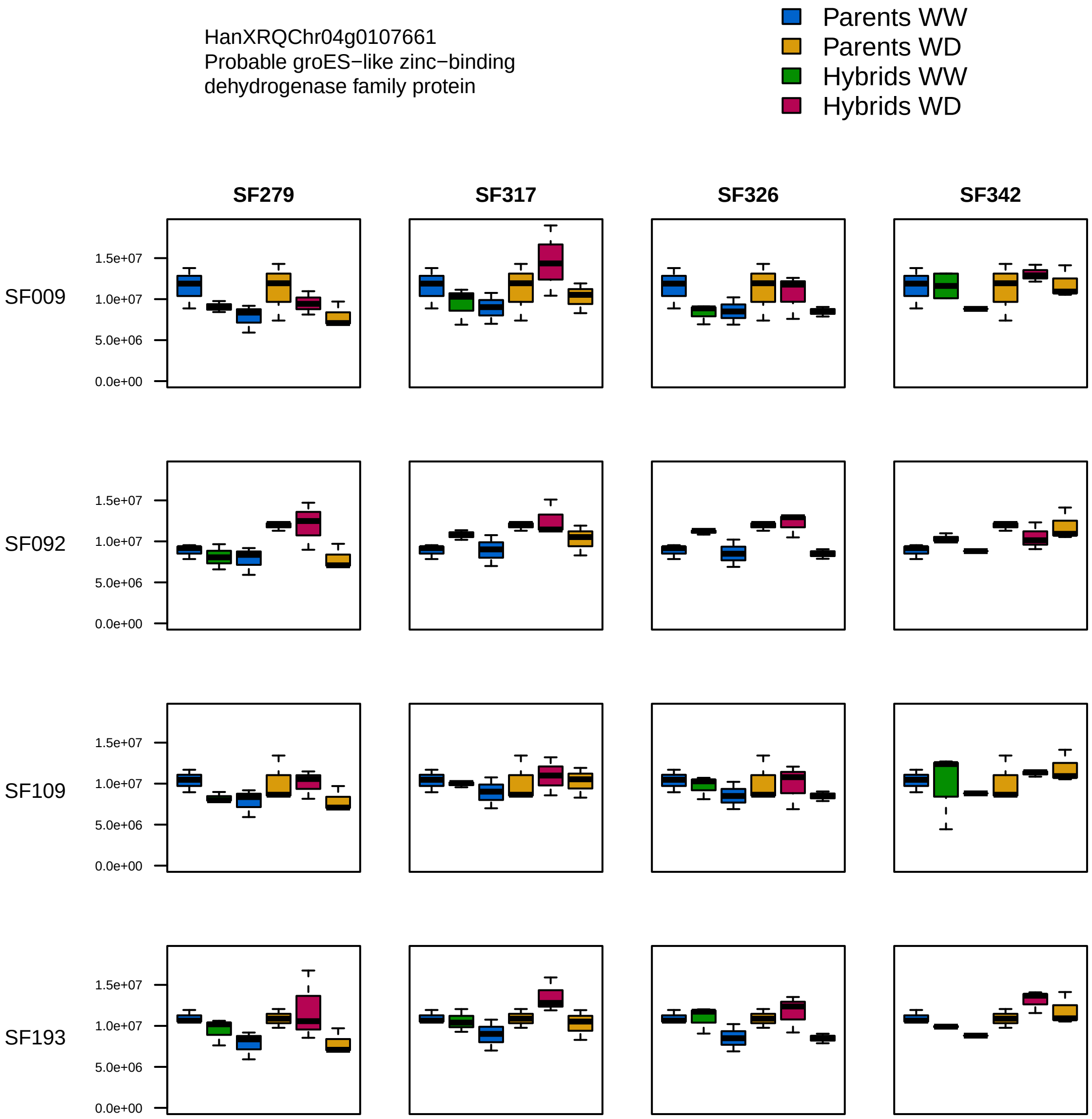

HanXRQChr04g0107661
Probable groES−like zinc−binding
dehydrogenase family protein
Parents WW
Parents WD
Hybrids WW
Hybrids WD
SF279
SF317
SF326
SF342
SF009
SF092
SF109
SF193
0.0e+00
5.0e+06
1.0e+07
1.5e+07

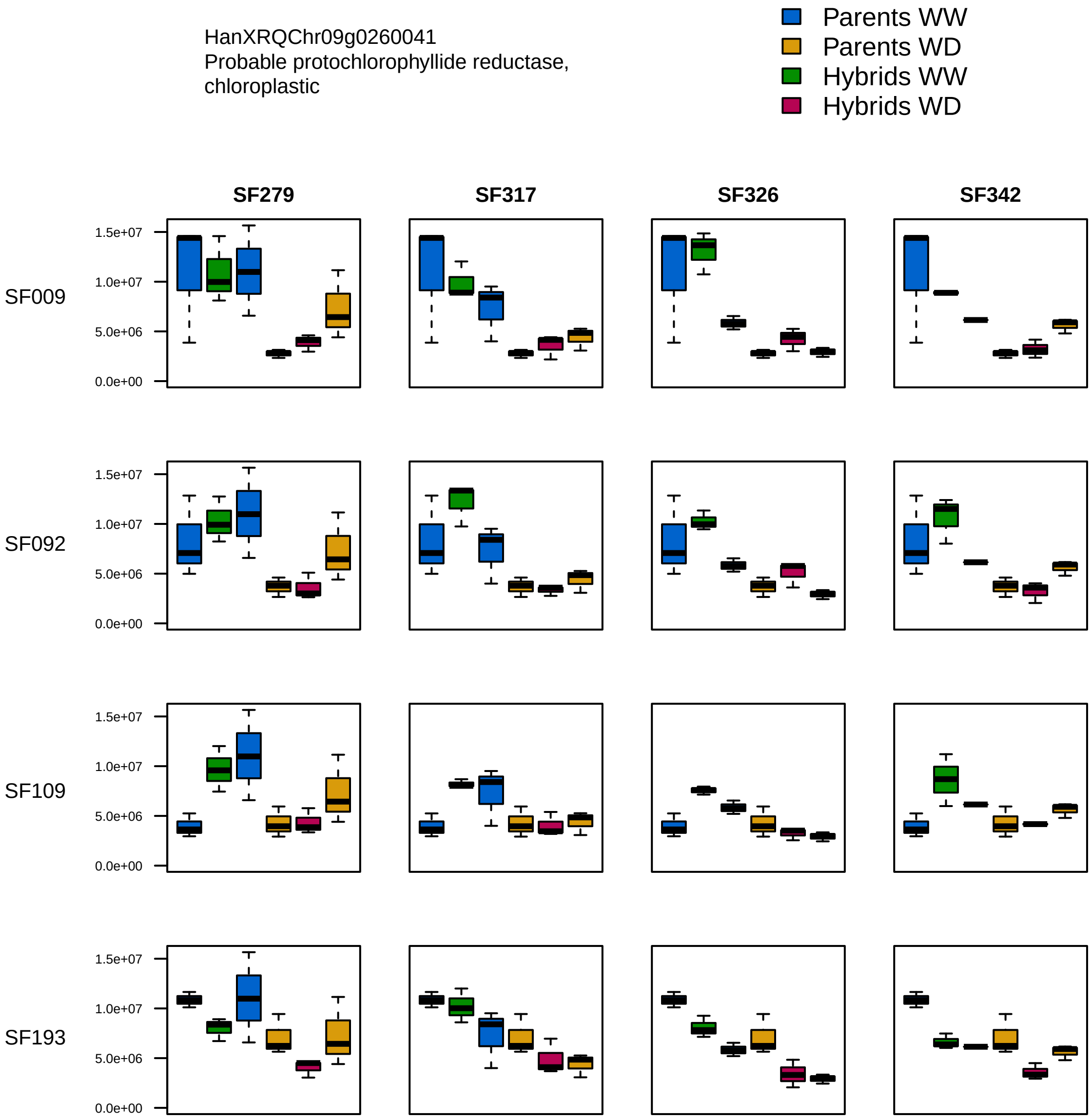
HanXRQChr09g0260041
Probable protochlorophyllide reductase,
chloroplastic
Parents WW
Parents WD
Hybrids WW
Hybrids WD
SF279
SF317
SF326
SF342
SF009
SF092
SF109
SF193
1.5e+07
1.0e+07
5.0e+06
0.0e+00

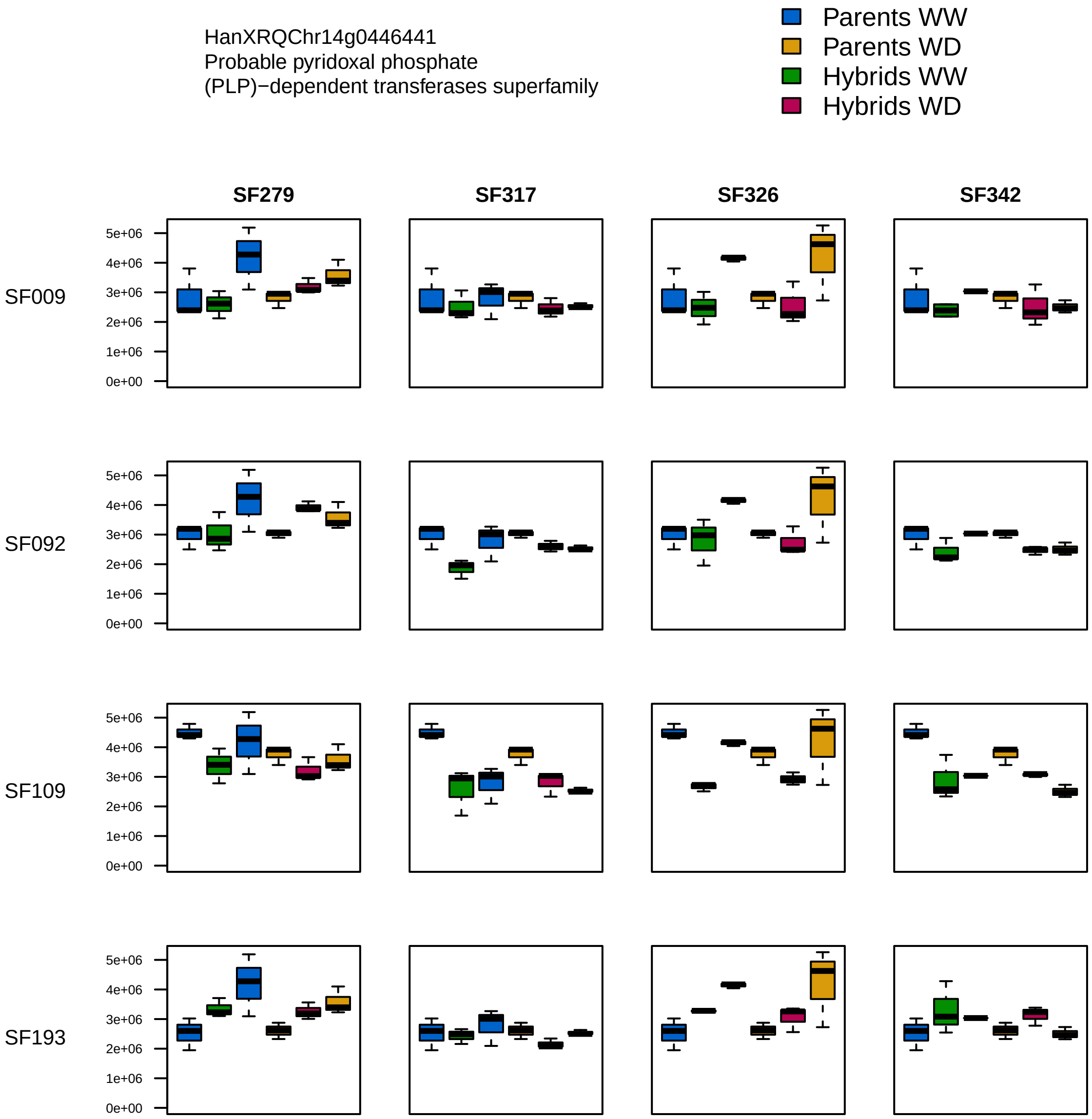
HanXRQChr14g0446441
Probable pyridoxal phosphate
(PLP)−dependent transferases superfamily
Parents WW
Parents WD
Hybrids WW
Hybrids WD
SF279
SF317
SF326
SF342
SF009
SF092
SF109
SF193
0e+00
1e+06
2e+06
3e+06
4e+06
5e+06

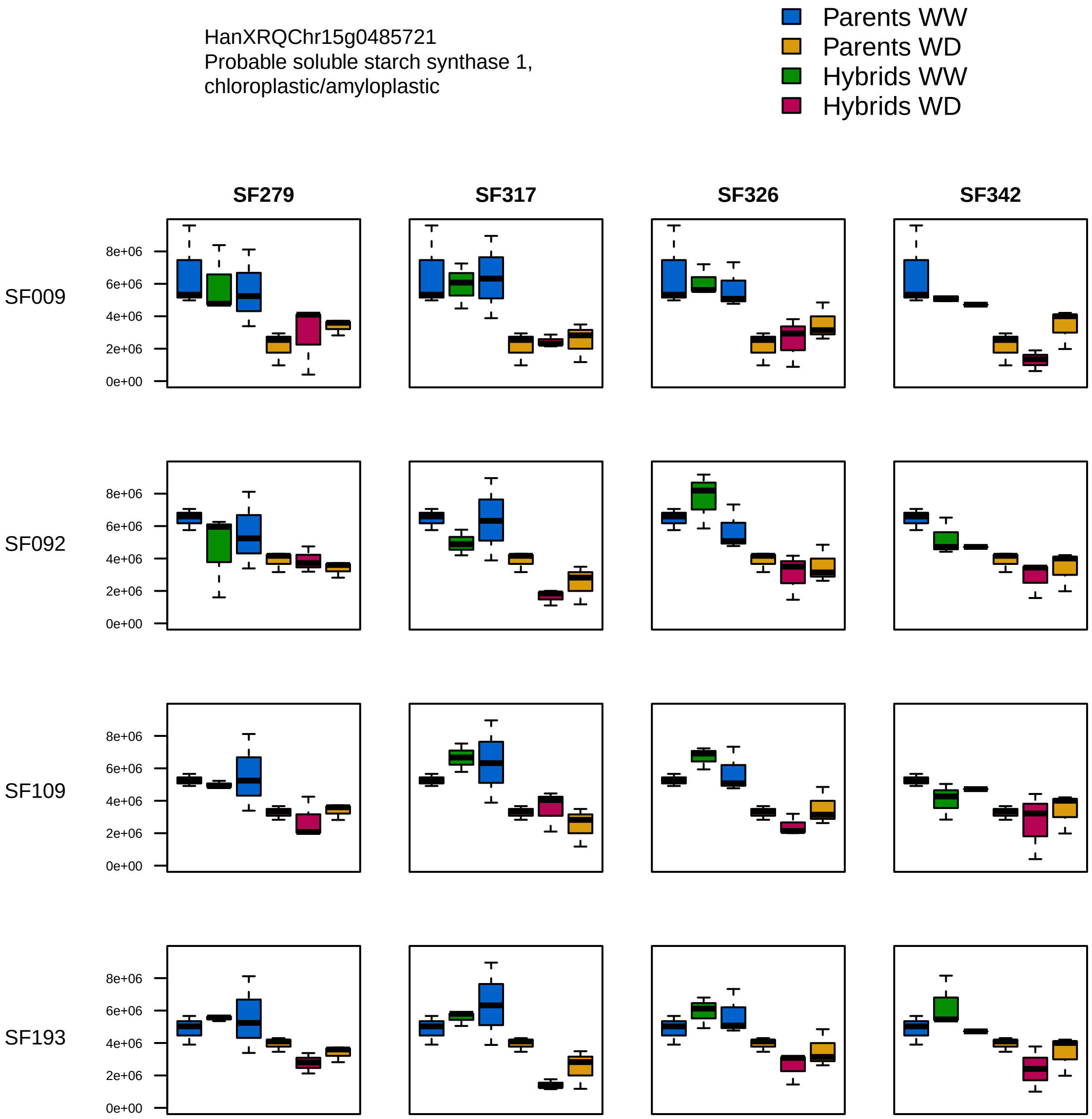

HanXRQChr15g0485721
Probable soluble starch synthase 1, chloroplastic/amyloplastic
Parents WW
Parents WD
Hybrids WW
Hybrids WD
SF279
SF317
SF326
SF342
SF009
SF092
SF109
SF193
0e+00
2e+06
4e+06
6e+06
8e+06

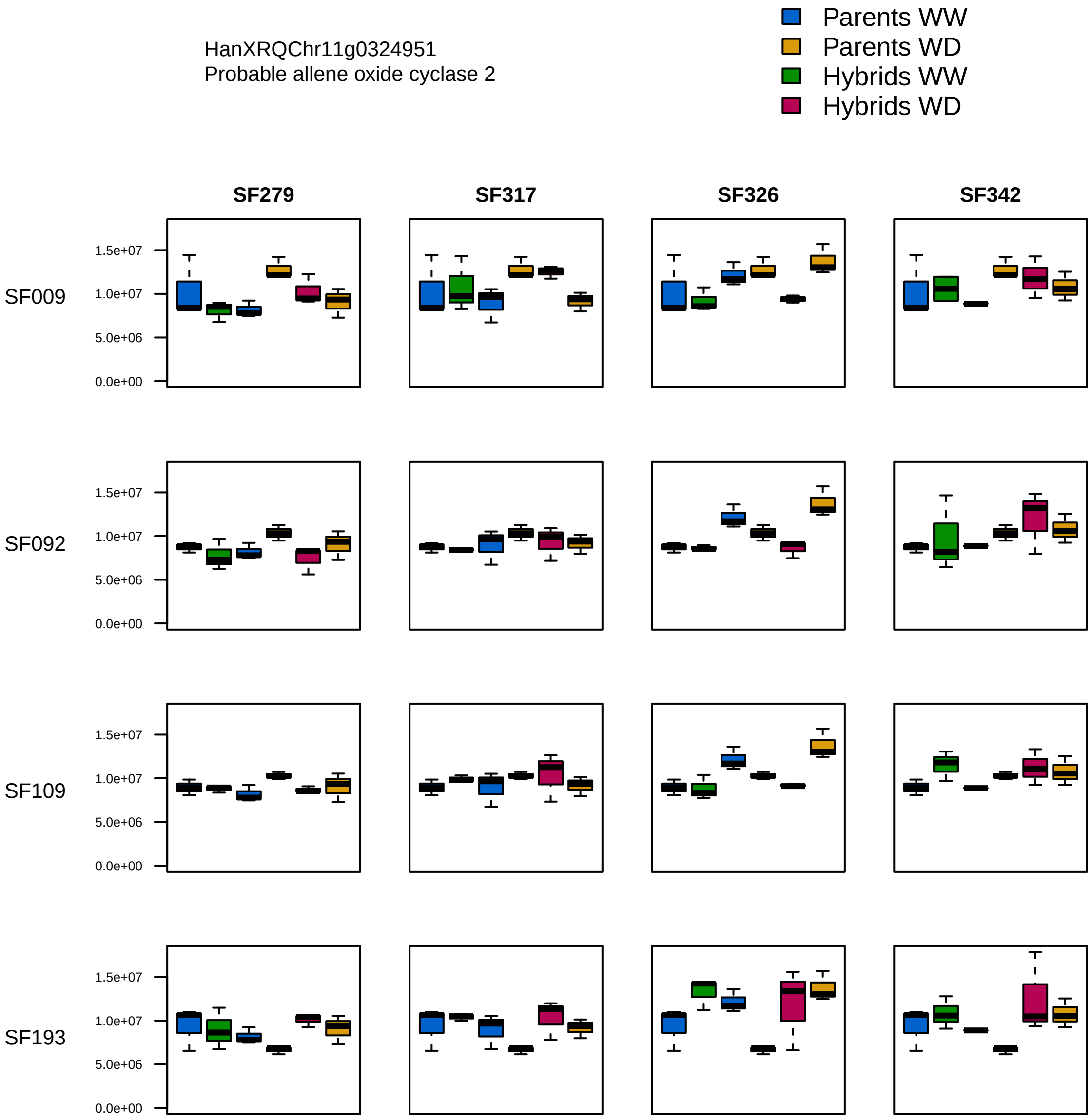
HanXRQChr11g0324951
Probable allene oxide cyclase 2
Parents WW
Parents WD
Hybrids WW
Hybrids WD
SF279
SF317
SF326
SF342
SF009
SF092
SF109
SF193
1.5e+07
1.0e+07
5.0e+06
0.0e+00

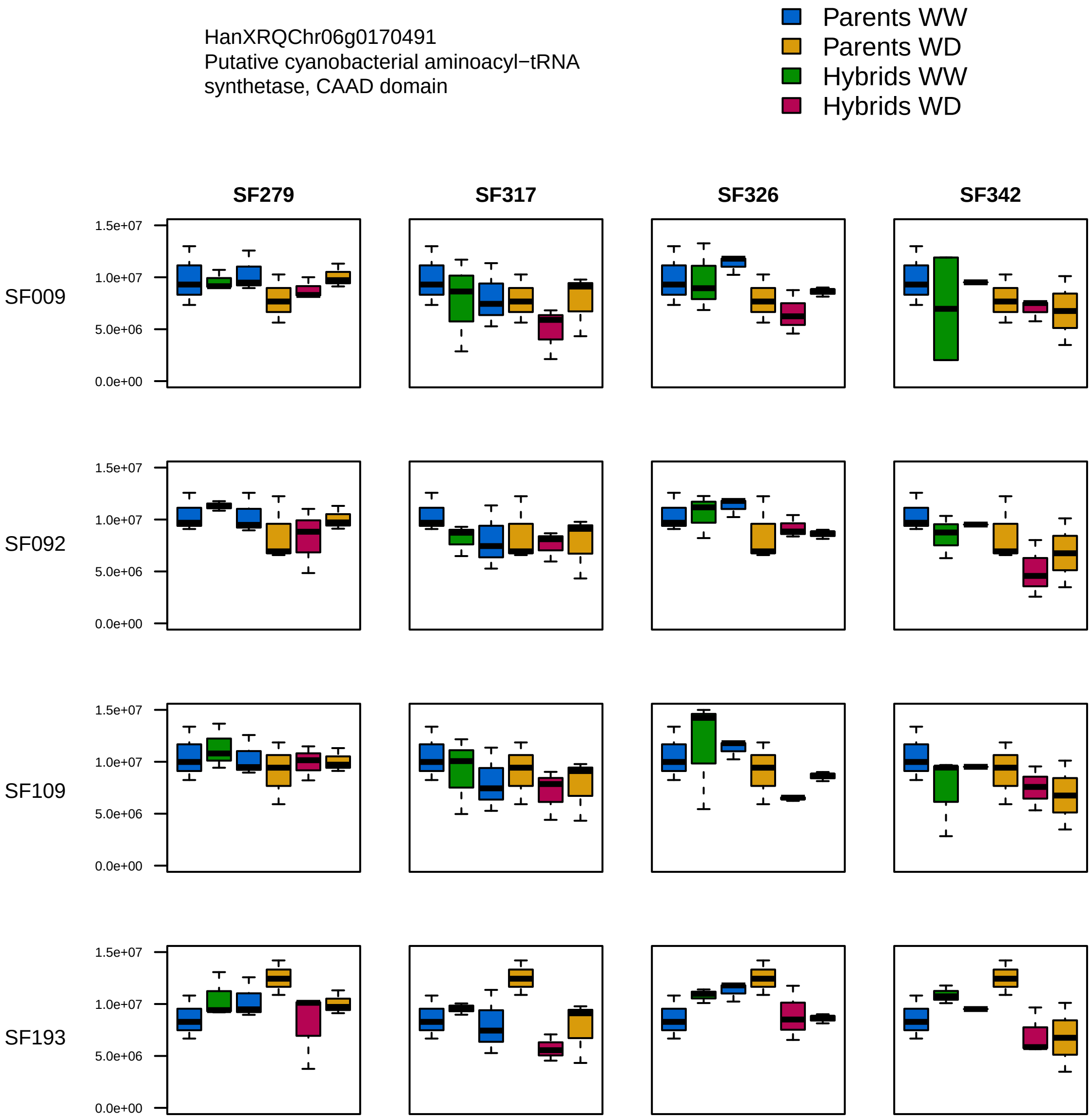
HanXRQChr06g0170491
Putative cyanobacterial aminoacyl−tRNA synthetase, CAAD domain
Parents WW
Parents WD
Hybrids WW
Hybrids WD
SF279
SF317
SF326
SF342
SF009
SF092
SF109
SF193
1.5e+07
1.0e+07
5.0e+06
0.0e+00

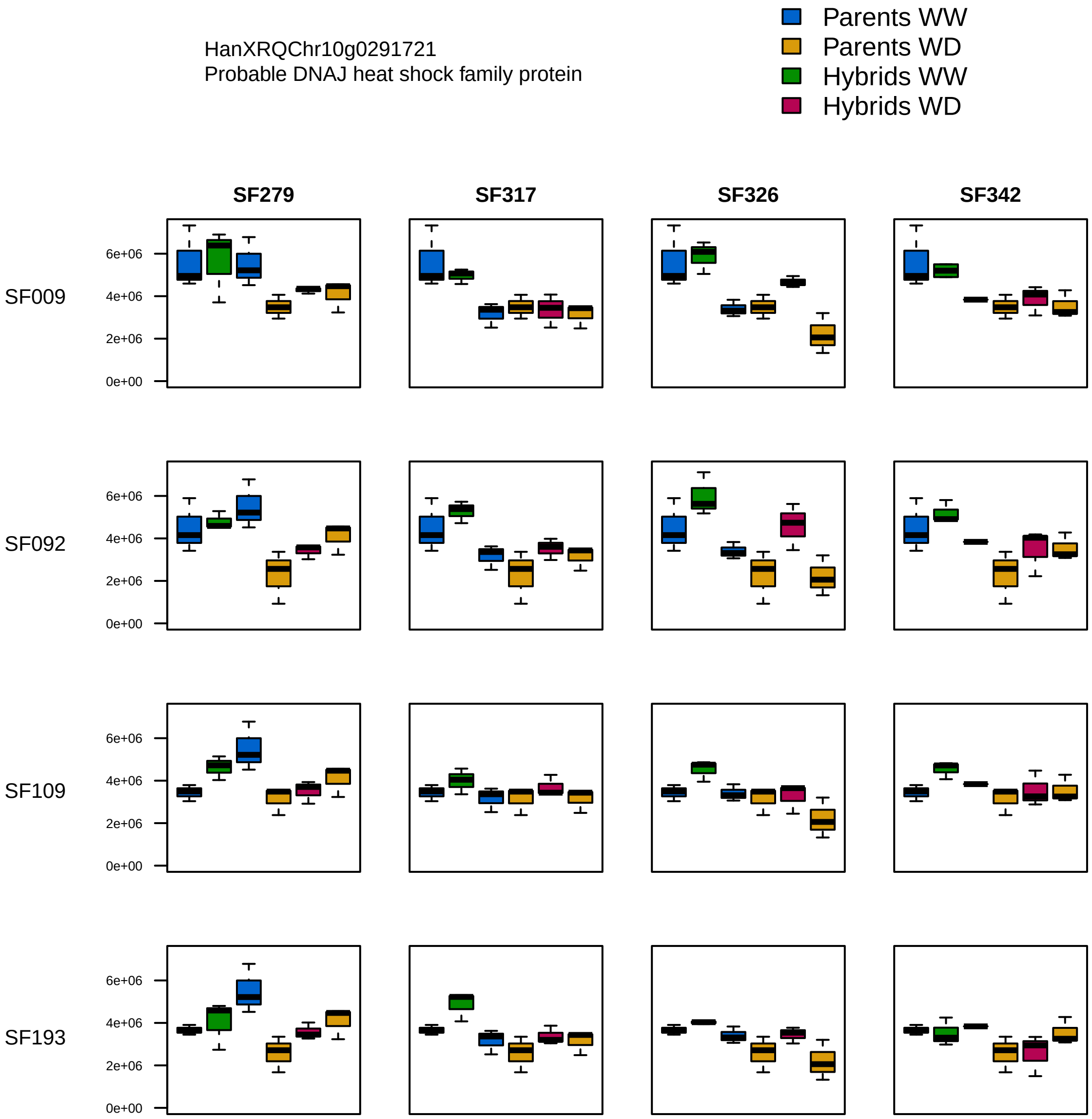
HanXRQChr10g0291721
Probable DNAJ heat shock family protein
Parents WW
Parents WD
Hybrids WW
Hybrids WD
SF279
SF317
SF326
SF342
SF009
SF092
SF109
SF193
6e+06
4e+06
2e+06
0e+00

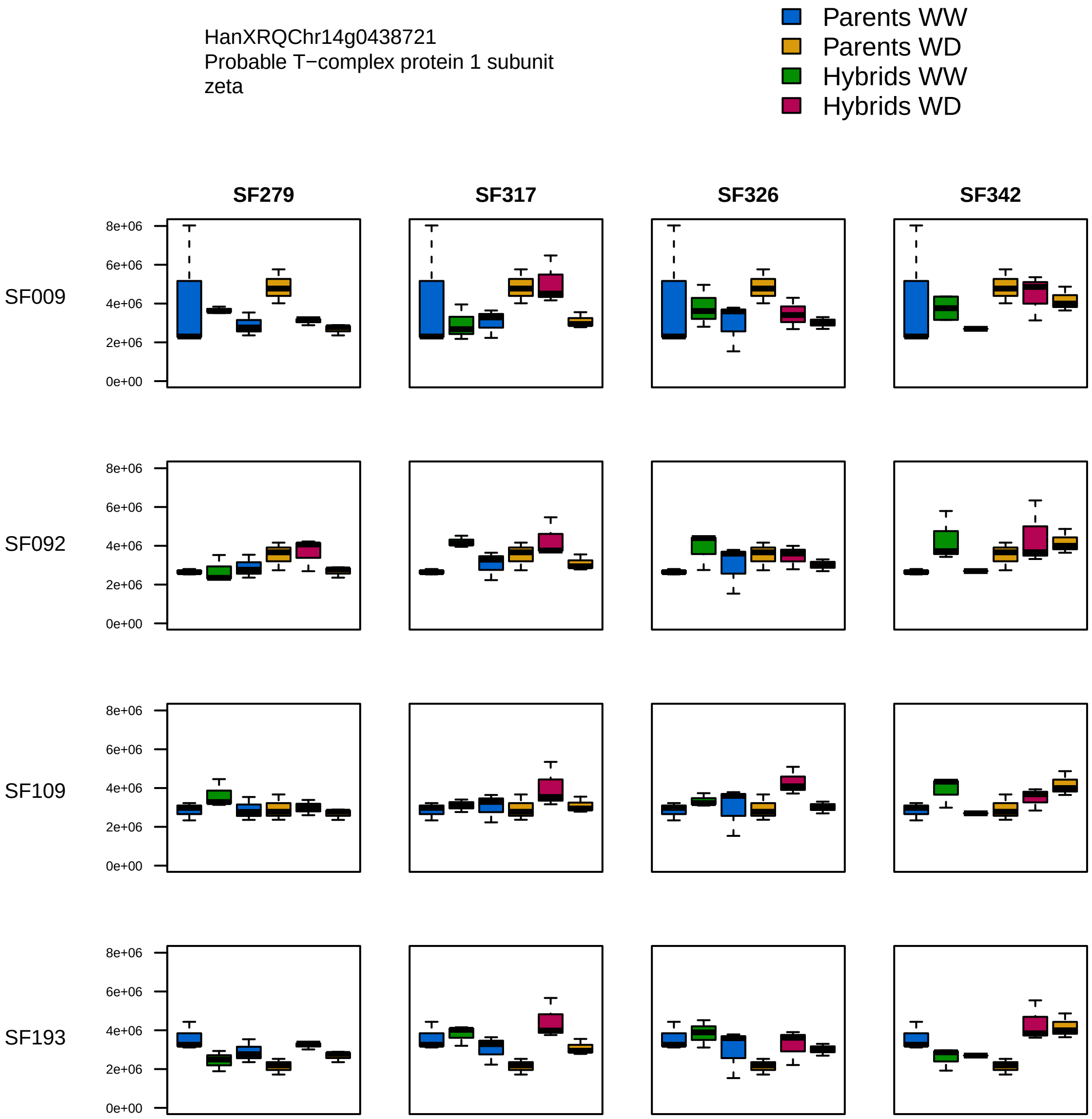
HanXRQChr14g0438721
Probable T−complex protein 1 subunit
zeta
Parents WW
Parents WD
Hybrids WW
Hybrids WD
SF279
SF317
SF326
SF342
SF009
SF092
SF109
SF193
0e+00
2e+06
4e+06
6e+06
8e+06

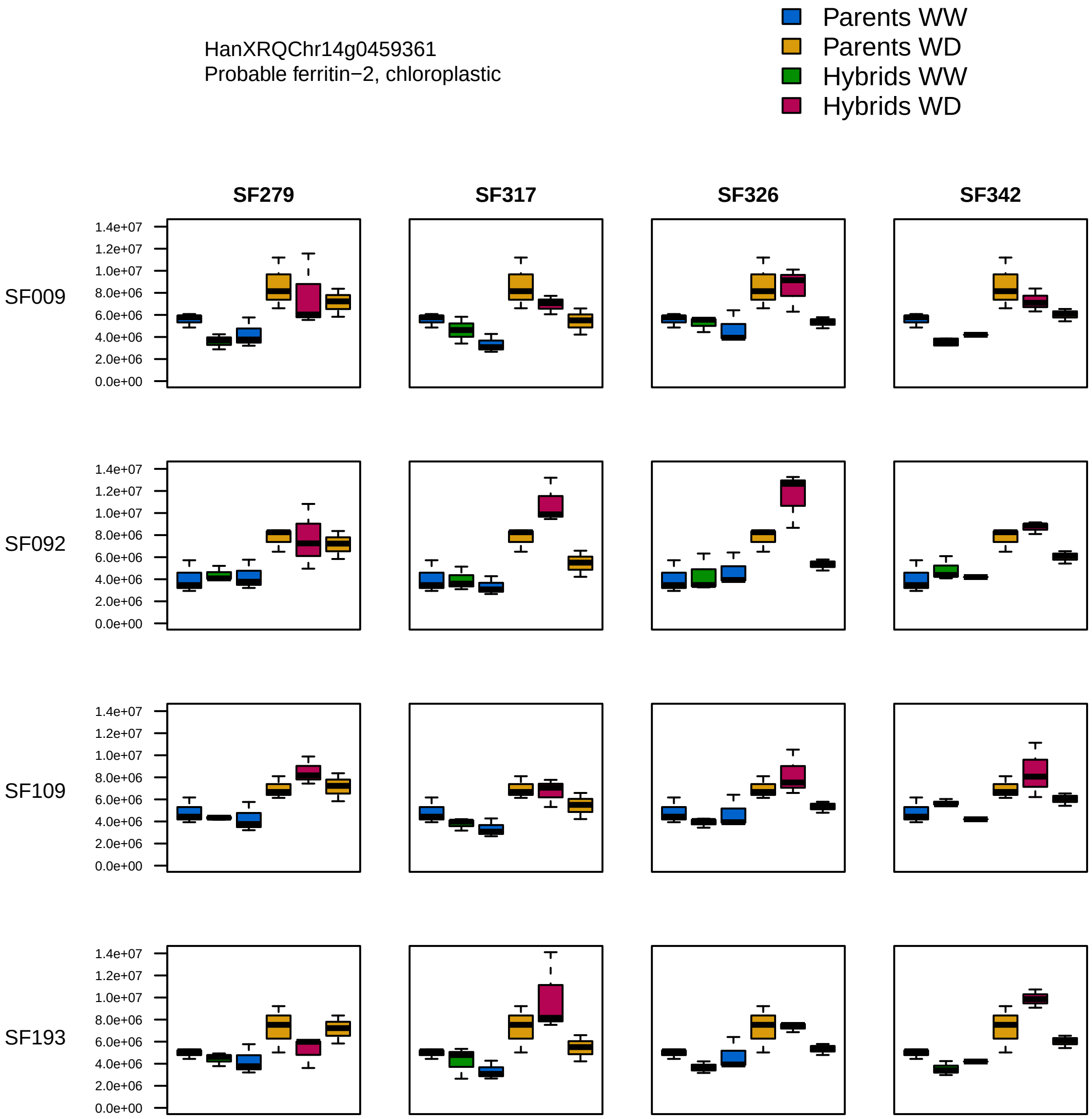

HanXRQChr14g0459361
Probable ferritin−2, chloroplastic
Parents WW
Parents WD
Hybrids WW
Hybrids WD
SF279
SF317
SF326
SF342
SF009
SF092
SF109
SF193
1.4e+07
1.2e+07
1.0e+07
8.0e+06
6.0e+06
4.0e+06
2.0e+06
0.0e+00

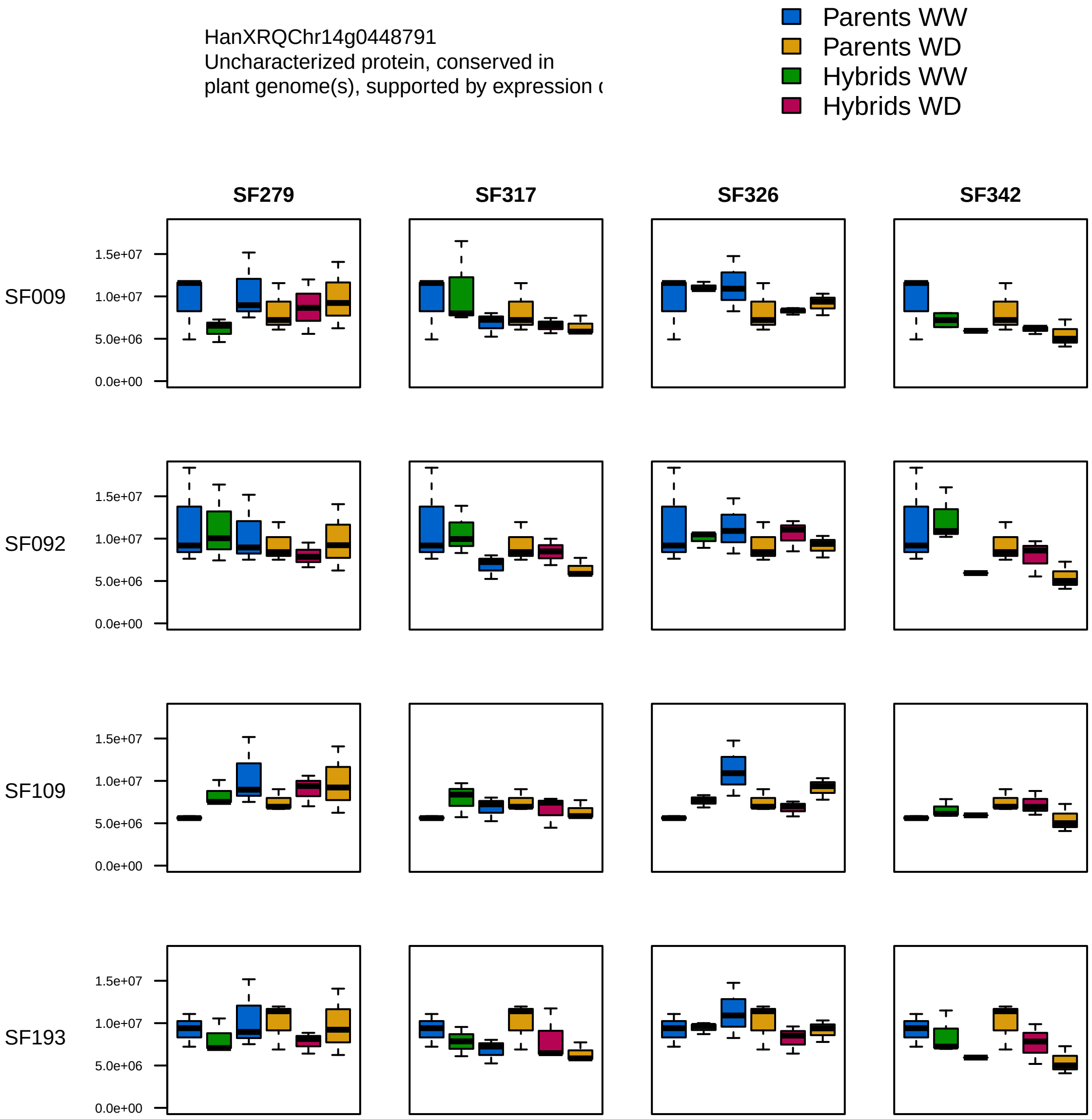
HanXRQChr14g0448791
Uncharacterized protein, conserved in
plant genome(s), supported by expression
Parents WW
Parents WD
Hybrids WW
Hybrids WD
SF279
SF317
SF326
SF342
SF009
SF092
SF109
SF193
1.5e+07
1.0e+07
5.0e+06
0.0e+00

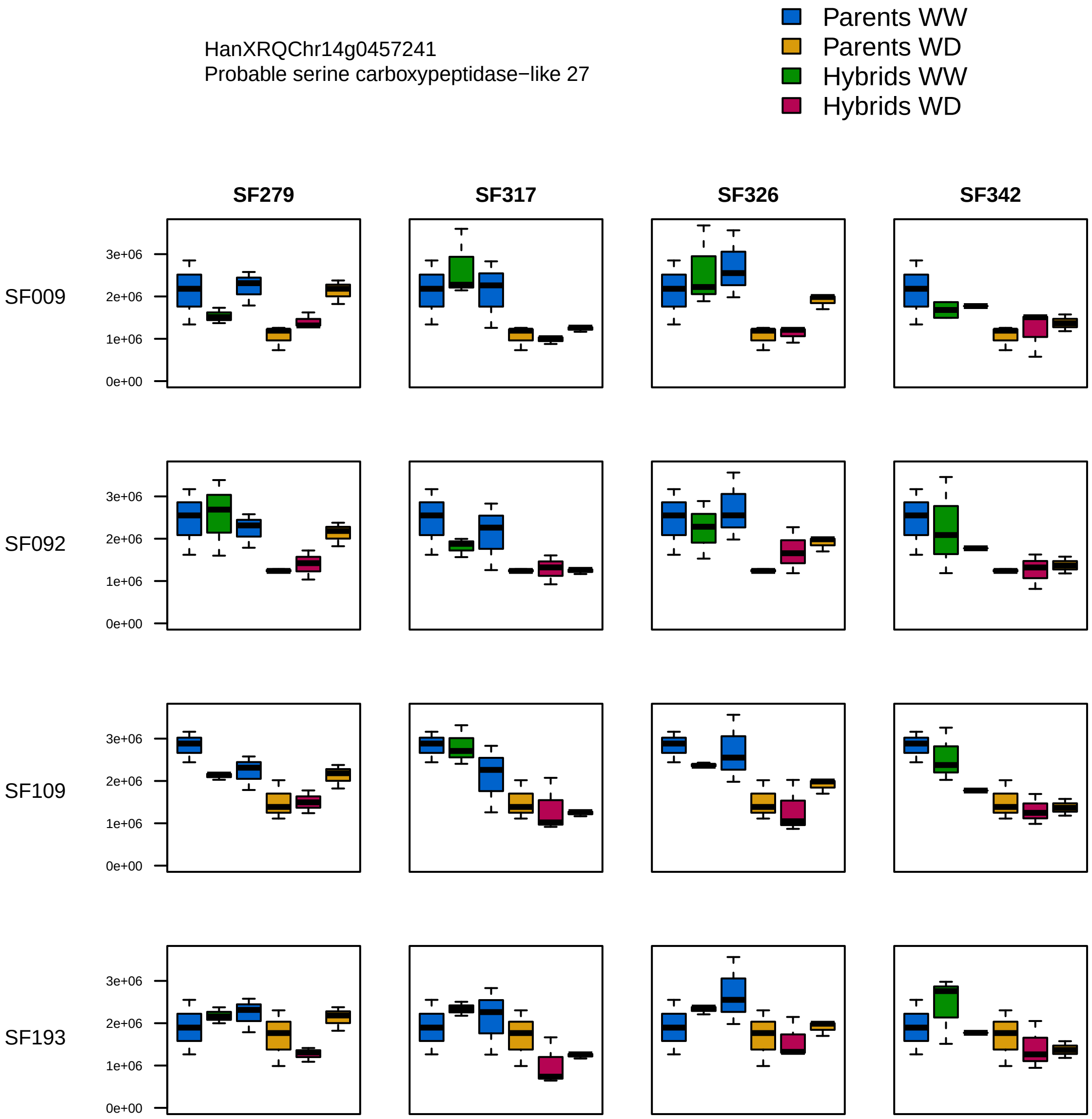
HanXRQChr14g0457241
Probable serine carboxypeptidase−like 27
Parents WW
Parents WD
Hybrids WW
Hybrids WD
SF279
SF317
SF326
SF342
SF009
SF092
SF109
SF193
3e+06
2e+06
1e+06
0e+00

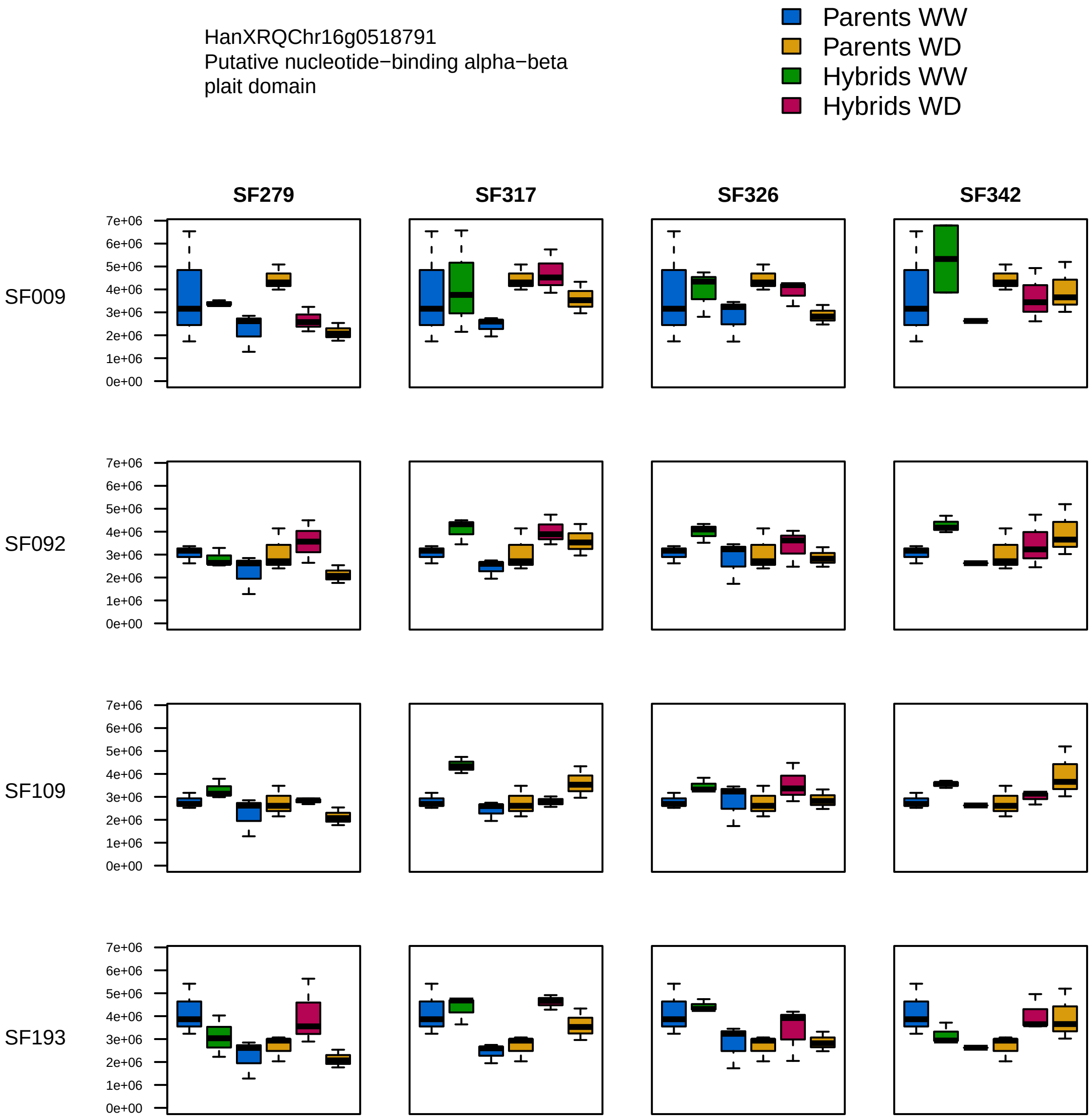
HanXRQChr16g0518791
Putative nucleotide−binding alpha−beta plait domain
Parents WW
Parents WD
Hybrids WW
Hybrids WD
SF279
SF317
SF326
SF342
SF009
SF092
SF109
SF193
7e+06
6e+06
5e+06
4e+06
3e+06
2e+06
1e+06
0e+00

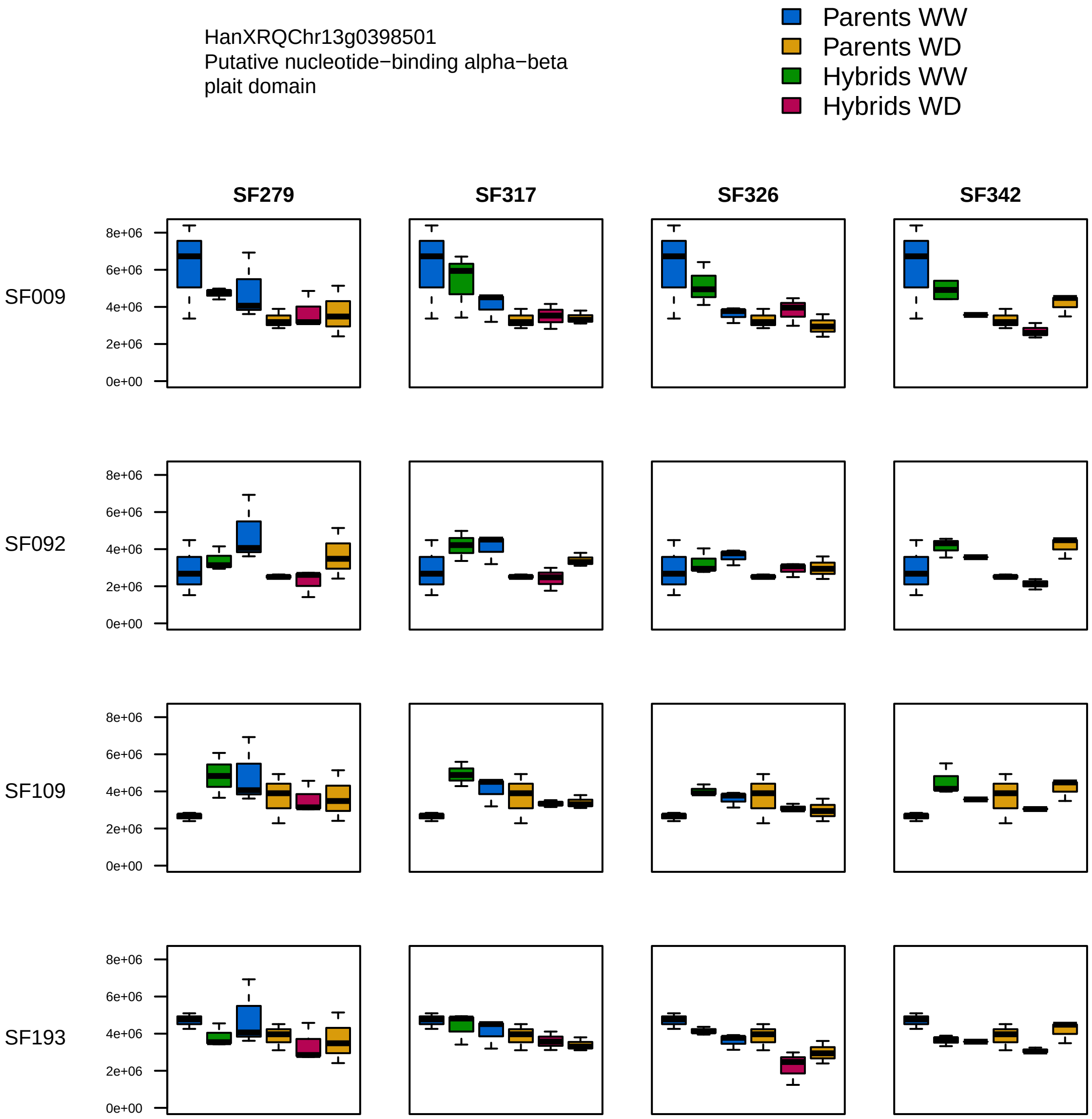

HanXRQChr13g0398501
Putative nucleotide−binding alpha−beta plait domain
Parents WW
Parents WD
Hybrids WW
Hybrids WD
SF279
SF317
SF326
SF342
SF009
SF092
SF109
SF193
0e+00
2e+06
4e+06
6e+06
8e+06

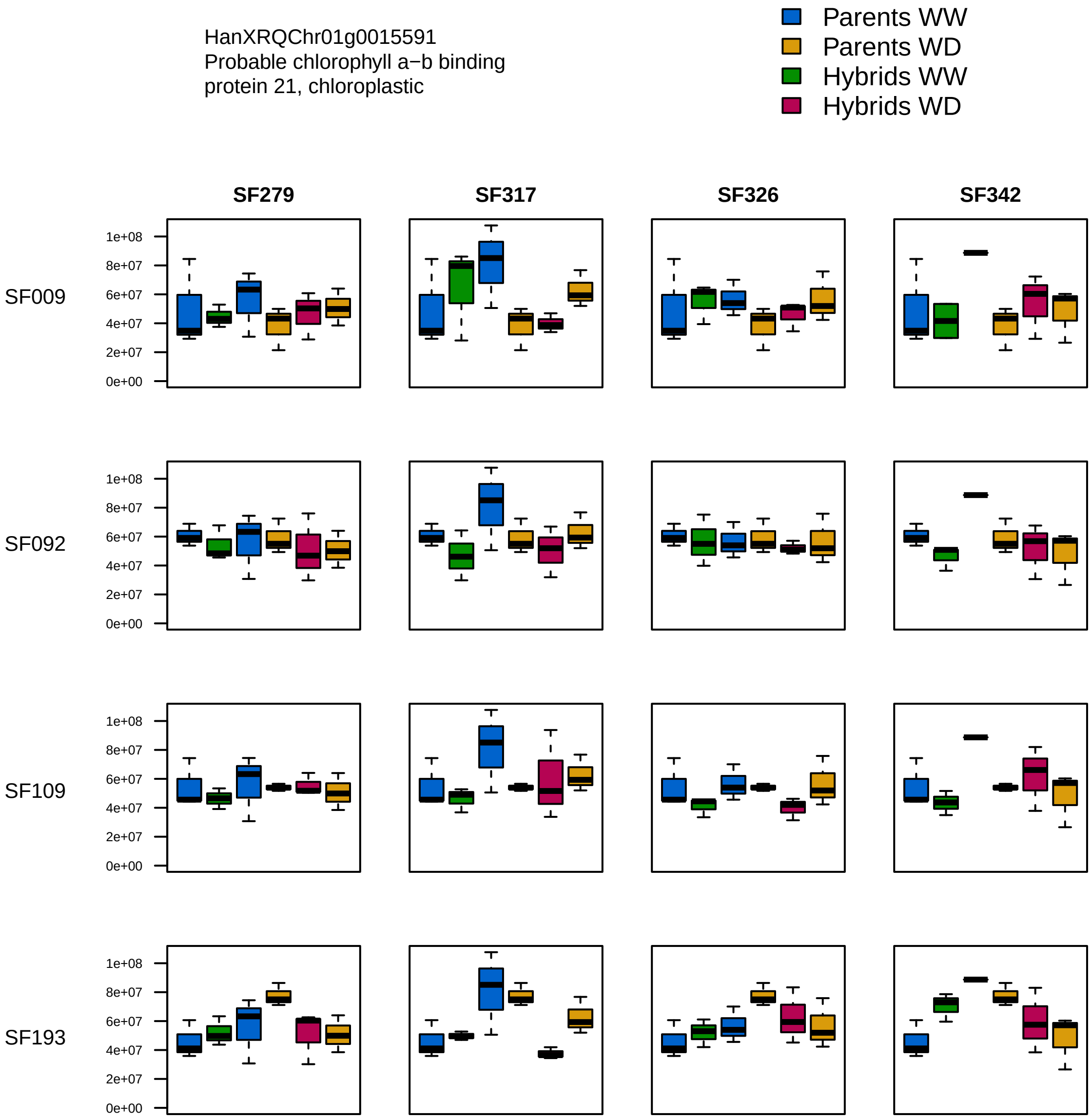
HanXRQChr01g0015591
Probable chlorophyll a−b binding
protein 21, chloroplastic
Parents WW
Parents WD
Hybrids WW
Hybrids WD
SF279
SF317
SF326
SF342
SF009
SF092
SF109
SF193
1e+08
8e+07
6e+07
4e+07
2e+07
0e+00

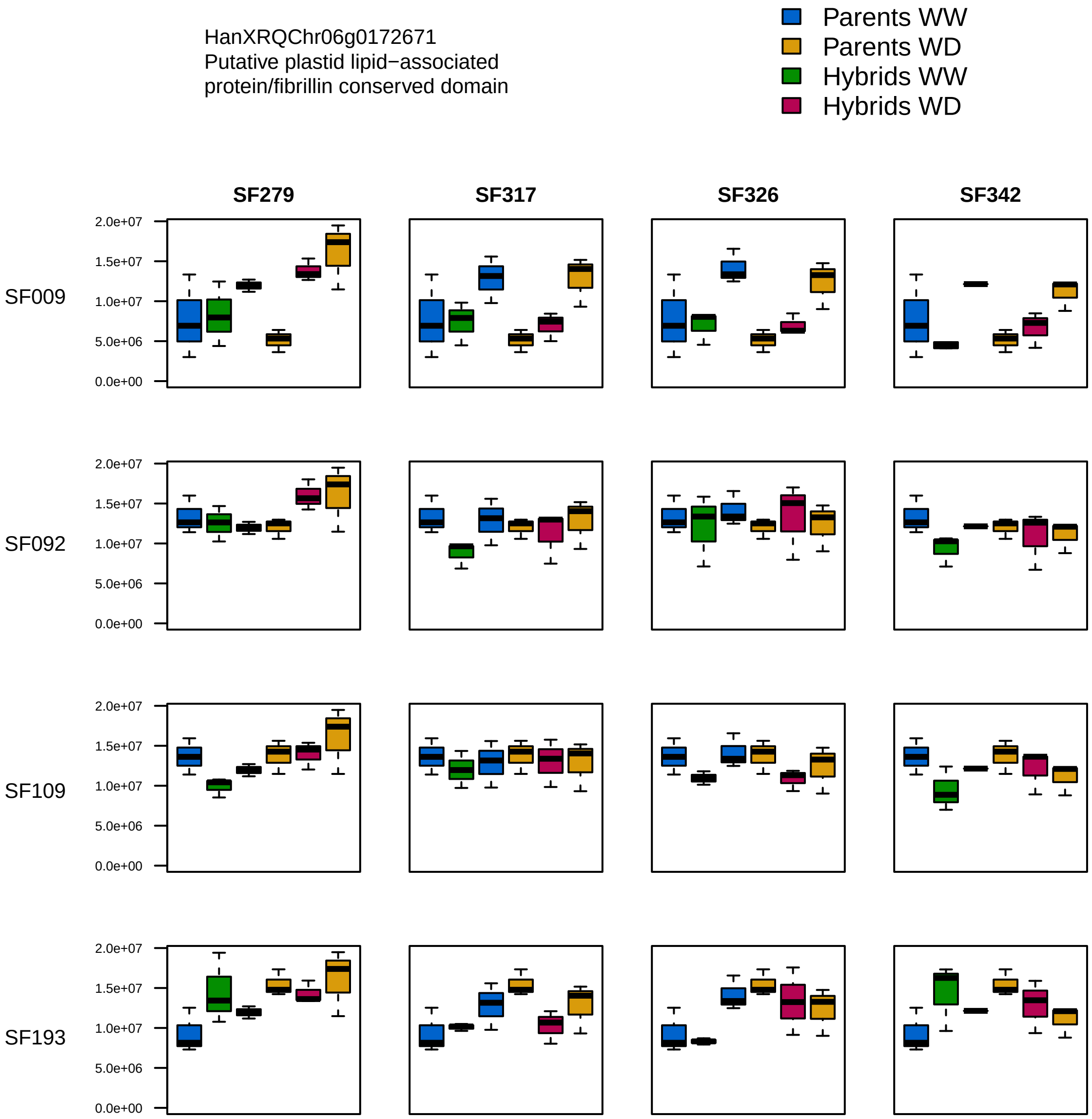

HanXRQChr06g0172671
Putative plastid lipid−associated
protein/fibrillin conserved domain
Parents WW
Parents WD
Hybrids WW
Hybrids WD
SF279
SF317
SF326
SF342
SF009
SF092
SF109
SF193
2.0e+07
1.5e+07
1.0e+07
5.0e+06
0.0e+00

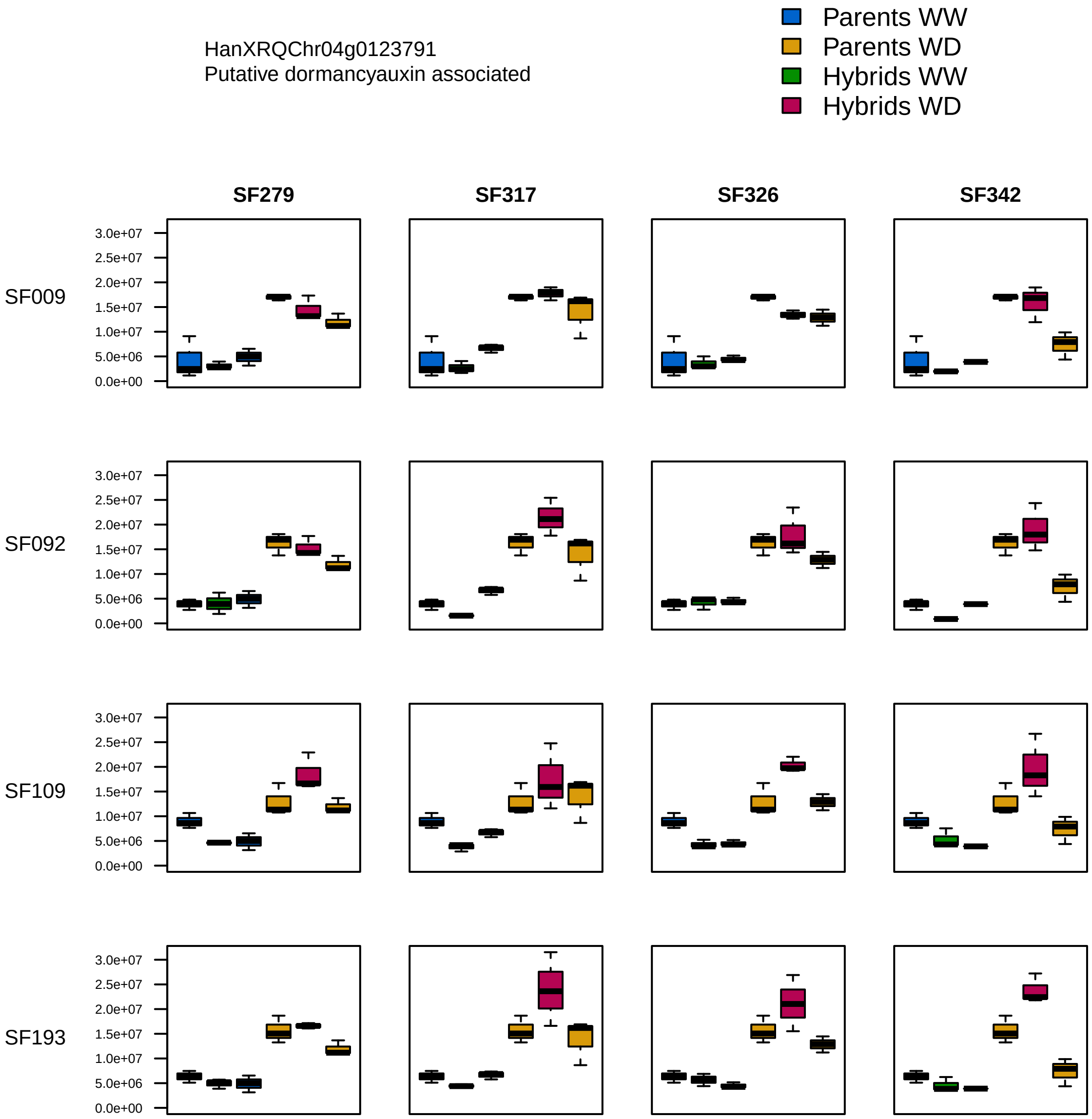
HanXRQChr04g0123791
Putative dormancyauxin associated
Parents WW
Parents WD
Hybrids WW
Hybrids WD
SF279
SF317
SF326
SF342
SF009
SF092
SF109
SF193
3.0e+07
2.5e+07
2.0e+07
1.5e+07
1.0e+07
5.0e+06
0.0e+00

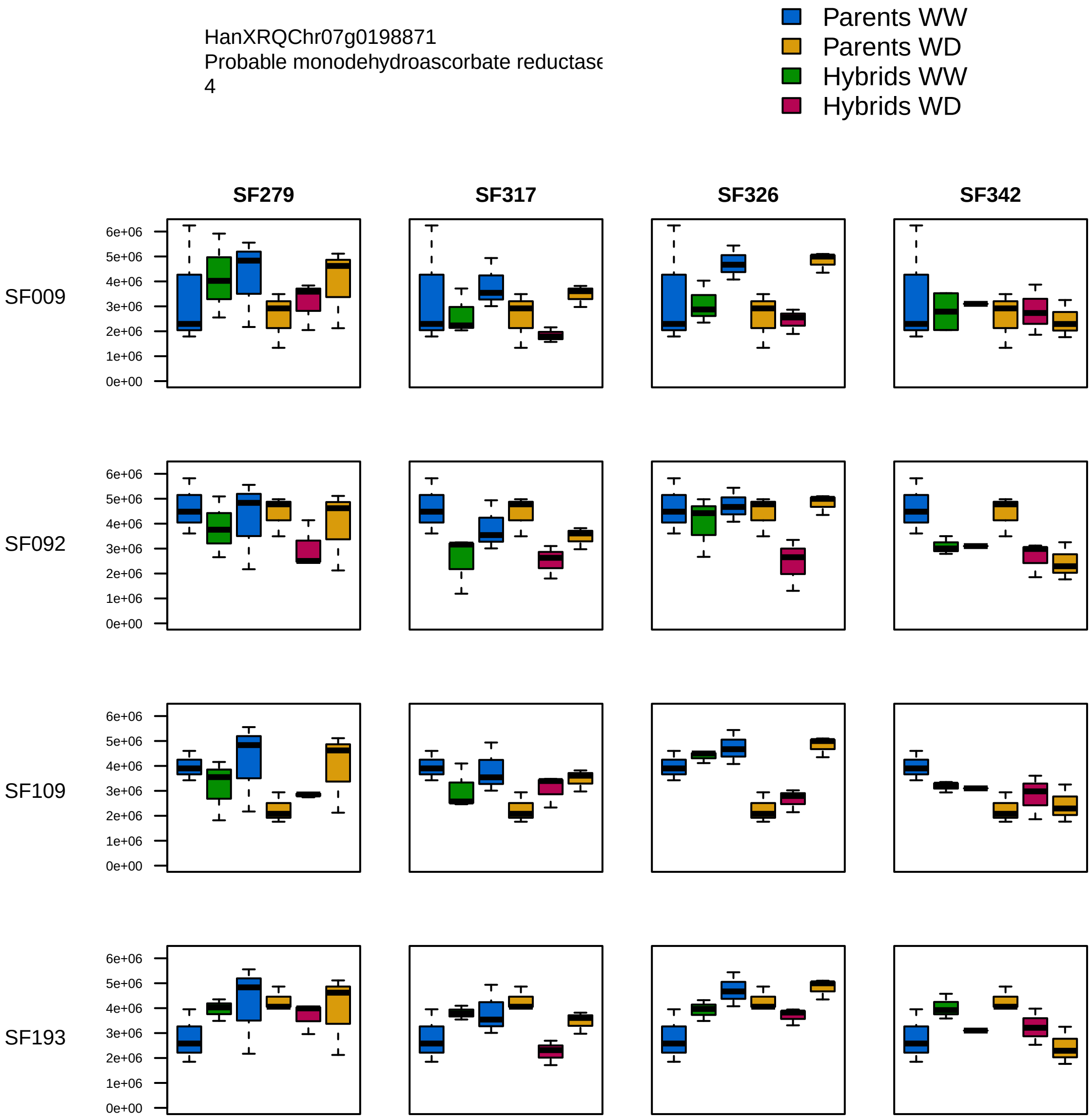
HanXRQChr07g0198871
Probable monodehydroascorbate reductase
4
Parents WW
Parents WD
Hybrids WW
Hybrids WD
SF279
SF317
SF326
SF342
SF009
SF092
SF109
SF193
6e+06
5e+06
4e+06
3e+06
2e+06
1e+06
0e+00

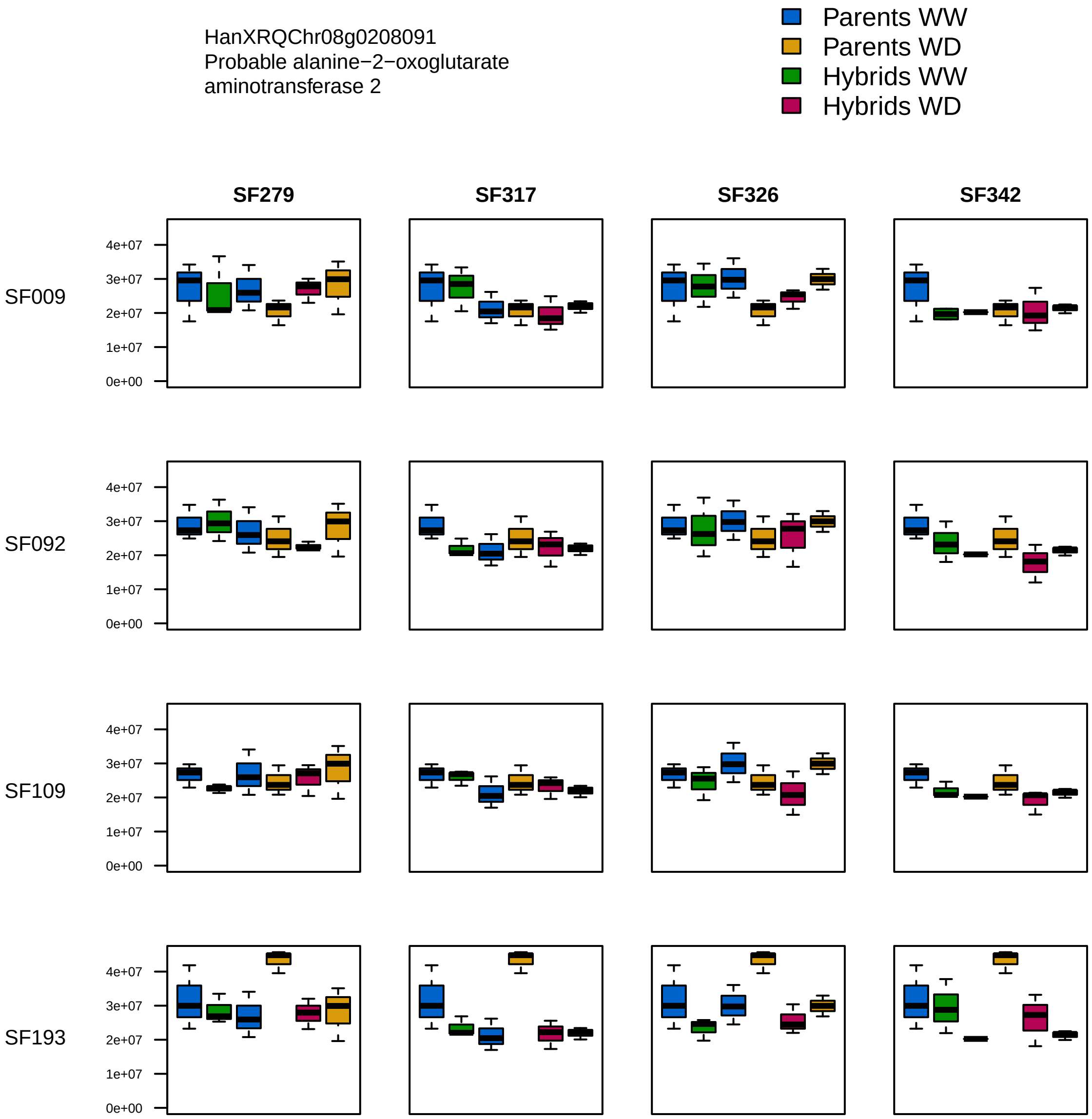

HanXRQChr08g0208091
Probable alanine−2−oxoglutarate
aminotransferase 2
Parents WW
Parents WD
Hybrids WW
Hybrids WD
SF279
SF317
SF326
SF342
SF009
SF092
SF109
SF193
4e+07
3e+07
2e+07
1e+07
0e+00

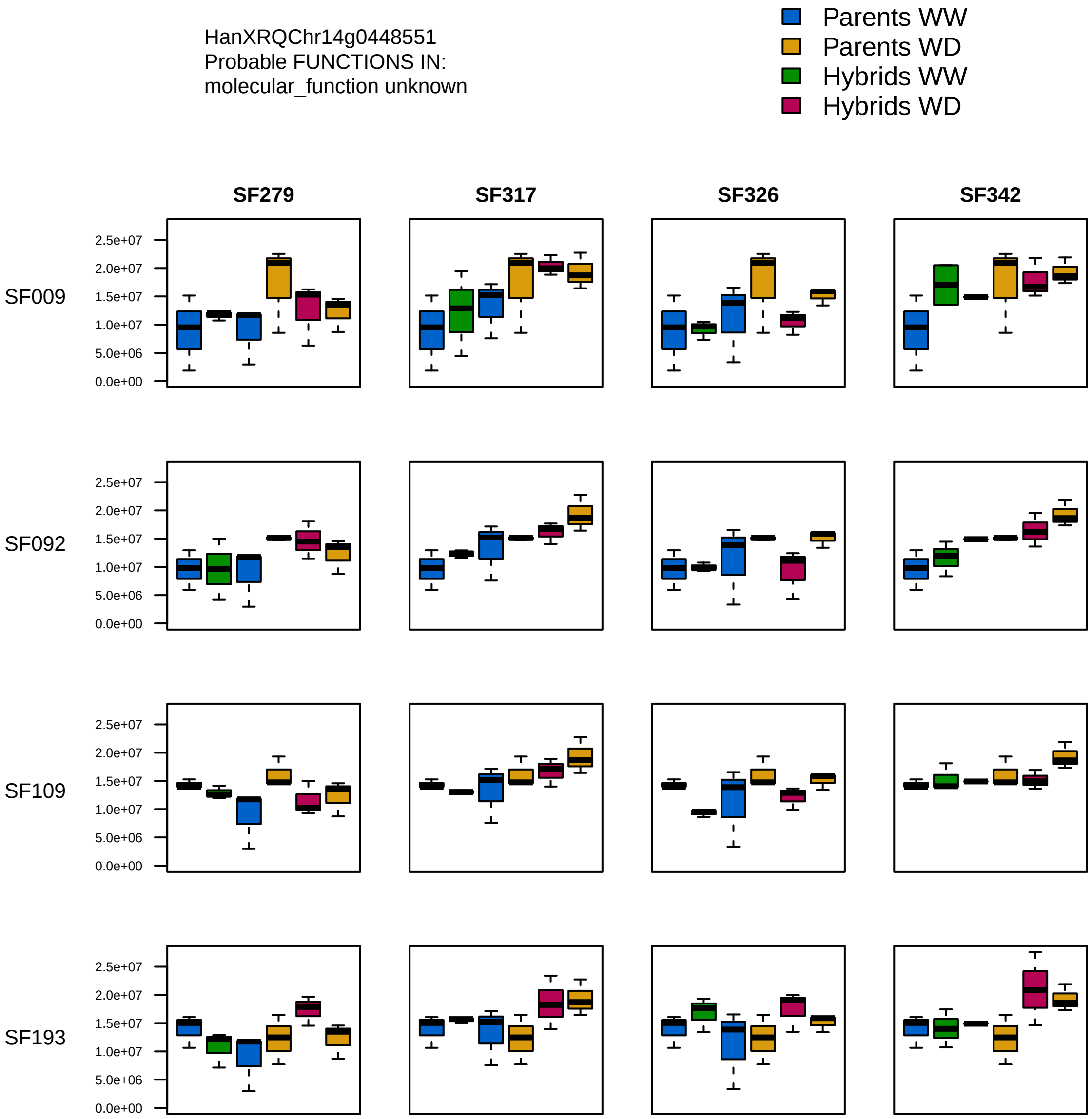
HanXRQChr14g0448551
Probable FUNCTIONS IN:
molecular_function unknown
Parents WW
Parents WD
Hybrids WW
Hybrids WD
SF279
SF317
SF326
SF342
SF009
SF092
SF109
SF193
2.5e+07
2.0e+07
1.5e+07
1.0e+07
5.0e+06
0.0e+00

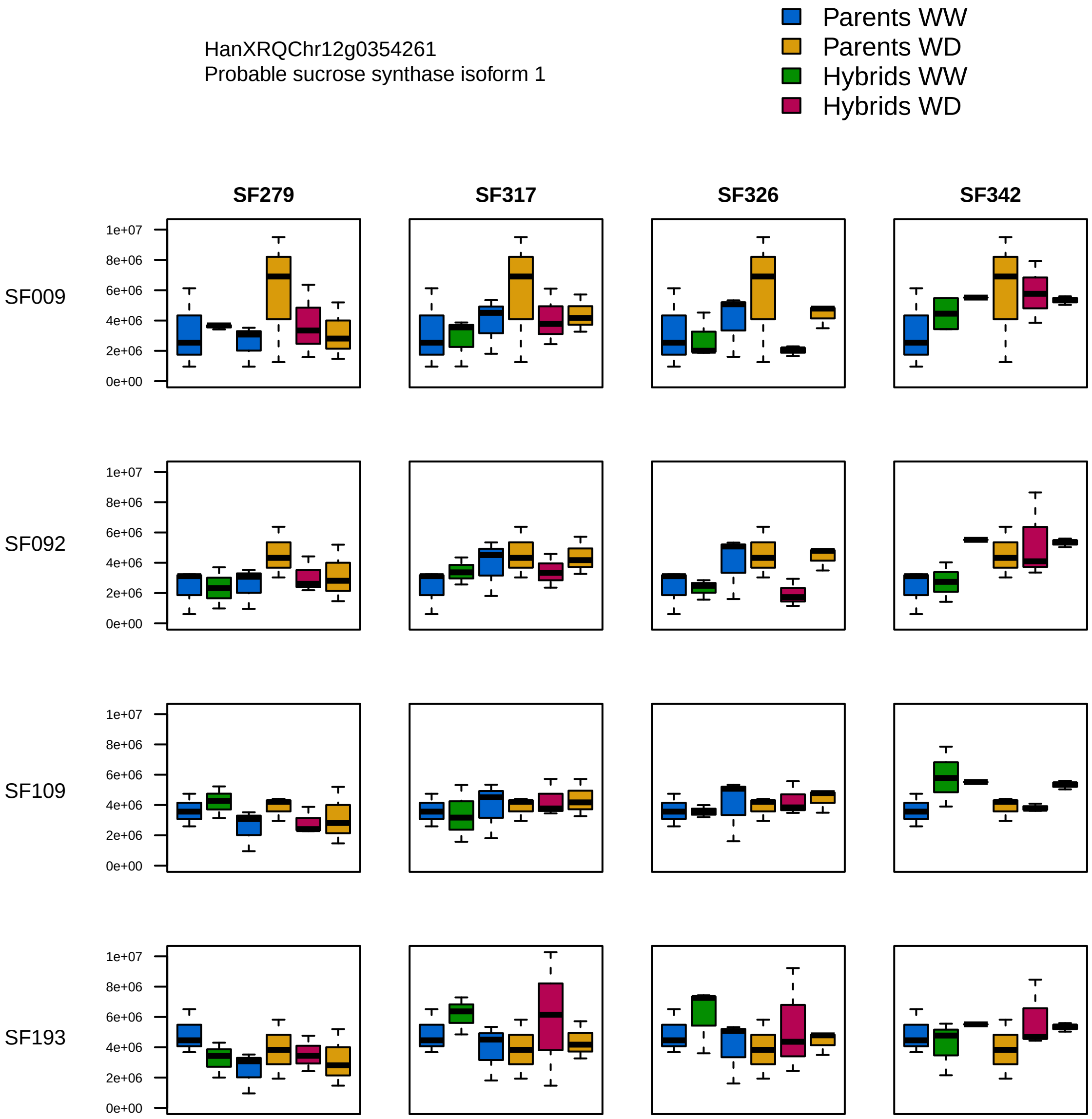
HanXRQChr12g0354261
Probable sucrose synthase isoform 1
Parents WW
Parents WD
Hybrids WW
Hybrids WD
SF279
SF317
SF326
SF342
SF009
SF092
SF109
SF193
0e+00
2e+06
4e+06
6e+06
8e+06
1e+07

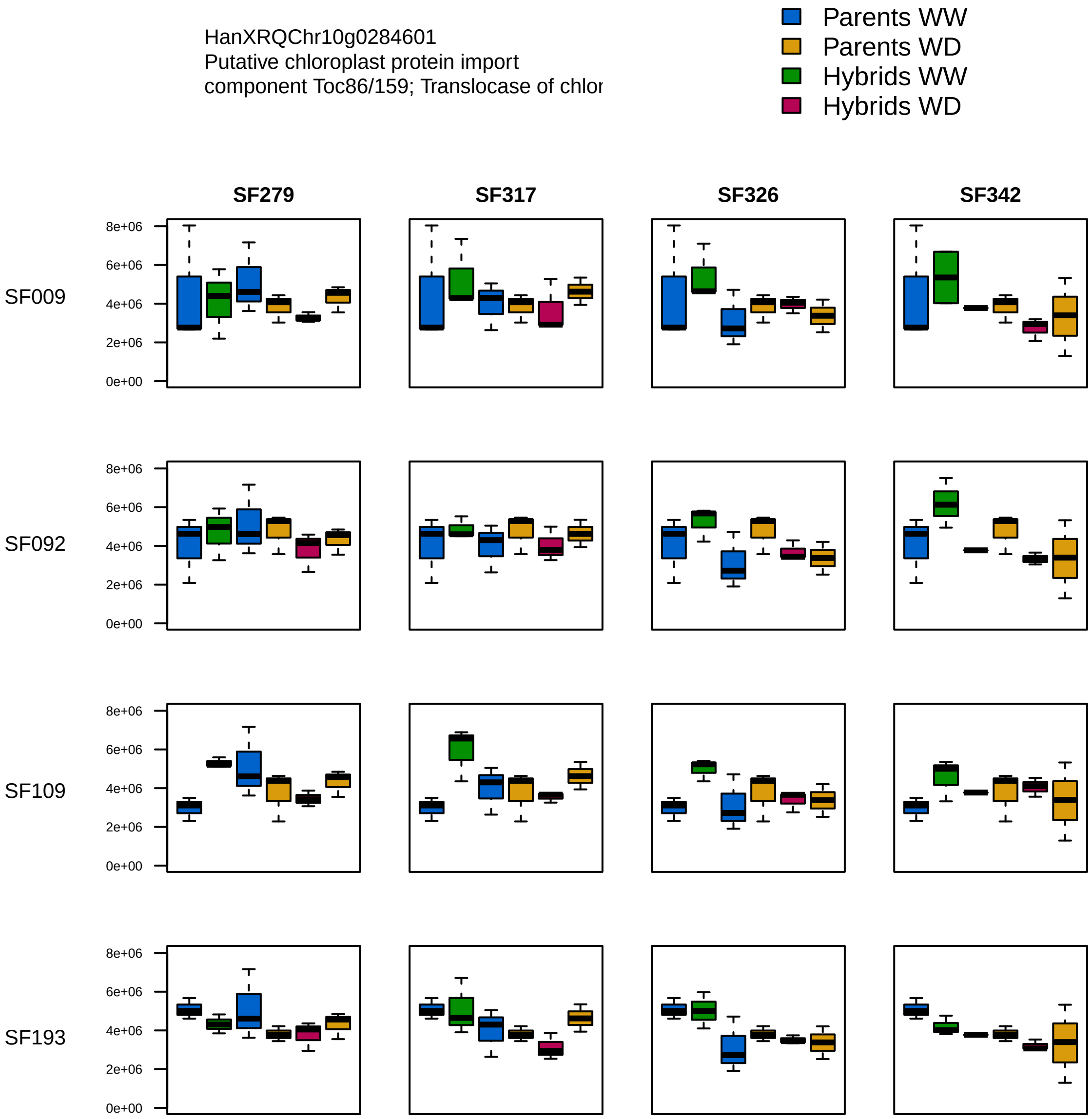

HanXRQChr10g0284601
Putative chloroplast protein import
component Toc86/159; Translocase of chlor
Parents WW
Parents WD
Hybrids WW
Hybrids WD
SF279
SF317
SF326
SF342
SF009
SF092
SF109
SF193
8e+06
6e+06
4e+06
2e+06
0e+00

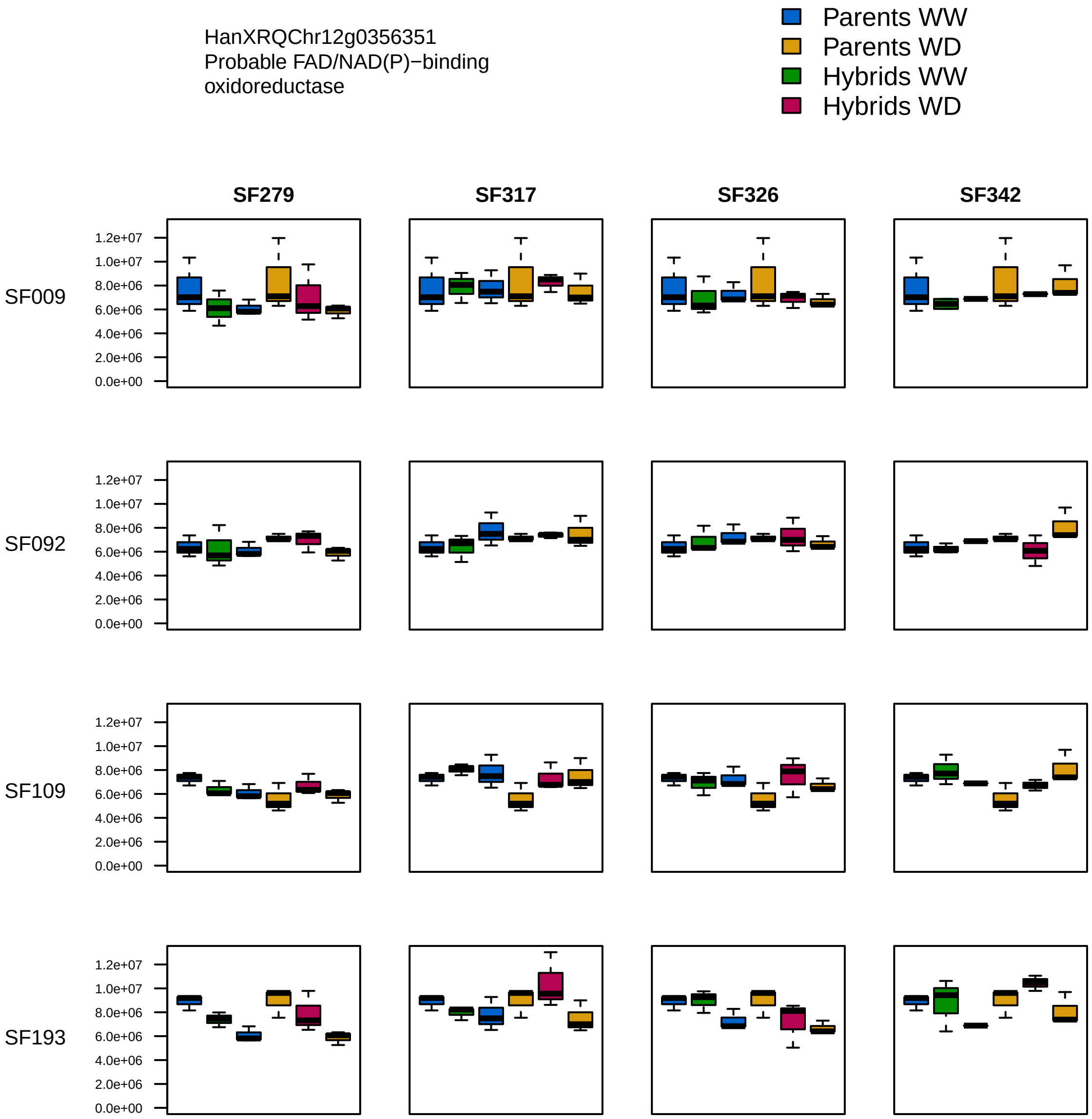
HanXRQChr12g0356351
Probable FAD/NAD(P)−binding
oxidoreductase
Parents WW
Parents WD
Hybrids WW
Hybrids WD
SF279
SF317
SF326
SF342
SF009
SF092
SF109
SF193
1.2e+07
1.0e+07
8.0e+06
6.0e+06
4.0e+06
2.0e+06
0.0e+00

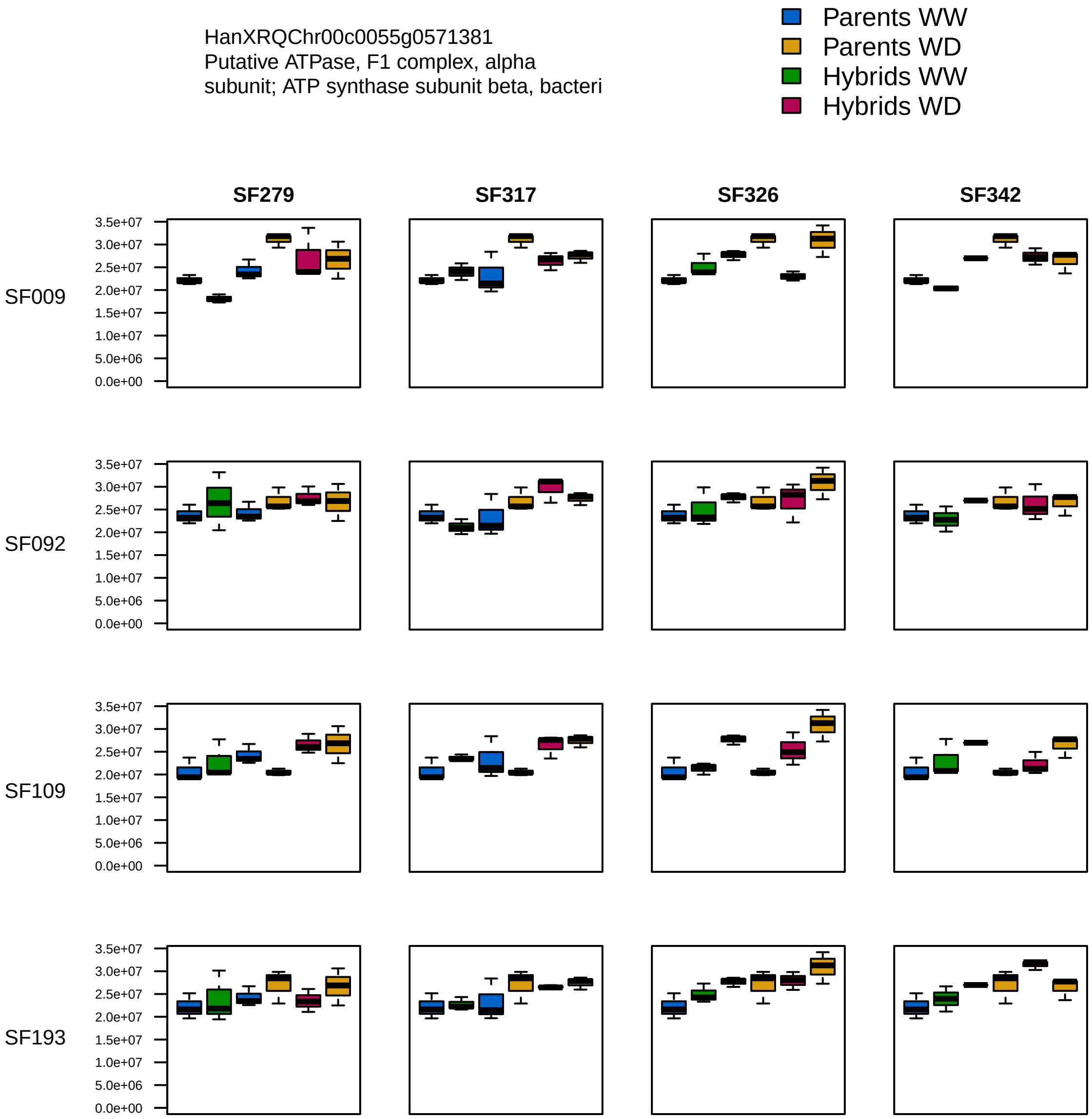
HanXRQChr00c0055g0571381
Putative ATPase, F1 complex, alpha
subunit; ATP synthase subunit beta, bacteri
Parents WW
Parents WD
Hybrids WW
Hybrids WD
SF279
SF317
SF326
SF342
SF009
SF092
SF109
SF193
3.5e+07
3.0e+07
2.5e+07
2.0e+07
1.5e+07
1.0e+07
5.0e+06
0.0e+00

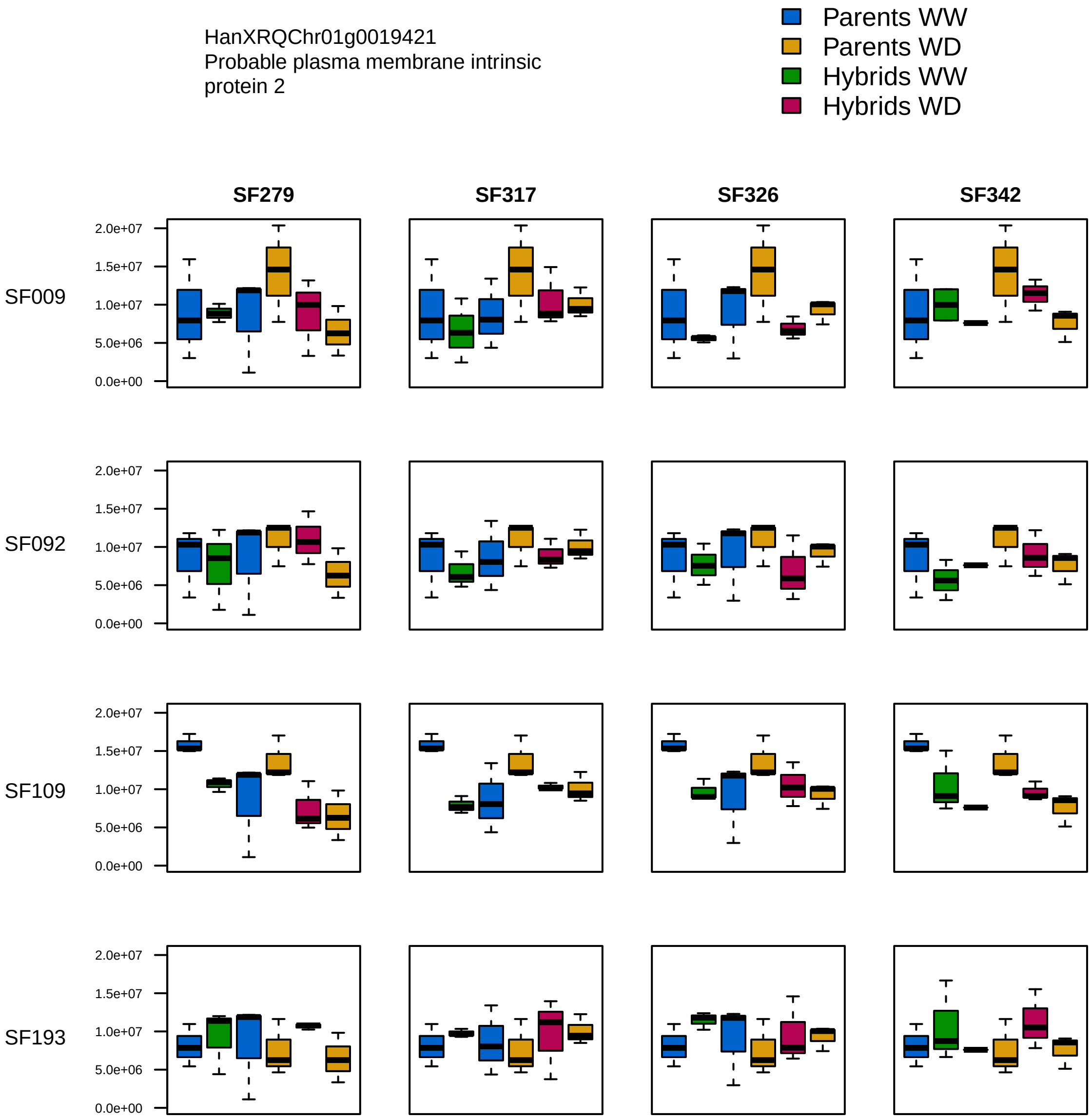
HanXRQChr01g0019421
Probable plasma membrane intrinsic
protein 2
Parents WW
Parents WD
Hybrids WW
Hybrids WD
SF279
SF317
SF326
SF342
SF009
SF092
SF109
SF193
2.0e+07
1.5e+07
1.0e+07
5.0e+06
0.0e+00

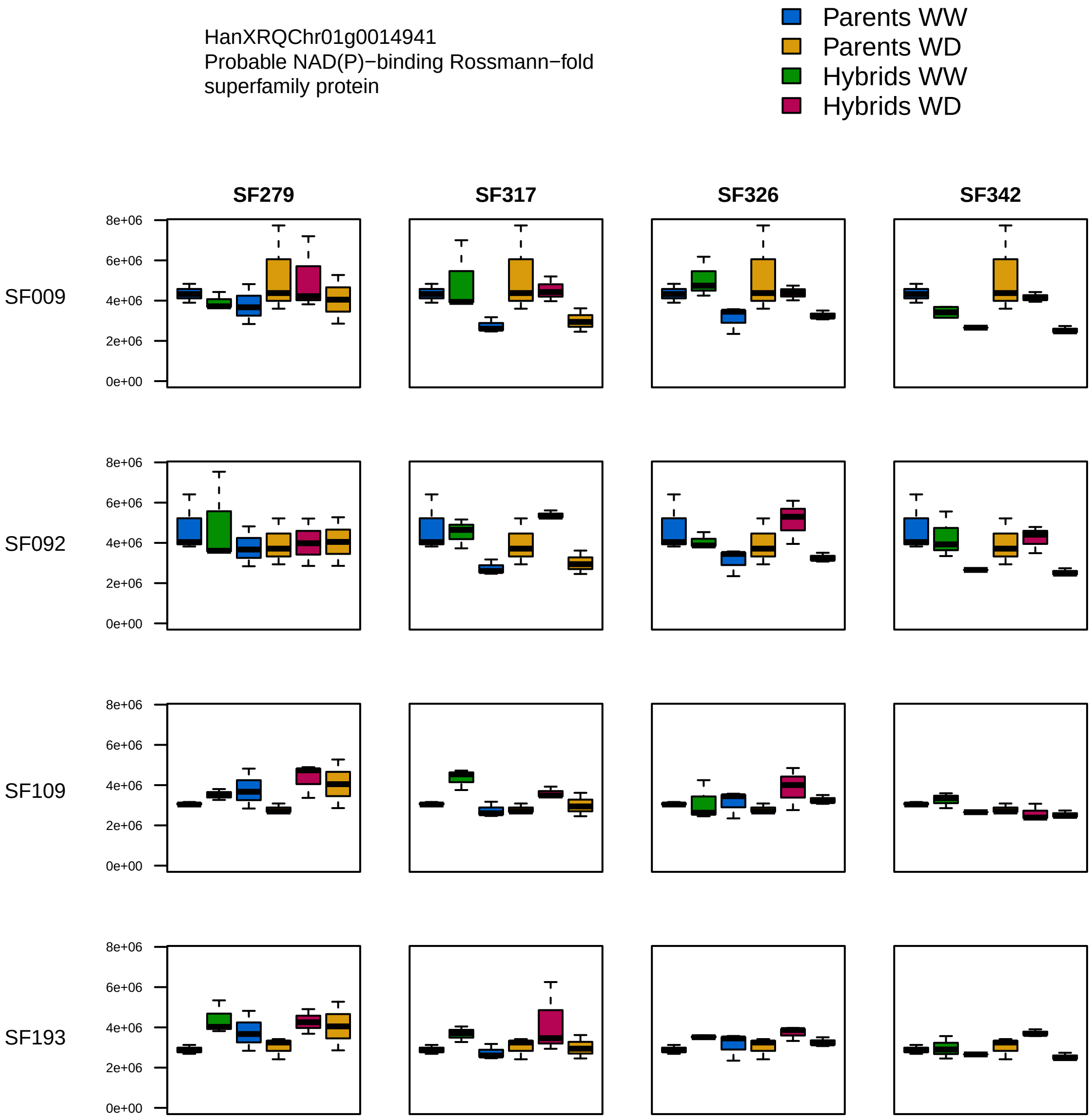
HanXRQChr01g0014941
Probable NAD(P)−binding Rossmann−fold
superfamily protein
Parents WW
Parents WD
Hybrids WW
Hybrids WD
SF279
SF317
SF326
SF342
SF009
SF092
SF109
SF193
8e+06
6e+06
4e+06
2e+06
0e+00

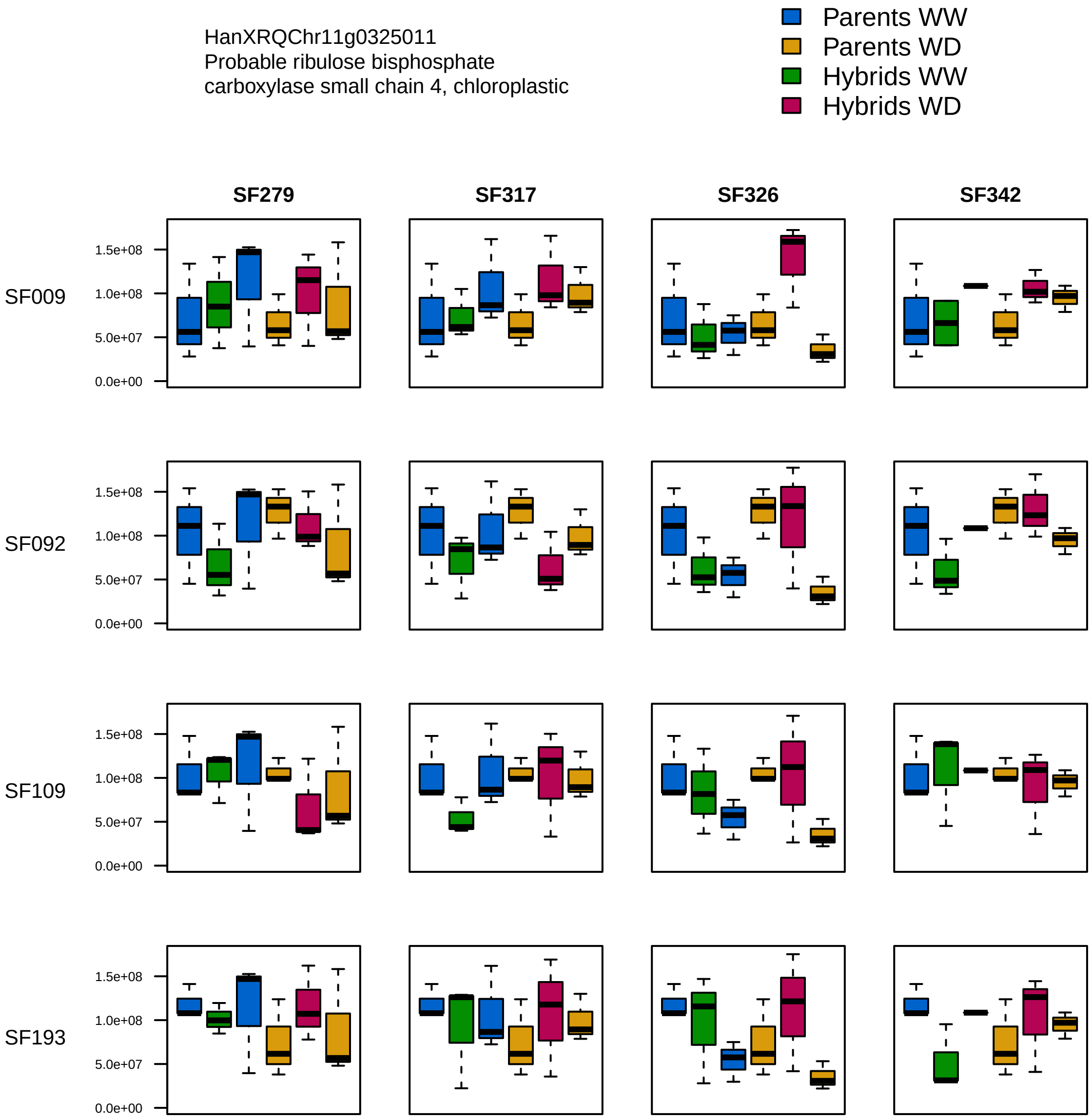
HanXRQChr11g0325011
Probable ribulose bisphosphate
carboxylase small chain 4, chloroplastic
Parents WW
Parents WD
Hybrids WW
Hybrids WD
SF279
SF317
SF326
SF342
SF009
SF092
SF109
SF193
1.5e+08
1.0e+08
5.0e+07
0.0e+00

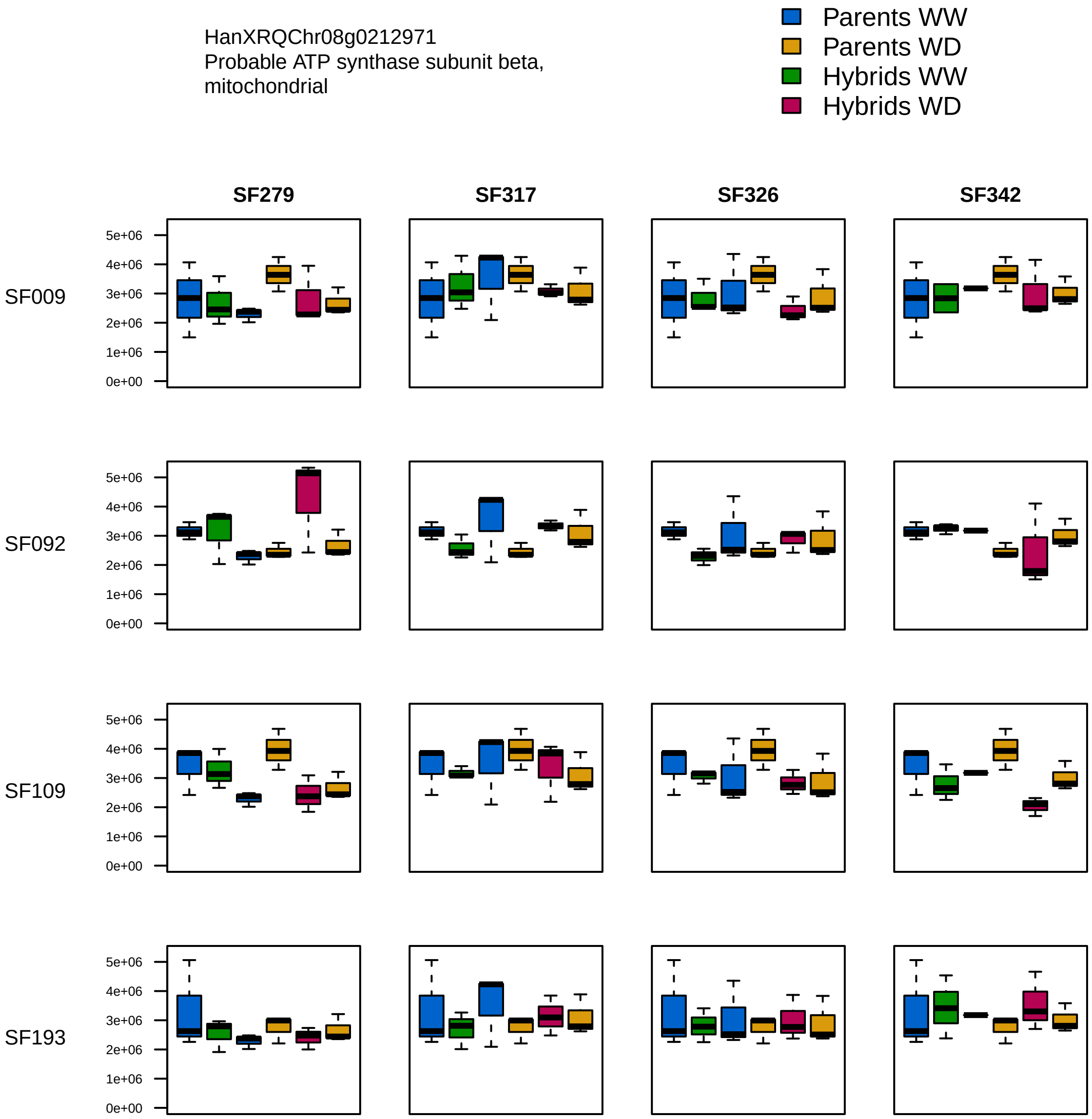

HanXRQChr08g0212971
Probable ATP synthase subunit beta,
mitochondrial
Parents WW
Parents WD
Hybrids WW
Hybrids WD
SF279
SF317
SF326
SF342
SF009
SF092
SF109
SF193
5e+06
4e+06
3e+06
2e+06
1e+06
0e+00

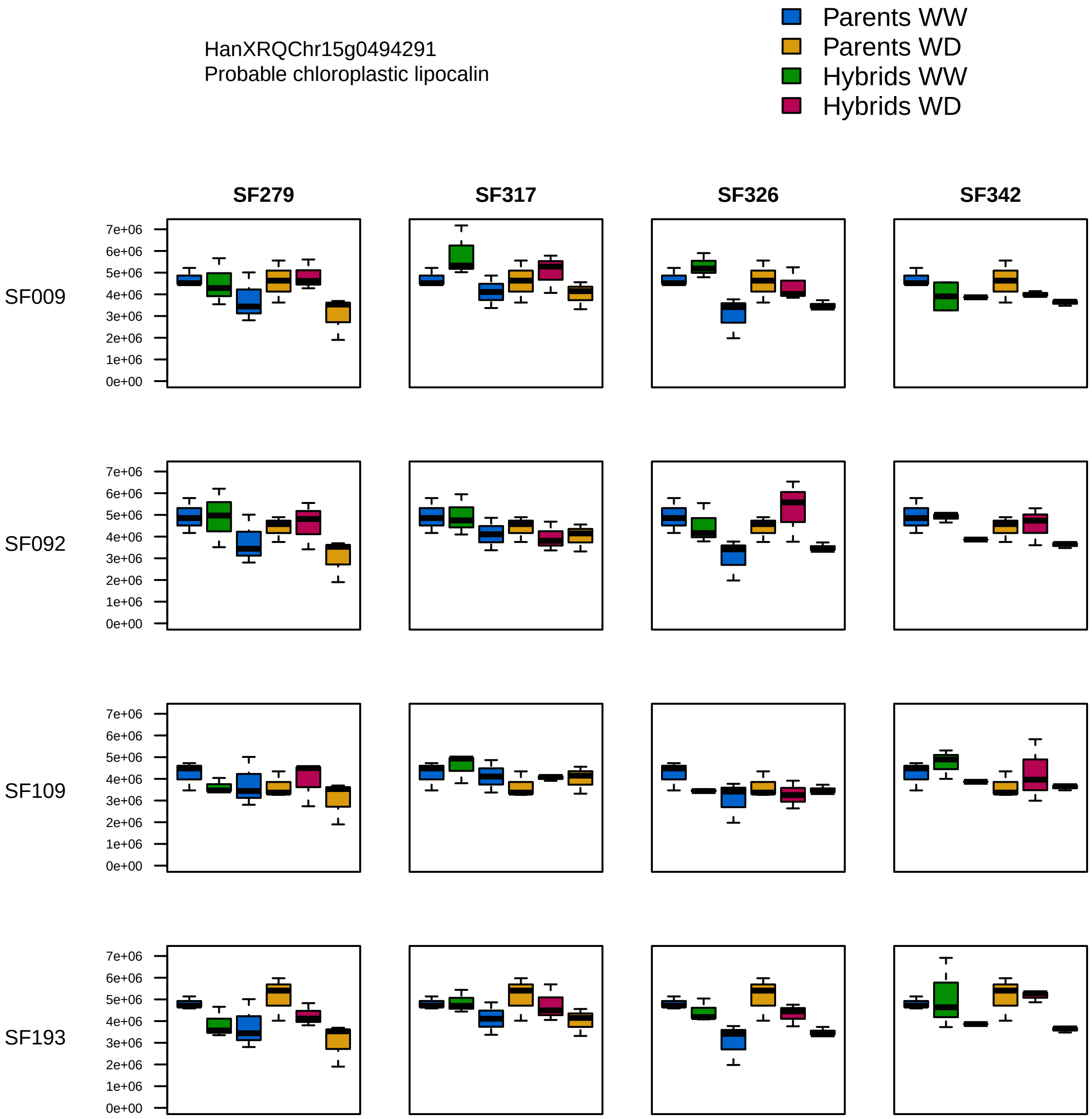
HanXRQChr15g0494291
Probable chloroplastic lipocalin
Parents WW
Parents WD
Hybrids WW
Hybrids WD
SF279
SF317
SF326
SF342
SF009
SF092
SF109
SF193
7e+06
6e+06
5e+06
4e+06
3e+06
2e+06
1e+06
0e+00

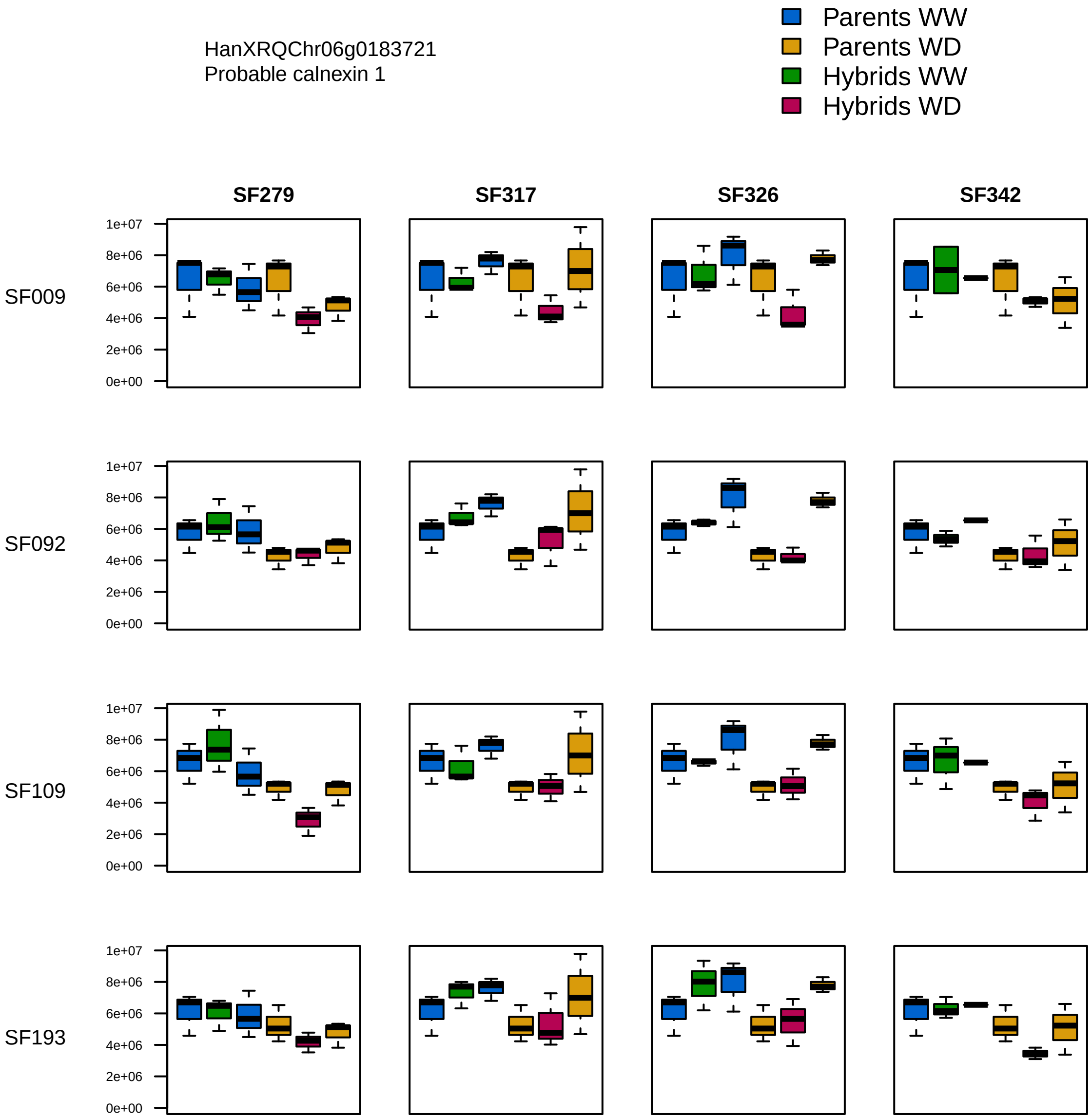
HanXRQChr06g0183721
Probable calnexin 1
Parents WW
Parents WD
Hybrids WW
Hybrids WD
SF279
SF317
SF326
SF342
SF009
SF092
SF109
SF193
1e+07
8e+06
6e+06
4e+06
2e+06
0e+00

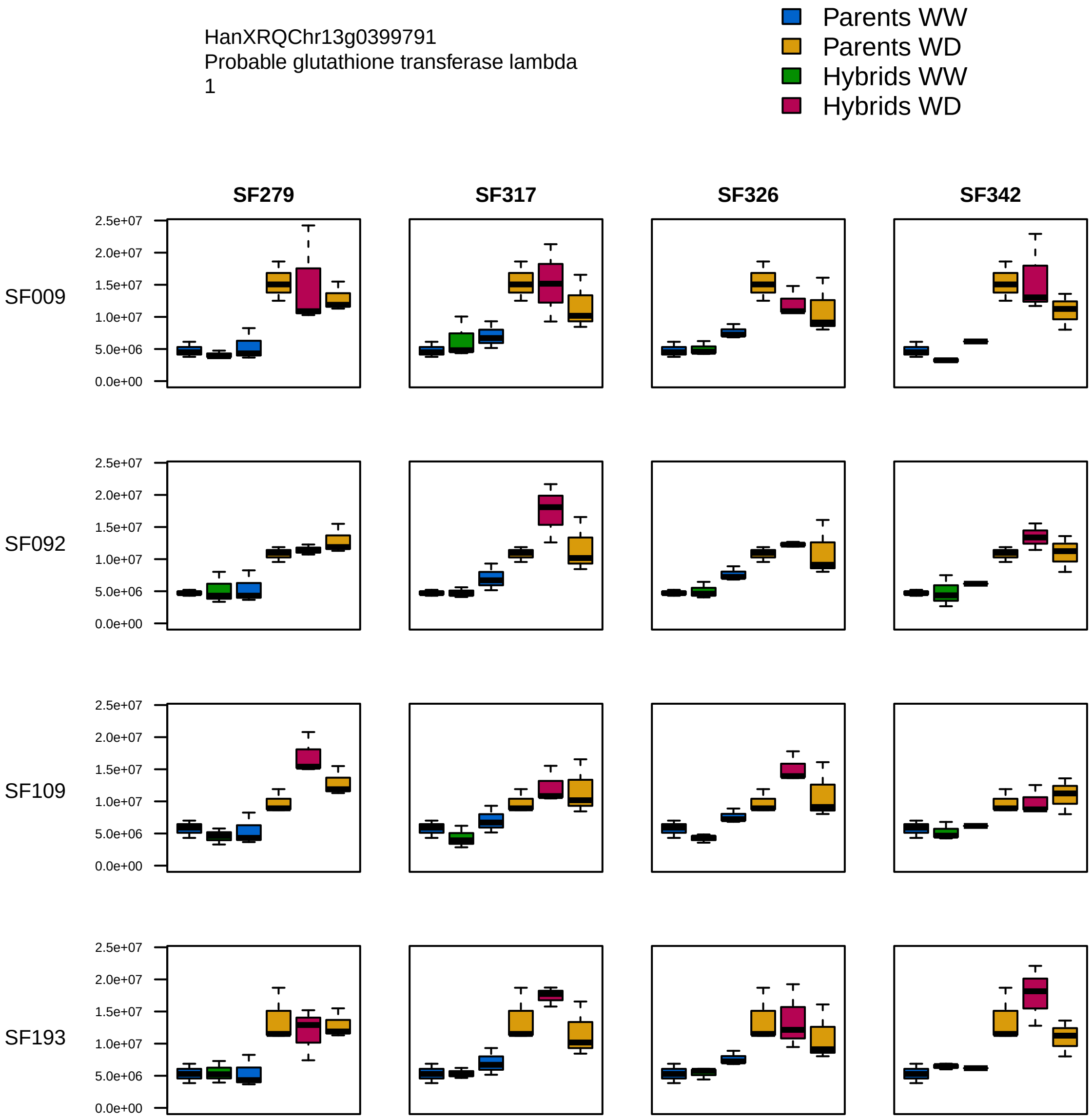
HanXRQChr13g0399791
Probable glutathione transferase lambda
1
Parents WW
Parents WD
Hybrids WW
Hybrids WD
SF279
SF317
SF326
SF342
SF009
SF092
SF109
SF193
2.5e+07
2.0e+07
1.5e+07
1.0e+07
5.0e+06
0.0e+00

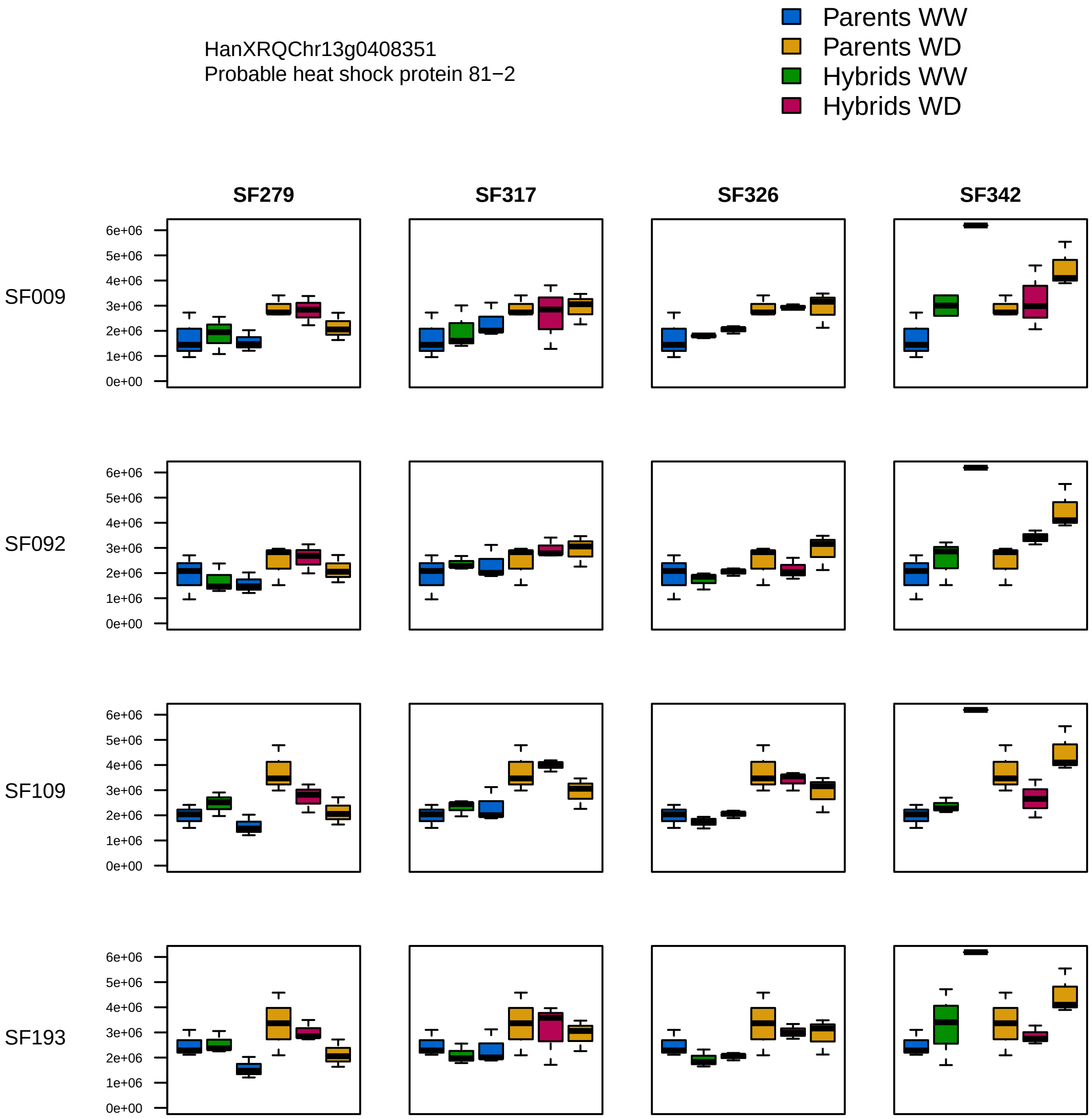
HanXRQChr13g0408351
Probable heat shock protein 81−2
Parents WW
Parents WD
Hybrids WW
Hybrids WD
SF279
SF317
SF326
SF342
SF009
SF092
SF109
SF193
0e+00
1e+06
2e+06
3e+06
4e+06
5e+06
6e+06

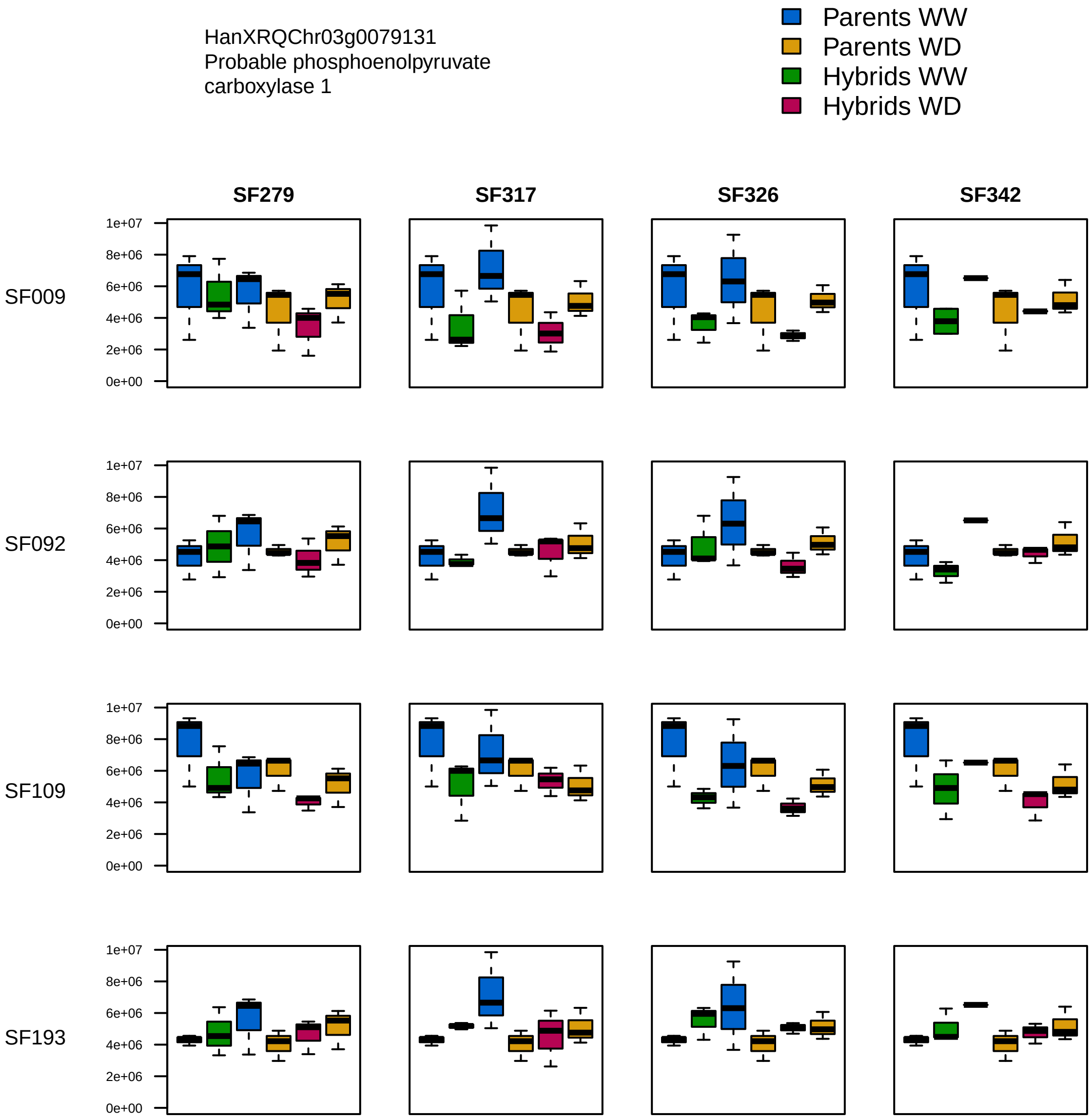
HanXRQChr03g0079131
Probable phosphoenolpyruvate
carboxylase 1
Parents WW
Parents WD
Hybrids WW
Hybrids WD
SF279
SF317
SF326
SF342
SF009
SF092
SF109
SF193
1e+07
8e+06
6e+06
4e+06
2e+06
0e+00

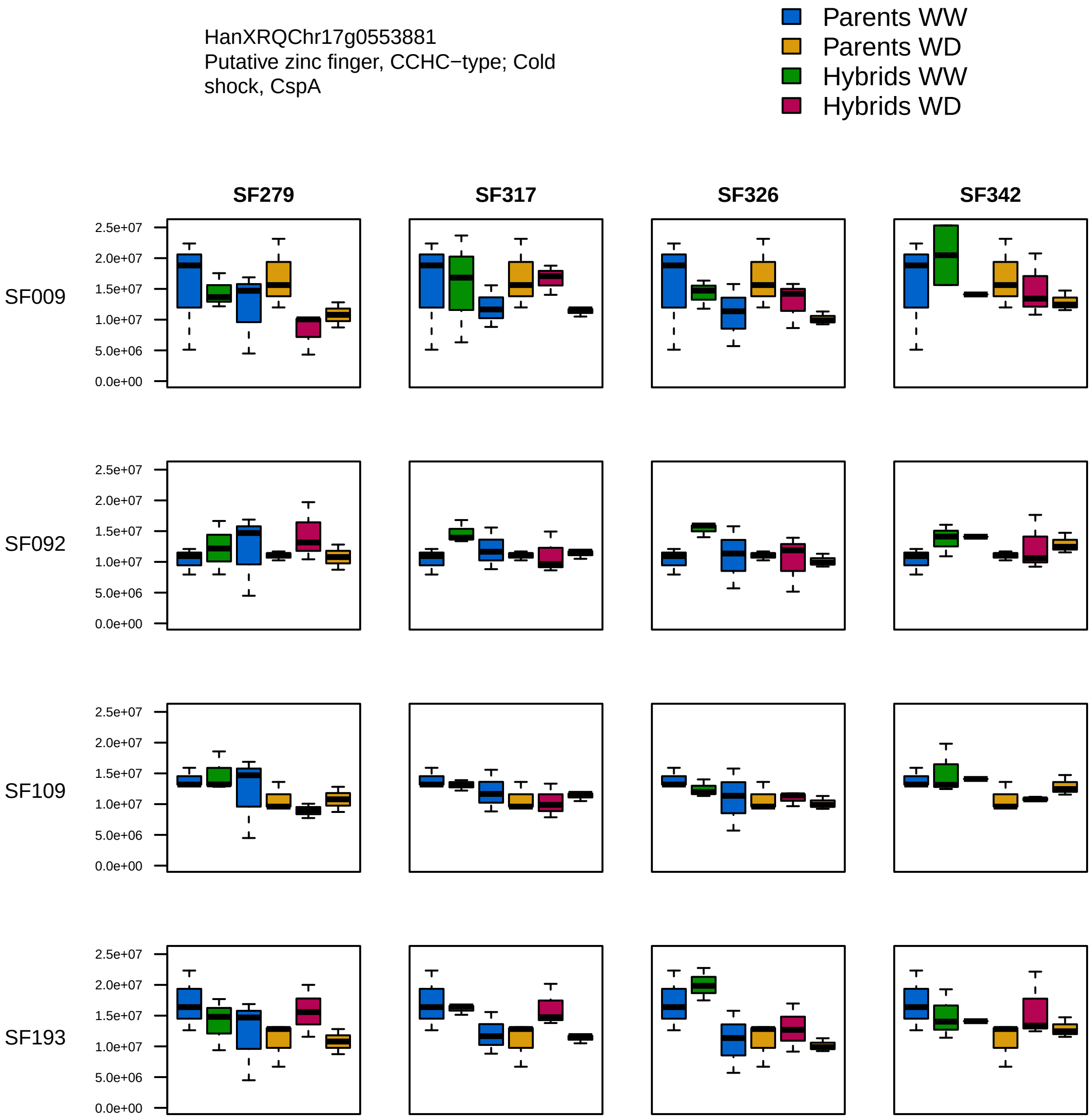
HanXRQChr17g0553881
Putative zinc finger, CCHC−type; Cold shock, CspA
Parents WW
Parents WD
Hybrids WW
Hybrids WD
SF279
SF317
SF326
SF342
SF009
SF092
SF109
SF193
0.0e+00
5.0e+06
1.0e+07
1.5e+07
2.0e+07
2.5e+07

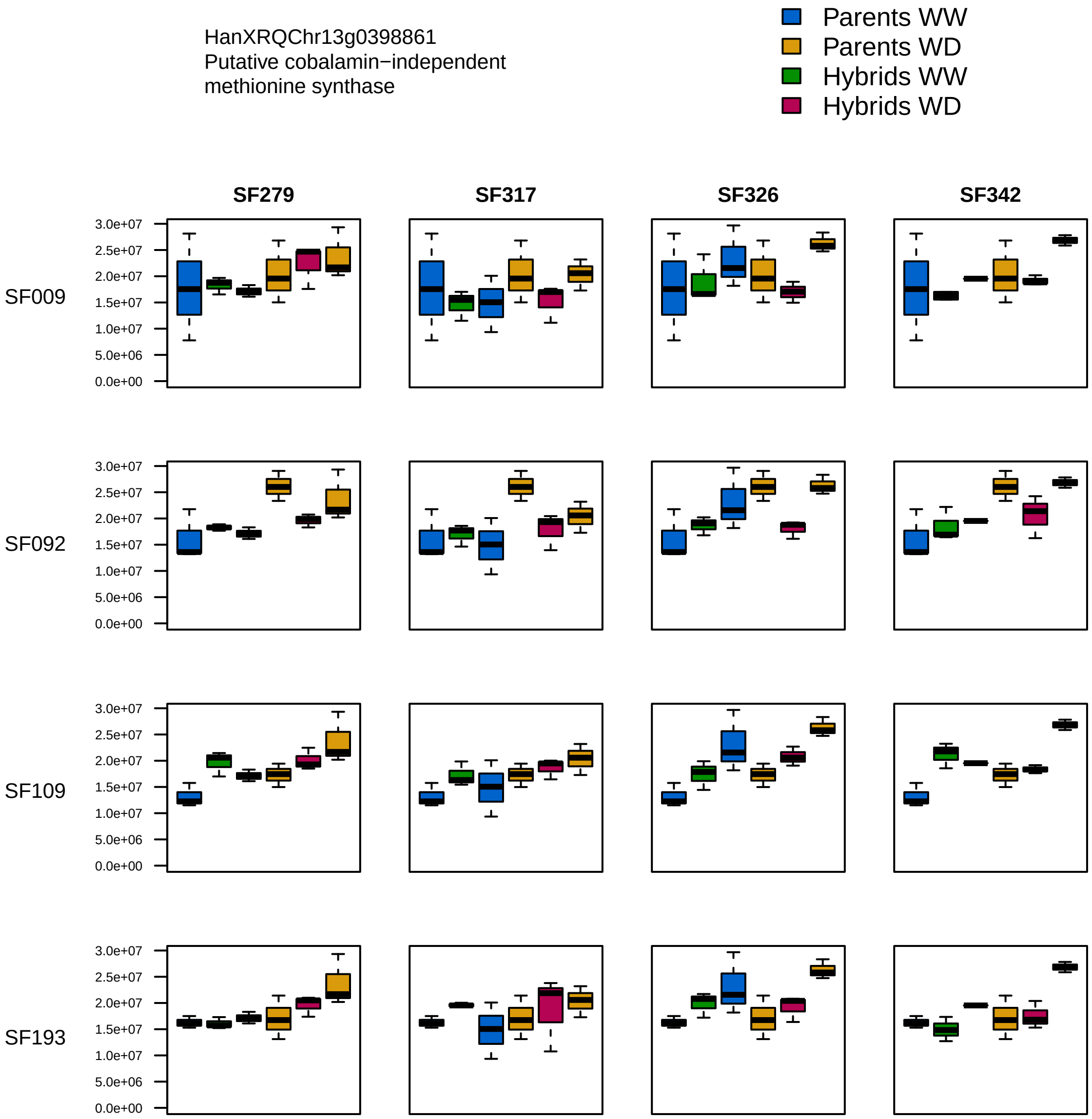
HanXRQChr13g0398861
Putative cobalamin−independent
methionine synthase
Parents WW
Parents WD
Hybrids WW
Hybrids WD
SF279
SF317
SF326
SF342
SF009
SF092
SF109
SF193
3.0e+07
2.5e+07
2.0e+07
1.5e+07
1.0e+07
5.0e+06
0.0e+00

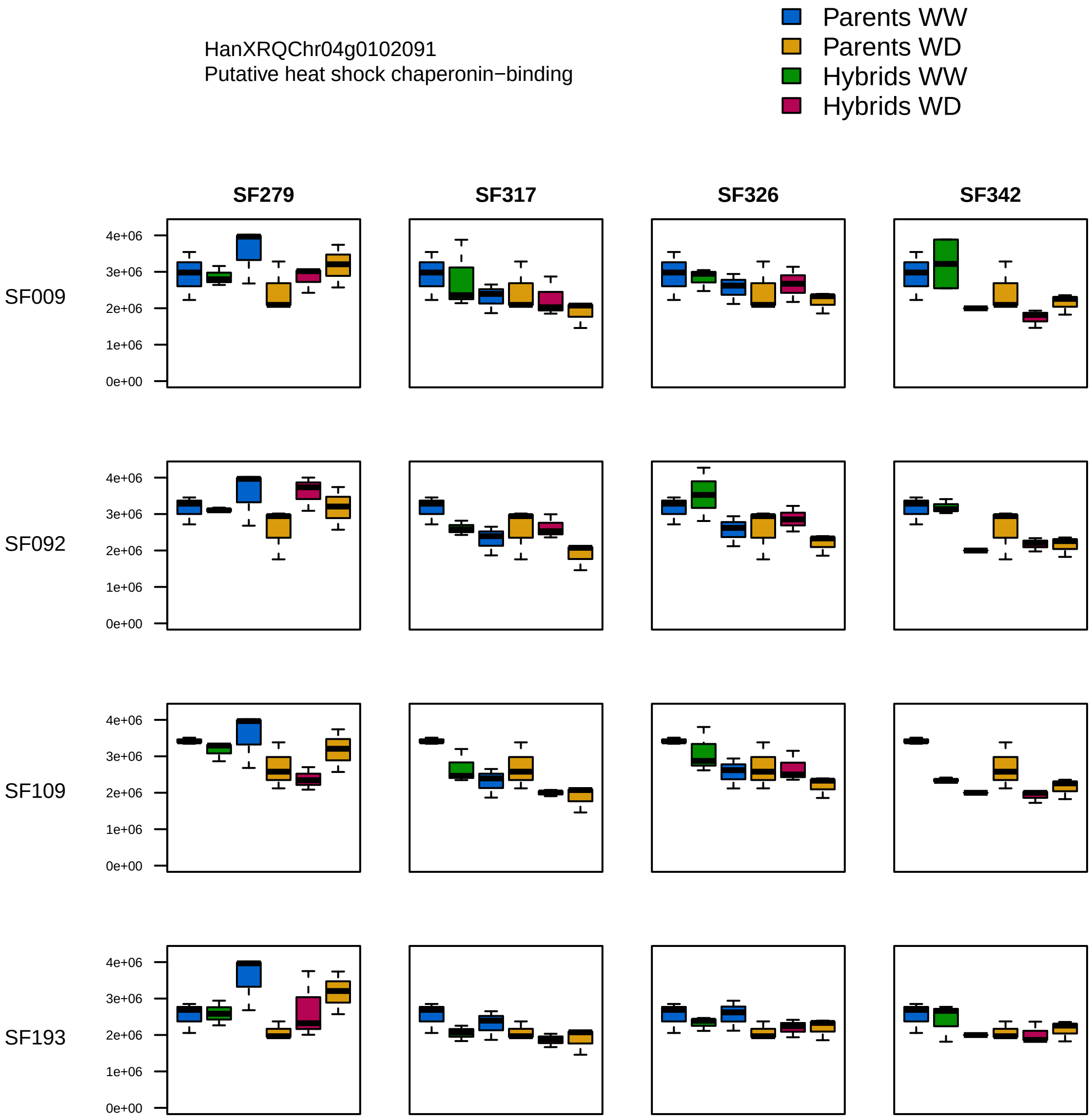

HanXRQChr04g0102091
Putative heat shock chaperonin−binding
Parents WW
Parents WD
Hybrids WW
Hybrids WD
SF279
SF317
SF326
SF342
SF009
SF092
SF109
SF193
4e+06
3e+06
2e+06
1e+06
0e+00

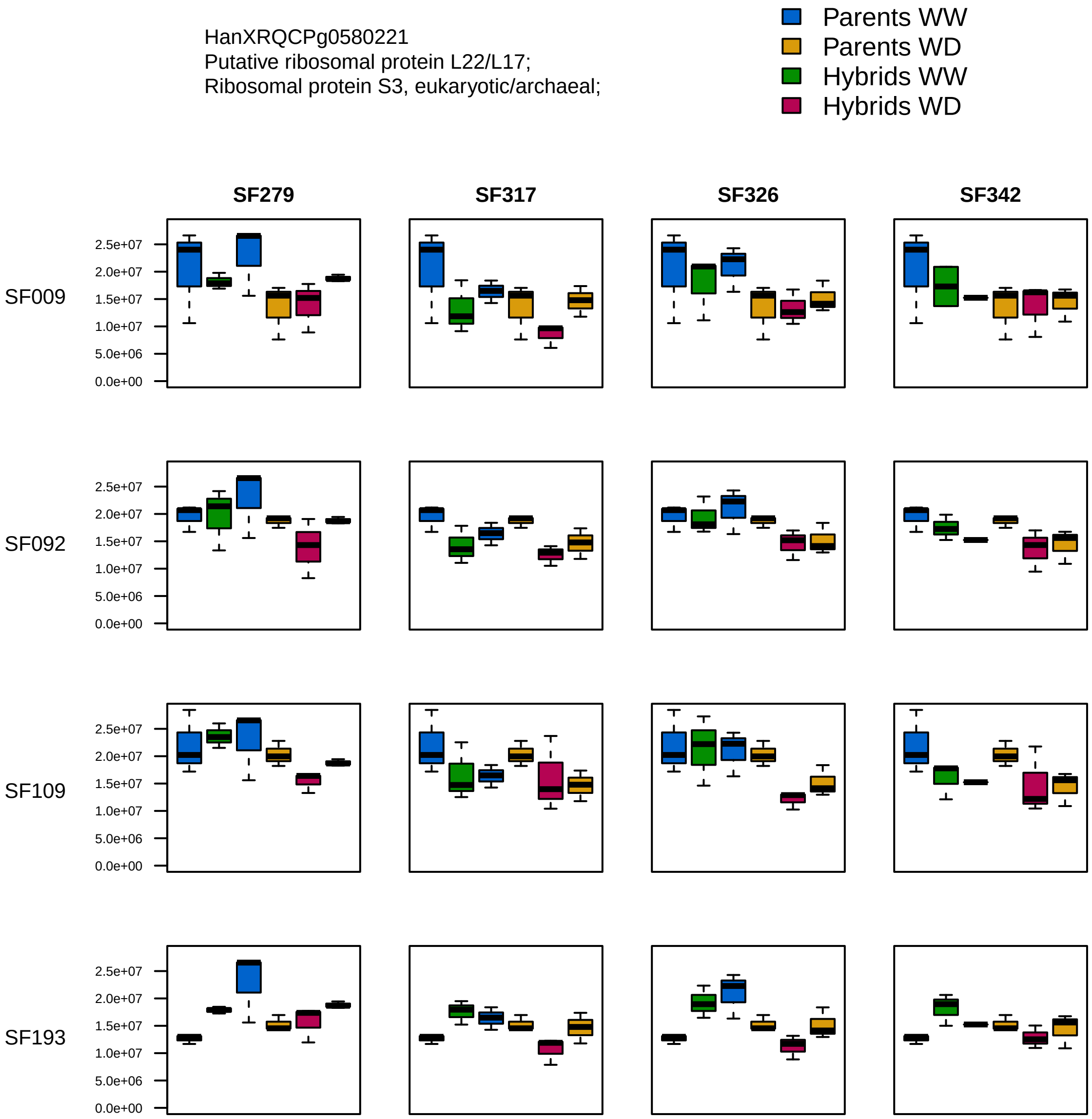

HanXRQCPg0580221
Putative ribosomal protein L22/L17;
Ribosomal protein S3, eukaryotic/archaeal;
Parents WW
Parents WD
Hybrids WW
Hybrids WD
SF279
SF317
SF326
SF342
SF009
SF092
SF109
SF193
0.0e+00
5.0e+06
1.0e+07
1.5e+07
2.0e+07
2.5e+07

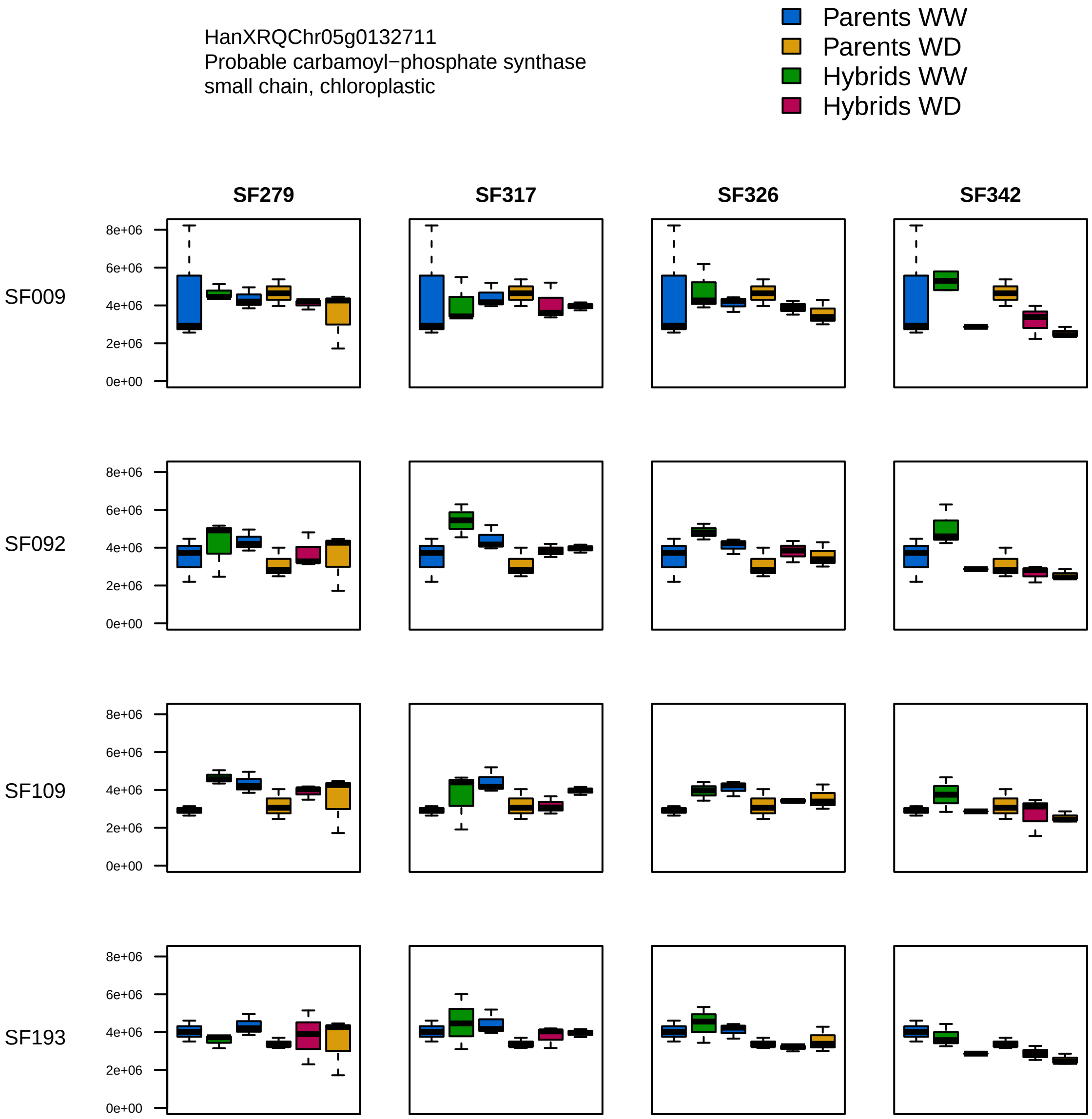
HanXRQChr05g0132711
Probable carbamoyl−phosphate synthase
small chain, chloroplastic
Parents WW
Parents WD
Hybrids WW
Hybrids WD
SF279
SF317
SF326
SF342
SF009
SF092
SF109
SF193
0e+00
2e+06
4e+06
6e+06
8e+06

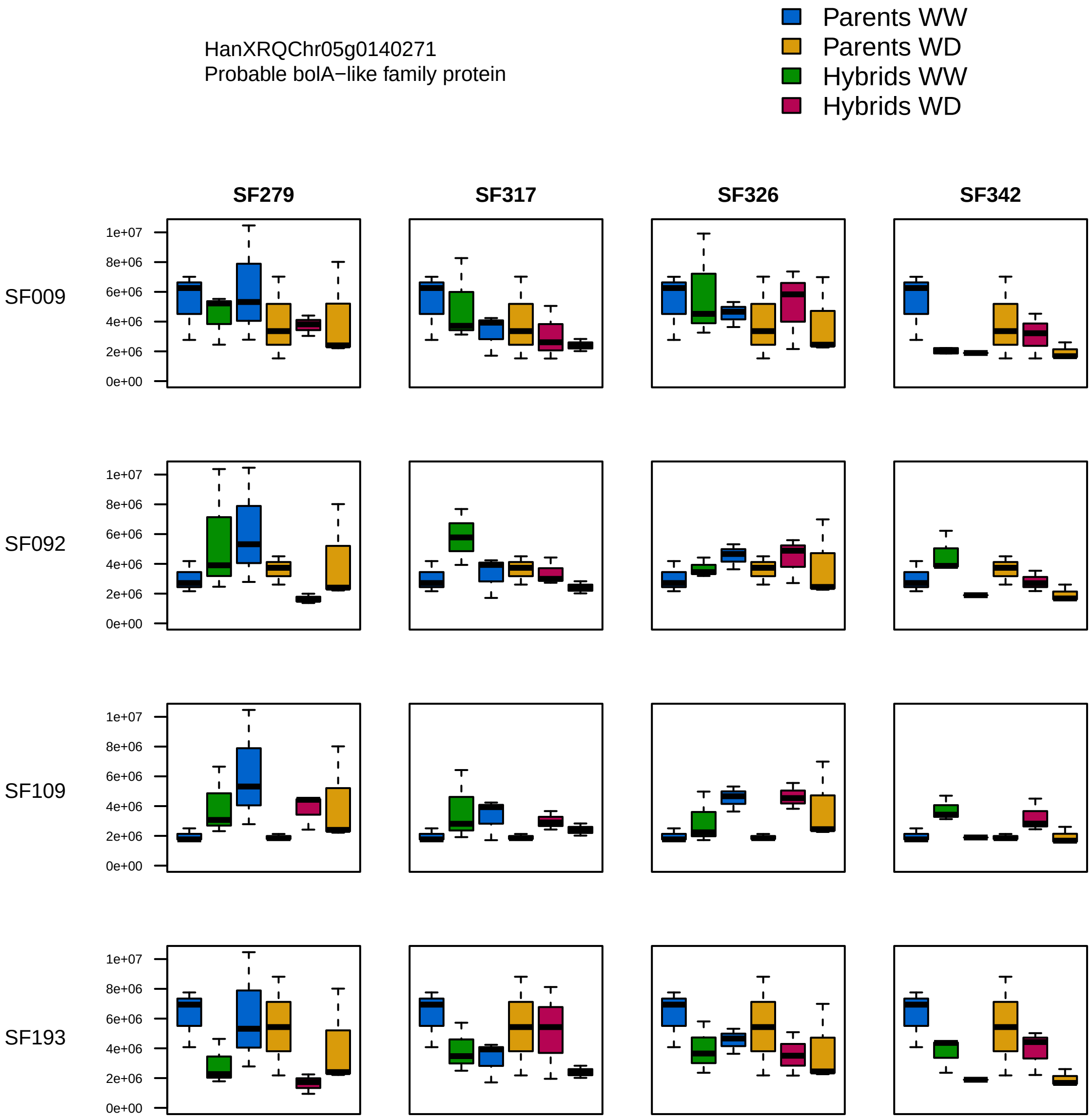
HanXRQChr05g0140271
Probable bolA−like family protein
Parents WW
Parents WD
Hybrids WW
Hybrids WD
SF279
SF317
SF326
SF342
SF009
SF092
SF109
SF193
1e+07
8e+06
6e+06
4e+06
2e+06
0e+00

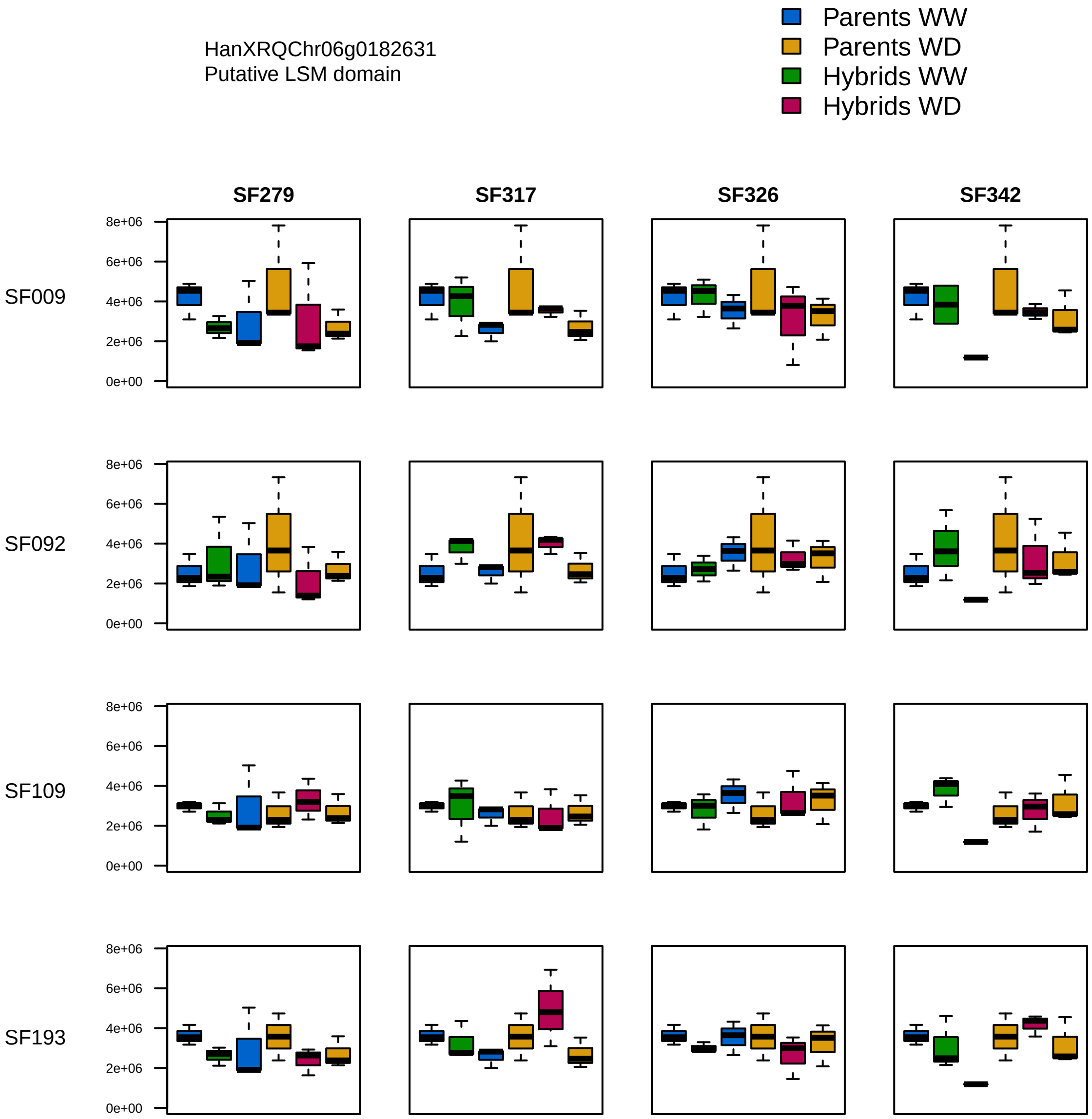

HanXRQChr06g0182631
Putative LSM domain
Parents WW
Parents WD
Hybrids WW
Hybrids WD
SF279
SF317
SF326
SF342
SF009
SF092
SF109
SF193
8e+06
6e+06
4e+06
2e+06
0e+00

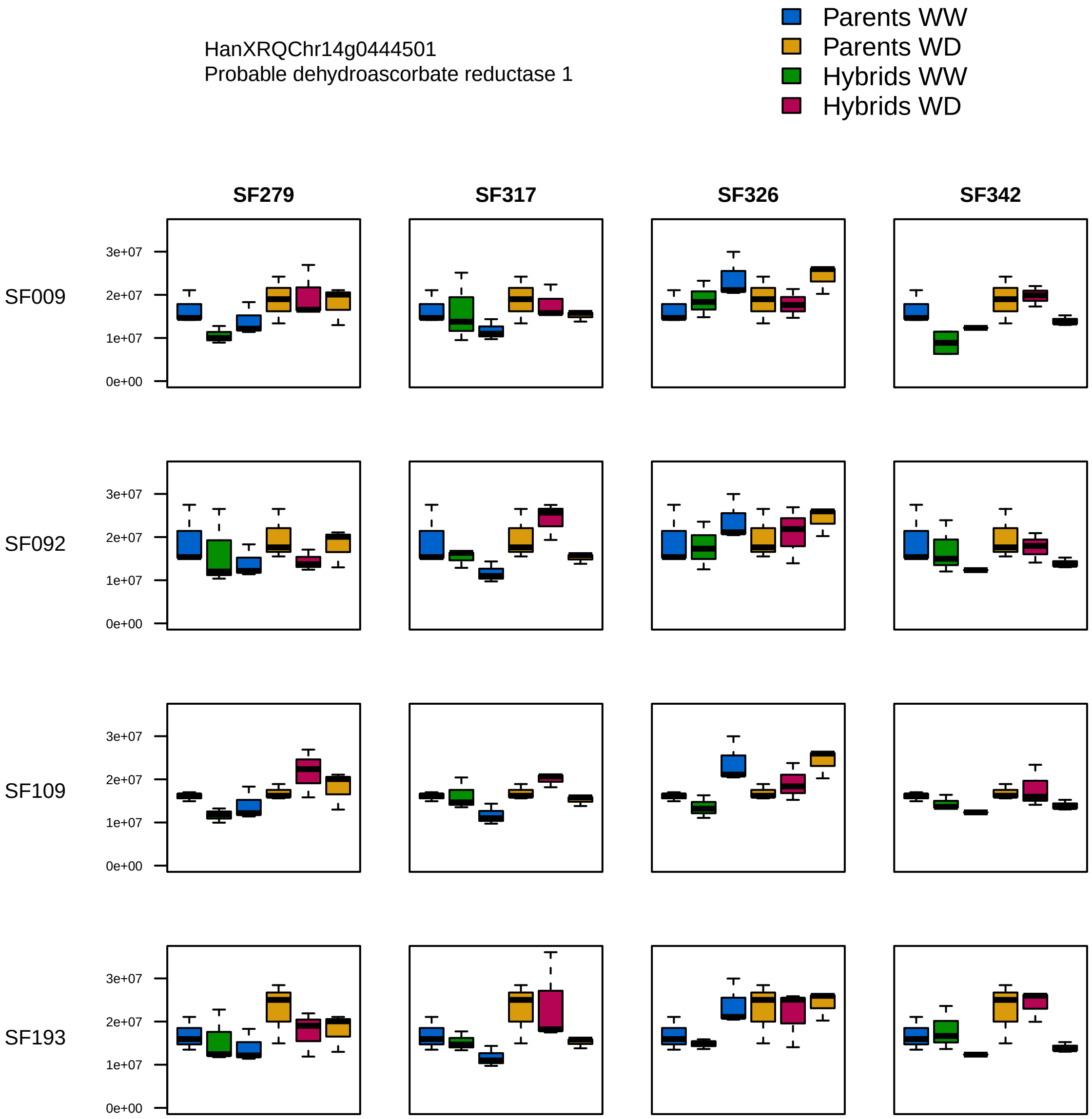
HanXRQChr14g0444501
Probable dehydroascorbate reductase 1
Parents WW
Parents WD
Hybrids WW
Hybrids WD
SF279
SF317
SF326
SF342
SF009
SF092
SF109
SF193
0e+00
1e+07
2e+07
3e+07

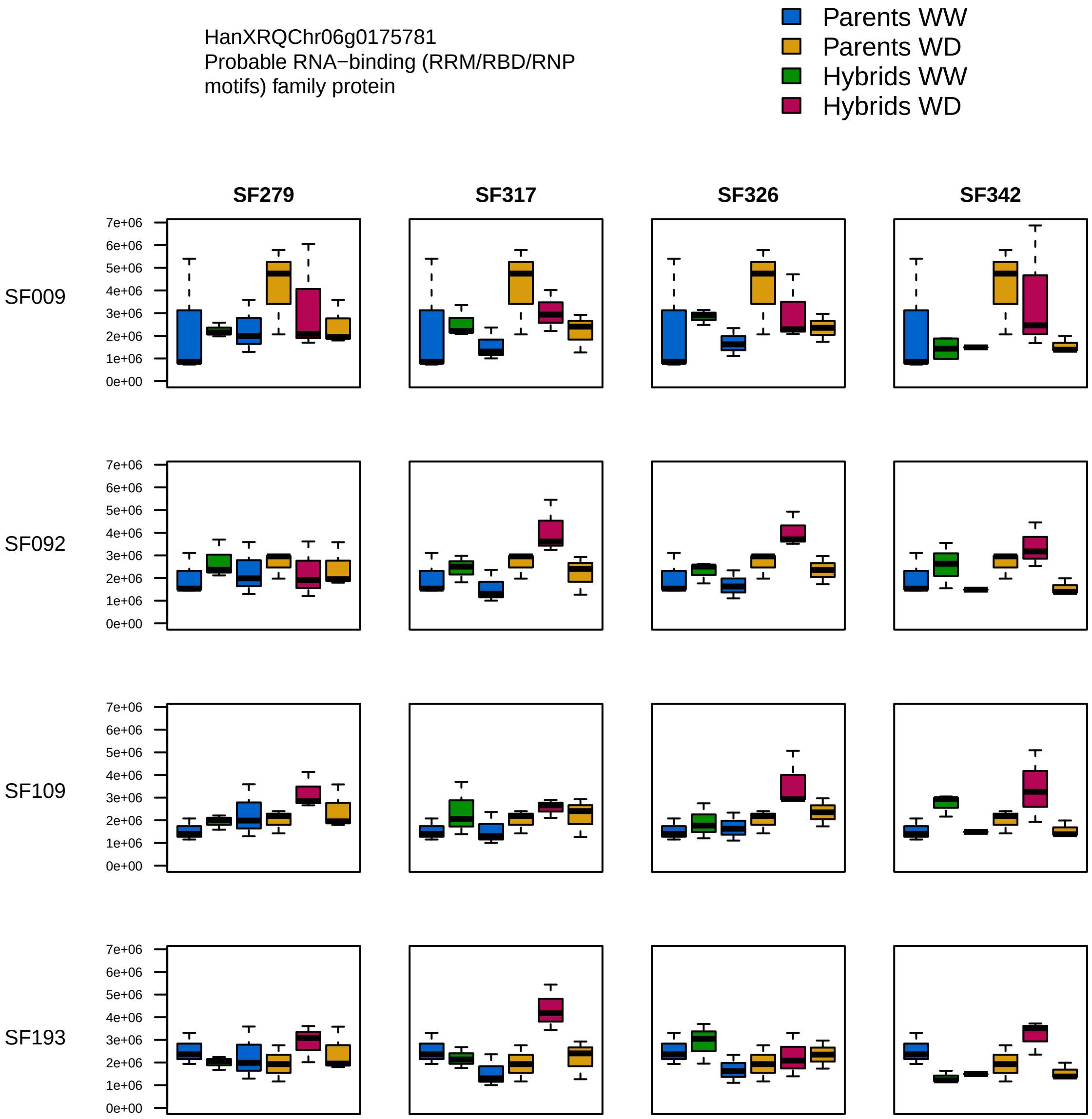

HanXRQChr06g0175781
Probable RNA−binding (RRM/RBD/RNP motifs) family protein
Parents WW
Parents WD
Hybrids WW
Hybrids WD
SF279
SF317
SF326
SF342
SF009
SF092
SF109
SF193
0e+00
1e+06
2e+06
3e+06
4e+06
5e+06
6e+06
7e+06

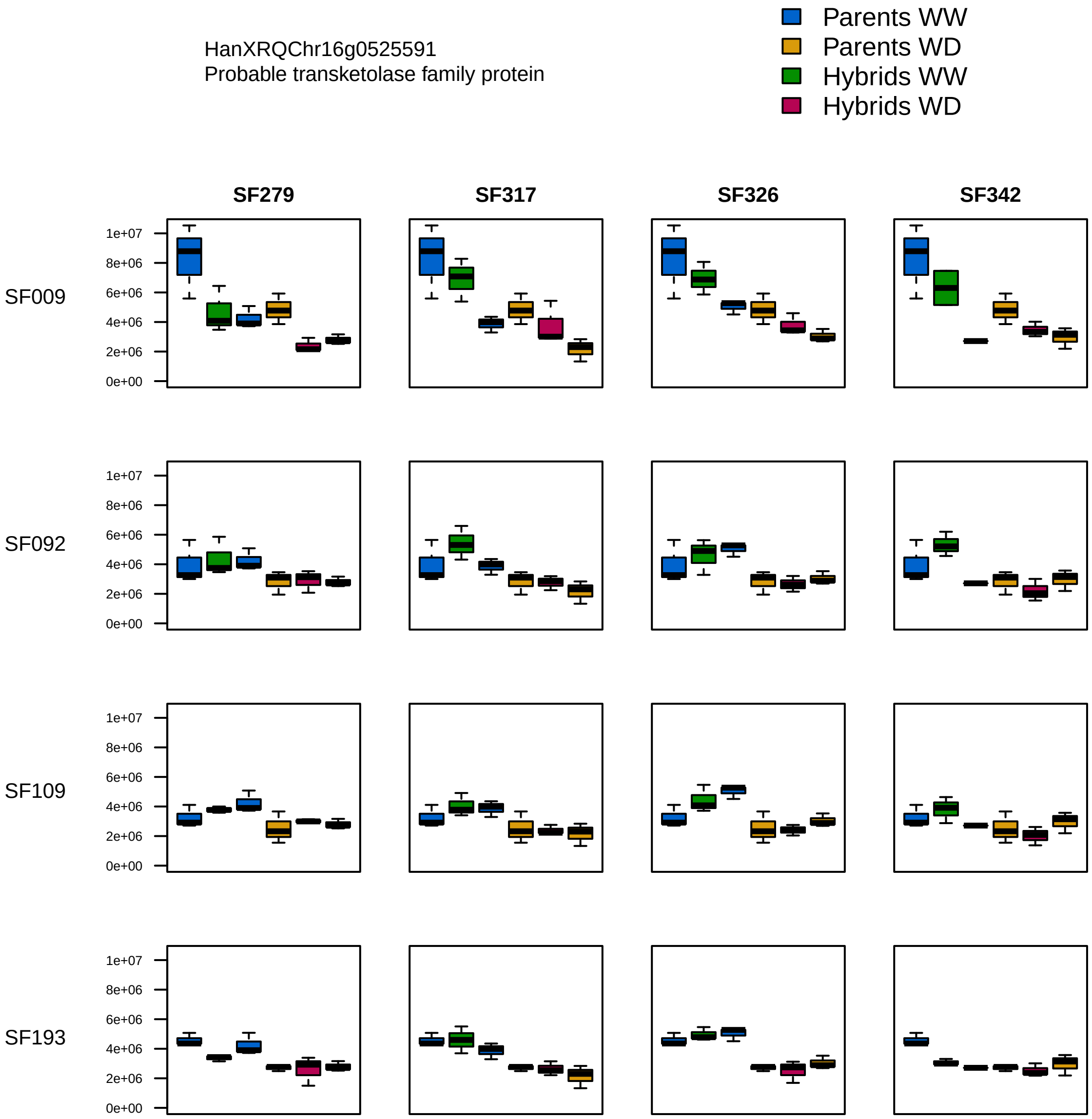
HanXRQChr16g0525591
Probable transketolase family protein
Parents WW
Parents WD
Hybrids WW
Hybrids WD
SF279
SF317
SF326
SF342
SF009
SF092
SF109
SF193
1e+07
8e+06
6e+06
4e+06
2e+06
0e+00

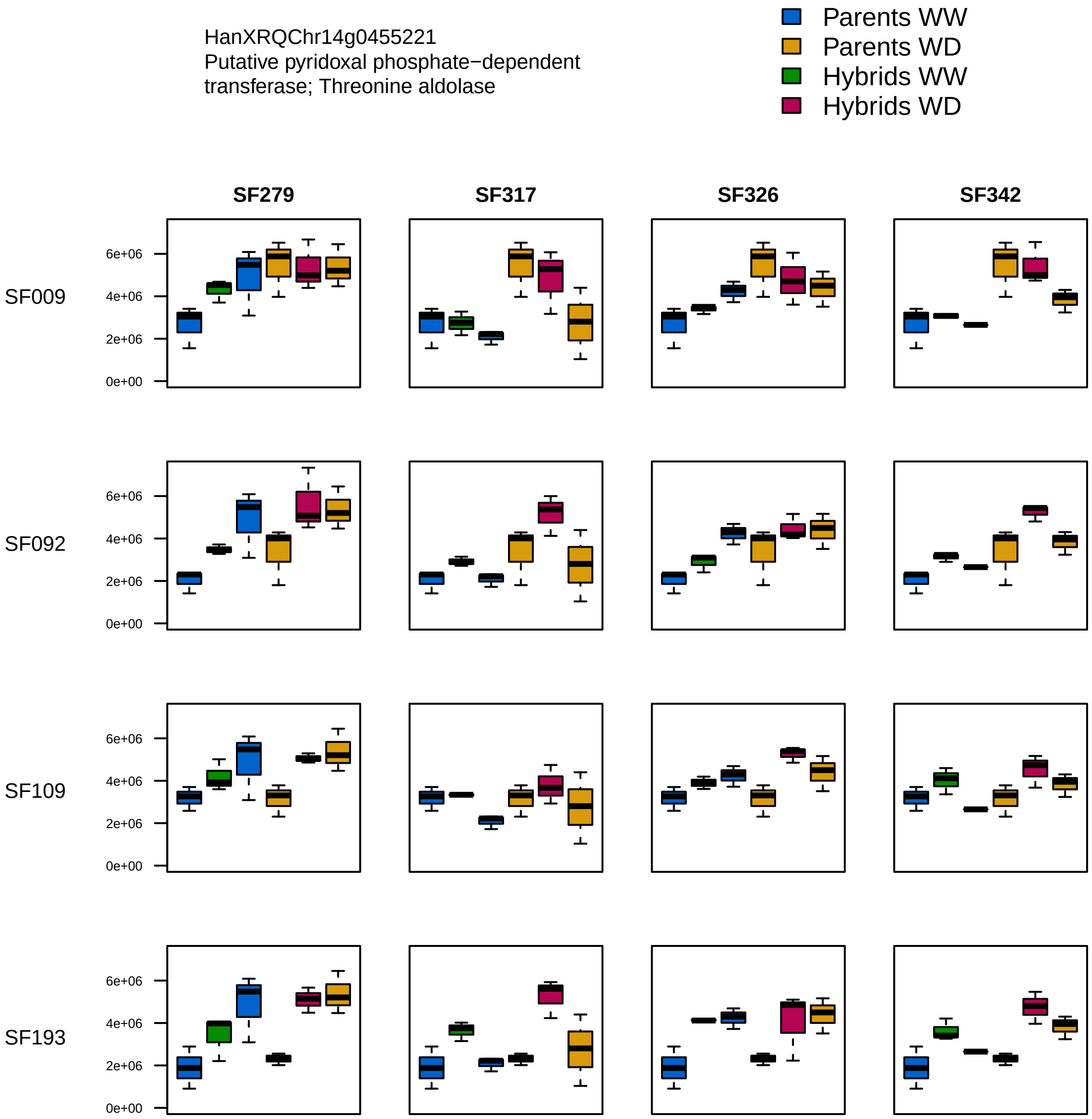
HanXRQChr14g0455221
Putative pyridoxal phosphate−dependent
transferase; Threonine aldolase
Parents WW
Parents WD
Hybrids WW
Hybrids WD
SF279
SF317
SF326
SF342
SF009
SF092
SF109
SF193
6e+06
4e+06
2e+06
0e+00

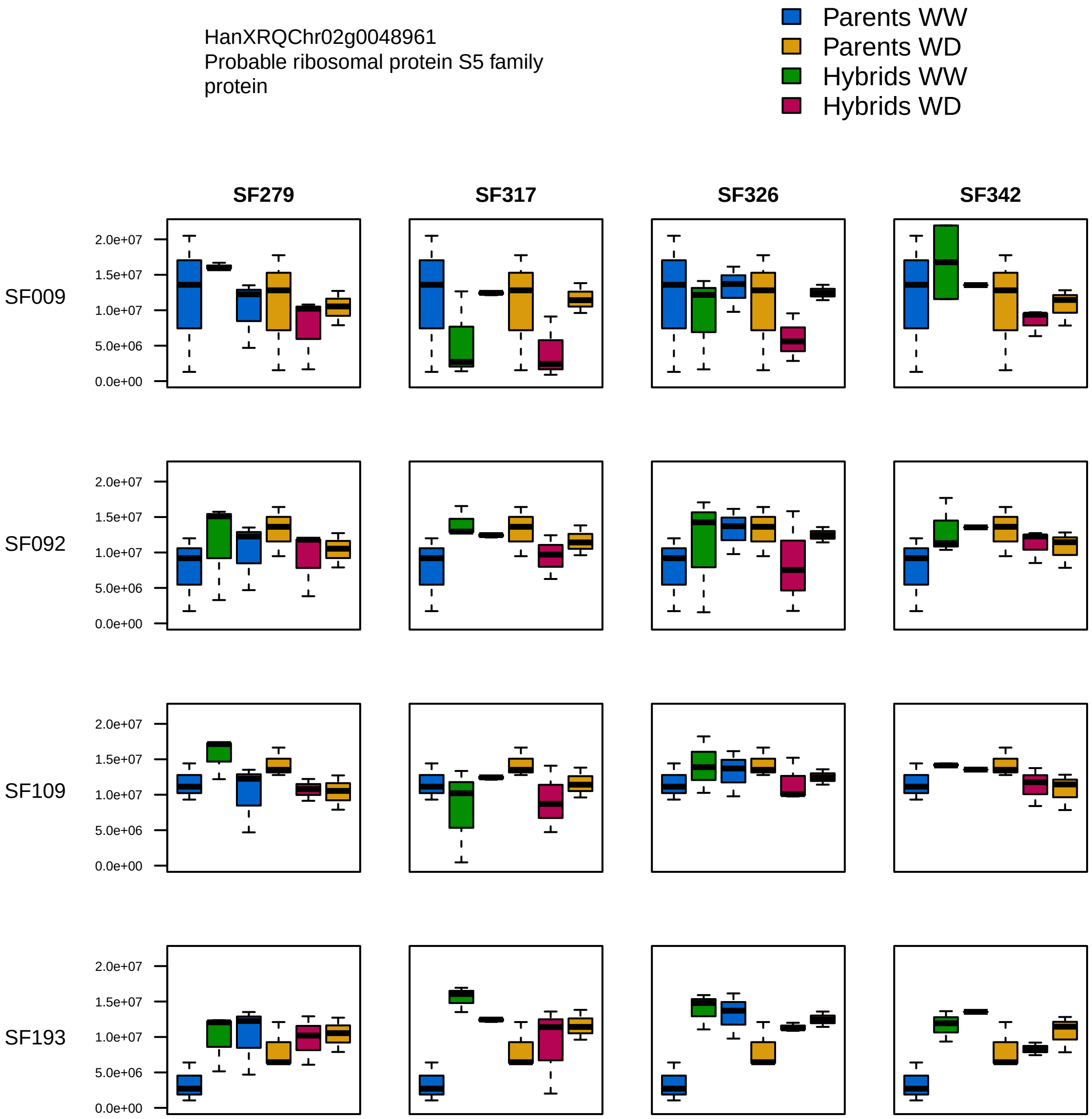
HanXRQChr02g0048961
Probable ribosomal protein S5 family
protein
Parents WW
Parents WD
Hybrids WW
Hybrids WD
SF279
SF317
SF326
SF342
SF009
SF092
SF109
SF193
2.0e+07
1.5e+07
1.0e+07
5.0e+06
0.0e+00

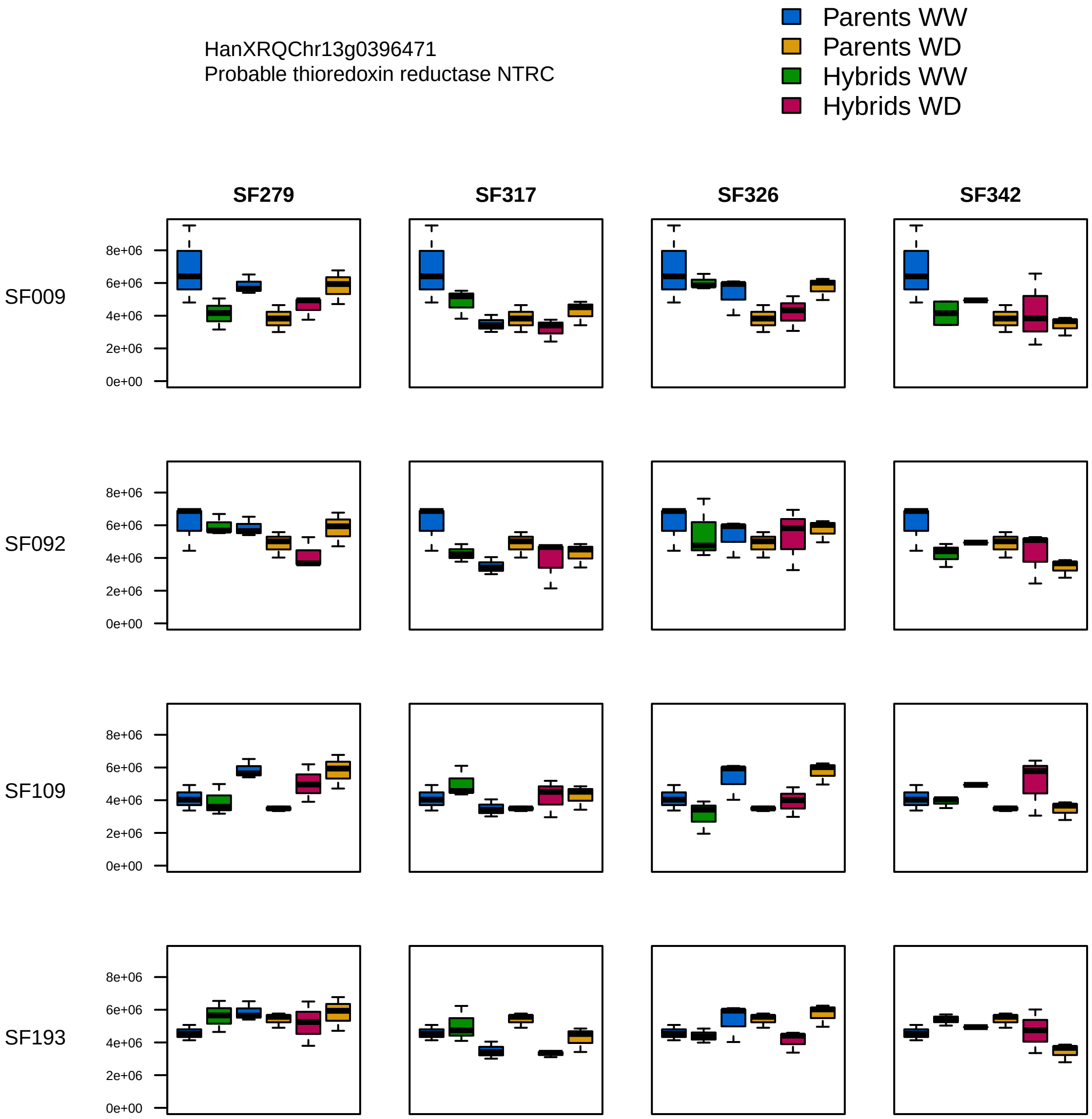
HanXRQChr13g0396471
Probable thioredoxin reductase NTRC
Parents WW
Parents WD
Hybrids WW
Hybrids WD
SF279
SF317
SF326
SF342
SF009
SF092
SF109
SF193
8e+06
6e+06
4e+06
2e+06
0e+00

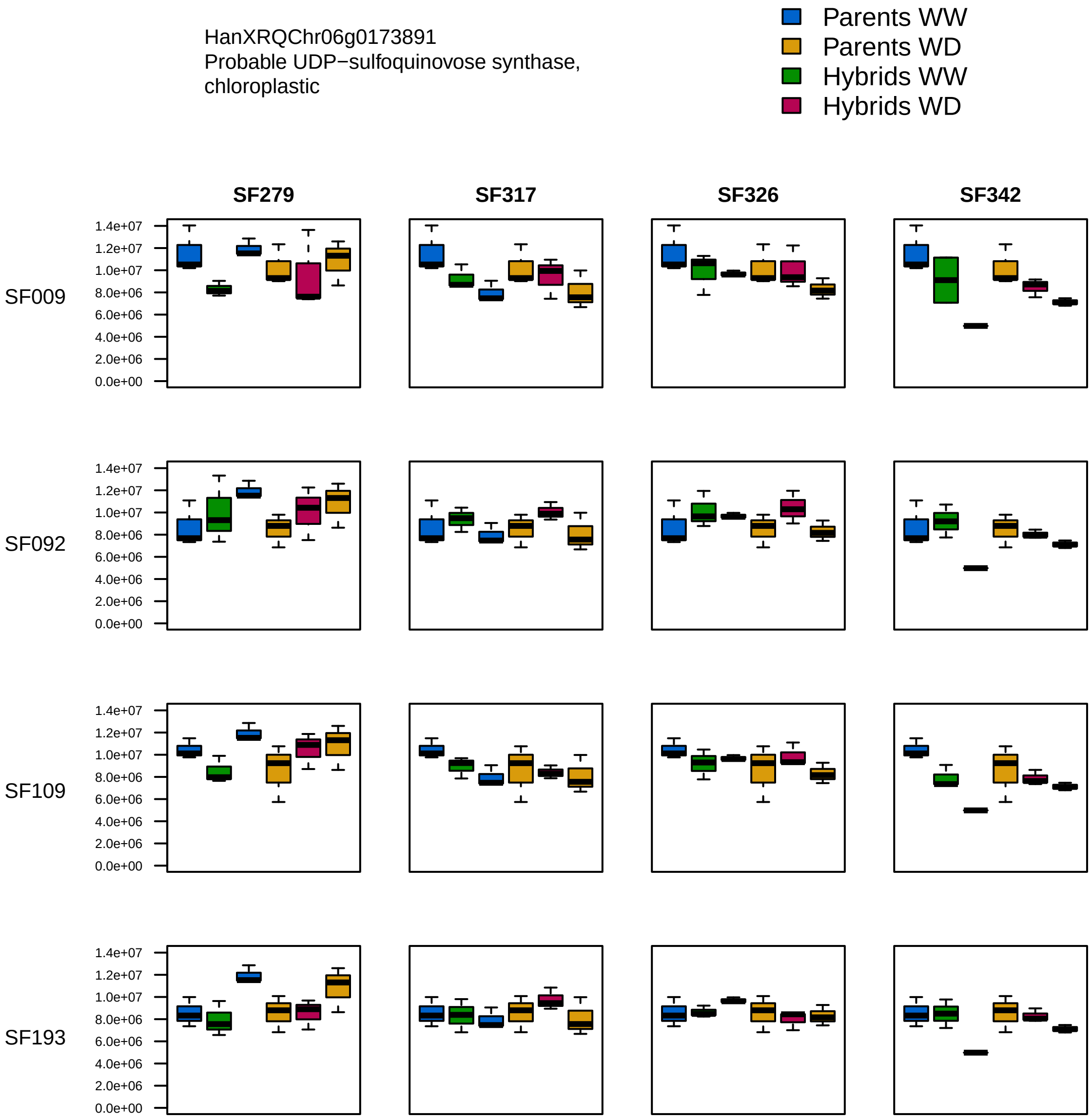
HanXRQChr06g0173891
Probable UDP−sulfoquinovose synthase, chloroplastic
Parents WW
Parents WD
Hybrids WW
Hybrids WD
SF279
SF317
SF326
SF342
SF009
SF092
SF109
SF193
0.0e+00
2.0e+06
4.0e+06
6.0e+06
8.0e+06
1.0e+07
1.2e+07
1.4e+07

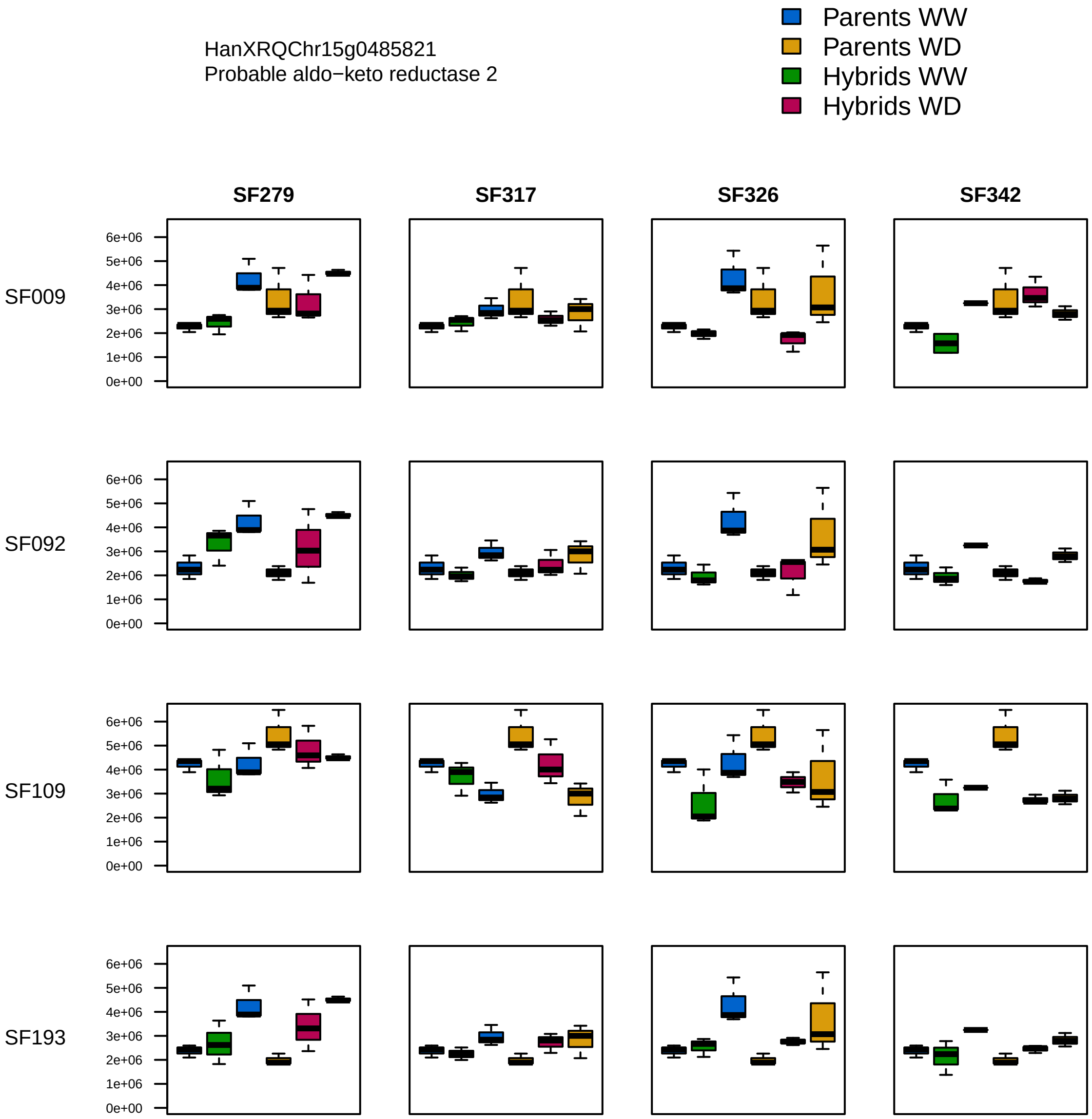
HanXRQChr15g0485821
Probable aldo−keto reductase 2
Parents WW
Parents WD
Hybrids WW
Hybrids WD
SF279
SF317
SF326
SF342
SF009
SF092
SF109
SF193
6e+06
5e+06
4e+06
3e+06
2e+06
1e+06
0e+00

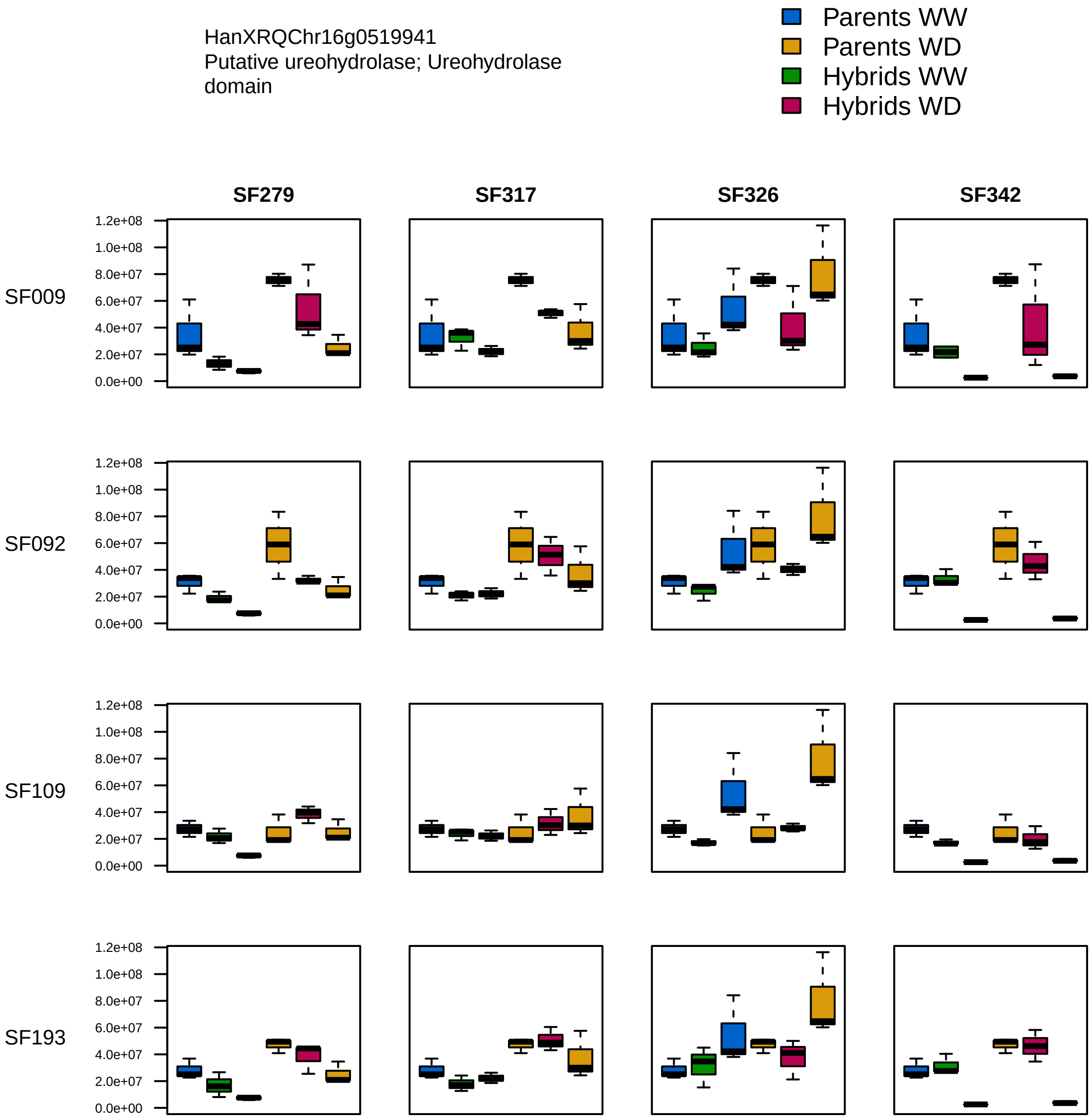
HanXRQChr16g0519941
Putative ureohydrolase; Ureohydrolase domain
Parents WW
Parents WD
Hybrids WW
Hybrids WD
SF279
SF317
SF326
SF342
SF009
SF092
SF109
SF193
1.2e+08
1.0e+08
8.0e+07
6.0e+07
4.0e+07
2.0e+07
0.0e+00

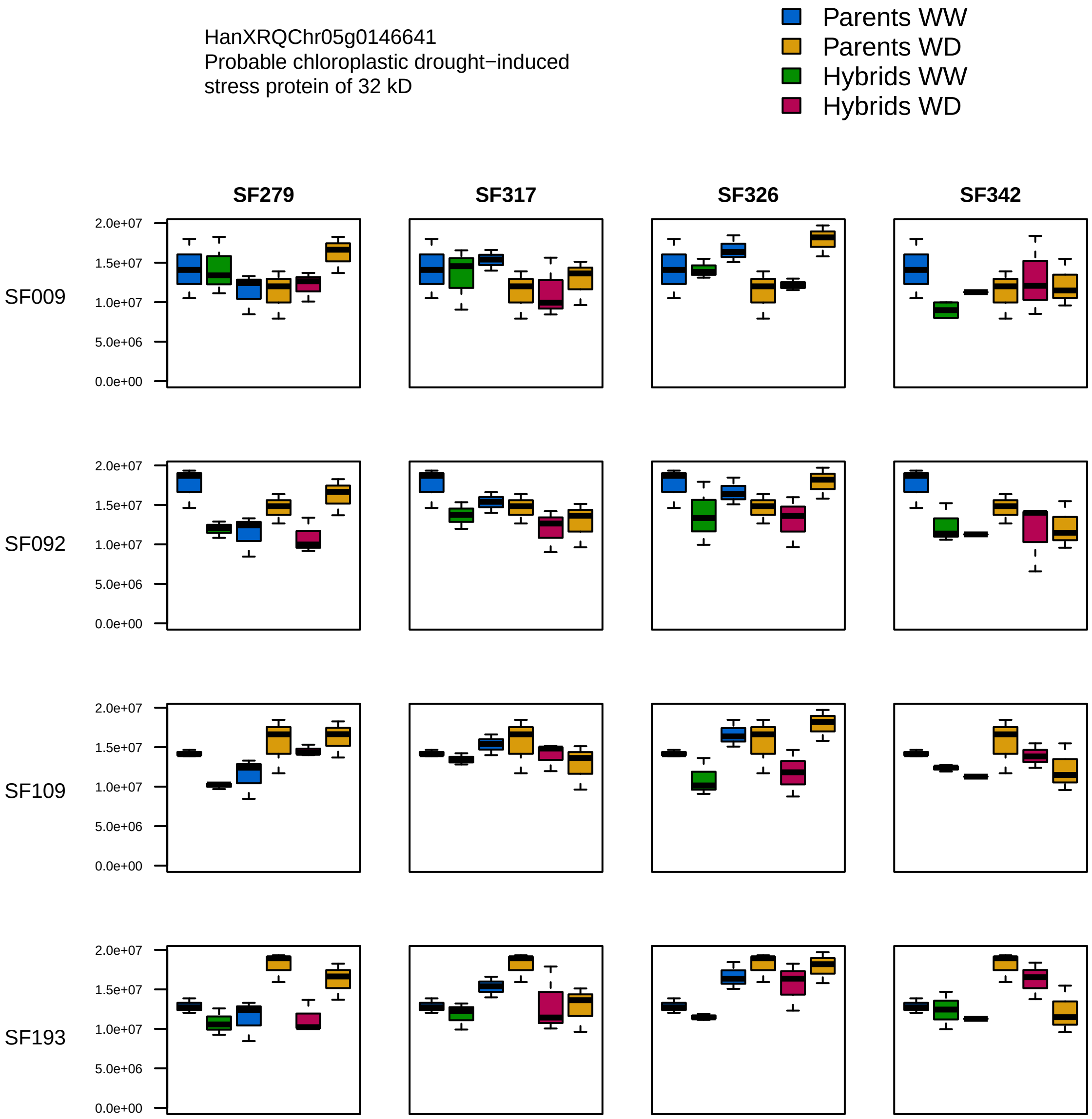

HanXRQChr05g0146641
Probable chloroplastic drought−induced
stress protein of 32 kD
Parents WW
Parents WD
Hybrids WW
Hybrids WD
SF279
SF317
SF326
SF342
SF009
SF092
SF109
SF193
2.0e+07
1.5e+07
1.0e+07
5.0e+06
0.0e+00

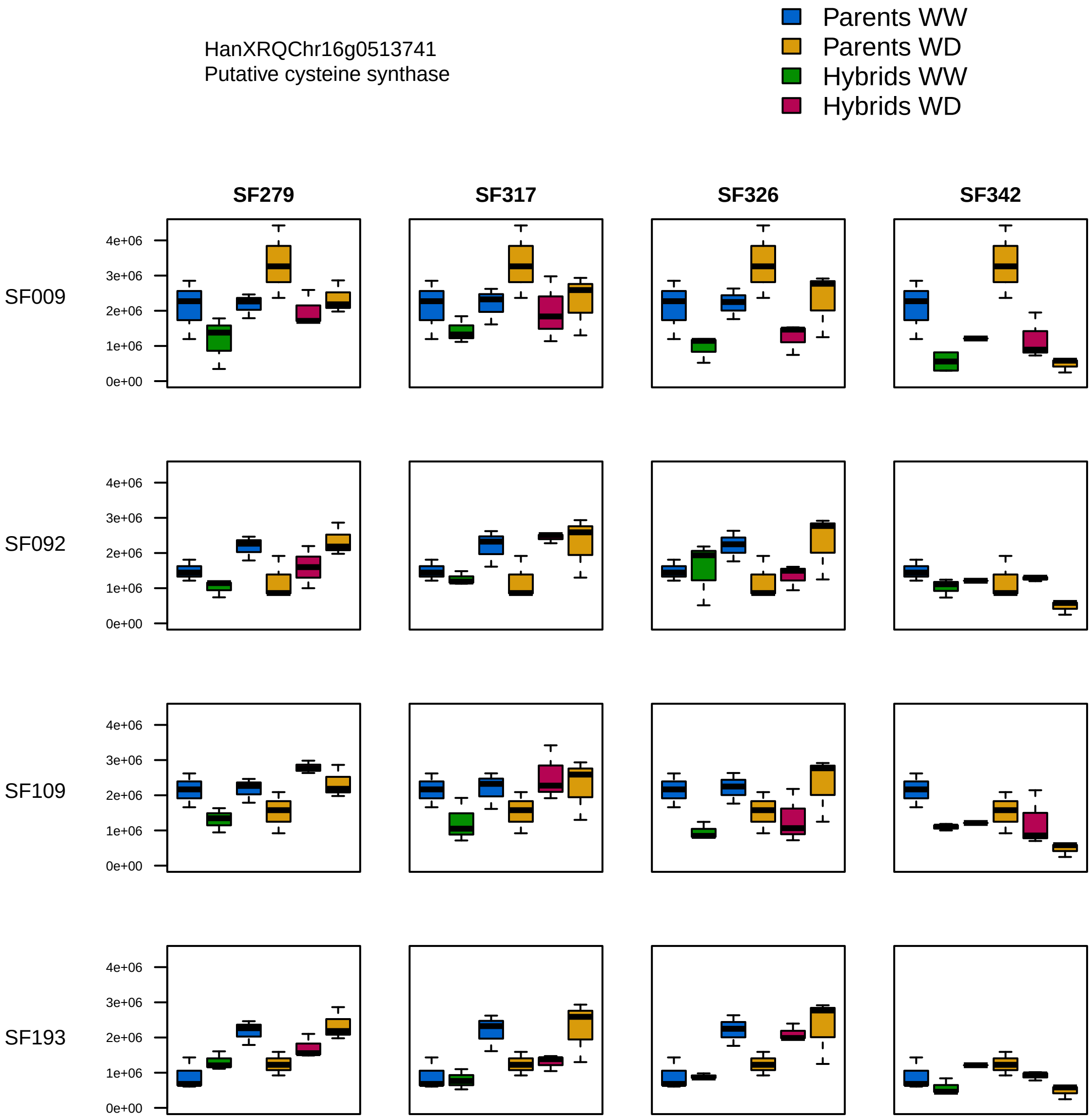
HanXRQChr16g0513741
Putative cysteine synthase
Parents WW
Parents WD
Hybrids WW
Hybrids WD
SF279
SF317
SF326
SF342
SF009
SF092
SF109
SF193
4e+06
3e+06
2e+06
1e+06
0e+00

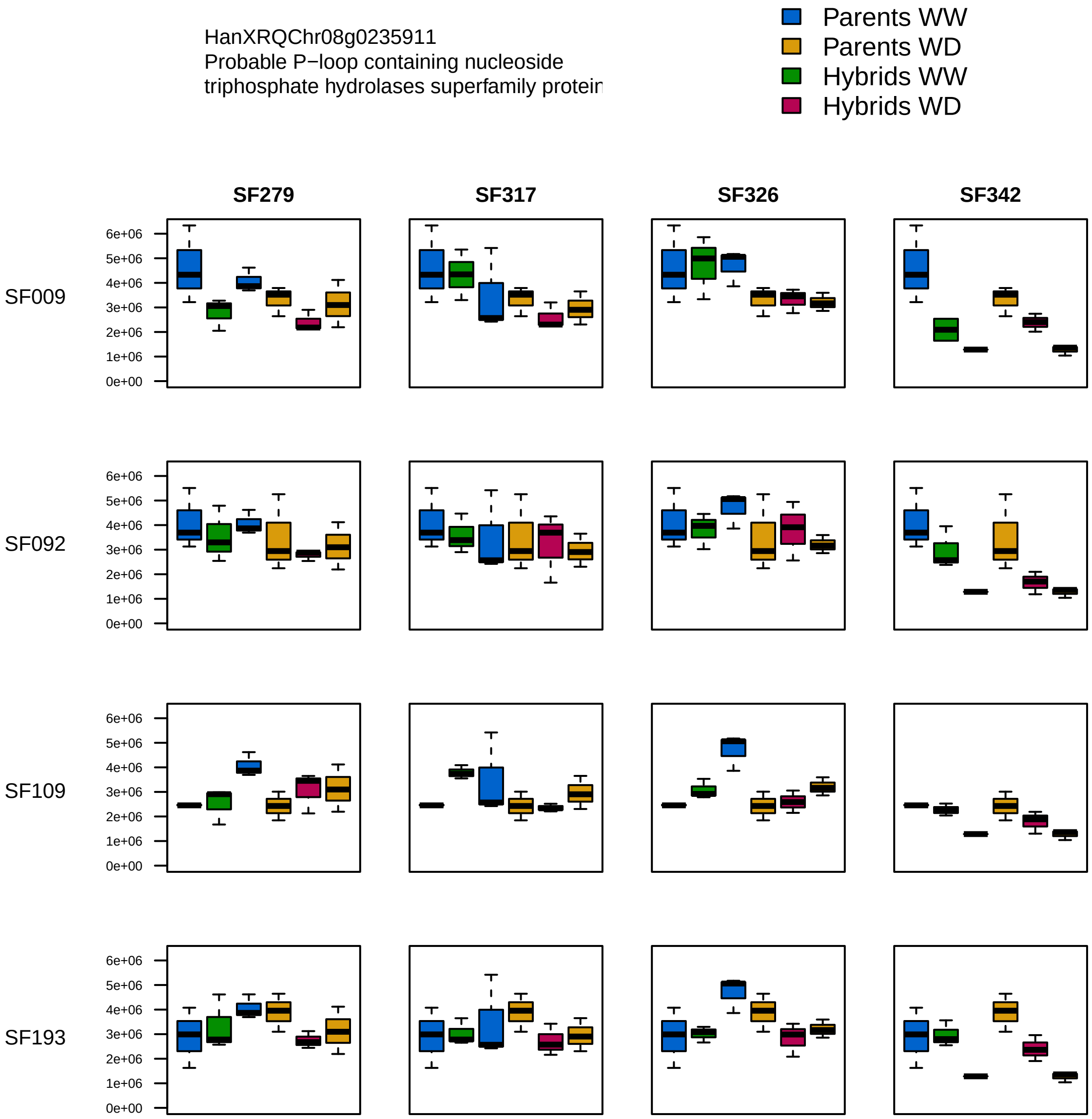

HanXRQChr08g0235911
Probable P−loop containing nucleoside
triphosphate hydrolases superfamily protein
Parents WW
Parents WD
Hybrids WW
Hybrids WD
SF279
SF317
SF326
SF342
SF009
SF092
SF109
SF193
0e+00
1e+06
2e+06
3e+06
4e+06
5e+06
6e+06

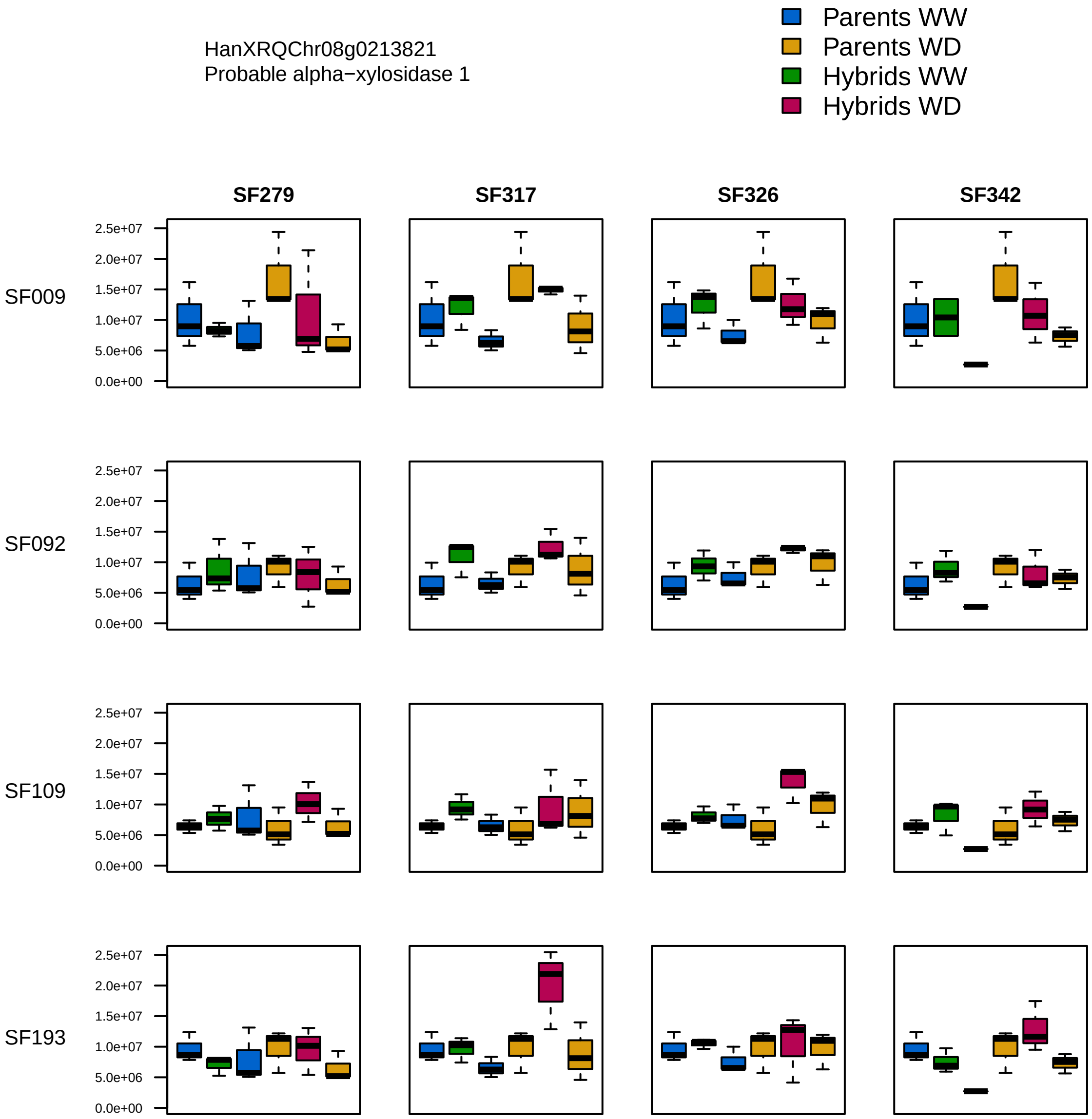
HanXRQChr08g0213821
Probable alpha−xylosidase 1
Parents WW
Parents WD
Hybrids WW
Hybrids WD
SF279
SF317
SF326
SF342
SF009
SF092
SF109
SF193
2.5e+07
2.0e+07
1.5e+07
1.0e+07
5.0e+06
0.0e+00

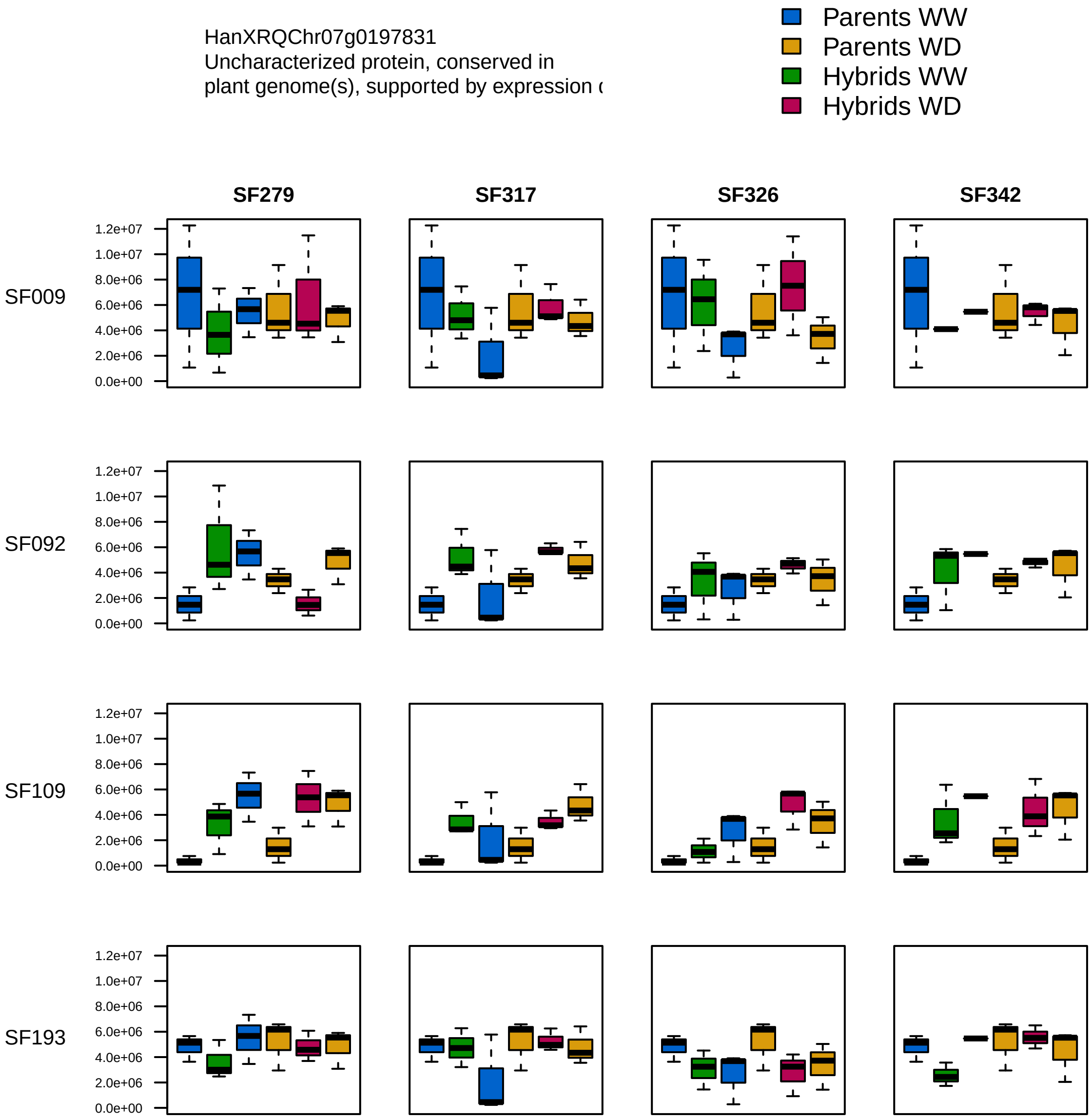

HanXRQChr07g0197831
Uncharacterized protein, conserved in
plant genome(s), supported by expression
Parents WW
Parents WD
Hybrids WW
Hybrids WD
SF279
SF317
SF326
SF342
SF009
SF092
SF109
SF193
1.2e+07
1.0e+07
8.0e+06
6.0e+06
4.0e+06
2.0e+06
0.0e+00

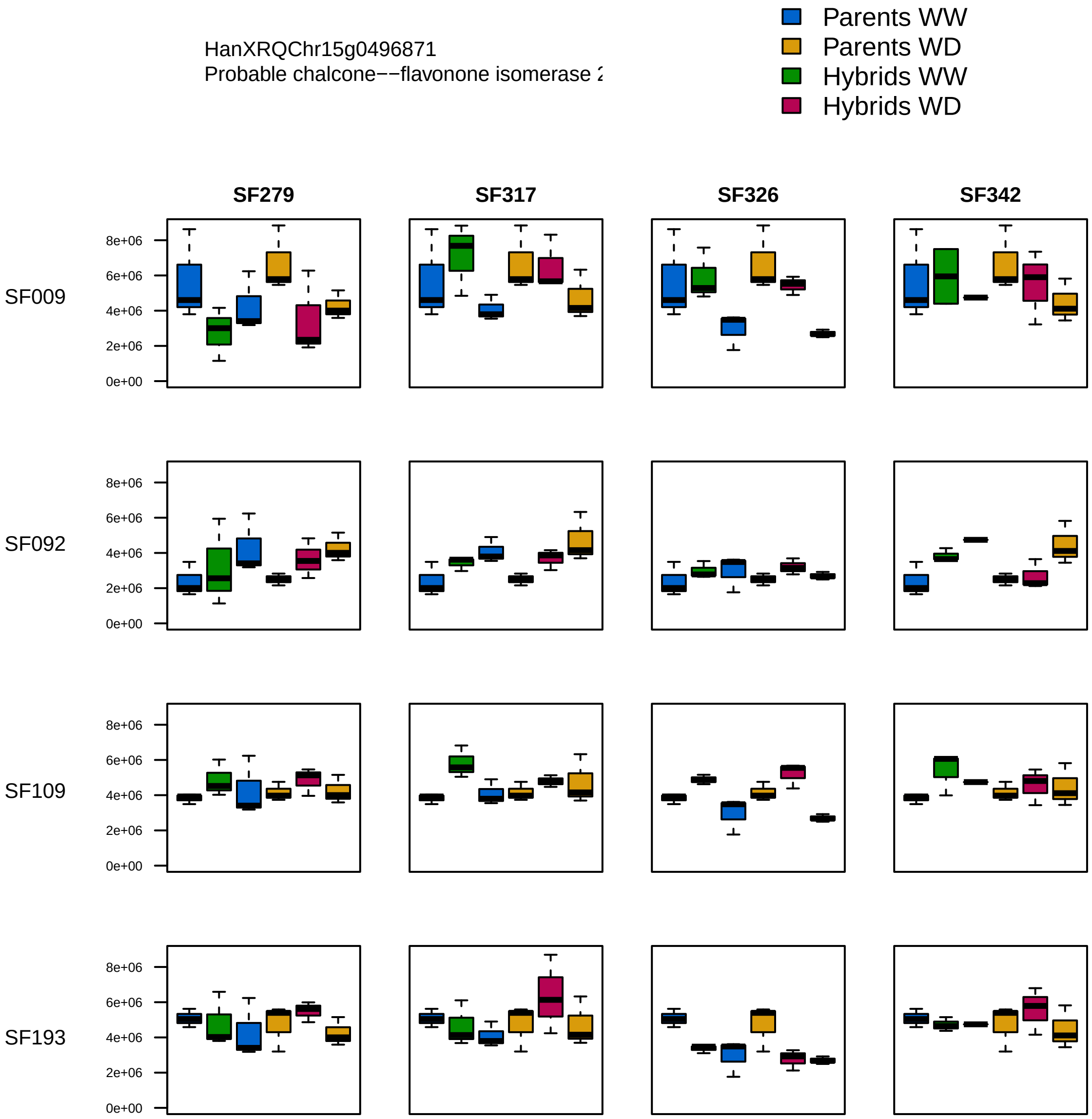

HanXRQChr15g0496871
Probable chalcone−−flavonone isomerase 2
Parents WW
Parents WD
Hybrids WW
Hybrids WD
SF279
SF317
SF326
SF342
SF009
SF092
SF109
SF193
8e+06
6e+06
4e+06
2e+06
0e+00

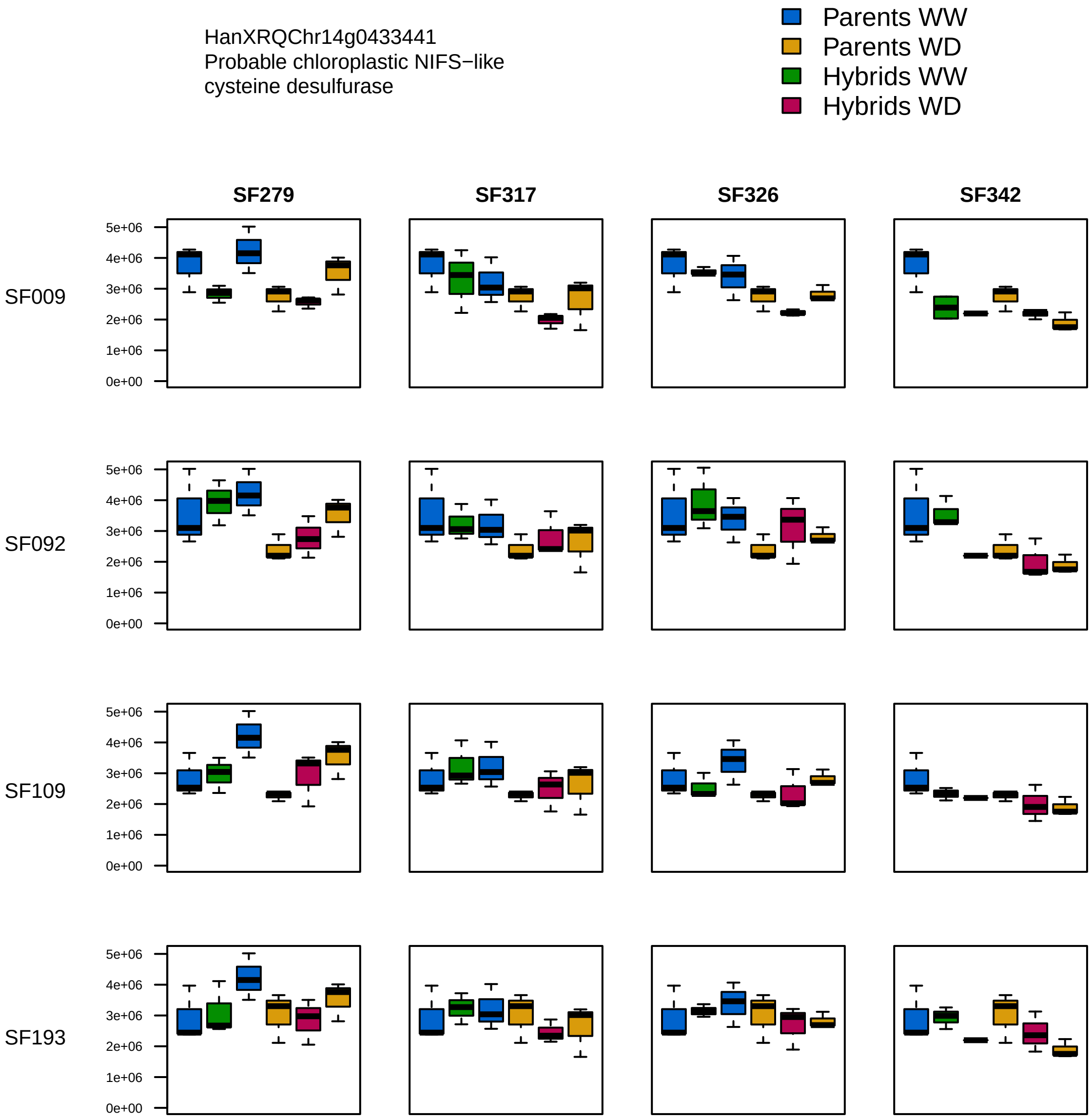
HanXRQChr14g0433441
Probable chloroplastic NIFS−like
cysteine desulfurase
Parents WW
Parents WD
Hybrids WW
Hybrids WD
SF279
SF317
SF326
SF342
SF009
SF092
SF109
SF193
5e+06
4e+06
3e+06
2e+06
1e+06
0e+00

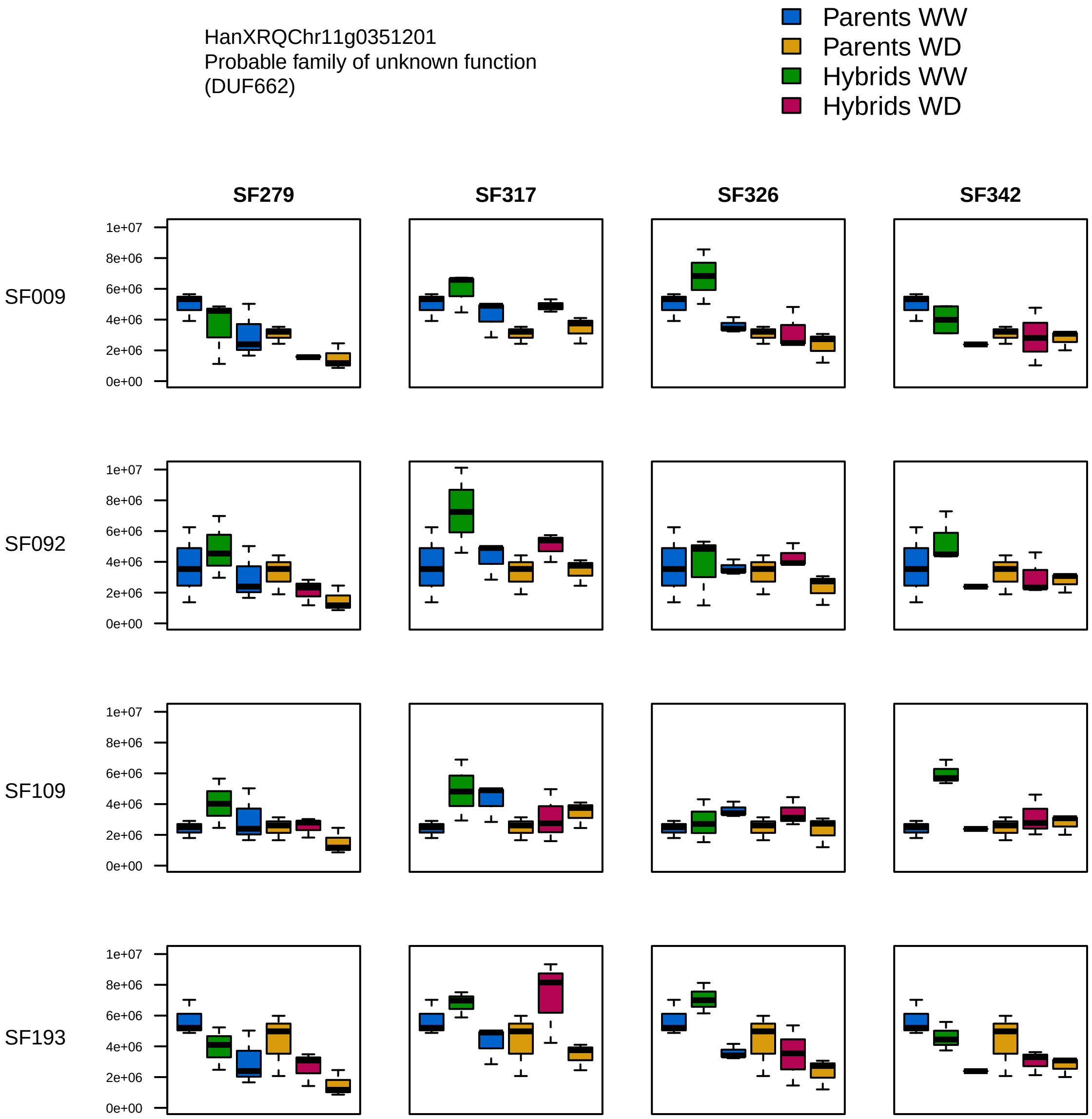
HanXRQChr11g0351201
Probable family of unknown function
(DUF662)
Parents WW
Parents WD
Hybrids WW
Hybrids WD
SF279
SF317
SF326
SF342
SF009
SF092
SF109
SF193
1e+07
8e+06
6e+06
4e+06
2e+06
0e+00

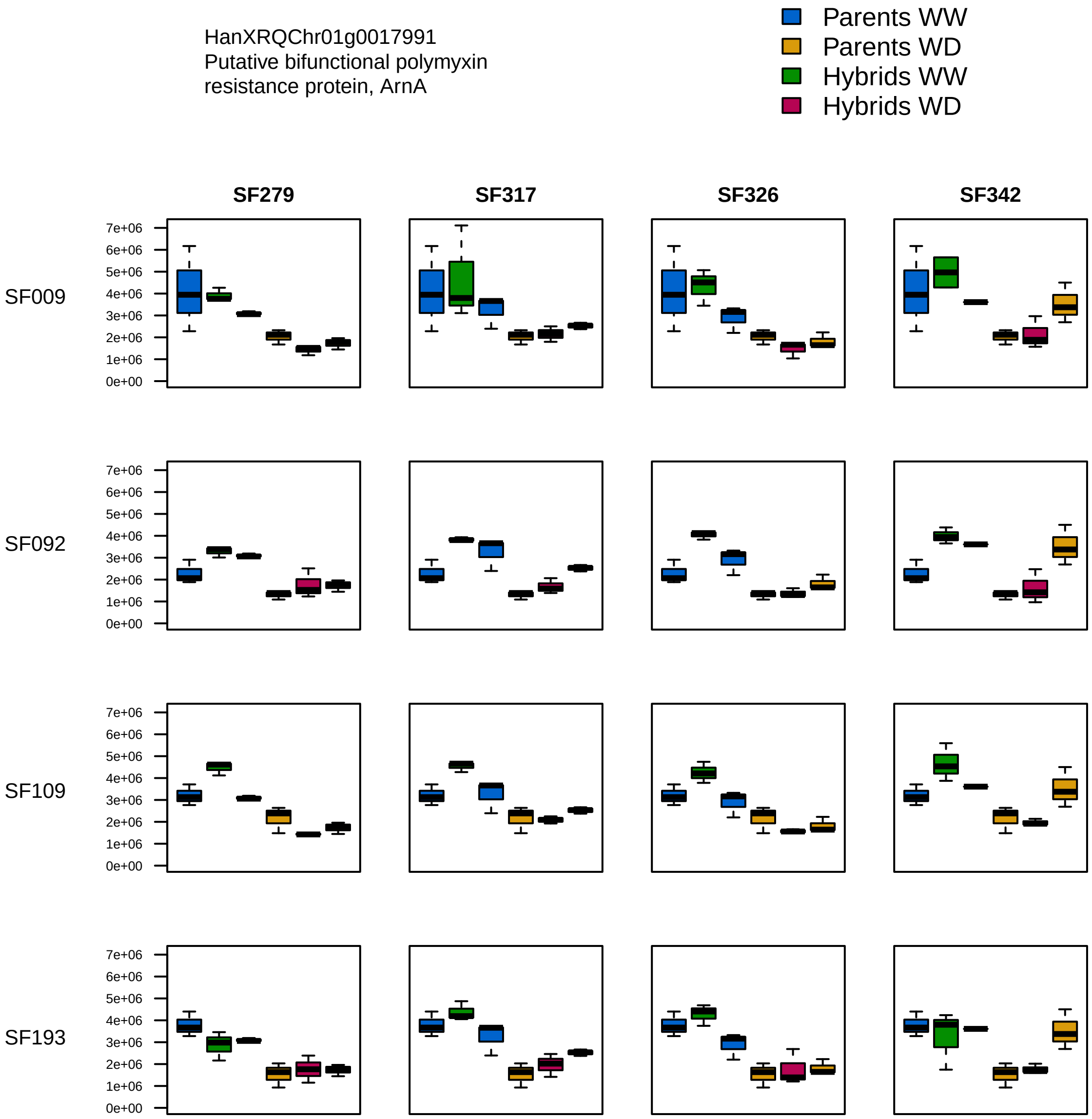
HanXRQChr01g0017991
Putative bifunctional polymyxin
resistance protein, ArnA
Parents WW
Parents WD
Hybrids WW
Hybrids WD
SF279
SF317
SF326
SF342
SF009
SF092
SF109
SF193
7e+06
6e+06
5e+06
4e+06
3e+06
2e+06
1e+06
0e+00

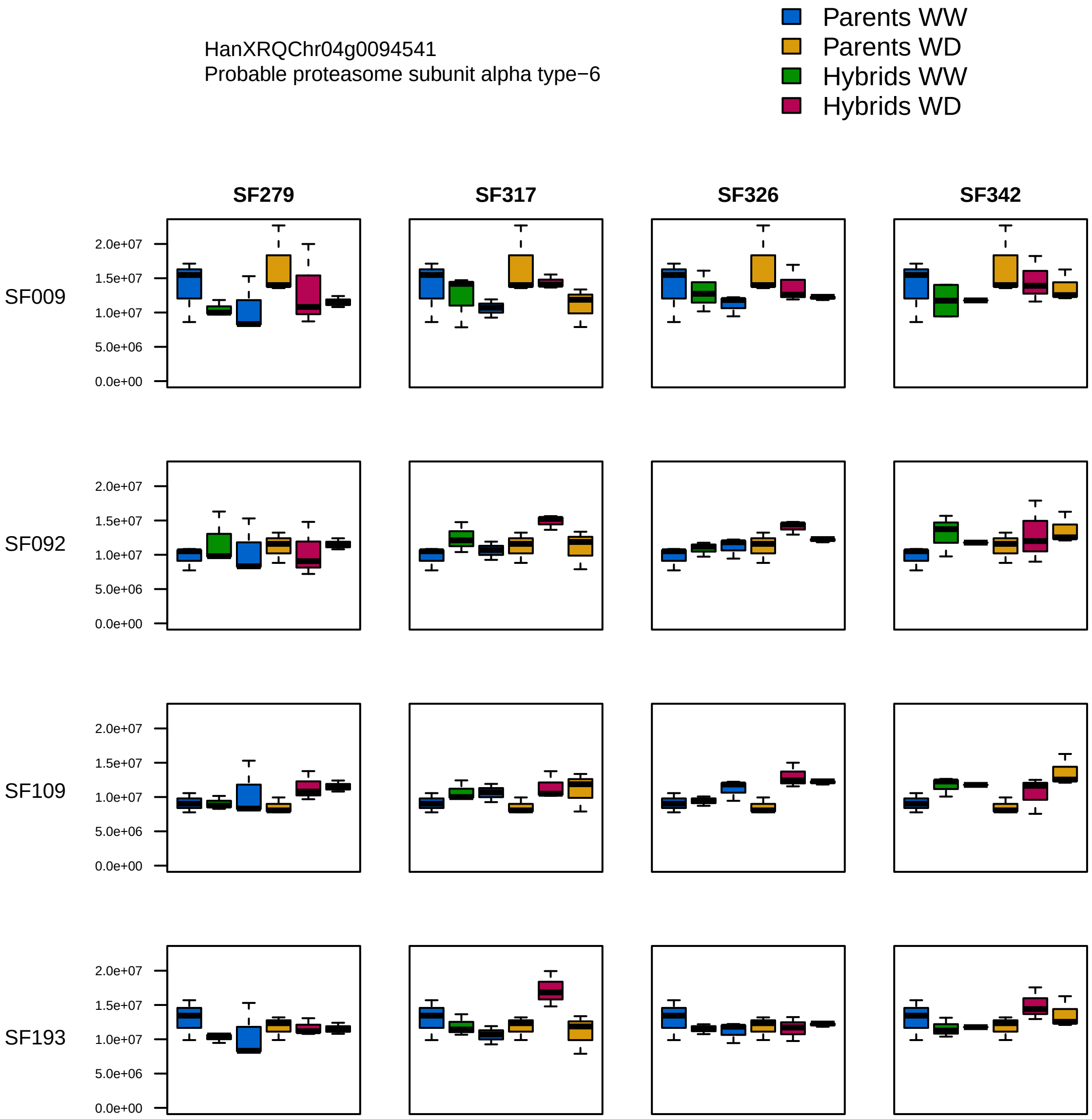
HanXRQChr04g0094541
Probable proteasome subunit alpha type−6
Parents WW
Parents WD
Hybrids WW
Hybrids WD
SF279
SF317
SF326
SF342
SF009
SF092
SF109
SF193
0.0e+00
5.0e+06
1.0e+07
1.5e+07
2.0e+07

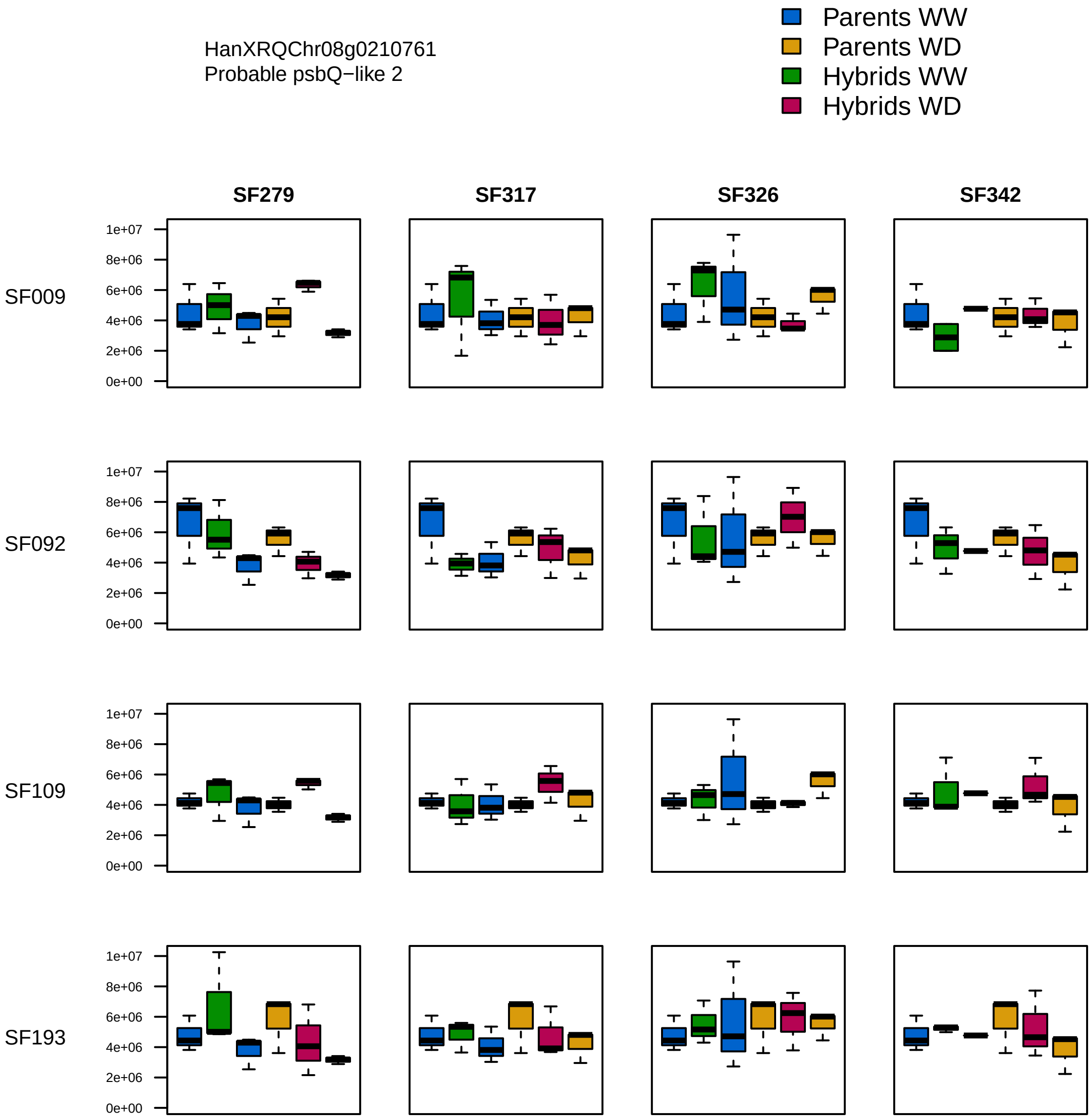
HanXRQChr08g0210761
Probable psbQ−like 2
Parents WW
Parents WD
Hybrids WW
Hybrids WD
SF279
SF317
SF326
SF342
SF009
SF092
SF109
SF193
1e+07
8e+06
6e+06
4e+06
2e+06
0e+00

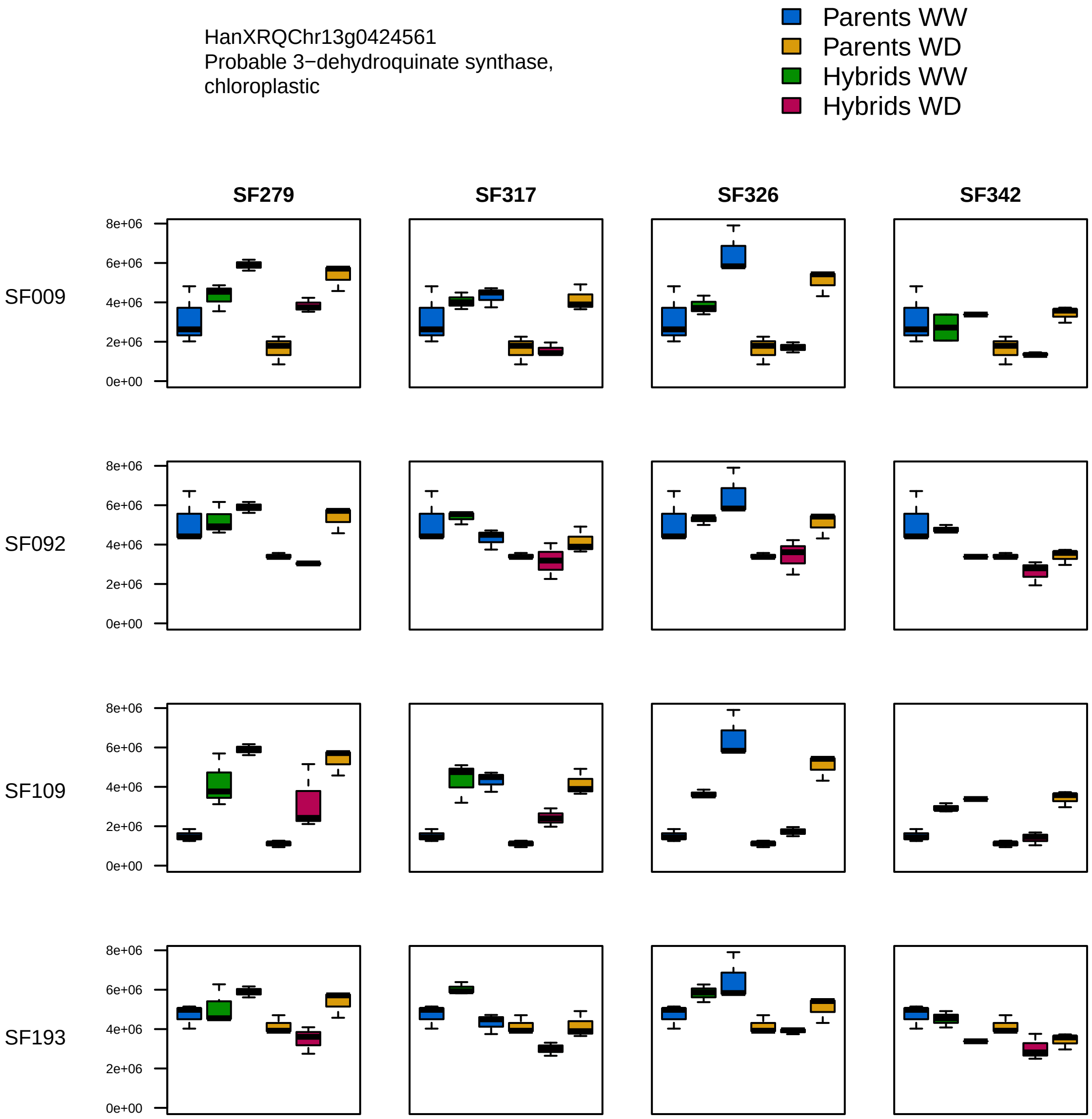
HanXRQChr13g0424561
Probable 3−dehydroquinate synthase, chloroplastic
Parents WW
Parents WD
Hybrids WW
Hybrids WD
SF279
SF317
SF326
SF342
SF009
SF092
SF109
SF193
0e+00
2e+06
4e+06
6e+06
8e+06

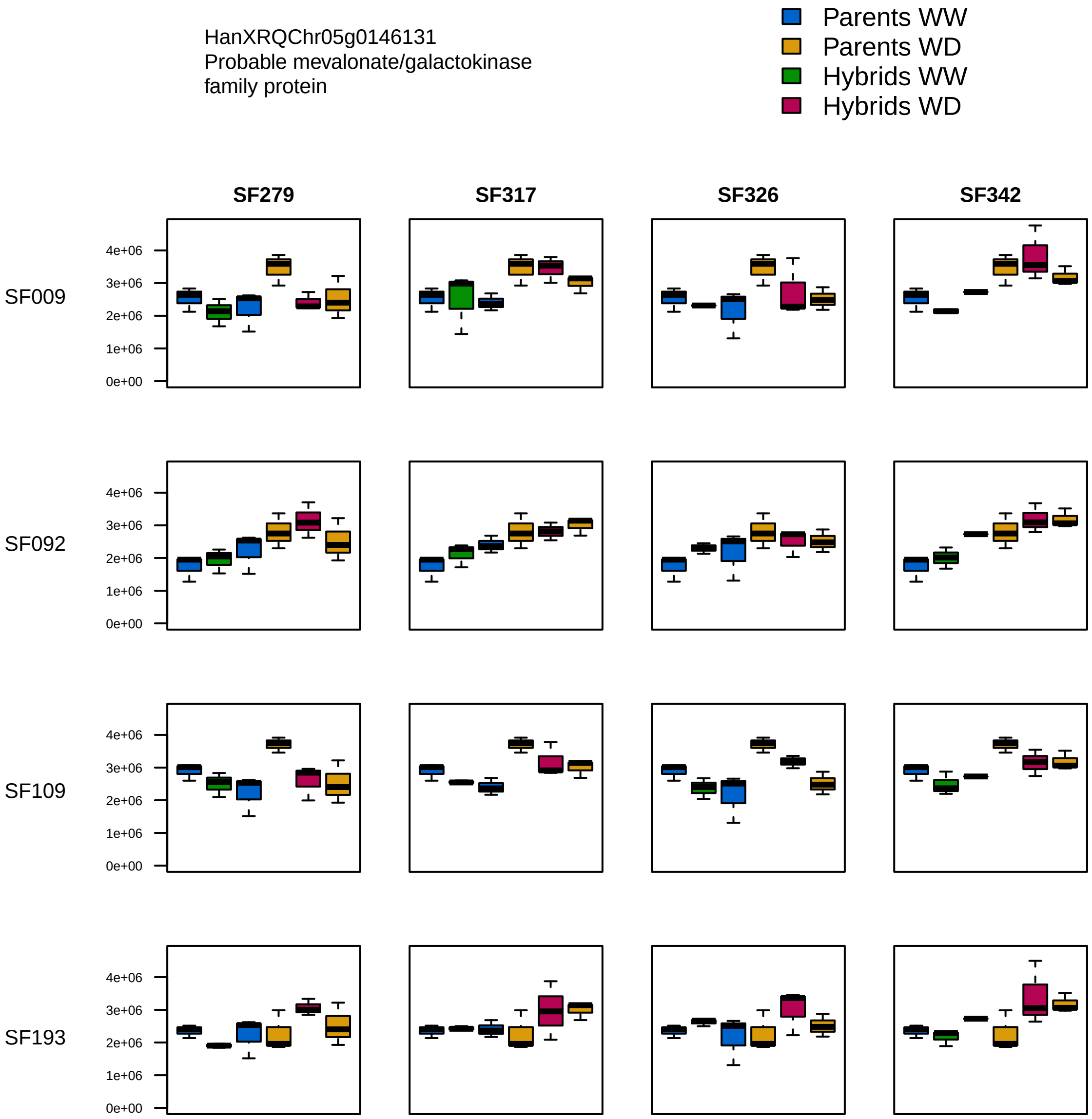
HanXRQChr05g0146131
Probable mevalonate/galactokinase
family protein
Parents WW
Parents WD
Hybrids WW
Hybrids WD
SF279
SF317
SF326
SF342
SF009
SF092
SF109
SF193
4e+06
3e+06
2e+06
1e+06
0e+00

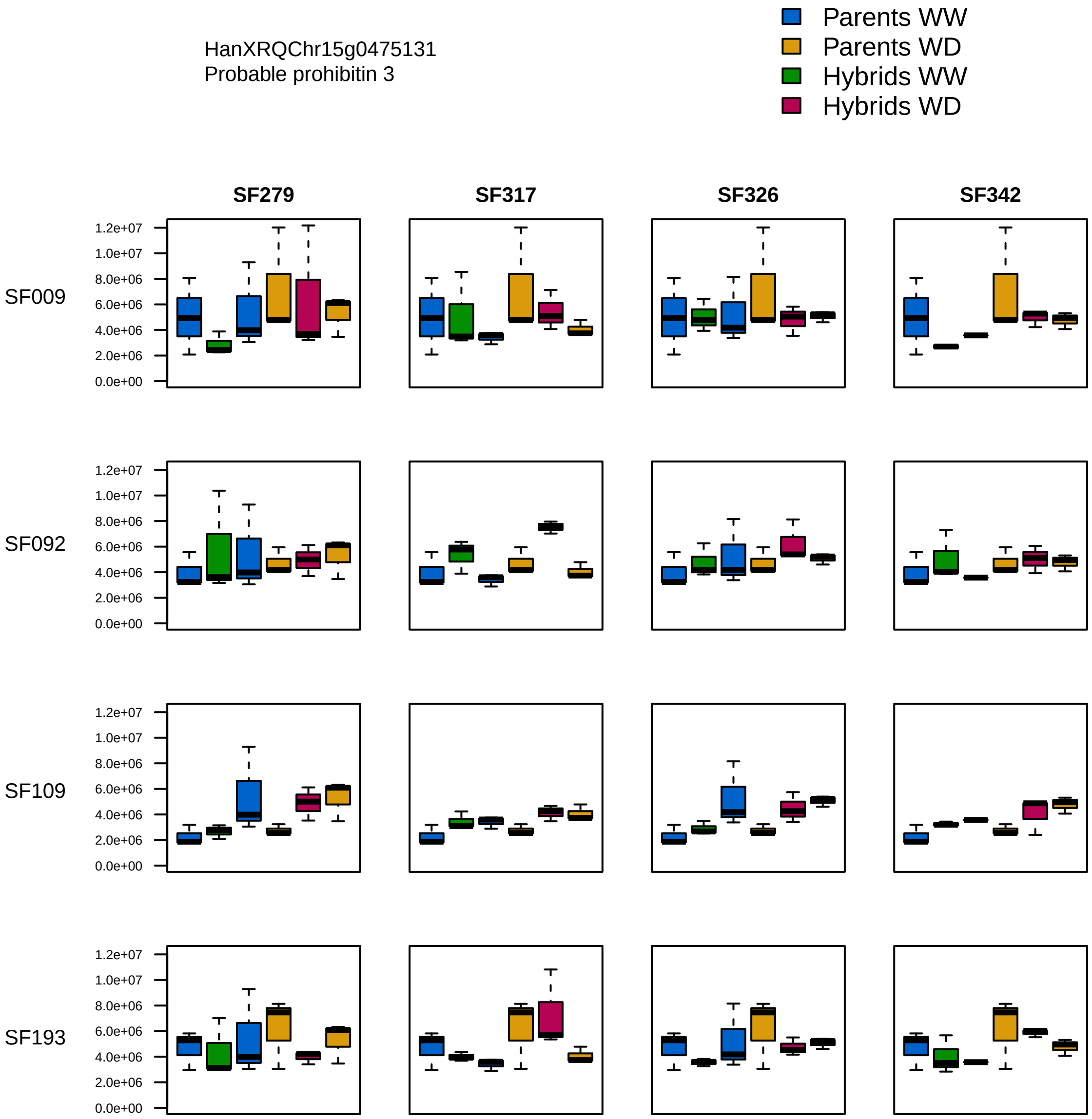

HanXRQChr15g0475131
Probable prohibitin 3
Parents WW
Parents WD
Hybrids WW
Hybrids WD
SF279
SF317
SF326
SF342
SF009
SF092
SF109
SF193
1.2e+07
1.0e+07
8.0e+06
6.0e+06
4.0e+06
2.0e+06
0.0e+00

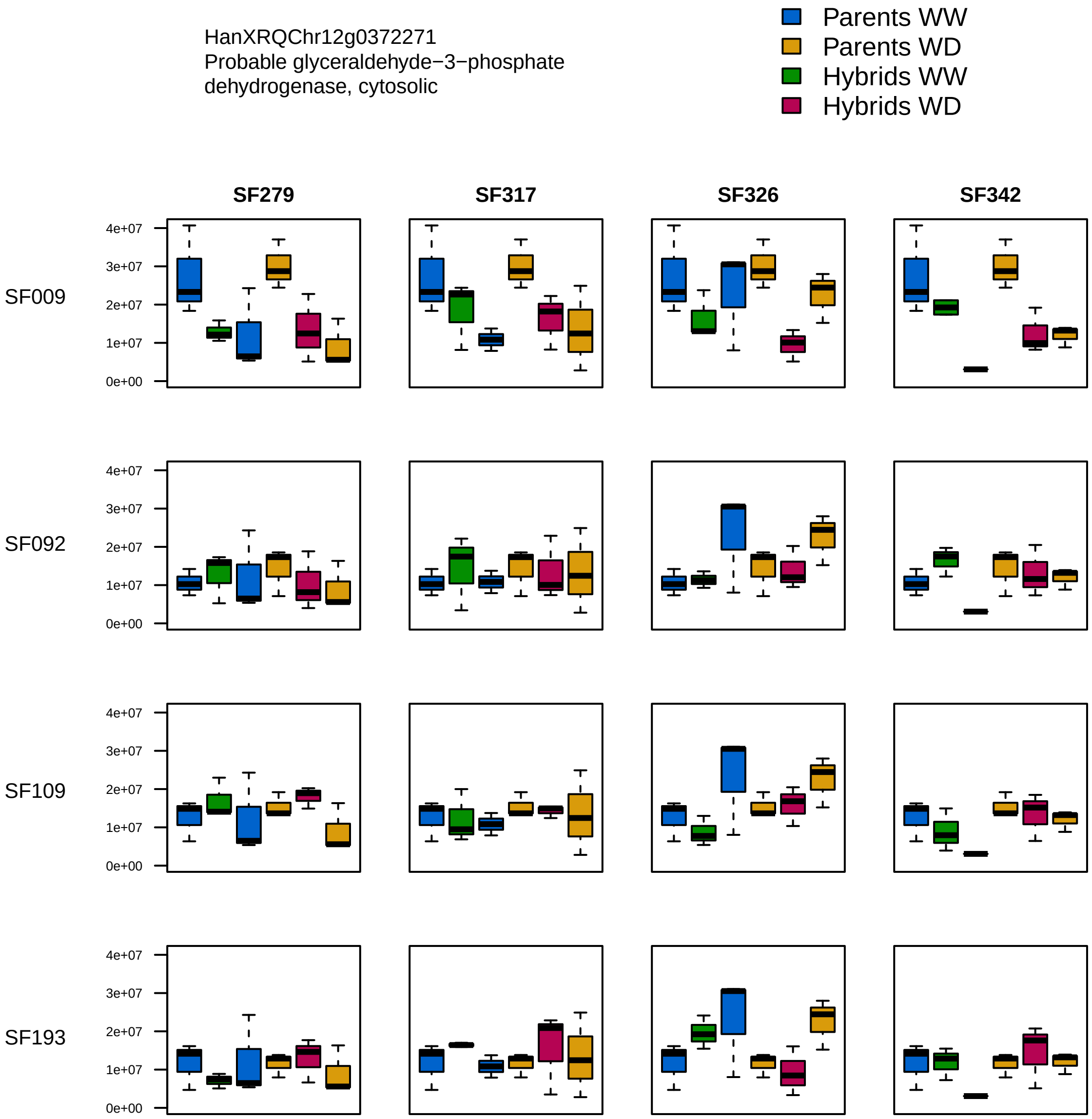

HanXRQChr12g0372271
Probable glyceraldehyde−3−phosphate
dehydrogenase, cytosolic
Parents WW
Parents WD
Hybrids WW
Hybrids WD
SF279
SF317
SF326
SF342
SF009
SF092
SF109
SF193
4e+07
3e+07
2e+07
1e+07
0e+00

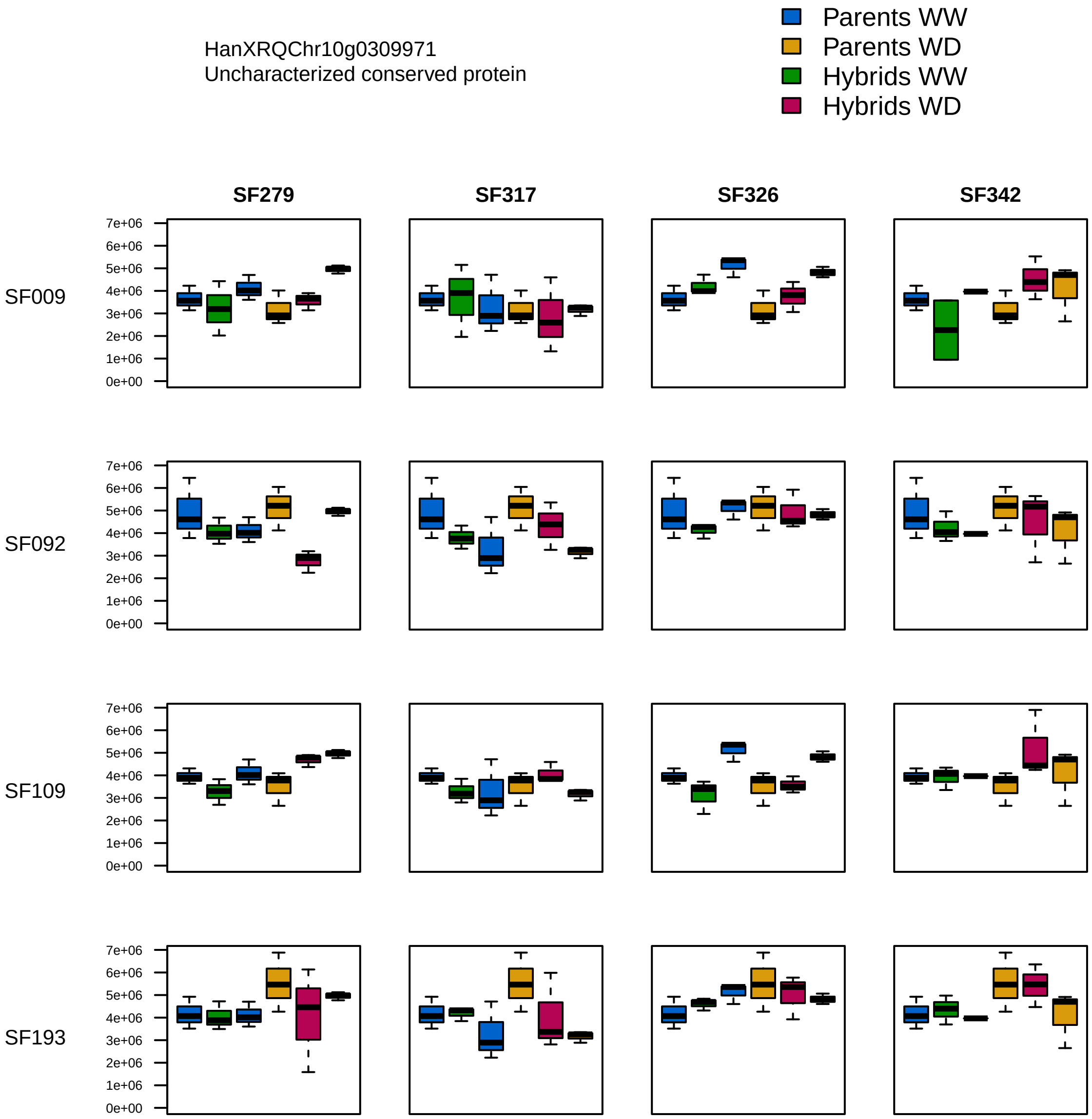

HanXRQChr10g0309971
Uncharacterized conserved protein
Parents WW
Parents WD
Hybrids WW
Hybrids WD
SF279
SF317
SF326
SF342
SF009
SF092
SF109
SF193
0e+00
1e+06
2e+06
3e+06
4e+06
5e+06
6e+06
7e+06

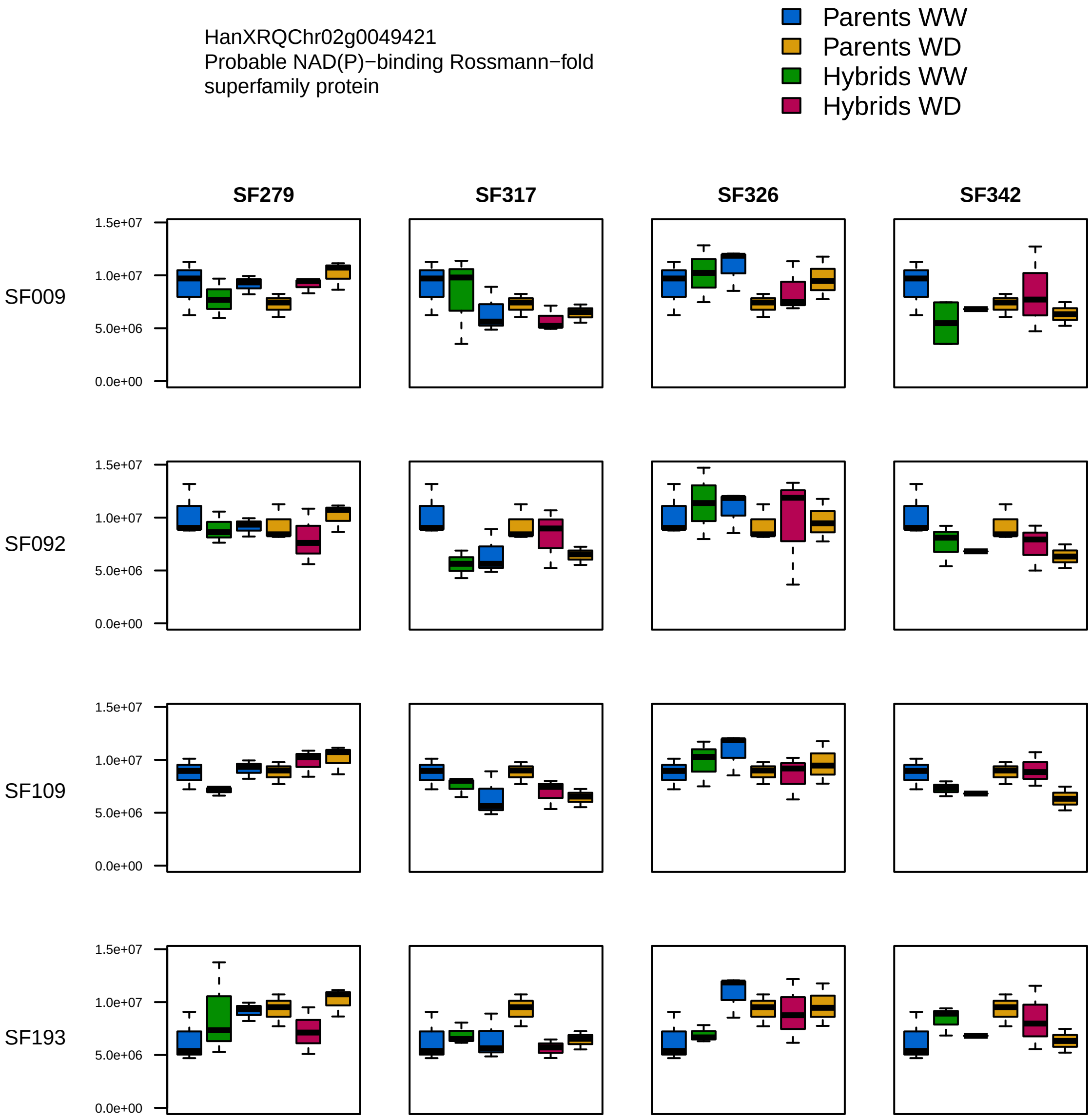

HanXRQChr02g0049421
Probable NAD(P)−binding Rossmann−fold
superfamily protein
Parents WW
Parents WD
Hybrids WW
Hybrids WD
SF279
SF317
SF326
SF342
SF009
SF092
SF109
SF193
0.0e+00
5.0e+06
1.0e+07
1.5e+07

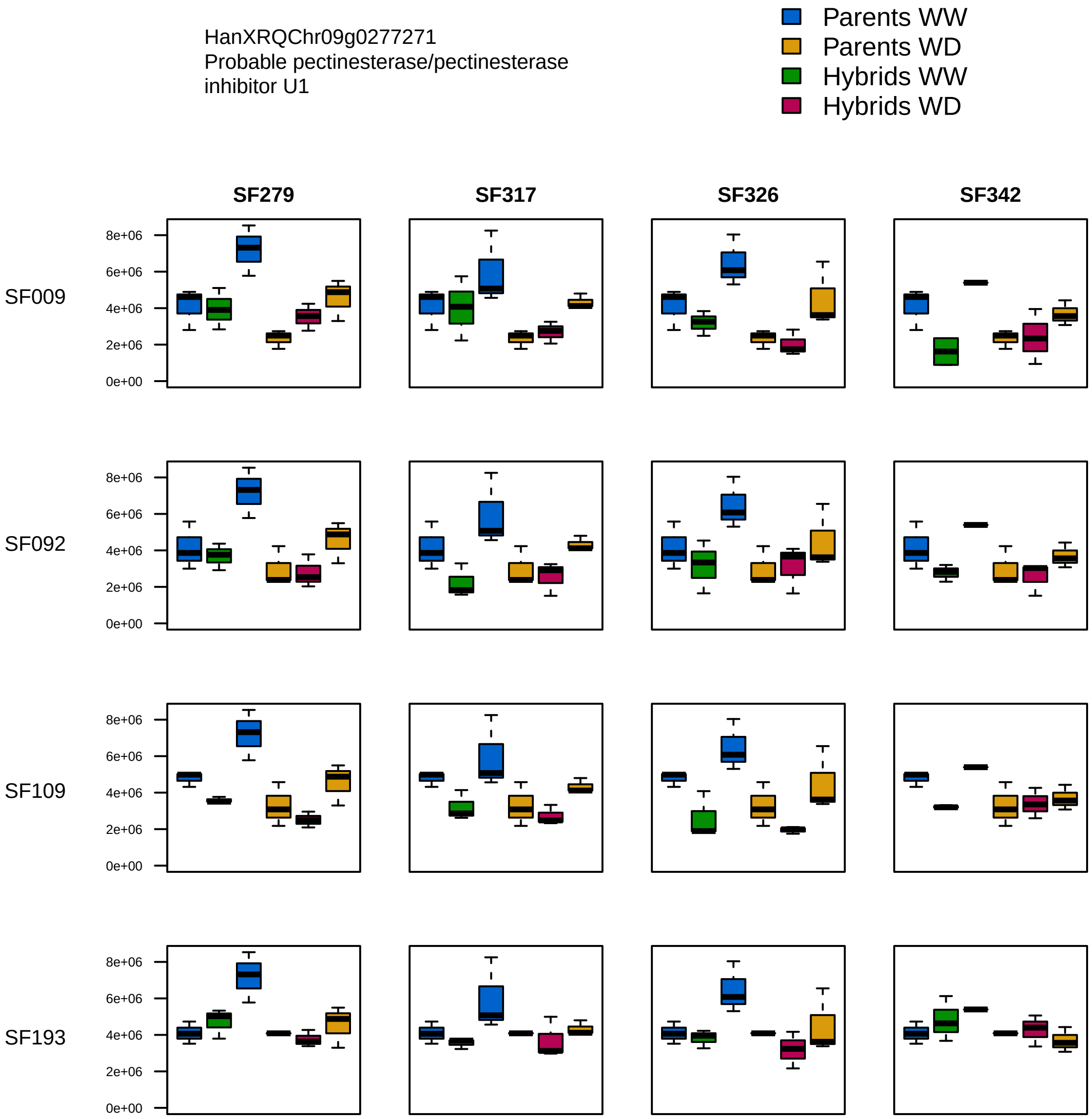
HanXRQChr09g0277271
Probable pectinesterase/pectinesterase inhibitor U1
Parents WW
Parents WD
Hybrids WW
Hybrids WD
SF279
SF317
SF326
SF342
SF009
SF092
SF109
SF193
0e+00
2e+06
4e+06
6e+06
8e+06

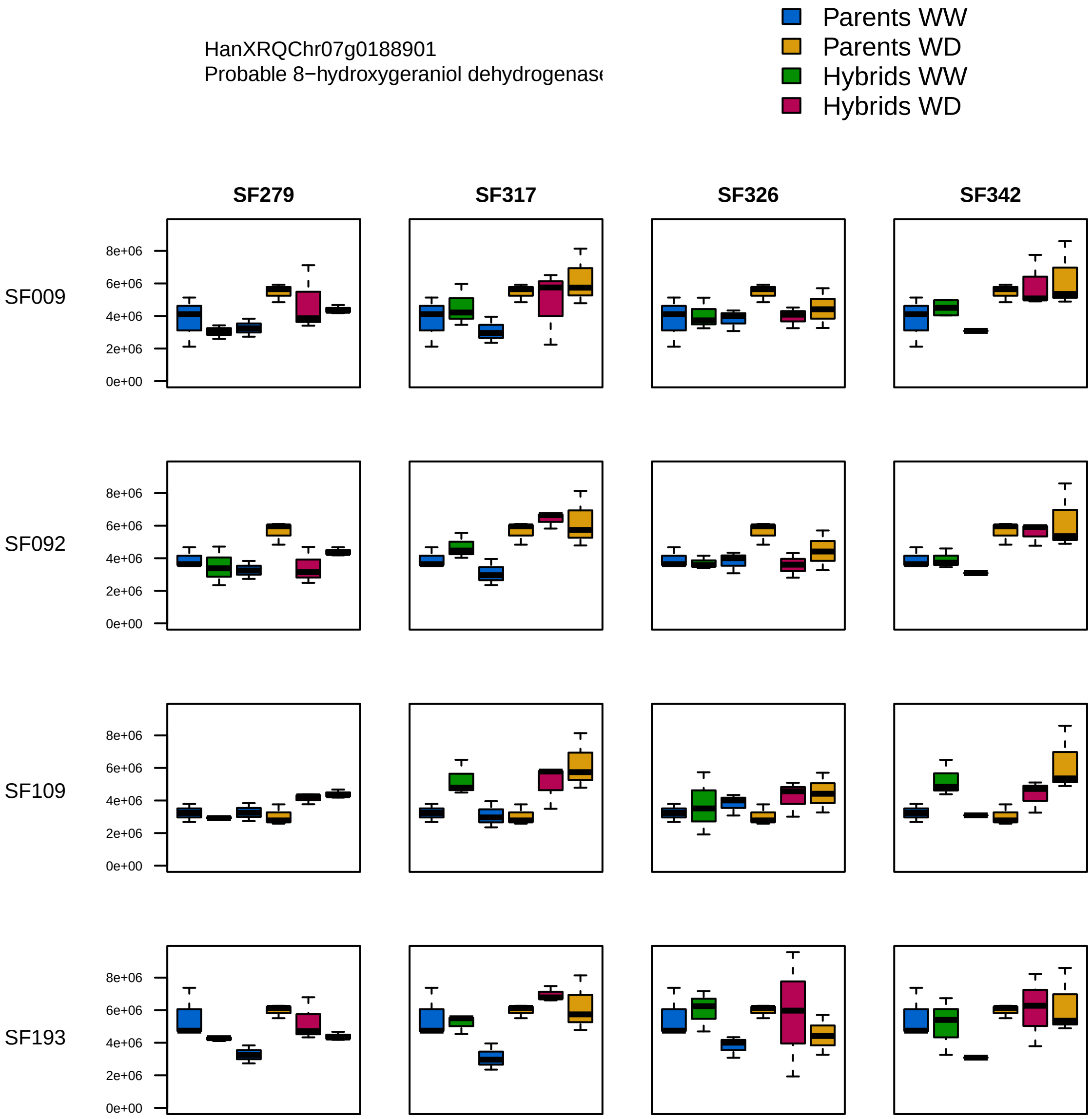

HanXRQChr07g0188901
Probable 8−hydroxygeraniol dehydrogenase
Parents WW
Parents WD
Hybrids WW
Hybrids WD
SF279
SF317
SF326
SF342
SF009
SF092
SF109
SF193
0e+00
2e+06
4e+06
6e+06
8e+06

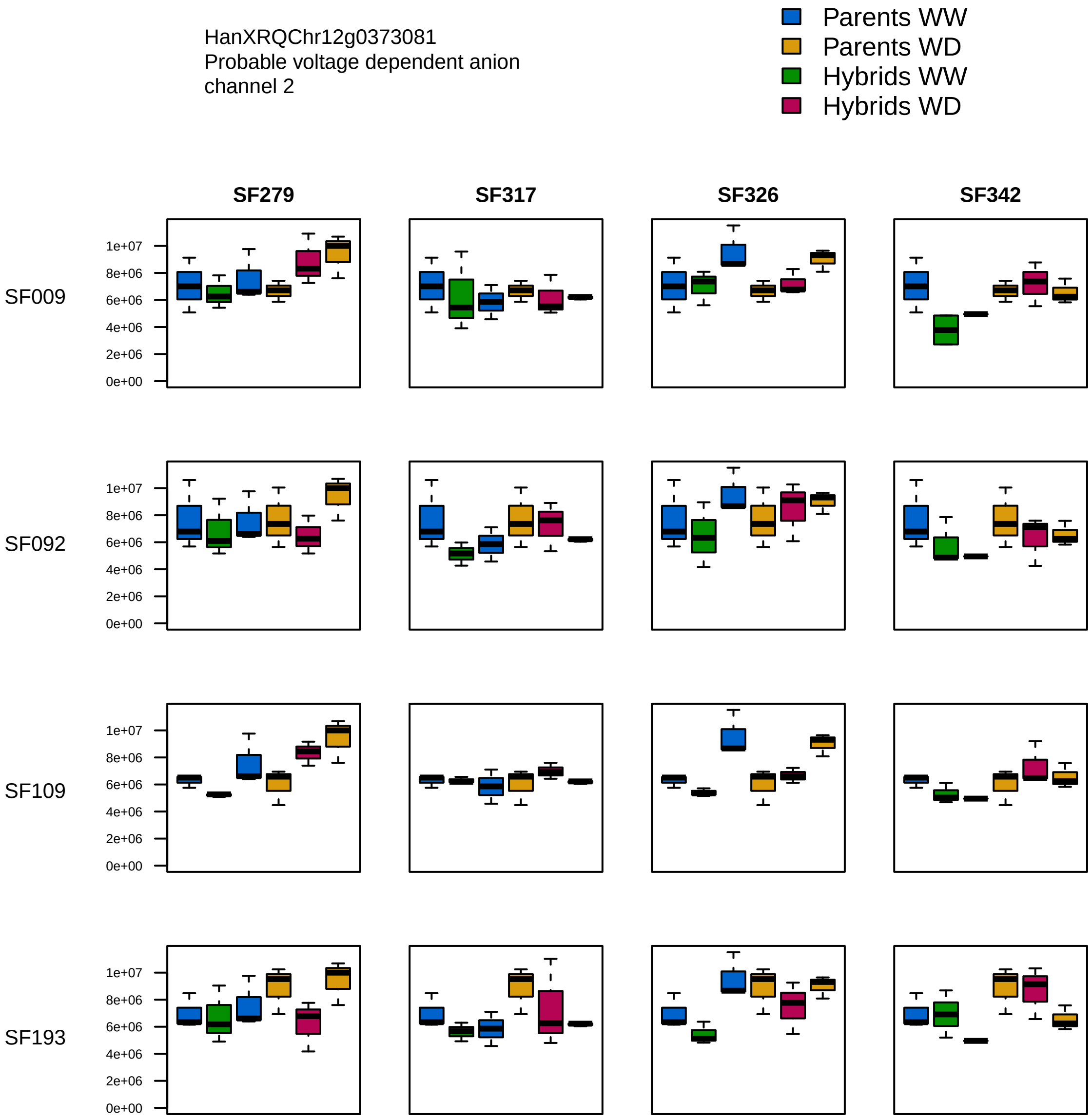

HanXRQChr12g0373081
Probable voltage dependent anion
channel 2
Parents WW
Parents WD
Hybrids WW
Hybrids WD
SF279
SF317
SF326
SF342
SF009
SF092
SF109
SF193
1e+07
8e+06
6e+06
4e+06
2e+06
0e+00

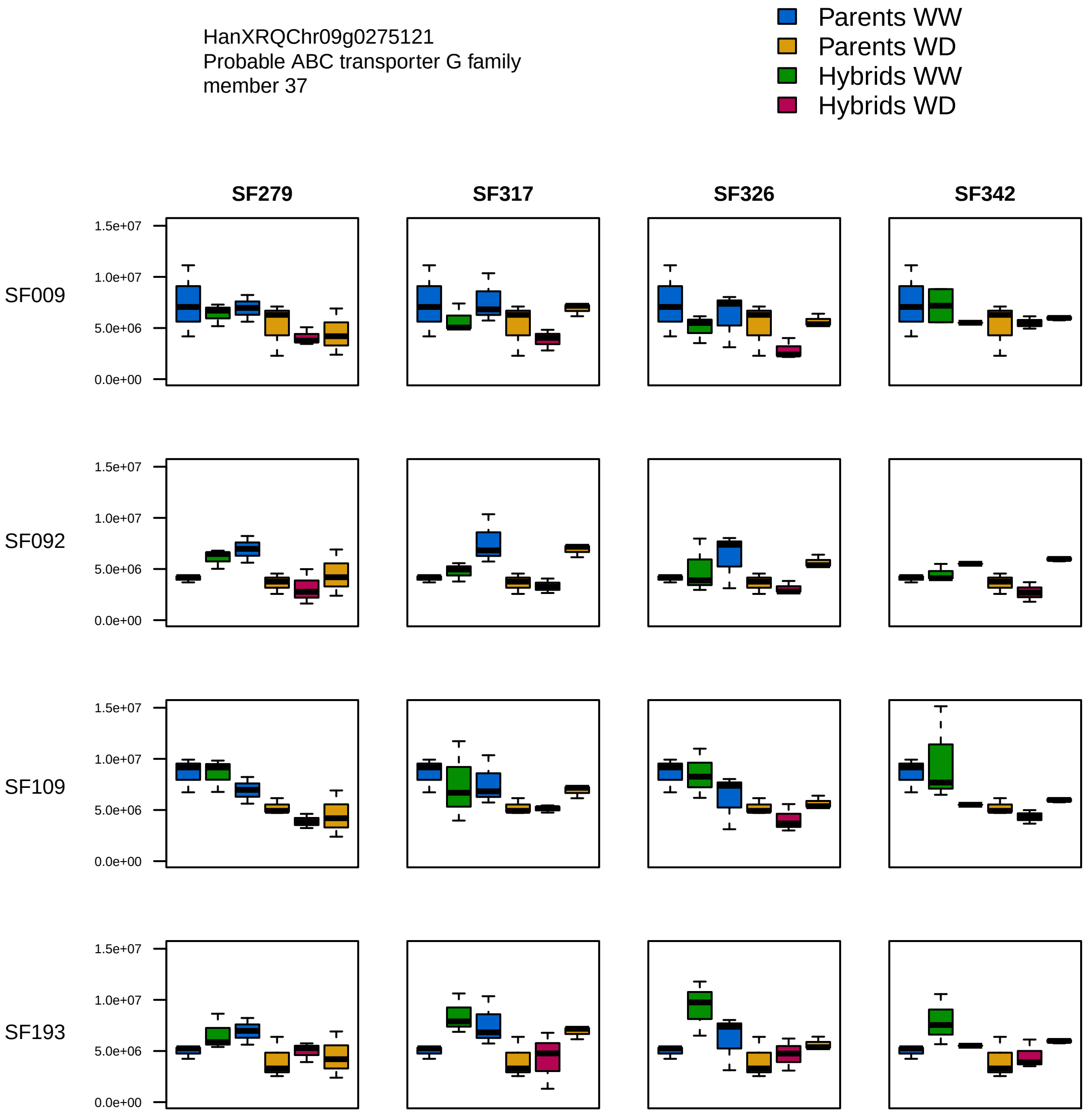
HanXRQChr09g0275121
Probable ABC transporter G family
member 37
Parents WW
Parents WD
Hybrids WW
Hybrids WD
SF279
SF317
SF326
SF342
SF009
SF092
SF109
SF193
1.5e+07
1.0e+07
5.0e+06
0.0e+00

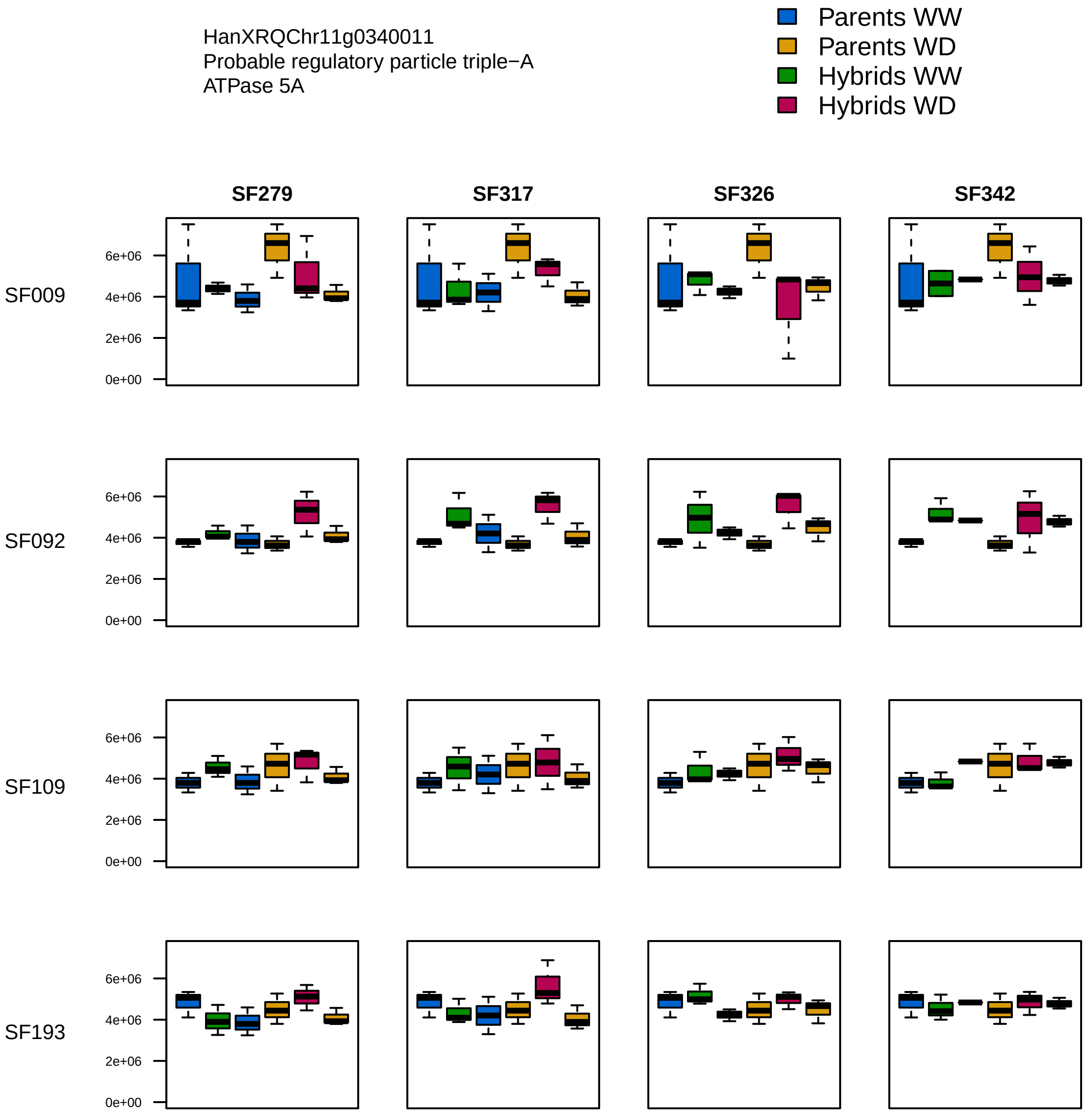
HanXRQChr11g0340011
Probable regulatory particle triple−A
ATPase 5A
Parents WW
Parents WD
Hybrids WW
Hybrids WD
SF279
SF317
SF326
SF342
SF009
SF092
SF109
SF193
6e+06
4e+06
2e+06
0e+00

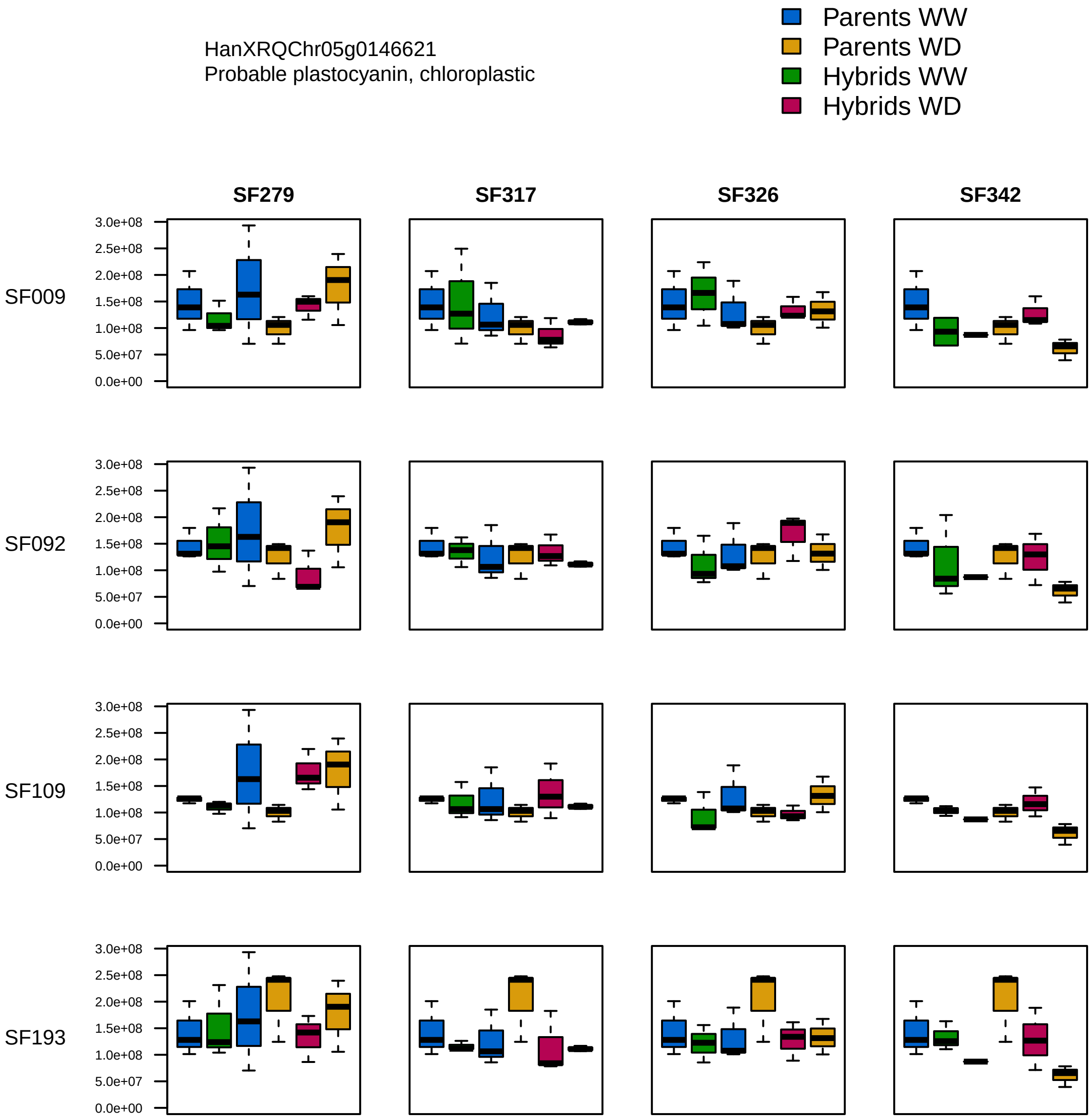
HanXRQChr05g0146621
Probable plastocyanin, chloroplastic
Parents WW
Parents WD
Hybrids WW
Hybrids WD
SF279
SF317
SF326
SF342
SF009
SF092
SF109
SF193
3.0e+08
2.5e+08
2.0e+08
1.5e+08
1.0e+08
5.0e+07
0.0e+00

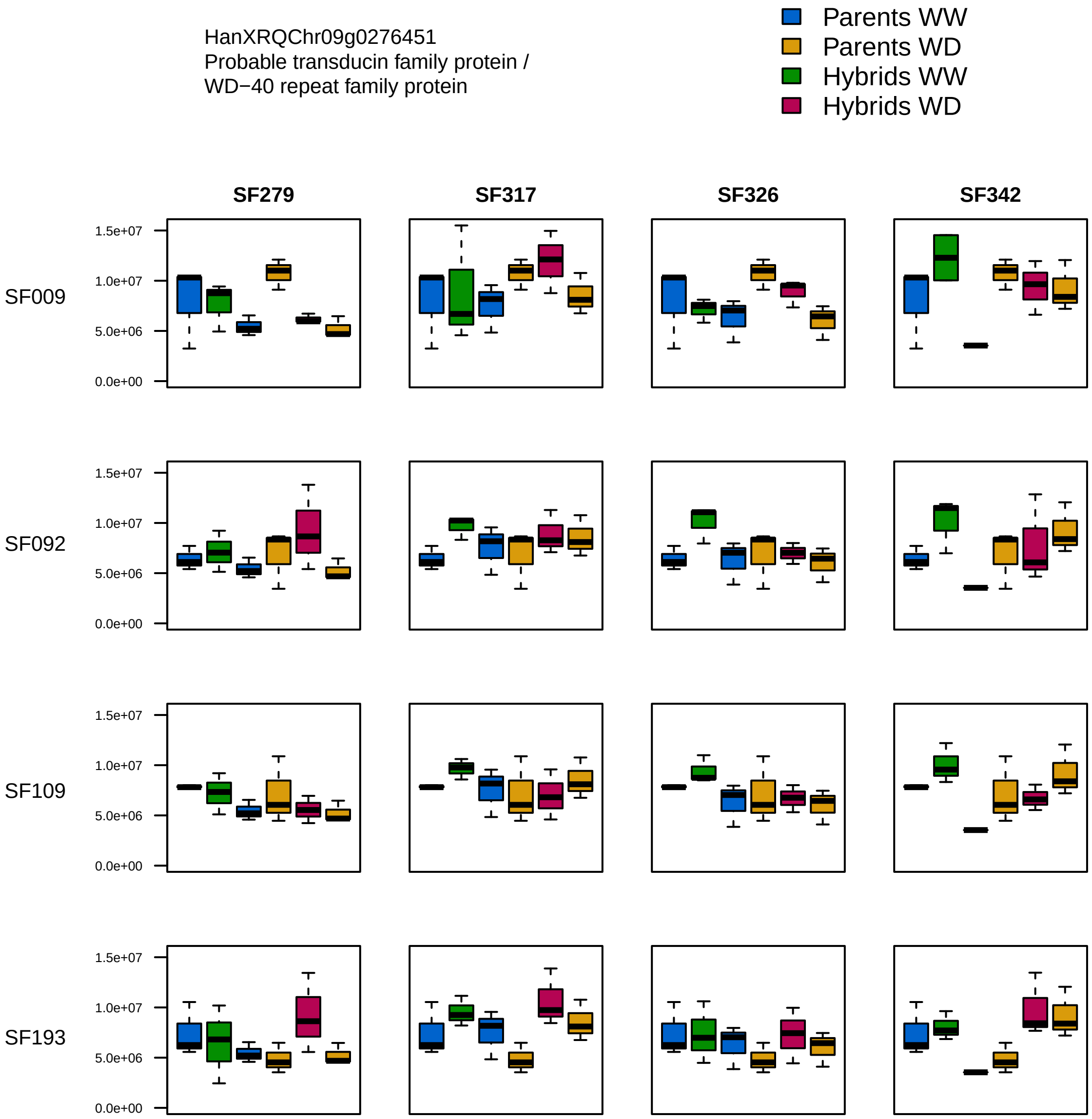
HanXRQChr09g0276451
Probable transducin family protein /
WD−40 repeat family protein
Parents WW
Parents WD
Hybrids WW
Hybrids WD
SF279
SF317
SF326
SF342
SF009
SF092
SF109
SF193
1.5e+07
1.0e+07
5.0e+06
0.0e+00

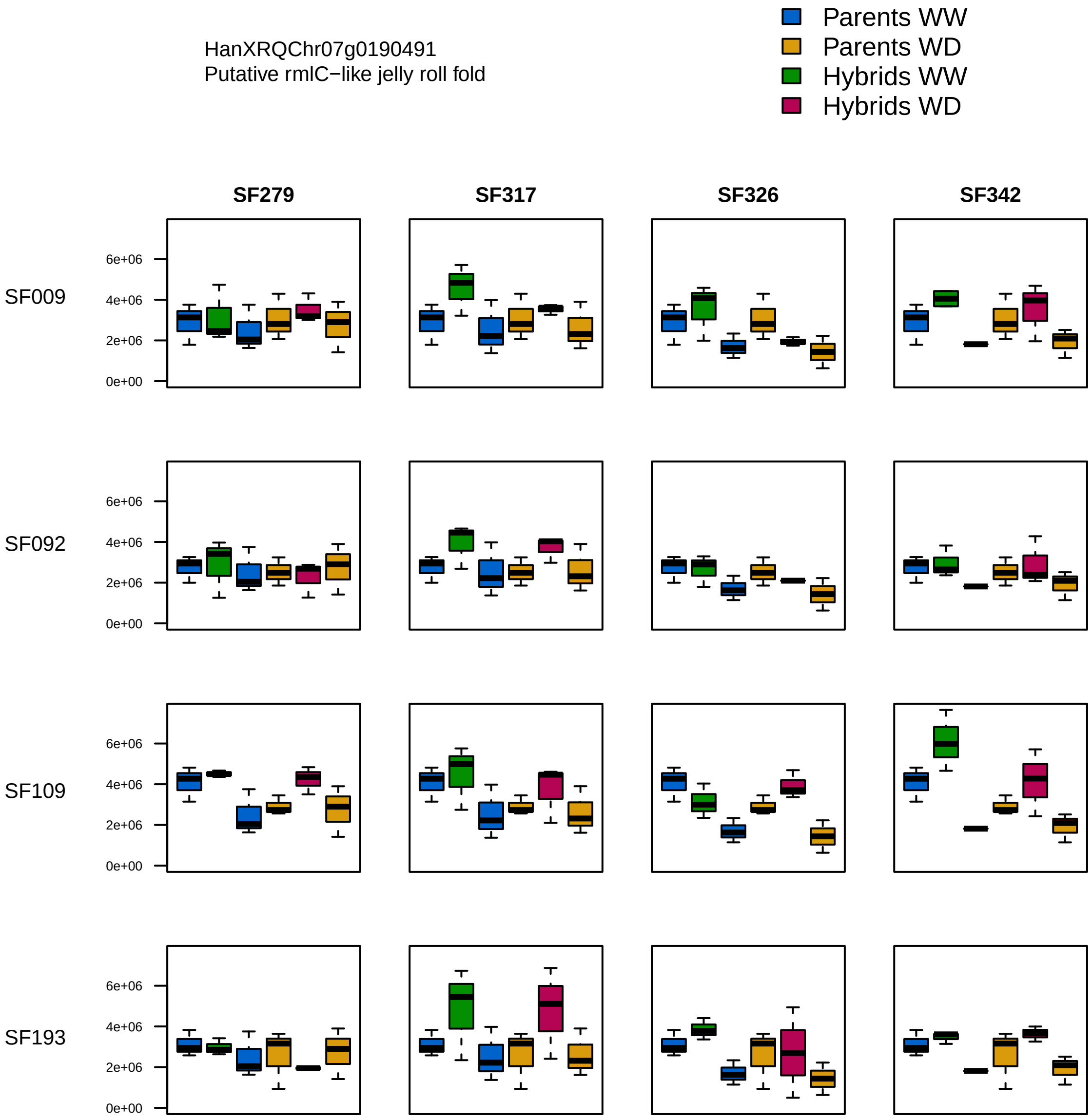

HanXRQChr07g0190491
Putative rmlC−like jelly roll fold
Parents WW
Parents WD
Hybrids WW
Hybrids WD
SF279
SF317
SF326
SF342
SF009
SF092
SF109
SF193
0e+00
2e+06
4e+06
6e+06

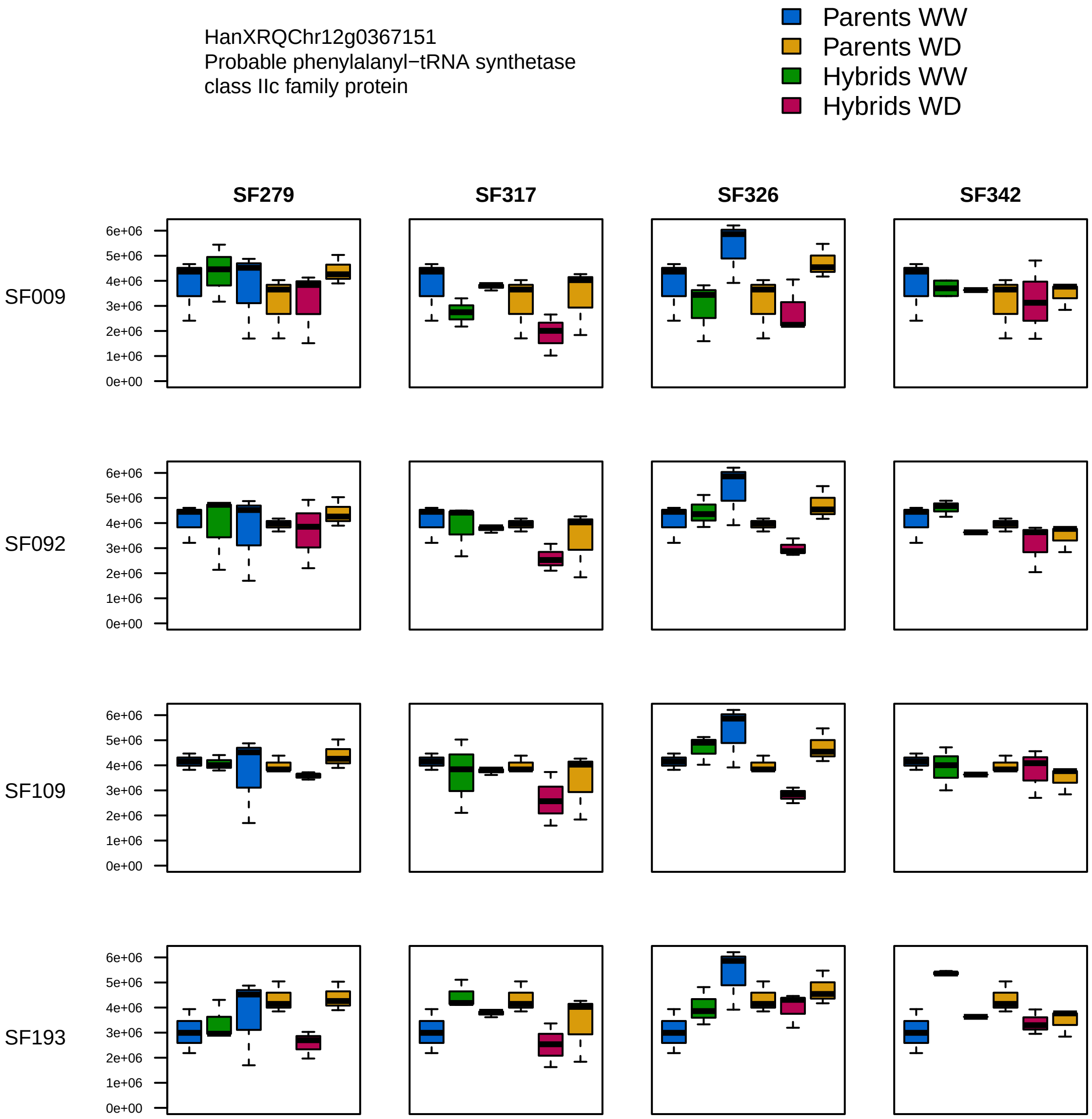
HanXRQChr12g0367151
Probable phenylalanyl−tRNA synthetase
class IIc family protein
Parents WW
Parents WD
Hybrids WW
Hybrids WD
SF279
SF317
SF326
SF342
SF009
SF092
SF109
SF193
6e+06
5e+06
4e+06
3e+06
2e+06
1e+06
0e+00

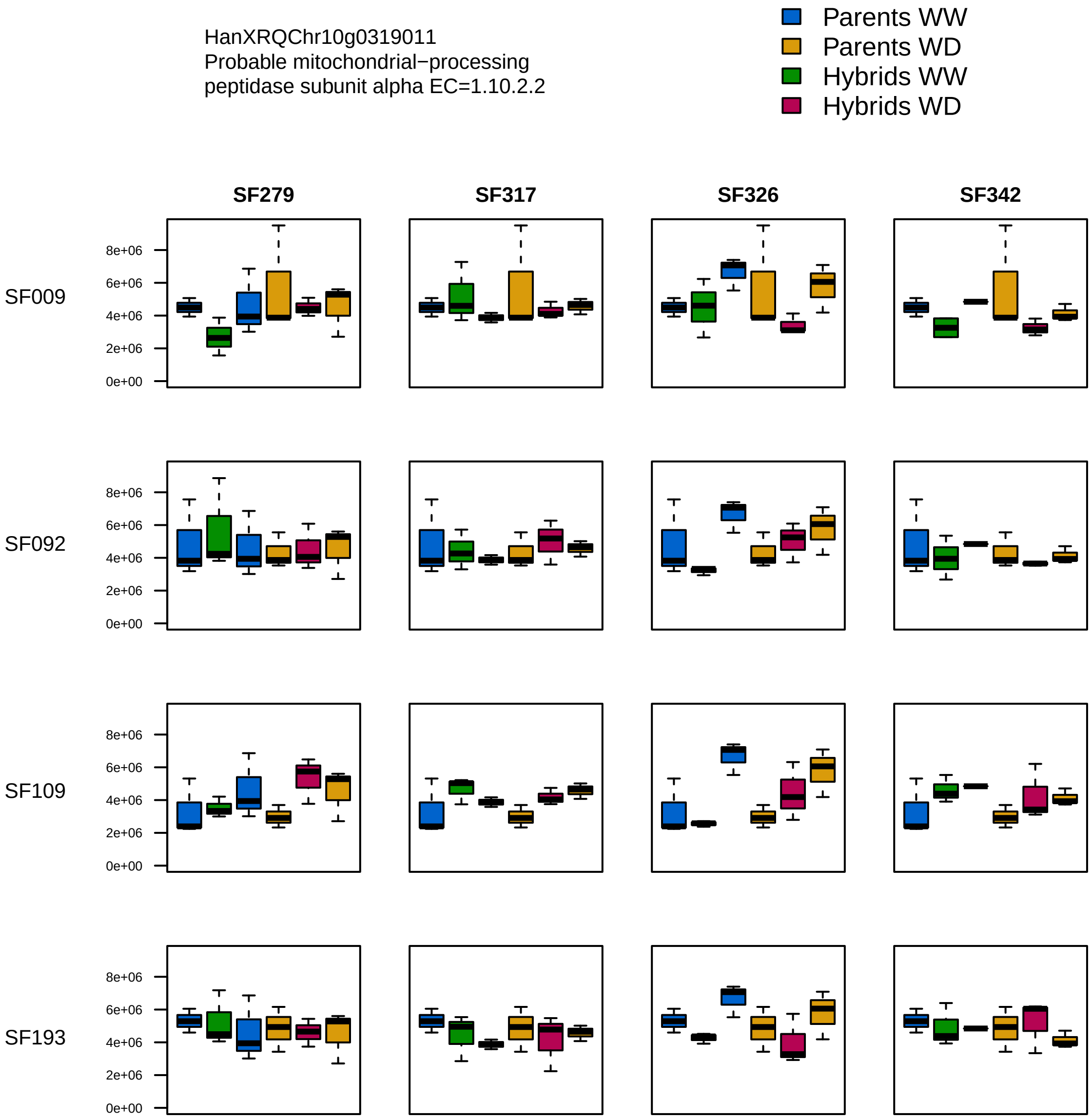
HanXRQChr10g0319011
Probable mitochondrial−processing
peptidase subunit alpha EC=1.10.2.2
Parents WW
Parents WD
Hybrids WW
Hybrids WD
SF279
SF317
SF326
SF342
SF009
SF092
SF109
SF193
8e+06
6e+06
4e+06
2e+06
0e+00

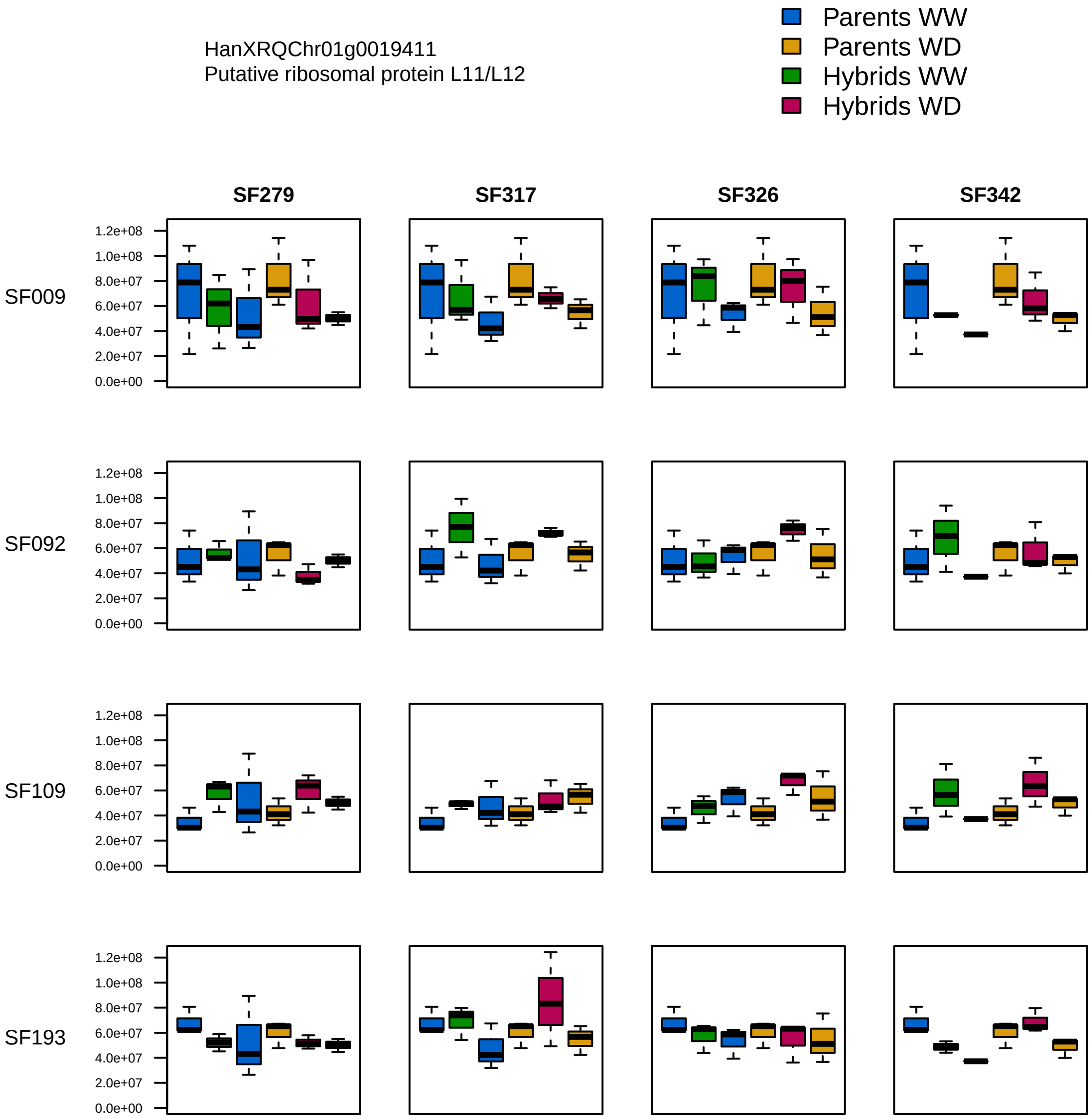
HanXRQChr01g0019411
Putative ribosomal protein L11/L12
Parents WW
Parents WD
Hybrids WW
Hybrids WD
SF279
SF317
SF326
SF342
SF009
SF092
SF109
SF193
1.2e+08
1.0e+08
8.0e+07
6.0e+07
4.0e+07
2.0e+07
0.0e+00

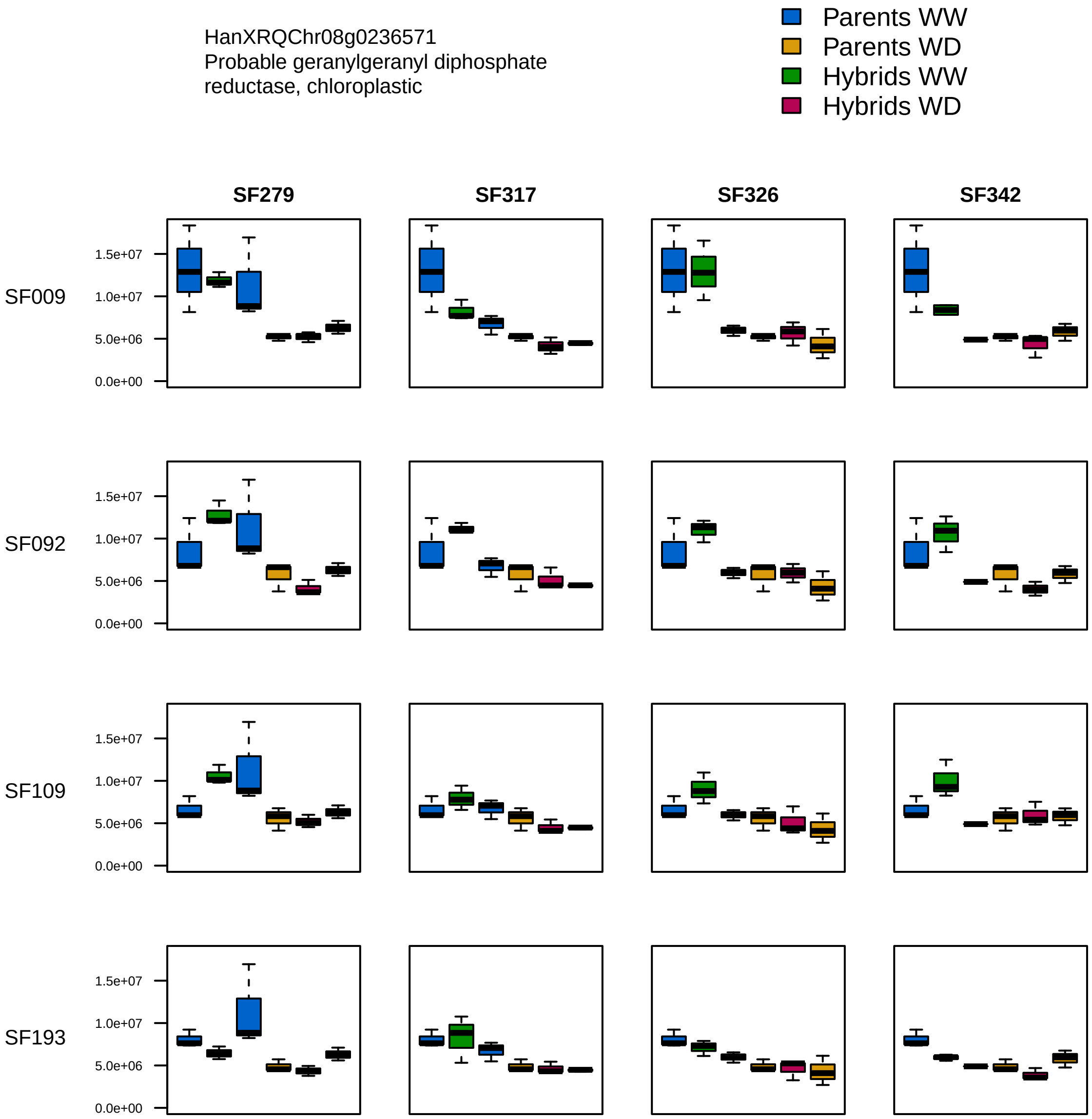

HanXRQChr08g0236571
Probable geranylgeranyl diphosphate reductase, chloroplastic
Parents WW
Parents WD
Hybrids WW
Hybrids WD
SF279
SF317
SF326
SF342
SF009
SF092
SF109
SF193
1.5e+07
1.0e+07
5.0e+06
0.0e+00

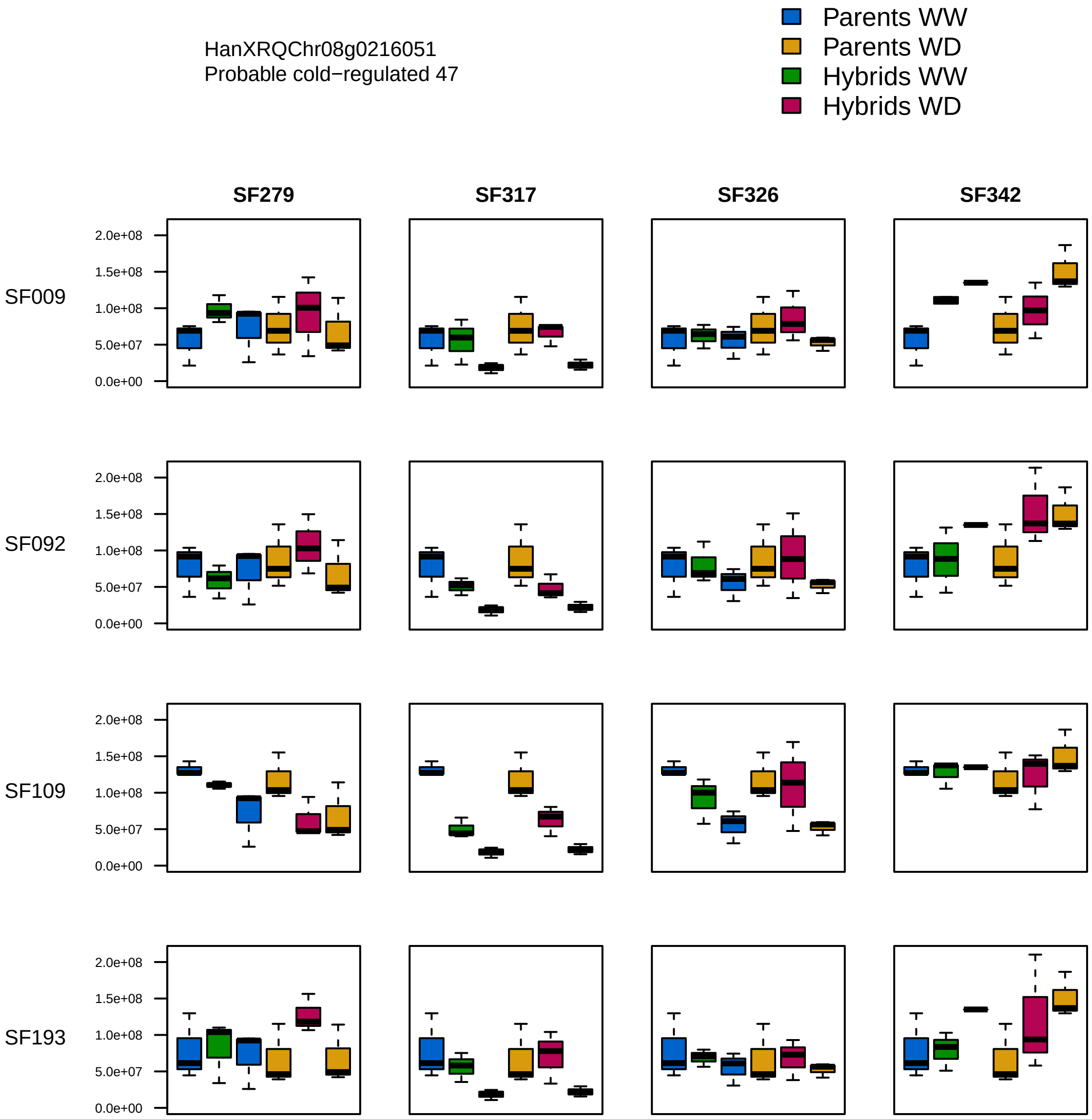
HanXRQChr08g0216051
Probable cold−regulated 47
Parents WW
Parents WD
Hybrids WW
Hybrids WD
SF279
SF317
SF326
SF342
SF009
SF092
SF109
SF193
0.0e+00
5.0e+07
1.0e+08
1.5e+08
2.0e+08

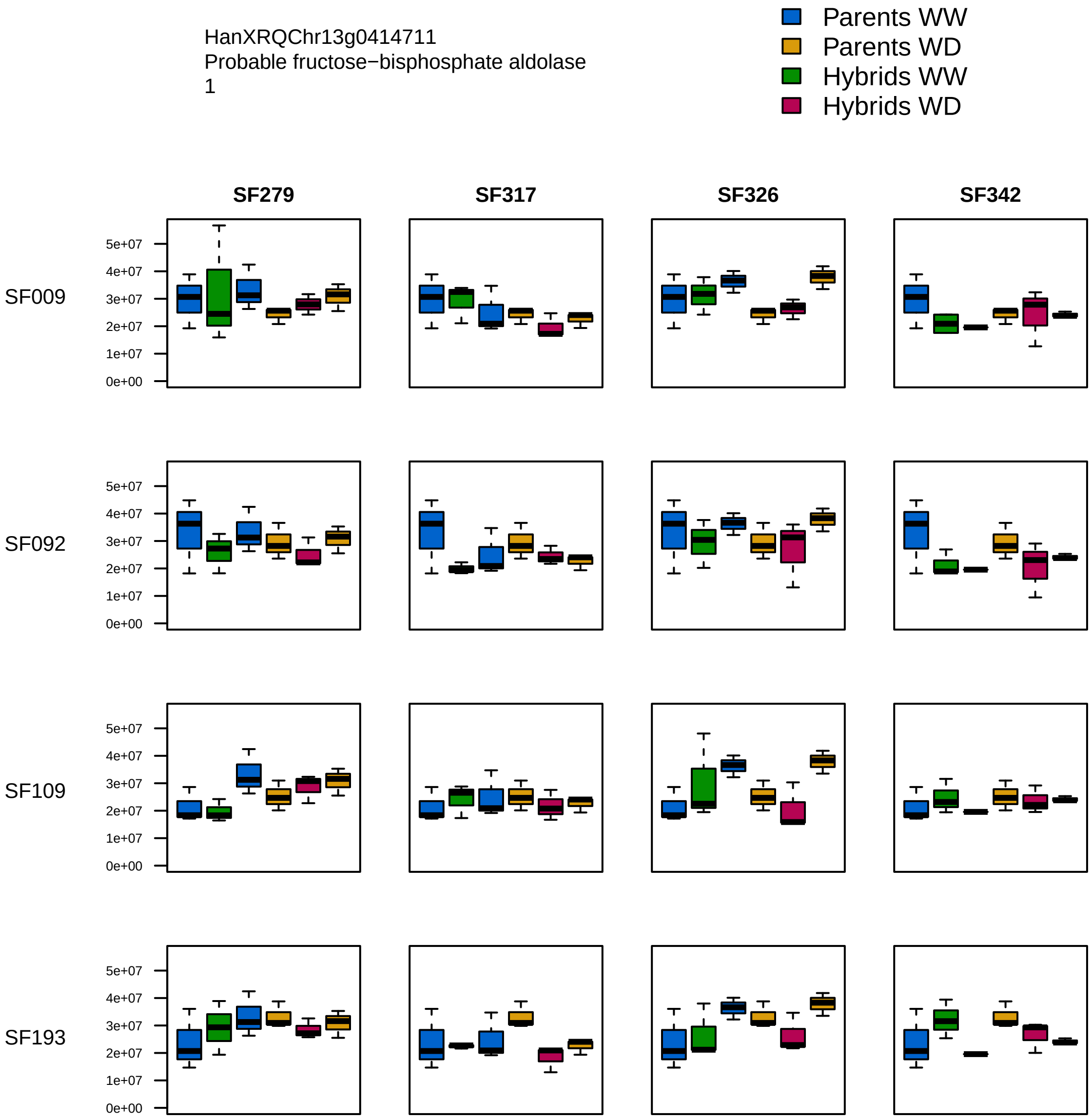
HanXRQChr13g0414711
Probable fructose−bisphosphate aldolase
1
Parents WW
Parents WD
Hybrids WW
Hybrids WD
SF279
SF317
SF326
SF342
SF009
SF092
SF109
SF193
5e+07
4e+07
3e+07
2e+07
1e+07
0e+00

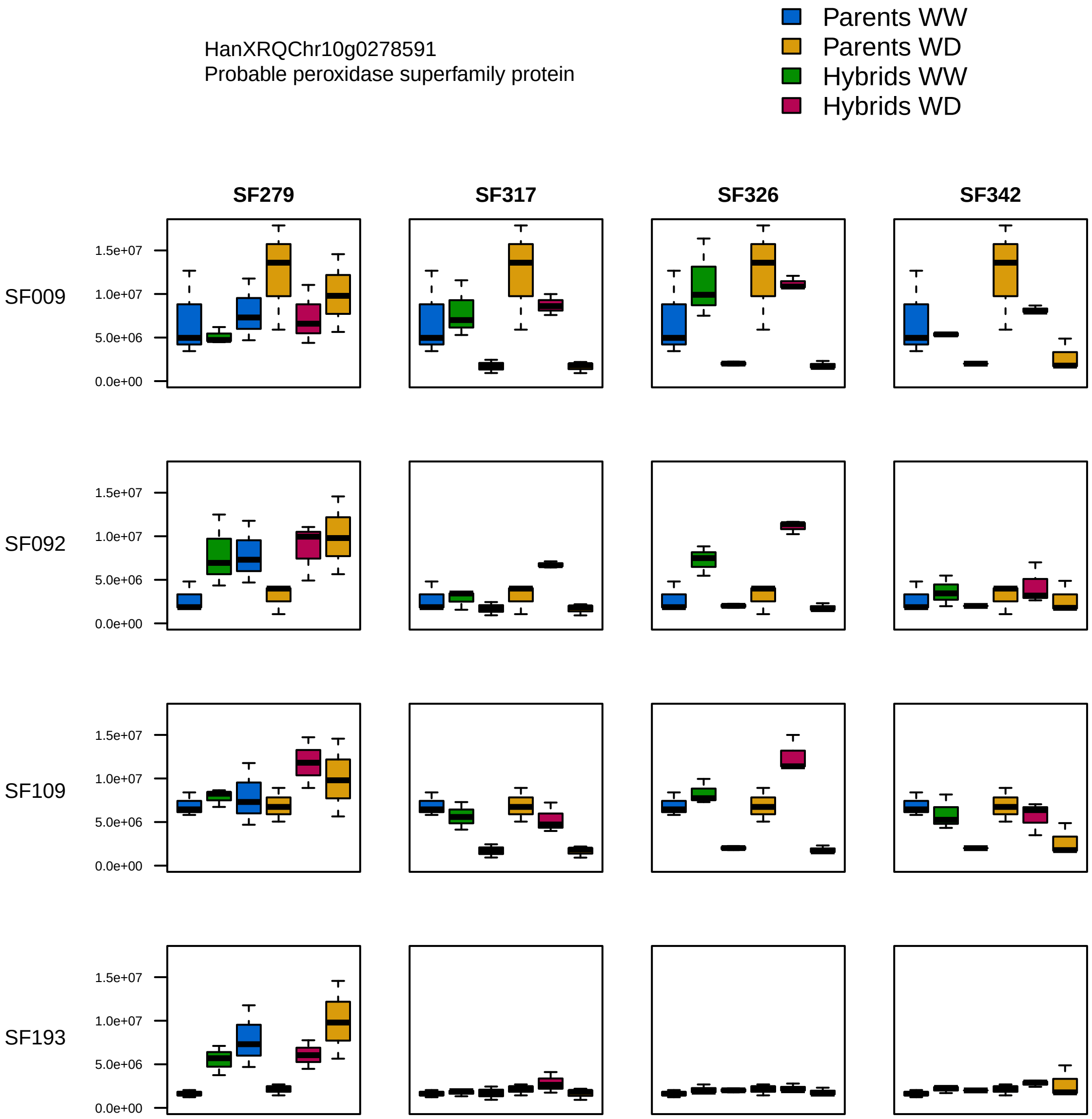
HanXRQChr10g0278591
Probable peroxidase superfamily protein
Parents WW
Parents WD
Hybrids WW
Hybrids WD
SF279
SF317
SF326
SF342
SF009
SF092
SF109
SF193
1.5e+07
1.0e+07
5.0e+06
0.0e+00

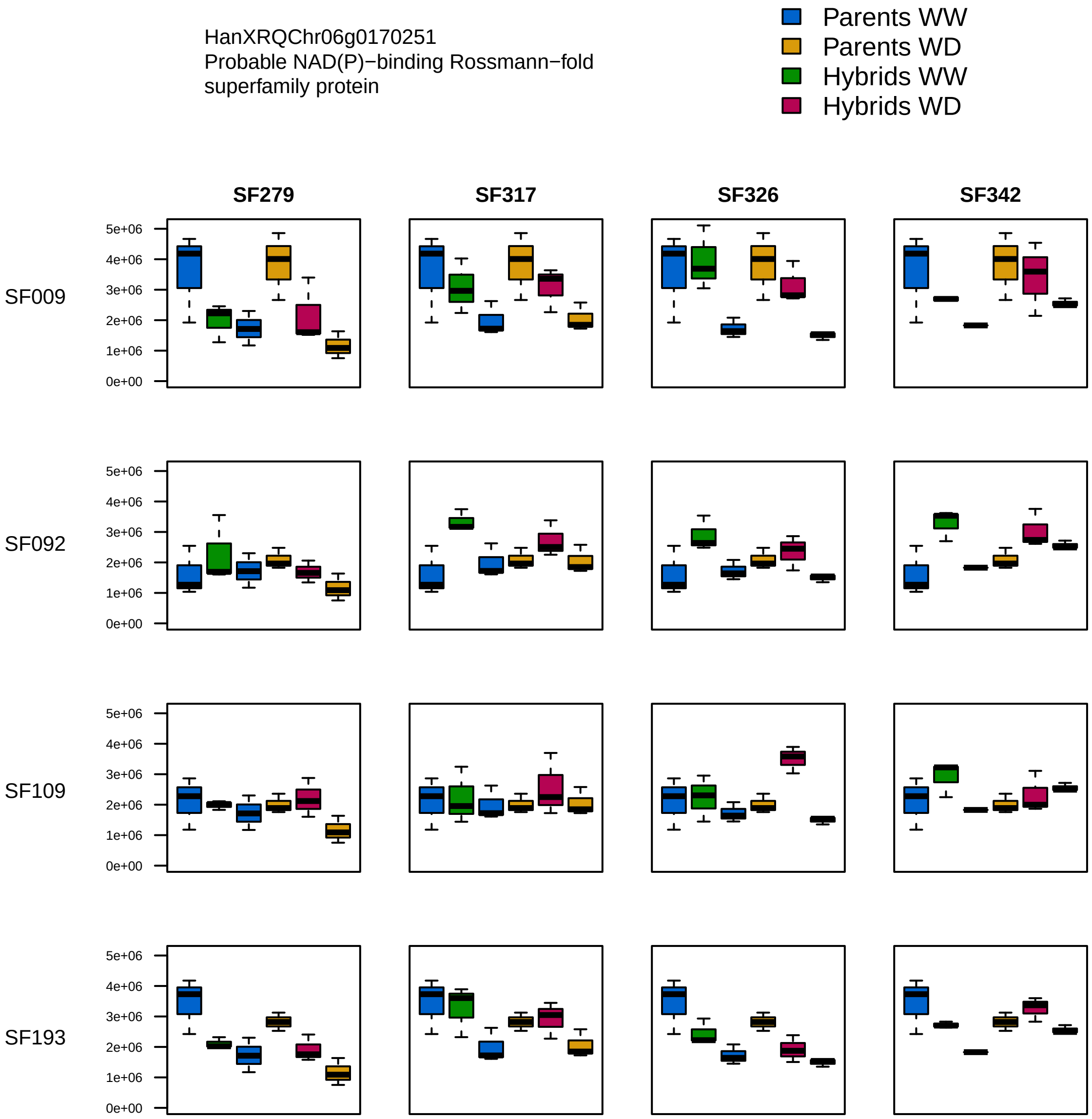

HanXRQChr06g0170251
Probable NAD(P)−binding Rossmann−fold superfamily protein
Parents WW
Parents WD
Hybrids WW
Hybrids WD
SF279
SF317
SF326
SF342
SF009
SF092
SF109
SF193
0e+00
1e+06
2e+06
3e+06
4e+06
5e+06

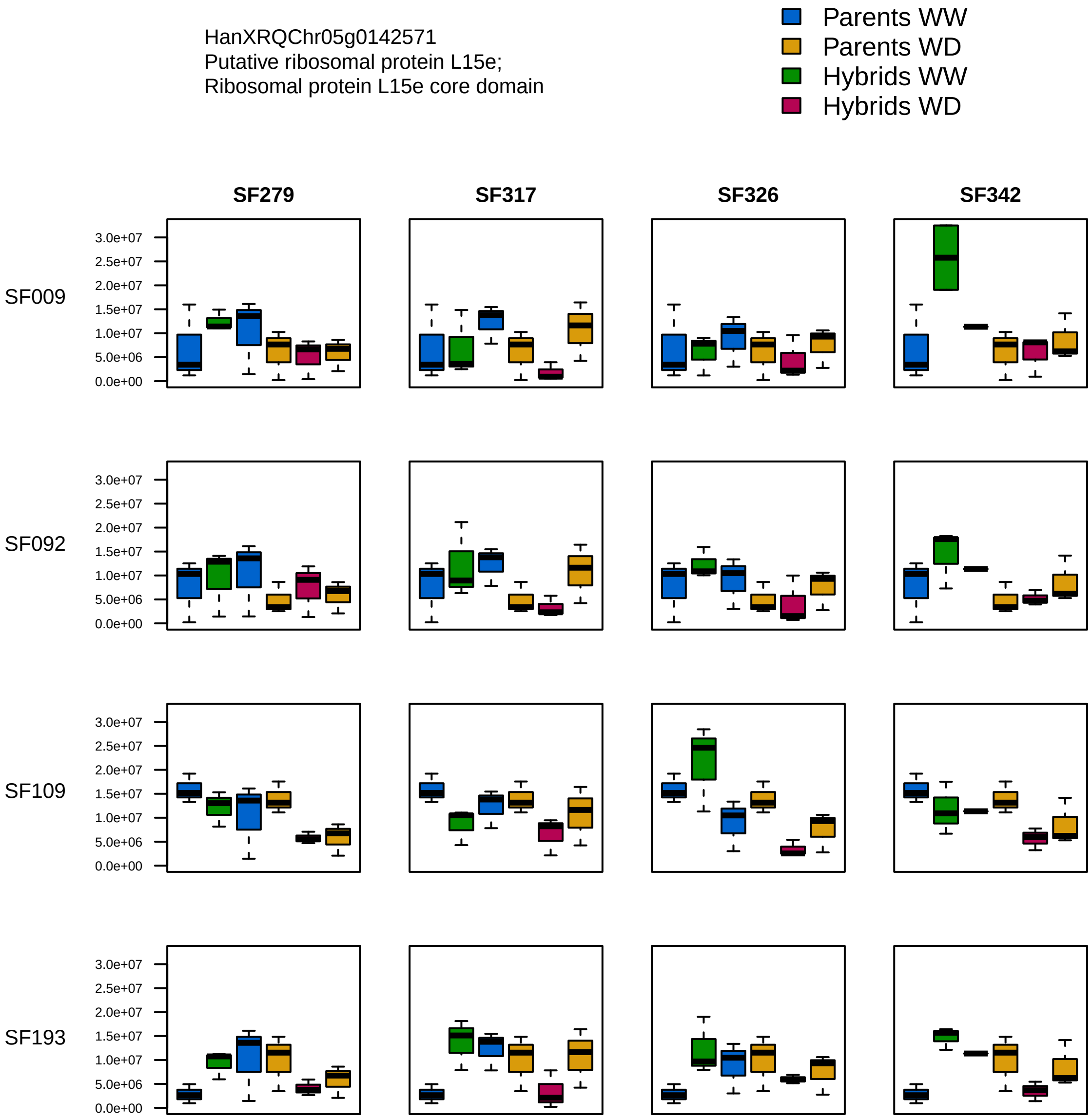
HanXRQChr05g0142571
Putative ribosomal protein L15e;
Ribosomal protein L15e core domain
Parents WW
Parents WD
Hybrids WW
Hybrids WD
SF279
SF317
SF326
SF342
SF009
SF092
SF109
SF193
3.0e+07
2.5e+07
2.0e+07
1.5e+07
1.0e+07
5.0e+06
0.0e+00

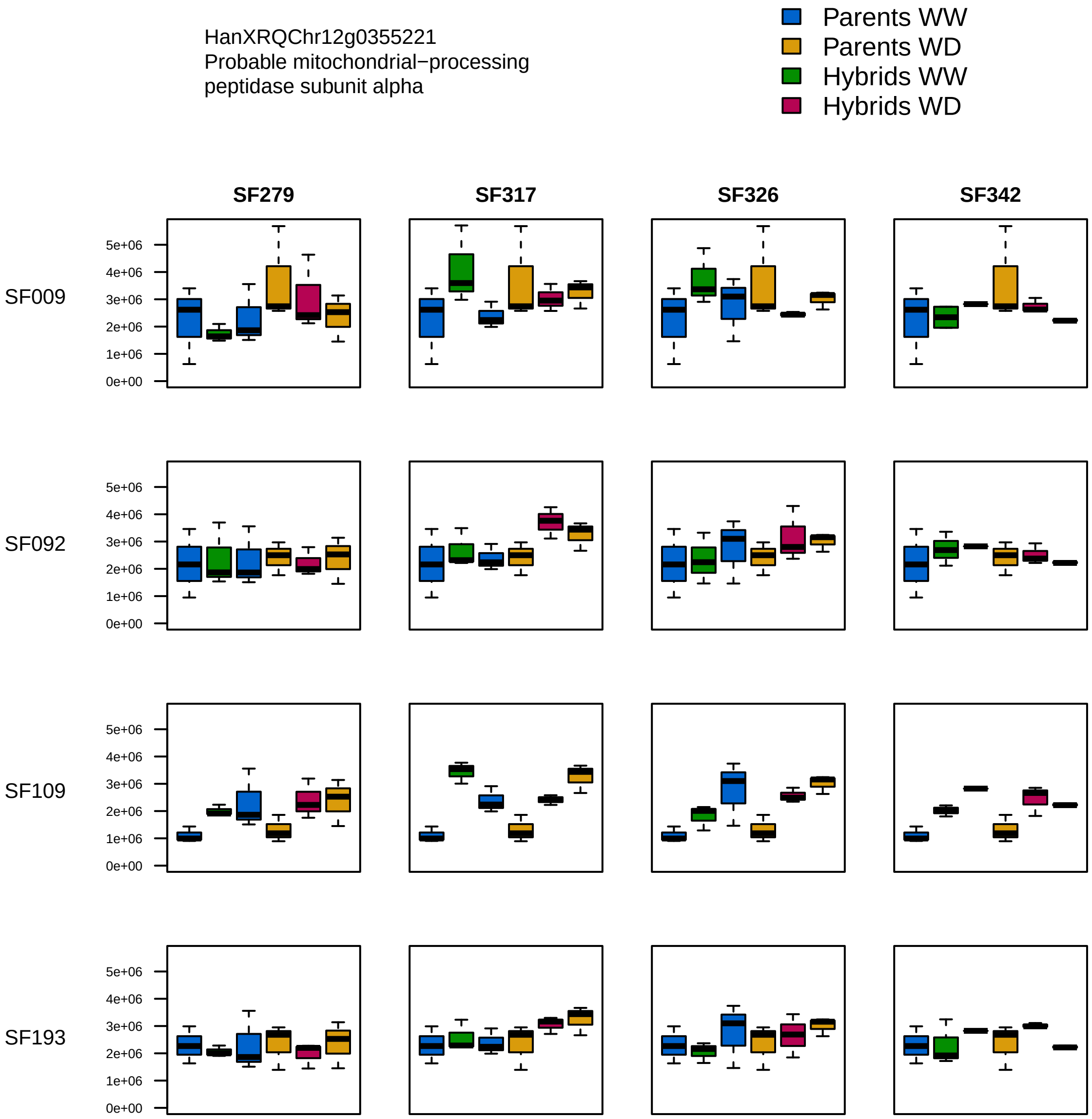
HanXRQChr12g0355221
Probable mitochondrial−processing
peptidase subunit alpha
Parents WW
Parents WD
Hybrids WW
Hybrids WD
SF279
SF317
SF326
SF342
SF009
SF092
SF109
SF193
5e+06
4e+06
3e+06
2e+06
1e+06
0e+00

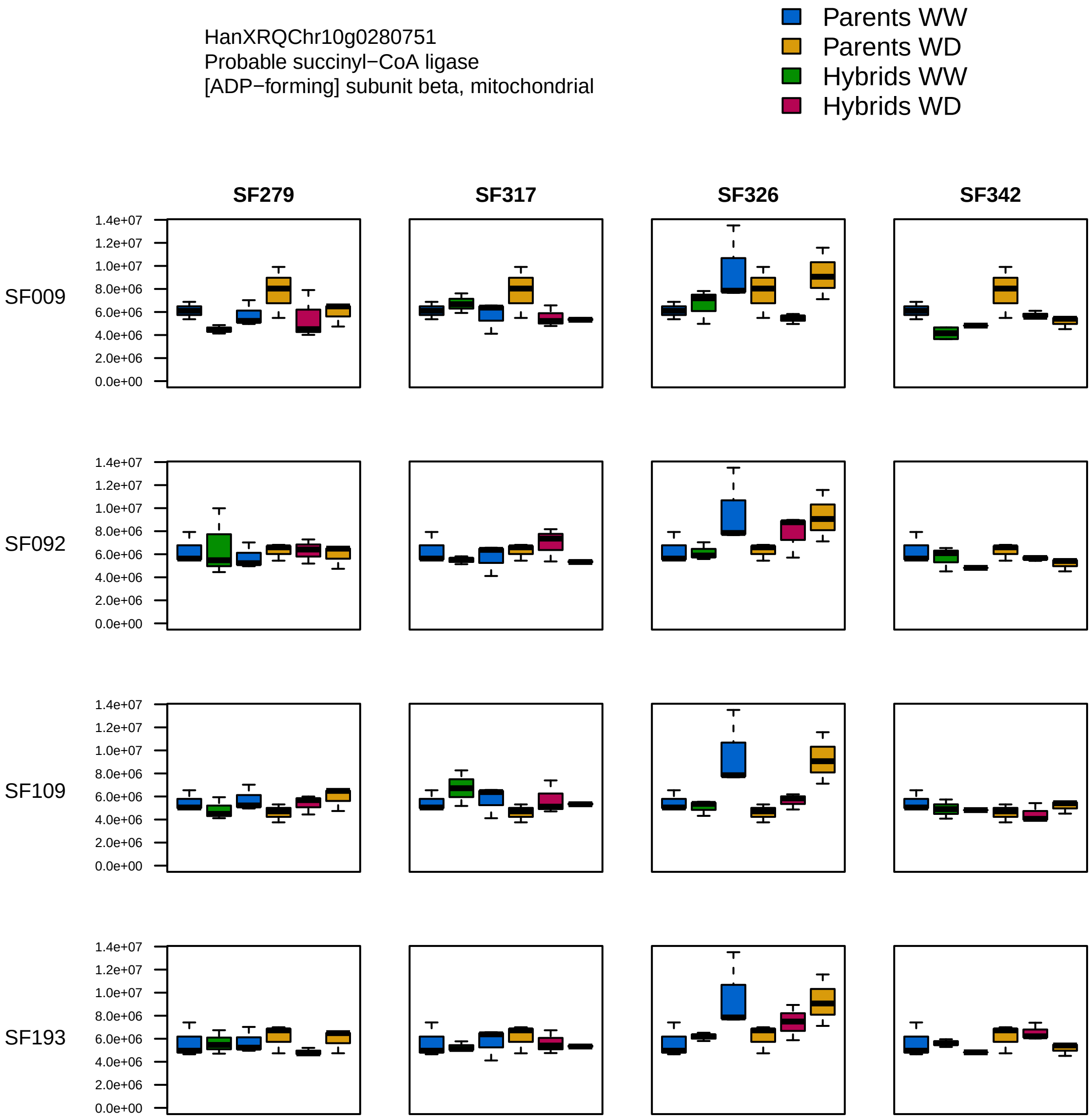
HanXRQChr10g0280751
Probable succinyl−CoA ligase
[ADP−forming] subunit beta, mitochondrial
Parents WW
Parents WD
Hybrids WW
Hybrids WD
SF279
SF317
SF326
SF342
SF009
SF092
SF109
SF193
0.0e+00
2.0e+06
4.0e+06
6.0e+06
8.0e+06
1.0e+07
1.2e+07
1.4e+07

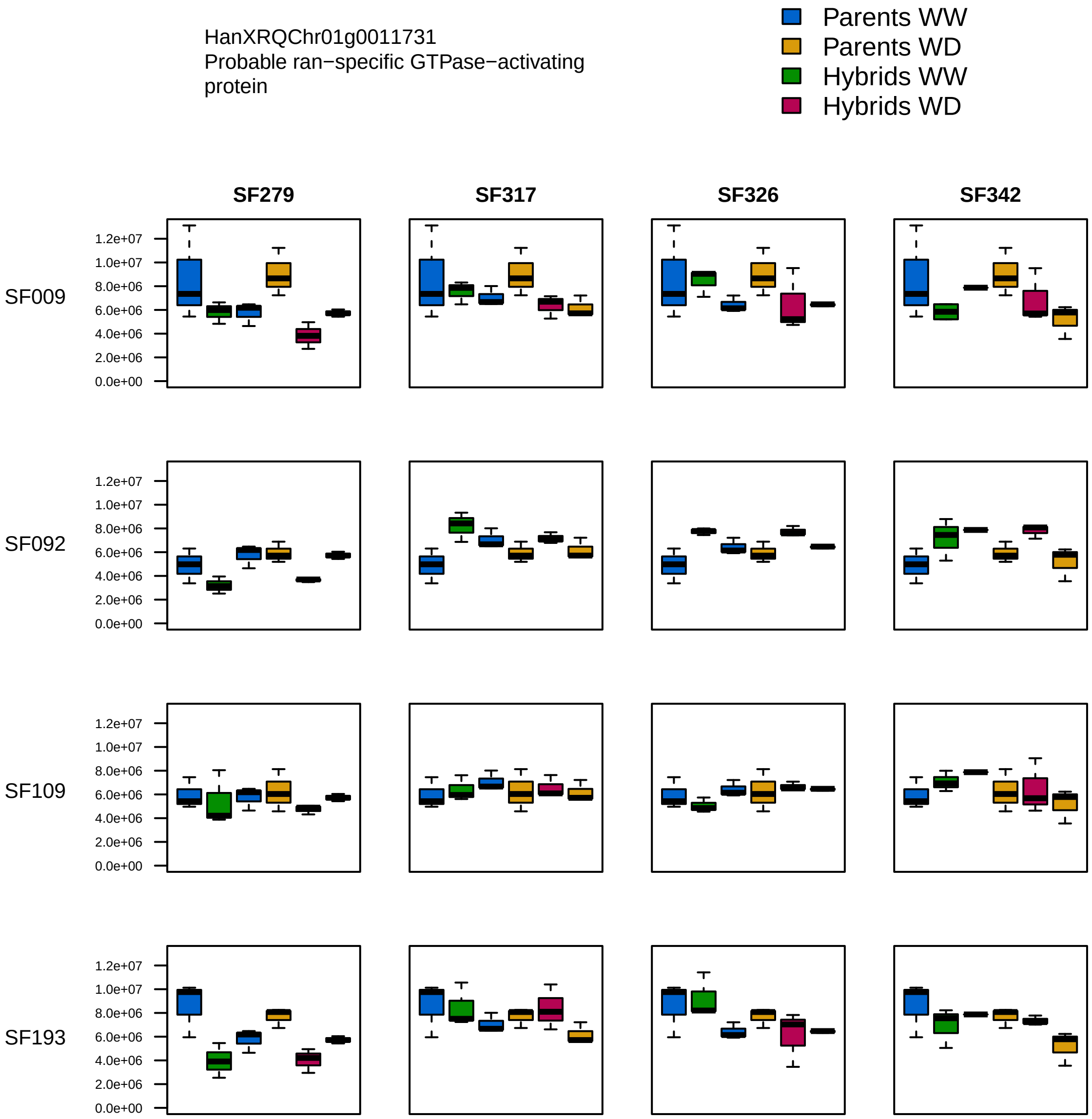
HanXRQChr01g0011731
Probable ran−specific GTPase−activating
protein
Parents WW
Parents WD
Hybrids WW
Hybrids WD
SF279
SF317
SF326
SF342
SF009
SF092
SF109
SF193
0.0e+00
2.0e+06
4.0e+06
6.0e+06
8.0e+06
1.0e+07
1.2e+07

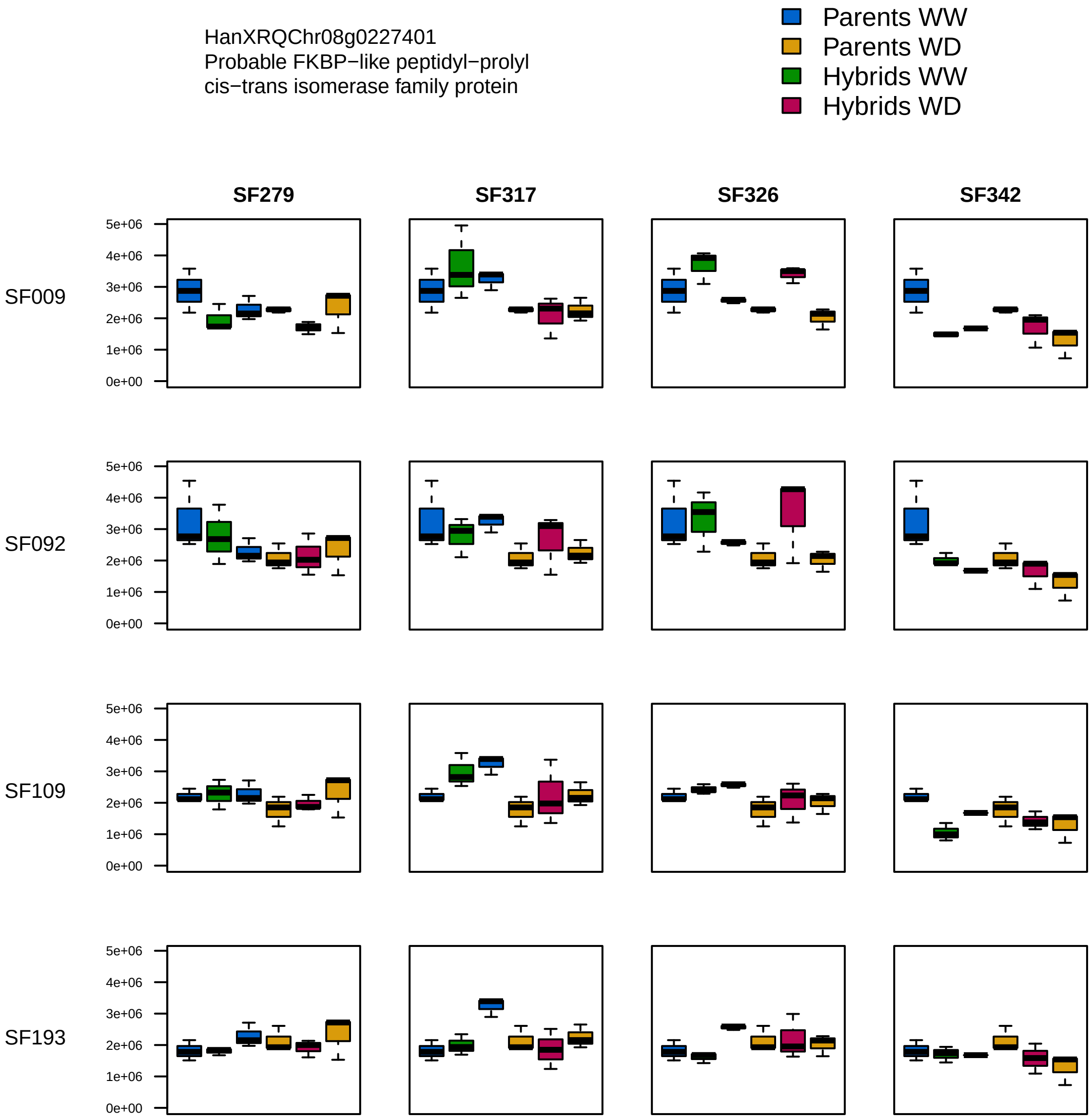
HanXRQChr08g0227401
Probable FKBP−like peptidyl−prolyl
cis−trans isomerase family protein
Parents WW
Parents WD
Hybrids WW
Hybrids WD
SF279
SF317
SF326
SF342
SF009
SF092
SF109
SF193
5e+06
4e+06
3e+06
2e+06
1e+06
0e+00

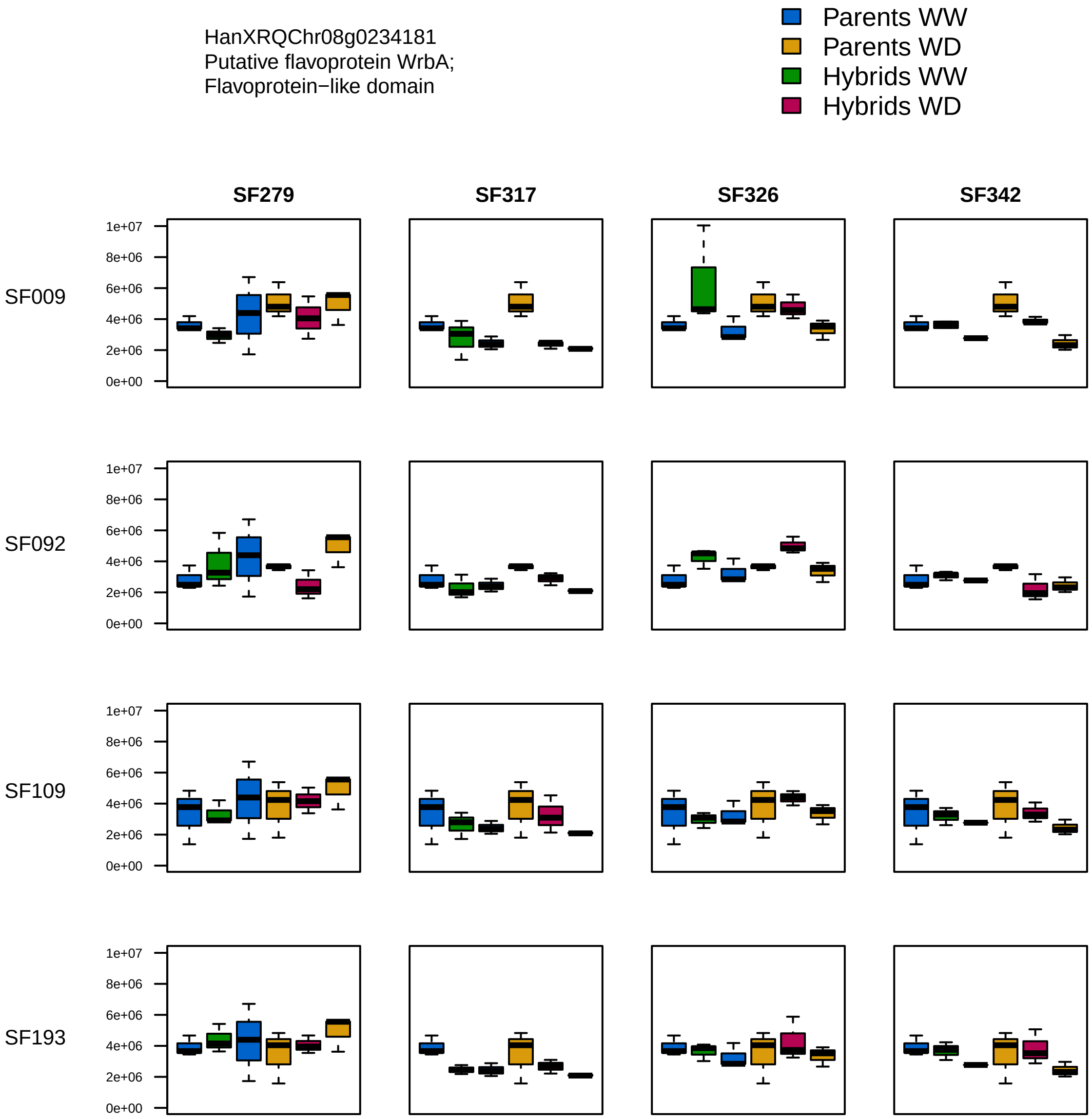
HanXRQChr08g0234181
Putative flavoprotein WrbA;
Flavoprotein−like domain
Parents WW
Parents WD
Hybrids WW
Hybrids WD
SF279
SF317
SF326
SF342
SF009
SF092
SF109
SF193
1e+07
8e+06
6e+06
4e+06
2e+06
0e+00

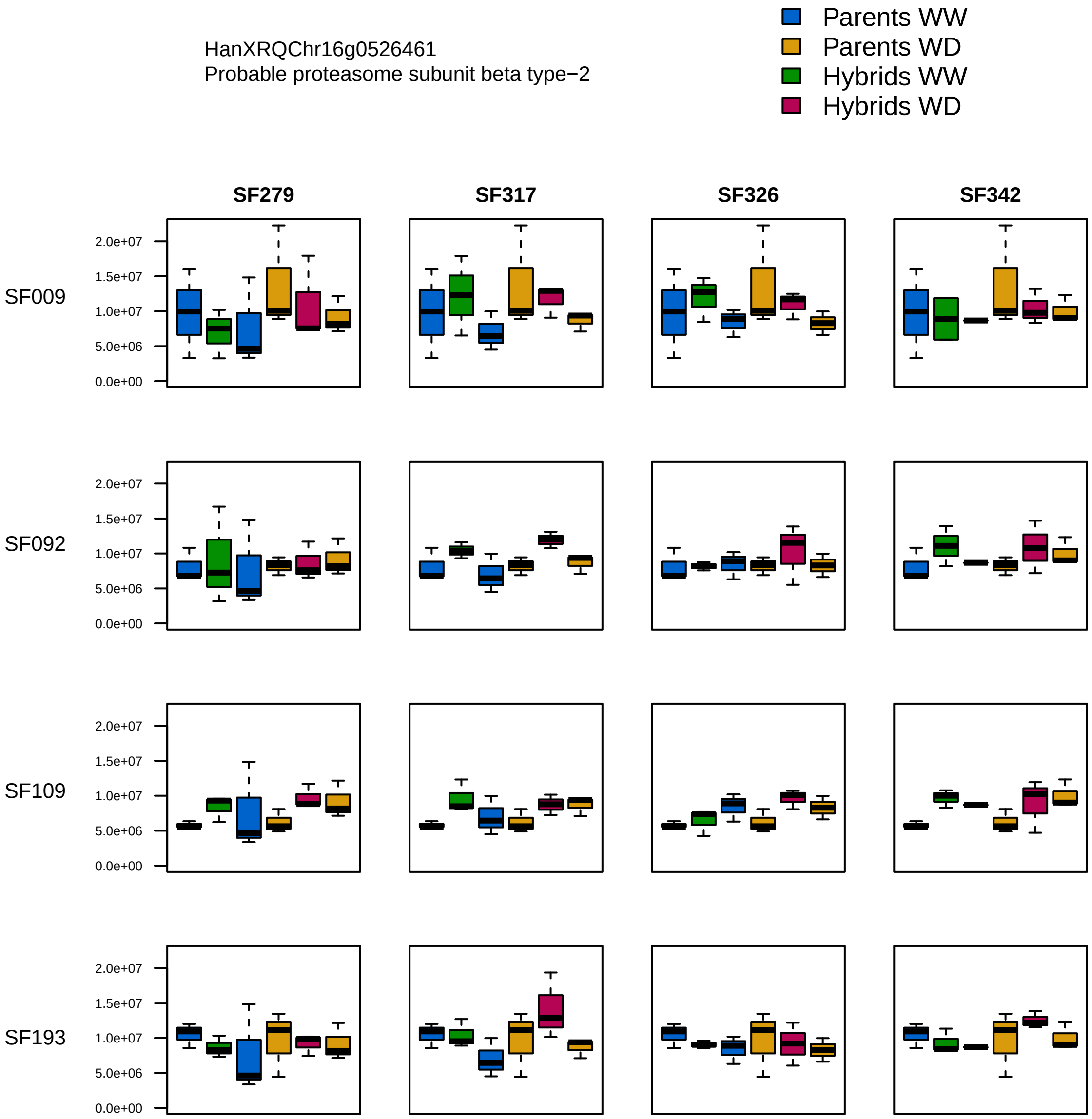
HanXRQChr16g0526461
Probable proteasome subunit beta type−2
Parents WW
Parents WD
Hybrids WW
Hybrids WD
SF279
SF317
SF326
SF342
SF009
SF092
SF109
SF193
0.0e+00
5.0e+06
1.0e+07
1.5e+07
2.0e+07

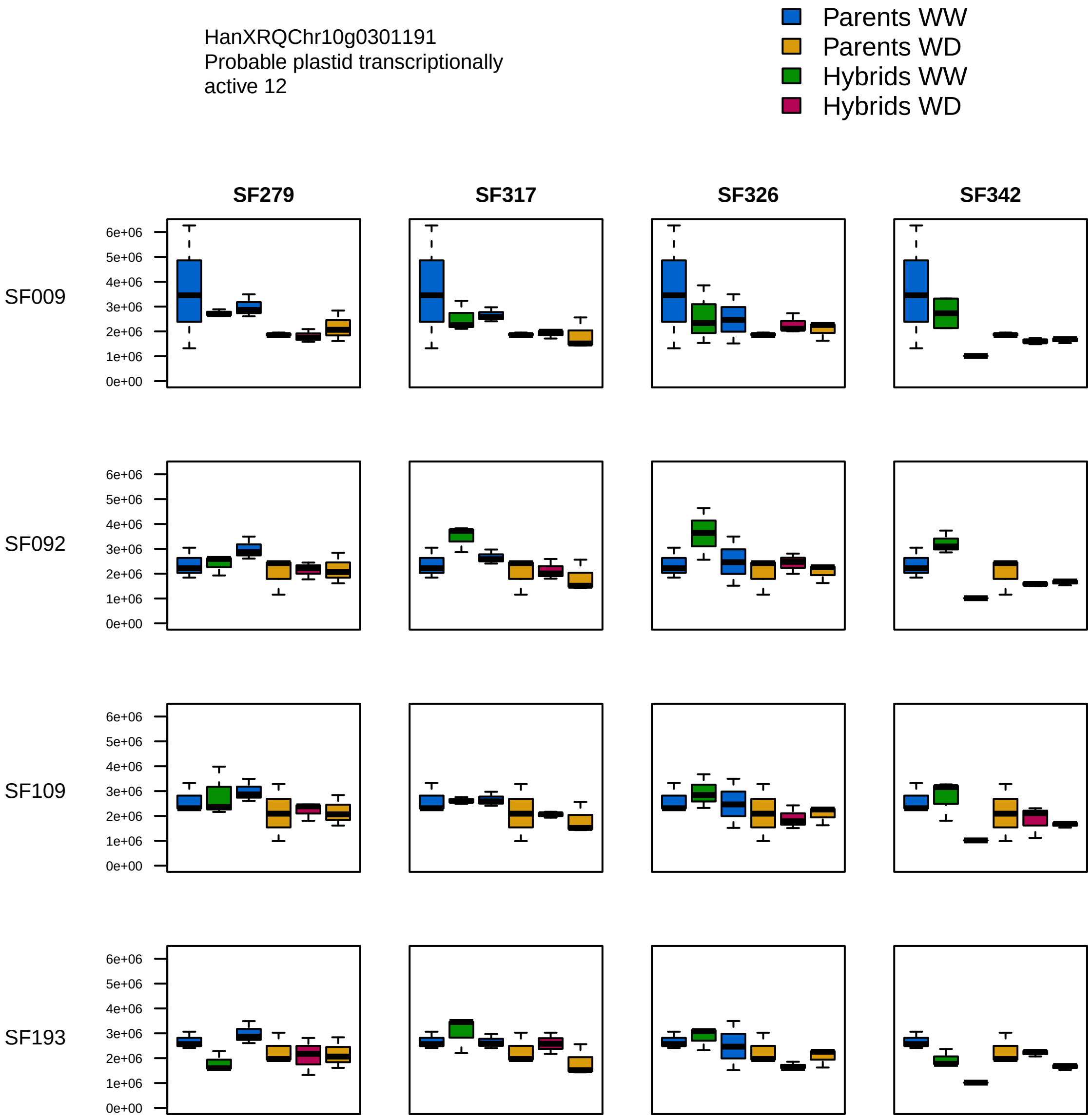

HanXRQChr10g0301191
Probable plastid transcriptionally active 12
Parents WW
Parents WD
Hybrids WW
Hybrids WD
SF279
SF317
SF326
SF342
SF009
SF092
SF109
SF193
0e+00
1e+06
2e+06
3e+06
4e+06
5e+06
6e+06

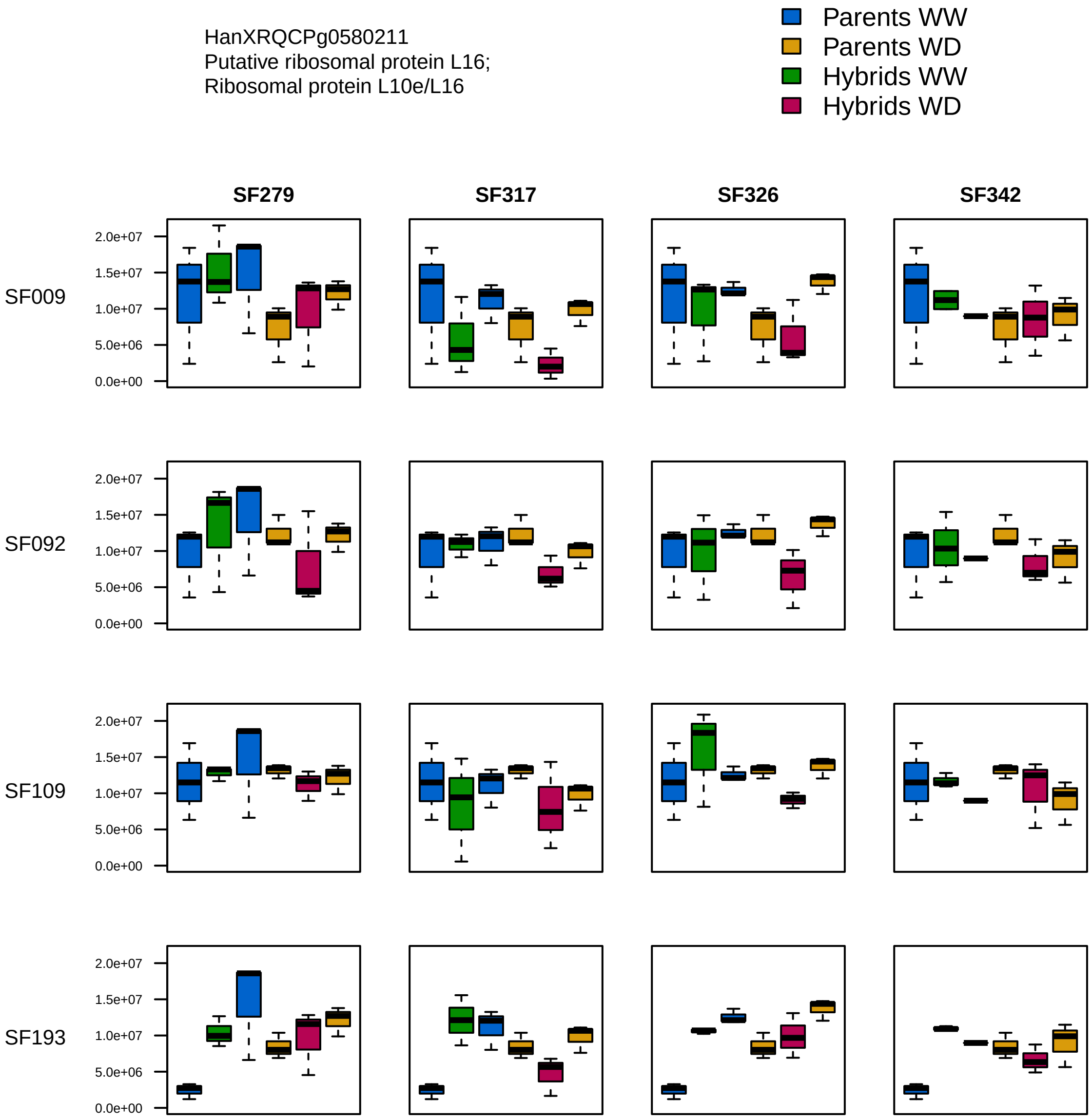
HanXRQCPg0580211
Putative ribosomal protein L16;
Ribosomal protein L10e/L16
Parents WW
Parents WD
Hybrids WW
Hybrids WD
SF279
SF317
SF326
SF342
SF009
SF092
SF109
SF193
2.0e+07
1.5e+07
1.0e+07
5.0e+06
0.0e+00

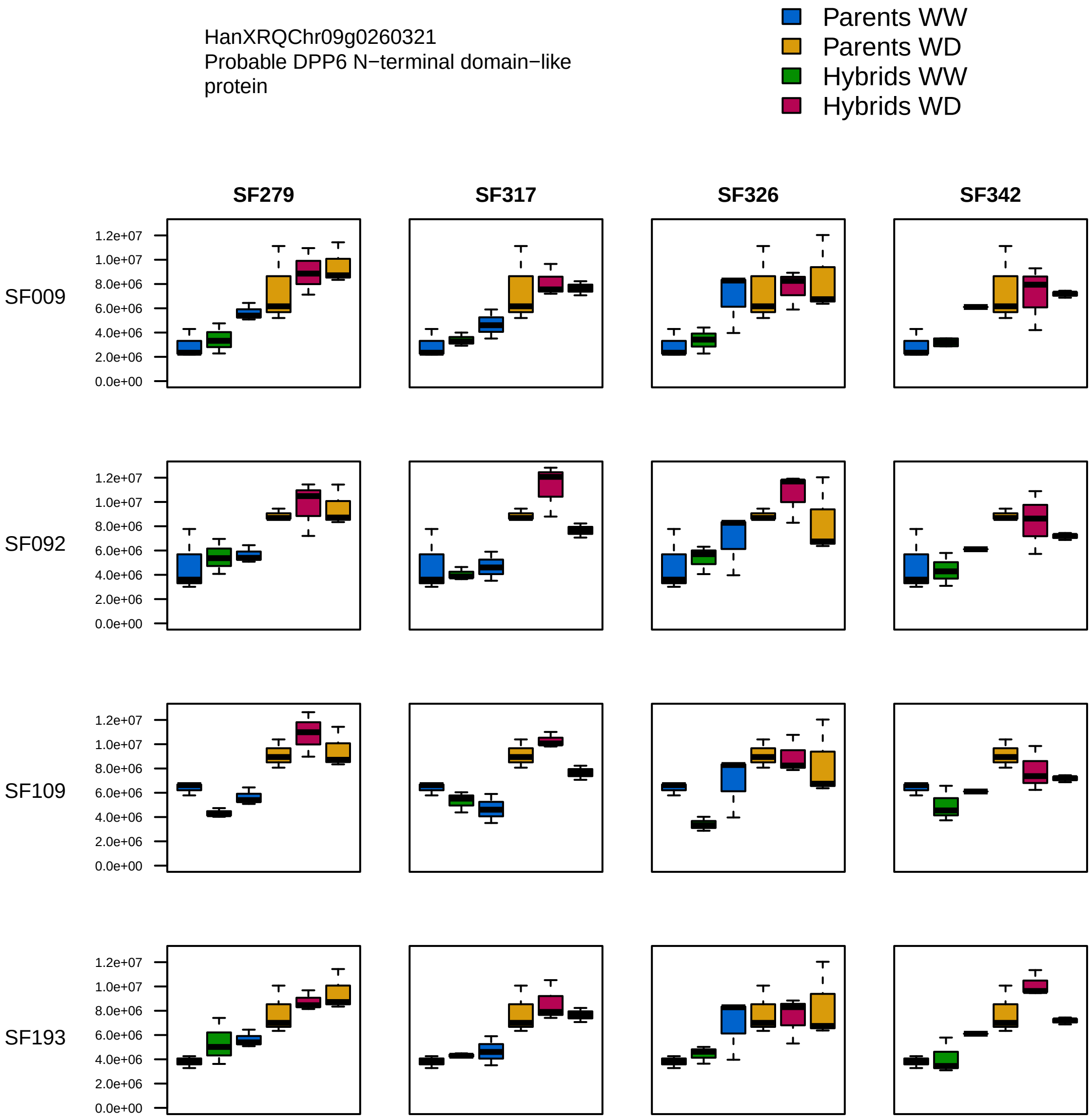

HanXRQChr09g0260321
Probable DPP6 N−terminal domain−like protein
Parents WW
Parents WD
Hybrids WW
Hybrids WD
SF279
SF317
SF326
SF342
SF009
SF092
SF109
SF193
1.2e+07
1.0e+07
8.0e+06
6.0e+06
4.0e+06
2.0e+06
0.0e+00

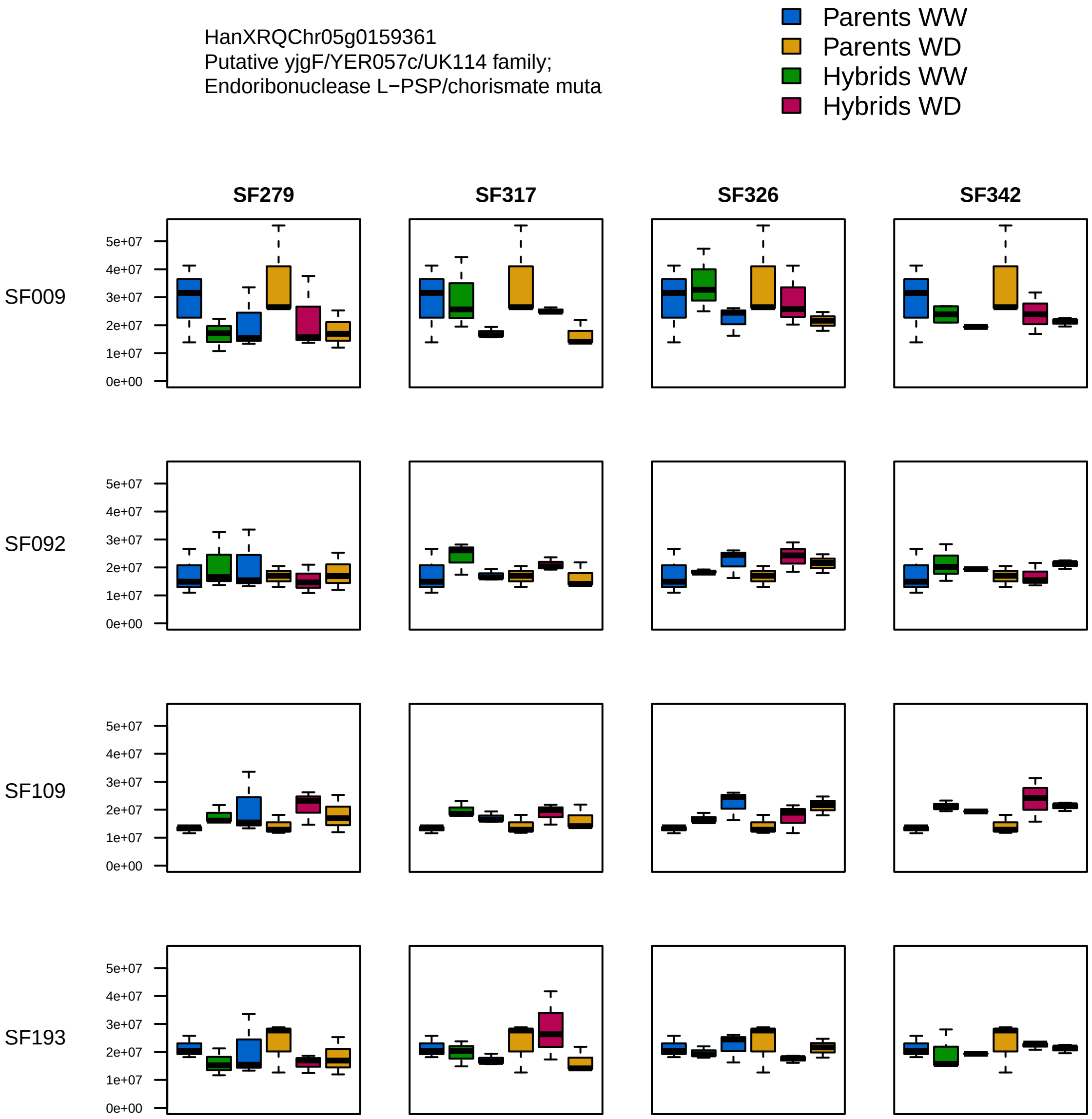
HanXRQChr05g0159361
Putative yjgF/YER057c/UK114 family;
Endoribonuclease L−PSP/chorismate muta
Parents WW
Parents WD
Hybrids WW
Hybrids WD
SF279
SF317
SF326
SF342
SF009
SF092
SF109
SF193
5e+07
4e+07
3e+07
2e+07
1e+07
0e+00

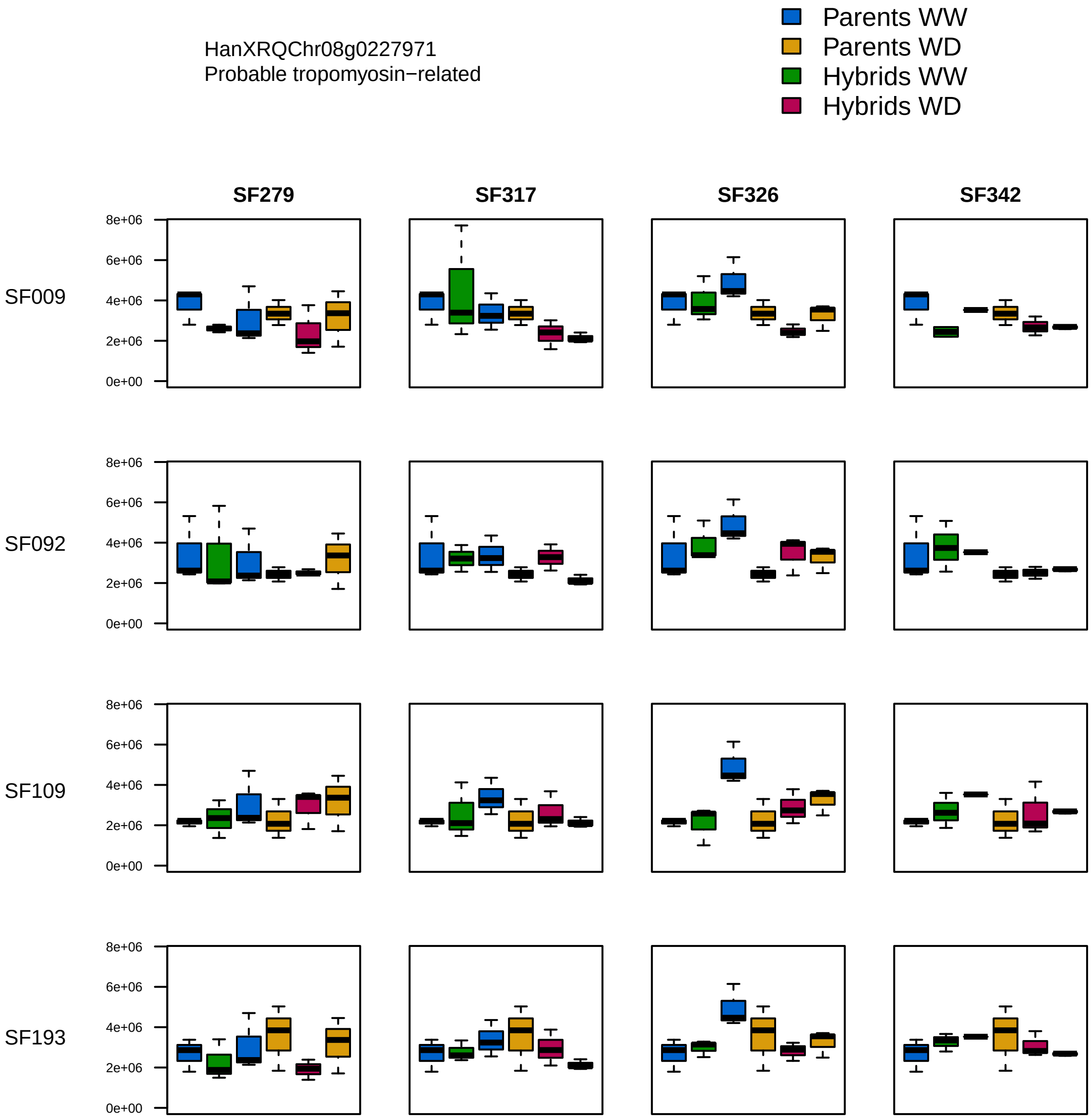
HanXRQChr08g0227971
Probable tropomyosin−related
Parents WW
Parents WD
Hybrids WW
Hybrids WD
SF279
SF317
SF326
SF342
SF009
SF092
SF109
SF193
8e+06
6e+06
4e+06
2e+06
0e+00

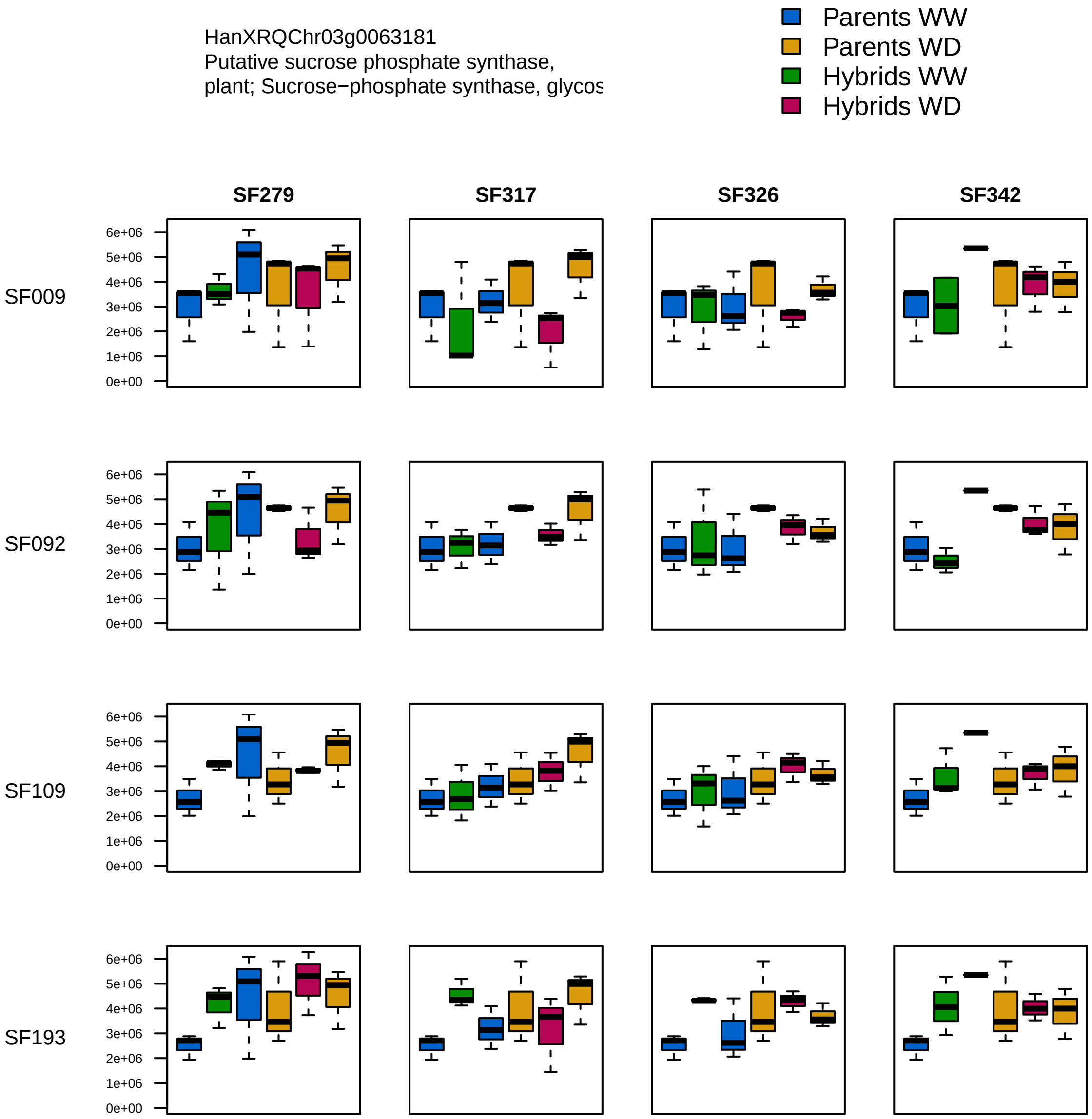
HanXRQChr03g0063181
Putative sucrose phosphate synthase,
plant; Sucrose−phosphate synthase, glycos
Parents WW
Parents WD
Hybrids WW
Hybrids WD
SF279
SF317
SF326
SF342
SF009
SF092
SF109
SF193
6e+06
5e+06
4e+06
3e+06
2e+06
1e+06
0e+00

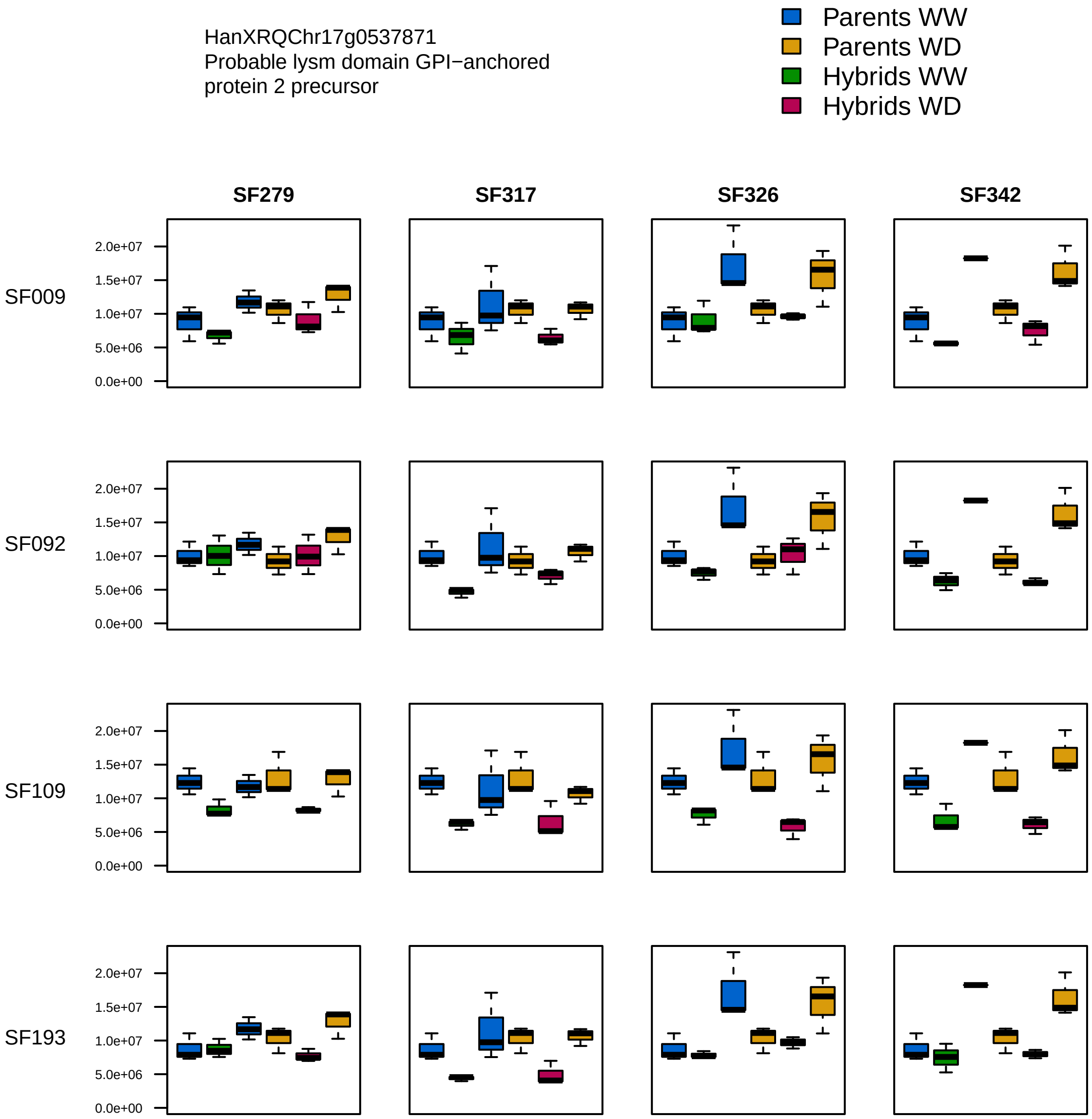
HanXRQChr17g0537871
Probable lysm domain GPI−anchored
protein 2 precursor
Parents WW
Parents WD
Hybrids WW
Hybrids WD
SF279
SF317
SF326
SF342
SF009
SF092
SF109
SF193
0.0e+00
5.0e+06
1.0e+07
1.5e+07
2.0e+07

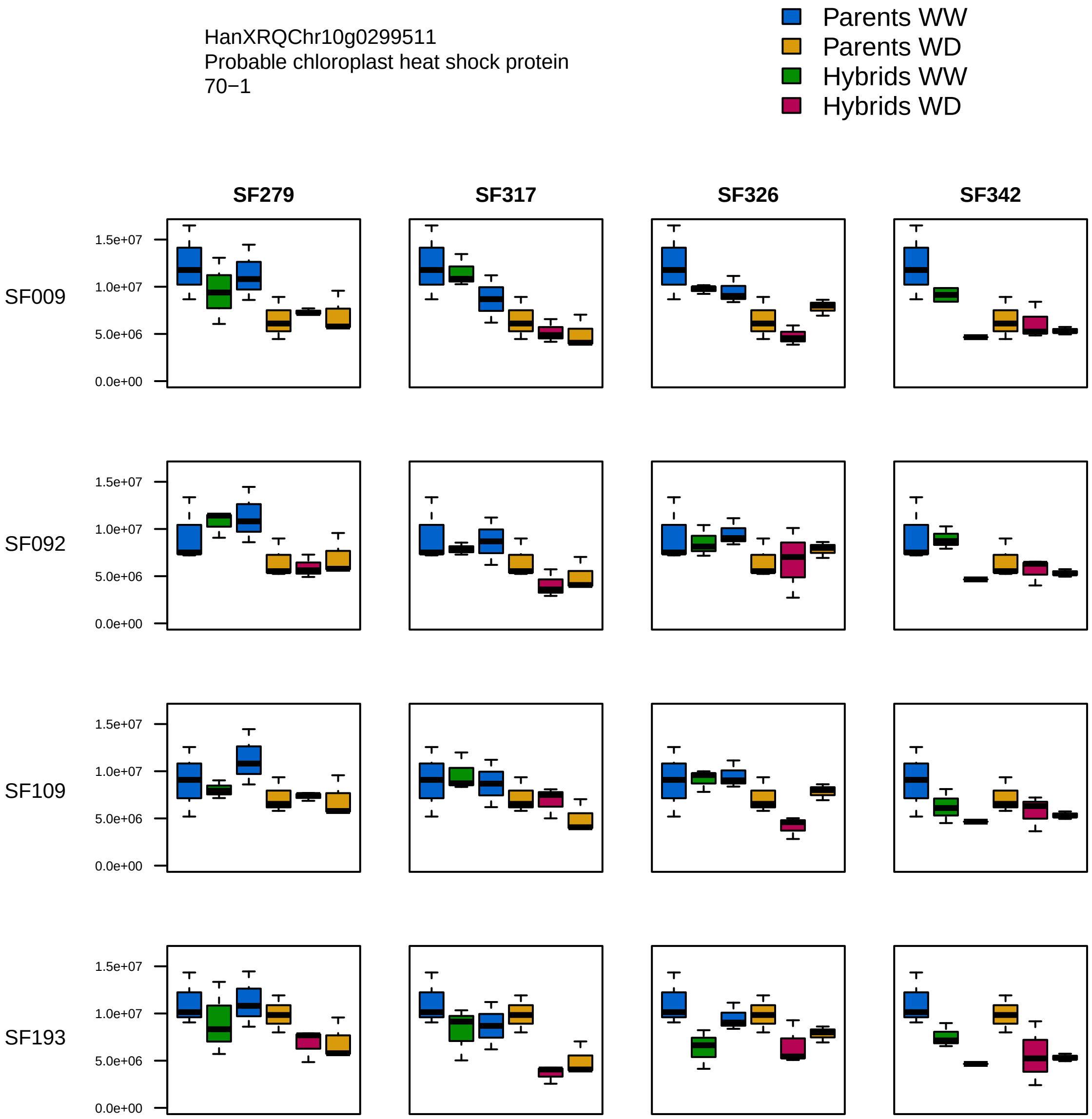

HanXRQChr10g0299511
Probable chloroplast heat shock protein 70−1
Parents WW
Parents WD
Hybrids WW
Hybrids WD
SF279
SF317
SF326
SF342
SF009
SF092
SF109
SF193
1.5e+07
1.0e+07
5.0e+06
0.0e+00

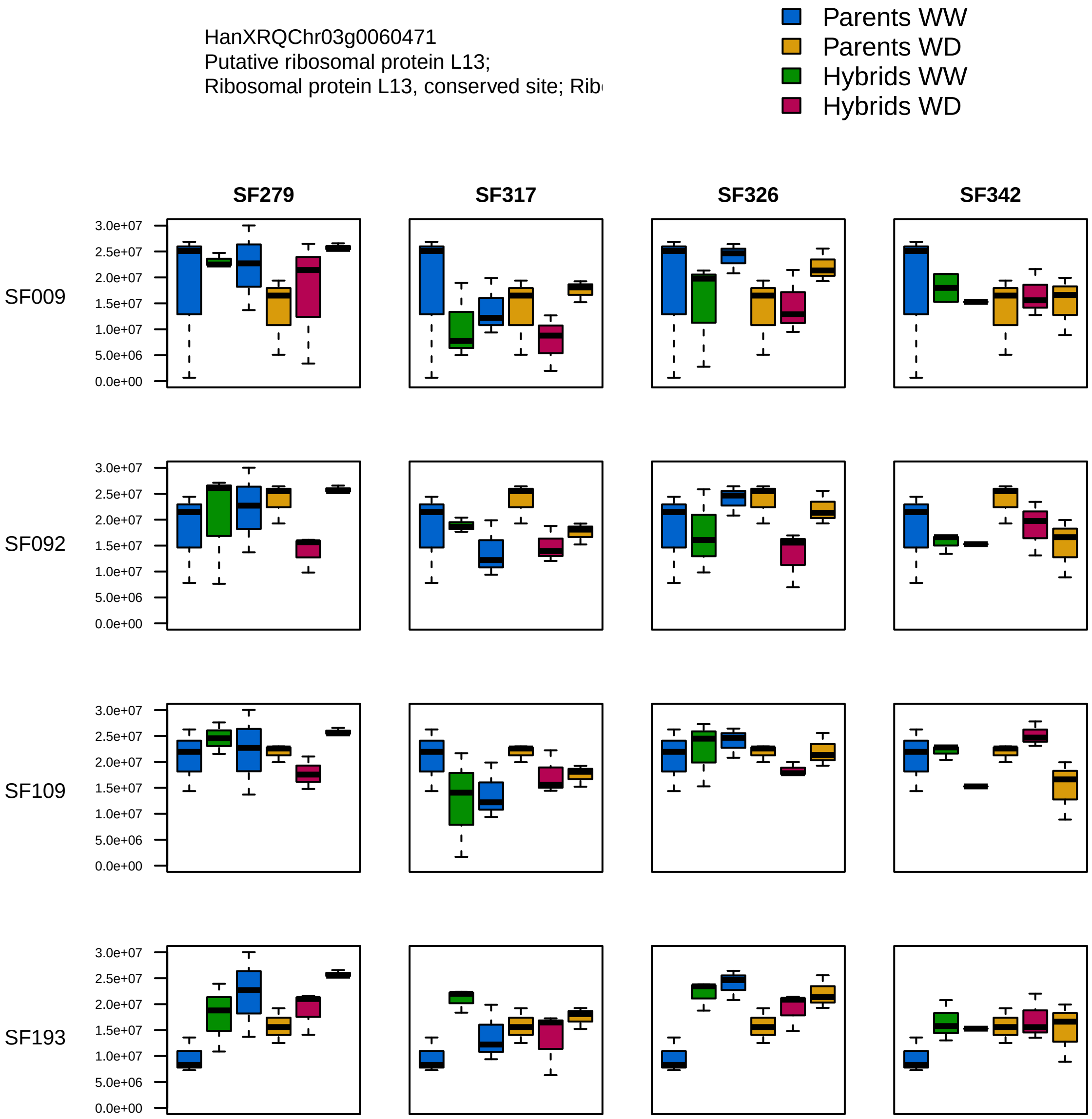
HanXRQChr03g0060471
Putative ribosomal protein L13;
Ribosomal protein L13, conserved site; Rib
Parents WW
Parents WD
Hybrids WW
Hybrids WD
SF279
SF317
SF326
SF342
SF009
SF092
SF109
SF193
3.0e+07
2.5e+07
2.0e+07
1.5e+07
1.0e+07
5.0e+06
0.0e+00

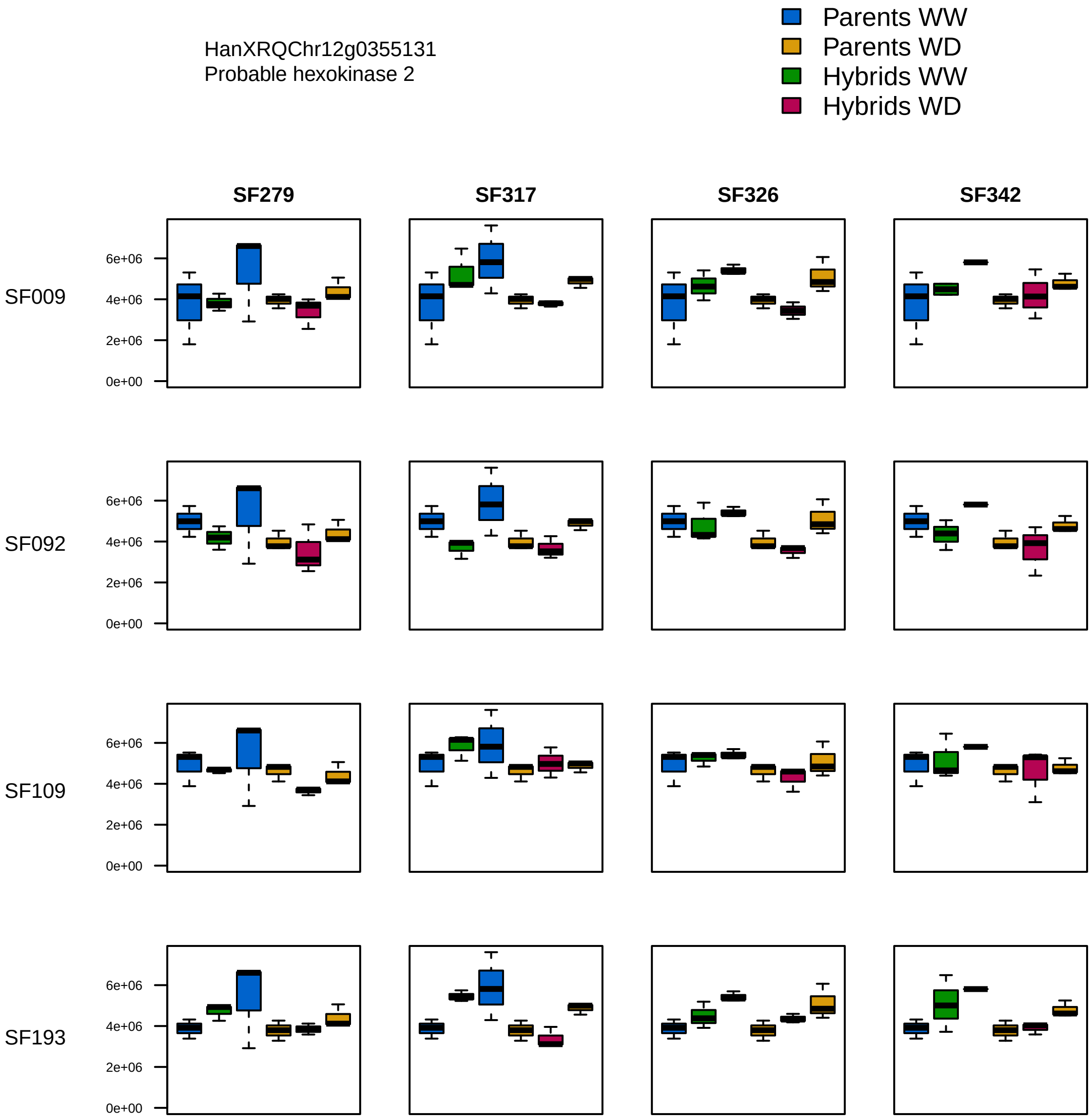
HanXRQChr12g0355131
Probable hexokinase 2
Parents WW
Parents WD
Hybrids WW
Hybrids WD
SF279
SF317
SF326
SF342
SF009
SF092
SF109
SF193
6e+06
4e+06
2e+06
0e+00

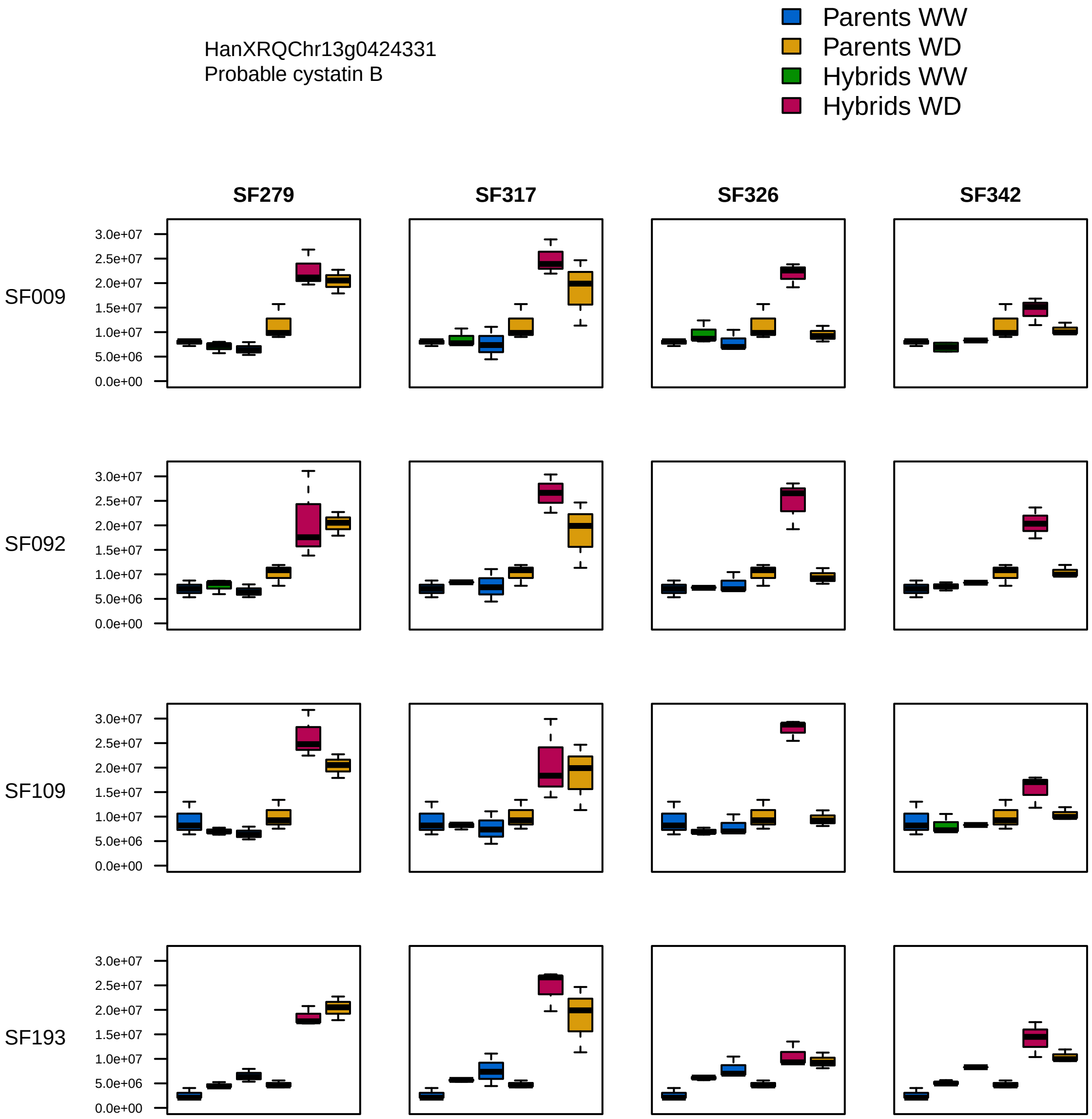

HanXRQChr13g0424331
Probable cystatin B
Parents WW
Parents WD
Hybrids WW
Hybrids WD
SF279
SF317
SF326
SF342
SF009
SF092
SF109
SF193
3.0e+07
2.5e+07
2.0e+07
1.5e+07
1.0e+07
5.0e+06
0.0e+00

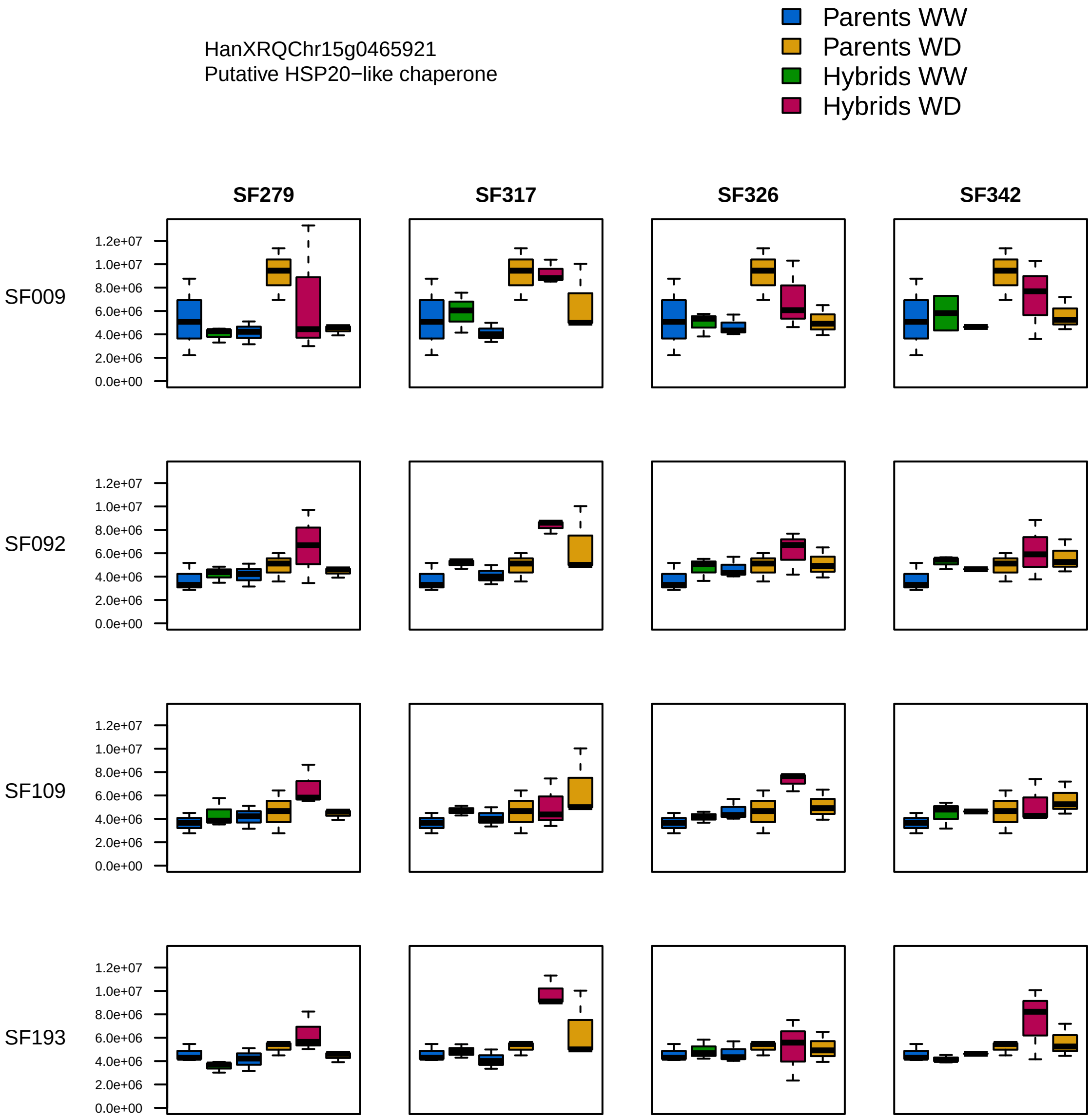
HanXRQChr15g0465921
Putative HSP20−like chaperone
Parents WW
Parents WD
Hybrids WW
Hybrids WD
SF279
SF317
SF326
SF342
SF009
SF092
SF109
SF193
1.2e+07
1.0e+07
8.0e+06
6.0e+06
4.0e+06
2.0e+06
0.0e+00

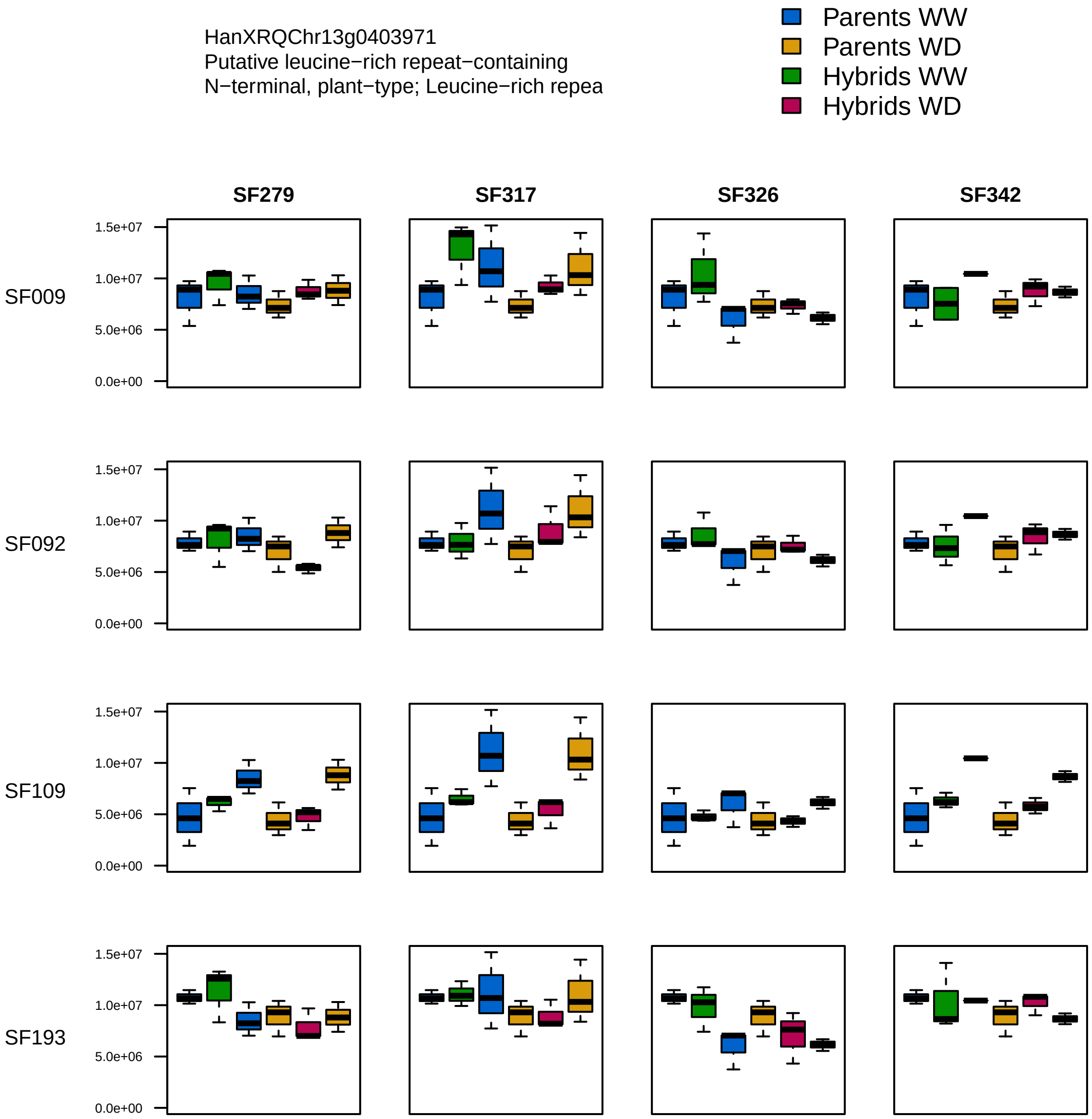
HanXRQChr13g0403971
Putative leucine−rich repeat−containing
N−terminal, plant−type; Leucine−rich repea
Parents WW
Parents WD
Hybrids WW
Hybrids WD
SF279
SF317
SF326
SF342
SF009
SF092
SF109
SF193
1.5e+07
1.0e+07
5.0e+06
0.0e+00

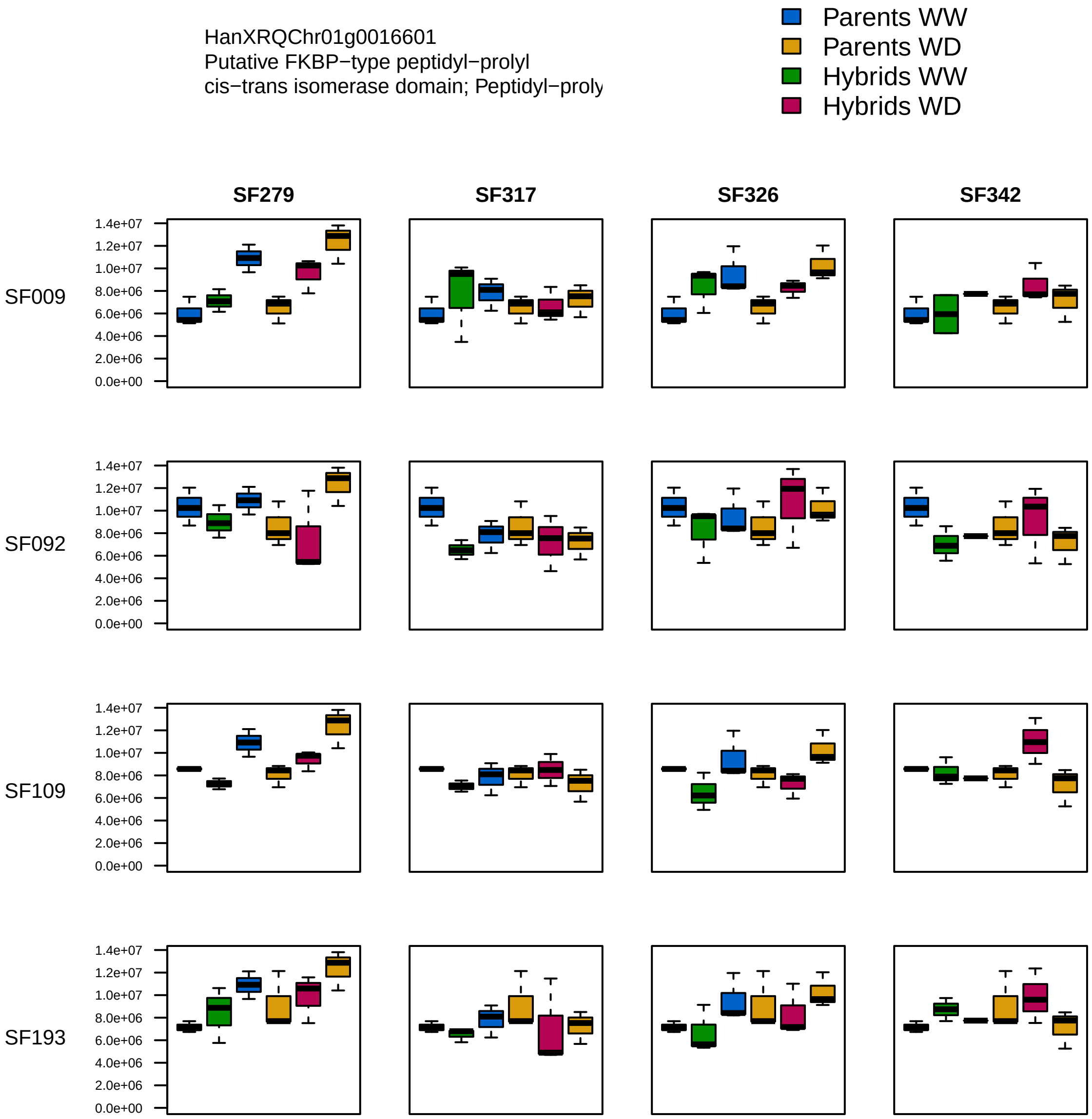
HanXRQChr01g0016601
Putative FKBP−type peptidyl−prolyl
cis−trans isomerase domain; Peptidyl−proly
Parents WW
Parents WD
Hybrids WW
Hybrids WD
SF279
SF317
SF326
SF342
SF009
SF092
SF109
SF193
1.4e+07
1.2e+07
1.0e+07
8.0e+06
6.0e+06
4.0e+06
2.0e+06
0.0e+00

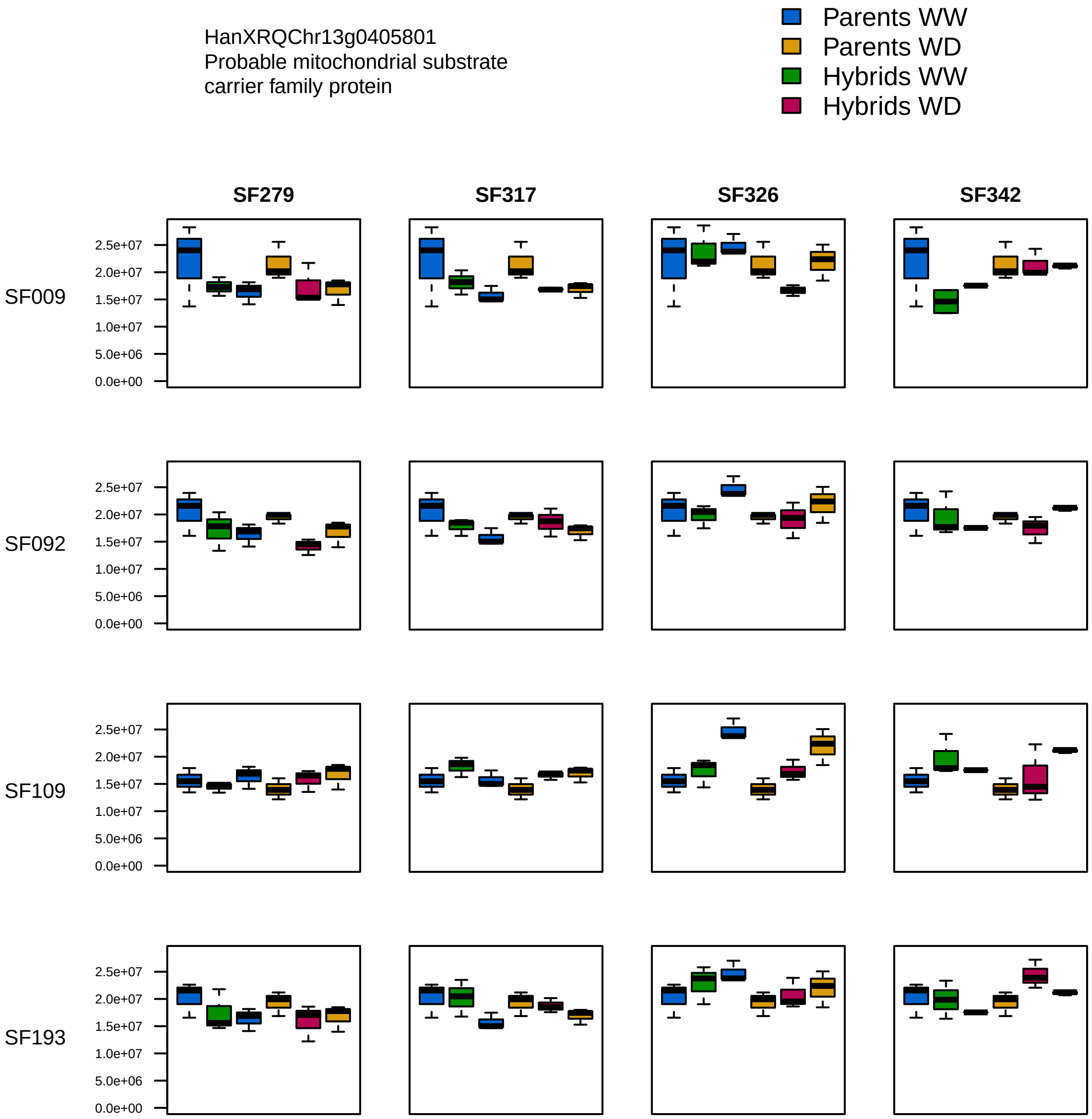
HanXRQChr13g0405801
Probable mitochondrial substrate
carrier family protein
Parents WW
Parents WD
Hybrids WW
Hybrids WD
SF279
SF317
SF326
SF342
SF009
SF092
SF109
SF193
0.0e+00
5.0e+06
1.0e+07
1.5e+07
2.0e+07
2.5e+07

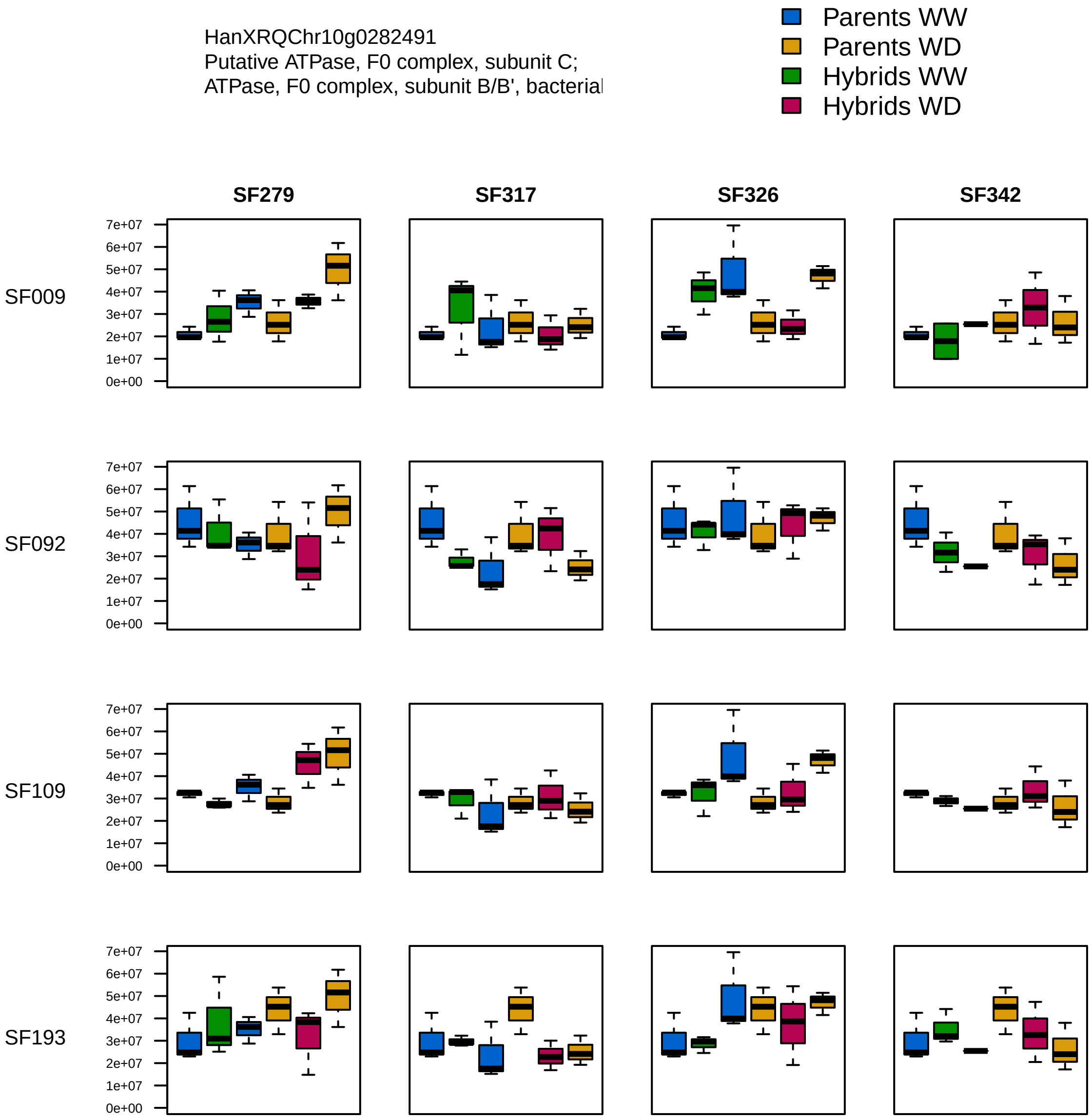
HanXRQChr10g0282491
Putative ATPase, F0 complex, subunit C;
ATPase, F0 complex, subunit B/B', bacterial
Parents WW
Parents WD
Hybrids WW
Hybrids WD
SF279
SF317
SF326
SF342
SF009
SF092
SF109
SF193
7e+07
6e+07
5e+07
4e+07
3e+07
2e+07
1e+07
0e+00

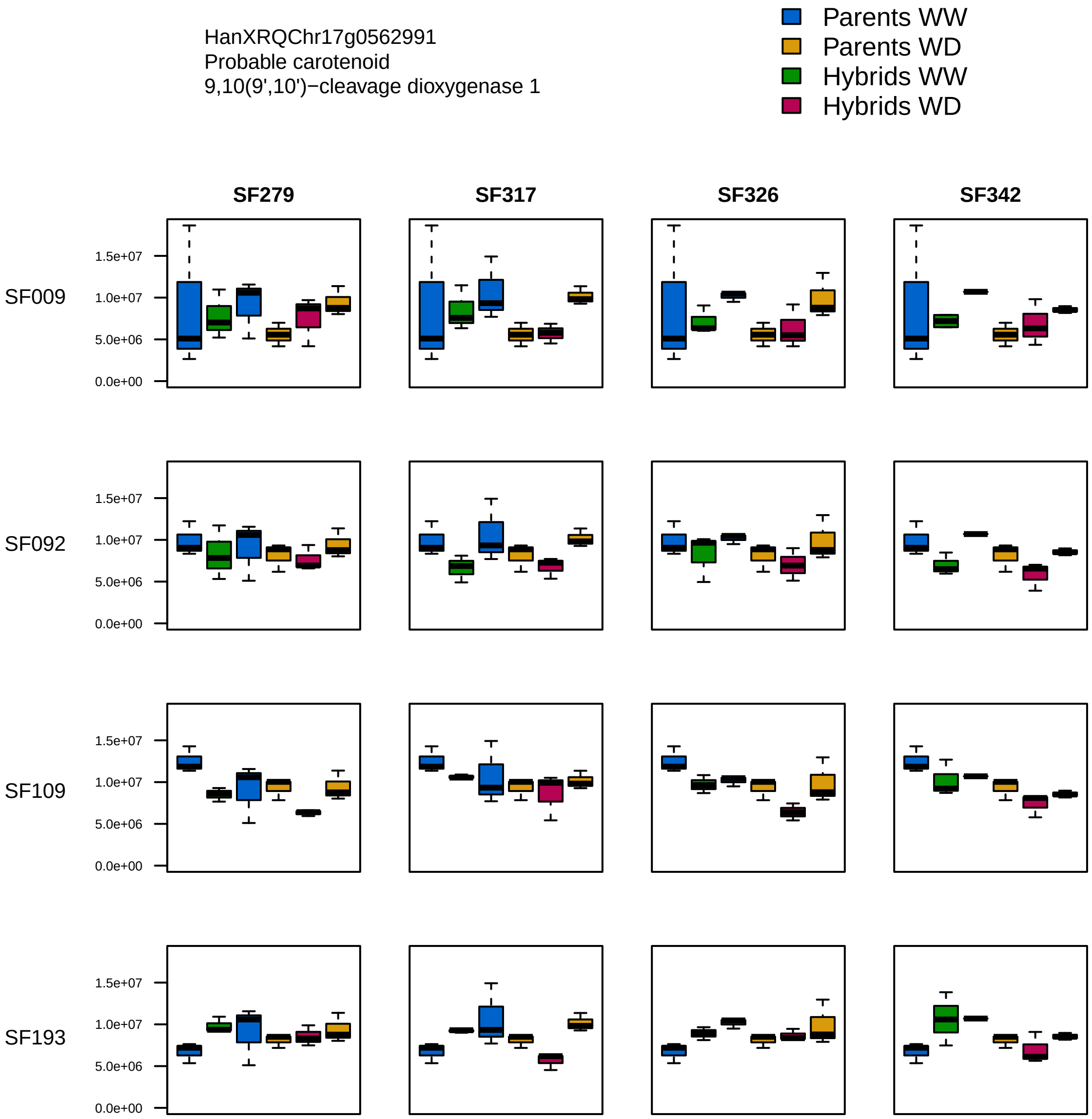

HanXRQChr17g0562991
Probable carotenoid
9,10(9',10')−cleavage dioxygenase 1
Parents WW
Parents WD
Hybrids WW
Hybrids WD
SF279
SF317
SF326
SF342
SF009
SF092
SF109
SF193
1.5e+07
1.0e+07
5.0e+06
0.0e+00

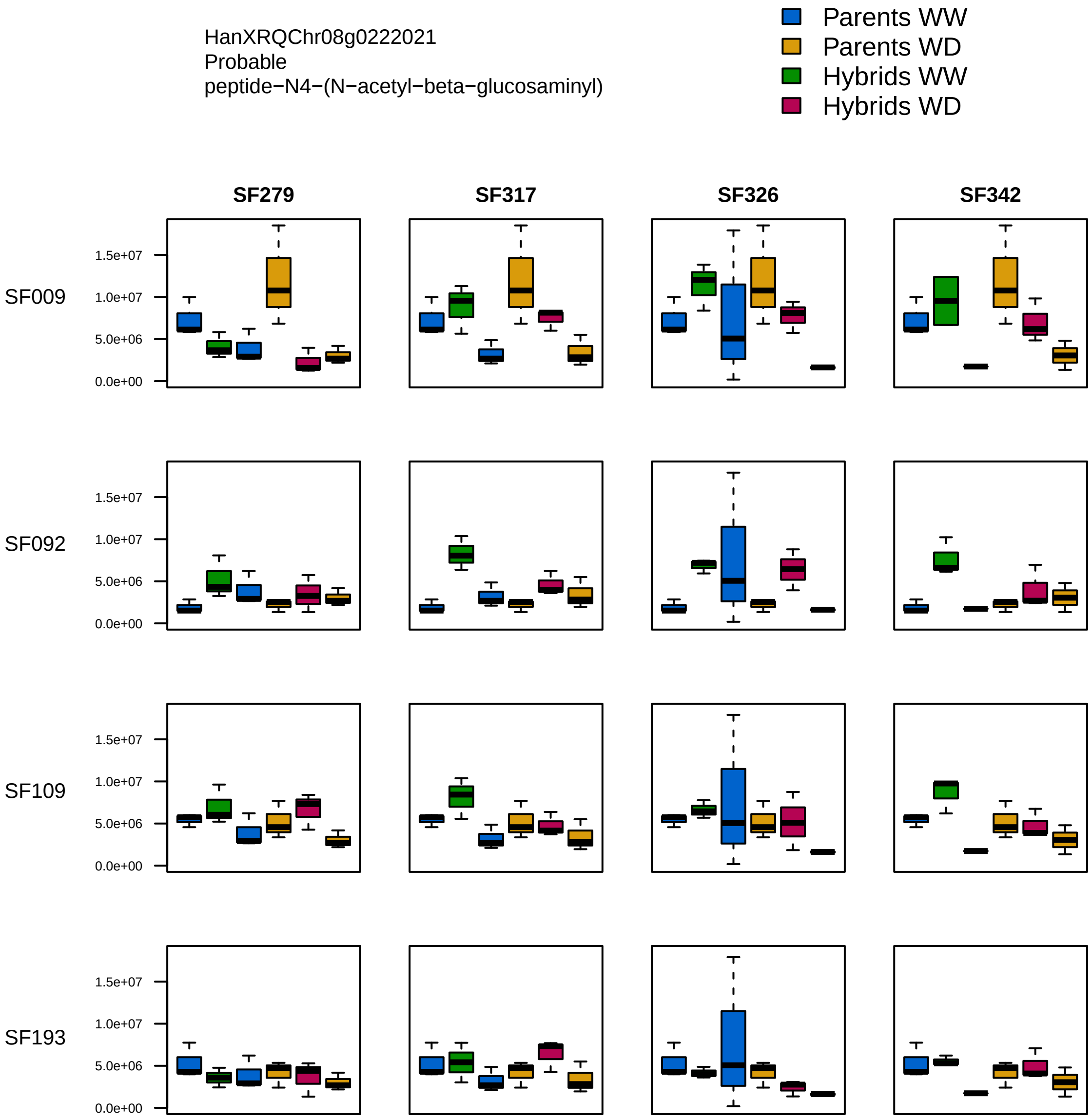
HanXRQChr08g0222021
Probable
peptide−N4−(N−acetyl−beta−glucosaminyl)
Parents WW
Parents WD
Hybrids WW
Hybrids WD
SF279
SF317
SF326
SF342
SF009
SF092
SF109
SF193
1.5e+07
1.0e+07
5.0e+06
0.0e+00

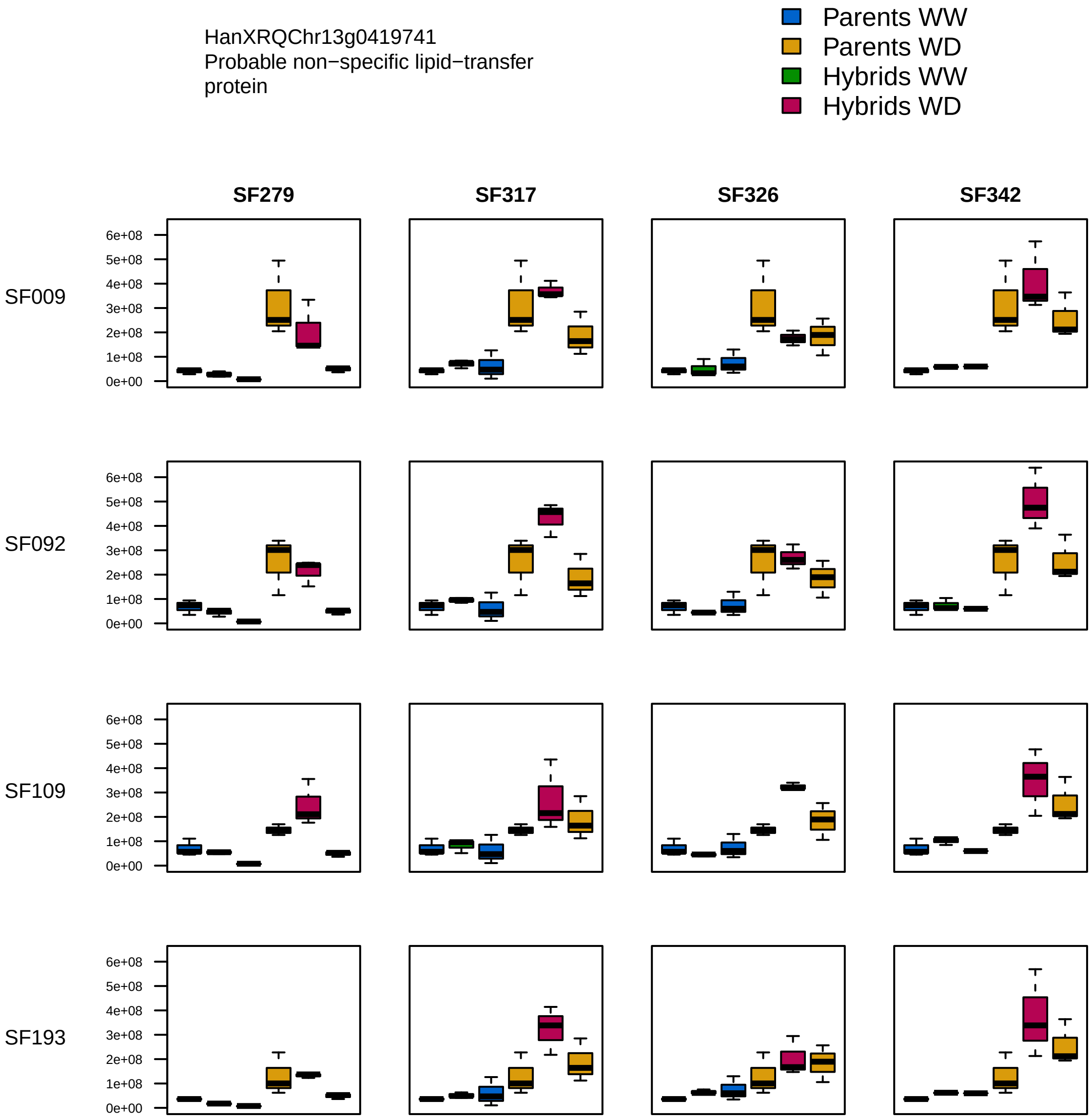
HanXRQChr13g0419741
Probable non−specific lipid−transfer protein
Parents WW
Parents WD
Hybrids WW
Hybrids WD
SF279
SF317
SF326
SF342
SF009
SF092
SF109
SF193
6e+08
5e+08
4e+08
3e+08
2e+08
1e+08
0e+00

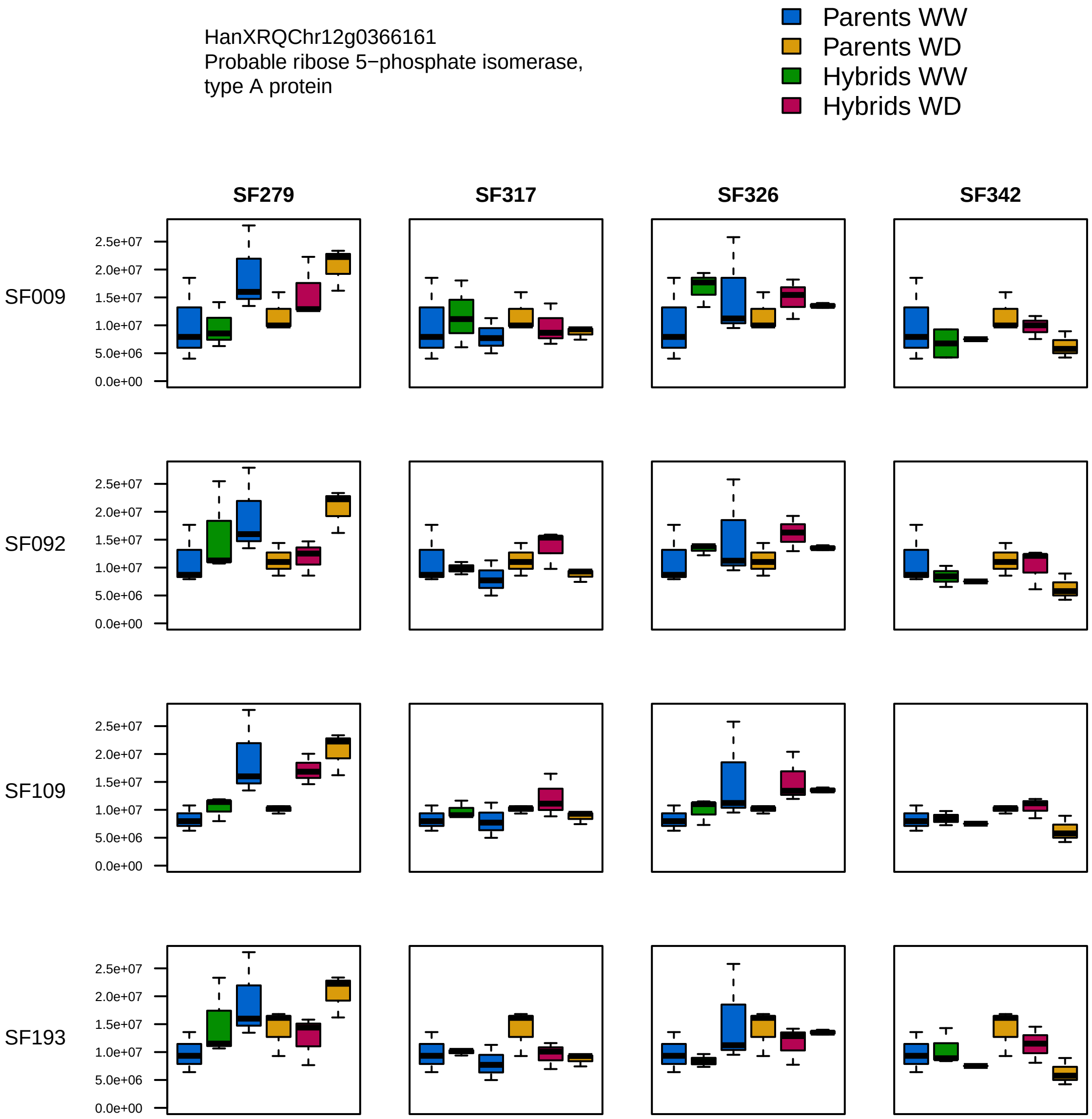

HanXRQChr12g0366161
Probable ribose 5−phosphate isomerase,
type A protein
Parents WW
Parents WD
Hybrids WW
Hybrids WD
SF279
SF317
SF326
SF342
SF009
SF092
SF109
SF193
0.0e+00
5.0e+06
1.0e+07
1.5e+07
2.0e+07
2.5e+07

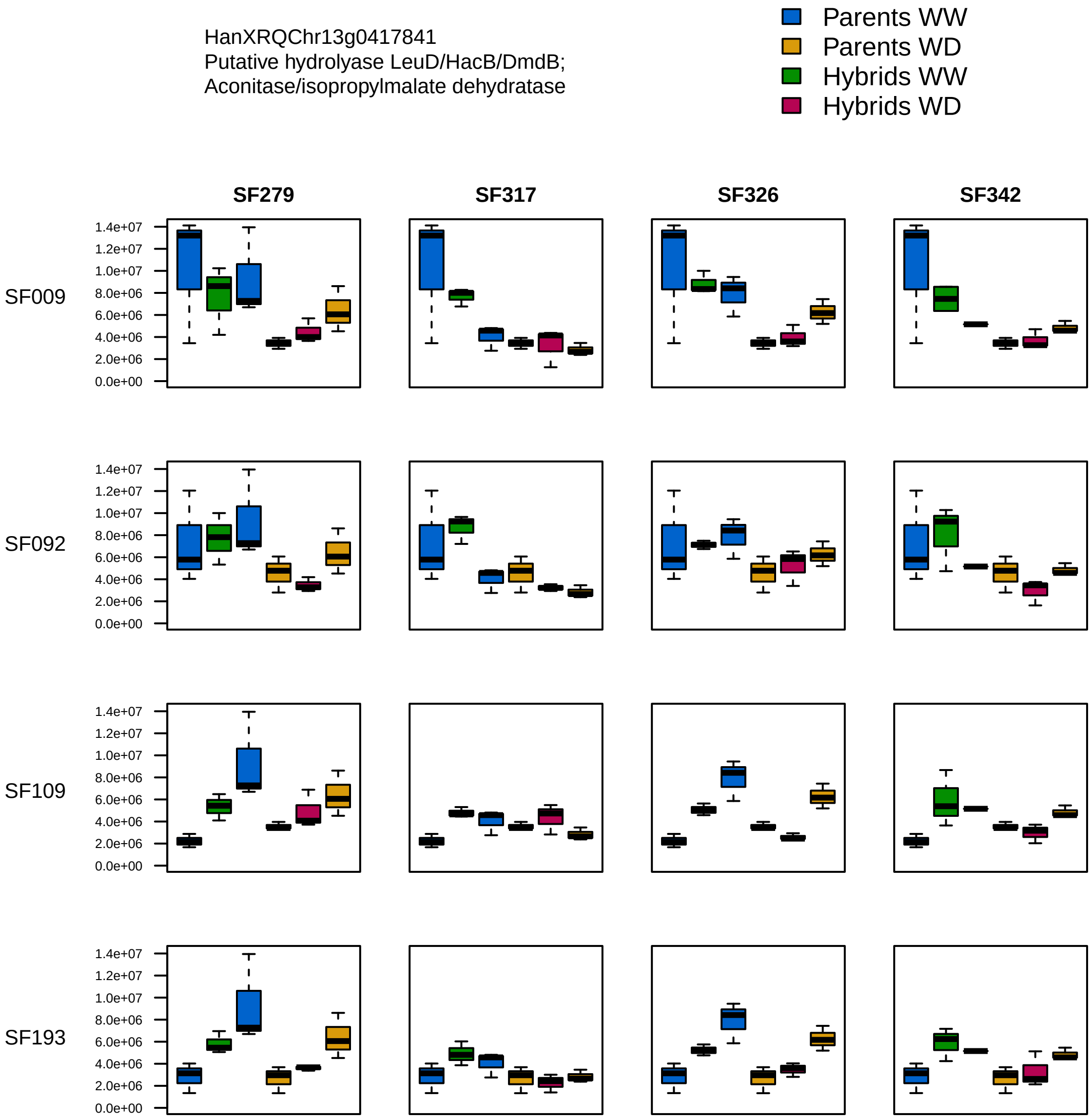
HanXRQChr13g0417841
Putative hydrolyase LeuD/HacB/DmdB;
Aconitase/isopropylmalate dehydratase
Parents WW
Parents WD
Hybrids WW
Hybrids WD
SF279
SF317
SF326
SF342
SF009
SF092
SF109
SF193
1.4e+07
1.2e+07
1.0e+07
8.0e+06
6.0e+06
4.0e+06
2.0e+06
0.0e+00

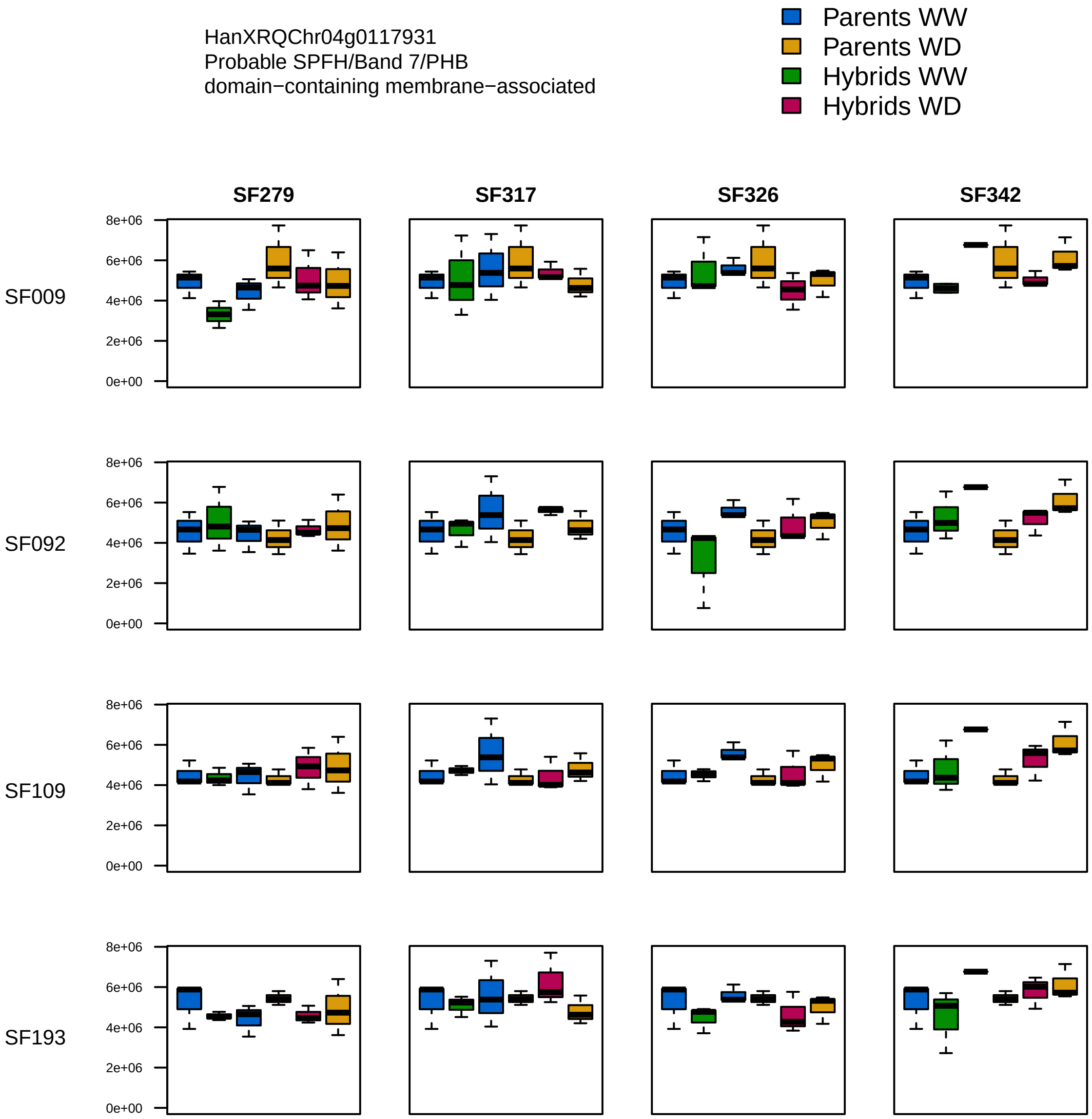
HanXRQChr04g0117931
Probable SPFH/Band 7/PHB
domain−containing membrane−associated
Parents WW
Parents WD
Hybrids WW
Hybrids WD
SF279
SF317
SF326
SF342
SF009
SF092
SF109
SF193
8e+06
6e+06
4e+06
2e+06
0e+00

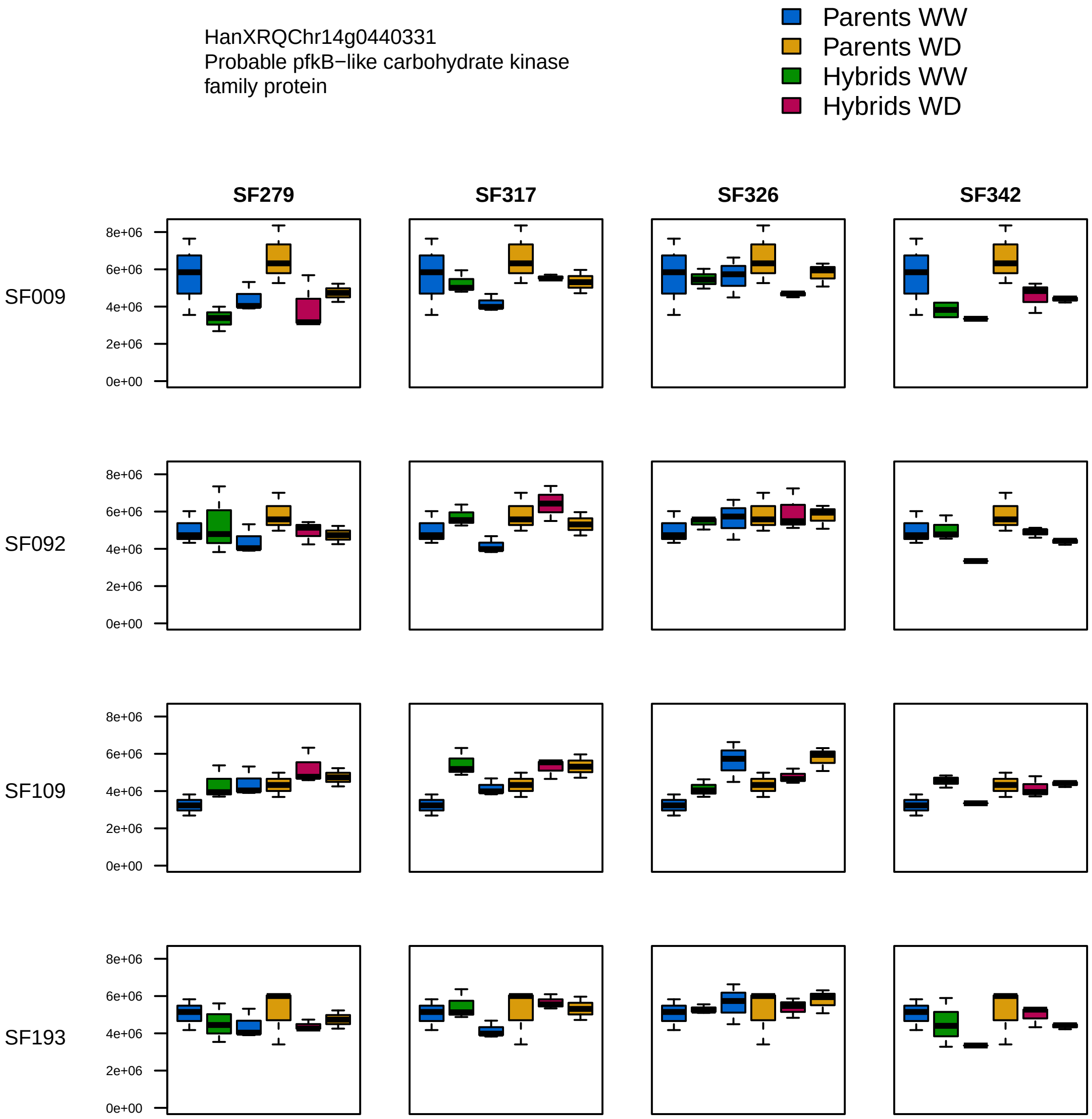

HanXRQChr14g0440331
Probable pfkB−like carbohydrate kinase
family protein
Parents WW
Parents WD
Hybrids WW
Hybrids WD
SF279
SF317
SF326
SF342
SF009
SF092
SF109
SF193
8e+06
6e+06
4e+06
2e+06
0e+00

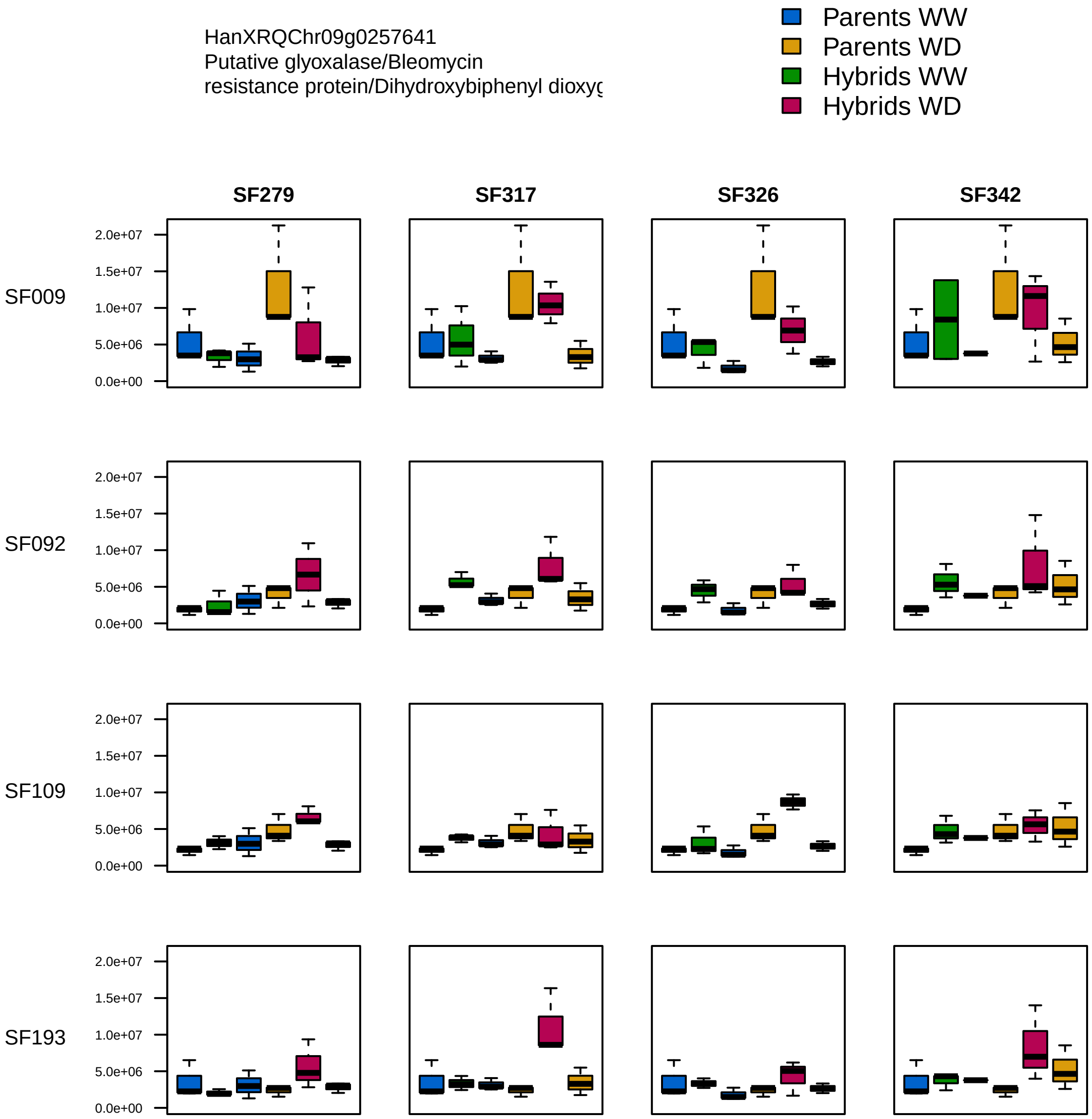

HanXRQChr09g0257641
Putative glyoxalase/Bleomycin
resistance protein/Dihydroxybiphenyl dioxyg
Parents WW
Parents WD
Hybrids WW
Hybrids WD
SF279
SF317
SF326
SF342
SF009
SF092
SF109
SF193
0.0e+00
5.0e+06
1.0e+07
1.5e+07
2.0e+07

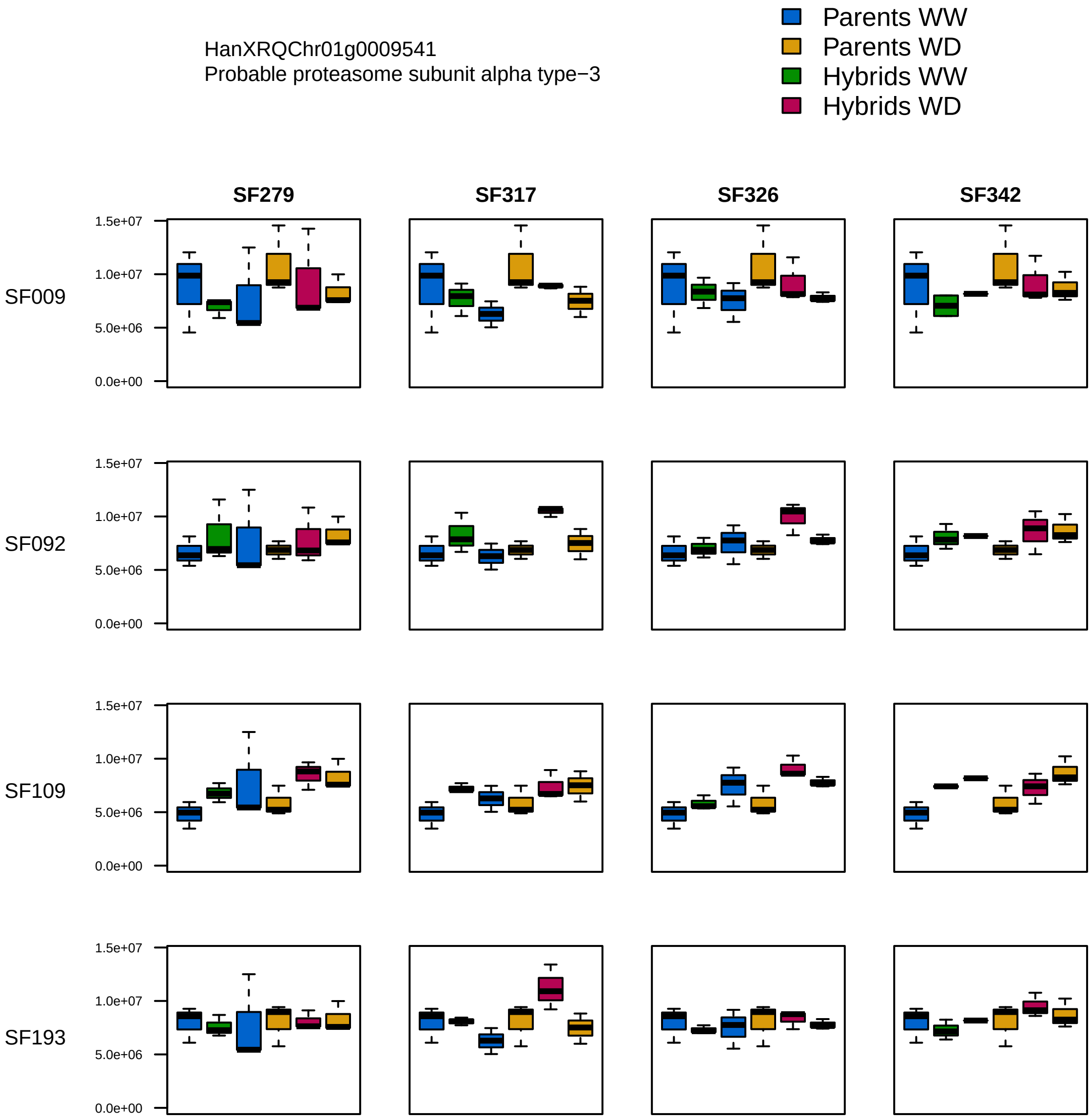

HanXRQChr01g0009541
Probable proteasome subunit alpha type−3
Parents WW
Parents WD
Hybrids WW
Hybrids WD
SF279
SF317
SF326
SF342
SF009
SF092
SF109
SF193
1.5e+07
1.0e+07
5.0e+06
0.0e+00

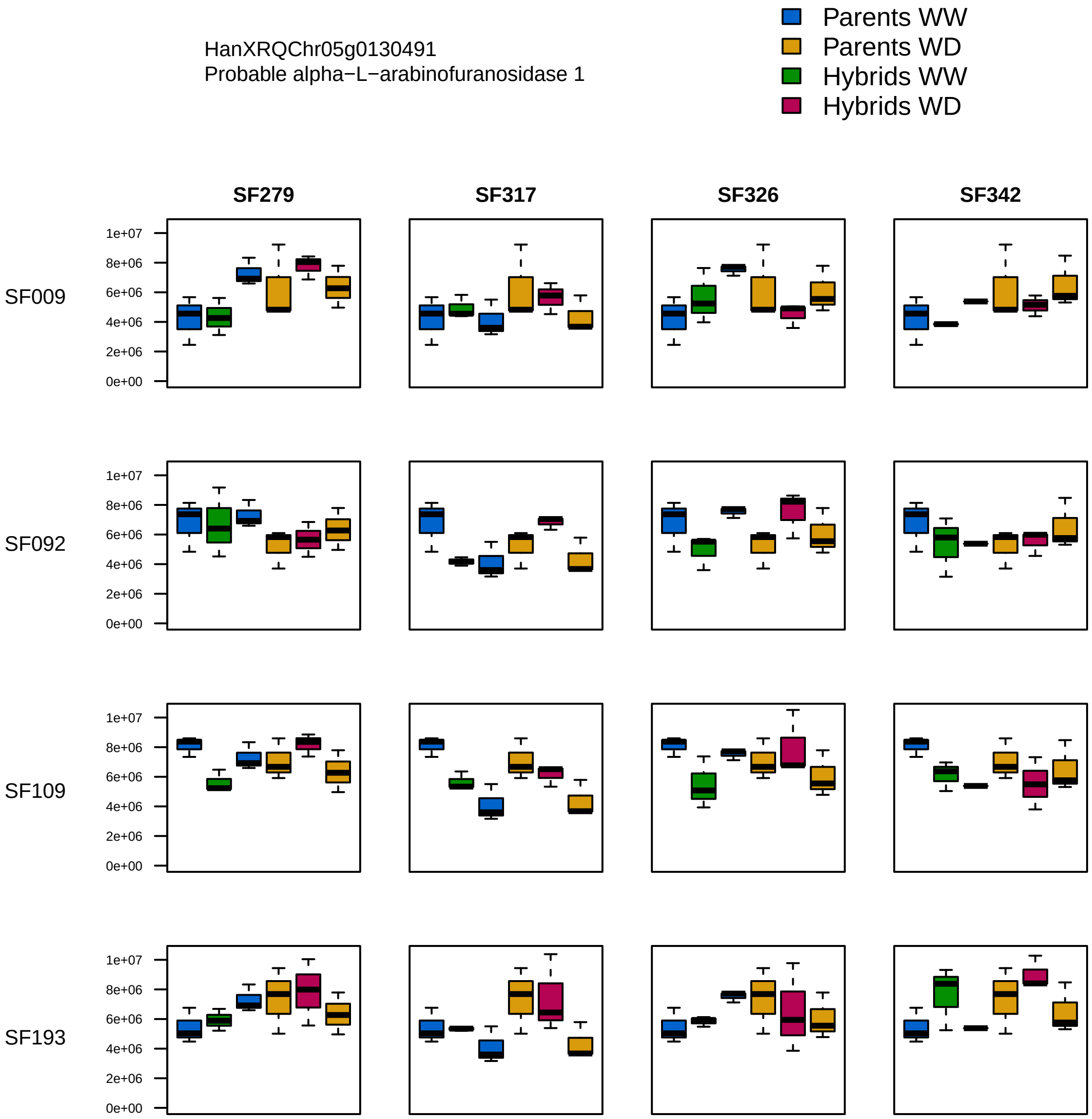

HanXRQChr05g0130491
Probable alpha−L−arabinofuranosidase 1
Parents WW
Parents WD
Hybrids WW
Hybrids WD
SF279
SF317
SF326
SF342
SF009
SF092
SF109
SF193
1e+07
8e+06
6e+06
4e+06
2e+06
0e+00

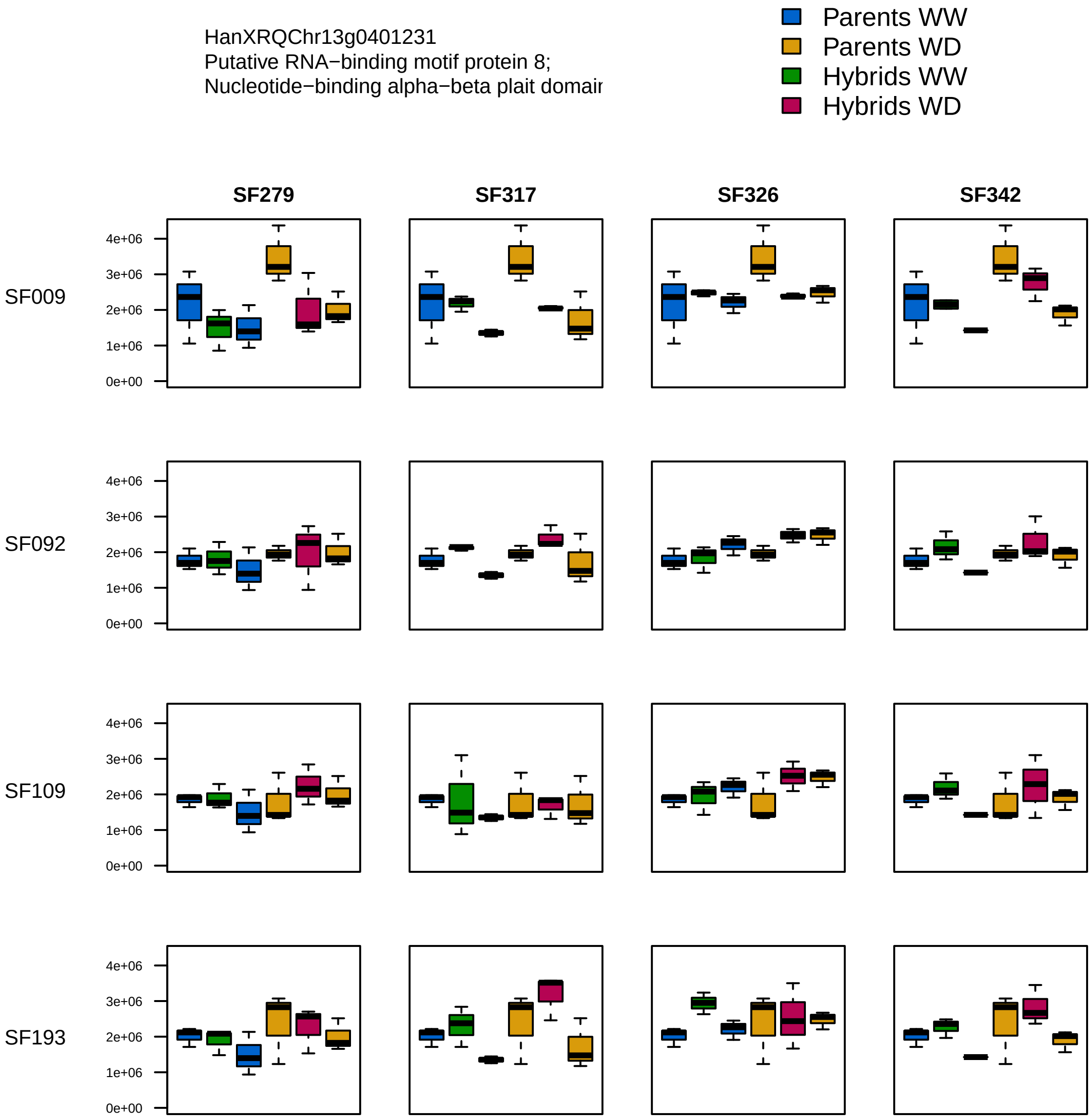

HanXRQChr13g0401231
Putative RNA−binding motif protein 8;
Nucleotide−binding alpha−beta plait domair
Parents WW
Parents WD
Hybrids WW
Hybrids WD
SF279
SF317
SF326
SF342
SF009
SF092
SF109
SF193
0e+00
1e+06
2e+06
3e+06
4e+06

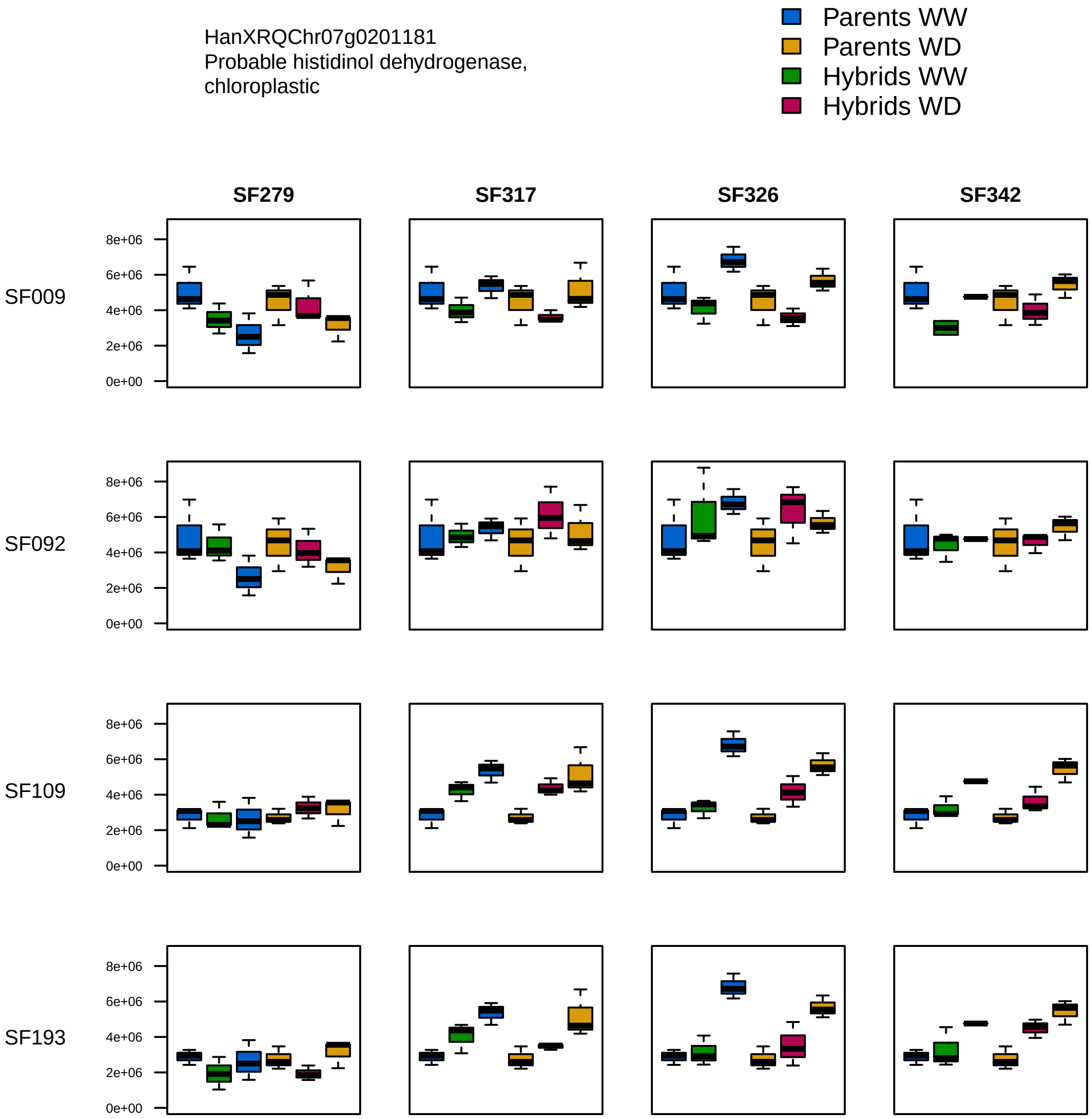

HanXRQChr07g0201181
Probable histidinol dehydrogenase,
chloroplastic
Parents WW
Parents WD
Hybrids WW
Hybrids WD
SF279
SF317
SF326
SF342
SF009
SF092
SF109
SF193
8e+06
6e+06
4e+06
2e+06
0e+00

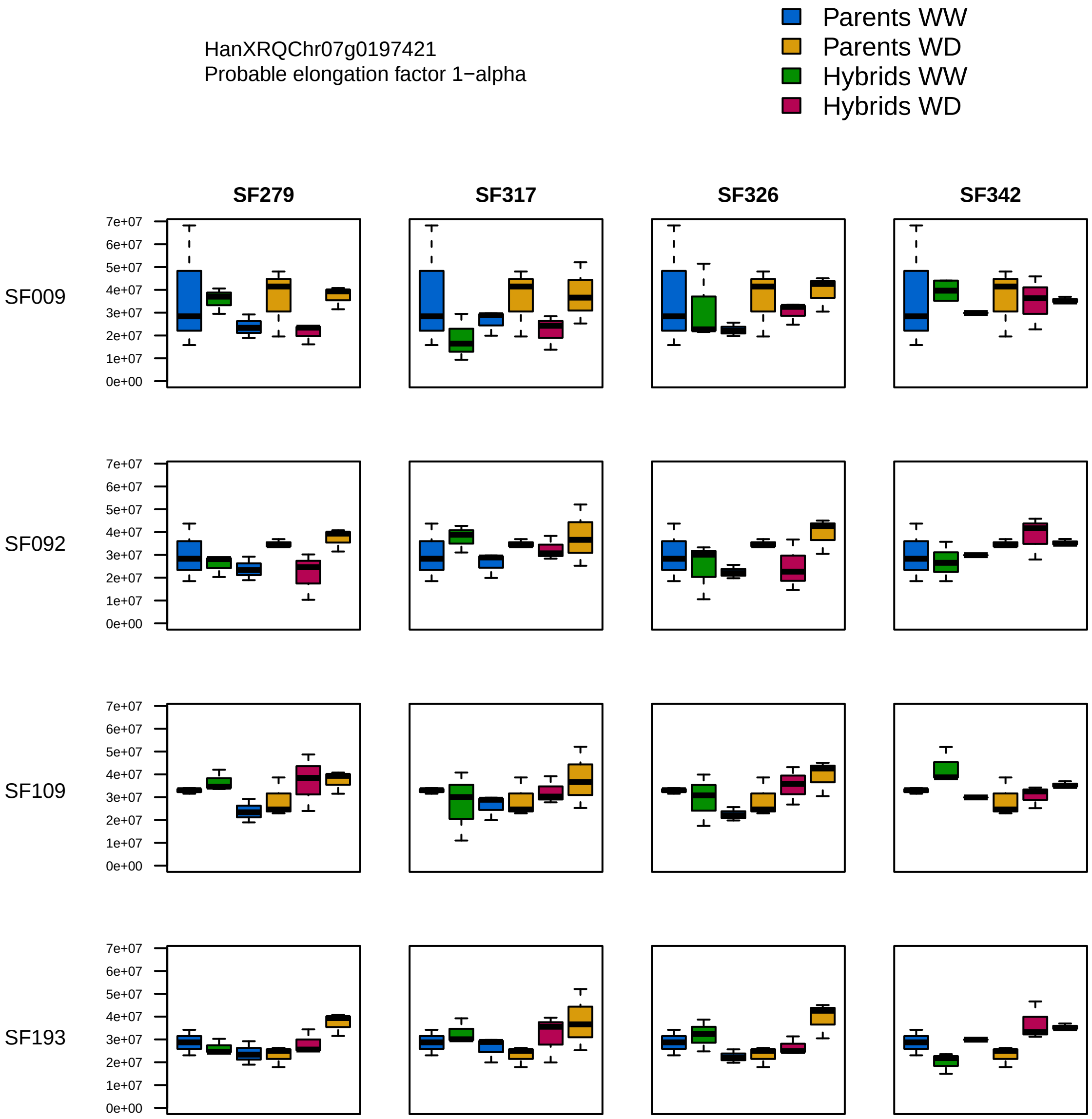

HanXRQChr07g0197421
Probable elongation factor 1−alpha
Parents WW
Parents WD
Hybrids WW
Hybrids WD
SF279
SF317
SF326
SF342
SF009
SF092
SF109
SF193
7e+07
6e+07
5e+07
4e+07
3e+07
2e+07
1e+07
0e+00

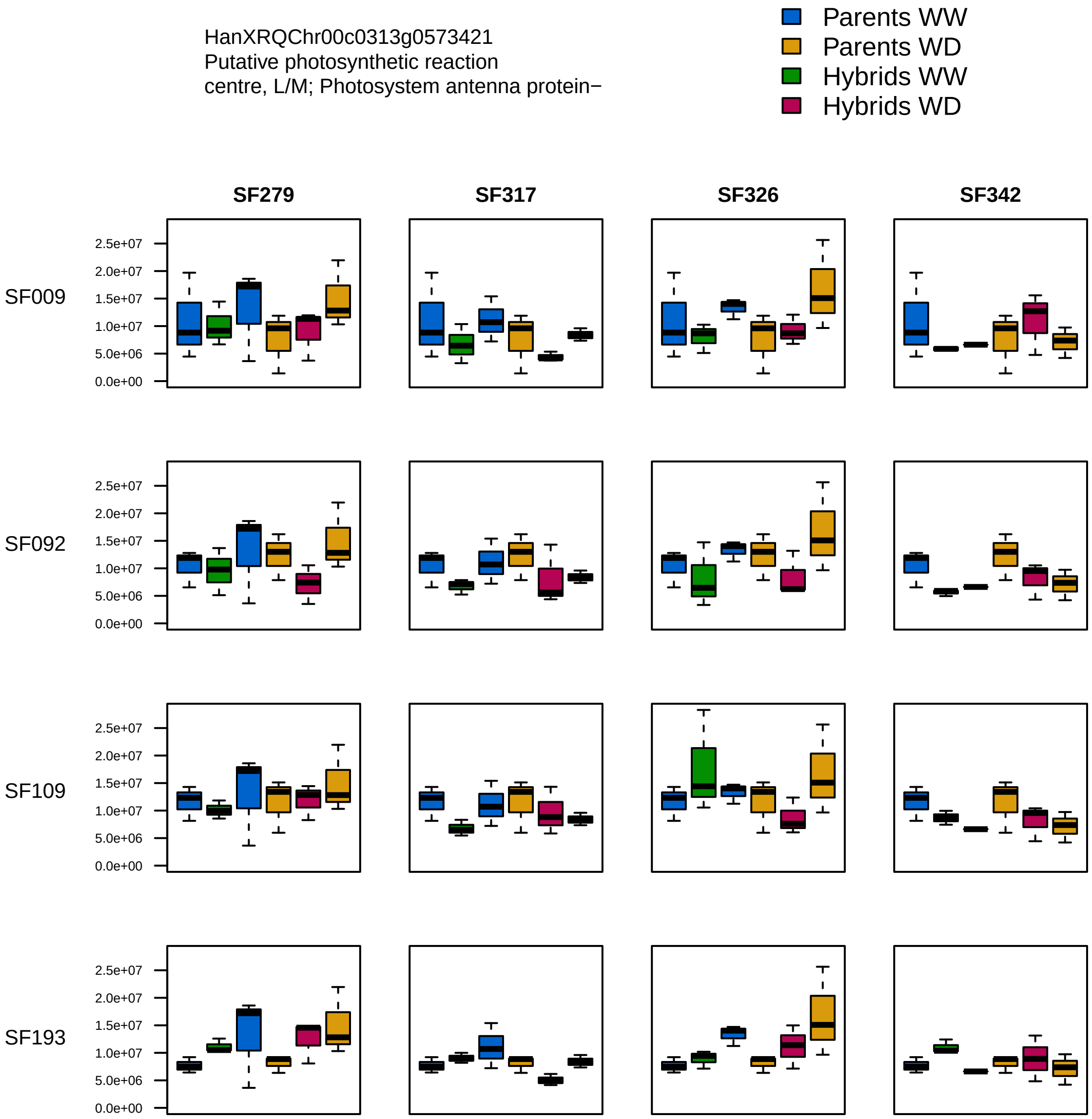

HanXRQChr00c0313g0573421
Putative photosynthetic reaction
centre, L/M; Photosystem antenna protein−
Parents WW
Parents WD
Hybrids WW
Hybrids WD
SF279
SF317
SF326
SF342
SF009
SF092
SF109
SF193
2.5e+07
2.0e+07
1.5e+07
1.0e+07
5.0e+06
0.0e+00

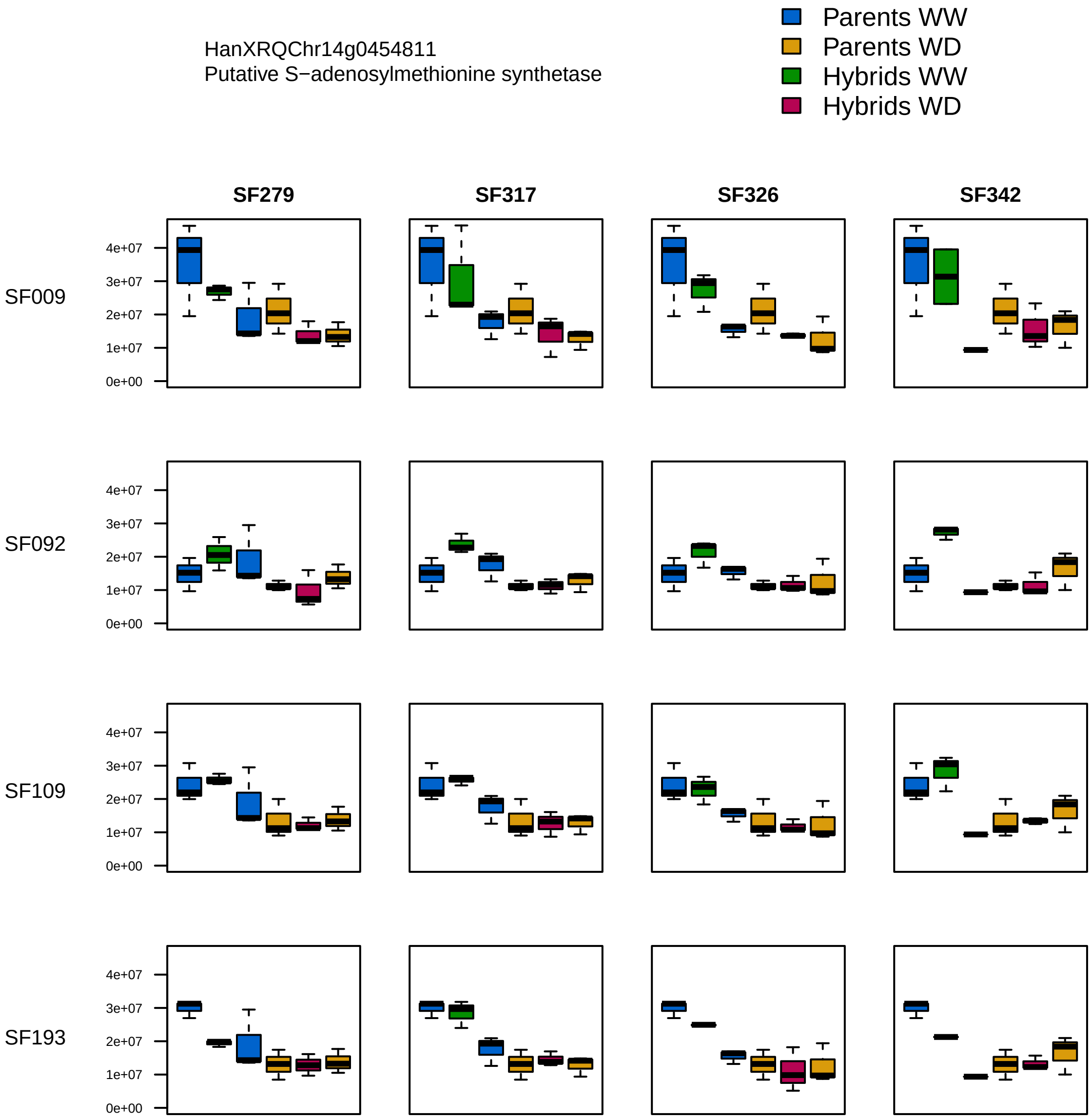
HanXRQChr14g0454811
Putative S−adenosylmethionine synthetase
Parents WW
Parents WD
Hybrids WW
Hybrids WD
SF279
SF317
SF326
SF342
SF009
SF092
SF109
SF193
0e+00
1e+07
2e+07
3e+07
4e+07

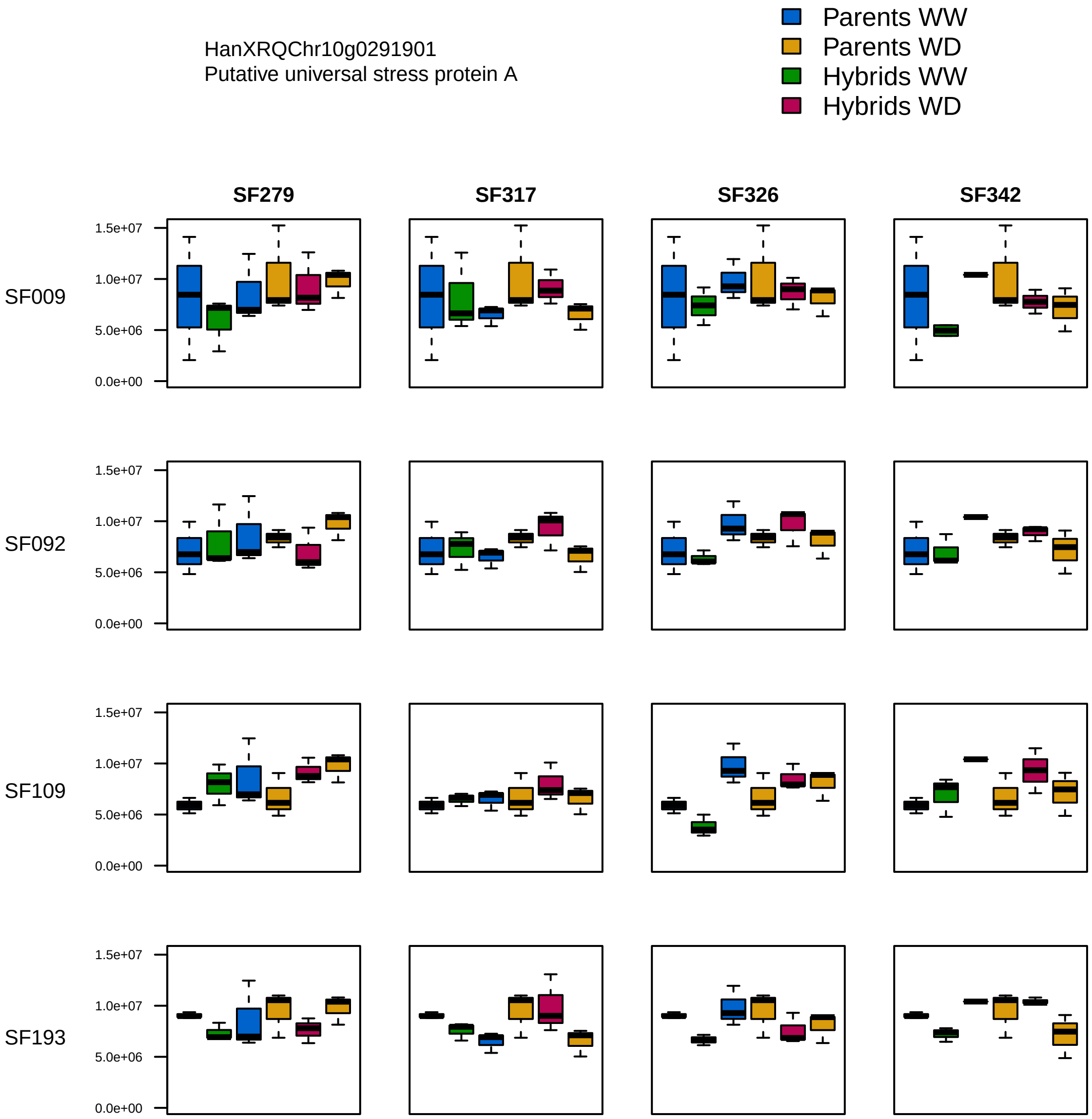
HanXRQChr10g0291901
Putative universal stress protein A
Parents WW
Parents WD
Hybrids WW
Hybrids WD
SF279
SF317
SF326
SF342
SF009
SF092
SF109
SF193
1.5e+07
1.0e+07
5.0e+06
0.0e+00

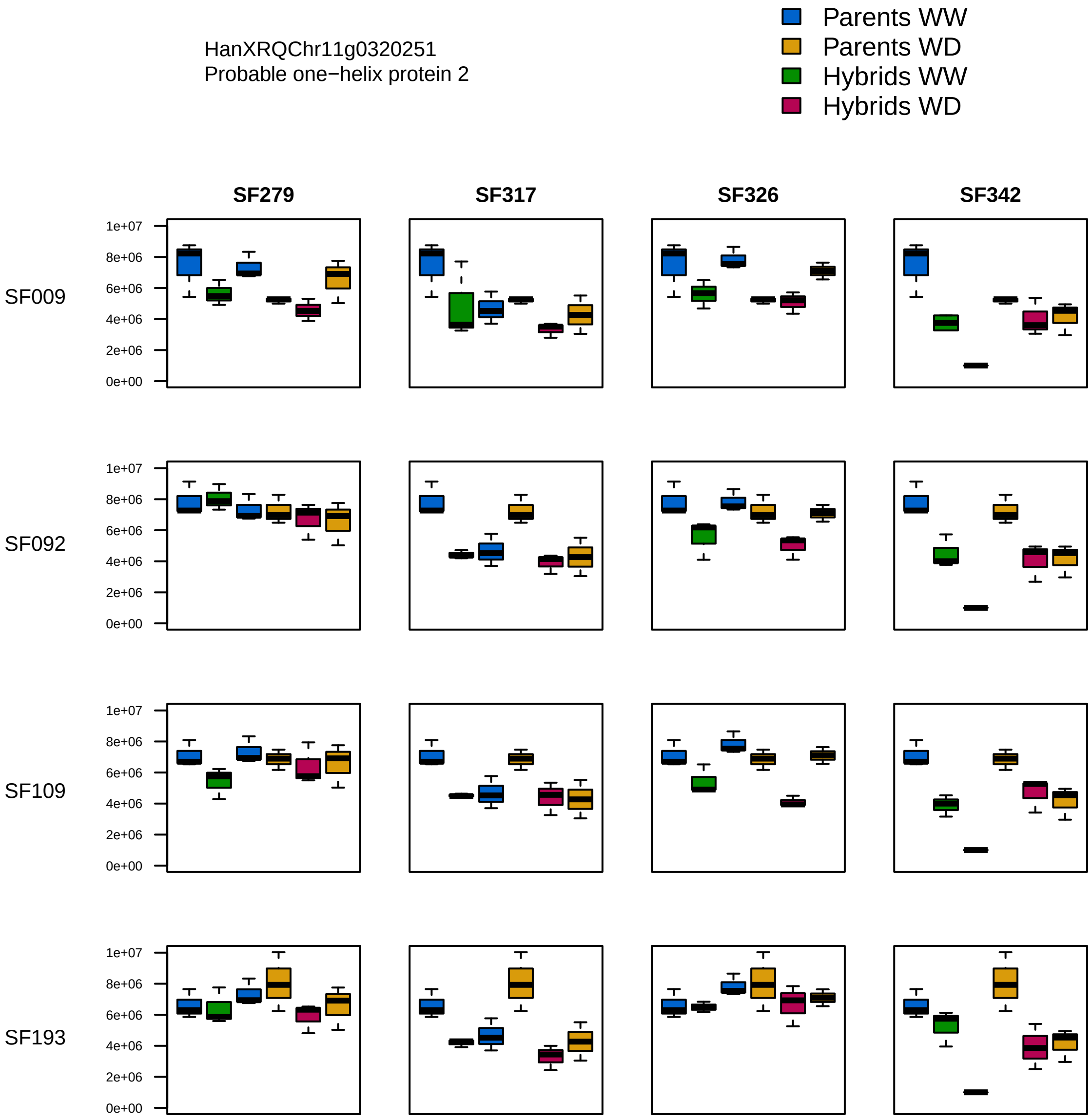
HanXRQChr11g0320251
Probable one−helix protein 2
Parents WW
Parents WD
Hybrids WW
Hybrids WD
SF279
SF317
SF326
SF342
SF009
SF092
SF109
SF193
1e+07
8e+06
6e+06
4e+06
2e+06
0e+00

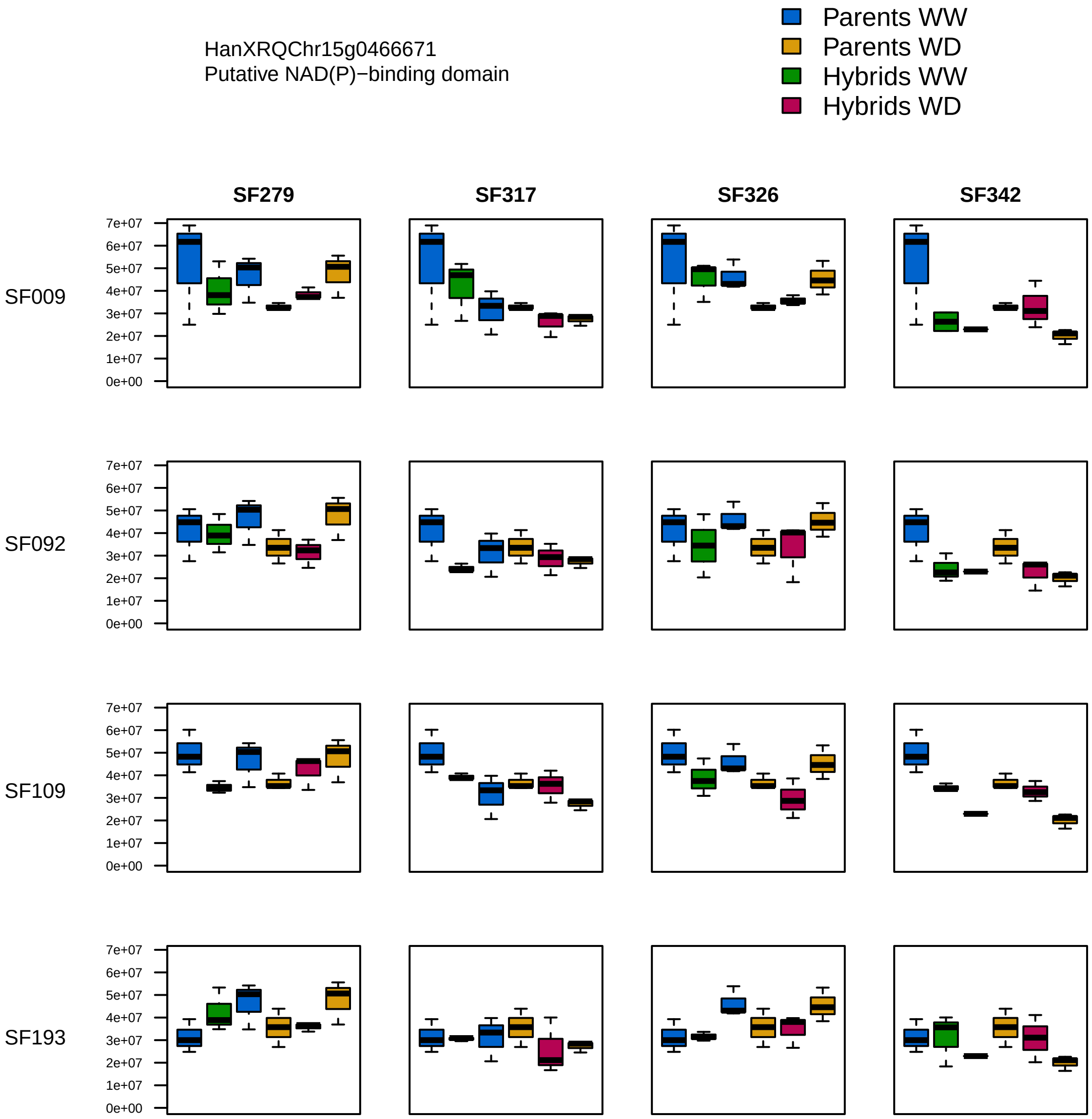
HanXRQChr15g0466671
Putative NAD(P)−binding domain
Parents WW
Parents WD
Hybrids WW
Hybrids WD
SF279
SF317
SF326
SF342
SF009
SF092
SF109
SF193
7e+07
6e+07
5e+07
4e+07
3e+07
2e+07
1e+07
0e+00

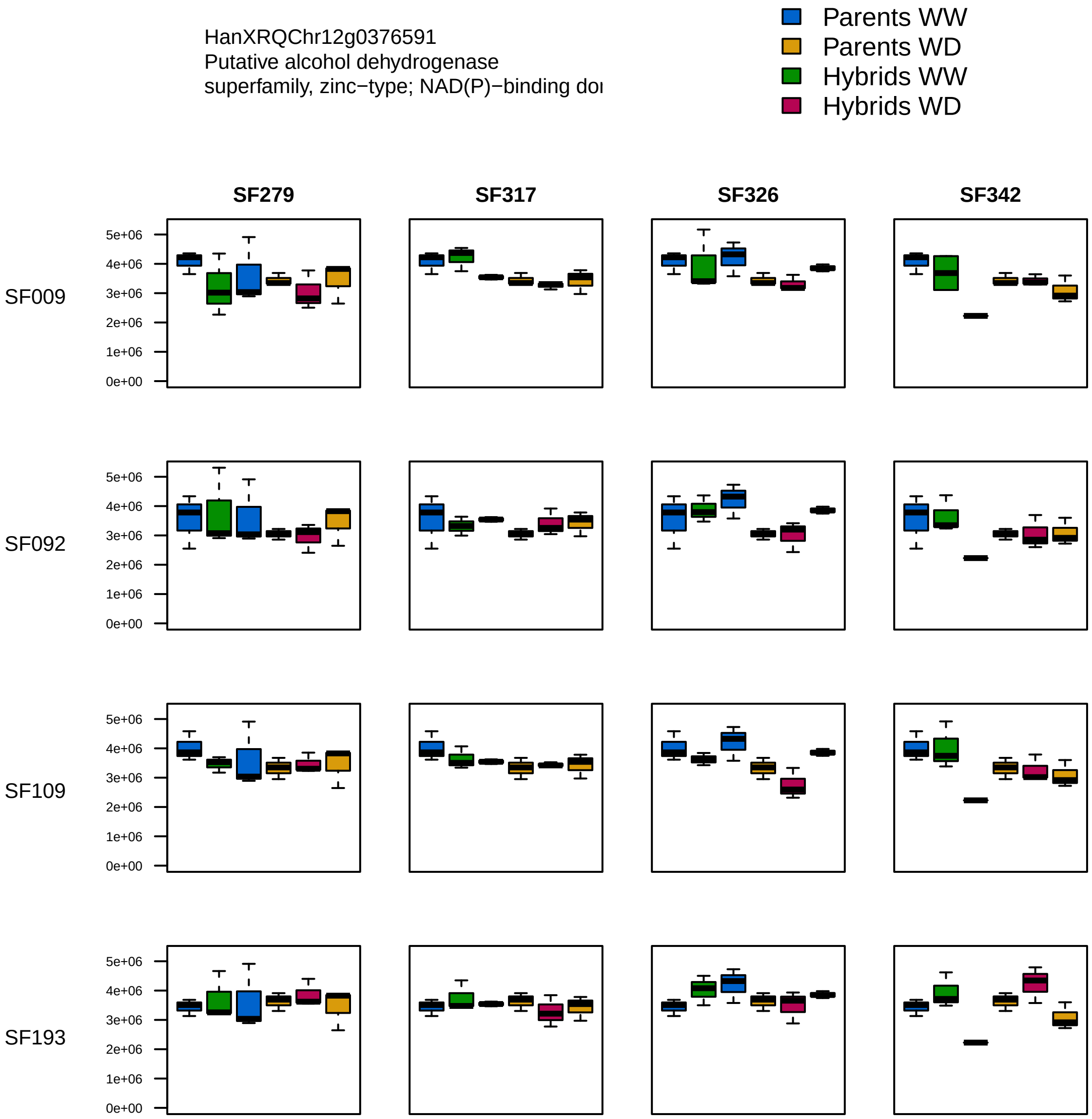
HanXRQChr12g0376591
Putative alcohol dehydrogenase
superfamily, zinc−type; NAD(P)−binding doı
Parents WW
Parents WD
Hybrids WW
Hybrids WD
SF279
SF317
SF326
SF342
SF009
SF092
SF109
SF193
5e+06
4e+06
3e+06
2e+06
1e+06
0e+00

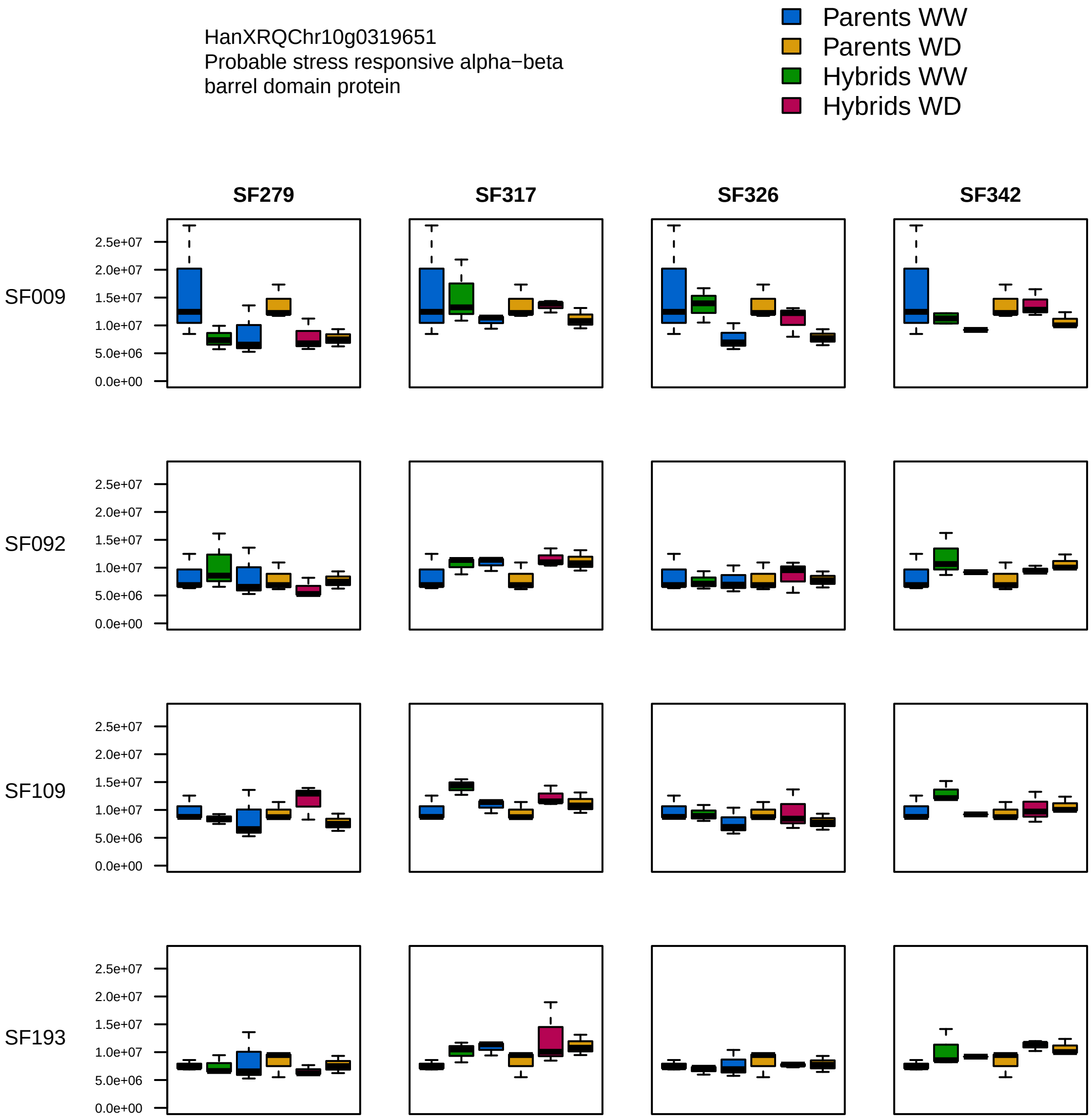
HanXRQChr10g0319651
Probable stress responsive alpha−beta
barrel domain protein
Parents WW
Parents WD
Hybrids WW
Hybrids WD
SF279
SF317
SF326
SF342
SF009
SF092
SF109
SF193
0.0e+00
5.0e+06
1.0e+07
1.5e+07
2.0e+07
2.5e+07

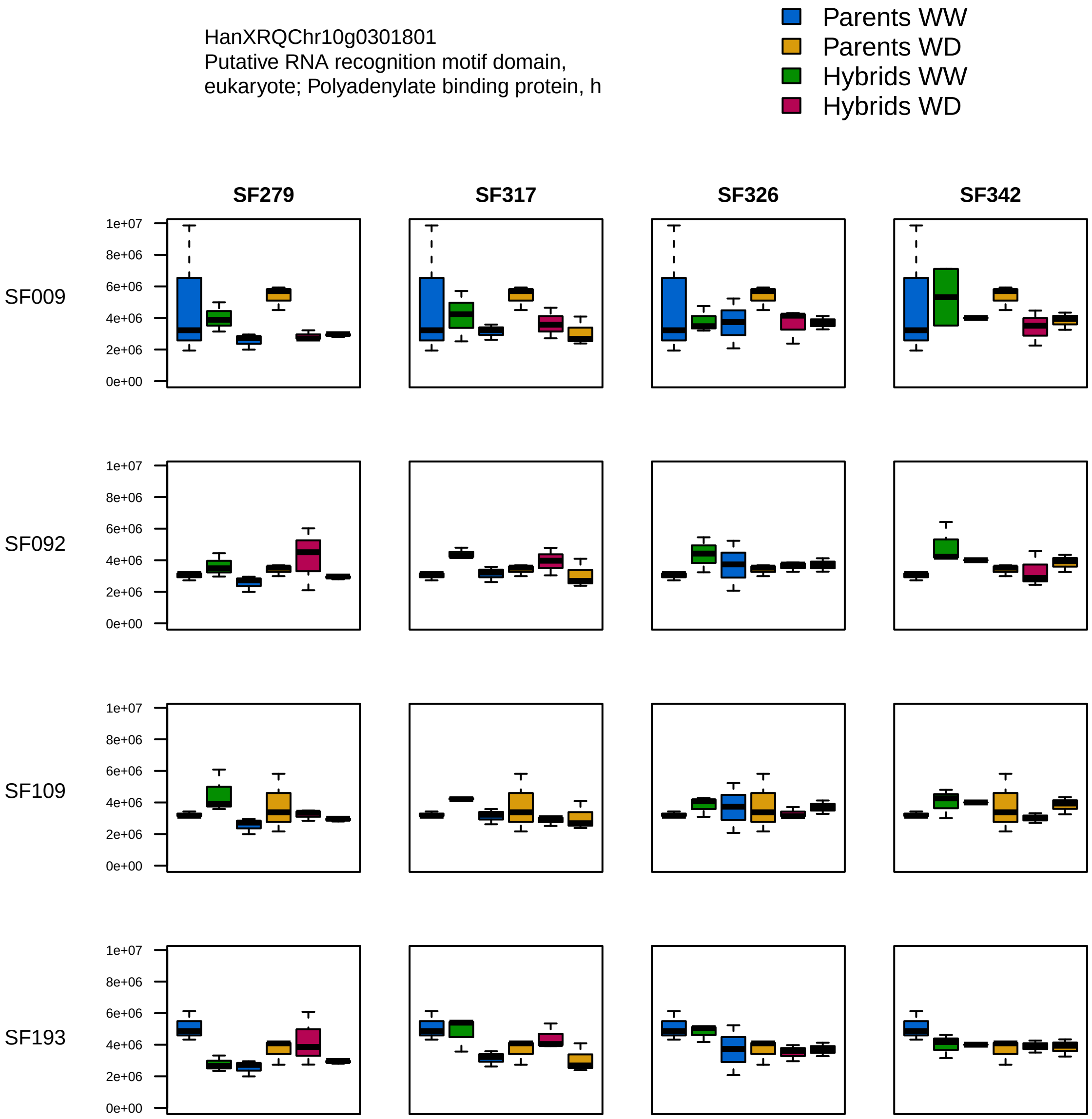
HanXRQChr10g0301801
Putative RNA recognition motif domain,
eukaryote; Polyadenylate binding protein, h
Parents WW
Parents WD
Hybrids WW
Hybrids WD
SF279
SF317
SF326
SF342
SF009
SF092
SF109
SF193
1e+07
8e+06
6e+06
4e+06
2e+06
0e+00

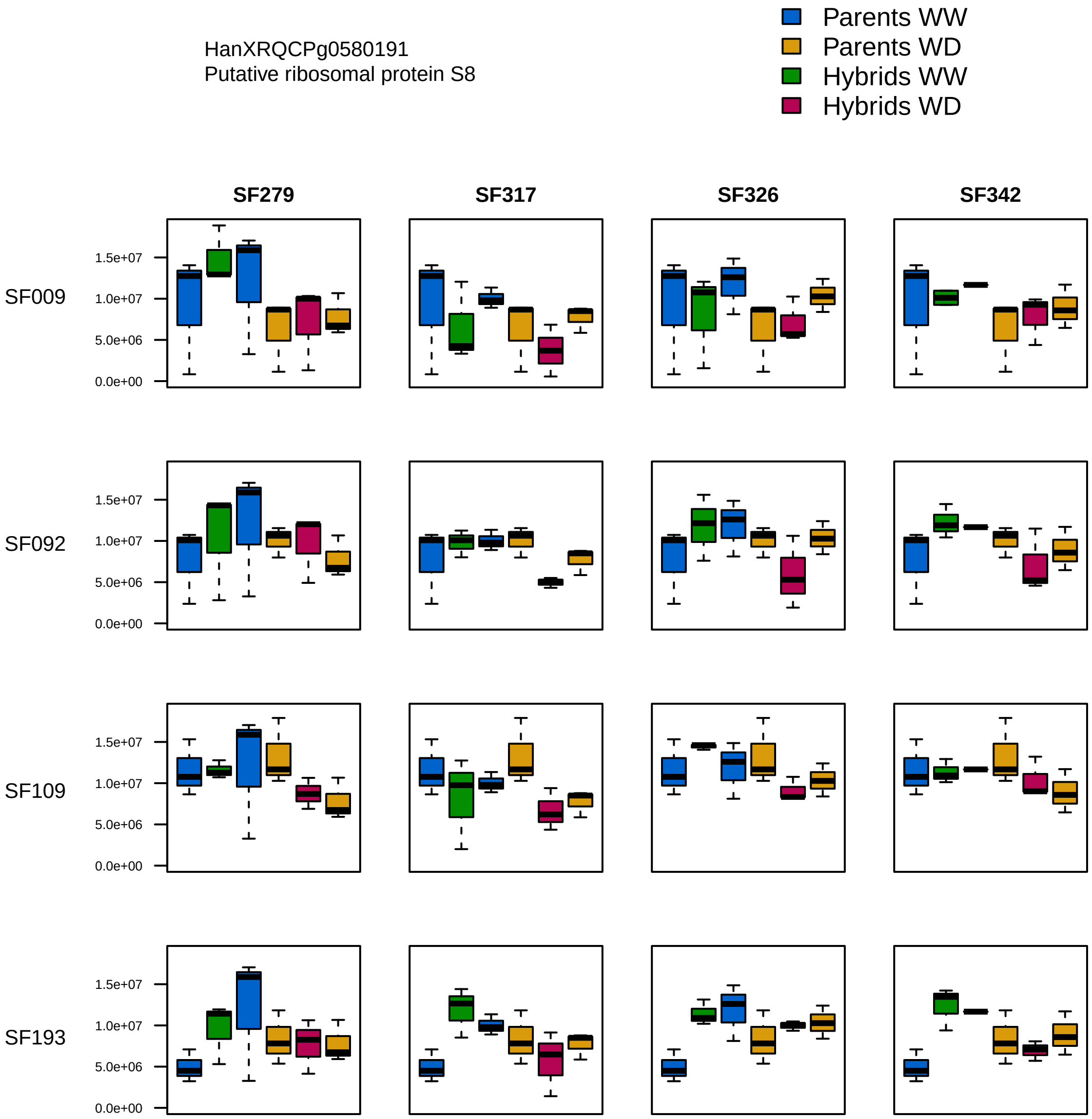
HanXRQCPg0580191
Putative ribosomal protein S8
Parents WW
Parents WD
Hybrids WW
Hybrids WD
SF279
SF317
SF326
SF342
SF009
SF092
SF109
SF193
1.5e+07
1.0e+07
5.0e+06
0.0e+00

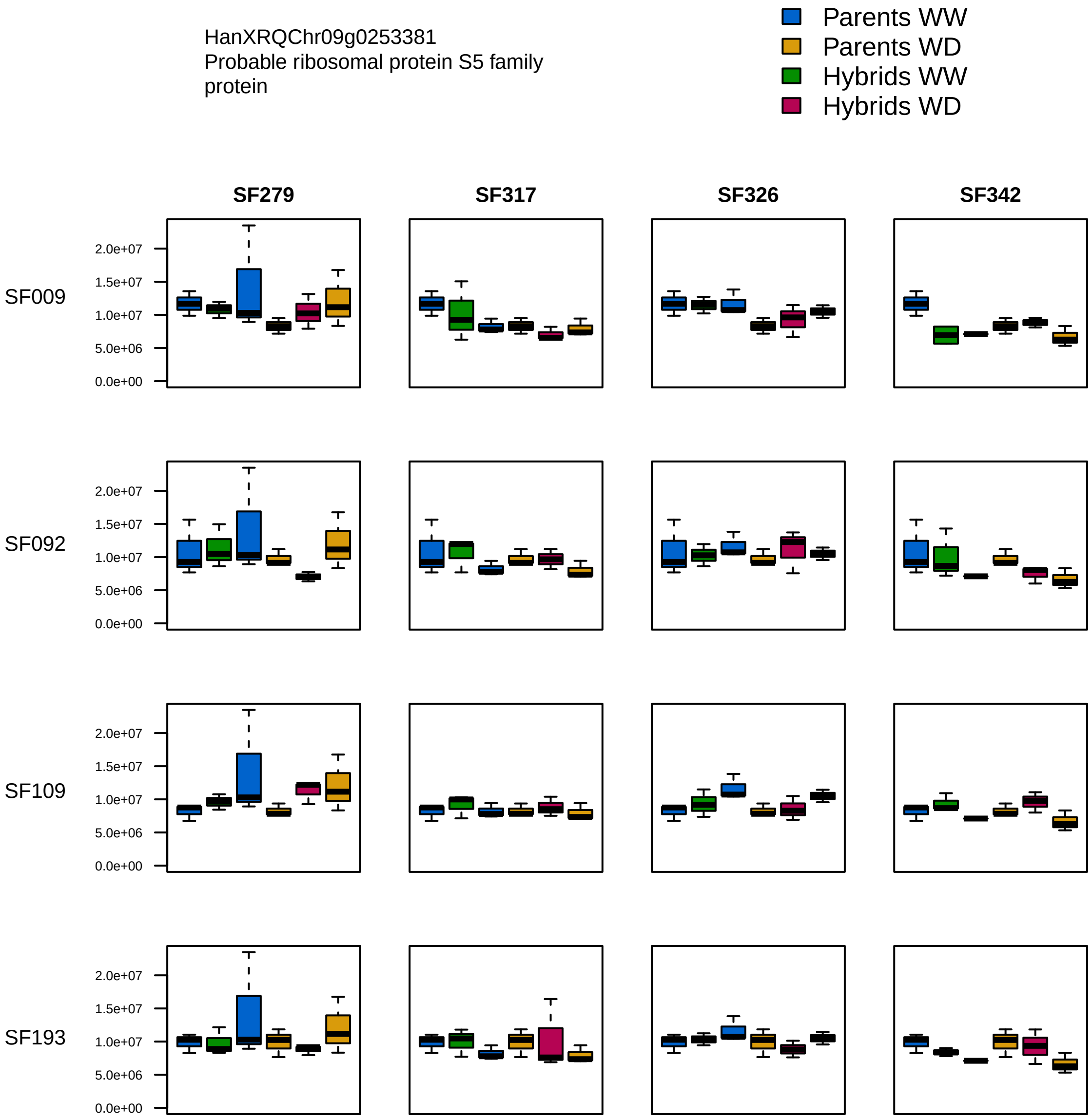

HanXRQChr09g0253381
Probable ribosomal protein S5 family protein
Parents WW
Parents WD
Hybrids WW
Hybrids WD
SF279
SF317
SF326
SF342
SF009
SF092
SF109
SF193
0.0e+00
5.0e+06
1.0e+07
1.5e+07
2.0e+07

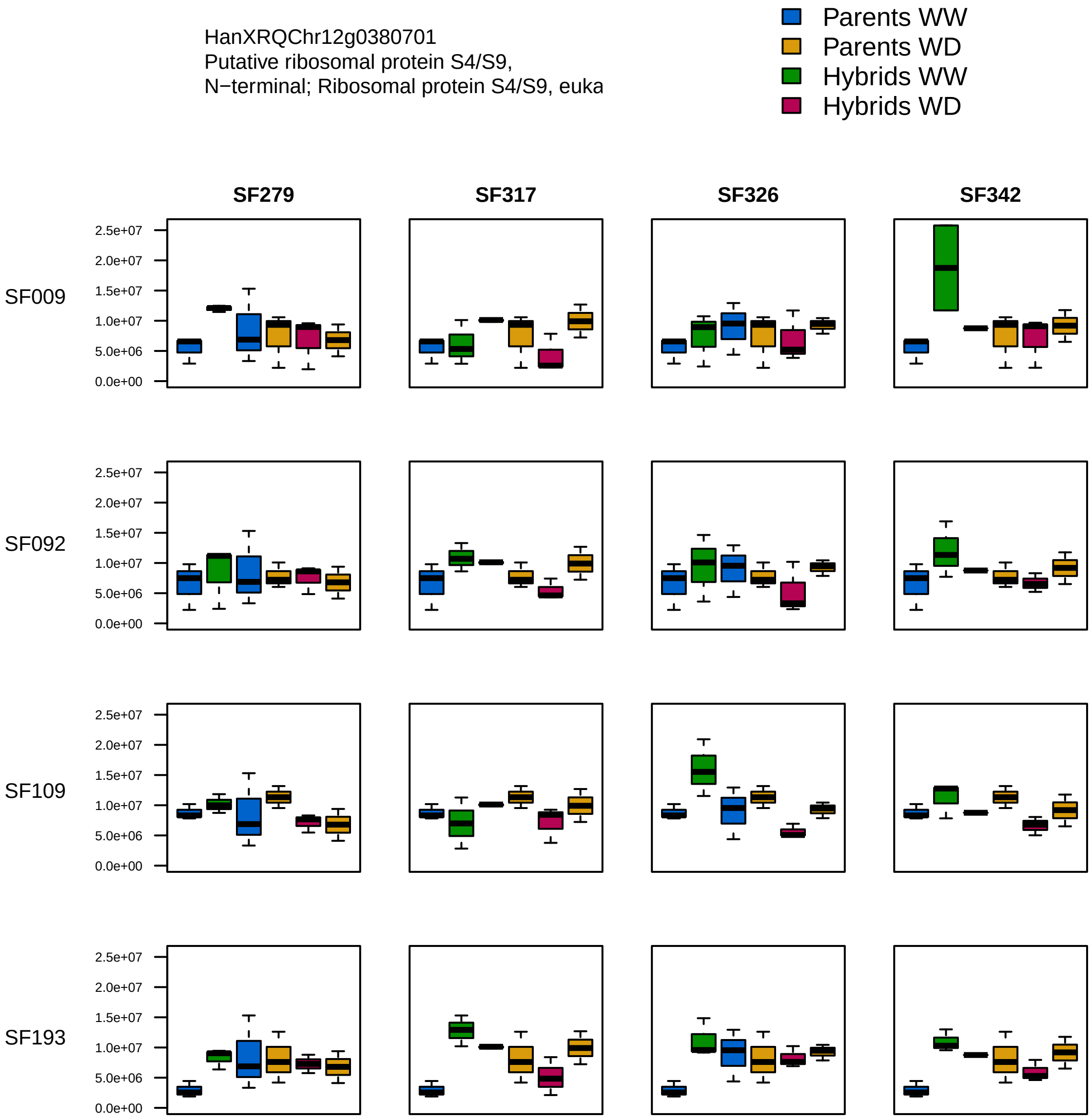
HanXRQChr12g0380701
Putative ribosomal protein S4/S9,
N−terminal; Ribosomal protein S4/S9, euka
Parents WW
Parents WD
Hybrids WW
Hybrids WD
SF279
SF317
SF326
SF342
SF009
SF092
SF109
SF193
2.5e+07
2.0e+07
1.5e+07
1.0e+07
5.0e+06
0.0e+00

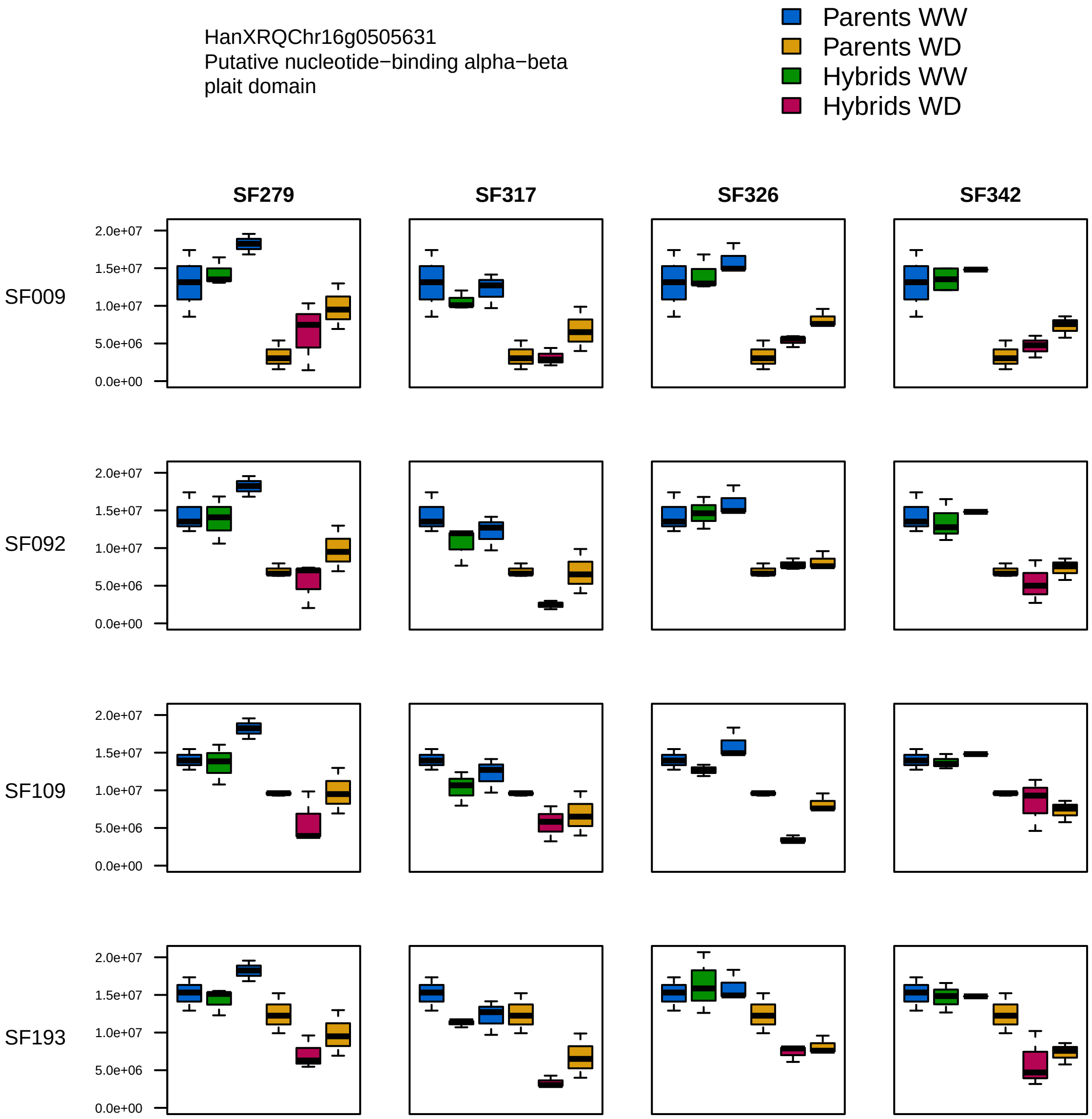

HanXRQChr16g0505631
Putative nucleotide−binding alpha−beta
plait domain
Parents WW
Parents WD
Hybrids WW
Hybrids WD
SF279
SF317
SF326
SF342
SF009
SF092
SF109
SF193
2.0e+07
1.5e+07
1.0e+07
5.0e+06
0.0e+00

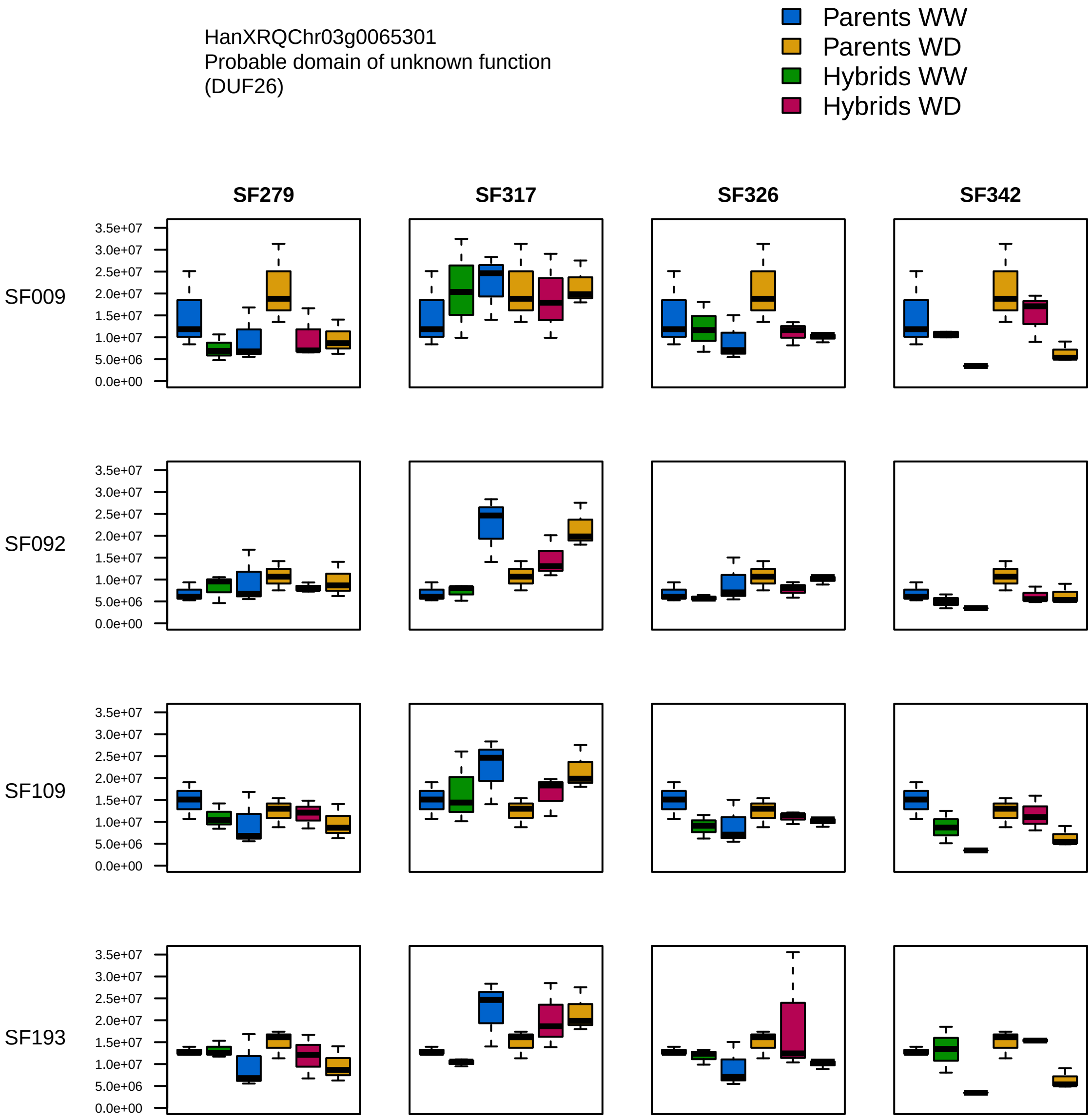

HanXRQChr03g0065301
Probable domain of unknown function
(DUF26)
Parents WW
Parents WD
Hybrids WW
Hybrids WD
SF279
SF317
SF326
SF342
SF009
SF092
SF109
SF193
3.5e+07
3.0e+07
2.5e+07
2.0e+07
1.5e+07
1.0e+07
5.0e+06
0.0e+00

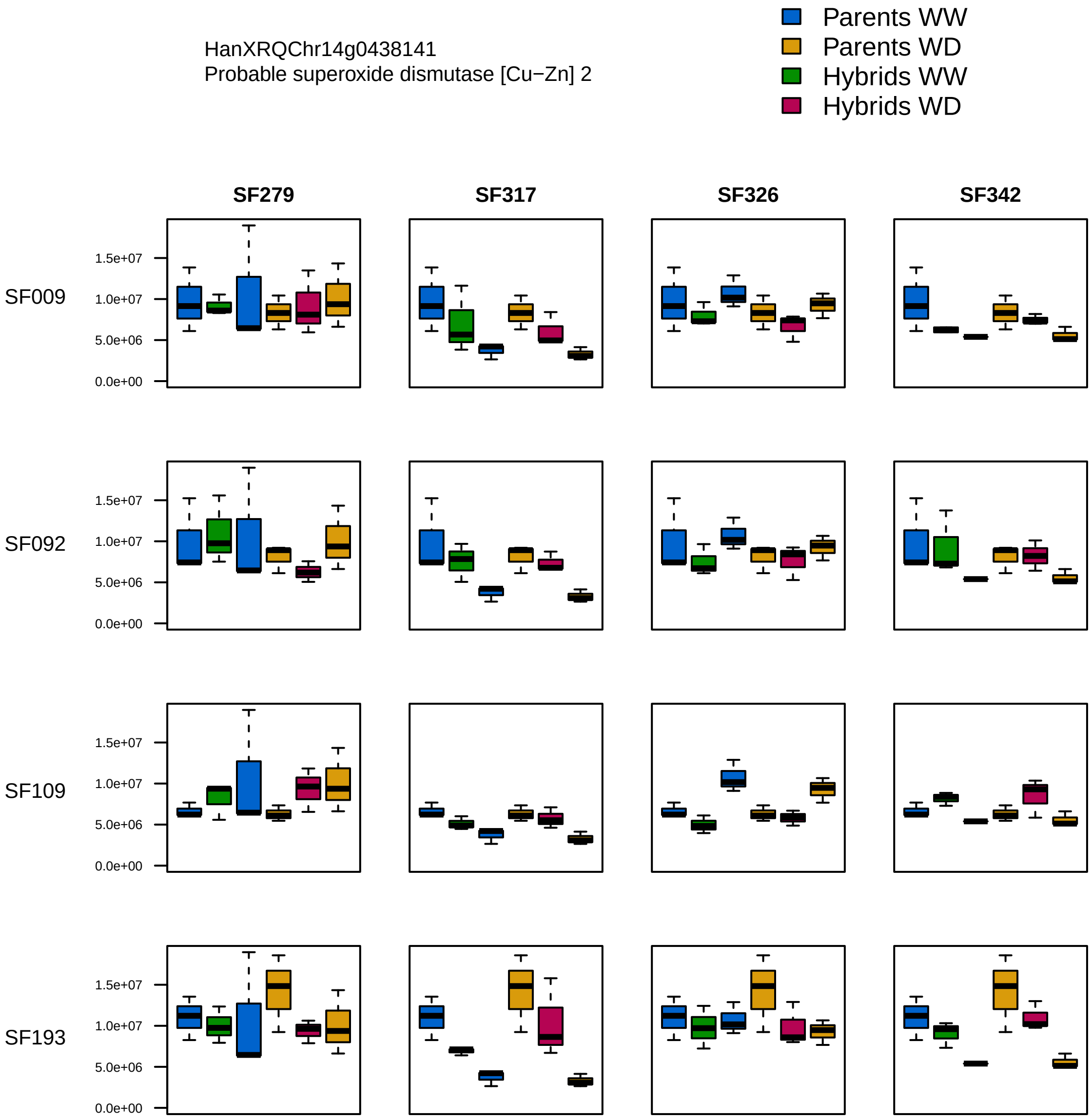
HanXRQChr14g0438141
Probable superoxide dismutase [Cu−Zn] 2
Parents WW
Parents WD
Hybrids WW
Hybrids WD
SF279
SF317
SF326
SF342
SF009
SF092
SF109
SF193
1.5e+07
1.0e+07
5.0e+06
0.0e+00

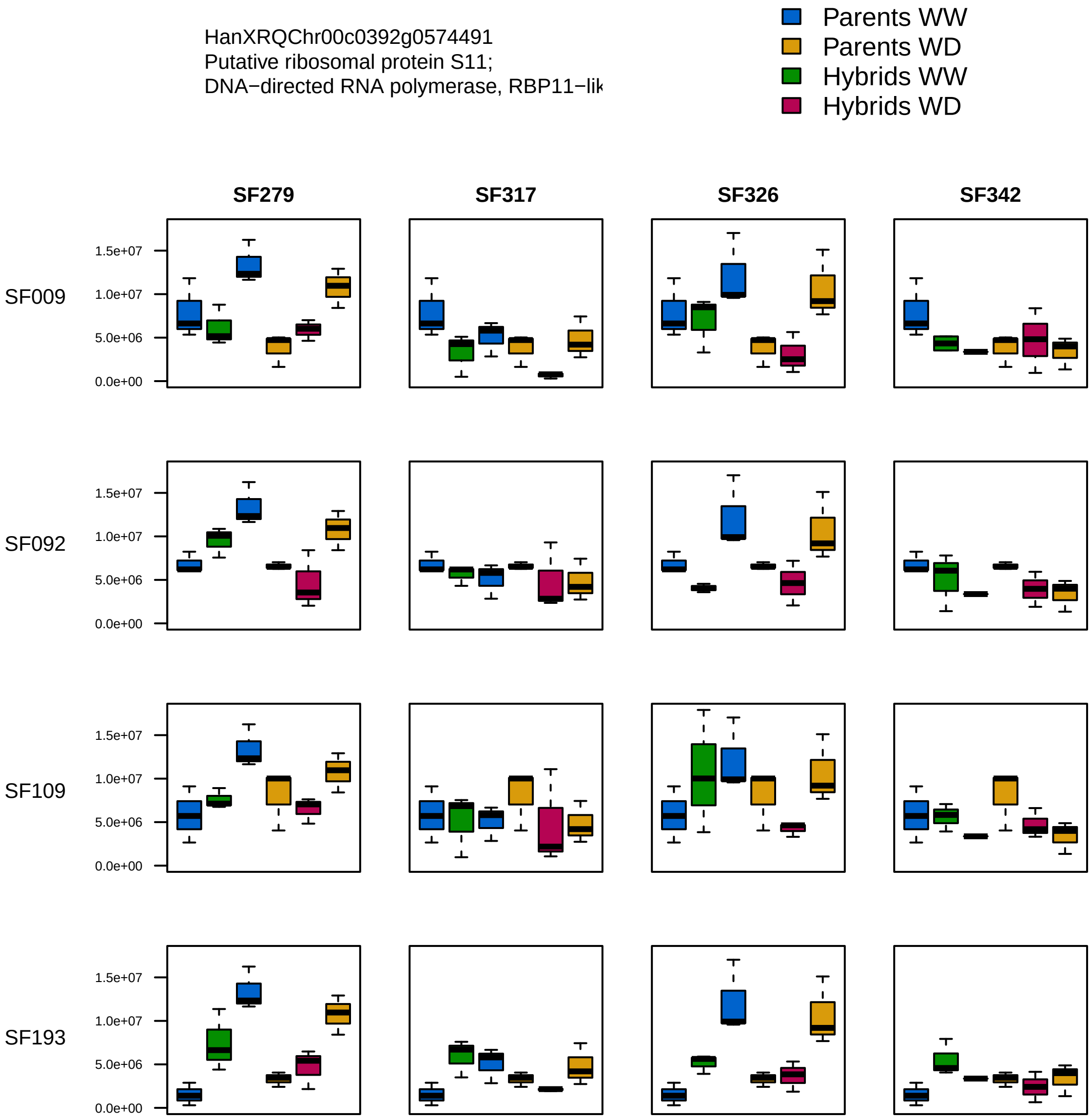
HanXRQChr00c0392g0574491
Putative ribosomal protein S11;
DNA−directed RNA polymerase, RBP11−lik
Parents WW
Parents WD
Hybrids WW
Hybrids WD
SF279
SF317
SF326
SF342
SF009
SF092
SF109
SF193
1.5e+07
1.0e+07
5.0e+06
0.0e+00

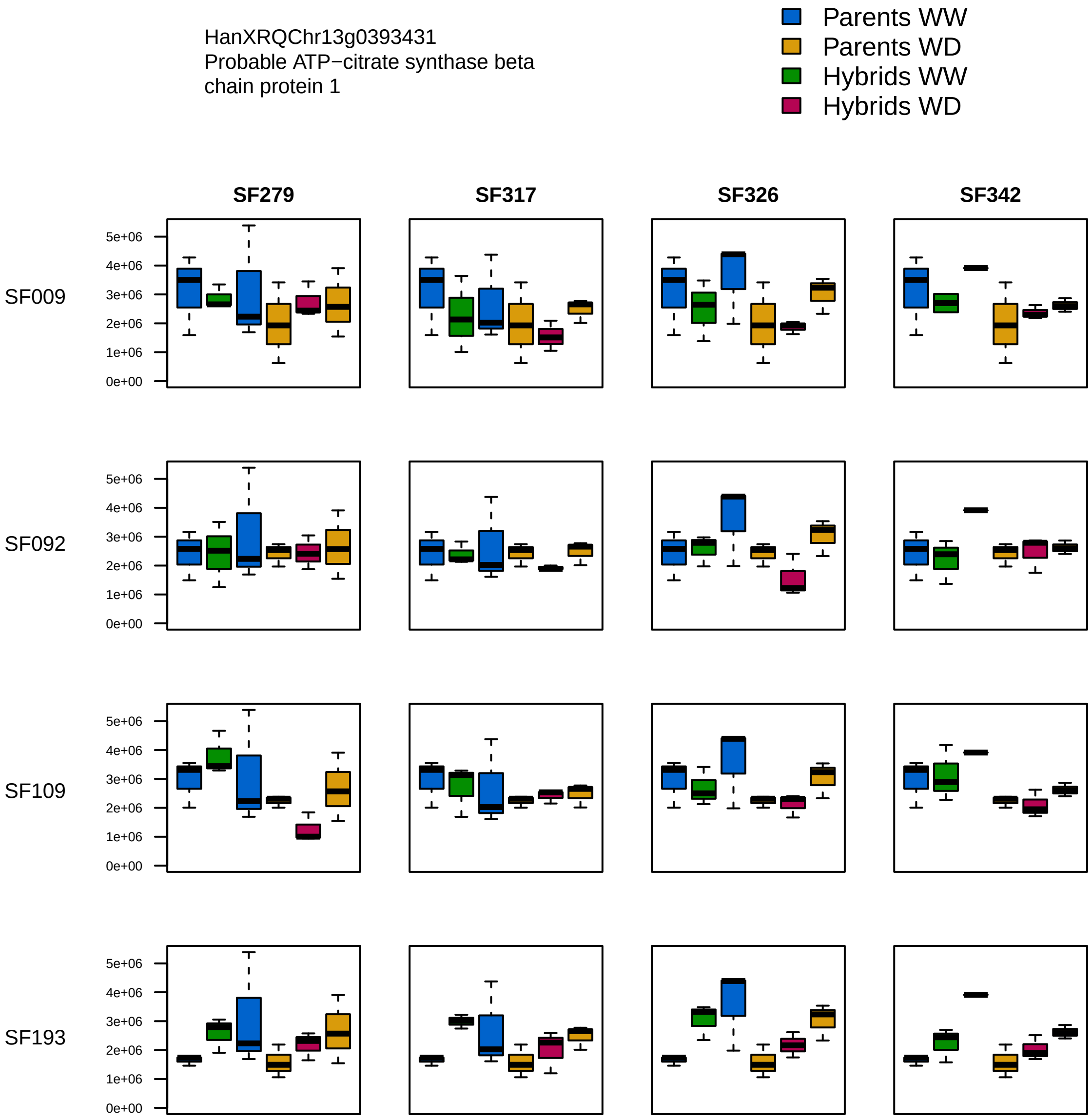
HanXRQChr13g0393431
Probable ATP−citrate synthase beta
chain protein 1
Parents WW
Parents WD
Hybrids WW
Hybrids WD
SF279
SF317
SF326
SF342
SF009
SF092
SF109
SF193
5e+06
4e+06
3e+06
2e+06
1e+06
0e+00

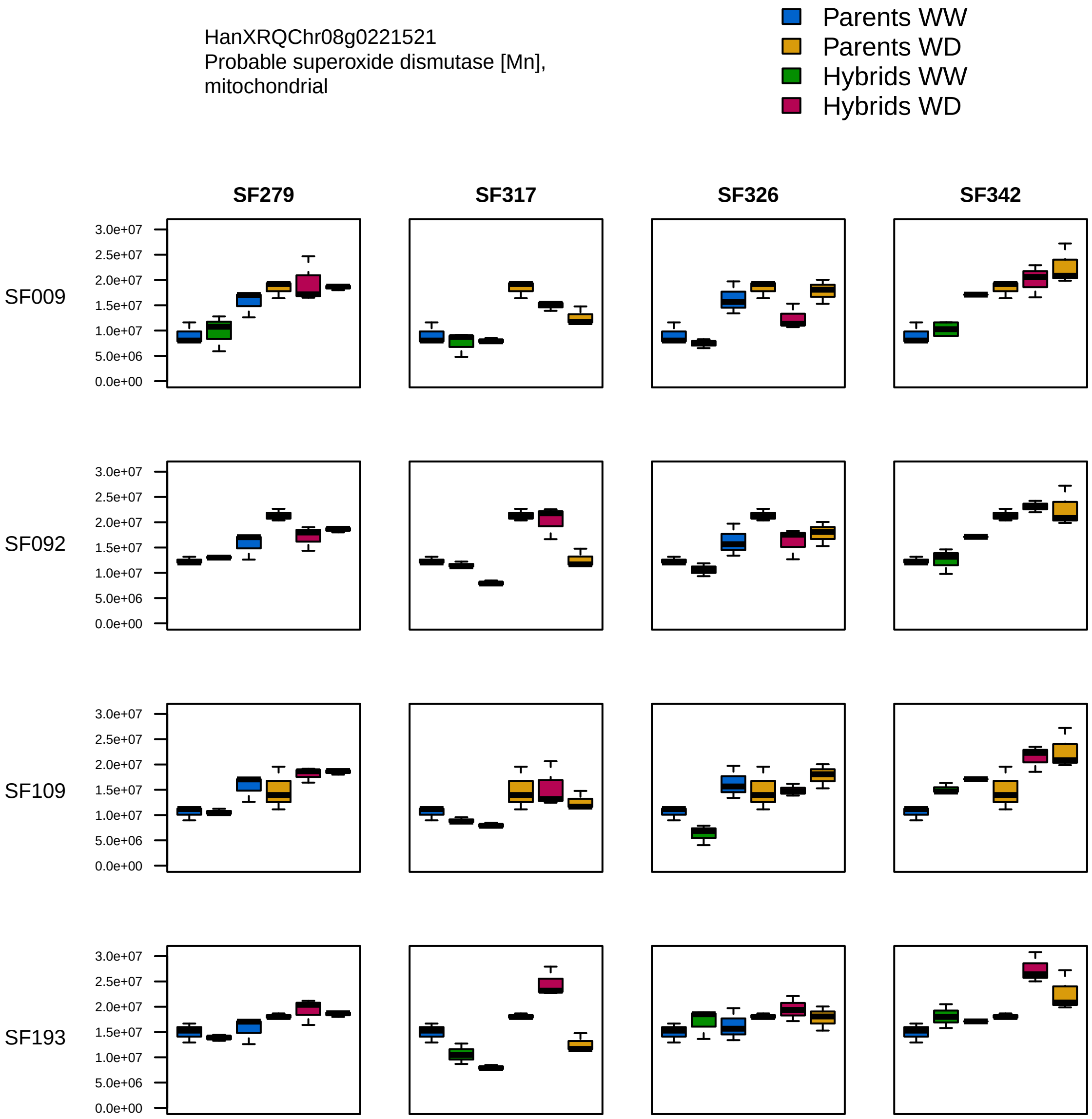
HanXRQChr08g0221521
Probable superoxide dismutase [Mn], mitochondrial
Parents WW
Parents WD
Hybrids WW
Hybrids WD
SF279
SF317
SF326
SF342
SF009
SF092
SF109
SF193
3.0e+07
2.5e+07
2.0e+07
1.5e+07
1.0e+07
5.0e+06
0.0e+00

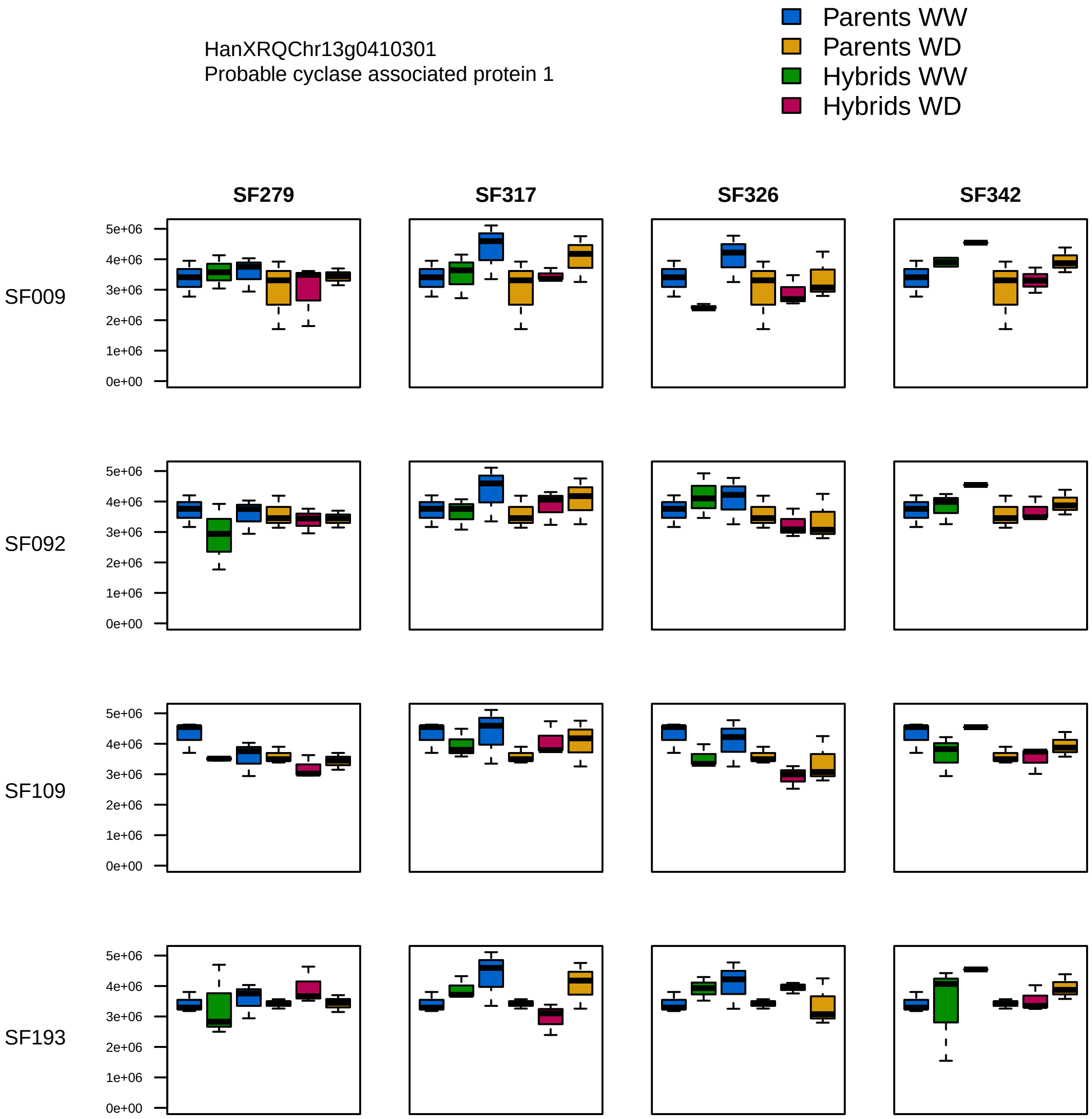
HanXRQChr13g0410301
Probable cyclase associated protein 1
Parents WW
Parents WD
Hybrids WW
Hybrids WD
SF279
SF317
SF326
SF342
SF009
SF092
SF109
SF193
5e+06
4e+06
3e+06
2e+06
1e+06
0e+00

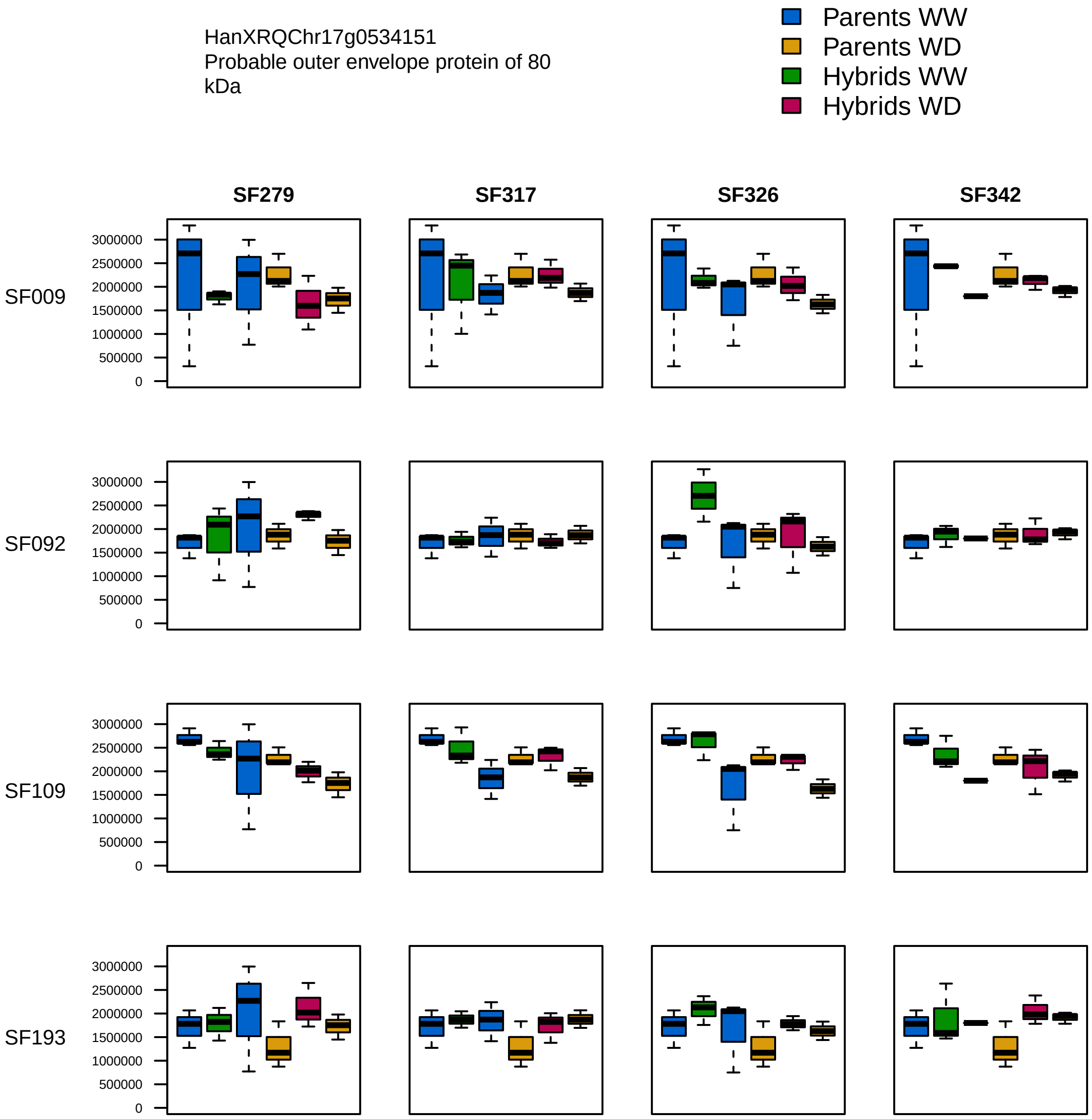
HanXRQChr17g0534151
Probable outer envelope protein of 80 kDa
Parents WW
Parents WD
Hybrids WW
Hybrids WD
SF279
SF317
SF326
SF342
SF009
SF092
SF109
SF193
3000000
2500000
2000000
1500000
1000000
500000
0

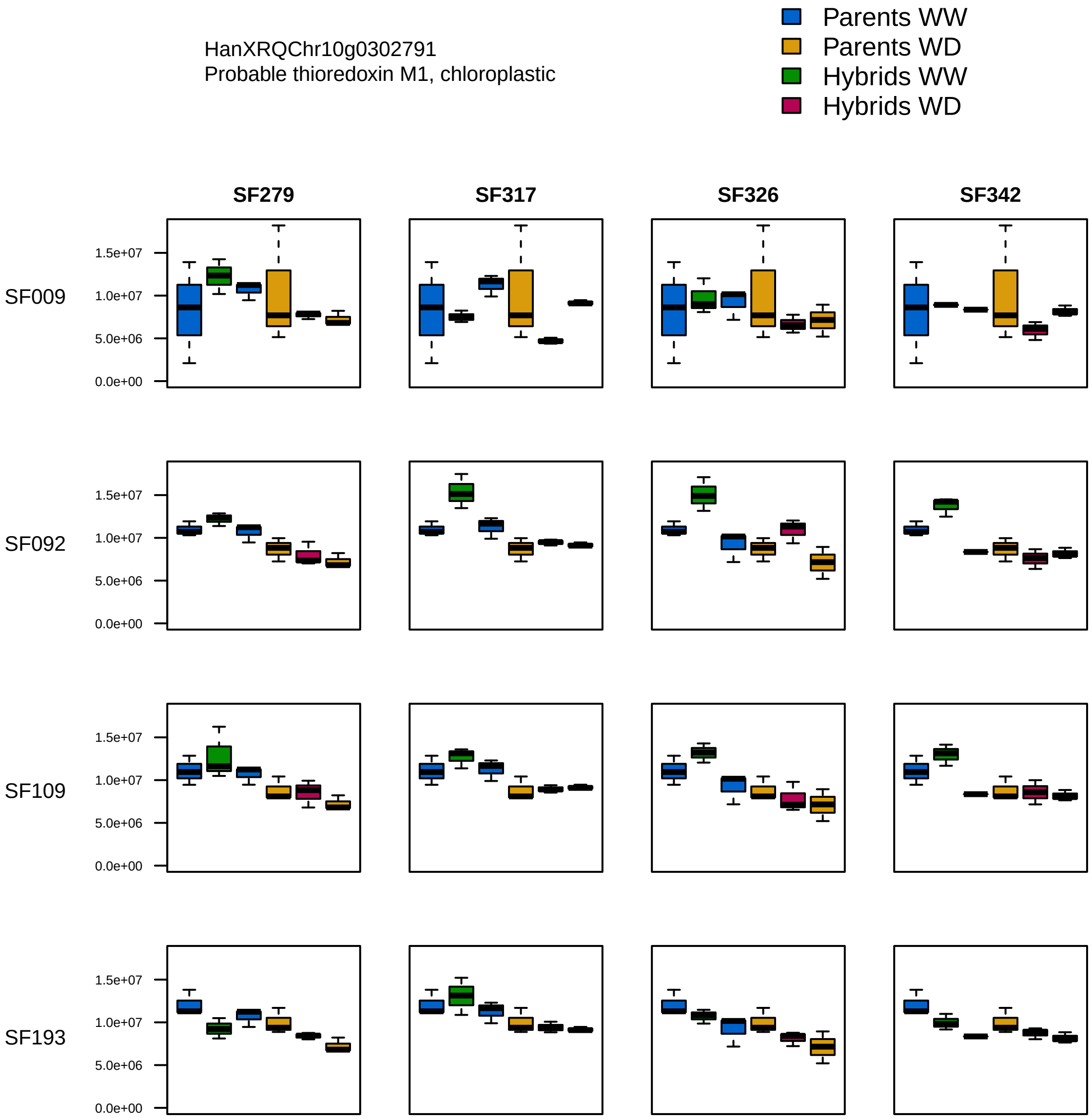
HanXRQChr10g0302791
Probable thioredoxin M1, chloroplastic
Parents WW
Parents WD
Hybrids WW
Hybrids WD
SF279
SF317
SF326
SF342
SF009
SF092
SF109
SF193
1.5e+07
1.0e+07
5.0e+06
0.0e+00

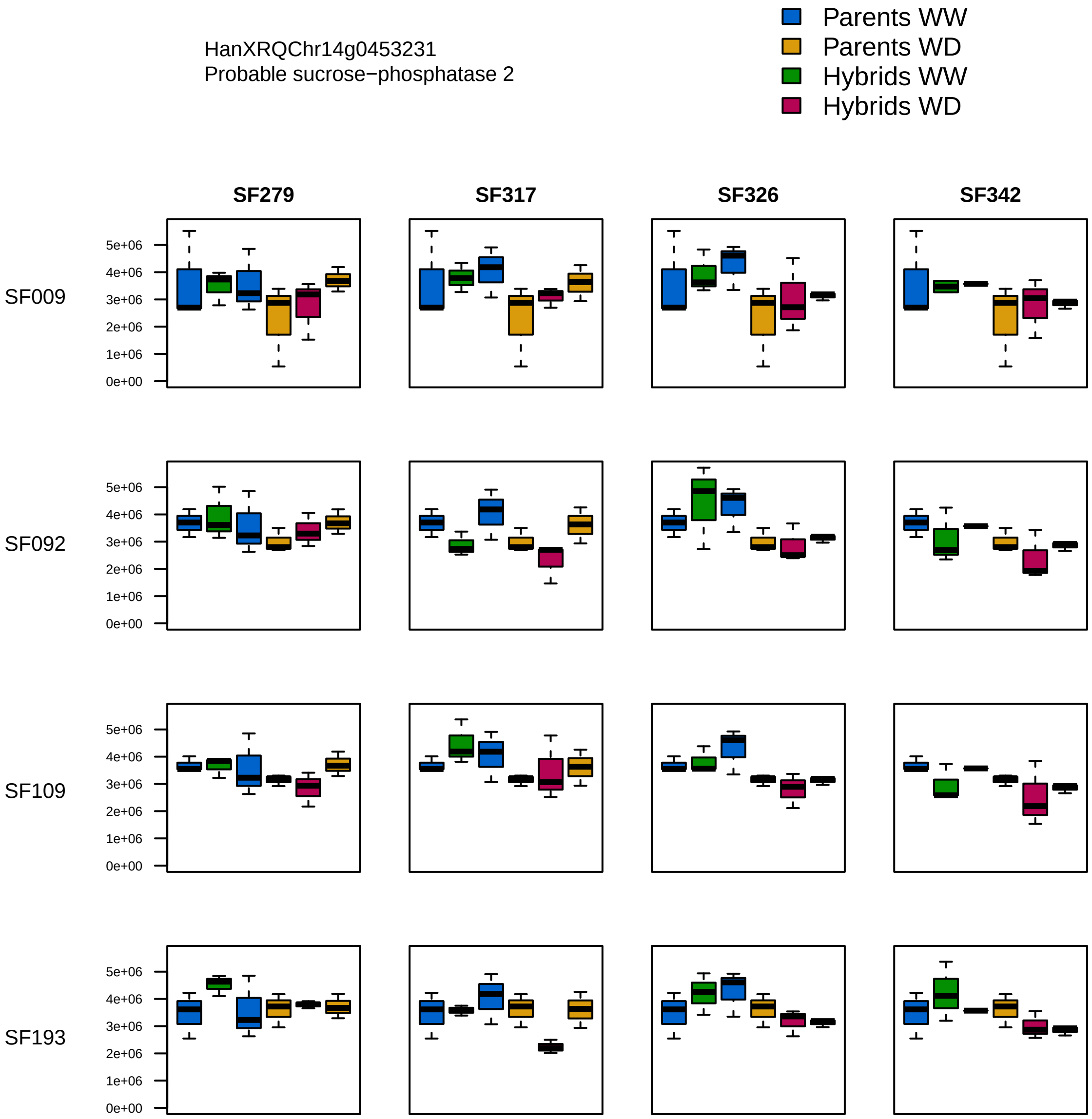
HanXRQChr14g0453231
Probable sucrose−phosphatase 2
Parents WW
Parents WD
Hybrids WW
Hybrids WD
SF279
SF317
SF326
SF342
SF009
SF092
SF109
SF193
5e+06
4e+06
3e+06
2e+06
1e+06
0e+00

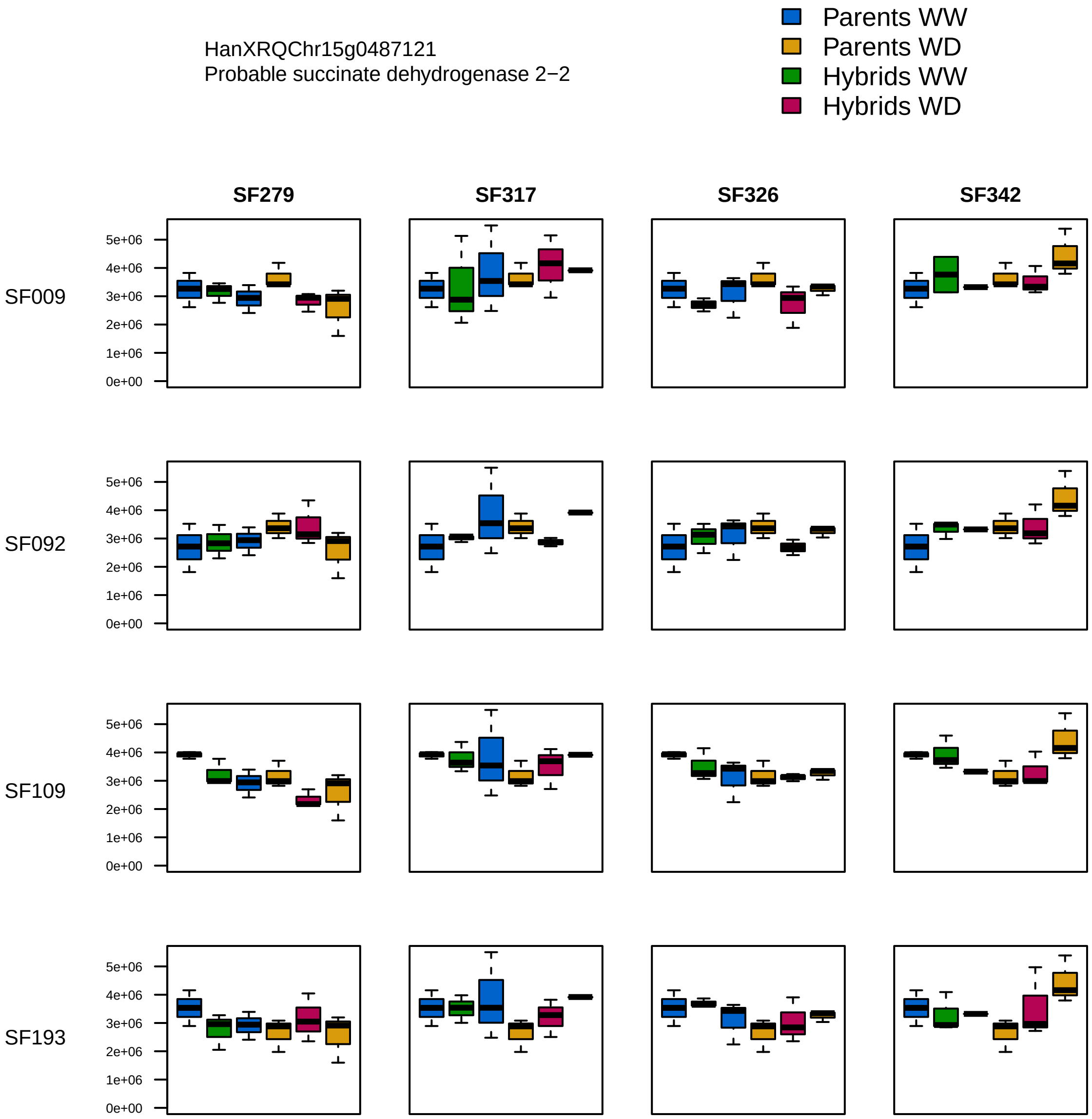
HanXRQChr15g0487121
Probable succinate dehydrogenase 2−2
Parents WW
Parents WD
Hybrids WW
Hybrids WD
SF279
SF317
SF326
SF342
SF009
SF092
SF109
SF193
0e+00
1e+06
2e+06
3e+06
4e+06
5e+06

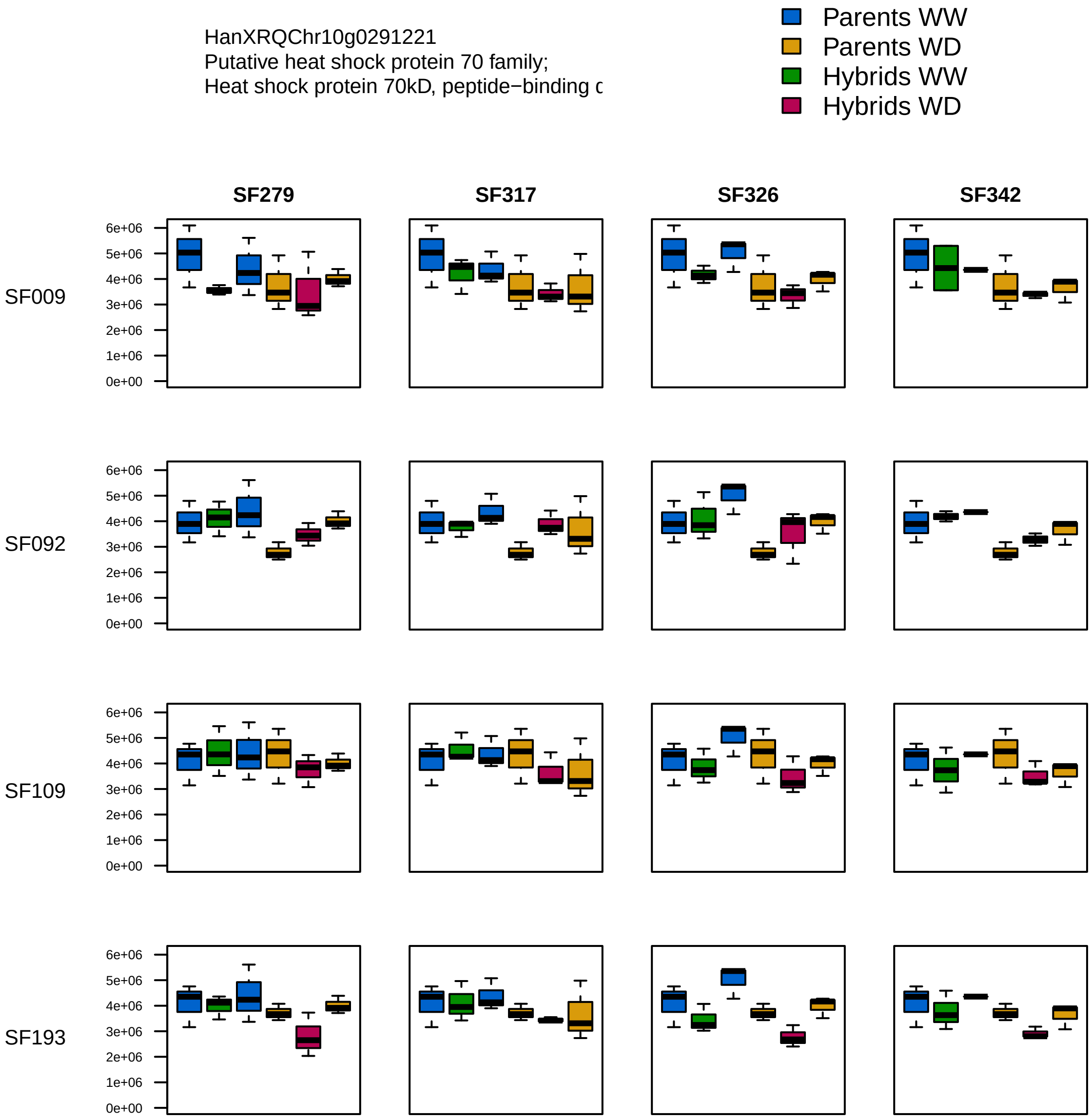

HanXRQChr10g0291221
Putative heat shock protein 70 family;
Heat shock protein 70kD, peptide−binding c
Parents WW
Parents WD
Hybrids WW
Hybrids WD
SF279
SF317
SF326
SF342
SF009
SF092
SF109
SF193
6e+06
5e+06
4e+06
3e+06
2e+06
1e+06
0e+00

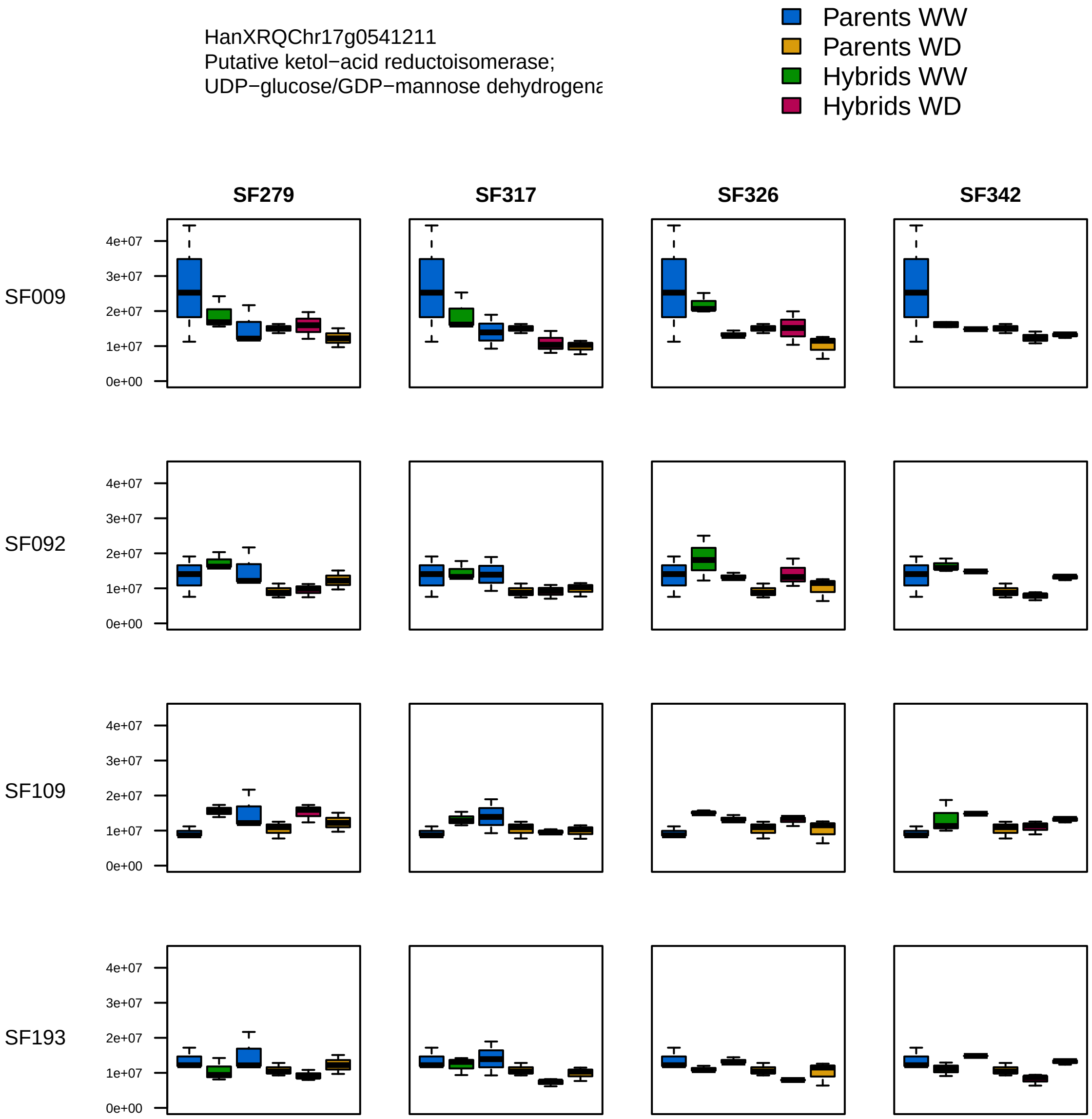
HanXRQChr17g0541211
Putative ketol−acid reductoisomerase;
UDP−glucose/GDP−mannose dehydrogena
Parents WW
Parents WD
Hybrids WW
Hybrids WD
SF279
SF317
SF326
SF342
SF009
SF092
SF109
SF193
4e+07
3e+07
2e+07
1e+07
0e+00

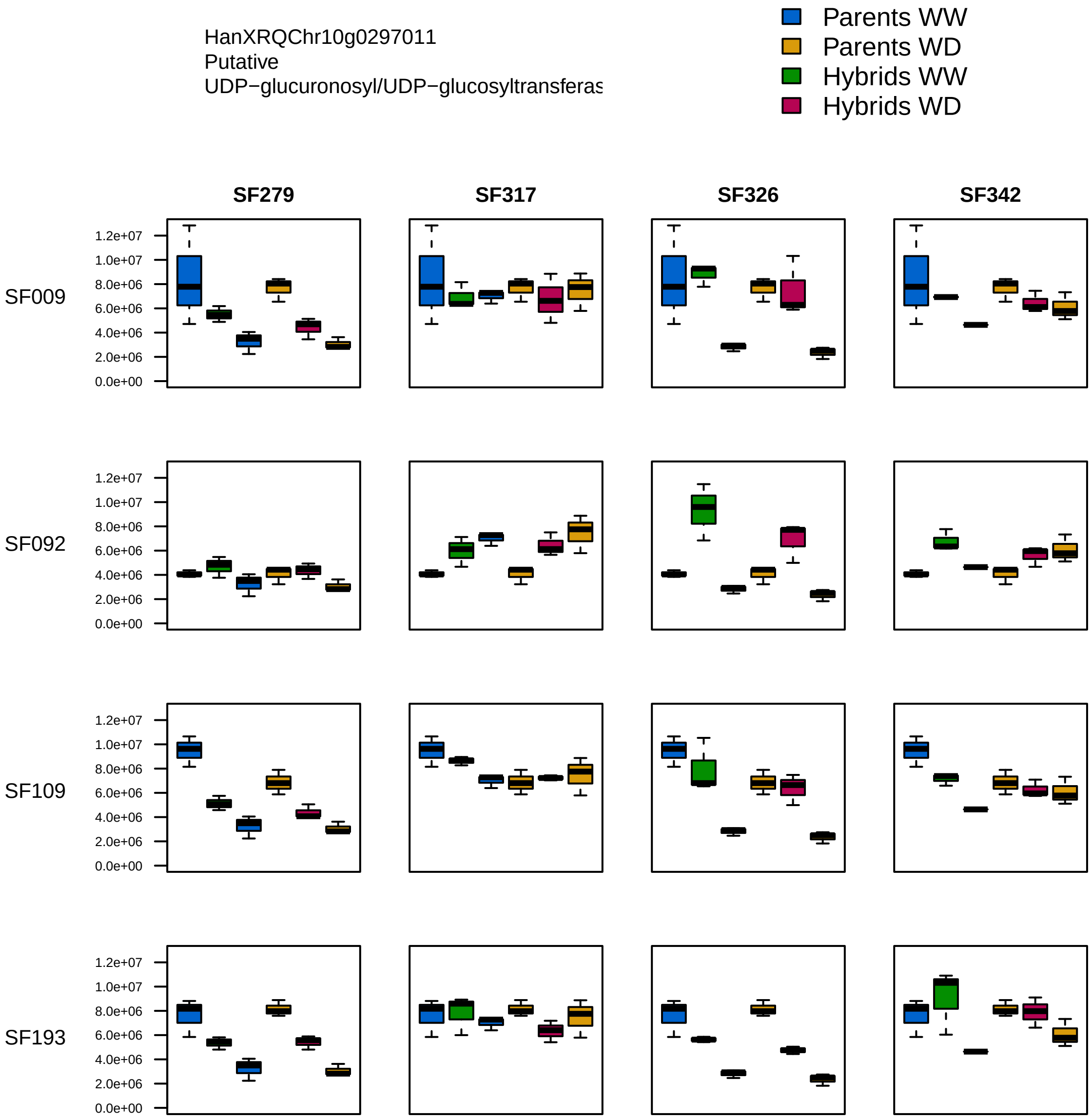
HanXRQChr10g0297011
Putative
UDP−glucuronosyl/UDP−glucosyltransferas
Parents WW
Parents WD
Hybrids WW
Hybrids WD
SF279
SF317
SF326
SF342
SF009
SF092
SF109
SF193
0.0e+00
2.0e+06
4.0e+06
6.0e+06
8.0e+06
1.0e+07
1.2e+07

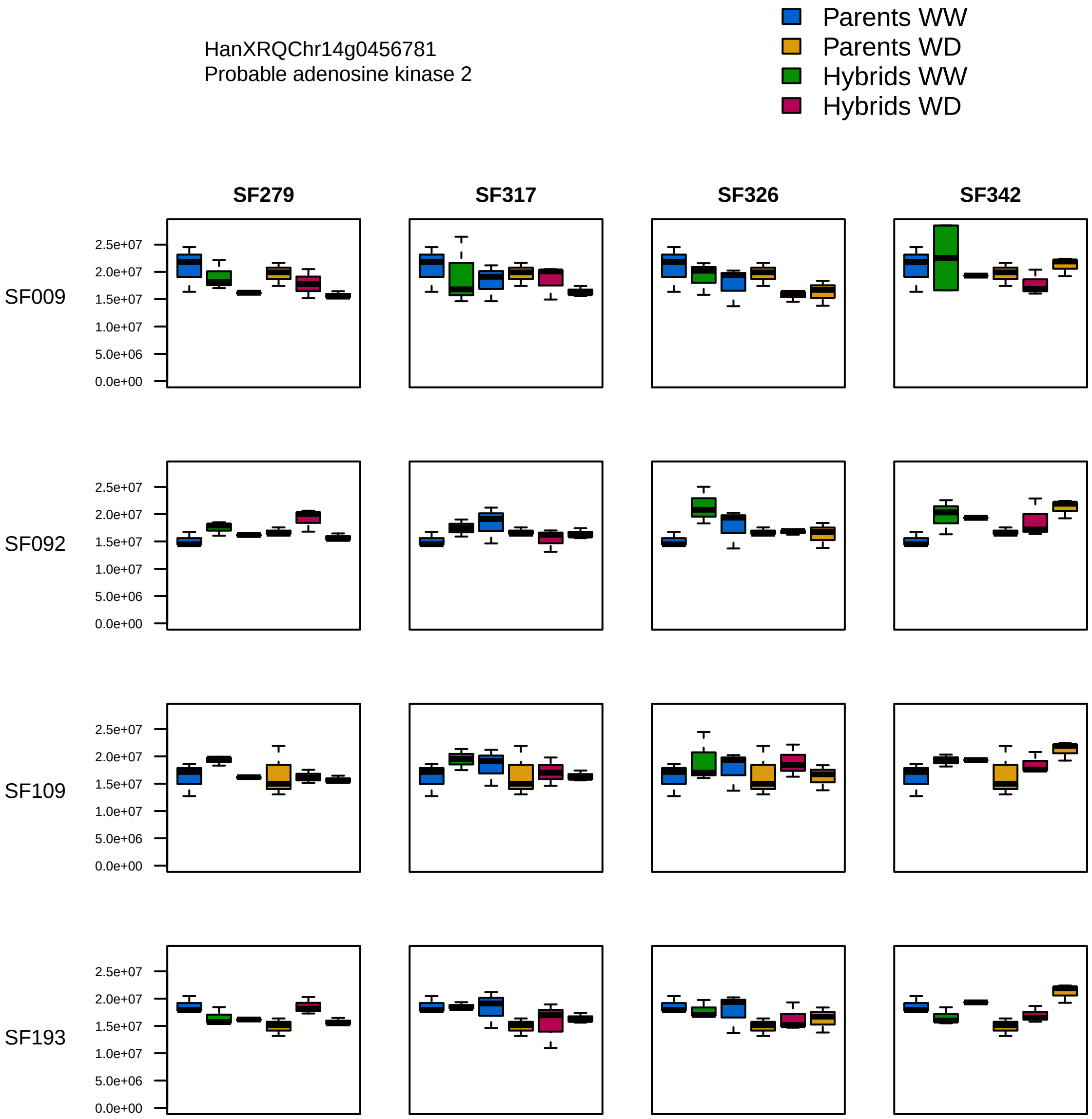
HanXRQChr14g0456781
Probable adenosine kinase 2
Parents WW
Parents WD
Hybrids WW
Hybrids WD
SF279
SF317
SF326
SF342
SF009
SF092
SF109
SF193
2.5e+07
2.0e+07
1.5e+07
1.0e+07
5.0e+06
0.0e+00

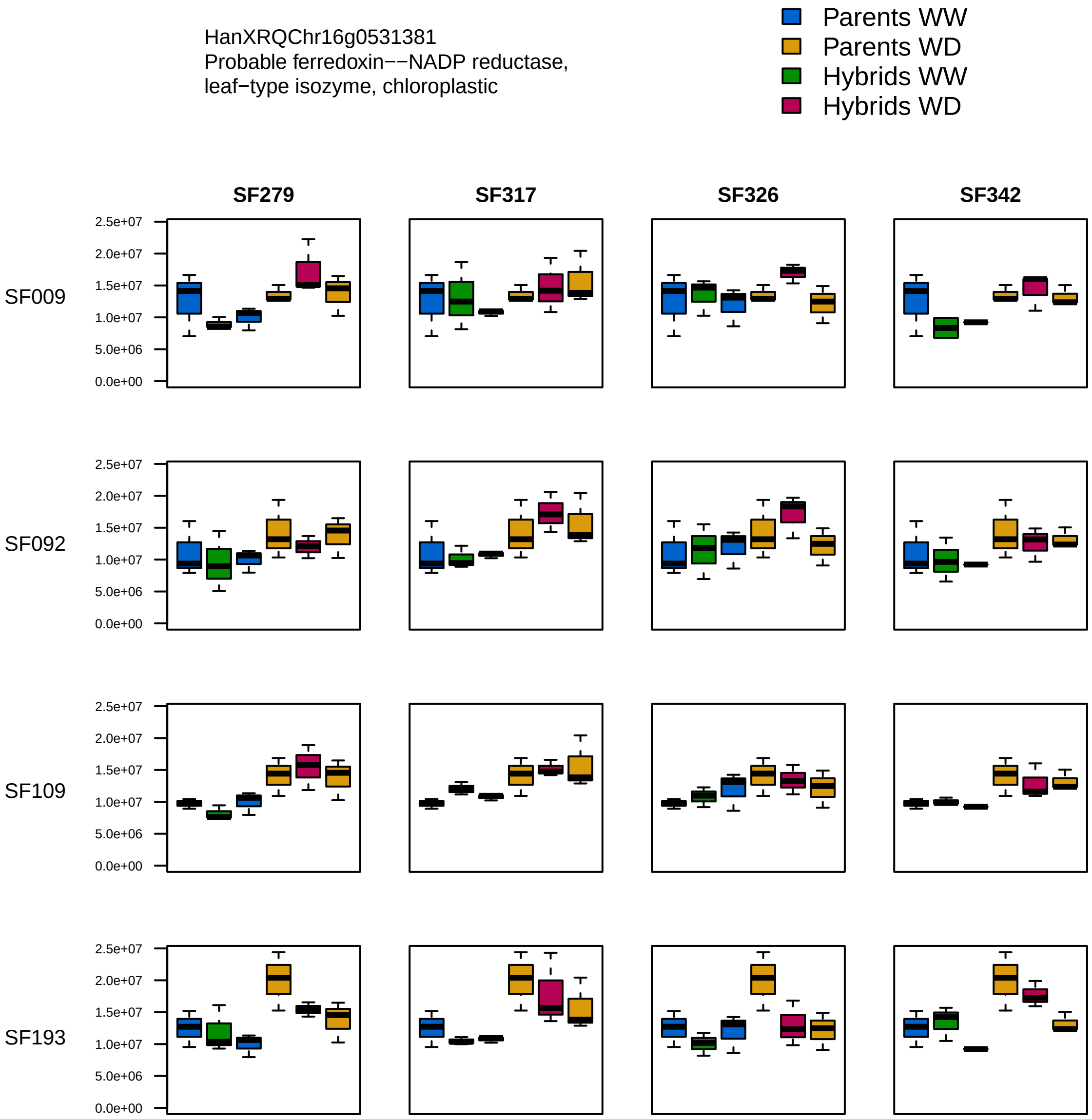
HanXRQChr16g0531381
Probable ferredoxin−−NADP reductase,
leaf−type isozyme, chloroplastic
Parents WW
Parents WD
Hybrids WW
Hybrids WD
SF279
SF317
SF326
SF342
SF009
SF092
SF109
SF193
2.5e+07
2.0e+07
1.5e+07
1.0e+07
5.0e+06
0.0e+00

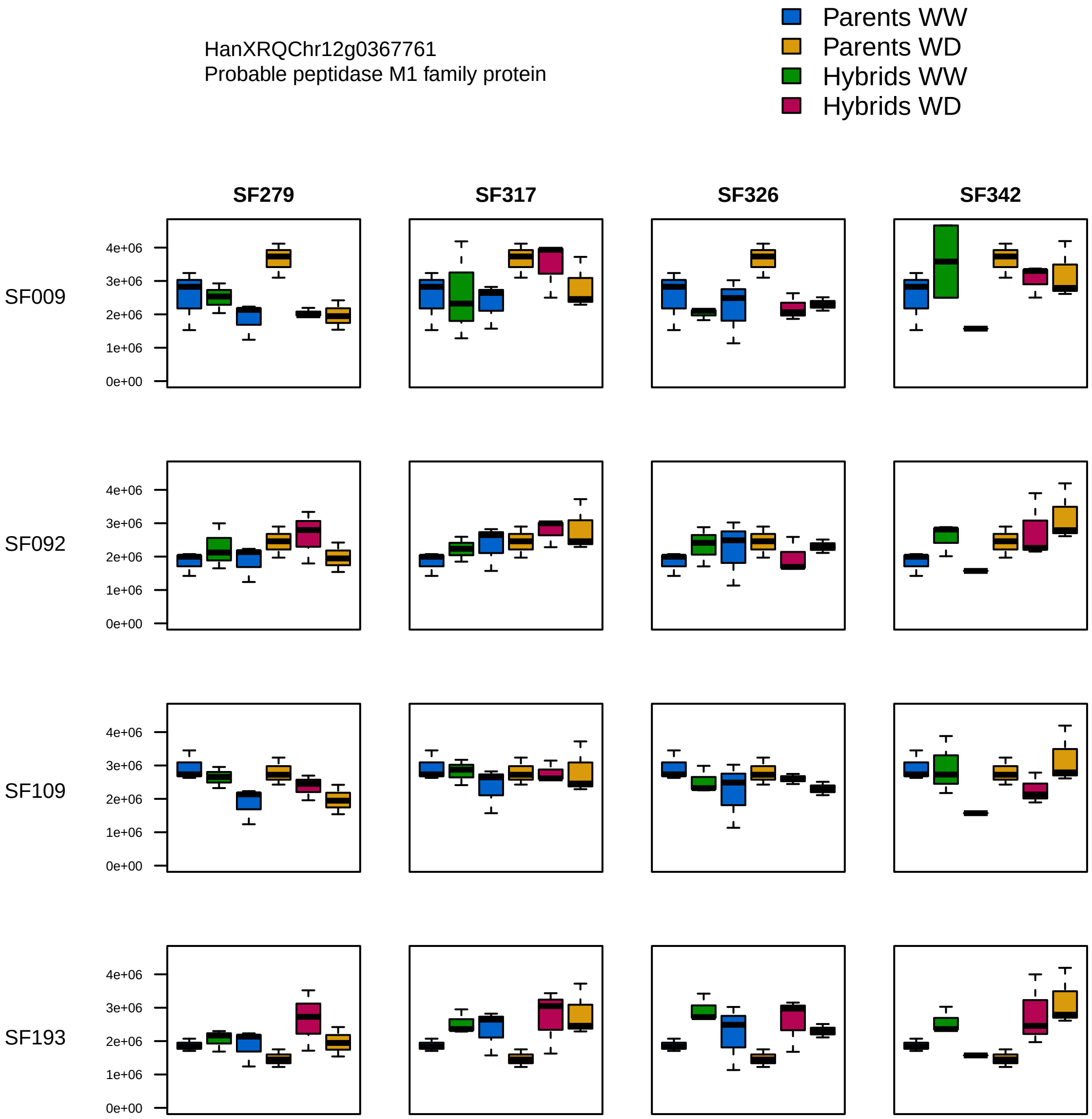

HanXRQChr12g0367761
Probable peptidase M1 family protein
Parents WW
Parents WD
Hybrids WW
Hybrids WD
SF279
SF317
SF326
SF342
SF009
SF092
SF109
SF193
0e+00
1e+06
2e+06
3e+06
4e+06

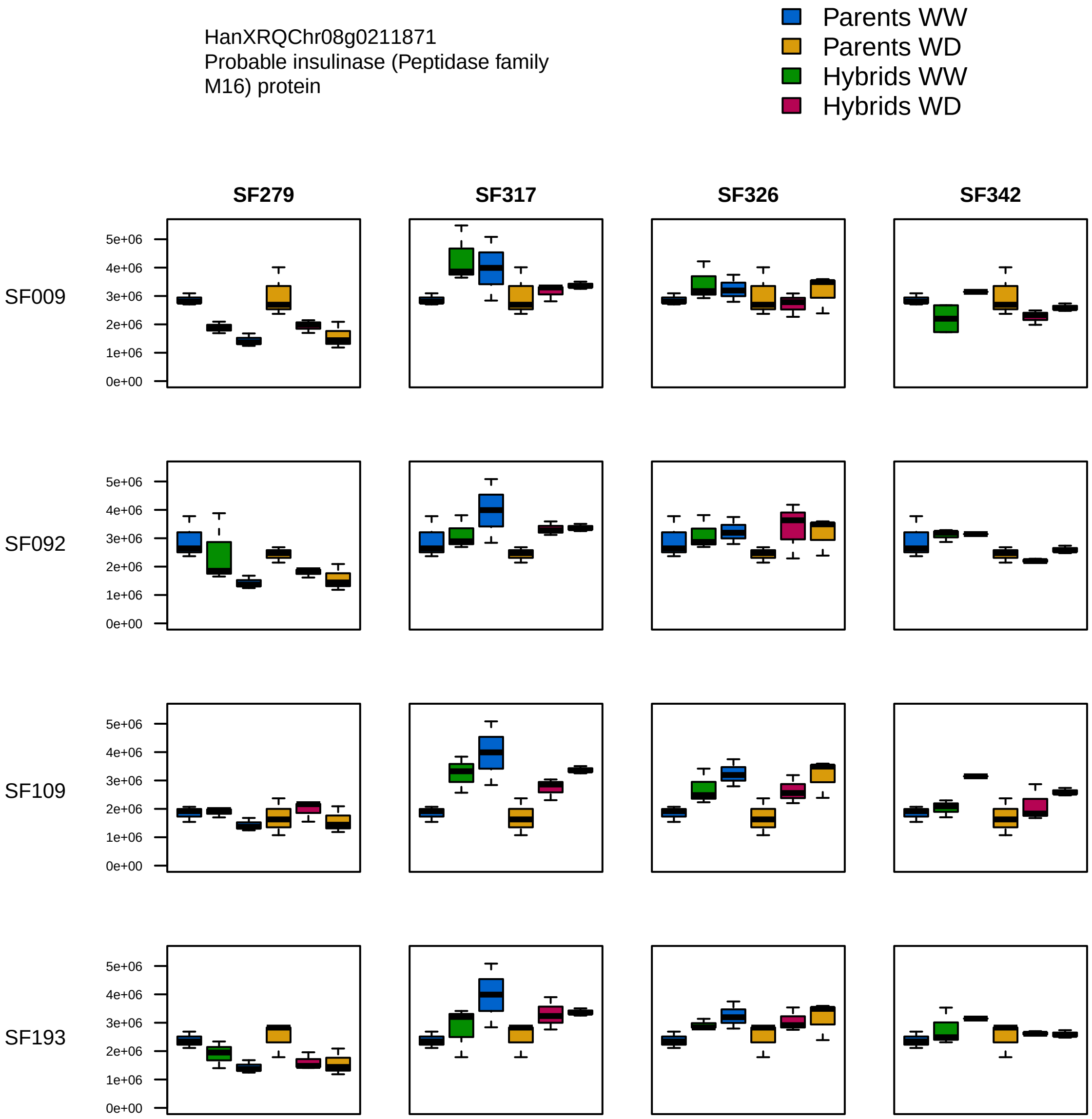
HanXRQChr08g0211871
Probable insulinase (Peptidase family M16) protein
Parents WW
Parents WD
Hybrids WW
Hybrids WD
SF279
SF317
SF326
SF342
SF009
SF092
SF109
SF193
0e+00
1e+06
2e+06
3e+06
4e+06
5e+06

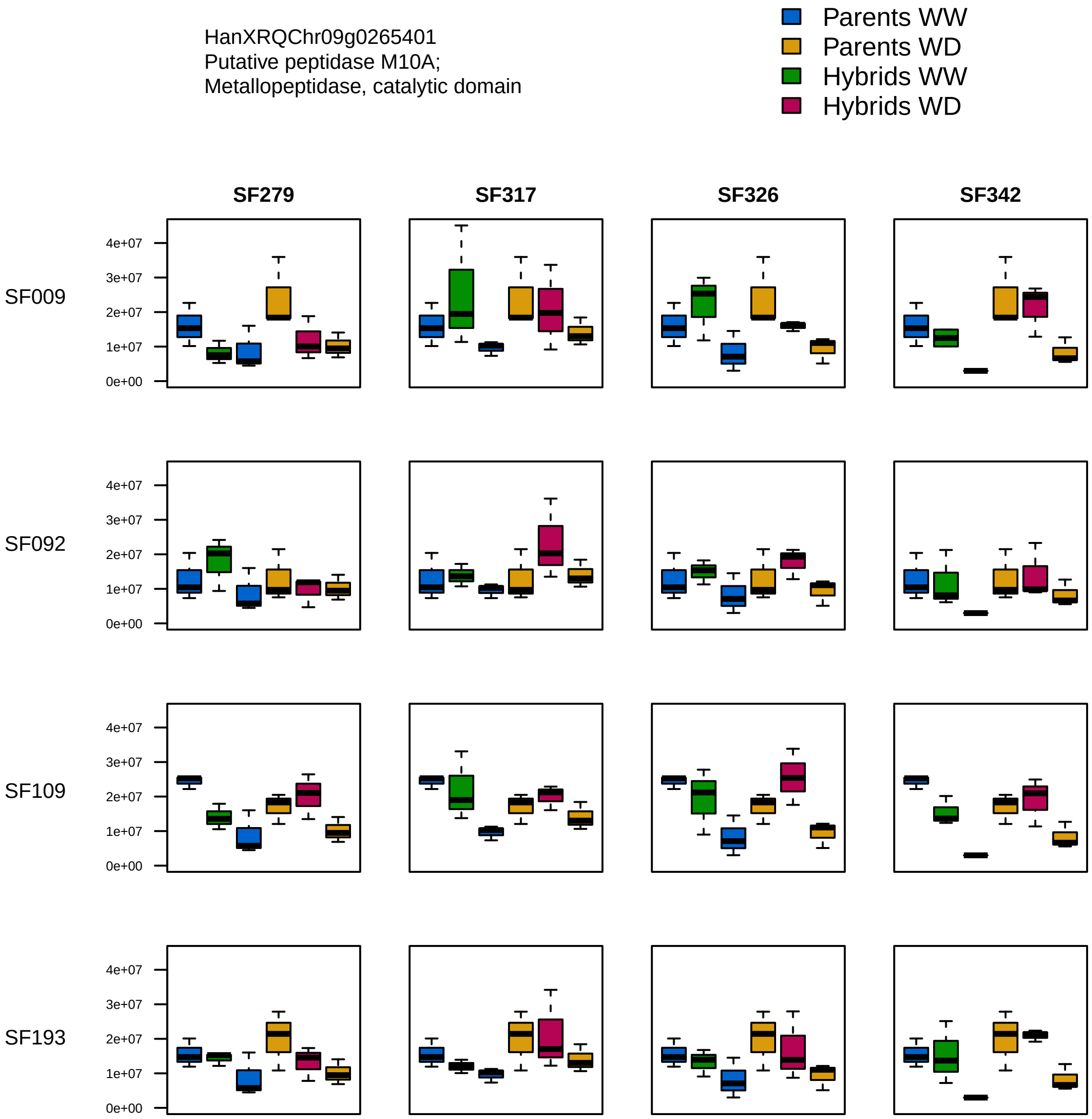

HanXRQChr09g0265401
Putative peptidase M10A;
Metallopeptidase, catalytic domain
Parents WW
Parents WD
Hybrids WW
Hybrids WD
SF279
SF317
SF326
SF342
SF009
SF092
SF109
SF193
0e+00
1e+07
2e+07
3e+07
4e+07

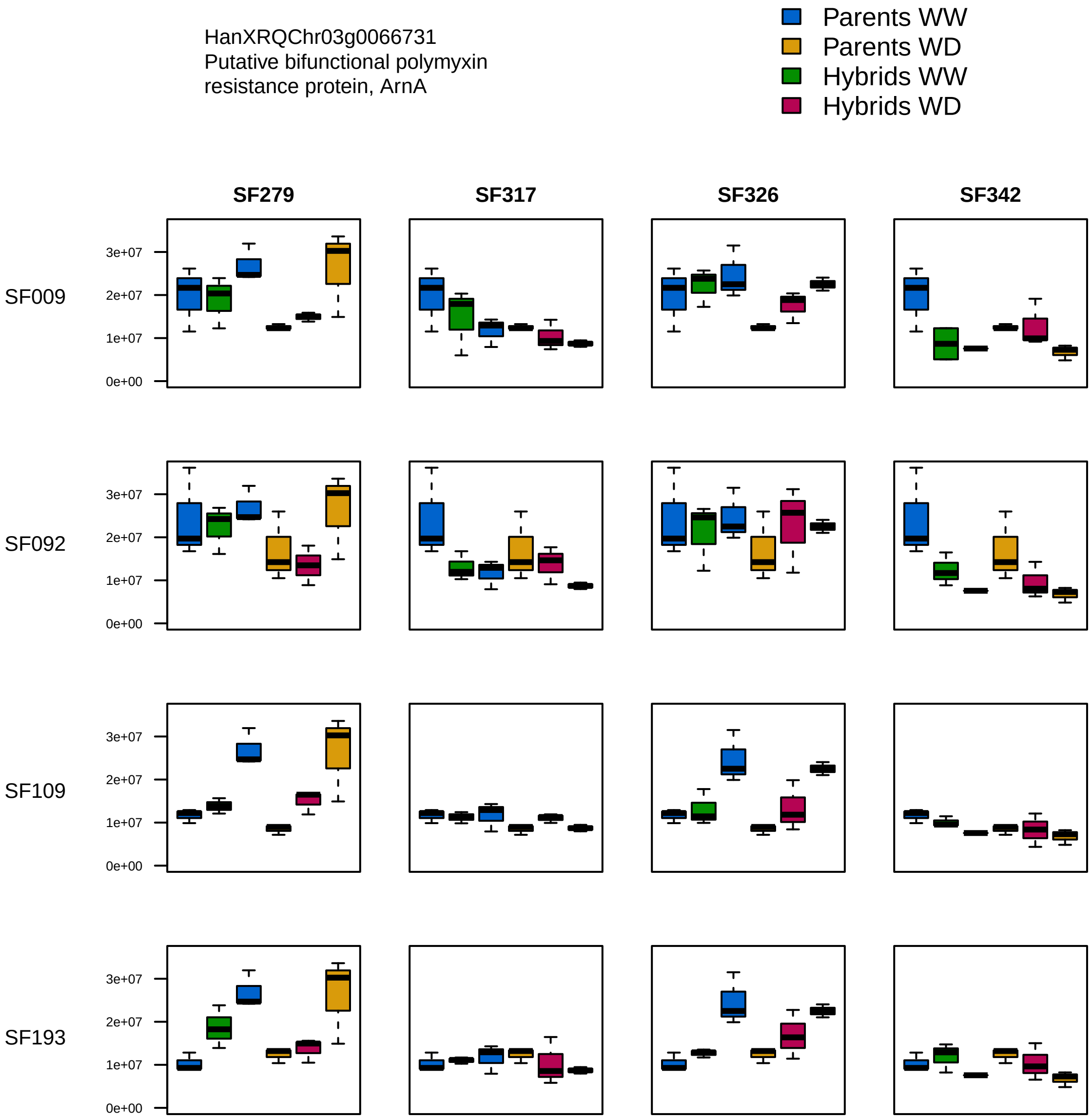

HanXRQChr03g0066731
Putative bifunctional polymyxin
resistance protein, ArnA
Parents WW
Parents WD
Hybrids WW
Hybrids WD
SF279
SF317
SF326
SF342
SF009
SF092
SF109
SF193
3e+07
2e+07
1e+07
0e+00

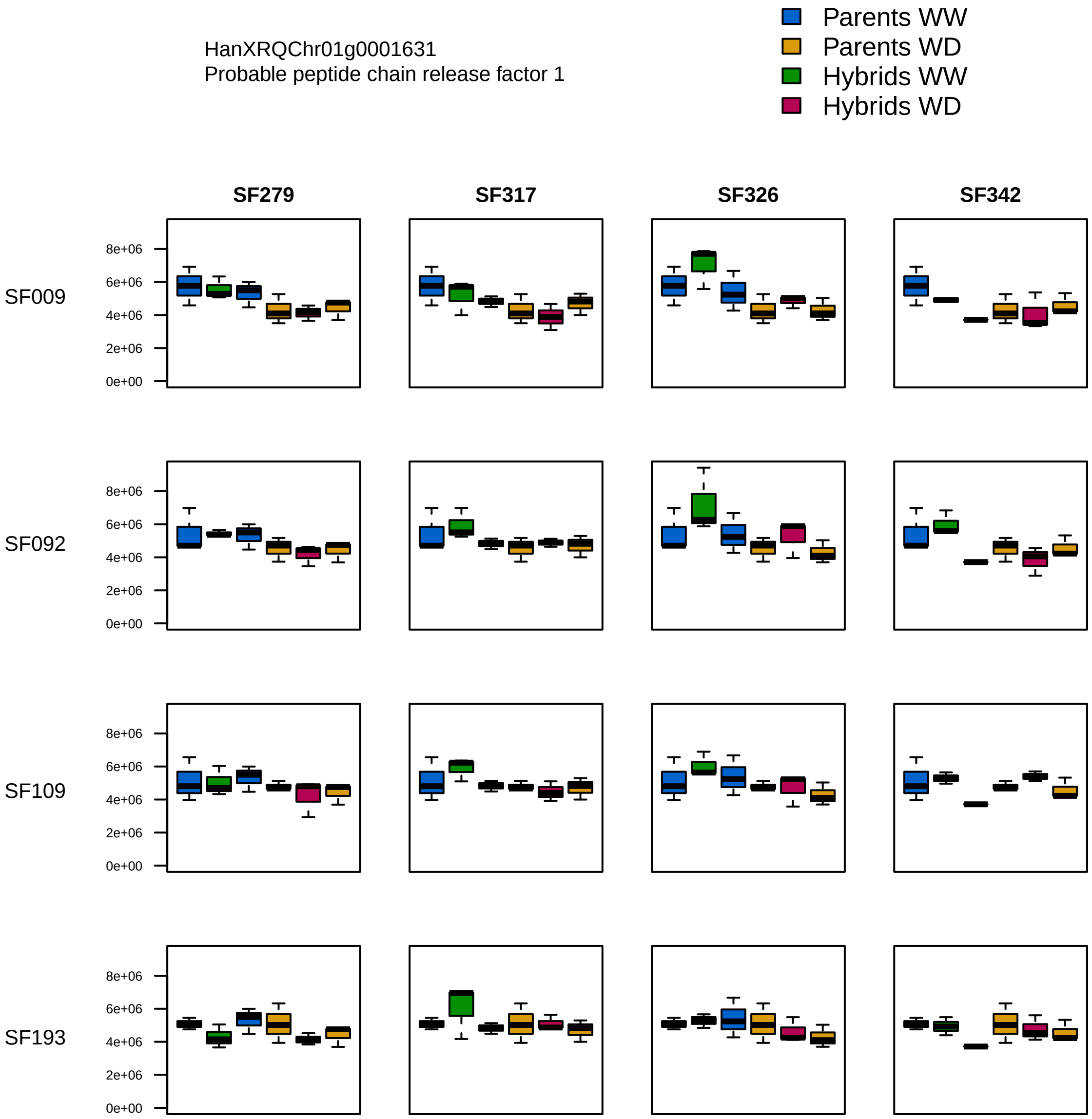
HanXRQChr01g0001631
Probable peptide chain release factor 1
Parents WW
Parents WD
Hybrids WW
Hybrids WD
SF279
SF317
SF326
SF342
SF009
SF092
SF109
SF193
8e+06
6e+06
4e+06
2e+06
0e+00

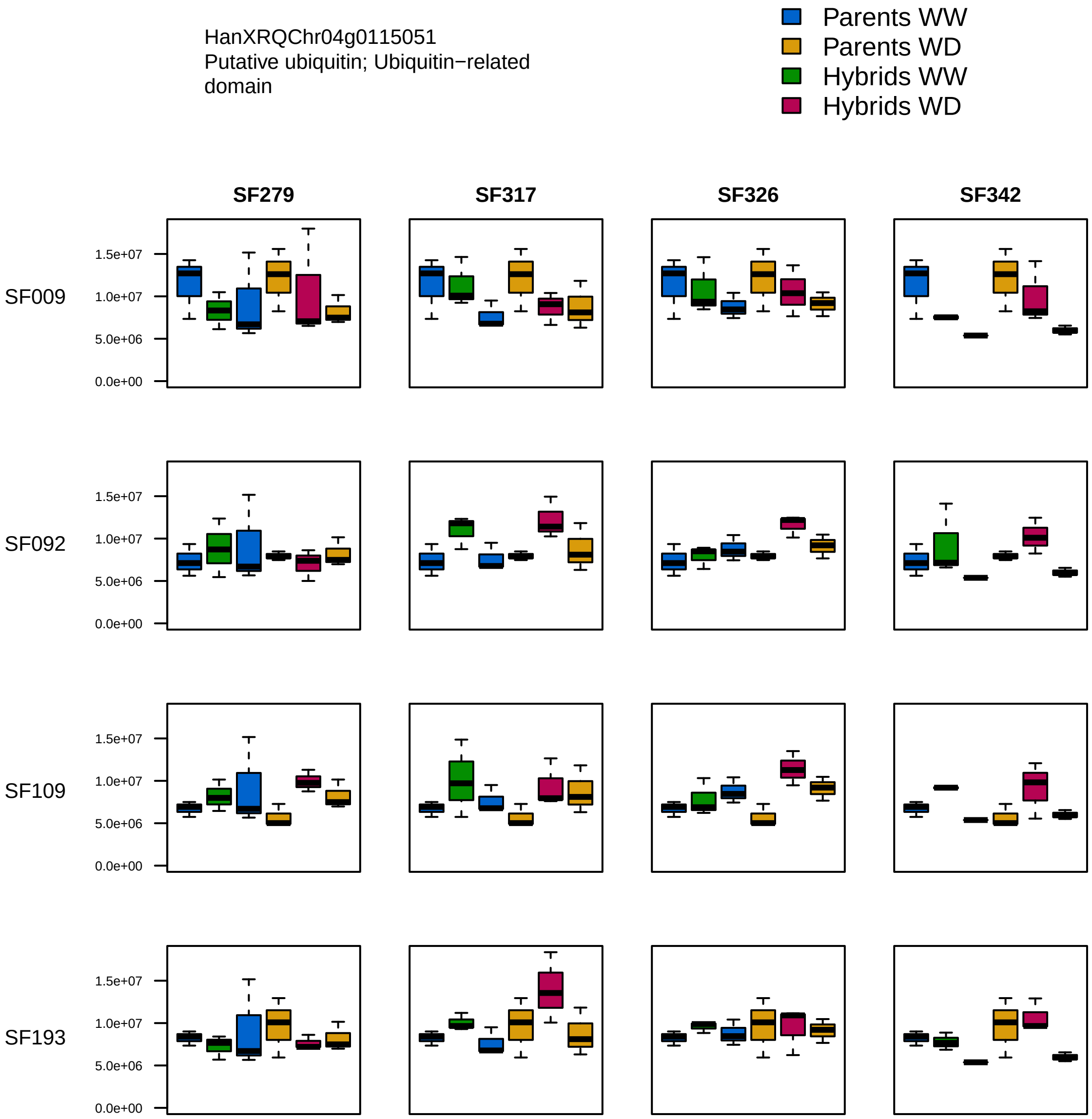
HanXRQChr04g0115051
Putative ubiquitin; Ubiquitin−related domain
Parents WW
Parents WD
Hybrids WW
Hybrids WD
SF279
SF317
SF326
SF342
SF009
SF092
SF109
SF193
1.5e+07
1.0e+07
5.0e+06
0.0e+00

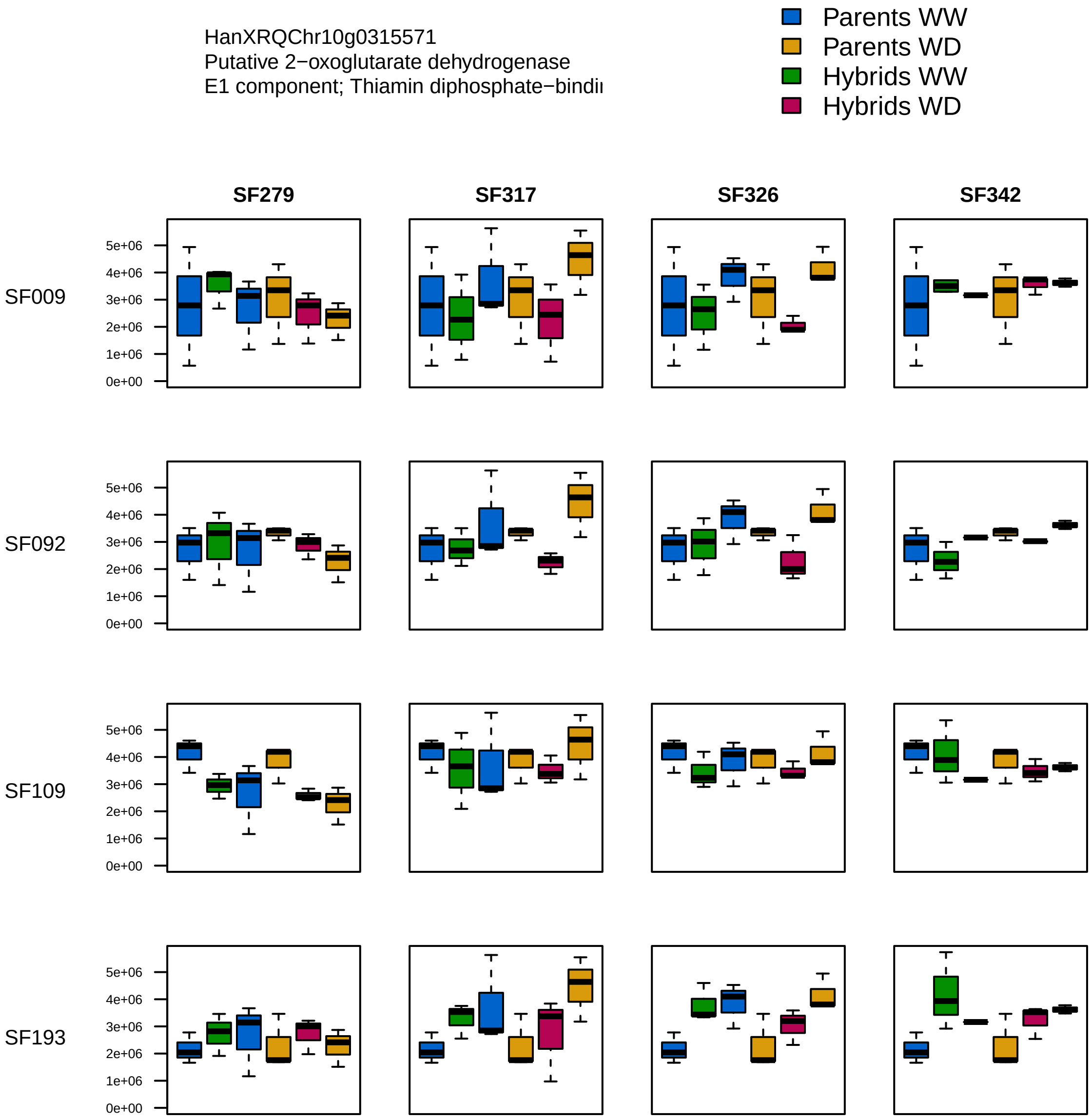

HanXRQChr10g0315571
Putative 2−oxoglutarate dehydrogenase
E1 component; Thiamin diphosphate−bindi
Parents WW
Parents WD
Hybrids WW
Hybrids WD
SF279
SF317
SF326
SF342
SF009
SF092
SF109
SF193
0e+00
1e+06
2e+06
3e+06
4e+06
5e+06

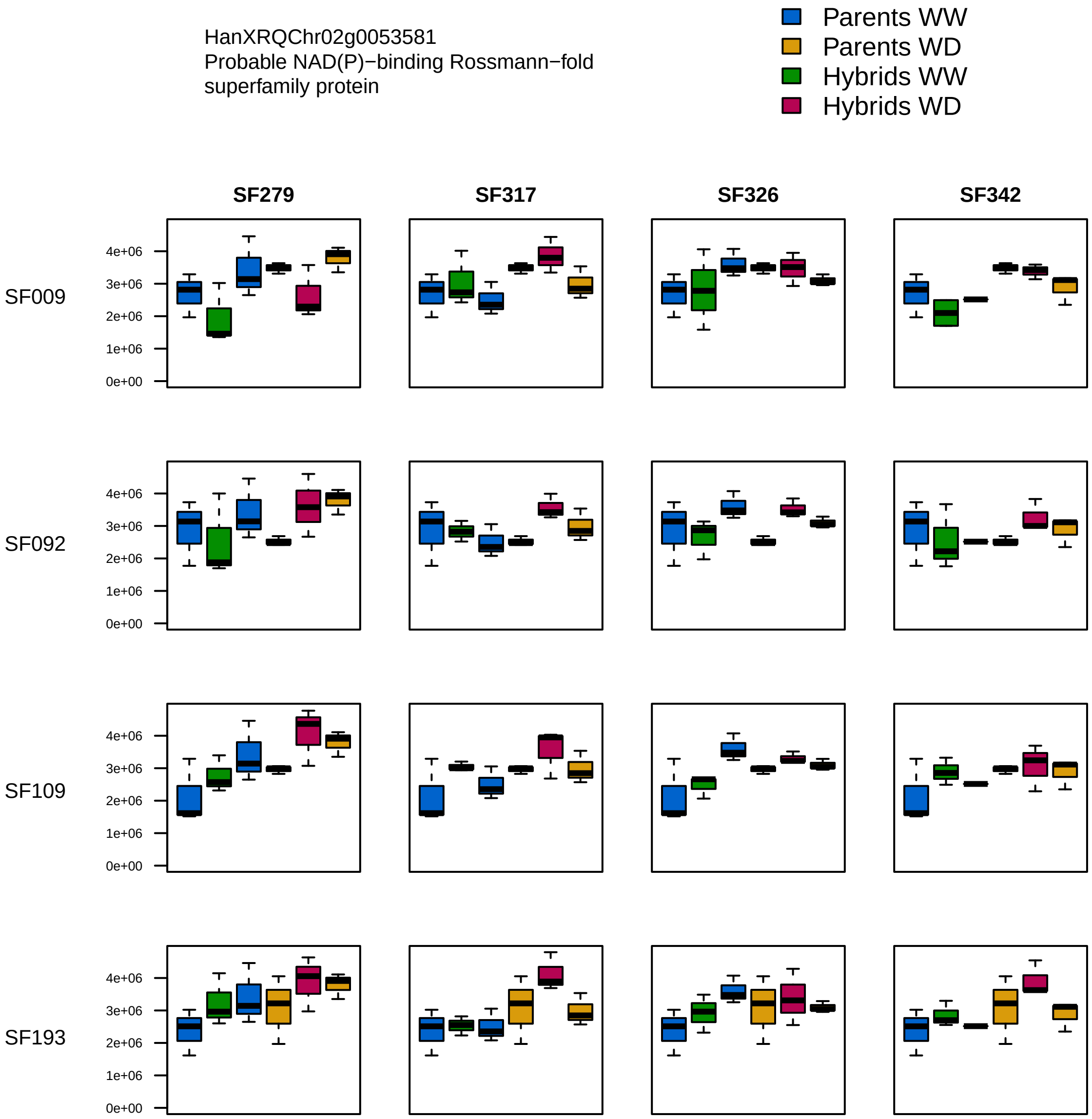
HanXRQChr02g0053581
Probable NAD(P)−binding Rossmann−fold
superfamily protein
Parents WW
Parents WD
Hybrids WW
Hybrids WD
SF279
SF317
SF326
SF342
SF009
SF092
SF109
SF193
0e+00
1e+06
2e+06
3e+06
4e+06

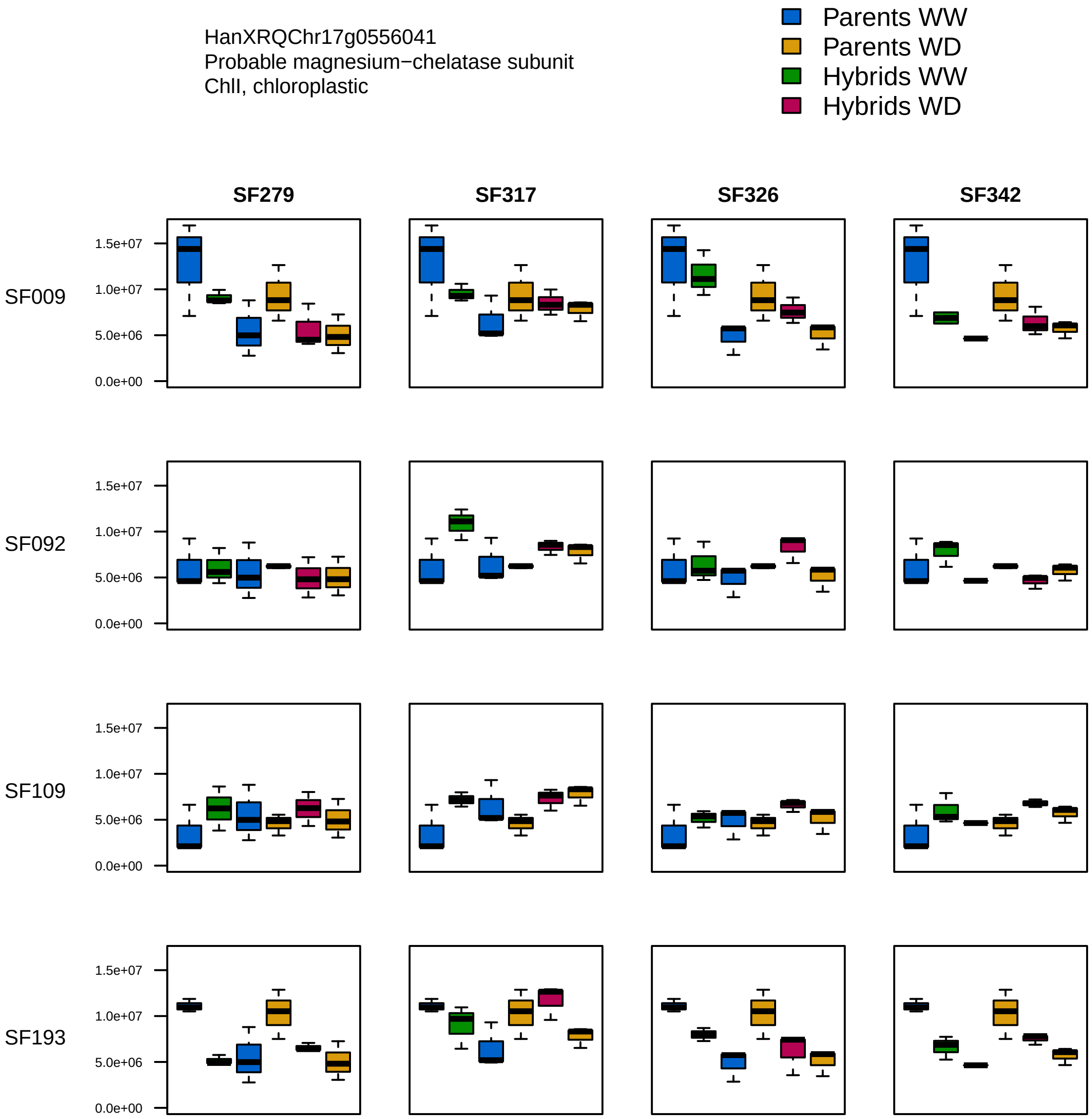

HanXRQChr17g0556041
Probable magnesium−chelatase subunit
ChlI, chloroplastic
Parents WW
Parents WD
Hybrids WW
Hybrids WD
SF279
SF317
SF326
SF342
SF009
SF092
SF109
SF193
1.5e+07
1.0e+07
5.0e+06
0.0e+00

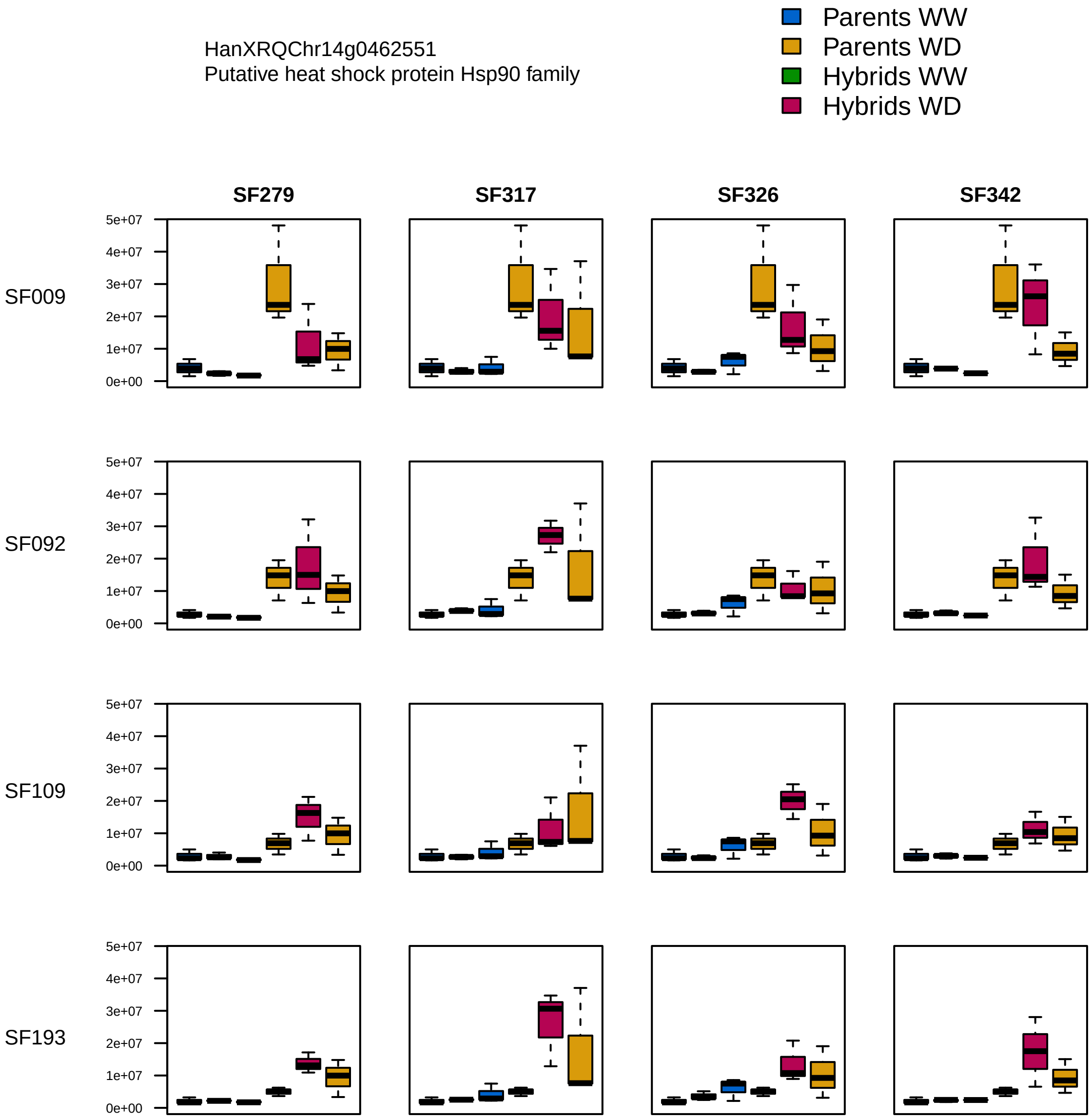
HanXRQChr14g0462551
Putative heat shock protein Hsp90 family
Parents WW
Parents WD
Hybrids WW
Hybrids WD
SF279
SF317
SF326
SF342
SF009
SF092
SF109
SF193
5e+07
4e+07
3e+07
2e+07
1e+07
0e+00

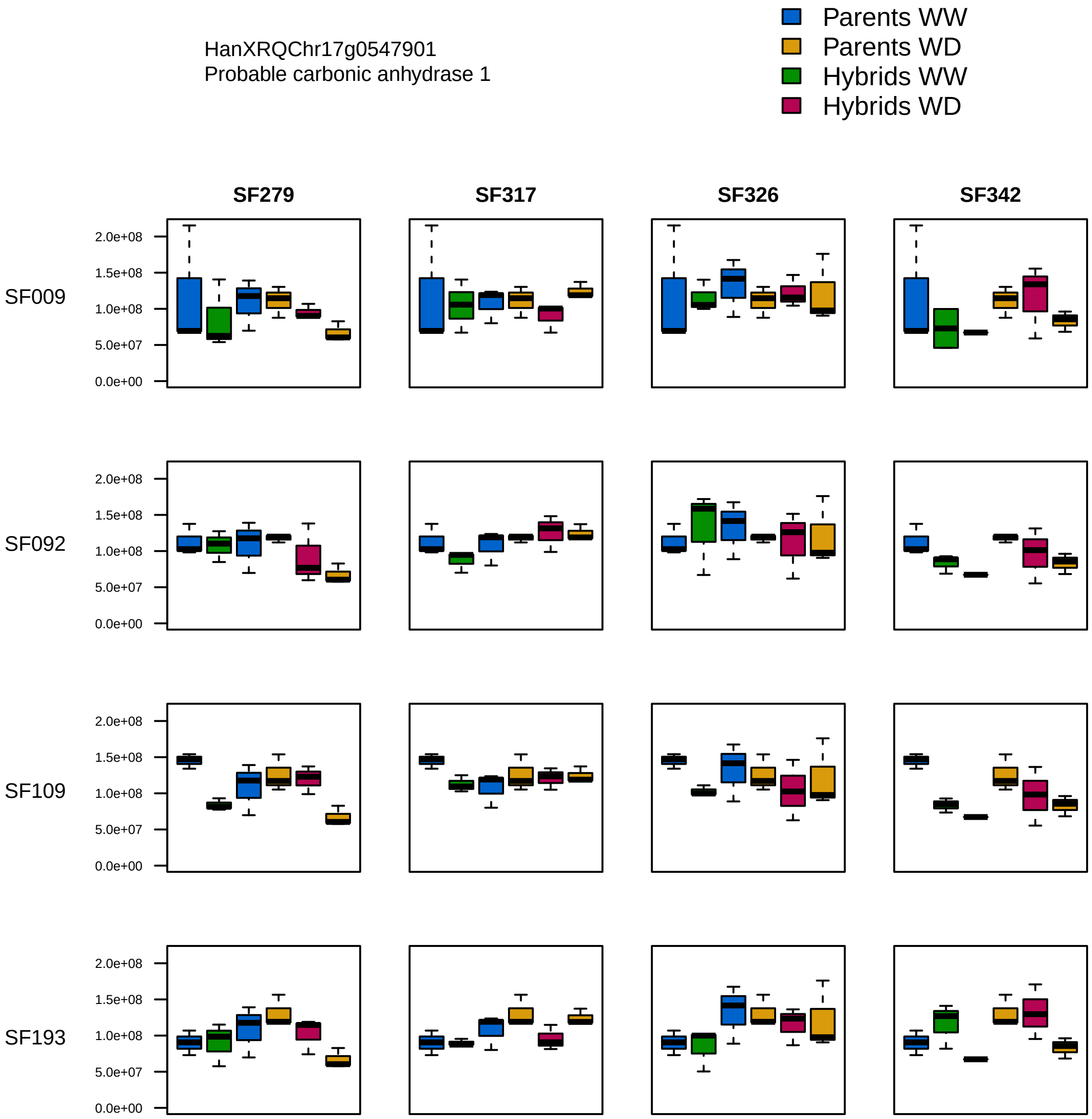
HanXRQChr17g0547901
Probable carbonic anhydrase 1
Parents WW
Parents WD
Hybrids WW
Hybrids WD
SF279
SF317
SF326
SF342
SF009
SF092
SF109
SF193
2.0e+08
1.5e+08
1.0e+08
5.0e+07
0.0e+00

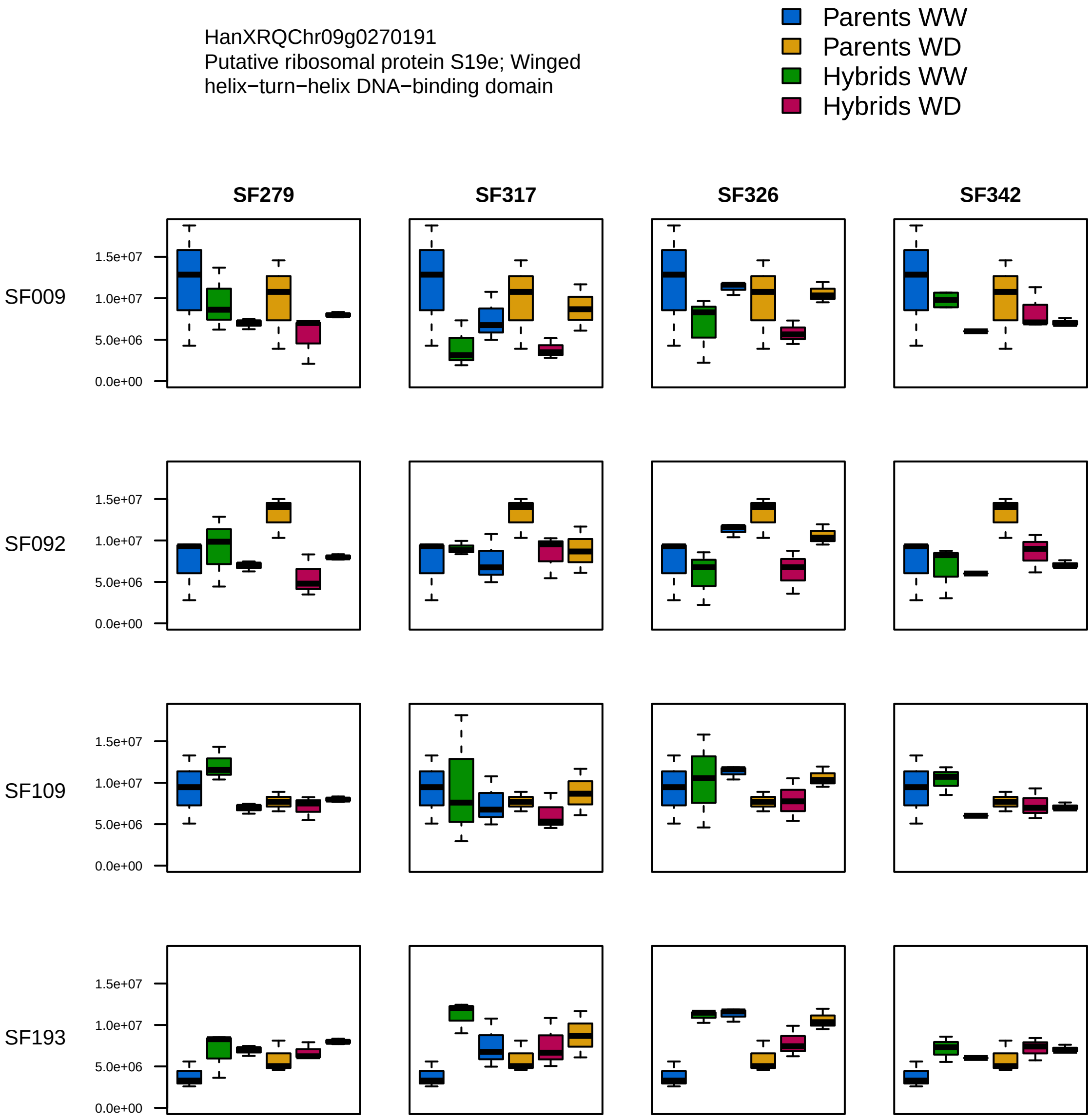
HanXRQChr09g0270191
Putative ribosomal protein S19e; Winged helix−turn−helix DNA−binding domain
Parents WW
Parents WD
Hybrids WW
Hybrids WD
SF279
SF317
SF326
SF342
SF009
SF092
SF109
SF193
1.5e+07
1.0e+07
5.0e+06
0.0e+00

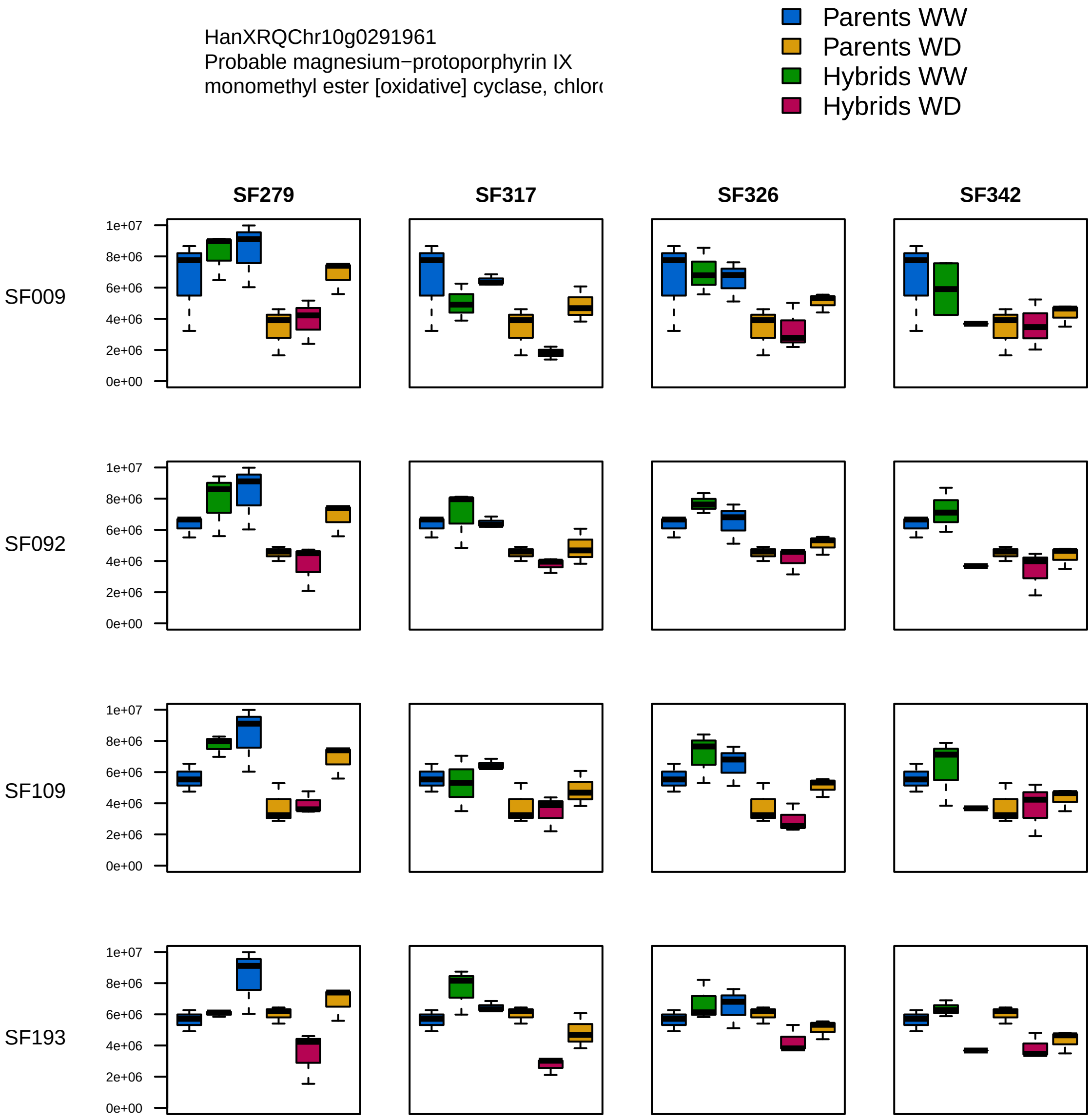

HanXRQChr10g0291961
Probable magnesium−protoporphyrin IX
monomethyl ester [oxidative] cyclase, chlor
Parents WW
Parents WD
Hybrids WW
Hybrids WD
SF279
SF317
SF326
SF342
SF009
SF092
SF109
SF193
1e+07
8e+06
6e+06
4e+06
2e+06
0e+00

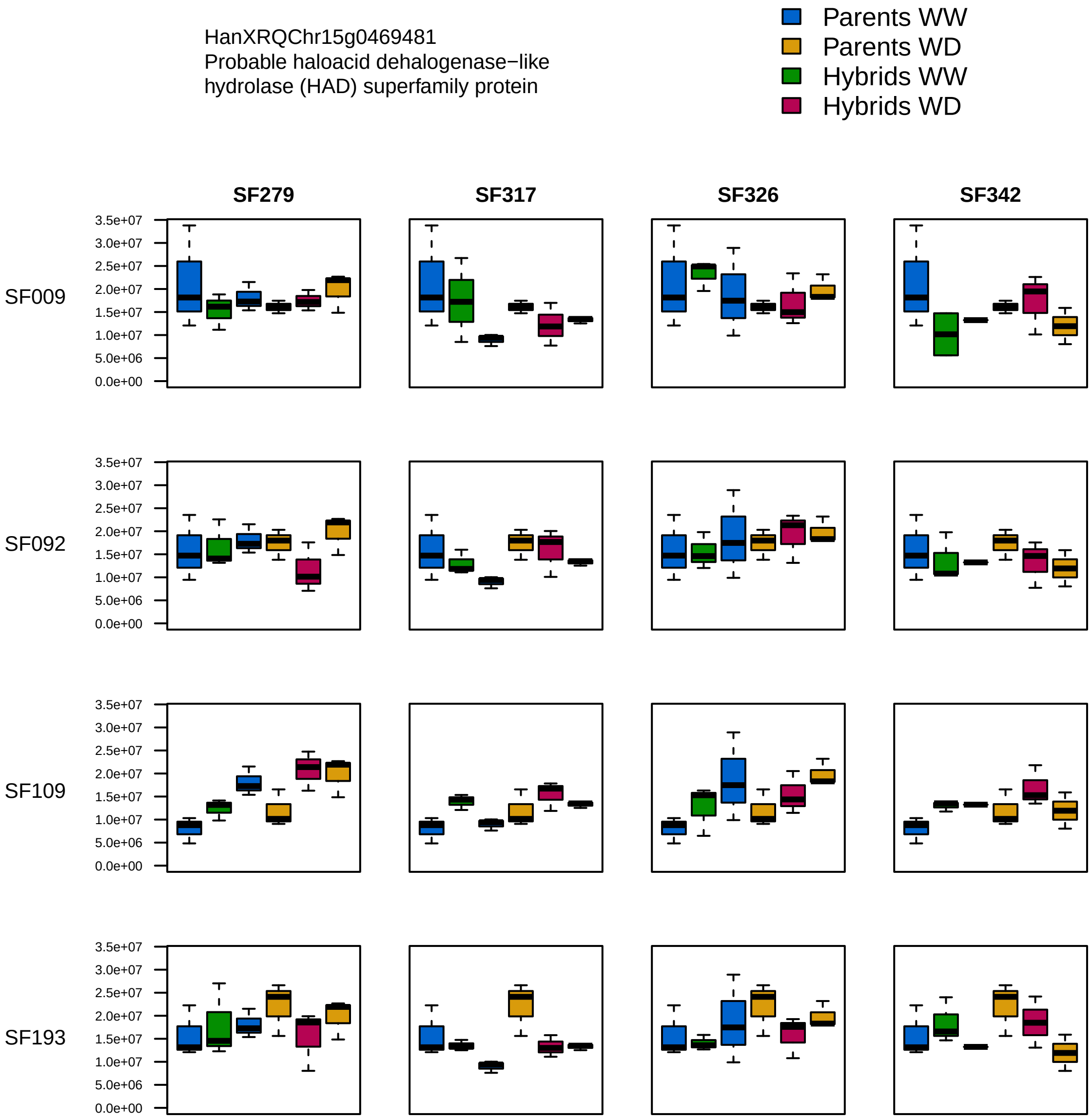

HanXRQChr15g0469481
Probable haloacid dehalogenase−like
hydrolase (HAD) superfamily protein
Parents WW
Parents WD
Hybrids WW
Hybrids WD
SF279
SF317
SF326
SF342
SF009
SF092
SF109
SF193
3.5e+07
3.0e+07
2.5e+07
2.0e+07
1.5e+07
1.0e+07
5.0e+06
0.0e+00

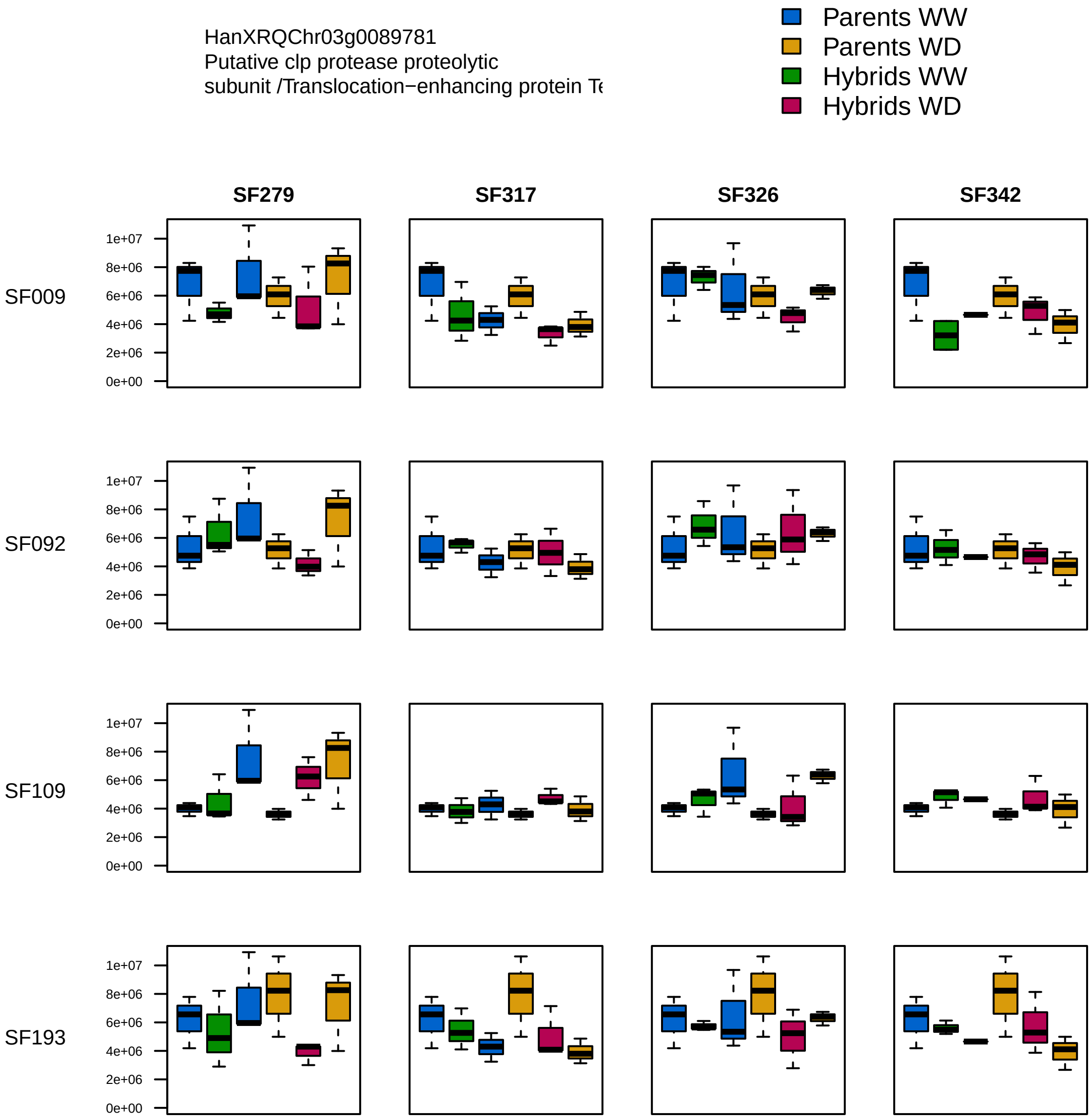

HanXRQChr03g0089781
Putative clp protease proteolytic
subunit /Translocation−enhancing protein Te
Parents WW
Parents WD
Hybrids WW
Hybrids WD
SF279
SF317
SF326
SF342
SF009
SF092
SF109
SF193
1e+07
8e+06
6e+06
4e+06
2e+06
0e+00

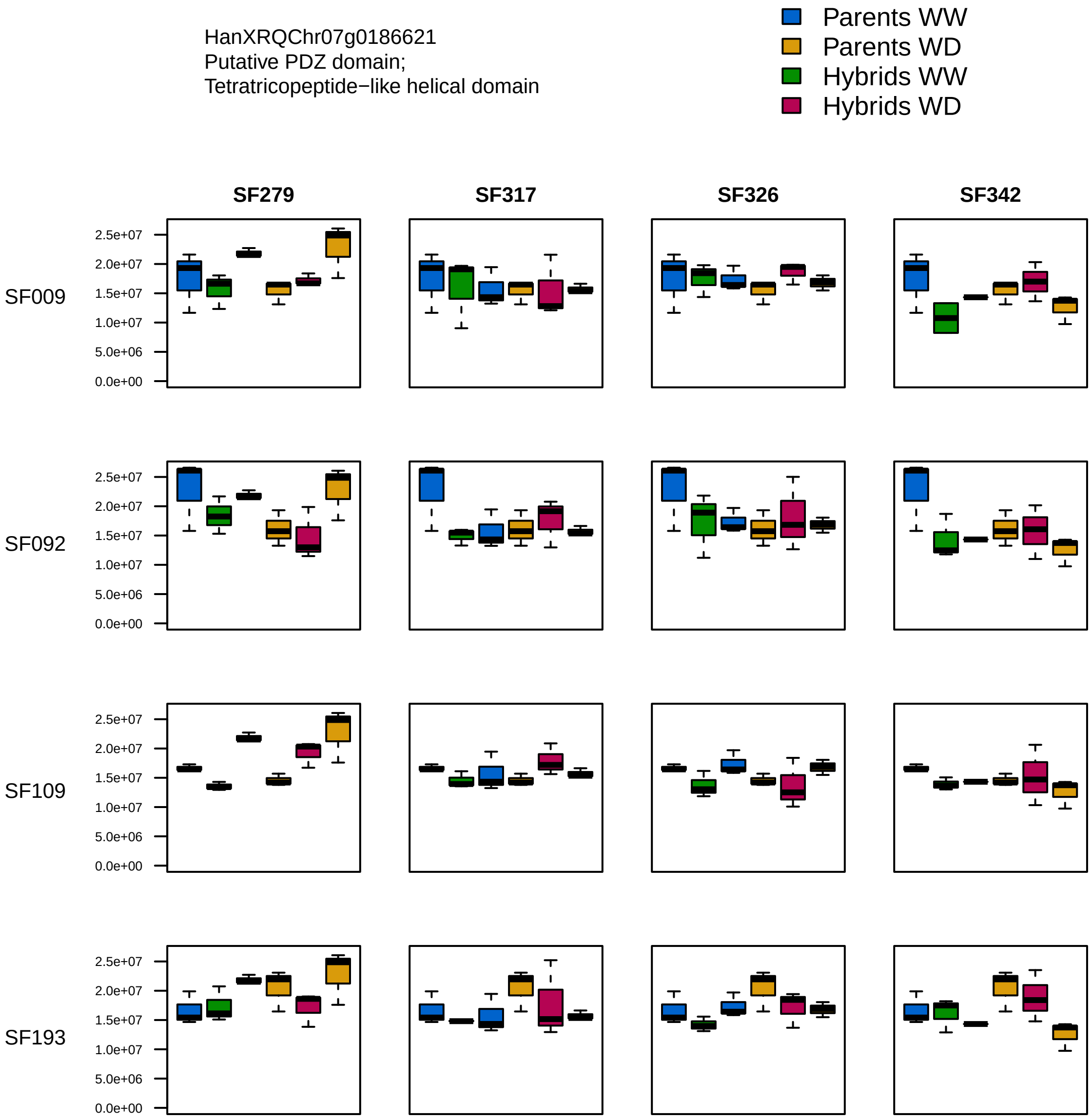

HanXRQChr07g0186621
Putative PDZ domain;
Tetratricopeptide−like helical domain
Parents WW
Parents WD
Hybrids WW
Hybrids WD
SF279
SF317
SF326
SF342
SF009
SF092
SF109
SF193
2.5e+07
2.0e+07
1.5e+07
1.0e+07
5.0e+06
0.0e+00

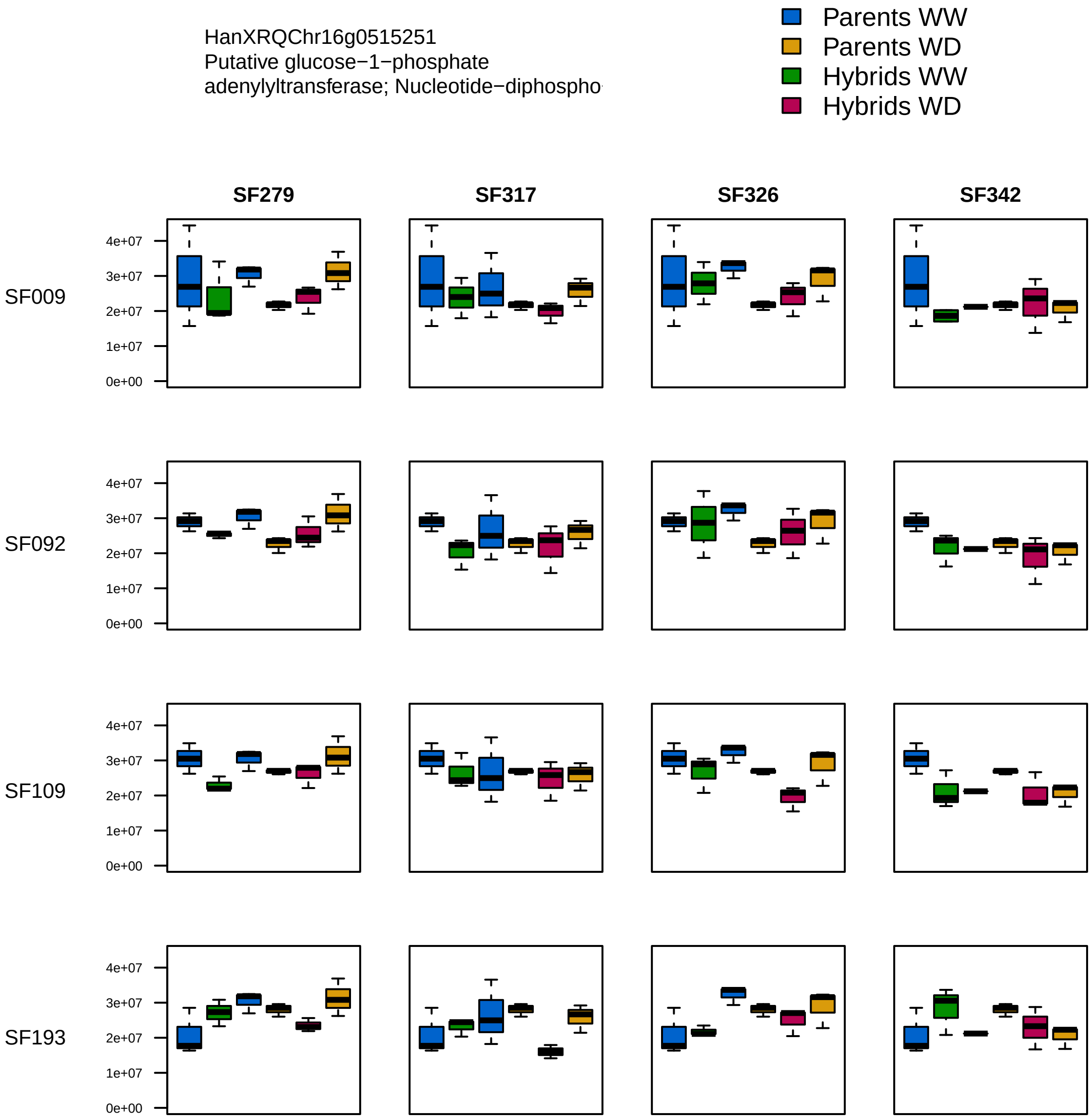
HanXRQChr16g0515251
Putative glucose−1−phosphate
adenylyltransferase; Nucleotide−diphospho
Parents WW
Parents WD
Hybrids WW
Hybrids WD
SF279
SF317
SF326
SF342
SF009
SF092
SF109
SF193
4e+07
3e+07
2e+07
1e+07
0e+00

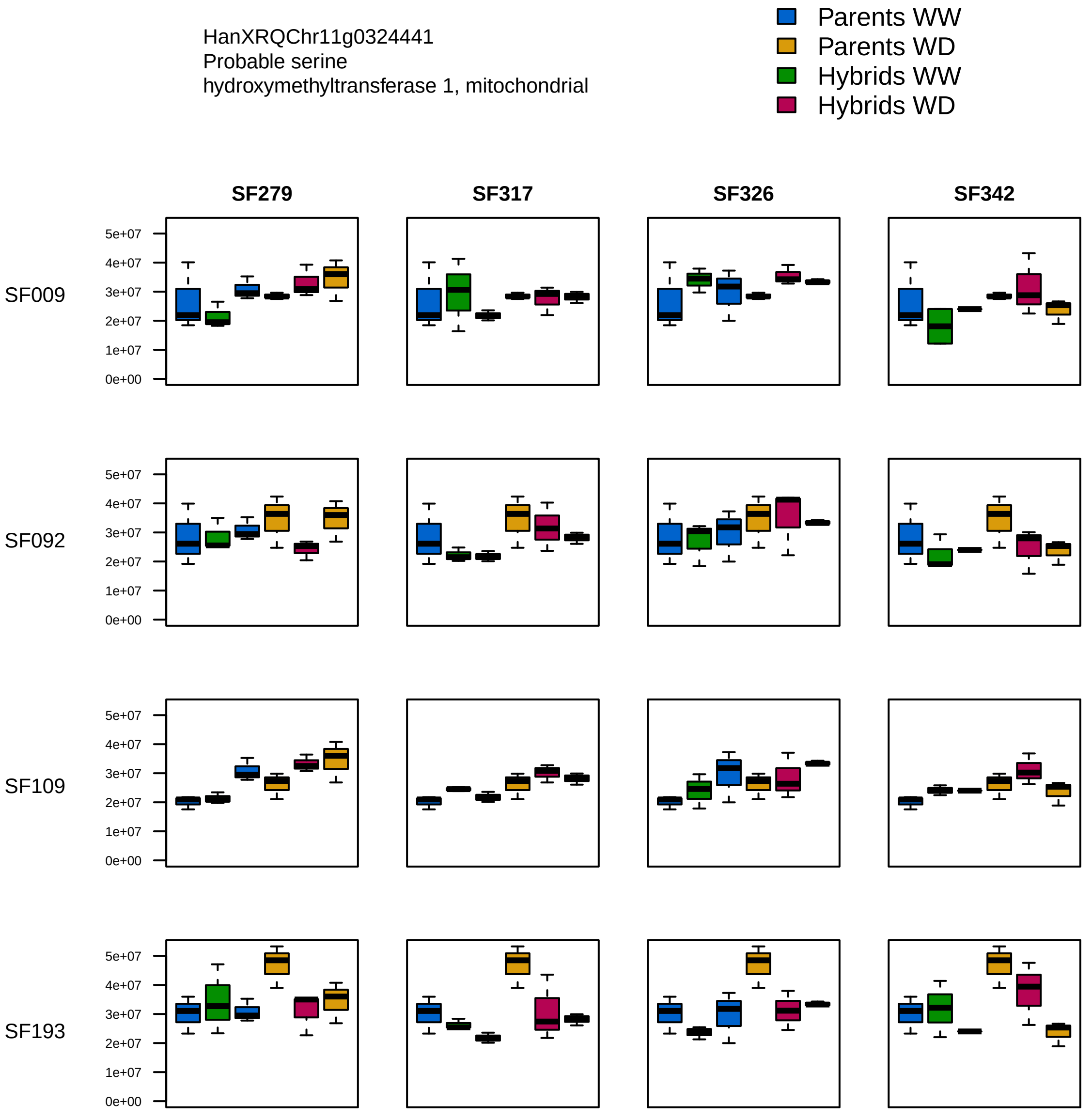
HanXRQChr11g0324441
Probable serine
hydroxymethyltransferase 1, mitochondrial
Parents WW
Parents WD
Hybrids WW
Hybrids WD
SF279
SF317
SF326
SF342
SF009
SF092
SF109
SF193
5e+07
4e+07
3e+07
2e+07
1e+07
0e+00

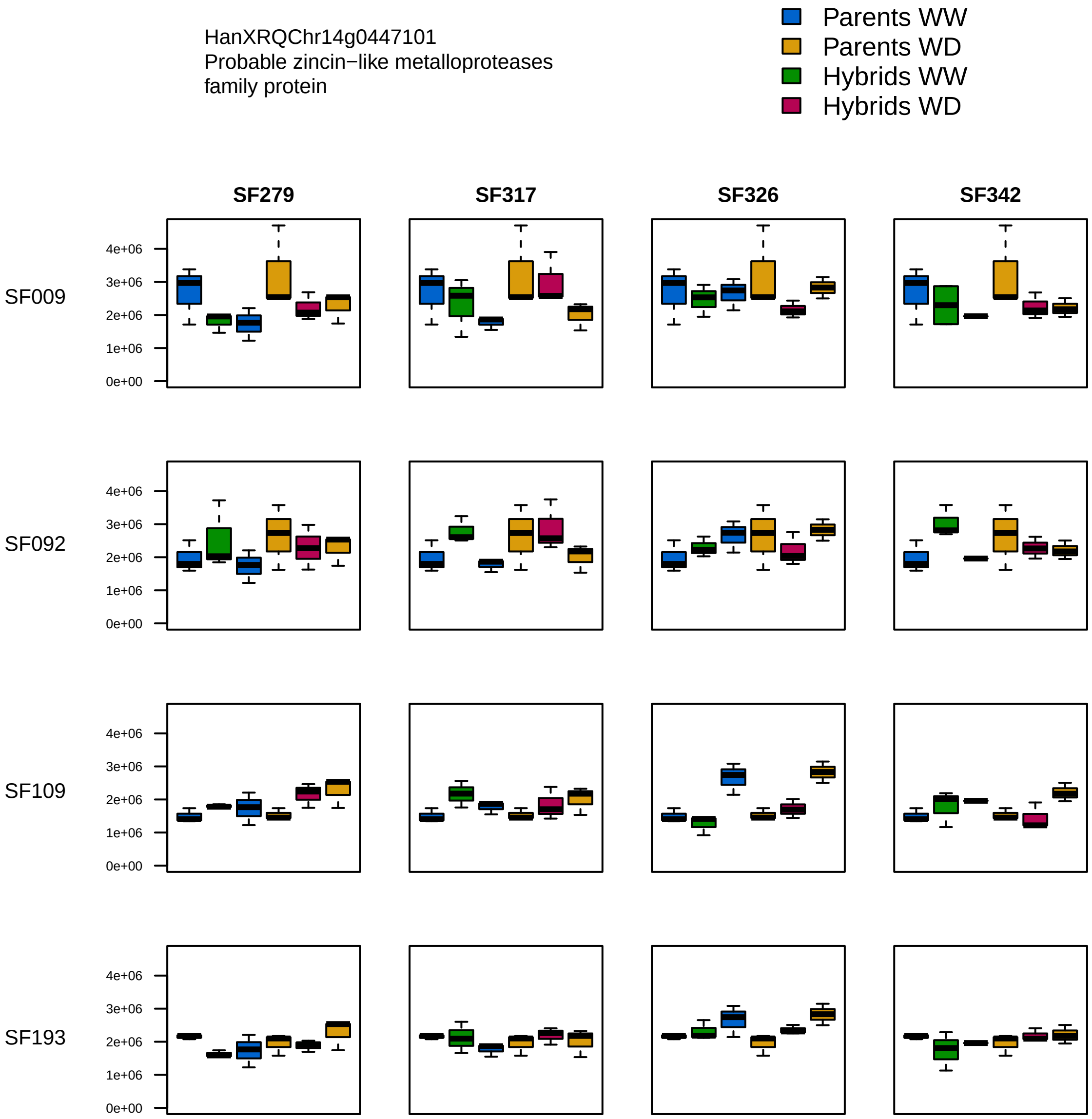
HanXRQChr14g0447101
Probable zincin−like metalloproteases
family protein
Parents WW
Parents WD
Hybrids WW
Hybrids WD
SF279
SF317
SF326
SF342
SF009
SF092
SF109
SF193
0e+00
1e+06
2e+06
3e+06
4e+06

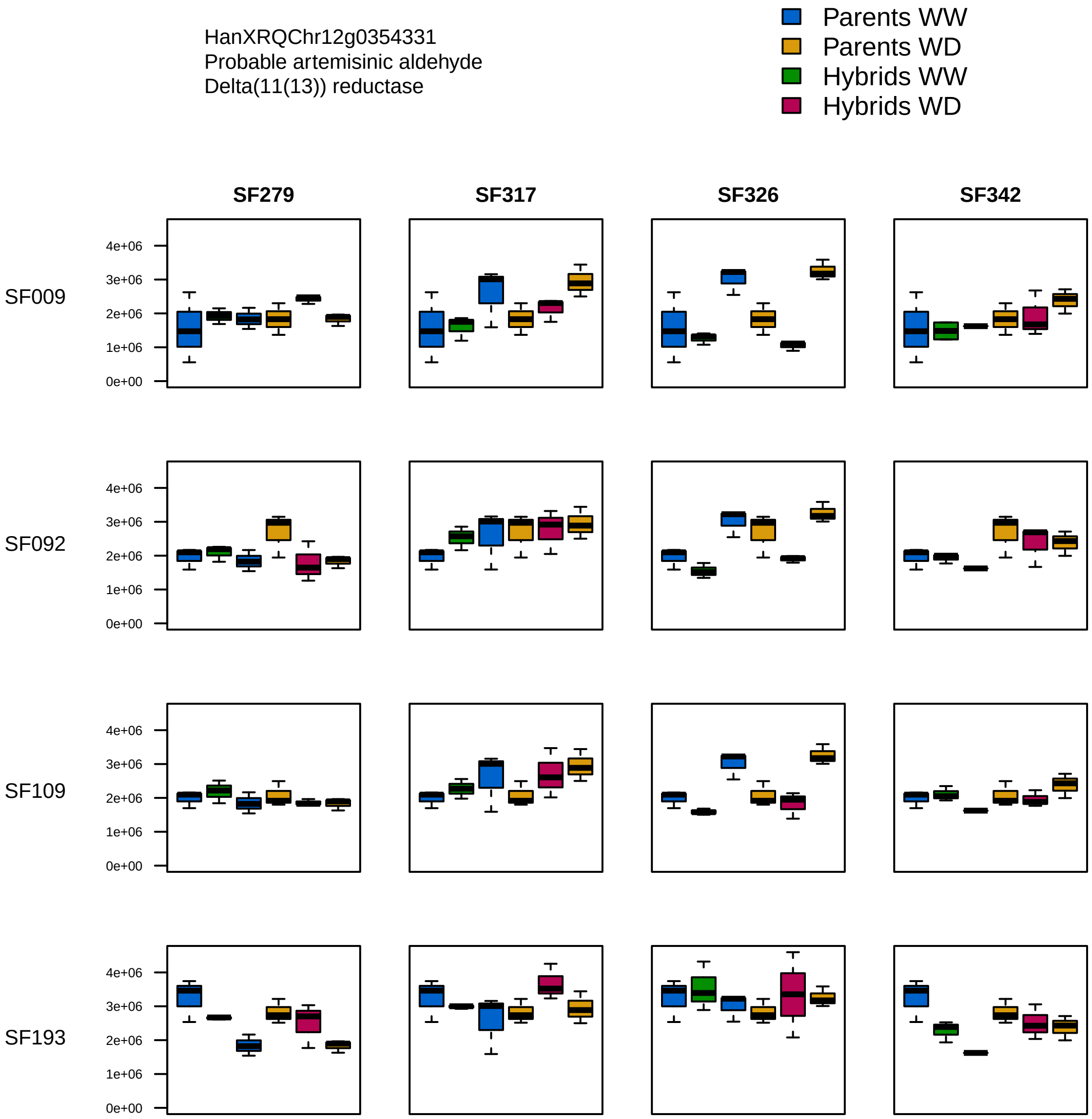

HanXRQChr12g0354331
Probable artemisinic aldehyde
Delta(11(13)) reductase
Parents WW
Parents WD
Hybrids WW
Hybrids WD
SF279
SF317
SF326
SF342
SF009
SF092
SF109
SF193
4e+06
3e+06
2e+06
1e+06
0e+00

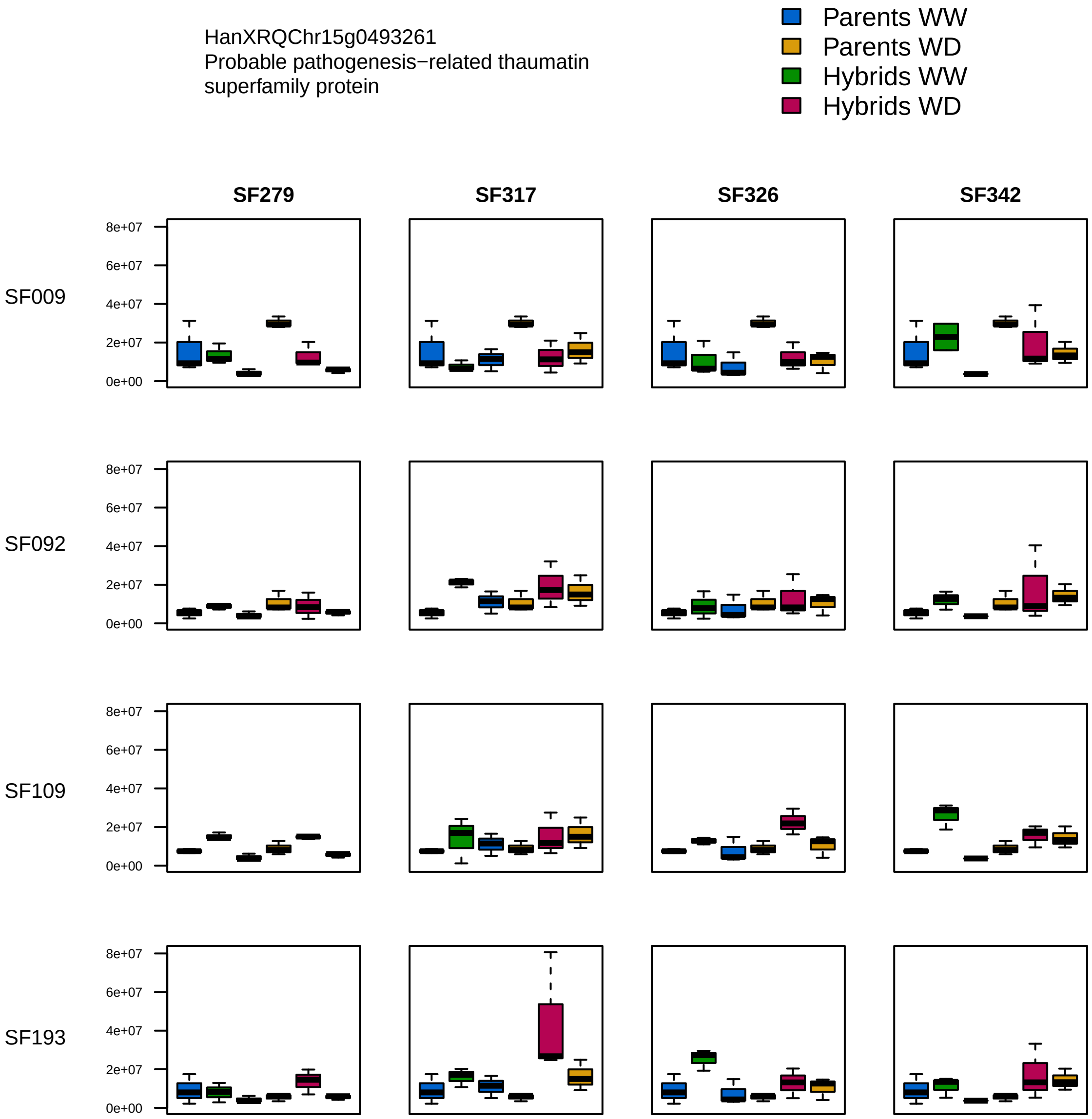
HanXRQChr15g0493261
Probable pathogenesis−related thaumatin superfamily protein
Parents WW
Parents WD
Hybrids WW
Hybrids WD
SF279
SF317
SF326
SF342
SF009
SF092
SF109
SF193
8e+07
6e+07
4e+07
2e+07
0e+00

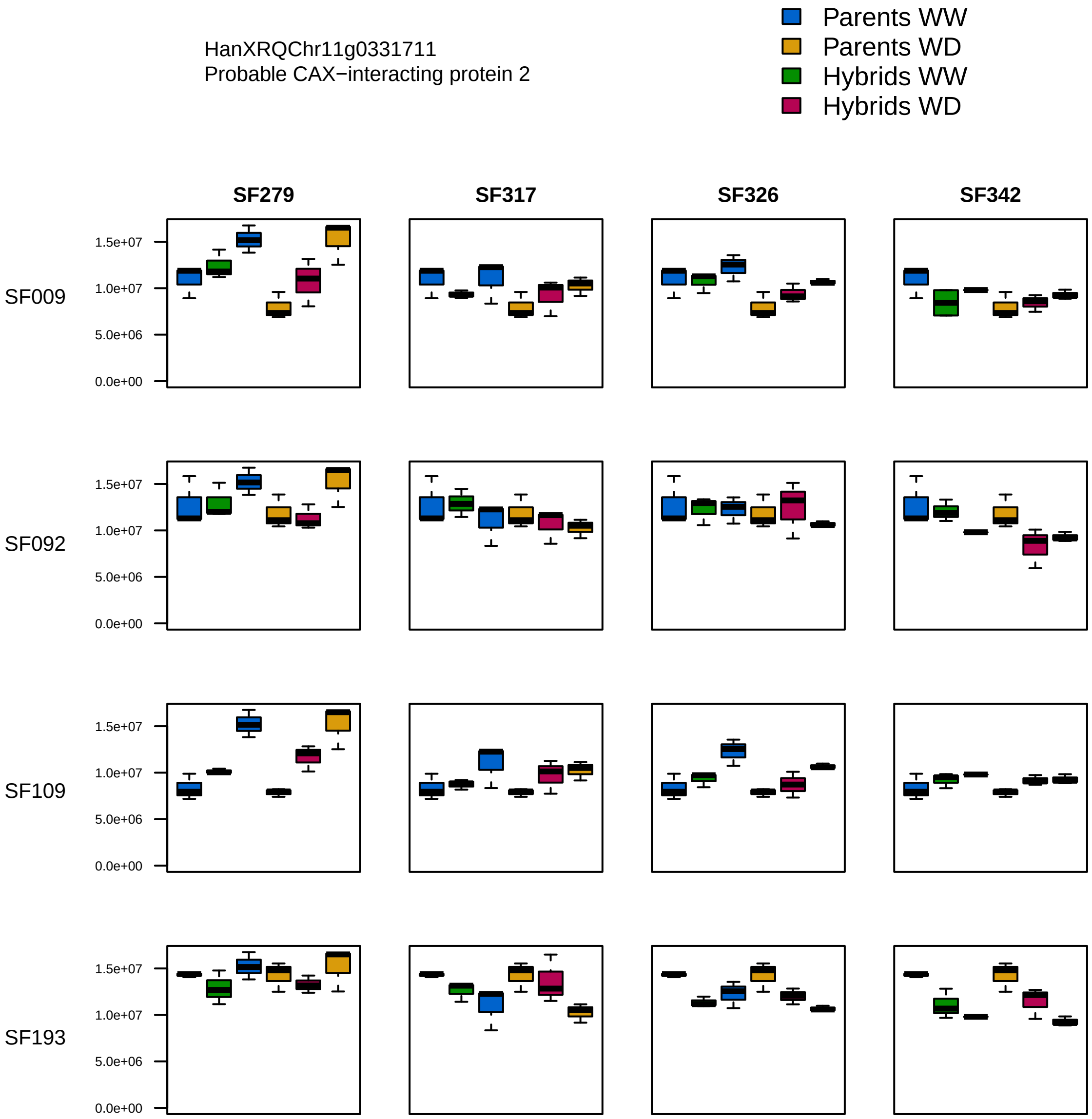

HanXRQChr11g0331711
Probable CAX−interacting protein 2
Parents WW
Parents WD
Hybrids WW
Hybrids WD
SF279
SF317
SF326
SF342
SF009
SF092
SF109
SF193
1.5e+07
1.0e+07
5.0e+06
0.0e+00

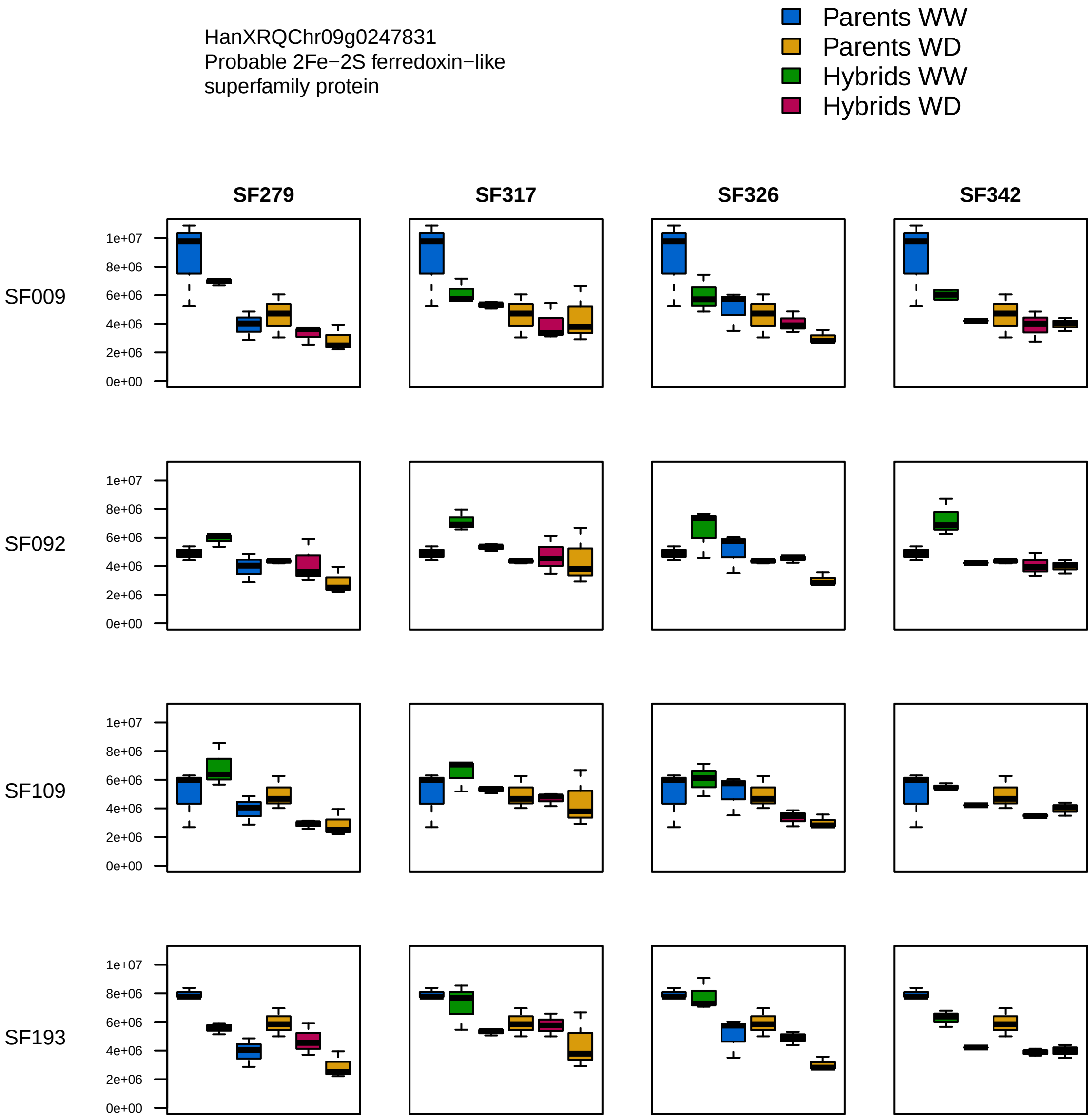
HanXRQChr09g0247831
Probable 2Fe−2S ferredoxin−like
superfamily protein
Parents WW
Parents WD
Hybrids WW
Hybrids WD
SF279
SF317
SF326
SF342
SF009
SF092
SF109
SF193
1e+07
8e+06
6e+06
4e+06
2e+06
0e+00

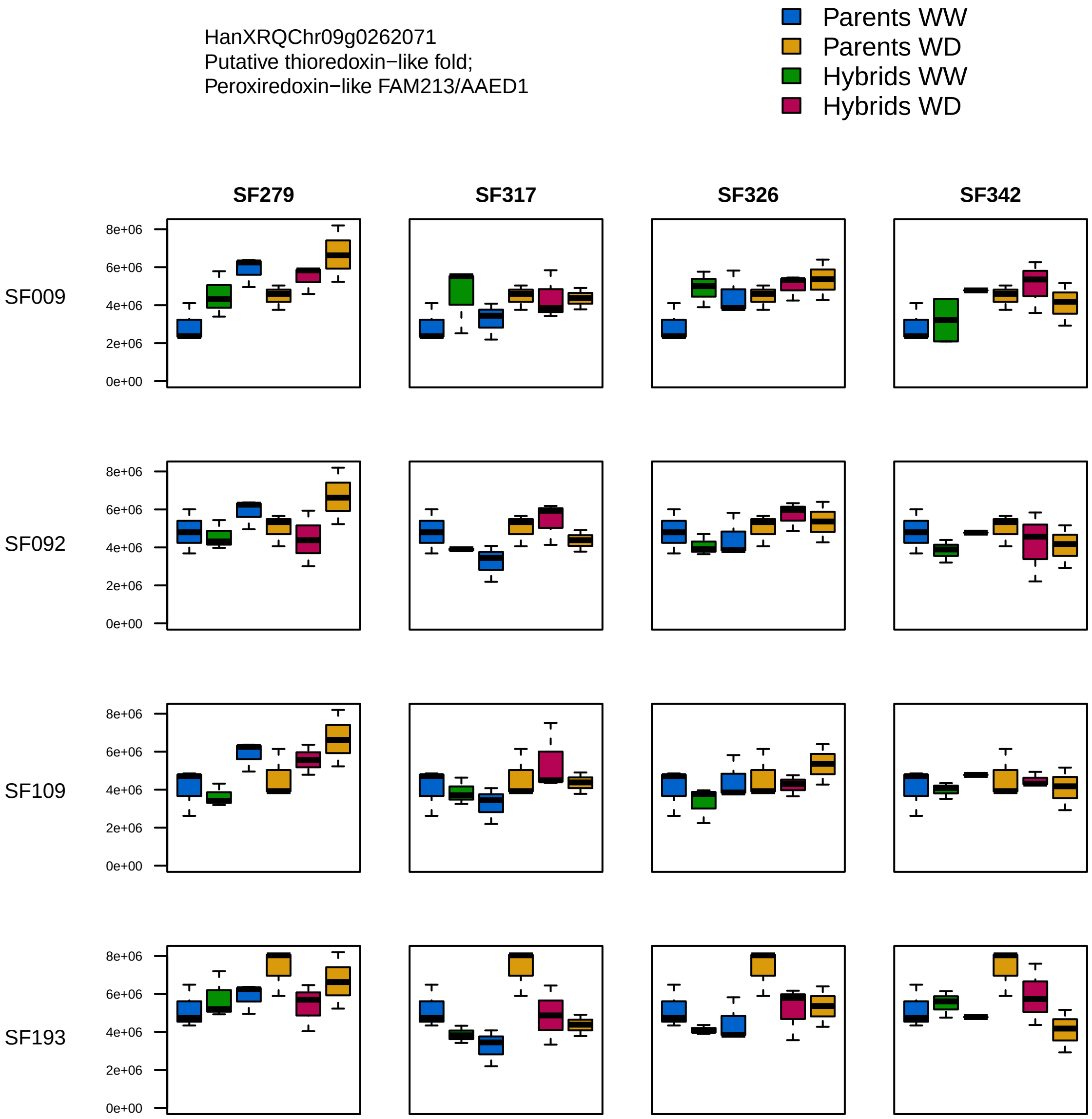
HanXRQChr09g0262071
Putative thioredoxin−like fold;
Peroxiredoxin−like FAM213/AAED1
Parents WW
Parents WD
Hybrids WW
Hybrids WD
SF279
SF317
SF326
SF342
SF009
SF092
SF109
SF193
0e+00
2e+06
4e+06
6e+06
8e+06

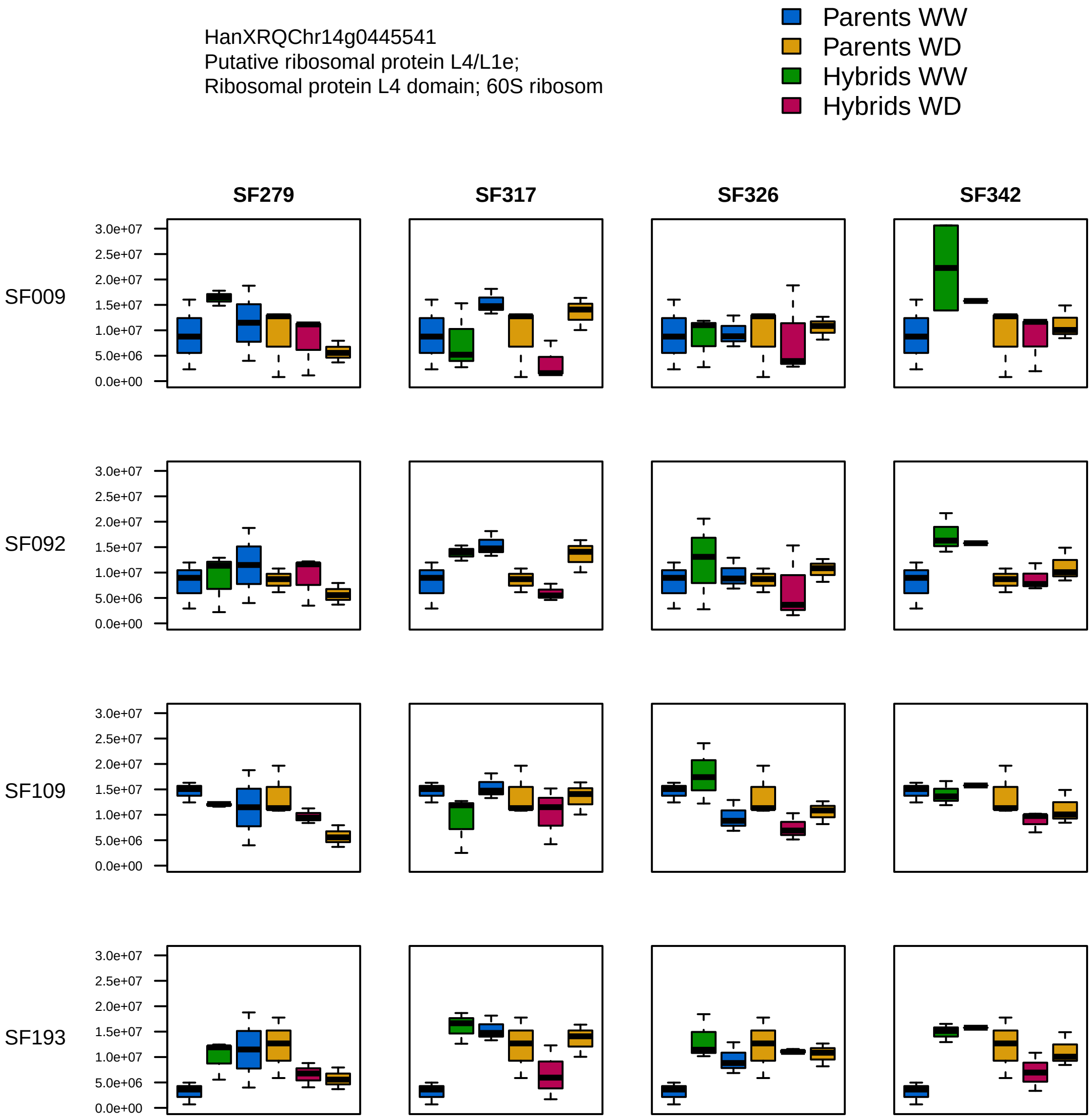
HanXRQChr14g0445541
Putative ribosomal protein L4/L1e;
Ribosomal protein L4 domain; 60S ribosom
Parents WW
Parents WD
Hybrids WW
Hybrids WD
SF279
SF317
SF326
SF342
SF009
SF092
SF109
SF193
3.0e+07
2.5e+07
2.0e+07
1.5e+07
1.0e+07
5.0e+06
0.0e+00

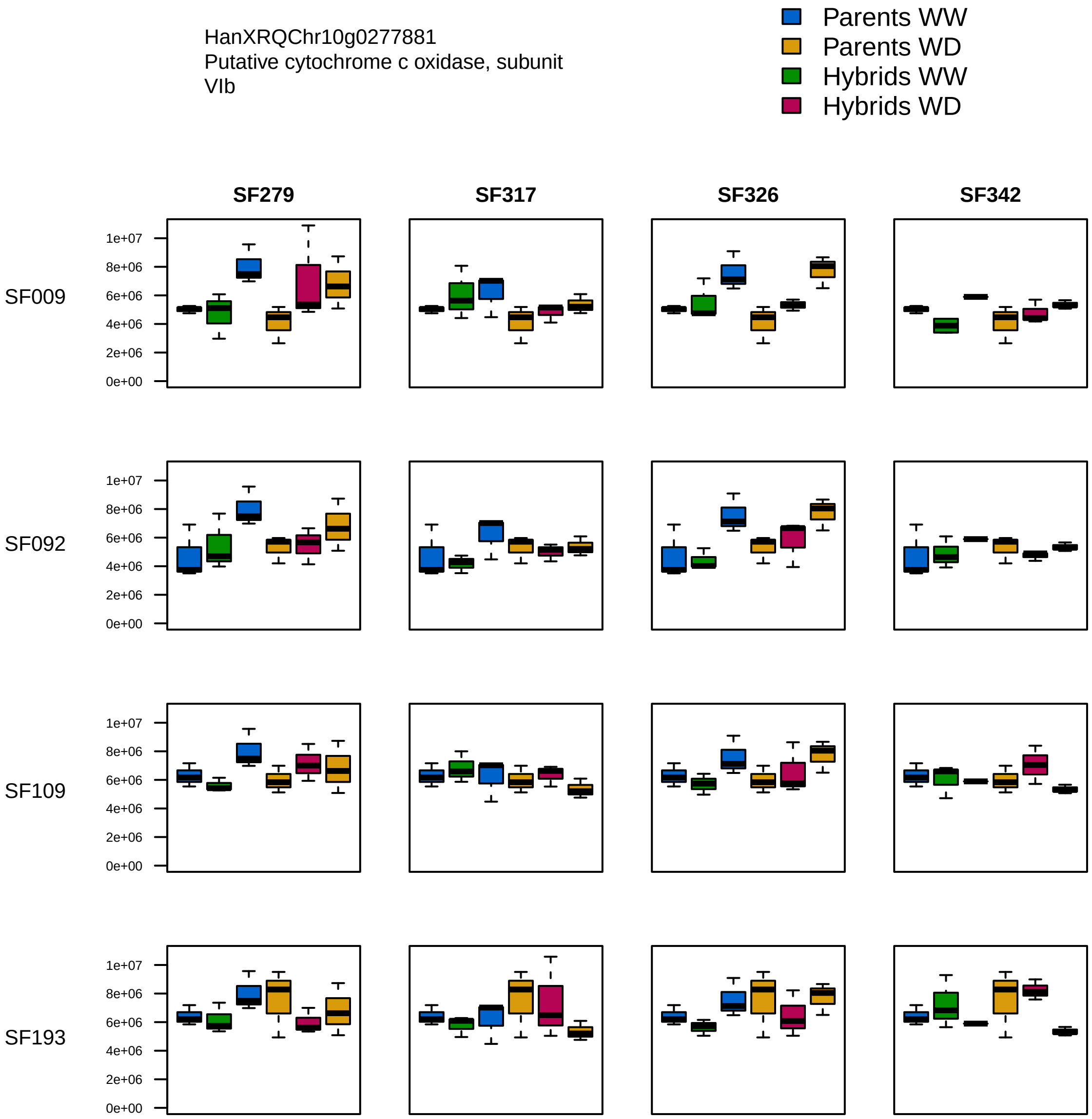
HanXRQChr10g0277881
Putative cytochrome c oxidase, subunit VIb
Parents WW
Parents WD
Hybrids WW
Hybrids WD
SF279
SF317
SF326
SF342
SF009
SF092
SF109
SF193
1e+07
8e+06
6e+06
4e+06
2e+06
0e+00

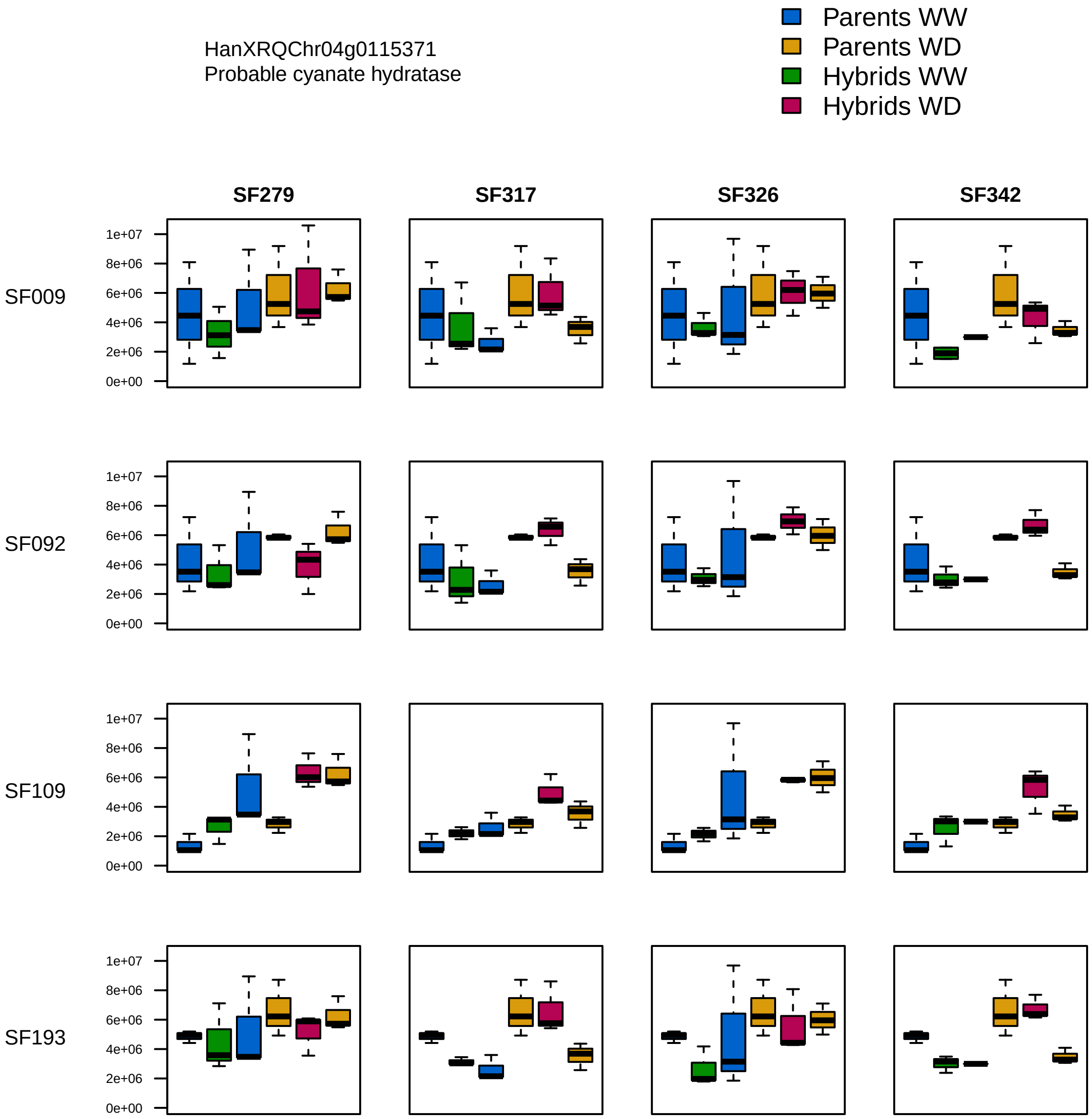

HanXRQChr04g0115371
Probable cyanate hydratase
Parents WW
Parents WD
Hybrids WW
Hybrids WD
SF279
SF317
SF326
SF342
SF009
SF092
SF109
SF193
1e+07
8e+06
6e+06
4e+06
2e+06
0e+00

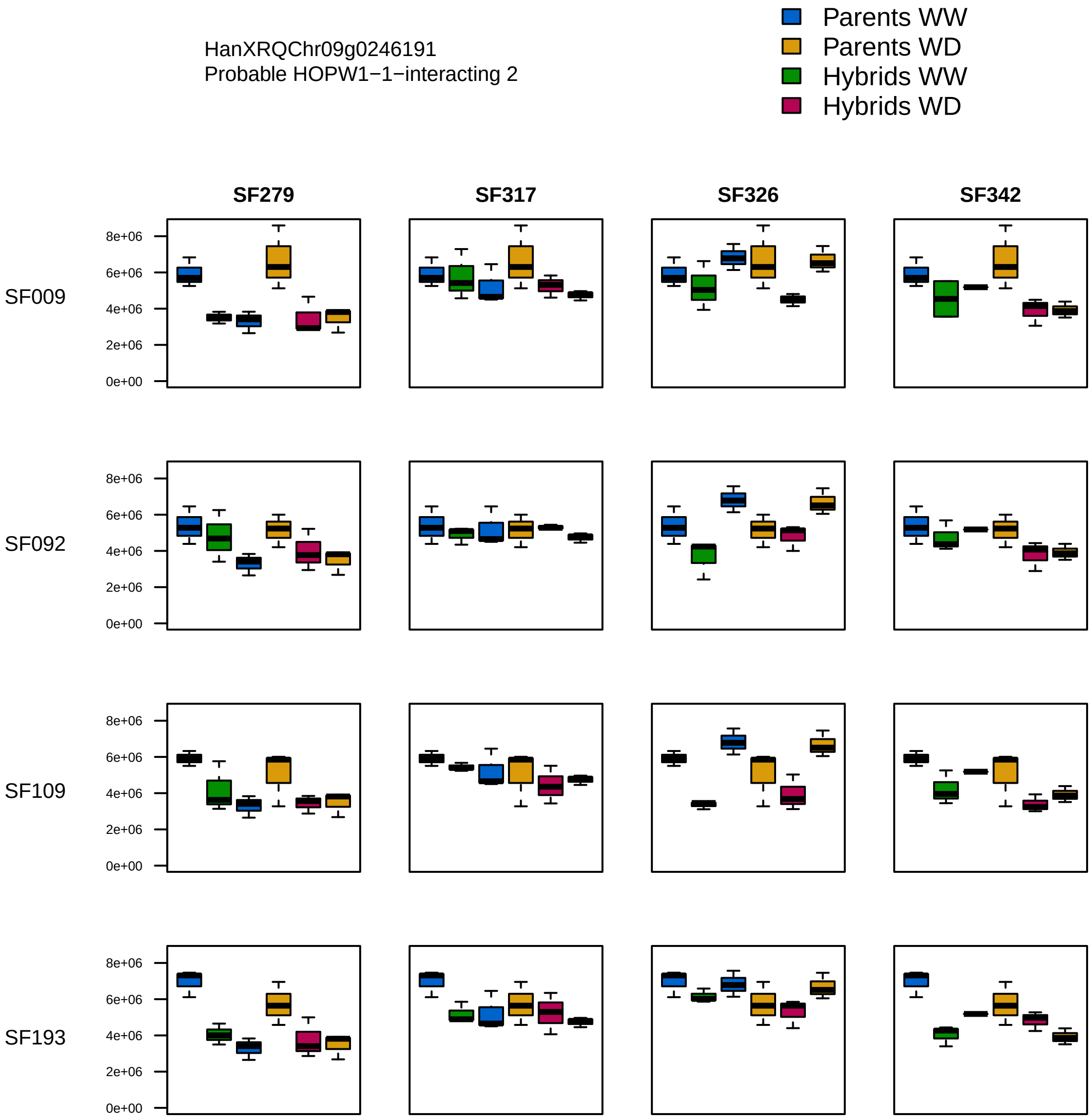
HanXRQChr09g0246191
Probable HOPW1−1−interacting 2
Parents WW
Parents WD
Hybrids WW
Hybrids WD
SF279
SF317
SF326
SF342
SF009
SF092
SF109
SF193
0e+00
2e+06
4e+06
6e+06
8e+06

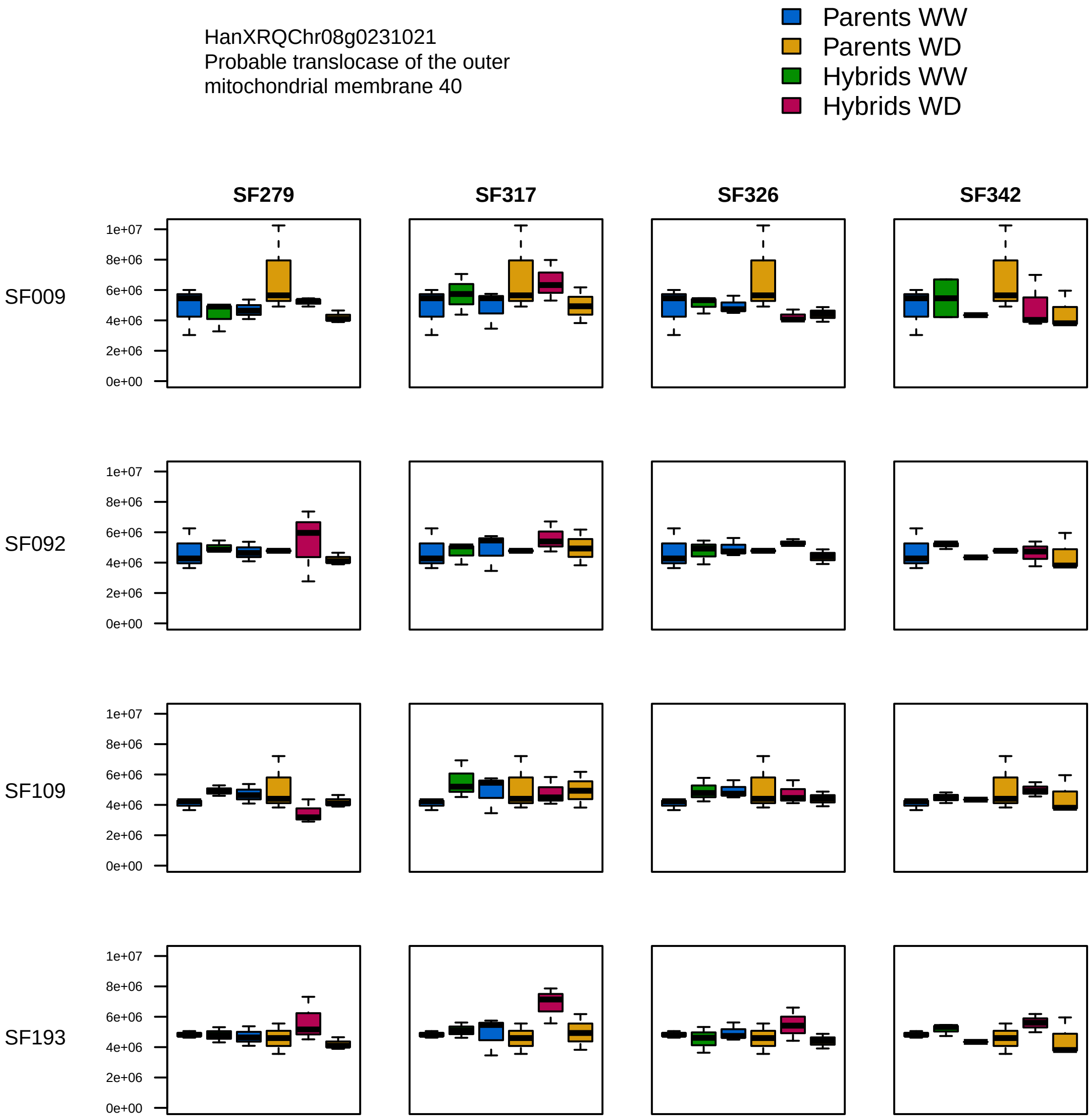
HanXRQChr08g0231021
Probable translocase of the outer
mitochondrial membrane 40
Parents WW
Parents WD
Hybrids WW
Hybrids WD
SF279
SF317
SF326
SF342
SF009
SF092
SF109
SF193
1e+07
8e+06
6e+06
4e+06
2e+06
0e+00

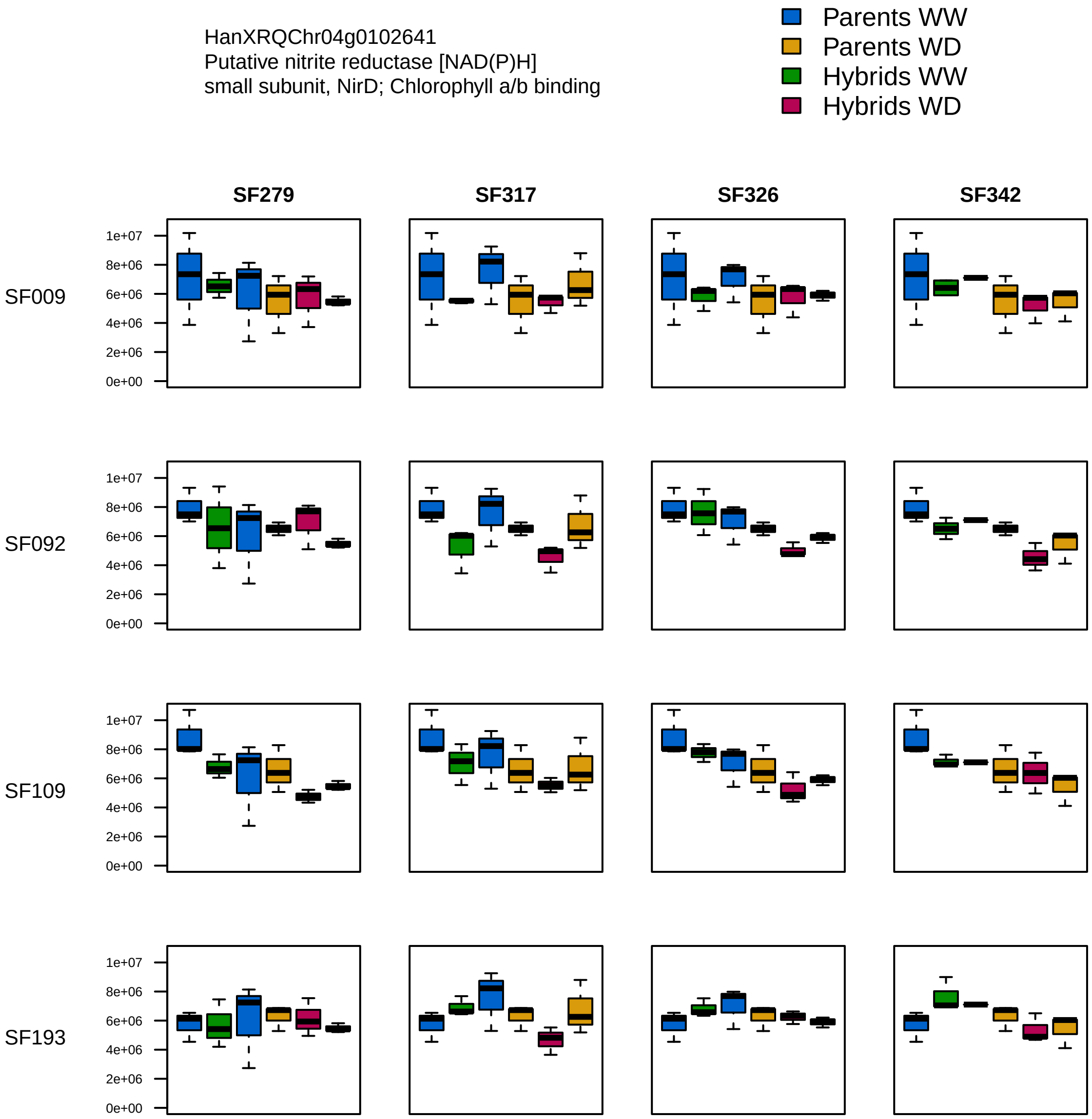

HanXRQChr04g0102641
Putative nitrite reductase [NAD(P)H]
small subunit, NirD; Chlorophyll a/b binding
Parents WW
Parents WD
Hybrids WW
Hybrids WD
SF279
SF317
SF326
SF342
SF009
SF092
SF109
SF193
1e+07
8e+06
6e+06
4e+06
2e+06
0e+00

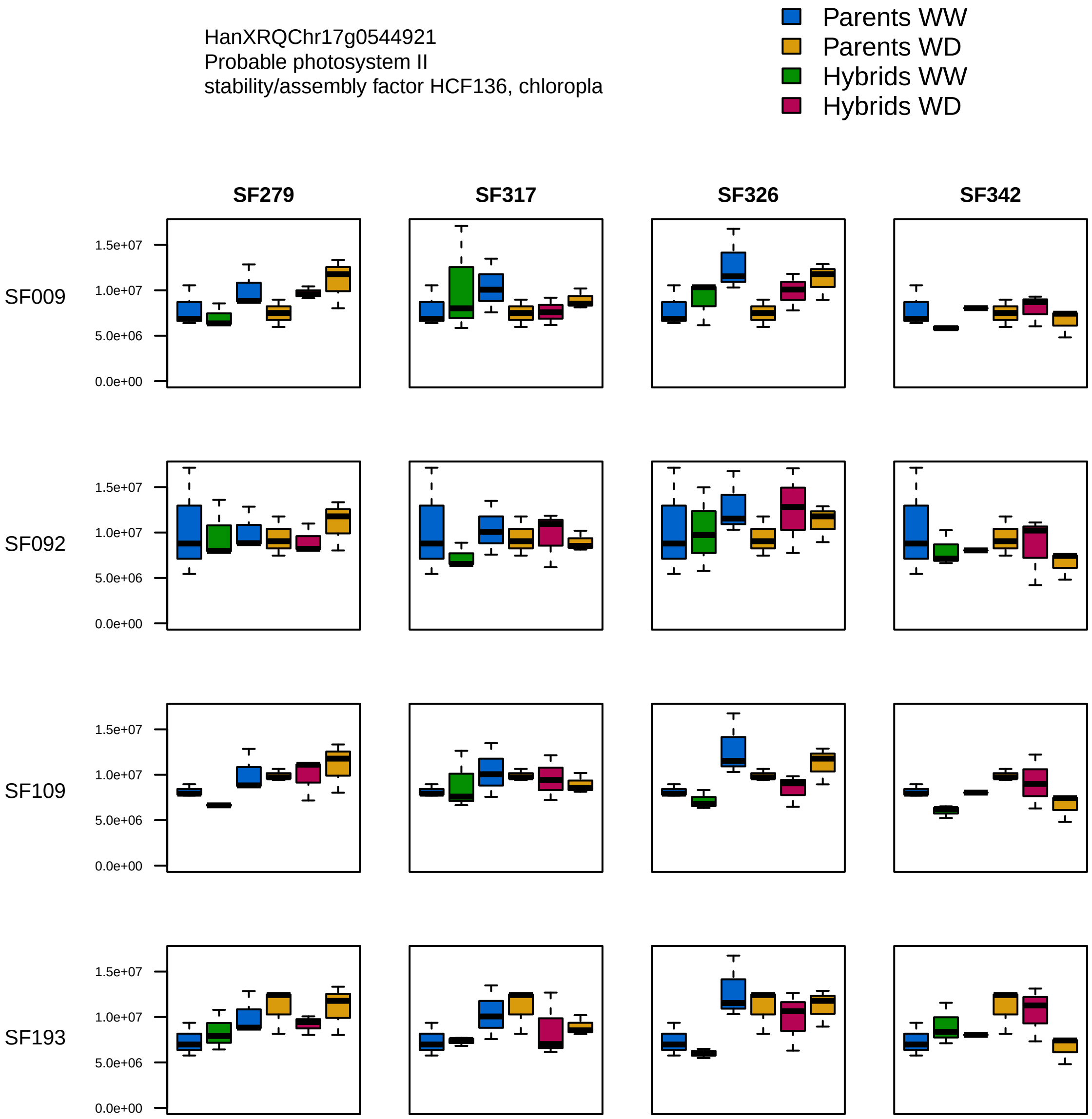
HanXRQChr17g0544921
Probable photosystem II
stability/assembly factor HCF136, chloropla
Parents WW
Parents WD
Hybrids WW
Hybrids WD
SF279
SF317
SF326
SF342
SF009
SF092
SF109
SF193
1.5e+07
1.0e+07
5.0e+06
0.0e+00

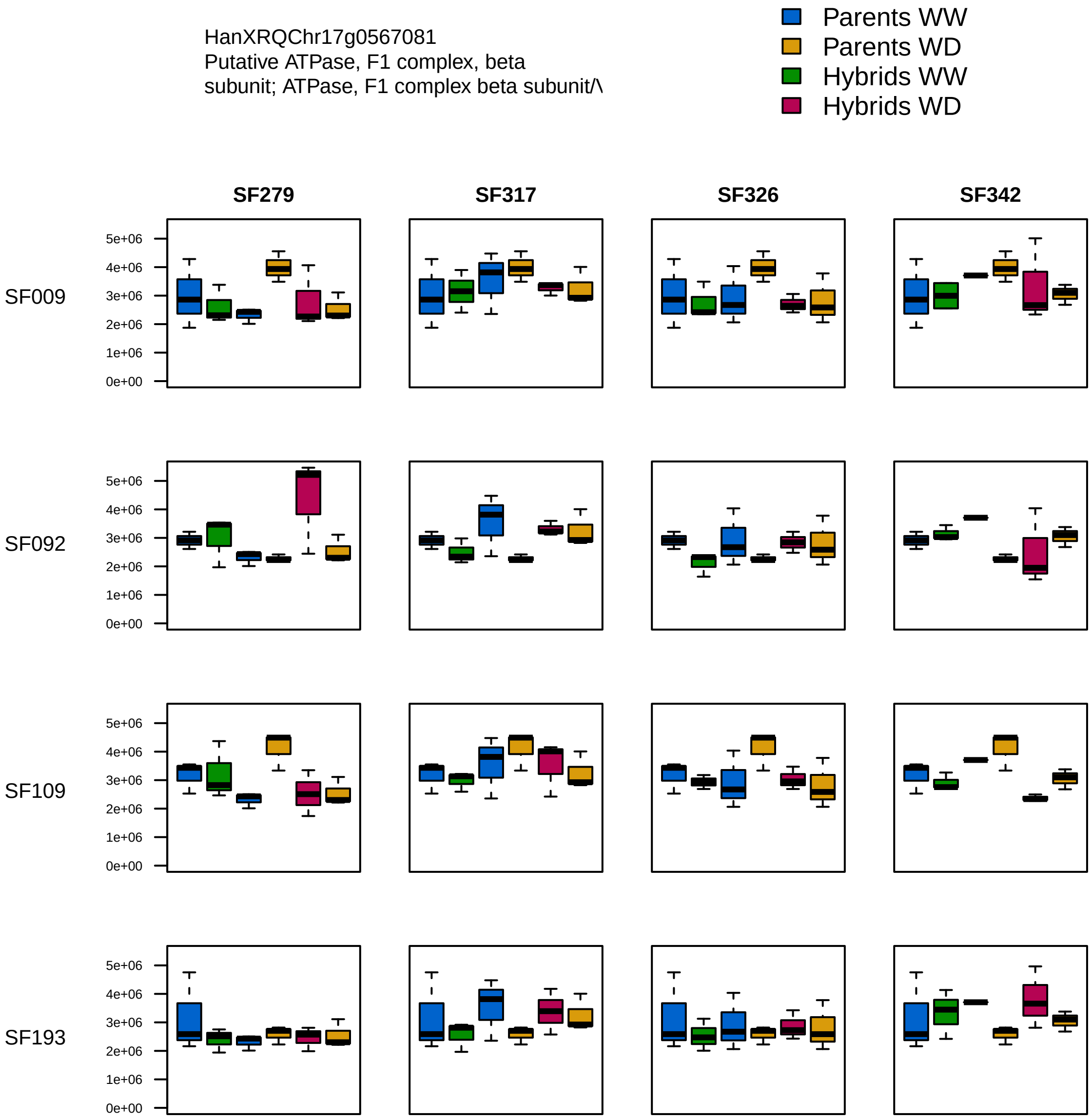
HanXRQChr17g0567081
Putative ATPase, F1 complex, beta
subunit; ATPase, F1 complex beta subunit/\
Parents WW
Parents WD
Hybrids WW
Hybrids WD
SF279
SF317
SF326
SF342
SF009
SF092
SF109
SF193
5e+06
4e+06
3e+06
2e+06
1e+06
0e+00

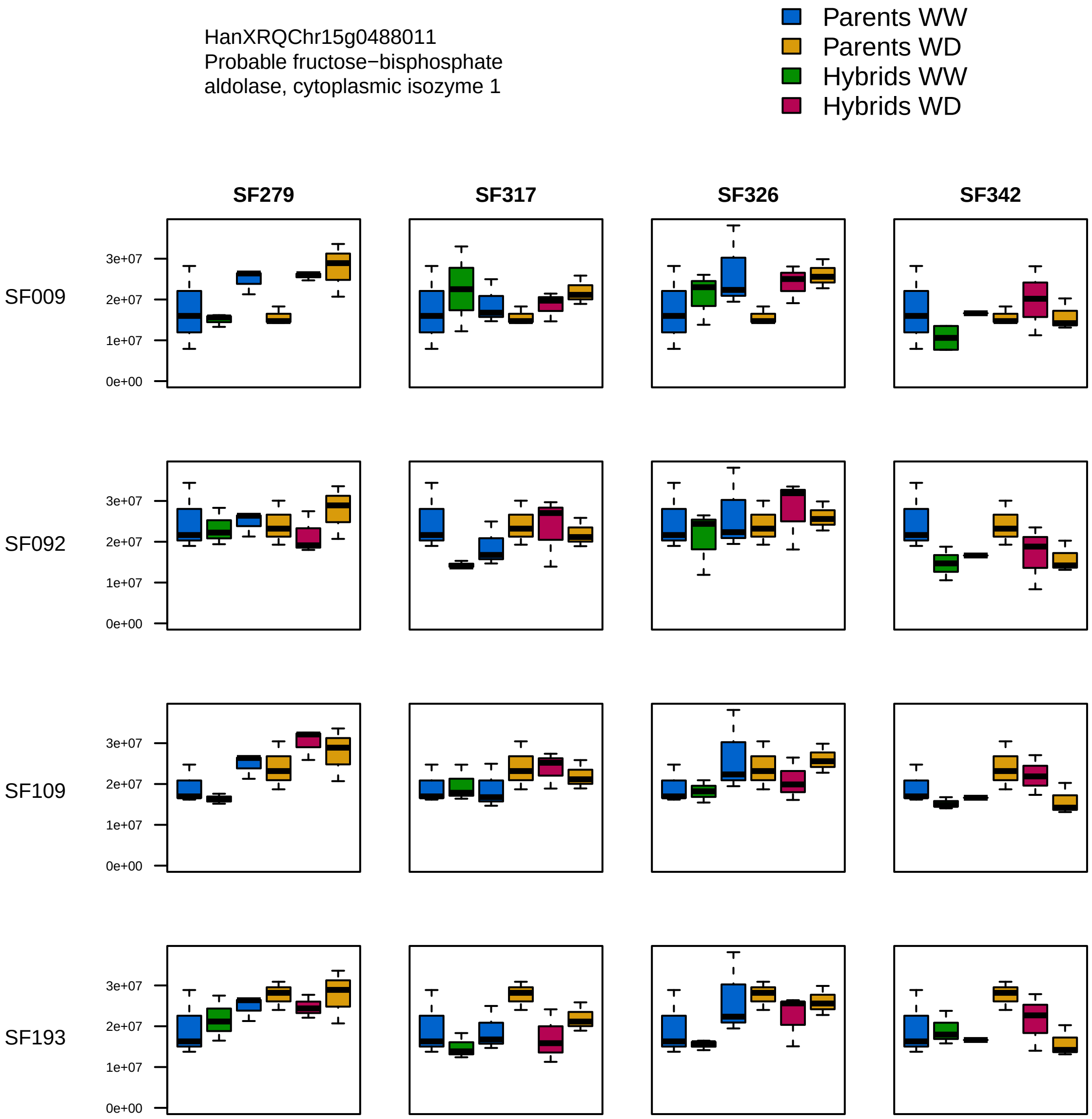
HanXRQChr15g0488011
Probable fructose−bisphosphate
aldolase, cytoplasmic isozyme 1
Parents WW
Parents WD
Hybrids WW
Hybrids WD
SF279
SF317
SF326
SF342
SF009
SF092
SF109
SF193
0e+00
1e+07
2e+07
3e+07

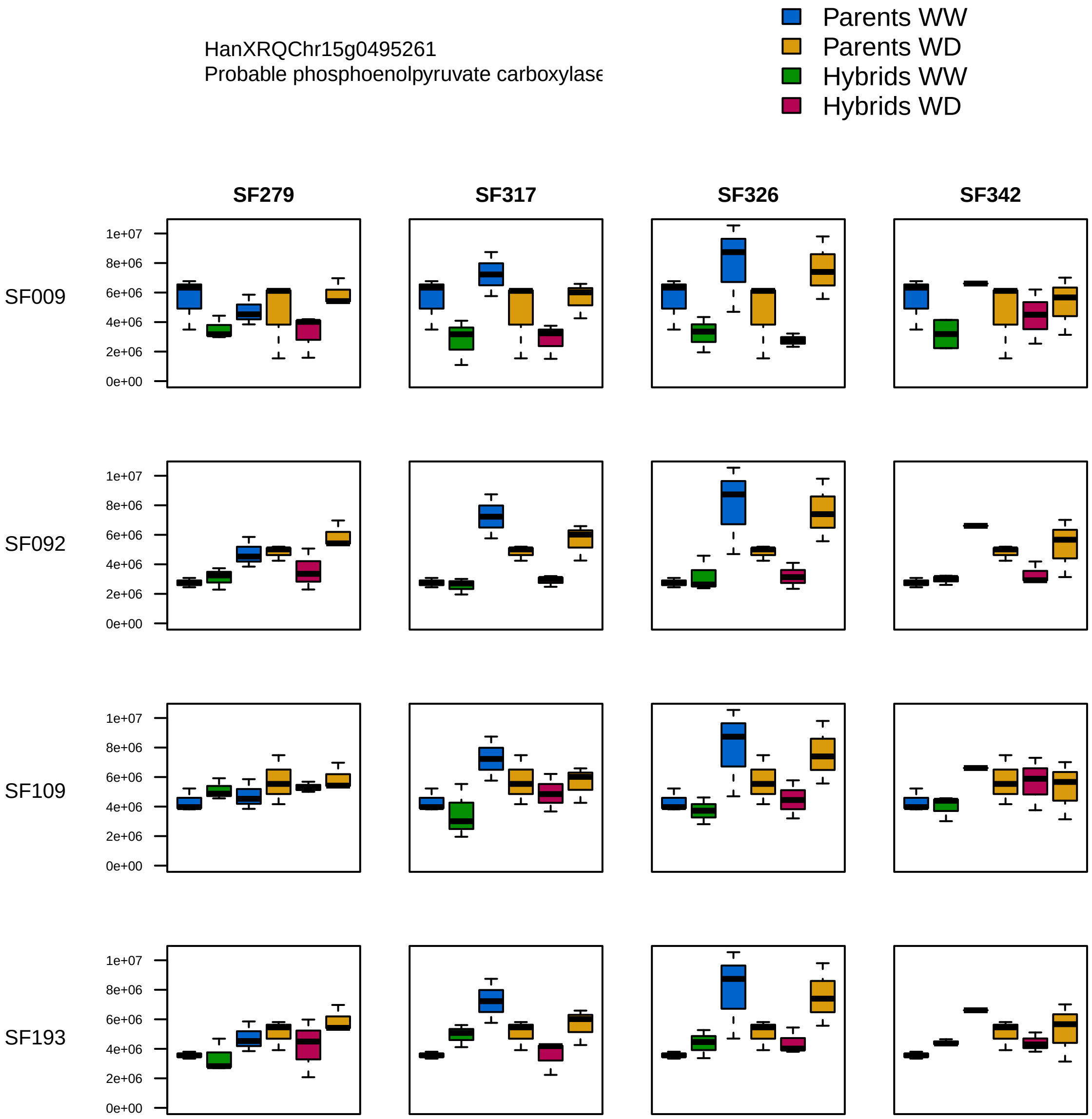
HanXRQChr15g0495261
Probable phosphoenolpyruvate carboxylase
Parents WW
Parents WD
Hybrids WW
Hybrids WD
SF279
SF317
SF326
SF342
SF009
SF092
SF109
SF193
1e+07
8e+06
6e+06
4e+06
2e+06
0e+00

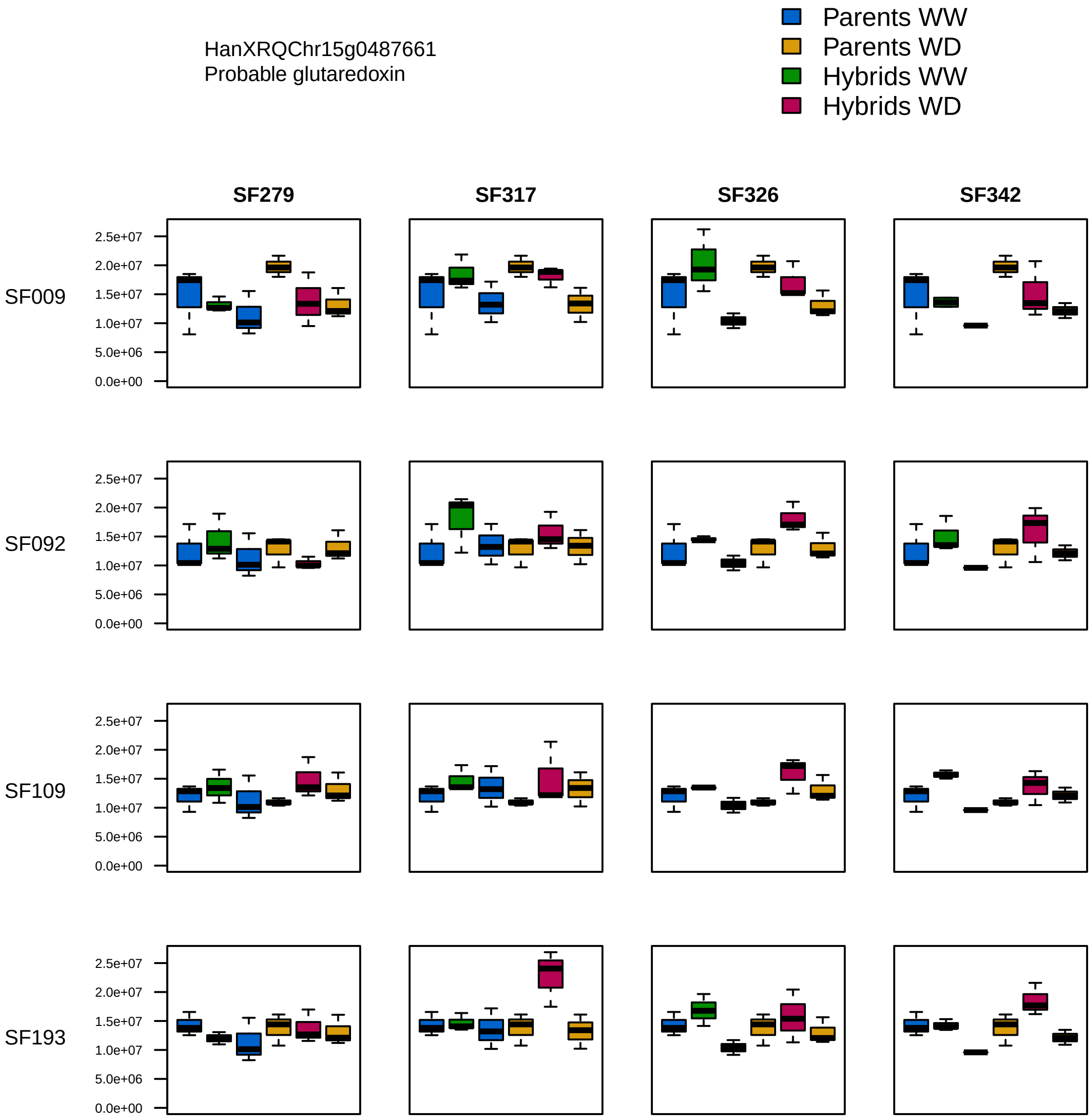
HanXRQChr15g0487661
Probable glutaredoxin
Parents WW
Parents WD
Hybrids WW
Hybrids WD
SF279
SF317
SF326
SF342
SF009
SF092
SF109
SF193
2.5e+07
2.0e+07
1.5e+07
1.0e+07
5.0e+06
0.0e+00

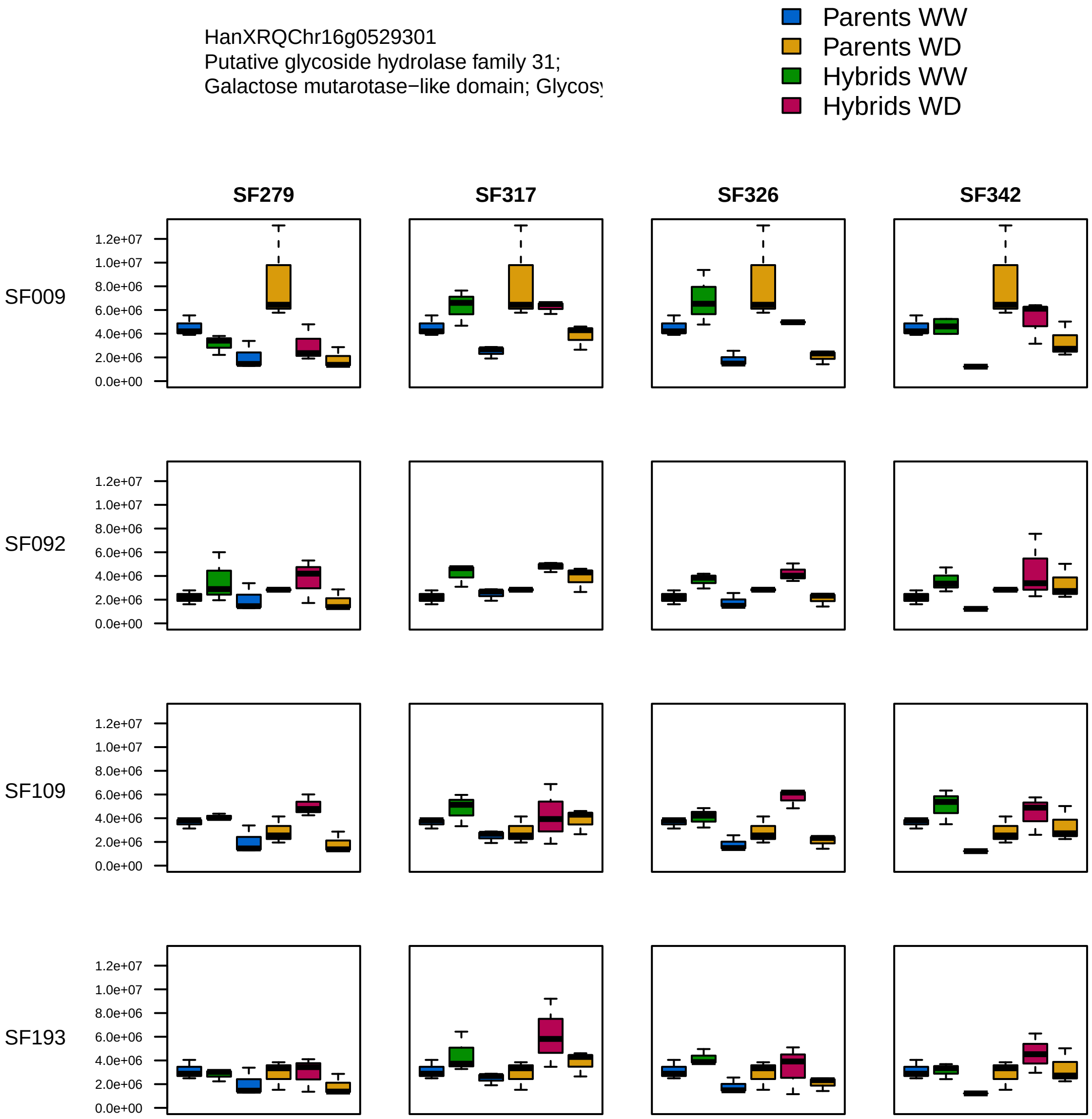

HanXRQChr16g0529301
Putative glycoside hydrolase family 31;
Galactose mutarotase−like domain; Glycos
Parents WW
Parents WD
Hybrids WW
Hybrids WD
SF279
SF317
SF326
SF342
SF009
SF092
SF109
SF193
1.2e+07
1.0e+07
8.0e+06
6.0e+06
4.0e+06
2.0e+06
0.0e+00

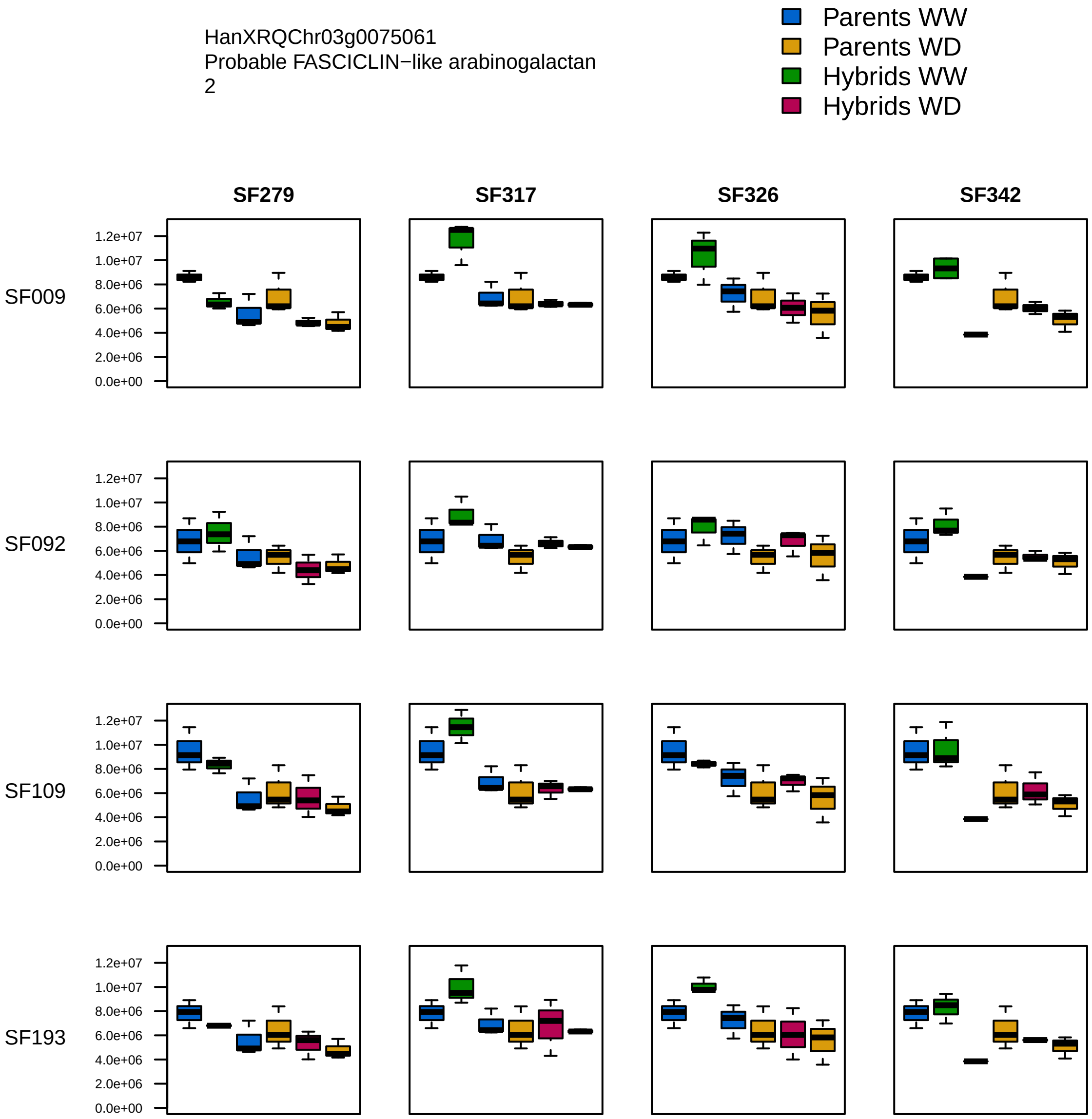
HanXRQChr03g0075061
Probable FASCICLIN−like arabinogalactan
2
Parents WW
Parents WD
Hybrids WW
Hybrids WD
SF279
SF317
SF326
SF342
SF009
SF092
SF109
SF193
1.2e+07
1.0e+07
8.0e+06
6.0e+06
4.0e+06
2.0e+06
0.0e+00

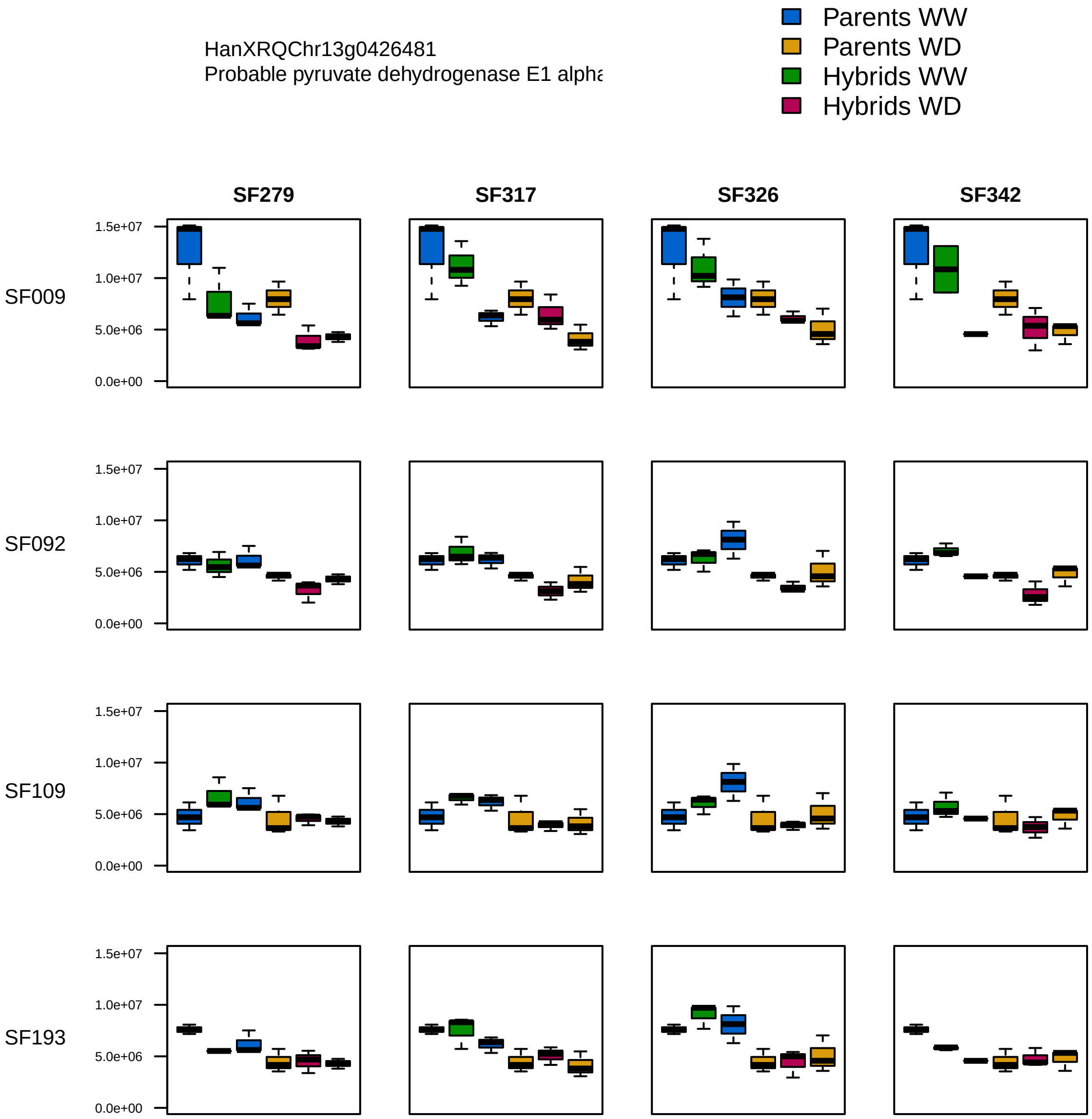

HanXRQChr13g0426481
Probable pyruvate dehydrogenase E1 alpha
Parents WW
Parents WD
Hybrids WW
Hybrids WD
SF279
SF317
SF326
SF342
SF009
SF092
SF109
SF193
1.5e+07
1.0e+07
5.0e+06
0.0e+00

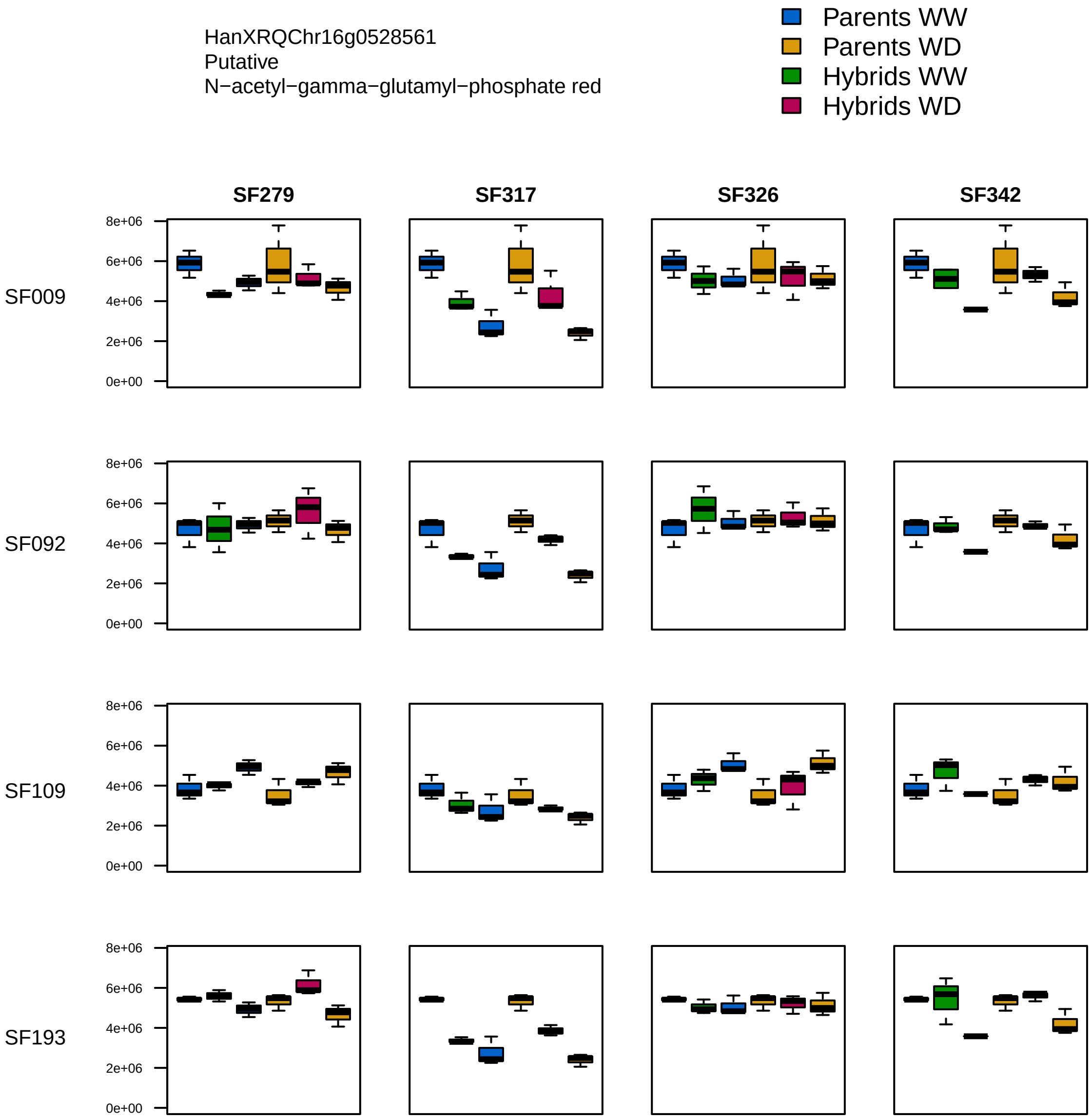

HanXRQChr16g0528561
Putative
N−acetyl−gamma−glutamyl−phosphate red
Parents WW
Parents WD
Hybrids WW
Hybrids WD
SF279
SF317
SF326
SF342
SF009
SF092
SF109
SF193
8e+06
6e+06
4e+06
2e+06
0e+00

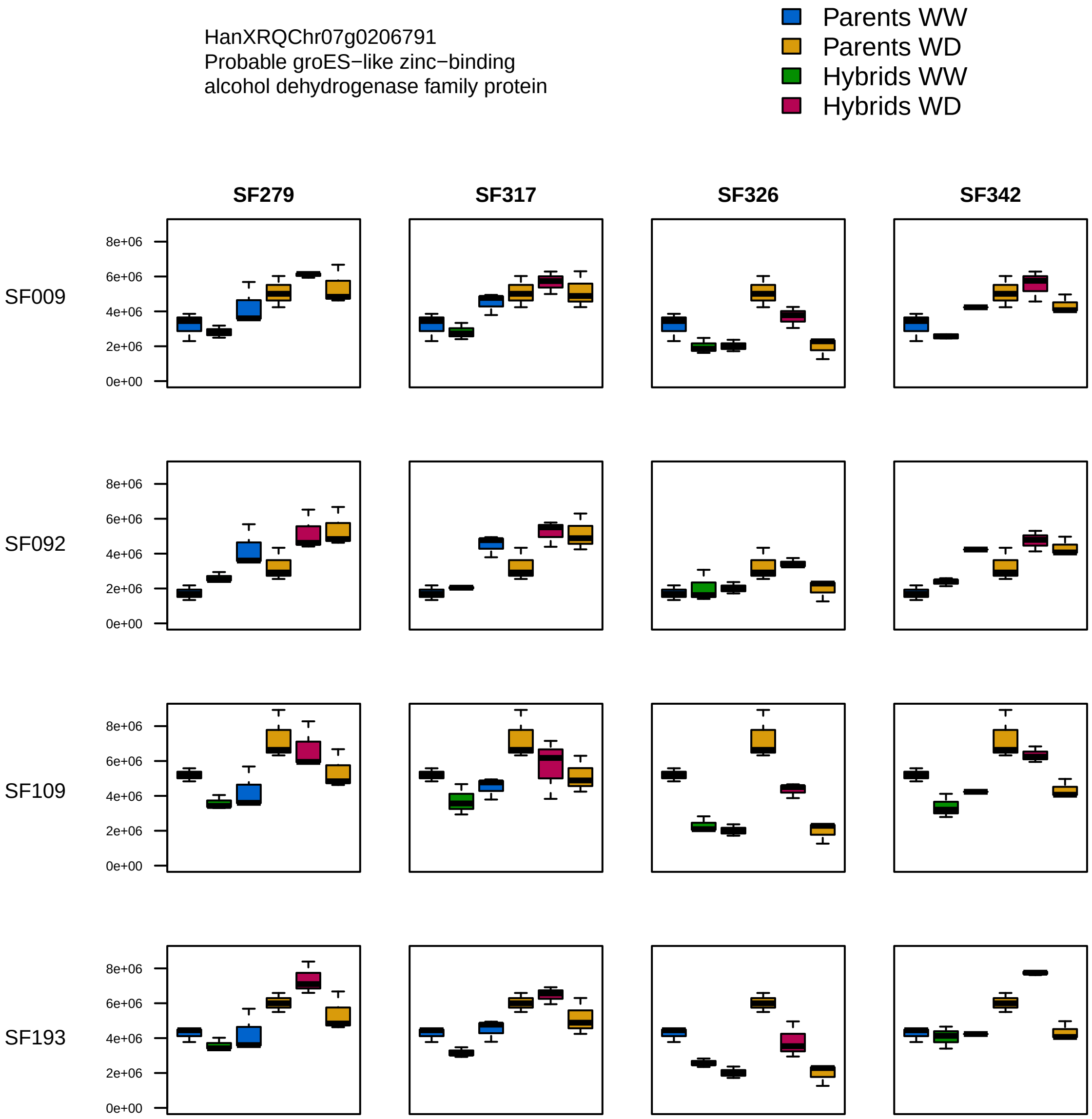

HanXRQChr07g0206791
Probable groES−like zinc−binding
alcohol dehydrogenase family protein
Parents WW
Parents WD
Hybrids WW
Hybrids WD
SF279
SF317
SF326
SF342
SF009
SF092
SF109
SF193
8e+06
6e+06
4e+06
2e+06
0e+00

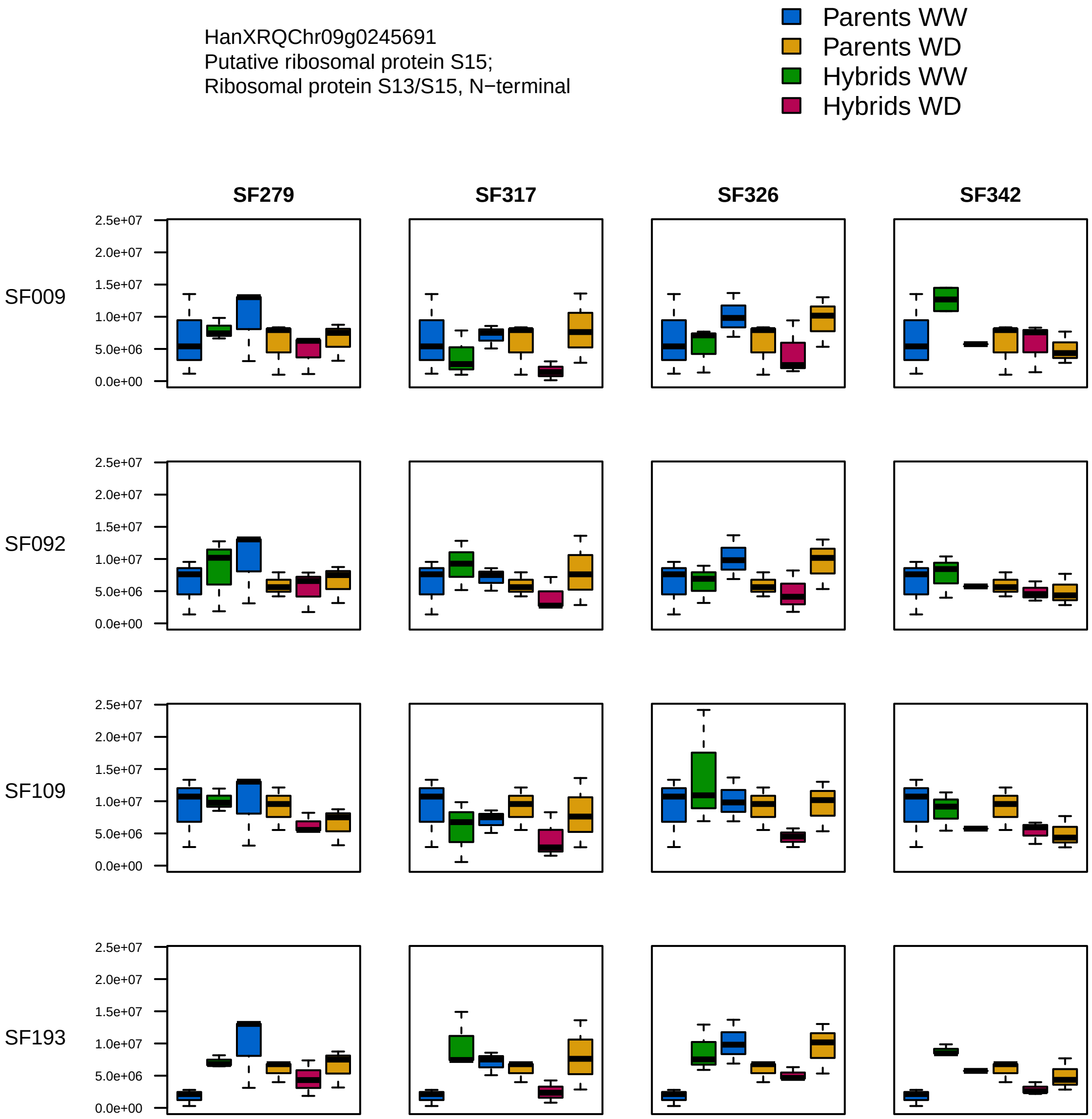
HanXRQChr09g0245691
Putative ribosomal protein S15;
Ribosomal protein S13/S15, N−terminal
Parents WW
Parents WD
Hybrids WW
Hybrids WD
SF279
SF317
SF326
SF342
SF009
SF092
SF109
SF193
2.5e+07
2.0e+07
1.5e+07
1.0e+07
5.0e+06
0.0e+00

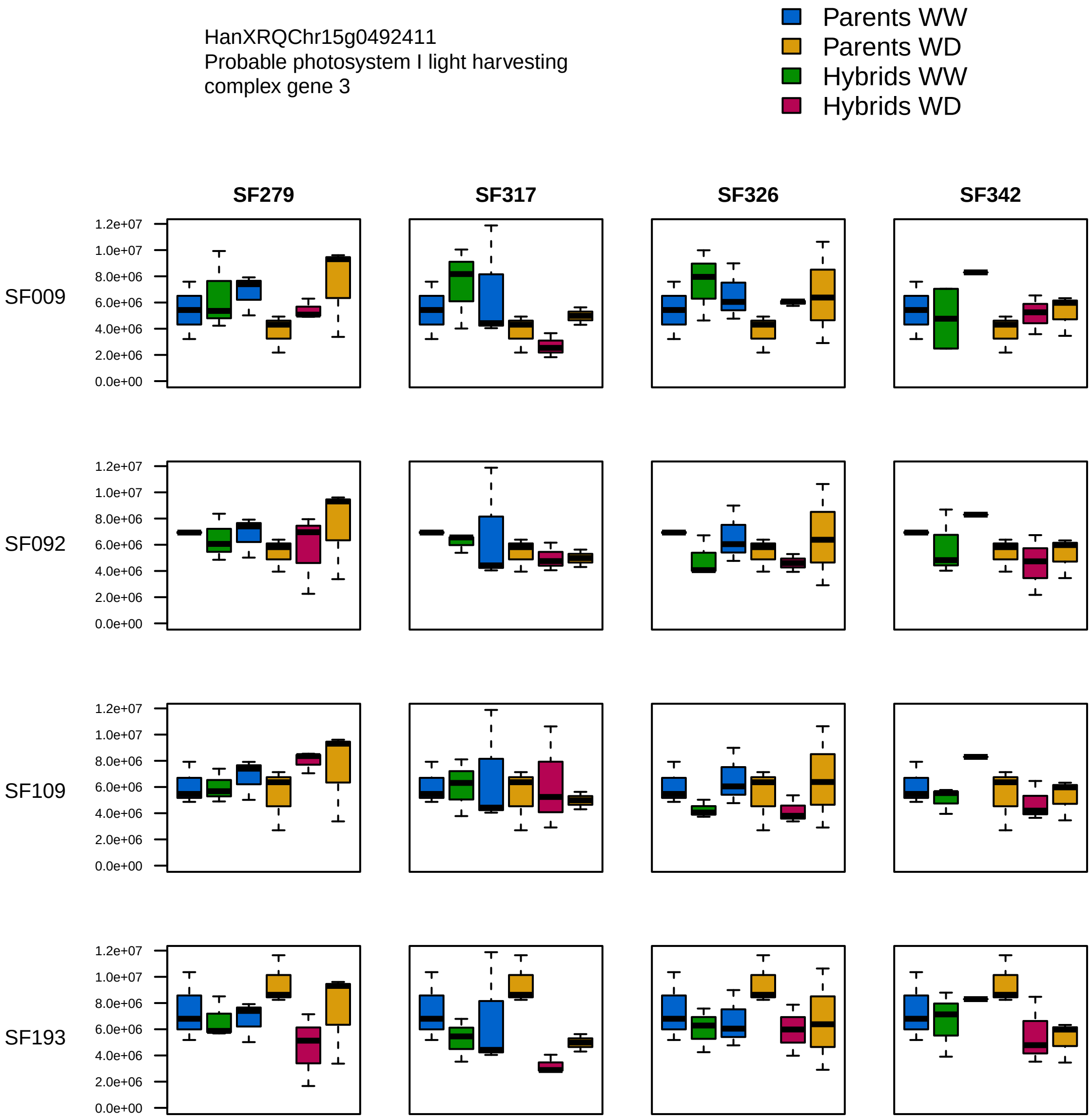
HanXRQChr15g0492411
Probable photosystem I light harvesting complex gene 3
Parents WW
Parents WD
Hybrids WW
Hybrids WD
SF279
SF317
SF326
SF342
SF009
SF092
SF109
SF193
1.2e+07
1.0e+07
8.0e+06
6.0e+06
4.0e+06
2.0e+06
0.0e+00

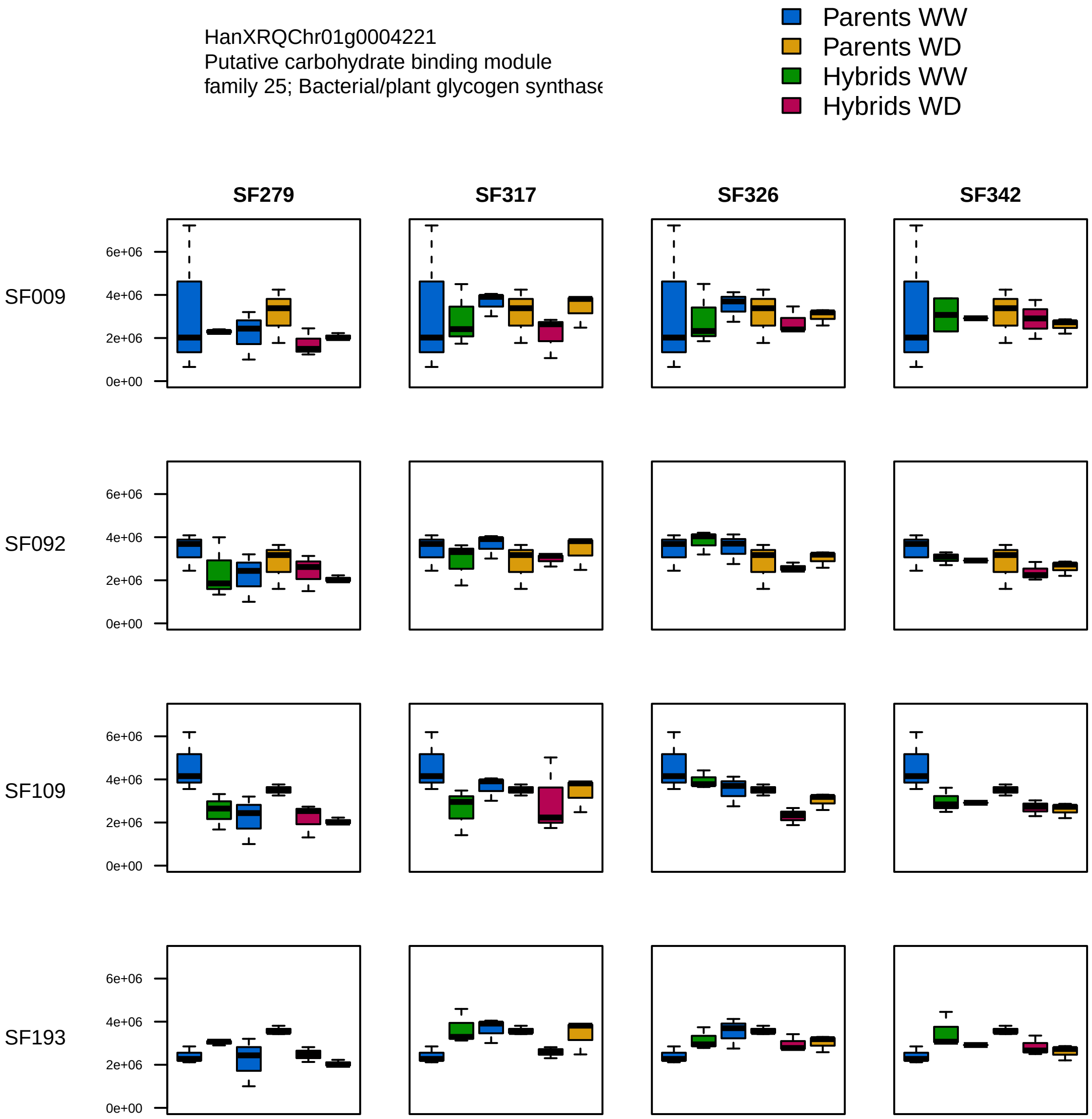

HanXRQChr01g0004221
Putative carbohydrate binding module
family 25; Bacterial/plant glycogen synthase
Parents WW
Parents WD
Hybrids WW
Hybrids WD
SF279
SF317
SF326
SF342
SF009
SF092
SF109
SF193
0e+00
2e+06
4e+06
6e+06

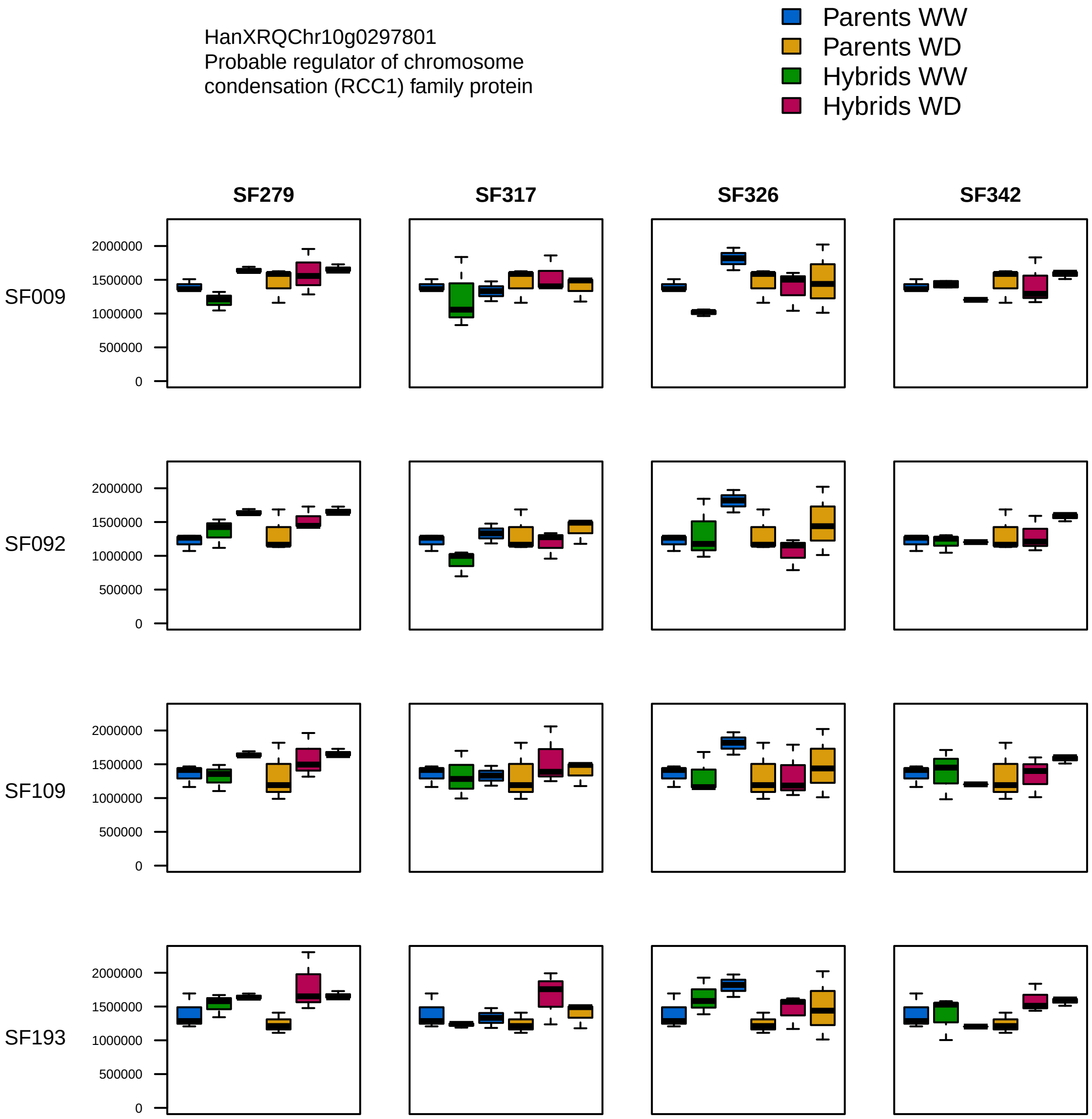
HanXRQChr10g0297801
Probable regulator of chromosome
condensation (RCC1) family protein
Parents WW
Parents WD
Hybrids WW
Hybrids WD
SF279
SF317
SF326
SF342
SF009
SF092
SF109
SF193
2000000
1500000
1000000
500000
0

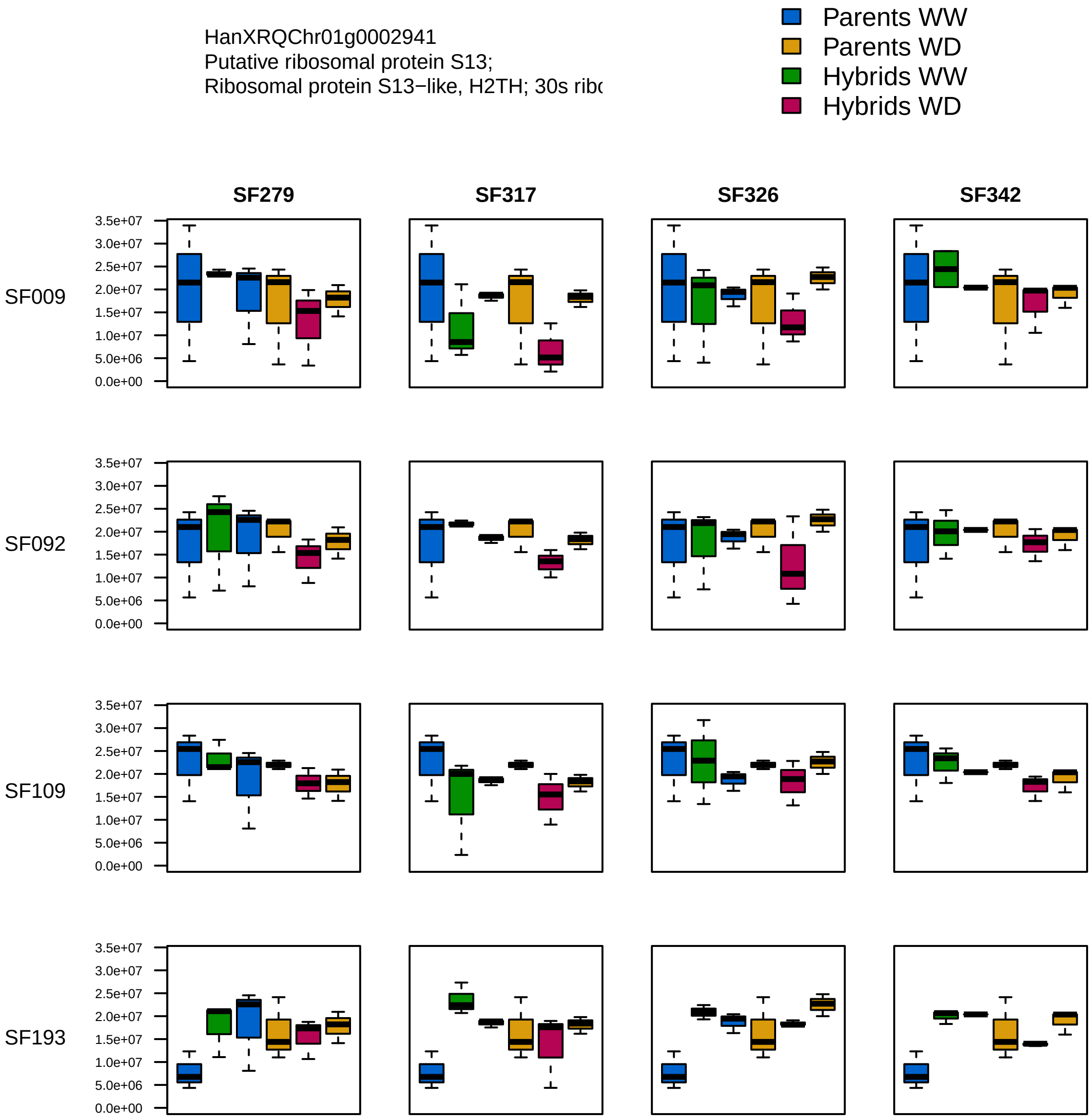
HanXRQChr01g0002941
Putative ribosomal protein S13;
Ribosomal protein S13−like, H2TH; 30s ribo
Parents WW
Parents WD
Hybrids WW
Hybrids WD
SF279
SF317
SF326
SF342
SF009
SF092
SF109
SF193
3.5e+07
3.0e+07
2.5e+07
2.0e+07
1.5e+07
1.0e+07
5.0e+06
0.0e+00

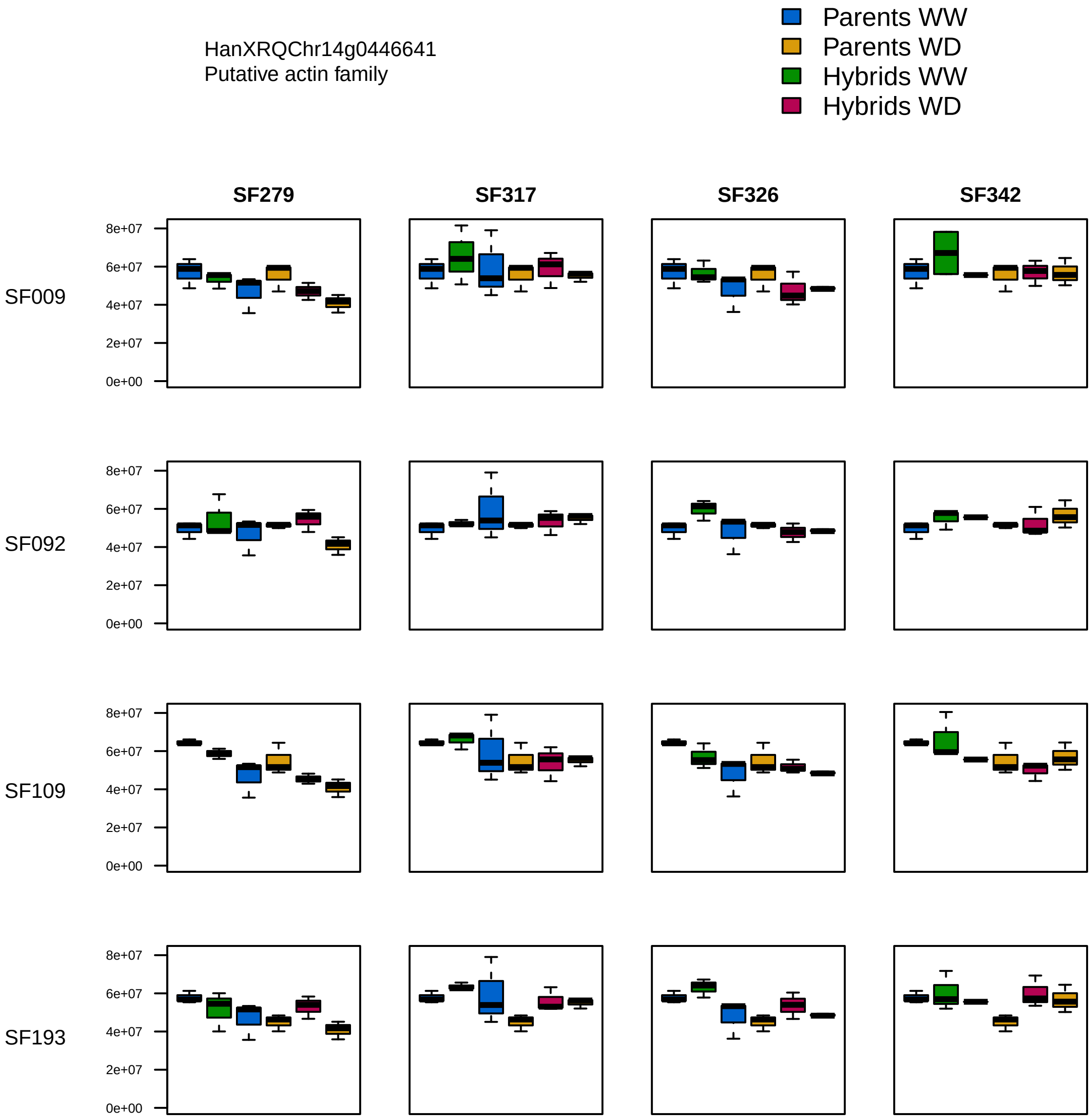
HanXRQChr14g0446641
Putative actin family
Parents WW
Parents WD
Hybrids WW
Hybrids WD
SF279
SF317
SF326
SF342
SF009
SF092
SF109
SF193
8e+07
6e+07
4e+07
2e+07
0e+00

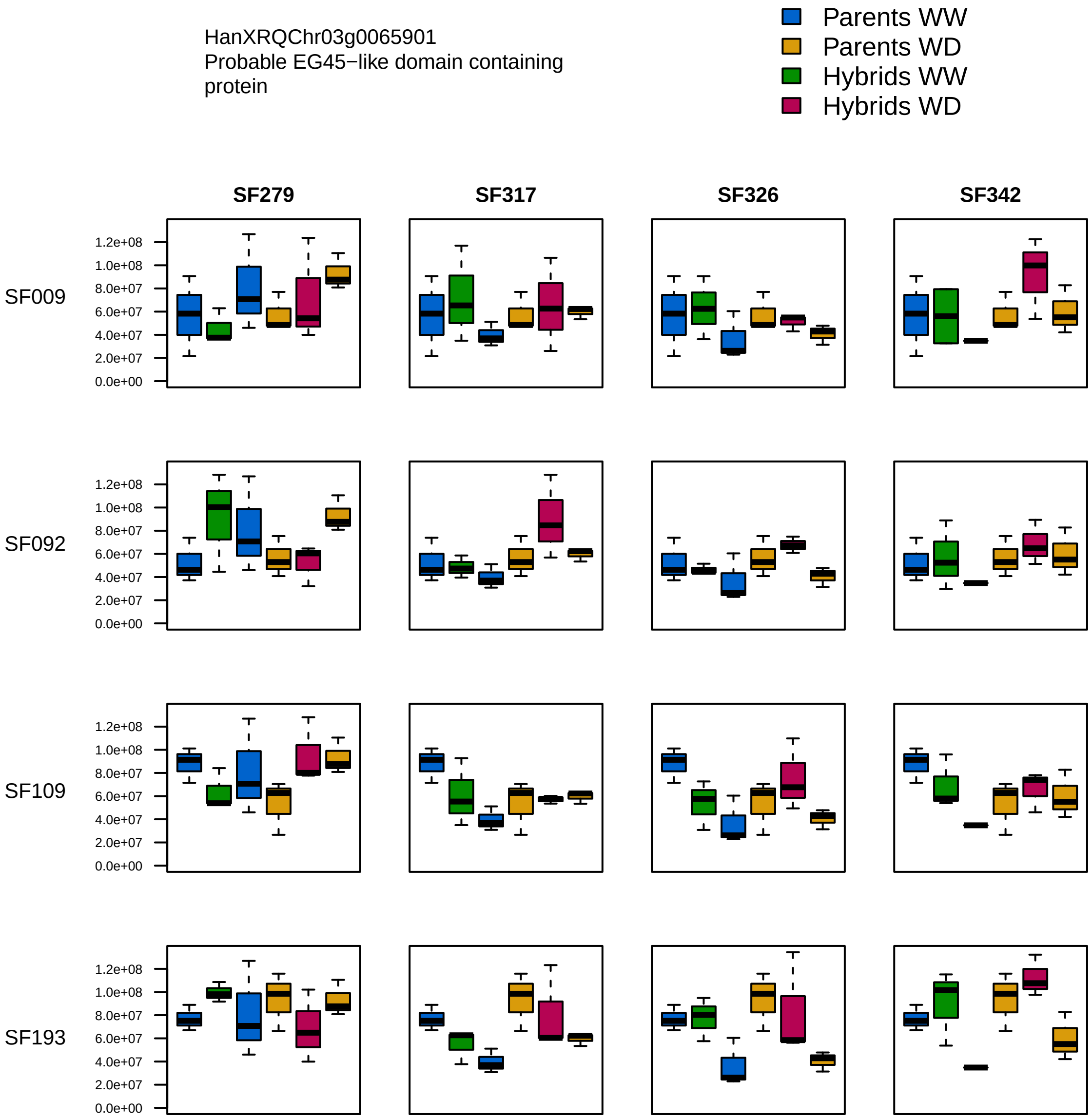

HanXRQChr03g0065901
Probable EG45−like domain containing protein
Parents WW
Parents WD
Hybrids WW
Hybrids WD
SF279
SF317
SF326
SF342
SF009
SF092
SF109
SF193
0.0e+00
2.0e+07
4.0e+07
6.0e+07
8.0e+07
1.0e+08
1.2e+08

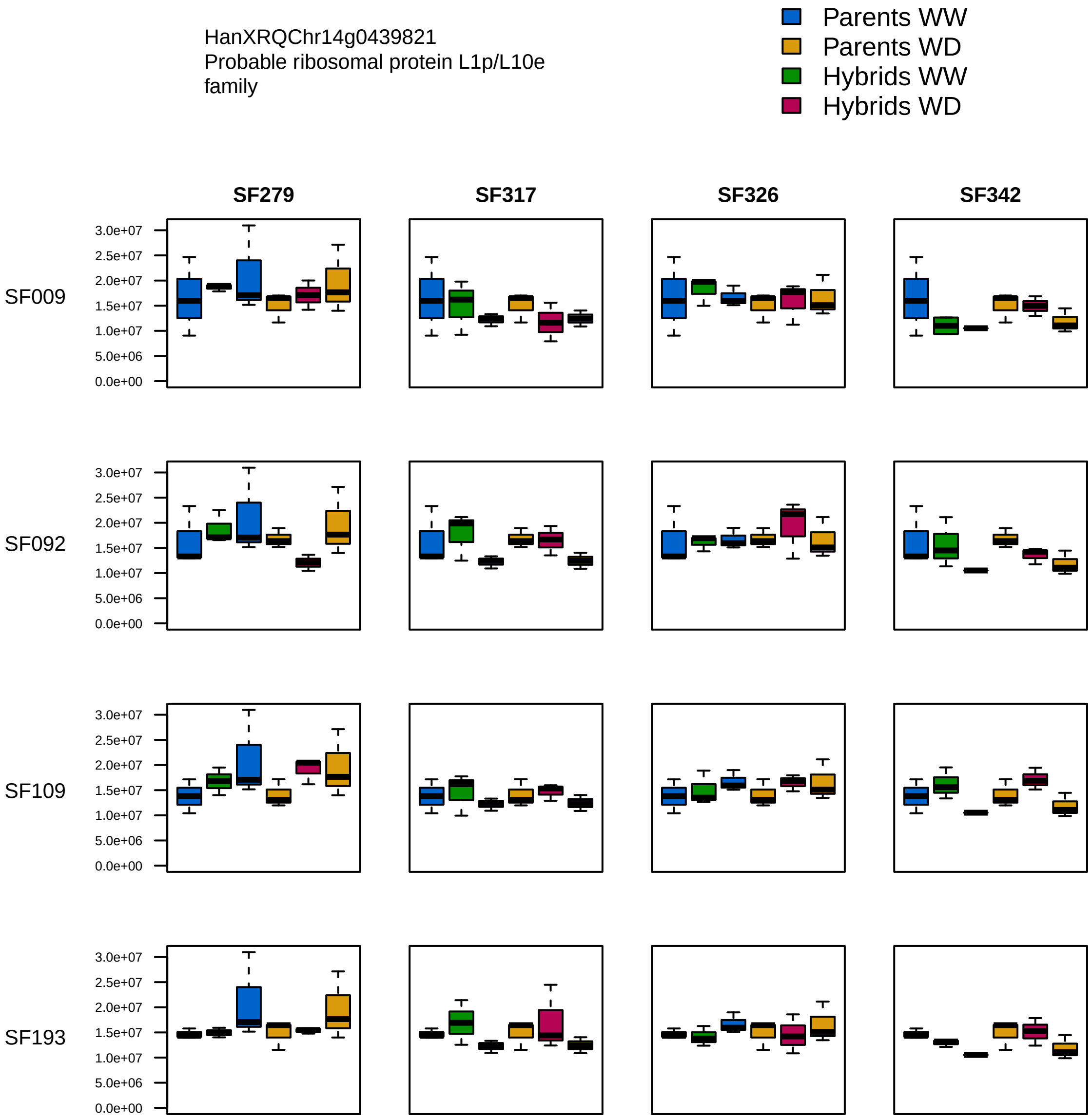
HanXRQChr14g0439821
Probable ribosomal protein L1p/L10e family
Parents WW
Parents WD
Hybrids WW
Hybrids WD
SF279
SF317
SF326
SF342
SF009
SF092
SF109
SF193
0.0e+00
5.0e+06
1.0e+07
1.5e+07
2.0e+07
2.5e+07
3.0e+07

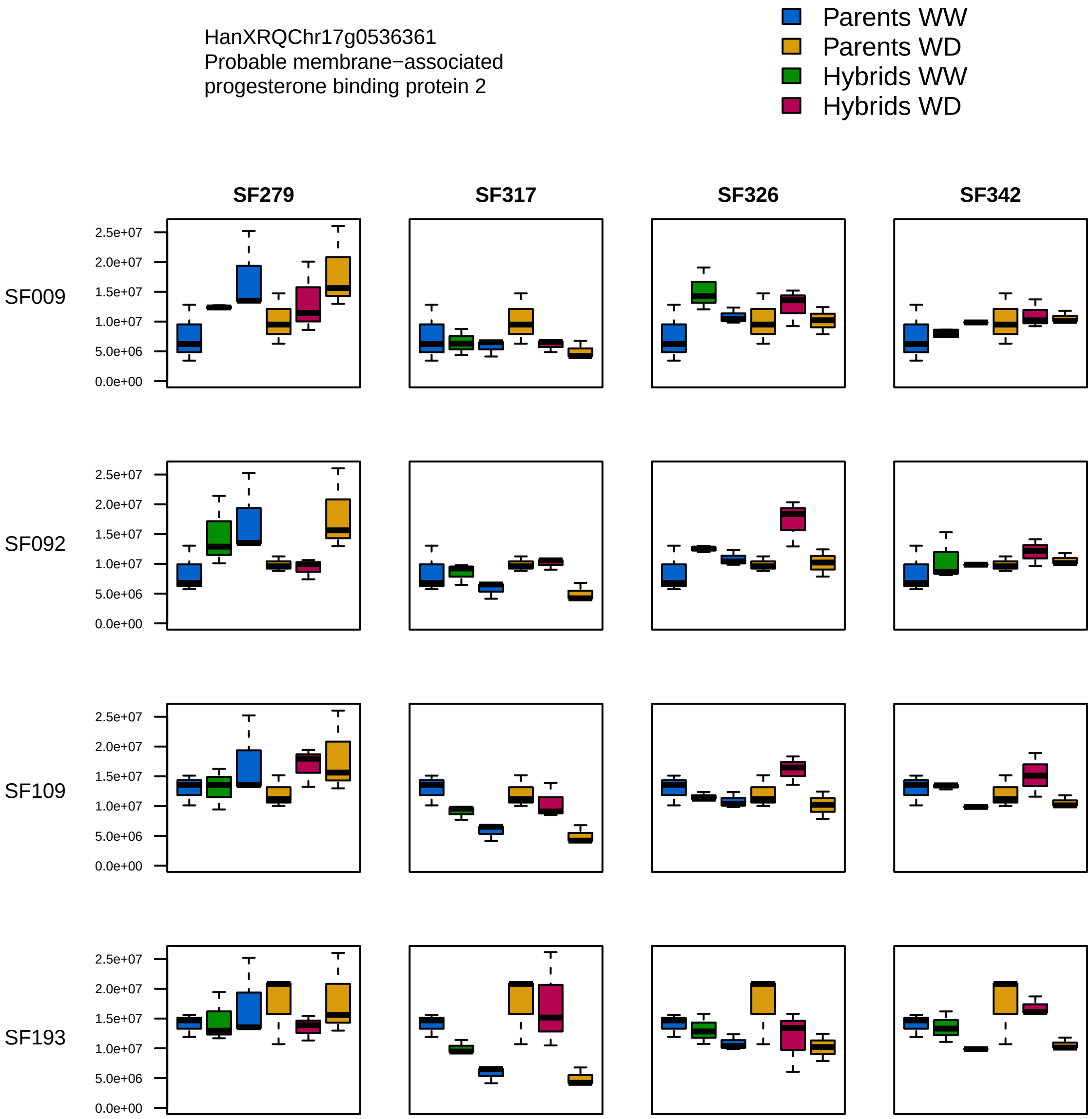
HanXRQChr17g0536361
Probable membrane−associated
progesterone binding protein 2
Parents WW
Parents WD
Hybrids WW
Hybrids WD
SF279
SF317
SF326
SF342
SF009
SF092
SF109
SF193
2.5e+07
2.0e+07
1.5e+07
1.0e+07
5.0e+06
0.0e+00

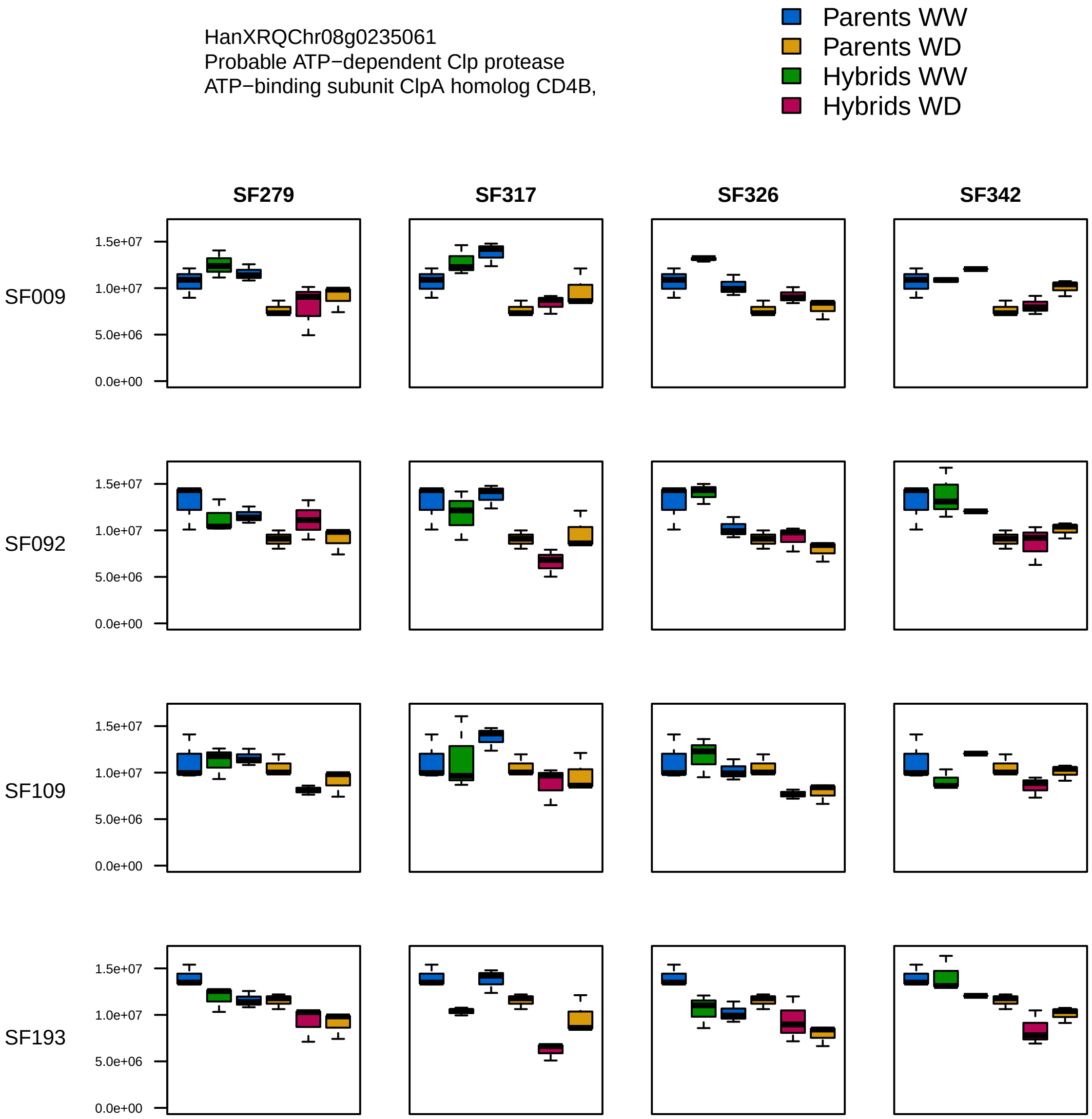
HanXRQChr08g0235061
Probable ATP−dependent Clp protease
ATP−binding subunit ClpA homolog CD4B,
Parents WW
Parents WD
Hybrids WW
Hybrids WD
SF279
SF317
SF326
SF342
SF009
SF092
SF109
SF193
1.5e+07
1.0e+07
5.0e+06
0.0e+00

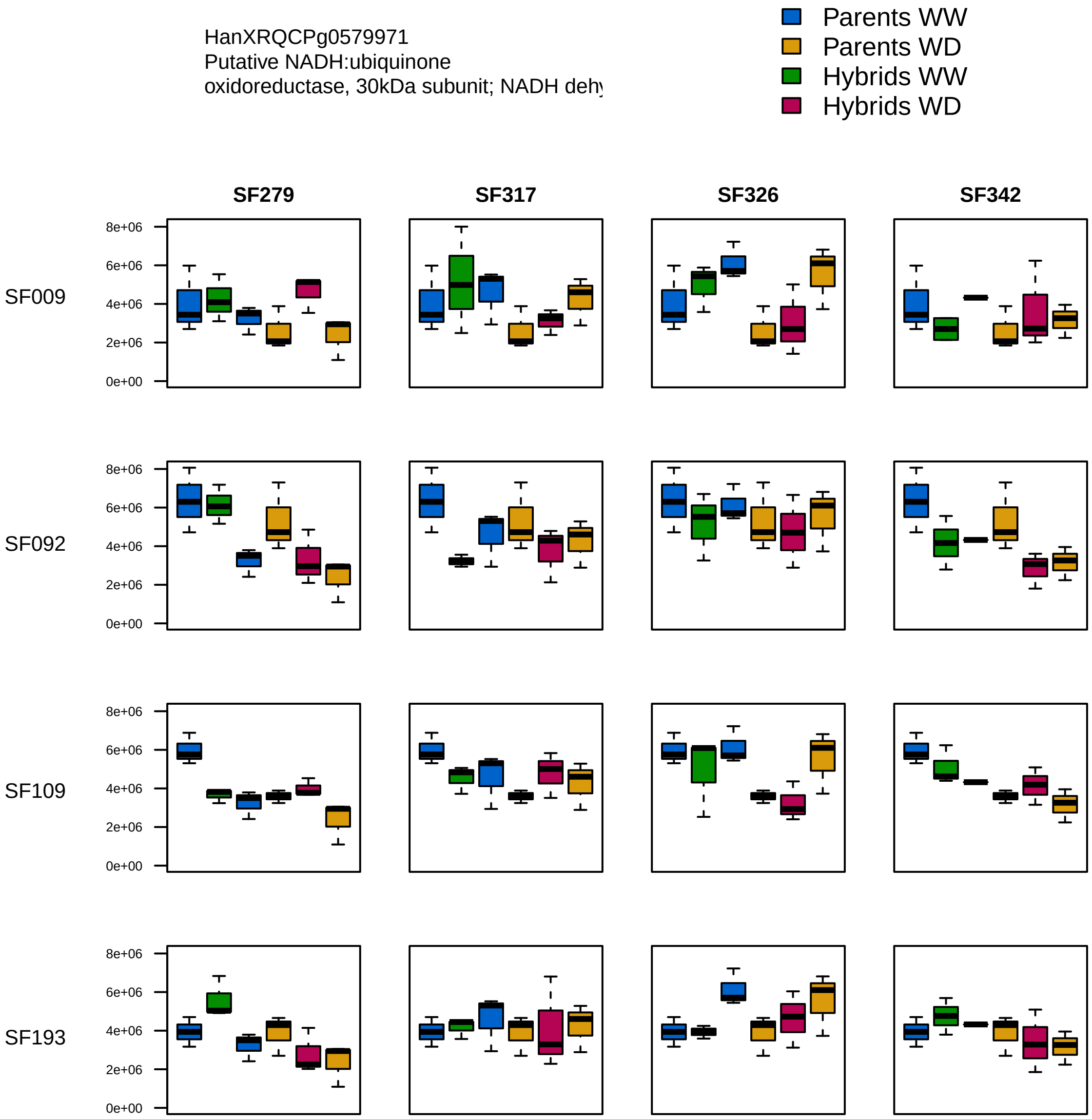
HanXRQCPg0579971
Putative NADH:ubiquinone
oxidoreductase, 30kDa subunit; NADH deh
Parents WW
Parents WD
Hybrids WW
Hybrids WD
SF279
SF317
SF326
SF342
SF009
SF092
SF109
SF193
8e+06
6e+06
4e+06
2e+06
0e+00

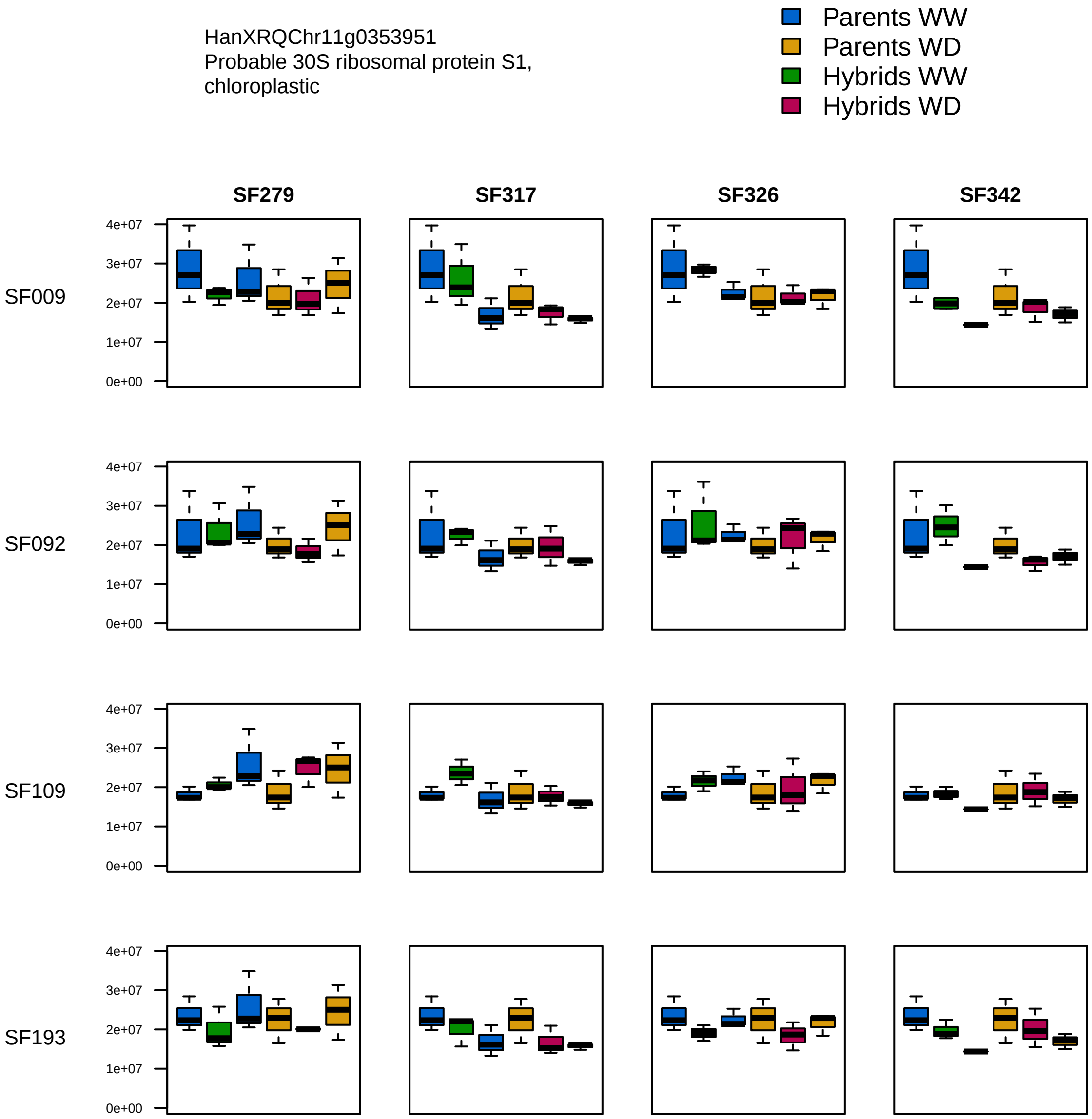
HanXRQChr11g0353951
Probable 30S ribosomal protein S1, chloroplastic
Parents WW
Parents WD
Hybrids WW
Hybrids WD
SF279
SF317
SF326
SF342
SF009
SF092
SF109
SF193
4e+07
3e+07
2e+07
1e+07
0e+00

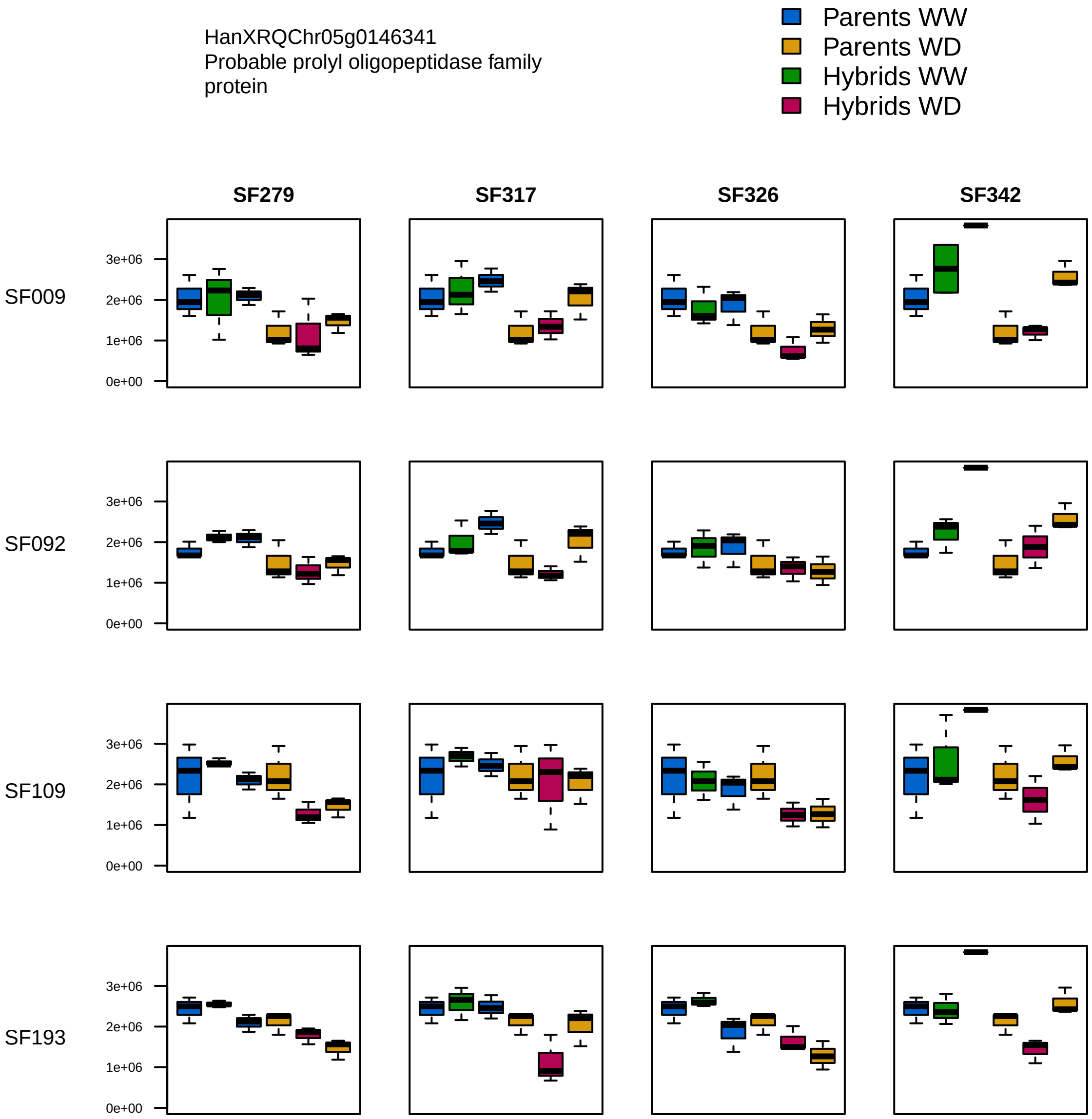
HanXRQChr05g0146341
Probable prolyl oligopeptidase family
protein
Parents WW
Parents WD
Hybrids WW
Hybrids WD
SF279
SF317
SF326
SF342
SF009
SF092
SF109
SF193
0e+00
1e+06
2e+06
3e+06

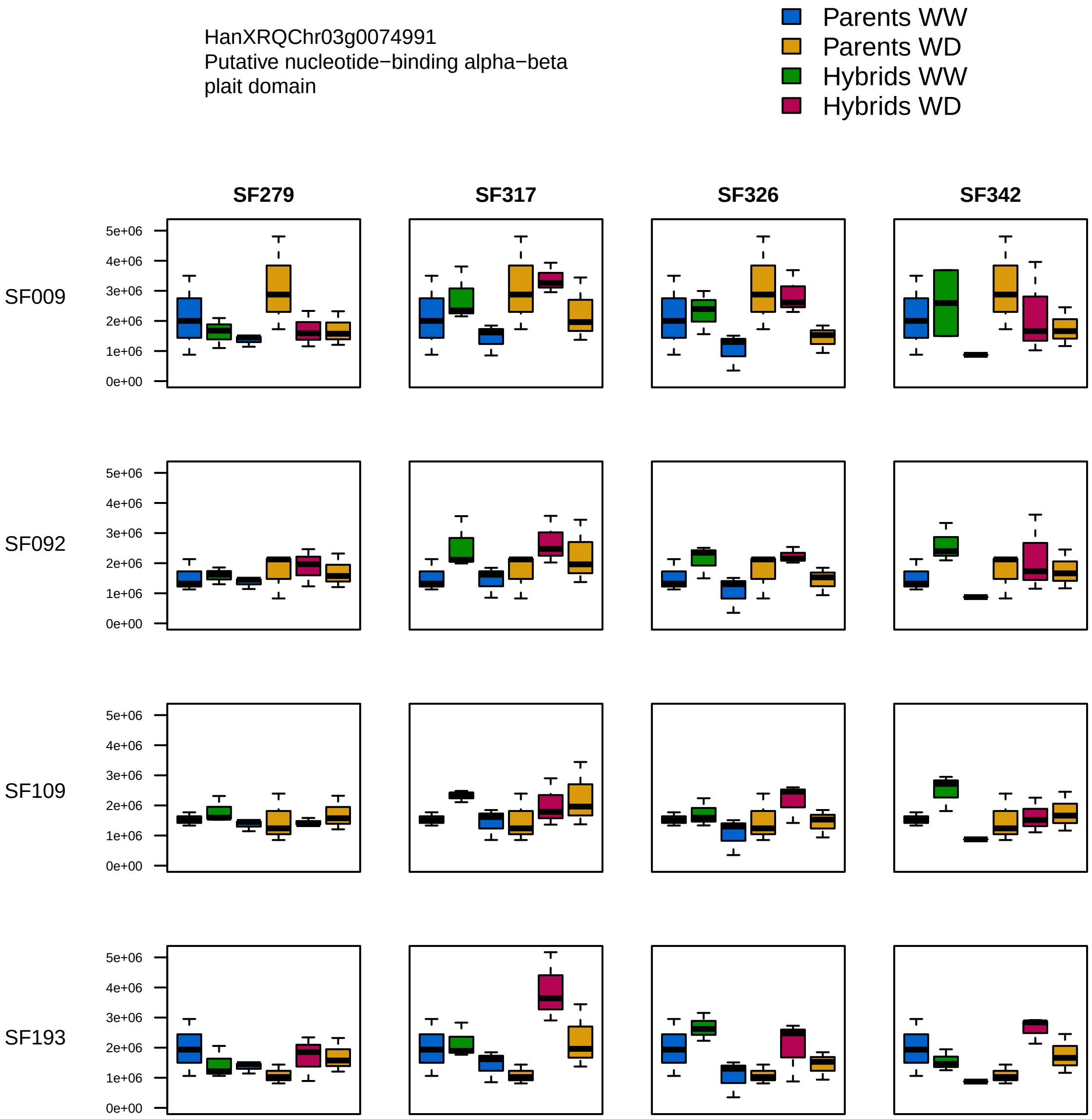
HanXRQChr03g0074991
Putative nucleotide−binding alpha−beta
plait domain
Parents WW
Parents WD
Hybrids WW
Hybrids WD
SF279
SF317
SF326
SF342
SF009
SF092
SF109
SF193
5e+06
4e+06
3e+06
2e+06
1e+06
0e+00

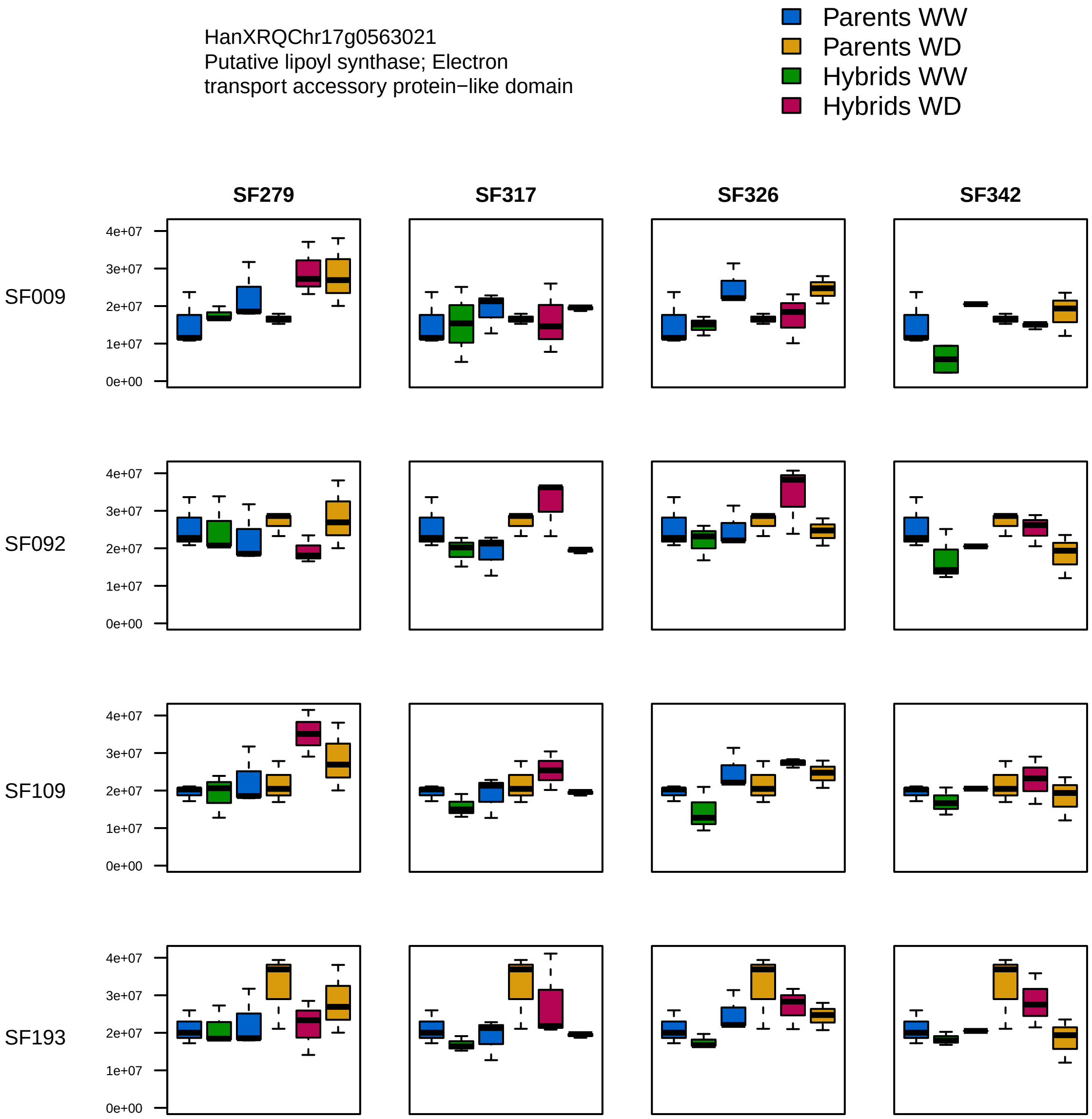
HanXRQChr17g0563021
Putative lipoyl synthase; Electron transport accessory protein−like domain
Parents WW
Parents WD
Hybrids WW
Hybrids WD
SF279
SF317
SF326
SF342
SF009
SF092
SF109
SF193
4e+07
3e+07
2e+07
1e+07
0e+00

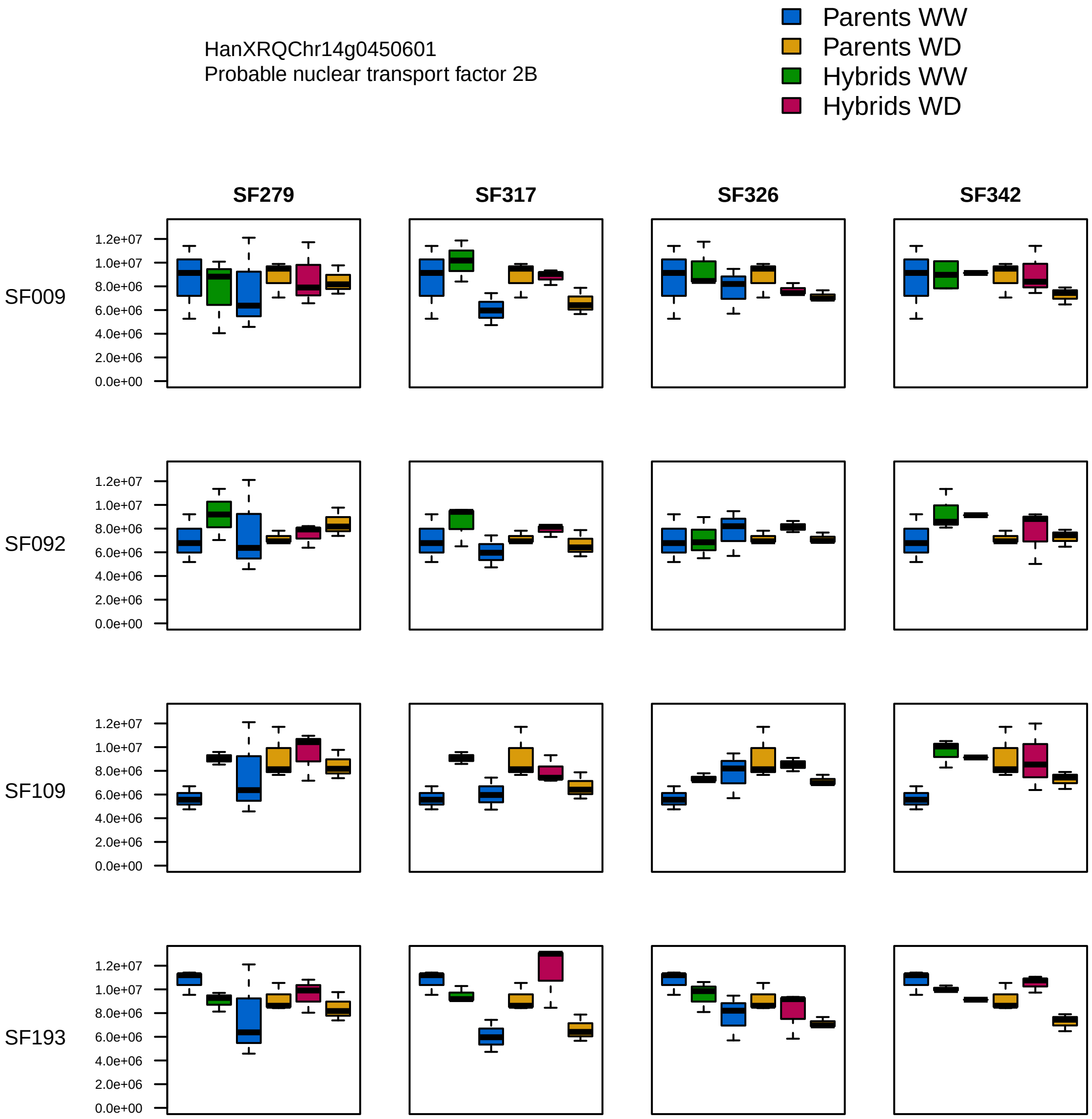

HanXRQChr14g0450601
Probable nuclear transport factor 2B
Parents WW
Parents WD
Hybrids WW
Hybrids WD
SF279
SF317
SF326
SF342
SF009
SF092
SF109
SF193
0.0e+00
2.0e+06
4.0e+06
6.0e+06
8.0e+06
1.0e+07
1.2e+07

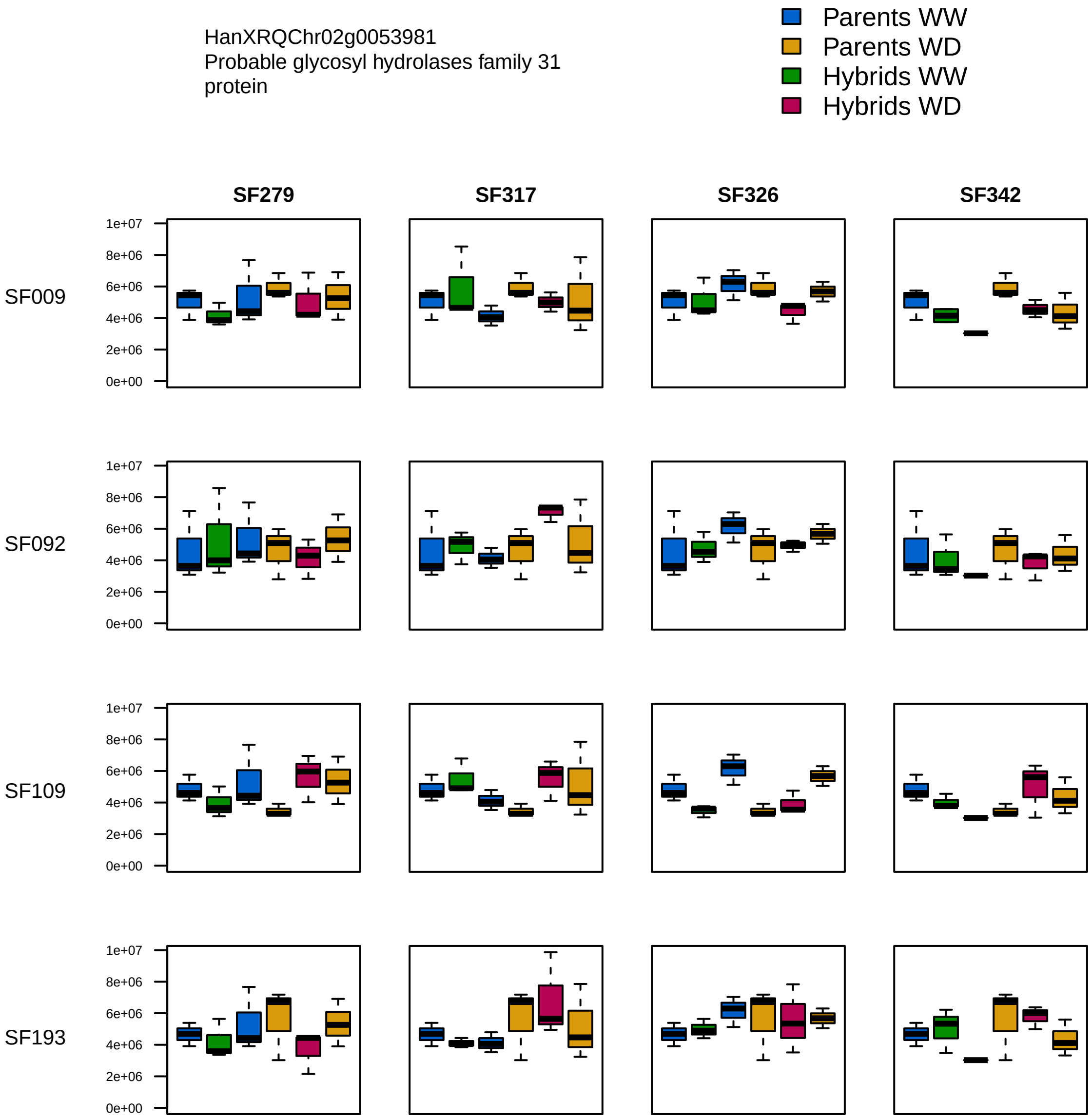
HanXRQChr02g0053981
Probable glycosyl hydrolases family 31
protein
Parents WW
Parents WD
Hybrids WW
Hybrids WD
SF279
SF317
SF326
SF342
SF009
SF092
SF109
SF193
1e+07
8e+06
6e+06
4e+06
2e+06
0e+00

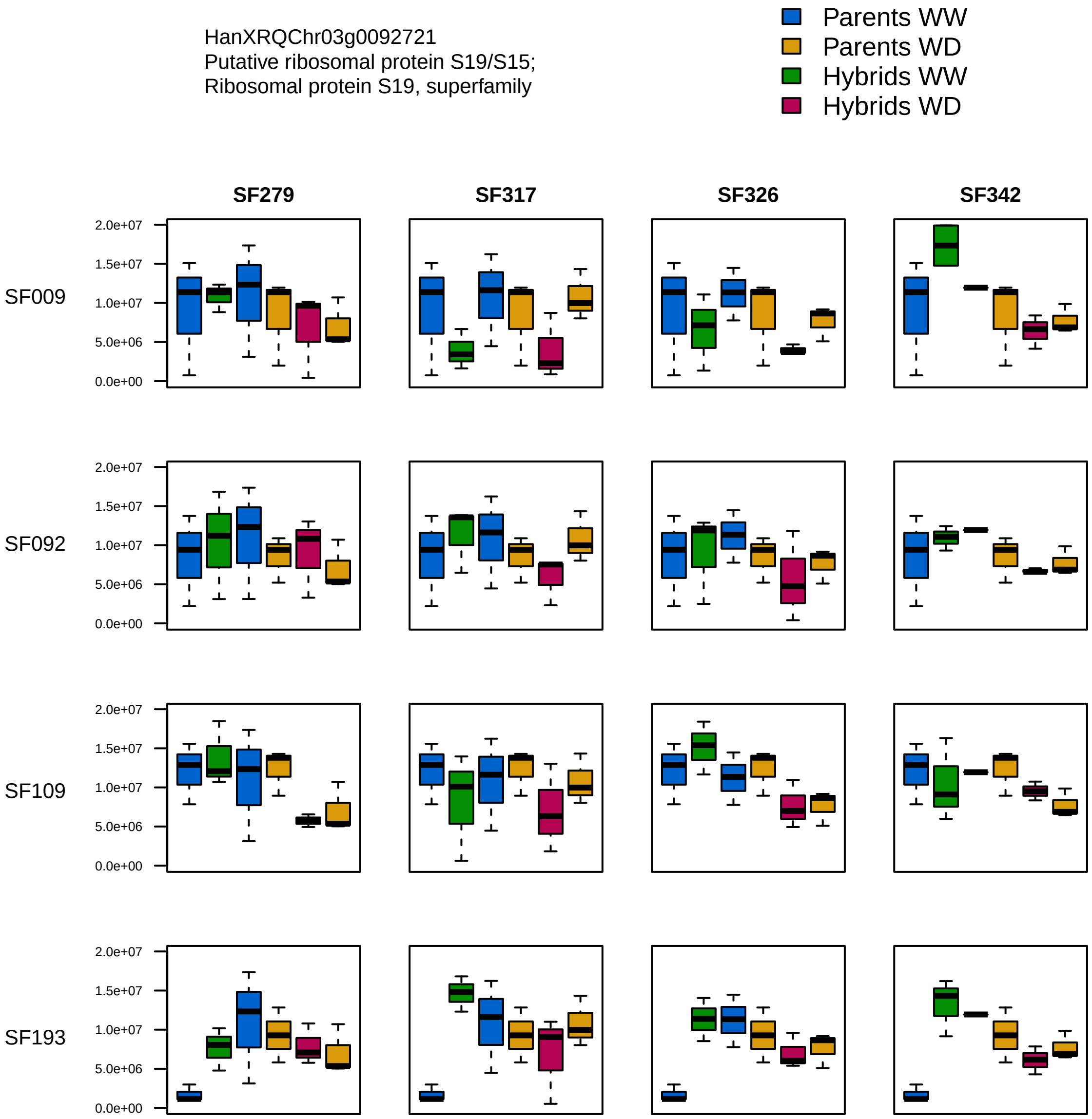
HanXRQChr03g0092721
Putative ribosomal protein S19/S15;
Ribosomal protein S19, superfamily
Parents WW
Parents WD
Hybrids WW
Hybrids WD
SF279
SF317
SF326
SF342
SF009
SF092
SF109
SF193
0.0e+00
5.0e+06
1.0e+07
1.5e+07
2.0e+07

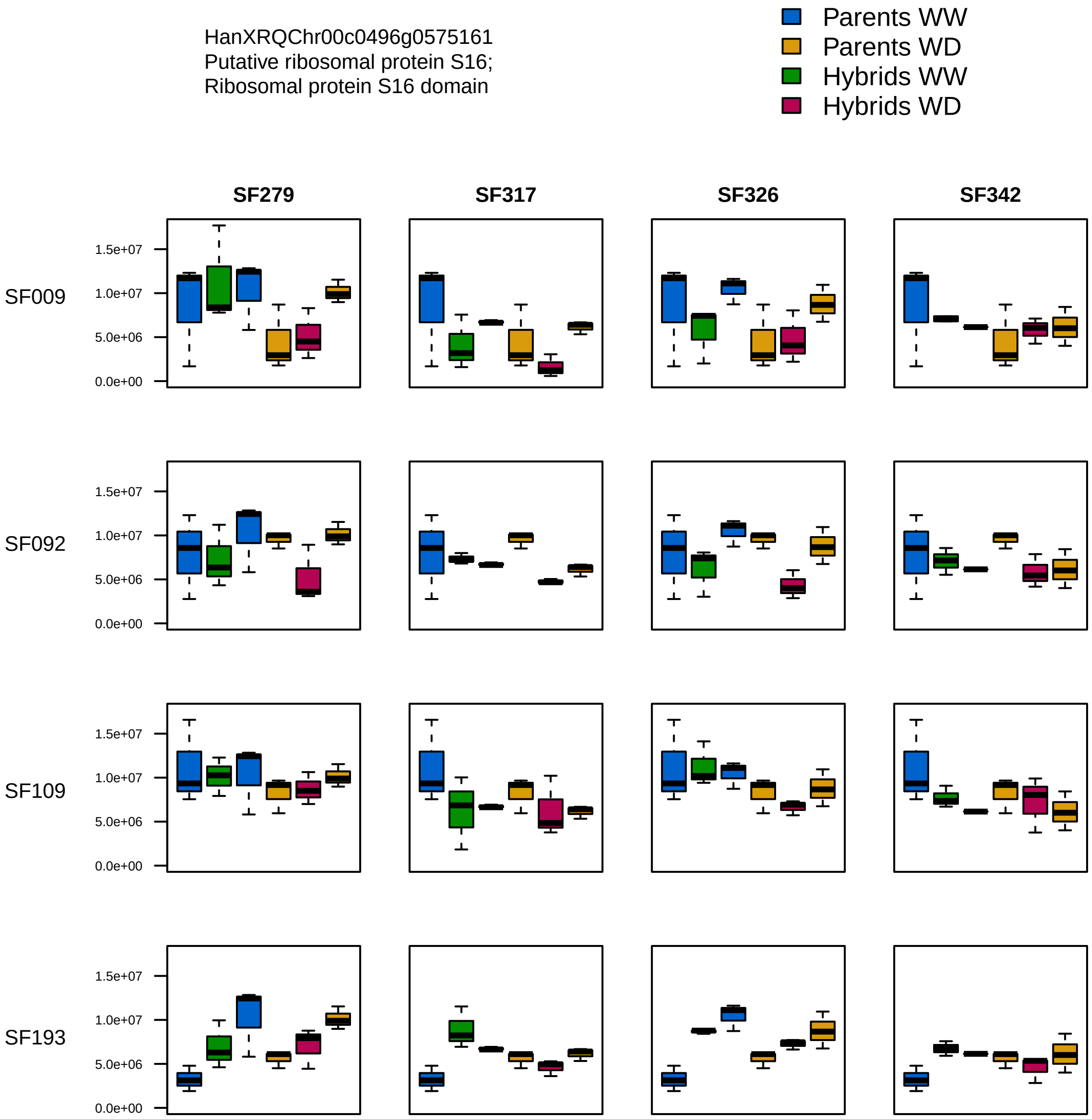
HanXRQChr00c0496g0575161
Putative ribosomal protein S16;
Ribosomal protein S16 domain
Parents WW
Parents WD
Hybrids WW
Hybrids WD
SF279
SF317
SF326
SF342
SF009
SF092
SF109
SF193
1.5e+07
1.0e+07
5.0e+06
0.0e+00

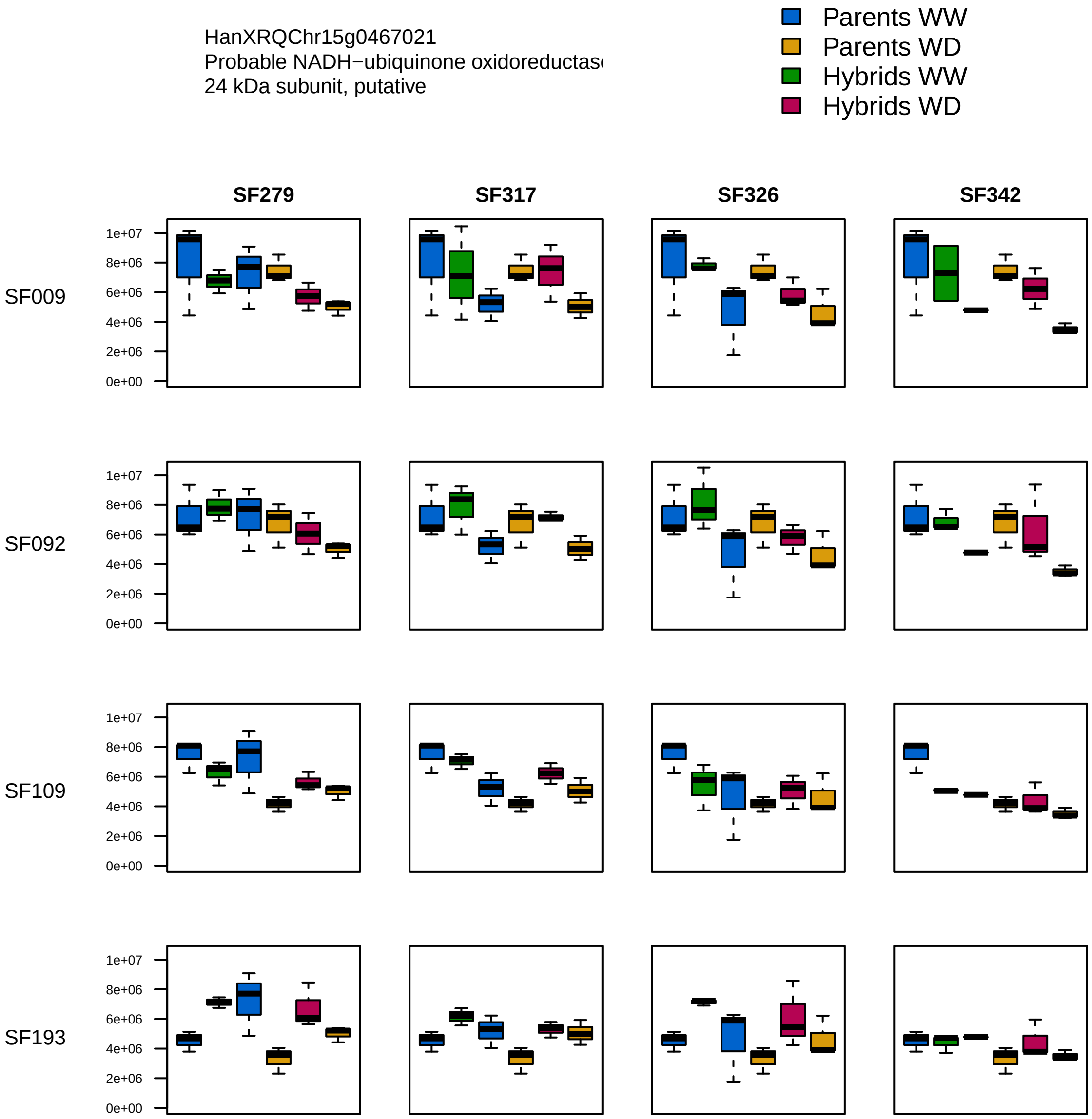

HanXRQChr15g0467021
Probable NADH−ubiquinone oxidoreductas
24 kDa subunit, putative
Parents WW
Parents WD
Hybrids WW
Hybrids WD
SF279
SF317
SF326
SF342
SF009
SF092
SF109
SF193
1e+07
8e+06
6e+06
4e+06
2e+06
0e+00

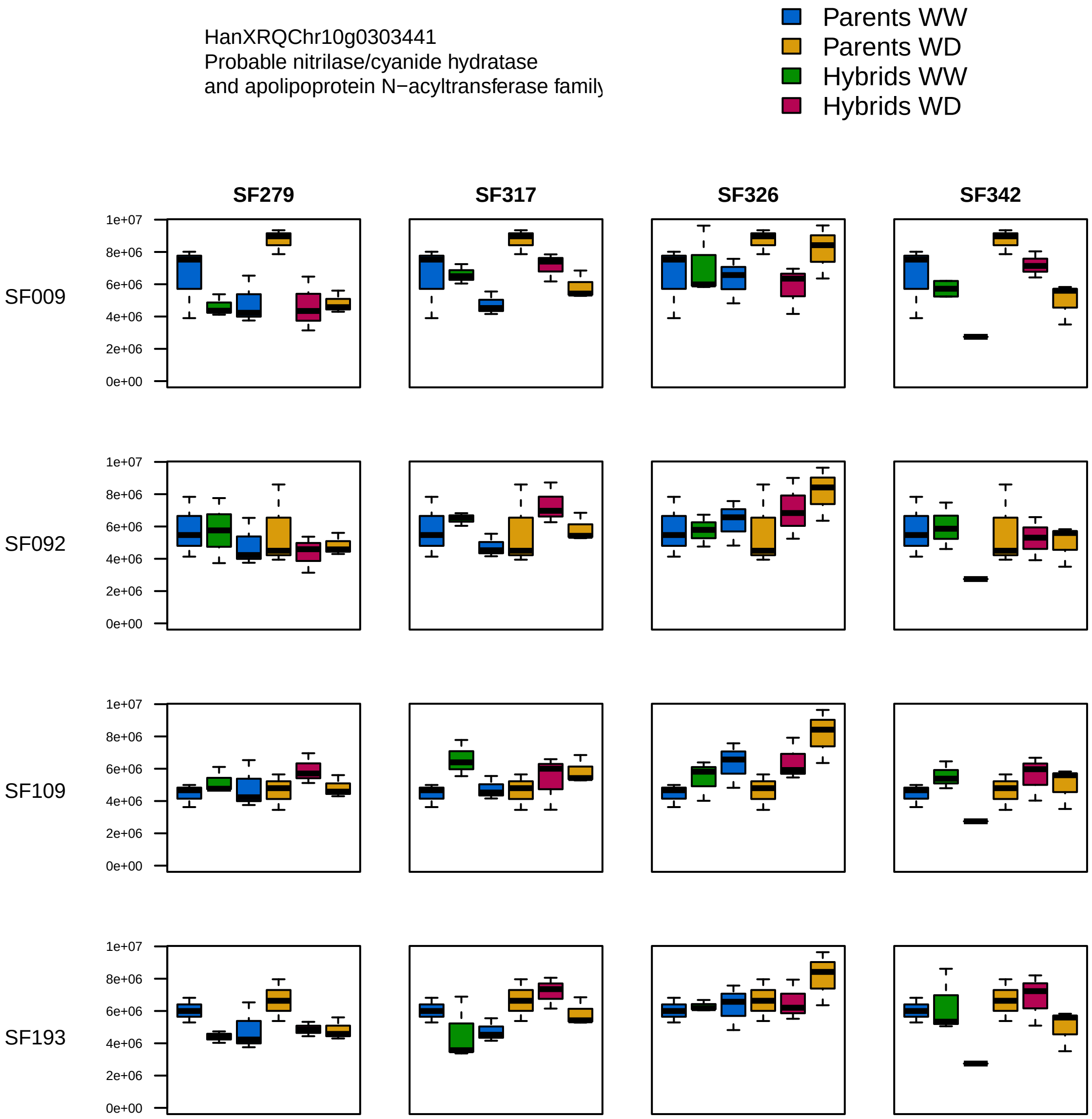

HanXRQChr10g0303441
Probable nitrilase/cyanide hydratase
and apolipoprotein N−acyltransferase family
Parents WW
Parents WD
Hybrids WW
Hybrids WD
SF279
SF317
SF326
SF342
SF009
SF092
SF109
SF193
1e+07
8e+06
6e+06
4e+06
2e+06
0e+00

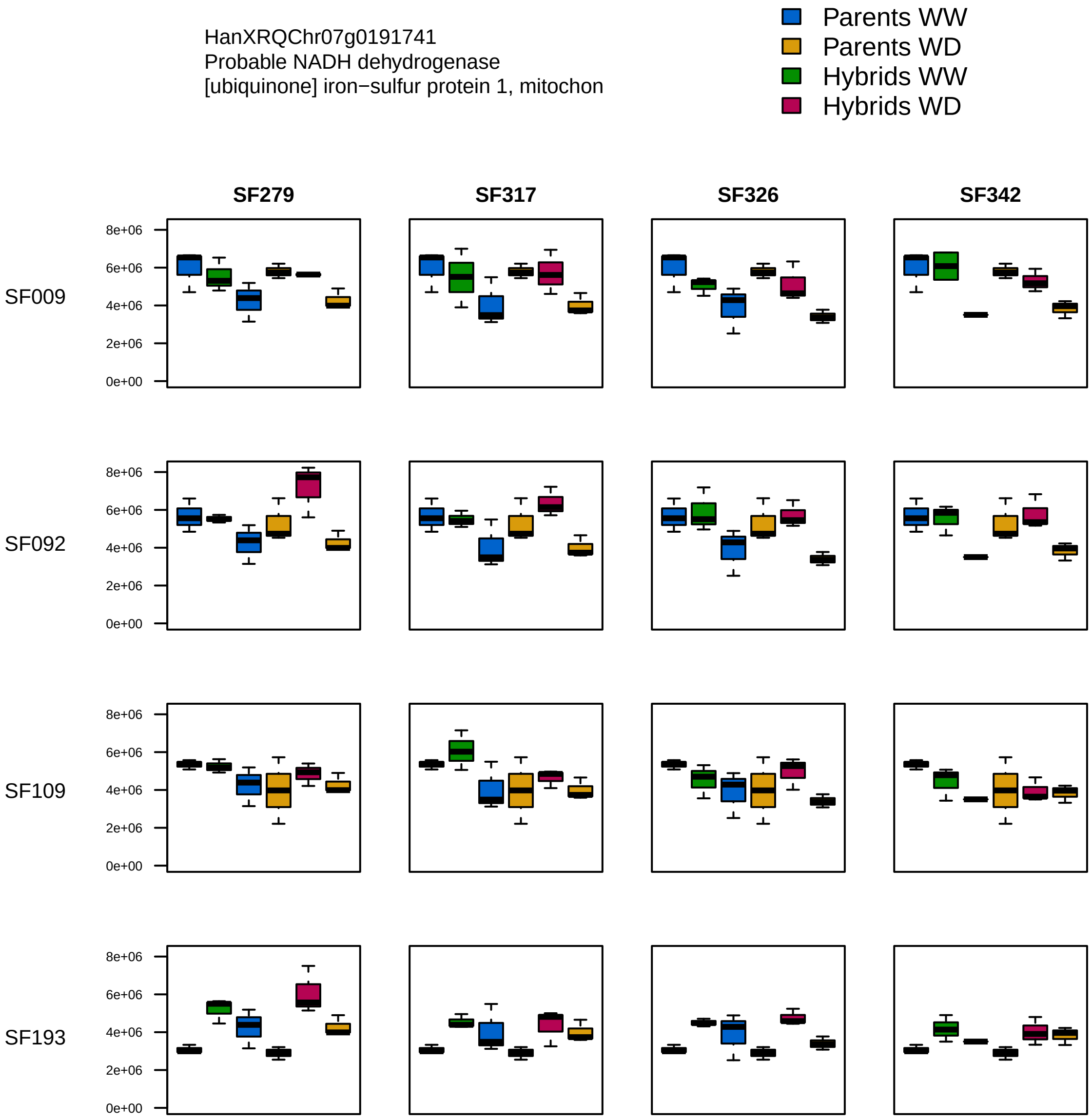

HanXRQChr07g0191741
Probable NADH dehydrogenase
[ubiquinone] iron−sulfur protein 1, mitochon
Parents WW
Parents WD
Hybrids WW
Hybrids WD
SF279
SF317
SF326
SF342
SF009
SF092
SF109
SF193
0e+00
2e+06
4e+06
6e+06
8e+06

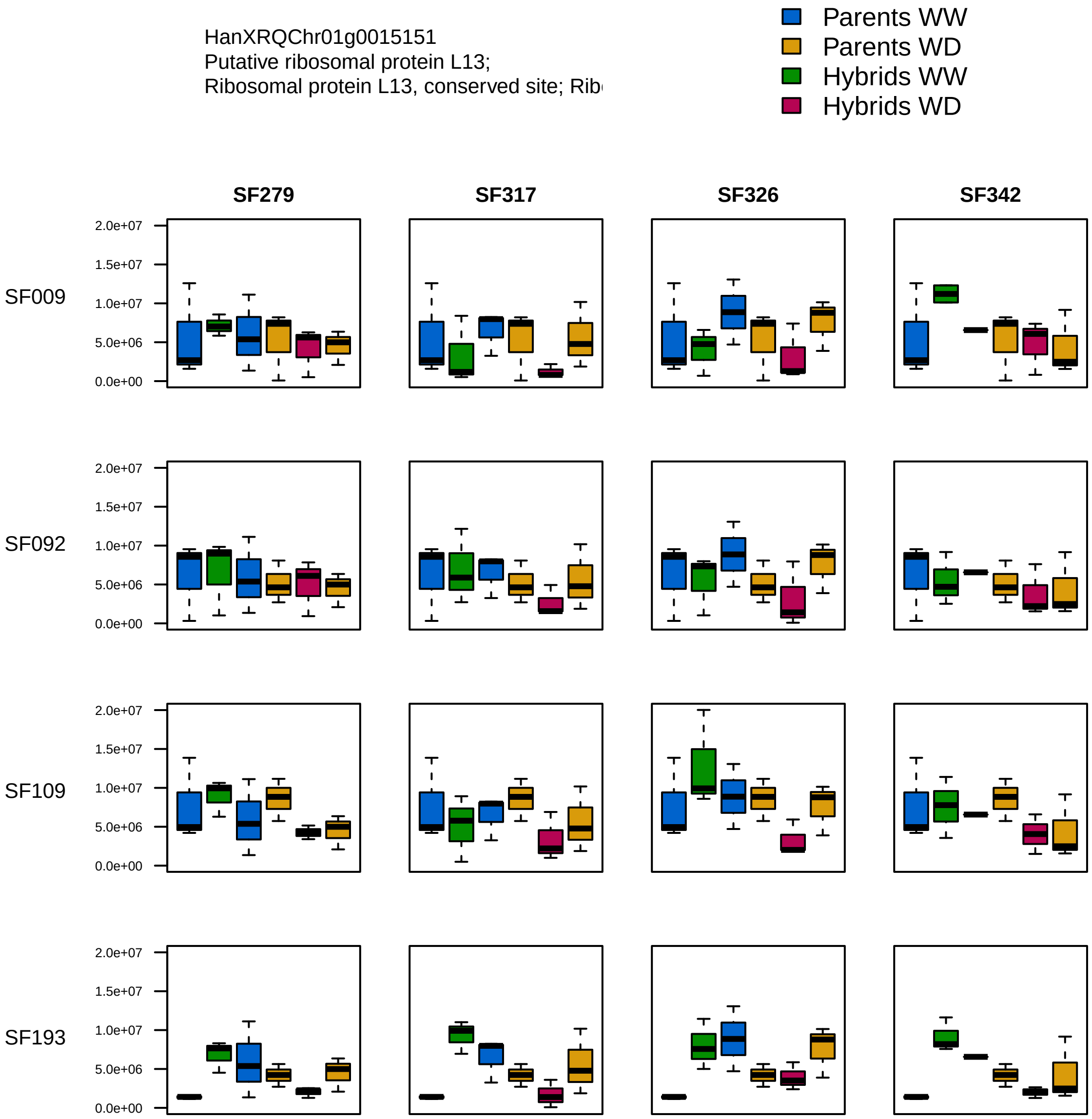
HanXRQChr01g0015151
Putative ribosomal protein L13;
Ribosomal protein L13, conserved site; Rib
Parents WW
Parents WD
Hybrids WW
Hybrids WD
SF279
SF317
SF326
SF342
SF009
SF092
SF109
SF193
2.0e+07
1.5e+07
1.0e+07
5.0e+06
0.0e+00

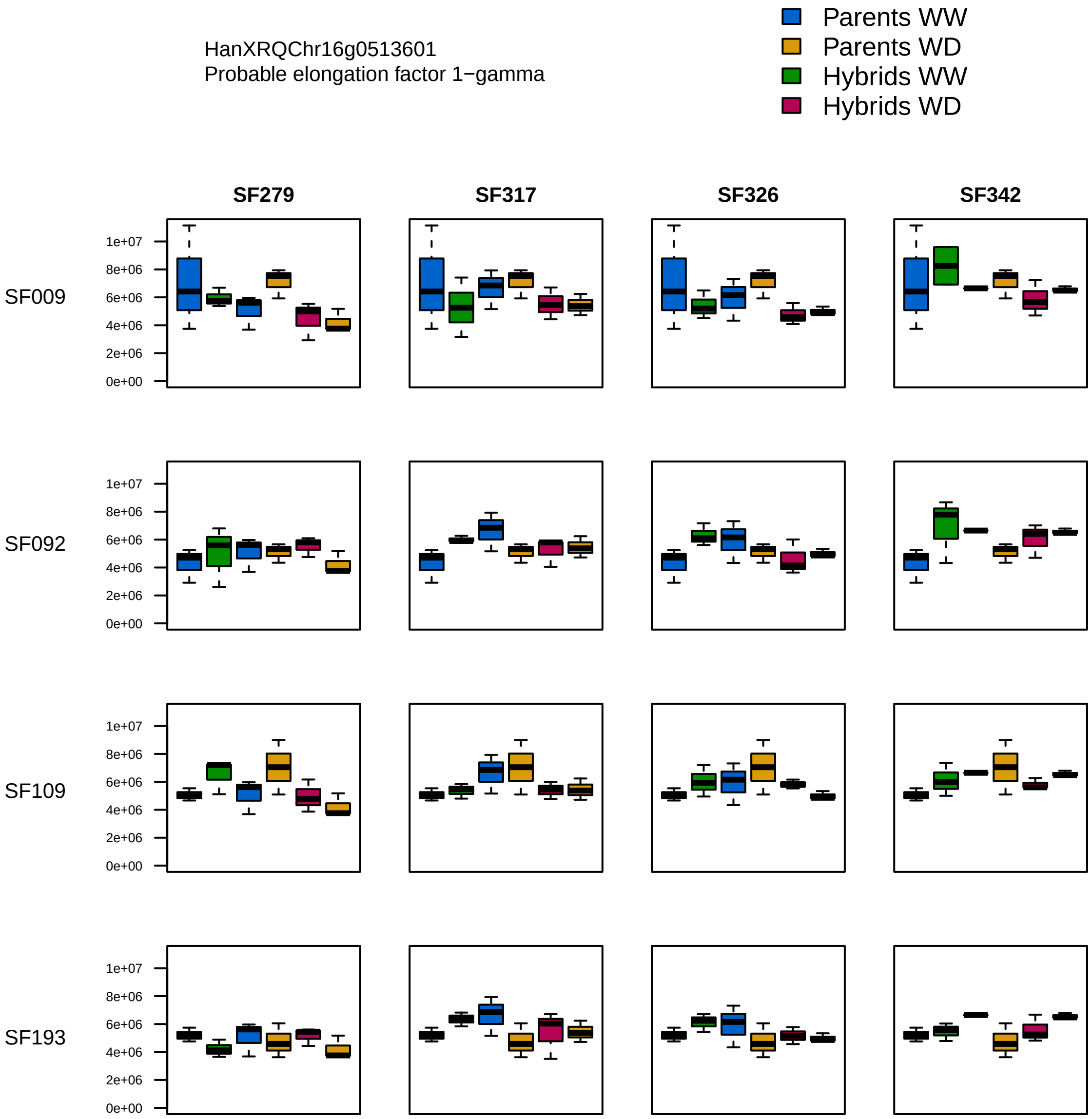
HanXRQChr16g0513601
Probable elongation factor 1−gamma
Parents WW
Parents WD
Hybrids WW
Hybrids WD
SF279
SF317
SF326
SF342
SF009
SF092
SF109
SF193
1e+07
8e+06
6e+06
4e+06
2e+06
0e+00

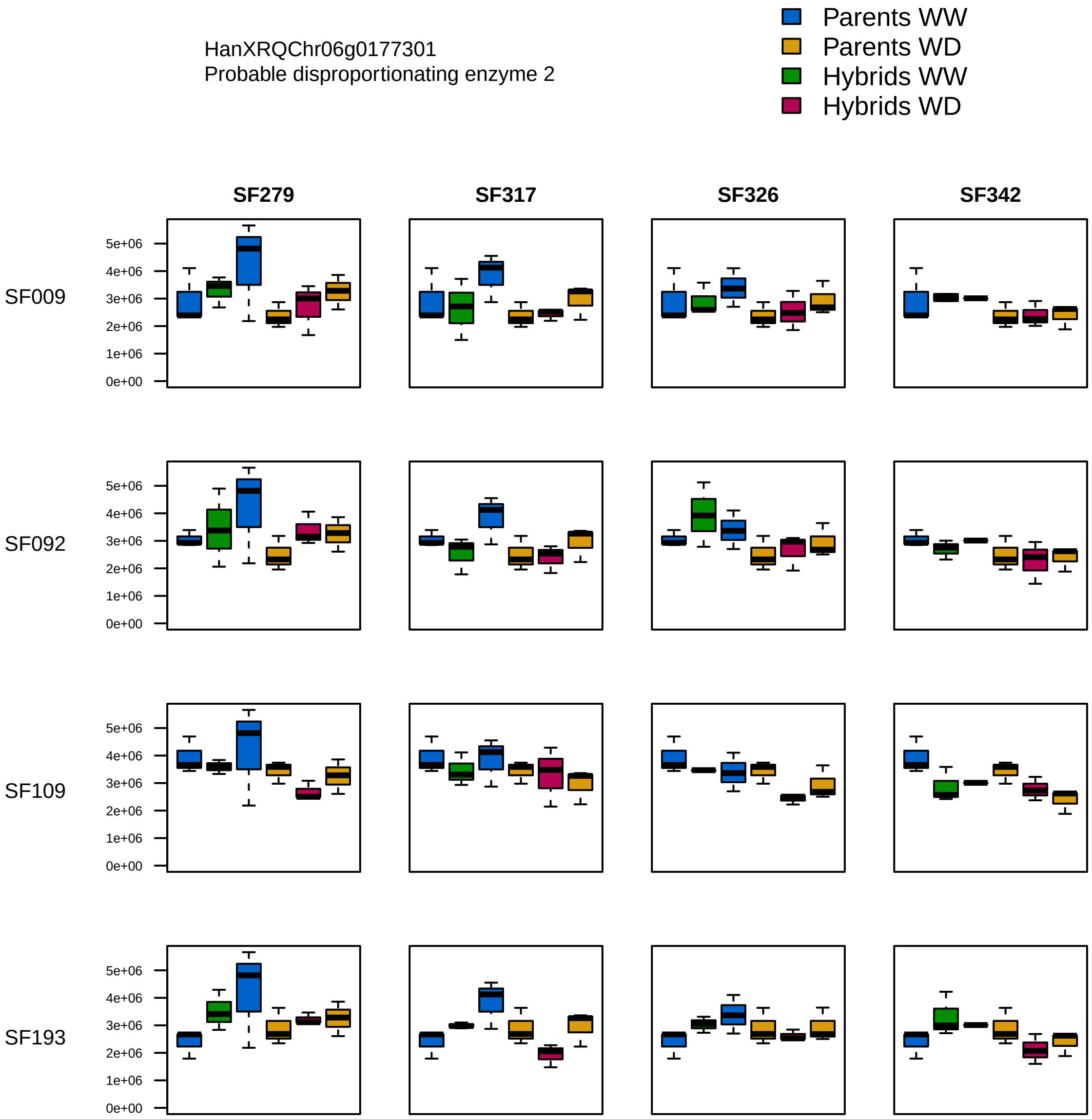
HanXRQChr06g0177301
Probable disproportionating enzyme 2
Parents WW
Parents WD
Hybrids WW
Hybrids WD
SF279
SF317
SF326
SF342
SF009
SF092
SF109
SF193
0e+00
1e+06
2e+06
3e+06
4e+06
5e+06

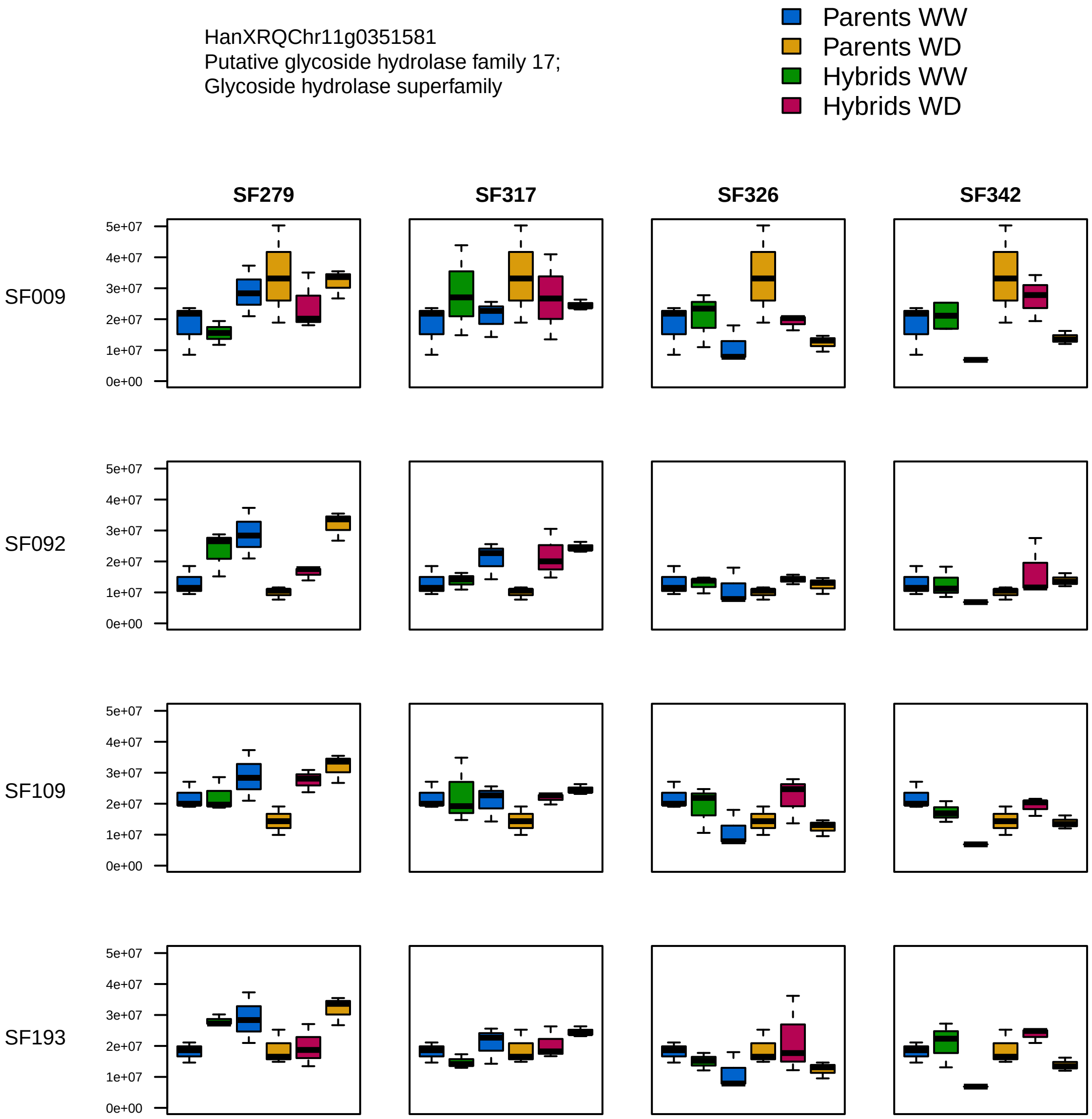

HanXRQChr11g0351581
Putative glycoside hydrolase family 17;
Glycoside hydrolase superfamily
Parents WW
Parents WD
Hybrids WW
Hybrids WD
SF279
SF317
SF326
SF342
SF009
SF092
SF109
SF193
5e+07
4e+07
3e+07
2e+07
1e+07
0e+00

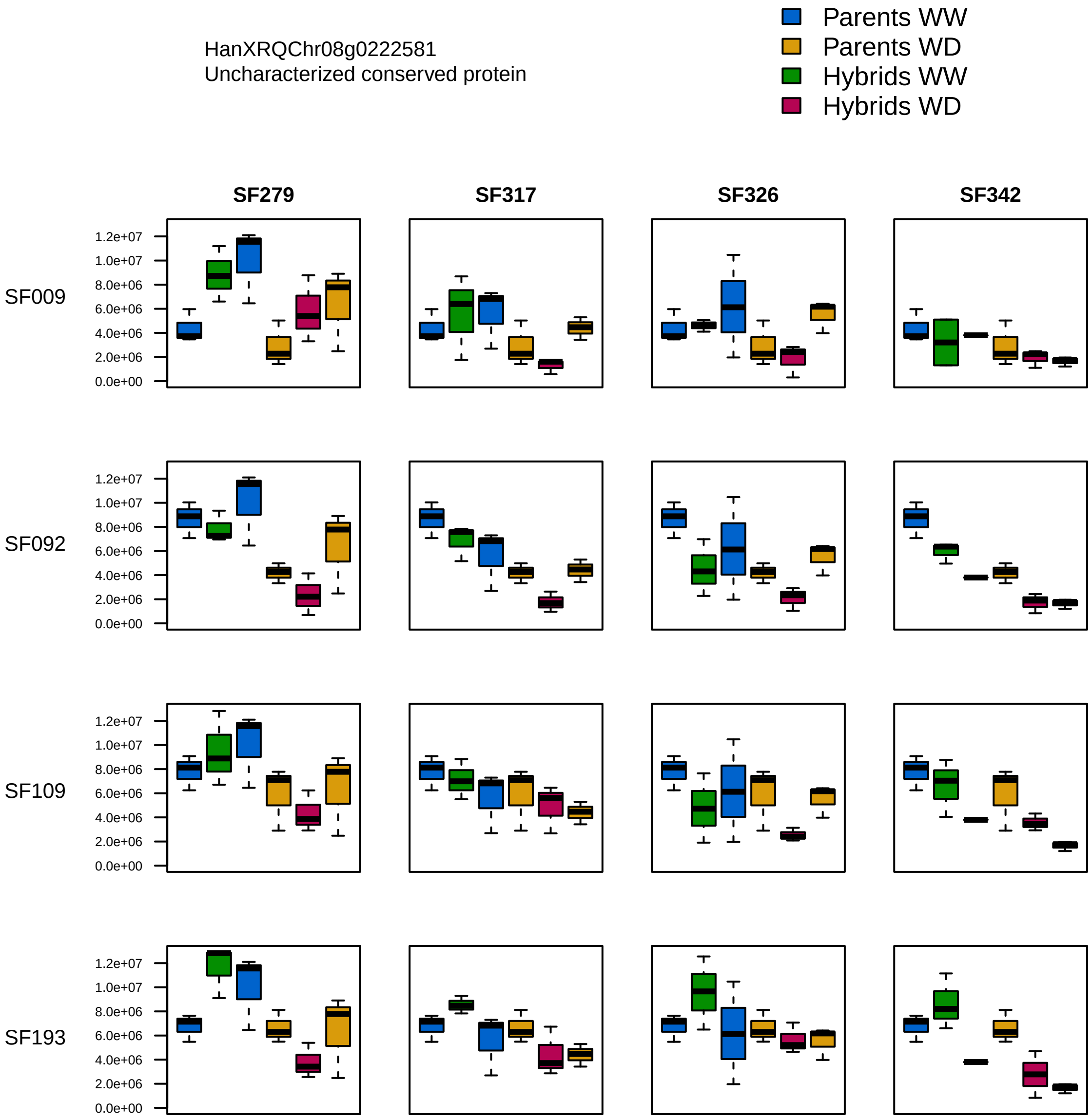

HanXRQChr08g0222581
Uncharacterized conserved protein
Parents WW
Parents WD
Hybrids WW
Hybrids WD
SF279
SF317
SF326
SF342
SF009
SF092
SF109
SF193
0.0e+00
2.0e+06
4.0e+06
6.0e+06
8.0e+06
1.0e+07
1.2e+07

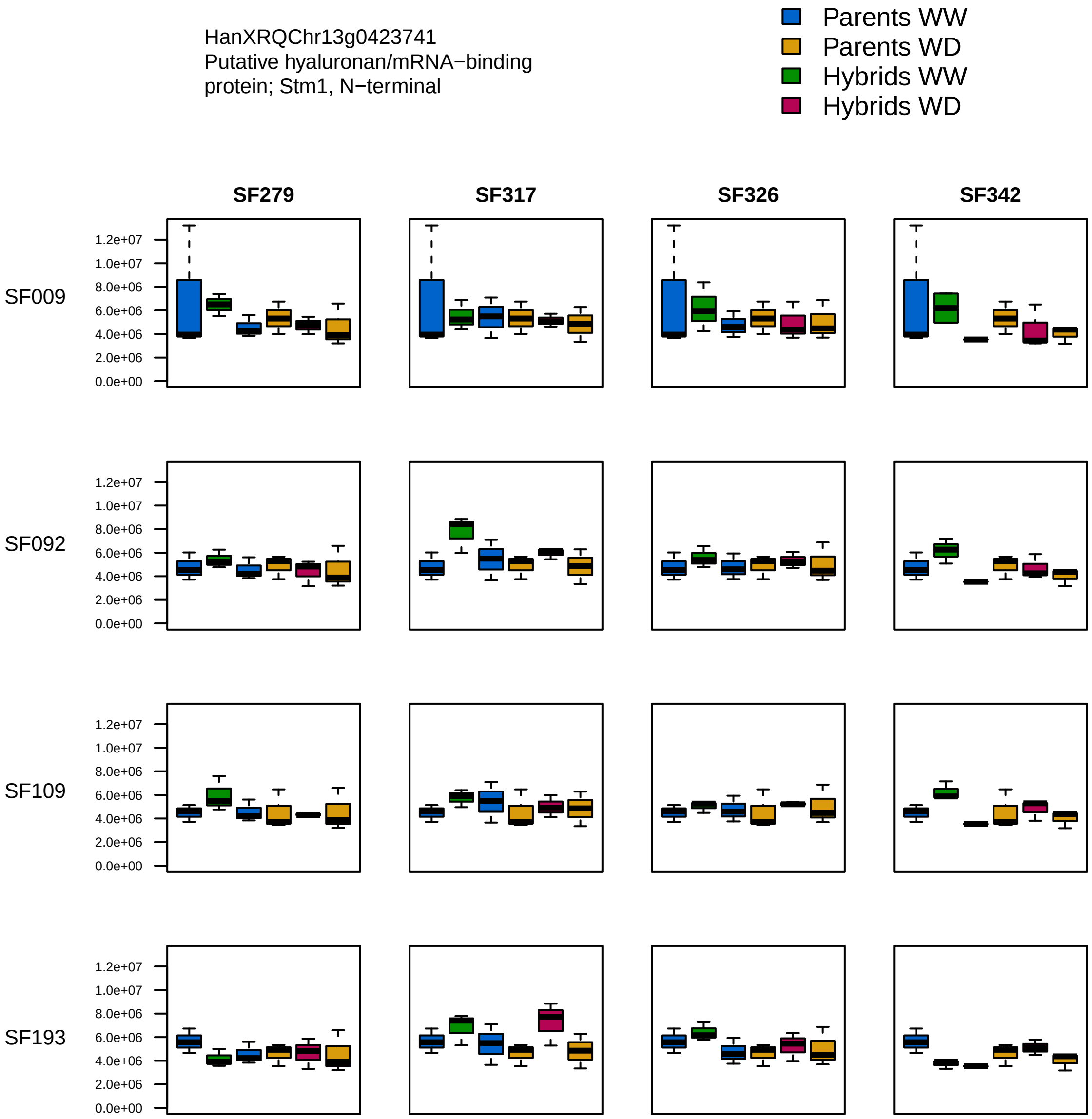
HanXRQChr13g0423741
Putative hyaluronan/mRNA−binding
protein; Stm1, N−terminal
Parents WW
Parents WD
Hybrids WW
Hybrids WD
SF279
SF317
SF326
SF342
SF009
SF092
SF109
SF193
1.2e+07
1.0e+07
8.0e+06
6.0e+06
4.0e+06
2.0e+06
0.0e+00

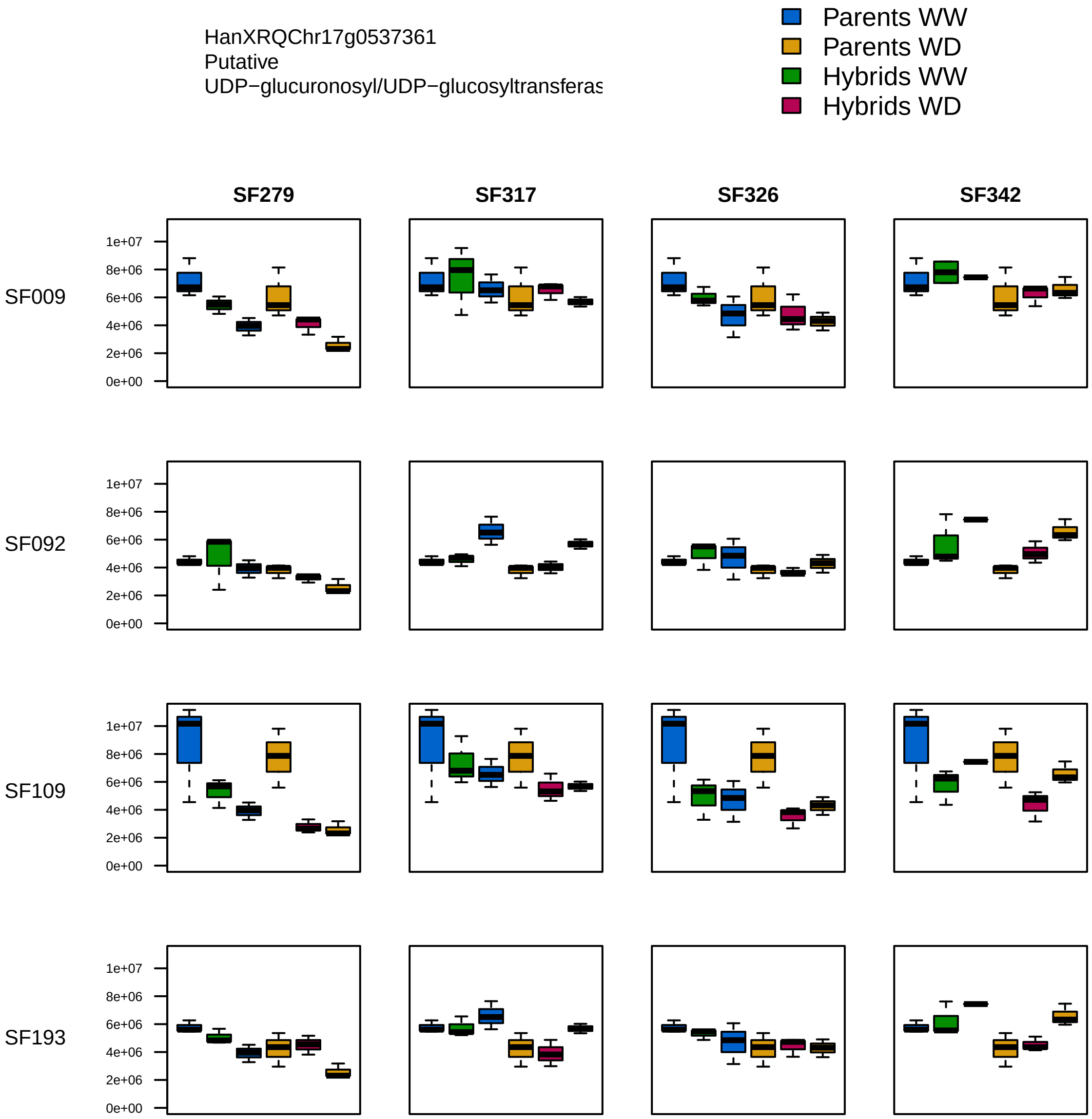
HanXRQChr17g0537361
Putative
UDP−glucuronosyl/UDP−glucosyltransferas
Parents WW
Parents WD
Hybrids WW
Hybrids WD
SF279
SF317
SF326
SF342
SF009
SF092
SF109
SF193
1e+07
8e+06
6e+06
4e+06
2e+06
0e+00

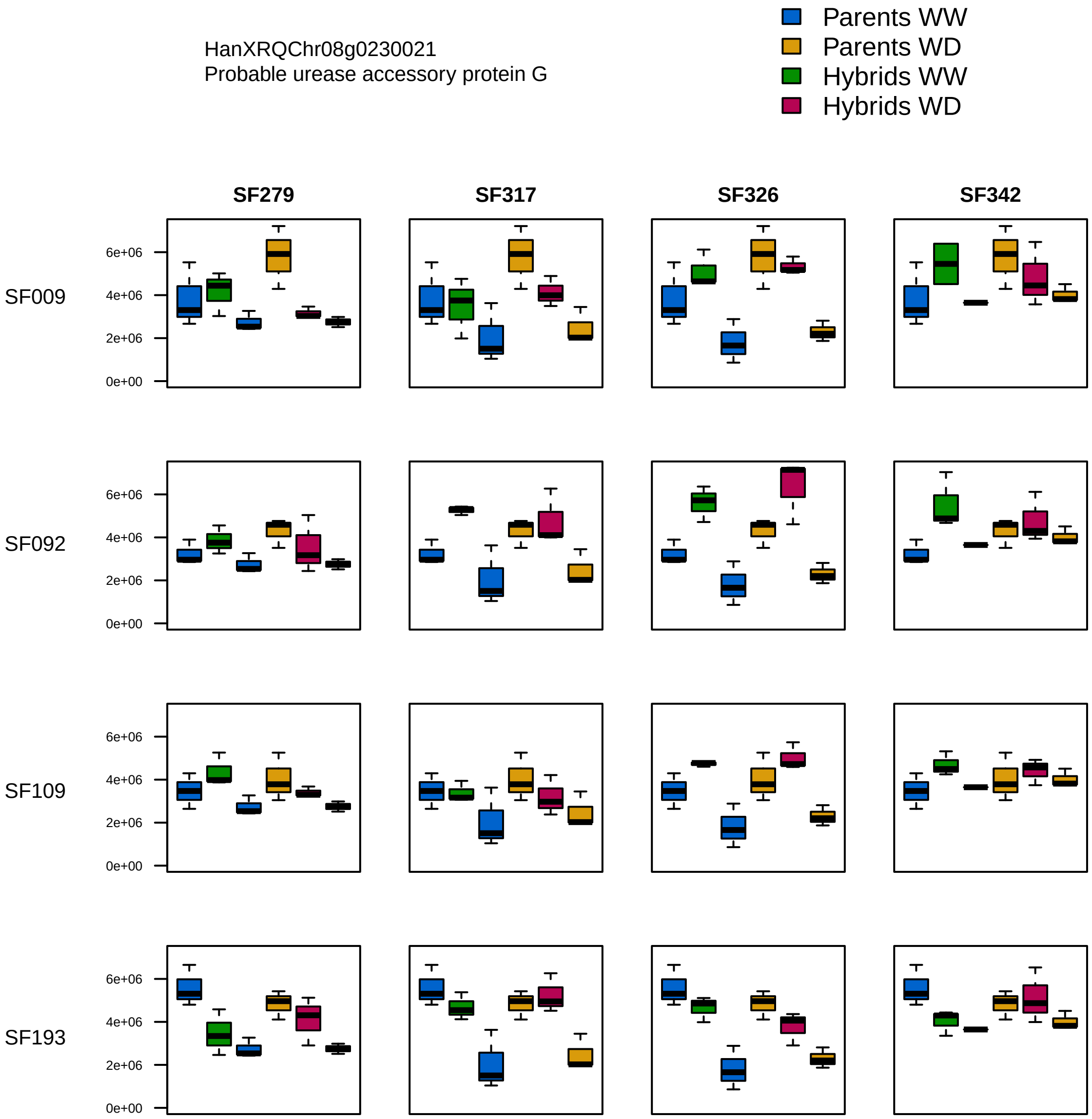
HanXRQChr08g0230021
Probable urease accessory protein G
Parents WW
Parents WD
Hybrids WW
Hybrids WD
SF279
SF317
SF326
SF342
SF009
SF092
SF109
SF193
0e+00
2e+06
4e+06
6e+06

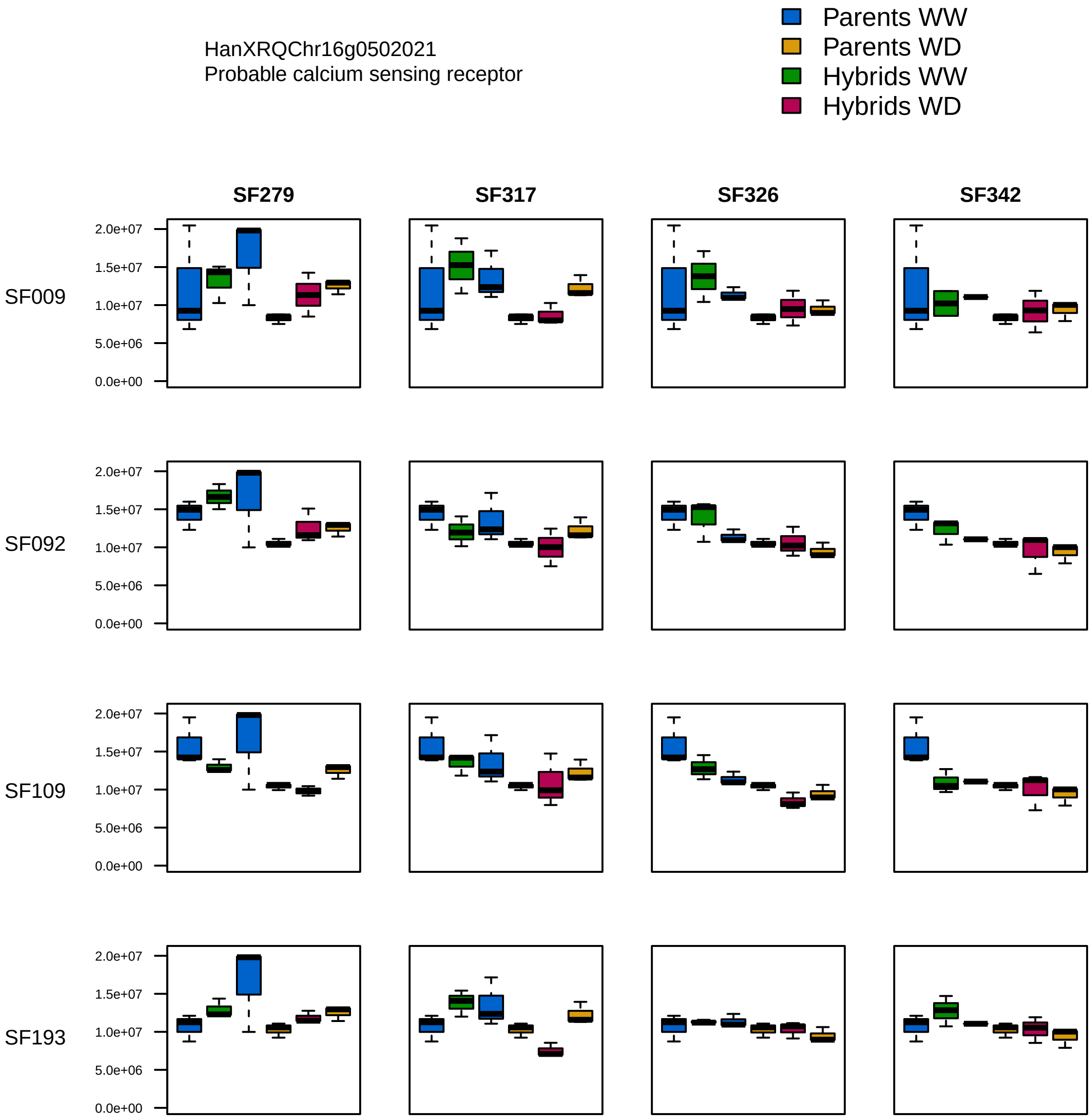
HanXRQChr16g0502021
Probable calcium sensing receptor
Parents WW
Parents WD
Hybrids WW
Hybrids WD
SF279
SF317
SF326
SF342
SF009
SF092
SF109
SF193
2.0e+07
1.5e+07
1.0e+07
5.0e+06
0.0e+00

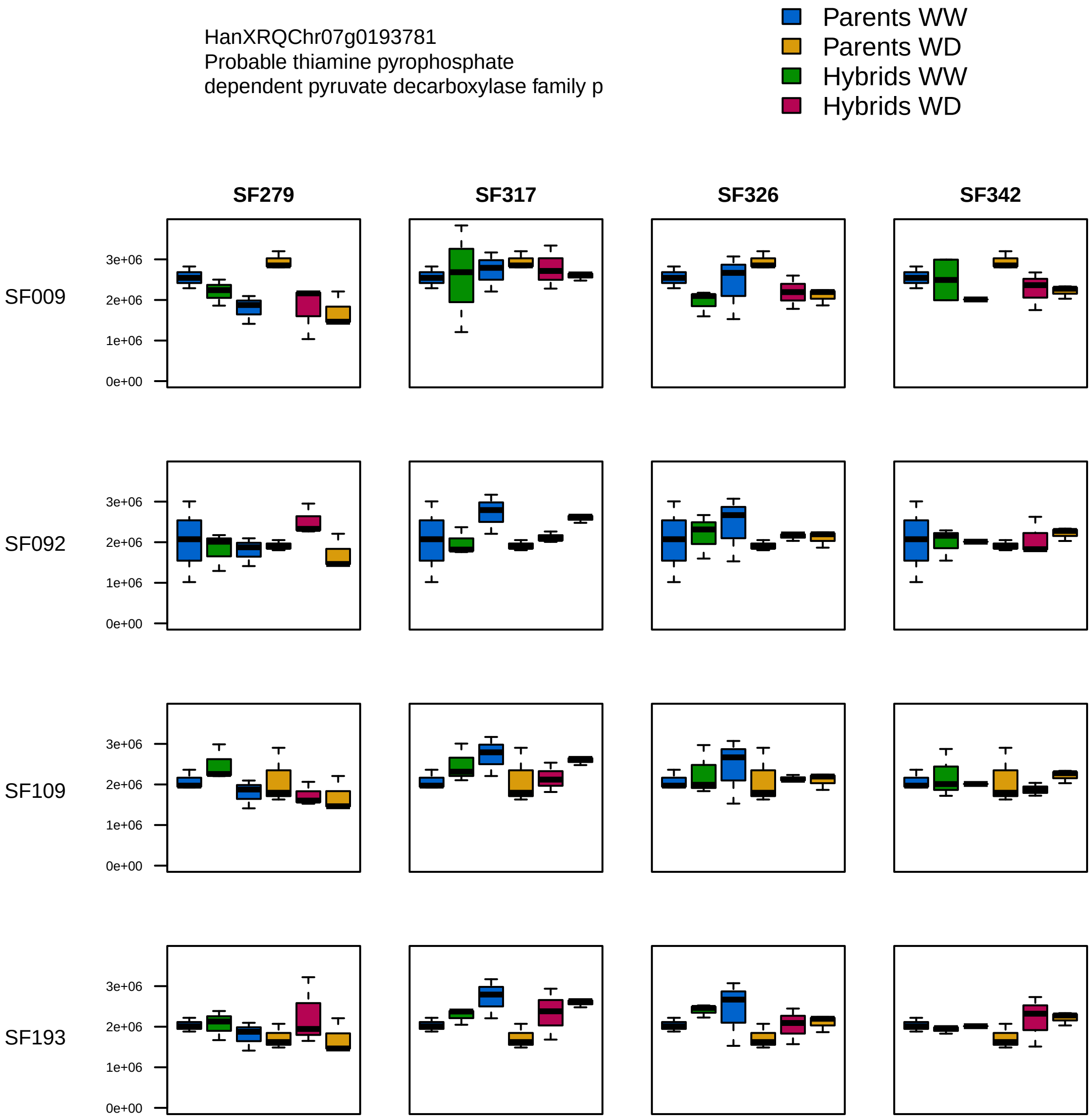
HanXRQChr07g0193781
Probable thiamine pyrophosphate
dependent pyruvate decarboxylase family p
Parents WW
Parents WD
Hybrids WW
Hybrids WD
SF279
SF317
SF326
SF342
SF009
SF092
SF109
SF193
0e+00
1e+06
2e+06
3e+06

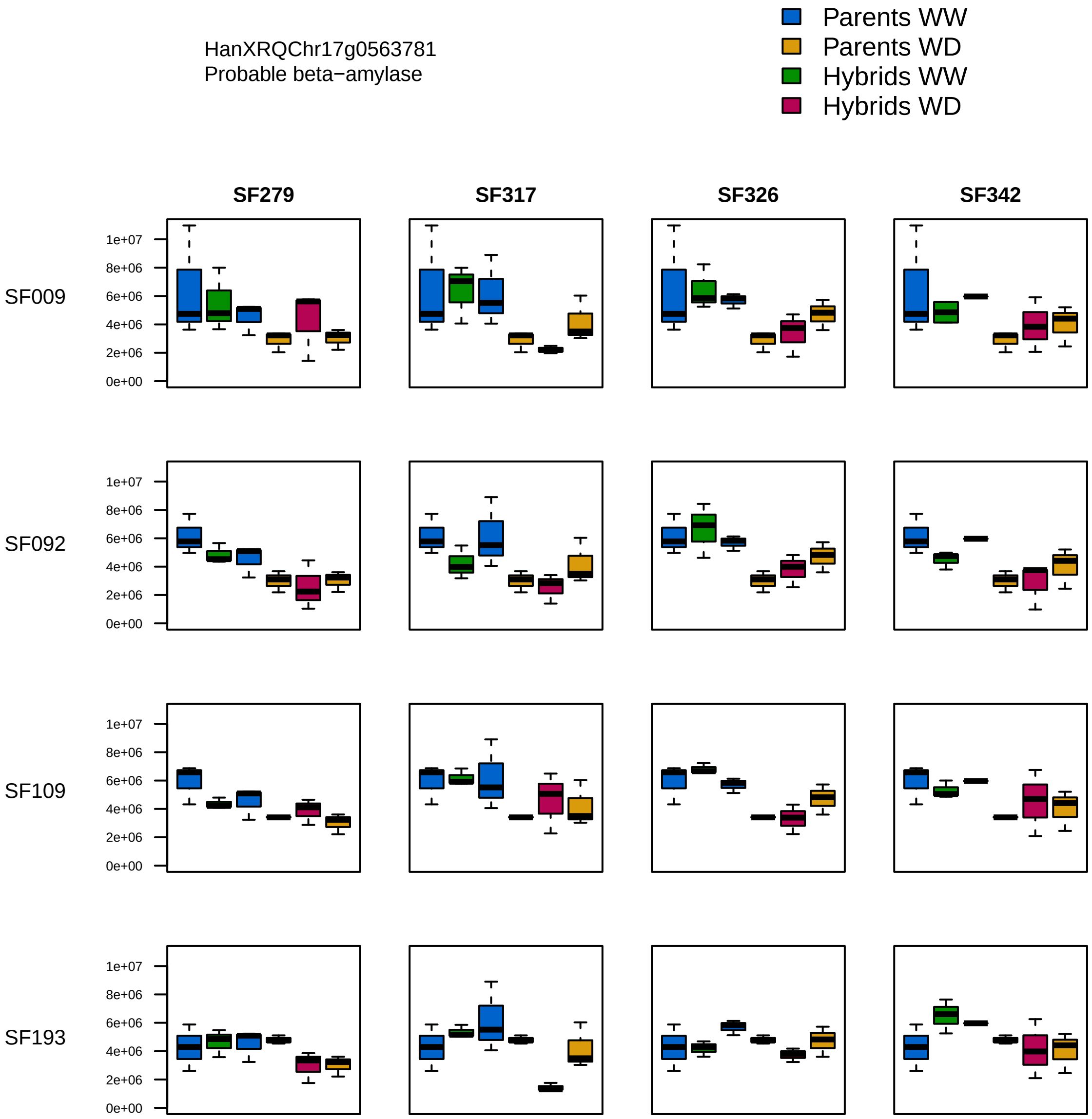
HanXRQChr17g0563781
Probable beta−amylase
Parents WW
Parents WD
Hybrids WW
Hybrids WD
SF279
SF317
SF326
SF342
SF009
SF092
SF109
SF193
1e+07
8e+06
6e+06
4e+06
2e+06
0e+00

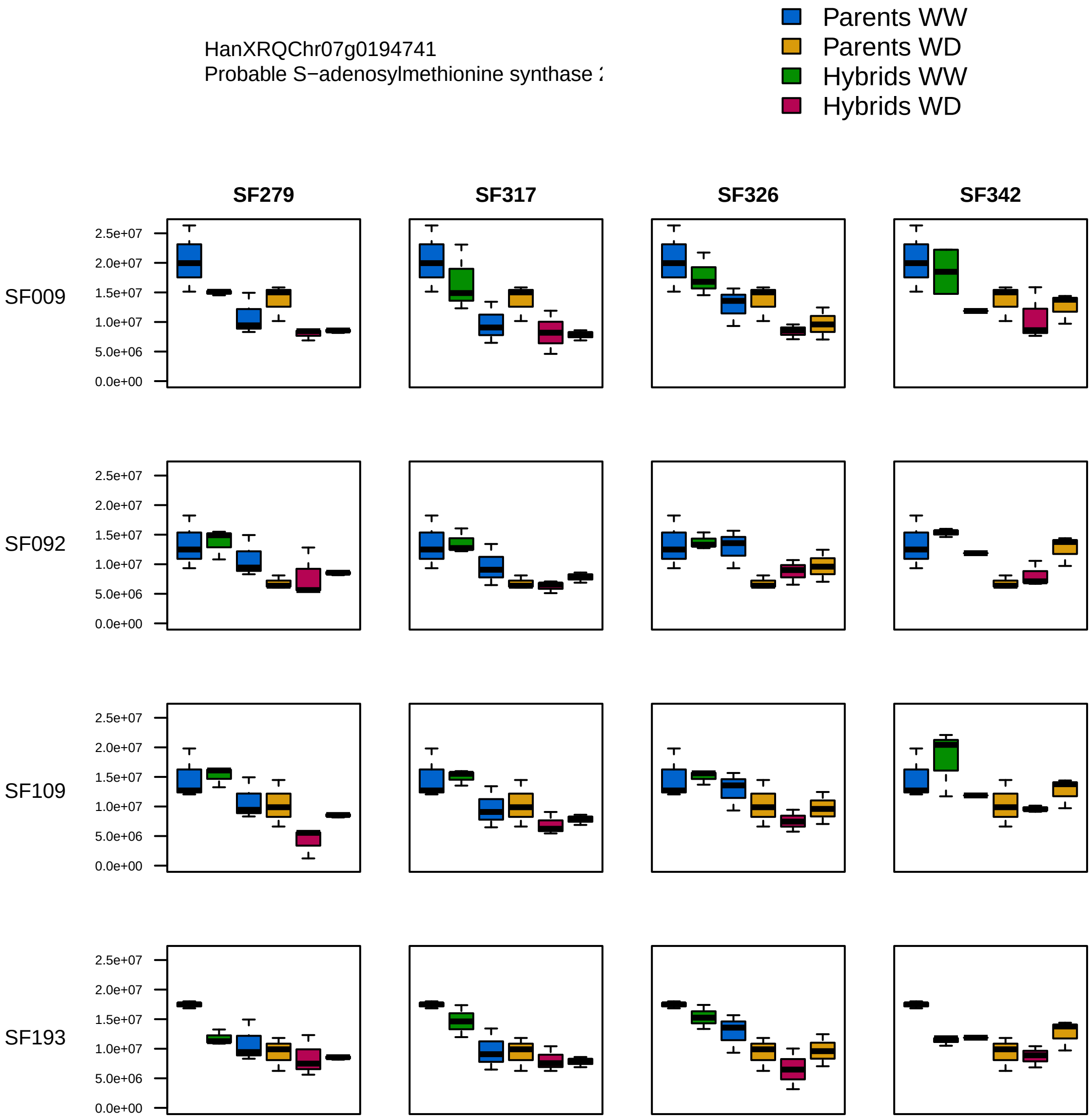
HanXRQChr07g0194741
Probable S−adenosylmethionine synthase 2
Parents WW
Parents WD
Hybrids WW
Hybrids WD
SF279
SF317
SF326
SF342
SF009
SF092
SF109
SF193
2.5e+07
2.0e+07
1.5e+07
1.0e+07
5.0e+06
0.0e+00

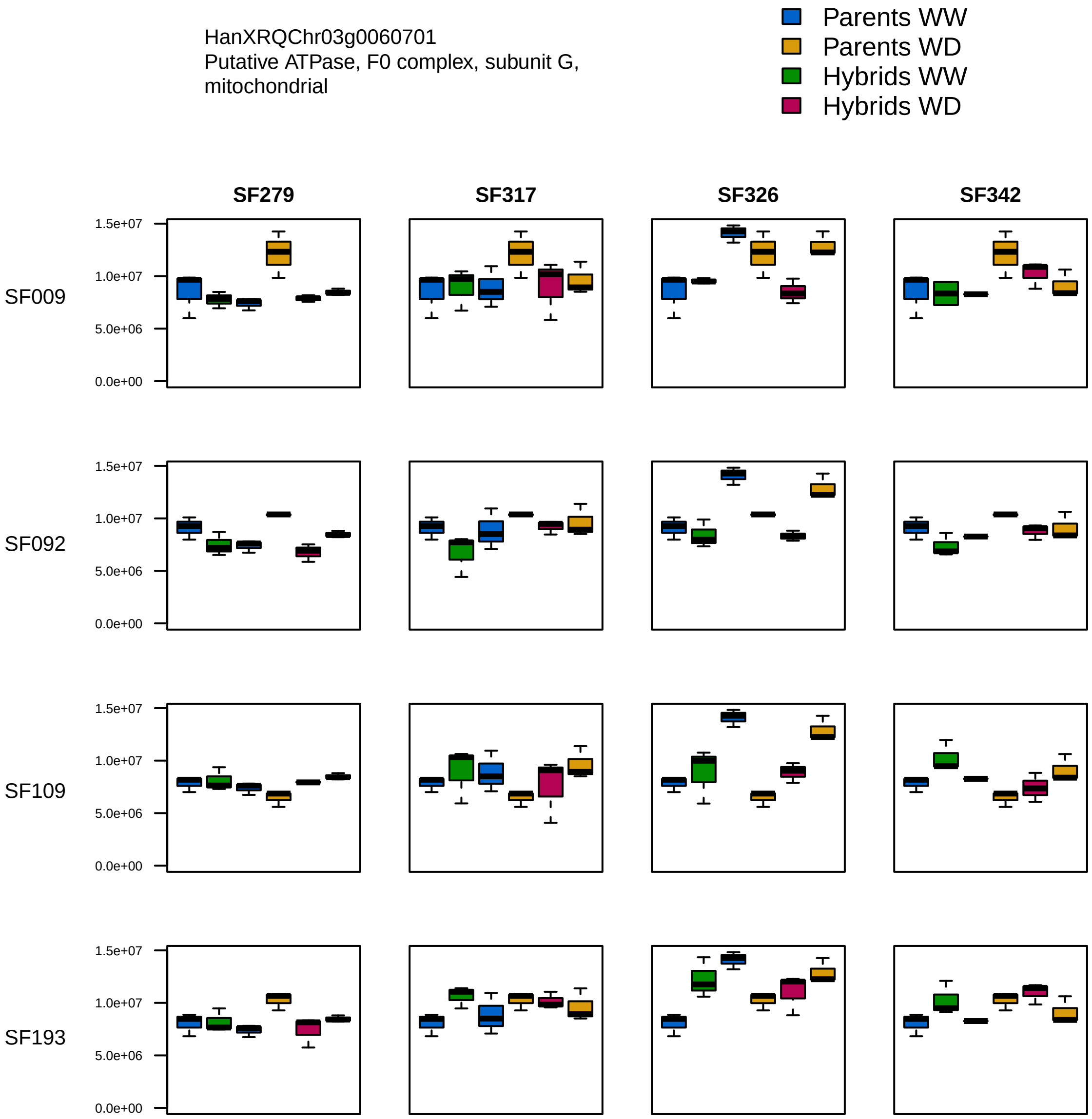

HanXRQChr03g0060701
Putative ATPase, F0 complex, subunit G,
mitochondrial
Parents WW
Parents WD
Hybrids WW
Hybrids WD
SF279
SF317
SF326
SF342
SF009
SF092
SF109
SF193
1.5e+07
1.0e+07
5.0e+06
0.0e+00

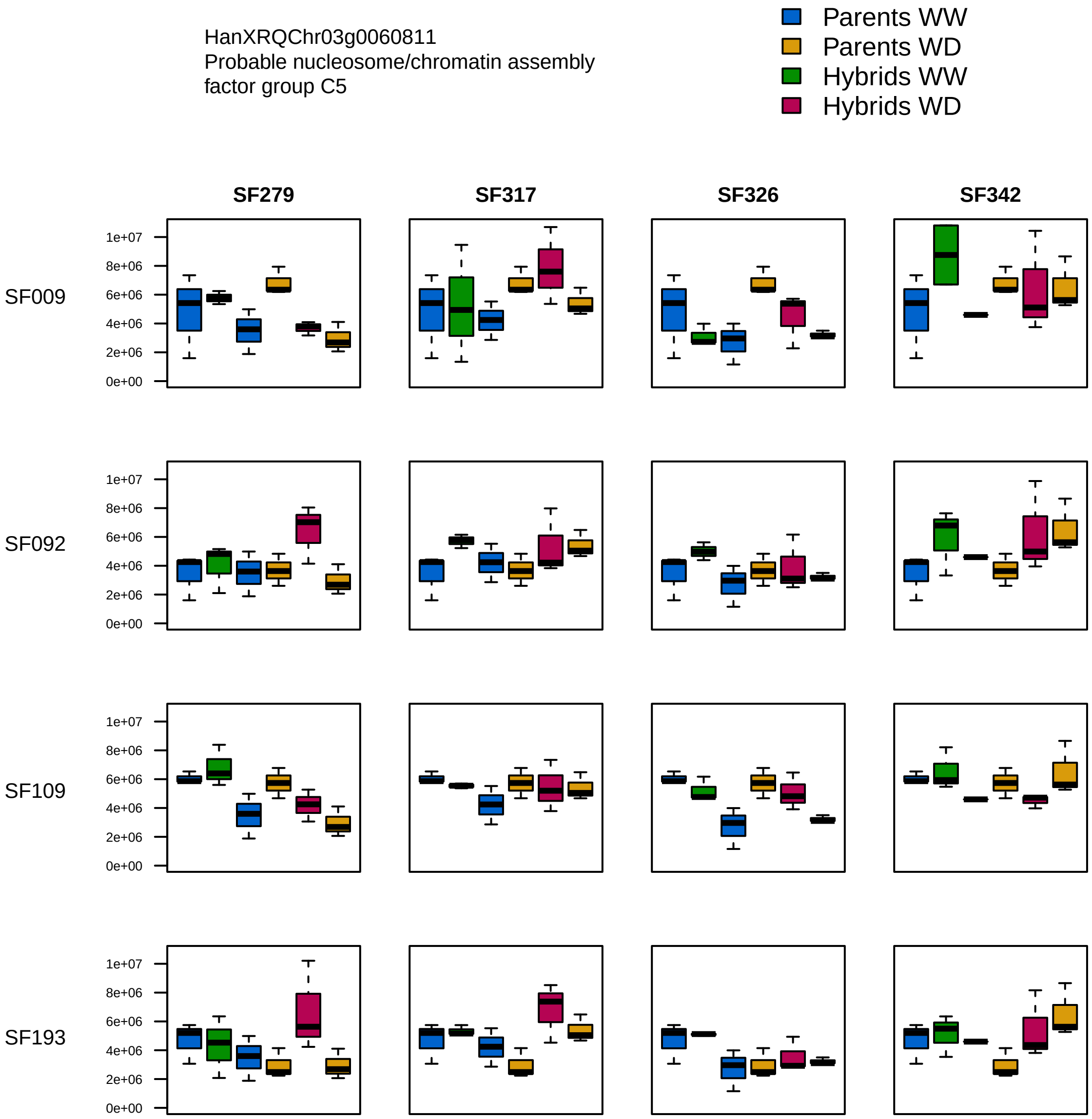
HanXRQChr03g0060811
Probable nucleosome/chromatin assembly factor group C5
Parents WW
Parents WD
Hybrids WW
Hybrids WD
SF279
SF317
SF326
SF342
SF009
SF092
SF109
SF193
1e+07
8e+06
6e+06
4e+06
2e+06
0e+00

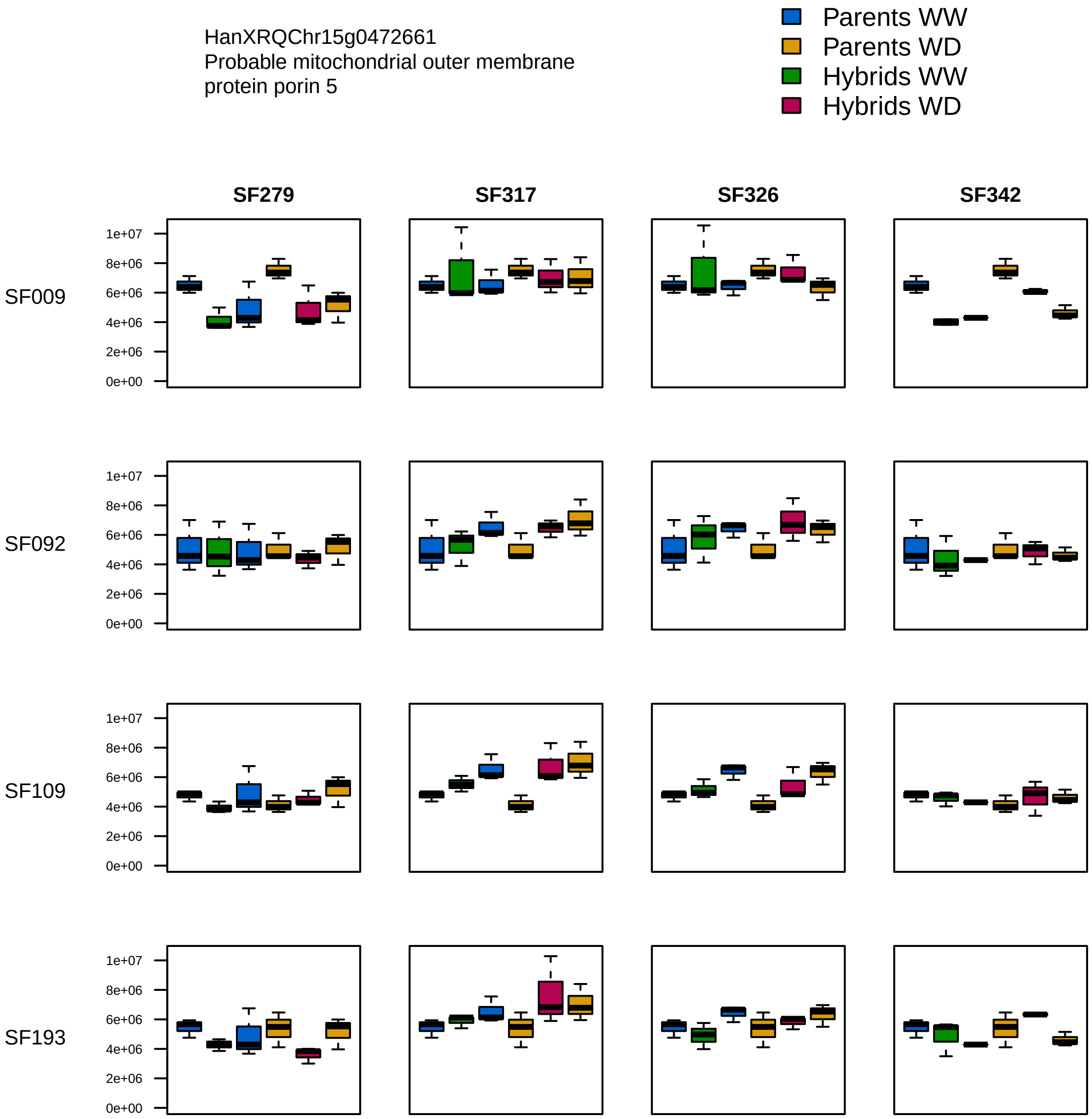
HanXRQChr15g0472661
Probable mitochondrial outer membrane protein porin 5
Parents WW
Parents WD
Hybrids WW
Hybrids WD
SF279
SF317
SF326
SF342
SF009
SF092
SF109
SF193
1e+07
8e+06
6e+06
4e+06
2e+06
0e+00

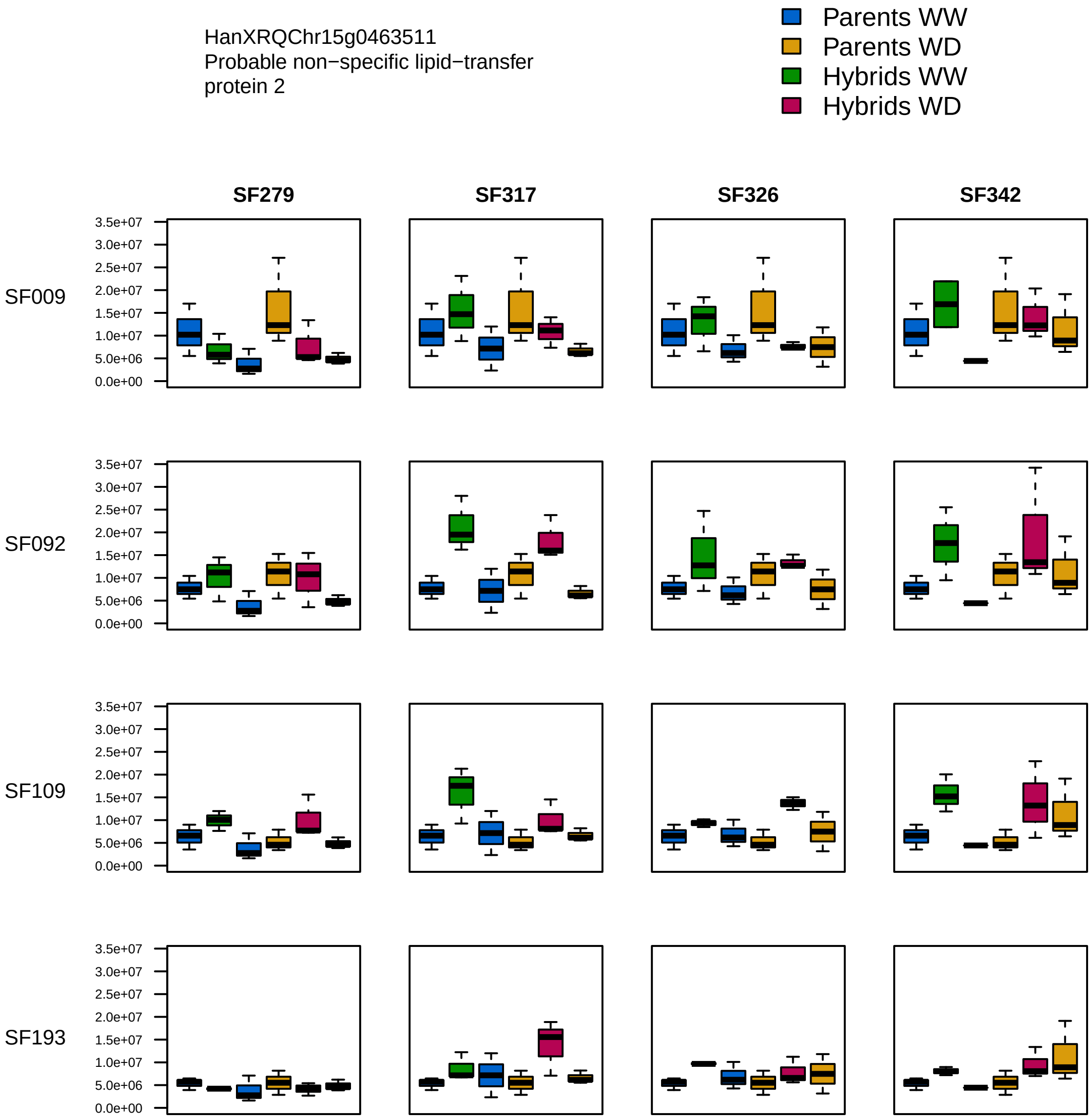
HanXRQChr15g0463511
Probable non−specific lipid−transfer protein 2
Parents WW
Parents WD
Hybrids WW
Hybrids WD
SF279
SF317
SF326
SF342
SF009
SF092
SF109
SF193
3.5e+07
3.0e+07
2.5e+07
2.0e+07
1.5e+07
1.0e+07
5.0e+06
0.0e+00

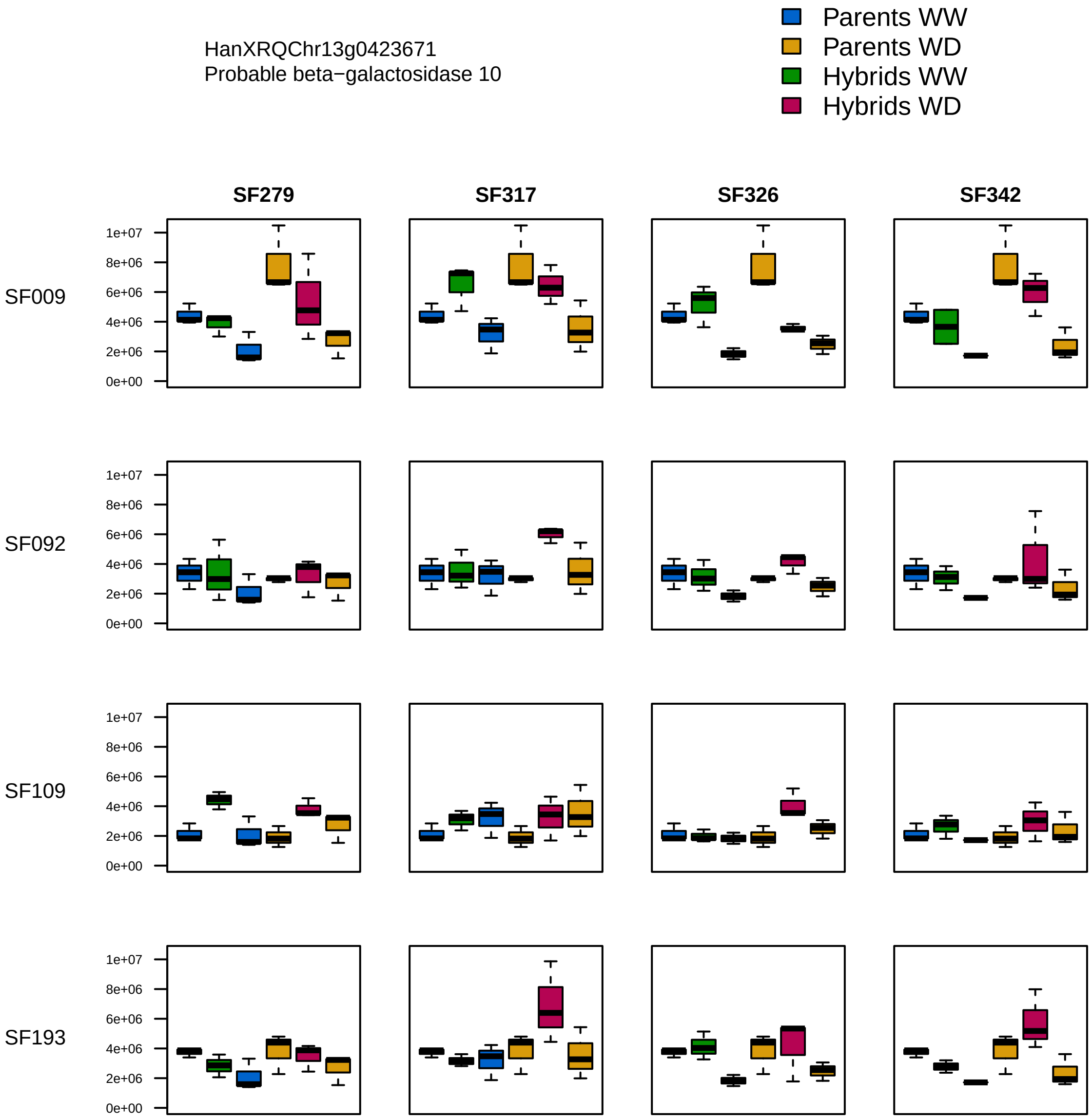
HanXRQChr13g0423671
Probable beta−galactosidase 10
Parents WW
Parents WD
Hybrids WW
Hybrids WD
SF279
SF317
SF326
SF342
SF009
SF092
SF109
SF193
1e+07
8e+06
6e+06
4e+06
2e+06
0e+00

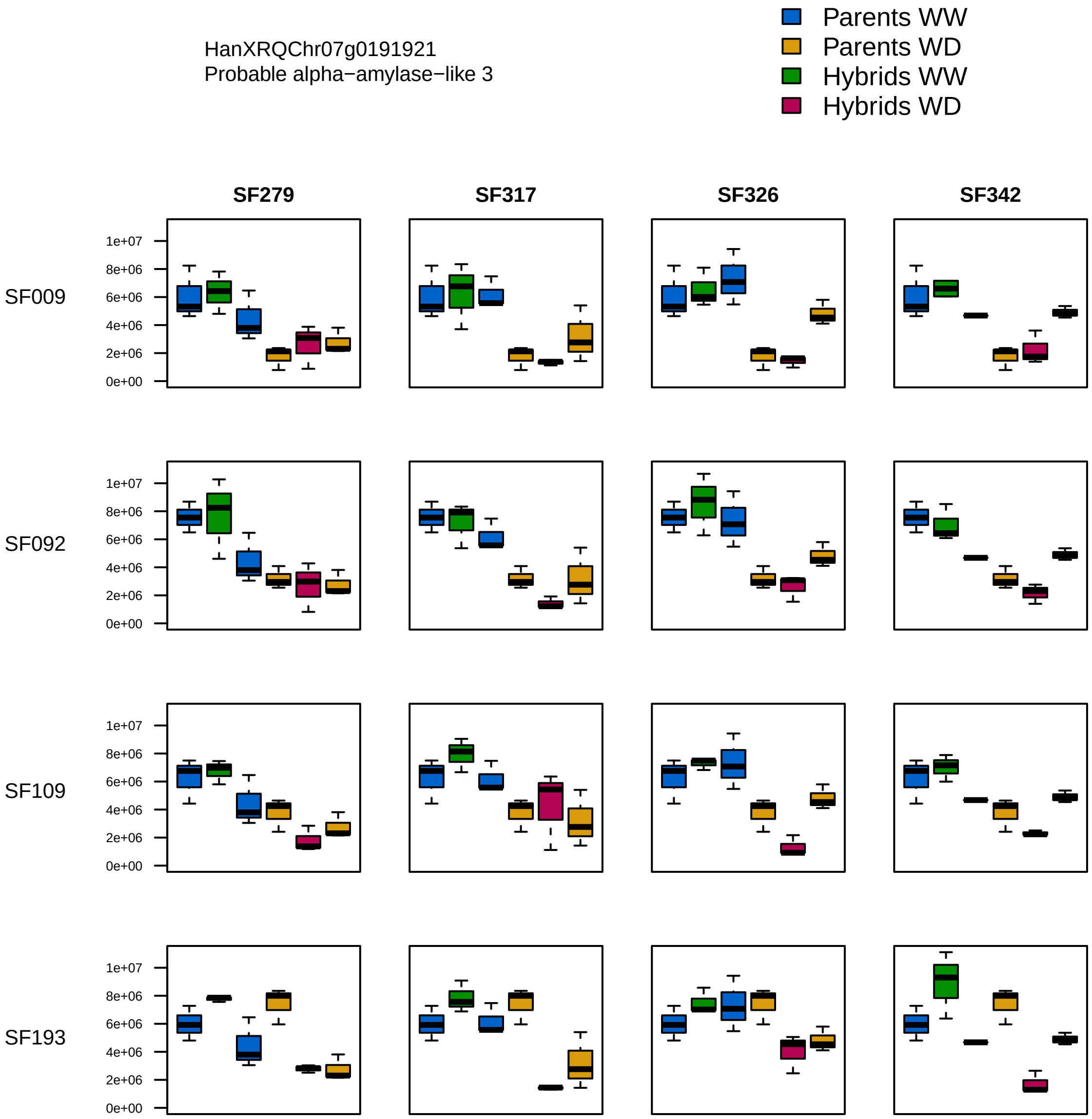
HanXRQChr07g0191921
Probable alpha−amylase−like 3
Parents WW
Parents WD
Hybrids WW
Hybrids WD
SF279
SF317
SF326
SF342
SF009
SF092
SF109
SF193
1e+07
8e+06
6e+06
4e+06
2e+06
0e+00

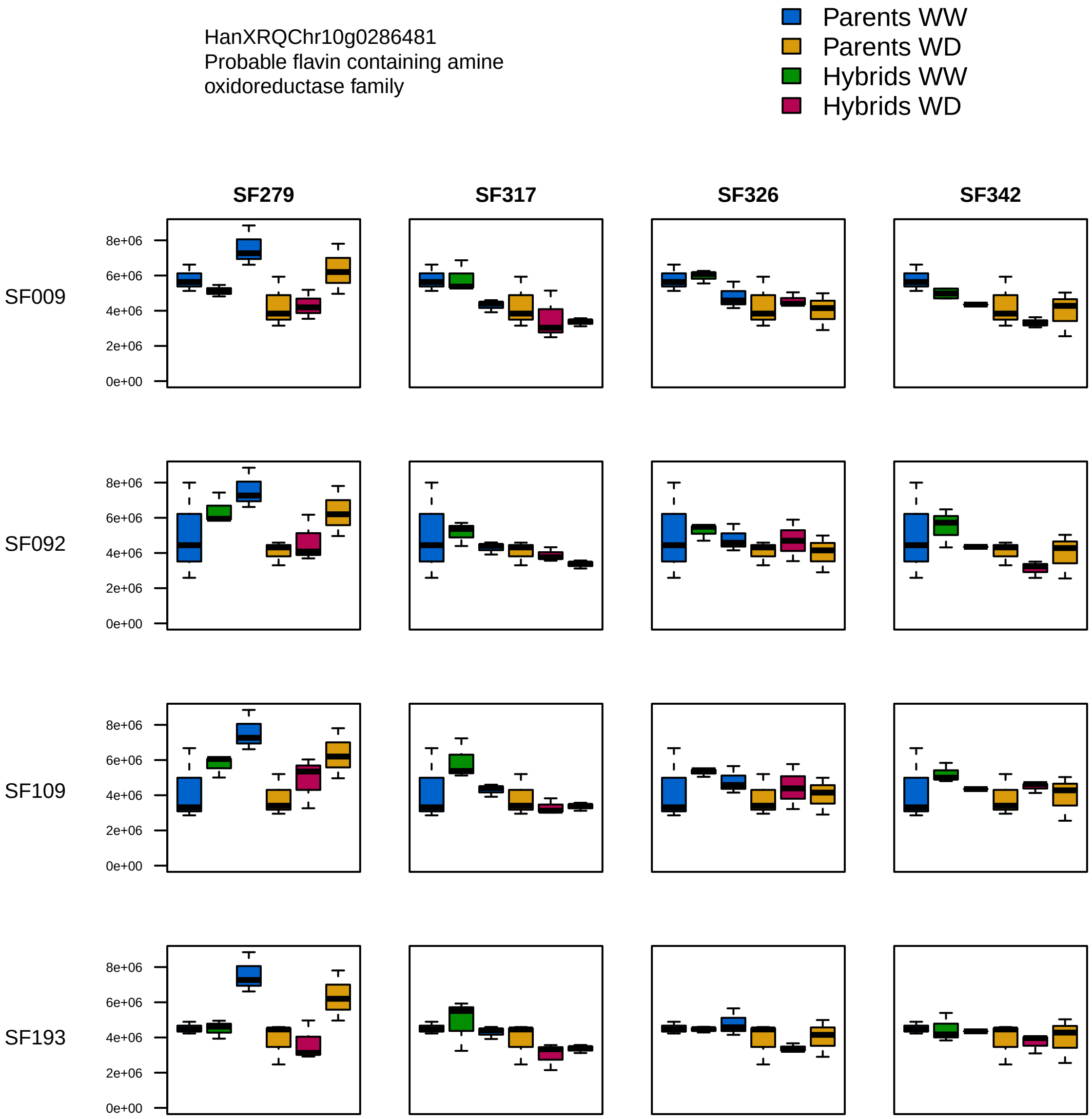
HanXRQChr10g0286481
Probable flavin containing amine
oxidoreductase family
Parents WW
Parents WD
Hybrids WW
Hybrids WD
SF279
SF317
SF326
SF342
SF009
SF092
SF109
SF193
8e+06
6e+06
4e+06
2e+06
0e+00

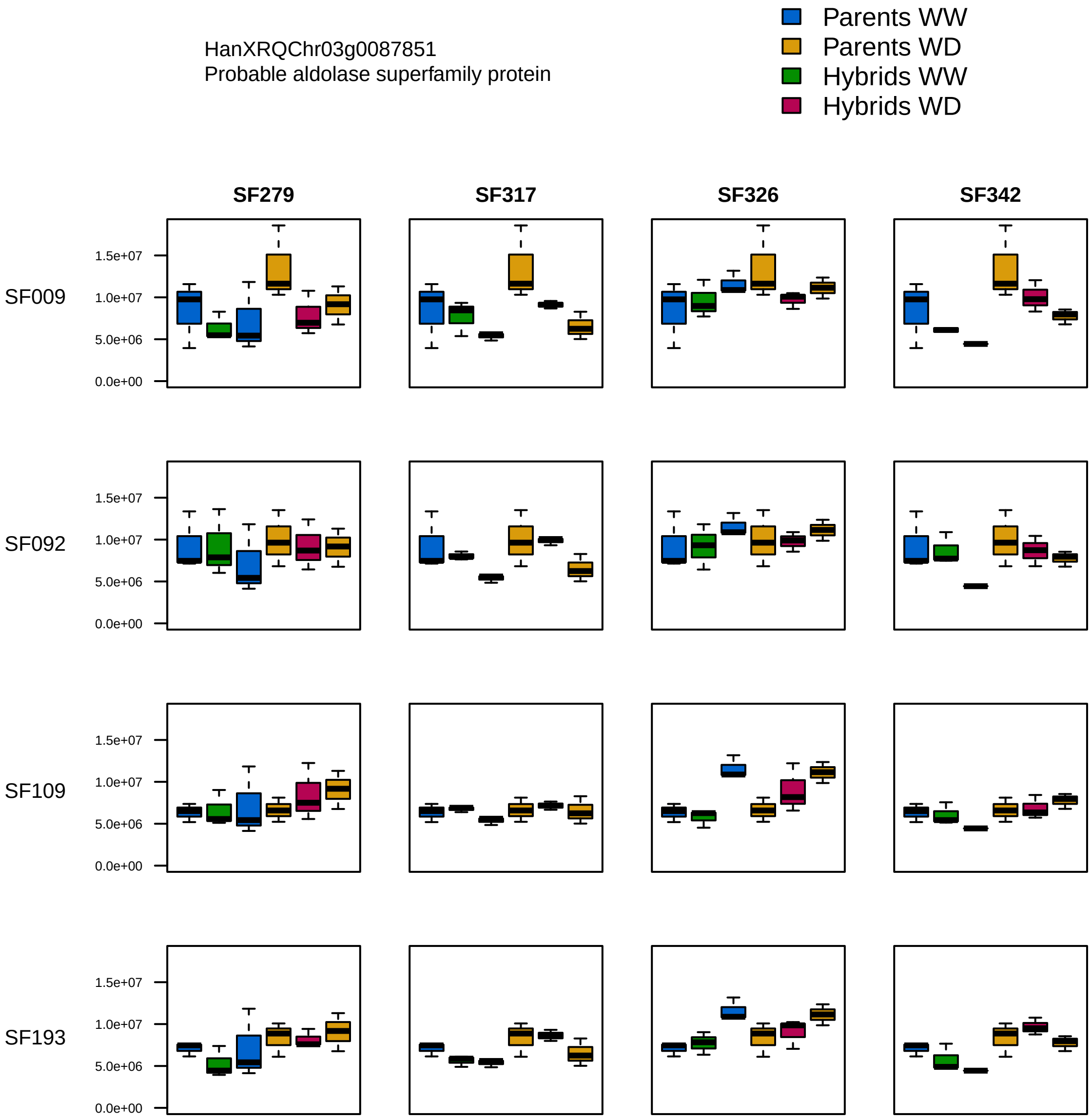

HanXRQChr03g0087851
Probable aldolase superfamily protein
Parents WW
Parents WD
Hybrids WW
Hybrids WD
SF279
SF317
SF326
SF342
SF009
SF092
SF109
SF193
1.5e+07
1.0e+07
5.0e+06
0.0e+00

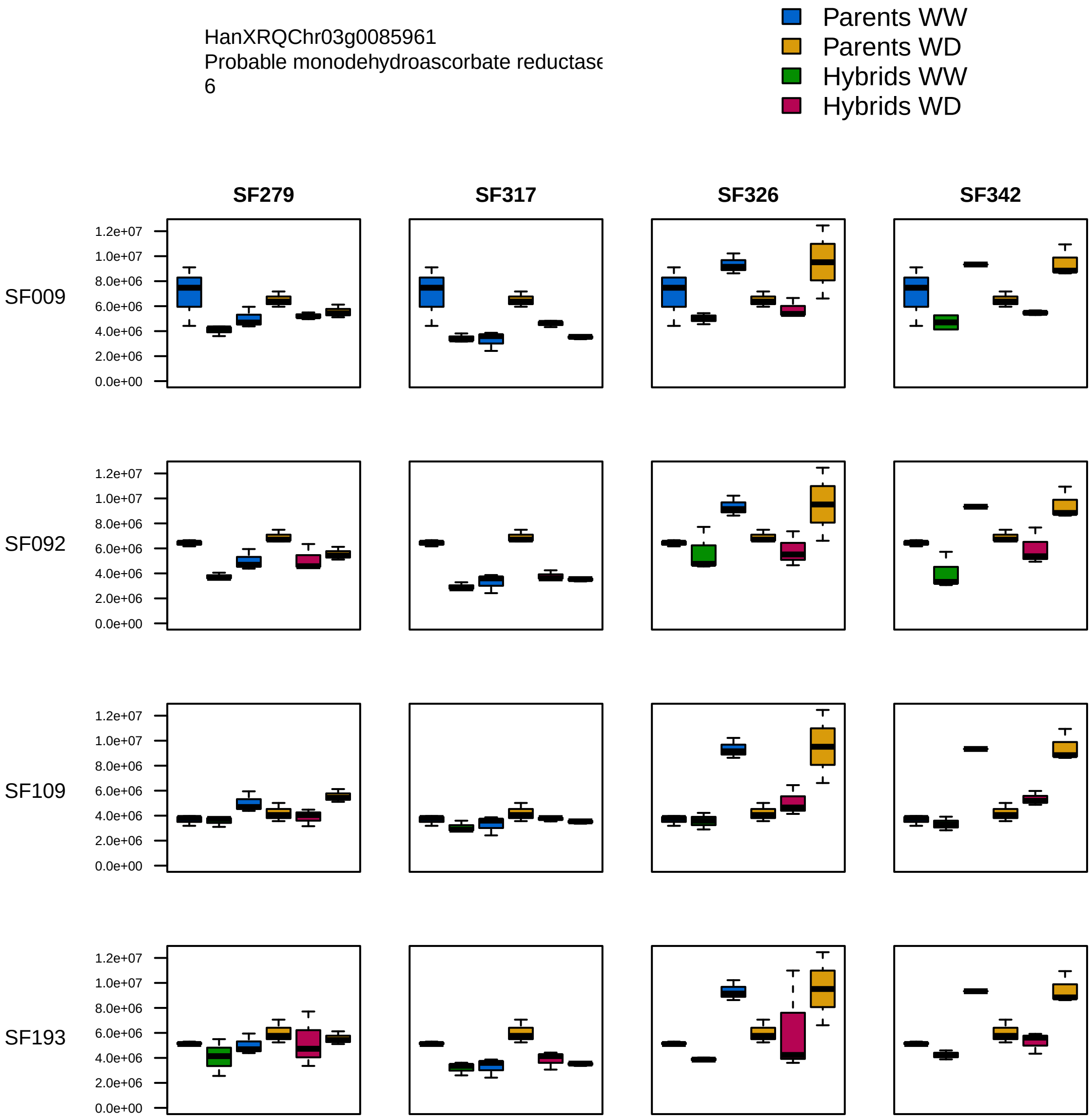
HanXRQChr03g0085961
Probable monodehydroascorbate reductase
6
Parents WW
Parents WD
Hybrids WW
Hybrids WD
SF279
SF317
SF326
SF342
SF009
SF092
SF109
SF193
1.2e+07
1.0e+07
8.0e+06
6.0e+06
4.0e+06
2.0e+06
0.0e+00

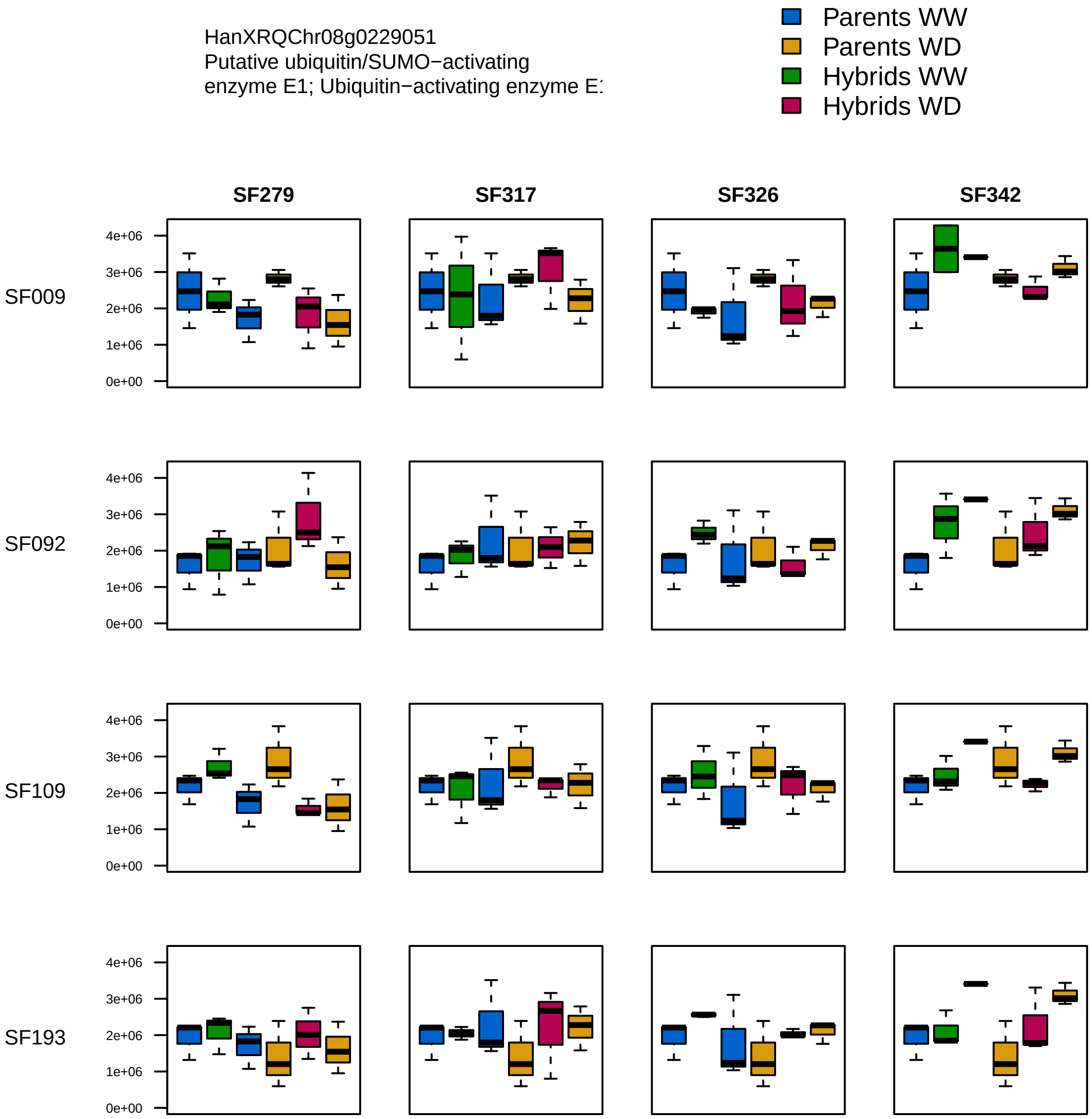
HanXRQChr08g0229051
Putative ubiquitin/SUMO−activating
enzyme E1; Ubiquitin−activating enzyme E:
Parents WW
Parents WD
Hybrids WW
Hybrids WD
SF279
SF317
SF326
SF342
SF009
SF092
SF109
SF193
4e+06
3e+06
2e+06
1e+06
0e+00

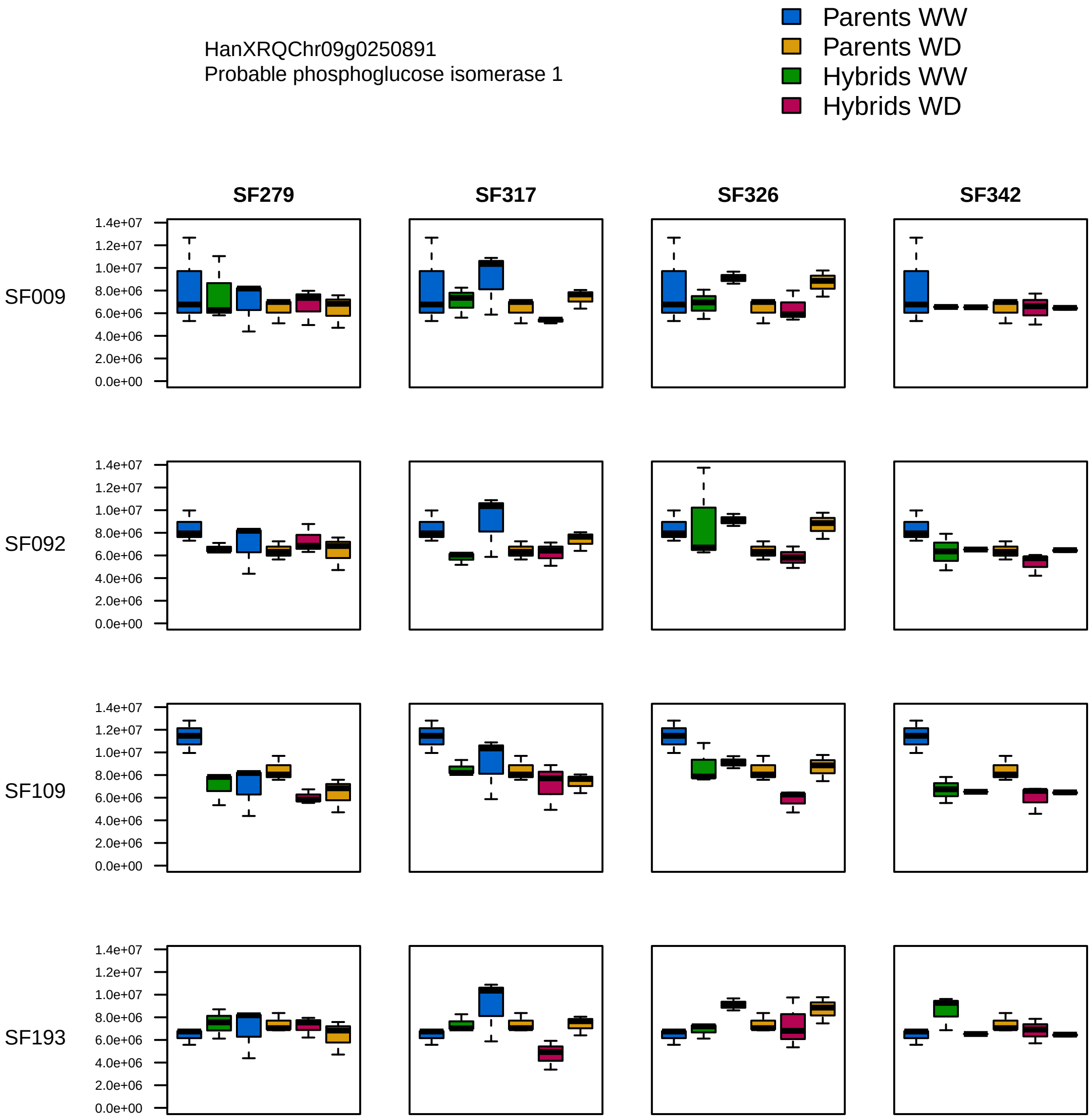
HanXRQChr09g0250891
Probable phosphoglucose isomerase 1
Parents WW
Parents WD
Hybrids WW
Hybrids WD
SF279
SF317
SF326
SF342
SF009
SF092
SF109
SF193
1.4e+07
1.2e+07
1.0e+07
8.0e+06
6.0e+06
4.0e+06
2.0e+06
0.0e+00

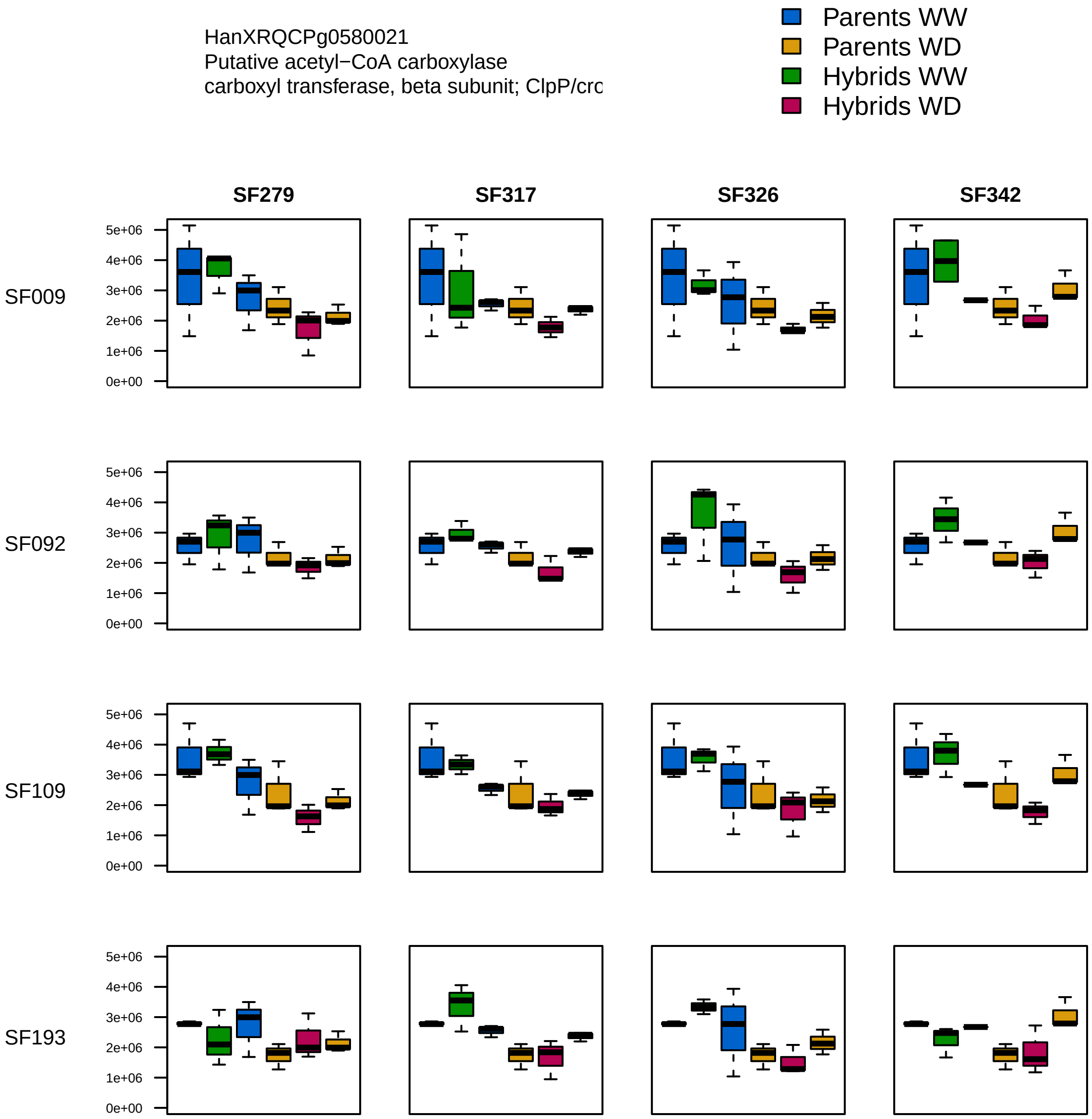
HanXRQCPg0580021
Putative acetyl−CoA carboxylase
carboxyl transferase, beta subunit; ClpP/crc
Parents WW
Parents WD
Hybrids WW
Hybrids WD
SF279
SF317
SF326
SF342
SF009
SF092
SF109
SF193
0e+00
1e+06
2e+06
3e+06
4e+06
5e+06

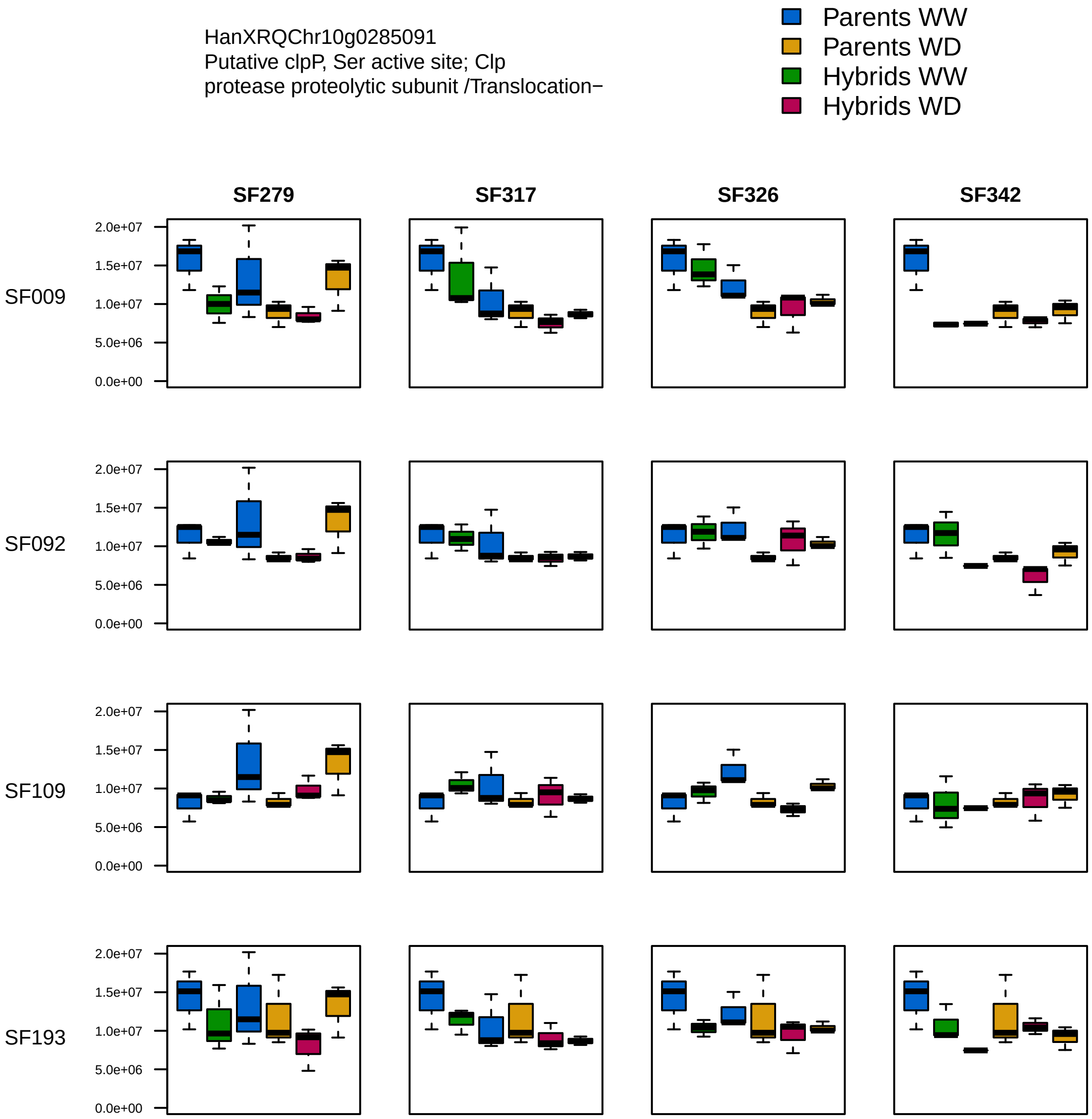
HanXRQChr10g0285091
Putative clpP, Ser active site; Clp
protease proteolytic subunit /Translocation−
Parents WW
Parents WD
Hybrids WW
Hybrids WD
SF279
SF317
SF326
SF342
SF009
SF092
SF109
SF193
2.0e+07
1.5e+07
1.0e+07
5.0e+06
0.0e+00

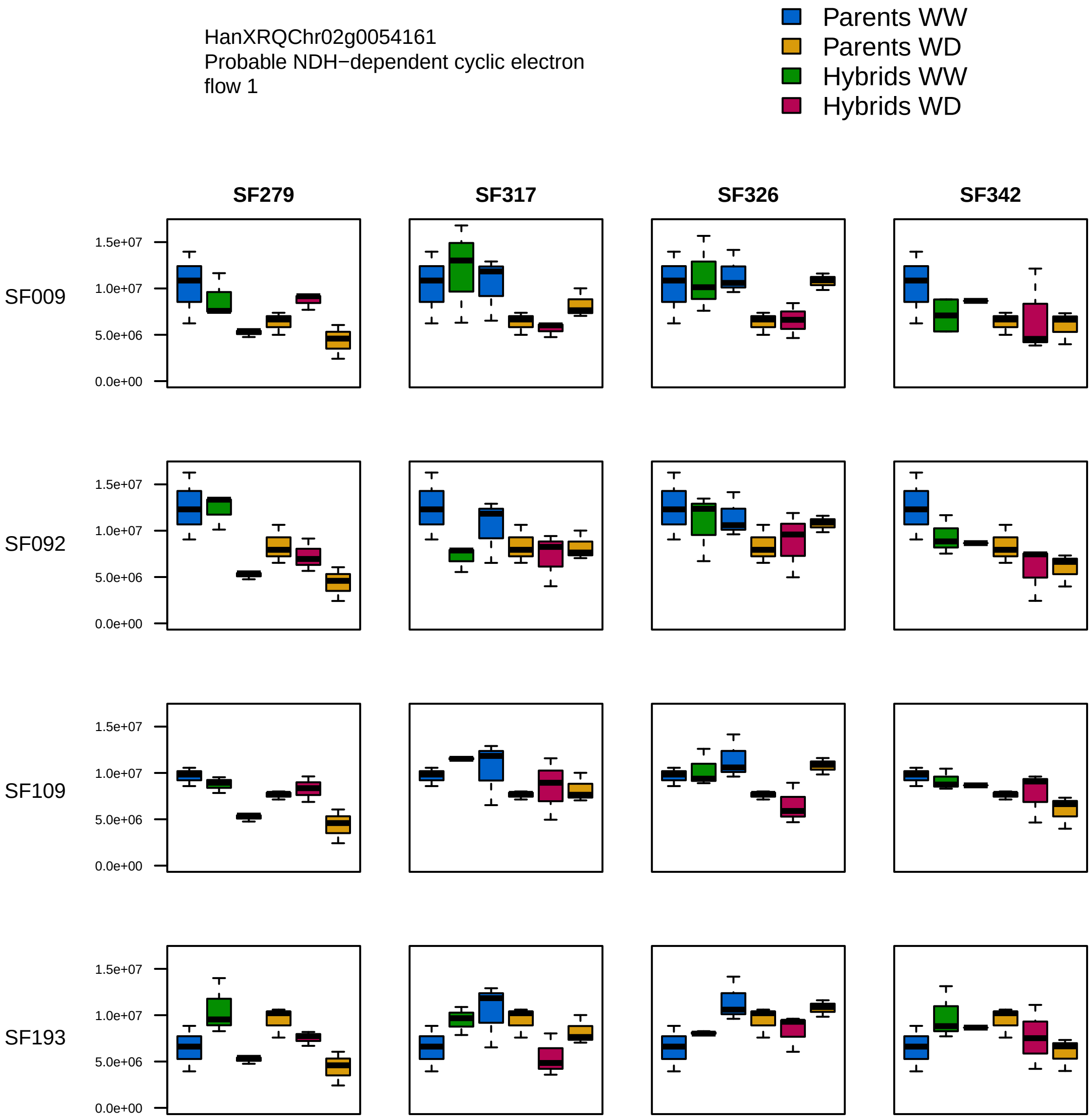

HanXRQChr02g0054161
Probable NDH−dependent cyclic electron flow 1
Parents WW
Parents WD
Hybrids WW
Hybrids WD
SF279
SF317
SF326
SF342
SF009
SF092
SF109
SF193
1.5e+07
1.0e+07
5.0e+06
0.0e+00

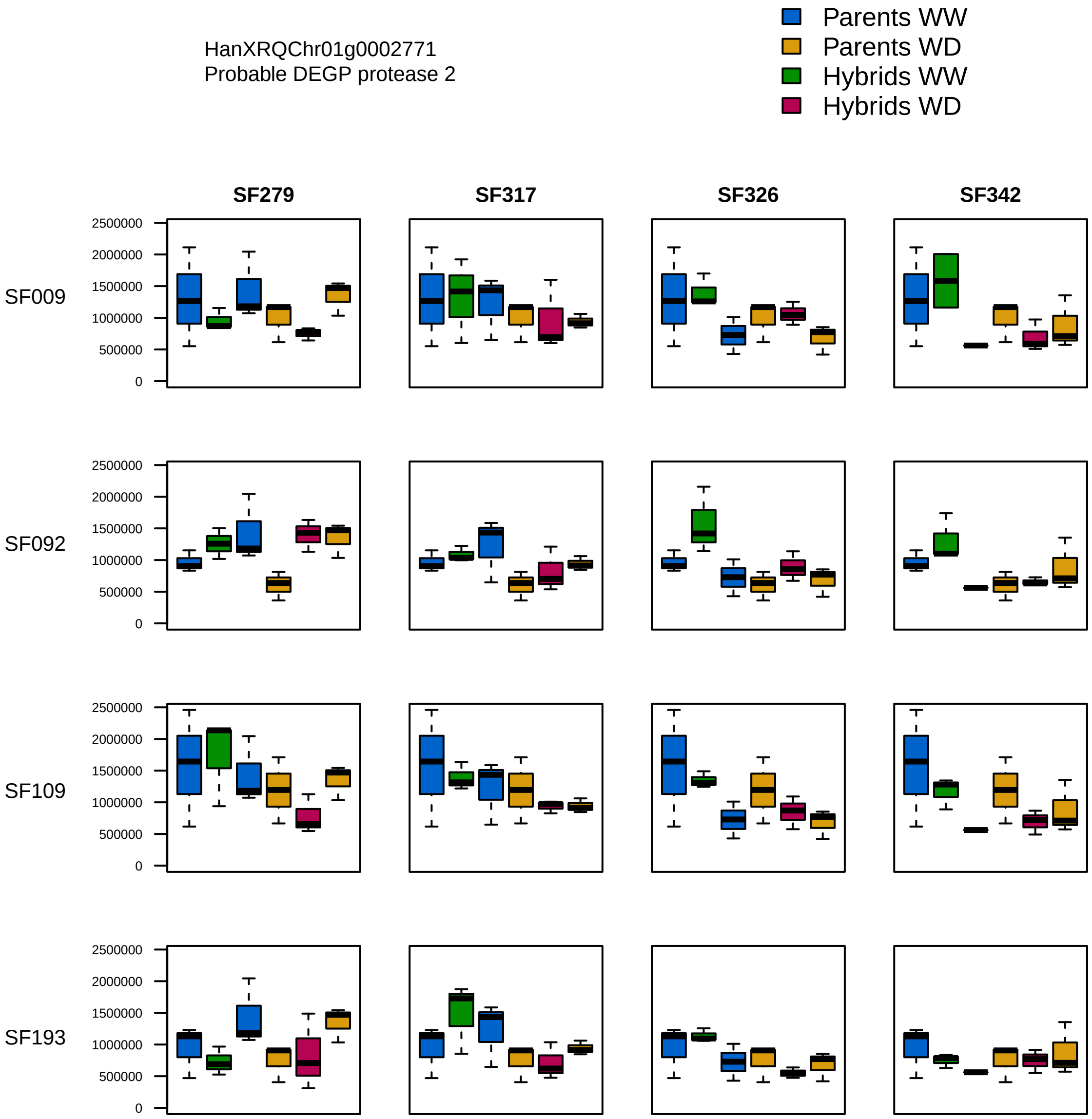
HanXRQChr01g0002771
Probable DEGP protease 2
Parents WW
Parents WD
Hybrids WW
Hybrids WD
SF279
SF317
SF326
SF342
SF009
SF092
SF109
SF193
2500000
2000000
1500000
1000000
500000
0

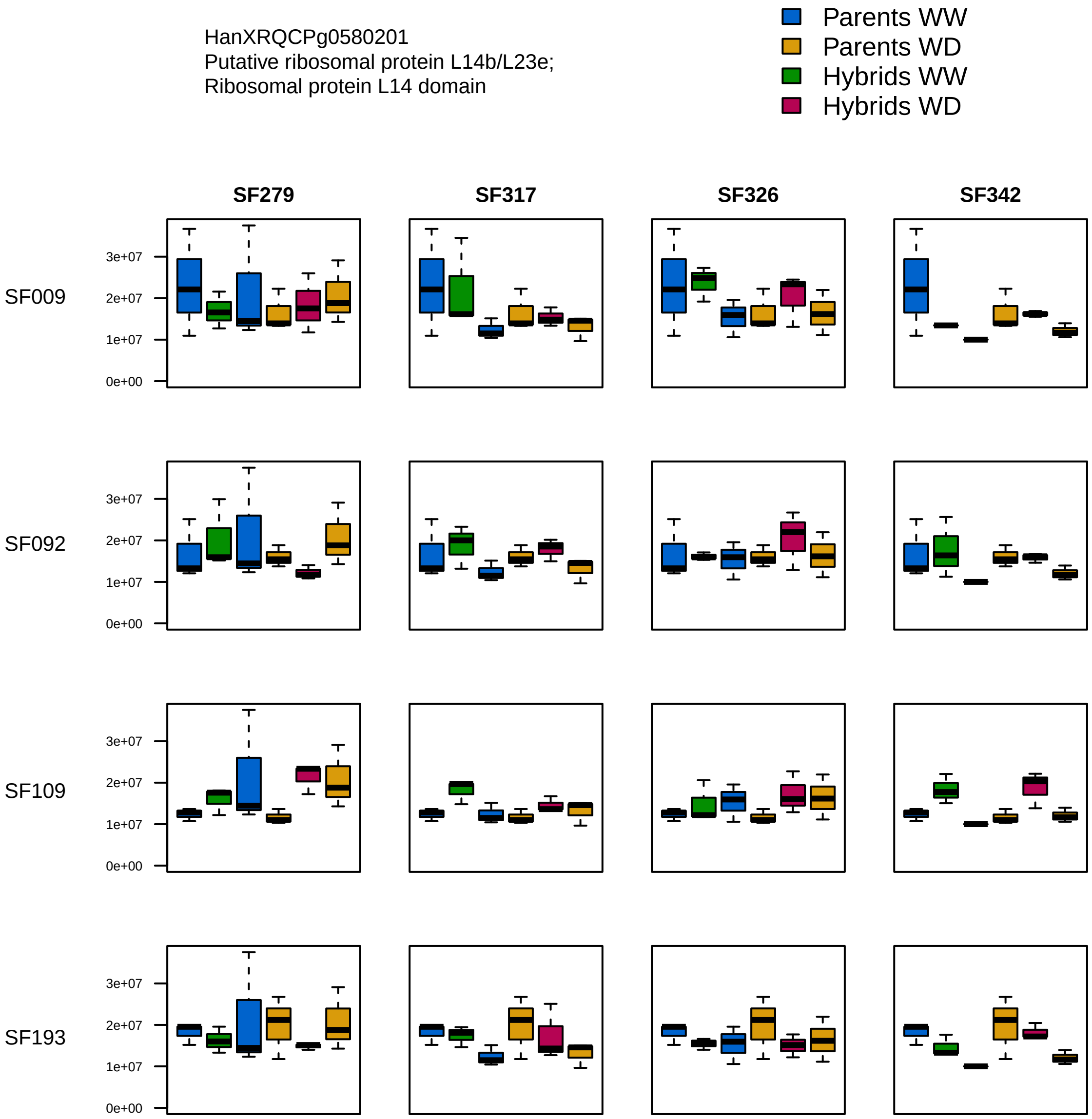
HanXRQCPg0580201
Putative ribosomal protein L14b/L23e;
Ribosomal protein L14 domain
Parents WW
Parents WD
Hybrids WW
Hybrids WD
SF279
SF317
SF326
SF342
SF009
SF092
SF109
SF193
0e+00
1e+07
2e+07
3e+07

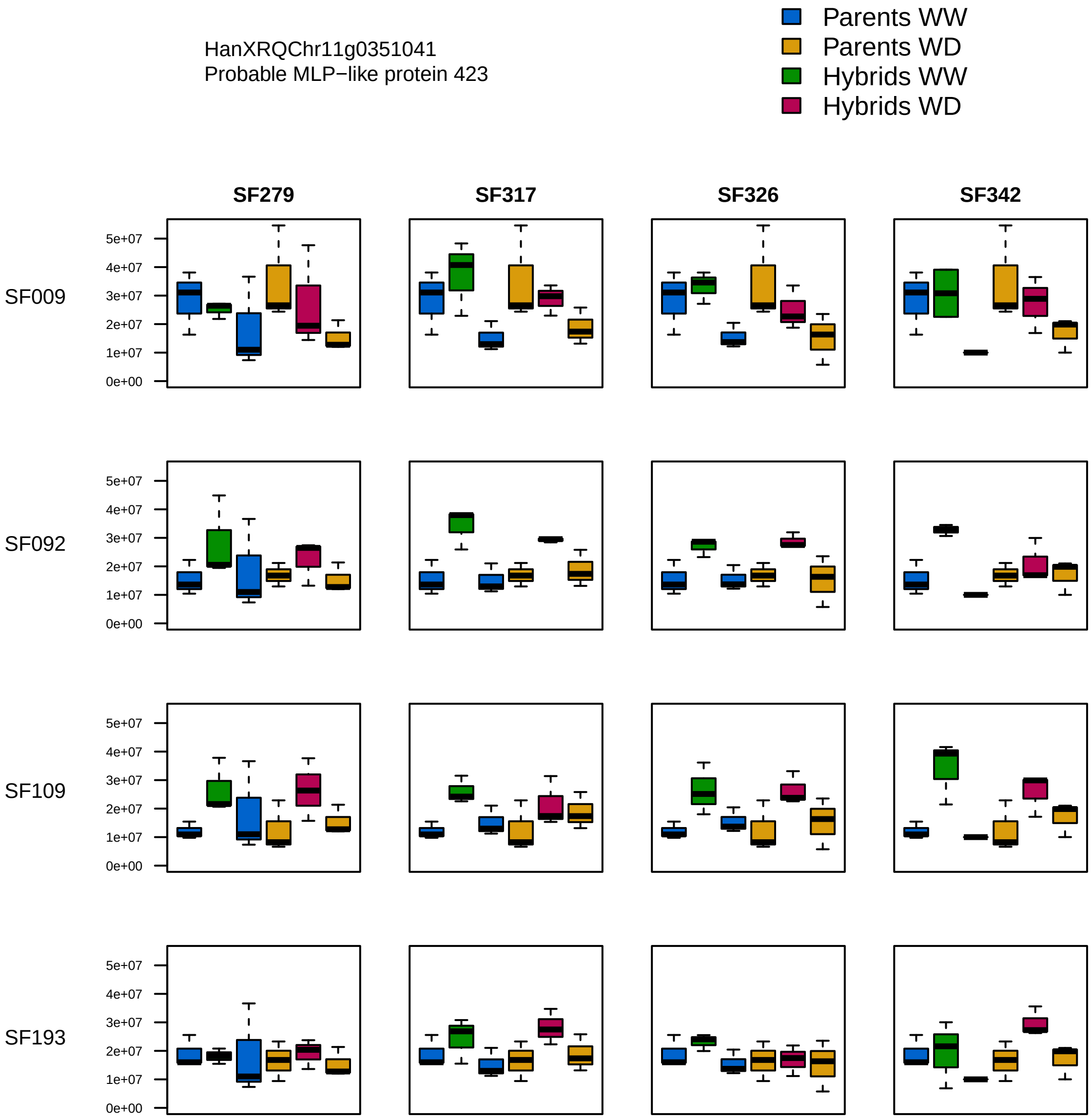
HanXRQChr11g0351041
Probable MLP−like protein 423
Parents WW
Parents WD
Hybrids WW
Hybrids WD
SF279
SF317
SF326
SF342
SF009
SF092
SF109
SF193
5e+07
4e+07
3e+07
2e+07
1e+07
0e+00

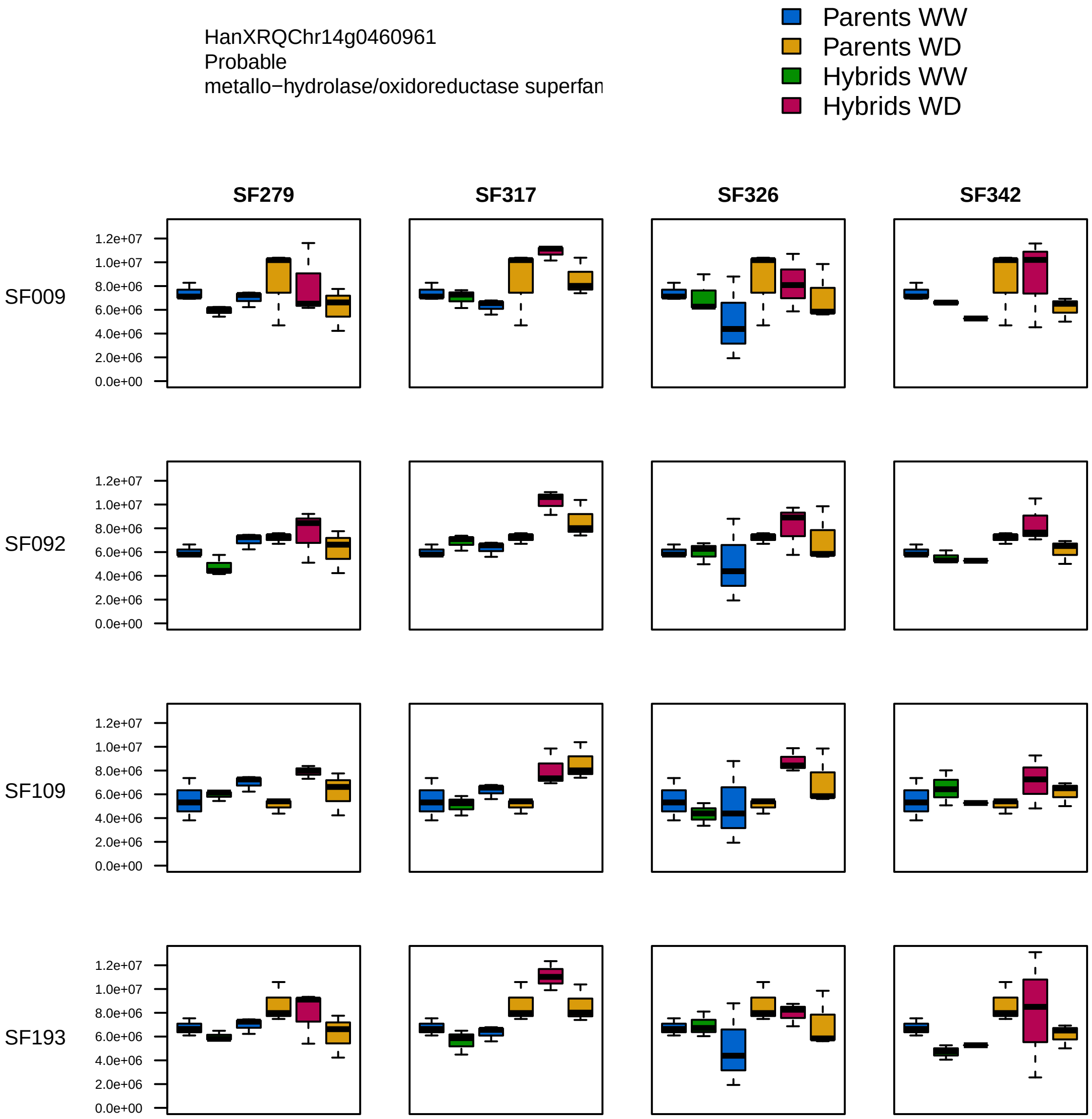

HanXRQChr14g0460961
Probable
metallo−hydrolase/oxidoreductase superfan
Parents WW
Parents WD
Hybrids WW
Hybrids WD
SF279
SF317
SF326
SF342
SF009
SF092
SF109
SF193
1.2e+07
1.0e+07
8.0e+06
6.0e+06
4.0e+06
2.0e+06
0.0e+00

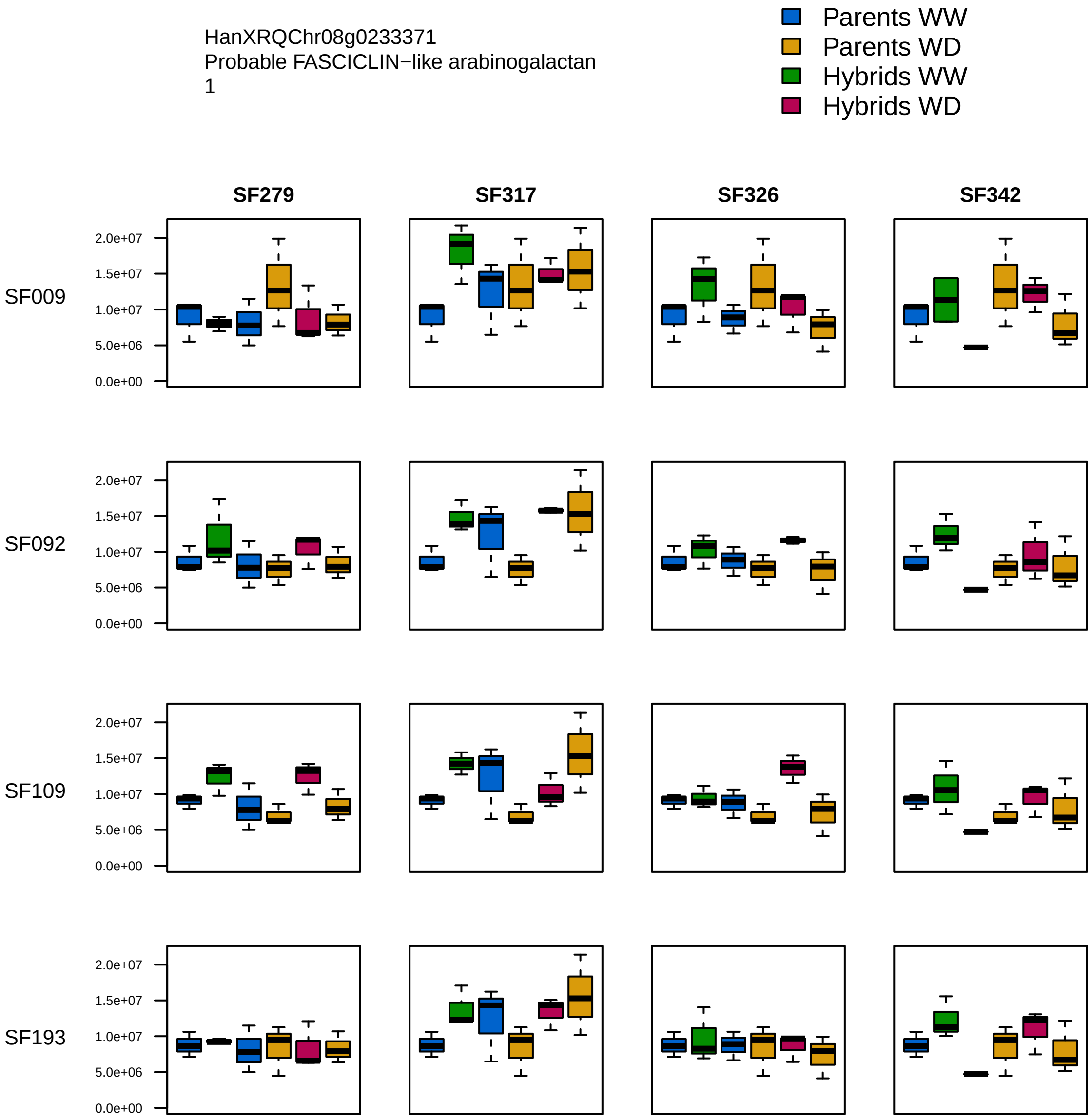
HanXRQChr08g0233371
Probable FASCICLIN−like arabinogalactan
1
Parents WW
Parents WD
Hybrids WW
Hybrids WD
SF279
SF317
SF326
SF342
SF009
SF092
SF109
SF193
2.0e+07
1.5e+07
1.0e+07
5.0e+06
0.0e+00

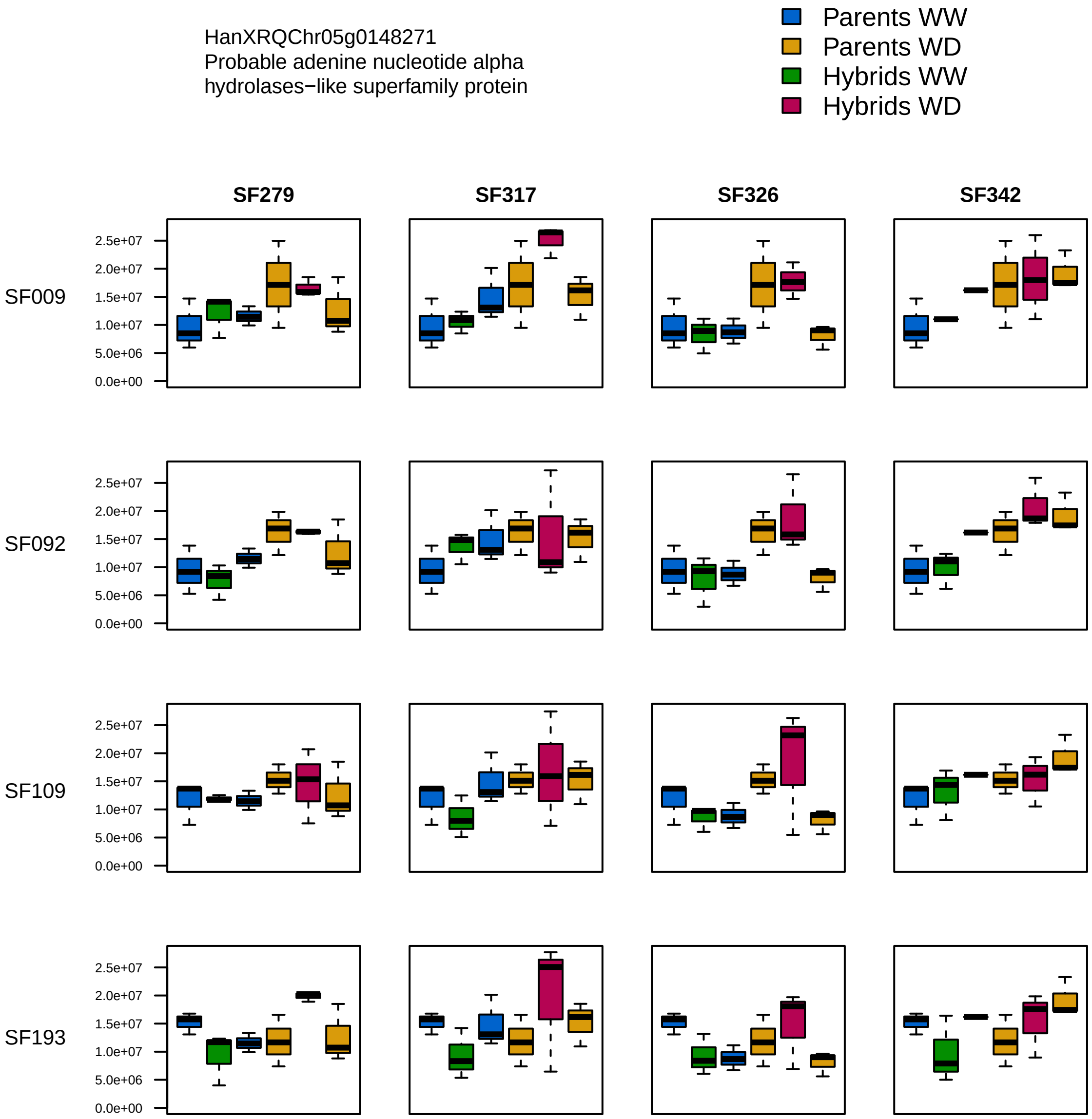
HanXRQChr05g0148271
Probable adenine nucleotide alpha
hydrolases−like superfamily protein
Parents WW
Parents WD
Hybrids WW
Hybrids WD
SF279
SF317
SF326
SF342
SF009
SF092
SF109
SF193
2.5e+07
2.0e+07
1.5e+07
1.0e+07
5.0e+06
0.0e+00

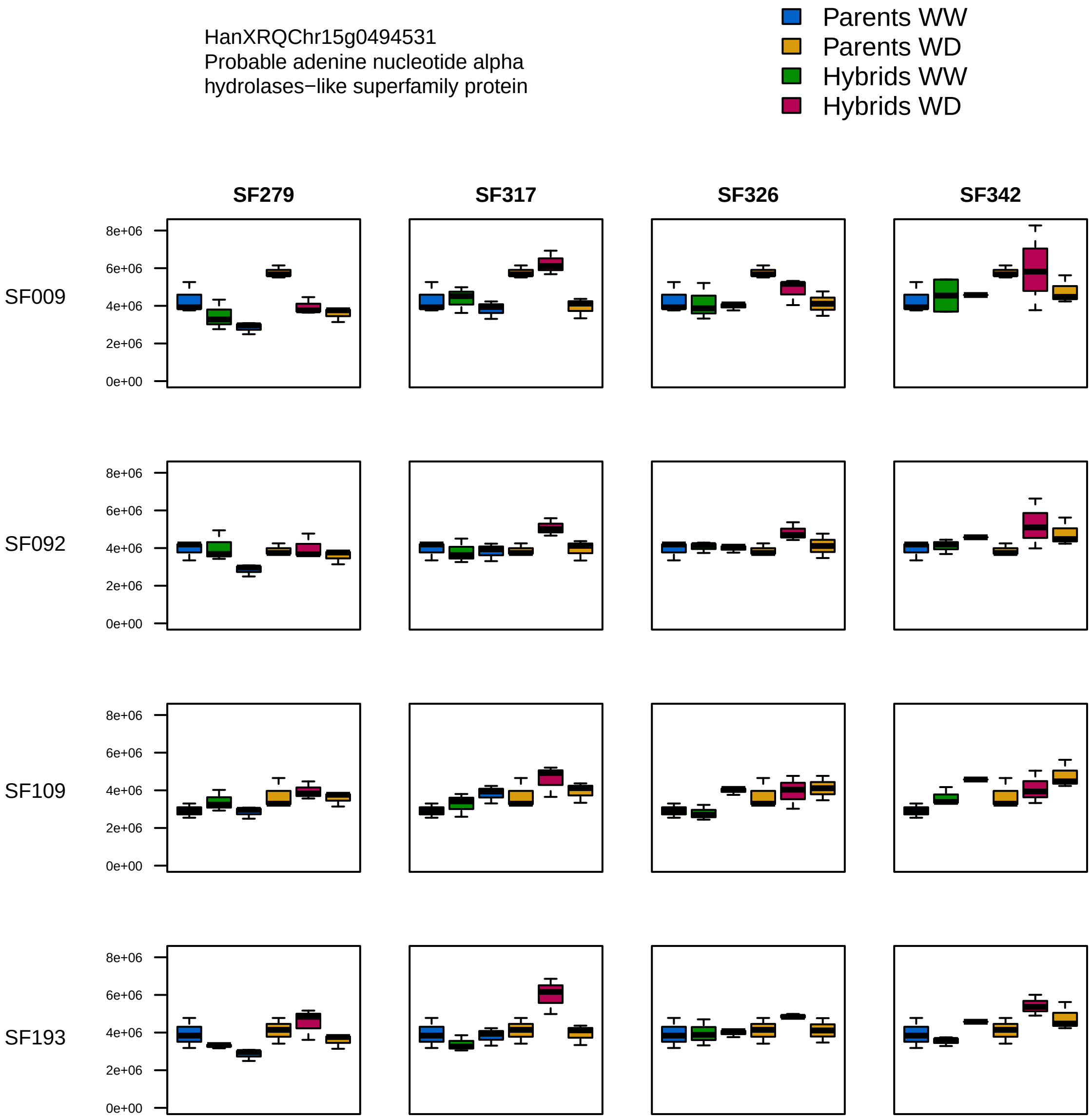
HanXRQChr15g0494531
Probable adenine nucleotide alpha
hydrolases−like superfamily protein
Parents WW
Parents WD
Hybrids WW
Hybrids WD
SF279
SF317
SF326
SF342
SF009
SF092
SF109
SF193
0e+00
2e+06
4e+06
6e+06
8e+06

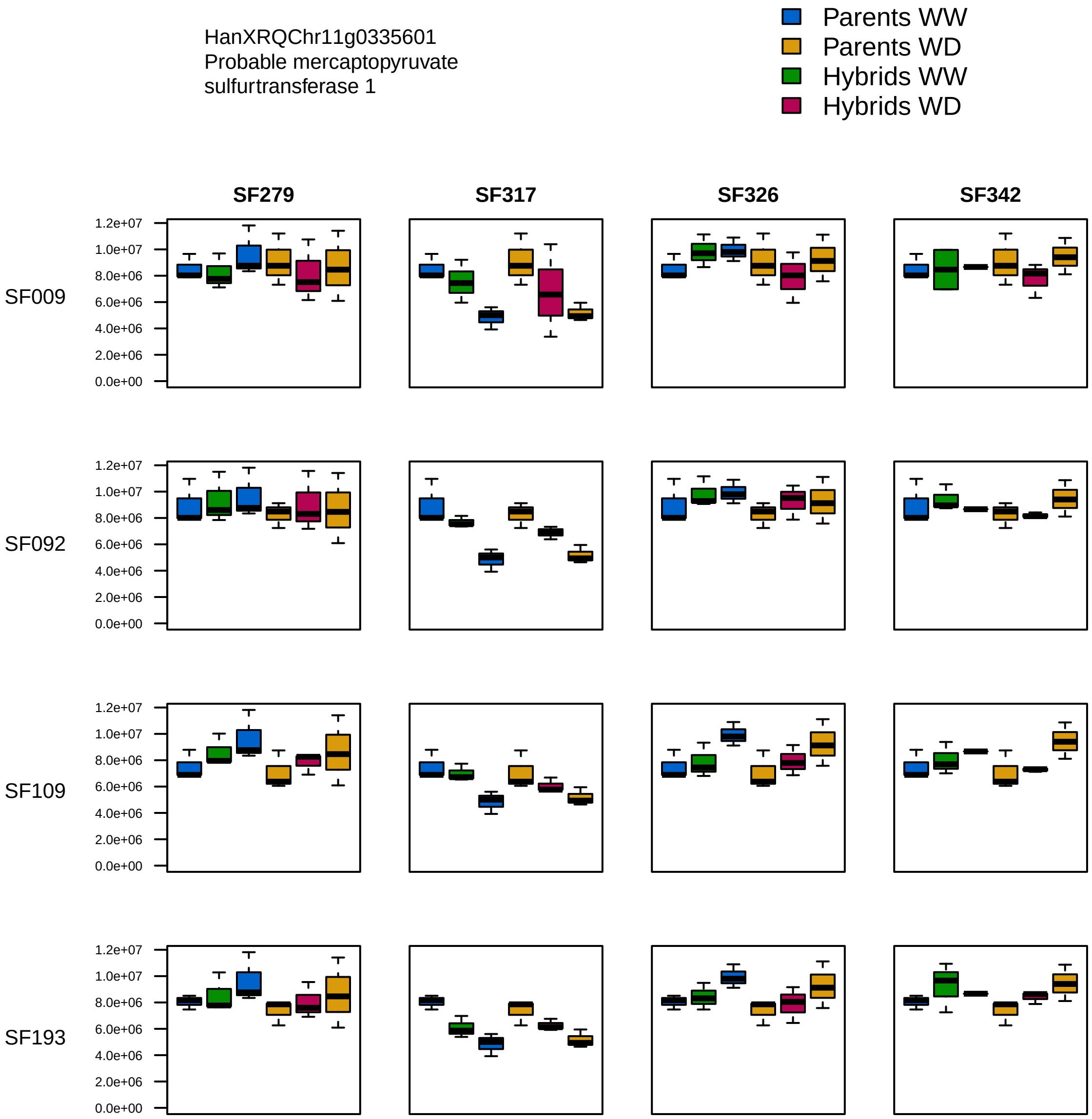
HanXRQChr11g0335601
Probable mercaptopyruvate
sulfurtransferase 1
Parents WW
Parents WD
Hybrids WW
Hybrids WD
SF279
SF317
SF326
SF342
SF009
SF092
SF109
SF193
0.0e+00
2.0e+06
4.0e+06
6.0e+06
8.0e+06
1.0e+07
1.2e+07

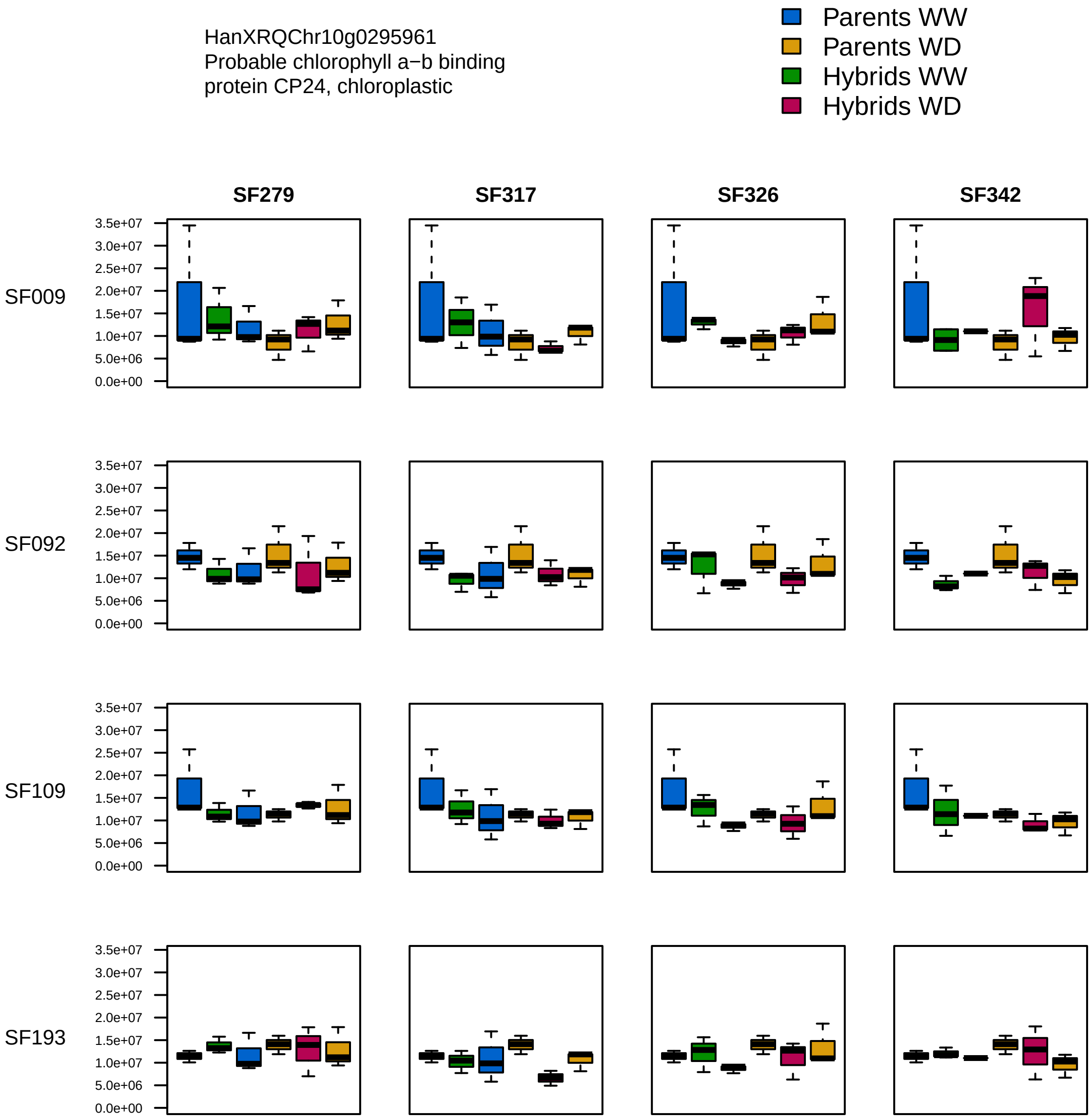

HanXRQChr10g0295961
Probable chlorophyll a−b binding
protein CP24, chloroplastic
Parents WW
Parents WD
Hybrids WW
Hybrids WD
SF279
SF317
SF326
SF342
SF009
SF092
SF109
SF193
3.5e+07
3.0e+07
2.5e+07
2.0e+07
1.5e+07
1.0e+07
5.0e+06
0.0e+00

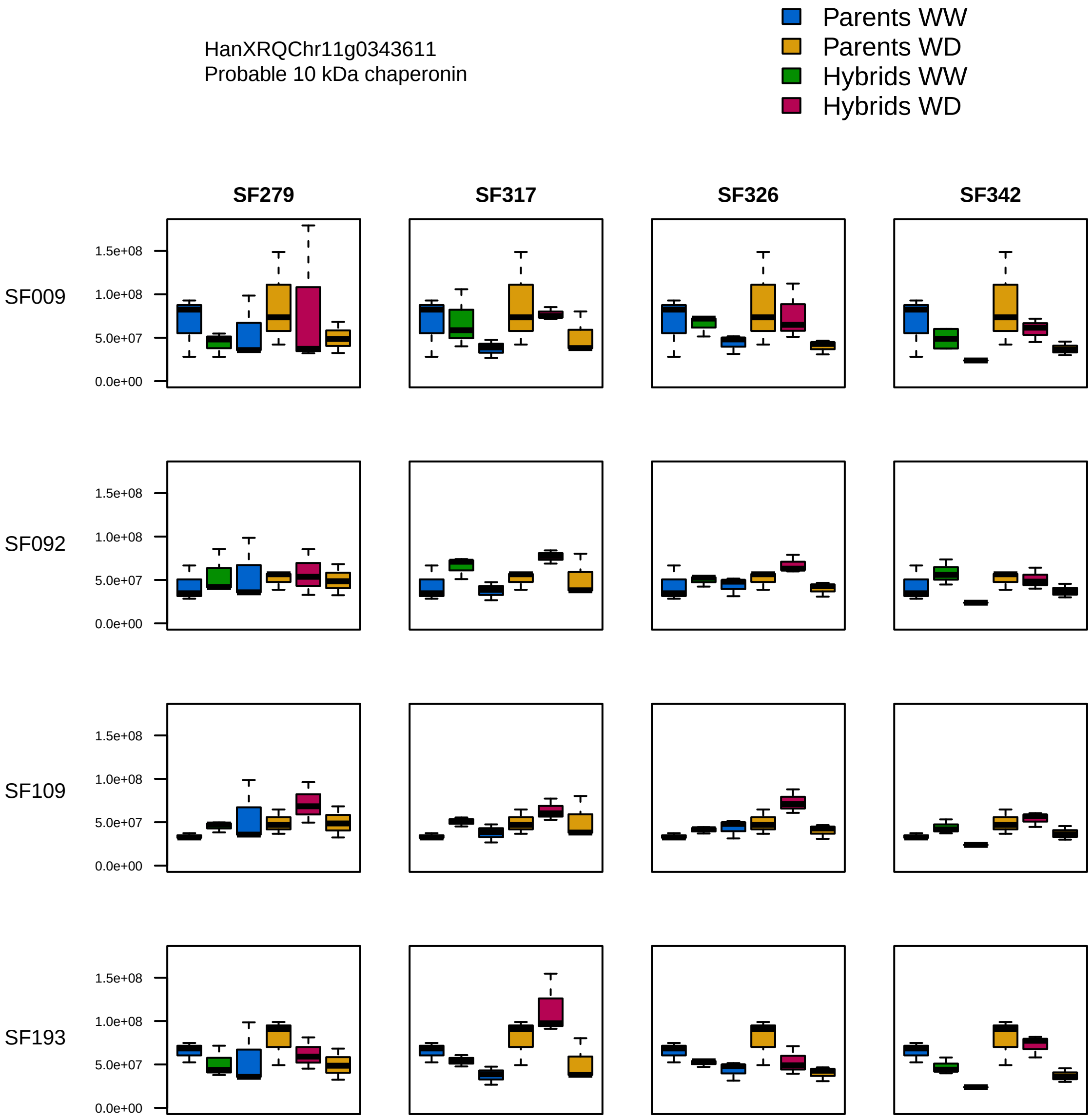
HanXRQChr11g0343611
Probable 10 kDa chaperonin
Parents WW
Parents WD
Hybrids WW
Hybrids WD
SF279
SF317
SF326
SF342
SF009
SF092
SF109
SF193
0.0e+00
5.0e+07
1.0e+08
1.5e+08

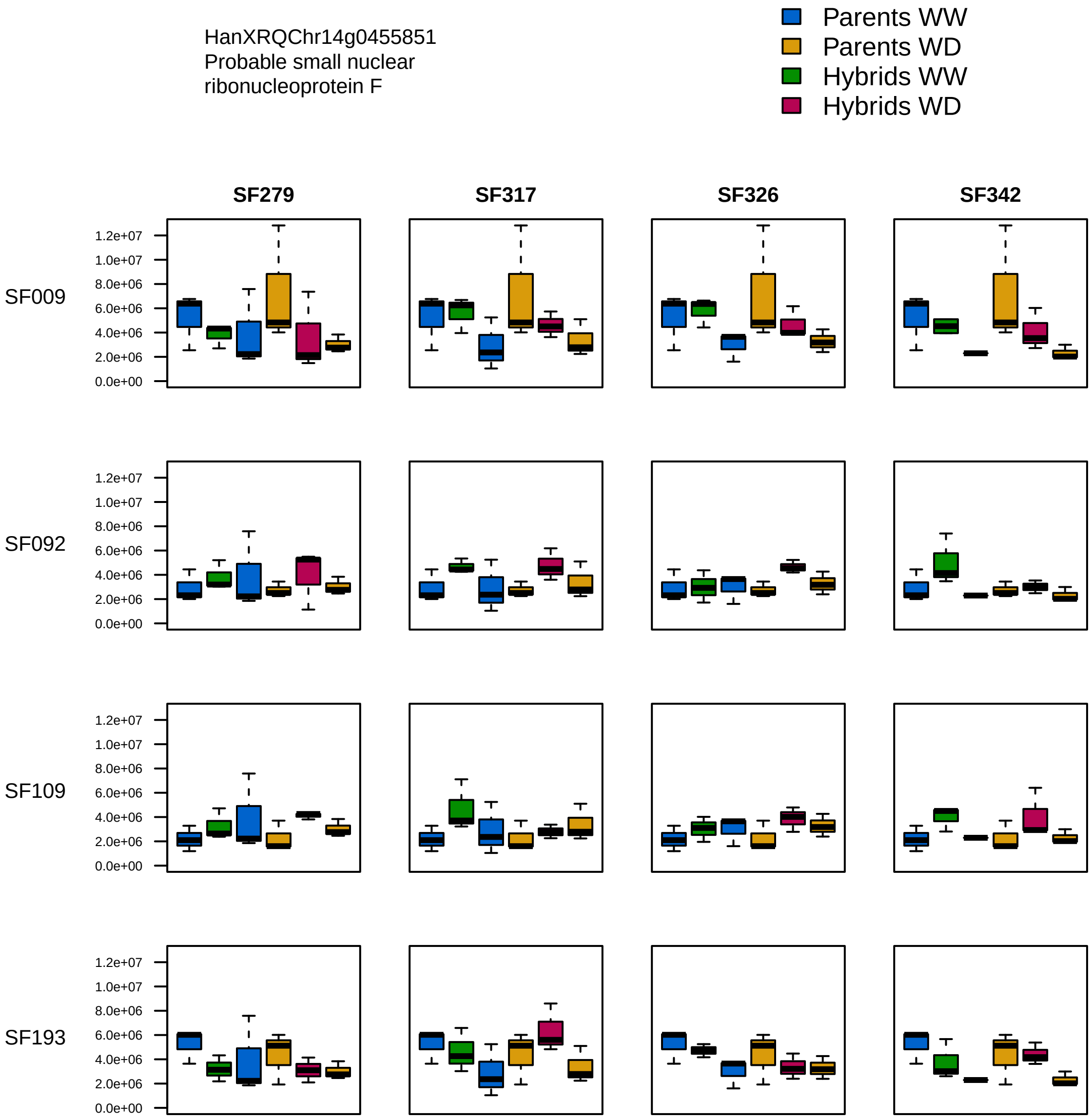

HanXRQChr14g0455851
Probable small nuclear
ribonucleoprotein F
Parents WW
Parents WD
Hybrids WW
Hybrids WD
SF279
SF317
SF326
SF342
SF009
SF092
SF109
SF193
1.2e+07
1.0e+07
8.0e+06
6.0e+06
4.0e+06
2.0e+06
0.0e+00

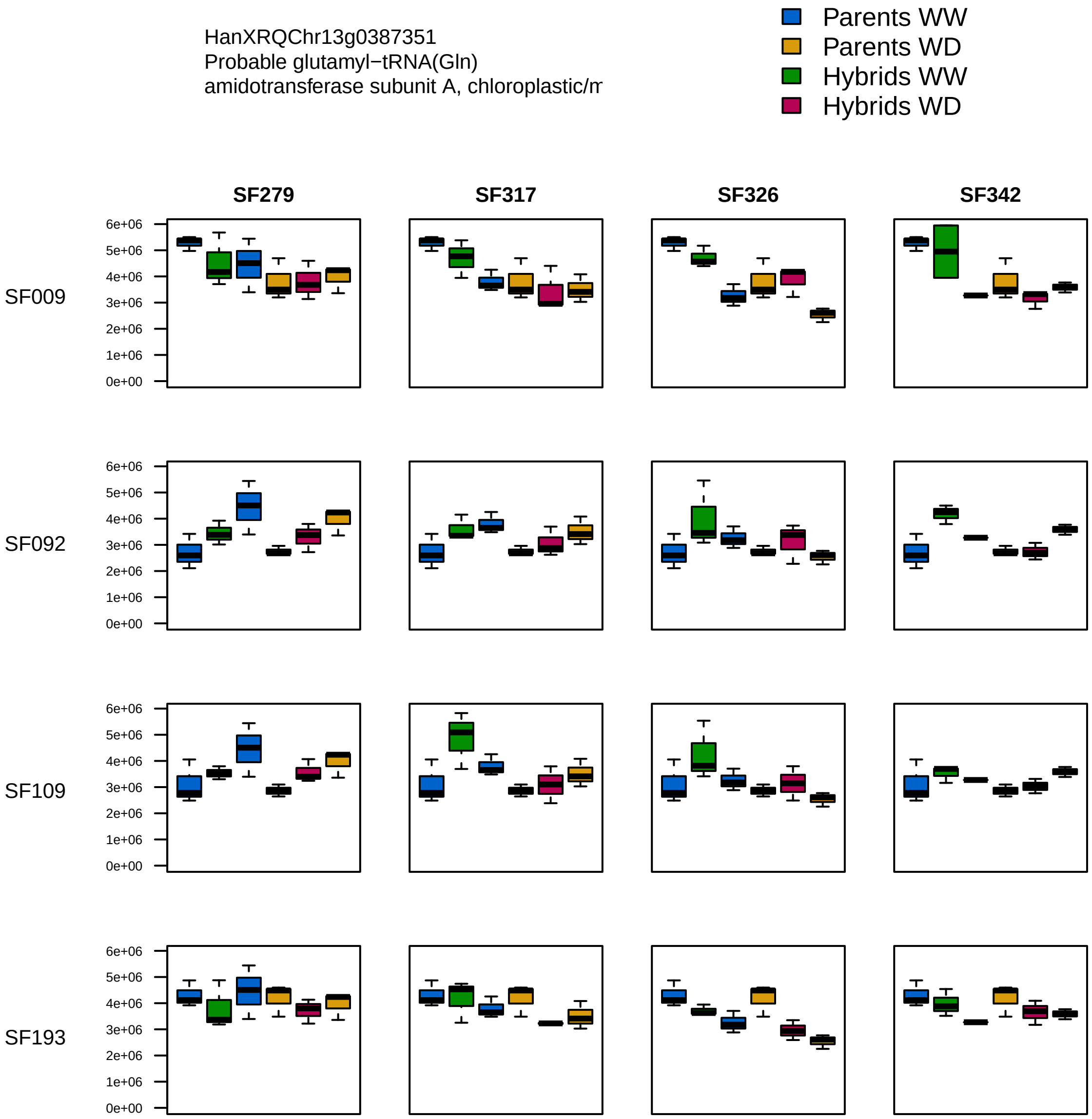

HanXRQChr13g0387351
Probable glutamyl−tRNA(Gln)
amidotransferase subunit A, chloroplastic/m
Parents WW
Parents WD
Hybrids WW
Hybrids WD
SF279
SF317
SF326
SF342
SF009
SF092
SF109
SF193
0e+00
1e+06
2e+06
3e+06
4e+06
5e+06
6e+06

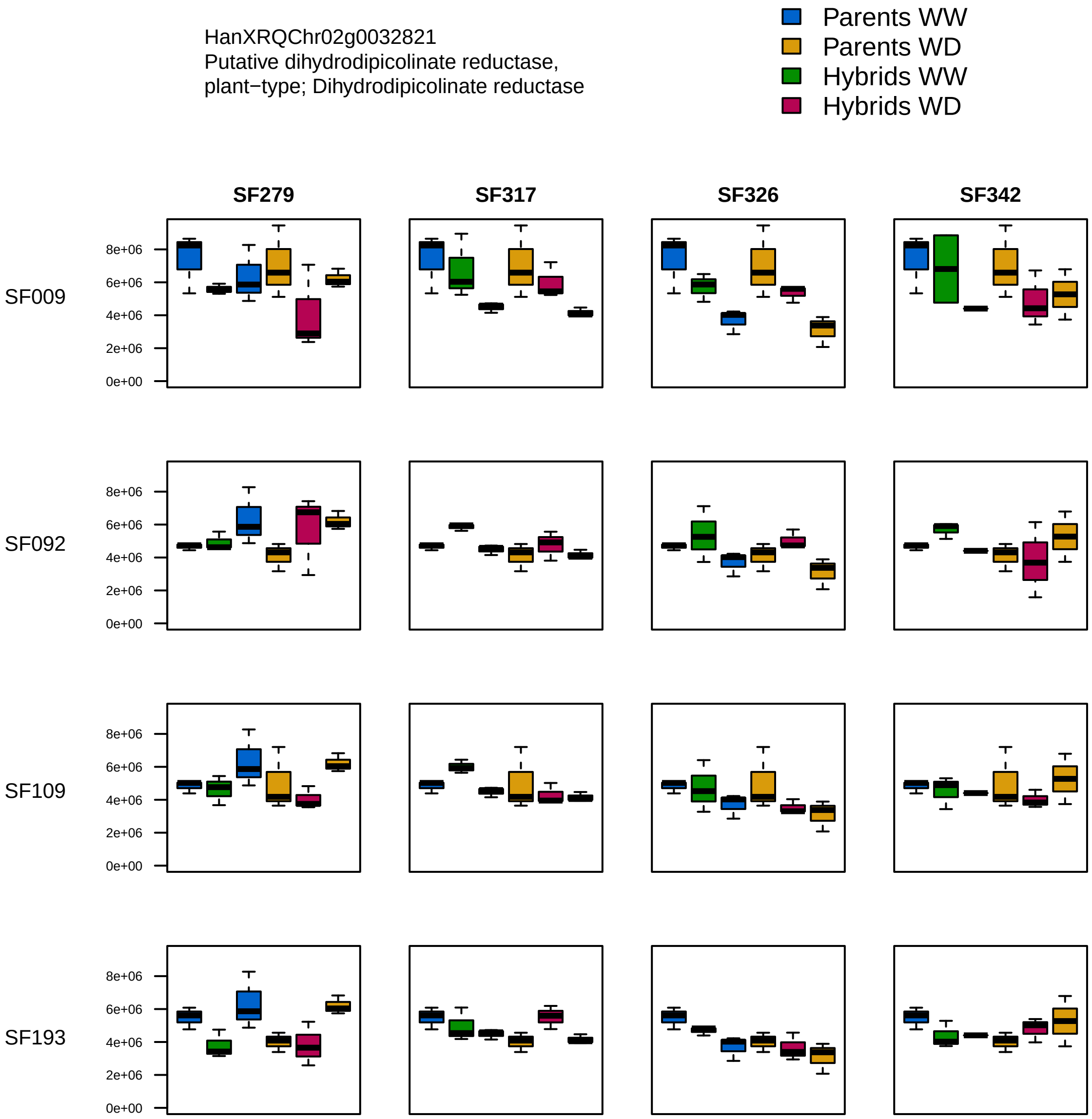
HanXRQChr02g0032821
Putative dihydrodipicolinate reductase,
plant−type; Dihydrodipicolinate reductase
Parents WW
Parents WD
Hybrids WW
Hybrids WD
SF279
SF317
SF326
SF342
SF009
SF092
SF109
SF193
8e+06
6e+06
4e+06
2e+06
0e+00

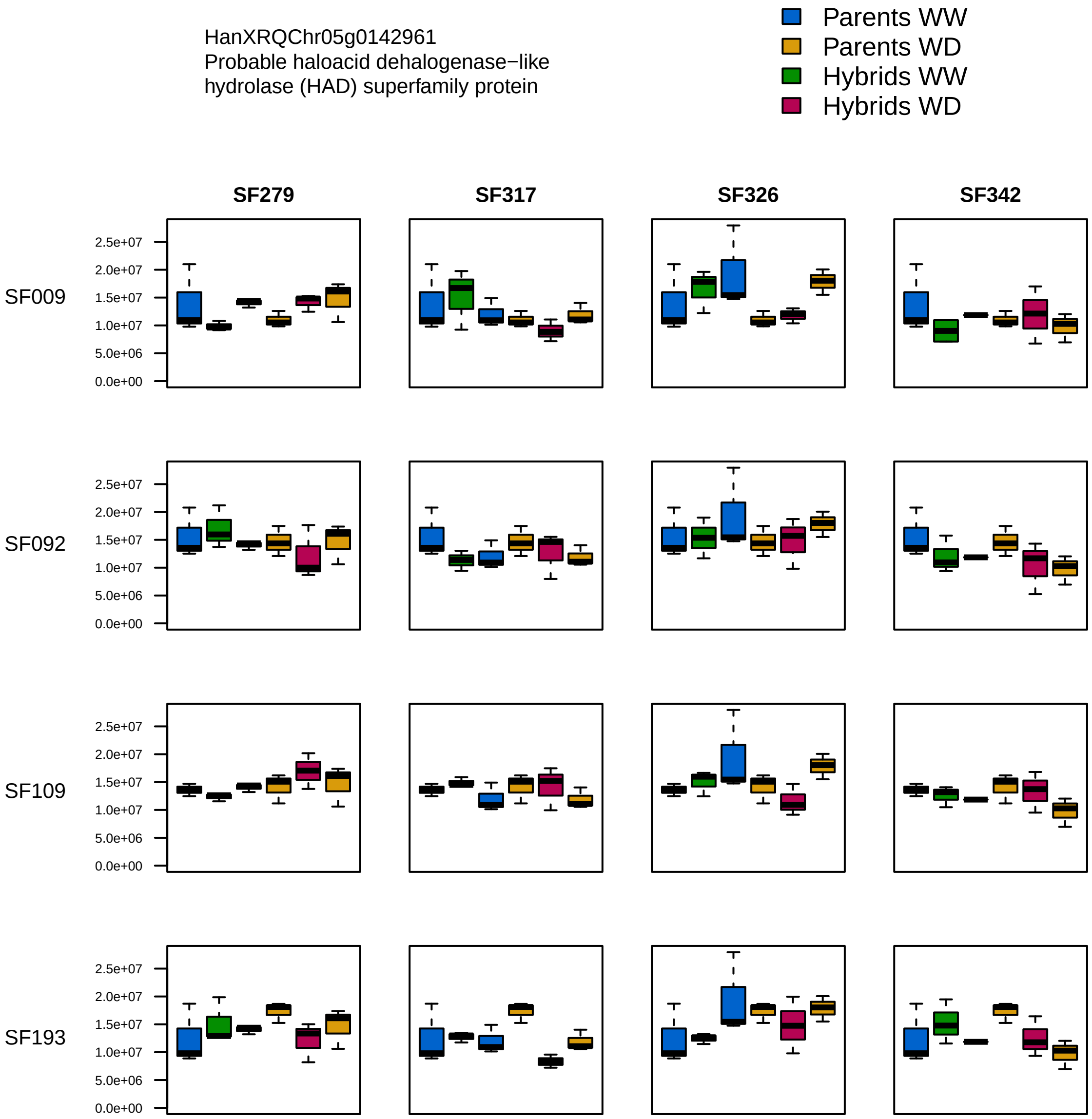

HanXRQChr05g0142961
Probable haloacid dehalogenase−like
hydrolase (HAD) superfamily protein
Parents WW
Parents WD
Hybrids WW
Hybrids WD
SF279
SF317
SF326
SF342
SF009
SF092
SF109
SF193
2.5e+07
2.0e+07
1.5e+07
1.0e+07
5.0e+06
0.0e+00

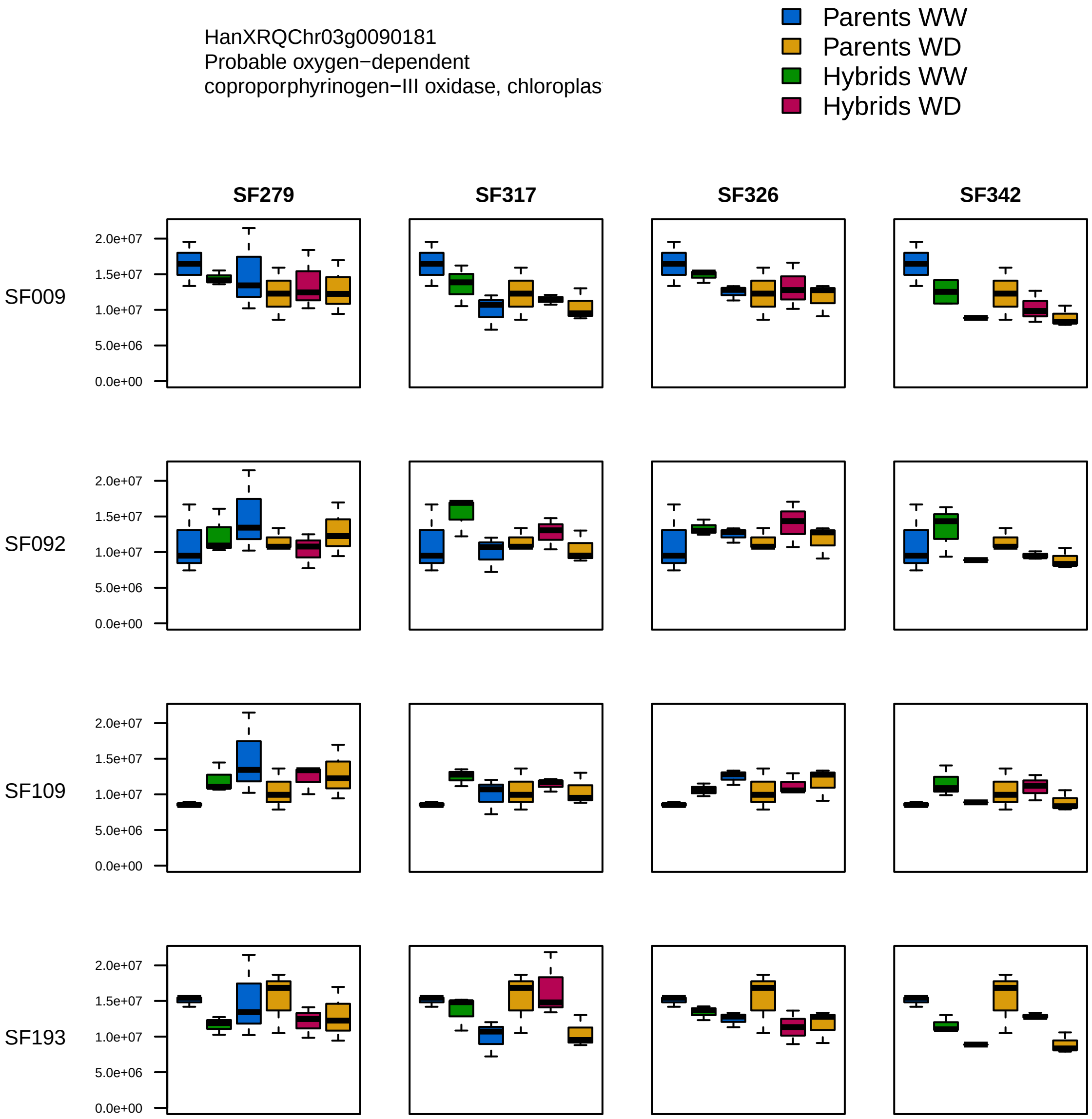
HanXRQChr03g0090181
Probable oxygen−dependent
coproporphyrinogen−III oxidase, chloroplas
Parents WW
Parents WD
Hybrids WW
Hybrids WD
SF279
SF317
SF326
SF342
SF009
SF092
SF109
SF193
2.0e+07
1.5e+07
1.0e+07
5.0e+06
0.0e+00

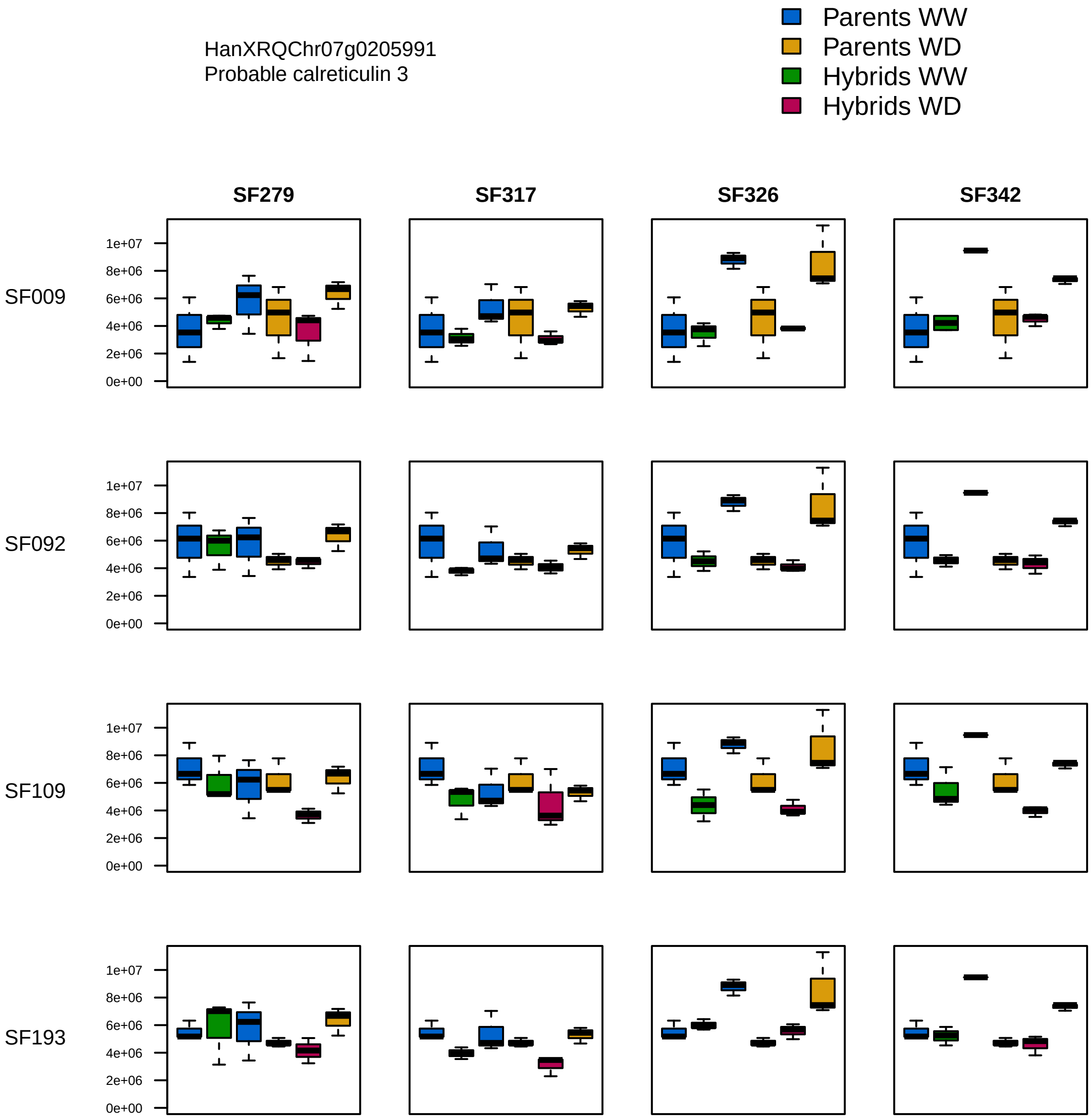

HanXRQChr07g0205991
Probable calreticulin 3
Parents WW
Parents WD
Hybrids WW
Hybrids WD
SF279
SF317
SF326
SF342
SF009
SF092
SF109
SF193
1e+07
8e+06
6e+06
4e+06
2e+06
0e+00

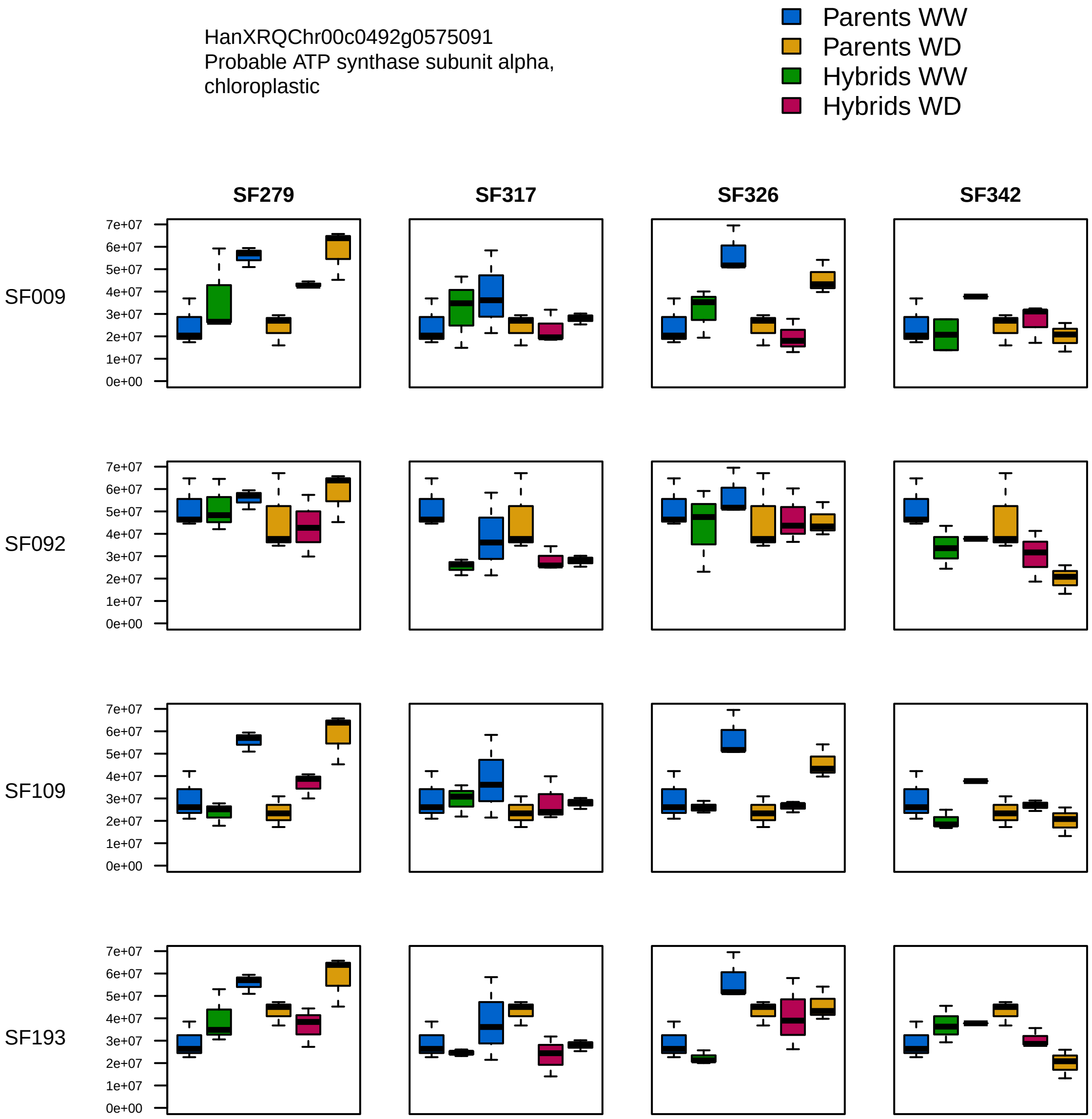

HanXRQChr00c0492g0575091
Probable ATP synthase subunit alpha, chloroplastic
Parents WW
Parents WD
Hybrids WW
Hybrids WD
SF279
SF317
SF326
SF342
SF009
SF092
SF109
SF193
7e+07
6e+07
5e+07
4e+07
3e+07
2e+07
1e+07
0e+00

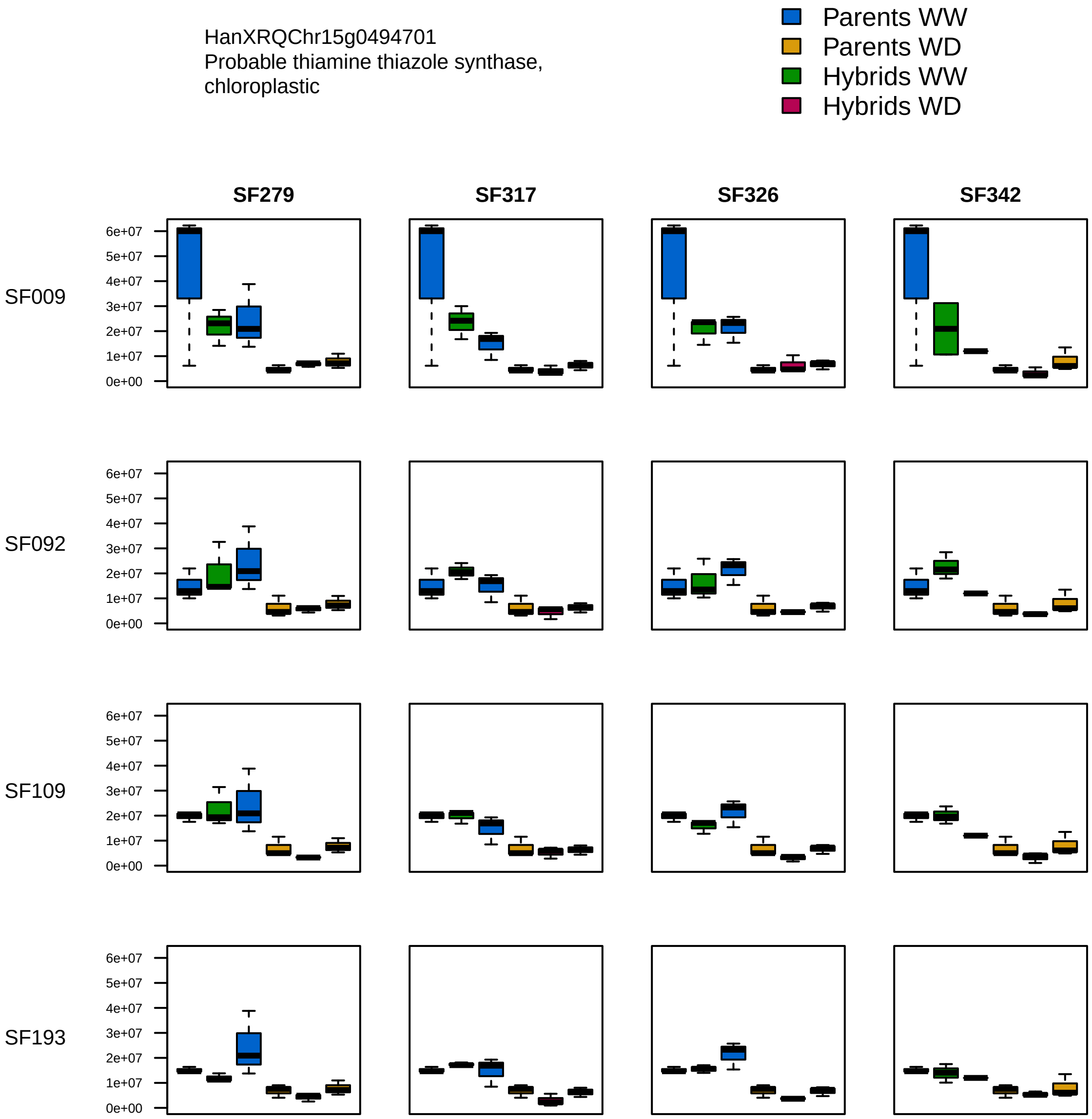
HanXRQChr15g0494701
Probable thiamine thiazole synthase, chloroplastic
Parents WW
Parents WD
Hybrids WW
Hybrids WD
SF279
SF317
SF326
SF342
SF009
SF092
SF109
SF193
6e+07
5e+07
4e+07
3e+07
2e+07
1e+07
0e+00

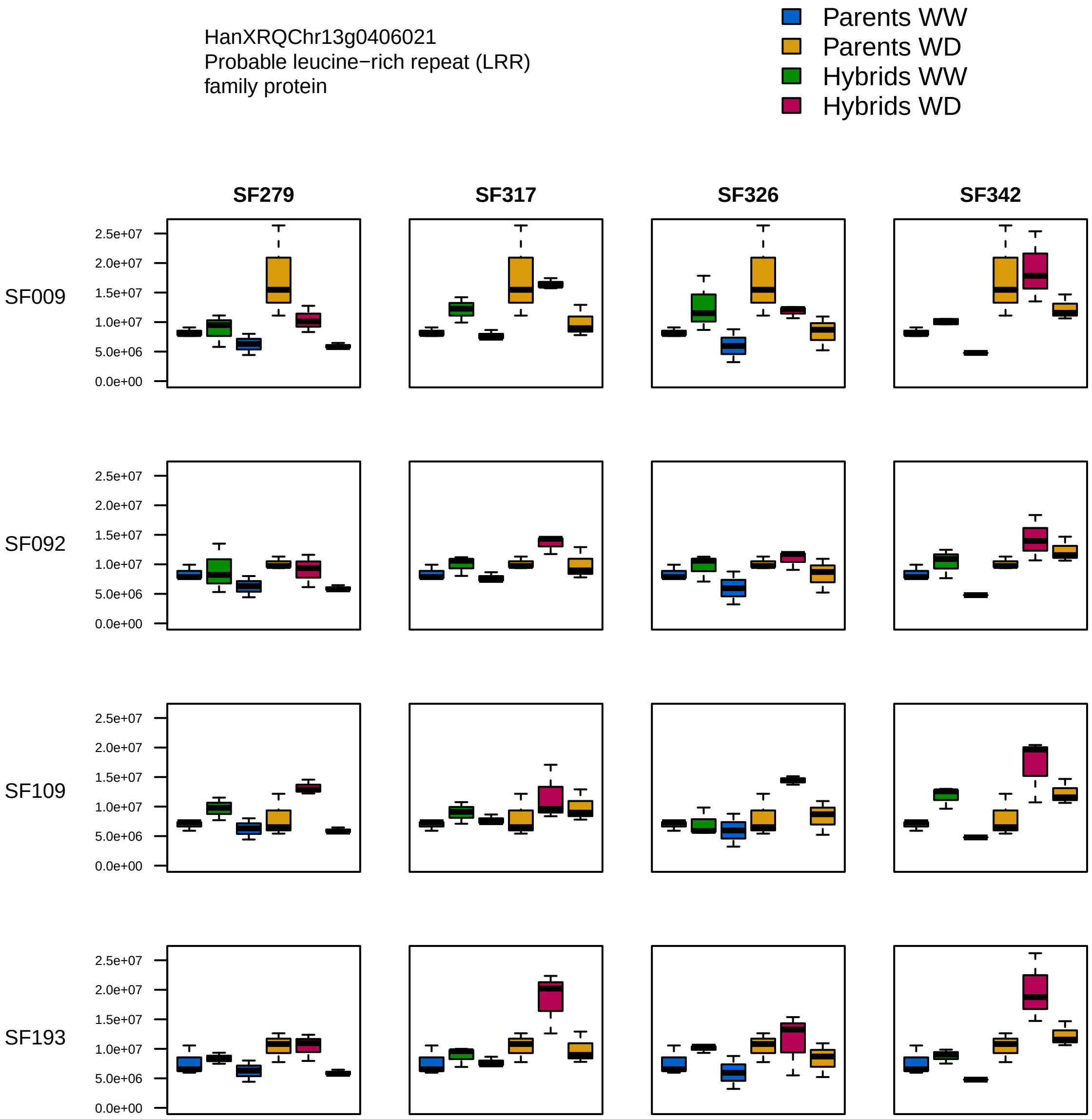
HanXRQChr13g0406021
Probable leucine−rich repeat (LRR)
family protein
Parents WW
Parents WD
Hybrids WW
Hybrids WD
SF279
SF317
SF326
SF342
SF009
SF092
SF109
SF193
2.5e+07
2.0e+07
1.5e+07
1.0e+07
5.0e+06
0.0e+00

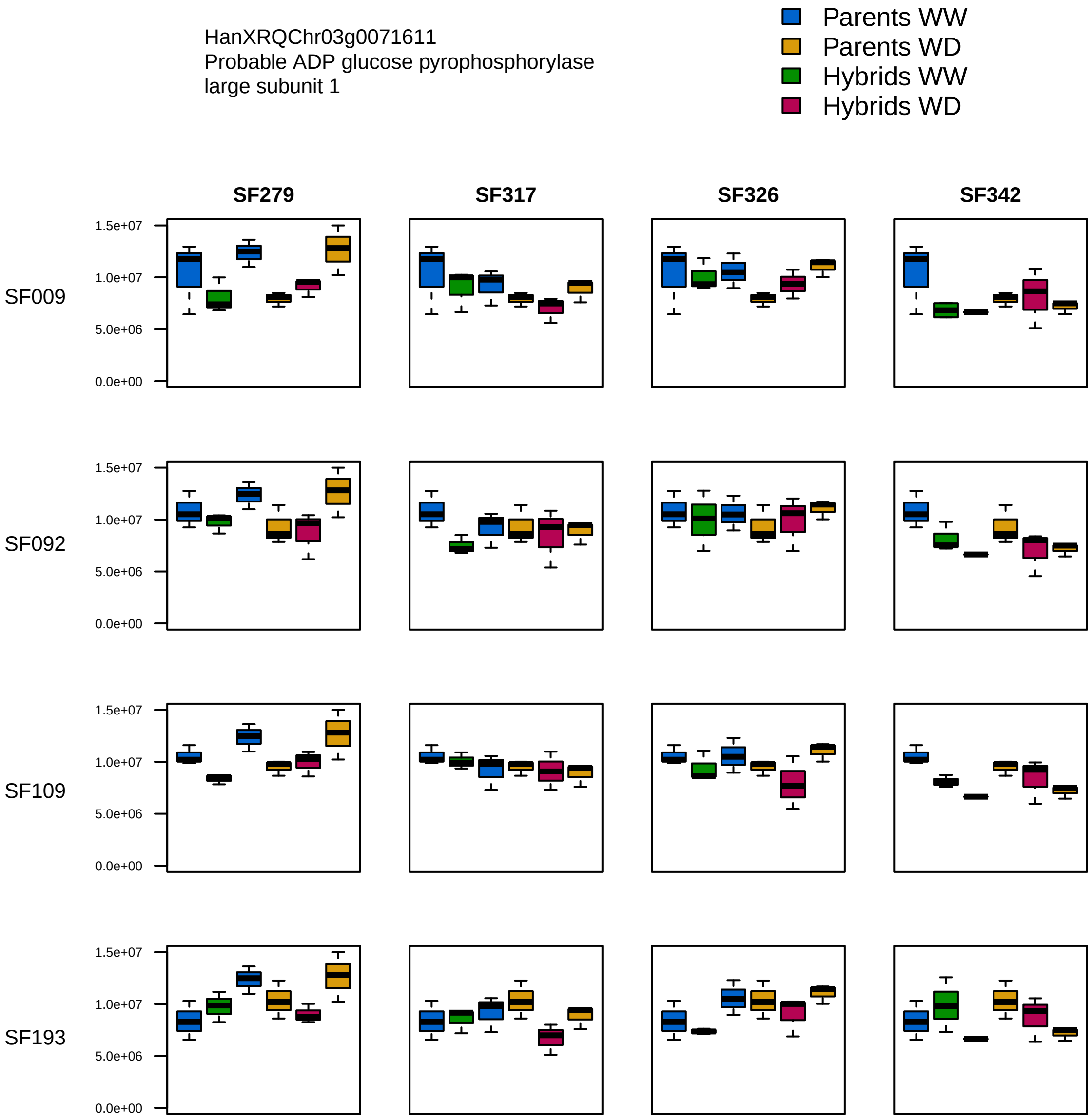

HanXRQChr03g0071611
Probable ADP glucose pyrophosphorylase
large subunit 1
Parents WW
Parents WD
Hybrids WW
Hybrids WD
SF279
SF317
SF326
SF342
SF009
SF092
SF109
SF193
1.5e+07
1.0e+07
5.0e+06
0.0e+00

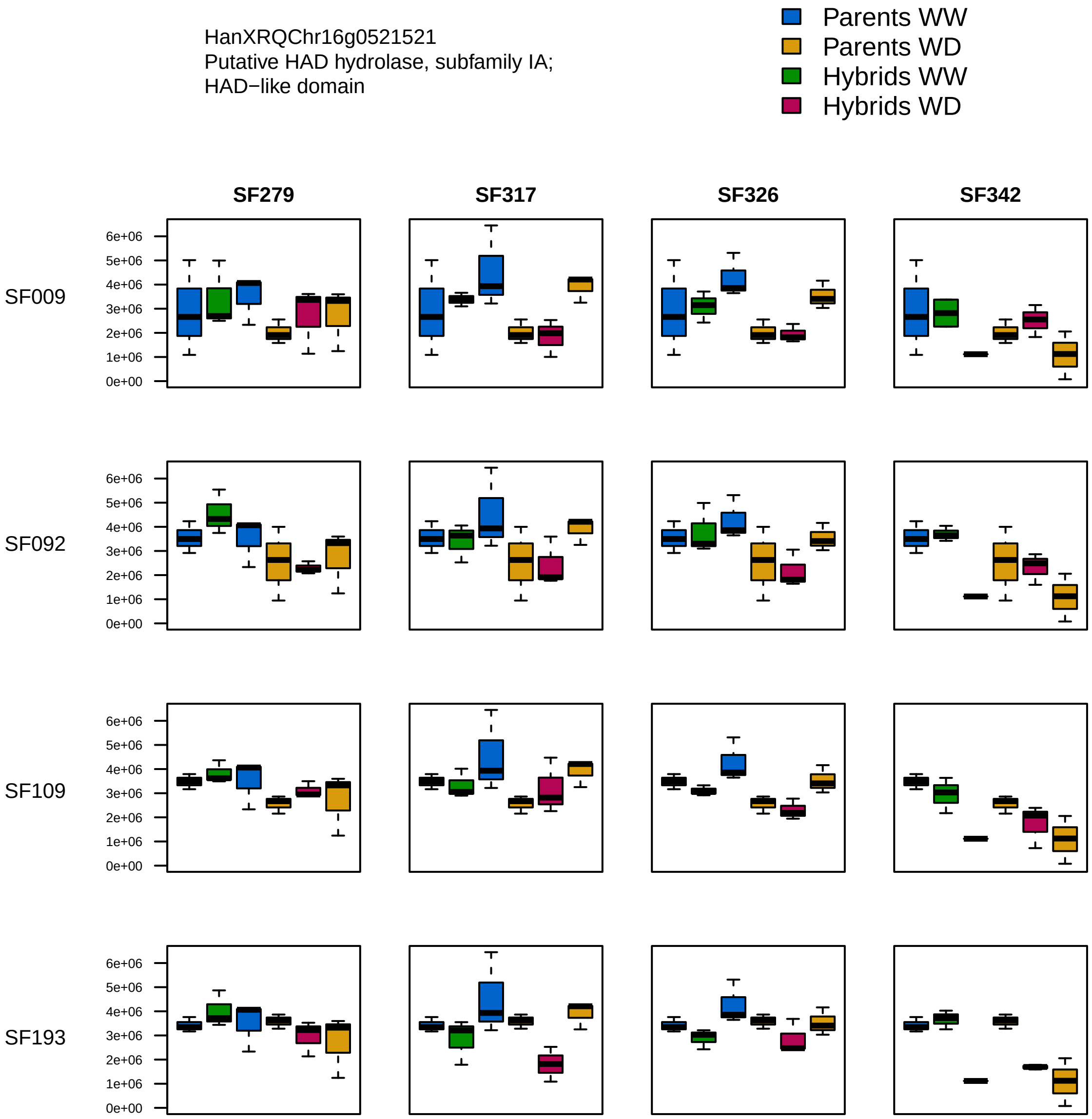
HanXRQChr16g0521521
Putative HAD hydrolase, subfamily IA;
HAD−like domain
Parents WW
Parents WD
Hybrids WW
Hybrids WD
SF279
SF317
SF326
SF342
SF009
SF092
SF109
SF193
0e+00
1e+06
2e+06
3e+06
4e+06
5e+06
6e+06

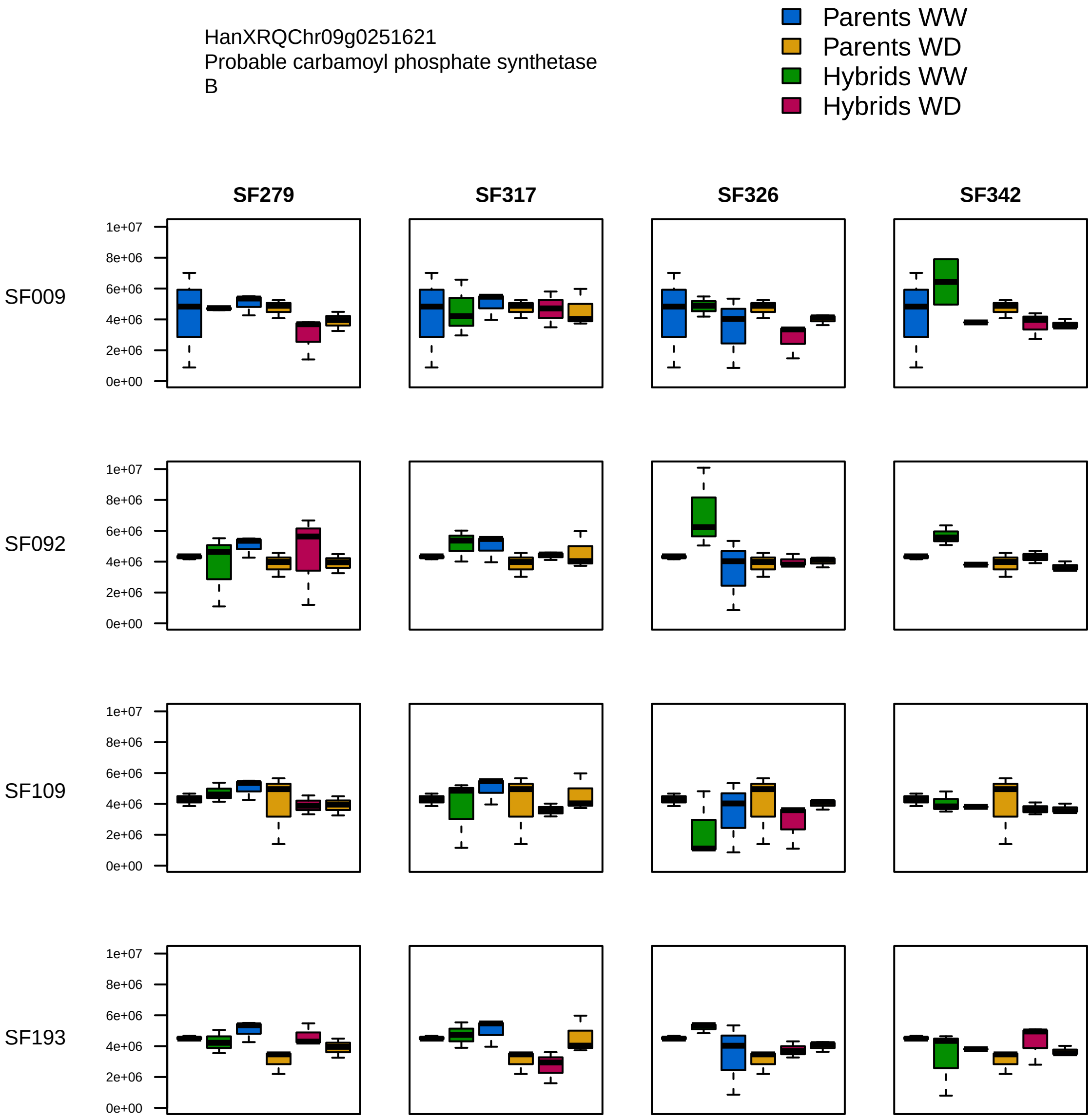
HanXRQChr09g0251621
Probable carbamoyl phosphate synthetase
B
Parents WW
Parents WD
Hybrids WW
Hybrids WD
SF279
SF317
SF326
SF342
SF009
SF092
SF109
SF193
1e+07
8e+06
6e+06
4e+06
2e+06
0e+00

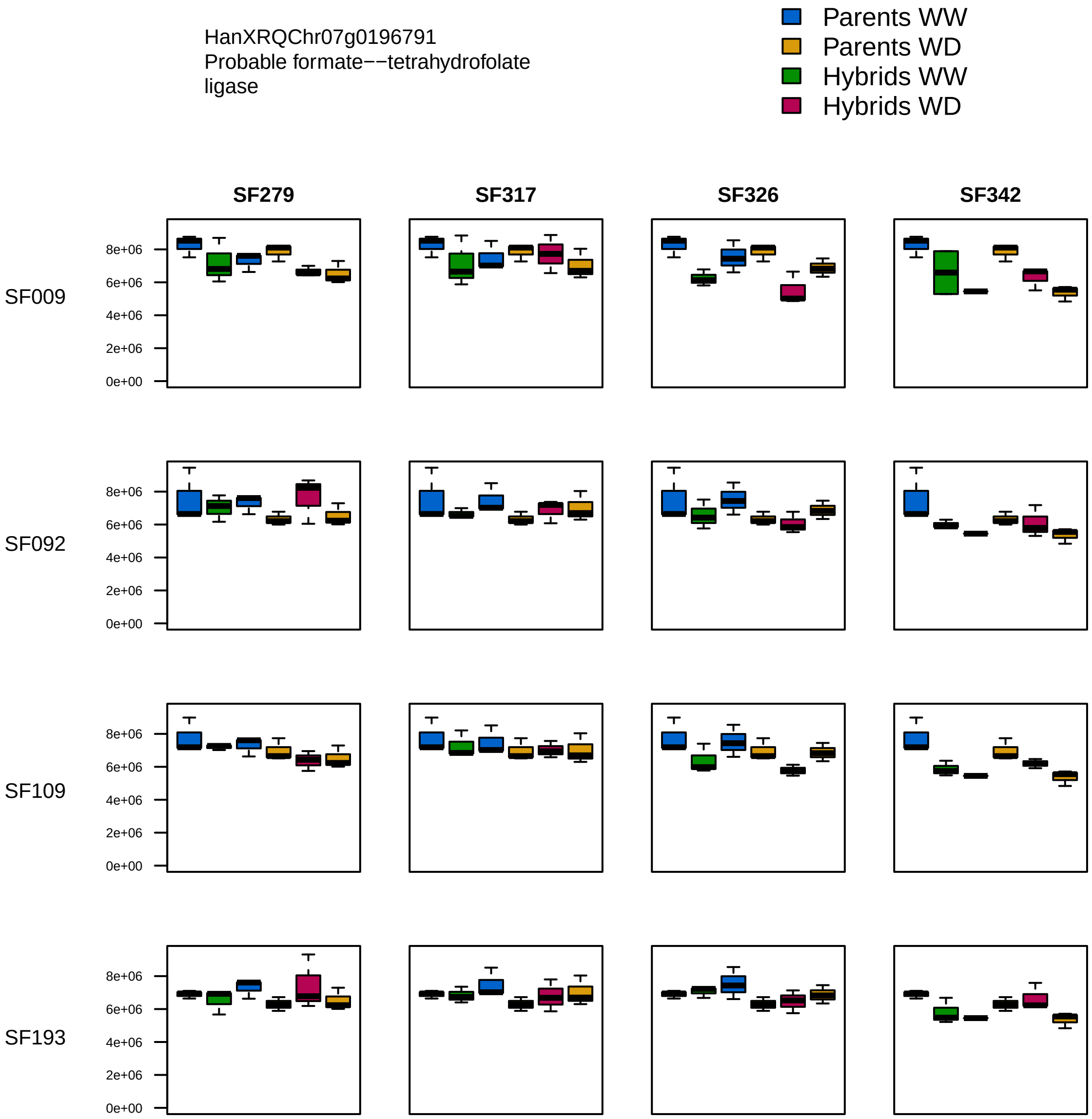
HanXRQChr07g0196791
Probable formate−−tetrahydrofolate
ligase
Parents WW
Parents WD
Hybrids WW
Hybrids WD
SF279
SF317
SF326
SF342
SF009
SF092
SF109
SF193
0e+00
2e+06
4e+06
6e+06
8e+06

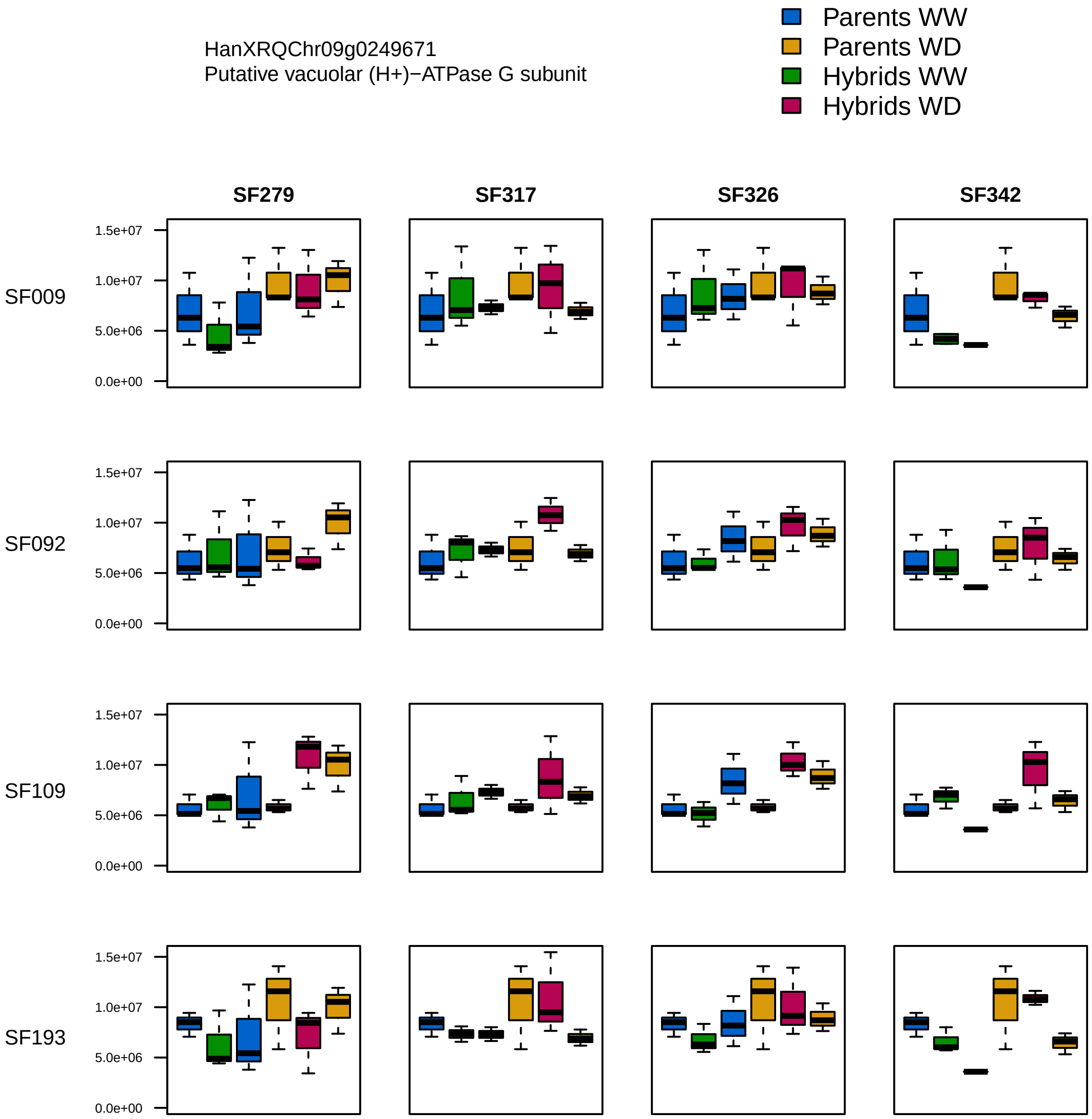
HanXRQChr09g0249671
Putative vacuolar (H+)−ATPase G subunit
Parents WW
Parents WD
Hybrids WW
Hybrids WD
SF279
SF317
SF326
SF342
SF009
SF092
SF109
SF193
1.5e+07
1.0e+07
5.0e+06
0.0e+00

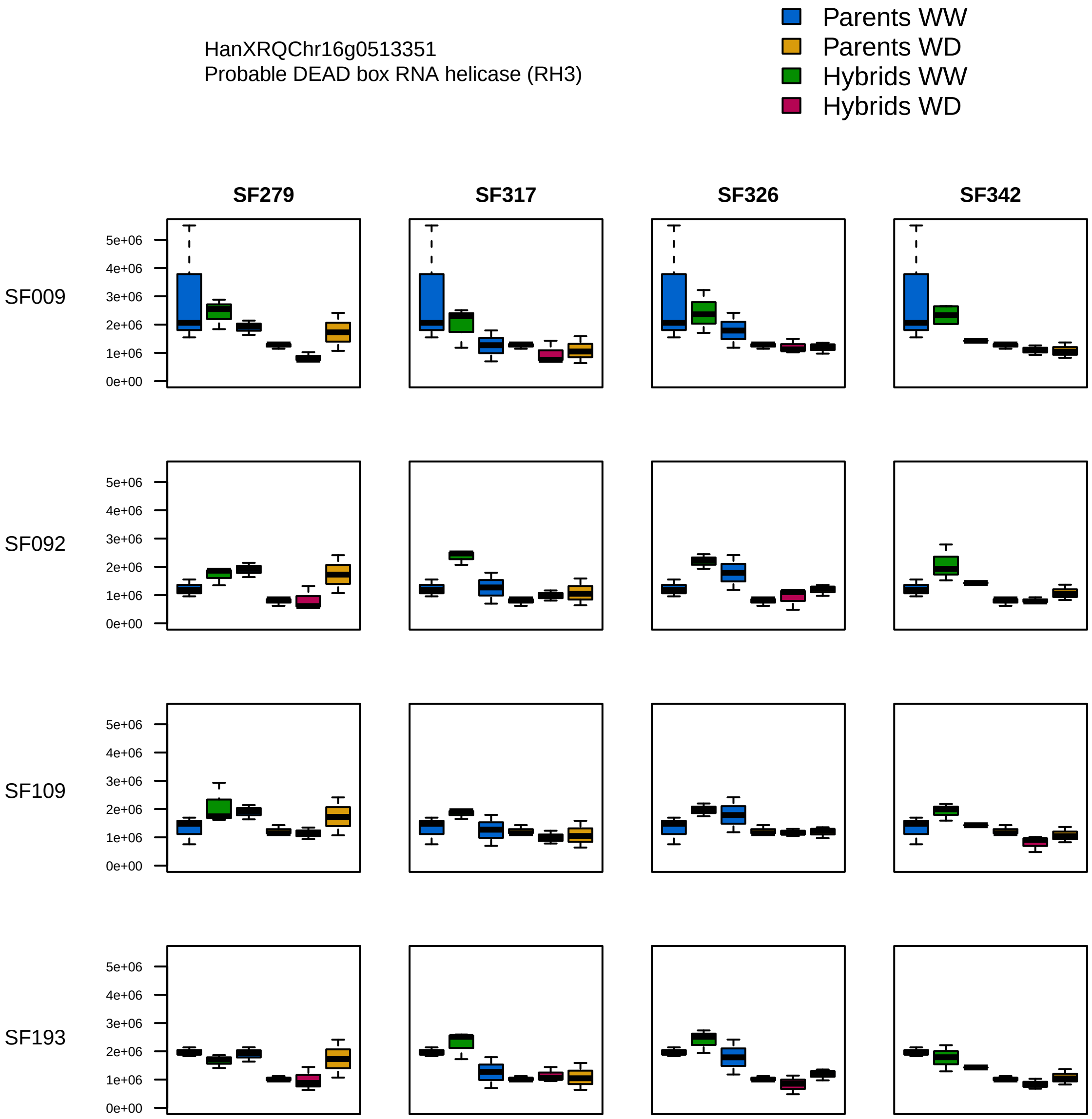
HanXRQChr16g0513351
Probable DEAD box RNA helicase (RH3)
Parents WW
Parents WD
Hybrids WW
Hybrids WD
SF279
SF317
SF326
SF342
SF009
SF092
SF109
SF193
0e+00
1e+06
2e+06
3e+06
4e+06
5e+06

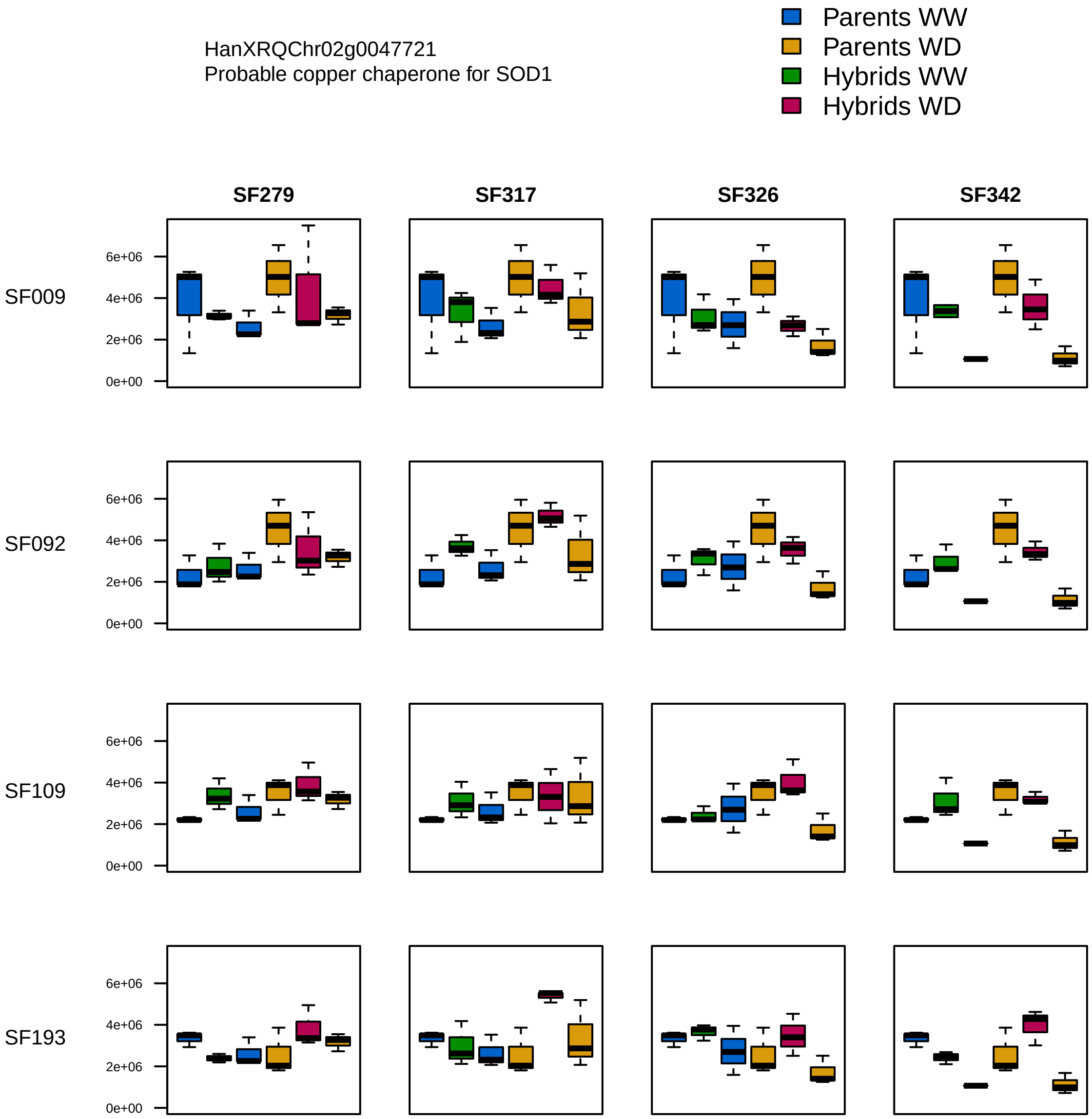

HanXRQChr02g0047721
Probable copper chaperone for SOD1
Parents WW
Parents WD
Hybrids WW
Hybrids WD
SF279
SF317
SF326
SF342
SF009
SF092
SF109
SF193
0e+00
2e+06
4e+06
6e+06

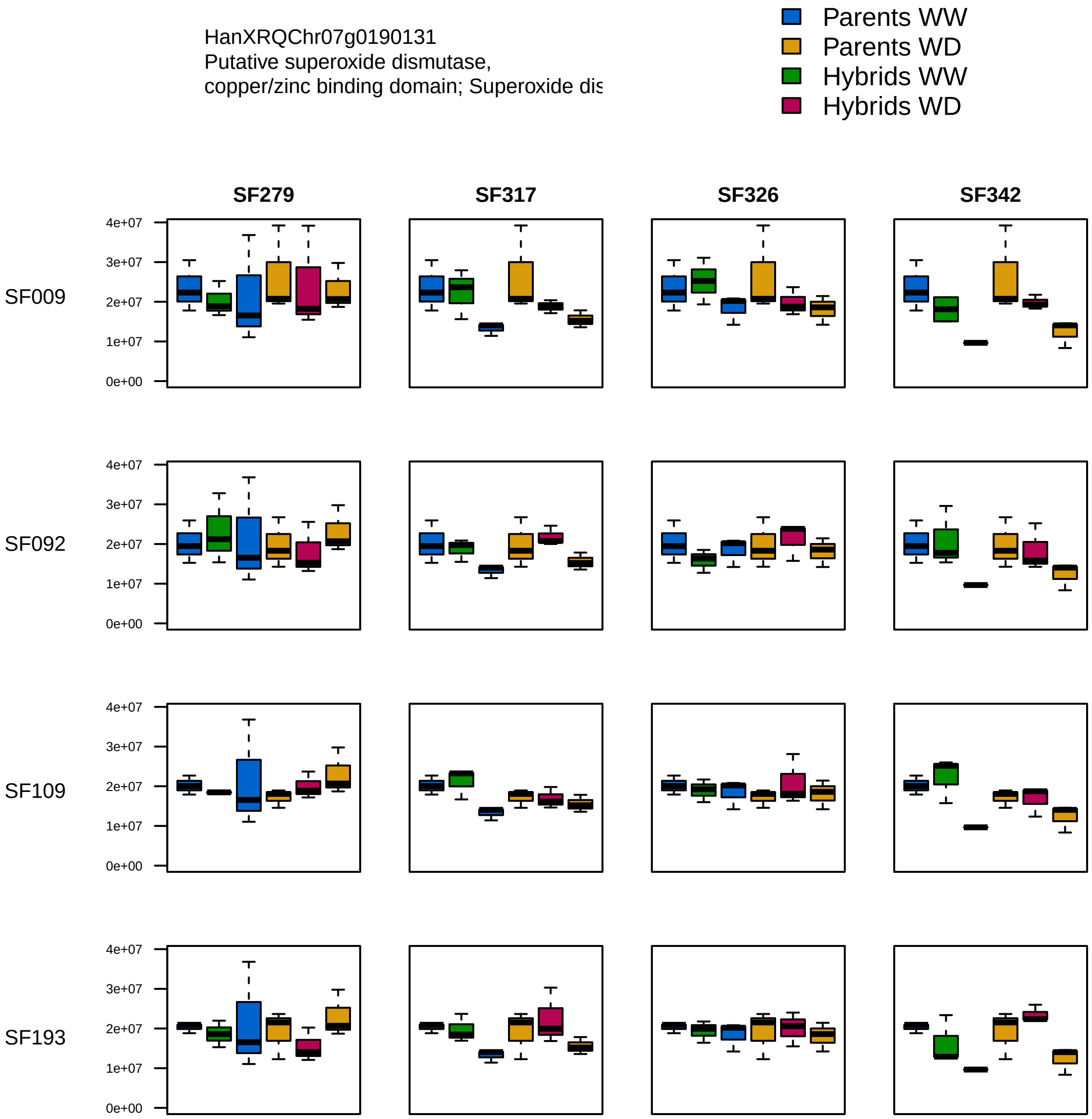

HanXRQChr07g0190131
Putative superoxide dismutase,
copper/zinc binding domain; Superoxide dis
Parents WW
Parents WD
Hybrids WW
Hybrids WD
SF279
SF317
SF326
SF342
SF009
SF092
SF109
SF193
4e+07
3e+07
2e+07
1e+07
0e+00

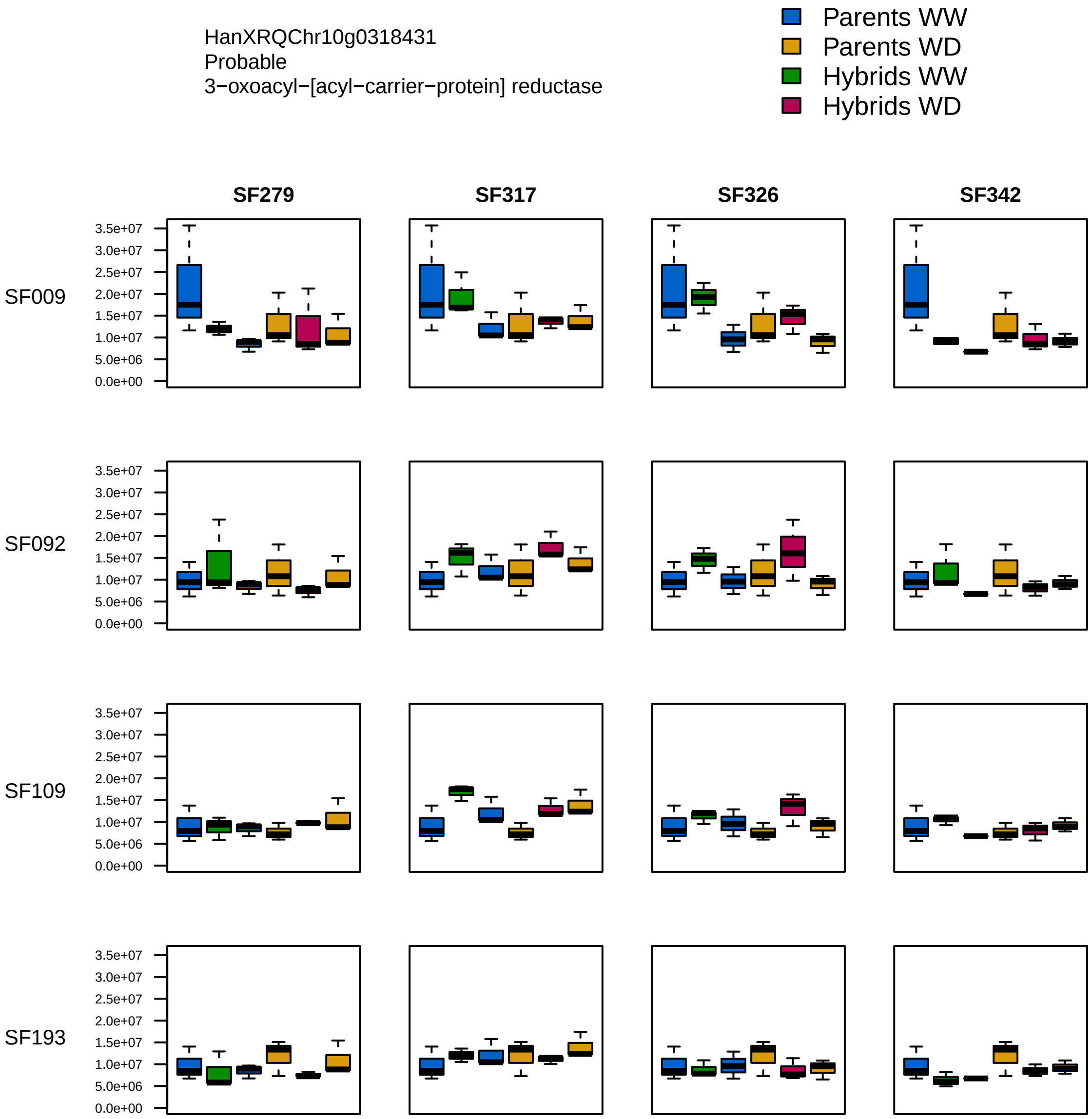

HanXRQChr10g0318431
Probable
3−oxoacyl−[acyl−carrier−protein] reductase
Parents WW
Parents WD
Hybrids WW
Hybrids WD
SF279
SF317
SF326
SF342
SF009
SF092
SF109
SF193
3.5e+07
3.0e+07
2.5e+07
2.0e+07
1.5e+07
1.0e+07
5.0e+06
0.0e+00

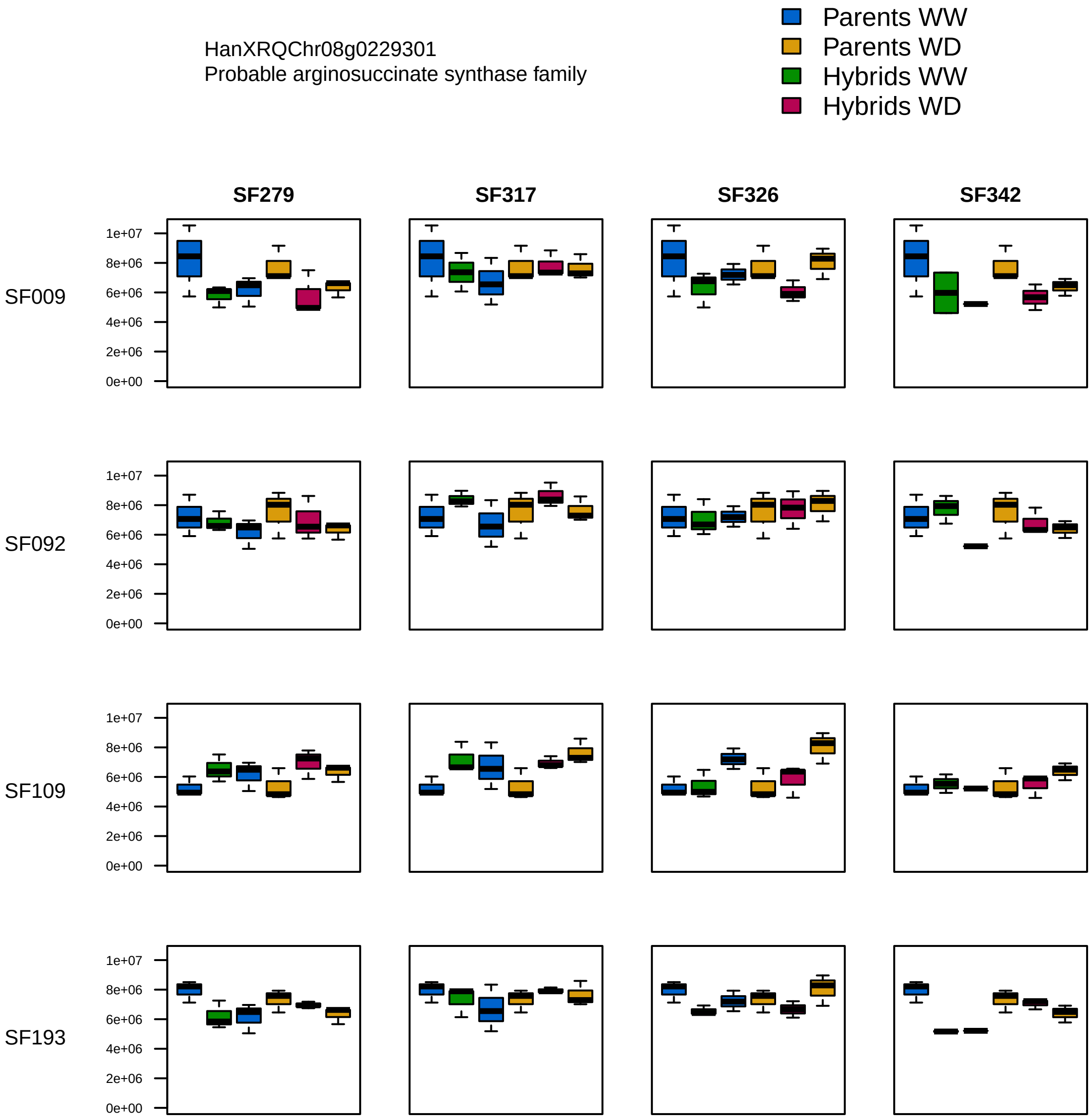

HanXRQChr08g0229301
Probable arginosuccinate synthase family
Parents WW
Parents WD
Hybrids WW
Hybrids WD
SF279
SF317
SF326
SF342
SF009
SF092
SF109
SF193
1e+07
8e+06
6e+06
4e+06
2e+06
0e+00

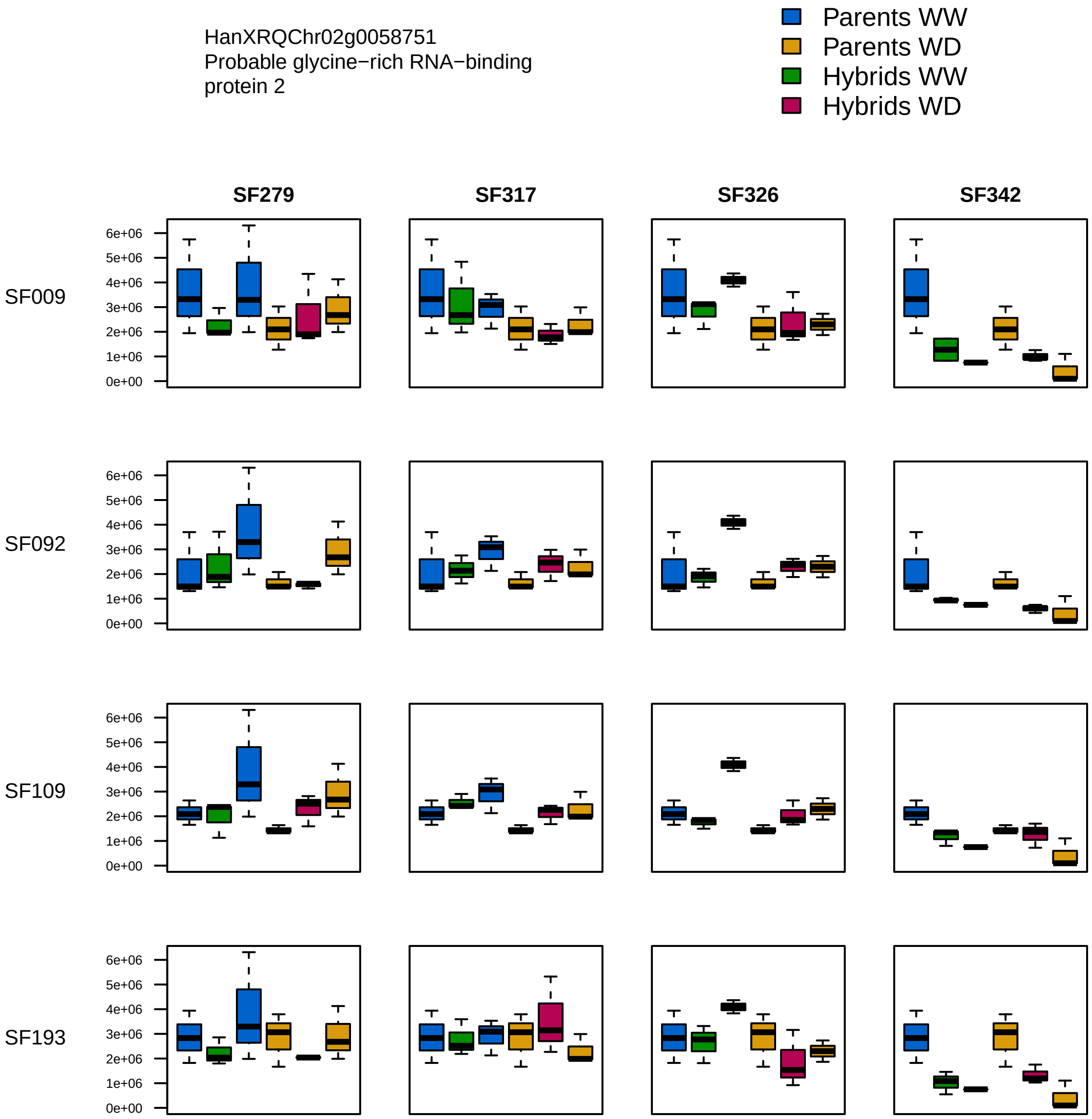
HanXRQChr02g0058751
Probable glycine−rich RNA−binding
protein 2
Parents WW
Parents WD
Hybrids WW
Hybrids WD
SF279
SF317
SF326
SF342
SF009
SF092
SF109
SF193
0e+00
1e+06
2e+06
3e+06
4e+06
5e+06
6e+06

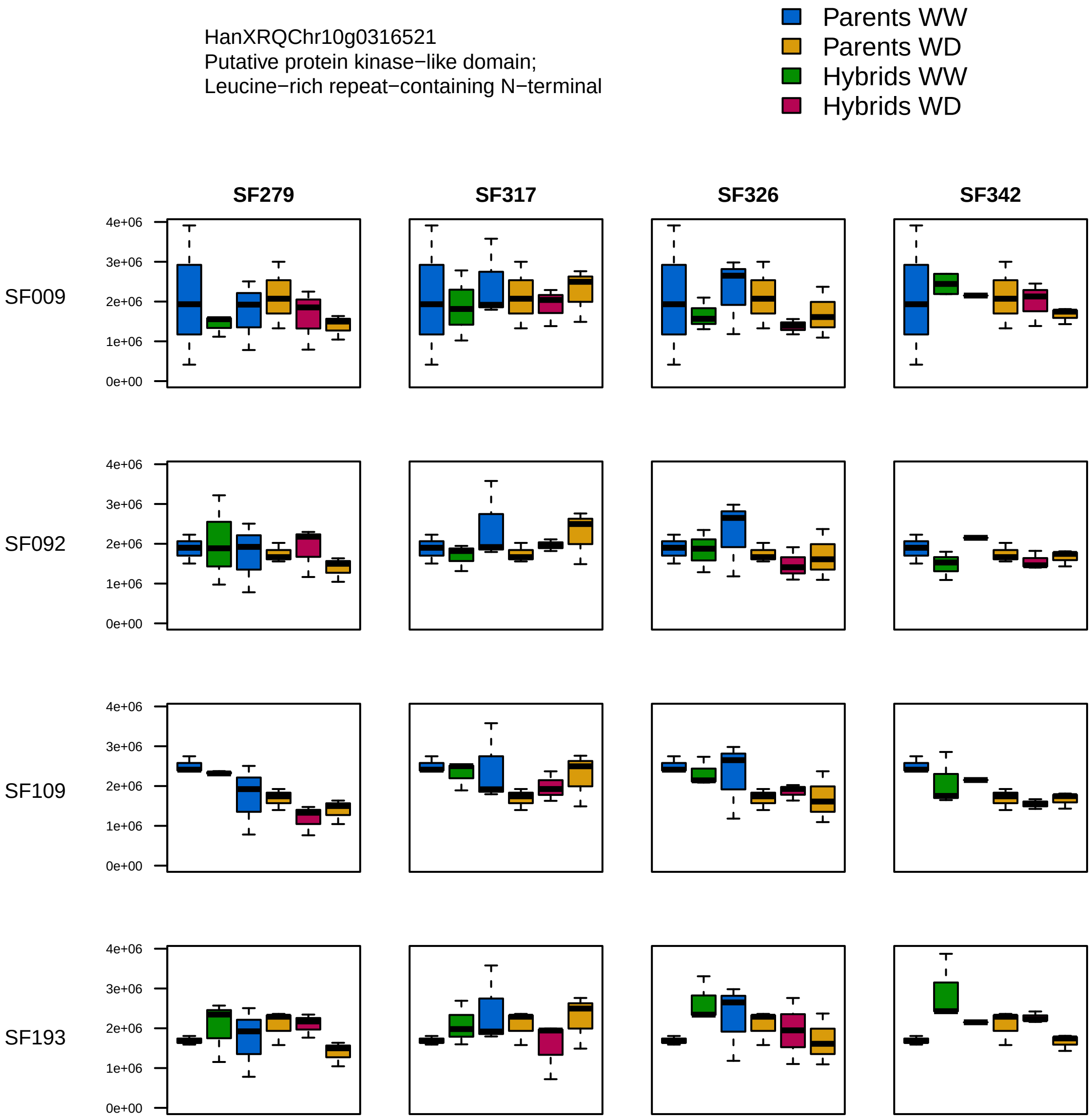

HanXRQChr10g0316521
Putative protein kinase−like domain;
Leucine−rich repeat−containing N−terminal
Parents WW
Parents WD
Hybrids WW
Hybrids WD
SF279
SF317
SF326
SF342
SF009
SF092
SF109
SF193
0e+00
1e+06
2e+06
3e+06
4e+06

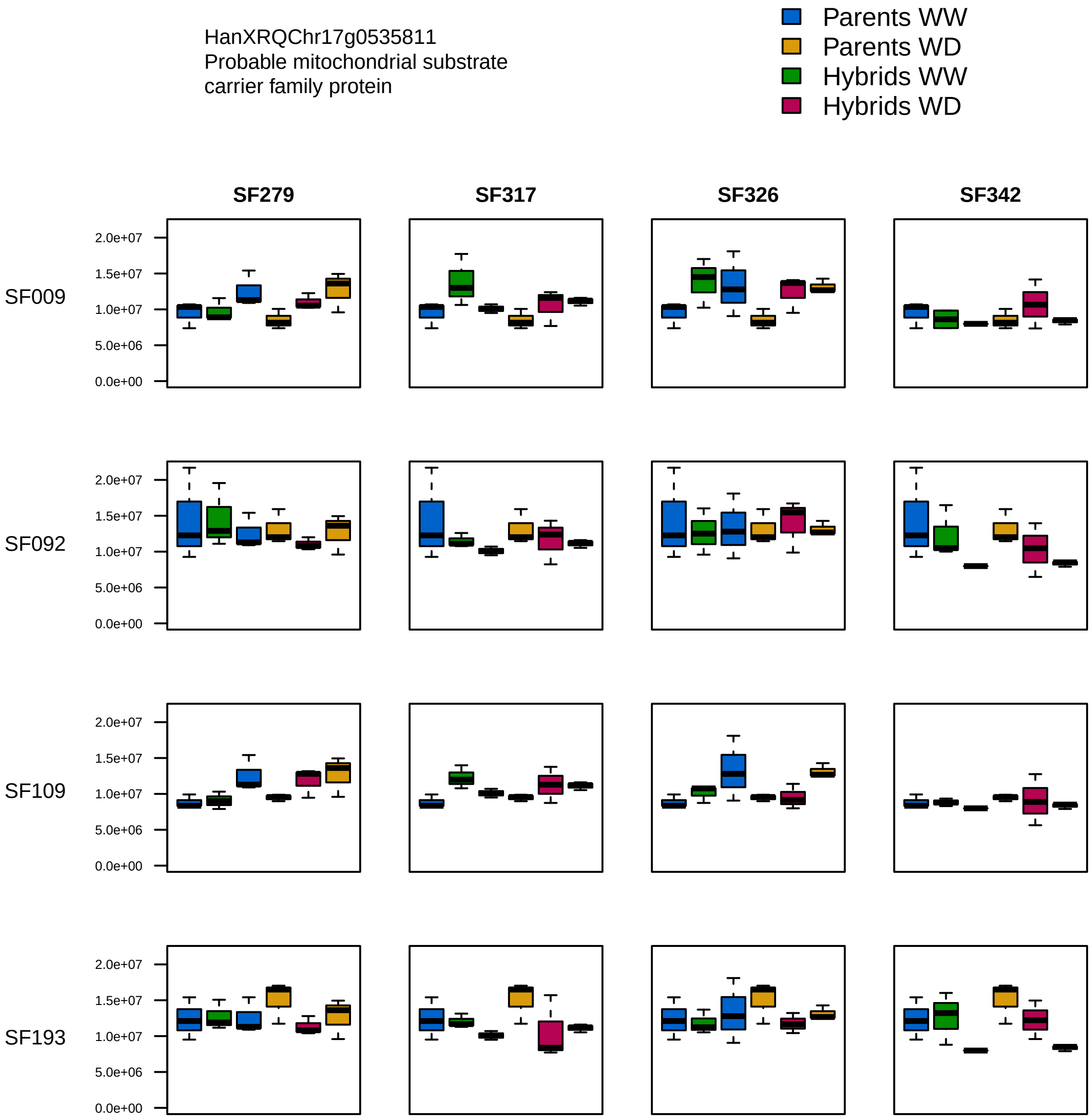

HanXRQChr17g0535811
Probable mitochondrial substrate
carrier family protein
Parents WW
Parents WD
Hybrids WW
Hybrids WD
SF279
SF317
SF326
SF342
SF009
SF092
SF109
SF193
2.0e+07
1.5e+07
1.0e+07
5.0e+06
0.0e+00

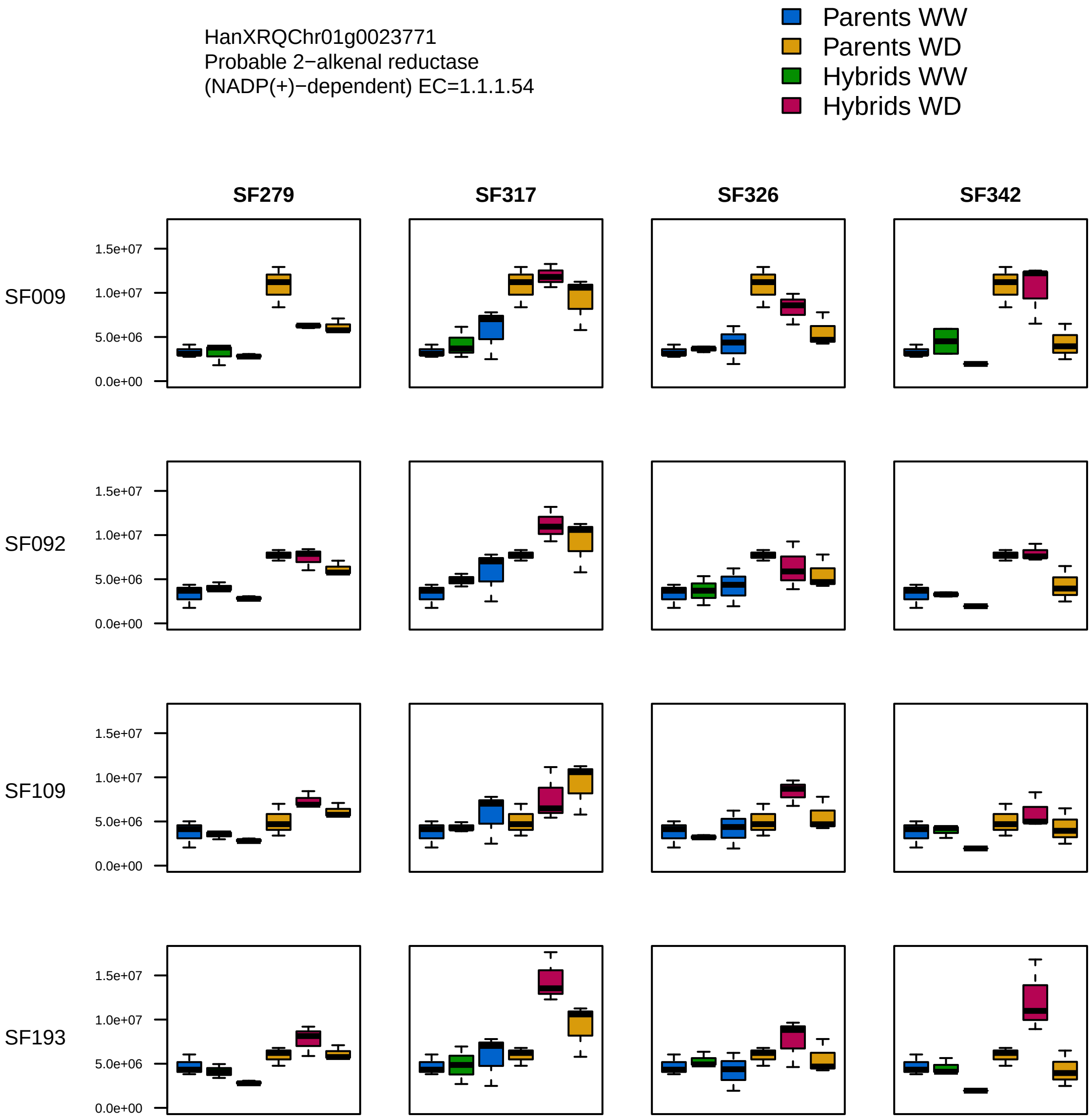
HanXRQChr01g0023771
Probable 2−alkenal reductase
(NADP(+)−dependent) EC=1.1.1.54
Parents WW
Parents WD
Hybrids WW
Hybrids WD
SF279
SF317
SF326
SF342
SF009
SF092
SF109
SF193
1.5e+07
1.0e+07
5.0e+06
0.0e+00

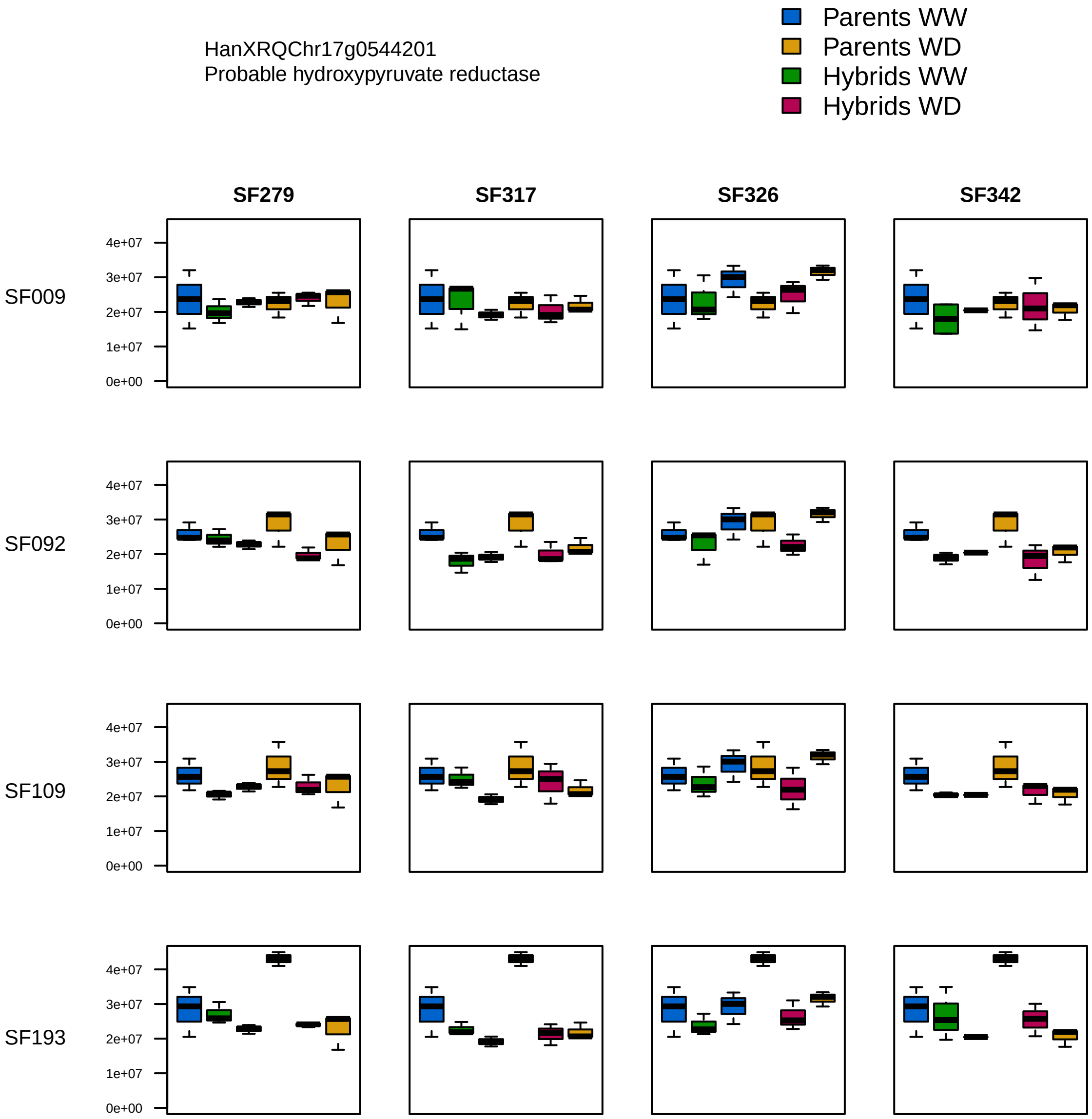
HanXRQChr17g0544201
Probable hydroxypyruvate reductase
Parents WW
Parents WD
Hybrids WW
Hybrids WD
SF279
SF317
SF326
SF342
SF009
SF092
SF109
SF193
0e+00
1e+07
2e+07
3e+07
4e+07

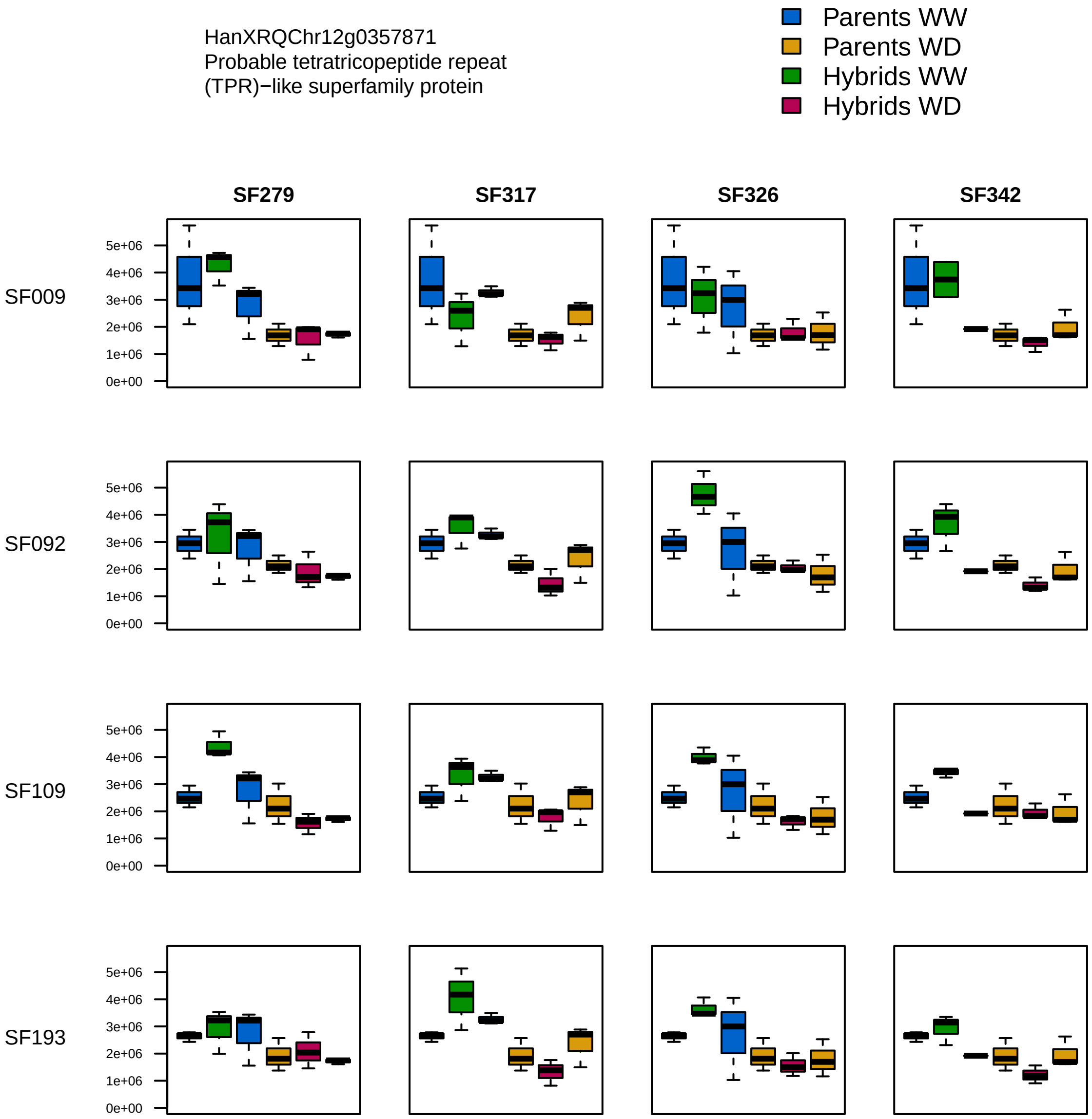
HanXRQChr12g0357871
Probable tetratricopeptide repeat
(TPR)−like superfamily protein
Parents WW
Parents WD
Hybrids WW
Hybrids WD
SF279
SF317
SF326
SF342
SF009
SF092
SF109
SF193
0e+00
1e+06
2e+06
3e+06
4e+06
5e+06

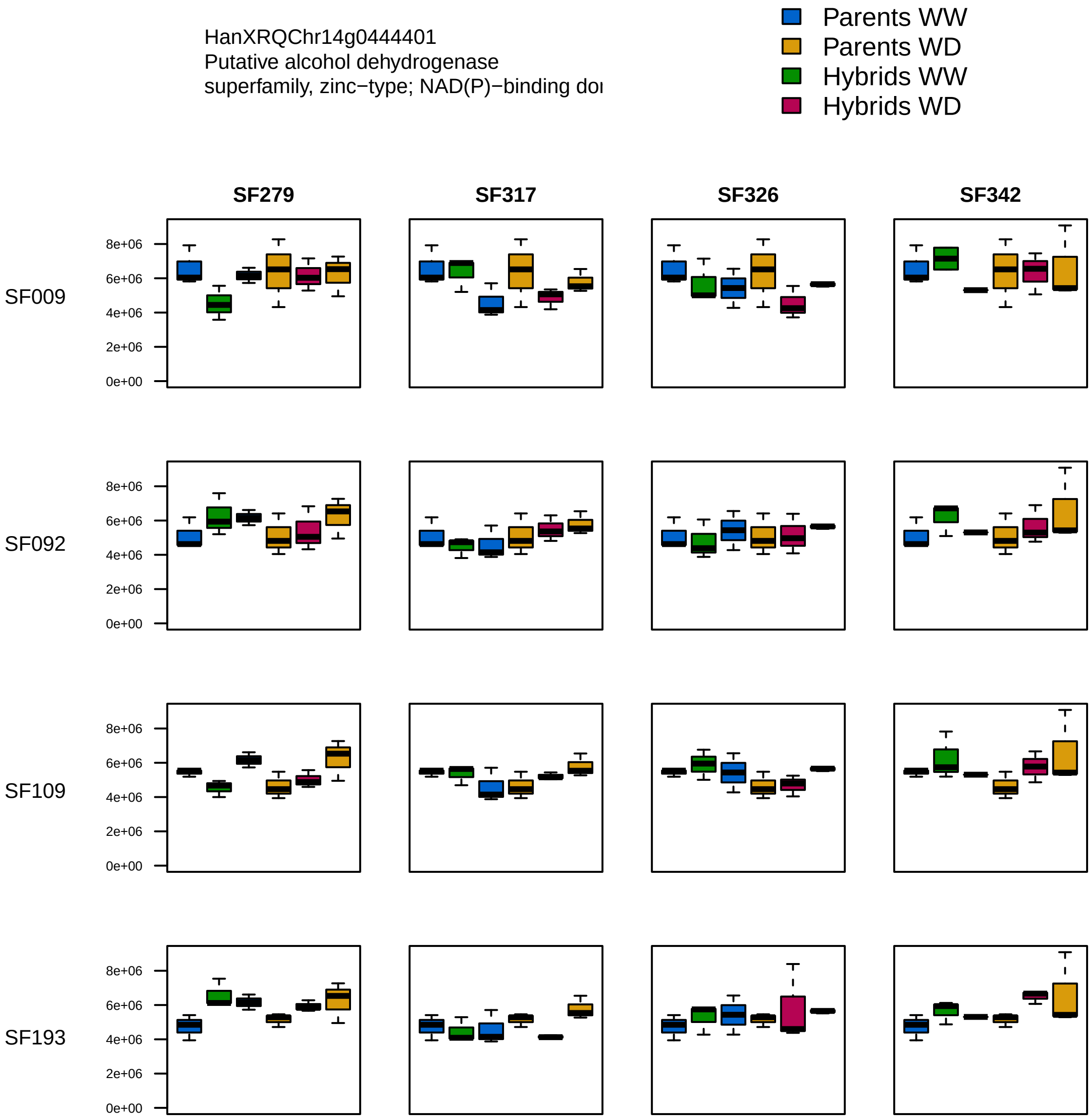
HanXRQChr14g0444401
Putative alcohol dehydrogenase
superfamily, zinc−type; NAD(P)−binding do
Parents WW
Parents WD
Hybrids WW
Hybrids WD
SF279
SF317
SF326
SF342
SF009
SF092
SF109
SF193
0e+00
2e+06
4e+06
6e+06
8e+06

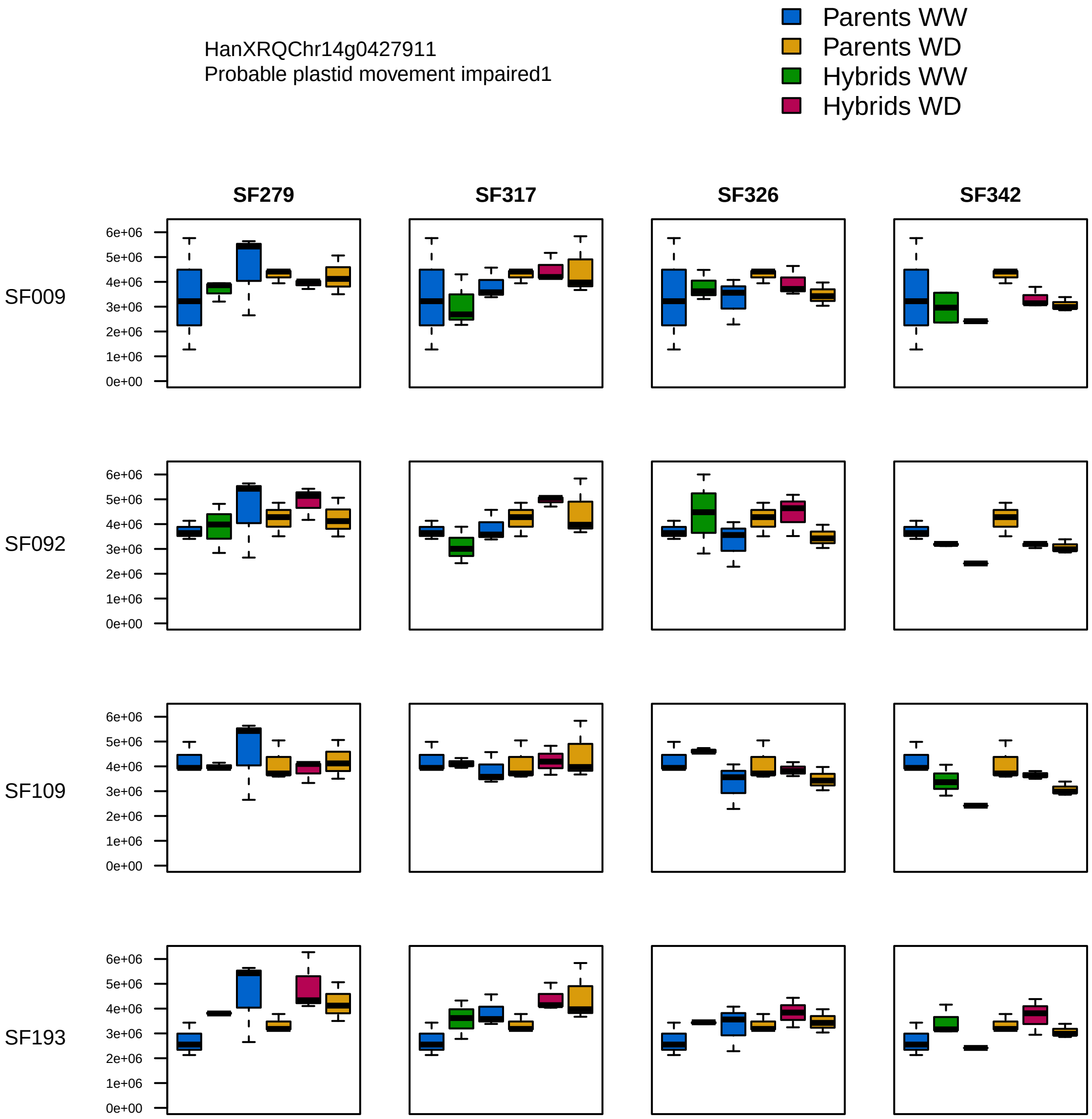
HanXRQChr14g0427911
Probable plastid movement impaired1
Parents WW
Parents WD
Hybrids WW
Hybrids WD
SF279
SF317
SF326
SF342
SF009
SF092
SF109
SF193
0e+00
1e+06
2e+06
3e+06
4e+06
5e+06
6e+06

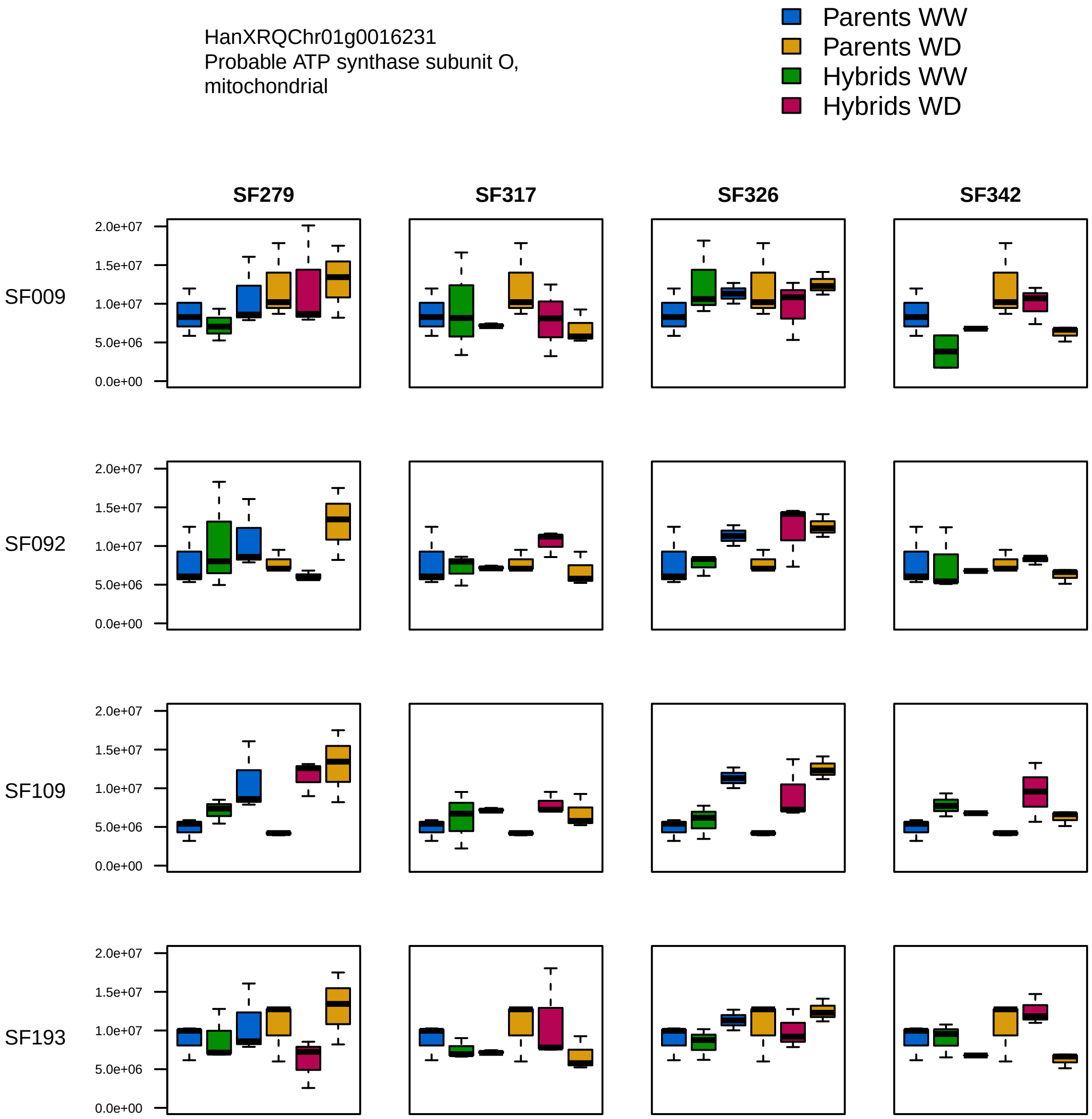
HanXRQChr01g0016231
Probable ATP synthase subunit O,
mitochondrial
Parents WW
Parents WD
Hybrids WW
Hybrids WD
SF279
SF317
SF326
SF342
SF009
SF092
SF109
SF193
2.0e+07
1.5e+07
1.0e+07
5.0e+06
0.0e+00

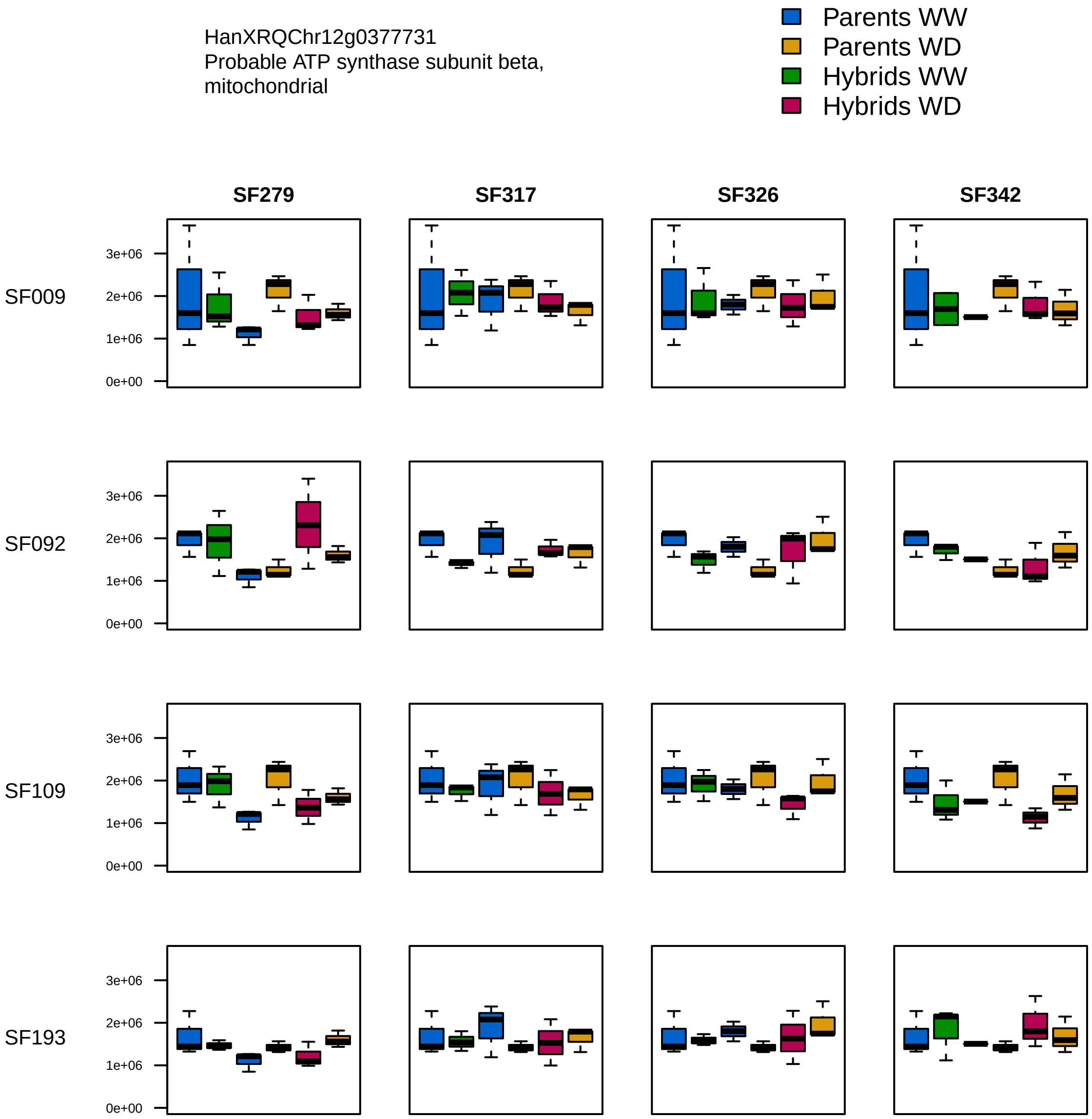
HanXRQChr12g0377731
Probable ATP synthase subunit beta, mitochondrial
Parents WW
Parents WD
Hybrids WW
Hybrids WD
SF279
SF317
SF326
SF342
SF009
SF092
SF109
SF193
0e+00
1e+06
2e+06
3e+06

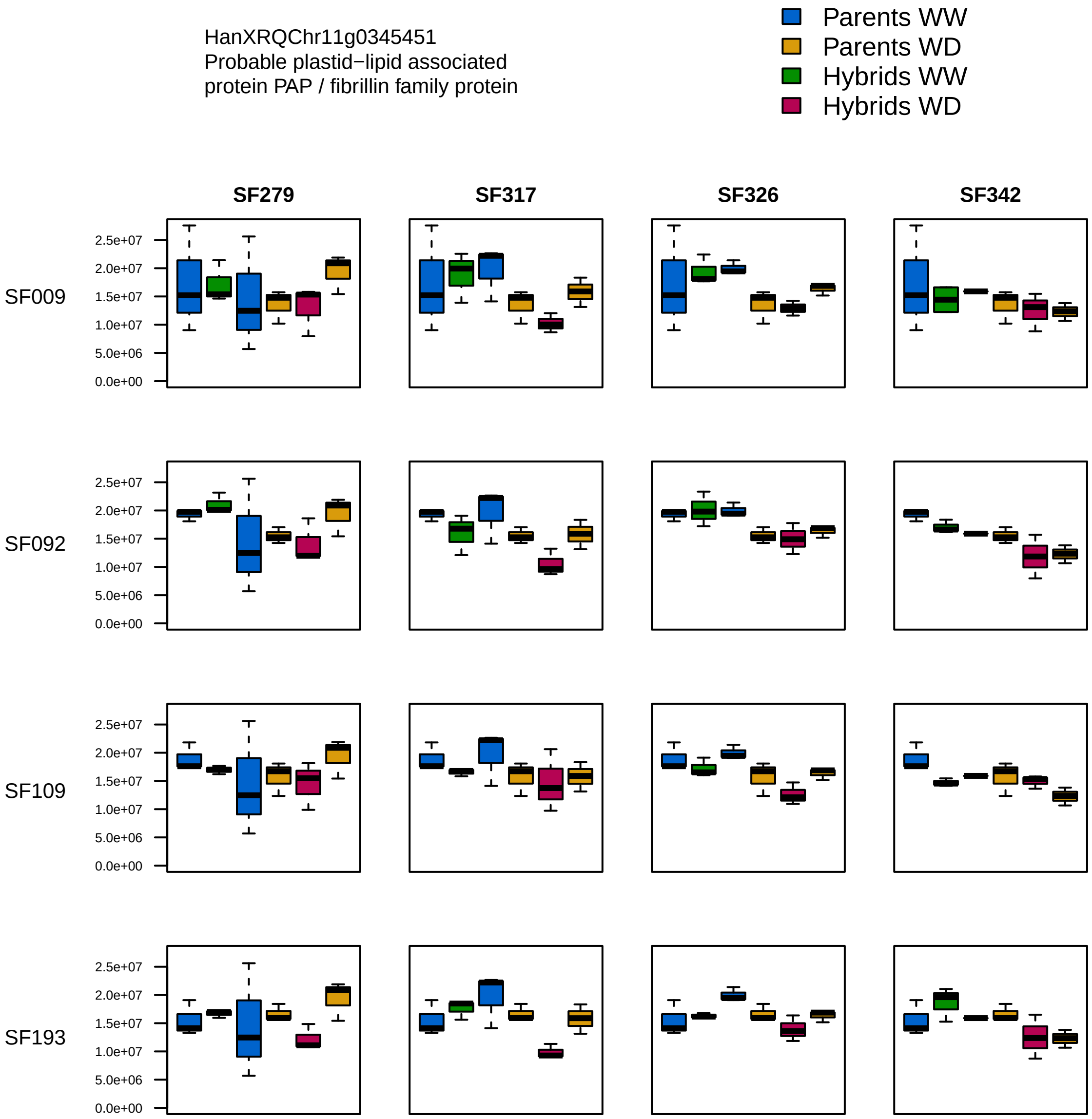

HanXRQChr11g0345451
Probable plastid−lipid associated
protein PAP / fibrillin family protein
Parents WW
Parents WD
Hybrids WW
Hybrids WD
SF279
SF317
SF326
SF342
SF009
SF092
SF109
SF193
0.0e+00
5.0e+06
1.0e+07
1.5e+07
2.0e+07
2.5e+07

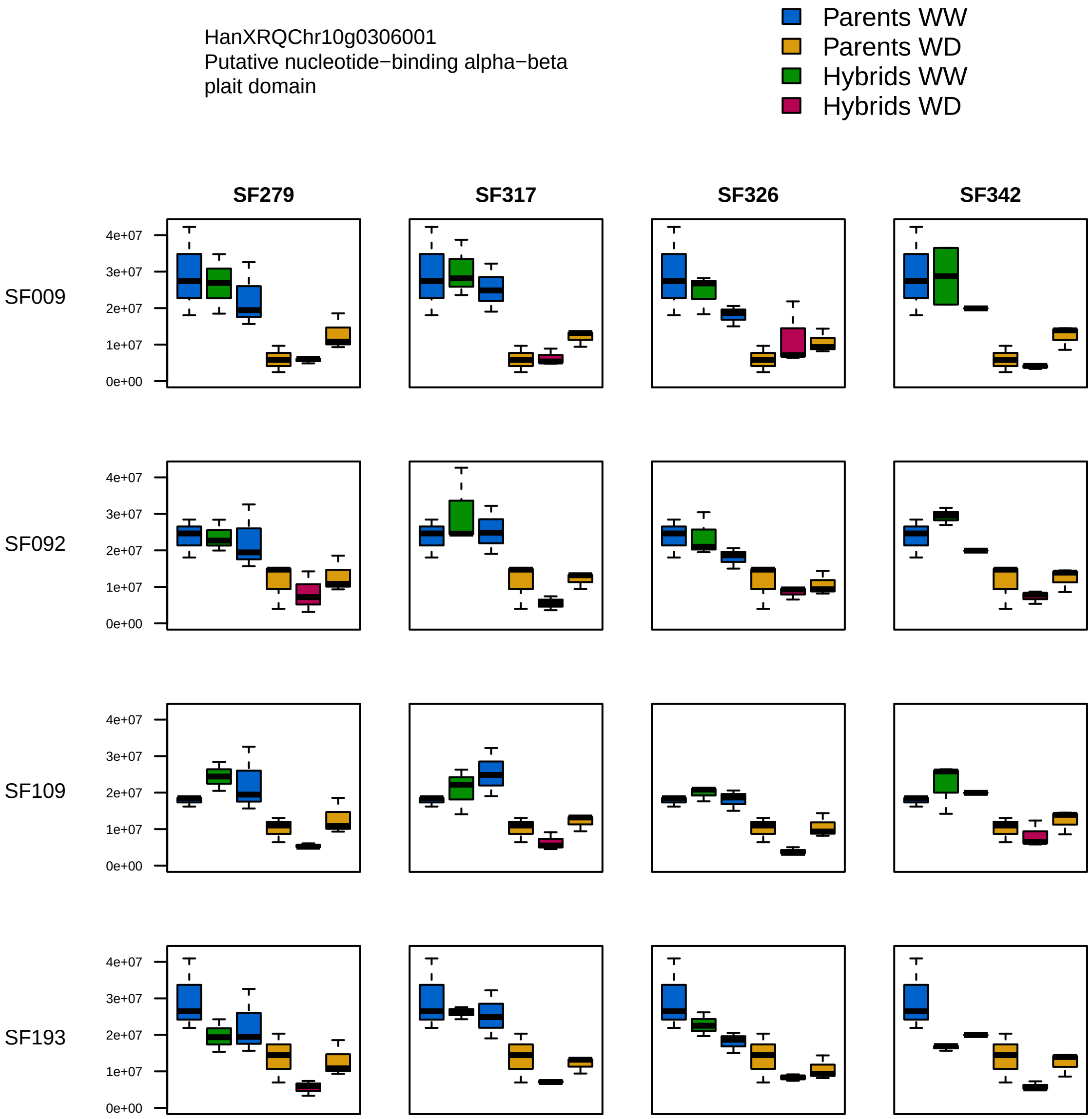

HanXRQChr10g0306001
Putative nucleotide−binding alpha−beta
plait domain
Parents WW
Parents WD
Hybrids WW
Hybrids WD
SF279
SF317
SF326
SF342
SF009
SF092
SF109
SF193
4e+07
3e+07
2e+07
1e+07
0e+00

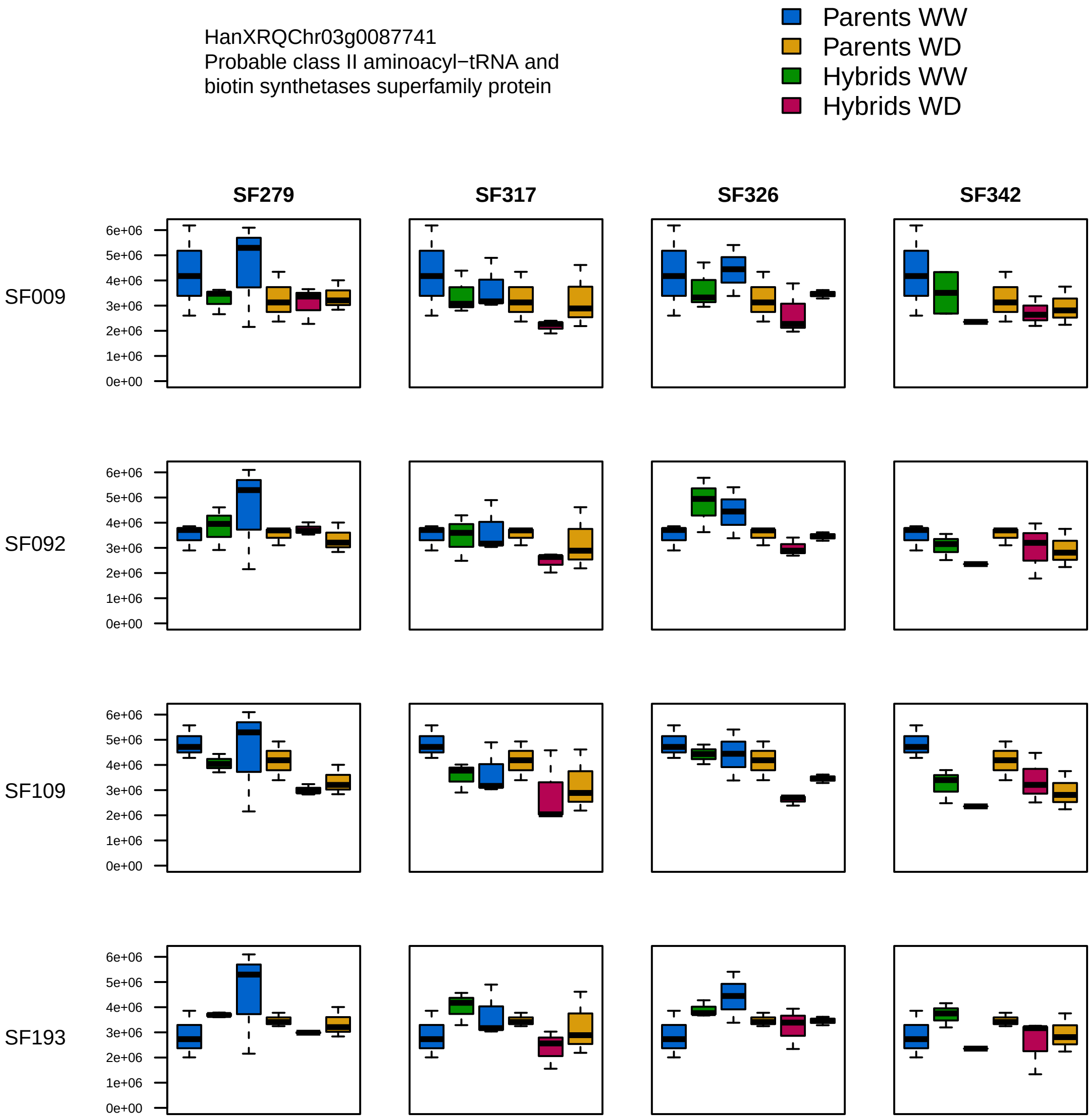
HanXRQChr03g0087741
Probable class II aminoacyl−tRNA and
biotin synthetases superfamily protein
Parents WW
Parents WD
Hybrids WW
Hybrids WD
SF279
SF317
SF326
SF342
SF009
SF092
SF109
SF193
6e+06
5e+06
4e+06
3e+06
2e+06
1e+06
0e+00

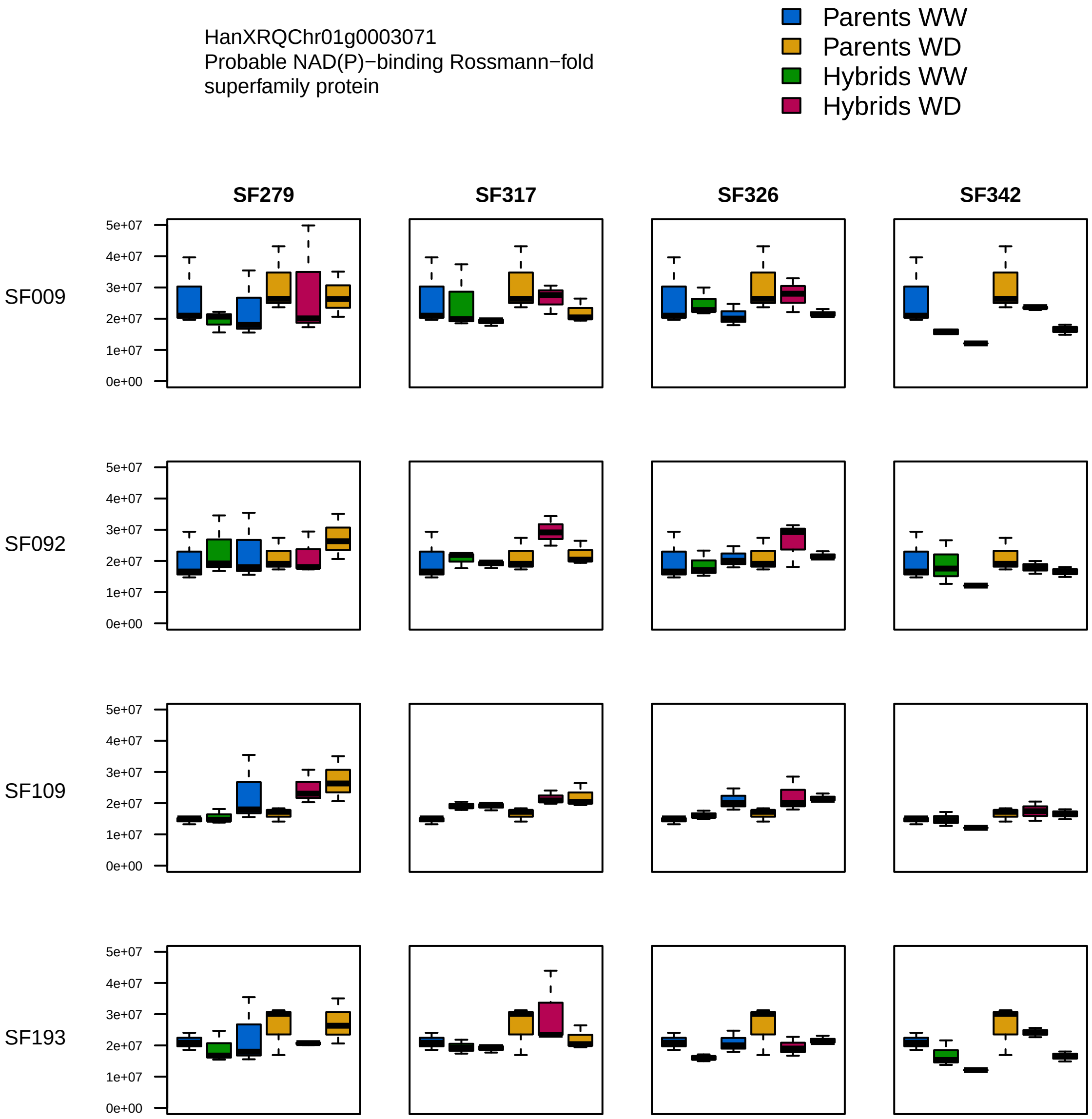
HanXRQChr01g0003071
Probable NAD(P)−binding Rossmann−fold
superfamily protein
Parents WW
Parents WD
Hybrids WW
Hybrids WD
SF279
SF317
SF326
SF342
SF009
SF092
SF109
SF193
5e+07
4e+07
3e+07
2e+07
1e+07
0e+00

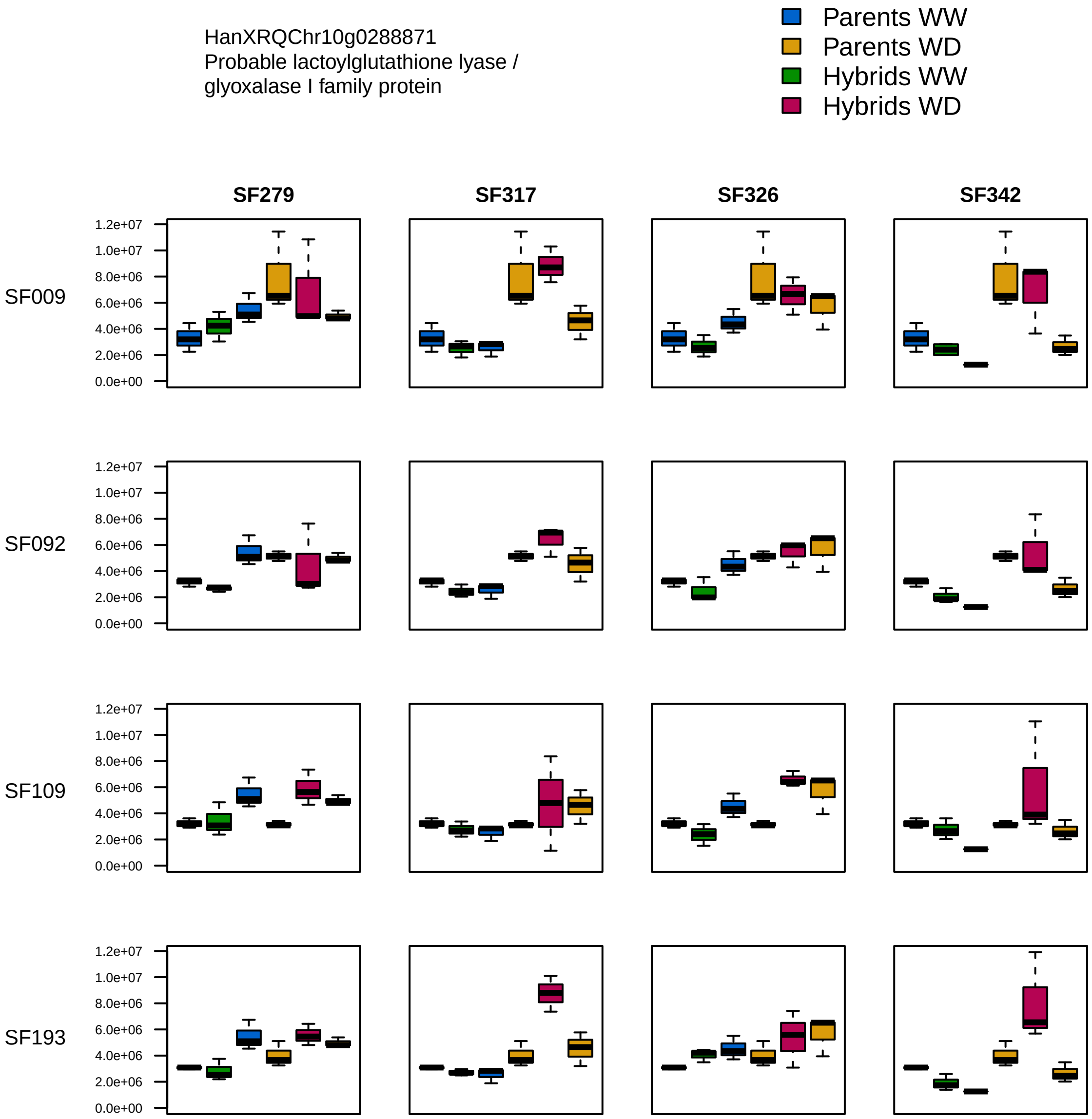
HanXRQChr10g0288871
Probable lactoylglutathione lyase /
glyoxalase I family protein
Parents WW
Parents WD
Hybrids WW
Hybrids WD
SF279
SF317
SF326
SF342
SF009
SF092
SF109
SF193
0.0e+00
2.0e+06
4.0e+06
6.0e+06
8.0e+06
1.0e+07
1.2e+07

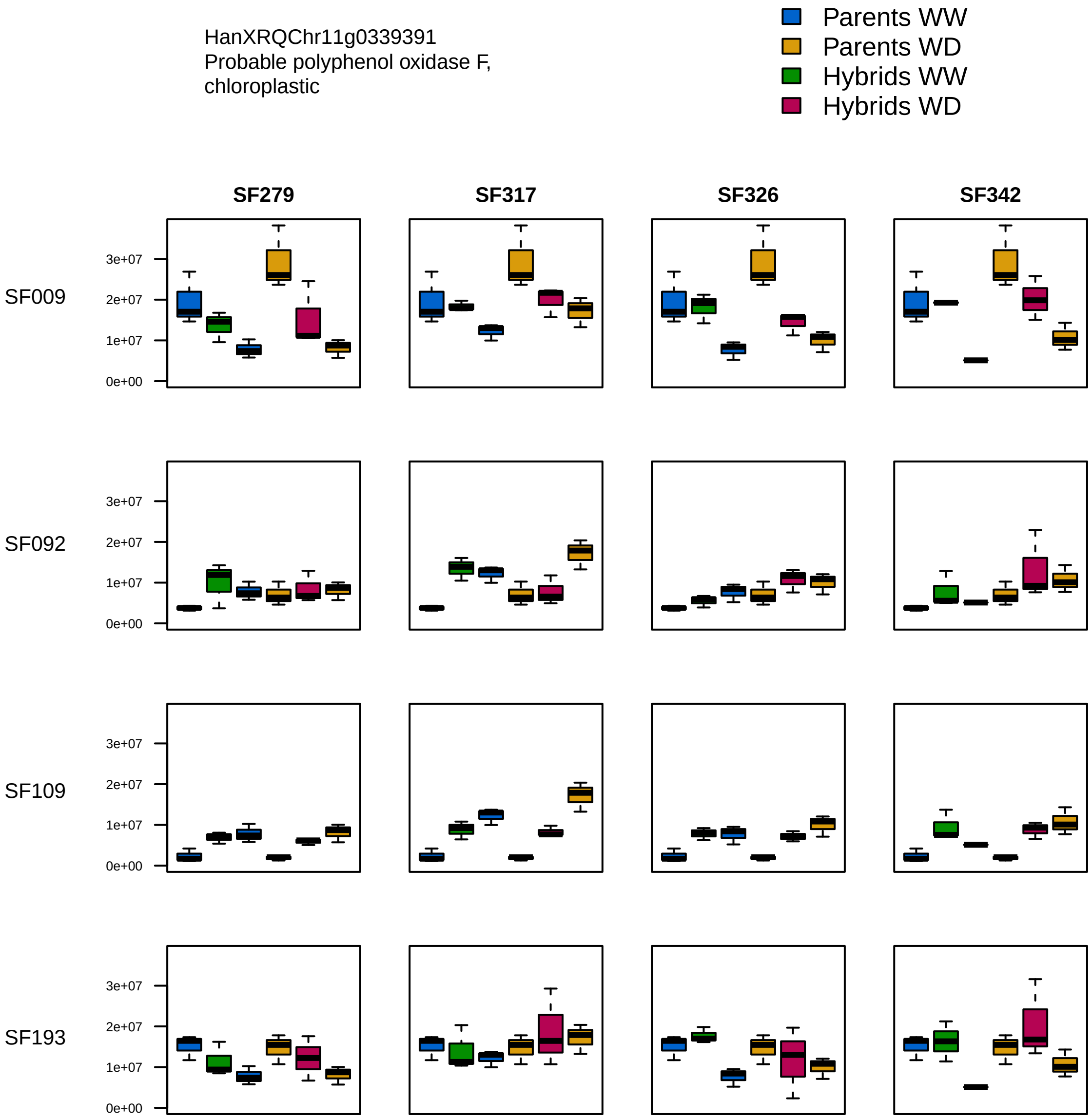

HanXRQChr11g0339391
Probable polyphenol oxidase F,
chloroplastic
Parents WW
Parents WD
Hybrids WW
Hybrids WD
SF279
SF317
SF326
SF342
SF009
SF092
SF109
SF193
0e+00
1e+07
2e+07
3e+07

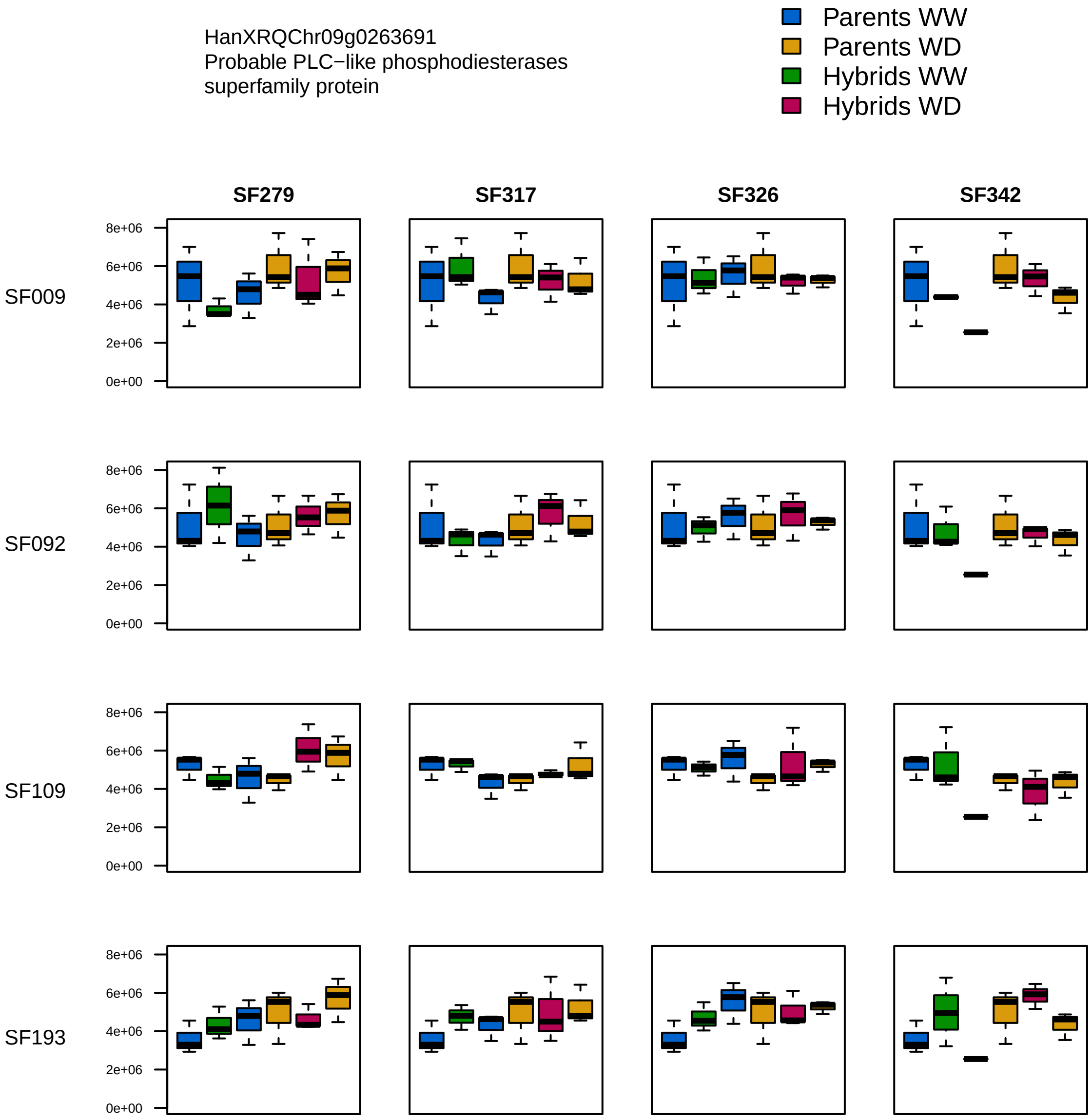
HanXRQChr09g0263691
Probable PLC−like phosphodiesterases
superfamily protein
Parents WW
Parents WD
Hybrids WW
Hybrids WD
SF279
SF317
SF326
SF342
SF009
SF092
SF109
SF193
8e+06
6e+06
4e+06
2e+06
0e+00

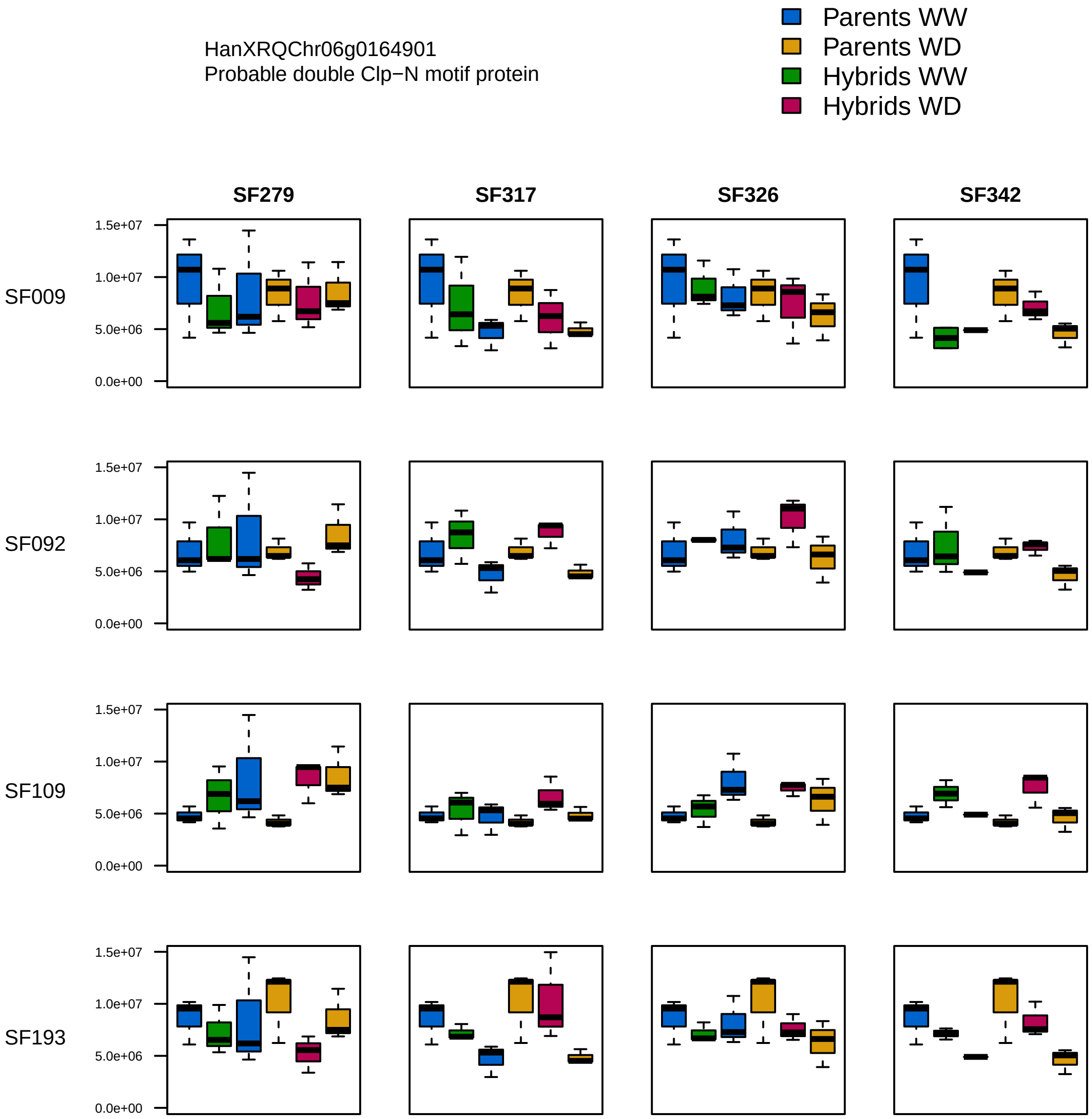
HanXRQChr06g0164901
Probable double Clp−N motif protein
Parents WW
Parents WD
Hybrids WW
Hybrids WD
SF279
SF317
SF326
SF342
SF009
SF092
SF109
SF193
1.5e+07
1.0e+07
5.0e+06
0.0e+00

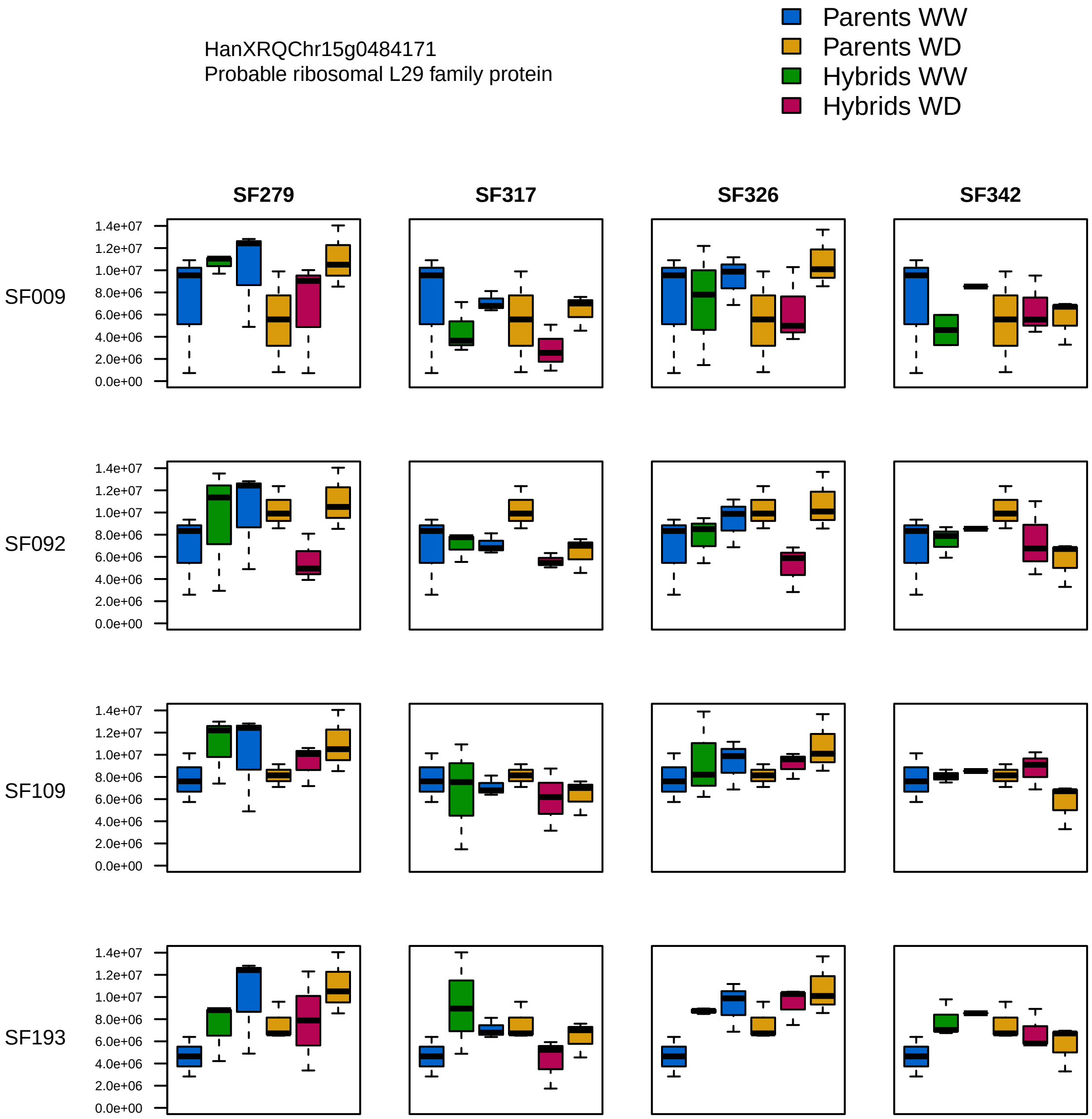
HanXRQChr15g0484171
Probable ribosomal L29 family protein
Parents WW
Parents WD
Hybrids WW
Hybrids WD
SF279
SF317
SF326
SF342
SF009
SF092
SF109
SF193
1.4e+07
1.2e+07
1.0e+07
8.0e+06
6.0e+06
4.0e+06
2.0e+06
0.0e+00

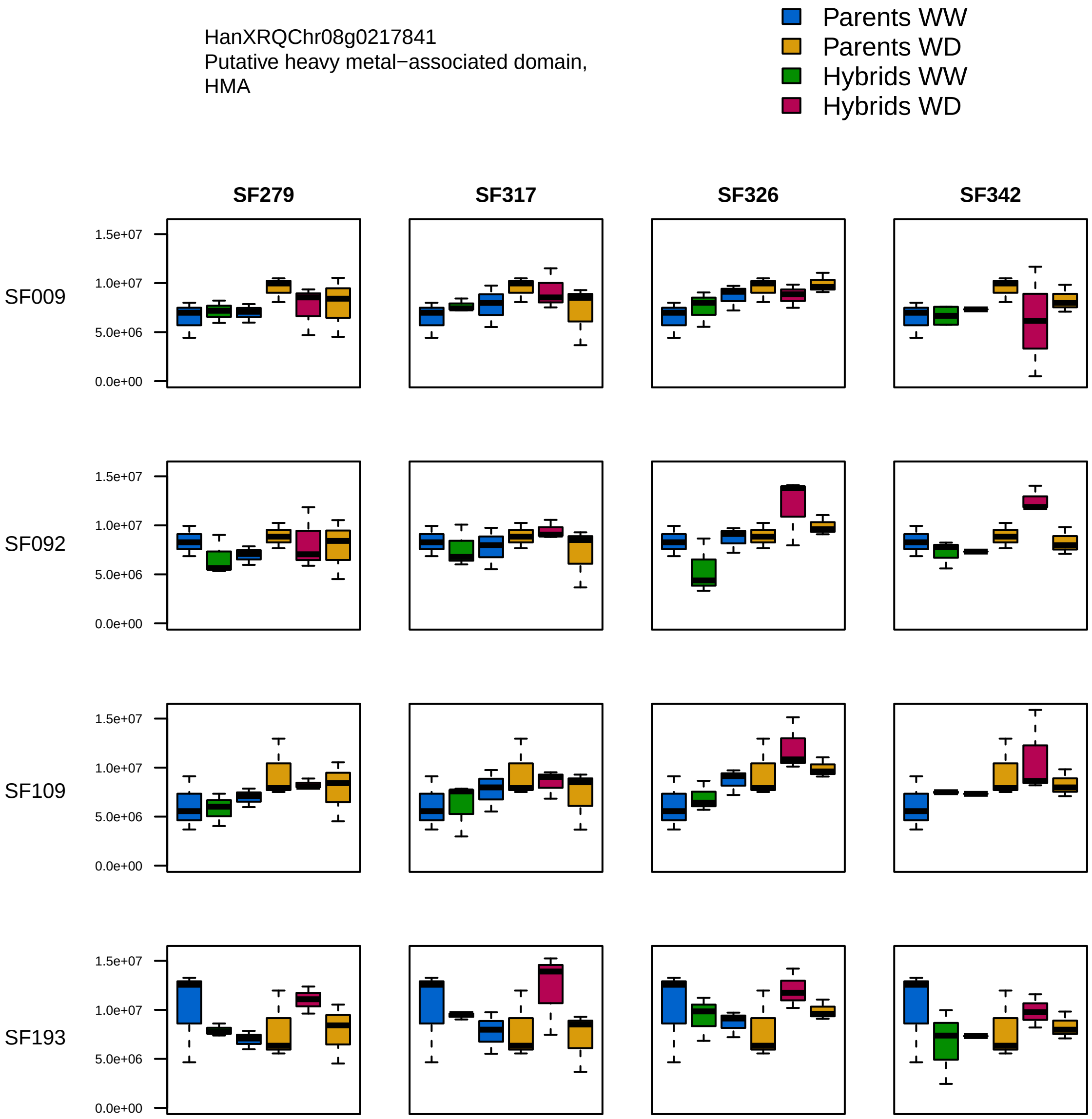

HanXRQChr08g0217841
Putative heavy metal−associated domain,
HMA
Parents WW
Parents WD
Hybrids WW
Hybrids WD
SF279
SF317
SF326
SF342
SF009
SF092
SF109
SF193
1.5e+07
1.0e+07
5.0e+06
0.0e+00

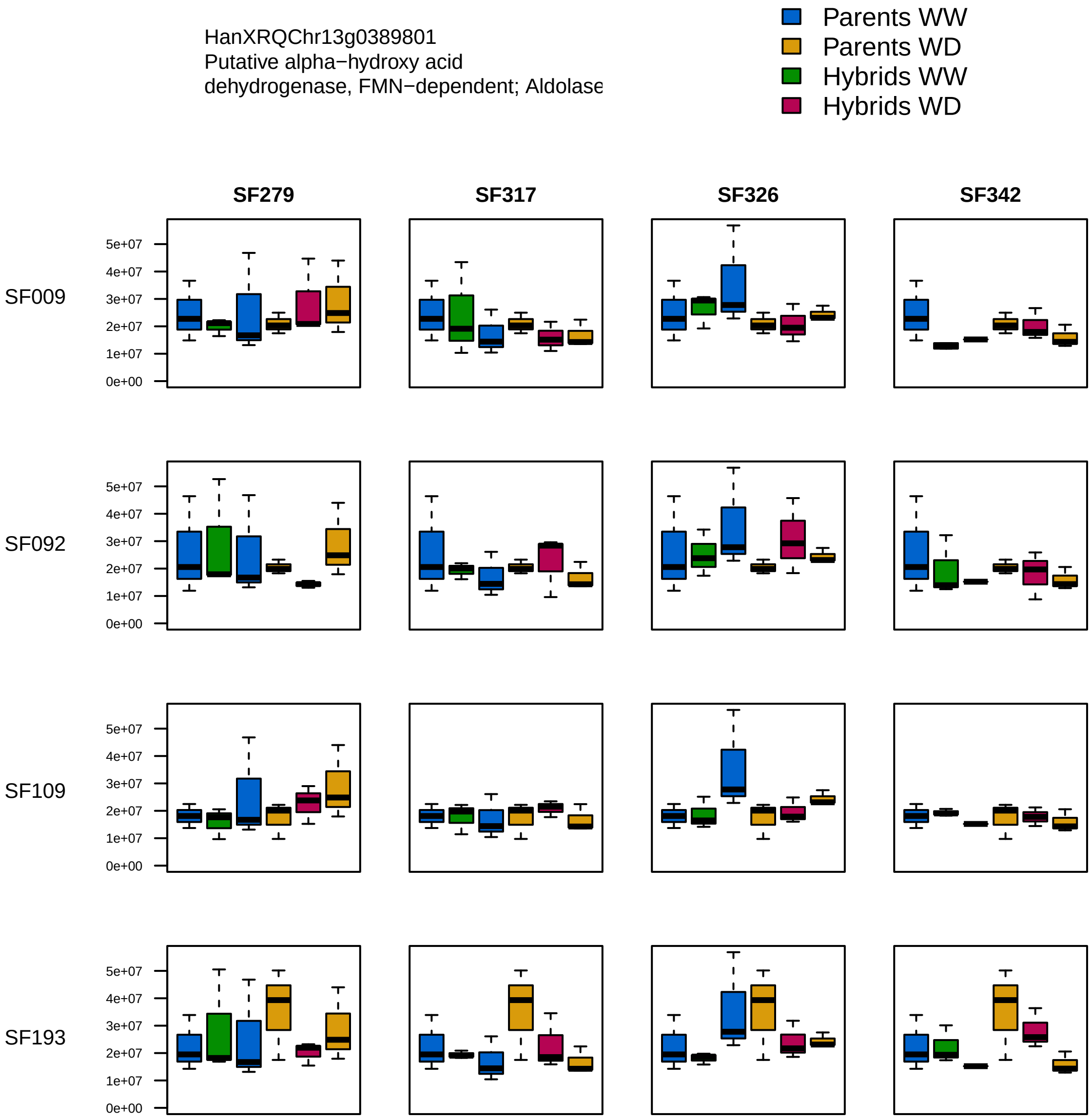

HanXRQChr13g0389801
Putative alpha−hydroxy acid
dehydrogenase, FMN−dependent; Aldolase
Parents WW
Parents WD
Hybrids WW
Hybrids WD
SF279
SF317
SF326
SF342
SF009
SF092
SF109
SF193
5e+07
4e+07
3e+07
2e+07
1e+07
0e+00

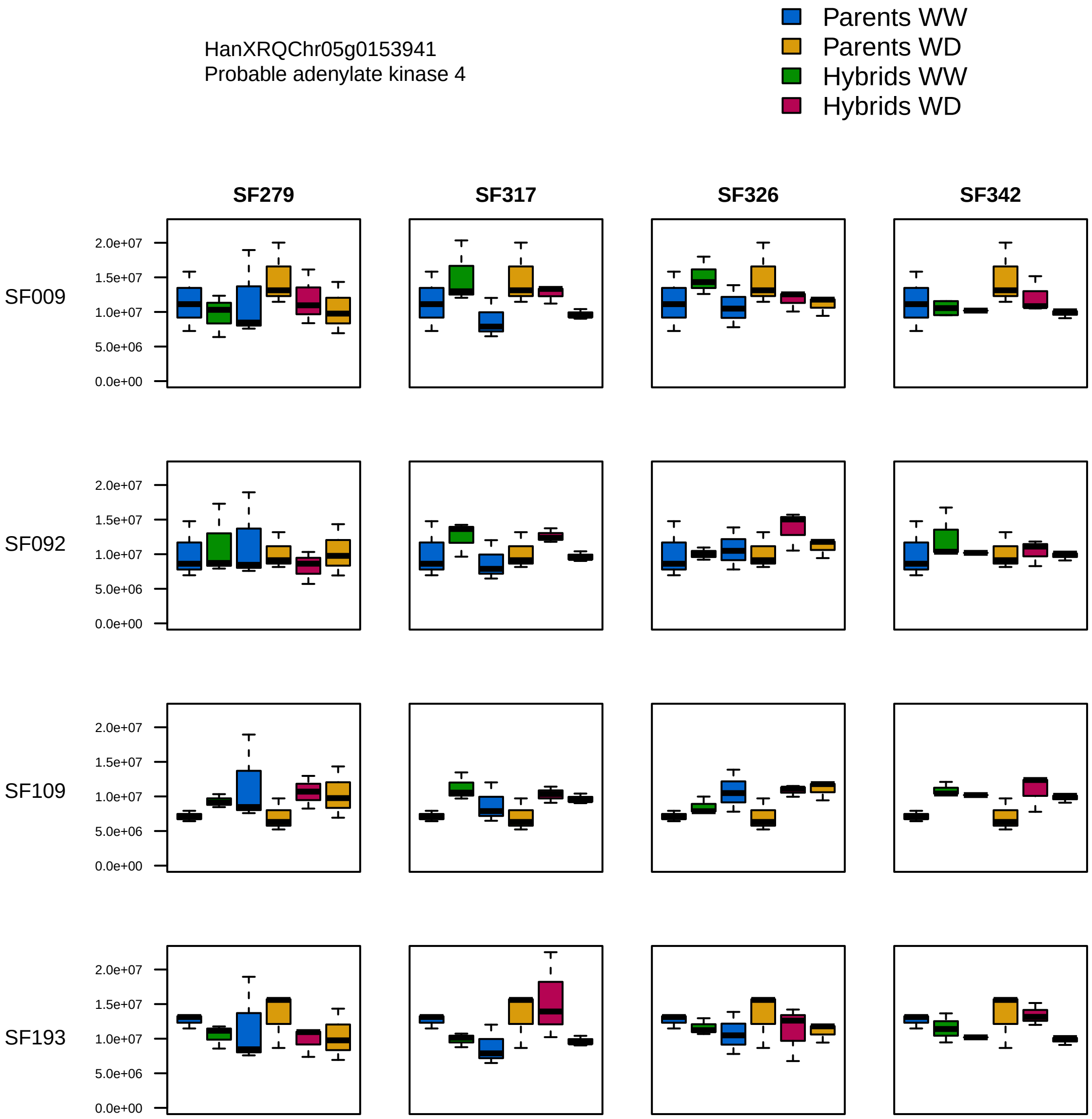
HanXRQChr05g0153941
Probable adenylate kinase 4
Parents WW
Parents WD
Hybrids WW
Hybrids WD
SF279
SF317
SF326
SF342
SF009
SF092
SF109
SF193
2.0e+07
1.5e+07
1.0e+07
5.0e+06
0.0e+00

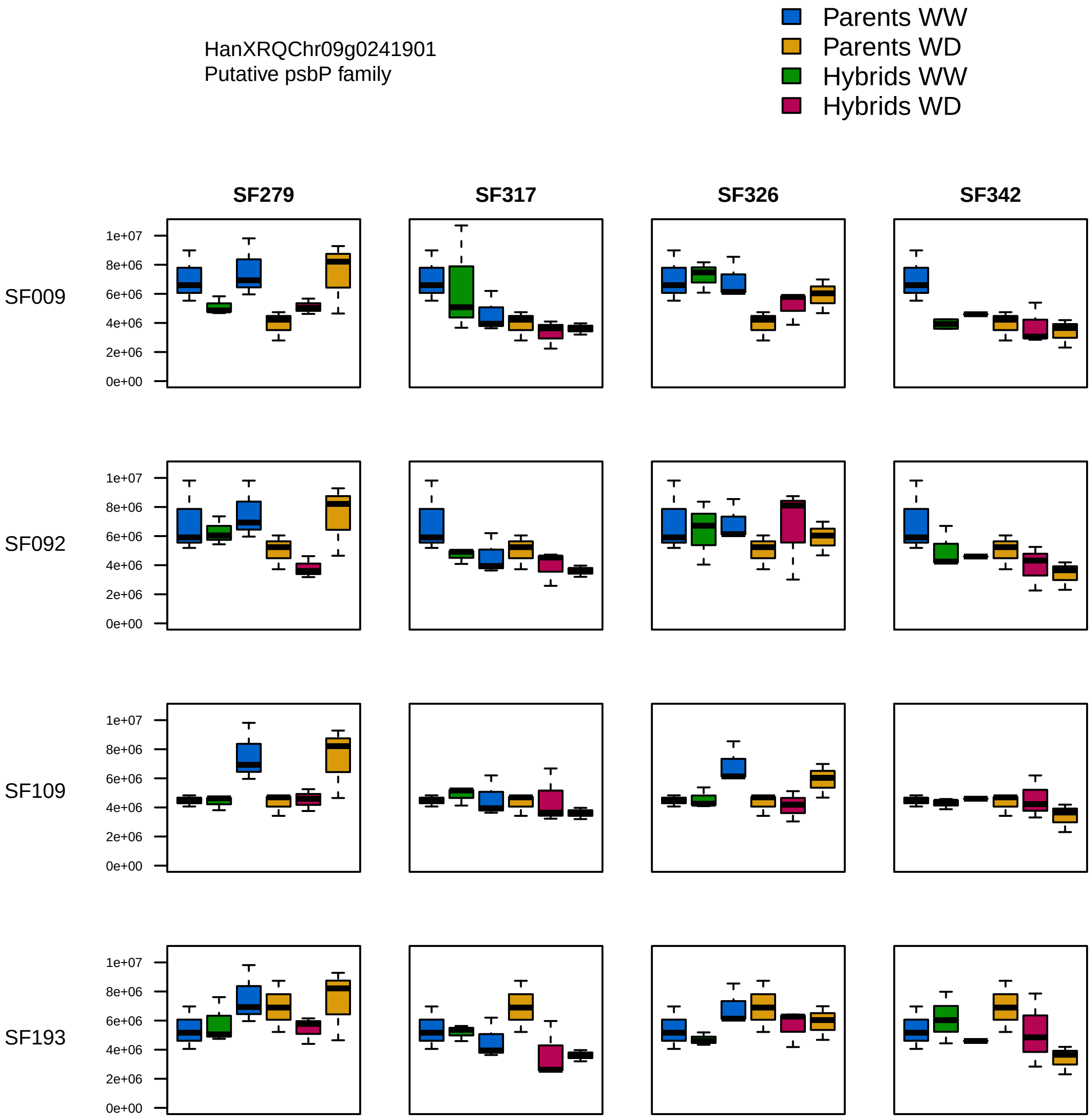
HanXRQChr09g0241901
Putative psbP family
Parents WW
Parents WD
Hybrids WW
Hybrids WD
SF279
SF317
SF326
SF342
SF009
SF092
SF109
SF193
1e+07
8e+06
6e+06
4e+06
2e+06
0e+00

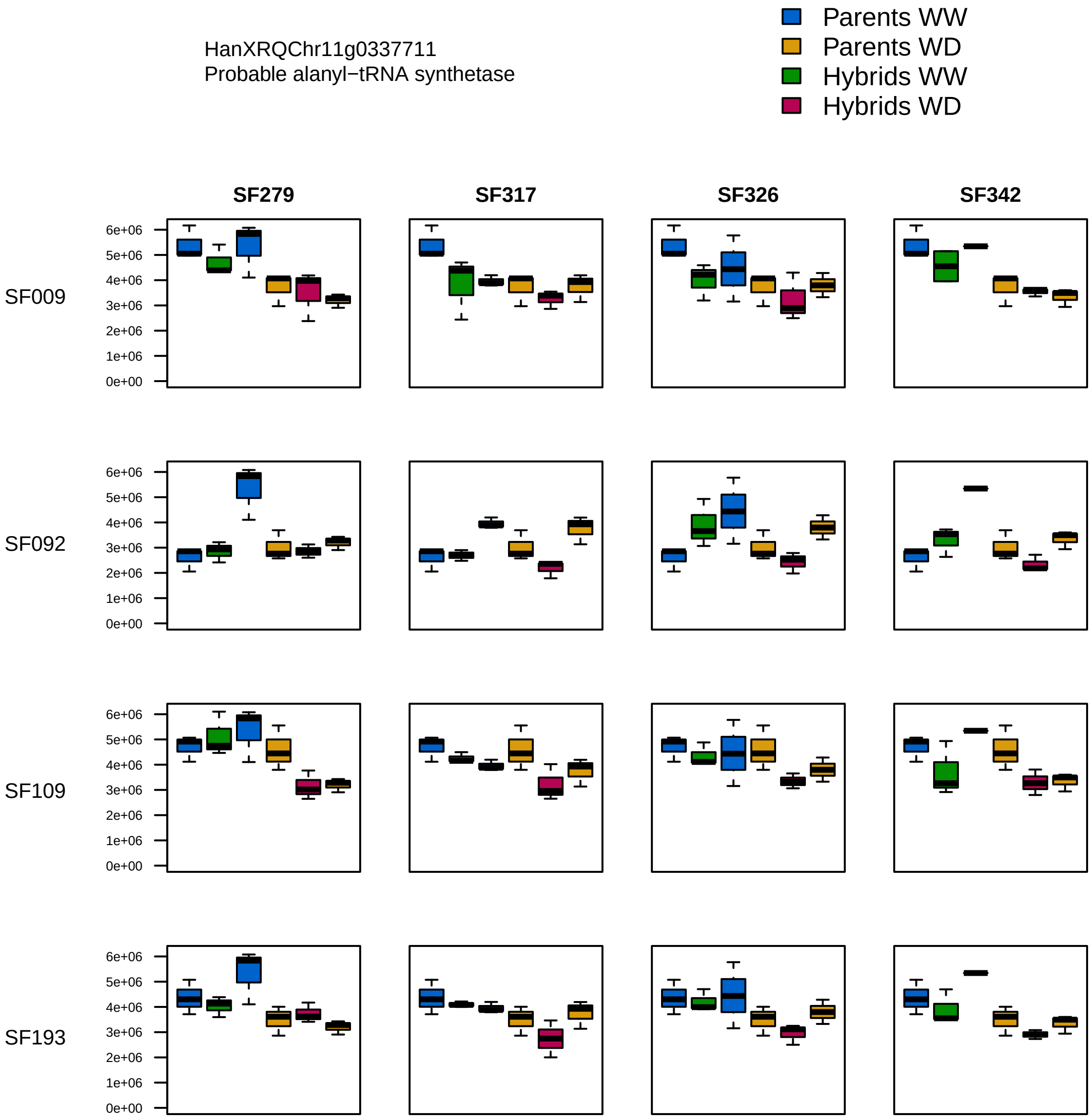

HanXRQChr11g0337711
Probable alanyl−tRNA synthetase
Parents WW
Parents WD
Hybrids WW
Hybrids WD
SF279
SF317
SF326
SF342
SF009
SF092
SF109
SF193
6e+06
5e+06
4e+06
3e+06
2e+06
1e+06
0e+00

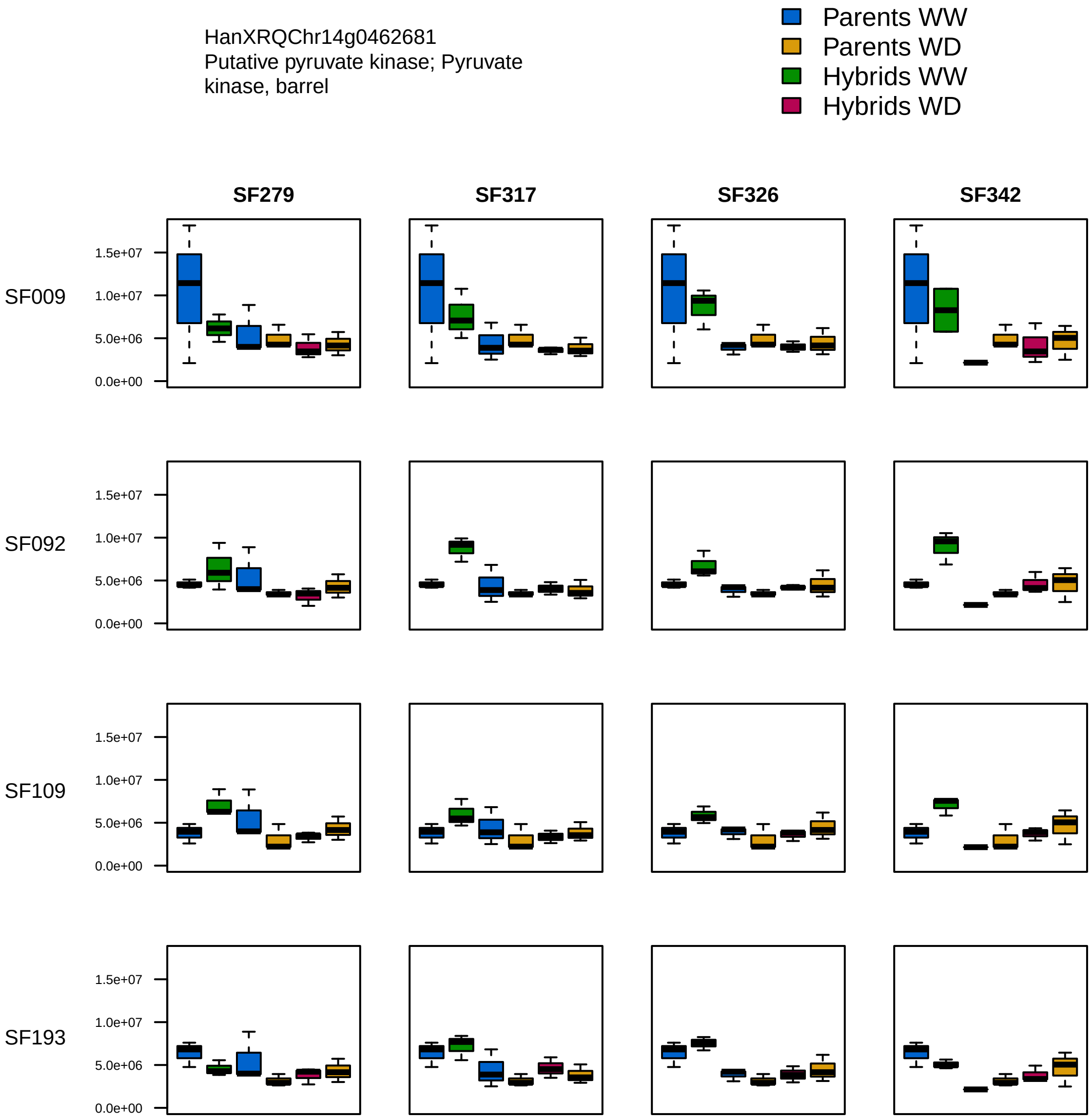
HanXRQChr14g0462681
Putative pyruvate kinase; Pyruvate kinase, barrel
Parents WW
Parents WD
Hybrids WW
Hybrids WD
SF279
SF317
SF326
SF342
SF009
SF092
SF109
SF193
1.5e+07
1.0e+07
5.0e+06
0.0e+00

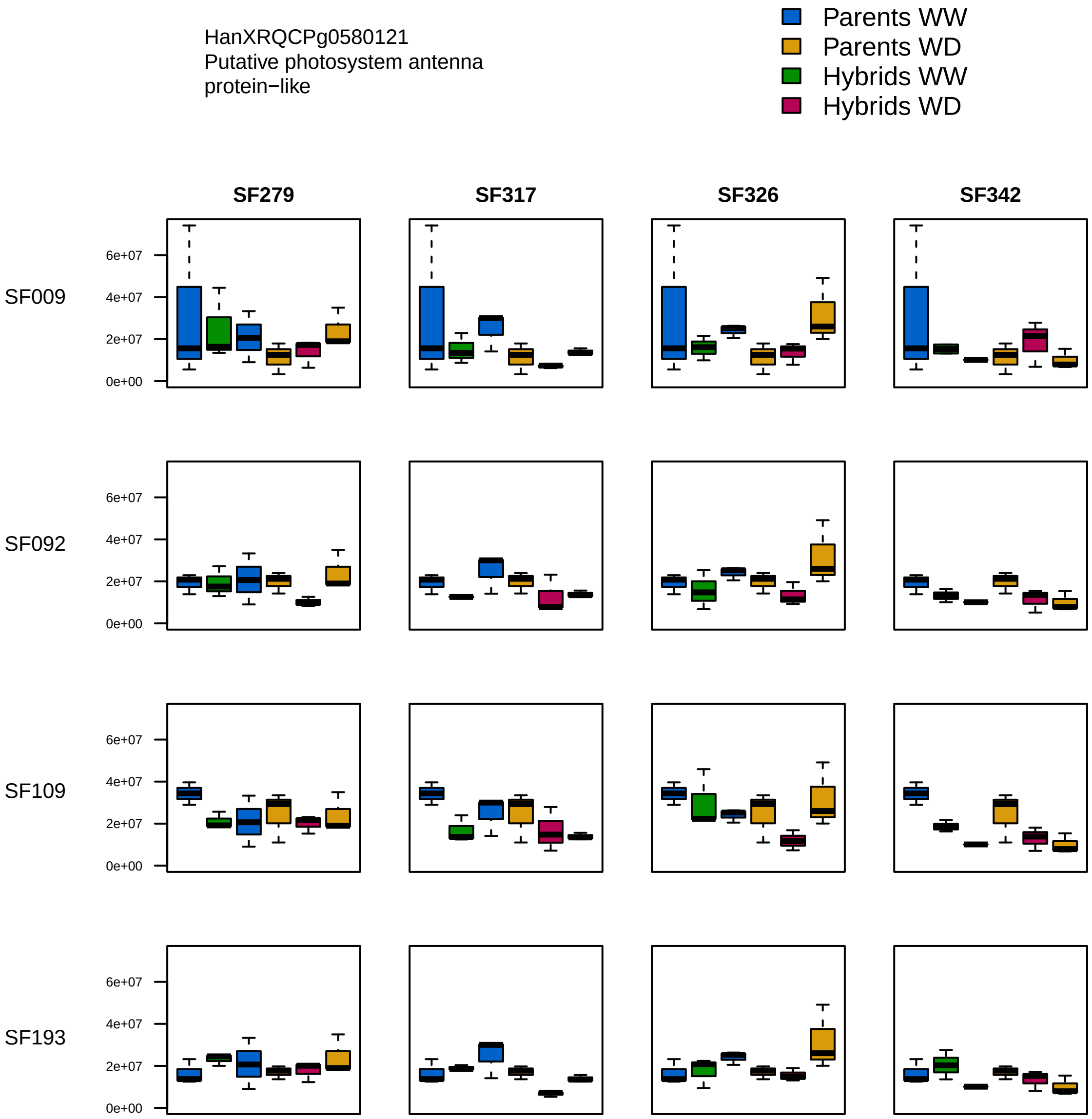

HanXRQCPg0580121
Putative photosystem antenna protein−like
Parents WW
Parents WD
Hybrids WW
Hybrids WD
SF279
SF317
SF326
SF342
SF009
SF092
SF109
SF193
6e+07
4e+07
2e+07
0e+00

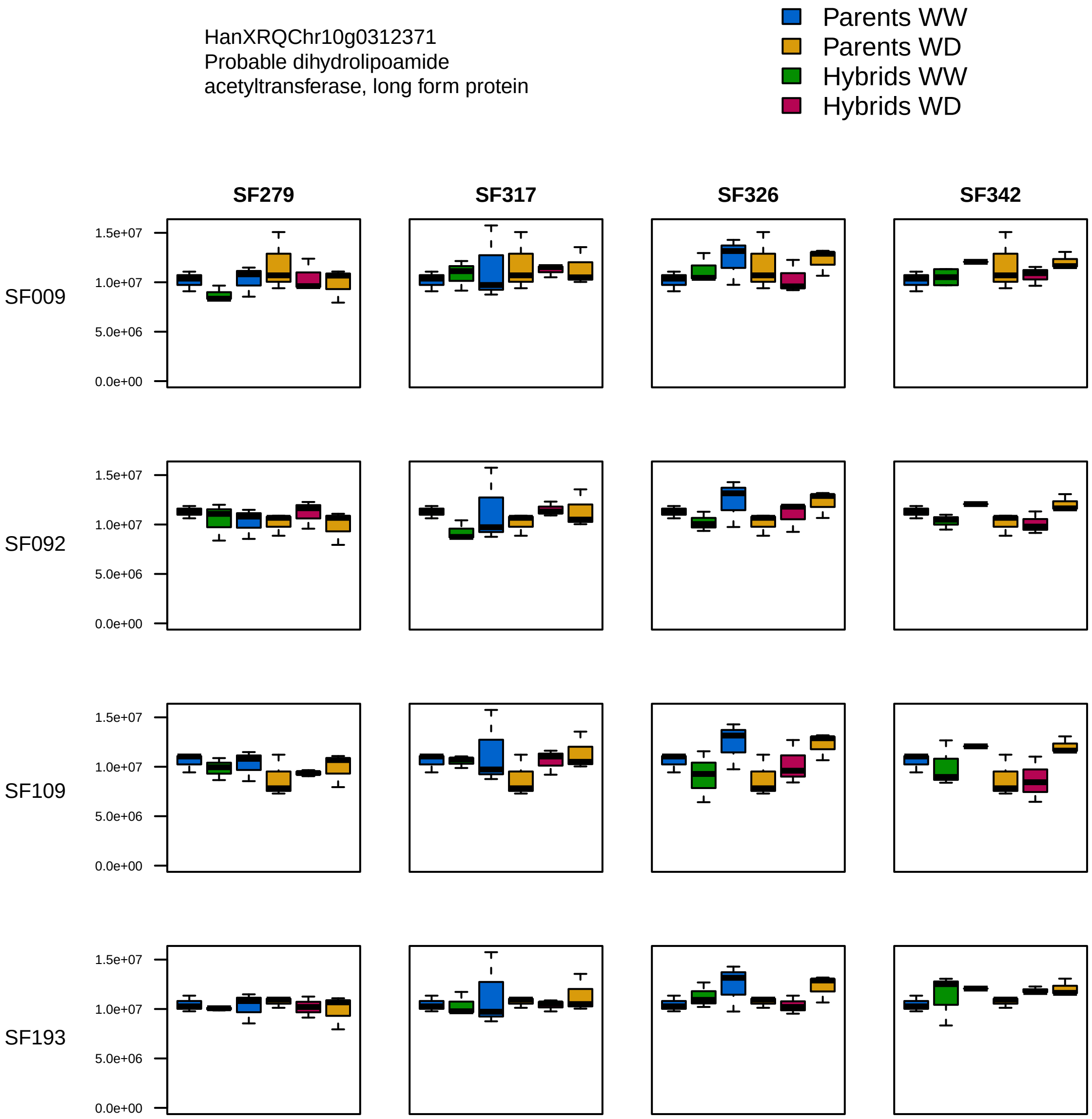

HanXRQChr10g0312371
Probable dihydrolipoamide
acetyltransferase, long form protein
Parents WW
Parents WD
Hybrids WW
Hybrids WD
SF279
SF317
SF326
SF342
SF009
SF092
SF109
SF193
1.5e+07
1.0e+07
5.0e+06
0.0e+00

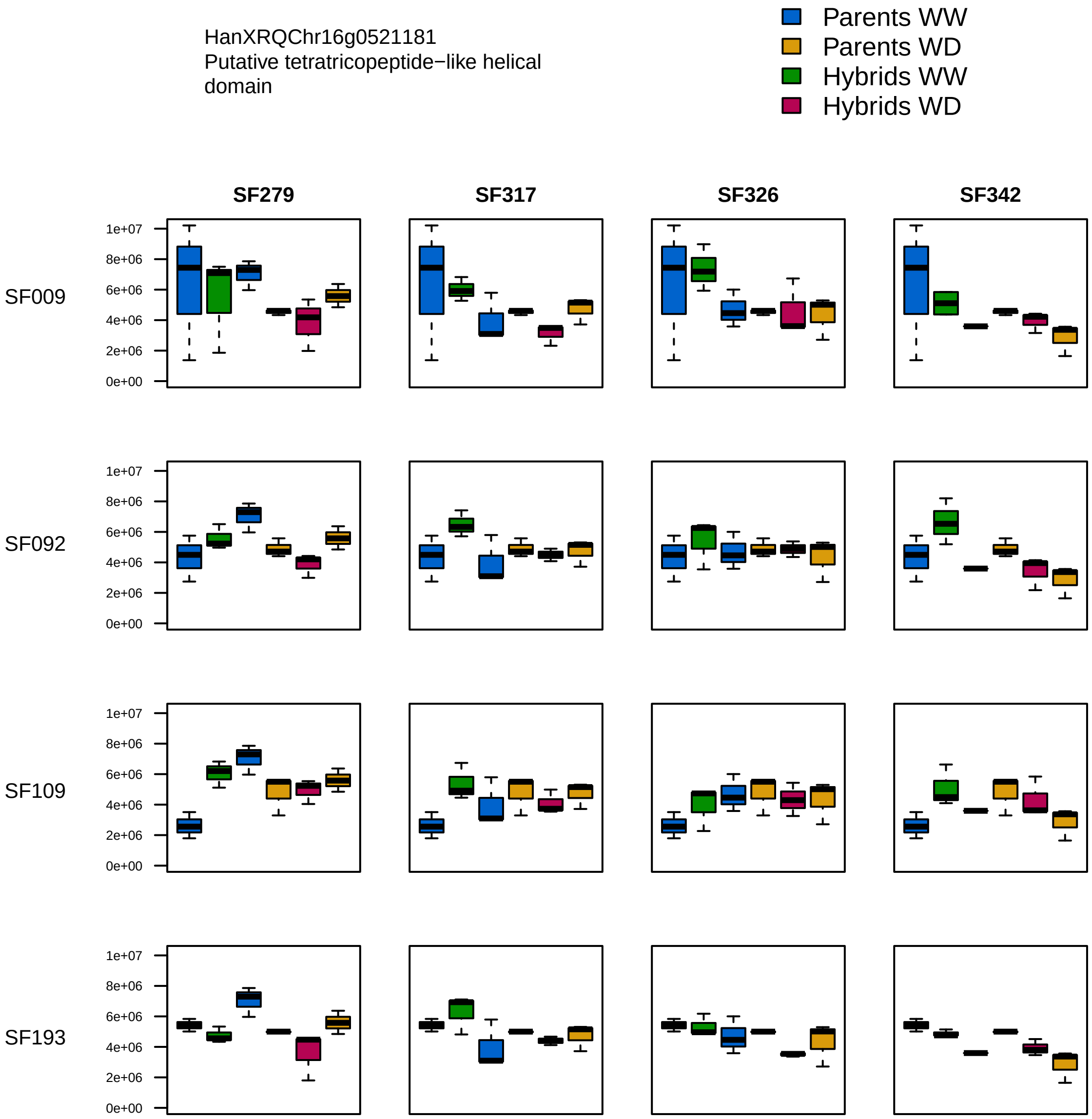

HanXRQChr16g0521181
Putative tetratricopeptide−like helical domain
Parents WW
Parents WD
Hybrids WW
Hybrids WD
SF279
SF317
SF326
SF342
SF009
SF092
SF109
SF193
0e+00
2e+06
4e+06
6e+06
8e+06
1e+07

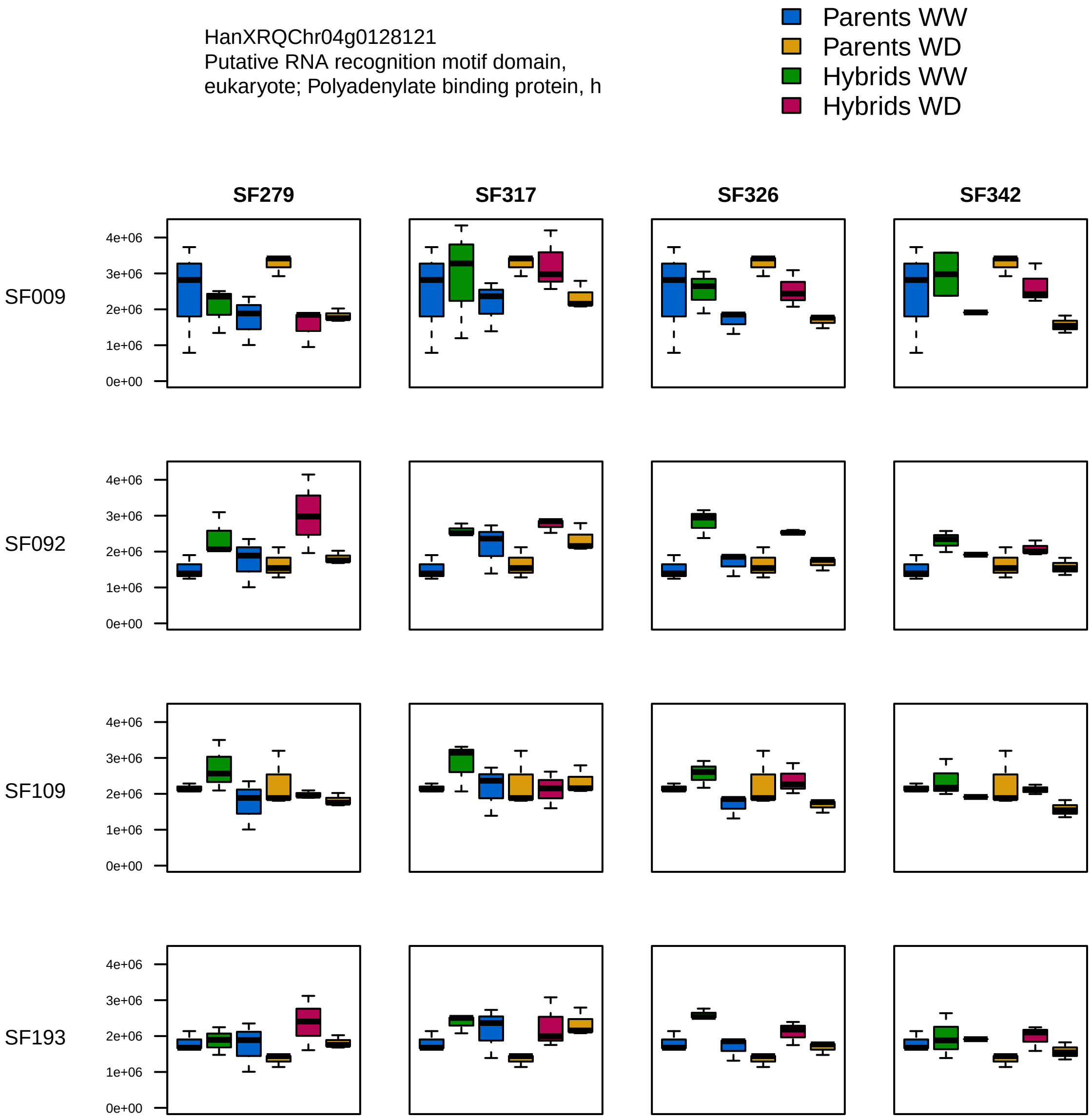
HanXRQChr04g0128121
Putative RNA recognition motif domain,
eukaryote; Polyadenylate binding protein, h
Parents WW
Parents WD
Hybrids WW
Hybrids WD
SF279
SF317
SF326
SF342
SF009
SF092
SF109
SF193
0e+00
1e+06
2e+06
3e+06
4e+06

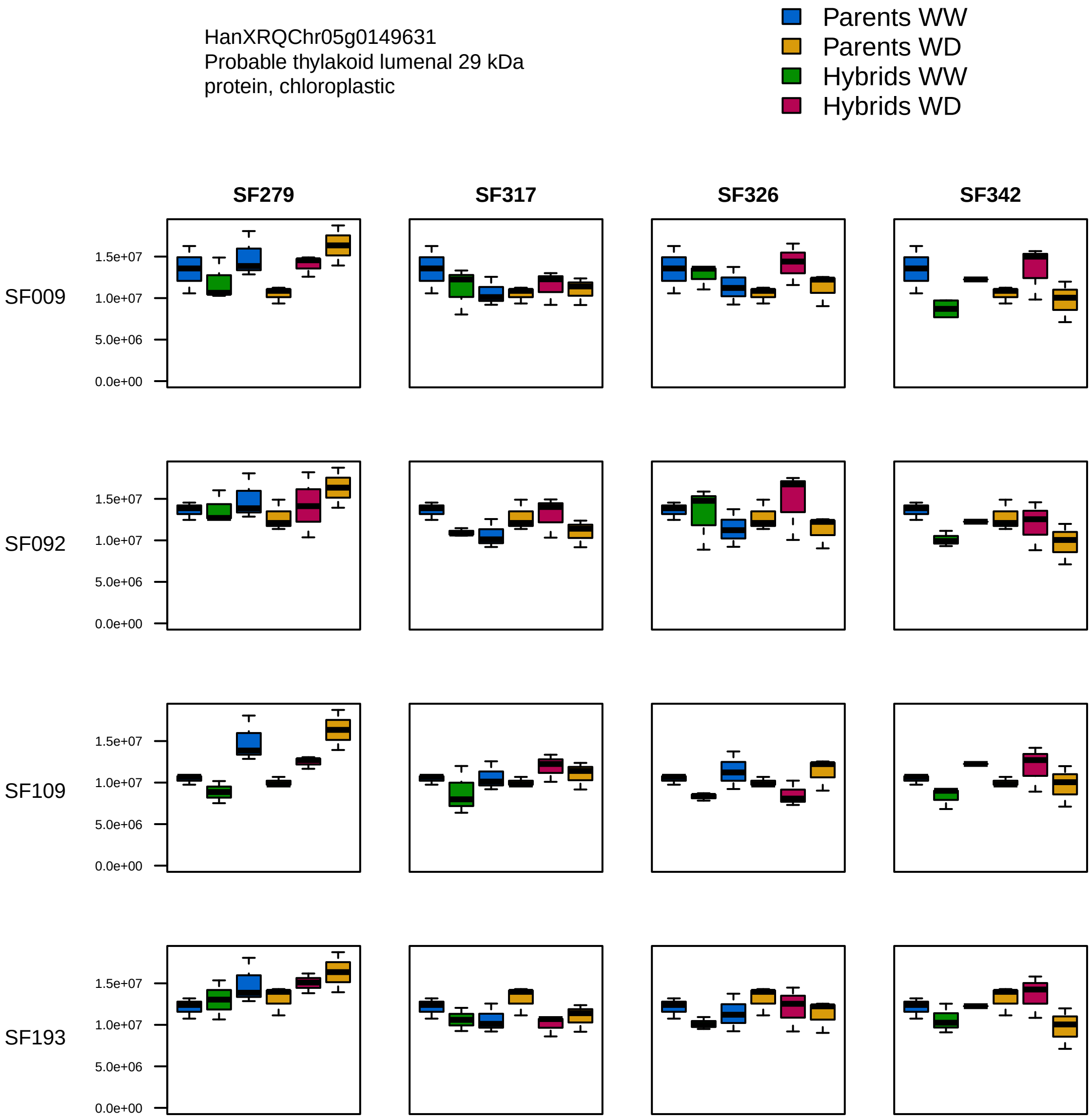
HanXRQChr05g0149631
Probable thylakoid lumenal 29 kDa protein, chloroplastic
Parents WW
Parents WD
Hybrids WW
Hybrids WD
SF279
SF317
SF326
SF342
SF009
SF092
SF109
SF193
0.0e+00
5.0e+06
1.0e+07
1.5e+07

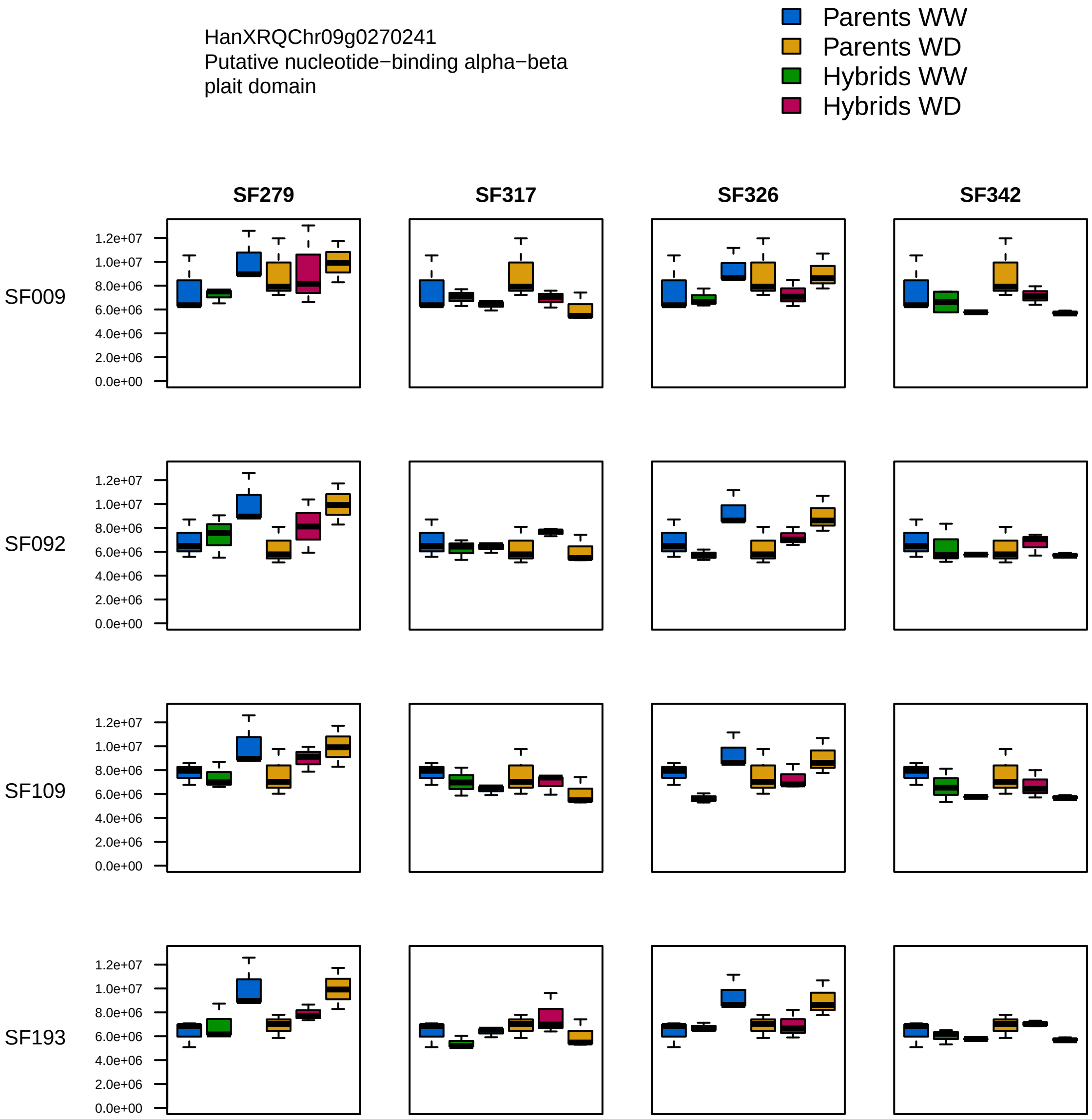
HanXRQChr09g0270241
Putative nucleotide−binding alpha−beta
plait domain
Parents WW
Parents WD
Hybrids WW
Hybrids WD
SF279
SF317
SF326
SF342
SF009
SF092
SF109
SF193
0.0e+00
2.0e+06
4.0e+06
6.0e+06
8.0e+06
1.0e+07
1.2e+07

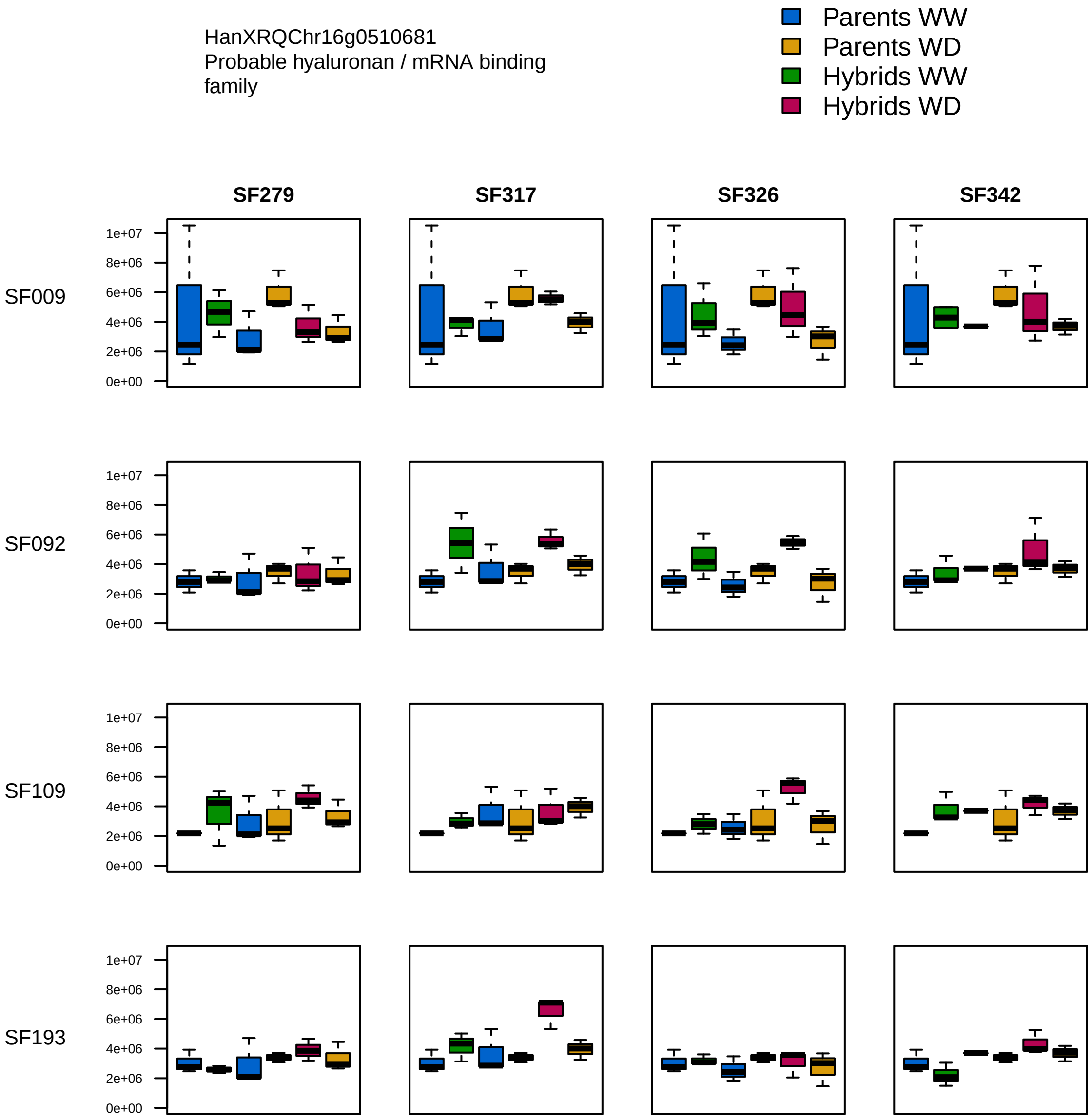
HanXRQChr16g0510681
Probable hyaluronan / mRNA binding
family
Parents WW
Parents WD
Hybrids WW
Hybrids WD
SF279
SF317
SF326
SF342
SF009
SF092
SF109
SF193
1e+07
8e+06
6e+06
4e+06
2e+06
0e+00

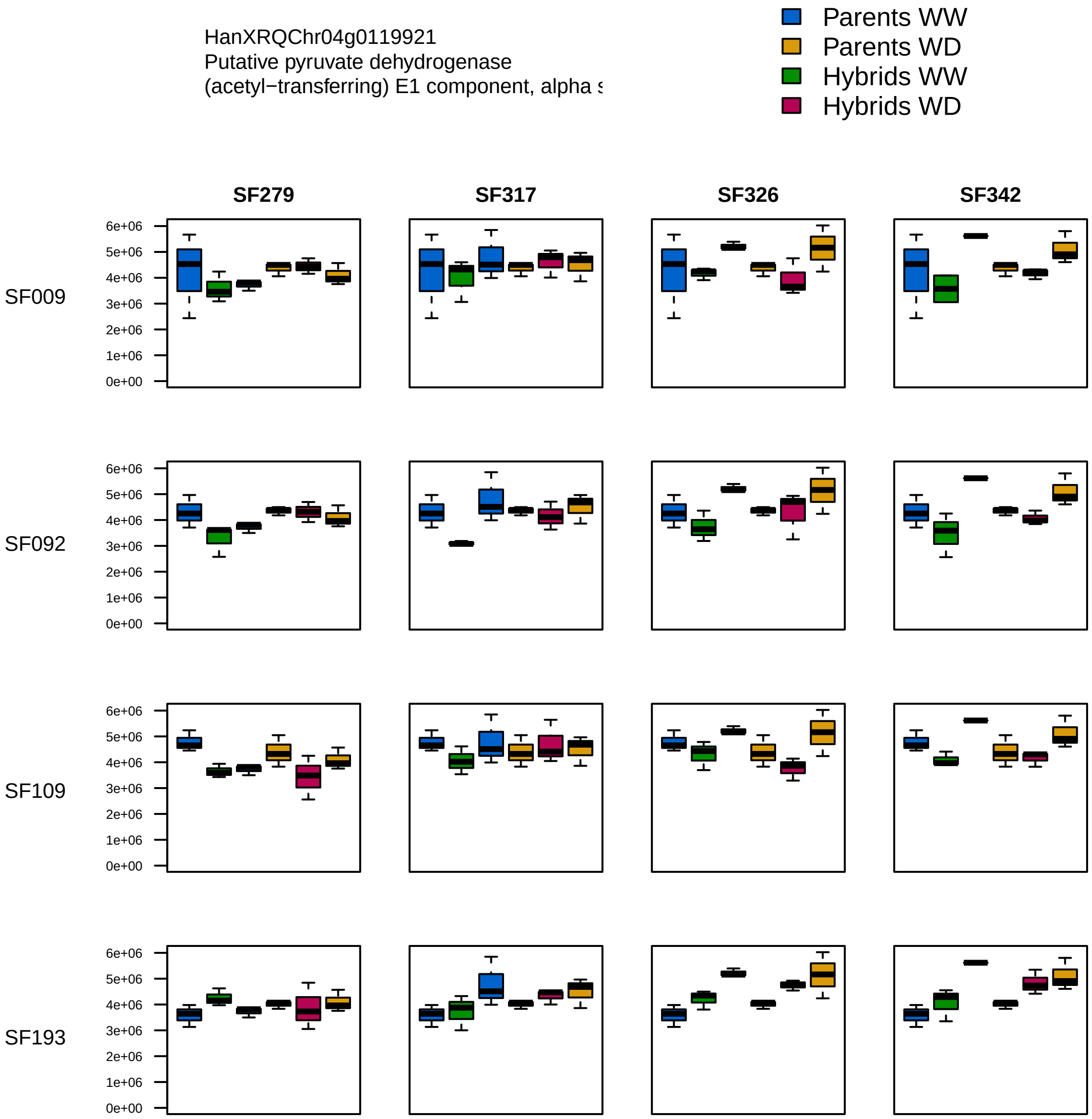
HanXRQChr04g0119921
Putative pyruvate dehydrogenase
(acetyl−transferring) E1 component, alpha s
Parents WW
Parents WD
Hybrids WW
Hybrids WD
SF279
SF317
SF326
SF342
SF009
SF092
SF109
SF193
6e+06
5e+06
4e+06
3e+06
2e+06
1e+06
0e+00

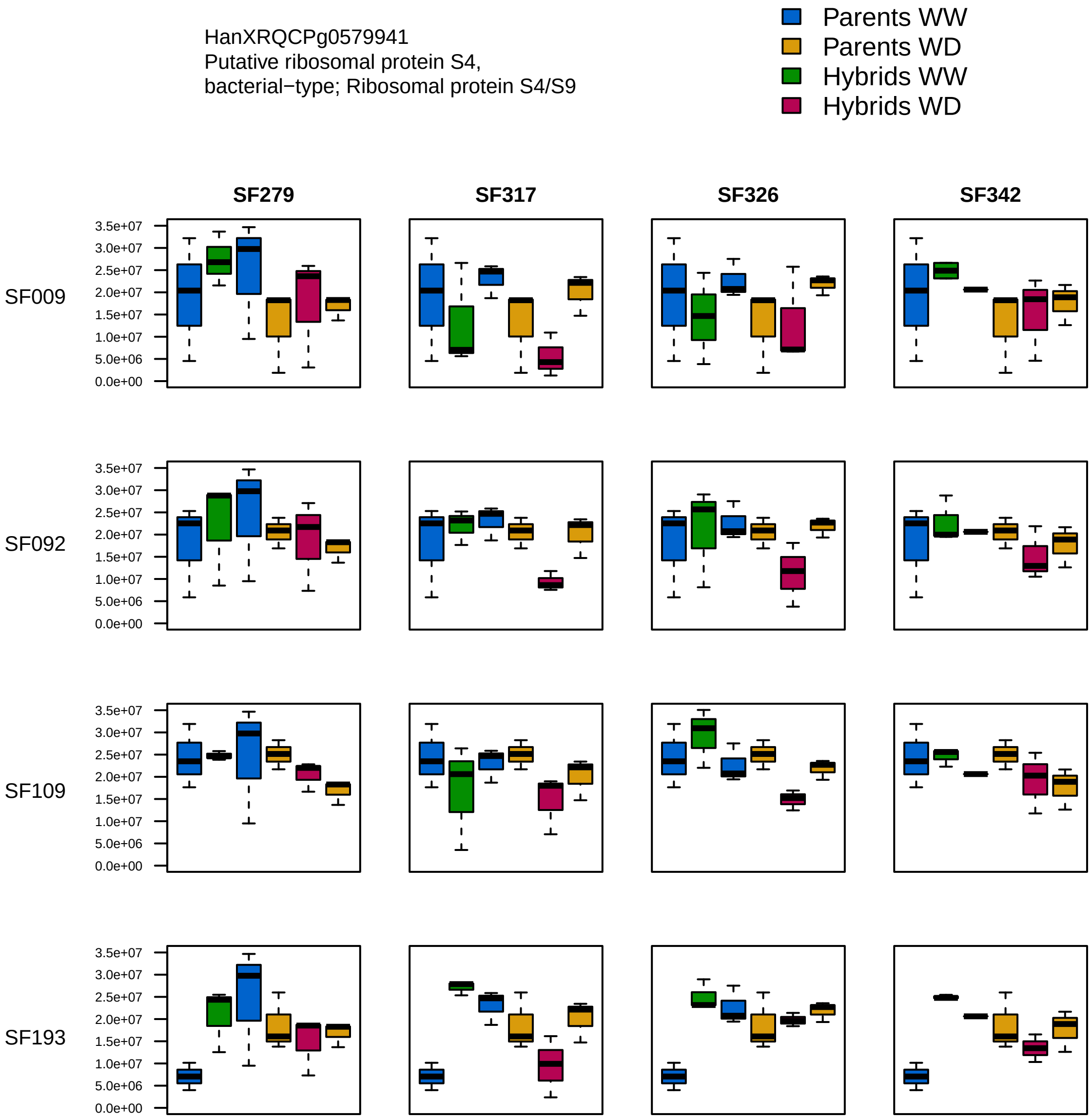
HanXRQCPg0579941
Putative ribosomal protein S4,
bacterial−type; Ribosomal protein S4/S9
Parents WW
Parents WD
Hybrids WW
Hybrids WD
SF279
SF317
SF326
SF342
SF009
SF092
SF109
SF193
3.5e+07
3.0e+07
2.5e+07
2.0e+07
1.5e+07
1.0e+07
5.0e+06
0.0e+00

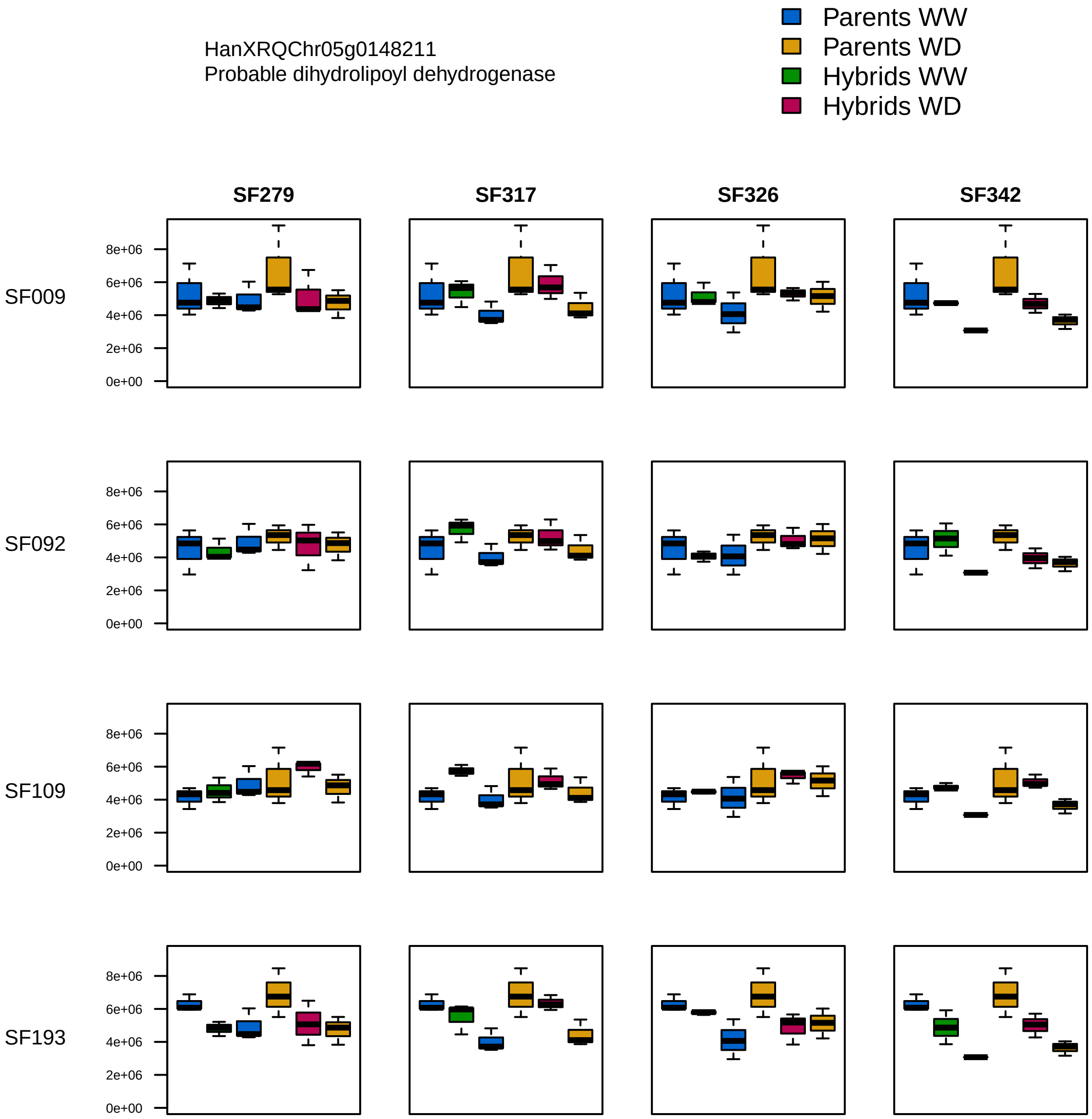

HanXRQChr05g0148211
Probable dihydrolipoyl dehydrogenase
Parents WW
Parents WD
Hybrids WW
Hybrids WD
SF279
SF317
SF326
SF342
SF009
SF092
SF109
SF193
8e+06
6e+06
4e+06
2e+06
0e+00

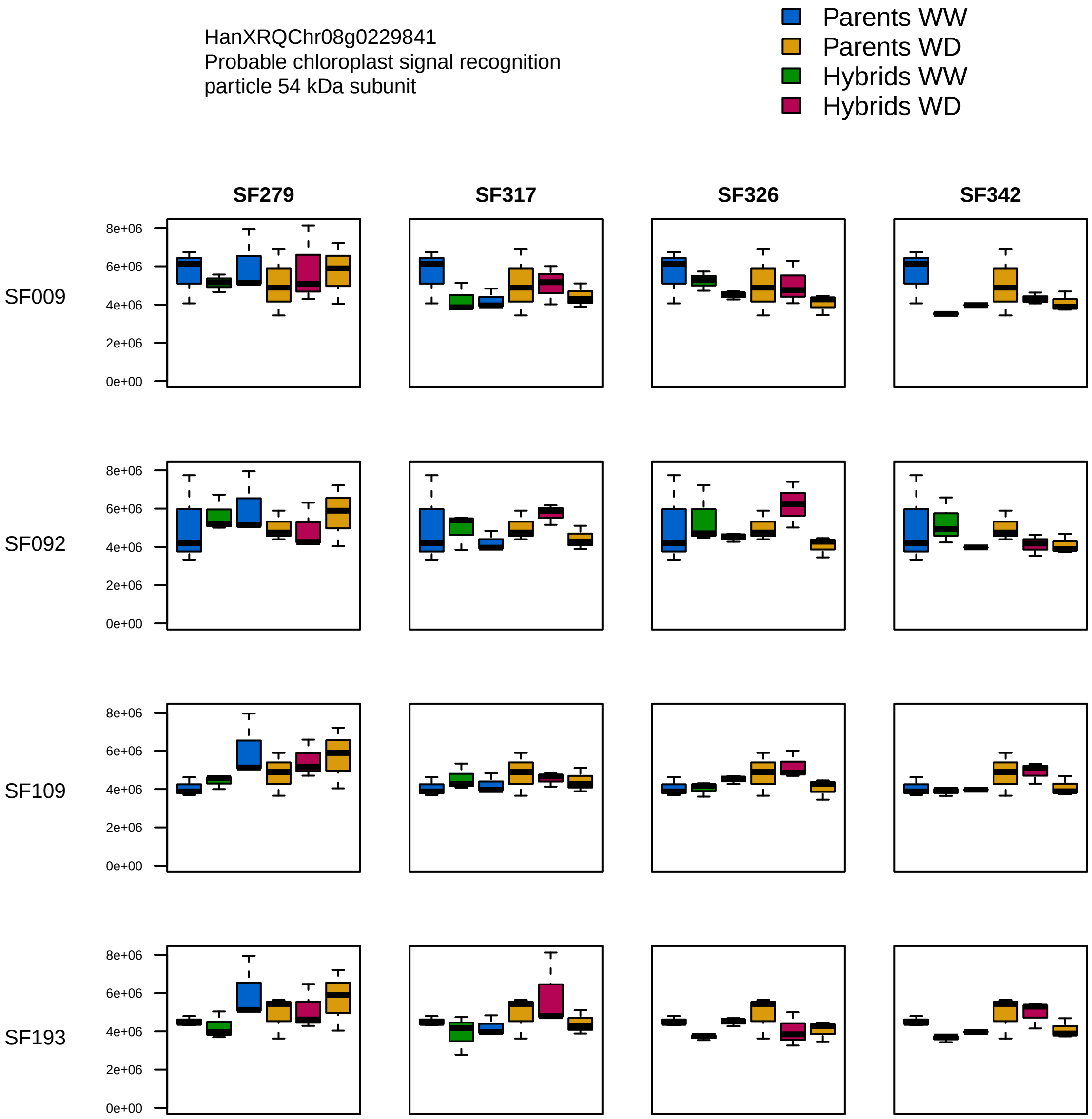
HanXRQChr08g0229841
Probable chloroplast signal recognition particle 54 kDa subunit
Parents WW
Parents WD
Hybrids WW
Hybrids WD
SF279
SF317
SF326
SF342
SF009
SF092
SF109
SF193
0e+00
2e+06
4e+06
6e+06
8e+06

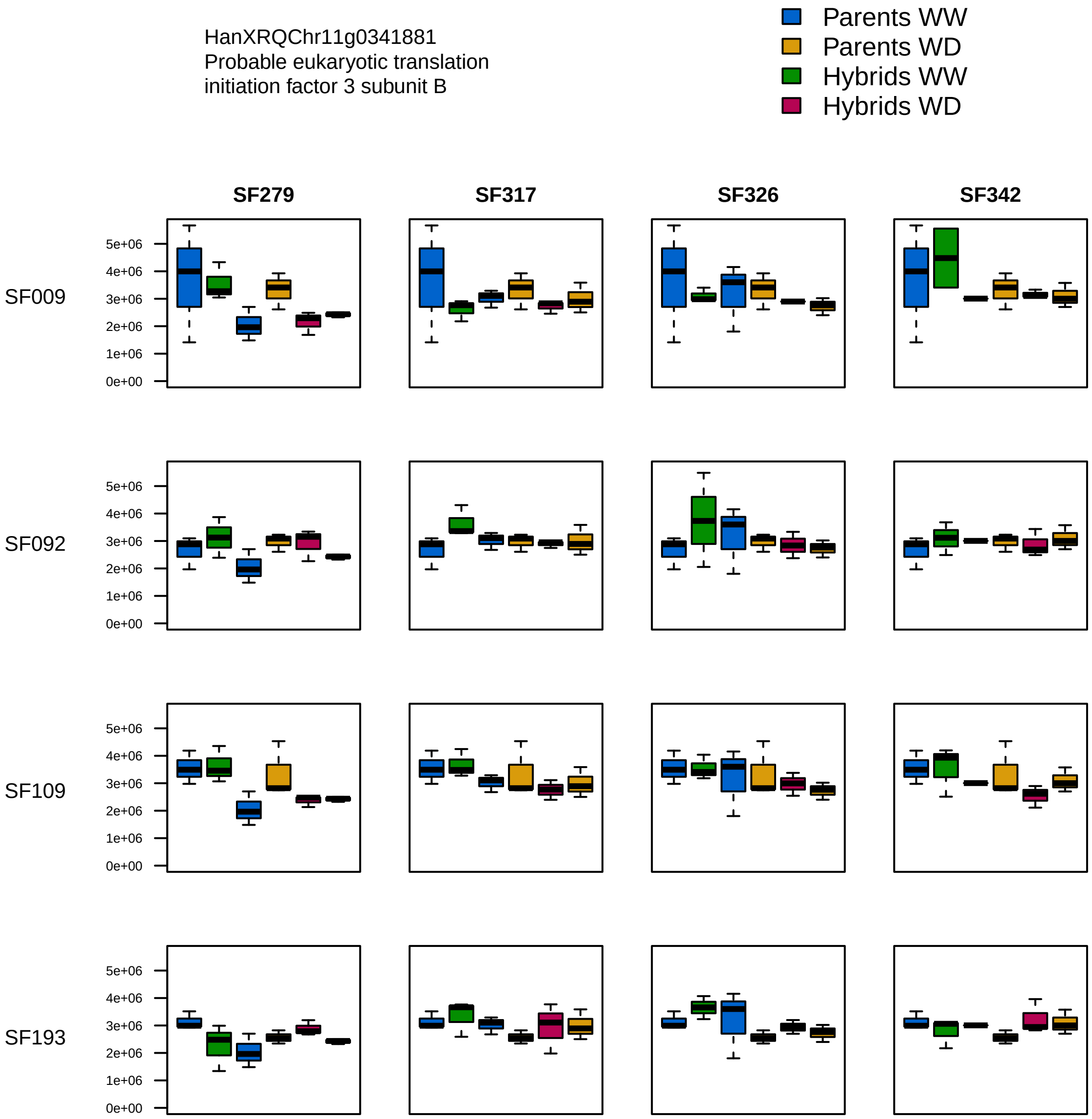
HanXRQChr11g0341881
Probable eukaryotic translation
initiation factor 3 subunit B
Parents WW
Parents WD
Hybrids WW
Hybrids WD
SF279
SF317
SF326
SF342
SF009
SF092
SF109
SF193
0e+00
1e+06
2e+06
3e+06
4e+06
5e+06

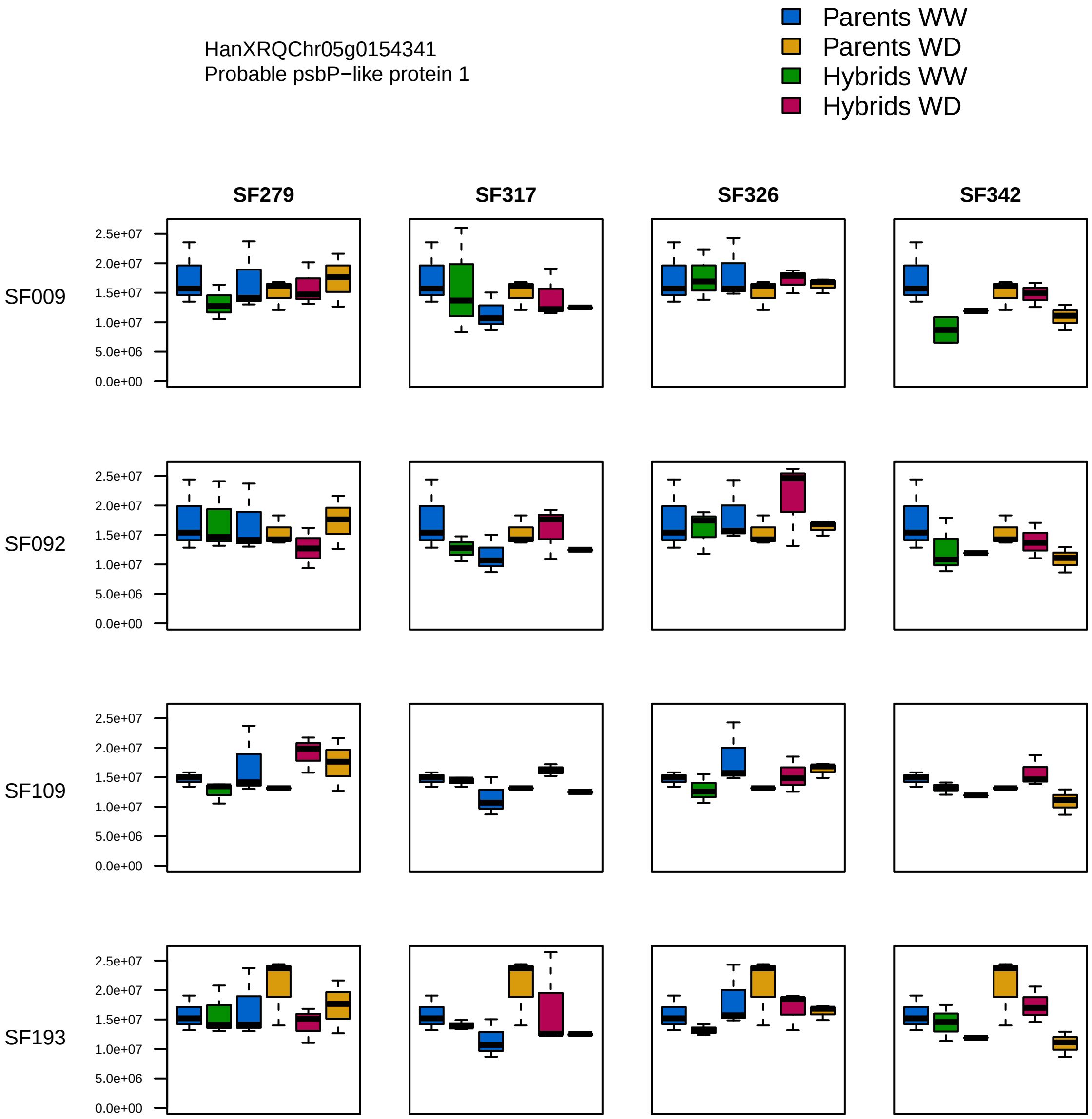
HanXRQChr05g0154341
Probable psbP−like protein 1
Parents WW
Parents WD
Hybrids WW
Hybrids WD
SF279
SF317
SF326
SF342
SF009
SF092
SF109
SF193
2.5e+07
2.0e+07
1.5e+07
1.0e+07
5.0e+06
0.0e+00

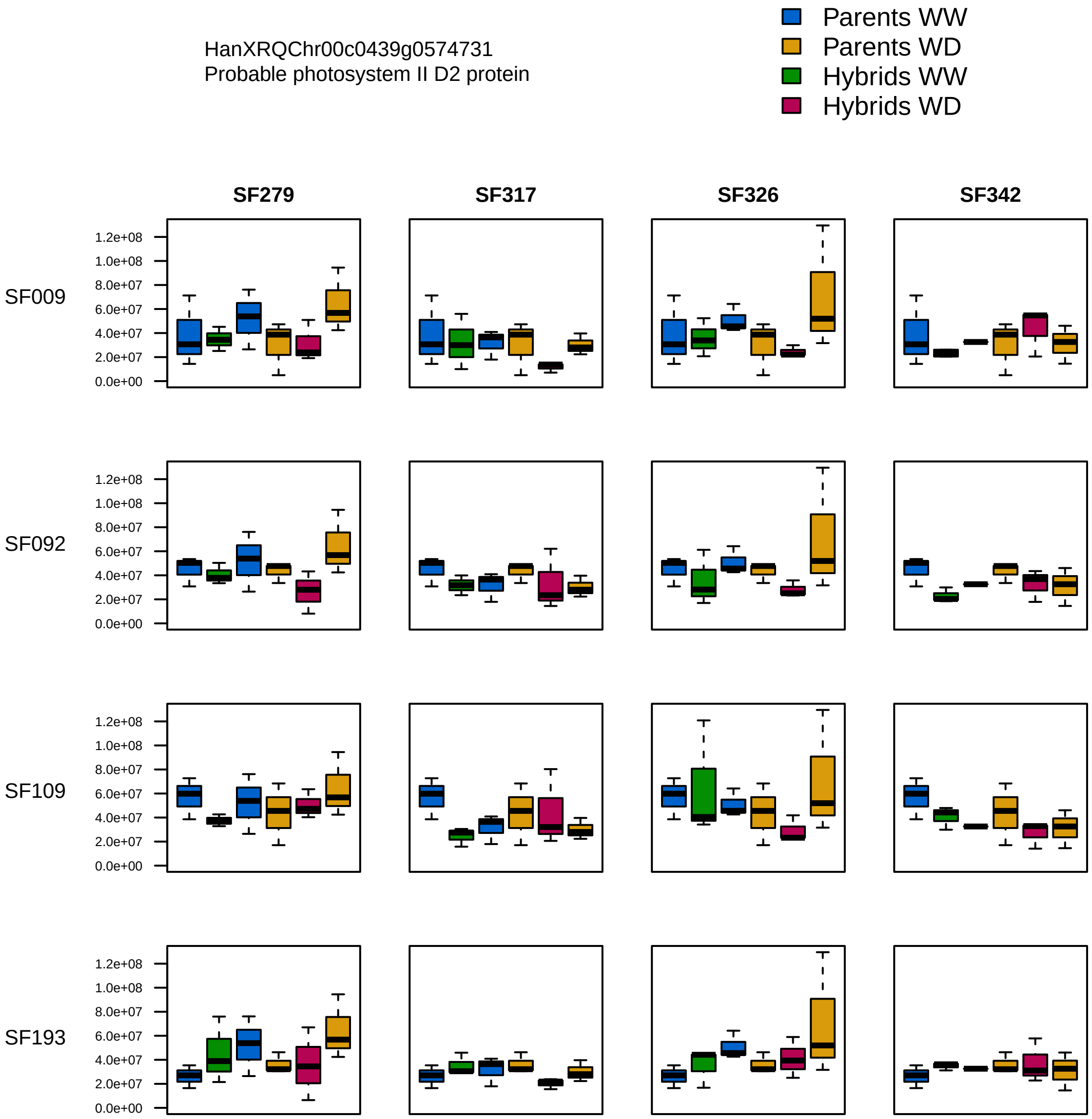
HanXRQChr00c0439g0574731
Probable photosystem II D2 protein
Parents WW
Parents WD
Hybrids WW
Hybrids WD
SF279
SF317
SF326
SF342
SF009
SF092
SF109
SF193
1.2e+08
1.0e+08
8.0e+07
6.0e+07
4.0e+07
2.0e+07
0.0e+00

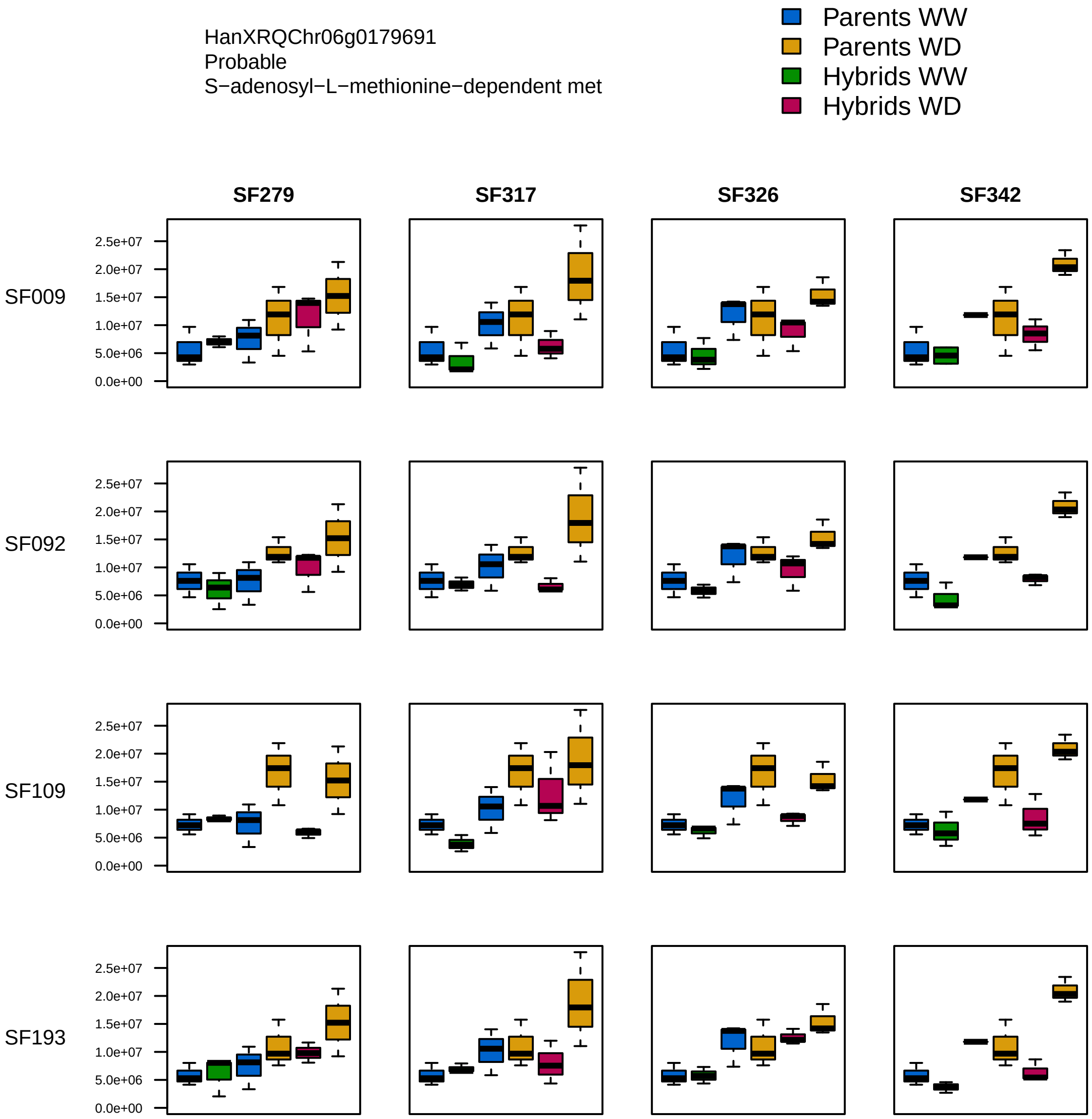

HanXRQChr06g0179691
Probable
S−adenosyl−L−methionine−dependent met
Parents WW
Parents WD
Hybrids WW
Hybrids WD
SF279
SF317
SF326
SF342
SF009
SF092
SF109
SF193
2.5e+07
2.0e+07
1.5e+07
1.0e+07
5.0e+06
0.0e+00

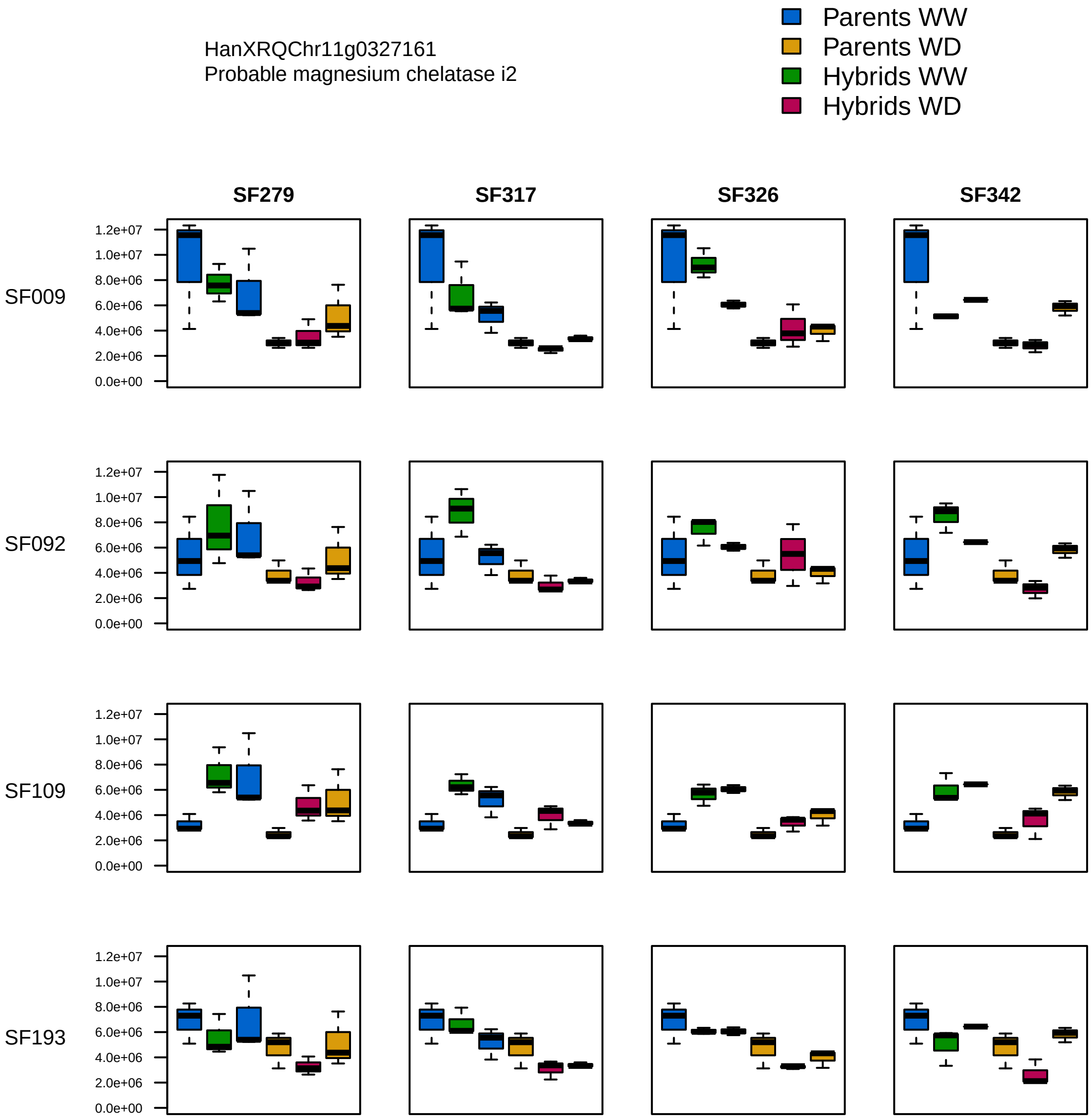

HanXRQChr11g0327161
Probable magnesium chelatase i2
Parents WW
Parents WD
Hybrids WW
Hybrids WD
SF279
SF317
SF326
SF342
SF009
SF092
SF109
SF193
0.0e+00
2.0e+06
4.0e+06
6.0e+06
8.0e+06
1.0e+07
1.2e+07

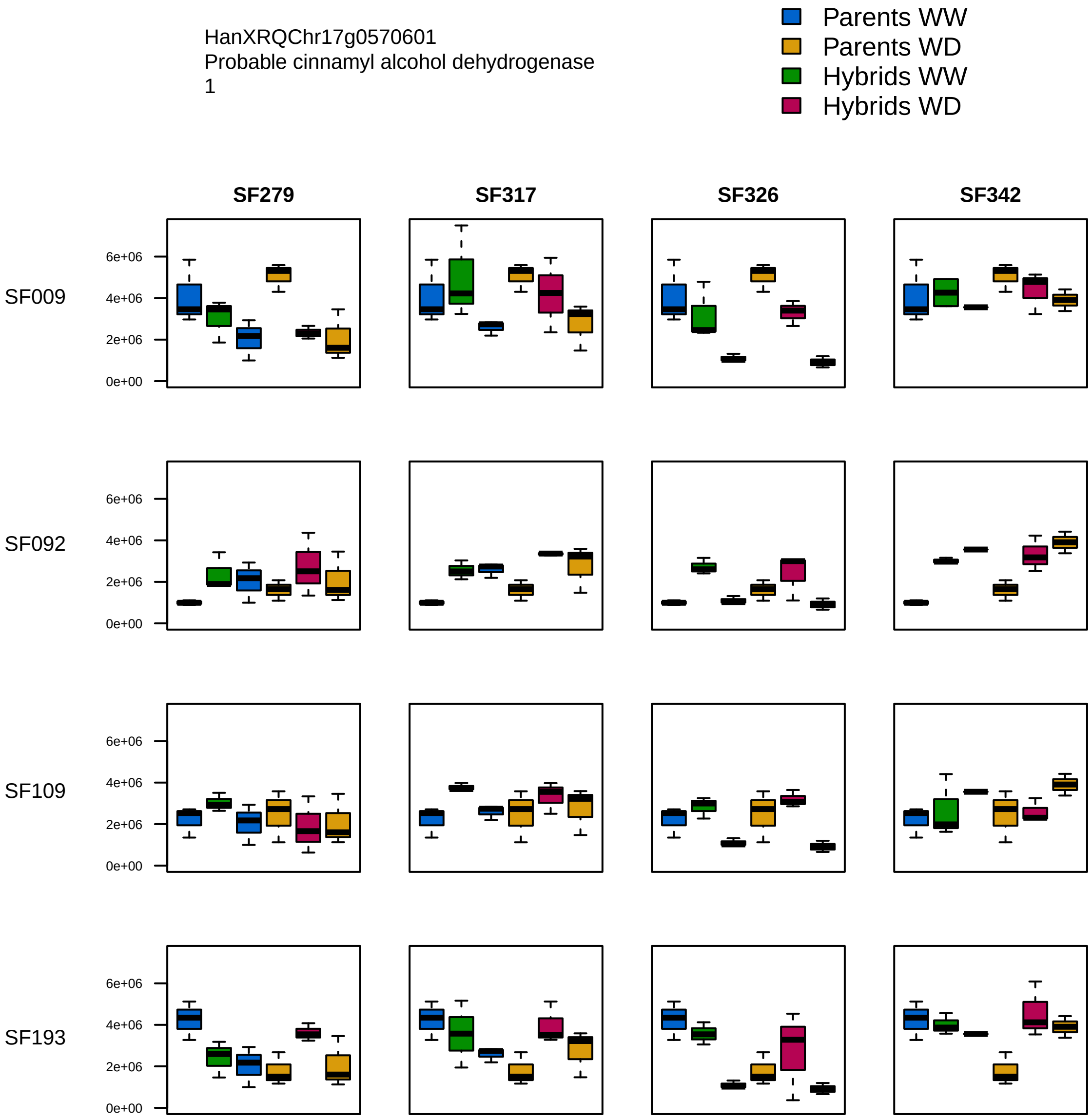
HanXRQChr17g0570601
Probable cinnamyl alcohol dehydrogenase
1
Parents WW
Parents WD
Hybrids WW
Hybrids WD
SF279
SF317
SF326
SF342
SF009
SF092
SF109
SF193
0e+00
2e+06
4e+06
6e+06

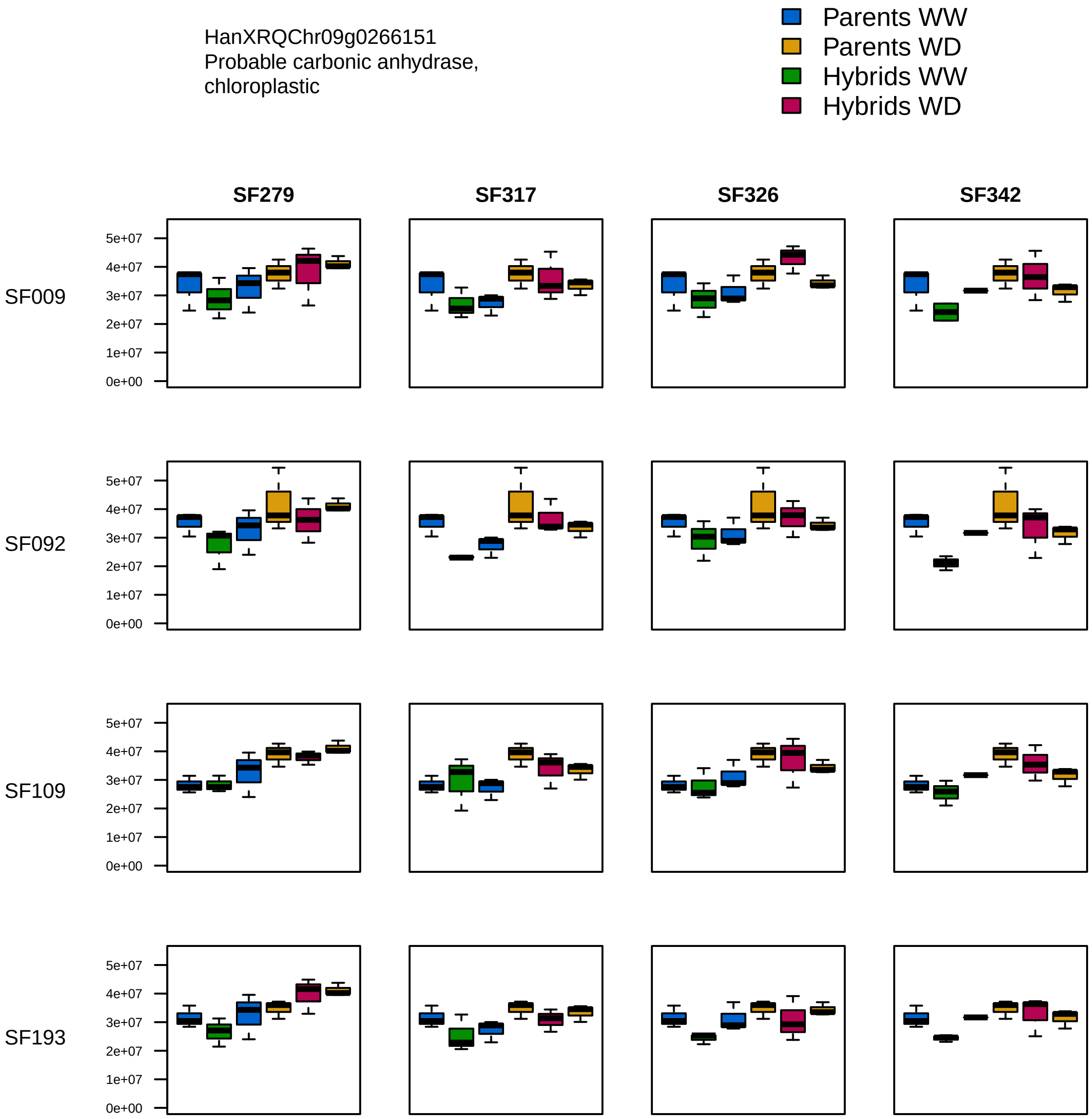
HanXRQChr09g0266151
Probable carbonic anhydrase, chloroplastic
Parents WW
Parents WD
Hybrids WW
Hybrids WD
SF279
SF317
SF326
SF342
SF009
SF092
SF109
SF193
0e+00
1e+07
2e+07
3e+07
4e+07
5e+07

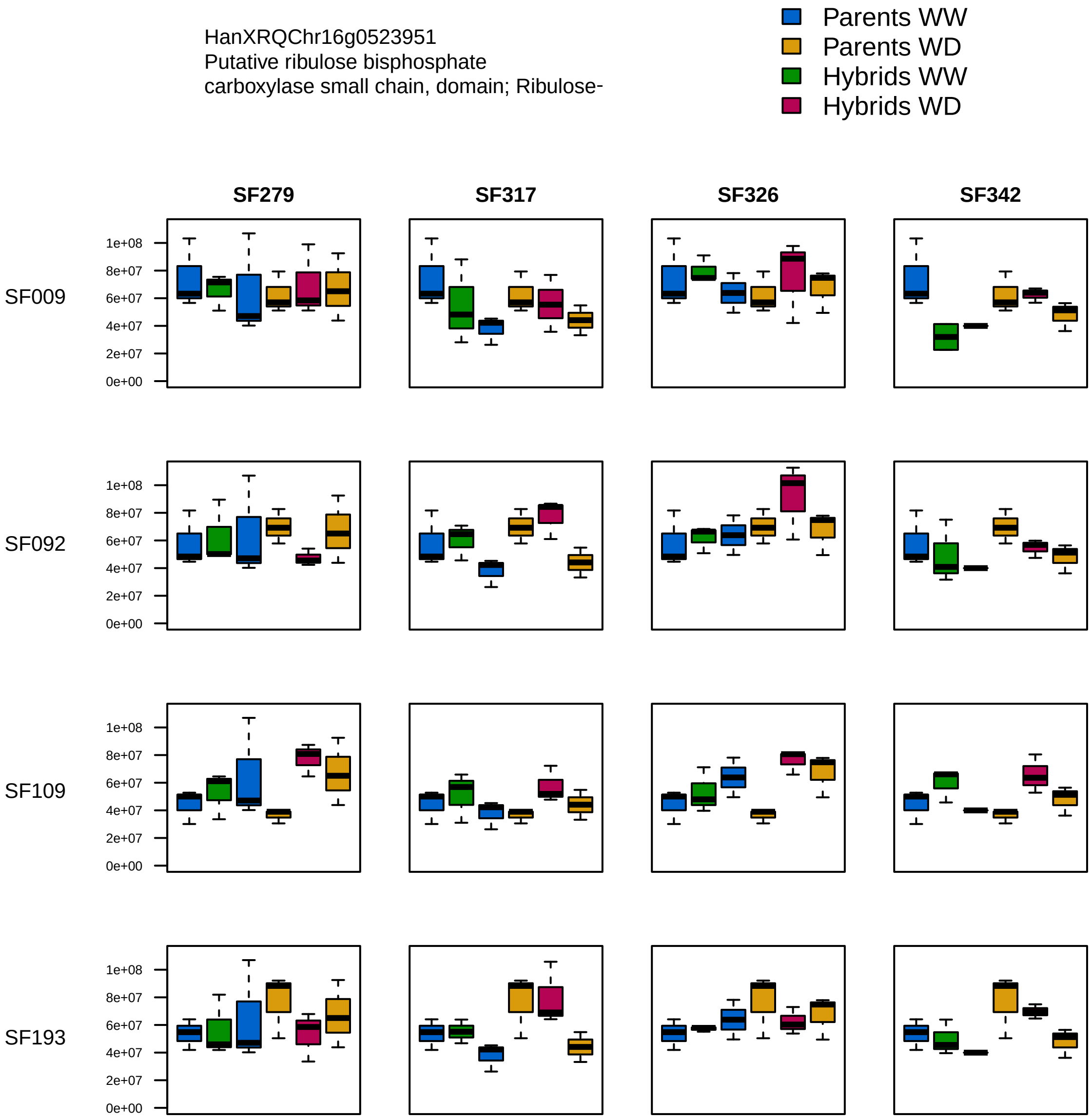
HanXRQChr16g0523951
Putative ribulose bisphosphate
carboxylase small chain, domain; Ribulose-
Parents WW
Parents WD
Hybrids WW
Hybrids WD
SF279
SF317
SF326
SF342
SF009
SF092
SF109
SF193
1e+08
8e+07
6e+07
4e+07
2e+07
0e+00

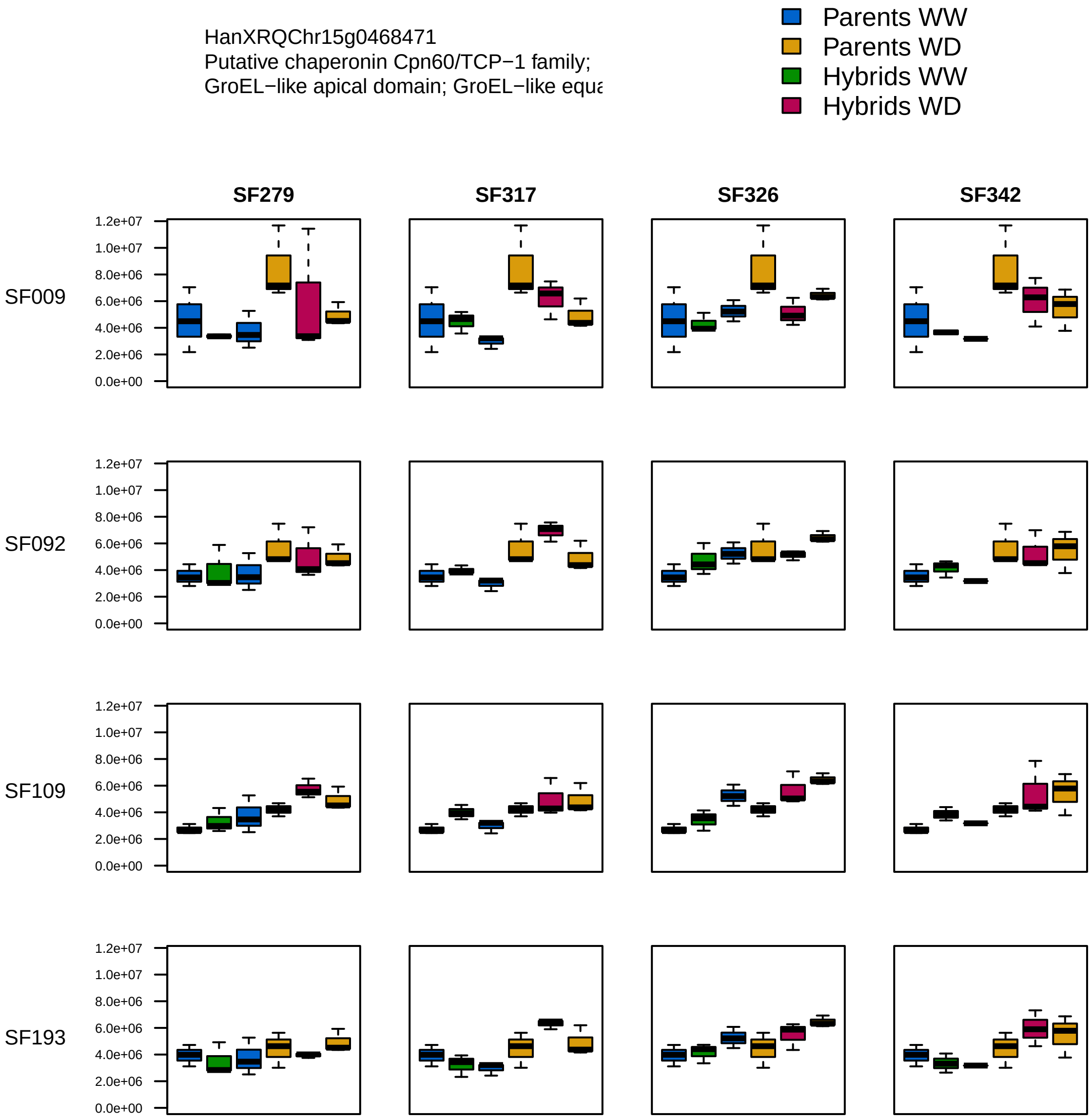
HanXRQChr15g0468471
Putative chaperonin Cpn60/TCP−1 family;
GroEL−like apical domain; GroEL−like equa
Parents WW
Parents WD
Hybrids WW
Hybrids WD
SF279
SF317
SF326
SF342
SF009
SF092
SF109
SF193
1.2e+07
1.0e+07
8.0e+06
6.0e+06
4.0e+06
2.0e+06
0.0e+00

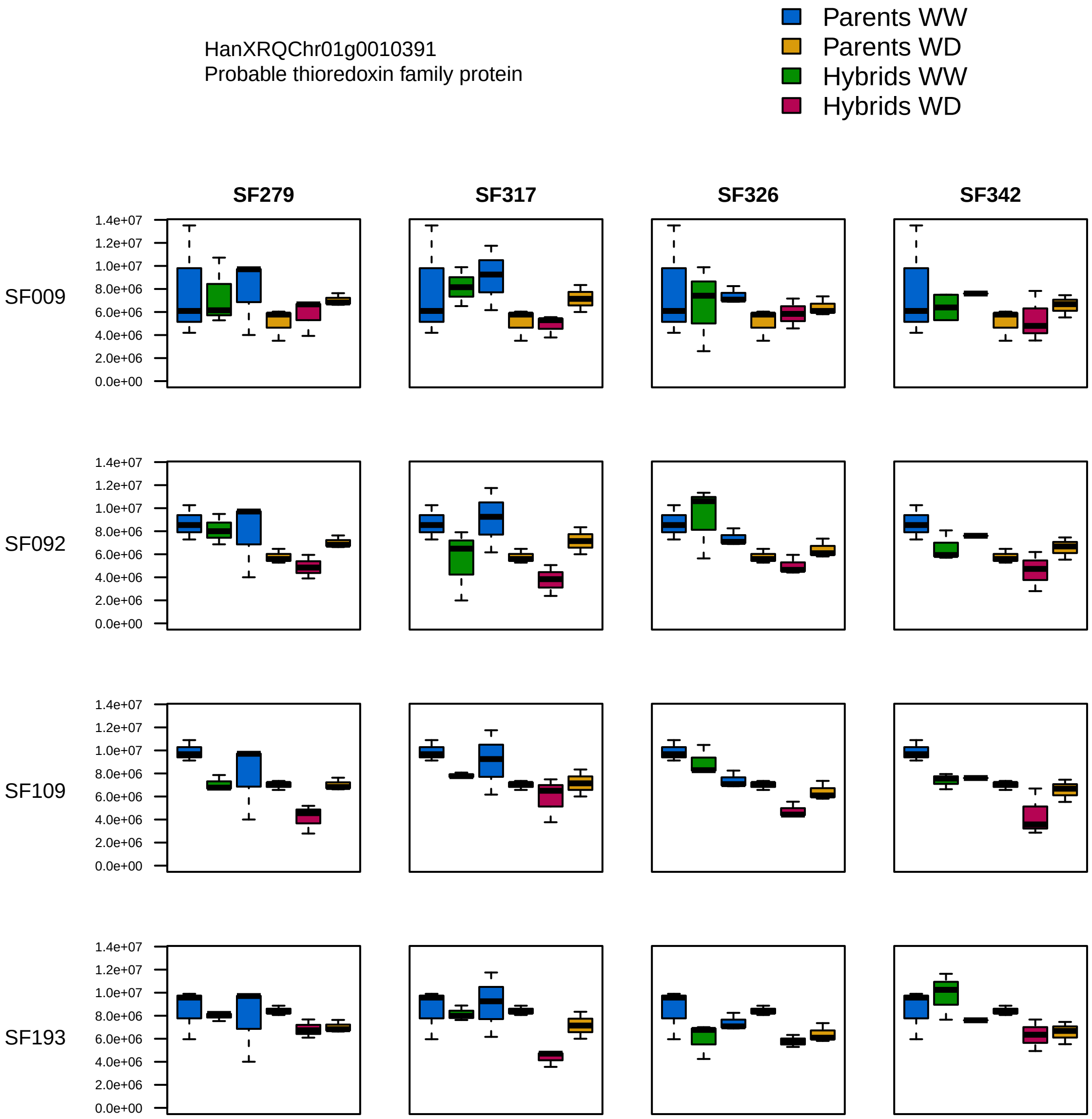
HanXRQChr01g0010391
Probable thioredoxin family protein
Parents WW
Parents WD
Hybrids WW
Hybrids WD
SF279
SF317
SF326
SF342
SF009
SF092
SF109
SF193
1.4e+07
1.2e+07
1.0e+07
8.0e+06
6.0e+06
4.0e+06
2.0e+06
0.0e+00

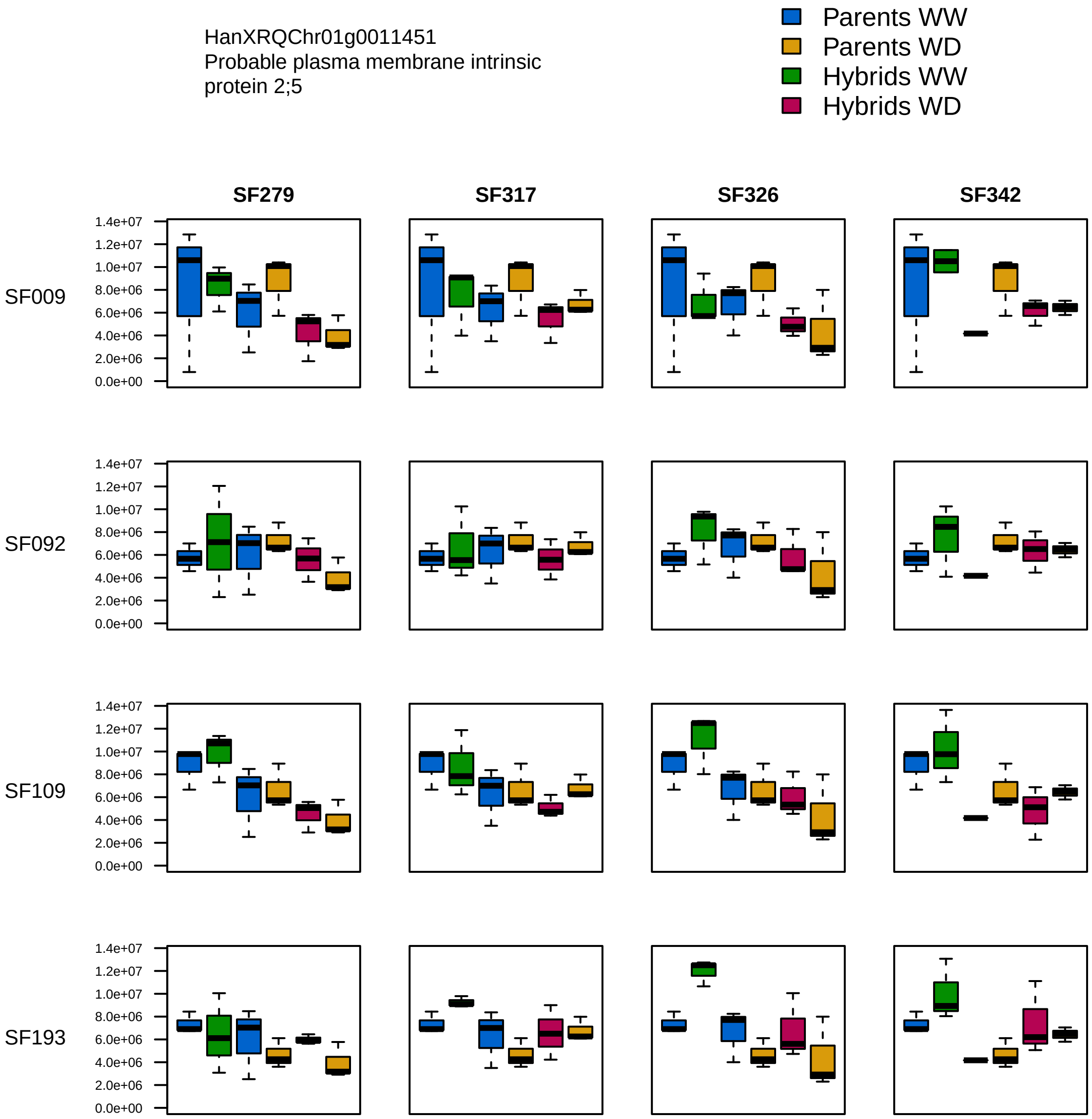
HanXRQChr01g0011451
Probable plasma membrane intrinsic protein 2;5
Parents WW
Parents WD
Hybrids WW
Hybrids WD
SF279
SF317
SF326
SF342
SF009
SF092
SF109
SF193
1.4e+07
1.2e+07
1.0e+07
8.0e+06
6.0e+06
4.0e+06
2.0e+06
0.0e+00

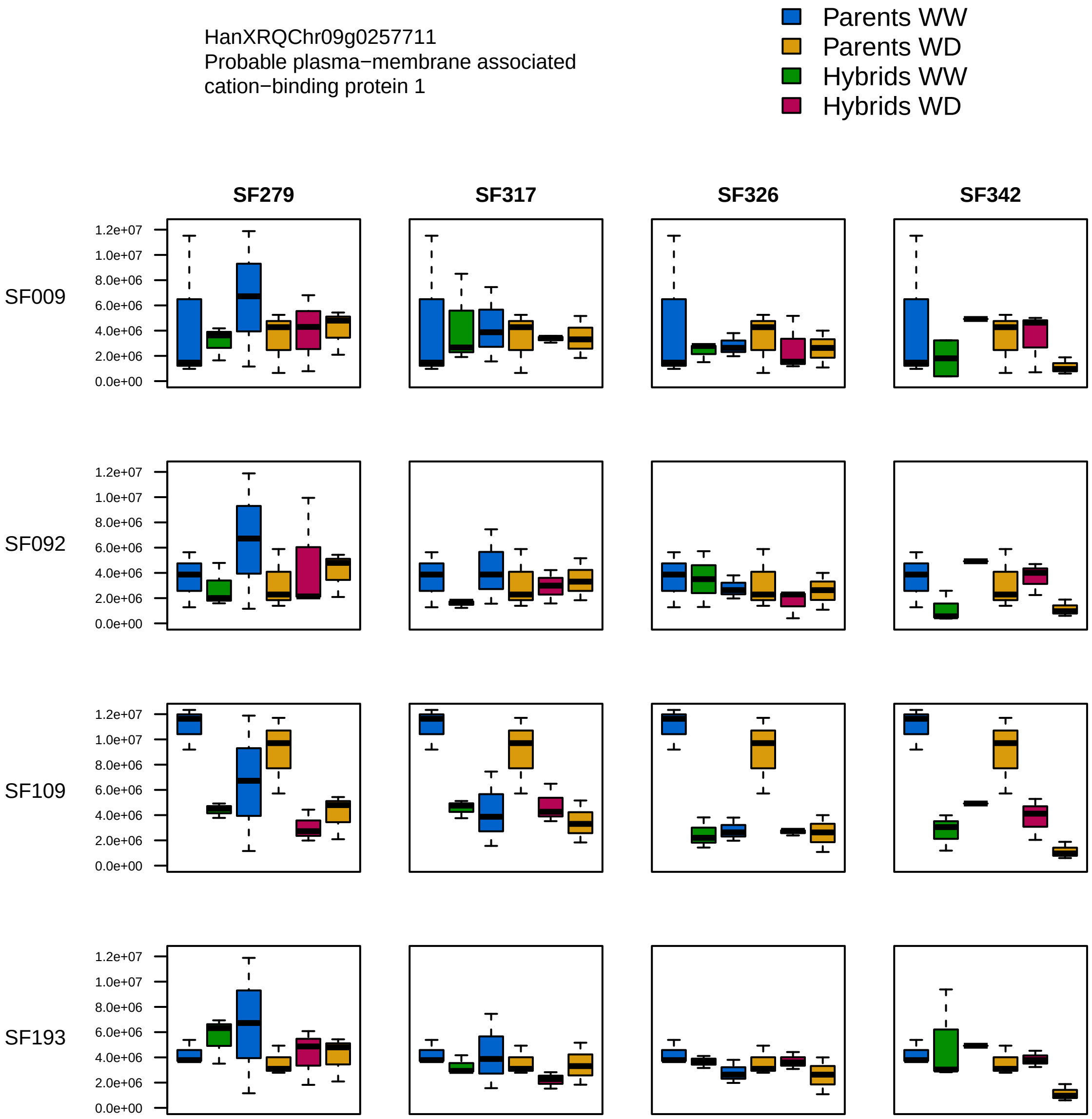
HanXRQChr09g0257711
Probable plasma−membrane associated
cation−binding protein 1
Parents WW
Parents WD
Hybrids WW
Hybrids WD
SF279
SF317
SF326
SF342
SF009
SF092
SF109
SF193
1.2e+07
1.0e+07
8.0e+06
6.0e+06
4.0e+06
2.0e+06
0.0e+00

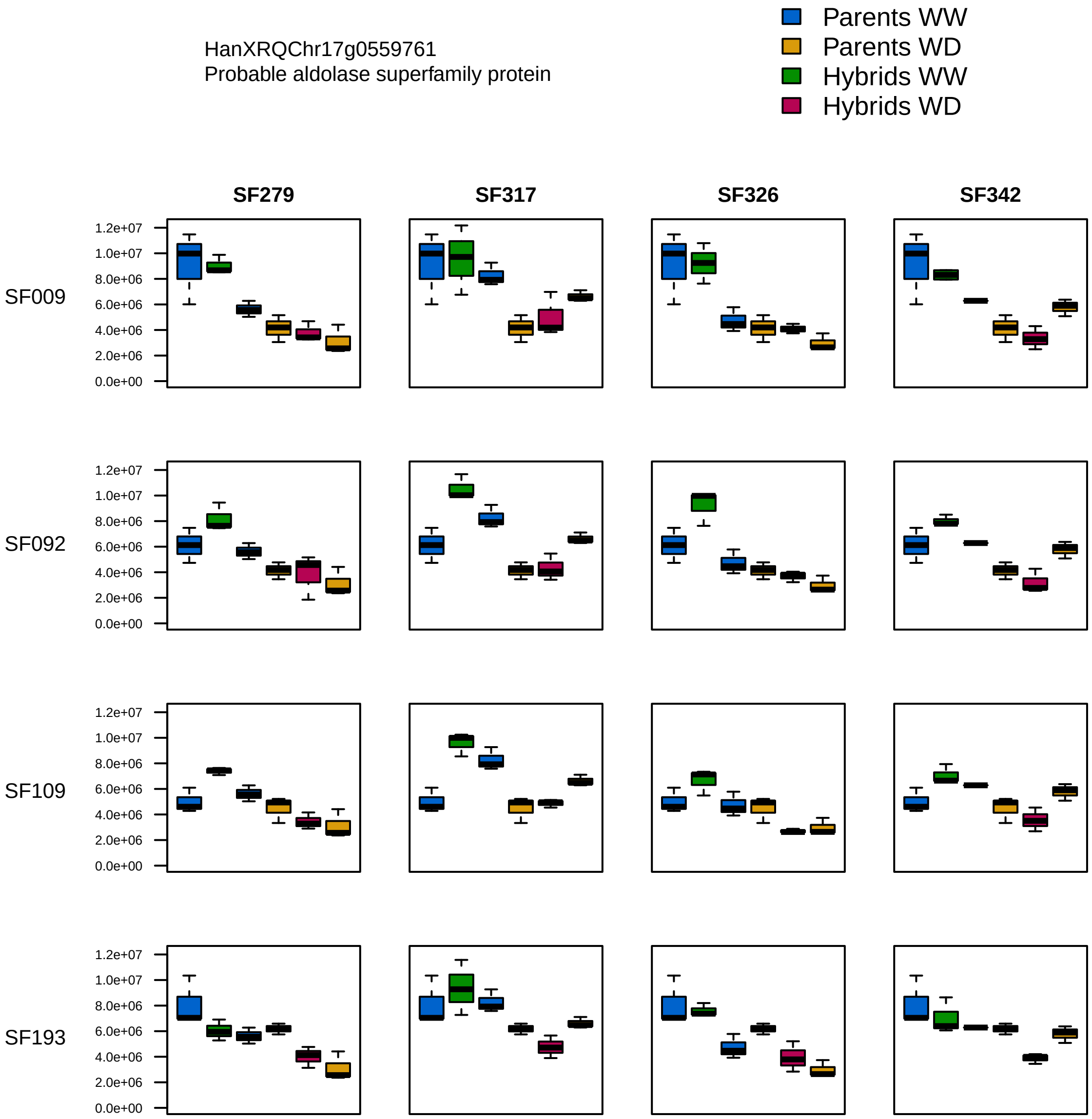

HanXRQChr17g0559761
Probable aldolase superfamily protein
Parents WW
Parents WD
Hybrids WW
Hybrids WD
SF279
SF317
SF326
SF342
SF009
SF092
SF109
SF193
1.2e+07
1.0e+07
8.0e+06
6.0e+06
4.0e+06
2.0e+06
0.0e+00

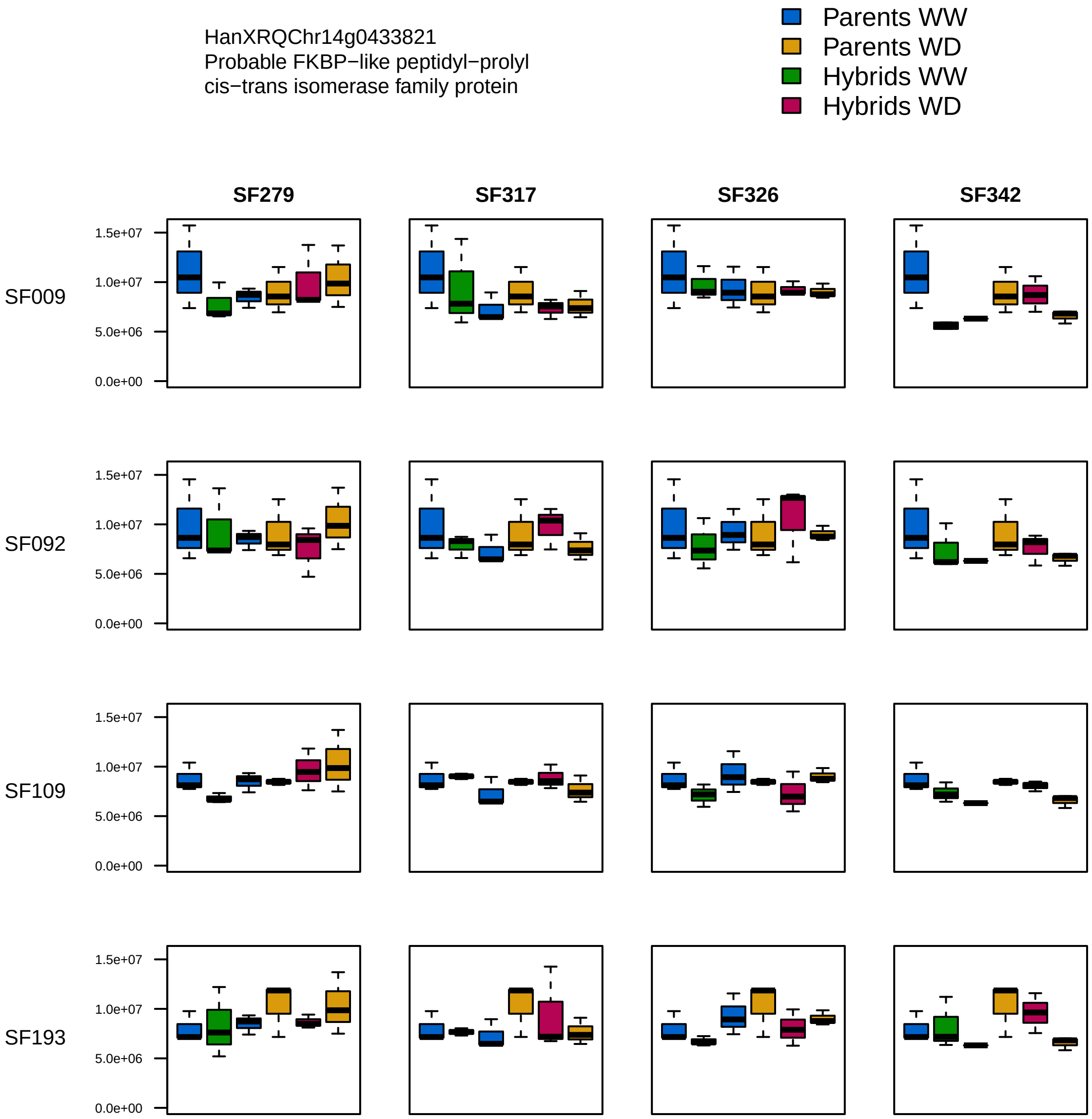
HanXRQChr14g0433821
Probable FKBP−like peptidyl−prolyl
cis−trans isomerase family protein
Parents WW
Parents WD
Hybrids WW
Hybrids WD
SF279
SF317
SF326
SF342
SF009
SF092
SF109
SF193
1.5e+07
1.0e+07
5.0e+06
0.0e+00

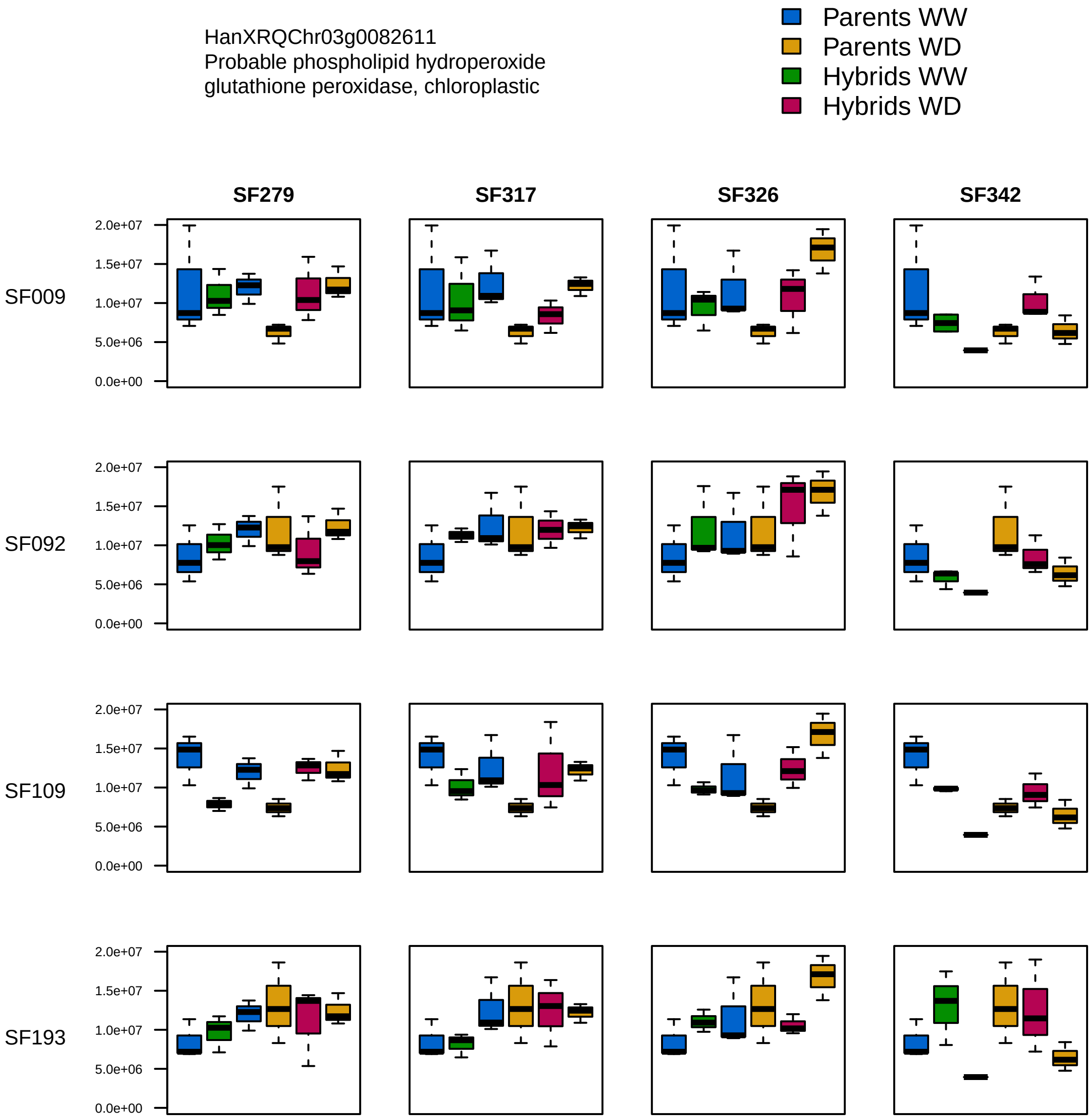

HanXRQChr03g0082611
Probable phospholipid hydroperoxide
glutathione peroxidase, chloroplastic
Parents WW
Parents WD
Hybrids WW
Hybrids WD
SF279
SF317
SF326
SF342
SF009
SF092
SF109
SF193
2.0e+07
1.5e+07
1.0e+07
5.0e+06
0.0e+00

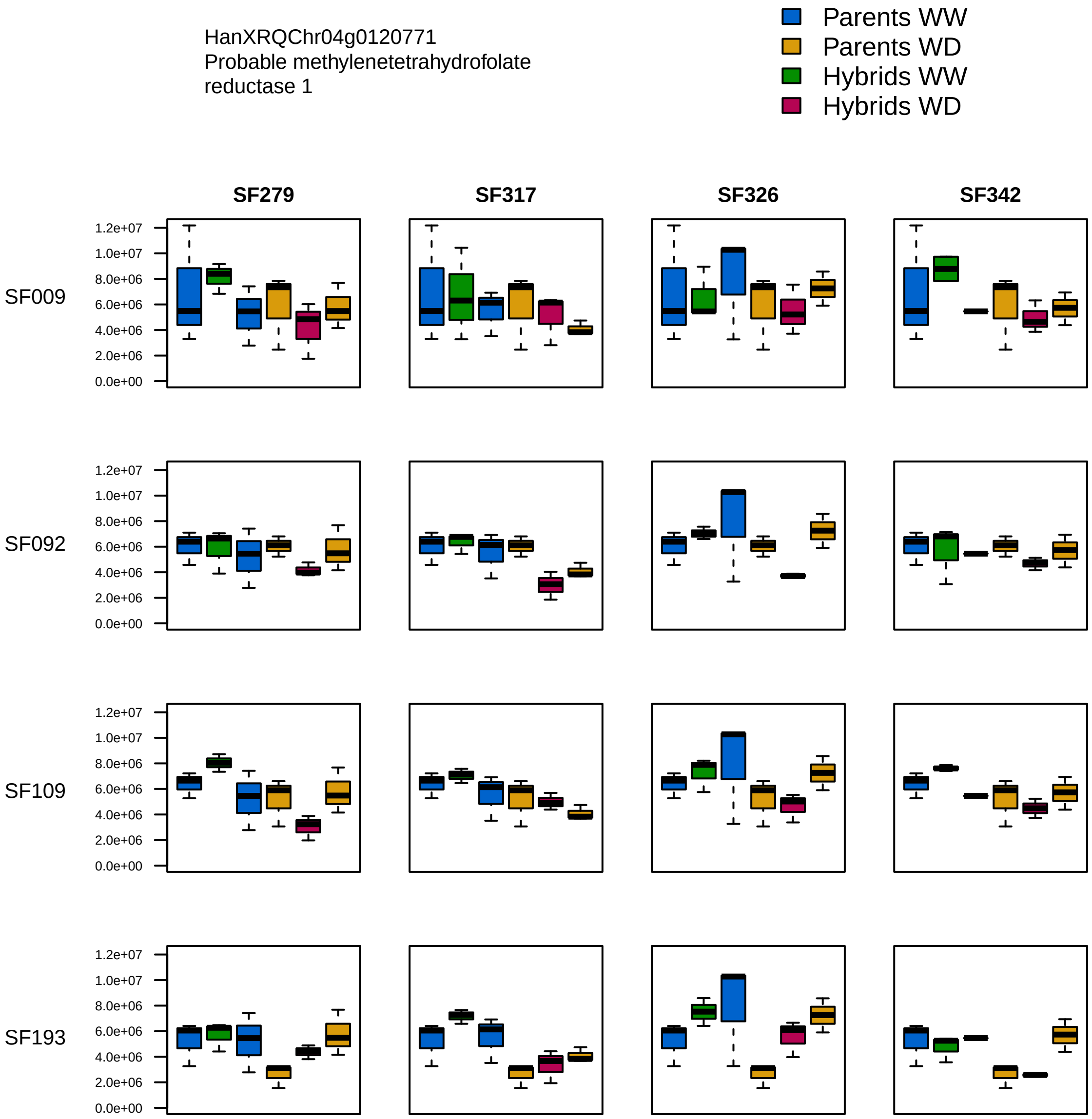
HanXRQChr04g0120771
Probable methylenetetrahydrofolate
reductase 1
Parents WW
Parents WD
Hybrids WW
Hybrids WD
SF279
SF317
SF326
SF342
SF009
SF092
SF109
SF193
1.2e+07
1.0e+07
8.0e+06
6.0e+06
4.0e+06
2.0e+06
0.0e+00

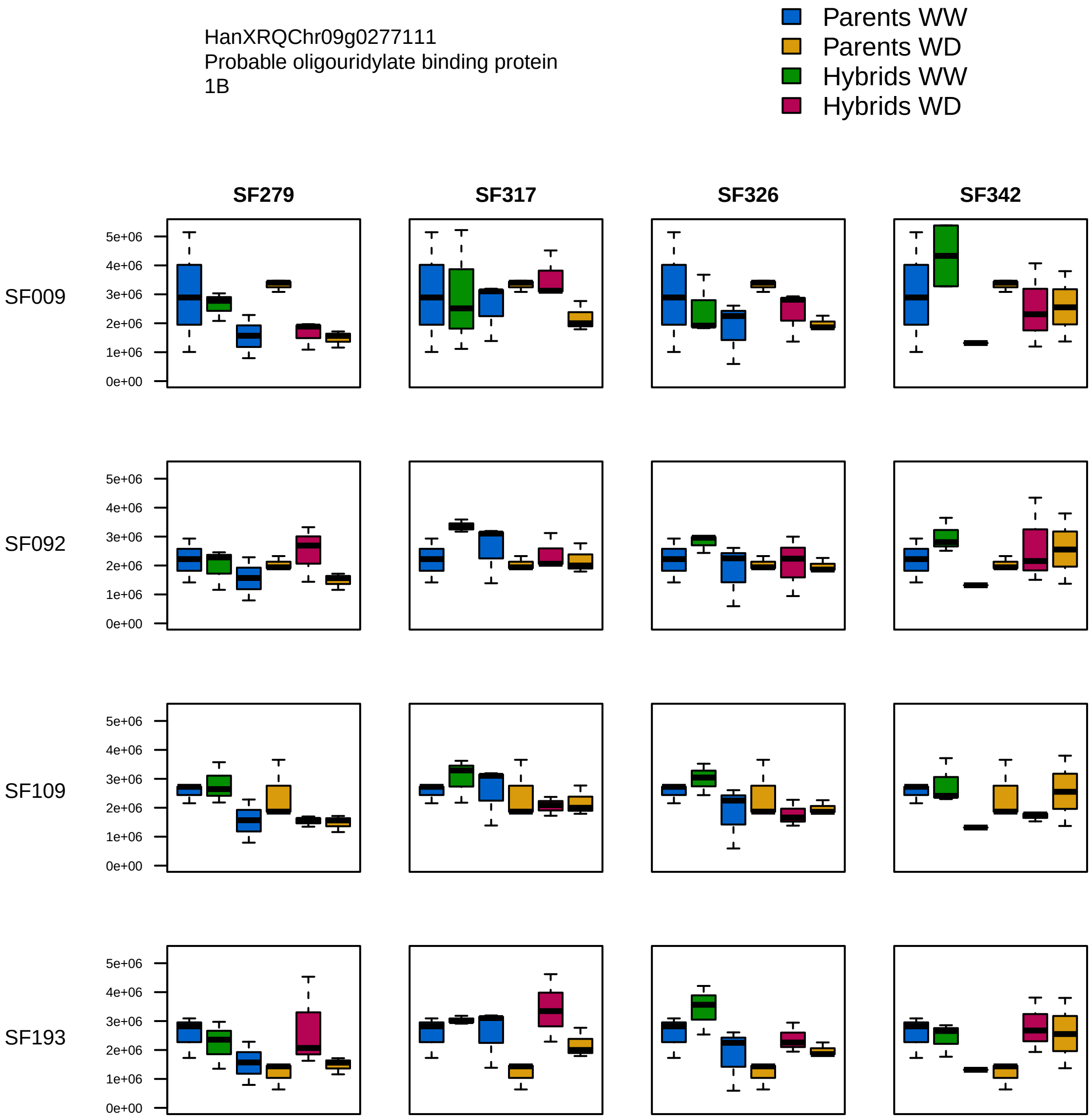

HanXRQChr09g0277111
Probable oligouridylate binding protein
1B
Parents WW
Parents WD
Hybrids WW
Hybrids WD
SF279
SF317
SF326
SF342
SF009
SF092
SF109
SF193
5e+06
4e+06
3e+06
2e+06
1e+06
0e+00

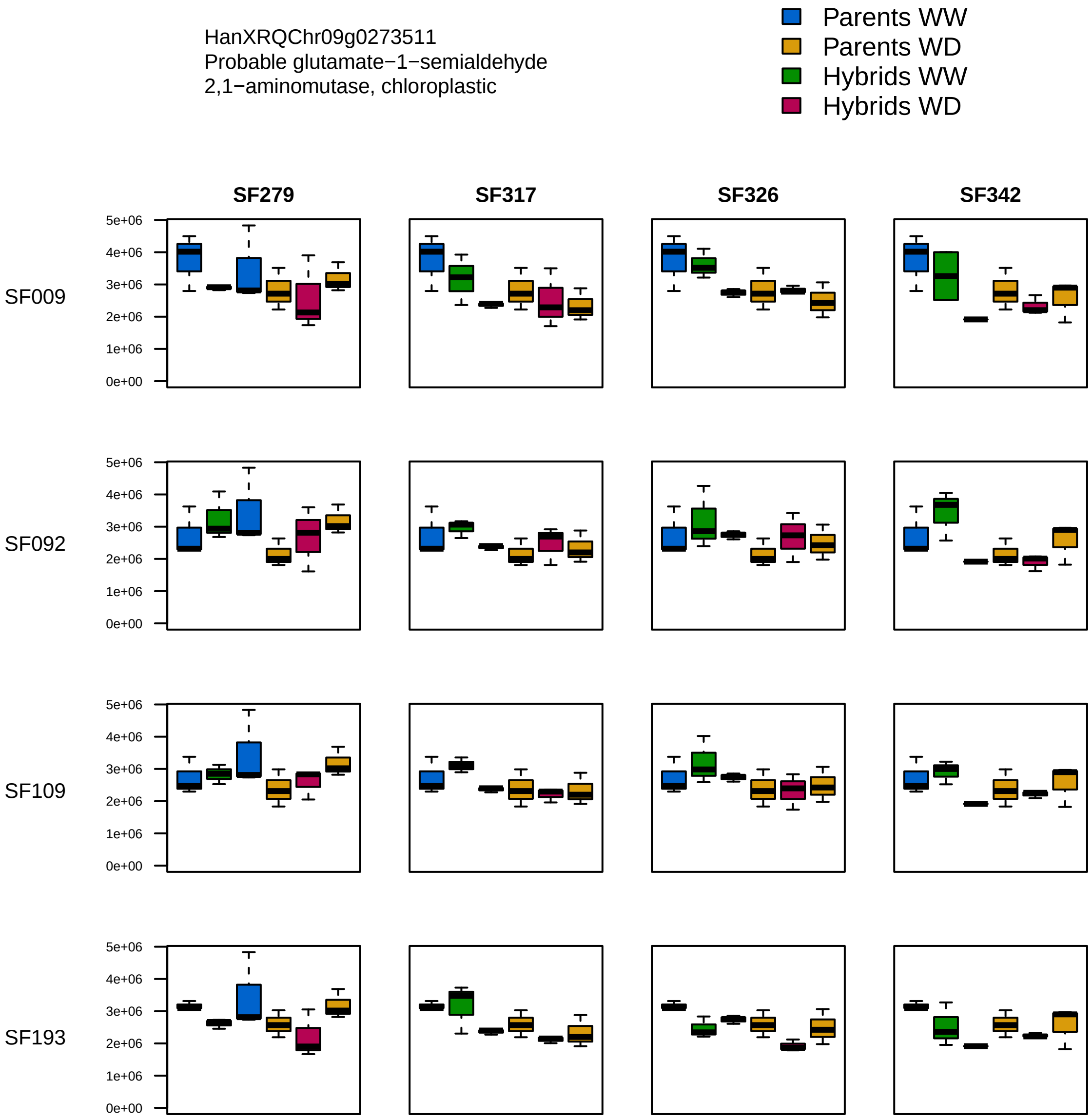
HanXRQChr09g0273511
Probable glutamate−1−semialdehyde
2,1−aminomutase, chloroplastic
Parents WW
Parents WD
Hybrids WW
Hybrids WD
SF279
SF317
SF326
SF342
SF009
SF092
SF109
SF193
5e+06
4e+06
3e+06
2e+06
1e+06
0e+00

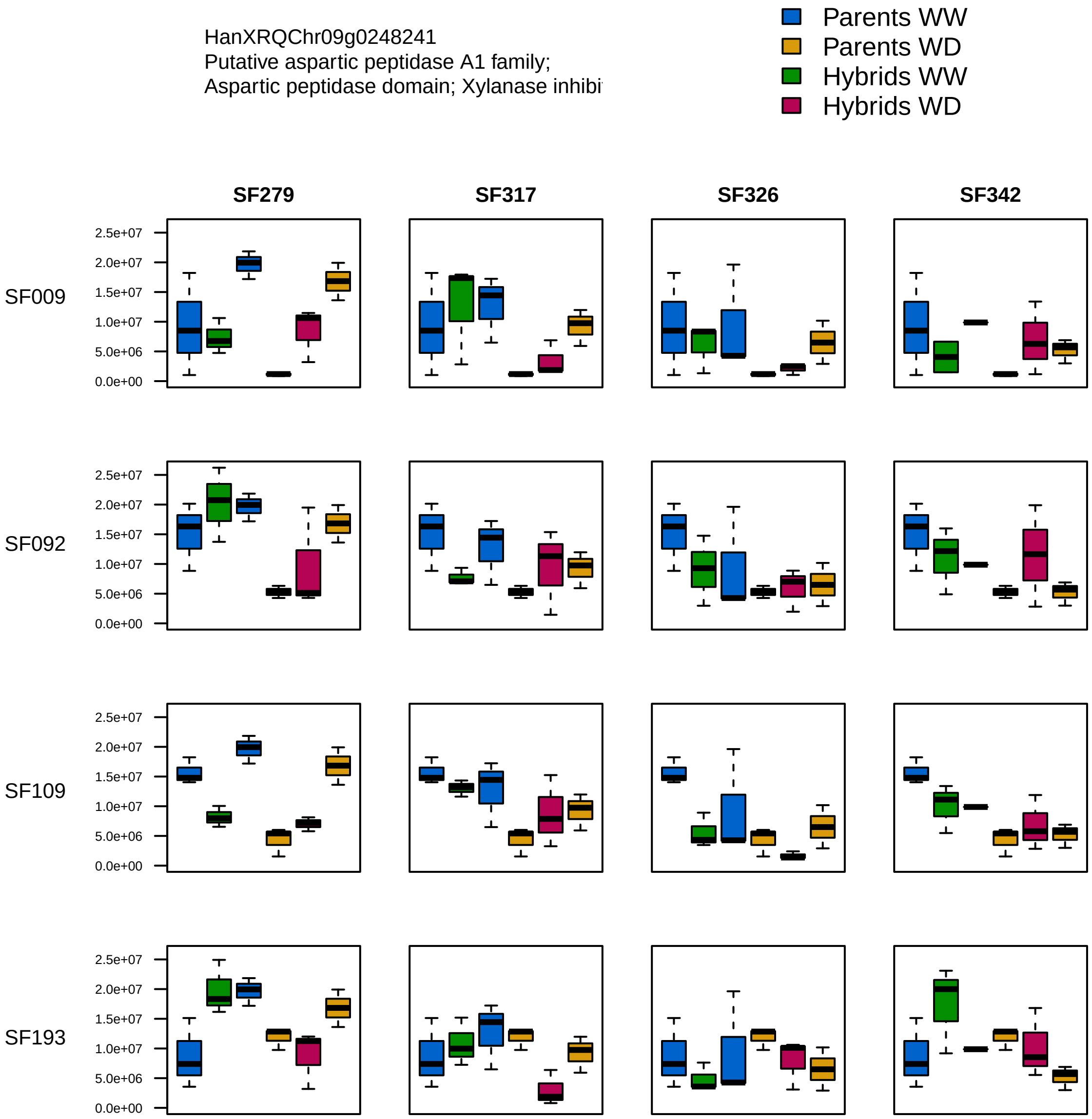
HanXRQChr09g0248241
Putative aspartic peptidase A1 family;
Aspartic peptidase domain; Xylanase inhibi
Parents WW
Parents WD
Hybrids WW
Hybrids WD
SF279
SF317
SF326
SF342
SF009
SF092
SF109
SF193
2.5e+07
2.0e+07
1.5e+07
1.0e+07
5.0e+06
0.0e+00

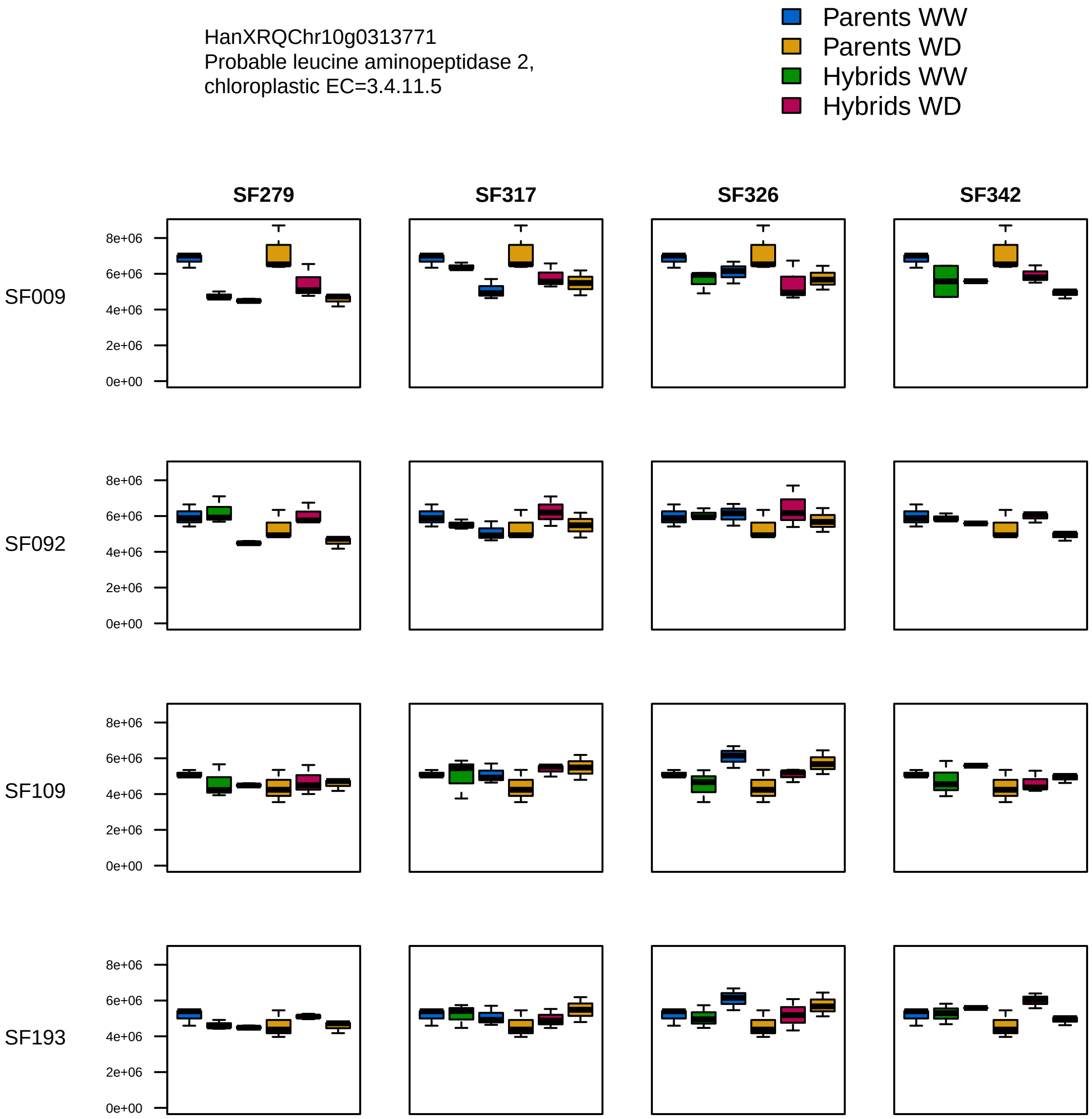
HanXRQChr10g0313771
Probable leucine aminopeptidase 2,
chloroplastic EC=3.4.11.5
Parents WW
Parents WD
Hybrids WW
Hybrids WD
SF279
SF317
SF326
SF342
SF009
SF092
SF109
SF193
8e+06
6e+06
4e+06
2e+06
0e+00

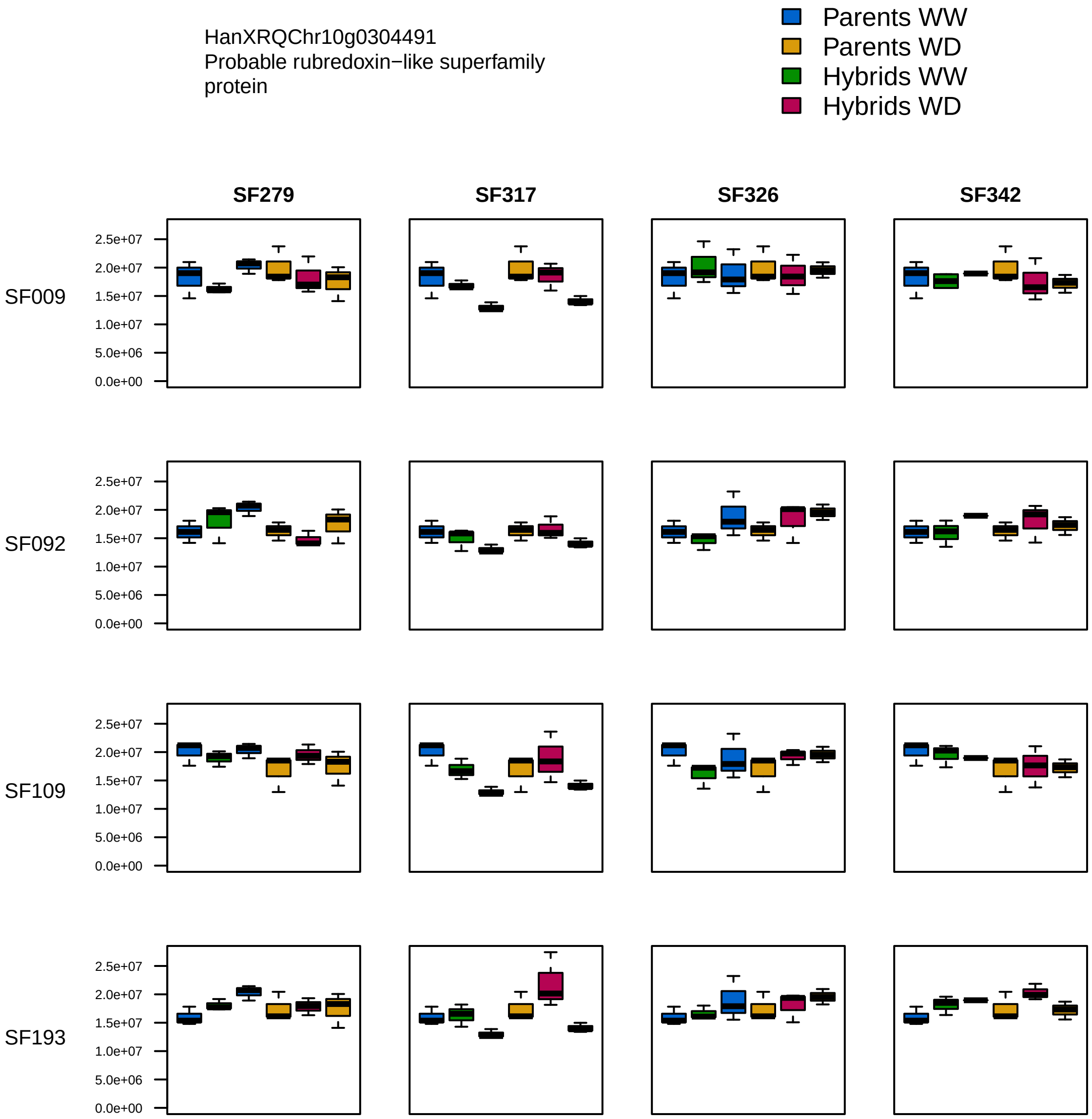

HanXRQChr10g0304491
Probable rubredoxin−like superfamily protein
Parents WW
Parents WD
Hybrids WW
Hybrids WD
SF279
SF317
SF326
SF342
SF009
SF092
SF109
SF193
2.5e+07
2.0e+07
1.5e+07
1.0e+07
5.0e+06
0.0e+00

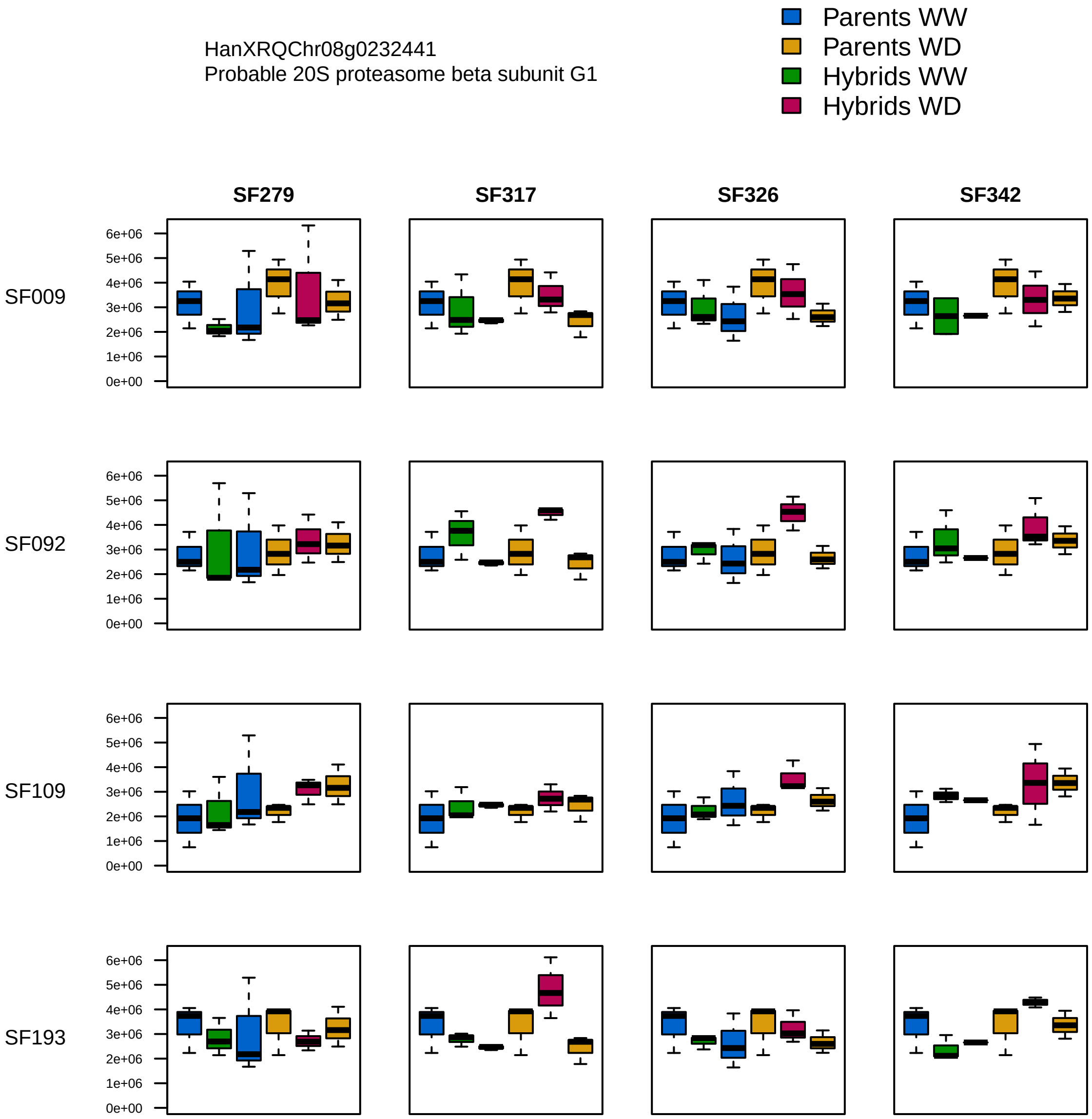
HanXRQChr08g0232441
Probable 20S proteasome beta subunit G1
Parents WW
Parents WD
Hybrids WW
Hybrids WD
SF279
SF317
SF326
SF342
SF009
SF092
SF109
SF193
0e+00
1e+06
2e+06
3e+06
4e+06
5e+06
6e+06

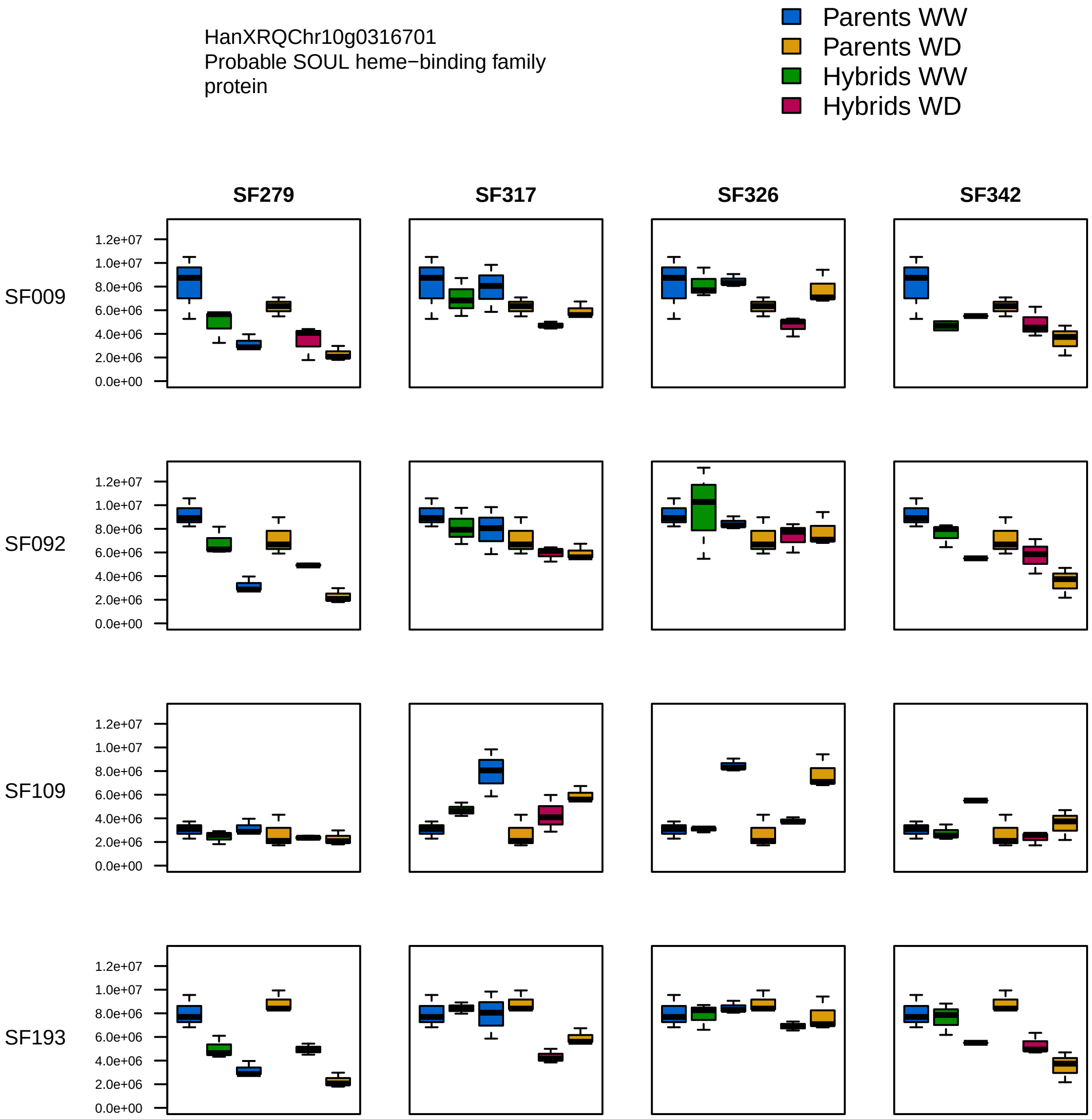
HanXRQChr10g0316701
Probable SOUL heme−binding family
protein
Parents WW
Parents WD
Hybrids WW
Hybrids WD
SF279
SF317
SF326
SF342
SF009
SF092
SF109
SF193
1.2e+07
1.0e+07
8.0e+06
6.0e+06
4.0e+06
2.0e+06
0.0e+00

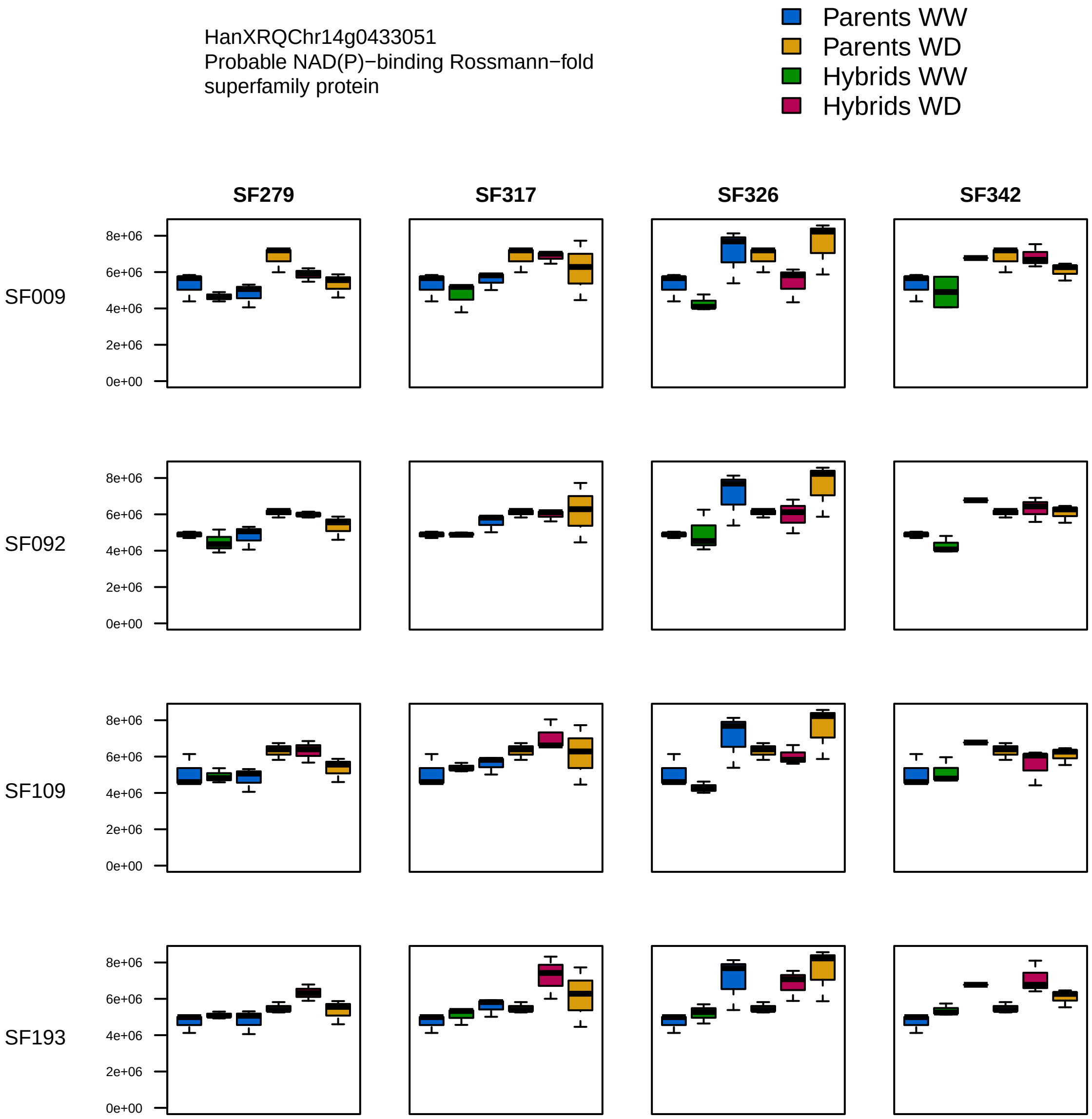
HanXRQChr14g0433051
Probable NAD(P)−binding Rossmann−fold
superfamily protein
Parents WW
Parents WD
Hybrids WW
Hybrids WD
SF279
SF317
SF326
SF342
SF009
SF092
SF109
SF193
8e+06
6e+06
4e+06
2e+06
0e+00

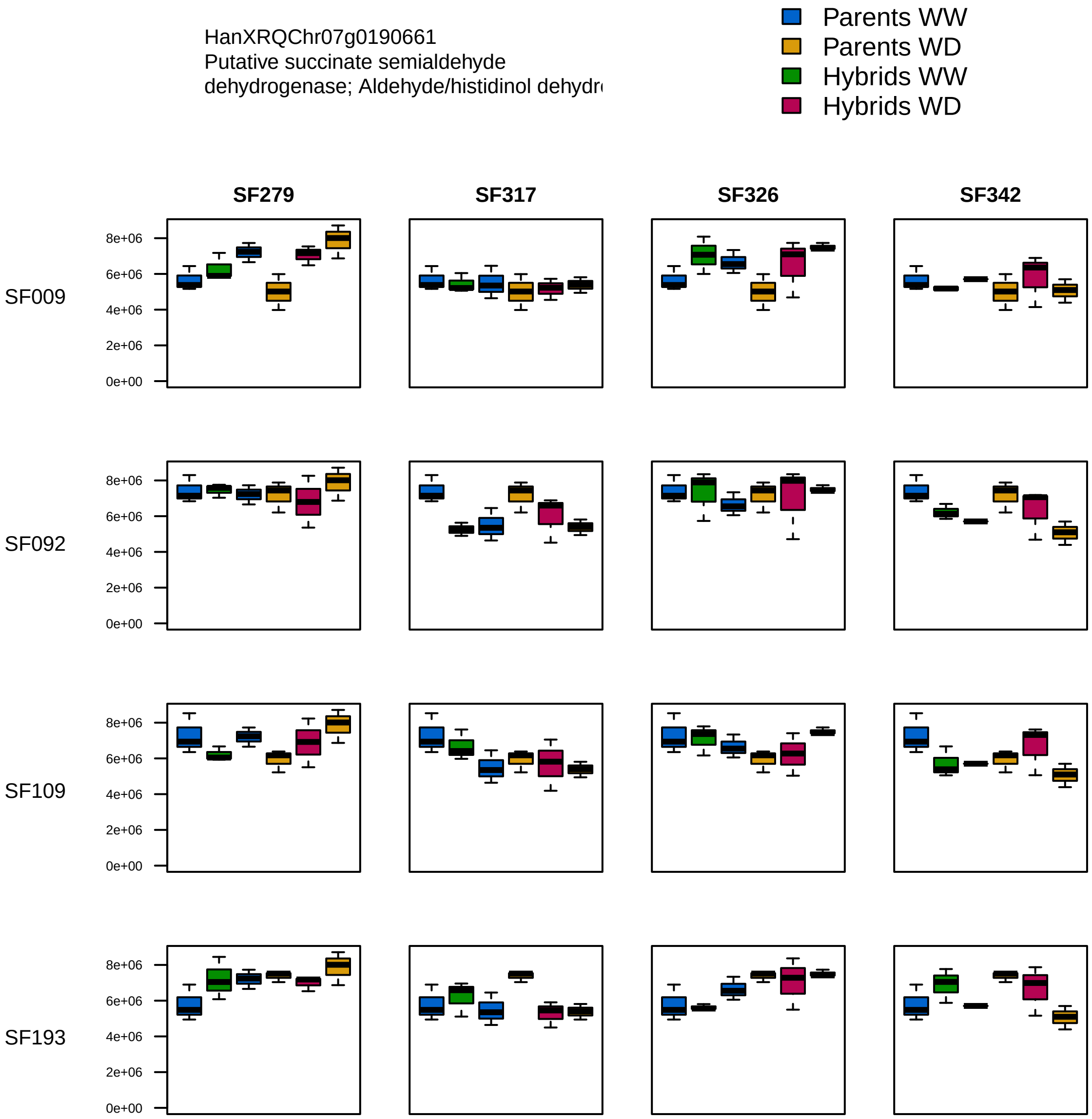

HanXRQChr07g0190661
Putative succinate semialdehyde
dehydrogenase; Aldehyde/histidinol dehydr
Parents WW
Parents WD
Hybrids WW
Hybrids WD
SF279
SF317
SF326
SF342
SF009
SF092
SF109
SF193
8e+06
6e+06
4e+06
2e+06
0e+00

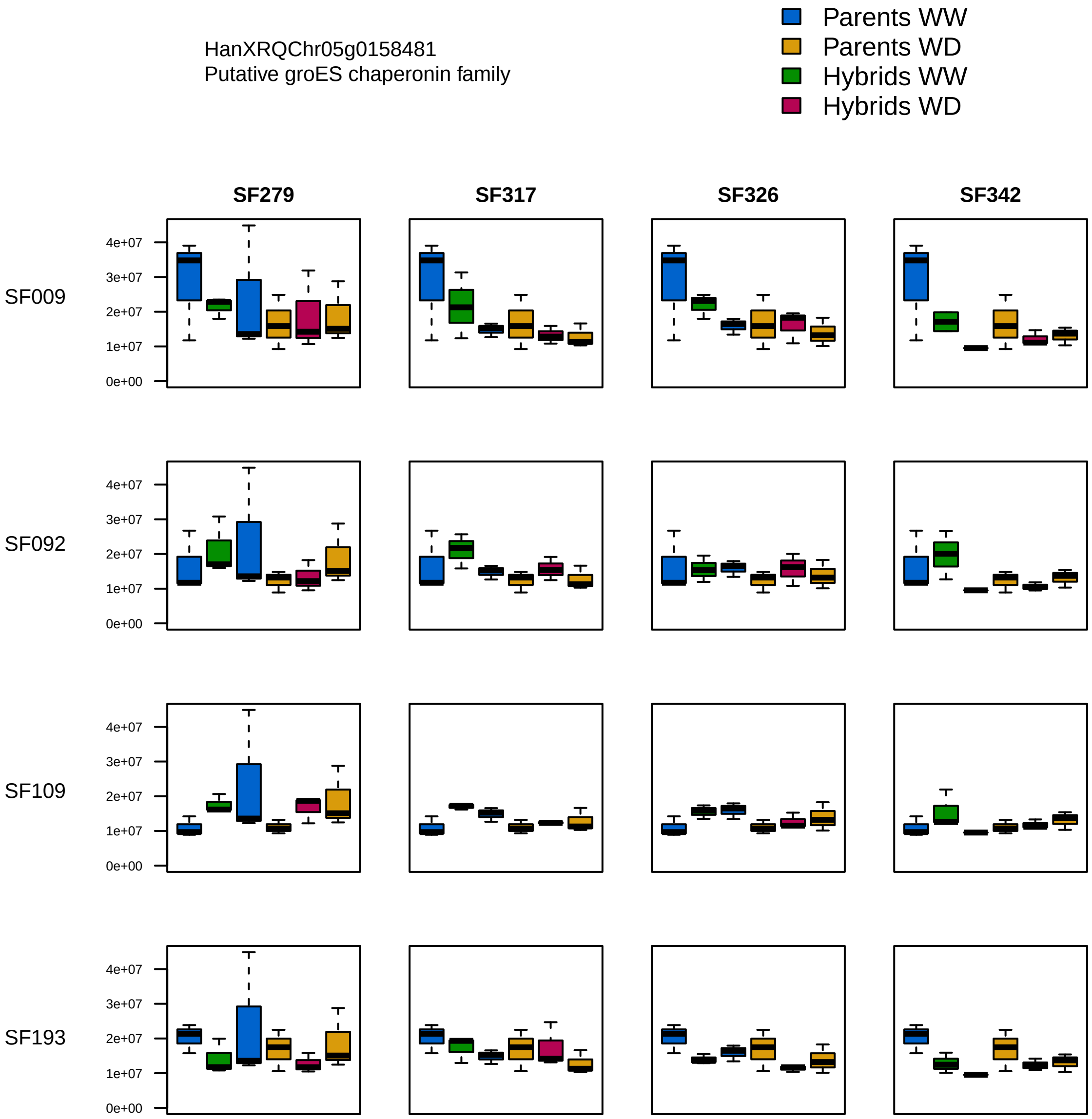
HanXRQChr05g0158481
Putative groES chaperonin family
Parents WW
Parents WD
Hybrids WW
Hybrids WD
SF279
SF317
SF326
SF342
SF009
SF092
SF109
SF193
4e+07
3e+07
2e+07
1e+07
0e+00

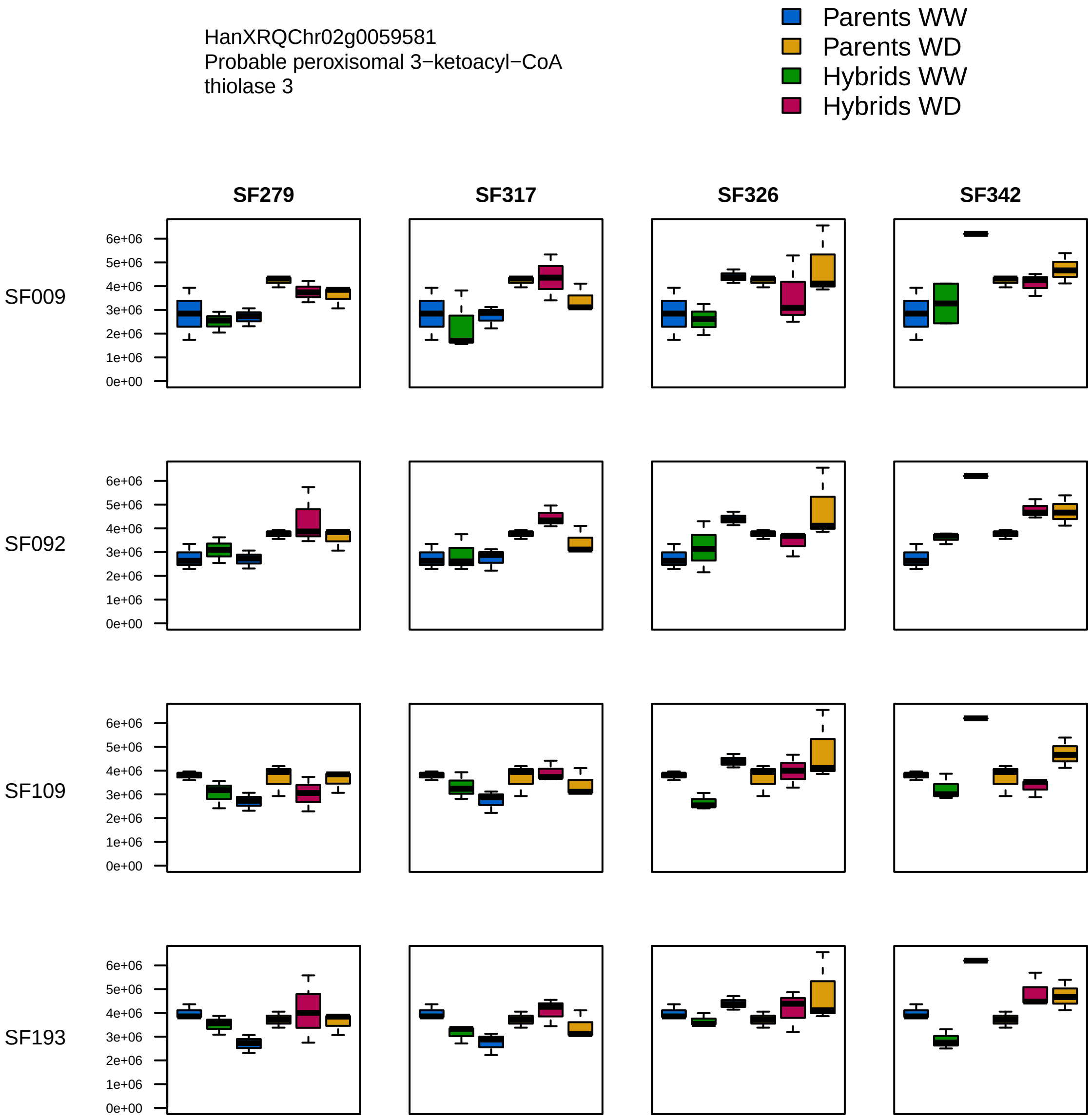

HanXRQChr02g0059581
Probable peroxisomal 3−ketoacyl−CoA
thiolase 3
Parents WW
Parents WD
Hybrids WW
Hybrids WD
SF279
SF317
SF326
SF342
SF009
SF092
SF109
SF193
0e+00
1e+06
2e+06
3e+06
4e+06
5e+06
6e+06

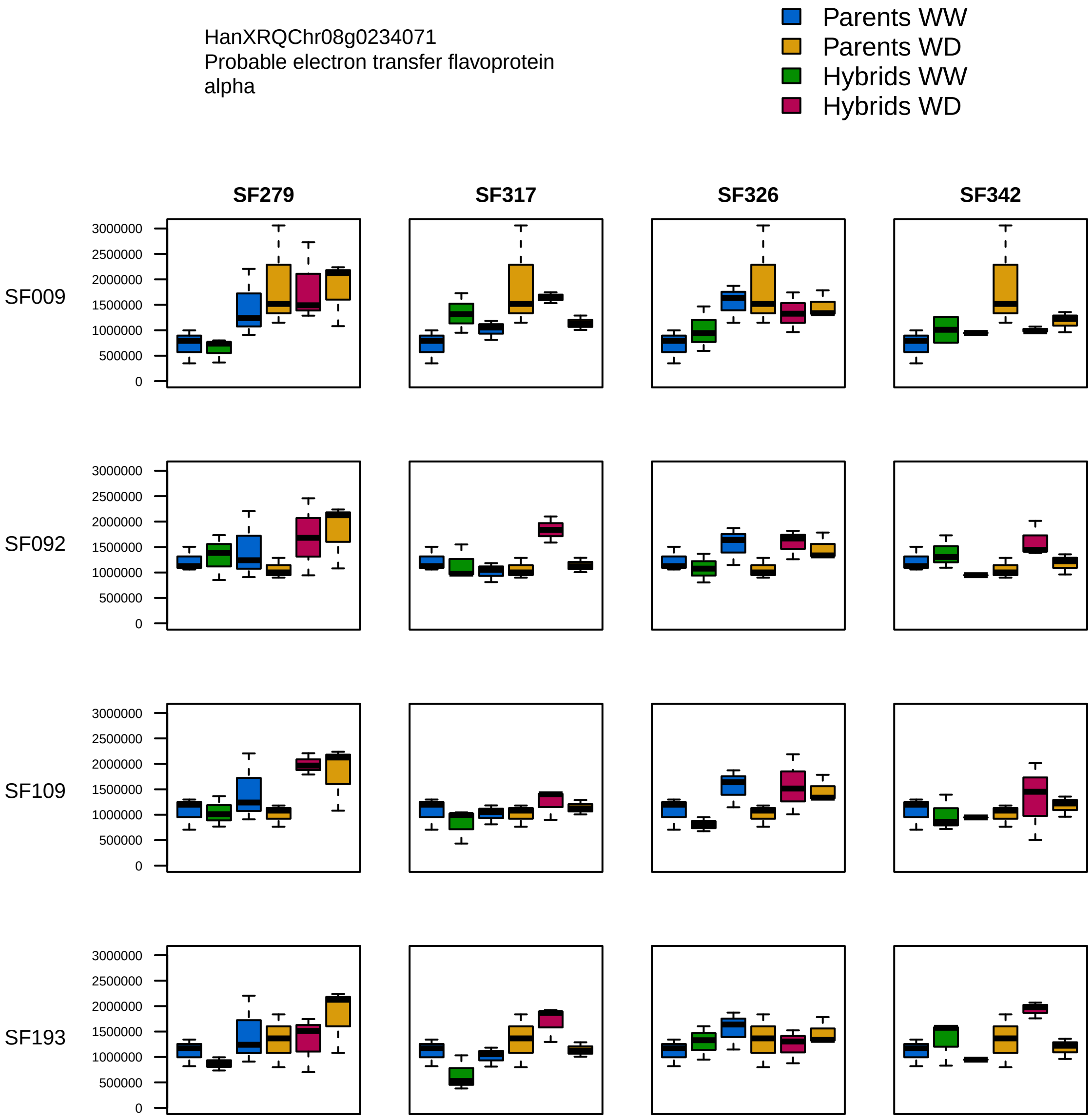
HanXRQChr08g0234071
Probable electron transfer flavoprotein
alpha
Parents WW
Parents WD
Hybrids WW
Hybrids WD
SF279
SF317
SF326
SF342
SF009
SF092
SF109
SF193
3000000
2500000
2000000
1500000
1000000
500000
0

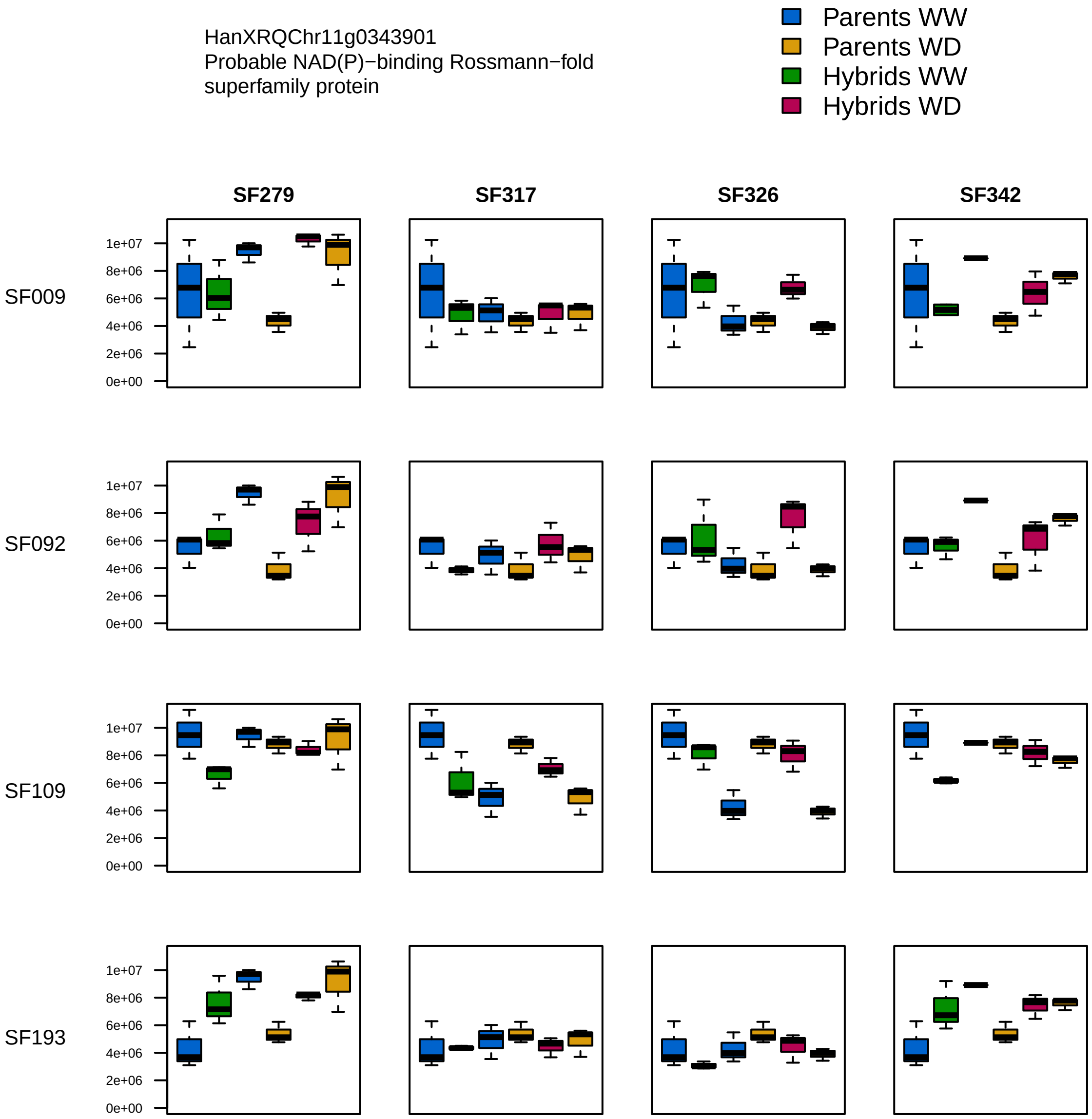
HanXRQChr11g0343901
Probable NAD(P)−binding Rossmann−fold
superfamily protein
Parents WW
Parents WD
Hybrids WW
Hybrids WD
SF279
SF317
SF326
SF342
SF009
SF092
SF109
SF193
0e+00
2e+06
4e+06
6e+06
8e+06
1e+07

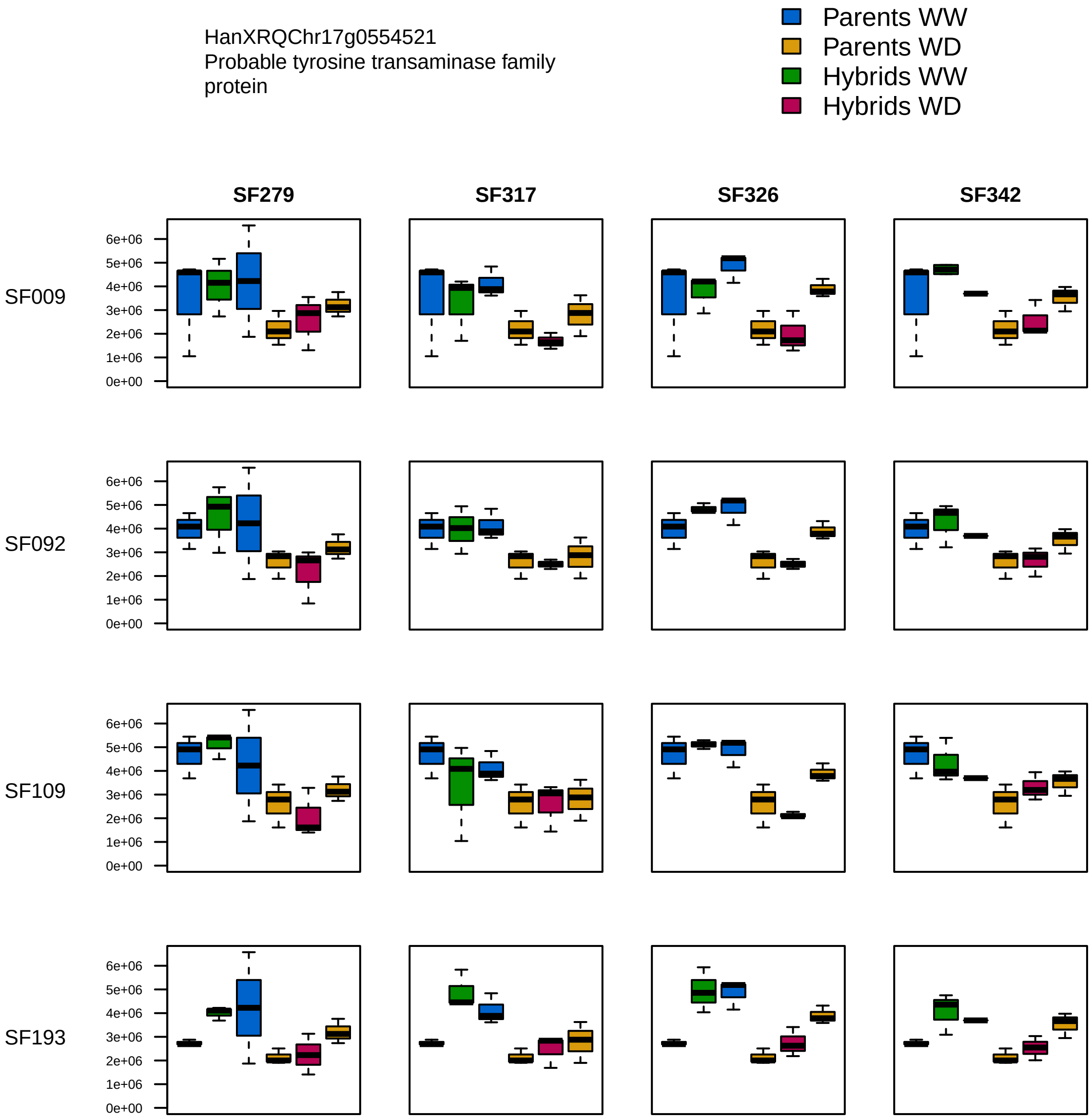
HanXRQChr17g0554521
Probable tyrosine transaminase family protein
Parents WW
Parents WD
Hybrids WW
Hybrids WD
SF279
SF317
SF326
SF342
SF009
SF092
SF109
SF193
6e+06
5e+06
4e+06
3e+06
2e+06
1e+06
0e+00

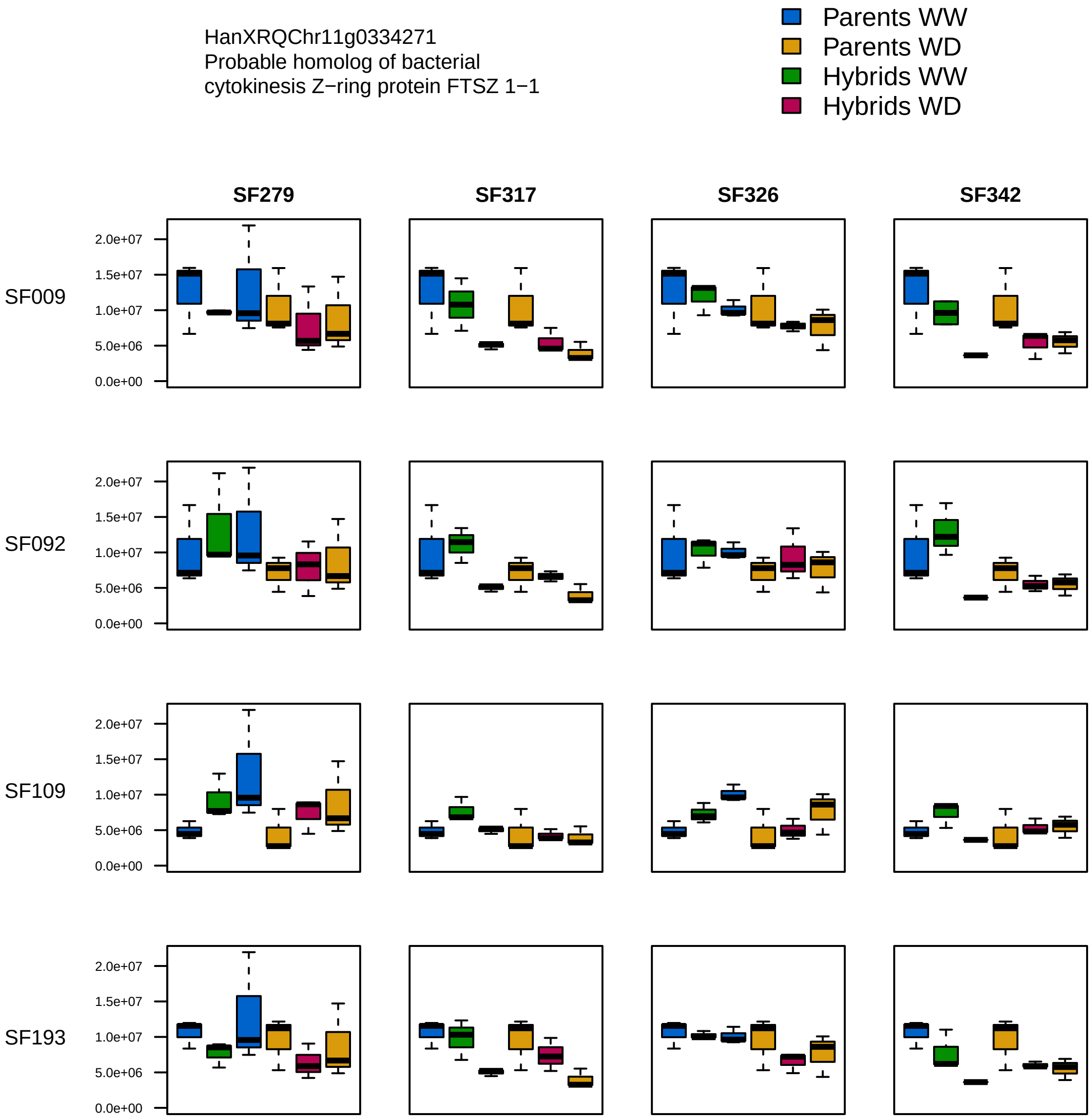
HanXRQChr11g0334271
Probable homolog of bacterial
cytokinesis Z−ring protein FTSZ 1−1
Parents WW
Parents WD
Hybrids WW
Hybrids WD
SF279
SF317
SF326
SF342
SF009
SF092
SF109
SF193
2.0e+07
1.5e+07
1.0e+07
5.0e+06
0.0e+00

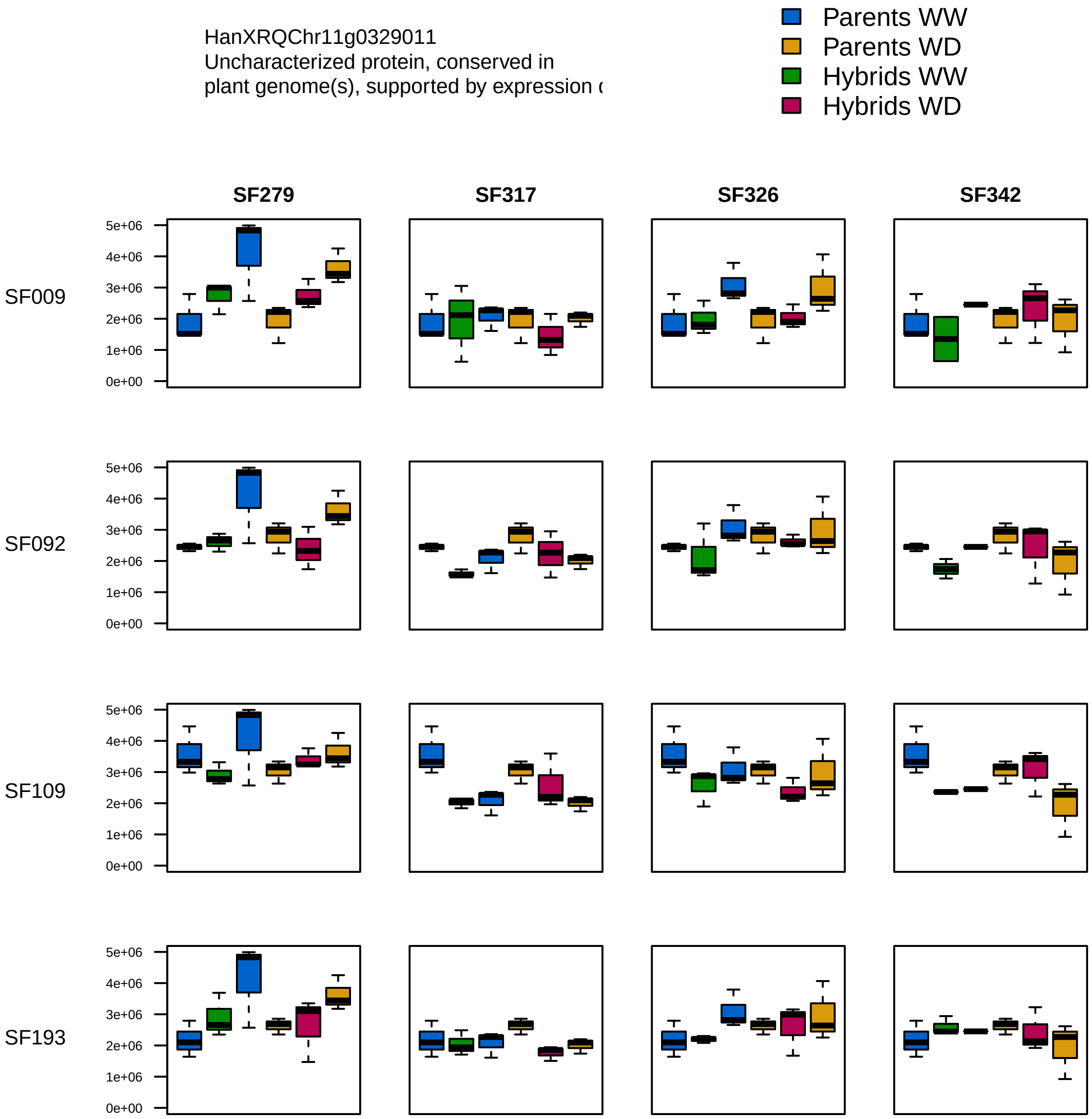
HanXRQChr11g0329011
Uncharacterized protein, conserved in
plant genome(s), supported by expression
Parents WW
Parents WD
Hybrids WW
Hybrids WD
SF279
SF317
SF326
SF342
SF009
SF092
SF109
SF193
5e+06
4e+06
3e+06
2e+06
1e+06
0e+00

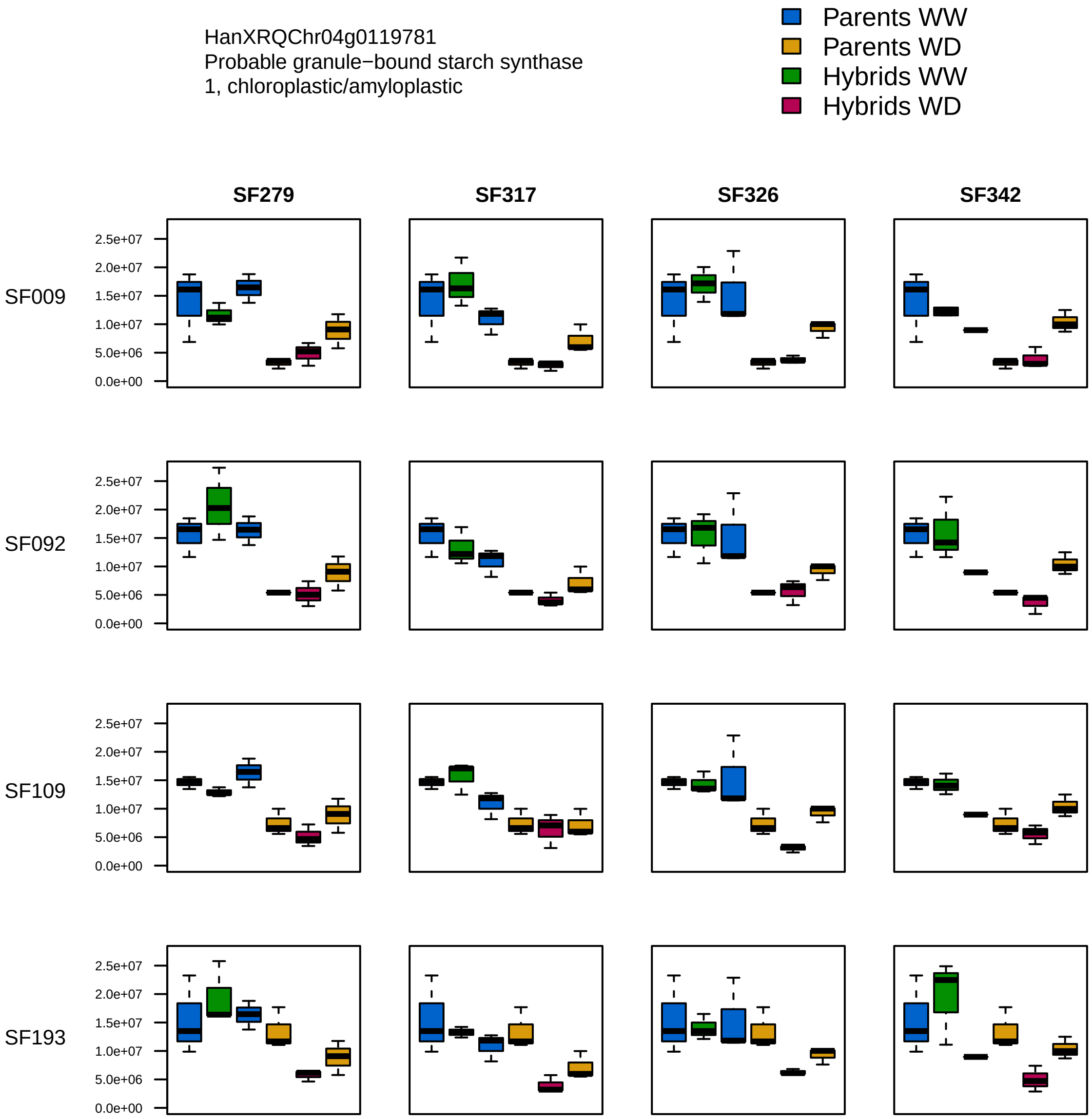
HanXRQChr04g0119781
Probable granule−bound starch synthase
1, chloroplastic/amyloplastic
Parents WW
Parents WD
Hybrids WW
Hybrids WD
SF279
SF317
SF326
SF342
SF009
SF092
SF109
SF193
2.5e+07
2.0e+07
1.5e+07
1.0e+07
5.0e+06
0.0e+00

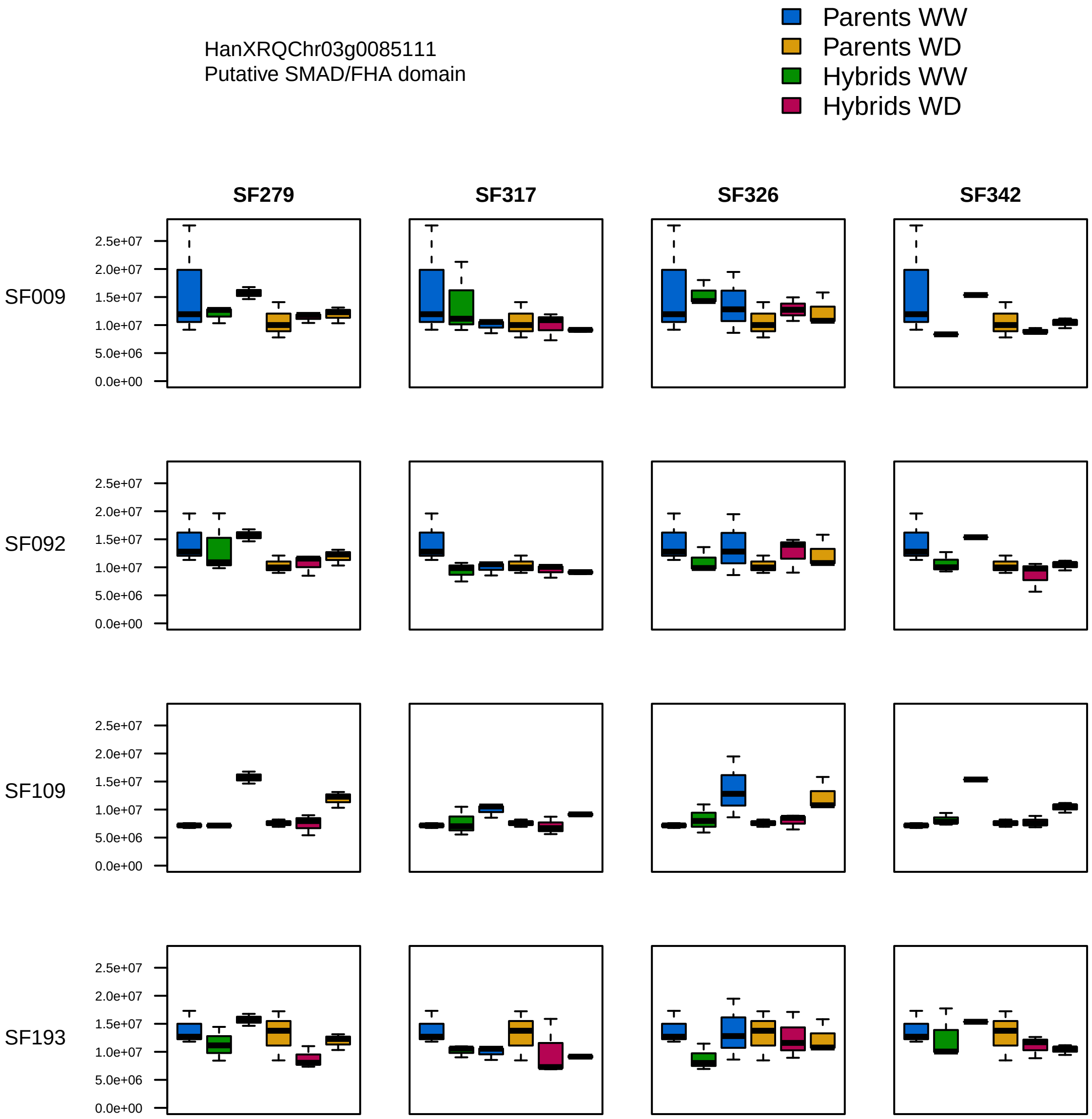
HanXRQChr03g0085111
Putative SMAD/FHA domain
Parents WW
Parents WD
Hybrids WW
Hybrids WD
SF279
SF317
SF326
SF342
SF009
SF092
SF109
SF193
2.5e+07
2.0e+07
1.5e+07
1.0e+07
5.0e+06
0.0e+00

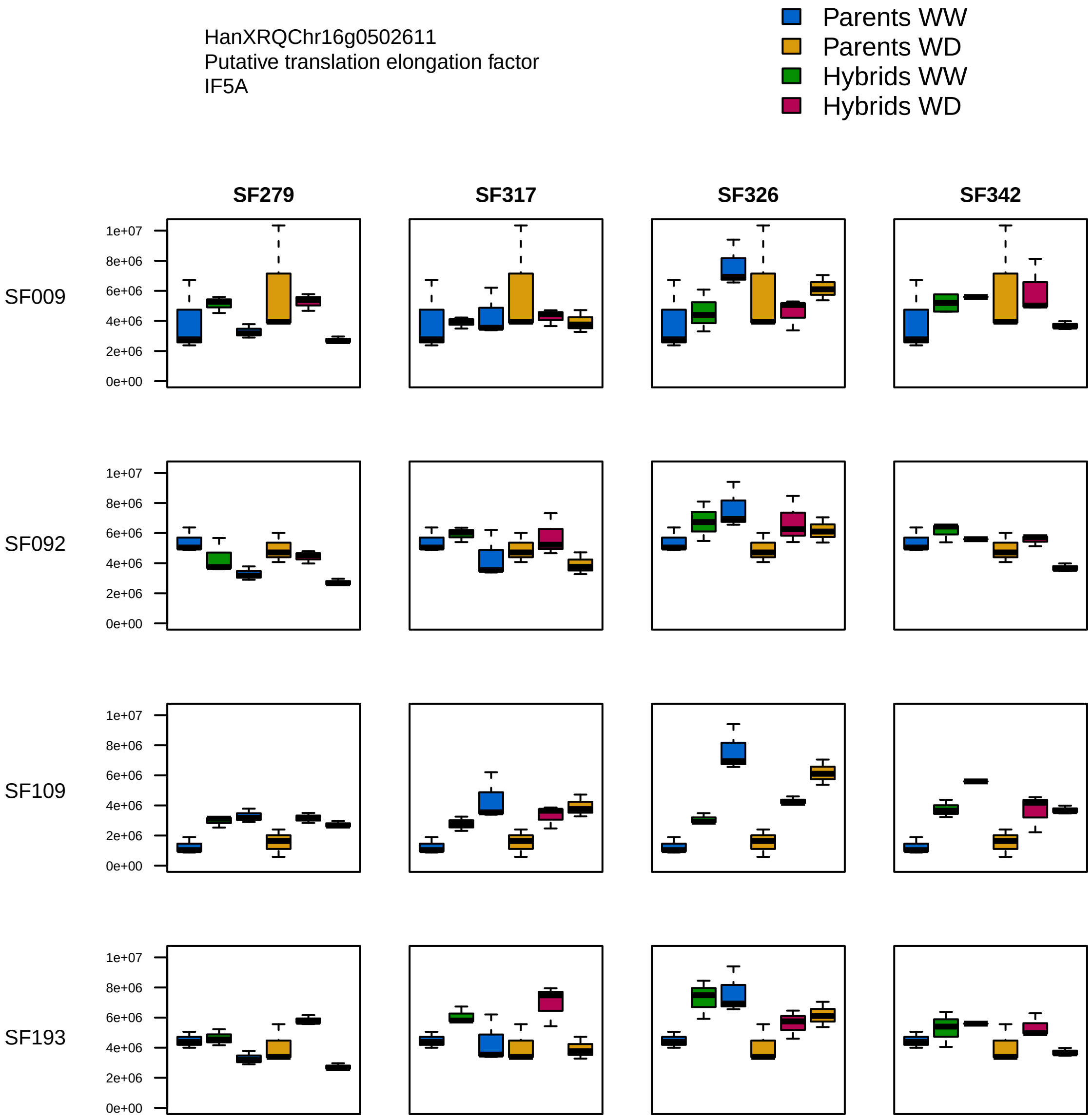
HanXRQChr16g0502611
Putative translation elongation factor
IF5A
Parents WW
Parents WD
Hybrids WW
Hybrids WD
SF279
SF317
SF326
SF342
SF009
SF092
SF109
SF193
1e+07
8e+06
6e+06
4e+06
2e+06
0e+00

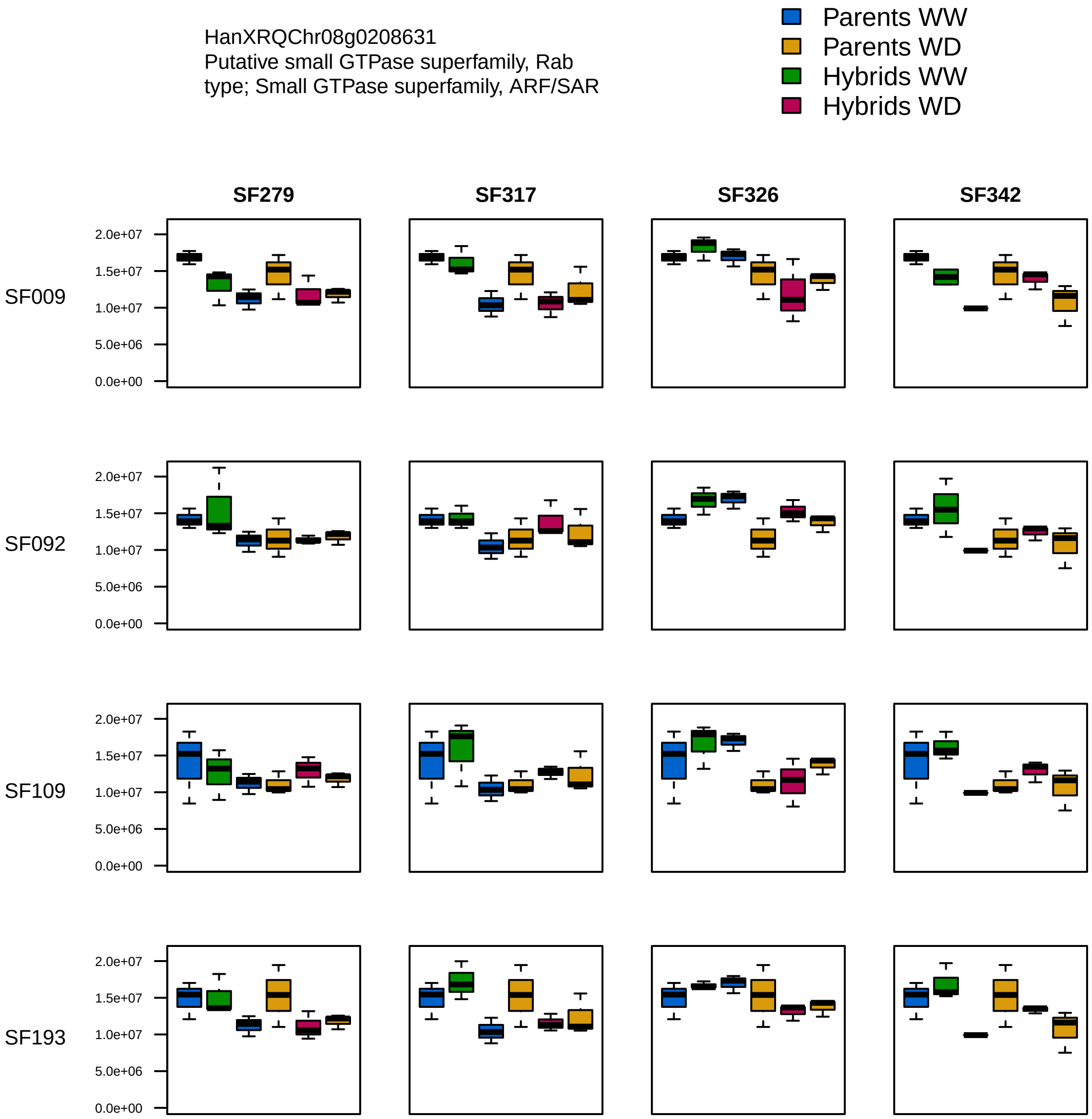

HanXRQChr08g0208631
Putative small GTPase superfamily, Rab type; Small GTPase superfamily, ARF/SAR
Parents WW
Parents WD
Hybrids WW
Hybrids WD
SF279
SF317
SF326
SF342
SF009
SF092
SF109
SF193
2.0e+07
1.5e+07
1.0e+07
5.0e+06
0.0e+00

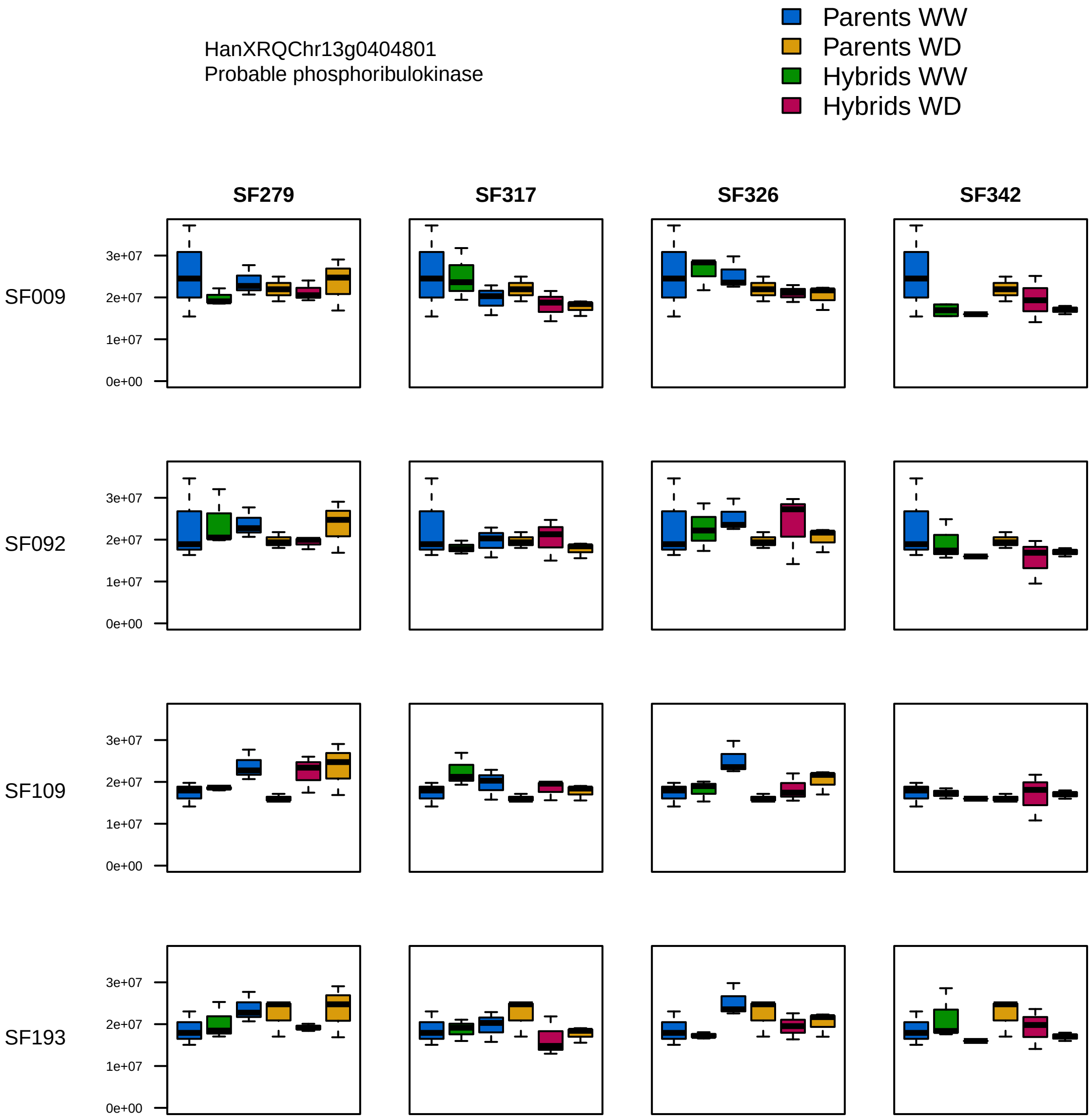

HanXRQChr13g0404801
Probable phosphoribulokinase
Parents WW
Parents WD
Hybrids WW
Hybrids WD
SF279
SF317
SF326
SF342
SF009
SF092
SF109
SF193
3e+07
2e+07
1e+07
0e+00

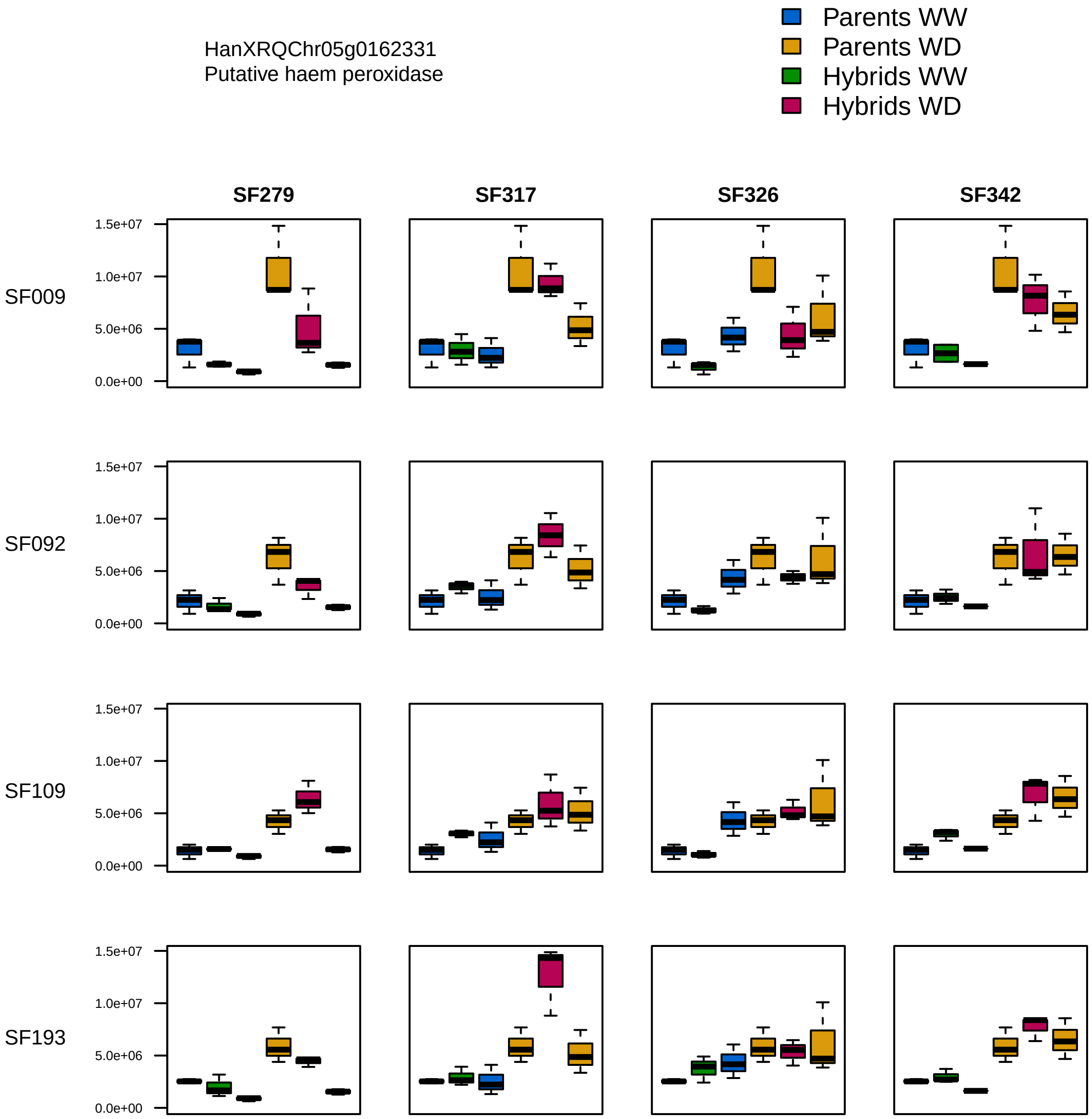

HanXRQChr05g0162331
Putative haem peroxidase
Parents WW
Parents WD
Hybrids WW
Hybrids WD
SF279
SF317
SF326
SF342
SF009
SF092
SF109
SF193
1.5e+07
1.0e+07
5.0e+06
0.0e+00

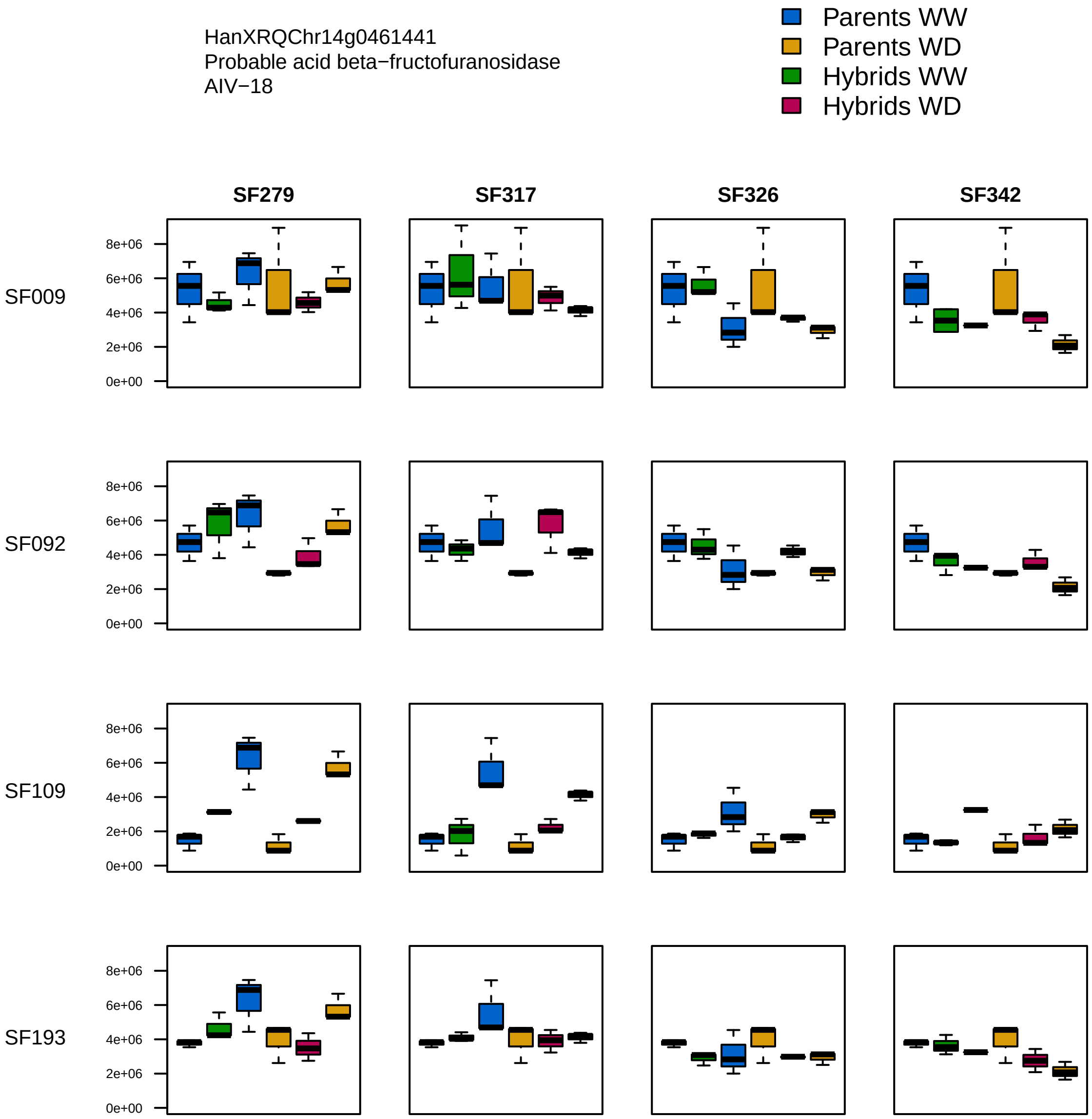
HanXRQChr14g0461441
Probable acid beta−fructofuranosidase
AIV−18
Parents WW
Parents WD
Hybrids WW
Hybrids WD
SF279
SF317
SF326
SF342
SF009
SF092
SF109
SF193
8e+06
6e+06
4e+06
2e+06
0e+00

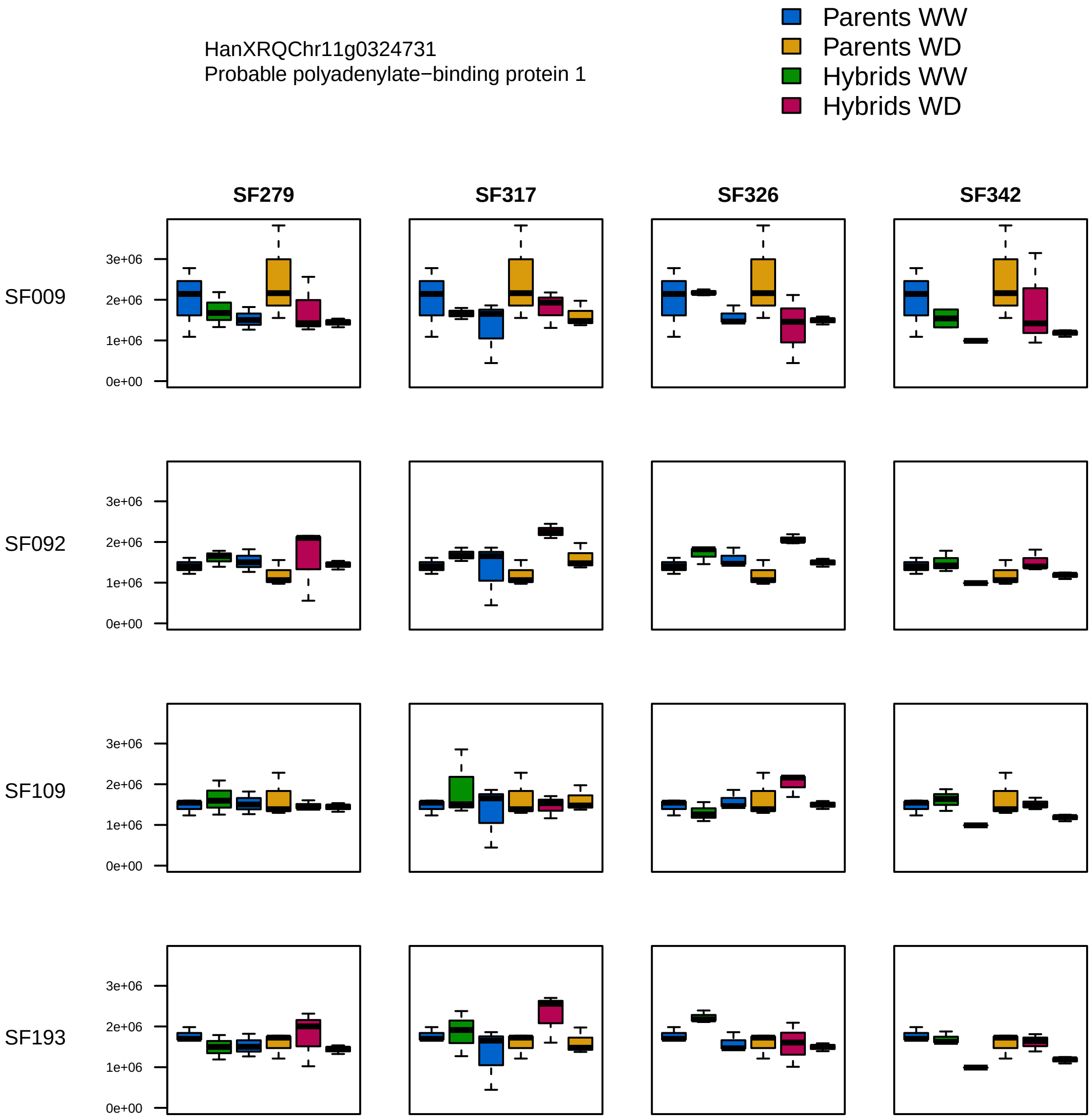
HanXRQChr11g0324731
Probable polyadenylate−binding protein 1
Parents WW
Parents WD
Hybrids WW
Hybrids WD
SF279
SF317
SF326
SF342
SF009
SF092
SF109
SF193
0e+00
1e+06
2e+06
3e+06

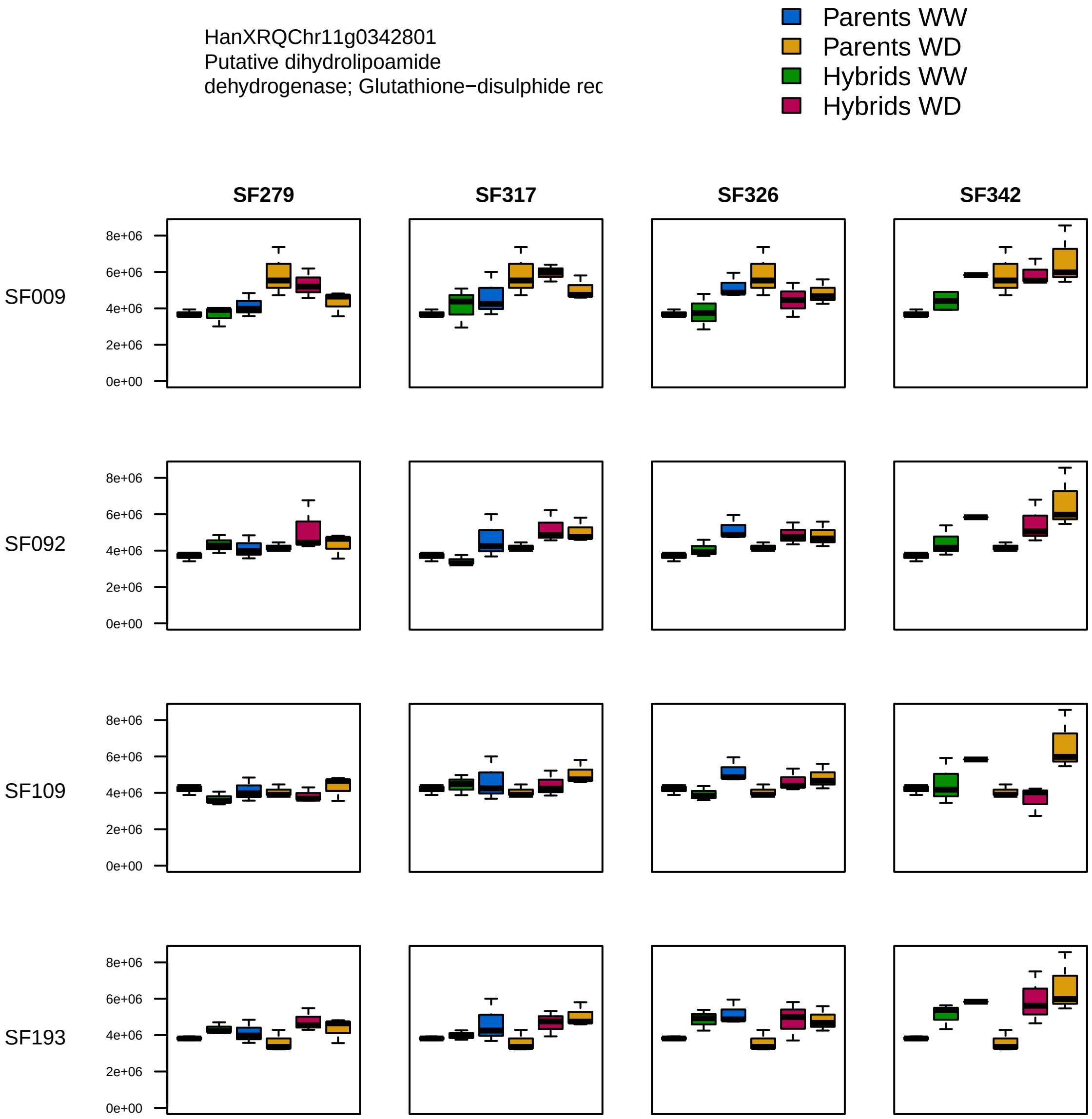

HanXRQChr11g0342801
Putative dihydrolipoamide
dehydrogenase; Glutathione−disulphide rec
Parents WW
Parents WD
Hybrids WW
Hybrids WD
SF279
SF317
SF326
SF342
SF009
SF092
SF109
SF193
0e+00
2e+06
4e+06
6e+06
8e+06

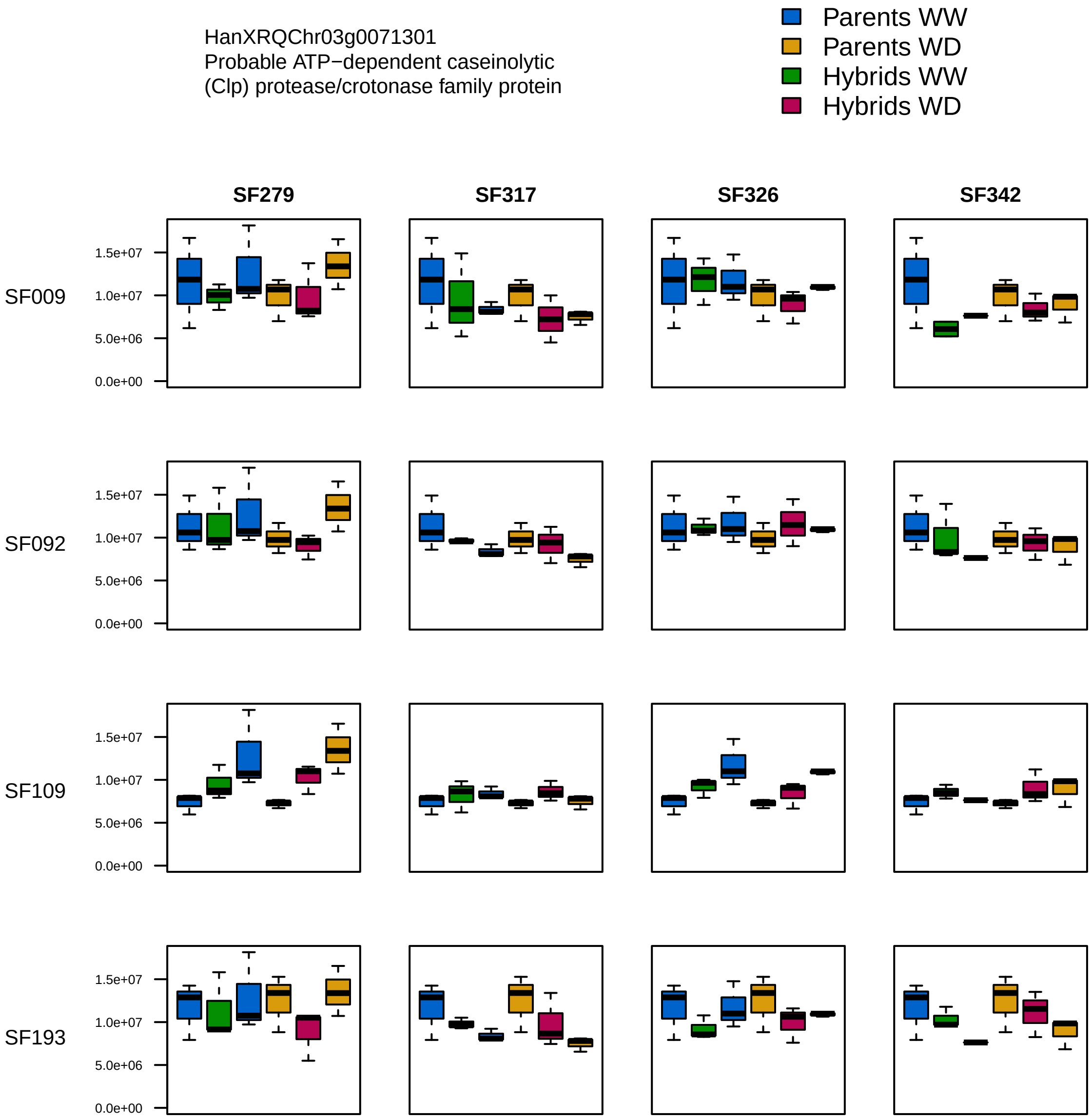

HanXRQChr03g0071301
Probable ATP−dependent caseinolytic
(Clp) protease/crotonase family protein
Parents WW
Parents WD
Hybrids WW
Hybrids WD
SF279
SF317
SF326
SF342
SF009
SF092
SF109
SF193
1.5e+07
1.0e+07
5.0e+06
0.0e+00

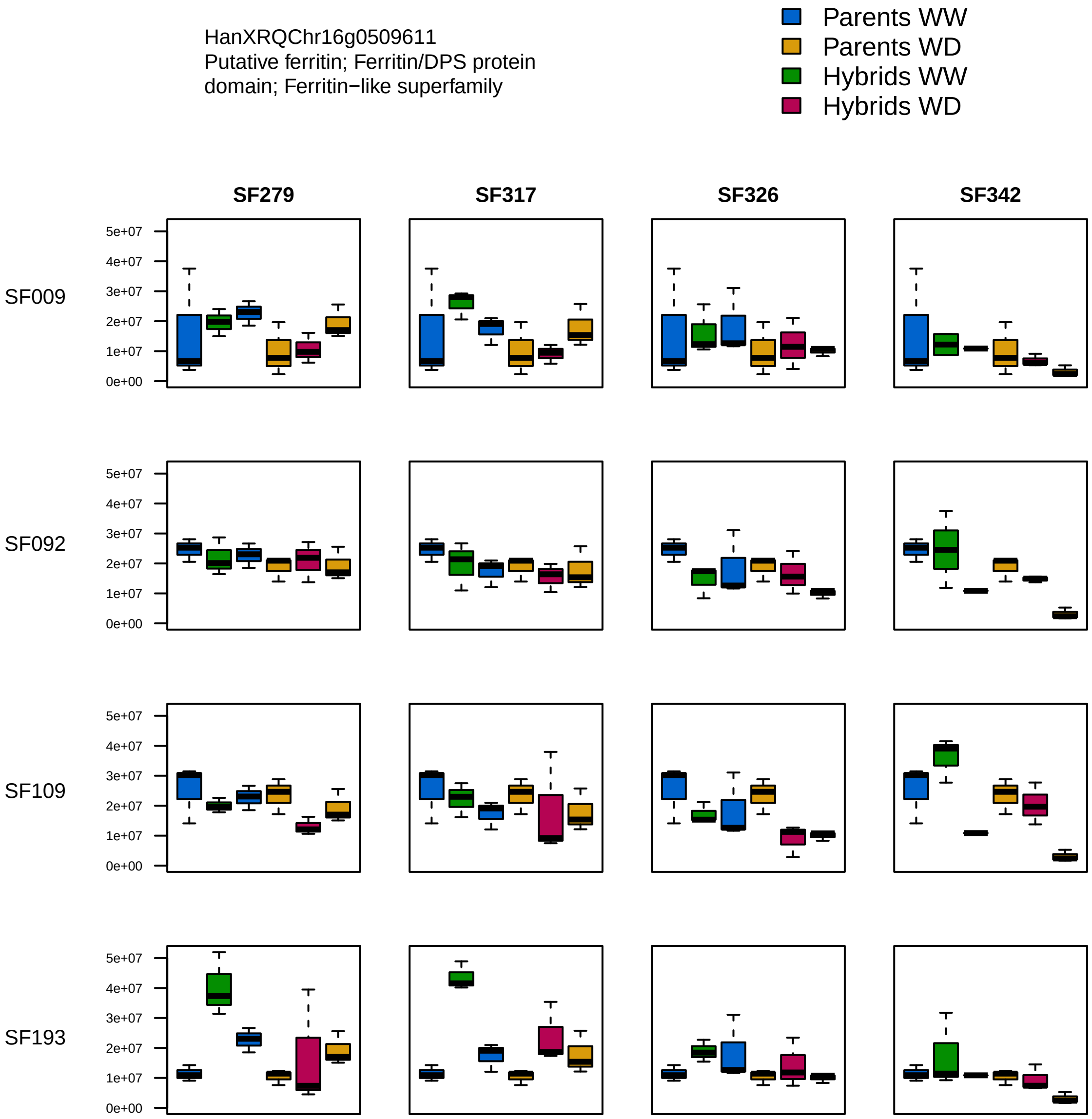

HanXRQChr16g0509611
Putative ferritin; Ferritin/DPS protein
domain; Ferritin−like superfamily
Parents WW
Parents WD
Hybrids WW
Hybrids WD
SF279
SF317
SF326
SF342
SF009
SF092
SF109
SF193
5e+07
4e+07
3e+07
2e+07
1e+07
0e+00

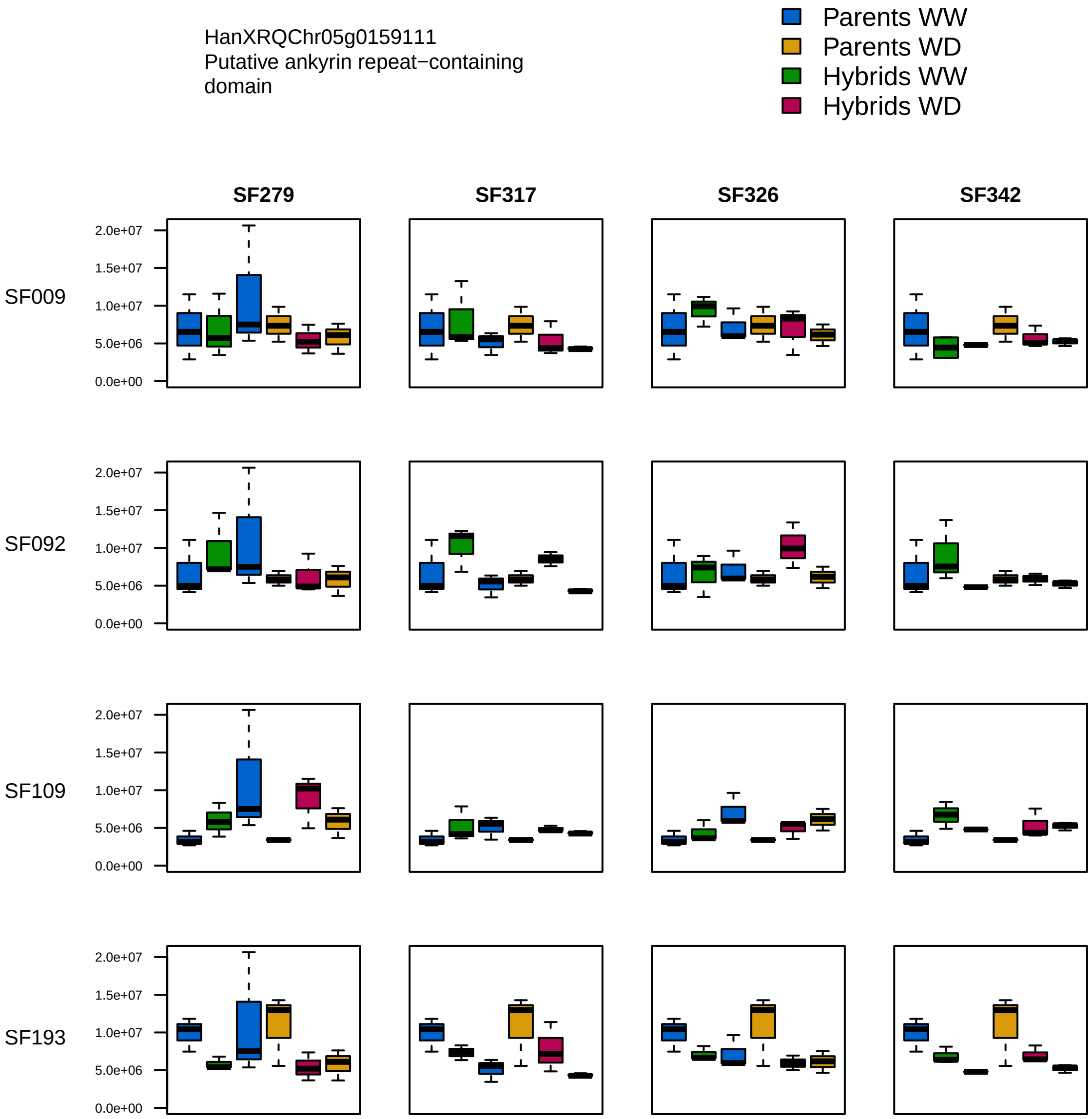
HanXRQChr05g0159111
Putative ankyrin repeat−containing
domain
Parents WW
Parents WD
Hybrids WW
Hybrids WD
SF279
SF317
SF326
SF342
SF009
SF092
SF109
SF193
2.0e+07
1.5e+07
1.0e+07
5.0e+06
0.0e+00

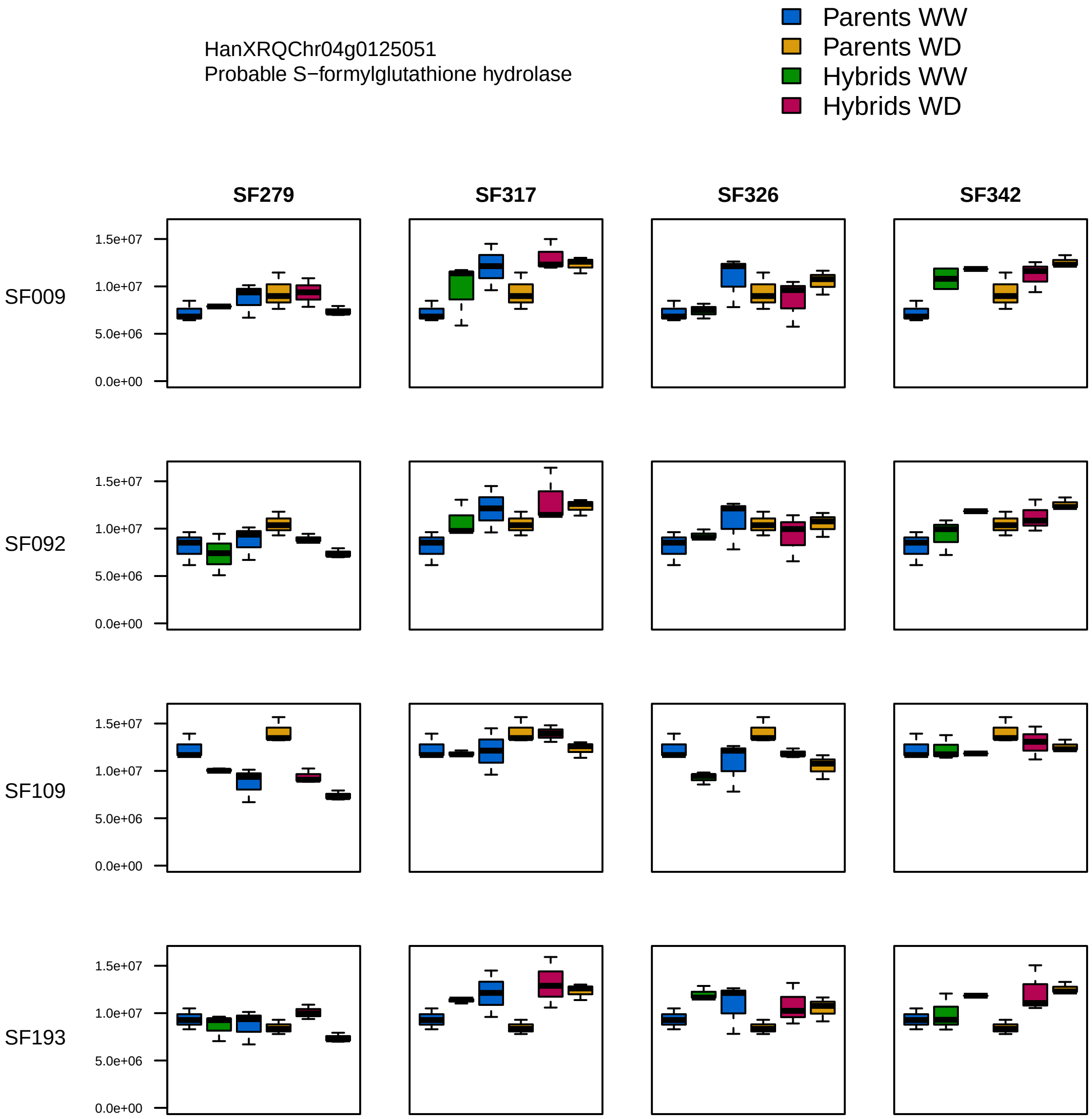
HanXRQChr04g0125051
Probable S−formylglutathione hydrolase
Parents WW
Parents WD
Hybrids WW
Hybrids WD
SF279
SF317
SF326
SF342
SF009
SF092
SF109
SF193
1.5e+07
1.0e+07
5.0e+06
0.0e+00

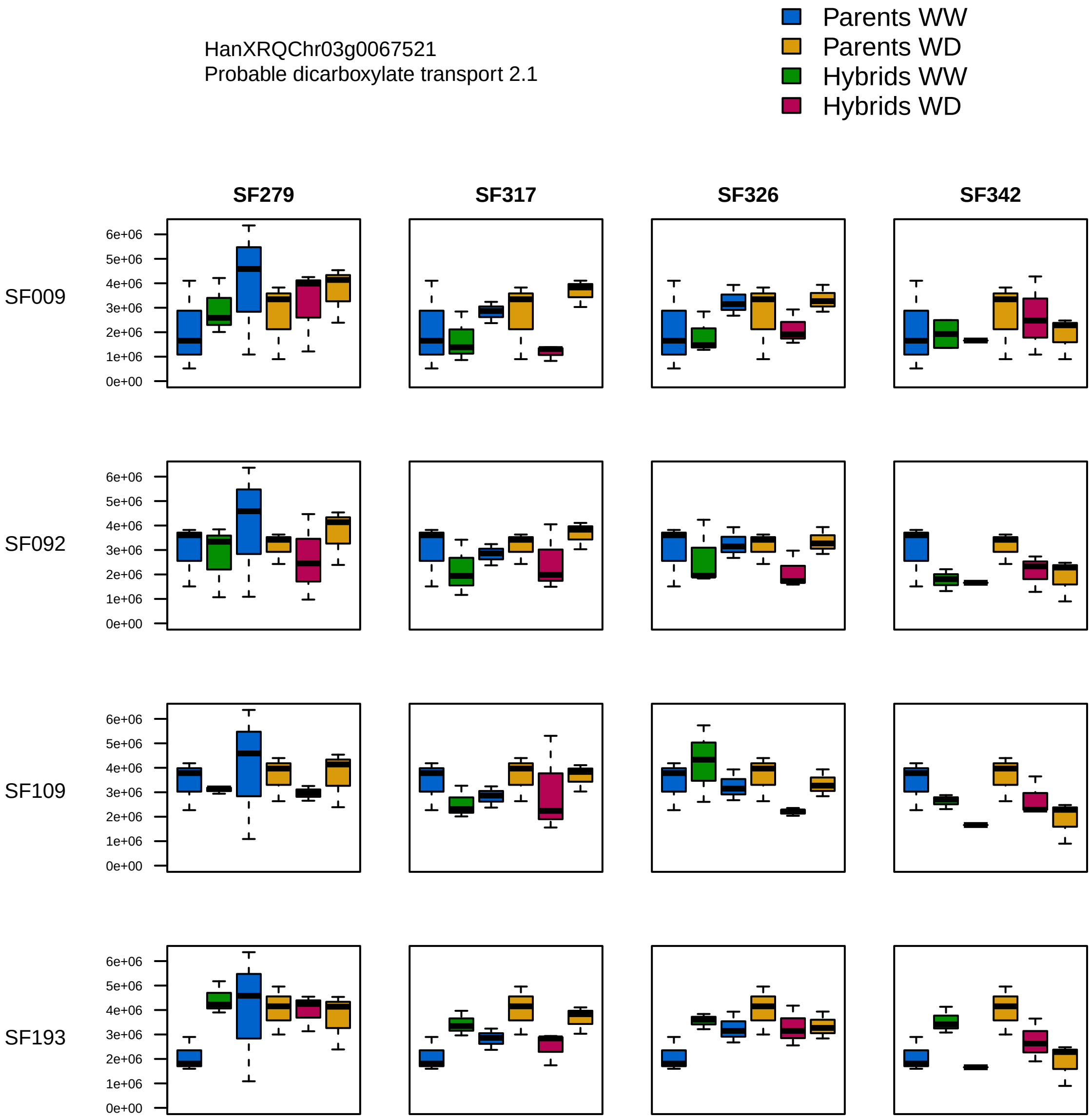
HanXRQChr03g0067521
Probable dicarboxylate transport 2.1
Parents WW
Parents WD
Hybrids WW
Hybrids WD
SF279
SF317
SF326
SF342
SF009
SF092
SF109
SF193
0e+00
1e+06
2e+06
3e+06
4e+06
5e+06
6e+06

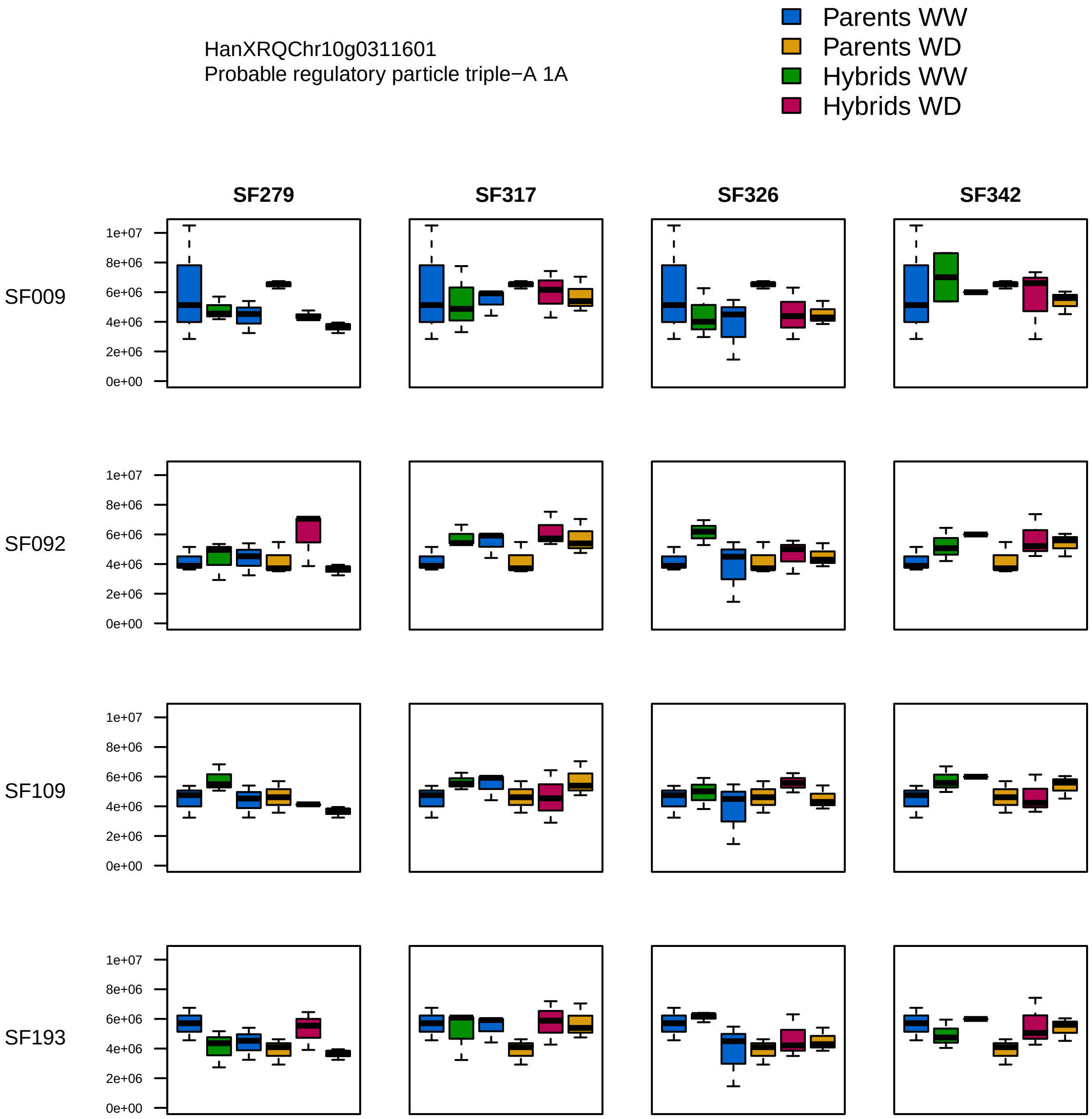
HanXRQChr10g0311601
Probable regulatory particle triple−A 1A
Parents WW
Parents WD
Hybrids WW
Hybrids WD
SF279
SF317
SF326
SF342
SF009
SF092
SF109
SF193
1e+07
8e+06
6e+06
4e+06
2e+06
0e+00

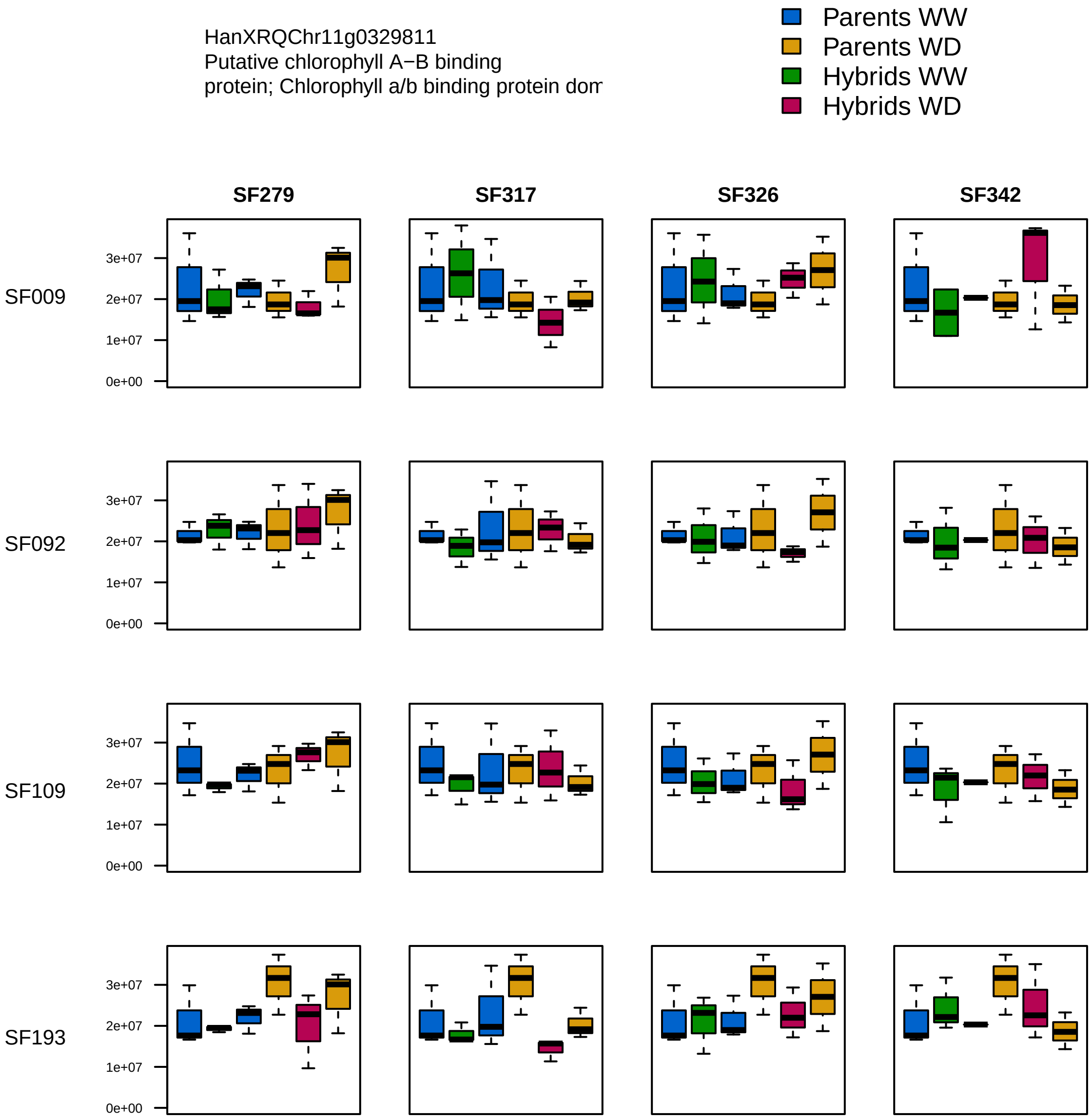

HanXRQChr11g0329811
Putative chlorophyll A−B binding
protein; Chlorophyll a/b binding protein dom
Parents WW
Parents WD
Hybrids WW
Hybrids WD
SF279
SF317
SF326
SF342
SF009
SF092
SF109
SF193
0e+00
1e+07
2e+07
3e+07

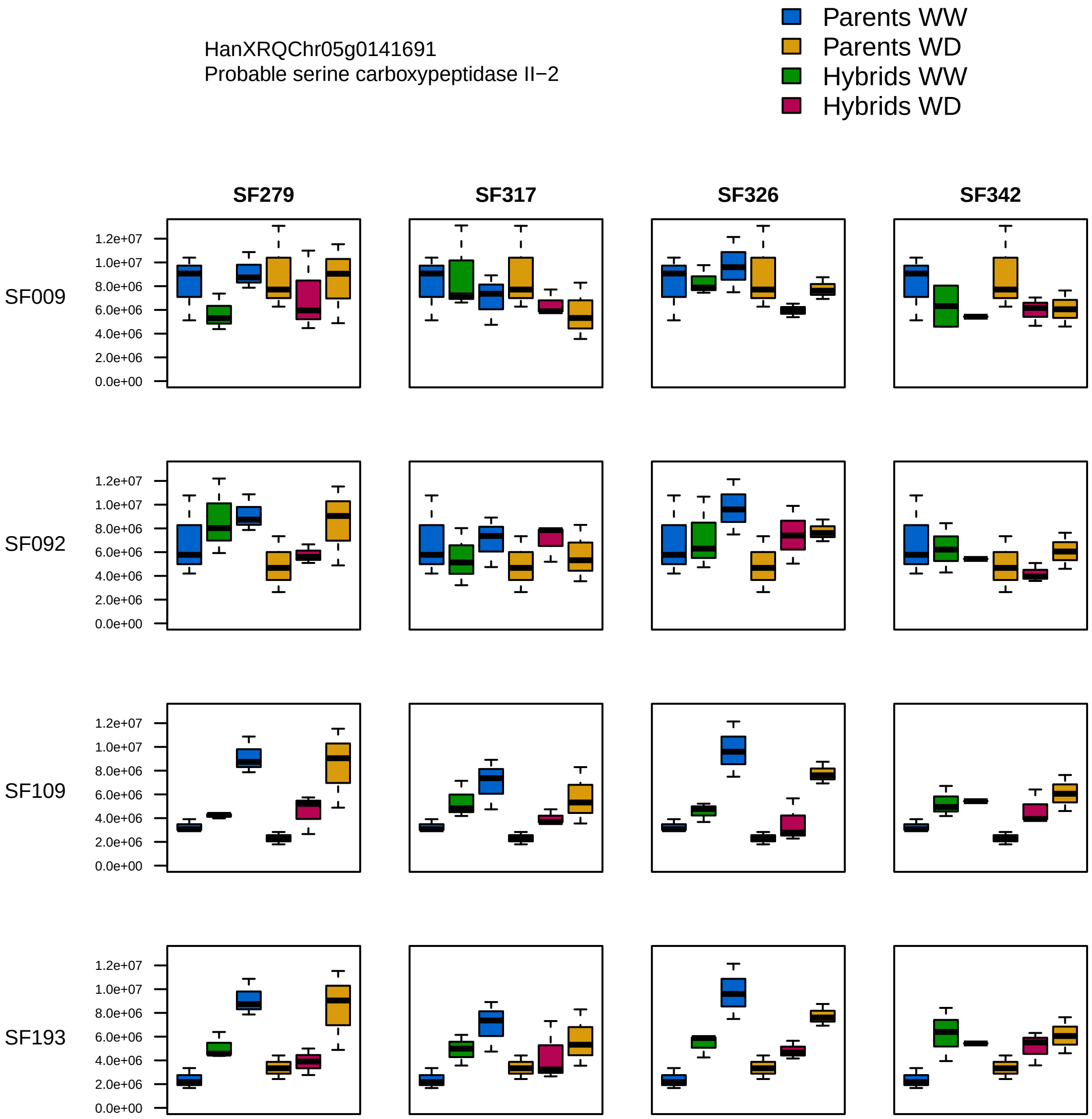

HanXRQChr05g0141691
Probable serine carboxypeptidase II−2
Parents WW
Parents WD
Hybrids WW
Hybrids WD
SF279
SF317
SF326
SF342
SF009
SF092
SF109
SF193
1.2e+07
1.0e+07
8.0e+06
6.0e+06
4.0e+06
2.0e+06
0.0e+00

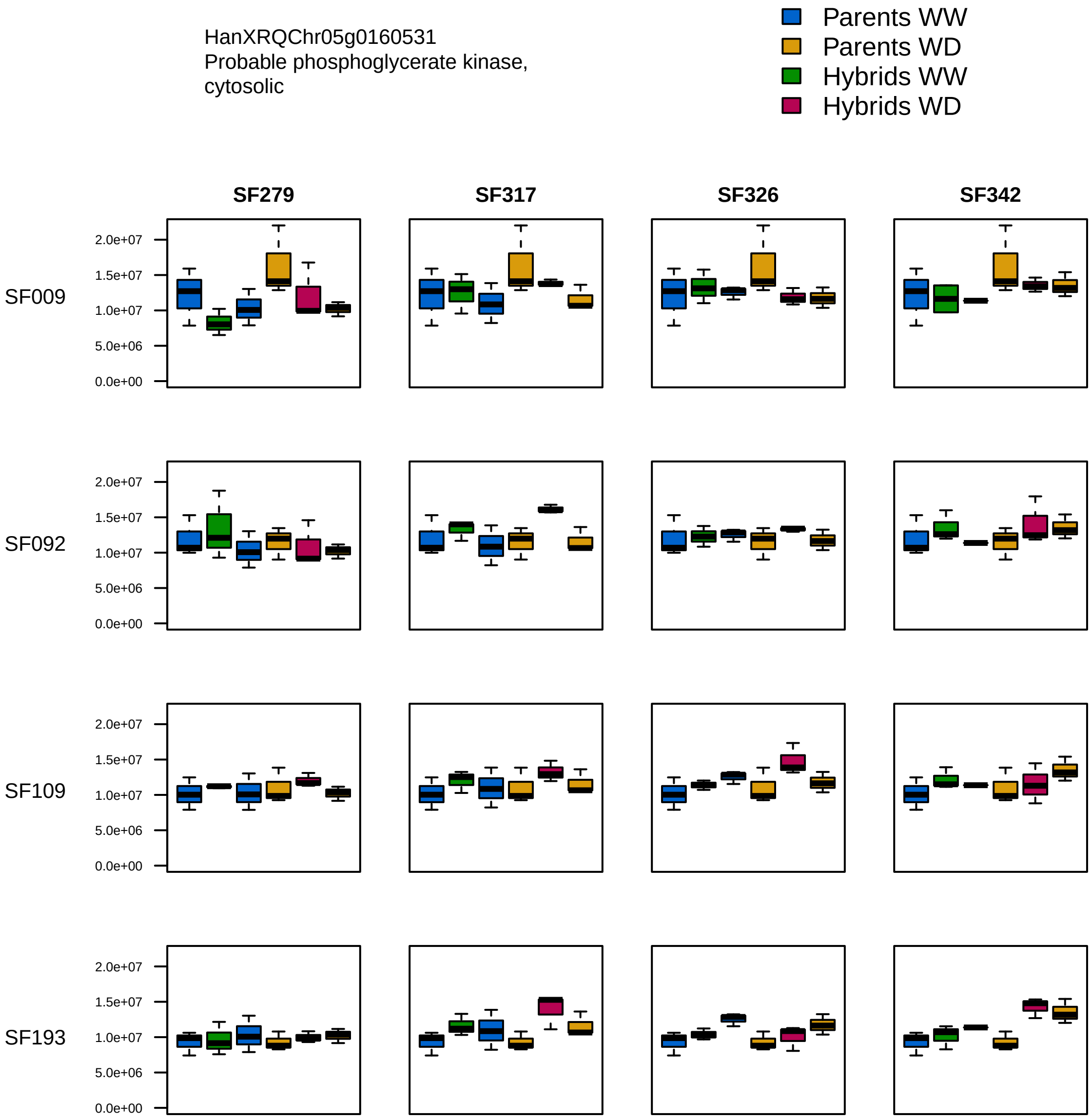

HanXRQChr05g0160531
Probable phosphoglycerate kinase, cytosolic
Parents WW
Parents WD
Hybrids WW
Hybrids WD
SF279
SF317
SF326
SF342
SF009
SF092
SF109
SF193
0.0e+00
5.0e+06
1.0e+07
1.5e+07
2.0e+07

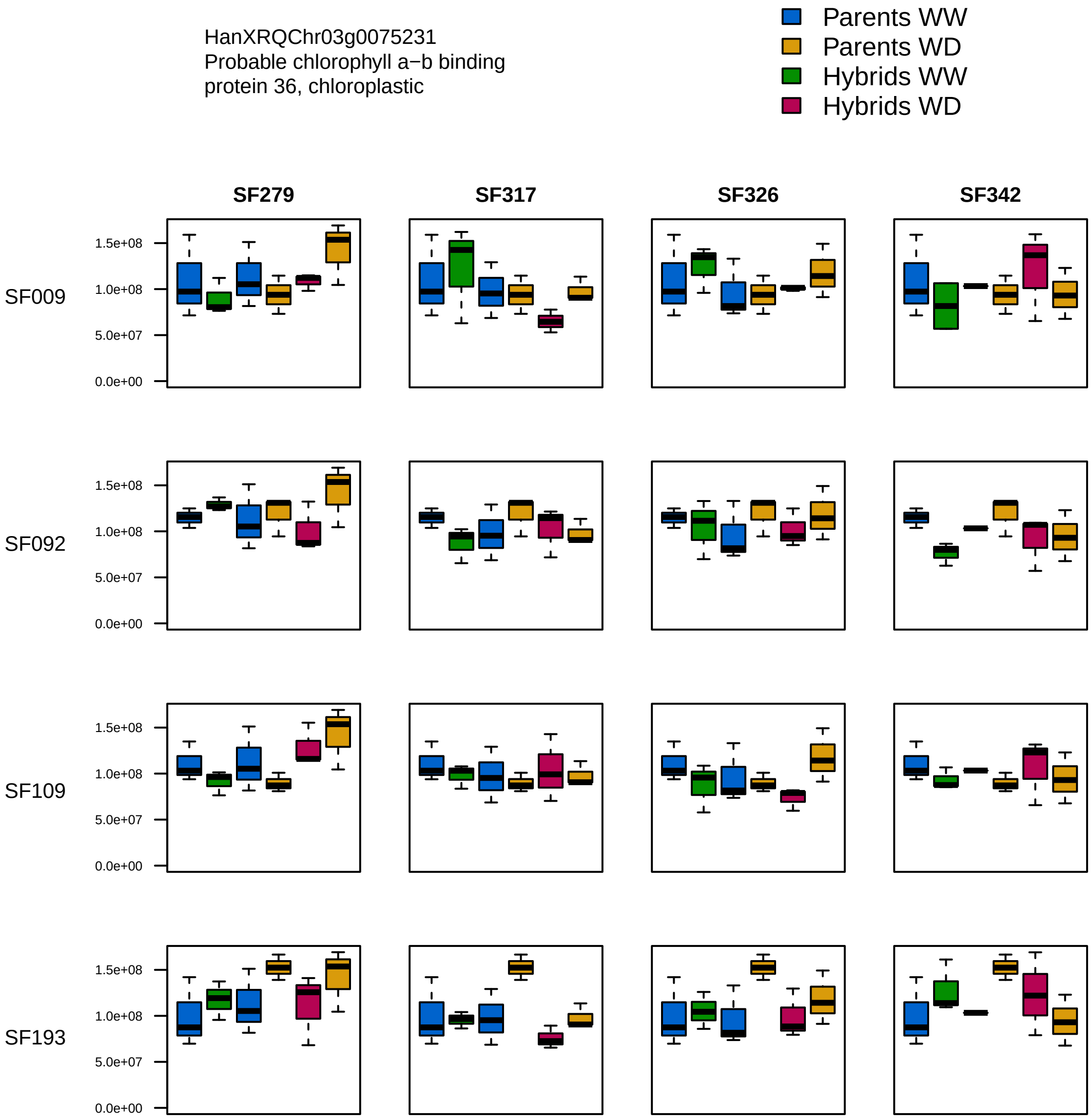
HanXRQChr03g0075231
Probable chlorophyll a−b binding
protein 36, chloroplastic
Parents WW
Parents WD
Hybrids WW
Hybrids WD
SF279
SF317
SF326
SF342
SF009
SF092
SF109
SF193
1.5e+08
1.0e+08
5.0e+07
0.0e+00

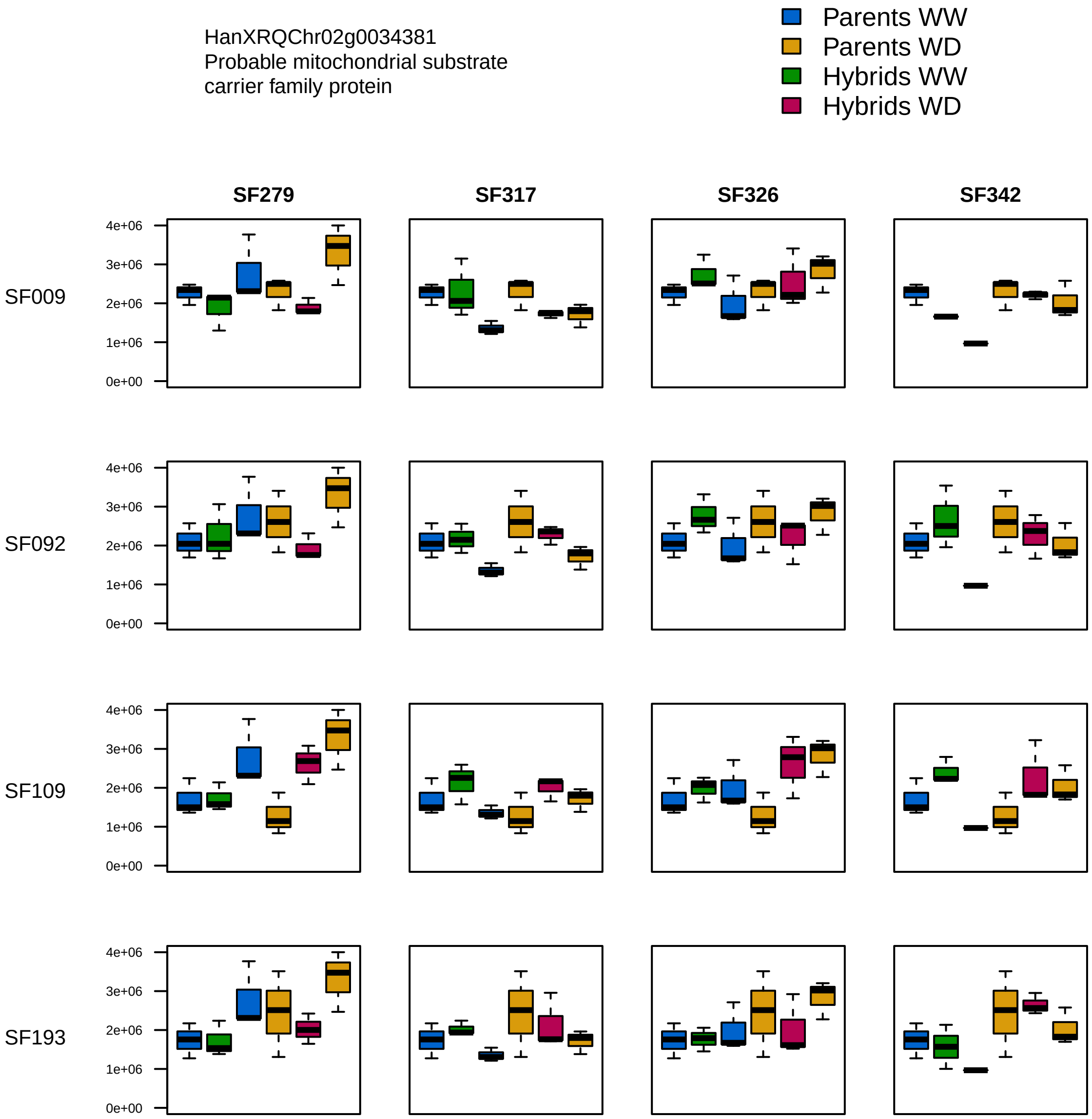

HanXRQChr02g0034381
Probable mitochondrial substrate
carrier family protein
Parents WW
Parents WD
Hybrids WW
Hybrids WD
SF279
SF317
SF326
SF342
SF009
SF092
SF109
SF193
4e+06
3e+06
2e+06
1e+06
0e+00

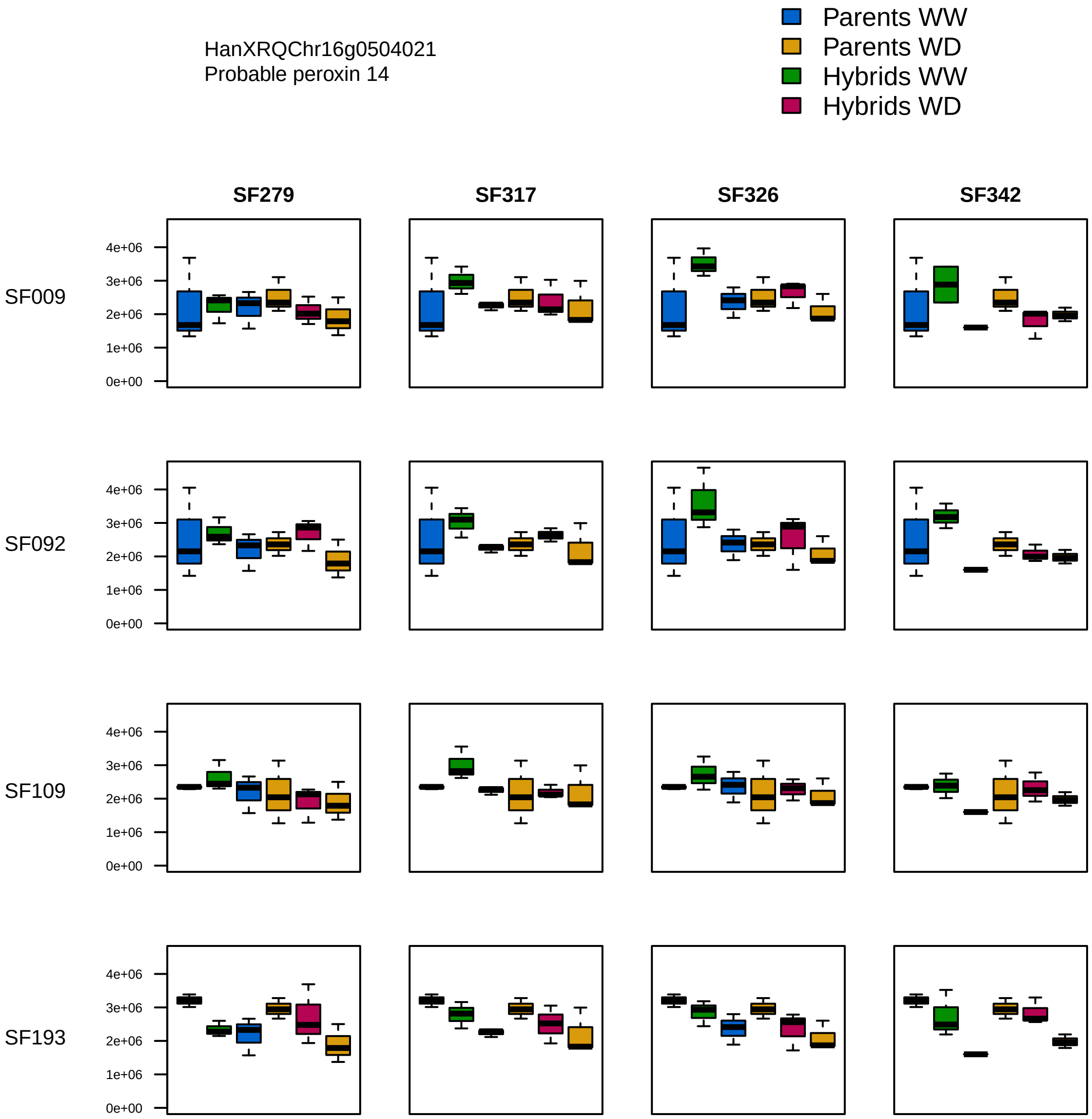
HanXRQChr16g0504021
Probable peroxin 14
Parents WW
Parents WD
Hybrids WW
Hybrids WD
SF279
SF317
SF326
SF342
SF009
SF092
SF109
SF193
4e+06
3e+06
2e+06
1e+06
0e+00

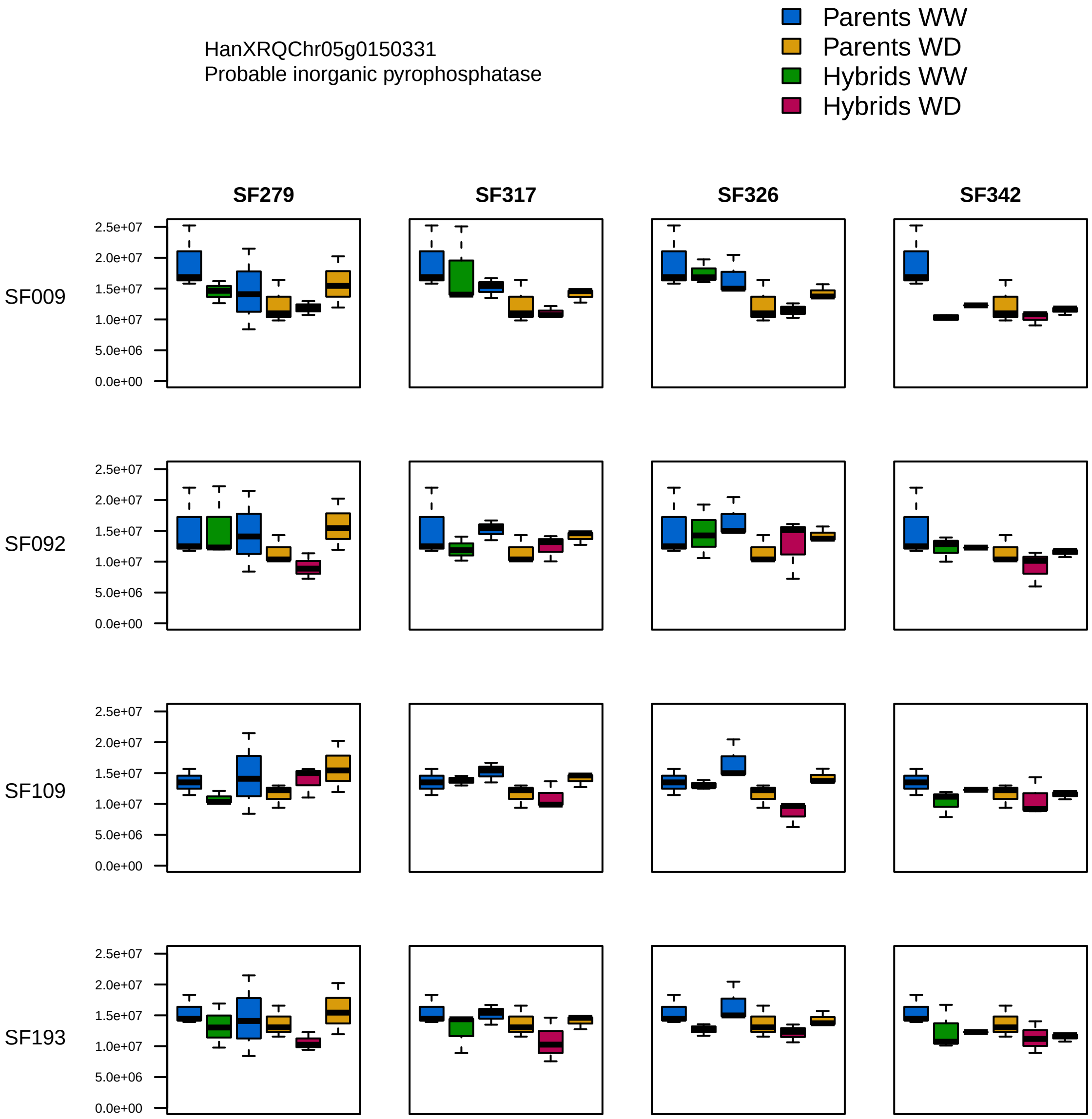

HanXRQChr05g0150331
Probable inorganic pyrophosphatase
Parents WW
Parents WD
Hybrids WW
Hybrids WD
SF279
SF317
SF326
SF342
SF009
SF092
SF109
SF193
2.5e+07
2.0e+07
1.5e+07
1.0e+07
5.0e+06
0.0e+00

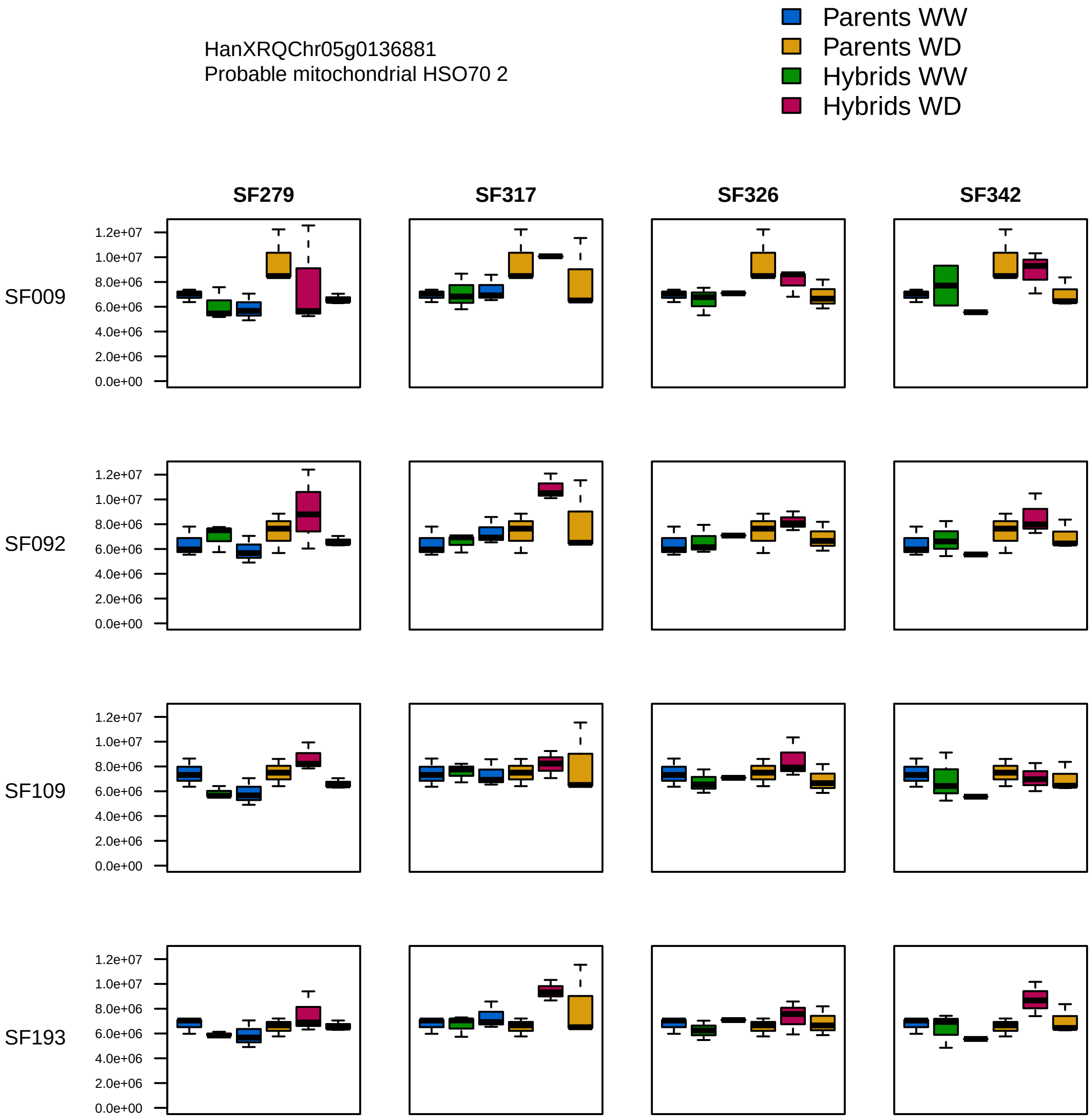

HanXRQChr05g0136881
Probable mitochondrial HSO70 2
Parents WW
Parents WD
Hybrids WW
Hybrids WD
SF279
SF317
SF326
SF342
SF009
SF092
SF109
SF193
0.0e+00
2.0e+06
4.0e+06
6.0e+06
8.0e+06
1.0e+07
1.2e+07

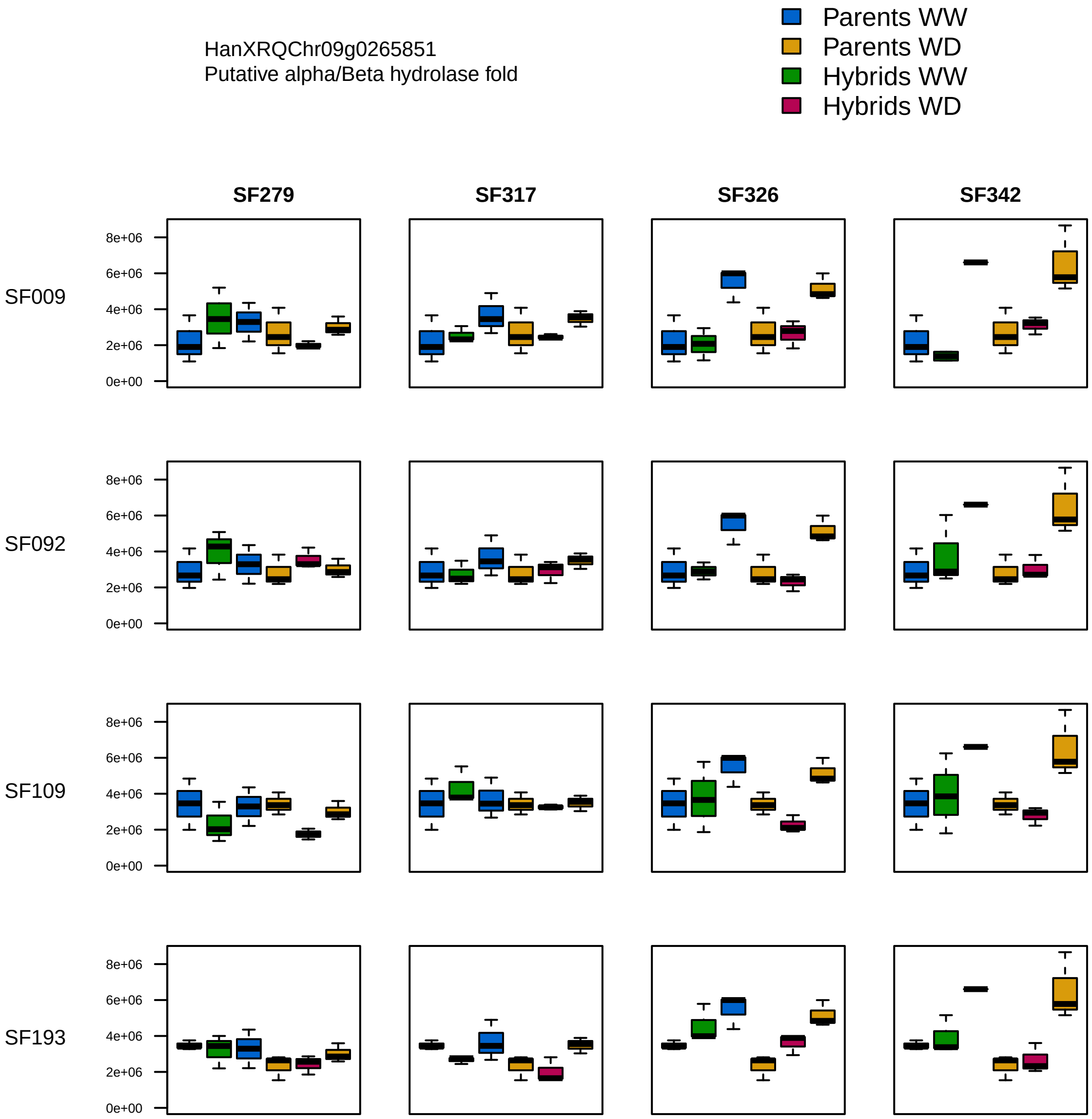
HanXRQChr09g0265851
Putative alpha/Beta hydrolase fold
Parents WW
Parents WD
Hybrids WW
Hybrids WD
SF279
SF317
SF326
SF342
SF009
SF092
SF109
SF193
8e+06
6e+06
4e+06
2e+06
0e+00

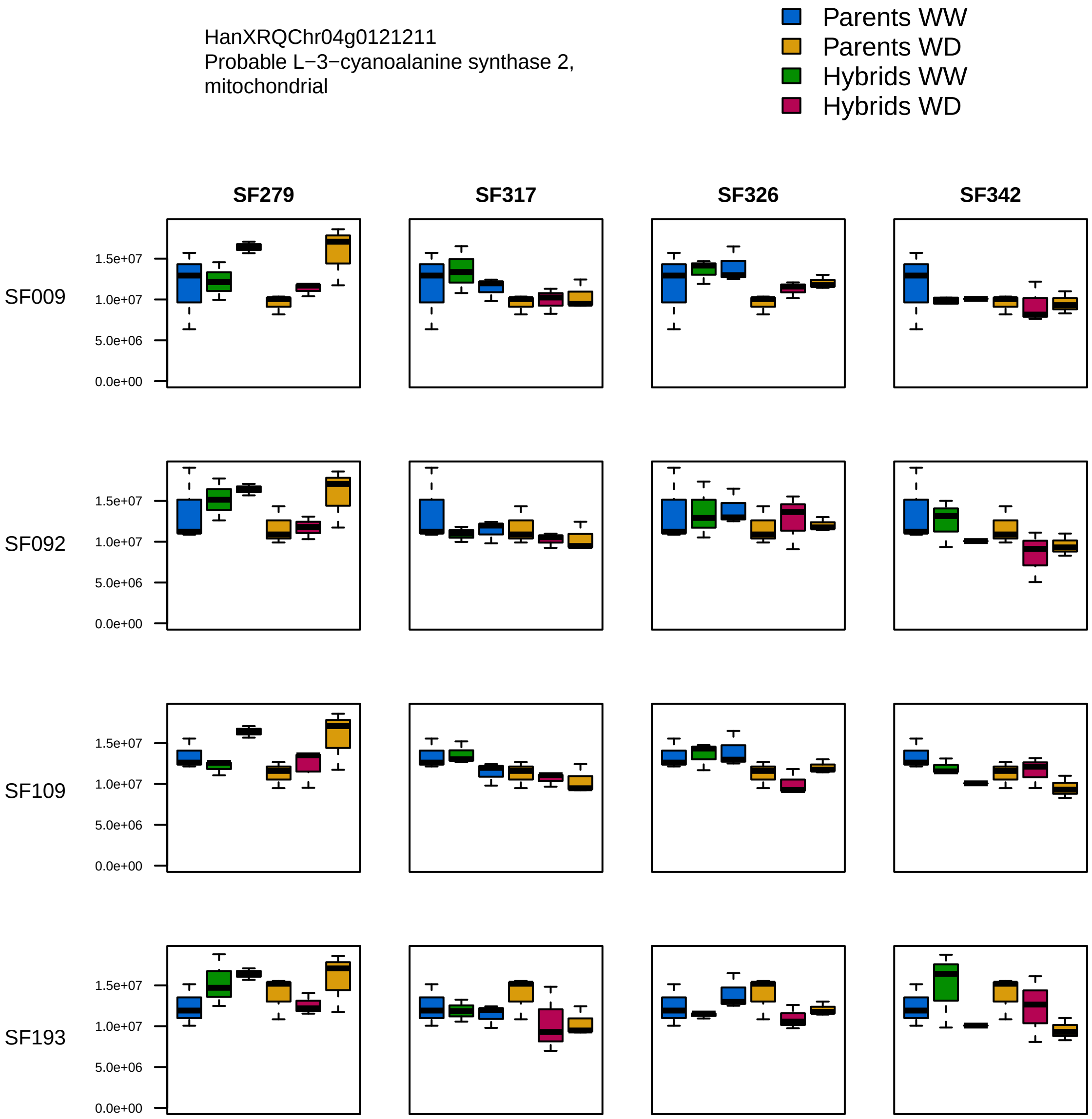

HanXRQChr04g0121211
Probable L−3−cyanoalanine synthase 2, mitochondrial
Parents WW
Parents WD
Hybrids WW
Hybrids WD
SF279
SF317
SF326
SF342
SF009
SF092
SF109
SF193
1.5e+07
1.0e+07
5.0e+06
0.0e+00

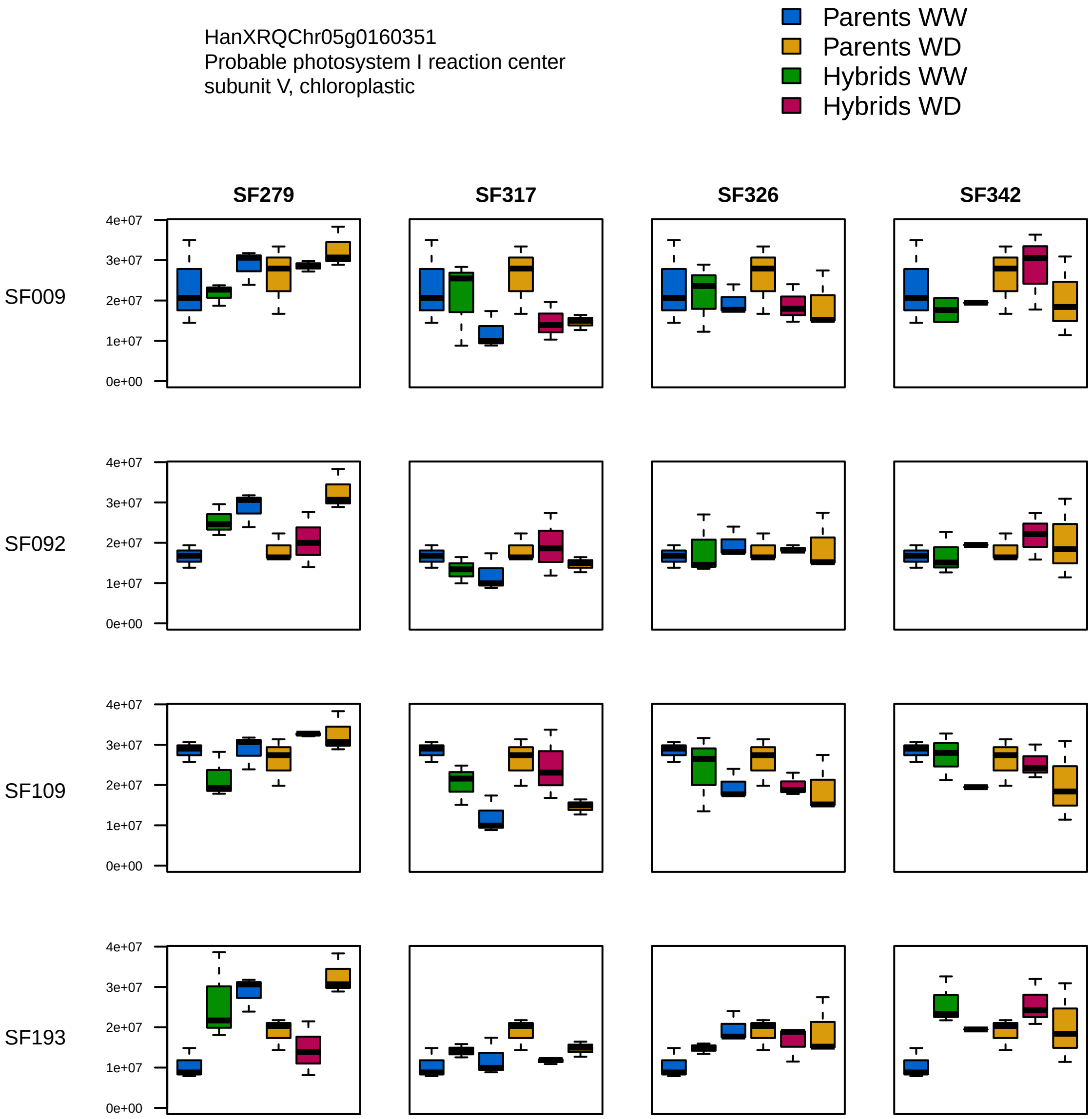

HanXRQChr05g0160351
Probable photosystem I reaction center
subunit V, chloroplastic
Parents WW
Parents WD
Hybrids WW
Hybrids WD
SF279
SF317
SF326
SF342
SF009
SF092
SF109
SF193
0e+00
1e+07
2e+07
3e+07
4e+07

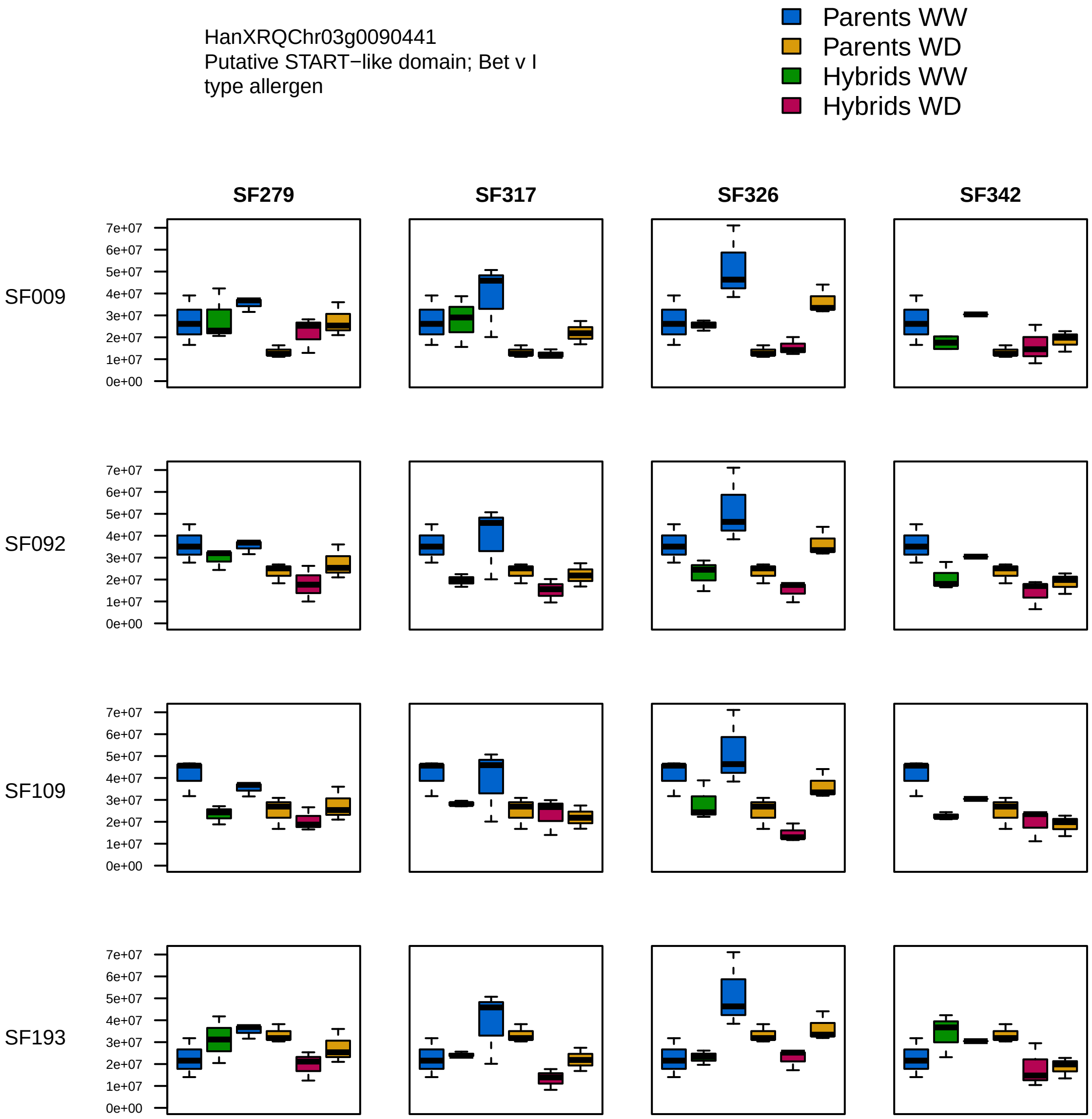
HanXRQChr03g0090441
Putative START−like domain; Bet v I
type allergen
Parents WW
Parents WD
Hybrids WW
Hybrids WD
SF279
SF317
SF326
SF342
SF009
SF092
SF109
SF193
7e+07
6e+07
5e+07
4e+07
3e+07
2e+07
1e+07
0e+00

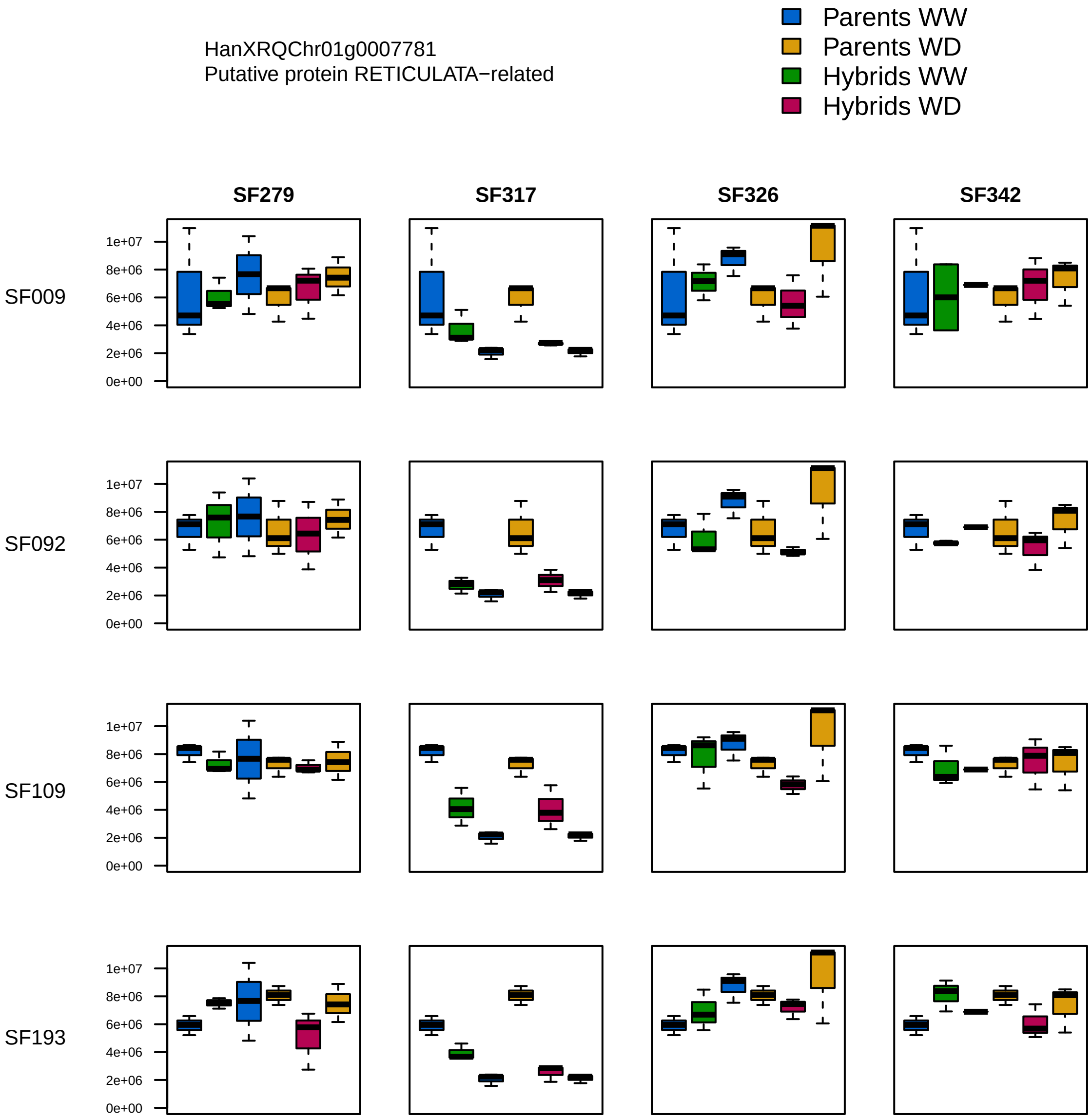
HanXRQChr01g0007781
Putative protein RETICULATA−related
Parents WW
Parents WD
Hybrids WW
Hybrids WD
SF279
SF317
SF326
SF342
SF009
SF092
SF109
SF193
1e+07
8e+06
6e+06
4e+06
2e+06
0e+00

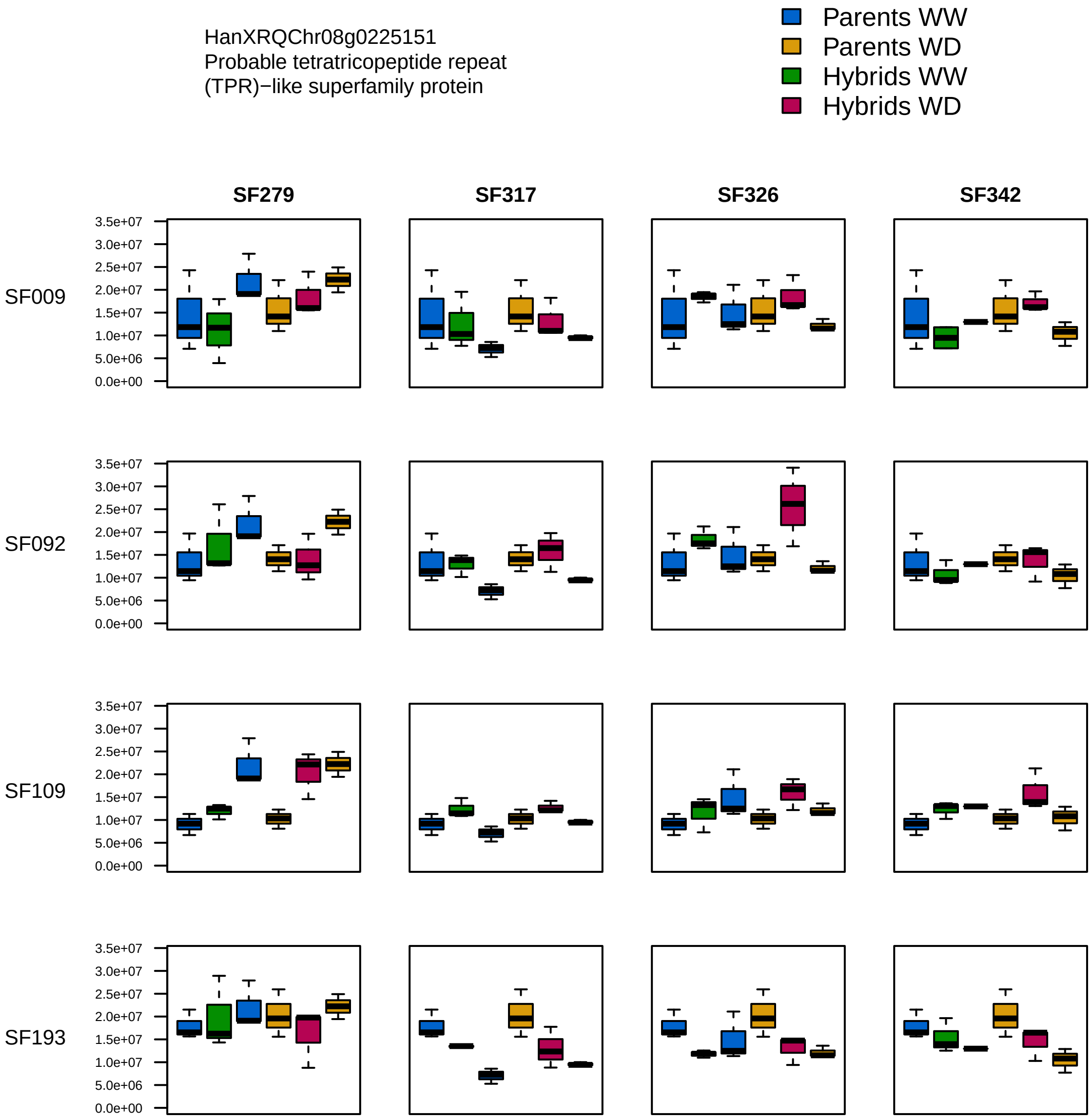
HanXRQChr08g0225151
Probable tetratricopeptide repeat
(TPR)−like superfamily protein
Parents WW
Parents WD
Hybrids WW
Hybrids WD
SF279
SF317
SF326
SF342
SF009
SF092
SF109
SF193
3.5e+07
3.0e+07
2.5e+07
2.0e+07
1.5e+07
1.0e+07
5.0e+06
0.0e+00

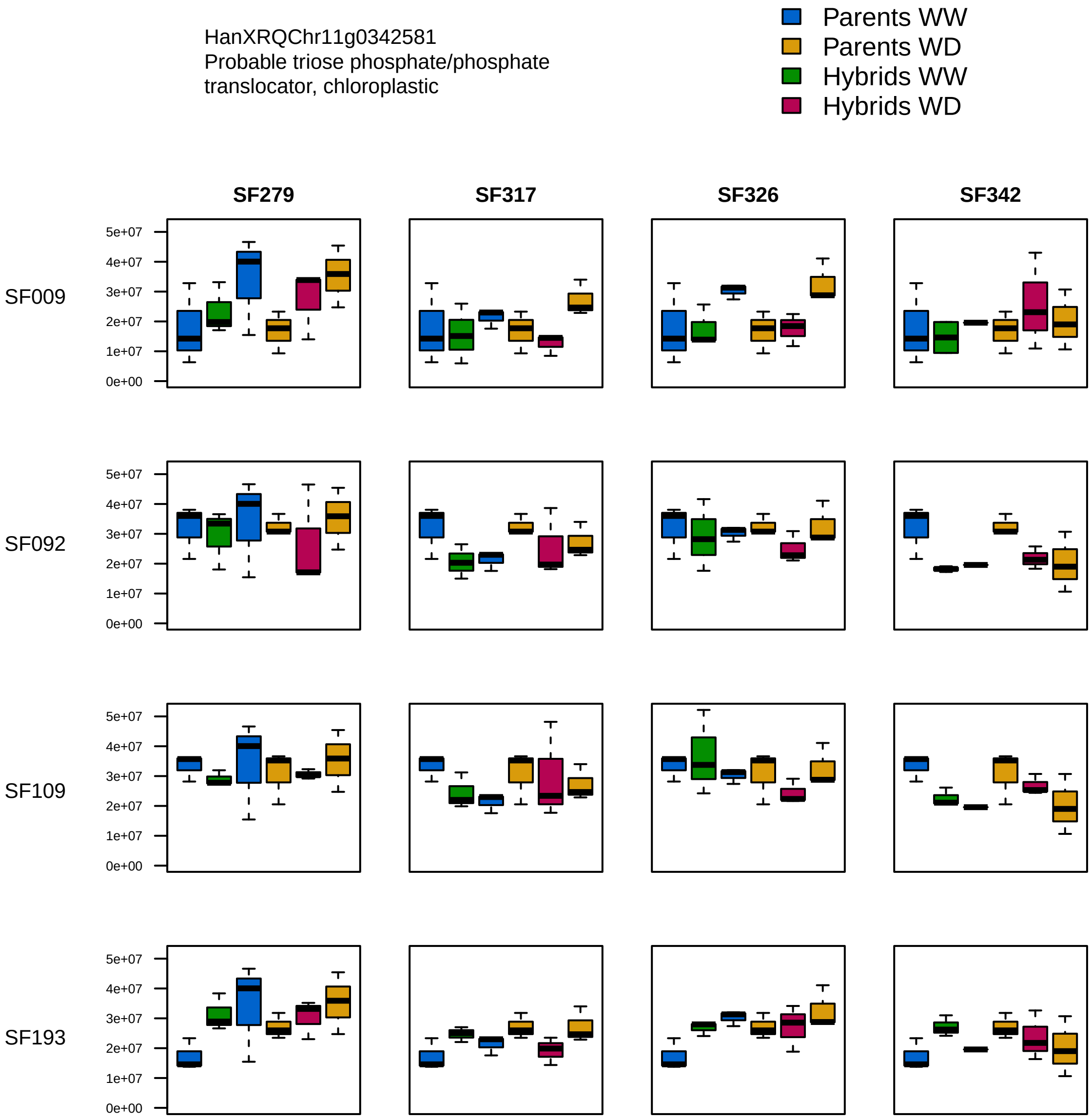

HanXRQChr11g0342581
Probable triose phosphate/phosphate translocator, chloroplastic
Parents WW
Parents WD
Hybrids WW
Hybrids WD
SF279
SF317
SF326
SF342
SF009
SF092
SF109
SF193
0e+00
1e+07
2e+07
3e+07
4e+07
5e+07

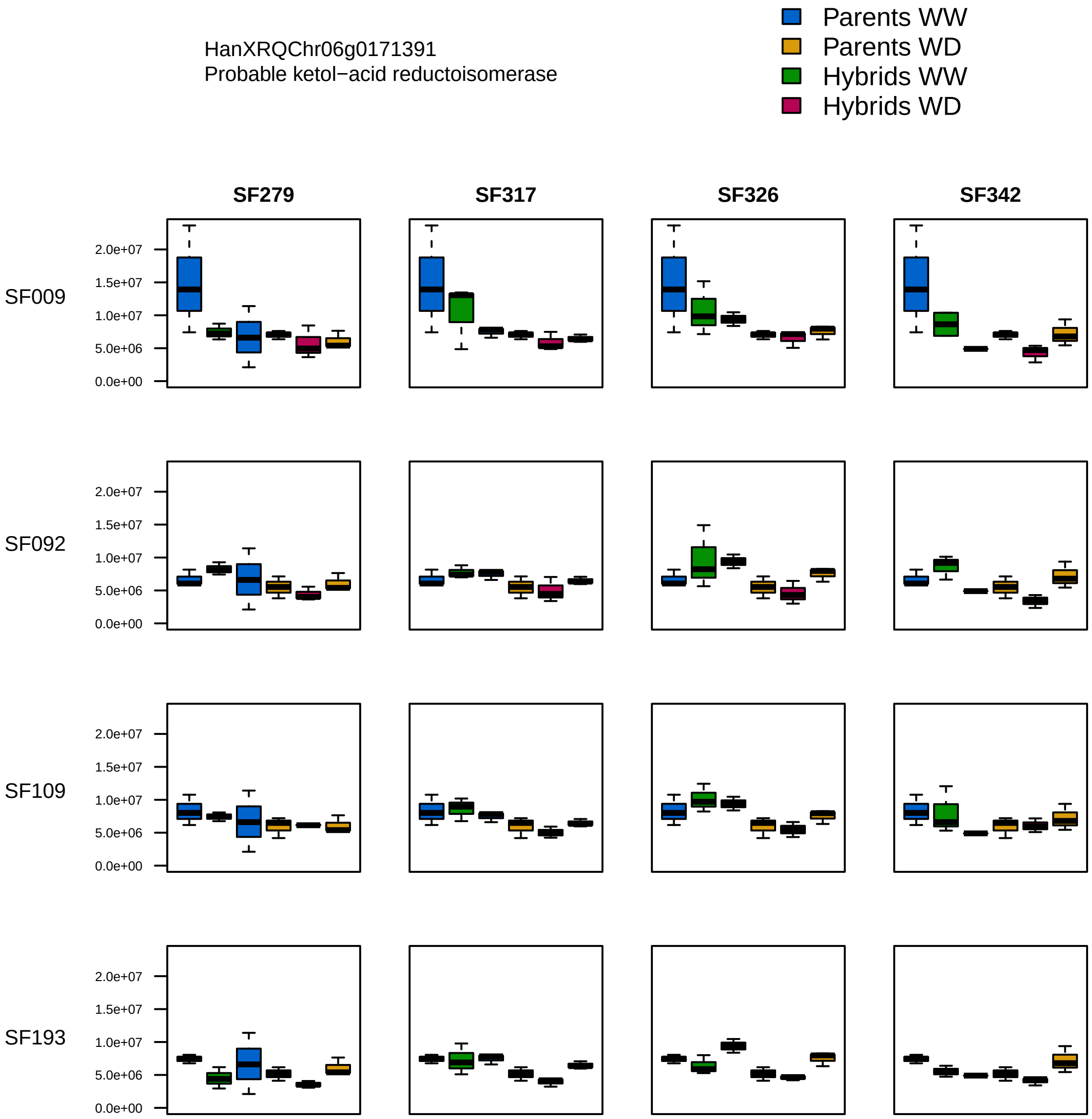
HanXRQChr06g0171391
Probable ketol−acid reductoisomerase
Parents WW
Parents WD
Hybrids WW
Hybrids WD
SF279
SF317
SF326
SF342
SF009
SF092
SF109
SF193
0.0e+00
5.0e+06
1.0e+07
1.5e+07
2.0e+07

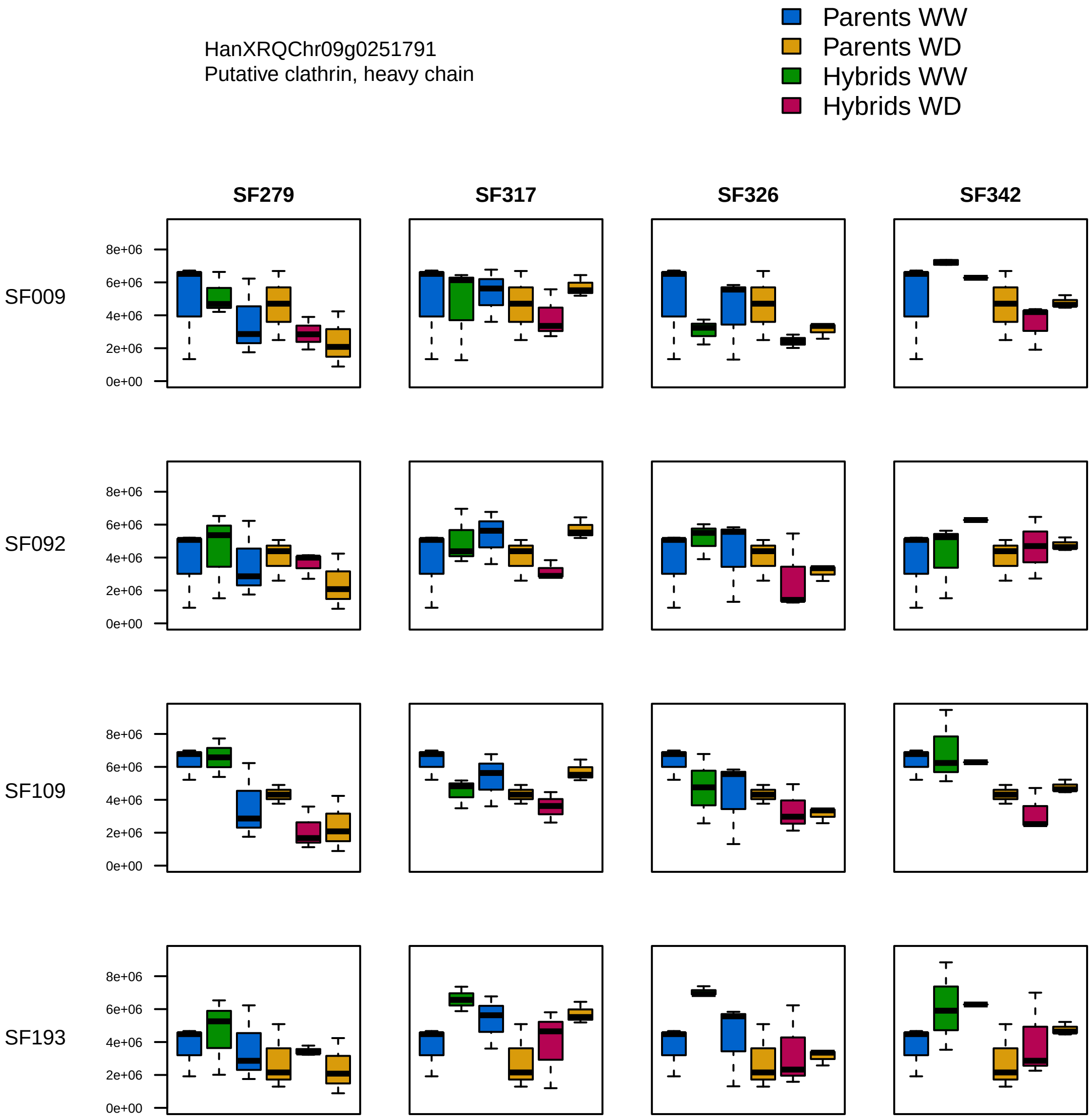
HanXRQChr09g0251791
Putative clathrin, heavy chain
Parents WW
Parents WD
Hybrids WW
Hybrids WD
SF279
SF317
SF326
SF342
SF009
SF092
SF109
SF193
8e+06
6e+06
4e+06
2e+06
0e+00

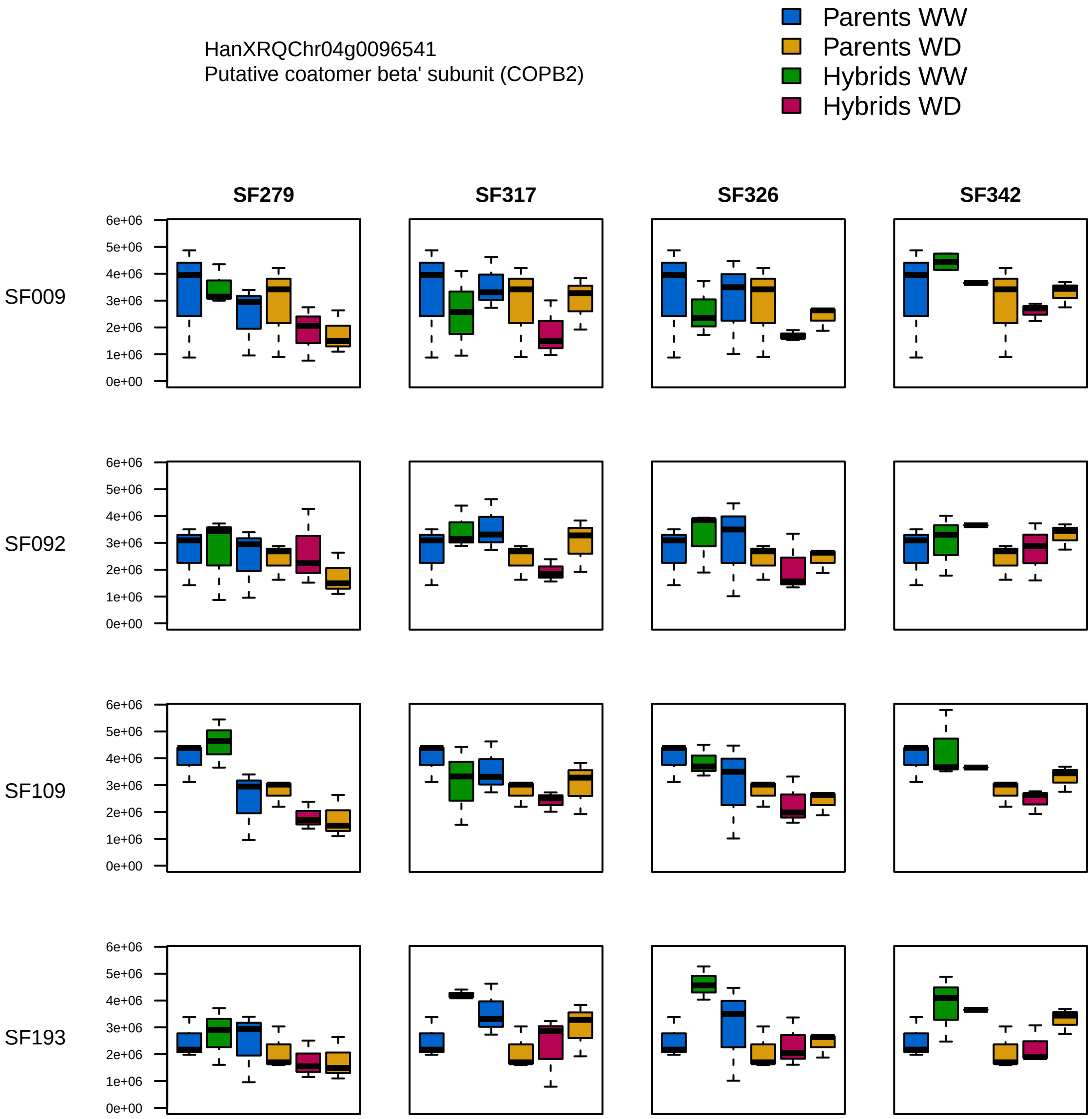
HanXRQChr04g0096541
Putative coatomer beta' subunit (COPB2)
Parents WW
Parents WD
Hybrids WW
Hybrids WD
SF279
SF317
SF326
SF342
SF009
SF092
SF109
SF193
0e+00
1e+06
2e+06
3e+06
4e+06
5e+06
6e+06

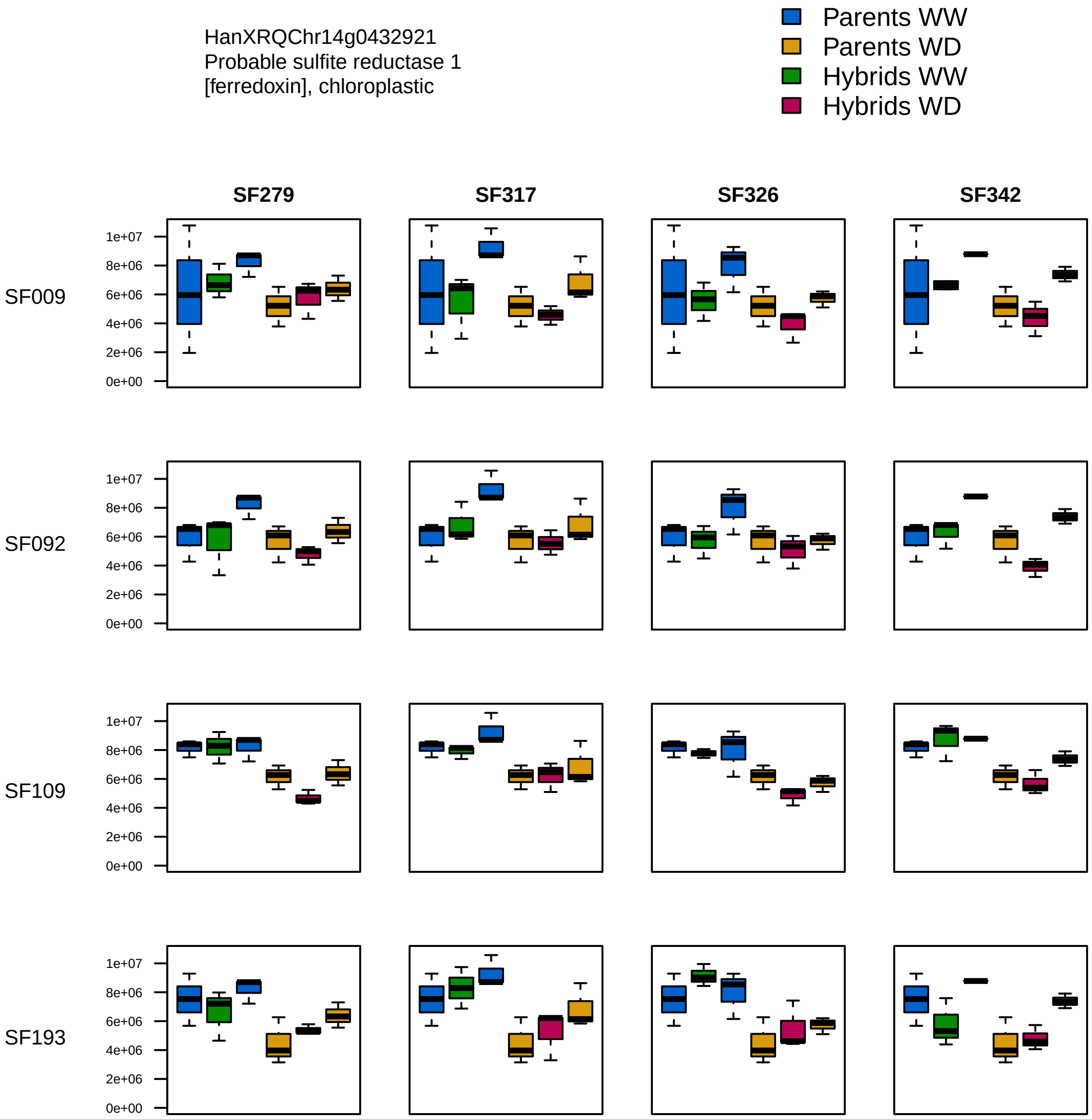

HanXRQChr14g0432921
Probable sulfite reductase 1
[ferredoxin], chloroplastic
Parents WW
Parents WD
Hybrids WW
Hybrids WD
SF279
SF317
SF326
SF342
SF009
SF092
SF109
SF193
1e+07
8e+06
6e+06
4e+06
2e+06
0e+00

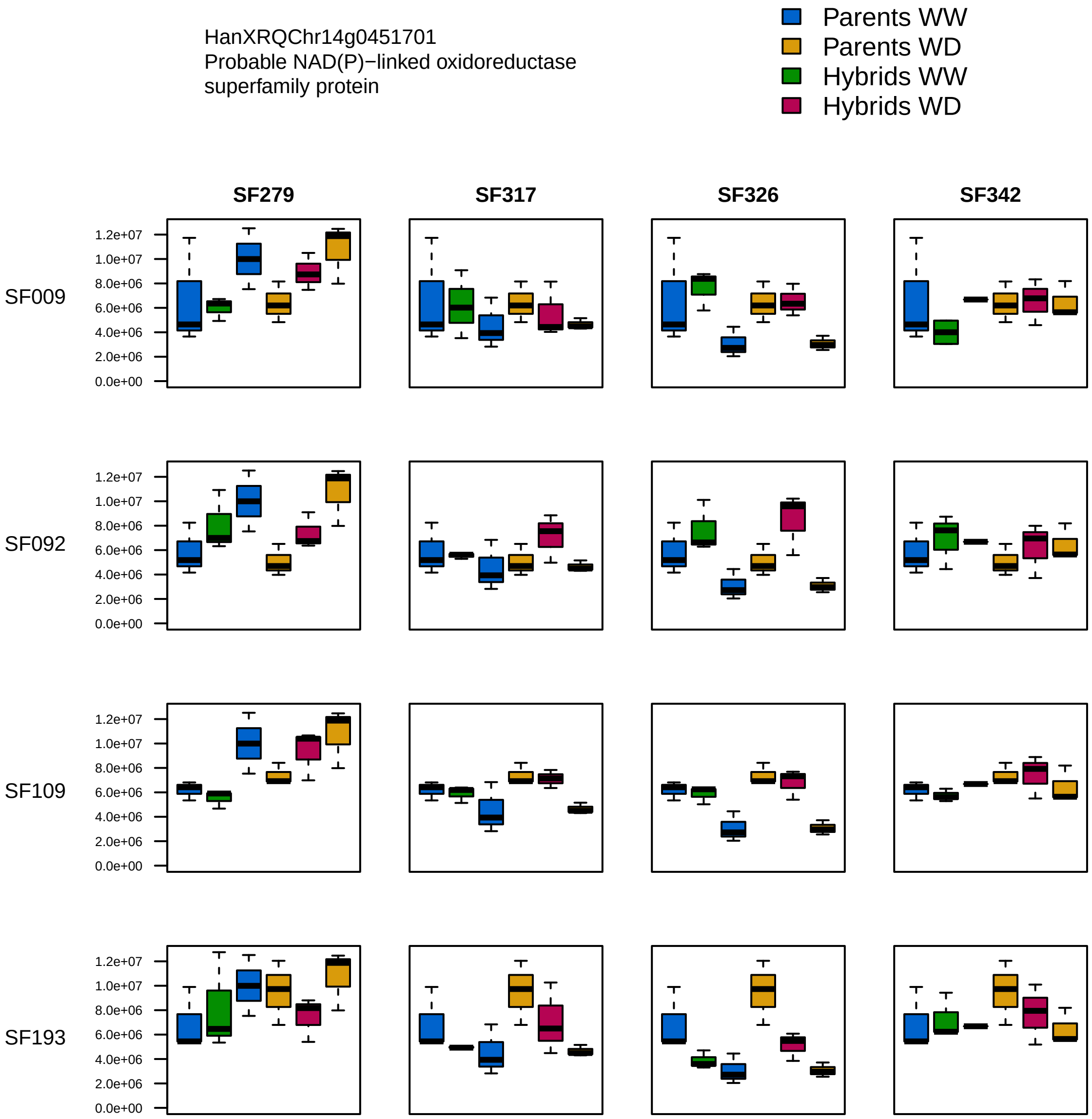
HanXRQChr14g0451701
Probable NAD(P)−linked oxidoreductase
superfamily protein
Parents WW
Parents WD
Hybrids WW
Hybrids WD
SF279
SF317
SF326
SF342
SF009
SF092
SF109
SF193
0.0e+00
2.0e+06
4.0e+06
6.0e+06
8.0e+06
1.0e+07
1.2e+07

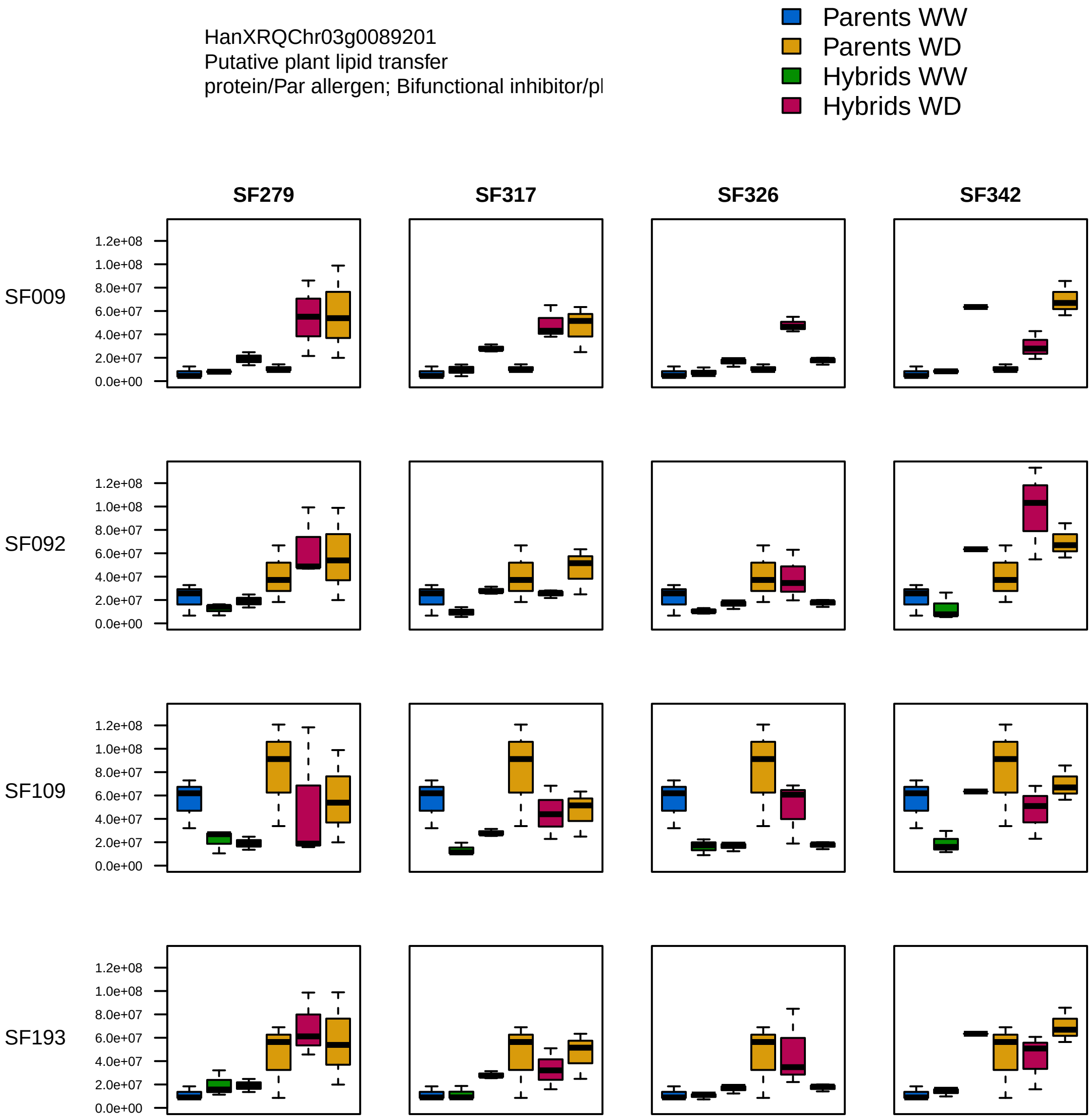

HanXRQChr03g0089201
Putative plant lipid transfer
protein/Par allergen; Bifunctional inhibitor/pl
Parents WW
Parents WD
Hybrids WW
Hybrids WD
SF279
SF317
SF326
SF342
SF009
SF092
SF109
SF193
1.2e+08
1.0e+08
8.0e+07
6.0e+07
4.0e+07
2.0e+07
0.0e+00

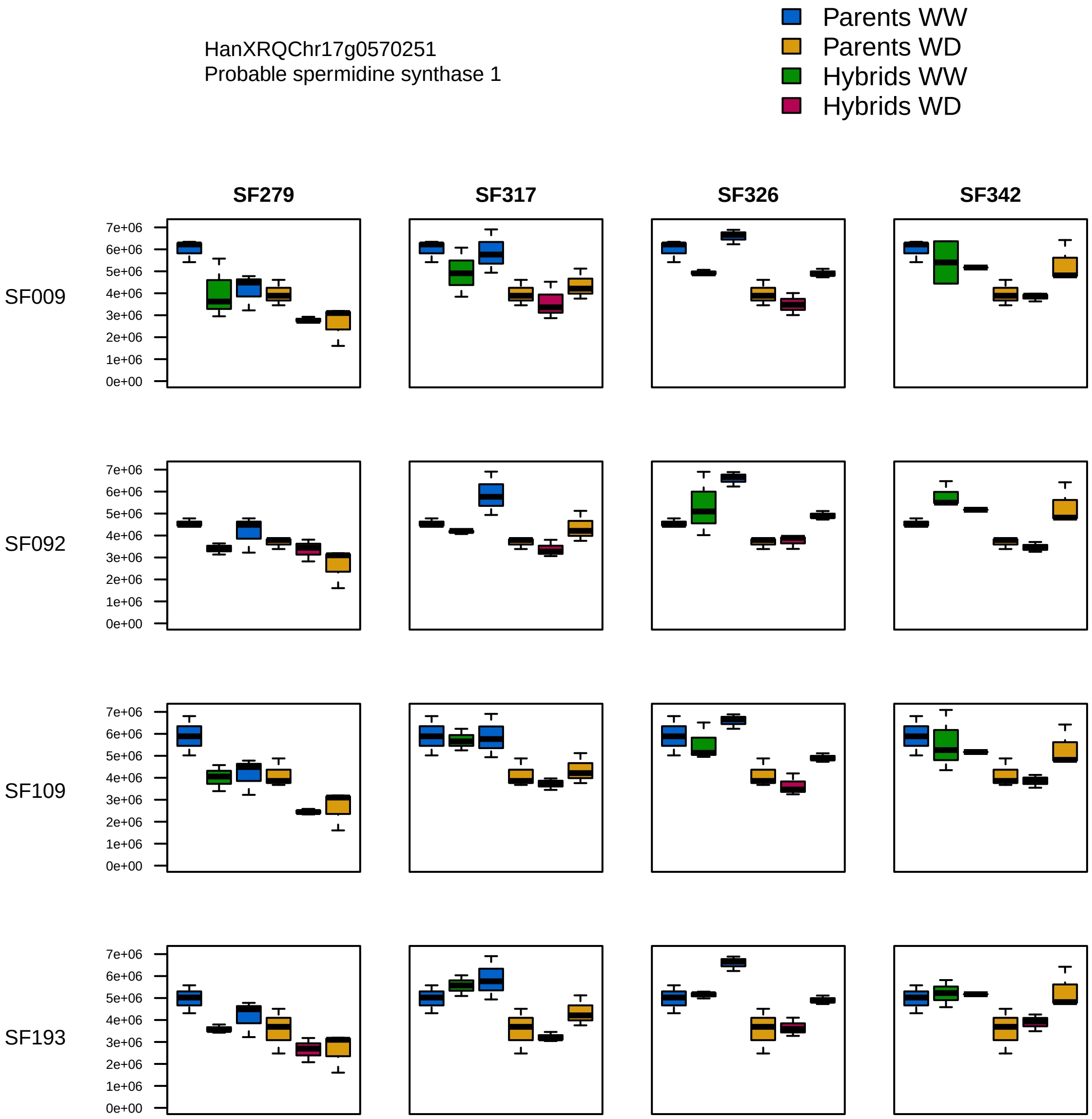

HanXRQChr17g0570251
Probable spermidine synthase 1
Parents WW
Parents WD
Hybrids WW
Hybrids WD
SF279
SF317
SF326
SF342
SF009
SF092
SF109
SF193
7e+06
6e+06
5e+06
4e+06
3e+06
2e+06
1e+06
0e+00

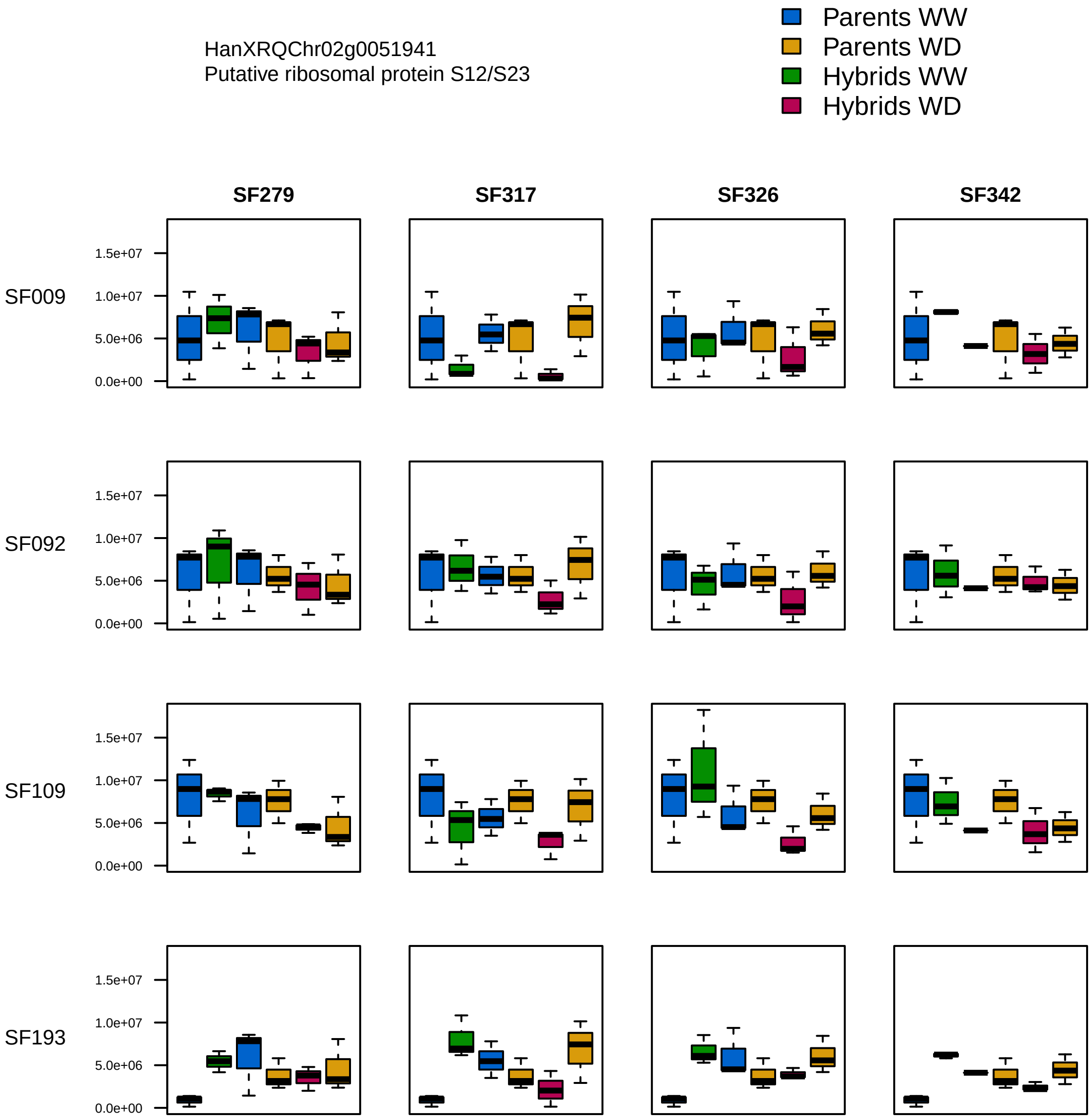

HanXRQChr02g0051941
Putative ribosomal protein S12/S23
Parents WW
Parents WD
Hybrids WW
Hybrids WD
SF279
SF317
SF326
SF342
SF009
SF092
SF109
SF193
1.5e+07
1.0e+07
5.0e+06
0.0e+00

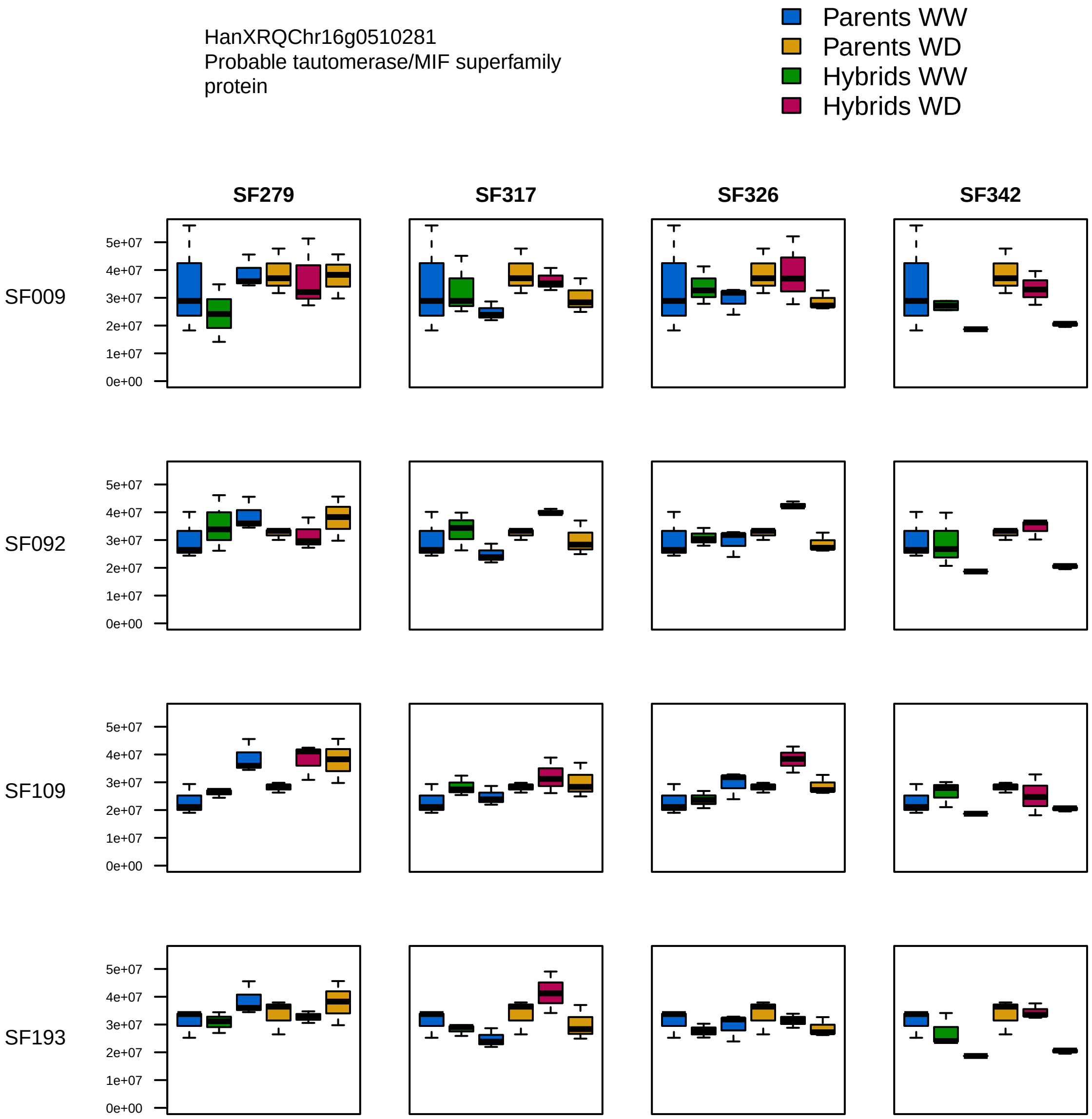

HanXRQChr16g0510281
Probable tautomerase/MIF superfamily protein
Parents WW
Parents WD
Hybrids WW
Hybrids WD
SF279
SF317
SF326
SF342
SF009
SF092
SF109
SF193
0e+00
1e+07
2e+07
3e+07
4e+07
5e+07

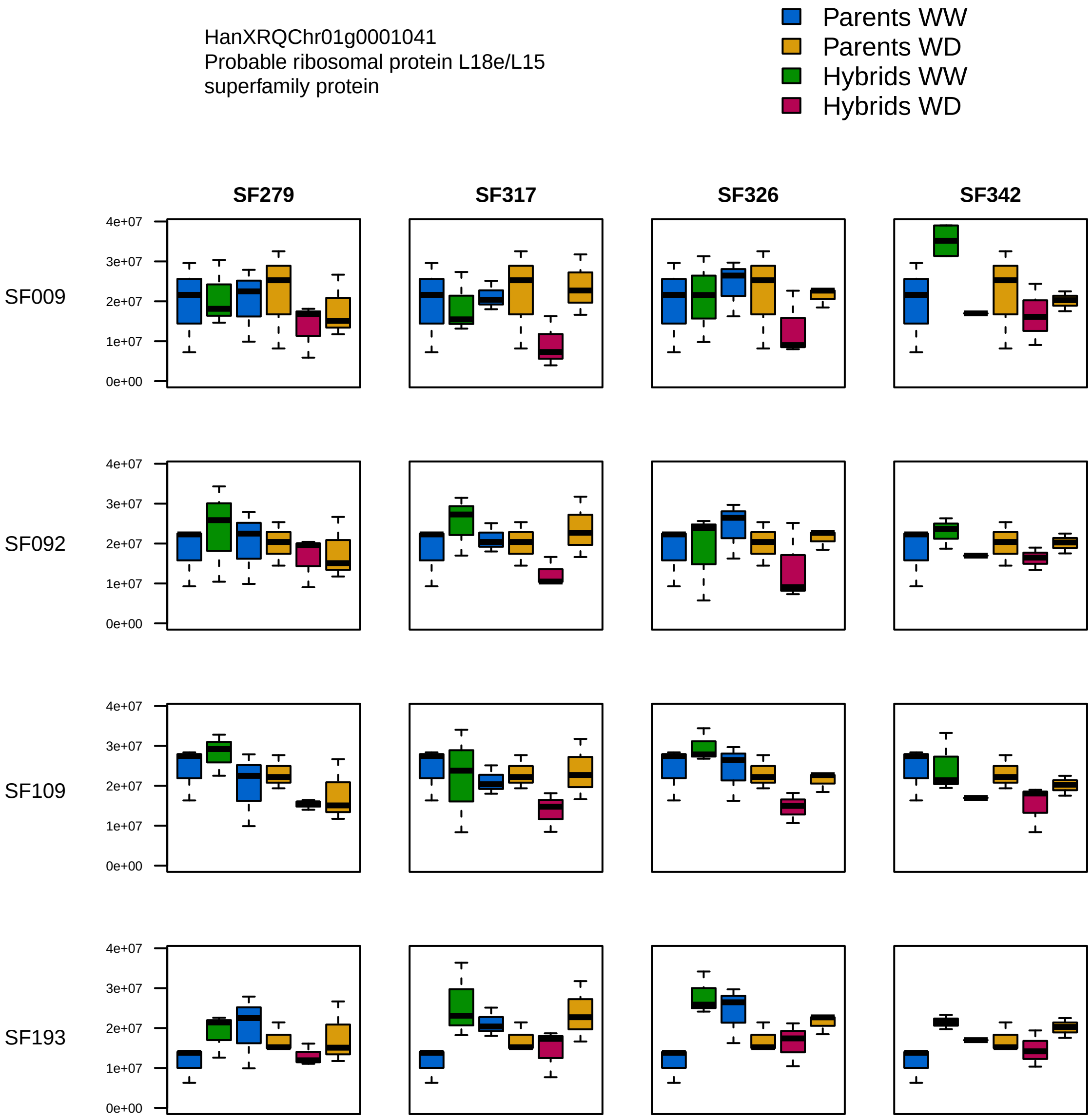
HanXRQChr01g0001041
Probable ribosomal protein L18e/L15 superfamily protein
Parents WW
Parents WD
Hybrids WW
Hybrids WD
SF279
SF317
SF326
SF342
SF009
SF092
SF109
SF193
4e+07
3e+07
2e+07
1e+07
0e+00

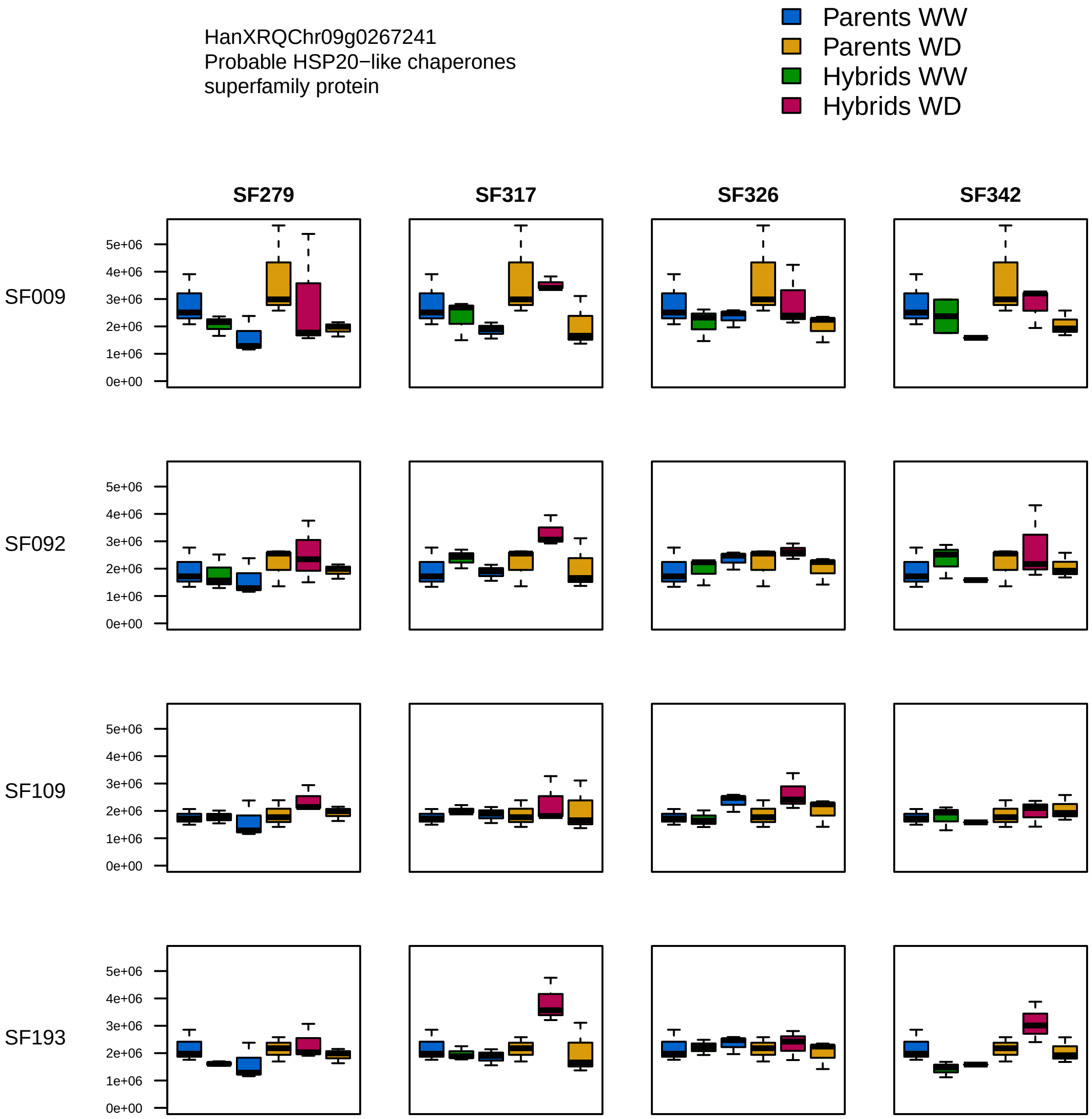
HanXRQChr09g0267241
Probable HSP20−like chaperones
superfamily protein
Parents WW
Parents WD
Hybrids WW
Hybrids WD
SF279
SF317
SF326
SF342
SF009
SF092
SF109
SF193
0e+00
1e+06
2e+06
3e+06
4e+06
5e+06

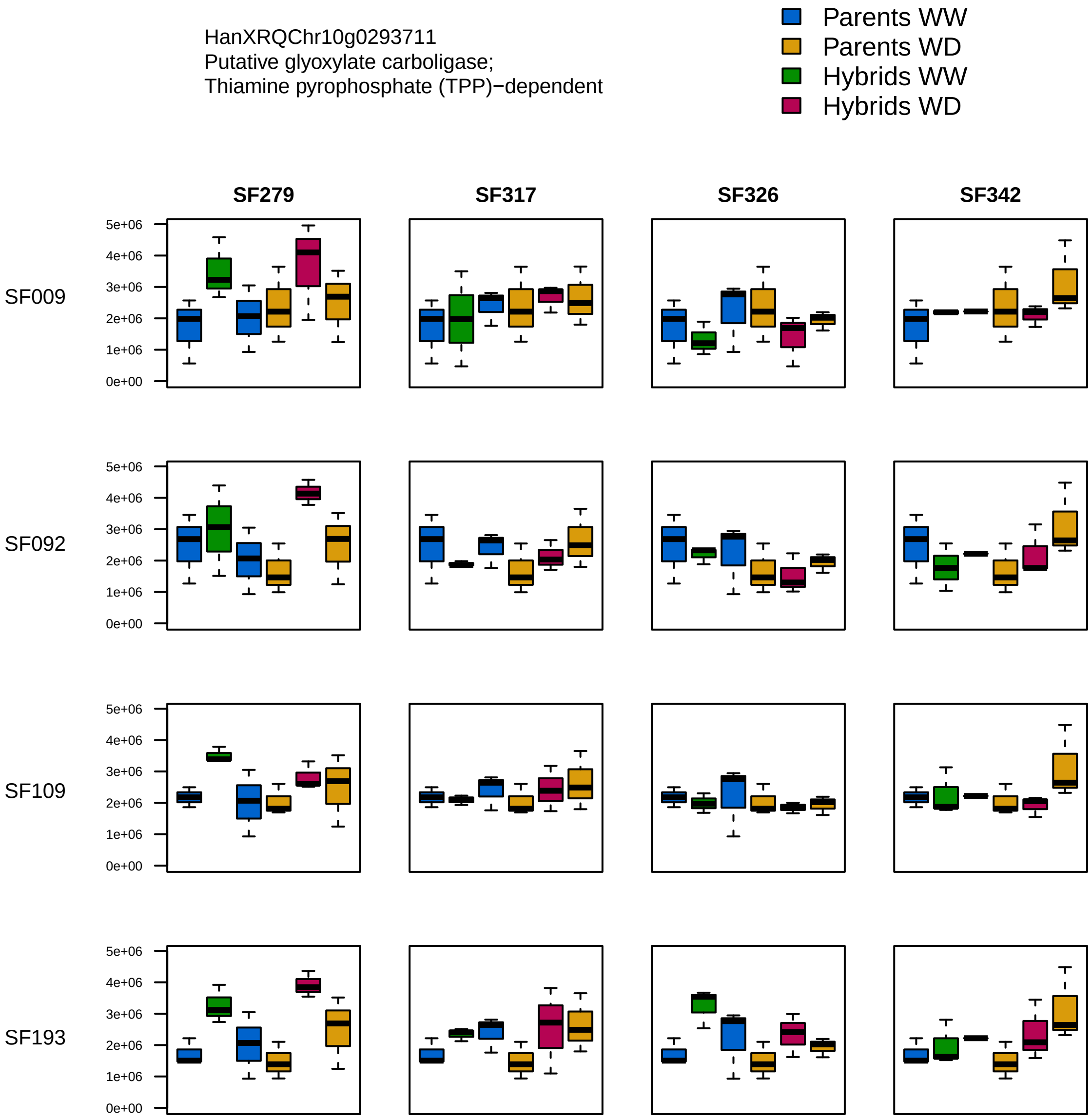
HanXRQChr10g0293711
Putative glyoxylate carboligase;
Thiamine pyrophosphate (TPP)−dependent
Parents WW
Parents WD
Hybrids WW
Hybrids WD
SF279
SF317
SF326
SF342
SF009
SF092
SF109
SF193
0e+00
1e+06
2e+06
3e+06
4e+06
5e+06

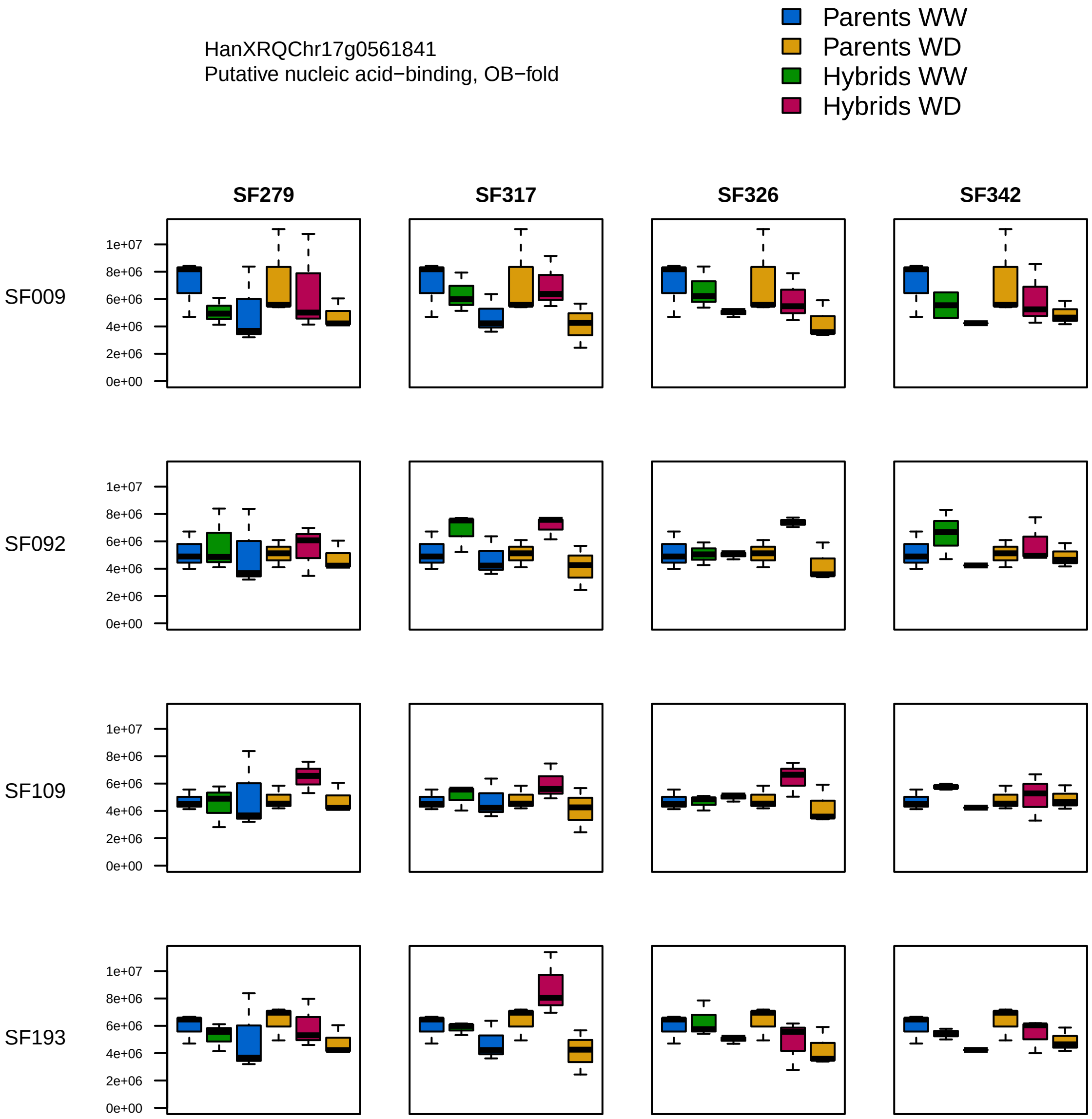

HanXRQChr17g0561841
Putative nucleic acid−binding, OB−fold
Parents WW
Parents WD
Hybrids WW
Hybrids WD
SF279
SF317
SF326
SF342
SF009
SF092
SF109
SF193
1e+07
8e+06
6e+06
4e+06
2e+06
0e+00

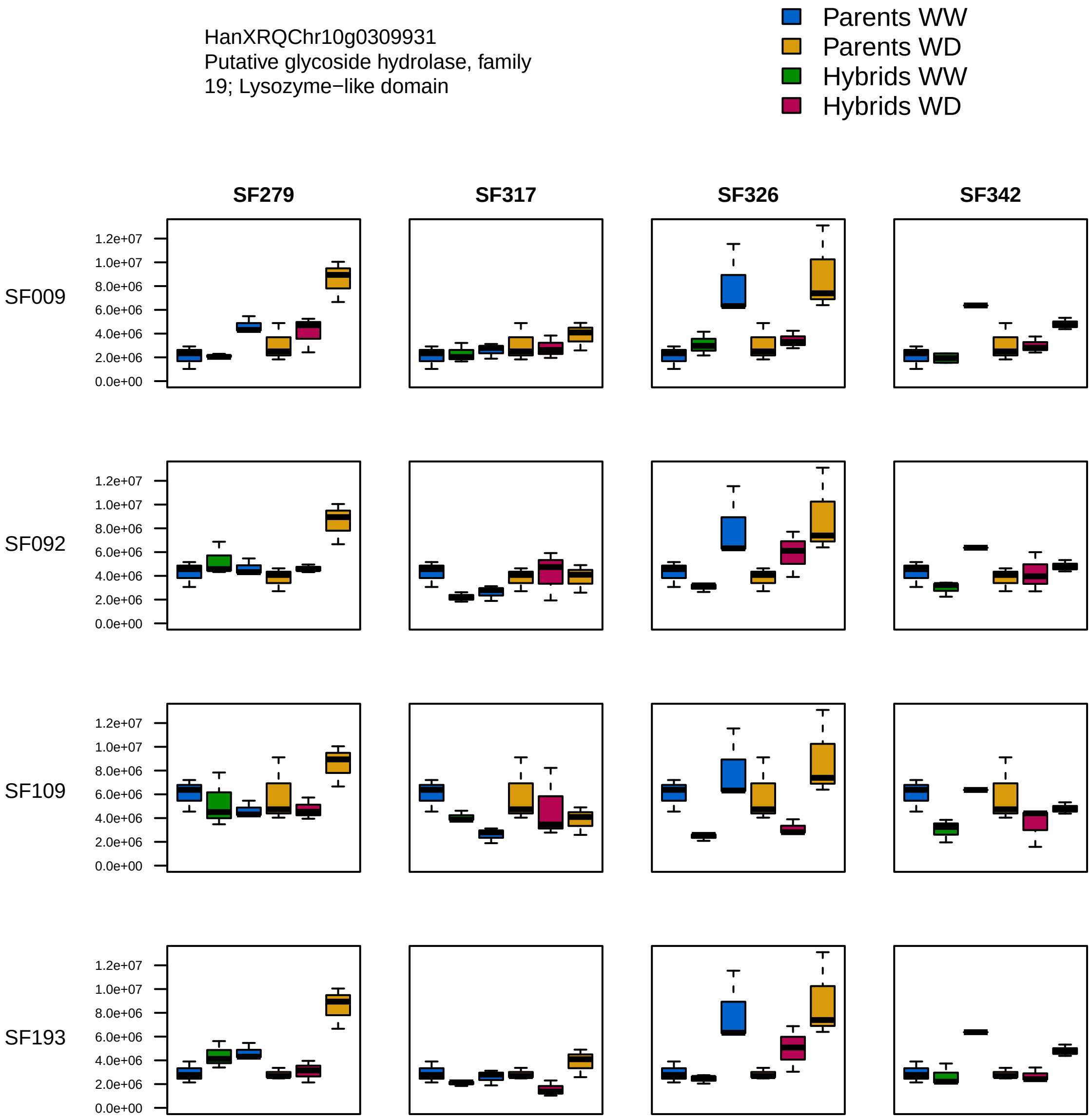

HanXRQChr10g0309931
Putative glycoside hydrolase, family 19; Lysozyme−like domain
Parents WW
Parents WD
Hybrids WW
Hybrids WD
SF279
SF317
SF326
SF342
SF009
SF092
SF109
SF193
1.2e+07
1.0e+07
8.0e+06
6.0e+06
4.0e+06
2.0e+06
0.0e+00

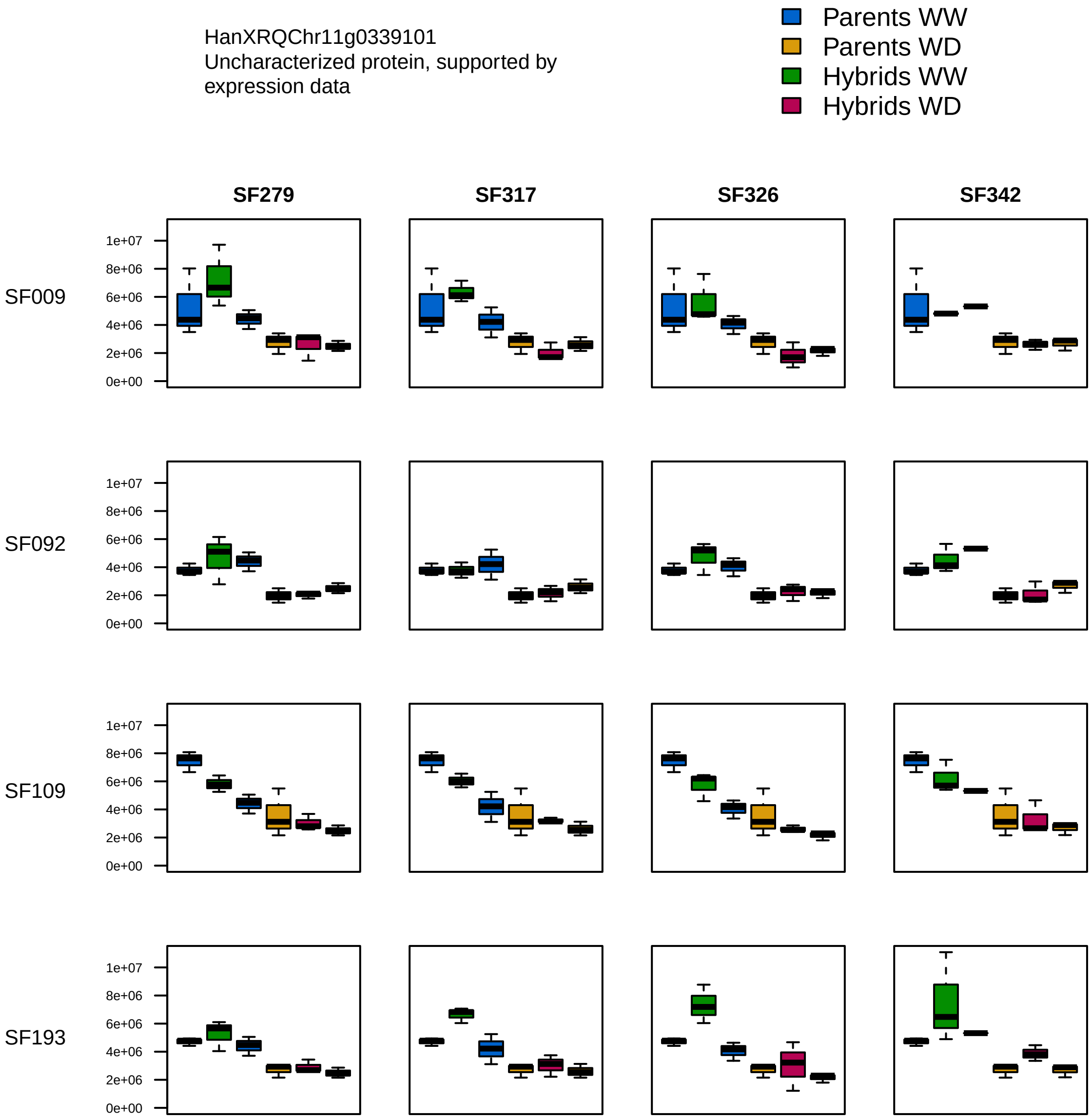
HanXRQChr11g0339101
Uncharacterized protein, supported by
expression data
Parents WW
Parents WD
Hybrids WW
Hybrids WD
SF279
SF317
SF326
SF342
SF009
SF092
SF109
SF193
1e+07
8e+06
6e+06
4e+06
2e+06
0e+00

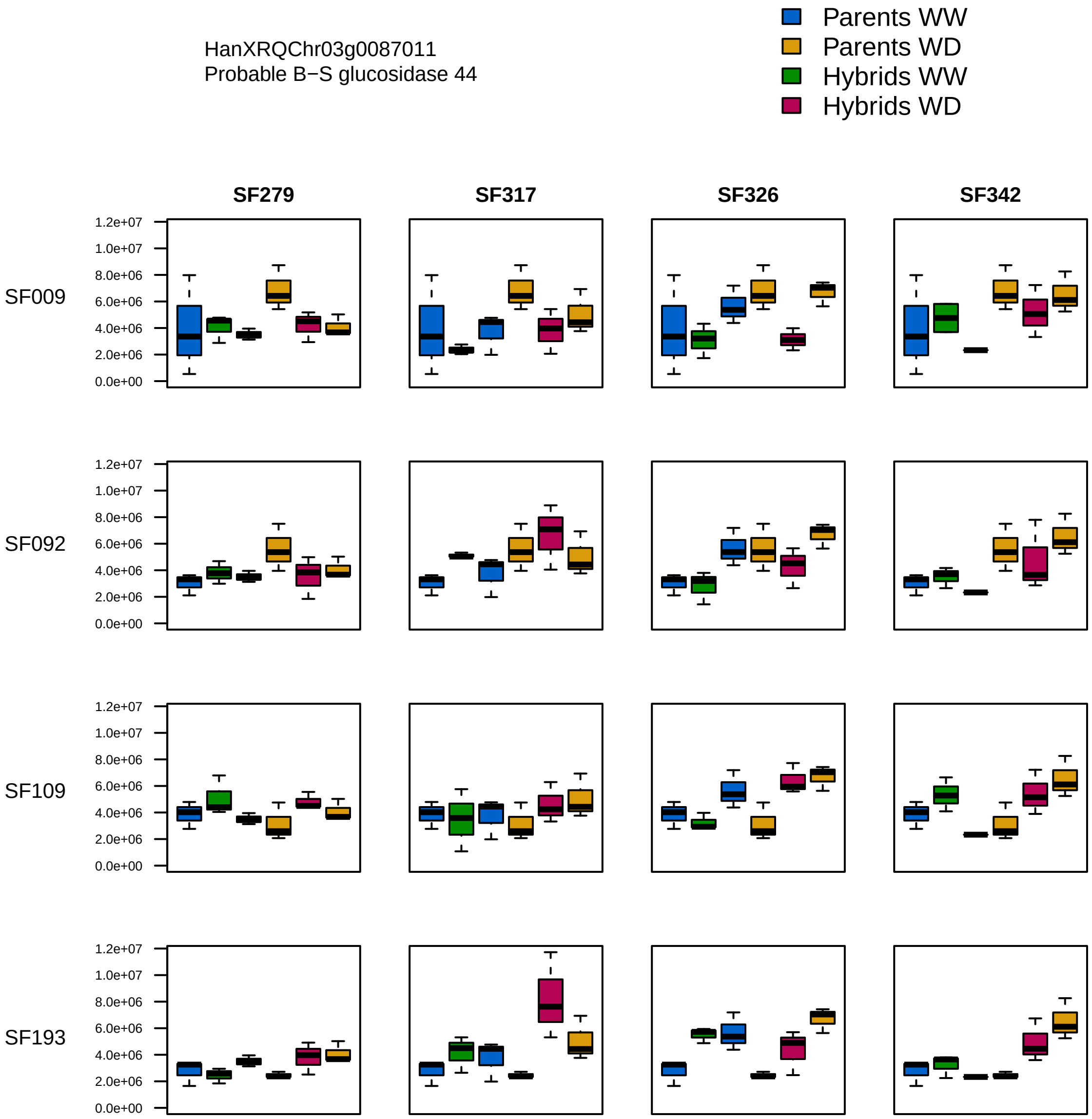
HanXRQChr03g0087011
Probable B−S glucosidase 44
Parents WW
Parents WD
Hybrids WW
Hybrids WD
SF279
SF317
SF326
SF342
SF009
SF092
SF109
SF193
1.2e+07
1.0e+07
8.0e+06
6.0e+06
4.0e+06
2.0e+06
0.0e+00

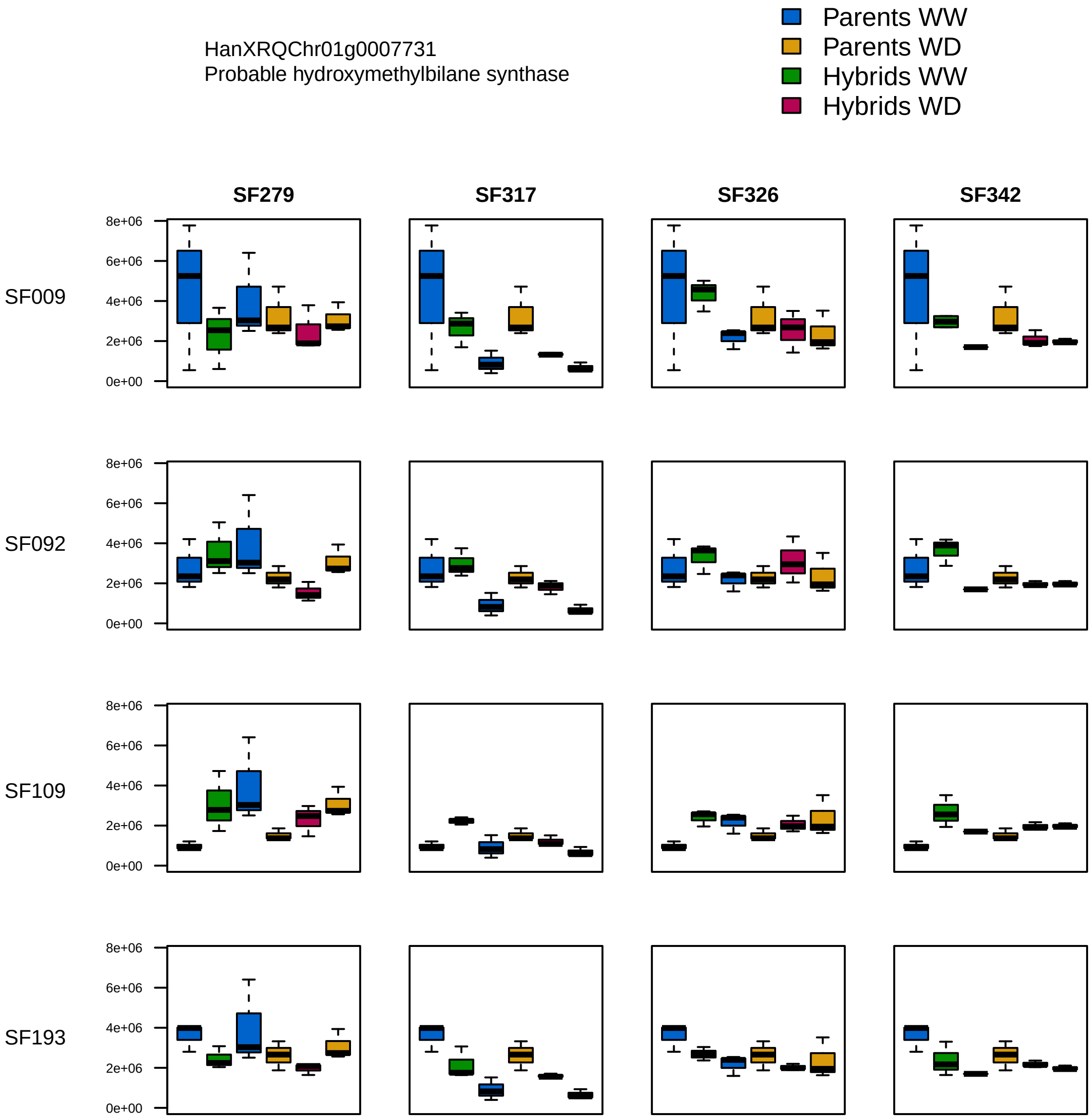
HanXRQChr01g0007731
Probable hydroxymethylbilane synthase
Parents WW
Parents WD
Hybrids WW
Hybrids WD
SF279
SF317
SF326
SF342
SF009
SF092
SF109
SF193
0e+00
2e+06
4e+06
6e+06
8e+06

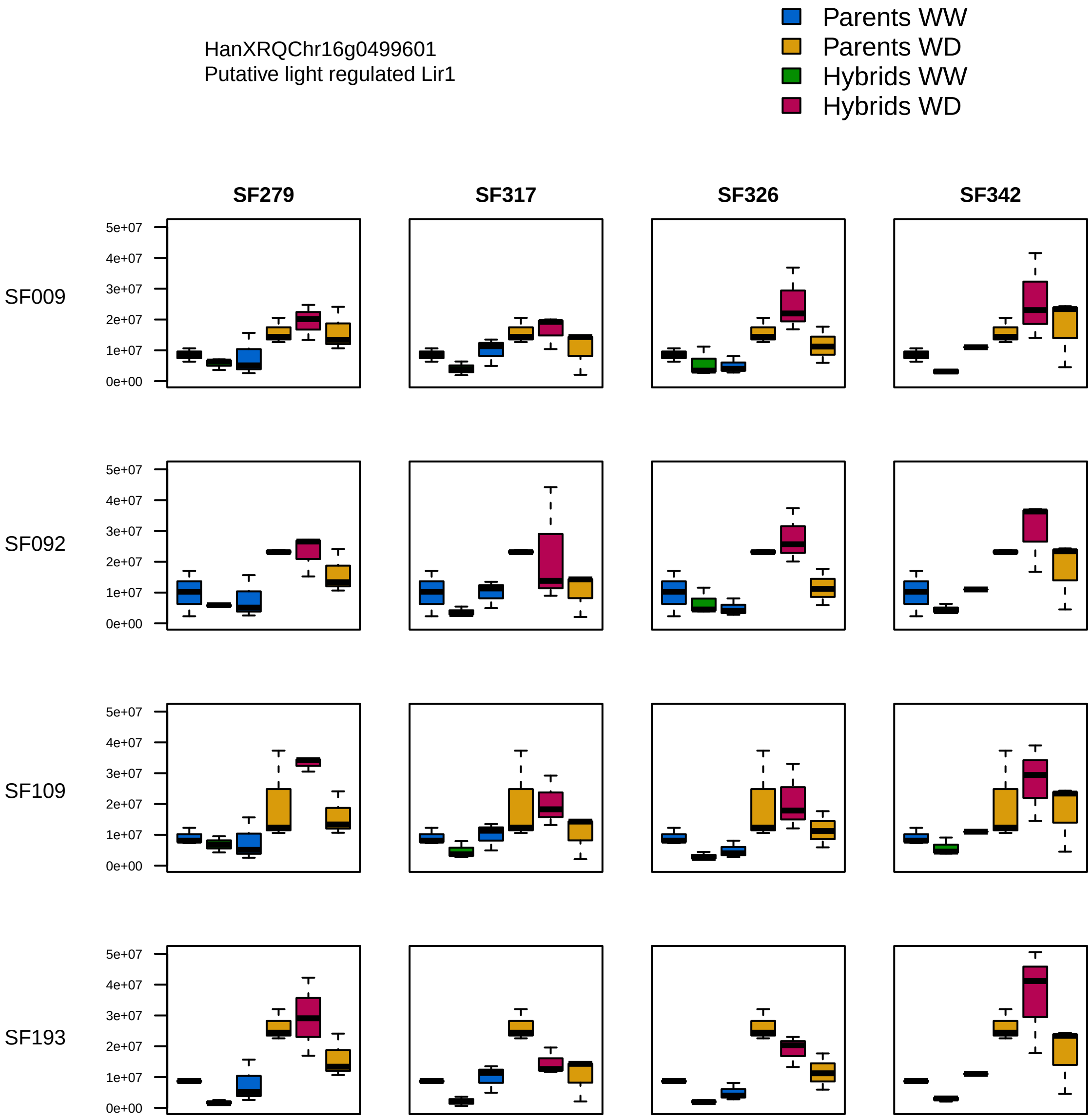

HanXRQChr16g0499601
Putative light regulated Lir1
Parents WW
Parents WD
Hybrids WW
Hybrids WD
SF279
SF317
SF326
SF342
SF009
SF092
SF109
SF193
5e+07
4e+07
3e+07
2e+07
1e+07
0e+00

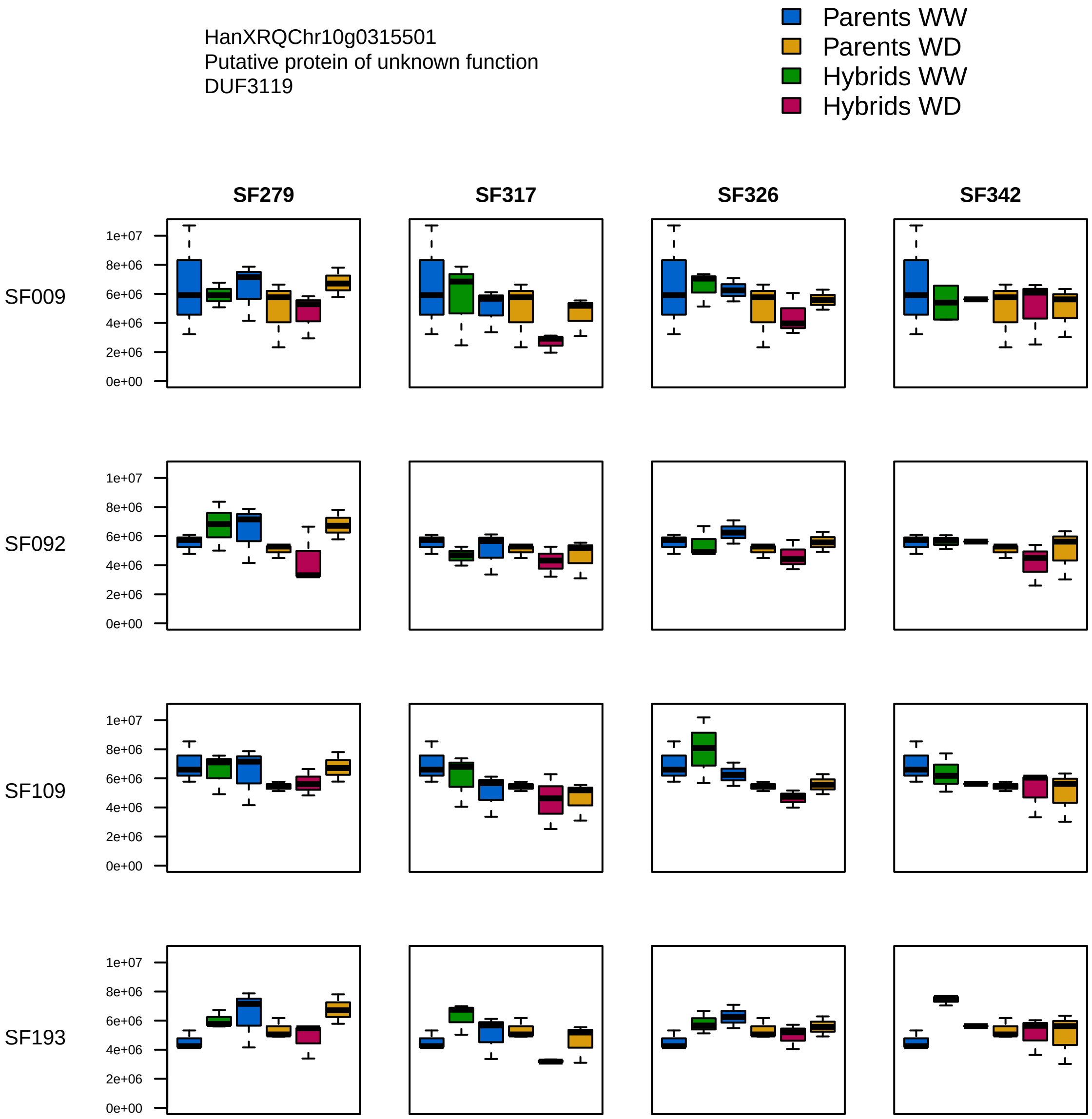
HanXRQChr10g0315501
Putative protein of unknown function
DUF3119
Parents WW
Parents WD
Hybrids WW
Hybrids WD
SF279
SF317
SF326
SF342
SF009
SF092
SF109
SF193
1e+07
8e+06
6e+06
4e+06
2e+06
0e+00

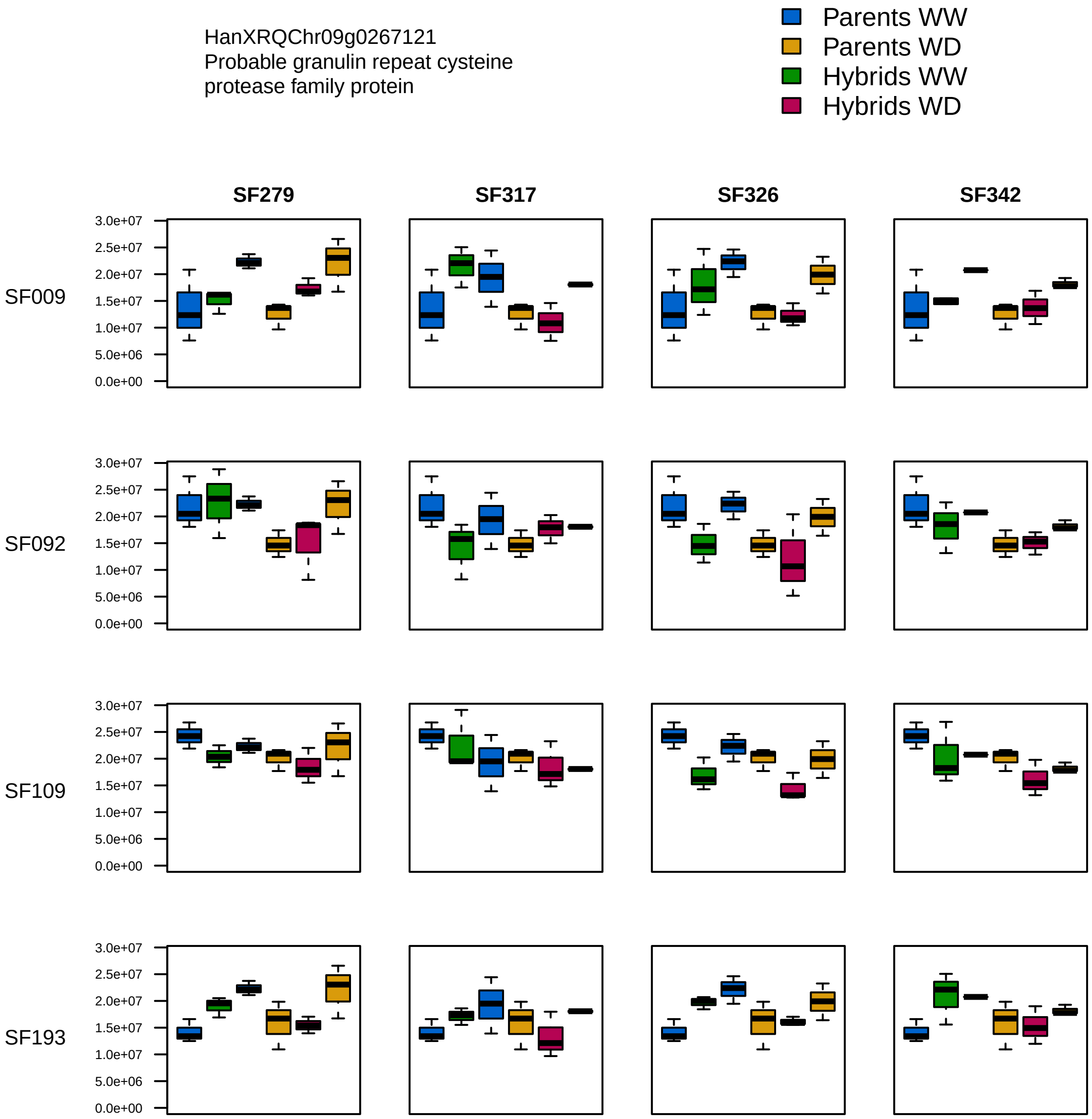
HanXRQChr09g0267121
Probable granulin repeat cysteine protease family protein
Parents WW
Parents WD
Hybrids WW
Hybrids WD
SF279
SF317
SF326
SF342
SF009
SF092
SF109
SF193
3.0e+07
2.5e+07
2.0e+07
1.5e+07
1.0e+07
5.0e+06
0.0e+00

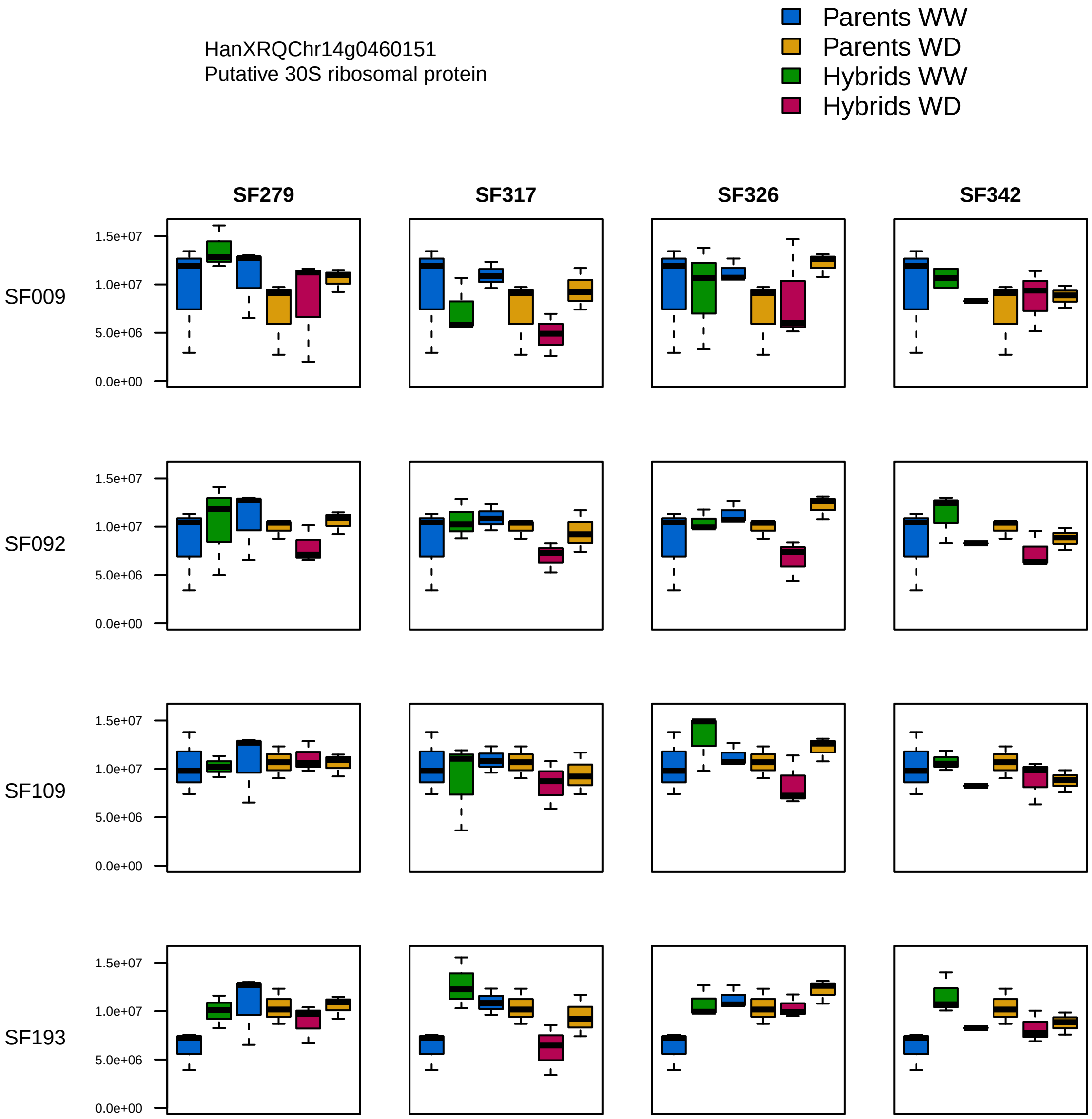
HanXRQChr14g0460151
Putative 30S ribosomal protein
Parents WW
Parents WD
Hybrids WW
Hybrids WD
SF279
SF317
SF326
SF342
SF009
SF092
SF109
SF193
1.5e+07
1.0e+07
5.0e+06
0.0e+00

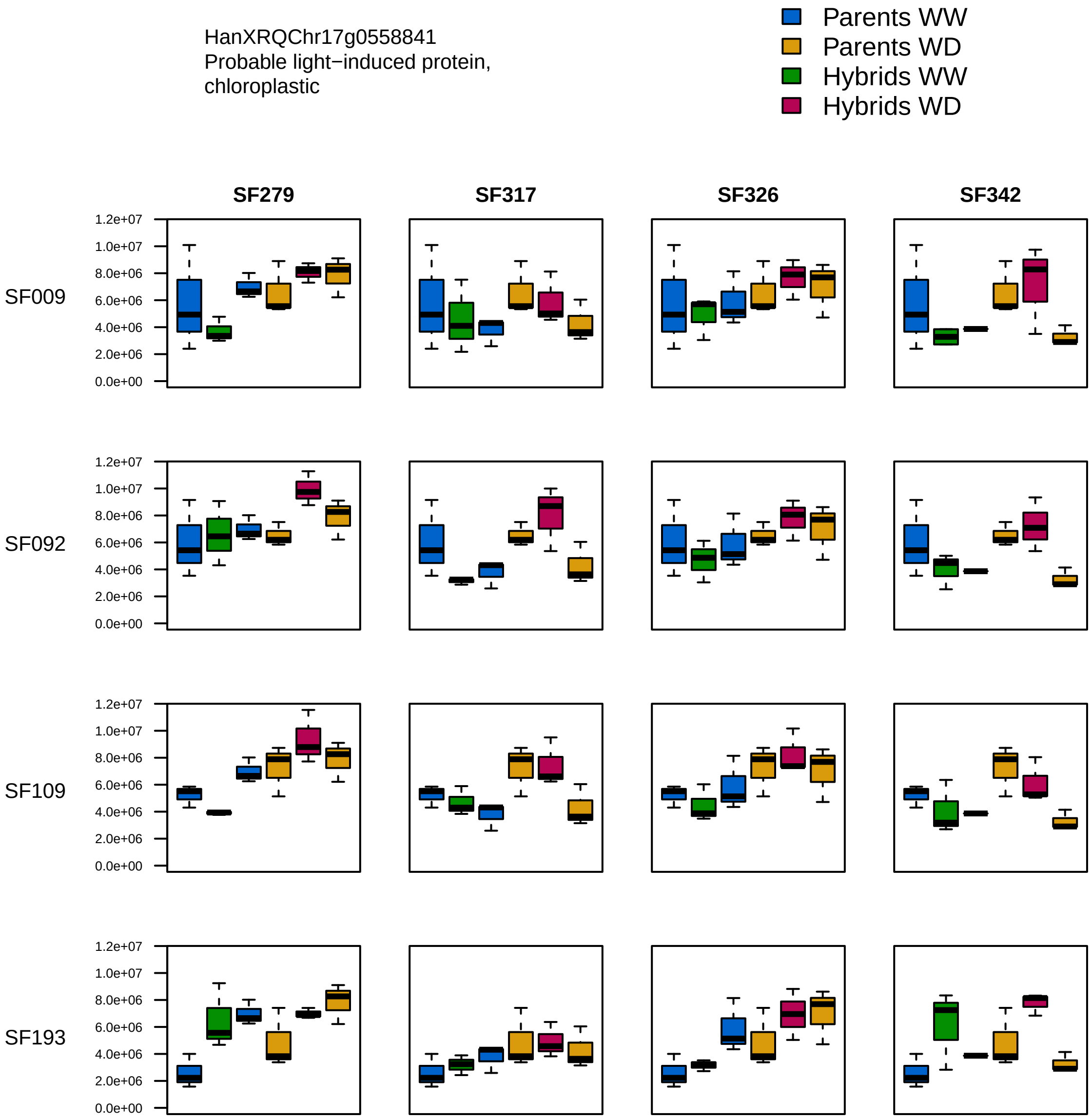

HanXRQChr17g0558841
Probable light−induced protein,
chloroplastic
Parents WW
Parents WD
Hybrids WW
Hybrids WD
SF279
SF317
SF326
SF342
SF009
SF092
SF109
SF193
1.2e+07
1.0e+07
8.0e+06
6.0e+06
4.0e+06
2.0e+06
0.0e+00

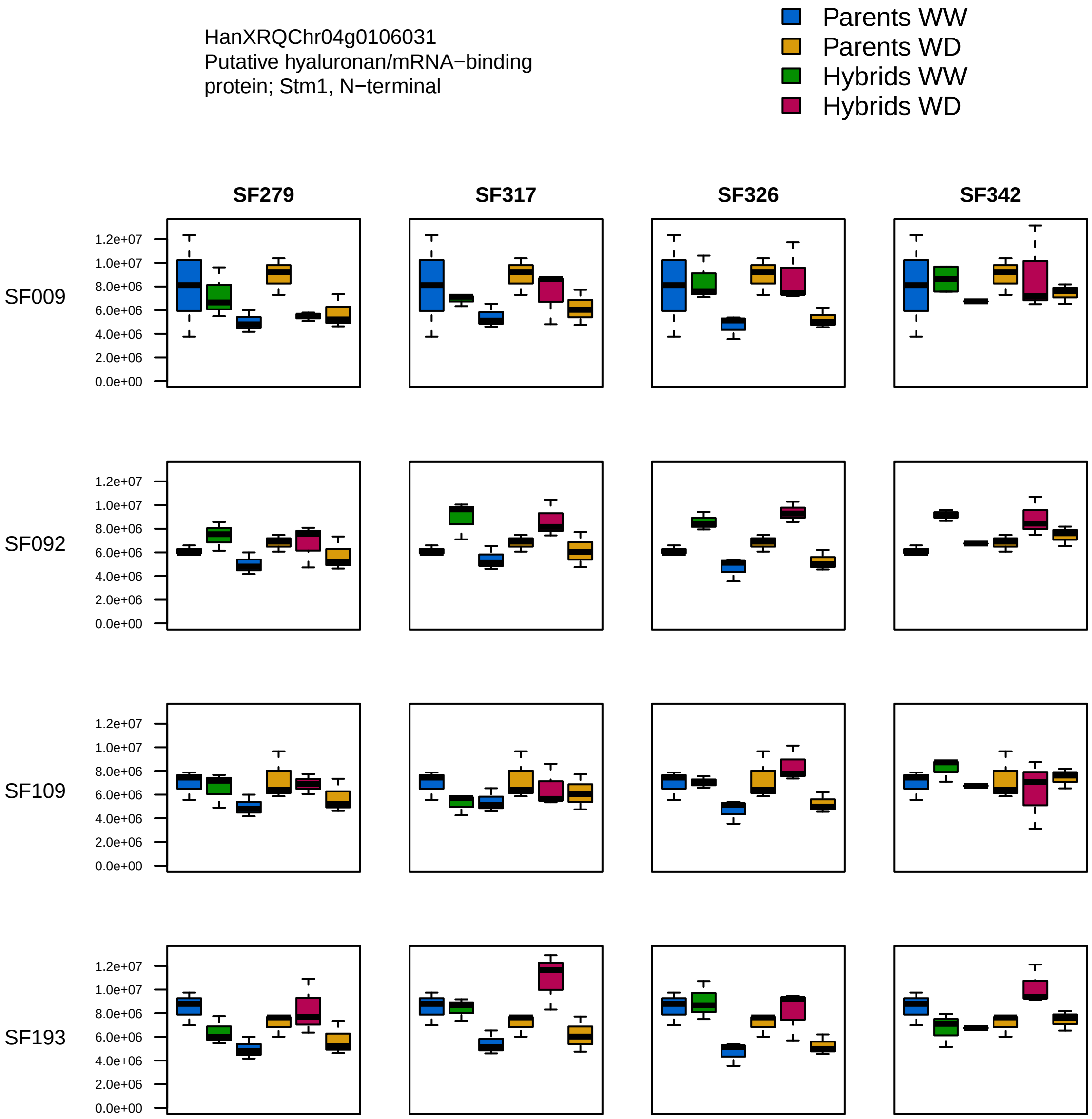
HanXRQChr04g0106031
Putative hyaluronan/mRNA−binding
protein; Stm1, N−terminal
Parents WW
Parents WD
Hybrids WW
Hybrids WD
SF279
SF317
SF326
SF342
SF009
SF092
SF109
SF193
1.2e+07
1.0e+07
8.0e+06
6.0e+06
4.0e+06
2.0e+06
0.0e+00

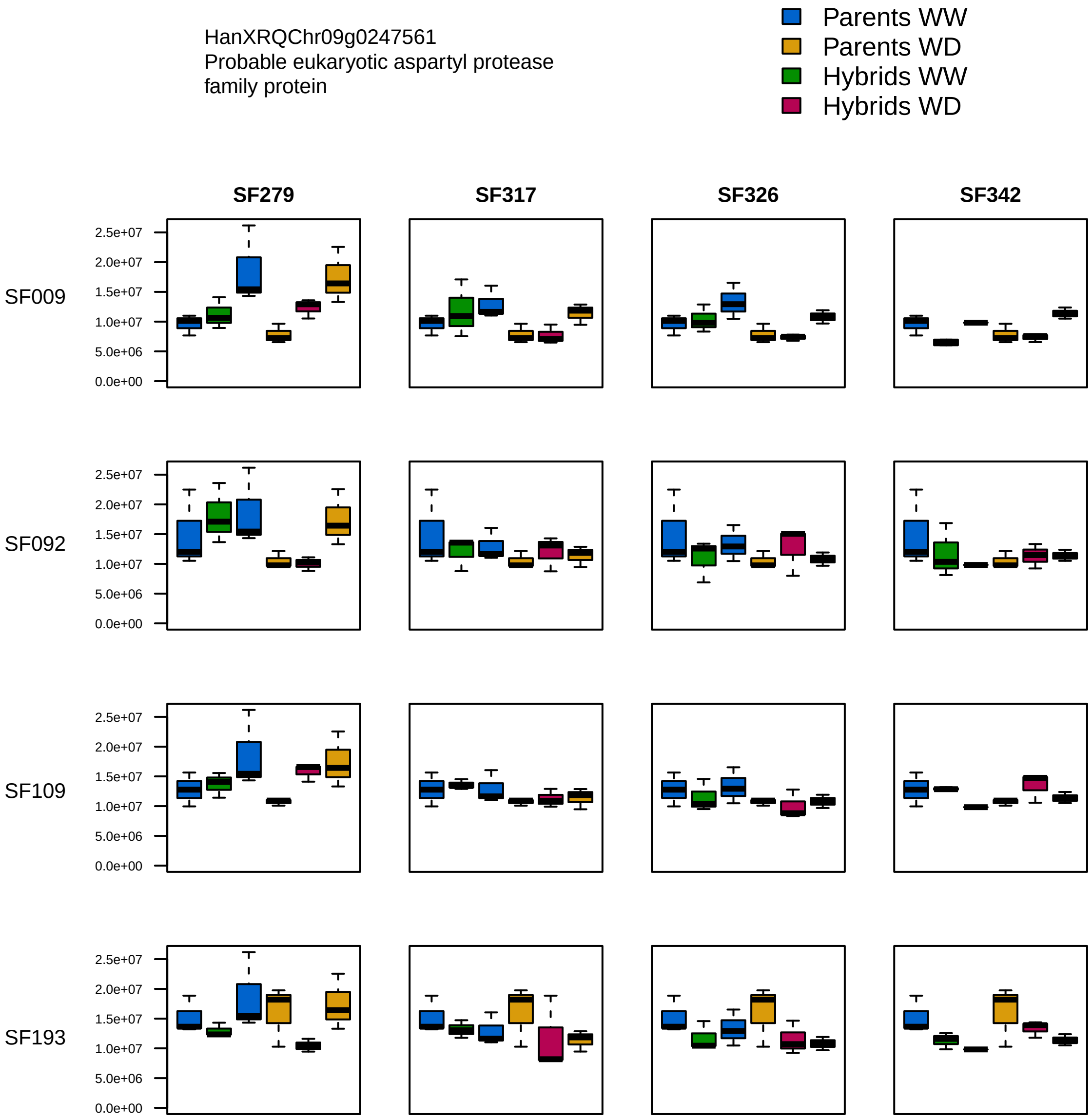

HanXRQChr09g0247561
Probable eukaryotic aspartyl protease
family protein
Parents WW
Parents WD
Hybrids WW
Hybrids WD
SF279
SF317
SF326
SF342
SF009
SF092
SF109
SF193
2.5e+07
2.0e+07
1.5e+07
1.0e+07
5.0e+06
0.0e+00

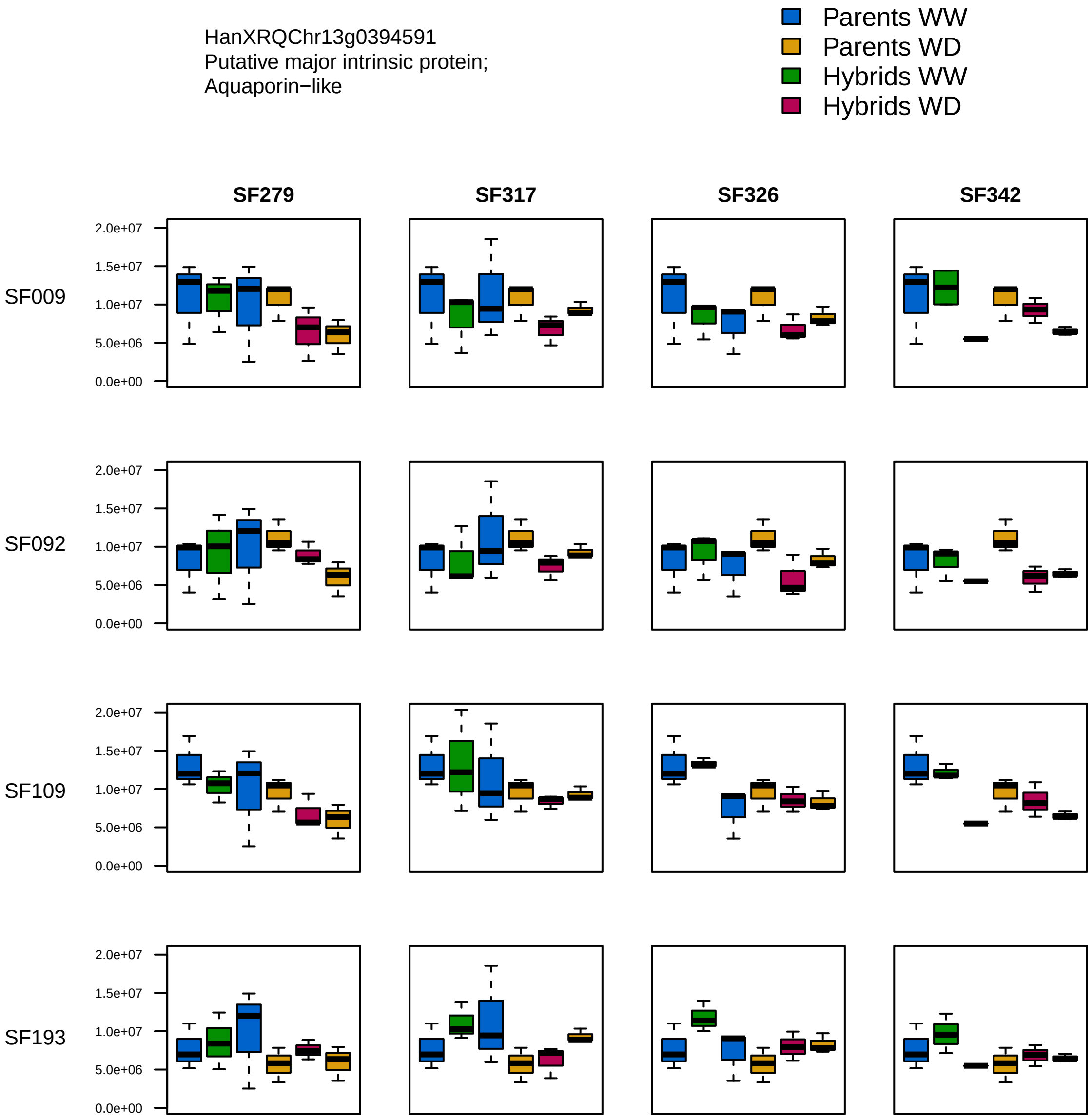
HanXRQChr13g0394591
Putative major intrinsic protein;
Aquaporin−like
Parents WW
Parents WD
Hybrids WW
Hybrids WD
SF279
SF317
SF326
SF342
SF009
SF092
SF109
SF193
2.0e+07
1.5e+07
1.0e+07
5.0e+06
0.0e+00

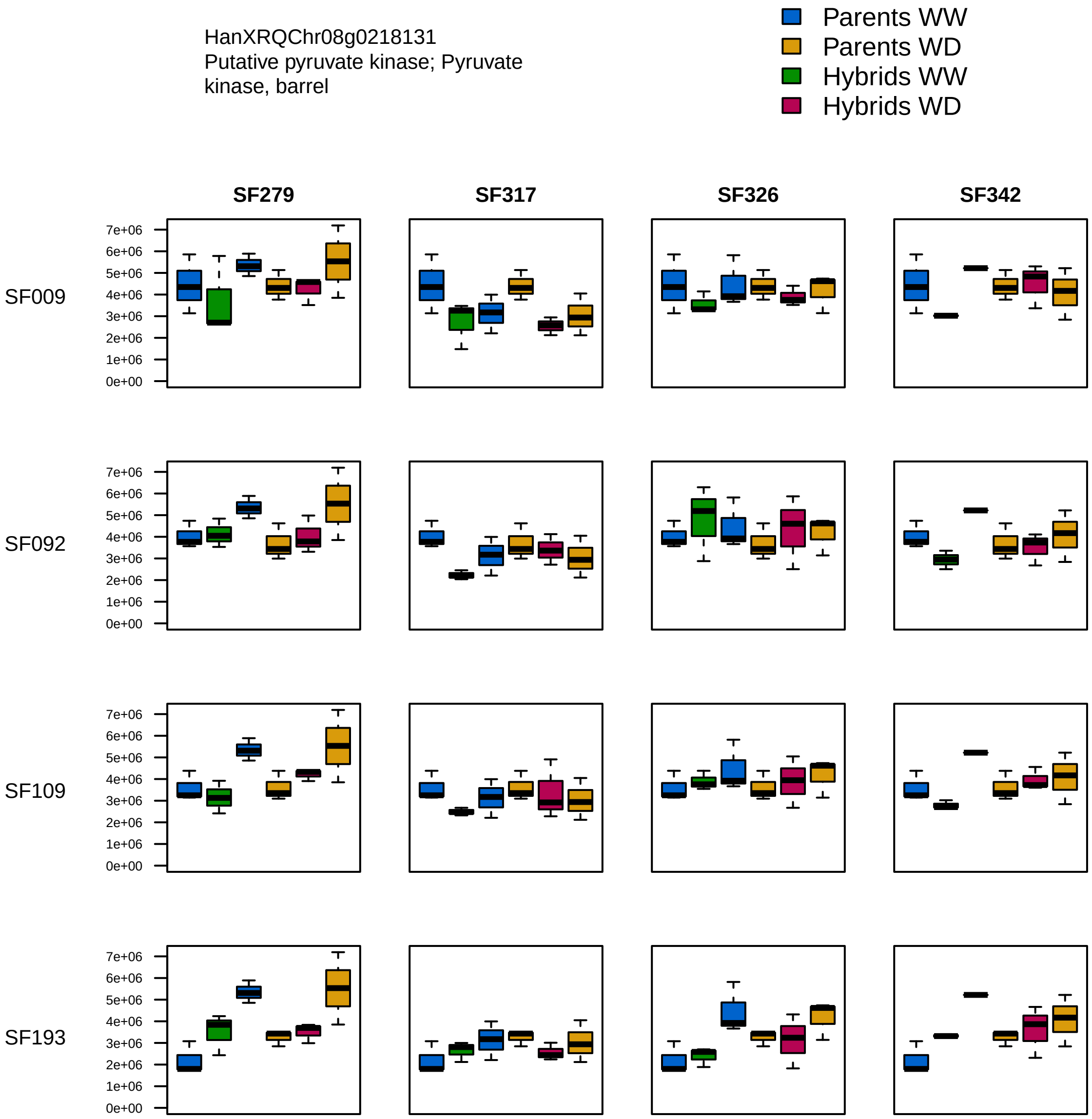
HanXRQChr08g0218131
Putative pyruvate kinase; Pyruvate kinase, barrel
Parents WW
Parents WD
Hybrids WW
Hybrids WD
SF279
SF317
SF326
SF342
SF009
SF092
SF109
SF193
7e+06
6e+06
5e+06
4e+06
3e+06
2e+06
1e+06
0e+00

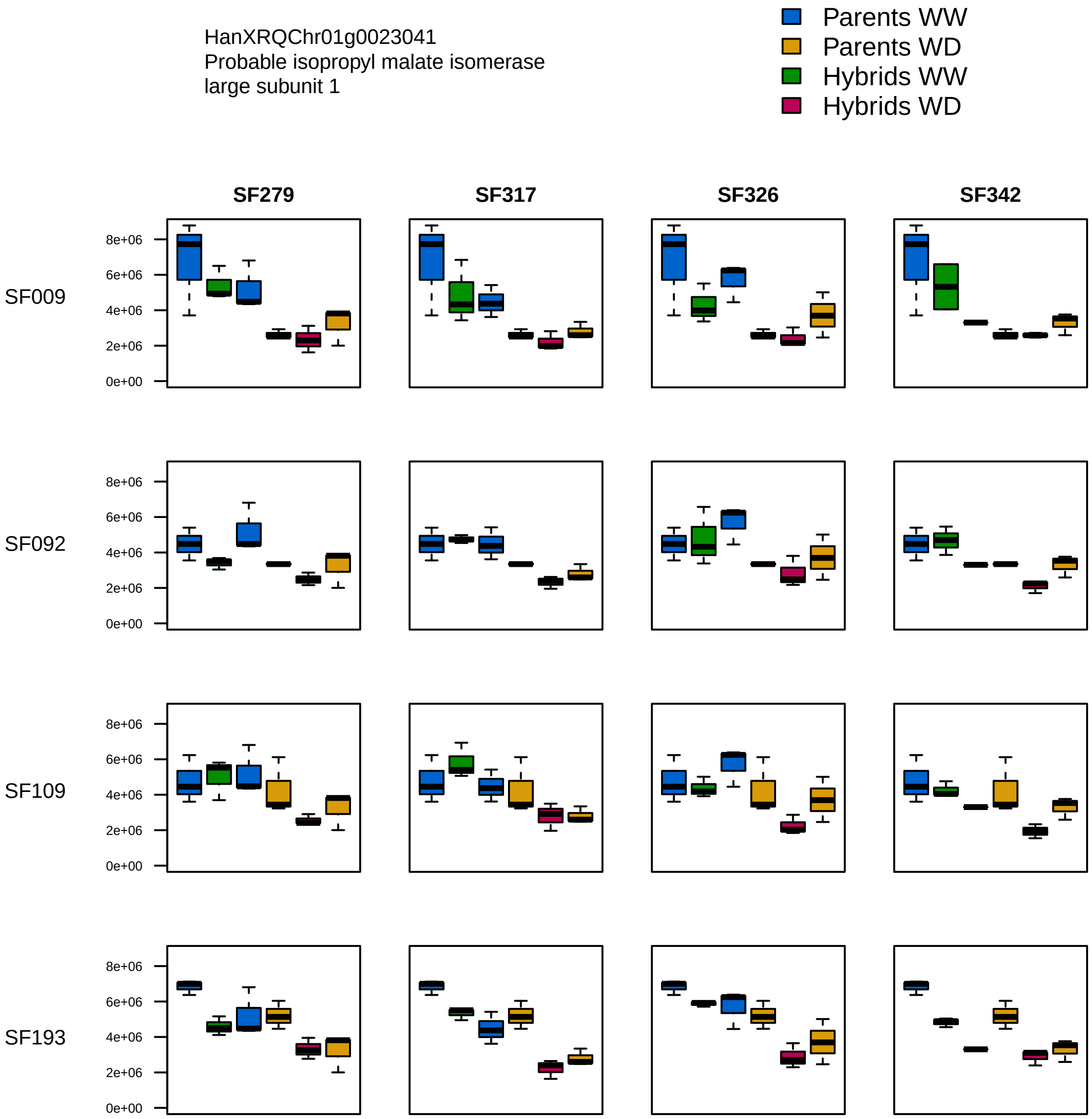
HanXRQChr01g0023041
Probable isopropyl malate isomerase
large subunit 1
Parents WW
Parents WD
Hybrids WW
Hybrids WD
SF279
SF317
SF326
SF342
SF009
SF092
SF109
SF193
0e+00
2e+06
4e+06
6e+06
8e+06

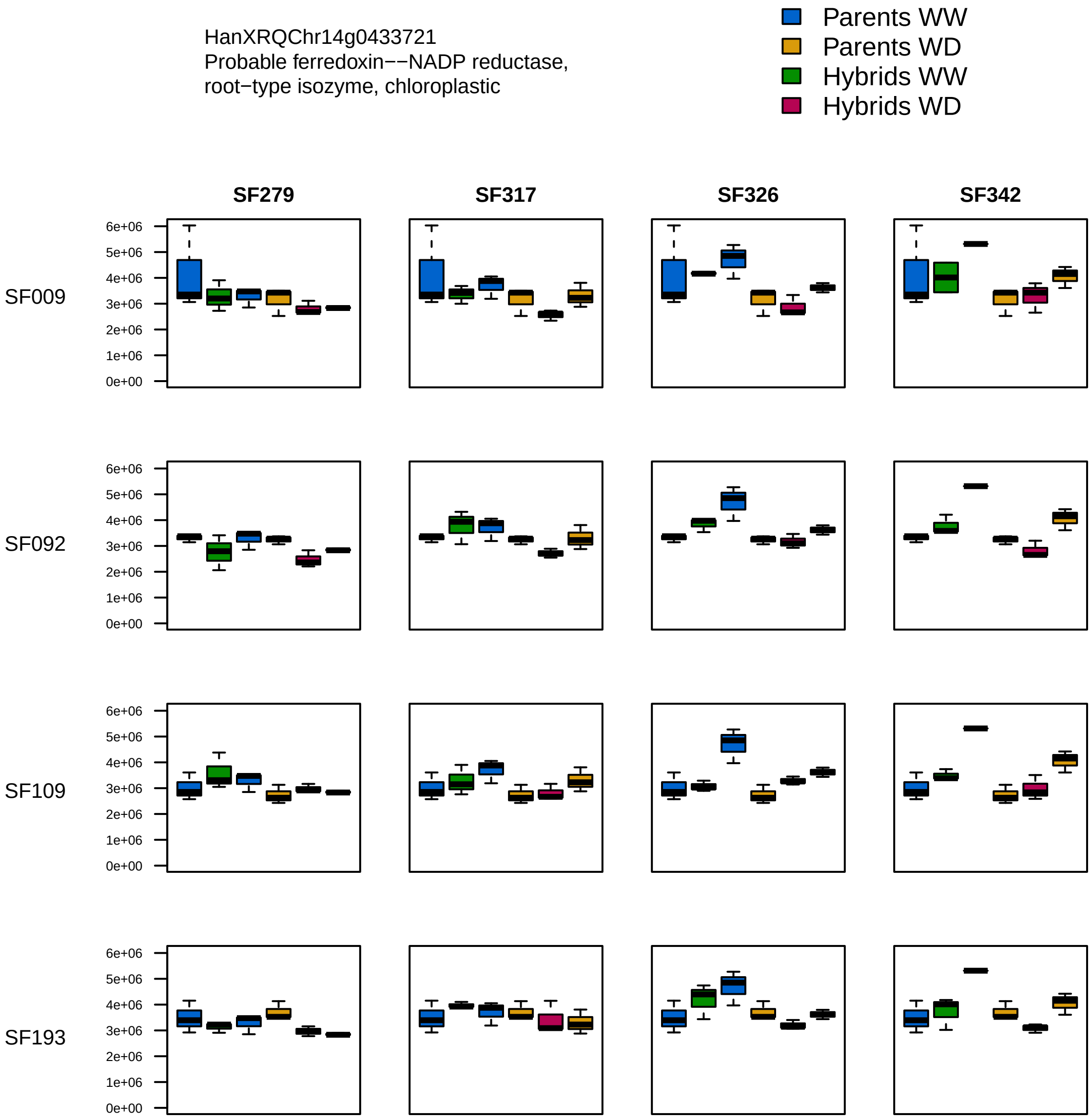

HanXRQChr14g0433721
Probable ferredoxin−−NADP reductase,
root−type isozyme, chloroplastic
Parents WW
Parents WD
Hybrids WW
Hybrids WD
SF279
SF317
SF326
SF342
SF009
SF092
SF109
SF193
6e+06
5e+06
4e+06
3e+06
2e+06
1e+06
0e+00

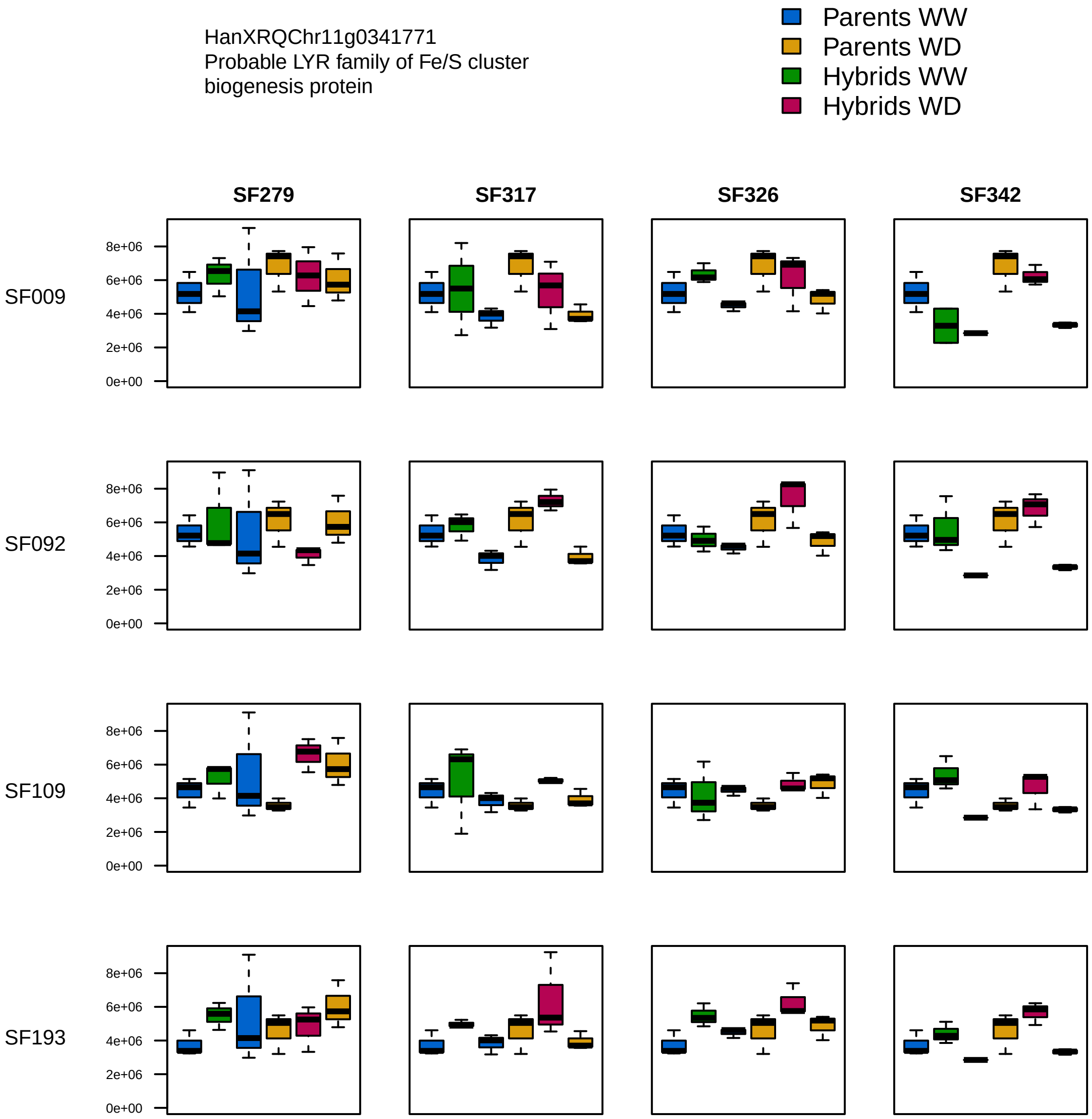

HanXRQChr11g0341771
Probable LYR family of Fe/S cluster biogenesis protein
Parents WW
Parents WD
Hybrids WW
Hybrids WD
SF279
SF317
SF326
SF342
SF009
SF092
SF109
SF193
8e+06
6e+06
4e+06
2e+06
0e+00

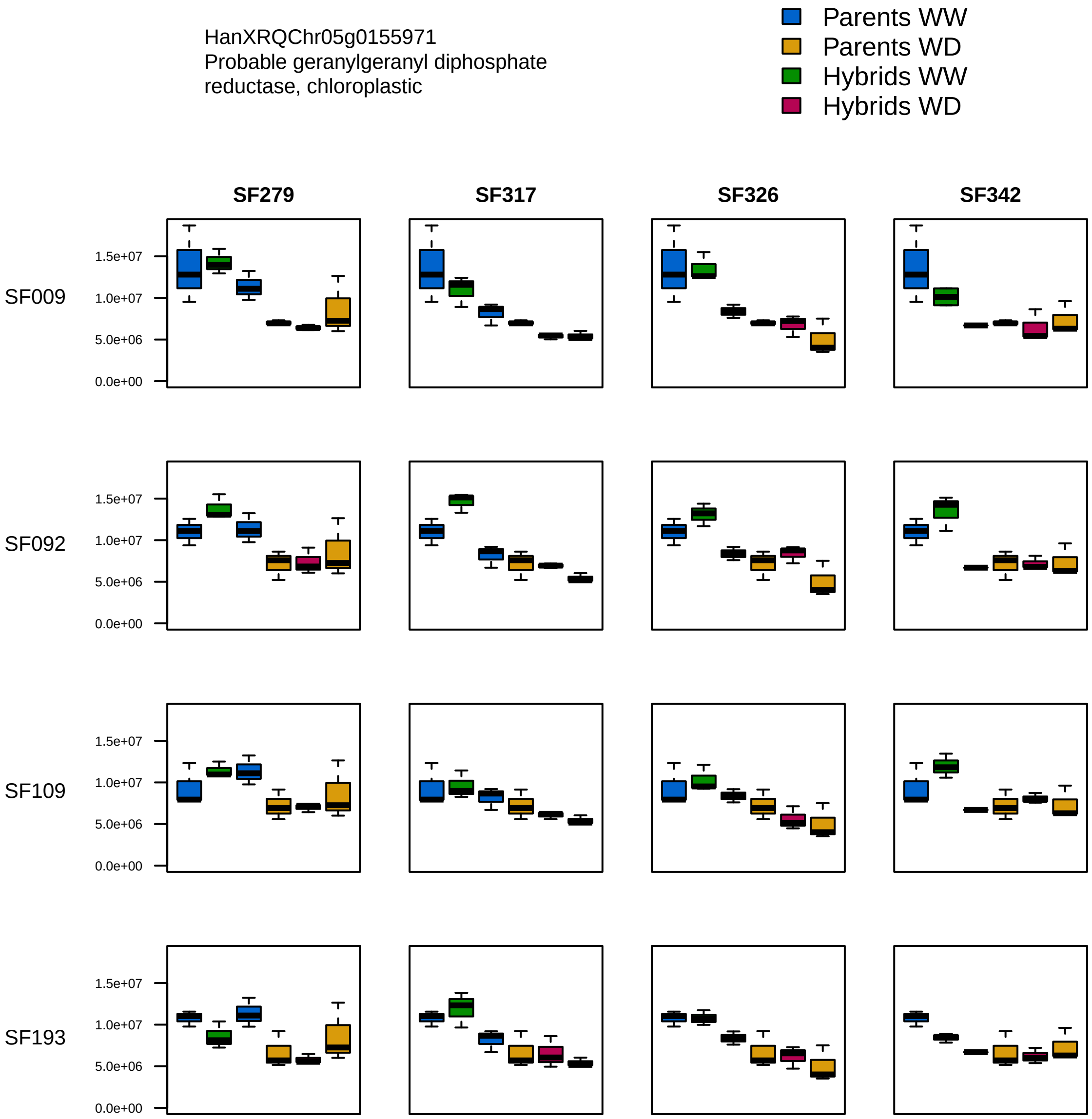
HanXRQChr05g0155971
Probable geranylgeranyl diphosphate
reductase, chloroplastic
Parents WW
Parents WD
Hybrids WW
Hybrids WD
SF279
SF317
SF326
SF342
SF009
SF092
SF109
SF193
1.5e+07
1.0e+07
5.0e+06
0.0e+00

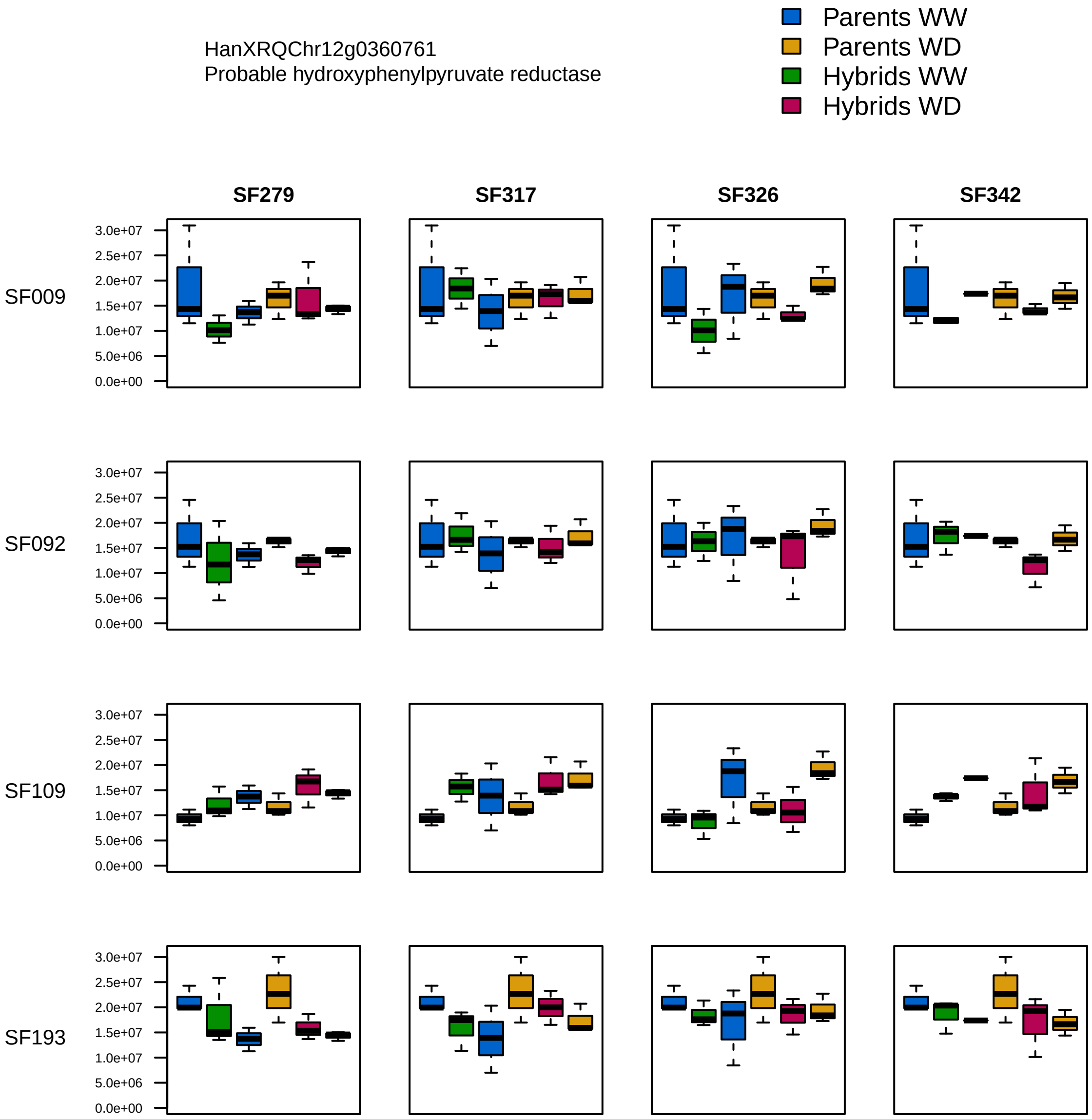

HanXRQChr12g0360761
Probable hydroxyphenylpyruvate reductase
Parents WW
Parents WD
Hybrids WW
Hybrids WD
SF279
SF317
SF326
SF342
SF009
SF092
SF109
SF193
3.0e+07
2.5e+07
2.0e+07
1.5e+07
1.0e+07
5.0e+06
0.0e+00

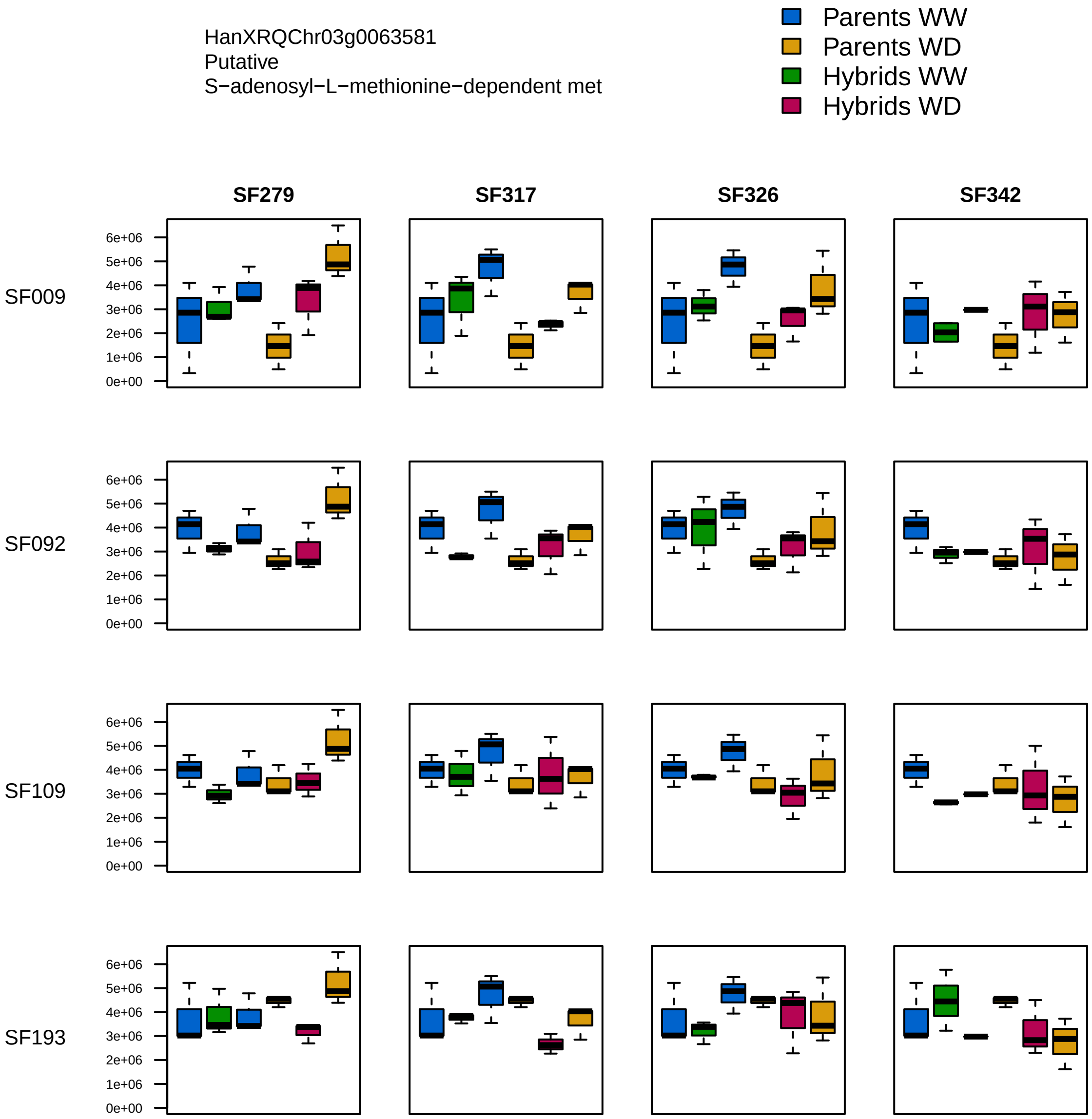

HanXRQChr03g0063581
Putative
S−adenosyl−L−methionine−dependent met
Parents WW
Parents WD
Hybrids WW
Hybrids WD
SF279
SF317
SF326
SF342
SF009
SF092
SF109
SF193
0e+00
1e+06
2e+06
3e+06
4e+06
5e+06
6e+06

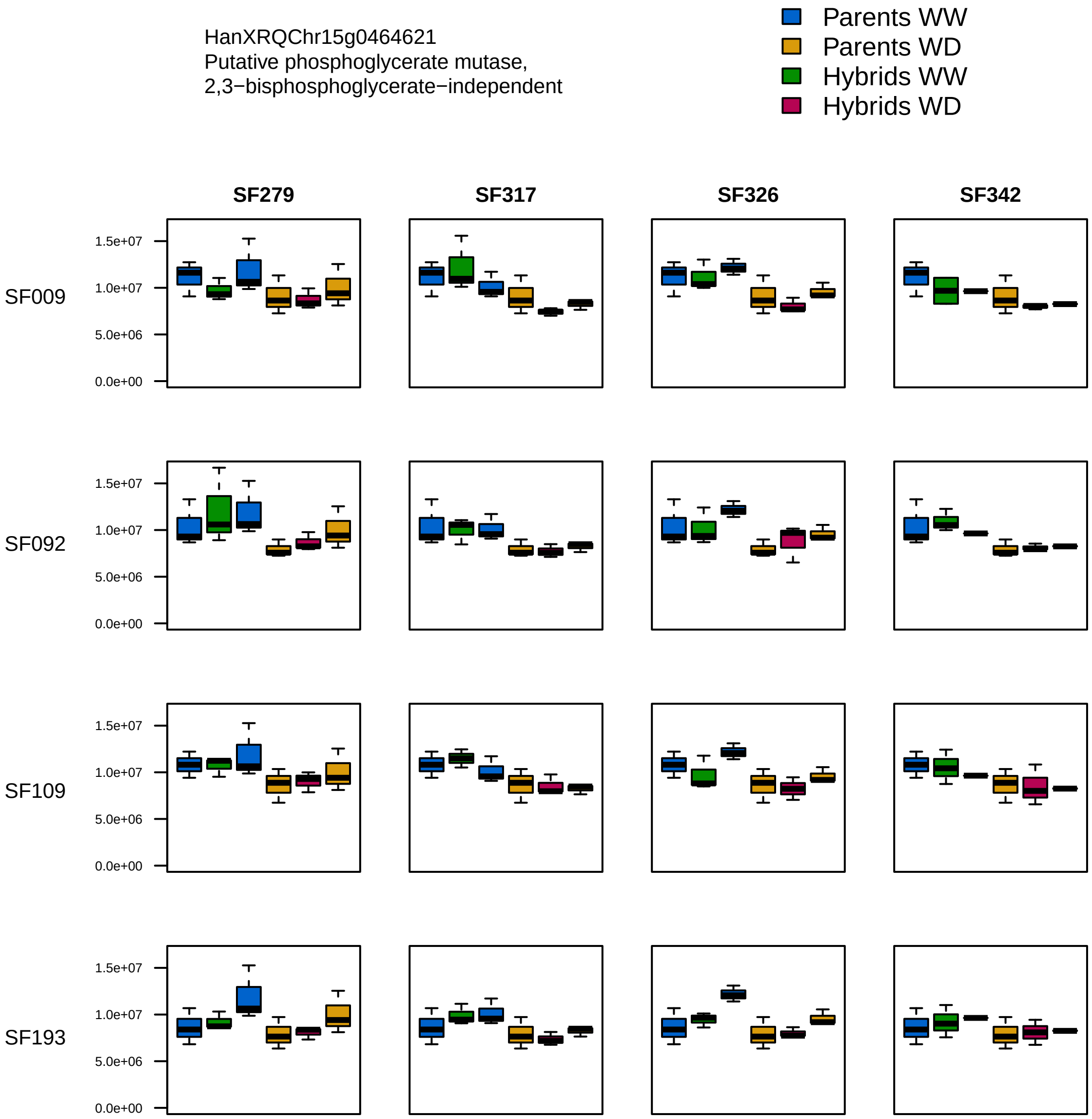
HanXRQChr15g0464621
Putative phosphoglycerate mutase,
2,3−bisphosphoglycerate−independent
Parents WW
Parents WD
Hybrids WW
Hybrids WD
SF279
SF317
SF326
SF342
SF009
SF092
SF109
SF193
1.5e+07
1.0e+07
5.0e+06
0.0e+00

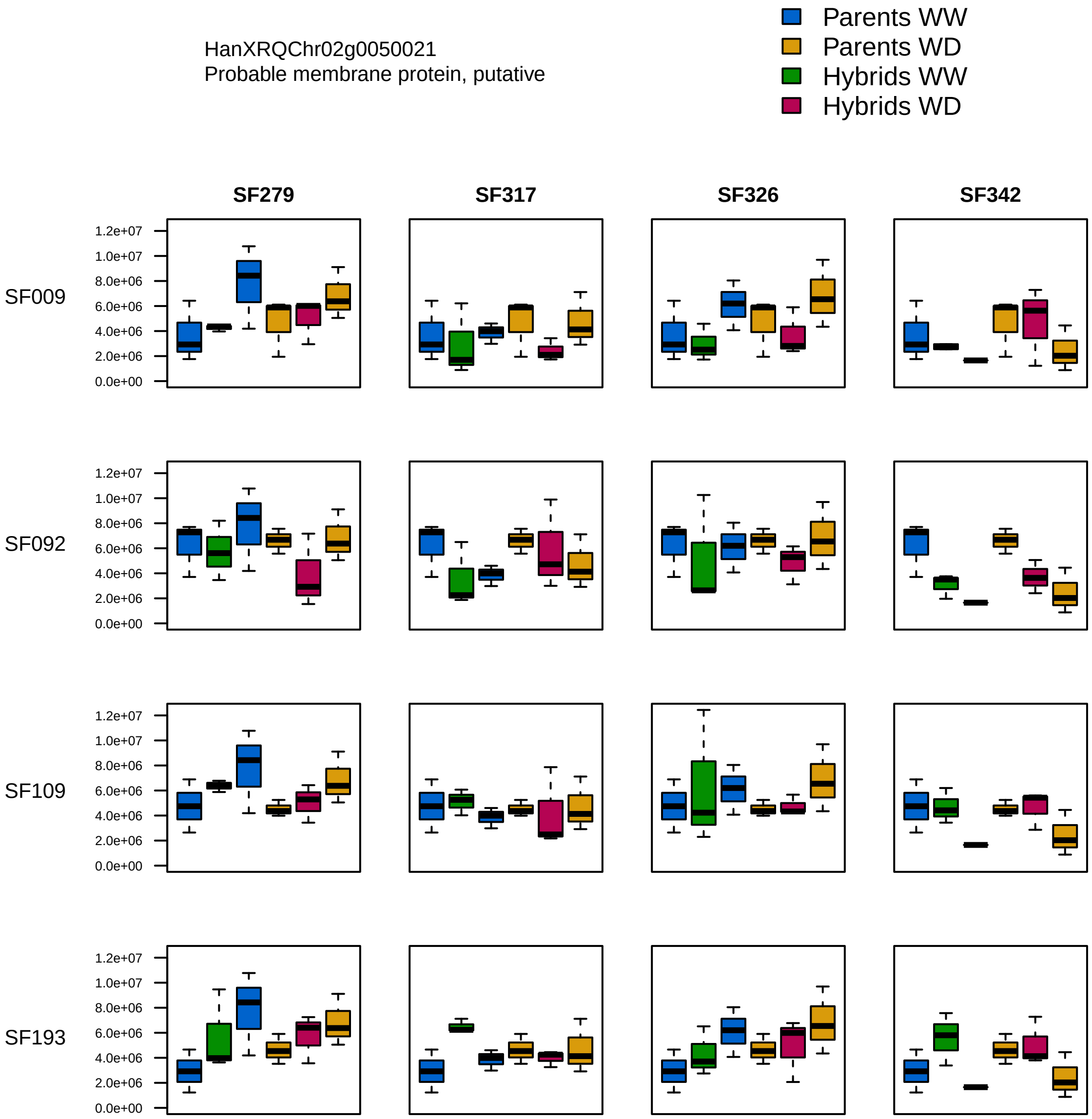

HanXRQChr02g0050021
Probable membrane protein, putative
Parents WW
Parents WD
Hybrids WW
Hybrids WD
SF279
SF317
SF326
SF342
SF009
SF092
SF109
SF193
0.0e+00
2.0e+06
4.0e+06
6.0e+06
8.0e+06
1.0e+07
1.2e+07

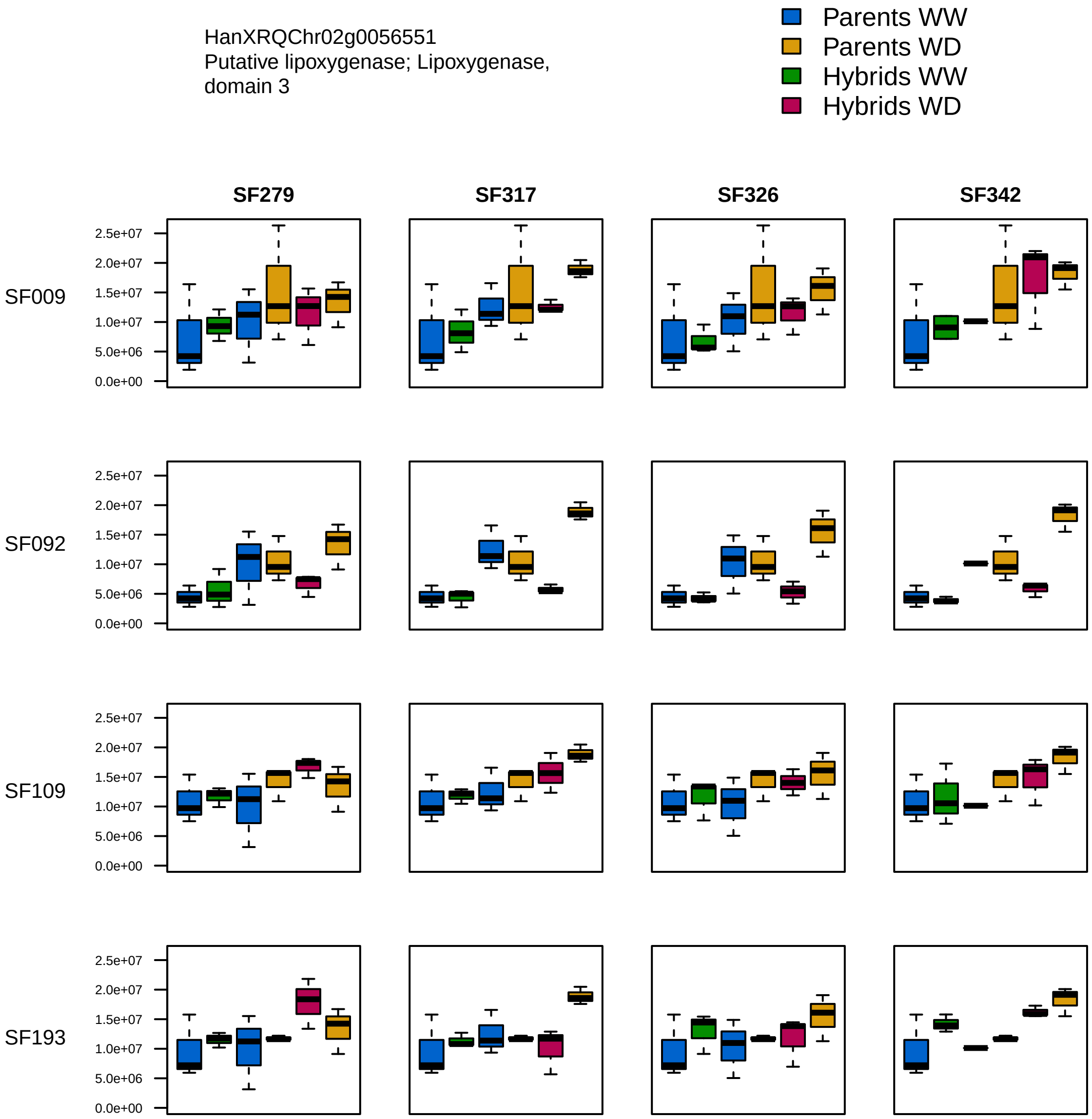
HanXRQChr02g0056551
Putative lipoxygenase; Lipoxygenase, domain 3
Parents WW
Parents WD
Hybrids WW
Hybrids WD
SF279
SF317
SF326
SF342
SF009
SF092
SF109
SF193
2.5e+07
2.0e+07
1.5e+07
1.0e+07
5.0e+06
0.0e+00

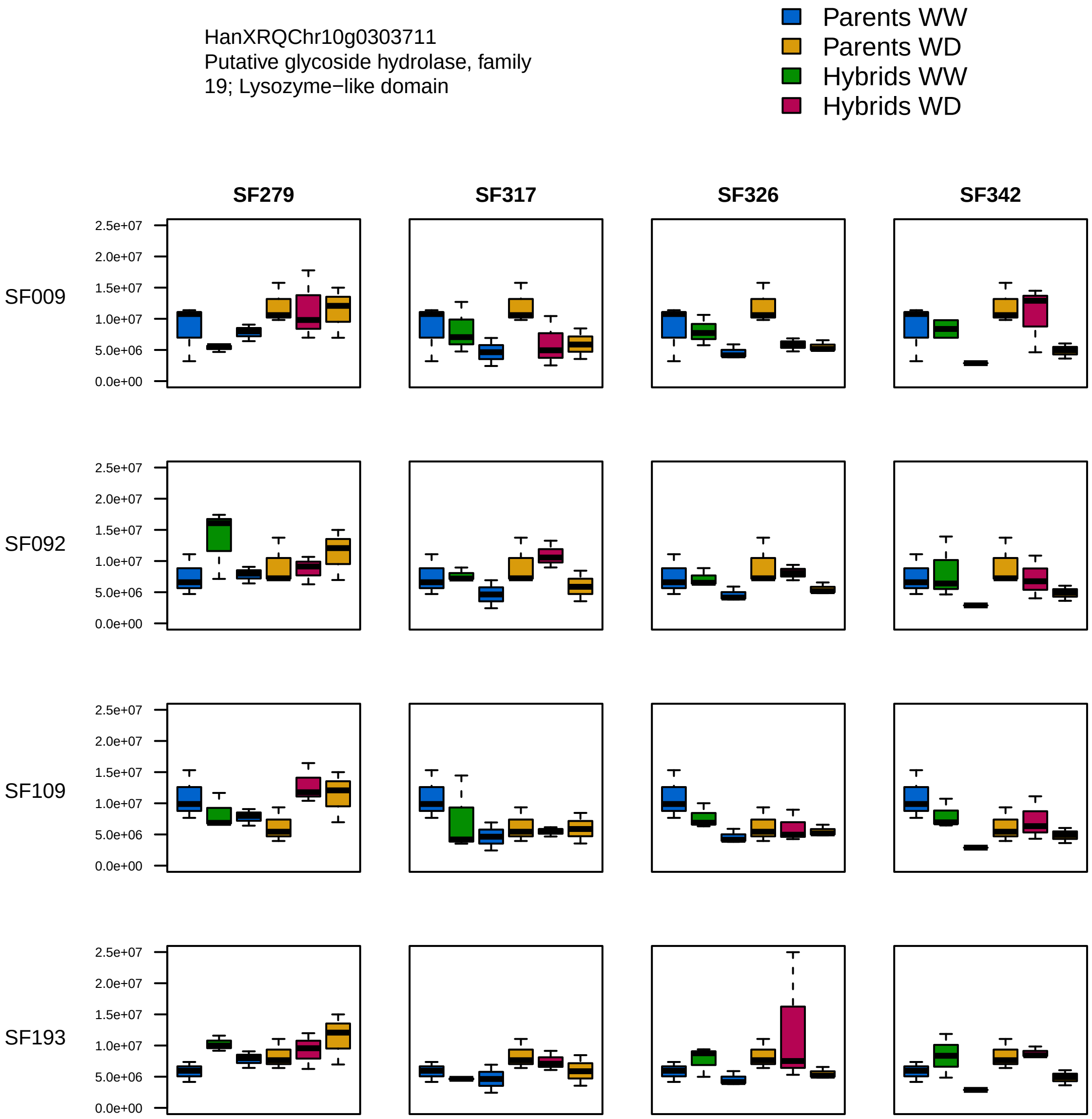
HanXRQChr10g0303711
Putative glycoside hydrolase, family 19; Lysozyme−like domain
Parents WW
Parents WD
Hybrids WW
Hybrids WD
SF279
SF317
SF326
SF342
SF009
SF092
SF109
SF193
2.5e+07
2.0e+07
1.5e+07
1.0e+07
5.0e+06
0.0e+00

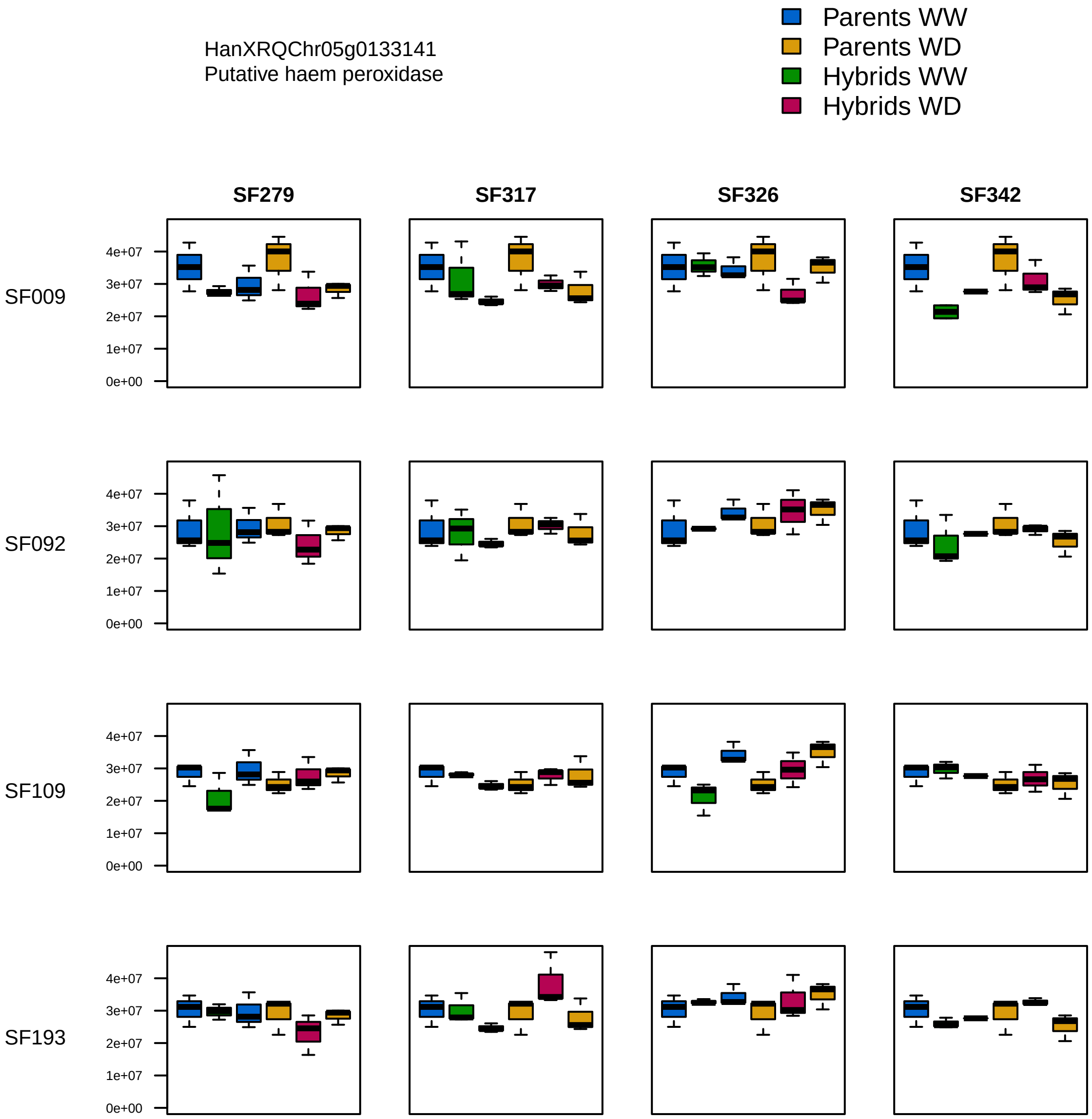

HanXRQChr05g0133141
Putative haem peroxidase
Parents WW
Parents WD
Hybrids WW
Hybrids WD
SF279
SF317
SF326
SF342
SF009
SF092
SF109
SF193
0e+00
1e+07
2e+07
3e+07
4e+07

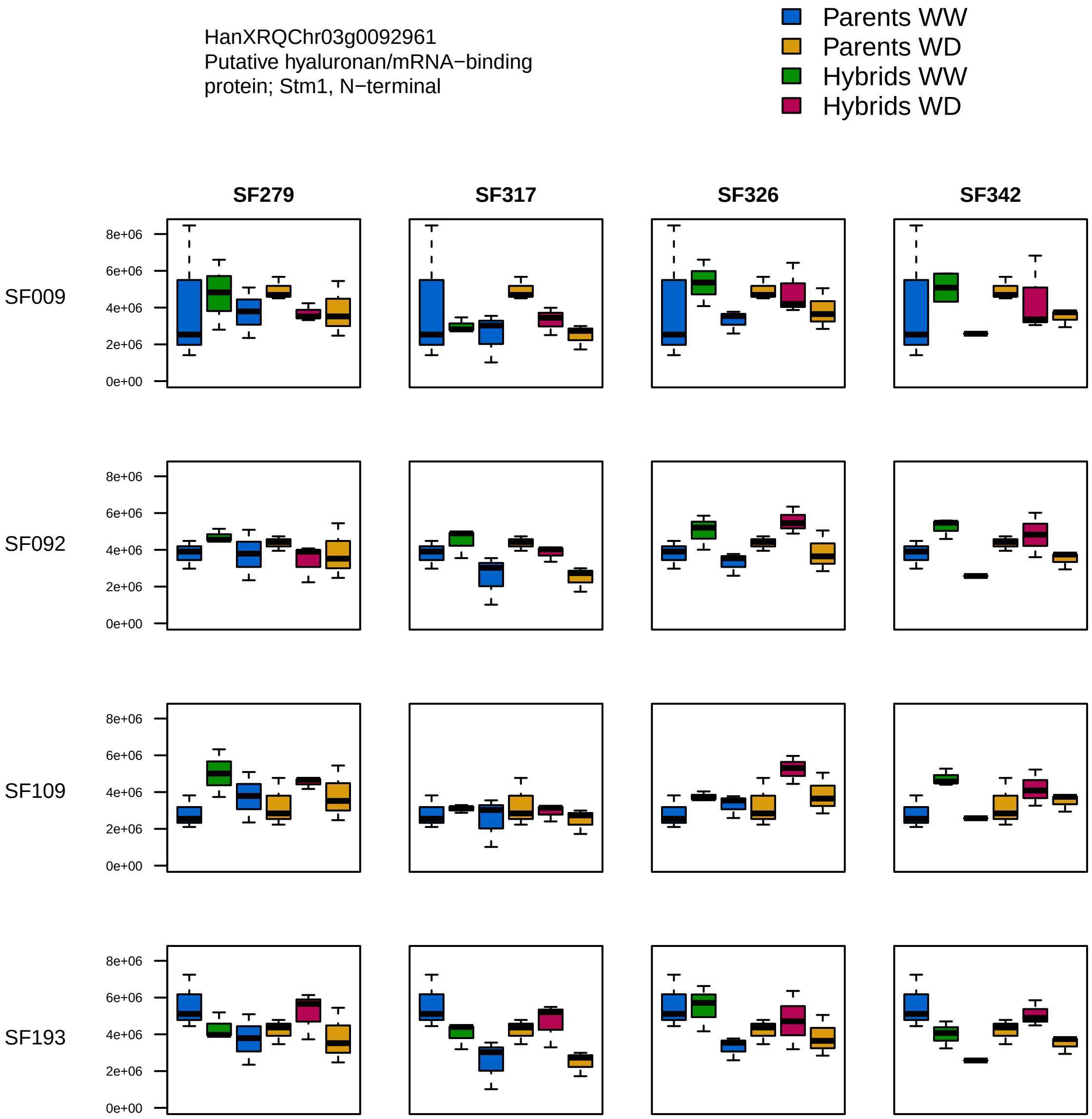
HanXRQChr03g0092961
Putative hyaluronan/mRNA−binding
protein; Stm1, N−terminal
Parents WW
Parents WD
Hybrids WW
Hybrids WD
SF279
SF317
SF326
SF342
SF009
SF092
SF109
SF193
8e+06
6e+06
4e+06
2e+06
0e+00

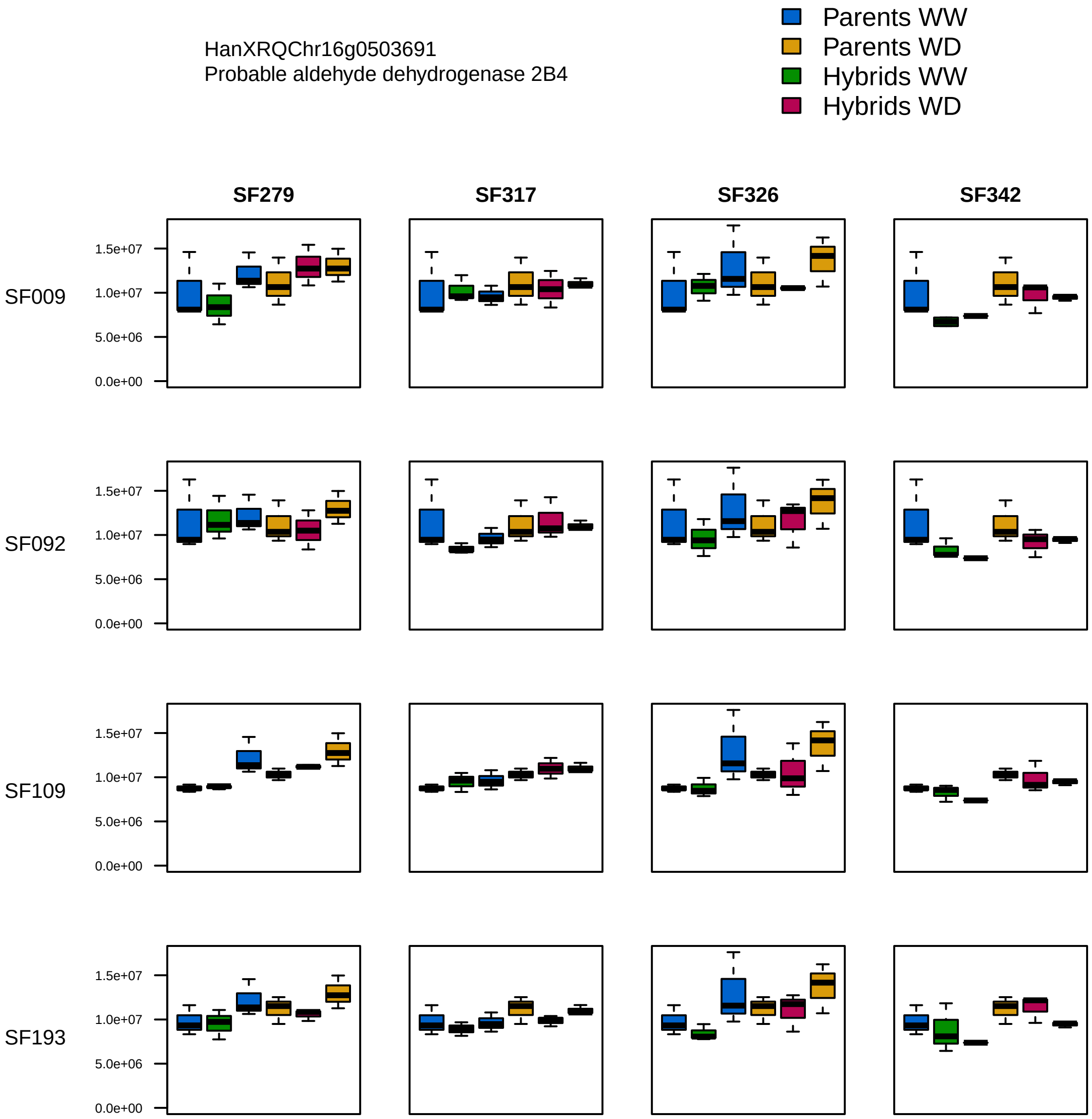

HanXRQChr16g0503691
Probable aldehyde dehydrogenase 2B4
Parents WW
Parents WD
Hybrids WW
Hybrids WD
SF279
SF317
SF326
SF342
SF009
SF092
SF109
SF193
1.5e+07
1.0e+07
5.0e+06
0.0e+00

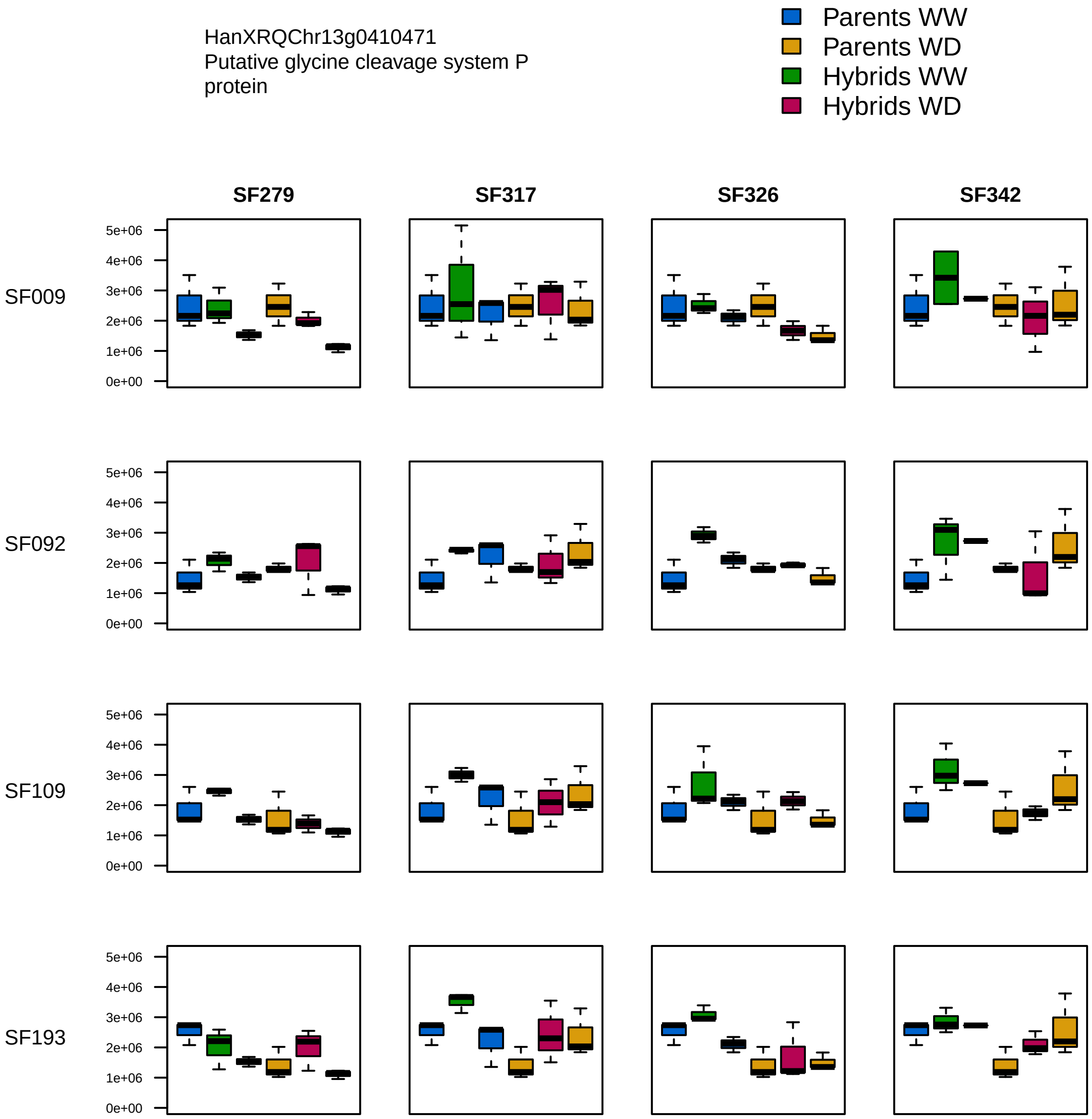
HanXRQChr13g0410471
Putative glycine cleavage system P protein
Parents WW
Parents WD
Hybrids WW
Hybrids WD
SF279
SF317
SF326
SF342
SF009
SF092
SF109
SF193
0e+00
1e+06
2e+06
3e+06
4e+06
5e+06

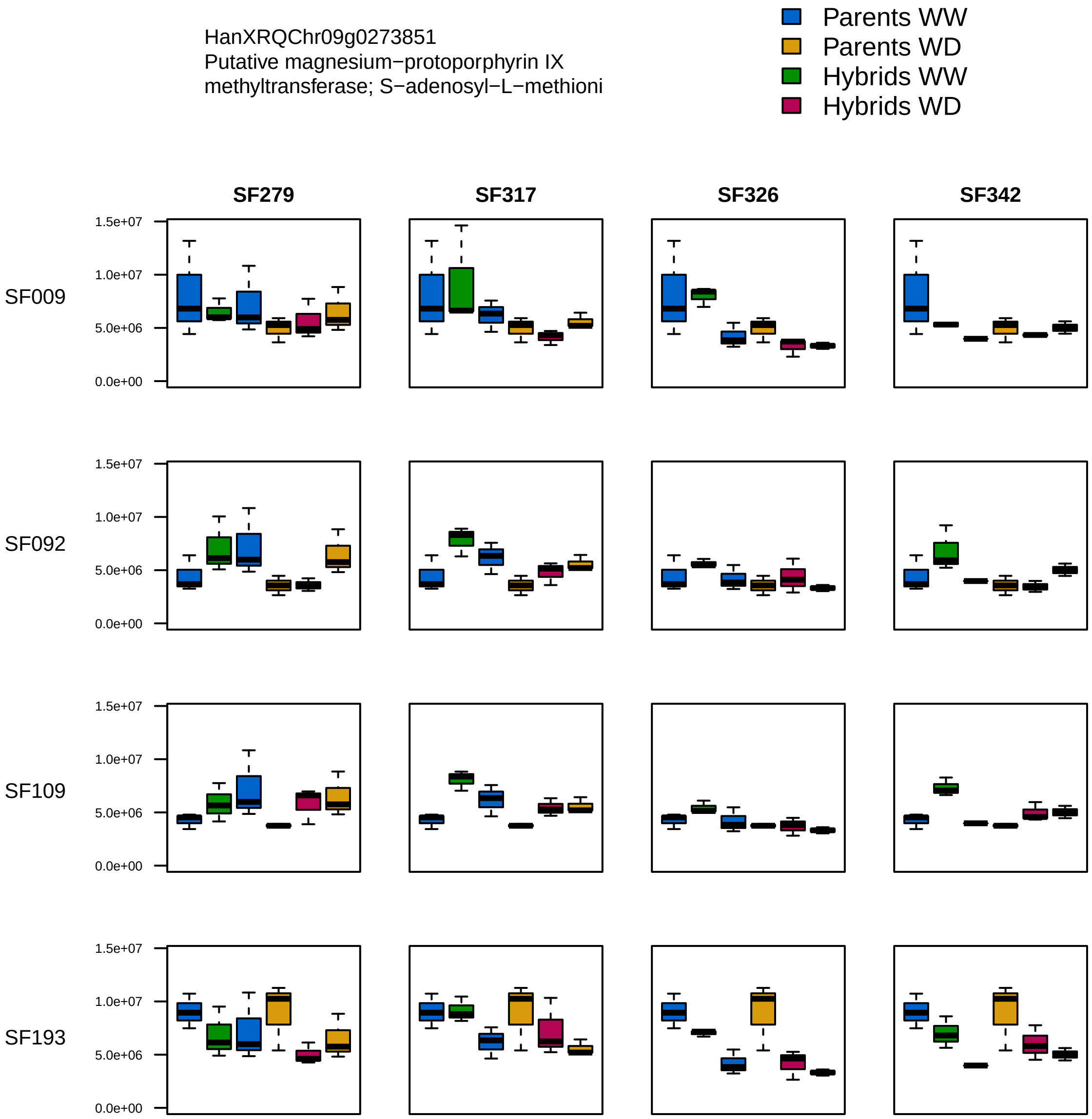
HanXRQChr09g0273851
Putative magnesium−protoporphyrin IX
methyltransferase; S−adenosyl−L−methioni
Parents WW
Parents WD
Hybrids WW
Hybrids WD
SF279
SF317
SF326
SF342
SF009
SF092
SF109
SF193
1.5e+07
1.0e+07
5.0e+06
0.0e+00

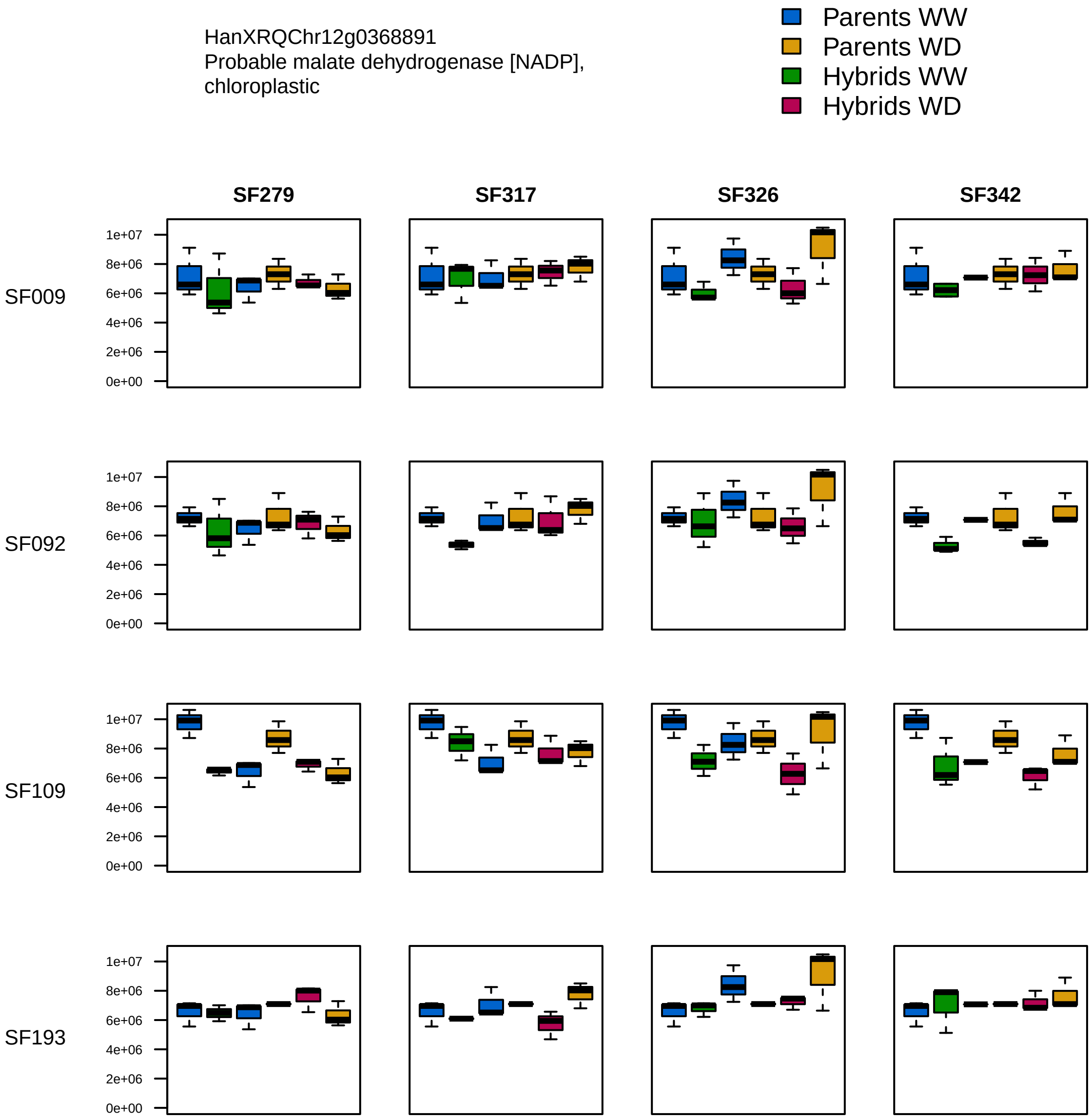
HanXRQChr12g0368891
Probable malate dehydrogenase [NADP], chloroplastic
Parents WW
Parents WD
Hybrids WW
Hybrids WD
SF279
SF317
SF326
SF342
SF009
SF092
SF109
SF193
1e+07
8e+06
6e+06
4e+06
2e+06
0e+00

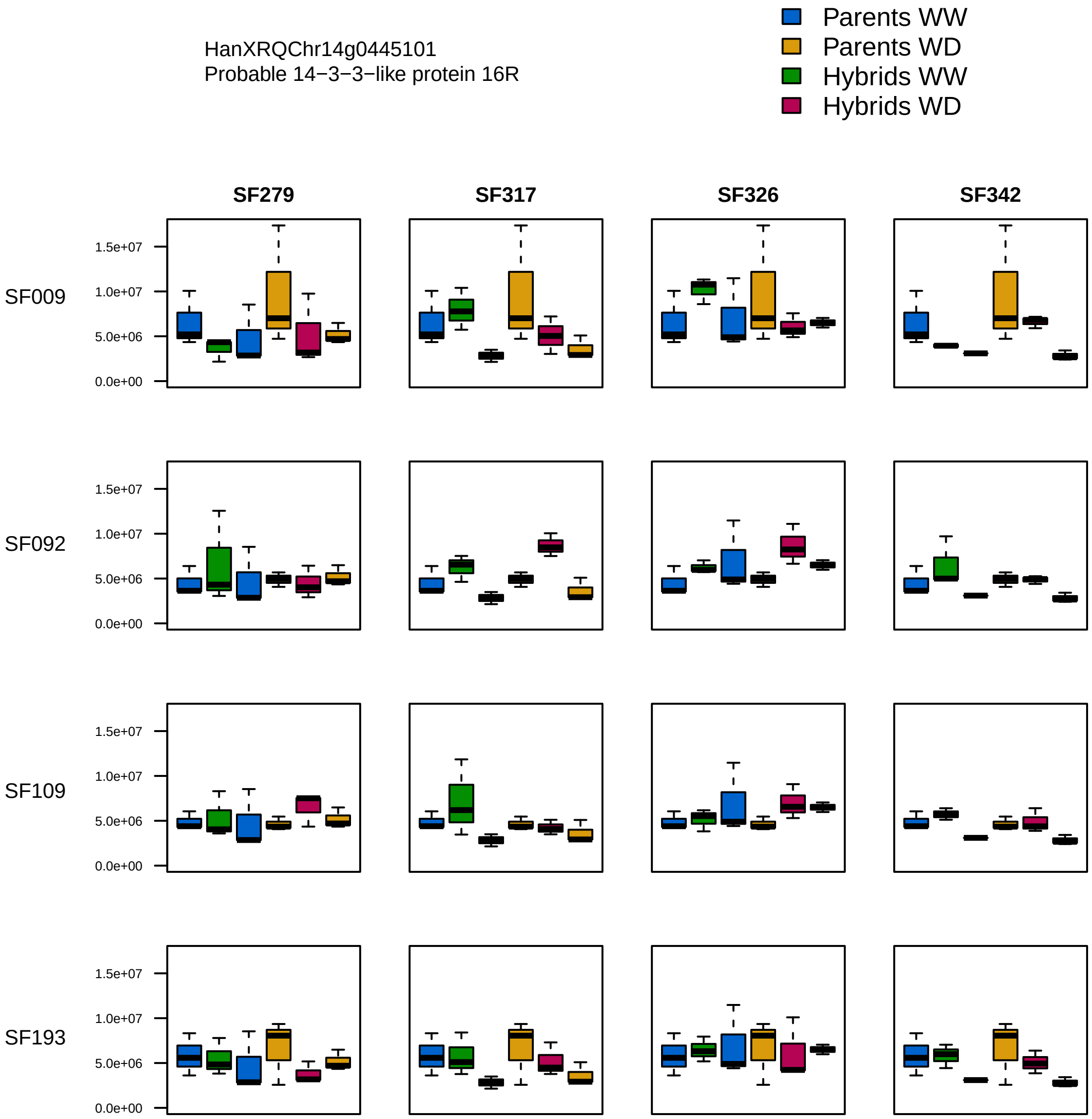
HanXRQChr14g0445101
Probable 14−3−3−like protein 16R
Parents WW
Parents WD
Hybrids WW
Hybrids WD
SF279
SF317
SF326
SF342
SF009
SF092
SF109
SF193
1.5e+07
1.0e+07
5.0e+06
0.0e+00

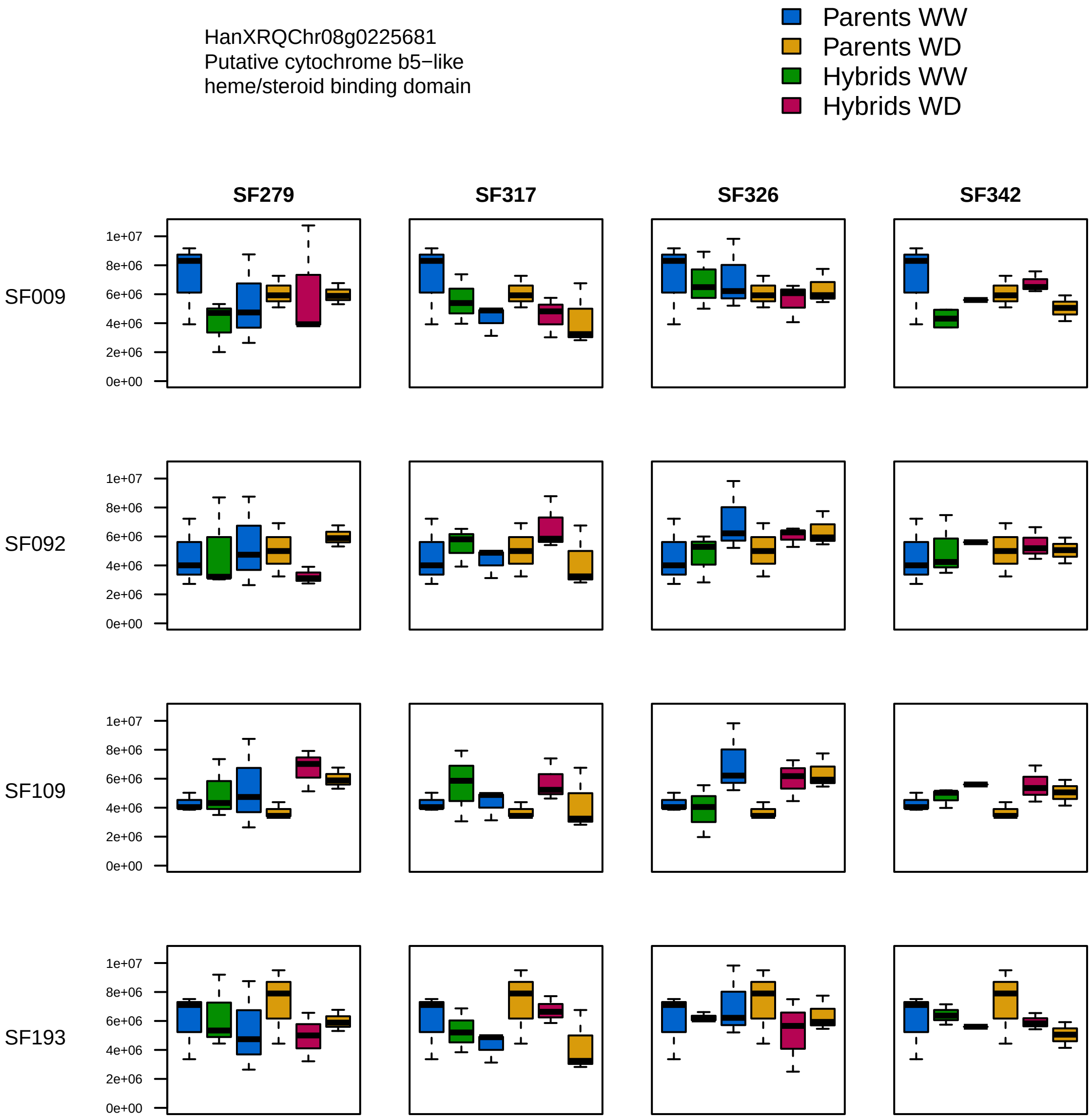

HanXRQChr08g0225681
Putative cytochrome b5−like
heme/steroid binding domain
Parents WW
Parents WD
Hybrids WW
Hybrids WD
SF279
SF317
SF326
SF342
SF009
SF092
SF109
SF193
1e+07
8e+06
6e+06
4e+06
2e+06
0e+00

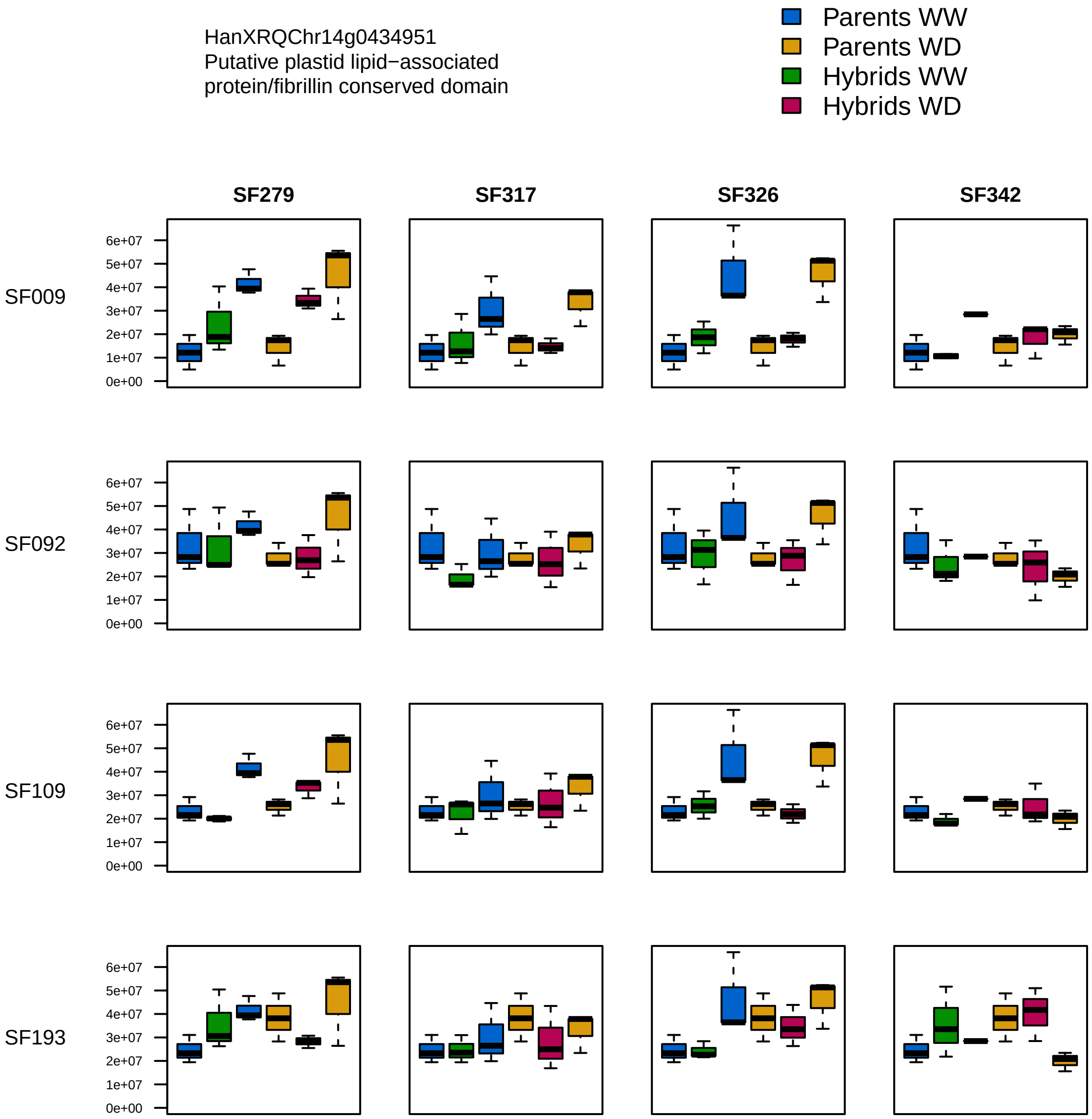

HanXRQChr14g0434951
Putative plastid lipid−associated
protein/fibrillin conserved domain
Parents WW
Parents WD
Hybrids WW
Hybrids WD
SF279
SF317
SF326
SF342
SF009
SF092
SF109
SF193
0e+00
1e+07
2e+07
3e+07
4e+07
5e+07
6e+07

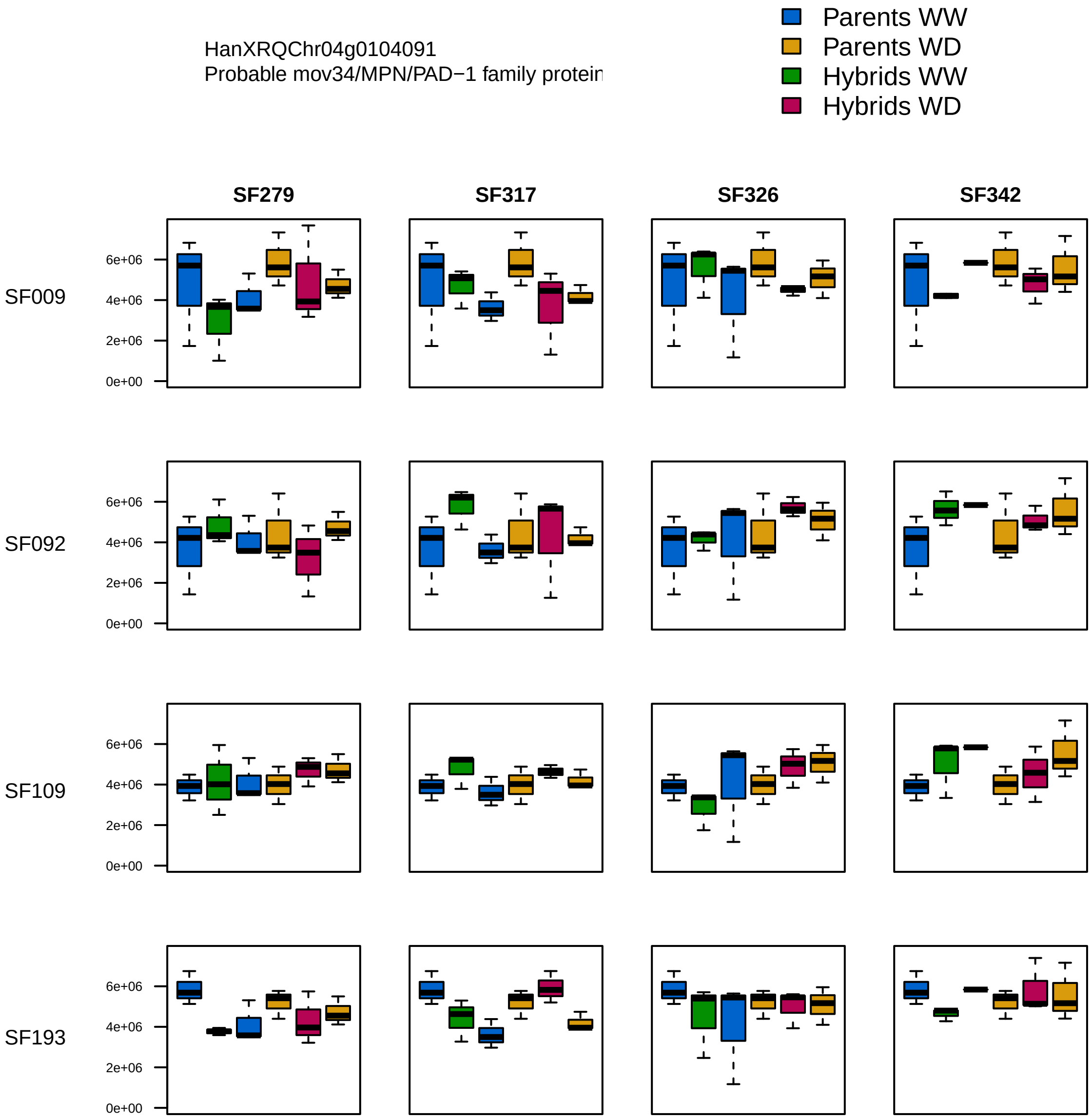
HanXRQChr04g0104091
Probable mov34/MPN/PAD−1 family protein
Parents WW
Parents WD
Hybrids WW
Hybrids WD
SF279
SF317
SF326
SF342
SF009
SF092
SF109
SF193
0e+00
2e+06
4e+06
6e+06

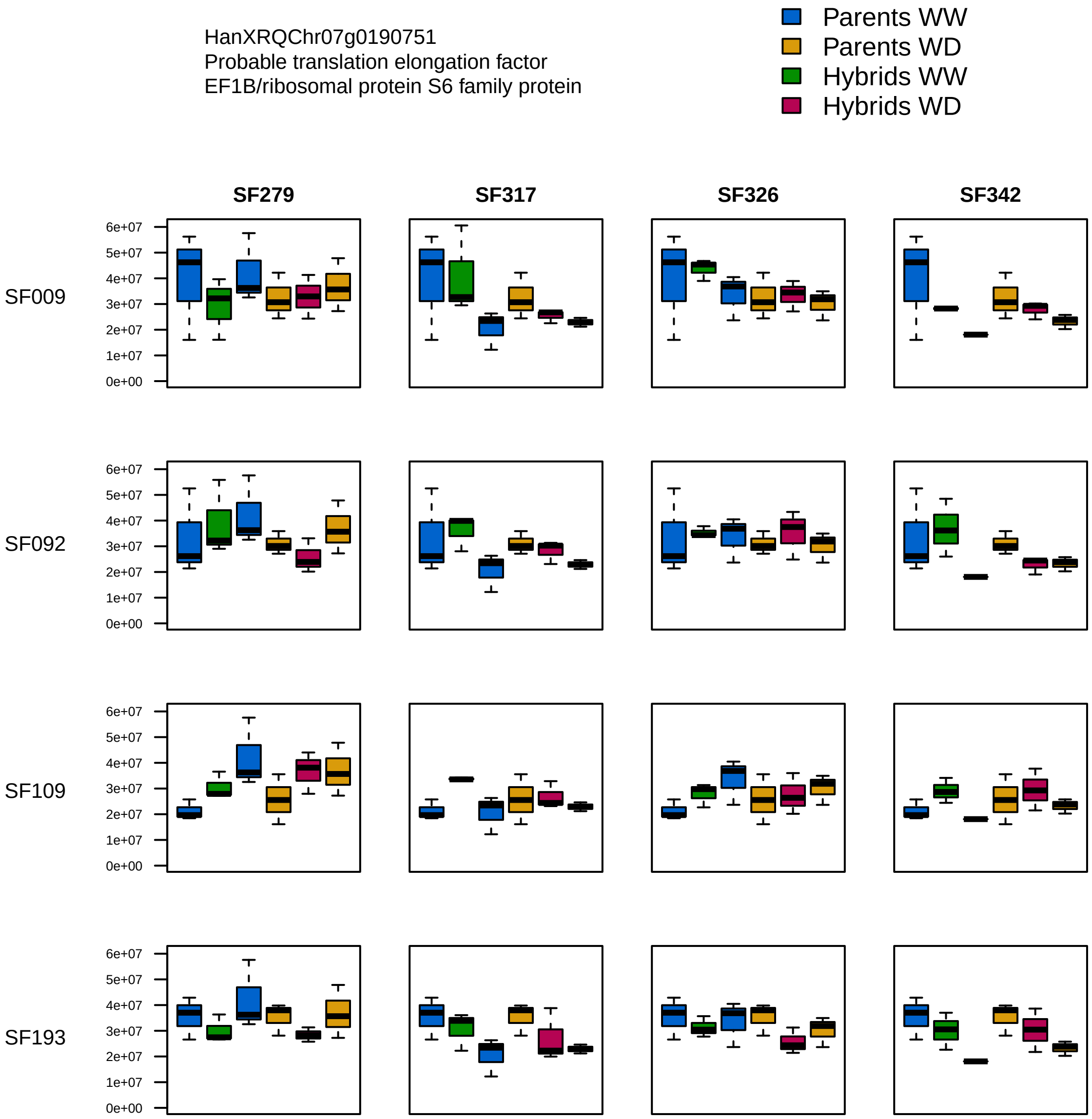
HanXRQChr07g0190751
Probable translation elongation factor
EF1B/ribosomal protein S6 family protein
Parents WW
Parents WD
Hybrids WW
Hybrids WD
SF279
SF317
SF326
SF342
SF009
SF092
SF109
SF193
6e+07
5e+07
4e+07
3e+07
2e+07
1e+07
0e+00

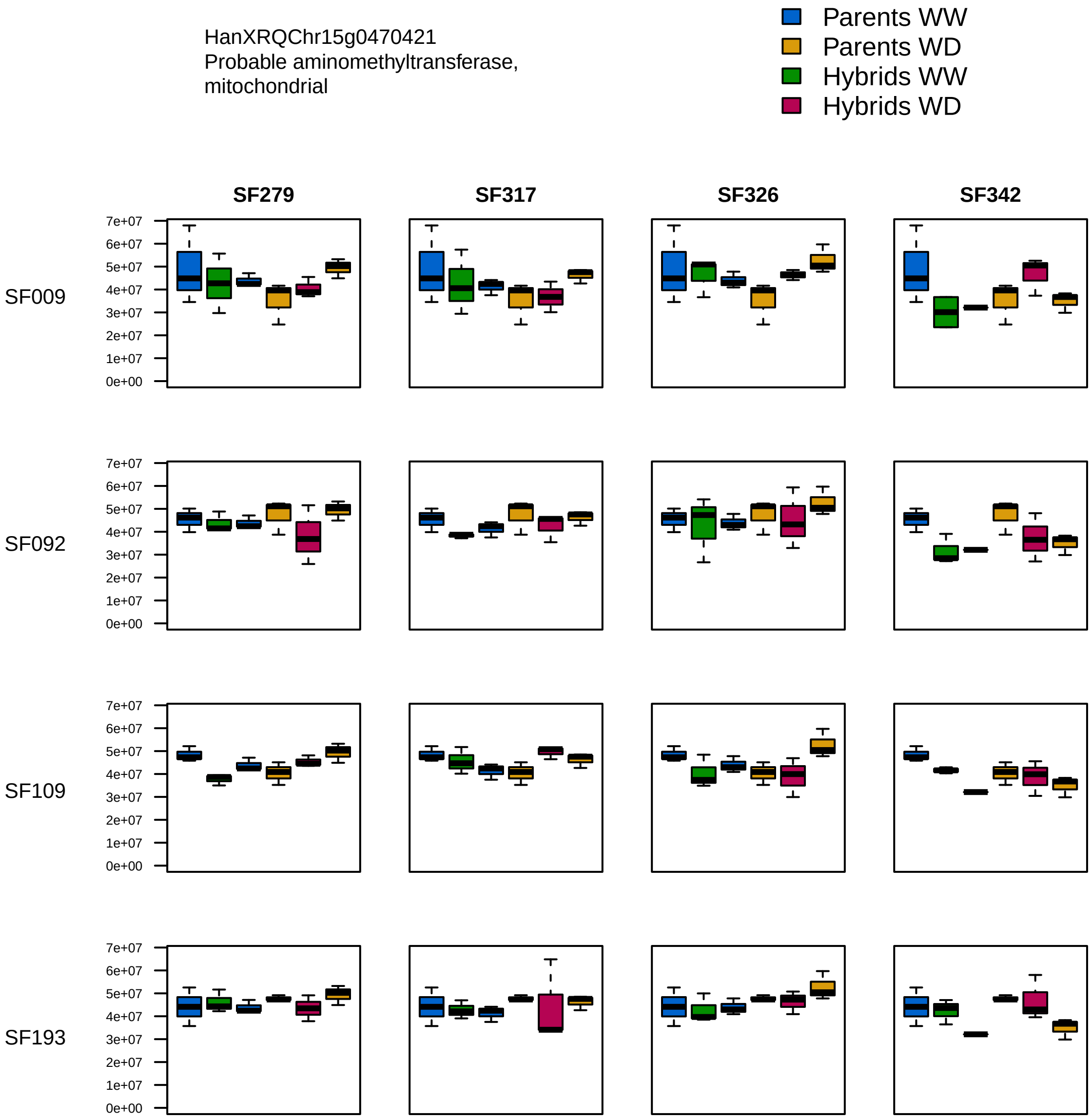

HanXRQChr15g0470421
Probable aminomethyltransferase,
mitochondrial
Parents WW
Parents WD
Hybrids WW
Hybrids WD
SF279
SF317
SF326
SF342
SF009
SF092
SF109
SF193
7e+07
6e+07
5e+07
4e+07
3e+07
2e+07
1e+07
0e+00

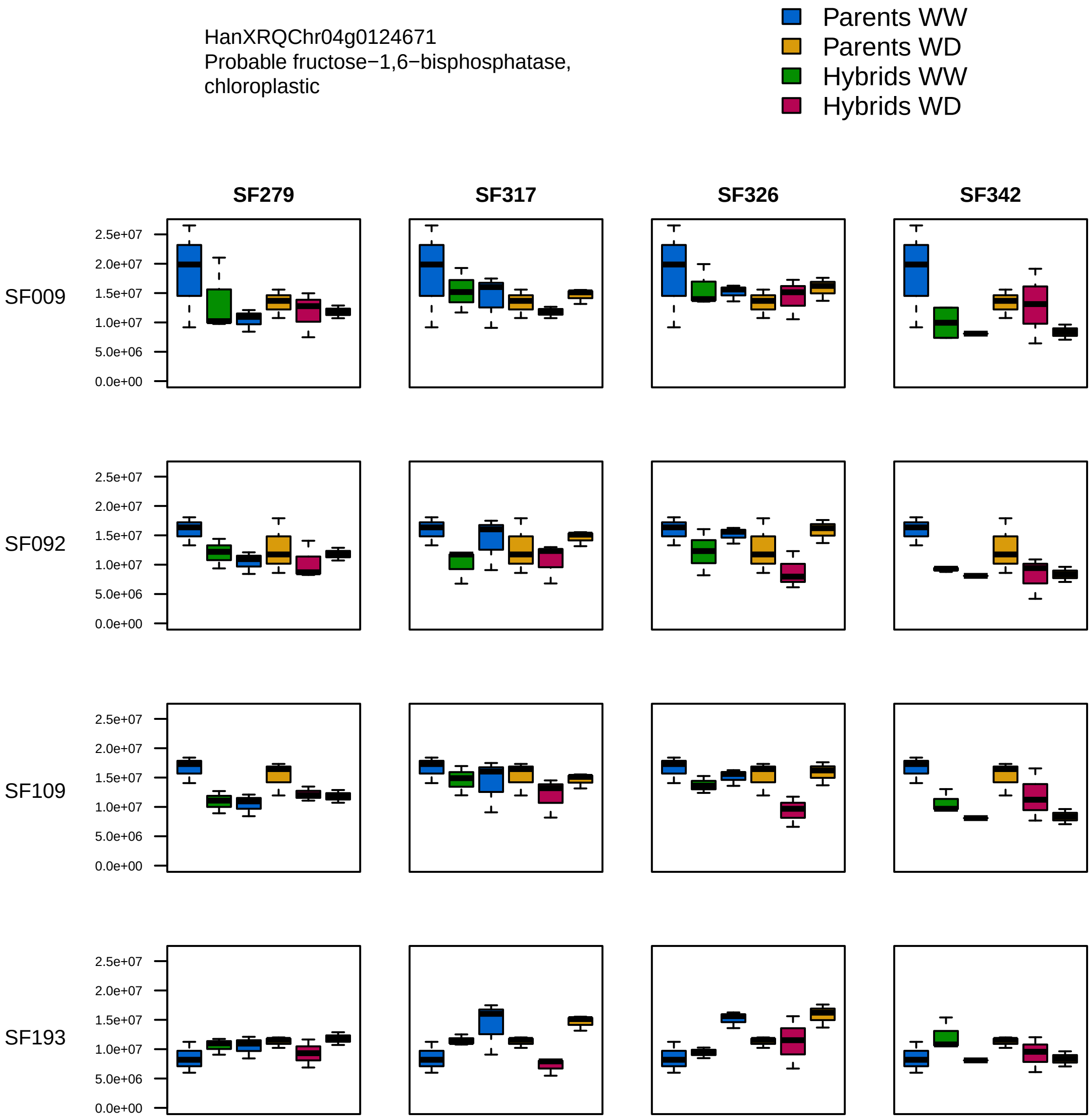
HanXRQChr04g0124671
Probable fructose−1,6−bisphosphatase, chloroplastic
Parents WW
Parents WD
Hybrids WW
Hybrids WD
SF279
SF317
SF326
SF342
SF009
SF092
SF109
SF193
0.0e+00
5.0e+06
1.0e+07
1.5e+07
2.0e+07
2.5e+07

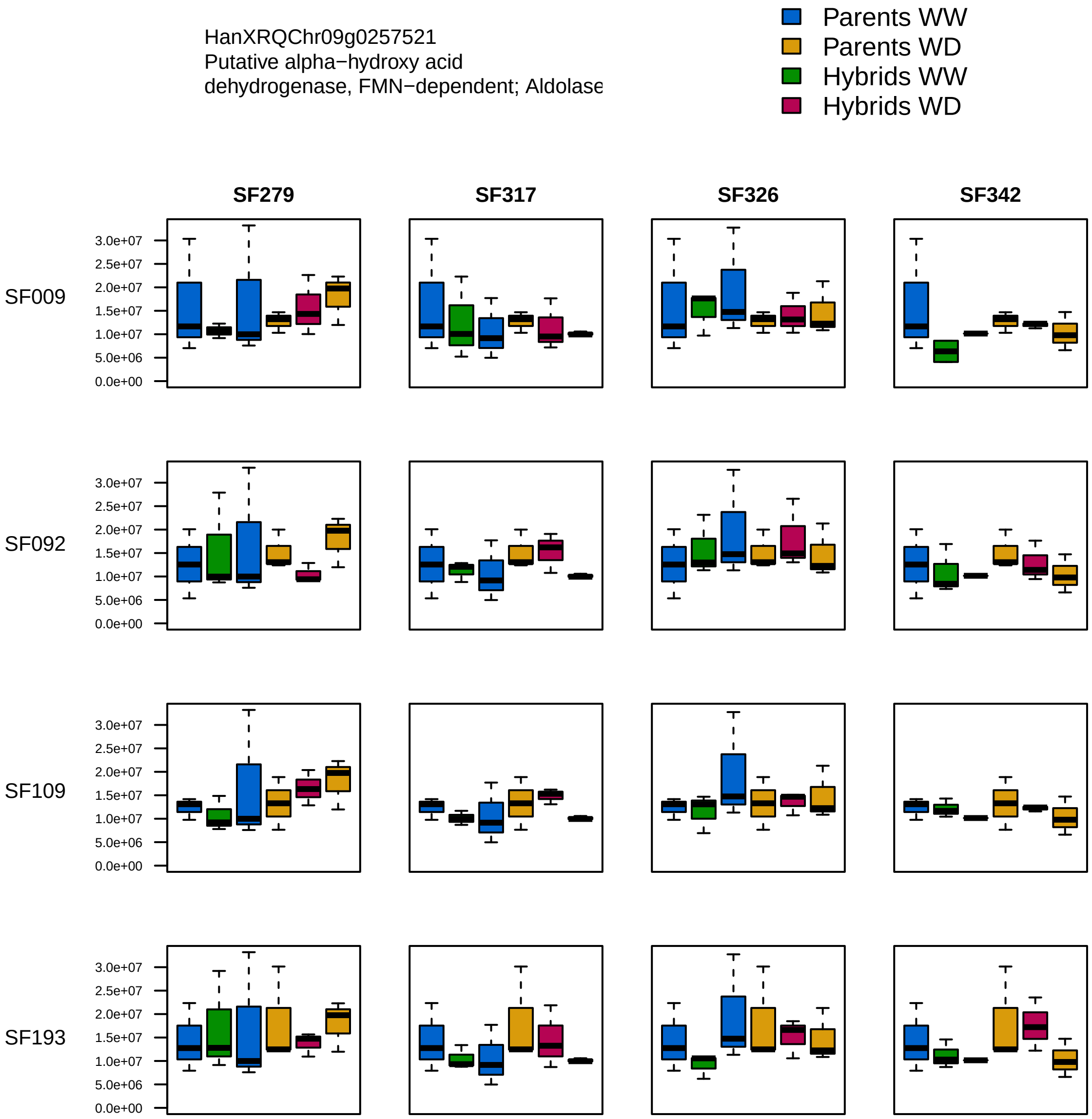
HanXRQChr09g0257521
Putative alpha−hydroxy acid
dehydrogenase, FMN−dependent; Aldolase
Parents WW
Parents WD
Hybrids WW
Hybrids WD
SF279
SF317
SF326
SF342
SF009
SF092
SF109
SF193
0.0e+00
5.0e+06
1.0e+07
1.5e+07
2.0e+07
2.5e+07
3.0e+07

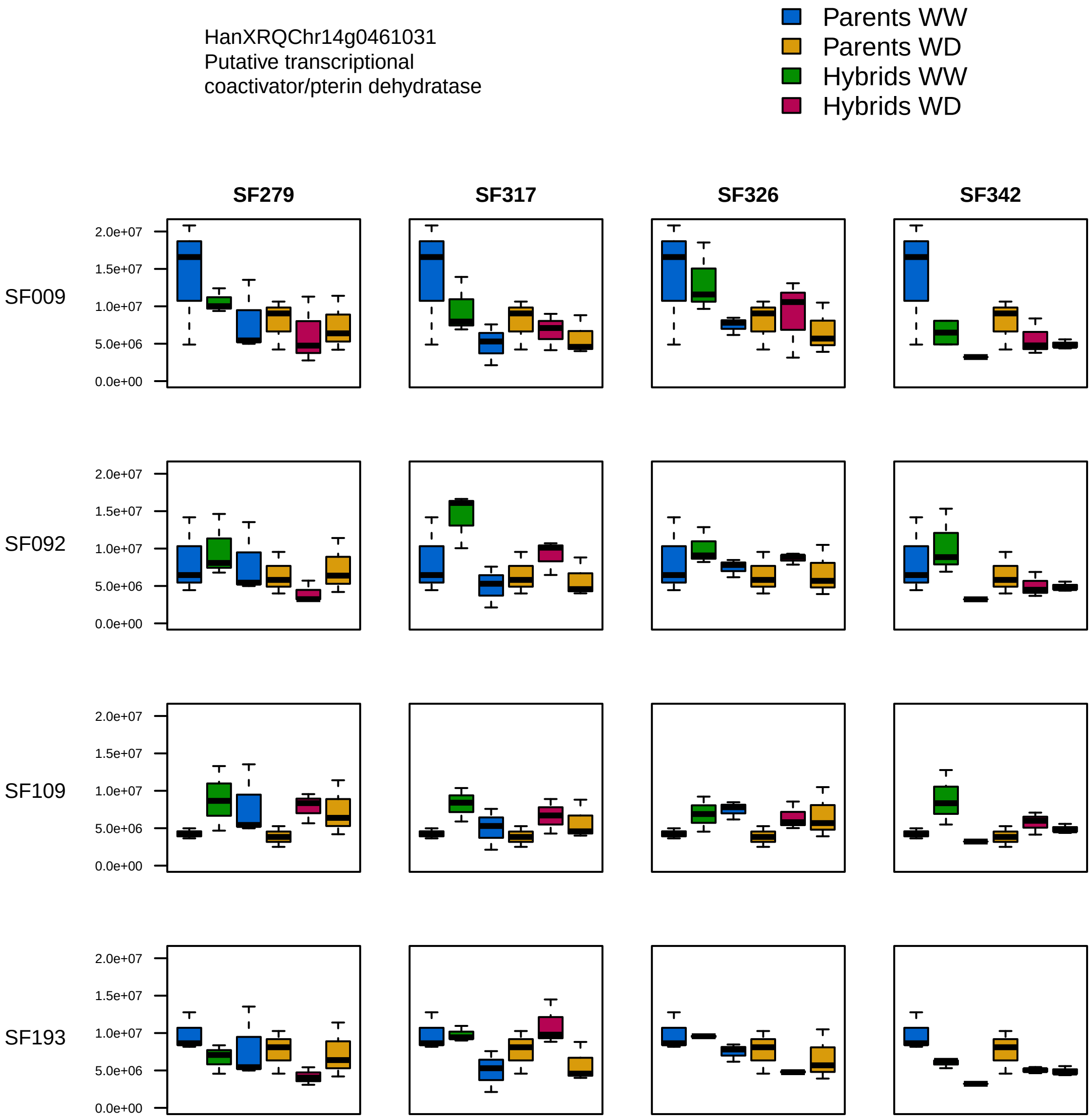
HanXRQChr14g0461031
Putative transcriptional
coactivator/pterin dehydratase
Parents WW
Parents WD
Hybrids WW
Hybrids WD
SF279
SF317
SF326
SF342
SF009
SF092
SF109
SF193
2.0e+07
1.5e+07
1.0e+07
5.0e+06
0.0e+00

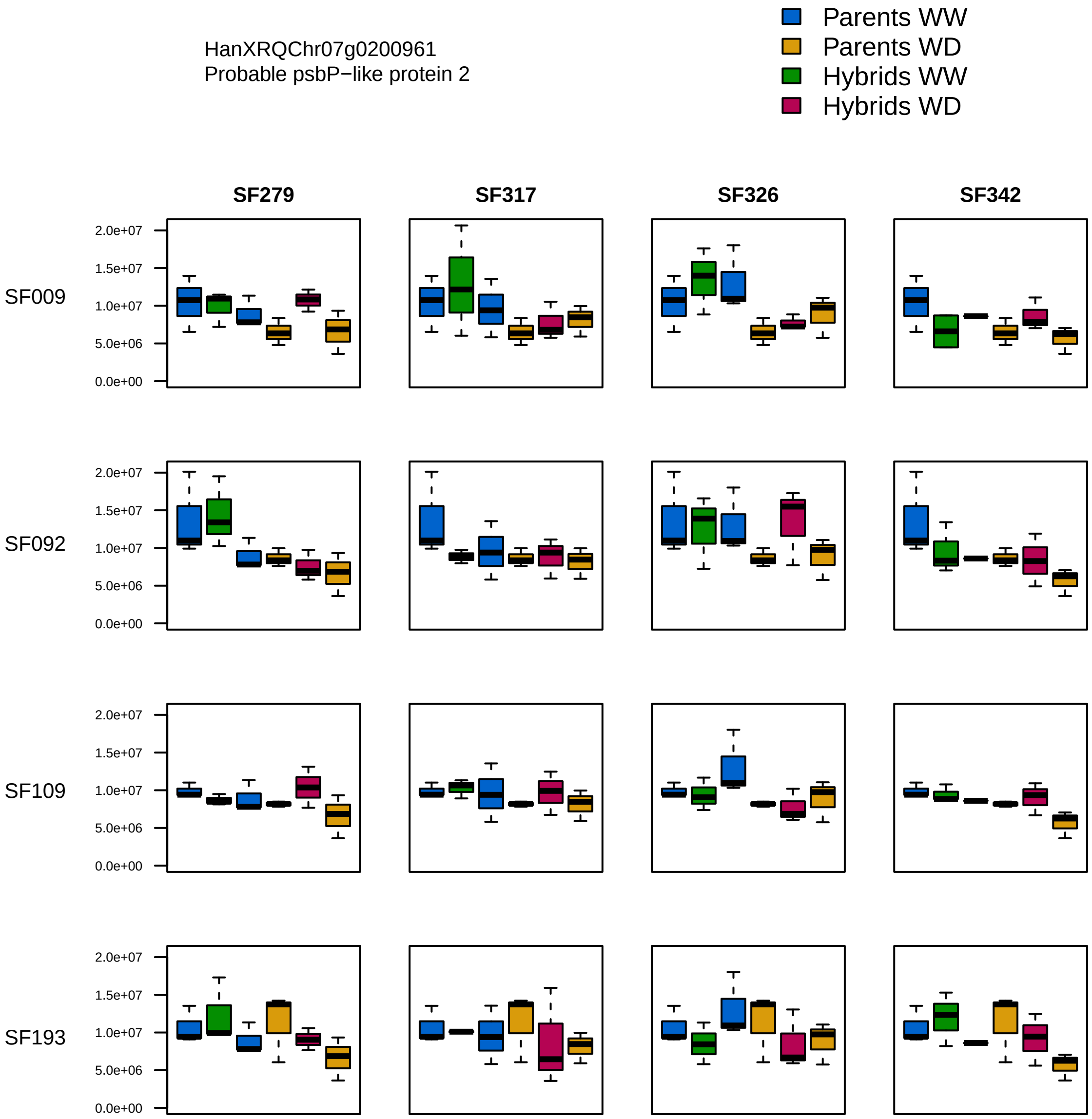

HanXRQChr07g0200961
Probable psbP−like protein 2
Parents WW
Parents WD
Hybrids WW
Hybrids WD
SF279
SF317
SF326
SF342
SF009
SF092
SF109
SF193
2.0e+07
1.5e+07
1.0e+07
5.0e+06
0.0e+00

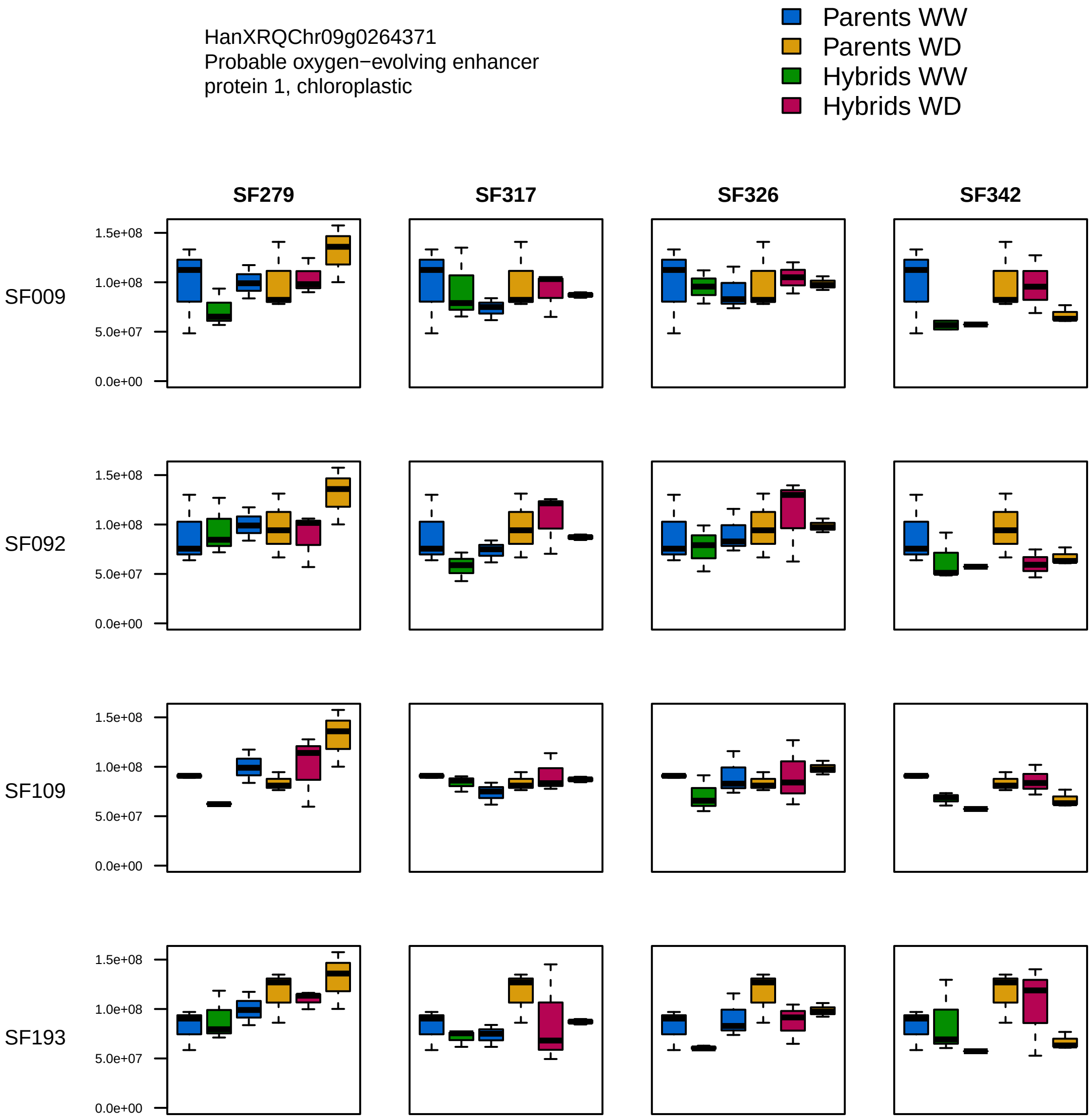
HanXRQChr09g0264371
Probable oxygen−evolving enhancer
protein 1, chloroplastic
Parents WW
Parents WD
Hybrids WW
Hybrids WD
SF279
SF317
SF326
SF342
SF009
SF092
SF109
SF193
1.5e+08
1.0e+08
5.0e+07
0.0e+00

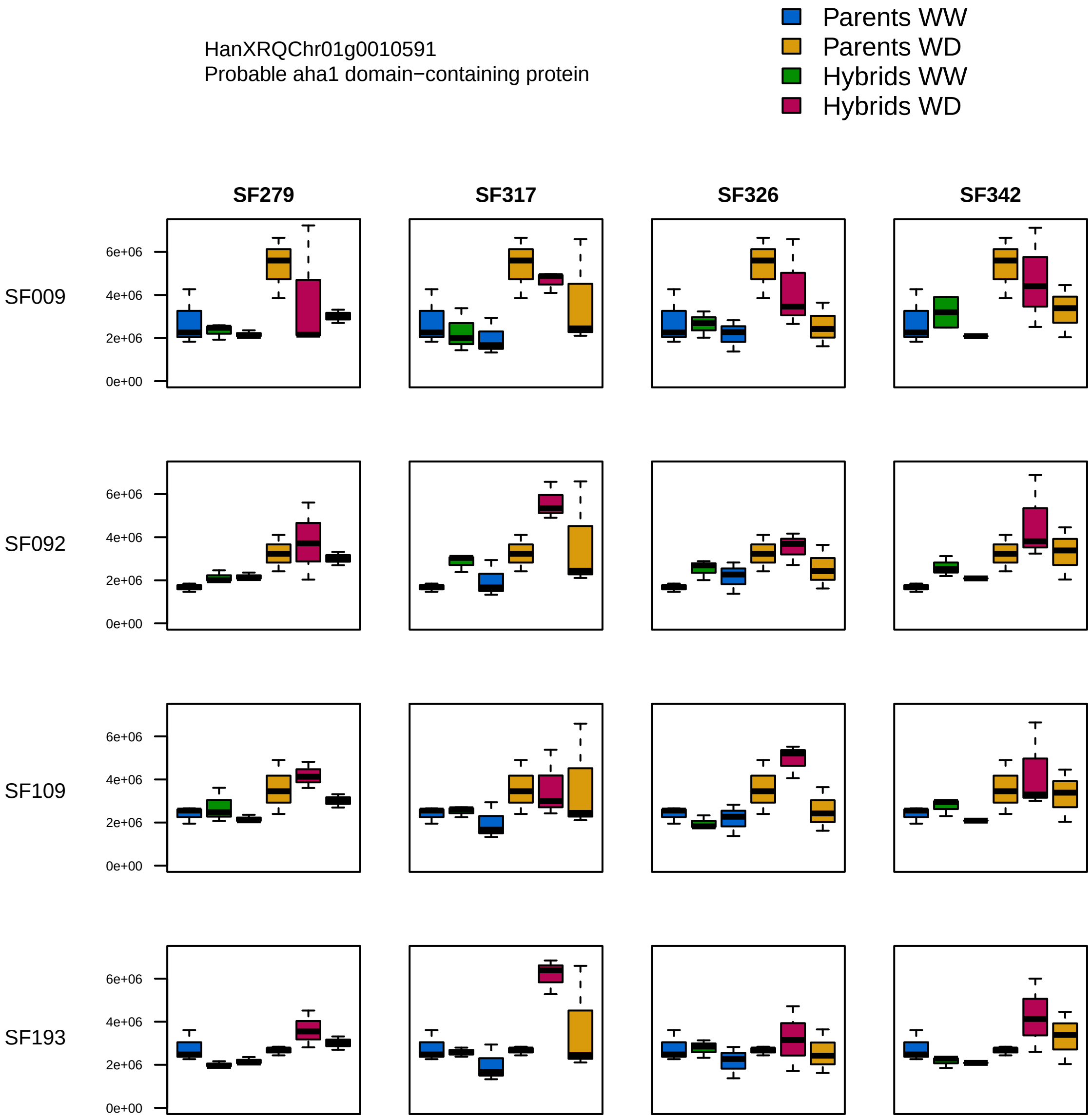

HanXRQChr01g0010591
Probable aha1 domain−containing protein
Parents WW
Parents WD
Hybrids WW
Hybrids WD
SF279
SF317
SF326
SF342
SF009
SF092
SF109
SF193
0e+00
2e+06
4e+06
6e+06

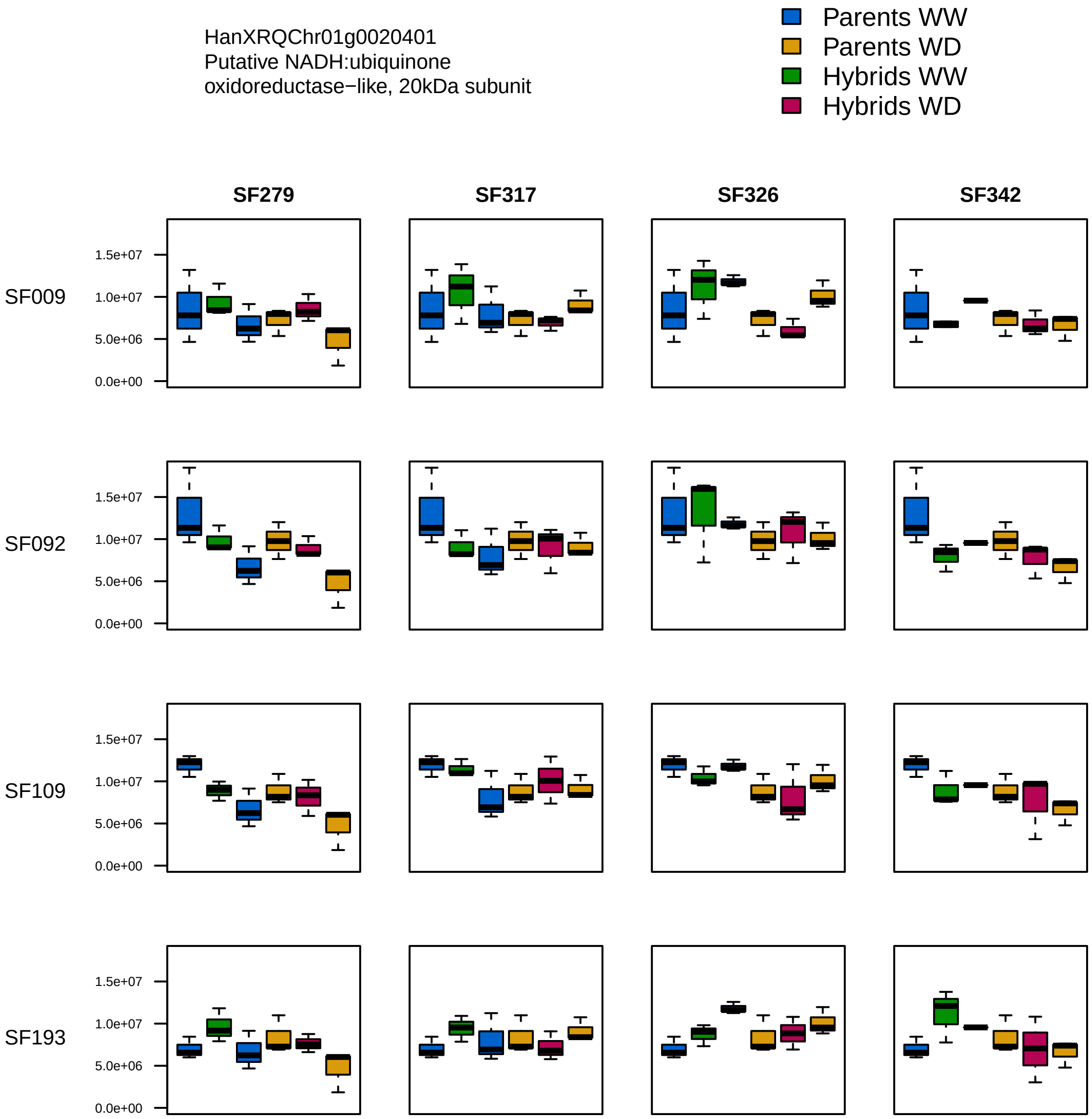

HanXRQChr01g0020401
Putative NADH:ubiquinone
oxidoreductase−like, 20kDa subunit
Parents WW
Parents WD
Hybrids WW
Hybrids WD
SF279
SF317
SF326
SF342
SF009
SF092
SF109
SF193
1.5e+07
1.0e+07
5.0e+06
0.0e+00

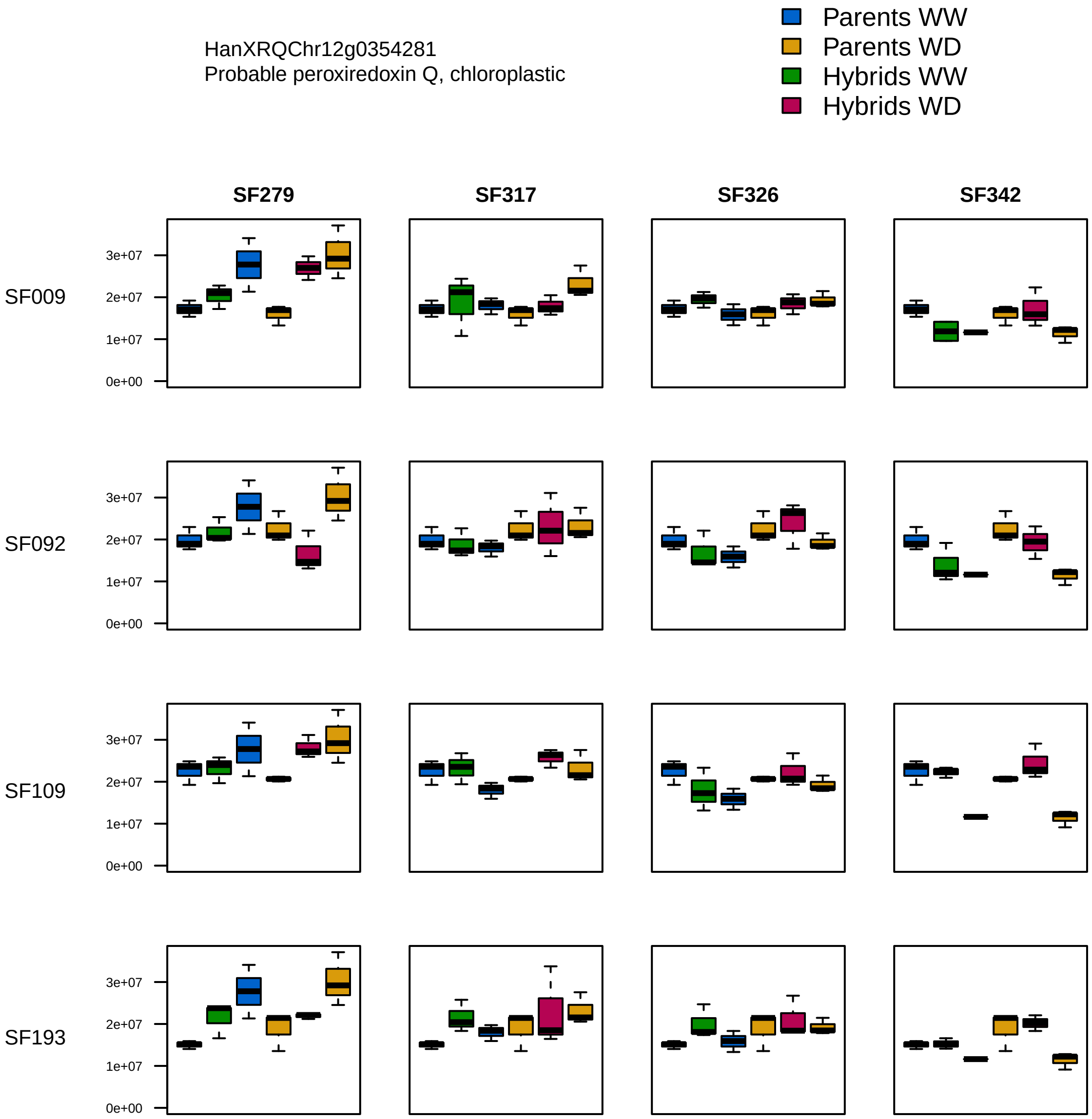
HanXRQChr12g0354281
Probable peroxiredoxin Q, chloroplastic
Parents WW
Parents WD
Hybrids WW
Hybrids WD
SF279
SF317
SF326
SF342
SF009
SF092
SF109
SF193
0e+00
1e+07
2e+07
3e+07

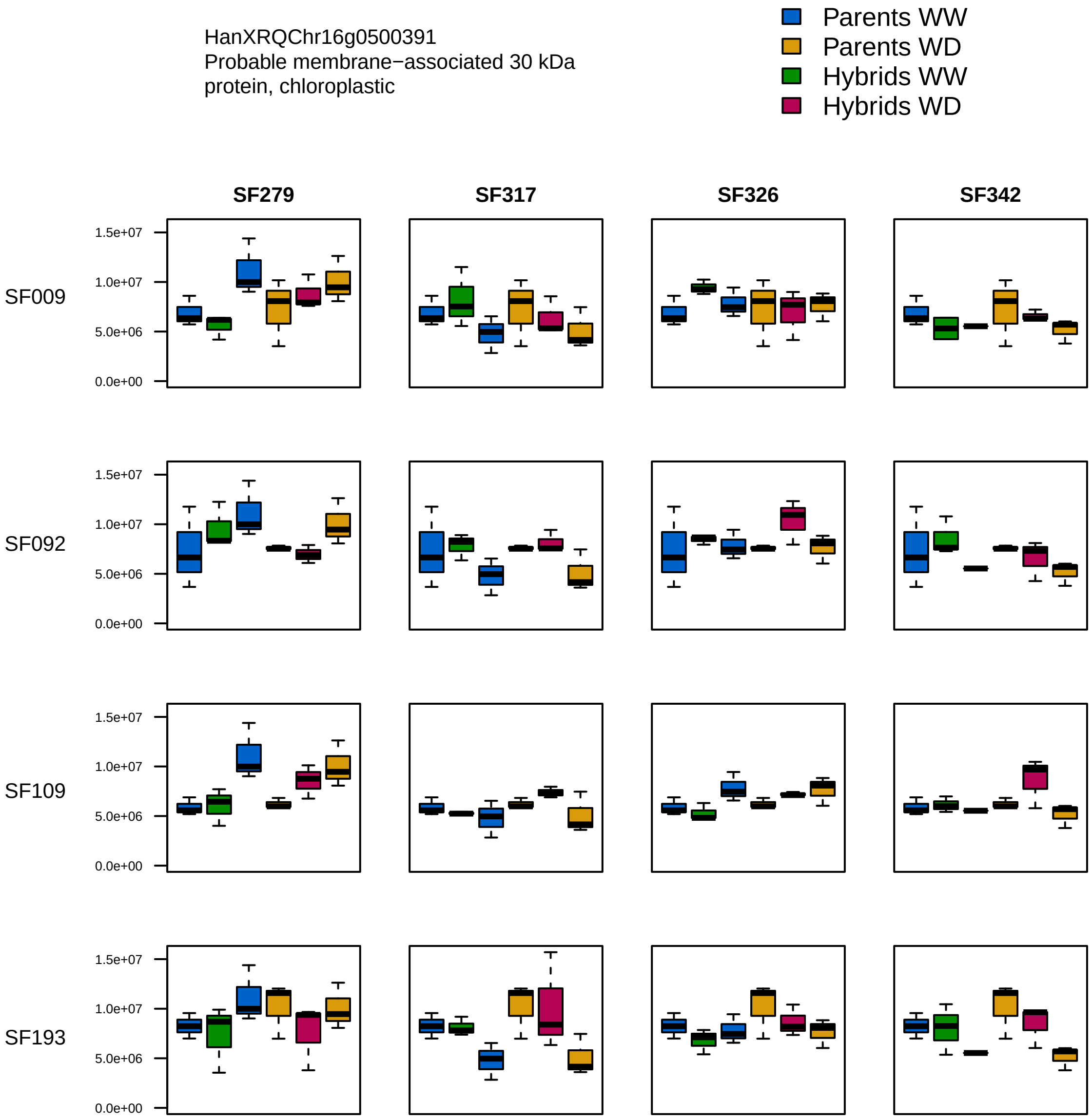
HanXRQChr16g0500391
Probable membrane−associated 30 kDa protein, chloroplastic
Parents WW
Parents WD
Hybrids WW
Hybrids WD
SF279
SF317
SF326
SF342
SF009
SF092
SF109
SF193
1.5e+07
1.0e+07
5.0e+06
0.0e+00

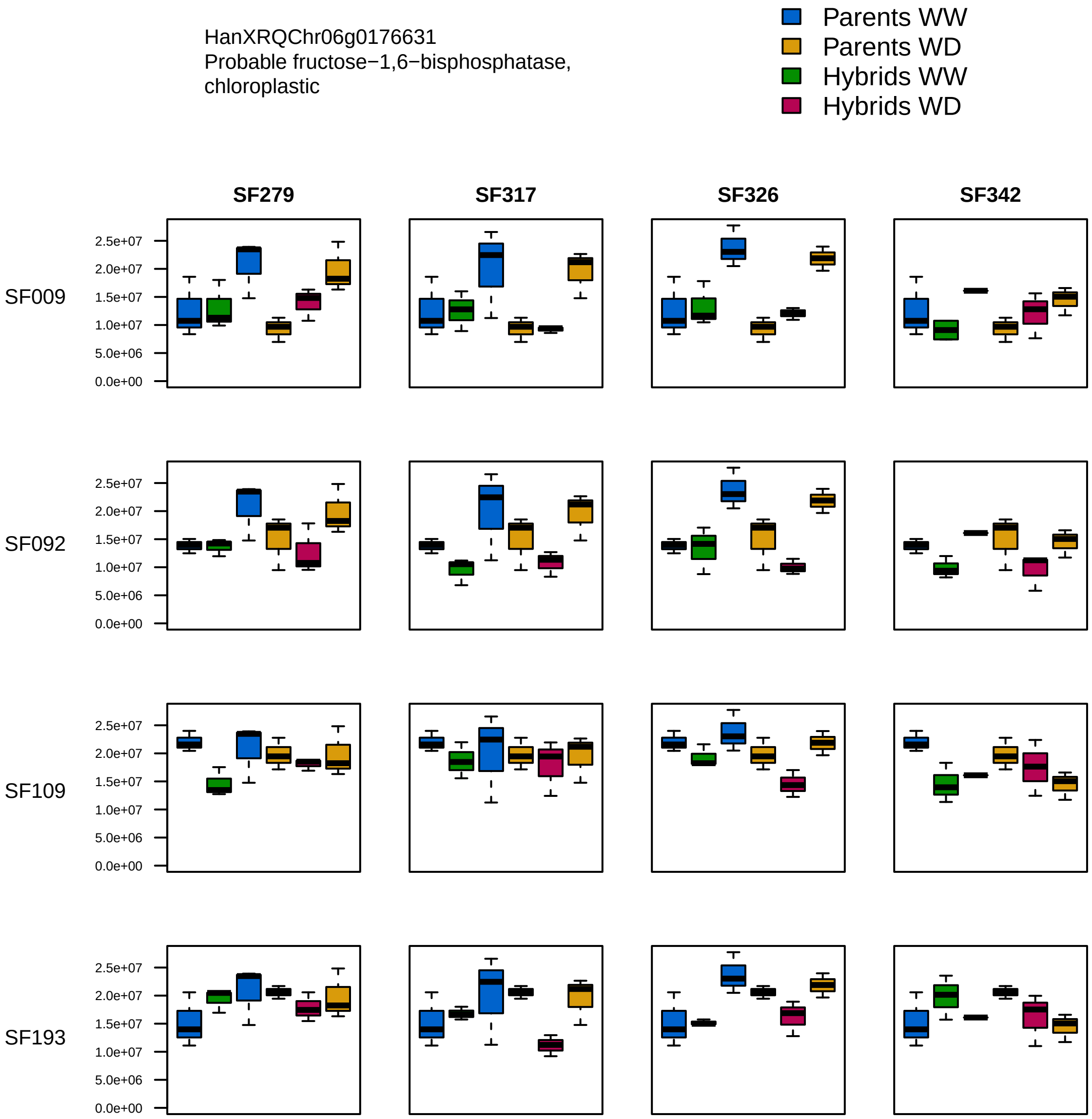
HanXRQChr06g0176631
Probable fructose−1,6−bisphosphatase, chloroplastic
Parents WW
Parents WD
Hybrids WW
Hybrids WD
SF279
SF317
SF326
SF342
SF009
SF092
SF109
SF193
0.0e+00
5.0e+06
1.0e+07
1.5e+07
2.0e+07
2.5e+07

HanXRQChr03g0092611
Putative chaperonin Cpn60/TCP−1 family;
GroEL−like apical domain; GroEL−like equa

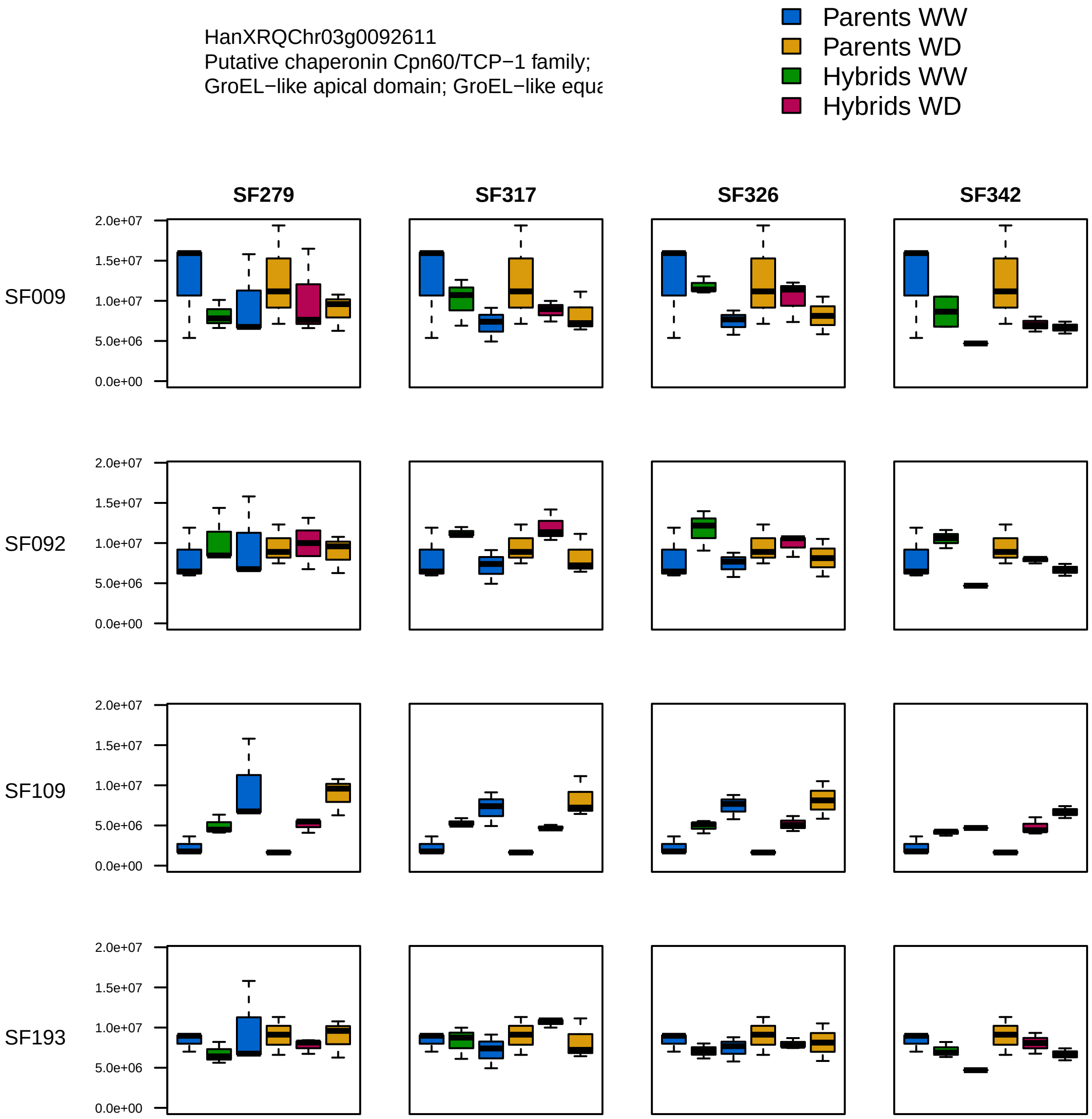

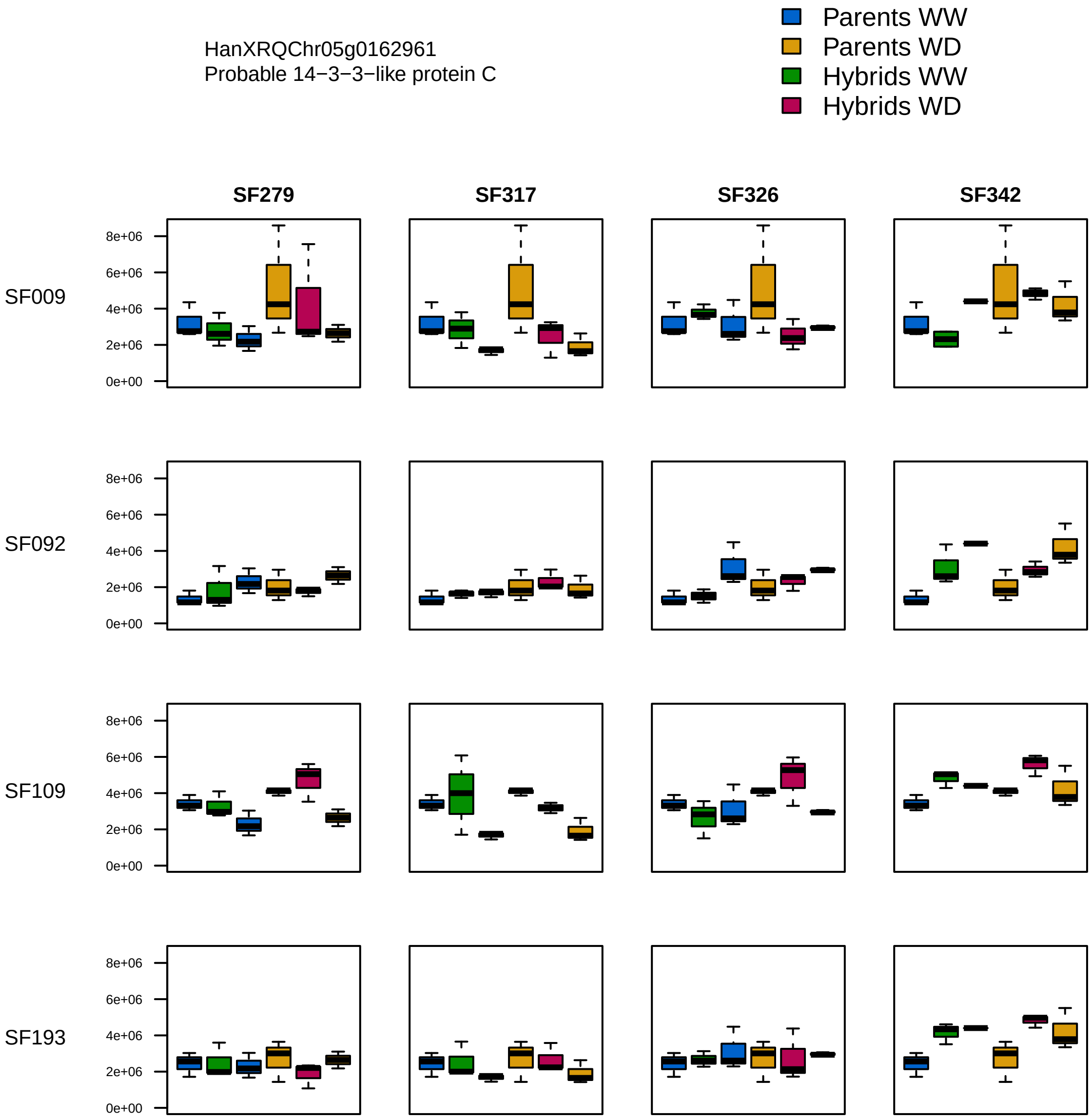
HanXRQChr05g0162961
Probable 14−3−3−like protein C
Parents WW
Parents WD
Hybrids WW
Hybrids WD
SF279
SF317
SF326
SF342
SF009
SF092
SF109
SF193
8e+06
6e+06
4e+06
2e+06
0e+00

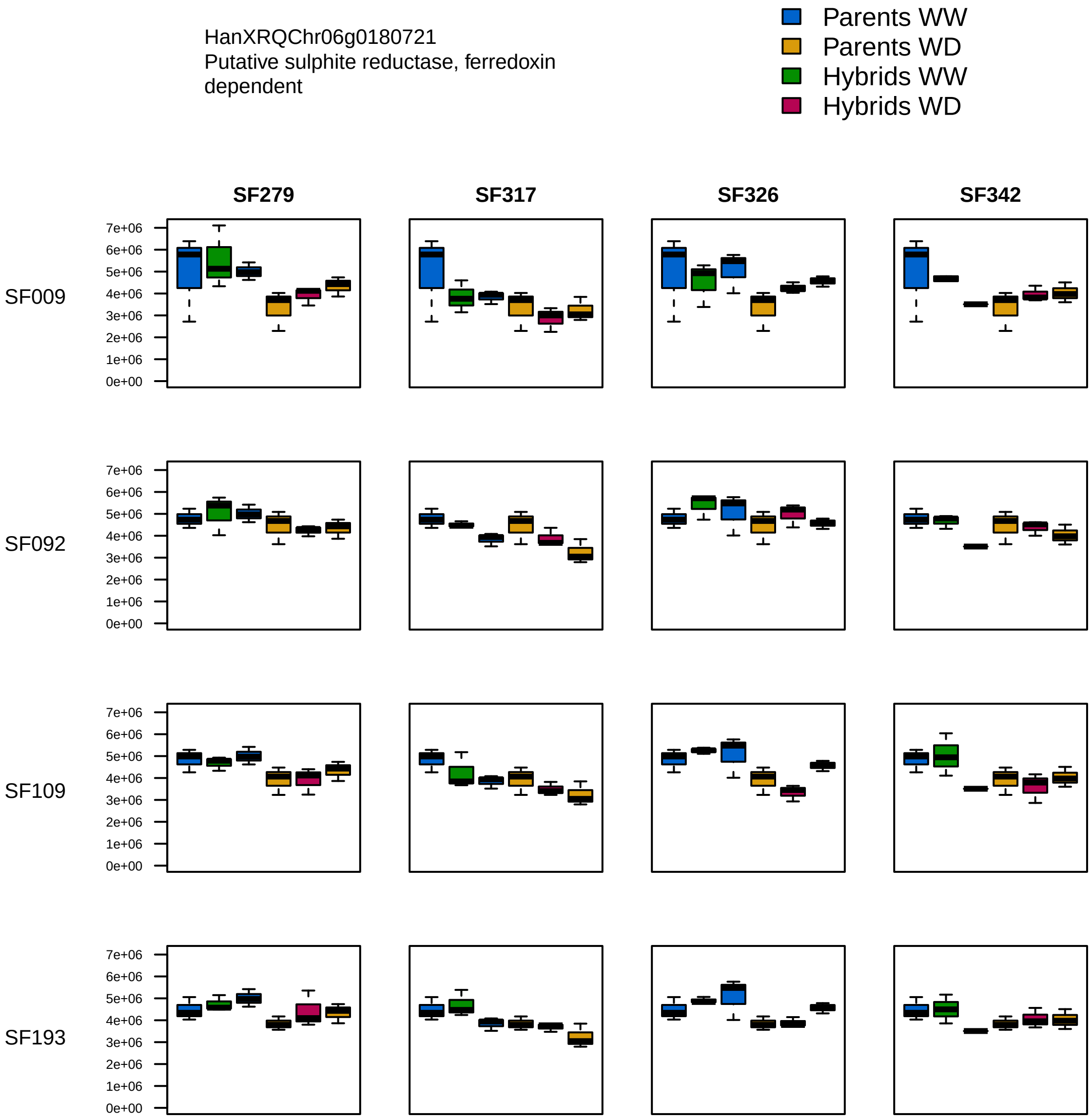

HanXRQChr06g0180721
Putative sulphite reductase, ferredoxin dependent
Parents WW
Parents WD
Hybrids WW
Hybrids WD
SF279
SF317
SF326
SF342
SF009
SF092
SF109
SF193
7e+06
6e+06
5e+06
4e+06
3e+06
2e+06
1e+06
0e+00

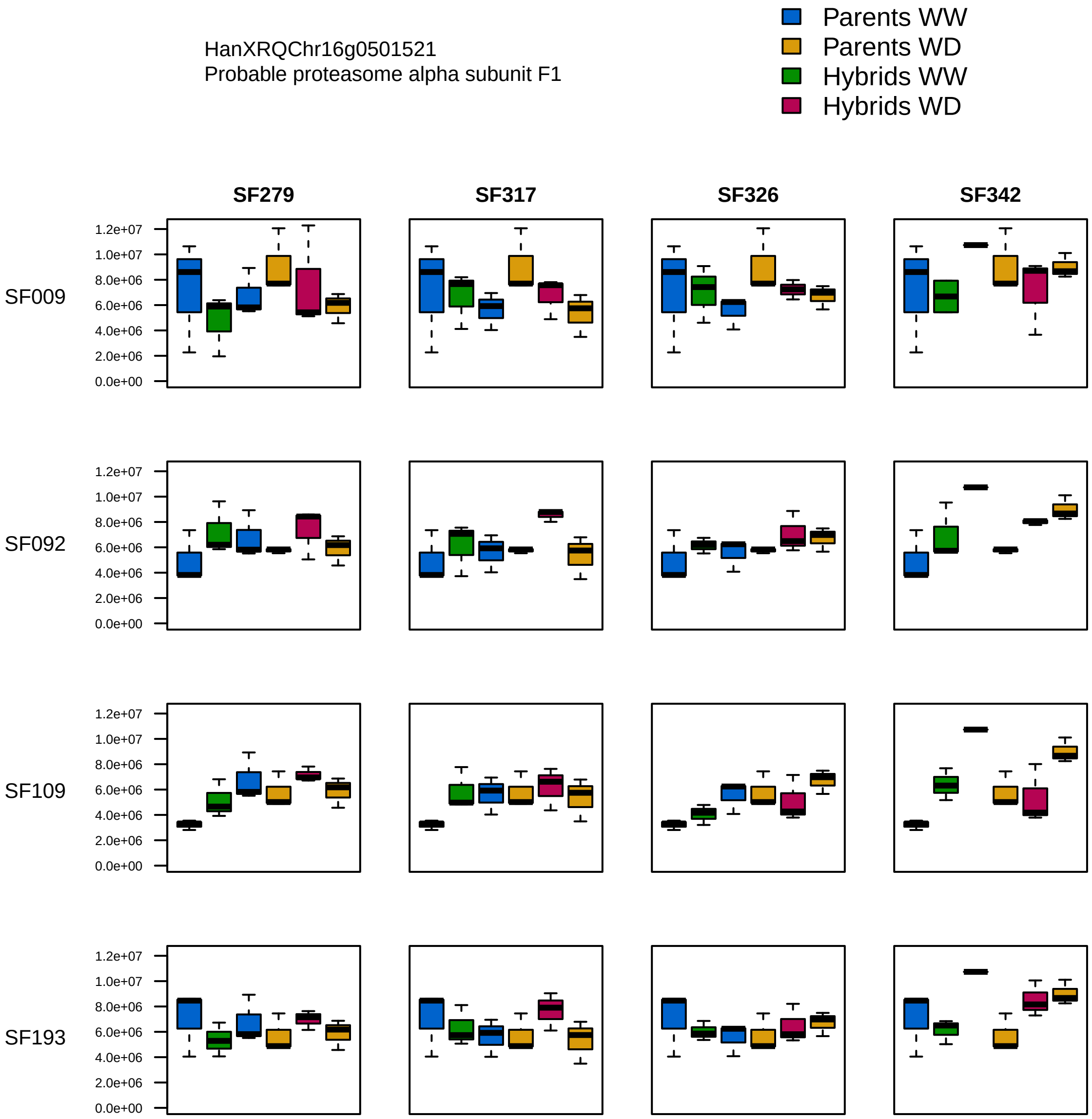
HanXRQChr16g0501521
Probable proteasome alpha subunit F1
Parents WW
Parents WD
Hybrids WW
Hybrids WD
SF279
SF317
SF326
SF342
SF009
SF092
SF109
SF193
0.0e+00
2.0e+06
4.0e+06
6.0e+06
8.0e+06
1.0e+07
1.2e+07

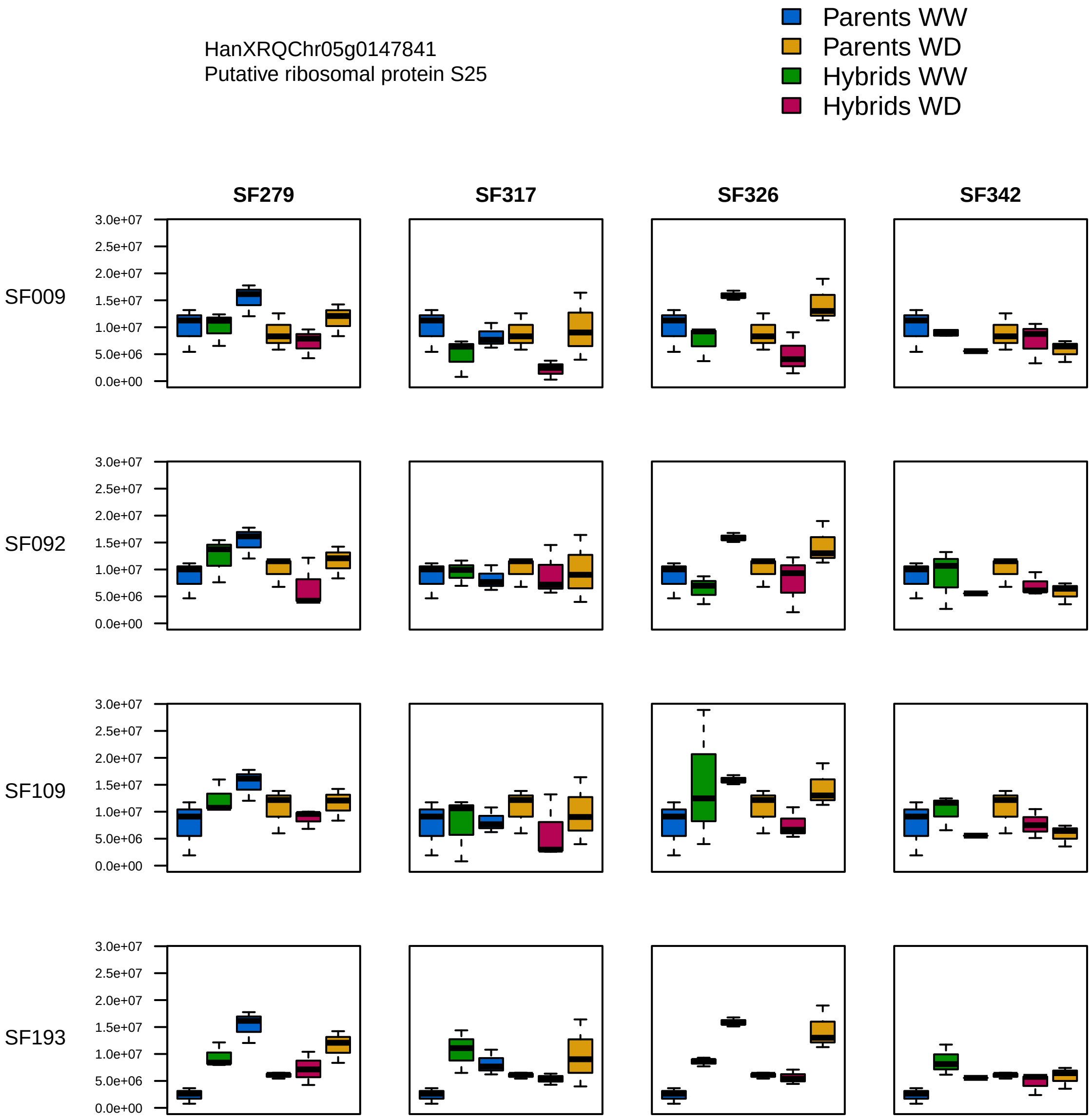

HanXRQChr05g0147841
Putative ribosomal protein S25
Parents WW
Parents WD
Hybrids WW
Hybrids WD
SF279
SF317
SF326
SF342
SF009
SF092
SF109
SF193
3.0e+07
2.5e+07
2.0e+07
1.5e+07
1.0e+07
5.0e+06
0.0e+00

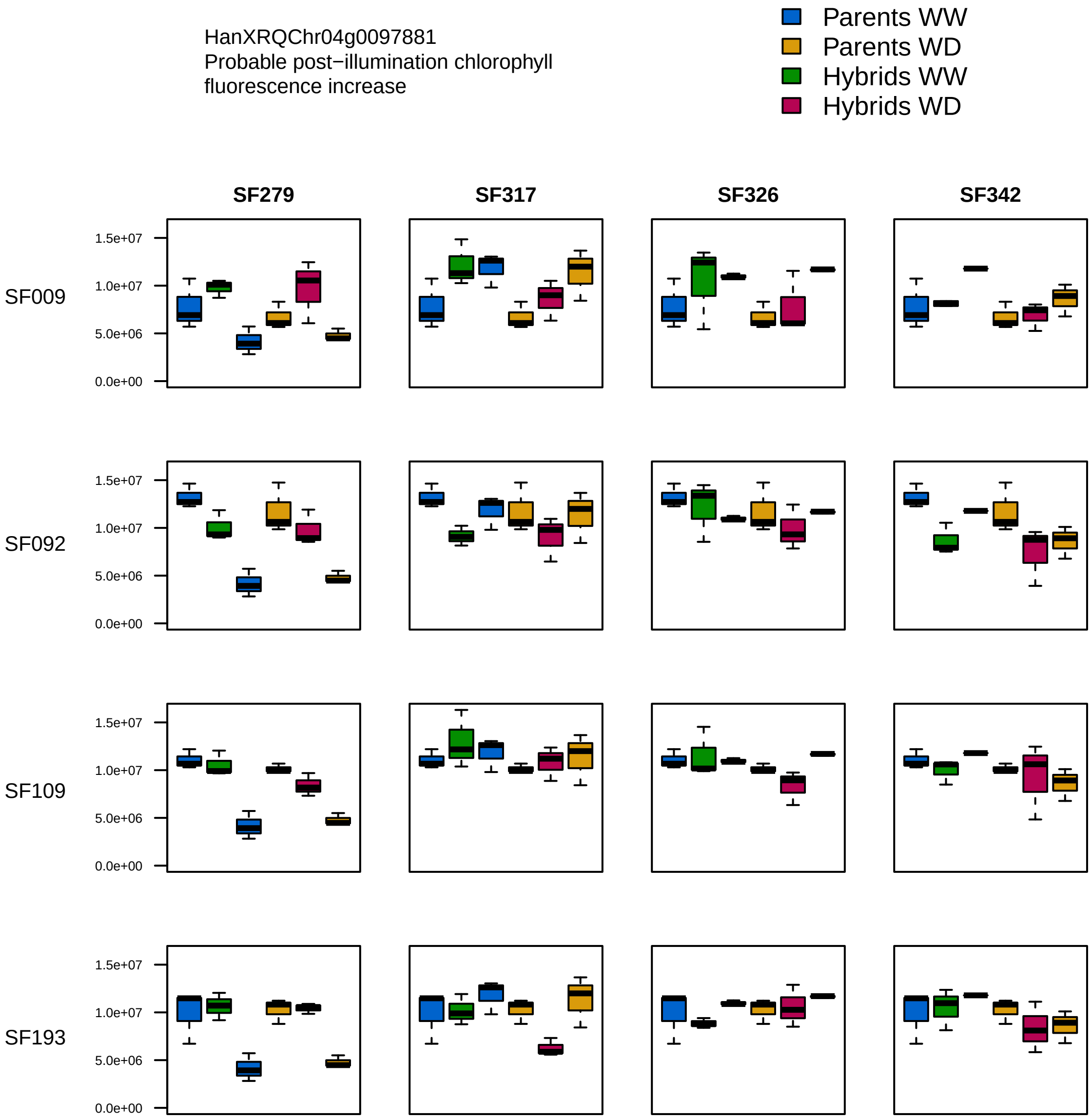
HanXRQChr04g0097881
Probable post−illumination chlorophyll
fluorescence increase
Parents WW
Parents WD
Hybrids WW
Hybrids WD
SF279
SF317
SF326
SF342
SF009
SF092
SF109
SF193
1.5e+07
1.0e+07
5.0e+06
0.0e+00

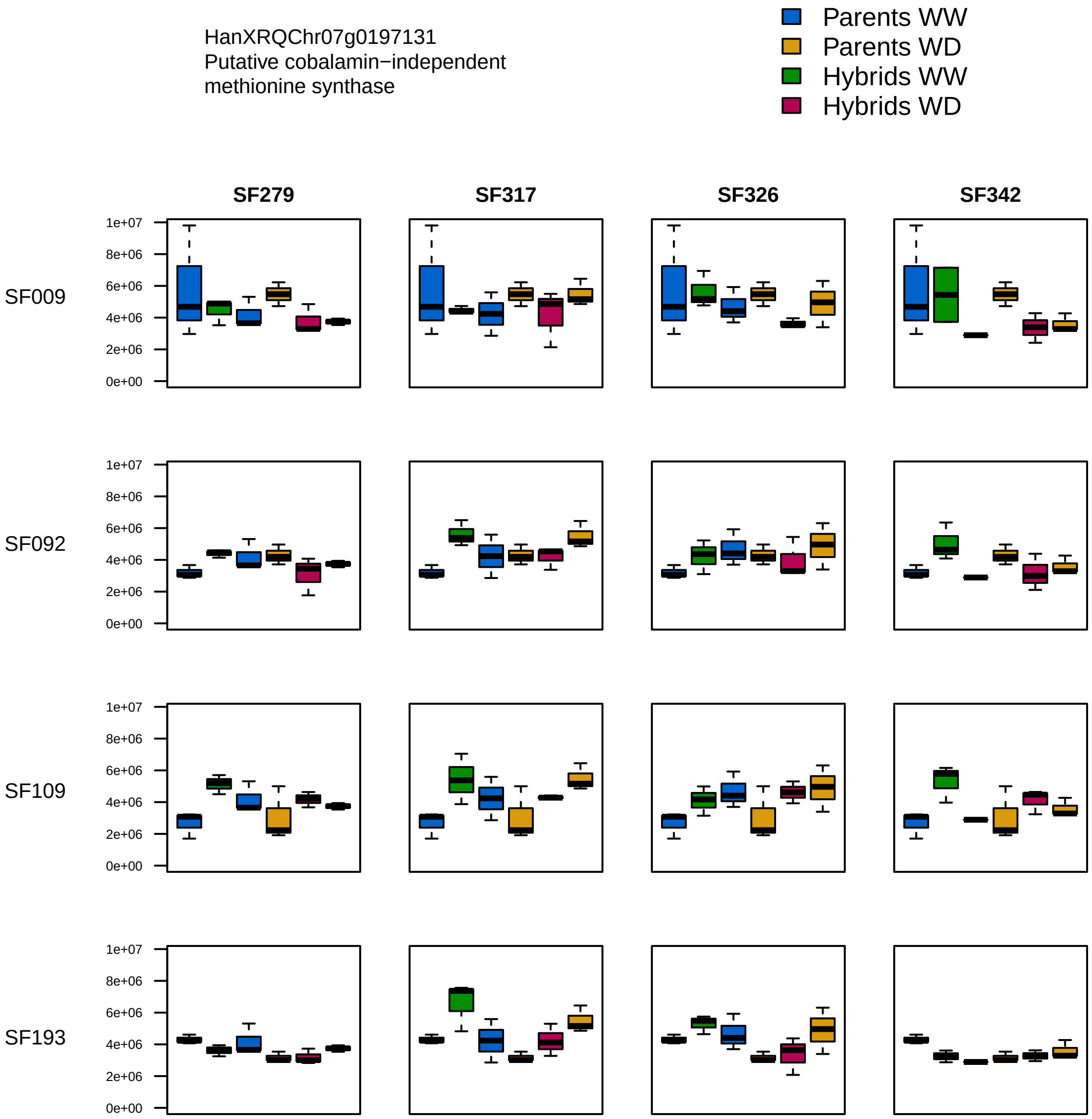
HanXRQChr07g0197131
Putative cobalamin−independent
methionine synthase
Parents WW
Parents WD
Hybrids WW
Hybrids WD
SF279
SF317
SF326
SF342
SF009
SF092
SF109
SF193
1e+07
8e+06
6e+06
4e+06
2e+06
0e+00

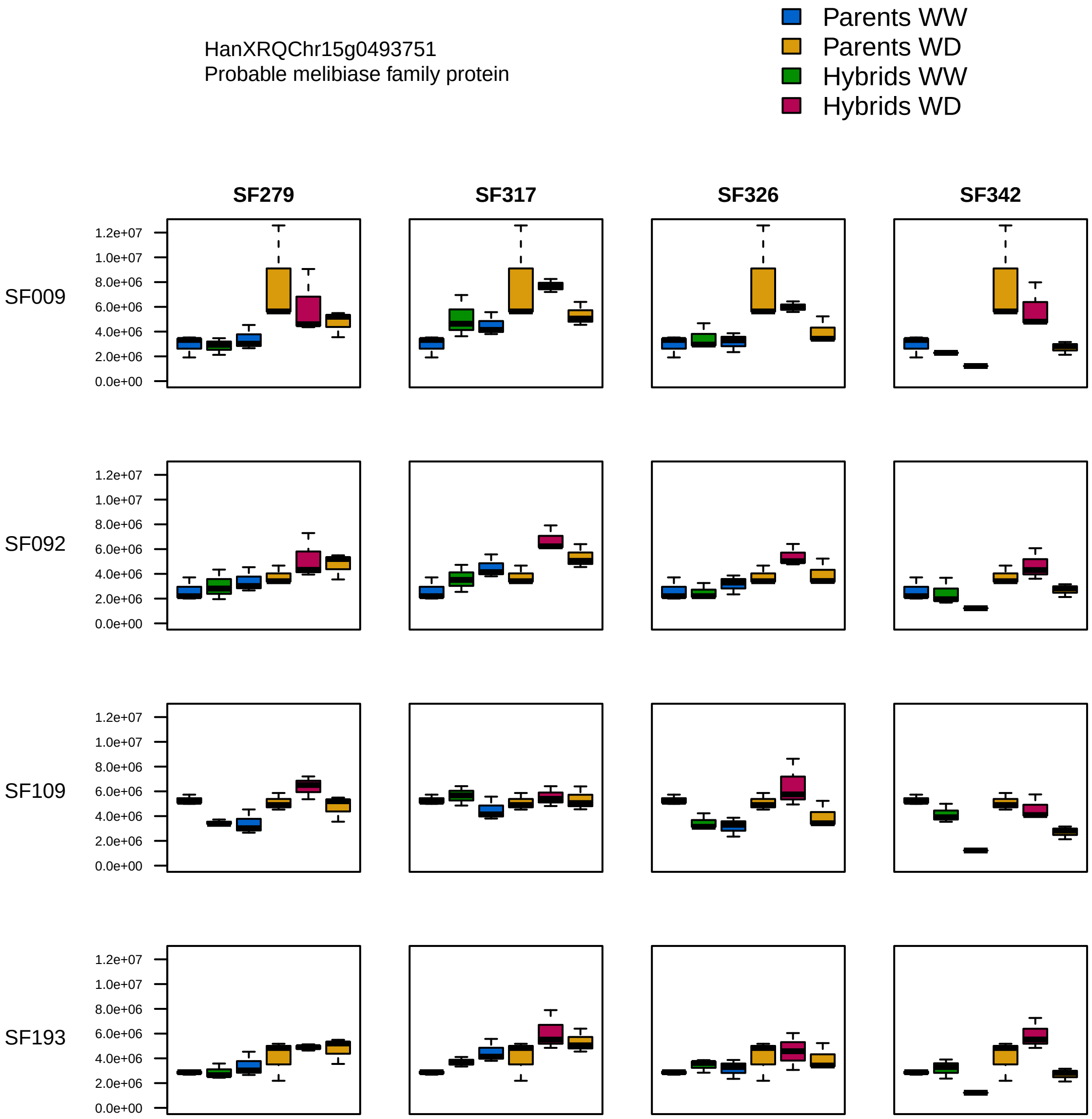
HanXRQChr15g0493751
Probable melibiase family protein
Parents WW
Parents WD
Hybrids WW
Hybrids WD
SF279
SF317
SF326
SF342
SF009
SF092
SF109
SF193
1.2e+07
1.0e+07
8.0e+06
6.0e+06
4.0e+06
2.0e+06
0.0e+00

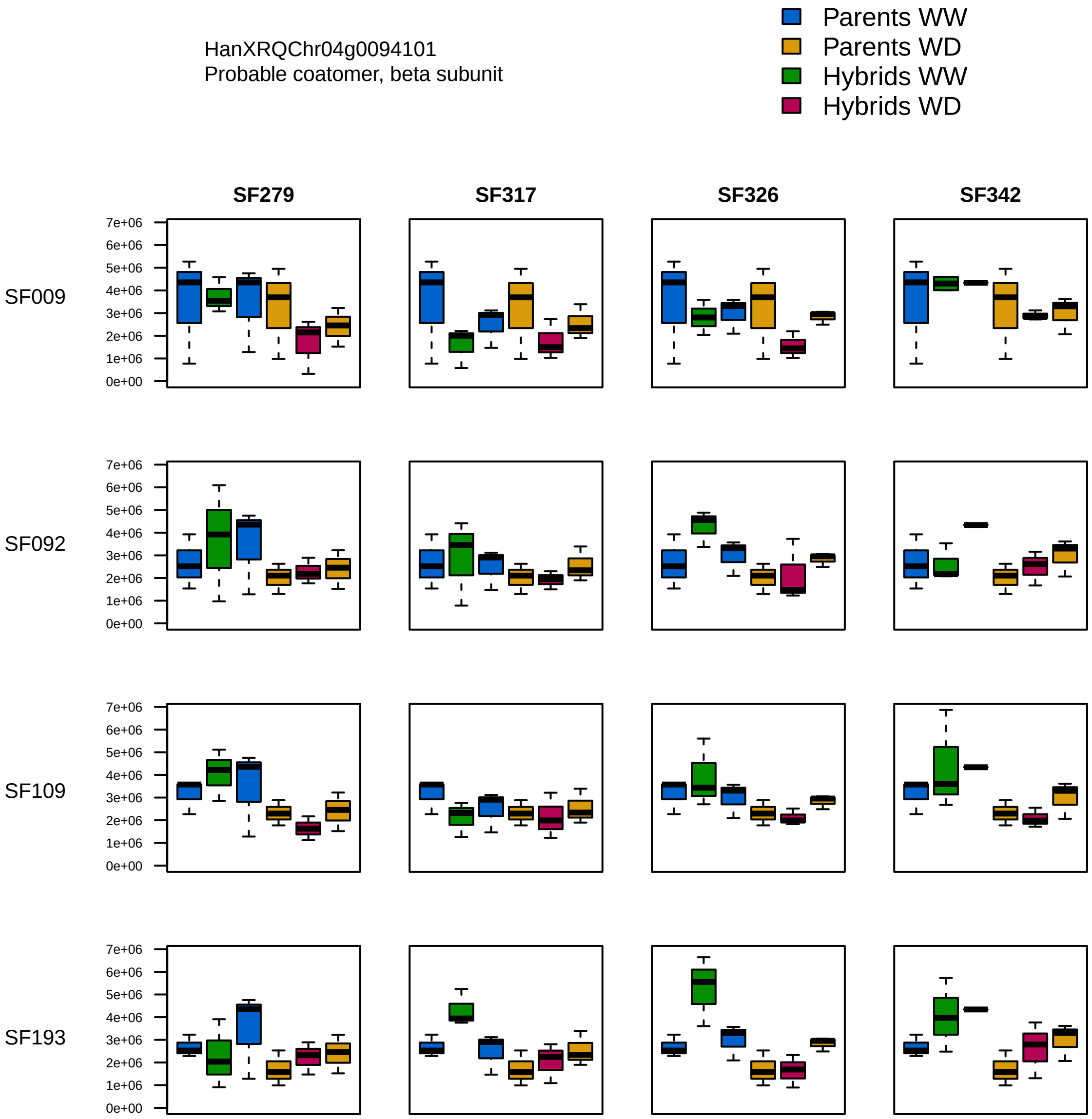

HanXRQChr04g0094101
Probable coatomer, beta subunit
Parents WW
Parents WD
Hybrids WW
Hybrids WD
SF279
SF317
SF326
SF342
SF009
SF092
SF109
SF193
7e+06
6e+06
5e+06
4e+06
3e+06
2e+06
1e+06
0e+00

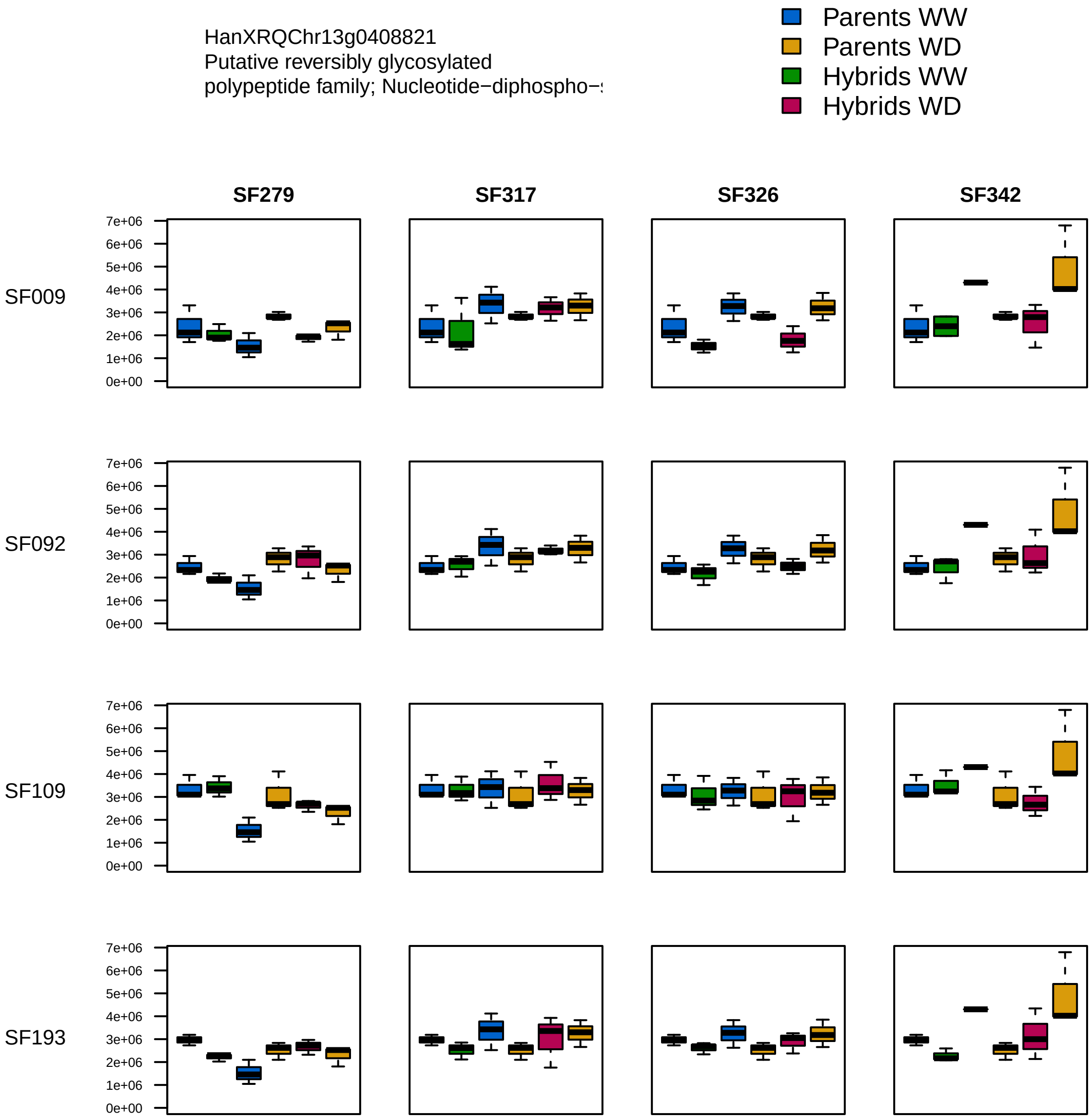

HanXRQChr13g0408821
Putative reversibly glycosylated
polypeptide family; Nucleotide−diphospho−s
Parents WW
Parents WD
Hybrids WW
Hybrids WD
SF279
SF317
SF326
SF342
SF009
SF092
SF109
SF193
7e+06
6e+06
5e+06
4e+06
3e+06
2e+06
1e+06
0e+00

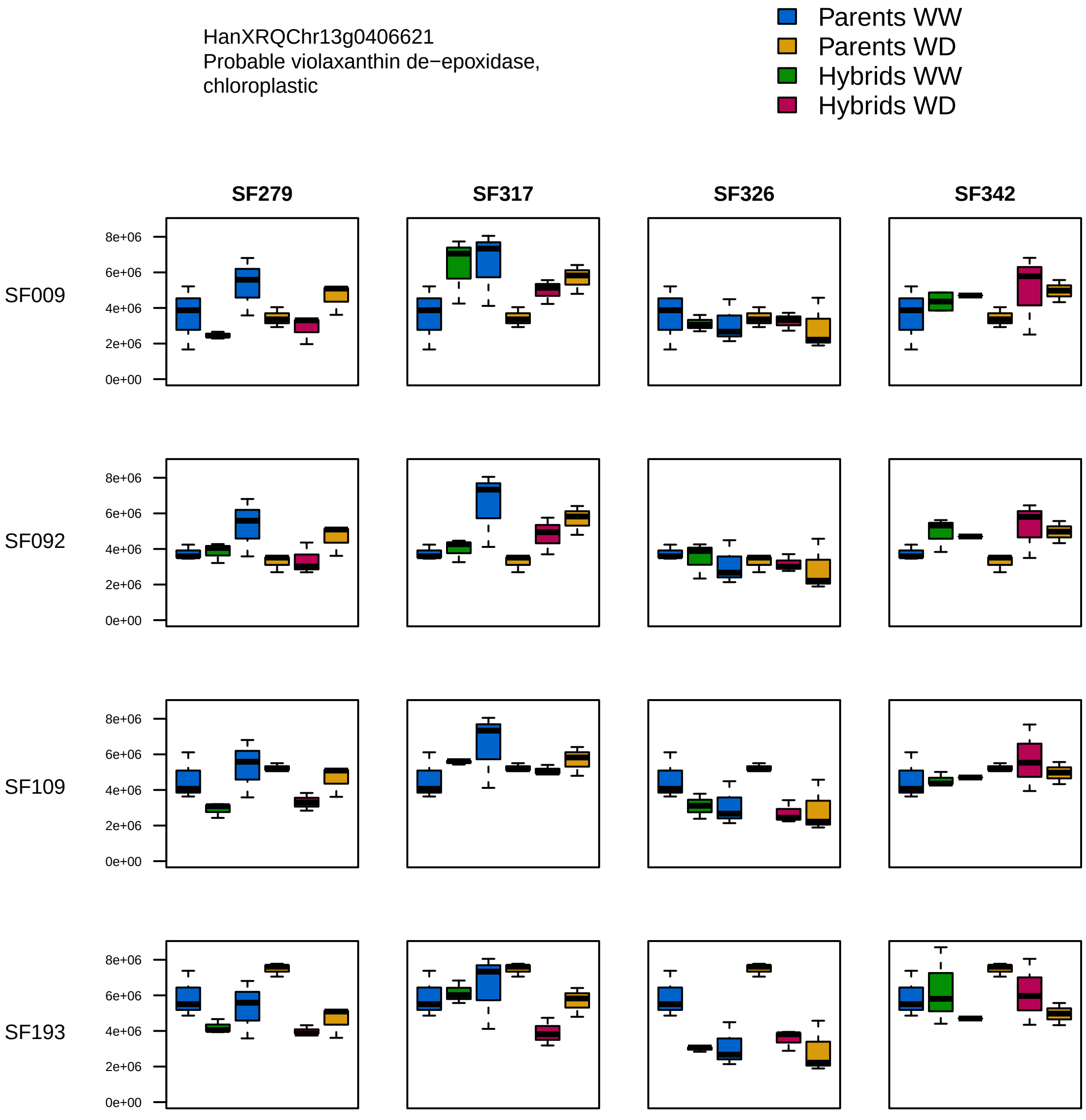
HanXRQChr13g0406621
Probable violaxanthin de−epoxidase,
chloroplastic
Parents WW
Parents WD
Hybrids WW
Hybrids WD
SF279
SF317
SF326
SF342
SF009
SF092
SF109
SF193
8e+06
6e+06
4e+06
2e+06
0e+00

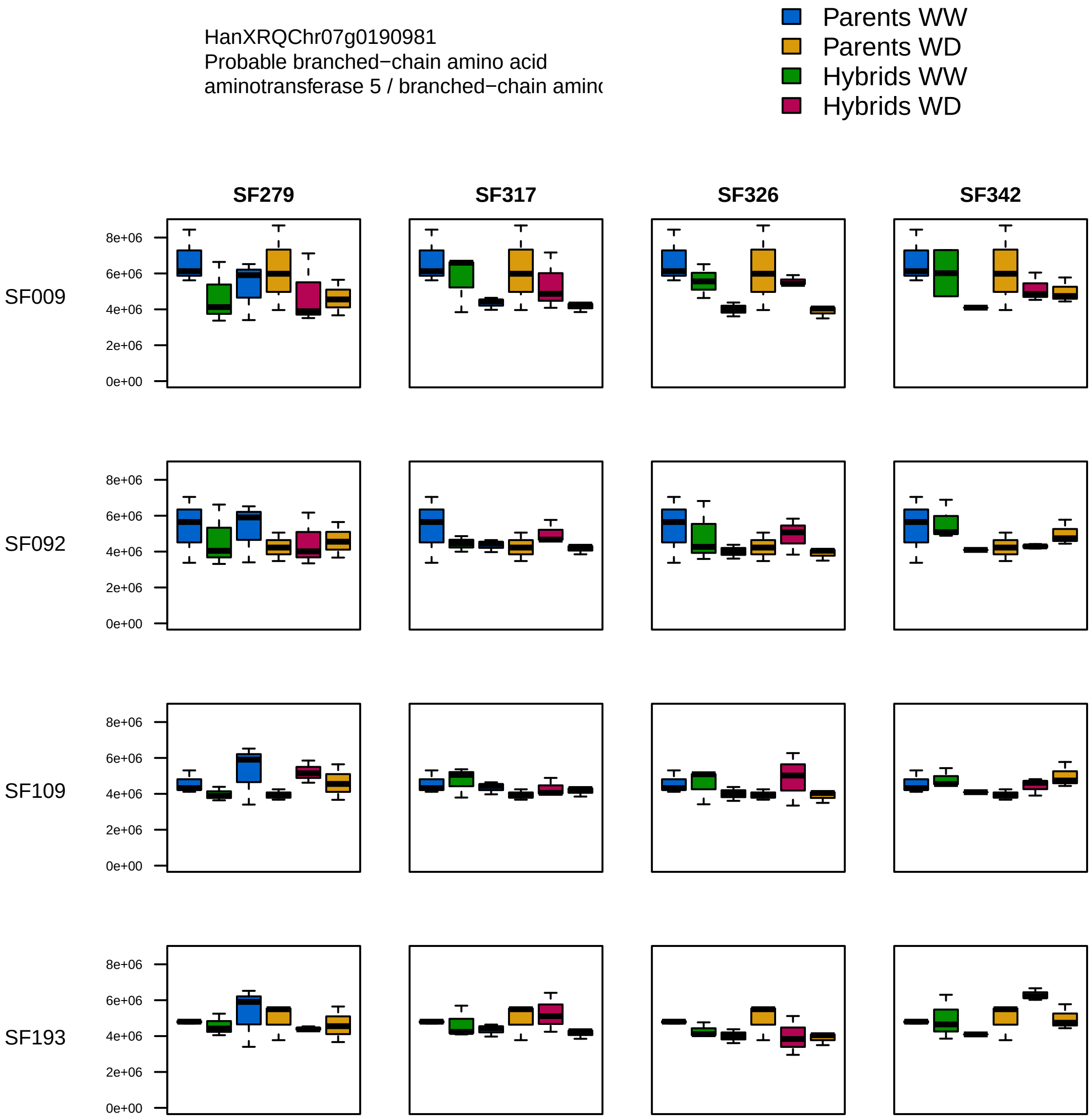

HanXRQChr07g0190981
Probable branched−chain amino acid
aminotransferase 5 / branched−chain aminc
Parents WW
Parents WD
Hybrids WW
Hybrids WD
SF279
SF317
SF326
SF342
SF009
SF092
SF109
SF193
8e+06
6e+06
4e+06
2e+06
0e+00

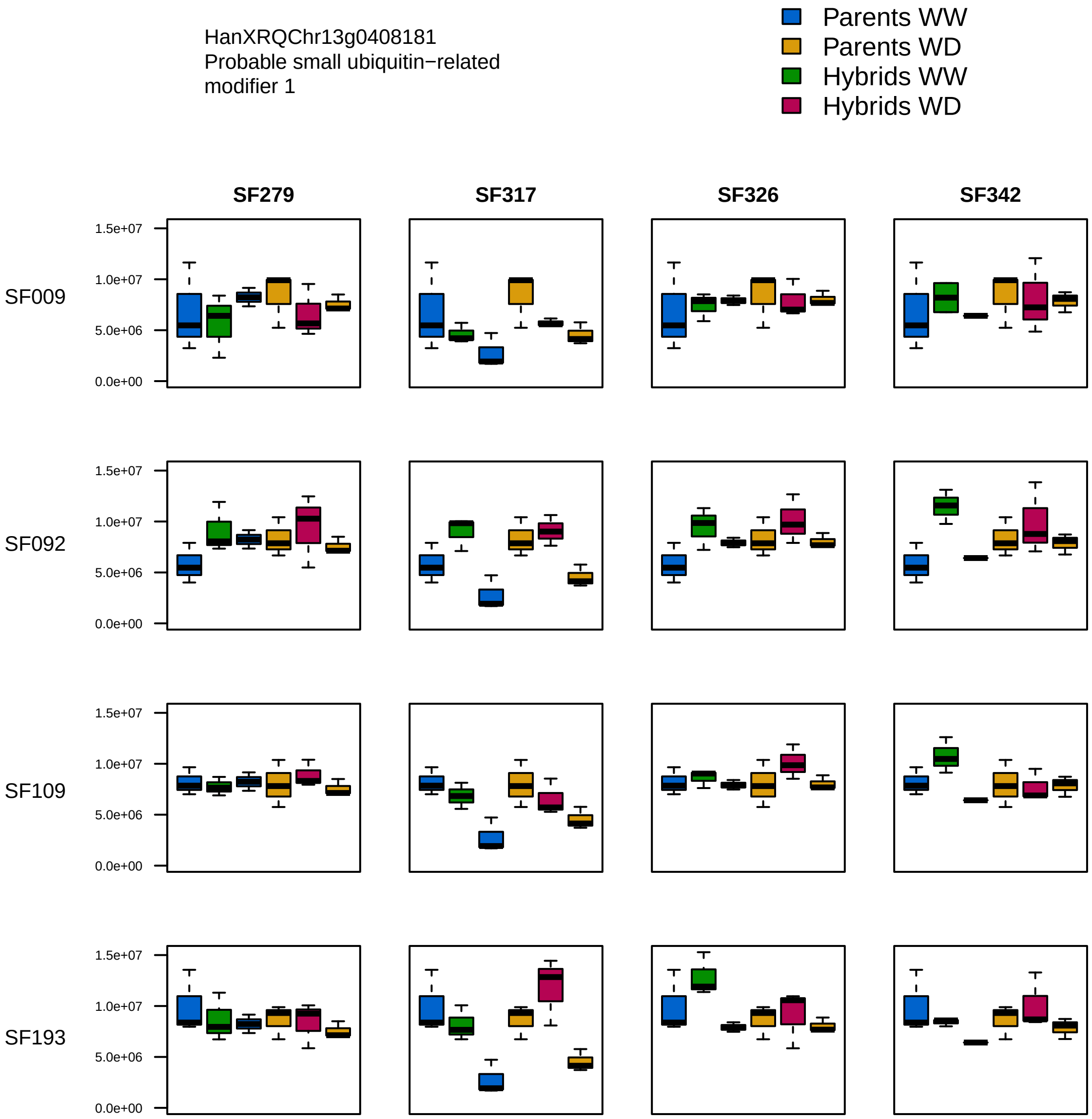
HanXRQChr13g0408181
Probable small ubiquitin−related
modifier 1
Parents WW
Parents WD
Hybrids WW
Hybrids WD
SF279
SF317
SF326
SF342
SF009
SF092
SF109
SF193
1.5e+07
1.0e+07
5.0e+06
0.0e+00

HanXRQChr16g0509271
Probable CLP protease proteolytic subunit 1

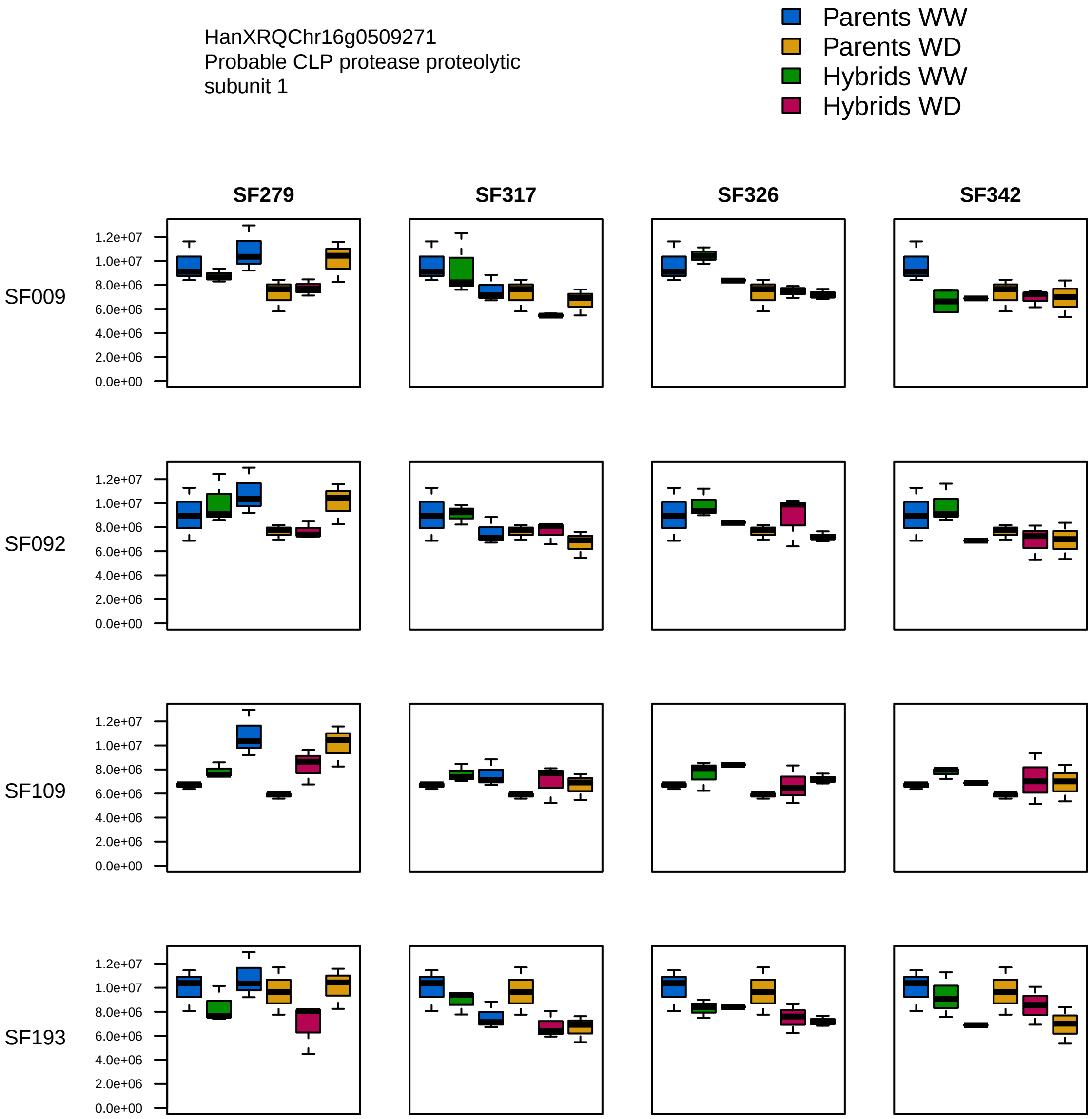

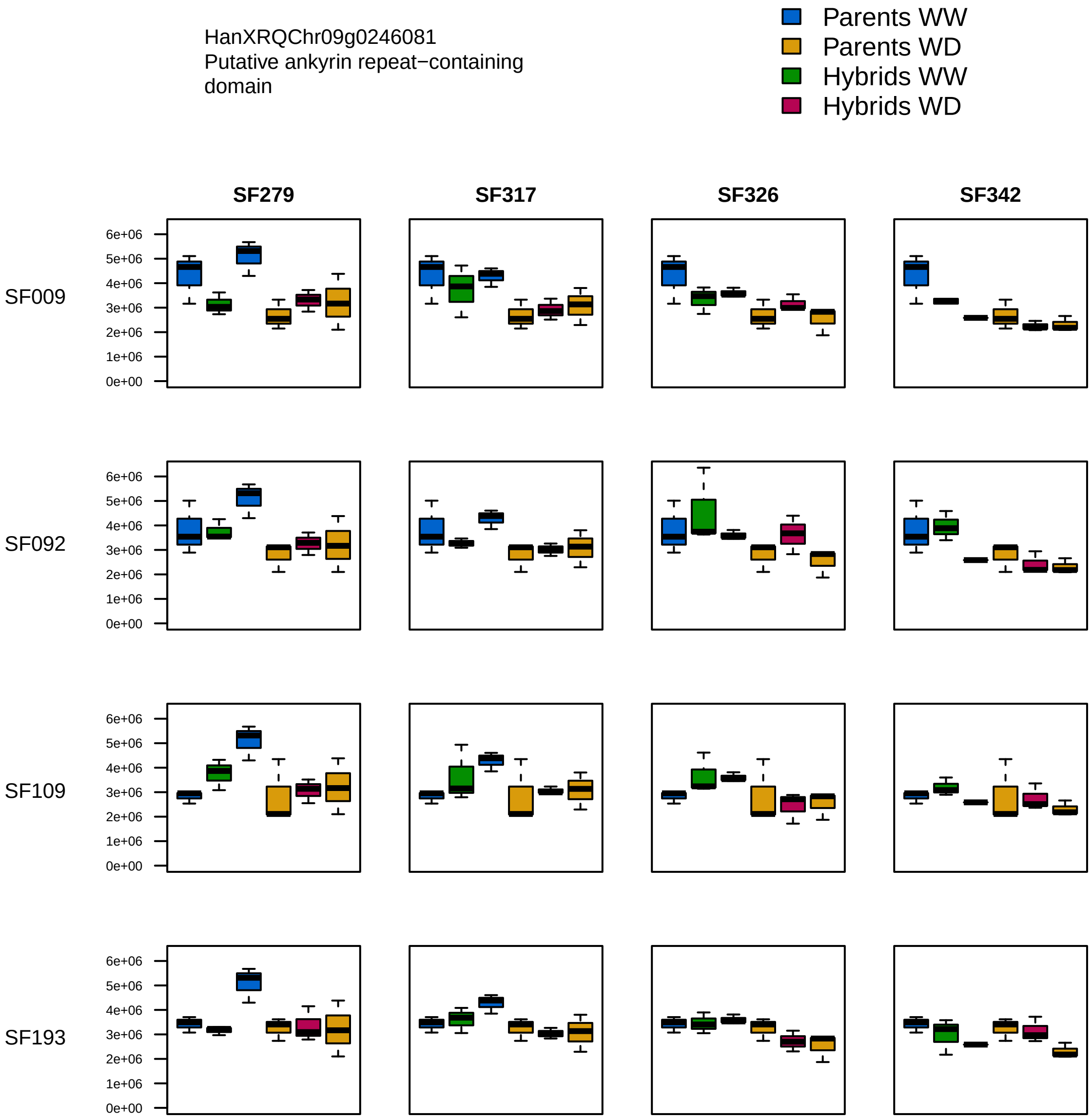

HanXRQChr09g0246081
Putative ankyrin repeat−containing
domain
Parents WW
Parents WD
Hybrids WW
Hybrids WD
SF279
SF317
SF326
SF342
SF009
SF092
SF109
SF193
6e+06
5e+06
4e+06
3e+06
2e+06
1e+06
0e+00

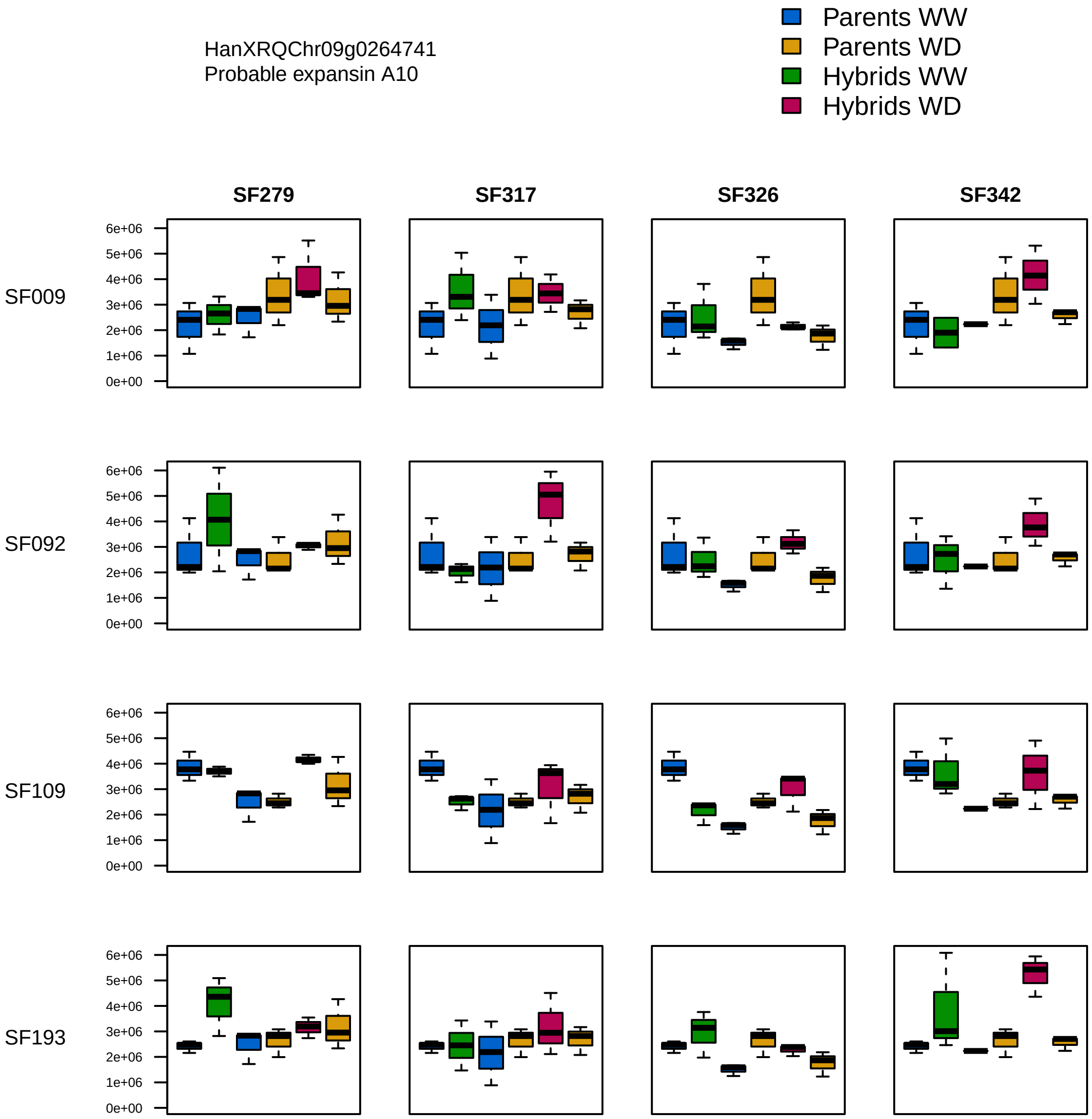
HanXRQChr09g0264741
Probable expansin A10
Parents WW
Parents WD
Hybrids WW
Hybrids WD
SF279
SF317
SF326
SF342
SF009
SF092
SF109
SF193
0e+00
1e+06
2e+06
3e+06
4e+06
5e+06
6e+06

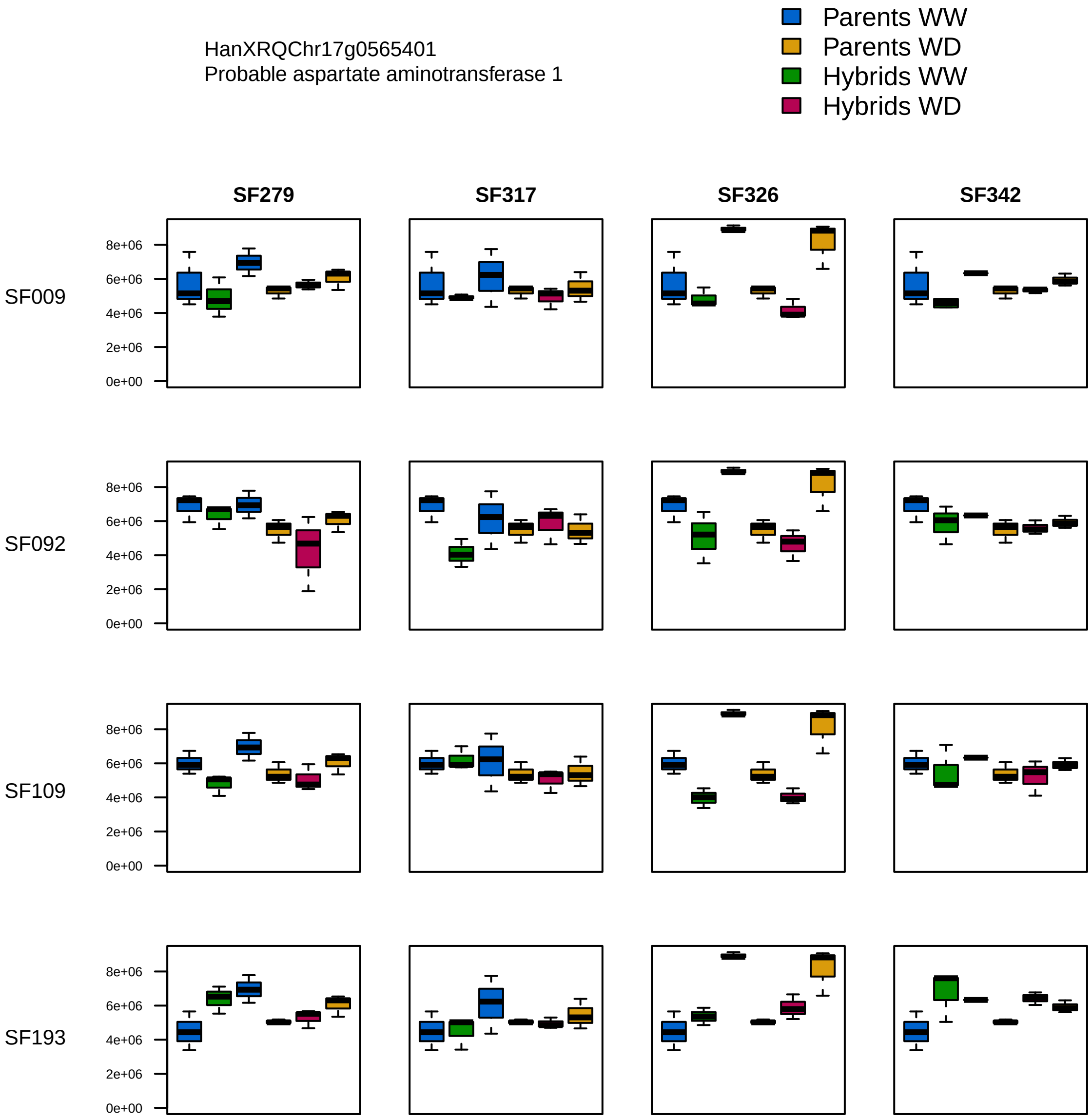
HanXRQChr17g0565401
Probable aspartate aminotransferase 1
Parents WW
Parents WD
Hybrids WW
Hybrids WD
SF279
SF317
SF326
SF342
SF009
SF092
SF109
SF193
8e+06
6e+06
4e+06
2e+06
0e+00

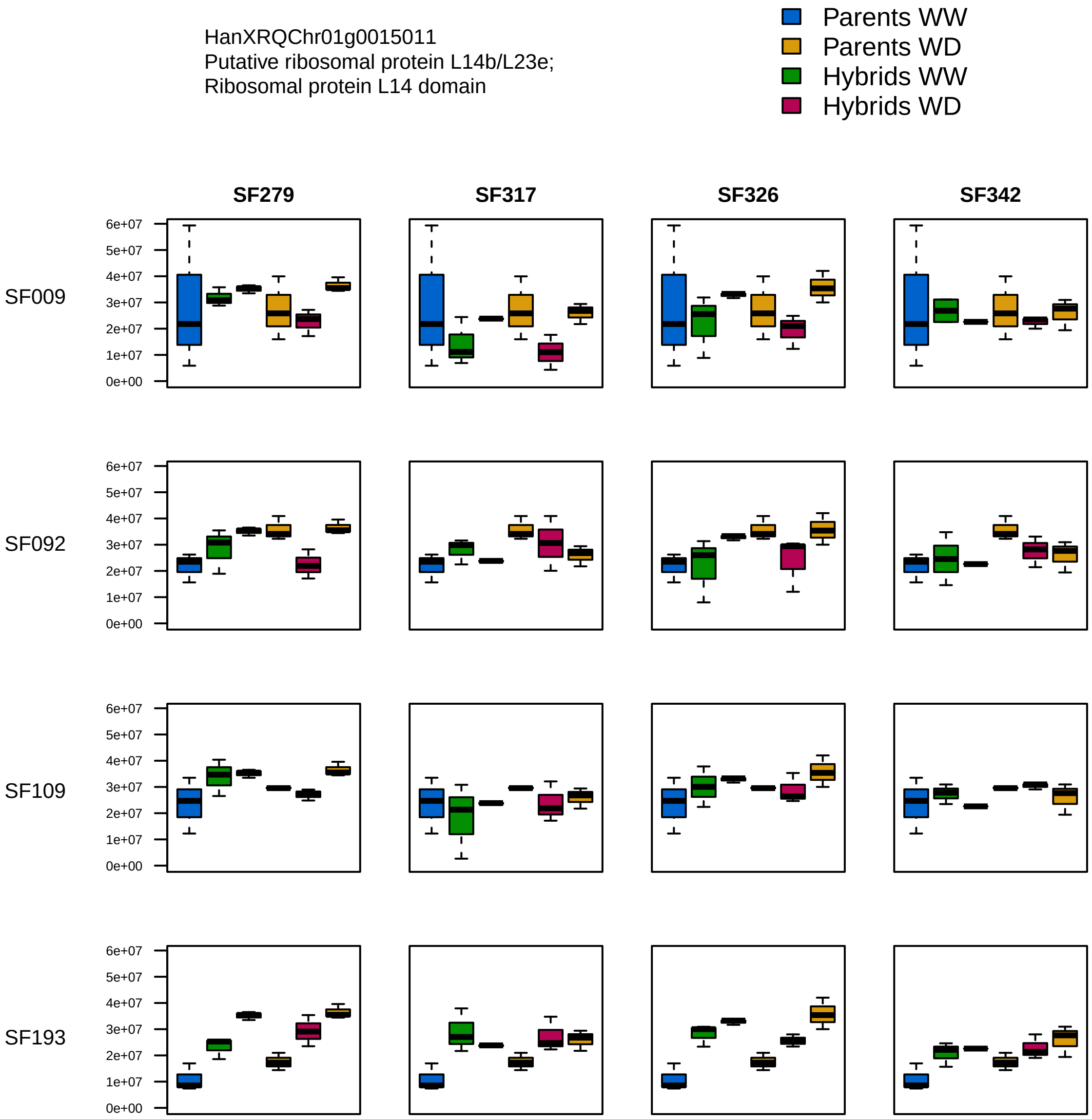

HanXRQChr01g0015011
Putative ribosomal protein L14b/L23e;
Ribosomal protein L14 domain
Parents WW
Parents WD
Hybrids WW
Hybrids WD
SF279
SF317
SF326
SF342
SF009
SF092
SF109
SF193
0e+00
1e+07
2e+07
3e+07
4e+07
5e+07
6e+07

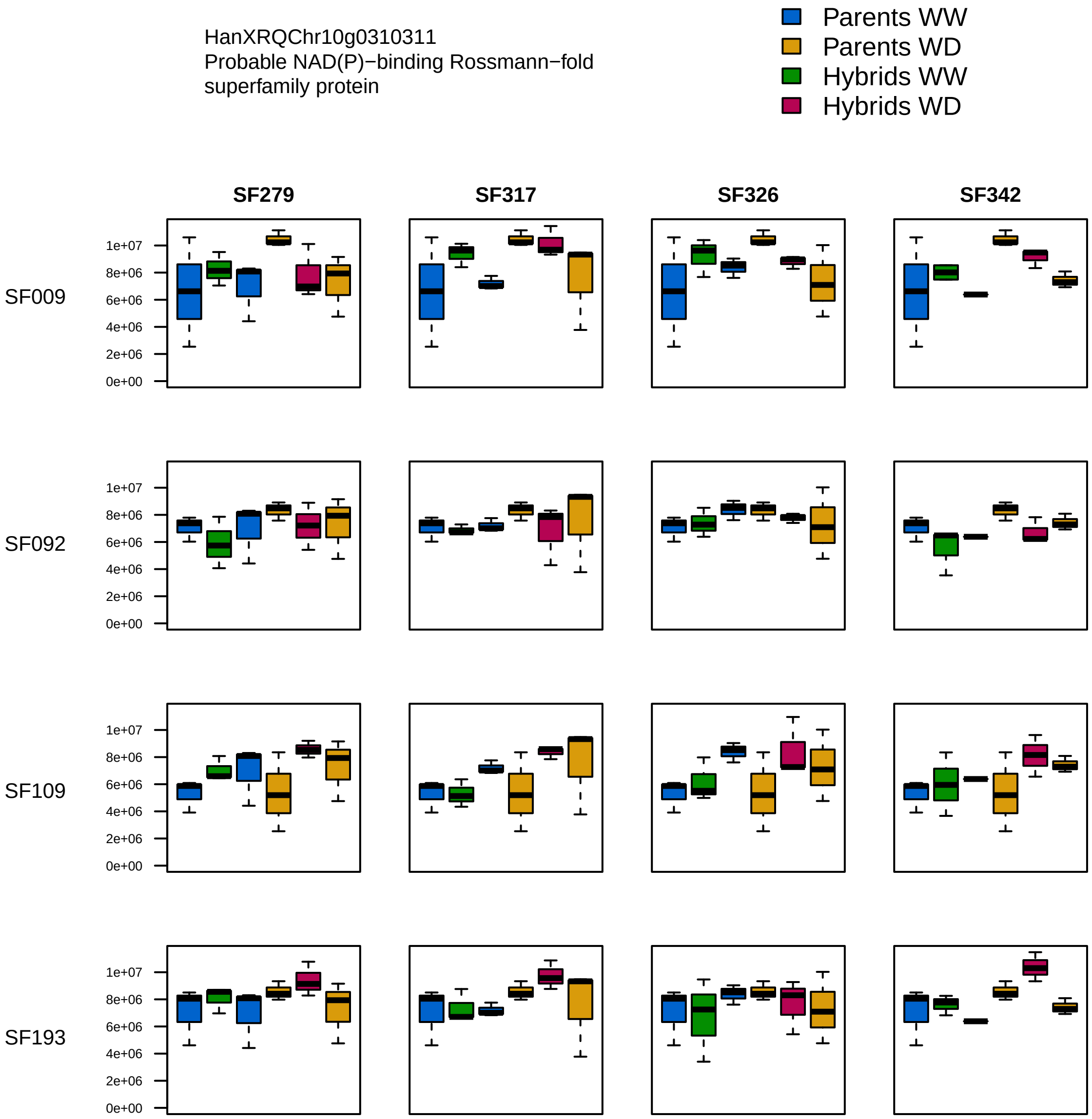
HanXRQChr10g0310311
Probable NAD(P)−binding Rossmann−fold
superfamily protein
Parents WW
Parents WD
Hybrids WW
Hybrids WD
SF279
SF317
SF326
SF342
SF009
SF092
SF109
SF193
0e+00
2e+06
4e+06
6e+06
8e+06
1e+07

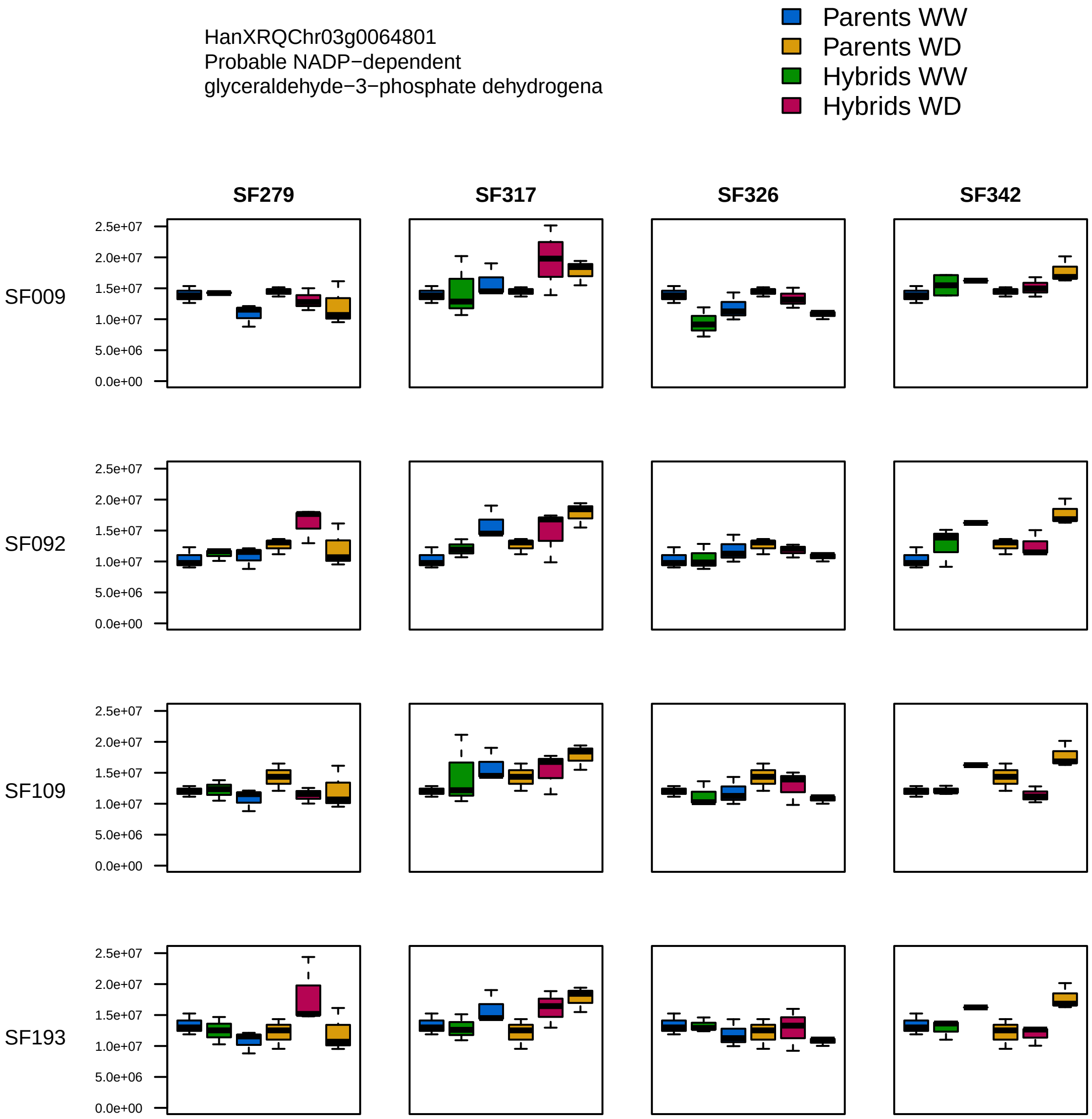
HanXRQChr03g0064801
Probable NADP−dependent
glyceraldehyde−3−phosphate dehydrogena
Parents WW
Parents WD
Hybrids WW
Hybrids WD
SF279
SF317
SF326
SF342
SF009
SF092
SF109
SF193
2.5e+07
2.0e+07
1.5e+07
1.0e+07
5.0e+06
0.0e+00

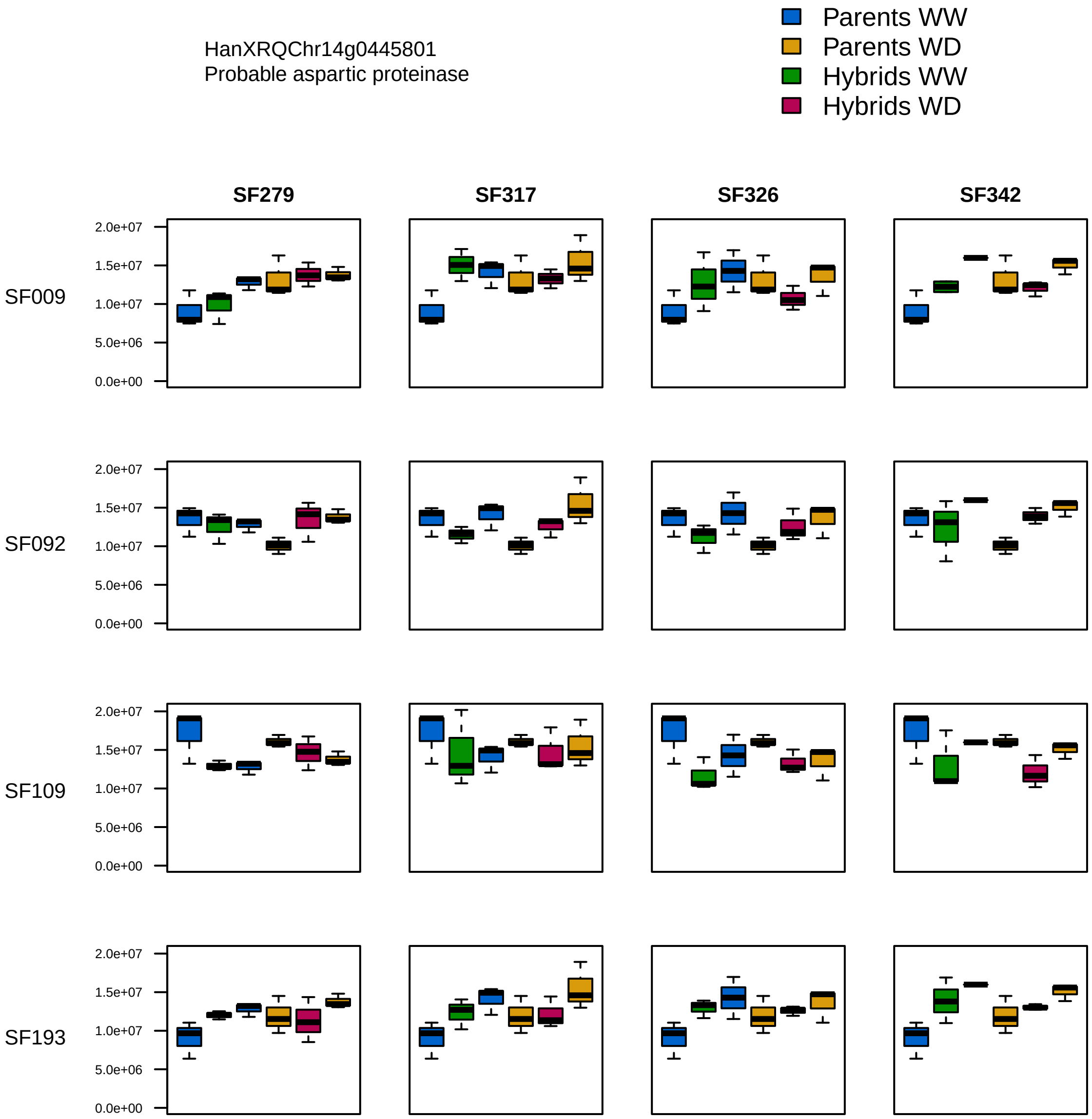

HanXRQChr14g0445801
Probable aspartic proteinase
Parents WW
Parents WD
Hybrids WW
Hybrids WD
SF279
SF317
SF326
SF342
SF009
SF092
SF109
SF193
2.0e+07
1.5e+07
1.0e+07
5.0e+06
0.0e+00

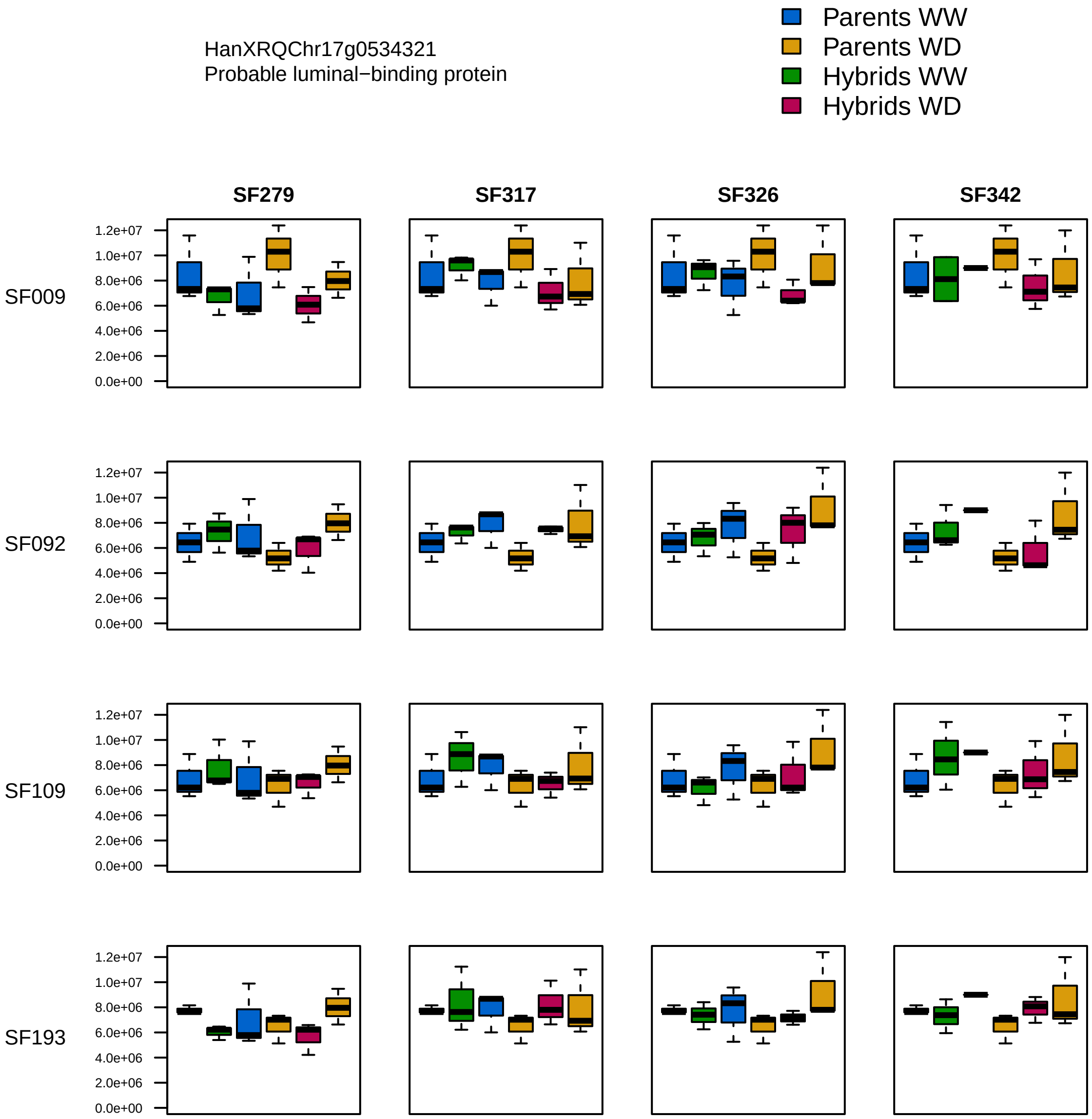

HanXRQChr17g0534321
Probable luminal−binding protein
Parents WW
Parents WD
Hybrids WW
Hybrids WD
SF279
SF317
SF326
SF342
SF009
SF092
SF109
SF193
1.2e+07
1.0e+07
8.0e+06
6.0e+06
4.0e+06
2.0e+06
0.0e+00

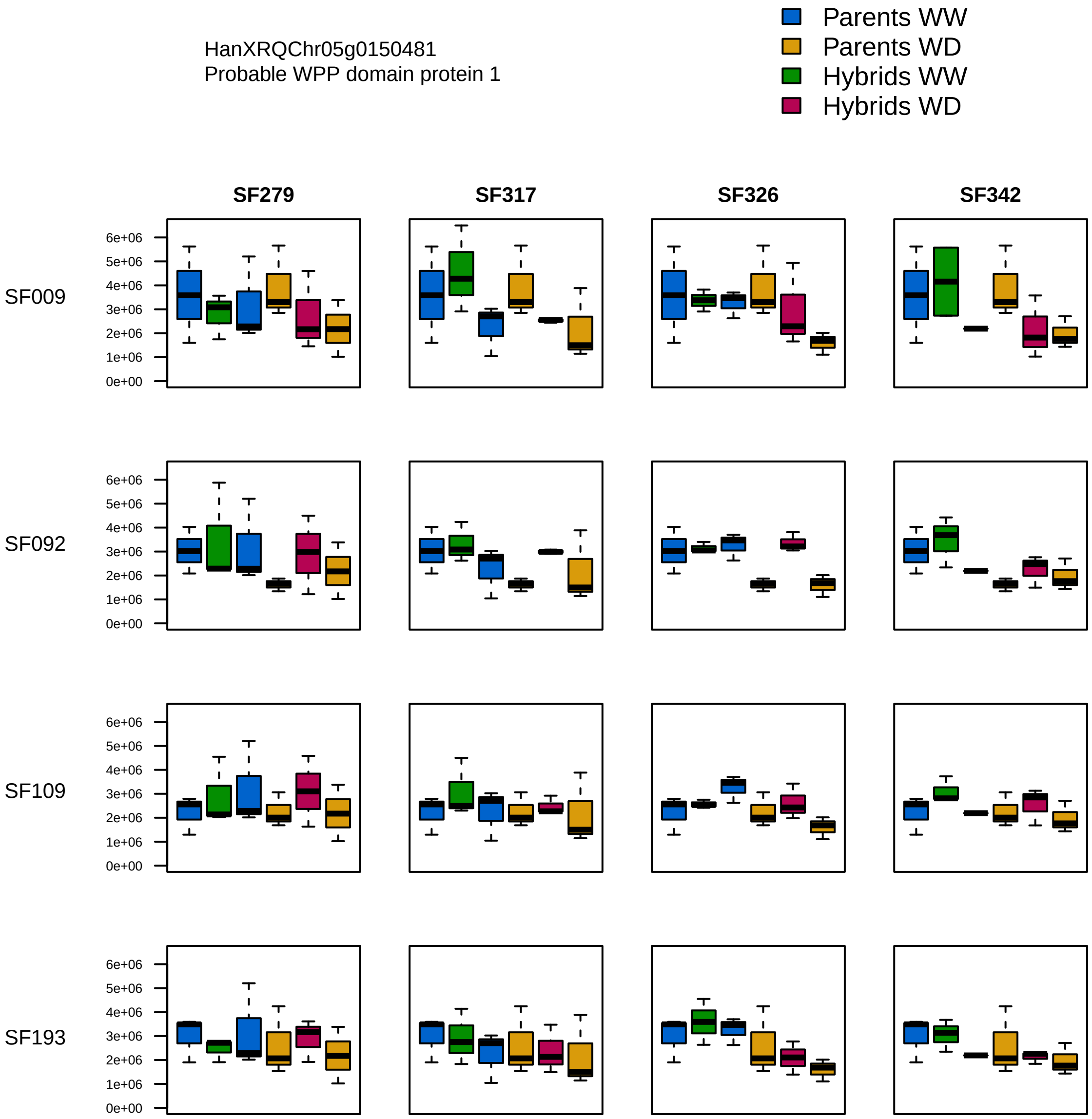
HanXRQChr05g0150481
Probable WPP domain protein 1
Parents WW
Parents WD
Hybrids WW
Hybrids WD
SF279
SF317
SF326
SF342
SF009
SF092
SF109
SF193
0e+00
1e+06
2e+06
3e+06
4e+06
5e+06
6e+06

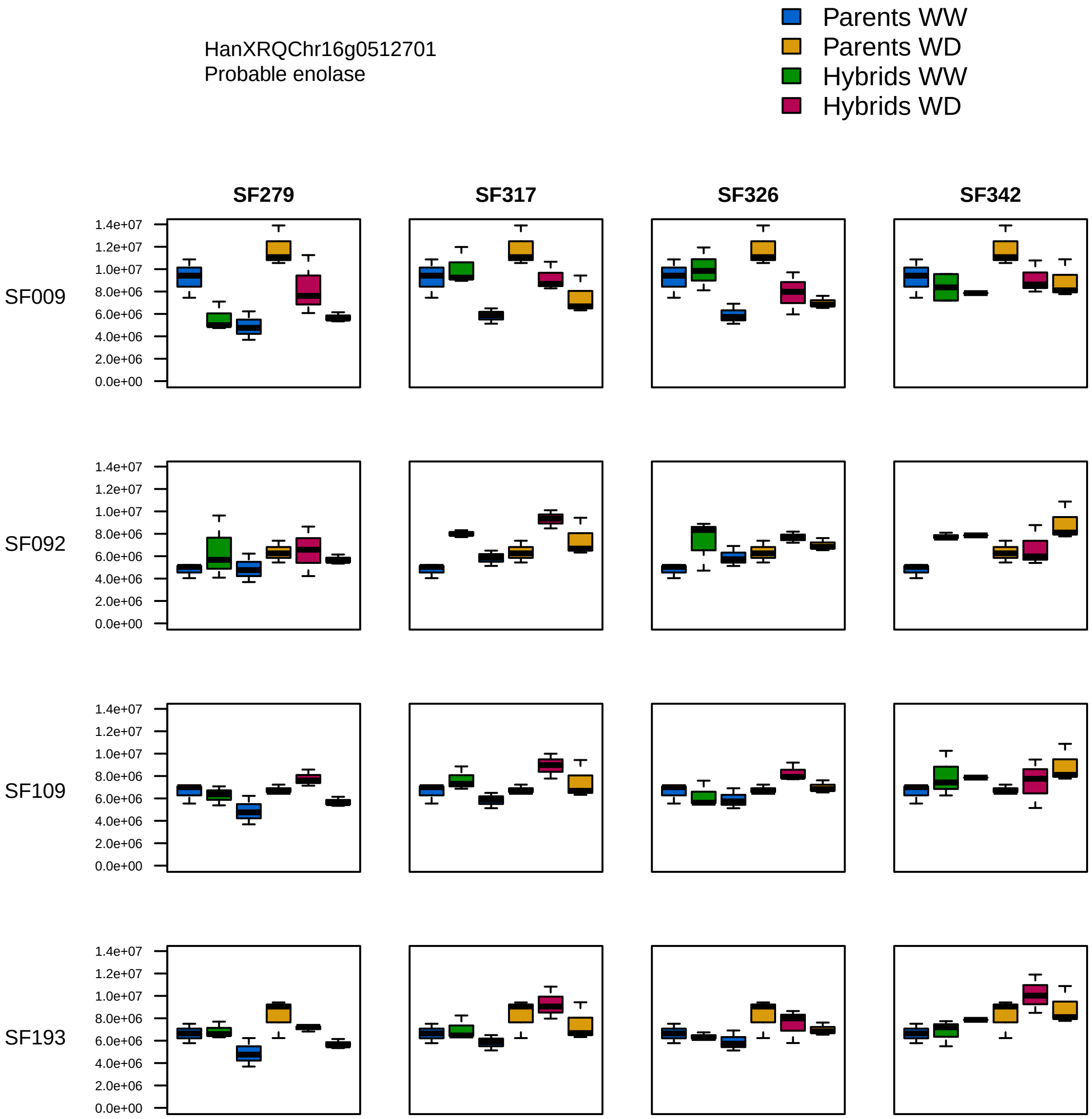

HanXRQChr16g0512701
Probable enolase
Parents WW
Parents WD
Hybrids WW
Hybrids WD
SF279
SF317
SF326
SF342
SF009
SF092
SF109
SF193
1.4e+07
1.2e+07
1.0e+07
8.0e+06
6.0e+06
4.0e+06
2.0e+06
0.0e+00

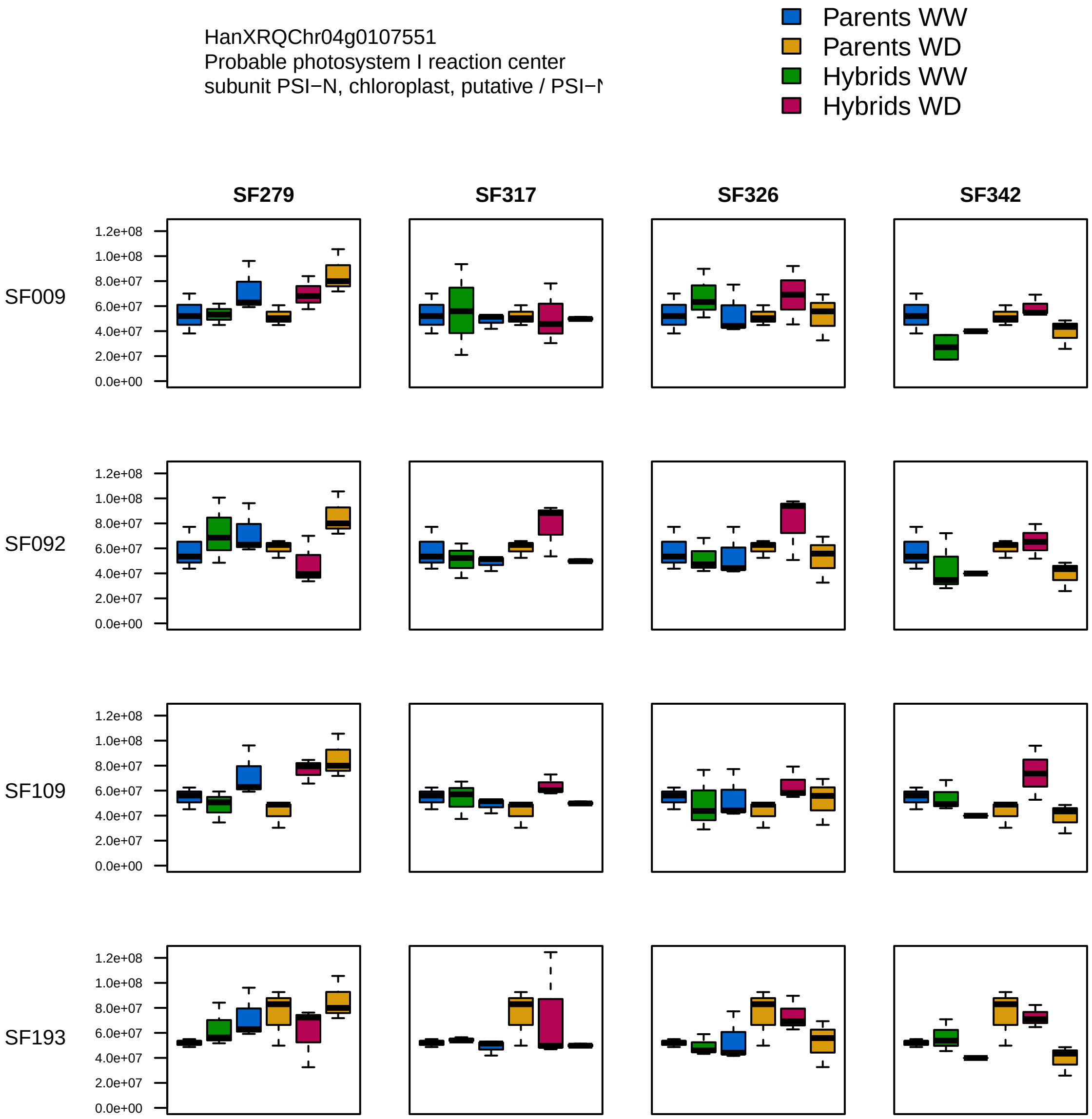

HanXRQChr04g0107551
Probable photosystem I reaction center
subunit PSI−N, chloroplast, putative / PSI−N
Parents WW
Parents WD
Hybrids WW
Hybrids WD
SF279
SF317
SF326
SF342
SF009
SF092
SF109
SF193
1.2e+08
1.0e+08
8.0e+07
6.0e+07
4.0e+07
2.0e+07
0.0e+00

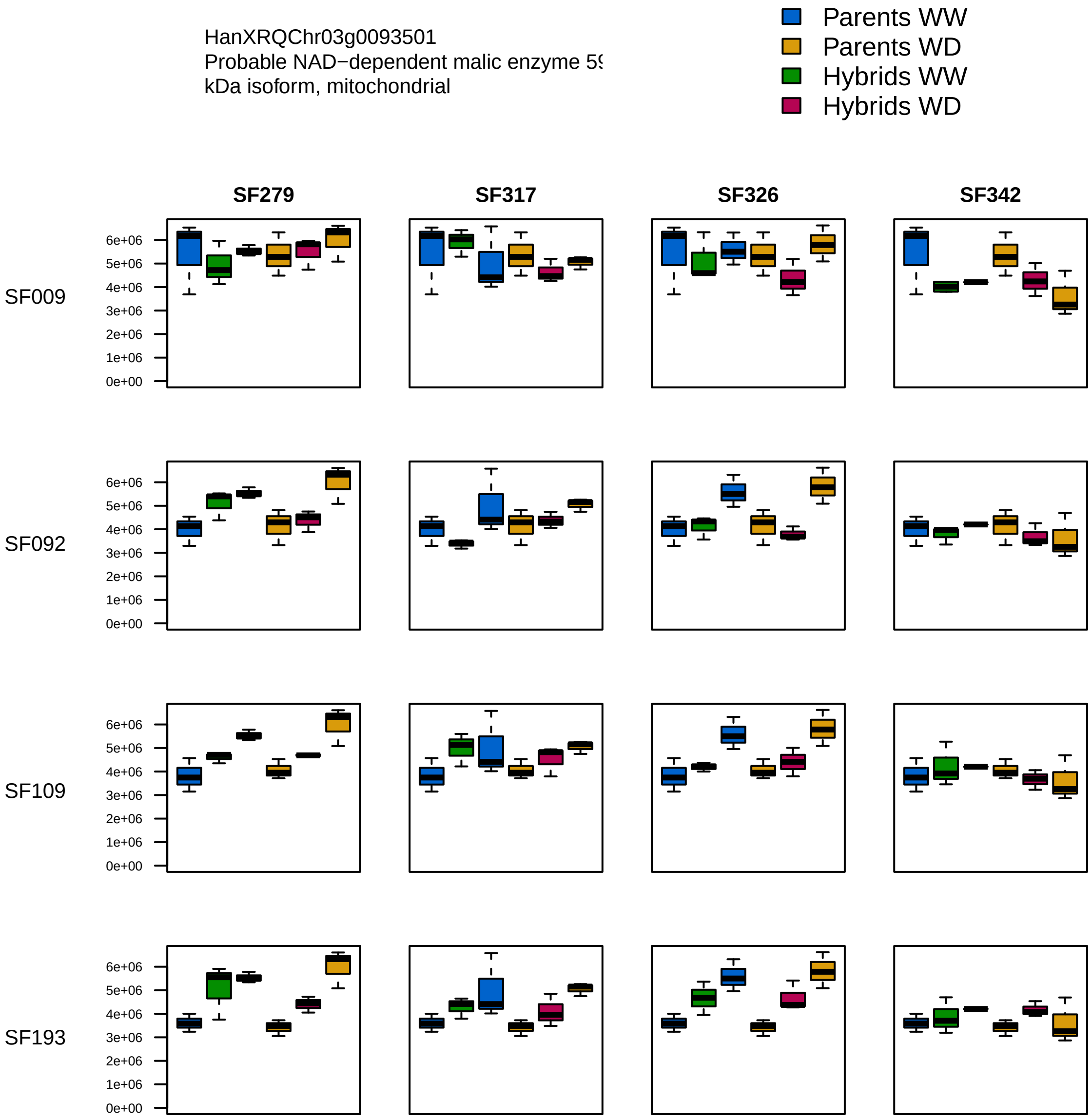

HanXRQChr03g0093501
Probable NAD−dependent malic enzyme 59
kDa isoform, mitochondrial
Parents WW
Parents WD
Hybrids WW
Hybrids WD
SF279
SF317
SF326
SF342
SF009
SF092
SF109
SF193
0e+00
1e+06
2e+06
3e+06
4e+06
5e+06
6e+06

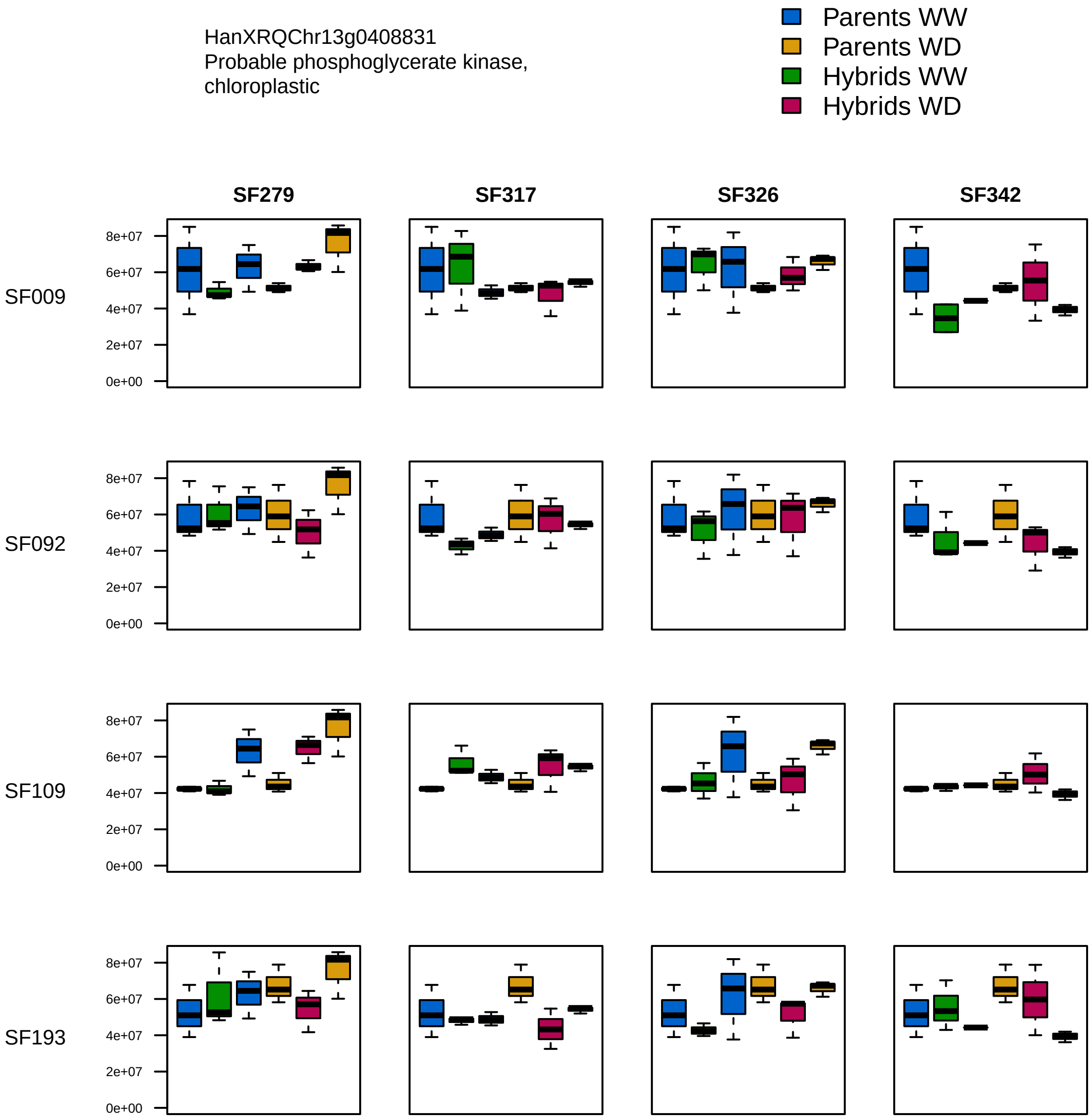

HanXRQChr13g0408831
Probable phosphoglycerate kinase,
chloroplastic
Parents WW
Parents WD
Hybrids WW
Hybrids WD
SF279
SF317
SF326
SF342
SF009
SF092
SF109
SF193
8e+07
6e+07
4e+07
2e+07
0e+00

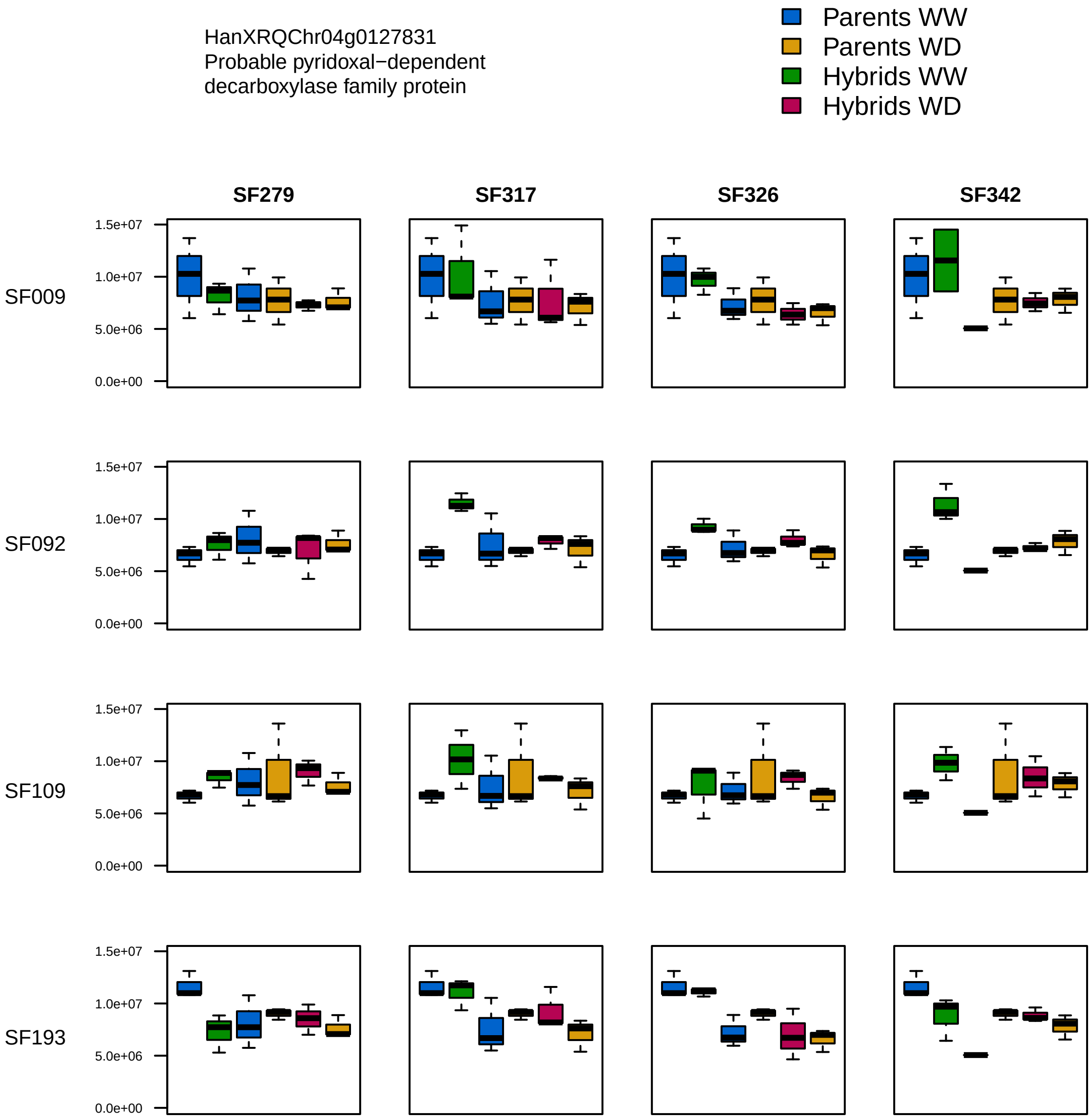
HanXRQChr04g0127831
Probable pyridoxal−dependent
decarboxylase family protein
Parents WW
Parents WD
Hybrids WW
Hybrids WD
SF279
SF317
SF326
SF342
SF009
SF092
SF109
SF193
1.5e+07
1.0e+07
5.0e+06
0.0e+00

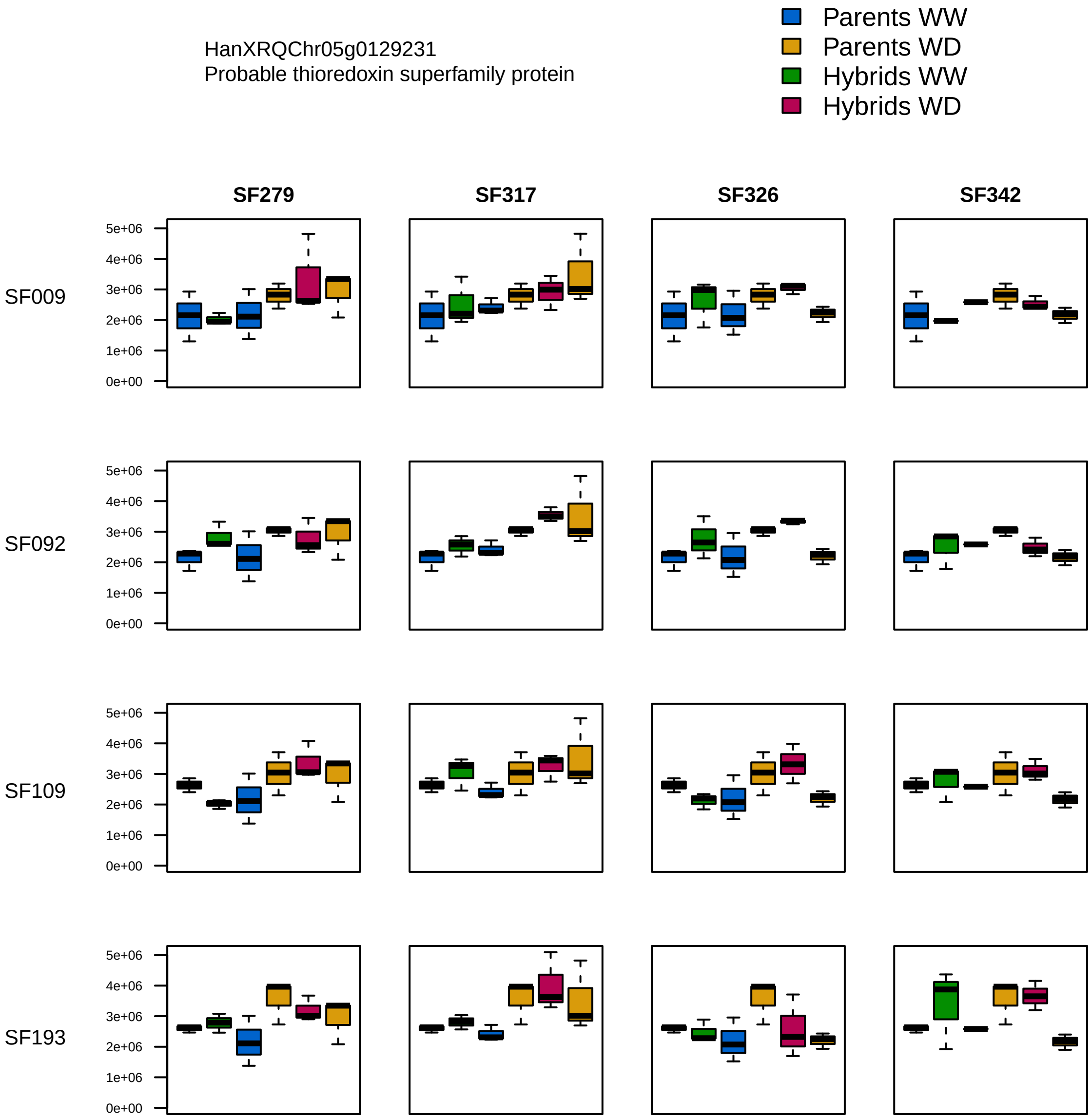
HanXRQChr05g0129231
Probable thioredoxin superfamily protein
Parents WW
Parents WD
Hybrids WW
Hybrids WD
SF279
SF317
SF326
SF342
SF009
SF092
SF109
SF193
0e+00
1e+06
2e+06
3e+06
4e+06
5e+06

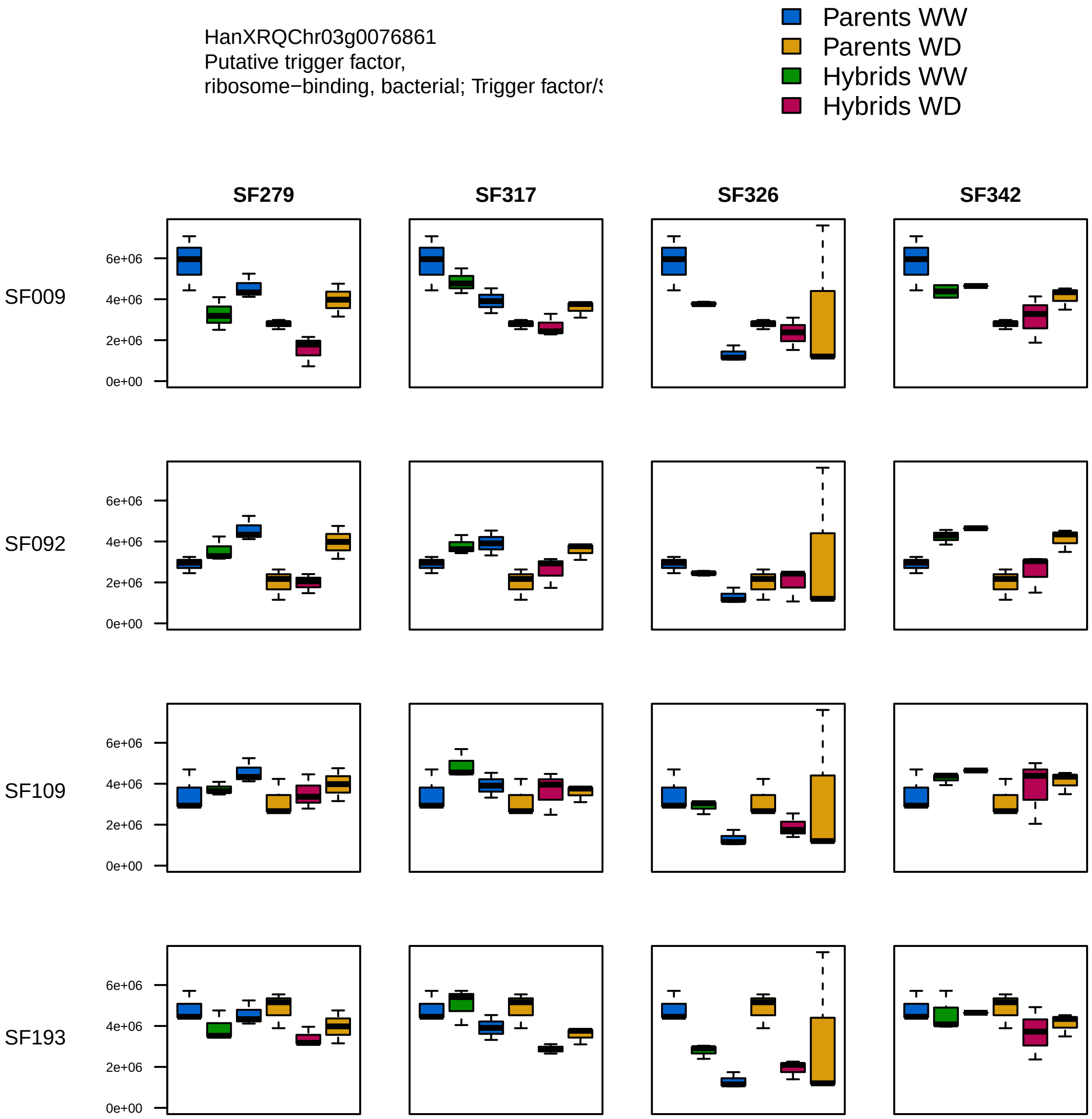
HanXRQChr03g0076861
Putative trigger factor,
ribosome−binding, bacterial; Trigger factor/S
Parents WW
Parents WD
Hybrids WW
Hybrids WD
SF279
SF317
SF326
SF342
SF009
SF092
SF109
SF193
6e+06
4e+06
2e+06
0e+00

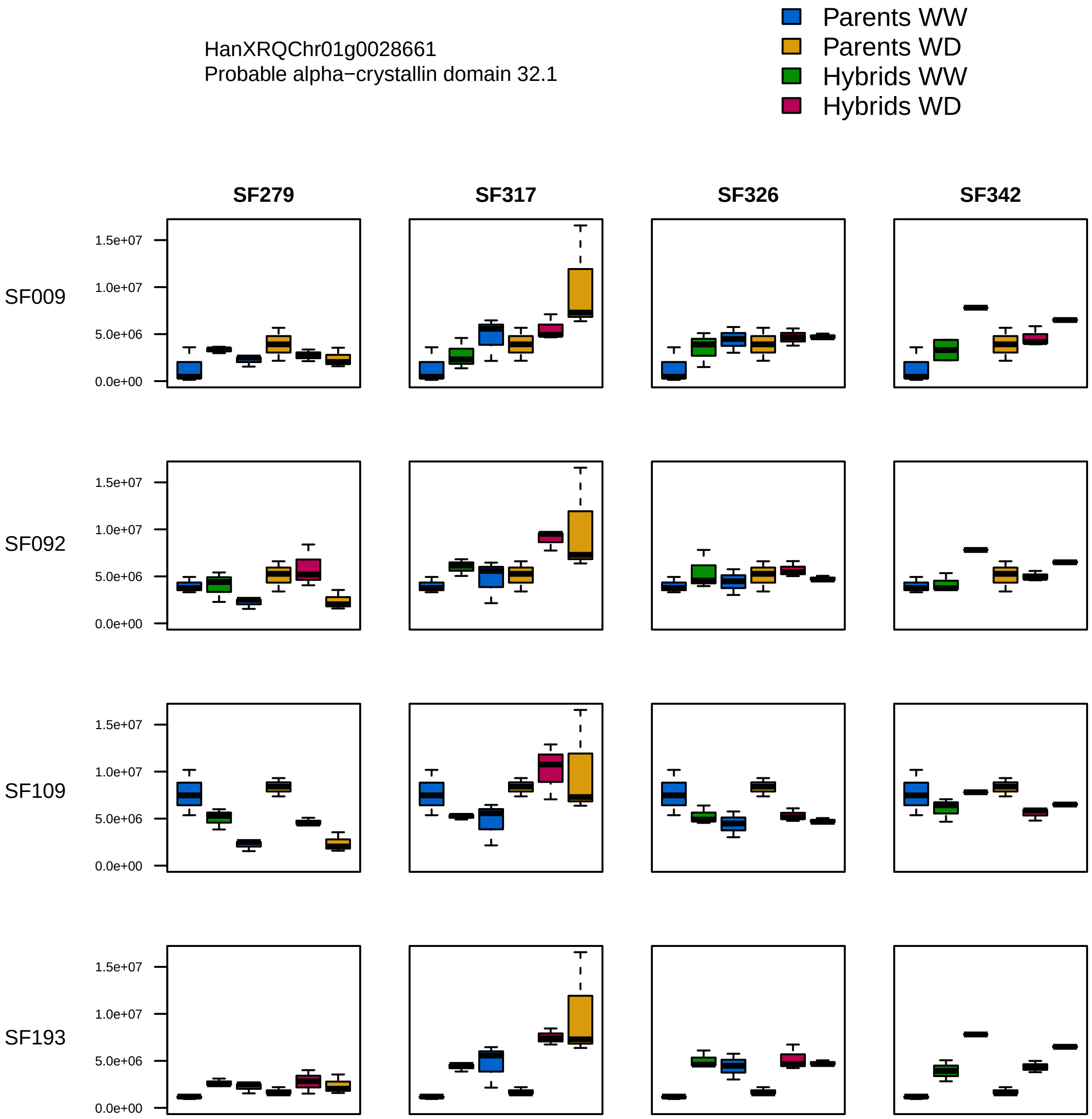

HanXRQChr01g0028661
Probable alpha−crystallin domain 32.1
Parents WW
Parents WD
Hybrids WW
Hybrids WD
SF279
SF317
SF326
SF342
SF009
SF092
SF109
SF193
0.0e+00
5.0e+06
1.0e+07
1.5e+07

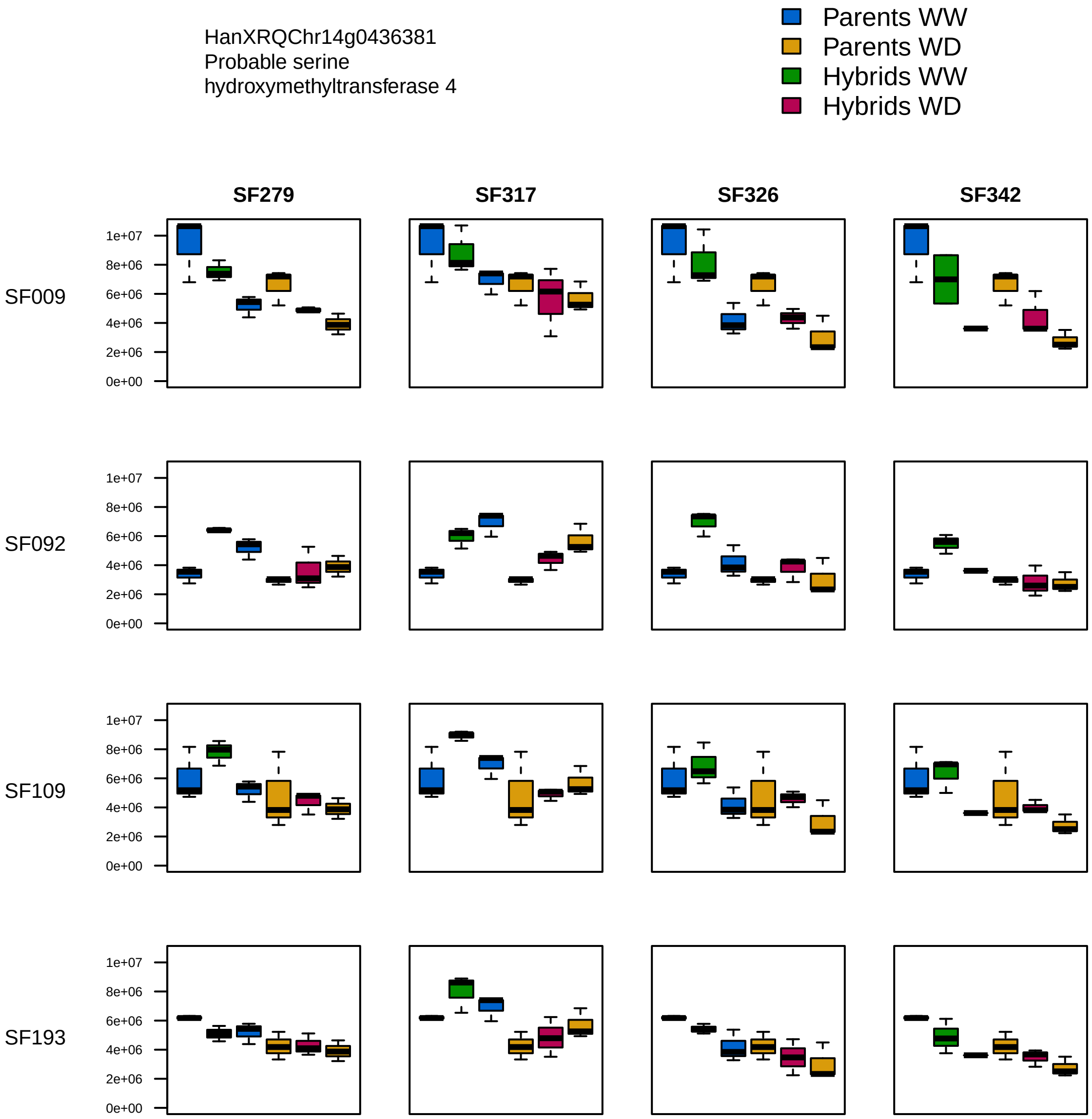
HanXRQChr14g0436381
Probable serine
hydroxymethyltransferase 4
Parents WW
Parents WD
Hybrids WW
Hybrids WD
SF279
SF317
SF326
SF342
SF009
SF092
SF109
SF193
1e+07
8e+06
6e+06
4e+06
2e+06
0e+00

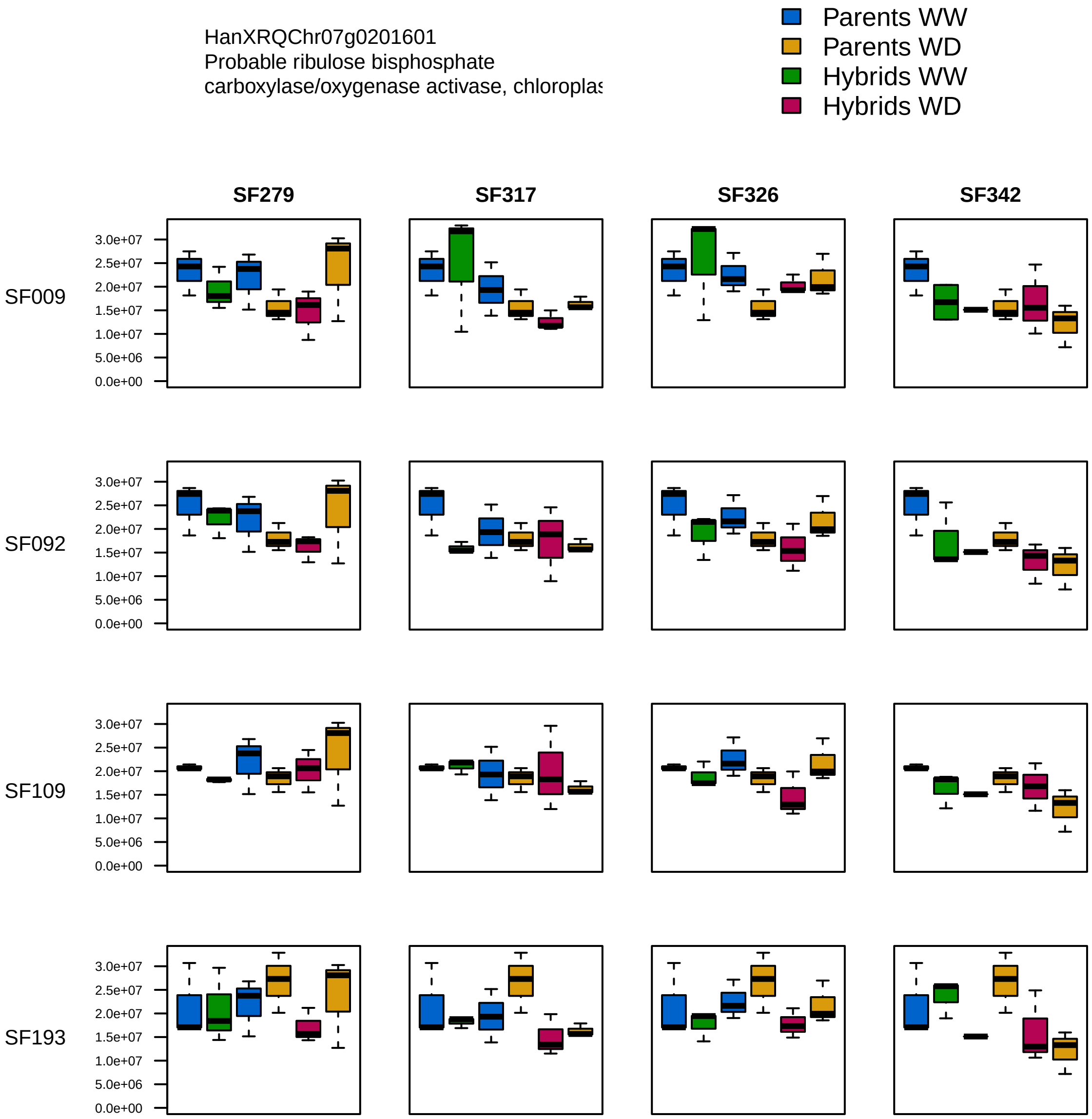
HanXRQChr07g0201601
Probable ribulose bisphosphate
carboxylase/oxygenase activase, chloroplas
Parents WW
Parents WD
Hybrids WW
Hybrids WD
SF279
SF317
SF326
SF342
SF009
SF092
SF109
SF193
0.0e+00
5.0e+06
1.0e+07
1.5e+07
2.0e+07
2.5e+07
3.0e+07

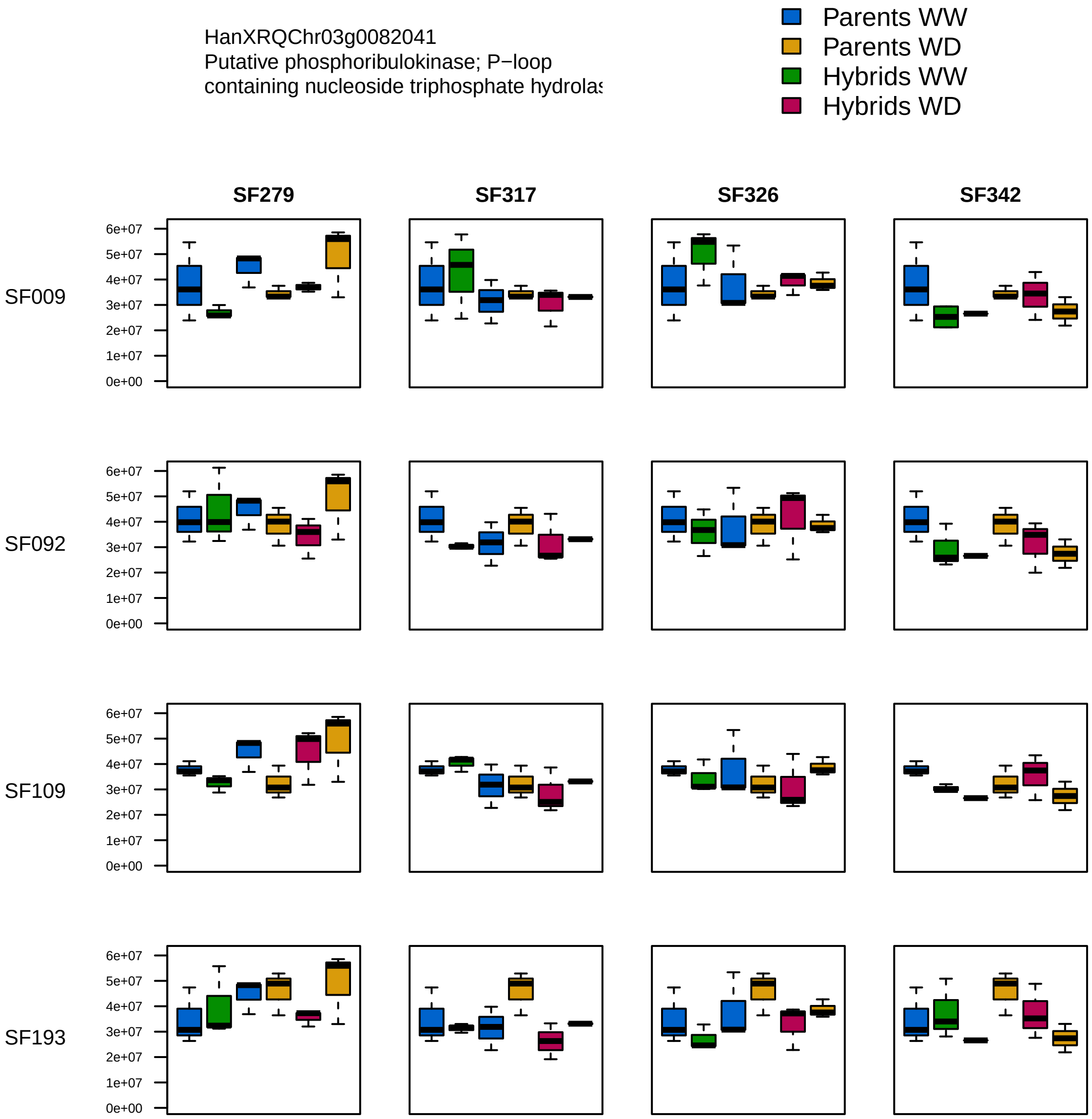
HanXRQChr03g0082041
Putative phosphoribulokinase; P−loop
containing nucleoside triphosphate hydrolas
Parents WW
Parents WD
Hybrids WW
Hybrids WD
SF279
SF317
SF326
SF342
SF009
SF092
SF109
SF193
6e+07
5e+07
4e+07
3e+07
2e+07
1e+07
0e+00

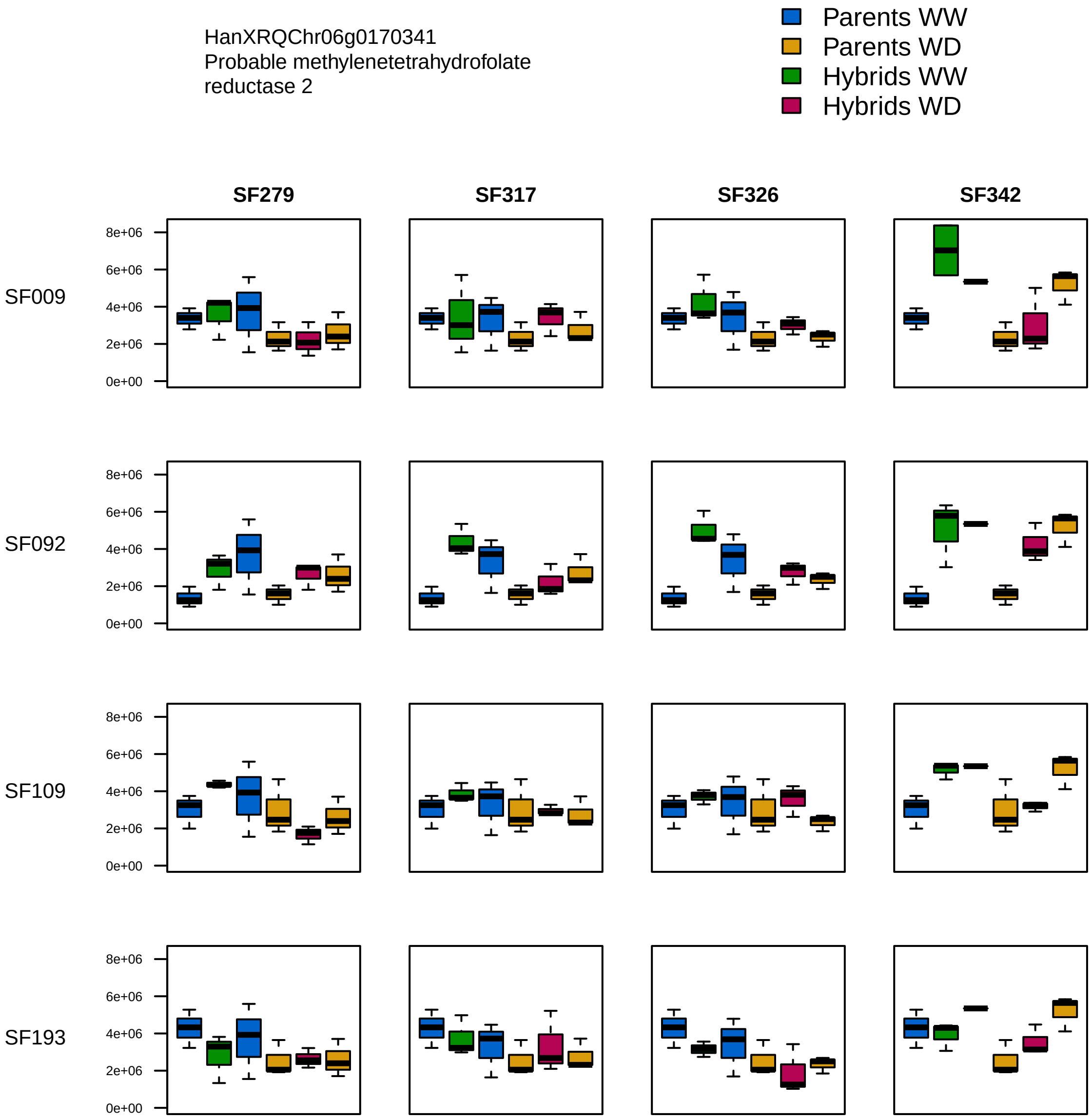
HanXRQChr06g0170341
Probable methylenetetrahydrofolate
reductase 2
Parents WW
Parents WD
Hybrids WW
Hybrids WD
SF279
SF317
SF326
SF342
SF009
SF092
SF109
SF193
0e+00
2e+06
4e+06
6e+06
8e+06

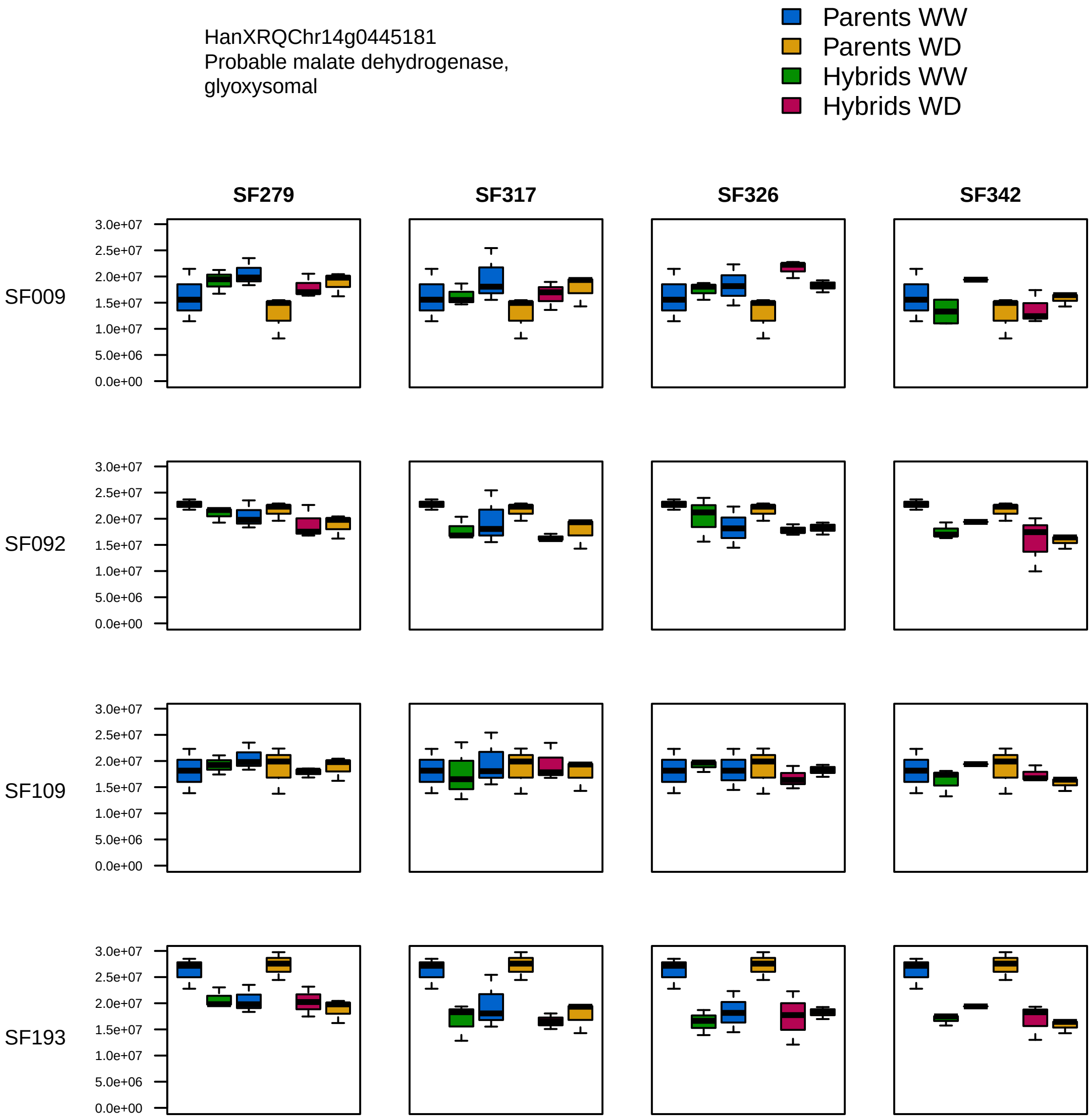
HanXRQChr14g0445181
Probable malate dehydrogenase,
glyoxysomal
Parents WW
Parents WD
Hybrids WW
Hybrids WD
SF279
SF317
SF326
SF342
SF009
SF092
SF109
SF193
3.0e+07
2.5e+07
2.0e+07
1.5e+07
1.0e+07
5.0e+06
0.0e+00

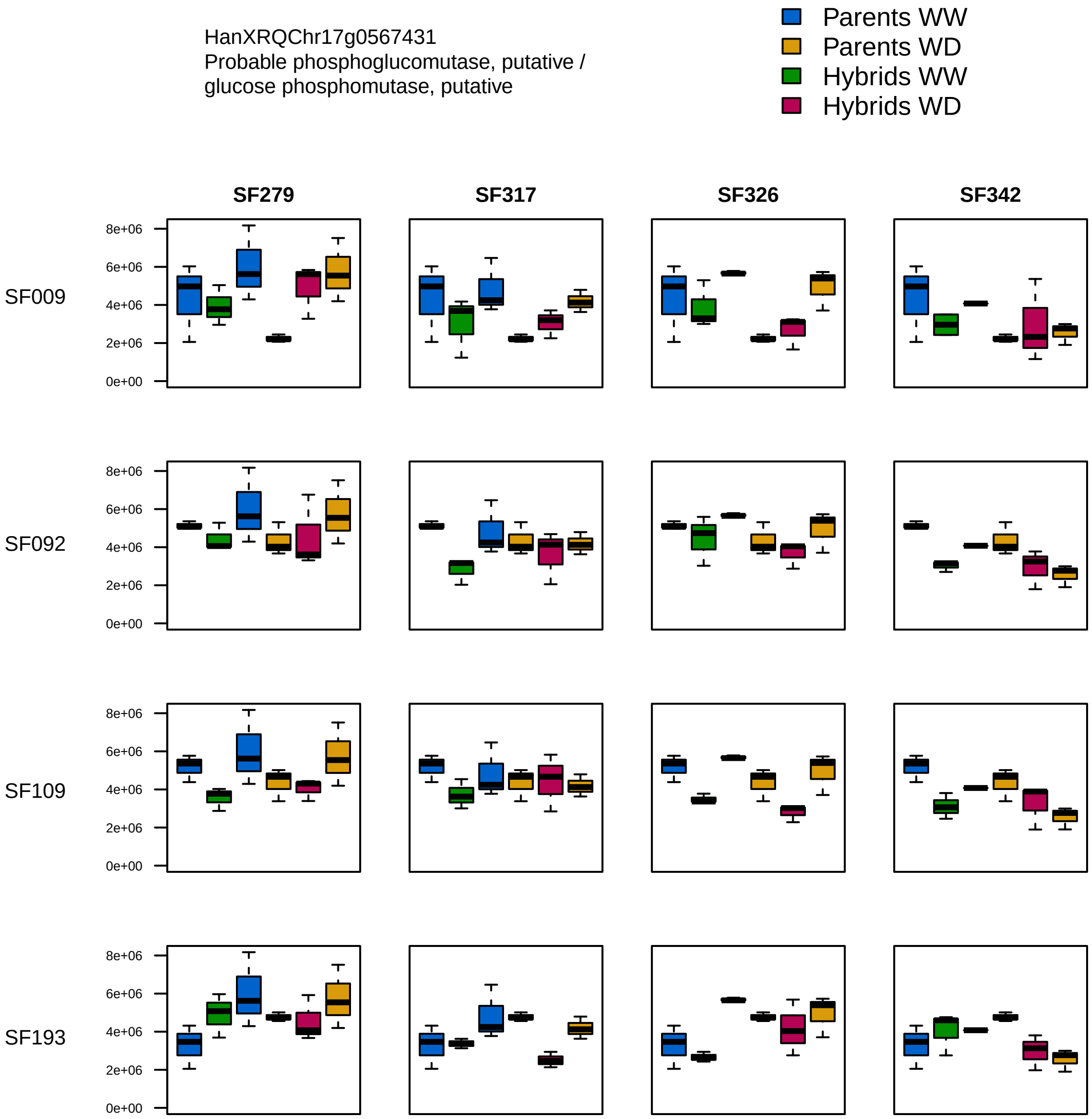
HanXRQChr17g0567431
Probable phosphoglucomutase, putative /
glucose phosphomutase, putative
Parents WW
Parents WD
Hybrids WW
Hybrids WD
SF279
SF317
SF326
SF342
SF009
SF092
SF109
SF193
0e+00
2e+06
4e+06
6e+06
8e+06

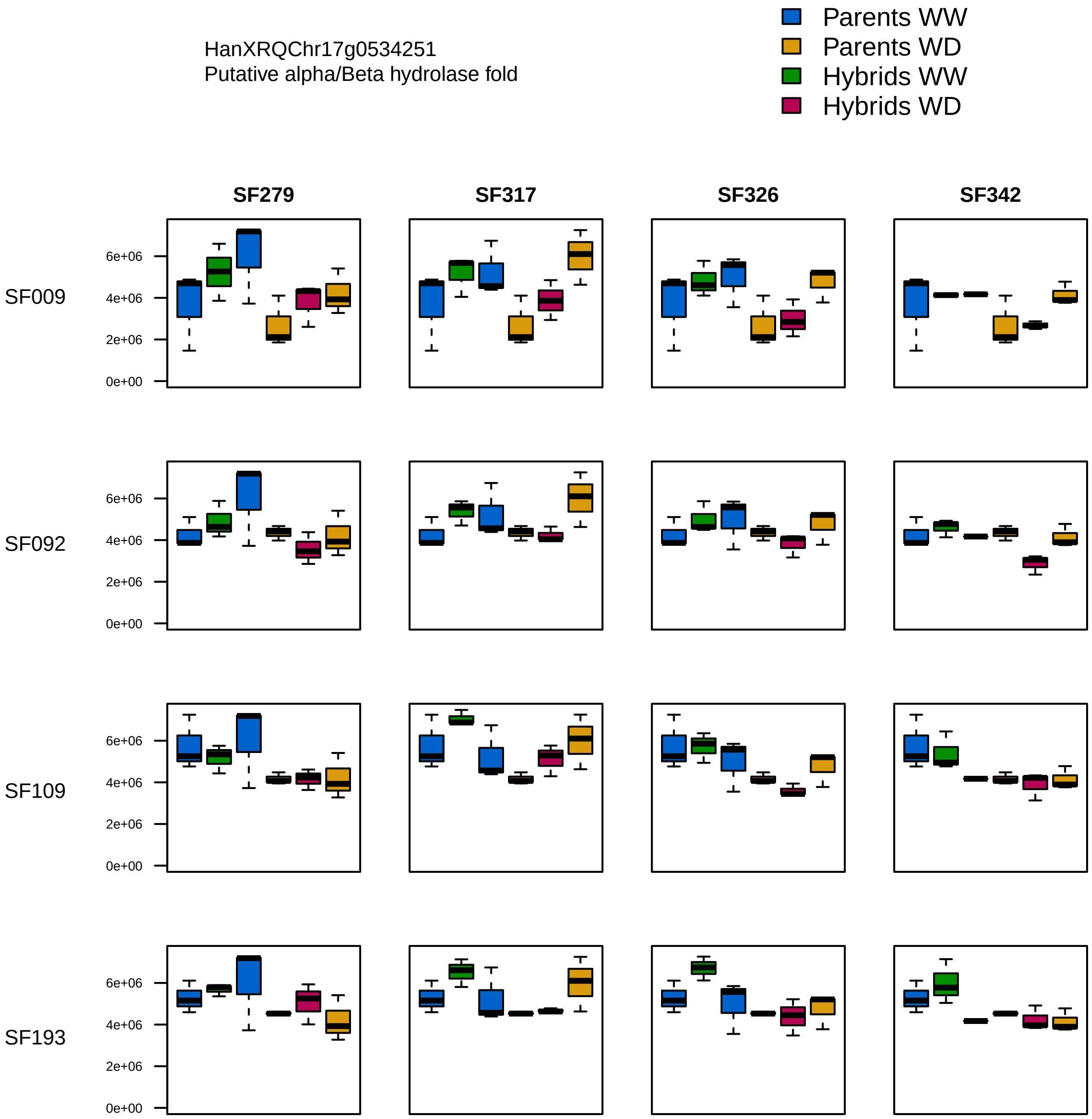
HanXRQChr17g0534251
Putative alpha/Beta hydrolase fold
Parents WW
Parents WD
Hybrids WW
Hybrids WD
SF279
SF317
SF326
SF342
SF009
SF092
SF109
SF193
0e+00
2e+06
4e+06
6e+06

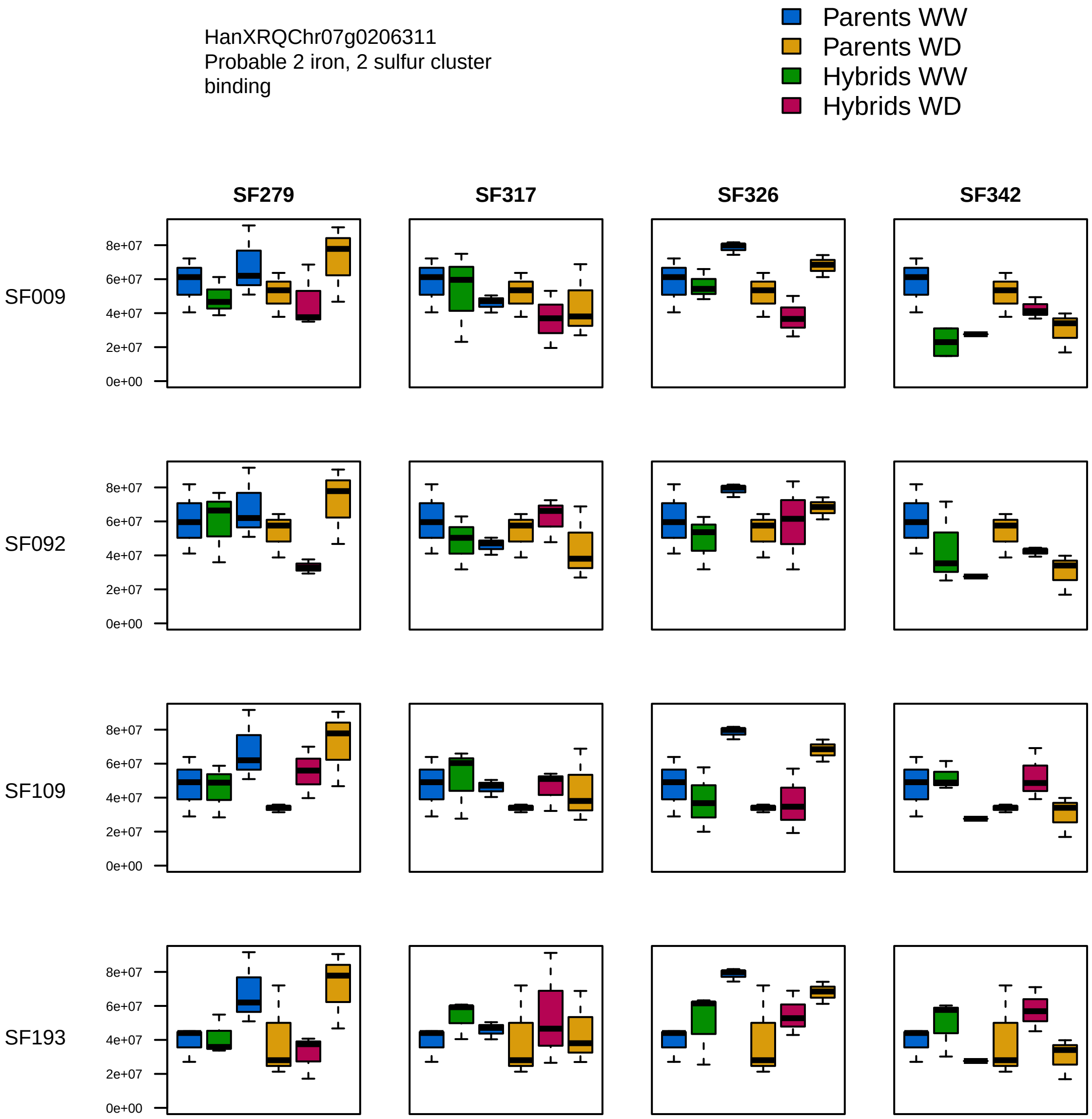

HanXRQChr07g0206311
Probable 2 iron, 2 sulfur cluster binding
Parents WW
Parents WD
Hybrids WW
Hybrids WD
SF279
SF317
SF326
SF342
SF009
SF092
SF109
SF193
0e+00
2e+07
4e+07
6e+07
8e+07

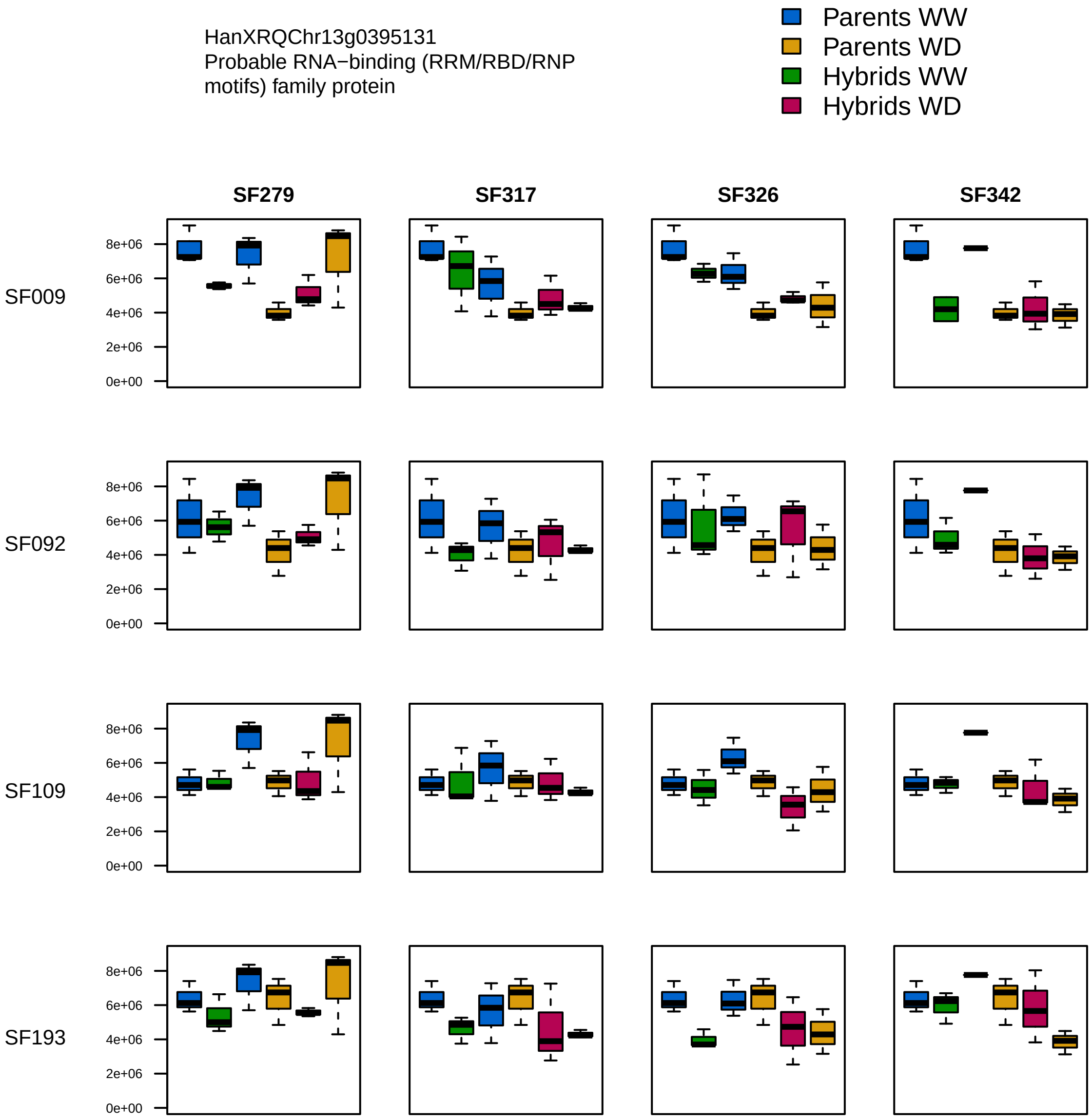

HanXRQChr13g0395131
Probable RNA−binding (RRM/RBD/RNP motifs) family protein
Parents WW
Parents WD
Hybrids WW
Hybrids WD
SF279
SF317
SF326
SF342
SF009
SF092
SF109
SF193
8e+06
6e+06
4e+06
2e+06
0e+00

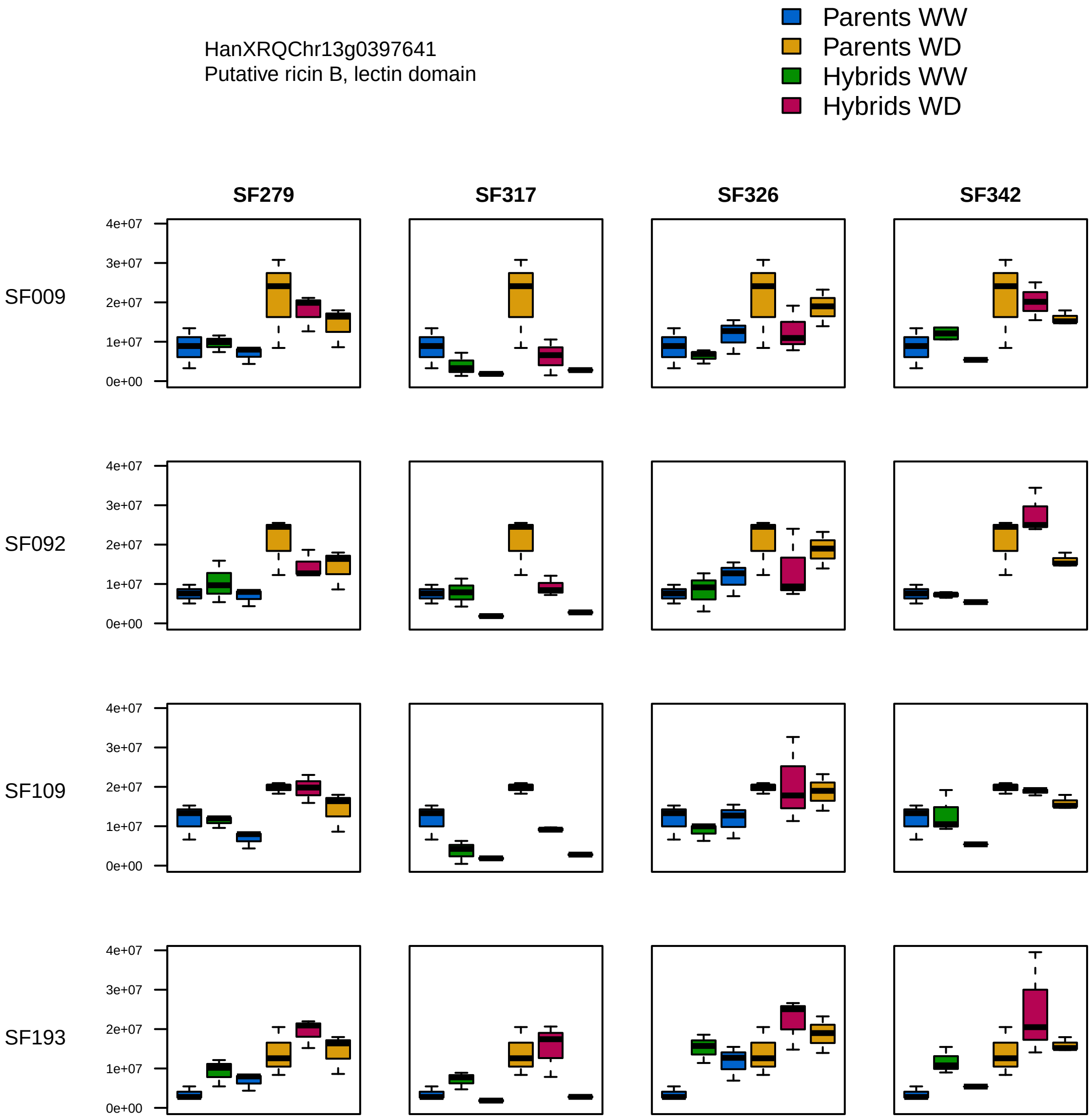

HanXRQChr13g0397641
Putative ricin B, lectin domain
Parents WW
Parents WD
Hybrids WW
Hybrids WD
SF279
SF317
SF326
SF342
SF009
SF092
SF109
SF193
4e+07
3e+07
2e+07
1e+07
0e+00

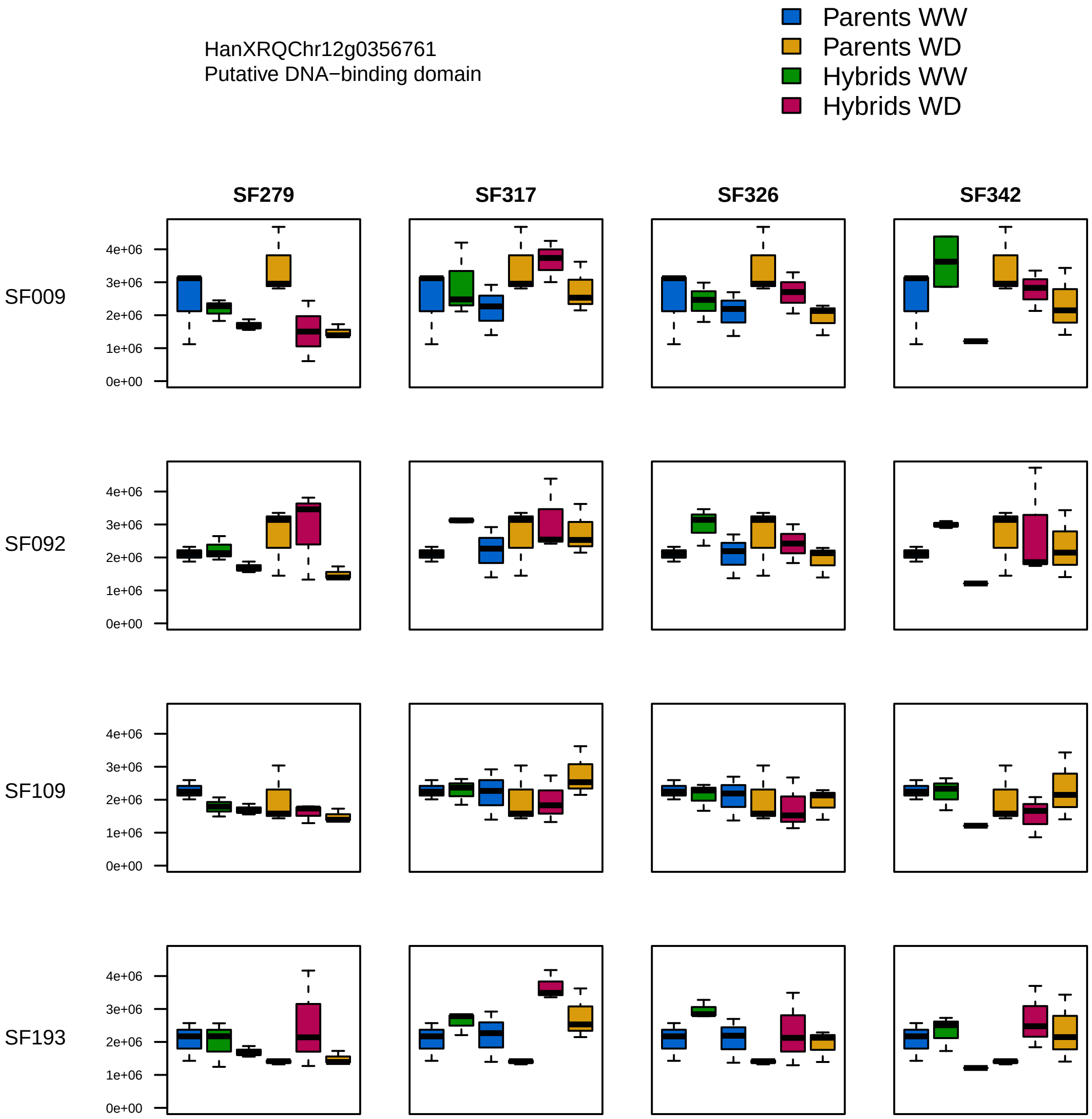

HanXRQChr12g0356761
Putative DNA−binding domain
Parents WW
Parents WD
Hybrids WW
Hybrids WD
SF279
SF317
SF326
SF342
SF009
SF092
SF109
SF193
4e+06
3e+06
2e+06
1e+06
0e+00

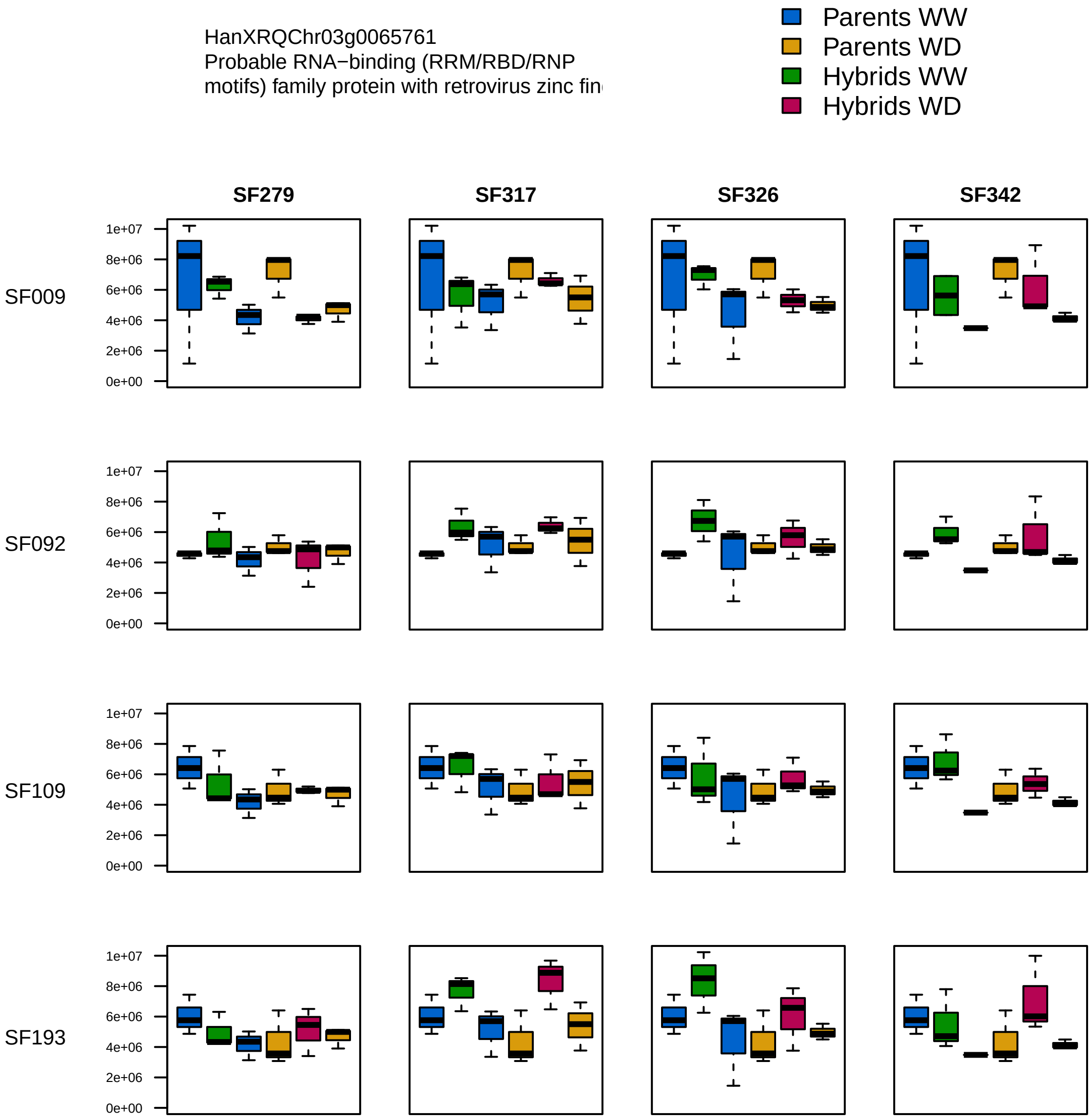
HanXRQChr03g0065761
Probable RNA−binding (RRM/RBD/RNP
motifs) family protein with retrovirus zinc fin
Parents WW
Parents WD
Hybrids WW
Hybrids WD
SF279
SF317
SF326
SF342
SF009
SF092
SF109
SF193
1e+07
8e+06
6e+06
4e+06
2e+06
0e+00

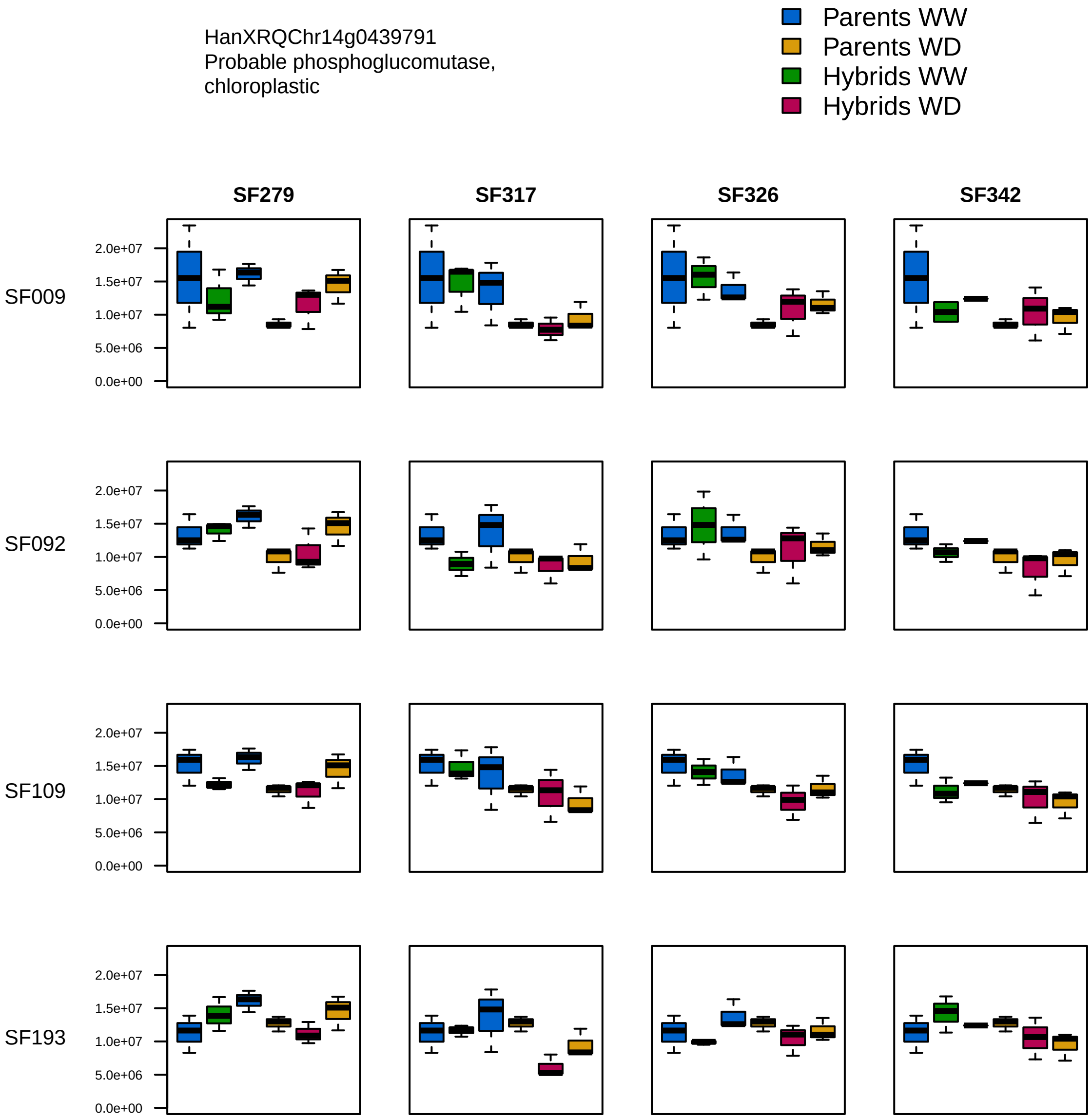
HanXRQChr14g0439791
Probable phosphoglucomutase, chloroplastic
Parents WW
Parents WD
Hybrids WW
Hybrids WD
SF279
SF317
SF326
SF342
SF009
SF092
SF109
SF193
0.0e+00
5.0e+06
1.0e+07
1.5e+07
2.0e+07

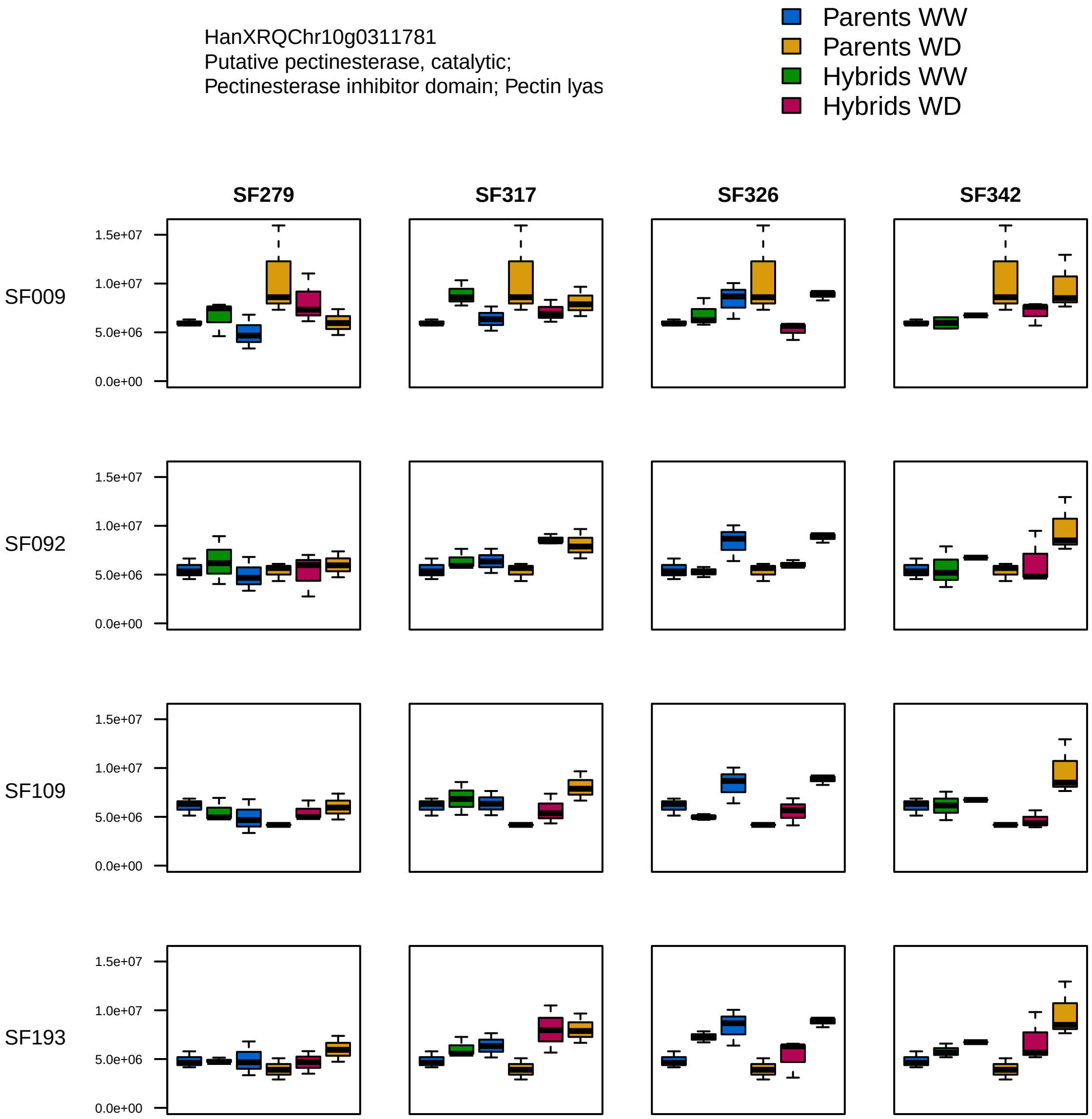
HanXRQChr10g0311781
Putative pectinesterase, catalytic;
Pectinesterase inhibitor domain; Pectin lyas
Parents WW
Parents WD
Hybrids WW
Hybrids WD
SF279
SF317
SF326
SF342
SF009
SF092
SF109
SF193
1.5e+07
1.0e+07
5.0e+06
0.0e+00

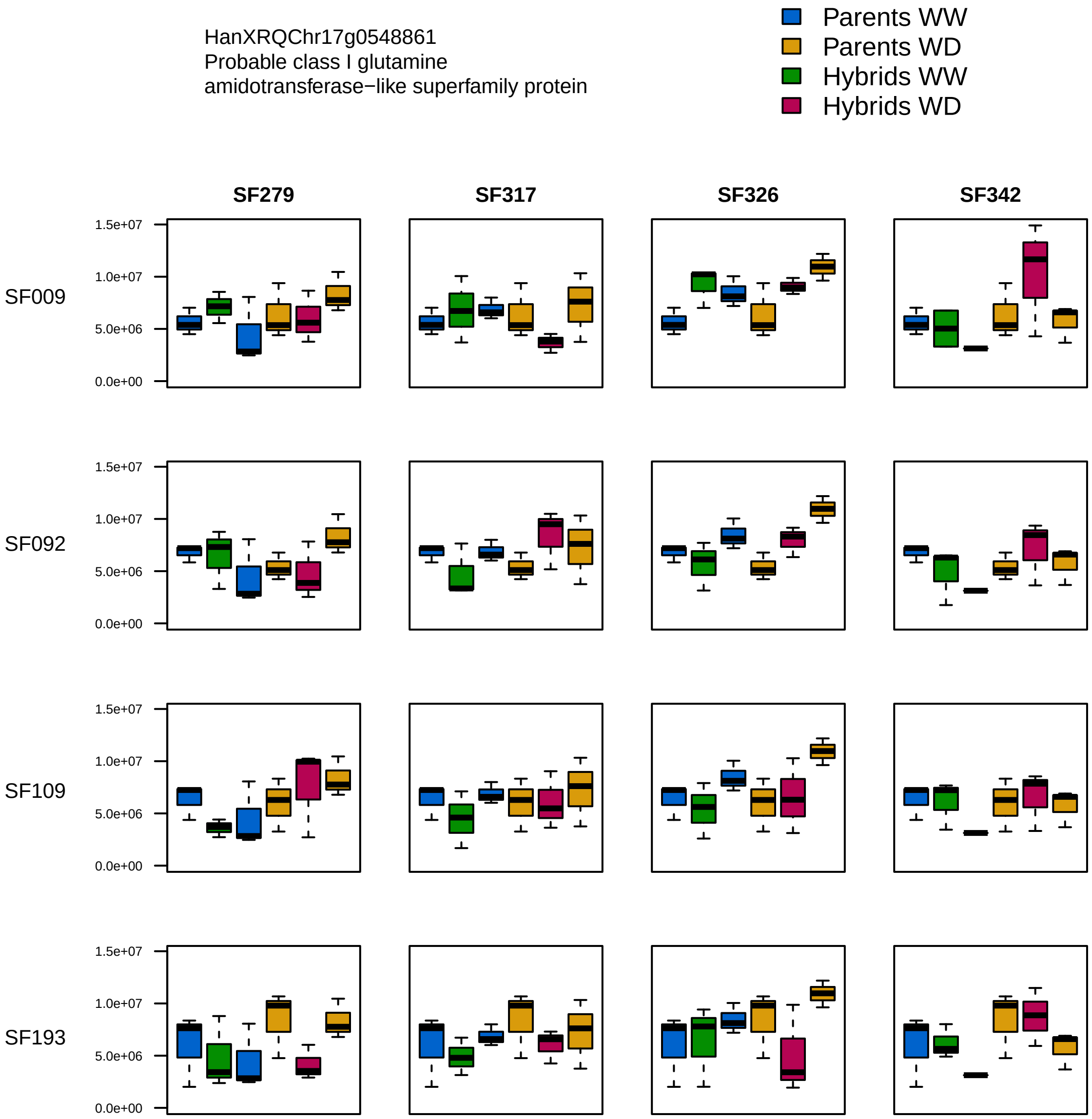
HanXRQChr17g0548861
Probable class I glutamine
amidotransferase−like superfamily protein
Parents WW
Parents WD
Hybrids WW
Hybrids WD
SF279
SF317
SF326
SF342
SF009
SF092
SF109
SF193
1.5e+07
1.0e+07
5.0e+06
0.0e+00

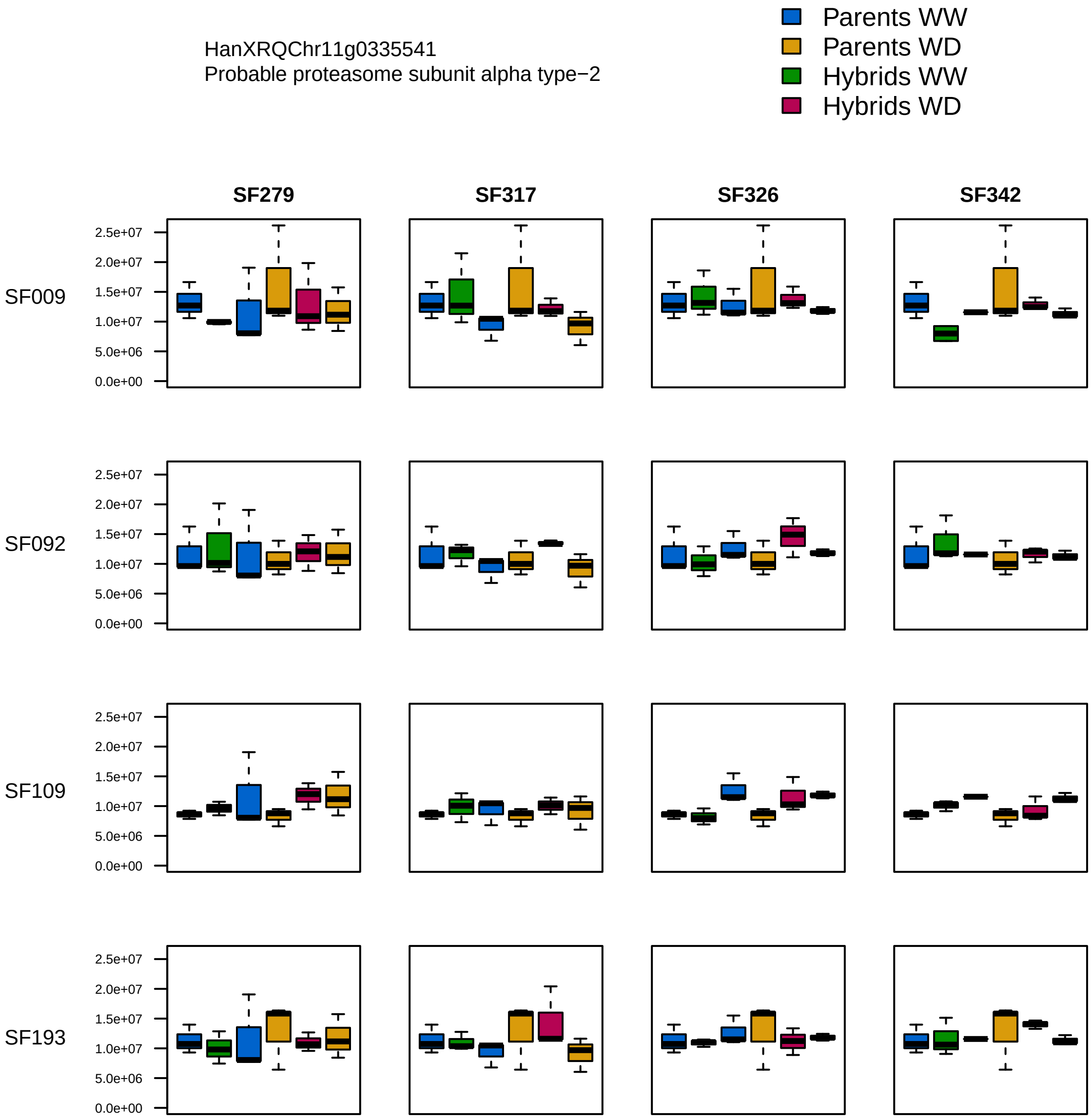
HanXRQChr11g0335541
Probable proteasome subunit alpha type−2
Parents WW
Parents WD
Hybrids WW
Hybrids WD
SF279
SF317
SF326
SF342
SF009
SF092
SF109
SF193
2.5e+07
2.0e+07
1.5e+07
1.0e+07
5.0e+06
0.0e+00

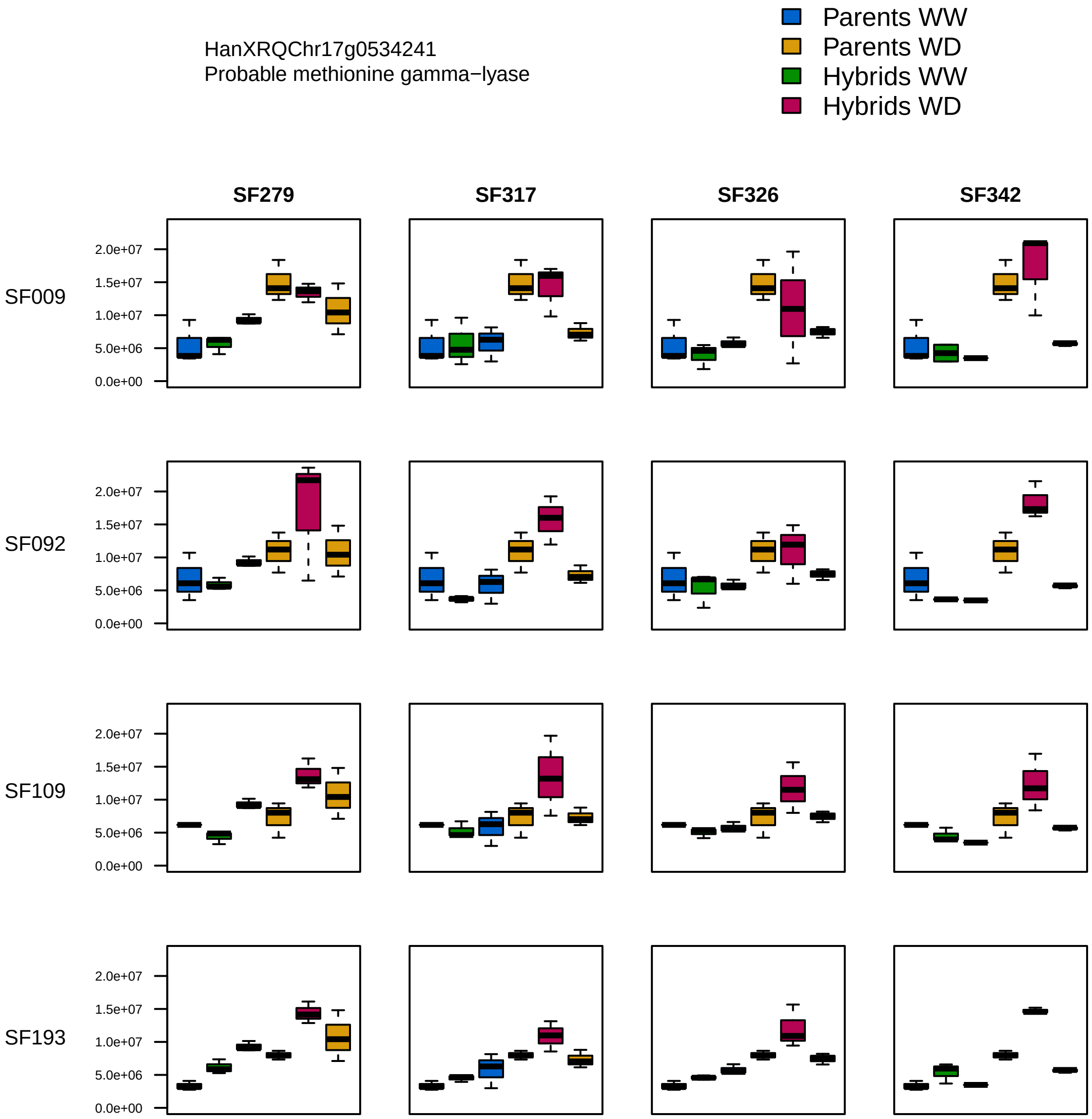
HanXRQChr17g0534241
Probable methionine gamma−lyase
Parents WW
Parents WD
Hybrids WW
Hybrids WD
SF279
SF317
SF326
SF342
SF009
SF092
SF109
SF193
0.0e+00
5.0e+06
1.0e+07
1.5e+07
2.0e+07

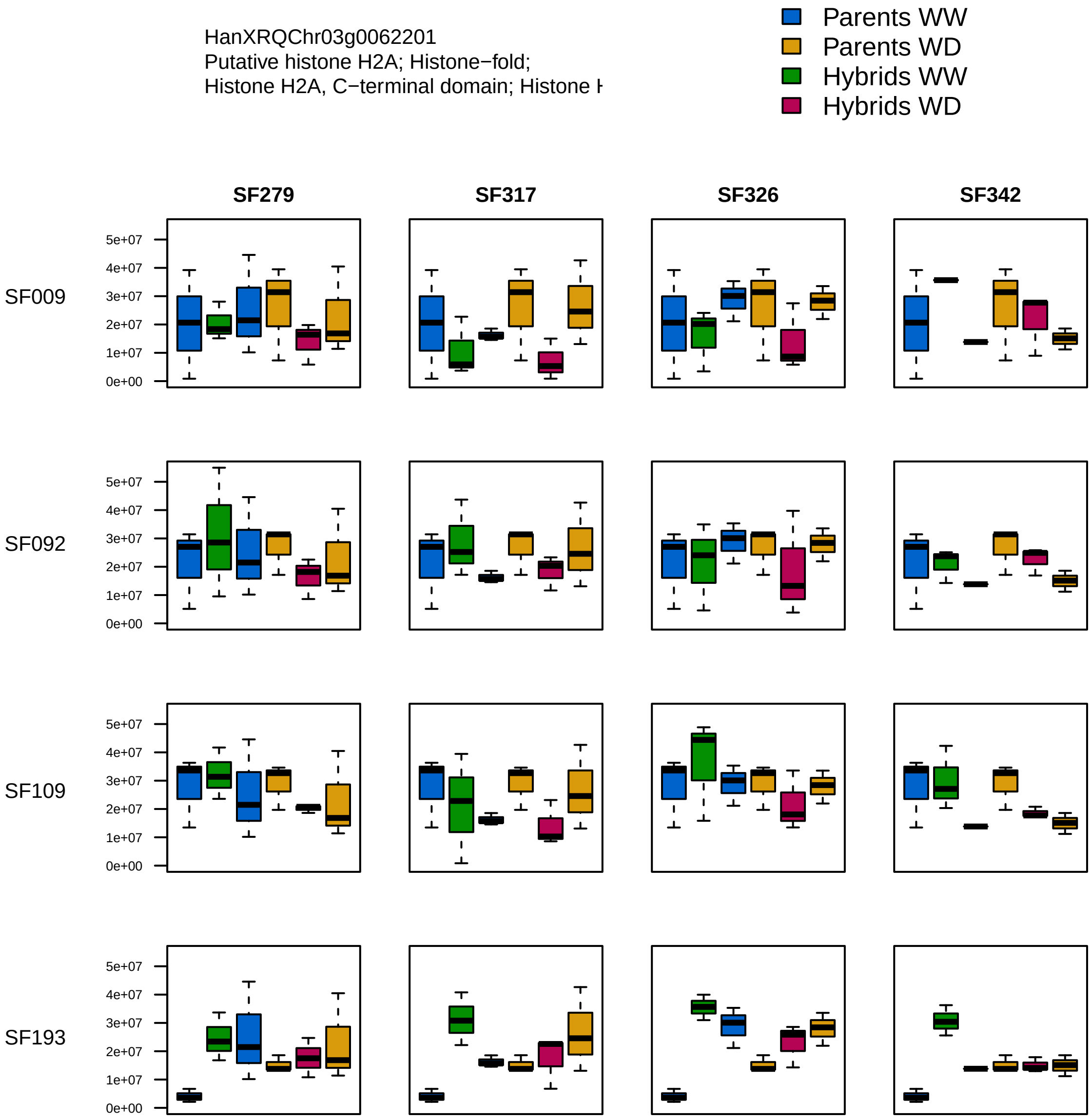
HanXRQChr03g0062201
Putative histone H2A; Histone−fold;
Histone H2A, C−terminal domain; Histone H
Parents WW
Parents WD
Hybrids WW
Hybrids WD
SF279
SF317
SF326
SF342
SF009
SF092
SF109
SF193
5e+07
4e+07
3e+07
2e+07
1e+07
0e+00

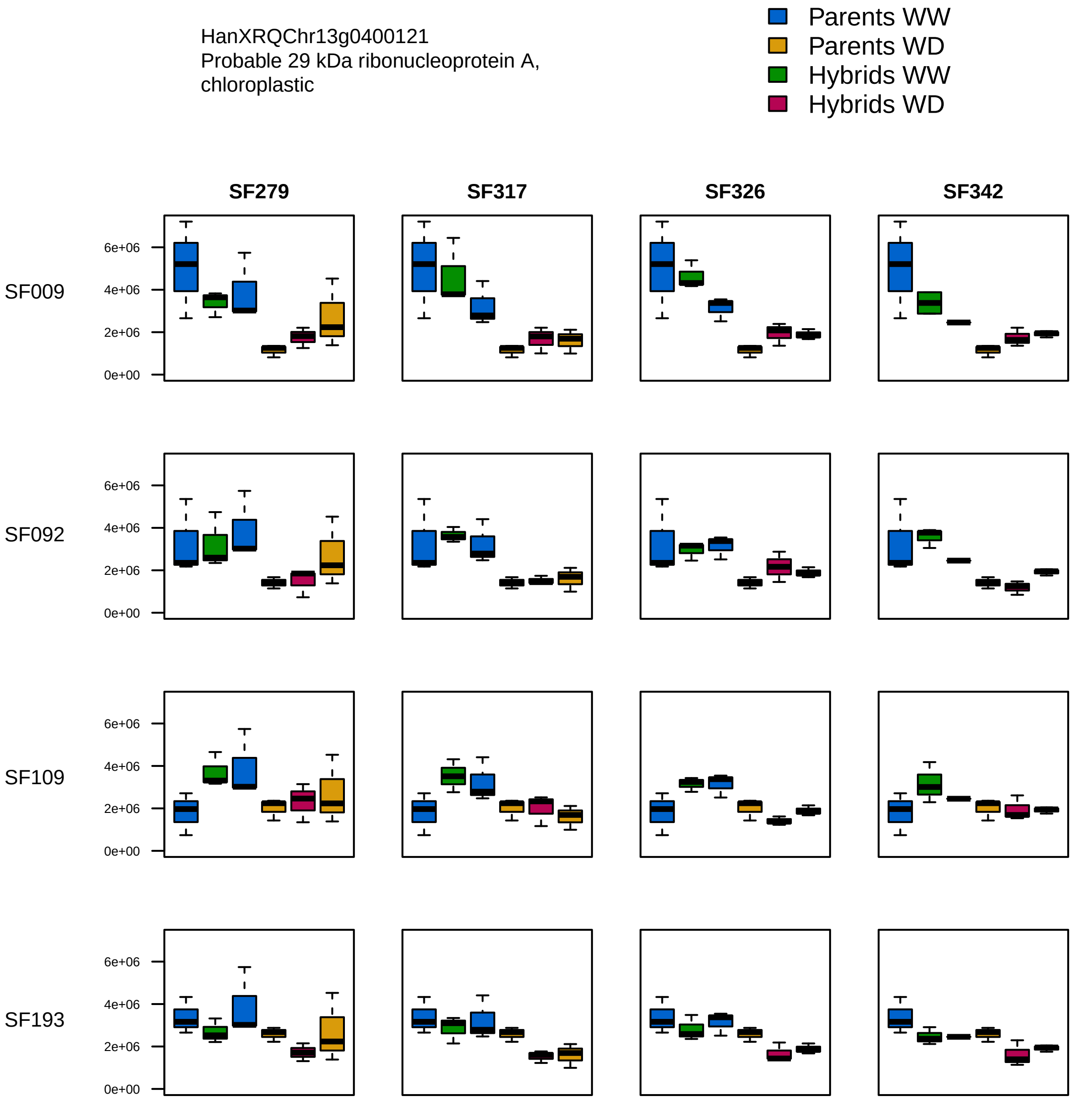
HanXRQChr13g0400121
Probable 29 kDa ribonucleoprotein A,
chloroplastic
Parents WW
Parents WD
Hybrids WW
Hybrids WD
SF279
SF317
SF326
SF342
SF009
SF092
SF109
SF193
0e+00
2e+06
4e+06
6e+06

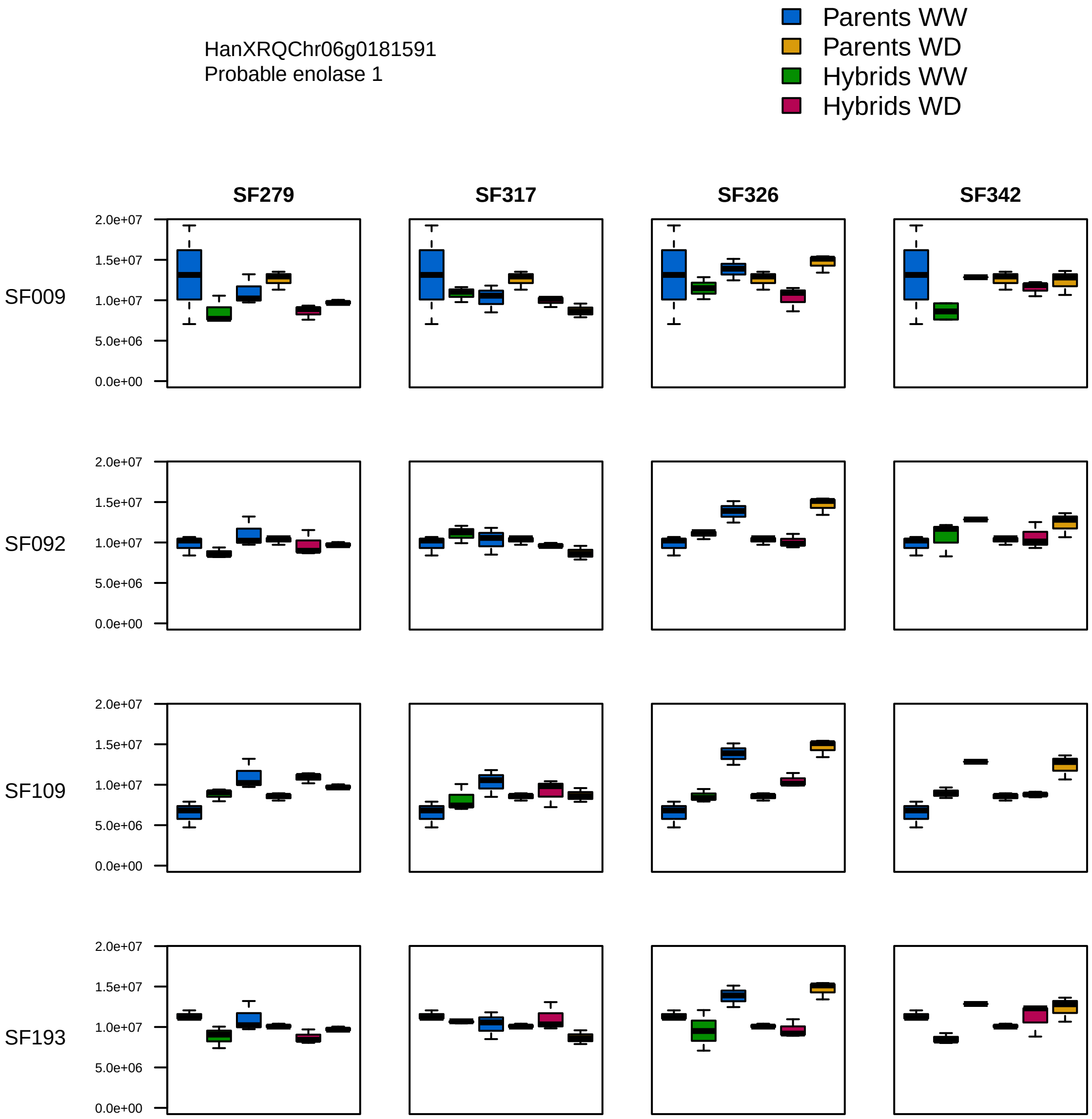

HanXRQChr06g0181591
Probable enolase 1
Parents WW
Parents WD
Hybrids WW
Hybrids WD
SF279
SF317
SF326
SF342
SF009
SF092
SF109
SF193
2.0e+07
1.5e+07
1.0e+07
5.0e+06
0.0e+00

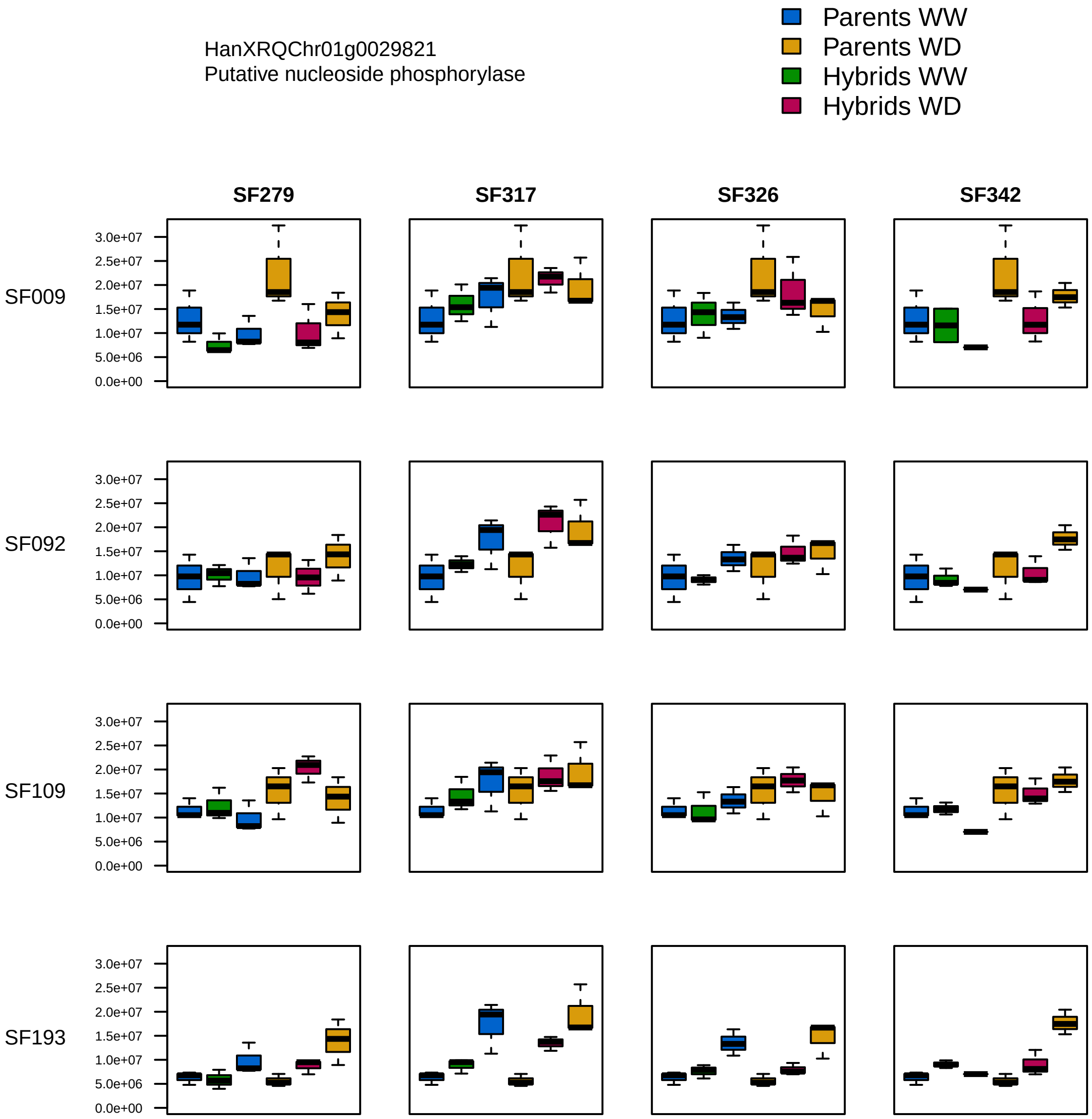
HanXRQChr01g0029821
Putative nucleoside phosphorylase
Parents WW
Parents WD
Hybrids WW
Hybrids WD
SF279
SF317
SF326
SF342
SF009
SF092
SF109
SF193
3.0e+07
2.5e+07
2.0e+07
1.5e+07
1.0e+07
5.0e+06
0.0e+00

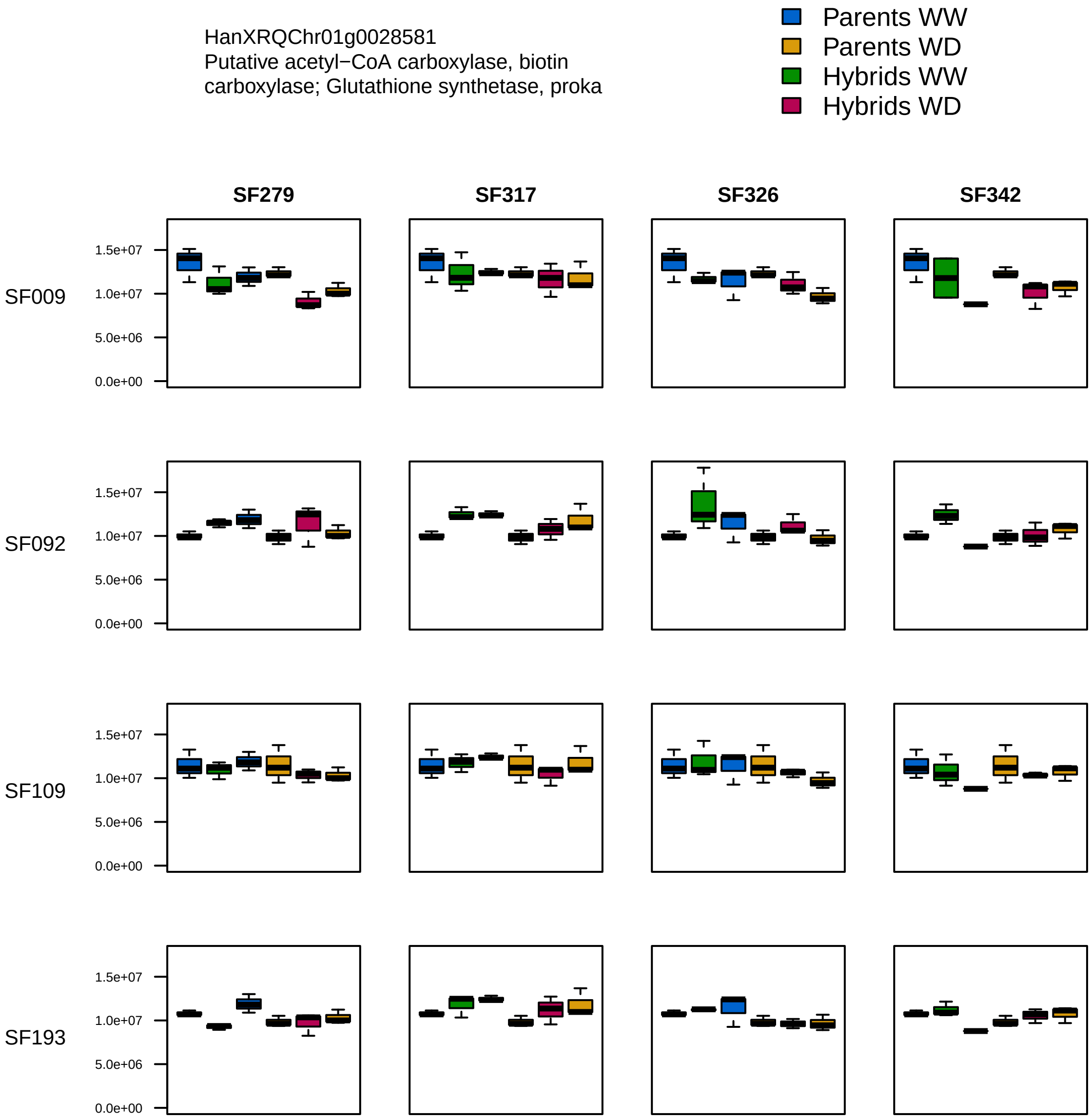
HanXRQChr01g0028581
Putative acetyl−CoA carboxylase, biotin
carboxylase; Glutathione synthetase, proka
Parents WW
Parents WD
Hybrids WW
Hybrids WD
SF279
SF317
SF326
SF342
SF009
SF092
SF109
SF193
0.0e+00
5.0e+06
1.0e+07
1.5e+07

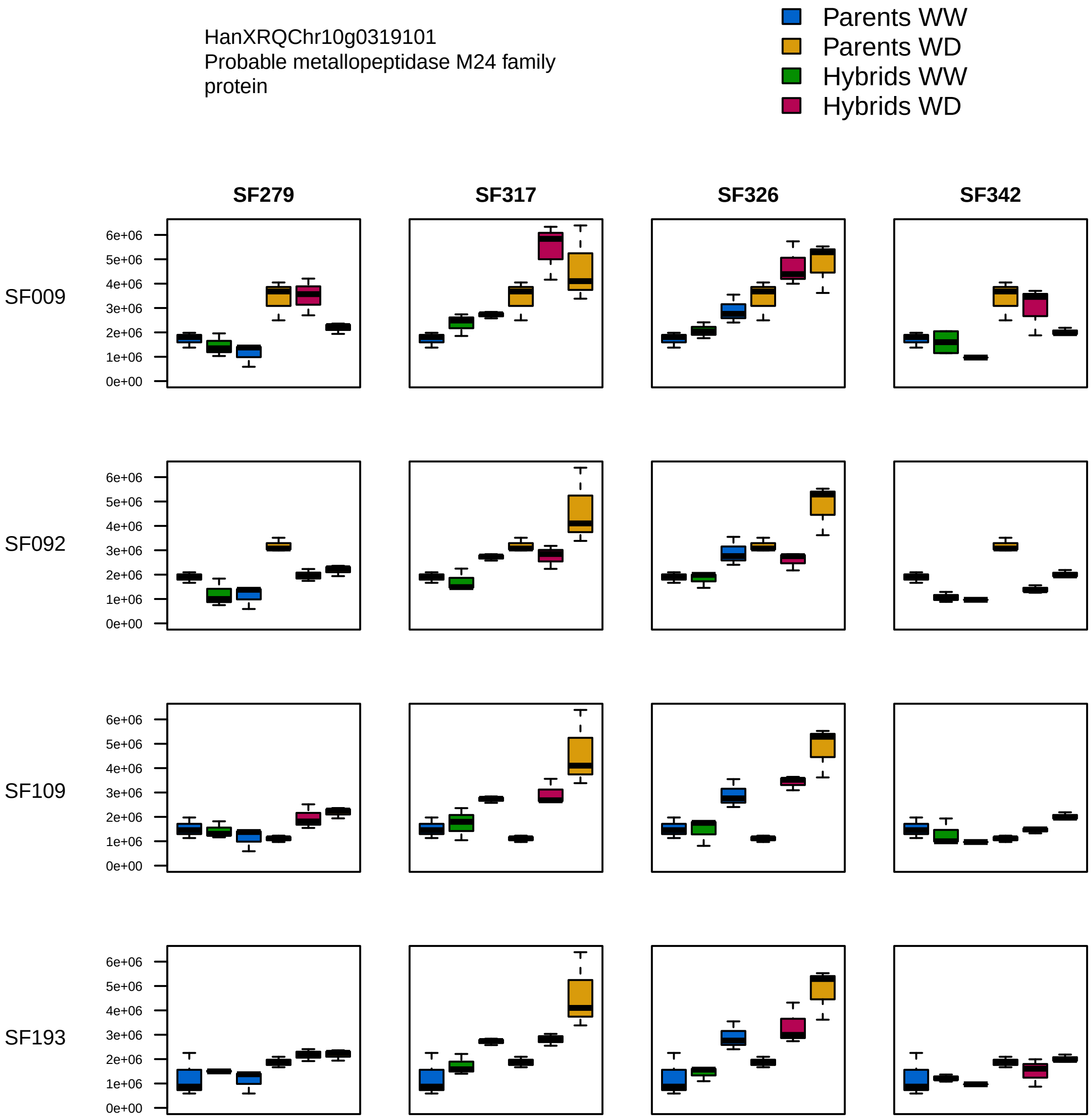

HanXRQChr10g0319101
Probable metallopeptidase M24 family protein
Parents WW
Parents WD
Hybrids WW
Hybrids WD
SF279
SF317
SF326
SF342
SF009
SF092
SF109
SF193
0e+00
1e+06
2e+06
3e+06
4e+06
5e+06
6e+06

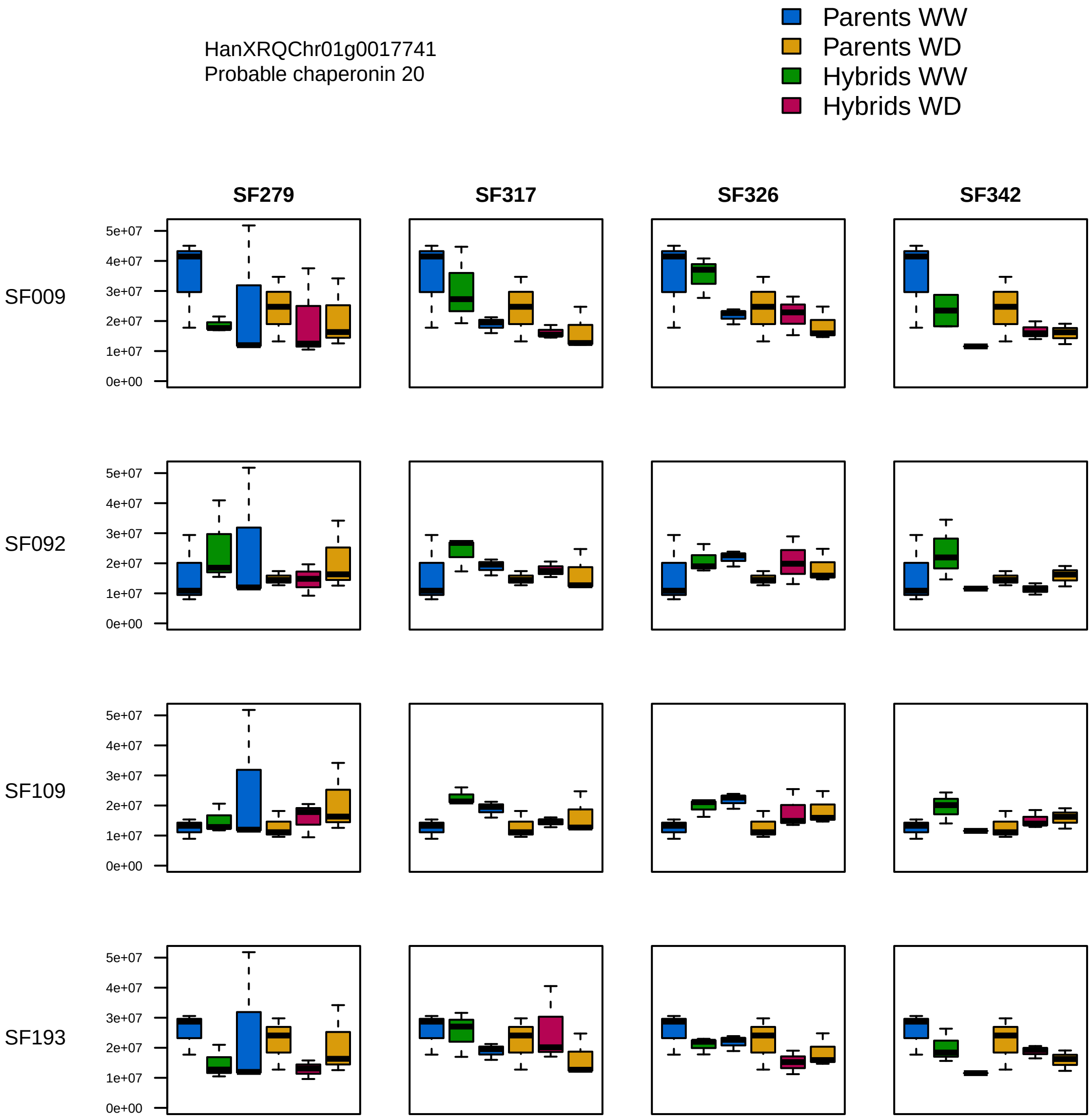
HanXRQChr01g0017741
Probable chaperonin 20
Parents WW
Parents WD
Hybrids WW
Hybrids WD
SF279
SF317
SF326
SF342
SF009
SF092
SF109
SF193
5e+07
4e+07
3e+07
2e+07
1e+07
0e+00

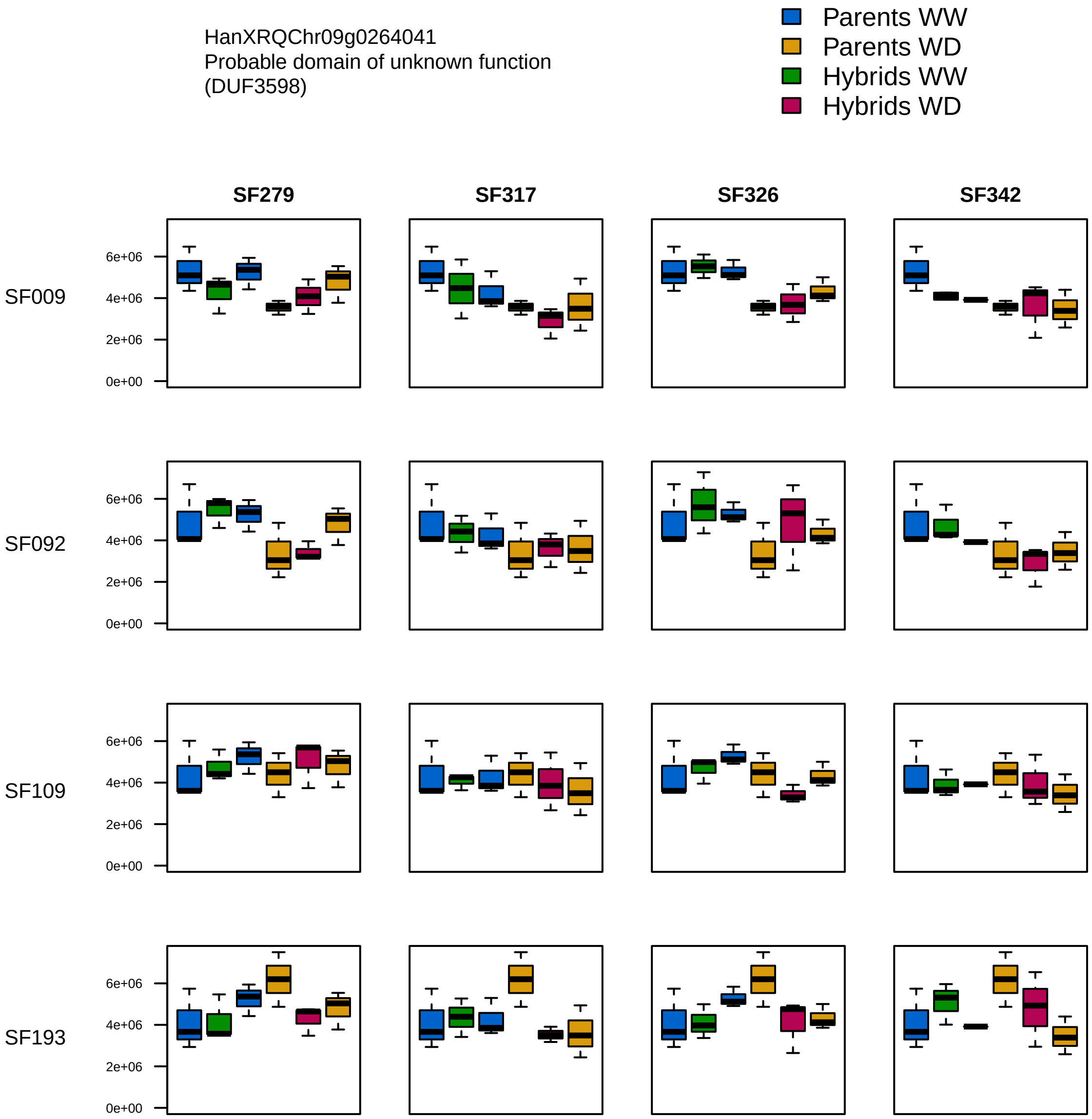
HanXRQChr09g0264041
Probable domain of unknown function
(DUF3598)
Parents WW
Parents WD
Hybrids WW
Hybrids WD
SF279
SF317
SF326
SF342
SF009
SF092
SF109
SF193
6e+06
4e+06
2e+06
0e+00

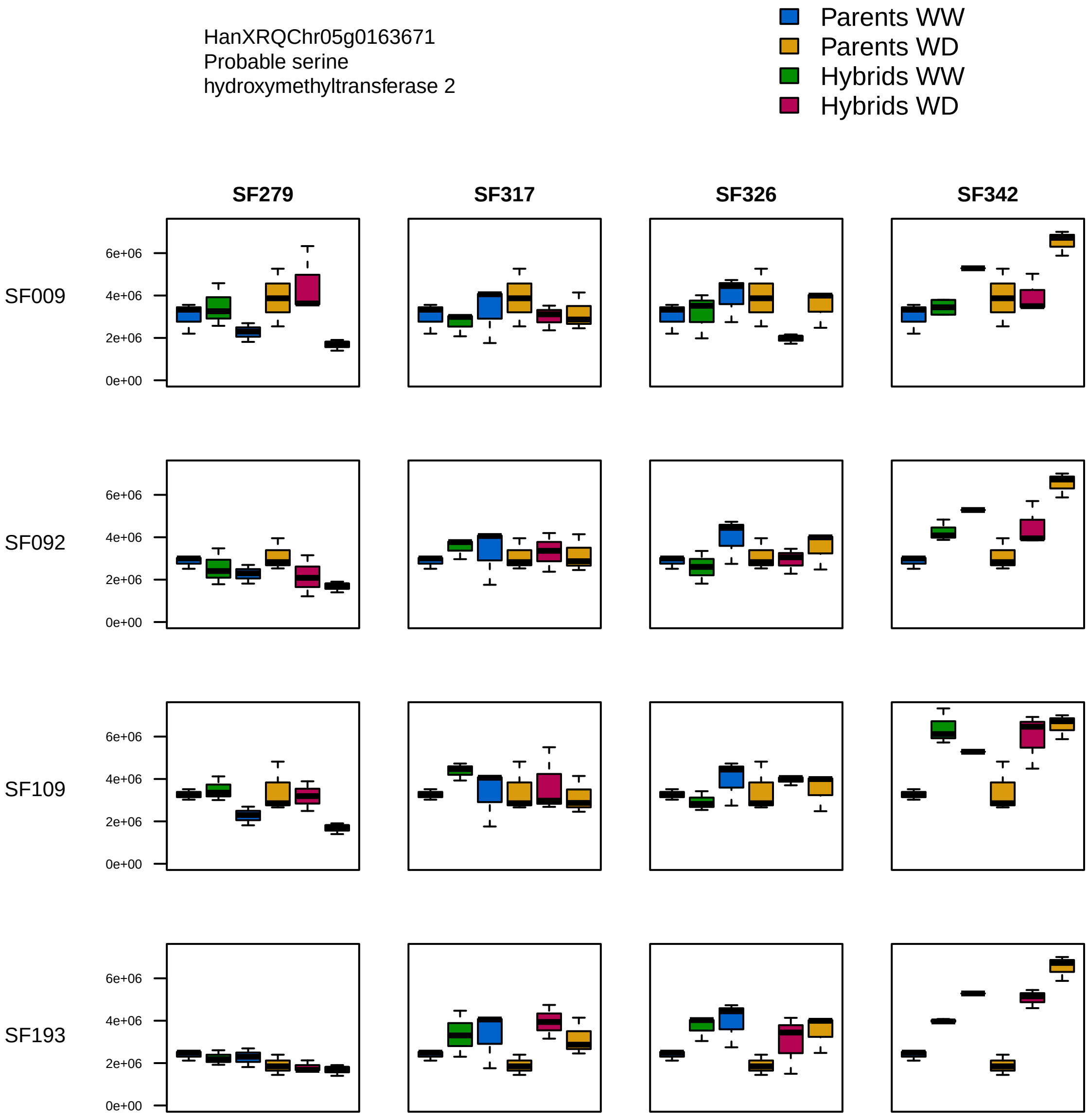
HanXRQChr05g0163671
Probable serine
hydroxymethyltransferase 2
Parents WW
Parents WD
Hybrids WW
Hybrids WD
SF279
SF317
SF326
SF342
SF009
SF092
SF109
SF193
6e+06
4e+06
2e+06
0e+00

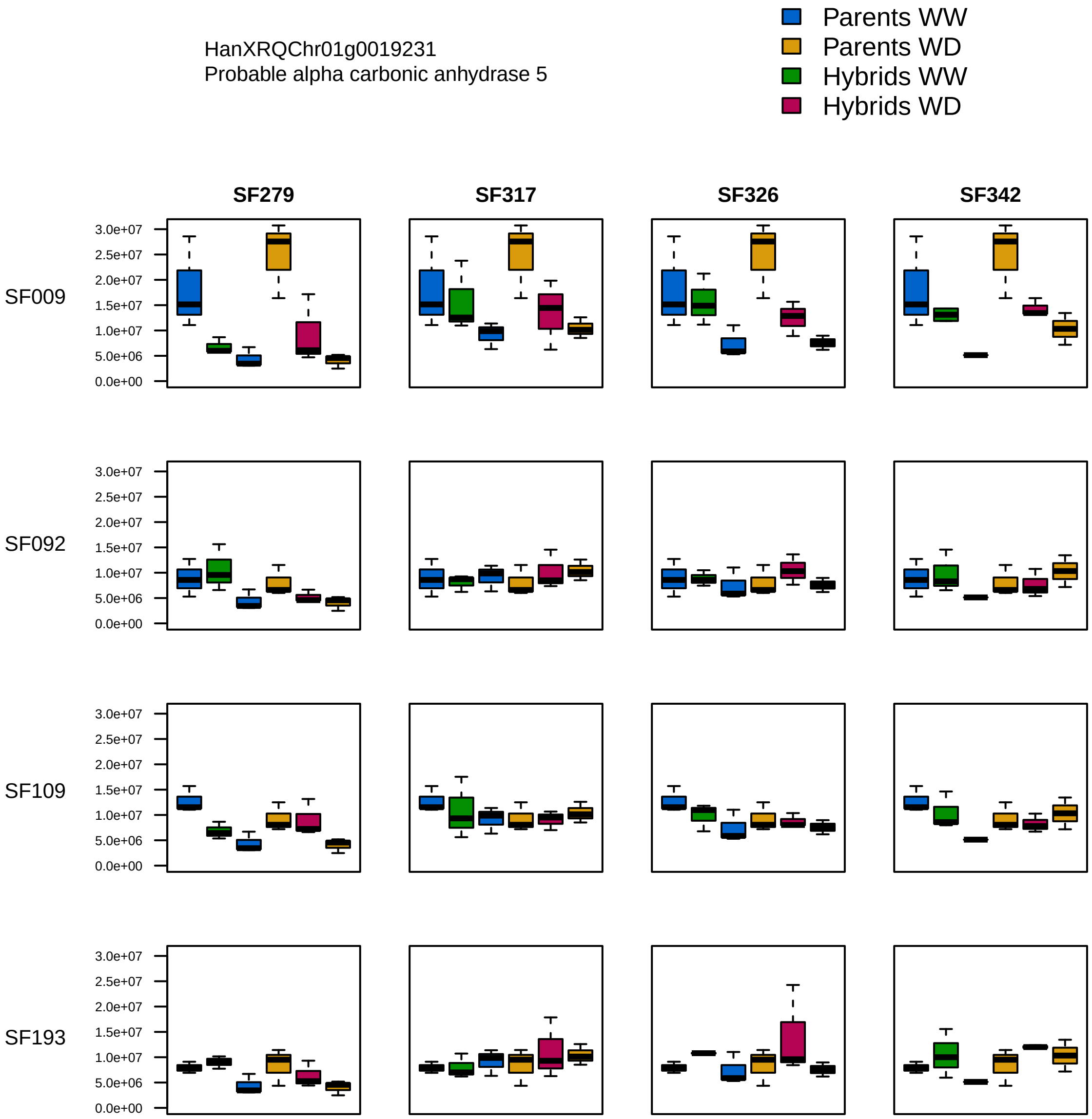

HanXRQChr01g0019231
Probable alpha carbonic anhydrase 5
Parents WW
Parents WD
Hybrids WW
Hybrids WD
SF279
SF317
SF326
SF342
SF009
SF092
SF109
SF193
3.0e+07
2.5e+07
2.0e+07
1.5e+07
1.0e+07
5.0e+06
0.0e+00

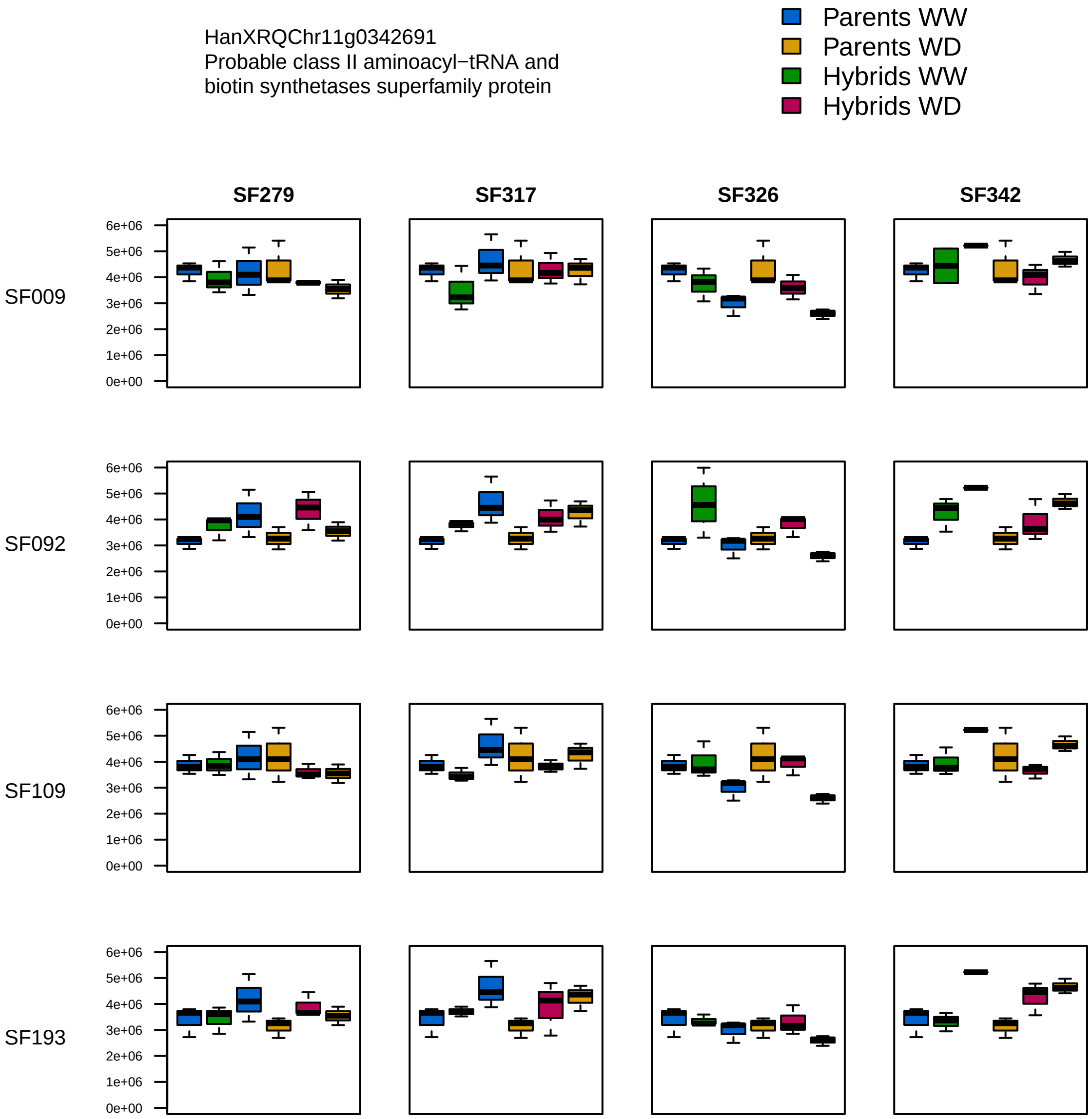

HanXRQChr11g0342691
Probable class II aminoacyl−tRNA and
biotin synthetases superfamily protein
Parents WW
Parents WD
Hybrids WW
Hybrids WD
SF279
SF317
SF326
SF342
SF009
SF092
SF109
SF193
0e+00
1e+06
2e+06
3e+06
4e+06
5e+06
6e+06

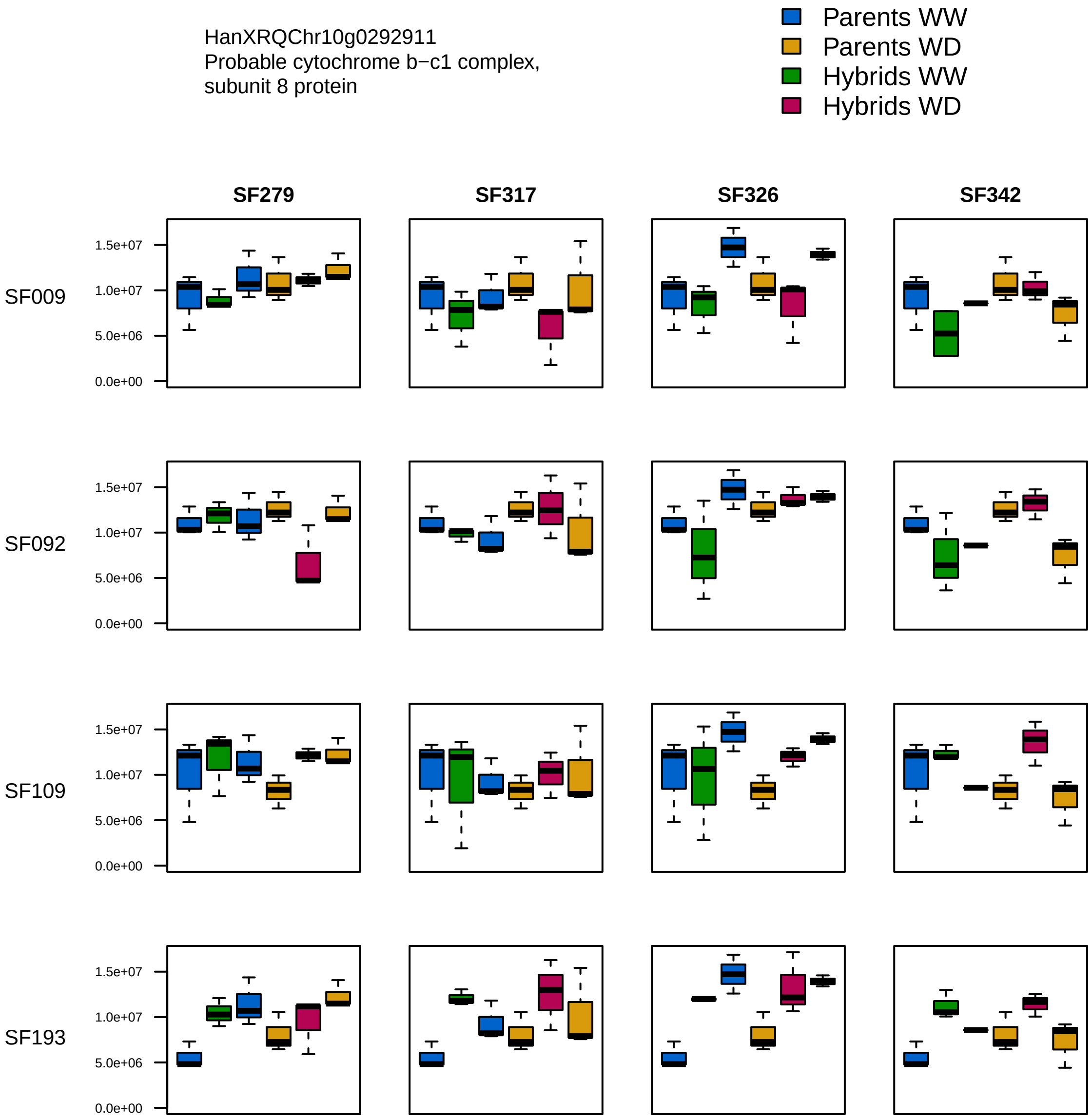
HanXRQChr10g0292911
Probable cytochrome b−c1 complex,
subunit 8 protein
Parents WW
Parents WD
Hybrids WW
Hybrids WD
SF279
SF317
SF326
SF342
SF009
SF092
SF109
SF193
1.5e+07
1.0e+07
5.0e+06
0.0e+00

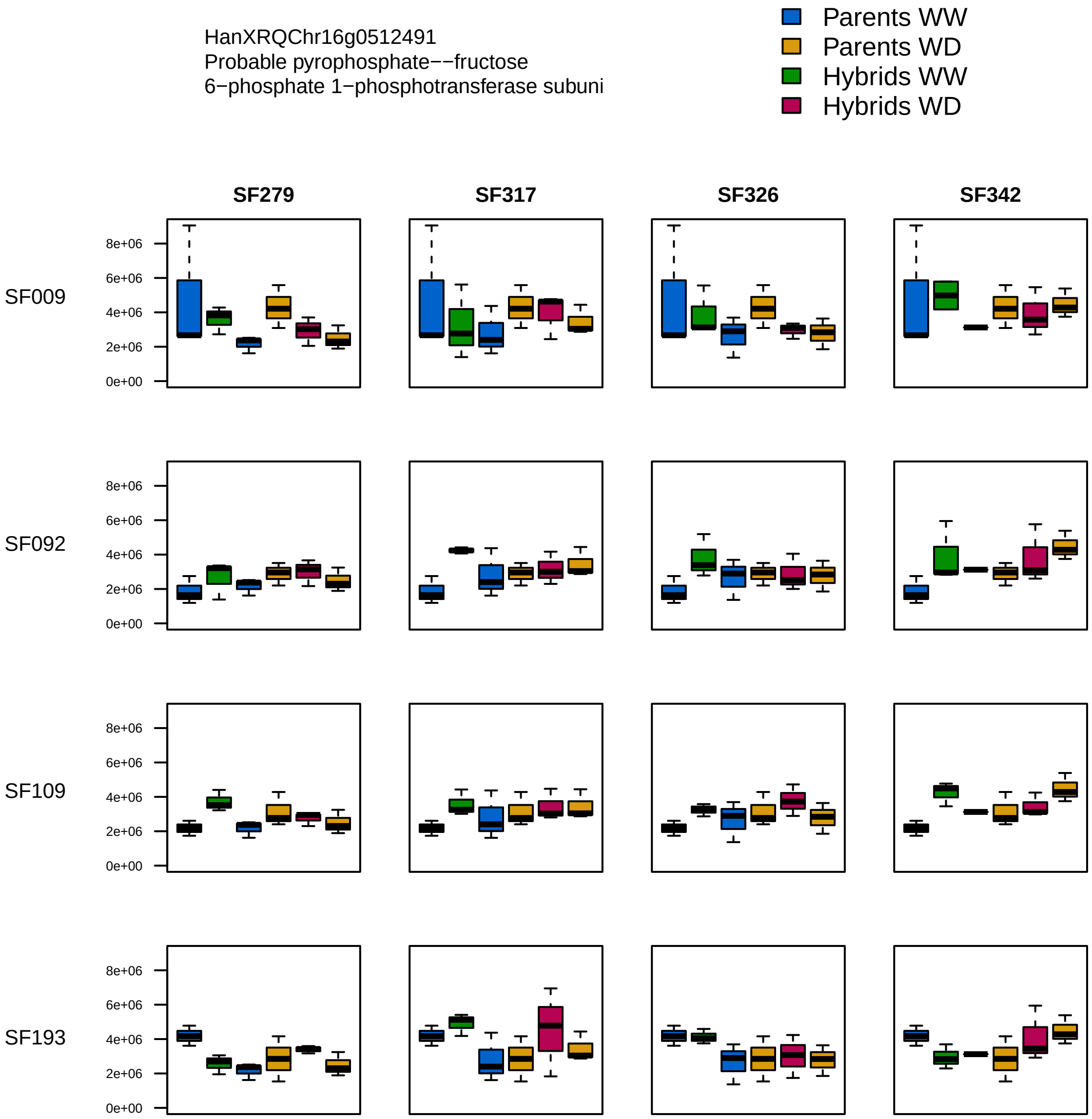
HanXRQChr16g0512491
Probable pyrophosphate−−fructose
6−phosphate 1−phosphotransferase subuni
Parents WW
Parents WD
Hybrids WW
Hybrids WD
SF279
SF317
SF326
SF342
SF009
SF092
SF109
SF193
8e+06
6e+06
4e+06
2e+06
0e+00

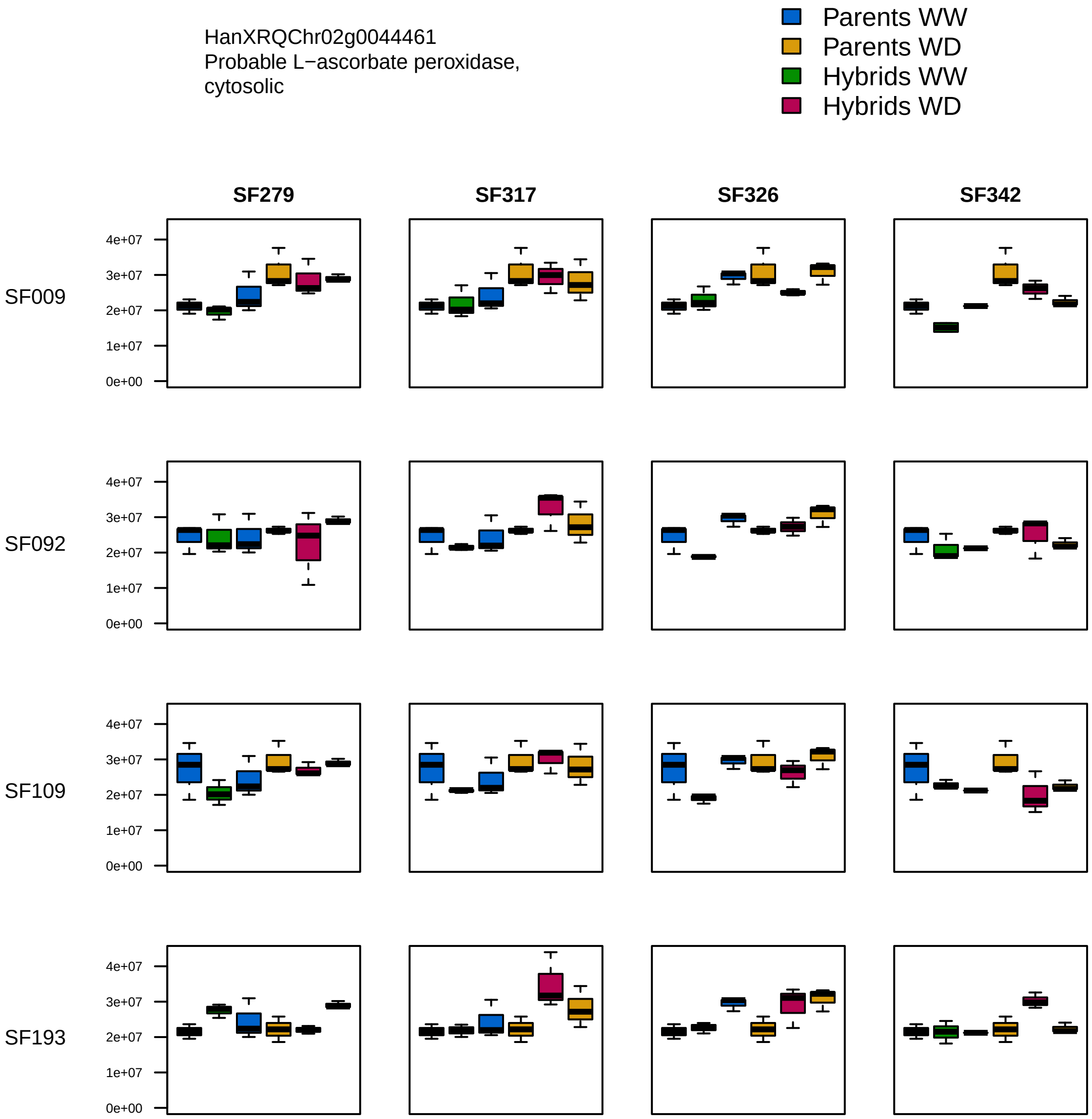
HanXRQChr02g0044461
Probable L−ascorbate peroxidase,
cytosolic
Parents WW
Parents WD
Hybrids WW
Hybrids WD
SF279
SF317
SF326
SF342
SF009
SF092
SF109
SF193
0e+00
1e+07
2e+07
3e+07
4e+07

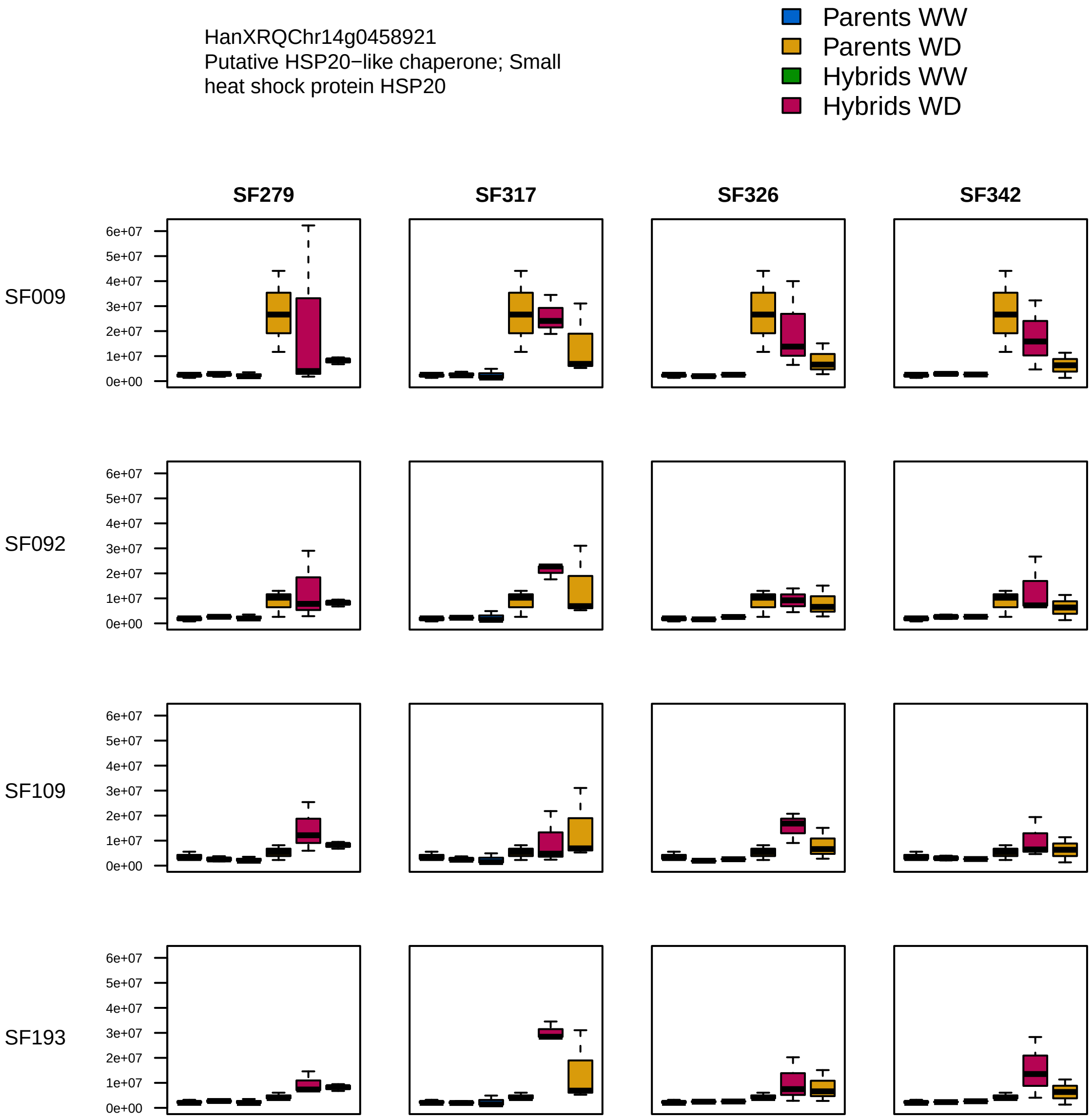
HanXRQChr14g0458921
Putative HSP20−like chaperone; Small heat shock protein HSP20
Parents WW
Parents WD
Hybrids WW
Hybrids WD
SF279
SF317
SF326
SF342
SF009
SF092
SF109
SF193
6e+07
5e+07
4e+07
3e+07
2e+07
1e+07
0e+00

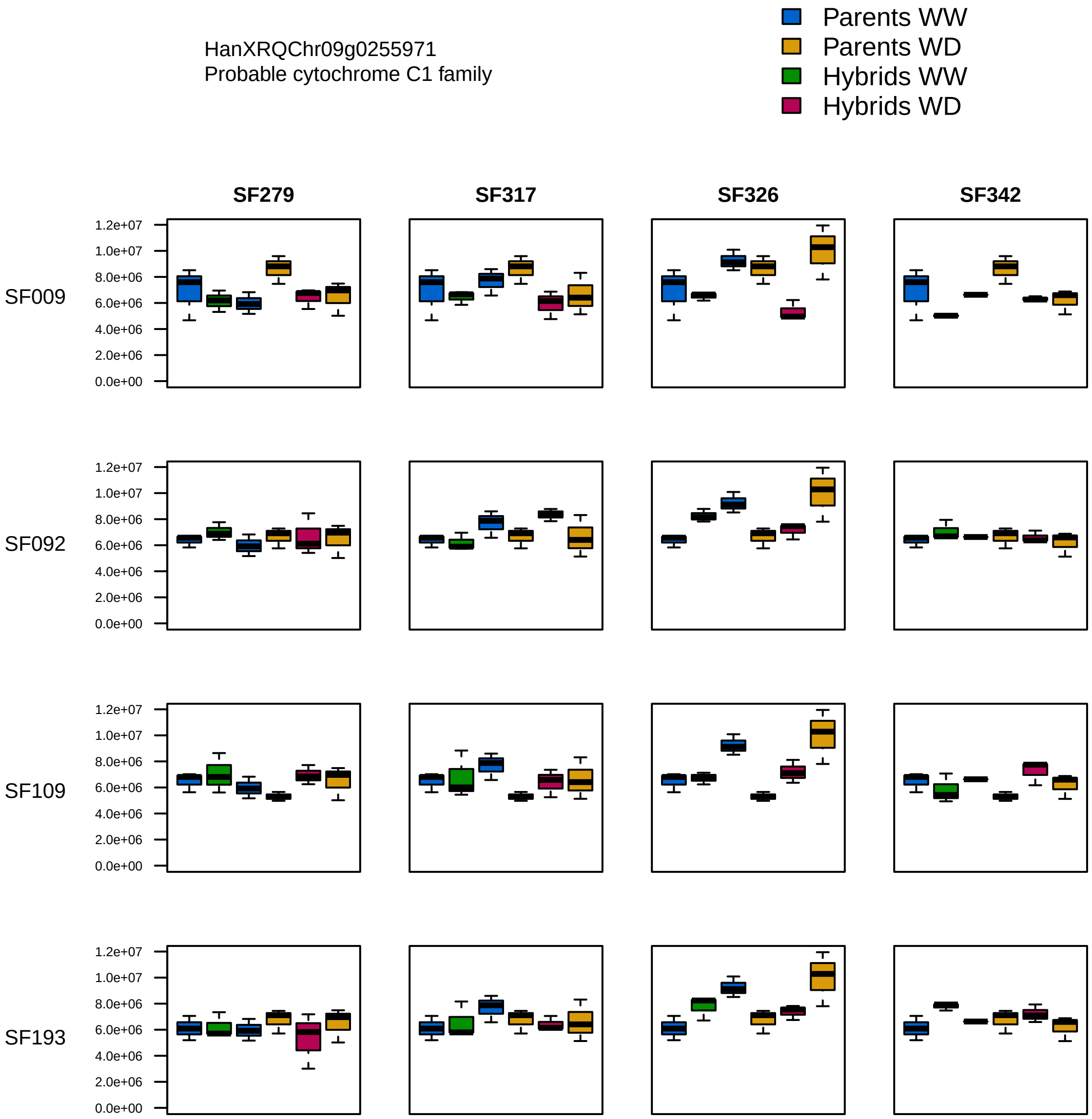
HanXRQChr09g0255971
Probable cytochrome C1 family
Parents WW
Parents WD
Hybrids WW
Hybrids WD
SF279
SF317
SF326
SF342
SF009
SF092
SF109
SF193
1.2e+07
1.0e+07
8.0e+06
6.0e+06
4.0e+06
2.0e+06
0.0e+00

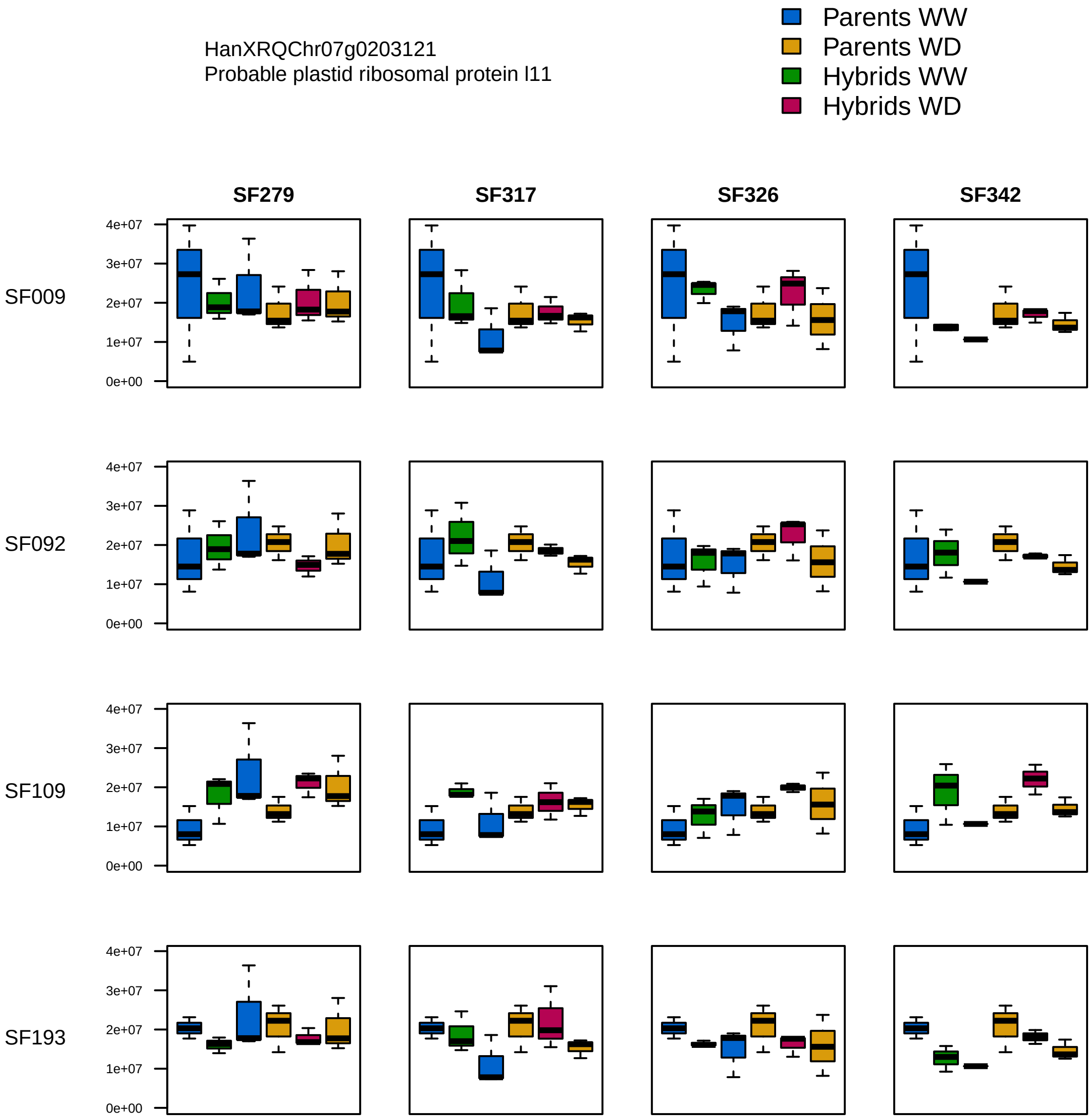

HanXRQChr07g0203121
Probable plastid ribosomal protein l11
Parents WW
Parents WD
Hybrids WW
Hybrids WD
SF279
SF317
SF326
SF342
SF009
SF092
SF109
SF193
4e+07
3e+07
2e+07
1e+07
0e+00

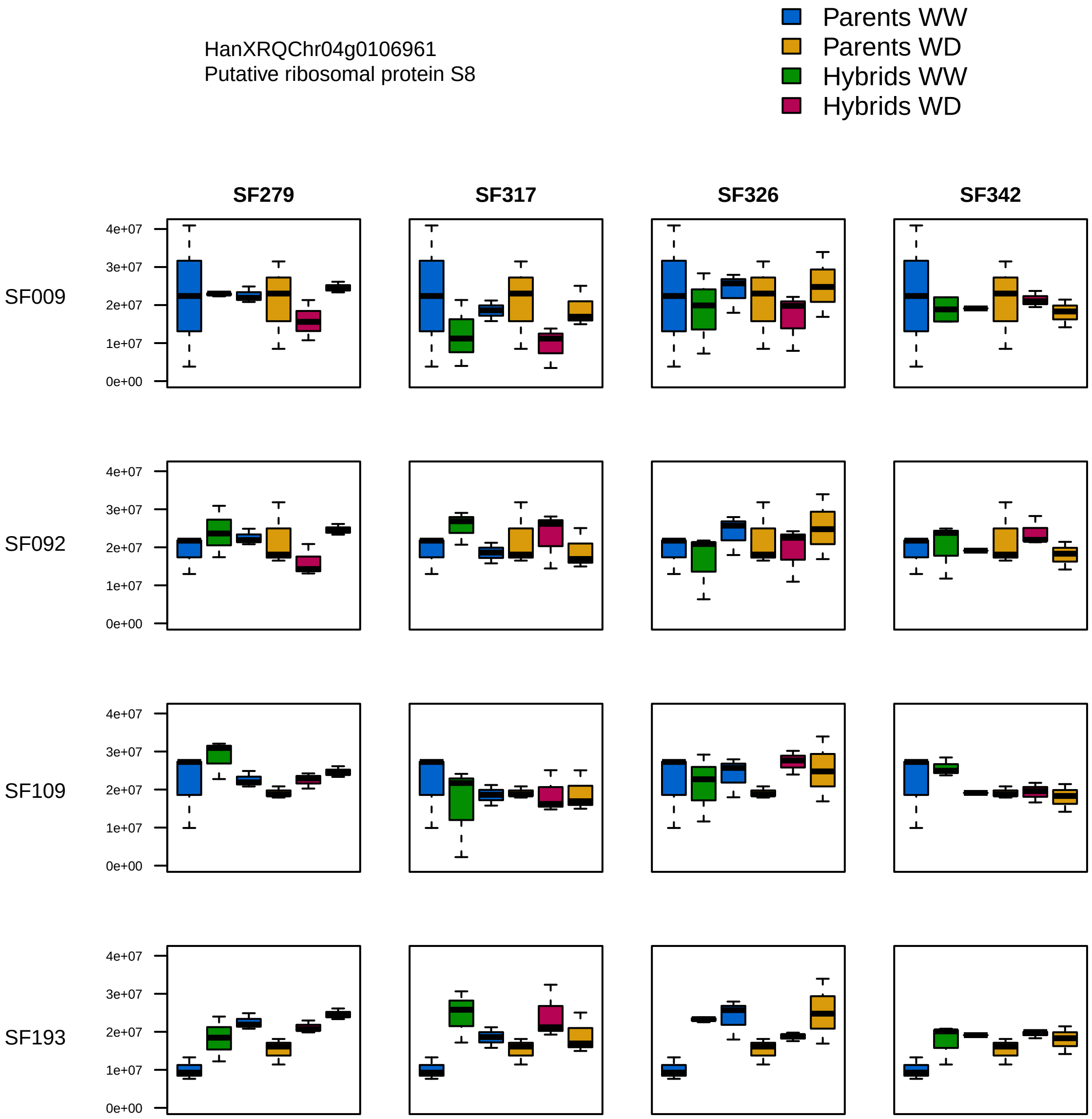
HanXRQChr04g0106961
Putative ribosomal protein S8
Parents WW
Parents WD
Hybrids WW
Hybrids WD
SF279
SF317
SF326
SF342
SF009
SF092
SF109
SF193
4e+07
3e+07
2e+07
1e+07
0e+00

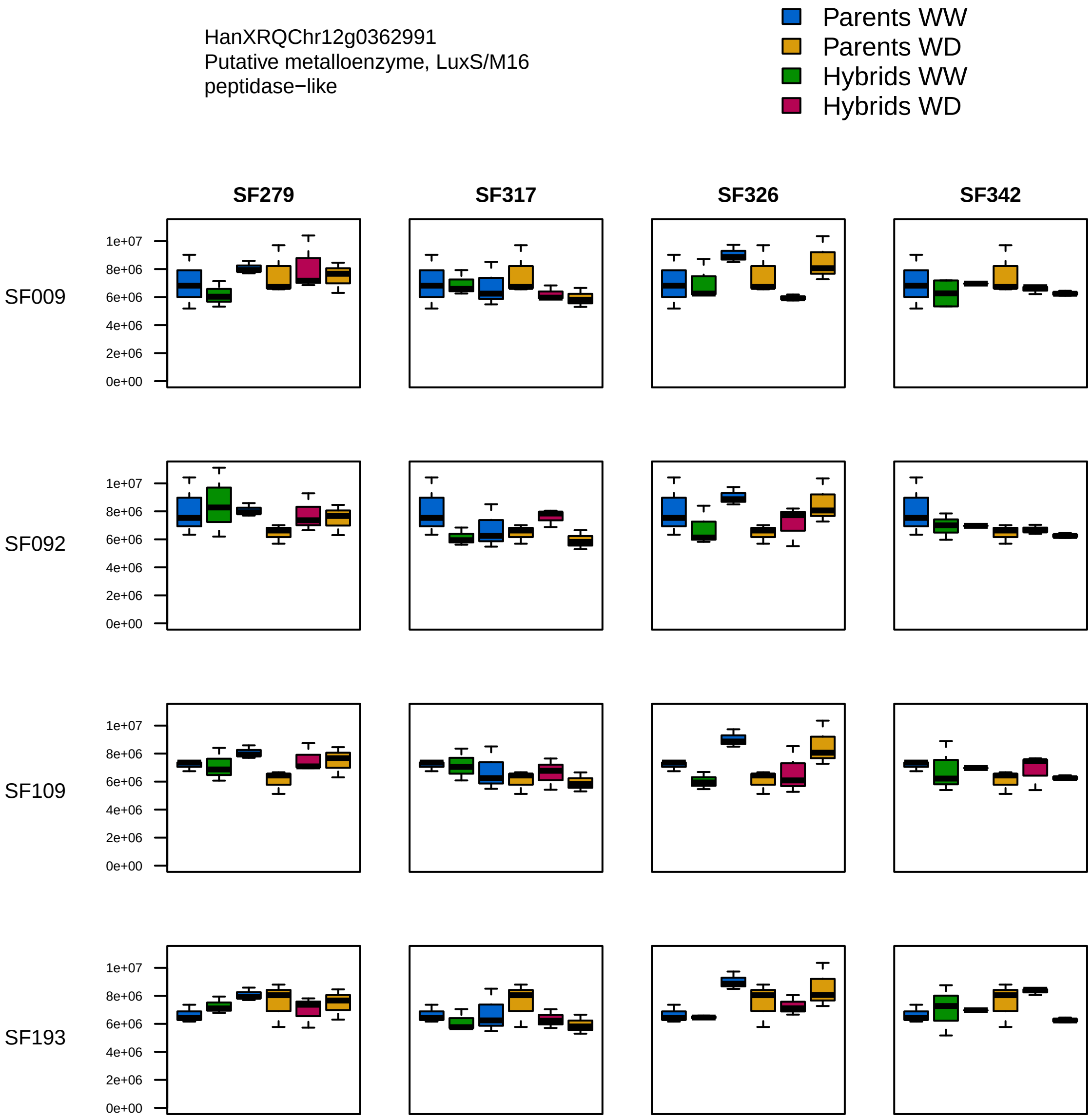
HanXRQChr12g0362991
Putative metalloenzyme, LuxS/M16
peptidase−like
Parents WW
Parents WD
Hybrids WW
Hybrids WD
SF279
SF317
SF326
SF342
SF009
SF092
SF109
SF193
1e+07
8e+06
6e+06
4e+06
2e+06
0e+00

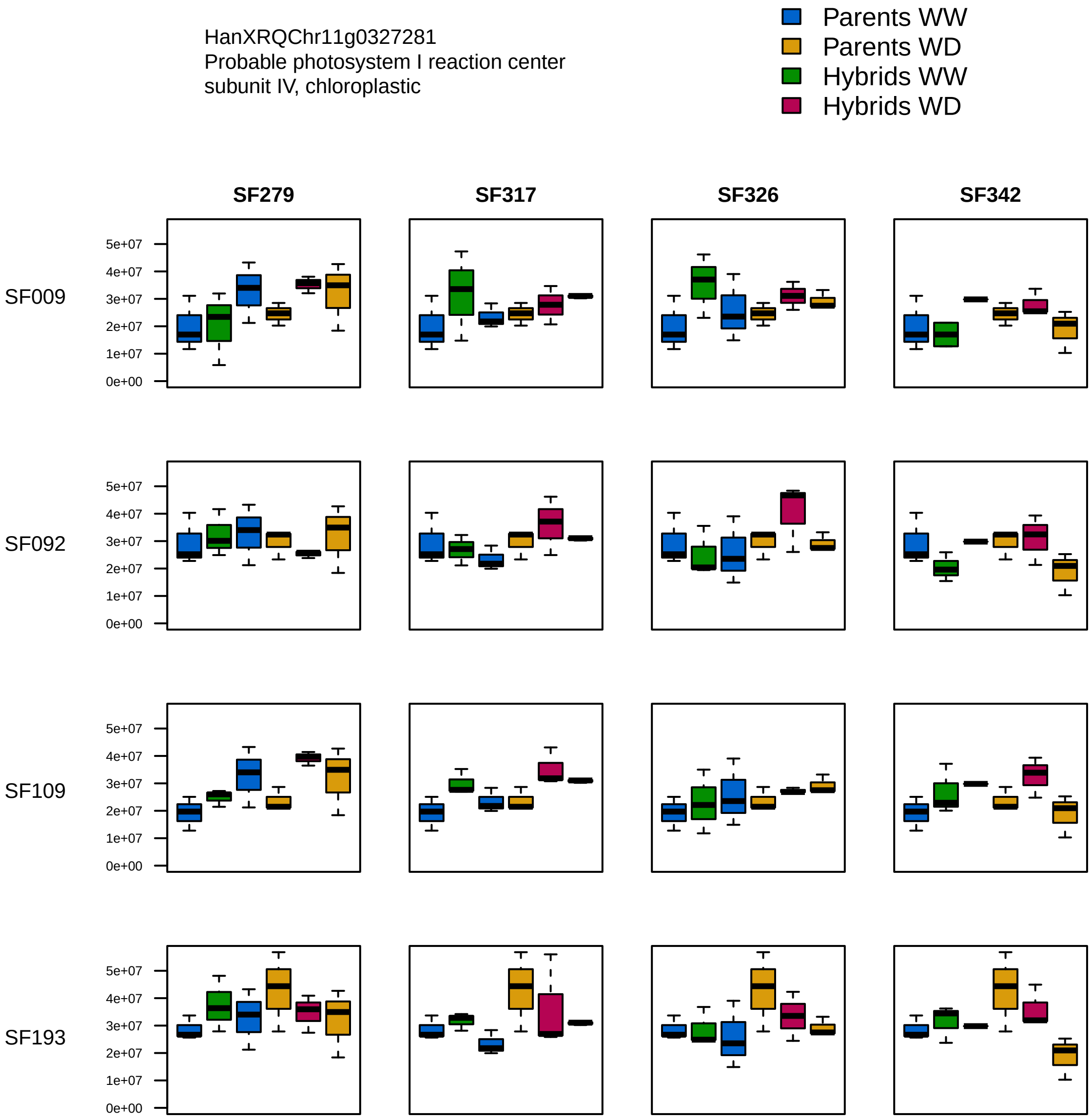

HanXRQChr11g0327281
Probable photosystem I reaction center
subunit IV, chloroplastic
Parents WW
Parents WD
Hybrids WW
Hybrids WD
SF279
SF317
SF326
SF342
SF009
SF092
SF109
SF193
5e+07
4e+07
3e+07
2e+07
1e+07
0e+00

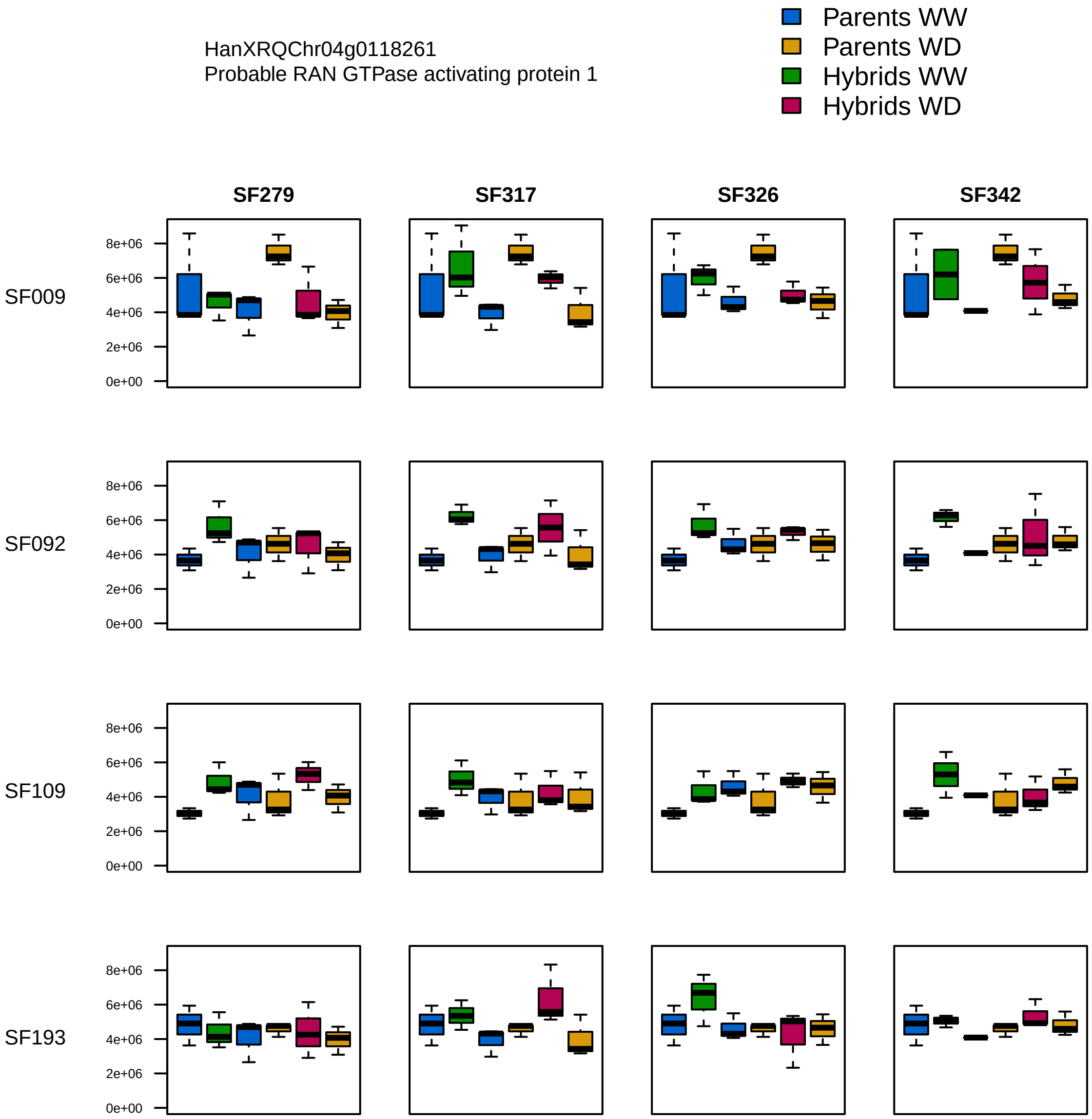

HanXRQChr04g0118261
Probable RAN GTPase activating protein 1
Parents WW
Parents WD
Hybrids WW
Hybrids WD
SF279
SF317
SF326
SF342
SF009
SF092
SF109
SF193
8e+06
6e+06
4e+06
2e+06
0e+00

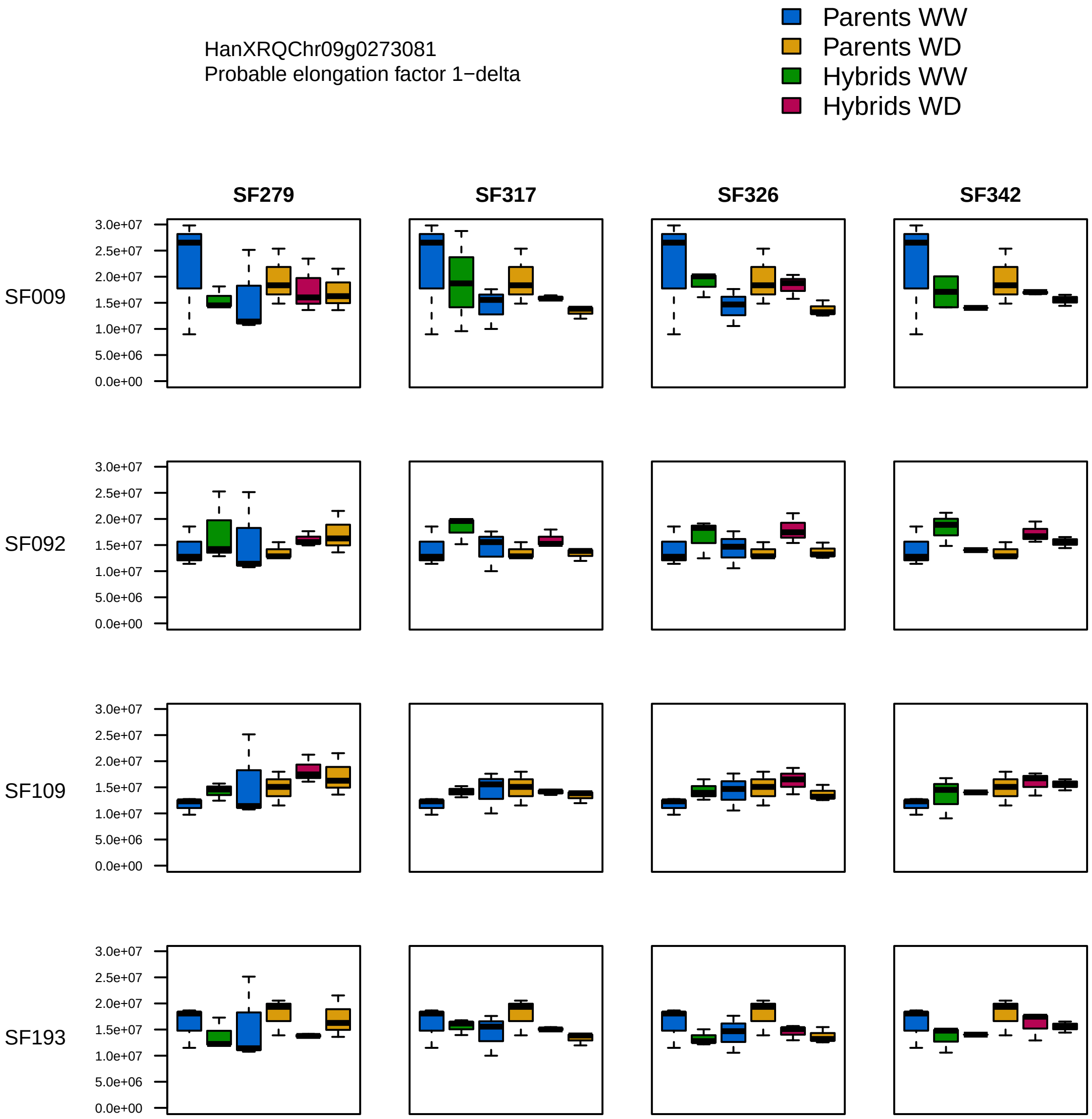

HanXRQChr09g0273081
Probable elongation factor 1−delta
Parents WW
Parents WD
Hybrids WW
Hybrids WD
SF279
SF317
SF326
SF342
SF009
SF092
SF109
SF193
3.0e+07
2.5e+07
2.0e+07
1.5e+07
1.0e+07
5.0e+06
0.0e+00

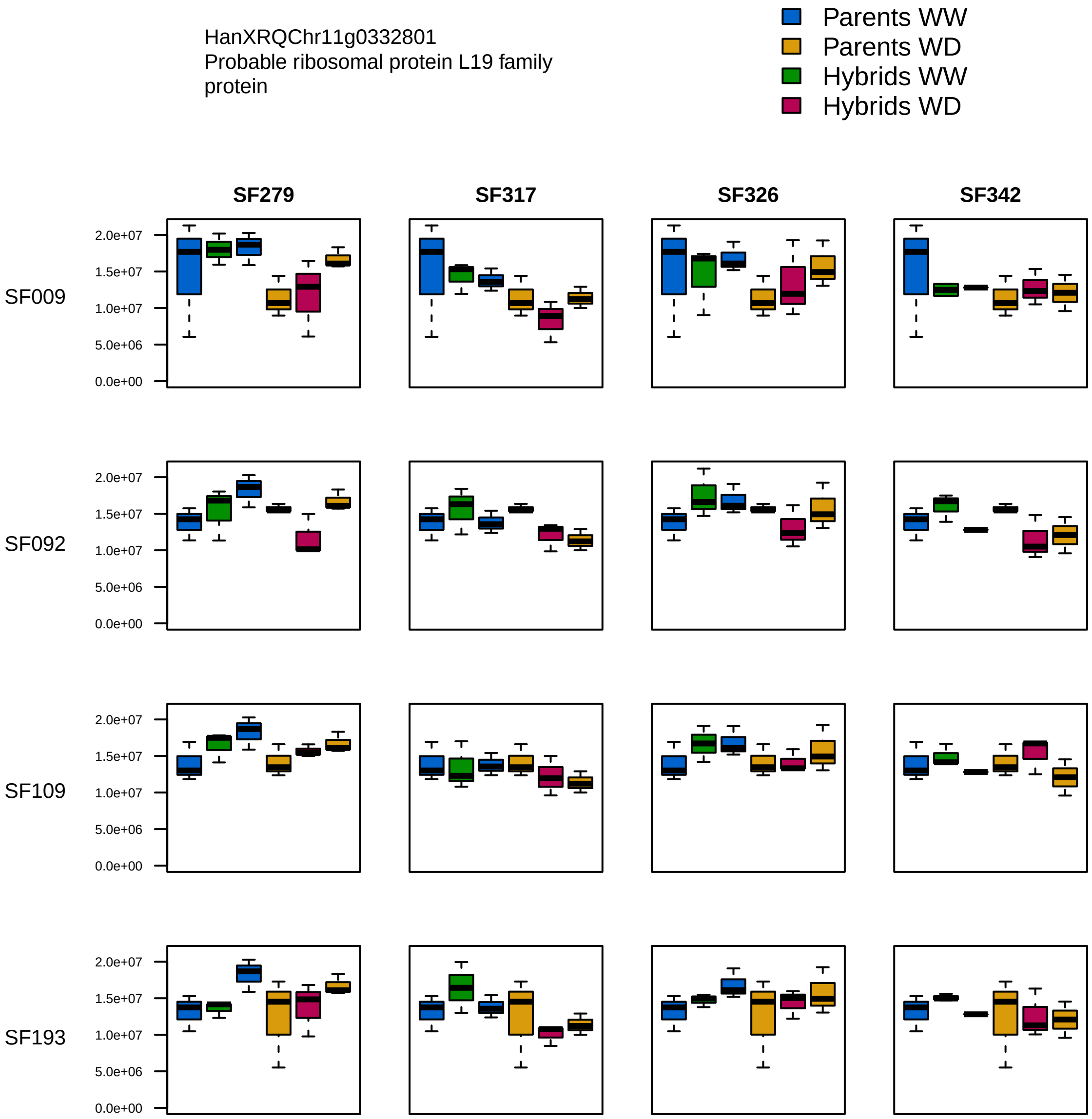

HanXRQChr11g0332801
Probable ribosomal protein L19 family protein
Parents WW
Parents WD
Hybrids WW
Hybrids WD
SF279
SF317
SF326
SF342
SF009
SF092
SF109
SF193
2.0e+07
1.5e+07
1.0e+07
5.0e+06
0.0e+00

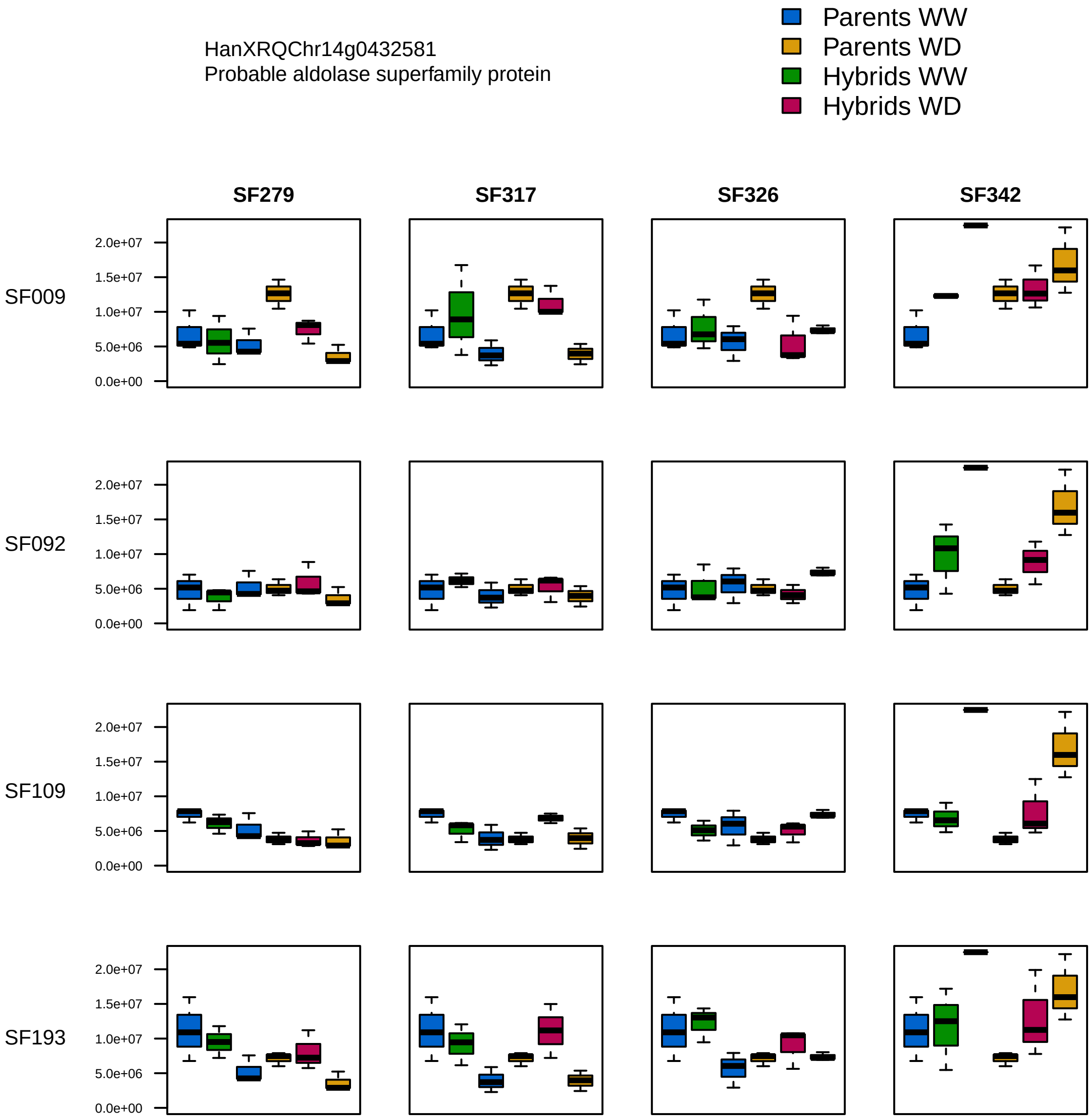
HanXRQChr14g0432581
Probable aldolase superfamily protein
Parents WW
Parents WD
Hybrids WW
Hybrids WD
SF279
SF317
SF326
SF342
SF009
SF092
SF109
SF193
2.0e+07
1.5e+07
1.0e+07
5.0e+06
0.0e+00

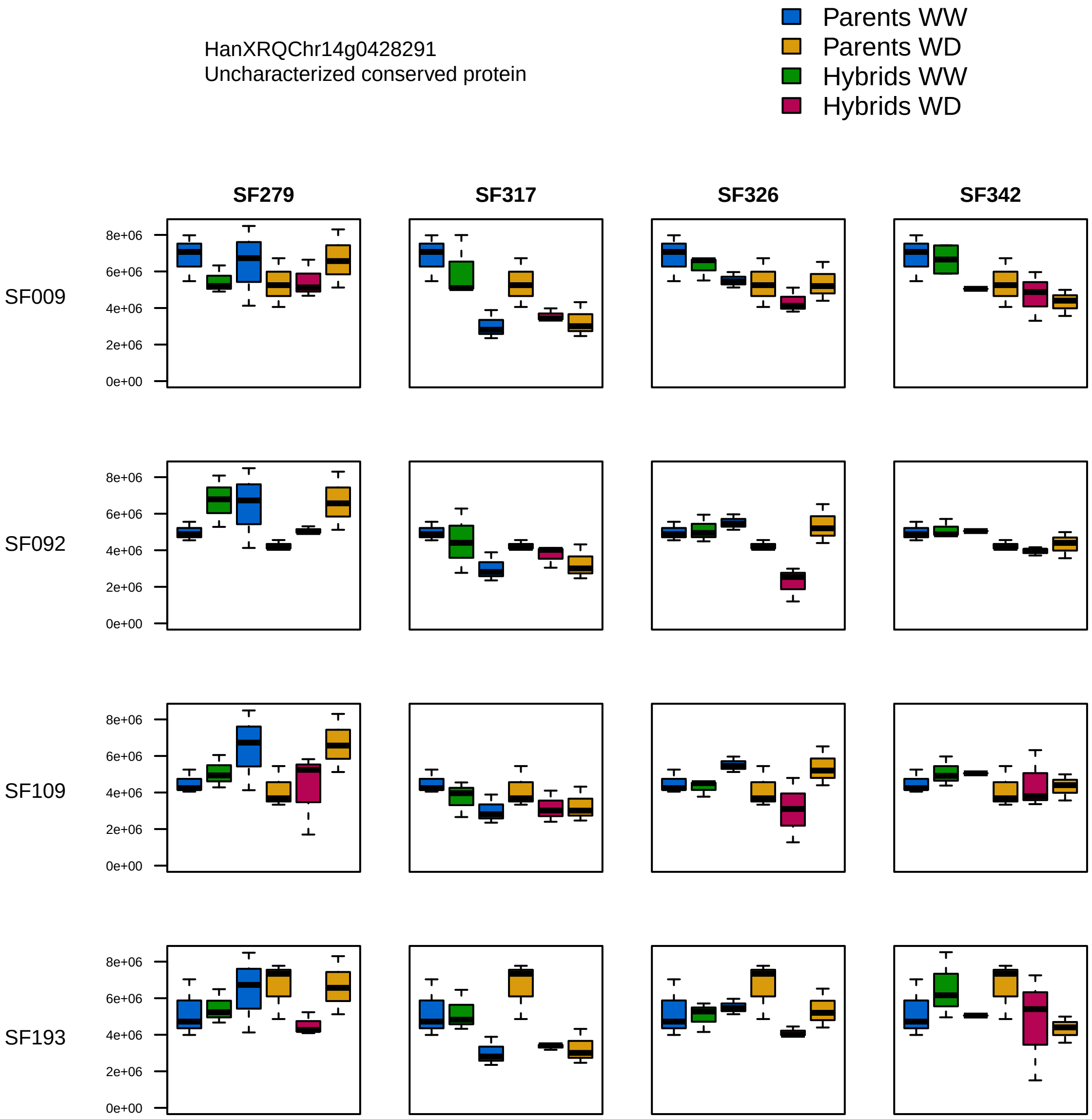
HanXRQChr14g0428291
Uncharacterized conserved protein
Parents WW
Parents WD
Hybrids WW
Hybrids WD
SF279
SF317
SF326
SF342
SF009
SF092
SF109
SF193
8e+06
6e+06
4e+06
2e+06
0e+00

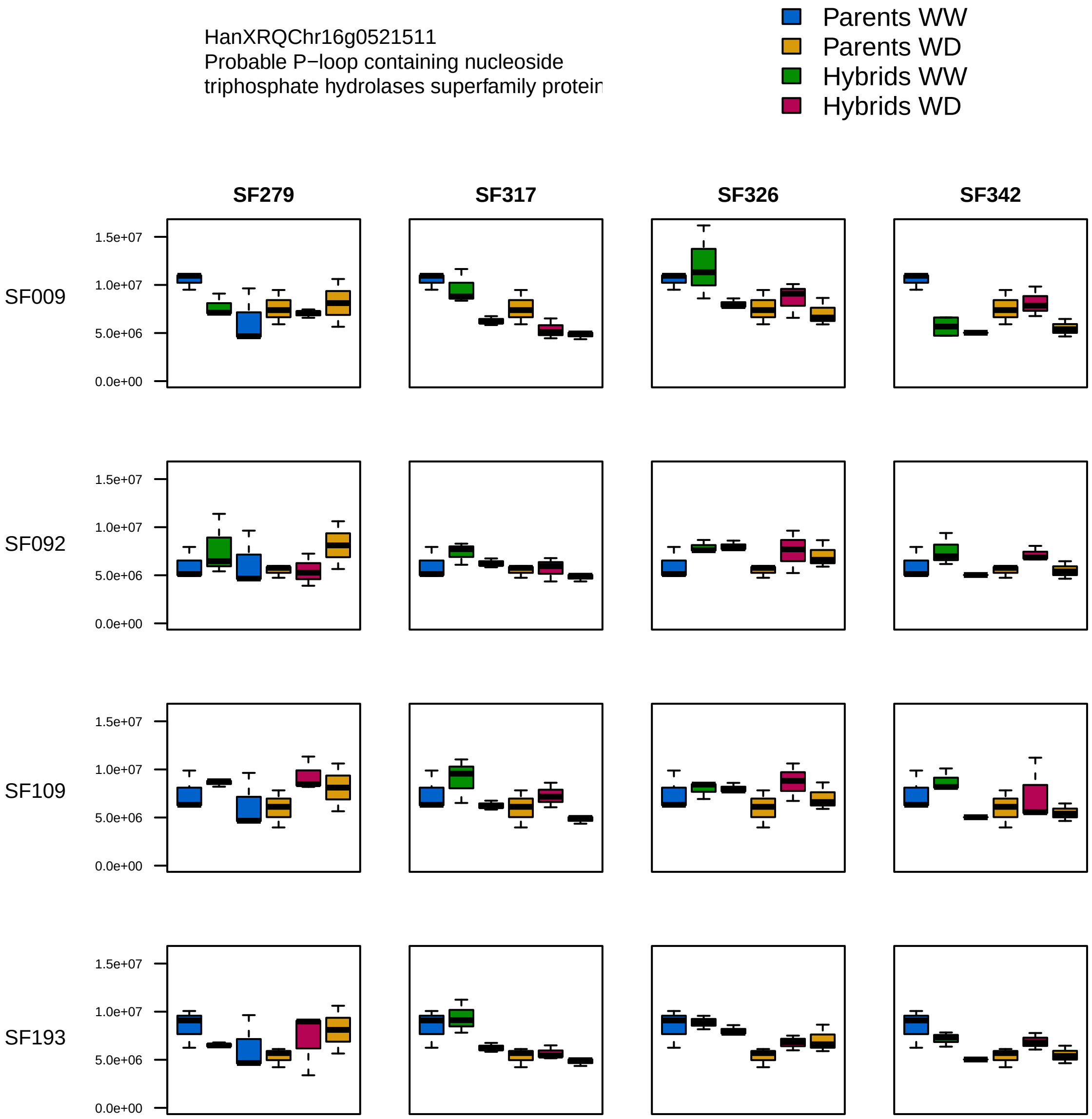

HanXRQChr16g0521511
Probable P−loop containing nucleoside triphosphate hydrolases superfamily protein
Parents WW
Parents WD
Hybrids WW
Hybrids WD
SF279
SF317
SF326
SF342
SF009
SF092
SF109
SF193
1.5e+07
1.0e+07
5.0e+06
0.0e+00

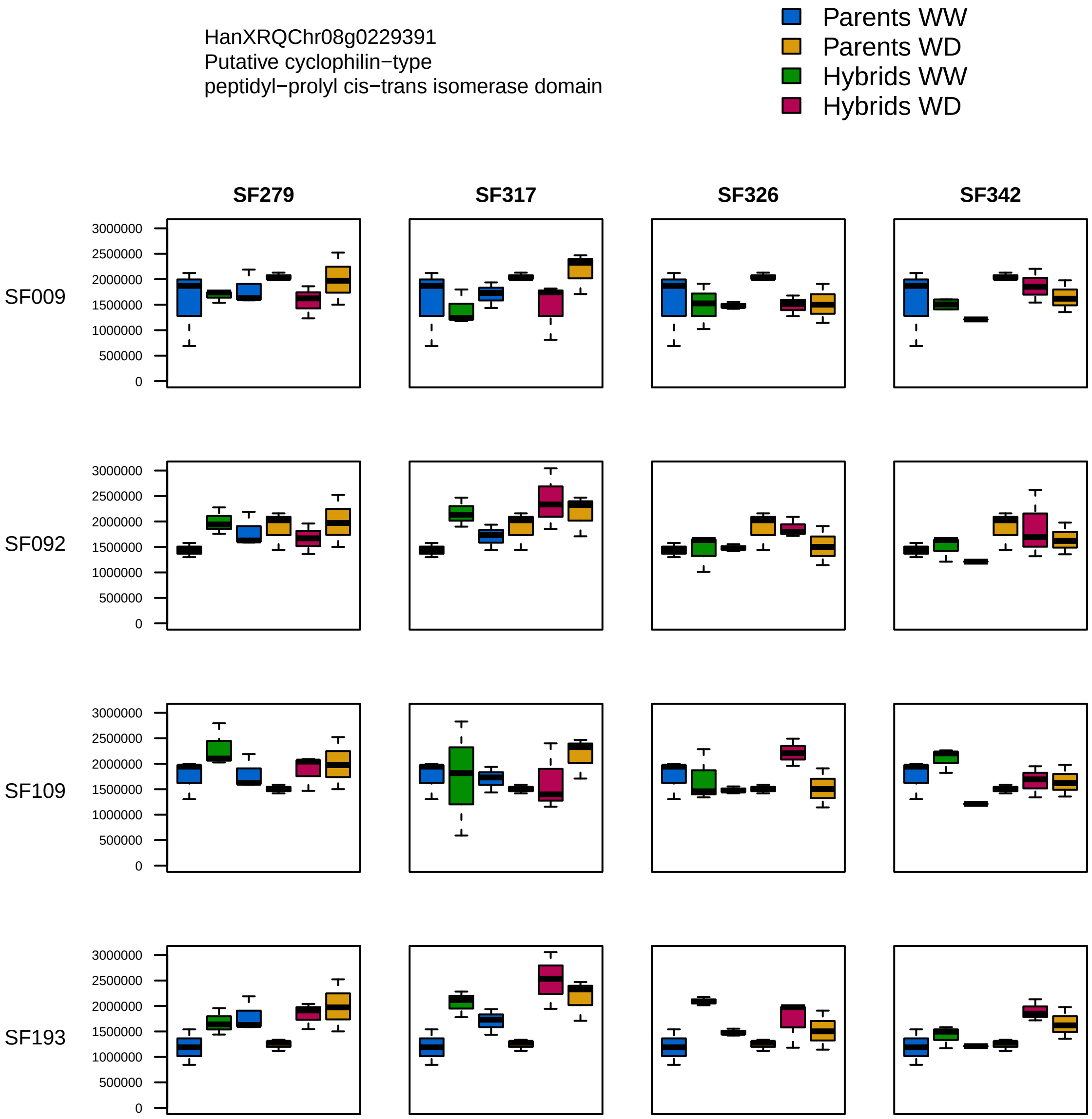
HanXRQChr08g0229391
Putative cyclophilin−type
peptidyl−prolyl cis−trans isomerase domain
Parents WW
Parents WD
Hybrids WW
Hybrids WD
SF279
SF317
SF326
SF342
SF009
SF092
SF109
SF193
3000000
2500000
2000000
1500000
1000000
500000
0

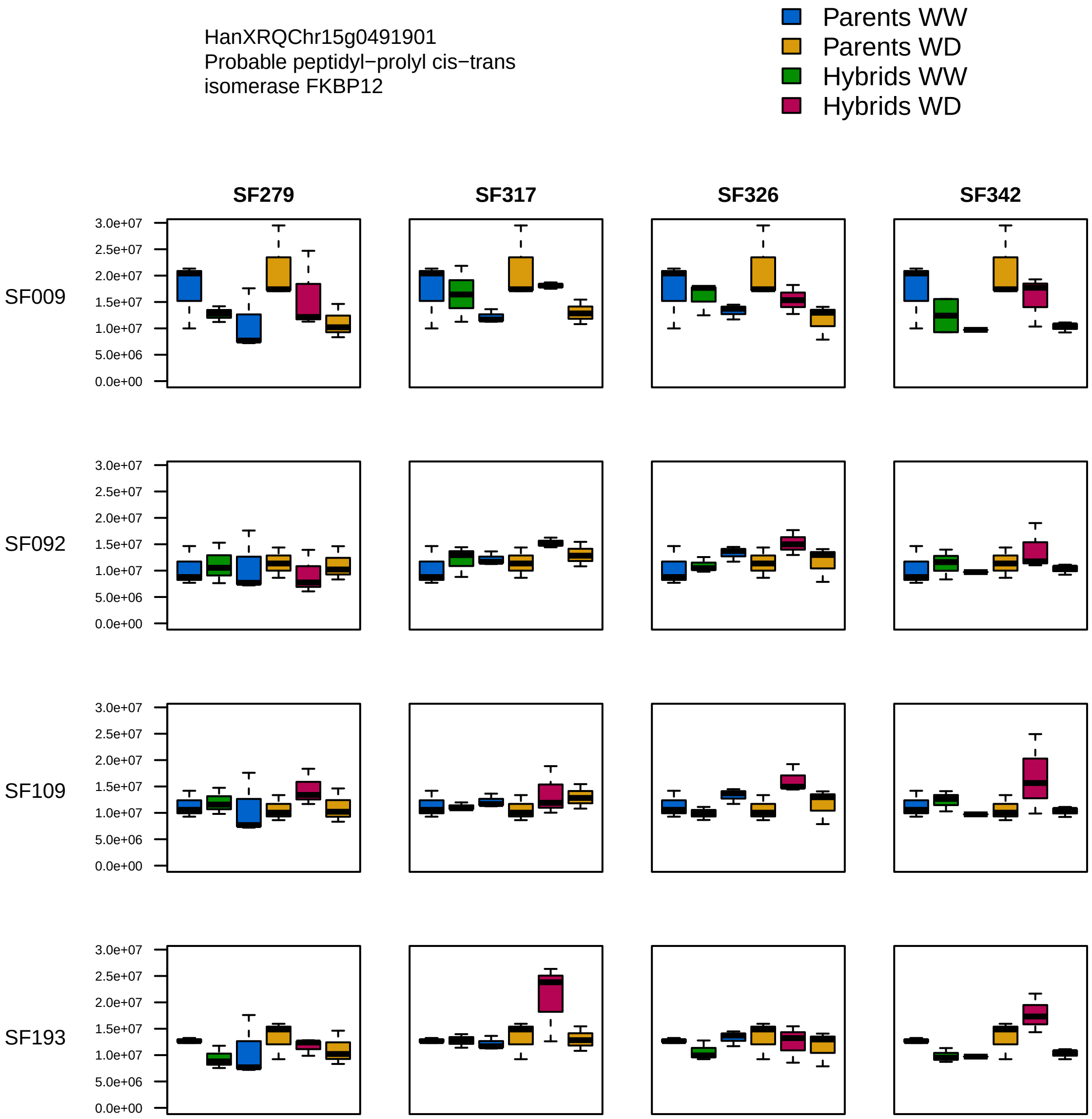
HanXRQChr15g0491901
Probable peptidyl−prolyl cis−trans
isomerase FKBP12
Parents WW
Parents WD
Hybrids WW
Hybrids WD
SF279
SF317
SF326
SF342
SF009
SF092
SF109
SF193
3.0e+07
2.5e+07
2.0e+07
1.5e+07
1.0e+07
5.0e+06
0.0e+00

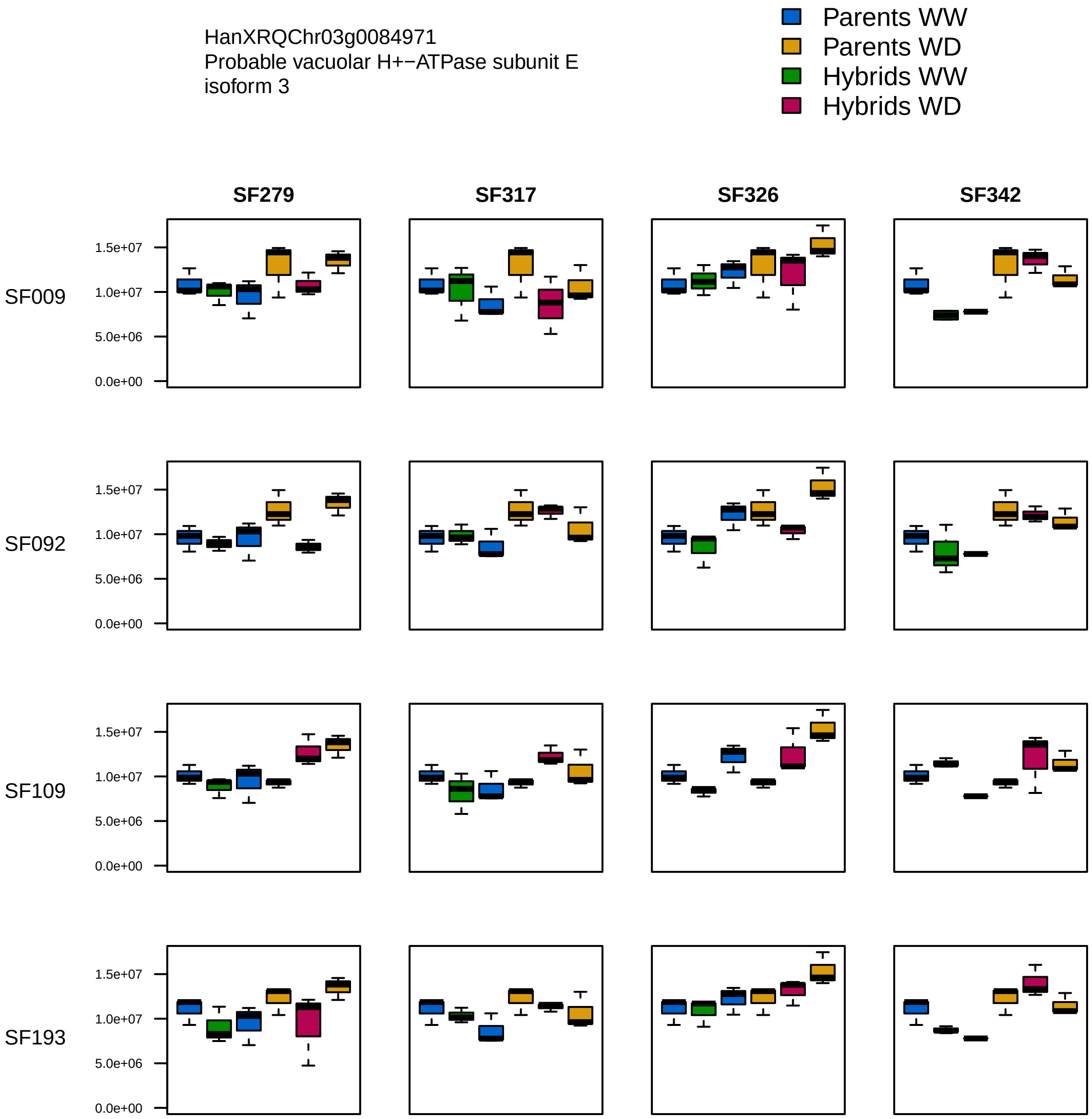

HanXRQChr03g0084971
Probable vacuolar H+−ATPase subunit E
isoform 3
Parents WW
Parents WD
Hybrids WW
Hybrids WD
SF279
SF317
SF326
SF342
SF009
SF092
SF109
SF193
1.5e+07
1.0e+07
5.0e+06
0.0e+00

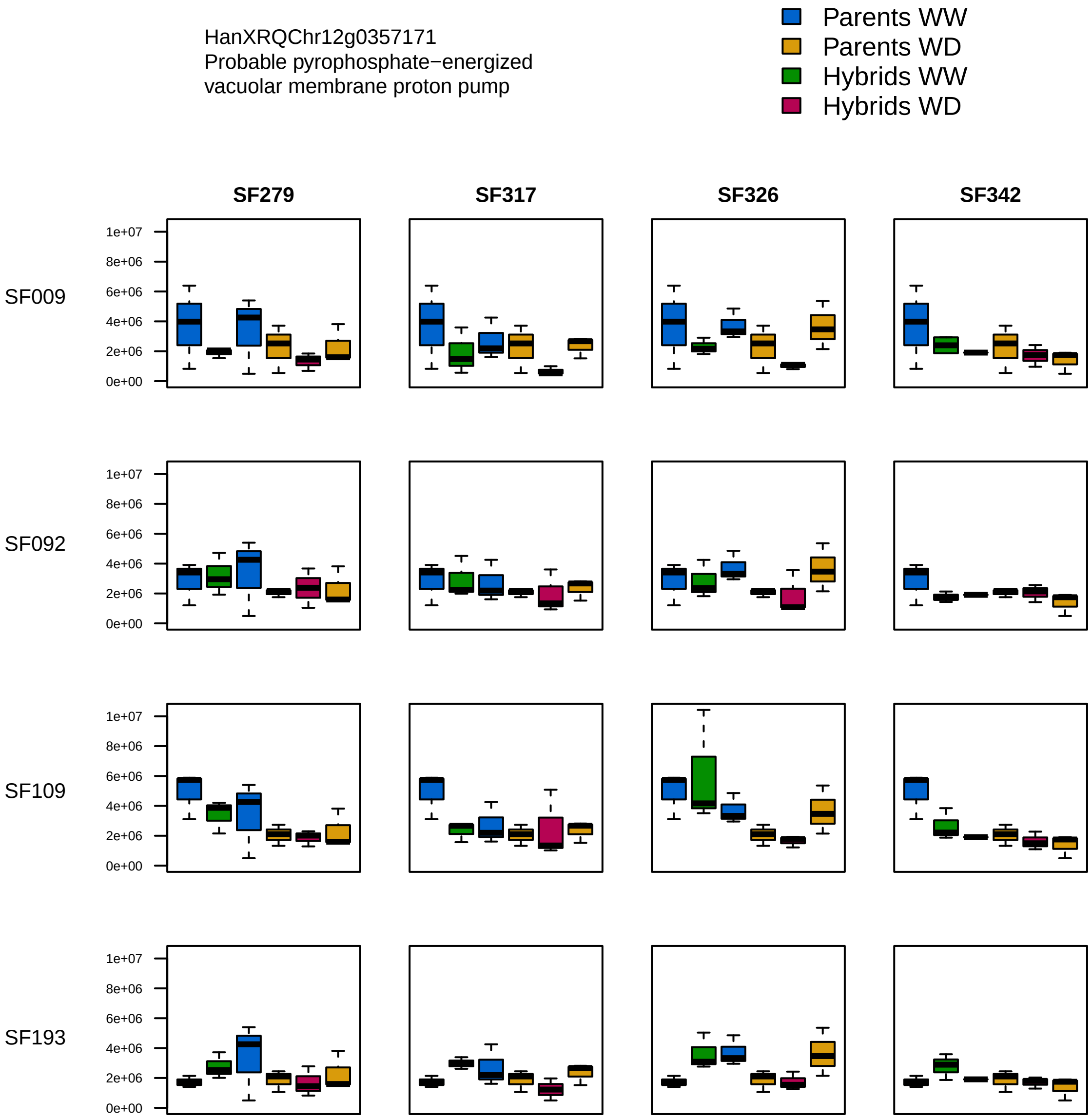
HanXRQChr12g0357171
Probable pyrophosphate−energized
vacuolar membrane proton pump
Parents WW
Parents WD
Hybrids WW
Hybrids WD
SF279
SF317
SF326
SF342
SF009
SF092
SF109
SF193
1e+07
8e+06
6e+06
4e+06
2e+06
0e+00

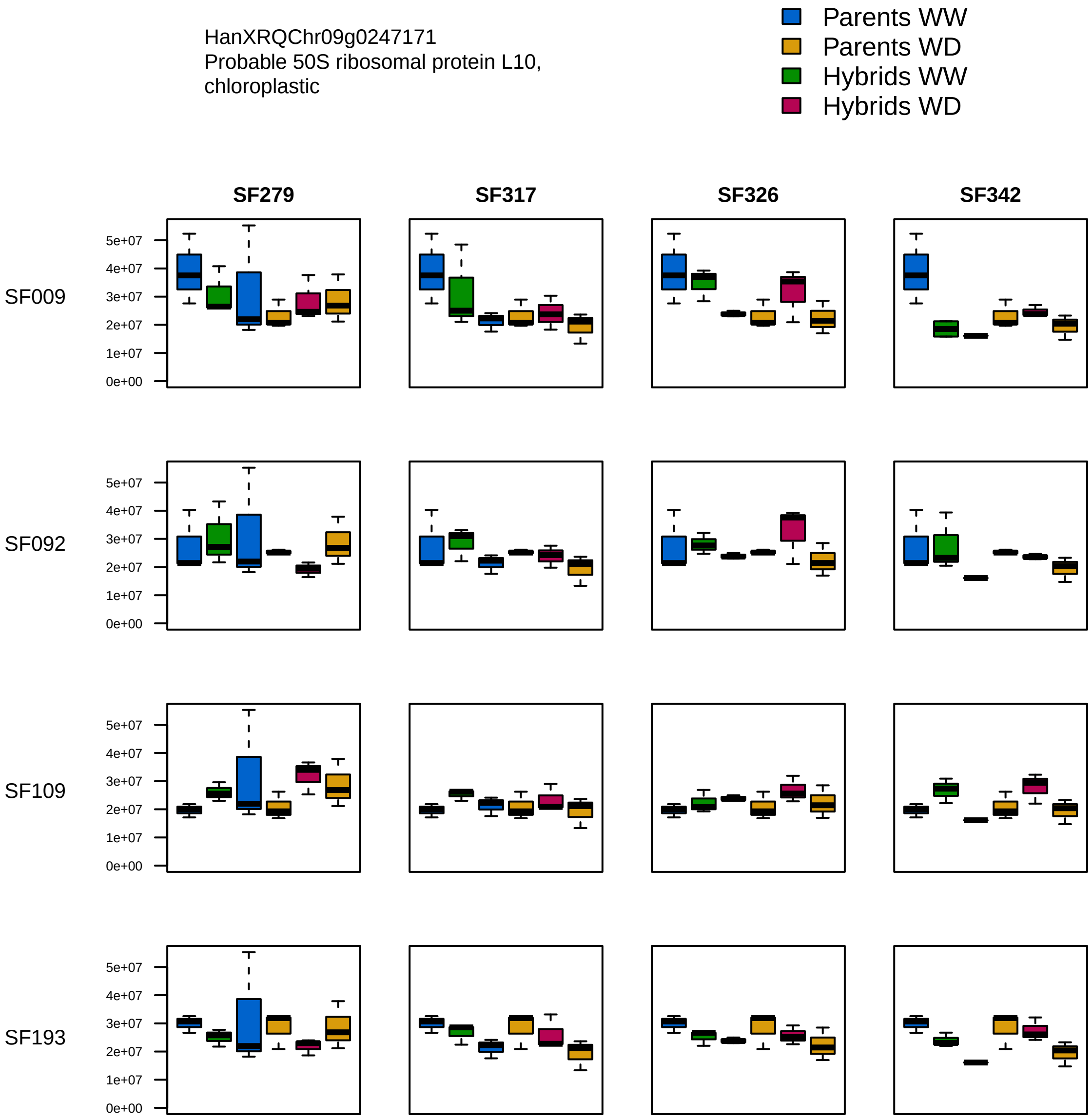

HanXRQChr09g0247171
Probable 50S ribosomal protein L10, chloroplastic
Parents WW
Parents WD
Hybrids WW
Hybrids WD
SF279
SF317
SF326
SF342
SF009
SF092
SF109
SF193
0e+00
1e+07
2e+07
3e+07
4e+07
5e+07

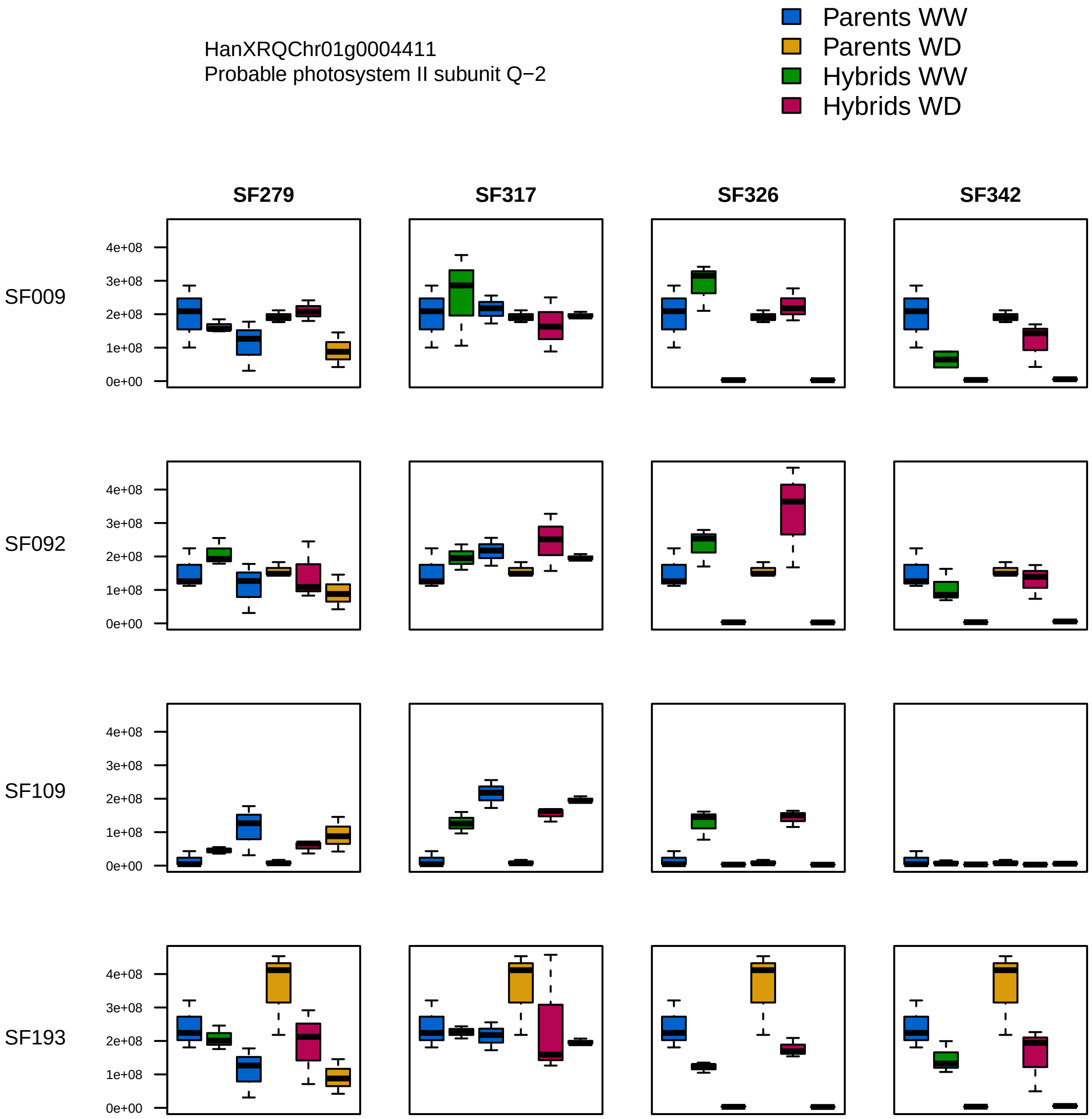
HanXRQChr01g0004411
Probable photosystem II subunit Q−2
Parents WW
Parents WD
Hybrids WW
Hybrids WD
SF279
SF317
SF326
SF342
SF009
SF092
SF109
SF193
0e+00
1e+08
2e+08
3e+08
4e+08

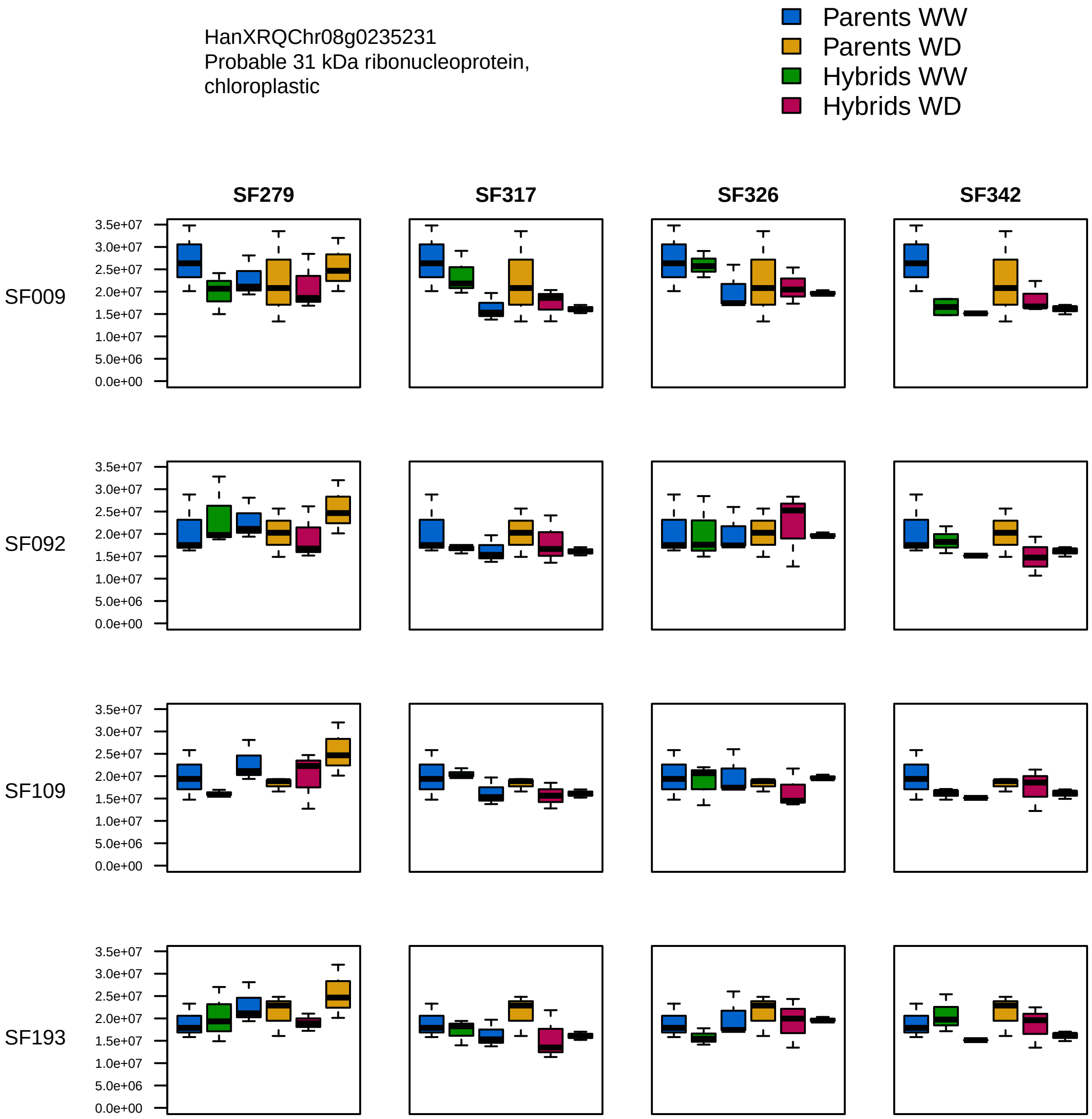
HanXRQChr08g0235231
Probable 31 kDa ribonucleoprotein, chloroplastic
Parents WW
Parents WD
Hybrids WW
Hybrids WD
SF279
SF317
SF326
SF342
SF009
SF092
SF109
SF193
3.5e+07
3.0e+07
2.5e+07
2.0e+07
1.5e+07
1.0e+07
5.0e+06
0.0e+00

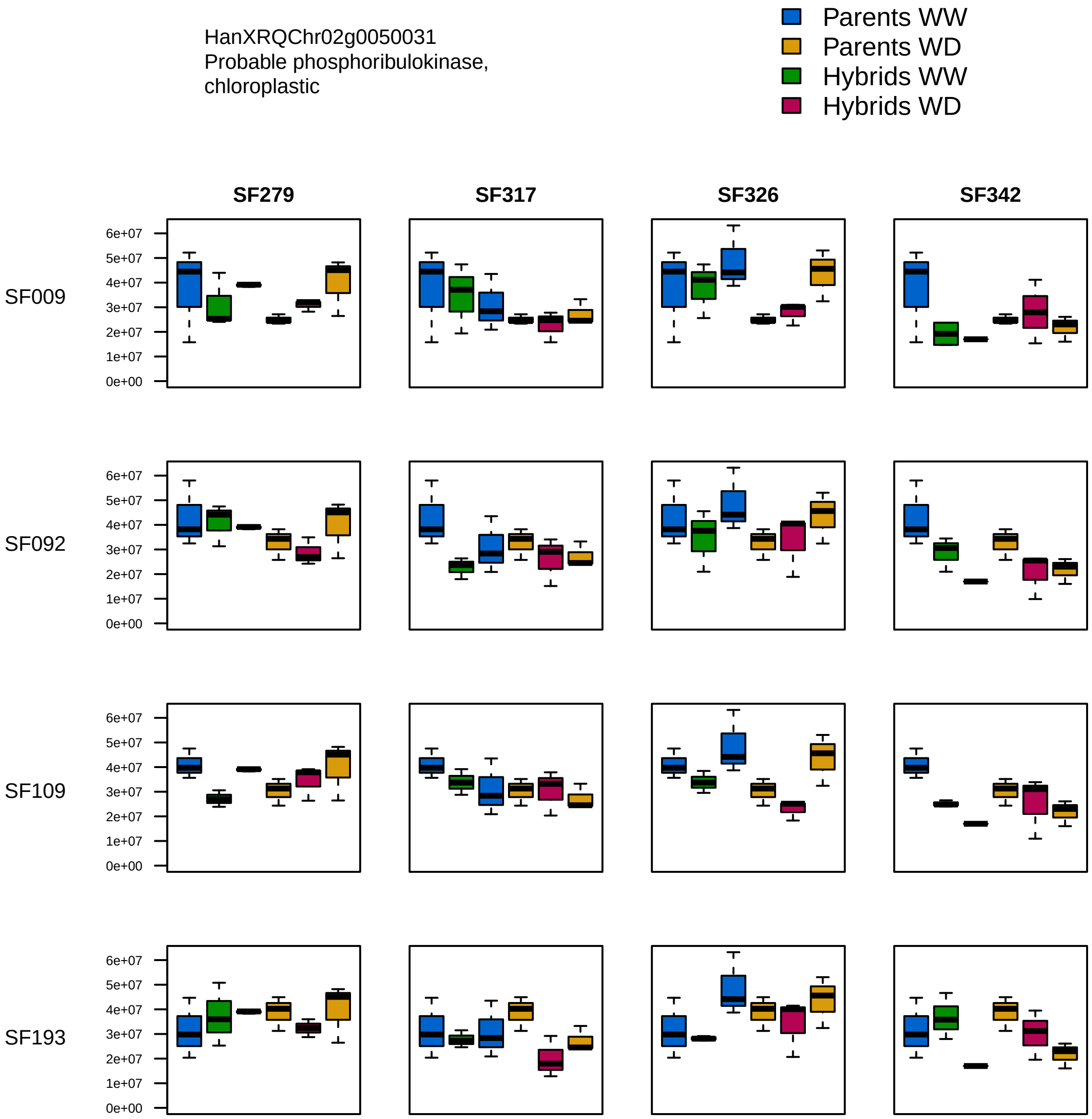

HanXRQChr02g0050031
Probable phosphoribulokinase, chloroplastic
Parents WW
Parents WD
Hybrids WW
Hybrids WD
SF279
SF317
SF326
SF342
SF009
SF092
SF109
SF193
6e+07
5e+07
4e+07
3e+07
2e+07
1e+07
0e+00

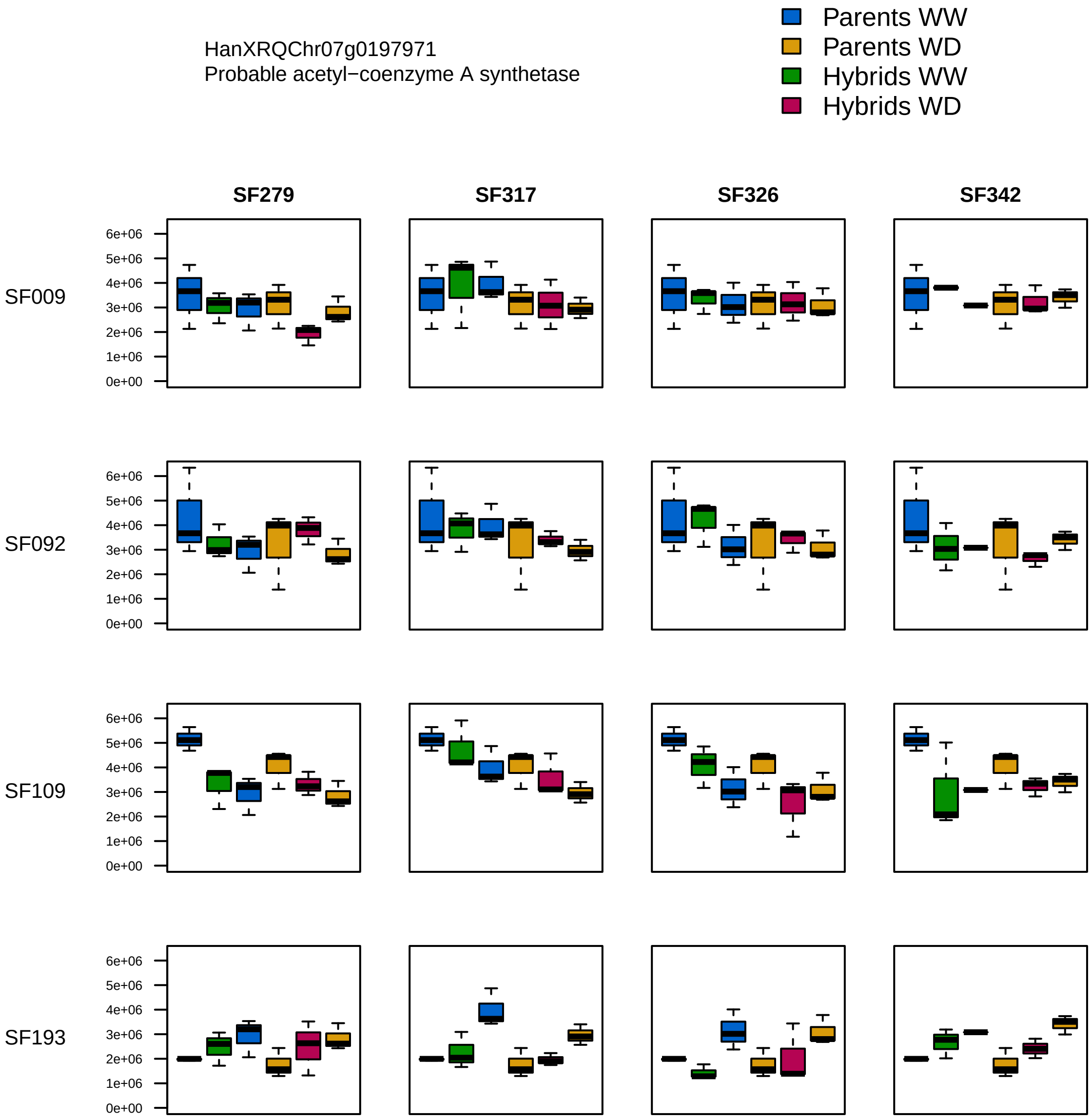

HanXRQChr07g0197971
Probable acetyl−coenzyme A synthetase
Parents WW
Parents WD
Hybrids WW
Hybrids WD
SF279
SF317
SF326
SF342
SF009
SF092
SF109
SF193
6e+06
5e+06
4e+06
3e+06
2e+06
1e+06
0e+00

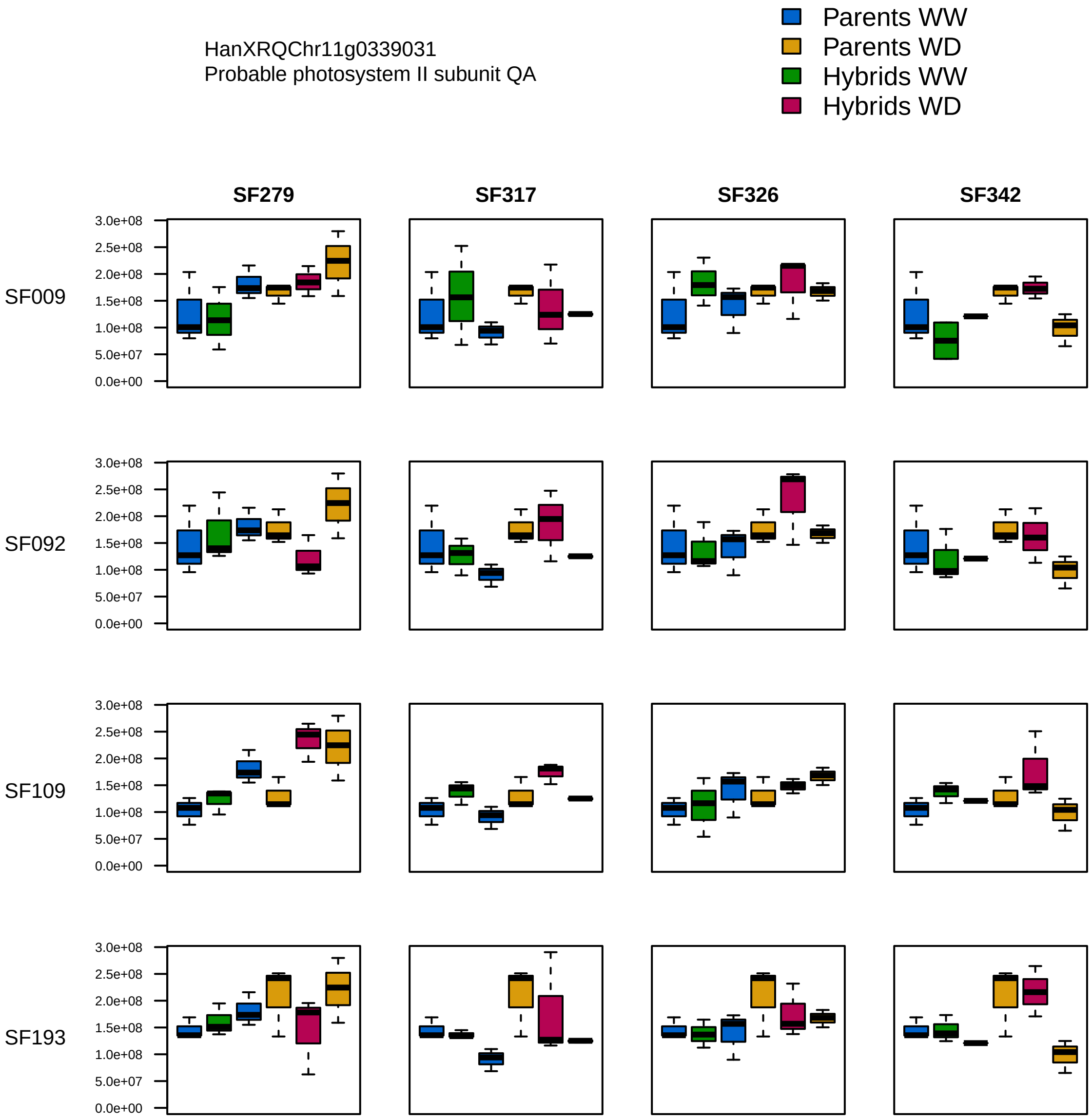
HanXRQChr11g0339031
Probable photosystem II subunit QA
Parents WW
Parents WD
Hybrids WW
Hybrids WD
SF279
SF317
SF326
SF342
SF009
SF092
SF109
SF193
3.0e+08
2.5e+08
2.0e+08
1.5e+08
1.0e+08
5.0e+07
0.0e+00

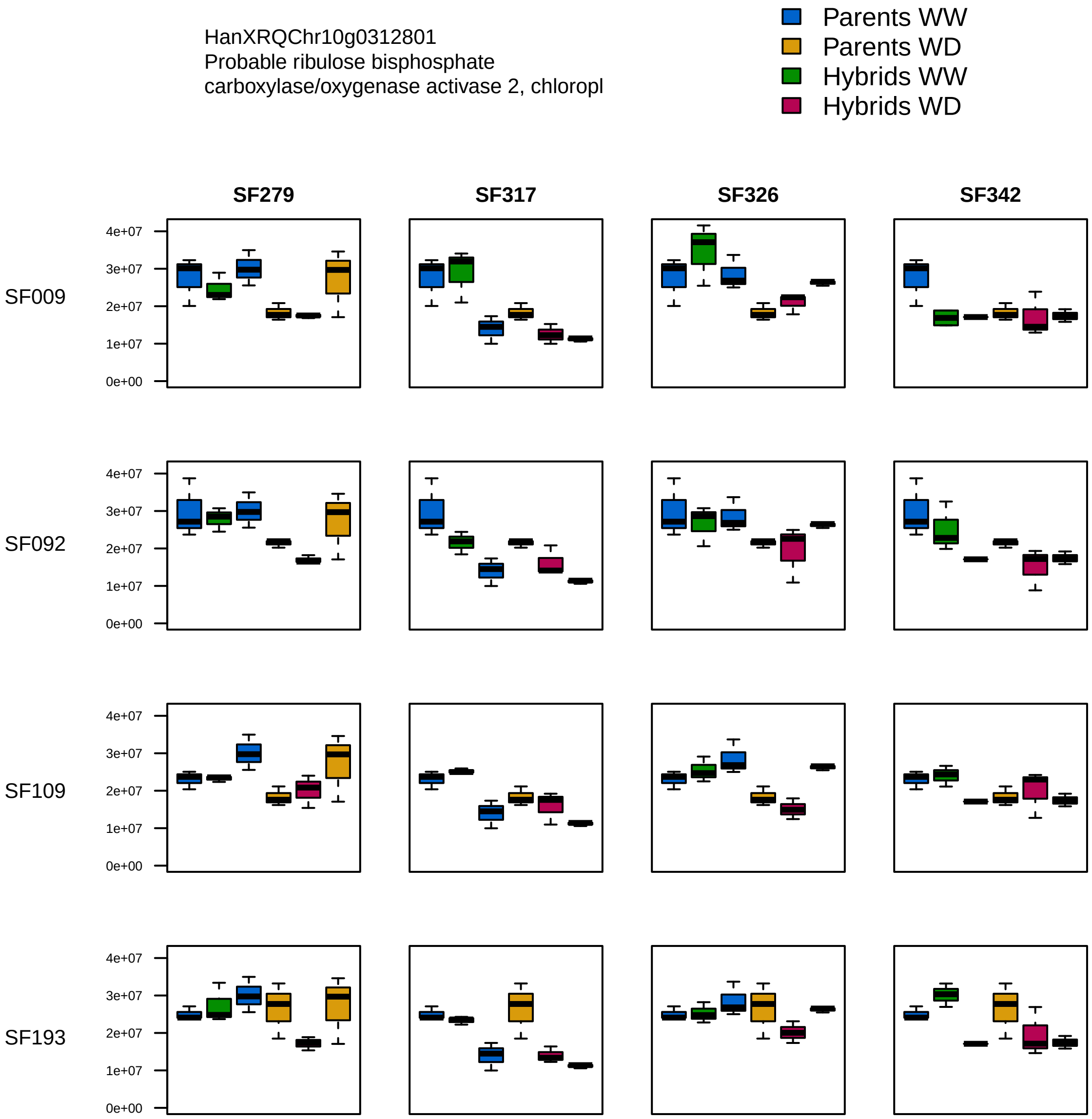

HanXRQChr10g0312801
Probable ribulose bisphosphate
carboxylase/oxygenase activase 2, chloropl
Parents WW
Parents WD
Hybrids WW
Hybrids WD
SF279
SF317
SF326
SF342
SF009
SF092
SF109
SF193
4e+07
3e+07
2e+07
1e+07
0e+00

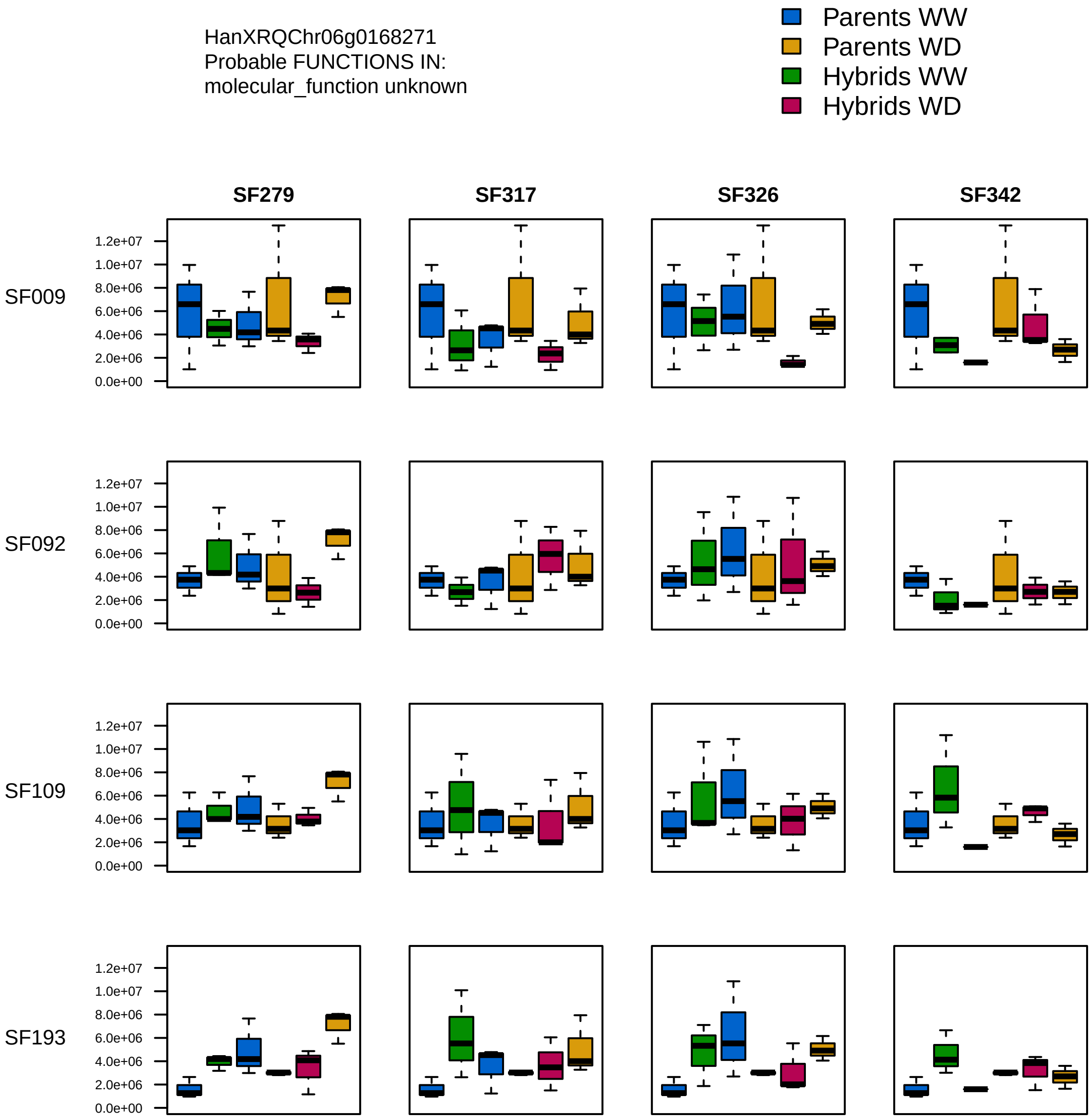

HanXRQChr06g0168271
Probable FUNCTIONS IN:
molecular_function unknown
Parents WW
Parents WD
Hybrids WW
Hybrids WD
SF279
SF317
SF326
SF342
SF009
SF092
SF109
SF193
1.2e+07
1.0e+07
8.0e+06
6.0e+06
4.0e+06
2.0e+06
0.0e+00

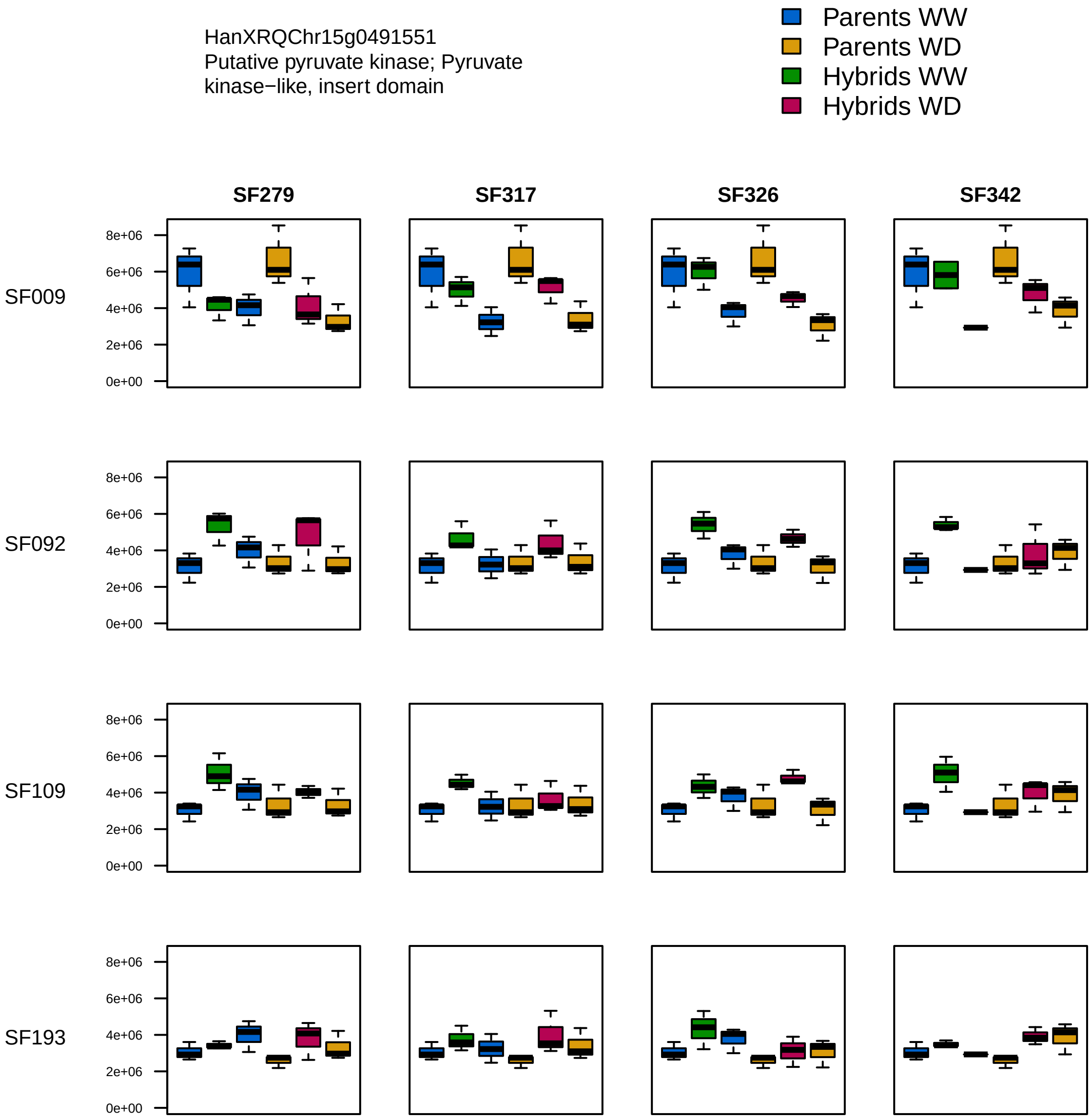
HanXRQChr15g0491551
Putative pyruvate kinase; Pyruvate kinase−like, insert domain
Parents WW
Parents WD
Hybrids WW
Hybrids WD
SF279
SF317
SF326
SF342
SF009
SF092
SF109
SF193
0e+00
2e+06
4e+06
6e+06
8e+06

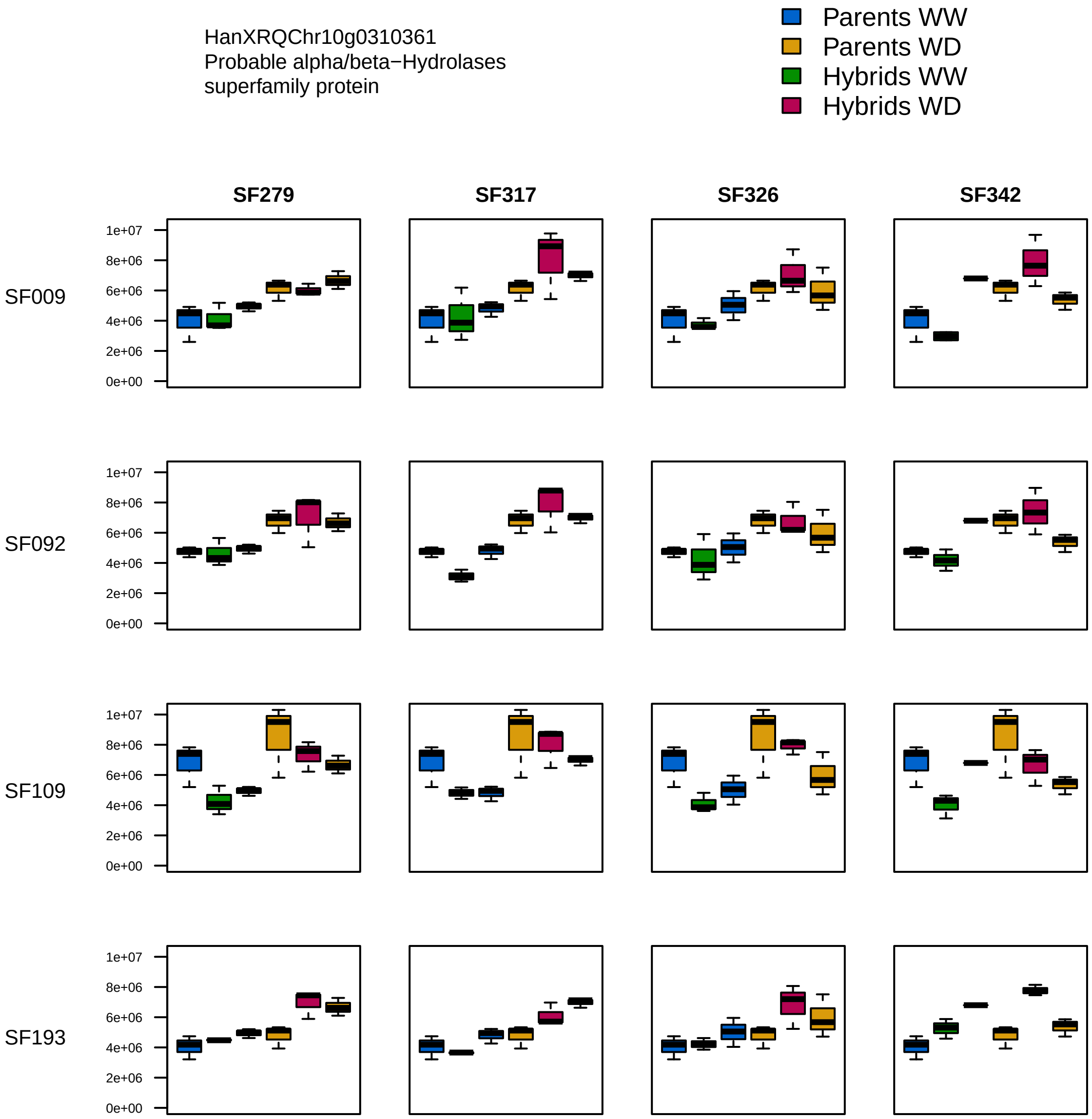
HanXRQChr10g0310361
Probable alpha/beta−Hydrolases
superfamily protein
Parents WW
Parents WD
Hybrids WW
Hybrids WD
SF279
SF317
SF326
SF342
SF009
SF092
SF109
SF193
0e+00
2e+06
4e+06
6e+06
8e+06
1e+07

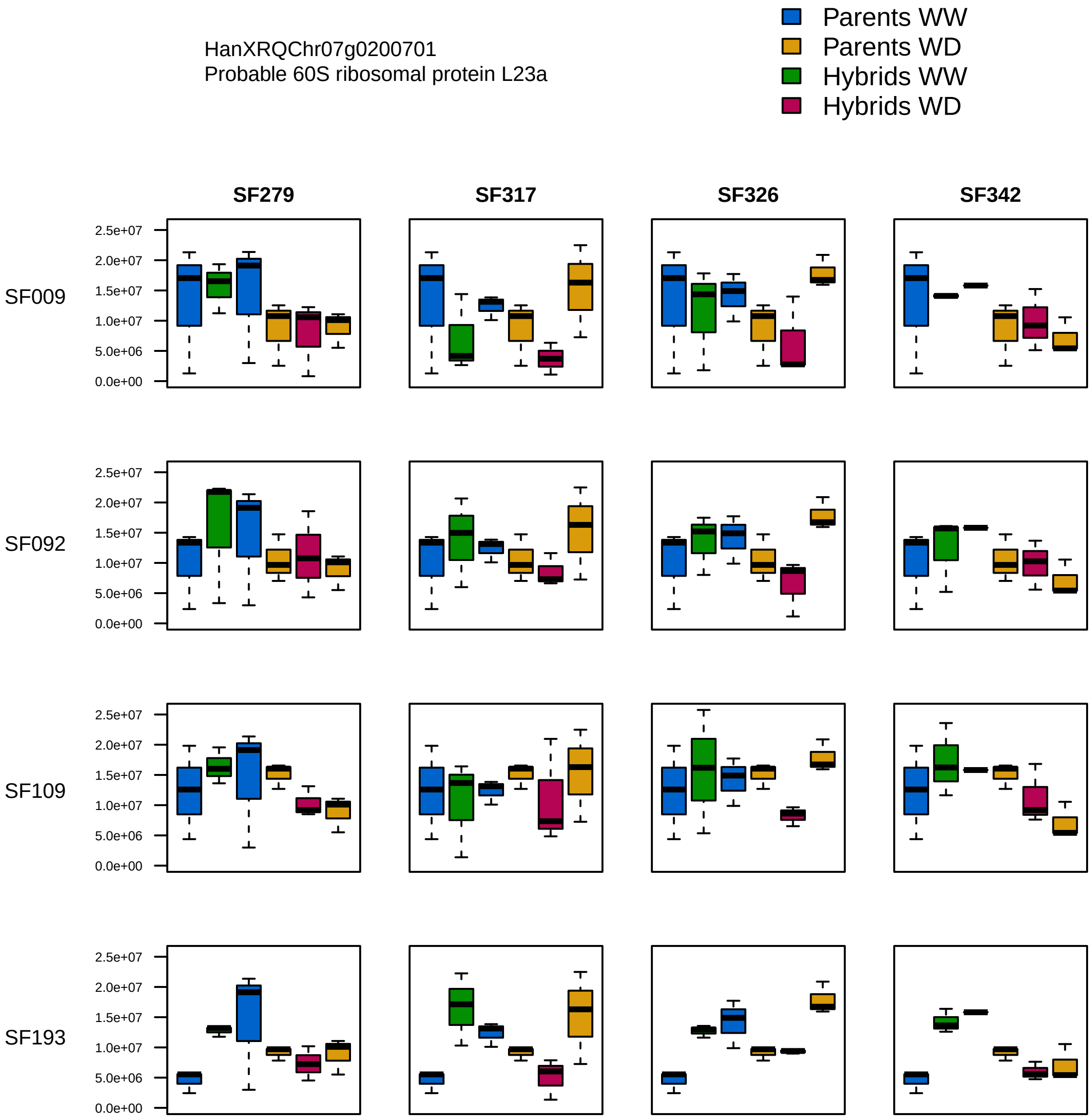
HanXRQChr07g0200701
Probable 60S ribosomal protein L23a
Parents WW
Parents WD
Hybrids WW
Hybrids WD
SF279
SF317
SF326
SF342
SF009
SF092
SF109
SF193
0.0e+00
5.0e+06
1.0e+07
1.5e+07
2.0e+07
2.5e+07

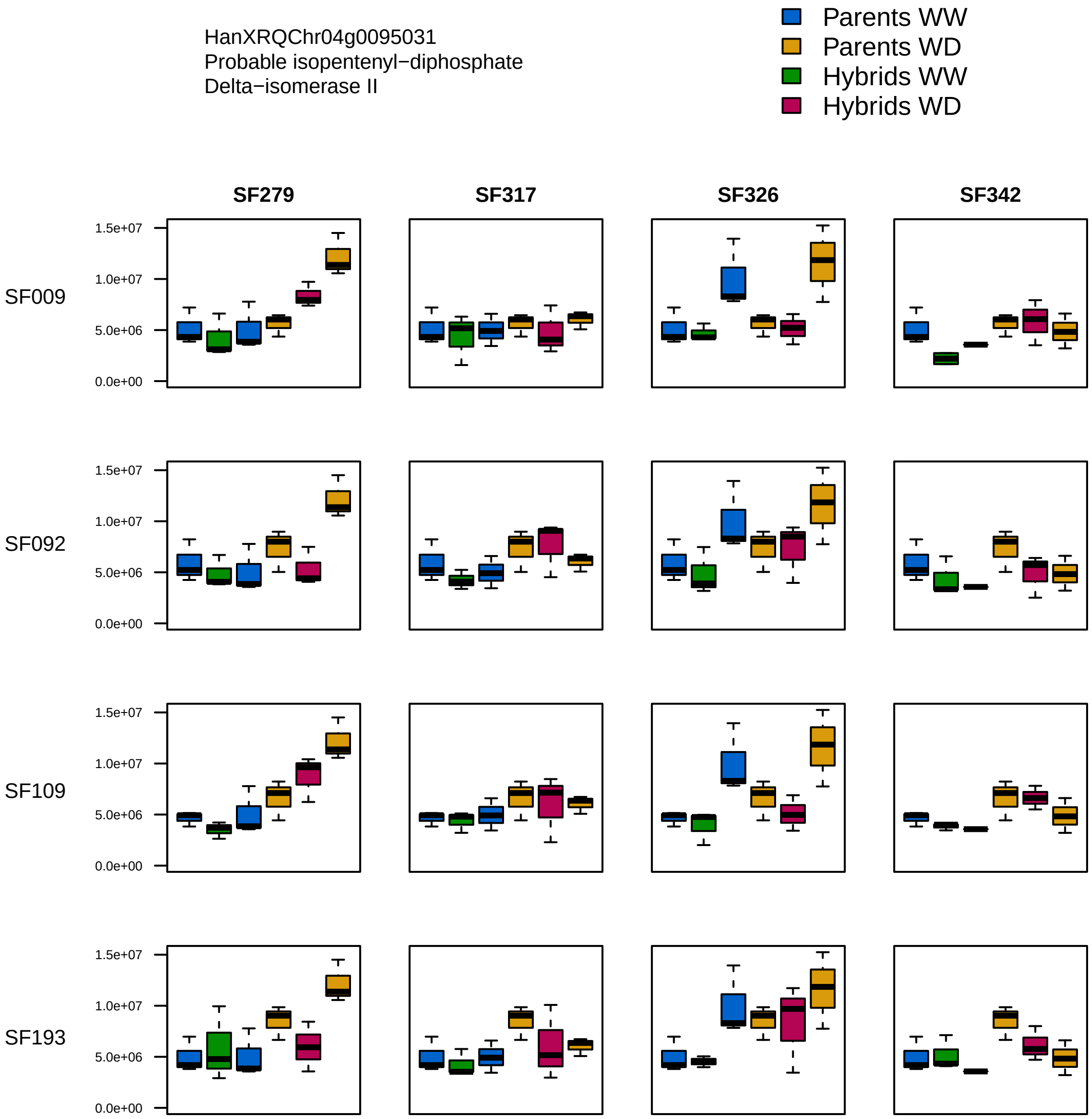

HanXRQChr04g0095031
Probable isopentenyl−diphosphate
Delta−isomerase II
Parents WW
Parents WD
Hybrids WW
Hybrids WD
SF279
SF317
SF326
SF342
SF009
SF092
SF109
SF193
1.5e+07
1.0e+07
5.0e+06
0.0e+00

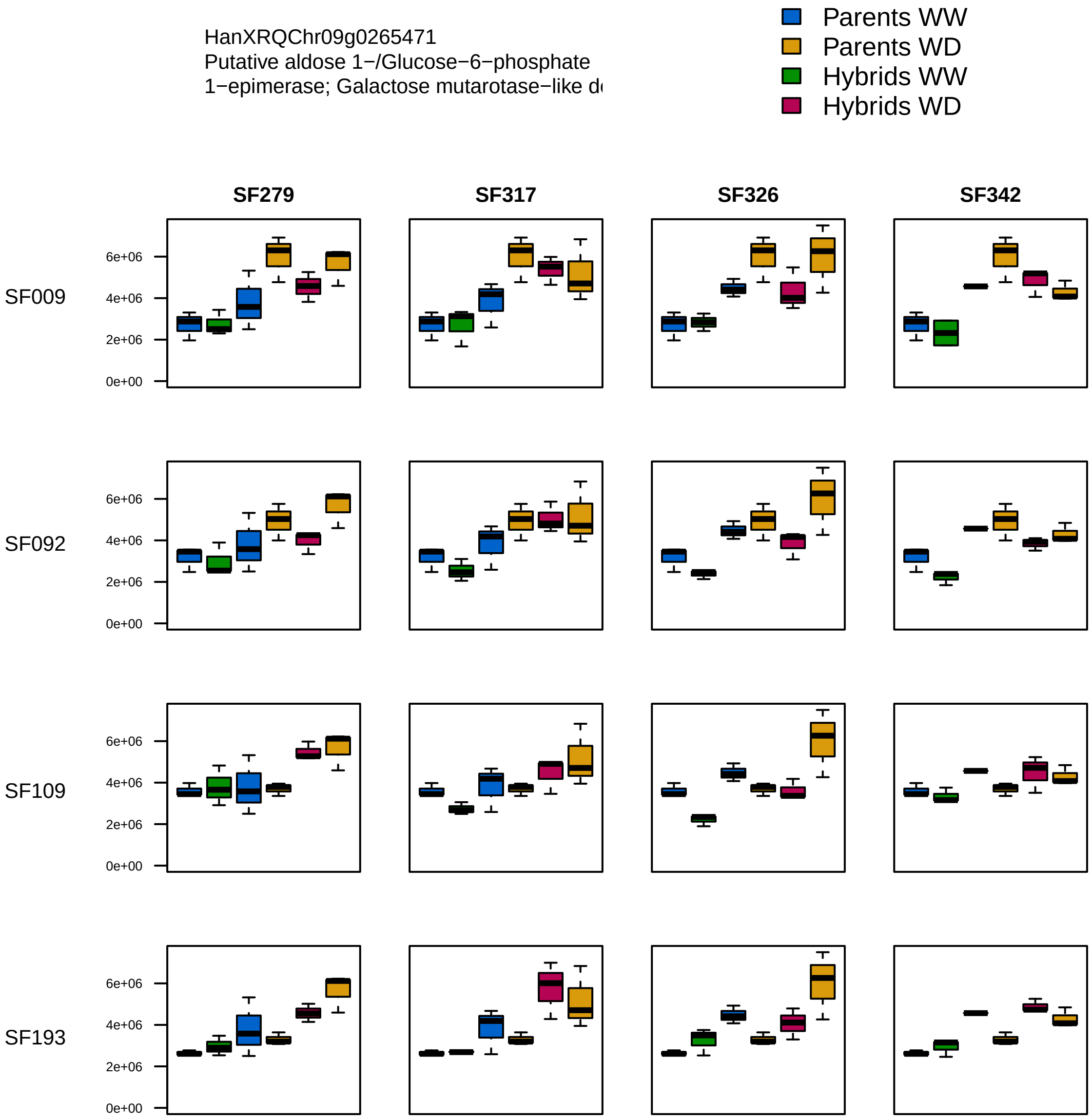
HanXRQChr09g0265471
Putative aldose 1−/Glucose−6−phosphate
1−epimerase; Galactose mutarotase−like d
Parents WW
Parents WD
Hybrids WW
Hybrids WD
SF279
SF317
SF326
SF342
SF009
SF092
SF109
SF193
0e+00
2e+06
4e+06
6e+06

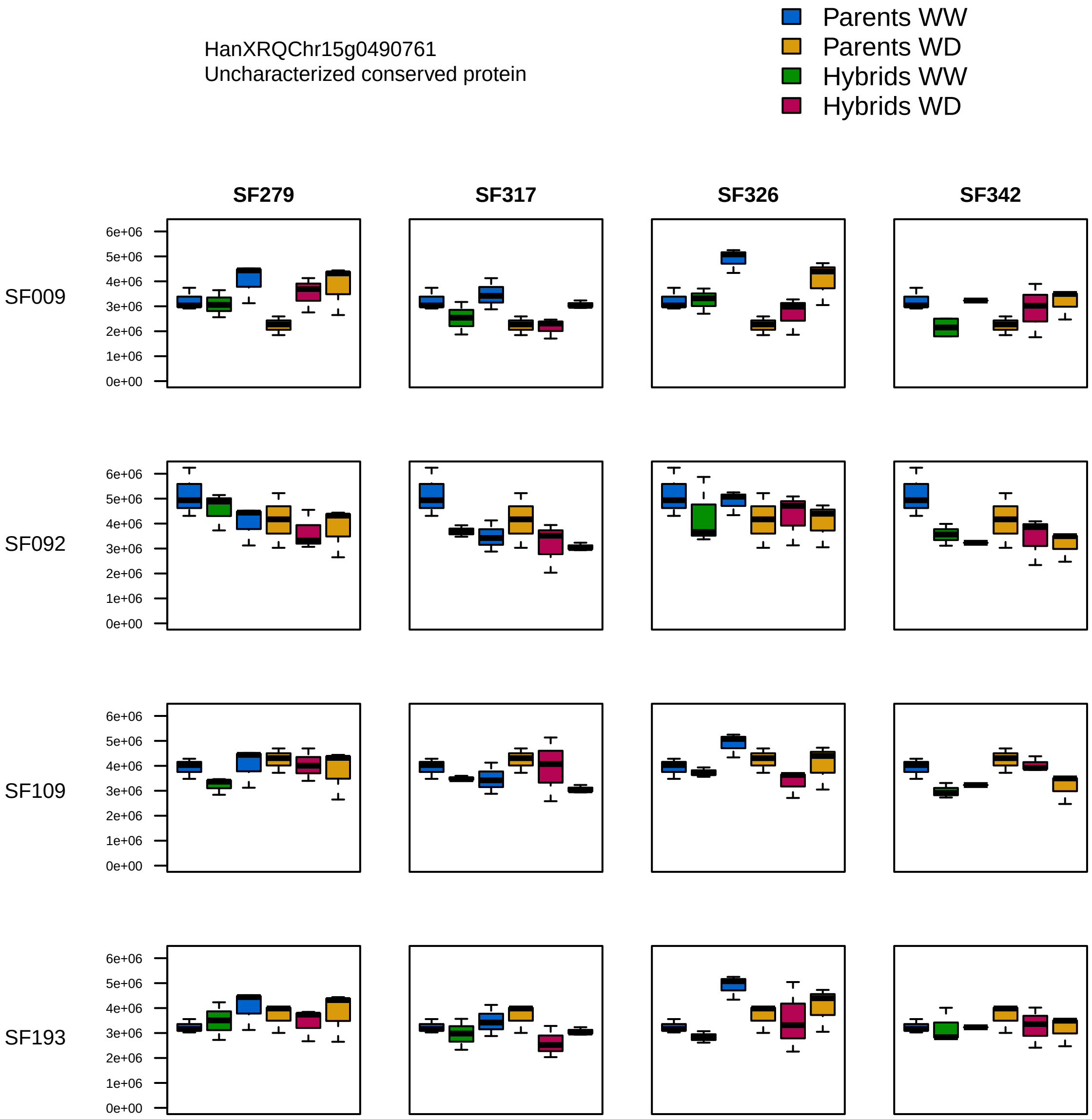
HanXRQChr15g0490761
Uncharacterized conserved protein
Parents WW
Parents WD
Hybrids WW
Hybrids WD
SF279
SF317
SF326
SF342
SF009
SF092
SF109
SF193
6e+06
5e+06
4e+06
3e+06
2e+06
1e+06
0e+00

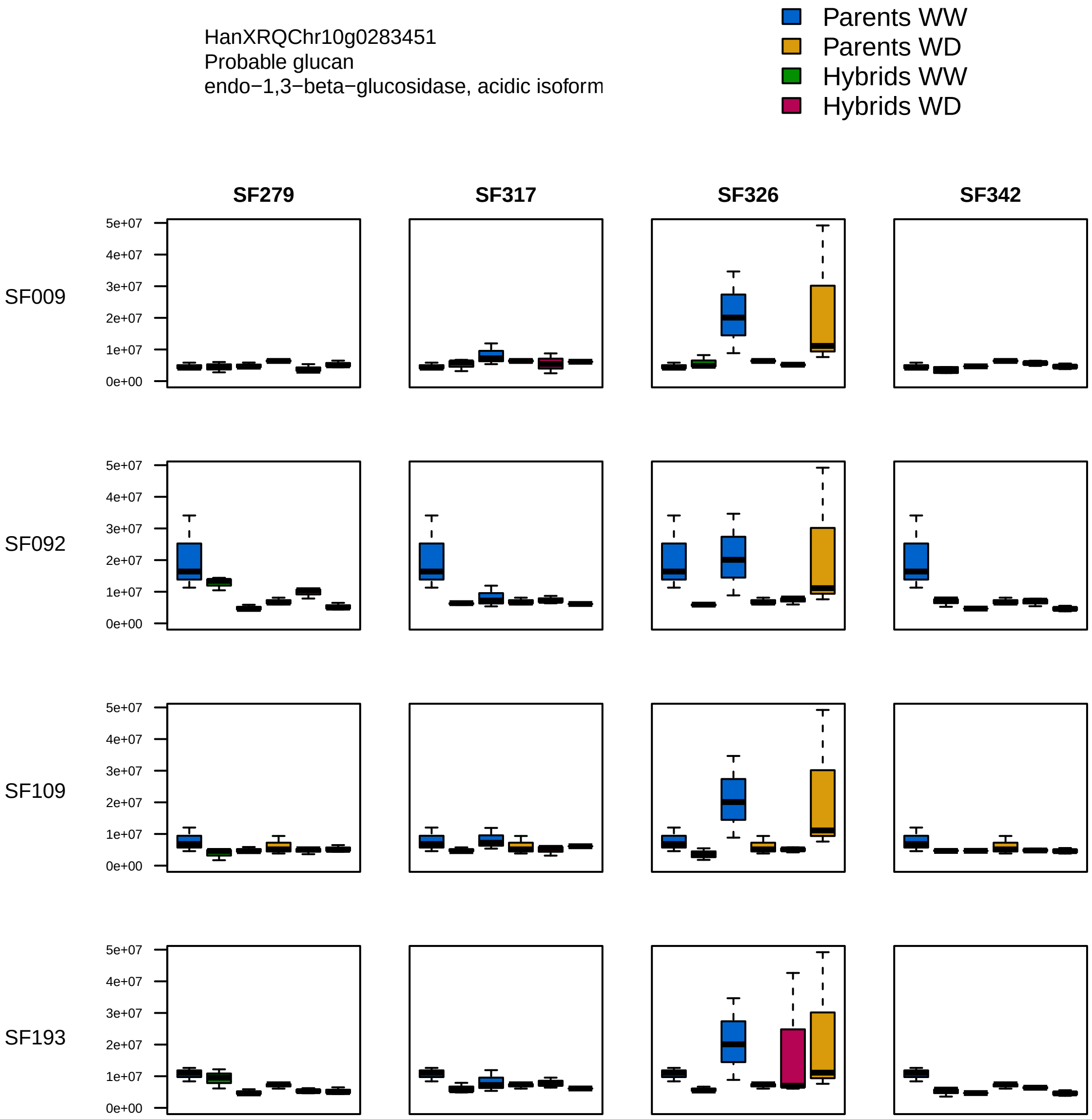
HanXRQChr10g0283451
Probable glucan
endo−1,3−beta−glucosidase, acidic isoform
Parents WW
Parents WD
Hybrids WW
Hybrids WD
SF279
SF317
SF326
SF342
SF009
SF092
SF109
SF193
5e+07
4e+07
3e+07
2e+07
1e+07
0e+00

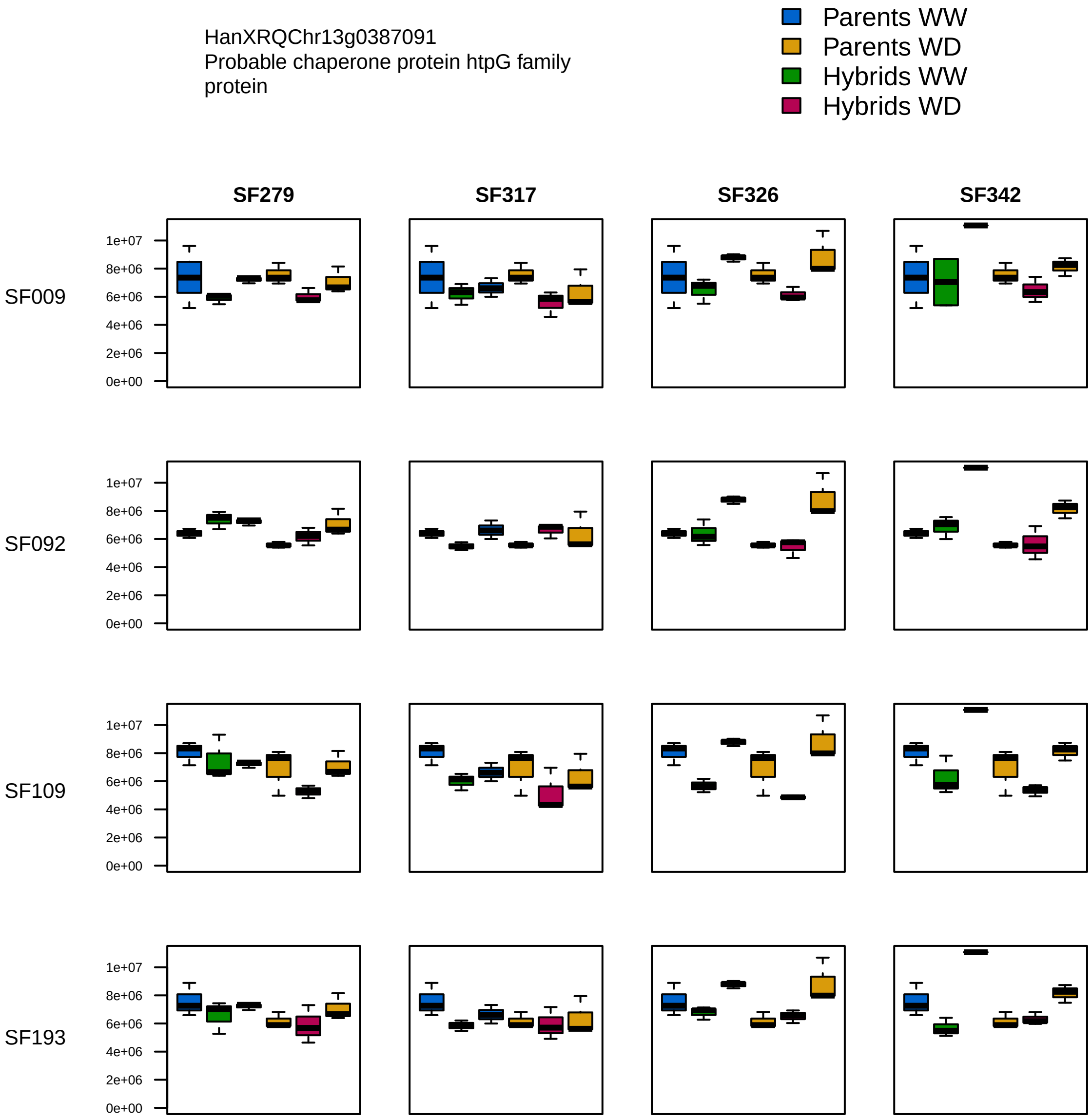
HanXRQChr13g0387091
Probable chaperone protein htpG family protein
Parents WW
Parents WD
Hybrids WW
Hybrids WD
SF279
SF317
SF326
SF342
SF009
SF092
SF109
SF193
1e+07
8e+06
6e+06
4e+06
2e+06
0e+00

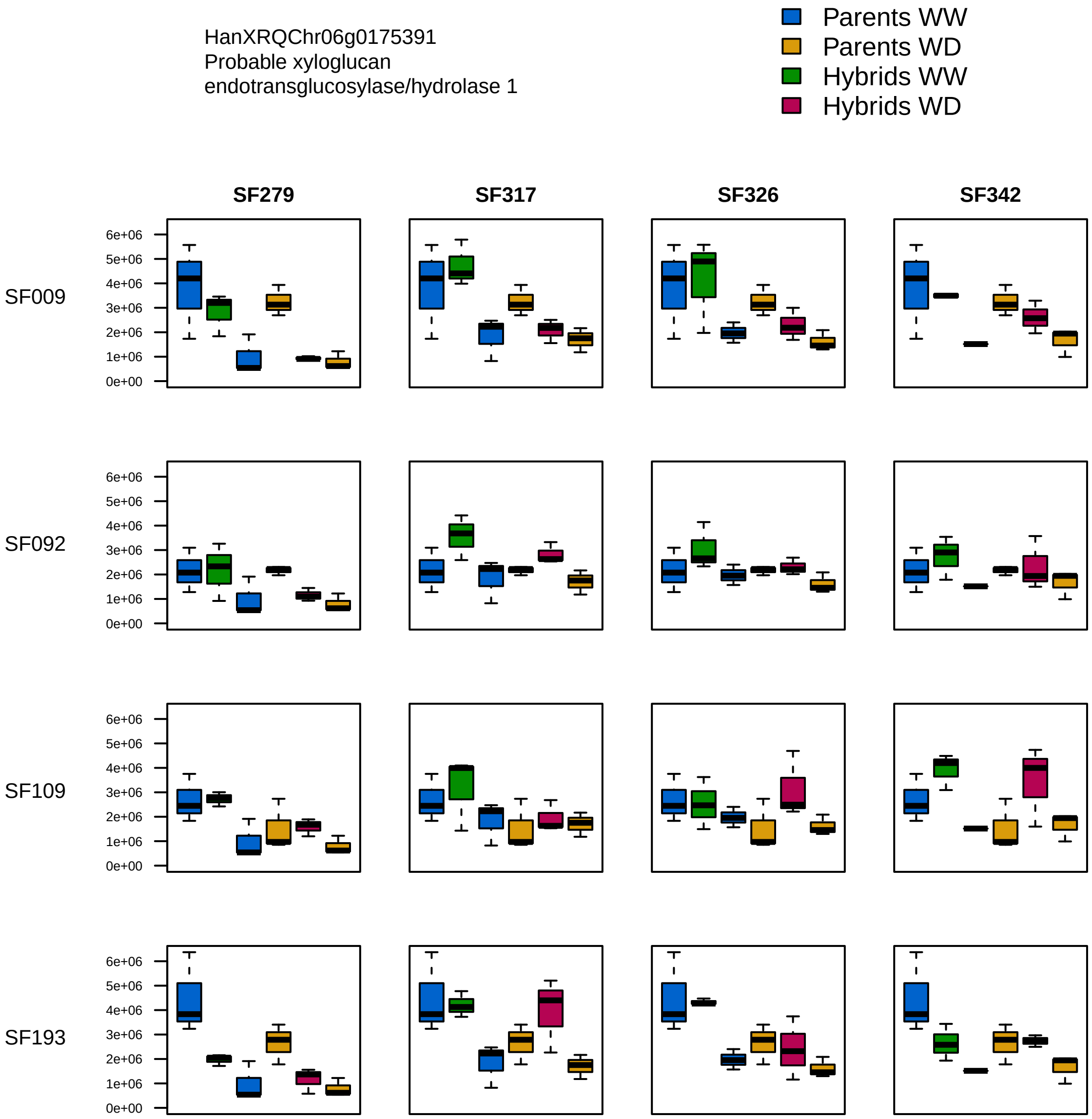
HanXRQChr06g0175391
Probable xyloglucan
endotransglucosylase/hydrolase 1
Parents WW
Parents WD
Hybrids WW
Hybrids WD
SF279
SF317
SF326
SF342
SF009
SF092
SF109
SF193
0e+00
1e+06
2e+06
3e+06
4e+06
5e+06
6e+06

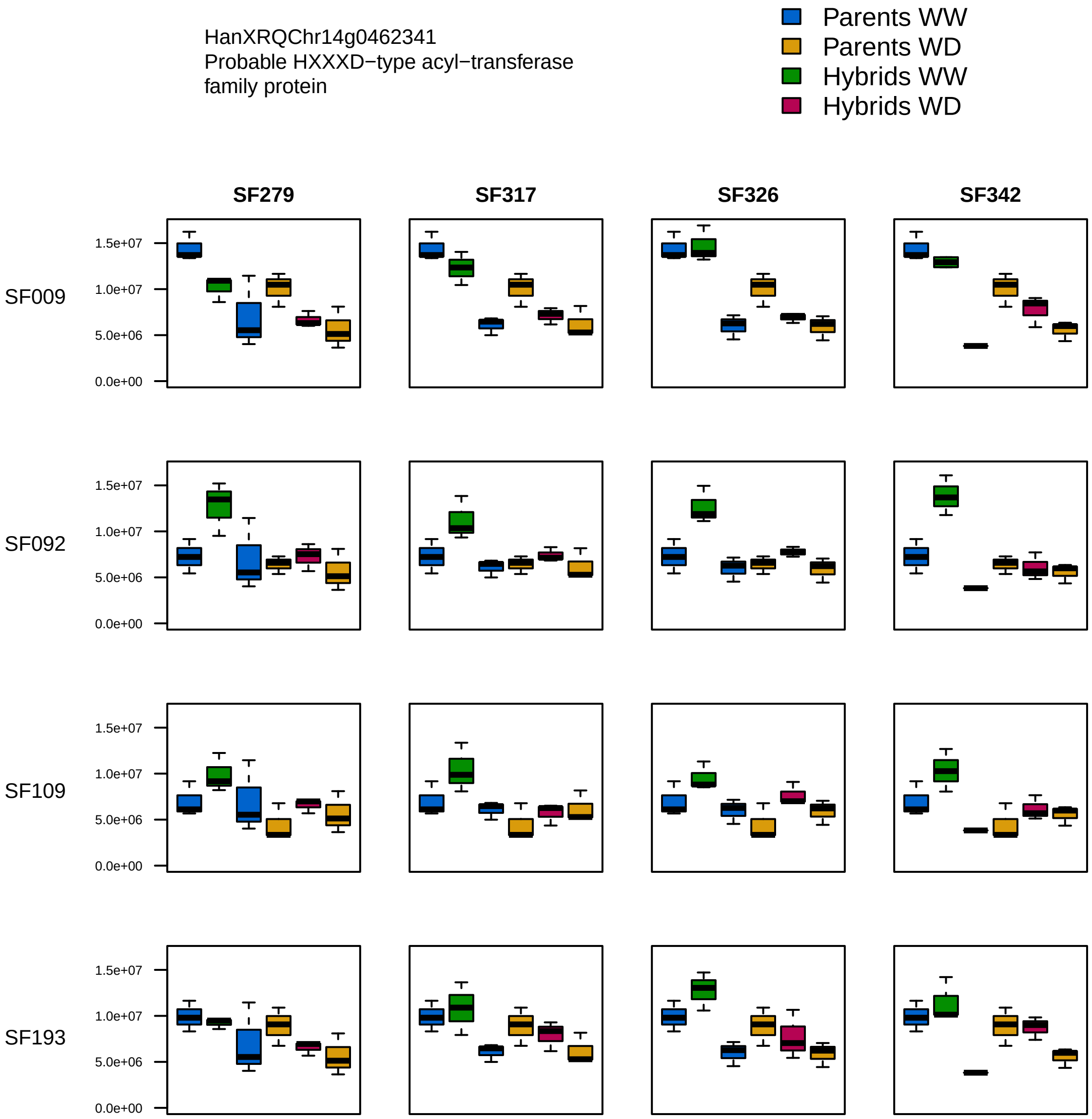

HanXRQChr14g0462341
Probable HXXXD−type acyl−transferase family protein
Parents WW
Parents WD
Hybrids WW
Hybrids WD
SF279
SF317
SF326
SF342
SF009
SF092
SF109
SF193
1.5e+07
1.0e+07
5.0e+06
0.0e+00

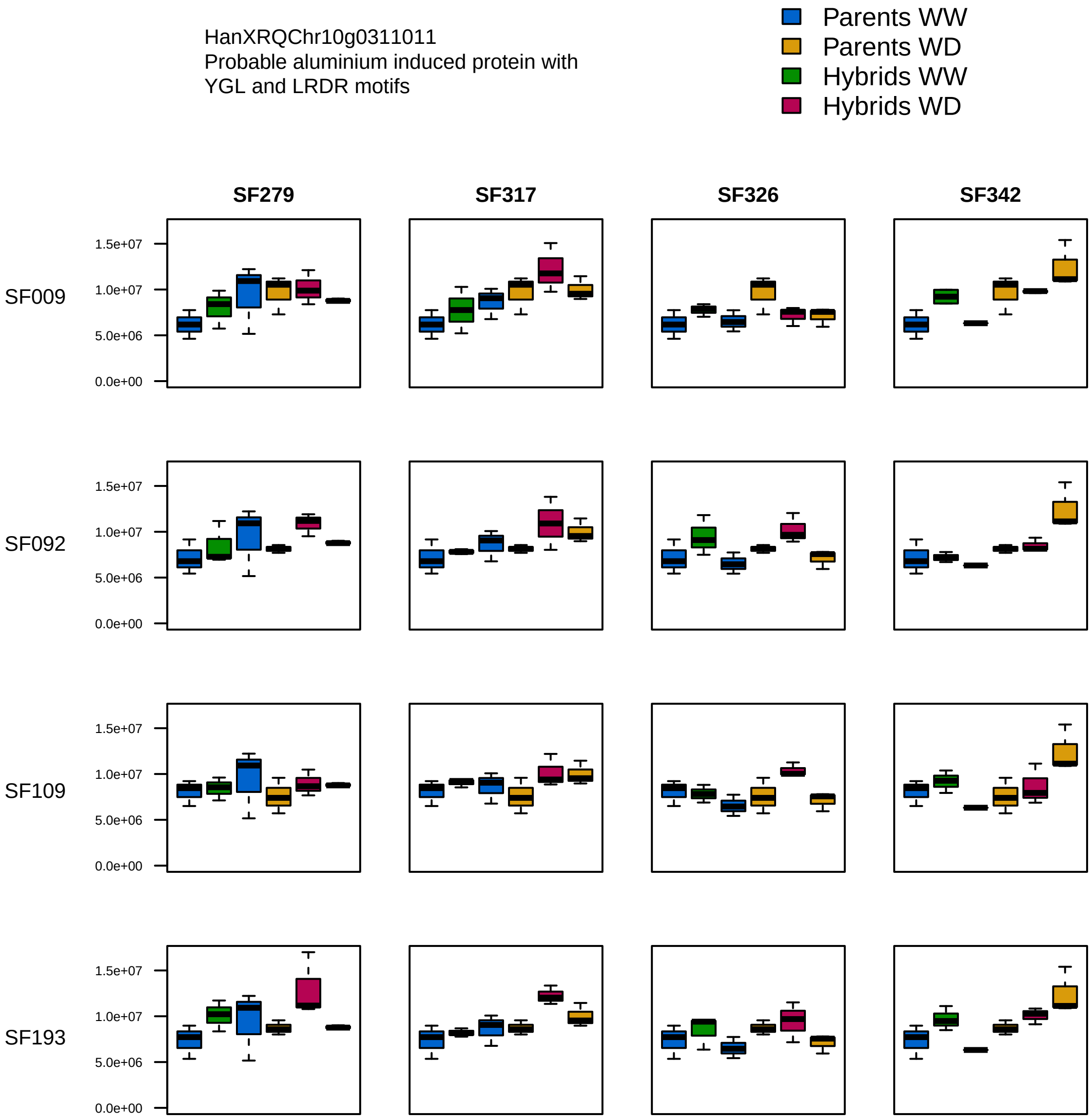
HanXRQChr10g0311011
Probable aluminium induced protein with
YGL and LRDR motifs
Parents WW
Parents WD
Hybrids WW
Hybrids WD
SF279
SF317
SF326
SF342
SF009
SF092
SF109
SF193
1.5e+07
1.0e+07
5.0e+06
0.0e+00

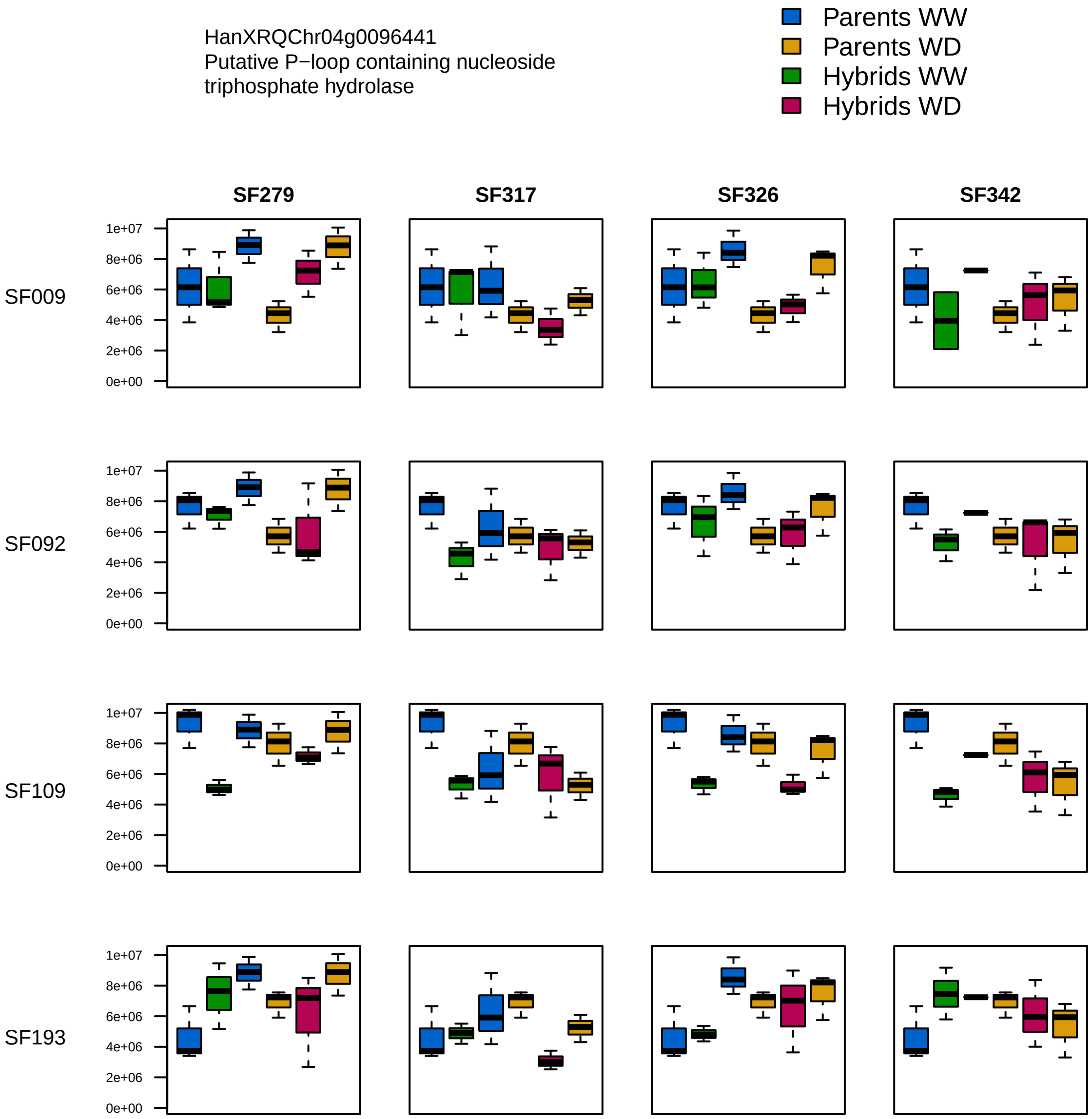
HanXRQChr04g0096441
Putative P−loop containing nucleoside triphosphate hydrolase
Parents WW
Parents WD
Hybrids WW
Hybrids WD
SF279
SF317
SF326
SF342
SF009
SF092
SF109
SF193
1e+07
8e+06
6e+06
4e+06
2e+06
0e+00

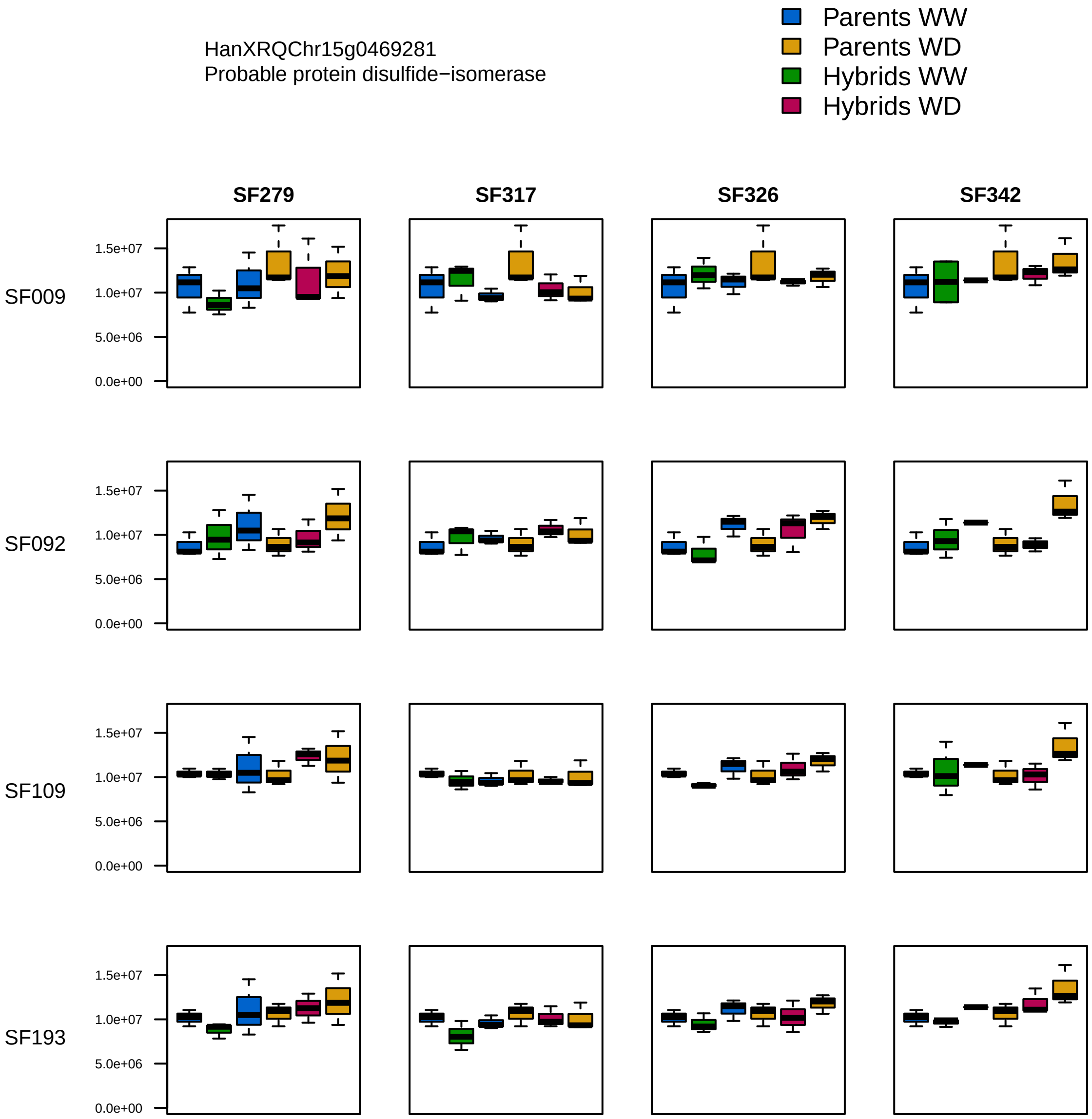
HanXRQChr15g0469281
Probable protein disulfide−isomerase
Parents WW
Parents WD
Hybrids WW
Hybrids WD
SF279
SF317
SF326
SF342
SF009
SF092
SF109
SF193
1.5e+07
1.0e+07
5.0e+06
0.0e+00

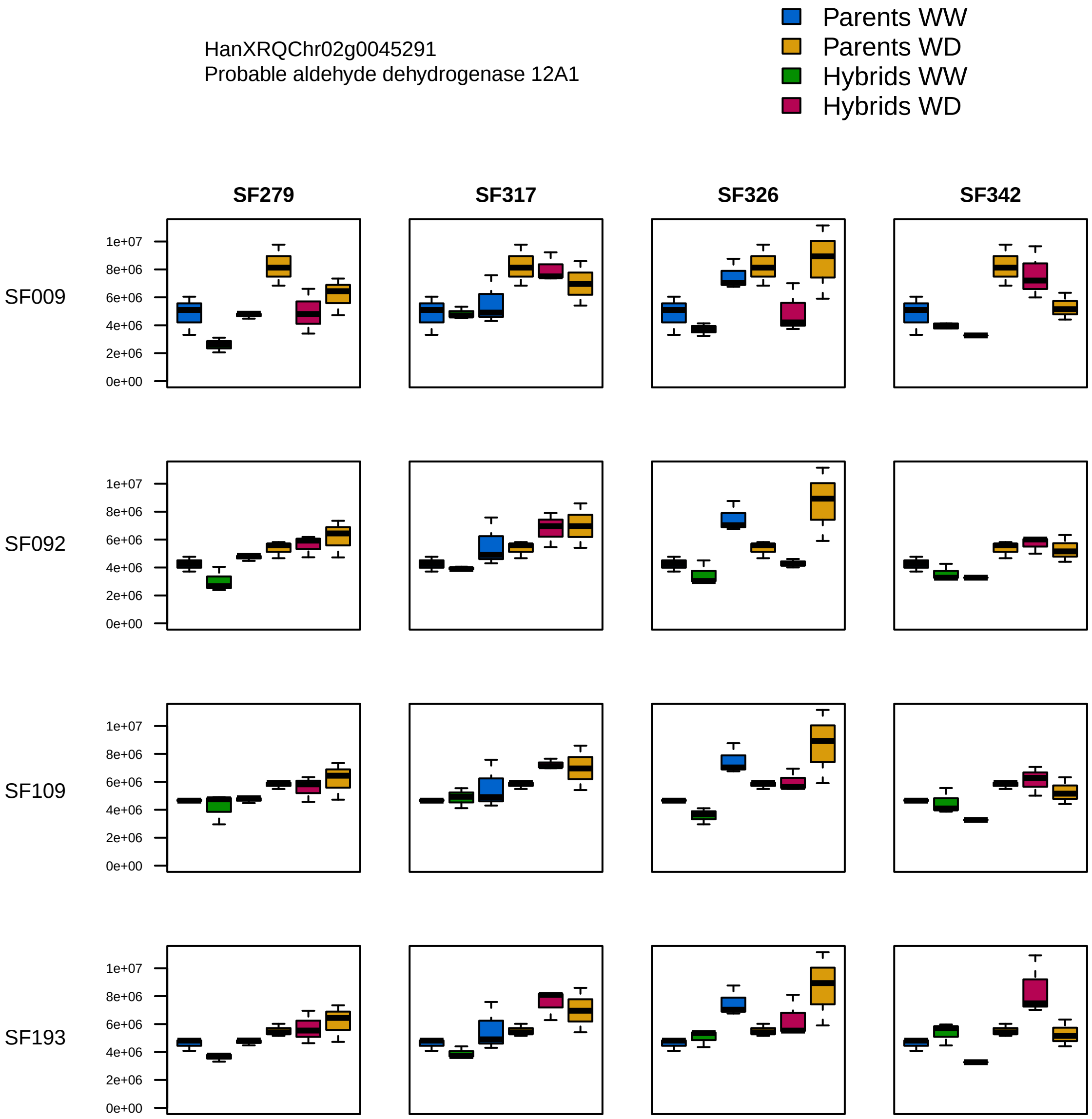
HanXRQChr02g0045291
Probable aldehyde dehydrogenase 12A1
Parents WW
Parents WD
Hybrids WW
Hybrids WD
SF279
SF317
SF326
SF342
SF009
SF092
SF109
SF193
1e+07
8e+06
6e+06
4e+06
2e+06
0e+00

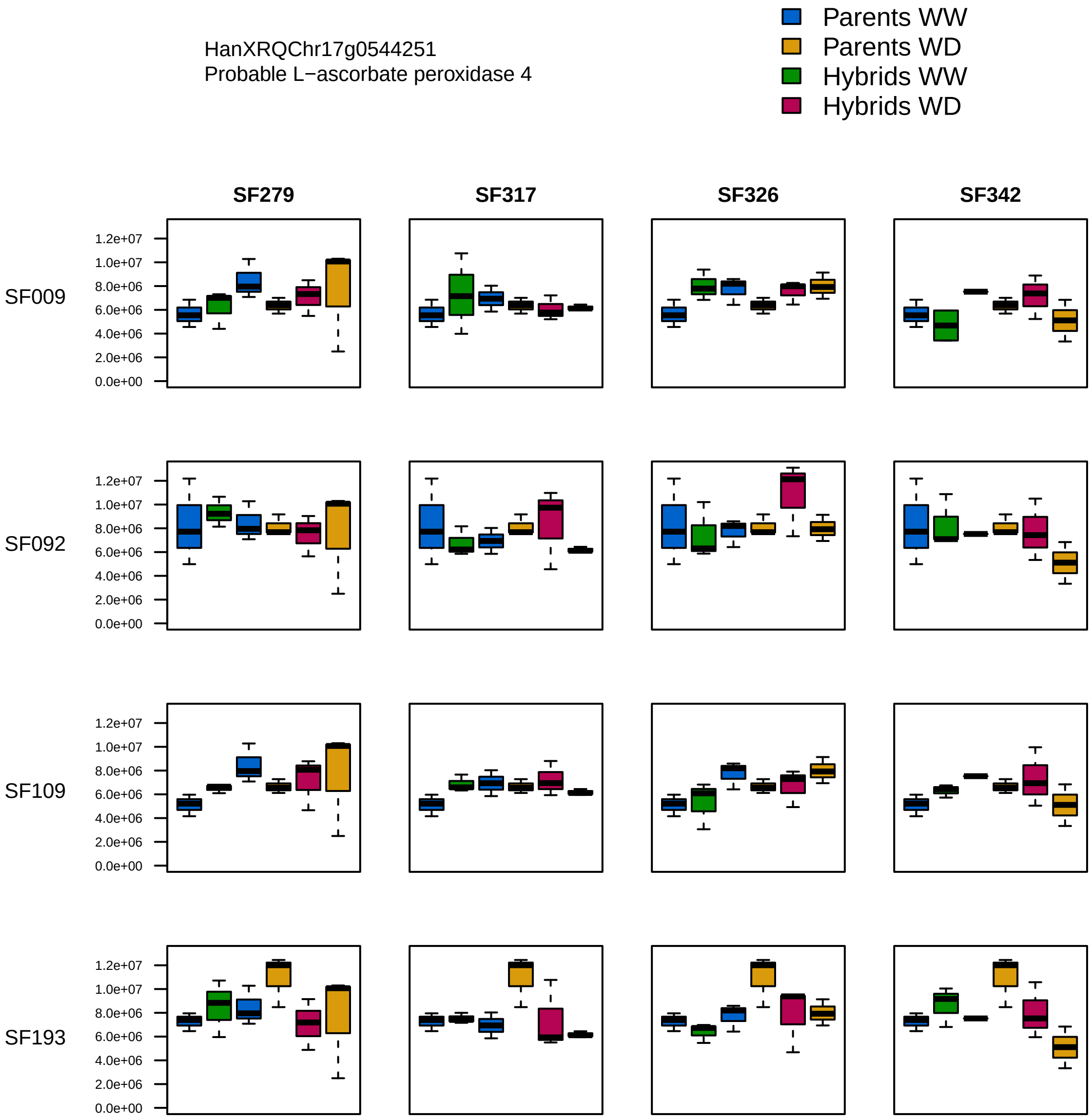
HanXRQChr17g0544251
Probable L−ascorbate peroxidase 4
Parents WW
Parents WD
Hybrids WW
Hybrids WD
SF279
SF317
SF326
SF342
SF009
SF092
SF109
SF193
1.2e+07
1.0e+07
8.0e+06
6.0e+06
4.0e+06
2.0e+06
0.0e+00

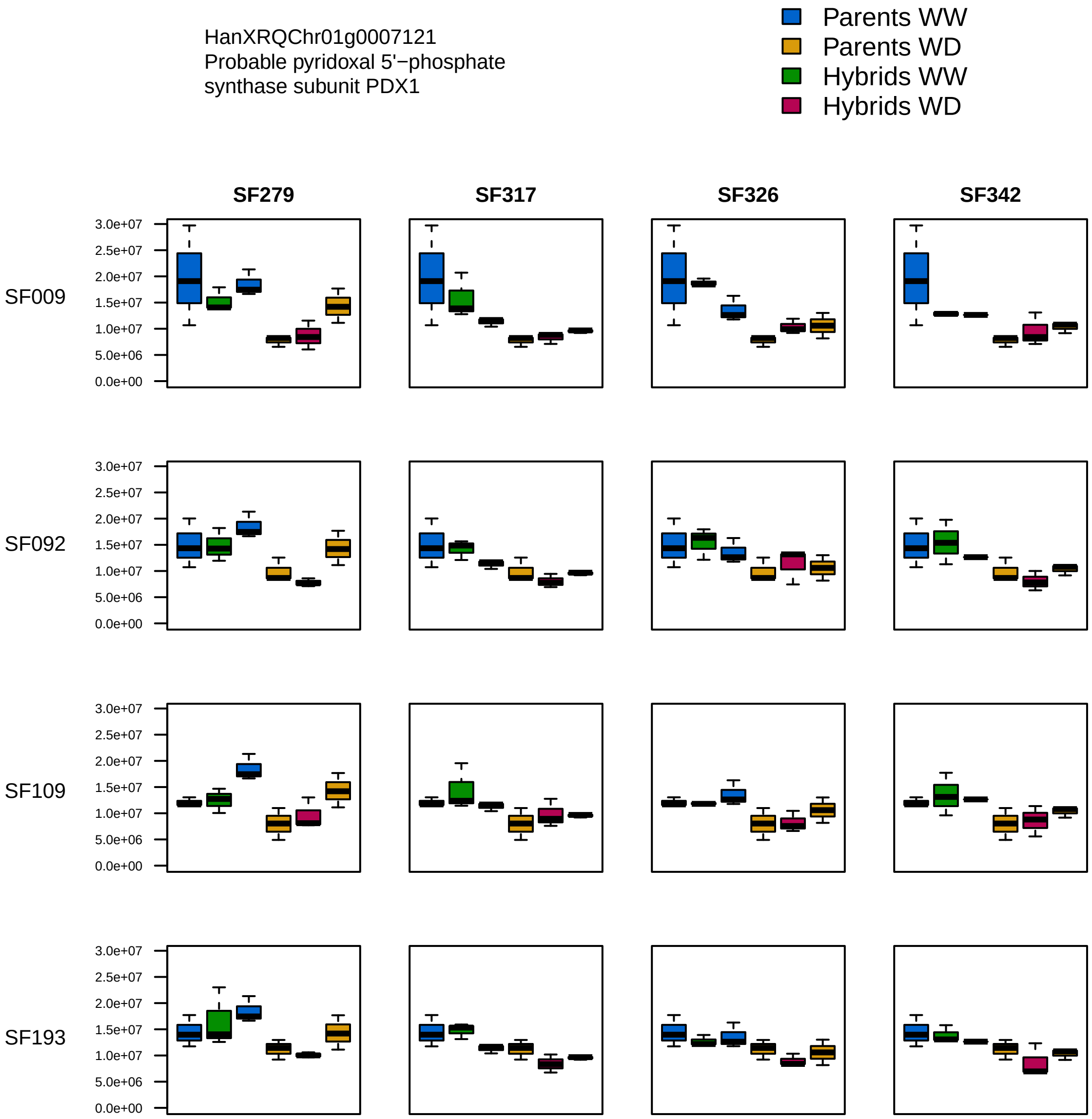
HanXRQChr01g0007121
Probable pyridoxal 5'−phosphate
synthase subunit PDX1
Parents WW
Parents WD
Hybrids WW
Hybrids WD
SF279
SF317
SF326
SF342
SF009
SF092
SF109
SF193
3.0e+07
2.5e+07
2.0e+07
1.5e+07
1.0e+07
5.0e+06
0.0e+00

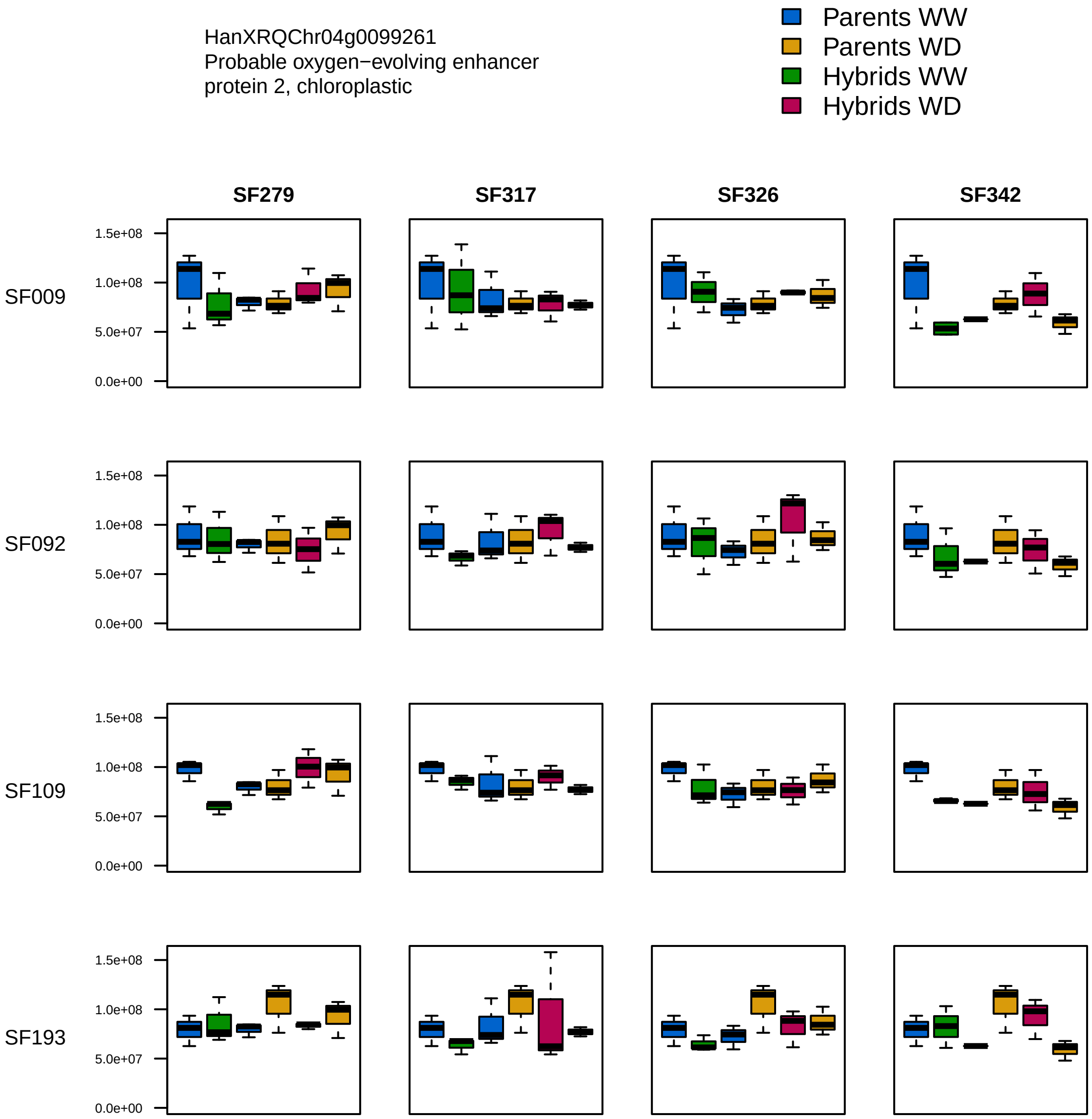
HanXRQChr04g0099261
Probable oxygen−evolving enhancer
protein 2, chloroplastic
Parents WW
Parents WD
Hybrids WW
Hybrids WD
SF279
SF317
SF326
SF342
SF009
SF092
SF109
SF193
1.5e+08
1.0e+08
5.0e+07
0.0e+00

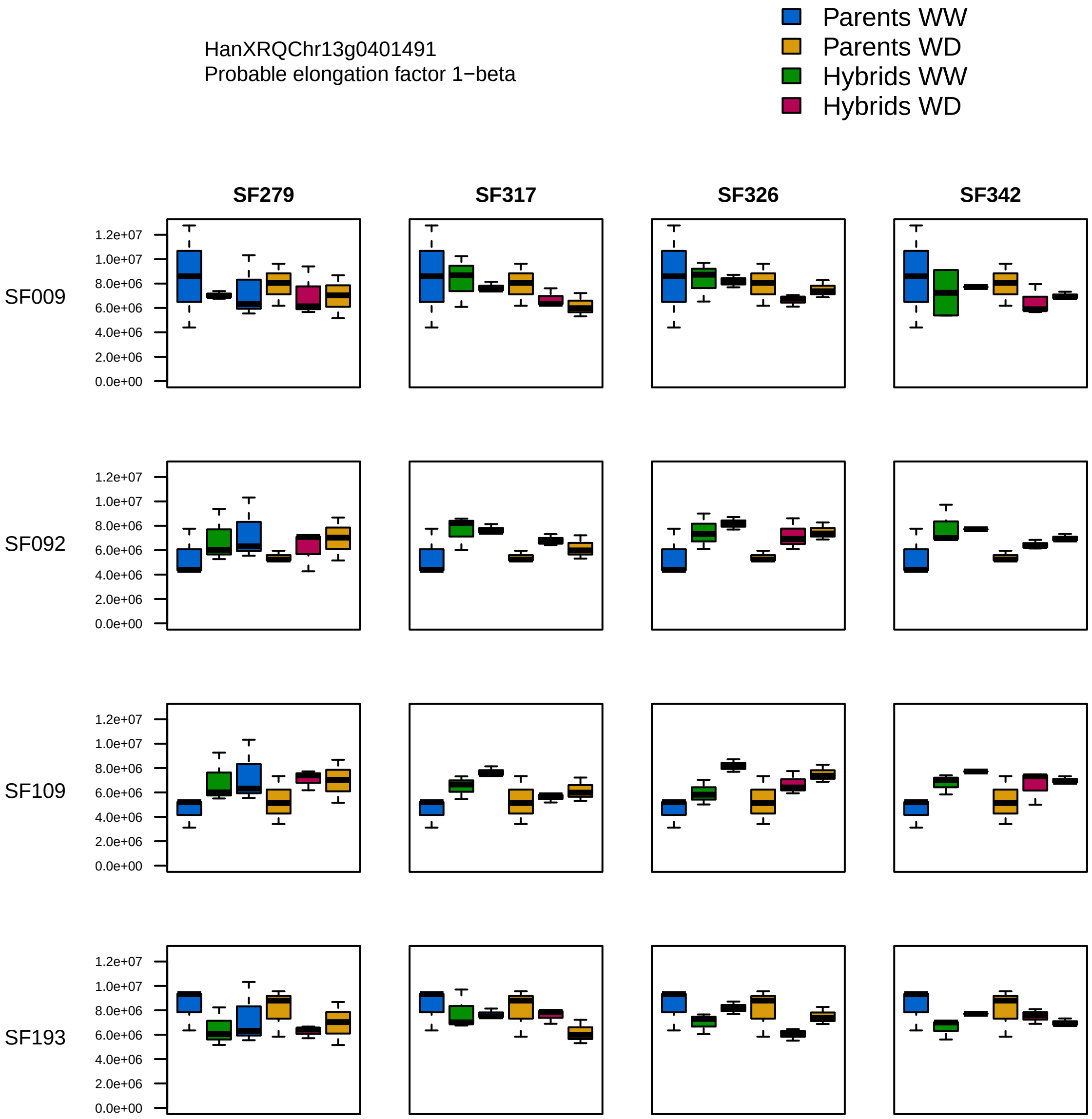
HanXRQChr13g0401491
Probable elongation factor 1−beta
Parents WW
Parents WD
Hybrids WW
Hybrids WD
SF279
SF317
SF326
SF342
SF009
SF092
SF109
SF193
1.2e+07
1.0e+07
8.0e+06
6.0e+06
4.0e+06
2.0e+06
0.0e+00

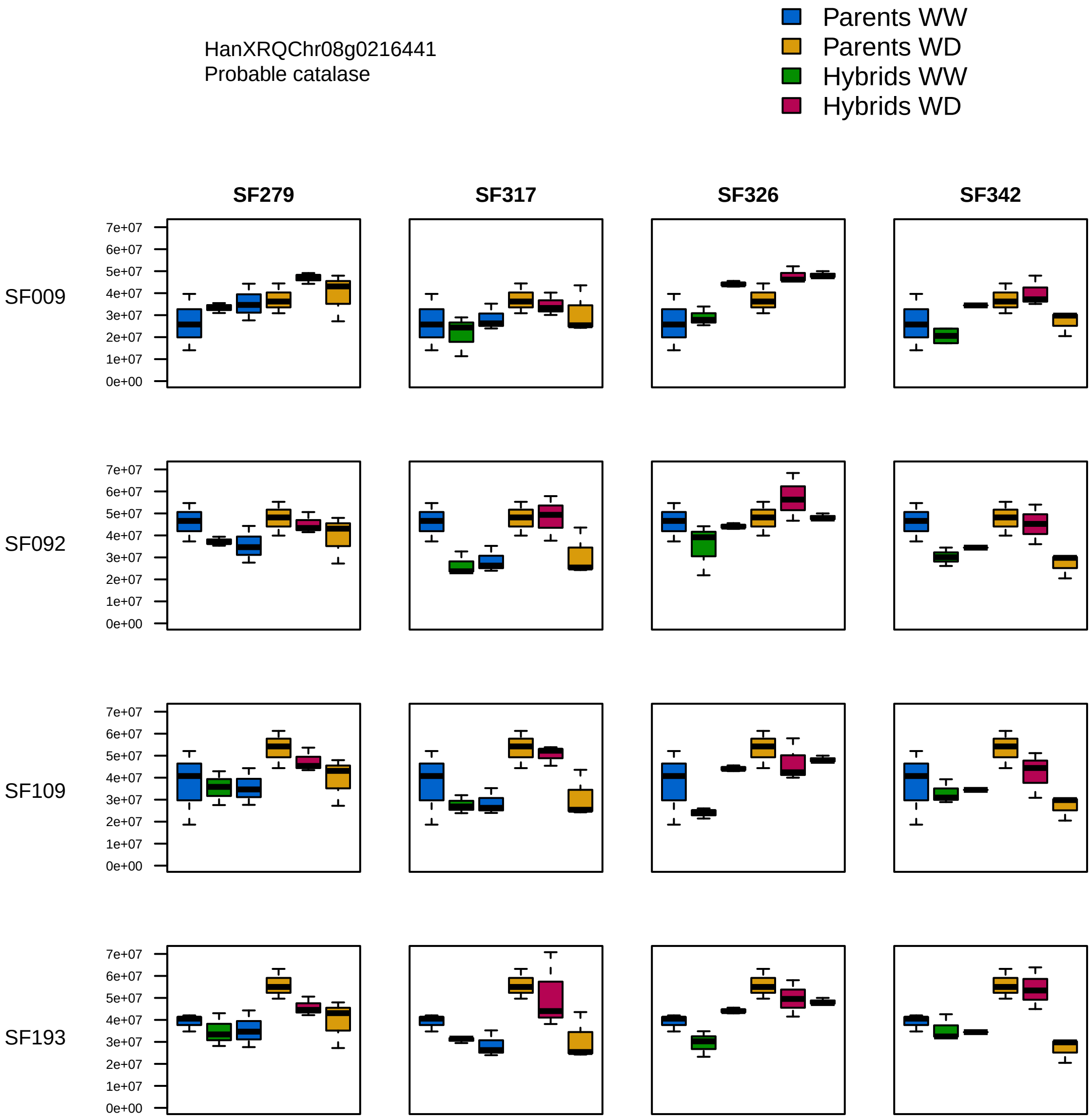
HanXRQChr08g0216441
Probable catalase
Parents WW
Parents WD
Hybrids WW
Hybrids WD
SF279
SF317
SF326
SF342
SF009
SF092
SF109
SF193
7e+07
6e+07
5e+07
4e+07
3e+07
2e+07
1e+07
0e+00

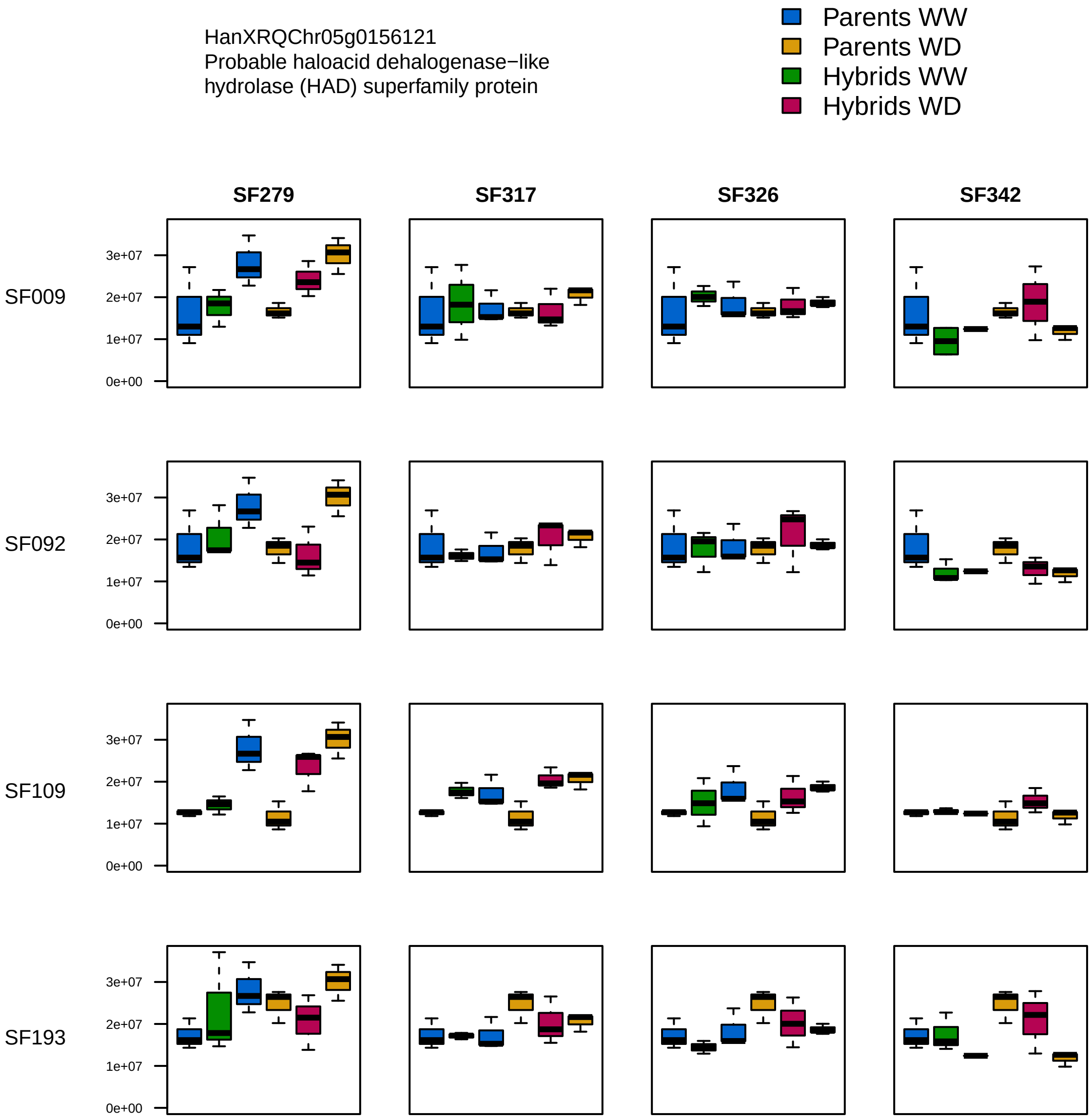

HanXRQChr05g0156121
Probable haloacid dehalogenase−like
hydrolase (HAD) superfamily protein
Parents WW
Parents WD
Hybrids WW
Hybrids WD
SF279
SF317
SF326
SF342
SF009
SF092
SF109
SF193
0e+00
1e+07
2e+07
3e+07

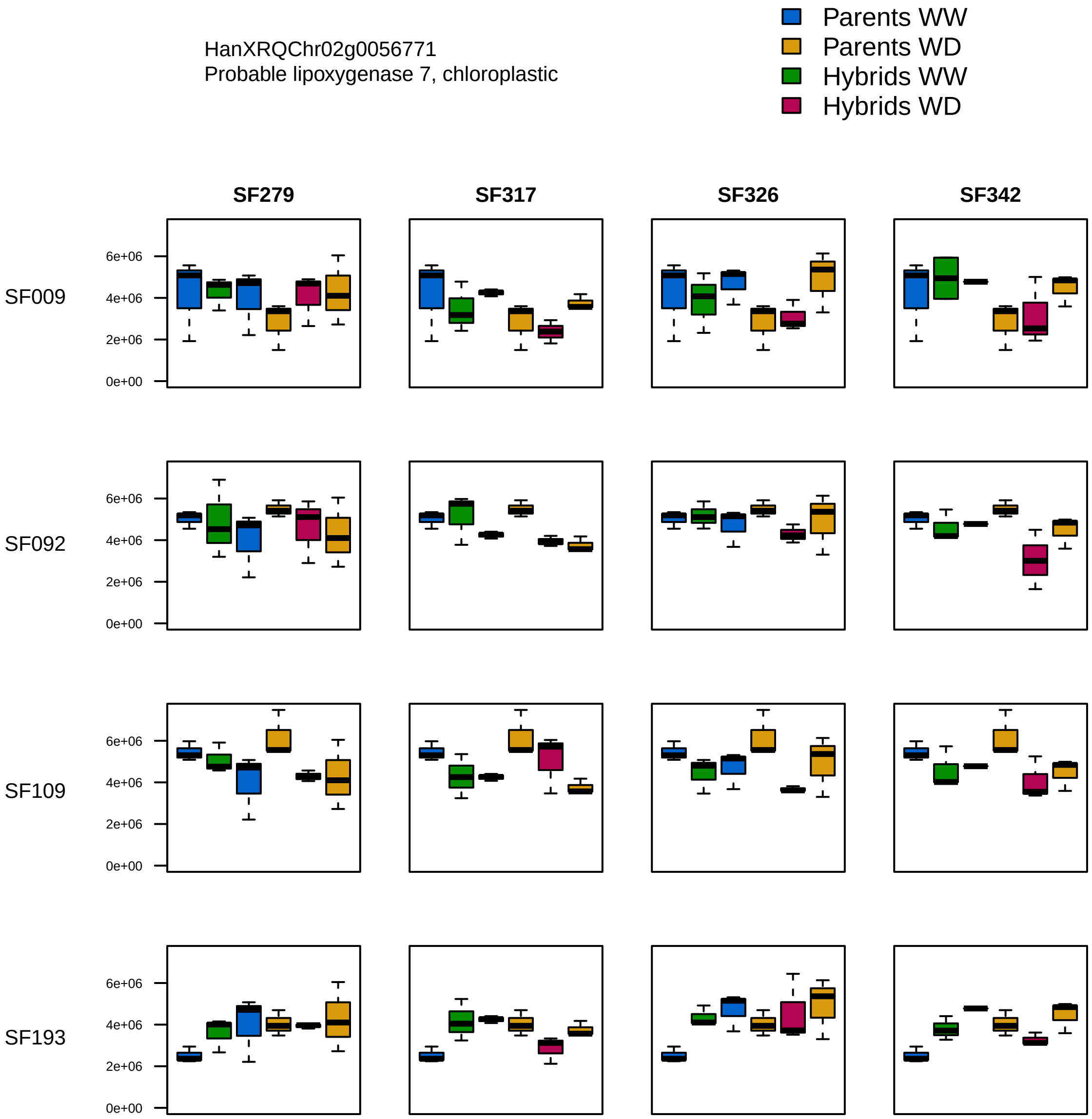

HanXRQChr02g0056771
Probable lipoxygenase 7, chloroplastic
Parents WW
Parents WD
Hybrids WW
Hybrids WD
SF279
SF317
SF326
SF342
SF009
SF092
SF109
SF193
0e+00
2e+06
4e+06
6e+06

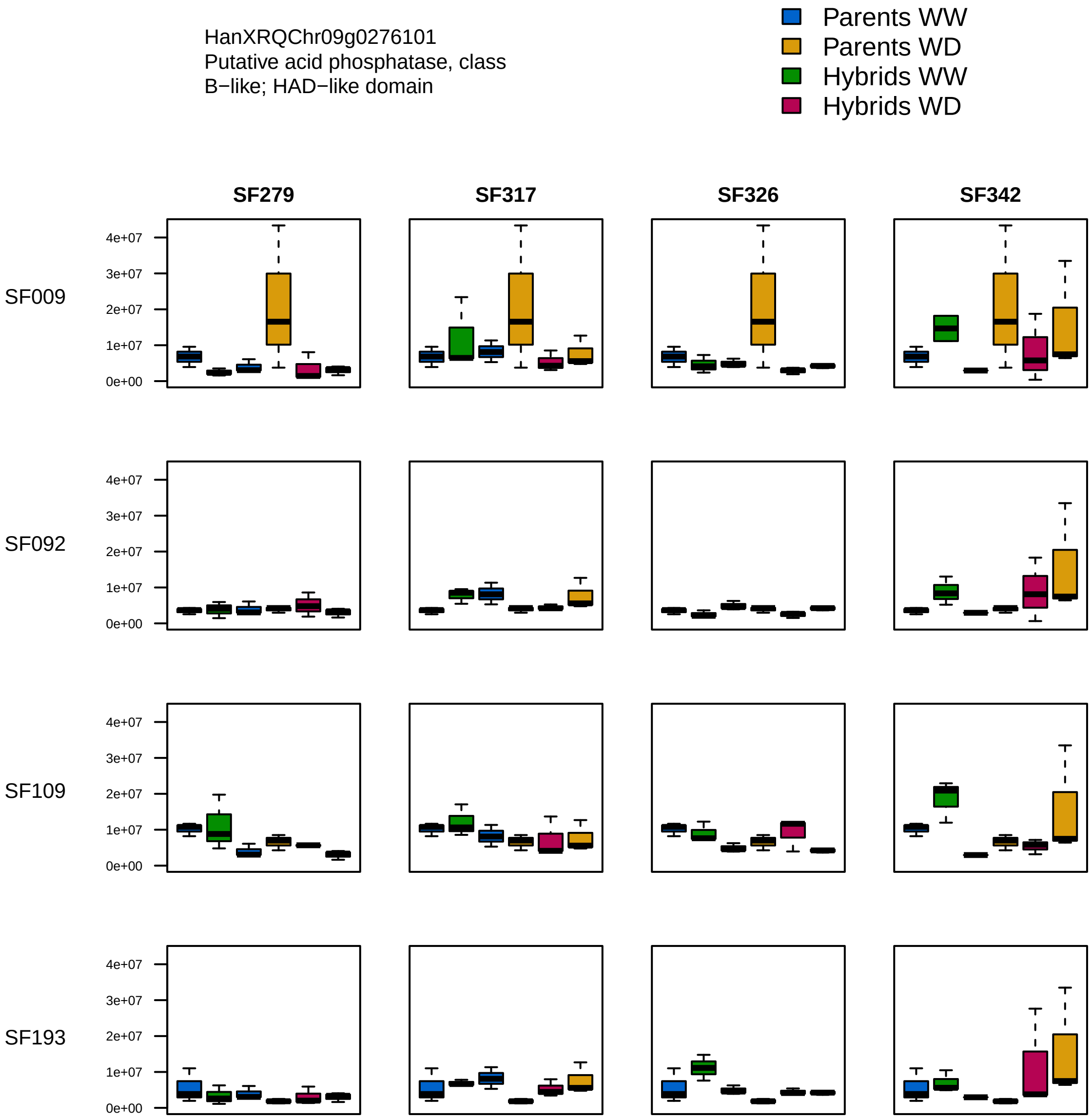

HanXRQChr09g0276101
Putative acid phosphatase, class
B−like; HAD−like domain
Parents WW
Parents WD
Hybrids WW
Hybrids WD
SF279
SF317
SF326
SF342
SF009
SF092
SF109
SF193
0e+00
1e+07
2e+07
3e+07
4e+07

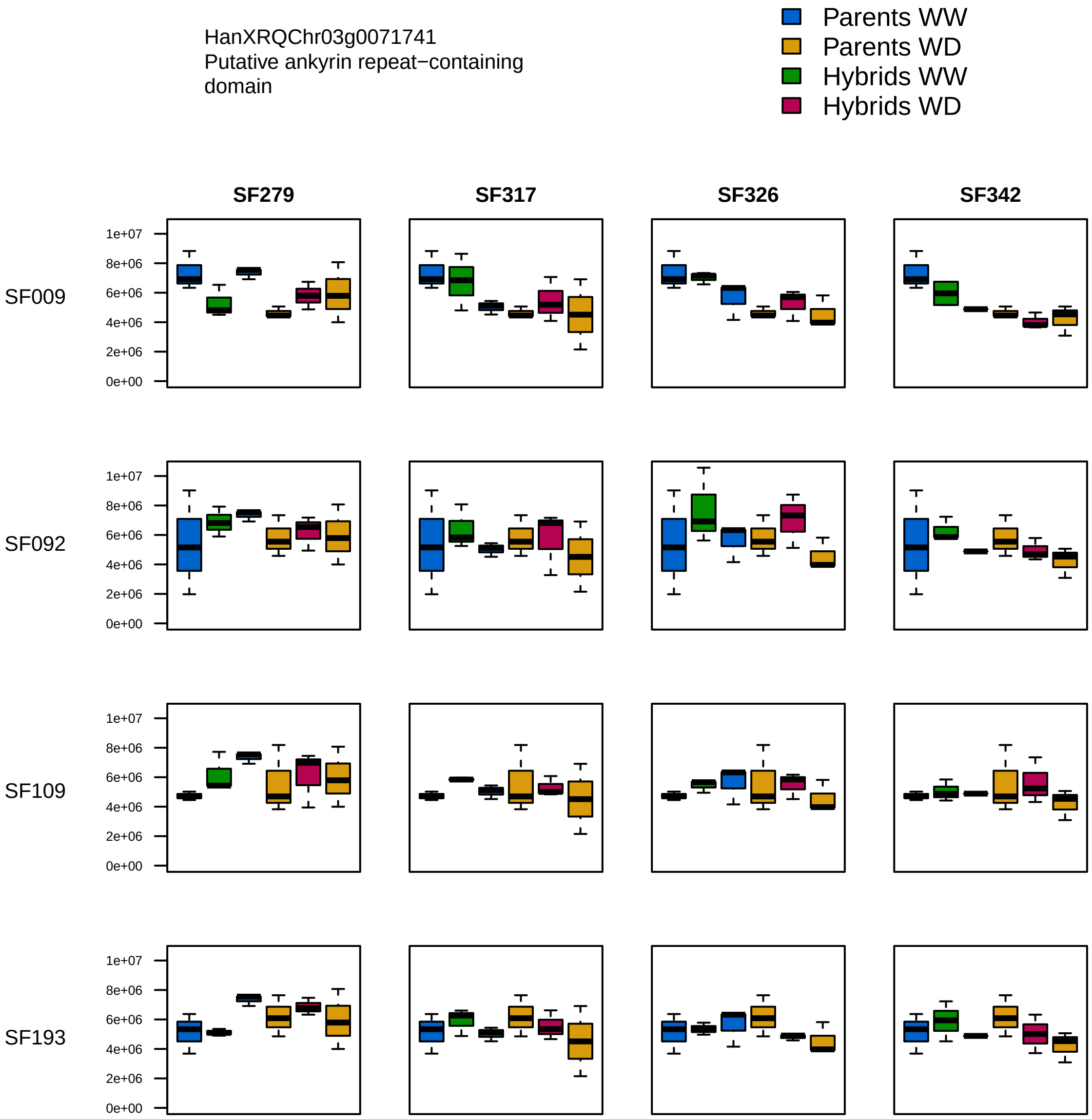
HanXRQChr03g0071741
Putative ankyrin repeat−containing domain
Parents WW
Parents WD
Hybrids WW
Hybrids WD
SF279
SF317
SF326
SF342
SF009
SF092
SF109
SF193
0e+00
2e+06
4e+06
6e+06
8e+06
1e+07

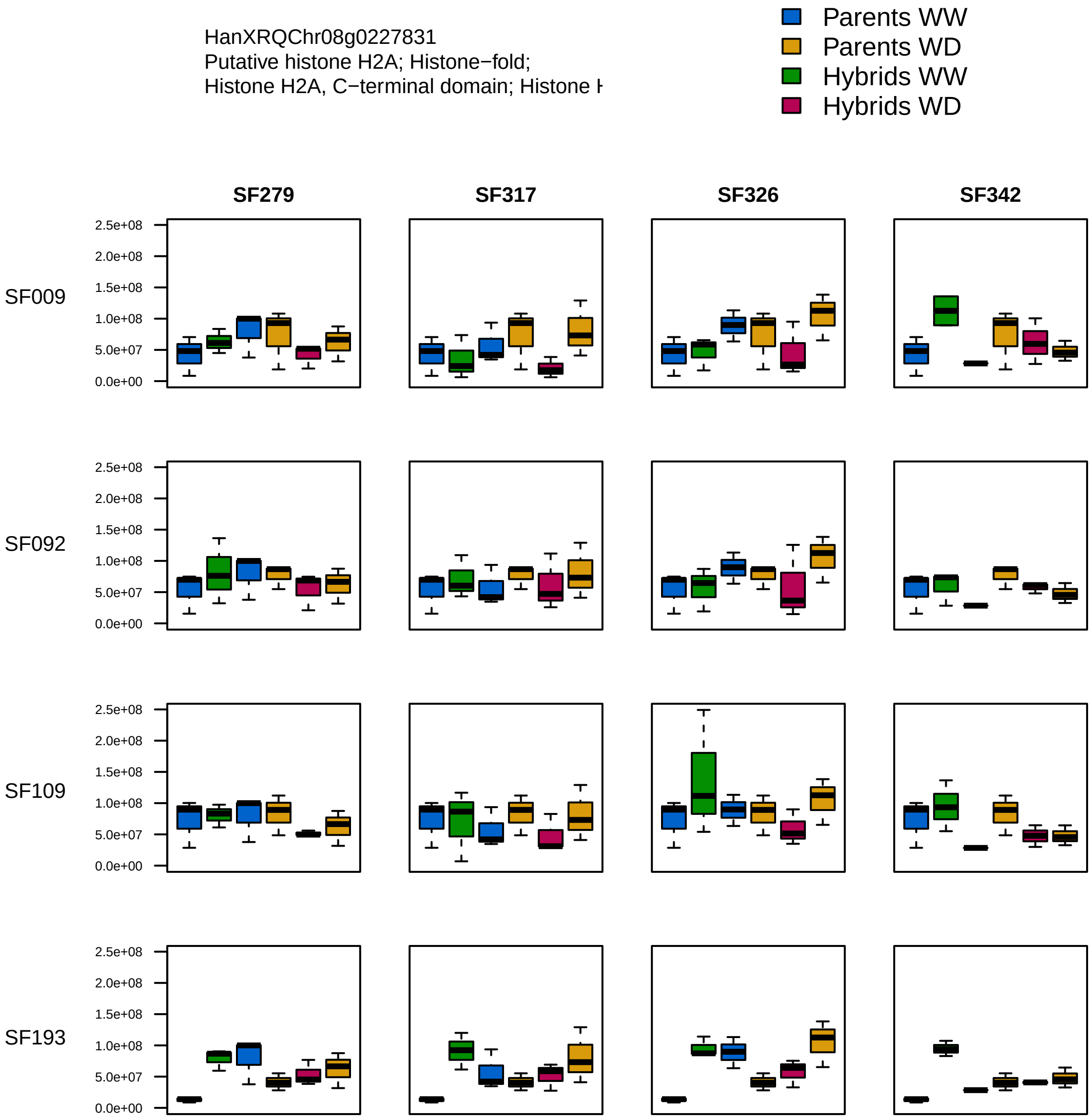
HanXRQChr08g0227831
Putative histone H2A; Histone−fold;
Histone H2A, C−terminal domain; Histone H
Parents WW
Parents WD
Hybrids WW
Hybrids WD
SF279
SF317
SF326
SF342
SF009
SF092
SF109
SF193
2.5e+08
2.0e+08
1.5e+08
1.0e+08
5.0e+07
0.0e+00

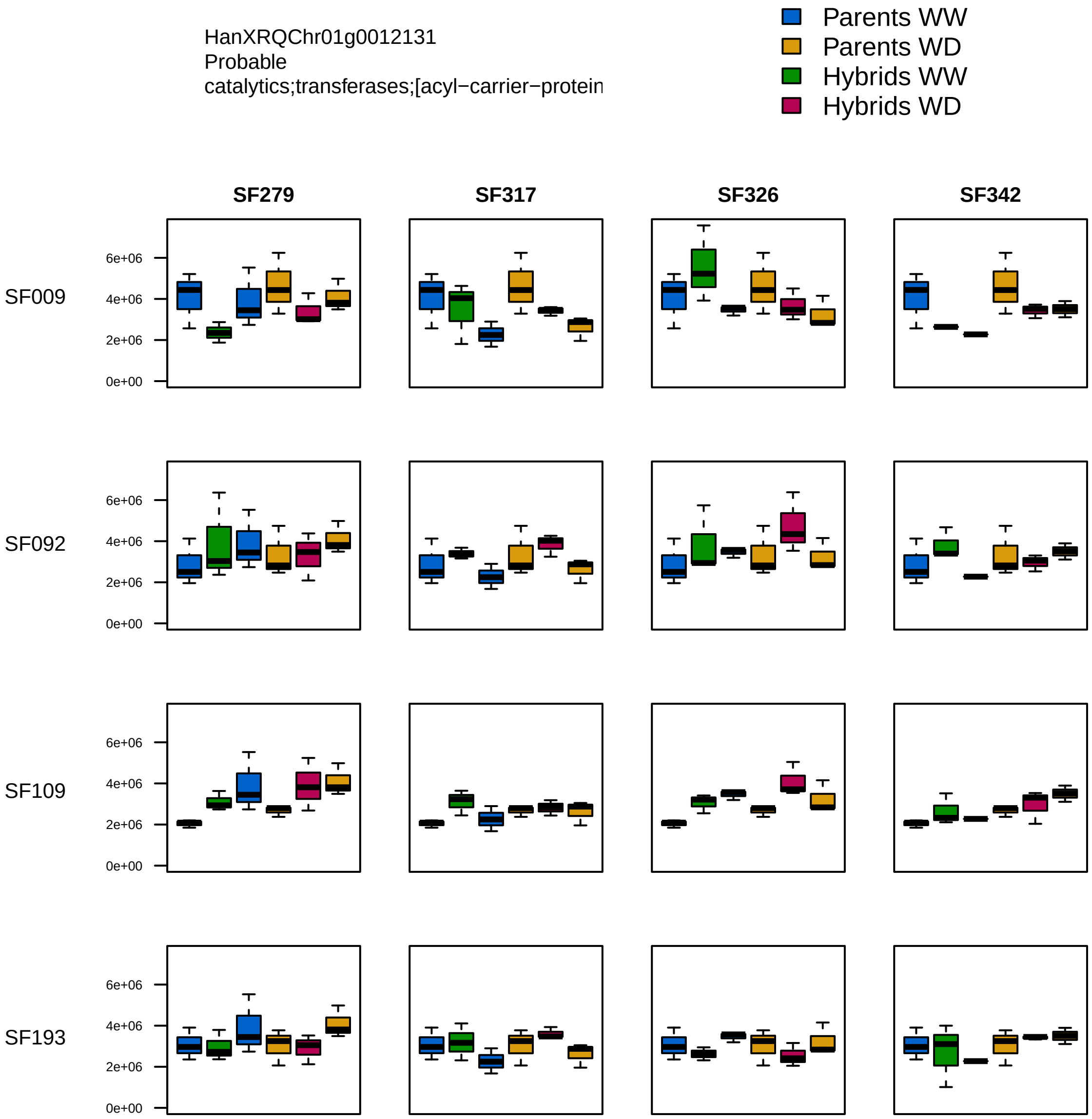
HanXRQChr01g0012131
Probable
catalytics;transferases;[acyl−carrier−protein
Parents WW
Parents WD
Hybrids WW
Hybrids WD
SF279
SF317
SF326
SF342
SF009
SF092
SF109
SF193
0e+00
2e+06
4e+06
6e+06

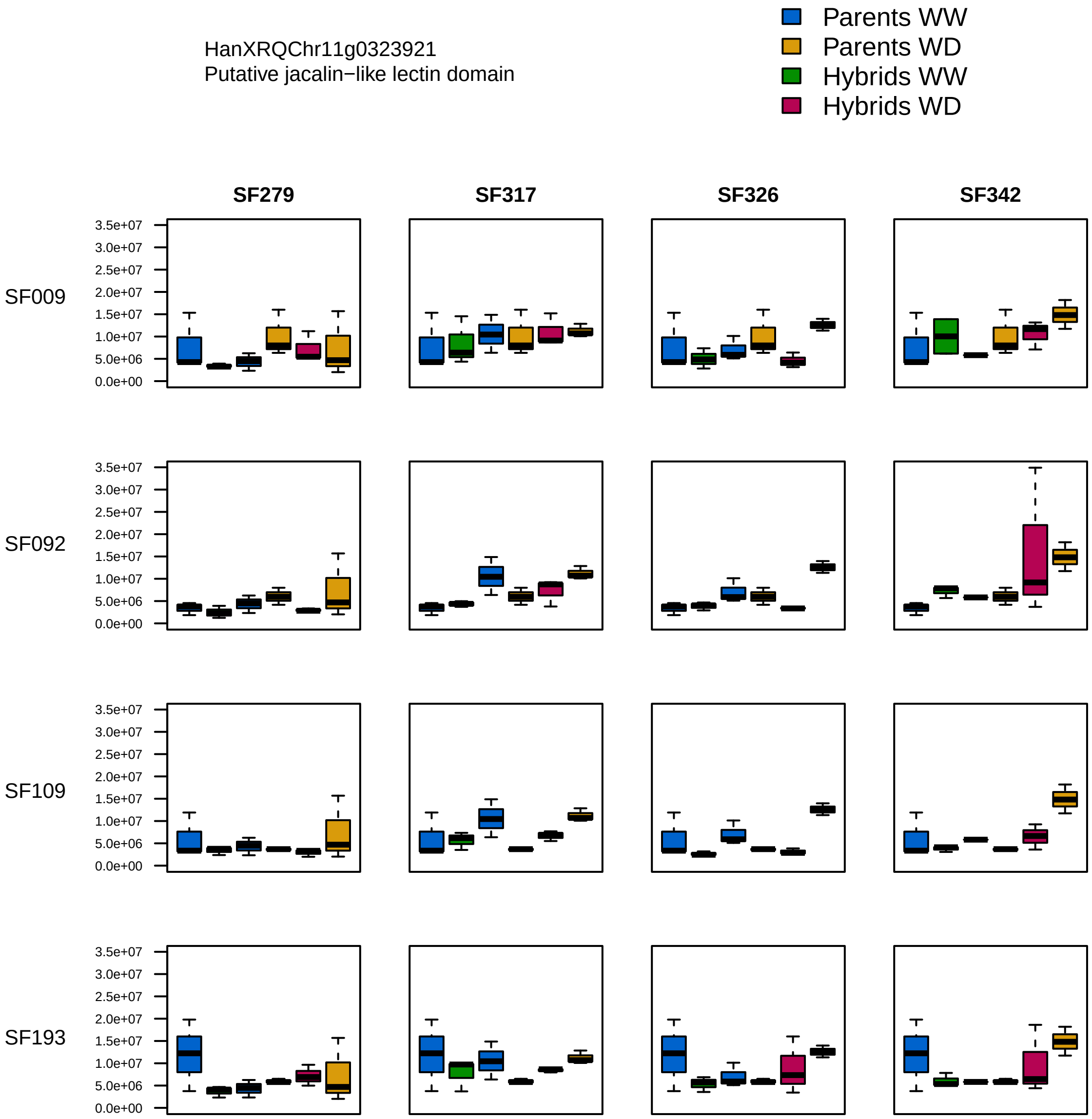

HanXRQChr11g0323921
Putative jacalin−like lectin domain
Parents WW
Parents WD
Hybrids WW
Hybrids WD
SF279
SF317
SF326
SF342
SF009
SF092
SF109
SF193
3.5e+07
3.0e+07
2.5e+07
2.0e+07
1.5e+07
1.0e+07
5.0e+06
0.0e+00

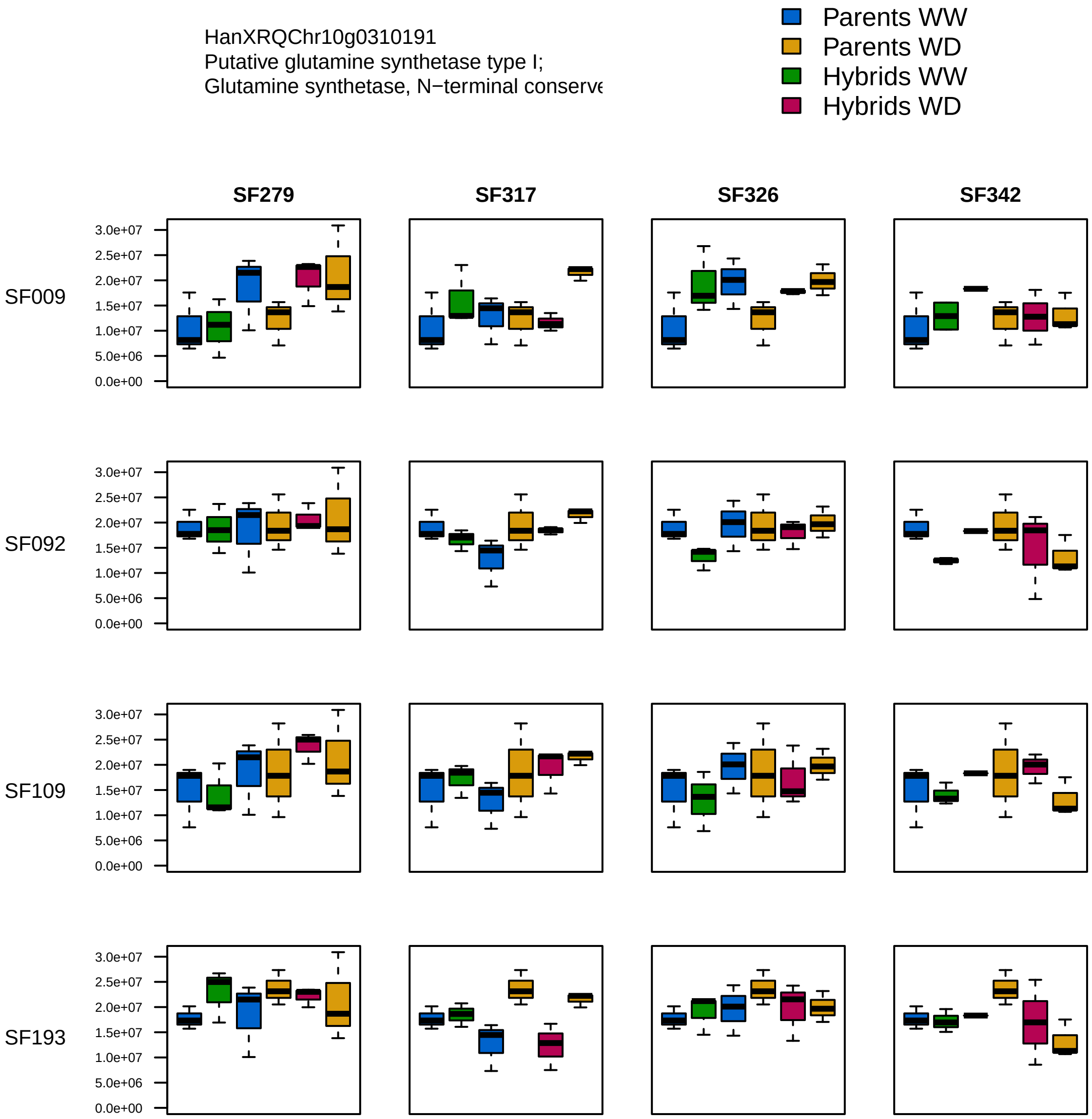
HanXRQChr10g0310191
Putative glutamine synthetase type I;
Glutamine synthetase, N−terminal conserve
Parents WW
Parents WD
Hybrids WW
Hybrids WD
SF279
SF317
SF326
SF342
SF009
SF092
SF109
SF193
3.0e+07
2.5e+07
2.0e+07
1.5e+07
1.0e+07
5.0e+06
0.0e+00

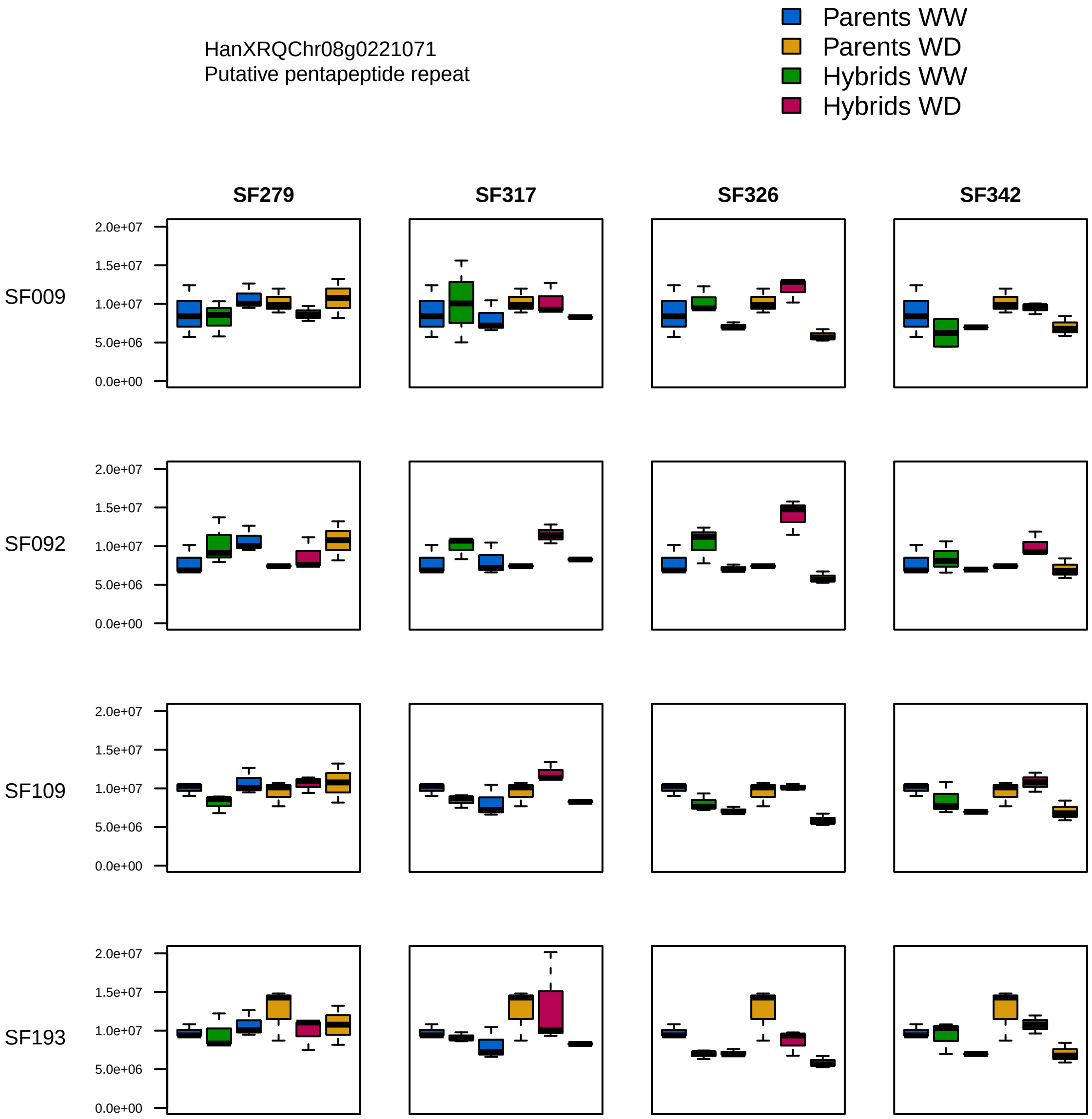
HanXRQChr08g0221071
Putative pentapeptide repeat
Parents WW
Parents WD
Hybrids WW
Hybrids WD
SF279
SF317
SF326
SF342
SF009
SF092
SF109
SF193
2.0e+07
1.5e+07
1.0e+07
5.0e+06
0.0e+00

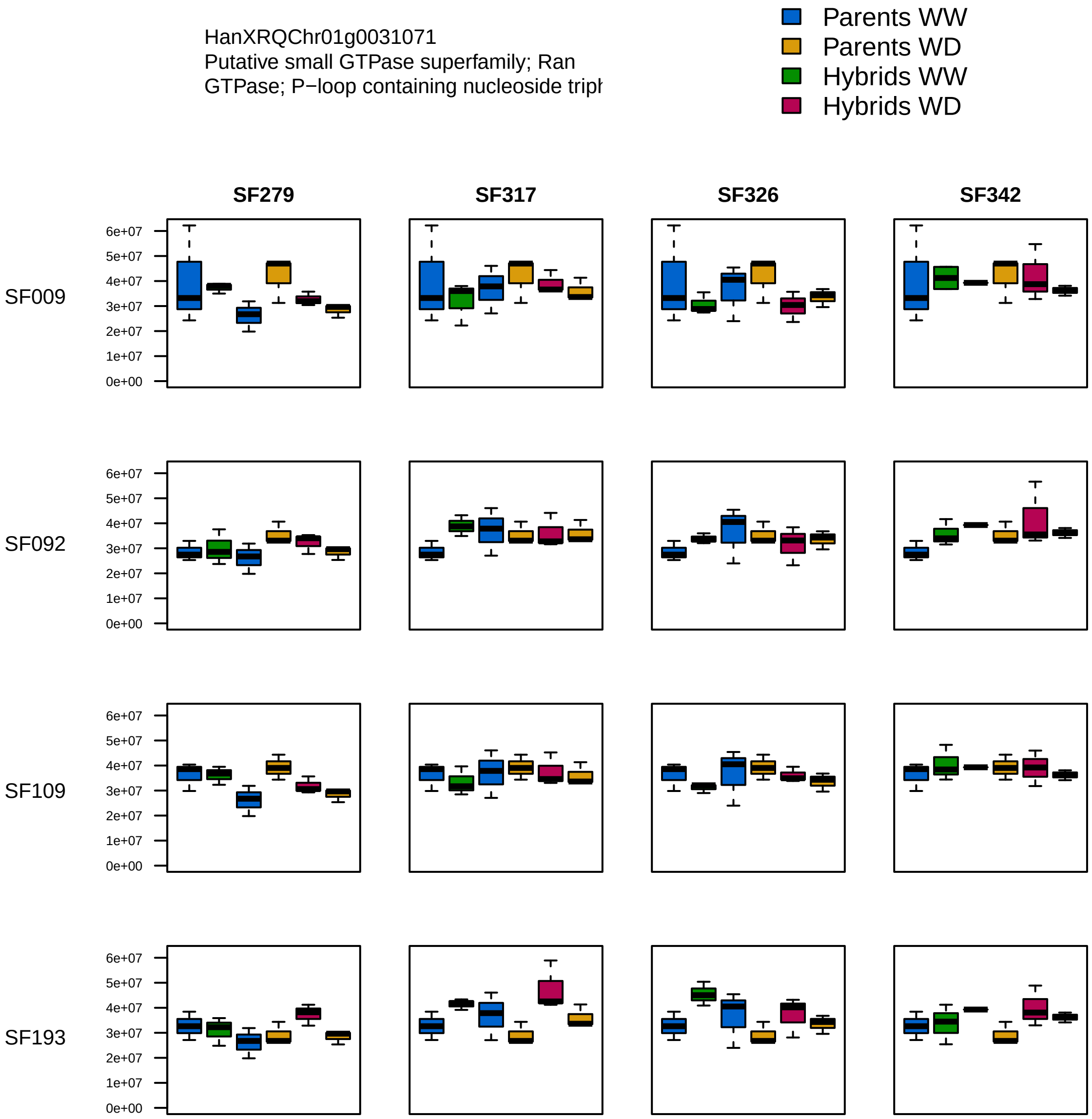

HanXRQChr01g0031071
Putative small GTPase superfamily; Ran
GTPase; P−loop containing nucleoside tripl
Parents WW
Parents WD
Hybrids WW
Hybrids WD
SF279
SF317
SF326
SF342
SF009
SF092
SF109
SF193
6e+07
5e+07
4e+07
3e+07
2e+07
1e+07
0e+00

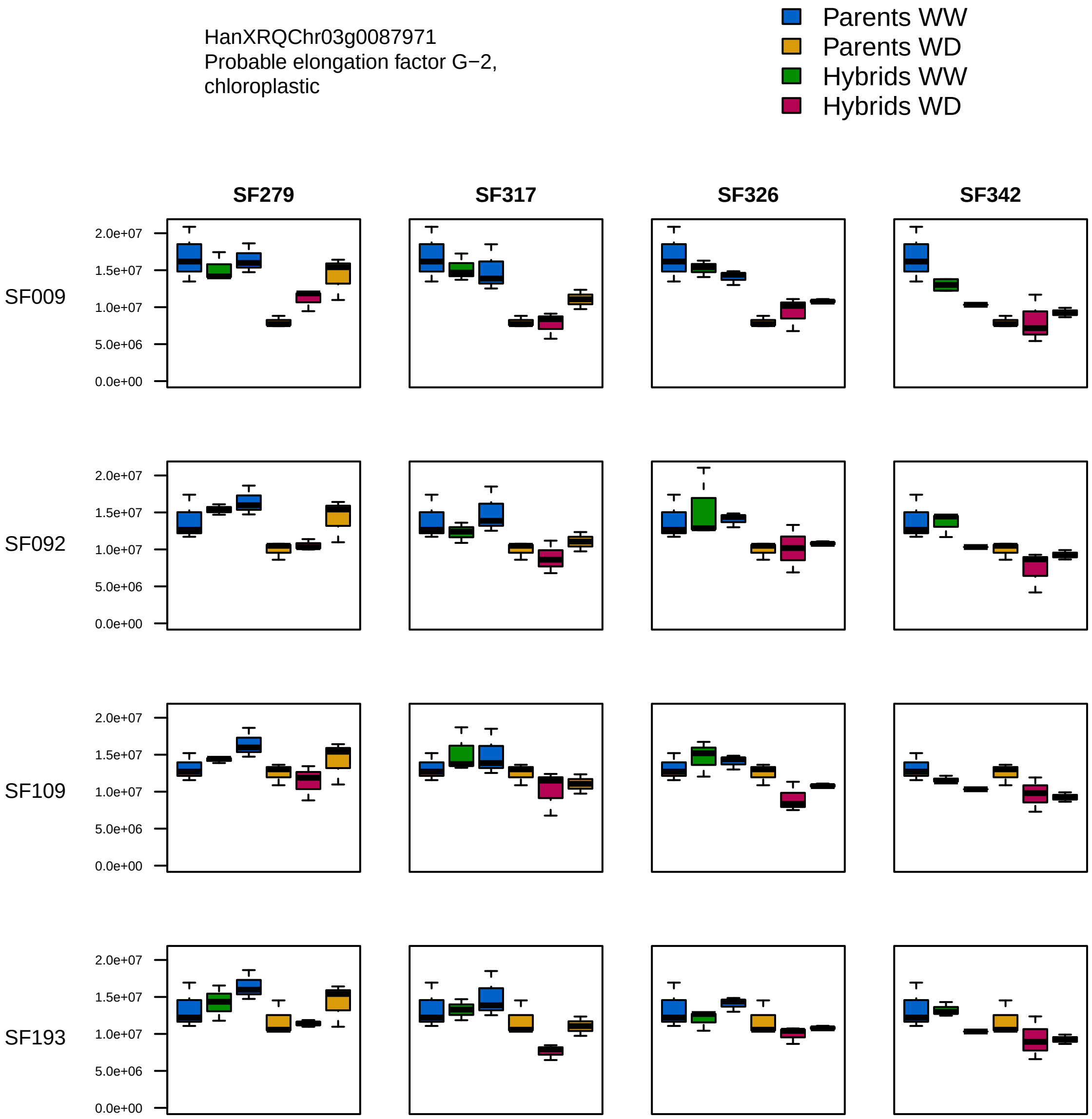
HanXRQChr03g0087971
Probable elongation factor G−2, chloroplastic
Parents WW
Parents WD
Hybrids WW
Hybrids WD
SF279
SF317
SF326
SF342
SF009
SF092
SF109
SF193
2.0e+07
1.5e+07
1.0e+07
5.0e+06
0.0e+00

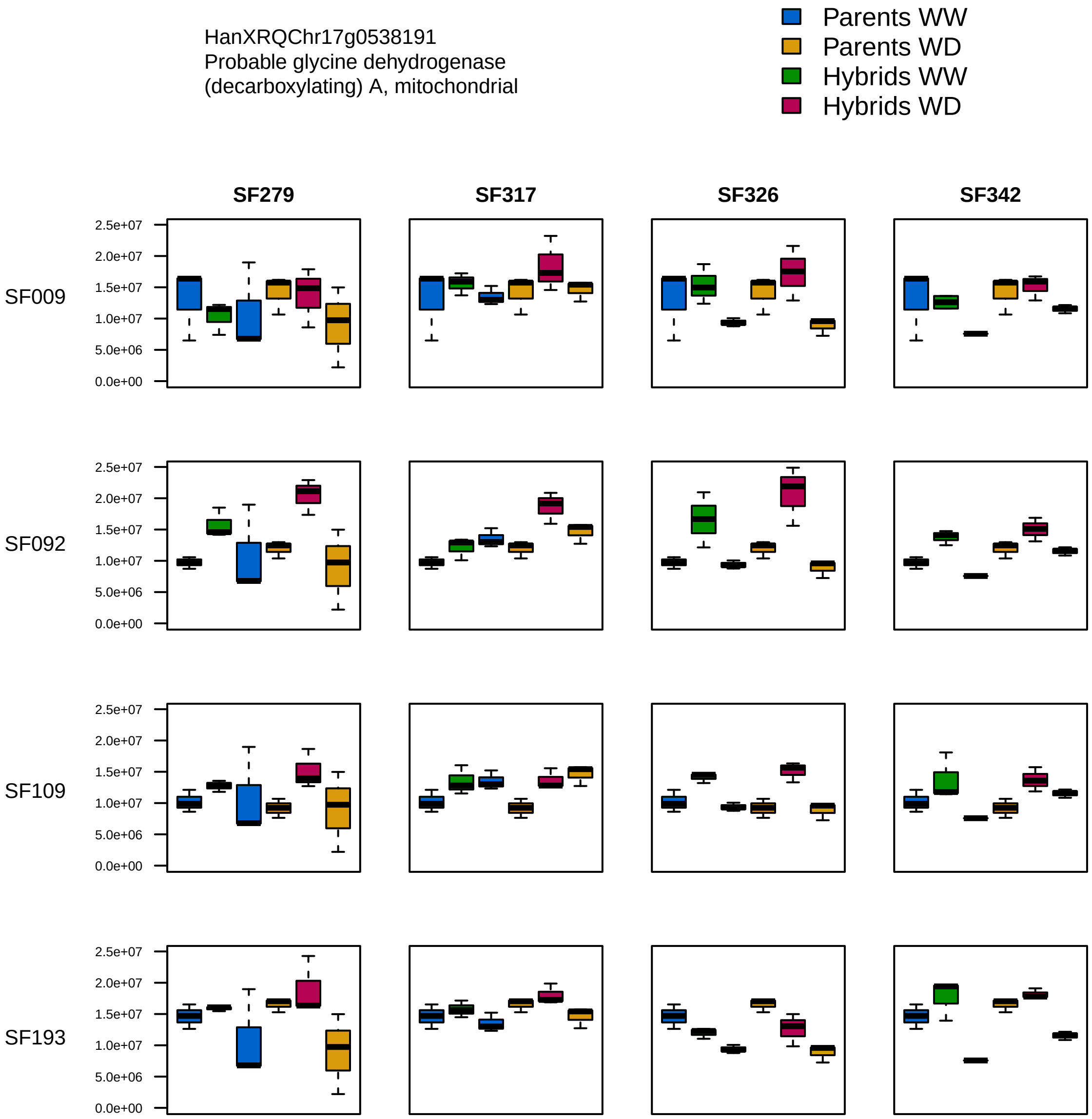

HanXRQChr17g0538191
Probable glycine dehydrogenase
(decarboxylating) A, mitochondrial
Parents WW
Parents WD
Hybrids WW
Hybrids WD
SF279
SF317
SF326
SF342
SF009
SF092
SF109
SF193
2.5e+07
2.0e+07
1.5e+07
1.0e+07
5.0e+06
0.0e+00

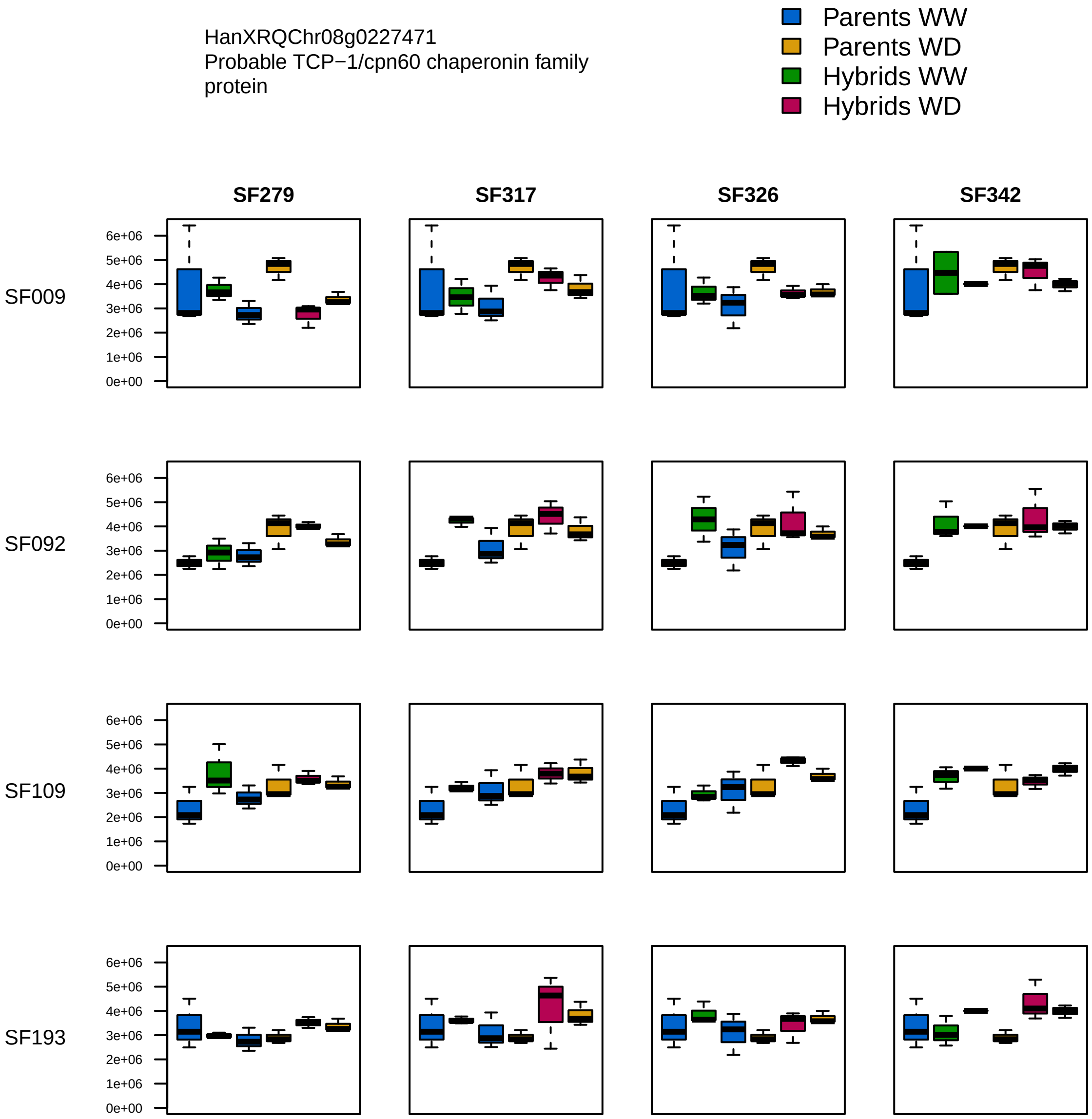

HanXRQChr08g0227471
Probable TCP−1/cpn60 chaperonin family protein
Parents WW
Parents WD
Hybrids WW
Hybrids WD
SF279
SF317
SF326
SF342
SF009
SF092
SF109
SF193
6e+06
5e+06
4e+06
3e+06
2e+06
1e+06
0e+00

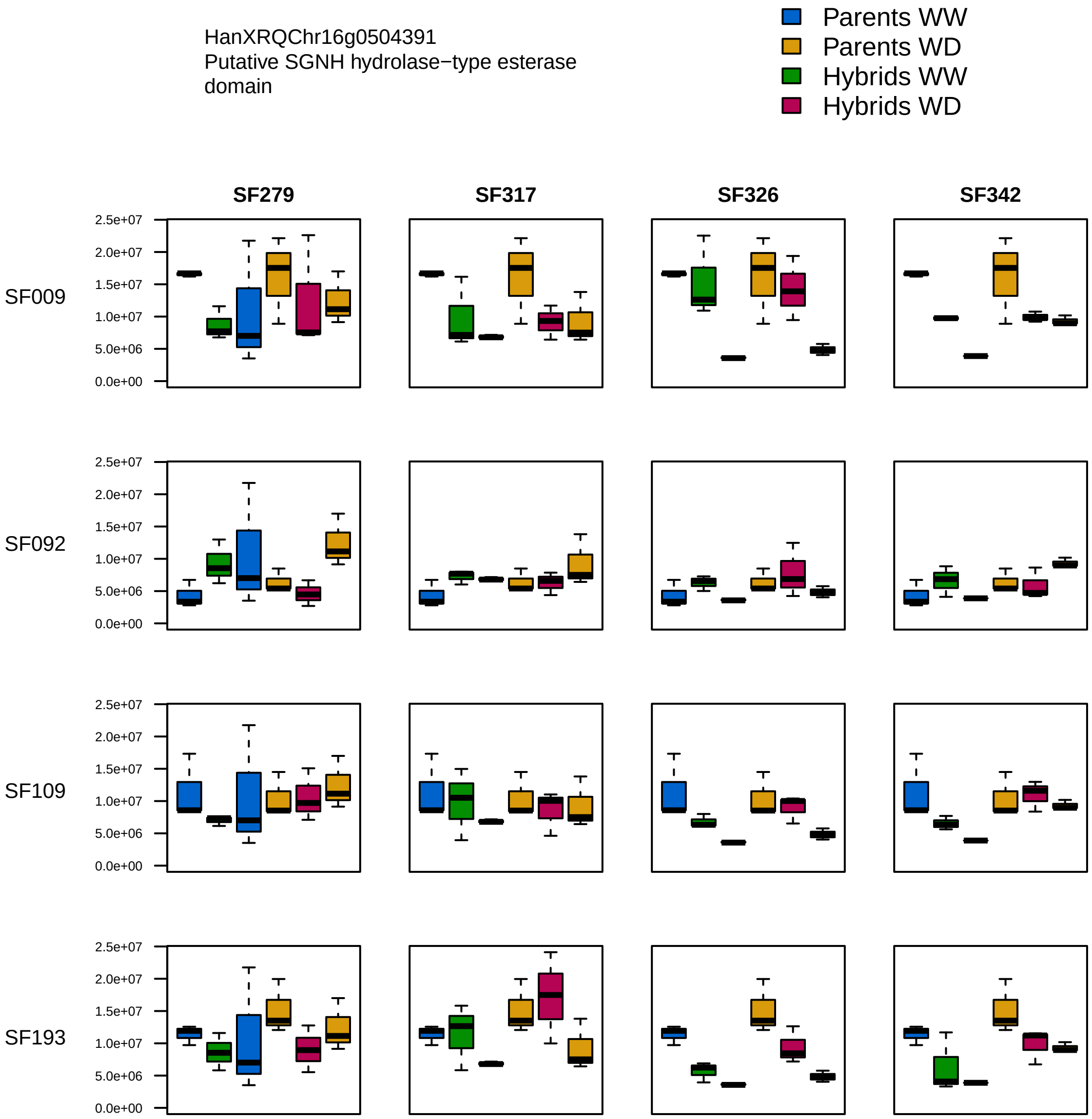
HanXRQChr16g0504391
Putative SGNH hydrolase−type esterase domain
Parents WW
Parents WD
Hybrids WW
Hybrids WD
SF279
SF317
SF326
SF342
SF009
SF092
SF109
SF193
2.5e+07
2.0e+07
1.5e+07
1.0e+07
5.0e+06
0.0e+00

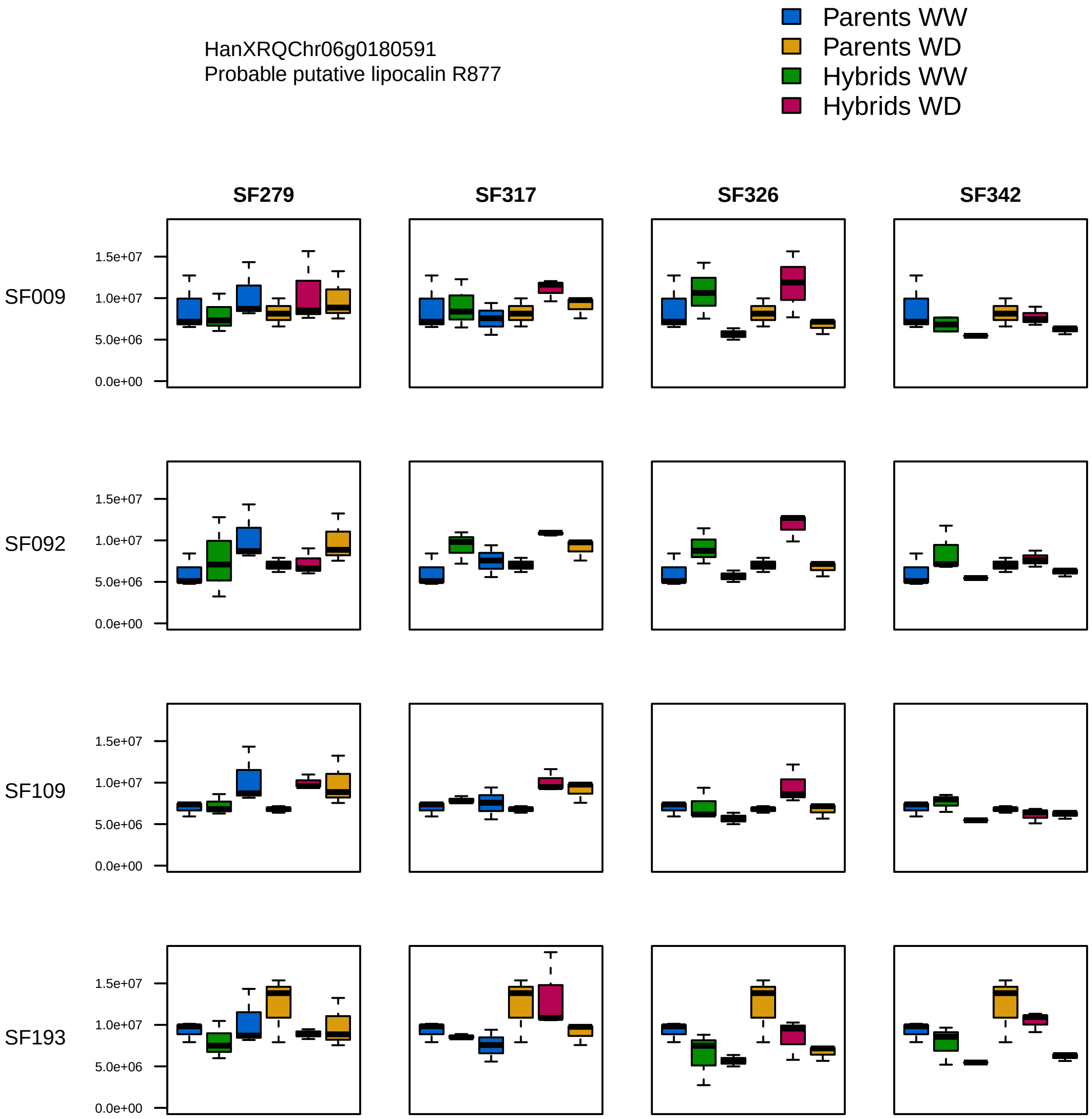

HanXRQChr06g0180591
Probable putative lipocalin R877
Parents WW
Parents WD
Hybrids WW
Hybrids WD
SF279
SF317
SF326
SF342
SF009
SF092
SF109
SF193
1.5e+07
1.0e+07
5.0e+06
0.0e+00

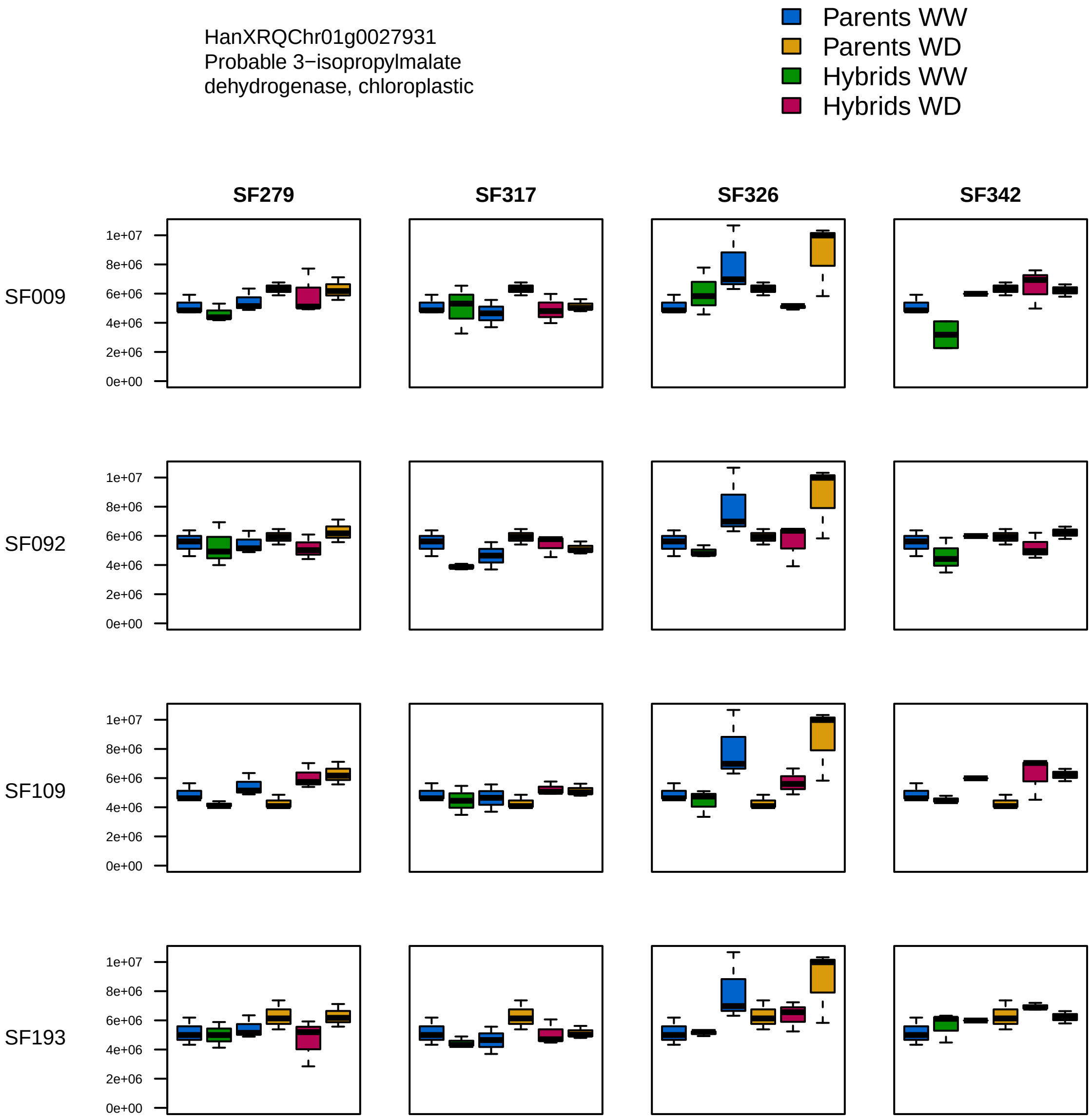
HanXRQChr01g0027931
Probable 3−isopropylmalate
dehydrogenase, chloroplastic
Parents WW
Parents WD
Hybrids WW
Hybrids WD
SF279
SF317
SF326
SF342
SF009
SF092
SF109
SF193
1e+07
8e+06
6e+06
4e+06
2e+06
0e+00

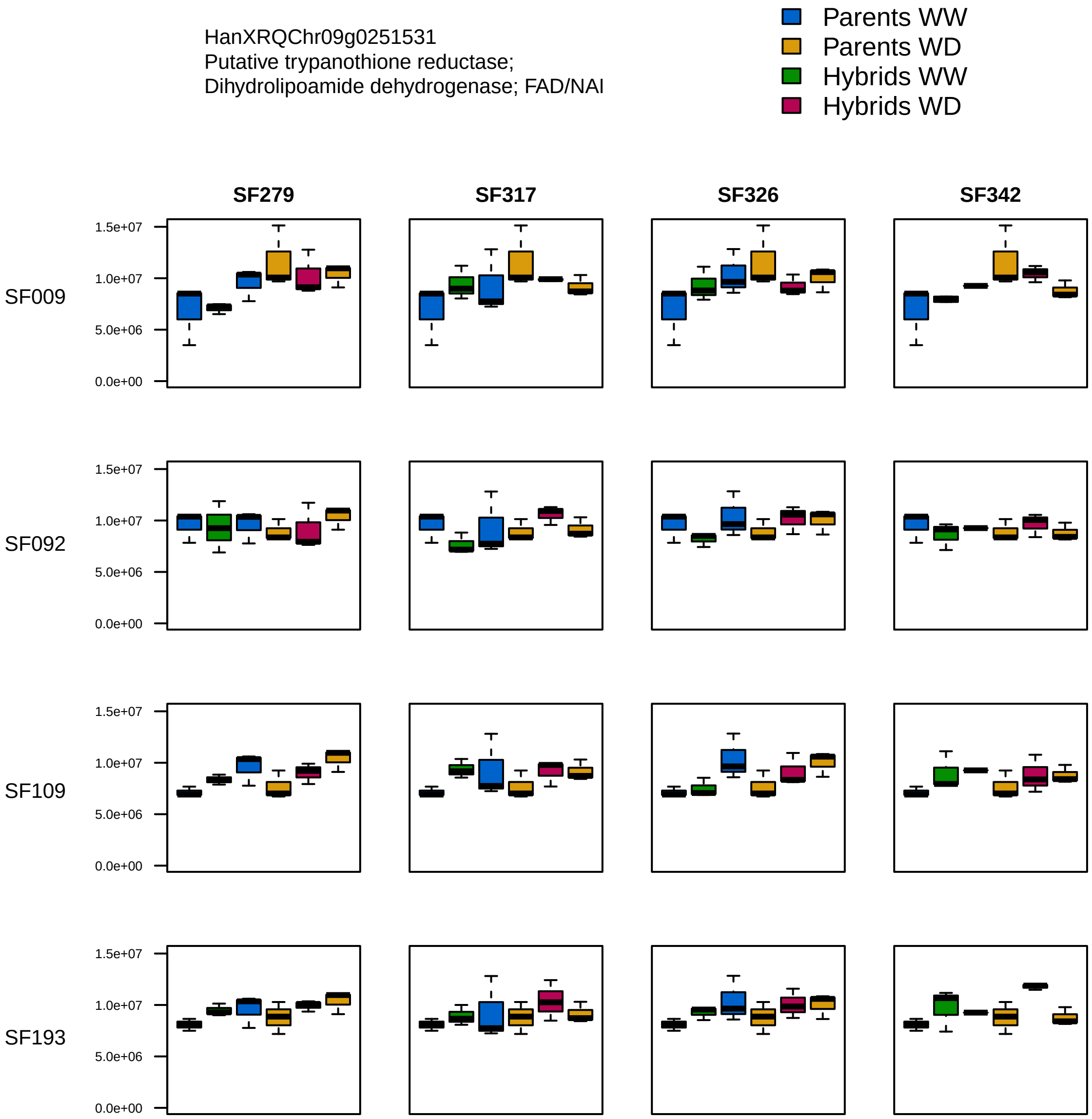
HanXRQChr09g0251531
Putative trypanothione reductase;
Dihydrolipoamide dehydrogenase; FAD/NAI
Parents WW
Parents WD
Hybrids WW
Hybrids WD
SF279
SF317
SF326
SF342
SF009
SF092
SF109
SF193
1.5e+07
1.0e+07
5.0e+06
0.0e+00

HanXRQChr10g0302621
Putative ribosomal protein S20

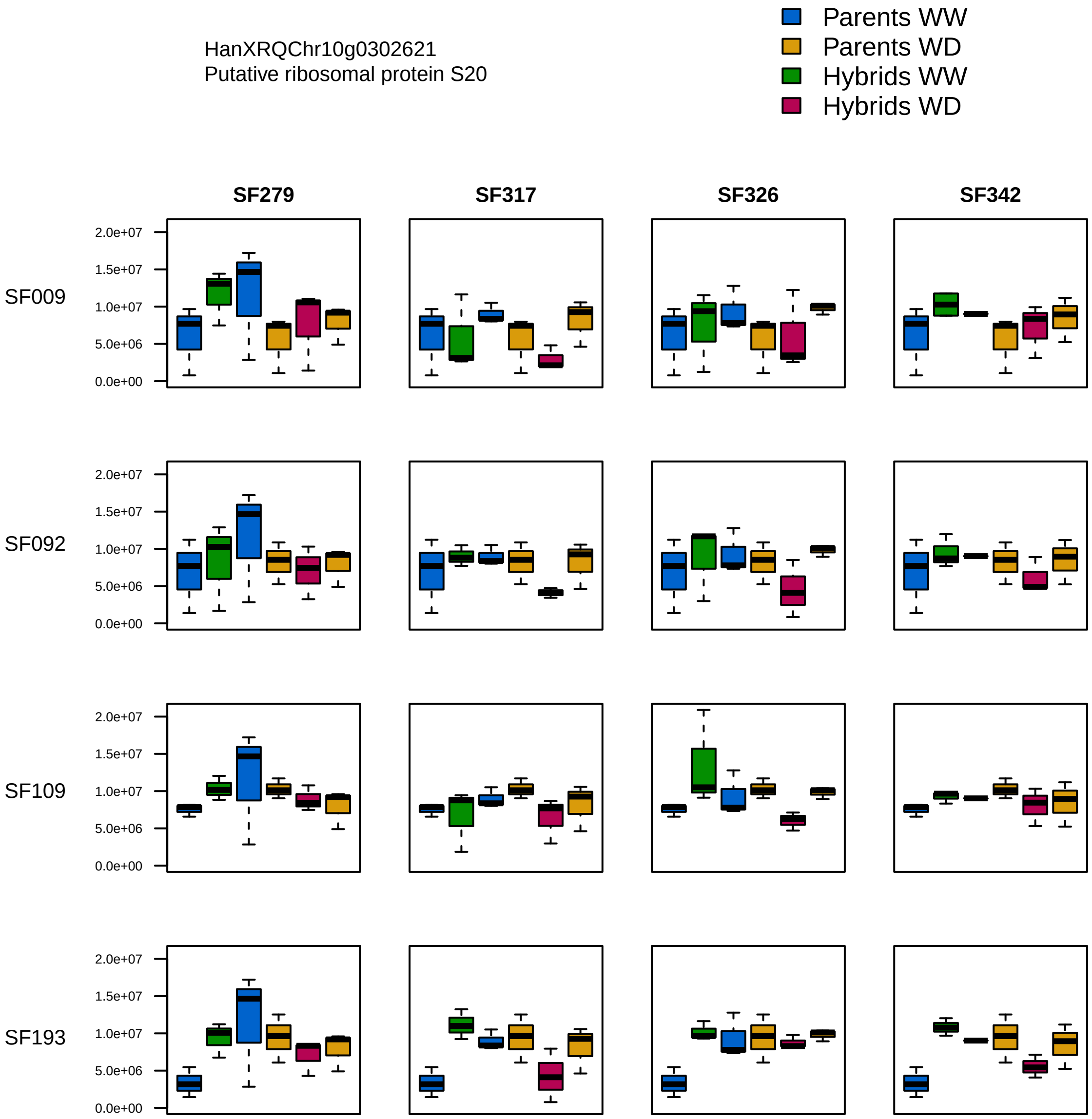

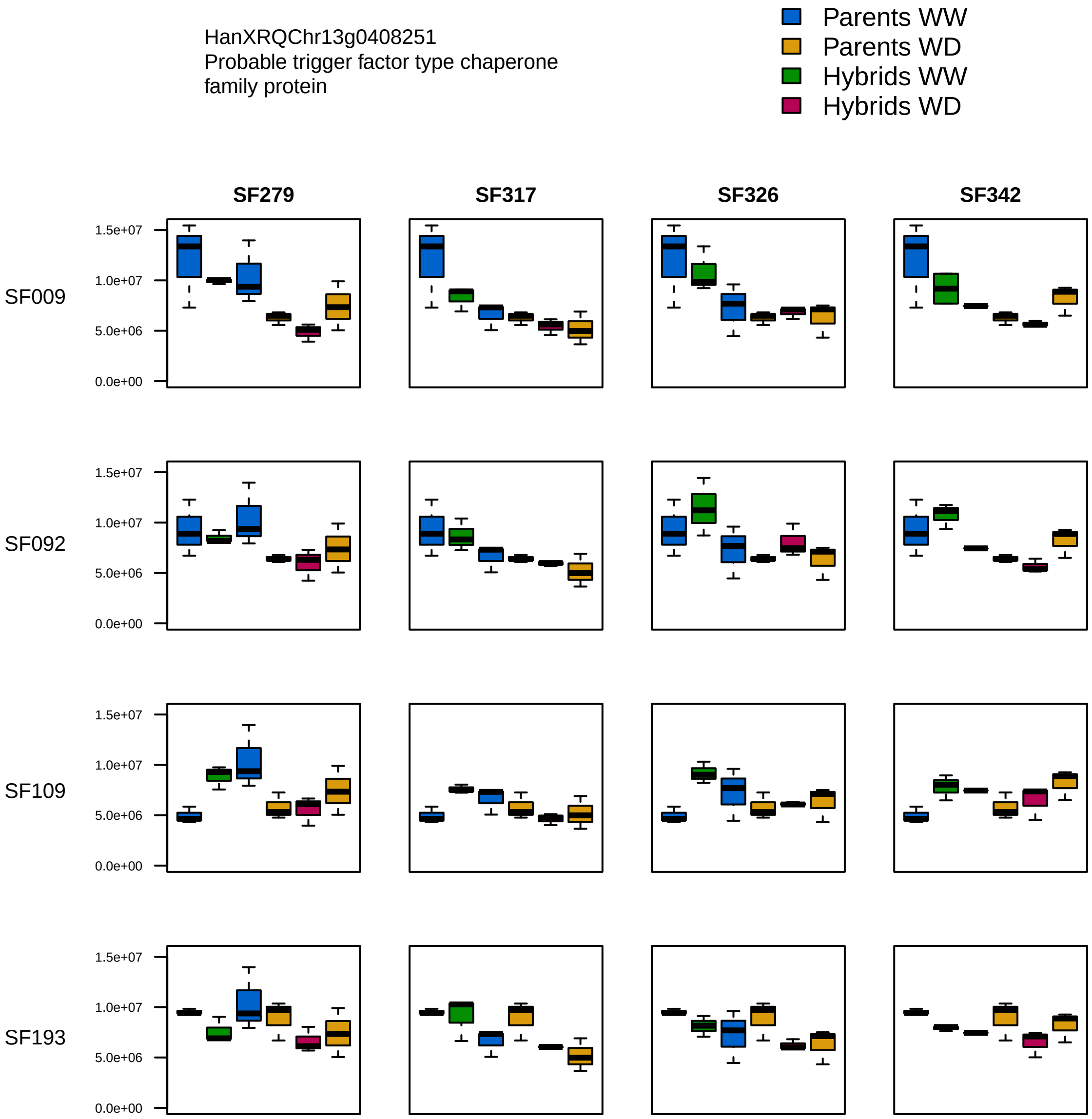
HanXRQChr13g0408251
Probable trigger factor type chaperone
family protein
Parents WW
Parents WD
Hybrids WW
Hybrids WD
SF279
SF317
SF326
SF342
SF009
SF092
SF109
SF193
1.5e+07
1.0e+07
5.0e+06
0.0e+00

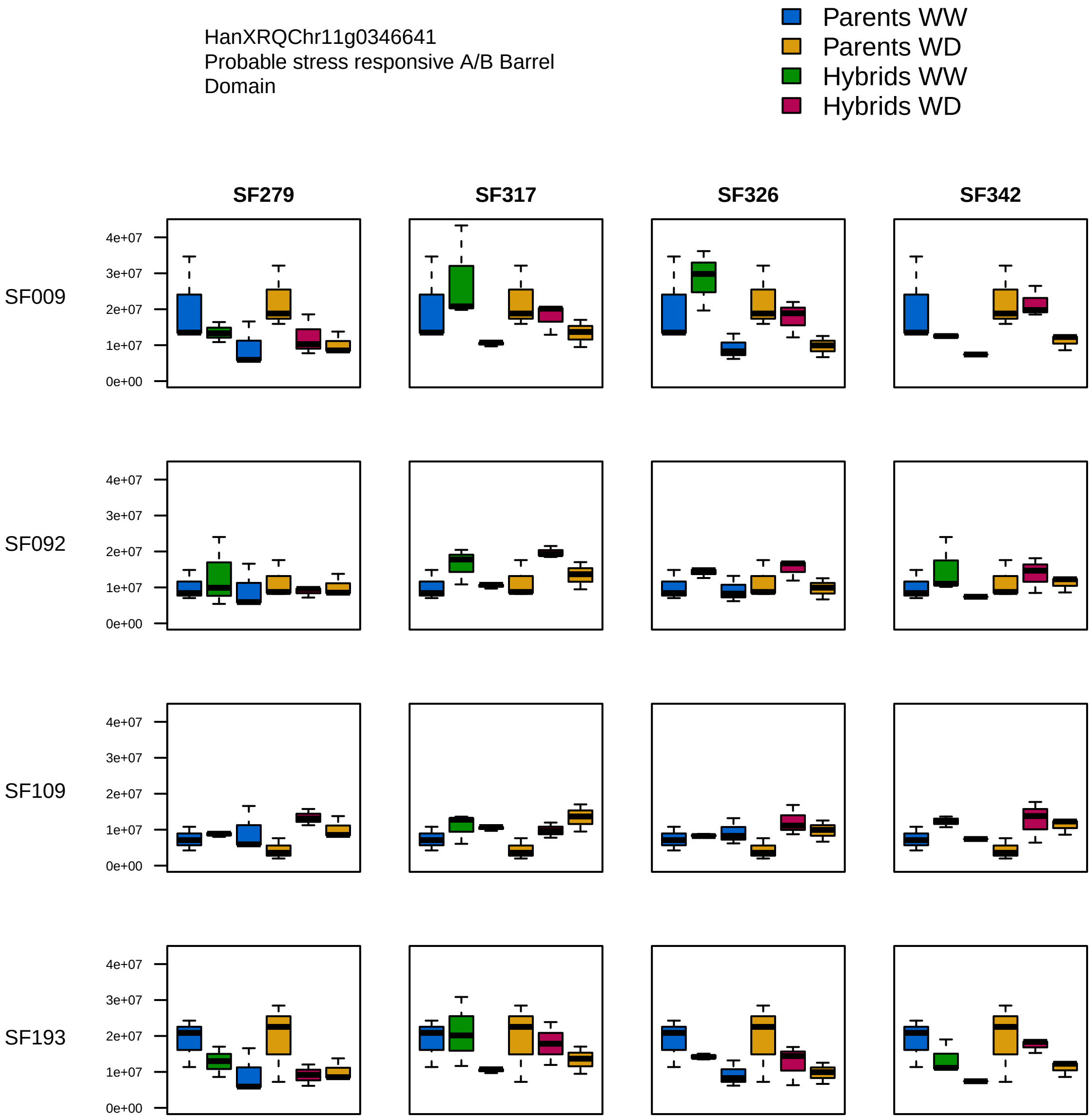
HanXRQChr11g0346641
Probable stress responsive A/B Barrel Domain
Parents WW
Parents WD
Hybrids WW
Hybrids WD
SF279
SF317
SF326
SF342
SF009
SF092
SF109
SF193
4e+07
3e+07
2e+07
1e+07
0e+00

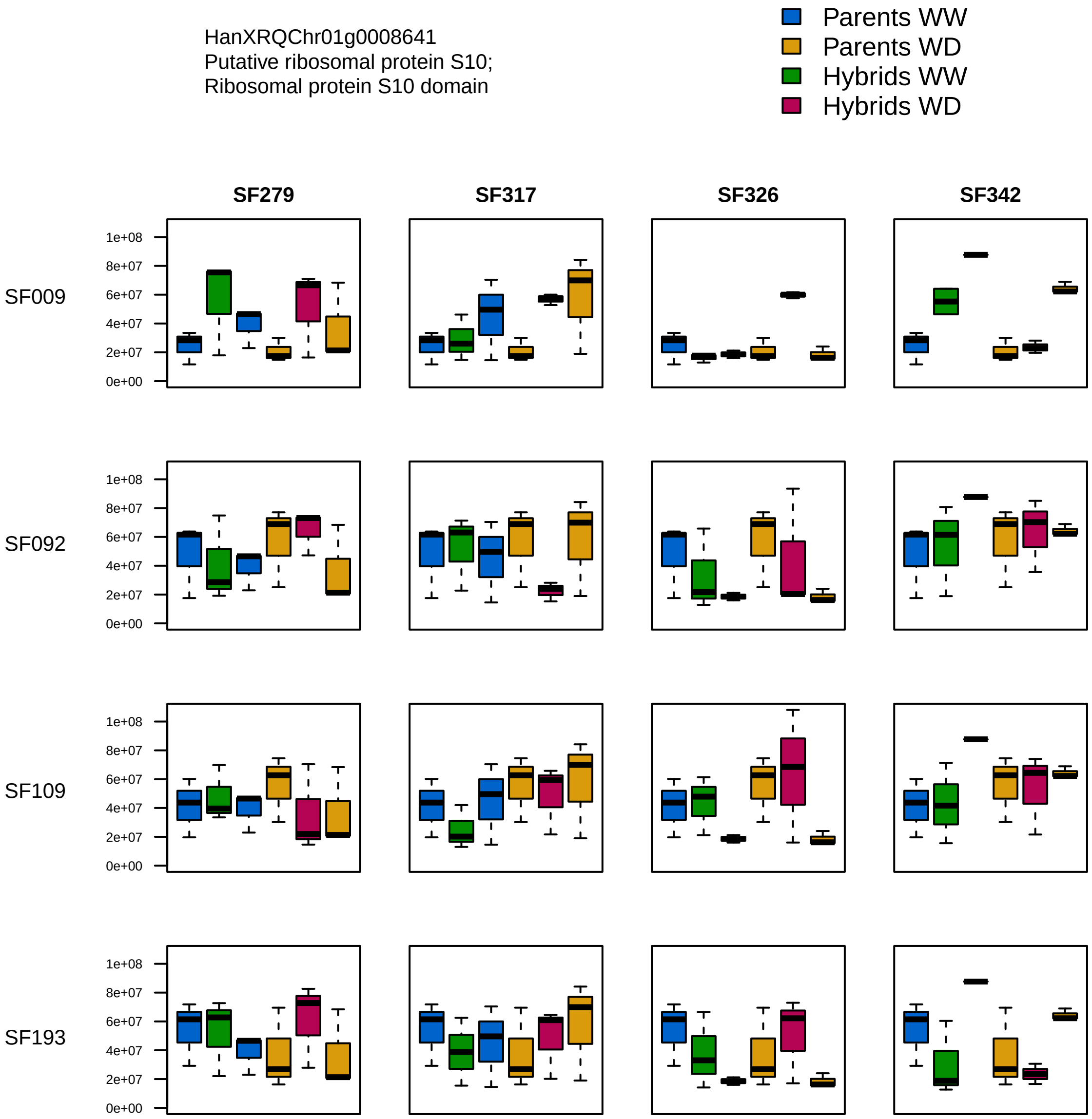
HanXRQChr01g0008641
Putative ribosomal protein S10;
Ribosomal protein S10 domain
Parents WW
Parents WD
Hybrids WW
Hybrids WD
SF279
SF317
SF326
SF342
SF009
SF092
SF109
SF193
1e+08
8e+07
6e+07
4e+07
2e+07
0e+00

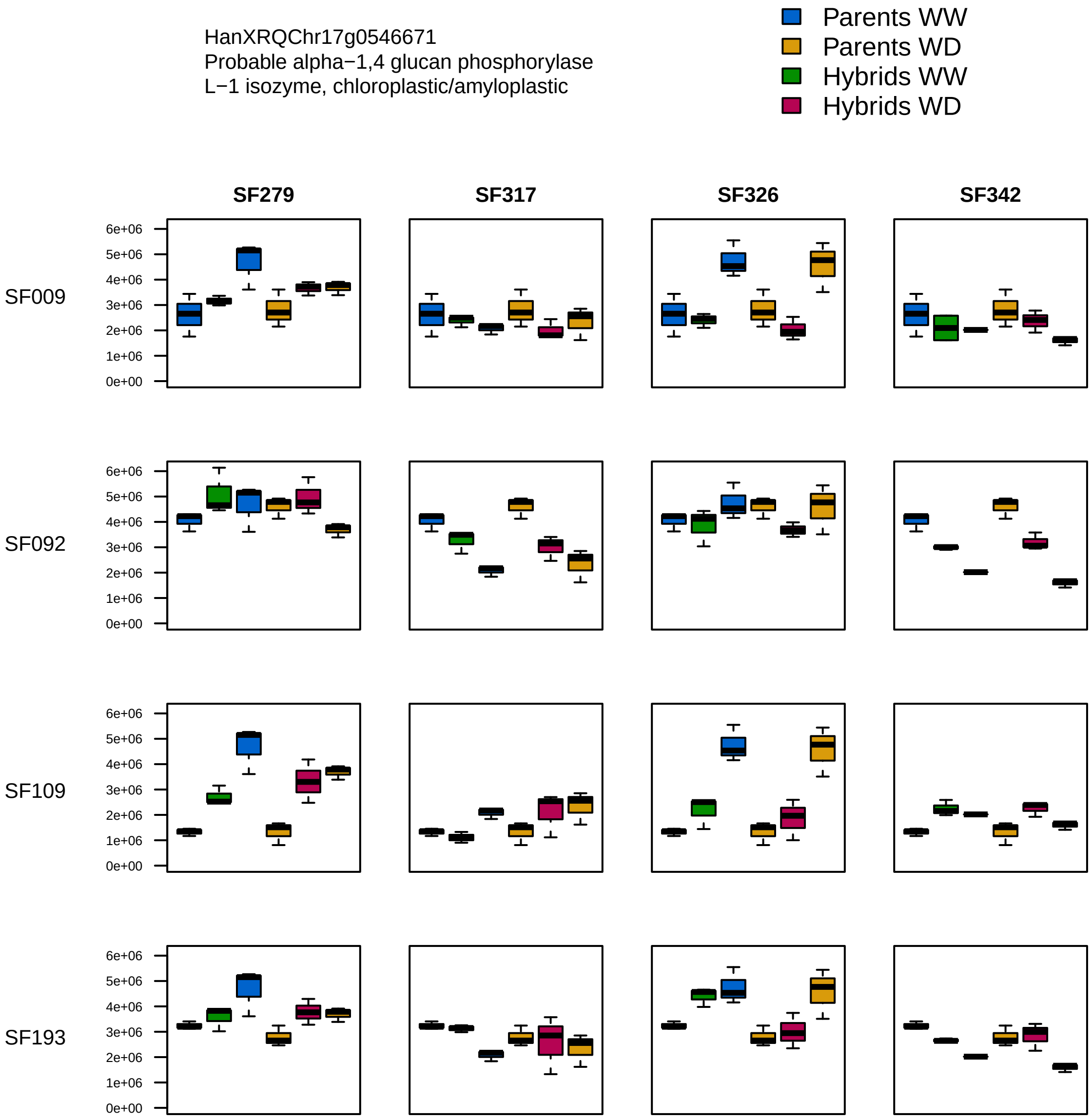
HanXRQChr17g0546671
Probable alpha−1,4 glucan phosphorylase
L−1 isozyme, chloroplastic/amyloplastic
Parents WW
Parents WD
Hybrids WW
Hybrids WD
SF279
SF317
SF326
SF342
SF009
SF092
SF109
SF193
0e+00
1e+06
2e+06
3e+06
4e+06
5e+06
6e+06

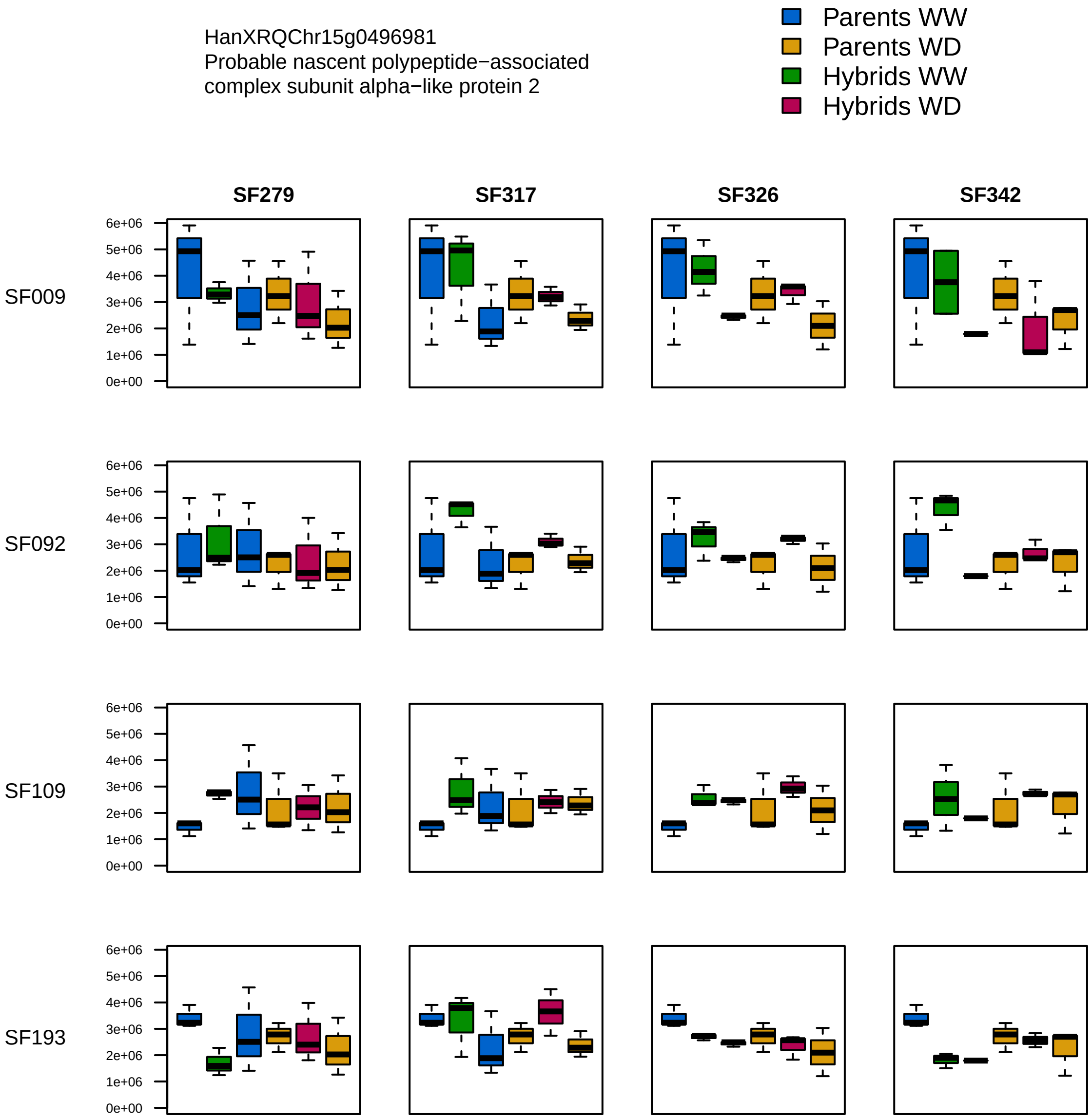
HanXRQChr15g0496981
Probable nascent polypeptide−associated
complex subunit alpha−like protein 2
Parents WW
Parents WD
Hybrids WW
Hybrids WD
SF279
SF317
SF326
SF342
SF009
SF092
SF109
SF193
6e+06
5e+06
4e+06
3e+06
2e+06
1e+06
0e+00

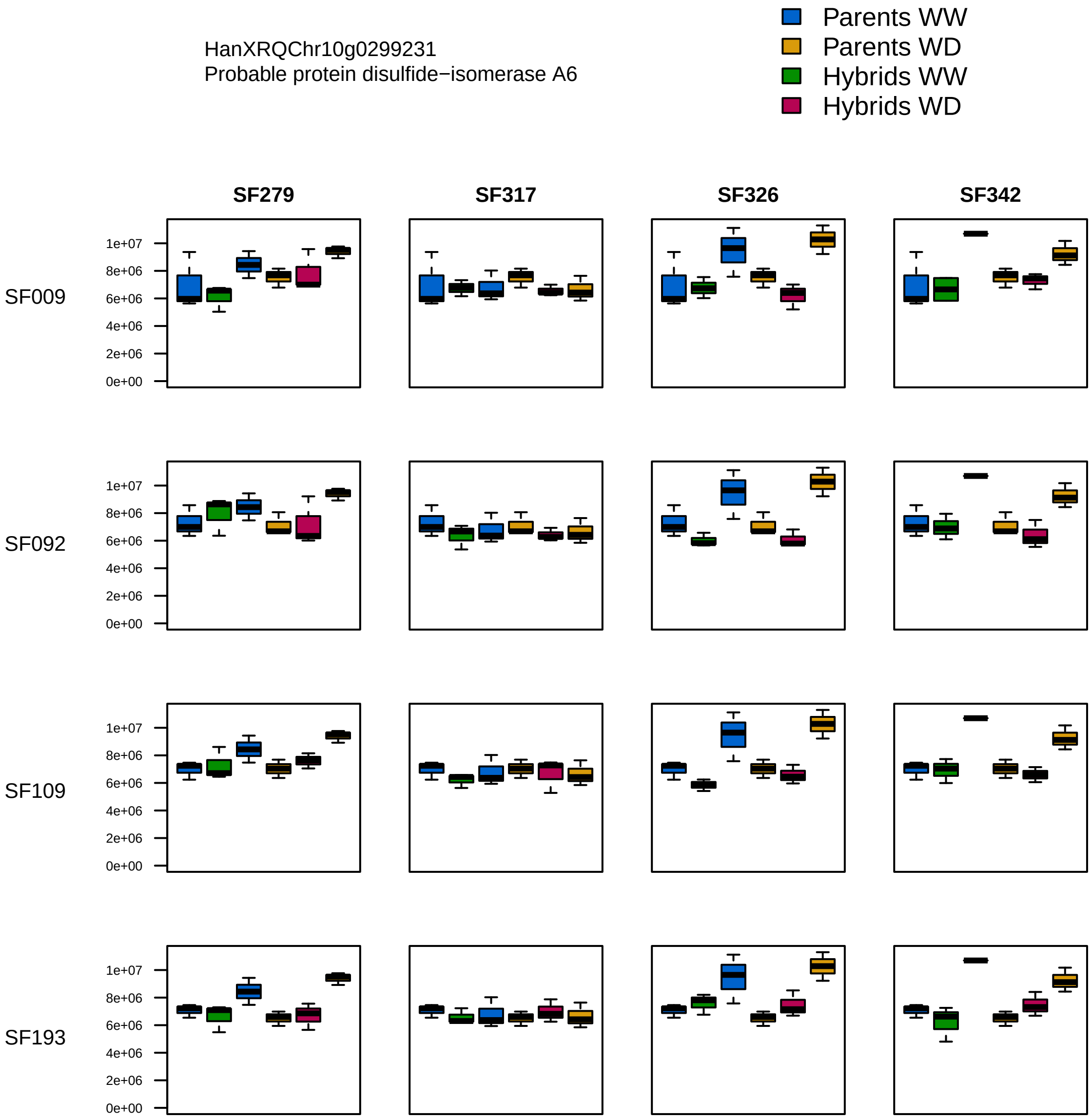
HanXRQChr10g0299231
Probable protein disulfide−isomerase A6
Parents WW
Parents WD
Hybrids WW
Hybrids WD
SF279
SF317
SF326
SF342
SF009
SF092
SF109
SF193
0e+00
2e+06
4e+06
6e+06
8e+06
1e+07

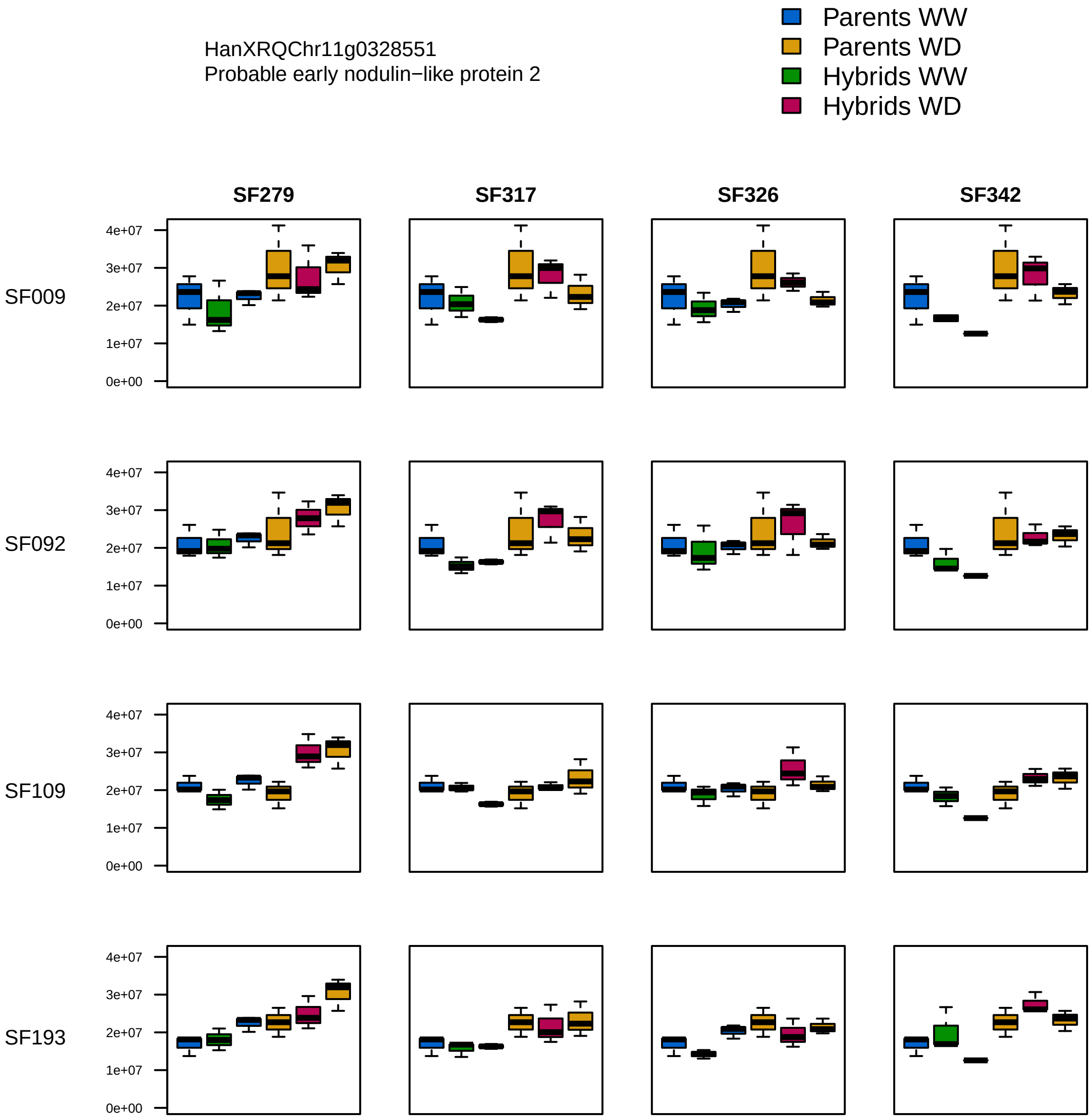
HanXRQChr11g0328551
Probable early nodulin−like protein 2
Parents WW
Parents WD
Hybrids WW
Hybrids WD
SF279
SF317
SF326
SF342
SF009
SF092
SF109
SF193
4e+07
3e+07
2e+07
1e+07
0e+00

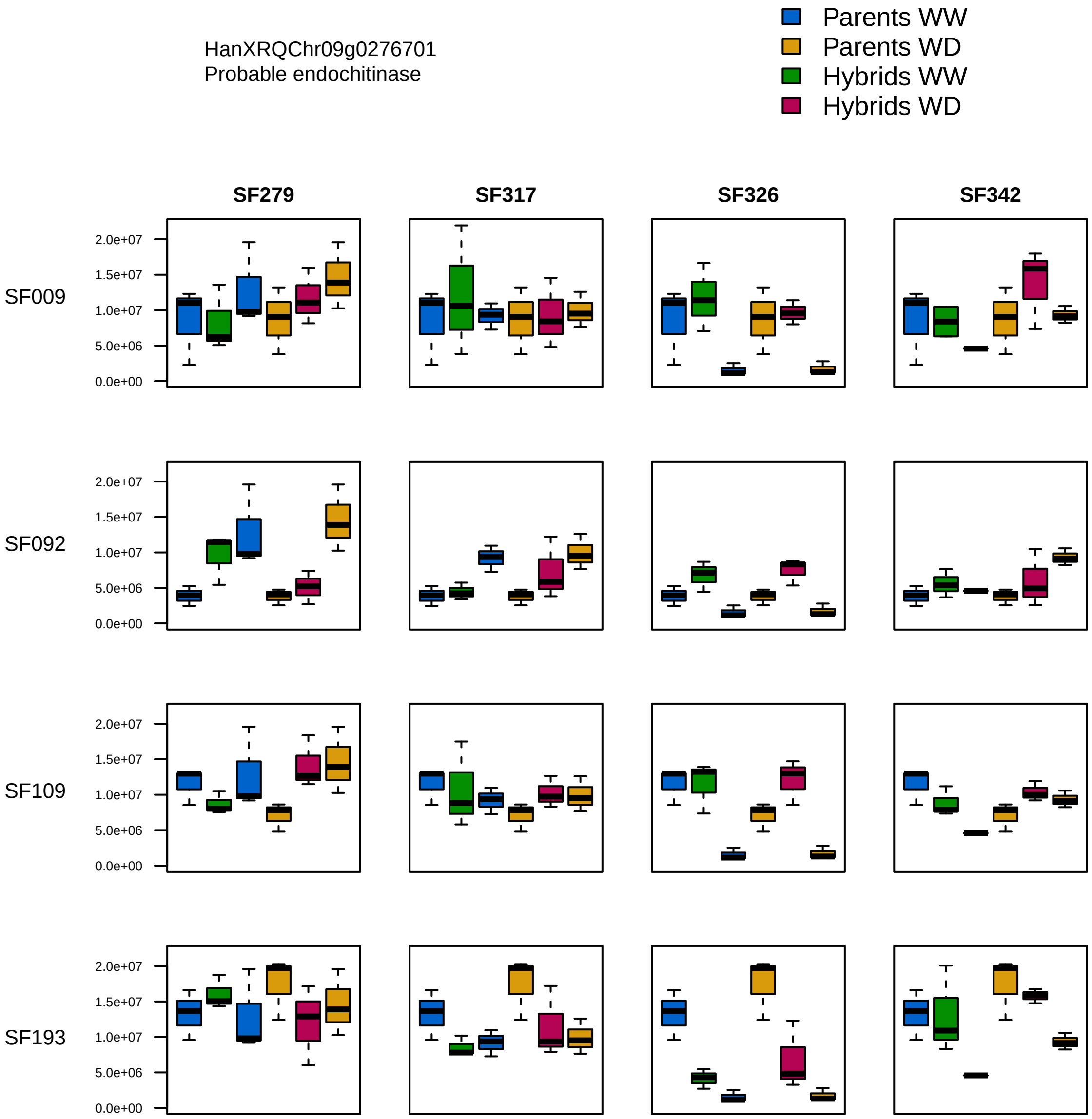

HanXRQChr09g0276701
Probable endochitinase
Parents WW
Parents WD
Hybrids WW
Hybrids WD
SF279
SF317
SF326
SF342
SF009
SF092
SF109
SF193
2.0e+07
1.5e+07
1.0e+07
5.0e+06
0.0e+00

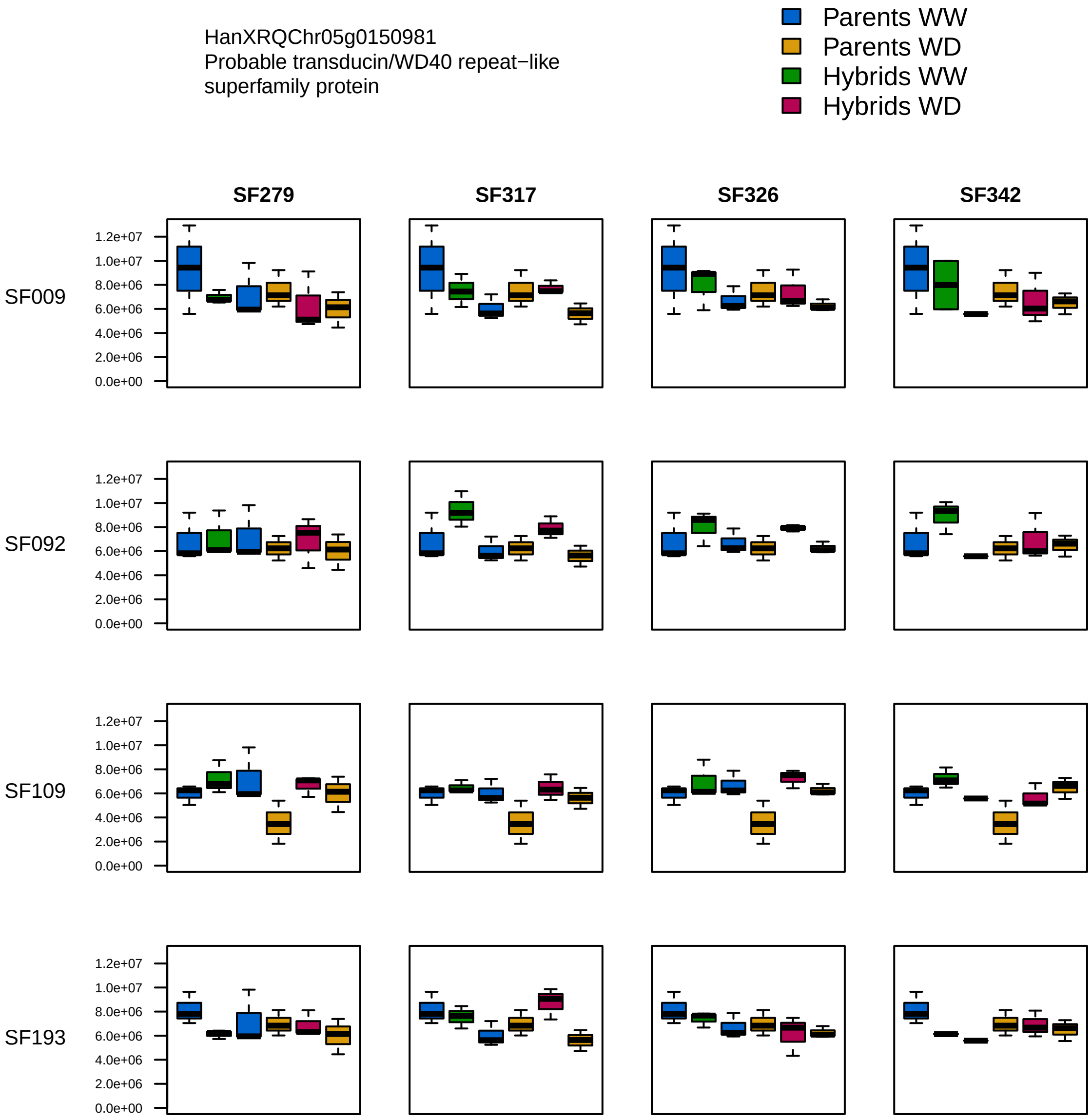
HanXRQChr05g0150981
Probable transducin/WD40 repeat−like
superfamily protein
Parents WW
Parents WD
Hybrids WW
Hybrids WD
SF279
SF317
SF326
SF342
SF009
SF092
SF109
SF193
1.2e+07
1.0e+07
8.0e+06
6.0e+06
4.0e+06
2.0e+06
0.0e+00

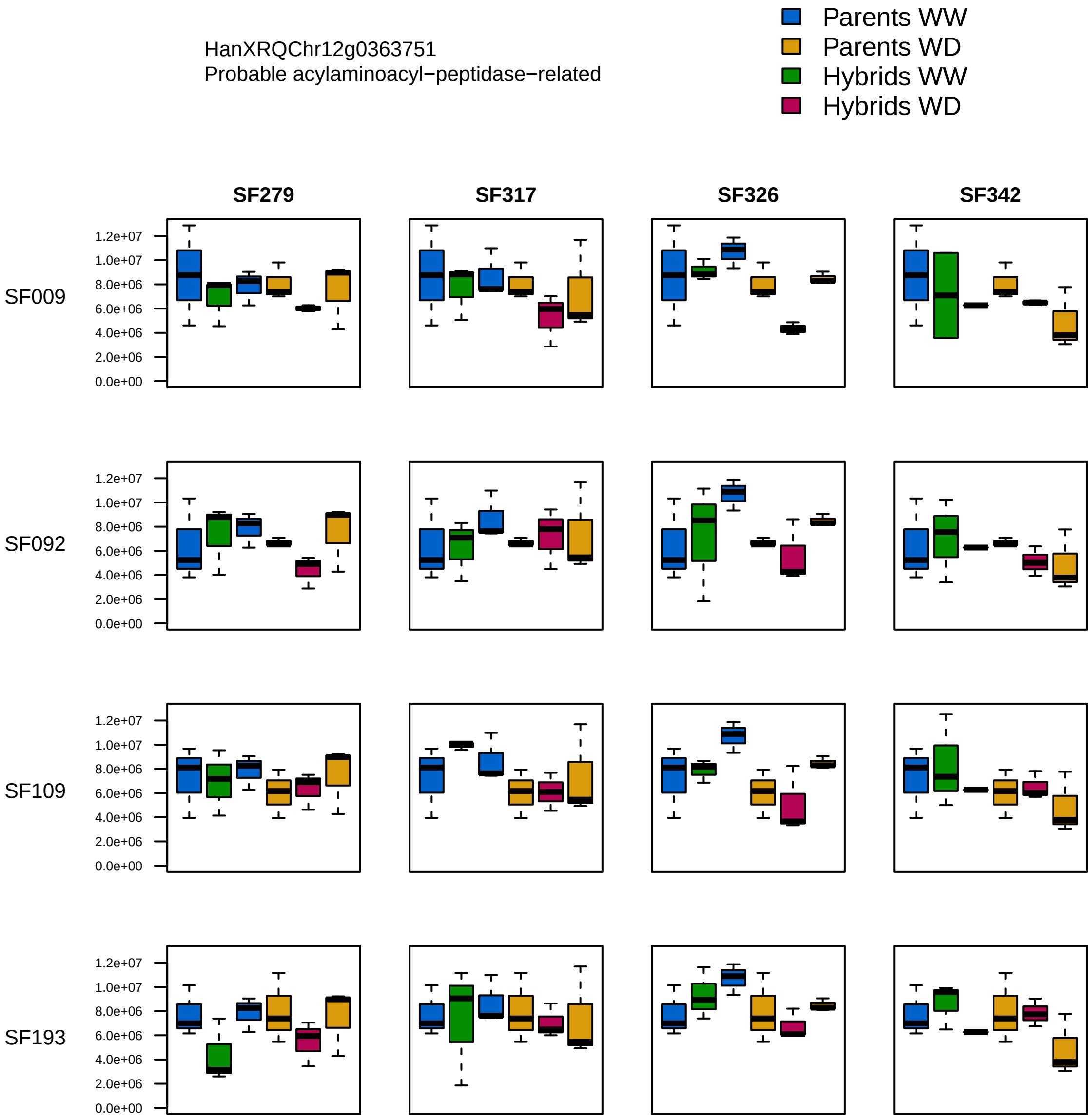
HanXRQChr12g0363751
Probable acylaminoacyl−peptidase−related
Parents WW
Parents WD
Hybrids WW
Hybrids WD
SF279
SF317
SF326
SF342
SF009
SF092
SF109
SF193
1.2e+07
1.0e+07
8.0e+06
6.0e+06
4.0e+06
2.0e+06
0.0e+00

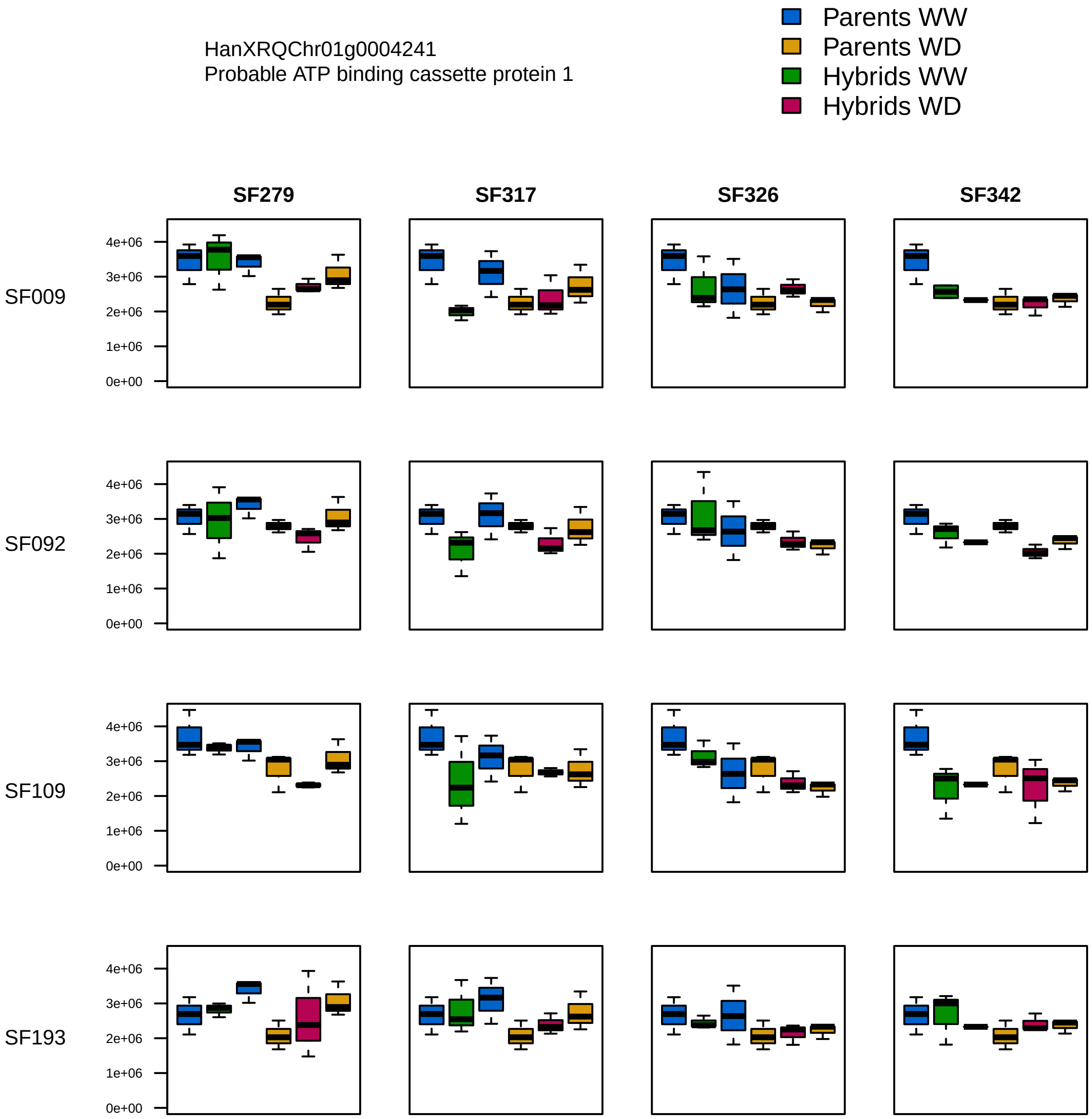
HanXRQChr01g0004241
Probable ATP binding cassette protein 1
Parents WW
Parents WD
Hybrids WW
Hybrids WD
SF279
SF317
SF326
SF342
SF009
SF092
SF109
SF193
4e+06
3e+06
2e+06
1e+06
0e+00

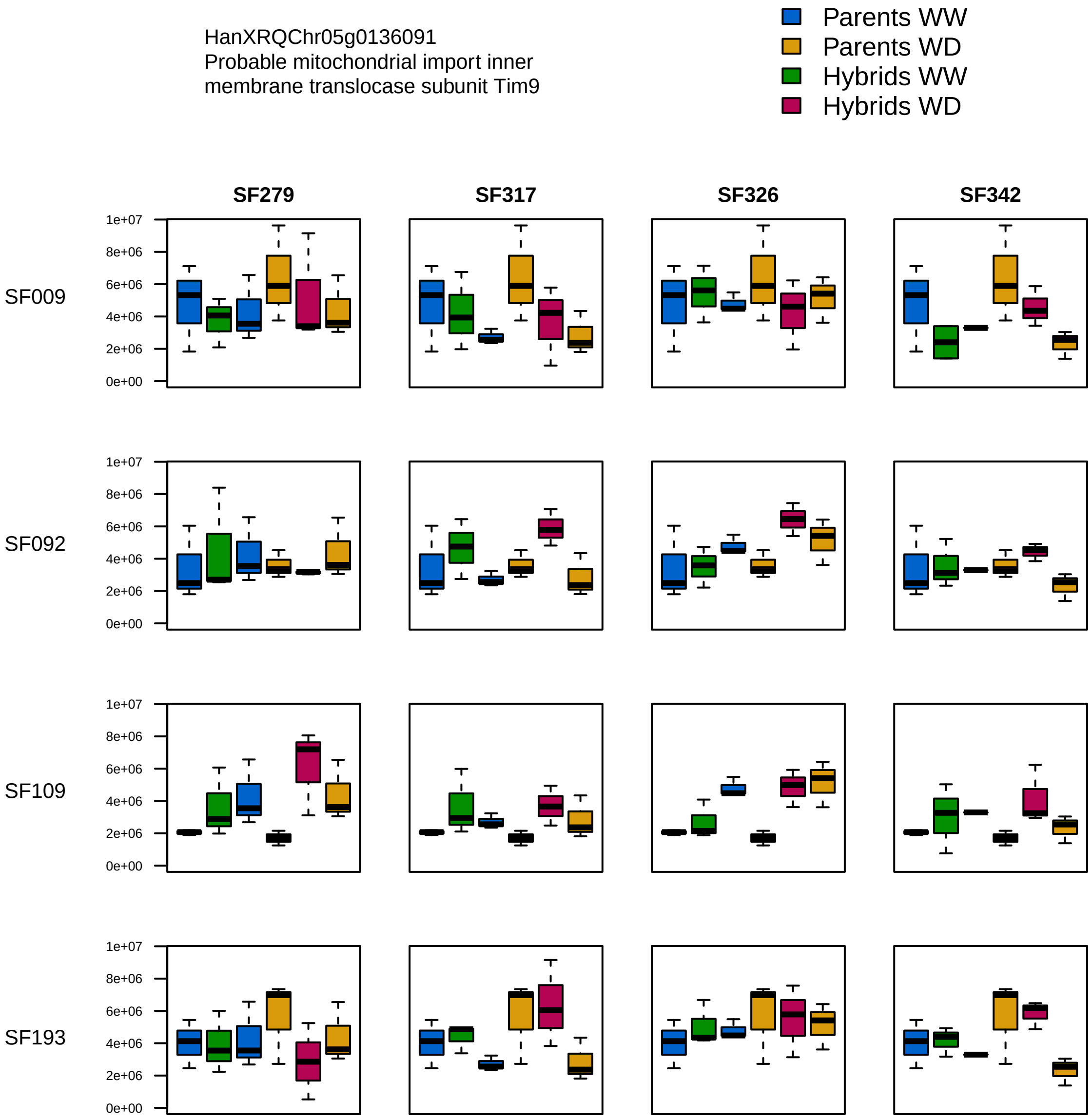
HanXRQChr05g0136091
Probable mitochondrial import inner
membrane translocase subunit Tim9
Parents WW
Parents WD
Hybrids WW
Hybrids WD
SF279
SF317
SF326
SF342
SF009
SF092
SF109
SF193
1e+07
8e+06
6e+06
4e+06
2e+06
0e+00

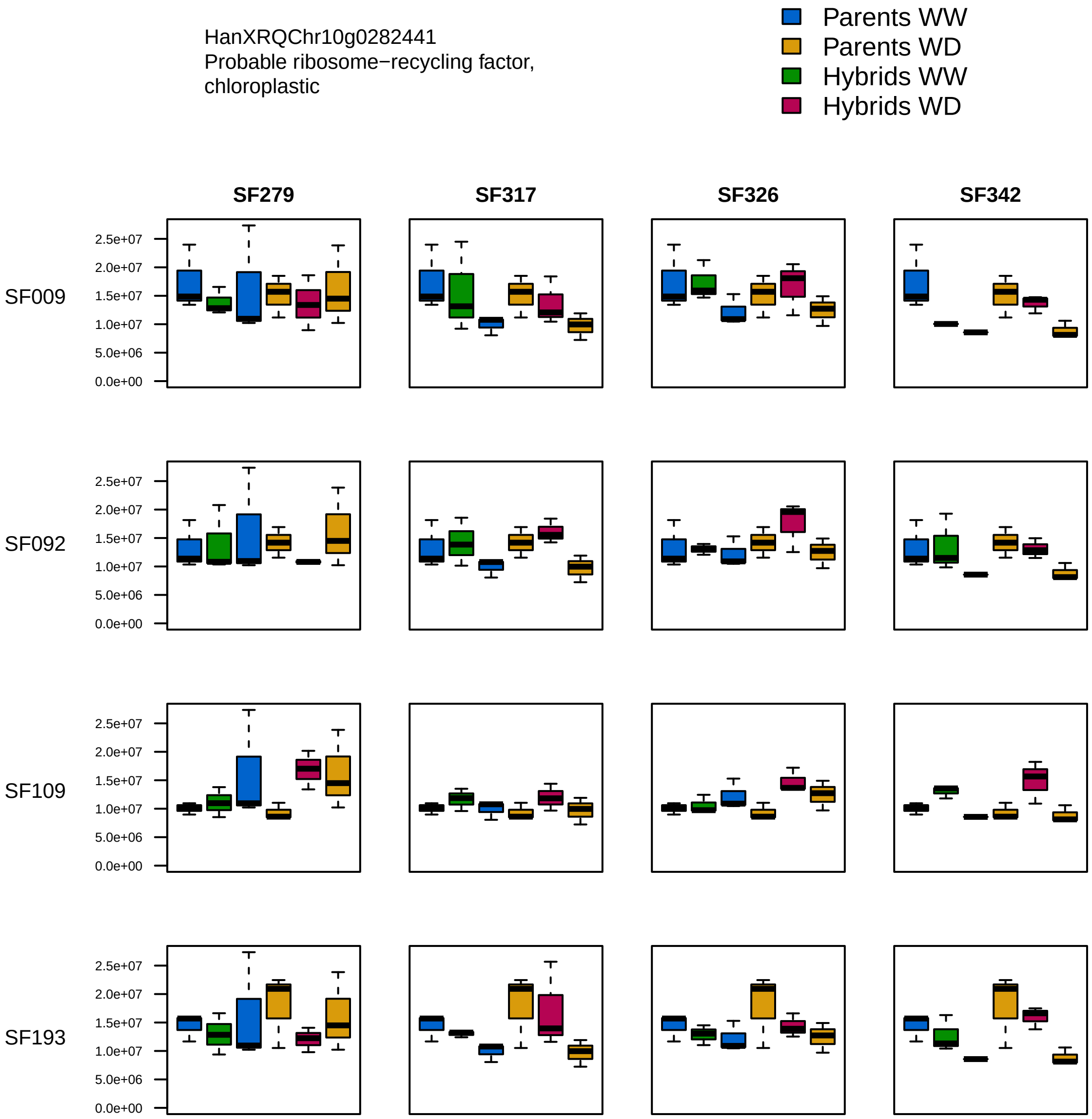
HanXRQChr10g0282441
Probable ribosome−recycling factor, chloroplastic
Parents WW
Parents WD
Hybrids WW
Hybrids WD
SF279
SF317
SF326
SF342
SF009
SF092
SF109
SF193
2.5e+07
2.0e+07
1.5e+07
1.0e+07
5.0e+06
0.0e+00

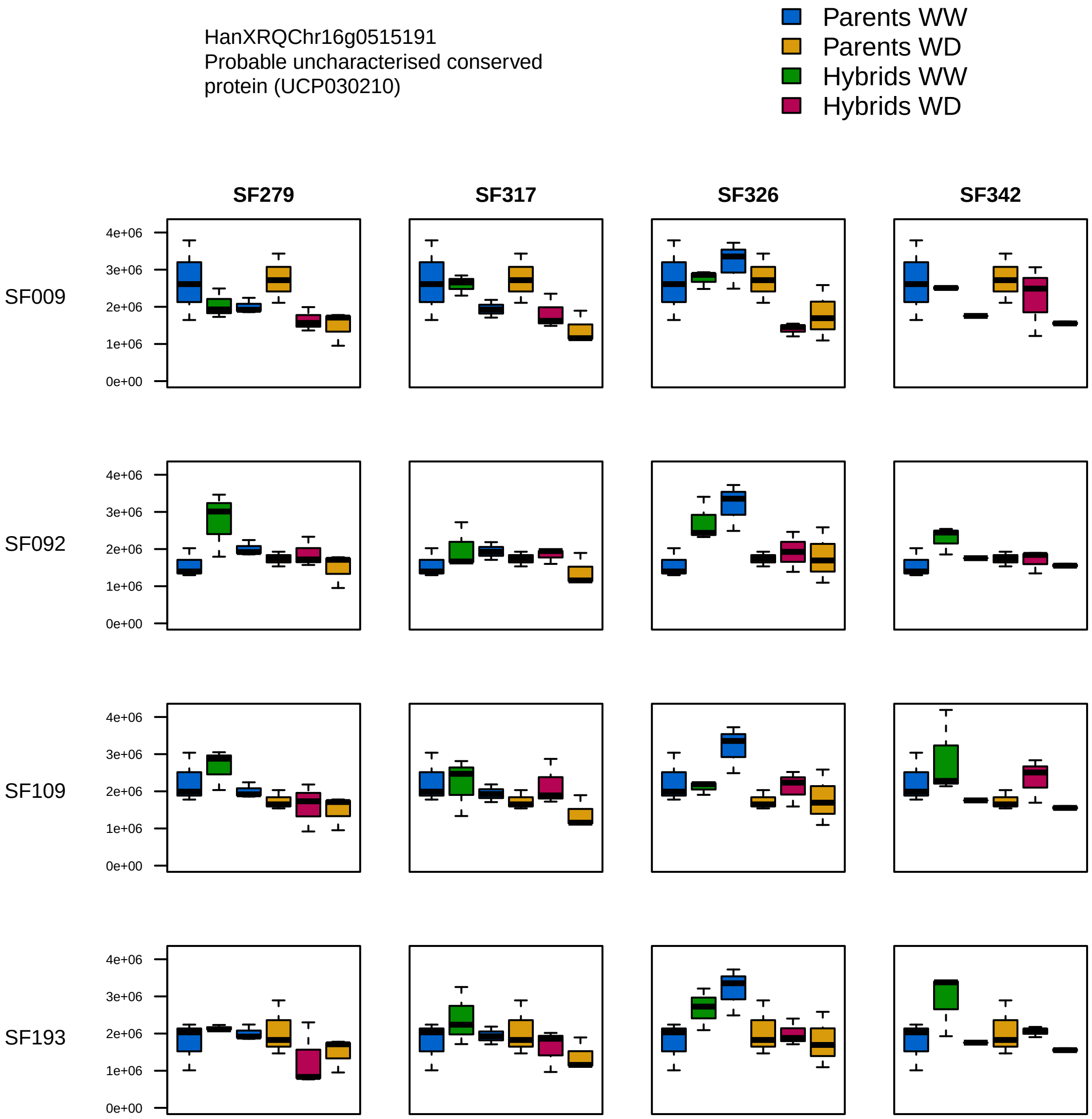
HanXRQChr16g0515191
Probable uncharacterised conserved protein (UCP030210)
Parents WW
Parents WD
Hybrids WW
Hybrids WD
SF279
SF317
SF326
SF342
SF009
SF092
SF109
SF193
0e+00
1e+06
2e+06
3e+06
4e+06

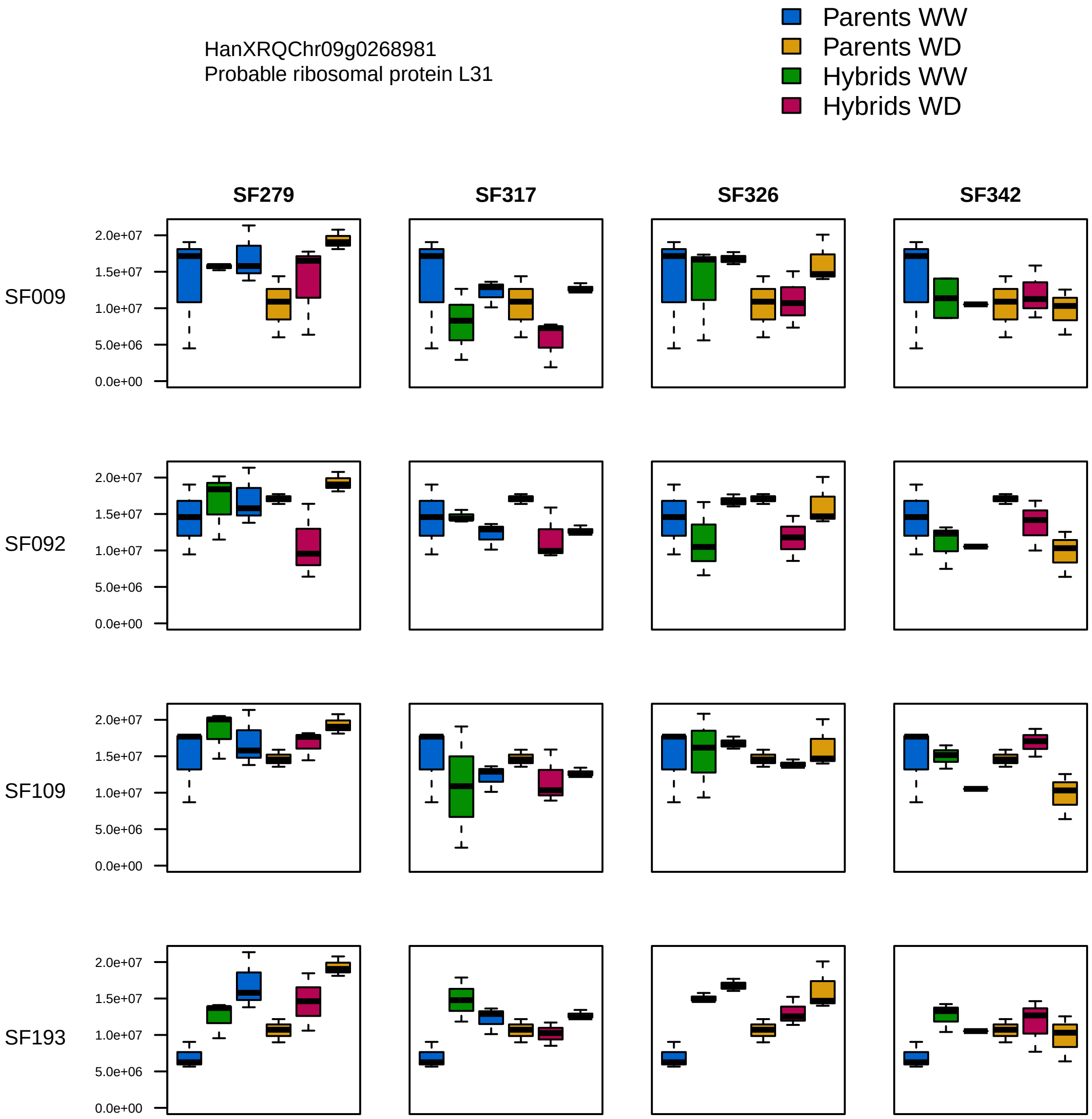

HanXRQChr09g0268981
Probable ribosomal protein L31
Parents WW
Parents WD
Hybrids WW
Hybrids WD
SF279
SF317
SF326
SF342
SF009
SF092
SF109
SF193
2.0e+07
1.5e+07
1.0e+07
5.0e+06
0.0e+00

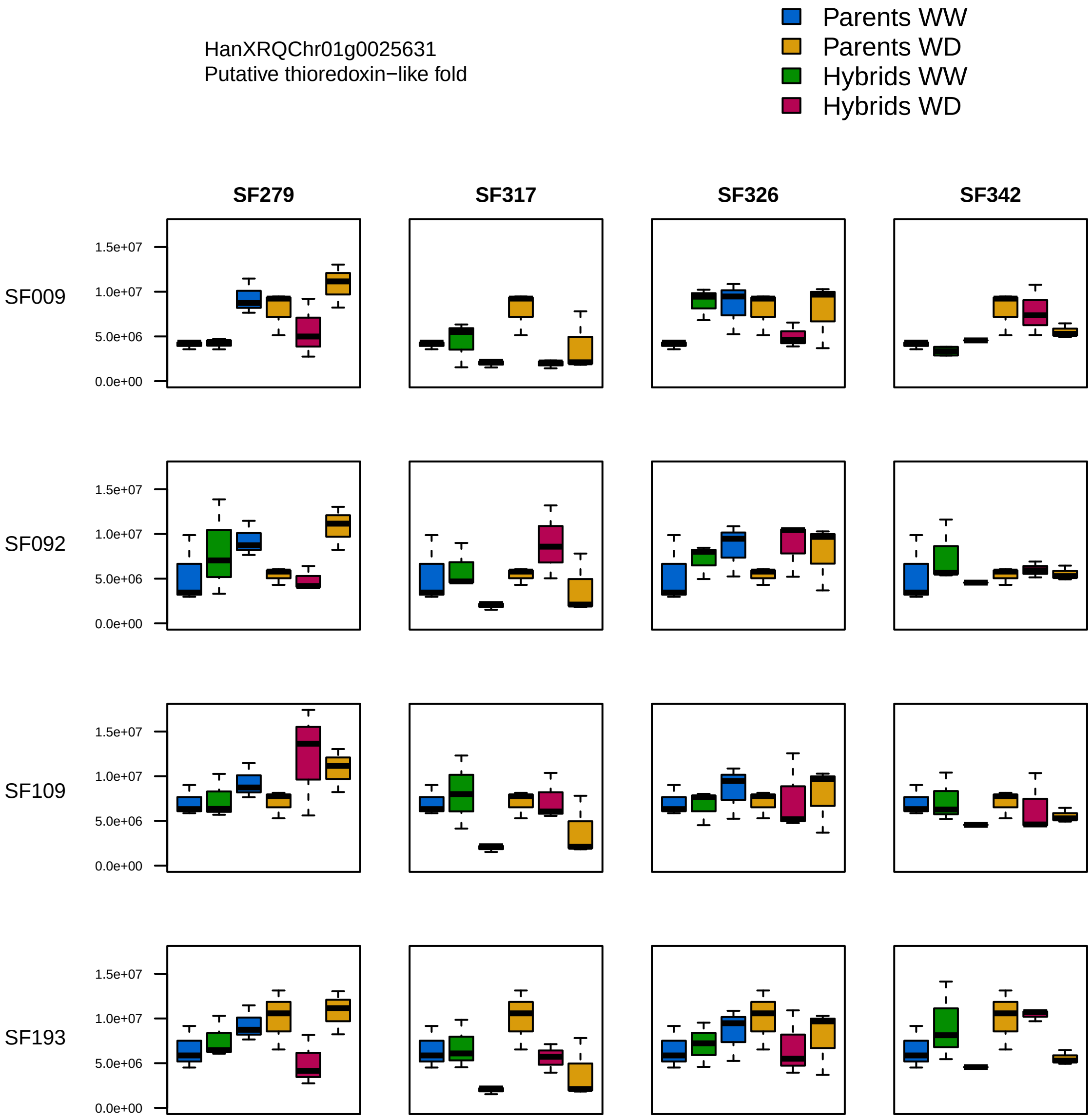

HanXRQChr01g0025631
Putative thioredoxin−like fold
Parents WW
Parents WD
Hybrids WW
Hybrids WD
SF279
SF317
SF326
SF342
SF009
SF092
SF109
SF193
1.5e+07
1.0e+07
5.0e+06
0.0e+00

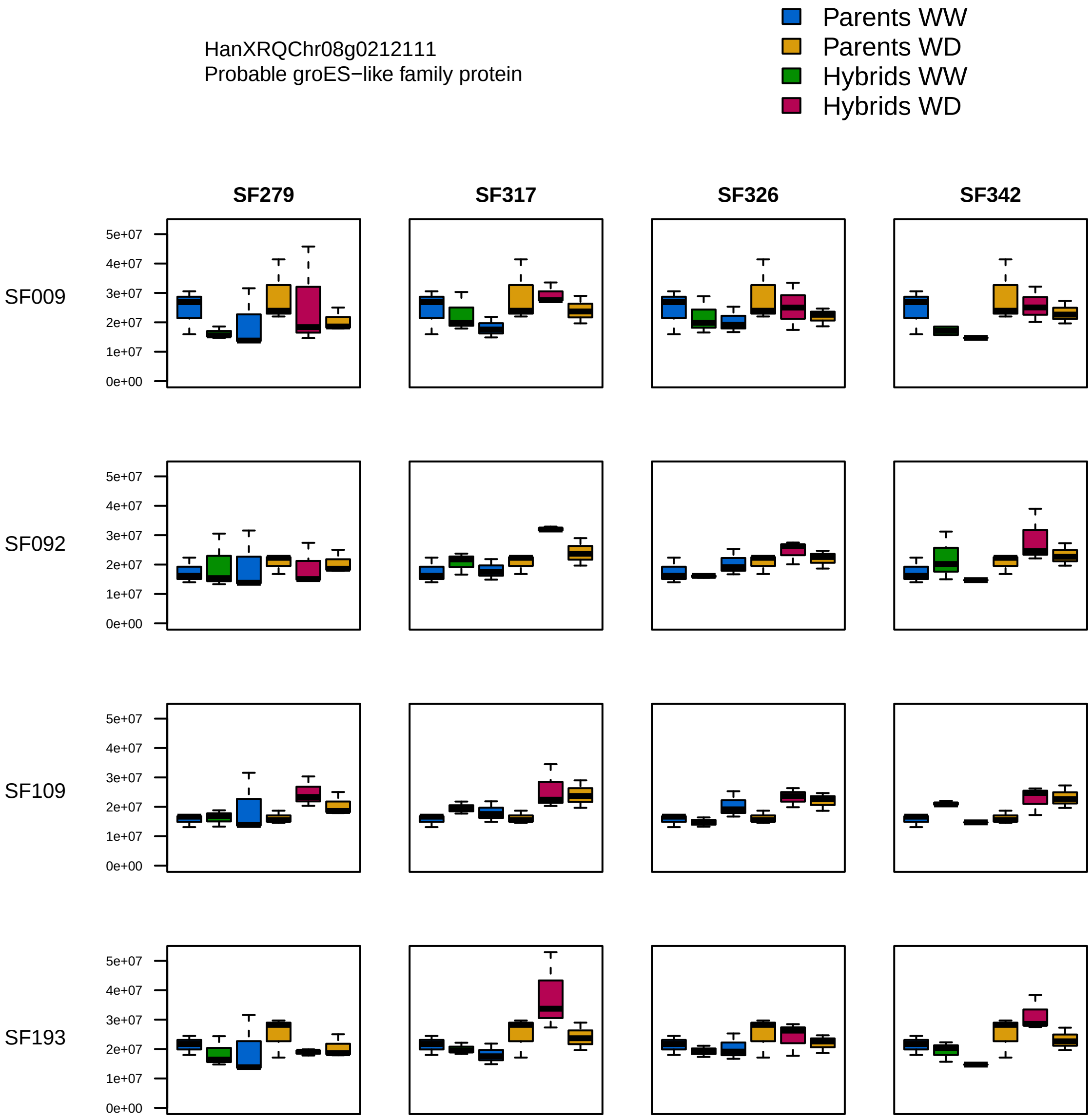
HanXRQChr08g0212111
Probable groES−like family protein
Parents WW
Parents WD
Hybrids WW
Hybrids WD
SF279
SF317
SF326
SF342
SF009
SF092
SF109
SF193
5e+07
4e+07
3e+07
2e+07
1e+07
0e+00

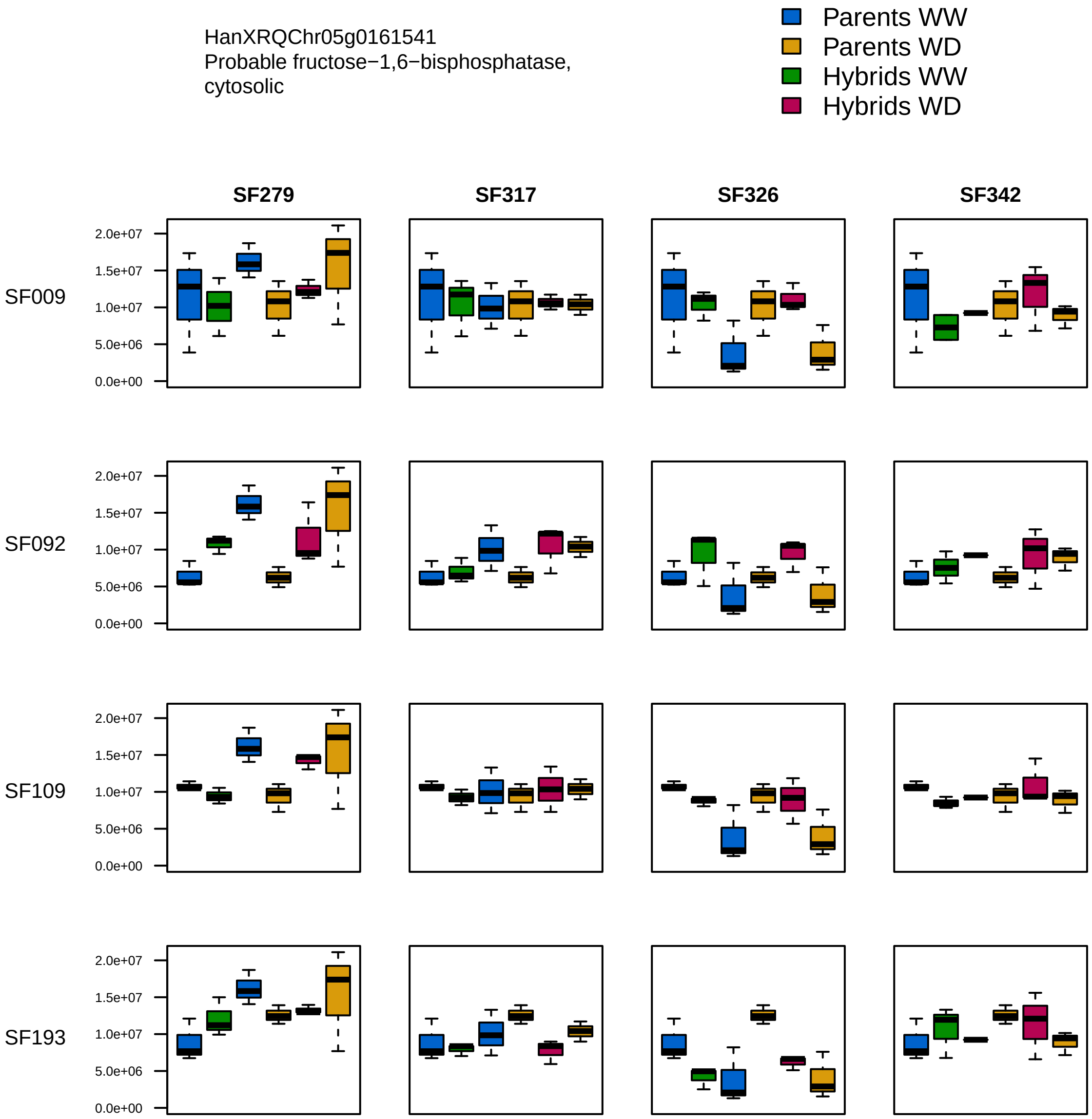
HanXRQChr05g0161541
Probable fructose−1,6−bisphosphatase, cytosolic
Parents WW
Parents WD
Hybrids WW
Hybrids WD
SF279
SF317
SF326
SF342
SF009
SF092
SF109
SF193
2.0e+07
1.5e+07
1.0e+07
5.0e+06
0.0e+00

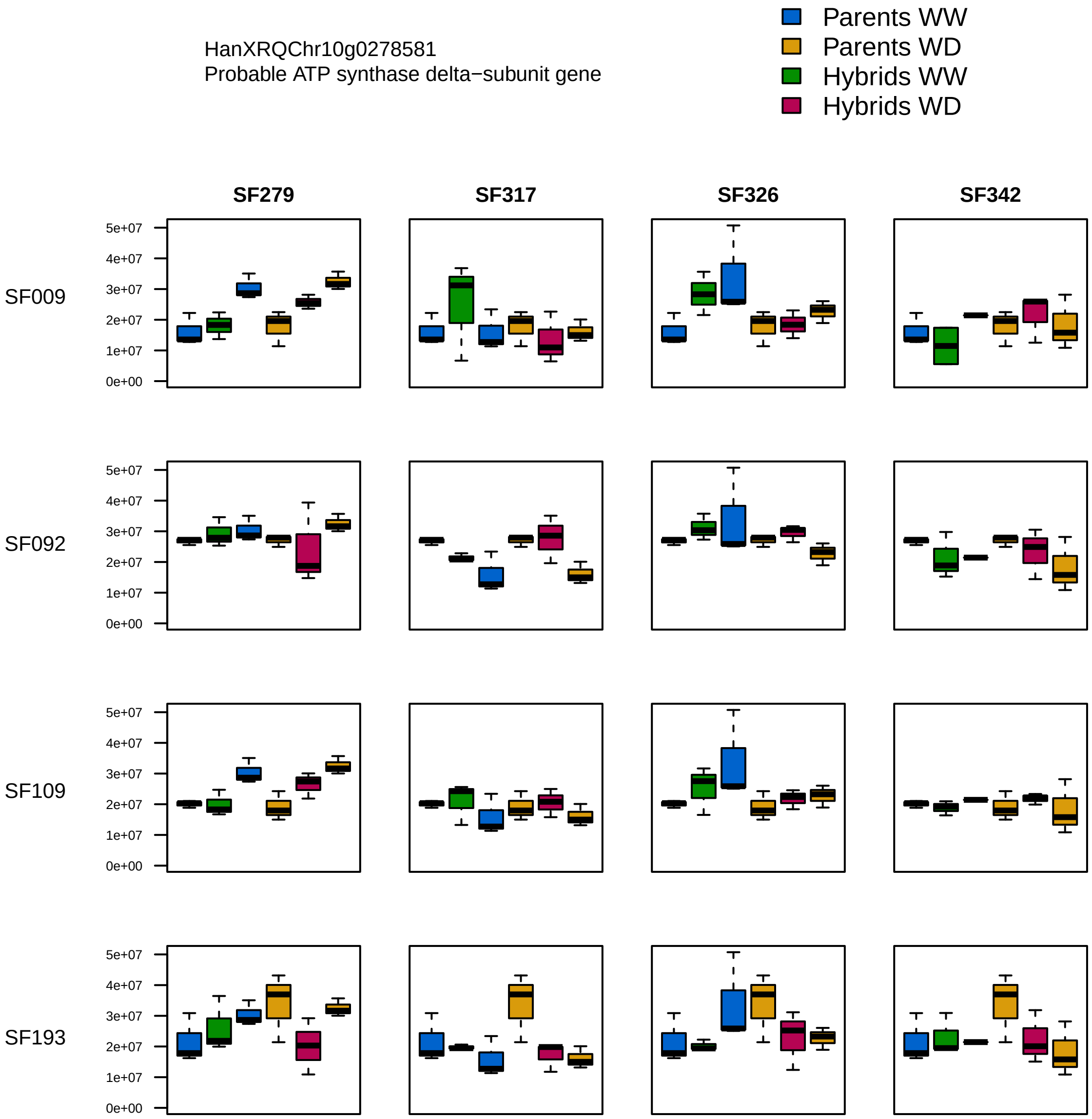
HanXRQChr10g0278581
Probable ATP synthase delta−subunit gene
Parents WW
Parents WD
Hybrids WW
Hybrids WD
SF279
SF317
SF326
SF342
SF009
SF092
SF109
SF193
5e+07
4e+07
3e+07
2e+07
1e+07
0e+00

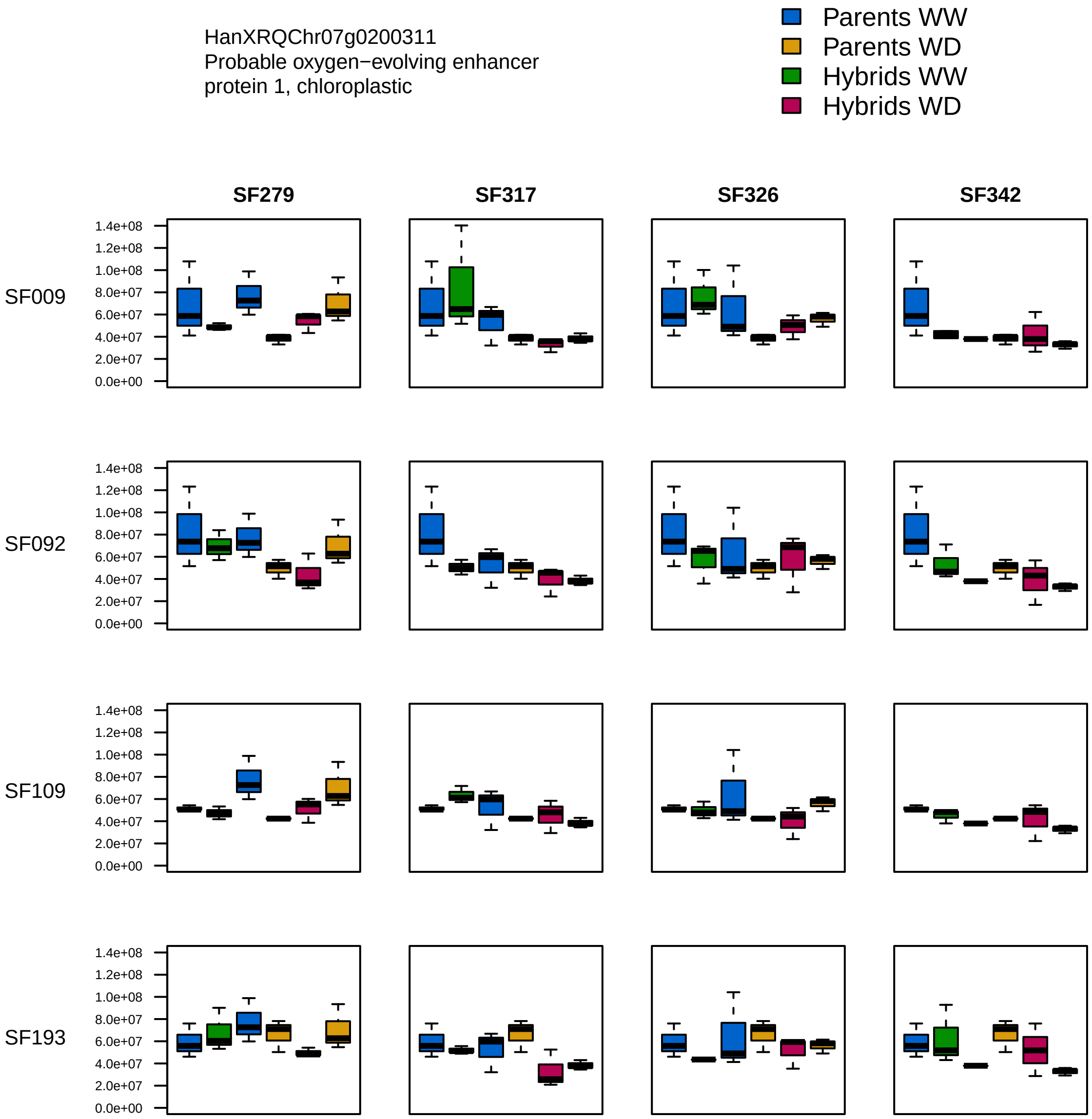
HanXRQChr07g0200311
Probable oxygen−evolving enhancer
protein 1, chloroplastic
Parents WW
Parents WD
Hybrids WW
Hybrids WD
SF279
SF317
SF326
SF342
SF009
SF092
SF109
SF193
1.4e+08
1.2e+08
1.0e+08
8.0e+07
6.0e+07
4.0e+07
2.0e+07
0.0e+00

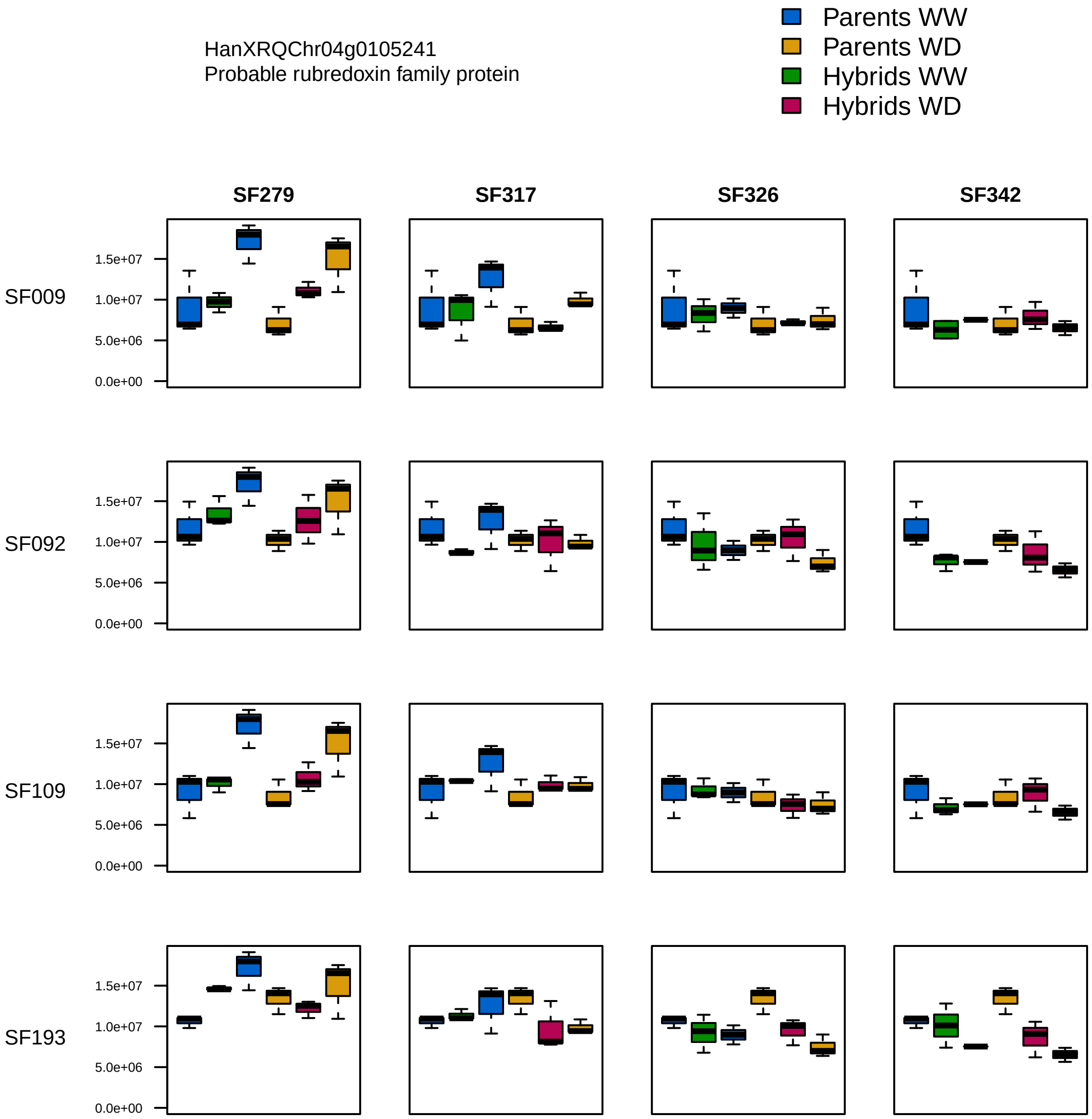
HanXRQChr04g0105241
Probable rubredoxin family protein
Parents WW
Parents WD
Hybrids WW
Hybrids WD
SF279
SF317
SF326
SF342
SF009
SF092
SF109
SF193
0.0e+00
5.0e+06
1.0e+07
1.5e+07

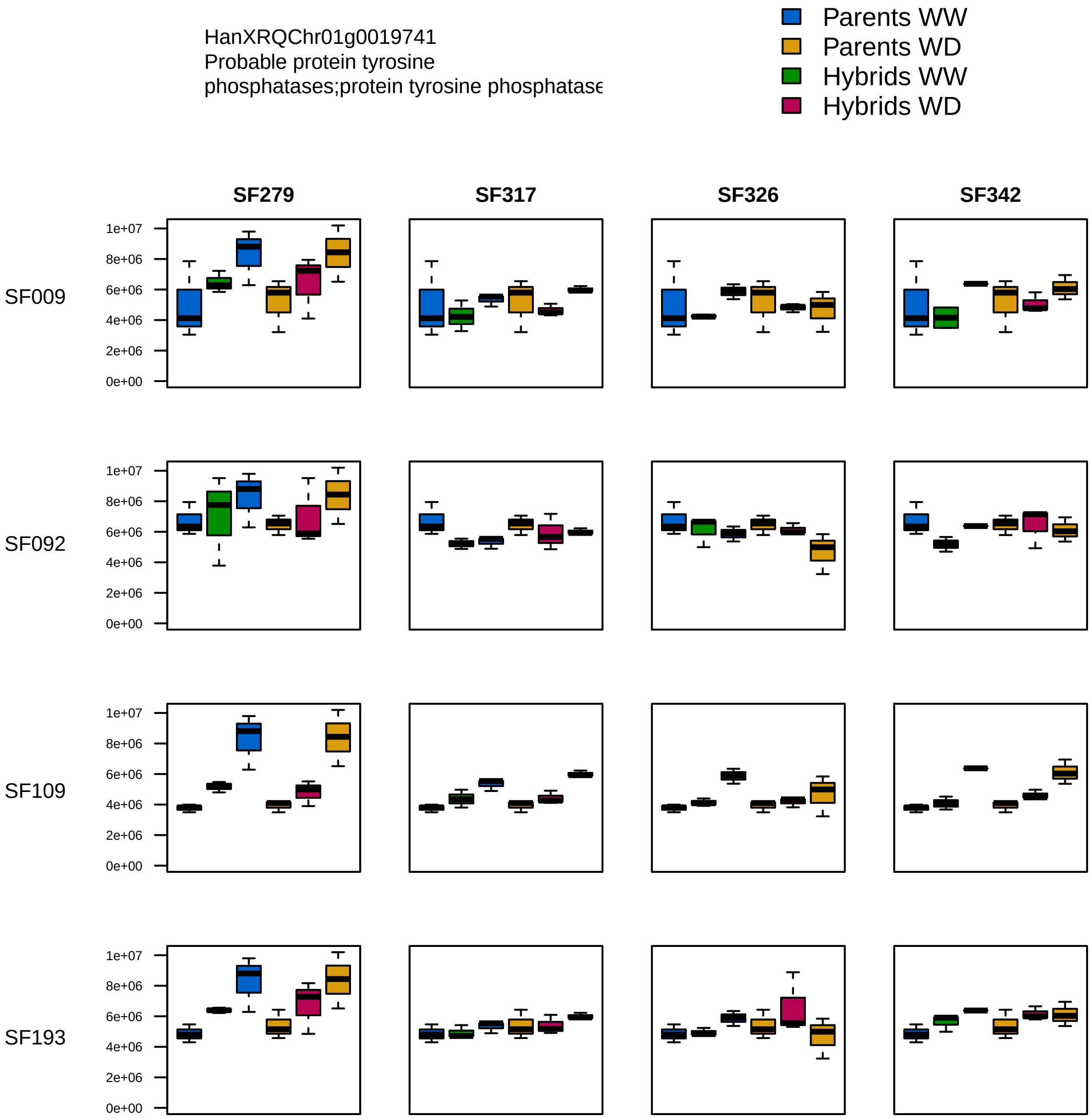
HanXRQChr01g0019741
Probable protein tyrosine
phosphatases;protein tyrosine phosphatase
Parents WW
Parents WD
Hybrids WW
Hybrids WD
SF279
SF317
SF326
SF342
SF009
SF092
SF109
SF193
1e+07
8e+06
6e+06
4e+06
2e+06
0e+00

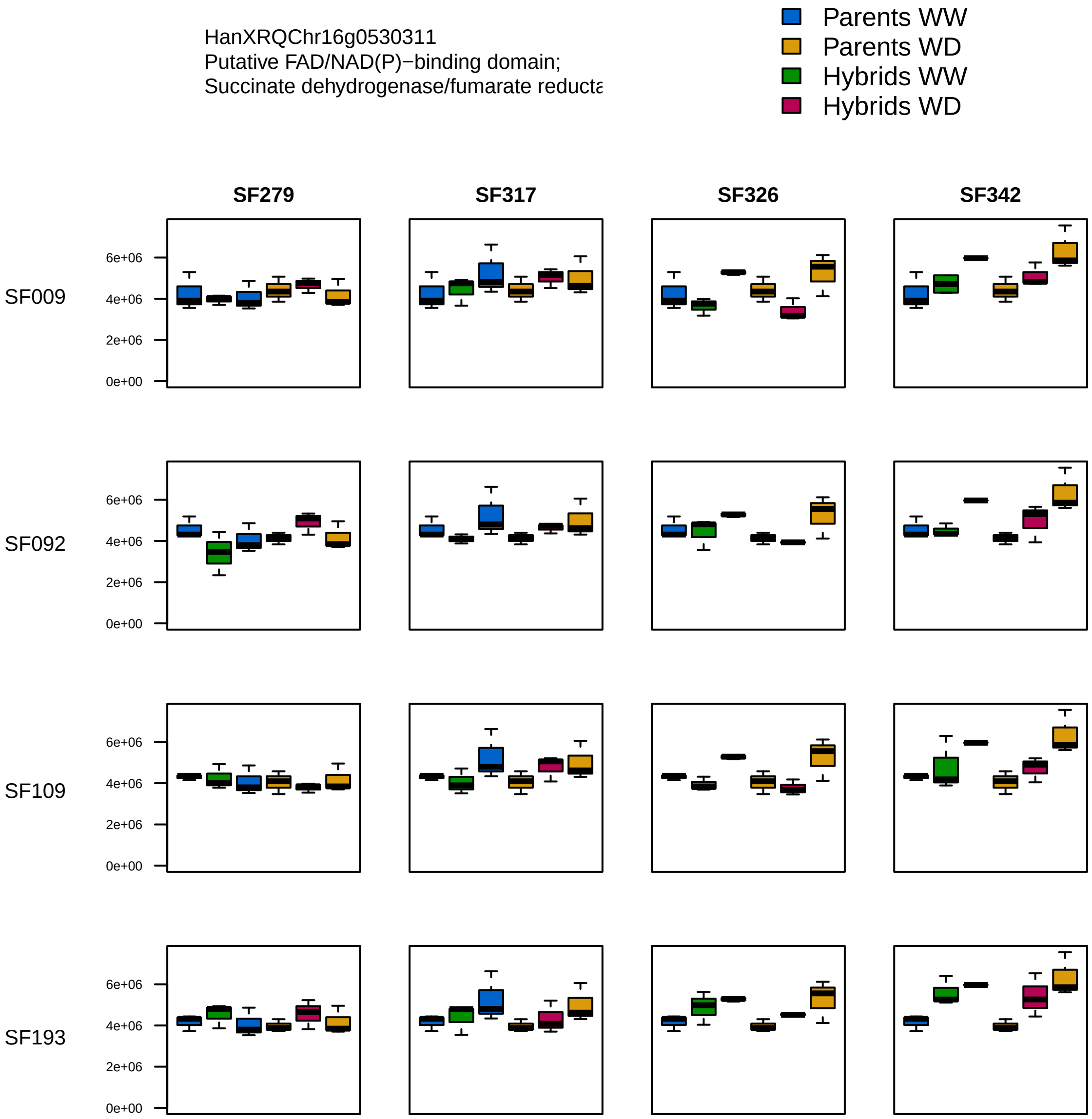
HanXRQChr16g0530311
Putative FAD/NAD(P)−binding domain;
Succinate dehydrogenase/fumarate reducta
Parents WW
Parents WD
Hybrids WW
Hybrids WD
SF279
SF317
SF326
SF342
SF009
SF092
SF109
SF193
6e+06
4e+06
2e+06
0e+00

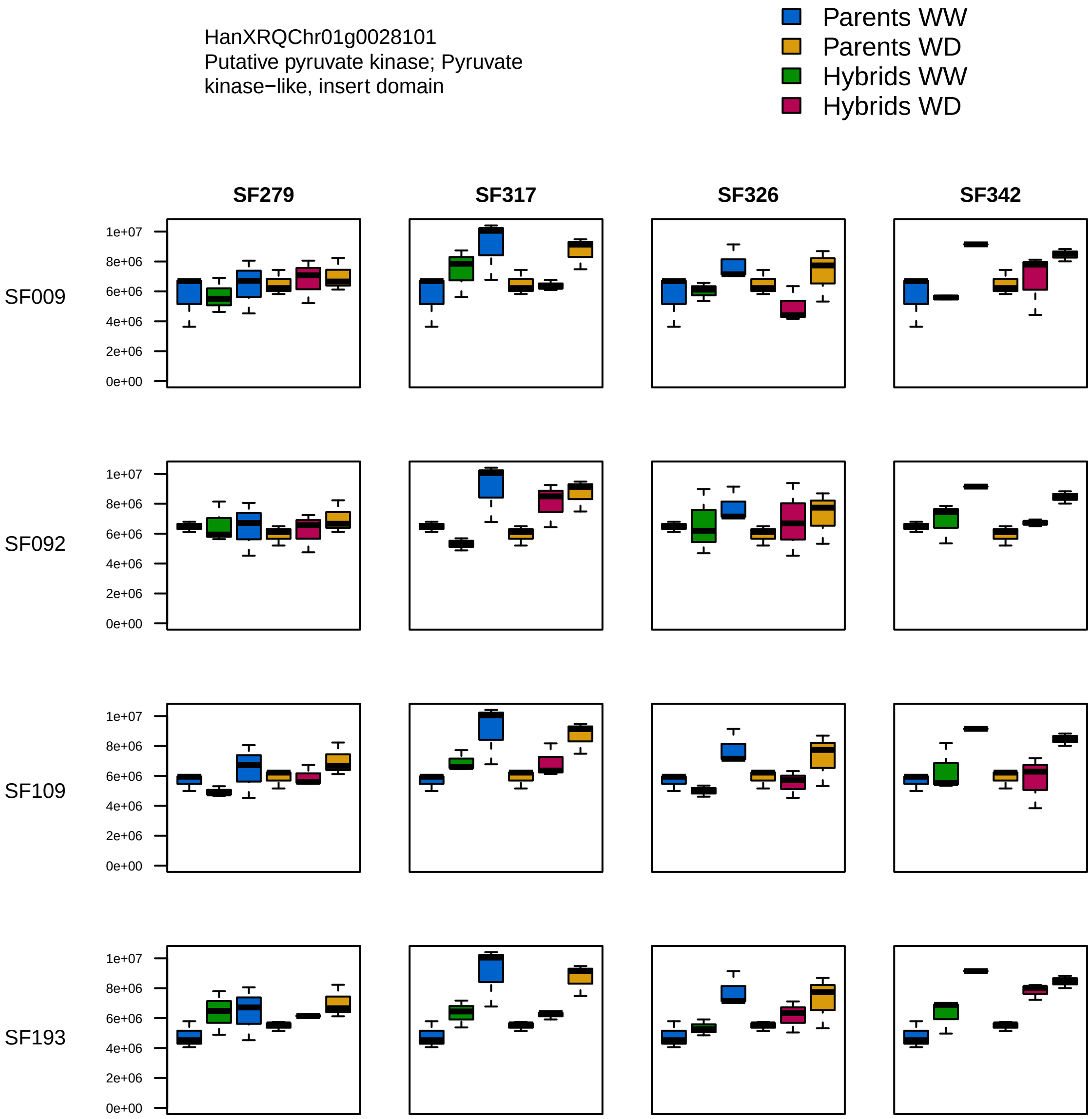
HanXRQChr01g0028101
Putative pyruvate kinase; Pyruvate
kinase−like, insert domain
Parents WW
Parents WD
Hybrids WW
Hybrids WD
SF279
SF317
SF326
SF342
SF009
SF092
SF109
SF193
1e+07
8e+06
6e+06
4e+06
2e+06
0e+00

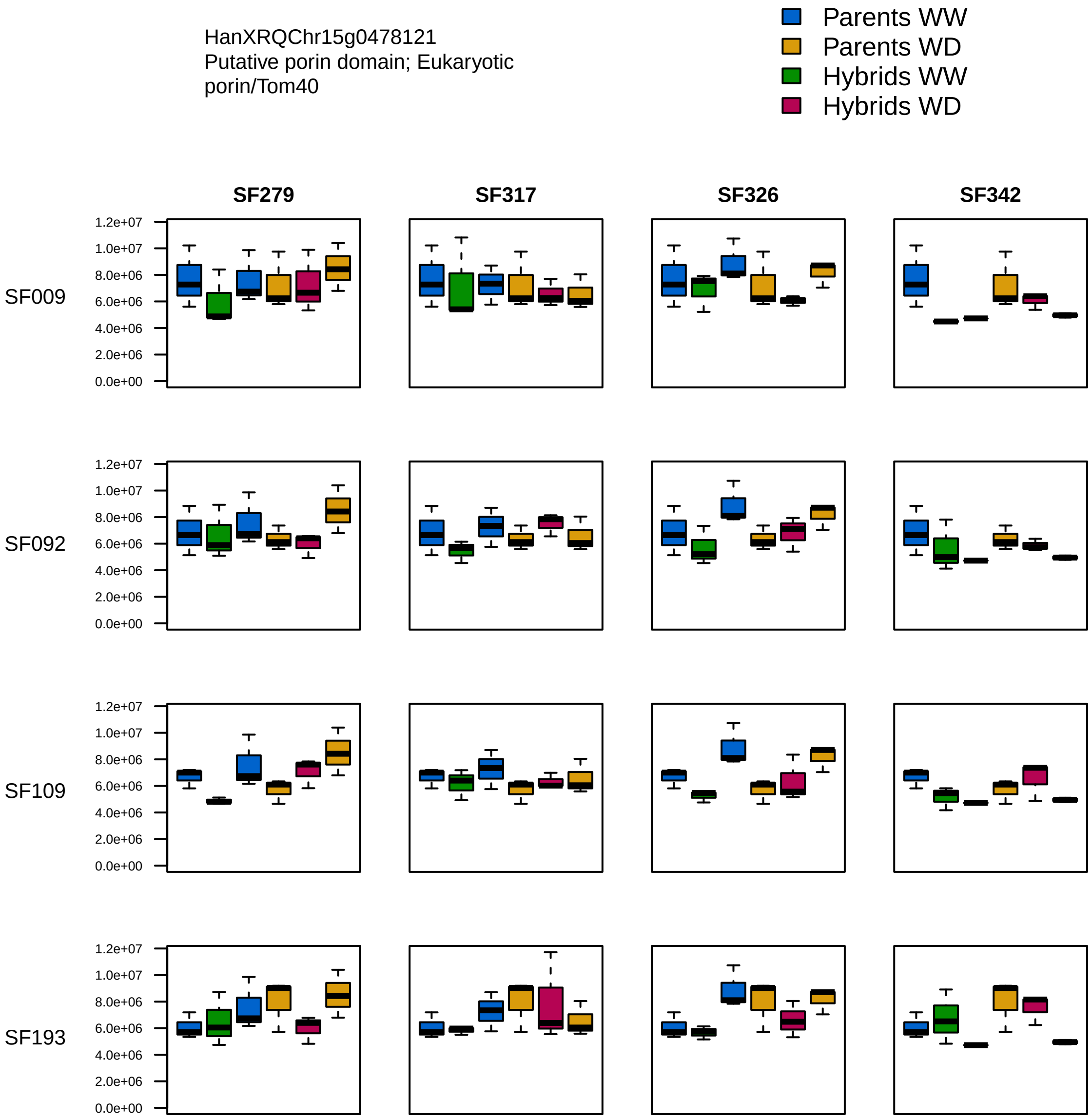
HanXRQChr15g0478121
Putative porin domain; Eukaryotic
porin/Tom40
Parents WW
Parents WD
Hybrids WW
Hybrids WD
SF279
SF317
SF326
SF342
SF009
SF092
SF109
SF193
1.2e+07
1.0e+07
8.0e+06
6.0e+06
4.0e+06
2.0e+06
0.0e+00

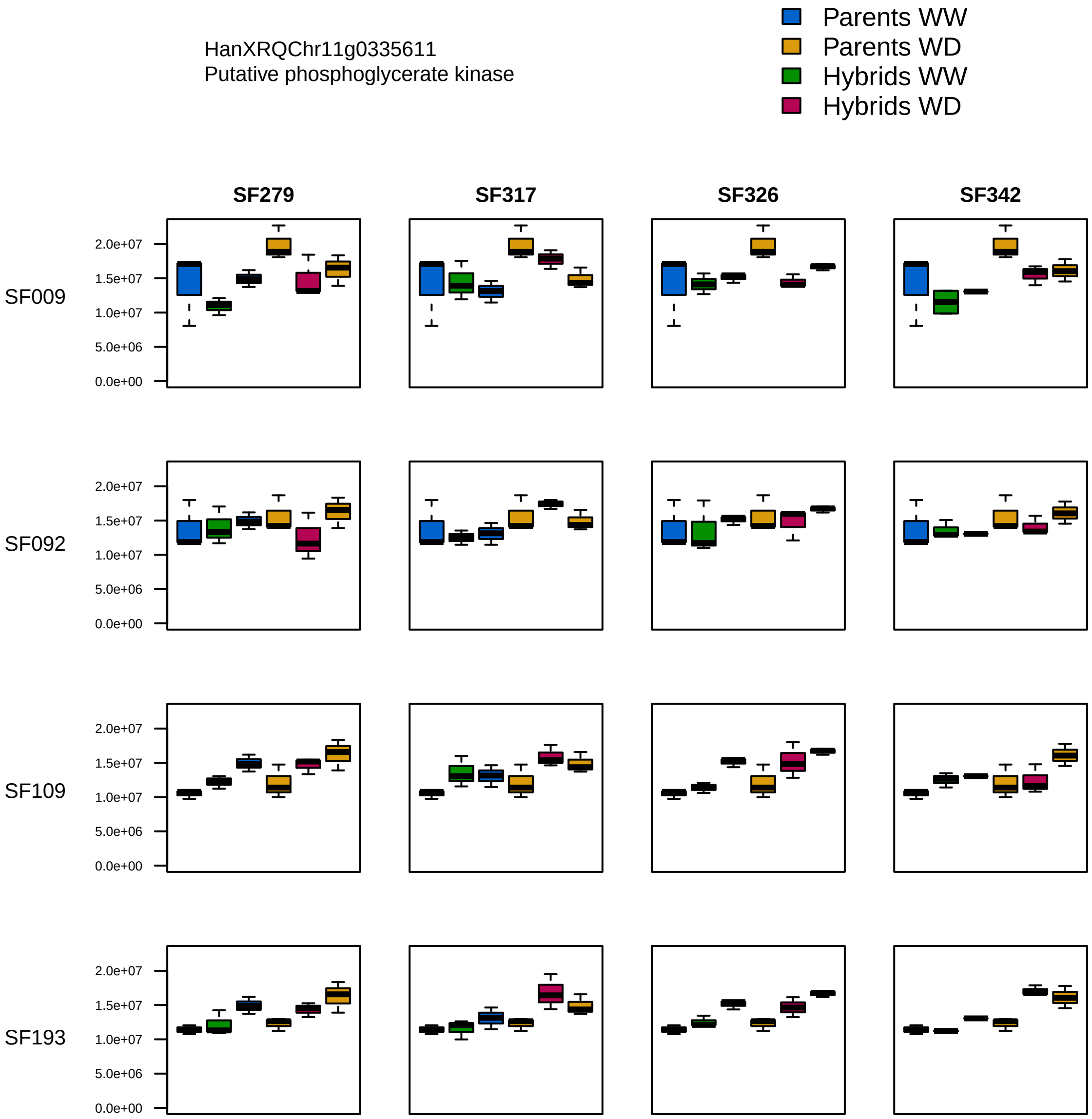
HanXRQChr11g0335611
Putative phosphoglycerate kinase
Parents WW
Parents WD
Hybrids WW
Hybrids WD
SF279
SF317
SF326
SF342
SF009
SF092
SF109
SF193
2.0e+07
1.5e+07
1.0e+07
5.0e+06
0.0e+00

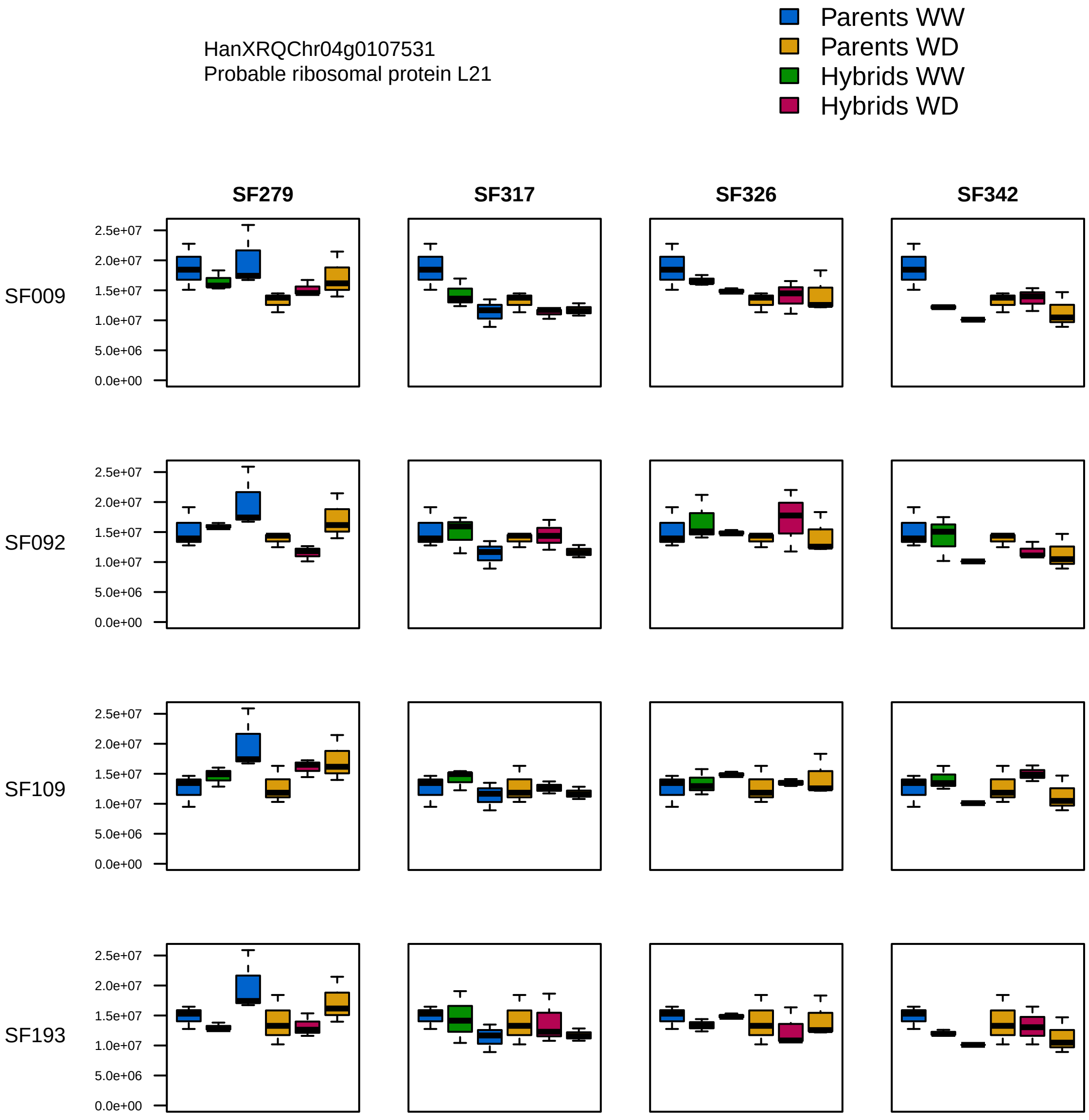

HanXRQChr04g0107531
Probable ribosomal protein L21
Parents WW
Parents WD
Hybrids WW
Hybrids WD
SF279
SF317
SF326
SF342
SF009
SF092
SF109
SF193
2.5e+07
2.0e+07
1.5e+07
1.0e+07
5.0e+06
0.0e+00

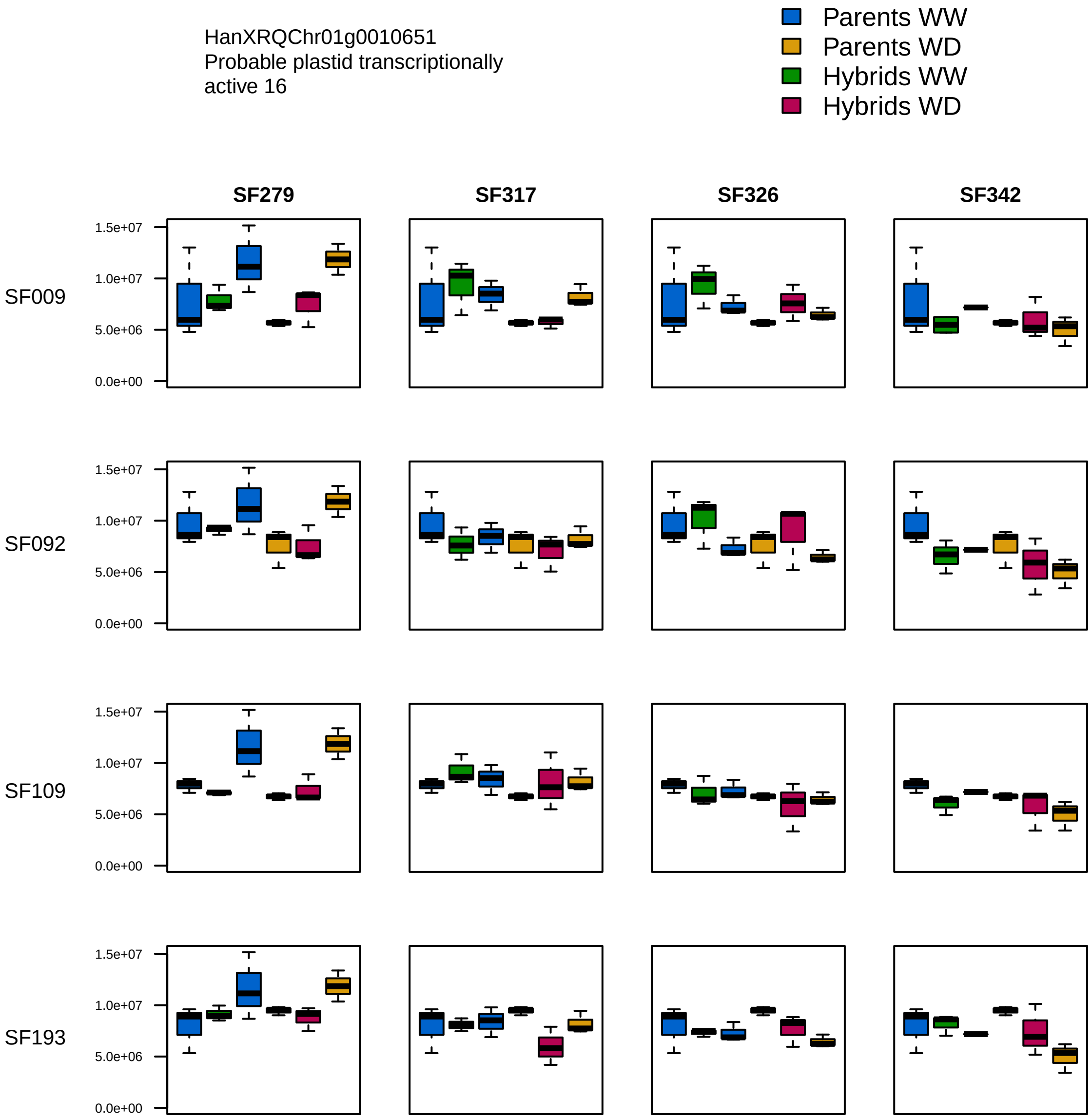

HanXRQChr01g0010651
Probable plastid transcriptionally
active 16
Parents WW
Parents WD
Hybrids WW
Hybrids WD
SF279
SF317
SF326
SF342
SF009
SF092
SF109
SF193
1.5e+07
1.0e+07
5.0e+06
0.0e+00

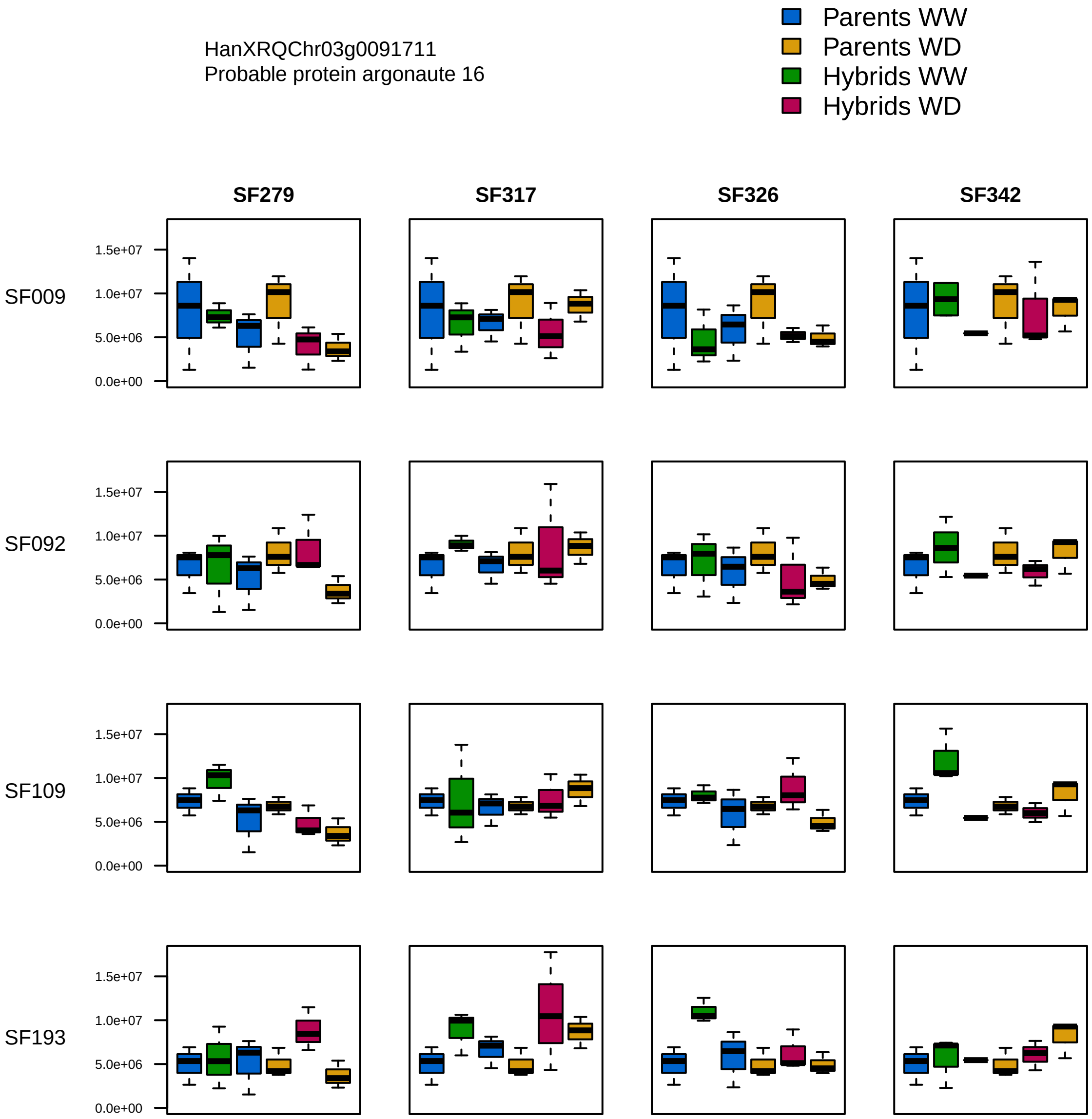

HanXRQChr03g0091711
Probable protein argonaute 16
Parents WW
Parents WD
Hybrids WW
Hybrids WD
SF279
SF317
SF326
SF342
SF009
SF092
SF109
SF193
1.5e+07
1.0e+07
5.0e+06
0.0e+00

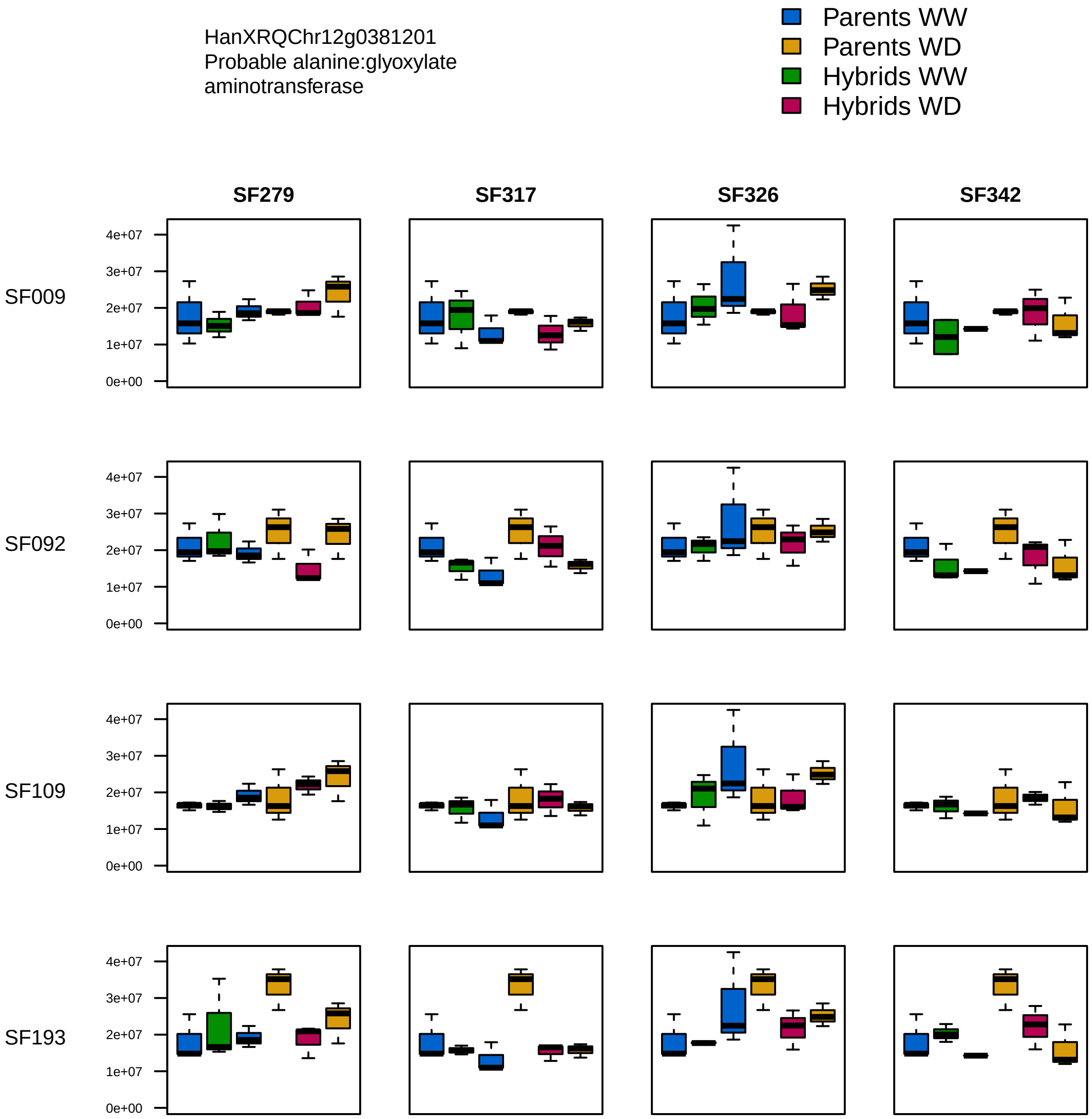
HanXRQChr12g0381201
Probable alanine:glyoxylate
aminotransferase
Parents WW
Parents WD
Hybrids WW
Hybrids WD
SF279
SF317
SF326
SF342
SF009
SF092
SF109
SF193
0e+00
1e+07
2e+07
3e+07
4e+07

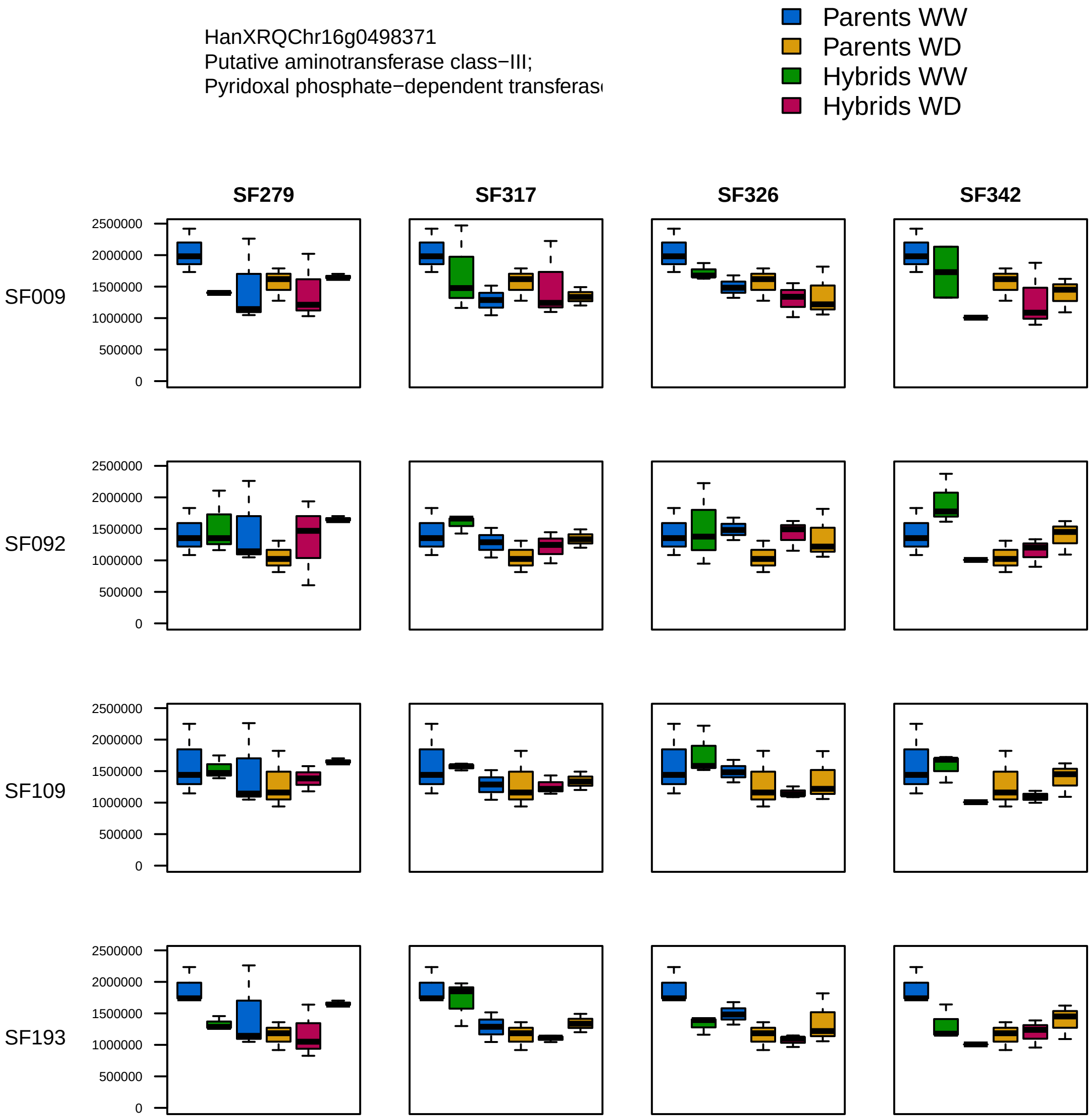
HanXRQChr16g0498371
Putative aminotransferase class−III;
Pyridoxal phosphate−dependent transferas
Parents WW
Parents WD
Hybrids WW
Hybrids WD
SF279
SF317
SF326
SF342
SF009
SF092
SF109
SF193
2500000
2000000
1500000
1000000
500000
0

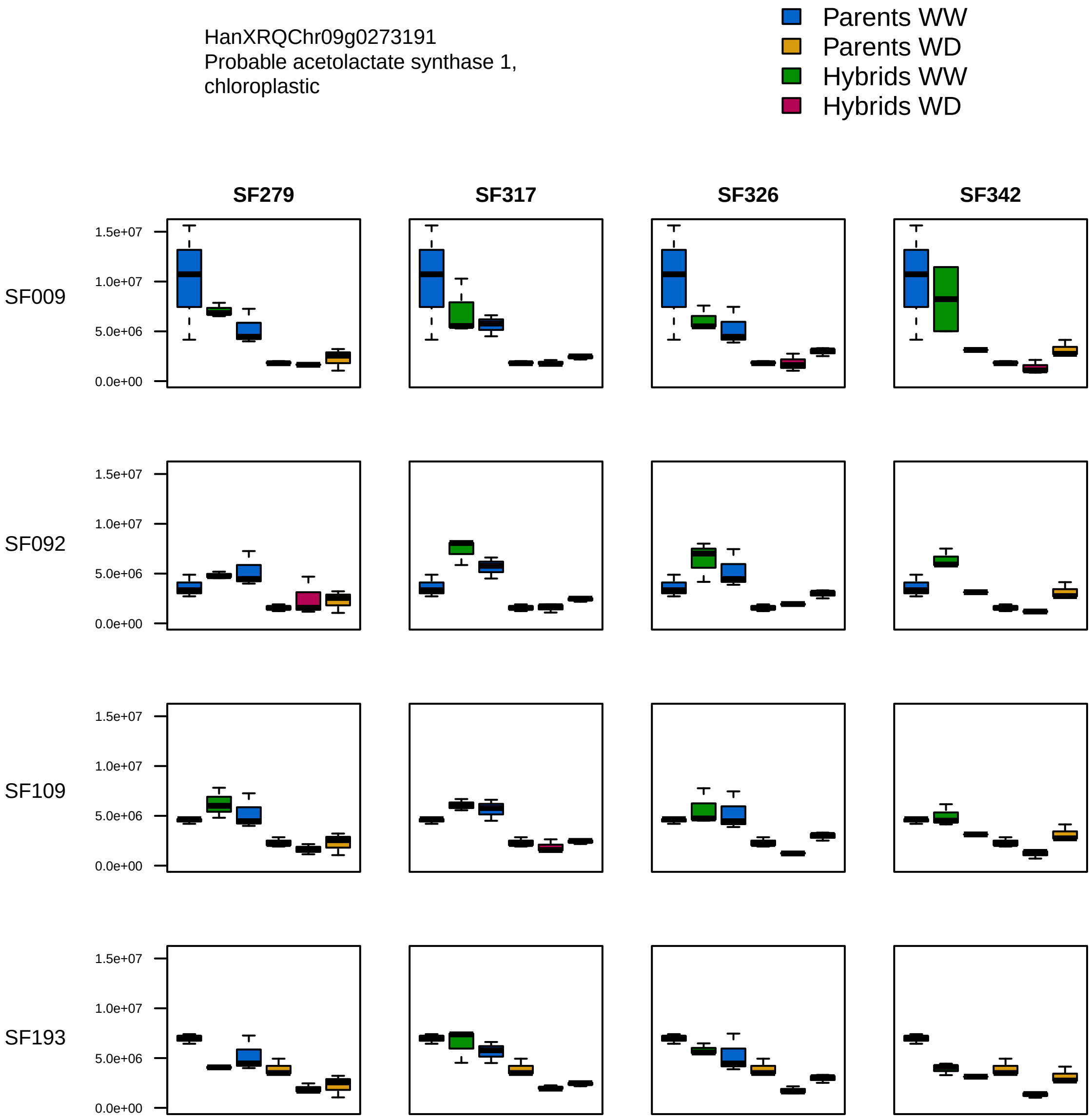

HanXRQChr09g0273191
Probable acetolactate synthase 1,
chloroplastic
Parents WW
Parents WD
Hybrids WW
Hybrids WD
SF279
SF317
SF326
SF342
SF009
SF092
SF109
SF193
1.5e+07
1.0e+07
5.0e+06
0.0e+00

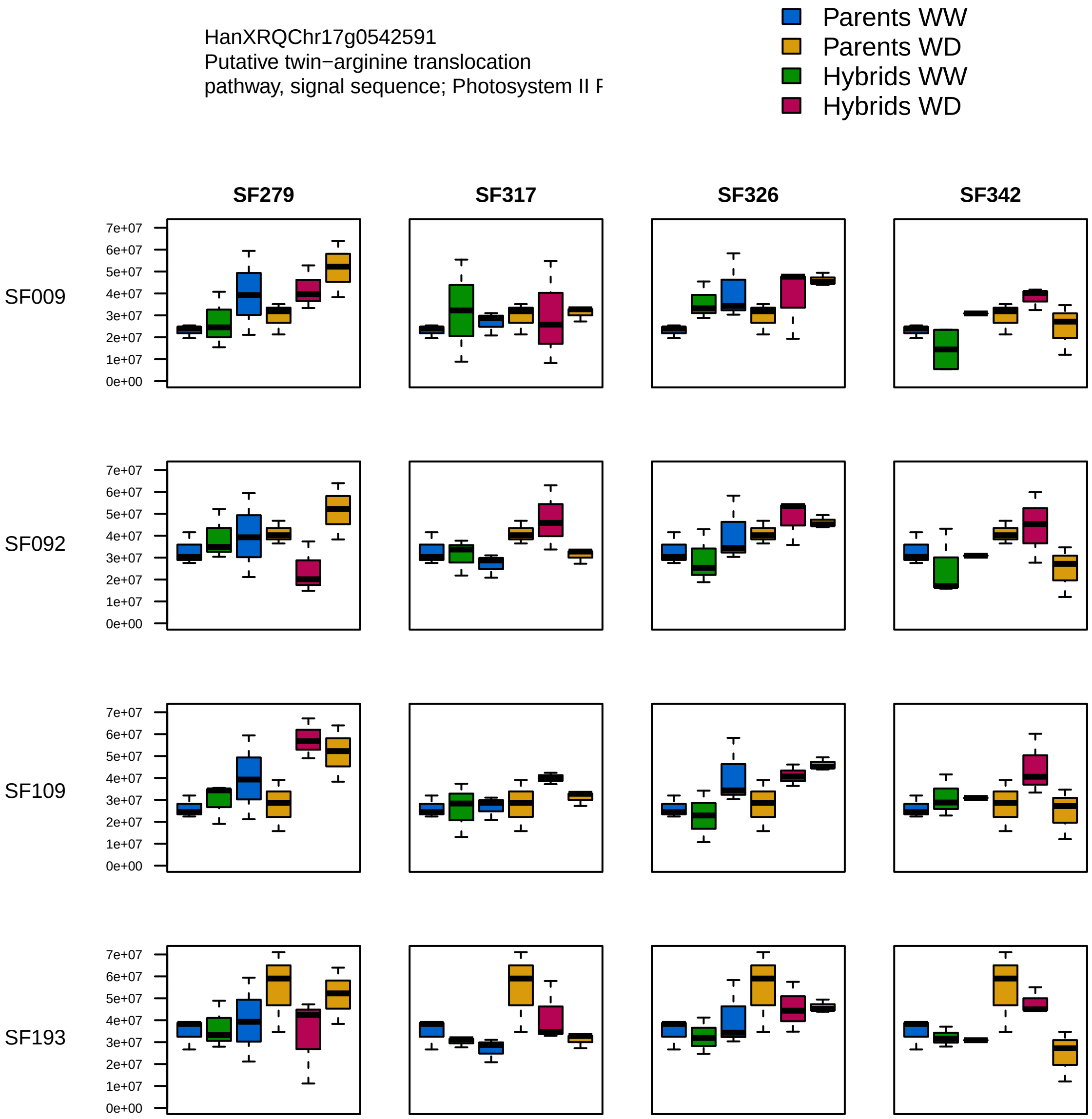

HanXRQChr17g0542591
Putative twin−arginine translocation
pathway, signal sequence; Photosystem II F
Parents WW
Parents WD
Hybrids WW
Hybrids WD
SF279
SF317
SF326
SF342
SF009
SF092
SF109
SF193
7e+07
6e+07
5e+07
4e+07
3e+07
2e+07
1e+07
0e+00

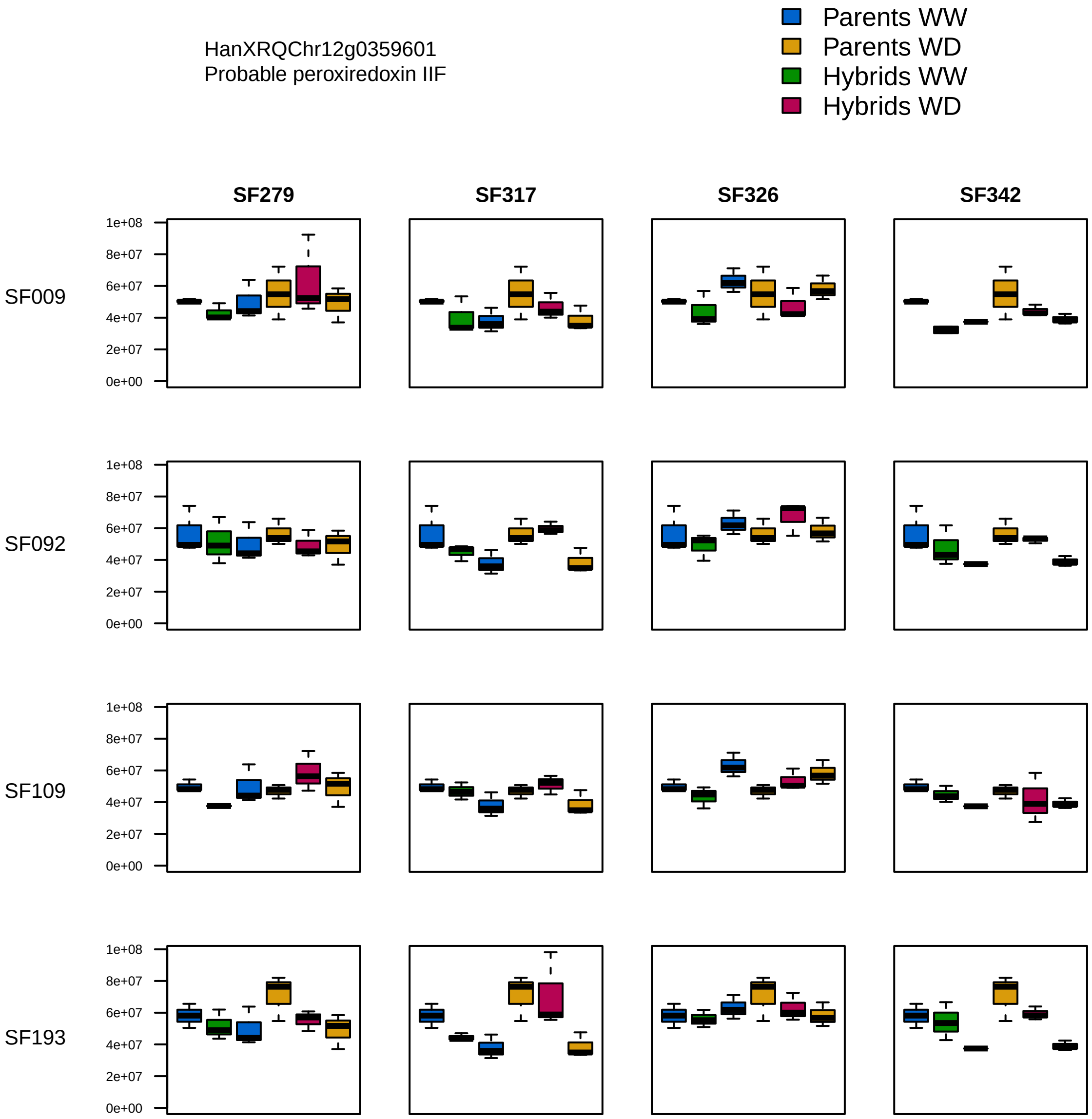
HanXRQChr12g0359601
Probable peroxiredoxin IIF
Parents WW
Parents WD
Hybrids WW
Hybrids WD
SF279
SF317
SF326
SF342
SF009
SF092
SF109
SF193
1e+08
8e+07
6e+07
4e+07
2e+07
0e+00

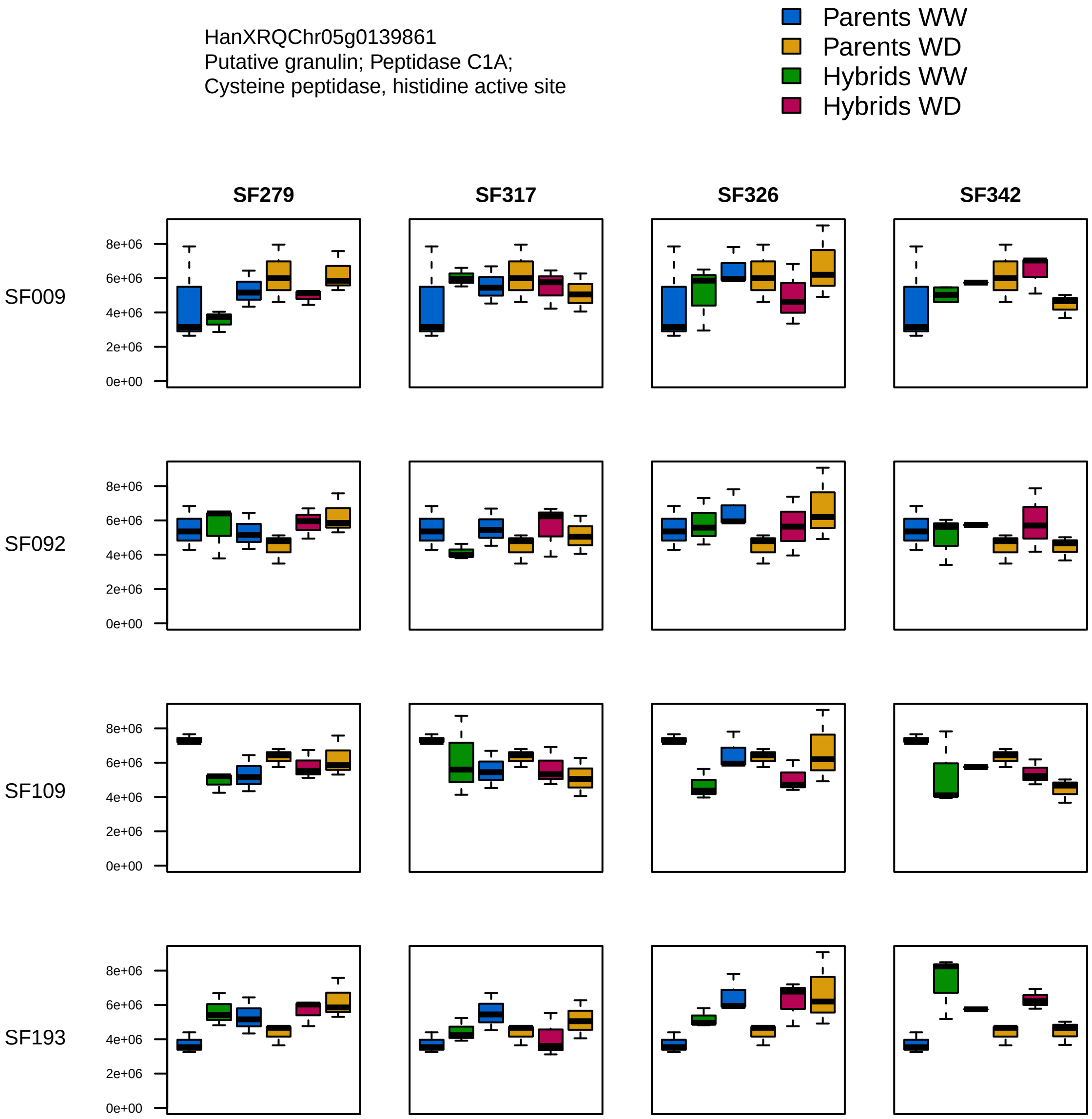
HanXRQChr05g0139861
Putative granulin; Peptidase C1A;
Cysteine peptidase, histidine active site
Parents WW
Parents WD
Hybrids WW
Hybrids WD
SF279
SF317
SF326
SF342
SF009
SF092
SF109
SF193
8e+06
6e+06
4e+06
2e+06
0e+00

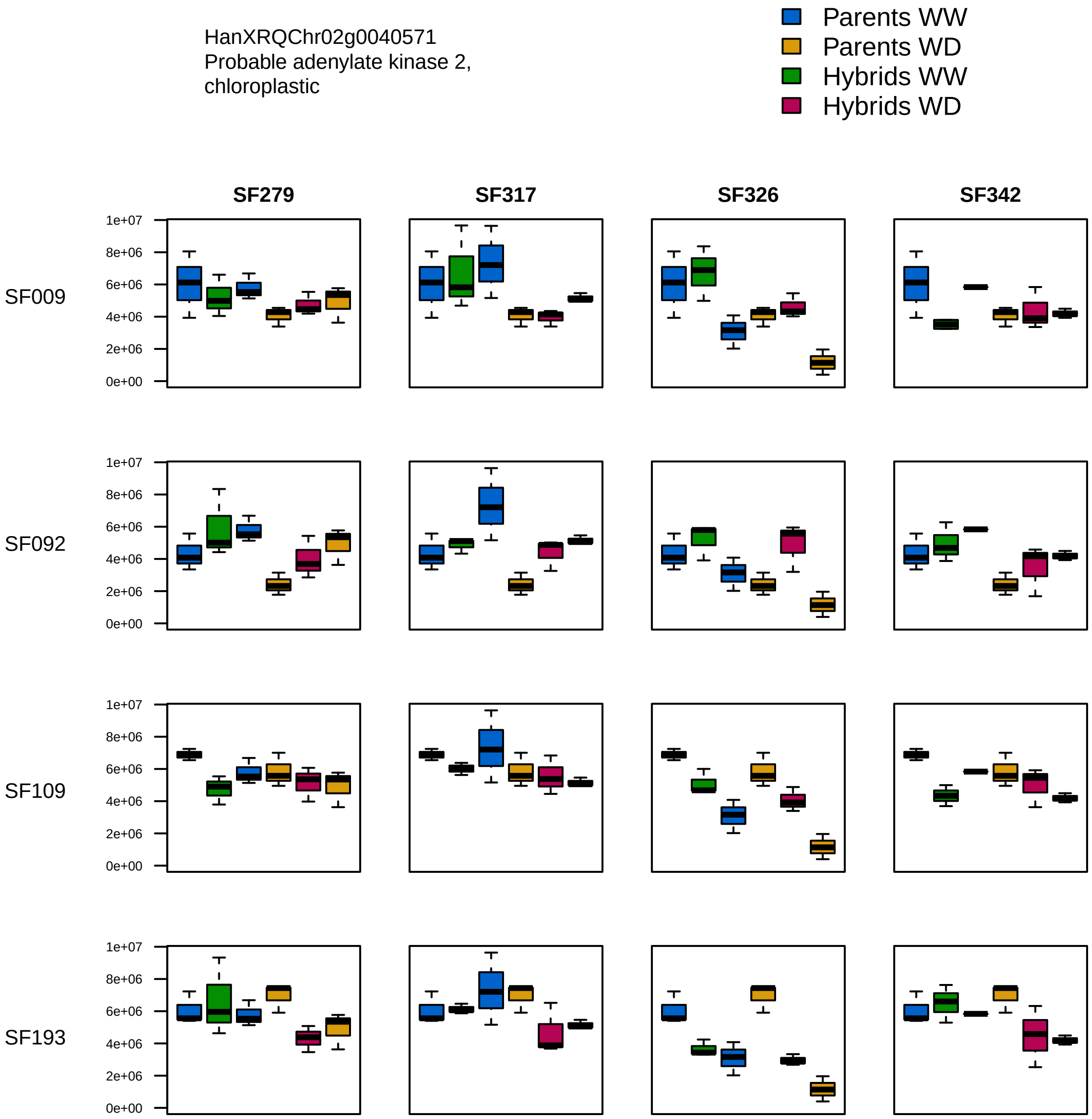
HanXRQChr02g0040571
Probable adenylate kinase 2,
chloroplastic
Parents WW
Parents WD
Hybrids WW
Hybrids WD
SF279
SF317
SF326
SF342
SF009
SF092
SF109
SF193
1e+07
8e+06
6e+06
4e+06
2e+06
0e+00

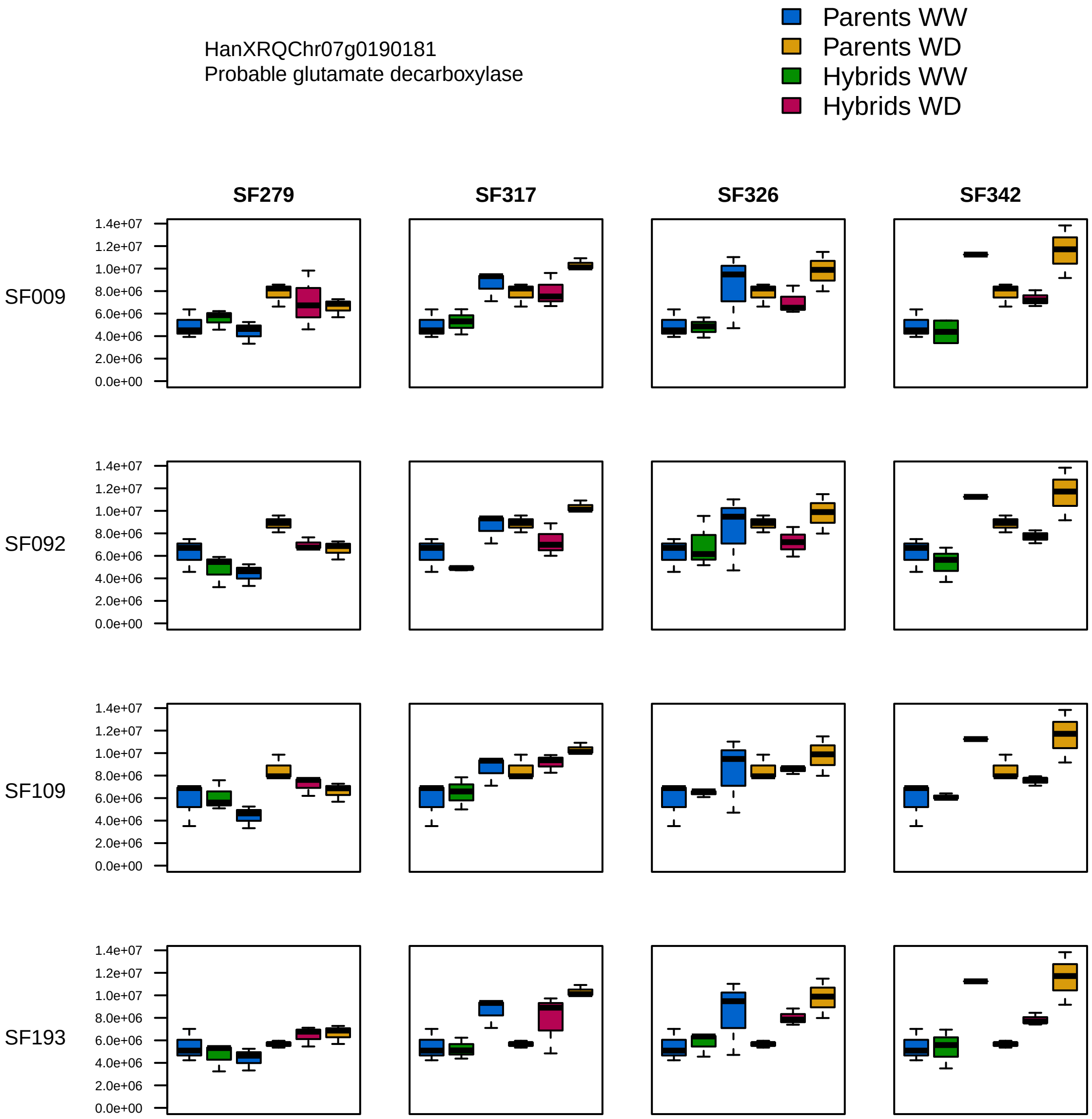
HanXRQChr07g0190181
Probable glutamate decarboxylase
Parents WW
Parents WD
Hybrids WW
Hybrids WD
SF279
SF317
SF326
SF342
SF009
SF092
SF109
SF193
1.4e+07
1.2e+07
1.0e+07
8.0e+06
6.0e+06
4.0e+06
2.0e+06
0.0e+00

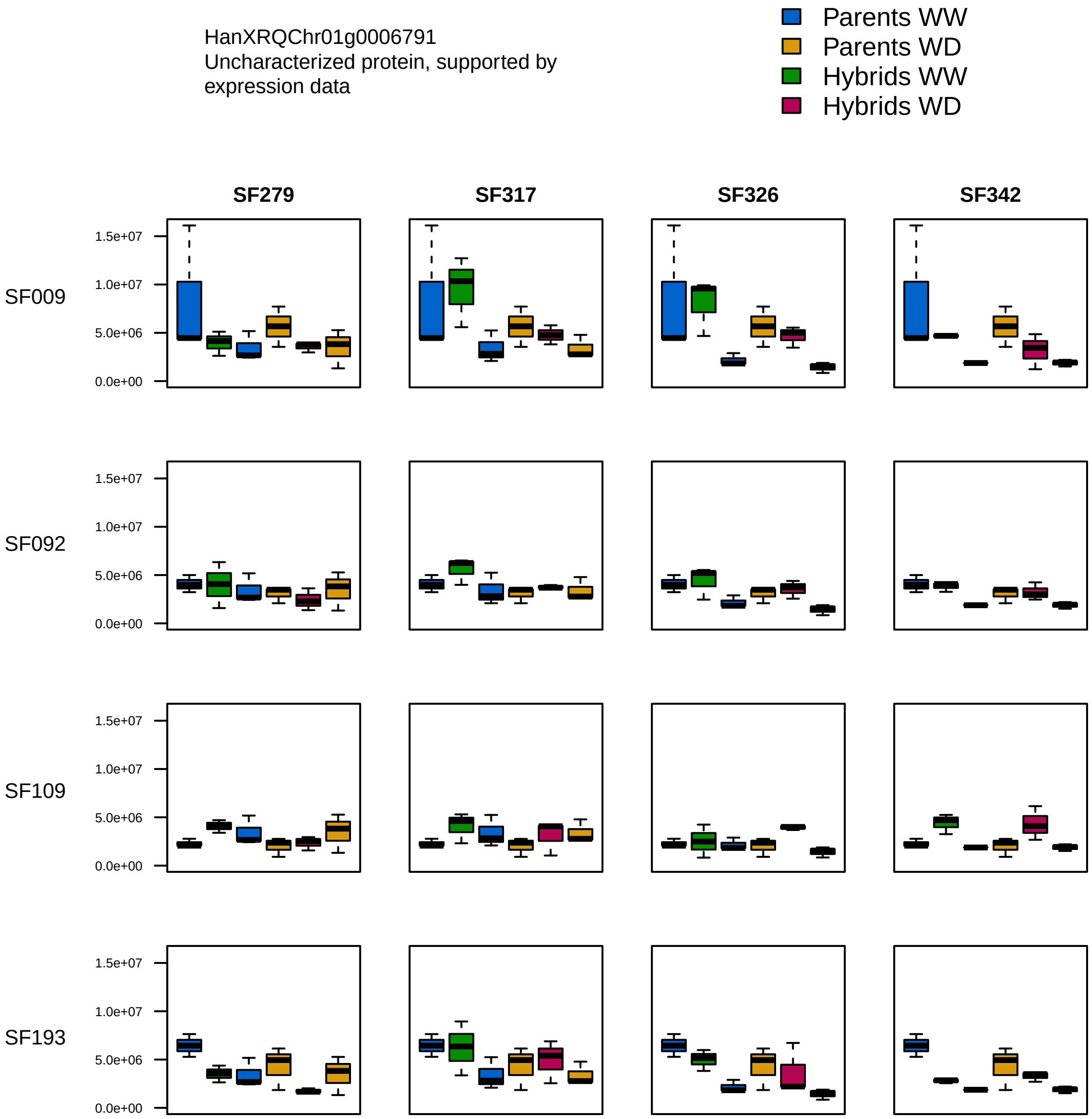
HanXRQChr01g0006791
Uncharacterized protein, supported by
expression data
Parents WW
Parents WD
Hybrids WW
Hybrids WD
SF279
SF317
SF326
SF342
SF009
SF092
SF109
SF193
1.5e+07
1.0e+07
5.0e+06
0.0e+00

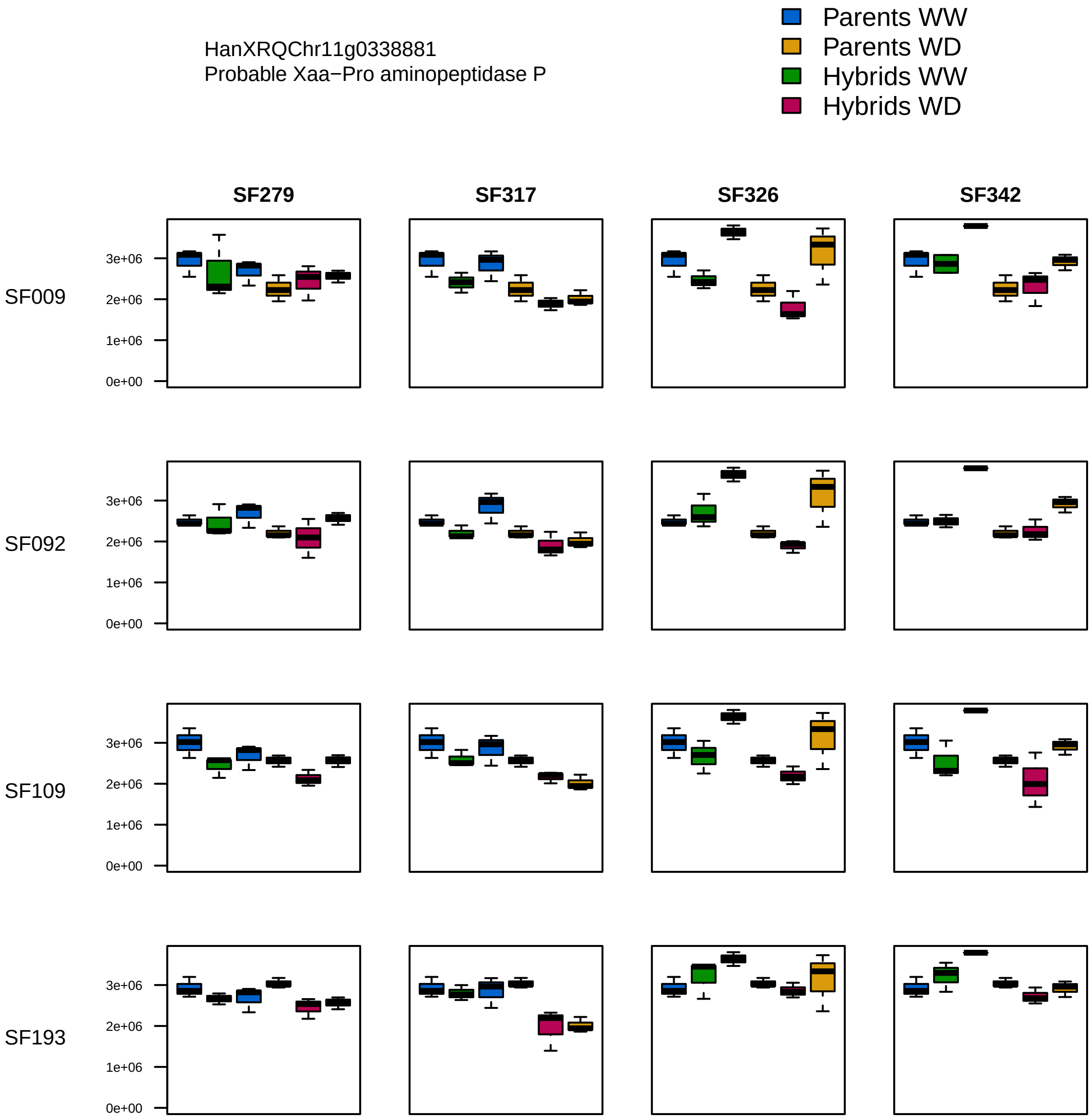
HanXRQChr11g0338881
Probable Xaa−Pro aminopeptidase P
Parents WW
Parents WD
Hybrids WW
Hybrids WD
SF279
SF317
SF326
SF342
SF009
SF092
SF109
SF193
0e+00
1e+06
2e+06
3e+06

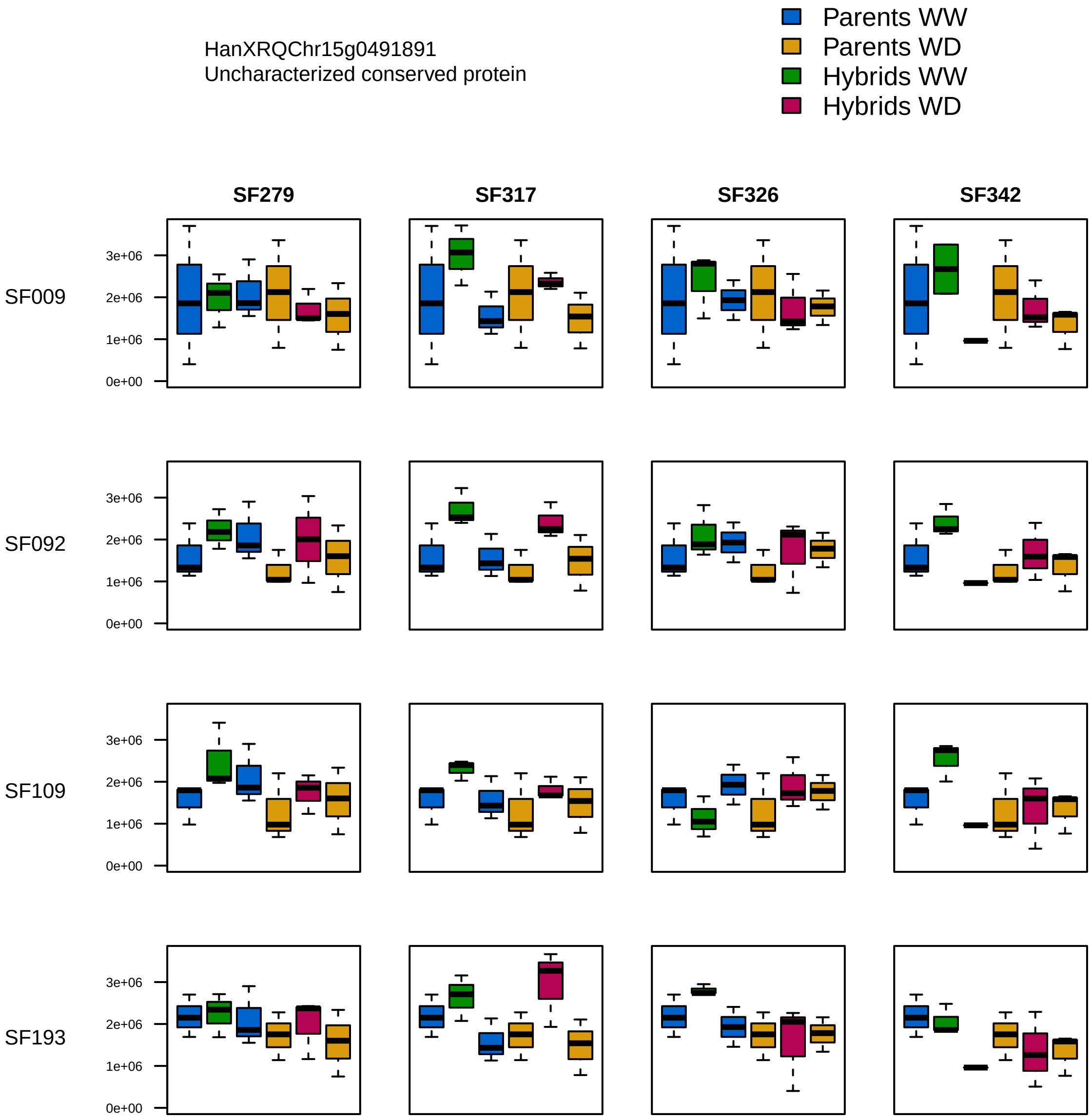

HanXRQChr15g0491891
Uncharacterized conserved protein
Parents WW
Parents WD
Hybrids WW
Hybrids WD
SF279
SF317
SF326
SF342
SF009
SF092
SF109
SF193
0e+00
1e+06
2e+06
3e+06

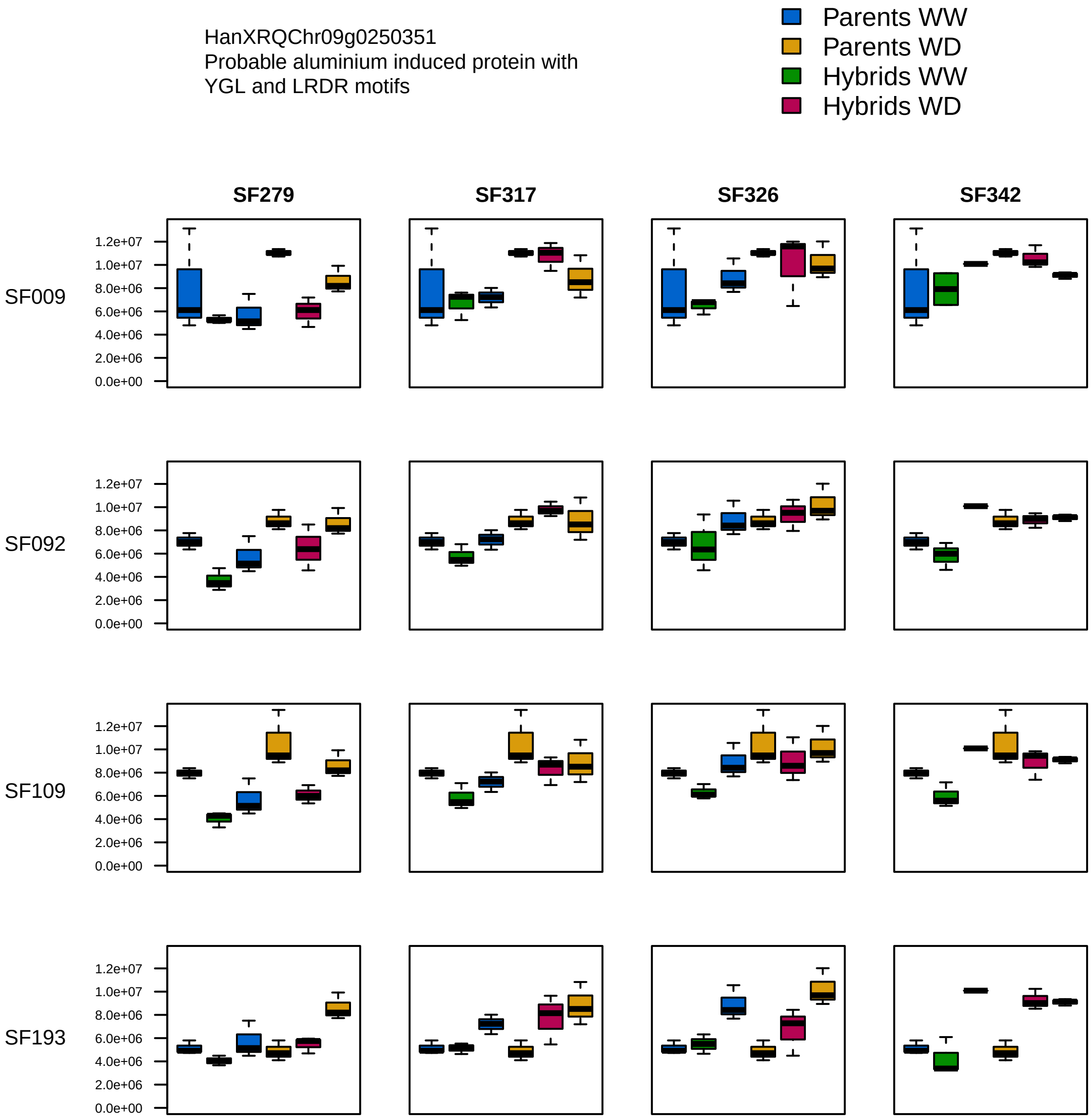
HanXRQChr09g0250351
Probable aluminium induced protein with
YGL and LRDR motifs
Parents WW
Parents WD
Hybrids WW
Hybrids WD
SF279
SF317
SF326
SF342
SF009
SF092
SF109
SF193
1.2e+07
1.0e+07
8.0e+06
6.0e+06
4.0e+06
2.0e+06
0.0e+00

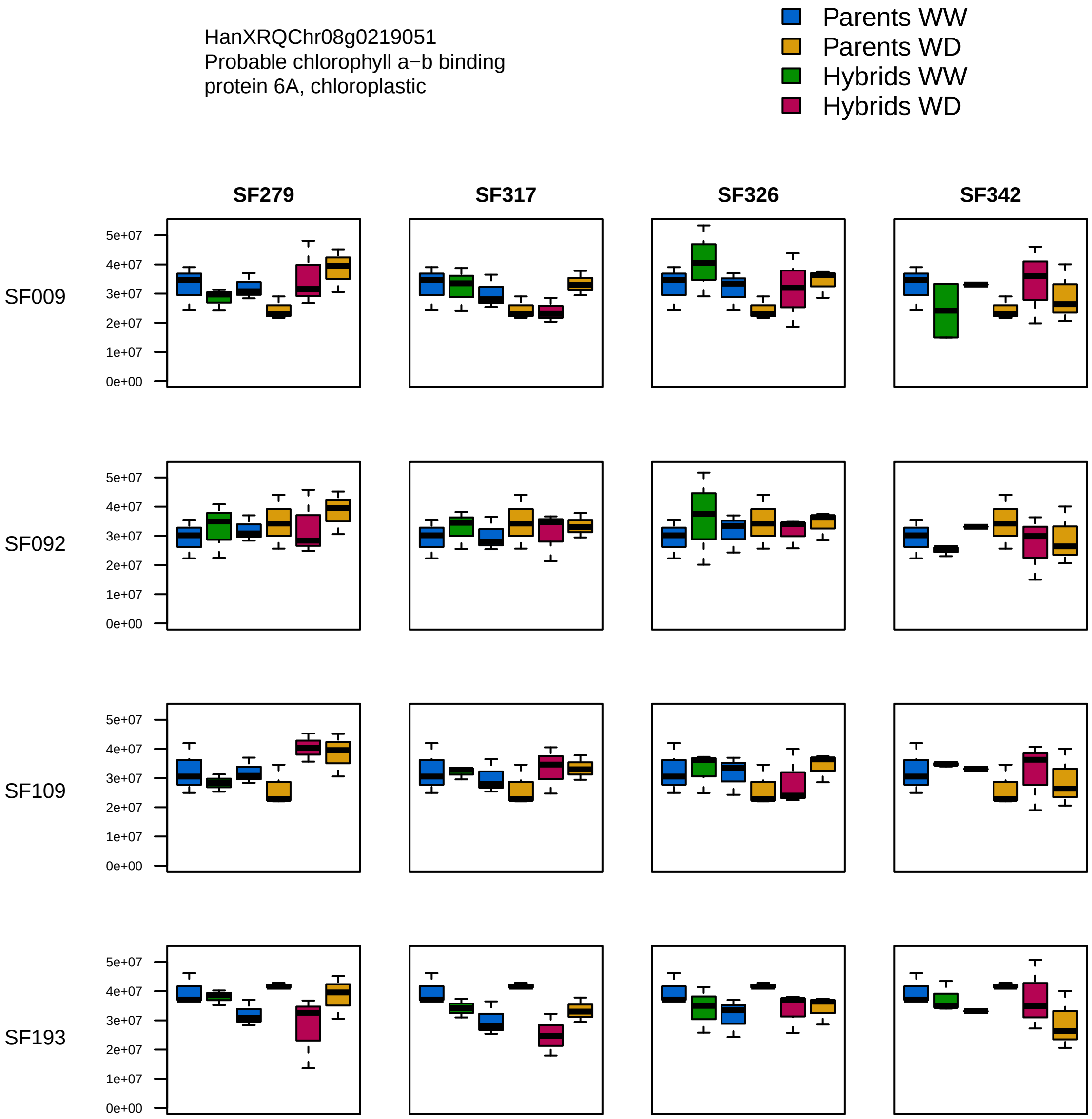
HanXRQChr08g0219051
Probable chlorophyll a−b binding
protein 6A, chloroplastic
Parents WW
Parents WD
Hybrids WW
Hybrids WD
SF279
SF317
SF326
SF342
SF009
SF092
SF109
SF193
5e+07
4e+07
3e+07
2e+07
1e+07
0e+00

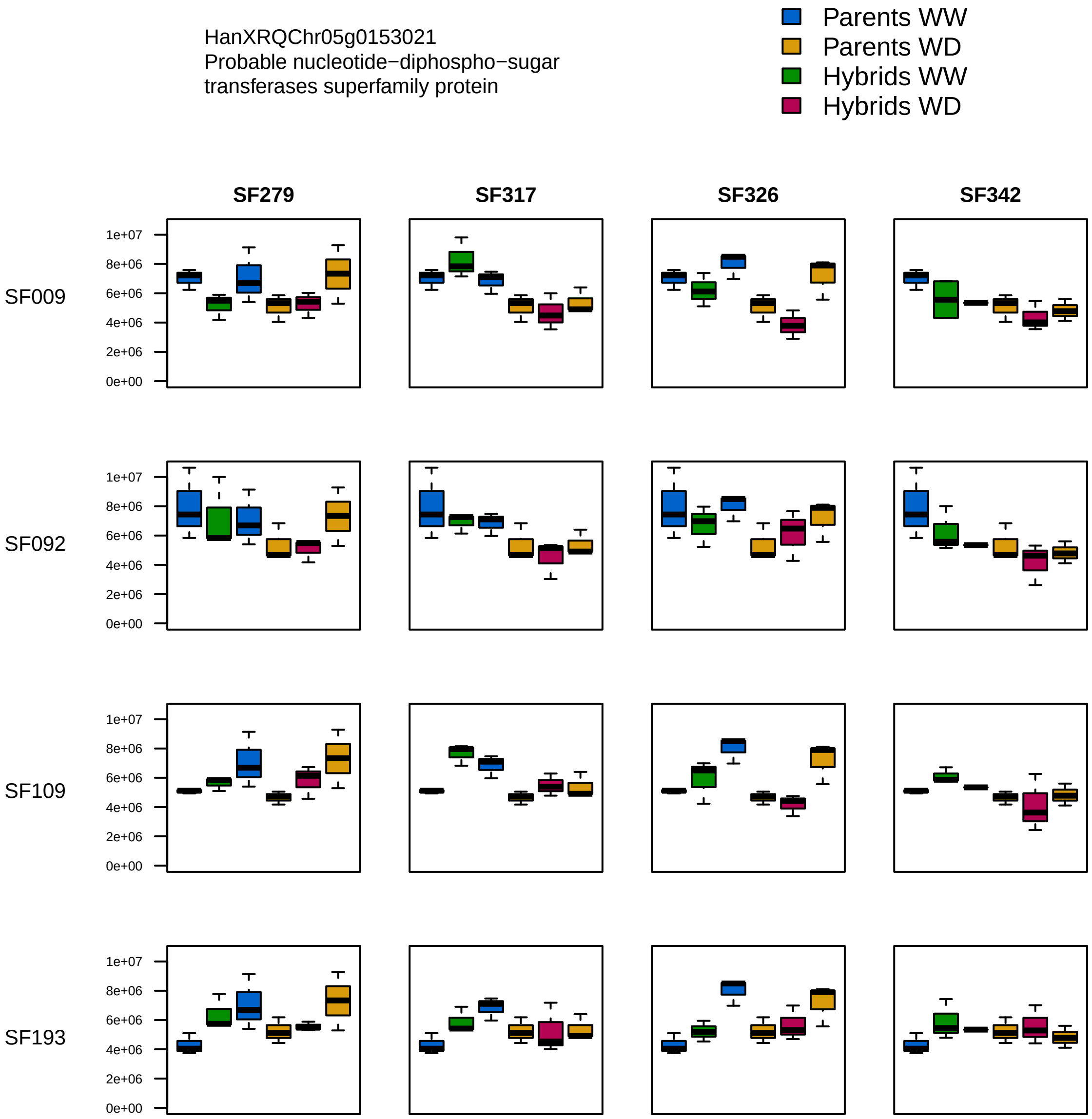
HanXRQChr05g0153021
Probable nucleotide−diphospho−sugar
transferases superfamily protein
Parents WW
Parents WD
Hybrids WW
Hybrids WD
SF279
SF317
SF326
SF342
SF009
SF092
SF109
SF193
1e+07
8e+06
6e+06
4e+06
2e+06
0e+00

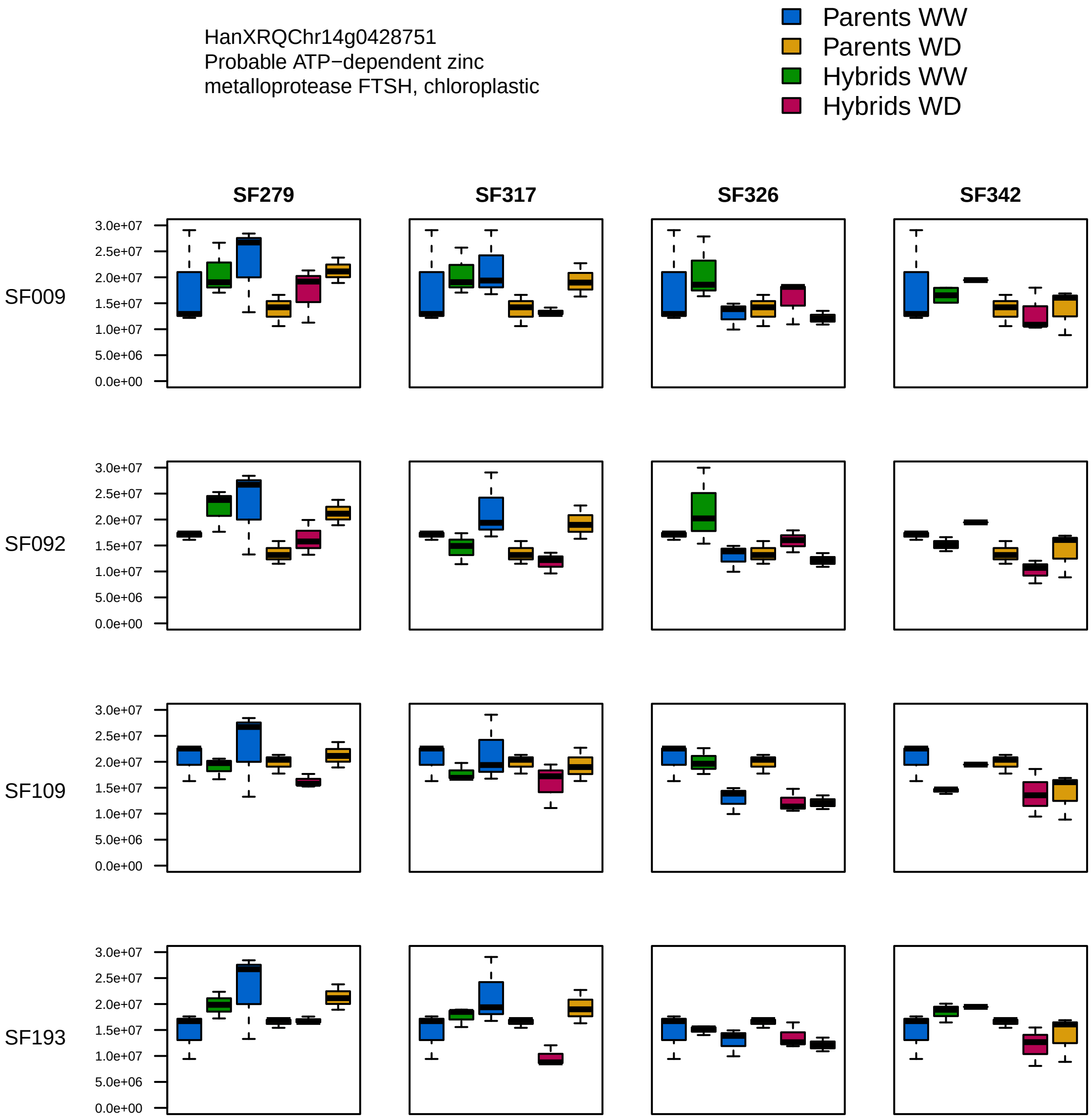
HanXRQChr14g0428751
Probable ATP−dependent zinc
metalloprotease FTSH, chloroplastic
Parents WW
Parents WD
Hybrids WW
Hybrids WD
SF279
SF317
SF326
SF342
SF009
SF092
SF109
SF193
3.0e+07
2.5e+07
2.0e+07
1.5e+07
1.0e+07
5.0e+06
0.0e+00

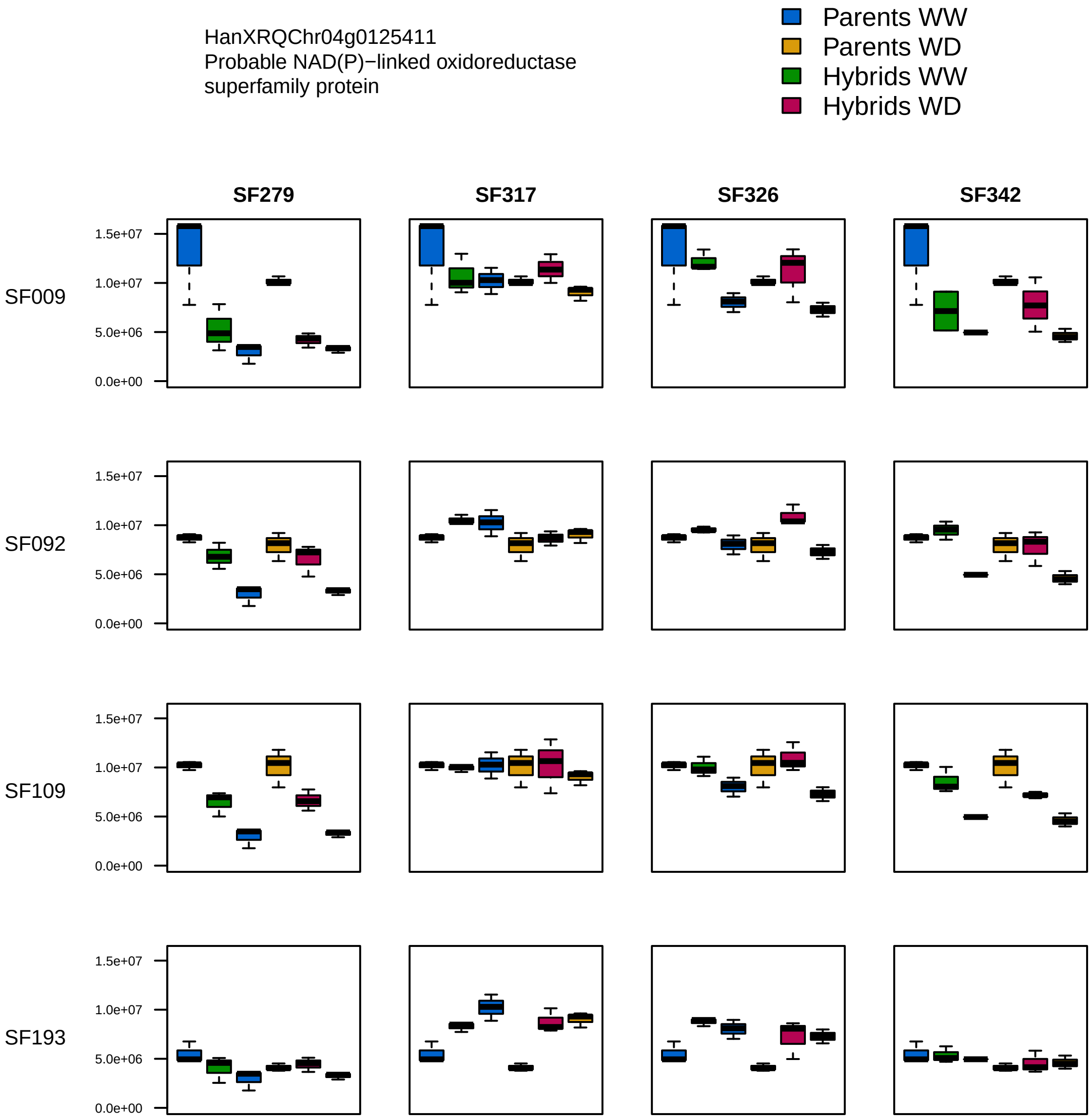

HanXRQChr04g0125411
Probable NAD(P)−linked oxidoreductase
superfamily protein
Parents WW
Parents WD
Hybrids WW
Hybrids WD
SF279
SF317
SF326
SF342
SF009
SF092
SF109
SF193
1.5e+07
1.0e+07
5.0e+06
0.0e+00

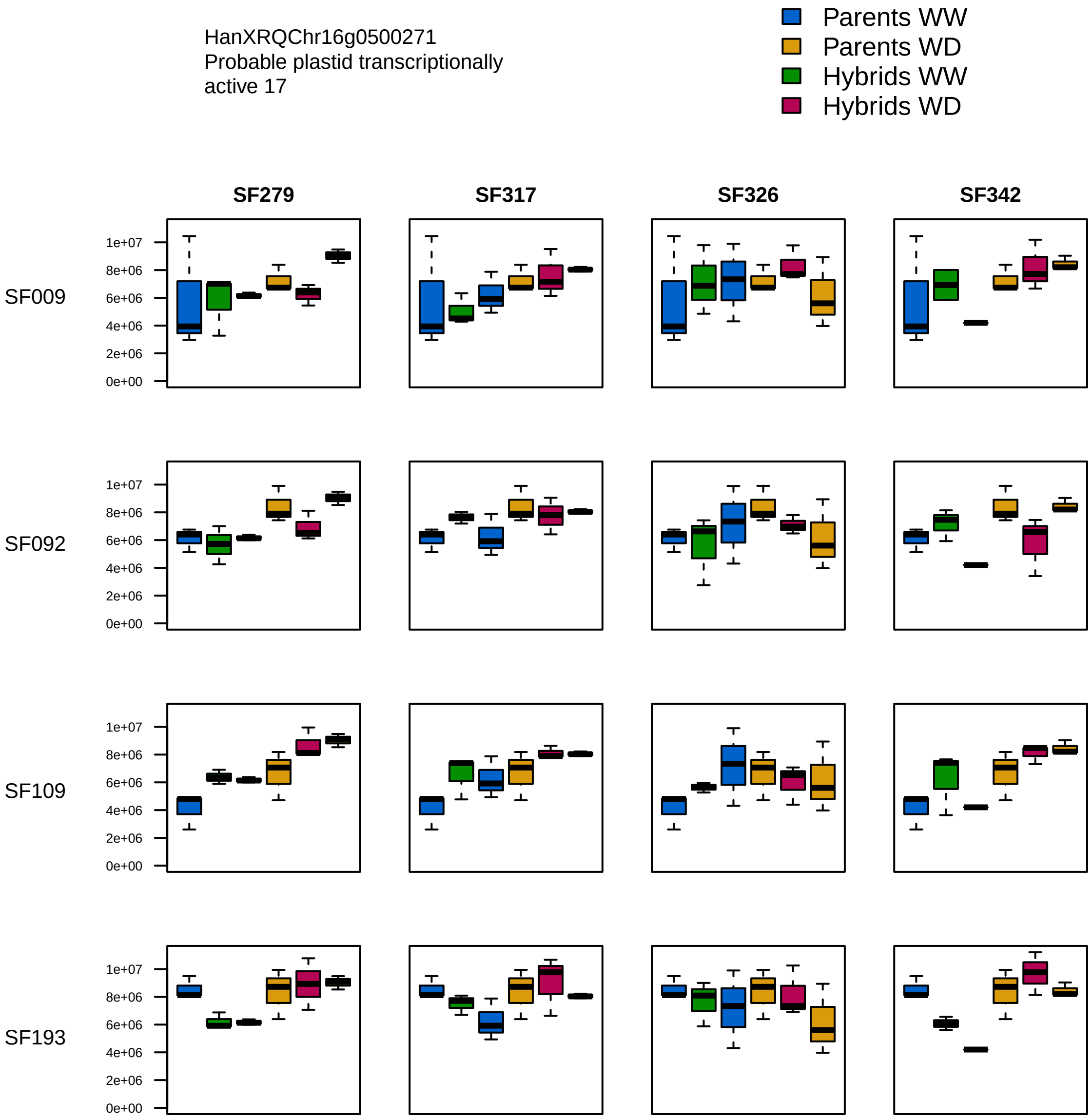

HanXRQChr16g0500271
Probable plastid transcriptionally active 17
Parents WW
Parents WD
Hybrids WW
Hybrids WD
SF279
SF317
SF326
SF342
SF009
SF092
SF109
SF193
0e+00
2e+06
4e+06
6e+06
8e+06
1e+07

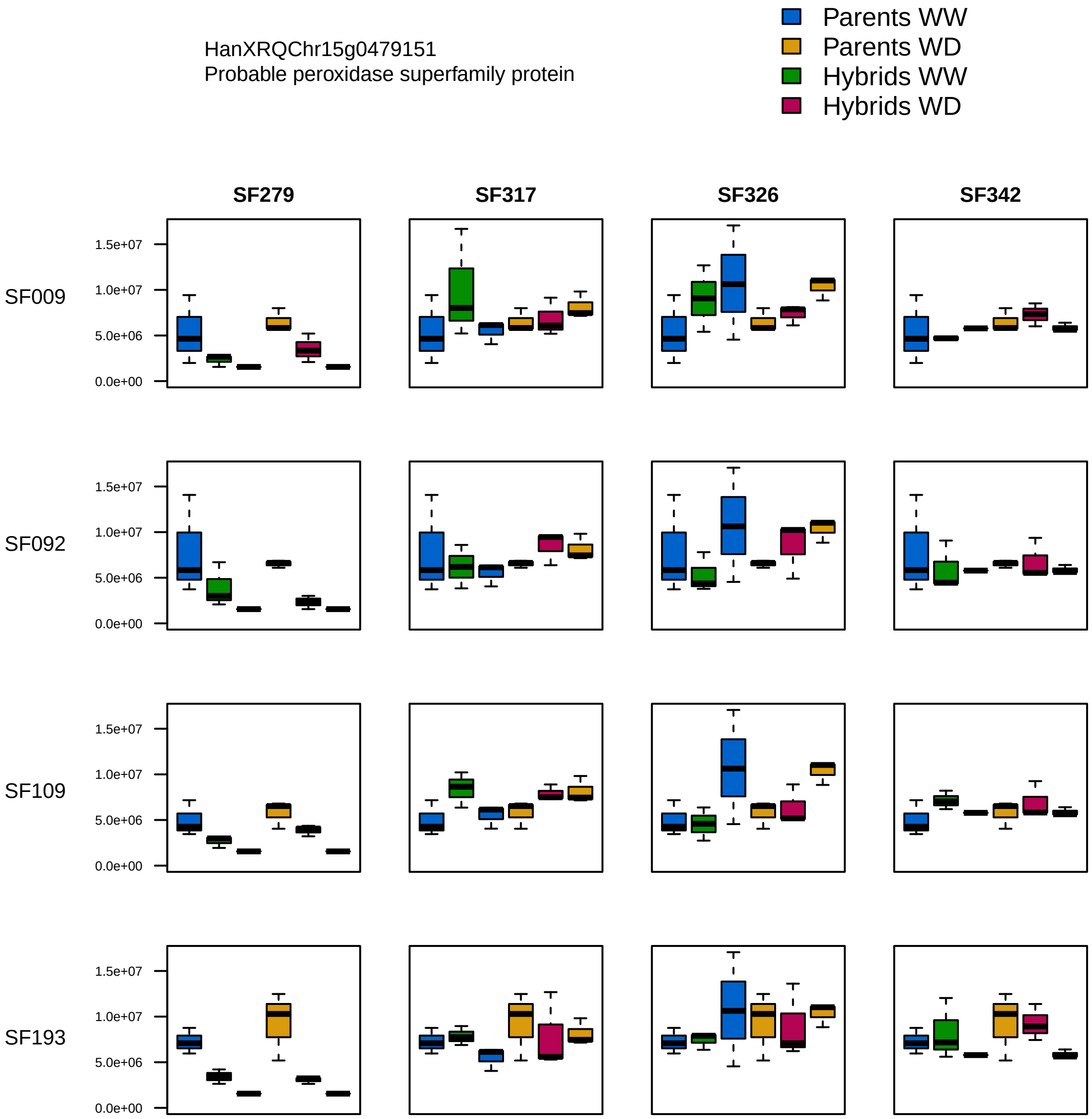

HanXRQChr15g0479151
Probable peroxidase superfamily protein
Parents WW
Parents WD
Hybrids WW
Hybrids WD
SF279
SF317
SF326
SF342
SF009
SF092
SF109
SF193
0.0e+00
5.0e+06
1.0e+07
1.5e+07

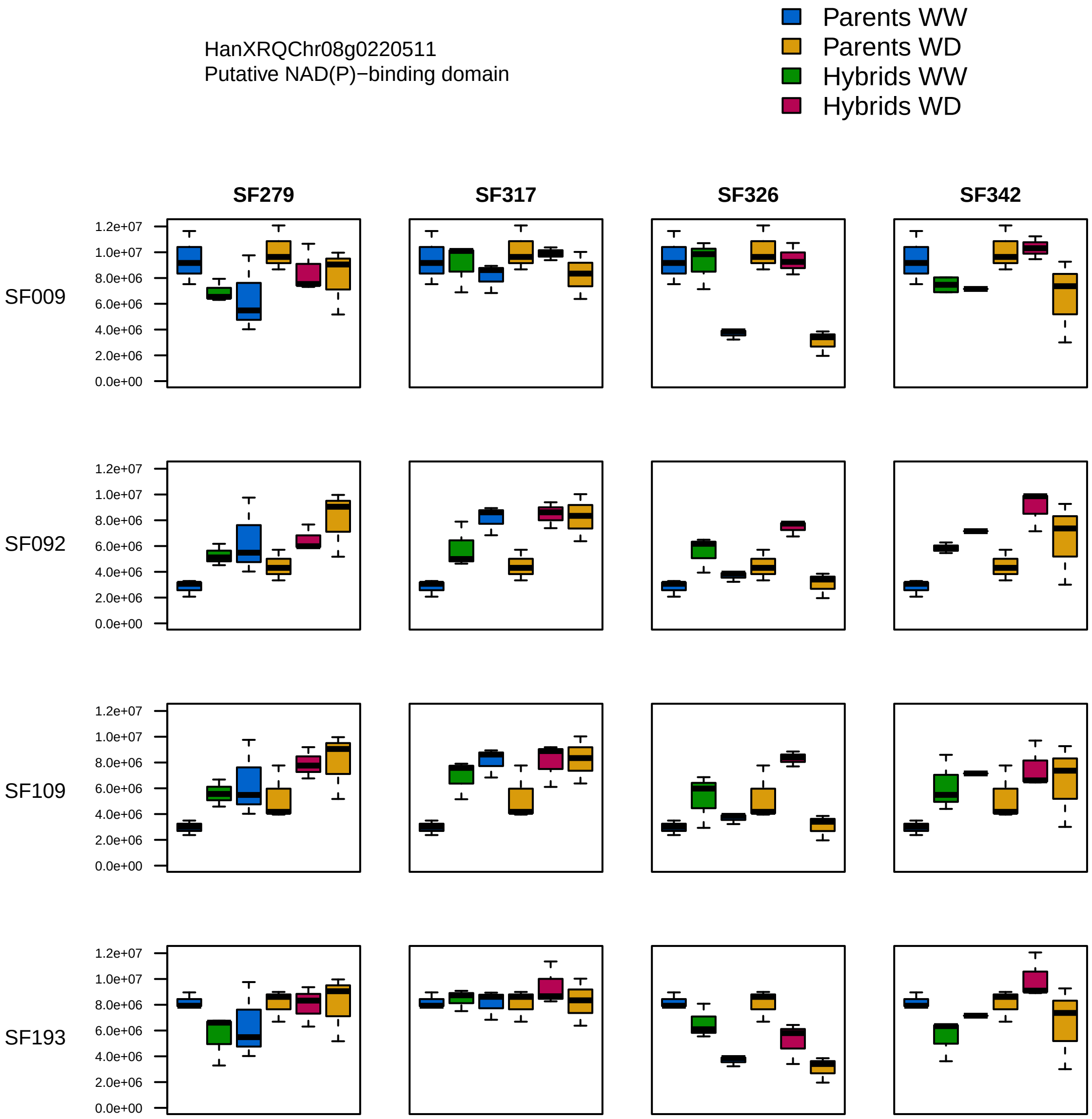
HanXRQChr08g0220511
Putative NAD(P)−binding domain
Parents WW
Parents WD
Hybrids WW
Hybrids WD
SF279
SF317
SF326
SF342
SF009
SF092
SF109
SF193
0.0e+00
2.0e+06
4.0e+06
6.0e+06
8.0e+06
1.0e+07
1.2e+07

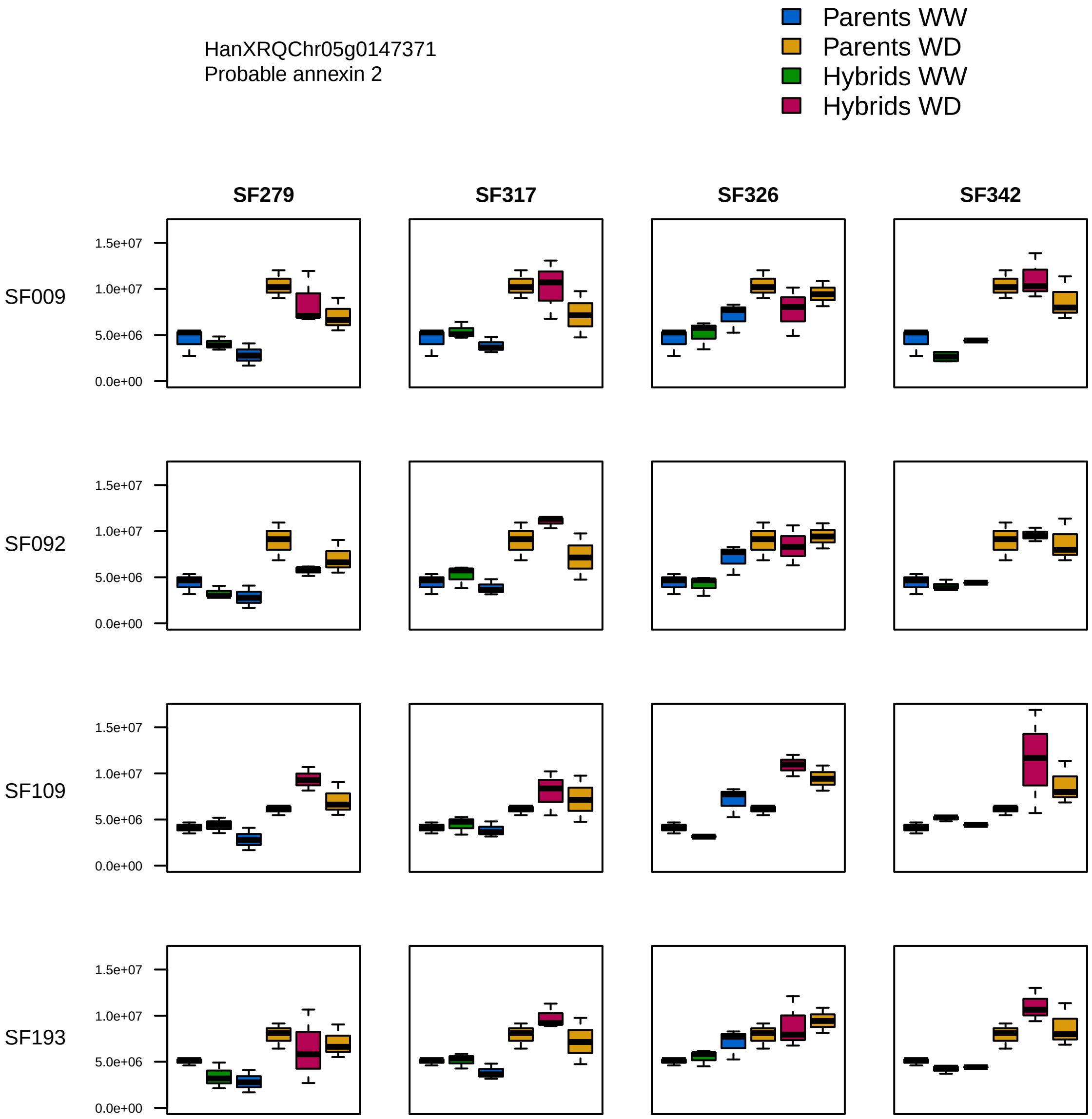

HanXRQChr05g0147371
Probable annexin 2
Parents WW
Parents WD
Hybrids WW
Hybrids WD
SF279
SF317
SF326
SF342
SF009
SF092
SF109
SF193
1.5e+07
1.0e+07
5.0e+06
0.0e+00

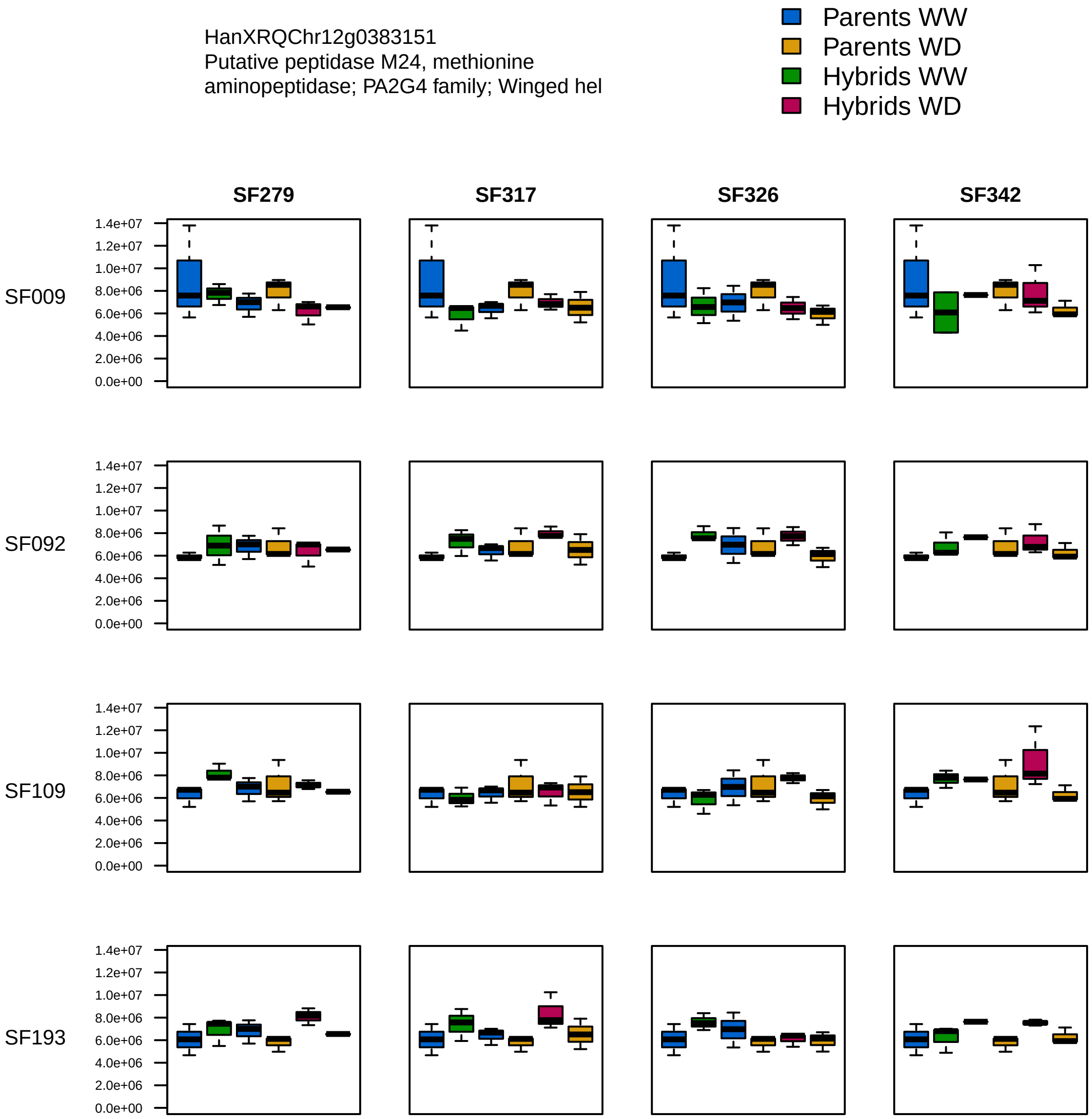
HanXRQChr12g0383151
Putative peptidase M24, methionine
aminopeptidase; PA2G4 family; Winged hel
Parents WW
Parents WD
Hybrids WW
Hybrids WD
SF279
SF317
SF326
SF342
SF009
SF092
SF109
SF193
1.4e+07
1.2e+07
1.0e+07
8.0e+06
6.0e+06
4.0e+06
2.0e+06
0.0e+00

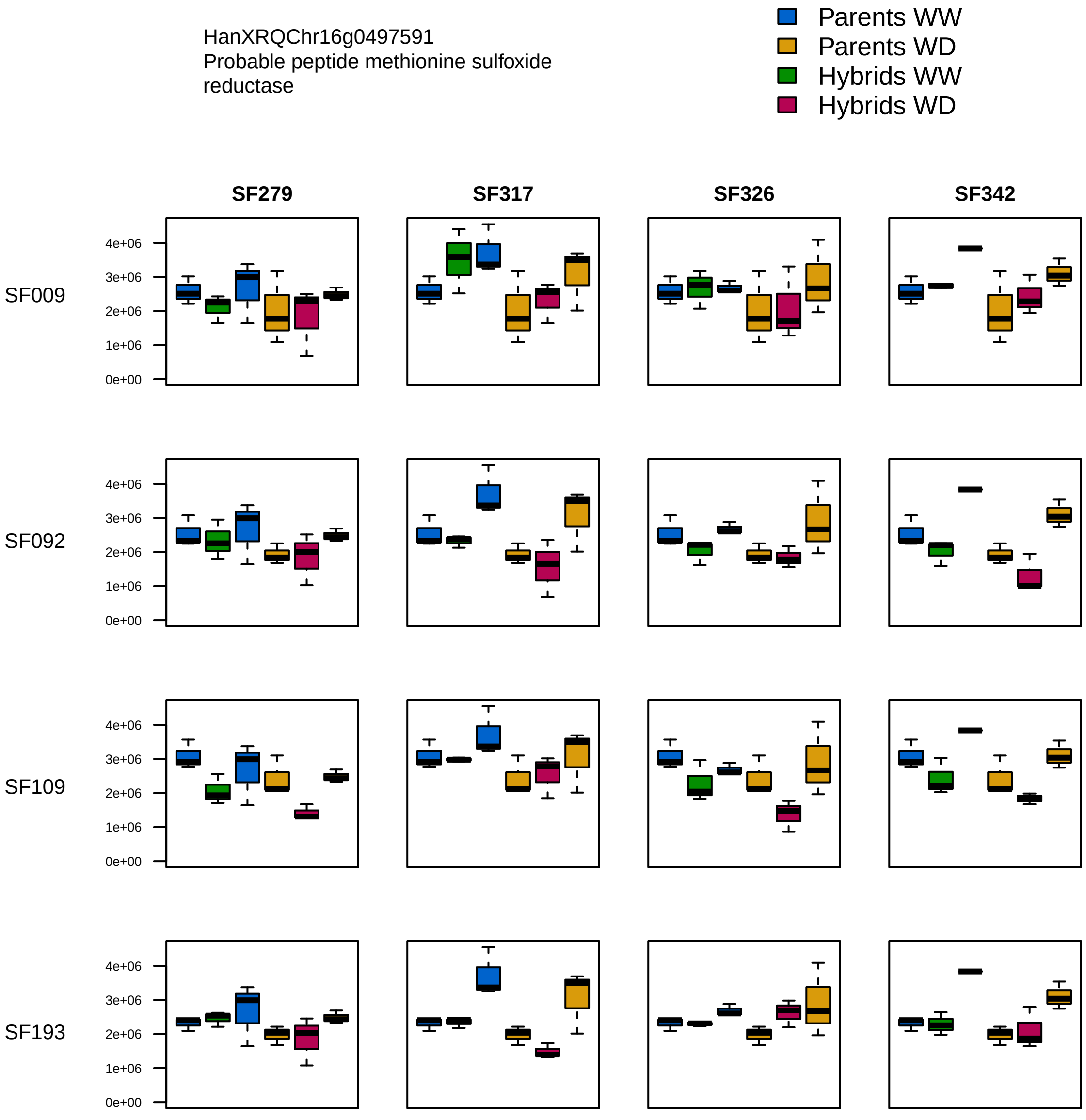
HanXRQChr16g0497591
Probable peptide methionine sulfoxide reductase
Parents WW
Parents WD
Hybrids WW
Hybrids WD
SF279
SF317
SF326
SF342
SF009
SF092
SF109
SF193
0e+00
1e+06
2e+06
3e+06
4e+06

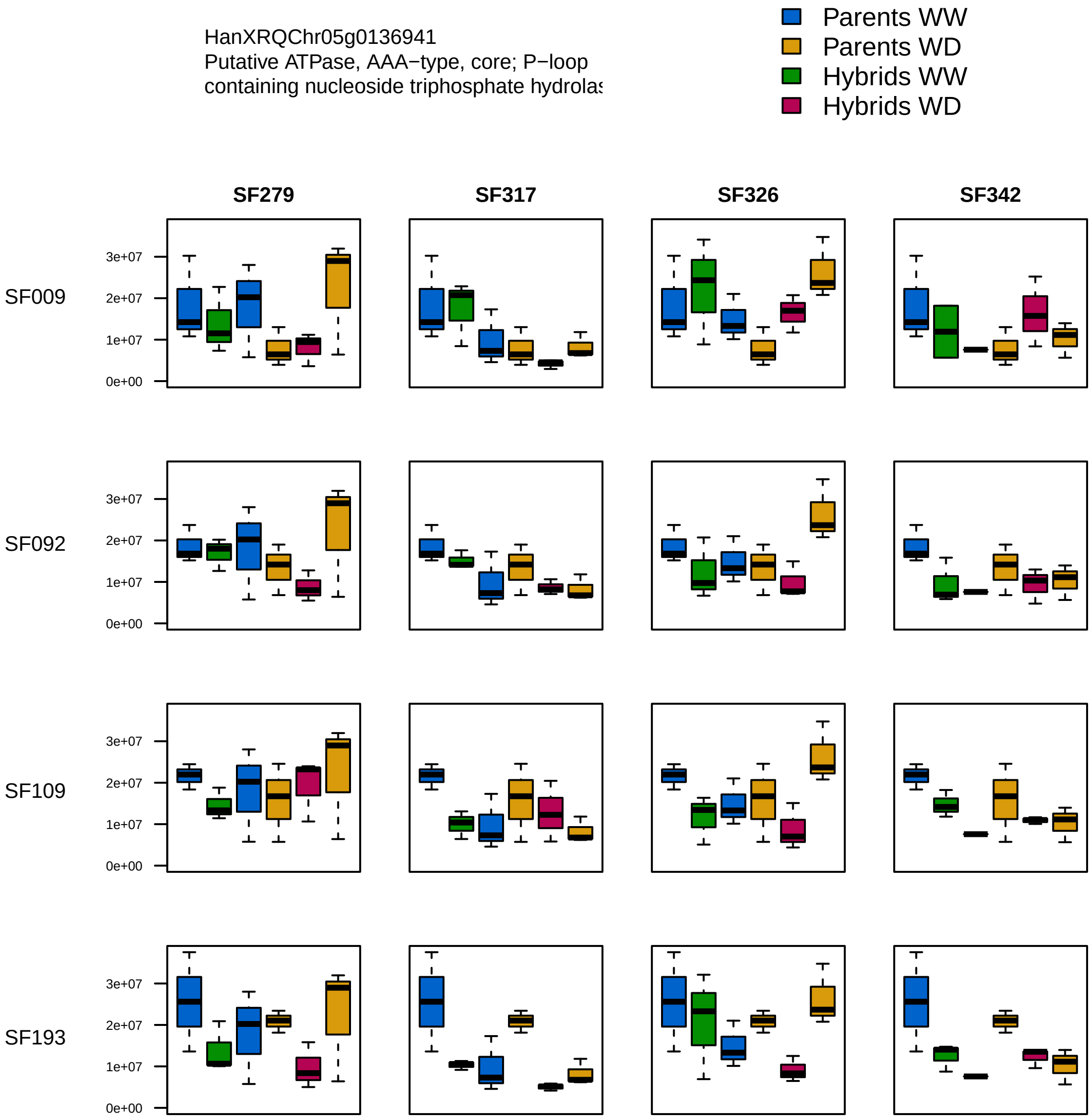
HanXRQChr05g0136941
Putative ATPase, AAA−type, core; P−loop
containing nucleoside triphosphate hydrolas
Parents WW
Parents WD
Hybrids WW
Hybrids WD
SF279
SF317
SF326
SF342
SF009
SF092
SF109
SF193
3e+07
2e+07
1e+07
0e+00

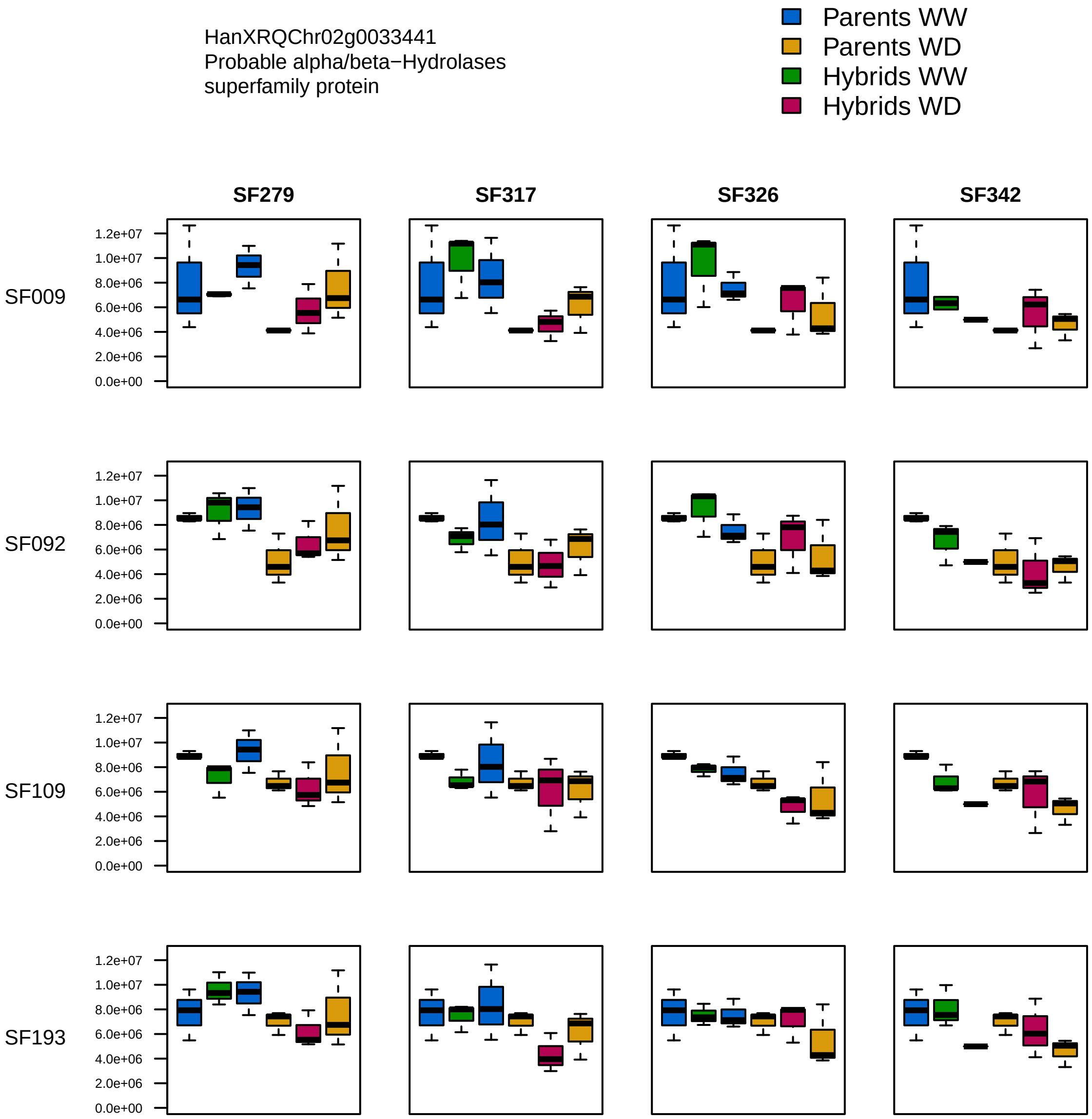
HanXRQChr02g0033441
Probable alpha/beta−Hydrolases
superfamily protein
Parents WW
Parents WD
Hybrids WW
Hybrids WD
SF279
SF317
SF326
SF342
SF009
SF092
SF109
SF193
1.2e+07
1.0e+07
8.0e+06
6.0e+06
4.0e+06
2.0e+06
0.0e+00

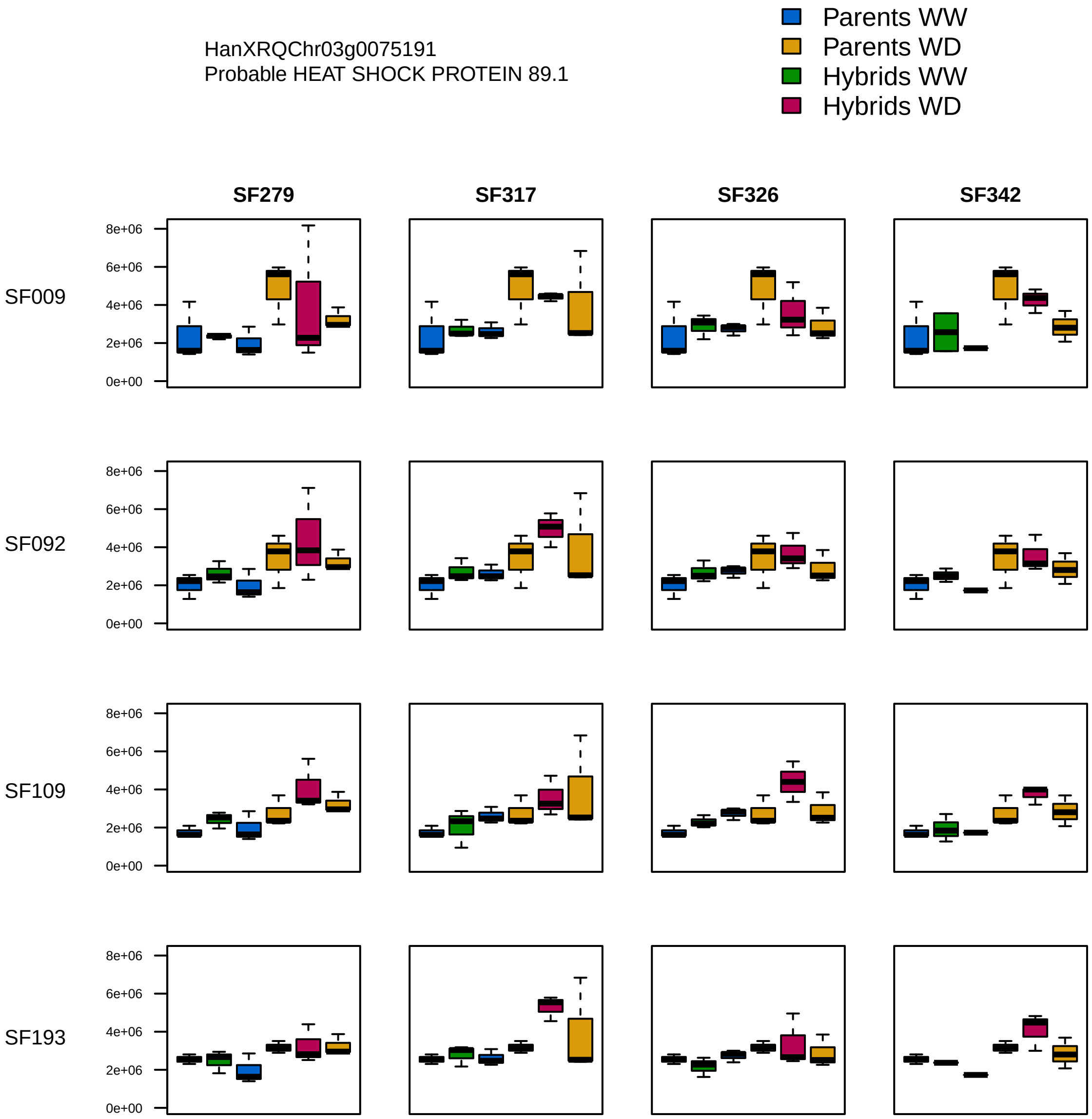

HanXRQChr03g0075191
Probable HEAT SHOCK PROTEIN 89.1
Parents WW
Parents WD
Hybrids WW
Hybrids WD
SF279
SF317
SF326
SF342
SF009
SF092
SF109
SF193
8e+06
6e+06
4e+06
2e+06
0e+00

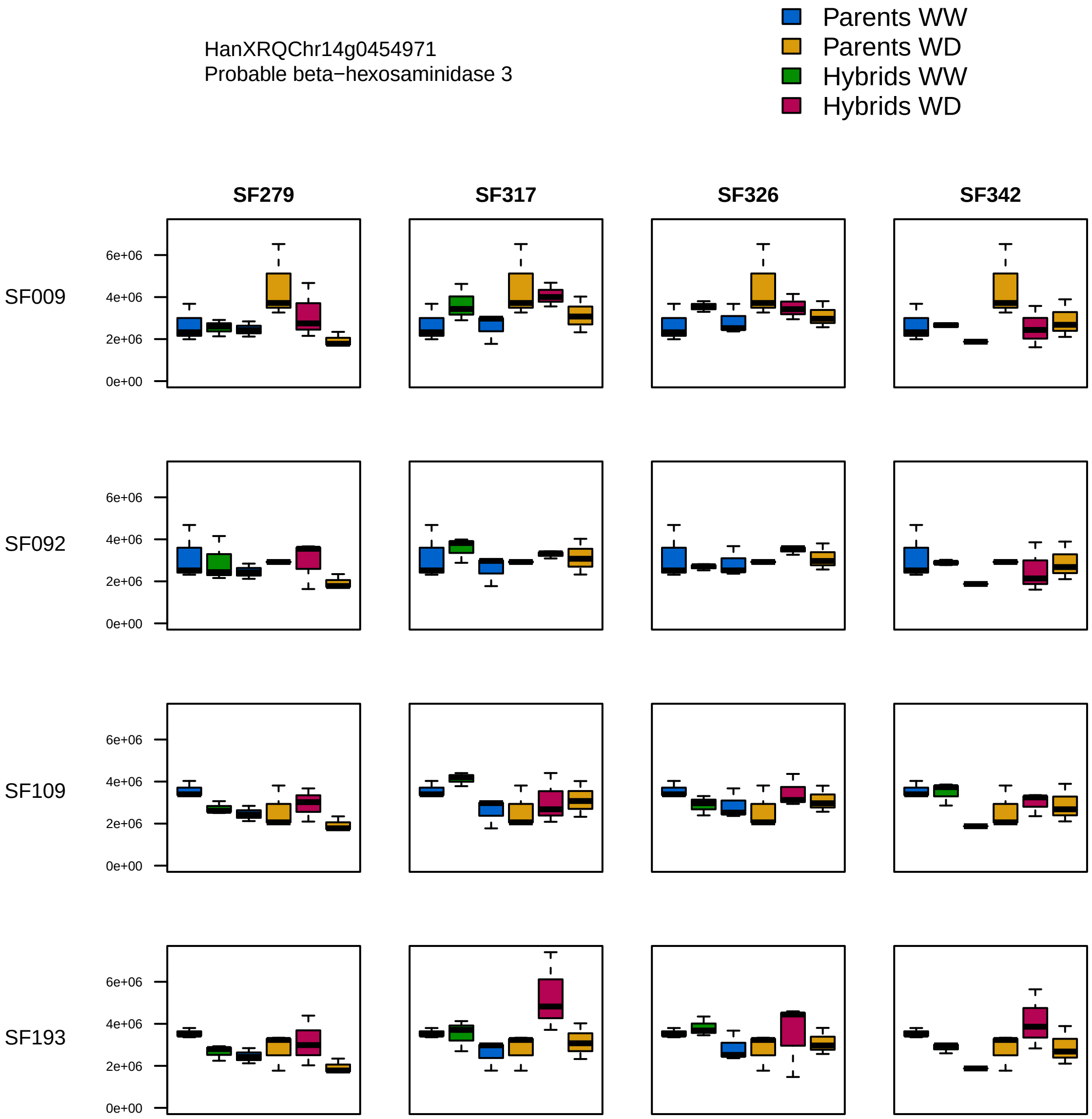
HanXRQChr14g0454971
Probable beta−hexosaminidase 3
Parents WW
Parents WD
Hybrids WW
Hybrids WD
SF279
SF317
SF326
SF342
SF009
SF092
SF109
SF193
0e+00
2e+06
4e+06
6e+06

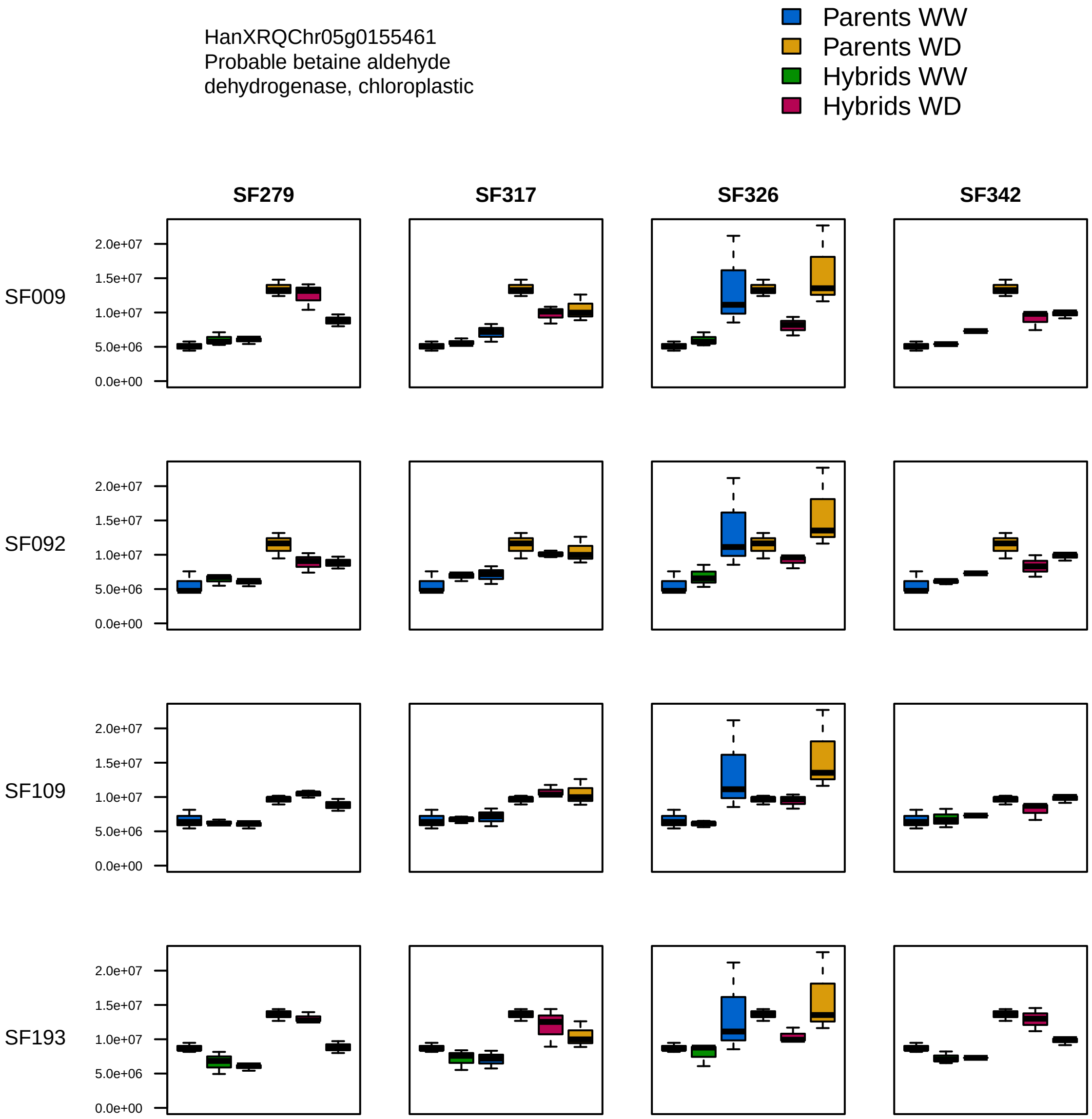
HanXRQChr05g0155461
Probable betaine aldehyde
dehydrogenase, chloroplastic
Parents WW
Parents WD
Hybrids WW
Hybrids WD
SF279
SF317
SF326
SF342
SF009
SF092
SF109
SF193
2.0e+07
1.5e+07
1.0e+07
5.0e+06
0.0e+00

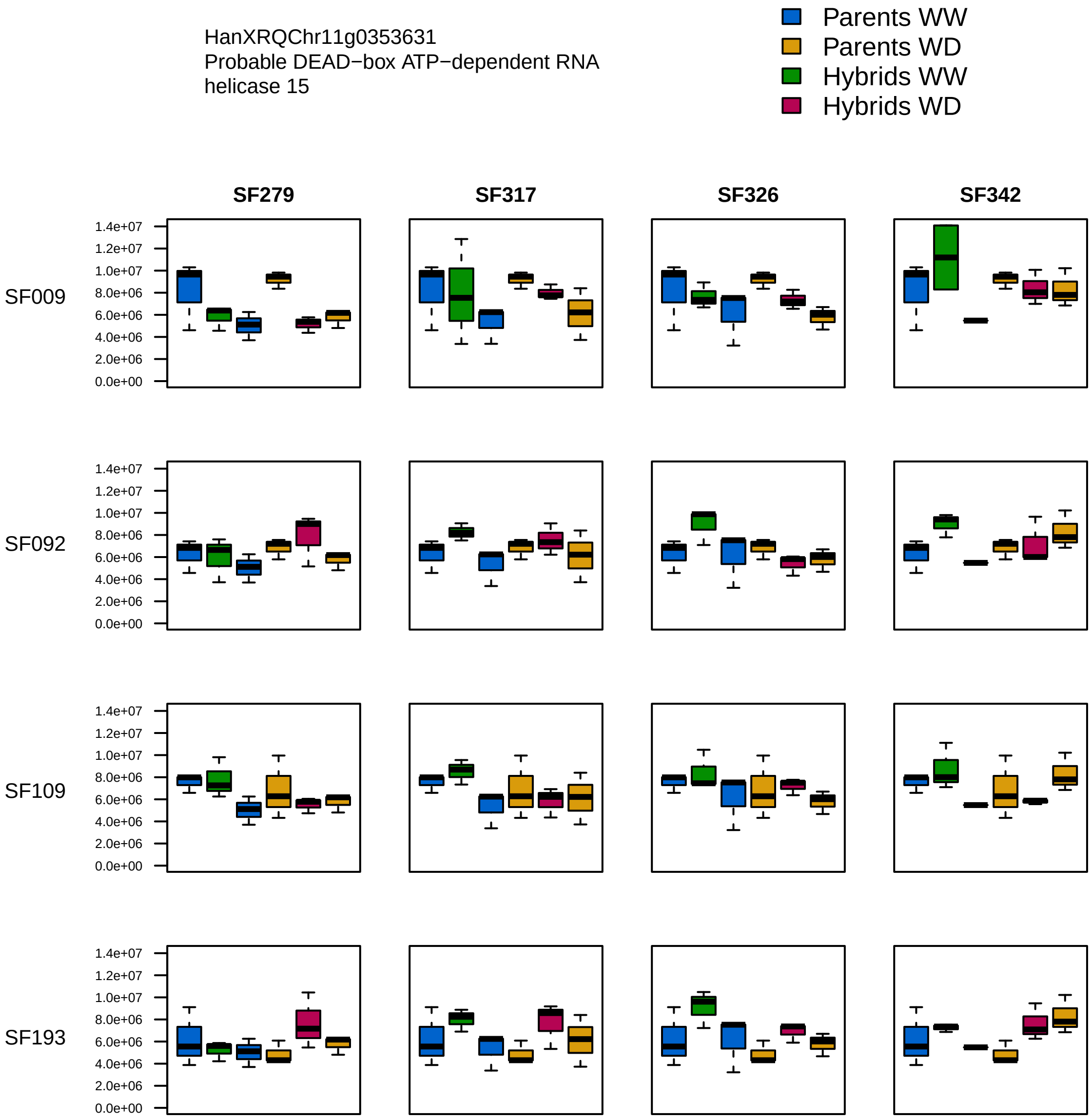

HanXRQChr11g0353631
Probable DEAD−box ATP−dependent RNA
helicase 15
Parents WW
Parents WD
Hybrids WW
Hybrids WD
SF279
SF317
SF326
SF342
SF009
SF092
SF109
SF193
1.4e+07
1.2e+07
1.0e+07
8.0e+06
6.0e+06
4.0e+06
2.0e+06
0.0e+00

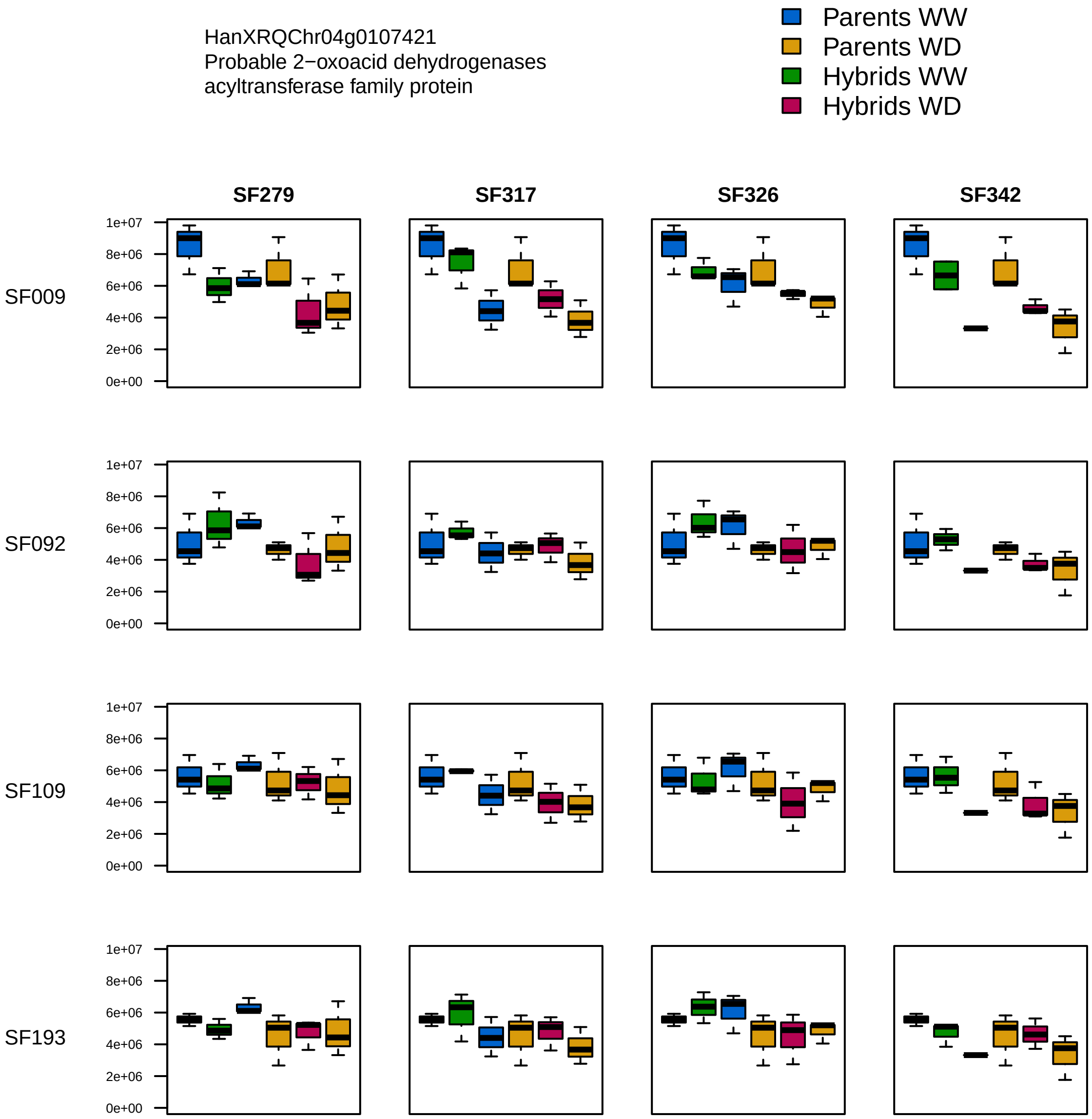
HanXRQChr04g0107421
Probable 2−oxoacid dehydrogenases
acyltransferase family protein
Parents WW
Parents WD
Hybrids WW
Hybrids WD
SF279
SF317
SF326
SF342
SF009
SF092
SF109
SF193
1e+07
8e+06
6e+06
4e+06
2e+06
0e+00

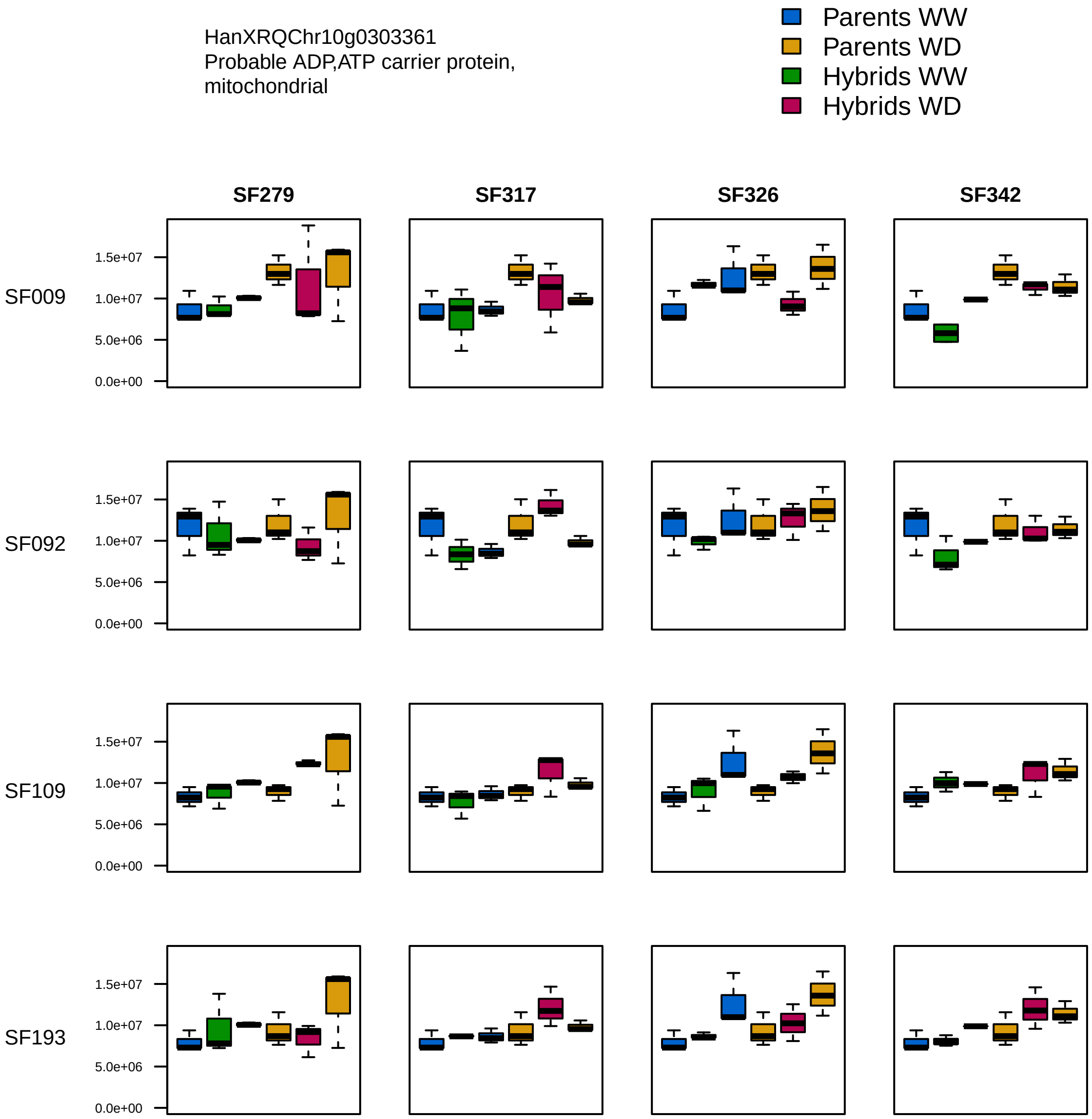

HanXRQChr10g0303361
Probable ADP,ATP carrier protein,
mitochondrial
Parents WW
Parents WD
Hybrids WW
Hybrids WD
SF279
SF317
SF326
SF342
SF009
SF092
SF109
SF193
1.5e+07
1.0e+07
5.0e+06
0.0e+00

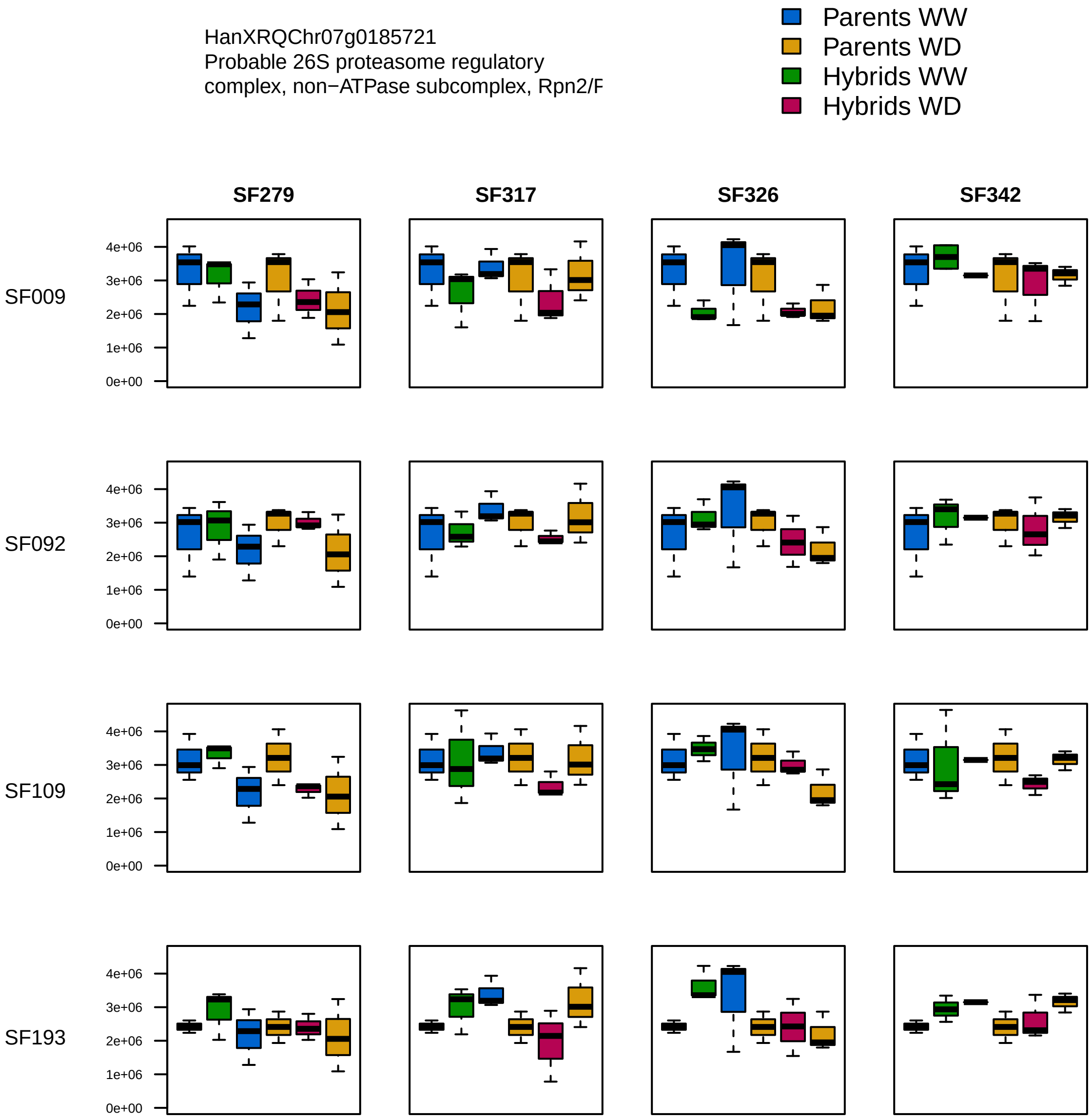
HanXRQChr07g0185721
Probable 26S proteasome regulatory
complex, non−ATPase subcomplex, Rpn2/F
Parents WW
Parents WD
Hybrids WW
Hybrids WD
SF279
SF317
SF326
SF342
SF009
SF092
SF109
SF193
0e+00
1e+06
2e+06
3e+06
4e+06

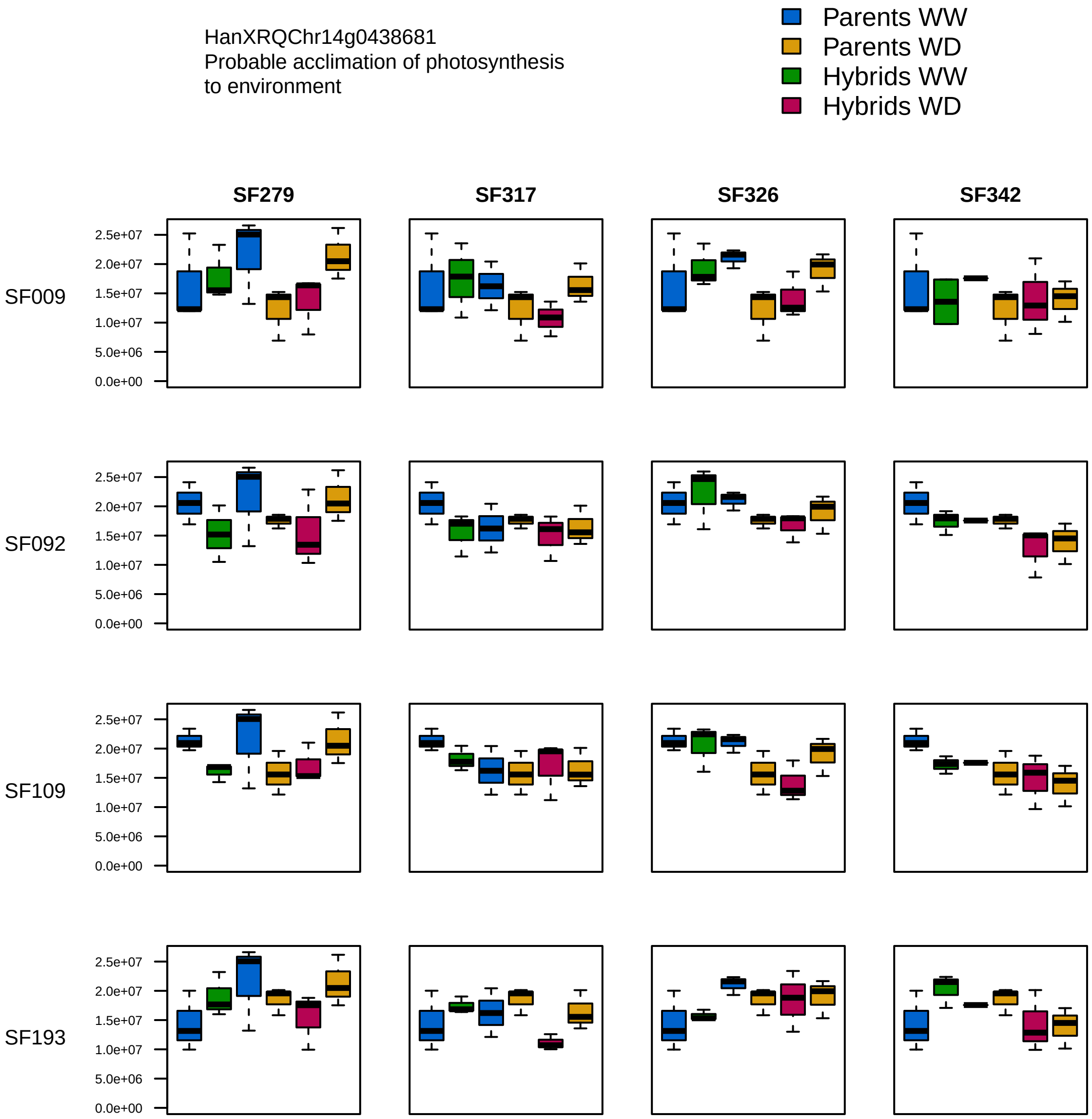
HanXRQChr14g0438681
Probable acclimation of photosynthesis
to environment
Parents WW
Parents WD
Hybrids WW
Hybrids WD
SF279
SF317
SF326
SF342
SF009
SF092
SF109
SF193
2.5e+07
2.0e+07
1.5e+07
1.0e+07
5.0e+06
0.0e+00

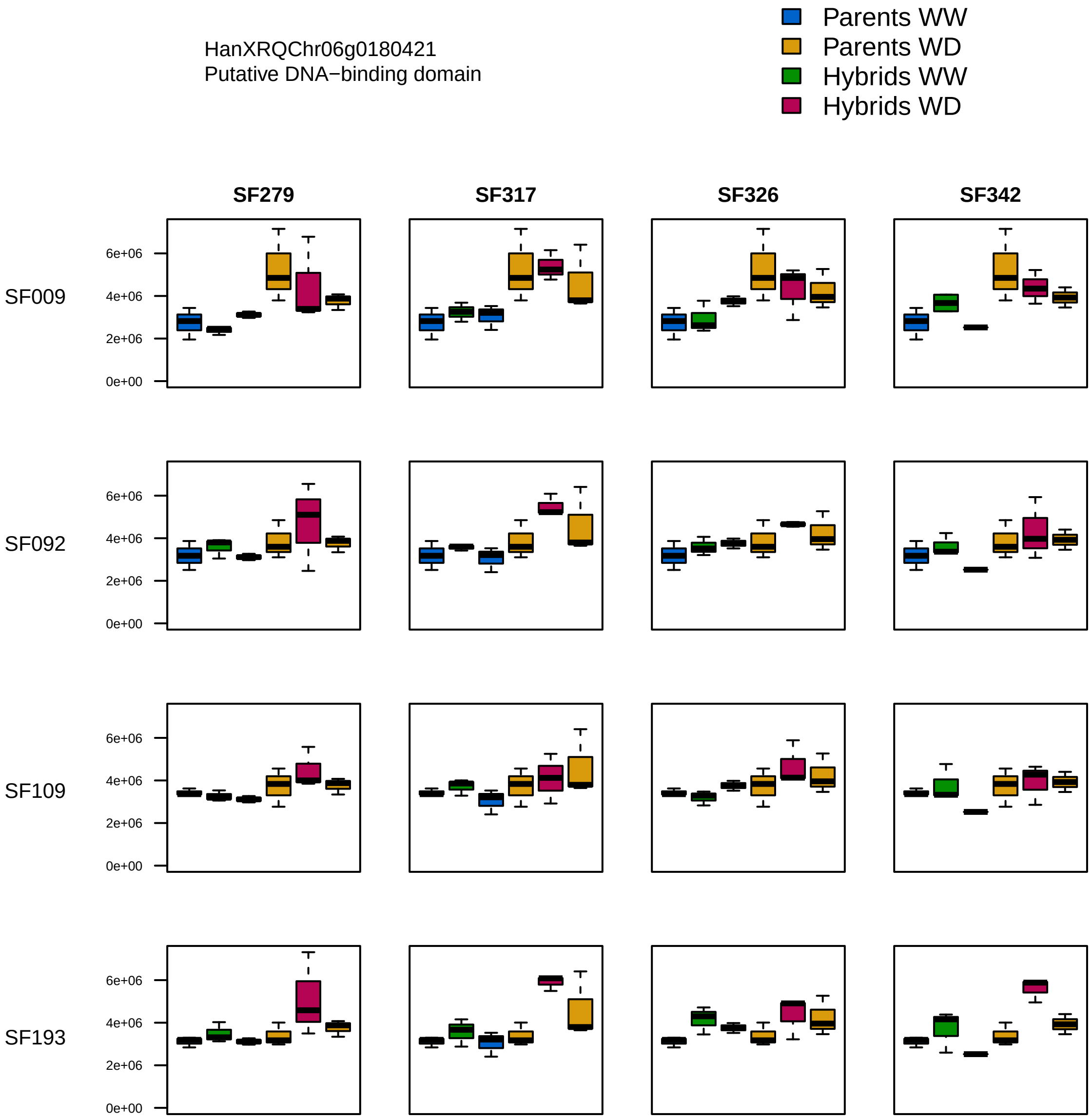
HanXRQChr06g0180421
Putative DNA−binding domain
Parents WW
Parents WD
Hybrids WW
Hybrids WD
SF279
SF317
SF326
SF342
SF009
SF092
SF109
SF193
6e+06
4e+06
2e+06
0e+00

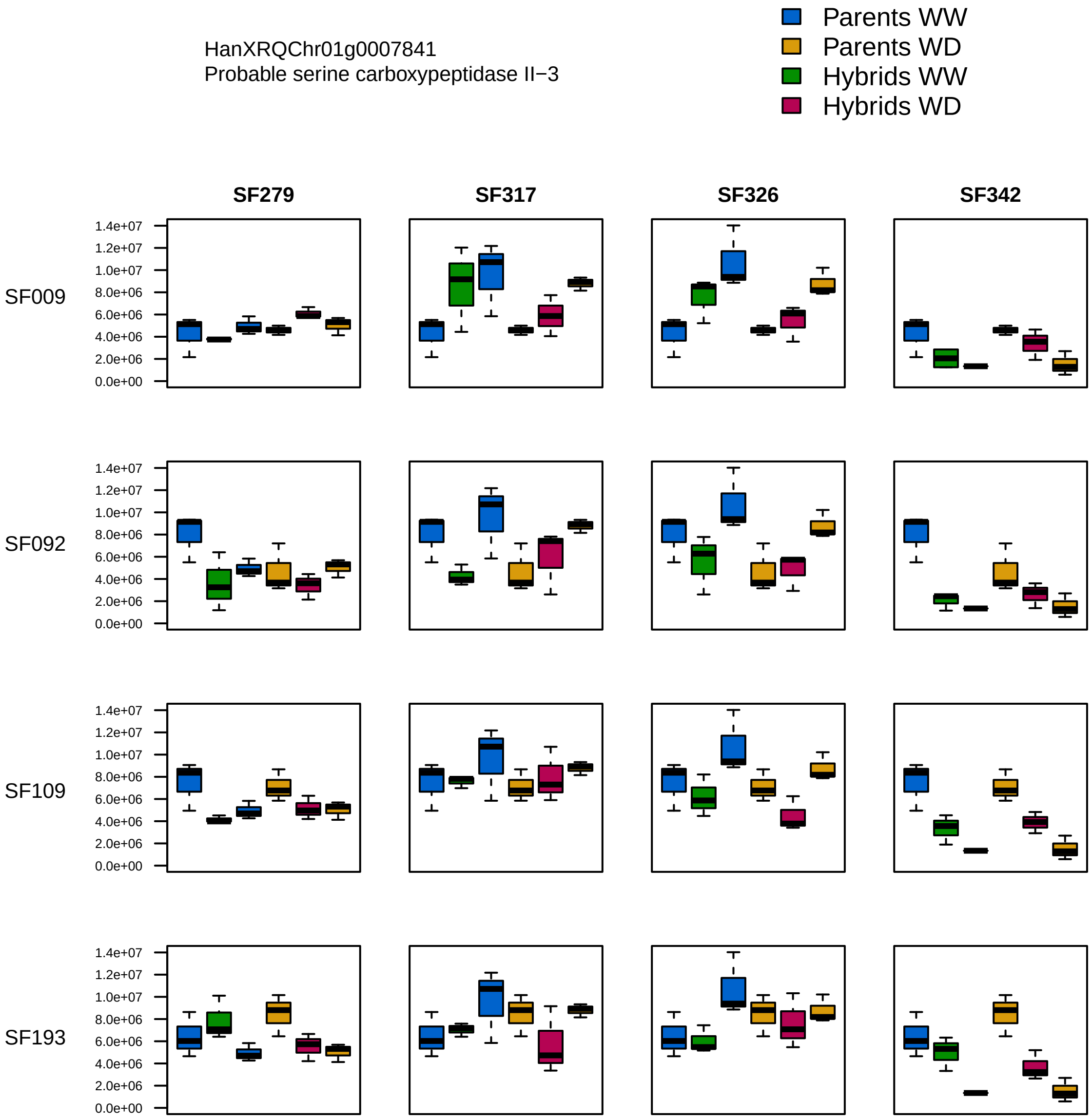
HanXRQChr01g0007841
Probable serine carboxypeptidase II−3
Parents WW
Parents WD
Hybrids WW
Hybrids WD
SF279
SF317
SF326
SF342
SF009
SF092
SF109
SF193
1.4e+07
1.2e+07
1.0e+07
8.0e+06
6.0e+06
4.0e+06
2.0e+06
0.0e+00

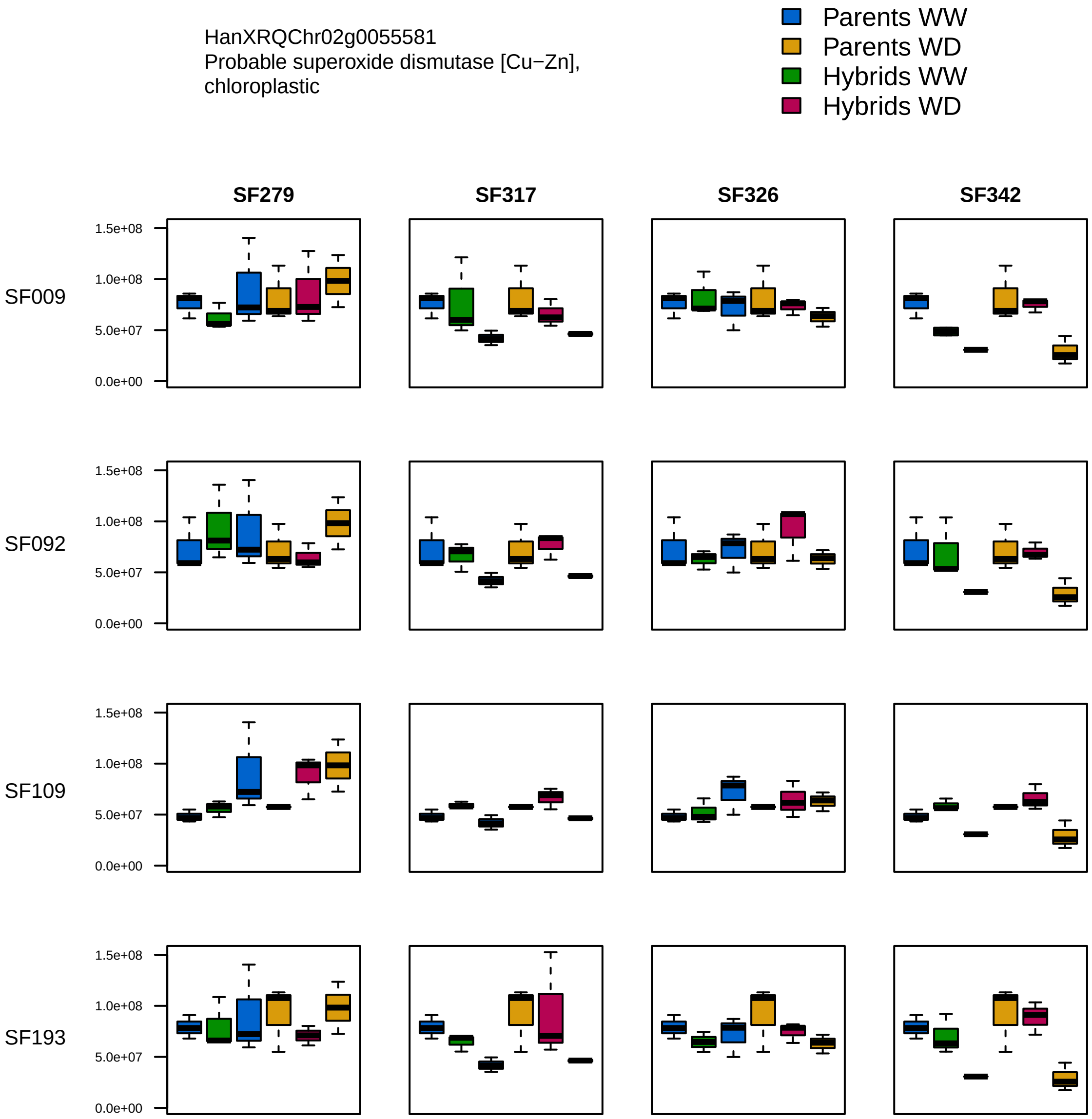
HanXRQChr02g0055581
Probable superoxide dismutase [Cu−Zn], chloroplastic
Parents WW
Parents WD
Hybrids WW
Hybrids WD
SF279
SF317
SF326
SF342
SF009
SF092
SF109
SF193
1.5e+08
1.0e+08
5.0e+07
0.0e+00

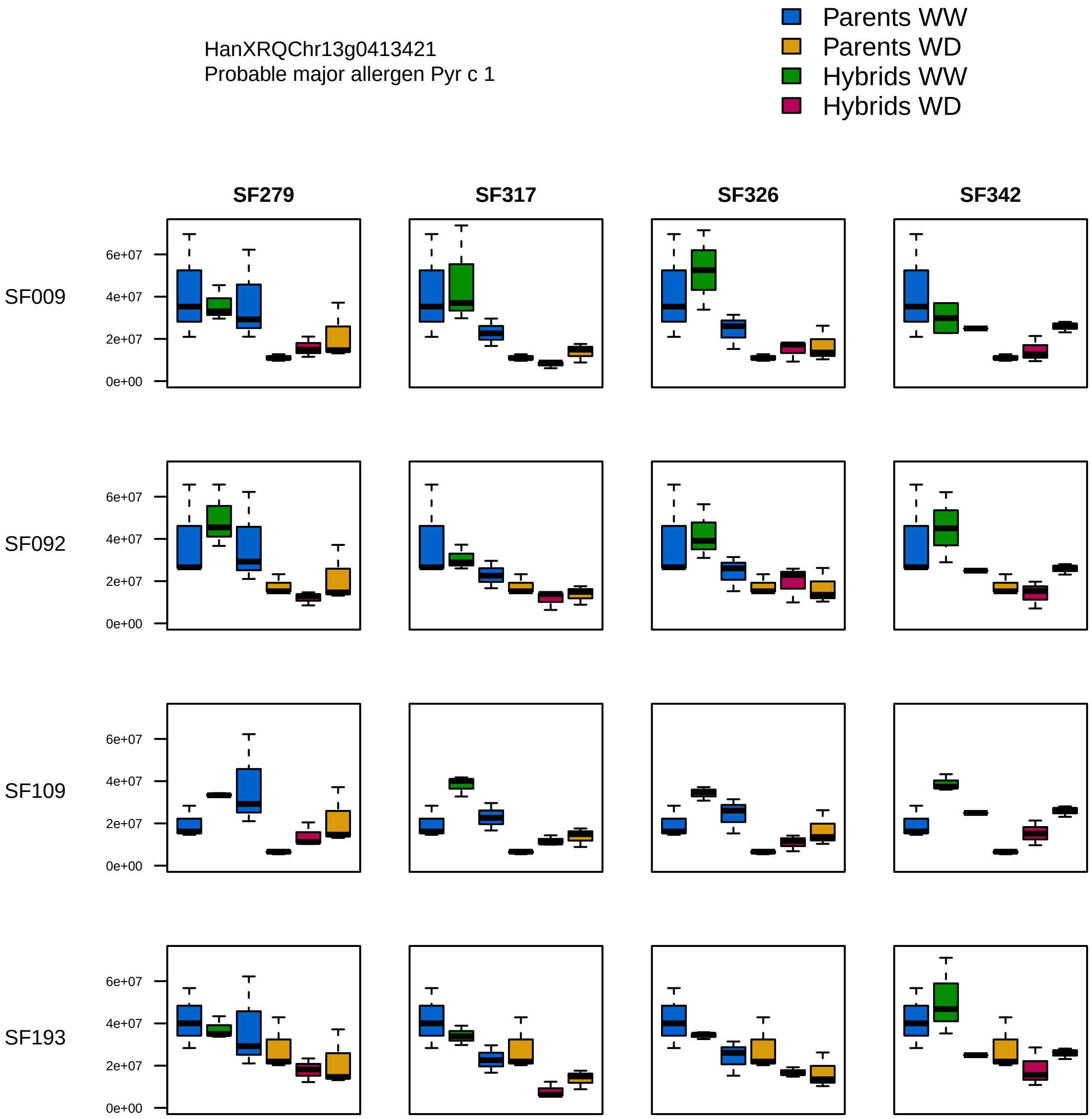
HanXRQChr13g0413421
Probable major allergen Pyr c 1
Parents WW
Parents WD
Hybrids WW
Hybrids WD
SF279
SF317
SF326
SF342
SF009
SF092
SF109
SF193
6e+07
4e+07
2e+07
0e+00

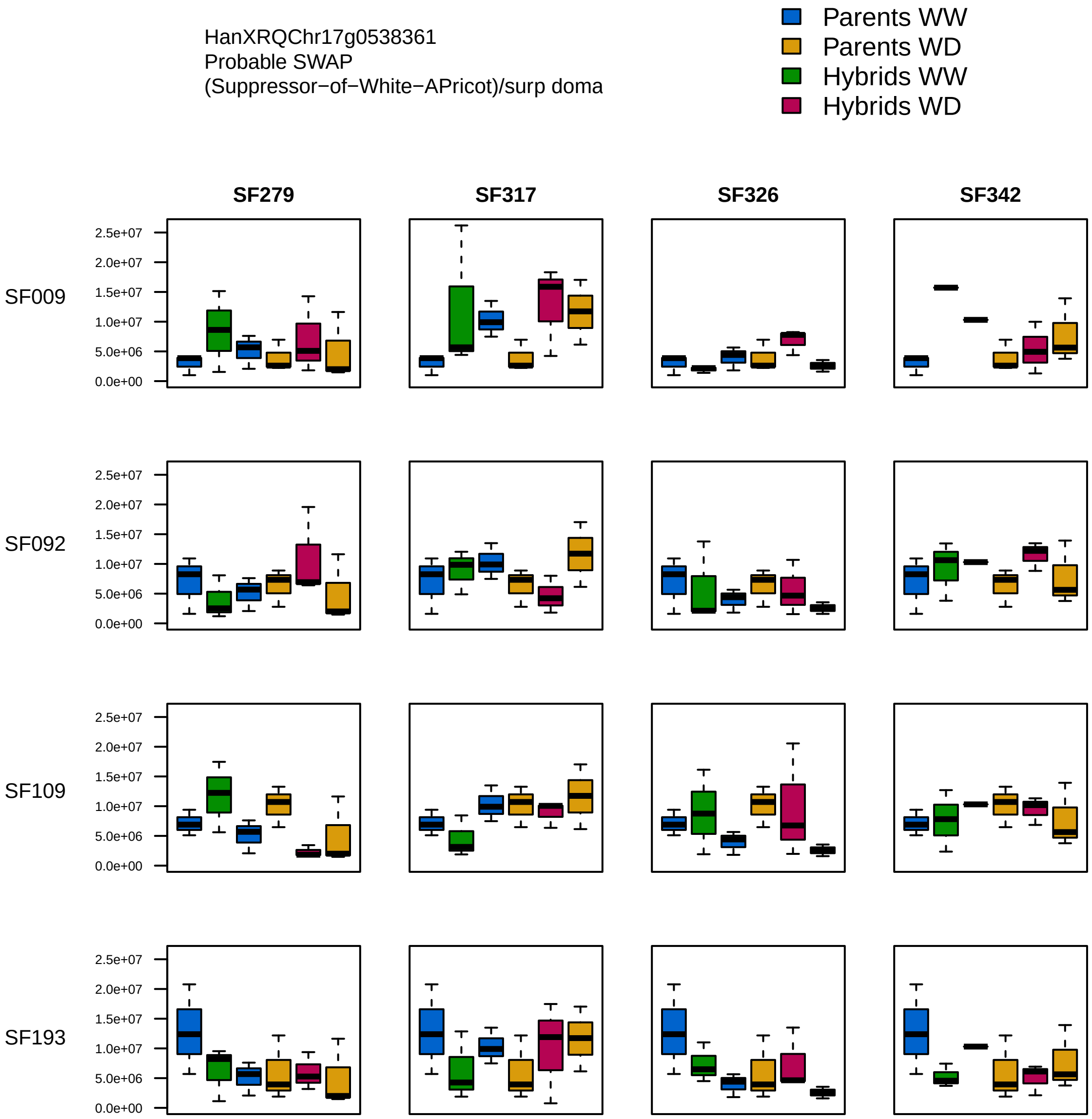
HanXRQChr17g0538361
Probable SWAP
(Suppressor−of−White−APricot)/surp doma
Parents WW
Parents WD
Hybrids WW
Hybrids WD
SF279
SF317
SF326
SF342
SF009
SF092
SF109
SF193
0.0e+00
5.0e+06
1.0e+07
1.5e+07
2.0e+07
2.5e+07

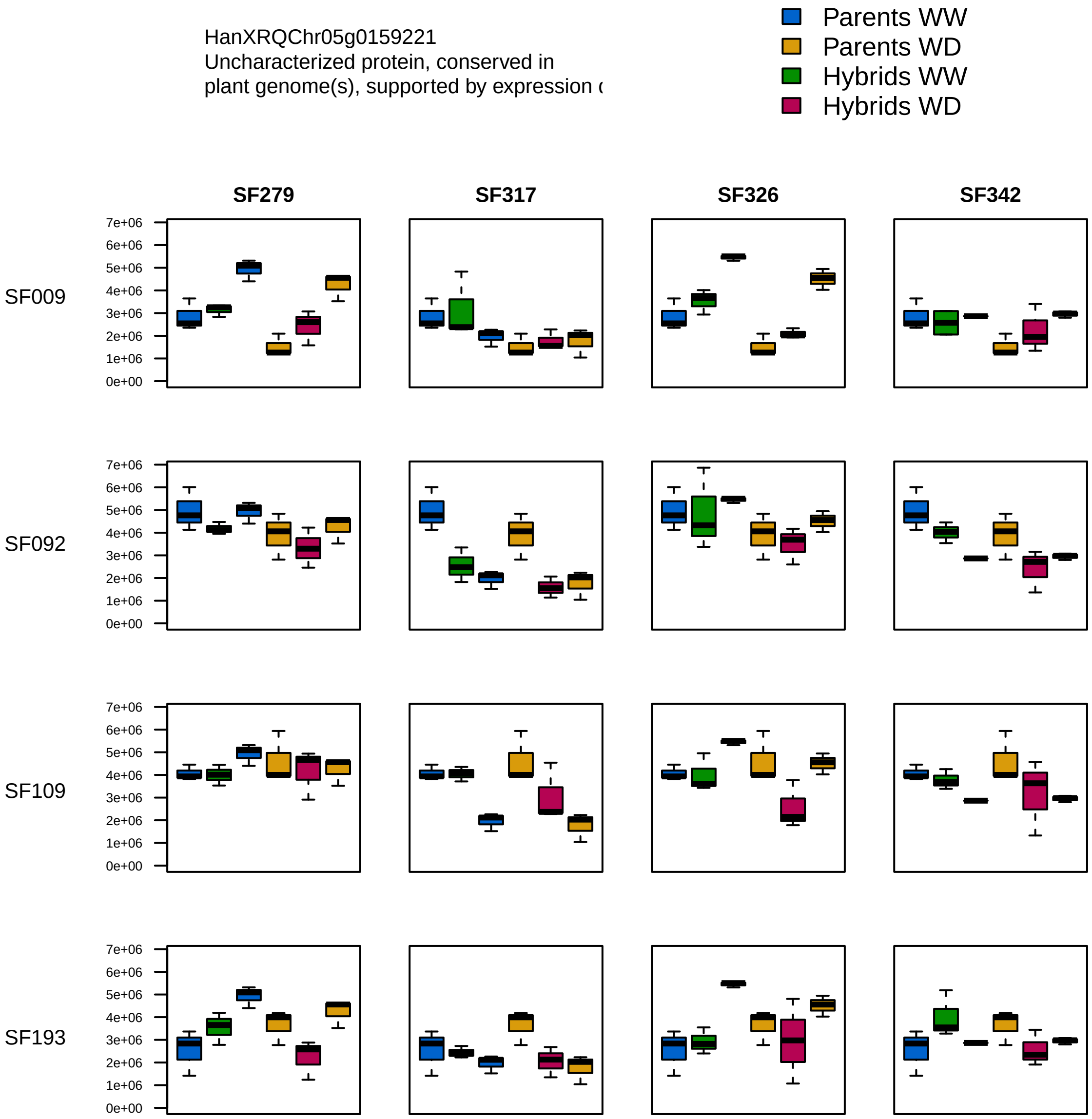
HanXRQChr05g0159221
Uncharacterized protein, conserved in
plant genome(s), supported by expression (
Parents WW
Parents WD
Hybrids WW
Hybrids WD
SF279
SF317
SF326
SF342
SF009
SF092
SF109
SF193
0e+00
1e+06
2e+06
3e+06
4e+06
5e+06
6e+06
7e+06

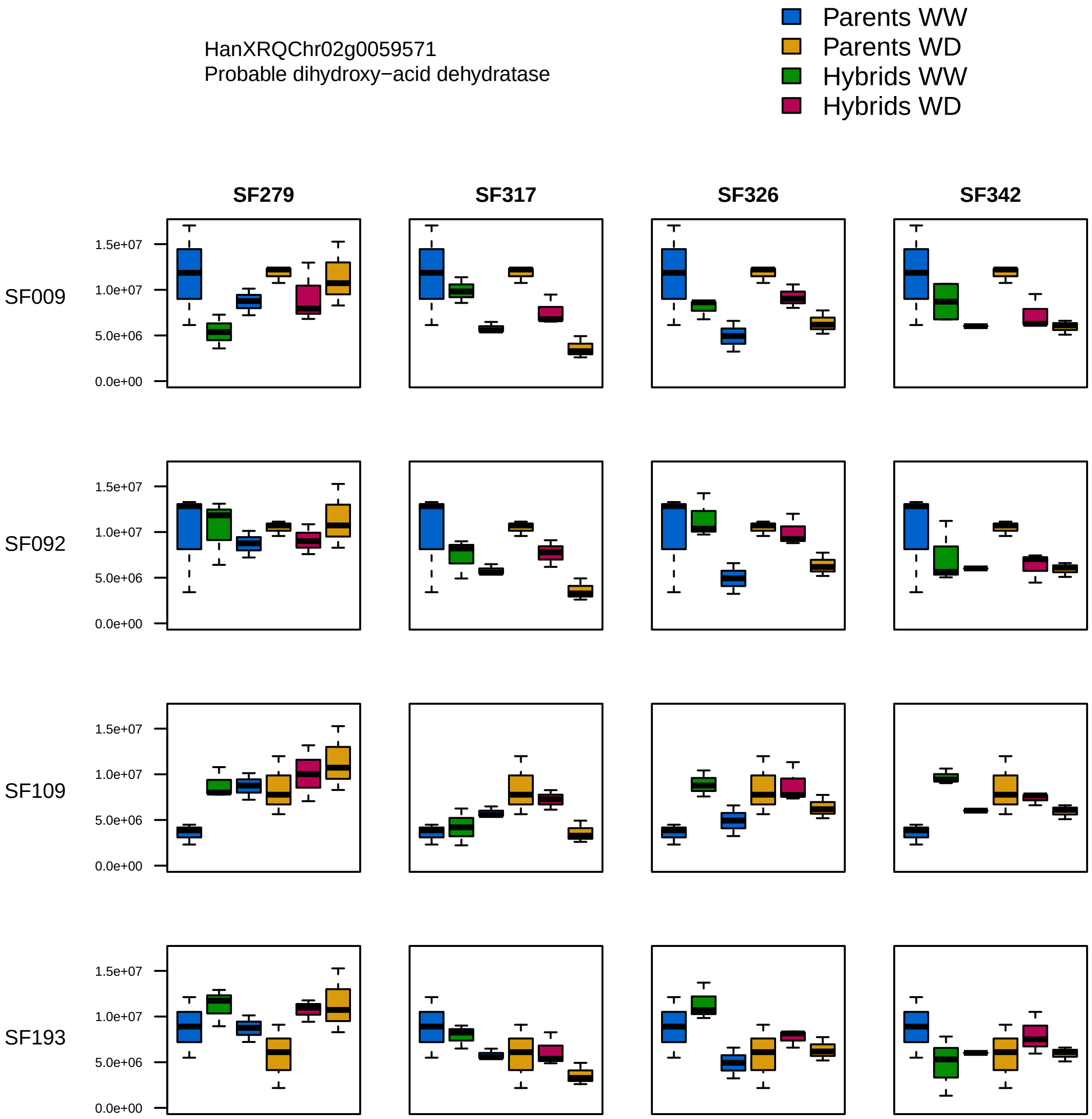
HanXRQChr02g0059571
Probable dihydroxy−acid dehydratase
Parents WW
Parents WD
Hybrids WW
Hybrids WD
SF279
SF317
SF326
SF342
SF009
SF092
SF109
SF193
1.5e+07
1.0e+07
5.0e+06
0.0e+00

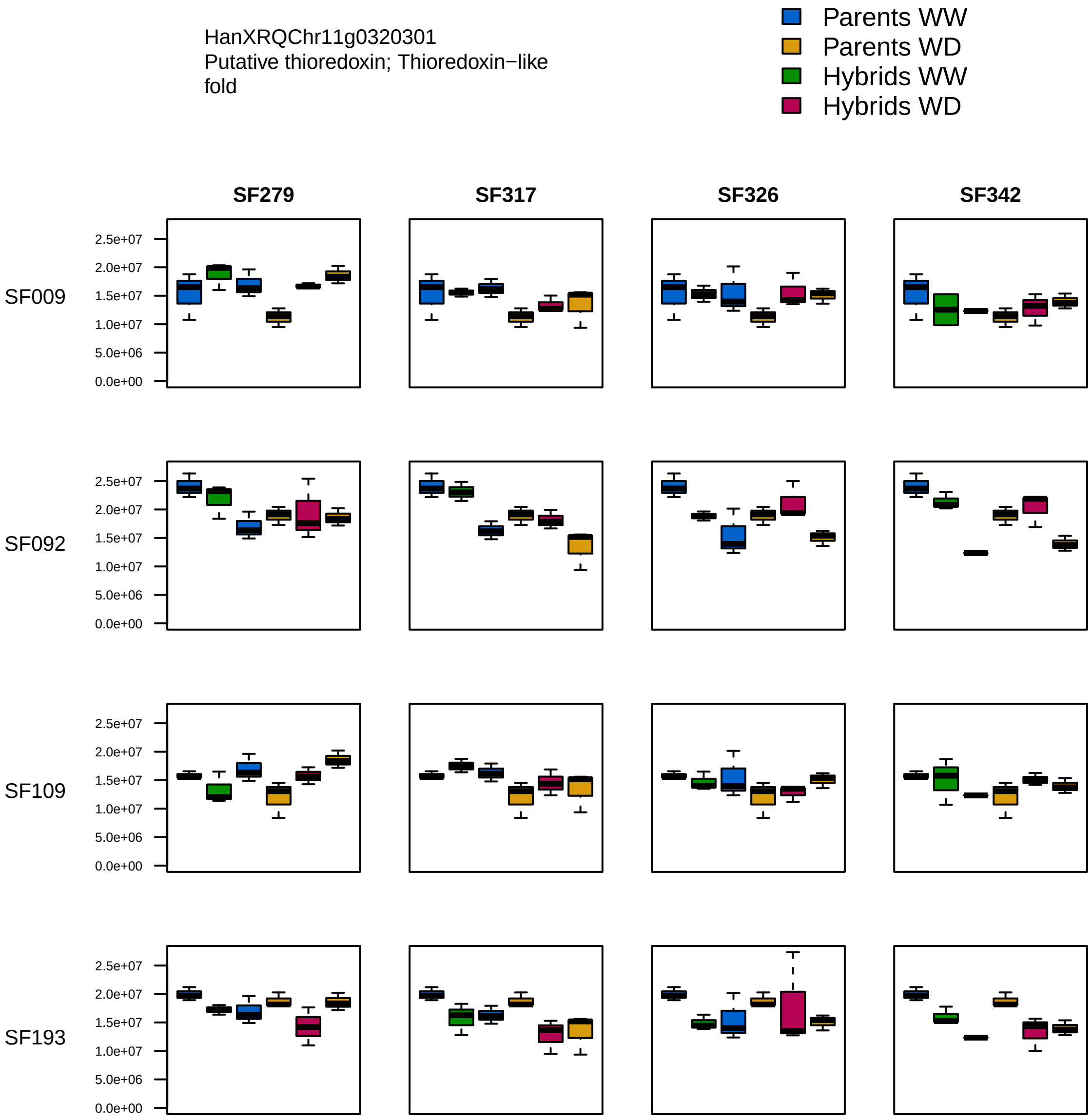
HanXRQChr11g0320301
Putative thioredoxin; Thioredoxin−like fold
Parents WW
Parents WD
Hybrids WW
Hybrids WD
SF279
SF317
SF326
SF342
SF009
SF092
SF109
SF193
2.5e+07
2.0e+07
1.5e+07
1.0e+07
5.0e+06
0.0e+00

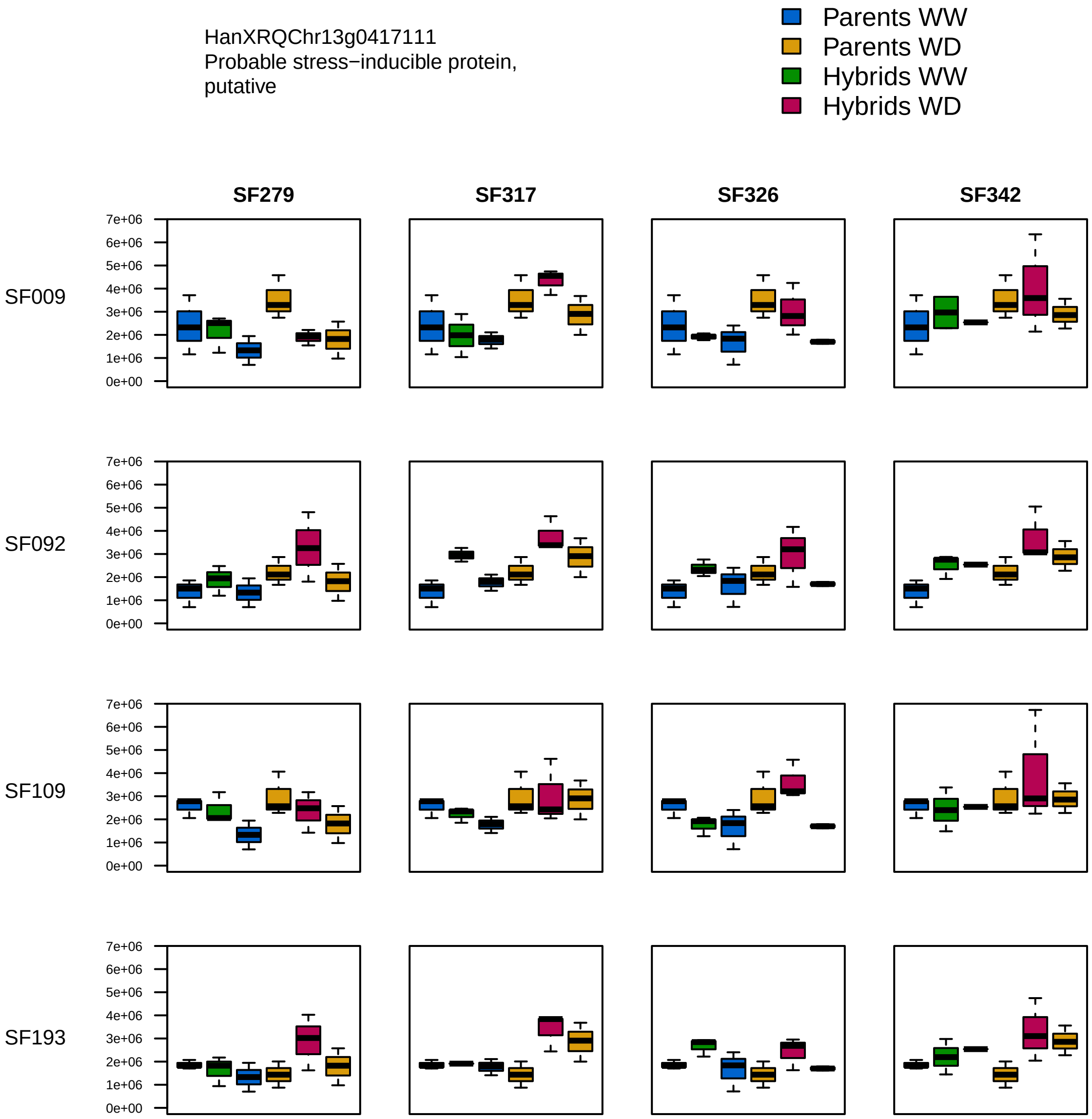

HanXRQChr13g0417111
Probable stress−inducible protein, putative
Parents WW
Parents WD
Hybrids WW
Hybrids WD
SF279
SF317
SF326
SF342
SF009
SF092
SF109
SF193
7e+06
6e+06
5e+06
4e+06
3e+06
2e+06
1e+06
0e+00

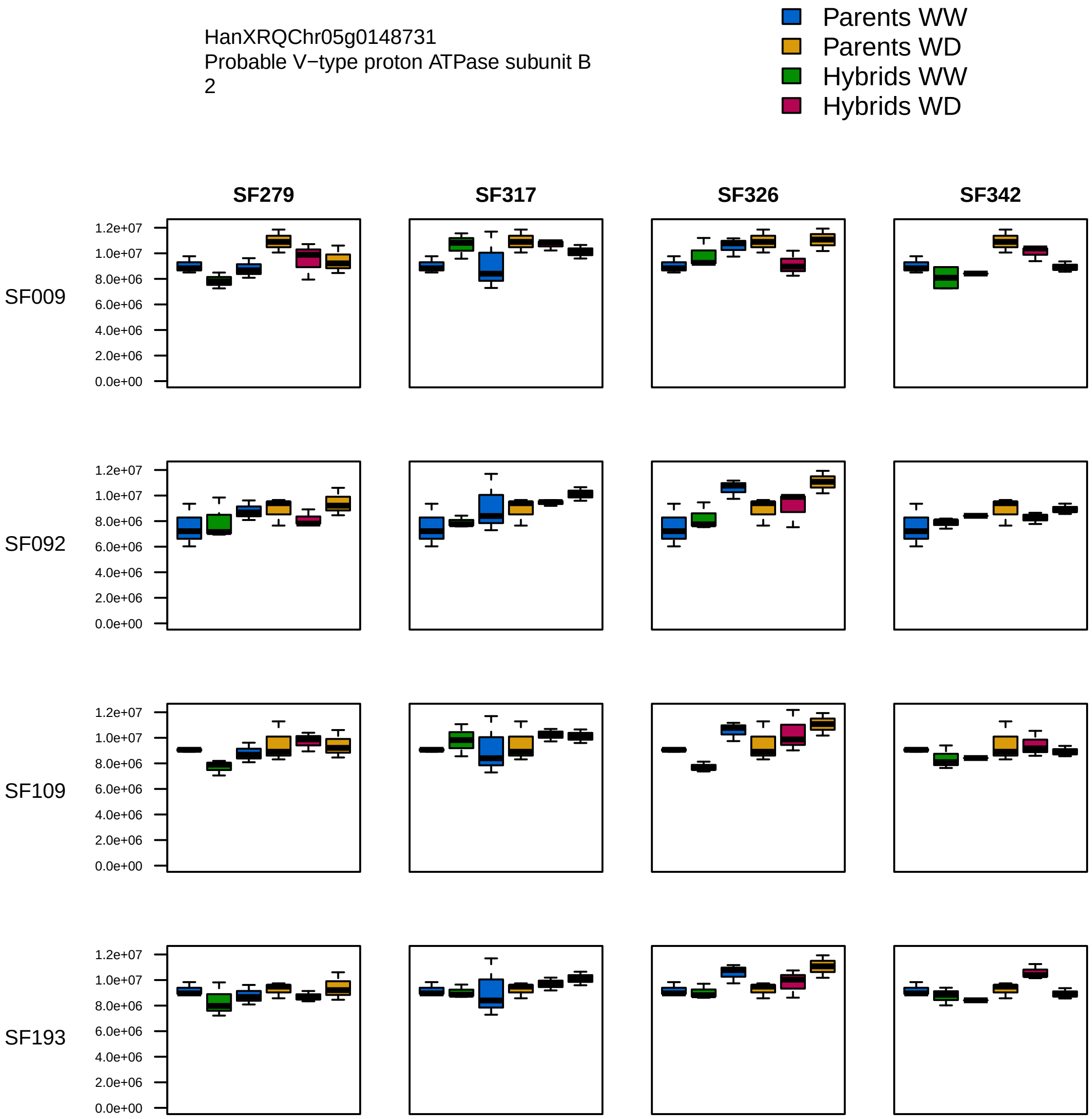
HanXRQChr05g0148731
Probable V−type proton ATPase subunit B
2
Parents WW
Parents WD
Hybrids WW
Hybrids WD
SF279
SF317
SF326
SF342
SF009
SF092
SF109
SF193
1.2e+07
1.0e+07
8.0e+06
6.0e+06
4.0e+06
2.0e+06
0.0e+00

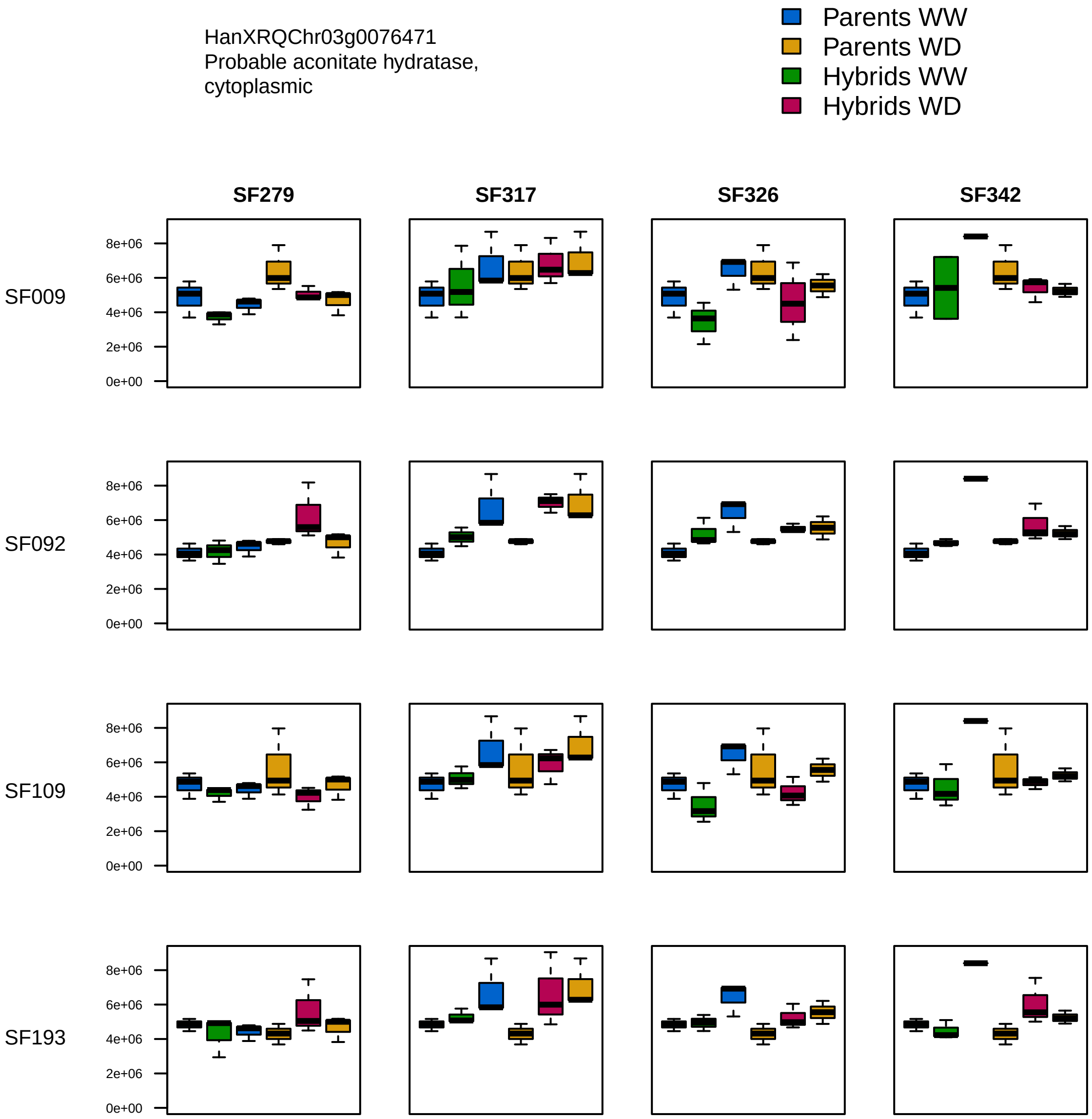
HanXRQChr03g0076471
Probable aconitate hydratase,
cytoplasmic
Parents WW
Parents WD
Hybrids WW
Hybrids WD
SF279
SF317
SF326
SF342
SF009
SF092
SF109
SF193
0e+00
2e+06
4e+06
6e+06
8e+06

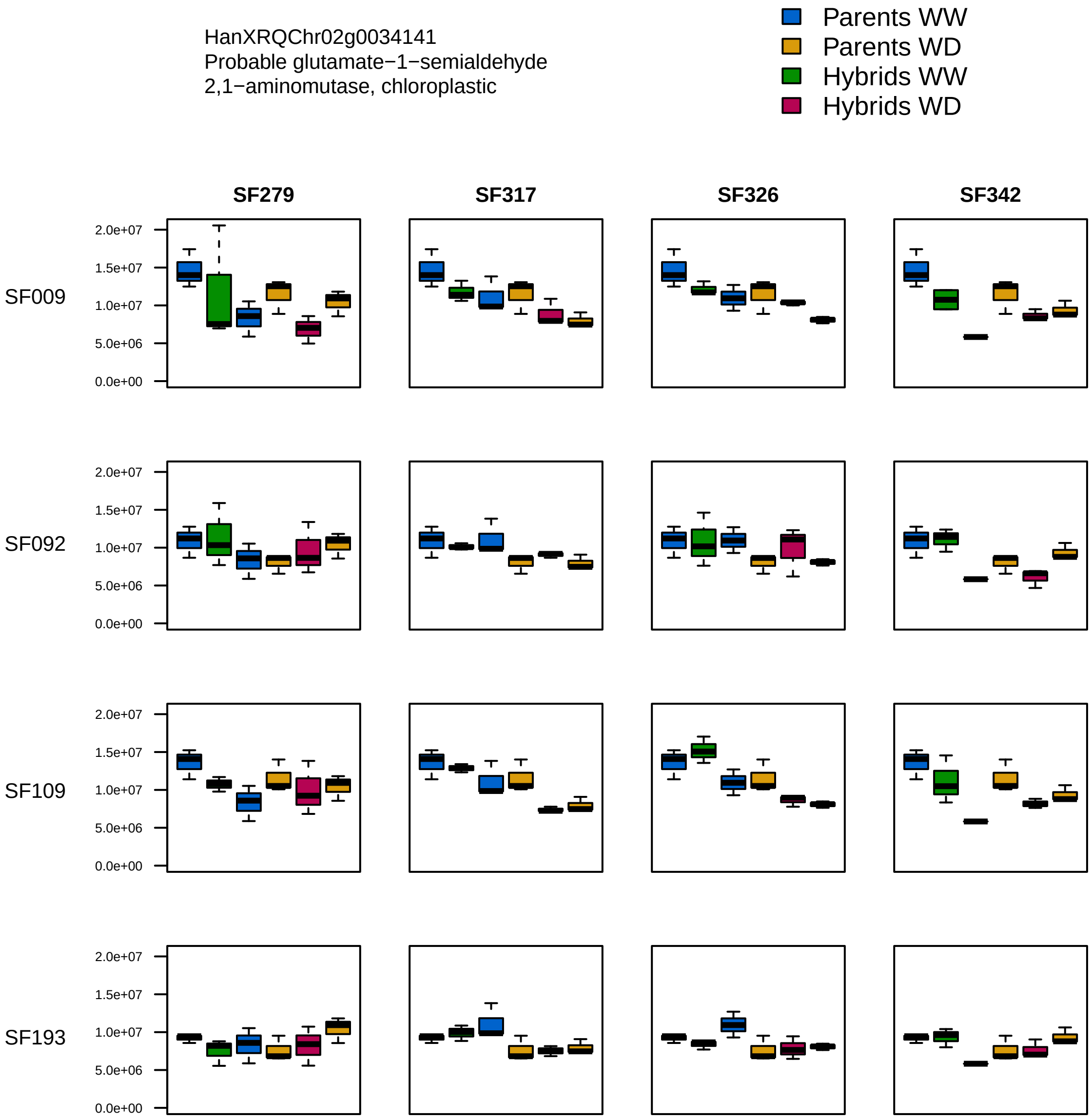

HanXRQChr02g0034141
Probable glutamate−1−semialdehyde
2,1−aminomutase, chloroplastic
Parents WW
Parents WD
Hybrids WW
Hybrids WD
SF279
SF317
SF326
SF342
SF009
SF092
SF109
SF193
2.0e+07
1.5e+07
1.0e+07
5.0e+06
0.0e+00

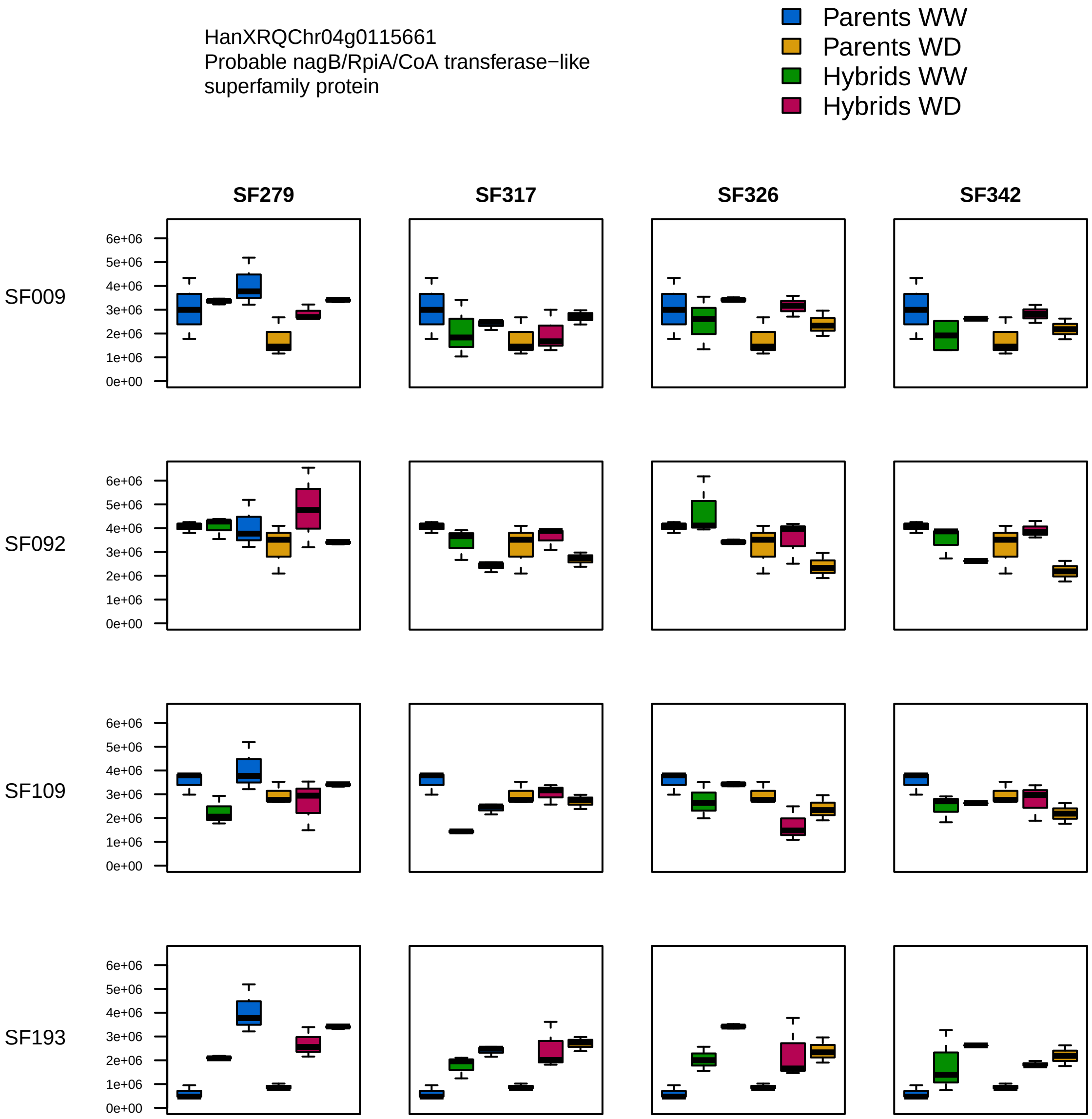
HanXRQChr04g0115661
Probable nagB/RpiA/CoA transferase−like
superfamily protein
Parents WW
Parents WD
Hybrids WW
Hybrids WD
SF279
SF317
SF326
SF342
SF009
SF092
SF109
SF193
0e+00
1e+06
2e+06
3e+06
4e+06
5e+06
6e+06

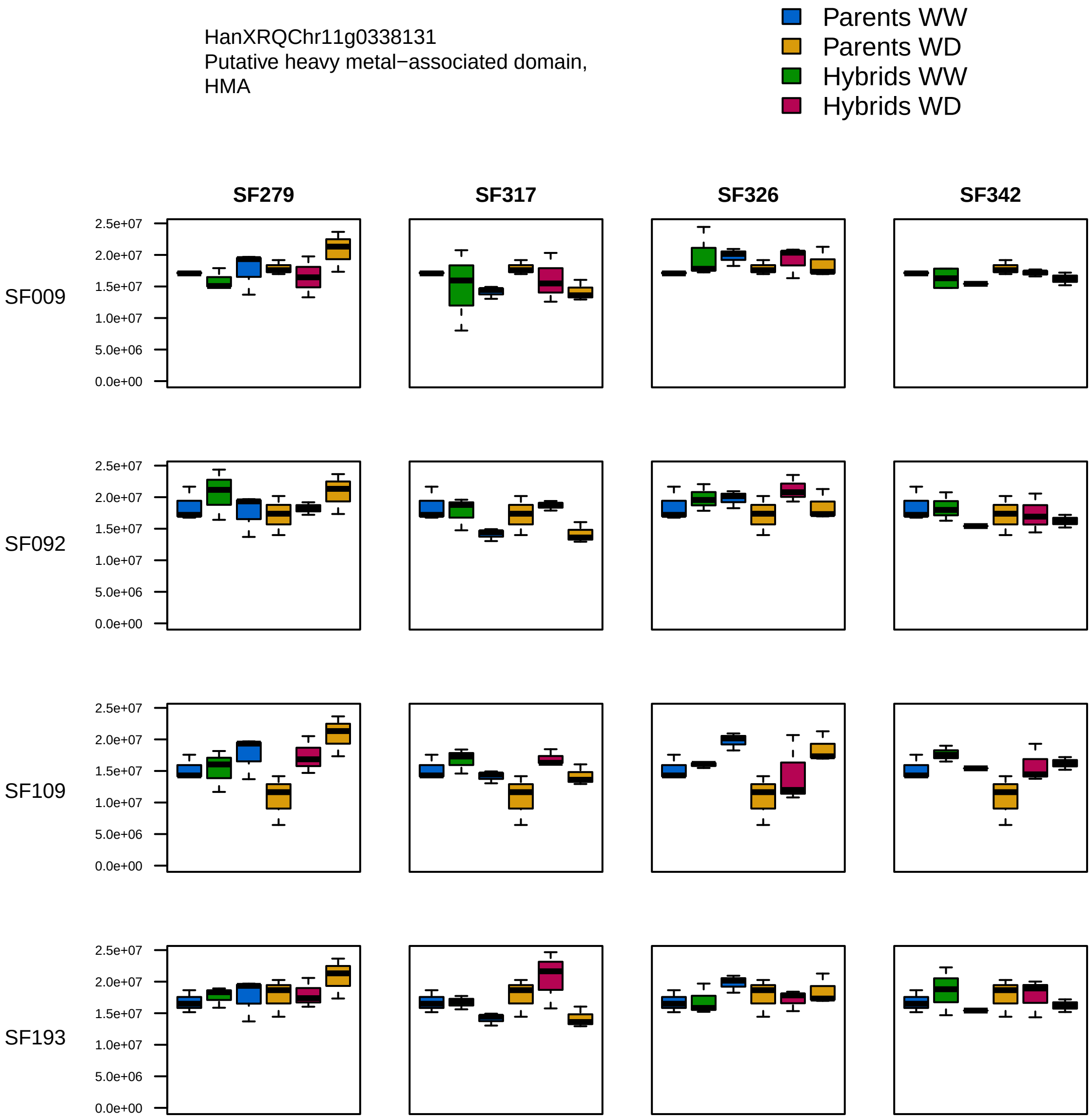
HanXRQChr11g0338131
Putative heavy metal−associated domain, HMA
Parents WW
Parents WD
Hybrids WW
Hybrids WD
SF279
SF317
SF326
SF342
SF009
SF092
SF109
SF193
2.5e+07
2.0e+07
1.5e+07
1.0e+07
5.0e+06
0.0e+00

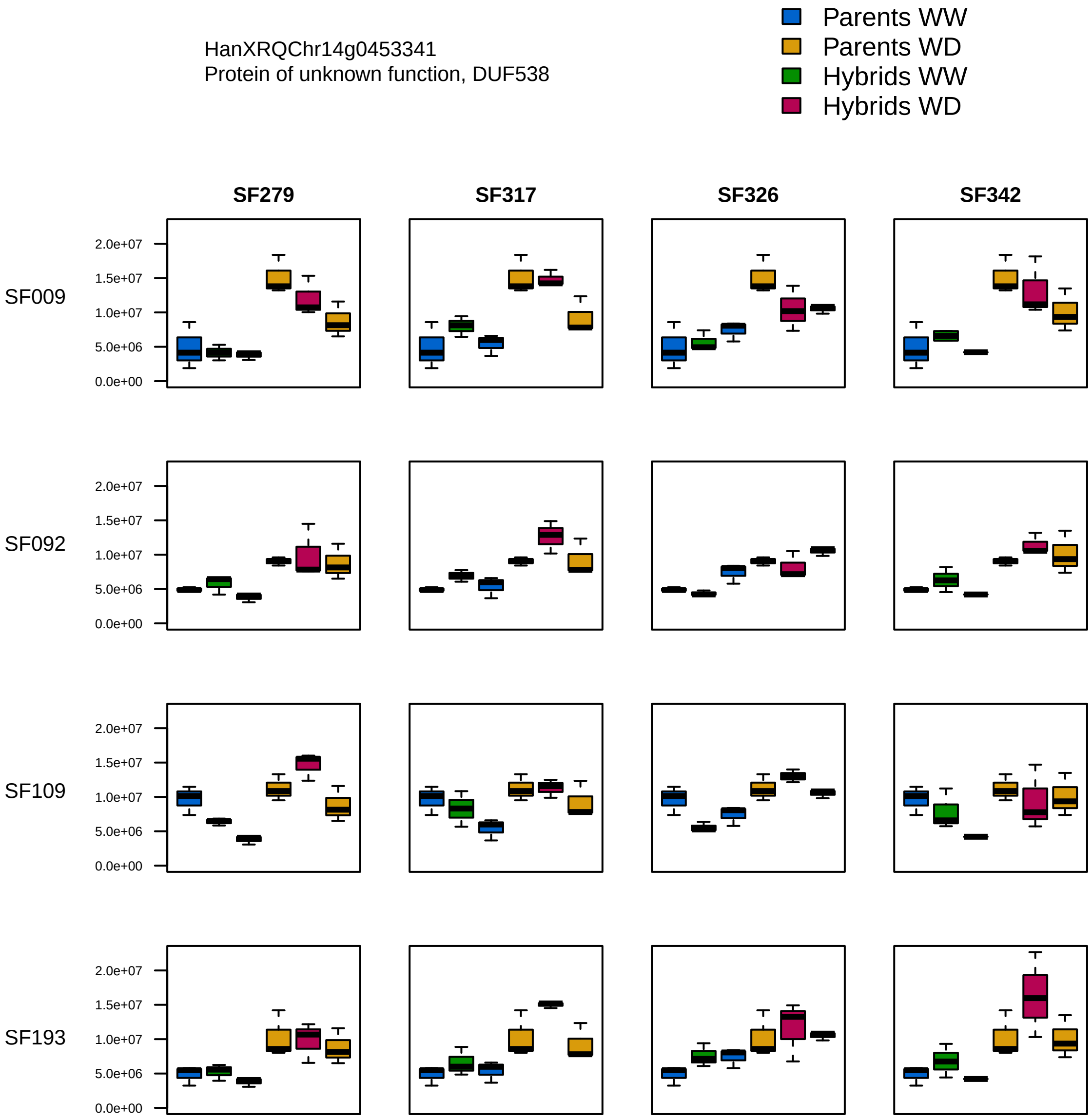
HanXRQChr14g0453341
Protein of unknown function, DUF538
Parents WW
Parents WD
Hybrids WW
Hybrids WD
SF279
SF317
SF326
SF342
SF009
SF092
SF109
SF193
2.0e+07
1.5e+07
1.0e+07
5.0e+06
0.0e+00

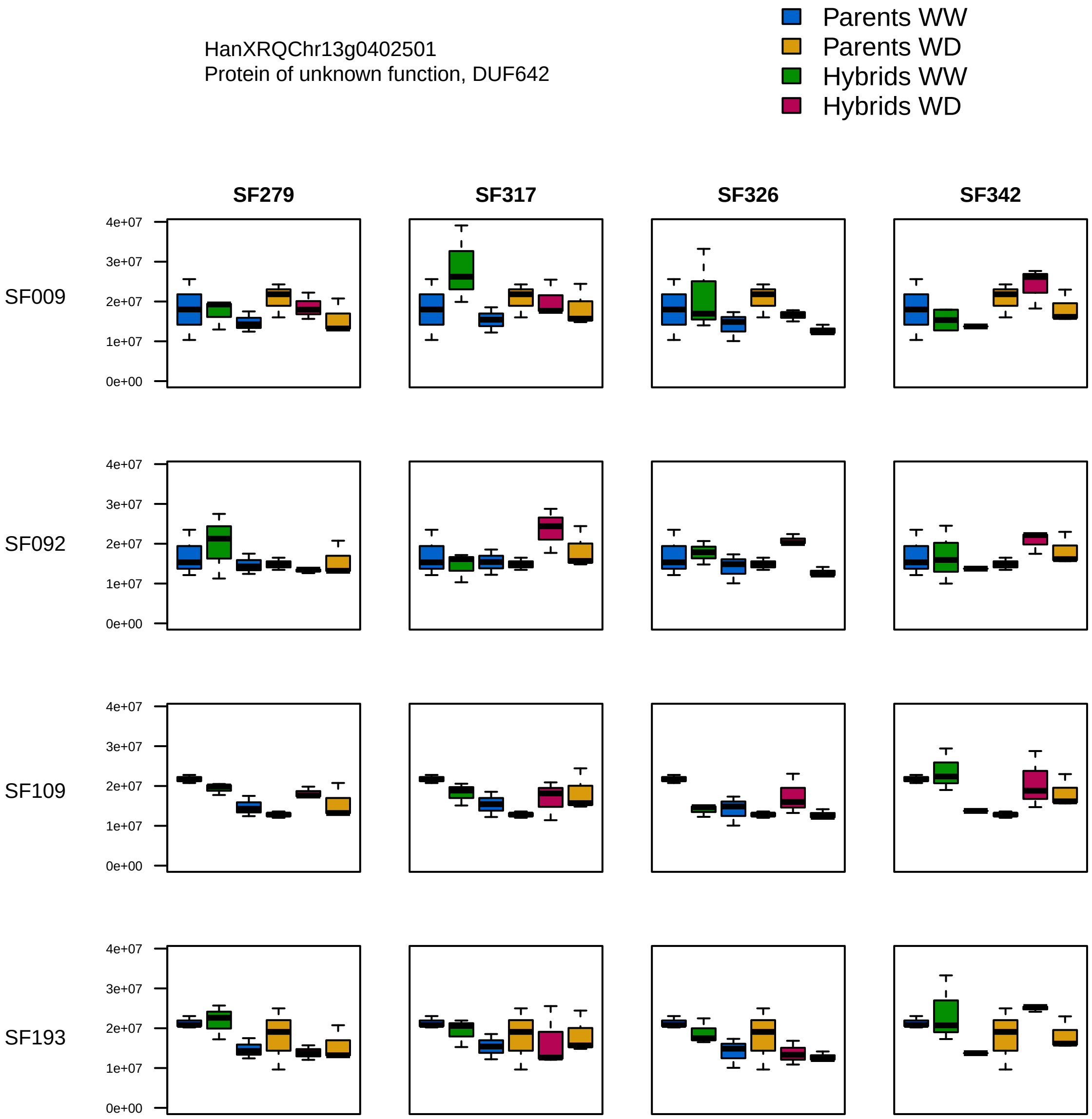

HanXRQChr13g0402501
Protein of unknown function, DUF642
Parents WW
Parents WD
Hybrids WW
Hybrids WD
SF279
SF317
SF326
SF342
SF009
SF092
SF109
SF193
4e+07
3e+07
2e+07
1e+07
0e+00

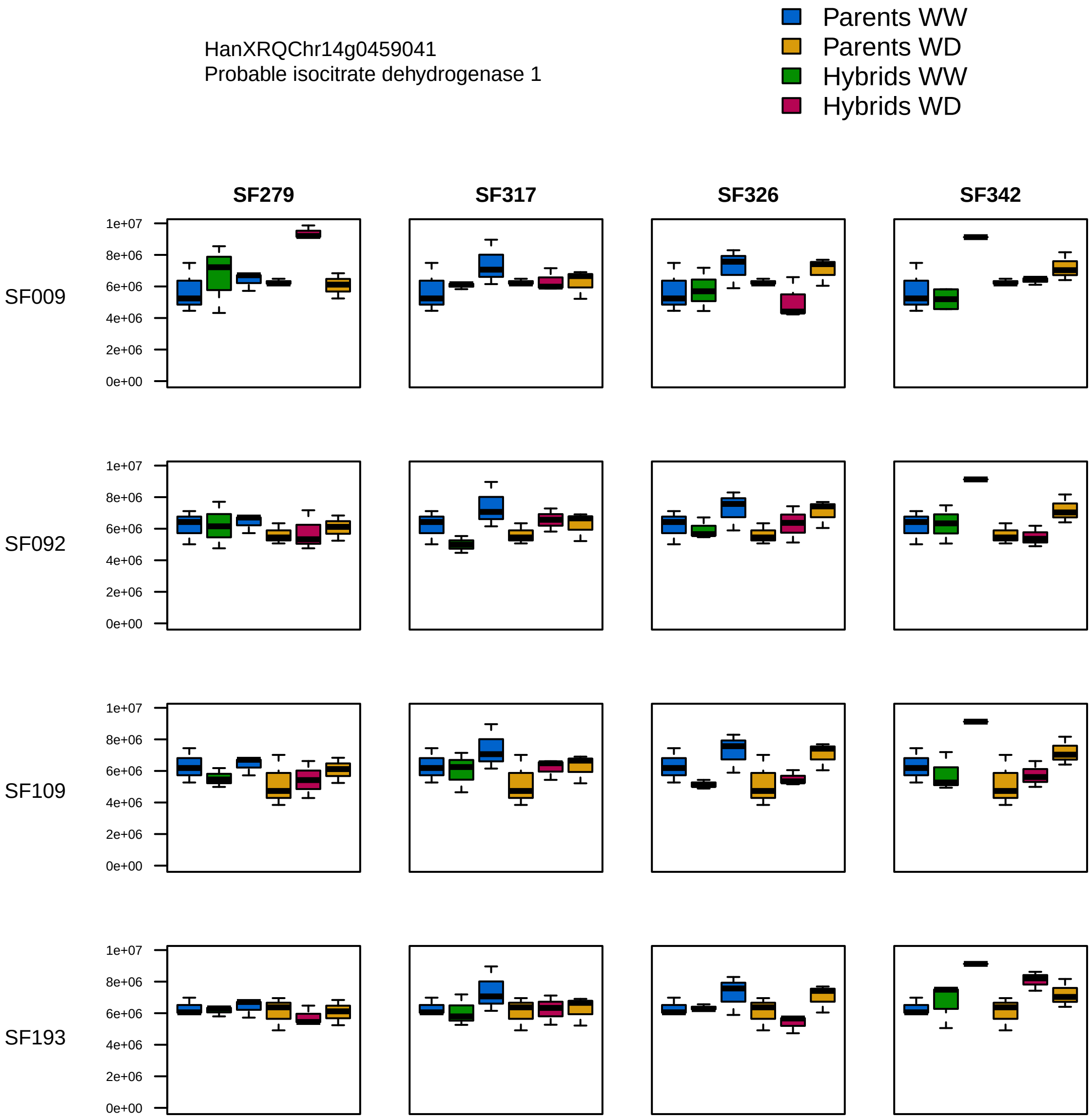
HanXRQChr14g0459041
Probable isocitrate dehydrogenase 1
Parents WW
Parents WD
Hybrids WW
Hybrids WD
SF279
SF317
SF326
SF342
SF009
SF092
SF109
SF193
1e+07
8e+06
6e+06
4e+06
2e+06
0e+00

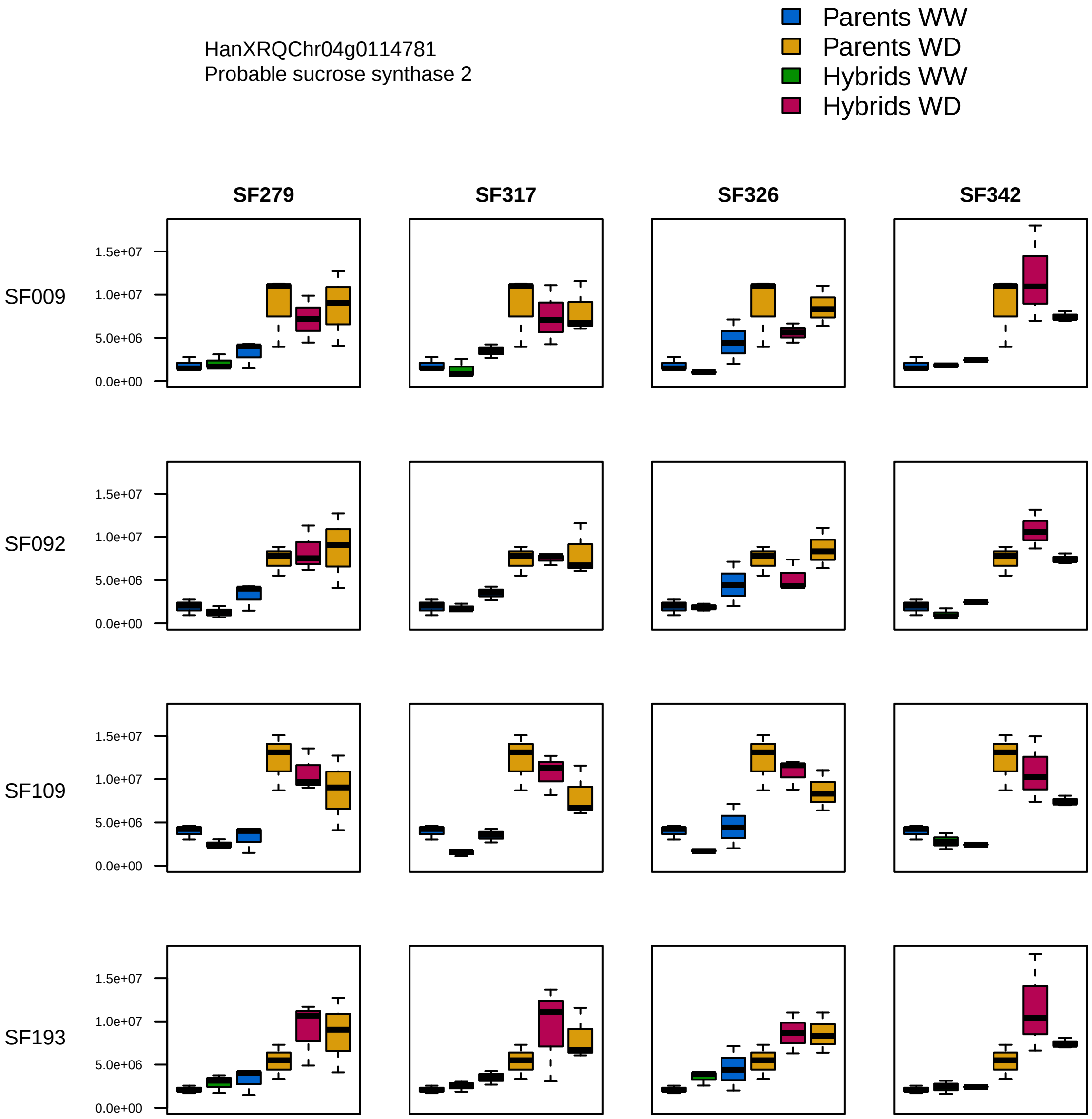

HanXRQChr04g0114781
Probable sucrose synthase 2
Parents WW
Parents WD
Hybrids WW
Hybrids WD
SF279
SF317
SF326
SF342
SF009
SF092
SF109
SF193
1.5e+07
1.0e+07
5.0e+06
0.0e+00

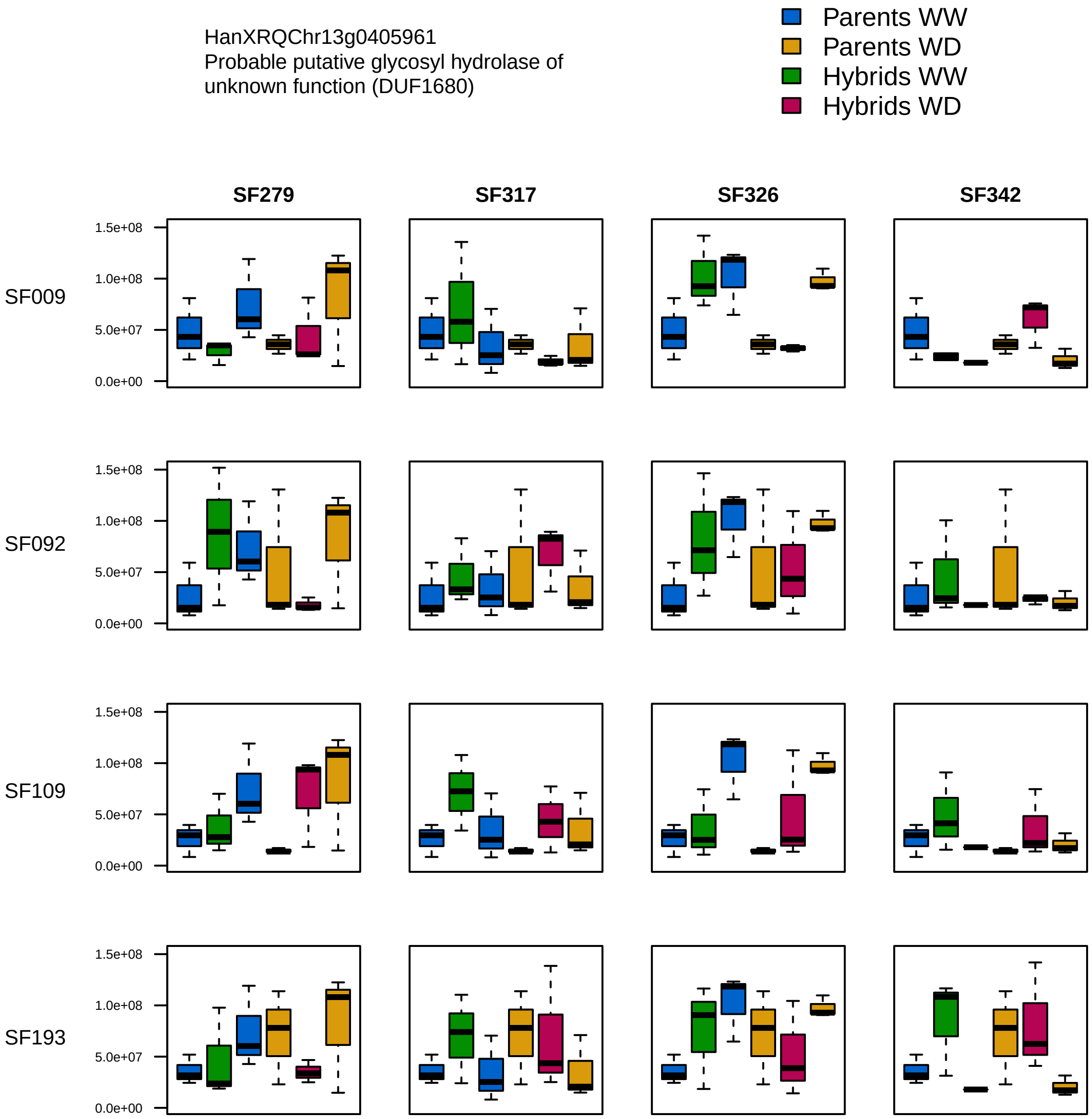
HanXRQChr13g0405961
Probable putative glycosyl hydrolase of
unknown function (DUF1680)
Parents WW
Parents WD
Hybrids WW
Hybrids WD
SF279
SF317
SF326
SF342
SF009
SF092
SF109
SF193
1.5e+08
1.0e+08
5.0e+07
0.0e+00

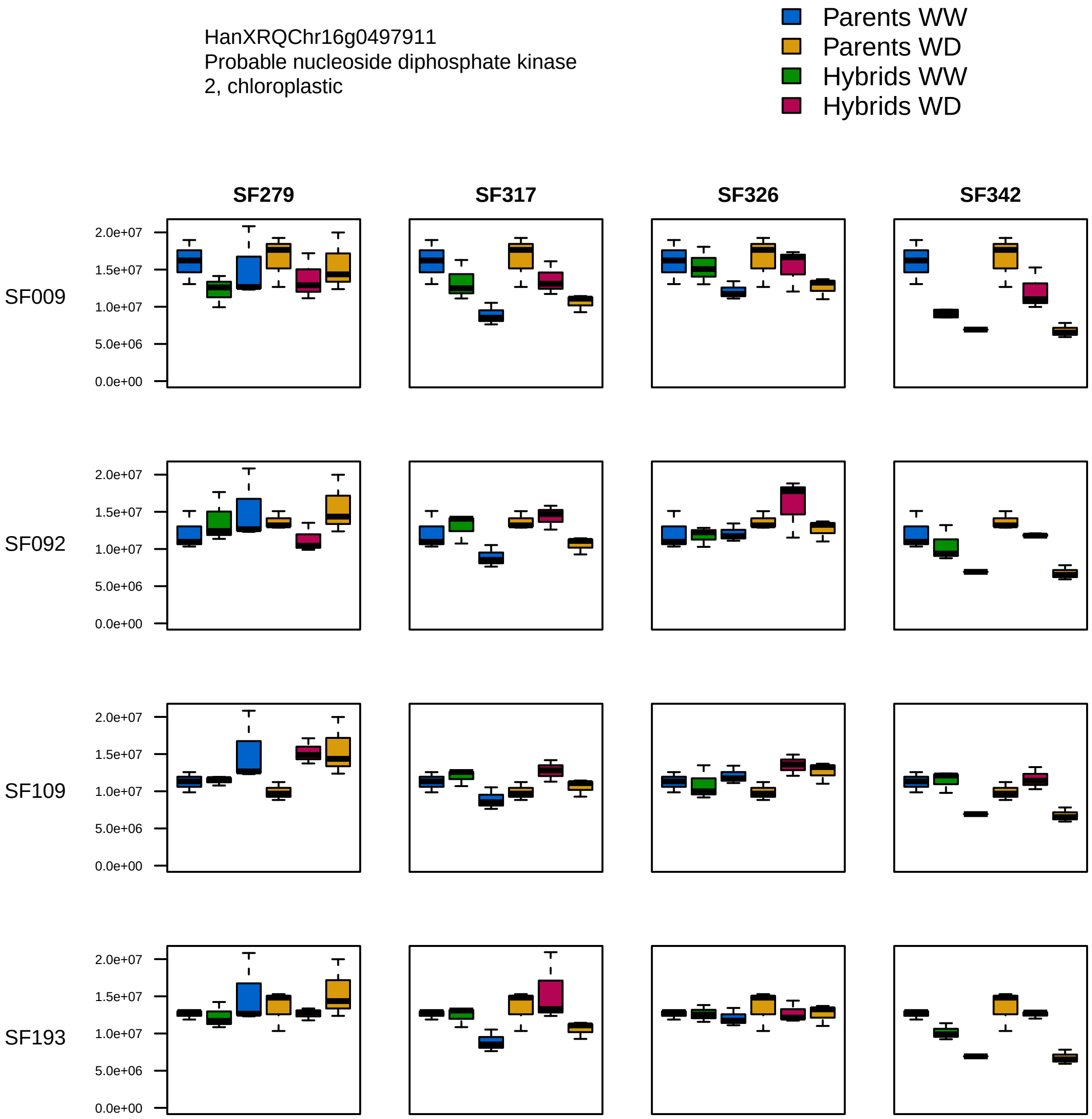
HanXRQChr16g0497911
Probable nucleoside diphosphate kinase
2, chloroplastic
Parents WW
Parents WD
Hybrids WW
Hybrids WD
SF279
SF317
SF326
SF342
SF009
SF092
SF109
SF193
2.0e+07
1.5e+07
1.0e+07
5.0e+06
0.0e+00

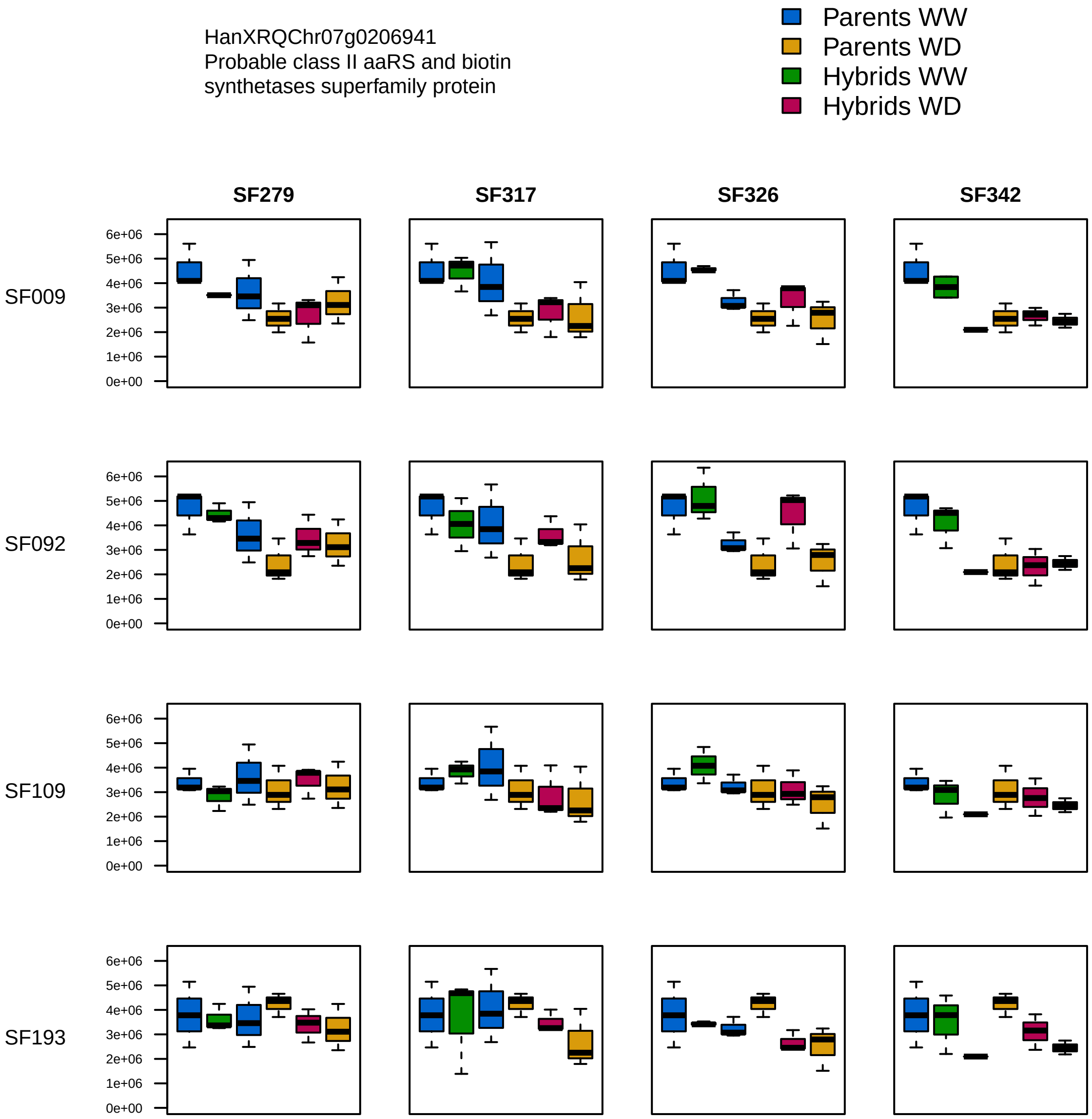

HanXRQChr07g0206941
Probable class II aaRS and biotin
synthetases superfamily protein
Parents WW
Parents WD
Hybrids WW
Hybrids WD
SF279
SF317
SF326
SF342
SF009
SF092
SF109
SF193
0e+00
1e+06
2e+06
3e+06
4e+06
5e+06
6e+06

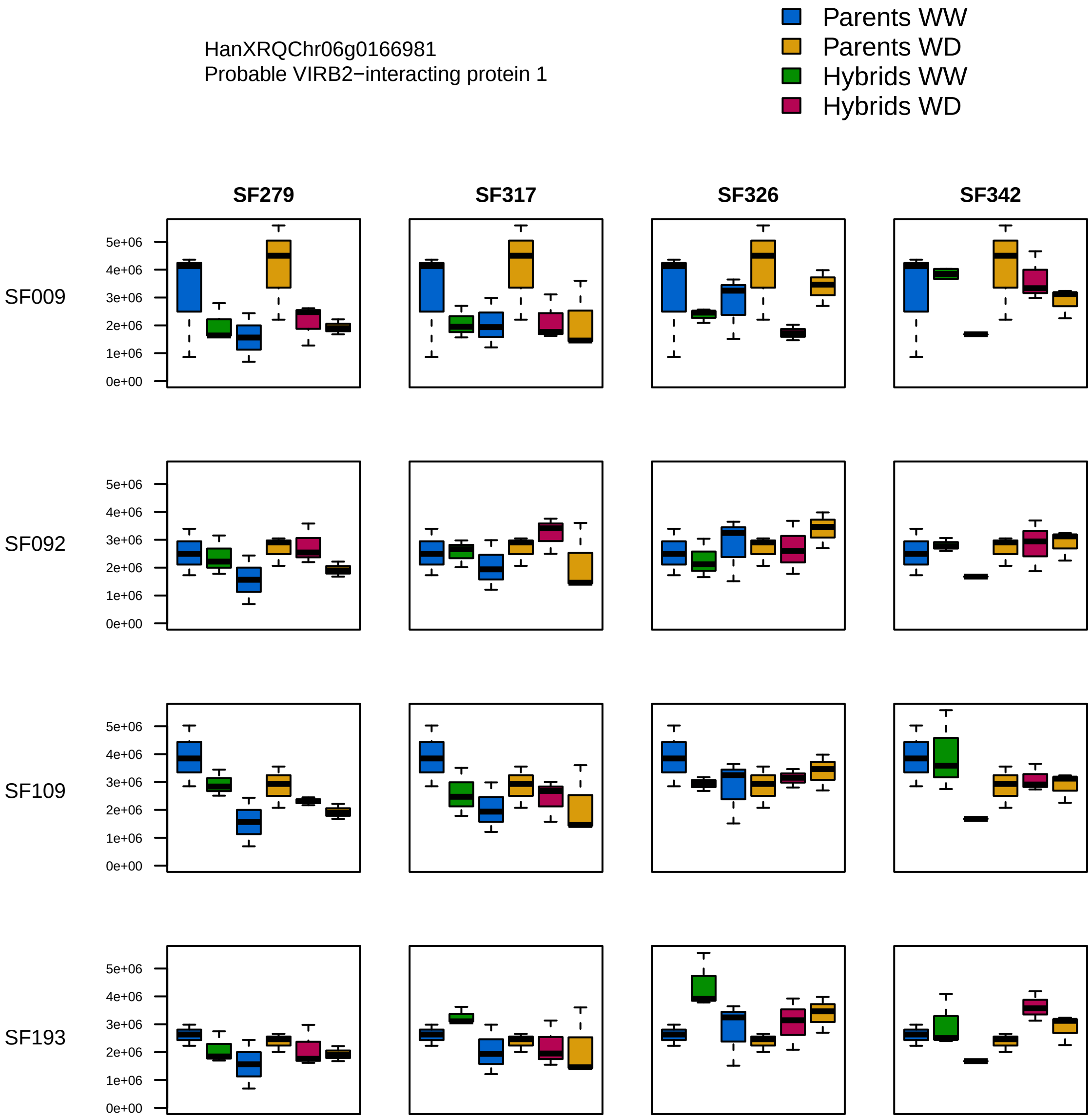
HanXRQChr06g0166981
Probable VIRB2−interacting protein 1
Parents WW
Parents WD
Hybrids WW
Hybrids WD
SF279
SF317
SF326
SF342
SF009
SF092
SF109
SF193
0e+00
1e+06
2e+06
3e+06
4e+06
5e+06

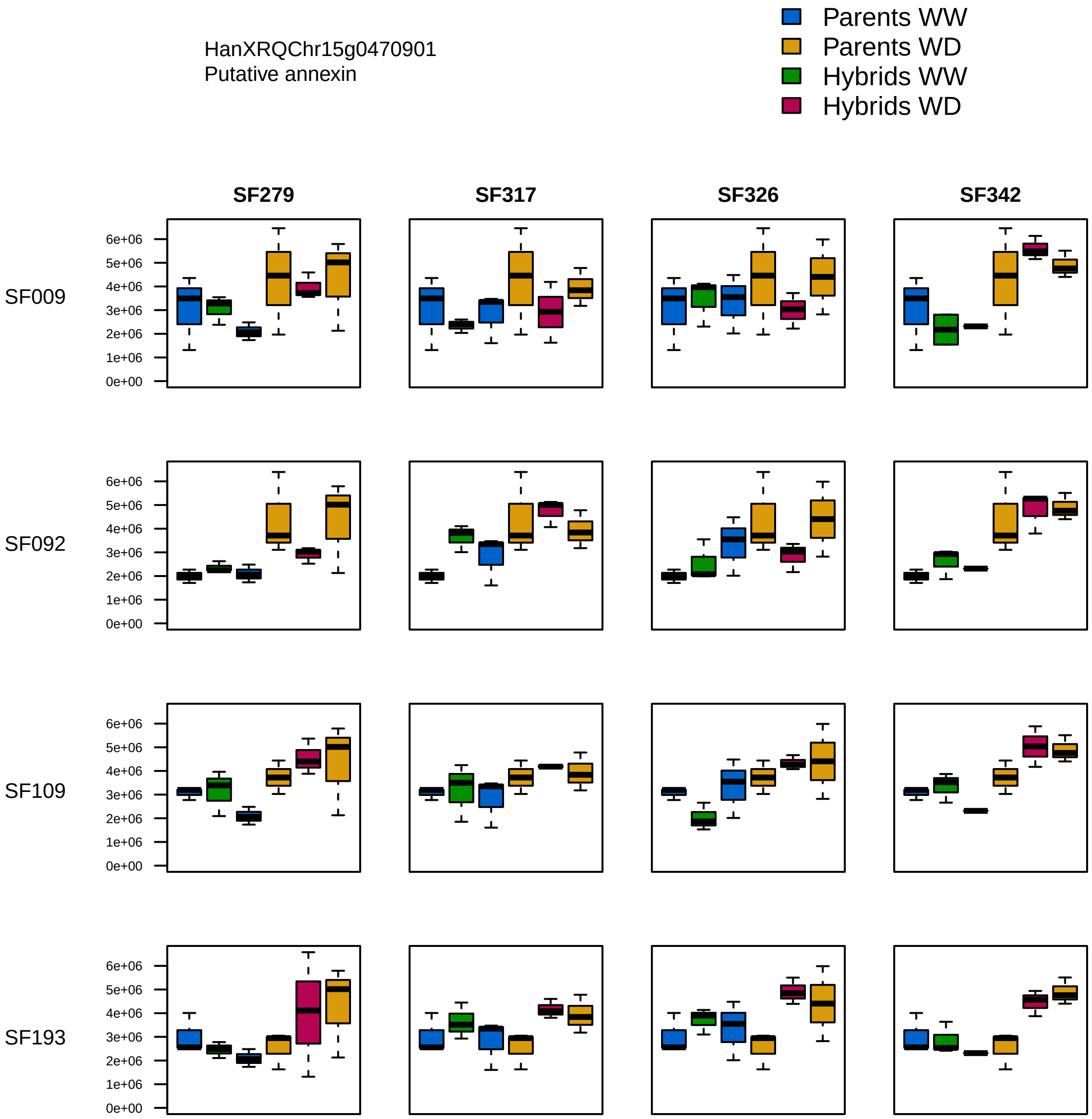

HanXRQChr15g0470901
Putative annexin
Parents WW
Parents WD
Hybrids WW
Hybrids WD
SF279
SF317
SF326
SF342
SF009
SF092
SF109
SF193
6e+06
5e+06
4e+06
3e+06
2e+06
1e+06
0e+00

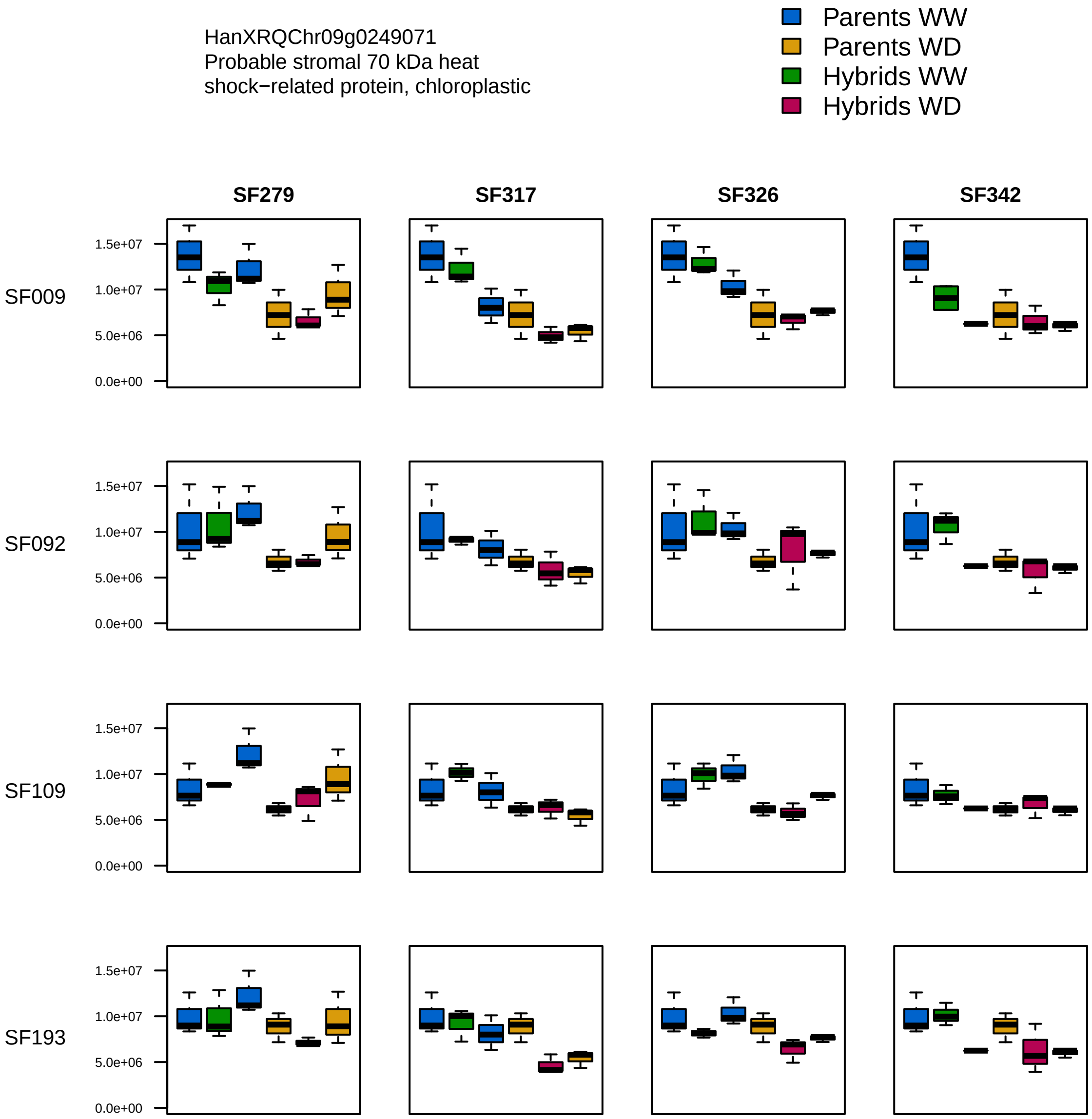
HanXRQChr09g0249071
Probable stromal 70 kDa heat
shock−related protein, chloroplastic
Parents WW
Parents WD
Hybrids WW
Hybrids WD
SF279
SF317
SF326
SF342
SF009
SF092
SF109
SF193
1.5e+07
1.0e+07
5.0e+06
0.0e+00

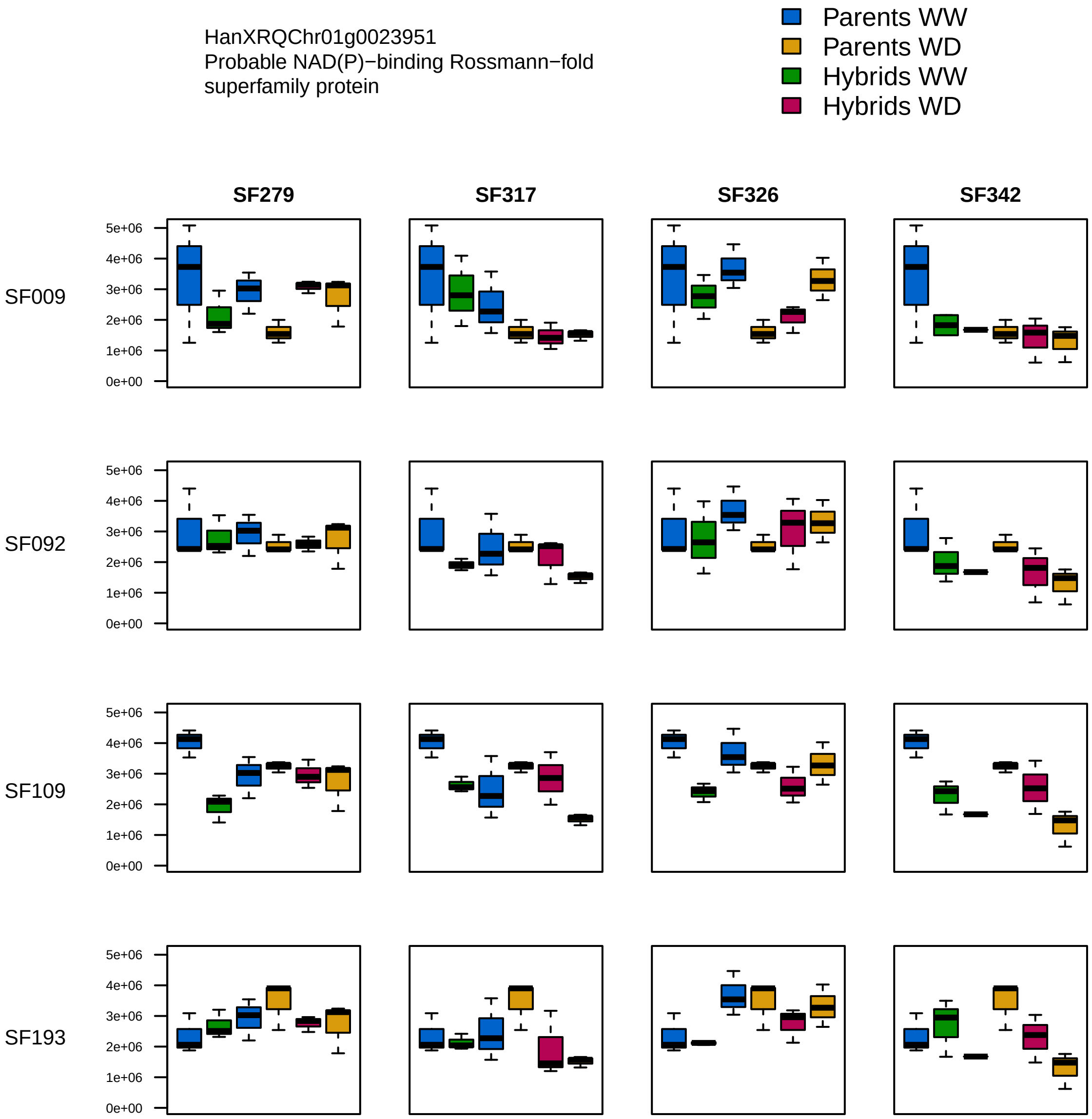
HanXRQChr01g0023951
Probable NAD(P)−binding Rossmann−fold
superfamily protein
Parents WW
Parents WD
Hybrids WW
Hybrids WD
SF279
SF317
SF326
SF342
SF009
SF092
SF109
SF193
5e+06
4e+06
3e+06
2e+06
1e+06
0e+00

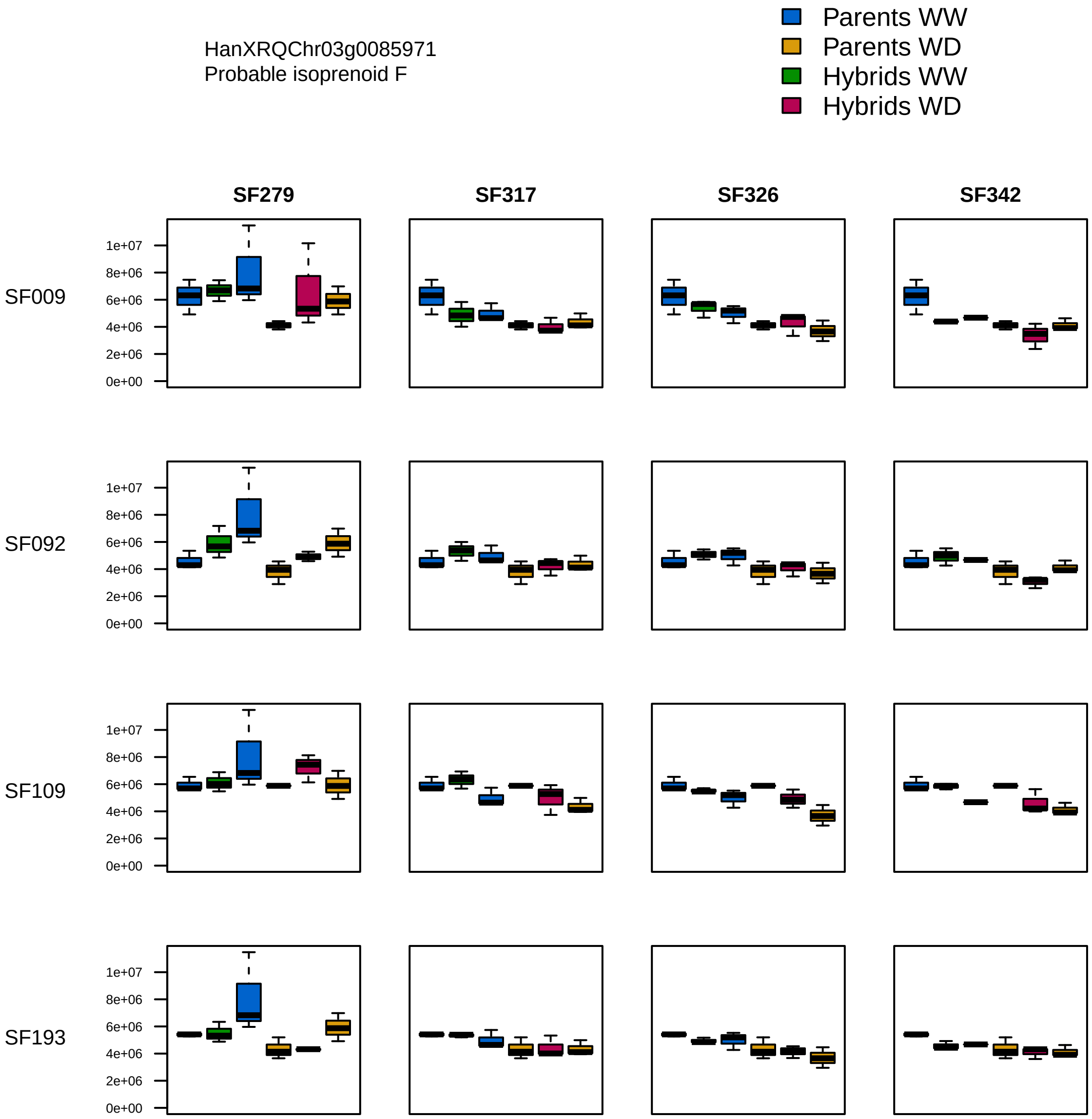
HanXRQChr03g0085971
Probable isoprenoid F
Parents WW
Parents WD
Hybrids WW
Hybrids WD
SF279
SF317
SF326
SF342
SF009
SF092
SF109
SF193
0e+00
2e+06
4e+06
6e+06
8e+06
1e+07

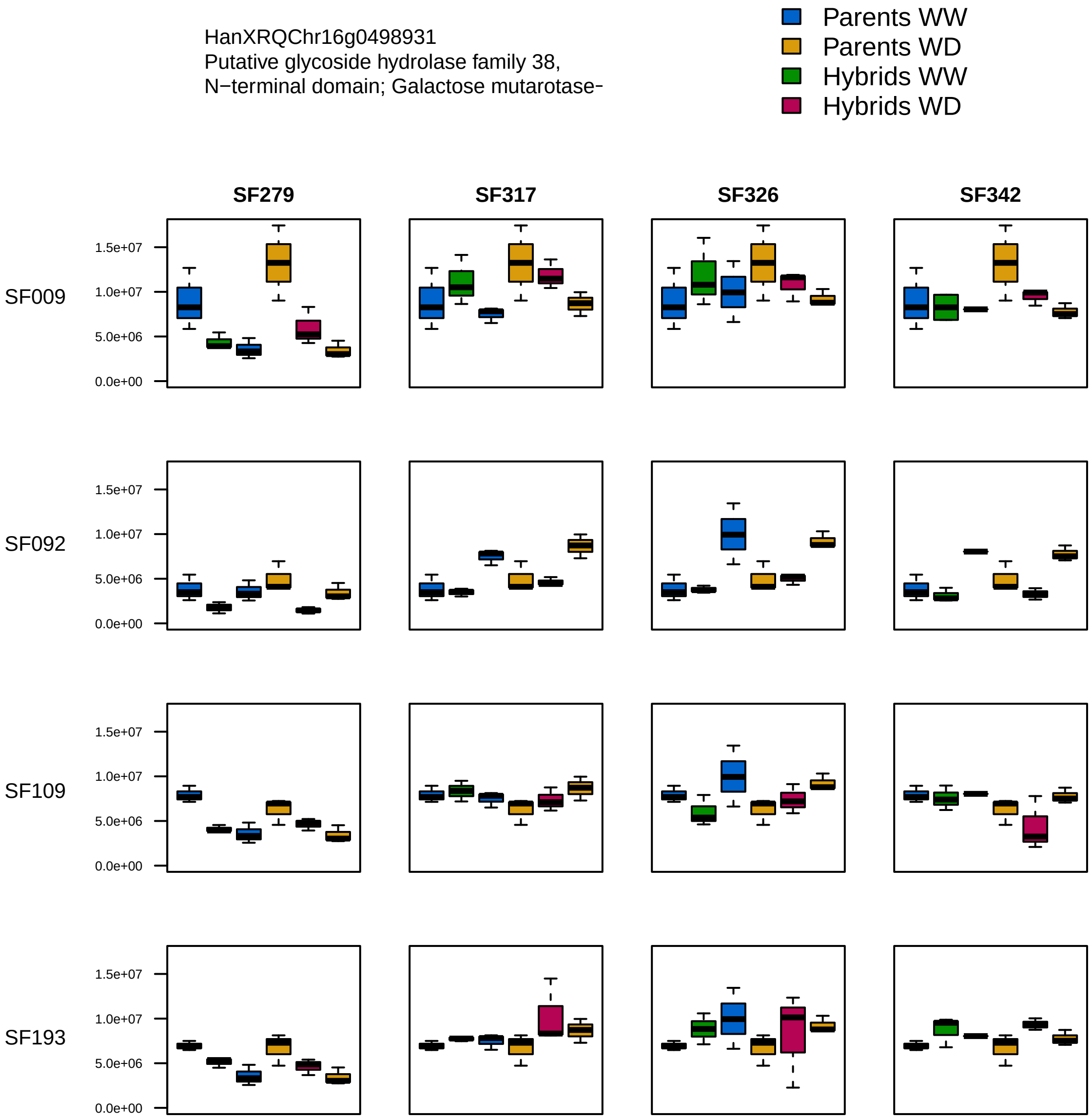
HanXRQChr16g0498931
Putative glycoside hydrolase family 38,
N−terminal domain; Galactose mutarotase-
Parents WW
Parents WD
Hybrids WW
Hybrids WD
SF279
SF317
SF326
SF342
SF009
SF092
SF109
SF193
1.5e+07
1.0e+07
5.0e+06
0.0e+00

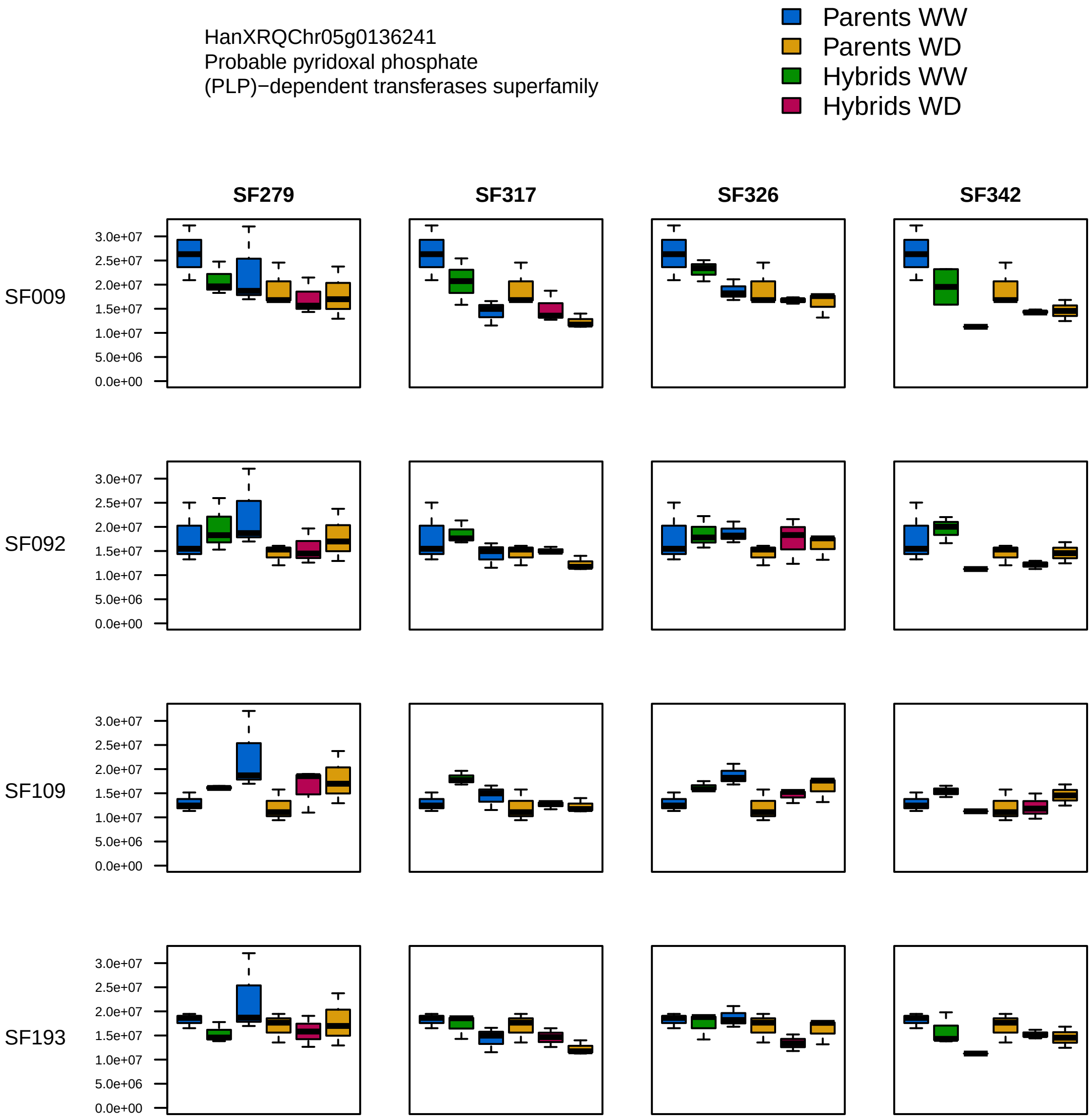

HanXRQChr05g0136241
Probable pyridoxal phosphate
(PLP)−dependent transferases superfamily
Parents WW
Parents WD
Hybrids WW
Hybrids WD
SF279
SF317
SF326
SF342
SF009
SF092
SF109
SF193
3.0e+07
2.5e+07
2.0e+07
1.5e+07
1.0e+07
5.0e+06
0.0e+00

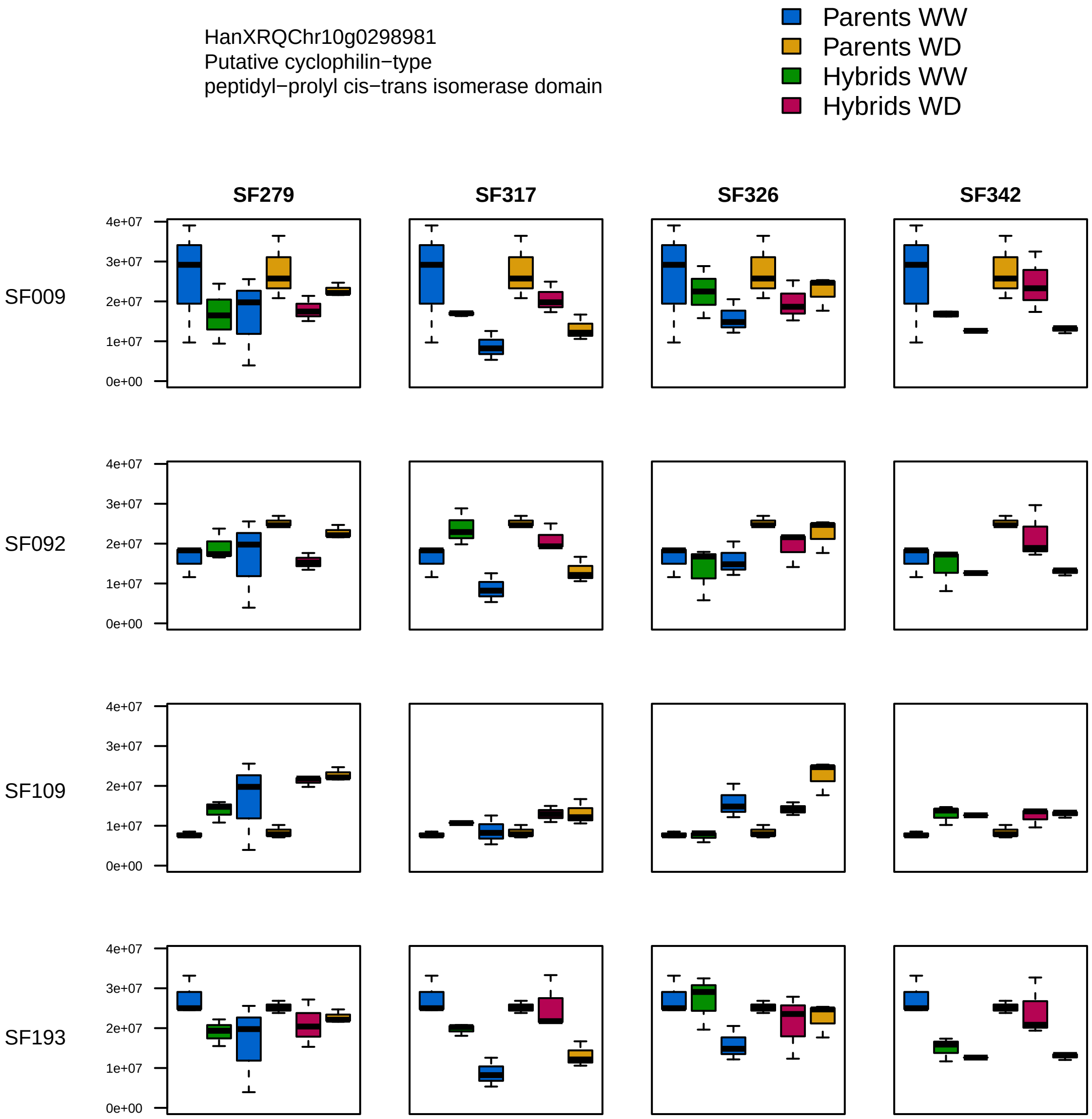
HanXRQChr10g0298981
Putative cyclophilin−type
peptidyl−prolyl cis−trans isomerase domain
Parents WW
Parents WD
Hybrids WW
Hybrids WD
SF279
SF317
SF326
SF342
SF009
SF092
SF109
SF193
4e+07
3e+07
2e+07
1e+07
0e+00

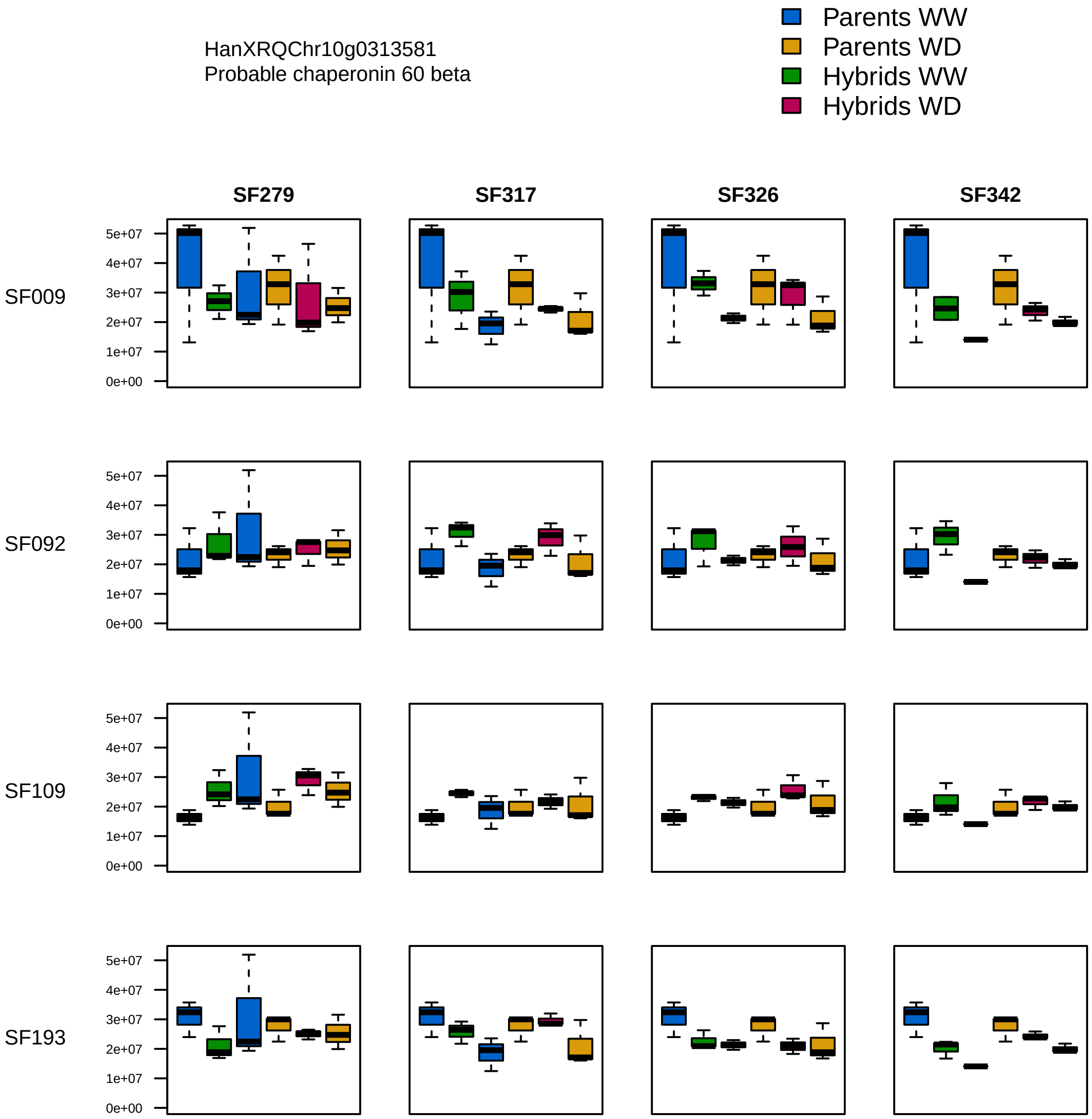
HanXRQChr10g0313581
Probable chaperonin 60 beta
Parents WW
Parents WD
Hybrids WW
Hybrids WD
SF279
SF317
SF326
SF342
SF009
SF092
SF109
SF193
5e+07
4e+07
3e+07
2e+07
1e+07
0e+00

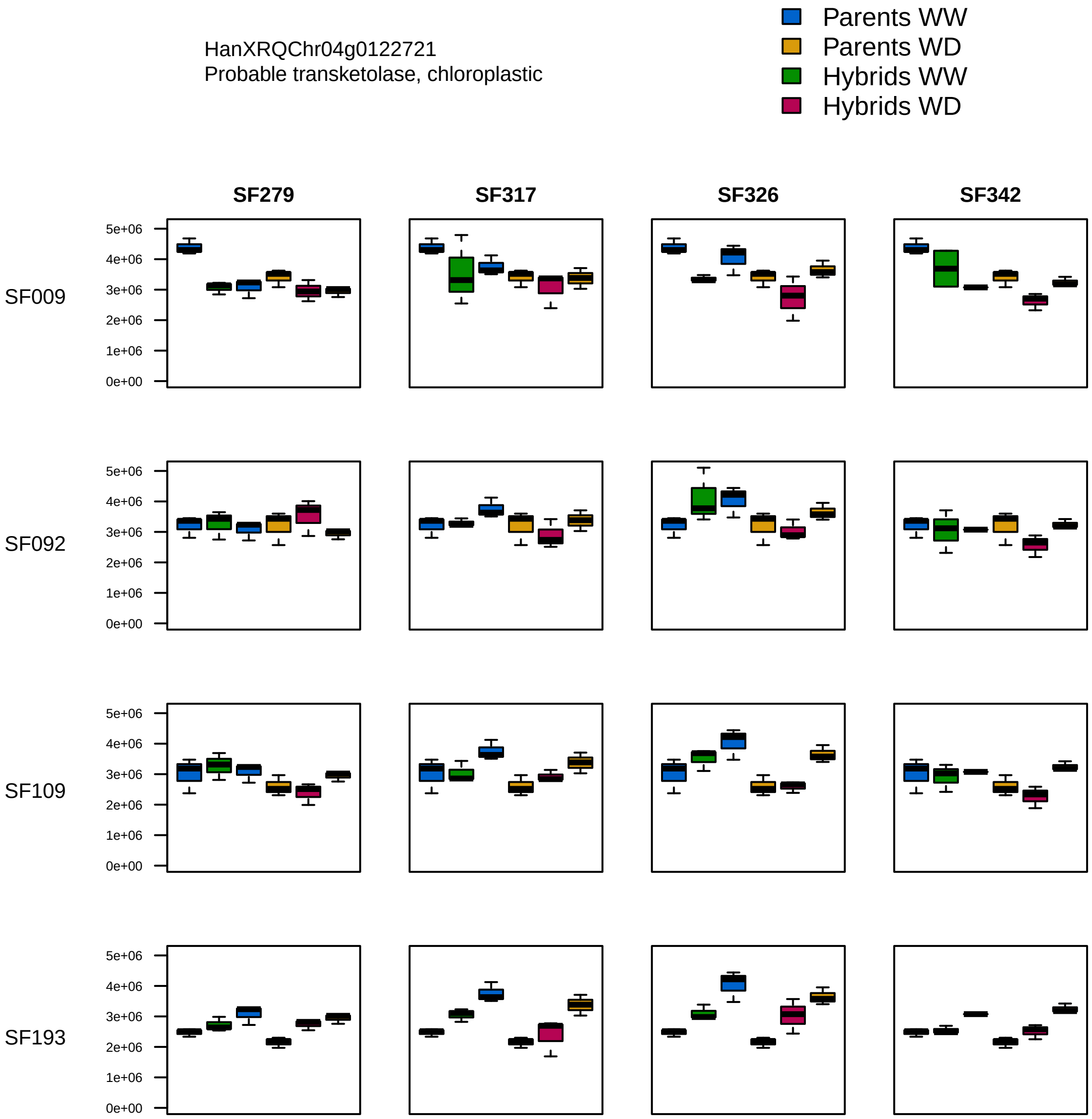
HanXRQChr04g0122721
Probable transketolase, chloroplastic
Parents WW
Parents WD
Hybrids WW
Hybrids WD
SF279
SF317
SF326
SF342
SF009
SF092
SF109
SF193
5e+06
4e+06
3e+06
2e+06
1e+06
0e+00

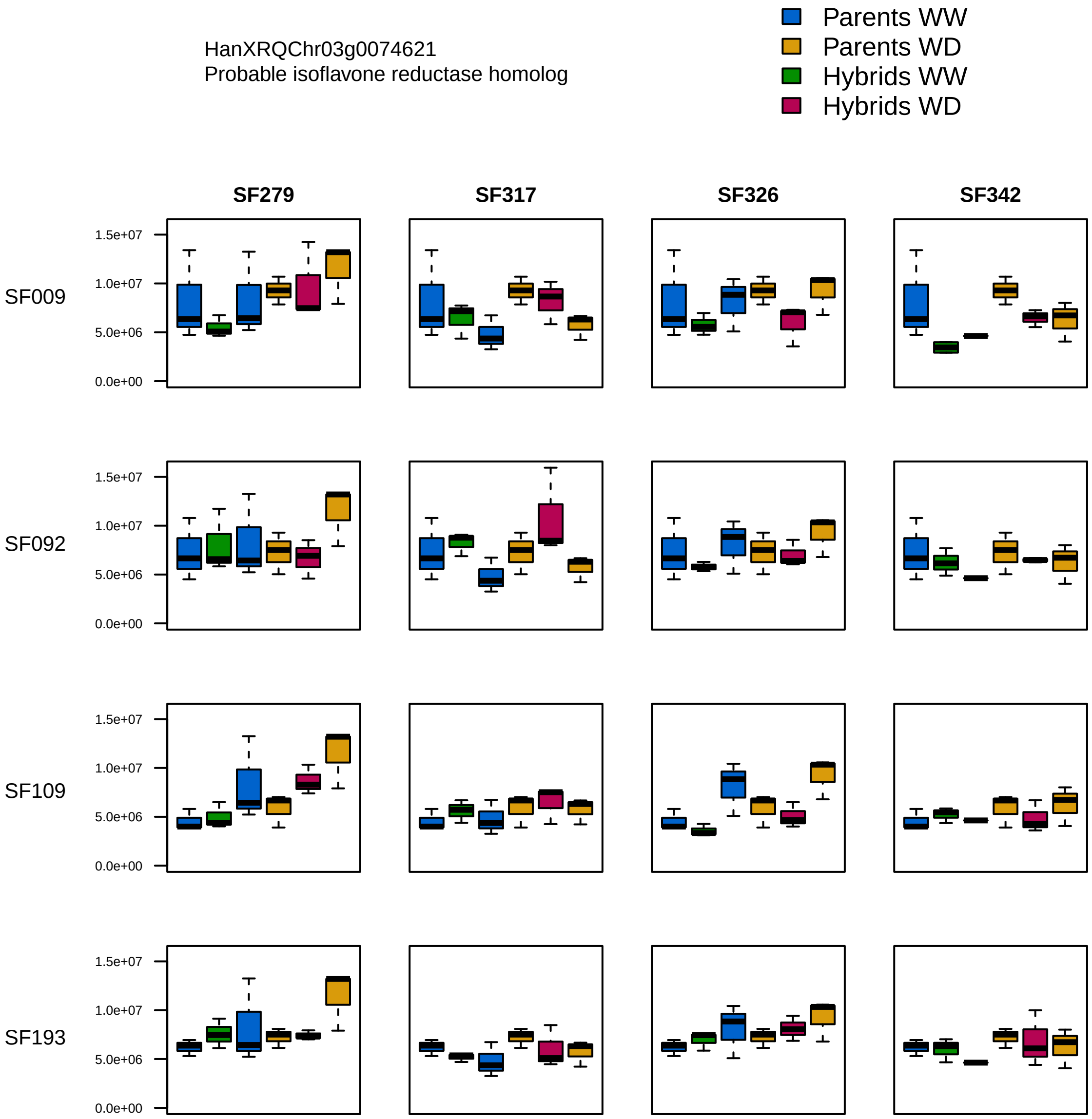

HanXRQChr03g0074621
Probable isoflavone reductase homolog
Parents WW
Parents WD
Hybrids WW
Hybrids WD
SF279
SF317
SF326
SF342
SF009
SF092
SF109
SF193
1.5e+07
1.0e+07
5.0e+06
0.0e+00

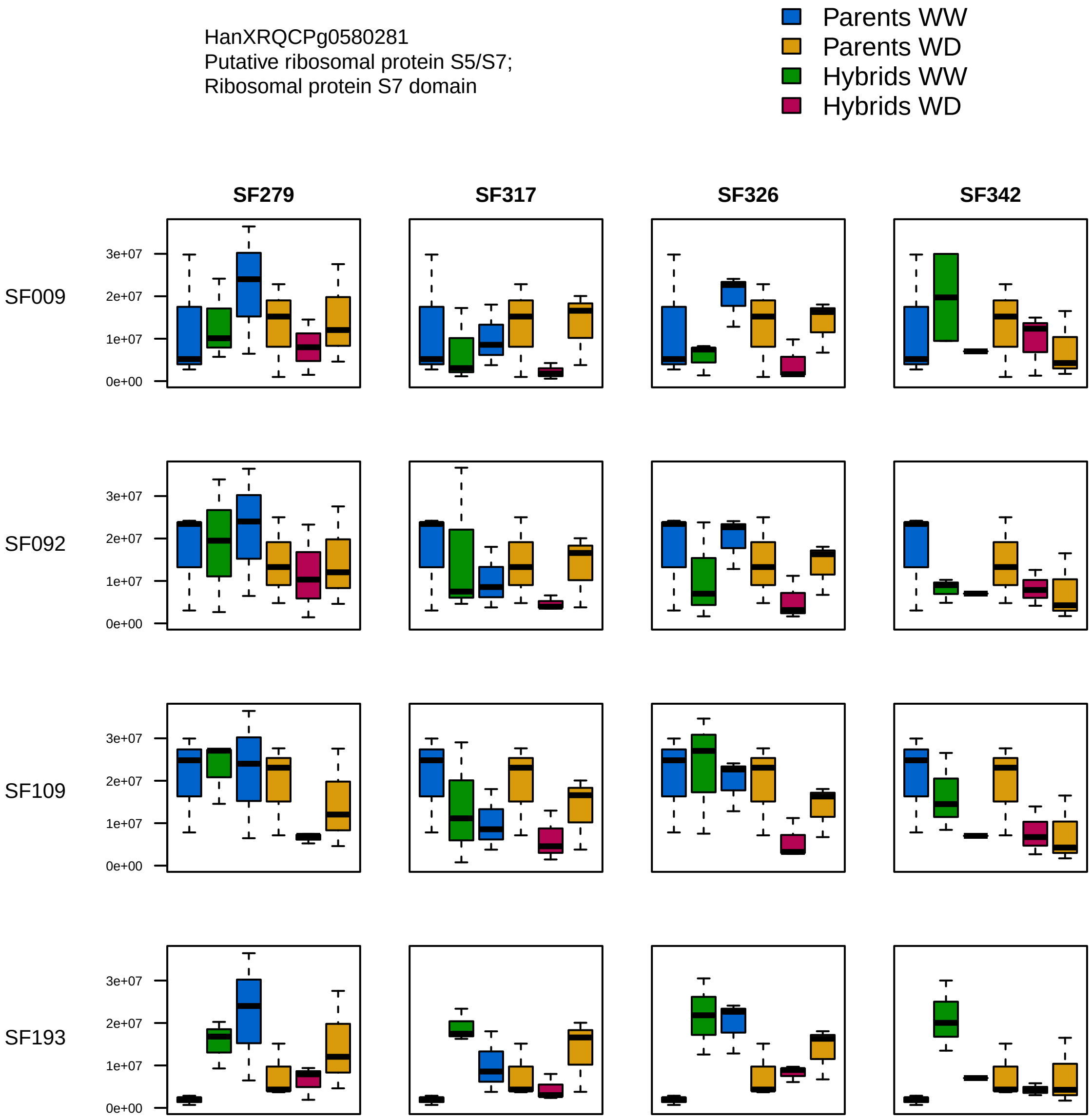
HanXRQCPg0580281
Putative ribosomal protein S5/S7;
Ribosomal protein S7 domain
Parents WW
Parents WD
Hybrids WW
Hybrids WD
SF279
SF317
SF326
SF342
SF009
SF092
SF109
SF193
0e+00
1e+07
2e+07
3e+07

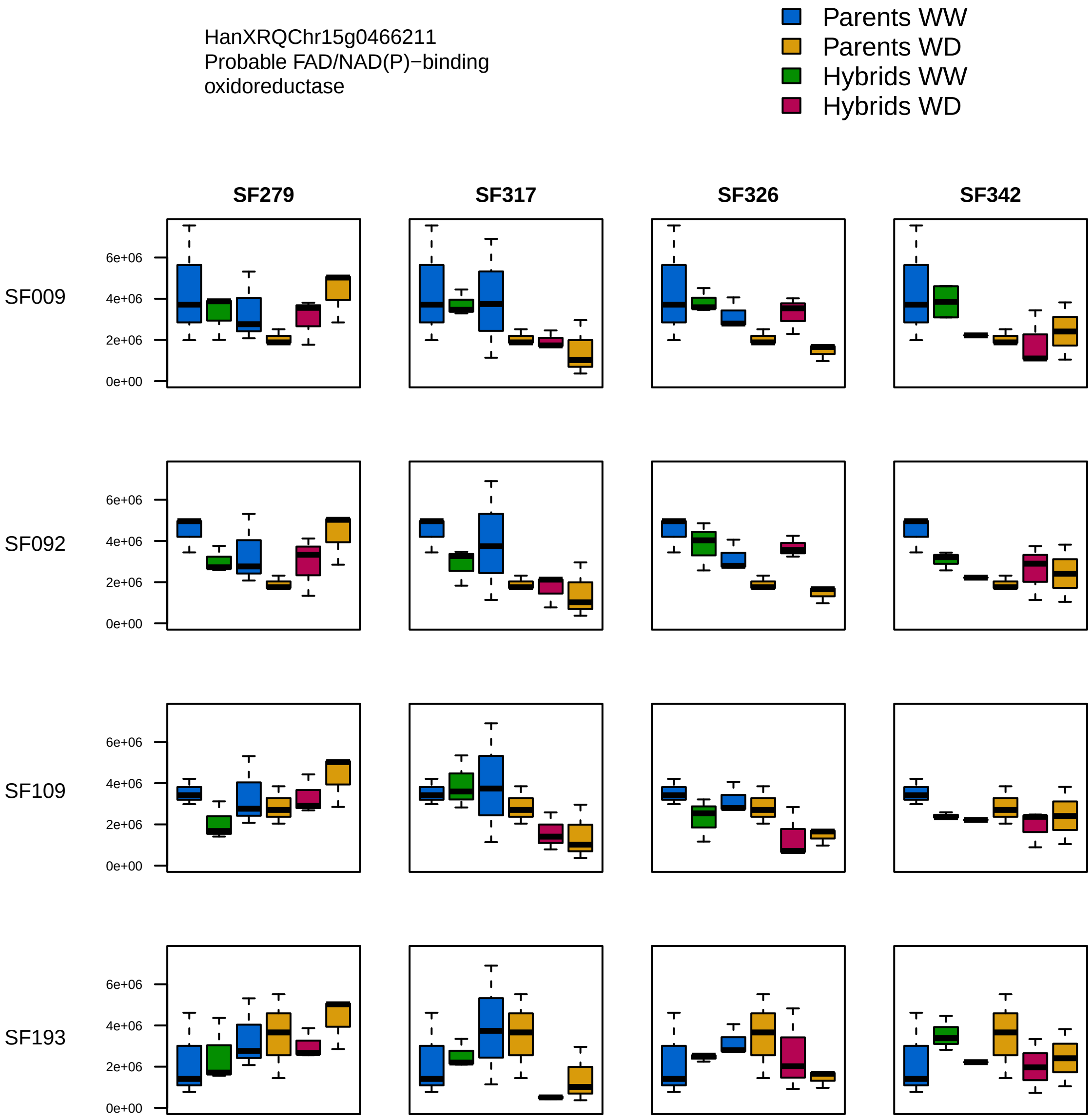

HanXRQChr15g0466211
Probable FAD/NAD(P)−binding
oxidoreductase
Parents WW
Parents WD
Hybrids WW
Hybrids WD
SF279
SF317
SF326
SF342
SF009
SF092
SF109
SF193
6e+06
4e+06
2e+06
0e+00

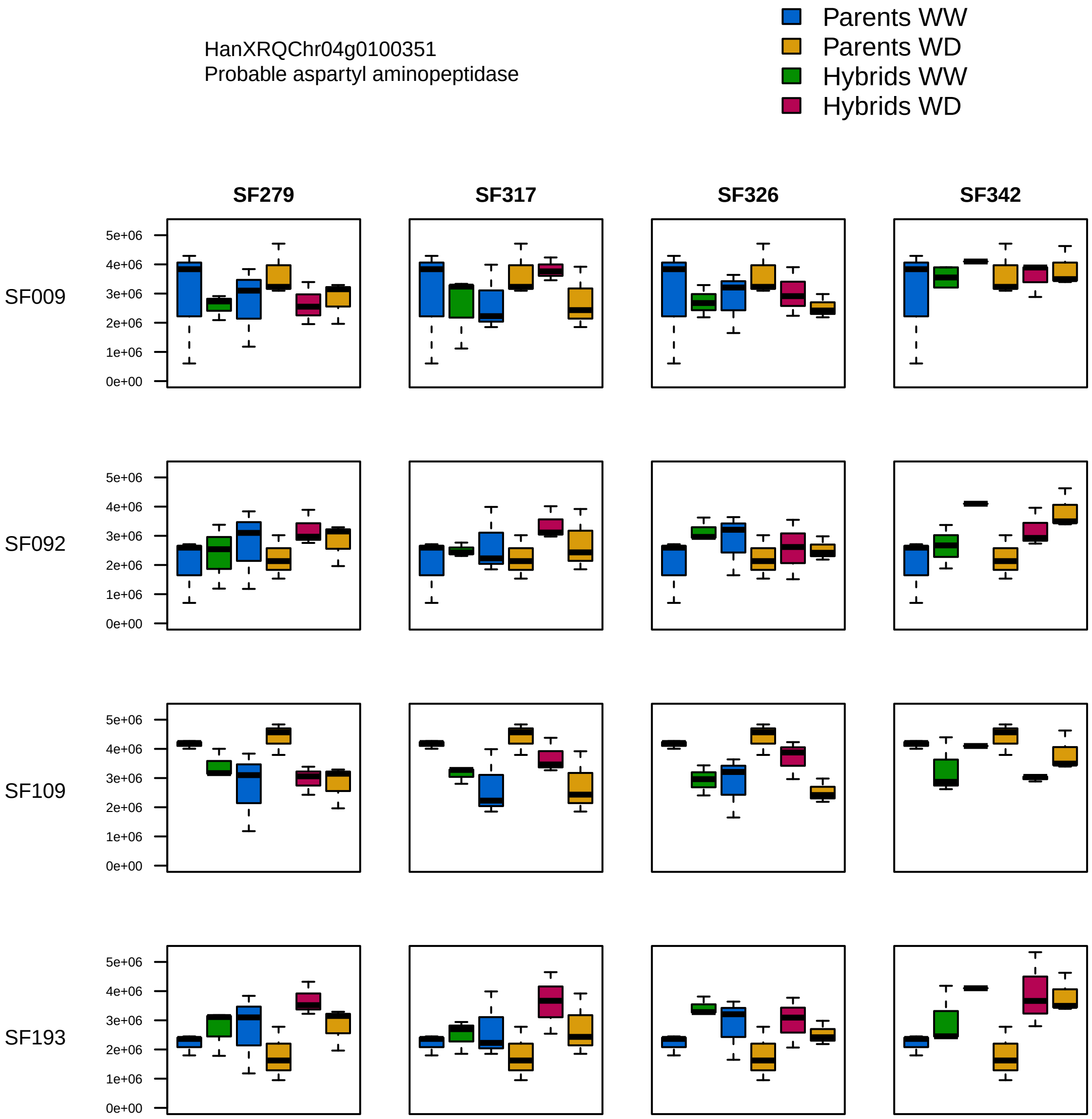

HanXRQChr04g0100351
Probable aspartyl aminopeptidase
Parents WW
Parents WD
Hybrids WW
Hybrids WD
SF279
SF317
SF326
SF342
SF009
SF092
SF109
SF193
5e+06
4e+06
3e+06
2e+06
1e+06
0e+00

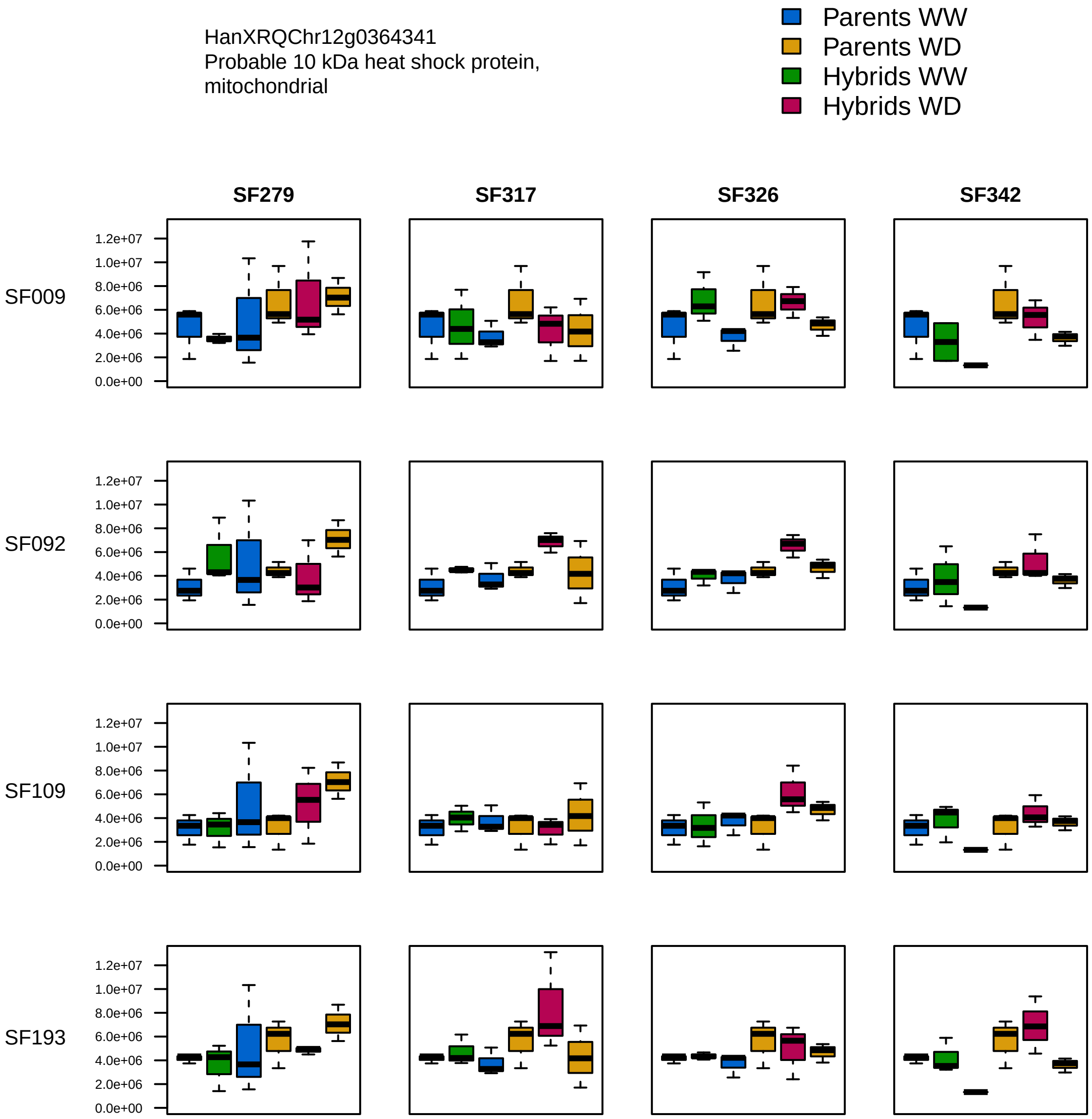
HanXRQChr12g0364341
Probable 10 kDa heat shock protein, mitochondrial
Parents WW
Parents WD
Hybrids WW
Hybrids WD
SF279
SF317
SF326
SF342
SF009
SF092
SF109
SF193
1.2e+07
1.0e+07
8.0e+06
6.0e+06
4.0e+06
2.0e+06
0.0e+00

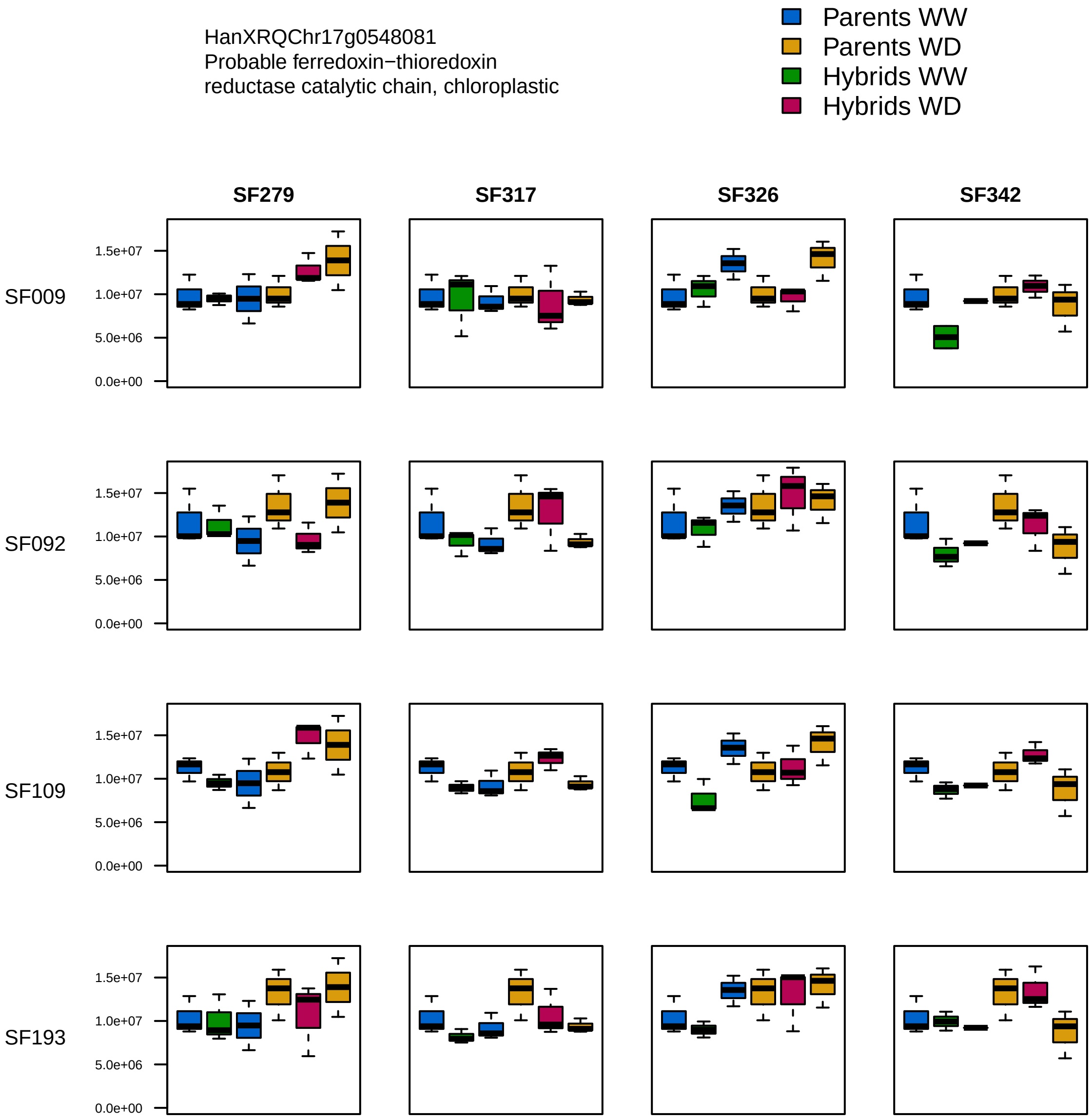
HanXRQChr17g0548081
Probable ferredoxin−thioredoxin
reductase catalytic chain, chloroplastic
Parents WW
Parents WD
Hybrids WW
Hybrids WD
SF279
SF317
SF326
SF342
SF009
SF092
SF109
SF193
1.5e+07
1.0e+07
5.0e+06
0.0e+00

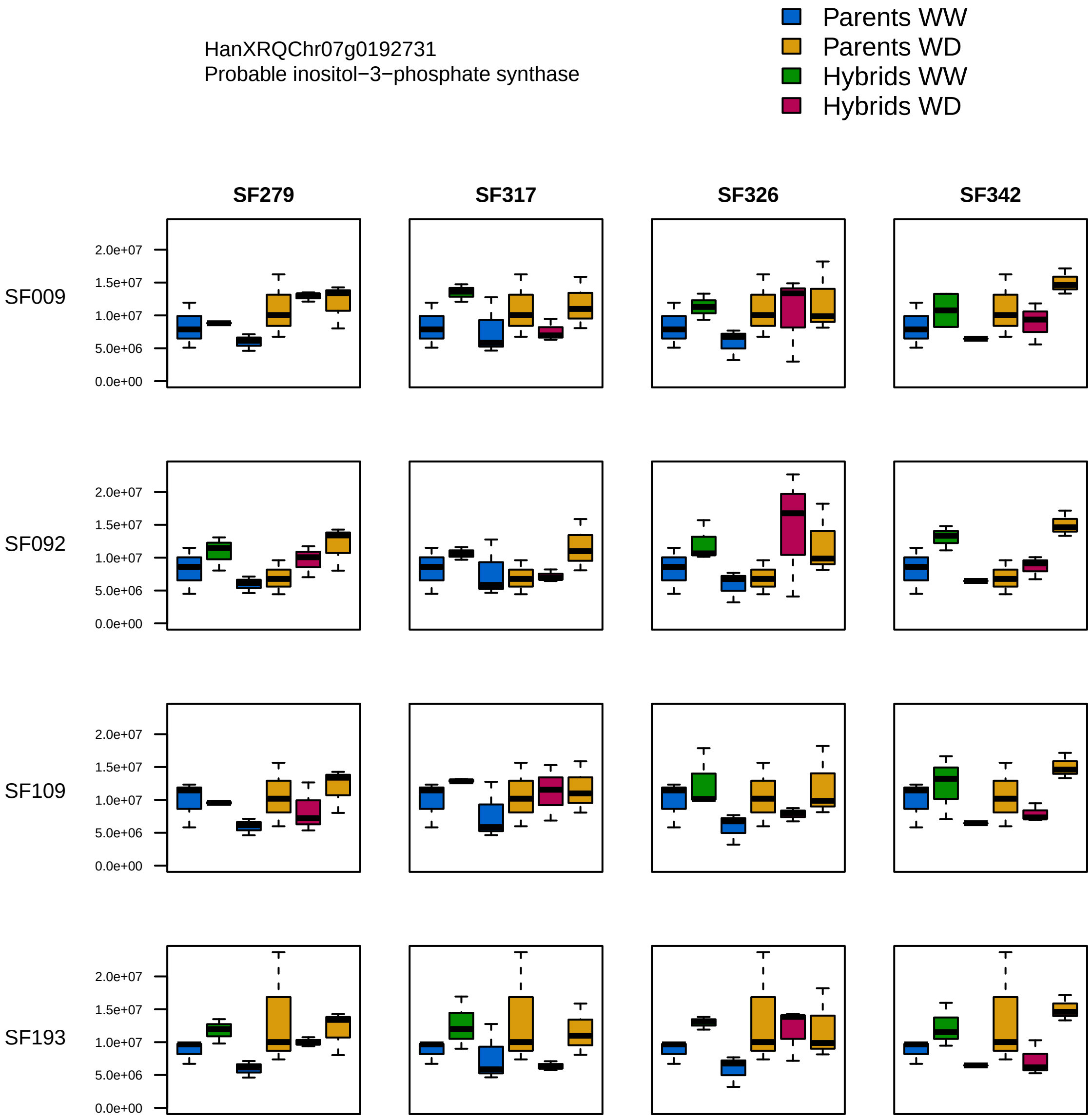
HanXRQChr07g0192731
Probable inositol−3−phosphate synthase
Parents WW
Parents WD
Hybrids WW
Hybrids WD
SF279
SF317
SF326
SF342
SF009
SF092
SF109
SF193
0.0e+00
5.0e+06
1.0e+07
1.5e+07
2.0e+07

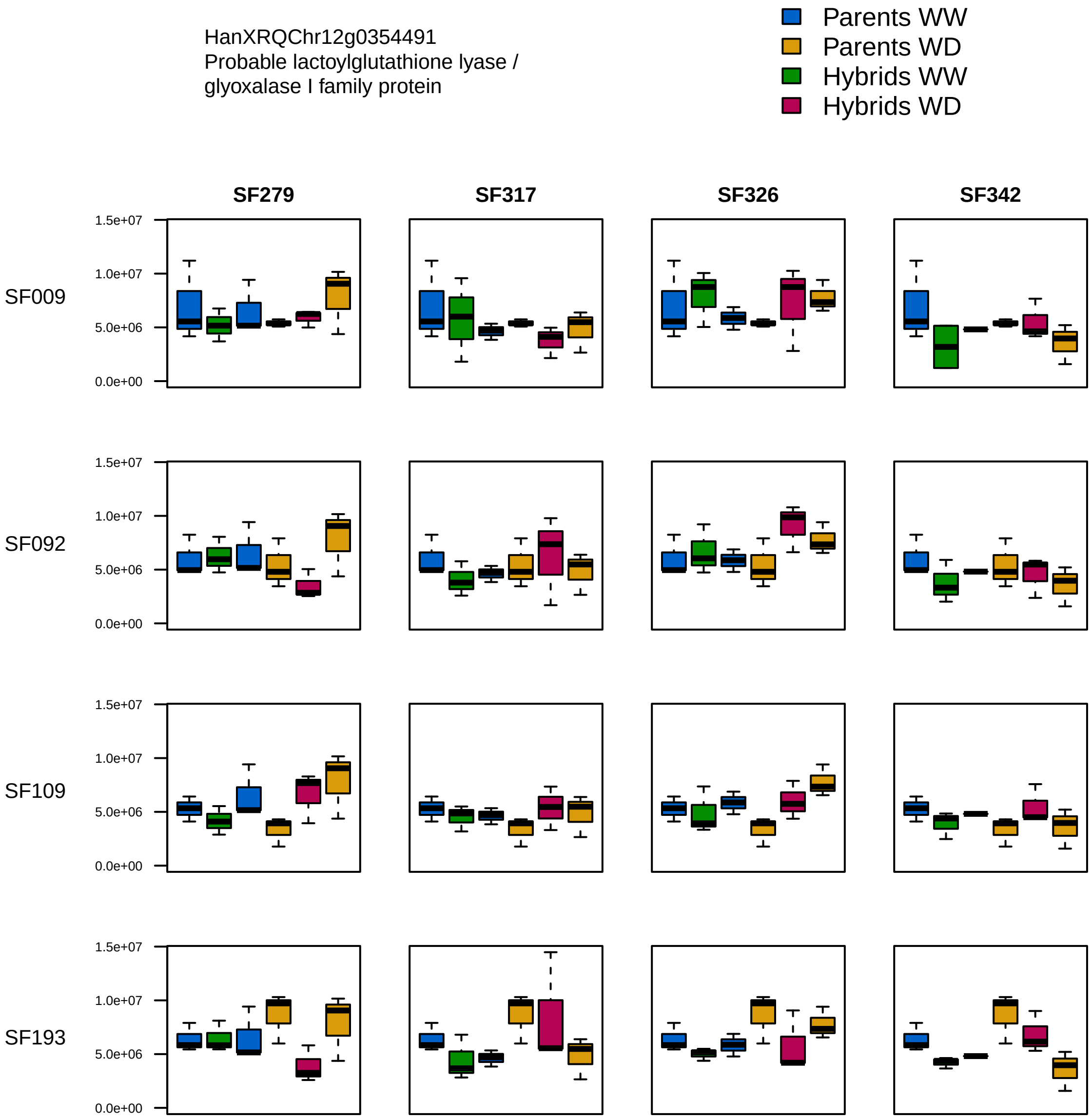
HanXRQChr12g0354491
Probable lactoylglutathione lyase /
glyoxalase I family protein
Parents WW
Parents WD
Hybrids WW
Hybrids WD
SF279
SF317
SF326
SF342
SF009
SF092
SF109
SF193
1.5e+07
1.0e+07
5.0e+06
0.0e+00

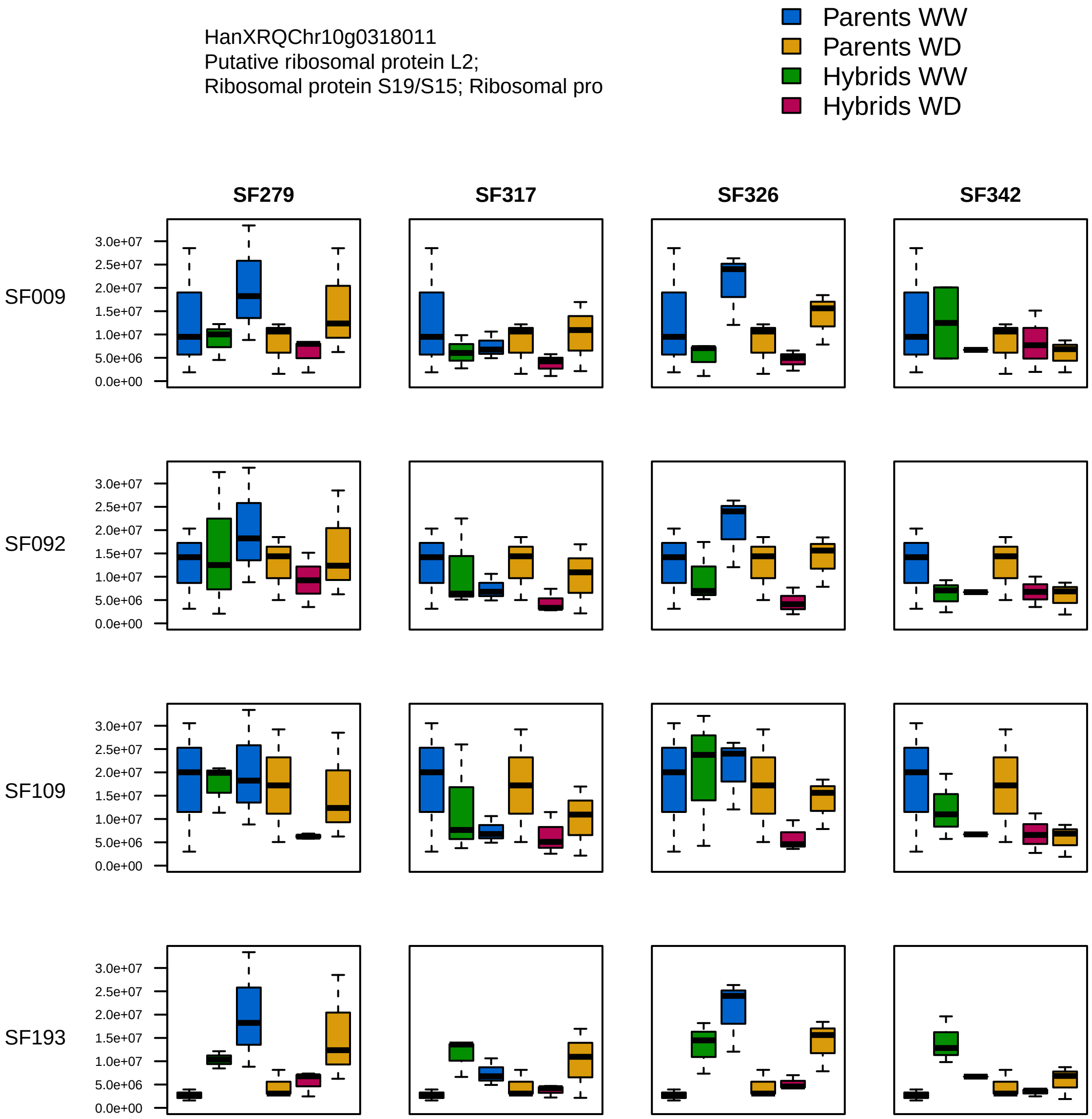

HanXRQChr10g0318011
Putative ribosomal protein L2;
Ribosomal protein S19/S15; Ribosomal pro
Parents WW
Parents WD
Hybrids WW
Hybrids WD
SF279
SF317
SF326
SF342
SF009
SF092
SF109
SF193
0.0e+00
5.0e+06
1.0e+07
1.5e+07
2.0e+07
2.5e+07
3.0e+07

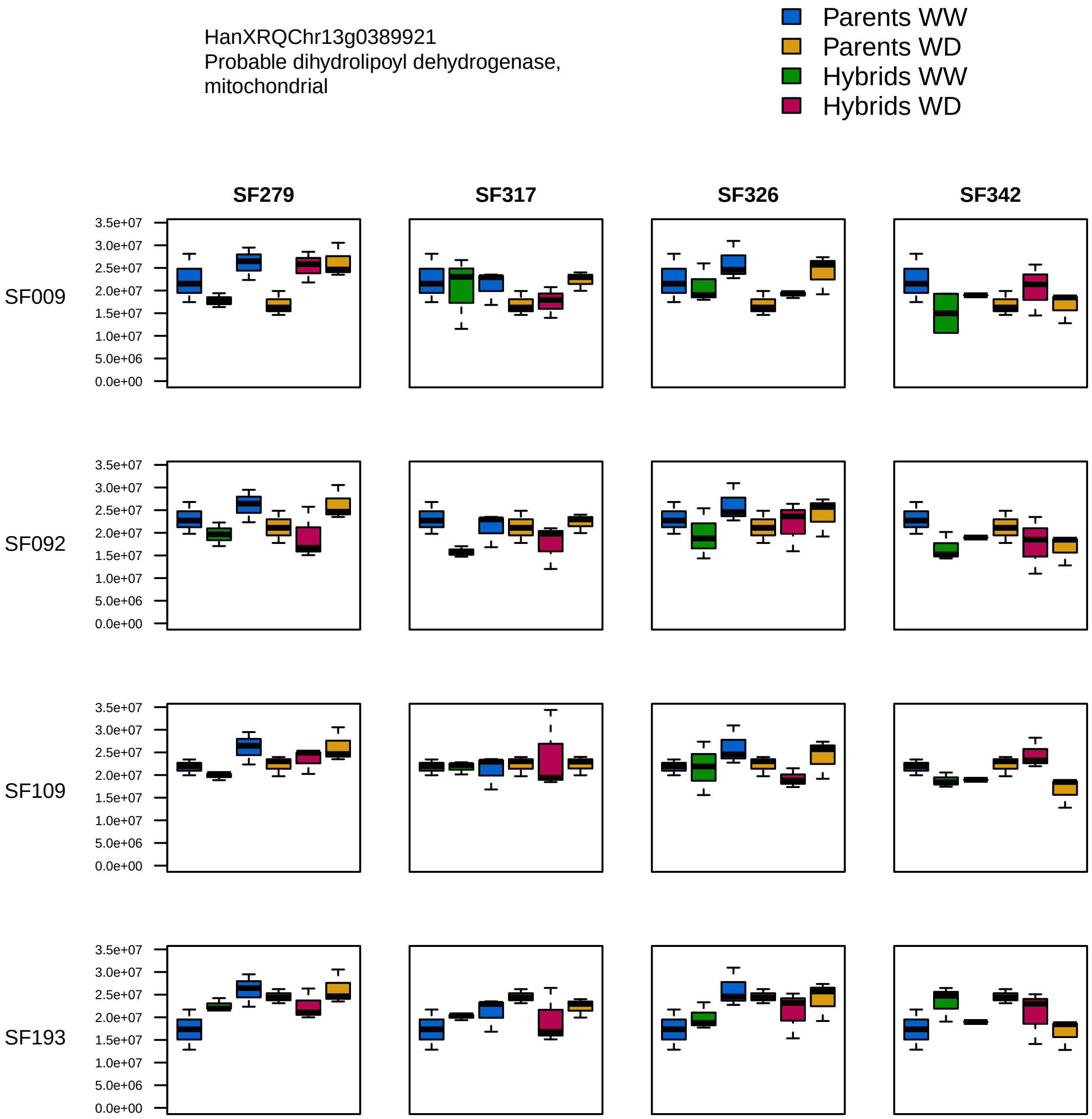
HanXRQChr13g0389921
Probable dihydrolipoyl dehydrogenase, mitochondrial
Parents WW
Parents WD
Hybrids WW
Hybrids WD
SF279
SF317
SF326
SF342
SF009
SF092
SF109
SF193
3.5e+07
3.0e+07
2.5e+07
2.0e+07
1.5e+07
1.0e+07
5.0e+06
0.0e+00

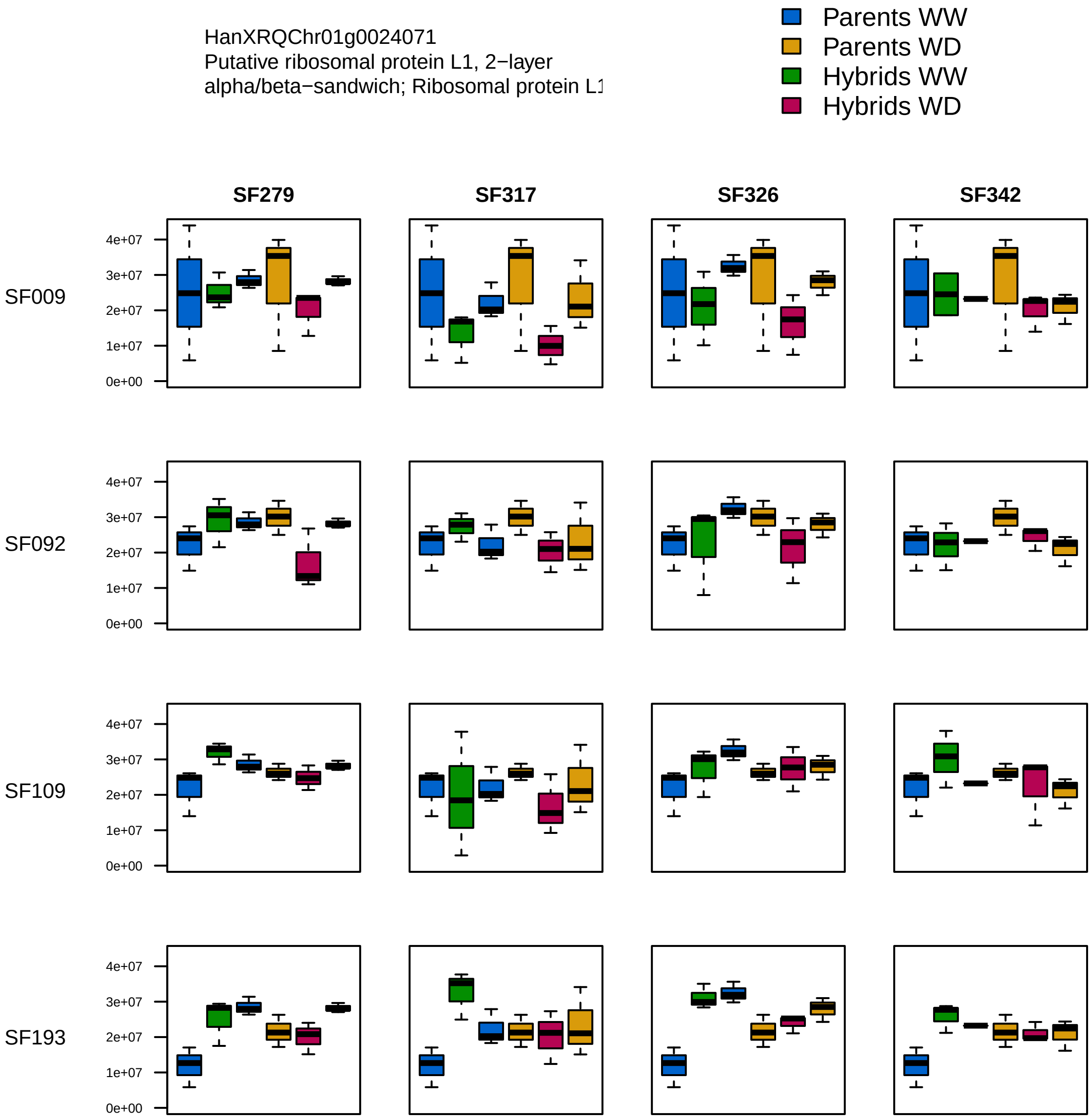

HanXRQChr01g0024071
Putative ribosomal protein L1, 2−layer
alpha/beta−sandwich; Ribosomal protein L1
Parents WW
Parents WD
Hybrids WW
Hybrids WD
SF279
SF317
SF326
SF342
SF009
SF092
SF109
SF193
4e+07
3e+07
2e+07
1e+07
0e+00

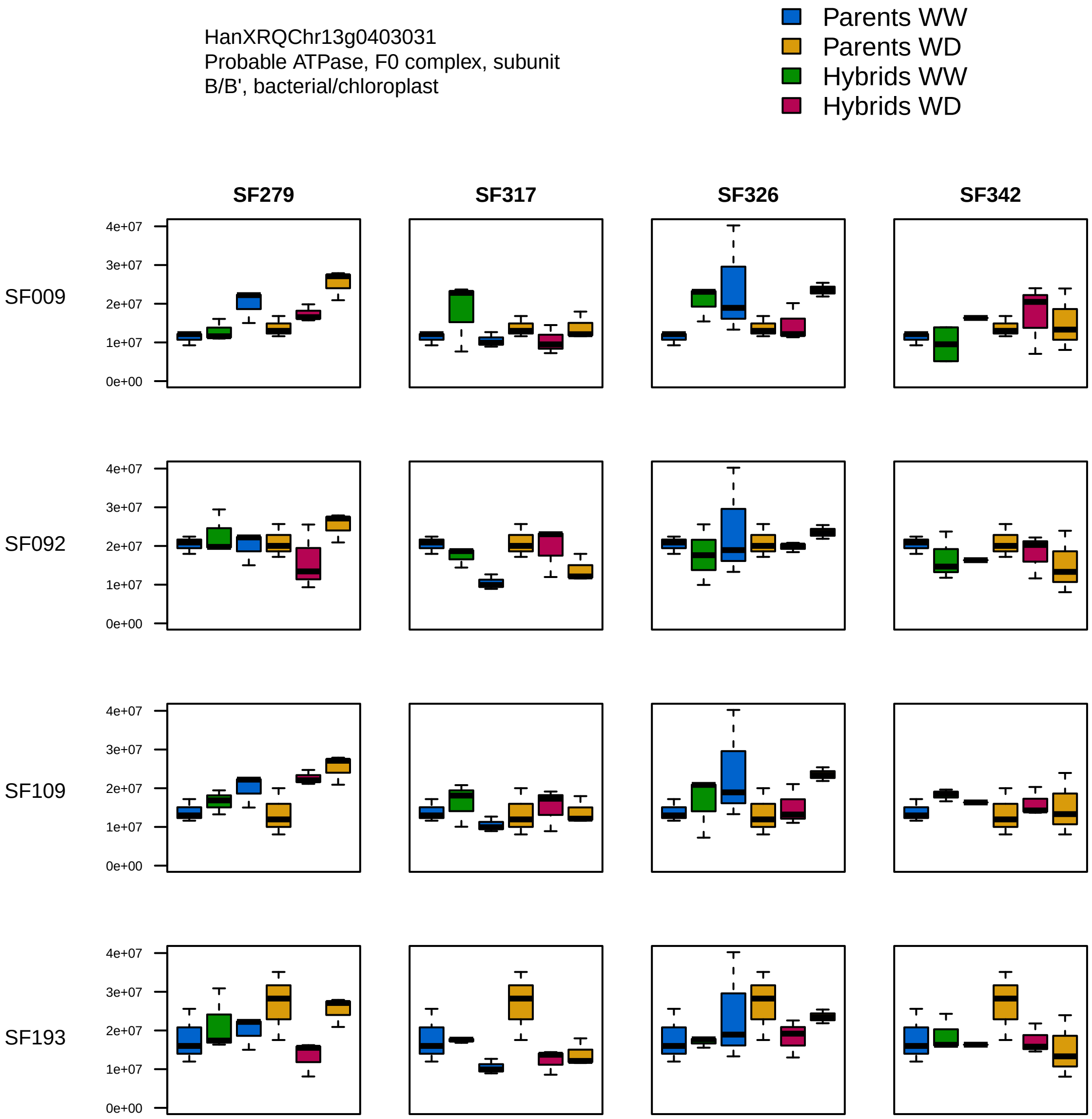

HanXRQChr13g0403031
Probable ATPase, F0 complex, subunit
B/B', bacterial/chloroplast
Parents WW
Parents WD
Hybrids WW
Hybrids WD
SF279
SF317
SF326
SF342
SF009
SF092
SF109
SF193
4e+07
3e+07
2e+07
1e+07
0e+00

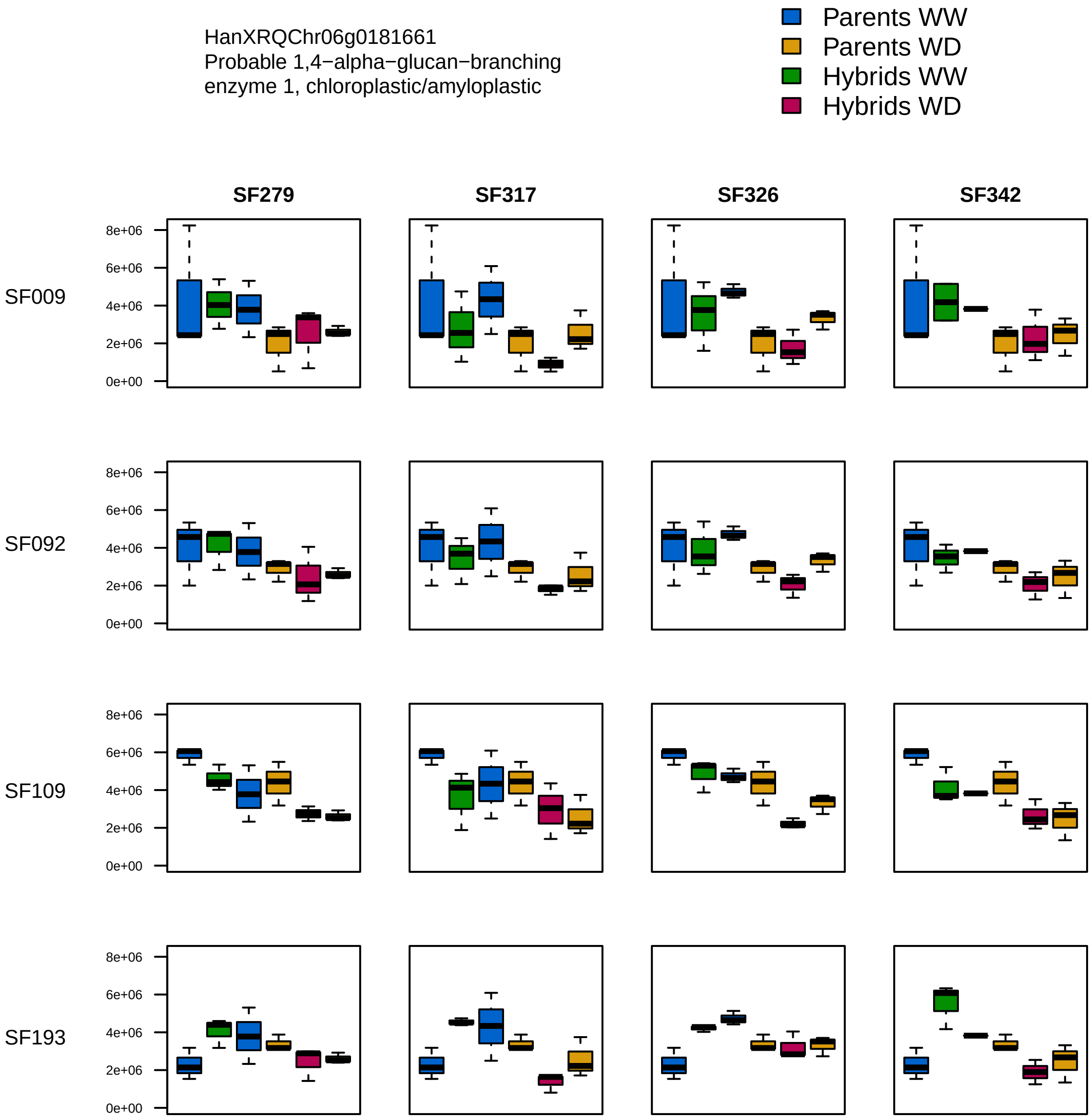
HanXRQChr06g0181661
Probable 1,4−alpha−glucan−branching
enzyme 1, chloroplastic/amyloplastic
Parents WW
Parents WD
Hybrids WW
Hybrids WD
SF279
SF317
SF326
SF342
SF009
SF092
SF109
SF193
8e+06
6e+06
4e+06
2e+06
0e+00

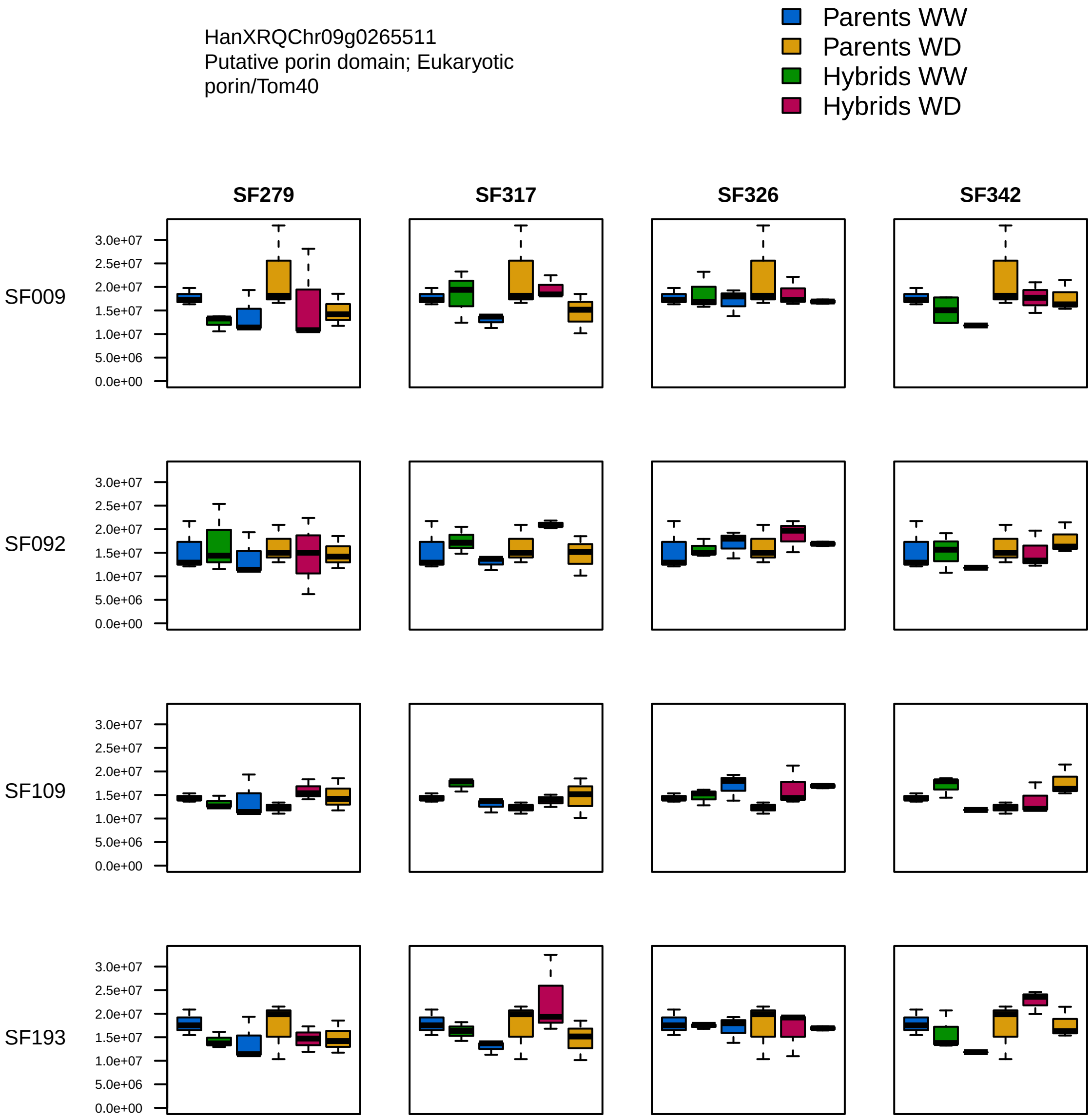
HanXRQChr09g0265511
Putative porin domain; Eukaryotic porin/Tom40
Parents WW
Parents WD
Hybrids WW
Hybrids WD
SF279
SF317
SF326
SF342
SF009
SF092
SF109
SF193
3.0e+07
2.5e+07
2.0e+07
1.5e+07
1.0e+07
5.0e+06
0.0e+00

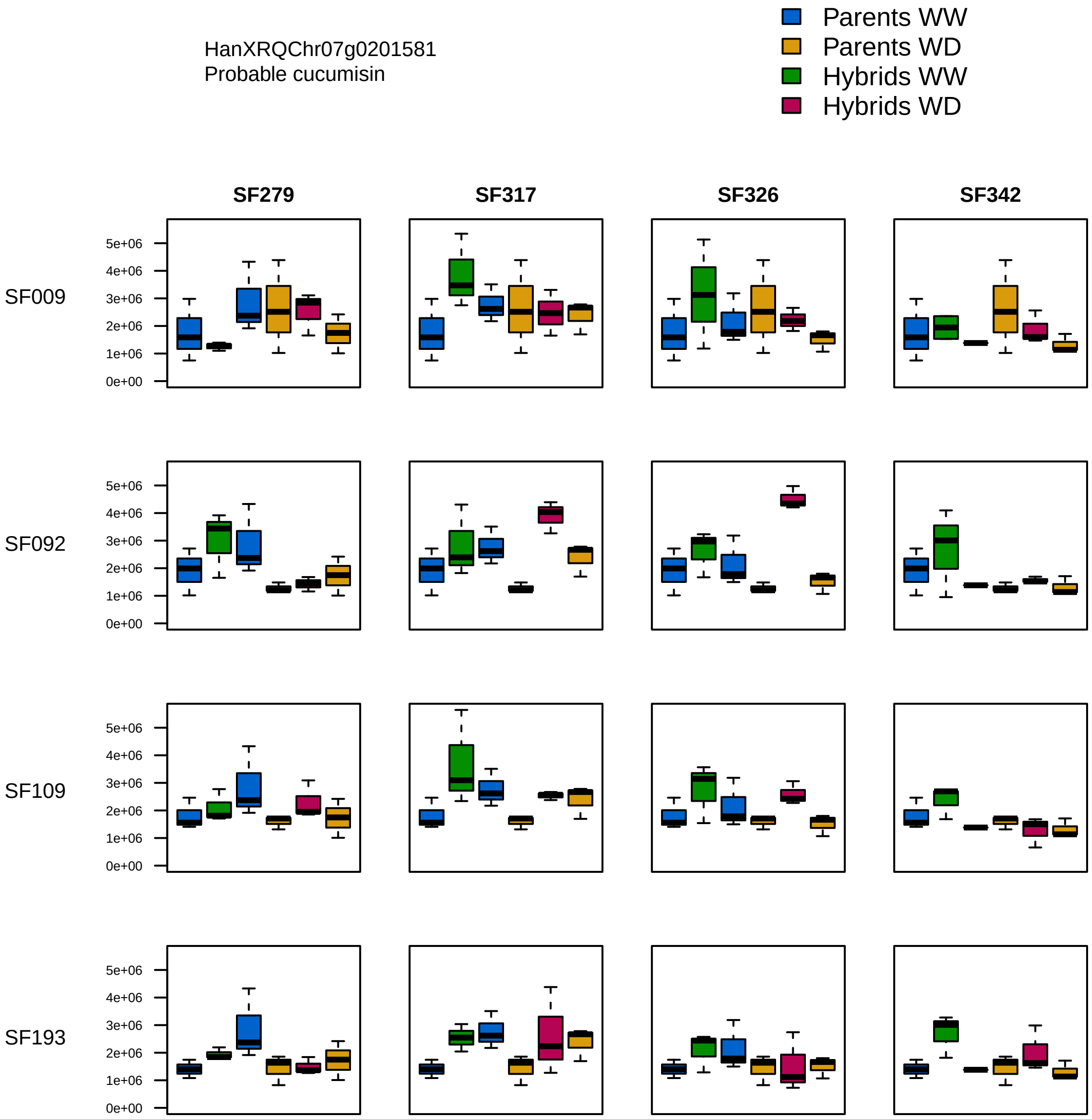

HanXRQChr07g0201581
Probable cucumisin
Parents WW
Parents WD
Hybrids WW
Hybrids WD
SF279
SF317
SF326
SF342
SF009
SF092
SF109
SF193
0e+00
1e+06
2e+06
3e+06
4e+06
5e+06

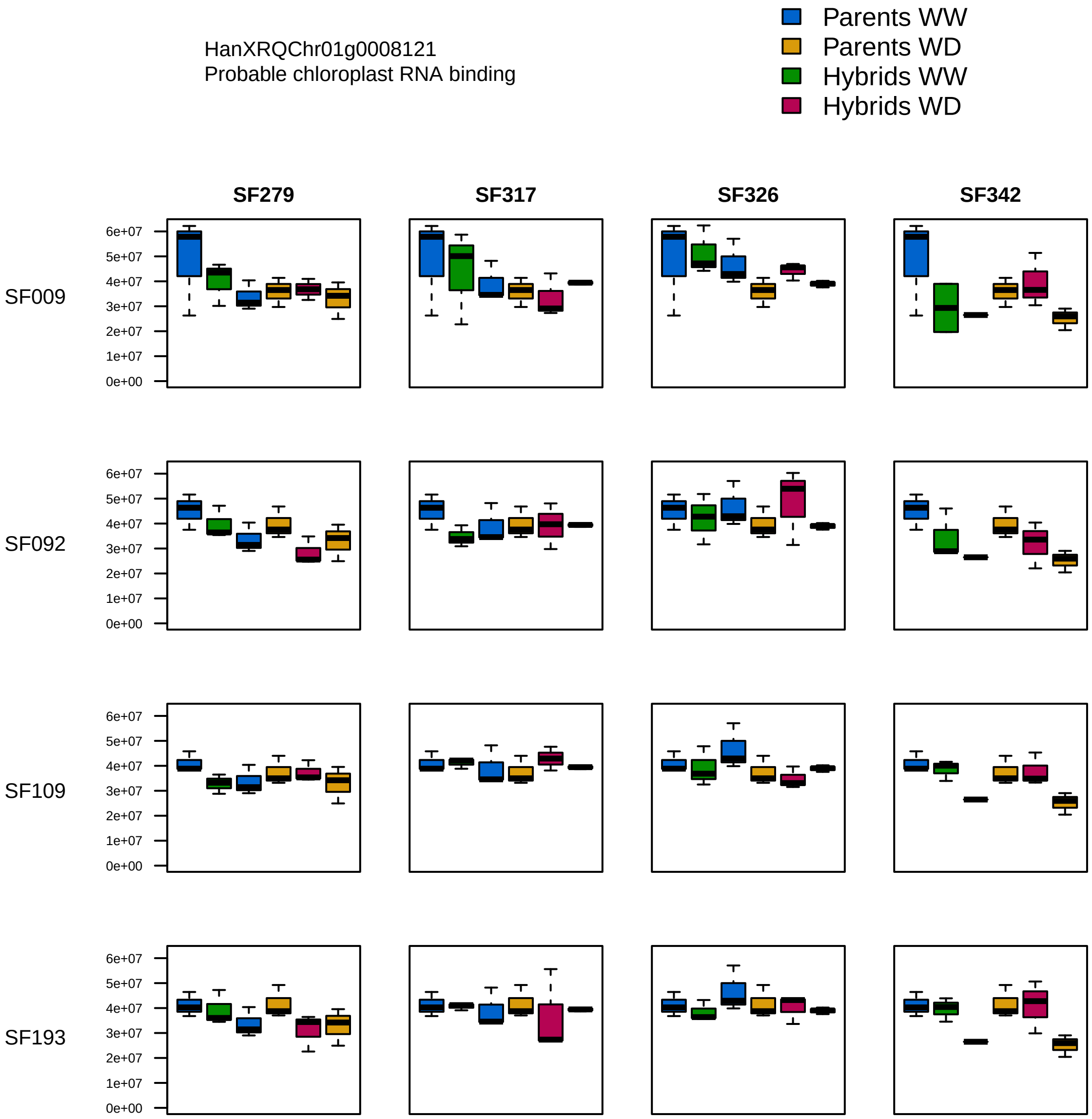
HanXRQChr01g0008121
Probable chloroplast RNA binding
Parents WW
Parents WD
Hybrids WW
Hybrids WD
SF279
SF317
SF326
SF342
SF009
SF092
SF109
SF193
6e+07
5e+07
4e+07
3e+07
2e+07
1e+07
0e+00

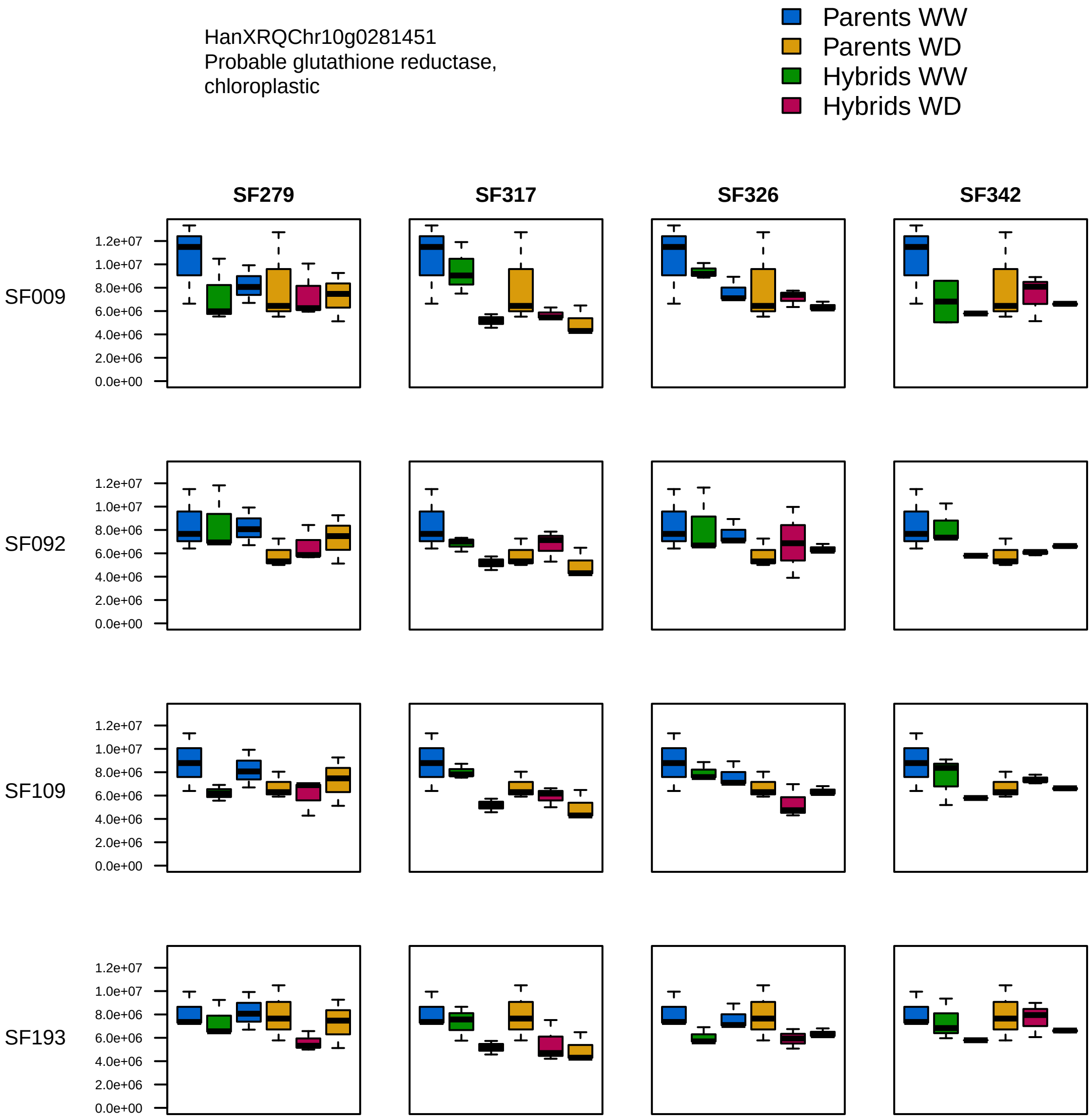
HanXRQChr10g0281451
Probable glutathione reductase,
chloroplastic
Parents WW
Parents WD
Hybrids WW
Hybrids WD
SF279
SF317
SF326
SF342
SF009
SF092
SF109
SF193
1.2e+07
1.0e+07
8.0e+06
6.0e+06
4.0e+06
2.0e+06
0.0e+00

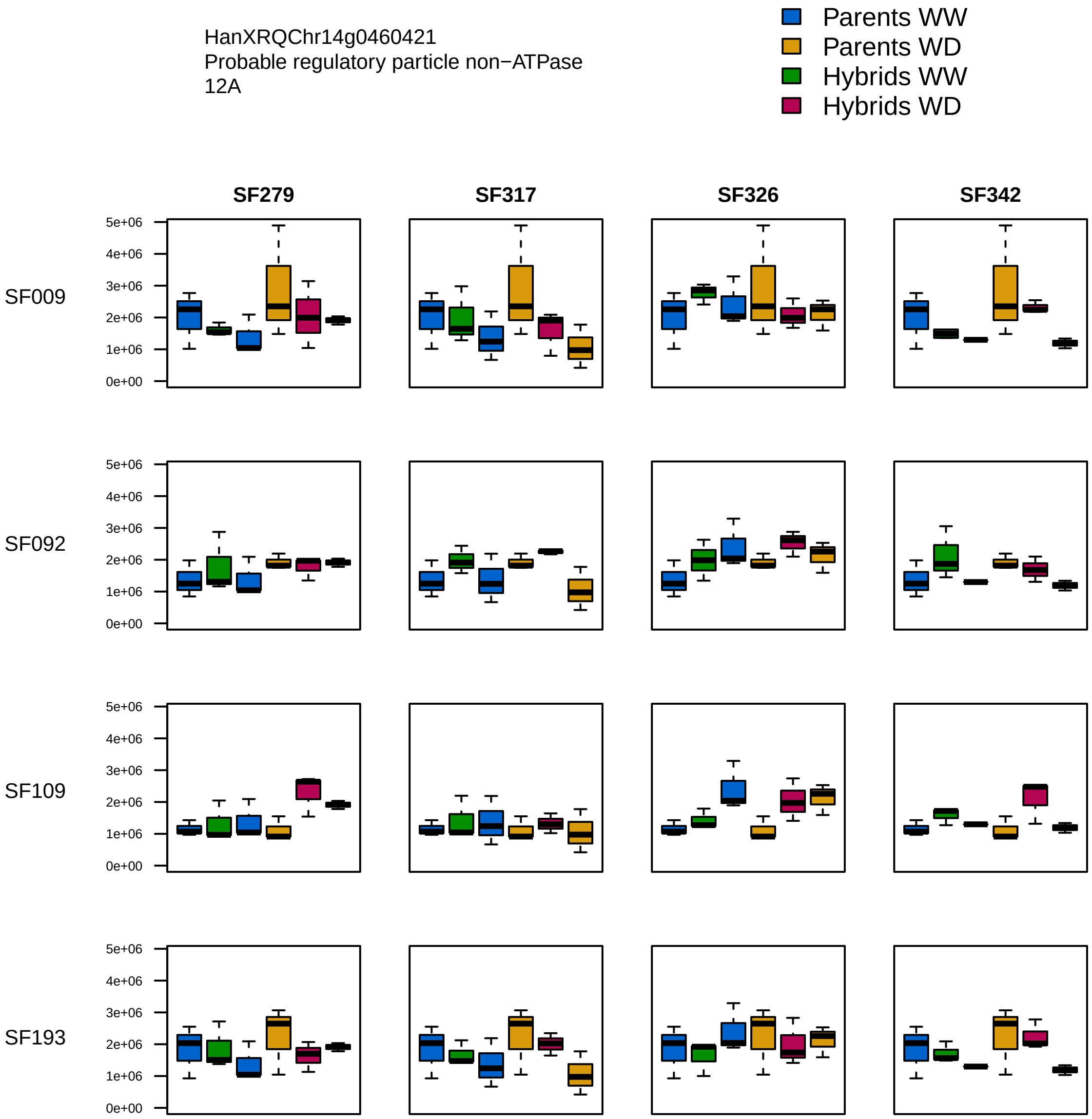
HanXRQChr14g0460421
Probable regulatory particle non−ATPase
12A
Parents WW
Parents WD
Hybrids WW
Hybrids WD
SF279
SF317
SF326
SF342
SF009
SF092
SF109
SF193
0e+00
1e+06
2e+06
3e+06
4e+06
5e+06

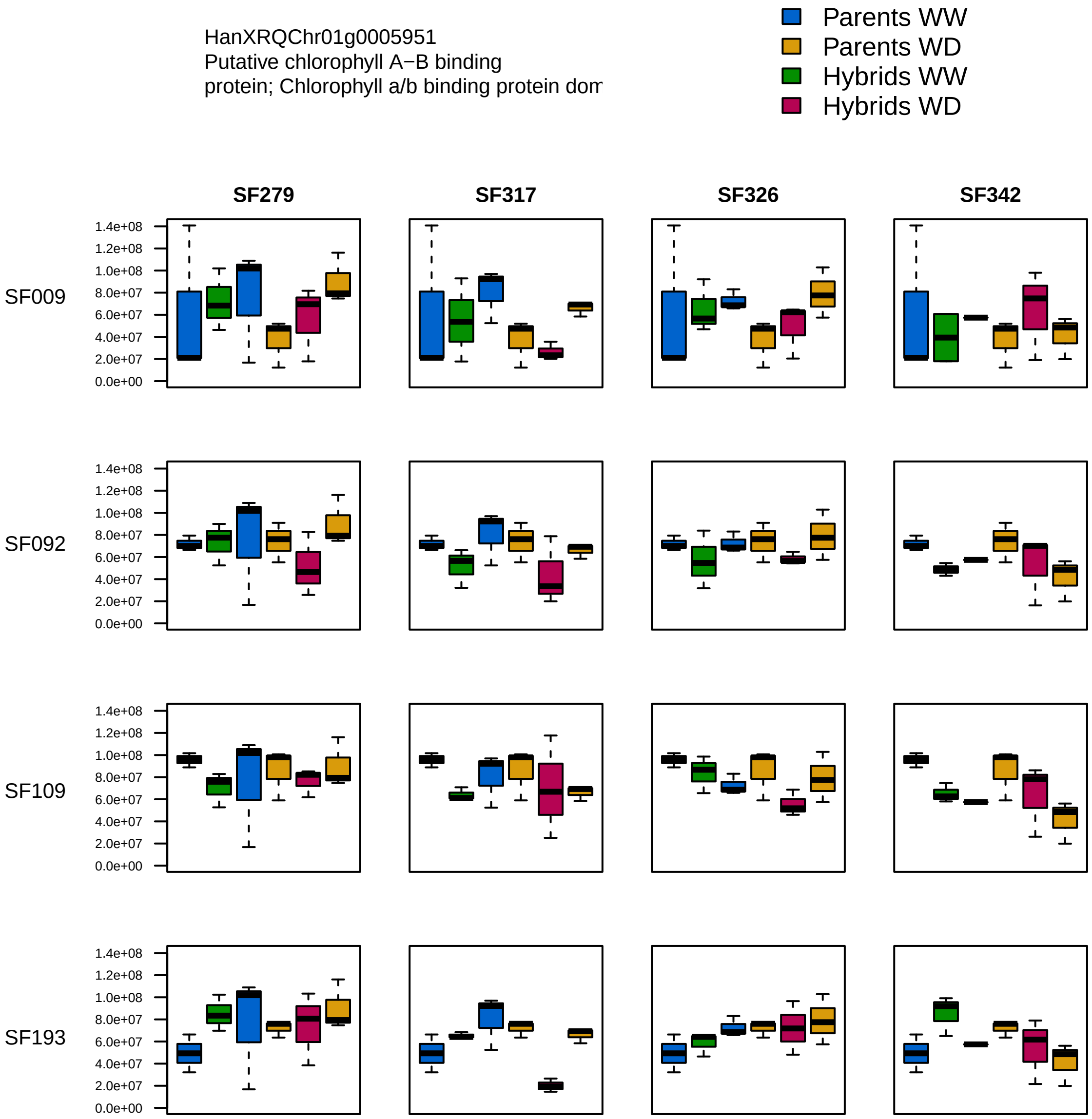
HanXRQChr01g0005951
Putative chlorophyll A−B binding
protein; Chlorophyll a/b binding protein dom
Parents WW
Parents WD
Hybrids WW
Hybrids WD
SF279
SF317
SF326
SF342
SF009
SF092
SF109
SF193
1.4e+08
1.2e+08
1.0e+08
8.0e+07
6.0e+07
4.0e+07
2.0e+07
0.0e+00

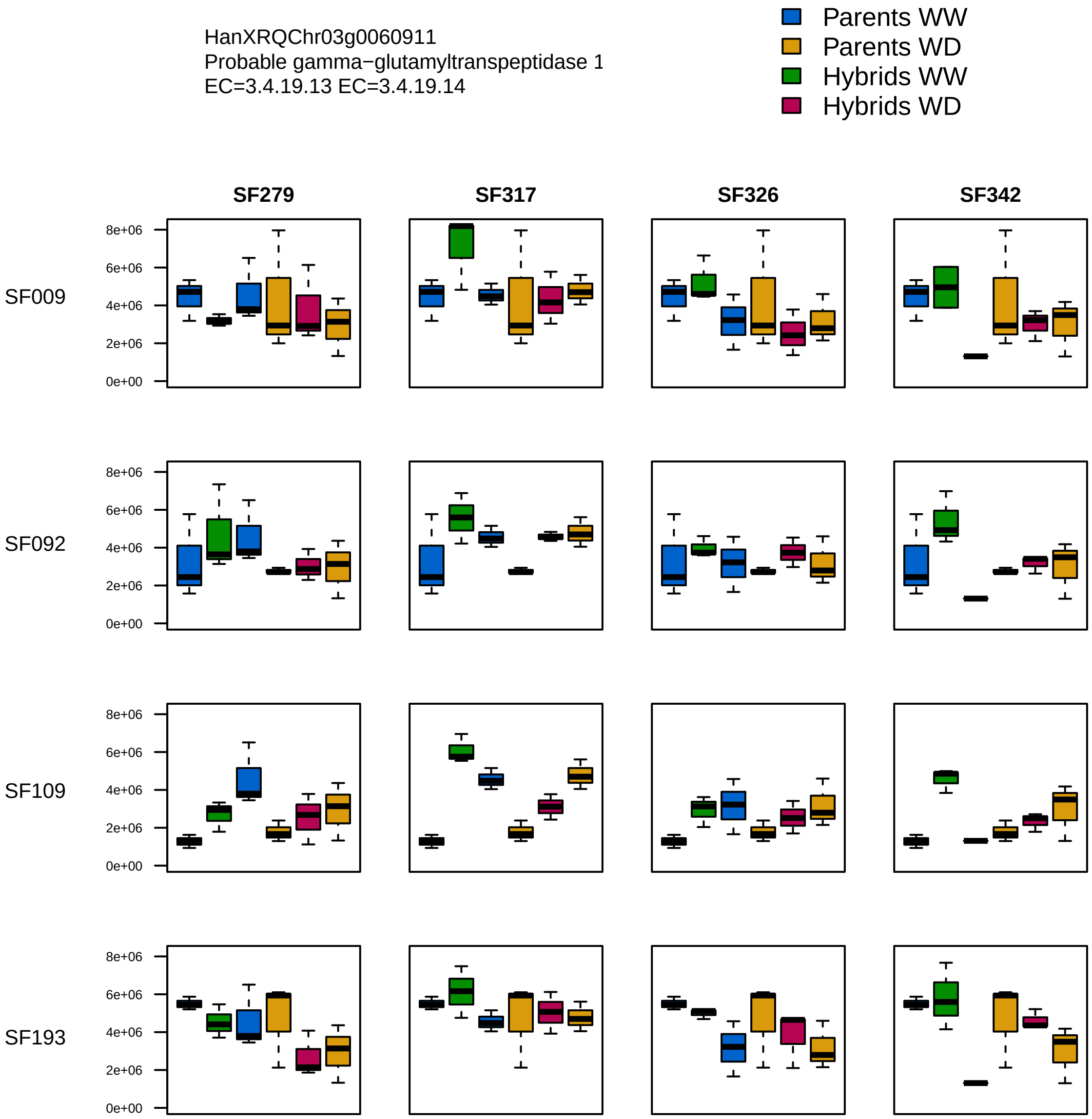
HanXRQChr03g0060911
Probable gamma−glutamyltranspeptidase 1
EC=3.4.19.13 EC=3.4.19.14
Parents WW
Parents WD
Hybrids WW
Hybrids WD
SF279
SF317
SF326
SF342
SF009
SF092
SF109
SF193
8e+06
6e+06
4e+06
2e+06
0e+00

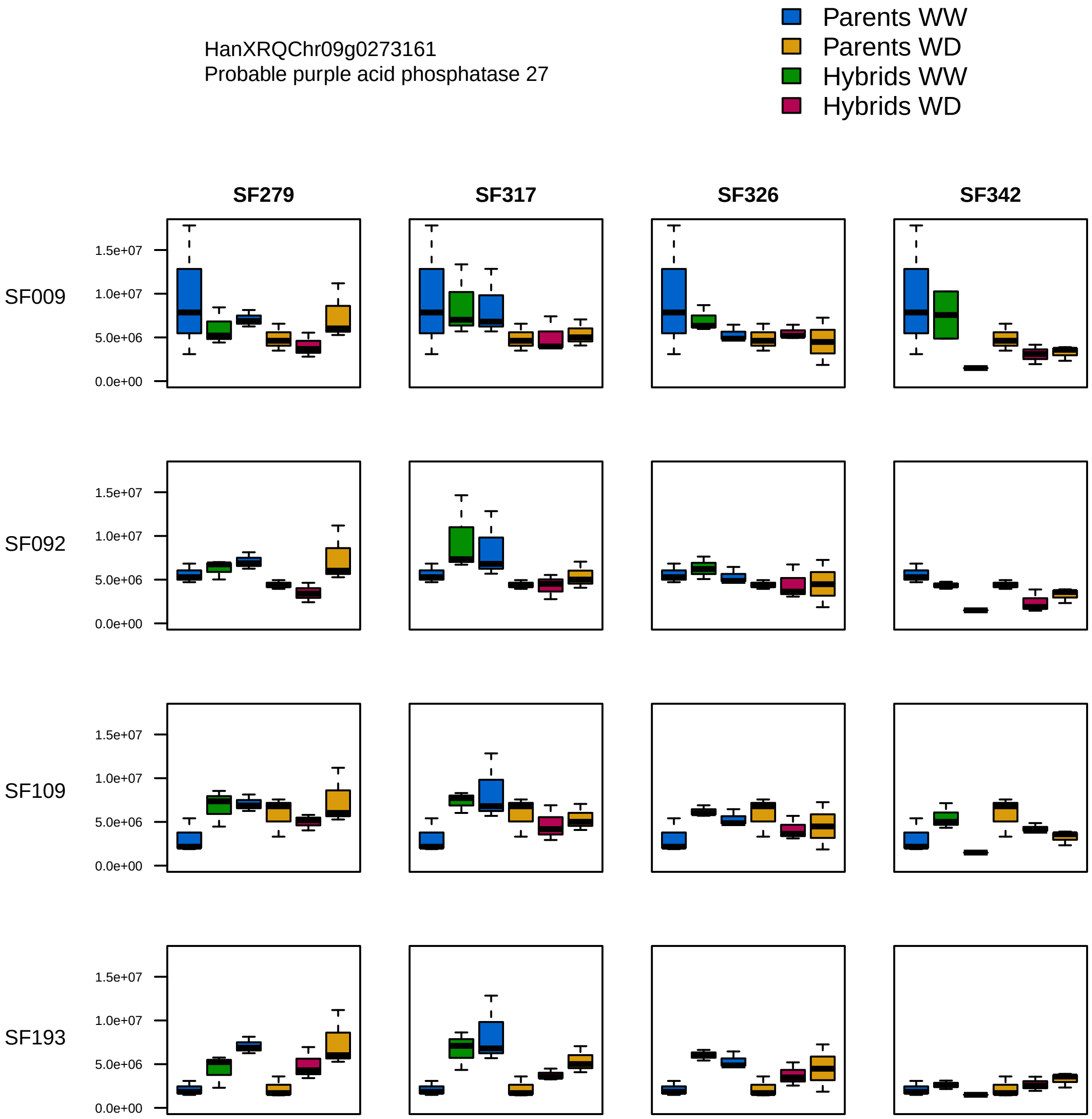

HanXRQChr09g0273161
Probable purple acid phosphatase 27
Parents WW
Parents WD
Hybrids WW
Hybrids WD
SF279
SF317
SF326
SF342
SF009
SF092
SF109
SF193
1.5e+07
1.0e+07
5.0e+06
0.0e+00

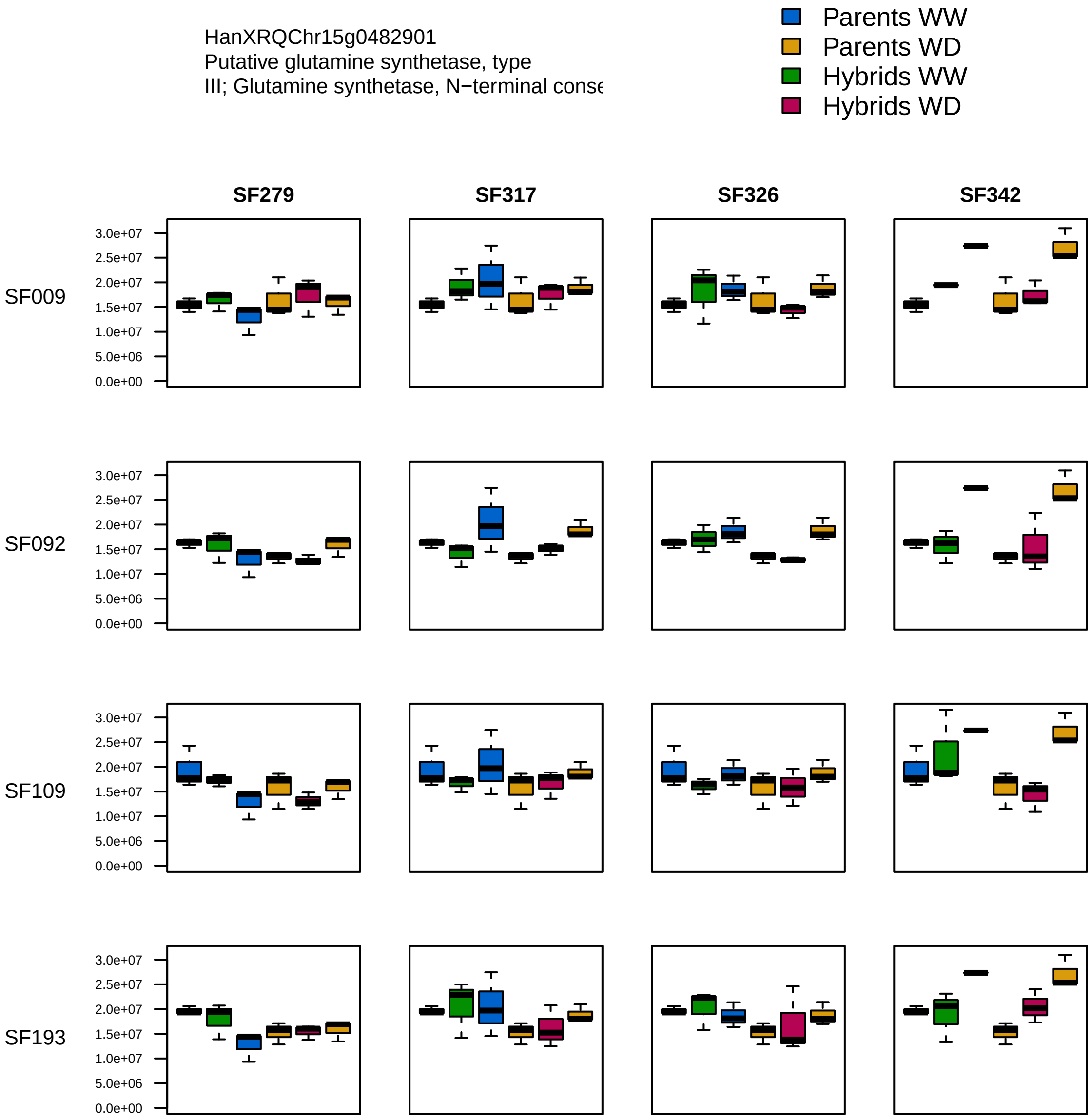
HanXRQChr15g0482901
Putative glutamine synthetase, type
III; Glutamine synthetase, N−terminal conse
Parents WW
Parents WD
Hybrids WW
Hybrids WD
SF279
SF317
SF326
SF342
SF009
SF092
SF109
SF193
3.0e+07
2.5e+07
2.0e+07
1.5e+07
1.0e+07
5.0e+06
0.0e+00

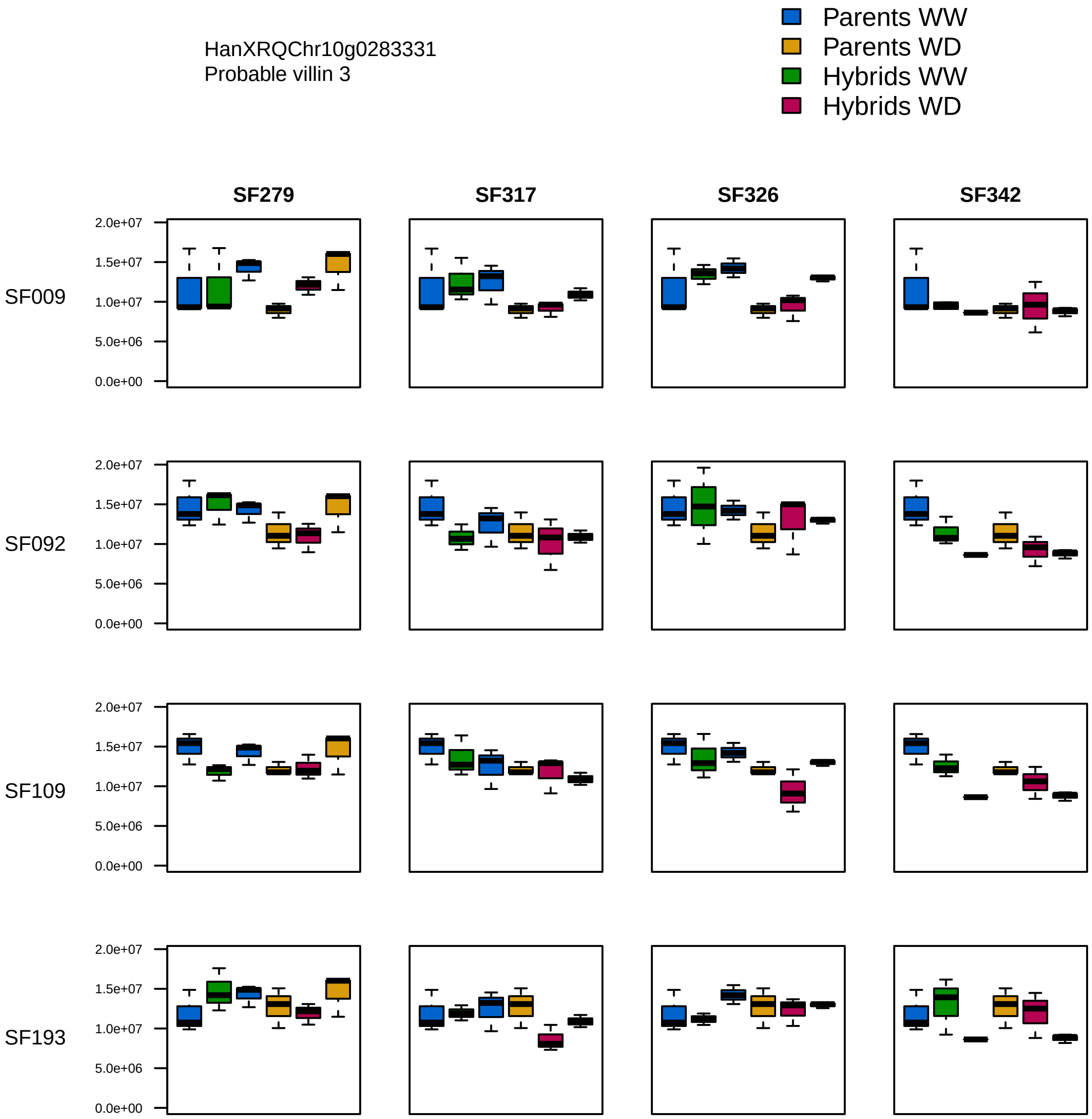
HanXRQChr10g0283331
Probable villin 3
Parents WW
Parents WD
Hybrids WW
Hybrids WD
SF279
SF317
SF326
SF342
SF009
SF092
SF109
SF193
2.0e+07
1.5e+07
1.0e+07
5.0e+06
0.0e+00

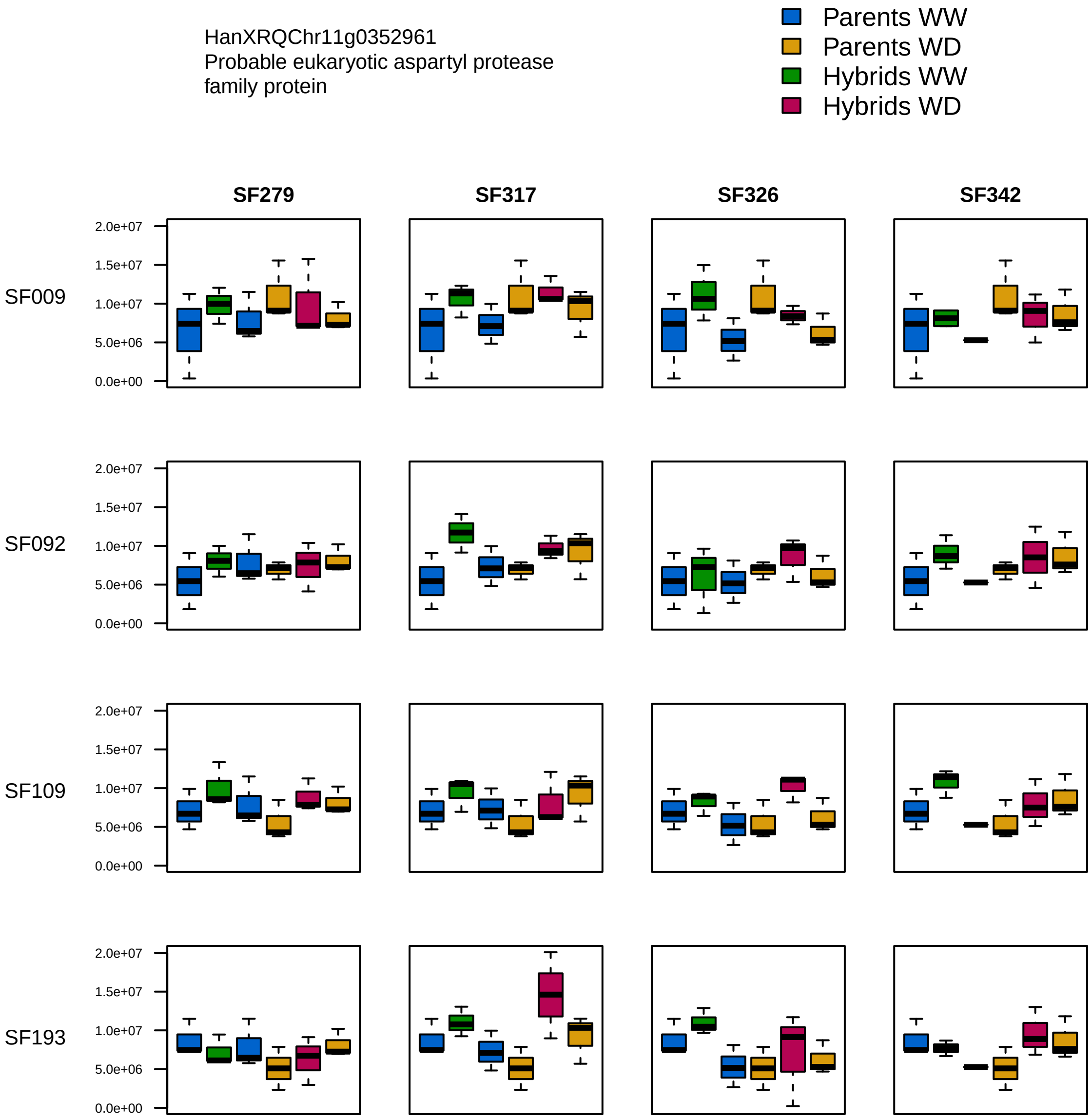
HanXRQChr11g0352961
Probable eukaryotic aspartyl protease
family protein
Parents WW
Parents WD
Hybrids WW
Hybrids WD
SF279
SF317
SF326
SF342
SF009
SF092
SF109
SF193
2.0e+07
1.5e+07
1.0e+07
5.0e+06
0.0e+00

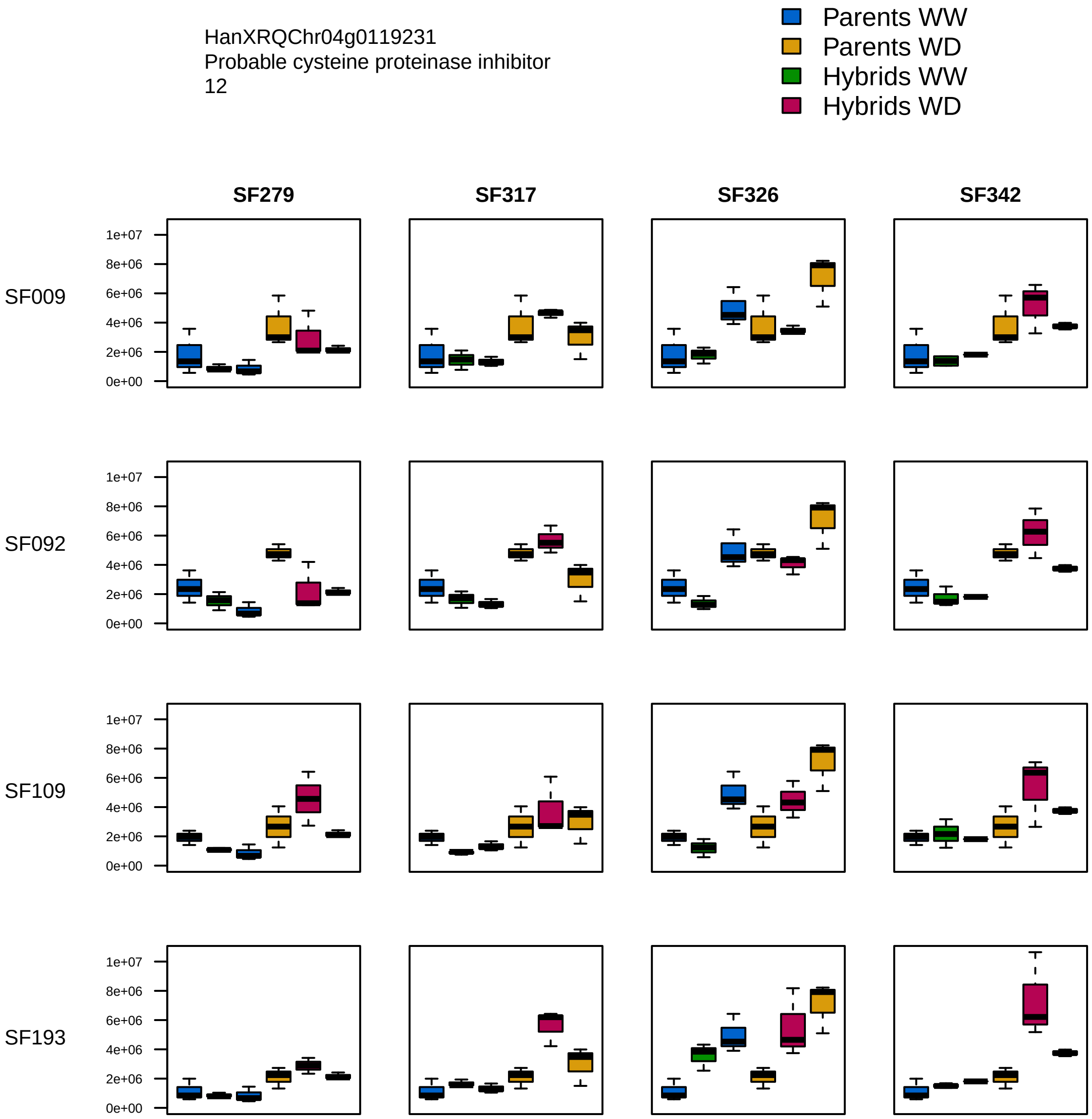
HanXRQChr04g0119231
Probable cysteine proteinase inhibitor
12
Parents WW
Parents WD
Hybrids WW
Hybrids WD
SF279
SF317
SF326
SF342
SF009
SF092
SF109
SF193
0e+00
2e+06
4e+06
6e+06
8e+06
1e+07

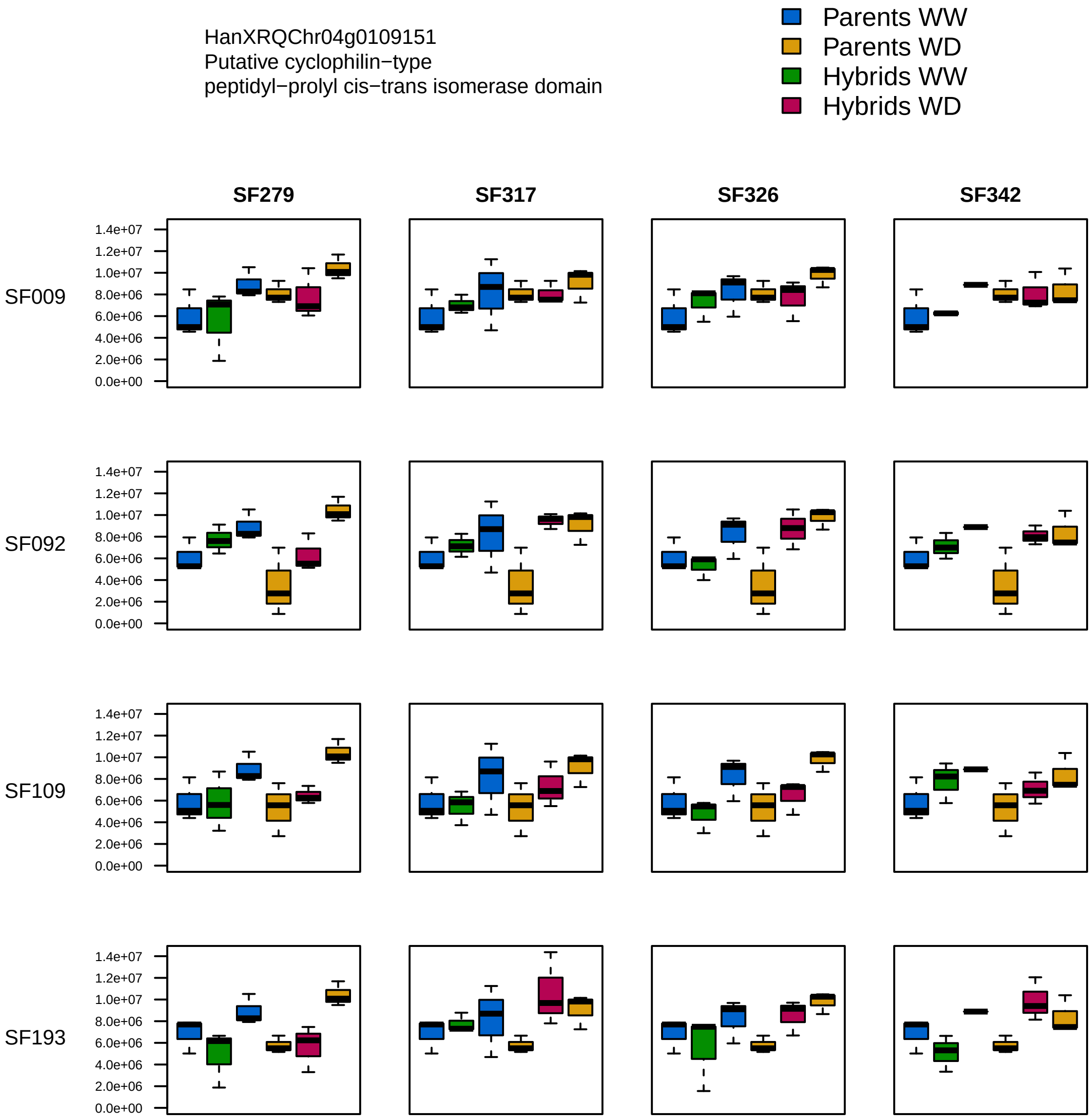
HanXRQChr04g0109151
Putative cyclophilin−type
peptidyl−prolyl cis−trans isomerase domain
Parents WW
Parents WD
Hybrids WW
Hybrids WD
SF279
SF317
SF326
SF342
SF009
SF092
SF109
SF193
1.4e+07
1.2e+07
1.0e+07
8.0e+06
6.0e+06
4.0e+06
2.0e+06
0.0e+00

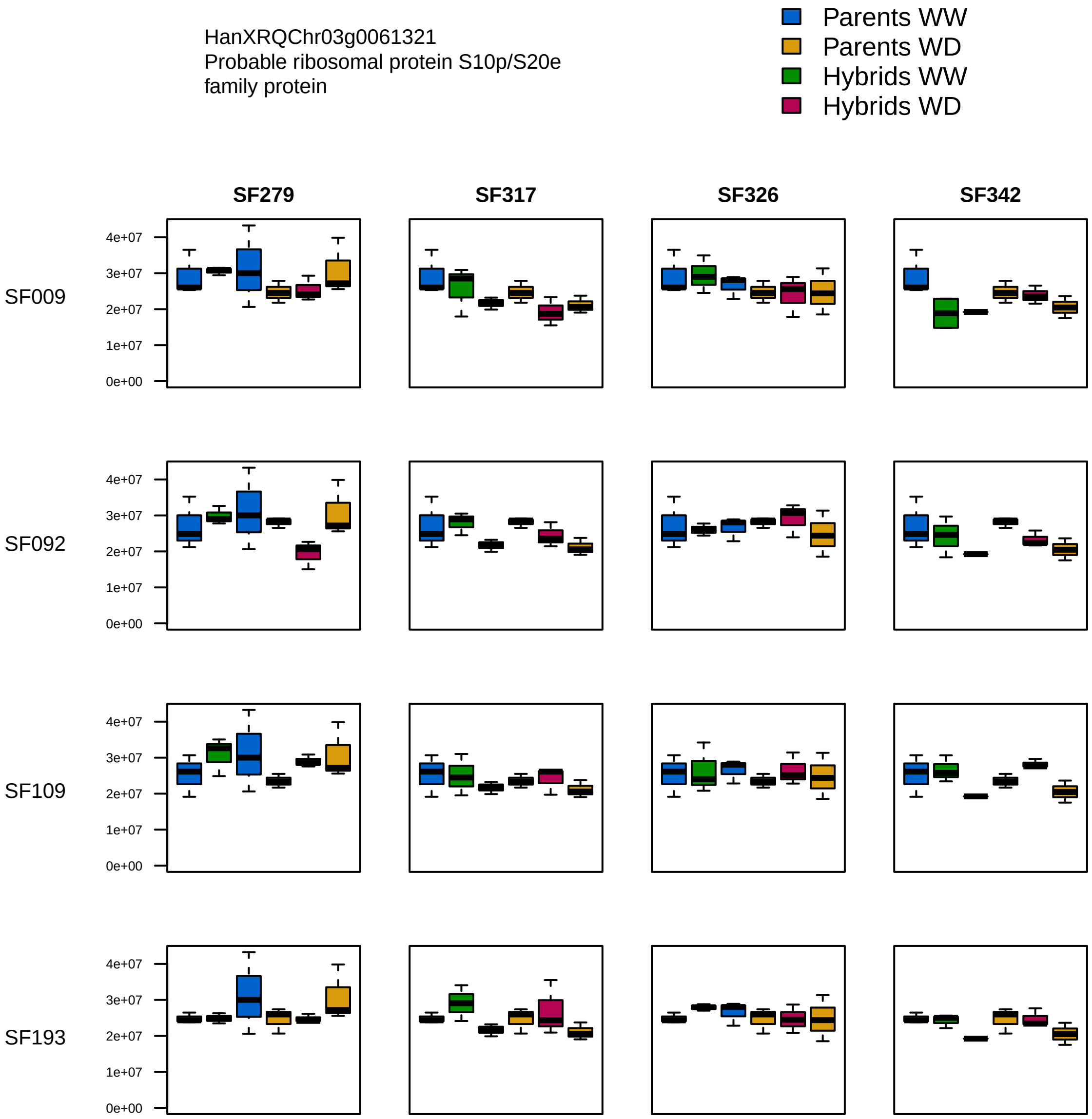
HanXRQChr03g0061321
Probable ribosomal protein S10p/S20e
family protein
Parents WW
Parents WD
Hybrids WW
Hybrids WD
SF279
SF317
SF326
SF342
SF009
SF092
SF109
SF193
0e+00
1e+07
2e+07
3e+07
4e+07

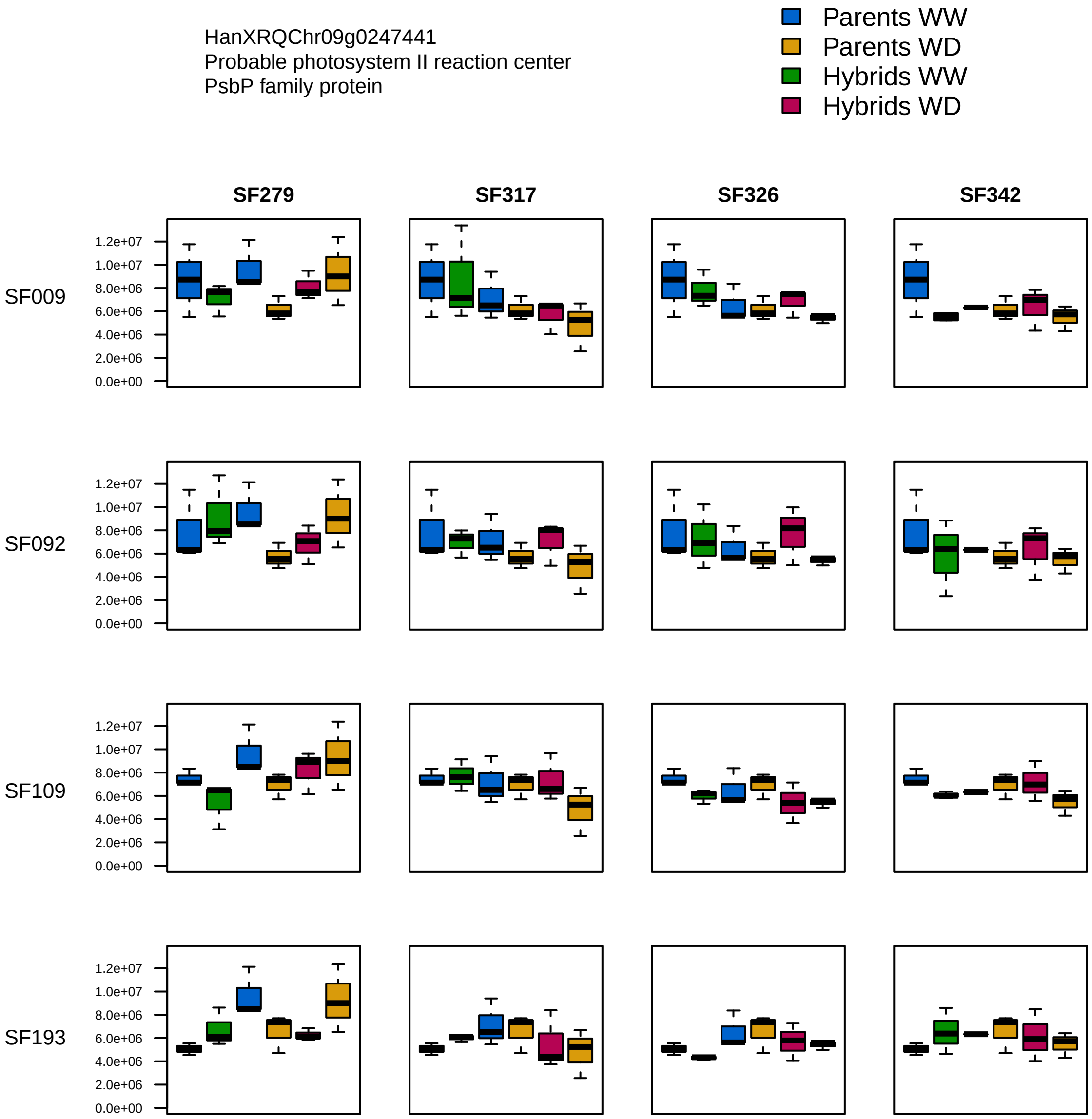
HanXRQChr09g0247441
Probable photosystem II reaction center
PsbP family protein
Parents WW
Parents WD
Hybrids WW
Hybrids WD
SF279
SF317
SF326
SF342
SF009
SF092
SF109
SF193
1.2e+07
1.0e+07
8.0e+06
6.0e+06
4.0e+06
2.0e+06
0.0e+00

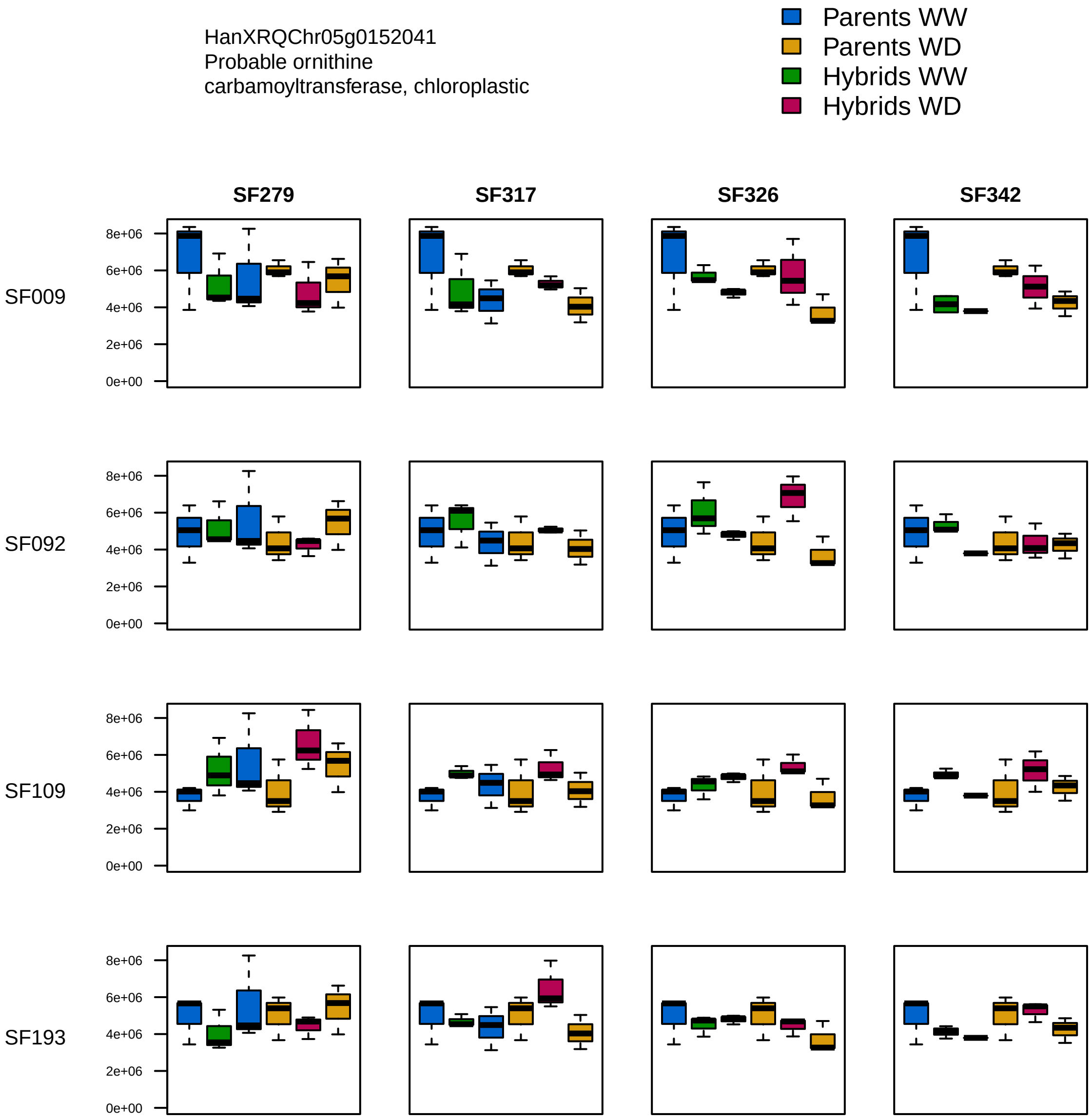
HanXRQChr05g0152041
Probable ornithine
carbamoyltransferase, chloroplastic
Parents WW
Parents WD
Hybrids WW
Hybrids WD
SF279
SF317
SF326
SF342
SF009
SF092
SF109
SF193
8e+06
6e+06
4e+06
2e+06
0e+00

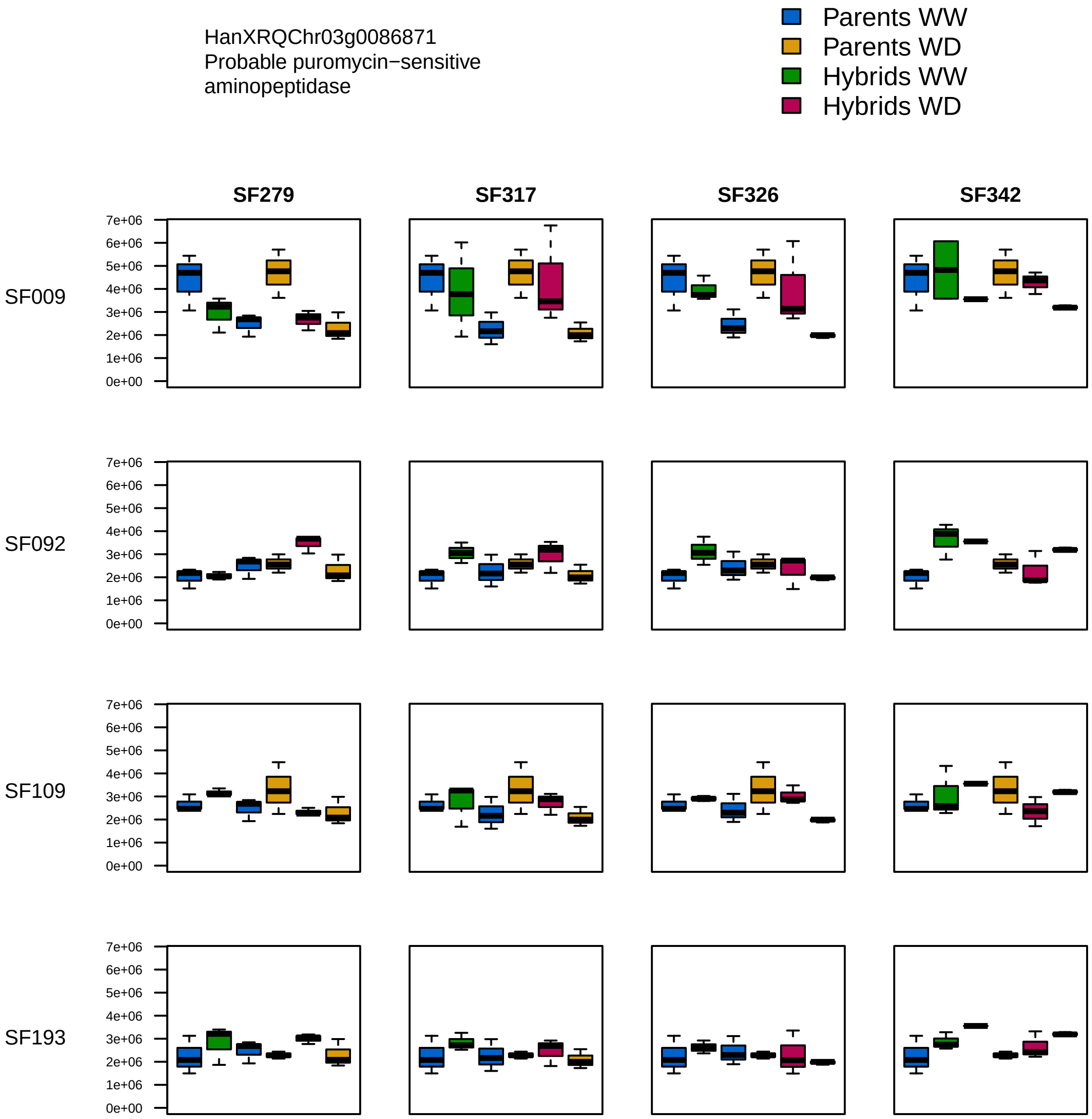

HanXRQChr03g0086871
Probable puromycin−sensitive
aminopeptidase
Parents WW
Parents WD
Hybrids WW
Hybrids WD
SF279
SF317
SF326
SF342
SF009
SF092
SF109
SF193
0e+00
1e+06
2e+06
3e+06
4e+06
5e+06
6e+06
7e+06

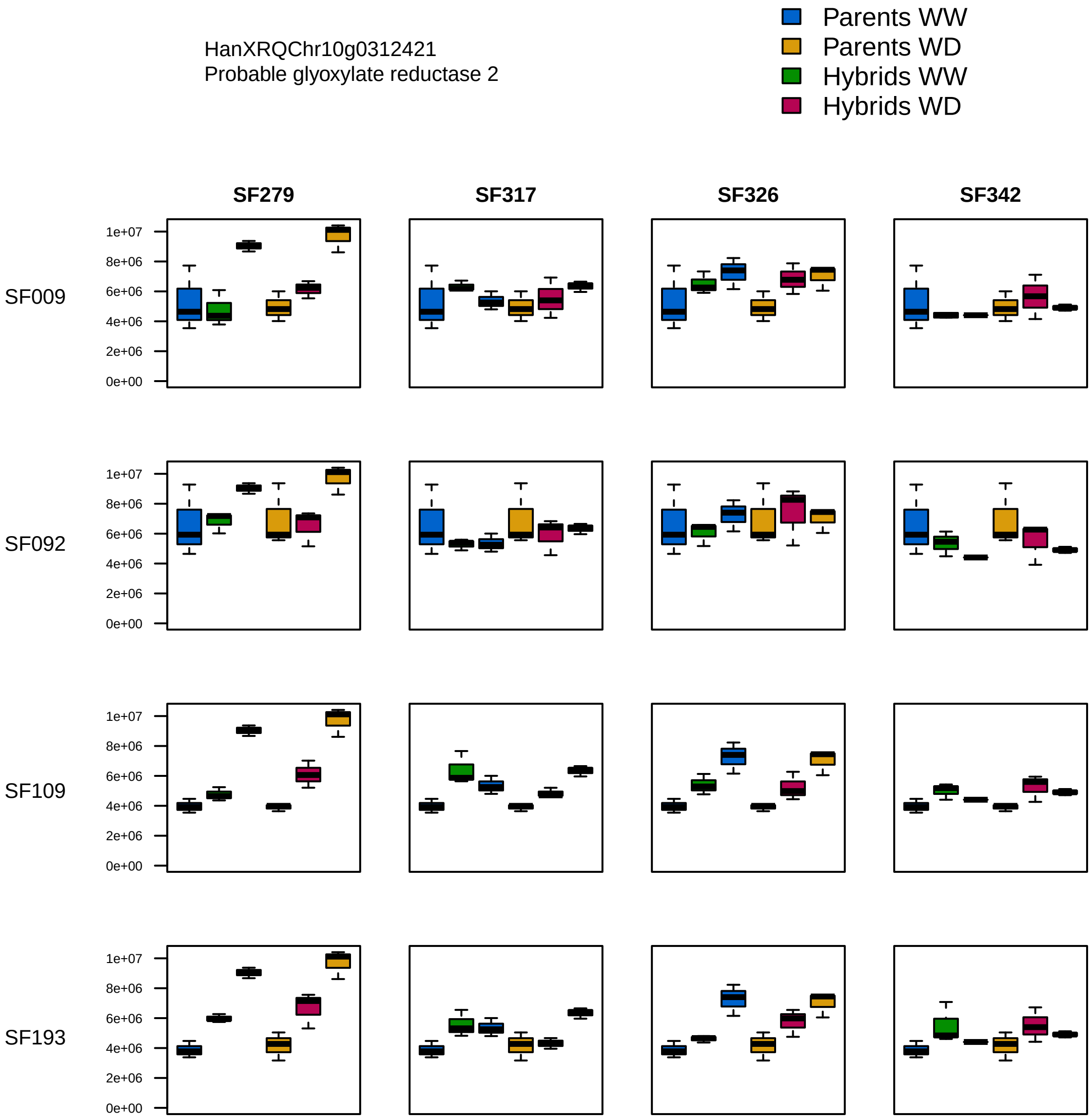

HanXRQChr10g0312421
Probable glyoxylate reductase 2
Parents WW
Parents WD
Hybrids WW
Hybrids WD
SF279
SF317
SF326
SF342
SF009
SF092
SF109
SF193
0e+00
2e+06
4e+06
6e+06
8e+06
1e+07

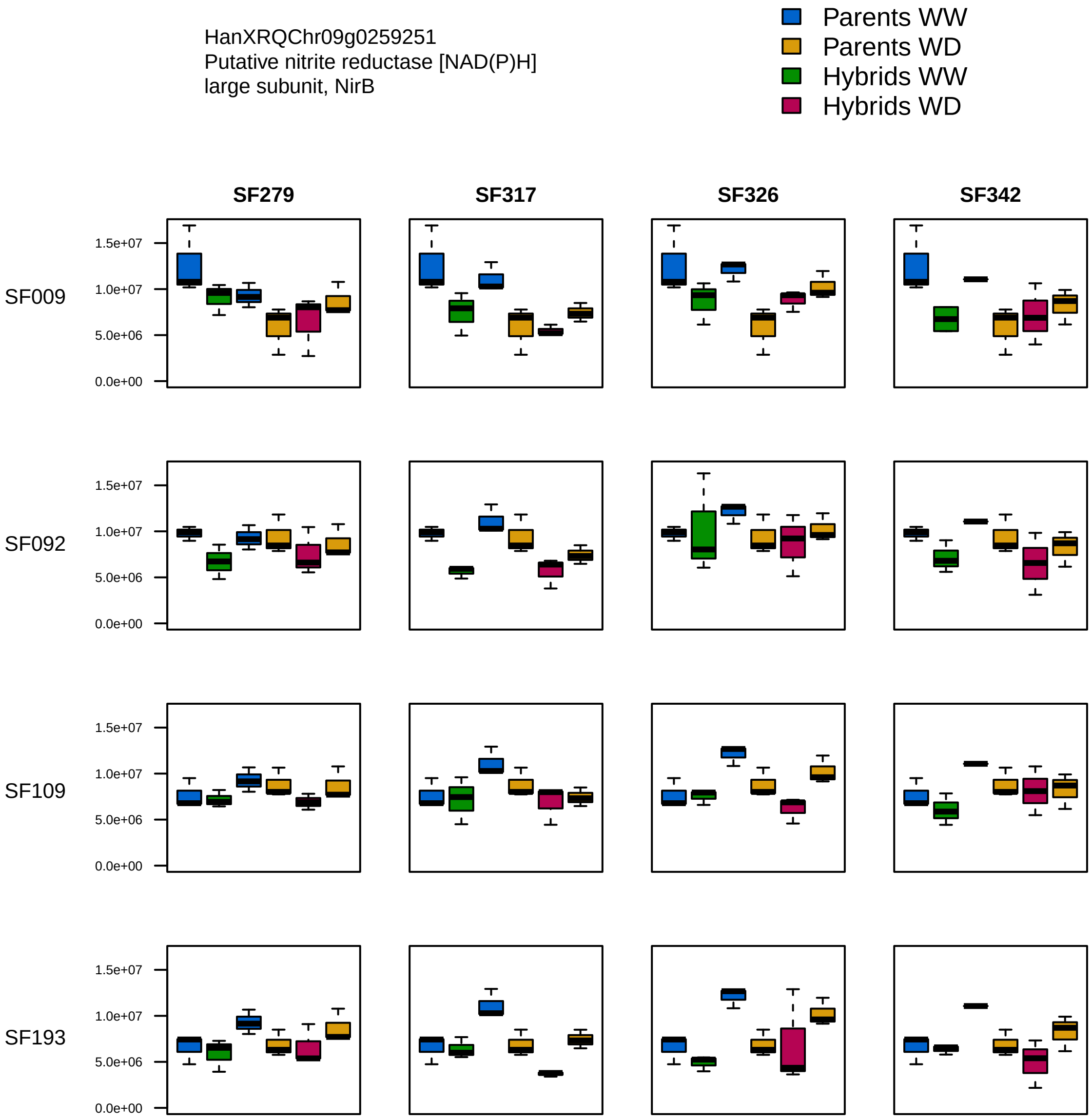

HanXRQChr09g0259251
Putative nitrite reductase [NAD(P)H]
large subunit, NirB
Parents WW
Parents WD
Hybrids WW
Hybrids WD
SF279
SF317
SF326
SF342
SF009
SF092
SF109
SF193
1.5e+07
1.0e+07
5.0e+06
0.0e+00

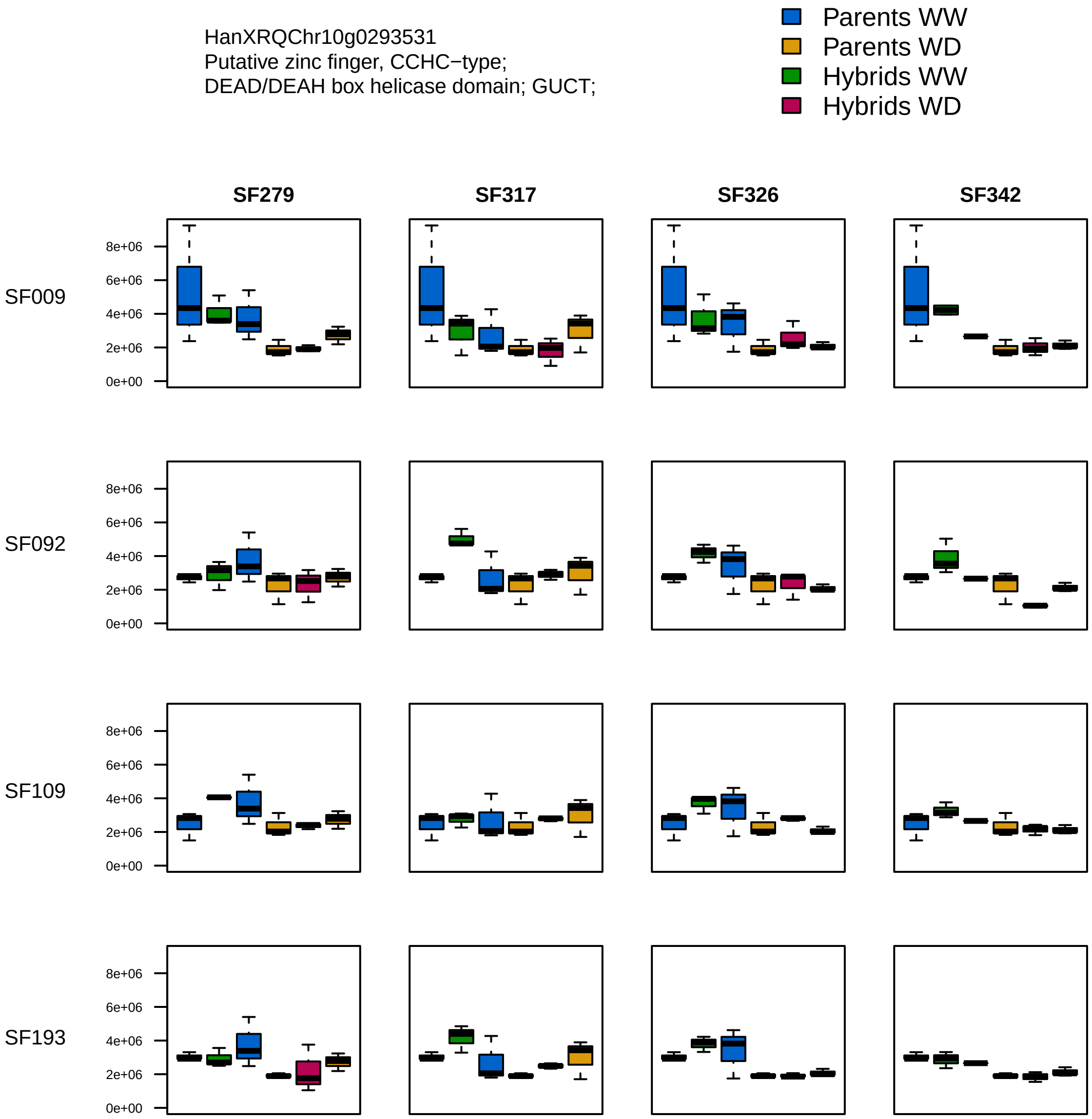

HanXRQChr10g0293531
Putative zinc finger, CCHC−type;
DEAD/DEAH box helicase domain; GUCT;
Parents WW
Parents WD
Hybrids WW
Hybrids WD
SF279
SF317
SF326
SF342
SF009
SF092
SF109
SF193
8e+06
6e+06
4e+06
2e+06
0e+00

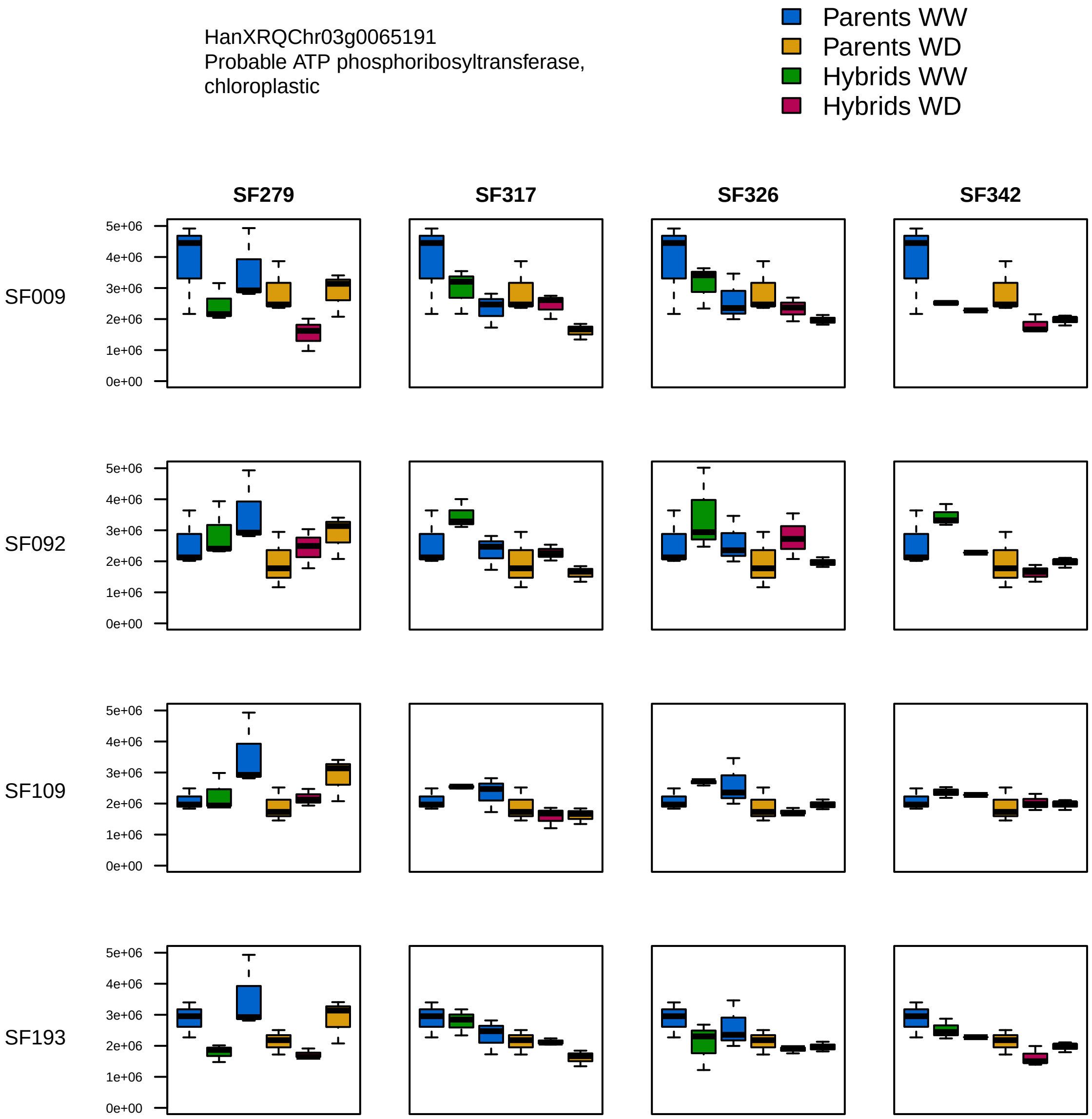

HanXRQChr03g0065191
Probable ATP phosphoribosyltransferase, chloroplastic
Parents WW
Parents WD
Hybrids WW
Hybrids WD
SF279
SF317
SF326
SF342
SF009
SF092
SF109
SF193
0e+00
1e+06
2e+06
3e+06
4e+06
5e+06

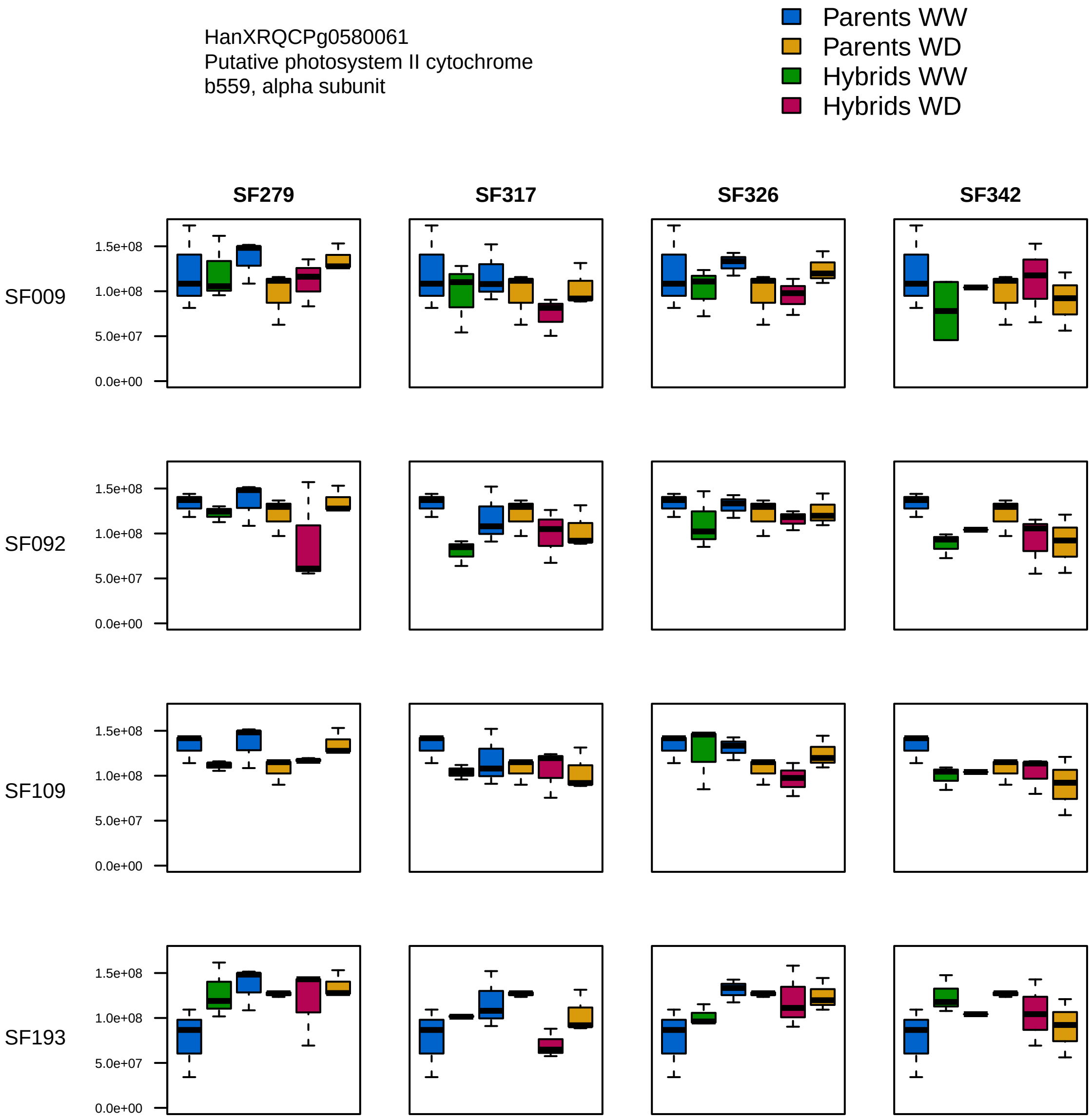
HanXRQCPg0580061
Putative photosystem II cytochrome
b559, alpha subunit
Parents WW
Parents WD
Hybrids WW
Hybrids WD
SF279
SF317
SF326
SF342
SF009
SF092
SF109
SF193
1.5e+08
1.0e+08
5.0e+07
0.0e+00

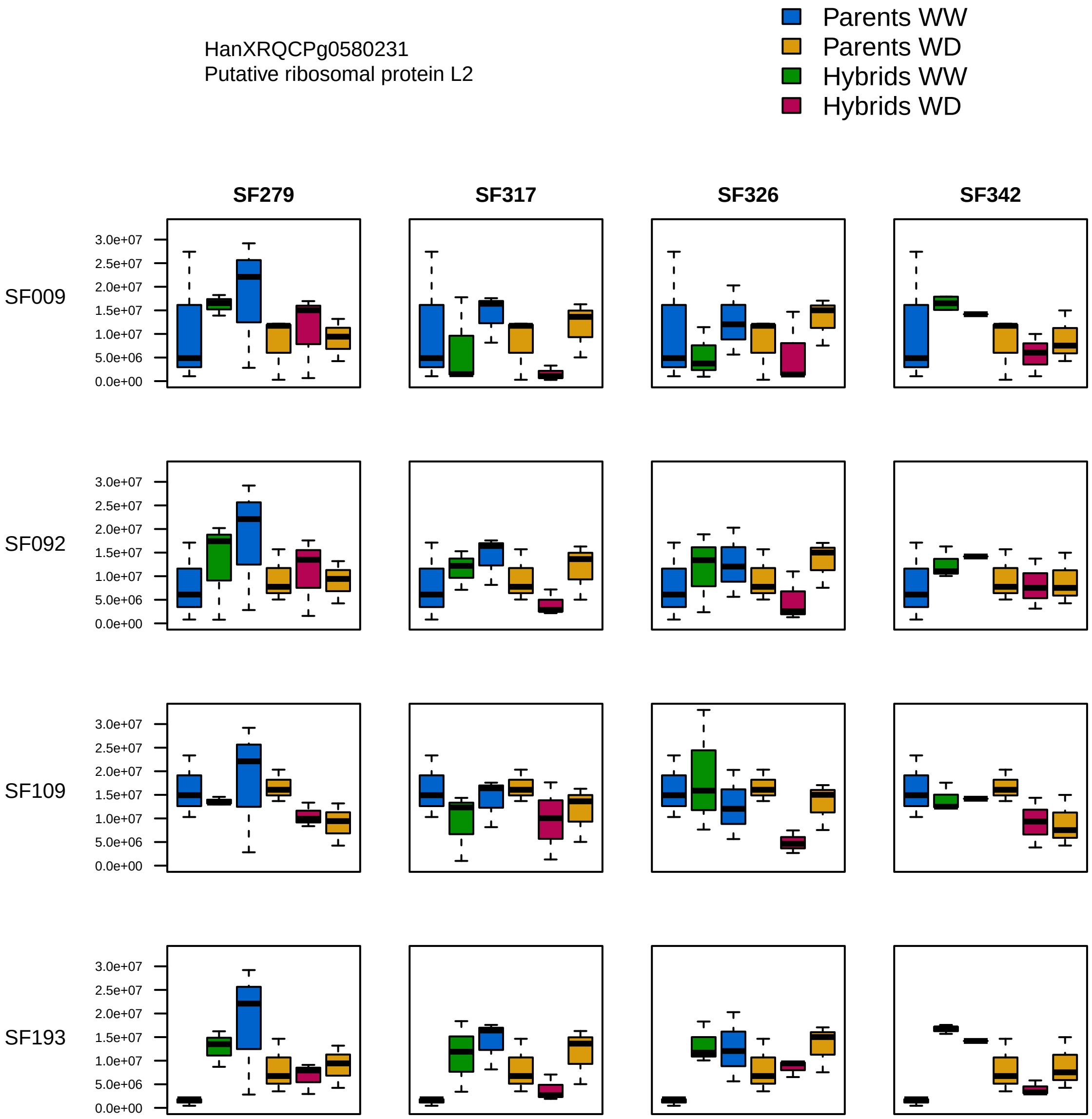
HanXRQCPg0580231
Putative ribosomal protein L2
Parents WW
Parents WD
Hybrids WW
Hybrids WD
SF279
SF317
SF326
SF342
SF009
SF092
SF109
SF193
3.0e+07
2.5e+07
2.0e+07
1.5e+07
1.0e+07
5.0e+06
0.0e+00

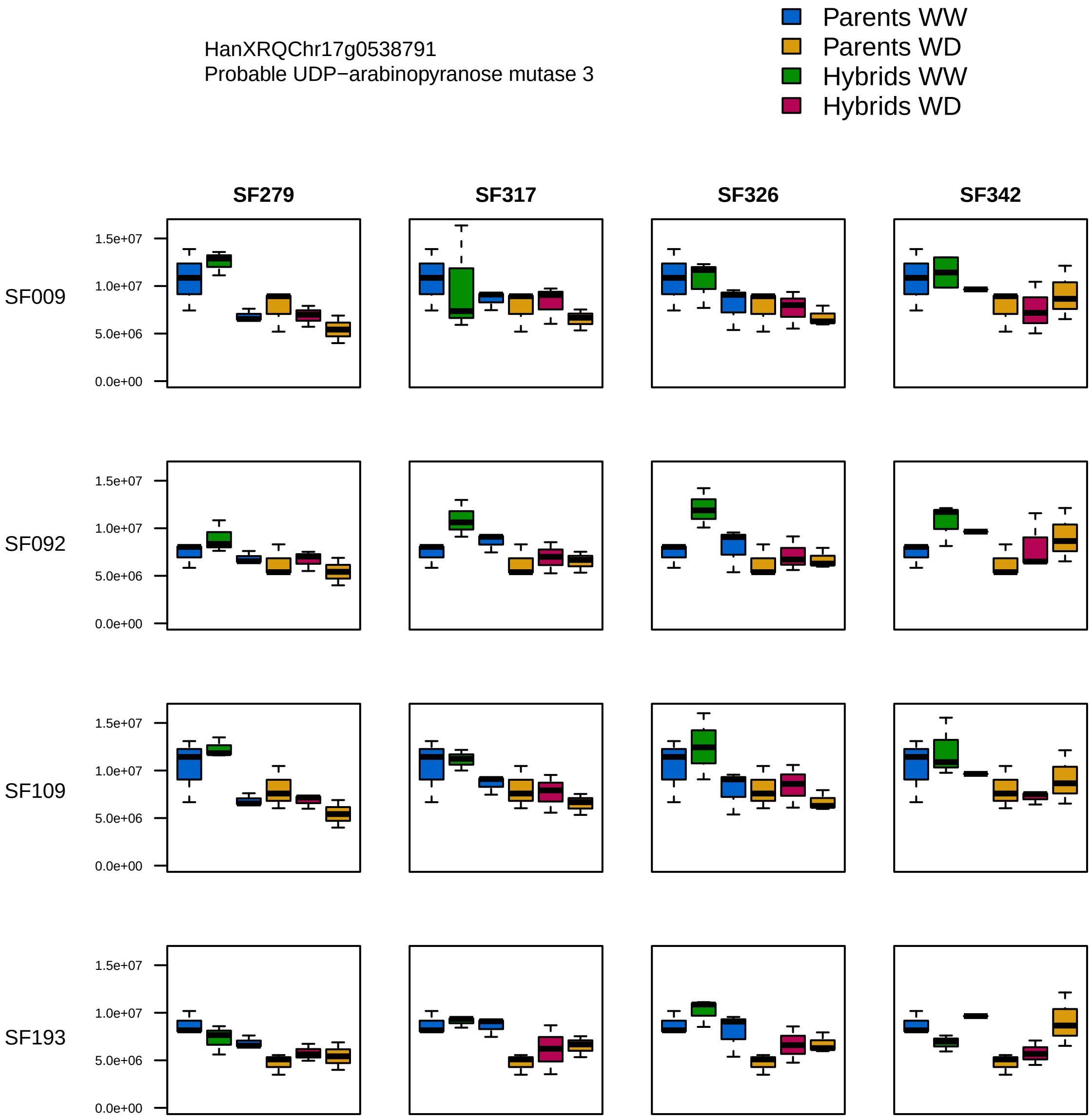
HanXRQChr17g0538791
Probable UDP−arabinopyranose mutase 3
Parents WW
Parents WD
Hybrids WW
Hybrids WD
SF279
SF317
SF326
SF342
SF009
SF092
SF109
SF193
1.5e+07
1.0e+07
5.0e+06
0.0e+00

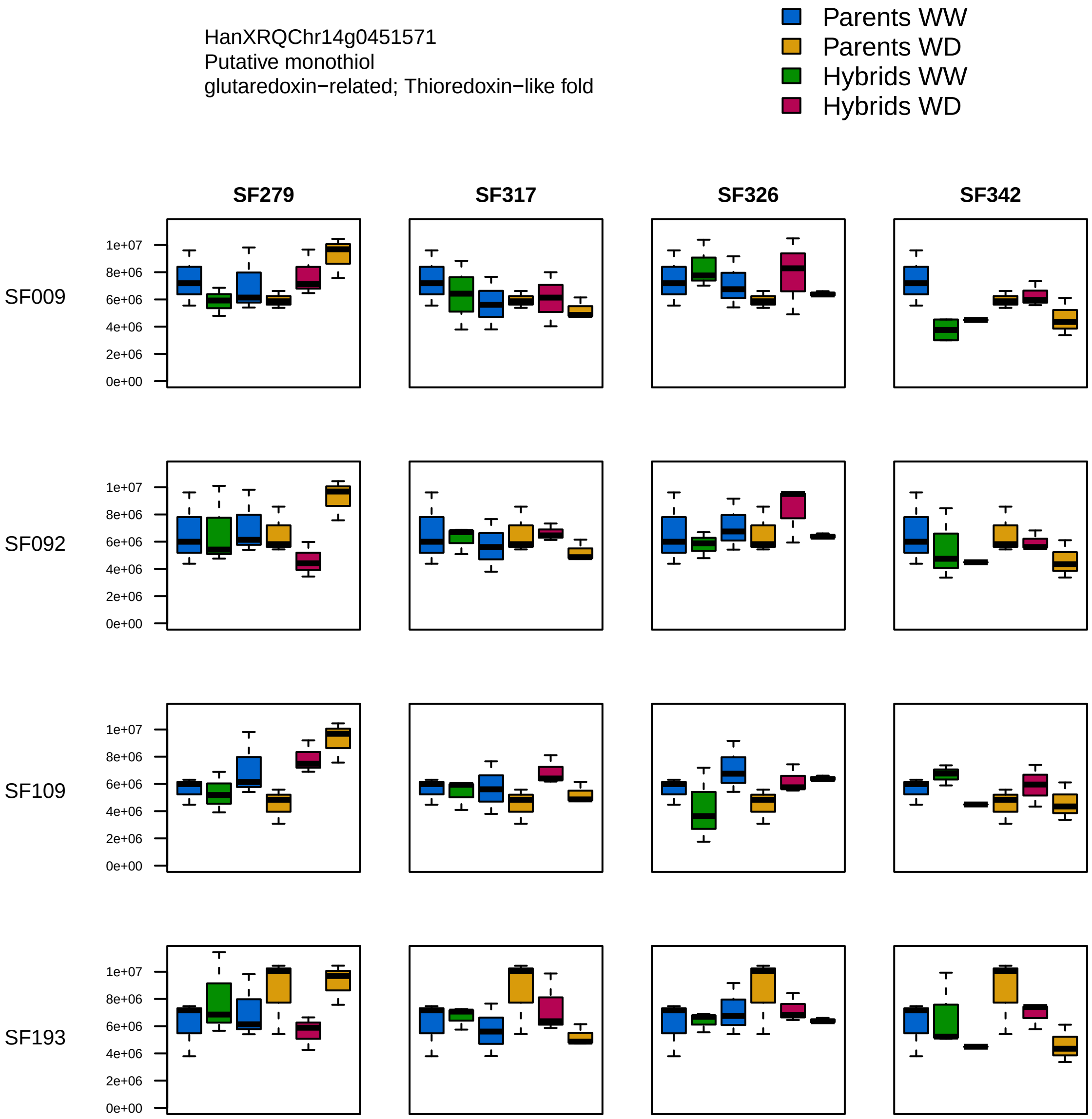
HanXRQChr14g0451571
Putative monothiol
glutaredoxin−related; Thioredoxin−like fold
Parents WW
Parents WD
Hybrids WW
Hybrids WD
SF279
SF317
SF326
SF342
SF009
SF092
SF109
SF193
1e+07
8e+06
6e+06
4e+06
2e+06
0e+00

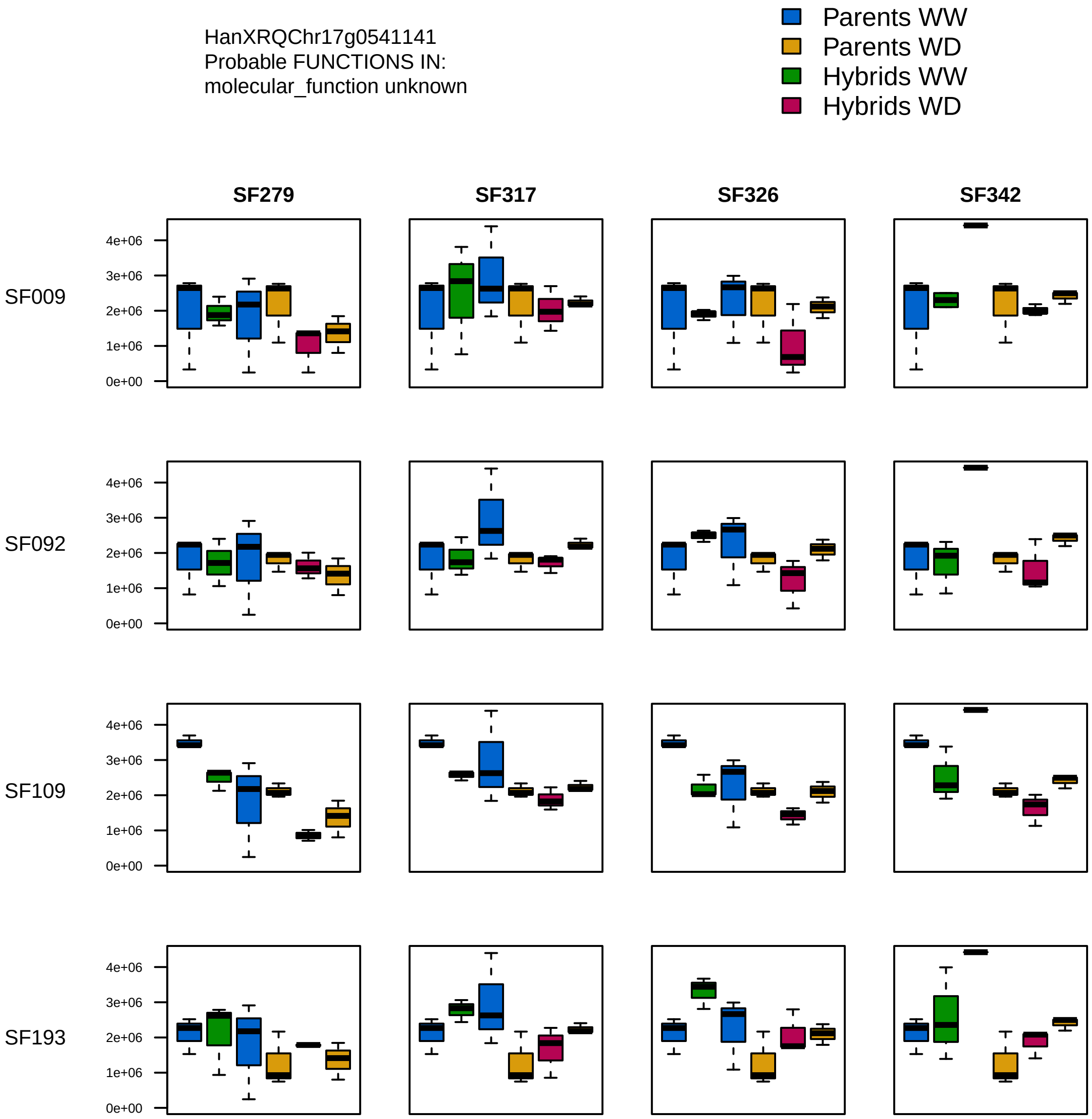

HanXRQChr17g0541141
Probable FUNCTIONS IN:
molecular_function unknown
Parents WW
Parents WD
Hybrids WW
Hybrids WD
SF279
SF317
SF326
SF342
SF009
SF092
SF109
SF193
0e+00
1e+06
2e+06
3e+06
4e+06

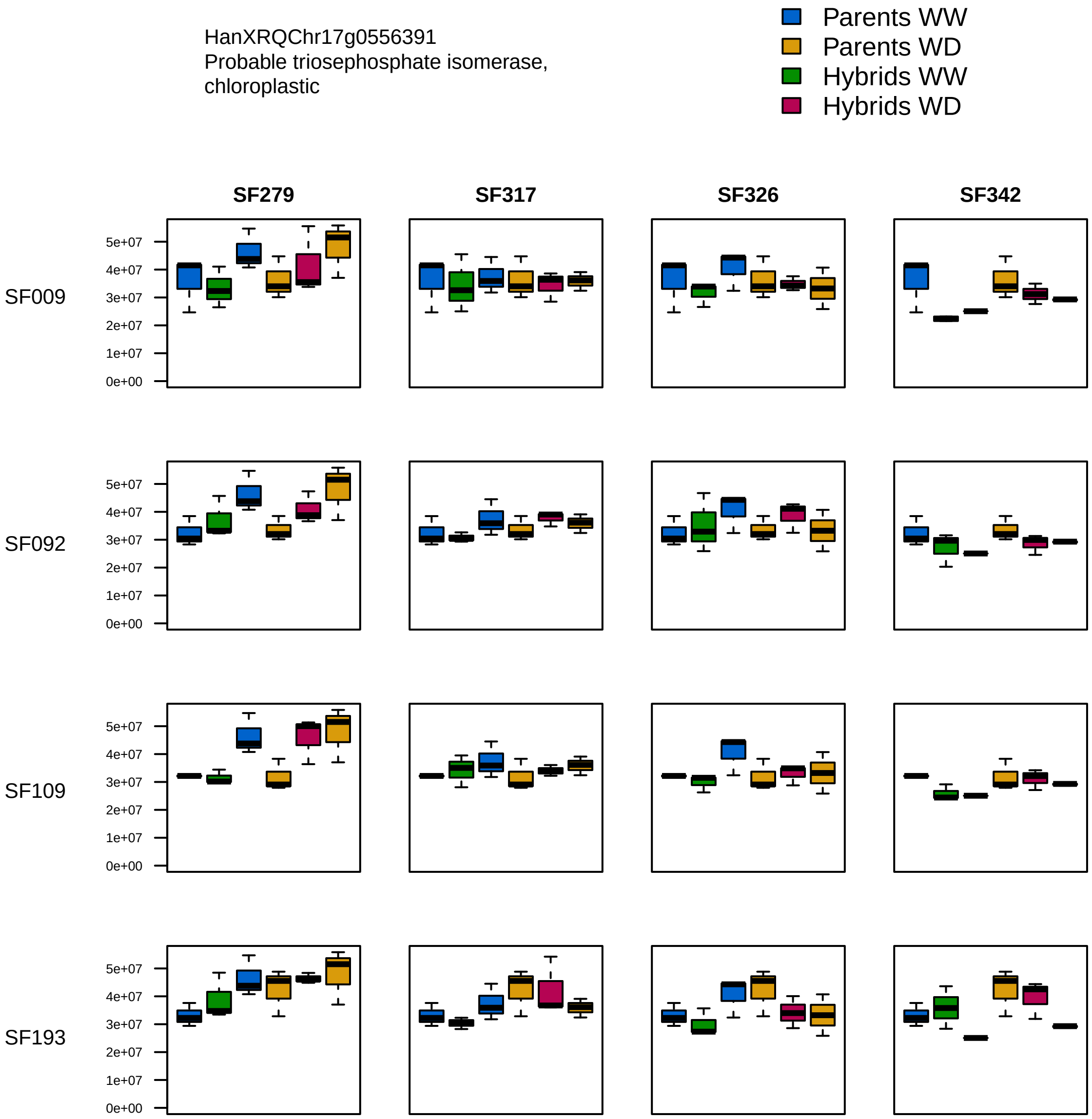
HanXRQChr17g0556391
Probable triosephosphate isomerase, chloroplastic
Parents WW
Parents WD
Hybrids WW
Hybrids WD
SF279
SF317
SF326
SF342
SF009
SF092
SF109
SF193
0e+00
1e+07
2e+07
3e+07
4e+07
5e+07

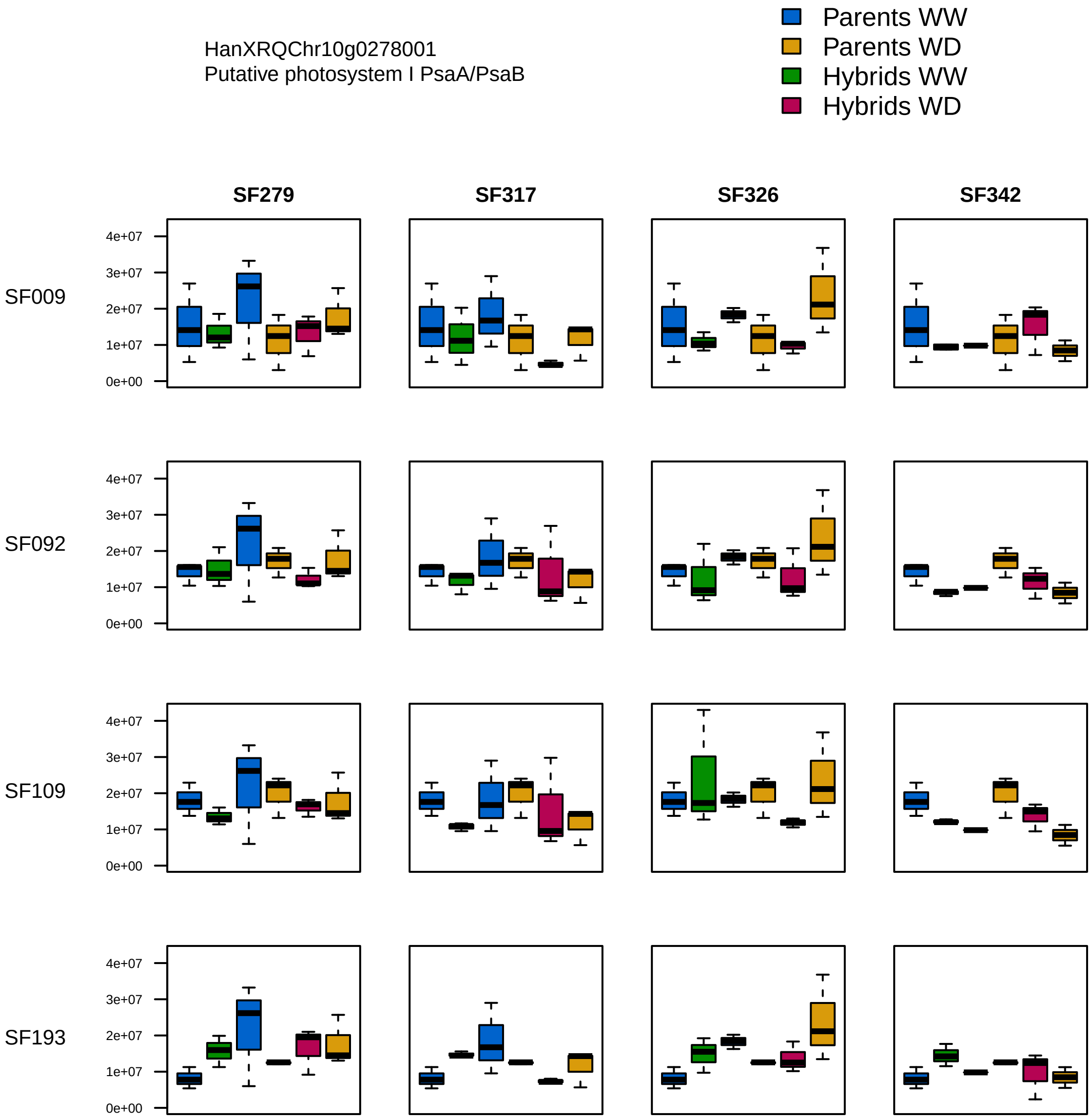
HanXRQChr10g0278001
Putative photosystem I PsaA/PsaB
Parents WW
Parents WD
Hybrids WW
Hybrids WD
SF279
SF317
SF326
SF342
SF009
SF092
SF109
SF193
4e+07
3e+07
2e+07
1e+07
0e+00

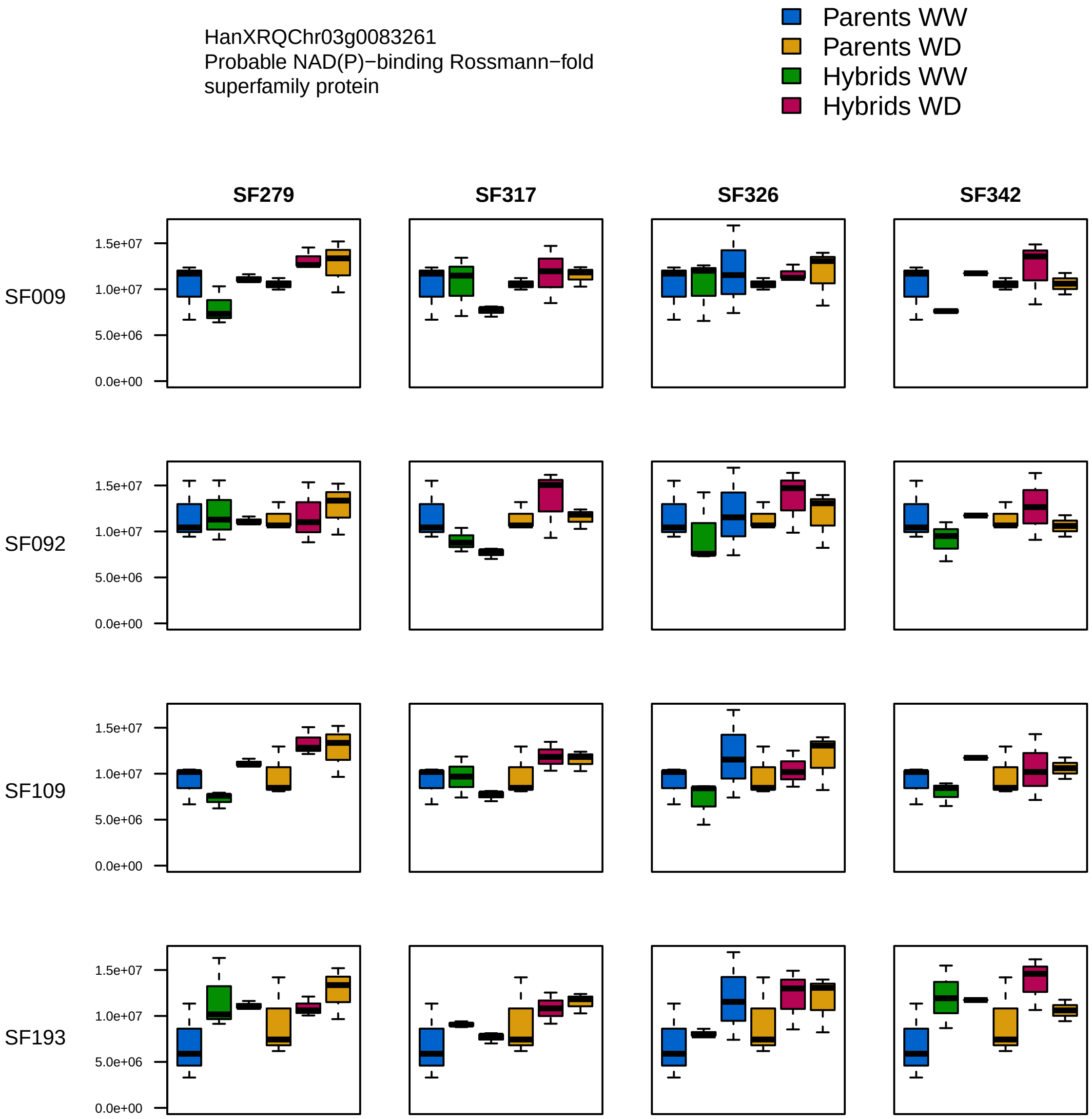
HanXRQChr03g0083261
Probable NAD(P)−binding Rossmann−fold
superfamily protein
Parents WW
Parents WD
Hybrids WW
Hybrids WD
SF279
SF317
SF326
SF342
SF009
SF092
SF109
SF193
1.5e+07
1.0e+07
5.0e+06
0.0e+00

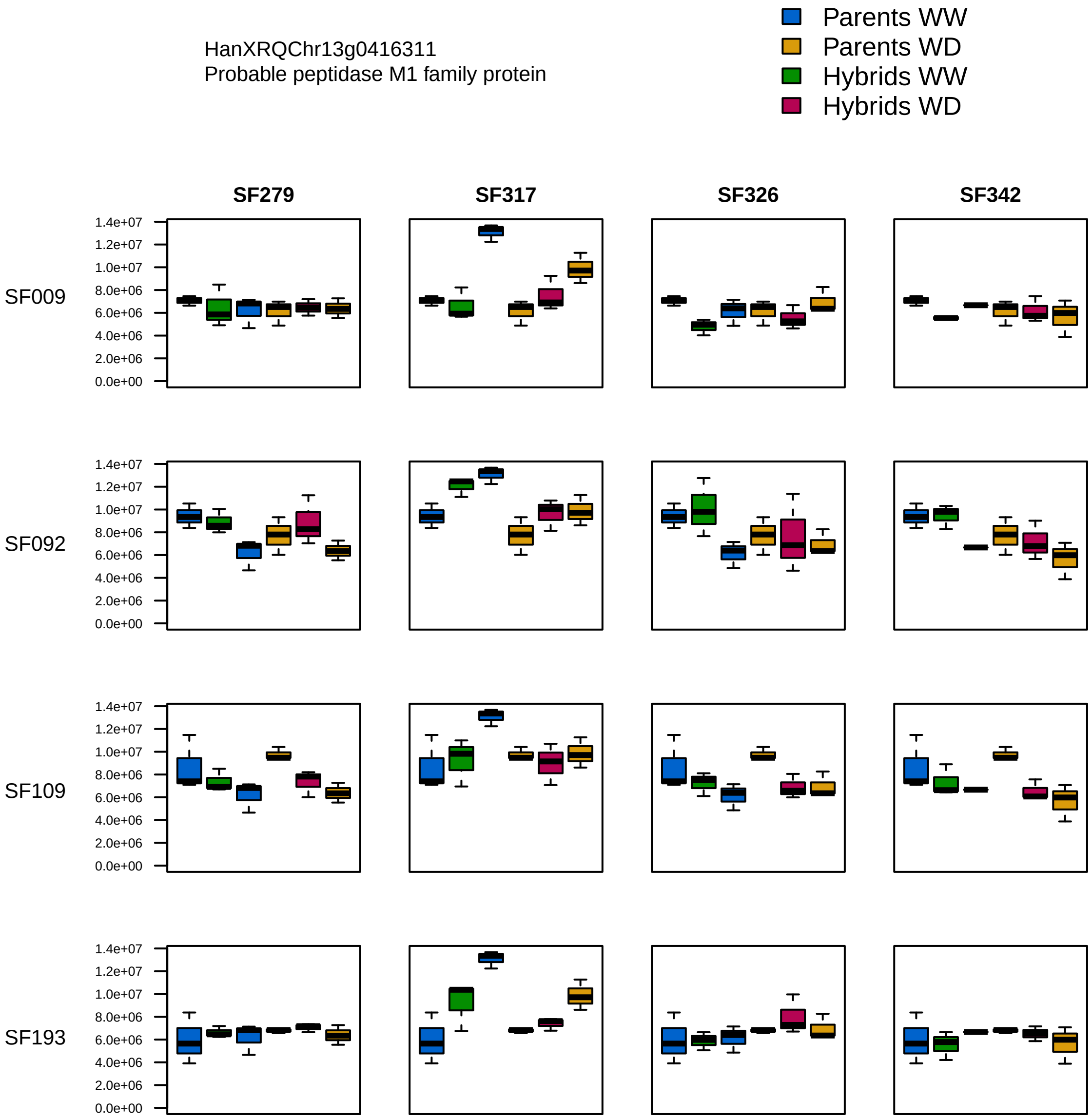

HanXRQChr13g0416311
Probable peptidase M1 family protein
Parents WW
Parents WD
Hybrids WW
Hybrids WD
SF279
SF317
SF326
SF342
SF009
SF092
SF109
SF193
1.4e+07
1.2e+07
1.0e+07
8.0e+06
6.0e+06
4.0e+06
2.0e+06
0.0e+00

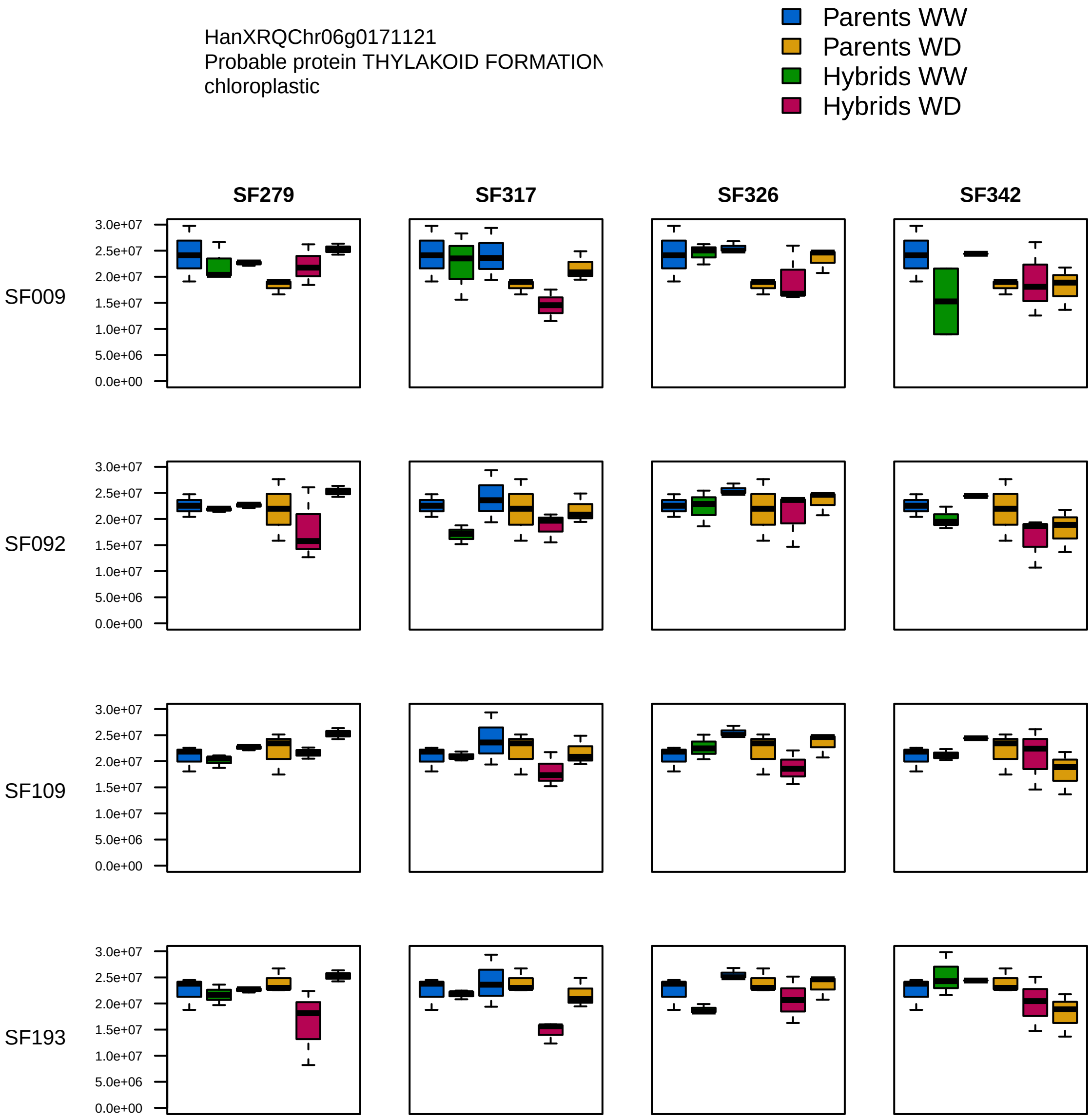

HanXRQChr06g0171121
Probable protein THYLAKOID FORMATION
chloroplastic
Parents WW
Parents WD
Hybrids WW
Hybrids WD
SF279
SF317
SF326
SF342
SF009
SF092
SF109
SF193
3.0e+07
2.5e+07
2.0e+07
1.5e+07
1.0e+07
5.0e+06
0.0e+00

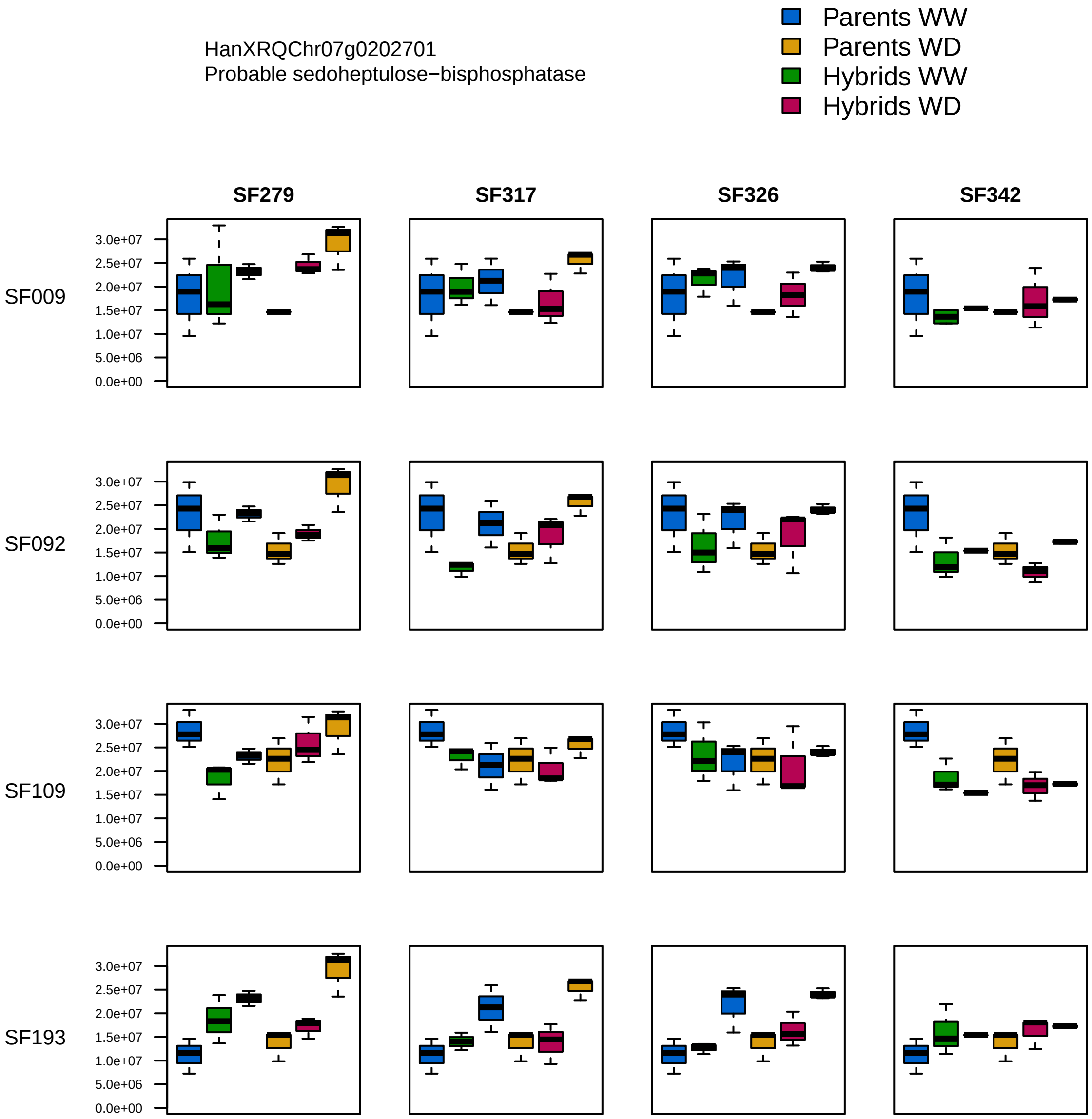
HanXRQChr07g0202701
Probable sedoheptulose−bisphosphatase
Parents WW
Parents WD
Hybrids WW
Hybrids WD
SF279
SF317
SF326
SF342
SF009
SF092
SF109
SF193
0.0e+00
5.0e+06
1.0e+07
1.5e+07
2.0e+07
2.5e+07
3.0e+07

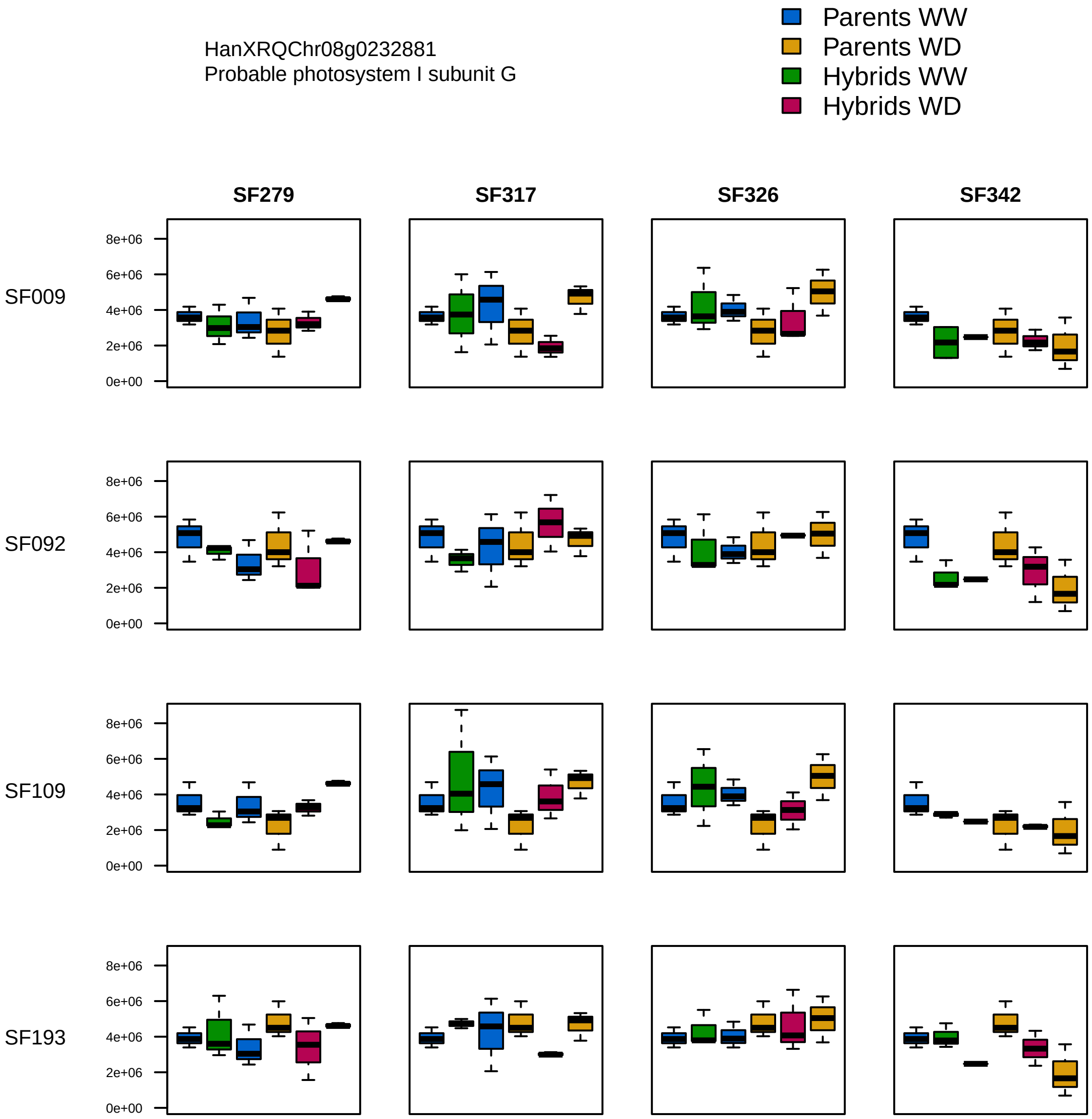
HanXRQChr08g0232881
Probable photosystem I subunit G
Parents WW
Parents WD
Hybrids WW
Hybrids WD
SF279
SF317
SF326
SF342
SF009
SF092
SF109
SF193
8e+06
6e+06
4e+06
2e+06
0e+00

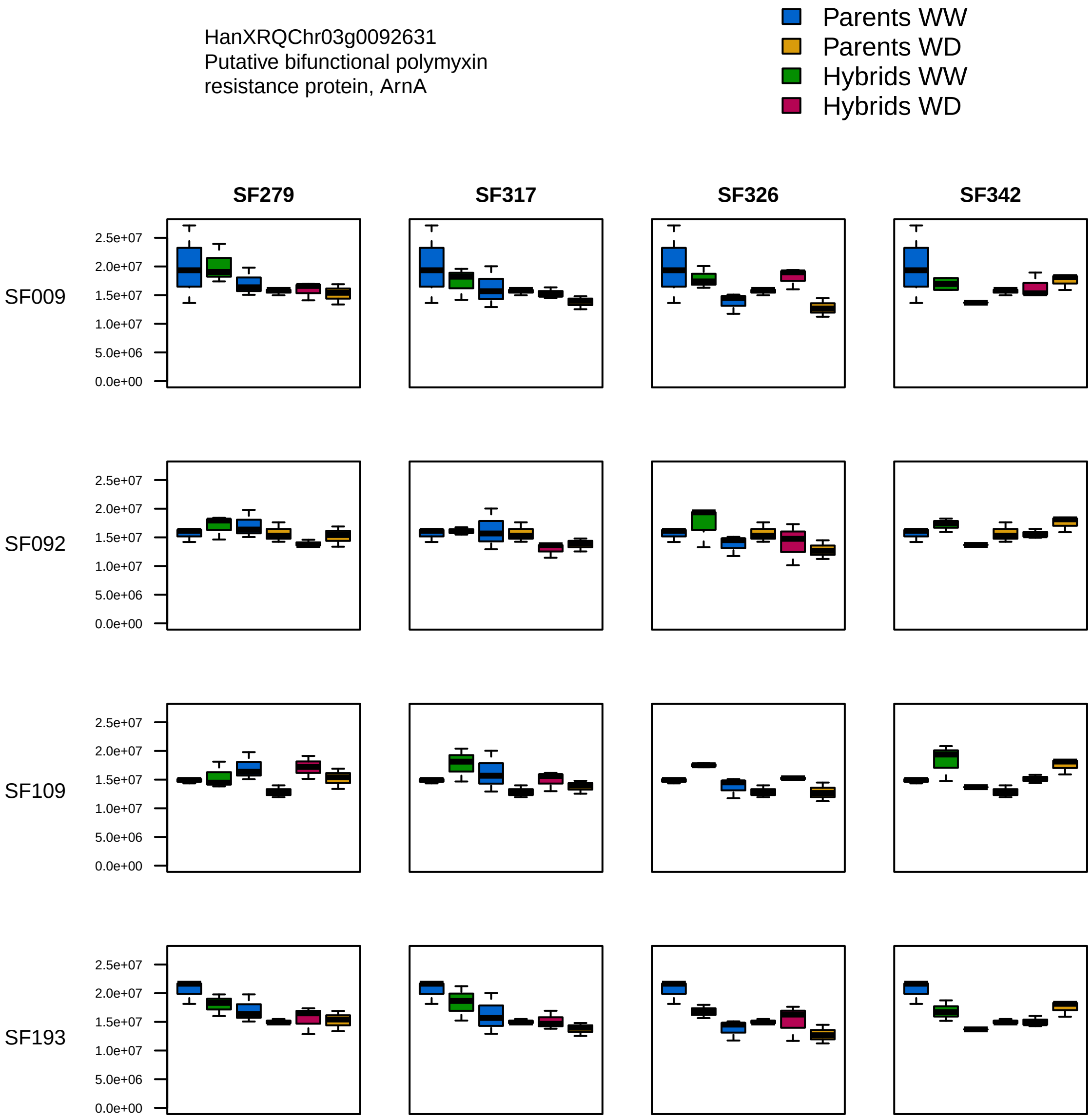

HanXRQChr03g0092631
Putative bifunctional polymyxin
resistance protein, ArnA
Parents WW
Parents WD
Hybrids WW
Hybrids WD
SF279
SF317
SF326
SF342
SF009
SF092
SF109
SF193
2.5e+07
2.0e+07
1.5e+07
1.0e+07
5.0e+06
0.0e+00

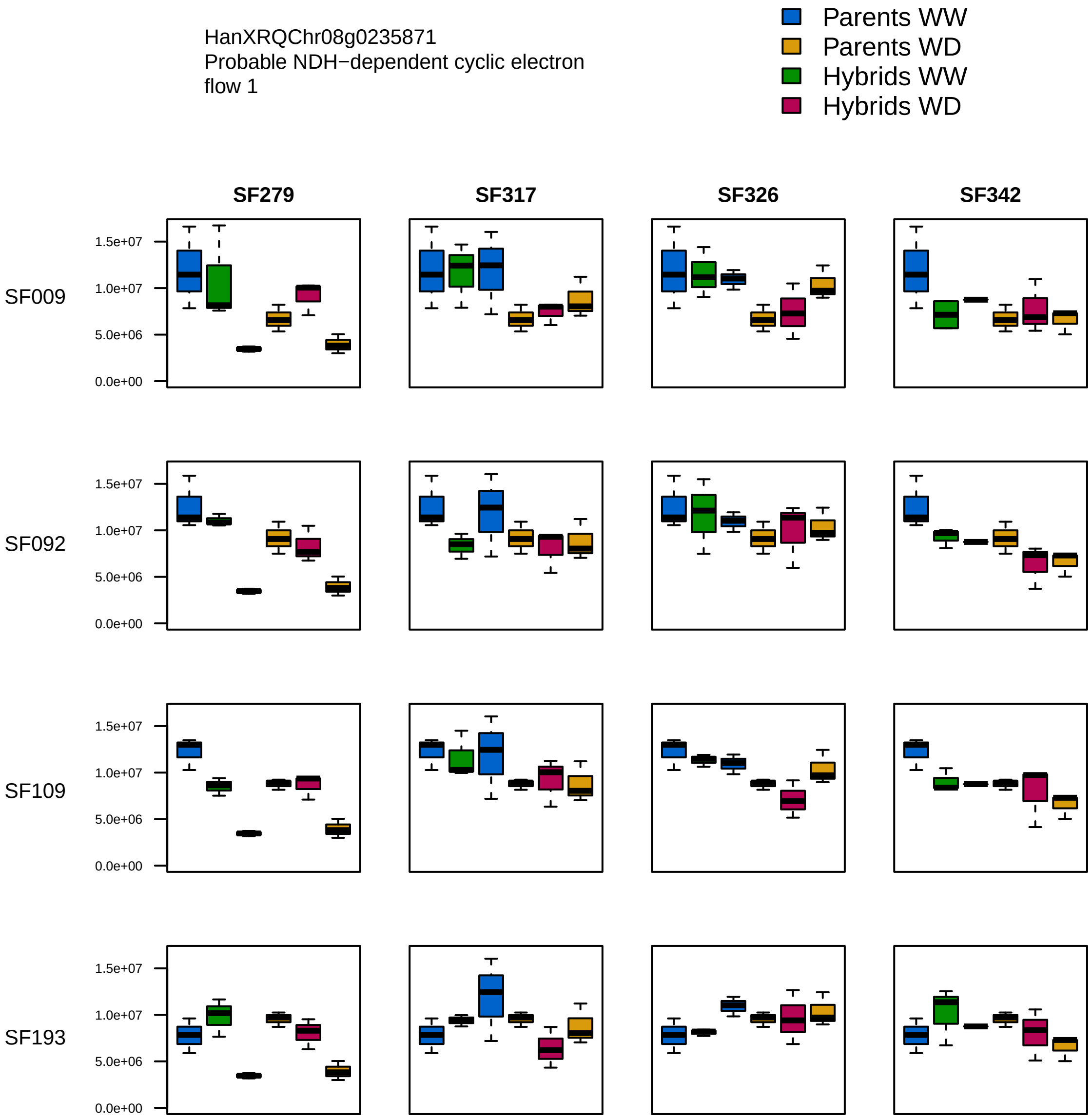

HanXRQChr08g0235871
Probable NDH−dependent cyclic electron flow 1
Parents WW
Parents WD
Hybrids WW
Hybrids WD
SF279
SF317
SF326
SF342
SF009
SF092
SF109
SF193
0.0e+00
5.0e+06
1.0e+07
1.5e+07

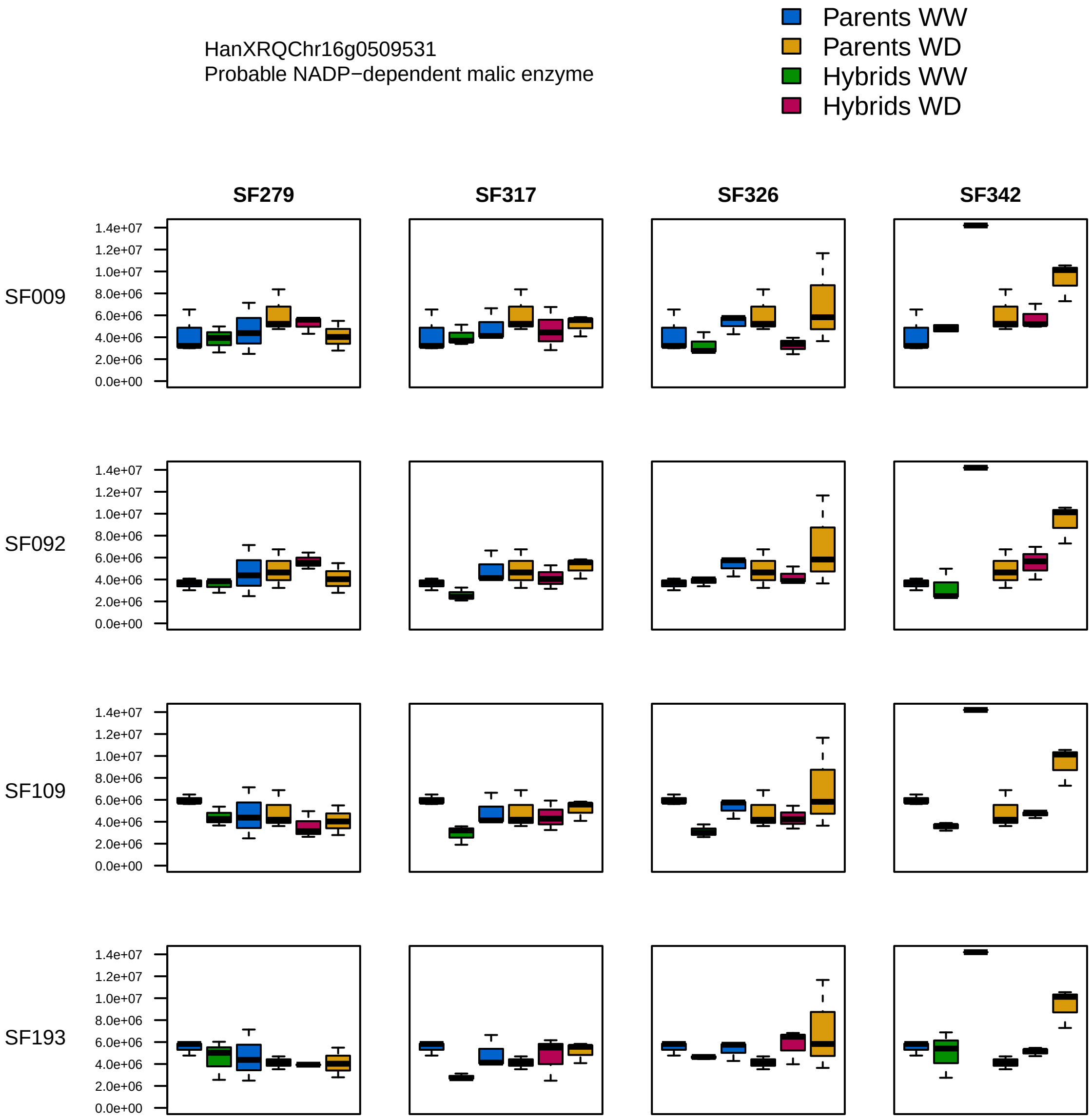

HanXRQChr16g0509531
Probable NADP−dependent malic enzyme
Parents WW
Parents WD
Hybrids WW
Hybrids WD
SF279
SF317
SF326
SF342
SF009
SF092
SF109
SF193
1.4e+07
1.2e+07
1.0e+07
8.0e+06
6.0e+06
4.0e+06
2.0e+06
0.0e+00

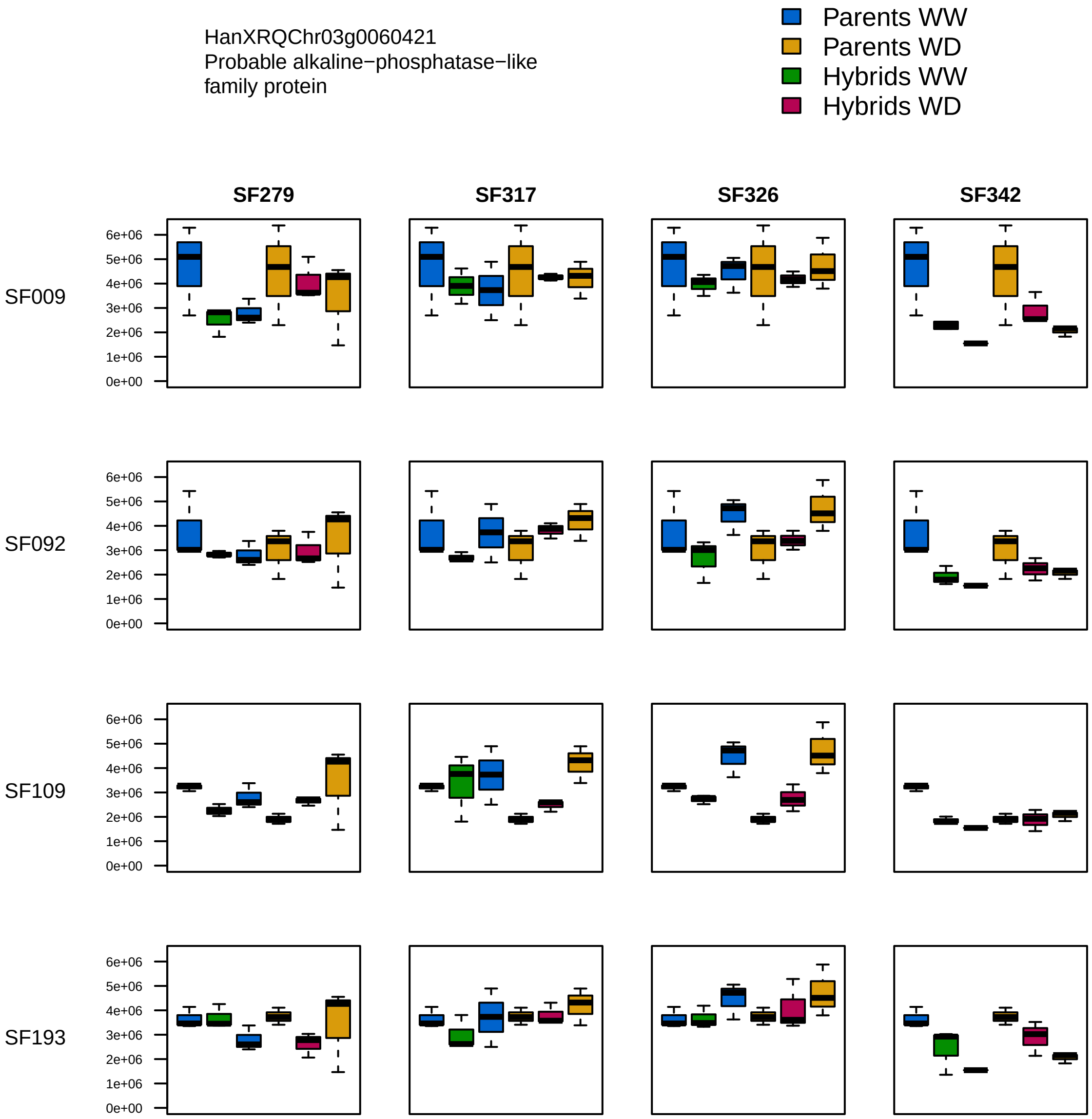
HanXRQChr03g0060421
Probable alkaline−phosphatase−like
family protein
Parents WW
Parents WD
Hybrids WW
Hybrids WD
SF279
SF317
SF326
SF342
SF009
SF092
SF109
SF193
6e+06
5e+06
4e+06
3e+06
2e+06
1e+06
0e+00

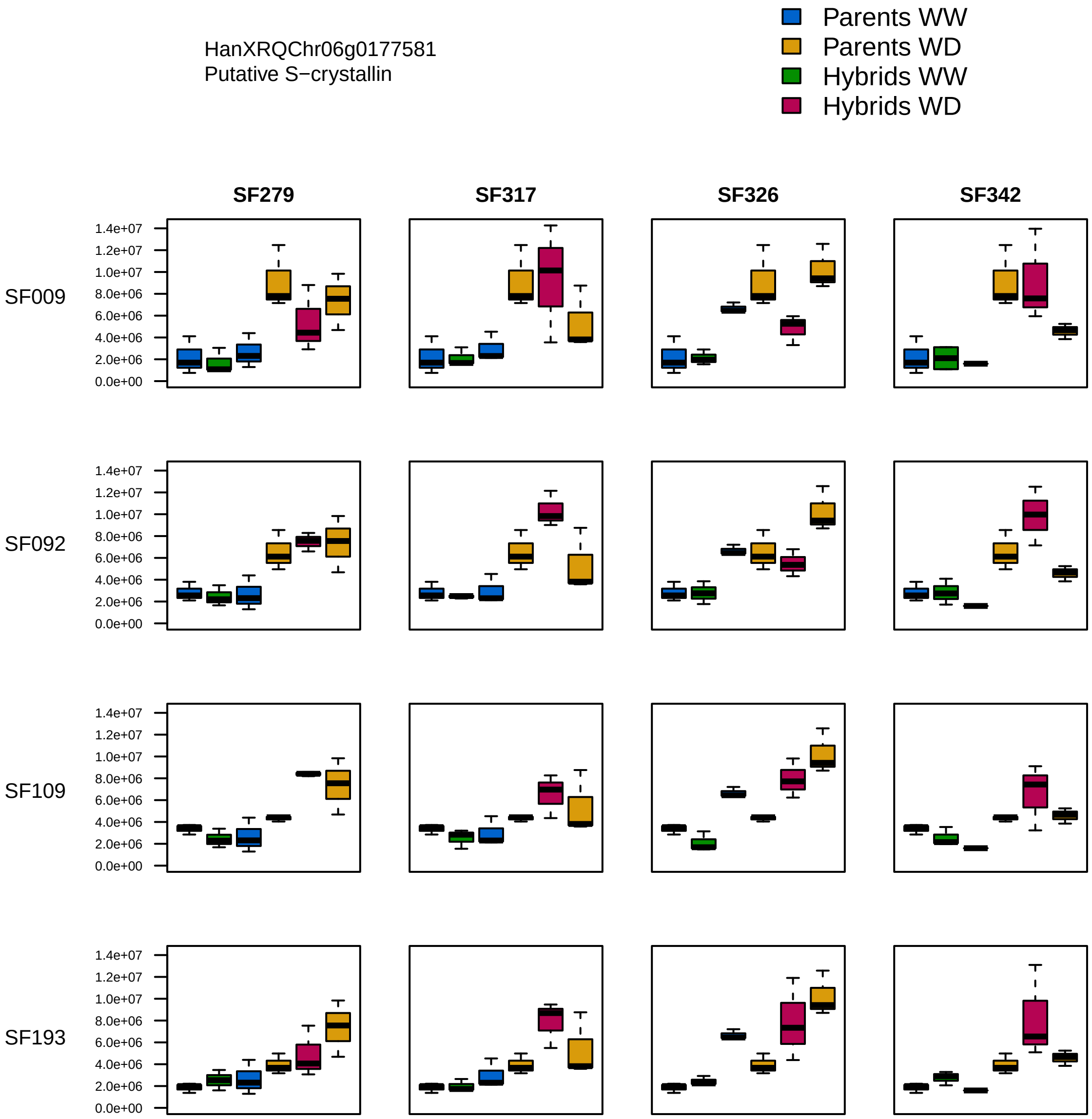
HanXRQChr06g0177581
Putative S−crystallin
Parents WW
Parents WD
Hybrids WW
Hybrids WD
SF279
SF317
SF326
SF342
SF009
SF092
SF109
SF193
1.4e+07
1.2e+07
1.0e+07
8.0e+06
6.0e+06
4.0e+06
2.0e+06
0.0e+00

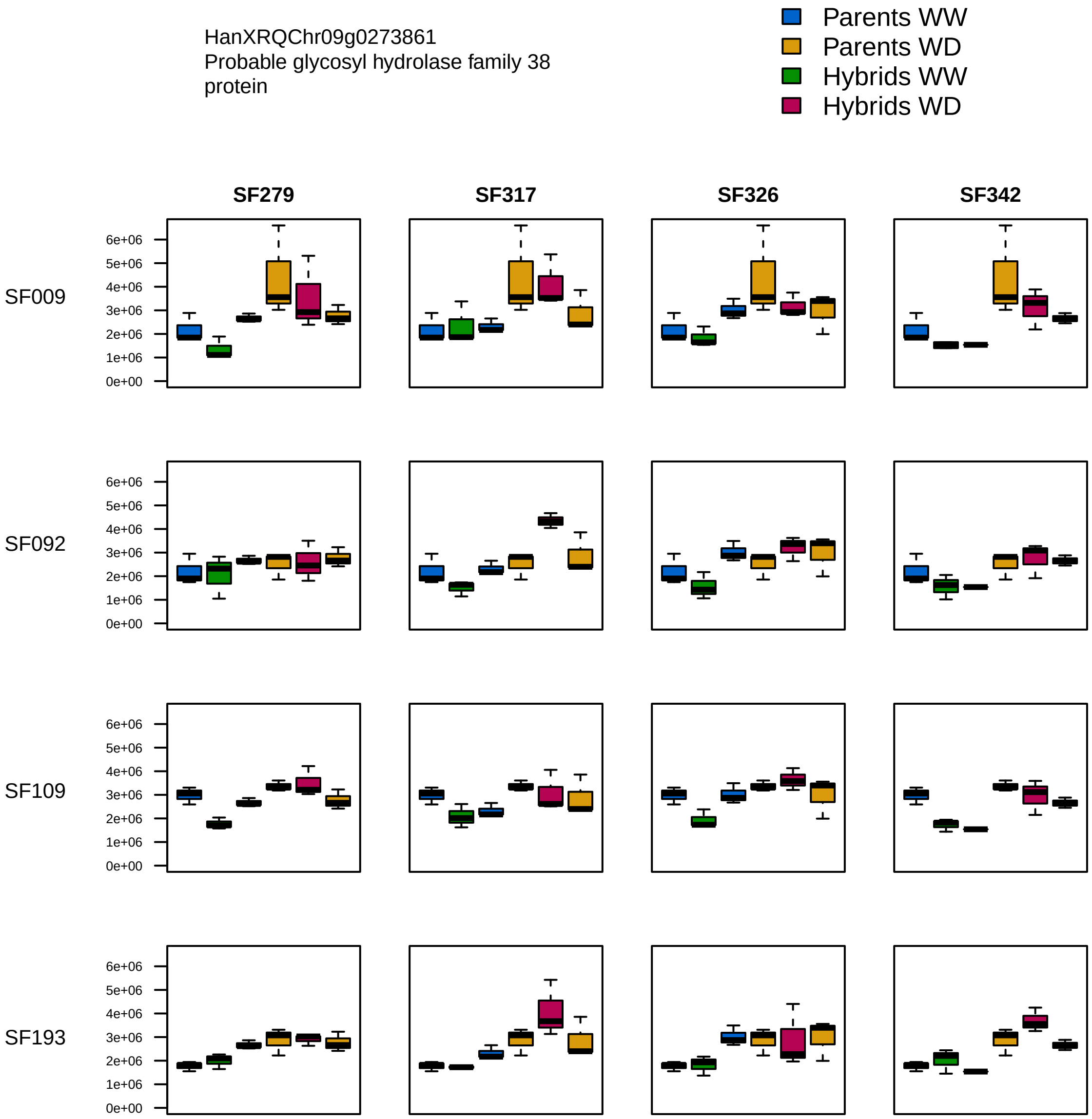
HanXRQChr09g0273861
Probable glycosyl hydrolase family 38
protein
Parents WW
Parents WD
Hybrids WW
Hybrids WD
SF279
SF317
SF326
SF342
SF009
SF092
SF109
SF193
0e+00
1e+06
2e+06
3e+06
4e+06
5e+06
6e+06

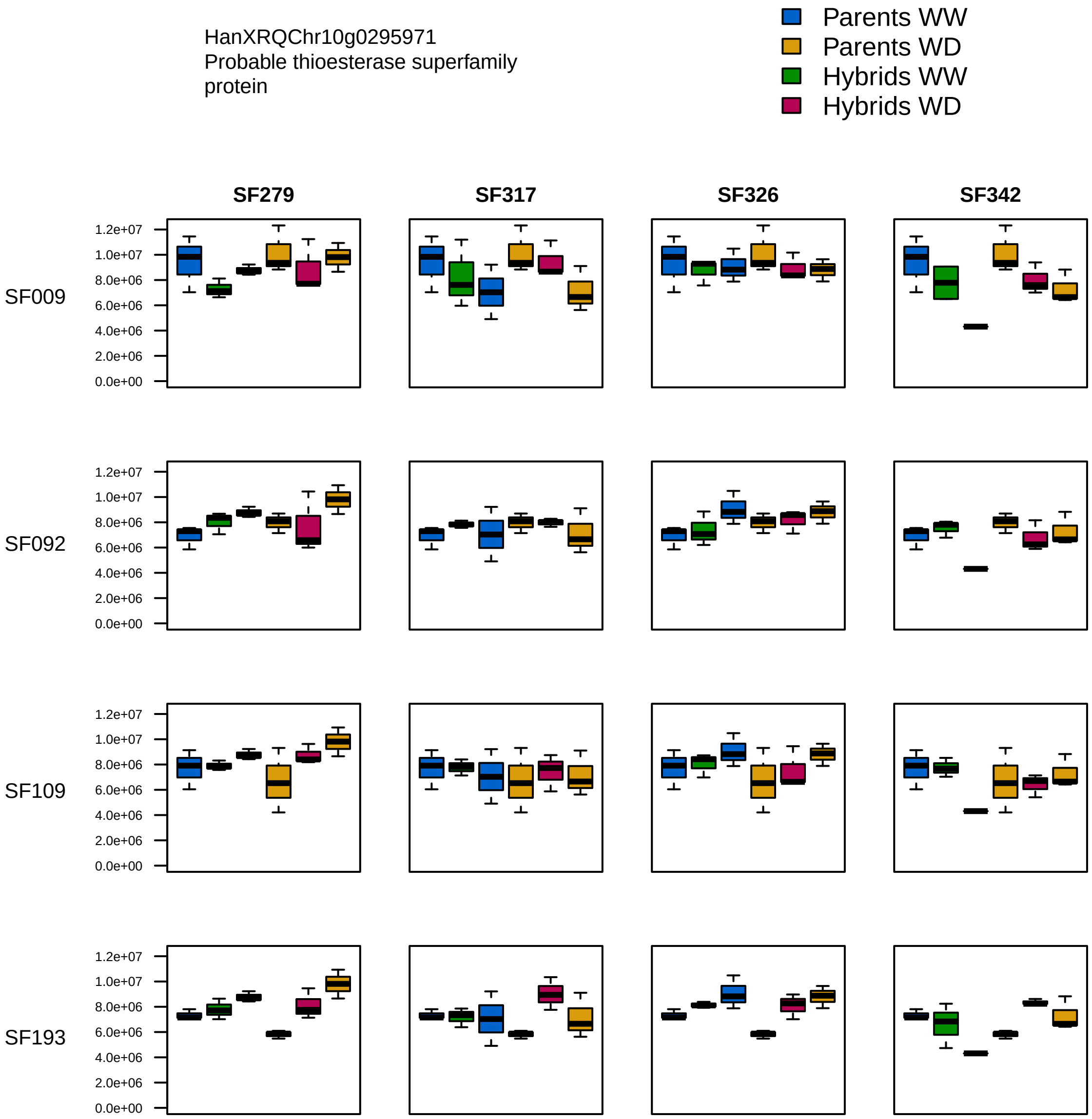
HanXRQChr10g0295971
Probable thioesterase superfamily protein
Parents WW
Parents WD
Hybrids WW
Hybrids WD
SF279
SF317
SF326
SF342
SF009
SF092
SF109
SF193
1.2e+07
1.0e+07
8.0e+06
6.0e+06
4.0e+06
2.0e+06
0.0e+00

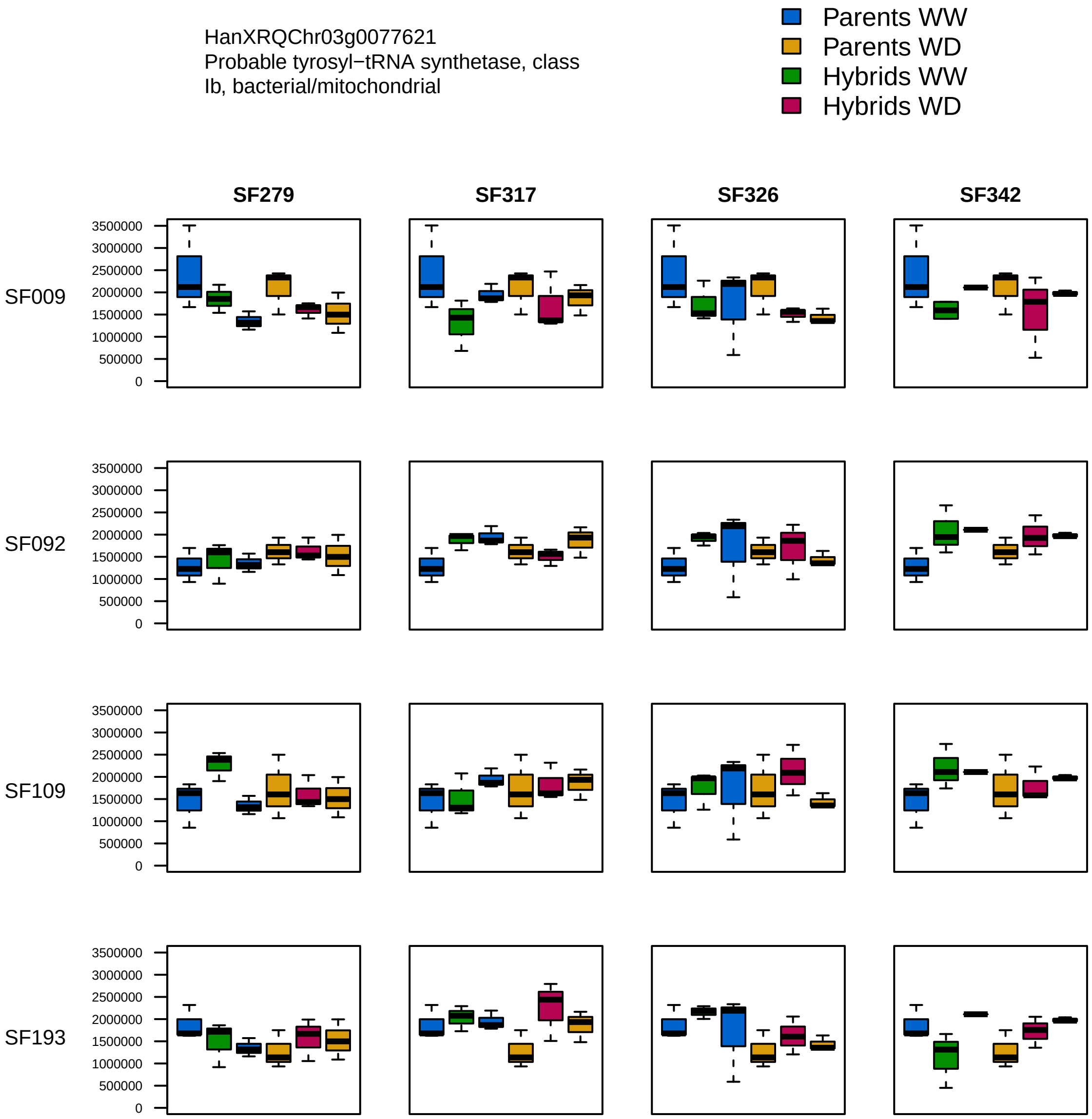

HanXRQChr03g0077621
Probable tyrosyl−tRNA synthetase, class Ib, bacterial/mitochondrial
Parents WW
Parents WD
Hybrids WW
Hybrids WD
SF279
SF317
SF326
SF342
SF009
SF092
SF109
SF193
0
500000
1000000
1500000
2000000
2500000
3000000
3500000

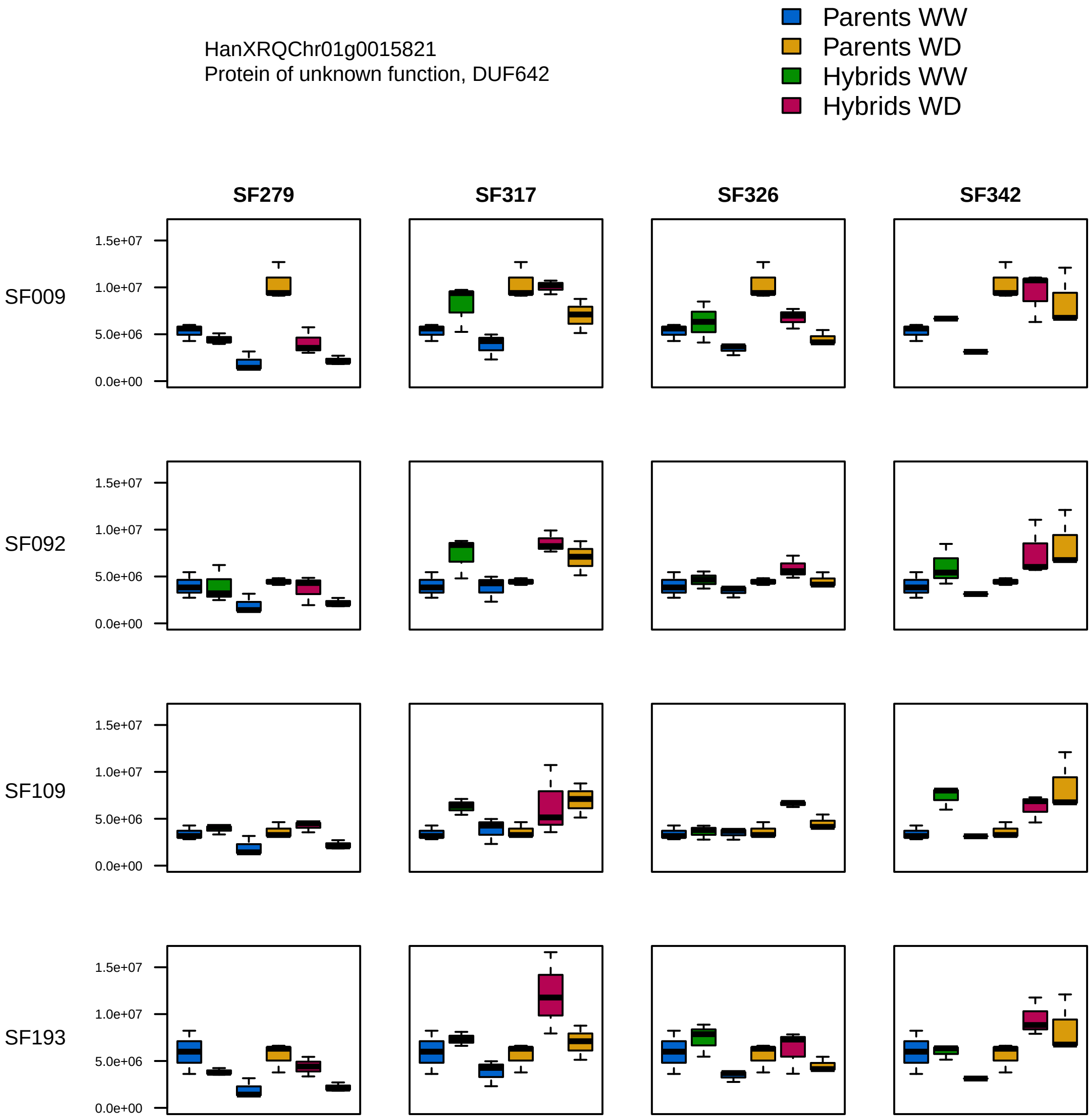
HanXRQChr01g0015821
Protein of unknown function, DUF642
Parents WW
Parents WD
Hybrids WW
Hybrids WD
SF279
SF317
SF326
SF342
SF009
SF092
SF109
SF193
1.5e+07
1.0e+07
5.0e+06
0.0e+00

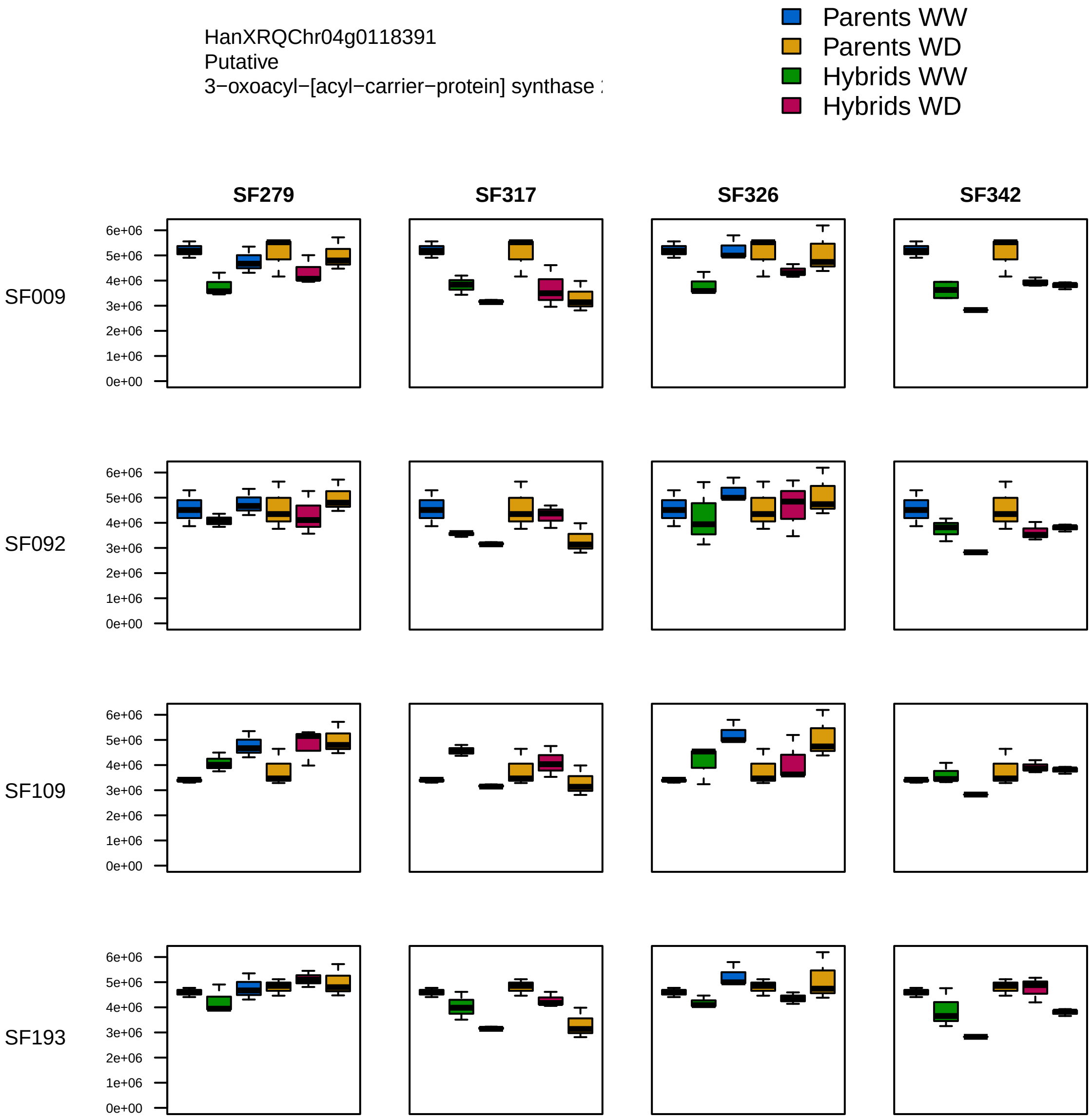

HanXRQChr04g0118391
Putative
3−oxoacyl−[acyl−carrier−protein] synthase
Parents WW
Parents WD
Hybrids WW
Hybrids WD
SF279
SF317
SF326
SF342
SF009
SF092
SF109
SF193
6e+06
5e+06
4e+06
3e+06
2e+06
1e+06
0e+00

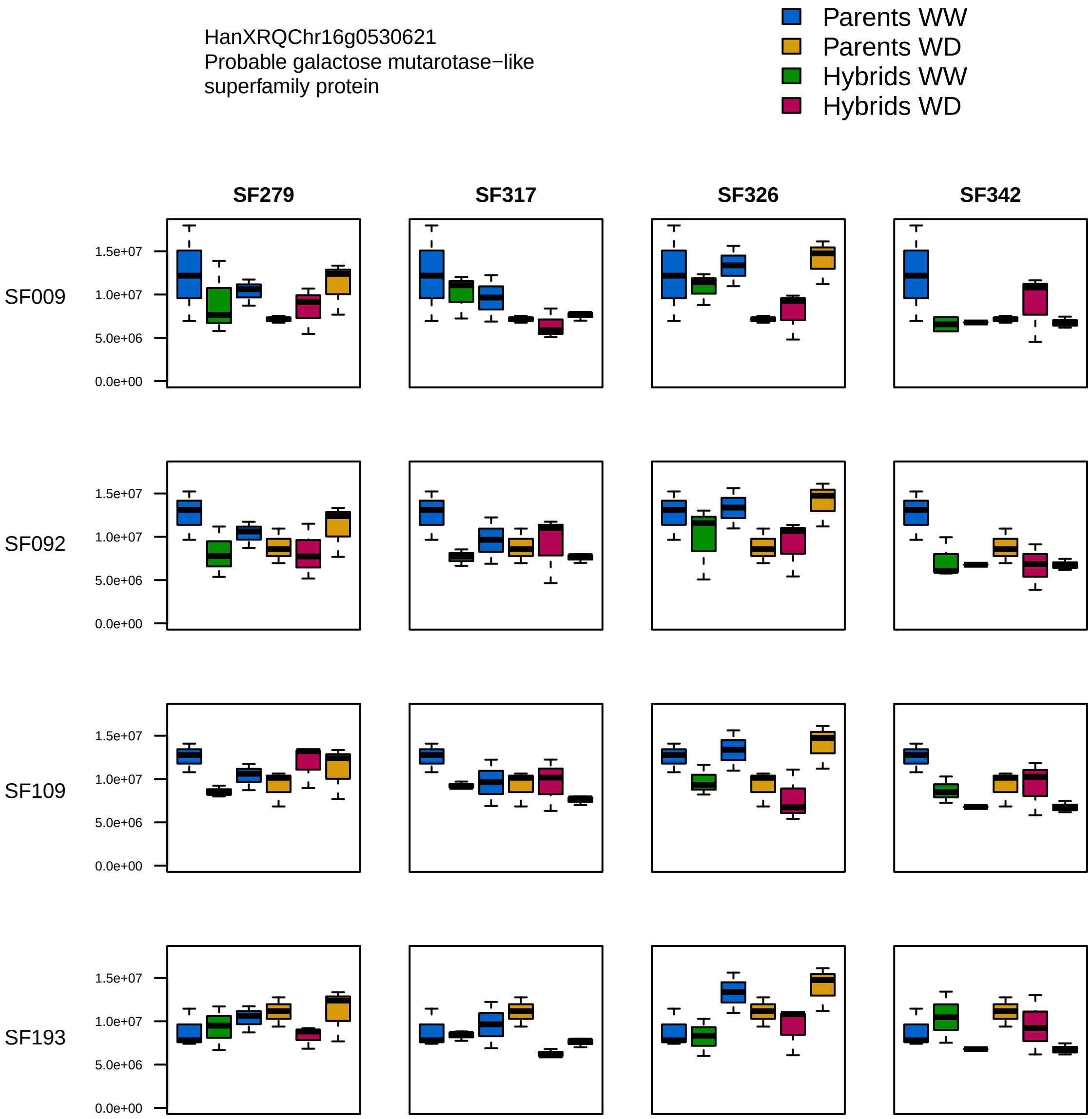
HanXRQChr16g0530621
Probable galactose mutarotase−like
superfamily protein
Parents WW
Parents WD
Hybrids WW
Hybrids WD
SF279
SF317
SF326
SF342
SF009
SF092
SF109
SF193
1.5e+07
1.0e+07
5.0e+06
0.0e+00

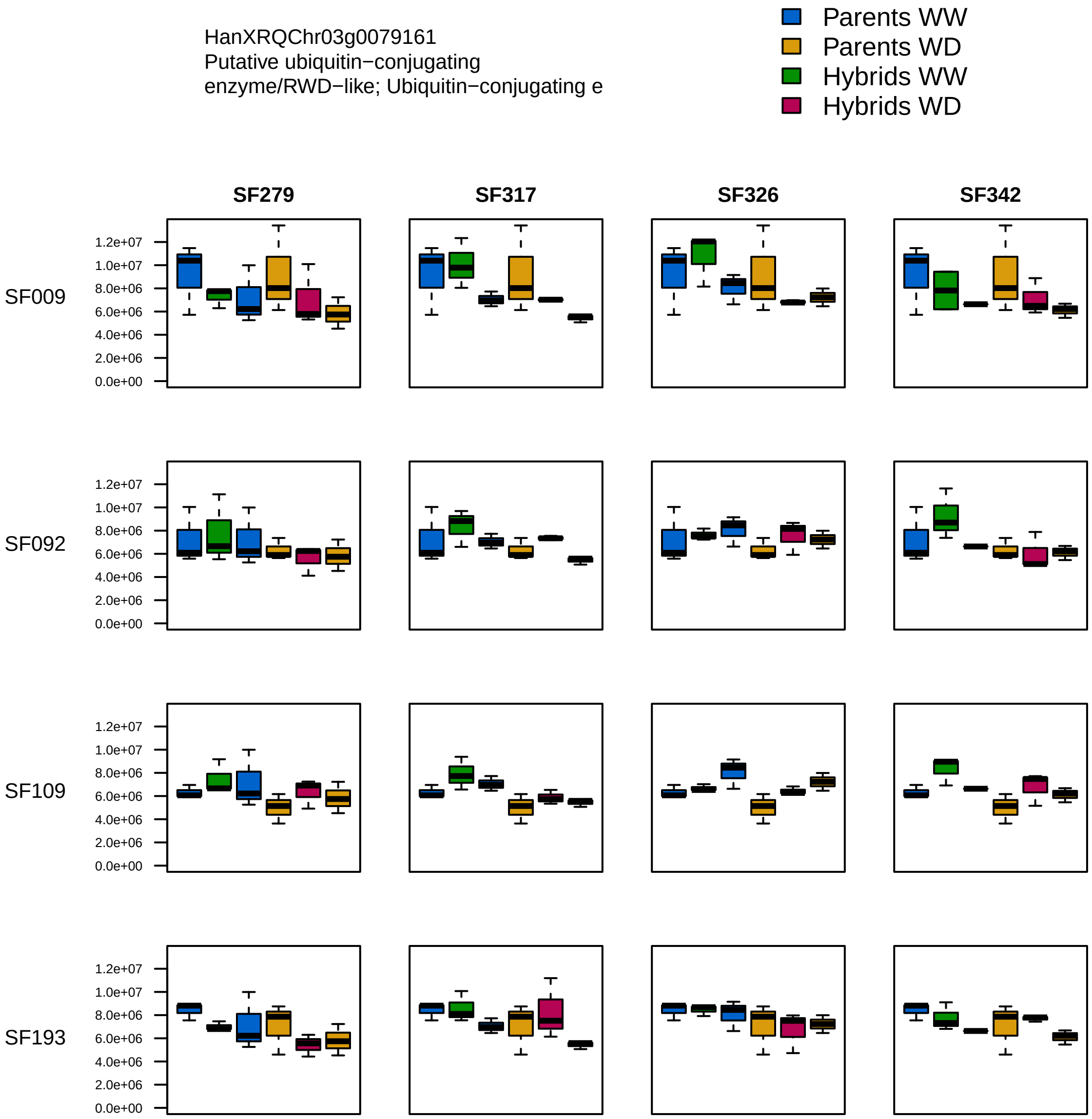
HanXRQChr03g0079161
Putative ubiquitin−conjugating
enzyme/RWD−like; Ubiquitin−conjugating e
Parents WW
Parents WD
Hybrids WW
Hybrids WD
SF279
SF317
SF326
SF342
SF009
SF092
SF109
SF193
1.2e+07
1.0e+07
8.0e+06
6.0e+06
4.0e+06
2.0e+06
0.0e+00

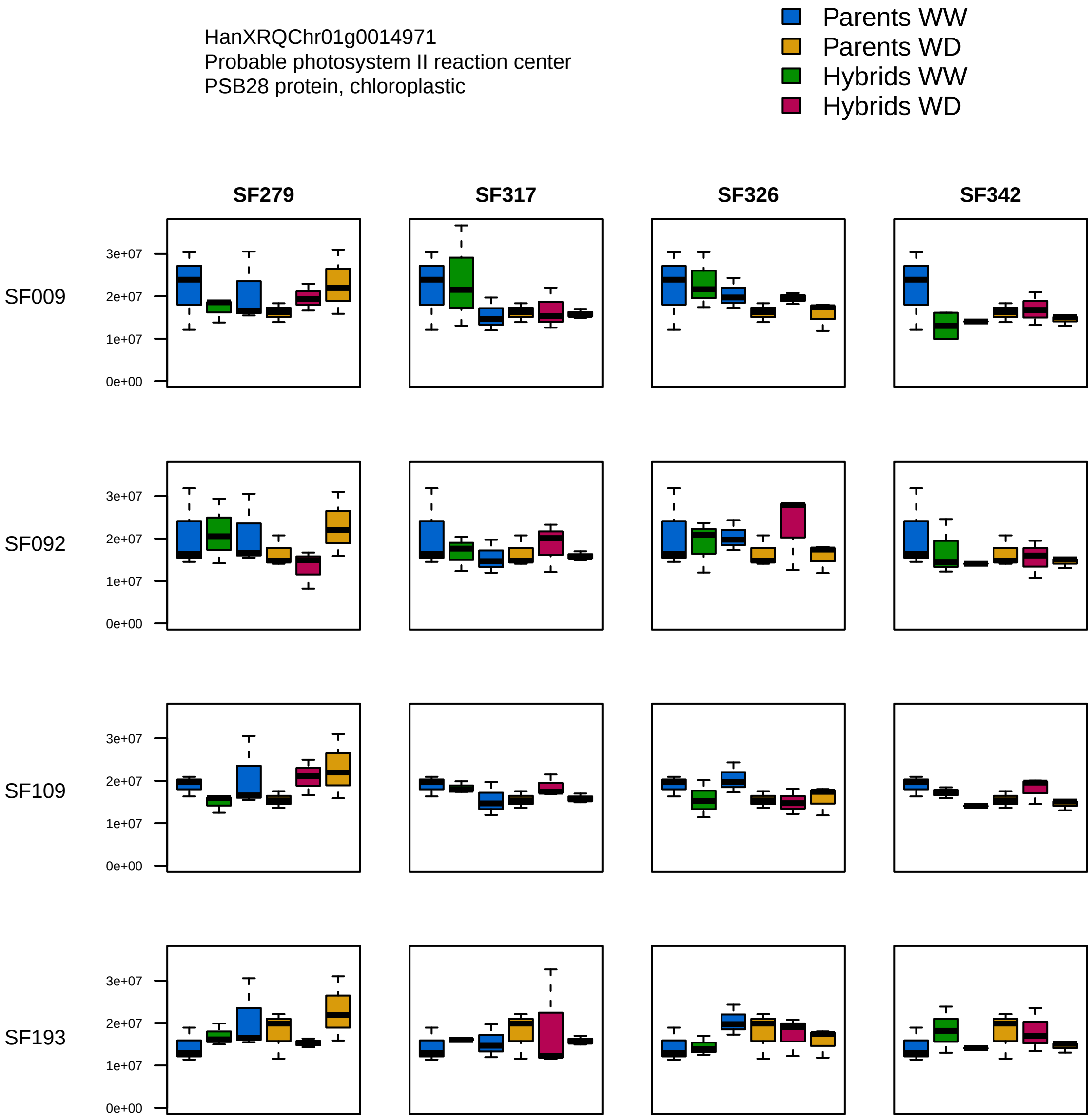
HanXRQChr01g0014971
Probable photosystem II reaction center
PSB28 protein, chloroplastic
Parents WW
Parents WD
Hybrids WW
Hybrids WD
SF279
SF317
SF326
SF342
SF009
SF092
SF109
SF193
0e+00
1e+07
2e+07
3e+07

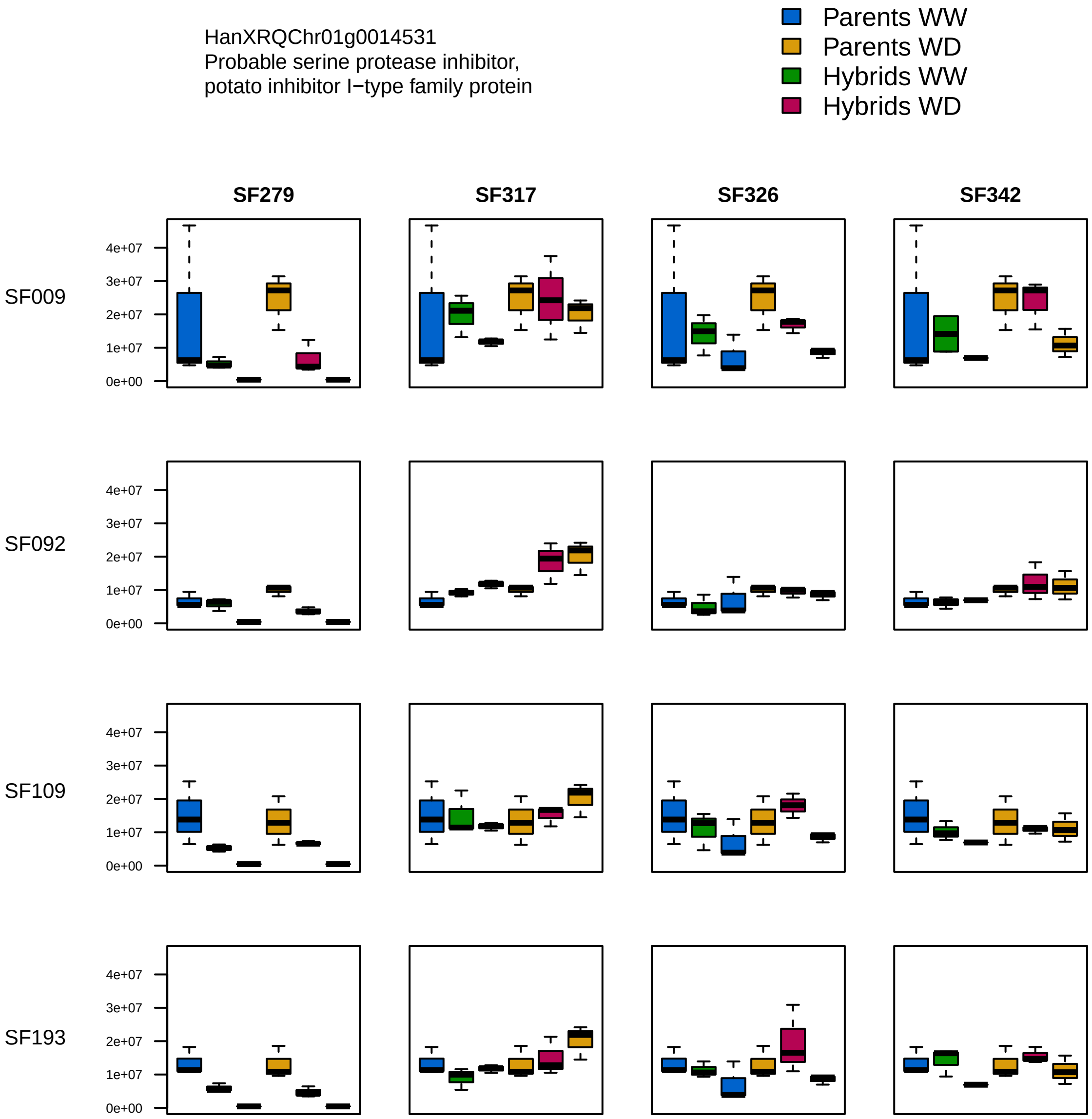
HanXRQChr01g0014531
Probable serine protease inhibitor,
potato inhibitor I−type family protein
Parents WW
Parents WD
Hybrids WW
Hybrids WD
SF279
SF317
SF326
SF342
SF009
SF092
SF109
SF193
0e+00
1e+07
2e+07
3e+07
4e+07

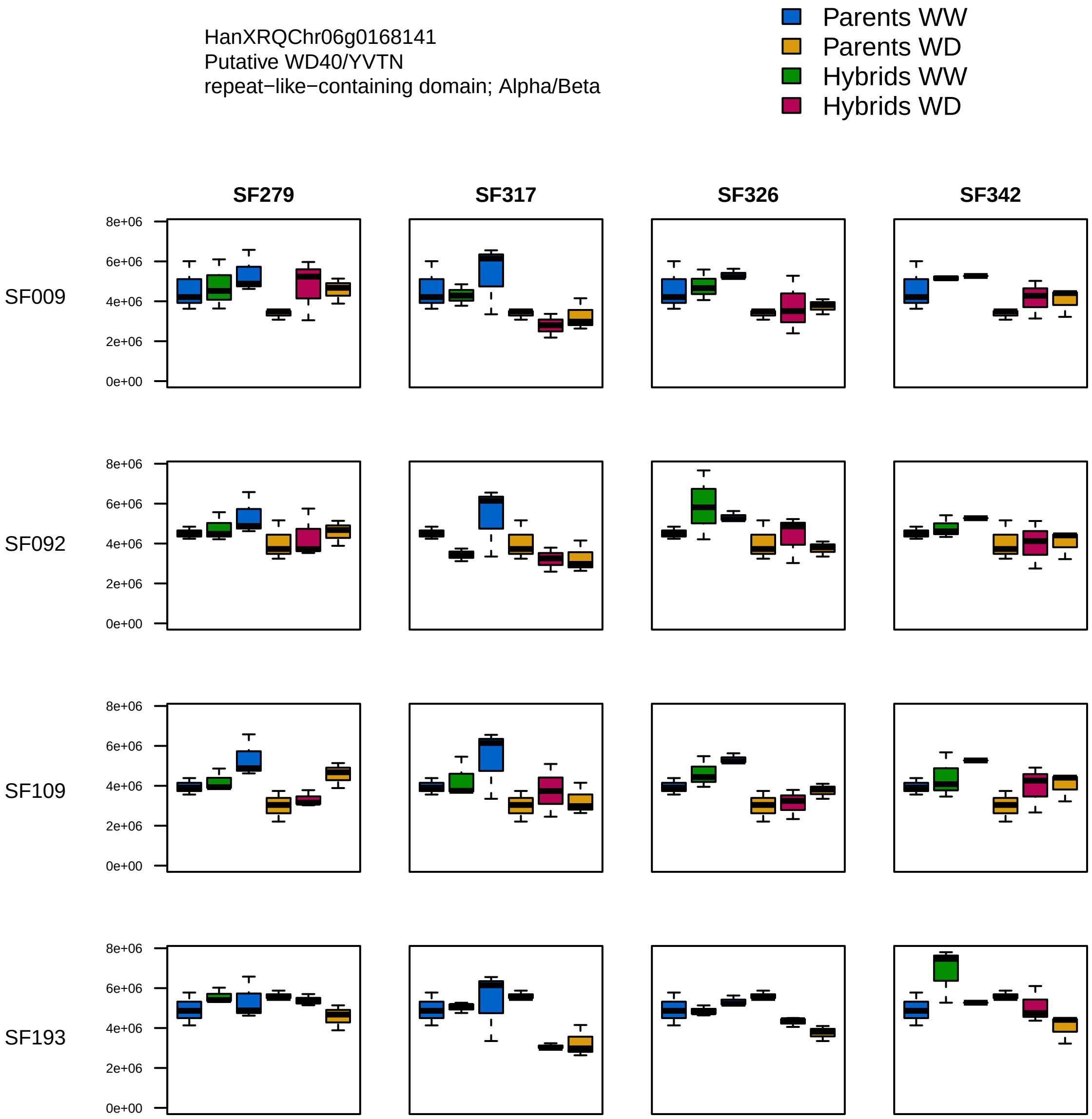
HanXRQChr06g0168141
Putative WD40/YVTN
repeat−like−containing domain; Alpha/Beta
Parents WW
Parents WD
Hybrids WW
Hybrids WD
SF279
SF317
SF326
SF342
SF009
SF092
SF109
SF193
0e+00
2e+06
4e+06
6e+06
8e+06

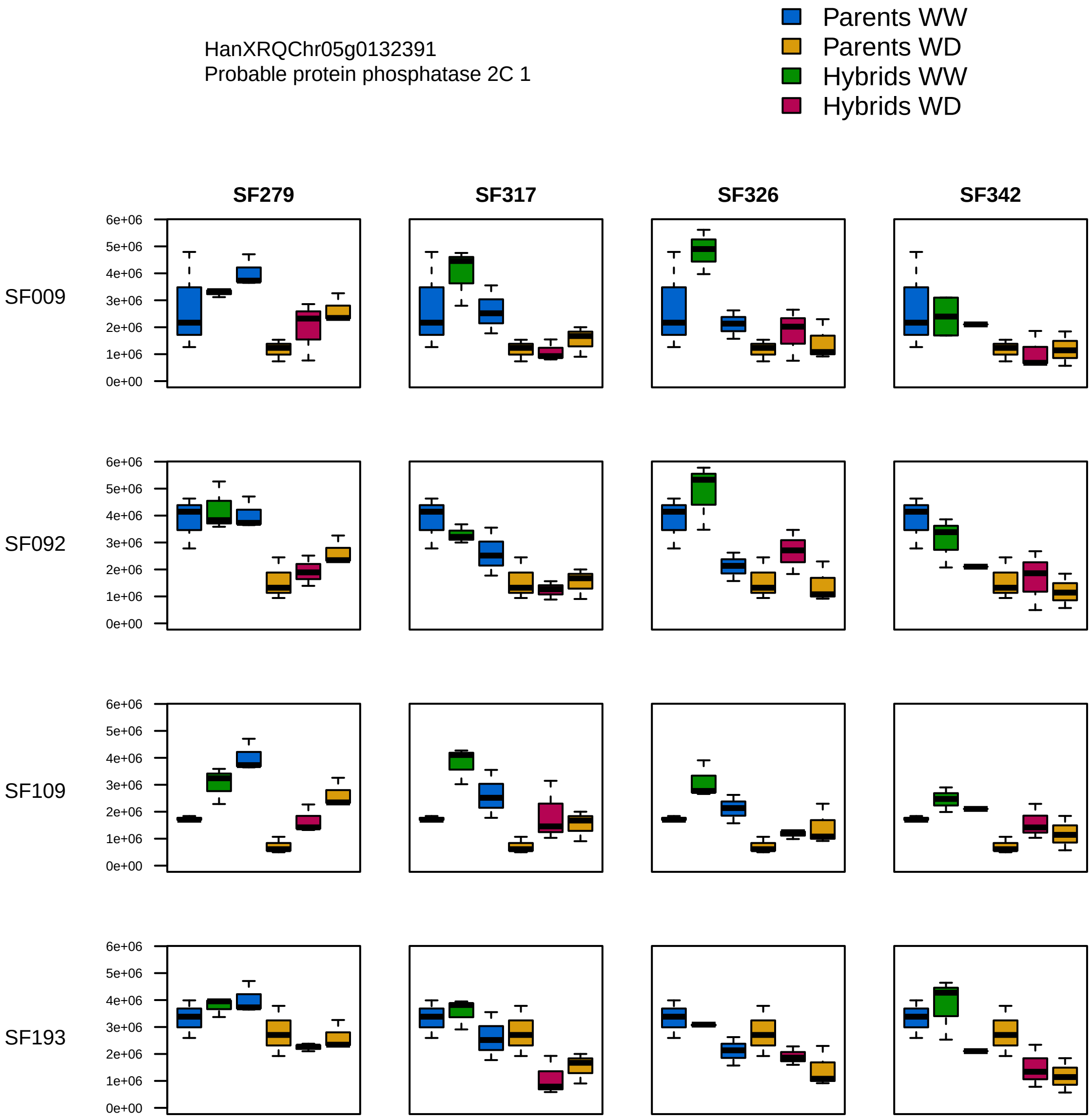
HanXRQChr05g0132391
Probable protein phosphatase 2C 1
Parents WW
Parents WD
Hybrids WW
Hybrids WD
SF279
SF317
SF326
SF342
SF009
SF092
SF109
SF193
6e+06
5e+06
4e+06
3e+06
2e+06
1e+06
0e+00

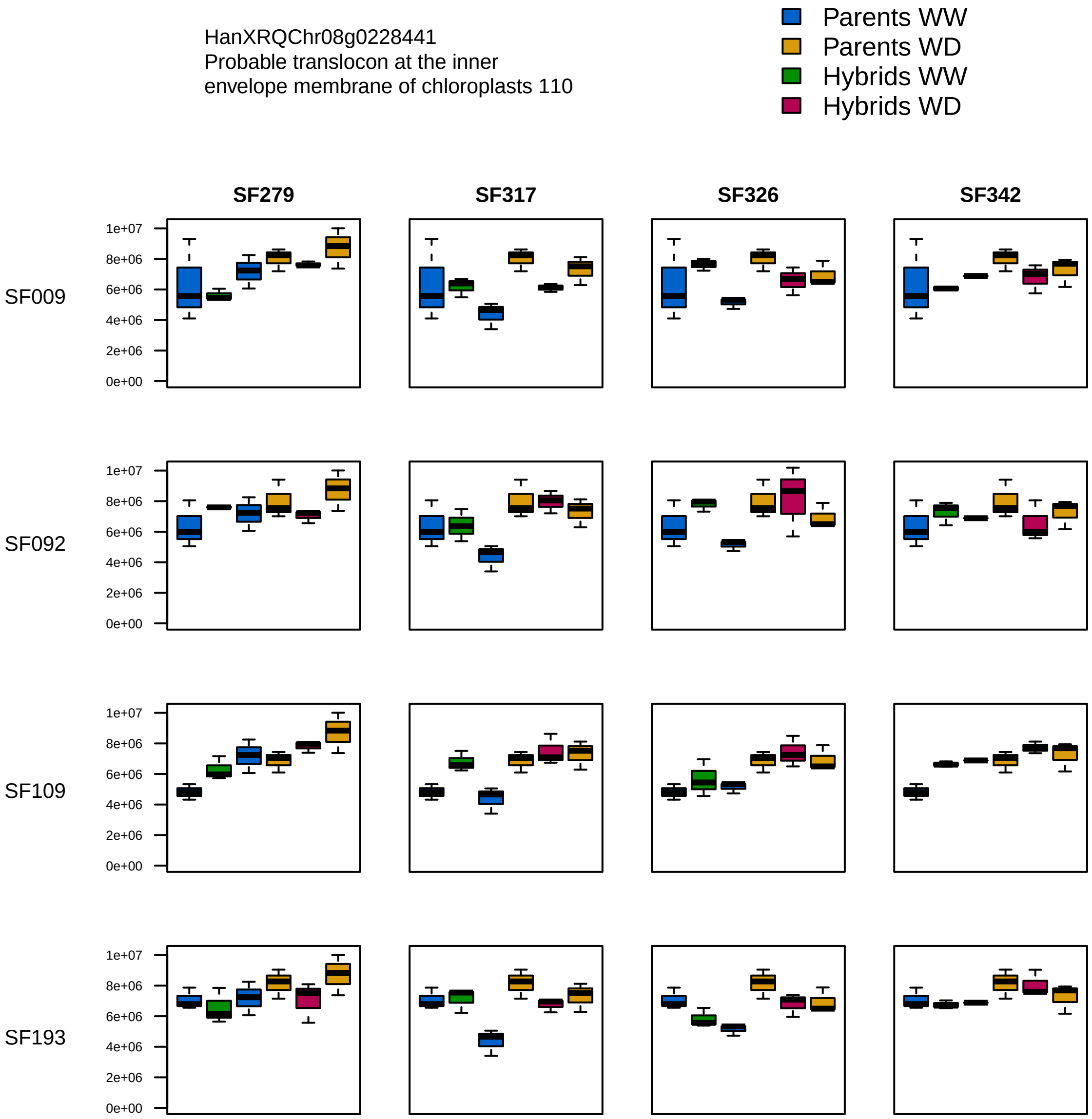
HanXRQChr08g0228441
Probable translocon at the inner
envelope membrane of chloroplasts 110
Parents WW
Parents WD
Hybrids WW
Hybrids WD
SF279
SF317
SF326
SF342
SF009
SF092
SF109
SF193
1e+07
8e+06
6e+06
4e+06
2e+06
0e+00

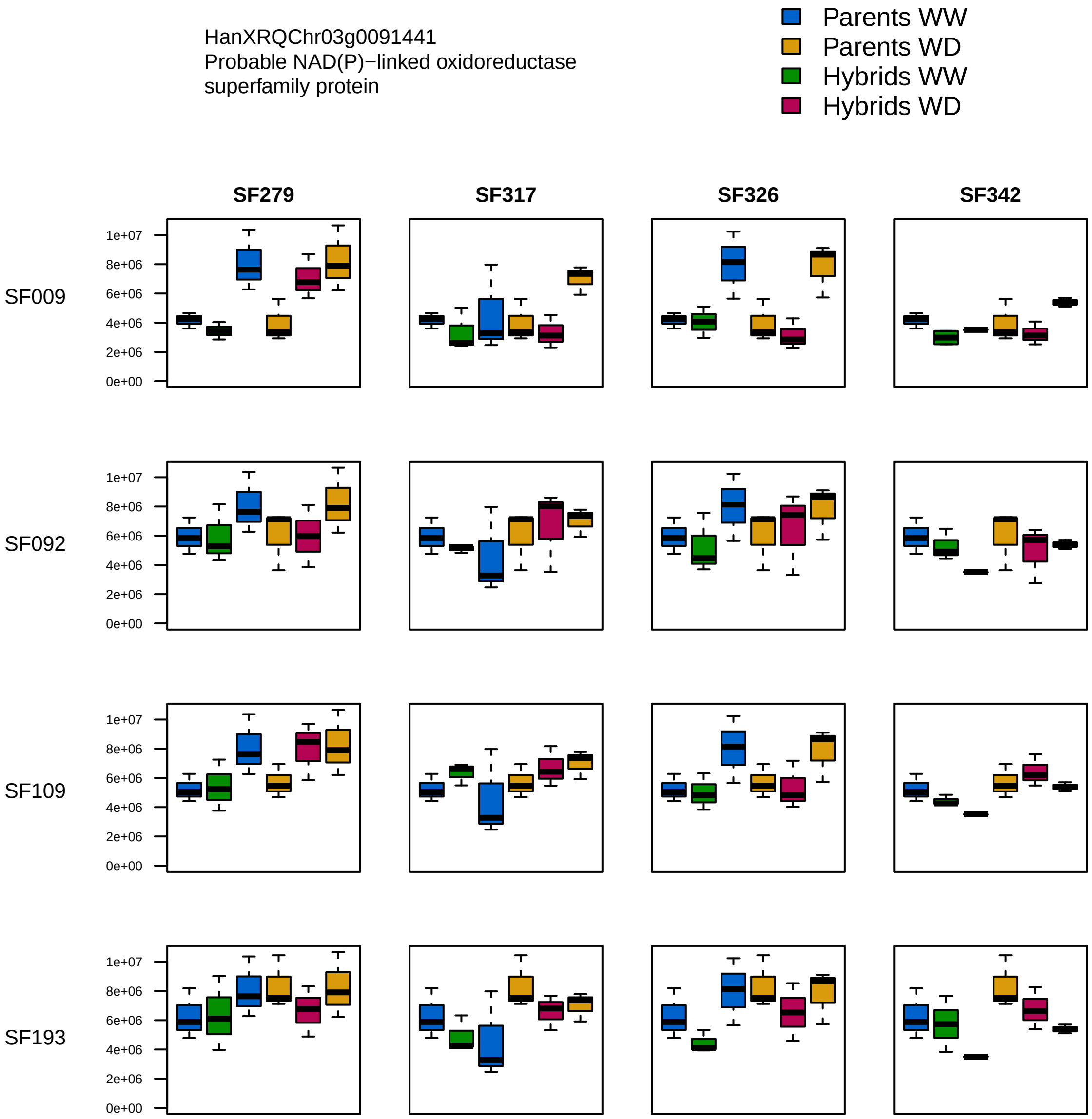
HanXRQChr03g0091441
Probable NAD(P)−linked oxidoreductase
superfamily protein
Parents WW
Parents WD
Hybrids WW
Hybrids WD
SF279
SF317
SF326
SF342
SF009
SF092
SF109
SF193
1e+07
8e+06
6e+06
4e+06
2e+06
0e+00

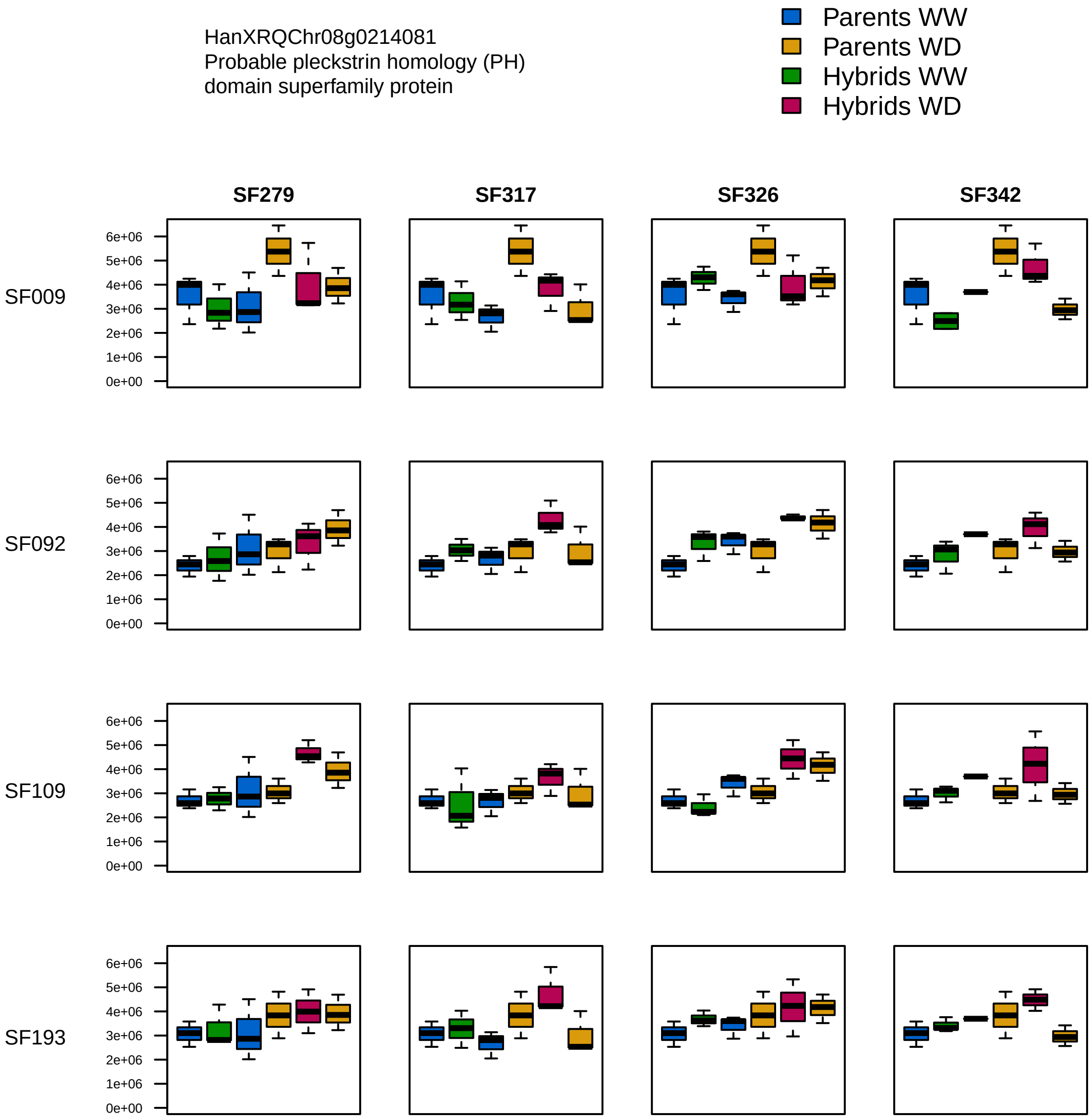
HanXRQChr08g0214081
Probable pleckstrin homology (PH)
domain superfamily protein
Parents WW
Parents WD
Hybrids WW
Hybrids WD
SF279
SF317
SF326
SF342
SF009
SF092
SF109
SF193
6e+06
5e+06
4e+06
3e+06
2e+06
1e+06
0e+00

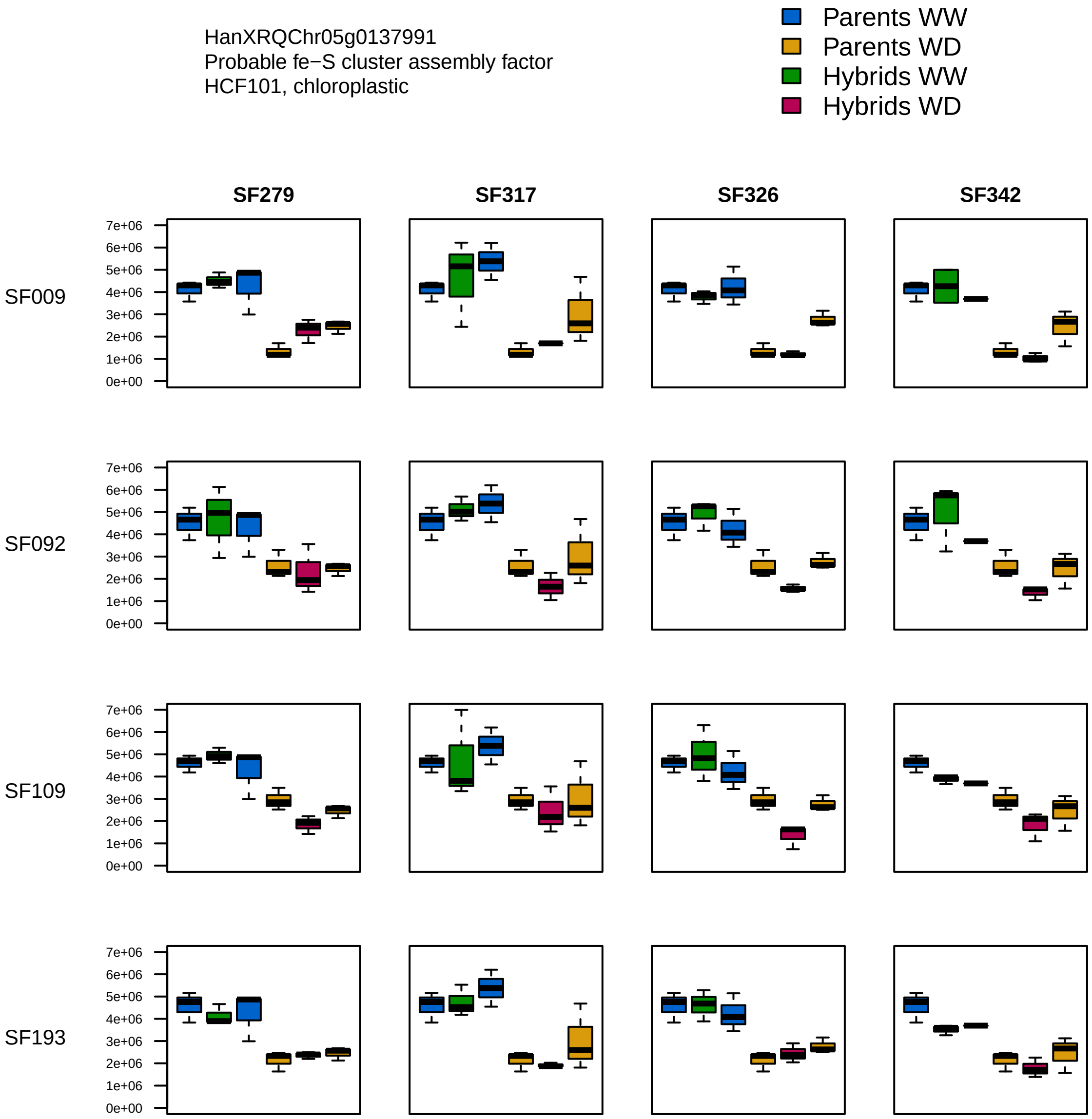
HanXRQChr05g0137991
Probable fe−S cluster assembly factor
HCF101, chloroplastic
Parents WW
Parents WD
Hybrids WW
Hybrids WD
SF279
SF317
SF326
SF342
SF009
SF092
SF109
SF193
7e+06
6e+06
5e+06
4e+06
3e+06
2e+06
1e+06
0e+00

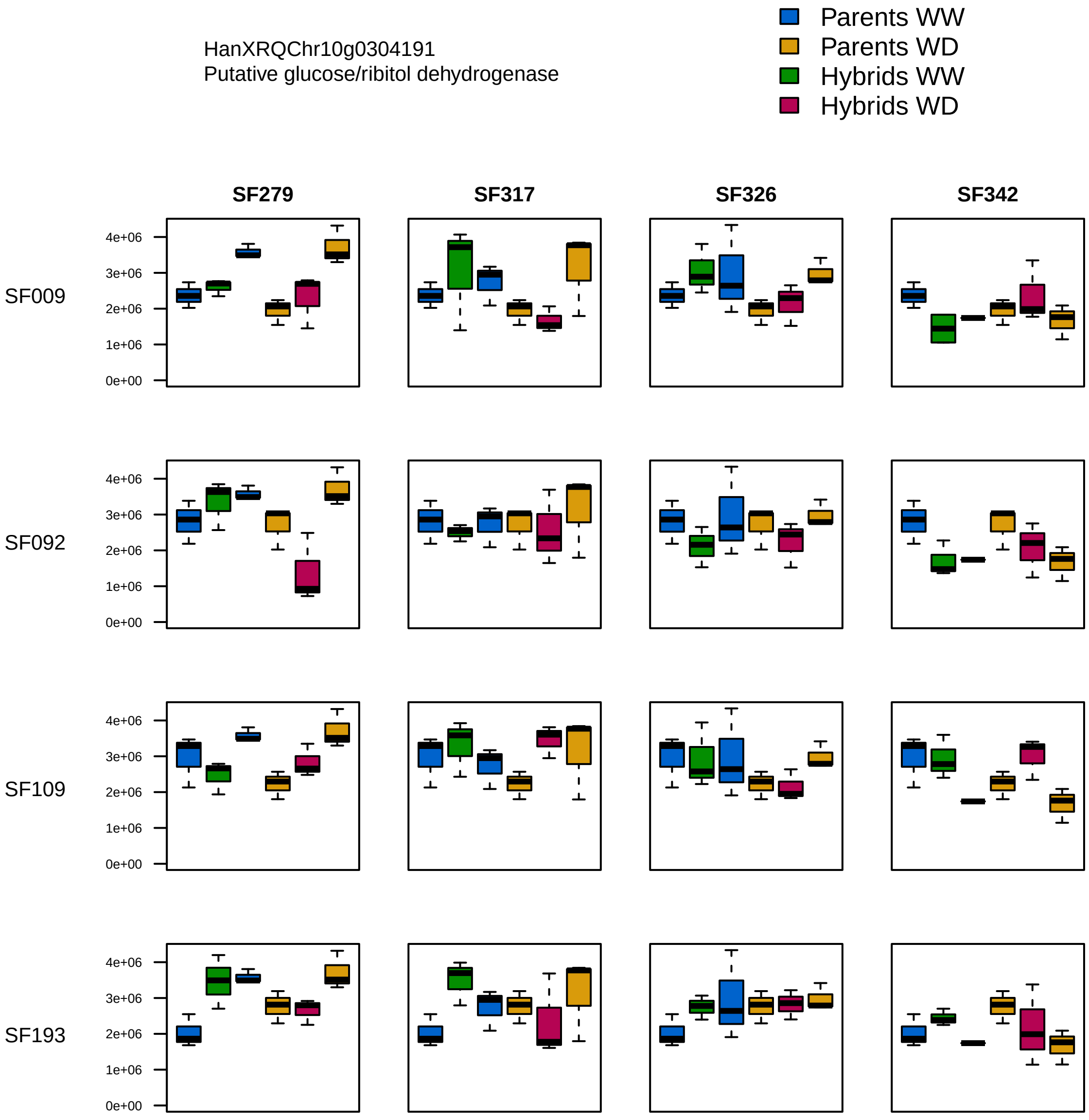
HanXRQChr10g0304191
Putative glucose/ribitol dehydrogenase
Parents WW
Parents WD
Hybrids WW
Hybrids WD
SF279
SF317
SF326
SF342
SF009
SF092
SF109
SF193
4e+06
3e+06
2e+06
1e+06
0e+00

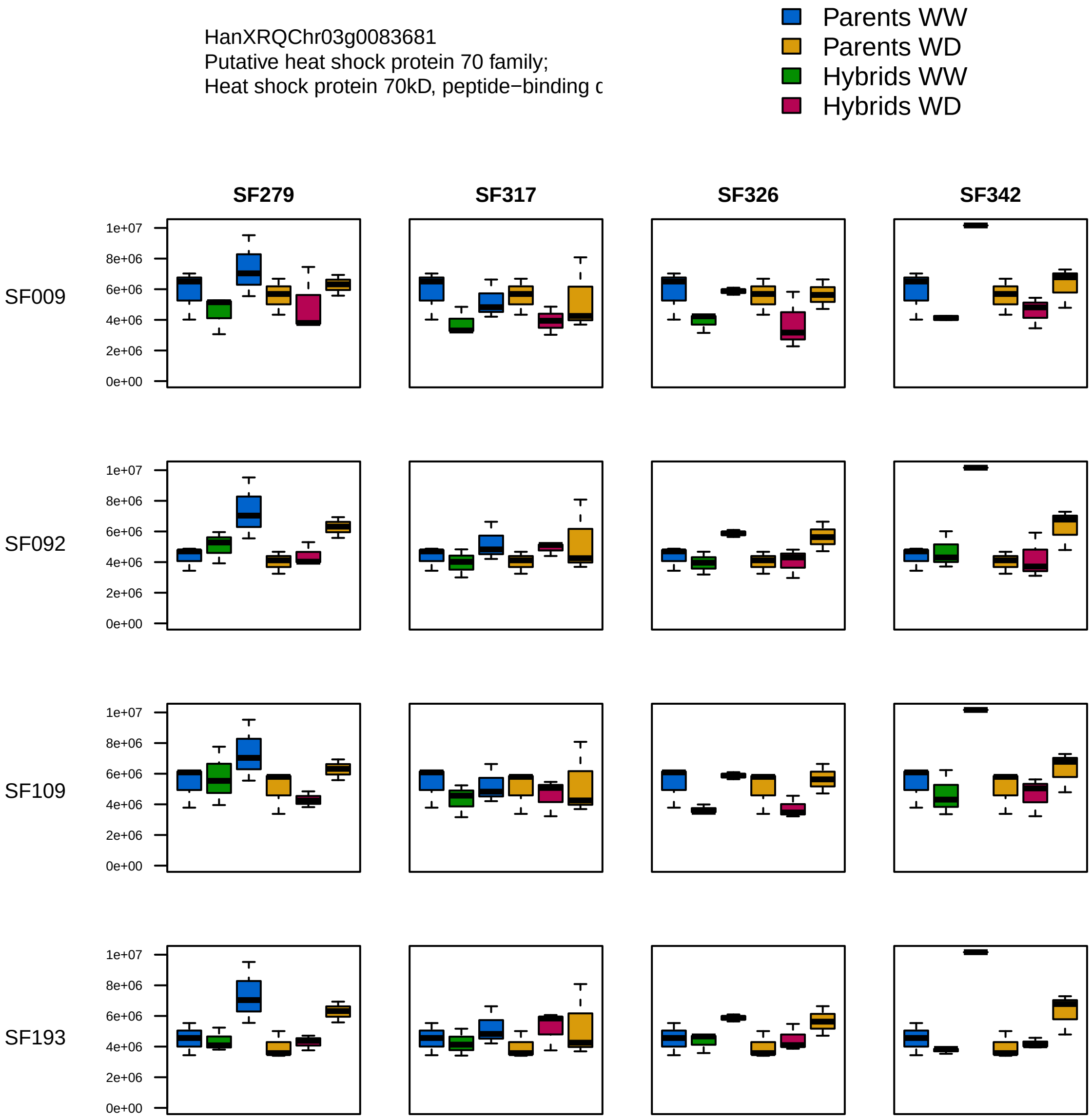
HanXRQChr03g0083681
Putative heat shock protein 70 family;
Heat shock protein 70kD, peptide−binding c
Parents WW
Parents WD
Hybrids WW
Hybrids WD
SF279
SF317
SF326
SF342
SF009
SF092
SF109
SF193
0e+00
2e+06
4e+06
6e+06
8e+06
1e+07

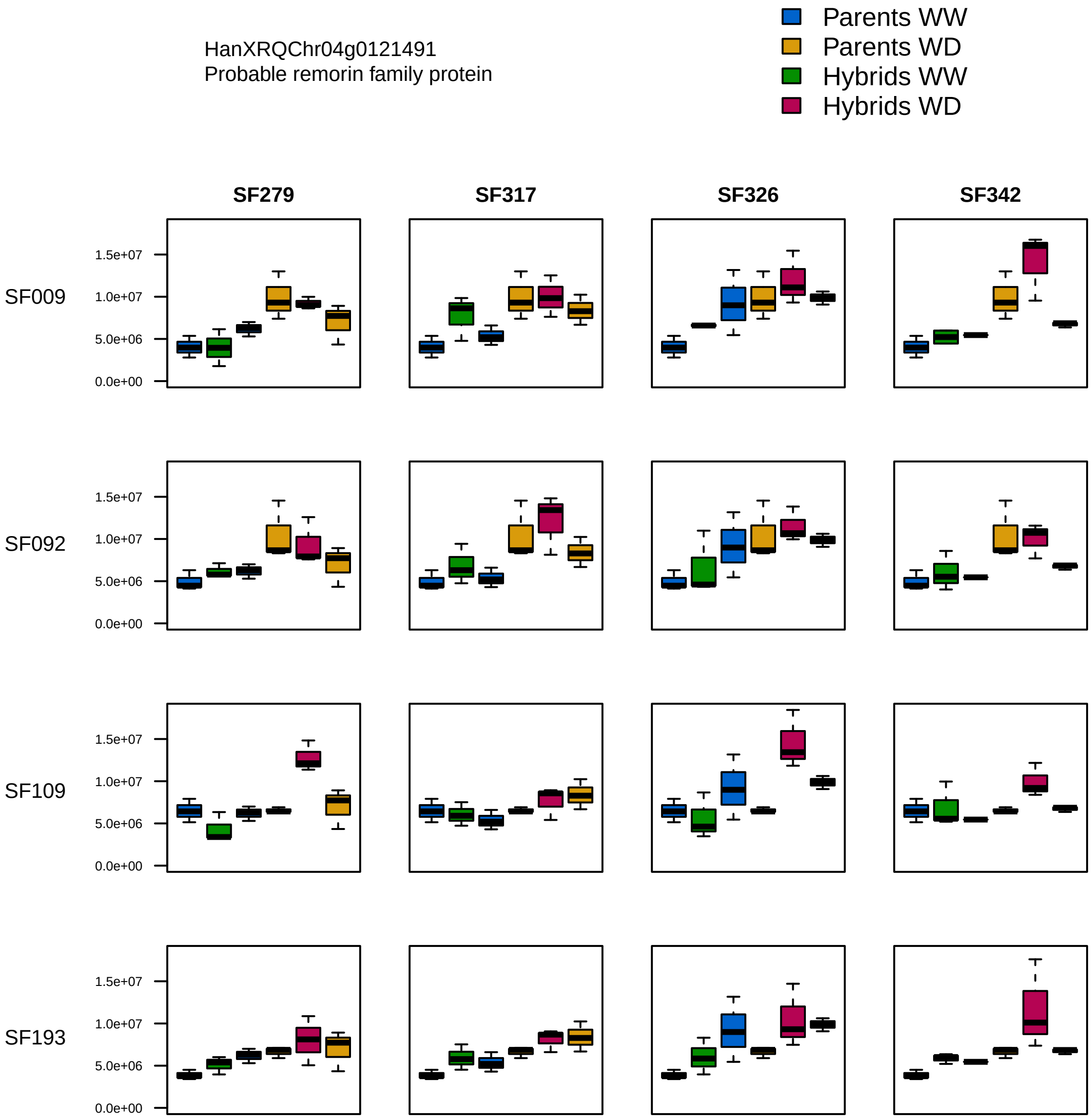

HanXRQChr04g0121491
Probable remorin family protein
Parents WW
Parents WD
Hybrids WW
Hybrids WD
SF279
SF317
SF326
SF342
SF009
SF092
SF109
SF193
1.5e+07
1.0e+07
5.0e+06
0.0e+00

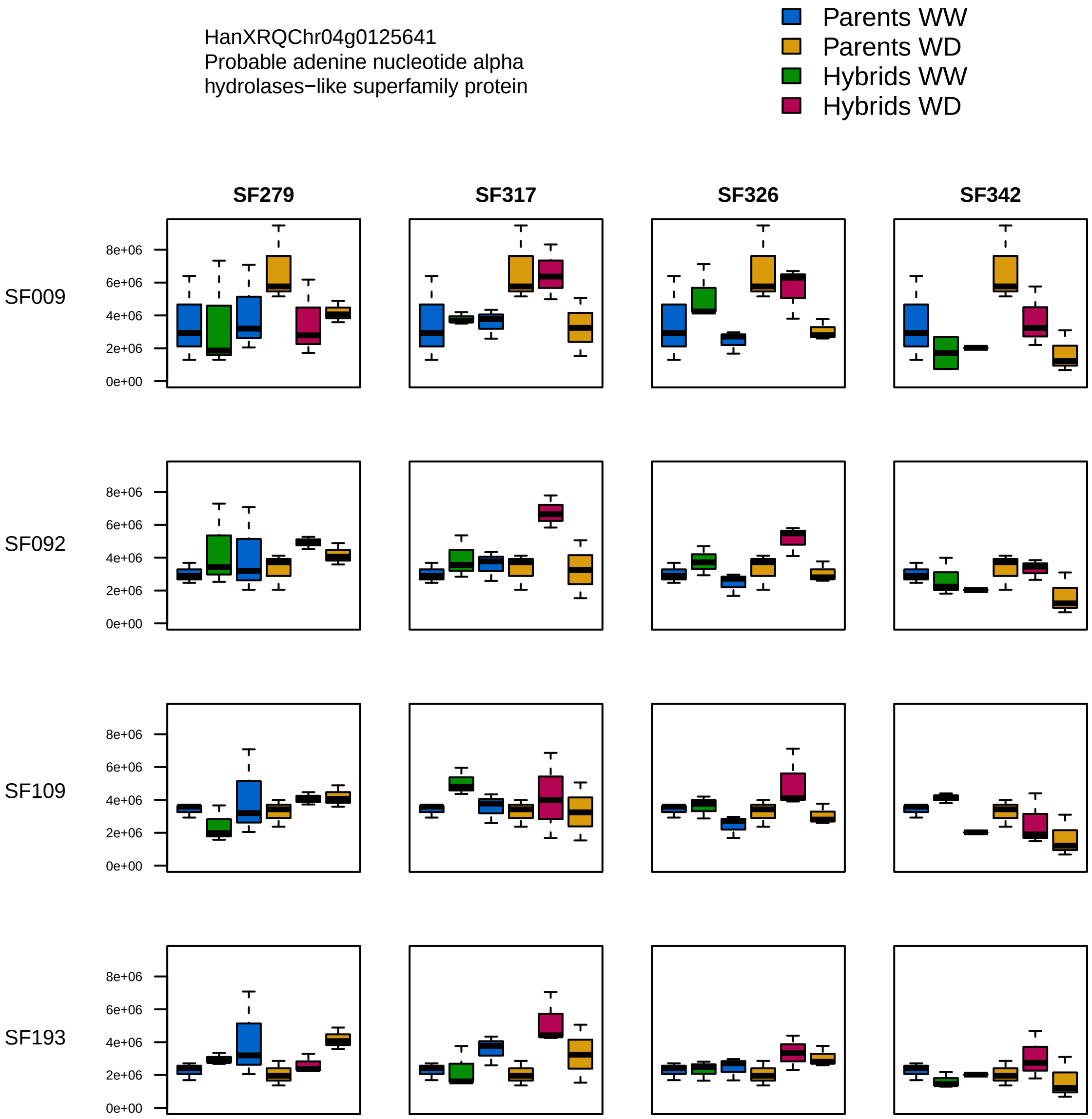
HanXRQChr04g0125641
Probable adenine nucleotide alpha
hydrolases−like superfamily protein
Parents WW
Parents WD
Hybrids WW
Hybrids WD
SF279
SF317
SF326
SF342
SF009
SF092
SF109
SF193
0e+00
2e+06
4e+06
6e+06
8e+06

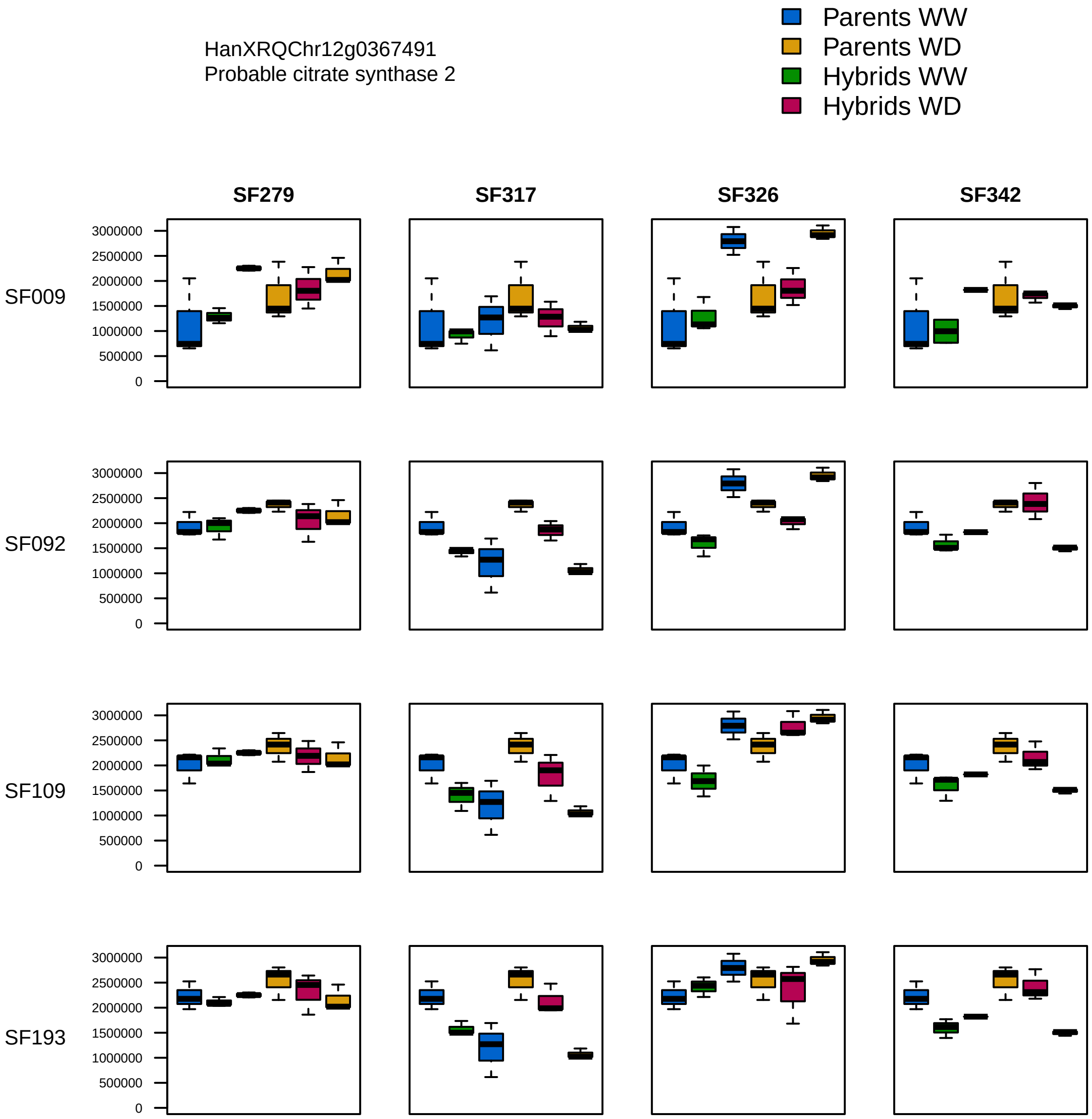
HanXRQChr12g0367491
Probable citrate synthase 2
Parents WW
Parents WD
Hybrids WW
Hybrids WD
SF279
SF317
SF326
SF342
SF009
SF092
SF109
SF193
3000000
2500000
2000000
1500000
1000000
500000
0

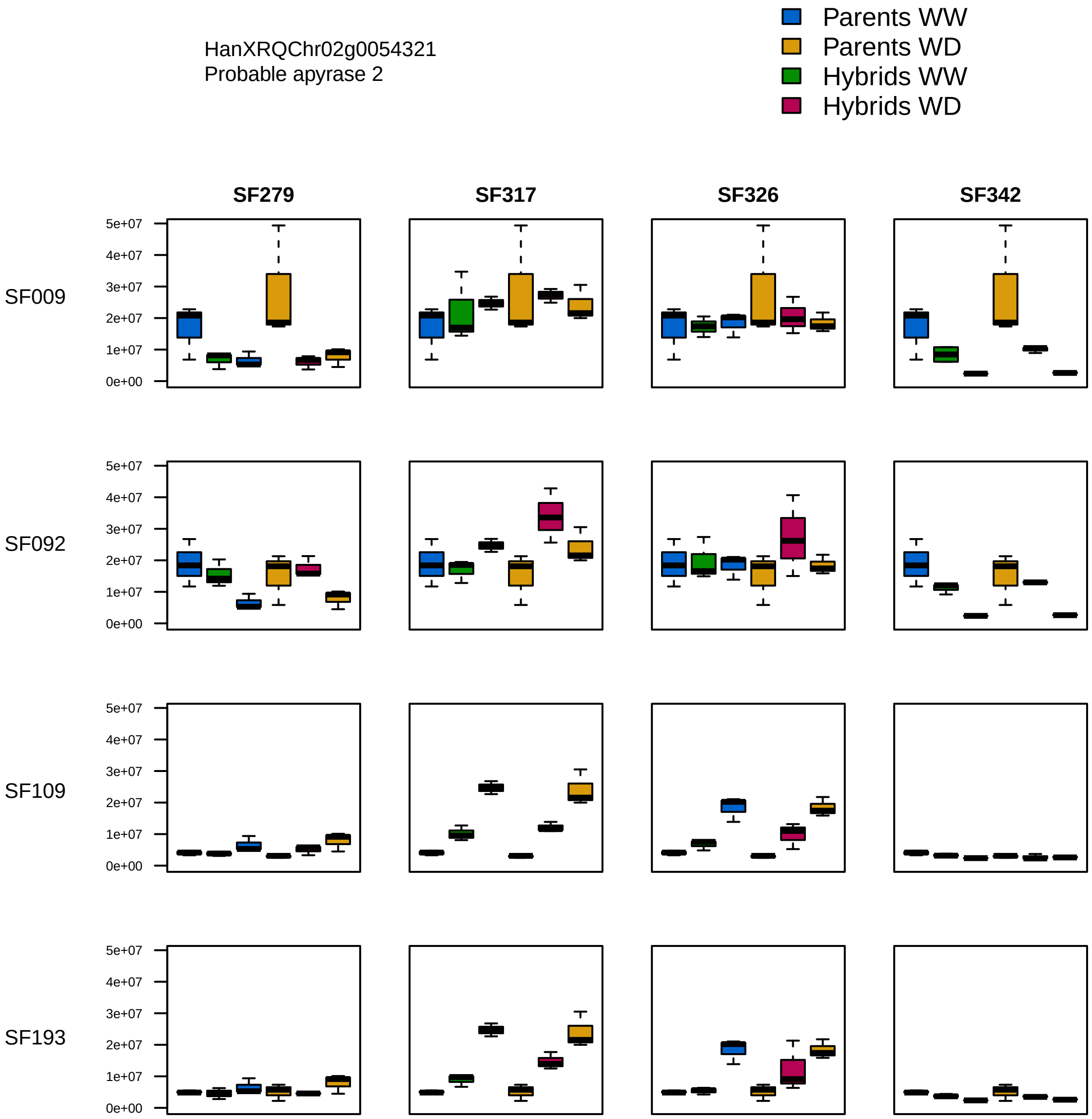
HanXRQChr02g0054321
Probable apyrase 2
Parents WW
Parents WD
Hybrids WW
Hybrids WD
SF279
SF317
SF326
SF342
SF009
SF092
SF109
SF193
5e+07
4e+07
3e+07
2e+07
1e+07
0e+00

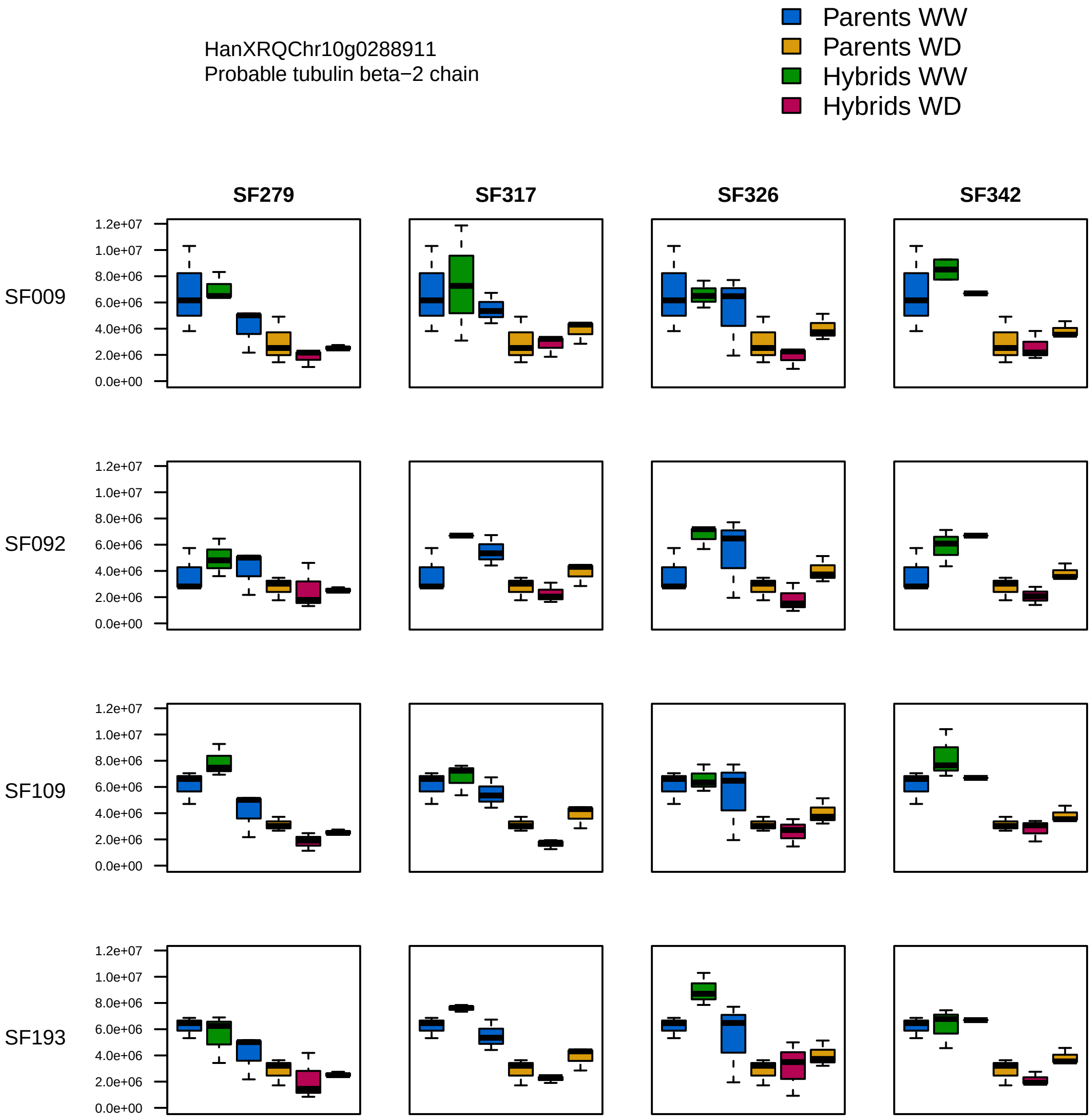

HanXRQChr10g0288911
Probable tubulin beta−2 chain
Parents WW
Parents WD
Hybrids WW
Hybrids WD
SF279
SF317
SF326
SF342
SF009
SF092
SF109
SF193
1.2e+07
1.0e+07
8.0e+06
6.0e+06
4.0e+06
2.0e+06
0.0e+00

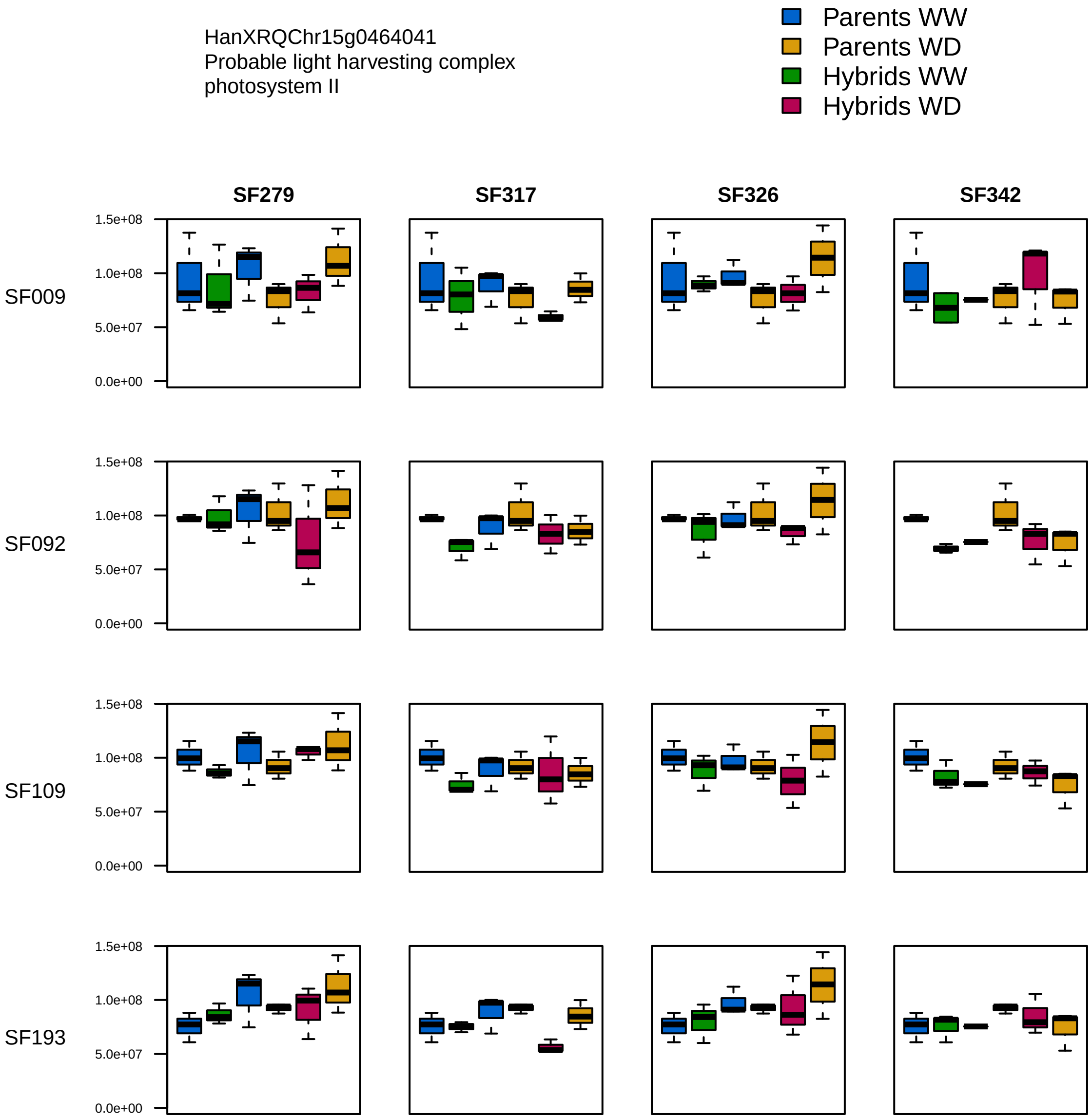
HanXRQChr15g0464041
Probable light harvesting complex
photosystem II
Parents WW
Parents WD
Hybrids WW
Hybrids WD
SF279
SF317
SF326
SF342
SF009
SF092
SF109
SF193
1.5e+08
1.0e+08
5.0e+07
0.0e+00

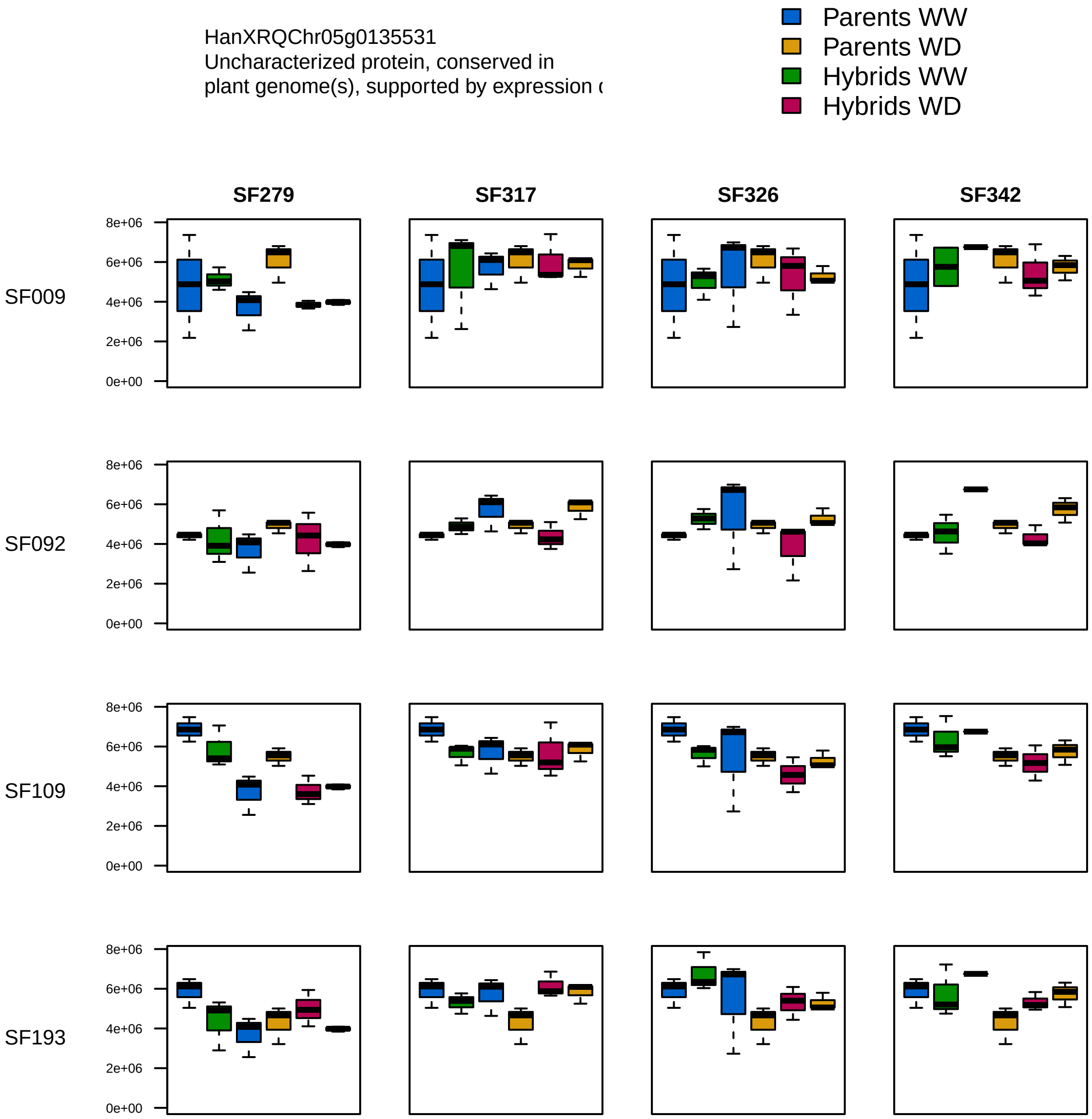

HanXRQChr05g0135531
Uncharacterized protein, conserved in
plant genome(s), supported by expression (
Parents WW
Parents WD
Hybrids WW
Hybrids WD
SF279
SF317
SF326
SF342
SF009
SF092
SF109
SF193
8e+06
6e+06
4e+06
2e+06
0e+00

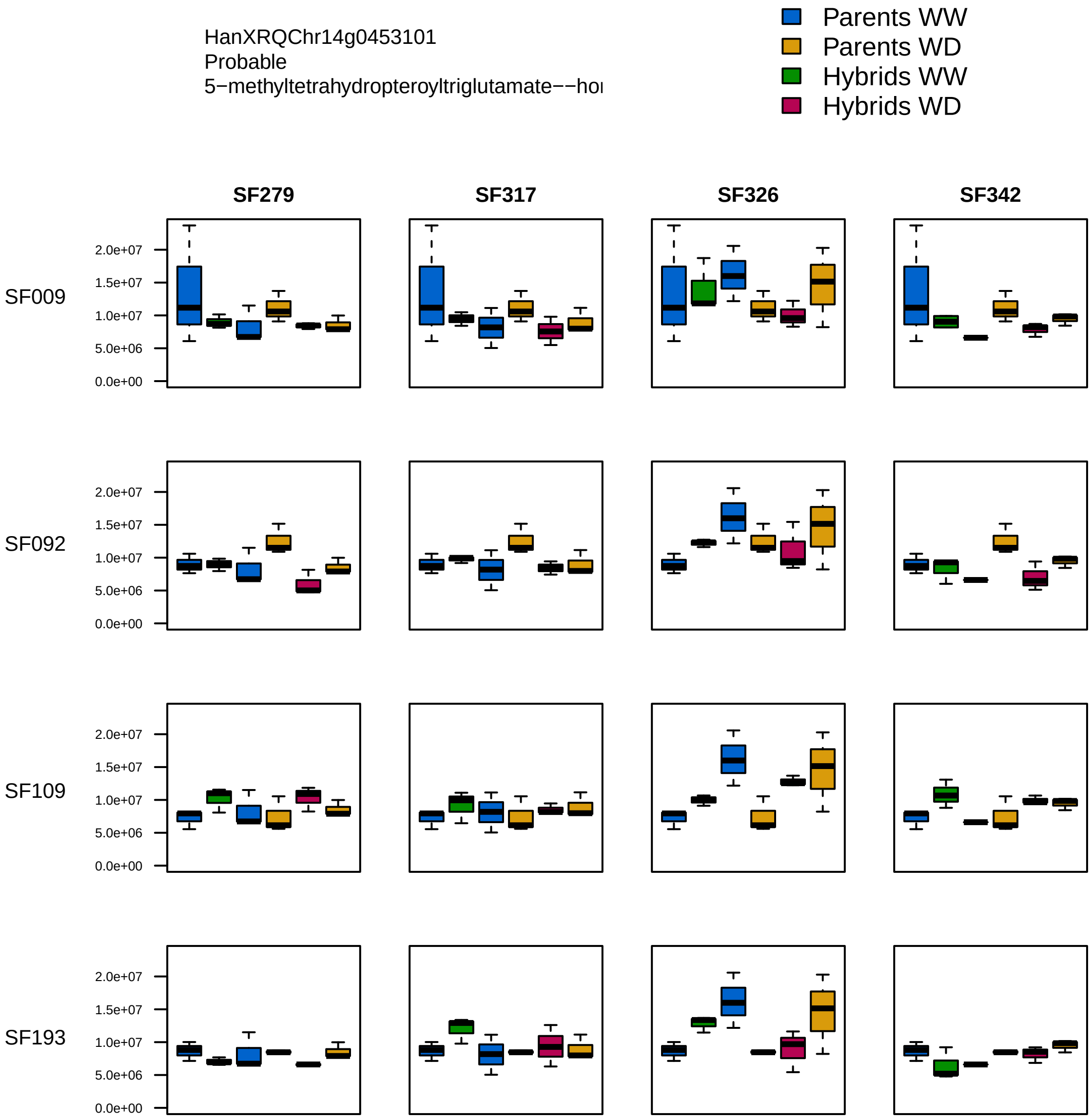
HanXRQChr14g0453101
Probable
5−methyltetrahydropteroyltriglutamate−−hoı
Parents WW
Parents WD
Hybrids WW
Hybrids WD
SF279
SF317
SF326
SF342
SF009
SF092
SF109
SF193
2.0e+07
1.5e+07
1.0e+07
5.0e+06
0.0e+00

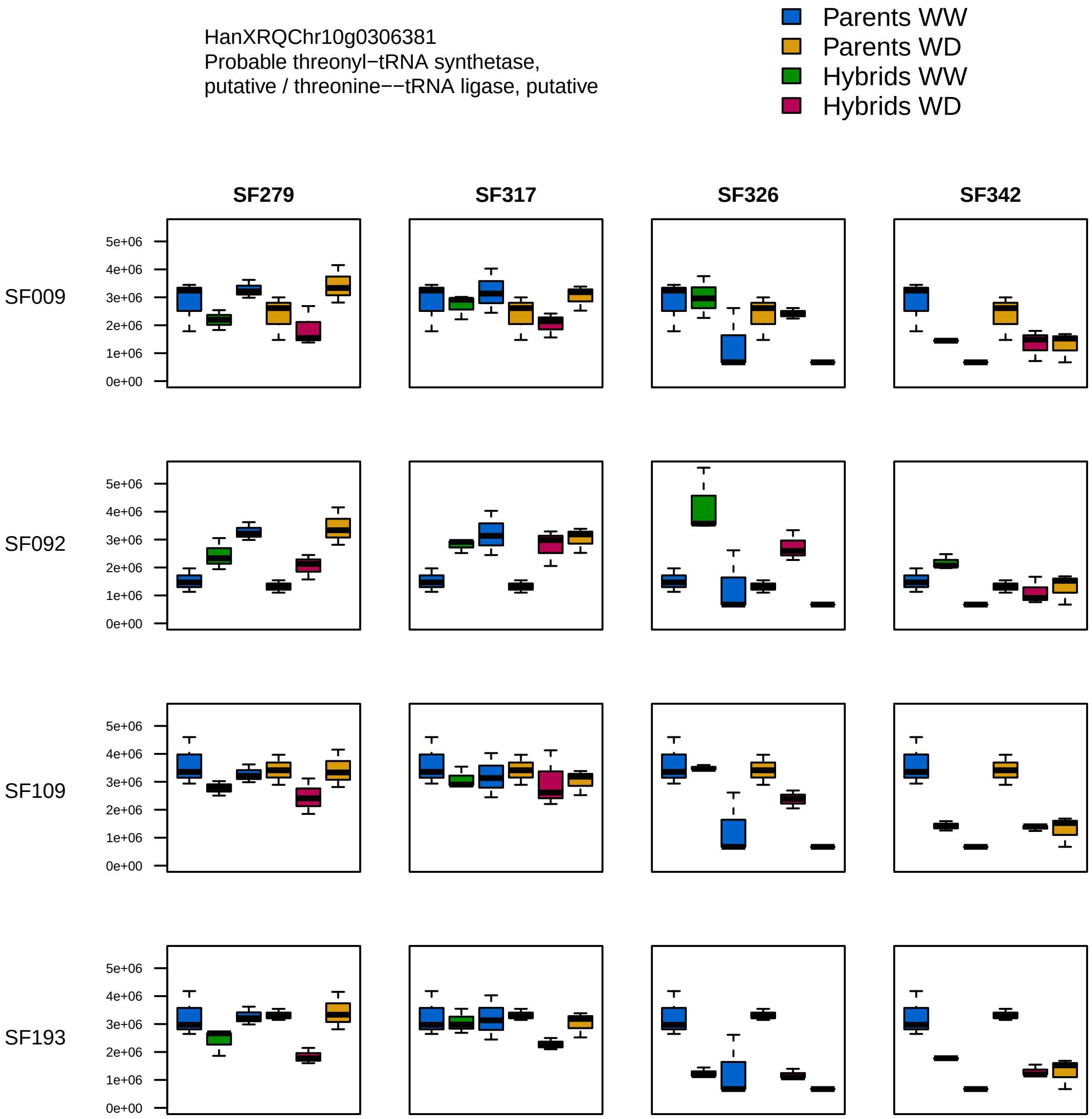
HanXRQChr10g0306381
Probable threonyl−tRNA synthetase,
putative / threonine−−tRNA ligase, putative
Parents WW
Parents WD
Hybrids WW
Hybrids WD
SF279
SF317
SF326
SF342
SF009
SF092
SF109
SF193
5e+06
4e+06
3e+06
2e+06
1e+06
0e+00

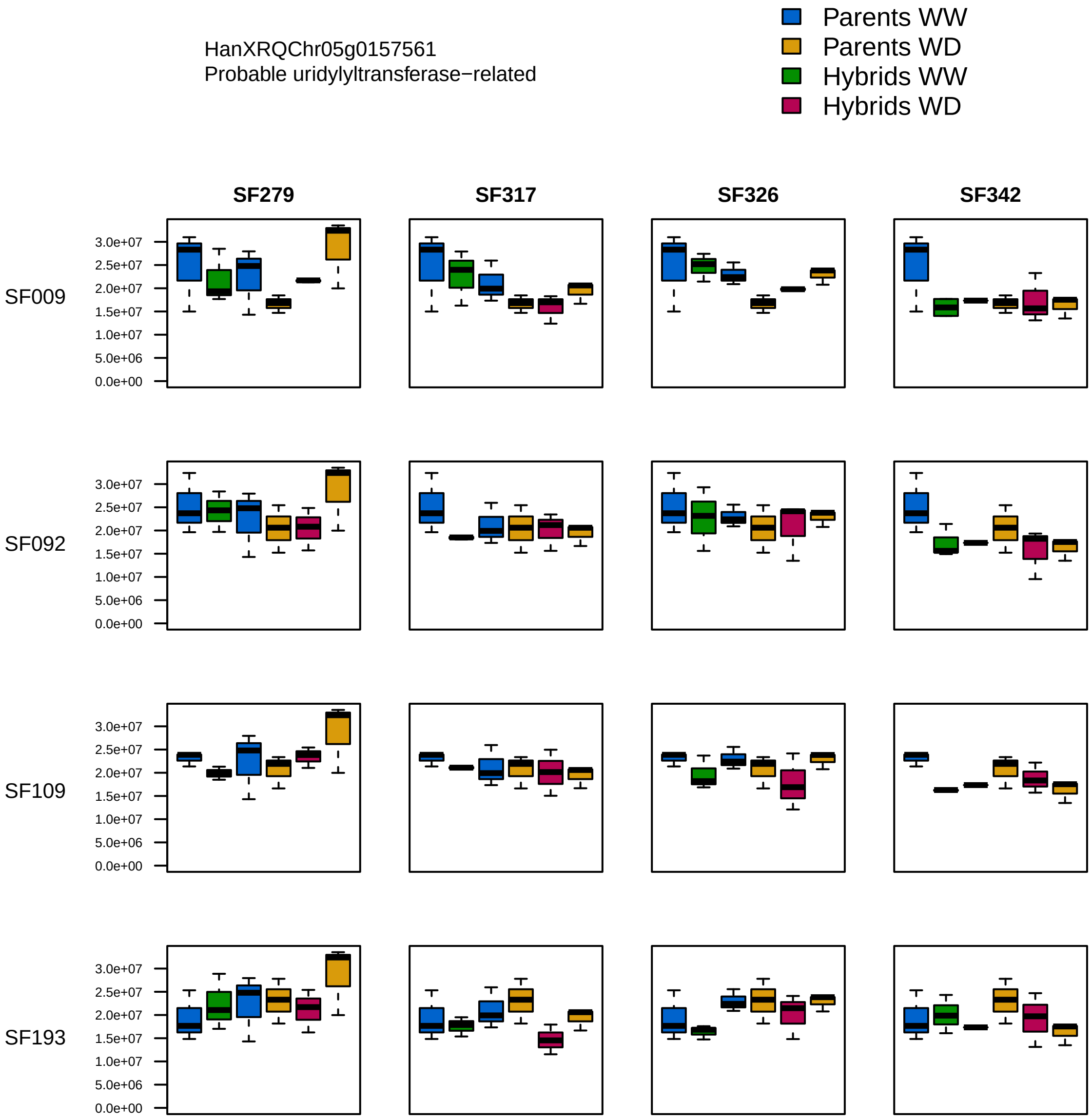
HanXRQChr05g0157561
Probable uridylyltransferase−related
Parents WW
Parents WD
Hybrids WW
Hybrids WD
SF279
SF317
SF326
SF342
SF009
SF092
SF109
SF193
0.0e+00
5.0e+06
1.0e+07
1.5e+07
2.0e+07
2.5e+07
3.0e+07

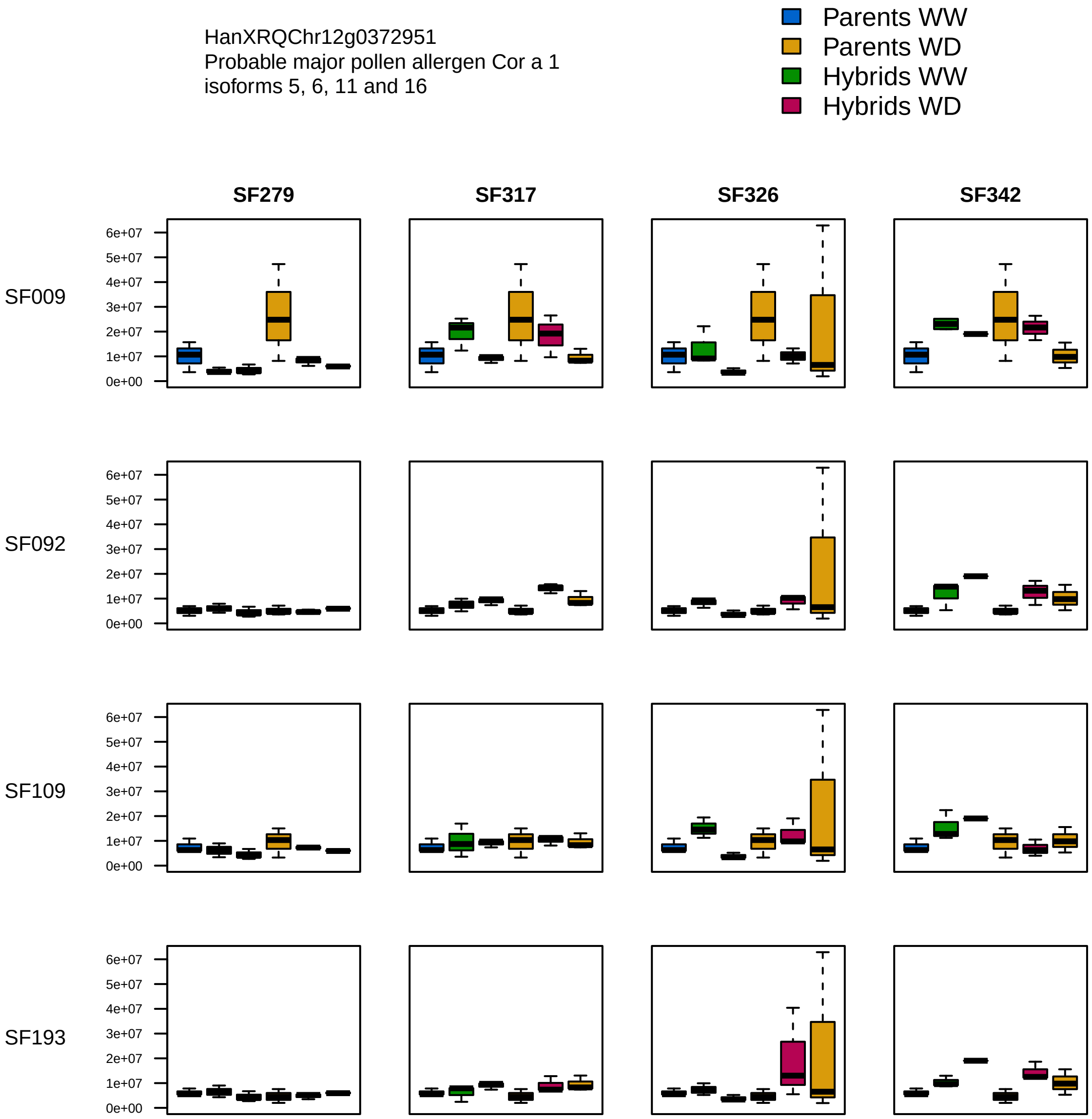
HanXRQChr12g0372951
Probable major pollen allergen Cor a 1
isoforms 5, 6, 11 and 16
Parents WW
Parents WD
Hybrids WW
Hybrids WD
SF279
SF317
SF326
SF342
SF009
SF092
SF109
SF193
6e+07
5e+07
4e+07
3e+07
2e+07
1e+07
0e+00

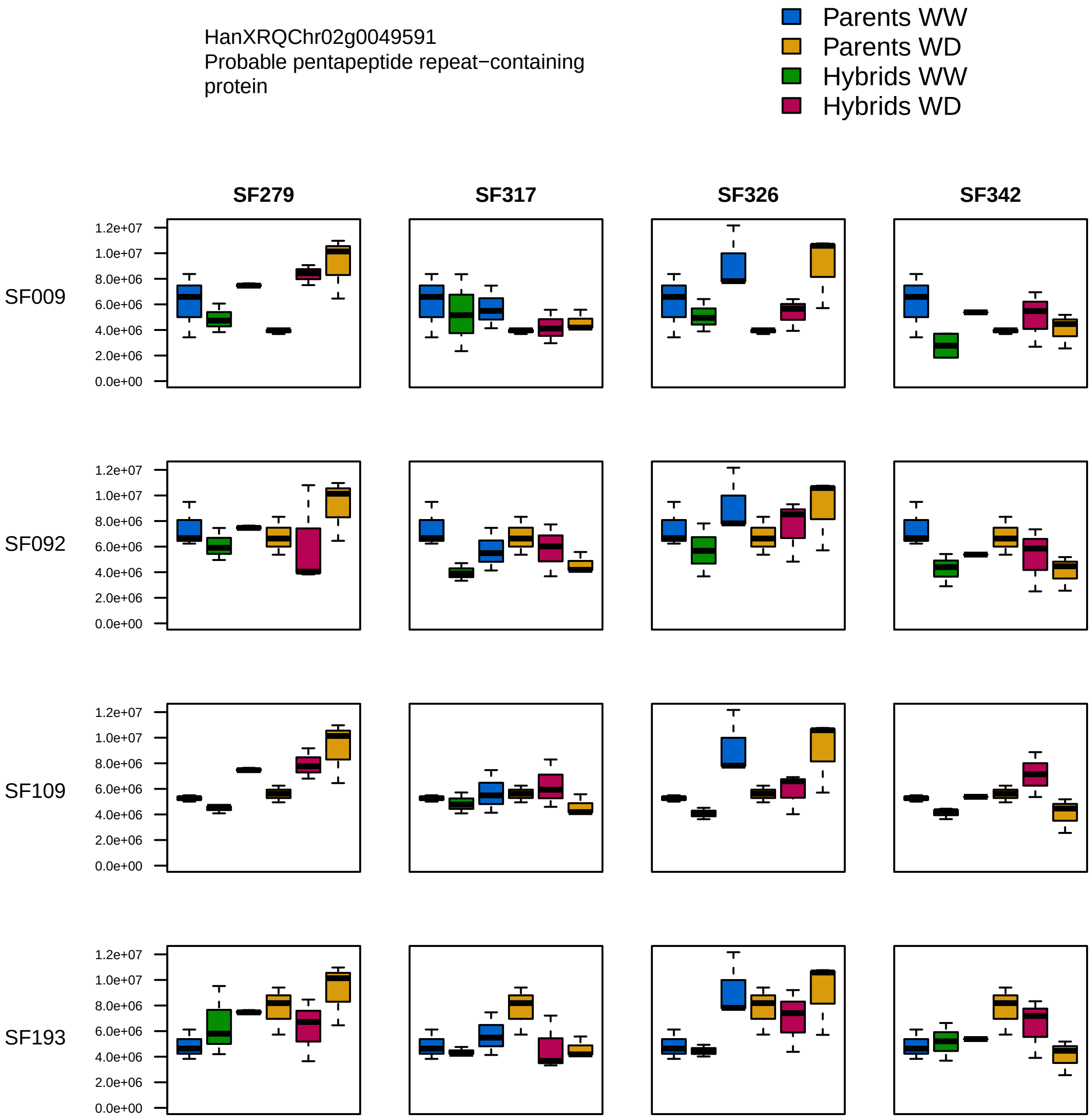
HanXRQChr02g0049591
Probable pentapeptide repeat−containing protein
Parents WW
Parents WD
Hybrids WW
Hybrids WD
SF279
SF317
SF326
SF342
SF009
SF092
SF109
SF193
1.2e+07
1.0e+07
8.0e+06
6.0e+06
4.0e+06
2.0e+06
0.0e+00

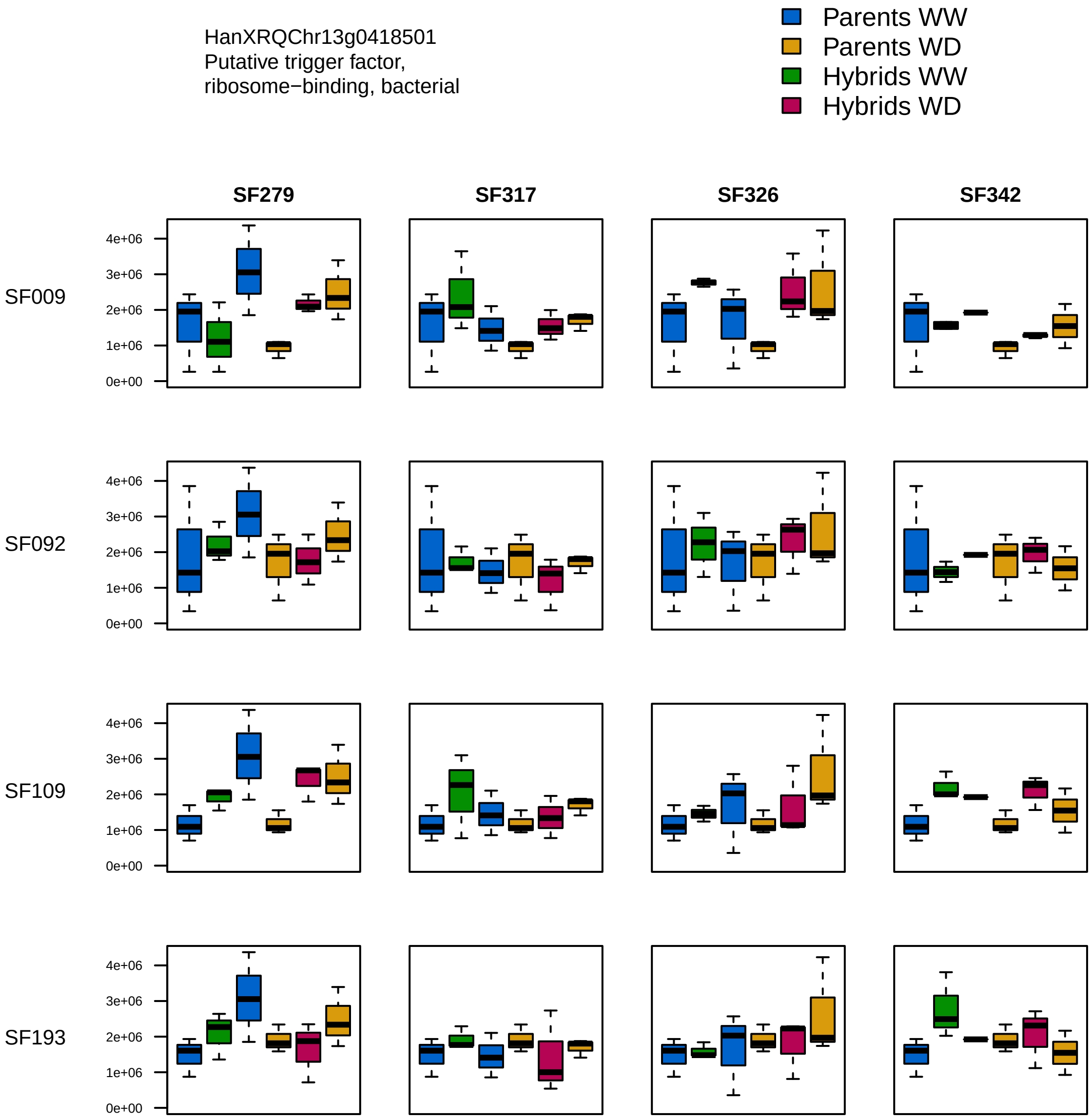
HanXRQChr13g0418501
Putative trigger factor,
ribosome−binding, bacterial
Parents WW
Parents WD
Hybrids WW
Hybrids WD
SF279
SF317
SF326
SF342
SF009
SF092
SF109
SF193
4e+06
3e+06
2e+06
1e+06
0e+00

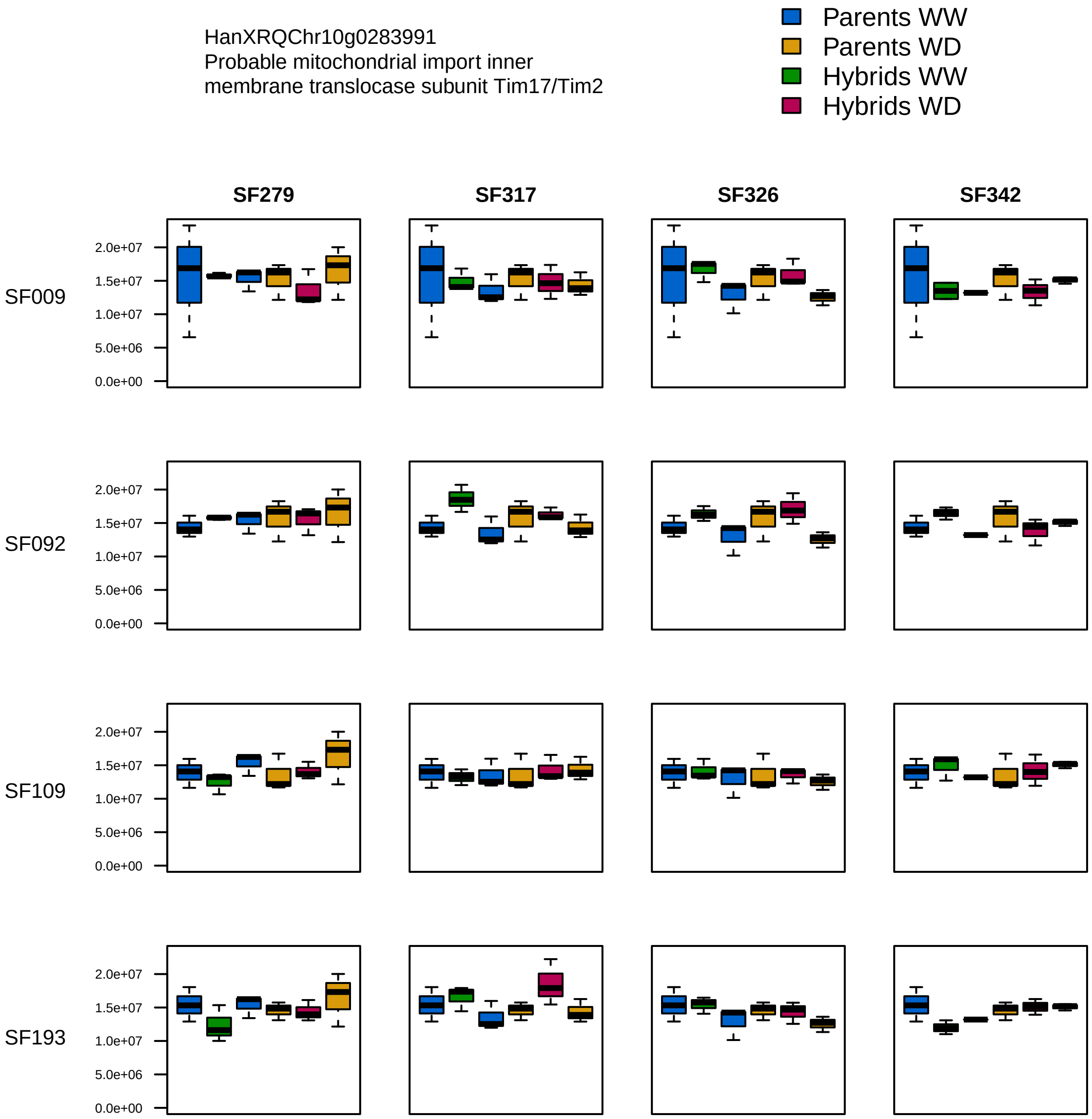
HanXRQChr10g0283991
Probable mitochondrial import inner
membrane translocase subunit Tim17/Tim2
Parents WW
Parents WD
Hybrids WW
Hybrids WD
SF279
SF317
SF326
SF342
SF009
SF092
SF109
SF193
0.0e+00
5.0e+06
1.0e+07
1.5e+07
2.0e+07

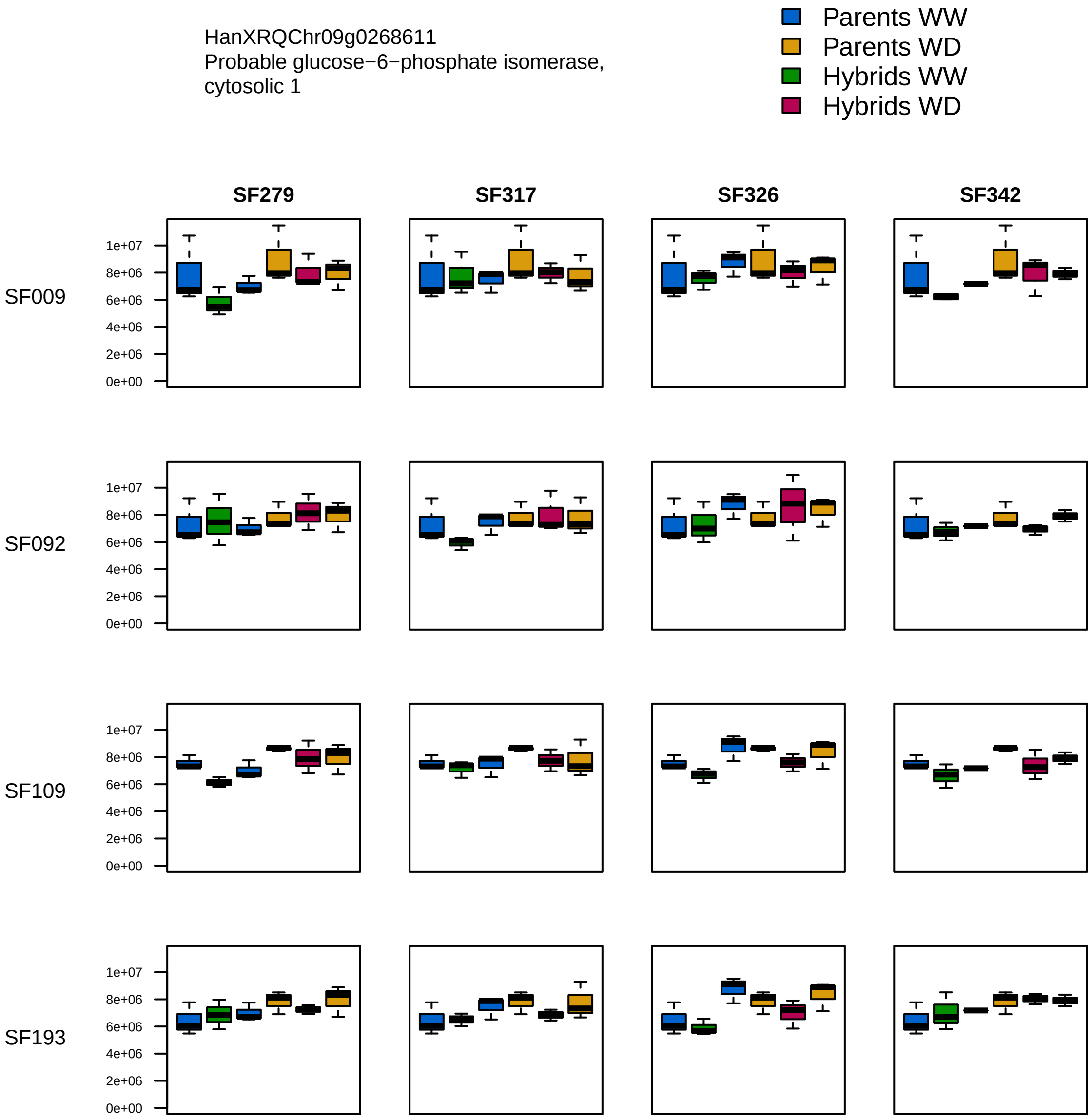

HanXRQChr09g0268611
Probable glucose−6−phosphate isomerase, cytosolic 1
Parents WW
Parents WD
Hybrids WW
Hybrids WD
SF279
SF317
SF326
SF342
SF009
SF092
SF109
SF193
1e+07
8e+06
6e+06
4e+06
2e+06
0e+00

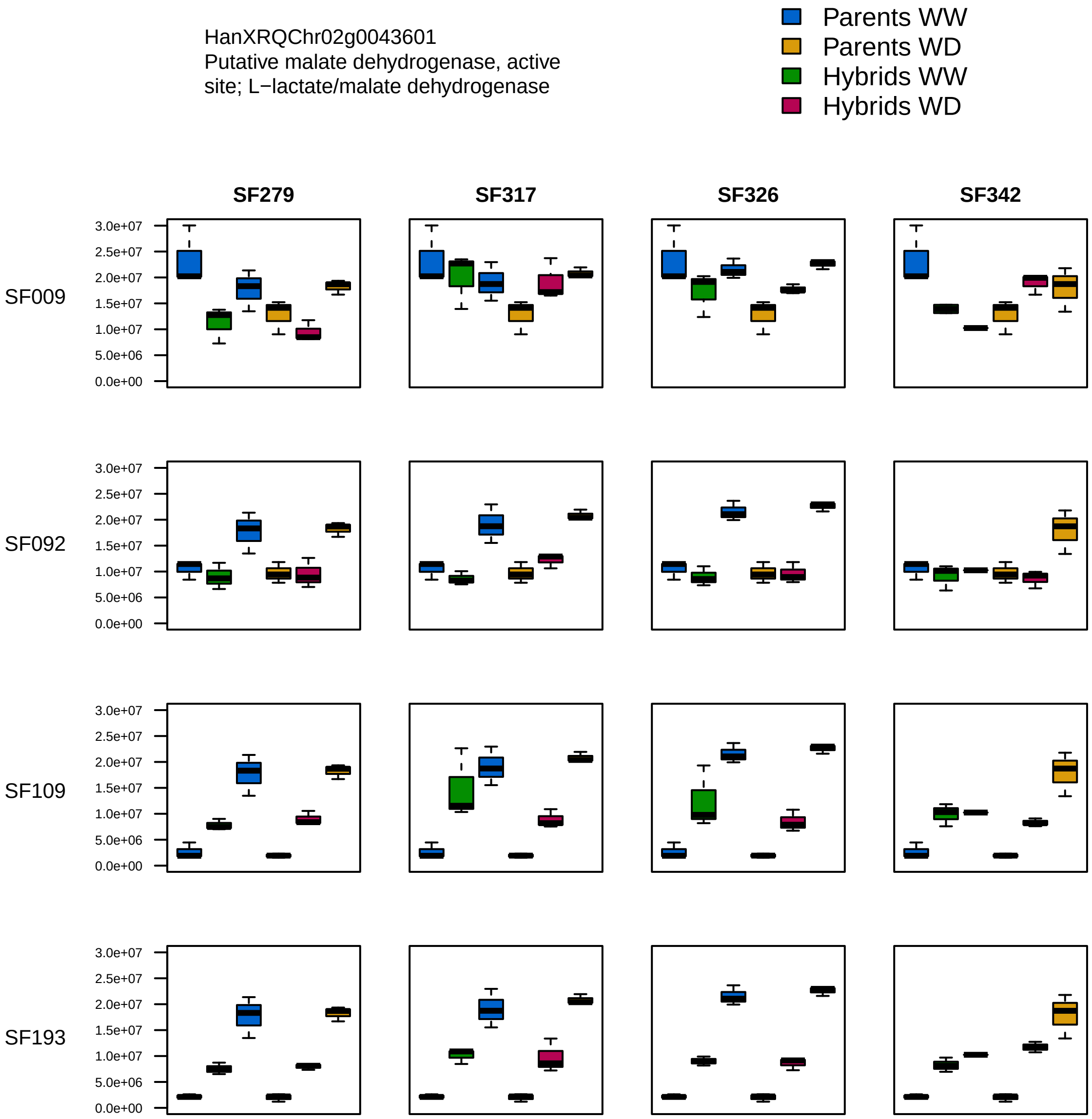
HanXRQChr02g0043601
Putative malate dehydrogenase, active site; L−lactate/malate dehydrogenase
Parents WW
Parents WD
Hybrids WW
Hybrids WD
SF279
SF317
SF326
SF342
SF009
SF092
SF109
SF193
3.0e+07
2.5e+07
2.0e+07
1.5e+07
1.0e+07
5.0e+06
0.0e+00

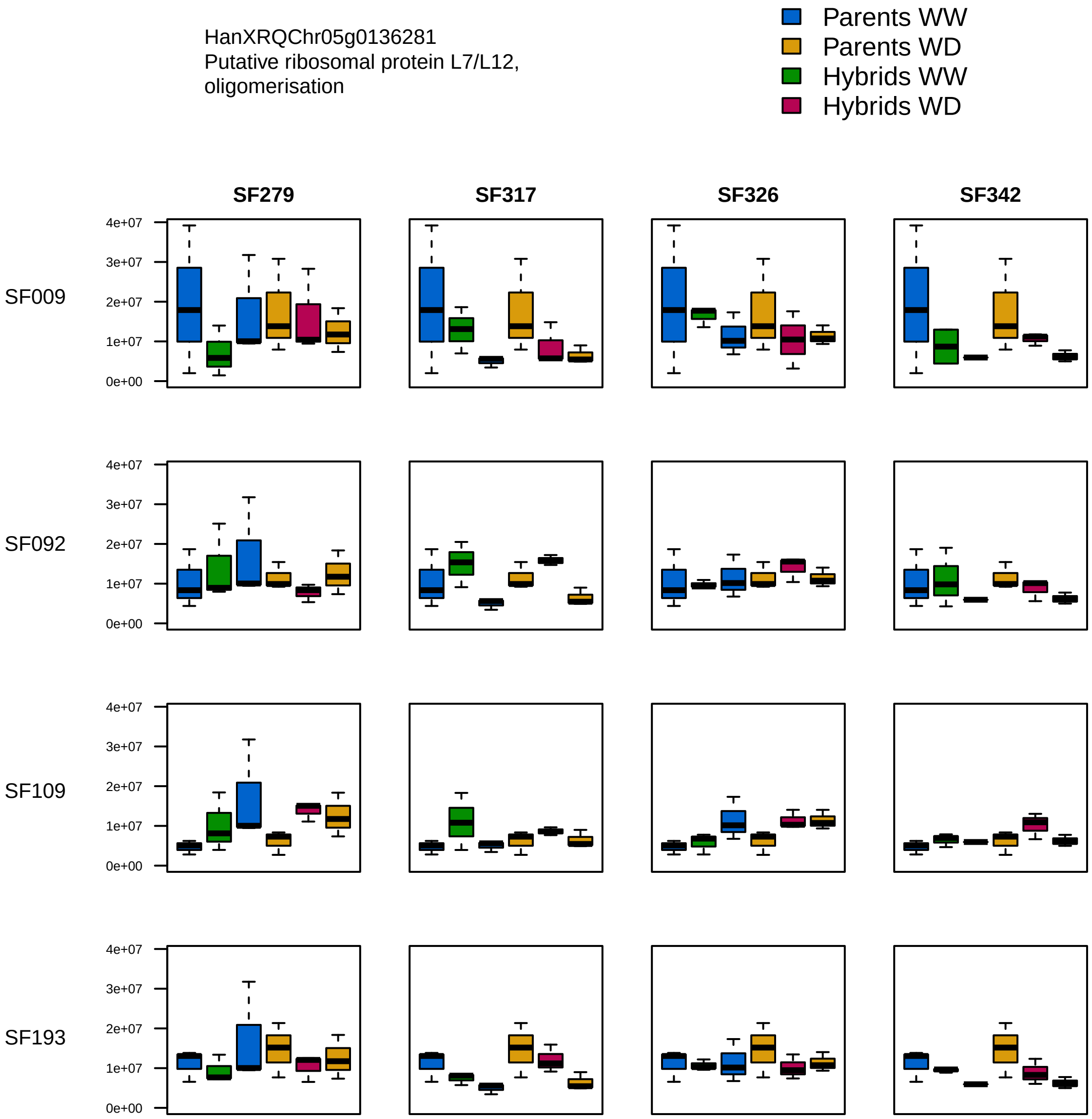

HanXRQChr05g0136281
Putative ribosomal protein L7/L12,
oligomerisation
Parents WW
Parents WD
Hybrids WW
Hybrids WD
SF279
SF317
SF326
SF342
SF009
SF092
SF109
SF193
4e+07
3e+07
2e+07
1e+07
0e+00

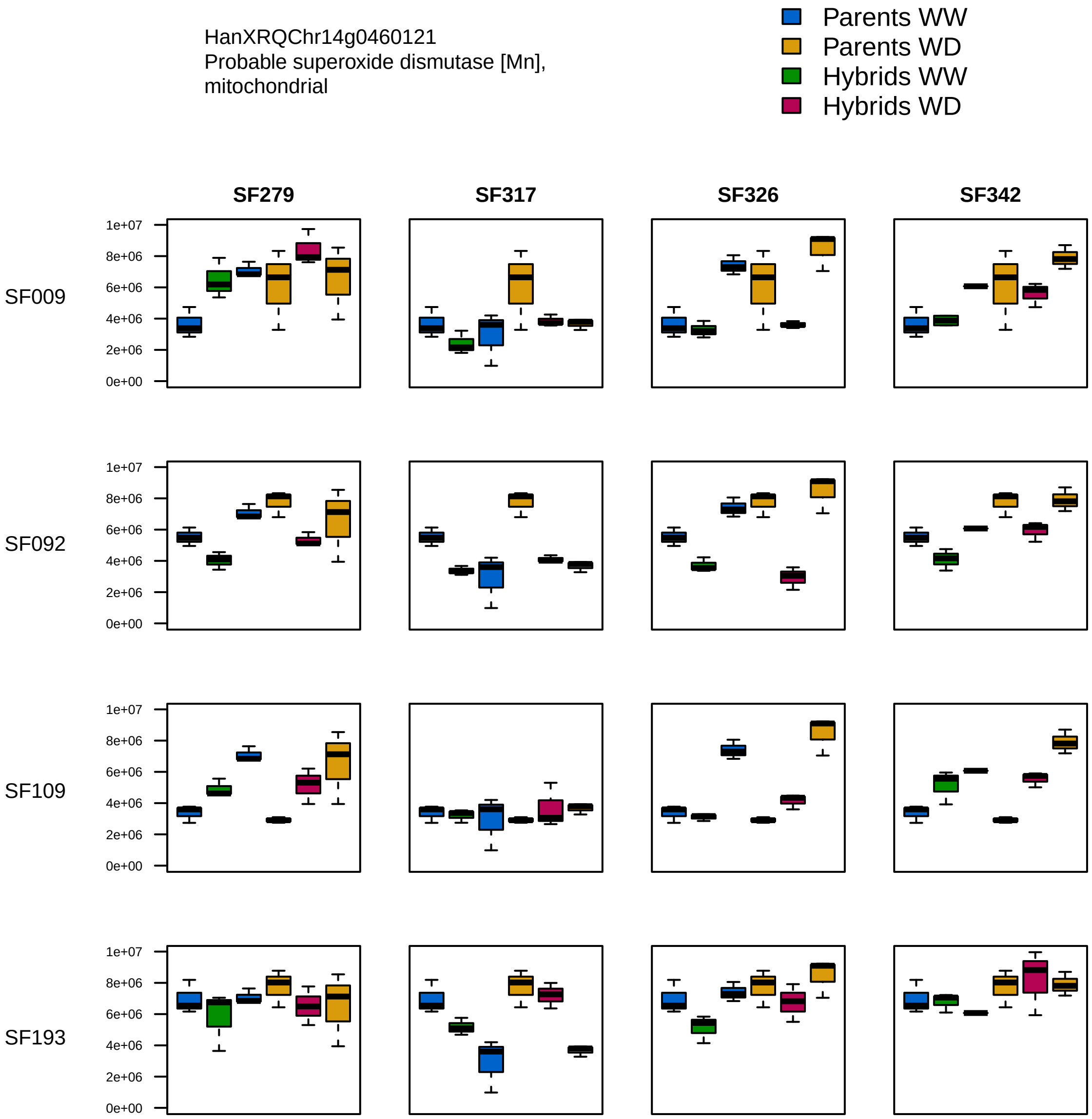
HanXRQChr14g0460121
Probable superoxide dismutase [Mn], mitochondrial
Parents WW
Parents WD
Hybrids WW
Hybrids WD
SF279
SF317
SF326
SF342
SF009
SF092
SF109
SF193
1e+07
8e+06
6e+06
4e+06
2e+06
0e+00

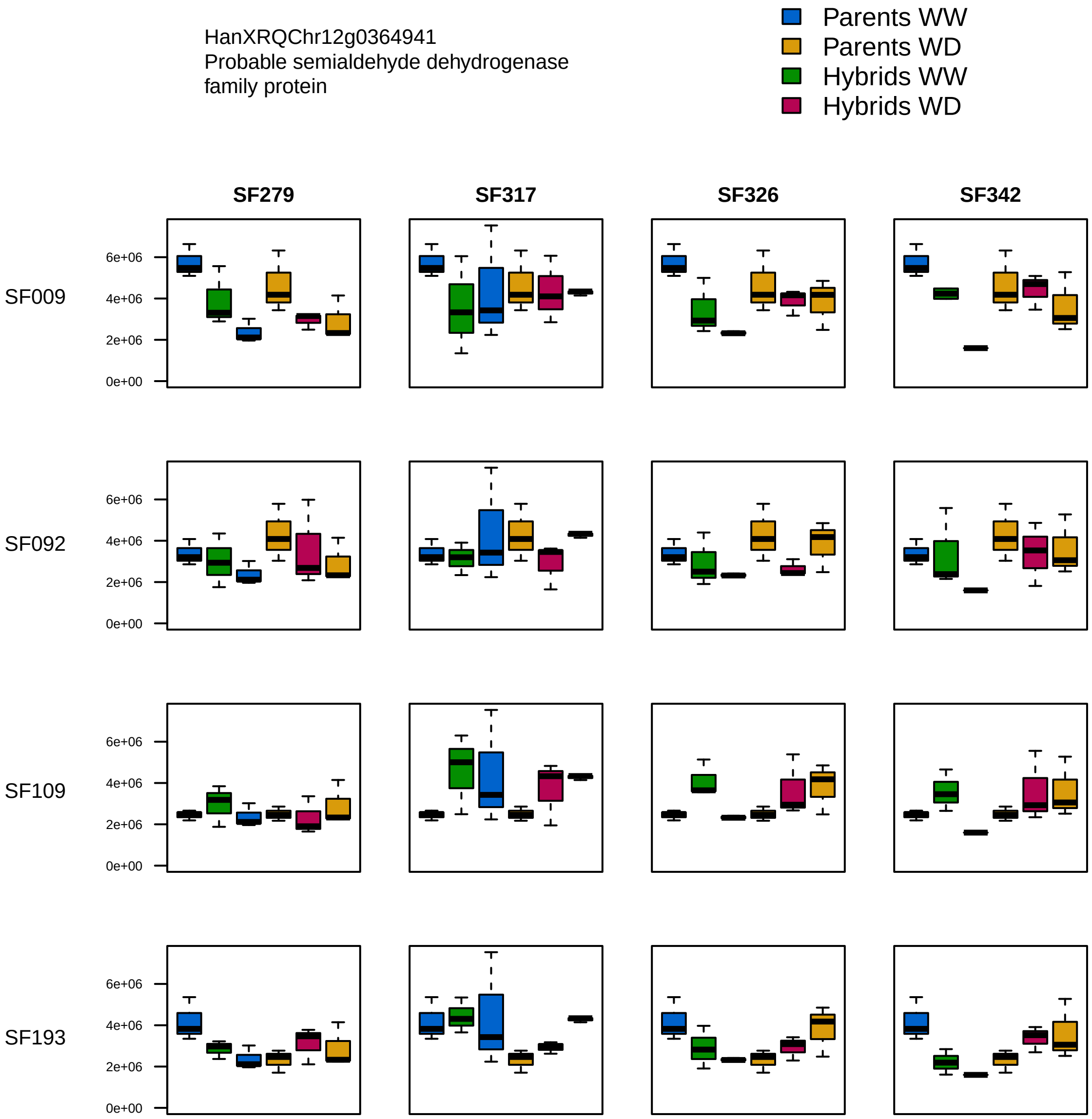
HanXRQChr12g0364941
Probable semialdehyde dehydrogenase
family protein
Parents WW
Parents WD
Hybrids WW
Hybrids WD
SF279
SF317
SF326
SF342
SF009
SF092
SF109
SF193
0e+00
2e+06
4e+06
6e+06

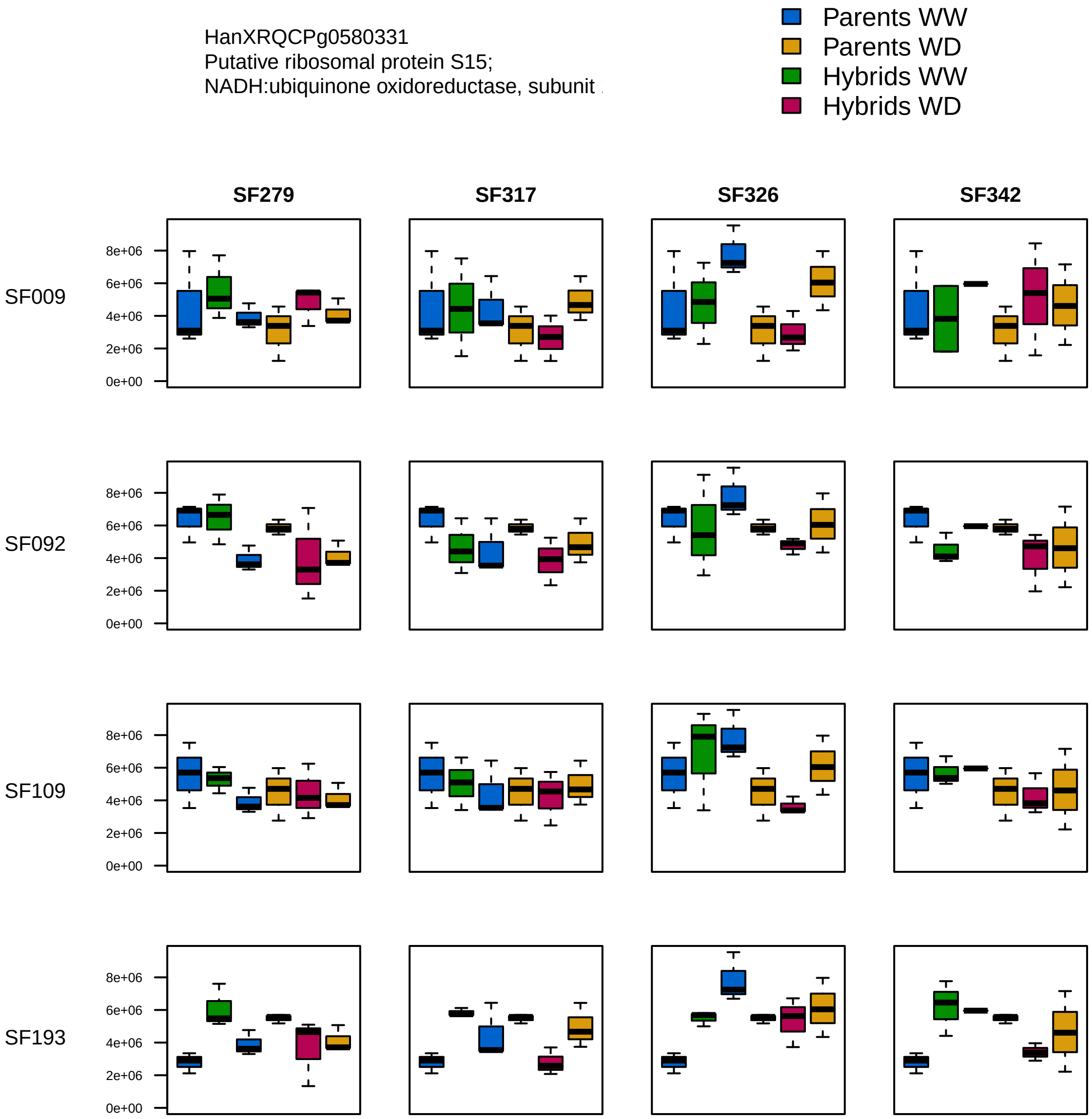
HanXRQCPg0580331
Putative ribosomal protein S15;
NADH:ubiquinone oxidoreductase, subunit
Parents WW
Parents WD
Hybrids WW
Hybrids WD
SF279
SF317
SF326
SF342
SF009
SF092
SF109
SF193
8e+06
6e+06
4e+06
2e+06
0e+00

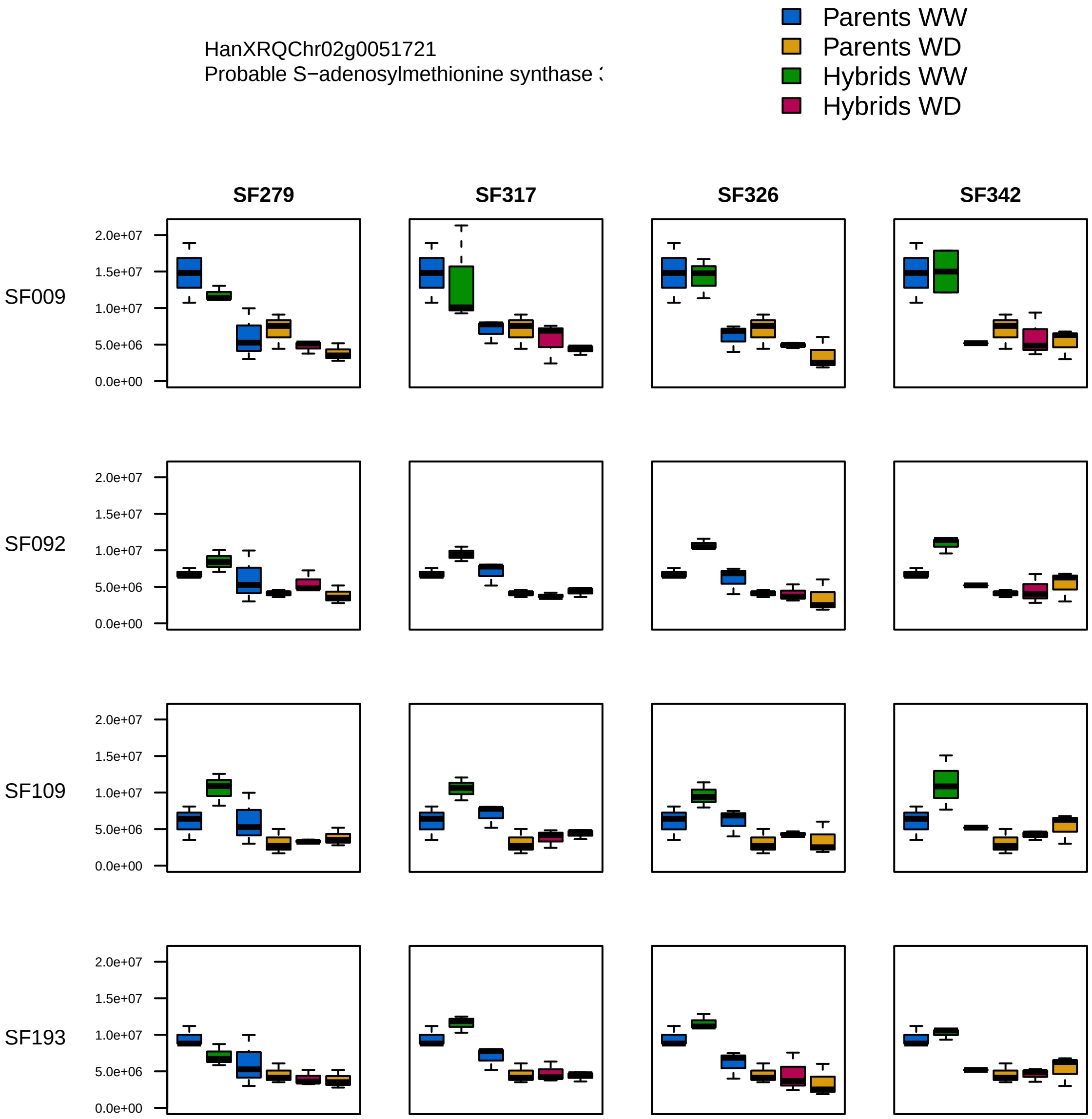
HanXRQChr02g0051721
Probable S−adenosylmethionine synthase
Parents WW
Parents WD
Hybrids WW
Hybrids WD
SF279
SF317
SF326
SF342
SF009
SF092
SF109
SF193
2.0e+07
1.5e+07
1.0e+07
5.0e+06
0.0e+00

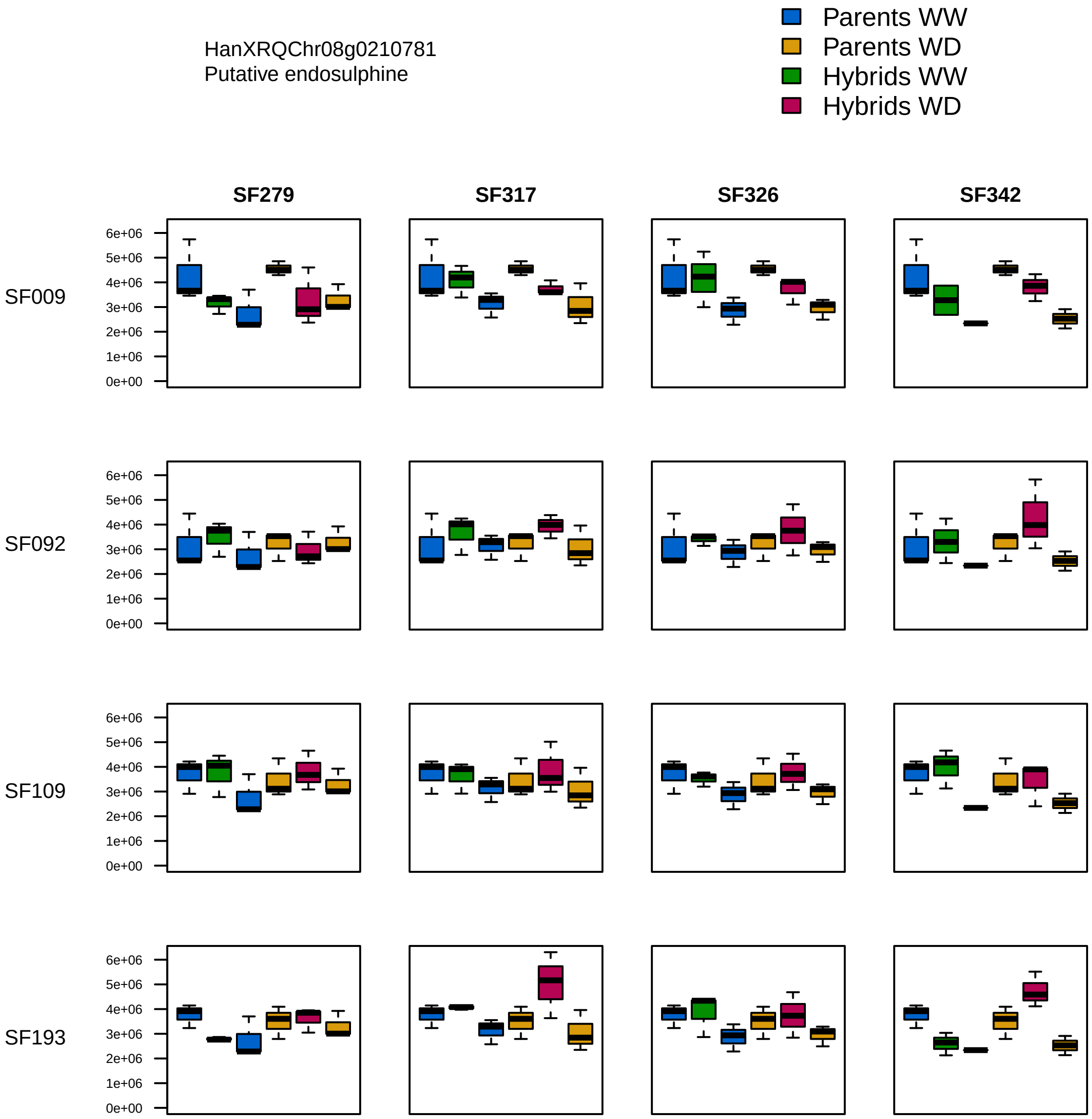
HanXRQChr08g0210781
Putative endosulphine
Parents WW
Parents WD
Hybrids WW
Hybrids WD
SF279
SF317
SF326
SF342
SF009
SF092
SF109
SF193
6e+06
5e+06
4e+06
3e+06
2e+06
1e+06
0e+00

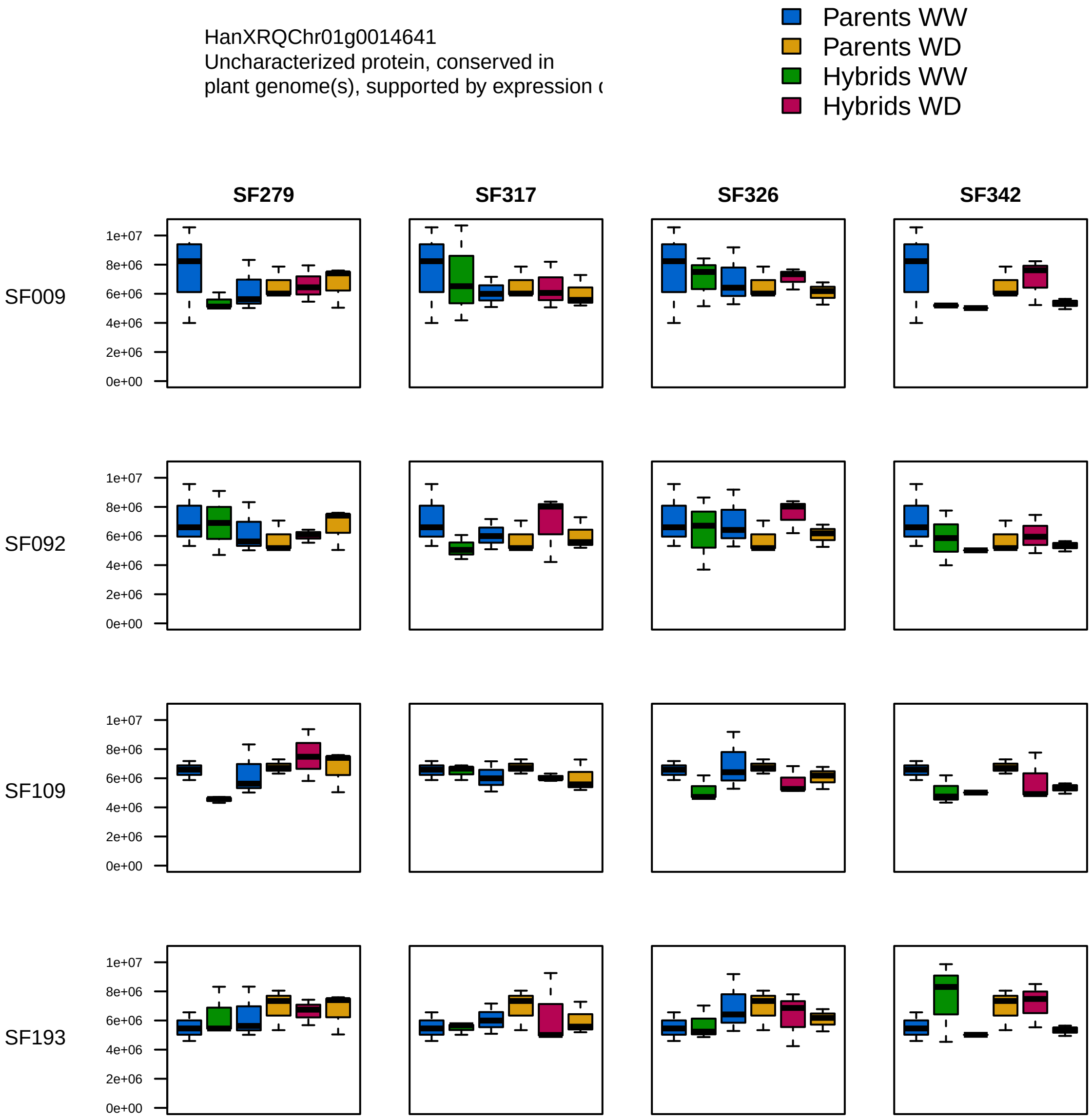
HanXRQChr01g0014641
Uncharacterized protein, conserved in
plant genome(s), supported by expression
Parents WW
Parents WD
Hybrids WW
Hybrids WD
SF279
SF317
SF326
SF342
SF009
SF092
SF109
SF193
1e+07
8e+06
6e+06
4e+06
2e+06
0e+00

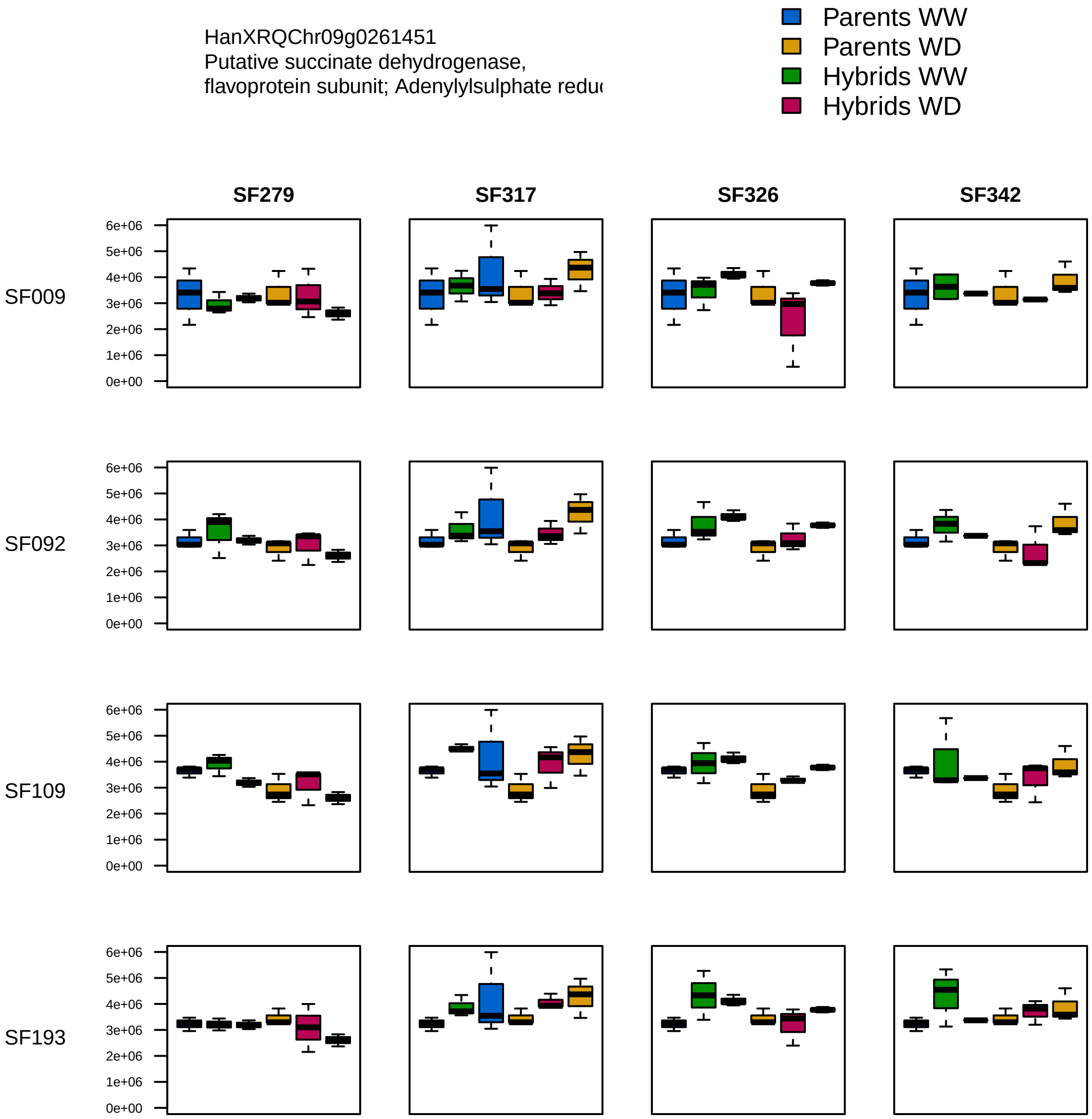
HanXRQChr09g0261451
Putative succinate dehydrogenase,
flavoprotein subunit; Adenylylsulphate redu
Parents WW
Parents WD
Hybrids WW
Hybrids WD
SF279
SF317
SF326
SF342
SF009
SF092
SF109
SF193
0e+00
1e+06
2e+06
3e+06
4e+06
5e+06
6e+06

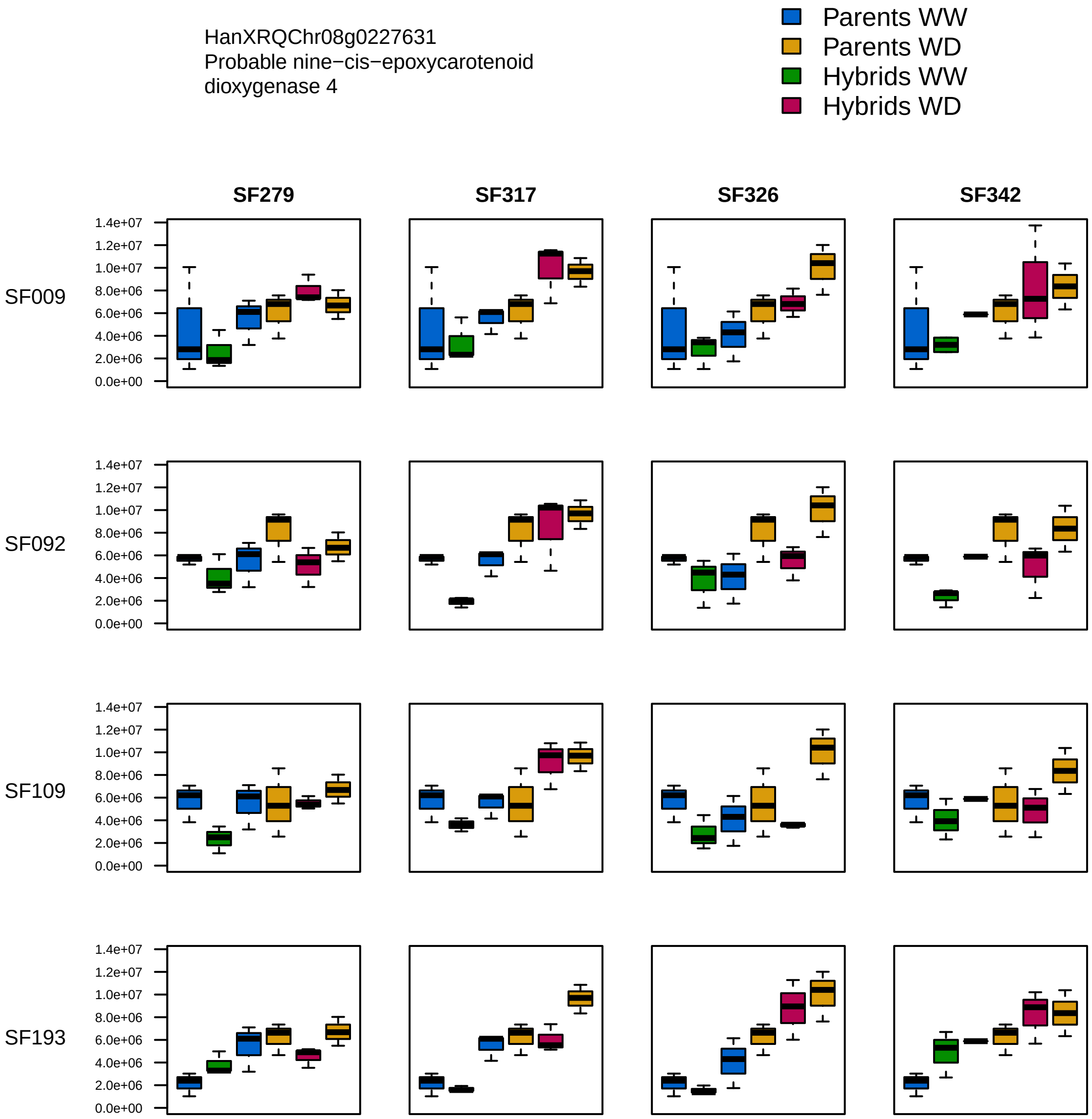
HanXRQChr08g0227631
Probable nine−cis−epoxycarotenoid
dioxygenase 4
Parents WW
Parents WD
Hybrids WW
Hybrids WD
SF279
SF317
SF326
SF342
SF009
SF092
SF109
SF193
1.4e+07
1.2e+07
1.0e+07
8.0e+06
6.0e+06
4.0e+06
2.0e+06
0.0e+00

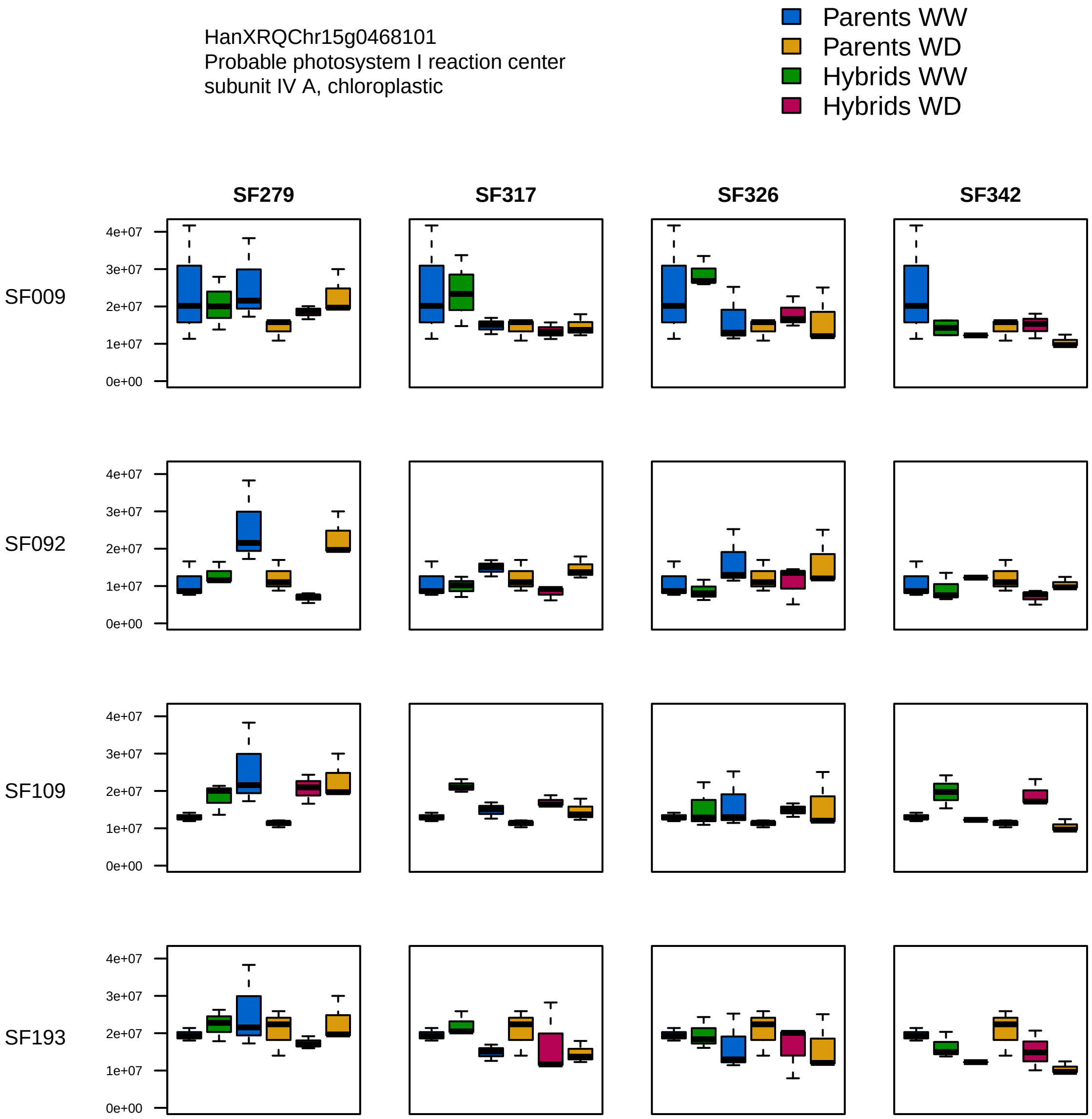
HanXRQChr15g0468101
Probable photosystem I reaction center
subunit IV A, chloroplastic
Parents WW
Parents WD
Hybrids WW
Hybrids WD
SF279
SF317
SF326
SF342
SF009
SF092
SF109
SF193
0e+00
1e+07
2e+07
3e+07
4e+07

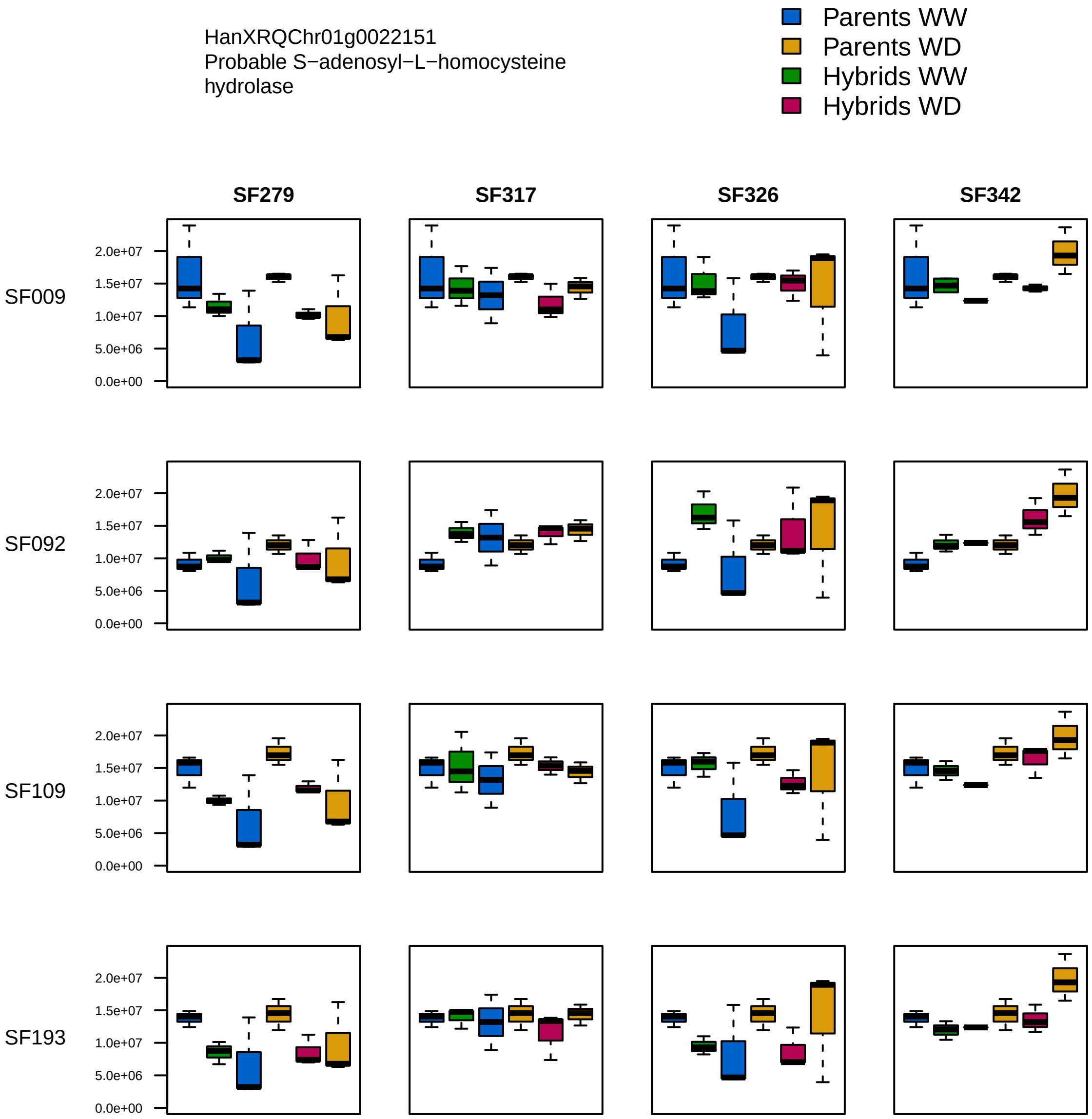
HanXRQChr01g0022151
Probable S−adenosyl−L−homocysteine
hydrolase
Parents WW
Parents WD
Hybrids WW
Hybrids WD
SF279
SF317
SF326
SF342
SF009
SF092
SF109
SF193
2.0e+07
1.5e+07
1.0e+07
5.0e+06
0.0e+00

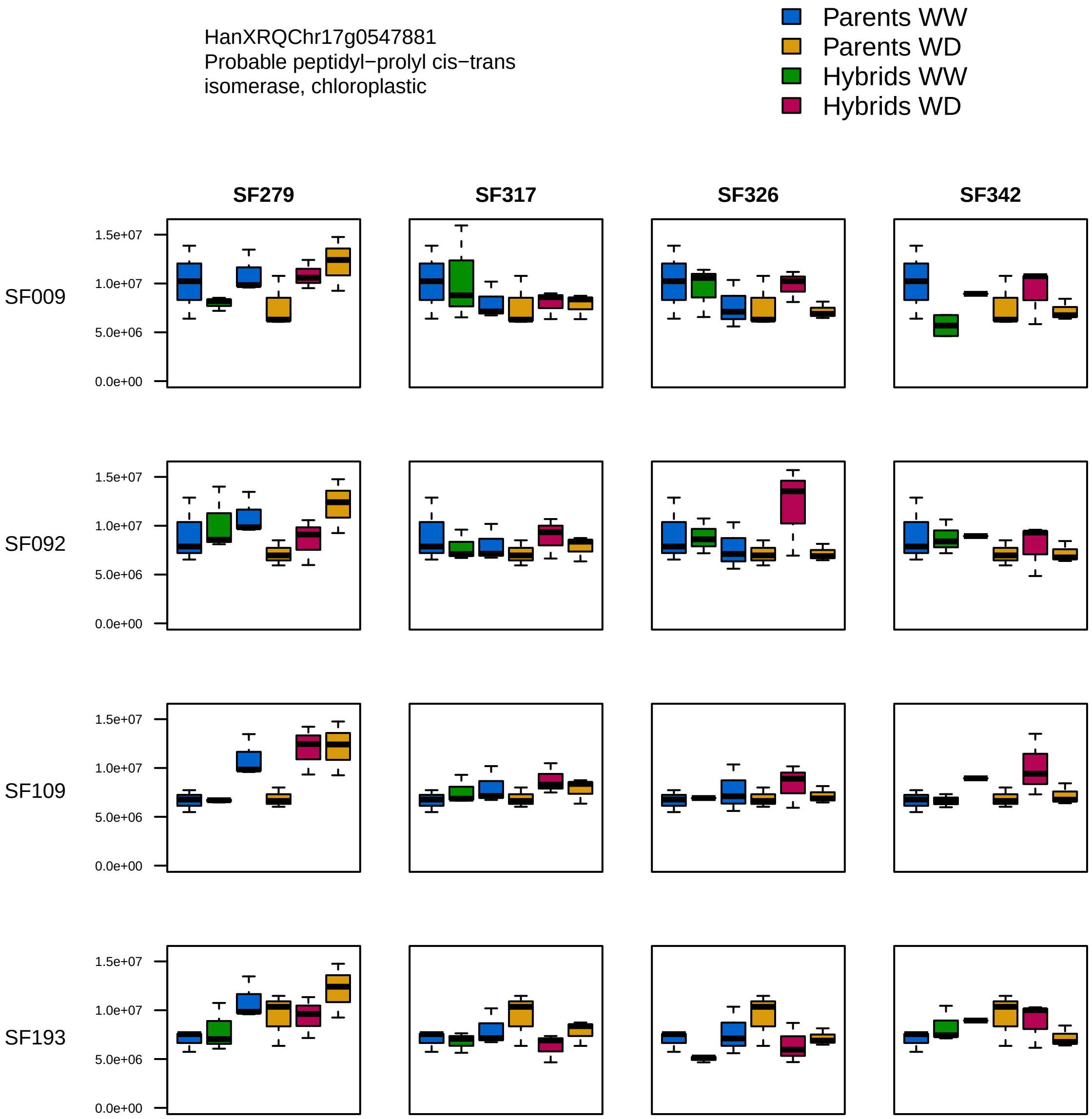
HanXRQChr17g0547881
Probable peptidyl−prolyl cis−trans
isomerase, chloroplastic
Parents WW
Parents WD
Hybrids WW
Hybrids WD
SF279
SF317
SF326
SF342
SF009
SF092
SF109
SF193
1.5e+07
1.0e+07
5.0e+06
0.0e+00

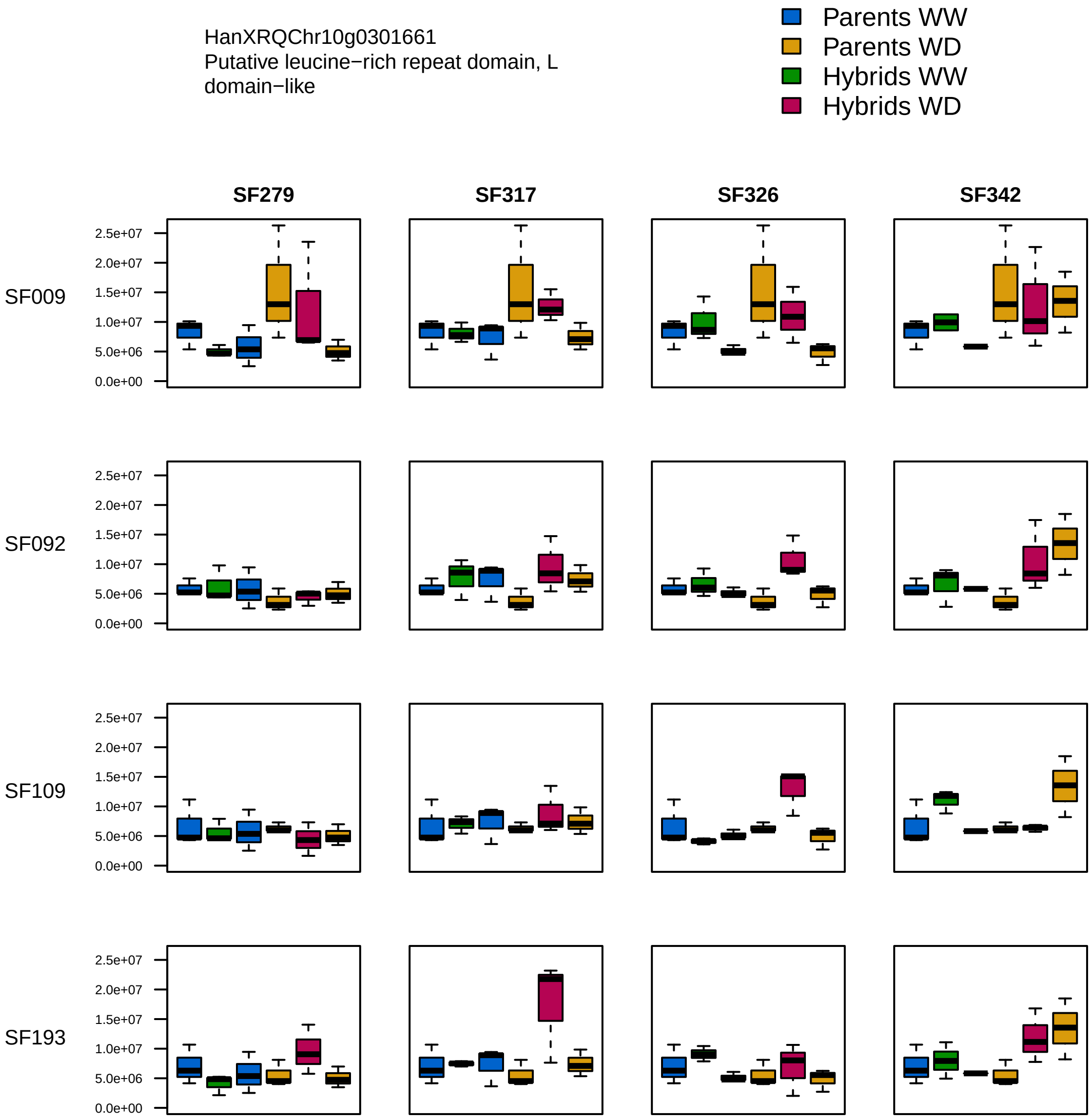

HanXRQChr10g0301661
Putative leucine−rich repeat domain, L domain−like
Parents WW
Parents WD
Hybrids WW
Hybrids WD
SF279
SF317
SF326
SF342
SF009
SF092
SF109
SF193
2.5e+07
2.0e+07
1.5e+07
1.0e+07
5.0e+06
0.0e+00

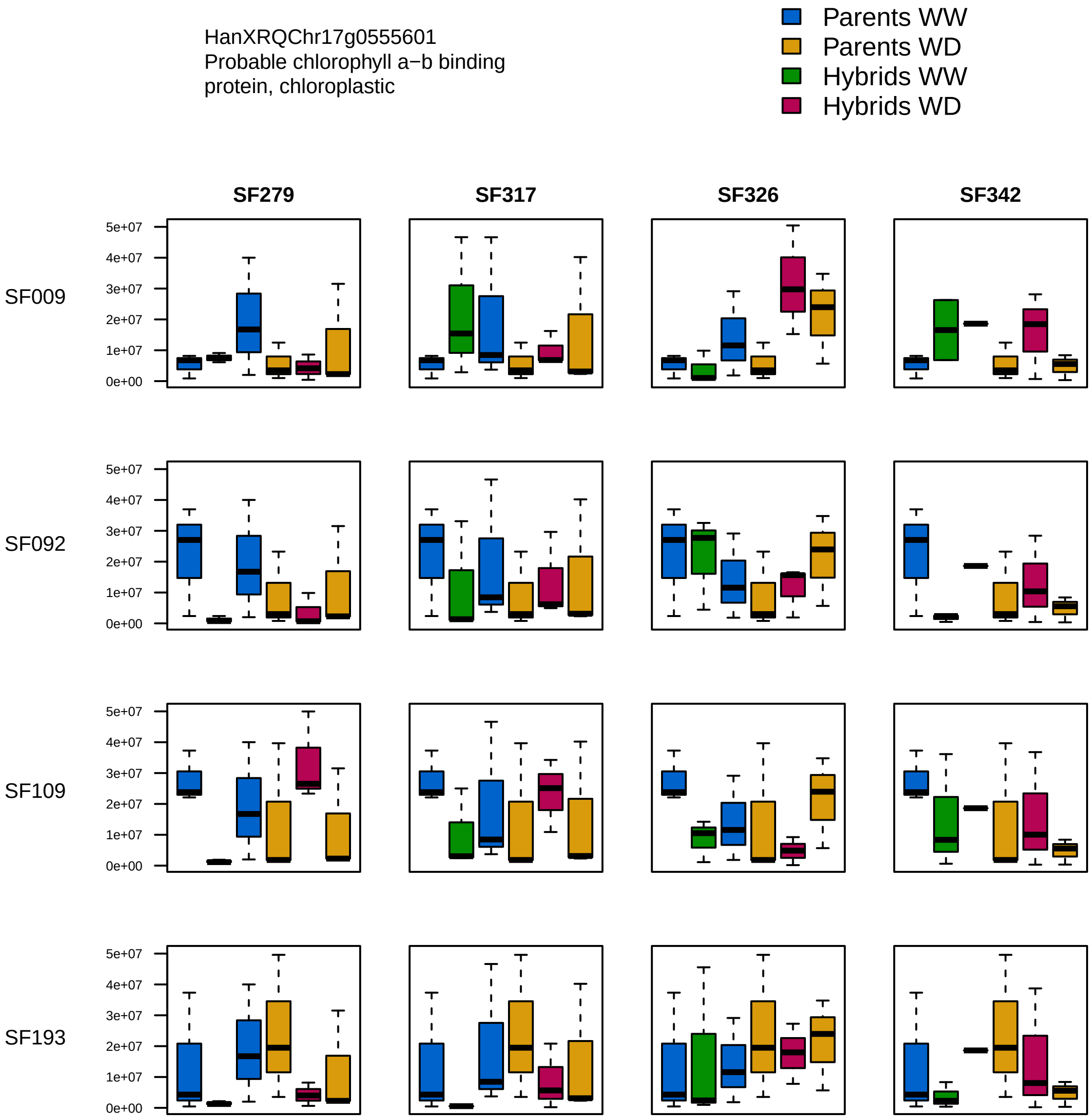
HanXRQChr17g0555601
Probable chlorophyll a−b binding
protein, chloroplastic
Parents WW
Parents WD
Hybrids WW
Hybrids WD
SF279
SF317
SF326
SF342
SF009
SF092
SF109
SF193
5e+07
4e+07
3e+07
2e+07
1e+07
0e+00

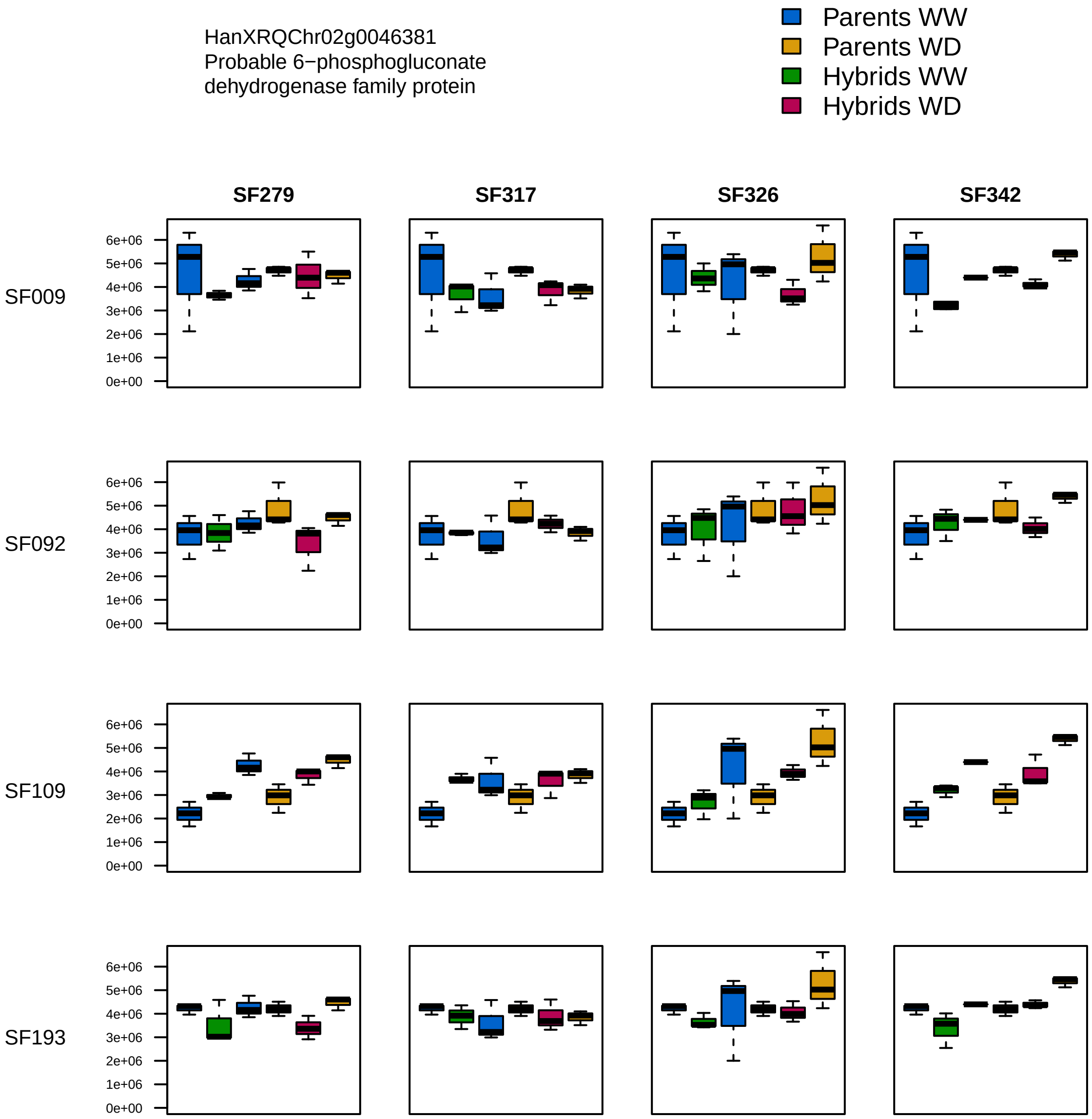
HanXRQChr02g0046381
Probable 6−phosphogluconate
dehydrogenase family protein
Parents WW
Parents WD
Hybrids WW
Hybrids WD
SF279
SF317
SF326
SF342
SF009
SF092
SF109
SF193
0e+00
1e+06
2e+06
3e+06
4e+06
5e+06
6e+06

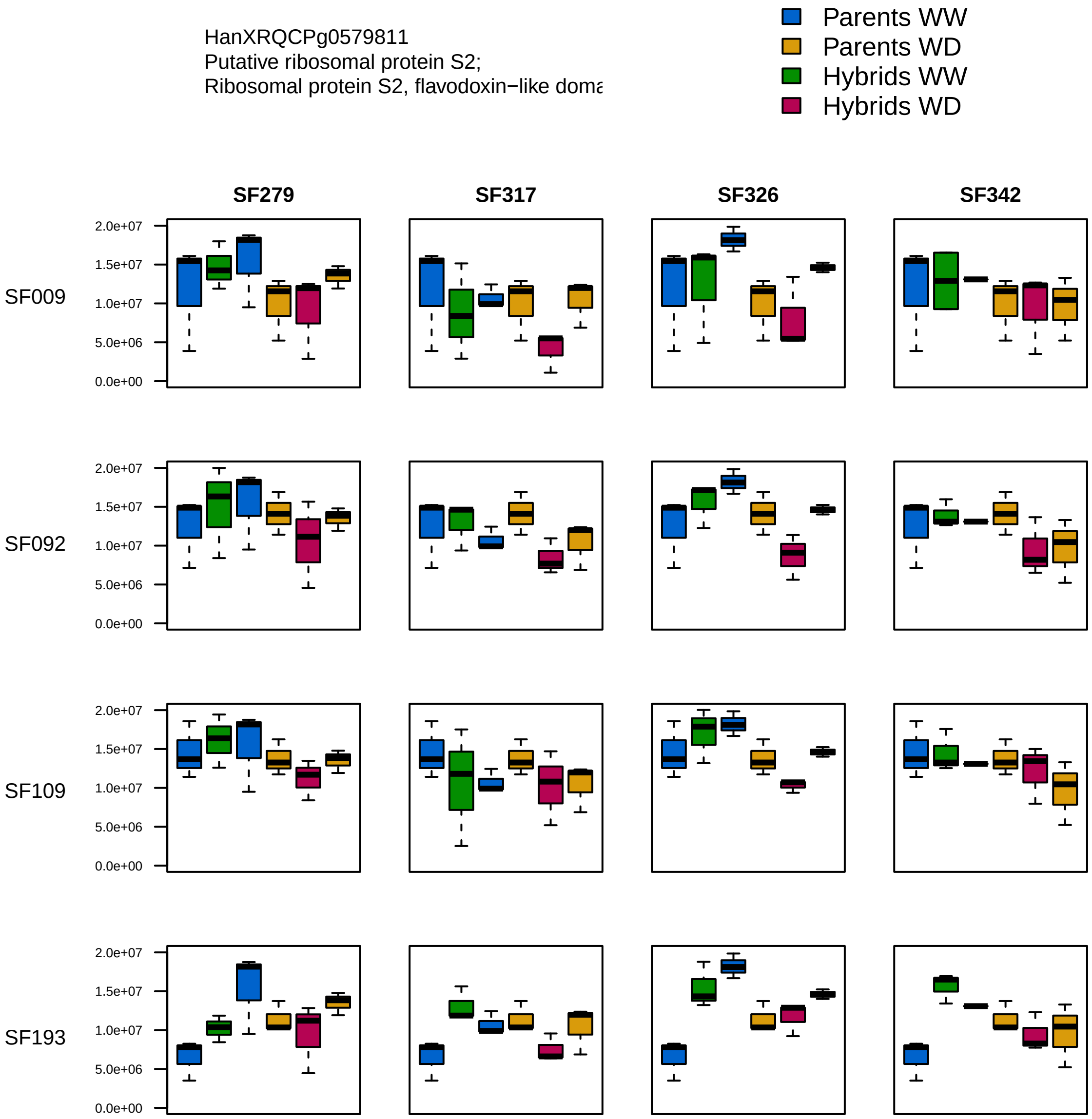

HanXRQCPg0579811
Putative ribosomal protein S2;
Ribosomal protein S2, flavodoxin−like doma
Parents WW
Parents WD
Hybrids WW
Hybrids WD
SF279
SF317
SF326
SF342
SF009
SF092
SF109
SF193
2.0e+07
1.5e+07
1.0e+07
5.0e+06
0.0e+00

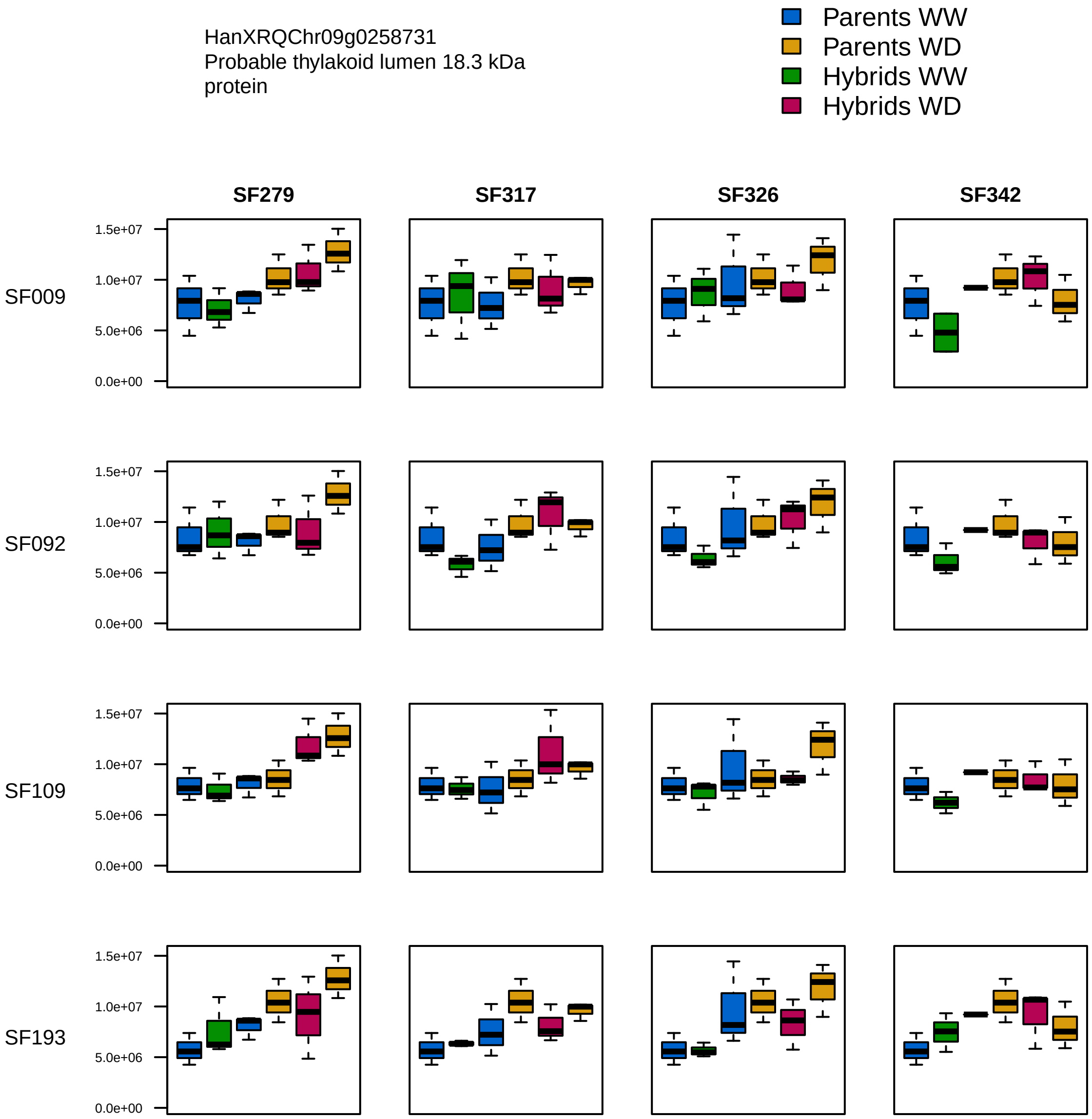
HanXRQChr09g0258731
Probable thylakoid lumen 18.3 kDa
protein
Parents WW
Parents WD
Hybrids WW
Hybrids WD
SF279
SF317
SF326
SF342
SF009
SF092
SF109
SF193
1.5e+07
1.0e+07
5.0e+06
0.0e+00

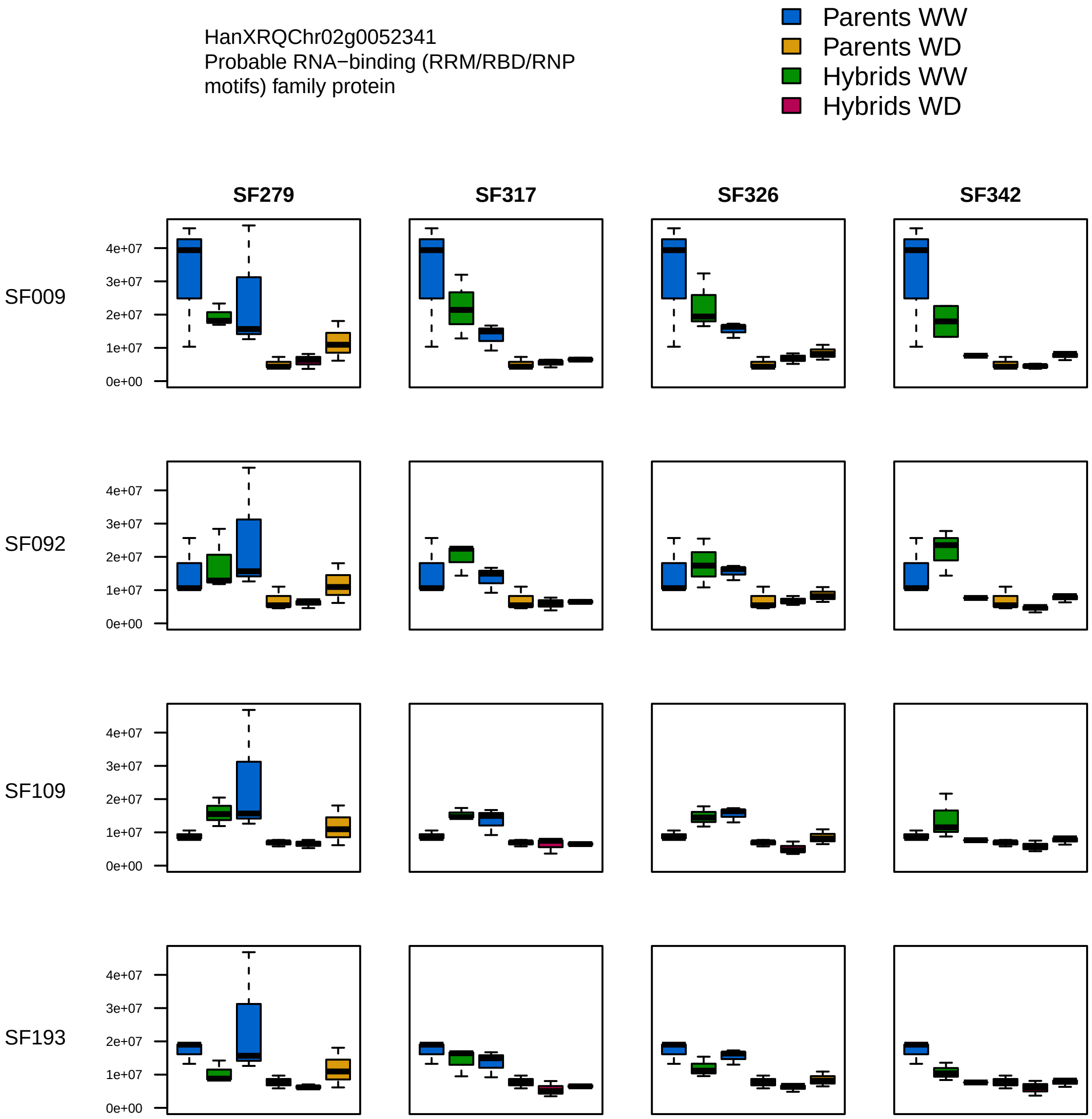

HanXRQChr02g0052341
Probable RNA−binding (RRM/RBD/RNP
motifs) family protein
Parents WW
Parents WD
Hybrids WW
Hybrids WD
SF279
SF317
SF326
SF342
SF009
SF092
SF109
SF193
0e+00
1e+07
2e+07
3e+07
4e+07

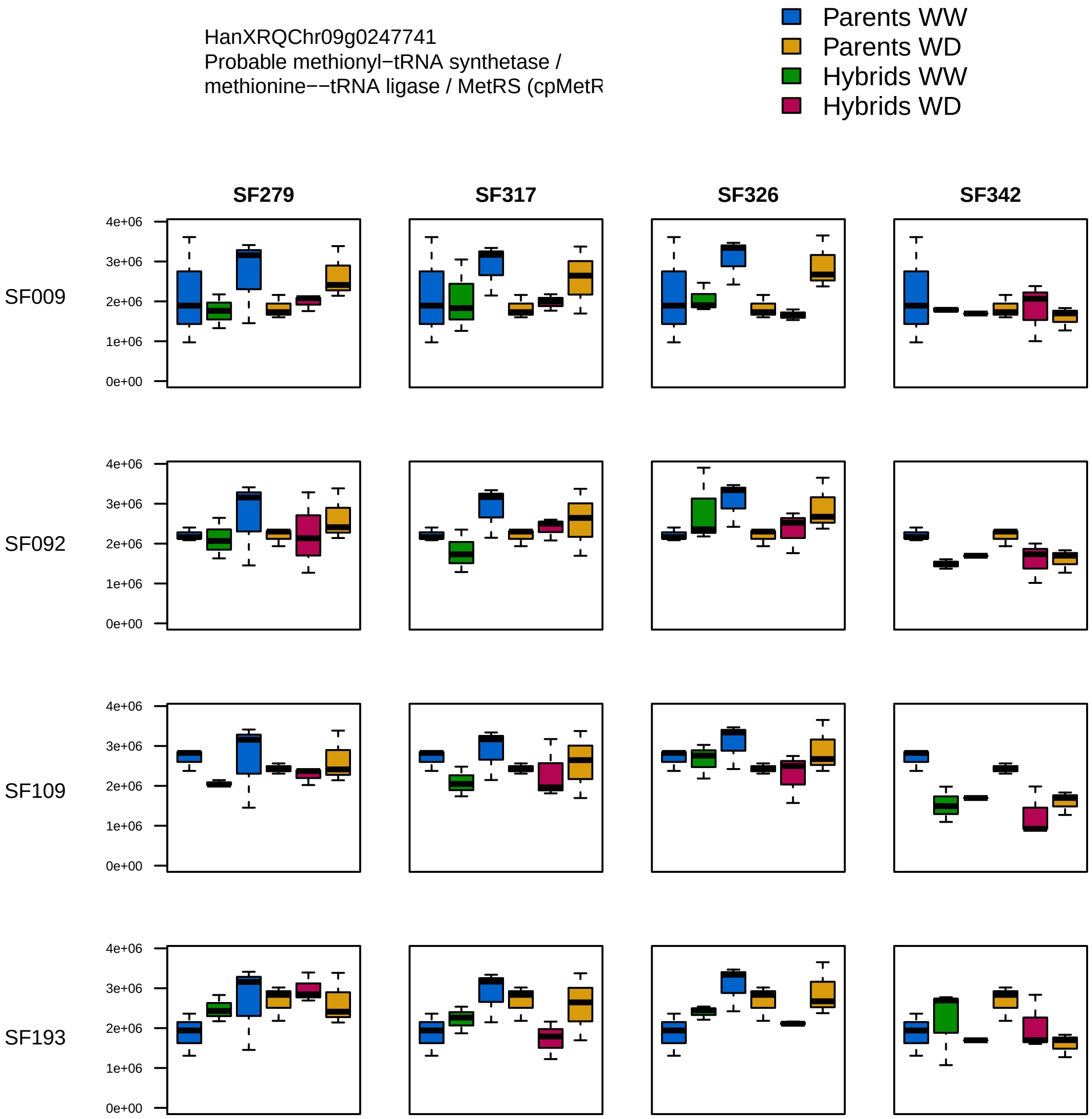
HanXRQChr09g0247741
Probable methionyl−tRNA synthetase /
methionine−−tRNA ligase / MetRS (cpMetR
Parents WW
Parents WD
Hybrids WW
Hybrids WD
SF279
SF317
SF326
SF342
SF009
SF092
SF109
SF193
0e+00
1e+06
2e+06
3e+06
4e+06

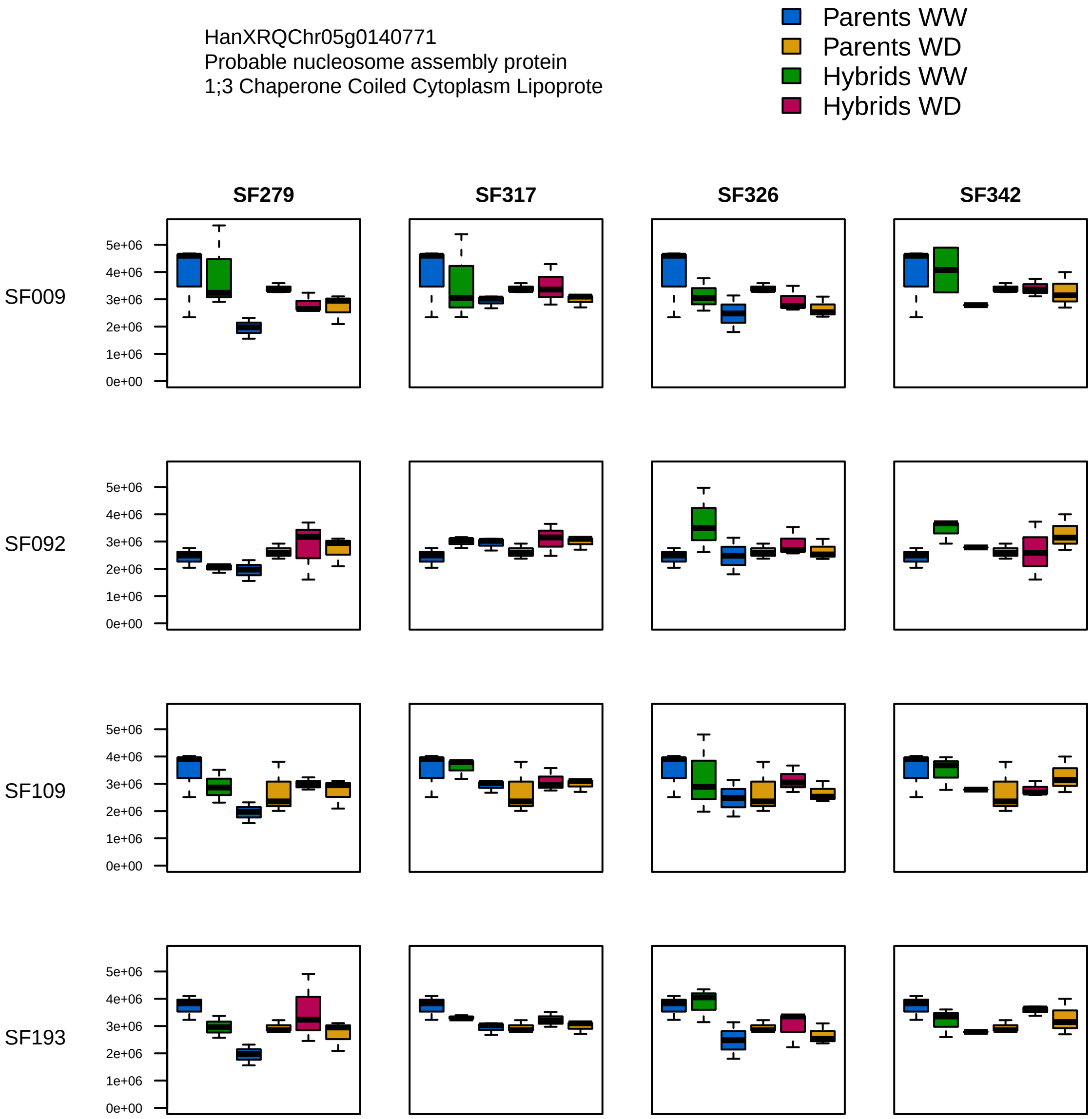

HanXRQChr05g0140771
Probable nucleosome assembly protein
1;3 Chaperone Coiled Cytoplasm Lipoprote
Parents WW
Parents WD
Hybrids WW
Hybrids WD
SF279
SF317
SF326
SF342
SF009
SF092
SF109
SF193
5e+06
4e+06
3e+06
2e+06
1e+06
0e+00

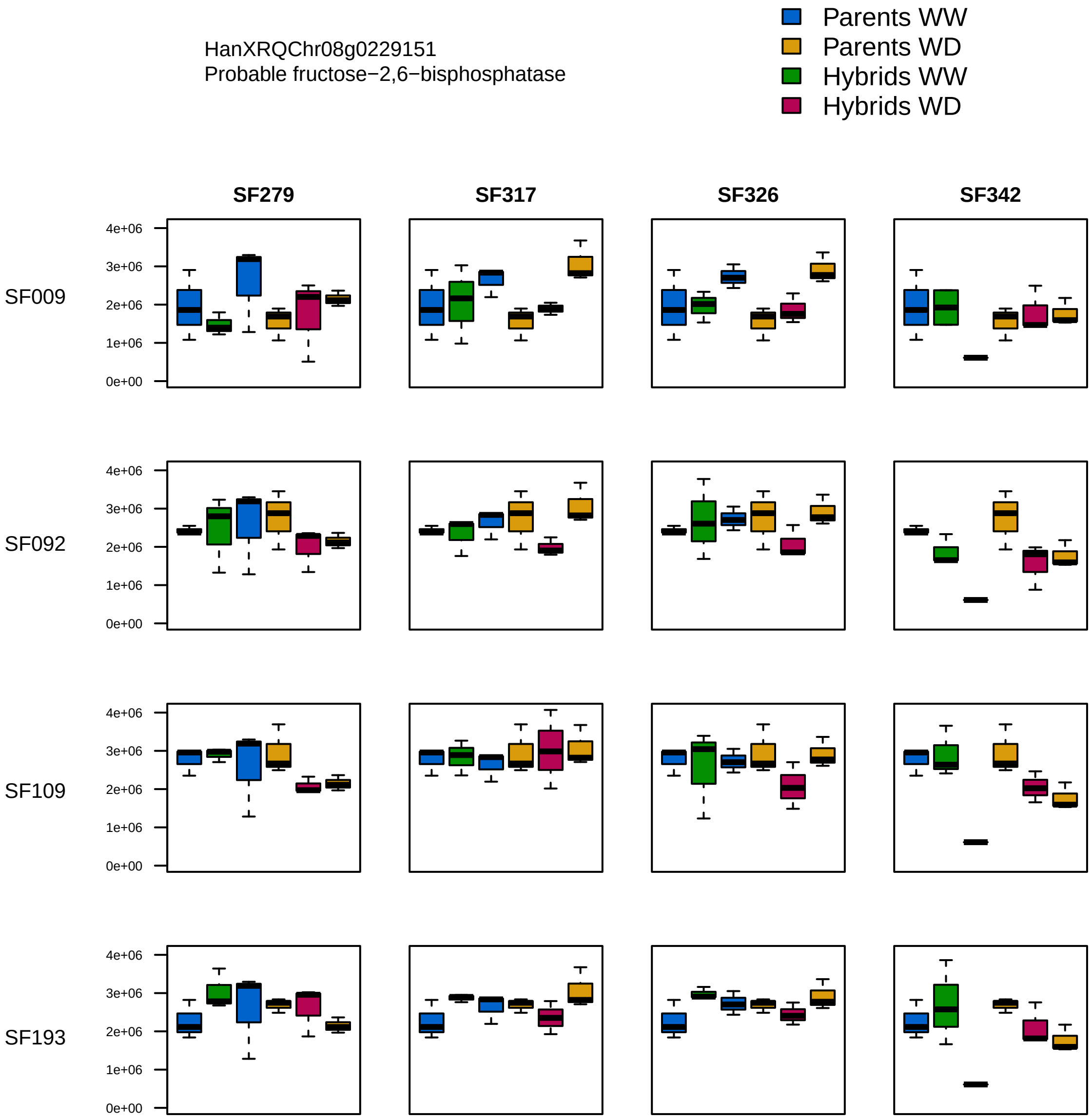
HanXRQChr08g0229151
Probable fructose−2,6−bisphosphatase
Parents WW
Parents WD
Hybrids WW
Hybrids WD
SF279
SF317
SF326
SF342
SF009
SF092
SF109
SF193
4e+06
3e+06
2e+06
1e+06
0e+00

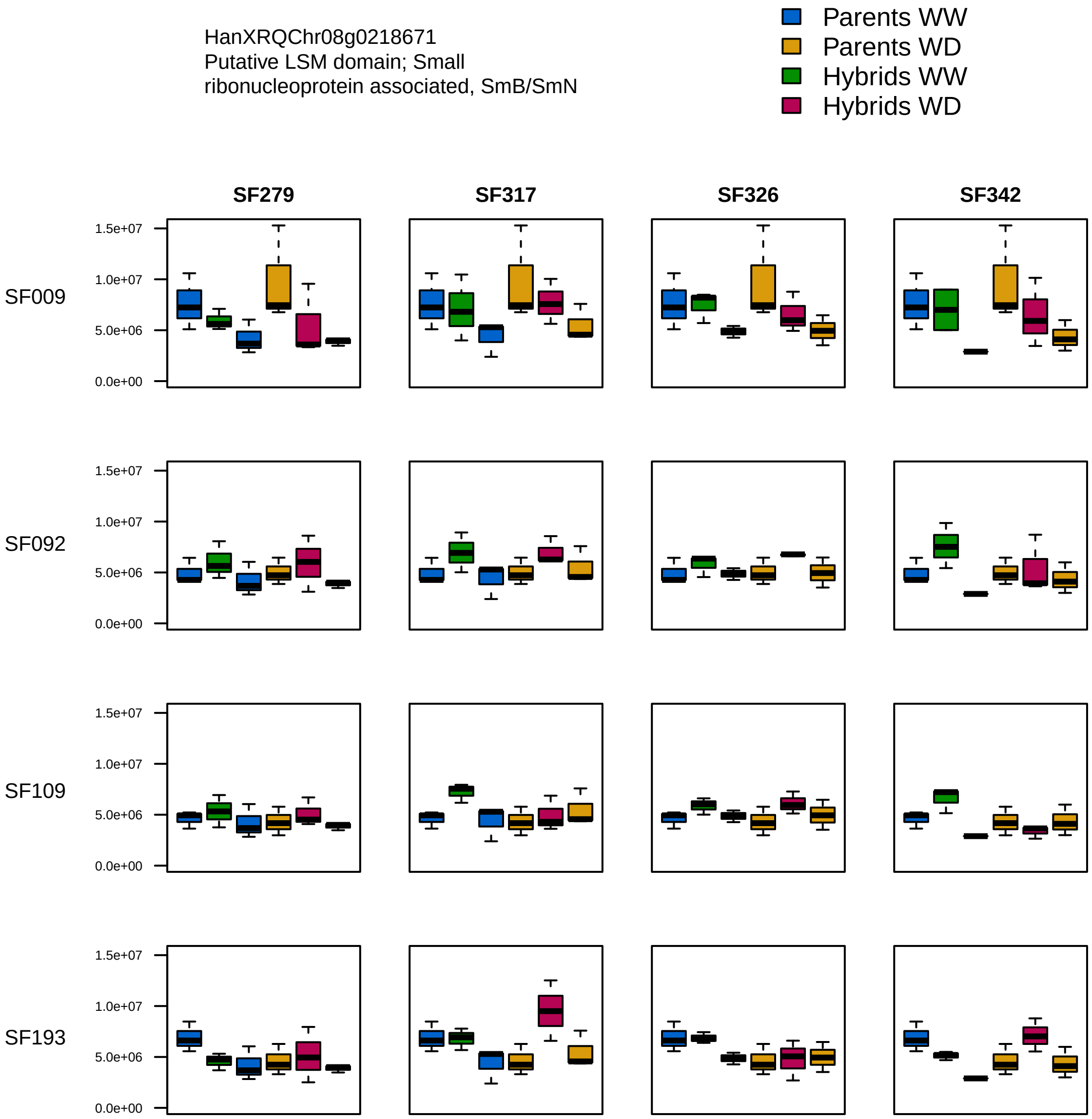
HanXRQChr08g0218671
Putative LSM domain; Small
ribonucleoprotein associated, SmB/SmN
Parents WW
Parents WD
Hybrids WW
Hybrids WD
SF279
SF317
SF326
SF342
SF009
SF092
SF109
SF193
1.5e+07
1.0e+07
5.0e+06
0.0e+00

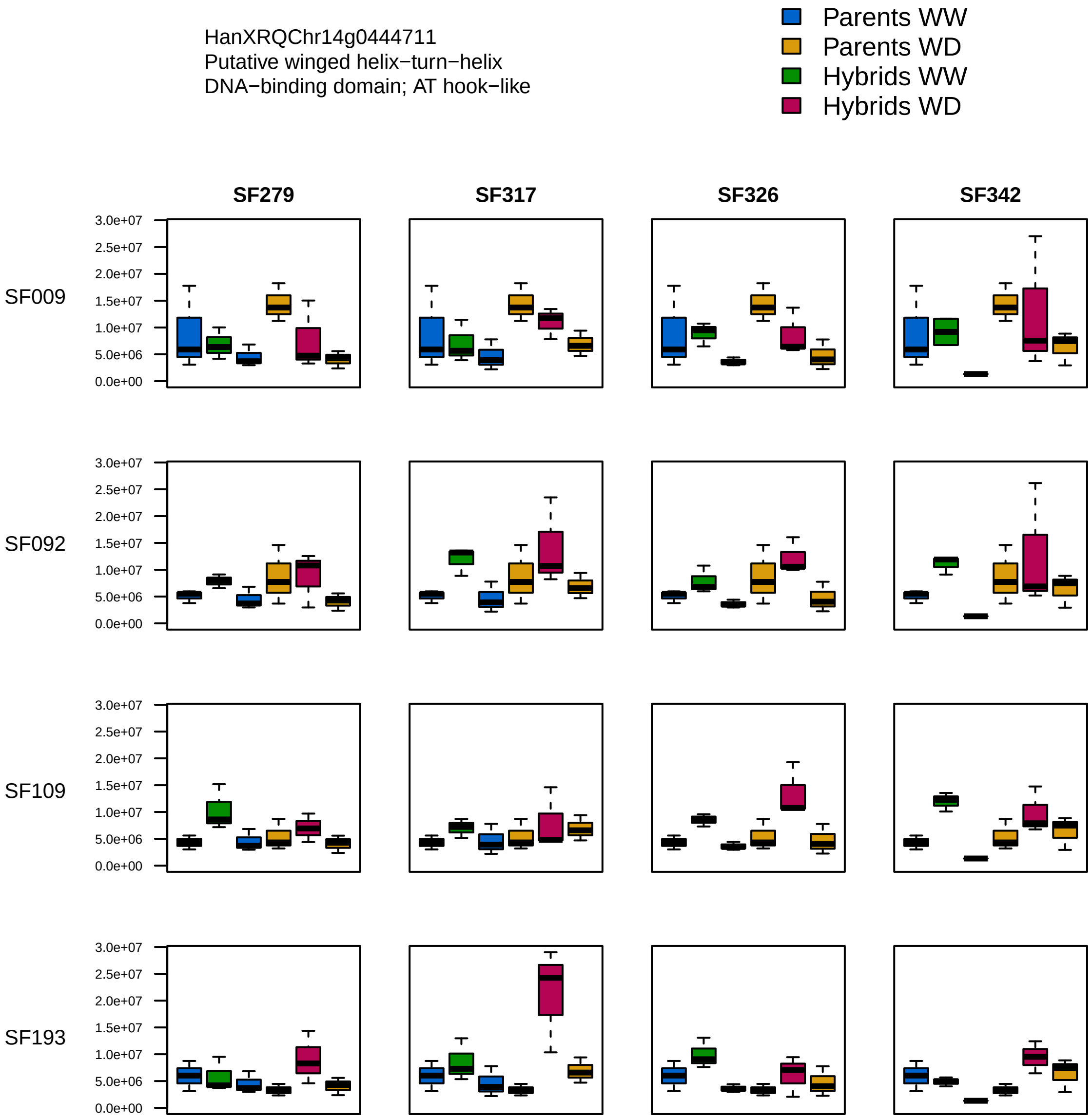
HanXRQChr14g0444711
Putative winged helix−turn−helix
DNA−binding domain; AT hook−like
Parents WW
Parents WD
Hybrids WW
Hybrids WD
SF279
SF317
SF326
SF342
SF009
SF092
SF109
SF193
0.0e+00
5.0e+06
1.0e+07
1.5e+07
2.0e+07
2.5e+07
3.0e+07

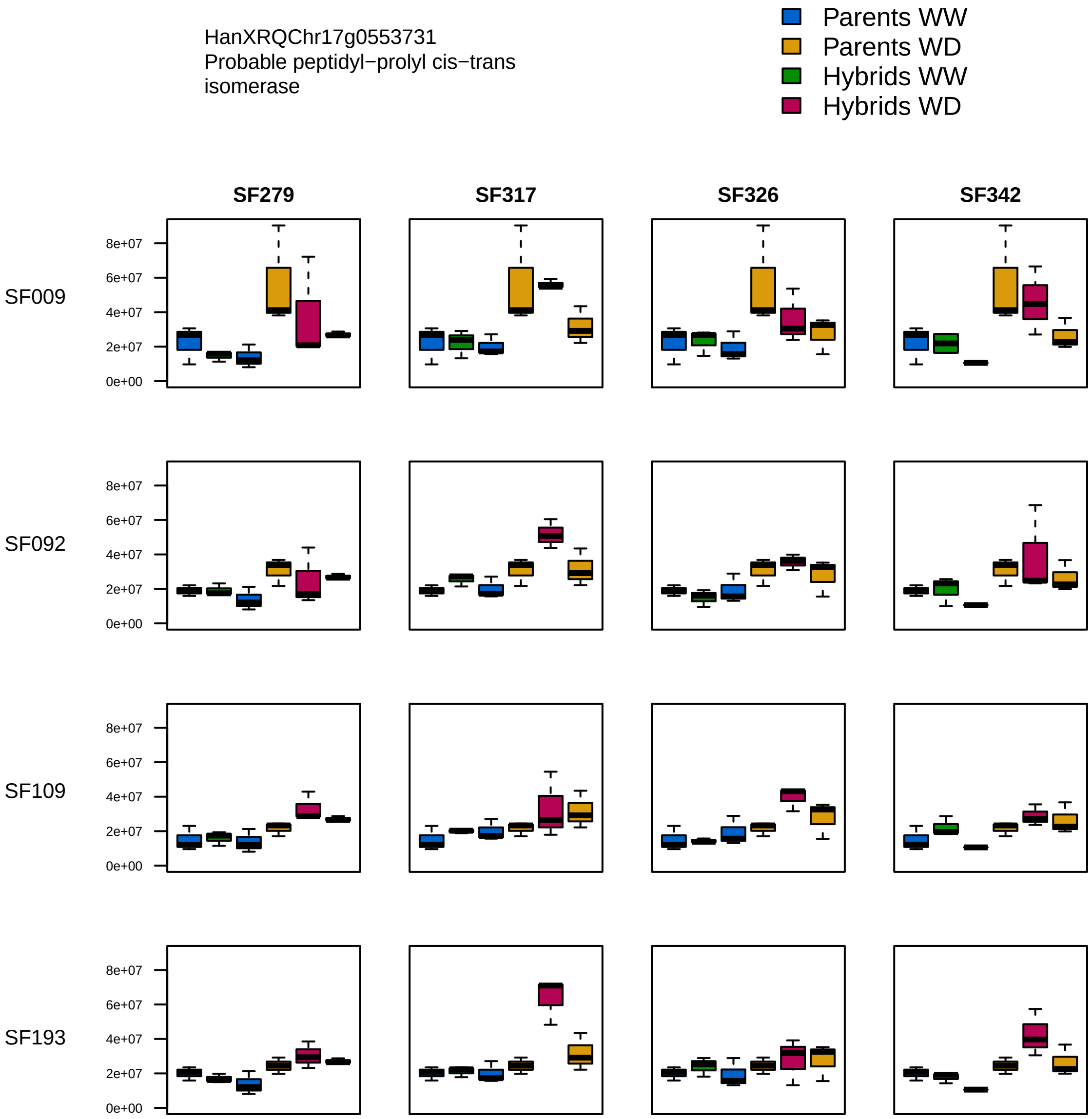
HanXRQChr17g0553731
Probable peptidyl−prolyl cis−trans
isomerase
Parents WW
Parents WD
Hybrids WW
Hybrids WD
SF279
SF317
SF326
SF342
SF009
SF092
SF109
SF193
8e+07
6e+07
4e+07
2e+07
0e+00

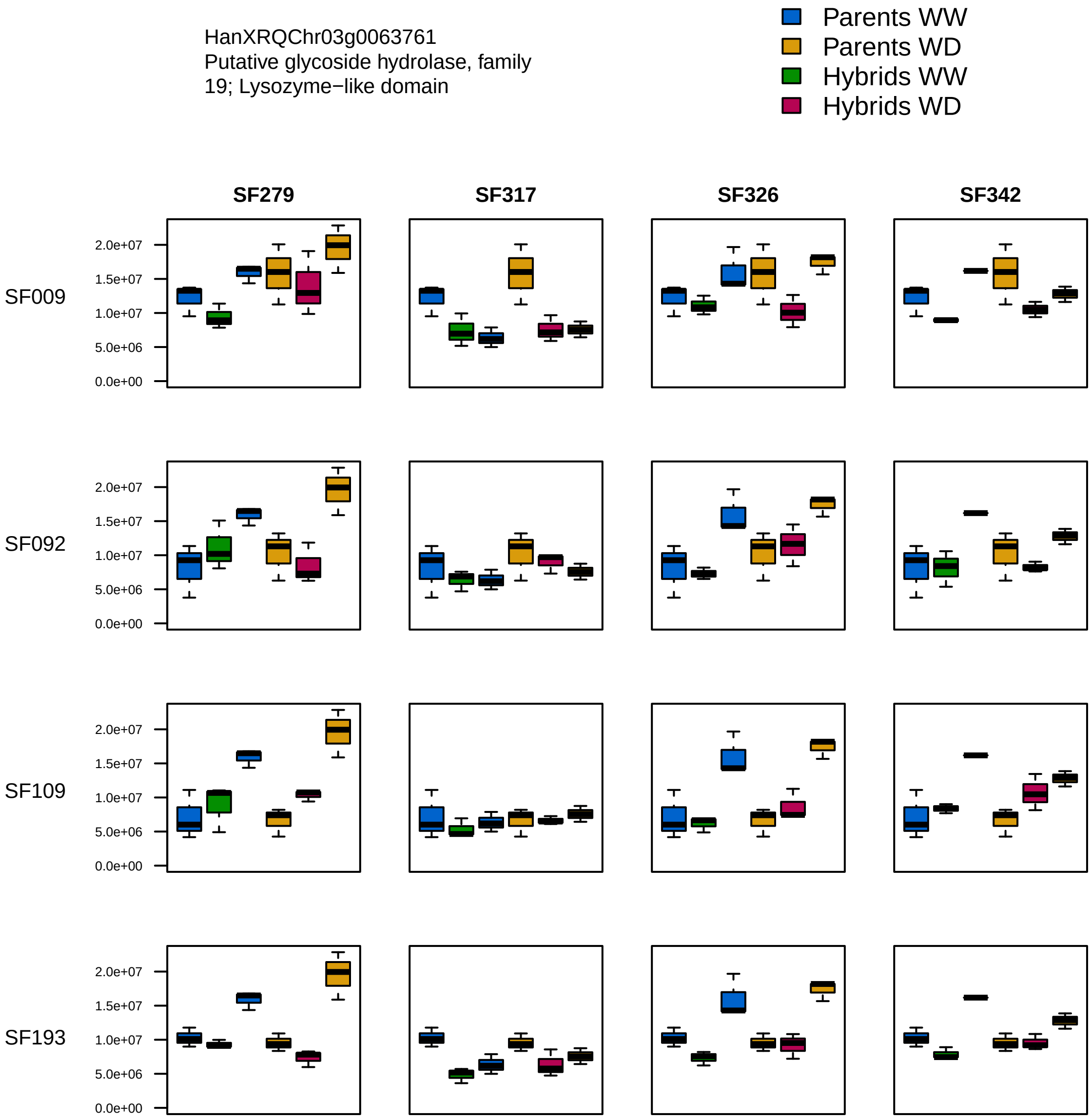
HanXRQChr03g0063761
Putative glycoside hydrolase, family
19; Lysozyme−like domain
Parents WW
Parents WD
Hybrids WW
Hybrids WD
SF279
SF317
SF326
SF342
SF009
SF092
SF109
SF193
2.0e+07
1.5e+07
1.0e+07
5.0e+06
0.0e+00

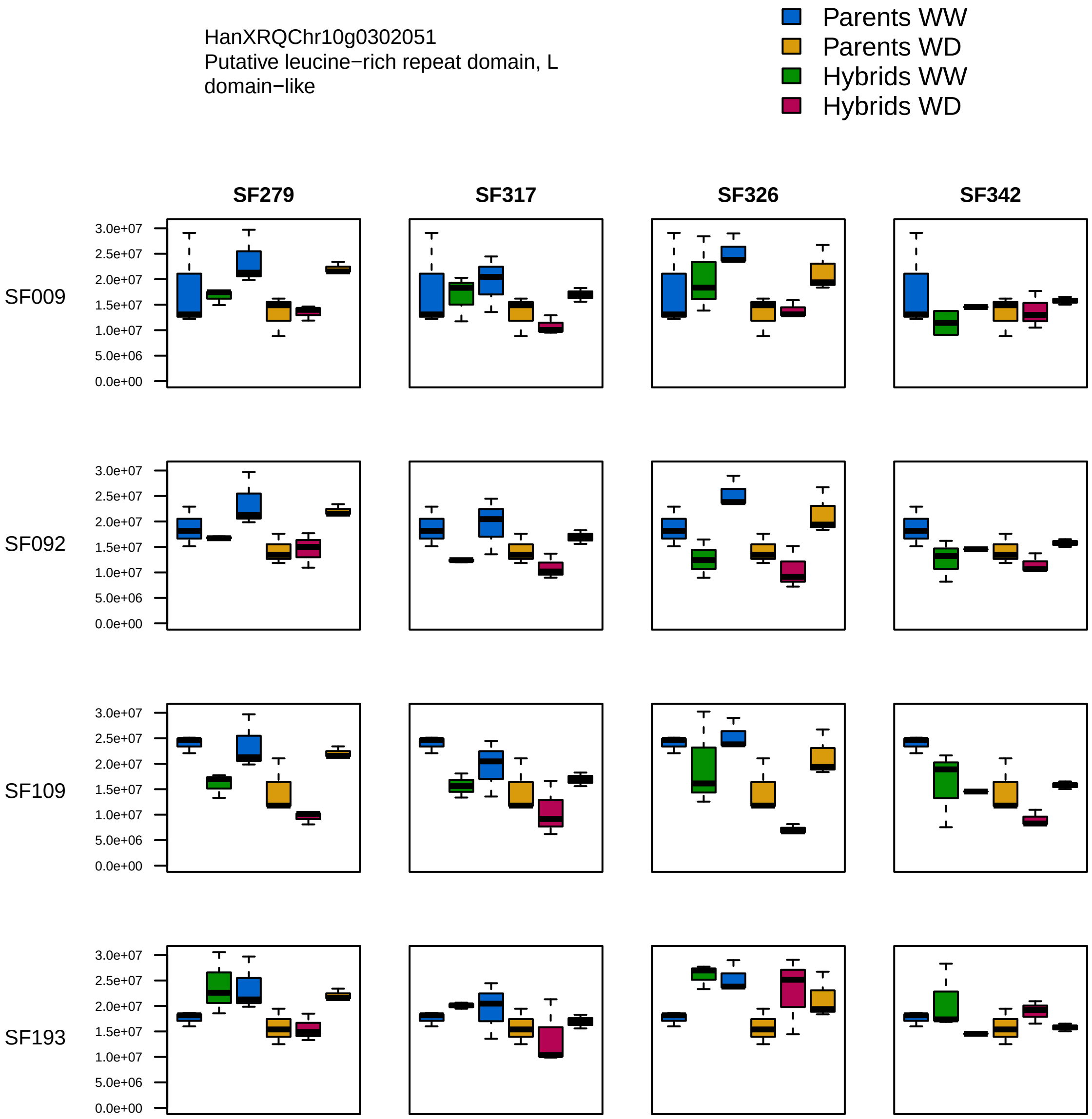

HanXRQChr10g0302051
Putative leucine−rich repeat domain, L
domain−like
Parents WW
Parents WD
Hybrids WW
Hybrids WD
SF279
SF317
SF326
SF342
SF009
SF092
SF109
SF193
0.0e+00
5.0e+06
1.0e+07
1.5e+07
2.0e+07
2.5e+07
3.0e+07

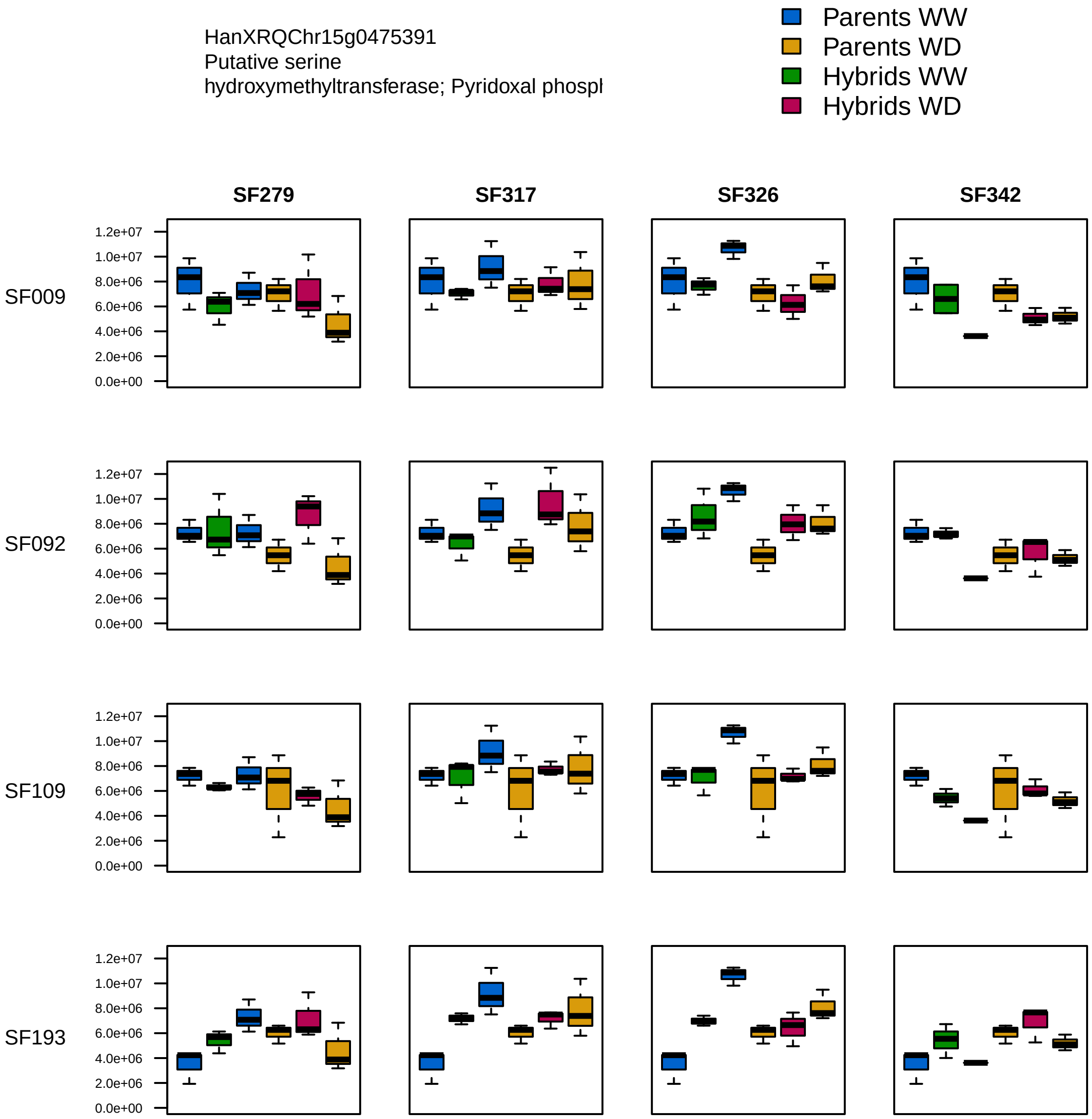
HanXRQChr15g0475391
Putative serine
hydroxymethyltransferase; Pyridoxal phospl
Parents WW
Parents WD
Hybrids WW
Hybrids WD
SF279
SF317
SF326
SF342
SF009
SF092
SF109
SF193
1.2e+07
1.0e+07
8.0e+06
6.0e+06
4.0e+06
2.0e+06
0.0e+00

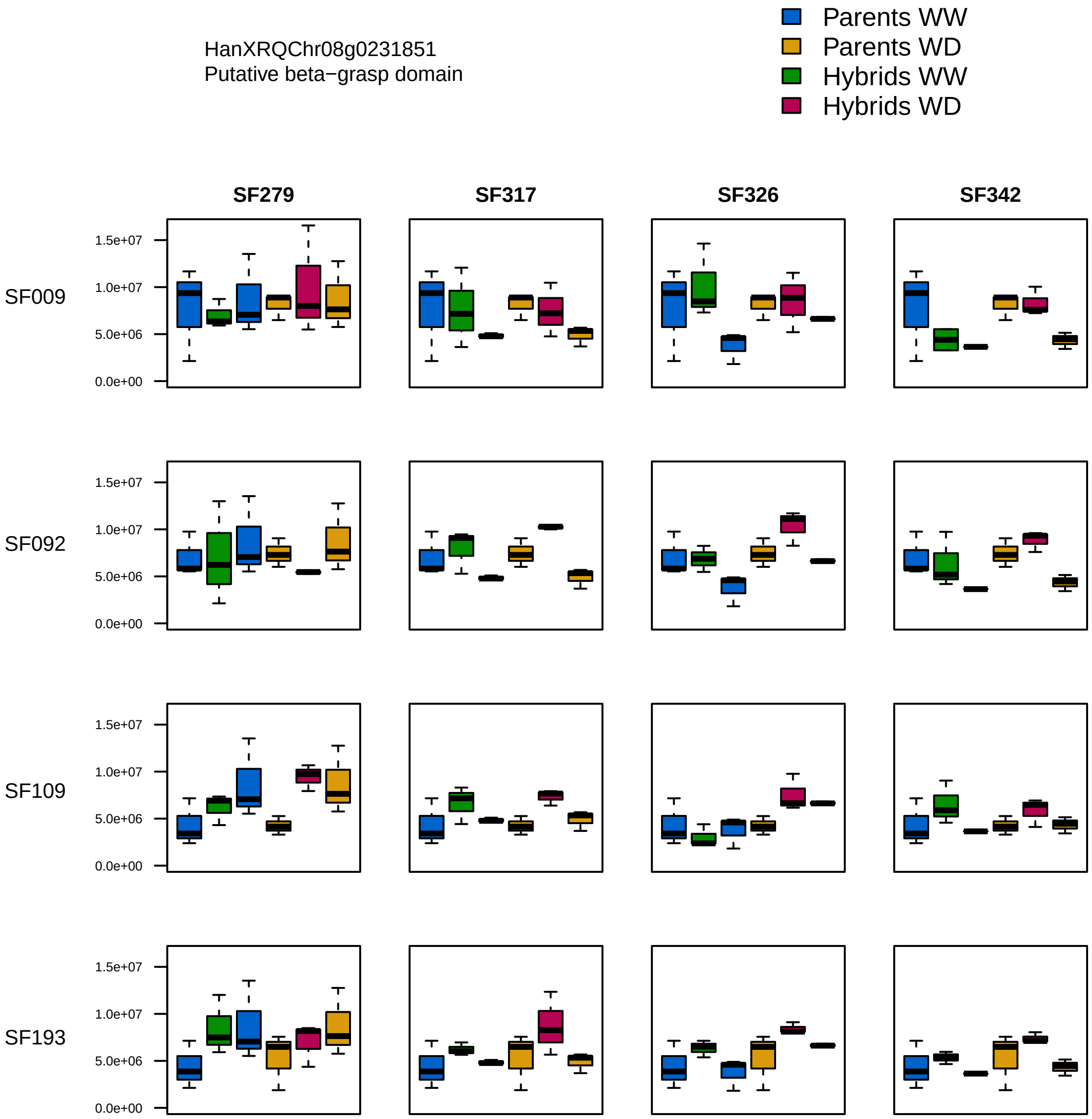
HanXRQChr08g0231851
Putative beta−grasp domain
Parents WW
Parents WD
Hybrids WW
Hybrids WD
SF279
SF317
SF326
SF342
SF009
SF092
SF109
SF193
1.5e+07
1.0e+07
5.0e+06
0.0e+00

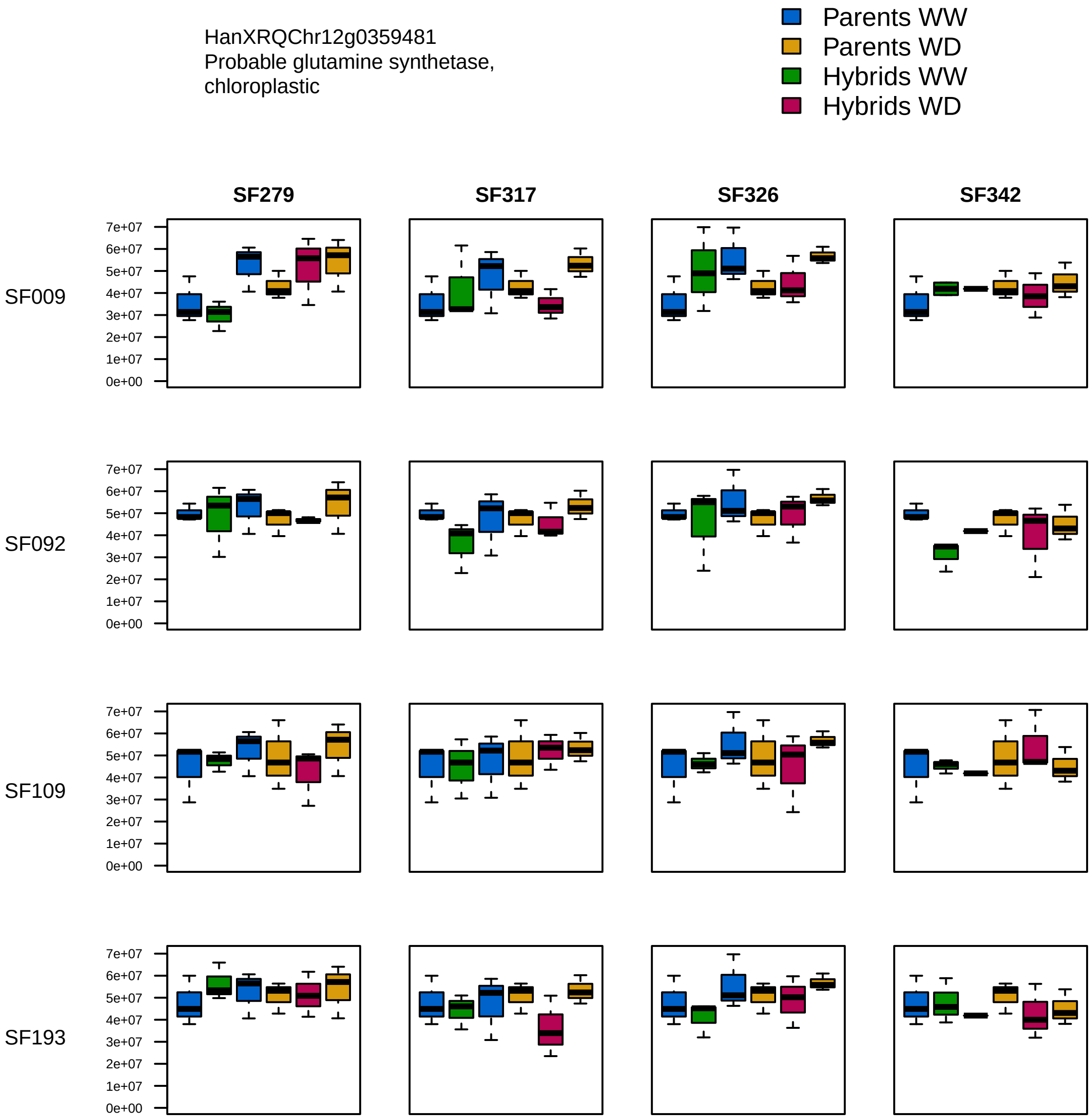
HanXRQChr12g0359481
Probable glutamine synthetase,
chloroplastic
Parents WW
Parents WD
Hybrids WW
Hybrids WD
SF279
SF317
SF326
SF342
SF009
SF092
SF109
SF193
7e+07
6e+07
5e+07
4e+07
3e+07
2e+07
1e+07
0e+00

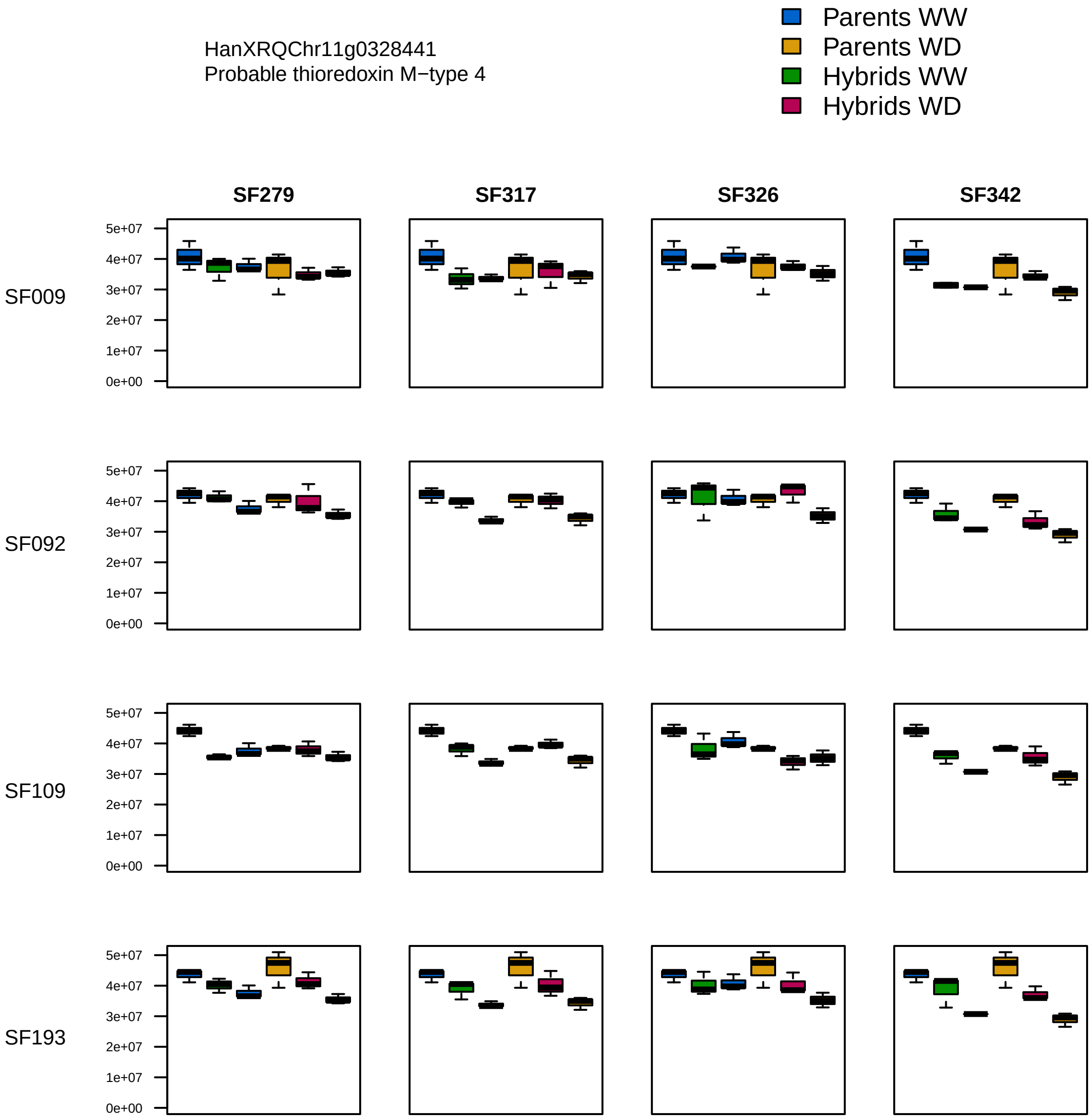

HanXRQChr11g0328441
Probable thioredoxin M−type 4
Parents WW
Parents WD
Hybrids WW
Hybrids WD
SF279
SF317
SF326
SF342
SF009
SF092
SF109
SF193
5e+07
4e+07
3e+07
2e+07
1e+07
0e+00

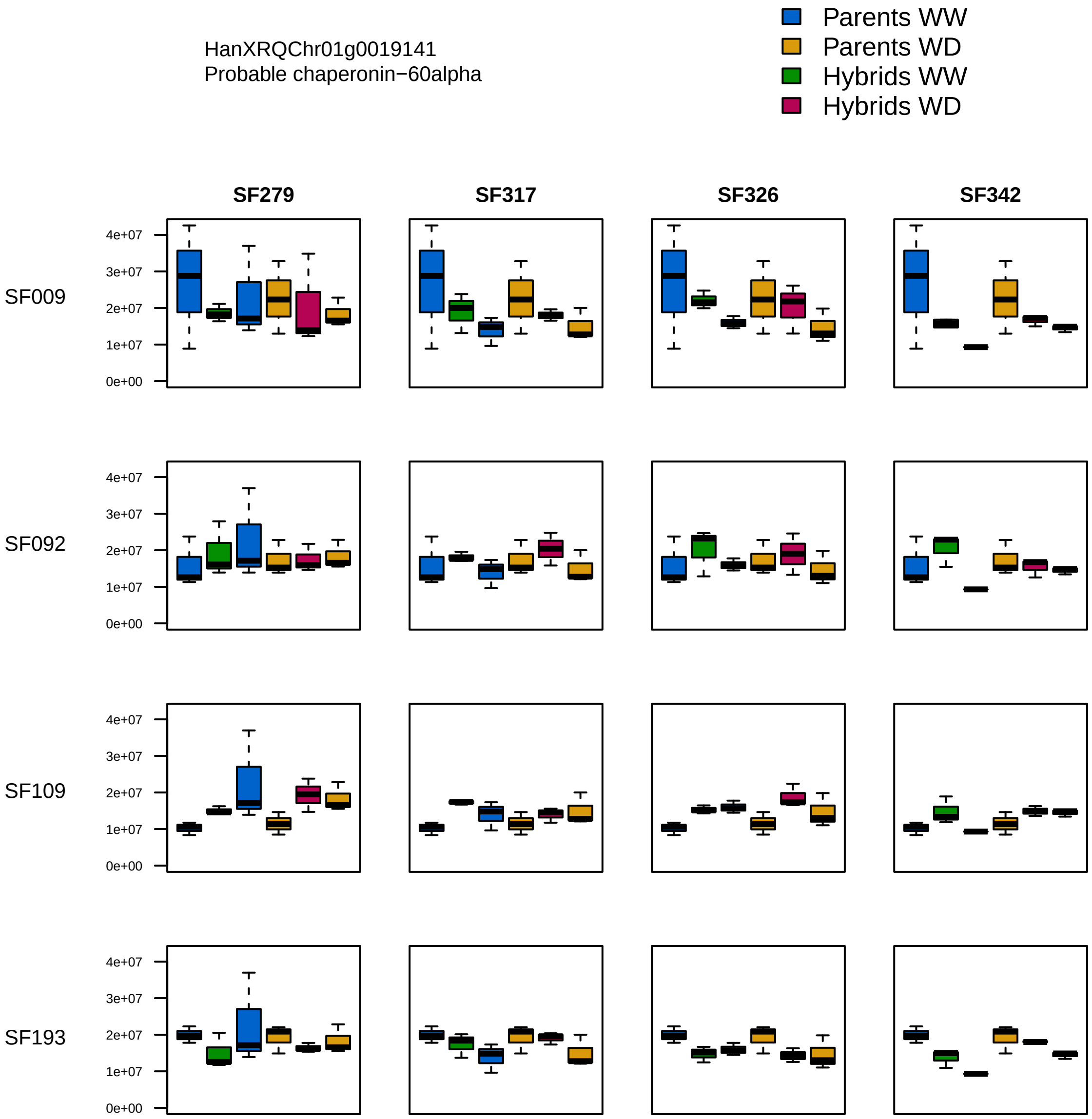
HanXRQChr01g0019141
Probable chaperonin−60alpha
Parents WW
Parents WD
Hybrids WW
Hybrids WD
SF279
SF317
SF326
SF342
SF009
SF092
SF109
SF193
4e+07
3e+07
2e+07
1e+07
0e+00

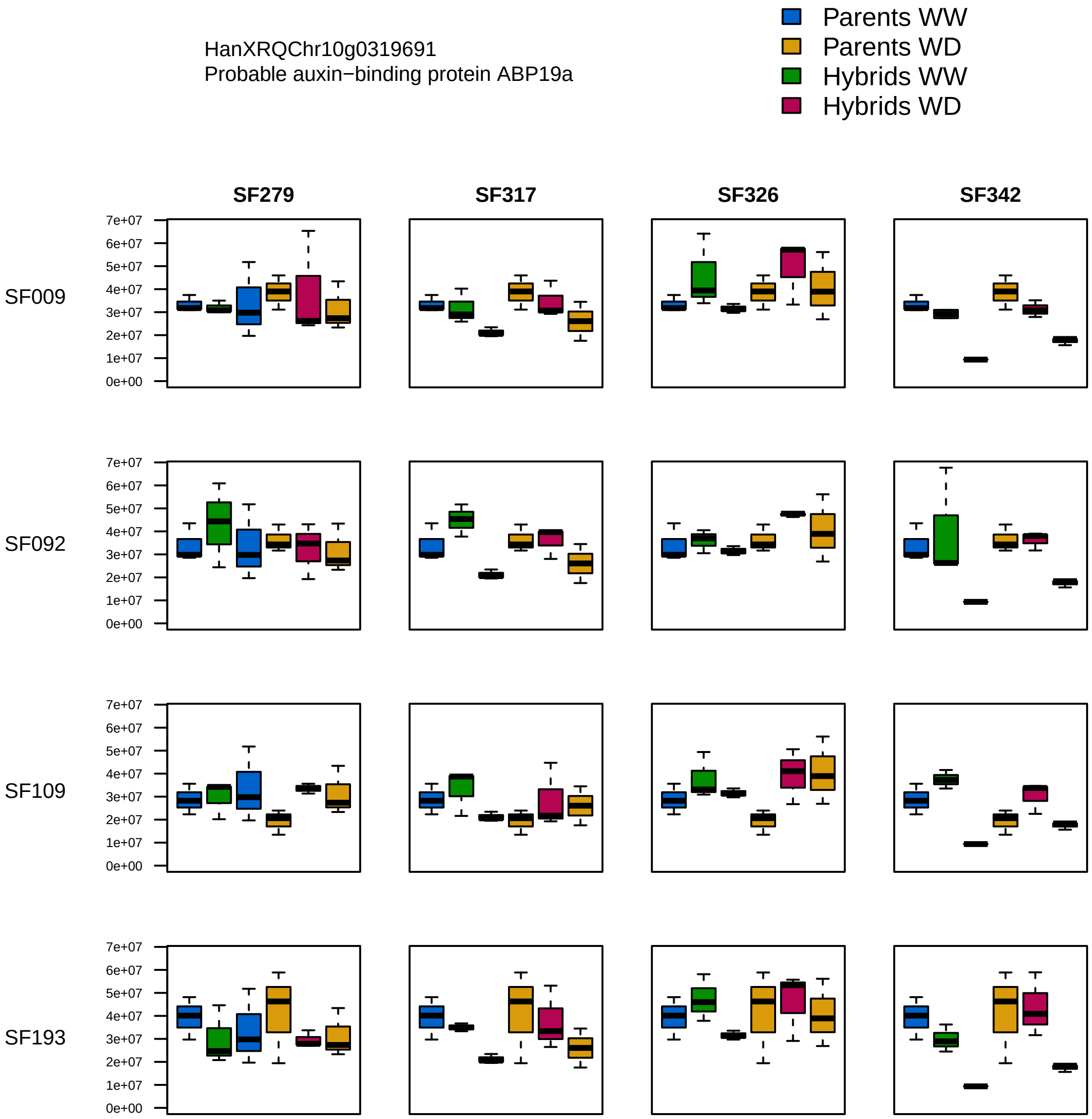

HanXRQChr10g0319691
Probable auxin−binding protein ABP19a
Parents WW
Parents WD
Hybrids WW
Hybrids WD
SF279
SF317
SF326
SF342
SF009
SF092
SF109
SF193
0e+00
1e+07
2e+07
3e+07
4e+07
5e+07
6e+07
7e+07

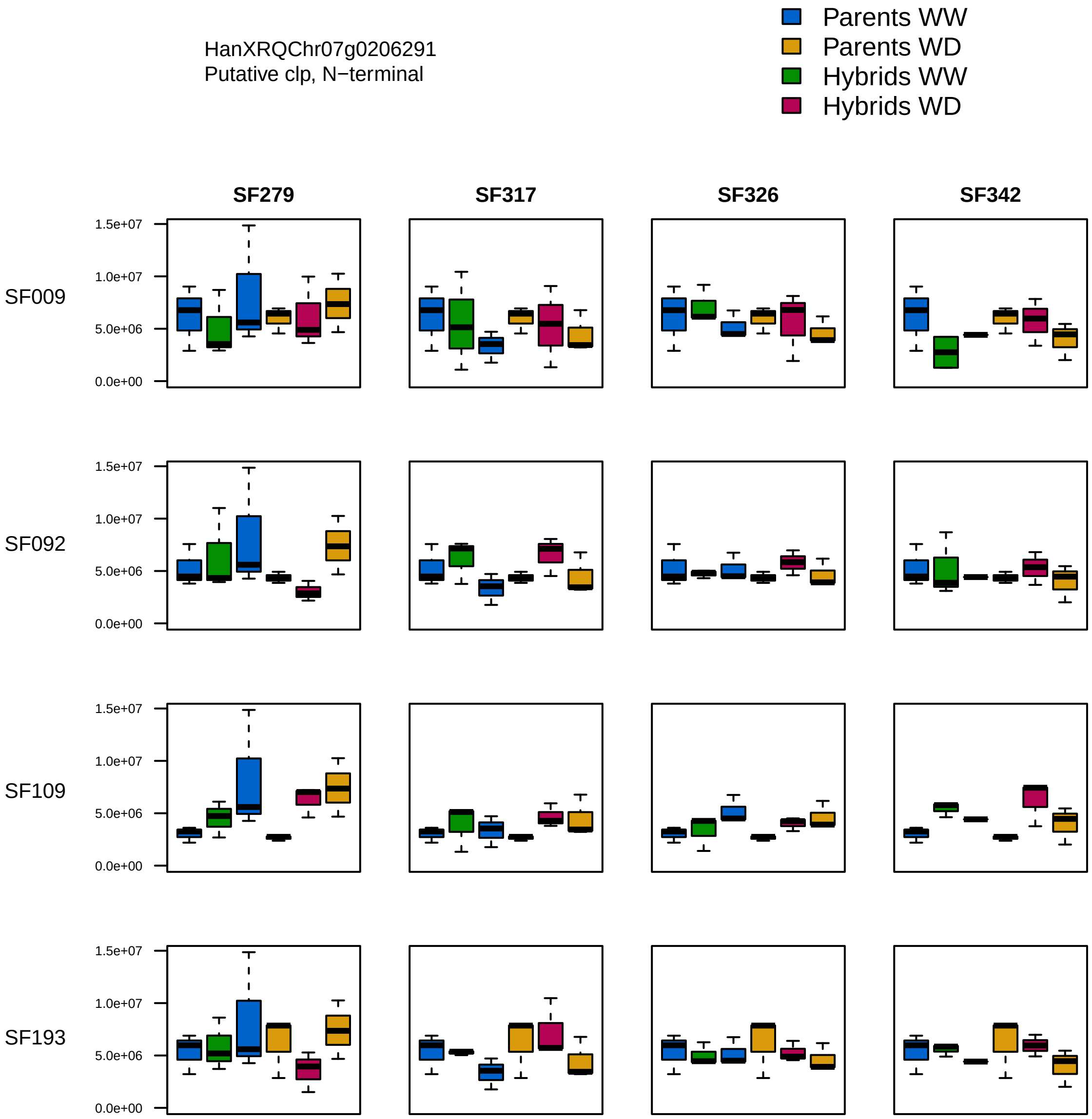
HanXRQChr07g0206291
Putative clp, N−terminal
Parents WW
Parents WD
Hybrids WW
Hybrids WD
SF279
SF317
SF326
SF342
SF009
SF092
SF109
SF193
1.5e+07
1.0e+07
5.0e+06
0.0e+00

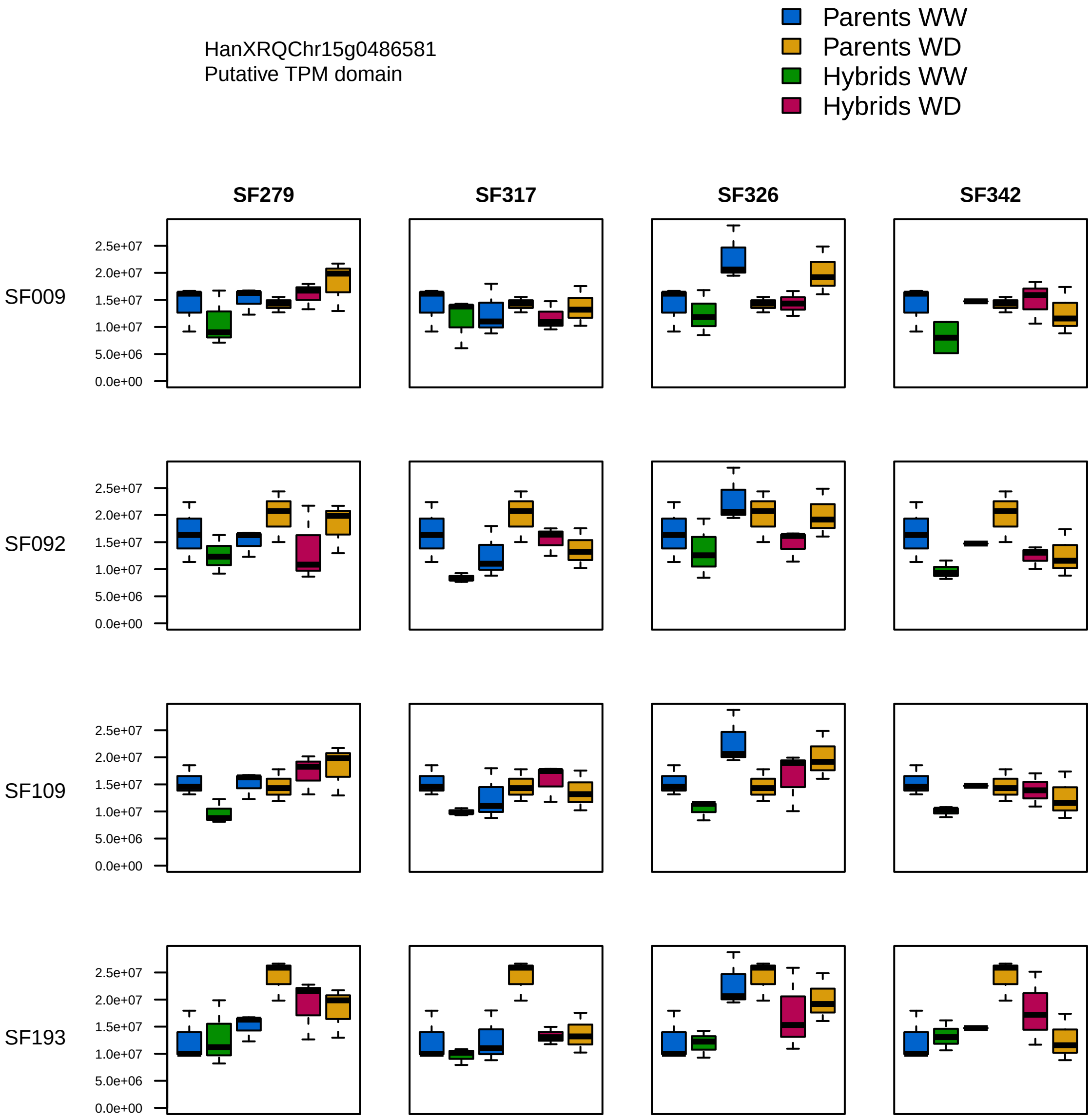
HanXRQChr15g0486581
Putative TPM domain
Parents WW
Parents WD
Hybrids WW
Hybrids WD
SF279
SF317
SF326
SF342
SF009
SF092
SF109
SF193
2.5e+07
2.0e+07
1.5e+07
1.0e+07
5.0e+06
0.0e+00

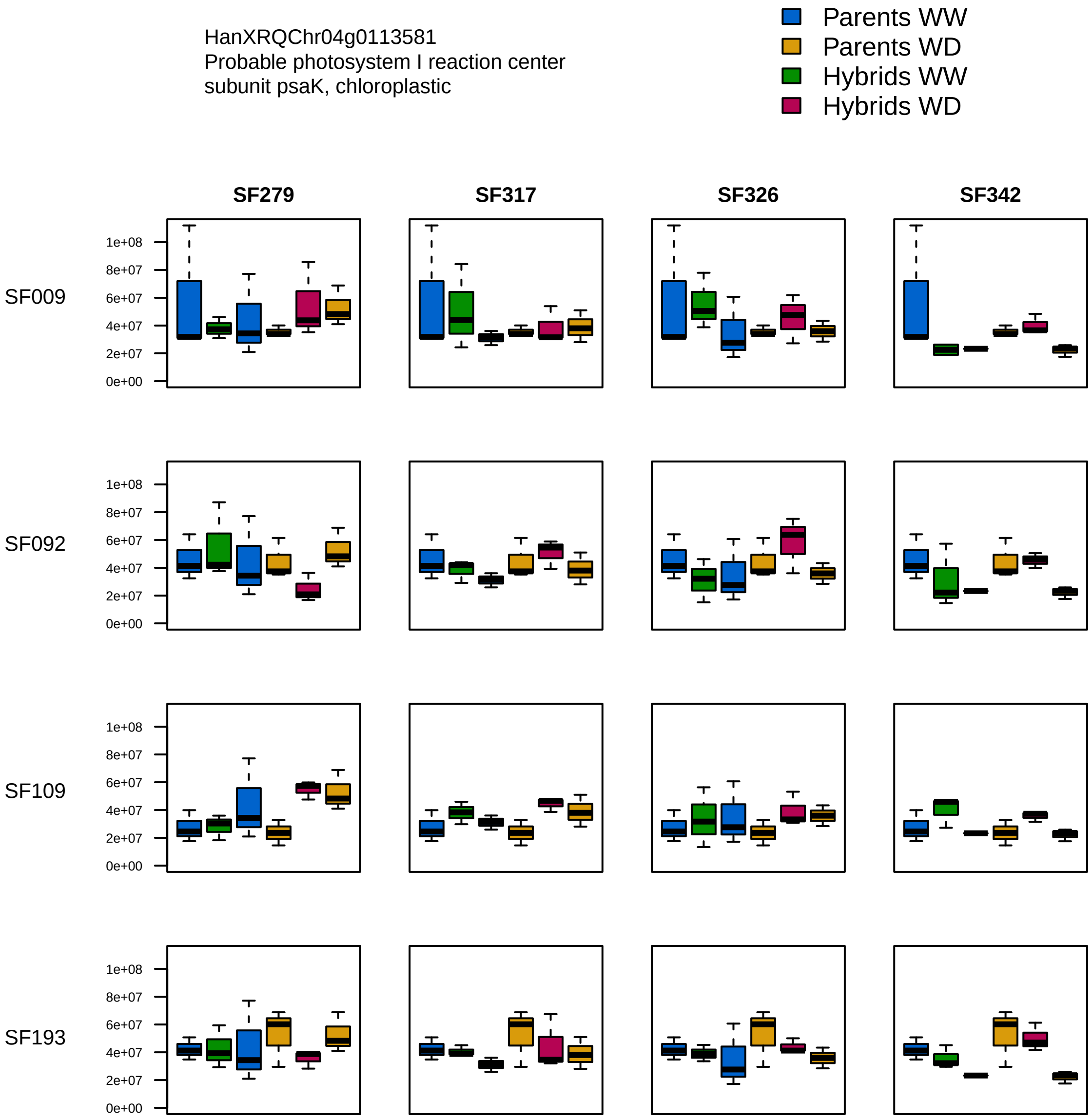

HanXRQChr04g0113581
Probable photosystem I reaction center
subunit psaK, chloroplastic
Parents WW
Parents WD
Hybrids WW
Hybrids WD
SF279
SF317
SF326
SF342
SF009
SF092
SF109
SF193
0e+00
2e+07
4e+07
6e+07
8e+07
1e+08

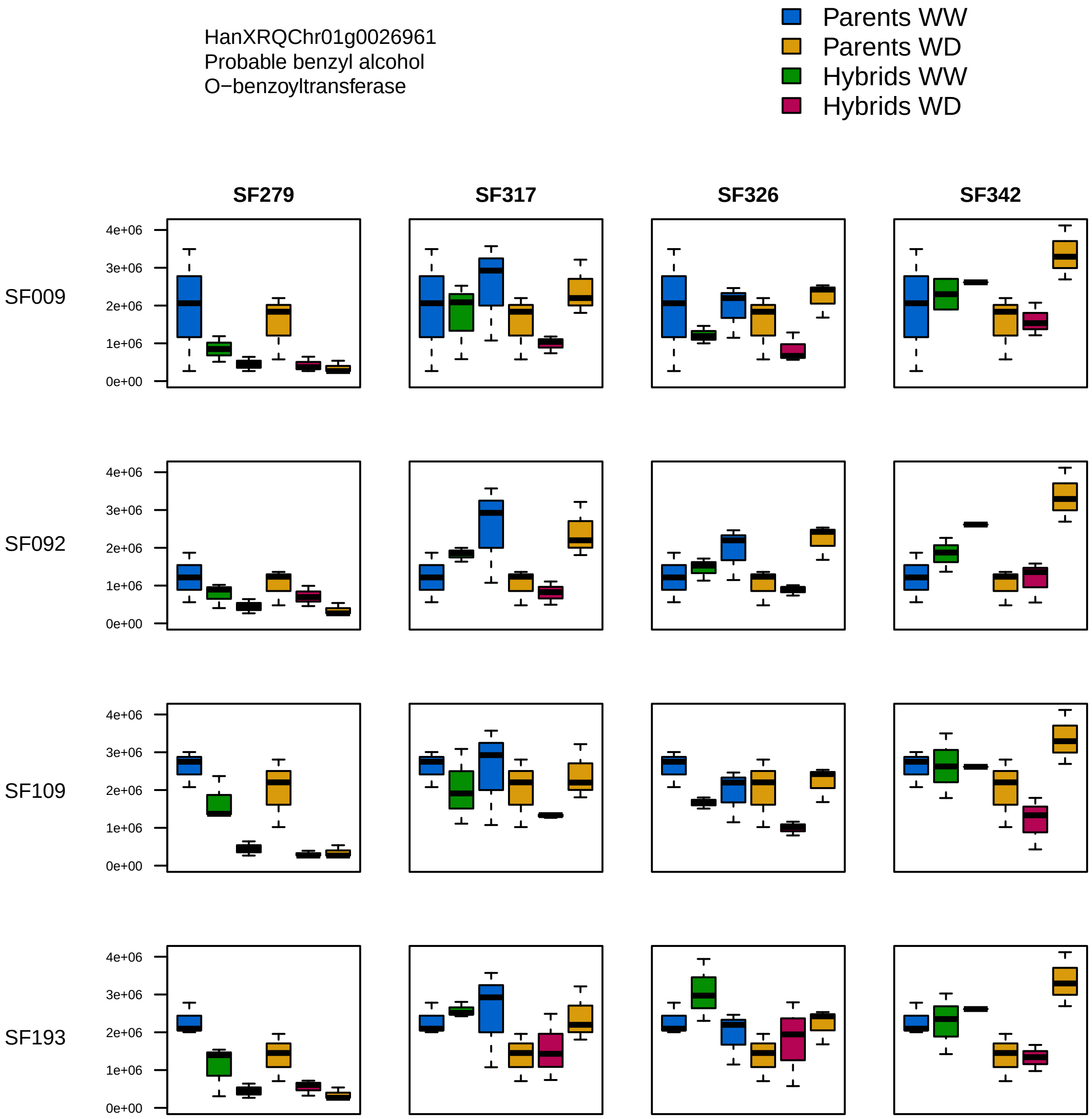
HanXRQChr01g0026961
Probable benzyl alcohol
O−benzoyltransferase
Parents WW
Parents WD
Hybrids WW
Hybrids WD
SF279
SF317
SF326
SF342
SF009
SF092
SF109
SF193
0e+00
1e+06
2e+06
3e+06
4e+06

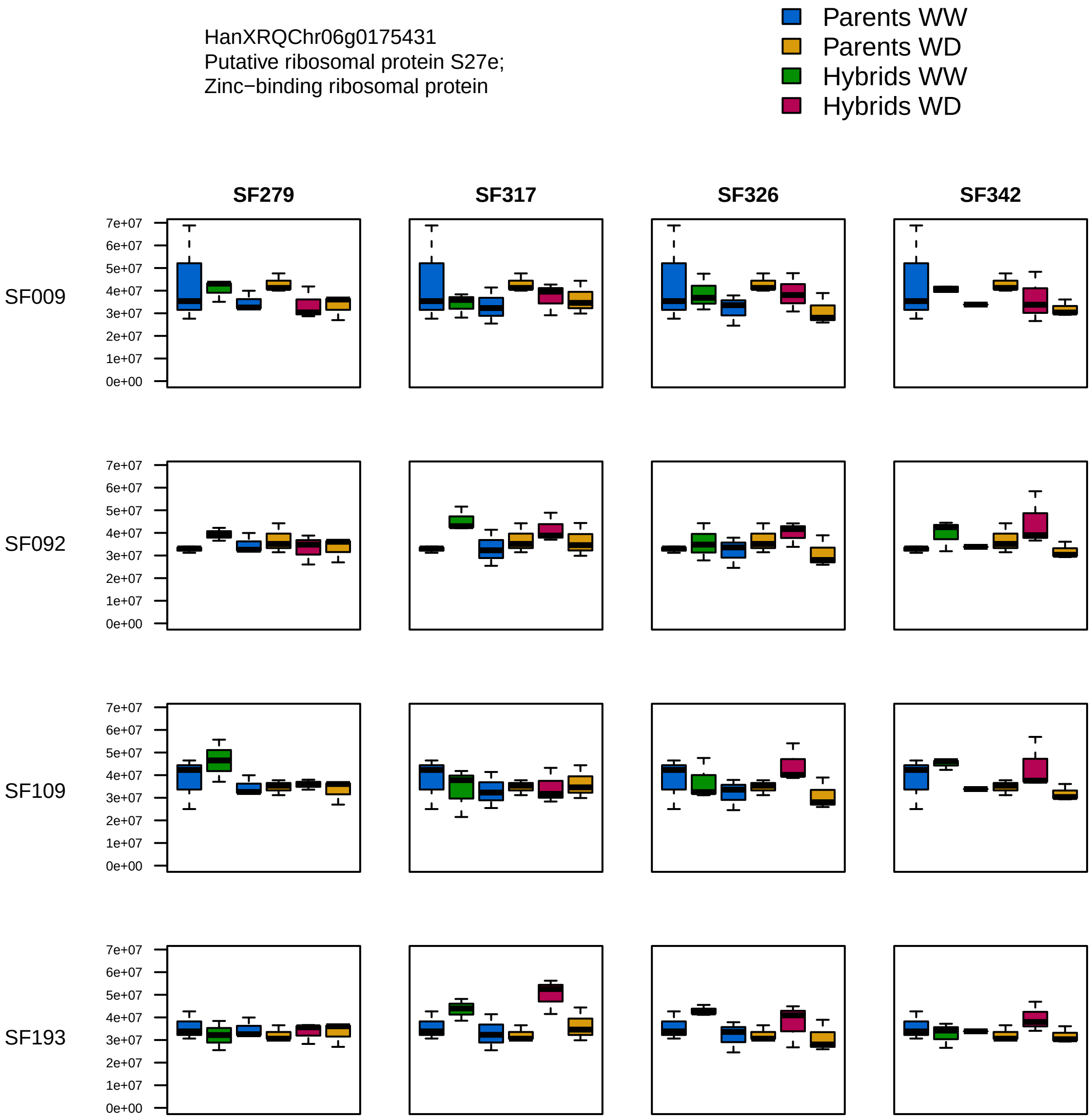
HanXRQChr06g0175431
Putative ribosomal protein S27e;
Zinc−binding ribosomal protein
Parents WW
Parents WD
Hybrids WW
Hybrids WD
SF279
SF317
SF326
SF342
SF009
SF092
SF109
SF193
7e+07
6e+07
5e+07
4e+07
3e+07
2e+07
1e+07
0e+00

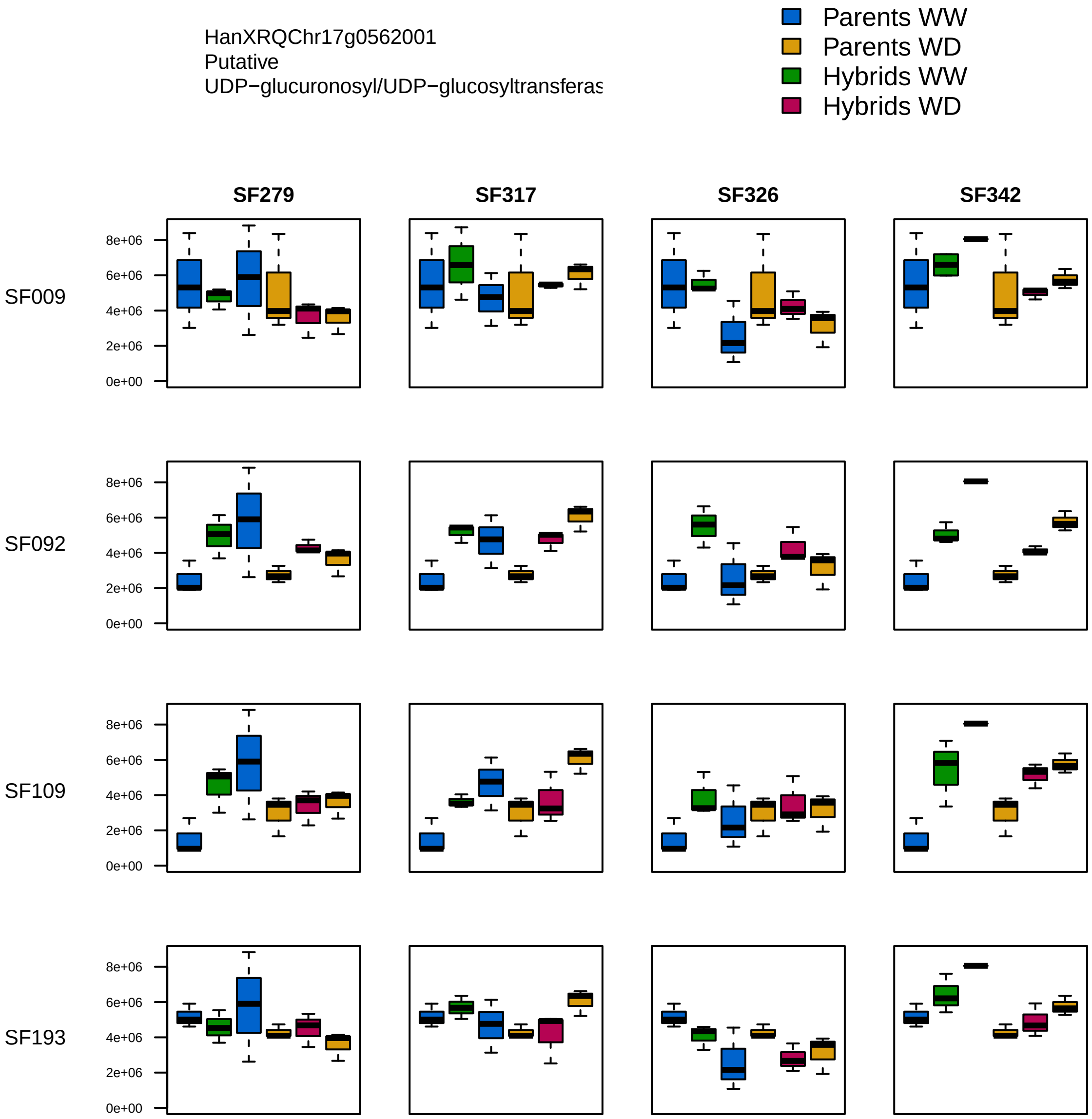
HanXRQChr17g0562001
Putative
UDP−glucuronosyl/UDP−glucosyltransferas
Parents WW
Parents WD
Hybrids WW
Hybrids WD
SF279
SF317
SF326
SF342
SF009
SF092
SF109
SF193
8e+06
6e+06
4e+06
2e+06
0e+00

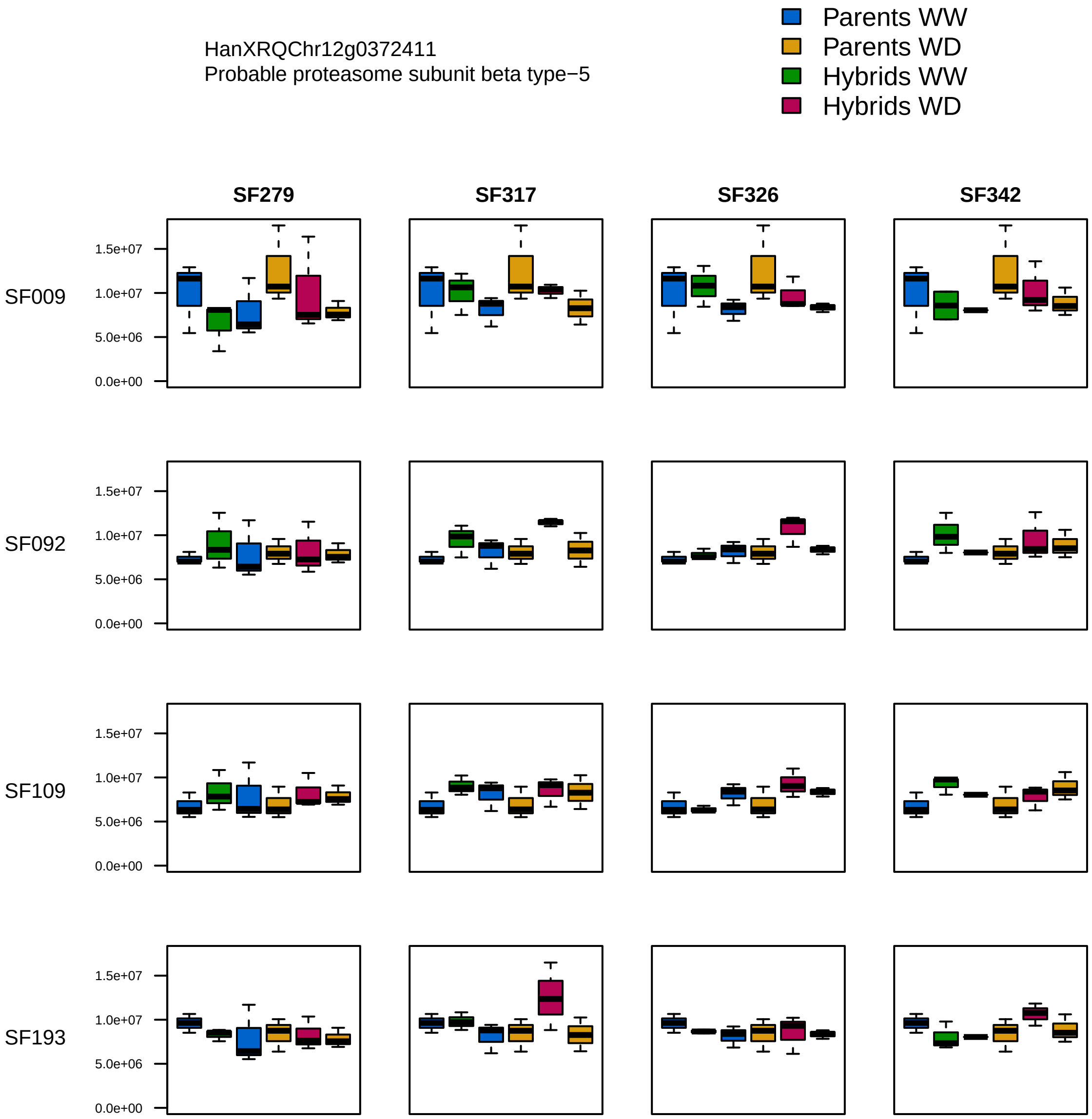
HanXRQChr12g0372411
Probable proteasome subunit beta type−5
Parents WW
Parents WD
Hybrids WW
Hybrids WD
SF279
SF317
SF326
SF342
SF009
SF092
SF109
SF193
1.5e+07
1.0e+07
5.0e+06
0.0e+00

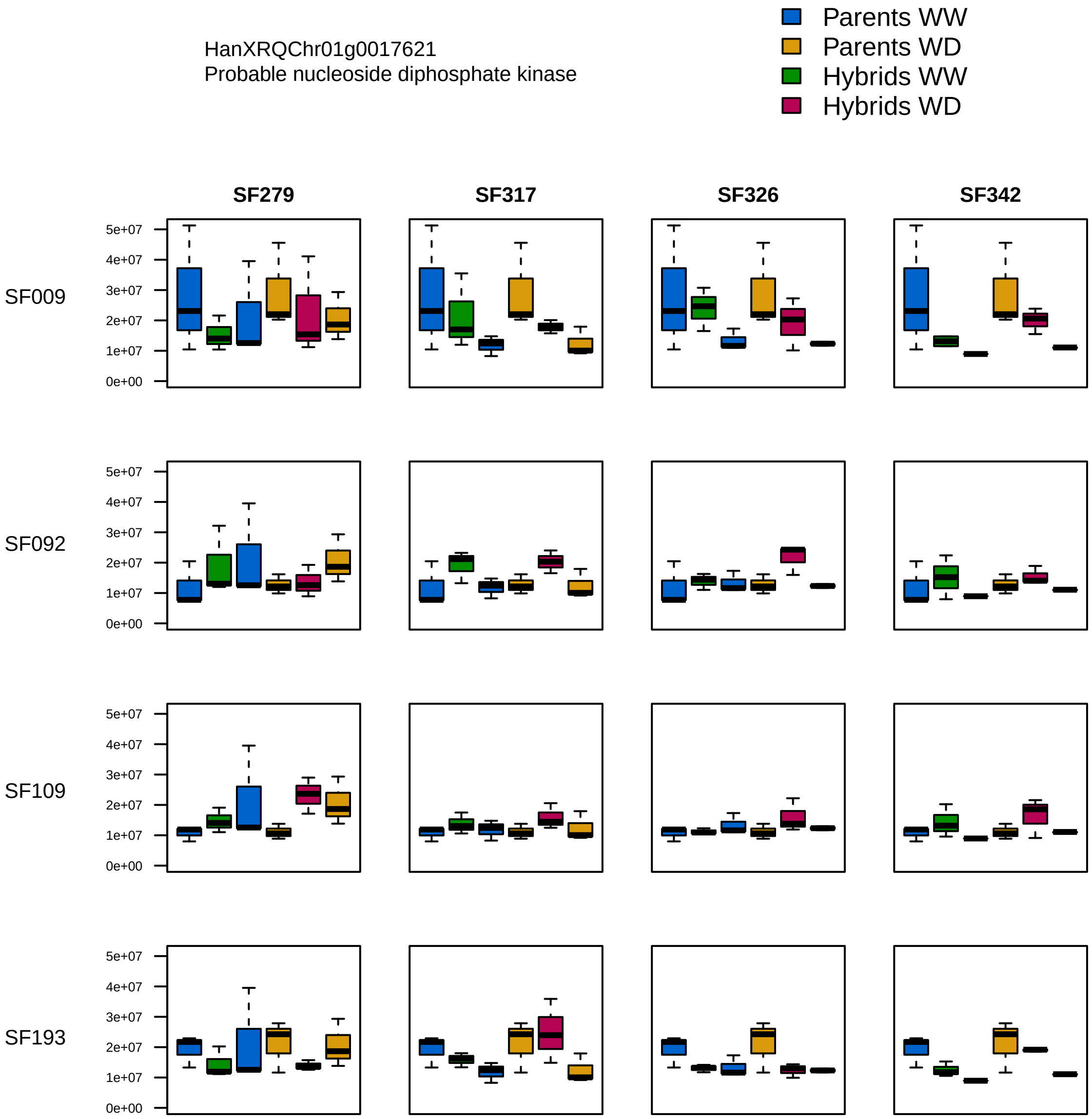
HanXRQChr01g0017621
Probable nucleoside diphosphate kinase
Parents WW
Parents WD
Hybrids WW
Hybrids WD
SF279
SF317
SF326
SF342
SF009
SF092
SF109
SF193
5e+07
4e+07
3e+07
2e+07
1e+07
0e+00

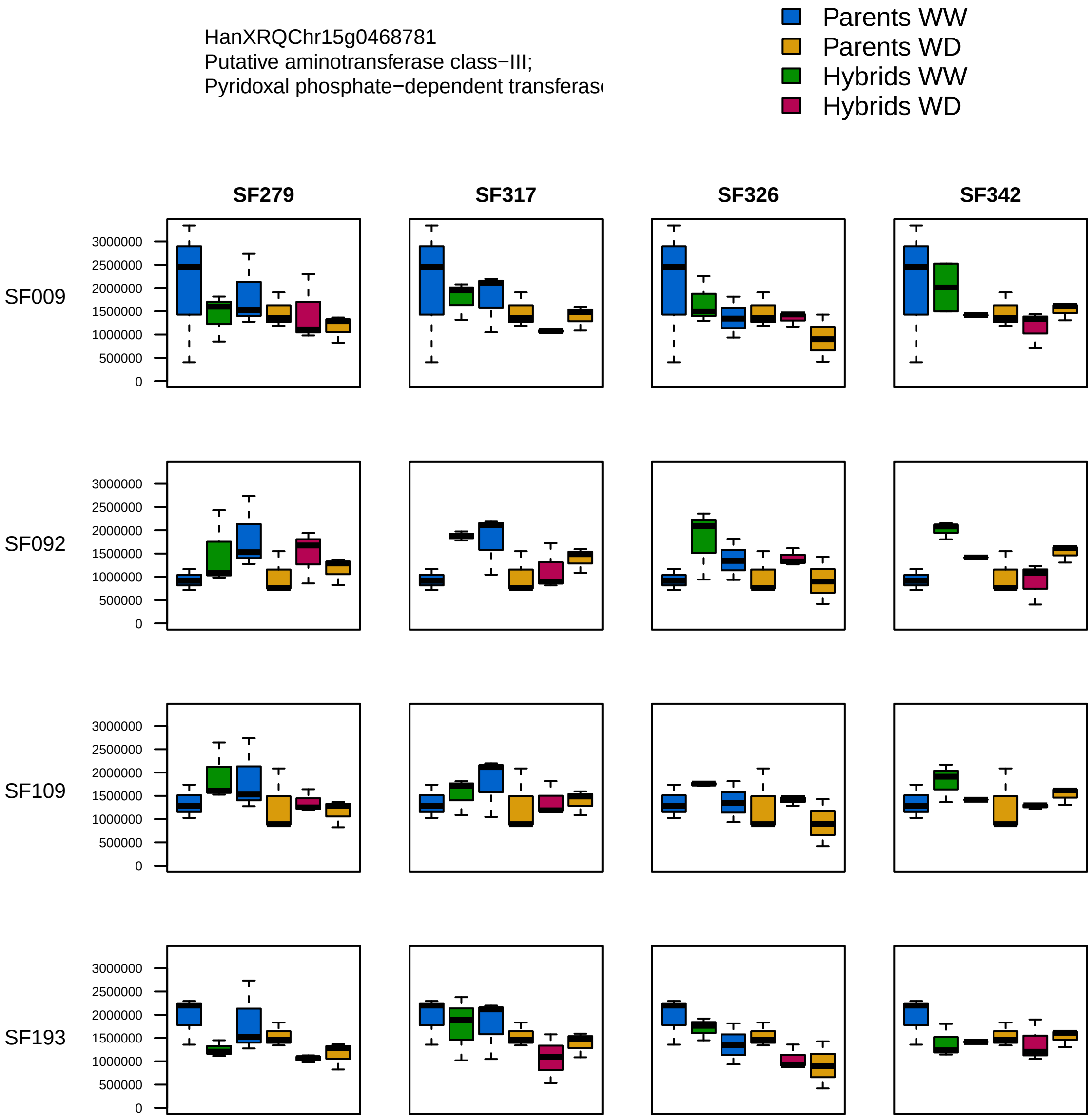
HanXRQChr15g0468781
Putative aminotransferase class−III;
Pyridoxal phosphate−dependent transferas
Parents WW
Parents WD
Hybrids WW
Hybrids WD
SF279
SF317
SF326
SF342
SF009
SF092
SF109
SF193
3000000
2500000
2000000
1500000
1000000
500000
0

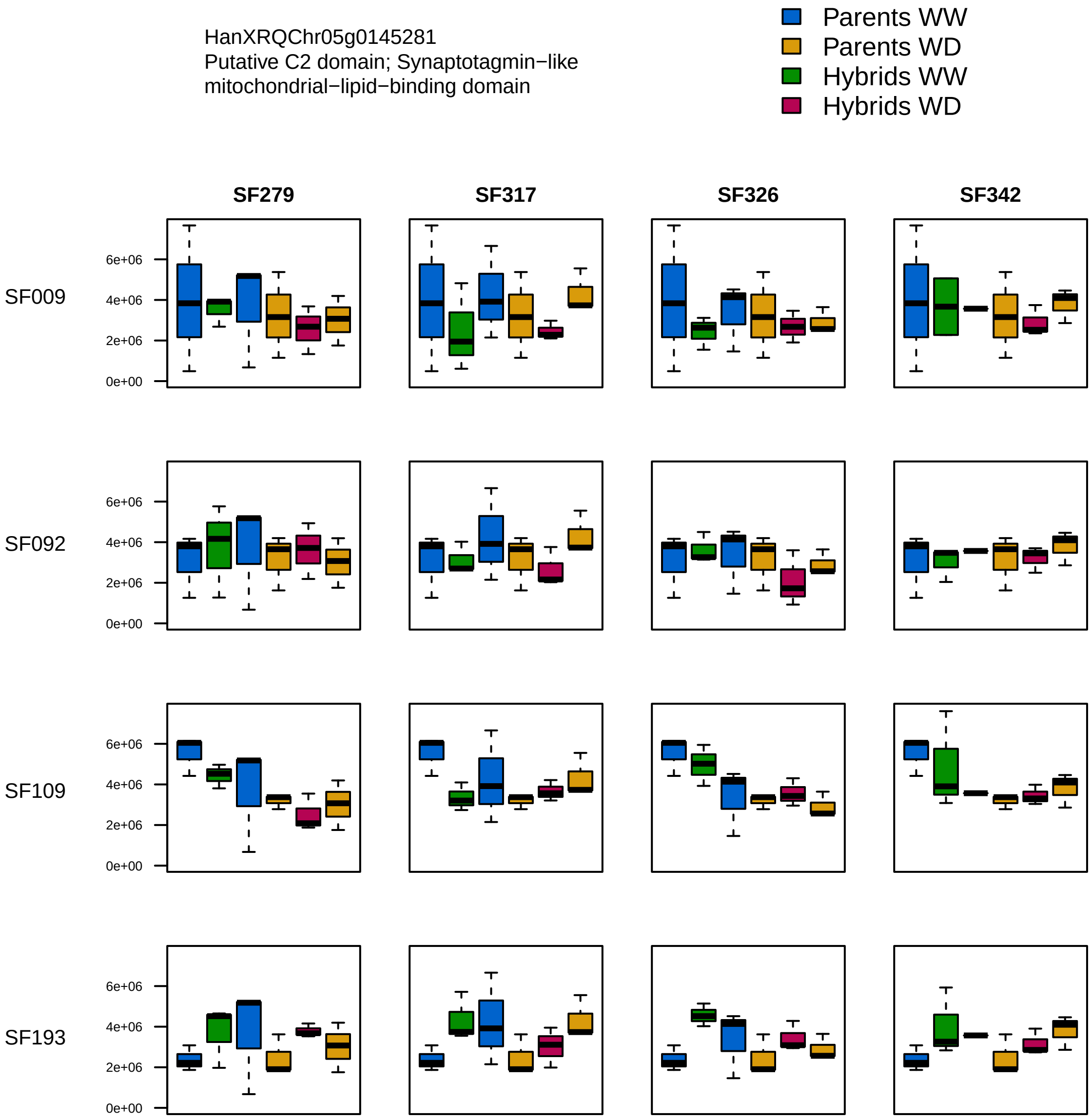

HanXRQChr05g0145281
Putative C2 domain; Synaptotagmin−like
mitochondrial−lipid−binding domain
Parents WW
Parents WD
Hybrids WW
Hybrids WD
SF279
SF317
SF326
SF342
SF009
SF092
SF109
SF193
6e+06
4e+06
2e+06
0e+00

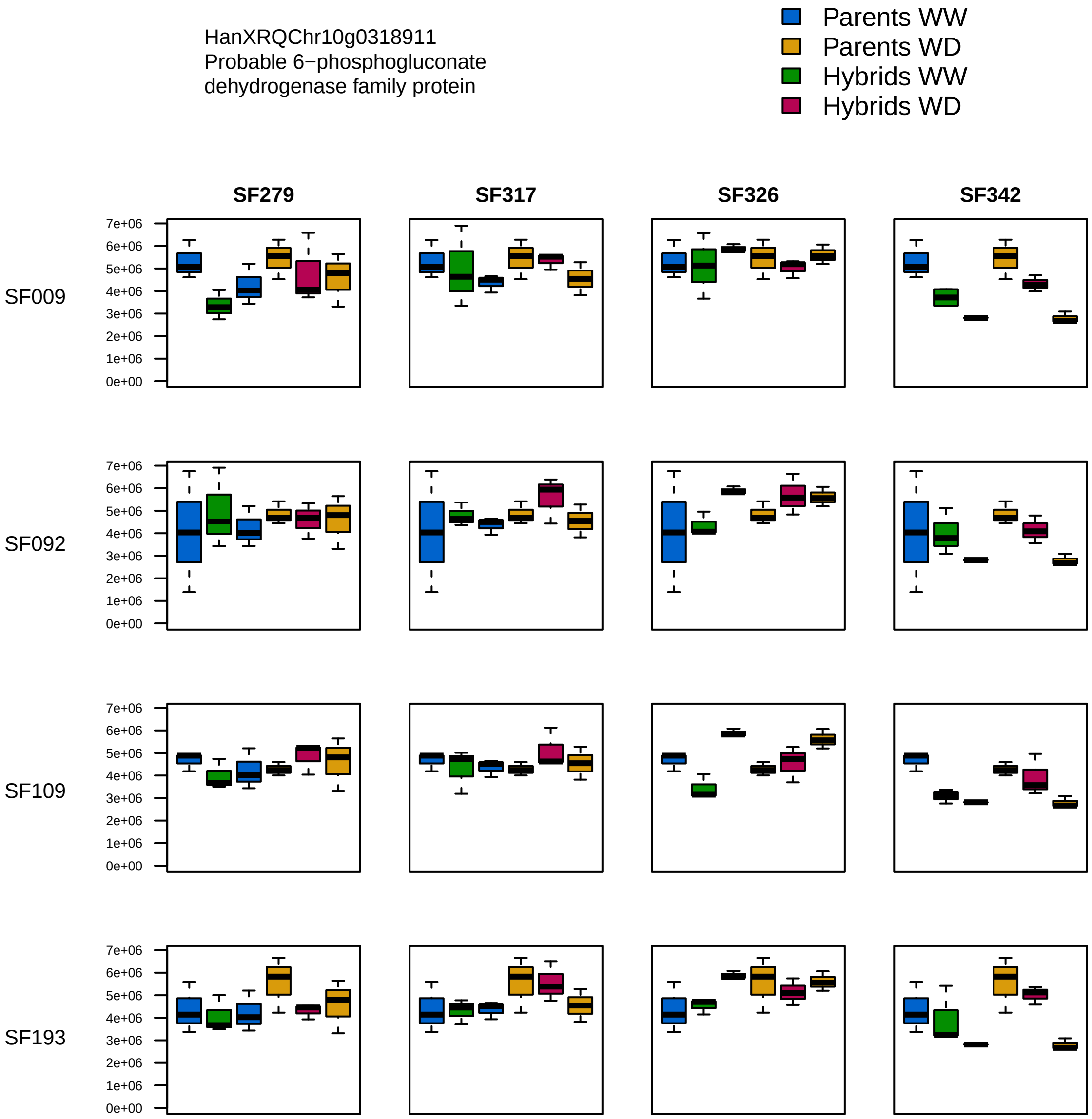
HanXRQChr10g0318911
Probable 6−phosphogluconate
dehydrogenase family protein
Parents WW
Parents WD
Hybrids WW
Hybrids WD
SF279
SF317
SF326
SF342
SF009
SF092
SF109
SF193
0e+00
1e+06
2e+06
3e+06
4e+06
5e+06
6e+06
7e+06

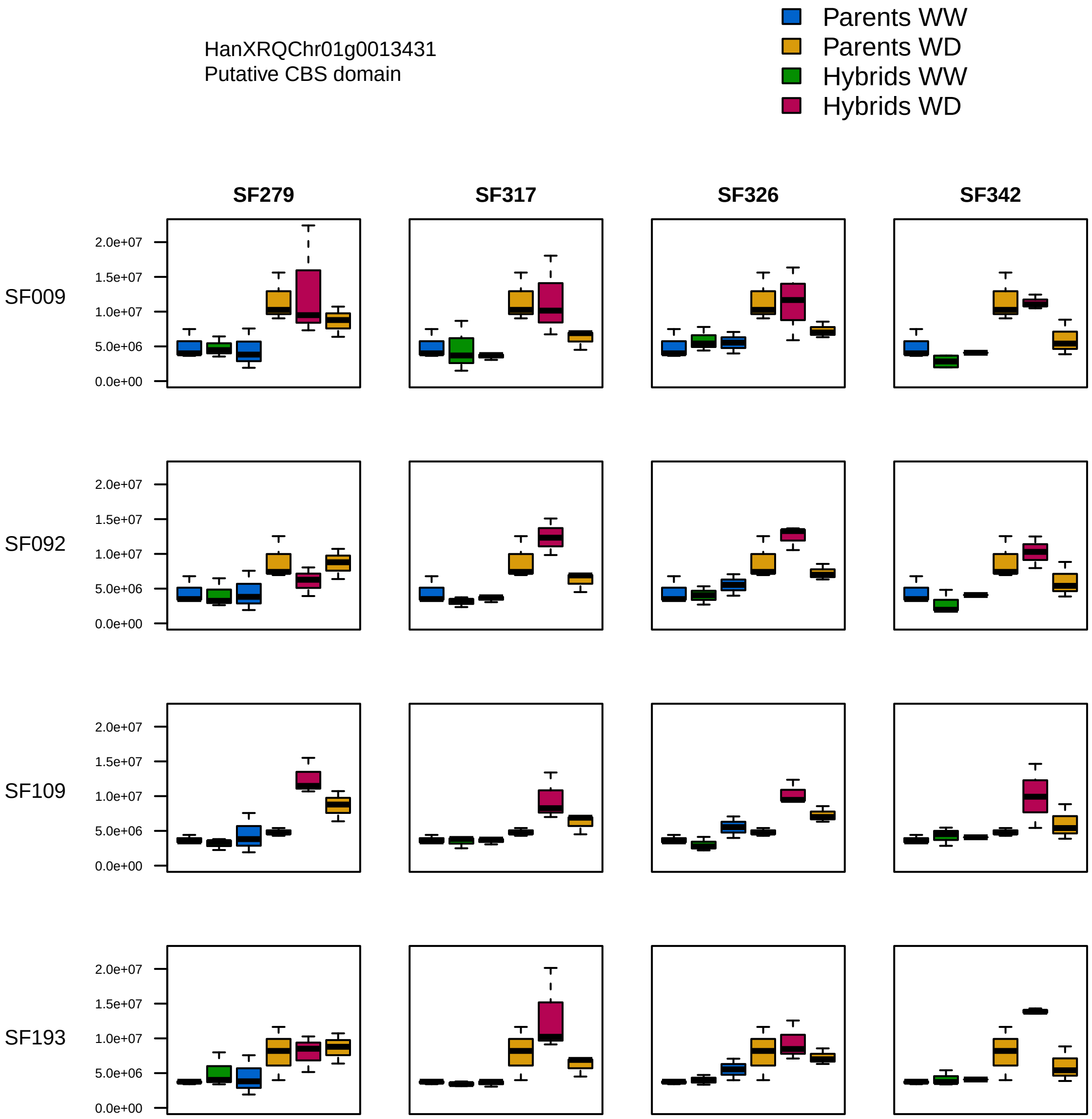
HanXRQChr01g0013431
Putative CBS domain
Parents WW
Parents WD
Hybrids WW
Hybrids WD
SF279
SF317
SF326
SF342
SF009
SF092
SF109
SF193
2.0e+07
1.5e+07
1.0e+07
5.0e+06
0.0e+00

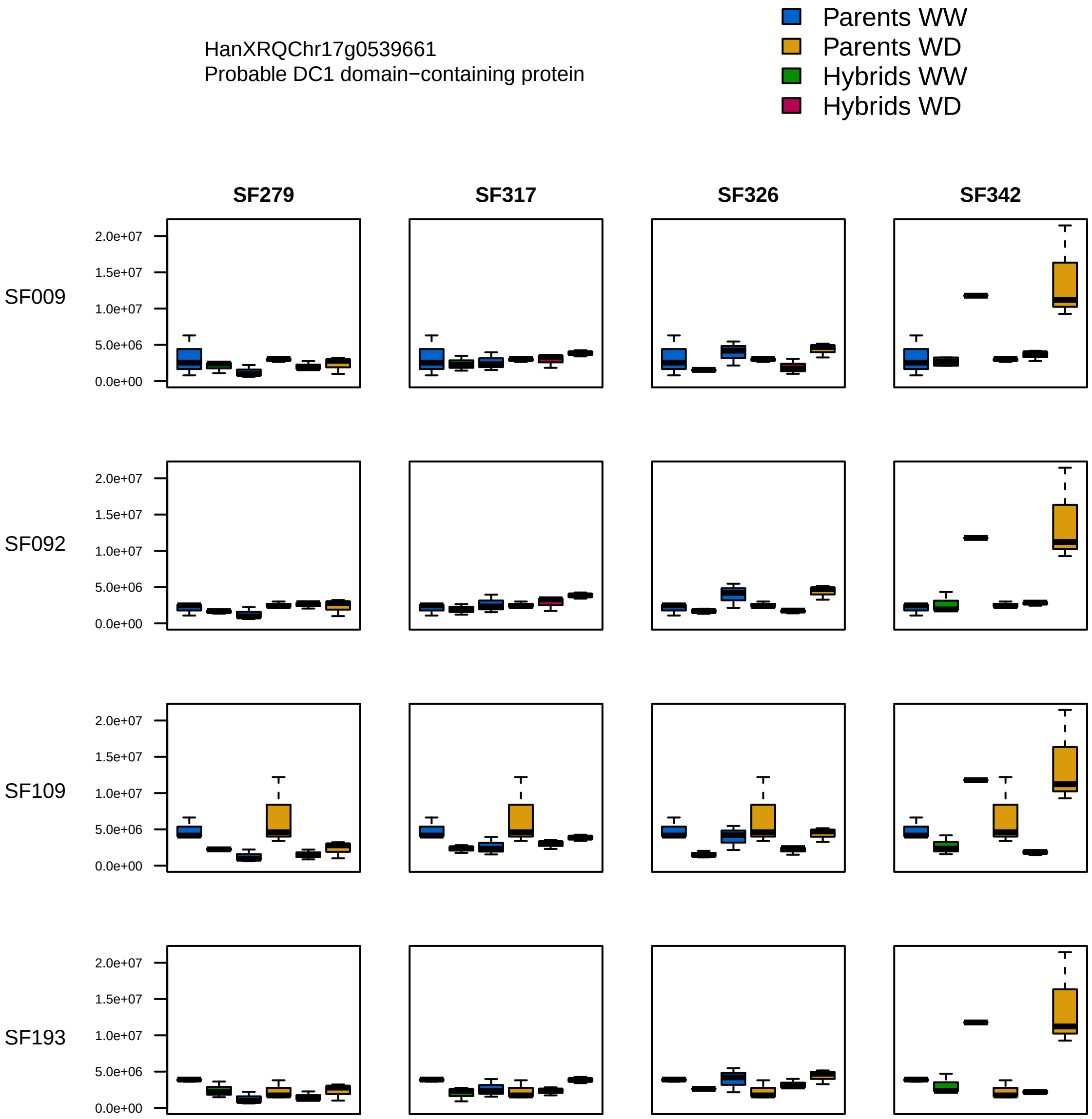
HanXRQChr17g0539661
Probable DC1 domain−containing protein
Parents WW
Parents WD
Hybrids WW
Hybrids WD
SF279
SF317
SF326
SF342
SF009
SF092
SF109
SF193
2.0e+07
1.5e+07
1.0e+07
5.0e+06
0.0e+00

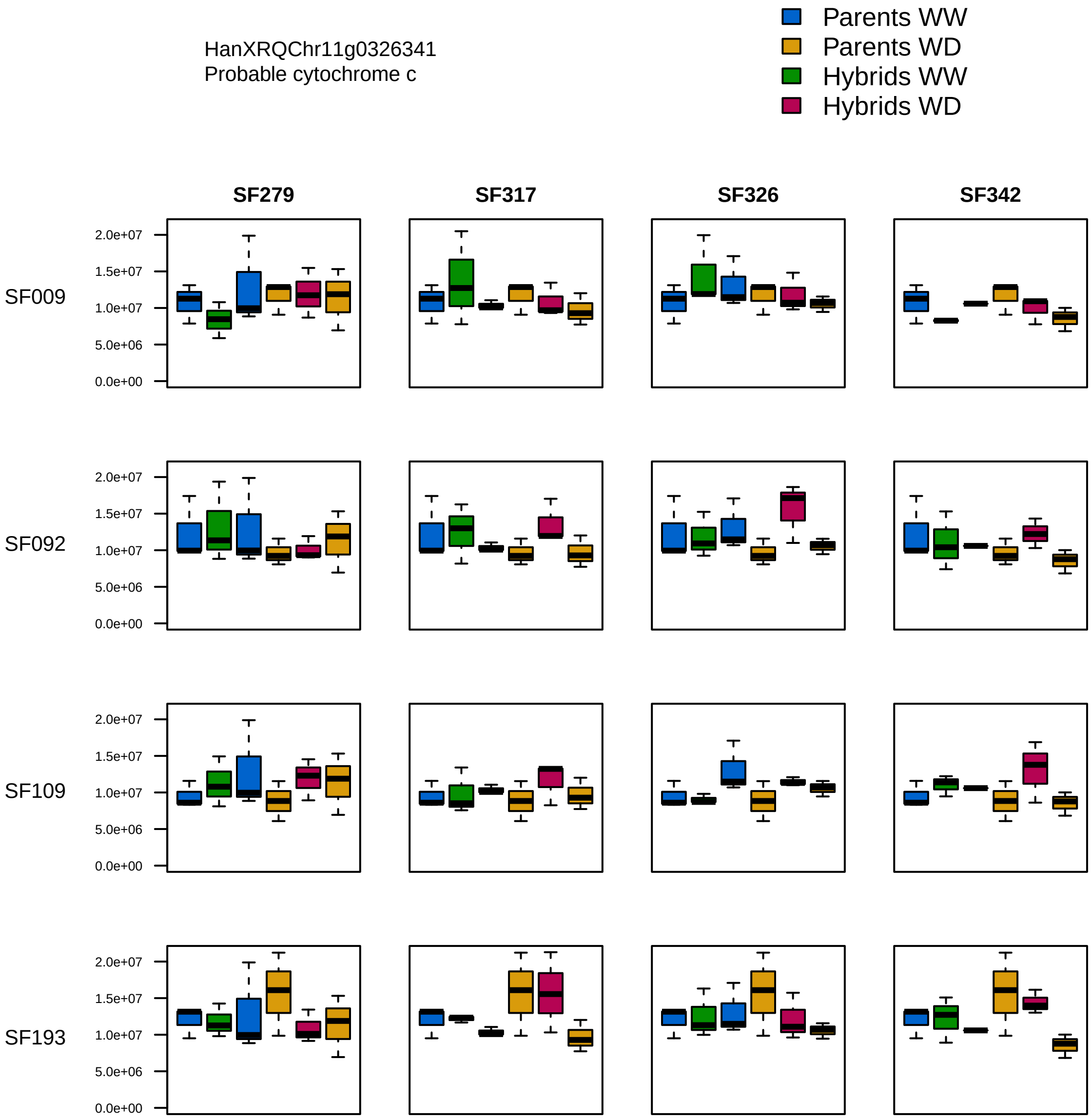
HanXRQChr11g0326341
Probable cytochrome c
Parents WW
Parents WD
Hybrids WW
Hybrids WD
SF279
SF317
SF326
SF342
SF009
SF092
SF109
SF193
2.0e+07
1.5e+07
1.0e+07
5.0e+06
0.0e+00

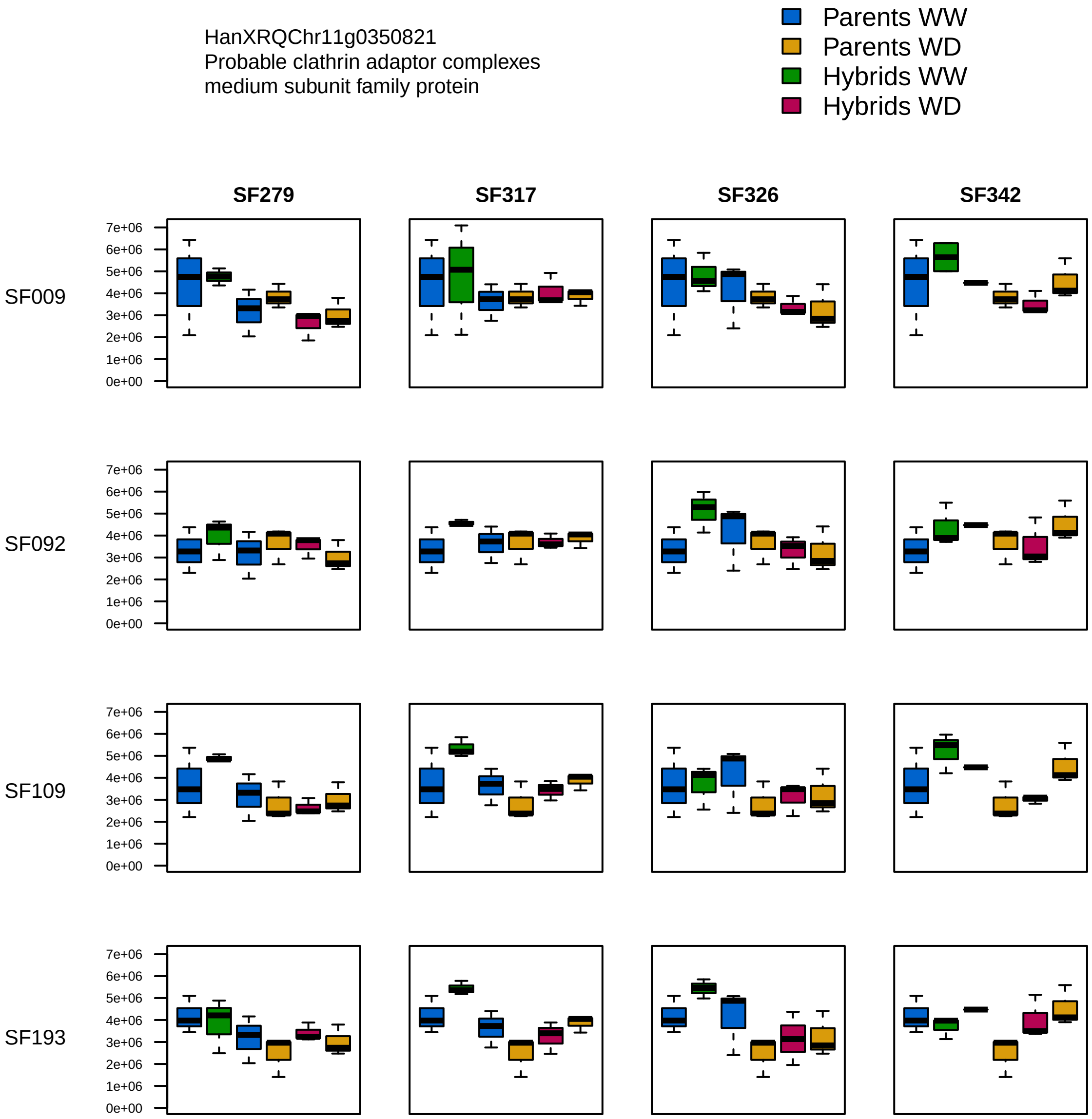
HanXRQChr11g0350821
Probable clathrin adaptor complexes
medium subunit family protein
Parents WW
Parents WD
Hybrids WW
Hybrids WD
SF279
SF317
SF326
SF342
SF009
SF092
SF109
SF193
0e+00
1e+06
2e+06
3e+06
4e+06
5e+06
6e+06
7e+06

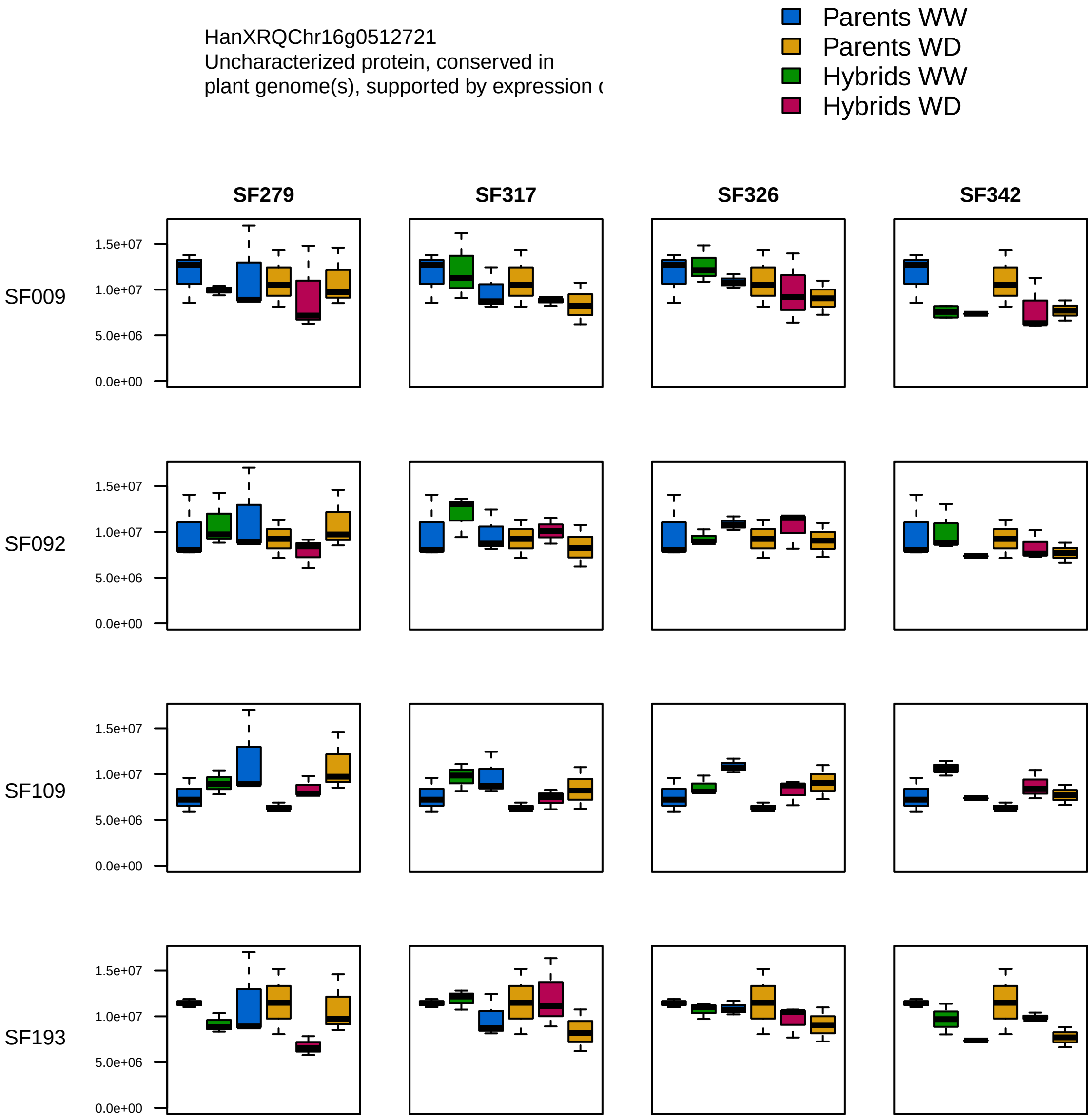
HanXRQChr16g0512721
Uncharacterized protein, conserved in
plant genome(s), supported by expression (
Parents WW
Parents WD
Hybrids WW
Hybrids WD
SF279
SF317
SF326
SF342
SF009
SF092
SF109
SF193
1.5e+07
1.0e+07
5.0e+06
0.0e+00

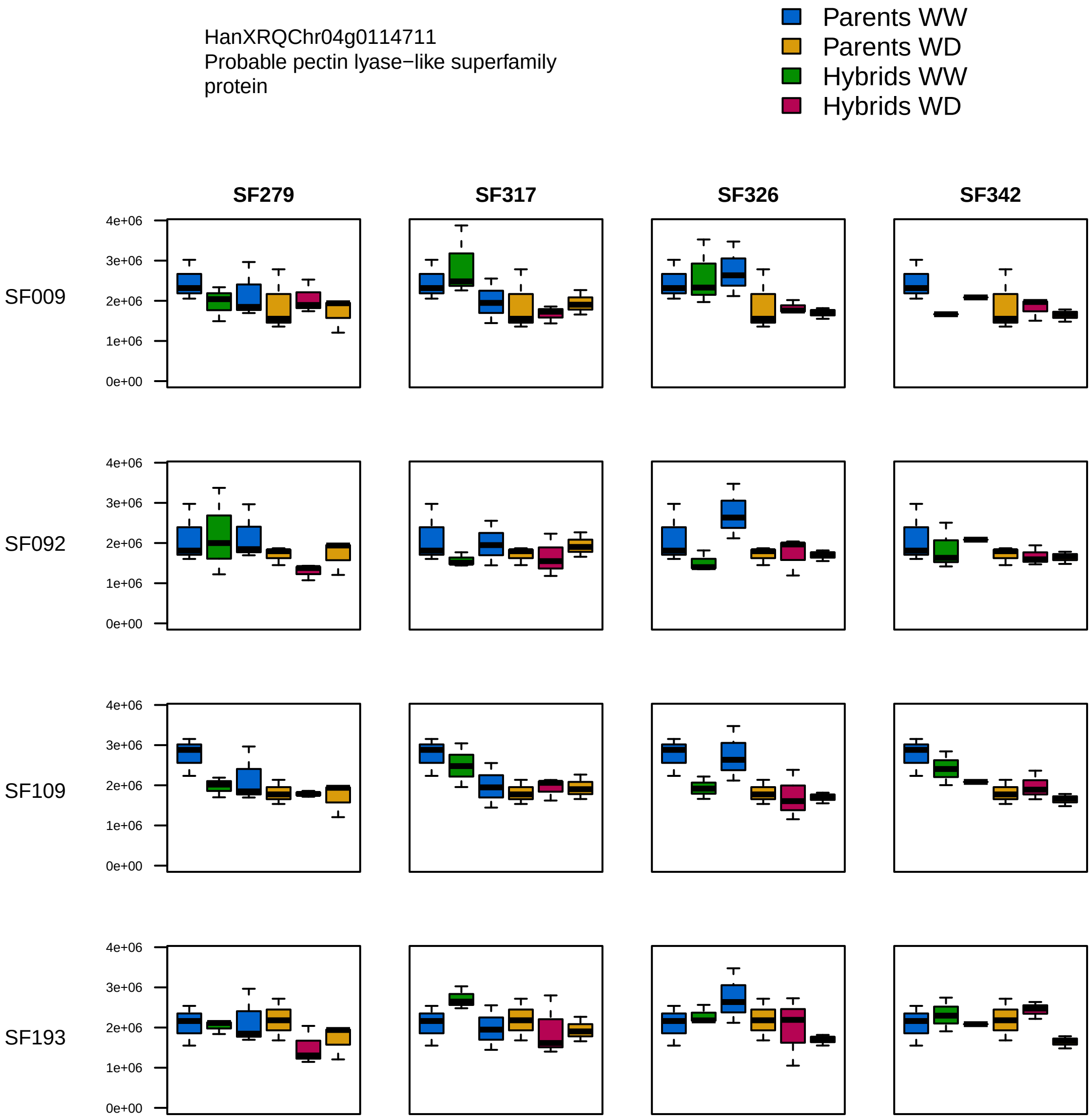

HanXRQChr04g0114711
Probable pectin lyase−like superfamily protein
Parents WW
Parents WD
Hybrids WW
Hybrids WD
SF279
SF317
SF326
SF342
SF009
SF092
SF109
SF193
0e+00
1e+06
2e+06
3e+06
4e+06

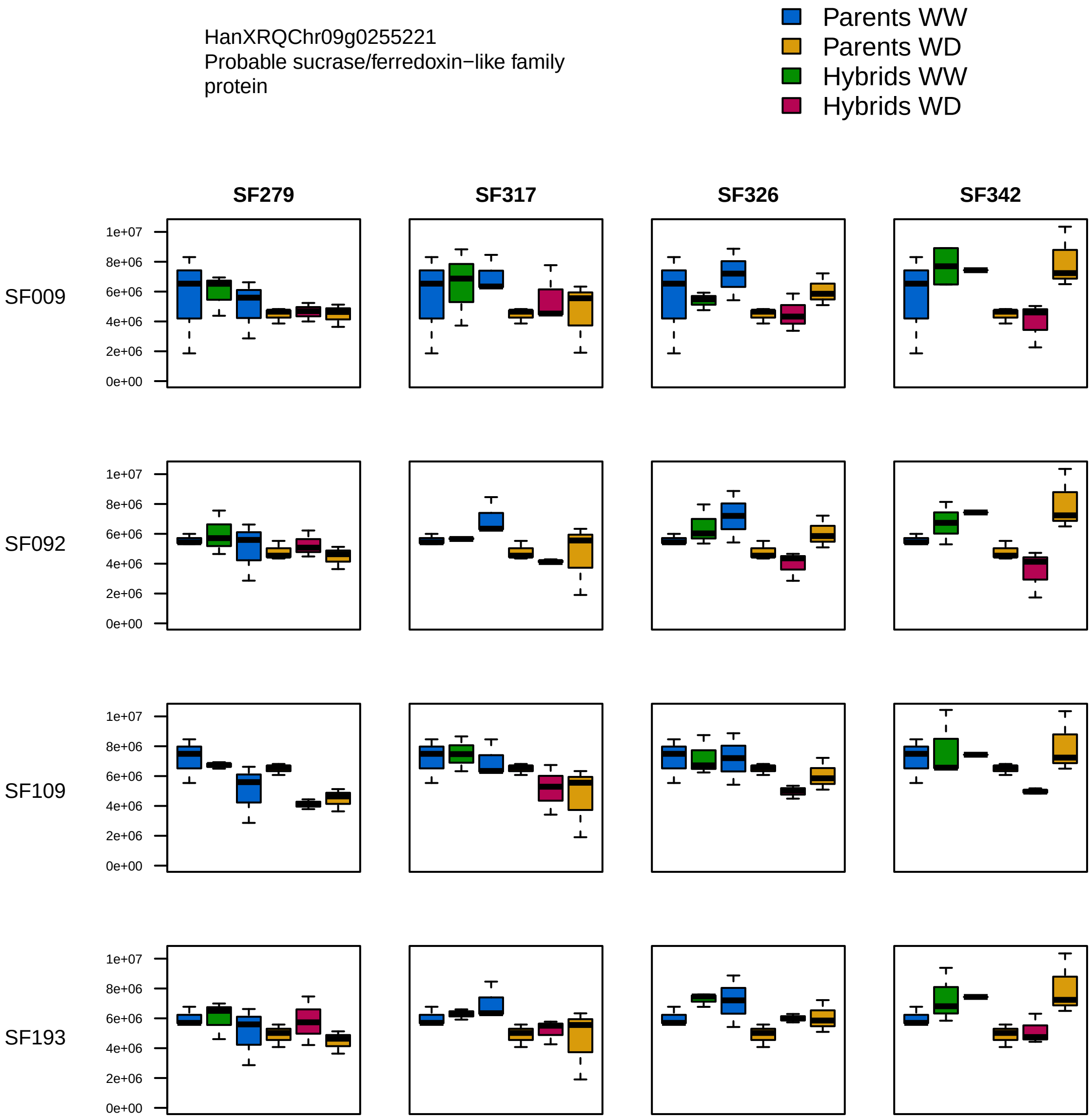
HanXRQChr09g0255221
Probable sucrase/ferredoxin−like family protein
Parents WW
Parents WD
Hybrids WW
Hybrids WD
SF279
SF317
SF326
SF342
SF009
SF092
SF109
SF193
1e+07
8e+06
6e+06
4e+06
2e+06
0e+00

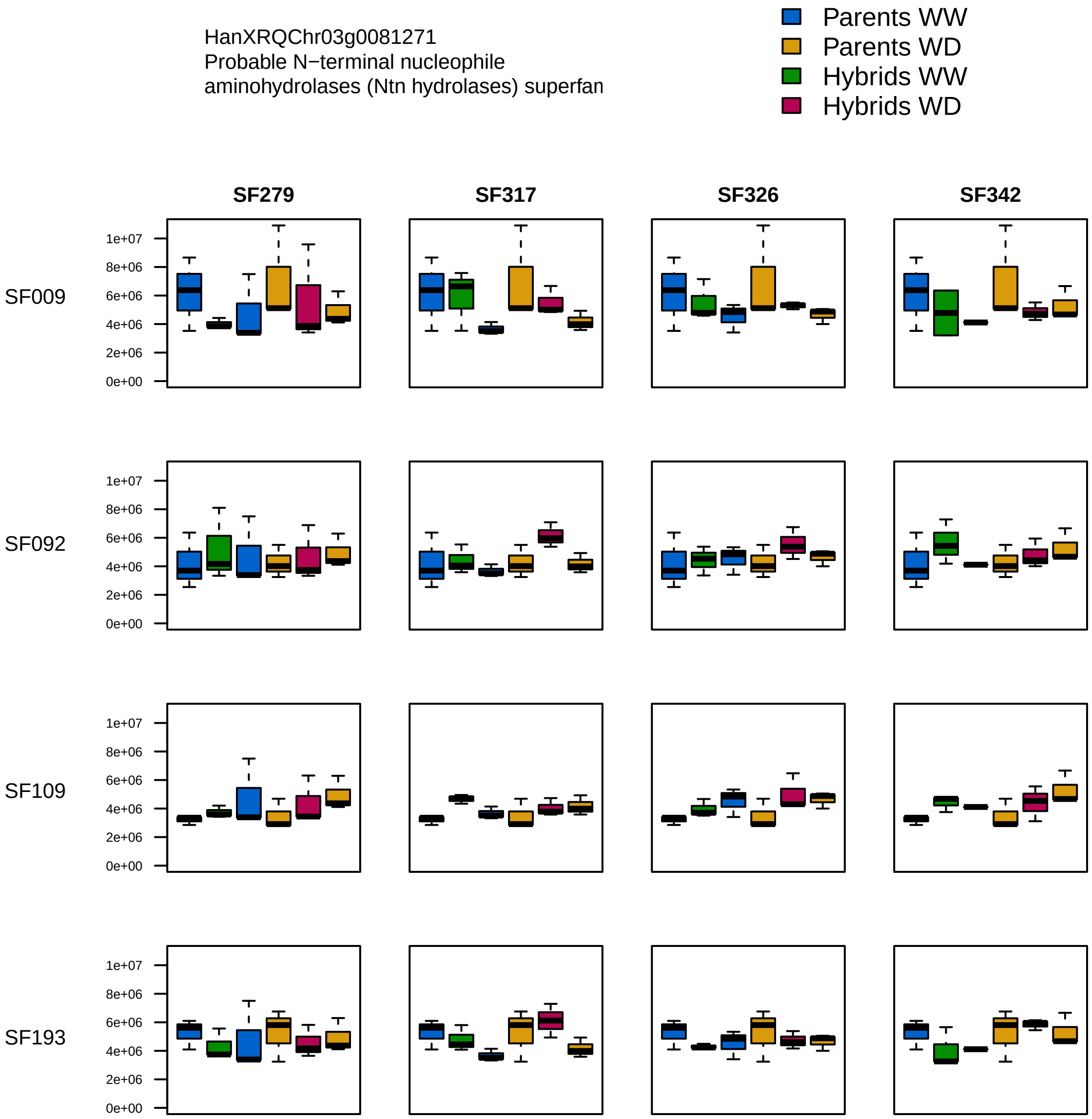
HanXRQChr03g0081271
Probable N−terminal nucleophile
aminohydrolases (Ntn hydrolases) superfan
Parents WW
Parents WD
Hybrids WW
Hybrids WD
SF279
SF317
SF326
SF342
SF009
SF092
SF109
SF193
1e+07
8e+06
6e+06
4e+06
2e+06
0e+00

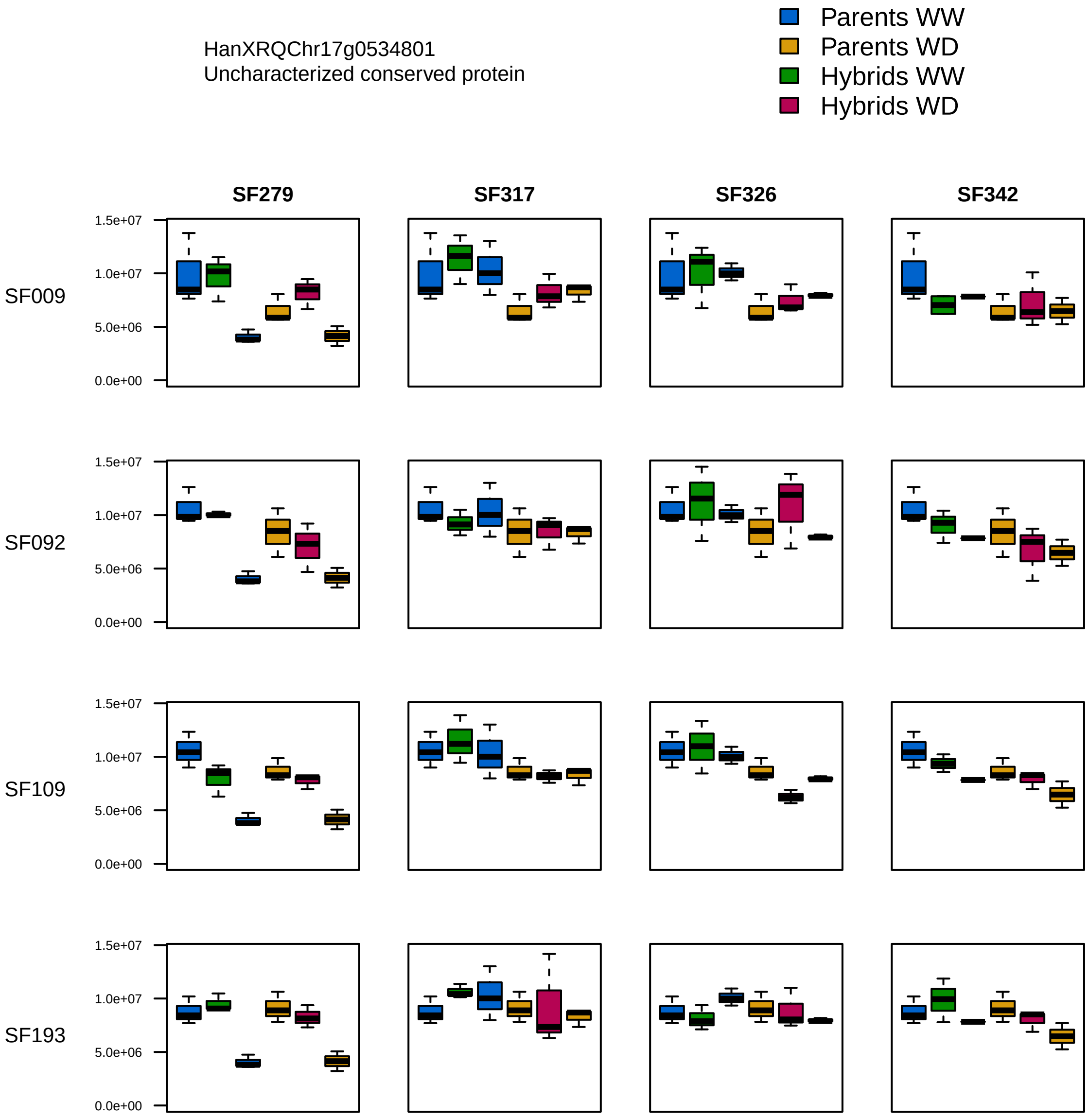

HanXRQChr17g0534801
Uncharacterized conserved protein
Parents WW
Parents WD
Hybrids WW
Hybrids WD
SF279
SF317
SF326
SF342
SF009
SF092
SF109
SF193
1.5e+07
1.0e+07
5.0e+06
0.0e+00

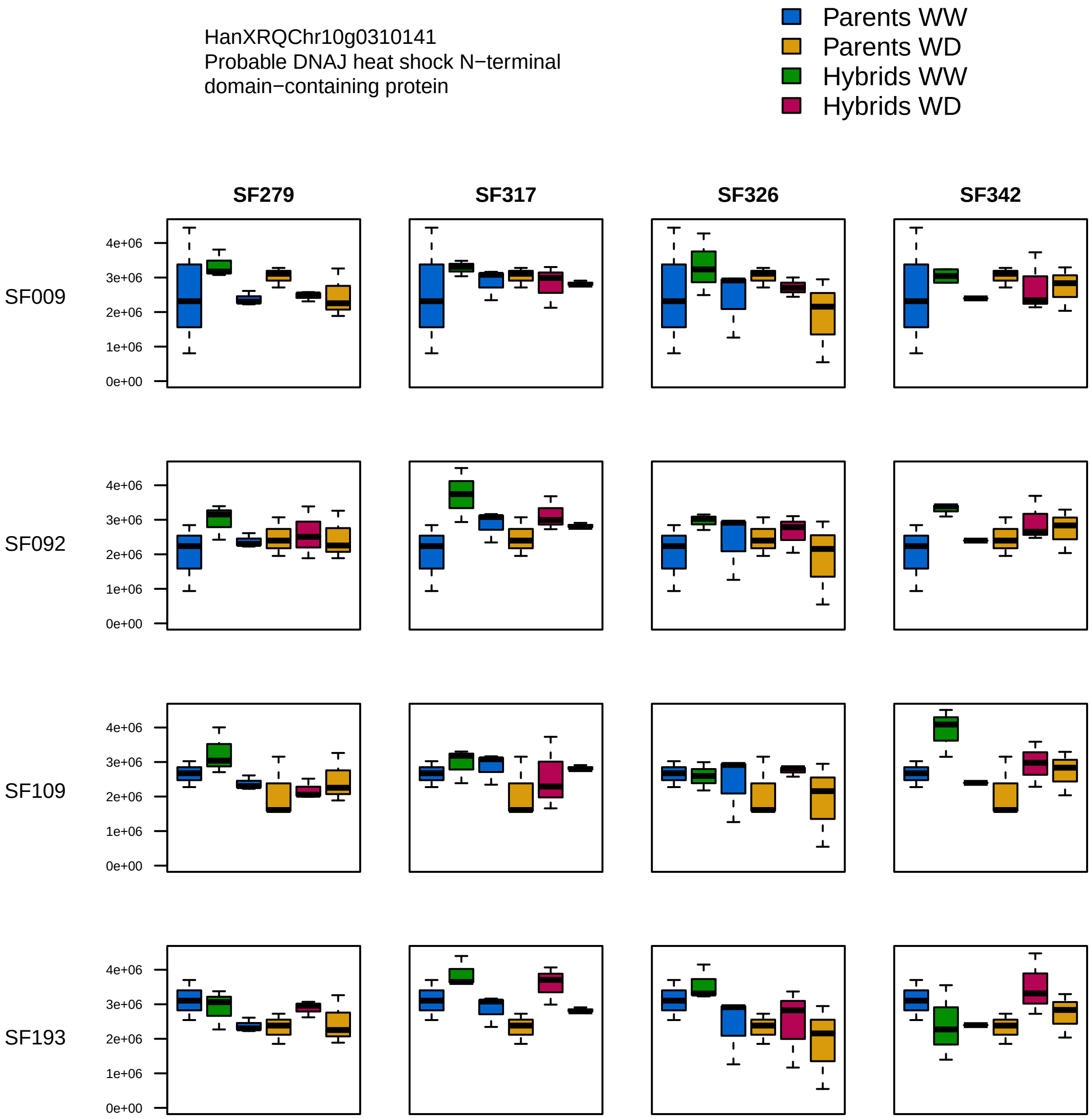
HanXRQChr10g0310141
Probable DNAJ heat shock N−terminal
domain−containing protein
Parents WW
Parents WD
Hybrids WW
Hybrids WD
SF279
SF317
SF326
SF342
SF009
SF092
SF109
SF193
0e+00
1e+06
2e+06
3e+06
4e+06

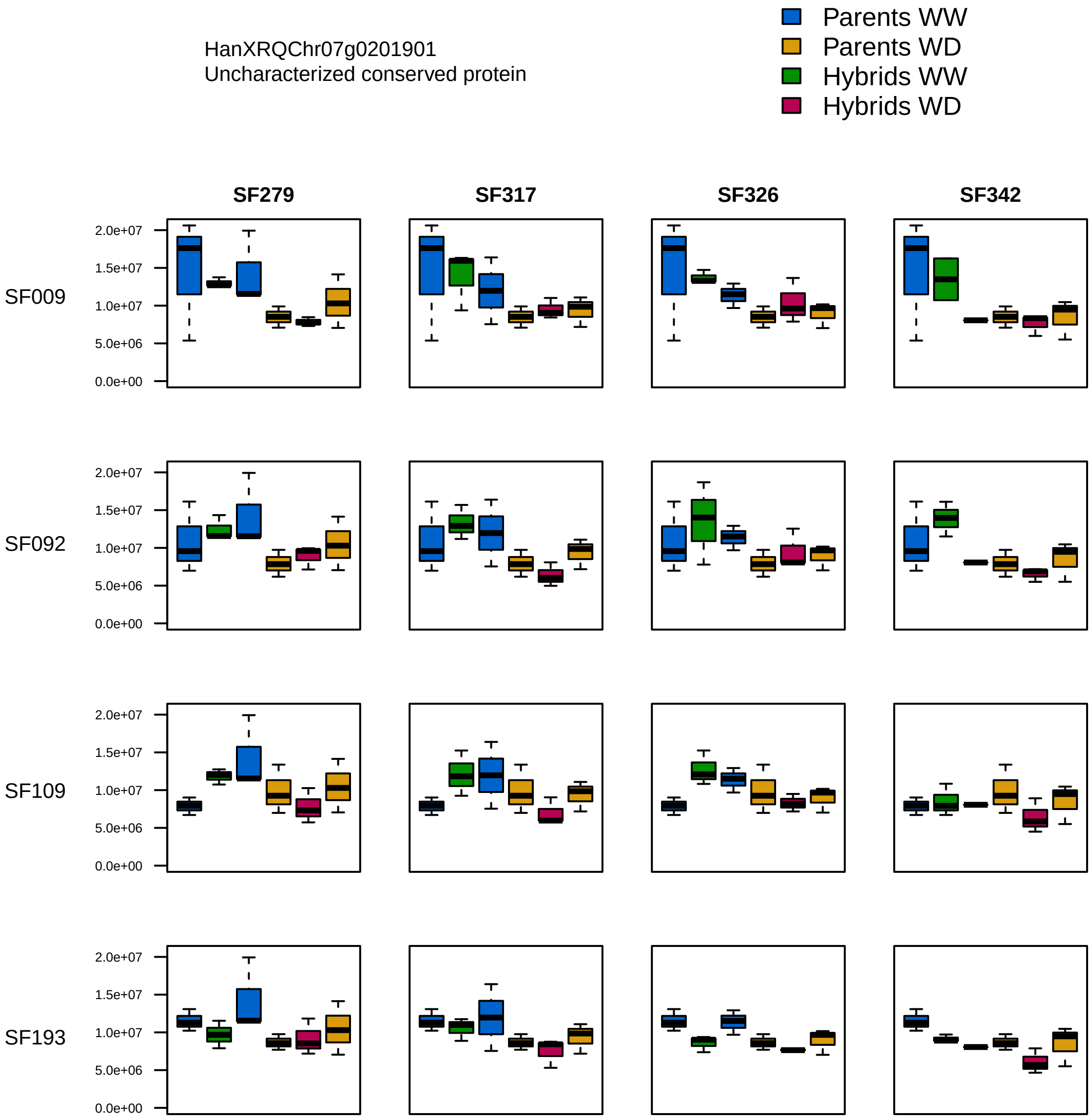
HanXRQChr07g0201901
Uncharacterized conserved protein
Parents WW
Parents WD
Hybrids WW
Hybrids WD
SF279
SF317
SF326
SF342
SF009
SF092
SF109
SF193
2.0e+07
1.5e+07
1.0e+07
5.0e+06
0.0e+00
